# Novel data dependent divider circuit block implementation for complex division and area critical applications

**DOI:** 10.1038/s41598-023-28343-3

**Published:** 2023-02-21

**Authors:** Udayan S. Patankar, Miguel E. Flores, Ants Koel

**Affiliations:** 1 Tallinn University of Technology, Tallinn, Estonia; 2 Electronics School, Don Bosco University, Soyapango, El Salvador

**Keywords:** Electrical and electronic engineering, Applied mathematics, Pure mathematics, Software, Mathematics and computing

## Abstract

This article elaborates on the state-of-the-art novel Udayan S. Patankar (USP)-Awadhoot algorithm for distinctive implementation area improvement for area-critical electronic applications. The proposed USP-Awadhoot divider is a digit recurrence class, but it can be flexibly implemented as a restoring or nonrestoring algorithm. The implementation example indicates the use of the Baudhayan-Pythagoras triplet method in association with the proposed USP-Awadhoot divider. The triplet method provides an easy way to generate Mat_Term1, Mat_Term2, and T_Term, which are further utilized with the proposed USP-Awadhoot divider. The USP-Awadhoot divider is implemented in three parts. First is preprocessing circuit stage for executing a dynamic separate scaling operation on input operands, ensuring the inputs are in the correct form. Second is the processing circuit stage for implementing the conversion logic expressed by the Awadhoot matrix, and third is the postprocessing circuit stage for recombining the individual results into the final result. The proposed divider works upto 285 MHz frequency with a power estimation of 3.366 W, also significantly improves the chip area requirements over those of the commercially and noncommercially implemented solutions.

## Introduction

Enhancement in the semiconductor manufacturing industry has proven valuable and innovative for existing applications such as communications, transport, signal processing, and computation, where mathematics plays a vital role and enables the evolution of new fields of work and study, mainly data protection, statistical data analysis, computational processing, signal processing, artificial intelligence, image processing, high-performance graphics rendering systems (such as graphic processing units (GPUs), complex systems on chips, central processing units, biomedical equipment, fuzzy control, and space engineering^1–15^. Performance evaluations of division operation implementations typically fall into the latency range of tens of clock cycles to hundreds of clock cycles^16–22^. Researchers have focused more on creating better adders and multipliers instead of developing dedicated algorithms for division operations to improve the divider circuit's implementation performance. Therefore, the prospect of improving or developing a new algorithm is plausible. This article elaborates on the state-of-the-art novel Udayan S. Patankar (USP)-Awadhoot algorithm to achieve distinctive implementation area improvement. Furthermore, the sections below describe a divider implementation based on the state-of-the-art novel USP-Awadhoot algorithm; a statistical analysis of its implementation resources; a comparative discussion with different dividers, complex division operations, and area-critical application followed by the conclusion and the future work directions.

### Division circuit block taxonomy

**Figure 1:**
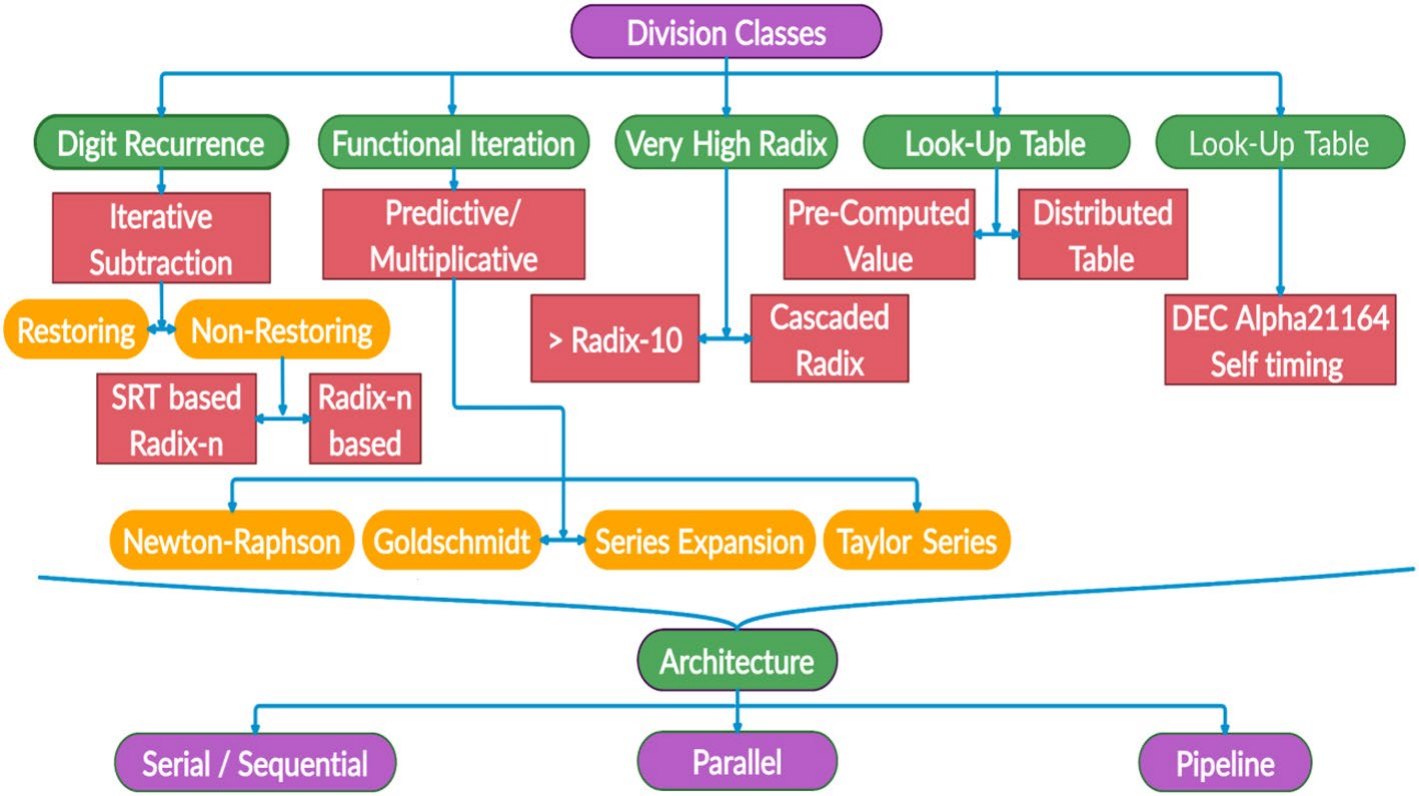
Division algorithm taxonomy.

A study presented in^23^ indicated the performance dependency of a sophisticated system on a division circuit block implementation. It stated that the slightest improvement, such as a 1% improvement in a division circuit block, can increase the original system performance by up to 20%. The hierarchical distribution of various classes of division algorithms is expressed as a division algorithm taxonomy in Fig. 1 based on conversion logic, hardware architecture, performance, and execution type^6,16,24–30^.

Digit recurrence is one of the most trusted, implemented, researched, and commercially utilized division algorithms among all divider implementation classes. Restoring algorithms and some nonrestoring algorithms implement simple conversion logic but requiring longer conversion time and large areas. Although the conversion logic is simple, it is not suited for high-frequency applications due to latency problems. Functional iterative class dividers compute a quotient bit based on the estimation or approximation of series expansion functions, such as the Newton-Rapson algorithm^31,32^, Goldschmidt algorithm^11,33–36^, Taylor series algorithm^11,37–40^. This approach utilizes multiplication instead of subtraction operations, reducing the number of required iterations, and can generate multiple quotient digits in one iteration with low latency, but the area required for a multiplier is higher than that of an adder or subtractor. This type of divider has a major drawback regarding quotient bit inaccuracy because of the direct rounding off of approximate solution values rather than infinitely precise values. The induced error depends on the accuracy of the initial estimation.

In the Newton–Raphson iteration method, which is limited to two multiplications and must proceed in series, a large error is generated due to the rounding off to approximate value. Reducing this error requires the introduction of a trade-off between the additional chip area required for the LUT and the latency of the divider. The Goldschmidt algorithm is another functional iterative divider whose major drawback is that it does not provide a remainder, making it useful only for floating-point division^36^. Another drawback is that 1's complement can prevent carry propagation delay, but it adds a new approximation error in each iteration. In Taylor series dividers, Taylor series expansion is used to calculate accurate anti-divisors (reciprocals) to reduce the error in the least-important bits of quotient precision with a parallel powering section that computes high-order terms, leading to extra hardware overhead and increased area requirements.

Variable-latency class^41,42^ dividers are very rare due to their complexity and area constraints. A high radix divider^43^ reduces the latency but requires a high-capacity LUT, which is impractical for implementation. The LUT class^27,44^ requires storage such as read-only memory (ROM), which increases the area requirements of its implementations. For better performance, either optimized area and hardware resources are needed, or the latency cycles must be interrelated. Three types of hardware architectures can be used for divider implementations. A serial hardware architecture^25,45,46^ requires higher latency and a larger conversion time, making it inappropriate for highly critical applications. A parallel hardware architecture^6,10,13^ contrasts with serial architectures, requires multiple cores to work together simultaneously, makes synchronization critical, and has high area requirements, leading to increased implementation costs. A pipelined architecture^9,25,45,47^ is the best choice for achieving parallelism in a sequential architecture with parallel processing. Some or all processes of division algorithms can be pipelined to achieve partial parallel processing. The radix based SRT division algorithm is one of the most implemented nonrestoring digit recurrence algorithms. The SRT algorithm named after Sweeney, Robertson, and Tocher is used in serial, parallel, pipelined, and cascaded architectures and various applications^16,25,28,48–64^. Although the SRT algorithm was the first choice for commercial implementations of the majority of soft and modern processors, such as Intel's Pentium processor^65^, Xilinx's FPGA controllers^66^, and the arithmetic logic units (ALUs) of complex hardware, it is restricted to specific low radix values (significantly less than 10). Radix-2 and radix-4 are the most implementable formats of the SRT algorithm. The main reasons for limiting the implementations of the SRT algorithm to low radix values are the increase in the quotient selection logic's criticality and the enormous increase in the area requirements of storing LUTs for this logic. It results in the failure to follow the execution cycle, as it requires multiple clock cycles for execution.

### Complex divider

A software or hardware divider forms complex numbers based on the conventional formula using a complex conjugate, where ${z}_{1}$, and ${z}_{2}$ are two complex numbers consisting of real and imaginary parts. The divide-and-conquer concept must be used to implement two separate dividers for a complex number's real and imaginary parts. In the end, these two parts must be connected into one part to represent a complex quotient and the remainder as a final result. Many different approaches have recommended alternate number system for complex number representation as a single entity instead of separate real and imaginary parts, but it increases the complexity of conversion logic, resulting in area overhead^67–71^. A software or hardware divider forms complex numbers based on the conventional formula mentioned in (2), where ${z}_{1}$, and ${z}_{2}$ are two complex numbers that may lead to overflow or underflow conditions when the operands are near the extreme ends of the representable range^72^.

1 $${z}_{1}={x}_{1}+{iy}_{1} \; and \; {z}_{2}={x}_{2}+{iy}_{2}$$

2 $$\frac{{z}_{1}}{{z}_{2}}=\frac{\left({x}_{1}{x}_{2}+{y}_{1}{y}_{2}\right)}{\left({x}_{2}^{2}+{y}_{2}^{2}\right)}+\frac{\left({x}_{2}{y}_{1}-{x}_{1}{y}_{2}\right)i}{\left({x}_{2}^{2}+{y}_{2}^{2}\right)}$$

Smith’s algorithm solved this drawback by providing a more robust calculation, which was further enhanced by the Stewart process. Ultimately, the Stewart process makes the algorithm more complex rather than making it more robust. It also lacks a guarantee regarding the correctness of the rounding process for the quotient's real and imaginary parts during complex division. The hardware implementation of Smith’s division algorithm is unsuitable because of the overhead caused by the costliest hardware components needed^72^. The hardware implementation of a single divider could be critical and in the complex divider, we must implement two sets of dividers for the real and imaginary parts of the given complex number, restricting the SRT divider to low radix to keep low area overhead and less critical conversion logic. In the case of a functional iteration divider, the correctness of the obtained result depends on the closeness of the reciprocal value selected in the initial iteration. Among several approaches for high-radix complex dividers, the best is a prescaled divider^72,73^, where the divisor and dividend are multiplied by the same scaling factor so the resultant divisor must be close to unity. The main drawback of this method is that it requires an extra full-width divider for calculating the scaling factor.

### Research questions

Many researchers have worked on various parameter improvement techniques, such as prescaling operands, carry-save remainders, array implementations, truncations, and differential LUTs, leading to the possibility of developing a new technique or combination of fast or moderate methods in terms of time and area efficiency. The current article is concerned with the following research problems/questions.

- Investigate the theory of conversion logic to develop a dynamic separate scaling operation/factor for input operands. Here separate scaling operations/factors mean one for the dividend and another for the divisor. Also, dynamic means different values for separate scaling operations/factors for different combinations of input operands.
- Improve the implementation area requirements to realize a dedicated divider circuit.
- Reduce the criticality of conversion logic by avoiding overlapping regions in quotient selection.

We propose a digit recurrence divider based on a state-of-the-art novel USP-Awadhoot algorithm for improving distinctive divider implementations with moderate operation speeds suitable for complex division and area-critical applications. In the following sections, we discuss the implementation of a Baudhayan-Pythagoras triplet method using a novel state-of-the-art USP-Awadhoot algorithm-based divider developed according to the ancient theories provided by Vedic mathematics during the early centuries. We also discuss the statistical analysis of implementation resources and elaborate on a comparative discussion with different dividers, followed by a conclusion and future work directions.

### Complex division via the Baudhayan-Pythagoras triplet algorithm using a novel state-of-the-art USP-Awadhoot divider circuit block

In the present article, we discussed the unique way of complex division based on the Baudhayan-Pythagoras triplet method and the proposed novel state-of-the-art USP-Awadhoot divider circuit block. The use of the Baudhayan-Pythagoras triplet algorithm is possible because of the geometric properties of the complex numbers, which can be used to represent them via real and imaginary axis. The proposed complex division implementation is partitioned into three parts. The Baudhayan-Pythagoras triplet algorithm is used for the input circuit stage, ensuring the separation of the real and imaginary parts of complex number for further calculation. The second stage consists of a novel state-of-the-art USP-Awadhoot divider circuit block, which actually performs the division in real and imaginary parts of the complex number. The third stage consists of the recombination stage representing the final results in complex numbers. The Pythagorean theorem was known long before Pythagoras (570–500/490 BCE); Baudhayan (800–740 BCE) is said to be the pioneer of the Pythagorean theorem. Baudhayan formulated the relation between the hypotenuse and other sides of a triangle in terms of the area of the triangle in his book titled Baudhāyan Śulbasûtra, and in contrast, Pythagoras presented proof of the relationship between the hypotenuse and other sides of a triangle in terms of length^74,75^ giving the equation

3 $$Baudhayan{\text{-}}Pythagoras \; Triplet (x, y, z) = T (x, y, z)$$

As $i=\sqrt{-1}$ or ${i}^{2}=-1$, we can correlate the Baudhayan-Pythagoras triplet function $T (x, y, z)$ with the complex number, and we can represent a given complex number in terms of $T (x, y, z)$. The first two variables of the triplet are considered the real and imaginary coefficients of a given complex number. The following equations are used to develop the input circuit stage.

4 $${r}_{1}={x}_{1}+i{y}_{1} \; and \; {r}_{2}={x}_{2}+i{y}_{2}$$

5 $$T({r}_{1})=f ( {x}_{1},{y}_{1},{z}_{1})$$

6 $$T({r}_{2})=f ( {x}_{2},{y}_{2},{z}_{2})$$

7 $$\frac{T({r}_{1})}{T({r}_{2})}=\frac{f ( {x}_{1},{y}_{1},{z}_{1})}{f ( {x}_{2},{y}_{2},{z}_{2})}=\left[\left({x}_{1}{x}_{2}+{y}_{1}{y}_{2}\right), \left({x}_{2}{y}_{1}-{x}_{1}{y}_{2}\right), {z}_{2}^{2}\right]$$

**Figure 2:**
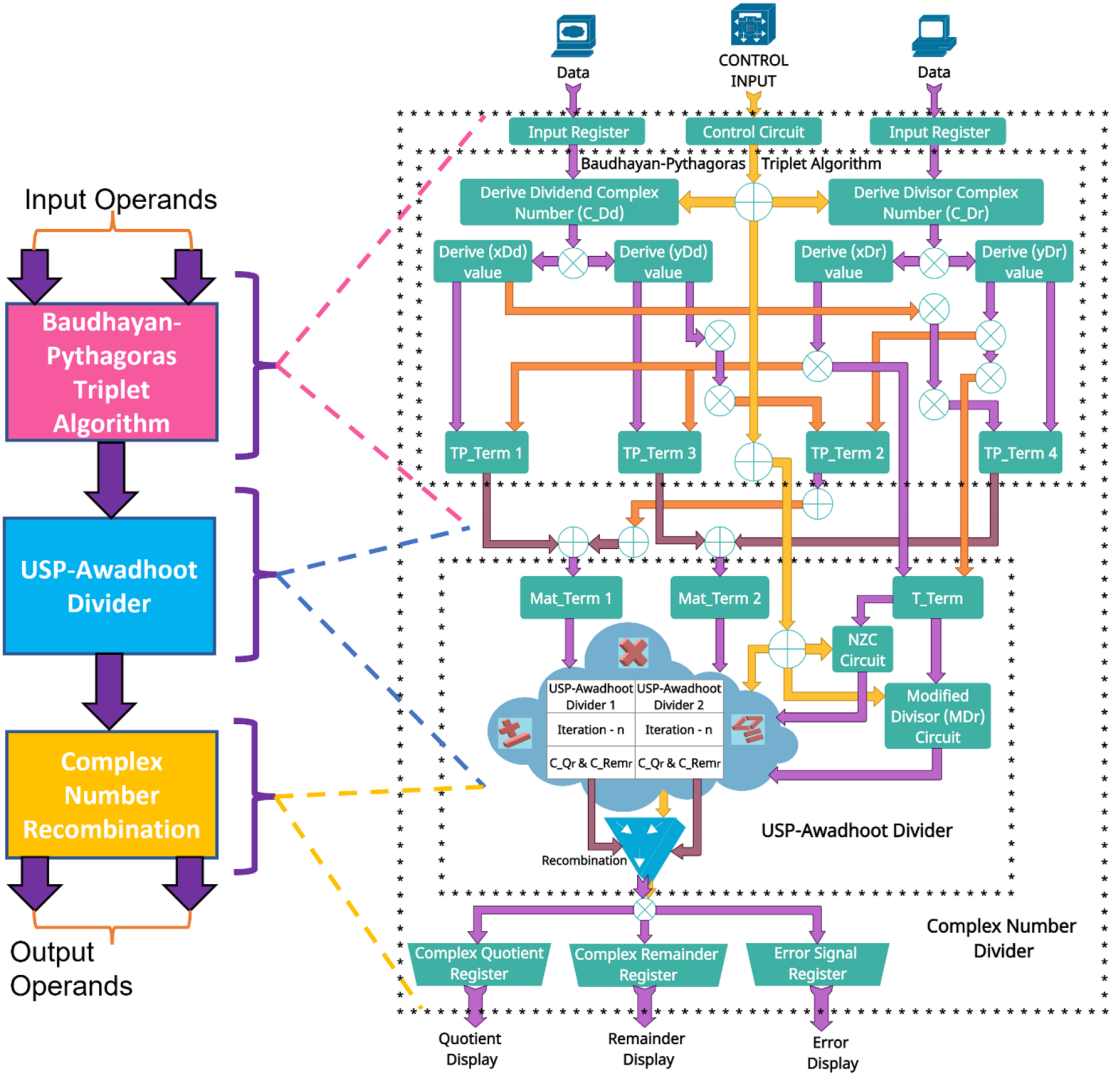
Schematic block diagram of the complex divider.

A detailed list of essential terms associated with the input circuit stage of complex divider implementation, as shown in Fig. 2, given below:

- Complex number one is termed ${r}_{1}={x}_{1}+{y}_{1}i$.
- Complex number two is termed ${r}_{2}={x}_{2}+{y}_{2}i$.
- The dividend of a complex number is termed “${C\_D}_{d}$”.
- The divisor of a complex number is termed “${C\_D}_{r}$”.
- The real number coefficient of the dividend of a complex number is termed “${xD}_{d}$”.
- The imaginary number coefficient of the dividend of a complex number is termed “${yD}_{d}$”.
- The real number coefficient of the divisor of a complex number is termed “${xD}_{r}$”.
- The imaginary number coefficient of the divisor of a complex number is termed “${yD}_{r}$”.
- The first triplet product term is named “TP_Term1”.
- The second triplet product term is named “TP_Term2”.
- The third triplet product term is named “TP_Term3”.
- The fourth triplet product term is named “TP_Term4”.
- The triplet term is named “T_Term”.
- The first triplet matrix term is named “Mat_Term1”.
- The second triplet matrix term is named “Mat_Term2”.
- The USP-Awadhoot dividend of a complex number is termed “${C\_D}_{d1}$”.
- The USP-Awadhoot divisor of a complex number is termed “${C\_D}_{r1}$”.
- The real number coefficient of the USP-Awadhoot quotient is termed “${C\_Q}_{r}$”.
- The real number coefficient of the USP-Awadhoot remainder is termed “$C\_{Rem}_{r}$”.
- The imaginary number coefficient of the USP-Awadhoot quotient is termed “${C\_Q}_{i}$”.
- The imaginary number coefficient of the USP-Awadhoot remainder is termed “${C\_Rem}_{i}$”.
- The final quotient of a complex number is termed “$C\_Q"$.
- The final remainder of a complex number is termed “$C\_Rem$”.

Figure 2 illustrates the process of complex division implementation based on the Baudhayan-Pythagoras triplet algorithm and the proposed novel state-of-the-art USP-Awadhoot divider circuit block. Smith and Stewart's algorithm^72,73^, represented by Eqs. (8) and (9), is generally used for software implementation of complex division, but the underflow and overflow conditions could occur during extreme distance between divisor and dividend, which results in the incorrect recombination of the real and imaginary part of the quotient and remainder^37^. We use the Baudhayan-Pythagoras triplet algorithm as an input circuit stage of the complex divider to eliminate this drawback.

8 $$\frac{{r}_{1}}{{r}_{2}}=\frac{{x}_{1}+i{y}_{1}}{{x}_{2}+i{y}_{2}}=\frac{\frac{{y}_{2}}{{x}_{2}}\left({x}_{1}+{y}_{1}\right)}{\frac{{y}_{2}}{{x}_{2}}\left({x}_{2}+{y}_{2}\right)}+i\frac{\frac{{y}_{2}}{{x}_{2}}\left({y}_{1}-{x}_{1}\right)}{\frac{{y}_{2}}{{x}_{2}}\left({x}_{2}+{y}_{2}\right)} \;\; if \;\; ({x}_{2}\ge {y}_{2})$$

9 $$\frac{{r}_{1}}{{r}_{2}}=\frac{{x}_{1}+i{y}_{1}}{{x}_{2}+i{y}_{2}}=\frac{\frac{{x}_{2}}{{y}_{2}}\left({x}_{1}+{y}_{1}\right)}{\frac{{x}_{2}}{{y}_{2}}\left({x}_{2}+{y}_{2}\right)} i\frac{\frac{{x}_{2}}{{y}_{2}}\left({x}_{1}-{y}_{1}\right)}{\frac{{x}_{2}}{{y}_{2}}\left({x}_{2}+{y}_{2}\right)} \;\; if \;\; ({x}_{2}\le {y}_{2})$$

**Figure 3:**
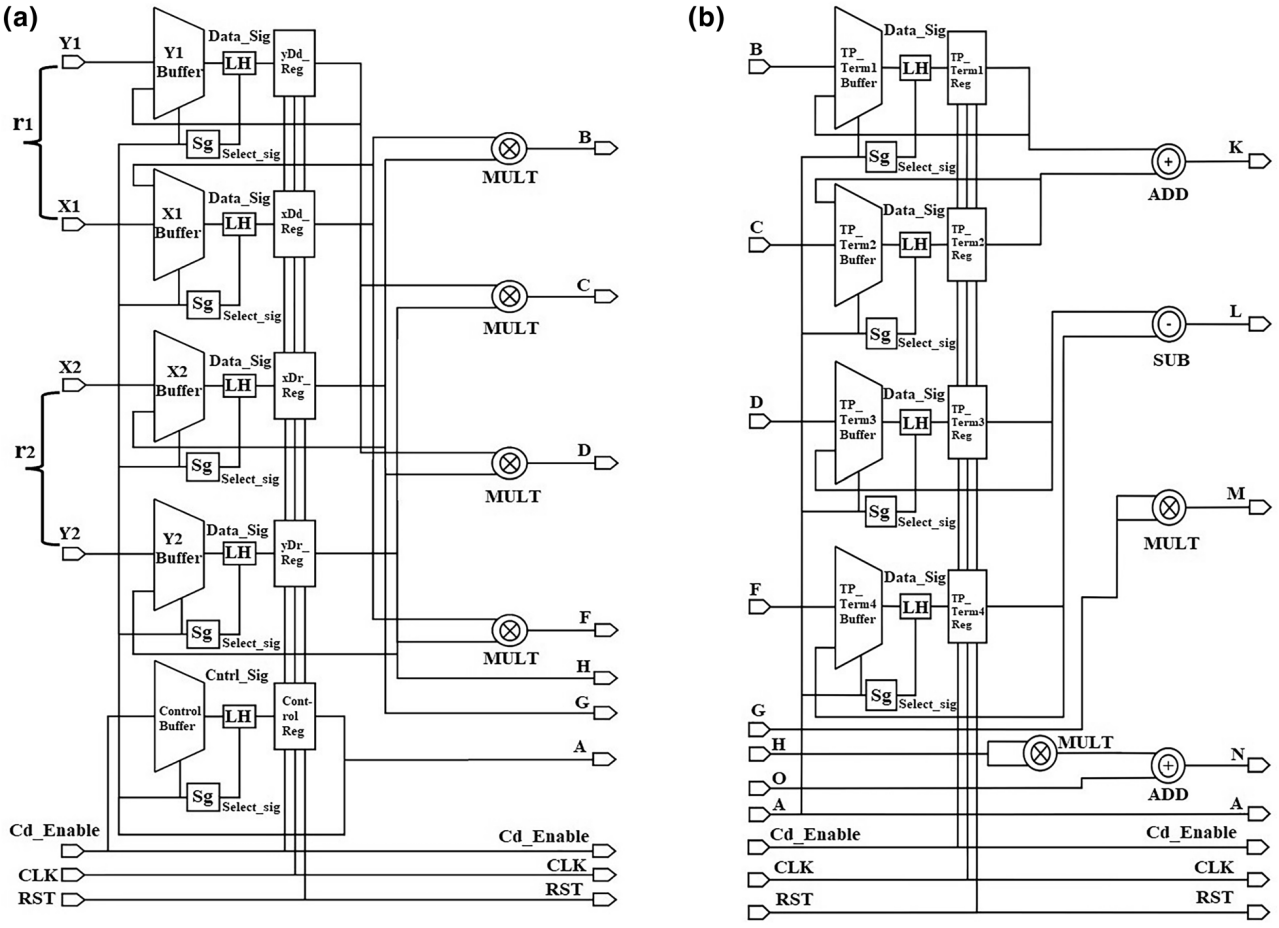

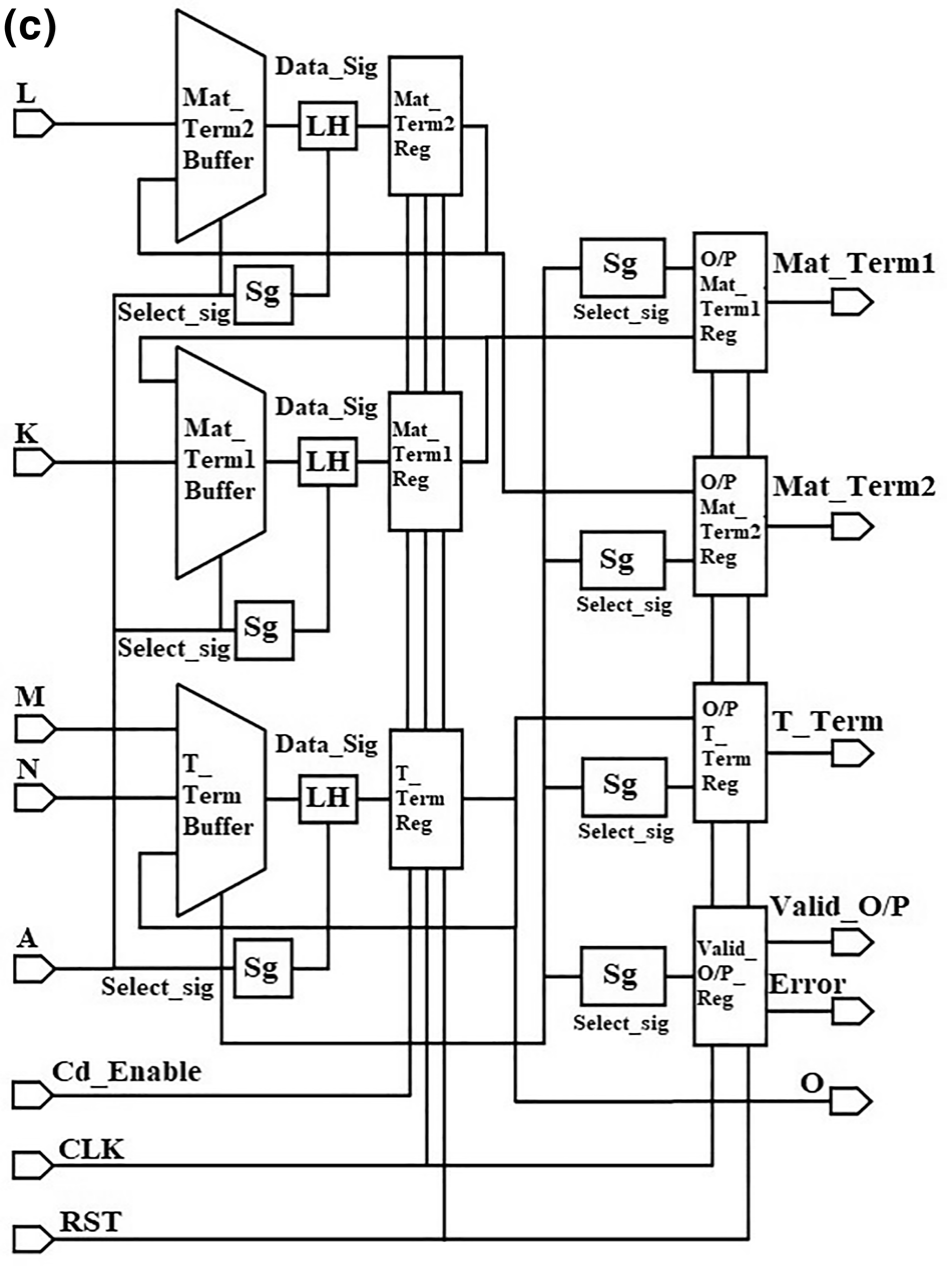
Schematic diagram of the proposed Baudhayan-Pythagoras triplet algorithm circuit block of the complex divider.

The first stage of the Baudhayan-Pythagoras triplet algorithm circuit block of the proposed divider separates the real and imaginary parts of the input operands and considers their real numeric values, especially the imaginary number’s real numeric value without considering the imaginary unit ($i$), which is recombined at the final recombination circuit block. During the Baudhayan-Pythagoras triplet algorithm circuit block, all operands are processed to develop the Mat_Term1, Mat_Term2, and T_Term values/signals as an output of the first stage of the proposed divider. The logic behind Mat_Term1, Mat_Term2, and T_Term is explained in the next section with Fig. 3. In the second stage, the USP-Awadhoot divider circuit block receives Mat_Term1, Mat_Term2, and T_Term values/signals from the first stage. The Mat_Term1, Mat_Term2, and T_Term values/signals are essential to keep the operations under bounded conditions and avoid underflow and overflow conditions. During the second stage, the USP-Awadhoot divider circuit block generates two sets of the quotient and remainder values/signals separately as an output. In the final stage, the recombination circuit block rearranges quotients and remainders into complex numbers by adding an imaginary unit ($i$) with the real numeric value of the imaginary coefficient. It provides resultant quantities for display, storage, or further communication. The detailed work is explained in the next section.

### Working theory of the Baudhayan-Pythagoras triplet algorithm

Figure 3a–c illustrates the schematic diagram of the proposed Baudhayan-Pythagoras triplet algorithm circuit block implementation. It consists of three circuit stages: input, intermediate, and output. The different signals used in the Baudhayan-Pythagoras triplet algorithm circuit block implementation are grouped into input operand, control, output, and indicator signal groups.

The signals ${r}_{1} \; \mathrm{ and} \; {r}_{2}$ are the input operand data signals; Mat_Term1, Mat_Term2, and T_Term are output signals; Valid_O/P and Error are indicator signals; and cd_enable, CLK, and RST are considered control signals. the control group CLK signal provides the timing reference signal for computation execution. The reference clock signal’s period value is dependent on the working frequency. When the CLK signal continues generating the reference signal and the control group signals (cd_enable and RST) both possess low logic values, then the operation of the proposed circuit is in an idle state. The values of the input operand, output, and indicator group signals are in a high-impedance tri-state condition during the idle state. As shown in Fig. 3a, the input operand signals ${r}_{1} \; and \; {r}_{2}$ provide two complex numbers used to perform division operations based on the current statuses of the cd_enable, CLK, and RST control signals. In the input circuit stage, all real and imaginary parts of input operands are separated and stored in the input buffer and wait until the cd_enable signal is high (1) and reset (RST) is low (0), and the output circuit stage initializes the Mat_Term1, Mat_Term2, and T_Term signals from the output and indicator signal group to 00, assuring that the previous computation results are not involved in the current computation. Once the cd_enable signal is applied, this signal is used to develop a select signal and is stored in the control register to connect with further circuit stages. The input operand data is provided for further computation in the input circuit stage. The input operand data is stored into ${x}_{1}, {x}_{2}, {y}_{1} \; and \; {y}_{2}$ buffers to extract ${xD}_{d}, {yD}_{d}, {xD}_{r}, \; and \; {yD}_{r}$ values, respectively, for the generation of signals B to G.

Figure 3b illustrates the intermediate circuit stage of the proposed Baudhayan-Pythagoras triplet algorithm implementation. The intermediate circuit stage receives signal B to signal G data from the input circuit stage and the O signal data from the output circuit stage. The forward signals B, C, D, F, H, and G are generated from the computation of TP_Term1 to TP_Term4, as

10 $$\mathrm{TP}\_\mathrm{Term}1= ({xD}_{d} \times {xD}_{r})$$

11 $$\mathrm{TP}\_\mathrm{Term}2= ({yD}_{d} \times {yD}_{r})$$

12 $$\mathrm{TP}\_\mathrm{Term}3=({xD}_{r} \times {yD}_{d})$$

13 $$\mathrm{TP}\_\mathrm{Term}4= ({xD}_{d} \times {yD}_{r})$$

The computed data are stored in separate buffers and provided for further computation based on the selected signal data to calculate partial Mat_Term1, partial Mat_Term2, and partial T_Term values. Signals K, L, M, and N, indicate the partial Mat_Term1, partial Mat_Term2, and partial T_Term values and transfer the respective data to the next circuit stage.

14 $$\mathrm{T}\_\mathrm{Term}= ({{xD}_{r})}^{2} + {({yD}_{r})}^{2}$$

15 $$\mathrm{Mat}\_\mathrm{Term}1=\mathrm{ TP}\_\mathrm{Term}1 +\mathrm{ TP}\_\mathrm{Term}2$$

16 $$\mathrm{Mat}\_\mathrm{Term}2=\mathrm{TP}\_\mathrm{Term}3 -\mathrm{ TP}\_\mathrm{Term}4$$

Figure 3c illustrates the output circuit stage of the proposed Baudhayan-Pythagoras triplet algorithm implementation. It receives signals K, L, M, and N from the intermediate circuit stage and stores the respective data in Mat_Term1, Mat_Term2, and T_Term buffers. The output circuit initializes the Valid_O/P and Error signals of the indicator group to 00 to ensure that no previously computed data are included. When the RST signal is deactivated, the cd_enable signal is activated during the initial state. Partial values are converted into the final Mat_Term1, Mat_Term2, and T_Term values based on the selected signal logic, and they are utilized as the Mat_Term1, Mat_Term2, and T_Term output group signals. These signals are connected with the proposed USP-Awadhoot divider circuit block and complex number recombination circuit to receive the final division result of complex input operands. The Valid_O/P and Error signal indicates computation completion and invalid working conditions, respectively. After completing the computation operation, depending on the completion of data computation, the Valid_O/P and Error are updated and validate the computation and O/P results, i.e., whether the obtained values are correct or incorrect. If a high logic signal activates an RST signal, then the proposed divider circuit suspends its current computation operation state and resets it to the initialization state.

### Working theory of the proposed novel state-of-the-art USP-Awadhoot divider circuit block

As discussed in the previous section, the novel state-of-the-art USP-Awadhoot Divider Circuit Block is the second major part of the proposed complex divider. The Baudhayan-Pythagoras triplet algorithm circuit is used as an input stage to arrange given operands in a required format to be supplied for the next stage of a complex divider to reduce the criticality of calculation and reduce the area overhead due to complex conversion logic. The major requirement of using novel state-of-the-art USP-Awadhoot divider circuit block is to reduce the implementation area for conversion logic. As we know, we have to use separate dividers for real and imaginary parts in maximum complex dividers. The SRT dividers are restricted to low radix as the implementation area increases with high radix, making conversion logic very complex due to overlapping regions. Functional iterative dividers take more area than SRT dividers, and sometimes the final results contain round-off errors. Thus, we proposed to use novel-state-of-the-art USP-Awadhoot Divider Circuit Block developed on the novel concept of a dynamic separate scaling operation/factor for input operands to reduce the implementation area for conversion logic and eliminate the overlapping region, which can simplify the conversion logic.

**Figure 4:**
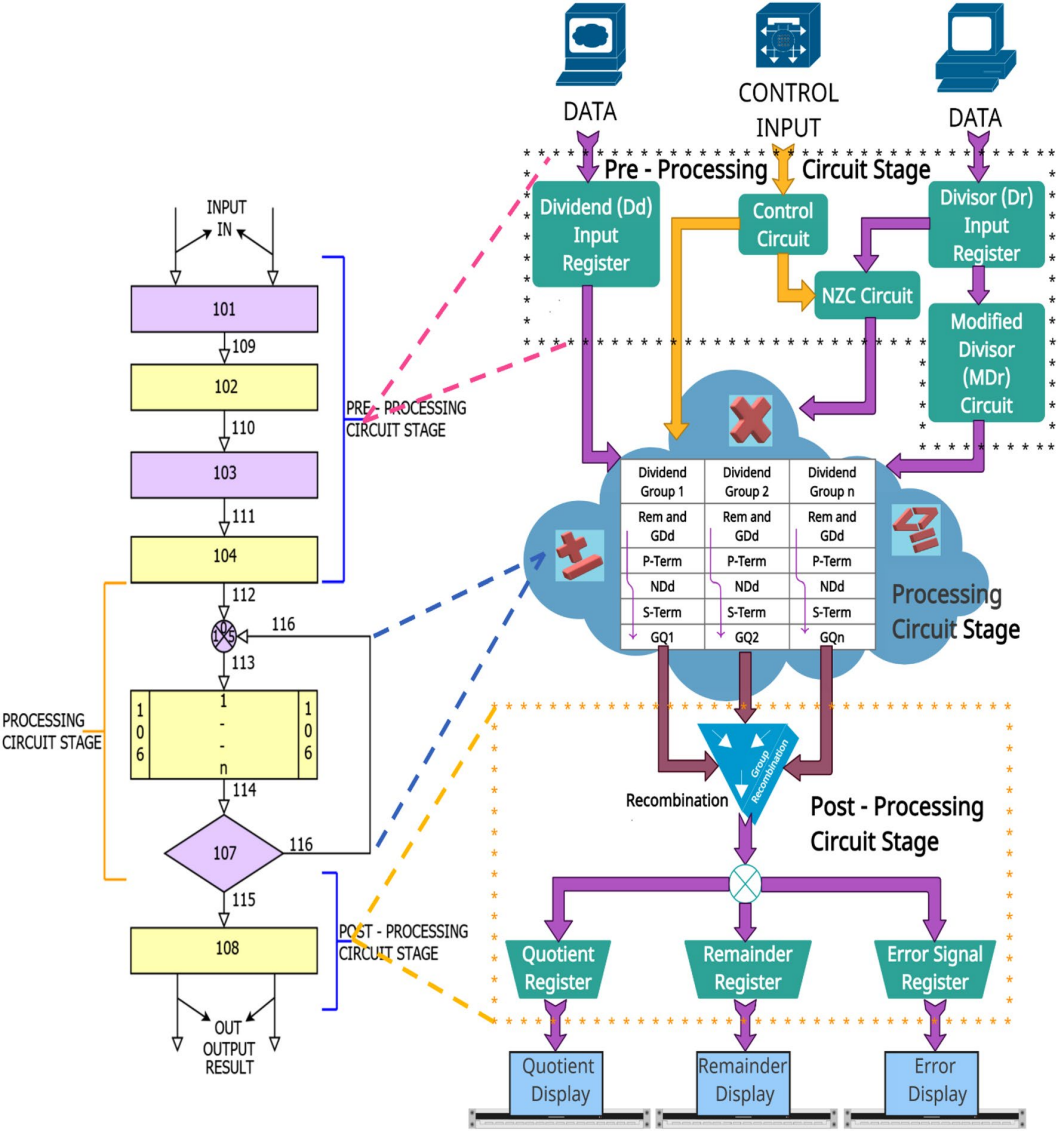
Schematic block diagram of the proposed USP-Awadhoot algorithm-based divider.

The working theory of the proposed novel state-of-the-art USP-Awadhoot divider circuit block is based on a three-stage algorithm developed and built on the ancient Indian mathematics (Vedic mathematics) rules. The detailed embodiments of the state-of-the-art USP-Awadhoot divider circuit block are presented herein with reference to the accompanying results, facts, and figures that describe a circuit implementation for achieving an area-effective implementation of the divider circuit with moderate time and power consumption. Figure 4 illustrates the functional block diagram of the proposed divider circuit block and is expressed in three circuit stages: Preprocessing circuit stage, Processing circuit stage, and Postprocessing circuit stage. Numbered blocks {101 to 104} stipulate preprocessing circuit stage components/elements as per the proposed algorithm. The preprocessing circuit stage accepts the input data (here, the dividend and divisor values) from the external channel, performing initial input processing and confirming that the data are in their correct form for the primary processing circuit stage. Arrows between the numbered blocks indicate data flow directions. Numbered blocks {105 to 107} stipulate processing circuit stage components/elements as per the proposed algorithm. This stage accepts the input data from the preprocessing circuit, performs iterations to implement the steps involved in the Awadhoot matrix, and provides separate group quotient bits that are further supplied to the postprocessing circuit stage. Numbered blocks {107 to 108} stipulate the postprocessing circuit stage components. This stage recombines separate quotient bits (hereafter termed group quotient bits) and presents the quotient and remainder data separately; output on the controlling signal verifies the correctness of the division operation performed by the circuit.

The first number is Divisor (${D}_{r}$), and the second number, Dividend (${D}_{d}$), are provided as input data signals for the proposed divider circuit, and Enable (E), Clock (CLK), Reset (RST), and Fd_Enable are provided as control input signals for the proposed divider circuit. The preprocessing circuit element covers the input data storage, control circuit, NZC circuit, and modified divisor circuit. The multiple outputs yielded by the preprocessing circuit stage are further fed to the processing circuit stage. The processing circuit stage iteratively constructs the core conversion logic, consisting of a dividend group, a p-term, an ${ND}_{d}$ S-term and group quotients are arranged in a particular sequence of steps in one iterative circuit stage. The same element structure is used in all iterative circuit blocks of the processing circuit stage. At the end of the processing circuit stage, all individual dividend group quotients and the remainder are passed to the postprocessing stage. The individual dividend group quotients are recombined to form the final quotient and remainder in the postprocessing stage. In the end, confirmation of the completion of a successful conversion is validated by the present value of the valid output signal and the error signal in the postprocessing circuit stage. Detailed descriptions of the three stages are explained further, but it is essential to understand the vital terms or elements used in these three circuit stages of the USP-Awadhoot technique for the hardware and circuit implementation of a divider (or simply the USP-Awadhoot division technique). This approach utilizes a variable conversion time based on the dividend grouping technique. It has the following important terminology.

- ${D}_{d} = \left[{d}_{1} {d}_{2}\ldots \; \ldots \; \ldots \; \ldots {d}_{k}\right]$, where "${D}_{d}$" represents dividends with a maximum size of "k" digits.
- $Q = \left[{q}_{1} {q}_{2}\ldots \;\ldots \; \ldots {q}_{k-1}\right]$, where "Q" represents a quotient with a maximum size of "$k-1$" digits. Except for the condition in which both operands are single digits, the maximum digit size is "k" digits.
- ${D}_{r} = \left[{d}_{1} {d}_{2}\ldots \; \ldots \; \ldots {d}_{k}\right]$, where "${D}_{r}$" represents a divisor with a maximum size of "k" digits.
- $R = \left[{r}_{1} {r}_{2}\ldots \; \ldots \; \ldots {r}_{k-1}\right]$, where "R" represents a remainder with a maximum size of "$k-1$" digits.
- ${ND}_{r}= \left[{ndr}_{1} {ndr}_{2}\ldots \; \ldots \; \ldots \; \ldots {ndr}_{m}\right]$, where "${ND}_{r}$" represents a "New Divisor" with a maximum size of "m" digits. The range is defined as "$k-1 \le m \le k+1$".
- $FD = \left[{fd}_{1}\right]$, where "FD" represents a "Flag Digit" with a maximum size of a single digit with a fixed range of [1,2……9].
- ${MD}_{r} = \left[{md}_{1}{md}_{2}\ldots \; \ldots \; \ldots {md}_{p}\right]$, where "${MD}_{r}$" represents a "Modified Divisor" with a maximum size of "p" digits. The range is defined as "$p \le k-1$".
- $NZC = \left[{nzc}_{1} {nzc}_{2}\ldots \; \ldots \; \ldots {nzc}_{p}\right]$, where "NZC" represents the "Number of Zeroes Cancelled" with a maximum size of "p" digits. The range is defined as "$p \le k-1$".
- ${ND}_{d} = \left[{ndd}_{1} {ndd}_{2}\ldots \; \ldots \; \ldots {ndd}_{k}\right]$, where "${ND}_{d}$" represents a "Net Dividend" with a maximum size of "k" digits.
- ${{G}_{r}D}_{d}= \left[{gdd}_{1} {gdd}_{2}\ldots \; \ldots \; \ldots {gdd}_{k}\right]$, where "${{G}_{r}D}_{d}$" represents a "Gross Dividend" with a maximum size of "k" digits.

After providing all inputs, at numbered block 101, the preprocessing circuit stage obtains a divisor (${D}_{r}$) and dividend (${D}_{d}$) with maximum word sizes of "k" digits. The widths of the dividend and divisor determine the circuit hardware requirements. Prior to storing the input in the operand registers, i.e., the dividend register and divisor register, the divisor (${D}_{r}$) and dividend (${D}_{d}$) operands undergo an input normalization process that confirms that the input operands' widths are within the permissible limits and are in the required frame format. The use of a hex number system aids in keep this stage simple in terms of the implementation. This condition is not a restriction. In the SRT divider implementation, multiple number systems were used to reduce the criticality in the input circuitry. One of the best examples for expressing this involves binary-coded decimal (BCD) numbers, as explained in^76^, where BCD numbers are used to implement the radix-10 SRT divider. The proposed algorithm is represented with a hexadecimal number system to provide a robust frame structure for electronic implementation. It is not restricted to hexadecimal number systems and can be used with other number systems, such as binary, decimal, and octal systems. The controlling signal circuit generates reference signals to control individual elements of the proposed divider's three circuit stages; then, the divisor (${D}_{r}$) undergoes a check for invalid conditions, i.e., division by zero. This would indicate an error signal at numbered block 108 derived by signal 115 and redistribute further for display or transmission in the postprocessing circuit stage upon detecting the invalid condition. In the false case, the divisor (${D}_{r}$) is passed by 109 to numbered block 102, where the circuit obtains a flag digit (FD) and a new divisor (${ND}_{r}$); this step follows the basic concept of obtaining the FD and ${ND}_{r}$.

Later, at numbered block 103, the FD and ${ND}_{r}$ are used to obtain the modified divisor (${MD}_{r}$) and the number of zeros canceled (NZC); this step follows the basic concept of obtaining an ${MD}_{r}$ and the NZC with respect to Fig. 4. Furthermore, the values of the FD and NZC are supplied to numbered block 104 by the 111 path. At this stage, dividend sectioning/regrouping is performed, and dividend groups are given out based on the NZC value provided by the previous step. Unlike the various SRT implementations that utilize operand prescaling or truncation^65,77^, a fixed number of dividend sectioning or partitioning operations are performed to enhance the implementation; the proposed divider performs a cross combination of divisor prescaling and dividend sectioning or partitioning, giving us the upper hand to achieve area efficiency in the division implementation.

As discussed, the hardware requirements depend on the operand size; the maximum number of iterative circuit elements never exceeds the maximum operand size. This suggests that if the operand size is 8 bits, then a maximum of 8 iterative circuit stages is needed. Nevertheless, the number of iterative circuit stages used in a particular conversion depends on the value of the NZC. Similar to the variable-latency class algorithms, the dynamic nature of iterative circuit stages provides flexible conversion clock cycles for every dividend-divisor combination with the possibility of a variable quotient bit retiring rate in different iterations or some iterations requiring less execution time, resulting in different conversion times in different sets of dividends and divisors. Once the NZC value is determined, the circuit completes the preprocessing circuit stage and arranges the dividends ${MD}_{r}$ and FD in separate dividend groups as per the arrangement shown in the Awadhoot matrix by sending data to numbered blocks 105–107. The proposed divider's processing circuit stage performs a computational process on the Awadhoot matrix (following Fig. 4) to obtain the group quotient's value and the remainder. Block number 106 shows the iterative circuit stages; as discussed earlier, the maximum number of iterative circuit stages is not greater than the operand width size. Numbered block 107 is the condition checker, which confirms that the computation ends in the iterative circuit stage. Once block 107 ensures the completion of the computation, the data are passed to the postprocessing circuit stage at block 108 of Fig. 4. Figure 4 represents the group recombination circuit followed by a distribution circuit for the separate visualization or transmission of the quotient (Q) and the remainder (R). After computing the Awadhoot matrix, the individual group quotients are recombined as per the relative weights and form a final quotient (Q). The final residue or remainder is obtained from the last iterative circuit stage, depending upon the conversion status.

- First: Net Dividend = 0. This shows that the dividend (${D}_{d}$) is completely divisible by the divisor (${D}_{r}$), where the remainder (R) = 0 and the quotient (Q) = the partial quotient (${PQ}_{n}$) formed by concatenating the individual group quotients ${(GQ}_{n})$.
- Second: Net Dividend = Divisor (${D}_{r}$). This shows that the dividend (${D}_{d}$) is completely divisible by the divisor (${D}_{r}$), where the remainder (R) = 0 and the quotient (Q) = the partial quotient (${PQ}_{n}$) + 1.
- Third: Net Dividend ($${ND}_{d}$$) > Divisor ($${D}_{r}$$). The remainder (R) = $${R}_{AQ}$$ the value obtained during the calculation of the additional quotient ($$AQ$$), and the quotient (Q) = the partial quotient ($${PQ}_{n}$$) + the additional quotient ($$AQ$$), where the $$AQ$$ is derived by initializing the count to zero, subtracting the divisor ($${D}_{r}$$) from the last iteration of the net dividend ($${ND}_{d}$$), and incrementing the count by one until we obtain a sub-result that is zero or less than the divisor ($${D}_{r})$$.
- Fourth: Net Dividend (${ND}_{d}$) < Divisor (${D}_{r}$). The remainder = the value of the last iteration of the ${ND}_{r},$ and the quotient (Q) = the partial quotient (${PQ}_{n}$).

The last step of the proposed divider recombines the individual group quotient ${(GQ}_{n})$ in the postprocessing circuit stage. Upon completing the conversion process, the final quotient and remainder are available, along with an error signal indicating the conversion's correctness and the presented data.

17 $${D}_{d} = \left[{d}_{1} {d}_{2} \ldots \;\ldots \; \ldots \ldots {d}_{k}\right] \;\; and \;\; {D}_{r} = \left[{d}_{1} {d}_{2}\ldots \; \ldots \; \ldots {d}_{k}\right]$$

18 $$Division(Q,R)=f\left({D}_{d},{D}_{r}\right)=f\left(Awadhoot \;\;matrix, Condition\right)$$

19 $$Awadhoot \;\; matrix\left({GQ}_{n},{R}_{n}\right)=f\left({GD}_{d},{MD}_{r}, FD\right)$$

20 $$f\left({GD}_{d},{MD}_{r}, FD\right)={\sum }_{n=1}^{k}\left[\left({R}_{n-1}|{GD}_{dn}\right)+{(P-Term)}_{n}-{\left(S-Term\right)}_{n}\right]$$

21 $$Condition(Q,R)=f\left({ND}_{dn}, PQ, AQ\right)$$

Therefore, after the last iteration of the Awadhoot matrix, based on the condition function, the final quotient and remainder value are calculated and represented as follows:

22 $$Division(Q,R)=(PQ,0) \;\;\,\,\,\, if \;\; { ND}_{dn}=0$$

23 $$Division(Q,R)= [(PQ+1), 0] \,\,\,\,\;\; if \;\; { ND}_{dn}={D}_{r}$$

24 $$Division(Q,R)=(PQ,{ND}_{dn}) \,\,\,\,\,\;\; if \;\; { ND}_{dn}<{D}_{r}$$

25 $$Division(Q,R)=[(PQ+AQ), {R}_{AQ}] \,\,\,\,\,\;\; if \;\; { ND}_{dn}>{D}_{r}$$

where PQ is the partial quotient, AQ is the additional quotient and ${R}_{AQ}$ is the remainder generated during the calculation of the additional quotient.

In most of the performance enhancement schemes utilized in divider circuit block implementations, scaling down the operands with a common static scaling factor is considered a preliminary option. Different methods may be available for calculating the scaling factor, but the same factor scales down both operands (divisor and dividend). Thus, even after scaling down, the relationship between the divisor and dividend remains the same. Considering that the dividend is (x), the divisor is (y), and both operands are scaled down by a common scaling factor (m), the relation between the dividend and divisor is expressed as

26 $$\left(x \to y\right) = \left({x}_{m}\to {y}_{m}\right)$$

Example: If the dividend = 500, the divisor = 50, and they are scaled down by common factor 5, then

27 $$\left(\frac{500}{50}\times 100\right)=1000\%$$

where the relationship between the scaled-down values is presented as

28 $$\left(\frac{100}{10}\times 100\right)=1000\%$$

Several ways of finalizing the scaling factor may be available, but the same scaling factor is used to scale down both operands. By performing scaling, we can reduce the values of operands, which can reduce the number of iterations required to calculate the quotient bits; however, this does not allow us to reduce the divisor quantity beyond the preliminary relation. Even though it is possible to further scale down the dividend or divisor, it is not executed because of the divisor has reached its limits. Nevertheless, doing so increases the area overhead. As explained in Fig. 4, the preprocessing circuit consists of the control circuit, NZC circuit, and modified divisor circuit, confirming the use of a dynamic separate scaling operation/factor, reducing the number of iterations required for the quotient calculation and ensuring that different factors scale down the divisor and dividend. Partitioning the original dividend value into several group dividends is also considered to ease the process of designing the quotient bit selection logic. The preprocessing circuit provides a key improvement to achieve performance enhancement. The processing circuit stage provides restoring and nonrestoring functionality for executing the division steps described in the Awadhoot matrix. It also provides a second improvement in the form of clearer and simpler quotient selection logic without overlapping conditions, unlike in the SRT divider, where the overlapping region is critical and causes severe losses in the case of misalignment of the overlapping area.

### The Awadhoot matrix circuit elements of the processing circuit stage

**Figure 5:**
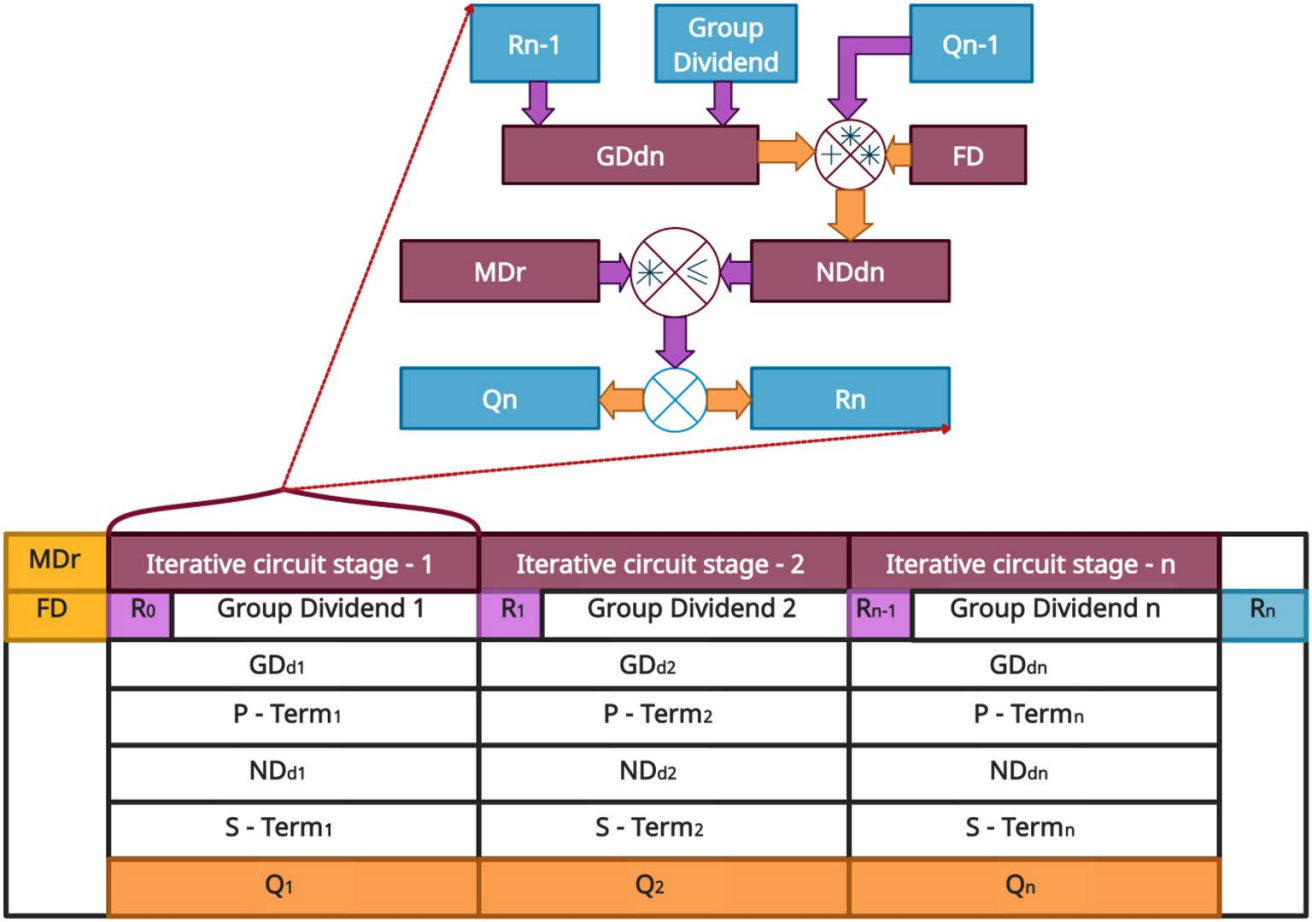
The Awadhoot matrix.

Figure 5 illustrates the particular arrangement of the processing circuit stage elements of the proposed algorithm. The structure is termed the Awadhoot matrix. The Awadhoot matrix provides a computational arrangement of various aspects of the processing circuit stage of a proposed divider. Figure 5 shows that each column represents an individual iterative circuit stage, and each row represents the elements of the corresponding iterative circuit stage. We can use a single set or multiple sets of iterative circuit elements depending on which hardware architecture is considered for implementation.

The Awadhoot matrix arrangement is composed of the previous remainder ($${R}_{n-1})$$, the group dividend $$({GD}_{dn})$$, the group quotient of the previous iteration ($$G{Q}_{n-1})$$, the gross dividend ($${{G}_{r}D}_{dn})$$, the flag digit ($$FD)$$, the modified divisor ($${MD}_{r})$$, the net dividend ($${ND}_{dn})$$, the present quotient ($${Q}_{n})$$ and the present remainder ($${R}_{n})$$. The proposed divider circuit hardware requirement depends on the number of dividend groups made in the preprocessing stage of the divider, and the maximum possible number of dividend groups is related to the maximum width among the available operands. This Awadhoot matrix arrangement provides a detailed structure of the processing circuit, which can be realized by serial, parallel, or pipeline hardware architectures. In the present article, we compare the sequential combinational circuit implementation of the Awadhoot matrix. Under the idle condition, the values of the previous remainder ($${R}_{n-1})$$ and the previous iteration group quotient ($${Q}_{n-1})$$ are considered zero to avoid any computational errors in an iterative circuit. Upon the execution value of the first iteration circuit stage, the group quotient and remainder are used as the previous iteration group quotient ($${Q}_{n-1})$$ and the previous remainder ($${R}_{n-1})$$, respectively, for the next iteration circuit stage and depend on the number of dividend groups.

A detailed description of the Awadhoot matrix is given in the patent application. In short, a gross dividend ($${{G}_{r}D}_{dn})$$ is derived from the previous remainder ($${R}_{n-1})$$ and the present value of the group dividend at the first level of the iterative circuit stage of the Awadhoot matrix. Further simple addition and multiplication operations are performed with the gross dividend ($${{G}_{r}D}_{dn})$$, previous iteration group quotient ($${GQ}_{n-1})$$ and flag digit ($$FD)$$ to derive the value of the net dividend ($${ND}_{dn})$$, which is indicated by the p-term terminology in the Awadhoot matrix. Additional multiplication operations are performed depending on the condition of the present net dividend ($${ND}_{dn})$$ value in comparison with the value of the modified divisor ($${MD}_{r})$$ to obtain the value of the s-term. Depending on the comparison, the final value of the group quotient ($$GQ)$$ and the present remainder ($${R}_{n})$$ are calculated and presented for the next iterative circuit stage or postprocessing circuit stage. During the execution of the postprocessing circuit stage, all individual group quotient values are recombined together with the associative weights to form the final quotient value. Later, this final quotient and remainder value are displayed or transmitted to other circuits if necessary. We consider the hexadecimal number system to implement the proposed system due to its ease of use in digital systems and computer applications. Hexadecimal numbers in digital electronics result in better readability and provide a frame structure with a fixed bit size to represent each decimal number in a digital form.

We obtain several representation forms in the binary representations of decimal numbers or digits. Sometimes they can be represented by one-bit equivalent binary numbers or multiple-bit binary numbers, whereas in the case of hexadecimal representation, every hexadecimal digit is defined as a frame of four binary bits. The fixed frame used for representing hexadecimal numbers in digital or binary form provides better support for performing operations such as shifting, comparing, and giving a simple logic for quotient bit selection in the processing circuit stage.

The use of a hexadecimal system also simplifies internal operations, such as concatenating digits in digital computations. A binary system could also be used, but hexadecimal numbers also provide the advantage of working with four bits per digit each time. In the binary system, the minimal number of bits to be considered for computation is one; in the case of a hexadecimal system, four binary bits are used, providing more clarity for understanding the computation process performed on a digital system with long bitstream data. The conversion of any digit value of any number system into a single digit by repetitively adding all digits is called a beejank or digital root. The beejank operation (digital root), alternate beejank operation, deviation, and alternate representation of addition and subtraction, which we perform during the iteration of the processing circuit stage, exhibit great ease of use in the quotient bit selection logic developed with the hexadecimal system.

The beejank approach is used to verify answers except for the binary number system; hence, we consider a hexadecimal number system in the quotient bit selection logic to confirm the correct selection of the quotient bit in a particular iteration. The same operations are performed with a number and its beejank; if both results are found to be the same, the answer is verified. The beejank does not indicate a deficiency in the minimum (zero) and maximum numbers. If the placement(s) of a digit (digits) in a number is/are interchanged, then this change is not indicated by the beejank. If the beejank is negative, then ‘9’ is added to convert it to a positive value.

29 $${(F89A0BCD)}_{16}={(F+8+9+A+0+B+C+D)}_{16}$$

30 $${(F89A0BCD)}_{16}={(4E)}_{16}={(4+E)}_{16}={(12)}_{16}={(3)}_{16}$$

The difference between a number and its nearest base is called the deviation.

31 $$Number={(100F)}_{16} \;\; Base=1000 \;\; Deviation=00F$$

During computation, the deviation also supports the representation of the addition or subtraction of two numbers as one hexadecimal number, which can reduce the number of steps required in the quotient bit selection logic and helps to reduce the area requirement and complexity of the quotient bit selection logic.

### Implementation statistics and performance results analysis

The very high-speed integrated circuit (VHDL) hardware description language is used to develop the implementation idea based on the functional block diagram of the proposed USP-Awadhoot algorithm-based divider. To realize the theoretical concept and idea of the proposed state-of-the-art novel USP-Awadhoot algorithm-based divider, we develop a synthesizable architecture. This synthesizable architecture implementation provides a unified way of comparing and testing the proposed divider. To develop and implement the proposed USP-Awadhoot algorithm-based divider, we use the Vivado 2016 simulation tool with the Zybo development board based on Xilinx Zynq XC7Z010, XCZU7EV-FFVC1156-2-E with Zynq UltraScale + MPSoC and the Quartus Prime Lite simulation software with the Cyclone IV development board based on the EP4CE6E22C8N Cyclone IV FPGA manufactured by Altera to generate a truth table and cross-verify the simulation results by comparing the outputs separately. Here two different FPGA’s (Xilinx and Altera) were used to test the correctness of the logical results when implemented with different structured FPGAs. Xilinx implementation and simulation statistics of the proposed divider considered further for comparison. The overall performance of the divider depends on the proposed algorithm for the data-dependent divider, which determines the latency for a particular input operand combination. To gain complete control over each implementation detail and make the synthesis as technology independent as possible, we create a set of components based exclusively on register transfer level (RTL) descriptions. In other words, each component is described by some structure composed of basic gates, and their connectivity is similar to that of the various implementations studied during the review.

**Figure 6:**
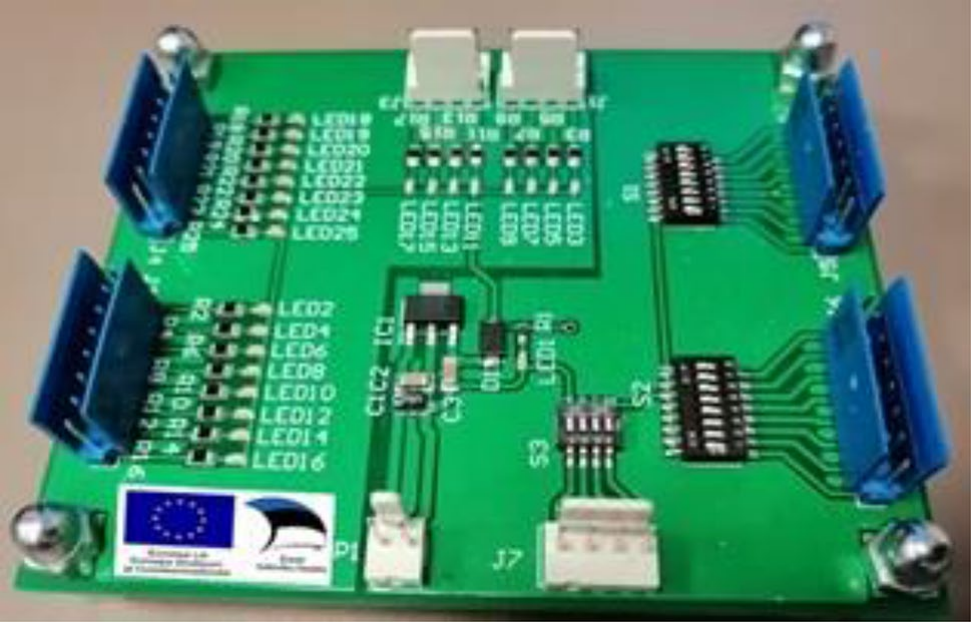
Logic test bench board.

We verify the simulation output of the proposed divider, which depends on the random number generator (RNG) outputs for the random and sequential input operand combinations covering complete bit or digit range operands, as proposed by the truth table. To verify whether the generated output is valid, we prepare a truth table for every possible operand combination, including the true or theoretical results. We execute this process for every possible input operand combination and compare its results with the truth table. Any differences in the results indicate the incorrectness of the calculation and are rectified via corrective actions. Also, all the possible input operand conditions are sequentially and randomly checked on both experimental test boards. We created one logic test bench board, as shown in Fig. 6, that can provide operand values with multiple word sizes. The logic test bench board is designed so that it can check minuscule bit size combinations and offer the flexibility to add extra bits to the input operands in cases with extended bit sizes. We tested the operation of the proposed USP-Awadhoot algorithm-based divider with static inputs supplied via separate single-pole single-throw rocker switches and a continuous sequence generator. We executed simulations with different clock frequencies and also tested by implementing them in both development boards to determine the working latency and conversion speed.

After verifying the simulations and hardware implementations of different versions of the proposed divider circuit, the implementation statistics of every version are mapped to the LUTs, flip-flops, registers, multiplexers, latches, adder, subtractor, multiplier, basic gates, clock frequency, and power. In short, the number of transistors or gates used indicates the implemented area, hardware resource utilization, working frequency, and power consumption needed to realize the proposed USP-Awadhoot algorithm-based divider. Thus, the behavior of the proposed USP-Awadhoot algorithm-based divider is mapped to the latency performance function designed to keep track of the required clock cycles for a given pair of input operands. It is the best way to compare implementation statistics based on area, and the other approach for performance comparison is based on the latency time.

The physical implementation of the proposed divider based on the USP-Awadhoot division algorithm is performed with various hardware implementation techniques, referred to as the implementation versions. A study of different implementation versions indicates the total trade-offs between area, time, and power, so depending on the application, one must decide which implementation version should be utilized. Implementation versions V8.1 to V8.9 are based on 8-bit operands, whereas V16.1, V24.1 and V31.1 are the 16-bit operand, 24-bit operand, and 31-bit operand implementations, respectively. The variances in the resulting parameters for different versions of the proposed divider circuit indicate the effectiveness of the corresponding versions.

As mentioned above, version V8.1 is the first implementation version of the proposed USP-Awadhoot divider with 8-bit operands. All the steps involved in the preprocessing, processing, and postprocessing circuit stages are implemented sequentially, and the execution of each step is concluded in a separate clock cycle. However, it requires less implementation area (explained in a further section), resulting in the slowest implementation of the proposed divider circuit because storing the intermediate values generated during execution is not needed, as the next step is executed only after the completion of the first step. Version V8.2 is the successor to version V8.1; it includes storing intermediate values generated during the execution process, causing the area overhead to increase compared to that of the predecessor version (V8.1). Version V8.3 is the third version of the 8-bit implementation of the proposed USP-Awadhoot divider in the series utilizing separate clock cycle executions for each step of the preprocessing, processing, and postprocessing circuit stages. While implementing these versions, we change the ${ND}_{d}$, the remainder and the error signal calculation circuit and provide an extra buffer to hold the values. This improves the accuracy of the output by providing the correct remainder value. Version V8.4 initiates the concurrent execution concept to implement the proposed USP-Awadhoot divider. In this version, the circuit allows the data available at the input data lines to be stored at input registers, the most significant bits (MSB) are stored as separate hexadecimal integers, and the least significant bits (LSB) are stored as additional hexadecimal integers in an array of hexadecimal integer elements for the dividend. The same process is applied to the divisor. Concurrently, the preprocessing circuit stage formulates the FD and ${ND}_{r}$ values by working only on the least significant hexadecimal part of the divisor.

V8.5, V8.6, V8.7, V8.8 and V8.9 are the successors of the V8.4 version of the proposed USP-Awadhoot divider implementation. In the V8.5 version of the implementation, the processing circuit stage concurrently executes the ${ND}_{d}$ and ${GQ}_{n}$ calculation steps. In the V8.6 version of the implementation, the processing circuit stage utilizes the predefined values for error conditions during the concurrent execution of the ${ND}_{d}$ and ${GQ}_{n}$ calculations. It reduces the area overhead and power consumption while increasing the execution speed. In the V8.7 version of the implementation, the processing circuit stage concurrently executes the residue/remainder and additional quotient calculations. Based on the concurrent execution process, the ${ND}_{d}$ values are compared, and the condition selection circuit is activated in the postprocessing circuit stage to compute the final values for the quotient and residue as per the display requirements. In the V8.8 version of the implementation, the processing circuit stage introduces an extra buffer and counter in addition to those in the V8.7 version to improve the expected group residue/remainder calculations. This version improves the working clock cycle requirements and maintains the same area requirements as the V8.7 version of the proposed USP-Awadhoot divider implementation. In the V8.9 version of the implementation, the processing circuit stage introduces different logic to implement alternate conditions to the residue/remainder and additional quotient calculations. This version improves the implementation area or resource utilization by keeping same clock cycle requirements as the V8.8 version of the proposed USP-Awadhoot divider implementation. Versions V16.1, V24.1 and V31.1 are the 16-bit operand, 24-bit operand, and 31-bit operand implementations, respectively, based on the modifications performed in the V8.9 version. V31.1 implementation uses 31 bit operands to avoid overflow condition during conversion.  

**Figure 7:**
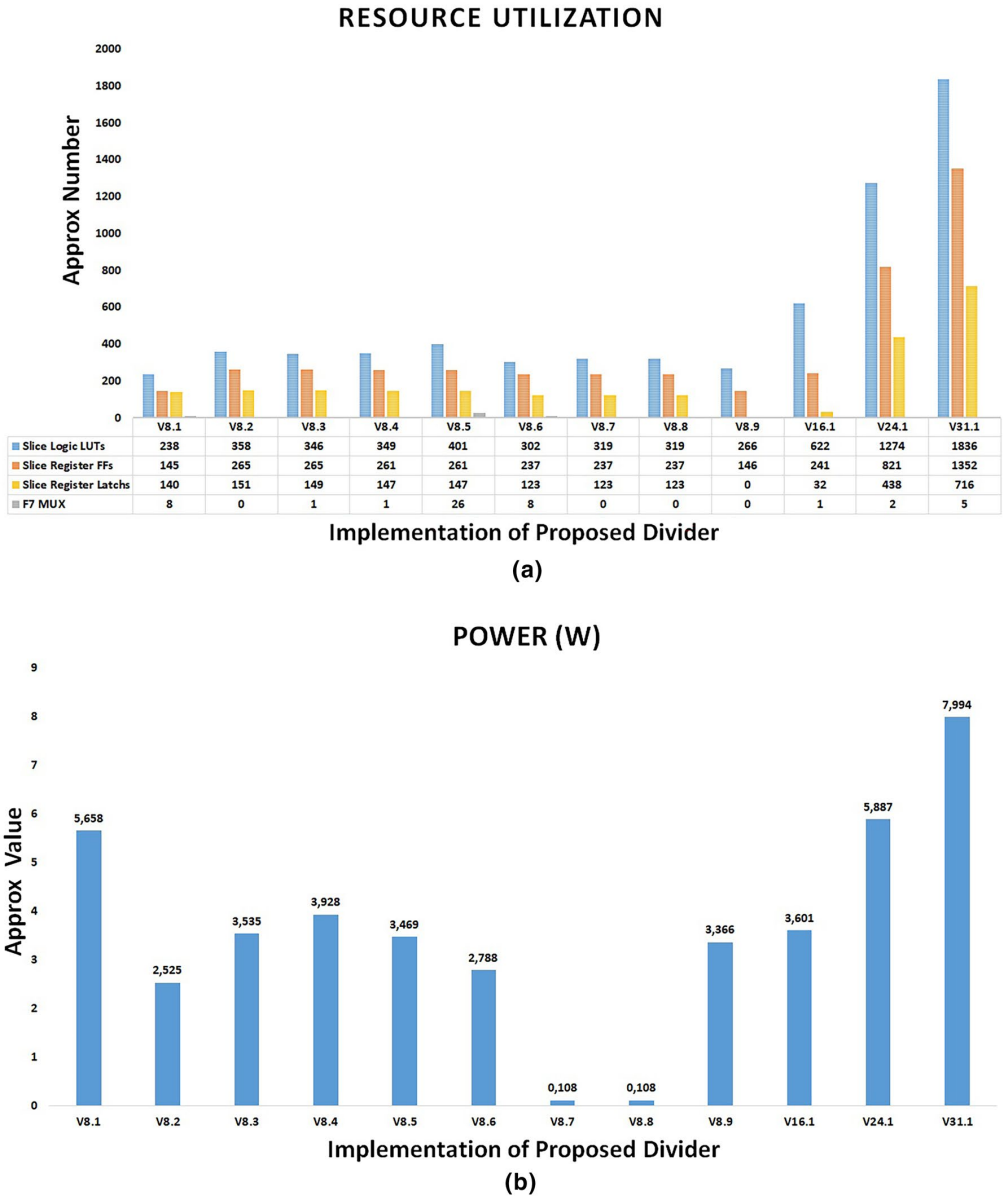

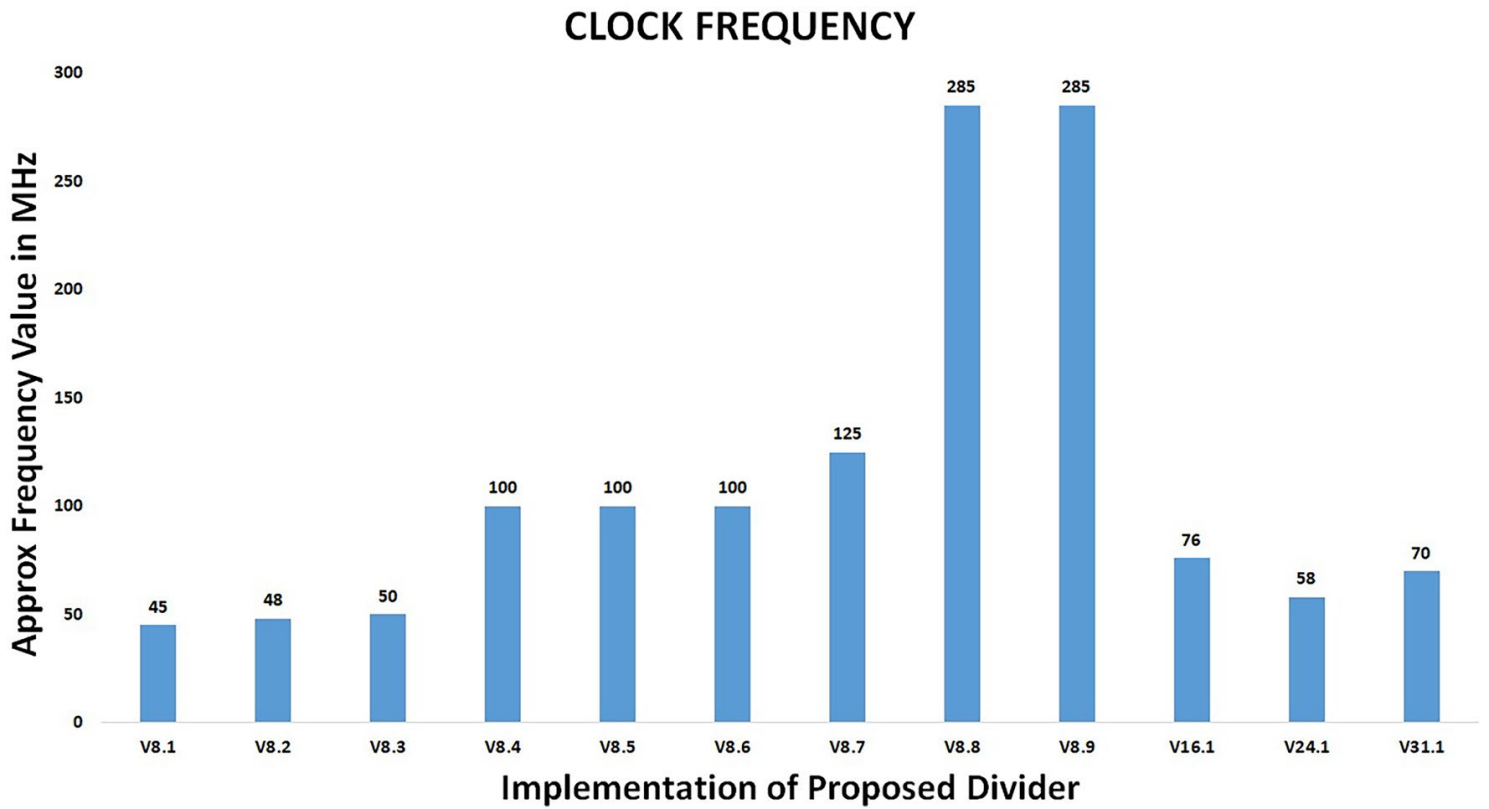
Hardware resource utilization of the proposed USP-Awadhoot algorithm-based divider.

Figure 7a–c shows hardware resource utilizations required by multiple versions of the proposed USP-Awadhoot algorithm-based divider implementations. The results shown in Fig. 7 are the actual data for the proposed circuit implementation based on Xilinx FPGA and Vivado 2016 simulation tool, which is considered as a baseline to compare with several other implementations to draw a comparative analysis. The circuit arrangements are different in terms of how they execute the various states of the logic flow state diagram of the proposed divider to improve the implementation area and enhance the operational performance with respect to the space, latency time, and power of the proposed divider circuit based on the USP-Awadhoot division algorithm. While implementing these versions, we must consider that area is an essential point when working with embedded systems. Based on the slice logic LUT graph of the hardware resource utilization, we confirm that each implementation requires a minimum of 238 counts of slice logic LUTs after comparing the different 8-bit implementation versions.

Slice logic represent the group of hardware resources necessary to create a configurable logic block. Every slice logic contains a fixed numbers of LUTs and slice register flip-flops; sometimes, they are accompanied by slice register latches and multiplexers. A LUT is a collection of logic gates that are hard-wired on an FPGA. LUTs store a predefined list of outputs for every combination of inputs and provide a fast way to retrieve a logic operation's output. A flip-flop is a circuit that is capable of two stable states and represents a single bit. A multiplexer, also known as a mux, is a circuit that selects between two or more inputs and outputs the selected input. Different FPGA families implement slices and LUTs differently. For example, a logic slice on a Virtex-II FPGA has two LUTs and two flip-flops, but a logic slice on a Virtex-5 FPGA has four LUTs and four flip-flops. Additionally, the number of inputs to a LUT, commonly two to six, depends on the selected FPGA family. A register is a group of flip-flops that stores a bit pattern. A register on an FPGA has a clock, input data, output data, and enabled signal ports. Every clock cycle, the input data are latched and stored internally, and the output data are updated to match the internally stored data.

While implementing the proposed divider, an approximately one hundred and forty-six slice register flip-flops, and 37 bounded input outputs are required in the case of an 8-bit implementation. The bounded I/O is divided into two groups of data operands, which are input, and output data lines and control lines used to control the divider's operation and indicate an error if it occurs during computation. Some implementation versions require additional seven-input multiplexers or eight-input multiplexers. Versions V8.1 and V8.6 require eight seven-input multiplexers, V8.3 and V8.4 require one seven-input multiplexer, and V8.5 requires twenty-six seven-input multiplexers. Along with the area and power analyses, frequency or cycle time calculations also form an important part of divider circuit analysis.

The proposed divider's implementation uses variable power (approximately from a minimum of 0.108 watts to a maximum of 7.9941 W). It works at up to 285 MHz depending on the implementation version selected, i.e., the 8-bit, 16-bit, 24-bit, or 31-bit implementation. Depending on the best suitable hardware resource utilization combination and a better performance analysis of the proposed USP-Awadhoot algorithm-based divider implementation, version V8.9 is the most suitable option for implementation in an application with a lower-to-moderate range of resource requirements. This version requires 266 slice logic LUTs, 146 slice register flip-flops, zero latches and 37 bounded input–outputs with no seven-input multiplexers, DSP, or eight-input multiplexers. Simulation confirms it works at a moderate frequency up to 285 MHz and requires 3.366 watts estimated power to run. For all the comparative studies we consider version V8.9 of the proposed divider circuit.

During each clock cycle, the processing unit can perform basic operations such as fetching instructions or data, accessing memory, and reading or writing data. The processing unit often requires multiple clock cycles to complete a single process. The processing unit's frequency is calculated depending on the clock cycles, also termed the cycles per second or frequency. Here, the clock frequency is considered the system frequency or the divider's working frequency. The clock frequency is also considered a reference point for executing different instructions during the implementation of a particular operation. The frequency of a processing unit is also known as the processor's clock speed. Clock speed is essential for determining the processor's overall performance. Since processors have different instruction sets, they may differ in the number of cycles needed to complete each instruction (or cycles per instruction (CPI)). Some processors can perform faster than others, even at slower clock speeds. This indicates that studying the clock cycles required for a particular conversion is essential.

Latency analysis is conducted in terms of clock cycles as the behaviour of the proposed USP-Awadhoot algorithm-based divider is mapped with a latency time performance function designed to keep a record of the number of clock cycles required given a pair of input operands. Simulations obtain the minimum and maximum performance of the proposed divider with analytically determining best and worst cases. The use of a latency time calculation in terms of clock cycles for determining the best and worst cases is rational when comparing divider performance. To consider the latency time performance of the proposed divider, we choose two options; the first is a sequential truth table that assures that each combination of input operands is considered during the execution, and its related data are stored.

The second option is RNG, is suitable for evaluating operands possessing larger word sizes with more significance. In the case of the data-dependent divider, the execution time depends not on how large the dividend is but on how far the divisor is from the dividend. The larger the distance between the dividend and divisor, the more execution time is needed. We conduct a clock performance analysis of the comprehensive range of dividends and divisors for 8-bit operands with the RNG and the sequential truth table, where the number of possible combinations is 65K; for higher bit sizes, only the RNG method is considered due to the possibility of billions of combinations. There are two possible ways to achieve variable-latency time in the division operation: one is by varying the frequency for performing the iteration process, and the second is by varying the number of iterations. One can provide a variable conversion rate or time by having variable latency. Latency is defined as the total time taken by an operation to generate the first output after providing an input; in other words, it is the total number of clock cycles required after providing inputs to develop the first result. We conduct a divider circuit clock performance analysis to understand the nature of the proposed divider.

The proposed division circuit based on the USP-Awadhoot algorithm executes all possible input operand combinations while performing the divider circuit's clock performance analysis. Every possible combination of dividend and divisor values is executed, giving us the details regarding the number of clock cycles required to compute a dividend–divisor combination. In the present article, we provide the implementation data for the eight-bit operands, suggesting that the divisor's width and the dividend's width are eight bits. The eight bits of each operand are expressed as two hexadecimal numbers, making it easier to execute the computation. We perform 65K combinations of input operands.

To represent the data in a standard format, we divide the dividend range into three sections with a low range of dividend values in the first region named dividend range 00, suggesting that the dividend has half-filled four-bit values or one-digit-lower hexadecimal values; a middle range of dividend values in the second region named dividend range 80, suggesting a range of half-filled four-bit values to six-bit values or lower two-digit hexadecimal values; and a high range of dividend values in the third region named dividend range FF, suggesting a range from six-bit values or lower two-digit hexadecimal values to full eight-bit values or higher two-digit hexadecimal values. The execution of the proposed divider is performed based on the USP-Awadhoot division algorithm by considering different divisor values starting from half-filled four-bit values or lower values of single hexadecimal digits to full eight-bit values or two-digit hexadecimal digit values. A relative presentation between the various combinations of operands with their required clock cycles is provided to compute the results.

The results generated from this clock performance analysis state that the proposed divider circuit based on the USP-Awadhoot algorithm requires a variable number of clock cycles to perform division operations on the particular operands provided at specific times. This shows that the proposed divider circuit requires the fewest clock cycles (0 clock cycles) when the operands exhibit invalid conditions. An invalid condition suggests that the divisor value is zero, and division by zero yields an indefinite condition that causes the generation of the invalid condition. An exception to the invalid condition exists with a dividend value of zero; when the input operand value indicates that the dividend and divisor values are both zero, then the proposed divider circuit takes slightly more clock cycles (17 clock cycles) to finish the execution process. The important reason behind this is that the circuit first detects of dividend value to indicate it as a nonzero value. If it detects a dividend value of zero, it sets the temporary output to zero and checks the divisor for a nonzero value. If it detects a nonzero value, then the final answer is zero. In a case with zero, the result must be changed to an invalid result generating an error signal, which requires extra clock cycles to execute the error signal generation process. The most clock cycles required for computing operands are two hundred and seventy-five clock cycles for the combination of the lowest divisor and the highest dividend value, indicating that more iterations must be completed before reaching the final result. When the divisor value is closer to the dividend value, the number of clock cycles required to execute the division step is lower.

**Figure 8:**
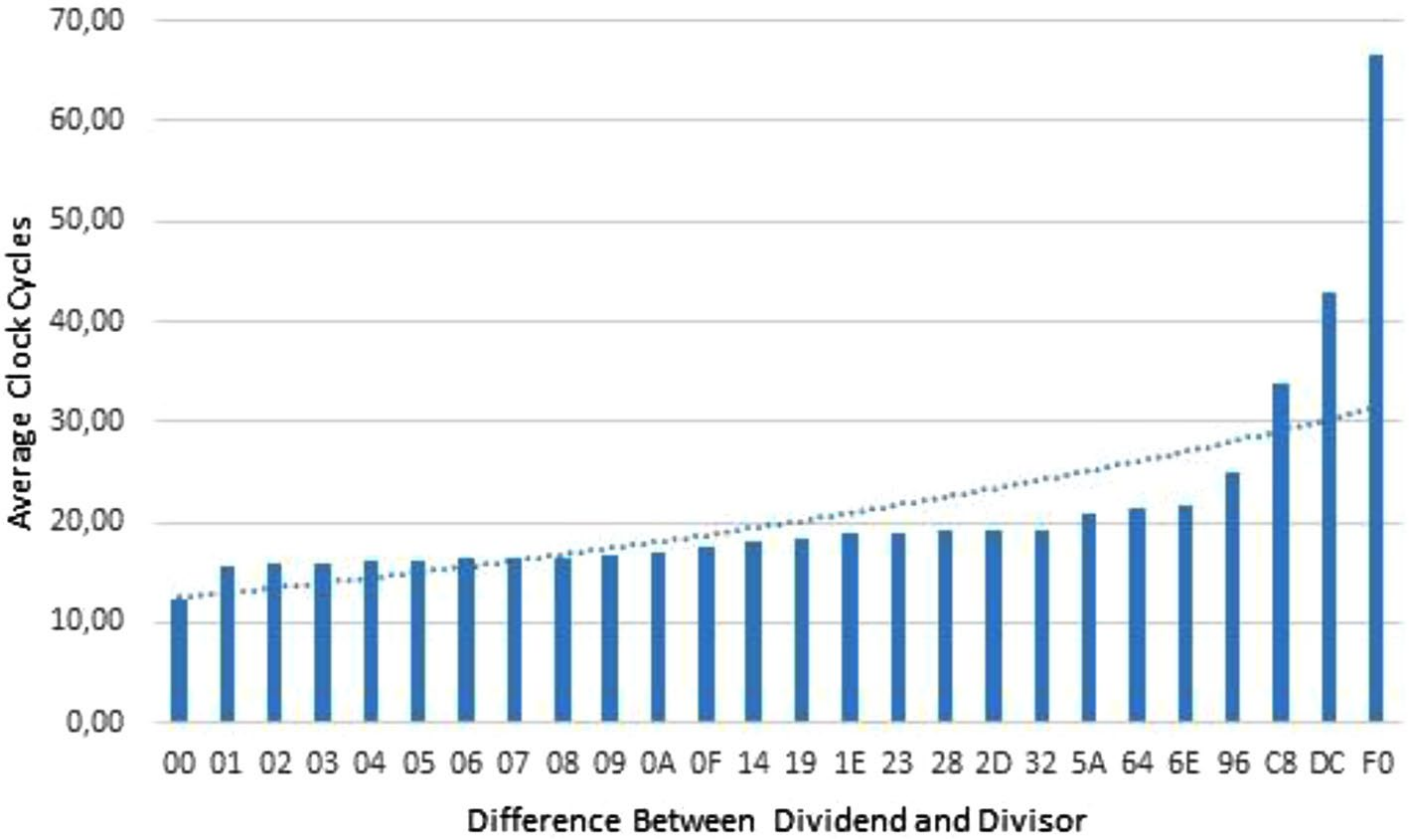
Clock performance analysis of the proposed USP-Awadhoot algorithm-based divider.

Figure 8 illustrates the behaviour of the proposed divider, based on the difference between the dividend and divisor values. It is clear that the performance of the proposed divider is data-dependent, the number of average clock cycles required over more than half the range of the difference between the dividend and divisor falls into an almost fixed range of clock cycles. The required values of the average maximum numbers of clock cycles stay in the range of twenty-eight to sixty-three clock cycles. When the distance between the divisor value and dividend value is less, the minimum required average number of clock cycles stays in the range of thirteen to twenty-four clock cycles. The lowest number of clock cycles (7 clock cycles) is required when the divisor value is unity.

Because the dividend value was previously confirmed to be a nonzero value and no iteration is performed, the final result value is calculated directly after confirming that the divisor value is unity. Midrange operand combinations possess clock requirements in the range of fifteen to thirty-five clock cycles. The divider circuit clock performance demonstrates the variable latency induced by the variable number of clock cycles required for different operand combinations in the proposed divider implementation and execution.

### Waveform analysis

This section discusses the functional waveform analysis of the proposed divider based on the USP-Awadhoot division algorithm. We present the working conditions of the various signals used or generated by implementations in different situations, such as idle/initial and off/on states. We consider various combinations of dividends and divisors, giving better insight into different signals and data at the time of a particular conversion process. A waveform analysis is used to study the nine signals data to provide a clear idea regarding the proposed divider's working conditions based on the USP-Awadhoot division algorithm. The nine signals, such as the reference CLK, dividend (${D}_{d}$), divisor (${D}_{r}$), enable (${F}_{d}\_enable$), quotient (Q_Result), remainder (Rem_Residue), Valid_O/P, Error and RST required for waveform analysis, are distributed into five different groups depending on the nature of each signal: the reference group, I/P operand group, control group, O/P results group and indicator group.

**Figure 9:**
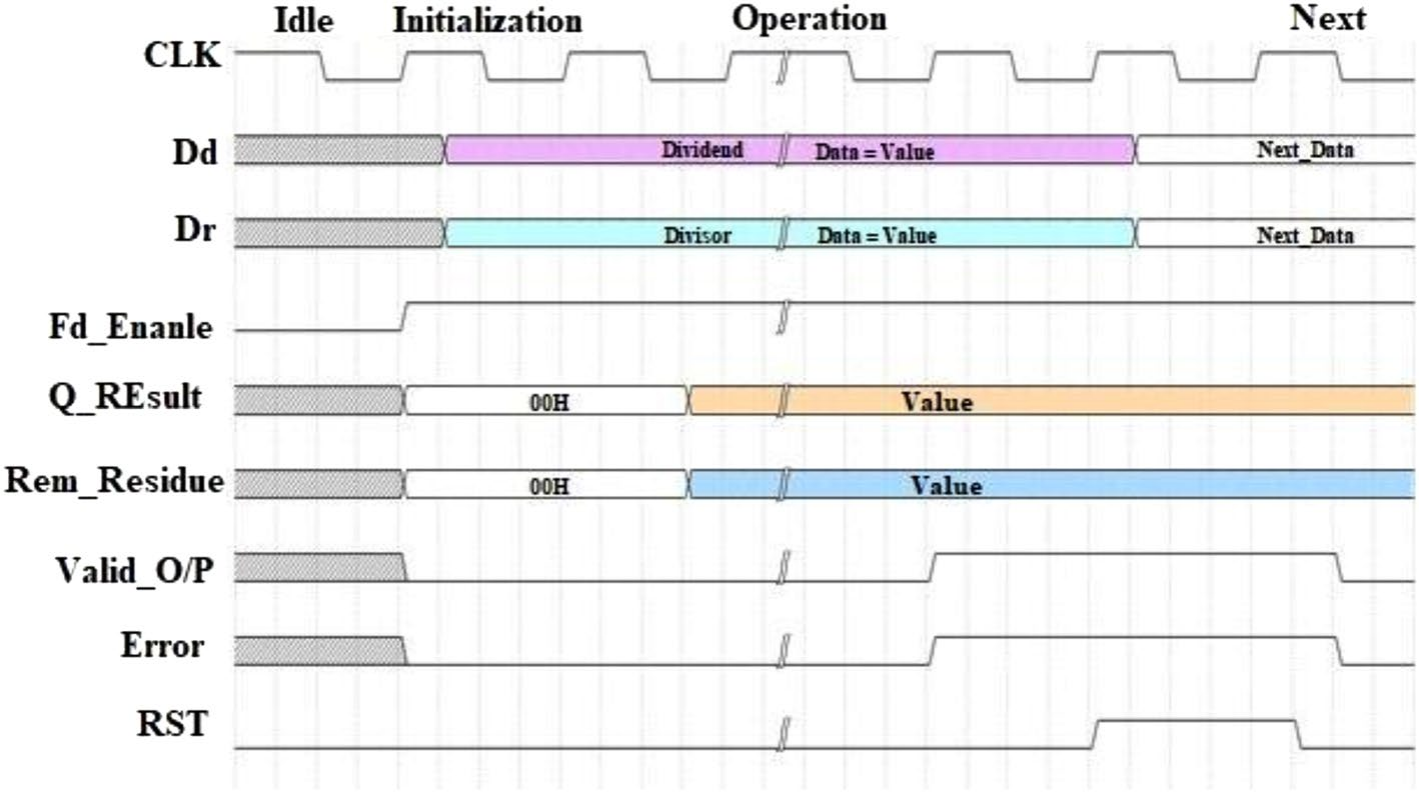
Waveform analysis of the proposed USP-Awadhoot algorithm-based divider.

Different dividend and divisor combinations require different clock cycles to operate; the reference group CLK signal provides the timing reference signal for computation execution. The reference clock signal's period value is dependent on the working frequency; thus, the higher the frequency is, the lower the clock period value. The I/P operand group consists of the dividend (${D}_{d}$) and the divisor (${D}_{r}$) signals, which indicate the dividend and divisor values, respectively. The control group consists of ${F}_{d}\_enable$ and RST signals to provide start and end control for the computation process. The indicator group consists of Valid_O/P and Error signals, indicating computation completion and alerting the system of any invalid working condition or incorrect execution. The last and essential O/P results group consists of the Q_Result and Rem_Residue signals that provide the values of the quotient and remainder, respectively, as results of the division operation performed by the proposed divider based on the USP-Awadhoot algorithm. Figure 9 indicates a reference working waveform diagram concerning the initial working condition. The initial working waveform is mainly sectorized into an idle state, an initialization state, an operation state, and the next state. The idle state shows the proposed divider circuit's nonworking or stationary condition and beginning state when only a power supply is provided to the circuit after a shutdown. In the idle state, the CLK signal continues generating the reference signal, and the values of the I/P operand group's dividend (${D}_{d}$) and divisor (${D}_{r}$) signals are in the high-impedance tri-state condition. The control group ${{F}_{d}}_{enable}$ and RST signals both possess low logic values, suggesting no operation. Similarly, the values of the indicator group and O/P result group's Q_Result and Rem_Residue signals are in a high-impedance tri-state condition, suggesting a stationary work condition. The initialization state indicates the next stage after applying the ${F}_{d}\_enable$ control signal to the proposed divider. In the initialization state, the proposed circuit resets the signal value of the O/P results group to the initial value of 00H, suggesting no result at the start. Later, it fetches the dividend (${D}_{d}$) and divisor (${D}_{r}$) data values from the input data lines that are stored in the input operand registers for further computation, as described in the previous sections. The indicator group's Valid_O/P and Error signals are set to logic low values, indicating that no computation operations have been performed yet.

The proposed divider circuit computes the Q_Result during the operation state, and the Rem_Residue signal provides the absolute value. After completing the computation operation, the Valid_O/P and Error are updated, and whether the computation and O/P result values are correct or incorrect is validated. At any instance, if a high-logic signal activates an RST signal, then the proposed divider circuit suspends its current computation operation state and resets it to the initialization state. The RST signal value is set to low logic, indicating an inactive reset signal that allows the continuing computation process to execute the division operation and compute the final O/P result values. Thus, depending on the control group signal values, the proposed divider circuit is ready to execute the next state.

### Comparative statistics

Due to strict conversion rules, the division is the most complex essential arithmetic operation and is difficult to implement. As discussed in the division circuit block taxonomy section, many ways to identify different divider circuits are available. Given the continued growth of industry and technological improvements, there is a demand for achieving an efficient trade-off between the area, latency time, and criticality of the conversion logic. Table 1 illustrates different dividers based on their mathematical formulations and theoretical backgrounds. Digit recurrence-based dividers are the most commercially implemented divider circuits that provide many ideas, processes, and hardware architectures. However, much room remains to improve digit recurrence algorithm-based divider circuits' areas, frequencies, and power levels. We develop a new state-of-the-art digit recurrence algorithm-based divider named the USP-Awadhoot algorithm divider.

**Table 1:** Summary of a comparative study on different division algorithm-based dividers.

Sr. no.	Algorithm	Equations	Important points
1	Restoring divider	For Jth iteration ${\mathrm{q}}_{\mathrm{j}} =0 \; \mathrm{ if } \; {\mathrm{R}}_{\mathrm{j}}^{\prime} < 0$ ${\mathrm{q}}_{\mathrm{j}} =1 \; \mathrm{ if }\;{\mathrm{R}}_{\mathrm{j }} ^{\prime} \ge 0$ ${\mathrm{R}}_{\mathrm{j}} =2{\mathrm{R}}_{\mathrm{j}-1 }\;\; \mathrm{ if } \;\;{\mathrm{q}}_{\mathrm{j}}=0$ ${R}_{j} ={R}_{j}^{\prime} \;\; if \;\; {q}_{j}=1$ ${\mathrm{R}}_{\mathrm{j}} ^{\prime}=2{\mathrm{R}}_{\mathrm{j}-1}-{\mathrm{D}}_{\mathrm{r}}$	It is similar to the long division algorithm
			Simple logic for implementation
			No requirement for a LUT
			Iterative subtraction is performed
			The nonredundant number system is used to write a quotient
			If the partial remainder value is not positive or zero, then the divisor is restored by the subtraction result performed in that iteration
			It requires a full-width comparator in each iteration, and the subtractor, shift register, and multiplier provide the approximate area requirement for algorithmic implementation
			Checks for possible MSB losses and overflow are needed
			Requires a full-width comparison at every iteration to obtain one bit of a quotient
			The quotient needs to be rearranged to obtain the actual quotient
2	Nonrestoring divider	For Jth iteration ${\mathrm{q}}_{\mathrm{j}}=-1 \; \mathrm{ if } \; {\mathrm{R}}_{\mathrm{j}-1 }<0$ ${\mathrm{q}}_{\mathrm{j}}=1 \; \mathrm{ if }\;{\mathrm{R}}_{\mathrm{j}-1 }\ge 0$ ${\mathrm{R}}_{\mathrm{j}}=2{\mathrm{R}}_{\mathrm{j}-1 }+ {\mathrm{D}}_{\mathrm{r}} \;\; \mathrm{ if } \;\; {\mathrm{q}}_{\mathrm{j}}= -1$ ${\mathrm{R}}_{\mathrm{j}}=2{\mathrm{R}}_{\mathrm{j}-1 }- {\mathrm{D}}_{\mathrm{r}} \;\; \mathrm{ if } \;\; {\mathrm{q}}_{\mathrm{j}}= +1$	Similar to the restoring algorithm, it does not require restoring the partial remainder if the subtraction result is negative
			No requirement for a LUT
			The operation in each iteration depends on the result of the previous iteration
			Only one addition or subtraction operation can be performed in each iteration, so separate hardware is needed
			The partial remainder is kept between − Dr and + Dr, and the quotient digit is − 1 or 1
			It requires a sign bit to decide whether to perform addition or subtraction; the adder, subtractor, and shift register give the approximate area requirement for algorithmic implementation
			Requires an extra bit to be added with the partial remainder to have a track on a sign
			Requires a separate adder and subtractor in each iteration
			The area utilization of the implementation is approximately equal to the area required to implement the adder, subtractor, and shift register
3	SRT divider	For Jth iteration ${q}_{j}=\overline{1} \;\; if \;\; {2R}_{j-1 }<-{D}_{r}$ ${q}_{j}=0 \;\; if \;\; {-{D}_{r} \le 2R}_{j-1 }\le {D}_{r}$ ${q}_{j}=1 \;\; if \;\; {2R}_{j-1 }\ge {D}_{r}$ Has one of the values *-m, −m* + *1… −1, 0,* + *1. . . m−1, m*, where *m* is an integer comprising k digits of radix-n as $\frac{1}{2} \left(n-1\right) \le m \le n-1$ $n={2}^{b} \;\; and \;\; k= x/b$ $Q=\sum_{j=1}^{k}{q}_{j}{n}^{-j}$ Quotient q is generated as a dividend through division by a divisor with the x most significant bits to remove b bits from the quotient in each iteration. Thus, radix-n performs k iterations to obtain the desired quotient	It is a nonrestoring algorithm based on radix-n
			Named after Dura W. Sweeney, James E. Robertson, and Keith D. Tocher
			For x bits, integer division requires k = x/b iterations, where b = the number of bits detected in each iteration
			n decides how many quotient bits are to be detected in each iteration; if n = 2, then one quotient bit is detected per iteration, and radix–n is typically selected as a power of base 2
			Each quotient digit has a value from {−m, −m + 1, ….., −1, 0, 1, ……., m−1, m}
			The algorithm implements 2’s complement value of ${D}_{r}$ instead of ${D}_{r}.$ This enables shifting over zeros to eliminate extra adders and subtractors
			It needs an extra subtractor to determine the next partial remainder
			Error results are obtained due to few MSBs being used to predict the quotient bits (as in the low radix case); the error decreases with the increase in the radix
			A quotient selection table plus a carry-saving adder (CSA) gives the approximate area requirement for algorithmic implementation. It requires a quotient selection LUT. This shows the iteration time of accessing the quotient selection table plus multiple additions and subtractions
			Selecting higher quotient bits causes complexity in the quotient selection logic, and higher radix implementations are complex due to the impractical multiples of the divisor
			It needs to convert the last remainder to a conventional representation to find the sign bit, and the quotient correction stage selection depends on the sign bit
4	Very high radix	*******	It removes more than ten quotient bits in one iteration; it requires a very large LUT with a large capacity for the quotient selection logic. A LUT is required for obtaining initial approximations of the reciprocal and the quotient digit selection logic
			It uses multiplication to form divisor multiples
			It differs from the regular radix-n divider in terms of the number and type of operations used in each iteration and the quotient digit selection logic
			A high radix makes quotient selection logic more complex and impractical to implement
5	Taylor series	$q={D}_{d}/{{D}_{r}} \; and \; {X}_{0}=1/{{D}_{r}}$ $q={D}_{d}{X}_{0}\left\{1+\left(1-{D}_{r}{X}_{0}\right)+ {\left(1-{D}_{r}{X}_{0}\right)}^{2}+{\left(1-{D}_{r}{X}_{0}\right)}^{3}\right\}$ ${D}_{d}=$ Dividend and ${D}_{r}=$ Divisor ${1/D}_{r}=Antidivisor$	It is a multiplicative iteration-based algorithm that thus requires a large area
			The precision depends upon the closeness with the antidivisor (reciprocal) estimation
			It provides a parallel powering section that computes high-order terms faster, with a minimal extension of the hardware overhead
			A quotient digit selection logic LUT and a three-full-word-length multiplier give the approximate area requirement for algorithmic implementation
6	Newton–Raphson	$Q= {D}_{d}/{D}_{r}=p\times {(q)}^{-1}$ $f(X)= 1/X-{q}^{-1}=0$ ${X}_{i+1}= {X}_{i}-\frac{f({X}_{i})}{f^{\prime}({X}_{i})}$ ${X}_{i+1}$= ${X}_{i}-\frac{(1/{X}_{i}-{q}^{-1})}{1/{X}_{i}^{2}}$ = ${X}_{i}\times (2-{q}^{-1}\times {X}_{i})$ ${\in }_{i+1}= {\in }_{i}^{2}({q}^{-1})$ $p=\mathrm{Dividend \;\; and } \;\; {(q)}^{-1}=Antidivisor$	The accuracy can be improved by selecting a proper root at the beginning
			The latency and error during convergence are directly dependent on the root selected at the beginning of the convergence process, the iteration time is approximately equal to the time required for two serial multiplications
			A multiplier, a quotient selection LUT, and control logic give the approximate area requirement for algorithmic implementation
			The final quotient is derived by multiplying the approximated reciprocal and dividend
			Shows the error induced due to the inaccuracy of the quotient digit prediction or estimation
			It requires multiplication and addition or subtraction at each iteration, and using 1's complement induces more error
7	Goldschmidt	${D}_{d}/{D}_{r}=N/D=A/B$ ${x}_{n+1}={x}_{n}\left(2-{y}_{n}\right)={x}_{n}{r}_{n}$ ${y}_{n+1}={y}_{n}\left(2-{y}_{n}\right)={y}_{n}{r}_{n}$	It is a convergence-based functional iterative class divider algorithm
			It multiplies both the dividend and divisor by the antidivisor or reciprocal
			It originates from the Taylor-Maclaurin series of $1/(x+1)$
			It does not provide a remainder
			1's complement can be used instead of (2 − ${y}_{n}$) to avoid carry propagation delays, but this adds a new approximation error in each iteration
			A quotient digit selection logic LUT, a one-full-word-length multiplier, and a one-full-word-length adder/subtractor logic give the approximate area requirement for algorithmic implementation
8	Variable-latency	******	Its variable execution time thus results in different conversion times for different sets of dividends and divisors
			Self-timing, result caching, and quotient digit speculation are some techniques used to provide variable latency
8	Variable latency	******	The DEC Alpha 21,164 is one of the best variable-latency class algorithm implementation examples, based on the concepts of the simple normalizing and nonrestoring division algorithm
9	Svoboda algorithm and Svoboda-Tung algorithm	$\left\{\frac{mn}{\left(m+1\right)\left(n-1\right)} < {D}_{r}< \frac{m(n-2)}{(n-1)(m-1)}\right\}$ $\left\{-m/n-1 <{R}_{j} < m/n-1\right\}$ $Range=\left\{0,\pm 1,\ldots \; \ldots .,\pm m\right\}$ $Boundry \; limit=\left\{n/2+1 \le m \le n-1\right\}$ $m=Range\; of \;SBD \;and \;n=Radix$	The quotient digit is predicted based on the partial remainder without considering the divisor; one or two MSBs of the partial remainder are used for generating quotient digit selection logic
			It can select a quotient digit out of the radix range if an overflow occurs due to compensation
			It requires prescaled operands and can work on conventional and signed digit ranges
			It is also a radix-n based algorithm with signed binary digit numbers, making it similar to the SRT algorithm
			It is applicable more than radix 4, and Prescaled operands are needed; it needs extra multipliers, resulting in more hardware overhead
10	Smaller dividend	${N}_{1}=\sum_{i=0}^{2n-1}{x}_{2n+i}{2}^{2n+i}$ ${N}_{2}=\sum_{i=0}^{2n-1}{x}_{i}{2}^{i}$ ${D}_{d}={N}_{1}+{N}_{2}$ ${D}_{d}/{D}_{r}=({N}_{1}+{N}_{2})/{D}_{r}={N}_{1}/{D}_{r}+{N}_{2}/{D}_{r}$	It is the simplest parallel computing algorithm
			The basic phenomenon behind this algorithm is to consider division as a fraction
			It requires an actual dividend greater than the divisor, i.e., a dividend bit count of 4n and divisor bit counts of n
			We can represent dividends in terms of fixed partitions based on their associated weights as per the dividers' radix values
			The area is directly dependent on the number of dividend partitions related to the dividers' radix values
11	Jebelean exact division	*D*_*d*_ = d *Q ${D}_{d}={D}_{dupk}{n}^{k}+{D}_{k}$ ${b}_{k}={\left({-n}^{-k}{D}_{dk}\right) }_{mod d}$ ${b}_{k}={\left({-n}_{mod d}^{-k}{D}_{dk}\right) }_{mod d}$ $modPower \; calls \; and \; ParallelPrefixSum \; call=O\left(\mathit{log}n\right)$	It is applied when complete division is performed on long integer operands in digital computation, even after knowing that the remainder is zero
			It starts from the least significant digits of the operands
			Remarkable performance is observed when the radix is a prime or power of 2
			It takes constant execution time to access a fixed-word-length LUT
			It takes O(log n) execution time, and for short division, O($n/\rho +\mathrm{log}\rho )$, where n is the word length of the dividend and $\rho$ is the number of processors
			It needs synchronization to execute calculations in parallel

During various simulations performed on the Vivado 2016 simulation tool, the results of different input operand combinations are collected, forming a truth table for all possible combinations of input operands. The collected simulation data are summarized into time values, current operand (dividend and divisor) values, the present values of the control signals, and the outputs (quotient, remainder, error state, and valid output) obtained by the computation of the algorithm. The data collected from the output of the Xilinxs simulation and hardware implementation on Xilinxs FPGAs are compared with their known solutions from the reference truth table prepared from theoretical calculations. Once the data obtained from the output of the hardware implementation are successfully compared, the resources and execution speeds obtained with various divider implementations are determined. However, the hardware resource utilization data presented in Fig. 7 of the proposed USP-Awadhoot algorithm-based divider is considered further for comparative analysis.

Bailey^1^ presented an article about statistical implementation data for restoring and nonrestoring algorithms in 2006. He presented a comparative analysis of the FPGA and Handel-C software implementation of restoring and nonrestoring division algorithms. These algorithms were implemented on RC-100 and RC-300 development boards produced by Celoxica using Xilinx's Spartan-II and a Virtex-II FPGA. A statistical comparison between the algorithms implemented as macro expressions with the Handel-C built-in integer divider is presented. For comparison, only restoring and nonrestoring algorithms based on the basic equations expressed in an earlier section are used without implementing the radix SRT algorithm.

The comparison presented in Table 2 concludes that Handel-C built-in divider is the slowest, as it can work on frequencies near 10 MHz. The chip area required in the FPGA is approximately more than double the area required by the proposed USP-Awadhoot algorithm-based divider. In Handel-C implementations, the use of subtraction for performing comparisons, its reuse as an input to a multiplexer, and the utilization of separate LUTs for addition and multiplexing requires extra hardware, limiting the speed improvement. The basic ideas of restoring and nonrestoring division algorithms can be implemented in a sufficiently small chip area, but the maximum working frequency is low. The number of LUTs may vary based on the hardware description languages (HDLs) used to implement the above algorithms.

**Table 2:** Comparative analysis of the resource utilization of the proposed USP-Awadhoot algorithm-based divider with those of the Handle-C and digit recurrence algorithm-based dividers.

Parameter/result	Slice logic LUTs	Clock frequency (MHz)	
		From	To
Handel–C	747	721	10,965
Restoring	115	13,716	20,345
Nonrestoring	144	24,175	40,073
Nonrestoring with pipeline	66	37,806	63,558
Proposed USP-Awadhoot divider version V8.9	266	50	285

In^5^, Matthews et al. discussed integer divider designs for the ascendancy of FPGA-based soft-processor over the adaptation of variable-latency execution units in their instruction pipeline. The implementation efforts were focused on the Quick-Div divider, which exhibits data dependency and variable latency in integer division. This divider was integrated into the FPGA-based Taiga RISC-V pipelined soft processor.

Taiga is a RISC-V open-source soft processor. Experimental implementations are performed over the Xilinx Virtex UltraScale + VCU118 board (XCVU9P-L2FLGA2104E) using Vivado 2018.3 synthesis. With ascendancy over the variable-latency execution unit's operation in the Taiga soft processor instruction pipeline, all dividers are realized with the RISC-V Taiga soft processor. A comparative statistic is derived between the implementations of the data-dependent variable-latency Quick-Div dividers and fixed- latency radix-n (n = 2, 4, 8, 16) dividers with the RISC-V Taiga soft processor. Quick-Div dividers are unsigned processes, so sign conversion is performed before and after conversion; completion is required depending on the instruction operands and types. Therefore, Quick-Div requires an additional three cycles for sign conversion. Table 3 illustrates the comprehensive results of the variable-latency Quick-Div dividers and the fixed-latency radix-n (n = 2, 4, 8, 16) dividers with the RISC-V Taiga soft processor compared with those of 8/16-bit USP-Awadhoot dividers. This indicates that the variable-latency Quick-Div dividers and fixed-latency radix-n (n = 2, 4, 8, 16) dividers require 5 to 7 times more chip area than the proposed USP-Awadhoot divider; this is represented by the numbers of slice logic LUTs and slice register flip-flops used for the implementation. In contrast, the maximum clock frequency is almost double the maximum clock frequency of the proposed USP-Awadhoot divider.

**Table 3:** Comparative analysis of the resource utilization of the proposed USP-Awadhoot algorithm-based divider with variable-latency Quick-Div dividers and fixed-latency radix-n dividers.

Name/parameter	Slice logic LUTs	Slice register flip-flop	Clock frequency (MHz)
Radix-2	1500	1100	375
Radix-4	1520	1200	350
Radix-8	1990	1000	350
Radix-16	2100	1200	300
Quick-Div initial	1600	1000	350
Quick-Div count leading zeros	1600	1150	375
Quick-Div CLZ-2BIT worst-case optimization	1700	1100	300
Proposed USP-Awadhoot divider—8 bits version V8.9	266	146	285
Proposed USP-Awadhoot divider—16 bits version V16.1	622	241	125

In^9^, Sorokin discussed the implementations of fixed-point dividers based on different algorithms on Xilinx’s common FPGA platform. Different divider modules have been compared with Xilinx's 32-bit IP core-pipelined divider. This indicates that a nonrestoring algorithm-based fixed-point divider module is much faster than the 32-bit Xilinx IP core-pipelined divider. This paper points out that the results are more approximations than exact values and demonstrate more practical division operations than digital operations. These approximated values can cause trouble in more critical applications, such as biomedical applications, sensor signal processing, coordinate computation for an item, etc.^9^. As we have discussed earlier, even for integer division, we must use a fractional divider, which includes a fixed-point or floating-point divider; thus, floating-point implementation is critical and complex, making it sometimes impracticable. Out of many theoretical concepts, one practicable solution was provided by Xilinx's IP core-pipelined divider^78^. 32-bit input operands produce 32-bit remainders in many cases, making them impossible to implement in applications where high calculation precision is needed. Another implementation-focused problem in this article concerns the chip area requirements of this solution. The fixed-point algorithm follows the basic principles of the simple paper-and-pencil division algorithm. A fixed-bit-length quotient is generated in every iteration of a fixed-point divider, similar to digit recurrence dividers. Much attention is given to improving the addition and multiplication operations, as speeding up addition operations reduces the computational time required in the actual division process. Replacing the divisor with its inverse value can allow the use of multiplication by an antidivider to obtain division results.

Speeding up dividers has been achieved by developing fast adders, carry look-ahead adders, matrix- or array-type adders, etc. Xilinx's Ip core divider has certain properties, such as the availability of drop-in modules for Virtex, Virtex-II, Virtex-II Pro, Virtex-4, Spartan-3, etc. The dividend can be up to 32 bits and has a fully pipelined structure. Table 4 compares 8/16/32-bit Xilinx IP core-pipelined dividers and the proposed USP-Awadhoot divider. This indicates that Xilinx’s IP core-pipelined dividers are bulkier, with three to five times more chip area, and the maximum clock frequency is almost the same as that of the proposed USP-Awadhoot dividers.

**Table 4:** Comparative analysis of the resource utilization of the proposed USP-Awadhoot algorithm-based divider with that of the Xilinx IP core-pipelined divider.

Name/parameter	Slice logic LUTs	Slice register flip-flops	Look-up tables	Frequency (MHz)
IP core-pipelined divider 8-bit	2247	4020	1400	204.3
IP core-pipelined divider 16-bit	2742	4904	1680	201.6
IP core-pipelined divider 32-bit	3843	6864	2240	193.1
Proposed USP-Awadhoot divider—8 bits version V8.9	266	146	0	285
Proposed USP-Awadhoot divider—16 bits version V16.1	622	241	0	125

**Figure 10:**
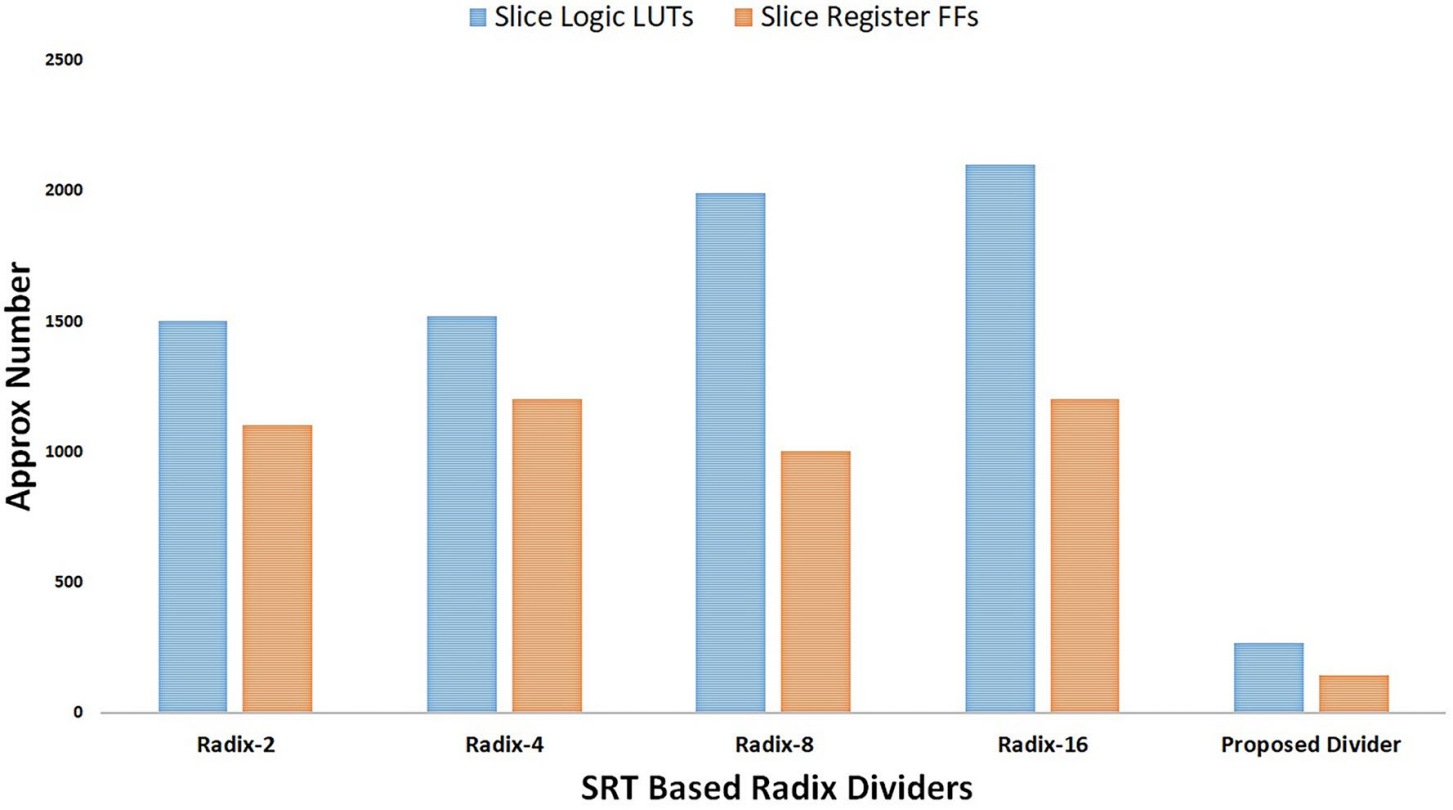
Comparative analysis regarding the hardware resource utilization of the proposed USP-Awadhoot algorithm-based divider and radix-n-based dividers.

Many nonrestoring algorithms have been designed and implemented, but the SRT algorithm is the most implemented approach. The basic SRT algorithm was implemented in^5,9,12,18,20,25,42,51,62,68,76–83^ for different applications utilizing different aspects of the algorithm. Figure 10 illustrates the comparative analysis regarding the hardware resource utilization of the USP-Awadhoot division algorithm-based divider and other SRT-based radix-n dividers. On average, the proposed USP-Awadhoot algorithm-based divider requires 266 slice logic LUTs, 146 slice register flip-flops with a power dissipation estimation of 3.366 watts. In contrast, the radix-2 to radix-16 divider implementations require 1500 to 2100 slice logic LUTs and 1100 to 1200 slice register flip-flops^5^. This indicates that the concept of different prescaling factors for the input operands used in the proposed USP-Awadhoot divider helps reduce its chip area requirements.

From the commercial and noncommercial implementation points of view, two classes of dividers are the main focus. One is the famous digit recurrence class, and the other is the functional iteration class of dividers. In^6^, Tatas et al. discussed different concepts involved in partitioning the main dividend into segments to represent an actual division of a numerator by a denominator as a series of smaller divisions with necessary requirement for numerator to meet (Numerator N= N1 + N2 +...). This concept of a series of divisions showcases a smaller-dividend division algorithm, where we must perform shifting, partial division, and accumulation operations. All intermediate operations are performed by considering the weights of the dividend bits.

32 $$\frac{N}{D}= \frac{N1}{D}+\frac{N2}{D}+ \frac{N3}{D}+\frac{N4}{D}+ \cdots$$

This algorithm can be implemented in both series and parallel architecture^8^, but a higher-radix system is critical and difficult to implement. The partitioned numerator's partial division process can be performed serially or in parallel due to the trend between cost and time. This algorithm is implemented with a length of N, D 32-bit dividends and a parallel-array divider, a sequential divider with two partitions, or a parallel divider with two partitions in the partial division stage. These implementations require 4316, 2136, and 3050 slices on a Xilinx Virtex-E 1000. From the above data, it is clear that the sequential implementation of the proposed algorithm is more area-efficient and moderate in terms of the time delay. If any corrective stage is required in a sequential divider, this will degrade the efficiency of serial dividers. In contrast, parallel implementation produces a slight reduction in the delay but insufficient decreases in the area and latency. The array implementation of this algorithm is inefficient as it increases the chip area by four times based on doubling the word length. Whereas the proposed USP-Awadhoot algorithm-based divider does not partition the given Numerator (Dividend) into smaller dividends like mentioned above (Numerator N = N1 + N2 +...). The USP-Awadhoot algorithm-based divider converts the Numerator (Dividend) into group dividends, which are not required to add up to the main Dividend value. The proposed USP-Awadhoot algorithm-based divider requires 266 slice logic LUTs, 146 slice register flip-flops with a power dissipation estimation of 3.366 watts indicating better implementation area performance.

In^27^, Kasim et al. discussed a divider block with precomputed values stored in ROM in terms of a LUT. This divider operation is similar to the dividers based on functional iteration algorithms such as Goldschmidt's algorithm and Newton's method^27^. The result of this divider is also an approximate value, unlike those of iterative subtraction class-based dividers. In^38^, Liu et al. discussed an algorithm that utilizes prescaling, series expansion, and Taylor series expansion together; hence, it is sometimes called a phase stretch transform (PST) algorithm. At the start, both operands are prescaled up to the suitable starting level. Operand prescaling is performed based on a scaling factor E0, stored in a LUT. In the second stage of the PST algorithm, series expansion is applied to the scaled operands to obtain an accurate antidivisor approximation. To calculate the partial quotient and the next remainder in the iteration stage, it utilizes 0-order Taylor series expansion. The iterative process must continue until a quotient is obtained with the required precision range of error. Three Taylor expansion iterations and a LUTs are needed to finish one operation.

**Figure 11:**
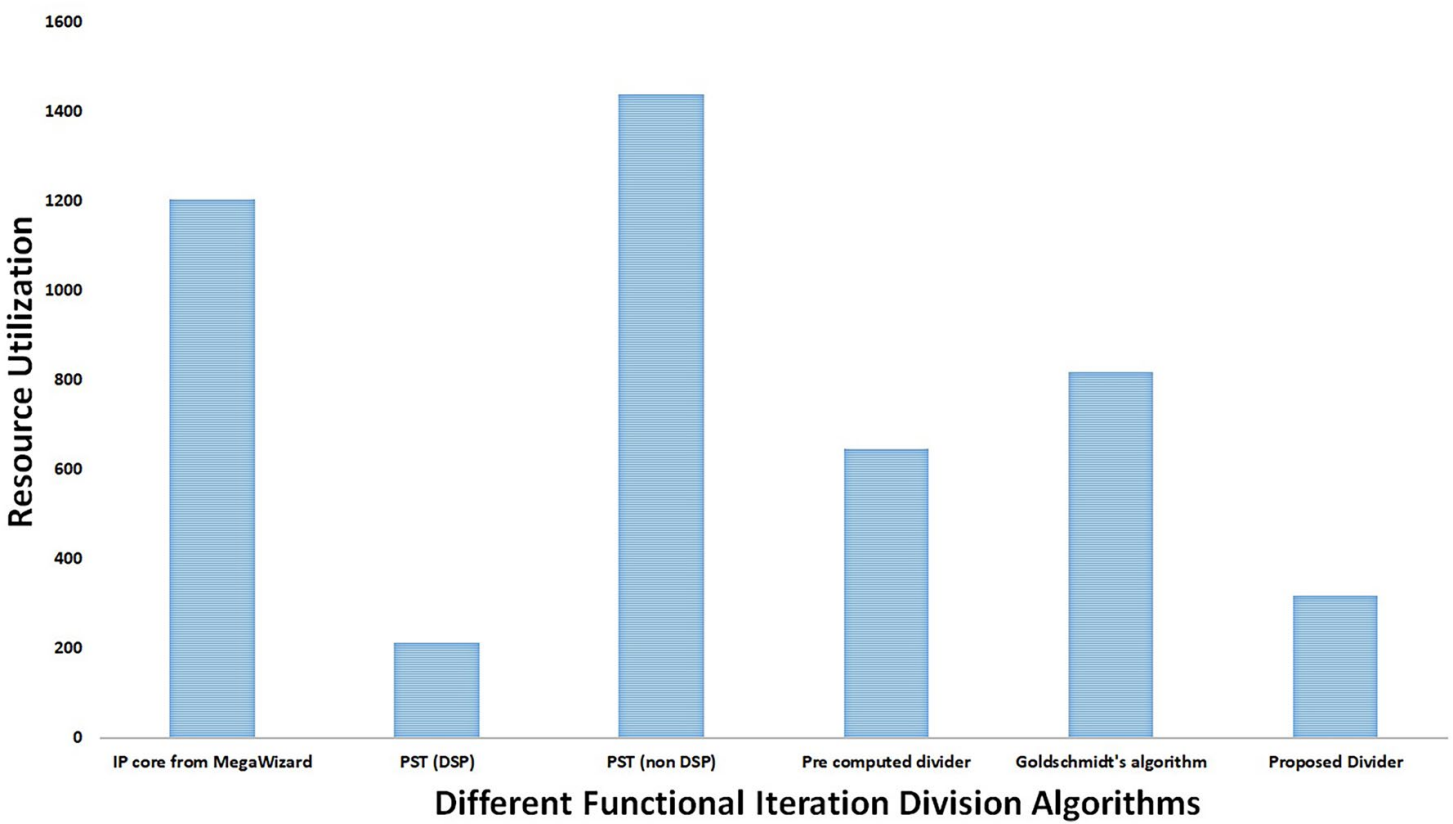
Comparative analysis regarding the hardware resource utilization of the proposed USP-Awadhoot algorithm-based divider and different functional dividers.

Figure 11 illustrates a comparative analysis regarding the hardware resource utilization of the proposed USP-Awadhoot algorithm-based divider implementation and that of different functional iteration divider implementations, as discussed above. As per the performance comparison between the proposed USP-Awadhoot divider and the Mega Wizard IP core, DSP, and non-DSP structures of the divider algorithm, the resource utilization of the proposed USP-Awadhoot algorithm-based divider includes 266 slice logic LUTs, 146 slice register flip-flops with a power dissipation estimation of 3.366 watts; whereas the PST algorithm-based divider requires 213 slice logic LUTs, 768 bytes of memory, and 28 DSP^84^, the PST algorithm-based divider without DSP needs 1437 slice logic LUTs and 768 bytes of memory. The precomputed divider and Goldschmidt's algorithm-based divider require 647 and 816 slice logic LUTs, respectively. The divider algorithm's Mega Wizard IP core, DSP, and non-DSP structures significantly delay the results, as their maximum clock frequencies are limited to 50 to 73 MHz. This framework does not save sufficient area relative to the proposed USP-Awadhoot divider. Additionally, the precomputed values introduce rounding errors in the calculation process. The proposed USP-Awadhoot divider displays better implementation area requirements and maximum clock frequency performance.

Table 5 illustrates a comparative analysis regarding the resource utilization of the proposed USP-Awadhoot division algorithm-based divider and the Xilinx LogiCORE IP Divider Generator V4.0^62^. The proposed USP-Awadhoot algorithm-based divider requires 266 slice logic LUTs, 146 slice register flip-flops, and a total of 37 bounded I/o's for providing input operands and reading the quotient and remainder outputs. The operating speed of the proposed divider is given in terms of its operating frequency, which is equal to 285 MHz, with a power dissipation estimation of 3.366 watts. The implementation statistics are derived for the proposed divider with a dividend width of eight bits, a divisor width of eight bits, a quotient width of eight bits, a remainder width of eight bits, and Error and Valid_O/P signals. The Valid_O/P signal indicates the computation's completion, whereas the Error signal indicates an invalid condition caused by a divisor value of zero, i.e., the divide-by-zero condition. The resource utilization analysis of the proposed divider implementation is conducted with Xilinx's LogiCORE IP Divider Generator V4.0. Xilinx is the leading candidate in the IC industry and has a wide range of Intellectual property (IPs). We consider Virtex 6 and 7, Kintex 7, and the Spartan 6 FPGA from Xilinx during the comparison. The number of slice register flip-flops used is constant at 288 for each FPGA IC, whereas the number of slice logic LUTs used ranges from 197 to 205 and the number of input LUT-FF pairs used slightly varies from 223 to 215 for Virtex 7, Kintex 7, Virtex 6 and Spartan 6. The document does not mention the power consumption of the LogiCORE IP Divider Generator V4.0, whereas the proposed divider based on the USP-Awadhoot division algorithm simulation estimates 3.366 Watts.

**Table 5:** Comparative analysis regarding the resource utilization of the proposed USP-Awadhoot algorithm-based divider and Xilinx's LogiCORE IP divider generator V4.0.

Parameter/result	Case 1	Case 2	Case 3	Case 4	Proposed divider version V8.9
Dividend width	8	8	8	8	8
Divisor width	8	8	8	8	8
Remainder and quotient width	8	8	8	8	8
LUT6-FF pairs	223	218	217	215	000
Slice logic LUTs	203	205	203	197	266
Slice register flip-flops	288	288	288	288	146
IC name	Virtex7	Kintex7	Virtex6	Spartan6	Xilinx Zynq XC7Z010

The proposed USP-Awadhoot algorithm-based divider implementation utilizes 64% less FPGA hardware resources than the Handel-C built-in divider but, it requires 57% and 75% more FPGA hardware resources than simple restoring and nonrestoring dividers. The proposed divider's working frequency based on the USP-Awadhoot algorithm implementation versions is approximatly 50% greater than other performances presented in^1^, indicating the FPGA resource utilization improvement achieved by the proposed USP-Awadhoot algorithm-based divider implementation. Based on the statistics presented in^9^, the proposed USP-Awadhoot algorithm-based divider implementation shows improvements in its FPGA resource utilization in terms of 86% to 88% improvements in the number of required slice logic LUTs (depending on the use of 8-bit or 16-bit operands) and 95% to 96% improvements in the number of slice register flip-flops required (depending on the use of 8-bit or 16-bit operands). Based on the statistics presented in^5^, the proposed USP-Awadhoot algorithm-based 8-bit divider implementation shows improvement in slice logic LUTs and slice register flip-flops requirement as the proposed divider implementation do not require any six input LUT-FF pairs. As compared to the variable-latency Quick-Div dividers and fixed-latency radix-n dividers but the results exhibit a comparatively lower working frequency of 285 MHz for the proposed divider. The power required for variable-latency Quick-Div dividers and fixed latency radix-n dividers is not mentioned, but the proposed divider simulation estimates 3.366 Watts.

As per the comparative statistics presented in^27^, the proposed USP-Awadhoot algorithm-based divider implementation exhibits an FPGA resource utilization improvement in terms of a 58% improvement in the number of required slice logic LUTs compared to that of the precomputed divider, a 67% improvement in the number of required slice logic LUTs compared to that of Goldschmidt's algorithm-based divider, a 76% improvement in the number of required slice logic LUTs compared to that of the divider developed from Quartus Mega functions. The proposed divider does not induce any errors in the computed results and simulations estimated required power of 3.366 watts. As per the statistics presented in^38^ It achieves a 82% improvement over the IP core from MegaWizard's operating frequency as it is upto 50.16 MHz and a 75% improvement over the PST-DSP and non-DSP dividers as it is upto 73 MHz. The proposed divider requires no extended memory or DSP compared to the IP core from the MegaWizard and the PST divider.

## Conclusion

The evaluation of addition and multiplication implementations typically falls into the latency range from a couple of clock cycles to less than ten clock cycles, while the performance evaluation of division operation implementations typically falls into the latency range from tens to hundreds of clock cycles and requires a high implementation area. The primary focus of the problem statement is to design and implement a reduced-area divider circuit block, providing straightforward dialectics between divisors, dividends, and quotients to avoid round-off errors. A design is developed by simulating the proposed technique and cross-verified by performing regular sequential and pseudorandom sequential analyses of the implementation against standard result tables generated by simulations and the theoretical study of the proposed idea. The main contributions highlighted in the article are as follows.

- Significant efforts were taken toward developing the state-of-the-art novel USP-Awadhoot algorithm-based divider circuit block implementation.
- The proposed USP-Awadhoot algorithm-based divider circuit block implementation substantially reduces the required implementation resources, resulting in better area efficiency.
- Successful implementation of a dynamic separate scaling operation/factor for input operands to reduce the conversion complexity.
- A novel divisor-dividend relationship is demonstrated with the proposed USP-Awadhoot algorithm-based divider circuit block implementation to derive dividend groups, modified divisors (${MD}_{r}$), and FD terms. This proves that the hypothesis of utilizing different scaling operation/factors for dividends and divisors can improve the area requirements by reducing resource utilization.
- A comparatively straightforward group quotient (${GQ}_{n}$) value selection logic is developed based on the unique relations derived between the dividend groups, modified divisors (${MD}_{r}$), and FD terms of the proposed technique or algorithm of the divider circuit block implementation; this is termed the Awadhoot matrix.
- A comparatively straightforward process for selecting the final quotient based on the group quotient (${GQ}_{n}$), partial quotient (${PQ}_{n}$), and additional quotient ($AQ$) values is developed.

The second-most significant contribution is the design and verification of complex division via the Baudhayan-Pythagoras triplet method using the novel state-of-the-art USP-Awadhoot divider circuit block implementation.

## Future work road map

- As the current implementation verifies the successful implementation of the proposed divider on different FPGAs, the next target is to design a dedicated integrated-circuit IP. The first step is to design a physical layout, starting from the floor plan, which determines which circuit component is placed in which area and extracts the parasitic values to prepare the final layout for fabrication.
- Another future work target is to improve the working frequency and conversion time. To do so, we must fuse some intermediate functional blocks such as separate addition, and multiplication can be performed in the fused mode such as fused-multiply-add (FMA). We need to test the implementation and verify the resource utilization of the proposed divider to validate these changes.
- The current implementation validates its successful implementation using combinational circuits. Thus, reducing the area and hardware resource utilization is also a future target. Some processes involved in the proposed divider can be represented as different hardware architectures, such as pipelined architectures, parallel architectures, array structures, and cascade structures. Thus, it is necessary to validate the usage of different architectures and compare their resource utilization levels to prove the usability of the proposed divider in different working environments with different requirements. Detailed implementation results will be utilized to choose the most suitable architecture implementation of the proposed divider in various applications based on their time, area, and power requirements.
- We must verify the performance of the proposed divider in various applications, such as image processing, particle detection, and signal processing. Complex number arithmetic is critical and requires a careful design and more hardware resources. It is also very helpful in various essential engineering applications, such as acoustic pulse reflectometry, astronomy, nonlinear radio frequency measurements, control theory applications such as finding root loci, Nyquist plots, and microwave system frequency responses.

## Methods

### Process of FPGA-based circuit integration

The circuit constructed based on the proposed USP-Awadhoot division algorithm is preliminarily implemented on an FPGA, and the computational results are studied and analyzed with the help of FPGA design simulation suites. We utilize the Xilinx Vivado design suite and the Altera Quartus II design suite for the proposed design. Before utilizing the design suites, we subdivide the complete computation into different FSM states. VHDL is utilized for the implementation, making it easier in design and performance simulations to analyze the resulting behavior. The tests and implementations of the circuits using FPGAs are parts of the design process for application-specific integrated circuit (ASIC) design. The manufacturing of the USP-Awadhoot division algorithm implemented in an ASIC is possible. We develop the proposed circuit implementation with the algorithm's established flow, which is tested in behavioral and implementation timing simulations to verify its performance and results. The post-simulation circuit is tested in the real world by implementing it in different FPGAs, especially those from Altera and Xilinx.

## Supplementary Information

Supplementary Information.

## Data Availability

All data generated or analyzed during this study are included in this published article [and its supplementary information files].
