# Supplementary material for: Novel data dependent divider circuit block implementation for complex division and area critical applications
Source: Sci Rep. 2023 Feb 21;13:3027. doi: 10.1038/s41598-023-28343-3 (PMC9944960; doi:10.1038/s41598-023-28343-3)
Supplement: Supplementary file 1 — Supplementary Information. [file 41598_2023_28343_MOESM1_ESM.pdf]

# Novel Data-Dependent Divider Circuit Block Implementation for Complex Division and Area-Critical Applications

Udayan S. Patankar[1*], Miguel E. Flores[2], and Ants Koel[3]

[1]PhD Researcher, TalTech University of Technology, Tallinn, Estonia

[2]Professor, Electronics School, Don Bosco University, El Salvador

[3]Associate Professor, TalTech University of Technology, Tallinn, Estonia

[1]udayan.patankar45@gmail.com and udayan.patankar@taltech.ee

[2]miguel.flores@udb.edu.sv

[3]ants.koel@taltech.ee

[1*]Orcid Id- 0000-0003-4167-6755,

[2]Orcid Id- 0000-0003-0514-6239,

[3]Orcid Id- 0000-0001-5635-0139

**[*]Corresponding author**

E-mail: udayan.patankar45@gmail.com and udayan.patankar@taltech.ee

| USP-Awadhoot algorithm based divider | | | | | | | Clk=125MHz | T= | 8,00E-09 | | | | | | | | |
|---|---|---|---|---|---|---|---|---|---|---|---|---|---|---|---|---|---|
| INPUT DATA | | | | THEORICAL DATA | | BEHAVIORAL SIM | | | | | | Sim timing | | | | | |
| DIVIDEND | | DIVISOR | | THEORICAL | RESIDUE_T | CLK PULSES | Time (nsec) | Result | Residue | Error | DT | time_ns | Result | Residue | Error S | Verification | Delta Time |
| 0 | 00 | 0 | 00 | ERROR | ERROR | 6 | 44 | 0 | 0 | 1 | 44 | 132 | 0 | 0 | 1 | 1 | 132 |
| 1 | 01 | 0 | 00 | ERROR | ERROR | 11 | 84 | 0 | 0 | 1 | 40 | 180 | 0 | 0 | 1 | 1 | 48 |
| 2 | 02 | 0 | 00 | ERROR | ERROR | 16 | 124 | 0 | 0 | 1 | 40 | 228 | 0 | 0 | 1 | 1 | 48 |
| 3 | 03 | 0 | 00 | ERROR | ERROR | 21 | 164 | 0 | 0 | 1 | 40 | 276 | 0 | 0 | 1 | 1 | 48 |
| 4 | 04 | 0 | 00 | ERROR | ERROR | 26 | 204 | 0 | 0 | 1 | 40 | 324 | 0 | 0 | 1 | 1 | 48 |
| 5 | 05 | 0 | 00 | ERROR | ERROR | 31 | 244 | 0 | 0 | 1 | 40 | 372 | 0 | 0 | 1 | 1 | 48 |
| 6 | 06 | 0 | 00 | ERROR | ERROR | 36 | 284 | 0 | 0 | 1 | 40 | 420 | 0 | 0 | 1 | 1 | 48 |
| 7 | 07 | 0 | 00 | ERROR | ERROR | 41 | 324 | 0 | 0 | 1 | 40 | 468 | 0 | 0 | 1 | 1 | 48 |
| 8 | 08 | 0 | 00 | ERROR | ERROR | 46 | 364 | 0 | 0 | 1 | 40 | 516 | 0 | 0 | 1 | 1 | 48 |
| 9 | 09 | 0 | 00 | ERROR | ERROR | 51 | 404 | 0 | 0 | 1 | 40 | 564 | 0 | 0 | 1 | 1 | 48 |
| 10 | 0A | 0 | 00 | ERROR | ERROR | 56 | 444 | 0 | 0 | 1 | 40 | 612 | 0 | 0 | 1 | 1 | 48 |
| 11 | 0B | 0 | 00 | ERROR | ERROR | 61 | 484 | 0 | 0 | 1 | 40 | 660 | 0 | 0 | 1 | 1 | 48 |
| 12 | 0C | 0 | 00 | ERROR | ERROR | 66 | 524 | 0 | 0 | 1 | 40 | 708 | 0 | 0 | 1 | 1 | 48 |
| 13 | 0D | 0 | 00 | ERROR | ERROR | 71 | 564 | 0 | 0 | 1 | 40 | 756 | 0 | 0 | 1 | 1 | 48 |
| 14 | 0E | 0 | 00 | ERROR | ERROR | 76 | 604 | 0 | 0 | 1 | 40 | 804 | 0 | 0 | 1 | 1 | 48 |
| 15 | 0F | 0 | 00 | ERROR | ERROR | 81 | 644 | 0 | 0 | 1 | 40 | 852 | 0 | 0 | 1 | 1 | 48 |
| 16 | 10 | 0 | 00 | ERROR | ERROR | 86 | 684 | 0 | 0 | 1 | 40 | 900 | 0 | 0 | 1 | 1 | 48 |
| 17 | 11 | 0 | 00 | ERROR | ERROR | 91 | 724 | 0 | 0 | 1 | 40 | 948 | 0 | 0 | 1 | 1 | 48 |
| 18 | 12 | 0 | 00 | ERROR | ERROR | 96 | 764 | 0 | 0 | 1 | 40 | 996 | 0 | 0 | 1 | 1 | 48 |
| 19 | 13 | 0 | 00 | ERROR | ERROR | 101 | 804 | 0 | 0 | 1 | 40 | 1044 | 0 | 0 | 1 | 1 | 48 |
| 20 | 14 | 0 | 00 | ERROR | ERROR | 106 | 844 | 0 | 0 | 1 | 40 | 1092 | 0 | 0 | 1 | 1 | 48 |
| 21 | 15 | 0 | 00 | ERROR | ERROR | 111 | 884 | 0 | 0 | 1 | 40 | 1140 | 0 | 0 | 1 | 1 | 48 |
| 22 | 16 | 0 | 00 | ERROR | ERROR | 116 | 924 | 0 | 0 | 1 | 40 | 1188 | 0 | 0 | 1 | 1 | 48 |
| 23 | 17 | 0 | 00 | ERROR | ERROR | 121 | 964 | 0 | 0 | 1 | 40 | 1236 | 0 | 0 | 1 | 1 | 48 |
| 24 | 18 | 0 | 00 | ERROR | ERROR | 126 | 1004 | 0 | 0 | 1 | 40 | 1284 | 0 | 0 | 1 | 1 | 48 |
| 25 | 19 | 0 | 00 | ERROR | ERROR | 131 | 1044 | 0 | 0 | 1 | 40 | 1332 | 0 | 0 | 1 | 1 | 48 |
| 26 | 1A | 0 | 00 | ERROR | ERROR | 136 | 1084 | 0 | 0 | 1 | 40 | 1380 | 0 | 0 | 1 | 1 | 48 |
| 27 | 1B | 0 | 00 | ERROR | ERROR | 141 | 1124 | 0 | 0 | 1 | 40 | 1428 | 0 | 0 | 1 | 1 | 48 |
| 28 | 1C | 0 | 00 | ERROR | ERROR | 146 | 1164 | 0 | 0 | 1 | 40 | 1476 | 0 | 0 | 1 | 1 | 48 |
| 29 | 1D | 0 | 00 | ERROR | ERROR | 151 | 1204 | 0 | 0 | 1 | 40 | 1524 | 0 | 0 | 1 | 1 | 48 |
| 30 | 1E | 0 | 00 | ERROR | ERROR | 156 | 1244 | 0 | 0 | 1 | 40 | 1572 | 0 | 0 | 1 | 1 | 48 |
| 31 | 1F | 0 | 00 | ERROR | ERROR | 161 | 1284 | 0 | 0 | 1 | 40 | 1620 | 0 | 0 | 1 | 1 | 48 |
| 32 | 20 | 0 | 00 | ERROR | ERROR | 166 | 1324 | 0 | 0 | 1 | 40 | 1668 | 0 | 0 | 1 | 1 | 48 |
| 33 | 21 | 0 | 00 | ERROR | ERROR | 171 | 1364 | 0 | 0 | 1 | 40 | 1716 | 0 | 0 | 1 | 1 | 48 |
| 34 | 22 | 0 | 00 | ERROR | ERROR | 176 | 1404 | 0 | 0 | 1 | 40 | 1764 | 0 | 0 | 1 | 1 | 48 |
| 35 | 23 | 0 | 00 | ERROR | ERROR | 181 | 1444 | 0 | 0 | 1 | 40 | 1812 | 0 | 0 | 1 | 1 | 48 |
| 36 | 24 | 0 | 00 | ERROR | ERROR | 186 | 1484 | 0 | 0 | 1 | 40 | 1860 | 0 | 0 | 1 | 1 | 48 |
| 37 | 25 | 0 | 00 | ERROR | ERROR | 191 | 1524 | 0 | 0 | 1 | 40 | 1908 | 0 | 0 | 1 | 1 | 48 |
| 38 | 26 | 0 | 00 | ERROR | ERROR | 196 | 1564 | 0 | 0 | 1 | 40 | 1956 | 0 | 0 | 1 | 1 | 48 |
| 39 | 27 | 0 | 00 | ERROR | ERROR | 201 | 1604 | 0 | 0 | 1 | 40 | 2004 | 0 | 0 | 1 | 1 | 48 |
| 40 | 28 | 0 | 00 | ERROR | ERROR | 206 | 1644 | 0 | 0 | 1 | 40 | 2052 | 0 | 0 | 1 | 1 | 48 |
| 41 | 29 | 0 | 00 | ERROR | ERROR | 211 | 1684 | 0 | 0 | 1 | 40 | 2100 | 0 | 0 | 1 | 1 | 48 |
| 42 | 2A | 0 | 00 | ERROR | ERROR | 216 | 1724 | 0 | 0 | 1 | 40 | 2148 | 0 | 0 | 1 | 1 | 48 |
| 43 | 2B | 0 | 00 | ERROR | ERROR | 221 | 1764 | 0 | 0 | 1 | 40 | 2196 | 0 | 0 | 1 | 1 | 48 |
| 44 | 2C | 0 | 00 | ERROR | ERROR | 226 | 1804 | 0 | 0 | 1 | 40 | 2244 | 0 | 0 | 1 | 1 | 48 |
| 45 | 2D | 0 | 00 | ERROR | ERROR | 231 | 1844 | 0 | 0 | 1 | 40 | 2292 | 0 | 0 | 1 | 1 | 48 |
| 46 | 2E | 0 | 00 | ERROR | ERROR | 236 | 1884 | 0 | 0 | 1 | 40 | 2340 | 0 | 0 | 1 | 1 | 48 |
| 47 | 2F | 0 | 00 | ERROR | ERROR | 241 | 1924 | 0 | 0 | 1 | 40 | 2388 | 0 | 0 | 1 | 1 | 48 |
| 48 | 30 | 0 | 00 | ERROR | ERROR | 246 | 1964 | 0 | 0 | 1 | 40 | 2436 | 0 | 0 | 1 | 1 | 48 |
| 49 | 31 | 0 | 00 | ERROR | ERROR | 251 | 2004 | 0 | 0 | 1 | 40 | 2484 | 0 | 0 | 1 | 1 | 48 |
| 50 | 32 | 0 | 00 | ERROR | ERROR | 256 | 2044 | 0 | 0 | 1 | 40 | 2532 | 0 | 0 | 1 | 1 | 48 |
| 51 | 33 | 0 | 00 | ERROR | ERROR | 261 | 2084 | 0 | 0 | 1 | 40 | 2580 | 0 | 0 | 1 | 1 | 48 |
| 52 | 34 | 0 | 00 | ERROR | ERROR | 266 | 2124 | 0 | 0 | 1 | 40 | 2628 | 0 | 0 | 1 | 1 | 48 |
| 53 | 35 | 0 | 00 | ERROR | ERROR | 271 | 2164 | 0 | 0 | 1 | 40 | 2676 | 0 | 0 | 1 | 1 | 48 |
| 54 | 36 | 0 | 00 | ERROR | ERROR | 276 | 2204 | 0 | 0 | 1 | 40 | 2724 | 0 | 0 | 1 | 1 | 48 |
| 55 | 37 | 0 | 00 | ERROR | ERROR | 281 | 2244 | 0 | 0 | 1 | 40 | 2772 | 0 | 0 | 1 | 1 | 48 |
| 56 | 38 | 0 | 00 | ERROR | ERROR | 286 | 2284 | 0 | 0 | 1 | 40 | 2820 | 0 | 0 | 1 | 1 | 48 |
| 57 | 39 | 0 | 00 | ERROR | ERROR | 291 | 2324 | 0 | 0 | 1 | 40 | 2868 | 0 | 0 | 1 | 1 | 48 |
| 58 | 3A | 0 | 00 | ERROR | ERROR | 296 | 2364 | 0 | 0 | 1 | 40 | 2916 | 0 | 0 | 1 | 1 | 48 |
| 59 | 3B | 0 | 00 | ERROR | ERROR | 301 | 2404 | 0 | 0 | 1 | 40 | 2964 | 0 | 0 | 1 | 1 | 48 |
| 60 | 3C | 0 | 00 | ERROR | ERROR | 306 | 2444 | 0 | 0 | 1 | 40 | 3012 | 0 | 0 | 1 | 1 | 48 |
| 61 | 3D | 0 | 00 | ERROR | ERROR | 311 | 2484 | 0 | 0 | 1 | 40 | 3060 | 0 | 0 | 1 | 1 | 48 |
| 62 | 3E | 0 | 00 | ERROR | ERROR | 316 | 2524 | 0 | 0 | 1 | 40 | 3108 | 0 | 0 | 1 | 1 | 48 |
| 63 | 3F | 0 | 00 | ERROR | ERROR | 321 | 2564 | 0 | 0 | 1 | 40 | 3156 | 0 | 0 | 1 | 1 | 48 |
| 64 | 40 | 0 | 00 | ERROR | ERROR | 326 | 2604 | 0 | 0 | 1 | 40 | 3204 | 0 | 0 | 1 | 1 | 48 |
| 65 | 41 | 0 | 00 | ERROR | ERROR | 331 | 2644 | 0 | 0 | 1 | 40 | 3252 | 0 | 0 | 1 | 1 | 48 |
| 66 | 42 | 0 | 00 | ERROR | ERROR | 336 | 2684 | 0 | 0 | 1 | 40 | 3300 | 0 | 0 | 1 | 1 | 48 |
| 67 | 43 | 0 | 00 | ERROR | ERROR | 341 | 2724 | 0 | 0 | 1 | 40 | 3348 | 0 | 0 | 1 | 1 | 48 |
| 68 | 44 | 0 | 00 | ERROR | ERROR | 346 | 2764 | 0 | 0 | 1 | 40 | 3396 | 0 | 0 | 1 | 1 | 48 |
| 69 | 45 | 0 | 00 | ERROR | ERROR | 351 | 2804 | 0 | 0 | 1 | 40 | 3444 | 0 | 0 | 1 | 1 | 48 |
| 70 | 46 | 0 | 00 | ERROR | ERROR | 356 | 2844 | 0 | 0 | 1 | 40 | 3492 | 0 | 0 | 1 | 1 | 48 |
| 71 | 47 | 0 | 00 | ERROR | ERROR | 361 | 2884 | 0 | 0 | 1 | 40 | 3540 | 0 | 0 | 1 | 1 | 48 |
| 72 | 48 | 0 | 00 | ERROR | ERROR | 366 | 2924 | 0 | 0 | 1 | 40 | 3588 | 0 | 0 | 1 | 1 | 48 |
| 73 | 49 | 0 | 00 | ERROR | ERROR | 371 | 2964 | 0 | 0 | 1 | 40 | 3636 | 0 | 0 | 1 | 1 | 48 |
| 74 | 4A | 0 | 00 | ERROR | ERROR | 376 | 3004 | 0 | 0 | 1 | 40 | 3684 | 0 | 0 | 1 | 1 | 48 |
| 75 | 4B | 0 | 00 | ERROR | ERROR | 381 | 3044 | 0 | 0 | 1 | 40 | 3732 | 0 | 0 | 1 | 1 | 48 |
| 76 | 4C | 0 | 00 | ERROR | ERROR | 386 | 3084 | 0 | 0 | 1 | 40 | 3780 | 0 | 0 | 1 | 1 | 48 |
| 77 | 4D | 0 | 00 | ERROR | ERROR | 391 | 3124 | 0 | 0 | 1 | 40 | 3828 | 0 | 0 | 1 | 1 | 48 |
| 78 | 4E | 0 | 00 | ERROR | ERROR | 396 | 3164 | 0 | 0 | 1 | 40 | 3876 | 0 | 0 | 1 | 1 | 48 |
| 79 | 4F | 0 | 00 | ERROR | ERROR | 401 | 3204 | 0 | 0 | 1 | 40 | 3924 | 0 | 0 | 1 | 1 | 48 |
| 80 | 50 | 0 | 00 | ERROR | ERROR | 406 | 3244 | 0 | 0 | 1 | 40 | 3972 | 0 | 0 | 1 | 1 | 48 |
| 81 | 51 | 0 | 00 | ERROR | ERROR | 411 | 3284 | 0 | 0 | 1 | 40 | 4020 | 0 | 0 | 1 | 1 | 48 |
| 82 | 52 | 0 | 00 | ERROR | ERROR | 416 | 3324 | 0 | 0 | 1 | 40 | 4068 | 0 | 0 | 1 | 1 | 48 |
| 83 | 53 | 0 | 00 | ERROR | ERROR | 421 | 3364 | 0 | 0 | 1 | 40 | 4116 | 0 | 0 | 1 | 1 | 48 |
| 84 | 54 | 0 | 00 | ERROR | ERROR | 426 | 3404 | 0 | 0 | 1 | 40 | 4164 | 0 | 0 | 1 | 1 | 48 |
| 85 | 55 | 0 | 00 | ERROR | ERROR | 431 | 3444 | 0 | 0 | 1 | 40 | 4212 | 0 | 0 | 1 | 1 | 48 |
| 86 | 56 | 0 | 00 | ERROR | ERROR | 436 | 3484 | 0 | 0 | 1 | 40 | 4260 | 0 | 0 | 1 | 1 | 48 |
| 87 | 57 | 0 | 00 | ERROR | ERROR | 441 | 3524 | 0 | 0 | 1 | 40 | 4308 | 0 | 0 | 1 | 1 | 48 |
| 88 | 58 | 0 | 00 | ERROR | ERROR | 446 | 3564 | 0 | 0 | 1 | 40 | 4356 | 0 | 0 | 1 | 1 | 48 |
| 89 | 59 | 0 | 00 | ERROR | ERROR | 451 | 3604 | 0 | 0 | 1 | 40 | 4404 | 0 | 0 | 1 | 1 | 48 |
| 90 | 5A | 0 | 00 | ERROR | ERROR | 456 | 3644 | 0 | 0 | 1 | 40 | 4452 | 0 | 0 | 1 | 1 | 48 |
| 91 | 5B | 0 | 00 | ERROR | ERROR | 461 | 3684 | 0 | 0 | 1 | 40 | 4500 | 0 | 0 | 1 | 1 | 48 |
| 92 | 5C | 0 | 00 | ERROR | ERROR | 466 | 3724 | 0 | 0 | 1 | 40 | 4548 | 0 | 0 | 1 | 1 | 48 |
| 93 | 5D | 0 | 00 | ERROR | ERROR | 471 | 3764 | 0 | 0 | 1 | 40 | 4596 | 0 | 0 | 1 | 1 | 48 |
| 94 | 5E | 0 | 00 | ERROR | ERROR | 476 | 3804 | 0 | 0 | 1 | 40 | 4644 | 0 | 0 | 1 | 1 | 48 |
| 95 | 5F | 0 | 00 | ERROR | ERROR | 481 | 3844 | 0 | 0 | 1 | 40 | 4692 | 0 | 0 | 1 | 1 | 48 |
| 96 | 60 | 0 | 00 | ERROR | ERROR | 486 | 3884 | 0 | 0 | 1 | 40 | 4740 | 0 | 0 | 1 | 1 | 48 |
| 97 | 61 | 0 | 00 | ERROR | ERROR | 491 | 3924 | 0 | 0 | 1 | 40 | 4788 | 0 | 0 | 1 | 1 | 48 |
| 98 | 62 | 0 | 00 | ERROR | ERROR | 496 | 3964 | 0 | 0 | 1 | 40 | 4836 | 0 | 0 | 1 | 1 | 48 |
| 99 | 63 | 0 | 00 | ERROR | ERROR | 501 | 4004 | 0 | 0 | 1 | 40 | 4884 | 0 | 0 | 1 | 1 | 48 |

| | | | | | | | | | | | | | | | | | |
|---|---|---|---|---|---|---|---|---|---|---|---|---|---|---|---|---|---|
| 100 | 64 | 0 | 00 | ERROR | ERROR | 506 | 4044 | 0 | 0 | 1 | 40 | 4932 | 0 | 0 | 1 | 1 | 48 |
| 101 | 65 | 0 | 00 | ERROR | ERROR | 511 | 4084 | 0 | 0 | 1 | 40 | 4980 | 0 | 0 | 1 | 1 | 48 |
| 102 | 66 | 0 | 00 | ERROR | ERROR | 516 | 4124 | 0 | 0 | 1 | 40 | 5028 | 0 | 0 | 1 | 1 | 48 |
| 103 | 67 | 0 | 00 | ERROR | ERROR | 521 | 4164 | 0 | 0 | 1 | 40 | 5076 | 0 | 0 | 1 | 1 | 48 |
| 104 | 68 | 0 | 00 | ERROR | ERROR | 526 | 4204 | 0 | 0 | 1 | 40 | 5124 | 0 | 0 | 1 | 1 | 48 |
| 105 | 69 | 0 | 00 | ERROR | ERROR | 531 | 4244 | 0 | 0 | 1 | 40 | 5172 | 0 | 0 | 1 | 1 | 48 |
| 106 | 6A | 0 | 00 | ERROR | ERROR | 536 | 4284 | 0 | 0 | 1 | 40 | 5220 | 0 | 0 | 1 | 1 | 48 |
| 107 | 6B | 0 | 00 | ERROR | ERROR | 541 | 4324 | 0 | 0 | 1 | 40 | 5268 | 0 | 0 | 1 | 1 | 48 |
| 108 | 6C | 0 | 00 | ERROR | ERROR | 546 | 4364 | 0 | 0 | 1 | 40 | 5316 | 0 | 0 | 1 | 1 | 48 |
| 109 | 6D | 0 | 00 | ERROR | ERROR | 551 | 4404 | 0 | 0 | 1 | 40 | 5364 | 0 | 0 | 1 | 1 | 48 |
| 110 | 6E | 0 | 00 | ERROR | ERROR | 556 | 4444 | 0 | 0 | 1 | 40 | 5412 | 0 | 0 | 1 | 1 | 48 |
| 111 | 6F | 0 | 00 | ERROR | ERROR | 561 | 4484 | 0 | 0 | 1 | 40 | 5460 | 0 | 0 | 1 | 1 | 48 |
| 112 | 70 | 0 | 00 | ERROR | ERROR | 566 | 4524 | 0 | 0 | 1 | 40 | 5508 | 0 | 0 | 1 | 1 | 48 |
| 113 | 71 | 0 | 00 | ERROR | ERROR | 571 | 4564 | 0 | 0 | 1 | 40 | 5556 | 0 | 0 | 1 | 1 | 48 |
| 114 | 72 | 0 | 00 | ERROR | ERROR | 576 | 4604 | 0 | 0 | 1 | 40 | 5604 | 0 | 0 | 1 | 1 | 48 |
| 115 | 73 | 0 | 00 | ERROR | ERROR | 581 | 4644 | 0 | 0 | 1 | 40 | 5652 | 0 | 0 | 1 | 1 | 48 |
| 116 | 74 | 0 | 00 | ERROR | ERROR | 586 | 4684 | 0 | 0 | 1 | 40 | 5700 | 0 | 0 | 1 | 1 | 48 |
| 117 | 75 | 0 | 00 | ERROR | ERROR | 591 | 4724 | 0 | 0 | 1 | 40 | 5748 | 0 | 0 | 1 | 1 | 48 |
| 118 | 76 | 0 | 00 | ERROR | ERROR | 596 | 4764 | 0 | 0 | 1 | 40 | 5796 | 0 | 0 | 1 | 1 | 48 |
| 119 | 77 | 0 | 00 | ERROR | ERROR | 601 | 4804 | 0 | 0 | 1 | 40 | 5844 | 0 | 0 | 1 | 1 | 48 |
| 120 | 78 | 0 | 00 | ERROR | ERROR | 606 | 4844 | 0 | 0 | 1 | 40 | 5892 | 0 | 0 | 1 | 1 | 48 |
| 121 | 79 | 0 | 00 | ERROR | ERROR | 611 | 4884 | 0 | 0 | 1 | 40 | 5940 | 0 | 0 | 1 | 1 | 48 |
| 122 | 7A | 0 | 00 | ERROR | ERROR | 616 | 4924 | 0 | 0 | 1 | 40 | 5988 | 0 | 0 | 1 | 1 | 48 |
| 123 | 7B | 0 | 00 | ERROR | ERROR | 621 | 4964 | 0 | 0 | 1 | 40 | 6036 | 0 | 0 | 1 | 1 | 48 |
| 124 | 7C | 0 | 00 | ERROR | ERROR | 626 | 5004 | 0 | 0 | 1 | 40 | 6084 | 0 | 0 | 1 | 1 | 48 |
| 125 | 7D | 0 | 00 | ERROR | ERROR | 631 | 5044 | 0 | 0 | 1 | 40 | 6132 | 0 | 0 | 1 | 1 | 48 |
| 126 | 7E | 0 | 00 | ERROR | ERROR | 636 | 5084 | 0 | 0 | 1 | 40 | 6180 | 0 | 0 | 1 | 1 | 48 |
| 127 | 7F | 0 | 00 | ERROR | ERROR | 641 | 5124 | 0 | 0 | 1 | 40 | 6228 | 0 | 0 | 1 | 1 | 48 |
| 128 | 80 | 0 | 00 | ERROR | ERROR | 646 | 5164 | 0 | 0 | 1 | 40 | 6276 | 0 | 0 | 1 | 1 | 48 |
| 129 | 81 | 0 | 00 | ERROR | ERROR | 651 | 5204 | 0 | 0 | 1 | 40 | 6324 | 0 | 0 | 1 | 1 | 48 |
| 130 | 82 | 0 | 00 | ERROR | ERROR | 656 | 5244 | 0 | 0 | 1 | 40 | 6372 | 0 | 0 | 1 | 1 | 48 |
| 131 | 83 | 0 | 00 | ERROR | ERROR | 661 | 5284 | 0 | 0 | 1 | 40 | 6420 | 0 | 0 | 1 | 1 | 48 |
| 132 | 84 | 0 | 00 | ERROR | ERROR | 666 | 5324 | 0 | 0 | 1 | 40 | 6468 | 0 | 0 | 1 | 1 | 48 |
| 133 | 85 | 0 | 00 | ERROR | ERROR | 671 | 5364 | 0 | 0 | 1 | 40 | 6516 | 0 | 0 | 1 | 1 | 48 |
| 134 | 86 | 0 | 00 | ERROR | ERROR | 676 | 5404 | 0 | 0 | 1 | 40 | 6564 | 0 | 0 | 1 | 1 | 48 |
| 135 | 87 | 0 | 00 | ERROR | ERROR | 681 | 5444 | 0 | 0 | 1 | 40 | 6612 | 0 | 0 | 1 | 1 | 48 |
| 136 | 88 | 0 | 00 | ERROR | ERROR | 686 | 5484 | 0 | 0 | 1 | 40 | 6660 | 0 | 0 | 1 | 1 | 48 |
| 137 | 89 | 0 | 00 | ERROR | ERROR | 691 | 5524 | 0 | 0 | 1 | 40 | 6708 | 0 | 0 | 1 | 1 | 48 |
| 138 | 8A | 0 | 00 | ERROR | ERROR | 696 | 5564 | 0 | 0 | 1 | 40 | 6756 | 0 | 0 | 1 | 1 | 48 |
| 139 | 8B | 0 | 00 | ERROR | ERROR | 701 | 5604 | 0 | 0 | 1 | 40 | 6804 | 0 | 0 | 1 | 1 | 48 |
| 140 | 8C | 0 | 00 | ERROR | ERROR | 706 | 5644 | 0 | 0 | 1 | 40 | 6852 | 0 | 0 | 1 | 1 | 48 |
| 141 | 8D | 0 | 00 | ERROR | ERROR | 711 | 5684 | 0 | 0 | 1 | 40 | 6900 | 0 | 0 | 1 | 1 | 48 |
| 142 | 8E | 0 | 00 | ERROR | ERROR | 716 | 5724 | 0 | 0 | 1 | 40 | 6948 | 0 | 0 | 1 | 1 | 48 |
| 143 | 8F | 0 | 00 | ERROR | ERROR | 721 | 5764 | 0 | 0 | 1 | 40 | 6996 | 0 | 0 | 1 | 1 | 48 |
| 144 | 90 | 0 | 00 | ERROR | ERROR | 726 | 5804 | 0 | 0 | 1 | 40 | 7044 | 0 | 0 | 1 | 1 | 48 |
| 145 | 91 | 0 | 00 | ERROR | ERROR | 731 | 5844 | 0 | 0 | 1 | 40 | 7092 | 0 | 0 | 1 | 1 | 48 |
| 146 | 92 | 0 | 00 | ERROR | ERROR | 736 | 5884 | 0 | 0 | 1 | 40 | 7140 | 0 | 0 | 1 | 1 | 48 |
| 147 | 93 | 0 | 00 | ERROR | ERROR | 741 | 5924 | 0 | 0 | 1 | 40 | 7188 | 0 | 0 | 1 | 1 | 48 |
| 148 | 94 | 0 | 00 | ERROR | ERROR | 746 | 5964 | 0 | 0 | 1 | 40 | 7236 | 0 | 0 | 1 | 1 | 48 |
| 149 | 95 | 0 | 00 | ERROR | ERROR | 751 | 6004 | 0 | 0 | 1 | 40 | 7284 | 0 | 0 | 1 | 1 | 48 |
| 150 | 96 | 0 | 00 | ERROR | ERROR | 756 | 6044 | 0 | 0 | 1 | 40 | 7332 | 0 | 0 | 1 | 1 | 48 |
| 151 | 97 | 0 | 00 | ERROR | ERROR | 761 | 6084 | 0 | 0 | 1 | 40 | 7380 | 0 | 0 | 1 | 1 | 48 |
| 152 | 98 | 0 | 00 | ERROR | ERROR | 766 | 6124 | 0 | 0 | 1 | 40 | 7428 | 0 | 0 | 1 | 1 | 48 |
| 153 | 99 | 0 | 00 | ERROR | ERROR | 771 | 6164 | 0 | 0 | 1 | 40 | 7476 | 0 | 0 | 1 | 1 | 48 |
| 154 | 9A | 0 | 00 | ERROR | ERROR | 776 | 6204 | 0 | 0 | 1 | 40 | 7524 | 0 | 0 | 1 | 1 | 48 |
| 155 | 9B | 0 | 00 | ERROR | ERROR | 781 | 6244 | 0 | 0 | 1 | 40 | 7572 | 0 | 0 | 1 | 1 | 48 |
| 156 | 9C | 0 | 00 | ERROR | ERROR | 786 | 6284 | 0 | 0 | 1 | 40 | 7620 | 0 | 0 | 1 | 1 | 48 |
| 157 | 9D | 0 | 00 | ERROR | ERROR | 791 | 6324 | 0 | 0 | 1 | 40 | 7668 | 0 | 0 | 1 | 1 | 48 |
| 158 | 9E | 0 | 00 | ERROR | ERROR | 796 | 6364 | 0 | 0 | 1 | 40 | 7716 | 0 | 0 | 1 | 1 | 48 |
| 159 | 9F | 0 | 00 | ERROR | ERROR | 801 | 6404 | 0 | 0 | 1 | 40 | 7764 | 0 | 0 | 1 | 1 | 48 |
| 160 | A0 | 0 | 00 | ERROR | ERROR | 806 | 6444 | 0 | 0 | 1 | 40 | 7812 | 0 | 0 | 1 | 1 | 48 |
| 161 | A1 | 0 | 00 | ERROR | ERROR | 811 | 6484 | 0 | 0 | 1 | 40 | 7860 | 0 | 0 | 1 | 1 | 48 |
| 162 | A2 | 0 | 00 | ERROR | ERROR | 816 | 6524 | 0 | 0 | 1 | 40 | 7908 | 0 | 0 | 1 | 1 | 48 |
| 163 | A3 | 0 | 00 | ERROR | ERROR | 821 | 6564 | 0 | 0 | 1 | 40 | 7956 | 0 | 0 | 1 | 1 | 48 |
| 164 | A4 | 0 | 00 | ERROR | ERROR | 826 | 6604 | 0 | 0 | 1 | 40 | 8004 | 0 | 0 | 1 | 1 | 48 |
| 165 | A5 | 0 | 00 | ERROR | ERROR | 831 | 6644 | 0 | 0 | 1 | 40 | 8052 | 0 | 0 | 1 | 1 | 48 |
| 166 | A6 | 0 | 00 | ERROR | ERROR | 836 | 6684 | 0 | 0 | 1 | 40 | 8100 | 0 | 0 | 1 | 1 | 48 |
| 167 | A7 | 0 | 00 | ERROR | ERROR | 841 | 6724 | 0 | 0 | 1 | 40 | 8148 | 0 | 0 | 1 | 1 | 48 |
| 168 | A8 | 0 | 00 | ERROR | ERROR | 846 | 6764 | 0 | 0 | 1 | 40 | 8196 | 0 | 0 | 1 | 1 | 48 |
| 169 | A9 | 0 | 00 | ERROR | ERROR | 851 | 6804 | 0 | 0 | 1 | 40 | 8244 | 0 | 0 | 1 | 1 | 48 |
| 170 | AA | 0 | 00 | ERROR | ERROR | 856 | 6844 | 0 | 0 | 1 | 40 | 8292 | 0 | 0 | 1 | 1 | 48 |
| 171 | AB | 0 | 00 | ERROR | ERROR | 861 | 6884 | 0 | 0 | 1 | 40 | 8340 | 0 | 0 | 1 | 1 | 48 |
| 172 | AC | 0 | 00 | ERROR | ERROR | 866 | 6924 | 0 | 0 | 1 | 40 | 8388 | 0 | 0 | 1 | 1 | 48 |
| 173 | AD | 0 | 00 | ERROR | ERROR | 871 | 6964 | 0 | 0 | 1 | 40 | 8436 | 0 | 0 | 1 | 1 | 48 |
| 174 | AE | 0 | 00 | ERROR | ERROR | 876 | 7004 | 0 | 0 | 1 | 40 | 8484 | 0 | 0 | 1 | 1 | 48 |
| 175 | AF | 0 | 00 | ERROR | ERROR | 881 | 7044 | 0 | 0 | 1 | 40 | 8532 | 0 | 0 | 1 | 1 | 48 |
| 176 | B0 | 0 | 00 | ERROR | ERROR | 886 | 7084 | 0 | 0 | 1 | 40 | 8580 | 0 | 0 | 1 | 1 | 48 |
| 177 | B1 | 0 | 00 | ERROR | ERROR | 891 | 7124 | 0 | 0 | 1 | 40 | 8628 | 0 | 0 | 1 | 1 | 48 |
| 178 | B2 | 0 | 00 | ERROR | ERROR | 896 | 7164 | 0 | 0 | 1 | 40 | 8676 | 0 | 0 | 1 | 1 | 48 |
| 179 | B3 | 0 | 00 | ERROR | ERROR | 901 | 7204 | 0 | 0 | 1 | 40 | 8724 | 0 | 0 | 1 | 1 | 48 |
| 180 | B4 | 0 | 00 | ERROR | ERROR | 906 | 7244 | 0 | 0 | 1 | 40 | 8772 | 0 | 0 | 1 | 1 | 48 |
| 181 | B5 | 0 | 00 | ERROR | ERROR | 911 | 7284 | 0 | 0 | 1 | 40 | 8820 | 0 | 0 | 1 | 1 | 48 |
| 182 | B6 | 0 | 00 | ERROR | ERROR | 916 | 7324 | 0 | 0 | 1 | 40 | 8868 | 0 | 0 | 1 | 1 | 48 |
| 183 | B7 | 0 | 00 | ERROR | ERROR | 921 | 7364 | 0 | 0 | 1 | 40 | 8916 | 0 | 0 | 1 | 1 | 48 |
| 184 | B8 | 0 | 00 | ERROR | ERROR | 926 | 7404 | 0 | 0 | 1 | 40 | 8964 | 0 | 0 | 1 | 1 | 48 |
| 185 | B9 | 0 | 00 | ERROR | ERROR | 931 | 7444 | 0 | 0 | 1 | 40 | 9012 | 0 | 0 | 1 | 1 | 48 |
| 186 | BA | 0 | 00 | ERROR | ERROR | 936 | 7484 | 0 | 0 | 1 | 40 | 9060 | 0 | 0 | 1 | 1 | 48 |
| 187 | BB | 0 | 00 | ERROR | ERROR | 941 | 7524 | 0 | 0 | 1 | 40 | 9108 | 0 | 0 | 1 | 1 | 48 |
| 188 | BC | 0 | 00 | ERROR | ERROR | 946 | 7564 | 0 | 0 | 1 | 40 | 9156 | 0 | 0 | 1 | 1 | 48 |
| 189 | BD | 0 | 00 | ERROR | ERROR | 951 | 7604 | 0 | 0 | 1 | 40 | 9204 | 0 | 0 | 1 | 1 | 48 |
| 190 | BE | 0 | 00 | ERROR | ERROR | 956 | 7644 | 0 | 0 | 1 | 40 | 9252 | 0 | 0 | 1 | 1 | 48 |
| 191 | BF | 0 | 00 | ERROR | ERROR | 961 | 7684 | 0 | 0 | 1 | 40 | 9300 | 0 | 0 | 1 | 1 | 48 |
| 192 | C0 | 0 | 00 | ERROR | ERROR | 966 | 7724 | 0 | 0 | 1 | 40 | 9348 | 0 | 0 | 1 | 1 | 48 |
| 193 | C1 | 0 | 00 | ERROR | ERROR | 971 | 7764 | 0 | 0 | 1 | 40 | 9396 | 0 | 0 | 1 | 1 | 48 |
| 194 | C2 | 0 | 00 | ERROR | ERROR | 976 | 7804 | 0 | 0 | 1 | 40 | 9444 | 0 | 0 | 1 | 1 | 48 |
| 195 | C3 | 0 | 00 | ERROR | ERROR | 981 | 7844 | 0 | 0 | 1 | 40 | 9492 | 0 | 0 | 1 | 1 | 48 |
| 196 | C4 | 0 | 00 | ERROR | ERROR | 986 | 7884 | 0 | 0 | 1 | 40 | 9540 | 0 | 0 | 1 | 1 | 48 |
| 197 | C5 | 0 | 00 | ERROR | ERROR | 991 | 7924 | 0 | 0 | 1 | 40 | 9588 | 0 | 0 | 1 | 1 | 48 |
| 198 | C6 | 0 | 00 | ERROR | ERROR | 996 | 7964 | 0 | 0 | 1 | 40 | 9636 | 0 | 0 | 1 | 1 | 48 |
| 199 | C7 | 0 | 00 | ERROR | ERROR | 1001 | 8004 | 0 | 0 | 1 | 40 | 9684 | 0 | 0 | 1 | 1 | 48 |
| 200 | C8 | 0 | 00 | ERROR | ERROR | 1006 | 8044 | 0 | 0 | 1 | 40 | 9732 | 0 | 0 | 1 | 1 | 48 |
| 201 | C9 | 0 | 00 | ERROR | ERROR | 1011 | 8084 | 0 | 0 | 1 | 40 | 9780 | 0 | 0 | 1 | 1 | 48 |
| 202 | CA | 0 | 00 | ERROR | ERROR | 1016 | 8124 | 0 | 0 | 1 | 40 | 9828 | 0 | 0 | 1 | 1 | 48 |

| | | | | | | | | | | | | | | | | | |
|---|---|---|---|---|---|---|---|---|---|---|---|---|---|---|---|---|---|
| 203 | CB | 0 | 00 | ERROR | ERROR | 1021 | 8164 | 0 | 0 | 1 | 40 | 9876 | 0 | 0 | 1 | 1 | 48 |
| 204 | CC | 0 | 00 | ERROR | ERROR | 1026 | 8204 | 0 | 0 | 1 | 40 | 9924 | 0 | 0 | 1 | 1 | 48 |
| 205 | CD | 0 | 00 | ERROR | ERROR | 1031 | 8244 | 0 | 0 | 1 | 40 | 9972 | 0 | 0 | 1 | 1 | 48 |
| 206 | CE | 0 | 00 | ERROR | ERROR | 1036 | 8284 | 0 | 0 | 1 | 40 | 10020 | 0 | 0 | 1 | 1 | 48 |
| 207 | CF | 0 | 00 | ERROR | ERROR | 1041 | 8324 | 0 | 0 | 1 | 40 | 10068 | 0 | 0 | 1 | 1 | 48 |
| 208 | D0 | 0 | 00 | ERROR | ERROR | 1046 | 8364 | 0 | 0 | 1 | 40 | 10116 | 0 | 0 | 1 | 1 | 48 |
| 209 | D1 | 0 | 00 | ERROR | ERROR | 1051 | 8404 | 0 | 0 | 1 | 40 | 10164 | 0 | 0 | 1 | 1 | 48 |
| 210 | D2 | 0 | 00 | ERROR | ERROR | 1056 | 8444 | 0 | 0 | 1 | 40 | 10212 | 0 | 0 | 1 | 1 | 48 |
| 211 | D3 | 0 | 00 | ERROR | ERROR | 1061 | 8484 | 0 | 0 | 1 | 40 | 10260 | 0 | 0 | 1 | 1 | 48 |
| 212 | D4 | 0 | 00 | ERROR | ERROR | 1066 | 8524 | 0 | 0 | 1 | 40 | 10308 | 0 | 0 | 1 | 1 | 48 |
| 213 | D5 | 0 | 00 | ERROR | ERROR | 1071 | 8564 | 0 | 0 | 1 | 40 | 10356 | 0 | 0 | 1 | 1 | 48 |
| 214 | D6 | 0 | 00 | ERROR | ERROR | 1076 | 8604 | 0 | 0 | 1 | 40 | 10404 | 0 | 0 | 1 | 1 | 48 |
| 215 | D7 | 0 | 00 | ERROR | ERROR | 1081 | 8644 | 0 | 0 | 1 | 40 | 10452 | 0 | 0 | 1 | 1 | 48 |
| 216 | D8 | 0 | 00 | ERROR | ERROR | 1086 | 8684 | 0 | 0 | 1 | 40 | 10500 | 0 | 0 | 1 | 1 | 48 |
| 217 | D9 | 0 | 00 | ERROR | ERROR | 1091 | 8724 | 0 | 0 | 1 | 40 | 10548 | 0 | 0 | 1 | 1 | 48 |
| 218 | DA | 0 | 00 | ERROR | ERROR | 1096 | 8764 | 0 | 0 | 1 | 40 | 10596 | 0 | 0 | 1 | 1 | 48 |
| 219 | DB | 0 | 00 | ERROR | ERROR | 1101 | 8804 | 0 | 0 | 1 | 40 | 10644 | 0 | 0 | 1 | 1 | 48 |
| 220 | DC | 0 | 00 | ERROR | ERROR | 1106 | 8844 | 0 | 0 | 1 | 40 | 10692 | 0 | 0 | 1 | 1 | 48 |
| 221 | DD | 0 | 00 | ERROR | ERROR | 1111 | 8884 | 0 | 0 | 1 | 40 | 10740 | 0 | 0 | 1 | 1 | 48 |
| 222 | DE | 0 | 00 | ERROR | ERROR | 1116 | 8924 | 0 | 0 | 1 | 40 | 10788 | 0 | 0 | 1 | 1 | 48 |
| 223 | DF | 0 | 00 | ERROR | ERROR | 1121 | 8964 | 0 | 0 | 1 | 40 | 10836 | 0 | 0 | 1 | 1 | 48 |
| 224 | E0 | 0 | 00 | ERROR | ERROR | 1126 | 9004 | 0 | 0 | 1 | 40 | 10884 | 0 | 0 | 1 | 1 | 48 |
| 225 | E1 | 0 | 00 | ERROR | ERROR | 1131 | 9044 | 0 | 0 | 1 | 40 | 10932 | 0 | 0 | 1 | 1 | 48 |
| 226 | E2 | 0 | 00 | ERROR | ERROR | 1136 | 9084 | 0 | 0 | 1 | 40 | 10980 | 0 | 0 | 1 | 1 | 48 |
| 227 | E3 | 0 | 00 | ERROR | ERROR | 1141 | 9124 | 0 | 0 | 1 | 40 | 11028 | 0 | 0 | 1 | 1 | 48 |
| 228 | E4 | 0 | 00 | ERROR | ERROR | 1146 | 9164 | 0 | 0 | 1 | 40 | 11076 | 0 | 0 | 1 | 1 | 48 |
| 229 | E5 | 0 | 00 | ERROR | ERROR | 1151 | 9204 | 0 | 0 | 1 | 40 | 11124 | 0 | 0 | 1 | 1 | 48 |
| 230 | E6 | 0 | 00 | ERROR | ERROR | 1156 | 9244 | 0 | 0 | 1 | 40 | 11172 | 0 | 0 | 1 | 1 | 48 |
| 231 | E7 | 0 | 00 | ERROR | ERROR | 1161 | 9284 | 0 | 0 | 1 | 40 | 11220 | 0 | 0 | 1 | 1 | 48 |
| 232 | E8 | 0 | 00 | ERROR | ERROR | 1166 | 9324 | 0 | 0 | 1 | 40 | 11268 | 0 | 0 | 1 | 1 | 48 |
| 233 | E9 | 0 | 00 | ERROR | ERROR | 1171 | 9364 | 0 | 0 | 1 | 40 | 11316 | 0 | 0 | 1 | 1 | 48 |
| 234 | EA | 0 | 00 | ERROR | ERROR | 1176 | 9404 | 0 | 0 | 1 | 40 | 11364 | 0 | 0 | 1 | 1 | 48 |
| 235 | EB | 0 | 00 | ERROR | ERROR | 1181 | 9444 | 0 | 0 | 1 | 40 | 11412 | 0 | 0 | 1 | 1 | 48 |
| 236 | EC | 0 | 00 | ERROR | ERROR | 1186 | 9484 | 0 | 0 | 1 | 40 | 11460 | 0 | 0 | 1 | 1 | 48 |
| 237 | ED | 0 | 00 | ERROR | ERROR | 1191 | 9524 | 0 | 0 | 1 | 40 | 11508 | 0 | 0 | 1 | 1 | 48 |
| 238 | EE | 0 | 00 | ERROR | ERROR | 1196 | 9564 | 0 | 0 | 1 | 40 | 11556 | 0 | 0 | 1 | 1 | 48 |
| 239 | EF | 0 | 00 | ERROR | ERROR | 1201 | 9604 | 0 | 0 | 1 | 40 | 11604 | 0 | 0 | 1 | 1 | 48 |
| 240 | F0 | 0 | 00 | ERROR | ERROR | 1206 | 9644 | 0 | 0 | 1 | 40 | 11652 | 0 | 0 | 1 | 1 | 48 |
| 241 | F1 | 0 | 00 | ERROR | ERROR | 1211 | 9684 | 0 | 0 | 1 | 40 | 11700 | 0 | 0 | 1 | 1 | 48 |
| 242 | F2 | 0 | 00 | ERROR | ERROR | 1216 | 9724 | 0 | 0 | 1 | 40 | 11748 | 0 | 0 | 1 | 1 | 48 |
| 243 | F3 | 0 | 00 | ERROR | ERROR | 1221 | 9764 | 0 | 0 | 1 | 40 | 11796 | 0 | 0 | 1 | 1 | 48 |
| 244 | F4 | 0 | 00 | ERROR | ERROR | 1226 | 9804 | 0 | 0 | 1 | 40 | 11844 | 0 | 0 | 1 | 1 | 48 |
| 245 | F5 | 0 | 00 | ERROR | ERROR | 1231 | 9844 | 0 | 0 | 1 | 40 | 11892 | 0 | 0 | 1 | 1 | 48 |
| 246 | F6 | 0 | 00 | ERROR | ERROR | 1236 | 9884 | 0 | 0 | 1 | 40 | 11940 | 0 | 0 | 1 | 1 | 48 |
| 247 | F7 | 0 | 00 | ERROR | ERROR | 1241 | 9924 | 0 | 0 | 1 | 40 | 11988 | 0 | 0 | 1 | 1 | 48 |
| 248 | F8 | 0 | 00 | ERROR | ERROR | 1246 | 9964 | 0 | 0 | 1 | 40 | 12036 | 0 | 0 | 1 | 1 | 48 |
| 249 | F9 | 0 | 00 | ERROR | ERROR | 1251 | 10004 | 0 | 0 | 1 | 40 | 12084 | 0 | 0 | 1 | 1 | 48 |
| 250 | FA | 0 | 00 | ERROR | ERROR | 1256 | 10044 | 0 | 0 | 1 | 40 | 12132 | 0 | 0 | 1 | 1 | 48 |
| 251 | FB | 0 | 00 | ERROR | ERROR | 1261 | 10084 | 0 | 0 | 1 | 40 | 12180 | 0 | 0 | 1 | 1 | 48 |
| 252 | FC | 0 | 00 | ERROR | ERROR | 1266 | 10124 | 0 | 0 | 1 | 40 | 12228 | 0 | 0 | 1 | 1 | 48 |
| 253 | FD | 0 | 00 | ERROR | ERROR | 1271 | 10164 | 0 | 0 | 1 | 40 | 12276 | 0 | 0 | 1 | 1 | 48 |
| 254 | FE | 0 | 00 | ERROR | ERROR | 1276 | 10204 | 0 | 0 | 1 | 40 | 12324 | 0 | 0 | 1 | 1 | 48 |
| 255 | FF | 0 | 00 | ERROR | ERROR | 1281 | 10244 | 0 | 0 | 1 | 40 | 12372 | 0 | 0 | 1 | 1 | 48 |
| 0 | 00 | 1 | 01 | 0 | 0 | 1293 | 10340 | 0 | 0 | 0 | 96 | 12476 | 0 | 0 | 0 | 1 | 104 |
| 1 | 01 | 1 | 01 | 1 | 0 | 1305 | 10436 | 1 | 0 | 0 | 96 | 12580 | 1 | 0 | 0 | 1 | 104 |
| 2 | 02 | 1 | 01 | 2 | 0 | 1324 | 10588 | 2 | 0 | 0 | 152 | 12740 | 2 | 0 | 0 | 1 | 160 |
| 3 | 03 | 1 | 01 | 3 | 0 | 1345 | 10756 | 3 | 0 | 0 | 168 | 12916 | 3 | 0 | 0 | 1 | 176 |
| 4 | 04 | 1 | 01 | 4 | 0 | 1368 | 10940 | 4 | 0 | 0 | 184 | 13108 | 4 | 0 | 0 | 1 | 192 |
| 5 | 05 | 1 | 01 | 5 | 0 | 1393 | 11140 | 5 | 0 | 0 | 200 | 13316 | 5 | 0 | 0 | 1 | 208 |
| 6 | 06 | 1 | 01 | 6 | 0 | 1420 | 11356 | 6 | 0 | 0 | 216 | 13540 | 6 | 0 | 0 | 1 | 224 |
| 7 | 07 | 1 | 01 | 7 | 0 | 1449 | 11588 | 7 | 0 | 0 | 232 | 13780 | 7 | 0 | 0 | 1 | 240 |
| 8 | 08 | 1 | 01 | 8 | 0 | 1480 | 11836 | 8 | 0 | 0 | 248 | 14036 | 8 | 0 | 0 | 1 | 256 |
| 9 | 09 | 1 | 01 | 9 | 0 | 1513 | 12100 | 9 | 0 | 0 | 264 | 14308 | 9 | 0 | 0 | 1 | 272 |
| 10 | 0A | 1 | 01 | 10 | 0 | 1548 | 12380 | 10 | 0 | 0 | 280 | 14596 | 10 | 0 | 0 | 1 | 288 |
| 11 | 0B | 1 | 01 | 11 | 0 | 1585 | 12676 | 11 | 0 | 0 | 296 | 14900 | 11 | 0 | 0 | 1 | 304 |
| 12 | 0C | 1 | 01 | 12 | 0 | 1624 | 12988 | 12 | 0 | 0 | 312 | 15220 | 12 | 0 | 0 | 1 | 320 |
| 13 | 0D | 1 | 01 | 13 | 0 | 1665 | 13316 | 13 | 0 | 0 | 328 | 15556 | 13 | 0 | 0 | 1 | 336 |
| 14 | 0E | 1 | 01 | 14 | 0 | 1708 | 13660 | 14 | 0 | 0 | 344 | 15908 | 14 | 0 | 0 | 1 | 352 |
| 15 | 0F | 1 | 01 | 15 | 0 | 1753 | 14020 | 15 | 0 | 0 | 360 | 16276 | 15 | 0 | 0 | 1 | 368 |
| 16 | 10 | 1 | 01 | 16 | 0 | 1805 | 14436 | 16 | 0 | 0 | 416 | 16700 | 16 | 0 | 0 | 1 | 424 |
| 17 | 11 | 1 | 01 | 17 | 0 | 1859 | 14868 | 17 | 0 | 0 | 432 | 17140 | 17 | 0 | 0 | 1 | 440 |
| 18 | 12 | 1 | 01 | 18 | 0 | 1915 | 15316 | 18 | 0 | 0 | 448 | 17596 | 18 | 0 | 0 | 1 | 456 |
| 19 | 13 | 1 | 01 | 19 | 0 | 1973 | 15780 | 19 | 0 | 0 | 464 | 18068 | 19 | 0 | 0 | 1 | 472 |
| 20 | 14 | 1 | 01 | 20 | 0 | 2033 | 16260 | 20 | 0 | 0 | 480 | 18556 | 20 | 0 | 0 | 1 | 488 |
| 21 | 15 | 1 | 01 | 21 | 0 | 2095 | 16756 | 21 | 0 | 0 | 496 | 19060 | 21 | 0 | 0 | 1 | 504 |
| 22 | 16 | 1 | 01 | 22 | 0 | 2159 | 17268 | 22 | 0 | 0 | 512 | 19580 | 22 | 0 | 0 | 1 | 520 |
| 23 | 17 | 1 | 01 | 23 | 0 | 2225 | 17796 | 23 | 0 | 0 | 528 | 20116 | 23 | 0 | 0 | 1 | 536 |
| 24 | 18 | 1 | 01 | 24 | 0 | 2293 | 18340 | 24 | 0 | 0 | 544 | 20668 | 24 | 0 | 0 | 1 | 552 |
| 25 | 19 | 1 | 01 | 25 | 0 | 2363 | 18900 | 25 | 0 | 0 | 560 | 21236 | 25 | 0 | 0 | 1 | 568 |
| 26 | 1A | 1 | 01 | 26 | 0 | 2435 | 19476 | 26 | 0 | 0 | 576 | 21820 | 26 | 0 | 0 | 1 | 584 |
| 27 | 1B | 1 | 01 | 27 | 0 | 2509 | 20068 | 27 | 0 | 0 | 592 | 22420 | 27 | 0 | 0 | 1 | 600 |
| 28 | 1C | 1 | 01 | 28 | 0 | 2585 | 20676 | 28 | 0 | 0 | 608 | 23036 | 28 | 0 | 0 | 1 | 616 |
| 29 | 1D | 1 | 01 | 29 | 0 | 2663 | 21300 | 29 | 0 | 0 | 624 | 23668 | 29 | 0 | 0 | 1 | 632 |
| 30 | 1E | 1 | 01 | 30 | 0 | 2743 | 21940 | 30 | 0 | 0 | 640 | 24316 | 30 | 0 | 0 | 1 | 648 |
| 31 | 1F | 1 | 01 | 31 | 0 | 2825 | 22596 | 31 | 0 | 0 | 656 | 24980 | 31 | 0 | 0 | 1 | 664 |
| 32 | 20 | 1 | 01 | 32 | 0 | 2909 | 23268 | 32 | 0 | 0 | 672 | 25660 | 32 | 0 | 0 | 1 | 680 |
| 33 | 21 | 1 | 01 | 33 | 0 | 2995 | 23956 | 33 | 0 | 0 | 688 | 26356 | 33 | 0 | 0 | 1 | 696 |
| 34 | 22 | 1 | 01 | 34 | 0 | 3083 | 24660 | 34 | 0 | 0 | 704 | 27068 | 34 | 0 | 0 | 1 | 712 |
| 35 | 23 | 1 | 01 | 35 | 0 | 3173 | 25380 | 35 | 0 | 0 | 720 | 27796 | 35 | 0 | 0 | 1 | 728 |
| 36 | 24 | 1 | 01 | 36 | 0 | 3265 | 26116 | 36 | 0 | 0 | 736 | 28540 | 36 | 0 | 0 | 1 | 744 |
| 37 | 25 | 1 | 01 | 37 | 0 | 3359 | 26868 | 37 | 0 | 0 | 752 | 29300 | 37 | 0 | 0 | 1 | 760 |
| 38 | 26 | 1 | 01 | 38 | 0 | 3455 | 27636 | 38 | 0 | 0 | 768 | 30076 | 38 | 0 | 0 | 1 | 776 |
| 39 | 27 | 1 | 01 | 39 | 0 | 3553 | 28420 | 39 | 0 | 0 | 784 | 30868 | 39 | 0 | 0 | 1 | 792 |
| 40 | 28 | 1 | 01 | 40 | 0 | 3653 | 29220 | 40 | 0 | 0 | 800 | 31676 | 40 | 0 | 0 | 1 | 808 |
| 41 | 29 | 1 | 01 | 41 | 0 | 3755 | 30036 | 41 | 0 | 0 | 816 | 32500 | 41 | 0 | 0 | 1 | 824 |
| 42 | 2A | 1 | 01 | 42 | 0 | 3859 | 30868 | 42 | 0 | 0 | 832 | 33340 | 42 | 0 | 0 | 1 | 840 |
| 43 | 2B | 1 | 01 | 43 | 0 | 3965 | 31716 | 43 | 0 | 0 | 848 | 34196 | 43 | 0 | 0 | 1 | 856 |
| 44 | 2C | 1 | 01 | 44 | 0 | 4073 | 32580 | 44 | 0 | 0 | 864 | 35068 | 44 | 0 | 0 | 1 | 872 |
| 45 | 2D | 1 | 01 | 45 | 0 | 4183 | 33460 | 45 | 0 | 0 | 880 | 35956 | 45 | 0 | 0 | 1 | 888 |
| 46 | 2E | 1 | 01 | 46 | 0 | 4295 | 34356 | 46 | 0 | 0 | 896 | 36860 | 46 | 0 | 0 | 1 | 904 |
| 47 | 2F | 1 | 01 | 47 | 0 | 4409 | 35268 | 47 | 0 | 0 | 912 | 37780 | 47 | 0 | 0 | 1 | 920 |
| 48 | 30 | 1 | 01 | 48 | 0 | 4525 | 36196 | 48 | 0 | 0 | 928 | 38716 | 48 | 0 | 0 | 1 | 936 |
| 49 | 31 | 1 | 01 | 49 | 0 | 4643 | 37140 | 49 | 0 | 0 | 944 | 39668 | 49 | 0 | 0 | 1 | 952 |

| | | | | | | | | | | | | | | | | | |
|---|---|---|---|---|---|---|---|---|---|---|---|---|---|---|---|---|---|
| 50 | 32 | 1 | 01 | 50 | 0 | 4763 | 38100 | 50 | 0 | 0 | 960 | 40636 | 50 | 0 | 0 | 1 | 968 |
| 51 | 33 | 1 | 01 | 51 | 0 | 4885 | 39076 | 51 | 0 | 0 | 976 | 41620 | 51 | 0 | 0 | 1 | 984 |
| 52 | 34 | 1 | 01 | 52 | 0 | 5009 | 40068 | 52 | 0 | 0 | 992 | 42620 | 52 | 0 | 0 | 1 | 1000 |
| 53 | 35 | 1 | 01 | 53 | 0 | 5135 | 41076 | 53 | 0 | 0 | 1008 | 43636 | 53 | 0 | 0 | 1 | 1016 |
| 54 | 36 | 1 | 01 | 54 | 0 | 5263 | 42100 | 54 | 0 | 0 | 1024 | 44668 | 54 | 0 | 0 | 1 | 1032 |
| 55 | 37 | 1 | 01 | 55 | 0 | 5393 | 43140 | 55 | 0 | 0 | 1040 | 45716 | 55 | 0 | 0 | 1 | 1048 |
| 56 | 38 | 1 | 01 | 56 | 0 | 5525 | 44196 | 56 | 0 | 0 | 1056 | 46780 | 56 | 0 | 0 | 1 | 1064 |
| 57 | 39 | 1 | 01 | 57 | 0 | 5659 | 45268 | 57 | 0 | 0 | 1072 | 47860 | 57 | 0 | 0 | 1 | 1080 |
| 58 | 3A | 1 | 01 | 58 | 0 | 5795 | 46356 | 58 | 0 | 0 | 1088 | 48956 | 58 | 0 | 0 | 1 | 1096 |
| 59 | 3B | 1 | 01 | 59 | 0 | 5933 | 47460 | 59 | 0 | 0 | 1104 | 50068 | 59 | 0 | 0 | 1 | 1112 |
| 60 | 3C | 1 | 01 | 60 | 0 | 6073 | 48580 | 60 | 0 | 0 | 1120 | 51196 | 60 | 0 | 0 | 1 | 1128 |
| 61 | 3D | 1 | 01 | 61 | 0 | 6215 | 49716 | 61 | 0 | 0 | 1136 | 52340 | 61 | 0 | 0 | 1 | 1144 |
| 62 | 3E | 1 | 01 | 62 | 0 | 6359 | 50868 | 62 | 0 | 0 | 1152 | 53500 | 62 | 0 | 0 | 1 | 1160 |
| 63 | 3F | 1 | 01 | 63 | 0 | 6505 | 52036 | 63 | 0 | 0 | 1168 | 54676 | 63 | 0 | 0 | 1 | 1176 |
| 64 | 40 | 1 | 01 | 64 | 0 | 6653 | 53220 | 64 | 0 | 0 | 1184 | 55868 | 64 | 0 | 0 | 1 | 1192 |
| 65 | 41 | 1 | 01 | 65 | 0 | 6803 | 54420 | 65 | 0 | 0 | 1200 | 57076 | 65 | 0 | 0 | 1 | 1208 |
| 66 | 42 | 1 | 01 | 66 | 0 | 6955 | 55636 | 66 | 0 | 0 | 1216 | 58300 | 66 | 0 | 0 | 1 | 1224 |
| 67 | 43 | 1 | 01 | 67 | 0 | 7109 | 56868 | 67 | 0 | 0 | 1232 | 59540 | 67 | 0 | 0 | 1 | 1240 |
| 68 | 44 | 1 | 01 | 68 | 0 | 7265 | 58116 | 68 | 0 | 0 | 1248 | 60796 | 68 | 0 | 0 | 1 | 1256 |
| 69 | 45 | 1 | 01 | 69 | 0 | 7423 | 59380 | 69 | 0 | 0 | 1264 | 62068 | 69 | 0 | 0 | 1 | 1272 |
| 70 | 46 | 1 | 01 | 70 | 0 | 7583 | 60660 | 70 | 0 | 0 | 1280 | 63356 | 70 | 0 | 0 | 1 | 1288 |
| 71 | 47 | 1 | 01 | 71 | 0 | 7745 | 61956 | 71 | 0 | 0 | 1296 | 64660 | 71 | 0 | 0 | 1 | 1304 |
| 72 | 48 | 1 | 01 | 72 | 0 | 7909 | 63268 | 72 | 0 | 0 | 1312 | 65980 | 72 | 0 | 0 | 1 | 1320 |
| 73 | 49 | 1 | 01 | 73 | 0 | 8075 | 64596 | 73 | 0 | 0 | 1328 | 67316 | 73 | 0 | 0 | 1 | 1336 |
| 74 | 4A | 1 | 01 | 74 | 0 | 8243 | 65940 | 74 | 0 | 0 | 1344 | 68668 | 74 | 0 | 0 | 1 | 1352 |
| 75 | 4B | 1 | 01 | 75 | 0 | 8413 | 67300 | 75 | 0 | 0 | 1360 | 70036 | 75 | 0 | 0 | 1 | 1368 |
| 76 | 4C | 1 | 01 | 76 | 0 | 8585 | 68676 | 76 | 0 | 0 | 1376 | 71420 | 76 | 0 | 0 | 1 | 1384 |
| 77 | 4D | 1 | 01 | 77 | 0 | 8759 | 70068 | 77 | 0 | 0 | 1392 | 72820 | 77 | 0 | 0 | 1 | 1400 |
| 78 | 4E | 1 | 01 | 78 | 0 | 8935 | 71476 | 78 | 0 | 0 | 1408 | 74236 | 78 | 0 | 0 | 1 | 1416 |
| 79 | 4F | 1 | 01 | 79 | 0 | 9113 | 72900 | 79 | 0 | 0 | 1424 | 75668 | 79 | 0 | 0 | 1 | 1432 |
| 80 | 50 | 1 | 01 | 80 | 0 | 9293 | 74340 | 80 | 0 | 0 | 1440 | 77116 | 80 | 0 | 0 | 1 | 1448 |
| 81 | 51 | 1 | 01 | 81 | 0 | 9475 | 75796 | 81 | 0 | 0 | 1456 | 78580 | 81 | 0 | 0 | 1 | 1464 |
| 82 | 52 | 1 | 01 | 82 | 0 | 9659 | 77268 | 82 | 0 | 0 | 1472 | 80060 | 82 | 0 | 0 | 1 | 1480 |
| 83 | 53 | 1 | 01 | 83 | 0 | 9845 | 78756 | 83 | 0 | 0 | 1488 | 81556 | 83 | 0 | 0 | 1 | 1496 |
| 84 | 54 | 1 | 01 | 84 | 0 | 10033 | 80260 | 84 | 0 | 0 | 1504 | 83068 | 84 | 0 | 0 | 1 | 1512 |
| 85 | 55 | 1 | 01 | 85 | 0 | 10223 | 81780 | 85 | 0 | 0 | 1520 | 84596 | 85 | 0 | 0 | 1 | 1528 |
| 86 | 56 | 1 | 01 | 86 | 0 | 10415 | 83316 | 86 | 0 | 0 | 1536 | 86140 | 86 | 0 | 0 | 1 | 1544 |
| 87 | 57 | 1 | 01 | 87 | 0 | 10609 | 84868 | 87 | 0 | 0 | 1552 | 87700 | 87 | 0 | 0 | 1 | 1560 |
| 88 | 58 | 1 | 01 | 88 | 0 | 10805 | 86436 | 88 | 0 | 0 | 1568 | 89276 | 88 | 0 | 0 | 1 | 1576 |
| 89 | 59 | 1 | 01 | 89 | 0 | 11003 | 88020 | 89 | 0 | 0 | 1584 | 90868 | 89 | 0 | 0 | 1 | 1592 |
| 90 | 5A | 1 | 01 | 90 | 0 | 11203 | 89620 | 90 | 0 | 0 | 1600 | 92476 | 90 | 0 | 0 | 1 | 1608 |
| 91 | 5B | 1 | 01 | 91 | 0 | 11405 | 91236 | 91 | 0 | 0 | 1616 | 94100 | 91 | 0 | 0 | 1 | 1624 |
| 92 | 5C | 1 | 01 | 92 | 0 | 11609 | 92868 | 92 | 0 | 0 | 1632 | 95740 | 92 | 0 | 0 | 1 | 1640 |
| 93 | 5D | 1 | 01 | 93 | 0 | 11815 | 94516 | 93 | 0 | 0 | 1648 | 97396 | 93 | 0 | 0 | 1 | 1656 |
| 94 | 5E | 1 | 01 | 94 | 0 | 12023 | 96180 | 94 | 0 | 0 | 1664 | 99068 | 94 | 0 | 0 | 1 | 1672 |
| 95 | 5F | 1 | 01 | 95 | 0 | 12233 | 97860 | 95 | 0 | 0 | 1680 | 100756 | 95 | 0 | 0 | 1 | 1688 |
| 96 | 60 | 1 | 01 | 96 | 0 | 12445 | 99556 | 96 | 0 | 0 | 1696 | 102460 | 96 | 0 | 0 | 1 | 1704 |
| 97 | 61 | 1 | 01 | 97 | 0 | 12659 | 101268 | 97 | 0 | 0 | 1712 | 104180 | 97 | 0 | 0 | 1 | 1720 |
| 98 | 62 | 1 | 01 | 98 | 0 | 12875 | 102996 | 98 | 0 | 0 | 1728 | 105916 | 98 | 0 | 0 | 1 | 1736 |
| 99 | 63 | 1 | 01 | 99 | 0 | 13093 | 104740 | 99 | 0 | 0 | 1744 | 107668 | 99 | 0 | 0 | 1 | 1752 |
| 100 | 64 | 1 | 01 | 100 | 0 | 13313 | 106500 | 100 | 0 | 0 | 1760 | 109436 | 100 | 0 | 0 | 1 | 1768 |
| 101 | 65 | 1 | 01 | 101 | 0 | 13535 | 108276 | 101 | 0 | 0 | 1776 | 111220 | 101 | 0 | 0 | 1 | 1784 |
| 102 | 66 | 1 | 01 | 102 | 0 | 13759 | 110068 | 102 | 0 | 0 | 1792 | 113020 | 102 | 0 | 0 | 1 | 1800 |
| 103 | 67 | 1 | 01 | 103 | 0 | 13985 | 111876 | 103 | 0 | 0 | 1808 | 114836 | 103 | 0 | 0 | 1 | 1816 |
| 104 | 68 | 1 | 01 | 104 | 0 | 14213 | 113700 | 104 | 0 | 0 | 1824 | 116668 | 104 | 0 | 0 | 1 | 1832 |
| 105 | 69 | 1 | 01 | 105 | 0 | 14443 | 115540 | 105 | 0 | 0 | 1840 | 118516 | 105 | 0 | 0 | 1 | 1848 |
| 106 | 6A | 1 | 01 | 106 | 0 | 14675 | 117396 | 106 | 0 | 0 | 1856 | 120380 | 106 | 0 | 0 | 1 | 1864 |
| 107 | 6B | 1 | 01 | 107 | 0 | 14909 | 119268 | 107 | 0 | 0 | 1872 | 122260 | 107 | 0 | 0 | 1 | 1880 |
| 108 | 6C | 1 | 01 | 108 | 0 | 15145 | 121156 | 108 | 0 | 0 | 1888 | 124156 | 108 | 0 | 0 | 1 | 1896 |
| 109 | 6D | 1 | 01 | 109 | 0 | 15383 | 123060 | 109 | 0 | 0 | 1904 | 126068 | 109 | 0 | 0 | 1 | 1912 |
| 110 | 6E | 1 | 01 | 110 | 0 | 15623 | 124980 | 110 | 0 | 0 | 1920 | 127996 | 110 | 0 | 0 | 1 | 1928 |
| 111 | 6F | 1 | 01 | 111 | 0 | 15865 | 126916 | 111 | 0 | 0 | 1936 | 129940 | 111 | 0 | 0 | 1 | 1944 |
| 112 | 70 | 1 | 01 | 112 | 0 | 16109 | 128868 | 112 | 0 | 0 | 1952 | 131900 | 112 | 0 | 0 | 1 | 1960 |
| 113 | 71 | 1 | 01 | 113 | 0 | 16355 | 130836 | 113 | 0 | 0 | 1968 | 133876 | 113 | 0 | 0 | 1 | 1976 |
| 114 | 72 | 1 | 01 | 114 | 0 | 16603 | 132820 | 114 | 0 | 0 | 1984 | 135868 | 114 | 0 | 0 | 1 | 1992 |
| 115 | 73 | 1 | 01 | 115 | 0 | 16853 | 134820 | 115 | 0 | 0 | 2000 | 137876 | 115 | 0 | 0 | 1 | 2008 |
| 116 | 74 | 1 | 01 | 116 | 0 | 17105 | 136836 | 116 | 0 | 0 | 2016 | 139900 | 116 | 0 | 0 | 1 | 2024 |
| 117 | 75 | 1 | 01 | 117 | 0 | 17359 | 138868 | 117 | 0 | 0 | 2032 | 141940 | 117 | 0 | 0 | 1 | 2040 |
| 118 | 76 | 1 | 01 | 118 | 0 | 17615 | 140916 | 118 | 0 | 0 | 2048 | 143996 | 118 | 0 | 0 | 1 | 2056 |
| 119 | 77 | 1 | 01 | 119 | 0 | 17873 | 142980 | 119 | 0 | 0 | 2064 | 146068 | 119 | 0 | 0 | 1 | 2072 |
| 120 | 78 | 1 | 01 | 120 | 0 | 18133 | 145060 | 120 | 0 | 0 | 2080 | 148156 | 120 | 0 | 0 | 1 | 2088 |
| 121 | 79 | 1 | 01 | 121 | 0 | 18395 | 147156 | 121 | 0 | 0 | 2096 | 150260 | 121 | 0 | 0 | 1 | 2104 |
| 122 | 7A | 1 | 01 | 122 | 0 | 18659 | 149268 | 122 | 0 | 0 | 2112 | 152380 | 122 | 0 | 0 | 1 | 2120 |
| 123 | 7B | 1 | 01 | 123 | 0 | 18925 | 151396 | 123 | 0 | 0 | 2128 | 154516 | 123 | 0 | 0 | 1 | 2136 |
| 124 | 7C | 1 | 01 | 124 | 0 | 19193 | 153540 | 124 | 0 | 0 | 2144 | 156668 | 124 | 0 | 0 | 1 | 2152 |
| 125 | 7D | 1 | 01 | 125 | 0 | 19463 | 155700 | 125 | 0 | 0 | 2160 | 158836 | 125 | 0 | 0 | 1 | 2168 |
| 126 | 7E | 1 | 01 | 126 | 0 | 19735 | 157876 | 126 | 0 | 0 | 2176 | 161020 | 126 | 0 | 0 | 1 | 2184 |
| 127 | 7F | 1 | 01 | 127 | 0 | 20009 | 160068 | 127 | 0 | 0 | 2192 | 163220 | 127 | 0 | 0 | 1 | 2200 |
| 128 | 80 | 1 | 01 | 128 | 0 | 20285 | 162276 | 128 | 0 | 0 | 2208 | 165436 | 128 | 0 | 0 | 1 | 2216 |
| 129 | 81 | 1 | 01 | 129 | 0 | 20563 | 164500 | 129 | 0 | 0 | 2224 | 167668 | 129 | 0 | 0 | 1 | 2232 |
| 130 | 82 | 1 | 01 | 130 | 0 | 20843 | 166740 | 130 | 0 | 0 | 2240 | 169916 | 130 | 0 | 0 | 1 | 2248 |
| 131 | 83 | 1 | 01 | 131 | 0 | 21125 | 168996 | 131 | 0 | 0 | 2256 | 172180 | 131 | 0 | 0 | 1 | 2264 |
| 132 | 84 | 1 | 01 | 132 | 0 | 21409 | 171268 | 132 | 0 | 0 | 2272 | 174460 | 132 | 0 | 0 | 1 | 2280 |
| 133 | 85 | 1 | 01 | 133 | 0 | 21695 | 173556 | 133 | 0 | 0 | 2288 | 176756 | 133 | 0 | 0 | 1 | 2296 |
| 134 | 86 | 1 | 01 | 134 | 0 | 21983 | 175860 | 134 | 0 | 0 | 2304 | 179068 | 134 | 0 | 0 | 1 | 2312 |
| 135 | 87 | 1 | 01 | 135 | 0 | 22273 | 178180 | 135 | 0 | 0 | 2320 | 181396 | 135 | 0 | 0 | 1 | 2328 |
| 136 | 88 | 1 | 01 | 136 | 0 | 22565 | 180516 | 136 | 0 | 0 | 2336 | 183740 | 136 | 0 | 0 | 1 | 2344 |
| 137 | 89 | 1 | 01 | 137 | 0 | 22859 | 182868 | 137 | 0 | 0 | 2352 | 186100 | 137 | 0 | 0 | 1 | 2360 |
| 138 | 8A | 1 | 01 | 138 | 0 | 23155 | 185236 | 138 | 0 | 0 | 2368 | 188476 | 138 | 0 | 0 | 1 | 2376 |
| 139 | 8B | 1 | 01 | 139 | 0 | 23453 | 187620 | 139 | 0 | 0 | 2384 | 190868 | 139 | 0 | 0 | 1 | 2392 |
| 140 | 8C | 1 | 01 | 140 | 0 | 23753 | 190020 | 140 | 0 | 0 | 2400 | 193276 | 140 | 0 | 0 | 1 | 2408 |
| 141 | 8D | 1 | 01 | 141 | 0 | 24055 | 192436 | 141 | 0 | 0 | 2416 | 195700 | 141 | 0 | 0 | 1 | 2424 |
| 142 | 8E | 1 | 01 | 142 | 0 | 24359 | 194868 | 142 | 0 | 0 | 2432 | 198140 | 142 | 0 | 0 | 1 | 2440 |
| 143 | 8F | 1 | 01 | 143 | 0 | 24665 | 197316 | 143 | 0 | 0 | 2448 | 200596 | 143 | 0 | 0 | 1 | 2456 |
| 144 | 90 | 1 | 01 | 144 | 0 | 24973 | 199780 | 144 | 0 | 0 | 2464 | 203068 | 144 | 0 | 0 | 1 | 2472 |
| 145 | 91 | 1 | 01 | 145 | 0 | 25283 | 202260 | 145 | 0 | 0 | 2480 | 205556 | 145 | 0 | 0 | 1 | 2488 |
| 146 | 92 | 1 | 01 | 146 | 0 | 25595 | 204756 | 146 | 0 | 0 | 2496 | 208060 | 146 | 0 | 0 | 1 | 2504 |
| 147 | 93 | 1 | 01 | 147 | 0 | 25909 | 207268 | 147 | 0 | 0 | 2512 | 210580 | 147 | 0 | 0 | 1 | 2520 |
| 148 | 94 | 1 | 01 | 148 | 0 | 26225 | 209796 | 148 | 0 | 0 | 2528 | 213116 | 148 | 0 | 0 | 1 | 2536 |
| 149 | 95 | 1 | 01 | 149 | 0 | 26543 | 212340 | 149 | 0 | 0 | 2544 | 215668 | 149 | 0 | 0 | 1 | 2552 |
| 150 | 96 | 1 | 01 | 150 | 0 | 26863 | 214900 | 150 | 0 | 0 | 2560 | 218236 | 150 | 0 | 0 | 1 | 2568 |
| 151 | 97 | 1 | 01 | 151 | 0 | 27185 | 217476 | 151 | 0 | 0 | 2576 | 220820 | 151 | 0 | 0 | 1 | 2584 |
| 152 | 98 | 1 | 01 | 152 | 0 | 27509 | 220068 | 152 | 0 | 0 | 2592 | 223420 | 152 | 0 | 0 | 1 | 2600 |

| | | | | | | | | | | | | | | | | | |
|---|---|---|---|---|---|---|---|---|---|---|---|---|---|---|---|---|---|
| 153 | 99 | 1 | 01 | 153 | 0 | 27835 | 222676 | 153 | 0 | 0 | 2608 | 226036 | 153 | 0 | 0 | 1 | 2616 |
| 154 | 9A | 1 | 01 | 154 | 0 | 28163 | 225300 | 154 | 0 | 0 | 2624 | 228668 | 154 | 0 | 0 | 1 | 2632 |
| 155 | 9B | 1 | 01 | 155 | 0 | 28493 | 227940 | 155 | 0 | 0 | 2640 | 231316 | 155 | 0 | 0 | 1 | 2648 |
| 156 | 9C | 1 | 01 | 156 | 0 | 28825 | 230596 | 156 | 0 | 0 | 2656 | 233980 | 156 | 0 | 0 | 1 | 2664 |
| 157 | 9D | 1 | 01 | 157 | 0 | 29159 | 233268 | 157 | 0 | 0 | 2672 | 236660 | 157 | 0 | 0 | 1 | 2680 |
| 158 | 9E | 1 | 01 | 158 | 0 | 29495 | 235956 | 158 | 0 | 0 | 2688 | 239356 | 158 | 0 | 0 | 1 | 2696 |
| 159 | 9F | 1 | 01 | 159 | 0 | 29833 | 238660 | 159 | 0 | 0 | 2704 | 242068 | 159 | 0 | 0 | 1 | 2712 |
| 160 | A0 | 1 | 01 | 160 | 0 | 30173 | 241380 | 160 | 0 | 0 | 2720 | 244796 | 160 | 0 | 0 | 1 | 2728 |
| 161 | A1 | 1 | 01 | 161 | 0 | 30515 | 244116 | 161 | 0 | 0 | 2736 | 247540 | 161 | 0 | 0 | 1 | 2744 |
| 162 | A2 | 1 | 01 | 162 | 0 | 30859 | 246868 | 162 | 0 | 0 | 2752 | 250300 | 162 | 0 | 0 | 1 | 2760 |
| 163 | A3 | 1 | 01 | 163 | 0 | 31205 | 249636 | 163 | 0 | 0 | 2768 | 253076 | 163 | 0 | 0 | 1 | 2776 |
| 164 | A4 | 1 | 01 | 164 | 0 | 31553 | 252420 | 164 | 0 | 0 | 2784 | 255868 | 164 | 0 | 0 | 1 | 2792 |
| 165 | A5 | 1 | 01 | 165 | 0 | 31903 | 255220 | 165 | 0 | 0 | 2800 | 258676 | 165 | 0 | 0 | 1 | 2808 |
| 166 | A6 | 1 | 01 | 166 | 0 | 32255 | 258036 | 166 | 0 | 0 | 2816 | 261500 | 166 | 0 | 0 | 1 | 2824 |
| 167 | A7 | 1 | 01 | 167 | 0 | 32609 | 260868 | 167 | 0 | 0 | 2832 | 264340 | 167 | 0 | 0 | 1 | 2840 |
| 168 | A8 | 1 | 01 | 168 | 0 | 32965 | 263716 | 168 | 0 | 0 | 2848 | 267196 | 168 | 0 | 0 | 1 | 2856 |
| 169 | A9 | 1 | 01 | 169 | 0 | 33323 | 266580 | 169 | 0 | 0 | 2864 | 270068 | 169 | 0 | 0 | 1 | 2872 |
| 170 | AA | 1 | 01 | 170 | 0 | 33683 | 269460 | 170 | 0 | 0 | 2880 | 272956 | 170 | 0 | 0 | 1 | 2888 |
| 171 | AB | 1 | 01 | 171 | 0 | 34045 | 272356 | 171 | 0 | 0 | 2896 | 275860 | 171 | 0 | 0 | 1 | 2904 |
| 172 | AC | 1 | 01 | 172 | 0 | 34409 | 275268 | 172 | 0 | 0 | 2912 | 278780 | 172 | 0 | 0 | 1 | 2920 |
| 173 | AD | 1 | 01 | 173 | 0 | 34775 | 278196 | 173 | 0 | 0 | 2928 | 281716 | 173 | 0 | 0 | 1 | 2936 |
| 174 | AE | 1 | 01 | 174 | 0 | 35143 | 281140 | 174 | 0 | 0 | 2944 | 284668 | 174 | 0 | 0 | 1 | 2952 |
| 175 | AF | 1 | 01 | 175 | 0 | 35513 | 284100 | 175 | 0 | 0 | 2960 | 287636 | 175 | 0 | 0 | 1 | 2968 |
| 176 | B0 | 1 | 01 | 176 | 0 | 35885 | 287076 | 176 | 0 | 0 | 2976 | 290620 | 176 | 0 | 0 | 1 | 2984 |
| 177 | B1 | 1 | 01 | 177 | 0 | 36259 | 290068 | 177 | 0 | 0 | 2992 | 293620 | 177 | 0 | 0 | 1 | 3000 |
| 178 | B2 | 1 | 01 | 178 | 0 | 36635 | 293076 | 178 | 0 | 0 | 3008 | 296636 | 178 | 0 | 0 | 1 | 3016 |
| 179 | B3 | 1 | 01 | 179 | 0 | 37013 | 296100 | 179 | 0 | 0 | 3024 | 299668 | 179 | 0 | 0 | 1 | 3032 |
| 180 | B4 | 1 | 01 | 180 | 0 | 37393 | 299140 | 180 | 0 | 0 | 3040 | 302716 | 180 | 0 | 0 | 1 | 3048 |
| 181 | B5 | 1 | 01 | 181 | 0 | 37775 | 302196 | 181 | 0 | 0 | 3056 | 305780 | 181 | 0 | 0 | 1 | 3064 |
| 182 | B6 | 1 | 01 | 182 | 0 | 38159 | 305268 | 182 | 0 | 0 | 3072 | 308860 | 182 | 0 | 0 | 1 | 3080 |
| 183 | B7 | 1 | 01 | 183 | 0 | 38545 | 308356 | 183 | 0 | 0 | 3088 | 311956 | 183 | 0 | 0 | 1 | 3096 |
| 184 | B8 | 1 | 01 | 184 | 0 | 38933 | 311460 | 184 | 0 | 0 | 3104 | 315068 | 184 | 0 | 0 | 1 | 3112 |
| 185 | B9 | 1 | 01 | 185 | 0 | 39323 | 314580 | 185 | 0 | 0 | 3120 | 318196 | 185 | 0 | 0 | 1 | 3128 |
| 186 | BA | 1 | 01 | 186 | 0 | 39715 | 317716 | 186 | 0 | 0 | 3136 | 321340 | 186 | 0 | 0 | 1 | 3144 |
| 187 | BB | 1 | 01 | 187 | 0 | 40109 | 320868 | 187 | 0 | 0 | 3152 | 324500 | 187 | 0 | 0 | 1 | 3160 |
| 188 | BC | 1 | 01 | 188 | 0 | 40505 | 324036 | 188 | 0 | 0 | 3168 | 327676 | 188 | 0 | 0 | 1 | 3176 |
| 189 | BD | 1 | 01 | 189 | 0 | 40903 | 327220 | 189 | 0 | 0 | 3184 | 330868 | 189 | 0 | 0 | 1 | 3192 |
| 190 | BE | 1 | 01 | 190 | 0 | 41303 | 330420 | 190 | 0 | 0 | 3200 | 334076 | 190 | 0 | 0 | 1 | 3208 |
| 191 | BF | 1 | 01 | 191 | 0 | 41705 | 333636 | 191 | 0 | 0 | 3216 | 337300 | 191 | 0 | 0 | 1 | 3224 |
| 192 | C0 | 1 | 01 | 192 | 0 | 42109 | 336868 | 192 | 0 | 0 | 3232 | 340540 | 192 | 0 | 0 | 1 | 3240 |
| 193 | C1 | 1 | 01 | 193 | 0 | 42515 | 340116 | 193 | 0 | 0 | 3248 | 343796 | 193 | 0 | 0 | 1 | 3256 |
| 194 | C2 | 1 | 01 | 194 | 0 | 42923 | 343380 | 194 | 0 | 0 | 3264 | 347068 | 194 | 0 | 0 | 1 | 3272 |
| 195 | C3 | 1 | 01 | 195 | 0 | 43333 | 346660 | 195 | 0 | 0 | 3280 | 350356 | 195 | 0 | 0 | 1 | 3288 |
| 196 | C4 | 1 | 01 | 196 | 0 | 43745 | 349956 | 196 | 0 | 0 | 3296 | 353660 | 196 | 0 | 0 | 1 | 3304 |
| 197 | C5 | 1 | 01 | 197 | 0 | 44159 | 353268 | 197 | 0 | 0 | 3312 | 356980 | 197 | 0 | 0 | 1 | 3320 |
| 198 | C6 | 1 | 01 | 198 | 0 | 44575 | 356596 | 198 | 0 | 0 | 3328 | 360316 | 198 | 0 | 0 | 1 | 3336 |
| 199 | C7 | 1 | 01 | 199 | 0 | 44993 | 359940 | 199 | 0 | 0 | 3344 | 363668 | 199 | 0 | 0 | 1 | 3352 |
| 200 | C8 | 1 | 01 | 200 | 0 | 45413 | 363300 | 200 | 0 | 0 | 3360 | 367036 | 200 | 0 | 0 | 1 | 3368 |
| 201 | C9 | 1 | 01 | 201 | 0 | 45835 | 366676 | 201 | 0 | 0 | 3376 | 370420 | 201 | 0 | 0 | 1 | 3384 |
| 202 | CA | 1 | 01 | 202 | 0 | 46259 | 370068 | 202 | 0 | 0 | 3392 | 373820 | 202 | 0 | 0 | 1 | 3400 |
| 203 | CB | 1 | 01 | 203 | 0 | 46685 | 373476 | 203 | 0 | 0 | 3408 | 377236 | 203 | 0 | 0 | 1 | 3416 |
| 204 | CC | 1 | 01 | 204 | 0 | 47113 | 376900 | 204 | 0 | 0 | 3424 | 380668 | 204 | 0 | 0 | 1 | 3432 |
| 205 | CD | 1 | 01 | 205 | 0 | 47543 | 380340 | 205 | 0 | 0 | 3440 | 384116 | 205 | 0 | 0 | 1 | 3448 |
| 206 | CE | 1 | 01 | 206 | 0 | 47975 | 383796 | 206 | 0 | 0 | 3456 | 387580 | 206 | 0 | 0 | 1 | 3464 |
| 207 | CF | 1 | 01 | 207 | 0 | 48409 | 387268 | 207 | 0 | 0 | 3472 | 391060 | 207 | 0 | 0 | 1 | 3480 |
| 208 | D0 | 1 | 01 | 208 | 0 | 48845 | 390756 | 208 | 0 | 0 | 3488 | 394556 | 208 | 0 | 0 | 1 | 3496 |
| 209 | D1 | 1 | 01 | 209 | 0 | 49283 | 394260 | 209 | 0 | 0 | 3504 | 398068 | 209 | 0 | 0 | 1 | 3512 |
| 210 | D2 | 1 | 01 | 210 | 0 | 49723 | 397780 | 210 | 0 | 0 | 3520 | 401596 | 210 | 0 | 0 | 1 | 3528 |
| 211 | D3 | 1 | 01 | 211 | 0 | 50165 | 401316 | 211 | 0 | 0 | 3536 | 405140 | 211 | 0 | 0 | 1 | 3544 |
| 212 | D4 | 1 | 01 | 212 | 0 | 50609 | 404868 | 212 | 0 | 0 | 3552 | 408700 | 212 | 0 | 0 | 1 | 3560 |
| 213 | D5 | 1 | 01 | 213 | 0 | 51055 | 408436 | 213 | 0 | 0 | 3568 | 412276 | 213 | 0 | 0 | 1 | 3576 |
| 214 | D6 | 1 | 01 | 214 | 0 | 51503 | 412020 | 214 | 0 | 0 | 3584 | 415868 | 214 | 0 | 0 | 1 | 3592 |
| 215 | D7 | 1 | 01 | 215 | 0 | 51953 | 415620 | 215 | 0 | 0 | 3600 | 419476 | 215 | 0 | 0 | 1 | 3608 |
| 216 | D8 | 1 | 01 | 216 | 0 | 52405 | 419236 | 216 | 0 | 0 | 3616 | 423100 | 216 | 0 | 0 | 1 | 3624 |
| 217 | D9 | 1 | 01 | 217 | 0 | 52859 | 422868 | 217 | 0 | 0 | 3632 | 426740 | 217 | 0 | 0 | 1 | 3640 |
| 218 | DA | 1 | 01 | 218 | 0 | 53315 | 426516 | 218 | 0 | 0 | 3648 | 430396 | 218 | 0 | 0 | 1 | 3656 |
| 219 | DB | 1 | 01 | 219 | 0 | 53773 | 430180 | 219 | 0 | 0 | 3664 | 434068 | 219 | 0 | 0 | 1 | 3672 |
| 220 | DC | 1 | 01 | 220 | 0 | 54233 | 433860 | 220 | 0 | 0 | 3680 | 437756 | 220 | 0 | 0 | 1 | 3688 |
| 221 | DD | 1 | 01 | 221 | 0 | 54695 | 437556 | 221 | 0 | 0 | 3696 | 441460 | 221 | 0 | 0 | 1 | 3704 |
| 222 | DE | 1 | 01 | 222 | 0 | 55159 | 441268 | 222 | 0 | 0 | 3712 | 445180 | 222 | 0 | 0 | 1 | 3720 |
| 223 | DF | 1 | 01 | 223 | 0 | 55625 | 444996 | 223 | 0 | 0 | 3728 | 448916 | 223 | 0 | 0 | 1 | 3736 |
| 224 | E0 | 1 | 01 | 224 | 0 | 56093 | 448740 | 224 | 0 | 0 | 3744 | 452668 | 224 | 0 | 0 | 1 | 3752 |
| 225 | E1 | 1 | 01 | 225 | 0 | 56563 | 452500 | 225 | 0 | 0 | 3760 | 456436 | 225 | 0 | 0 | 1 | 3768 |
| 226 | E2 | 1 | 01 | 226 | 0 | 57035 | 456276 | 226 | 0 | 0 | 3776 | 460220 | 226 | 0 | 0 | 1 | 3784 |
| 227 | E3 | 1 | 01 | 227 | 0 | 57509 | 460068 | 227 | 0 | 0 | 3792 | 464020 | 227 | 0 | 0 | 1 | 3800 |
| 228 | E4 | 1 | 01 | 228 | 0 | 57985 | 463876 | 228 | 0 | 0 | 3808 | 467836 | 228 | 0 | 0 | 1 | 3816 |
| 229 | E5 | 1 | 01 | 229 | 0 | 58463 | 467700 | 229 | 0 | 0 | 3824 | 471668 | 229 | 0 | 0 | 1 | 3832 |
| 230 | E6 | 1 | 01 | 230 | 0 | 58943 | 471540 | 230 | 0 | 0 | 3840 | 475516 | 230 | 0 | 0 | 1 | 3848 |
| 231 | E7 | 1 | 01 | 231 | 0 | 59425 | 475396 | 231 | 0 | 0 | 3856 | 479380 | 231 | 0 | 0 | 1 | 3864 |
| 232 | E8 | 1 | 01 | 232 | 0 | 59909 | 479268 | 232 | 0 | 0 | 3872 | 483260 | 232 | 0 | 0 | 1 | 3880 |
| 233 | E9 | 1 | 01 | 233 | 0 | 60395 | 483156 | 233 | 0 | 0 | 3888 | 487156 | 233 | 0 | 0 | 1 | 3896 |
| 234 | EA | 1 | 01 | 234 | 0 | 60883 | 487060 | 234 | 0 | 0 | 3904 | 491068 | 234 | 0 | 0 | 1 | 3912 |
| 235 | EB | 1 | 01 | 235 | 0 | 61373 | 490980 | 235 | 0 | 0 | 3920 | 494996 | 235 | 0 | 0 | 1 | 3928 |
| 236 | EC | 1 | 01 | 236 | 0 | 61865 | 494916 | 236 | 0 | 0 | 3936 | 498940 | 236 | 0 | 0 | 1 | 3944 |
| 237 | ED | 1 | 01 | 237 | 0 | 62359 | 498868 | 237 | 0 | 0 | 3952 | 502900 | 237 | 0 | 0 | 1 | 3960 |
| 238 | EE | 1 | 01 | 238 | 0 | 62855 | 502836 | 238 | 0 | 0 | 3968 | 506876 | 238 | 0 | 0 | 1 | 3976 |
| 239 | EF | 1 | 01 | 239 | 0 | 63353 | 506820 | 239 | 0 | 0 | 3984 | 510868 | 239 | 0 | 0 | 1 | 3992 |
| 240 | F0 | 1 | 01 | 240 | 0 | 63853 | 510820 | 240 | 0 | 0 | 4000 | 514876 | 240 | 0 | 0 | 1 | 4008 |
| 241 | F1 | 1 | 01 | 241 | 0 | 64355 | 514836 | 241 | 0 | 0 | 4016 | 518900 | 241 | 0 | 0 | 1 | 4024 |
| 242 | F2 | 1 | 01 | 242 | 0 | 64859 | 518868 | 242 | 0 | 0 | 4032 | 522940 | 242 | 0 | 0 | 1 | 4040 |
| 243 | F3 | 1 | 01 | 243 | 0 | 65365 | 522916 | 243 | 0 | 0 | 4048 | 526996 | 243 | 0 | 0 | 1 | 4056 |
| 244 | F4 | 1 | 01 | 244 | 0 | 65873 | 526980 | 244 | 0 | 0 | 4064 | 531068 | 244 | 0 | 0 | 1 | 4072 |
| 245 | F5 | 1 | 01 | 245 | 0 | 66383 | 531060 | 245 | 0 | 0 | 4080 | 535156 | 245 | 0 | 0 | 1 | 4088 |
| 246 | F6 | 1 | 01 | 246 | 0 | 66895 | 535156 | 246 | 0 | 0 | 4096 | 539260 | 246 | 0 | 0 | 1 | 4104 |
| 247 | F7 | 1 | 01 | 247 | 0 | 67409 | 539268 | 247 | 0 | 0 | 4112 | 543380 | 247 | 0 | 0 | 1 | 4120 |
| 248 | F8 | 1 | 01 | 248 | 0 | 67925 | 543396 | 248 | 0 | 0 | 4128 | 547516 | 248 | 0 | 0 | 1 | 4136 |
| 249 | F9 | 1 | 01 | 249 | 0 | 68443 | 547540 | 249 | 0 | 0 | 4144 | 551668 | 249 | 0 | 0 | 1 | 4152 |
| 250 | FA | 1 | 01 | 250 | 0 | 68963 | 551700 | 250 | 0 | 0 | 4160 | 555836 | 250 | 0 | 0 | 1 | 4168 |
| 251 | FB | 1 | 01 | 251 | 0 | 69485 | 555876 | 251 | 0 | 0 | 4176 | 560020 | 251 | 0 | 0 | 1 | 4184 |
| 252 | FC | 1 | 01 | 252 | 0 | 70009 | 560068 | 252 | 0 | 0 | 4192 | 564220 | 252 | 0 | 0 | 1 | 4200 |
| 253 | FD | 1 | 01 | 253 | 0 | 70535 | 564276 | 253 | 0 | 0 | 4208 | 568436 | 253 | 0 | 0 | 1 | 4216 |
| 254 | FE | 1 | 01 | 254 | 0 | 71063 | 568500 | 254 | 0 | 0 | 4224 | 572668 | 254 | 0 | 0 | 1 | 4232 |
| 255 | FF | 1 | 01 | 255 | 0 | 71593 | 572740 | 255 | 0 | 0 | 4240 | 576916 | 255 | 0 | 0 | 1 | 4248 |

| | | | | | | | | | | | | | | | | | |
|---|---|---|---|---|---|---|---|---|---|---|---|---|---|---|---|---|---|
| 0 | 00 | 2 | 02 | 0 | 0 | 71605 | 572836 | 0 | 0 | 0 | 96 | 577020 | 0 | 0 | 0 | 1 | 104 |
| 1 | 01 | 2 | 02 | 0 | 1 | 71619 | 572948 | 0 | 1 | 0 | 112 | 577140 | 0 | 1 | 0 | 1 | 120 |
| 2 | 02 | 2 | 02 | 1 | 0 | 71631 | 573044 | 1 | 0 | 0 | 96 | 577244 | 1 | 0 | 0 | 1 | 104 |
| 3 | 03 | 2 | 02 | 1 | 1 | 71648 | 573180 | 1 | 1 | 0 | 136 | 577388 | 1 | 1 | 0 | 1 | 144 |
| 4 | 04 | 2 | 02 | 2 | 0 | 71667 | 573332 | 2 | 0 | 0 | 152 | 577548 | 2 | 0 | 0 | 1 | 160 |
| 5 | 05 | 2 | 02 | 2 | 1 | 71686 | 573484 | 2 | 1 | 0 | 152 | 577708 | 2 | 1 | 0 | 1 | 160 |
| 6 | 06 | 2 | 02 | 3 | 0 | 71707 | 573652 | 3 | 0 | 0 | 168 | 577884 | 3 | 0 | 0 | 1 | 176 |
| 7 | 07 | 2 | 02 | 3 | 1 | 71728 | 573820 | 3 | 1 | 0 | 168 | 578060 | 3 | 1 | 0 | 1 | 176 |
| 8 | 08 | 2 | 02 | 4 | 0 | 71751 | 574004 | 4 | 0 | 0 | 184 | 578252 | 4 | 0 | 0 | 1 | 192 |
| 9 | 09 | 2 | 02 | 4 | 1 | 71774 | 574188 | 4 | 1 | 0 | 184 | 578444 | 4 | 1 | 0 | 1 | 192 |
| 10 | 0A | 2 | 02 | 5 | 0 | 71799 | 574388 | 5 | 0 | 0 | 200 | 578652 | 5 | 0 | 0 | 1 | 208 |
| 11 | 0B | 2 | 02 | 5 | 1 | 71824 | 574588 | 5 | 1 | 0 | 200 | 578860 | 5 | 1 | 0 | 1 | 208 |
| 12 | 0C | 2 | 02 | 6 | 0 | 71851 | 574804 | 6 | 0 | 0 | 216 | 579084 | 6 | 0 | 0 | 1 | 224 |
| 13 | 0D | 2 | 02 | 6 | 1 | 71878 | 575020 | 6 | 1 | 0 | 216 | 579308 | 6 | 1 | 0 | 1 | 224 |
| 14 | 0E | 2 | 02 | 7 | 0 | 71907 | 575252 | 7 | 0 | 0 | 232 | 579548 | 7 | 0 | 0 | 1 | 240 |
| 15 | 0F | 2 | 02 | 7 | 1 | 71936 | 575484 | 7 | 1 | 0 | 232 | 579788 | 7 | 1 | 0 | 1 | 240 |
| 16 | 10 | 2 | 02 | 8 | 0 | 71972 | 575772 | 8 | 0 | 0 | 288 | 580084 | 8 | 0 | 0 | 1 | 296 |
| 17 | 11 | 2 | 02 | 8 | 1 | 72008 | 576060 | 8 | 1 | 0 | 288 | 580380 | 8 | 1 | 0 | 1 | 296 |
| 18 | 12 | 2 | 02 | 9 | 0 | 72046 | 576364 | 9 | 0 | 0 | 304 | 580692 | 9 | 0 | 0 | 1 | 312 |
| 19 | 13 | 2 | 02 | 9 | 1 | 72084 | 576668 | 9 | 1 | 0 | 304 | 581004 | 9 | 1 | 0 | 1 | 312 |
| 20 | 14 | 2 | 02 | 10 | 0 | 72124 | 576988 | 10 | 0 | 0 | 320 | 581332 | 10 | 0 | 0 | 1 | 328 |
| 21 | 15 | 2 | 02 | 10 | 1 | 72164 | 577308 | 10 | 1 | 0 | 320 | 581660 | 10 | 1 | 0 | 1 | 328 |
| 22 | 16 | 2 | 02 | 11 | 0 | 72206 | 577644 | 11 | 0 | 0 | 336 | 582004 | 11 | 0 | 0 | 1 | 344 |
| 23 | 17 | 2 | 02 | 11 | 1 | 72248 | 577980 | 11 | 1 | 0 | 336 | 582348 | 11 | 1 | 0 | 1 | 344 |
| 24 | 18 | 2 | 02 | 12 | 0 | 72292 | 578332 | 12 | 0 | 0 | 352 | 582708 | 12 | 0 | 0 | 1 | 360 |
| 25 | 19 | 2 | 02 | 12 | 1 | 72336 | 578684 | 12 | 1 | 0 | 352 | 583068 | 12 | 1 | 0 | 1 | 360 |
| 26 | 1A | 2 | 02 | 13 | 0 | 72382 | 579052 | 13 | 0 | 0 | 368 | 583444 | 13 | 0 | 0 | 1 | 376 |
| 27 | 1B | 2 | 02 | 13 | 1 | 72428 | 579420 | 13 | 1 | 0 | 368 | 583820 | 13 | 1 | 0 | 1 | 376 |
| 28 | 1C | 2 | 02 | 14 | 0 | 72476 | 579804 | 14 | 0 | 0 | 384 | 584212 | 14 | 0 | 0 | 1 | 392 |
| 29 | 1D | 2 | 02 | 14 | 1 | 72524 | 580188 | 14 | 1 | 0 | 384 | 584604 | 14 | 1 | 0 | 1 | 392 |
| 30 | 1E | 2 | 02 | 15 | 0 | 72574 | 580588 | 15 | 0 | 0 | 400 | 585012 | 15 | 0 | 0 | 1 | 408 |
| 31 | 1F | 2 | 02 | 15 | 1 | 72624 | 580988 | 15 | 1 | 0 | 400 | 585420 | 15 | 1 | 0 | 1 | 408 |
| 32 | 20 | 2 | 02 | 16 | 0 | 72676 | 581404 | 16 | 0 | 0 | 416 | 585844 | 16 | 0 | 0 | 1 | 424 |
| 33 | 21 | 2 | 02 | 16 | 1 | 72728 | 581820 | 16 | 1 | 0 | 416 | 586268 | 16 | 1 | 0 | 1 | 424 |
| 34 | 22 | 2 | 02 | 17 | 0 | 72782 | 582252 | 17 | 0 | 0 | 432 | 586708 | 17 | 0 | 0 | 1 | 440 |
| 35 | 23 | 2 | 02 | 17 | 1 | 72836 | 582684 | 17 | 1 | 0 | 432 | 587148 | 17 | 1 | 0 | 1 | 440 |
| 36 | 24 | 2 | 02 | 18 | 0 | 72892 | 583132 | 18 | 0 | 0 | 448 | 587604 | 18 | 0 | 0 | 1 | 456 |
| 37 | 25 | 2 | 02 | 18 | 1 | 72948 | 583580 | 18 | 1 | 0 | 448 | 588060 | 18 | 1 | 0 | 1 | 456 |
| 38 | 26 | 2 | 02 | 19 | 0 | 73006 | 584044 | 19 | 0 | 0 | 464 | 588532 | 19 | 0 | 0 | 1 | 472 |
| 39 | 27 | 2 | 02 | 19 | 1 | 73064 | 584508 | 19 | 1 | 0 | 464 | 589004 | 19 | 1 | 0 | 1 | 472 |
| 40 | 28 | 2 | 02 | 20 | 0 | 73124 | 584988 | 20 | 0 | 0 | 480 | 589492 | 20 | 0 | 0 | 1 | 488 |
| 41 | 29 | 2 | 02 | 20 | 1 | 73184 | 585468 | 20 | 1 | 0 | 480 | 589980 | 20 | 1 | 0 | 1 | 488 |
| 42 | 2A | 2 | 02 | 21 | 0 | 73246 | 585964 | 21 | 0 | 0 | 496 | 590484 | 21 | 0 | 0 | 1 | 504 |
| 43 | 2B | 2 | 02 | 21 | 1 | 73308 | 586460 | 21 | 1 | 0 | 496 | 590988 | 21 | 1 | 0 | 1 | 504 |
| 44 | 2C | 2 | 02 | 22 | 0 | 73372 | 586972 | 22 | 0 | 0 | 512 | 591508 | 22 | 0 | 0 | 1 | 520 |
| 45 | 2D | 2 | 02 | 22 | 1 | 73436 | 587484 | 22 | 1 | 0 | 512 | 592028 | 22 | 1 | 0 | 1 | 520 |
| 46 | 2E | 2 | 02 | 23 | 0 | 73502 | 588012 | 23 | 0 | 0 | 528 | 592564 | 23 | 0 | 0 | 1 | 536 |
| 47 | 2F | 2 | 02 | 23 | 1 | 73568 | 588540 | 23 | 1 | 0 | 528 | 593100 | 23 | 1 | 0 | 1 | 536 |
| 48 | 30 | 2 | 02 | 24 | 0 | 73636 | 589084 | 24 | 0 | 0 | 544 | 593652 | 24 | 0 | 0 | 1 | 552 |
| 49 | 31 | 2 | 02 | 24 | 1 | 73704 | 589628 | 24 | 1 | 0 | 544 | 594204 | 24 | 1 | 0 | 1 | 552 |
| 50 | 32 | 2 | 02 | 25 | 0 | 73774 | 590188 | 25 | 0 | 0 | 560 | 594772 | 25 | 0 | 0 | 1 | 568 |
| 51 | 33 | 2 | 02 | 25 | 1 | 73844 | 590748 | 25 | 1 | 0 | 560 | 595340 | 25 | 1 | 0 | 1 | 568 |
| 52 | 34 | 2 | 02 | 26 | 0 | 73916 | 591324 | 26 | 0 | 0 | 576 | 595924 | 26 | 0 | 0 | 1 | 584 |
| 53 | 35 | 2 | 02 | 26 | 1 | 73988 | 591900 | 26 | 1 | 0 | 576 | 596508 | 26 | 1 | 0 | 1 | 584 |
| 54 | 36 | 2 | 02 | 27 | 0 | 74062 | 592492 | 27 | 0 | 0 | 592 | 597108 | 27 | 0 | 0 | 1 | 600 |
| 55 | 37 | 2 | 02 | 27 | 1 | 74136 | 593084 | 27 | 1 | 0 | 592 | 597708 | 27 | 1 | 0 | 1 | 600 |
| 56 | 38 | 2 | 02 | 28 | 0 | 74212 | 593692 | 28 | 0 | 0 | 608 | 598324 | 28 | 0 | 0 | 1 | 616 |
| 57 | 39 | 2 | 02 | 28 | 1 | 74288 | 594300 | 28 | 1 | 0 | 608 | 598940 | 28 | 1 | 0 | 1 | 616 |
| 58 | 3A | 2 | 02 | 29 | 0 | 74366 | 594924 | 29 | 0 | 0 | 624 | 599572 | 29 | 0 | 0 | 1 | 632 |
| 59 | 3B | 2 | 02 | 29 | 1 | 74444 | 595548 | 29 | 1 | 0 | 624 | 600204 | 29 | 1 | 0 | 1 | 632 |
| 60 | 3C | 2 | 02 | 30 | 0 | 74524 | 596188 | 30 | 0 | 0 | 640 | 600852 | 30 | 0 | 0 | 1 | 648 |
| 61 | 3D | 2 | 02 | 30 | 1 | 74604 | 596828 | 30 | 1 | 0 | 640 | 601500 | 30 | 1 | 0 | 1 | 648 |
| 62 | 3E | 2 | 02 | 31 | 0 | 74686 | 597484 | 31 | 0 | 0 | 656 | 602164 | 31 | 0 | 0 | 1 | 664 |
| 63 | 3F | 2 | 02 | 31 | 1 | 74768 | 598140 | 31 | 1 | 0 | 656 | 602828 | 31 | 1 | 0 | 1 | 664 |
| 64 | 40 | 2 | 02 | 32 | 0 | 74852 | 598812 | 32 | 0 | 0 | 672 | 603508 | 32 | 0 | 0 | 1 | 680 |
| 65 | 41 | 2 | 02 | 32 | 1 | 74936 | 599484 | 32 | 1 | 0 | 672 | 604188 | 32 | 1 | 0 | 1 | 680 |
| 66 | 42 | 2 | 02 | 33 | 0 | 75022 | 600172 | 33 | 0 | 0 | 688 | 604884 | 33 | 0 | 0 | 1 | 696 |
| 67 | 43 | 2 | 02 | 33 | 1 | 75108 | 600860 | 33 | 1 | 0 | 688 | 605580 | 33 | 1 | 0 | 1 | 696 |
| 68 | 44 | 2 | 02 | 34 | 0 | 75196 | 601564 | 34 | 0 | 0 | 704 | 606292 | 34 | 0 | 0 | 1 | 712 |
| 69 | 45 | 2 | 02 | 34 | 1 | 75284 | 602268 | 34 | 1 | 0 | 704 | 607004 | 34 | 1 | 0 | 1 | 712 |
| 70 | 46 | 2 | 02 | 35 | 0 | 75374 | 602988 | 35 | 0 | 0 | 720 | 607732 | 35 | 0 | 0 | 1 | 728 |
| 71 | 47 | 2 | 02 | 35 | 1 | 75464 | 603708 | 35 | 1 | 0 | 720 | 608460 | 35 | 1 | 0 | 1 | 728 |
| 72 | 48 | 2 | 02 | 36 | 0 | 75556 | 604444 | 36 | 0 | 0 | 736 | 609204 | 36 | 0 | 0 | 1 | 744 |
| 73 | 49 | 2 | 02 | 36 | 1 | 75648 | 605180 | 36 | 1 | 0 | 736 | 609948 | 36 | 1 | 0 | 1 | 744 |
| 74 | 4A | 2 | 02 | 37 | 0 | 75742 | 605932 | 37 | 0 | 0 | 752 | 610708 | 37 | 0 | 0 | 1 | 760 |
| 75 | 4B | 2 | 02 | 37 | 1 | 75836 | 606684 | 37 | 1 | 0 | 752 | 611468 | 37 | 1 | 0 | 1 | 760 |
| 76 | 4C | 2 | 02 | 38 | 0 | 75932 | 607452 | 38 | 0 | 0 | 768 | 612244 | 38 | 0 | 0 | 1 | 776 |
| 77 | 4D | 2 | 02 | 38 | 1 | 76028 | 608220 | 38 | 1 | 0 | 768 | 613020 | 38 | 1 | 0 | 1 | 776 |
| 78 | 4E | 2 | 02 | 39 | 0 | 76126 | 609004 | 39 | 0 | 0 | 784 | 613812 | 39 | 0 | 0 | 1 | 792 |
| 79 | 4F | 2 | 02 | 39 | 1 | 76224 | 609788 | 39 | 1 | 0 | 784 | 614604 | 39 | 1 | 0 | 1 | 792 |
| 80 | 50 | 2 | 02 | 40 | 0 | 76324 | 610588 | 40 | 0 | 0 | 800 | 615412 | 40 | 0 | 0 | 1 | 808 |
| 81 | 51 | 2 | 02 | 40 | 1 | 76424 | 611388 | 40 | 1 | 0 | 800 | 616220 | 40 | 1 | 0 | 1 | 808 |
| 82 | 52 | 2 | 02 | 41 | 0 | 76526 | 612204 | 41 | 0 | 0 | 816 | 617044 | 41 | 0 | 0 | 1 | 824 |
| 83 | 53 | 2 | 02 | 41 | 1 | 76628 | 613020 | 41 | 1 | 0 | 816 | 617868 | 41 | 1 | 0 | 1 | 824 |
| 84 | 54 | 2 | 02 | 42 | 0 | 76732 | 613852 | 42 | 0 | 0 | 832 | 618708 | 42 | 0 | 0 | 1 | 840 |
| 85 | 55 | 2 | 02 | 42 | 1 | 76836 | 614684 | 42 | 1 | 0 | 832 | 619548 | 42 | 1 | 0 | 1 | 840 |
| 86 | 56 | 2 | 02 | 43 | 0 | 76942 | 615532 | 43 | 0 | 0 | 848 | 620404 | 43 | 0 | 0 | 1 | 856 |
| 87 | 57 | 2 | 02 | 43 | 1 | 77048 | 616380 | 43 | 1 | 0 | 848 | 621260 | 43 | 1 | 0 | 1 | 856 |
| 88 | 58 | 2 | 02 | 44 | 0 | 77156 | 617244 | 44 | 0 | 0 | 864 | 622132 | 44 | 0 | 0 | 1 | 872 |
| 89 | 59 | 2 | 02 | 44 | 1 | 77264 | 618108 | 44 | 1 | 0 | 864 | 623004 | 44 | 1 | 0 | 1 | 872 |
| 90 | 5A | 2 | 02 | 45 | 0 | 77374 | 618988 | 45 | 0 | 0 | 880 | 623892 | 45 | 0 | 0 | 1 | 888 |
| 91 | 5B | 2 | 02 | 45 | 1 | 77484 | 619868 | 45 | 1 | 0 | 880 | 624780 | 45 | 1 | 0 | 1 | 888 |
| 92 | 5C | 2 | 02 | 46 | 0 | 77596 | 620764 | 46 | 0 | 0 | 896 | 625684 | 46 | 0 | 0 | 1 | 904 |
| 93 | 5D | 2 | 02 | 46 | 1 | 77708 | 621660 | 46 | 1 | 0 | 896 | 626588 | 46 | 1 | 0 | 1 | 904 |
| 94 | 5E | 2 | 02 | 47 | 0 | 77822 | 622572 | 47 | 0 | 0 | 912 | 627508 | 47 | 0 | 0 | 1 | 920 |
| 95 | 5F | 2 | 02 | 47 | 1 | 77936 | 623484 | 47 | 1 | 0 | 912 | 628428 | 47 | 1 | 0 | 1 | 920 |
| 96 | 60 | 2 | 02 | 48 | 0 | 78052 | 624412 | 48 | 0 | 0 | 928 | 629364 | 48 | 0 | 0 | 1 | 936 |
| 97 | 61 | 2 | 02 | 48 | 1 | 78168 | 625340 | 48 | 1 | 0 | 928 | 630300 | 48 | 1 | 0 | 1 | 936 |
| 98 | 62 | 2 | 02 | 49 | 0 | 78286 | 626284 | 49 | 0 | 0 | 944 | 631252 | 49 | 0 | 0 | 1 | 952 |
| 99 | 63 | 2 | 02 | 49 | 1 | 78404 | 627228 | 49 | 1 | 0 | 944 | 632204 | 49 | 1 | 0 | 1 | 952 |
| 100 | 64 | 2 | 02 | 50 | 0 | 78524 | 628188 | 50 | 0 | 0 | 960 | 633172 | 50 | 0 | 0 | 1 | 968 |
| 101 | 65 | 2 | 02 | 50 | 1 | 78644 | 629148 | 50 | 1 | 0 | 960 | 634140 | 50 | 1 | 0 | 1 | 968 |
| 102 | 66 | 2 | 02 | 51 | 0 | 78766 | 630124 | 51 | 0 | 0 | 976 | 635124 | 51 | 0 | 0 | 1 | 984 |

| | | | | | | | | | | | | | | | | | |
|---|---|---|---|---|---|---|---|---|---|---|---|---|---|---|---|---|---|
| 103 | 67 | 2 | 02 | 51 | 1 | 78888 | 631100 | 51 | 1 | 0 | 976 | 636108 | 51 | 1 | 0 | 1 | 984 |
| 104 | 68 | 2 | 02 | 52 | 0 | 79012 | 632092 | 52 | 0 | 0 | 992 | 637108 | 52 | 0 | 0 | 1 | 1000 |
| 105 | 69 | 2 | 02 | 52 | 1 | 79136 | 633084 | 52 | 1 | 0 | 992 | 638108 | 52 | 1 | 0 | 1 | 1000 |
| 106 | 6A | 2 | 02 | 53 | 0 | 79262 | 634092 | 53 | 0 | 0 | 1008 | 639124 | 53 | 0 | 0 | 1 | 1016 |
| 107 | 6B | 2 | 02 | 53 | 1 | 79388 | 635100 | 53 | 1 | 0 | 1008 | 640140 | 53 | 1 | 0 | 1 | 1016 |
| 108 | 6C | 2 | 02 | 54 | 0 | 79516 | 636124 | 54 | 0 | 0 | 1024 | 641172 | 54 | 0 | 0 | 1 | 1032 |
| 109 | 6D | 2 | 02 | 54 | 1 | 79644 | 637148 | 54 | 1 | 0 | 1024 | 642204 | 54 | 1 | 0 | 1 | 1032 |
| 110 | 6E | 2 | 02 | 55 | 0 | 79774 | 638188 | 55 | 0 | 0 | 1040 | 643252 | 55 | 0 | 0 | 1 | 1048 |
| 111 | 6F | 2 | 02 | 55 | 1 | 79904 | 639228 | 55 | 1 | 0 | 1040 | 644300 | 55 | 1 | 0 | 1 | 1048 |
| 112 | 70 | 2 | 02 | 56 | 0 | 80036 | 640284 | 56 | 0 | 0 | 1056 | 645364 | 56 | 0 | 0 | 1 | 1064 |
| 113 | 71 | 2 | 02 | 56 | 1 | 80168 | 641340 | 56 | 1 | 0 | 1056 | 646428 | 56 | 1 | 0 | 1 | 1064 |
| 114 | 72 | 2 | 02 | 57 | 0 | 80302 | 642412 | 57 | 0 | 0 | 1072 | 647508 | 57 | 0 | 0 | 1 | 1080 |
| 115 | 73 | 2 | 02 | 57 | 1 | 80436 | 643484 | 57 | 1 | 0 | 1072 | 648588 | 57 | 1 | 0 | 1 | 1080 |
| 116 | 74 | 2 | 02 | 58 | 0 | 80572 | 644572 | 58 | 0 | 0 | 1088 | 649684 | 58 | 0 | 0 | 1 | 1096 |
| 117 | 75 | 2 | 02 | 58 | 1 | 80708 | 645660 | 58 | 1 | 0 | 1088 | 650780 | 58 | 1 | 0 | 1 | 1096 |
| 118 | 76 | 2 | 02 | 59 | 0 | 80846 | 646764 | 59 | 0 | 0 | 1104 | 651892 | 59 | 0 | 0 | 1 | 1112 |
| 119 | 77 | 2 | 02 | 59 | 1 | 80984 | 647868 | 59 | 1 | 0 | 1104 | 653004 | 59 | 1 | 0 | 1 | 1112 |
| 120 | 78 | 2 | 02 | 60 | 0 | 81124 | 648988 | 60 | 0 | 0 | 1120 | 654132 | 60 | 0 | 0 | 1 | 1128 |
| 121 | 79 | 2 | 02 | 60 | 1 | 81264 | 650108 | 60 | 1 | 0 | 1120 | 655260 | 60 | 1 | 0 | 1 | 1128 |
| 122 | 7A | 2 | 02 | 61 | 0 | 81406 | 651244 | 61 | 0 | 0 | 1136 | 656404 | 61 | 0 | 0 | 1 | 1144 |
| 123 | 7B | 2 | 02 | 61 | 1 | 81548 | 652380 | 61 | 1 | 0 | 1136 | 657548 | 61 | 1 | 0 | 1 | 1144 |
| 124 | 7C | 2 | 02 | 62 | 0 | 81692 | 653532 | 62 | 0 | 0 | 1152 | 658708 | 62 | 0 | 0 | 1 | 1160 |
| 125 | 7D | 2 | 02 | 62 | 1 | 81836 | 654684 | 62 | 1 | 0 | 1152 | 659868 | 62 | 1 | 0 | 1 | 1160 |
| 126 | 7E | 2 | 02 | 63 | 0 | 81982 | 655852 | 63 | 0 | 0 | 1168 | 661044 | 63 | 0 | 0 | 1 | 1176 |
| 127 | 7F | 2 | 02 | 63 | 1 | 82128 | 657020 | 63 | 1 | 0 | 1168 | 662220 | 63 | 1 | 0 | 1 | 1176 |
| 128 | 80 | 2 | 02 | 64 | 0 | 82276 | 658204 | 64 | 0 | 0 | 1184 | 663412 | 64 | 0 | 0 | 1 | 1192 |
| 129 | 81 | 2 | 02 | 64 | 1 | 82424 | 659388 | 64 | 1 | 0 | 1184 | 664604 | 64 | 1 | 0 | 1 | 1192 |
| 130 | 82 | 2 | 02 | 65 | 0 | 82574 | 660588 | 65 | 0 | 0 | 1200 | 665812 | 65 | 0 | 0 | 1 | 1208 |
| 131 | 83 | 2 | 02 | 65 | 1 | 82724 | 661788 | 65 | 1 | 0 | 1200 | 667020 | 65 | 1 | 0 | 1 | 1208 |
| 132 | 84 | 2 | 02 | 66 | 0 | 82876 | 663004 | 66 | 0 | 0 | 1216 | 668244 | 66 | 0 | 0 | 1 | 1224 |
| 133 | 85 | 2 | 02 | 66 | 1 | 83028 | 664220 | 66 | 1 | 0 | 1216 | 669468 | 66 | 1 | 0 | 1 | 1224 |
| 134 | 86 | 2 | 02 | 67 | 0 | 83182 | 665452 | 67 | 0 | 0 | 1232 | 670708 | 67 | 0 | 0 | 1 | 1240 |
| 135 | 87 | 2 | 02 | 67 | 1 | 83336 | 666684 | 67 | 1 | 0 | 1232 | 671948 | 67 | 1 | 0 | 1 | 1240 |
| 136 | 88 | 2 | 02 | 68 | 0 | 83492 | 667932 | 68 | 0 | 0 | 1248 | 673204 | 68 | 0 | 0 | 1 | 1256 |
| 137 | 89 | 2 | 02 | 68 | 1 | 83648 | 669180 | 68 | 1 | 0 | 1248 | 674460 | 68 | 1 | 0 | 1 | 1256 |
| 138 | 8A | 2 | 02 | 69 | 0 | 83806 | 670444 | 69 | 0 | 0 | 1264 | 675732 | 69 | 0 | 0 | 1 | 1272 |
| 139 | 8B | 2 | 02 | 69 | 1 | 83964 | 671708 | 69 | 1 | 0 | 1264 | 677004 | 69 | 1 | 0 | 1 | 1272 |
| 140 | 8C | 2 | 02 | 70 | 0 | 84124 | 672988 | 70 | 0 | 0 | 1280 | 678292 | 70 | 0 | 0 | 1 | 1288 |
| 141 | 8D | 2 | 02 | 70 | 1 | 84284 | 674268 | 70 | 1 | 0 | 1280 | 679580 | 70 | 1 | 0 | 1 | 1288 |
| 142 | 8E | 2 | 02 | 71 | 0 | 84446 | 675564 | 71 | 0 | 0 | 1296 | 680884 | 71 | 0 | 0 | 1 | 1304 |
| 143 | 8F | 2 | 02 | 71 | 1 | 84608 | 676860 | 71 | 1 | 0 | 1296 | 682188 | 71 | 1 | 0 | 1 | 1304 |
| 144 | 90 | 2 | 02 | 72 | 0 | 84772 | 678172 | 72 | 0 | 0 | 1312 | 683508 | 72 | 0 | 0 | 1 | 1320 |
| 145 | 91 | 2 | 02 | 72 | 1 | 84936 | 679484 | 72 | 1 | 0 | 1312 | 684828 | 72 | 1 | 0 | 1 | 1320 |
| 146 | 92 | 2 | 02 | 73 | 0 | 85102 | 680812 | 73 | 0 | 0 | 1328 | 686164 | 73 | 0 | 0 | 1 | 1336 |
| 147 | 93 | 2 | 02 | 73 | 1 | 85268 | 682140 | 73 | 1 | 0 | 1328 | 687500 | 73 | 1 | 0 | 1 | 1336 |
| 148 | 94 | 2 | 02 | 74 | 0 | 85436 | 683484 | 74 | 0 | 0 | 1344 | 688852 | 74 | 0 | 0 | 1 | 1352 |
| 149 | 95 | 2 | 02 | 74 | 1 | 85604 | 684828 | 74 | 1 | 0 | 1344 | 690204 | 74 | 1 | 0 | 1 | 1352 |
| 150 | 96 | 2 | 02 | 75 | 0 | 85774 | 686188 | 75 | 0 | 0 | 1360 | 691572 | 75 | 0 | 0 | 1 | 1368 |
| 151 | 97 | 2 | 02 | 75 | 1 | 85944 | 687548 | 75 | 1 | 0 | 1360 | 692940 | 75 | 1 | 0 | 1 | 1368 |
| 152 | 98 | 2 | 02 | 76 | 0 | 86116 | 688924 | 76 | 0 | 0 | 1376 | 694324 | 76 | 0 | 0 | 1 | 1384 |
| 153 | 99 | 2 | 02 | 76 | 1 | 86288 | 690300 | 76 | 1 | 0 | 1376 | 695708 | 76 | 1 | 0 | 1 | 1384 |
| 154 | 9A | 2 | 02 | 77 | 0 | 86462 | 691692 | 77 | 0 | 0 | 1392 | 697108 | 77 | 0 | 0 | 1 | 1400 |
| 155 | 9B | 2 | 02 | 77 | 1 | 86636 | 693084 | 77 | 1 | 0 | 1392 | 698508 | 77 | 1 | 0 | 1 | 1400 |
| 156 | 9C | 2 | 02 | 78 | 0 | 86812 | 694492 | 78 | 0 | 0 | 1408 | 699924 | 78 | 0 | 0 | 1 | 1416 |
| 157 | 9D | 2 | 02 | 78 | 1 | 86988 | 695900 | 78 | 1 | 0 | 1408 | 701340 | 78 | 1 | 0 | 1 | 1416 |
| 158 | 9E | 2 | 02 | 79 | 0 | 87166 | 697324 | 79 | 0 | 0 | 1424 | 702772 | 79 | 0 | 0 | 1 | 1432 |
| 159 | 9F | 2 | 02 | 79 | 1 | 87344 | 698748 | 79 | 1 | 0 | 1424 | 704204 | 79 | 1 | 0 | 1 | 1432 |
| 160 | A0 | 2 | 02 | 80 | 0 | 87524 | 700188 | 80 | 0 | 0 | 1440 | 705652 | 80 | 0 | 0 | 1 | 1448 |
| 161 | A1 | 2 | 02 | 80 | 1 | 87704 | 701628 | 80 | 1 | 0 | 1440 | 707100 | 80 | 1 | 0 | 1 | 1448 |
| 162 | A2 | 2 | 02 | 81 | 0 | 87886 | 703084 | 81 | 0 | 0 | 1456 | 708564 | 81 | 0 | 0 | 1 | 1464 |
| 163 | A3 | 2 | 02 | 81 | 1 | 88068 | 704540 | 81 | 1 | 0 | 1456 | 710028 | 81 | 1 | 0 | 1 | 1464 |
| 164 | A4 | 2 | 02 | 82 | 0 | 88252 | 706012 | 82 | 0 | 0 | 1472 | 711508 | 82 | 0 | 0 | 1 | 1480 |
| 165 | A5 | 2 | 02 | 82 | 1 | 88436 | 707484 | 82 | 1 | 0 | 1472 | 712988 | 82 | 1 | 0 | 1 | 1480 |
| 166 | A6 | 2 | 02 | 83 | 0 | 88622 | 708972 | 83 | 0 | 0 | 1488 | 714484 | 83 | 0 | 0 | 1 | 1496 |
| 167 | A7 | 2 | 02 | 83 | 1 | 88808 | 710460 | 83 | 1 | 0 | 1488 | 715980 | 83 | 1 | 0 | 1 | 1496 |
| 168 | A8 | 2 | 02 | 84 | 0 | 88996 | 711964 | 84 | 0 | 0 | 1504 | 717492 | 84 | 0 | 0 | 1 | 1512 |
| 169 | A9 | 2 | 02 | 84 | 1 | 89184 | 713468 | 84 | 1 | 0 | 1504 | 719004 | 84 | 1 | 0 | 1 | 1512 |
| 170 | AA | 2 | 02 | 85 | 0 | 89374 | 714988 | 85 | 0 | 0 | 1520 | 720532 | 85 | 0 | 0 | 1 | 1528 |
| 171 | AB | 2 | 02 | 85 | 1 | 89564 | 716508 | 85 | 1 | 0 | 1520 | 722060 | 85 | 1 | 0 | 1 | 1528 |
| 172 | AC | 2 | 02 | 86 | 0 | 89756 | 718044 | 86 | 0 | 0 | 1536 | 723604 | 86 | 0 | 0 | 1 | 1544 |
| 173 | AD | 2 | 02 | 86 | 1 | 89948 | 719580 | 86 | 1 | 0 | 1536 | 725148 | 86 | 1 | 0 | 1 | 1544 |
| 174 | AE | 2 | 02 | 87 | 0 | 90142 | 721132 | 87 | 0 | 0 | 1552 | 726708 | 87 | 0 | 0 | 1 | 1560 |
| 175 | AF | 2 | 02 | 87 | 1 | 90336 | 722684 | 87 | 1 | 0 | 1552 | 728268 | 87 | 1 | 0 | 1 | 1560 |
| 176 | B0 | 2 | 02 | 88 | 0 | 90532 | 724252 | 88 | 0 | 0 | 1568 | 729844 | 88 | 0 | 0 | 1 | 1576 |
| 177 | B1 | 2 | 02 | 88 | 1 | 90728 | 725820 | 88 | 1 | 0 | 1568 | 731420 | 88 | 1 | 0 | 1 | 1576 |
| 178 | B2 | 2 | 02 | 89 | 0 | 90926 | 727404 | 89 | 0 | 0 | 1584 | 733012 | 89 | 0 | 0 | 1 | 1592 |
| 179 | B3 | 2 | 02 | 89 | 1 | 91124 | 728988 | 89 | 1 | 0 | 1584 | 734604 | 89 | 1 | 0 | 1 | 1592 |
| 180 | B4 | 2 | 02 | 90 | 0 | 91324 | 730588 | 90 | 0 | 0 | 1600 | 736212 | 90 | 0 | 0 | 1 | 1608 |
| 181 | B5 | 2 | 02 | 90 | 1 | 91524 | 732188 | 90 | 1 | 0 | 1600 | 737820 | 90 | 1 | 0 | 1 | 1608 |
| 182 | B6 | 2 | 02 | 91 | 0 | 91726 | 733804 | 91 | 0 | 0 | 1616 | 739444 | 91 | 0 | 0 | 1 | 1624 |
| 183 | B7 | 2 | 02 | 91 | 1 | 91928 | 735420 | 91 | 1 | 0 | 1616 | 741068 | 91 | 1 | 0 | 1 | 1624 |
| 184 | B8 | 2 | 02 | 92 | 0 | 92132 | 737052 | 92 | 0 | 0 | 1632 | 742708 | 92 | 0 | 0 | 1 | 1640 |
| 185 | B9 | 2 | 02 | 92 | 1 | 92336 | 738684 | 92 | 1 | 0 | 1632 | 744348 | 92 | 1 | 0 | 1 | 1640 |
| 186 | BA | 2 | 02 | 93 | 0 | 92542 | 740332 | 93 | 0 | 0 | 1648 | 746004 | 93 | 0 | 0 | 1 | 1656 |
| 187 | BB | 2 | 02 | 93 | 1 | 92748 | 741980 | 93 | 1 | 0 | 1648 | 747660 | 93 | 1 | 0 | 1 | 1656 |
| 188 | BC | 2 | 02 | 94 | 0 | 92956 | 743644 | 94 | 0 | 0 | 1664 | 749332 | 94 | 0 | 0 | 1 | 1672 |
| 189 | BD | 2 | 02 | 94 | 1 | 93164 | 745308 | 94 | 1 | 0 | 1664 | 751004 | 94 | 1 | 0 | 1 | 1672 |
| 190 | BE | 2 | 02 | 95 | 0 | 93374 | 746988 | 95 | 0 | 0 | 1680 | 752692 | 95 | 0 | 0 | 1 | 1688 |
| 191 | BF | 2 | 02 | 95 | 1 | 93584 | 748668 | 95 | 1 | 0 | 1680 | 754380 | 95 | 1 | 0 | 1 | 1688 |
| 192 | C0 | 2 | 02 | 96 | 0 | 93796 | 750364 | 96 | 0 | 0 | 1696 | 756084 | 96 | 0 | 0 | 1 | 1704 |
| 193 | C1 | 2 | 02 | 96 | 1 | 94008 | 752060 | 96 | 1 | 0 | 1696 | 757788 | 96 | 1 | 0 | 1 | 1704 |
| 194 | C2 | 2 | 02 | 97 | 0 | 94222 | 753772 | 97 | 0 | 0 | 1712 | 759508 | 97 | 0 | 0 | 1 | 1720 |
| 195 | C3 | 2 | 02 | 97 | 1 | 94436 | 755484 | 97 | 1 | 0 | 1712 | 761228 | 97 | 1 | 0 | 1 | 1720 |
| 196 | C4 | 2 | 02 | 98 | 0 | 94652 | 757212 | 98 | 0 | 0 | 1728 | 762964 | 98 | 0 | 0 | 1 | 1736 |
| 197 | C5 | 2 | 02 | 98 | 1 | 94868 | 758940 | 98 | 1 | 0 | 1728 | 764700 | 98 | 1 | 0 | 1 | 1736 |
| 198 | C6 | 2 | 02 | 99 | 0 | 95086 | 760684 | 99 | 0 | 0 | 1744 | 766452 | 99 | 0 | 0 | 1 | 1752 |
| 199 | C7 | 2 | 02 | 99 | 1 | 95304 | 762428 | 99 | 1 | 0 | 1744 | 768204 | 99 | 1 | 0 | 1 | 1752 |
| 200 | C8 | 2 | 02 | 100 | 0 | 95524 | 764188 | 100 | 0 | 0 | 1760 | 769972 | 100 | 0 | 0 | 1 | 1768 |
| 201 | C9 | 2 | 02 | 100 | 1 | 95744 | 765948 | 100 | 1 | 0 | 1760 | 771740 | 100 | 1 | 0 | 1 | 1768 |
| 202 | CA | 2 | 02 | 101 | 0 | 95966 | 767724 | 101 | 0 | 0 | 1776 | 773524 | 101 | 0 | 0 | 1 | 1784 |
| 203 | CB | 2 | 02 | 101 | 1 | 96188 | 769500 | 101 | 1 | 0 | 1776 | 775308 | 101 | 1 | 0 | 1 | 1784 |
| 204 | CC | 2 | 02 | 102 | 0 | 96412 | 771292 | 102 | 0 | 0 | 1792 | 777108 | 102 | 0 | 0 | 1 | 1800 |
| 205 | CD | 2 | 02 | 102 | 1 | 96636 | 773084 | 102 | 1 | 0 | 1792 | 778908 | 102 | 1 | 0 | 1 | 1800 |

| | | | | | | | | | | | | | | | | | |
|---|---|---|---|---|---|---|---|---|---|---|---|---|---|---|---|---|---|
| 206 | CE | 2 | 02 | 103 | 0 | 96862 | 774892 | 103 | 0 | 0 | 1808 | 780724 | 103 | 0 | 0 | 1 | 1816 |
| 207 | CF | 2 | 02 | 103 | 1 | 97088 | 776700 | 103 | 1 | 0 | 1808 | 782540 | 103 | 1 | 0 | 1 | 1816 |
| 208 | D0 | 2 | 02 | 104 | 0 | 97316 | 778524 | 104 | 0 | 0 | 1824 | 784372 | 104 | 0 | 0 | 1 | 1832 |
| 209 | D1 | 2 | 02 | 104 | 1 | 97544 | 780348 | 104 | 1 | 0 | 1824 | 786204 | 104 | 1 | 0 | 1 | 1832 |
| 210 | D2 | 2 | 02 | 105 | 0 | 97774 | 782188 | 105 | 0 | 0 | 1840 | 788052 | 105 | 0 | 0 | 1 | 1848 |
| 211 | D3 | 2 | 02 | 105 | 1 | 98004 | 784028 | 105 | 1 | 0 | 1840 | 789900 | 105 | 1 | 0 | 1 | 1848 |
| 212 | D4 | 2 | 02 | 106 | 0 | 98236 | 785884 | 106 | 0 | 0 | 1856 | 791764 | 106 | 0 | 0 | 1 | 1864 |
| 213 | D5 | 2 | 02 | 106 | 1 | 98468 | 787740 | 106 | 1 | 0 | 1856 | 793628 | 106 | 1 | 0 | 1 | 1864 |
| 214 | D6 | 2 | 02 | 107 | 0 | 98702 | 789612 | 107 | 0 | 0 | 1872 | 795508 | 107 | 0 | 0 | 1 | 1880 |
| 215 | D7 | 2 | 02 | 107 | 1 | 98936 | 791484 | 107 | 1 | 0 | 1872 | 797388 | 107 | 1 | 0 | 1 | 1880 |
| 216 | D8 | 2 | 02 | 108 | 0 | 99172 | 793372 | 108 | 0 | 0 | 1888 | 799284 | 108 | 0 | 0 | 1 | 1896 |
| 217 | D9 | 2 | 02 | 108 | 1 | 99408 | 795260 | 108 | 1 | 0 | 1888 | 801180 | 108 | 1 | 0 | 1 | 1896 |
| 218 | DA | 2 | 02 | 109 | 0 | 99646 | 797164 | 109 | 0 | 0 | 1904 | 803092 | 109 | 0 | 0 | 1 | 1912 |
| 219 | DB | 2 | 02 | 109 | 1 | 99884 | 799068 | 109 | 1 | 0 | 1904 | 805004 | 109 | 1 | 0 | 1 | 1912 |
| 220 | DC | 2 | 02 | 110 | 0 | 100124 | 800988 | 110 | 0 | 0 | 1920 | 806932 | 110 | 0 | 0 | 1 | 1928 |
| 221 | DD | 2 | 02 | 110 | 1 | 100364 | 802908 | 110 | 1 | 0 | 1920 | 808860 | 110 | 1 | 0 | 1 | 1928 |
| 222 | DE | 2 | 02 | 111 | 0 | 100606 | 804844 | 111 | 0 | 0 | 1936 | 810804 | 111 | 0 | 0 | 1 | 1944 |
| 223 | DF | 2 | 02 | 111 | 1 | 100848 | 806780 | 111 | 1 | 0 | 1936 | 812748 | 111 | 1 | 0 | 1 | 1944 |
| 224 | E0 | 2 | 02 | 112 | 0 | 101092 | 808732 | 112 | 0 | 0 | 1952 | 814708 | 112 | 0 | 0 | 1 | 1960 |
| 225 | E1 | 2 | 02 | 112 | 1 | 101336 | 810684 | 112 | 1 | 0 | 1952 | 816668 | 112 | 1 | 0 | 1 | 1960 |
| 226 | E2 | 2 | 02 | 113 | 0 | 101582 | 812652 | 113 | 0 | 0 | 1968 | 818644 | 113 | 0 | 0 | 1 | 1976 |
| 227 | E3 | 2 | 02 | 113 | 1 | 101828 | 814620 | 113 | 1 | 0 | 1968 | 820620 | 113 | 1 | 0 | 1 | 1976 |
| 228 | E4 | 2 | 02 | 114 | 0 | 102076 | 816604 | 114 | 0 | 0 | 1984 | 822612 | 114 | 0 | 0 | 1 | 1992 |
| 229 | E5 | 2 | 02 | 114 | 1 | 102324 | 818588 | 114 | 1 | 0 | 1984 | 824604 | 114 | 1 | 0 | 1 | 1992 |
| 230 | E6 | 2 | 02 | 115 | 0 | 102574 | 820588 | 115 | 0 | 0 | 2000 | 826612 | 115 | 0 | 0 | 1 | 2008 |
| 231 | E7 | 2 | 02 | 115 | 1 | 102824 | 822588 | 115 | 1 | 0 | 2000 | 828620 | 115 | 1 | 0 | 1 | 2008 |
| 232 | E8 | 2 | 02 | 116 | 0 | 103076 | 824604 | 116 | 0 | 0 | 2016 | 830644 | 116 | 0 | 0 | 1 | 2024 |
| 233 | E9 | 2 | 02 | 116 | 1 | 103328 | 826620 | 116 | 1 | 0 | 2016 | 832668 | 116 | 1 | 0 | 1 | 2024 |
| 234 | EA | 2 | 02 | 117 | 0 | 103582 | 828652 | 117 | 0 | 0 | 2032 | 834708 | 117 | 0 | 0 | 1 | 2040 |
| 235 | EB | 2 | 02 | 117 | 1 | 103836 | 830684 | 117 | 1 | 0 | 2032 | 836748 | 117 | 1 | 0 | 1 | 2040 |
| 236 | EC | 2 | 02 | 118 | 0 | 104092 | 832732 | 118 | 0 | 0 | 2048 | 838804 | 118 | 0 | 0 | 1 | 2056 |
| 237 | ED | 2 | 02 | 118 | 1 | 104348 | 834780 | 118 | 1 | 0 | 2048 | 840860 | 118 | 1 | 0 | 1 | 2056 |
| 238 | EE | 2 | 02 | 119 | 0 | 104606 | 836844 | 119 | 0 | 0 | 2064 | 842932 | 119 | 0 | 0 | 1 | 2072 |
| 239 | EF | 2 | 02 | 119 | 1 | 104864 | 838908 | 119 | 1 | 0 | 2064 | 845004 | 119 | 1 | 0 | 1 | 2072 |
| 240 | F0 | 2 | 02 | 120 | 0 | 105124 | 840988 | 120 | 0 | 0 | 2080 | 847092 | 120 | 0 | 0 | 1 | 2088 |
| 241 | F1 | 2 | 02 | 120 | 1 | 105384 | 843068 | 120 | 1 | 0 | 2080 | 849180 | 120 | 1 | 0 | 1 | 2088 |
| 242 | F2 | 2 | 02 | 121 | 0 | 105646 | 845164 | 121 | 0 | 0 | 2096 | 851284 | 121 | 0 | 0 | 1 | 2104 |
| 243 | F3 | 2 | 02 | 121 | 1 | 105908 | 847260 | 121 | 1 | 0 | 2096 | 853388 | 121 | 1 | 0 | 1 | 2104 |
| 244 | F4 | 2 | 02 | 122 | 0 | 106172 | 849372 | 122 | 0 | 0 | 2112 | 855508 | 122 | 0 | 0 | 1 | 2120 |
| 245 | F5 | 2 | 02 | 122 | 1 | 106436 | 851484 | 122 | 1 | 0 | 2112 | 857628 | 122 | 1 | 0 | 1 | 2120 |
| 246 | F6 | 2 | 02 | 123 | 0 | 106702 | 853612 | 123 | 0 | 0 | 2128 | 859764 | 123 | 0 | 0 | 1 | 2136 |
| 247 | F7 | 2 | 02 | 123 | 1 | 106968 | 855740 | 123 | 1 | 0 | 2128 | 861900 | 123 | 1 | 0 | 1 | 2136 |
| 248 | F8 | 2 | 02 | 124 | 0 | 107236 | 857884 | 124 | 0 | 0 | 2144 | 864052 | 124 | 0 | 0 | 1 | 2152 |
| 249 | F9 | 2 | 02 | 124 | 1 | 107504 | 860028 | 124 | 1 | 0 | 2144 | 866204 | 124 | 1 | 0 | 1 | 2152 |
| 250 | FA | 2 | 02 | 125 | 0 | 107774 | 862188 | 125 | 0 | 0 | 2160 | 868372 | 125 | 0 | 0 | 1 | 2168 |
| 251 | FB | 2 | 02 | 125 | 1 | 108044 | 864348 | 125 | 1 | 0 | 2160 | 870540 | 125 | 1 | 0 | 1 | 2168 |
| 252 | FC | 2 | 02 | 126 | 0 | 108316 | 866524 | 126 | 0 | 0 | 2176 | 872724 | 126 | 0 | 0 | 1 | 2184 |
| 253 | FD | 2 | 02 | 126 | 1 | 108588 | 868700 | 126 | 1 | 0 | 2176 | 874908 | 126 | 1 | 0 | 1 | 2184 |
| 254 | FE | 2 | 02 | 127 | 0 | 108862 | 870892 | 127 | 0 | 0 | 2192 | 877108 | 127 | 0 | 0 | 1 | 2200 |
| 255 | FF | 2 | 02 | 127 | 1 | 109136 | 873084 | 127 | 1 | 0 | 2192 | 879308 | 127 | 1 | 0 | 1 | 2200 |
| 0 | 00 | 3 | 03 | 0 | 0 | 109148 | 873180 | 0 | 0 | 0 | 96 | 879412 | 0 | 0 | 0 | 1 | 104 |
| 1 | 01 | 3 | 03 | 0 | 1 | 109162 | 873292 | 0 | 1 | 0 | 112 | 879532 | 0 | 1 | 0 | 1 | 120 |
| 2 | 02 | 3 | 03 | 0 | 2 | 109176 | 873404 | 0 | 2 | 0 | 112 | 879652 | 0 | 2 | 0 | 1 | 120 |
| 3 | 03 | 3 | 03 | 1 | 0 | 109188 | 873500 | 1 | 0 | 0 | 96 | 879756 | 1 | 0 | 0 | 1 | 104 |
| 4 | 04 | 3 | 03 | 1 | 1 | 109205 | 873636 | 1 | 1 | 0 | 136 | 879900 | 1 | 1 | 0 | 1 | 144 |
| 5 | 05 | 3 | 03 | 1 | 2 | 109222 | 873772 | 1 | 2 | 0 | 136 | 880044 | 1 | 2 | 0 | 1 | 144 |
| 6 | 06 | 3 | 03 | 2 | 0 | 109241 | 873924 | 2 | 0 | 0 | 152 | 880204 | 2 | 0 | 0 | 1 | 160 |
| 7 | 07 | 3 | 03 | 2 | 1 | 109260 | 874076 | 2 | 1 | 0 | 152 | 880364 | 2 | 1 | 0 | 1 | 160 |
| 8 | 08 | 3 | 03 | 2 | 2 | 109279 | 874228 | 2 | 2 | 0 | 152 | 880524 | 2 | 2 | 0 | 1 | 160 |
| 9 | 09 | 3 | 03 | 3 | 0 | 109300 | 874396 | 3 | 0 | 0 | 168 | 880700 | 3 | 0 | 0 | 1 | 176 |
| 10 | 0A | 3 | 03 | 3 | 1 | 109321 | 874564 | 3 | 1 | 0 | 168 | 880876 | 3 | 1 | 0 | 1 | 176 |
| 11 | 0B | 3 | 03 | 3 | 2 | 109342 | 874732 | 3 | 2 | 0 | 168 | 881052 | 3 | 2 | 0 | 1 | 176 |
| 12 | 0C | 3 | 03 | 4 | 0 | 109365 | 874916 | 4 | 0 | 0 | 184 | 881244 | 4 | 0 | 0 | 1 | 192 |
| 13 | 0D | 3 | 03 | 4 | 1 | 109388 | 875100 | 4 | 1 | 0 | 184 | 881436 | 4 | 1 | 0 | 1 | 192 |
| 14 | 0E | 3 | 03 | 4 | 2 | 109411 | 875284 | 4 | 2 | 0 | 184 | 881628 | 4 | 2 | 0 | 1 | 192 |
| 15 | 0F | 3 | 03 | 5 | 0 | 109436 | 875484 | 5 | 0 | 0 | 200 | 881836 | 5 | 0 | 0 | 1 | 208 |
| 16 | 10 | 3 | 03 | 5 | 1 | 109466 | 875724 | 5 | 1 | 0 | 240 | 882084 | 5 | 1 | 0 | 1 | 248 |
| 17 | 11 | 3 | 03 | 5 | 2 | 109496 | 875964 | 5 | 2 | 0 | 240 | 882332 | 5 | 2 | 0 | 1 | 248 |
| 18 | 12 | 3 | 03 | 6 | 0 | 109528 | 876220 | 6 | 0 | 0 | 256 | 882596 | 6 | 0 | 0 | 1 | 264 |
| 19 | 13 | 3 | 03 | 6 | 1 | 109560 | 876476 | 6 | 1 | 0 | 256 | 882860 | 6 | 1 | 0 | 1 | 264 |
| 20 | 14 | 3 | 03 | 6 | 2 | 109592 | 876732 | 6 | 2 | 0 | 256 | 883124 | 6 | 2 | 0 | 1 | 264 |
| 21 | 15 | 3 | 03 | 7 | 0 | 109626 | 877004 | 7 | 0 | 0 | 272 | 883404 | 7 | 0 | 0 | 1 | 280 |
| 22 | 16 | 3 | 03 | 7 | 1 | 109660 | 877276 | 7 | 1 | 0 | 272 | 883684 | 7 | 1 | 0 | 1 | 280 |
| 23 | 17 | 3 | 03 | 7 | 2 | 109694 | 877548 | 7 | 2 | 0 | 272 | 883964 | 7 | 2 | 0 | 1 | 280 |
| 24 | 18 | 3 | 03 | 8 | 0 | 109730 | 877836 | 8 | 0 | 0 | 288 | 884260 | 8 | 0 | 0 | 1 | 296 |
| 25 | 19 | 3 | 03 | 8 | 1 | 109766 | 878124 | 8 | 1 | 0 | 288 | 884556 | 8 | 1 | 0 | 1 | 296 |
| 26 | 1A | 3 | 03 | 8 | 2 | 109802 | 878412 | 8 | 2 | 0 | 288 | 884852 | 8 | 2 | 0 | 1 | 296 |
| 27 | 1B | 3 | 03 | 9 | 0 | 109840 | 878716 | 9 | 0 | 0 | 304 | 885164 | 9 | 0 | 0 | 1 | 312 |
| 28 | 1C | 3 | 03 | 9 | 1 | 109878 | 879020 | 9 | 1 | 0 | 304 | 885476 | 9 | 1 | 0 | 1 | 312 |
| 29 | 1D | 3 | 03 | 9 | 2 | 109916 | 879324 | 9 | 2 | 0 | 304 | 885788 | 9 | 2 | 0 | 1 | 312 |
| 30 | 1E | 3 | 03 | 10 | 0 | 109956 | 879644 | 10 | 0 | 0 | 320 | 886116 | 10 | 0 | 0 | 1 | 328 |
| 31 | 1F | 3 | 03 | 10 | 1 | 109996 | 879964 | 10 | 1 | 0 | 320 | 886444 | 10 | 1 | 0 | 1 | 328 |
| 32 | 20 | 3 | 03 | 10 | 2 | 110036 | 880284 | 10 | 2 | 0 | 320 | 886772 | 10 | 2 | 0 | 1 | 328 |
| 33 | 21 | 3 | 03 | 11 | 0 | 110078 | 880620 | 11 | 0 | 0 | 336 | 887116 | 11 | 0 | 0 | 1 | 344 |
| 34 | 22 | 3 | 03 | 11 | 1 | 110120 | 880956 | 11 | 1 | 0 | 336 | 887460 | 11 | 1 | 0 | 1 | 344 |
| 35 | 23 | 3 | 03 | 11 | 2 | 110162 | 881292 | 11 | 2 | 0 | 336 | 887804 | 11 | 2 | 0 | 1 | 344 |
| 36 | 24 | 3 | 03 | 12 | 0 | 110206 | 881644 | 12 | 0 | 0 | 352 | 888164 | 12 | 0 | 0 | 1 | 360 |
| 37 | 25 | 3 | 03 | 12 | 1 | 110250 | 881996 | 12 | 1 | 0 | 352 | 888524 | 12 | 1 | 0 | 1 | 360 |
| 38 | 26 | 3 | 03 | 12 | 2 | 110294 | 882348 | 12 | 2 | 0 | 352 | 888884 | 12 | 2 | 0 | 1 | 360 |
| 39 | 27 | 3 | 03 | 13 | 0 | 110340 | 882716 | 13 | 0 | 0 | 368 | 889260 | 13 | 0 | 0 | 1 | 376 |
| 40 | 28 | 3 | 03 | 13 | 1 | 110386 | 883084 | 13 | 1 | 0 | 368 | 889636 | 13 | 1 | 0 | 1 | 376 |
| 41 | 29 | 3 | 03 | 13 | 2 | 110432 | 883452 | 13 | 2 | 0 | 368 | 890012 | 13 | 2 | 0 | 1 | 376 |
| 42 | 2A | 3 | 03 | 14 | 0 | 110480 | 883836 | 14 | 0 | 0 | 384 | 890404 | 14 | 0 | 0 | 1 | 392 |
| 43 | 2B | 3 | 03 | 14 | 1 | 110528 | 884220 | 14 | 1 | 0 | 384 | 890796 | 14 | 1 | 0 | 1 | 392 |
| 44 | 2C | 3 | 03 | 14 | 2 | 110576 | 884604 | 14 | 2 | 0 | 384 | 891188 | 14 | 2 | 0 | 1 | 392 |
| 45 | 2D | 3 | 03 | 15 | 0 | 110626 | 885004 | 15 | 0 | 0 | 400 | 891596 | 15 | 0 | 0 | 1 | 408 |
| 46 | 2E | 3 | 03 | 15 | 1 | 110676 | 885404 | 15 | 1 | 0 | 400 | 892004 | 15 | 1 | 0 | 1 | 408 |
| 47 | 2F | 3 | 03 | 15 | 2 | 110726 | 885804 | 15 | 2 | 0 | 400 | 892412 | 15 | 2 | 0 | 1 | 408 |
| 48 | 30 | 3 | 03 | 16 | 0 | 110778 | 886220 | 16 | 0 | 0 | 416 | 892836 | 16 | 0 | 0 | 1 | 424 |
| 49 | 31 | 3 | 03 | 16 | 1 | 110830 | 886636 | 16 | 1 | 0 | 416 | 893260 | 16 | 1 | 0 | 1 | 424 |
| 50 | 32 | 3 | 03 | 16 | 2 | 110882 | 887052 | 16 | 2 | 0 | 416 | 893684 | 16 | 2 | 0 | 1 | 424 |
| 51 | 33 | 3 | 03 | 17 | 0 | 110936 | 887484 | 17 | 0 | 0 | 432 | 894124 | 17 | 0 | 0 | 1 | 440 |
| 52 | 34 | 3 | 03 | 17 | 1 | 110990 | 887916 | 17 | 1 | 0 | 432 | 894564 | 17 | 1 | 0 | 1 | 440 |

| | | | | | | | | | | | | | | | | | |
|---|---|---|---|---|---|---|---|---|---|---|---|---|---|---|---|---|---|
| 53 | 35 | 3 | 03 | 17 | 2 | 111044 | 888348 | 17 | 2 | 0 | 432 | 895004 | 17 | 2 | 0 | 1 | 440 |
| 54 | 36 | 3 | 03 | 18 | 0 | 111100 | 888796 | 18 | 0 | 0 | 448 | 895460 | 18 | 0 | 0 | 1 | 456 |
| 55 | 37 | 3 | 03 | 18 | 1 | 111156 | 889244 | 18 | 1 | 0 | 448 | 895916 | 18 | 1 | 0 | 1 | 456 |
| 56 | 38 | 3 | 03 | 18 | 2 | 111212 | 889692 | 18 | 2 | 0 | 448 | 896372 | 18 | 2 | 0 | 1 | 456 |
| 57 | 39 | 3 | 03 | 19 | 0 | 111270 | 890156 | 19 | 0 | 0 | 464 | 896844 | 19 | 0 | 0 | 1 | 472 |
| 58 | 3A | 3 | 03 | 19 | 1 | 111328 | 890620 | 19 | 1 | 0 | 464 | 897316 | 19 | 1 | 0 | 1 | 472 |
| 59 | 3B | 3 | 03 | 19 | 2 | 111386 | 891084 | 19 | 2 | 0 | 464 | 897788 | 19 | 2 | 0 | 1 | 472 |
| 60 | 3C | 3 | 03 | 20 | 0 | 111446 | 891564 | 20 | 0 | 0 | 480 | 898276 | 20 | 0 | 0 | 1 | 488 |
| 61 | 3D | 3 | 03 | 20 | 1 | 111506 | 892044 | 20 | 1 | 0 | 480 | 898764 | 20 | 1 | 0 | 1 | 488 |
| 62 | 3E | 3 | 03 | 20 | 2 | 111566 | 892524 | 20 | 2 | 0 | 480 | 899252 | 20 | 2 | 0 | 1 | 488 |
| 63 | 3F | 3 | 03 | 21 | 0 | 111628 | 893020 | 21 | 0 | 0 | 496 | 899756 | 21 | 0 | 0 | 1 | 504 |
| 64 | 40 | 3 | 03 | 21 | 1 | 111690 | 893516 | 21 | 1 | 0 | 496 | 900260 | 21 | 1 | 0 | 1 | 504 |
| 65 | 41 | 3 | 03 | 21 | 2 | 111752 | 894012 | 21 | 2 | 0 | 496 | 900764 | 21 | 2 | 0 | 1 | 504 |
| 66 | 42 | 3 | 03 | 22 | 0 | 111816 | 894524 | 22 | 0 | 0 | 512 | 901284 | 22 | 0 | 0 | 1 | 520 |
| 67 | 43 | 3 | 03 | 22 | 1 | 111880 | 895036 | 22 | 1 | 0 | 512 | 901804 | 22 | 1 | 0 | 1 | 520 |
| 68 | 44 | 3 | 03 | 22 | 2 | 111944 | 895548 | 22 | 2 | 0 | 512 | 902324 | 22 | 2 | 0 | 1 | 520 |
| 69 | 45 | 3 | 03 | 23 | 0 | 112010 | 896076 | 23 | 0 | 0 | 528 | 902860 | 23 | 0 | 0 | 1 | 536 |
| 70 | 46 | 3 | 03 | 23 | 1 | 112076 | 896604 | 23 | 1 | 0 | 528 | 903396 | 23 | 1 | 0 | 1 | 536 |
| 71 | 47 | 3 | 03 | 23 | 2 | 112142 | 897132 | 23 | 2 | 0 | 528 | 903932 | 23 | 2 | 0 | 1 | 536 |
| 72 | 48 | 3 | 03 | 24 | 0 | 112210 | 897676 | 24 | 0 | 0 | 544 | 904484 | 24 | 0 | 0 | 1 | 552 |
| 73 | 49 | 3 | 03 | 24 | 1 | 112278 | 898220 | 24 | 1 | 0 | 544 | 905036 | 24 | 1 | 0 | 1 | 552 |
| 74 | 4A | 3 | 03 | 24 | 2 | 112346 | 898764 | 24 | 2 | 0 | 544 | 905588 | 24 | 2 | 0 | 1 | 552 |
| 75 | 4B | 3 | 03 | 25 | 0 | 112416 | 899324 | 25 | 0 | 0 | 560 | 906156 | 25 | 0 | 0 | 1 | 568 |
| 76 | 4C | 3 | 03 | 25 | 1 | 112486 | 899884 | 25 | 1 | 0 | 560 | 906724 | 25 | 1 | 0 | 1 | 568 |
| 77 | 4D | 3 | 03 | 25 | 2 | 112556 | 900444 | 25 | 2 | 0 | 560 | 907292 | 25 | 2 | 0 | 1 | 568 |
| 78 | 4E | 3 | 03 | 26 | 0 | 112628 | 901020 | 26 | 0 | 0 | 576 | 907876 | 26 | 0 | 0 | 1 | 584 |
| 79 | 4F | 3 | 03 | 26 | 1 | 112700 | 901596 | 26 | 1 | 0 | 576 | 908460 | 26 | 1 | 0 | 1 | 584 |
| 80 | 50 | 3 | 03 | 26 | 2 | 112772 | 902172 | 26 | 2 | 0 | 576 | 909044 | 26 | 2 | 0 | 1 | 584 |
| 81 | 51 | 3 | 03 | 27 | 0 | 112846 | 902764 | 27 | 0 | 0 | 592 | 909644 | 27 | 0 | 0 | 1 | 600 |
| 82 | 52 | 3 | 03 | 27 | 1 | 112920 | 903356 | 27 | 1 | 0 | 592 | 910244 | 27 | 1 | 0 | 1 | 600 |
| 83 | 53 | 3 | 03 | 27 | 2 | 112994 | 903948 | 27 | 2 | 0 | 592 | 910844 | 27 | 2 | 0 | 1 | 600 |
| 84 | 54 | 3 | 03 | 28 | 0 | 113070 | 904556 | 28 | 0 | 0 | 608 | 911460 | 28 | 0 | 0 | 1 | 616 |
| 85 | 55 | 3 | 03 | 28 | 1 | 113146 | 905164 | 28 | 1 | 0 | 608 | 912076 | 28 | 1 | 0 | 1 | 616 |
| 86 | 56 | 3 | 03 | 28 | 2 | 113222 | 905772 | 28 | 2 | 0 | 608 | 912692 | 28 | 2 | 0 | 1 | 616 |
| 87 | 57 | 3 | 03 | 29 | 0 | 113300 | 906396 | 29 | 0 | 0 | 624 | 913324 | 29 | 0 | 0 | 1 | 632 |
| 88 | 58 | 3 | 03 | 29 | 1 | 113378 | 907020 | 29 | 1 | 0 | 624 | 913956 | 29 | 1 | 0 | 1 | 632 |
| 89 | 59 | 3 | 03 | 29 | 2 | 113456 | 907644 | 29 | 2 | 0 | 624 | 914588 | 29 | 2 | 0 | 1 | 632 |
| 90 | 5A | 3 | 03 | 30 | 0 | 113536 | 908284 | 30 | 0 | 0 | 640 | 915236 | 30 | 0 | 0 | 1 | 648 |
| 91 | 5B | 3 | 03 | 30 | 1 | 113616 | 908924 | 30 | 1 | 0 | 640 | 915884 | 30 | 1 | 0 | 1 | 648 |
| 92 | 5C | 3 | 03 | 30 | 2 | 113696 | 909564 | 30 | 2 | 0 | 640 | 916532 | 30 | 2 | 0 | 1 | 648 |
| 93 | 5D | 3 | 03 | 31 | 0 | 113778 | 910220 | 31 | 0 | 0 | 656 | 917196 | 31 | 0 | 0 | 1 | 664 |
| 94 | 5E | 3 | 03 | 31 | 1 | 113860 | 910876 | 31 | 1 | 0 | 656 | 917860 | 31 | 1 | 0 | 1 | 664 |
| 95 | 5F | 3 | 03 | 31 | 2 | 113942 | 911532 | 31 | 2 | 0 | 656 | 918524 | 31 | 2 | 0 | 1 | 664 |
| 96 | 60 | 3 | 03 | 32 | 0 | 114026 | 912204 | 32 | 0 | 0 | 672 | 919204 | 32 | 0 | 0 | 1 | 680 |
| 97 | 61 | 3 | 03 | 32 | 1 | 114110 | 912876 | 32 | 1 | 0 | 672 | 919884 | 32 | 1 | 0 | 1 | 680 |
| 98 | 62 | 3 | 03 | 32 | 2 | 114194 | 913548 | 32 | 2 | 0 | 672 | 920564 | 32 | 2 | 0 | 1 | 680 |
| 99 | 63 | 3 | 03 | 33 | 0 | 114280 | 914236 | 33 | 0 | 0 | 688 | 921260 | 33 | 0 | 0 | 1 | 696 |
| 100 | 64 | 3 | 03 | 33 | 1 | 114366 | 914924 | 33 | 1 | 0 | 688 | 921956 | 33 | 1 | 0 | 1 | 696 |
| 101 | 65 | 3 | 03 | 33 | 2 | 114452 | 915612 | 33 | 2 | 0 | 688 | 922652 | 33 | 2 | 0 | 1 | 696 |
| 102 | 66 | 3 | 03 | 34 | 0 | 114540 | 916316 | 34 | 0 | 0 | 704 | 923364 | 34 | 0 | 0 | 1 | 712 |
| 103 | 67 | 3 | 03 | 34 | 1 | 114628 | 917020 | 34 | 1 | 0 | 704 | 924076 | 34 | 1 | 0 | 1 | 712 |
| 104 | 68 | 3 | 03 | 34 | 2 | 114716 | 917724 | 34 | 2 | 0 | 704 | 924788 | 34 | 2 | 0 | 1 | 712 |
| 105 | 69 | 3 | 03 | 35 | 0 | 114806 | 918444 | 35 | 0 | 0 | 720 | 925516 | 35 | 0 | 0 | 1 | 728 |
| 106 | 6A | 3 | 03 | 35 | 1 | 114896 | 919164 | 35 | 1 | 0 | 720 | 926244 | 35 | 1 | 0 | 1 | 728 |
| 107 | 6B | 3 | 03 | 35 | 2 | 114986 | 919884 | 35 | 2 | 0 | 720 | 926972 | 35 | 2 | 0 | 1 | 728 |
| 108 | 6C | 3 | 03 | 36 | 0 | 115078 | 920620 | 36 | 0 | 0 | 736 | 927716 | 36 | 0 | 0 | 1 | 744 |
| 109 | 6D | 3 | 03 | 36 | 1 | 115170 | 921356 | 36 | 1 | 0 | 736 | 928460 | 36 | 1 | 0 | 1 | 744 |
| 110 | 6E | 3 | 03 | 36 | 2 | 115262 | 922092 | 36 | 2 | 0 | 736 | 929204 | 36 | 2 | 0 | 1 | 744 |
| 111 | 6F | 3 | 03 | 37 | 0 | 115356 | 922844 | 37 | 0 | 0 | 752 | 929964 | 37 | 0 | 0 | 1 | 760 |
| 112 | 70 | 3 | 03 | 37 | 1 | 115450 | 923596 | 37 | 1 | 0 | 752 | 930724 | 37 | 1 | 0 | 1 | 760 |
| 113 | 71 | 3 | 03 | 37 | 2 | 115544 | 924348 | 37 | 2 | 0 | 752 | 931484 | 37 | 2 | 0 | 1 | 760 |
| 114 | 72 | 3 | 03 | 38 | 0 | 115640 | 925116 | 38 | 0 | 0 | 768 | 932260 | 38 | 0 | 0 | 1 | 776 |
| 115 | 73 | 3 | 03 | 38 | 1 | 115736 | 925884 | 38 | 1 | 0 | 768 | 933036 | 38 | 1 | 0 | 1 | 776 |
| 116 | 74 | 3 | 03 | 38 | 2 | 115832 | 926652 | 38 | 2 | 0 | 768 | 933812 | 38 | 2 | 0 | 1 | 776 |
| 117 | 75 | 3 | 03 | 39 | 0 | 115930 | 927436 | 39 | 0 | 0 | 784 | 934604 | 39 | 0 | 0 | 1 | 792 |
| 118 | 76 | 3 | 03 | 39 | 1 | 116028 | 928220 | 39 | 1 | 0 | 784 | 935396 | 39 | 1 | 0 | 1 | 792 |
| 119 | 77 | 3 | 03 | 39 | 2 | 116126 | 929004 | 39 | 2 | 0 | 784 | 936188 | 39 | 2 | 0 | 1 | 792 |
| 120 | 78 | 3 | 03 | 40 | 0 | 116226 | 929804 | 40 | 0 | 0 | 800 | 936996 | 40 | 0 | 0 | 1 | 808 |
| 121 | 79 | 3 | 03 | 40 | 1 | 116326 | 930604 | 40 | 1 | 0 | 800 | 937804 | 40 | 1 | 0 | 1 | 808 |
| 122 | 7A | 3 | 03 | 40 | 2 | 116426 | 931404 | 40 | 2 | 0 | 800 | 938612 | 40 | 2 | 0 | 1 | 808 |
| 123 | 7B | 3 | 03 | 41 | 0 | 116528 | 932220 | 41 | 0 | 0 | 816 | 939436 | 41 | 0 | 0 | 1 | 824 |
| 124 | 7C | 3 | 03 | 41 | 1 | 116630 | 933036 | 41 | 1 | 0 | 816 | 940260 | 41 | 1 | 0 | 1 | 824 |
| 125 | 7D | 3 | 03 | 41 | 2 | 116732 | 933852 | 41 | 2 | 0 | 816 | 941084 | 41 | 2 | 0 | 1 | 824 |
| 126 | 7E | 3 | 03 | 42 | 0 | 116836 | 934684 | 42 | 0 | 0 | 832 | 941924 | 42 | 0 | 0 | 1 | 840 |
| 127 | 7F | 3 | 03 | 42 | 1 | 116940 | 935516 | 42 | 1 | 0 | 832 | 942764 | 42 | 1 | 0 | 1 | 840 |
| 128 | 80 | 3 | 03 | 42 | 2 | 117044 | 936348 | 42 | 2 | 0 | 832 | 943604 | 42 | 2 | 0 | 1 | 840 |
| 129 | 81 | 3 | 03 | 43 | 0 | 117150 | 937196 | 43 | 0 | 0 | 848 | 944460 | 43 | 0 | 0 | 1 | 856 |
| 130 | 82 | 3 | 03 | 43 | 1 | 117256 | 938044 | 43 | 1 | 0 | 848 | 945316 | 43 | 1 | 0 | 1 | 856 |
| 131 | 83 | 3 | 03 | 43 | 2 | 117362 | 938892 | 43 | 2 | 0 | 848 | 946172 | 43 | 2 | 0 | 1 | 856 |
| 132 | 84 | 3 | 03 | 44 | 0 | 117470 | 939756 | 44 | 0 | 0 | 864 | 947044 | 44 | 0 | 0 | 1 | 872 |
| 133 | 85 | 3 | 03 | 44 | 1 | 117578 | 940620 | 44 | 1 | 0 | 864 | 947916 | 44 | 1 | 0 | 1 | 872 |
| 134 | 86 | 3 | 03 | 44 | 2 | 117686 | 941484 | 44 | 2 | 0 | 864 | 948788 | 44 | 2 | 0 | 1 | 872 |
| 135 | 87 | 3 | 03 | 45 | 0 | 117796 | 942364 | 45 | 0 | 0 | 880 | 949676 | 45 | 0 | 0 | 1 | 888 |
| 136 | 88 | 3 | 03 | 45 | 1 | 117906 | 943244 | 45 | 1 | 0 | 880 | 950564 | 45 | 1 | 0 | 1 | 888 |
| 137 | 89 | 3 | 03 | 45 | 2 | 118016 | 944124 | 45 | 2 | 0 | 880 | 951452 | 45 | 2 | 0 | 1 | 888 |
| 138 | 8A | 3 | 03 | 46 | 0 | 118128 | 945020 | 46 | 0 | 0 | 896 | 952356 | 46 | 0 | 0 | 1 | 904 |
| 139 | 8B | 3 | 03 | 46 | 1 | 118240 | 945916 | 46 | 1 | 0 | 896 | 953260 | 46 | 1 | 0 | 1 | 904 |
| 140 | 8C | 3 | 03 | 46 | 2 | 118352 | 946812 | 46 | 2 | 0 | 896 | 954164 | 46 | 2 | 0 | 1 | 904 |
| 141 | 8D | 3 | 03 | 47 | 0 | 118466 | 947724 | 47 | 0 | 0 | 912 | 955084 | 47 | 0 | 0 | 1 | 920 |
| 142 | 8E | 3 | 03 | 47 | 1 | 118580 | 948636 | 47 | 1 | 0 | 912 | 956004 | 47 | 1 | 0 | 1 | 920 |
| 143 | 8F | 3 | 03 | 47 | 2 | 118694 | 949548 | 47 | 2 | 0 | 912 | 956924 | 47 | 2 | 0 | 1 | 920 |
| 144 | 90 | 3 | 03 | 48 | 0 | 118810 | 950476 | 48 | 0 | 0 | 928 | 957860 | 48 | 0 | 0 | 1 | 936 |
| 145 | 91 | 3 | 03 | 48 | 1 | 118926 | 951404 | 48 | 1 | 0 | 928 | 958796 | 48 | 1 | 0 | 1 | 936 |
| 146 | 92 | 3 | 03 | 48 | 2 | 119042 | 952332 | 48 | 2 | 0 | 928 | 959732 | 48 | 2 | 0 | 1 | 936 |
| 147 | 93 | 3 | 03 | 49 | 0 | 119160 | 953276 | 49 | 0 | 0 | 944 | 960684 | 49 | 0 | 0 | 1 | 952 |
| 148 | 94 | 3 | 03 | 49 | 1 | 119278 | 954220 | 49 | 1 | 0 | 944 | 961636 | 49 | 1 | 0 | 1 | 952 |
| 149 | 95 | 3 | 03 | 49 | 2 | 119396 | 955164 | 49 | 2 | 0 | 944 | 962588 | 49 | 2 | 0 | 1 | 952 |
| 150 | 96 | 3 | 03 | 50 | 0 | 119516 | 956124 | 50 | 0 | 0 | 960 | 963556 | 50 | 0 | 0 | 1 | 968 |
| 151 | 97 | 3 | 03 | 50 | 1 | 119636 | 957084 | 50 | 1 | 0 | 960 | 964524 | 50 | 1 | 0 | 1 | 968 |
| 152 | 98 | 3 | 03 | 50 | 2 | 119756 | 958044 | 50 | 2 | 0 | 960 | 965492 | 50 | 2 | 0 | 1 | 968 |
| 153 | 99 | 3 | 03 | 51 | 0 | 119878 | 959020 | 51 | 0 | 0 | 976 | 966476 | 51 | 0 | 0 | 1 | 984 |
| 154 | 9A | 3 | 03 | 51 | 1 | 120000 | 959996 | 51 | 1 | 0 | 976 | 967460 | 51 | 1 | 0 | 1 | 984 |
| 155 | 9B | 3 | 03 | 51 | 2 | 120122 | 960972 | 51 | 2 | 0 | 976 | 968444 | 51 | 2 | 0 | 1 | 984 |

| | | | | | | | | | | | | | | | | | |
|---|---|---|---|---|---|---|---|---|---|---|---|---|---|---|---|---|---|
| 156 | 9C | 3 | 03 | 52 | 0 | 120246 | 961964 | 52 | 0 | 0 | 992 | 969444 | 52 | 0 | 0 | 1 | 1000 |
| 157 | 9D | 3 | 03 | 52 | 1 | 120370 | 962956 | 52 | 1 | 0 | 992 | 970444 | 52 | 1 | 0 | 1 | 1000 |
| 158 | 9E | 3 | 03 | 52 | 2 | 120494 | 963948 | 52 | 2 | 0 | 992 | 971444 | 52 | 2 | 0 | 1 | 1000 |
| 159 | 9F | 3 | 03 | 53 | 0 | 120620 | 964956 | 53 | 0 | 0 | 1008 | 972460 | 53 | 0 | 0 | 1 | 1016 |
| 160 | A0 | 3 | 03 | 53 | 1 | 120746 | 965964 | 53 | 1 | 0 | 1008 | 973476 | 53 | 1 | 0 | 1 | 1016 |
| 161 | A1 | 3 | 03 | 53 | 2 | 120872 | 966972 | 53 | 2 | 0 | 1008 | 974492 | 53 | 2 | 0 | 1 | 1016 |
| 162 | A2 | 3 | 03 | 54 | 0 | 121000 | 967996 | 54 | 0 | 0 | 1024 | 975524 | 54 | 0 | 0 | 1 | 1032 |
| 163 | A3 | 3 | 03 | 54 | 1 | 121128 | 969020 | 54 | 1 | 0 | 1024 | 976556 | 54 | 1 | 0 | 1 | 1032 |
| 164 | A4 | 3 | 03 | 54 | 2 | 121256 | 970044 | 54 | 2 | 0 | 1024 | 977588 | 54 | 2 | 0 | 1 | 1032 |
| 165 | A5 | 3 | 03 | 55 | 0 | 121386 | 971084 | 55 | 0 | 0 | 1040 | 978636 | 55 | 0 | 0 | 1 | 1048 |
| 166 | A6 | 3 | 03 | 55 | 1 | 121516 | 972124 | 55 | 1 | 0 | 1040 | 979684 | 55 | 1 | 0 | 1 | 1048 |
| 167 | A7 | 3 | 03 | 55 | 2 | 121646 | 973164 | 55 | 2 | 0 | 1040 | 980732 | 55 | 2 | 0 | 1 | 1048 |
| 168 | A8 | 3 | 03 | 56 | 0 | 121778 | 974220 | 56 | 0 | 0 | 1056 | 981796 | 56 | 0 | 0 | 1 | 1064 |
| 169 | A9 | 3 | 03 | 56 | 1 | 121910 | 975276 | 56 | 1 | 0 | 1056 | 982860 | 56 | 1 | 0 | 1 | 1064 |
| 170 | AA | 3 | 03 | 56 | 2 | 122042 | 976332 | 56 | 2 | 0 | 1056 | 983924 | 56 | 2 | 0 | 1 | 1064 |
| 171 | AB | 3 | 03 | 57 | 0 | 122176 | 977404 | 57 | 0 | 0 | 1072 | 985004 | 57 | 0 | 0 | 1 | 1080 |
| 172 | AC | 3 | 03 | 57 | 1 | 122310 | 978476 | 57 | 1 | 0 | 1072 | 986084 | 57 | 1 | 0 | 1 | 1080 |
| 173 | AD | 3 | 03 | 57 | 2 | 122444 | 979548 | 57 | 2 | 0 | 1072 | 987164 | 57 | 2 | 0 | 1 | 1080 |
| 174 | AE | 3 | 03 | 58 | 0 | 122580 | 980636 | 58 | 0 | 0 | 1088 | 988260 | 58 | 0 | 0 | 1 | 1096 |
| 175 | AF | 3 | 03 | 58 | 1 | 122716 | 981724 | 58 | 1 | 0 | 1088 | 989356 | 58 | 1 | 0 | 1 | 1096 |
| 176 | B0 | 3 | 03 | 58 | 2 | 122852 | 982812 | 58 | 2 | 0 | 1088 | 990452 | 58 | 2 | 0 | 1 | 1096 |
| 177 | B1 | 3 | 03 | 59 | 0 | 122990 | 983916 | 59 | 0 | 0 | 1104 | 991564 | 59 | 0 | 0 | 1 | 1112 |
| 178 | B2 | 3 | 03 | 59 | 1 | 123128 | 985020 | 59 | 1 | 0 | 1104 | 992676 | 59 | 1 | 0 | 1 | 1112 |
| 179 | B3 | 3 | 03 | 59 | 2 | 123266 | 986124 | 59 | 2 | 0 | 1104 | 993788 | 59 | 2 | 0 | 1 | 1112 |
| 180 | B4 | 3 | 03 | 60 | 0 | 123406 | 987244 | 60 | 0 | 0 | 1120 | 994916 | 60 | 0 | 0 | 1 | 1128 |
| 181 | B5 | 3 | 03 | 60 | 1 | 123546 | 988364 | 60 | 1 | 0 | 1120 | 996044 | 60 | 1 | 0 | 1 | 1128 |
| 182 | B6 | 3 | 03 | 60 | 2 | 123686 | 989484 | 60 | 2 | 0 | 1120 | 997172 | 60 | 2 | 0 | 1 | 1128 |
| 183 | B7 | 3 | 03 | 61 | 0 | 123828 | 990620 | 61 | 0 | 0 | 1136 | 998316 | 61 | 0 | 0 | 1 | 1144 |
| 184 | B8 | 3 | 03 | 61 | 1 | 123970 | 991756 | 61 | 1 | 0 | 1136 | 999460 | 61 | 1 | 0 | 1 | 1144 |
| 185 | B9 | 3 | 03 | 61 | 2 | 124112 | 992892 | 61 | 2 | 0 | 1136 | 1000604 | 61 | 2 | 0 | 1 | 1144 |
| 186 | BA | 3 | 03 | 62 | 0 | 124256 | 994044 | 62 | 0 | 0 | 1152 | 1001764 | 62 | 0 | 0 | 1 | 1160 |
| 187 | BB | 3 | 03 | 62 | 1 | 124400 | 995196 | 62 | 1 | 0 | 1152 | 1002924 | 62 | 1 | 0 | 1 | 1160 |
| 188 | BC | 3 | 03 | 62 | 2 | 124544 | 996348 | 62 | 2 | 0 | 1152 | 1004084 | 62 | 2 | 0 | 1 | 1160 |
| 189 | BD | 3 | 03 | 63 | 0 | 124690 | 997516 | 63 | 0 | 0 | 1168 | 1005260 | 63 | 0 | 0 | 1 | 1176 |
| 190 | BE | 3 | 03 | 63 | 1 | 124836 | 998684 | 63 | 1 | 0 | 1168 | 1006436 | 63 | 1 | 0 | 1 | 1176 |
| 191 | BF | 3 | 03 | 63 | 2 | 124982 | 999852 | 63 | 2 | 0 | 1168 | 1007612 | 63 | 2 | 0 | 1 | 1176 |
| 192 | C0 | 3 | 03 | 64 | 0 | 125130 | 1001036 | 64 | 0 | 0 | 1184 | 1008804 | 64 | 0 | 0 | 1 | 1192 |
| 193 | C1 | 3 | 03 | 64 | 1 | 125278 | 1002220 | 64 | 1 | 0 | 1184 | 1009996 | 64 | 1 | 0 | 1 | 1192 |
| 194 | C2 | 3 | 03 | 64 | 2 | 125426 | 1003404 | 64 | 2 | 0 | 1184 | 1011188 | 64 | 2 | 0 | 1 | 1192 |
| 195 | C3 | 3 | 03 | 65 | 0 | 125576 | 1004604 | 65 | 0 | 0 | 1200 | 1012396 | 65 | 0 | 0 | 1 | 1208 |
| 196 | C4 | 3 | 03 | 65 | 1 | 125726 | 1005804 | 65 | 1 | 0 | 1200 | 1013604 | 65 | 1 | 0 | 1 | 1208 |
| 197 | C5 | 3 | 03 | 65 | 2 | 125876 | 1007004 | 65 | 2 | 0 | 1200 | 1014812 | 65 | 2 | 0 | 1 | 1208 |
| 198 | C6 | 3 | 03 | 66 | 0 | 126028 | 1008220 | 66 | 0 | 0 | 1216 | 1016036 | 66 | 0 | 0 | 1 | 1224 |
| 199 | C7 | 3 | 03 | 66 | 1 | 126180 | 1009436 | 66 | 1 | 0 | 1216 | 1017260 | 66 | 1 | 0 | 1 | 1224 |
| 200 | C8 | 3 | 03 | 66 | 2 | 126332 | 1010652 | 66 | 2 | 0 | 1216 | 1018484 | 66 | 2 | 0 | 1 | 1224 |
| 201 | C9 | 3 | 03 | 67 | 0 | 126486 | 1011884 | 67 | 0 | 0 | 1232 | 1019724 | 67 | 0 | 0 | 1 | 1240 |
| 202 | CA | 3 | 03 | 67 | 1 | 126640 | 1013116 | 67 | 1 | 0 | 1232 | 1020964 | 67 | 1 | 0 | 1 | 1240 |
| 203 | CB | 3 | 03 | 67 | 2 | 126794 | 1014348 | 67 | 2 | 0 | 1232 | 1022204 | 67 | 2 | 0 | 1 | 1240 |
| 204 | CC | 3 | 03 | 68 | 0 | 126950 | 1015596 | 68 | 0 | 0 | 1248 | 1023460 | 68 | 0 | 0 | 1 | 1256 |
| 205 | CD | 3 | 03 | 68 | 1 | 127106 | 1016844 | 68 | 1 | 0 | 1248 | 1024716 | 68 | 1 | 0 | 1 | 1256 |
| 206 | CE | 3 | 03 | 68 | 2 | 127262 | 1018092 | 68 | 2 | 0 | 1248 | 1025972 | 68 | 2 | 0 | 1 | 1256 |
| 207 | CF | 3 | 03 | 69 | 0 | 127420 | 1019356 | 69 | 0 | 0 | 1264 | 1027244 | 69 | 0 | 0 | 1 | 1272 |
| 208 | D0 | 3 | 03 | 69 | 1 | 127578 | 1020620 | 69 | 1 | 0 | 1264 | 1028516 | 69 | 1 | 0 | 1 | 1272 |
| 209 | D1 | 3 | 03 | 69 | 2 | 127736 | 1021884 | 69 | 2 | 0 | 1264 | 1029788 | 69 | 2 | 0 | 1 | 1272 |
| 210 | D2 | 3 | 03 | 70 | 0 | 127896 | 1023164 | 70 | 0 | 0 | 1280 | 1031076 | 70 | 0 | 0 | 1 | 1288 |
| 211 | D3 | 3 | 03 | 70 | 1 | 128056 | 1024444 | 70 | 1 | 0 | 1280 | 1032364 | 70 | 1 | 0 | 1 | 1288 |
| 212 | D4 | 3 | 03 | 70 | 2 | 128216 | 1025724 | 70 | 2 | 0 | 1280 | 1033652 | 70 | 2 | 0 | 1 | 1288 |
| 213 | D5 | 3 | 03 | 71 | 0 | 128378 | 1027020 | 71 | 0 | 0 | 1296 | 1034956 | 71 | 0 | 0 | 1 | 1304 |
| 214 | D6 | 3 | 03 | 71 | 1 | 128540 | 1028316 | 71 | 1 | 0 | 1296 | 1036260 | 71 | 1 | 0 | 1 | 1304 |
| 215 | D7 | 3 | 03 | 71 | 2 | 128702 | 1029612 | 71 | 2 | 0 | 1296 | 1037564 | 71 | 2 | 0 | 1 | 1304 |
| 216 | D8 | 3 | 03 | 72 | 0 | 128866 | 1030924 | 72 | 0 | 0 | 1312 | 1038884 | 72 | 0 | 0 | 1 | 1320 |
| 217 | D9 | 3 | 03 | 72 | 1 | 129030 | 1032236 | 72 | 1 | 0 | 1312 | 1040204 | 72 | 1 | 0 | 1 | 1320 |
| 218 | DA | 3 | 03 | 72 | 2 | 129194 | 1033548 | 72 | 2 | 0 | 1312 | 1041524 | 72 | 2 | 0 | 1 | 1320 |
| 219 | DB | 3 | 03 | 73 | 0 | 129360 | 1034876 | 73 | 0 | 0 | 1328 | 1042860 | 73 | 0 | 0 | 1 | 1336 |
| 220 | DC | 3 | 03 | 73 | 1 | 129526 | 1036204 | 73 | 1 | 0 | 1328 | 1044196 | 73 | 1 | 0 | 1 | 1336 |
| 221 | DD | 3 | 03 | 73 | 2 | 129692 | 1037532 | 73 | 2 | 0 | 1328 | 1045532 | 73 | 2 | 0 | 1 | 1336 |
| 222 | DE | 3 | 03 | 74 | 0 | 129860 | 1038876 | 74 | 0 | 0 | 1344 | 1046884 | 74 | 0 | 0 | 1 | 1352 |
| 223 | DF | 3 | 03 | 74 | 1 | 130028 | 1040220 | 74 | 1 | 0 | 1344 | 1048236 | 74 | 1 | 0 | 1 | 1352 |
| 224 | E0 | 3 | 03 | 74 | 2 | 130196 | 1041564 | 74 | 2 | 0 | 1344 | 1049588 | 74 | 2 | 0 | 1 | 1352 |
| 225 | E1 | 3 | 03 | 75 | 0 | 130366 | 1042924 | 75 | 0 | 0 | 1360 | 1050956 | 75 | 0 | 0 | 1 | 1368 |
| 226 | E2 | 3 | 03 | 75 | 1 | 130536 | 1044284 | 75 | 1 | 0 | 1360 | 1052324 | 75 | 1 | 0 | 1 | 1368 |
| 227 | E3 | 3 | 03 | 75 | 2 | 130706 | 1045644 | 75 | 2 | 0 | 1360 | 1053692 | 75 | 2 | 0 | 1 | 1368 |
| 228 | E4 | 3 | 03 | 76 | 0 | 130878 | 1047020 | 76 | 0 | 0 | 1376 | 1055076 | 76 | 0 | 0 | 1 | 1384 |
| 229 | E5 | 3 | 03 | 76 | 1 | 131050 | 1048396 | 76 | 1 | 0 | 1376 | 1056460 | 76 | 1 | 0 | 1 | 1384 |
| 230 | E6 | 3 | 03 | 76 | 2 | 131222 | 1049772 | 76 | 2 | 0 | 1376 | 1057844 | 76 | 2 | 0 | 1 | 1384 |
| 231 | E7 | 3 | 03 | 77 | 0 | 131396 | 1051164 | 77 | 0 | 0 | 1392 | 1059244 | 77 | 0 | 0 | 1 | 1400 |
| 232 | E8 | 3 | 03 | 77 | 1 | 131570 | 1052556 | 77 | 1 | 0 | 1392 | 1060644 | 77 | 1 | 0 | 1 | 1400 |
| 233 | E9 | 3 | 03 | 77 | 2 | 131744 | 1053948 | 77 | 2 | 0 | 1392 | 1062044 | 77 | 2 | 0 | 1 | 1400 |
| 234 | EA | 3 | 03 | 78 | 0 | 131920 | 1055356 | 78 | 0 | 0 | 1408 | 1063460 | 78 | 0 | 0 | 1 | 1416 |
| 235 | EB | 3 | 03 | 78 | 1 | 132096 | 1056764 | 78 | 1 | 0 | 1408 | 1064876 | 78 | 1 | 0 | 1 | 1416 |
| 236 | EC | 3 | 03 | 78 | 2 | 132272 | 1058172 | 78 | 2 | 0 | 1408 | 1066292 | 78 | 2 | 0 | 1 | 1416 |
| 237 | ED | 3 | 03 | 79 | 0 | 132450 | 1059596 | 79 | 0 | 0 | 1424 | 1067724 | 79 | 0 | 0 | 1 | 1432 |
| 238 | EE | 3 | 03 | 79 | 1 | 132628 | 1061020 | 79 | 1 | 0 | 1424 | 1069156 | 79 | 1 | 0 | 1 | 1432 |
| 239 | EF | 3 | 03 | 79 | 2 | 132806 | 1062444 | 79 | 2 | 0 | 1424 | 1070588 | 79 | 2 | 0 | 1 | 1432 |
| 240 | F0 | 3 | 03 | 80 | 0 | 132986 | 1063884 | 80 | 0 | 0 | 1440 | 1072036 | 80 | 0 | 0 | 1 | 1448 |
| 241 | F1 | 3 | 03 | 80 | 1 | 133166 | 1065324 | 80 | 1 | 0 | 1440 | 1073484 | 80 | 1 | 0 | 1 | 1448 |
| 242 | F2 | 3 | 03 | 80 | 2 | 133346 | 1066764 | 80 | 2 | 0 | 1440 | 1074932 | 80 | 2 | 0 | 1 | 1448 |
| 243 | F3 | 3 | 03 | 81 | 0 | 133528 | 1068220 | 81 | 0 | 0 | 1456 | 1076396 | 81 | 0 | 0 | 1 | 1464 |
| 244 | F4 | 3 | 03 | 81 | 1 | 133710 | 1069676 | 81 | 1 | 0 | 1456 | 1077860 | 81 | 1 | 0 | 1 | 1464 |
| 245 | F5 | 3 | 03 | 81 | 2 | 133892 | 1071132 | 81 | 2 | 0 | 1456 | 1079324 | 81 | 2 | 0 | 1 | 1464 |
| 246 | F6 | 3 | 03 | 82 | 0 | 134076 | 1072604 | 82 | 0 | 0 | 1472 | 1080804 | 82 | 0 | 0 | 1 | 1480 |
| 247 | F7 | 3 | 03 | 82 | 1 | 134260 | 1074076 | 82 | 1 | 0 | 1472 | 1082284 | 82 | 1 | 0 | 1 | 1480 |
| 248 | F8 | 3 | 03 | 82 | 2 | 134444 | 1075548 | 82 | 2 | 0 | 1472 | 1083764 | 82 | 2 | 0 | 1 | 1480 |
| 249 | F9 | 3 | 03 | 83 | 0 | 134630 | 1077036 | 83 | 0 | 0 | 1488 | 1085260 | 83 | 0 | 0 | 1 | 1496 |
| 250 | FA | 3 | 03 | 83 | 1 | 134816 | 1078524 | 83 | 1 | 0 | 1488 | 1086756 | 83 | 1 | 0 | 1 | 1496 |
| 251 | FB | 3 | 03 | 83 | 2 | 135002 | 1080012 | 83 | 2 | 0 | 1488 | 1088252 | 83 | 2 | 0 | 1 | 1496 |
| 252 | FC | 3 | 03 | 84 | 0 | 135190 | 1081516 | 84 | 0 | 0 | 1504 | 1089764 | 84 | 0 | 0 | 1 | 1512 |
| 253 | FD | 3 | 03 | 84 | 1 | 135378 | 1083020 | 84 | 1 | 0 | 1504 | 1091276 | 84 | 1 | 0 | 1 | 1512 |
| 254 | FE | 3 | 03 | 84 | 2 | 135566 | 1084524 | 84 | 2 | 0 | 1504 | 1092788 | 84 | 2 | 0 | 1 | 1512 |
| 255 | FF | 3 | 03 | 85 | 0 | 135756 | 1086044 | 85 | 0 | 0 | 1520 | 1094316 | 85 | 0 | 0 | 1 | 1528 |
| 0 | 00 | 4 | 04 | 0 | 0 | 135768 | 1086140 | 0 | 0 | 0 | 96 | 1094420 | 0 | 0 | 0 | 1 | 104 |
| 1 | 01 | 4 | 04 | 0 | 1 | 135782 | 1086252 | 0 | 1 | 0 | 112 | 1094540 | 0 | 1 | 0 | 1 | 120 |
| 2 | 02 | 4 | 04 | 0 | 2 | 135796 | 1086364 | 0 | 2 | 0 | 112 | 1094660 | 0 | 2 | 0 | 1 | 120 |

| | | | | | | | | | | | | | | | | | |
|---|---|---|---|---|---|---|---|---|---|---|---|---|---|---|---|---|---|
| 3 | 03 | 4 | 04 | 0 | 3 | 135810 | 1086476 | 0 | 3 | 0 | 112 | 1094780 | 0 | 3 | 0 | 1 | 120 |
| 4 | 04 | 4 | 04 | 1 | 0 | 135822 | 1086572 | 1 | 0 | 0 | 96 | 1094884 | 1 | 0 | 0 | 1 | 104 |
| 5 | 05 | 4 | 04 | 1 | 1 | 135839 | 1086708 | 1 | 1 | 0 | 136 | 1095028 | 1 | 1 | 0 | 1 | 144 |
| 6 | 06 | 4 | 04 | 1 | 2 | 135856 | 1086844 | 1 | 2 | 0 | 136 | 1095172 | 1 | 2 | 0 | 1 | 144 |
| 7 | 07 | 4 | 04 | 1 | 3 | 135873 | 1086980 | 1 | 3 | 0 | 136 | 1095316 | 1 | 3 | 0 | 1 | 144 |
| 8 | 08 | 4 | 04 | 2 | 0 | 135892 | 1087132 | 2 | 0 | 0 | 152 | 1095476 | 2 | 0 | 0 | 1 | 160 |
| 9 | 09 | 4 | 04 | 2 | 1 | 135911 | 1087284 | 2 | 1 | 0 | 152 | 1095636 | 2 | 1 | 0 | 1 | 160 |
| 10 | 0A | 4 | 04 | 2 | 2 | 135930 | 1087436 | 2 | 2 | 0 | 152 | 1095796 | 2 | 2 | 0 | 1 | 160 |
| 11 | 0B | 4 | 04 | 2 | 3 | 135949 | 1087588 | 2 | 3 | 0 | 152 | 1095956 | 2 | 3 | 0 | 1 | 160 |
| 12 | 0C | 4 | 04 | 3 | 0 | 135970 | 1087756 | 3 | 0 | 0 | 168 | 1096132 | 3 | 0 | 0 | 1 | 176 |
| 13 | 0D | 4 | 04 | 3 | 1 | 135991 | 1087924 | 3 | 1 | 0 | 168 | 1096308 | 3 | 1 | 0 | 1 | 176 |
| 14 | 0E | 4 | 04 | 3 | 2 | 136012 | 1088092 | 3 | 2 | 0 | 168 | 1096484 | 3 | 2 | 0 | 1 | 176 |
| 15 | 0F | 4 | 04 | 3 | 3 | 136033 | 1088260 | 3 | 3 | 0 | 168 | 1096660 | 3 | 3 | 0 | 1 | 176 |
| 16 | 10 | 4 | 04 | 4 | 0 | 136061 | 1088484 | 4 | 0 | 0 | 224 | 1096892 | 4 | 0 | 0 | 1 | 232 |
| 17 | 11 | 4 | 04 | 4 | 1 | 136089 | 1088708 | 4 | 1 | 0 | 224 | 1097124 | 4 | 1 | 0 | 1 | 232 |
| 18 | 12 | 4 | 04 | 4 | 2 | 136117 | 1088932 | 4 | 2 | 0 | 224 | 1097356 | 4 | 2 | 0 | 1 | 232 |
| 19 | 13 | 4 | 04 | 4 | 3 | 136145 | 1089156 | 4 | 3 | 0 | 224 | 1097588 | 4 | 3 | 0 | 1 | 232 |
| 20 | 14 | 4 | 04 | 5 | 0 | 136175 | 1089396 | 5 | 0 | 0 | 240 | 1097836 | 5 | 0 | 0 | 1 | 248 |
| 21 | 15 | 4 | 04 | 5 | 1 | 136205 | 1089636 | 5 | 1 | 0 | 240 | 1098084 | 5 | 1 | 0 | 1 | 248 |
| 22 | 16 | 4 | 04 | 5 | 2 | 136235 | 1089876 | 5 | 2 | 0 | 240 | 1098332 | 5 | 2 | 0 | 1 | 248 |
| 23 | 17 | 4 | 04 | 5 | 3 | 136265 | 1090116 | 5 | 3 | 0 | 240 | 1098580 | 5 | 3 | 0 | 1 | 248 |
| 24 | 18 | 4 | 04 | 6 | 0 | 136297 | 1090372 | 6 | 0 | 0 | 256 | 1098844 | 6 | 0 | 0 | 1 | 264 |
| 25 | 19 | 4 | 04 | 6 | 1 | 136329 | 1090628 | 6 | 1 | 0 | 256 | 1099108 | 6 | 1 | 0 | 1 | 264 |
| 26 | 1A | 4 | 04 | 6 | 2 | 136361 | 1090884 | 6 | 2 | 0 | 256 | 1099372 | 6 | 2 | 0 | 1 | 264 |
| 27 | 1B | 4 | 04 | 6 | 3 | 136393 | 1091140 | 6 | 3 | 0 | 256 | 1099636 | 6 | 3 | 0 | 1 | 264 |
| 28 | 1C | 4 | 04 | 7 | 0 | 136427 | 1091412 | 7 | 0 | 0 | 272 | 1099916 | 7 | 0 | 0 | 1 | 280 |
| 29 | 1D | 4 | 04 | 7 | 1 | 136461 | 1091684 | 7 | 1 | 0 | 272 | 1100196 | 7 | 1 | 0 | 1 | 280 |
| 30 | 1E | 4 | 04 | 7 | 2 | 136495 | 1091956 | 7 | 2 | 0 | 272 | 1100476 | 7 | 2 | 0 | 1 | 280 |
| 31 | 1F | 4 | 04 | 7 | 3 | 136529 | 1092228 | 7 | 3 | 0 | 272 | 1100756 | 7 | 3 | 0 | 1 | 280 |
| 32 | 20 | 4 | 04 | 8 | 0 | 136565 | 1092516 | 8 | 0 | 0 | 288 | 1101052 | 8 | 0 | 0 | 1 | 296 |
| 33 | 21 | 4 | 04 | 8 | 1 | 136601 | 1092804 | 8 | 1 | 0 | 288 | 1101348 | 8 | 1 | 0 | 1 | 296 |
| 34 | 22 | 4 | 04 | 8 | 2 | 136637 | 1093092 | 8 | 2 | 0 | 288 | 1101644 | 8 | 2 | 0 | 1 | 296 |
| 35 | 23 | 4 | 04 | 8 | 3 | 136673 | 1093380 | 8 | 3 | 0 | 288 | 1101940 | 8 | 3 | 0 | 1 | 296 |
| 36 | 24 | 4 | 04 | 9 | 0 | 136711 | 1093684 | 9 | 0 | 0 | 304 | 1102252 | 9 | 0 | 0 | 1 | 312 |
| 37 | 25 | 4 | 04 | 9 | 1 | 136749 | 1093988 | 9 | 1 | 0 | 304 | 1102564 | 9 | 1 | 0 | 1 | 312 |
| 38 | 26 | 4 | 04 | 9 | 2 | 136787 | 1094292 | 9 | 2 | 0 | 304 | 1102876 | 9 | 2 | 0 | 1 | 312 |
| 39 | 27 | 4 | 04 | 9 | 3 | 136825 | 1094596 | 9 | 3 | 0 | 304 | 1103188 | 9 | 3 | 0 | 1 | 312 |
| 40 | 28 | 4 | 04 | 10 | 0 | 136865 | 1094916 | 10 | 0 | 0 | 320 | 1103516 | 10 | 0 | 0 | 1 | 328 |
| 41 | 29 | 4 | 04 | 10 | 1 | 136905 | 1095236 | 10 | 1 | 0 | 320 | 1103844 | 10 | 1 | 0 | 1 | 328 |
| 42 | 2A | 4 | 04 | 10 | 2 | 136945 | 1095556 | 10 | 2 | 0 | 320 | 1104172 | 10 | 2 | 0 | 1 | 328 |
| 43 | 2B | 4 | 04 | 10 | 3 | 136985 | 1095876 | 10 | 3 | 0 | 320 | 1104500 | 10 | 3 | 0 | 1 | 328 |
| 44 | 2C | 4 | 04 | 11 | 0 | 137027 | 1096212 | 11 | 0 | 0 | 336 | 1104844 | 11 | 0 | 0 | 1 | 344 |
| 45 | 2D | 4 | 04 | 11 | 1 | 137069 | 1096548 | 11 | 1 | 0 | 336 | 1105188 | 11 | 1 | 0 | 1 | 344 |
| 46 | 2E | 4 | 04 | 11 | 2 | 137111 | 1096884 | 11 | 2 | 0 | 336 | 1105532 | 11 | 2 | 0 | 1 | 344 |
| 47 | 2F | 4 | 04 | 11 | 3 | 137153 | 1097220 | 11 | 3 | 0 | 336 | 1105876 | 11 | 3 | 0 | 1 | 344 |
| 48 | 30 | 4 | 04 | 12 | 0 | 137197 | 1097572 | 12 | 0 | 0 | 352 | 1106236 | 12 | 0 | 0 | 1 | 360 |
| 49 | 31 | 4 | 04 | 12 | 1 | 137241 | 1097924 | 12 | 1 | 0 | 352 | 1106596 | 12 | 1 | 0 | 1 | 360 |
| 50 | 32 | 4 | 04 | 12 | 2 | 137285 | 1098276 | 12 | 2 | 0 | 352 | 1106956 | 12 | 2 | 0 | 1 | 360 |
| 51 | 33 | 4 | 04 | 12 | 3 | 137329 | 1098628 | 12 | 3 | 0 | 352 | 1107316 | 12 | 3 | 0 | 1 | 360 |
| 52 | 34 | 4 | 04 | 13 | 0 | 137375 | 1098996 | 13 | 0 | 0 | 368 | 1107692 | 13 | 0 | 0 | 1 | 376 |
| 53 | 35 | 4 | 04 | 13 | 1 | 137421 | 1099364 | 13 | 1 | 0 | 368 | 1108068 | 13 | 1 | 0 | 1 | 376 |
| 54 | 36 | 4 | 04 | 13 | 2 | 137467 | 1099732 | 13 | 2 | 0 | 368 | 1108444 | 13 | 2 | 0 | 1 | 376 |
| 55 | 37 | 4 | 04 | 13 | 3 | 137513 | 1100100 | 13 | 3 | 0 | 368 | 1108820 | 13 | 3 | 0 | 1 | 376 |
| 56 | 38 | 4 | 04 | 14 | 0 | 137561 | 1100484 | 14 | 0 | 0 | 384 | 1109212 | 14 | 0 | 0 | 1 | 392 |
| 57 | 39 | 4 | 04 | 14 | 1 | 137609 | 1100868 | 14 | 1 | 0 | 384 | 1109604 | 14 | 1 | 0 | 1 | 392 |
| 58 | 3A | 4 | 04 | 14 | 2 | 137657 | 1101252 | 14 | 2 | 0 | 384 | 1109996 | 14 | 2 | 0 | 1 | 392 |
| 59 | 3B | 4 | 04 | 14 | 3 | 137705 | 1101636 | 14 | 3 | 0 | 384 | 1110388 | 14 | 3 | 0 | 1 | 392 |
| 60 | 3C | 4 | 04 | 15 | 0 | 137755 | 1102036 | 15 | 0 | 0 | 400 | 1110796 | 15 | 0 | 0 | 1 | 408 |
| 61 | 3D | 4 | 04 | 15 | 1 | 137805 | 1102436 | 15 | 1 | 0 | 400 | 1111204 | 15 | 1 | 0 | 1 | 408 |
| 62 | 3E | 4 | 04 | 15 | 2 | 137855 | 1102836 | 15 | 2 | 0 | 400 | 1111612 | 15 | 2 | 0 | 1 | 408 |
| 63 | 3F | 4 | 04 | 15 | 3 | 137905 | 1103236 | 15 | 3 | 0 | 400 | 1112020 | 15 | 3 | 0 | 1 | 408 |
| 64 | 40 | 4 | 04 | 16 | 0 | 137957 | 1103652 | 16 | 0 | 0 | 416 | 1112444 | 16 | 0 | 0 | 1 | 424 |
| 65 | 41 | 4 | 04 | 16 | 1 | 138009 | 1104068 | 16 | 1 | 0 | 416 | 1112868 | 16 | 1 | 0 | 1 | 424 |
| 66 | 42 | 4 | 04 | 16 | 2 | 138061 | 1104484 | 16 | 2 | 0 | 416 | 1113292 | 16 | 2 | 0 | 1 | 424 |
| 67 | 43 | 4 | 04 | 16 | 3 | 138113 | 1104900 | 16 | 3 | 0 | 416 | 1113716 | 16 | 3 | 0 | 1 | 424 |
| 68 | 44 | 4 | 04 | 17 | 0 | 138167 | 1105332 | 17 | 0 | 0 | 432 | 1114156 | 17 | 0 | 0 | 1 | 440 |
| 69 | 45 | 4 | 04 | 17 | 1 | 138221 | 1105764 | 17 | 1 | 0 | 432 | 1114596 | 17 | 1 | 0 | 1 | 440 |
| 70 | 46 | 4 | 04 | 17 | 2 | 138275 | 1106196 | 17 | 2 | 0 | 432 | 1115036 | 17 | 2 | 0 | 1 | 440 |
| 71 | 47 | 4 | 04 | 17 | 3 | 138329 | 1106628 | 17 | 3 | 0 | 432 | 1115476 | 17 | 3 | 0 | 1 | 440 |
| 72 | 48 | 4 | 04 | 18 | 0 | 138385 | 1107076 | 18 | 0 | 0 | 448 | 1115932 | 18 | 0 | 0 | 1 | 456 |
| 73 | 49 | 4 | 04 | 18 | 1 | 138441 | 1107524 | 18 | 1 | 0 | 448 | 1116388 | 18 | 1 | 0 | 1 | 456 |
| 74 | 4A | 4 | 04 | 18 | 2 | 138497 | 1107972 | 18 | 2 | 0 | 448 | 1116844 | 18 | 2 | 0 | 1 | 456 |
| 75 | 4B | 4 | 04 | 18 | 3 | 138553 | 1108420 | 18 | 3 | 0 | 448 | 1117300 | 18 | 3 | 0 | 1 | 456 |
| 76 | 4C | 4 | 04 | 19 | 0 | 138611 | 1108884 | 19 | 0 | 0 | 464 | 1117772 | 19 | 0 | 0 | 1 | 472 |
| 77 | 4D | 4 | 04 | 19 | 1 | 138669 | 1109348 | 19 | 1 | 0 | 464 | 1118244 | 19 | 1 | 0 | 1 | 472 |
| 78 | 4E | 4 | 04 | 19 | 2 | 138727 | 1109812 | 19 | 2 | 0 | 464 | 1118716 | 19 | 2 | 0 | 1 | 472 |
| 79 | 4F | 4 | 04 | 19 | 3 | 138785 | 1110276 | 19 | 3 | 0 | 464 | 1119188 | 19 | 3 | 0 | 1 | 472 |
| 80 | 50 | 4 | 04 | 20 | 0 | 138845 | 1110756 | 20 | 0 | 0 | 480 | 1119676 | 20 | 0 | 0 | 1 | 488 |
| 81 | 51 | 4 | 04 | 20 | 1 | 138905 | 1111236 | 20 | 1 | 0 | 480 | 1120164 | 20 | 1 | 0 | 1 | 488 |
| 82 | 52 | 4 | 04 | 20 | 2 | 138965 | 1111716 | 20 | 2 | 0 | 480 | 1120652 | 20 | 2 | 0 | 1 | 488 |
| 83 | 53 | 4 | 04 | 20 | 3 | 139025 | 1112196 | 20 | 3 | 0 | 480 | 1121140 | 20 | 3 | 0 | 1 | 488 |
| 84 | 54 | 4 | 04 | 21 | 0 | 139087 | 1112692 | 21 | 0 | 0 | 496 | 1121644 | 21 | 0 | 0 | 1 | 504 |
| 85 | 55 | 4 | 04 | 21 | 1 | 139149 | 1113188 | 21 | 1 | 0 | 496 | 1122148 | 21 | 1 | 0 | 1 | 504 |
| 86 | 56 | 4 | 04 | 21 | 2 | 139211 | 1113684 | 21 | 2 | 0 | 496 | 1122652 | 21 | 2 | 0 | 1 | 504 |
| 87 | 57 | 4 | 04 | 21 | 3 | 139273 | 1114180 | 21 | 3 | 0 | 496 | 1123156 | 21 | 3 | 0 | 1 | 504 |
| 88 | 58 | 4 | 04 | 22 | 0 | 139337 | 1114692 | 22 | 0 | 0 | 512 | 1123676 | 22 | 0 | 0 | 1 | 520 |
| 89 | 59 | 4 | 04 | 22 | 1 | 139401 | 1115204 | 22 | 1 | 0 | 512 | 1124196 | 22 | 1 | 0 | 1 | 520 |
| 90 | 5A | 4 | 04 | 22 | 2 | 139465 | 1115716 | 22 | 2 | 0 | 512 | 1124716 | 22 | 2 | 0 | 1 | 520 |
| 91 | 5B | 4 | 04 | 22 | 3 | 139529 | 1116228 | 22 | 3 | 0 | 512 | 1125236 | 22 | 3 | 0 | 1 | 520 |
| 92 | 5C | 4 | 04 | 23 | 0 | 139595 | 1116756 | 23 | 0 | 0 | 528 | 1125772 | 23 | 0 | 0 | 1 | 536 |
| 93 | 5D | 4 | 04 | 23 | 1 | 139661 | 1117284 | 23 | 1 | 0 | 528 | 1126308 | 23 | 1 | 0 | 1 | 536 |
| 94 | 5E | 4 | 04 | 23 | 2 | 139727 | 1117812 | 23 | 2 | 0 | 528 | 1126844 | 23 | 2 | 0 | 1 | 536 |
| 95 | 5F | 4 | 04 | 23 | 3 | 139793 | 1118340 | 23 | 3 | 0 | 528 | 1127380 | 23 | 3 | 0 | 1 | 536 |
| 96 | 60 | 4 | 04 | 24 | 0 | 139861 | 1118884 | 24 | 0 | 0 | 544 | 1127932 | 24 | 0 | 0 | 1 | 552 |
| 97 | 61 | 4 | 04 | 24 | 1 | 139929 | 1119428 | 24 | 1 | 0 | 544 | 1128484 | 24 | 1 | 0 | 1 | 552 |
| 98 | 62 | 4 | 04 | 24 | 2 | 139997 | 1119972 | 24 | 2 | 0 | 544 | 1129036 | 24 | 2 | 0 | 1 | 552 |
| 99 | 63 | 4 | 04 | 24 | 3 | 140065 | 1120516 | 24 | 3 | 0 | 544 | 1129588 | 24 | 3 | 0 | 1 | 552 |
| 100 | 64 | 4 | 04 | 25 | 0 | 140135 | 1121076 | 25 | 0 | 0 | 560 | 1130156 | 25 | 0 | 0 | 1 | 568 |
| 101 | 65 | 4 | 04 | 25 | 1 | 140205 | 1121636 | 25 | 1 | 0 | 560 | 1130724 | 25 | 1 | 0 | 1 | 568 |
| 102 | 66 | 4 | 04 | 25 | 2 | 140275 | 1122196 | 25 | 2 | 0 | 560 | 1131292 | 25 | 2 | 0 | 1 | 568 |
| 103 | 67 | 4 | 04 | 25 | 3 | 140345 | 1122756 | 25 | 3 | 0 | 560 | 1131860 | 25 | 3 | 0 | 1 | 568 |
| 104 | 68 | 4 | 04 | 26 | 0 | 140417 | 1123332 | 26 | 0 | 0 | 576 | 1132444 | 26 | 0 | 0 | 1 | 584 |
| 105 | 69 | 4 | 04 | 26 | 1 | 140489 | 1123908 | 26 | 1 | 0 | 576 | 1133028 | 26 | 1 | 0 | 1 | 584 |

| | | | | | | | | | | | | | | | | | |
|---|---|---|---|---|---|---|---|---|---|---|---|---|---|---|---|---|---|
| 106 | 6A | 4 | 04 | 26 | 2 | 140561 | 1124484 | 26 | 2 | 0 | 576 | 1133612 | 26 | 2 | 0 | 1 | 584 |
| 107 | 6B | 4 | 04 | 26 | 3 | 140633 | 1125060 | 26 | 3 | 0 | 576 | 1134196 | 26 | 3 | 0 | 1 | 584 |
| 108 | 6C | 4 | 04 | 27 | 0 | 140707 | 1125652 | 27 | 0 | 0 | 592 | 1134796 | 27 | 0 | 0 | 1 | 600 |
| 109 | 6D | 4 | 04 | 27 | 1 | 140781 | 1126244 | 27 | 1 | 0 | 592 | 1135396 | 27 | 1 | 0 | 1 | 600 |
| 110 | 6E | 4 | 04 | 27 | 2 | 140855 | 1126836 | 27 | 2 | 0 | 592 | 1135996 | 27 | 2 | 0 | 1 | 600 |
| 111 | 6F | 4 | 04 | 27 | 3 | 140929 | 1127428 | 27 | 3 | 0 | 592 | 1136596 | 27 | 3 | 0 | 1 | 600 |
| 112 | 70 | 4 | 04 | 28 | 0 | 141005 | 1128036 | 28 | 0 | 0 | 608 | 1137212 | 28 | 0 | 0 | 1 | 616 |
| 113 | 71 | 4 | 04 | 28 | 1 | 141081 | 1128644 | 28 | 1 | 0 | 608 | 1137828 | 28 | 1 | 0 | 1 | 616 |
| 114 | 72 | 4 | 04 | 28 | 2 | 141157 | 1129252 | 28 | 2 | 0 | 608 | 1138444 | 28 | 2 | 0 | 1 | 616 |
| 115 | 73 | 4 | 04 | 28 | 3 | 141233 | 1129860 | 28 | 3 | 0 | 608 | 1139060 | 28 | 3 | 0 | 1 | 616 |
| 116 | 74 | 4 | 04 | 29 | 0 | 141311 | 1130484 | 29 | 0 | 0 | 624 | 1139692 | 29 | 0 | 0 | 1 | 632 |
| 117 | 75 | 4 | 04 | 29 | 1 | 141389 | 1131108 | 29 | 1 | 0 | 624 | 1140324 | 29 | 1 | 0 | 1 | 632 |
| 118 | 76 | 4 | 04 | 29 | 2 | 141467 | 1131732 | 29 | 2 | 0 | 624 | 1140956 | 29 | 2 | 0 | 1 | 632 |
| 119 | 77 | 4 | 04 | 29 | 3 | 141545 | 1132356 | 29 | 3 | 0 | 624 | 1141588 | 29 | 3 | 0 | 1 | 632 |
| 120 | 78 | 4 | 04 | 30 | 0 | 141625 | 1132996 | 30 | 0 | 0 | 640 | 1142236 | 30 | 0 | 0 | 1 | 648 |
| 121 | 79 | 4 | 04 | 30 | 1 | 141705 | 1133636 | 30 | 1 | 0 | 640 | 1142884 | 30 | 1 | 0 | 1 | 648 |
| 122 | 7A | 4 | 04 | 30 | 2 | 141785 | 1134276 | 30 | 2 | 0 | 640 | 1143532 | 30 | 2 | 0 | 1 | 648 |
| 123 | 7B | 4 | 04 | 30 | 3 | 141865 | 1134916 | 30 | 3 | 0 | 640 | 1144180 | 30 | 3 | 0 | 1 | 648 |
| 124 | 7C | 4 | 04 | 31 | 0 | 141947 | 1135572 | 31 | 0 | 0 | 656 | 1144844 | 31 | 0 | 0 | 1 | 664 |
| 125 | 7D | 4 | 04 | 31 | 1 | 142029 | 1136228 | 31 | 1 | 0 | 656 | 1145508 | 31 | 1 | 0 | 1 | 664 |
| 126 | 7E | 4 | 04 | 31 | 2 | 142111 | 1136884 | 31 | 2 | 0 | 656 | 1146172 | 31 | 2 | 0 | 1 | 664 |
| 127 | 7F | 4 | 04 | 31 | 3 | 142193 | 1137540 | 31 | 3 | 0 | 656 | 1146836 | 31 | 3 | 0 | 1 | 664 |
| 128 | 80 | 4 | 04 | 32 | 0 | 142277 | 1138212 | 32 | 0 | 0 | 672 | 1147516 | 32 | 0 | 0 | 1 | 680 |
| 129 | 81 | 4 | 04 | 32 | 1 | 142361 | 1138884 | 32 | 1 | 0 | 672 | 1148196 | 32 | 1 | 0 | 1 | 680 |
| 130 | 82 | 4 | 04 | 32 | 2 | 142445 | 1139556 | 32 | 2 | 0 | 672 | 1148876 | 32 | 2 | 0 | 1 | 680 |
| 131 | 83 | 4 | 04 | 32 | 3 | 142529 | 1140228 | 32 | 3 | 0 | 672 | 1149556 | 32 | 3 | 0 | 1 | 680 |
| 132 | 84 | 4 | 04 | 33 | 0 | 142615 | 1140916 | 33 | 0 | 0 | 688 | 1150252 | 33 | 0 | 0 | 1 | 696 |
| 133 | 85 | 4 | 04 | 33 | 1 | 142701 | 1141604 | 33 | 1 | 0 | 688 | 1150948 | 33 | 1 | 0 | 1 | 696 |
| 134 | 86 | 4 | 04 | 33 | 2 | 142787 | 1142292 | 33 | 2 | 0 | 688 | 1151644 | 33 | 2 | 0 | 1 | 696 |
| 135 | 87 | 4 | 04 | 33 | 3 | 142873 | 1142980 | 33 | 3 | 0 | 688 | 1152340 | 33 | 3 | 0 | 1 | 696 |
| 136 | 88 | 4 | 04 | 34 | 0 | 142961 | 1143684 | 34 | 0 | 0 | 704 | 1153052 | 34 | 0 | 0 | 1 | 712 |
| 137 | 89 | 4 | 04 | 34 | 1 | 143049 | 1144388 | 34 | 1 | 0 | 704 | 1153764 | 34 | 1 | 0 | 1 | 712 |
| 138 | 8A | 4 | 04 | 34 | 2 | 143137 | 1145092 | 34 | 2 | 0 | 704 | 1154476 | 34 | 2 | 0 | 1 | 712 |
| 139 | 8B | 4 | 04 | 34 | 3 | 143225 | 1145796 | 34 | 3 | 0 | 704 | 1155188 | 34 | 3 | 0 | 1 | 712 |
| 140 | 8C | 4 | 04 | 35 | 0 | 143315 | 1146516 | 35 | 0 | 0 | 720 | 1155916 | 35 | 0 | 0 | 1 | 728 |
| 141 | 8D | 4 | 04 | 35 | 1 | 143405 | 1147236 | 35 | 1 | 0 | 720 | 1156644 | 35 | 1 | 0 | 1 | 728 |
| 142 | 8E | 4 | 04 | 35 | 2 | 143495 | 1147956 | 35 | 2 | 0 | 720 | 1157372 | 35 | 2 | 0 | 1 | 728 |
| 143 | 8F | 4 | 04 | 35 | 3 | 143585 | 1148676 | 35 | 3 | 0 | 720 | 1158100 | 35 | 3 | 0 | 1 | 728 |
| 144 | 90 | 4 | 04 | 36 | 0 | 143677 | 1149412 | 36 | 0 | 0 | 736 | 1158844 | 36 | 0 | 0 | 1 | 744 |
| 145 | 91 | 4 | 04 | 36 | 1 | 143769 | 1150148 | 36 | 1 | 0 | 736 | 1159588 | 36 | 1 | 0 | 1 | 744 |
| 146 | 92 | 4 | 04 | 36 | 2 | 143861 | 1150884 | 36 | 2 | 0 | 736 | 1160332 | 36 | 2 | 0 | 1 | 744 |
| 147 | 93 | 4 | 04 | 36 | 3 | 143953 | 1151620 | 36 | 3 | 0 | 736 | 1161076 | 36 | 3 | 0 | 1 | 744 |
| 148 | 94 | 4 | 04 | 37 | 0 | 144047 | 1152372 | 37 | 0 | 0 | 752 | 1161836 | 37 | 0 | 0 | 1 | 760 |
| 149 | 95 | 4 | 04 | 37 | 1 | 144141 | 1153124 | 37 | 1 | 0 | 752 | 1162596 | 37 | 1 | 0 | 1 | 760 |
| 150 | 96 | 4 | 04 | 37 | 2 | 144235 | 1153876 | 37 | 2 | 0 | 752 | 1163356 | 37 | 2 | 0 | 1 | 760 |
| 151 | 97 | 4 | 04 | 37 | 3 | 144329 | 1154628 | 37 | 3 | 0 | 752 | 1164116 | 37 | 3 | 0 | 1 | 760 |
| 152 | 98 | 4 | 04 | 38 | 0 | 144425 | 1155396 | 38 | 0 | 0 | 768 | 1164892 | 38 | 0 | 0 | 1 | 776 |
| 153 | 99 | 4 | 04 | 38 | 1 | 144521 | 1156164 | 38 | 1 | 0 | 768 | 1165668 | 38 | 1 | 0 | 1 | 776 |
| 154 | 9A | 4 | 04 | 38 | 2 | 144617 | 1156932 | 38 | 2 | 0 | 768 | 1166444 | 38 | 2 | 0 | 1 | 776 |
| 155 | 9B | 4 | 04 | 38 | 3 | 144713 | 1157700 | 38 | 3 | 0 | 768 | 1167220 | 38 | 3 | 0 | 1 | 776 |
| 156 | 9C | 4 | 04 | 39 | 0 | 144811 | 1158484 | 39 | 0 | 0 | 784 | 1168012 | 39 | 0 | 0 | 1 | 792 |
| 157 | 9D | 4 | 04 | 39 | 1 | 144909 | 1159268 | 39 | 1 | 0 | 784 | 1168804 | 39 | 1 | 0 | 1 | 792 |
| 158 | 9E | 4 | 04 | 39 | 2 | 145007 | 1160052 | 39 | 2 | 0 | 784 | 1169596 | 39 | 2 | 0 | 1 | 792 |
| 159 | 9F | 4 | 04 | 39 | 3 | 145105 | 1160836 | 39 | 3 | 0 | 784 | 1170388 | 39 | 3 | 0 | 1 | 792 |
| 160 | A0 | 4 | 04 | 40 | 0 | 145205 | 1161636 | 40 | 0 | 0 | 800 | 1171196 | 40 | 0 | 0 | 1 | 808 |
| 161 | A1 | 4 | 04 | 40 | 1 | 145305 | 1162436 | 40 | 1 | 0 | 800 | 1172004 | 40 | 1 | 0 | 1 | 808 |
| 162 | A2 | 4 | 04 | 40 | 2 | 145405 | 1163236 | 40 | 2 | 0 | 800 | 1172812 | 40 | 2 | 0 | 1 | 808 |
| 163 | A3 | 4 | 04 | 40 | 3 | 145505 | 1164036 | 40 | 3 | 0 | 800 | 1173620 | 40 | 3 | 0 | 1 | 808 |
| 164 | A4 | 4 | 04 | 41 | 0 | 145607 | 1164852 | 41 | 0 | 0 | 816 | 1174444 | 41 | 0 | 0 | 1 | 824 |
| 165 | A5 | 4 | 04 | 41 | 1 | 145709 | 1165668 | 41 | 1 | 0 | 816 | 1175268 | 41 | 1 | 0 | 1 | 824 |
| 166 | A6 | 4 | 04 | 41 | 2 | 145811 | 1166484 | 41 | 2 | 0 | 816 | 1176092 | 41 | 2 | 0 | 1 | 824 |
| 167 | A7 | 4 | 04 | 41 | 3 | 145913 | 1167300 | 41 | 3 | 0 | 816 | 1176916 | 41 | 3 | 0 | 1 | 824 |
| 168 | A8 | 4 | 04 | 42 | 0 | 146017 | 1168132 | 42 | 0 | 0 | 832 | 1177756 | 42 | 0 | 0 | 1 | 840 |
| 169 | A9 | 4 | 04 | 42 | 1 | 146121 | 1168964 | 42 | 1 | 0 | 832 | 1178596 | 42 | 1 | 0 | 1 | 840 |
| 170 | AA | 4 | 04 | 42 | 2 | 146225 | 1169796 | 42 | 2 | 0 | 832 | 1179436 | 42 | 2 | 0 | 1 | 840 |
| 171 | AB | 4 | 04 | 42 | 3 | 146329 | 1170628 | 42 | 3 | 0 | 832 | 1180276 | 42 | 3 | 0 | 1 | 840 |
| 172 | AC | 4 | 04 | 43 | 0 | 146435 | 1171476 | 43 | 0 | 0 | 848 | 1181132 | 43 | 0 | 0 | 1 | 856 |
| 173 | AD | 4 | 04 | 43 | 1 | 146541 | 1172324 | 43 | 1 | 0 | 848 | 1181988 | 43 | 1 | 0 | 1 | 856 |
| 174 | AE | 4 | 04 | 43 | 2 | 146647 | 1173172 | 43 | 2 | 0 | 848 | 1182844 | 43 | 2 | 0 | 1 | 856 |
| 175 | AF | 4 | 04 | 43 | 3 | 146753 | 1174020 | 43 | 3 | 0 | 848 | 1183700 | 43 | 3 | 0 | 1 | 856 |
| 176 | B0 | 4 | 04 | 44 | 0 | 146861 | 1174884 | 44 | 0 | 0 | 864 | 1184572 | 44 | 0 | 0 | 1 | 872 |
| 177 | B1 | 4 | 04 | 44 | 1 | 146969 | 1175748 | 44 | 1 | 0 | 864 | 1185444 | 44 | 1 | 0 | 1 | 872 |
| 178 | B2 | 4 | 04 | 44 | 2 | 147077 | 1176612 | 44 | 2 | 0 | 864 | 1186316 | 44 | 2 | 0 | 1 | 872 |
| 179 | B3 | 4 | 04 | 44 | 3 | 147185 | 1177476 | 44 | 3 | 0 | 864 | 1187188 | 44 | 3 | 0 | 1 | 872 |
| 180 | B4 | 4 | 04 | 45 | 0 | 147295 | 1178356 | 45 | 0 | 0 | 880 | 1188076 | 45 | 0 | 0 | 1 | 888 |
| 181 | B5 | 4 | 04 | 45 | 1 | 147405 | 1179236 | 45 | 1 | 0 | 880 | 1188964 | 45 | 1 | 0 | 1 | 888 |
| 182 | B6 | 4 | 04 | 45 | 2 | 147515 | 1180116 | 45 | 2 | 0 | 880 | 1189852 | 45 | 2 | 0 | 1 | 888 |
| 183 | B7 | 4 | 04 | 45 | 3 | 147625 | 1180996 | 45 | 3 | 0 | 880 | 1190740 | 45 | 3 | 0 | 1 | 888 |
| 184 | B8 | 4 | 04 | 46 | 0 | 147737 | 1181892 | 46 | 0 | 0 | 896 | 1191644 | 46 | 0 | 0 | 1 | 904 |
| 185 | B9 | 4 | 04 | 46 | 1 | 147849 | 1182788 | 46 | 1 | 0 | 896 | 1192548 | 46 | 1 | 0 | 1 | 904 |
| 186 | BA | 4 | 04 | 46 | 2 | 147961 | 1183684 | 46 | 2 | 0 | 896 | 1193452 | 46 | 2 | 0 | 1 | 904 |
| 187 | BB | 4 | 04 | 46 | 3 | 148073 | 1184580 | 46 | 3 | 0 | 896 | 1194356 | 46 | 3 | 0 | 1 | 904 |
| 188 | BC | 4 | 04 | 47 | 0 | 148187 | 1185492 | 47 | 0 | 0 | 912 | 1195276 | 47 | 0 | 0 | 1 | 920 |
| 189 | BD | 4 | 04 | 47 | 1 | 148301 | 1186404 | 47 | 1 | 0 | 912 | 1196196 | 47 | 1 | 0 | 1 | 920 |
| 190 | BE | 4 | 04 | 47 | 2 | 148415 | 1187316 | 47 | 2 | 0 | 912 | 1197116 | 47 | 2 | 0 | 1 | 920 |
| 191 | BF | 4 | 04 | 47 | 3 | 148529 | 1188228 | 47 | 3 | 0 | 912 | 1198036 | 47 | 3 | 0 | 1 | 920 |
| 192 | C0 | 4 | 04 | 48 | 0 | 148645 | 1189156 | 48 | 0 | 0 | 928 | 1198972 | 48 | 0 | 0 | 1 | 936 |
| 193 | C1 | 4 | 04 | 48 | 1 | 148761 | 1190084 | 48 | 1 | 0 | 928 | 1199908 | 48 | 1 | 0 | 1 | 936 |
| 194 | C2 | 4 | 04 | 48 | 2 | 148877 | 1191012 | 48 | 2 | 0 | 928 | 1200844 | 48 | 2 | 0 | 1 | 936 |
| 195 | C3 | 4 | 04 | 48 | 3 | 148993 | 1191940 | 48 | 3 | 0 | 928 | 1201780 | 48 | 3 | 0 | 1 | 936 |
| 196 | C4 | 4 | 04 | 49 | 0 | 149111 | 1192884 | 49 | 0 | 0 | 944 | 1202732 | 49 | 0 | 0 | 1 | 952 |
| 197 | C5 | 4 | 04 | 49 | 1 | 149229 | 1193828 | 49 | 1 | 0 | 944 | 1203684 | 49 | 1 | 0 | 1 | 952 |
| 198 | C6 | 4 | 04 | 49 | 2 | 149347 | 1194772 | 49 | 2 | 0 | 944 | 1204636 | 49 | 2 | 0 | 1 | 952 |
| 199 | C7 | 4 | 04 | 49 | 3 | 149465 | 1195716 | 49 | 3 | 0 | 944 | 1205588 | 49 | 3 | 0 | 1 | 952 |
| 200 | C8 | 4 | 04 | 50 | 0 | 149585 | 1196676 | 50 | 0 | 0 | 960 | 1206556 | 50 | 0 | 0 | 1 | 968 |
| 201 | C9 | 4 | 04 | 50 | 1 | 149705 | 1197636 | 50 | 1 | 0 | 960 | 1207524 | 50 | 1 | 0 | 1 | 968 |
| 202 | CA | 4 | 04 | 50 | 2 | 149825 | 1198596 | 50 | 2 | 0 | 960 | 1208492 | 50 | 2 | 0 | 1 | 968 |
| 203 | CB | 4 | 04 | 50 | 3 | 149945 | 1199556 | 50 | 3 | 0 | 960 | 1209460 | 50 | 3 | 0 | 1 | 968 |
| 204 | CC | 4 | 04 | 51 | 0 | 150067 | 1200532 | 51 | 0 | 0 | 976 | 1210444 | 51 | 0 | 0 | 1 | 984 |
| 205 | CD | 4 | 04 | 51 | 1 | 150189 | 1201508 | 51 | 1 | 0 | 976 | 1211428 | 51 | 1 | 0 | 1 | 984 |
| 206 | CE | 4 | 04 | 51 | 2 | 150311 | 1202484 | 51 | 2 | 0 | 976 | 1212412 | 51 | 2 | 0 | 1 | 984 |
| 207 | CF | 4 | 04 | 51 | 3 | 150433 | 1203460 | 51 | 3 | 0 | 976 | 1213396 | 51 | 3 | 0 | 1 | 984 |
| 208 | D0 | 4 | 04 | 52 | 0 | 150557 | 1204452 | 52 | 0 | 0 | 992 | 1214396 | 52 | 0 | 0 | 1 | 1000 |

| | | | | | | | | | | | | | | | | | |
|---|---|---|---|---|---|---|---|---|---|---|---|---|---|---|---|---|---|
| 209 | D1 | 4 | 04 | 52 | 1 | 150681 | 1205444 | 52 | 1 | 0 | 992 | 1215396 | 52 | 1 | 0 | 1 | 1000 |
| 210 | D2 | 4 | 04 | 52 | 2 | 150805 | 1206436 | 52 | 2 | 0 | 992 | 1216396 | 52 | 2 | 0 | 1 | 1000 |
| 211 | D3 | 4 | 04 | 52 | 3 | 150929 | 1207428 | 52 | 3 | 0 | 992 | 1217396 | 52 | 3 | 0 | 1 | 1000 |
| 212 | D4 | 4 | 04 | 53 | 0 | 151055 | 1208436 | 53 | 0 | 0 | 1008 | 1218412 | 53 | 0 | 0 | 1 | 1016 |
| 213 | D5 | 4 | 04 | 53 | 1 | 151181 | 1209444 | 53 | 1 | 0 | 1008 | 1219428 | 53 | 1 | 0 | 1 | 1016 |
| 214 | D6 | 4 | 04 | 53 | 2 | 151307 | 1210452 | 53 | 2 | 0 | 1008 | 1220444 | 53 | 2 | 0 | 1 | 1016 |
| 215 | D7 | 4 | 04 | 53 | 3 | 151433 | 1211460 | 53 | 3 | 0 | 1008 | 1221460 | 53 | 3 | 0 | 1 | 1016 |
| 216 | D8 | 4 | 04 | 54 | 0 | 151561 | 1212484 | 54 | 0 | 0 | 1024 | 1222492 | 54 | 0 | 0 | 1 | 1032 |
| 217 | D9 | 4 | 04 | 54 | 1 | 151689 | 1213508 | 54 | 1 | 0 | 1024 | 1223524 | 54 | 1 | 0 | 1 | 1032 |
| 218 | DA | 4 | 04 | 54 | 2 | 151817 | 1214532 | 54 | 2 | 0 | 1024 | 1224556 | 54 | 2 | 0 | 1 | 1032 |
| 219 | DB | 4 | 04 | 54 | 3 | 151945 | 1215556 | 54 | 3 | 0 | 1024 | 1225588 | 54 | 3 | 0 | 1 | 1032 |
| 220 | DC | 4 | 04 | 55 | 0 | 152075 | 1216596 | 55 | 0 | 0 | 1040 | 1226636 | 55 | 0 | 0 | 1 | 1048 |
| 221 | DD | 4 | 04 | 55 | 1 | 152205 | 1217636 | 55 | 1 | 0 | 1040 | 1227684 | 55 | 1 | 0 | 1 | 1048 |
| 222 | DE | 4 | 04 | 55 | 2 | 152335 | 1218676 | 55 | 2 | 0 | 1040 | 1228732 | 55 | 2 | 0 | 1 | 1048 |
| 223 | DF | 4 | 04 | 55 | 3 | 152465 | 1219716 | 55 | 3 | 0 | 1040 | 1229780 | 55 | 3 | 0 | 1 | 1048 |
| 224 | E0 | 4 | 04 | 56 | 0 | 152597 | 1220772 | 56 | 0 | 0 | 1056 | 1230844 | 56 | 0 | 0 | 1 | 1064 |
| 225 | E1 | 4 | 04 | 56 | 1 | 152729 | 1221828 | 56 | 1 | 0 | 1056 | 1231908 | 56 | 1 | 0 | 1 | 1064 |
| 226 | E2 | 4 | 04 | 56 | 2 | 152861 | 1222884 | 56 | 2 | 0 | 1056 | 1232972 | 56 | 2 | 0 | 1 | 1064 |
| 227 | E3 | 4 | 04 | 56 | 3 | 152993 | 1223940 | 56 | 3 | 0 | 1056 | 1234036 | 56 | 3 | 0 | 1 | 1064 |
| 228 | E4 | 4 | 04 | 57 | 0 | 153127 | 1225012 | 57 | 0 | 0 | 1072 | 1235116 | 57 | 0 | 0 | 1 | 1080 |
| 229 | E5 | 4 | 04 | 57 | 1 | 153261 | 1226084 | 57 | 1 | 0 | 1072 | 1236196 | 57 | 1 | 0 | 1 | 1080 |
| 230 | E6 | 4 | 04 | 57 | 2 | 153395 | 1227156 | 57 | 2 | 0 | 1072 | 1237276 | 57 | 2 | 0 | 1 | 1080 |
| 231 | E7 | 4 | 04 | 57 | 3 | 153529 | 1228228 | 57 | 3 | 0 | 1072 | 1238356 | 57 | 3 | 0 | 1 | 1080 |
| 232 | E8 | 4 | 04 | 58 | 0 | 153665 | 1229316 | 58 | 0 | 0 | 1088 | 1239452 | 58 | 0 | 0 | 1 | 1096 |
| 233 | E9 | 4 | 04 | 58 | 1 | 153801 | 1230404 | 58 | 1 | 0 | 1088 | 1240548 | 58 | 1 | 0 | 1 | 1096 |
| 234 | EA | 4 | 04 | 58 | 2 | 153937 | 1231492 | 58 | 2 | 0 | 1088 | 1241644 | 58 | 2 | 0 | 1 | 1096 |
| 235 | EB | 4 | 04 | 58 | 3 | 154073 | 1232580 | 58 | 3 | 0 | 1088 | 1242740 | 58 | 3 | 0 | 1 | 1096 |
| 236 | EC | 4 | 04 | 59 | 0 | 154211 | 1233684 | 59 | 0 | 0 | 1104 | 1243852 | 59 | 0 | 0 | 1 | 1112 |
| 237 | ED | 4 | 04 | 59 | 1 | 154349 | 1234788 | 59 | 1 | 0 | 1104 | 1244964 | 59 | 1 | 0 | 1 | 1112 |
| 238 | EE | 4 | 04 | 59 | 2 | 154487 | 1235892 | 59 | 2 | 0 | 1104 | 1246076 | 59 | 2 | 0 | 1 | 1112 |
| 239 | EF | 4 | 04 | 59 | 3 | 154625 | 1236996 | 59 | 3 | 0 | 1104 | 1247188 | 59 | 3 | 0 | 1 | 1112 |
| 240 | F0 | 4 | 04 | 60 | 0 | 154765 | 1238116 | 60 | 0 | 0 | 1120 | 1248316 | 60 | 0 | 0 | 1 | 1128 |
| 241 | F1 | 4 | 04 | 60 | 1 | 154905 | 1239236 | 60 | 1 | 0 | 1120 | 1249444 | 60 | 1 | 0 | 1 | 1128 |
| 242 | F2 | 4 | 04 | 60 | 2 | 155045 | 1240356 | 60 | 2 | 0 | 1120 | 1250572 | 60 | 2 | 0 | 1 | 1128 |
| 243 | F3 | 4 | 04 | 60 | 3 | 155185 | 1241476 | 60 | 3 | 0 | 1120 | 1251700 | 60 | 3 | 0 | 1 | 1128 |
| 244 | F4 | 4 | 04 | 61 | 0 | 155327 | 1242612 | 61 | 0 | 0 | 1136 | 1252844 | 61 | 0 | 0 | 1 | 1144 |
| 245 | F5 | 4 | 04 | 61 | 1 | 155469 | 1243748 | 61 | 1 | 0 | 1136 | 1253988 | 61 | 1 | 0 | 1 | 1144 |
| 246 | F6 | 4 | 04 | 61 | 2 | 155611 | 1244884 | 61 | 2 | 0 | 1136 | 1255132 | 61 | 2 | 0 | 1 | 1144 |
| 247 | F7 | 4 | 04 | 61 | 3 | 155753 | 1246020 | 61 | 3 | 0 | 1136 | 1256276 | 61 | 3 | 0 | 1 | 1144 |
| 248 | F8 | 4 | 04 | 62 | 0 | 155897 | 1247172 | 62 | 0 | 0 | 1152 | 1257436 | 62 | 0 | 0 | 1 | 1160 |
| 249 | F9 | 4 | 04 | 62 | 1 | 156041 | 1248324 | 62 | 1 | 0 | 1152 | 1258596 | 62 | 1 | 0 | 1 | 1160 |
| 250 | FA | 4 | 04 | 62 | 2 | 156185 | 1249476 | 62 | 2 | 0 | 1152 | 1259756 | 62 | 2 | 0 | 1 | 1160 |
| 251 | FB | 4 | 04 | 62 | 3 | 156329 | 1250628 | 62 | 3 | 0 | 1152 | 1260916 | 62 | 3 | 0 | 1 | 1160 |
| 252 | FC | 4 | 04 | 63 | 0 | 156475 | 1251796 | 63 | 0 | 0 | 1168 | 1262092 | 63 | 0 | 0 | 1 | 1176 |
| 253 | FD | 4 | 04 | 63 | 1 | 156621 | 1252964 | 63 | 1 | 0 | 1168 | 1263268 | 63 | 1 | 0 | 1 | 1176 |
| 254 | FE | 4 | 04 | 63 | 2 | 156767 | 1254132 | 63 | 2 | 0 | 1168 | 1264444 | 63 | 2 | 0 | 1 | 1176 |
| 255 | FF | 4 | 04 | 63 | 3 | 156913 | 1255300 | 63 | 3 | 0 | 1168 | 1265620 | 63 | 3 | 0 | 1 | 1176 |
| 0 | 00 | 5 | 05 | 0 | 0 | 156925 | 1255396 | 0 | 0 | 0 | 96 | 1265724 | 0 | 0 | 0 | 1 | 104 |
| 1 | 01 | 5 | 05 | 0 | 1 | 156939 | 1255508 | 0 | 1 | 0 | 112 | 1265844 | 0 | 1 | 0 | 1 | 120 |
| 2 | 02 | 5 | 05 | 0 | 2 | 156953 | 1255620 | 0 | 2 | 0 | 112 | 1265964 | 0 | 2 | 0 | 1 | 120 |
| 3 | 03 | 5 | 05 | 0 | 3 | 156967 | 1255732 | 0 | 3 | 0 | 112 | 1266084 | 0 | 3 | 0 | 1 | 120 |
| 4 | 04 | 5 | 05 | 0 | 4 | 156981 | 1255844 | 0 | 4 | 0 | 112 | 1266204 | 0 | 4 | 0 | 1 | 120 |
| 5 | 05 | 5 | 05 | 1 | 0 | 156993 | 1255940 | 1 | 0 | 0 | 96 | 1266308 | 1 | 0 | 0 | 1 | 104 |
| 6 | 06 | 5 | 05 | 1 | 1 | 157010 | 1256076 | 1 | 1 | 0 | 136 | 1266452 | 1 | 1 | 0 | 1 | 144 |
| 7 | 07 | 5 | 05 | 1 | 2 | 157027 | 1256212 | 1 | 2 | 0 | 136 | 1266596 | 1 | 2 | 0 | 1 | 144 |
| 8 | 08 | 5 | 05 | 1 | 3 | 157044 | 1256348 | 1 | 3 | 0 | 136 | 1266740 | 1 | 3 | 0 | 1 | 144 |
| 9 | 09 | 5 | 05 | 1 | 4 | 157061 | 1256484 | 1 | 4 | 0 | 136 | 1266884 | 1 | 4 | 0 | 1 | 144 |
| 10 | 0A | 5 | 05 | 2 | 0 | 157080 | 1256636 | 2 | 0 | 0 | 152 | 1267044 | 2 | 0 | 0 | 1 | 160 |
| 11 | 0B | 5 | 05 | 2 | 1 | 157099 | 1256788 | 2 | 1 | 0 | 152 | 1267204 | 2 | 1 | 0 | 1 | 160 |
| 12 | 0C | 5 | 05 | 2 | 2 | 157118 | 1256940 | 2 | 2 | 0 | 152 | 1267364 | 2 | 2 | 0 | 1 | 160 |
| 13 | 0D | 5 | 05 | 2 | 3 | 157137 | 1257092 | 2 | 3 | 0 | 152 | 1267524 | 2 | 3 | 0 | 1 | 160 |
| 14 | 0E | 5 | 05 | 2 | 4 | 157156 | 1257244 | 2 | 4 | 0 | 152 | 1267684 | 2 | 4 | 0 | 1 | 160 |
| 15 | 0F | 5 | 05 | 3 | 0 | 157177 | 1257412 | 3 | 0 | 0 | 168 | 1267860 | 3 | 0 | 0 | 1 | 176 |
| 16 | 10 | 5 | 05 | 3 | 1 | 157203 | 1257620 | 3 | 1 | 0 | 208 | 1268076 | 3 | 1 | 0 | 1 | 216 |
| 17 | 11 | 5 | 05 | 3 | 2 | 157229 | 1257828 | 3 | 2 | 0 | 208 | 1268292 | 3 | 2 | 0 | 1 | 216 |
| 18 | 12 | 5 | 05 | 3 | 3 | 157255 | 1258036 | 3 | 3 | 0 | 208 | 1268508 | 3 | 3 | 0 | 1 | 216 |
| 19 | 13 | 5 | 05 | 3 | 4 | 157281 | 1258244 | 3 | 4 | 0 | 208 | 1268724 | 3 | 4 | 0 | 1 | 216 |
| 20 | 14 | 5 | 05 | 4 | 0 | 157309 | 1258468 | 4 | 0 | 0 | 224 | 1268956 | 4 | 0 | 0 | 1 | 232 |
| 21 | 15 | 5 | 05 | 4 | 1 | 157337 | 1258692 | 4 | 1 | 0 | 224 | 1269188 | 4 | 1 | 0 | 1 | 232 |
| 22 | 16 | 5 | 05 | 4 | 2 | 157365 | 1258916 | 4 | 2 | 0 | 224 | 1269420 | 4 | 2 | 0 | 1 | 232 |
| 23 | 17 | 5 | 05 | 4 | 3 | 157393 | 1259140 | 4 | 3 | 0 | 224 | 1269652 | 4 | 3 | 0 | 1 | 232 |
| 24 | 18 | 5 | 05 | 4 | 4 | 157421 | 1259364 | 4 | 4 | 0 | 224 | 1269884 | 4 | 4 | 0 | 1 | 232 |
| 25 | 19 | 5 | 05 | 5 | 0 | 157451 | 1259604 | 5 | 0 | 0 | 240 | 1270132 | 5 | 0 | 0 | 1 | 248 |
| 26 | 1A | 5 | 05 | 5 | 1 | 157481 | 1259844 | 5 | 1 | 0 | 240 | 1270380 | 5 | 1 | 0 | 1 | 248 |
| 27 | 1B | 5 | 05 | 5 | 2 | 157511 | 1260084 | 5 | 2 | 0 | 240 | 1270628 | 5 | 2 | 0 | 1 | 248 |
| 28 | 1C | 5 | 05 | 5 | 3 | 157541 | 1260324 | 5 | 3 | 0 | 240 | 1270876 | 5 | 3 | 0 | 1 | 248 |
| 29 | 1D | 5 | 05 | 5 | 4 | 157571 | 1260564 | 5 | 4 | 0 | 240 | 1271124 | 5 | 4 | 0 | 1 | 248 |
| 30 | 1E | 5 | 05 | 6 | 0 | 157603 | 1260820 | 6 | 0 | 0 | 256 | 1271388 | 6 | 0 | 0 | 1 | 264 |
| 31 | 1F | 5 | 05 | 6 | 1 | 157635 | 1261076 | 6 | 1 | 0 | 256 | 1271652 | 6 | 1 | 0 | 1 | 264 |
| 32 | 20 | 5 | 05 | 6 | 2 | 157667 | 1261332 | 6 | 2 | 0 | 256 | 1271916 | 6 | 2 | 0 | 1 | 264 |
| 33 | 21 | 5 | 05 | 6 | 3 | 157699 | 1261588 | 6 | 3 | 0 | 256 | 1272180 | 6 | 3 | 0 | 1 | 264 |
| 34 | 22 | 5 | 05 | 6 | 4 | 157731 | 1261844 | 6 | 4 | 0 | 256 | 1272444 | 6 | 4 | 0 | 1 | 264 |
| 35 | 23 | 5 | 05 | 7 | 0 | 157765 | 1262116 | 7 | 0 | 0 | 272 | 1272724 | 7 | 0 | 0 | 1 | 280 |
| 36 | 24 | 5 | 05 | 7 | 1 | 157799 | 1262388 | 7 | 1 | 0 | 272 | 1273004 | 7 | 1 | 0 | 1 | 280 |
| 37 | 25 | 5 | 05 | 7 | 2 | 157833 | 1262660 | 7 | 2 | 0 | 272 | 1273284 | 7 | 2 | 0 | 1 | 280 |
| 38 | 26 | 5 | 05 | 7 | 3 | 157867 | 1262932 | 7 | 3 | 0 | 272 | 1273564 | 7 | 3 | 0 | 1 | 280 |
| 39 | 27 | 5 | 05 | 7 | 4 | 157901 | 1263204 | 7 | 4 | 0 | 272 | 1273844 | 7 | 4 | 0 | 1 | 280 |
| 40 | 28 | 5 | 05 | 8 | 0 | 157937 | 1263492 | 8 | 0 | 0 | 288 | 1274140 | 8 | 0 | 0 | 1 | 296 |
| 41 | 29 | 5 | 05 | 8 | 1 | 157973 | 1263780 | 8 | 1 | 0 | 288 | 1274436 | 8 | 1 | 0 | 1 | 296 |
| 42 | 2A | 5 | 05 | 8 | 2 | 158009 | 1264068 | 8 | 2 | 0 | 288 | 1274732 | 8 | 2 | 0 | 1 | 296 |
| 43 | 2B | 5 | 05 | 8 | 3 | 158045 | 1264356 | 8 | 3 | 0 | 288 | 1275028 | 8 | 3 | 0 | 1 | 296 |
| 44 | 2C | 5 | 05 | 8 | 4 | 158081 | 1264644 | 8 | 4 | 0 | 288 | 1275324 | 8 | 4 | 0 | 1 | 296 |
| 45 | 2D | 5 | 05 | 9 | 0 | 158119 | 1264948 | 9 | 0 | 0 | 304 | 1275636 | 9 | 0 | 0 | 1 | 312 |
| 46 | 2E | 5 | 05 | 9 | 1 | 158157 | 1265252 | 9 | 1 | 0 | 304 | 1275948 | 9 | 1 | 0 | 1 | 312 |
| 47 | 2F | 5 | 05 | 9 | 2 | 158195 | 1265556 | 9 | 2 | 0 | 304 | 1276260 | 9 | 2 | 0 | 1 | 312 |
| 48 | 30 | 5 | 05 | 9 | 3 | 158233 | 1265860 | 9 | 3 | 0 | 304 | 1276572 | 9 | 3 | 0 | 1 | 312 |
| 49 | 31 | 5 | 05 | 9 | 4 | 158271 | 1266164 | 9 | 4 | 0 | 304 | 1276884 | 9 | 4 | 0 | 1 | 312 |
| 50 | 32 | 5 | 05 | 10 | 0 | 158311 | 1266484 | 10 | 0 | 0 | 320 | 1277212 | 10 | 0 | 0 | 1 | 328 |
| 51 | 33 | 5 | 05 | 10 | 1 | 158351 | 1266804 | 10 | 1 | 0 | 320 | 1277540 | 10 | 1 | 0 | 1 | 328 |
| 52 | 34 | 5 | 05 | 10 | 2 | 158391 | 1267124 | 10 | 2 | 0 | 320 | 1277868 | 10 | 2 | 0 | 1 | 328 |
| 53 | 35 | 5 | 05 | 10 | 3 | 158431 | 1267444 | 10 | 3 | 0 | 320 | 1278196 | 10 | 3 | 0 | 1 | 328 |
| 54 | 36 | 5 | 05 | 10 | 4 | 158471 | 1267764 | 10 | 4 | 0 | 320 | 1278524 | 10 | 4 | 0 | 1 | 328 |
| 55 | 37 | 5 | 05 | 11 | 0 | 158513 | 1268100 | 11 | 0 | 0 | 336 | 1278868 | 11 | 0 | 0 | 1 | 344 |

| | | | | | | | | | | | | | | | | | |
|---|---|---|---|---|---|---|---|---|---|---|---|---|---|---|---|---|---|
| 56 | 38 | 5 | 05 | 11 | 1 | 158555 | 1268436 | 11 | 1 | 0 | 336 | 1279212 | 11 | 1 | 0 | 1 | 344 |
| 57 | 39 | 5 | 05 | 11 | 2 | 158597 | 1268772 | 11 | 2 | 0 | 336 | 1279556 | 11 | 2 | 0 | 1 | 344 |
| 58 | 3A | 5 | 05 | 11 | 3 | 158639 | 1269108 | 11 | 3 | 0 | 336 | 1279900 | 11 | 3 | 0 | 1 | 344 |
| 59 | 3B | 5 | 05 | 11 | 4 | 158681 | 1269444 | 11 | 4 | 0 | 336 | 1280244 | 11 | 4 | 0 | 1 | 344 |
| 60 | 3C | 5 | 05 | 12 | 0 | 158725 | 1269796 | 12 | 0 | 0 | 352 | 1280604 | 12 | 0 | 0 | 1 | 360 |
| 61 | 3D | 5 | 05 | 12 | 1 | 158769 | 1270148 | 12 | 1 | 0 | 352 | 1280964 | 12 | 1 | 0 | 1 | 360 |
| 62 | 3E | 5 | 05 | 12 | 2 | 158813 | 1270500 | 12 | 2 | 0 | 352 | 1281324 | 12 | 2 | 0 | 1 | 360 |
| 63 | 3F | 5 | 05 | 12 | 3 | 158857 | 1270852 | 12 | 3 | 0 | 352 | 1281684 | 12 | 3 | 0 | 1 | 360 |
| 64 | 40 | 5 | 05 | 12 | 4 | 158901 | 1271204 | 12 | 4 | 0 | 352 | 1282044 | 12 | 4 | 0 | 1 | 360 |
| 65 | 41 | 5 | 05 | 13 | 0 | 158947 | 1271572 | 13 | 0 | 0 | 368 | 1282420 | 13 | 0 | 0 | 1 | 376 |
| 66 | 42 | 5 | 05 | 13 | 1 | 158993 | 1271940 | 13 | 1 | 0 | 368 | 1282796 | 13 | 1 | 0 | 1 | 376 |
| 67 | 43 | 5 | 05 | 13 | 2 | 159039 | 1272308 | 13 | 2 | 0 | 368 | 1283172 | 13 | 2 | 0 | 1 | 376 |
| 68 | 44 | 5 | 05 | 13 | 3 | 159085 | 1272676 | 13 | 3 | 0 | 368 | 1283548 | 13 | 3 | 0 | 1 | 376 |
| 69 | 45 | 5 | 05 | 13 | 4 | 159131 | 1273044 | 13 | 4 | 0 | 368 | 1283924 | 13 | 4 | 0 | 1 | 376 |
| 70 | 46 | 5 | 05 | 14 | 0 | 159179 | 1273428 | 14 | 0 | 0 | 384 | 1284316 | 14 | 0 | 0 | 1 | 392 |
| 71 | 47 | 5 | 05 | 14 | 1 | 159227 | 1273812 | 14 | 1 | 0 | 384 | 1284708 | 14 | 1 | 0 | 1 | 392 |
| 72 | 48 | 5 | 05 | 14 | 2 | 159275 | 1274196 | 14 | 2 | 0 | 384 | 1285100 | 14 | 2 | 0 | 1 | 392 |
| 73 | 49 | 5 | 05 | 14 | 3 | 159323 | 1274580 | 14 | 3 | 0 | 384 | 1285492 | 14 | 3 | 0 | 1 | 392 |
| 74 | 4A | 5 | 05 | 14 | 4 | 159371 | 1274964 | 14 | 4 | 0 | 384 | 1285884 | 14 | 4 | 0 | 1 | 392 |
| 75 | 4B | 5 | 05 | 15 | 0 | 159421 | 1275364 | 15 | 0 | 0 | 400 | 1286292 | 15 | 0 | 0 | 1 | 408 |
| 76 | 4C | 5 | 05 | 15 | 1 | 159471 | 1275764 | 15 | 1 | 0 | 400 | 1286700 | 15 | 1 | 0 | 1 | 408 |
| 77 | 4D | 5 | 05 | 15 | 2 | 159521 | 1276164 | 15 | 2 | 0 | 400 | 1287108 | 15 | 2 | 0 | 1 | 408 |
| 78 | 4E | 5 | 05 | 15 | 3 | 159571 | 1276564 | 15 | 3 | 0 | 400 | 1287516 | 15 | 3 | 0 | 1 | 408 |
| 79 | 4F | 5 | 05 | 15 | 4 | 159621 | 1276964 | 15 | 4 | 0 | 400 | 1287924 | 15 | 4 | 0 | 1 | 408 |
| 80 | 50 | 5 | 05 | 16 | 0 | 159673 | 1277380 | 16 | 0 | 0 | 416 | 1288348 | 16 | 0 | 0 | 1 | 424 |
| 81 | 51 | 5 | 05 | 16 | 1 | 159725 | 1277796 | 16 | 1 | 0 | 416 | 1288772 | 16 | 1 | 0 | 1 | 424 |
| 82 | 52 | 5 | 05 | 16 | 2 | 159777 | 1278212 | 16 | 2 | 0 | 416 | 1289196 | 16 | 2 | 0 | 1 | 424 |
| 83 | 53 | 5 | 05 | 16 | 3 | 159829 | 1278628 | 16 | 3 | 0 | 416 | 1289620 | 16 | 3 | 0 | 1 | 424 |
| 84 | 54 | 5 | 05 | 16 | 4 | 159881 | 1279044 | 16 | 4 | 0 | 416 | 1290044 | 16 | 4 | 0 | 1 | 424 |
| 85 | 55 | 5 | 05 | 17 | 0 | 159935 | 1279476 | 17 | 0 | 0 | 432 | 1290484 | 17 | 0 | 0 | 1 | 440 |
| 86 | 56 | 5 | 05 | 17 | 1 | 159989 | 1279908 | 17 | 1 | 0 | 432 | 1290924 | 17 | 1 | 0 | 1 | 440 |
| 87 | 57 | 5 | 05 | 17 | 2 | 160043 | 1280340 | 17 | 2 | 0 | 432 | 1291364 | 17 | 2 | 0 | 1 | 440 |
| 88 | 58 | 5 | 05 | 17 | 3 | 160097 | 1280772 | 17 | 3 | 0 | 432 | 1291804 | 17 | 3 | 0 | 1 | 440 |
| 89 | 59 | 5 | 05 | 17 | 4 | 160151 | 1281204 | 17 | 4 | 0 | 432 | 1292244 | 17 | 4 | 0 | 1 | 440 |
| 90 | 5A | 5 | 05 | 18 | 0 | 160207 | 1281652 | 18 | 0 | 0 | 448 | 1292700 | 18 | 0 | 0 | 1 | 456 |
| 91 | 5B | 5 | 05 | 18 | 1 | 160263 | 1282100 | 18 | 1 | 0 | 448 | 1293156 | 18 | 1 | 0 | 1 | 456 |
| 92 | 5C | 5 | 05 | 18 | 2 | 160319 | 1282548 | 18 | 2 | 0 | 448 | 1293612 | 18 | 2 | 0 | 1 | 456 |
| 93 | 5D | 5 | 05 | 18 | 3 | 160375 | 1282996 | 18 | 3 | 0 | 448 | 1294068 | 18 | 3 | 0 | 1 | 456 |
| 94 | 5E | 5 | 05 | 18 | 4 | 160431 | 1283444 | 18 | 4 | 0 | 448 | 1294524 | 18 | 4 | 0 | 1 | 456 |
| 95 | 5F | 5 | 05 | 19 | 0 | 160489 | 1283908 | 19 | 0 | 0 | 464 | 1294996 | 19 | 0 | 0 | 1 | 472 |
| 96 | 60 | 5 | 05 | 19 | 1 | 160547 | 1284372 | 19 | 1 | 0 | 464 | 1295468 | 19 | 1 | 0 | 1 | 472 |
| 97 | 61 | 5 | 05 | 19 | 2 | 160605 | 1284836 | 19 | 2 | 0 | 464 | 1295940 | 19 | 2 | 0 | 1 | 472 |
| 98 | 62 | 5 | 05 | 19 | 3 | 160663 | 1285300 | 19 | 3 | 0 | 464 | 1296412 | 19 | 3 | 0 | 1 | 472 |
| 99 | 63 | 5 | 05 | 19 | 4 | 160721 | 1285764 | 19 | 4 | 0 | 464 | 1296884 | 19 | 4 | 0 | 1 | 472 |
| 100 | 64 | 5 | 05 | 20 | 0 | 160781 | 1286244 | 20 | 0 | 0 | 480 | 1297372 | 20 | 0 | 0 | 1 | 488 |
| 101 | 65 | 5 | 05 | 20 | 1 | 160841 | 1286724 | 20 | 1 | 0 | 480 | 1297860 | 20 | 1 | 0 | 1 | 488 |
| 102 | 66 | 5 | 05 | 20 | 2 | 160901 | 1287204 | 20 | 2 | 0 | 480 | 1298348 | 20 | 2 | 0 | 1 | 488 |
| 103 | 67 | 5 | 05 | 20 | 3 | 160961 | 1287684 | 20 | 3 | 0 | 480 | 1298836 | 20 | 3 | 0 | 1 | 488 |
| 104 | 68 | 5 | 05 | 20 | 4 | 161021 | 1288164 | 20 | 4 | 0 | 480 | 1299324 | 20 | 4 | 0 | 1 | 488 |
| 105 | 69 | 5 | 05 | 21 | 0 | 161083 | 1288660 | 21 | 0 | 0 | 496 | 1299828 | 21 | 0 | 0 | 1 | 504 |
| 106 | 6A | 5 | 05 | 21 | 1 | 161145 | 1289156 | 21 | 1 | 0 | 496 | 1300332 | 21 | 1 | 0 | 1 | 504 |
| 107 | 6B | 5 | 05 | 21 | 2 | 161207 | 1289652 | 21 | 2 | 0 | 496 | 1300836 | 21 | 2 | 0 | 1 | 504 |
| 108 | 6C | 5 | 05 | 21 | 3 | 161269 | 1290148 | 21 | 3 | 0 | 496 | 1301340 | 21 | 3 | 0 | 1 | 504 |
| 109 | 6D | 5 | 05 | 21 | 4 | 161331 | 1290644 | 21 | 4 | 0 | 496 | 1301844 | 21 | 4 | 0 | 1 | 504 |
| 110 | 6E | 5 | 05 | 22 | 0 | 161395 | 1291156 | 22 | 0 | 0 | 512 | 1302364 | 22 | 0 | 0 | 1 | 520 |
| 111 | 6F | 5 | 05 | 22 | 1 | 161459 | 1291668 | 22 | 1 | 0 | 512 | 1302884 | 22 | 1 | 0 | 1 | 520 |
| 112 | 70 | 5 | 05 | 22 | 2 | 161523 | 1292180 | 22 | 2 | 0 | 512 | 1303404 | 22 | 2 | 0 | 1 | 520 |
| 113 | 71 | 5 | 05 | 22 | 3 | 161587 | 1292692 | 22 | 3 | 0 | 512 | 1303924 | 22 | 3 | 0 | 1 | 520 |
| 114 | 72 | 5 | 05 | 22 | 4 | 161651 | 1293204 | 22 | 4 | 0 | 512 | 1304444 | 22 | 4 | 0 | 1 | 520 |
| 115 | 73 | 5 | 05 | 23 | 0 | 161717 | 1293732 | 23 | 0 | 0 | 528 | 1304980 | 23 | 0 | 0 | 1 | 536 |
| 116 | 74 | 5 | 05 | 23 | 1 | 161783 | 1294260 | 23 | 1 | 0 | 528 | 1305516 | 23 | 1 | 0 | 1 | 536 |
| 117 | 75 | 5 | 05 | 23 | 2 | 161849 | 1294788 | 23 | 2 | 0 | 528 | 1306052 | 23 | 2 | 0 | 1 | 536 |
| 118 | 76 | 5 | 05 | 23 | 3 | 161915 | 1295316 | 23 | 3 | 0 | 528 | 1306588 | 23 | 3 | 0 | 1 | 536 |
| 119 | 77 | 5 | 05 | 23 | 4 | 161981 | 1295844 | 23 | 4 | 0 | 528 | 1307124 | 23 | 4 | 0 | 1 | 536 |
| 120 | 78 | 5 | 05 | 24 | 0 | 162049 | 1296388 | 24 | 0 | 0 | 544 | 1307676 | 24 | 0 | 0 | 1 | 552 |
| 121 | 79 | 5 | 05 | 24 | 1 | 162117 | 1296932 | 24 | 1 | 0 | 544 | 1308228 | 24 | 1 | 0 | 1 | 552 |
| 122 | 7A | 5 | 05 | 24 | 2 | 162185 | 1297476 | 24 | 2 | 0 | 544 | 1308780 | 24 | 2 | 0 | 1 | 552 |
| 123 | 7B | 5 | 05 | 24 | 3 | 162253 | 1298020 | 24 | 3 | 0 | 544 | 1309332 | 24 | 3 | 0 | 1 | 552 |
| 124 | 7C | 5 | 05 | 24 | 4 | 162321 | 1298564 | 24 | 4 | 0 | 544 | 1309884 | 24 | 4 | 0 | 1 | 552 |
| 125 | 7D | 5 | 05 | 25 | 0 | 162391 | 1299124 | 25 | 0 | 0 | 560 | 1310452 | 25 | 0 | 0 | 1 | 568 |
| 126 | 7E | 5 | 05 | 25 | 1 | 162461 | 1299684 | 25 | 1 | 0 | 560 | 1311020 | 25 | 1 | 0 | 1 | 568 |
| 127 | 7F | 5 | 05 | 25 | 2 | 162531 | 1300244 | 25 | 2 | 0 | 560 | 1311588 | 25 | 2 | 0 | 1 | 568 |
| 128 | 80 | 5 | 05 | 25 | 3 | 162601 | 1300804 | 25 | 3 | 0 | 560 | 1312156 | 25 | 3 | 0 | 1 | 568 |
| 129 | 81 | 5 | 05 | 25 | 4 | 162671 | 1301364 | 25 | 4 | 0 | 560 | 1312724 | 25 | 4 | 0 | 1 | 568 |
| 130 | 82 | 5 | 05 | 26 | 0 | 162743 | 1301940 | 26 | 0 | 0 | 576 | 1313308 | 26 | 0 | 0 | 1 | 584 |
| 131 | 83 | 5 | 05 | 26 | 1 | 162815 | 1302516 | 26 | 1 | 0 | 576 | 1313892 | 26 | 1 | 0 | 1 | 584 |
| 132 | 84 | 5 | 05 | 26 | 2 | 162887 | 1303092 | 26 | 2 | 0 | 576 | 1314476 | 26 | 2 | 0 | 1 | 584 |
| 133 | 85 | 5 | 05 | 26 | 3 | 162959 | 1303668 | 26 | 3 | 0 | 576 | 1315060 | 26 | 3 | 0 | 1 | 584 |
| 134 | 86 | 5 | 05 | 26 | 4 | 163031 | 1304244 | 26 | 4 | 0 | 576 | 1315644 | 26 | 4 | 0 | 1 | 584 |
| 135 | 87 | 5 | 05 | 27 | 0 | 163105 | 1304836 | 27 | 0 | 0 | 592 | 1316244 | 27 | 0 | 0 | 1 | 600 |
| 136 | 88 | 5 | 05 | 27 | 1 | 163179 | 1305428 | 27 | 1 | 0 | 592 | 1316844 | 27 | 1 | 0 | 1 | 600 |
| 137 | 89 | 5 | 05 | 27 | 2 | 163253 | 1306020 | 27 | 2 | 0 | 592 | 1317444 | 27 | 2 | 0 | 1 | 600 |
| 138 | 8A | 5 | 05 | 27 | 3 | 163327 | 1306612 | 27 | 3 | 0 | 592 | 1318044 | 27 | 3 | 0 | 1 | 600 |
| 139 | 8B | 5 | 05 | 27 | 4 | 163401 | 1307204 | 27 | 4 | 0 | 592 | 1318644 | 27 | 4 | 0 | 1 | 600 |
| 140 | 8C | 5 | 05 | 28 | 0 | 163477 | 1307812 | 28 | 0 | 0 | 608 | 1319260 | 28 | 0 | 0 | 1 | 616 |
| 141 | 8D | 5 | 05 | 28 | 1 | 163553 | 1308420 | 28 | 1 | 0 | 608 | 1319876 | 28 | 1 | 0 | 1 | 616 |
| 142 | 8E | 5 | 05 | 28 | 2 | 163629 | 1309028 | 28 | 2 | 0 | 608 | 1320492 | 28 | 2 | 0 | 1 | 616 |
| 143 | 8F | 5 | 05 | 28 | 3 | 163705 | 1309636 | 28 | 3 | 0 | 608 | 1321108 | 28 | 3 | 0 | 1 | 616 |
| 144 | 90 | 5 | 05 | 28 | 4 | 163781 | 1310244 | 28 | 4 | 0 | 608 | 1321724 | 28 | 4 | 0 | 1 | 616 |
| 145 | 91 | 5 | 05 | 29 | 0 | 163859 | 1310868 | 29 | 0 | 0 | 624 | 1322356 | 29 | 0 | 0 | 1 | 632 |
| 146 | 92 | 5 | 05 | 29 | 1 | 163937 | 1311492 | 29 | 1 | 0 | 624 | 1322988 | 29 | 1 | 0 | 1 | 632 |
| 147 | 93 | 5 | 05 | 29 | 2 | 164015 | 1312116 | 29 | 2 | 0 | 624 | 1323620 | 29 | 2 | 0 | 1 | 632 |
| 148 | 94 | 5 | 05 | 29 | 3 | 164093 | 1312740 | 29 | 3 | 0 | 624 | 1324252 | 29 | 3 | 0 | 1 | 632 |
| 149 | 95 | 5 | 05 | 29 | 4 | 164171 | 1313364 | 29 | 4 | 0 | 624 | 1324884 | 29 | 4 | 0 | 1 | 632 |
| 150 | 96 | 5 | 05 | 30 | 0 | 164251 | 1314004 | 30 | 0 | 0 | 640 | 1325532 | 30 | 0 | 0 | 1 | 648 |
| 151 | 97 | 5 | 05 | 30 | 1 | 164331 | 1314644 | 30 | 1 | 0 | 640 | 1326180 | 30 | 1 | 0 | 1 | 648 |
| 152 | 98 | 5 | 05 | 30 | 2 | 164411 | 1315284 | 30 | 2 | 0 | 640 | 1326828 | 30 | 2 | 0 | 1 | 648 |
| 153 | 99 | 5 | 05 | 30 | 3 | 164491 | 1315924 | 30 | 3 | 0 | 640 | 1327476 | 30 | 3 | 0 | 1 | 648 |
| 154 | 9A | 5 | 05 | 30 | 4 | 164571 | 1316564 | 30 | 4 | 0 | 640 | 1328124 | 30 | 4 | 0 | 1 | 648 |
| 155 | 9B | 5 | 05 | 31 | 0 | 164653 | 1317220 | 31 | 0 | 0 | 656 | 1328788 | 31 | 0 | 0 | 1 | 664 |
| 156 | 9C | 5 | 05 | 31 | 1 | 164735 | 1317876 | 31 | 1 | 0 | 656 | 1329452 | 31 | 1 | 0 | 1 | 664 |
| 157 | 9D | 5 | 05 | 31 | 2 | 164817 | 1318532 | 31 | 2 | 0 | 656 | 1330116 | 31 | 2 | 0 | 1 | 664 |
| 158 | 9E | 5 | 05 | 31 | 3 | 164899 | 1319188 | 31 | 3 | 0 | 656 | 1330780 | 31 | 3 | 0 | 1 | 664 |

| | | | | | | | | | | | | | | | | | |
|---|---|---|---|---|---|---|---|---|---|---|---|---|---|---|---|---|---|
| 159 | 9F | 5 | 05 | 31 | 4 | 164981 | 1319844 | 31 | 4 | 0 | 656 | 1331444 | 31 | 4 | 0 | 1 | 664 |
| 160 | A0 | 5 | 05 | 32 | 0 | 165065 | 1320516 | 32 | 0 | 0 | 672 | 1332124 | 32 | 0 | 0 | 1 | 680 |
| 161 | A1 | 5 | 05 | 32 | 1 | 165149 | 1321188 | 32 | 1 | 0 | 672 | 1332804 | 32 | 1 | 0 | 1 | 680 |
| 162 | A2 | 5 | 05 | 32 | 2 | 165233 | 1321860 | 32 | 2 | 0 | 672 | 1333484 | 32 | 2 | 0 | 1 | 680 |
| 163 | A3 | 5 | 05 | 32 | 3 | 165317 | 1322532 | 32 | 3 | 0 | 672 | 1334164 | 32 | 3 | 0 | 1 | 680 |
| 164 | A4 | 5 | 05 | 32 | 4 | 165401 | 1323204 | 32 | 4 | 0 | 672 | 1334844 | 32 | 4 | 0 | 1 | 680 |
| 165 | A5 | 5 | 05 | 33 | 0 | 165487 | 1323892 | 33 | 0 | 0 | 688 | 1335540 | 33 | 0 | 0 | 1 | 696 |
| 166 | A6 | 5 | 05 | 33 | 1 | 165573 | 1324580 | 33 | 1 | 0 | 688 | 1336236 | 33 | 1 | 0 | 1 | 696 |
| 167 | A7 | 5 | 05 | 33 | 2 | 165659 | 1325268 | 33 | 2 | 0 | 688 | 1336932 | 33 | 2 | 0 | 1 | 696 |
| 168 | A8 | 5 | 05 | 33 | 3 | 165745 | 1325956 | 33 | 3 | 0 | 688 | 1337628 | 33 | 3 | 0 | 1 | 696 |
| 169 | A9 | 5 | 05 | 33 | 4 | 165831 | 1326644 | 33 | 4 | 0 | 688 | 1338324 | 33 | 4 | 0 | 1 | 696 |
| 170 | AA | 5 | 05 | 34 | 0 | 165919 | 1327348 | 34 | 0 | 0 | 704 | 1339036 | 34 | 0 | 0 | 1 | 712 |
| 171 | AB | 5 | 05 | 34 | 1 | 166007 | 1328052 | 34 | 1 | 0 | 704 | 1339748 | 34 | 1 | 0 | 1 | 712 |
| 172 | AC | 5 | 05 | 34 | 2 | 166095 | 1328756 | 34 | 2 | 0 | 704 | 1340460 | 34 | 2 | 0 | 1 | 712 |
| 173 | AD | 5 | 05 | 34 | 3 | 166183 | 1329460 | 34 | 3 | 0 | 704 | 1341172 | 34 | 3 | 0 | 1 | 712 |
| 174 | AE | 5 | 05 | 34 | 4 | 166271 | 1330164 | 34 | 4 | 0 | 704 | 1341884 | 34 | 4 | 0 | 1 | 712 |
| 175 | AF | 5 | 05 | 35 | 0 | 166361 | 1330884 | 35 | 0 | 0 | 720 | 1342612 | 35 | 0 | 0 | 1 | 728 |
| 176 | B0 | 5 | 05 | 35 | 1 | 166451 | 1331604 | 35 | 1 | 0 | 720 | 1343340 | 35 | 1 | 0 | 1 | 728 |
| 177 | B1 | 5 | 05 | 35 | 2 | 166541 | 1332324 | 35 | 2 | 0 | 720 | 1344068 | 35 | 2 | 0 | 1 | 728 |
| 178 | B2 | 5 | 05 | 35 | 3 | 166631 | 1333044 | 35 | 3 | 0 | 720 | 1344796 | 35 | 3 | 0 | 1 | 728 |
| 179 | B3 | 5 | 05 | 35 | 4 | 166721 | 1333764 | 35 | 4 | 0 | 720 | 1345524 | 35 | 4 | 0 | 1 | 728 |
| 180 | B4 | 5 | 05 | 36 | 0 | 166813 | 1334500 | 36 | 0 | 0 | 736 | 1346268 | 36 | 0 | 0 | 1 | 744 |
| 181 | B5 | 5 | 05 | 36 | 1 | 166905 | 1335236 | 36 | 1 | 0 | 736 | 1347012 | 36 | 1 | 0 | 1 | 744 |
| 182 | B6 | 5 | 05 | 36 | 2 | 166997 | 1335972 | 36 | 2 | 0 | 736 | 1347756 | 36 | 2 | 0 | 1 | 744 |
| 183 | B7 | 5 | 05 | 36 | 3 | 167089 | 1336708 | 36 | 3 | 0 | 736 | 1348500 | 36 | 3 | 0 | 1 | 744 |
| 184 | B8 | 5 | 05 | 36 | 4 | 167181 | 1337444 | 36 | 4 | 0 | 736 | 1349244 | 36 | 4 | 0 | 1 | 744 |
| 185 | B9 | 5 | 05 | 37 | 0 | 167275 | 1338196 | 37 | 0 | 0 | 752 | 1350004 | 37 | 0 | 0 | 1 | 760 |
| 186 | BA | 5 | 05 | 37 | 1 | 167369 | 1338948 | 37 | 1 | 0 | 752 | 1350764 | 37 | 1 | 0 | 1 | 760 |
| 187 | BB | 5 | 05 | 37 | 2 | 167463 | 1339700 | 37 | 2 | 0 | 752 | 1351524 | 37 | 2 | 0 | 1 | 760 |
| 188 | BC | 5 | 05 | 37 | 3 | 167557 | 1340452 | 37 | 3 | 0 | 752 | 1352284 | 37 | 3 | 0 | 1 | 760 |
| 189 | BD | 5 | 05 | 37 | 4 | 167651 | 1341204 | 37 | 4 | 0 | 752 | 1353044 | 37 | 4 | 0 | 1 | 760 |
| 190 | BE | 5 | 05 | 38 | 0 | 167747 | 1341972 | 38 | 0 | 0 | 768 | 1353820 | 38 | 0 | 0 | 1 | 776 |
| 191 | BF | 5 | 05 | 38 | 1 | 167843 | 1342740 | 38 | 1 | 0 | 768 | 1354596 | 38 | 1 | 0 | 1 | 776 |
| 192 | C0 | 5 | 05 | 38 | 2 | 167939 | 1343508 | 38 | 2 | 0 | 768 | 1355372 | 38 | 2 | 0 | 1 | 776 |
| 193 | C1 | 5 | 05 | 38 | 3 | 168035 | 1344276 | 38 | 3 | 0 | 768 | 1356148 | 38 | 3 | 0 | 1 | 776 |
| 194 | C2 | 5 | 05 | 38 | 4 | 168131 | 1345044 | 38 | 4 | 0 | 768 | 1356924 | 38 | 4 | 0 | 1 | 776 |
| 195 | C3 | 5 | 05 | 39 | 0 | 168229 | 1345828 | 39 | 0 | 0 | 784 | 1357716 | 39 | 0 | 0 | 1 | 792 |
| 196 | C4 | 5 | 05 | 39 | 1 | 168327 | 1346612 | 39 | 1 | 0 | 784 | 1358508 | 39 | 1 | 0 | 1 | 792 |
| 197 | C5 | 5 | 05 | 39 | 2 | 168425 | 1347396 | 39 | 2 | 0 | 784 | 1359300 | 39 | 2 | 0 | 1 | 792 |
| 198 | C6 | 5 | 05 | 39 | 3 | 168523 | 1348180 | 39 | 3 | 0 | 784 | 1360092 | 39 | 3 | 0 | 1 | 792 |
| 199 | C7 | 5 | 05 | 39 | 4 | 168621 | 1348964 | 39 | 4 | 0 | 784 | 1360884 | 39 | 4 | 0 | 1 | 792 |
| 200 | C8 | 5 | 05 | 40 | 0 | 168721 | 1349764 | 40 | 0 | 0 | 800 | 1361692 | 40 | 0 | 0 | 1 | 808 |
| 201 | C9 | 5 | 05 | 40 | 1 | 168821 | 1350564 | 40 | 1 | 0 | 800 | 1362500 | 40 | 1 | 0 | 1 | 808 |
| 202 | CA | 5 | 05 | 40 | 2 | 168921 | 1351364 | 40 | 2 | 0 | 800 | 1363308 | 40 | 2 | 0 | 1 | 808 |
| 203 | CB | 5 | 05 | 40 | 3 | 169021 | 1352164 | 40 | 3 | 0 | 800 | 1364116 | 40 | 3 | 0 | 1 | 808 |
| 204 | CC | 5 | 05 | 40 | 4 | 169121 | 1352964 | 40 | 4 | 0 | 800 | 1364924 | 40 | 4 | 0 | 1 | 808 |
| 205 | CD | 5 | 05 | 41 | 0 | 169223 | 1353780 | 41 | 0 | 0 | 816 | 1365748 | 41 | 0 | 0 | 1 | 824 |
| 206 | CE | 5 | 05 | 41 | 1 | 169325 | 1354596 | 41 | 1 | 0 | 816 | 1366572 | 41 | 1 | 0 | 1 | 824 |
| 207 | CF | 5 | 05 | 41 | 2 | 169427 | 1355412 | 41 | 2 | 0 | 816 | 1367396 | 41 | 2 | 0 | 1 | 824 |
| 208 | D0 | 5 | 05 | 41 | 3 | 169529 | 1356228 | 41 | 3 | 0 | 816 | 1368220 | 41 | 3 | 0 | 1 | 824 |
| 209 | D1 | 5 | 05 | 41 | 4 | 169631 | 1357044 | 41 | 4 | 0 | 816 | 1369044 | 41 | 4 | 0 | 1 | 824 |
| 210 | D2 | 5 | 05 | 42 | 0 | 169735 | 1357876 | 42 | 0 | 0 | 832 | 1369884 | 42 | 0 | 0 | 1 | 840 |
| 211 | D3 | 5 | 05 | 42 | 1 | 169839 | 1358708 | 42 | 1 | 0 | 832 | 1370724 | 42 | 1 | 0 | 1 | 840 |
| 212 | D4 | 5 | 05 | 42 | 2 | 169943 | 1359540 | 42 | 2 | 0 | 832 | 1371564 | 42 | 2 | 0 | 1 | 840 |
| 213 | D5 | 5 | 05 | 42 | 3 | 170047 | 1360372 | 42 | 3 | 0 | 832 | 1372404 | 42 | 3 | 0 | 1 | 840 |
| 214 | D6 | 5 | 05 | 42 | 4 | 170151 | 1361204 | 42 | 4 | 0 | 832 | 1373244 | 42 | 4 | 0 | 1 | 840 |
| 215 | D7 | 5 | 05 | 43 | 0 | 170257 | 1362052 | 43 | 0 | 0 | 848 | 1374100 | 43 | 0 | 0 | 1 | 856 |
| 216 | D8 | 5 | 05 | 43 | 1 | 170363 | 1362900 | 43 | 1 | 0 | 848 | 1374956 | 43 | 1 | 0 | 1 | 856 |
| 217 | D9 | 5 | 05 | 43 | 2 | 170469 | 1363748 | 43 | 2 | 0 | 848 | 1375812 | 43 | 2 | 0 | 1 | 856 |
| 218 | DA | 5 | 05 | 43 | 3 | 170575 | 1364596 | 43 | 3 | 0 | 848 | 1376668 | 43 | 3 | 0 | 1 | 856 |
| 219 | DB | 5 | 05 | 43 | 4 | 170681 | 1365444 | 43 | 4 | 0 | 848 | 1377524 | 43 | 4 | 0 | 1 | 856 |
| 220 | DC | 5 | 05 | 44 | 0 | 170789 | 1366308 | 44 | 0 | 0 | 864 | 1378396 | 44 | 0 | 0 | 1 | 872 |
| 221 | DD | 5 | 05 | 44 | 1 | 170897 | 1367172 | 44 | 1 | 0 | 864 | 1379268 | 44 | 1 | 0 | 1 | 872 |
| 222 | DE | 5 | 05 | 44 | 2 | 171005 | 1368036 | 44 | 2 | 0 | 864 | 1380140 | 44 | 2 | 0 | 1 | 872 |
| 223 | DF | 5 | 05 | 44 | 3 | 171113 | 1368900 | 44 | 3 | 0 | 864 | 1381012 | 44 | 3 | 0 | 1 | 872 |
| 224 | E0 | 5 | 05 | 44 | 4 | 171221 | 1369764 | 44 | 4 | 0 | 864 | 1381884 | 44 | 4 | 0 | 1 | 872 |
| 225 | E1 | 5 | 05 | 45 | 0 | 171331 | 1370644 | 45 | 0 | 0 | 880 | 1382772 | 45 | 0 | 0 | 1 | 888 |
| 226 | E2 | 5 | 05 | 45 | 1 | 171441 | 1371524 | 45 | 1 | 0 | 880 | 1383660 | 45 | 1 | 0 | 1 | 888 |
| 227 | E3 | 5 | 05 | 45 | 2 | 171551 | 1372404 | 45 | 2 | 0 | 880 | 1384548 | 45 | 2 | 0 | 1 | 888 |
| 228 | E4 | 5 | 05 | 45 | 3 | 171661 | 1373284 | 45 | 3 | 0 | 880 | 1385436 | 45 | 3 | 0 | 1 | 888 |
| 229 | E5 | 5 | 05 | 45 | 4 | 171771 | 1374164 | 45 | 4 | 0 | 880 | 1386324 | 45 | 4 | 0 | 1 | 888 |
| 230 | E6 | 5 | 05 | 46 | 0 | 171883 | 1375060 | 46 | 0 | 0 | 896 | 1387228 | 46 | 0 | 0 | 1 | 904 |
| 231 | E7 | 5 | 05 | 46 | 1 | 171995 | 1375956 | 46 | 1 | 0 | 896 | 1388132 | 46 | 1 | 0 | 1 | 904 |
| 232 | E8 | 5 | 05 | 46 | 2 | 172107 | 1376852 | 46 | 2 | 0 | 896 | 1389036 | 46 | 2 | 0 | 1 | 904 |
| 233 | E9 | 5 | 05 | 46 | 3 | 172219 | 1377748 | 46 | 3 | 0 | 896 | 1389940 | 46 | 3 | 0 | 1 | 904 |
| 234 | EA | 5 | 05 | 46 | 4 | 172331 | 1378644 | 46 | 4 | 0 | 896 | 1390844 | 46 | 4 | 0 | 1 | 904 |
| 235 | EB | 5 | 05 | 47 | 0 | 172445 | 1379556 | 47 | 0 | 0 | 912 | 1391764 | 47 | 0 | 0 | 1 | 920 |
| 236 | EC | 5 | 05 | 47 | 1 | 172559 | 1380468 | 47 | 1 | 0 | 912 | 1392684 | 47 | 1 | 0 | 1 | 920 |
| 237 | ED | 5 | 05 | 47 | 2 | 172673 | 1381380 | 47 | 2 | 0 | 912 | 1393604 | 47 | 2 | 0 | 1 | 920 |
| 238 | EE | 5 | 05 | 47 | 3 | 172787 | 1382292 | 47 | 3 | 0 | 912 | 1394524 | 47 | 3 | 0 | 1 | 920 |
| 239 | EF | 5 | 05 | 47 | 4 | 172901 | 1383204 | 47 | 4 | 0 | 912 | 1395444 | 47 | 4 | 0 | 1 | 920 |
| 240 | F0 | 5 | 05 | 48 | 0 | 173017 | 1384132 | 48 | 0 | 0 | 928 | 1396380 | 48 | 0 | 0 | 1 | 936 |
| 241 | F1 | 5 | 05 | 48 | 1 | 173133 | 1385060 | 48 | 1 | 0 | 928 | 1397316 | 48 | 1 | 0 | 1 | 936 |
| 242 | F2 | 5 | 05 | 48 | 2 | 173249 | 1385988 | 48 | 2 | 0 | 928 | 1398252 | 48 | 2 | 0 | 1 | 936 |
| 243 | F3 | 5 | 05 | 48 | 3 | 173365 | 1386916 | 48 | 3 | 0 | 928 | 1399188 | 48 | 3 | 0 | 1 | 936 |
| 244 | F4 | 5 | 05 | 48 | 4 | 173481 | 1387844 | 48 | 4 | 0 | 928 | 1400124 | 48 | 4 | 0 | 1 | 936 |
| 245 | F5 | 5 | 05 | 49 | 0 | 173599 | 1388788 | 49 | 0 | 0 | 944 | 1401076 | 49 | 0 | 0 | 1 | 952 |
| 246 | F6 | 5 | 05 | 49 | 1 | 173717 | 1389732 | 49 | 1 | 0 | 944 | 1402028 | 49 | 1 | 0 | 1 | 952 |
| 247 | F7 | 5 | 05 | 49 | 2 | 173835 | 1390676 | 49 | 2 | 0 | 944 | 1402980 | 49 | 2 | 0 | 1 | 952 |
| 248 | F8 | 5 | 05 | 49 | 3 | 173953 | 1391620 | 49 | 3 | 0 | 944 | 1403932 | 49 | 3 | 0 | 1 | 952 |
| 249 | F9 | 5 | 05 | 49 | 4 | 174071 | 1392564 | 49 | 4 | 0 | 944 | 1404884 | 49 | 4 | 0 | 1 | 952 |
| 250 | FA | 5 | 05 | 50 | 0 | 174191 | 1393524 | 50 | 0 | 0 | 960 | 1405852 | 50 | 0 | 0 | 1 | 968 |
| 251 | FB | 5 | 05 | 50 | 1 | 174311 | 1394484 | 50 | 1 | 0 | 960 | 1406820 | 50 | 1 | 0 | 1 | 968 |
| 252 | FC | 5 | 05 | 50 | 2 | 174431 | 1395444 | 50 | 2 | 0 | 960 | 1407788 | 50 | 2 | 0 | 1 | 968 |
| 253 | FD | 5 | 05 | 50 | 3 | 174551 | 1396404 | 50 | 3 | 0 | 960 | 1408756 | 50 | 3 | 0 | 1 | 968 |
| 254 | FE | 5 | 05 | 50 | 4 | 174671 | 1397364 | 50 | 4 | 0 | 960 | 1409724 | 50 | 4 | 0 | 1 | 968 |
| 255 | FF | 5 | 05 | 51 | 0 | 174793 | 1398340 | 51 | 0 | 0 | 976 | 1410708 | 51 | 0 | 0 | 1 | 984 |
| 0 | 00 | 6 | 06 | 0 | 0 | 174805 | 1398436 | 0 | 0 | 0 | 96 | 1410812 | 0 | 0 | 0 | 1 | 104 |
| 1 | 01 | 6 | 06 | 0 | 1 | 174819 | 1398548 | 0 | 1 | 0 | 112 | 1410932 | 0 | 1 | 0 | 1 | 120 |
| 2 | 02 | 6 | 06 | 0 | 2 | 174833 | 1398660 | 0 | 2 | 0 | 112 | 1411052 | 0 | 2 | 0 | 1 | 120 |
| 3 | 03 | 6 | 06 | 0 | 3 | 174847 | 1398772 | 0 | 3 | 0 | 112 | 1411172 | 0 | 3 | 0 | 1 | 120 |
| 4 | 04 | 6 | 06 | 0 | 4 | 174861 | 1398884 | 0 | 4 | 0 | 112 | 1411292 | 0 | 4 | 0 | 1 | 120 |
| 5 | 05 | 6 | 06 | 0 | 5 | 174875 | 1398996 | 0 | 5 | 0 | 112 | 1411412 | 0 | 5 | 0 | 1 | 120 |

| | | | | | | | | | | | | | | | | | |
|---|---|---|---|---|---|---|---|---|---|---|---|---|---|---|---|---|---|
| 6 | 06 | 6 | 06 | 1 | 0 | 174887 | 1399092 | 1 | 0 | 0 | 96 | 1411516 | 1 | 0 | 0 | 1 | 104 |
| 7 | 07 | 6 | 06 | 1 | 1 | 174904 | 1399228 | 1 | 1 | 0 | 136 | 1411660 | 1 | 1 | 0 | 1 | 144 |
| 8 | 08 | 6 | 06 | 1 | 2 | 174921 | 1399364 | 1 | 2 | 0 | 136 | 1411804 | 1 | 2 | 0 | 1 | 144 |
| 9 | 09 | 6 | 06 | 1 | 3 | 174938 | 1399500 | 1 | 3 | 0 | 136 | 1411948 | 1 | 3 | 0 | 1 | 144 |
| 10 | 0A | 6 | 06 | 1 | 4 | 174955 | 1399636 | 1 | 4 | 0 | 136 | 1412092 | 1 | 4 | 0 | 1 | 144 |
| 11 | 0B | 6 | 06 | 1 | 5 | 174972 | 1399772 | 1 | 5 | 0 | 136 | 1412236 | 1 | 5 | 0 | 1 | 144 |
| 12 | 0C | 6 | 06 | 2 | 0 | 174991 | 1399924 | 2 | 0 | 0 | 152 | 1412396 | 2 | 0 | 0 | 1 | 160 |
| 13 | 0D | 6 | 06 | 2 | 1 | 175010 | 1400076 | 2 | 1 | 0 | 152 | 1412556 | 2 | 1 | 0 | 1 | 160 |
| 14 | 0E | 6 | 06 | 2 | 2 | 175029 | 1400228 | 2 | 2 | 0 | 152 | 1412716 | 2 | 2 | 0 | 1 | 160 |
| 15 | 0F | 6 | 06 | 2 | 3 | 175048 | 1400380 | 2 | 3 | 0 | 152 | 1412876 | 2 | 3 | 0 | 1 | 160 |
| 16 | 10 | 6 | 06 | 2 | 4 | 175072 | 1400572 | 2 | 4 | 0 | 192 | 1413076 | 2 | 4 | 0 | 1 | 200 |
| 17 | 11 | 6 | 06 | 2 | 5 | 175096 | 1400764 | 2 | 5 | 0 | 192 | 1413276 | 2 | 5 | 0 | 1 | 200 |
| 18 | 12 | 6 | 06 | 3 | 0 | 175122 | 1400972 | 3 | 0 | 0 | 208 | 1413492 | 3 | 0 | 0 | 1 | 216 |
| 19 | 13 | 6 | 06 | 3 | 1 | 175148 | 1401180 | 3 | 1 | 0 | 208 | 1413708 | 3 | 1 | 0 | 1 | 216 |
| 20 | 14 | 6 | 06 | 3 | 2 | 175174 | 1401388 | 3 | 2 | 0 | 208 | 1413924 | 3 | 2 | 0 | 1 | 216 |
| 21 | 15 | 6 | 06 | 3 | 3 | 175200 | 1401596 | 3 | 3 | 0 | 208 | 1414140 | 3 | 3 | 0 | 1 | 216 |
| 22 | 16 | 6 | 06 | 3 | 4 | 175226 | 1401804 | 3 | 4 | 0 | 208 | 1414356 | 3 | 4 | 0 | 1 | 216 |
| 23 | 17 | 6 | 06 | 3 | 5 | 175252 | 1402012 | 3 | 5 | 0 | 208 | 1414572 | 3 | 5 | 0 | 1 | 216 |
| 24 | 18 | 6 | 06 | 4 | 0 | 175280 | 1402236 | 4 | 0 | 0 | 224 | 1414804 | 4 | 0 | 0 | 1 | 232 |
| 25 | 19 | 6 | 06 | 4 | 1 | 175308 | 1402460 | 4 | 1 | 0 | 224 | 1415036 | 4 | 1 | 0 | 1 | 232 |
| 26 | 1A | 6 | 06 | 4 | 2 | 175336 | 1402684 | 4 | 2 | 0 | 224 | 1415268 | 4 | 2 | 0 | 1 | 232 |
| 27 | 1B | 6 | 06 | 4 | 3 | 175364 | 1402908 | 4 | 3 | 0 | 224 | 1415500 | 4 | 3 | 0 | 1 | 232 |
| 28 | 1C | 6 | 06 | 4 | 4 | 175392 | 1403132 | 4 | 4 | 0 | 224 | 1415732 | 4 | 4 | 0 | 1 | 232 |
| 29 | 1D | 6 | 06 | 4 | 5 | 175420 | 1403356 | 4 | 5 | 0 | 224 | 1415964 | 4 | 5 | 0 | 1 | 232 |
| 30 | 1E | 6 | 06 | 5 | 0 | 175450 | 1403596 | 5 | 0 | 0 | 240 | 1416212 | 5 | 0 | 0 | 1 | 248 |
| 31 | 1F | 6 | 06 | 5 | 1 | 175480 | 1403836 | 5 | 1 | 0 | 240 | 1416460 | 5 | 1 | 0 | 1 | 248 |
| 32 | 20 | 6 | 06 | 5 | 2 | 175510 | 1404076 | 5 | 2 | 0 | 240 | 1416708 | 5 | 2 | 0 | 1 | 248 |
| 33 | 21 | 6 | 06 | 5 | 3 | 175540 | 1404316 | 5 | 3 | 0 | 240 | 1416956 | 5 | 3 | 0 | 1 | 248 |
| 34 | 22 | 6 | 06 | 5 | 4 | 175570 | 1404556 | 5 | 4 | 0 | 240 | 1417204 | 5 | 4 | 0 | 1 | 248 |
| 35 | 23 | 6 | 06 | 5 | 5 | 175600 | 1404796 | 5 | 5 | 0 | 240 | 1417452 | 5 | 5 | 0 | 1 | 248 |
| 36 | 24 | 6 | 06 | 6 | 0 | 175632 | 1405052 | 6 | 0 | 0 | 256 | 1417716 | 6 | 0 | 0 | 1 | 264 |
| 37 | 25 | 6 | 06 | 6 | 1 | 175664 | 1405308 | 6 | 1 | 0 | 256 | 1417980 | 6 | 1 | 0 | 1 | 264 |
| 38 | 26 | 6 | 06 | 6 | 2 | 175696 | 1405564 | 6 | 2 | 0 | 256 | 1418244 | 6 | 2 | 0 | 1 | 264 |
| 39 | 27 | 6 | 06 | 6 | 3 | 175728 | 1405820 | 6 | 3 | 0 | 256 | 1418508 | 6 | 3 | 0 | 1 | 264 |
| 40 | 28 | 6 | 06 | 6 | 4 | 175760 | 1406076 | 6 | 4 | 0 | 256 | 1418772 | 6 | 4 | 0 | 1 | 264 |
| 41 | 29 | 6 | 06 | 6 | 5 | 175792 | 1406332 | 6 | 5 | 0 | 256 | 1419036 | 6 | 5 | 0 | 1 | 264 |
| 42 | 2A | 6 | 06 | 7 | 0 | 175826 | 1406604 | 7 | 0 | 0 | 272 | 1419316 | 7 | 0 | 0 | 1 | 280 |
| 43 | 2B | 6 | 06 | 7 | 1 | 175860 | 1406876 | 7 | 1 | 0 | 272 | 1419596 | 7 | 1 | 0 | 1 | 280 |
| 44 | 2C | 6 | 06 | 7 | 2 | 175894 | 1407148 | 7 | 2 | 0 | 272 | 1419876 | 7 | 2 | 0 | 1 | 280 |
| 45 | 2D | 6 | 06 | 7 | 3 | 175928 | 1407420 | 7 | 3 | 0 | 272 | 1420156 | 7 | 3 | 0 | 1 | 280 |
| 46 | 2E | 6 | 06 | 7 | 4 | 175962 | 1407692 | 7 | 4 | 0 | 272 | 1420436 | 7 | 4 | 0 | 1 | 280 |
| 47 | 2F | 6 | 06 | 7 | 5 | 175996 | 1407964 | 7 | 5 | 0 | 272 | 1420716 | 7 | 5 | 0 | 1 | 280 |
| 48 | 30 | 6 | 06 | 8 | 0 | 176032 | 1408252 | 8 | 0 | 0 | 288 | 1421012 | 8 | 0 | 0 | 1 | 296 |
| 49 | 31 | 6 | 06 | 8 | 1 | 176068 | 1408540 | 8 | 1 | 0 | 288 | 1421308 | 8 | 1 | 0 | 1 | 296 |
| 50 | 32 | 6 | 06 | 8 | 2 | 176104 | 1408828 | 8 | 2 | 0 | 288 | 1421604 | 8 | 2 | 0 | 1 | 296 |
| 51 | 33 | 6 | 06 | 8 | 3 | 176140 | 1409116 | 8 | 3 | 0 | 288 | 1421900 | 8 | 3 | 0 | 1 | 296 |
| 52 | 34 | 6 | 06 | 8 | 4 | 176176 | 1409404 | 8 | 4 | 0 | 288 | 1422196 | 8 | 4 | 0 | 1 | 296 |
| 53 | 35 | 6 | 06 | 8 | 5 | 176212 | 1409692 | 8 | 5 | 0 | 288 | 1422492 | 8 | 5 | 0 | 1 | 296 |
| 54 | 36 | 6 | 06 | 9 | 0 | 176250 | 1409996 | 9 | 0 | 0 | 304 | 1422804 | 9 | 0 | 0 | 1 | 312 |
| 55 | 37 | 6 | 06 | 9 | 1 | 176288 | 1410300 | 9 | 1 | 0 | 304 | 1423116 | 9 | 1 | 0 | 1 | 312 |
| 56 | 38 | 6 | 06 | 9 | 2 | 176326 | 1410604 | 9 | 2 | 0 | 304 | 1423428 | 9 | 2 | 0 | 1 | 312 |
| 57 | 39 | 6 | 06 | 9 | 3 | 176364 | 1410908 | 9 | 3 | 0 | 304 | 1423740 | 9 | 3 | 0 | 1 | 312 |
| 58 | 3A | 6 | 06 | 9 | 4 | 176402 | 1411212 | 9 | 4 | 0 | 304 | 1424052 | 9 | 4 | 0 | 1 | 312 |
| 59 | 3B | 6 | 06 | 9 | 5 | 176440 | 1411516 | 9 | 5 | 0 | 304 | 1424364 | 9 | 5 | 0 | 1 | 312 |
| 60 | 3C | 6 | 06 | 10 | 0 | 176480 | 1411836 | 10 | 0 | 0 | 320 | 1424692 | 10 | 0 | 0 | 1 | 328 |
| 61 | 3D | 6 | 06 | 10 | 1 | 176520 | 1412156 | 10 | 1 | 0 | 320 | 1425020 | 10 | 1 | 0 | 1 | 328 |
| 62 | 3E | 6 | 06 | 10 | 2 | 176560 | 1412476 | 10 | 2 | 0 | 320 | 1425348 | 10 | 2 | 0 | 1 | 328 |
| 63 | 3F | 6 | 06 | 10 | 3 | 176600 | 1412796 | 10 | 3 | 0 | 320 | 1425676 | 10 | 3 | 0 | 1 | 328 |
| 64 | 40 | 6 | 06 | 10 | 4 | 176640 | 1413116 | 10 | 4 | 0 | 320 | 1426004 | 10 | 4 | 0 | 1 | 328 |
| 65 | 41 | 6 | 06 | 10 | 5 | 176680 | 1413436 | 10 | 5 | 0 | 320 | 1426332 | 10 | 5 | 0 | 1 | 328 |
| 66 | 42 | 6 | 06 | 11 | 0 | 176722 | 1413772 | 11 | 0 | 0 | 336 | 1426676 | 11 | 0 | 0 | 1 | 344 |
| 67 | 43 | 6 | 06 | 11 | 1 | 176764 | 1414108 | 11 | 1 | 0 | 336 | 1427020 | 11 | 1 | 0 | 1 | 344 |
| 68 | 44 | 6 | 06 | 11 | 2 | 176806 | 1414444 | 11 | 2 | 0 | 336 | 1427364 | 11 | 2 | 0 | 1 | 344 |
| 69 | 45 | 6 | 06 | 11 | 3 | 176848 | 1414780 | 11 | 3 | 0 | 336 | 1427708 | 11 | 3 | 0 | 1 | 344 |
| 70 | 46 | 6 | 06 | 11 | 4 | 176890 | 1415116 | 11 | 4 | 0 | 336 | 1428052 | 11 | 4 | 0 | 1 | 344 |
| 71 | 47 | 6 | 06 | 11 | 5 | 176932 | 1415452 | 11 | 5 | 0 | 336 | 1428396 | 11 | 5 | 0 | 1 | 344 |
| 72 | 48 | 6 | 06 | 12 | 0 | 176976 | 1415804 | 12 | 0 | 0 | 352 | 1428756 | 12 | 0 | 0 | 1 | 360 |
| 73 | 49 | 6 | 06 | 12 | 1 | 177020 | 1416156 | 12 | 1 | 0 | 352 | 1429116 | 12 | 1 | 0 | 1 | 360 |
| 74 | 4A | 6 | 06 | 12 | 2 | 177064 | 1416508 | 12 | 2 | 0 | 352 | 1429476 | 12 | 2 | 0 | 1 | 360 |
| 75 | 4B | 6 | 06 | 12 | 3 | 177108 | 1416860 | 12 | 3 | 0 | 352 | 1429836 | 12 | 3 | 0 | 1 | 360 |
| 76 | 4C | 6 | 06 | 12 | 4 | 177152 | 1417212 | 12 | 4 | 0 | 352 | 1430196 | 12 | 4 | 0 | 1 | 360 |
| 77 | 4D | 6 | 06 | 12 | 5 | 177196 | 1417564 | 12 | 5 | 0 | 352 | 1430556 | 12 | 5 | 0 | 1 | 360 |
| 78 | 4E | 6 | 06 | 13 | 0 | 177242 | 1417932 | 13 | 0 | 0 | 368 | 1430932 | 13 | 0 | 0 | 1 | 376 |
| 79 | 4F | 6 | 06 | 13 | 1 | 177288 | 1418300 | 13 | 1 | 0 | 368 | 1431308 | 13 | 1 | 0 | 1 | 376 |
| 80 | 50 | 6 | 06 | 13 | 2 | 177334 | 1418668 | 13 | 2 | 0 | 368 | 1431684 | 13 | 2 | 0 | 1 | 376 |
| 81 | 51 | 6 | 06 | 13 | 3 | 177380 | 1419036 | 13 | 3 | 0 | 368 | 1432060 | 13 | 3 | 0 | 1 | 376 |
| 82 | 52 | 6 | 06 | 13 | 4 | 177426 | 1419404 | 13 | 4 | 0 | 368 | 1432436 | 13 | 4 | 0 | 1 | 376 |
| 83 | 53 | 6 | 06 | 13 | 5 | 177472 | 1419772 | 13 | 5 | 0 | 368 | 1432812 | 13 | 5 | 0 | 1 | 376 |
| 84 | 54 | 6 | 06 | 14 | 0 | 177520 | 1420156 | 14 | 0 | 0 | 384 | 1433204 | 14 | 0 | 0 | 1 | 392 |
| 85 | 55 | 6 | 06 | 14 | 1 | 177568 | 1420540 | 14 | 1 | 0 | 384 | 1433596 | 14 | 1 | 0 | 1 | 392 |
| 86 | 56 | 6 | 06 | 14 | 2 | 177616 | 1420924 | 14 | 2 | 0 | 384 | 1433988 | 14 | 2 | 0 | 1 | 392 |
| 87 | 57 | 6 | 06 | 14 | 3 | 177664 | 1421308 | 14 | 3 | 0 | 384 | 1434380 | 14 | 3 | 0 | 1 | 392 |
| 88 | 58 | 6 | 06 | 14 | 4 | 177712 | 1421692 | 14 | 4 | 0 | 384 | 1434772 | 14 | 4 | 0 | 1 | 392 |
| 89 | 59 | 6 | 06 | 14 | 5 | 177760 | 1422076 | 14 | 5 | 0 | 384 | 1435164 | 14 | 5 | 0 | 1 | 392 |
| 90 | 5A | 6 | 06 | 15 | 0 | 177810 | 1422476 | 15 | 0 | 0 | 400 | 1435572 | 15 | 0 | 0 | 1 | 408 |
| 91 | 5B | 6 | 06 | 15 | 1 | 177860 | 1422876 | 15 | 1 | 0 | 400 | 1435980 | 15 | 1 | 0 | 1 | 408 |
| 92 | 5C | 6 | 06 | 15 | 2 | 177910 | 1423276 | 15 | 2 | 0 | 400 | 1436388 | 15 | 2 | 0 | 1 | 408 |
| 93 | 5D | 6 | 06 | 15 | 3 | 177960 | 1423676 | 15 | 3 | 0 | 400 | 1436796 | 15 | 3 | 0 | 1 | 408 |
| 94 | 5E | 6 | 06 | 15 | 4 | 178010 | 1424076 | 15 | 4 | 0 | 400 | 1437204 | 15 | 4 | 0 | 1 | 408 |
| 95 | 5F | 6 | 06 | 15 | 5 | 178060 | 1424476 | 15 | 5 | 0 | 400 | 1437612 | 15 | 5 | 0 | 1 | 408 |
| 96 | 60 | 6 | 06 | 16 | 0 | 178112 | 1424892 | 16 | 0 | 0 | 416 | 1438036 | 16 | 0 | 0 | 1 | 424 |
| 97 | 61 | 6 | 06 | 16 | 1 | 178164 | 1425308 | 16 | 1 | 0 | 416 | 1438460 | 16 | 1 | 0 | 1 | 424 |
| 98 | 62 | 6 | 06 | 16 | 2 | 178216 | 1425724 | 16 | 2 | 0 | 416 | 1438884 | 16 | 2 | 0 | 1 | 424 |
| 99 | 63 | 6 | 06 | 16 | 3 | 178268 | 1426140 | 16 | 3 | 0 | 416 | 1439308 | 16 | 3 | 0 | 1 | 424 |
| 100 | 64 | 6 | 06 | 16 | 4 | 178320 | 1426556 | 16 | 4 | 0 | 416 | 1439732 | 16 | 4 | 0 | 1 | 424 |
| 101 | 65 | 6 | 06 | 16 | 5 | 178372 | 1426972 | 16 | 5 | 0 | 416 | 1440156 | 16 | 5 | 0 | 1 | 424 |
| 102 | 66 | 6 | 06 | 17 | 0 | 178426 | 1427404 | 17 | 0 | 0 | 432 | 1440596 | 17 | 0 | 0 | 1 | 440 |
| 103 | 67 | 6 | 06 | 17 | 1 | 178480 | 1427836 | 17 | 1 | 0 | 432 | 1441036 | 17 | 1 | 0 | 1 | 440 |
| 104 | 68 | 6 | 06 | 17 | 2 | 178534 | 1428268 | 17 | 2 | 0 | 432 | 1441476 | 17 | 2 | 0 | 1 | 440 |
| 105 | 69 | 6 | 06 | 17 | 3 | 178588 | 1428700 | 17 | 3 | 0 | 432 | 1441916 | 17 | 3 | 0 | 1 | 440 |
| 106 | 6A | 6 | 06 | 17 | 4 | 178642 | 1429132 | 17 | 4 | 0 | 432 | 1442356 | 17 | 4 | 0 | 1 | 440 |
| 107 | 6B | 6 | 06 | 17 | 5 | 178696 | 1429564 | 17 | 5 | 0 | 432 | 1442796 | 17 | 5 | 0 | 1 | 440 |
| 108 | 6C | 6 | 06 | 18 | 0 | 178752 | 1430012 | 18 | 0 | 0 | 448 | 1443252 | 18 | 0 | 0 | 1 | 456 |

| | | | | | | | | | | | | | | | | | |
|---|---|---|---|---|---|---|---|---|---|---|---|---|---|---|---|---|---|
| 109 | 6D | 6 | 06 | 18 | 1 | 178808 | 1430460 | 18 | 1 | 0 | 448 | 1443708 | 18 | 1 | 0 | 1 | 456 |
| 110 | 6E | 6 | 06 | 18 | 2 | 178864 | 1430908 | 18 | 2 | 0 | 448 | 1444164 | 18 | 2 | 0 | 1 | 456 |
| 111 | 6F | 6 | 06 | 18 | 3 | 178920 | 1431356 | 18 | 3 | 0 | 448 | 1444620 | 18 | 3 | 0 | 1 | 456 |
| 112 | 70 | 6 | 06 | 18 | 4 | 178976 | 1431804 | 18 | 4 | 0 | 448 | 1445076 | 18 | 4 | 0 | 1 | 456 |
| 113 | 71 | 6 | 06 | 18 | 5 | 179032 | 1432252 | 18 | 5 | 0 | 448 | 1445532 | 18 | 5 | 0 | 1 | 456 |
| 114 | 72 | 6 | 06 | 19 | 0 | 179090 | 1432716 | 19 | 0 | 0 | 464 | 1446004 | 19 | 0 | 0 | 1 | 472 |
| 115 | 73 | 6 | 06 | 19 | 1 | 179148 | 1433180 | 19 | 1 | 0 | 464 | 1446476 | 19 | 1 | 0 | 1 | 472 |
| 116 | 74 | 6 | 06 | 19 | 2 | 179206 | 1433644 | 19 | 2 | 0 | 464 | 1446948 | 19 | 2 | 0 | 1 | 472 |
| 117 | 75 | 6 | 06 | 19 | 3 | 179264 | 1434108 | 19 | 3 | 0 | 464 | 1447420 | 19 | 3 | 0 | 1 | 472 |
| 118 | 76 | 6 | 06 | 19 | 4 | 179322 | 1434572 | 19 | 4 | 0 | 464 | 1447892 | 19 | 4 | 0 | 1 | 472 |
| 119 | 77 | 6 | 06 | 19 | 5 | 179380 | 1435036 | 19 | 5 | 0 | 464 | 1448364 | 19 | 5 | 0 | 1 | 472 |
| 120 | 78 | 6 | 06 | 20 | 0 | 179440 | 1435516 | 20 | 0 | 0 | 480 | 1448852 | 20 | 0 | 0 | 1 | 488 |
| 121 | 79 | 6 | 06 | 20 | 1 | 179500 | 1435996 | 20 | 1 | 0 | 480 | 1449340 | 20 | 1 | 0 | 1 | 488 |
| 122 | 7A | 6 | 06 | 20 | 2 | 179560 | 1436476 | 20 | 2 | 0 | 480 | 1449828 | 20 | 2 | 0 | 1 | 488 |
| 123 | 7B | 6 | 06 | 20 | 3 | 179620 | 1436956 | 20 | 3 | 0 | 480 | 1450316 | 20 | 3 | 0 | 1 | 488 |
| 124 | 7C | 6 | 06 | 20 | 4 | 179680 | 1437436 | 20 | 4 | 0 | 480 | 1450804 | 20 | 4 | 0 | 1 | 488 |
| 125 | 7D | 6 | 06 | 20 | 5 | 179740 | 1437916 | 20 | 5 | 0 | 480 | 1451292 | 20 | 5 | 0 | 1 | 488 |
| 126 | 7E | 6 | 06 | 21 | 0 | 179802 | 1438412 | 21 | 0 | 0 | 496 | 1451796 | 21 | 0 | 0 | 1 | 504 |
| 127 | 7F | 6 | 06 | 21 | 1 | 179864 | 1438908 | 21 | 1 | 0 | 496 | 1452300 | 21 | 1 | 0 | 1 | 504 |
| 128 | 80 | 6 | 06 | 21 | 2 | 179926 | 1439404 | 21 | 2 | 0 | 496 | 1452804 | 21 | 2 | 0 | 1 | 504 |
| 129 | 81 | 6 | 06 | 21 | 3 | 179988 | 1439900 | 21 | 3 | 0 | 496 | 1453308 | 21 | 3 | 0 | 1 | 504 |
| 130 | 82 | 6 | 06 | 21 | 4 | 180050 | 1440396 | 21 | 4 | 0 | 496 | 1453812 | 21 | 4 | 0 | 1 | 504 |
| 131 | 83 | 6 | 06 | 21 | 5 | 180112 | 1440892 | 21 | 5 | 0 | 496 | 1454316 | 21 | 5 | 0 | 1 | 504 |
| 132 | 84 | 6 | 06 | 22 | 0 | 180176 | 1441404 | 22 | 0 | 0 | 512 | 1454836 | 22 | 0 | 0 | 1 | 520 |
| 133 | 85 | 6 | 06 | 22 | 1 | 180240 | 1441916 | 22 | 1 | 0 | 512 | 1455356 | 22 | 1 | 0 | 1 | 520 |
| 134 | 86 | 6 | 06 | 22 | 2 | 180304 | 1442428 | 22 | 2 | 0 | 512 | 1455876 | 22 | 2 | 0 | 1 | 520 |
| 135 | 87 | 6 | 06 | 22 | 3 | 180368 | 1442940 | 22 | 3 | 0 | 512 | 1456396 | 22 | 3 | 0 | 1 | 520 |
| 136 | 88 | 6 | 06 | 22 | 4 | 180432 | 1443452 | 22 | 4 | 0 | 512 | 1456916 | 22 | 4 | 0 | 1 | 520 |
| 137 | 89 | 6 | 06 | 22 | 5 | 180496 | 1443964 | 22 | 5 | 0 | 512 | 1457436 | 22 | 5 | 0 | 1 | 520 |
| 138 | 8A | 6 | 06 | 23 | 0 | 180562 | 1444492 | 23 | 0 | 0 | 528 | 1457972 | 23 | 0 | 0 | 1 | 536 |
| 139 | 8B | 6 | 06 | 23 | 1 | 180628 | 1445020 | 23 | 1 | 0 | 528 | 1458508 | 23 | 1 | 0 | 1 | 536 |
| 140 | 8C | 6 | 06 | 23 | 2 | 180694 | 1445548 | 23 | 2 | 0 | 528 | 1459044 | 23 | 2 | 0 | 1 | 536 |
| 141 | 8D | 6 | 06 | 23 | 3 | 180760 | 1446076 | 23 | 3 | 0 | 528 | 1459580 | 23 | 3 | 0 | 1 | 536 |
| 142 | 8E | 6 | 06 | 23 | 4 | 180826 | 1446604 | 23 | 4 | 0 | 528 | 1460116 | 23 | 4 | 0 | 1 | 536 |
| 143 | 8F | 6 | 06 | 23 | 5 | 180892 | 1447132 | 23 | 5 | 0 | 528 | 1460652 | 23 | 5 | 0 | 1 | 536 |
| 144 | 90 | 6 | 06 | 24 | 0 | 180960 | 1447676 | 24 | 0 | 0 | 544 | 1461204 | 24 | 0 | 0 | 1 | 552 |
| 145 | 91 | 6 | 06 | 24 | 1 | 181028 | 1448220 | 24 | 1 | 0 | 544 | 1461756 | 24 | 1 | 0 | 1 | 552 |
| 146 | 92 | 6 | 06 | 24 | 2 | 181096 | 1448764 | 24 | 2 | 0 | 544 | 1462308 | 24 | 2 | 0 | 1 | 552 |
| 147 | 93 | 6 | 06 | 24 | 3 | 181164 | 1449308 | 24 | 3 | 0 | 544 | 1462860 | 24 | 3 | 0 | 1 | 552 |
| 148 | 94 | 6 | 06 | 24 | 4 | 181232 | 1449852 | 24 | 4 | 0 | 544 | 1463412 | 24 | 4 | 0 | 1 | 552 |
| 149 | 95 | 6 | 06 | 24 | 5 | 181300 | 1450396 | 24 | 5 | 0 | 544 | 1463964 | 24 | 5 | 0 | 1 | 552 |
| 150 | 96 | 6 | 06 | 25 | 0 | 181370 | 1450956 | 25 | 0 | 0 | 560 | 1464532 | 25 | 0 | 0 | 1 | 568 |
| 151 | 97 | 6 | 06 | 25 | 1 | 181440 | 1451516 | 25 | 1 | 0 | 560 | 1465100 | 25 | 1 | 0 | 1 | 568 |
| 152 | 98 | 6 | 06 | 25 | 2 | 181510 | 1452076 | 25 | 2 | 0 | 560 | 1465668 | 25 | 2 | 0 | 1 | 568 |
| 153 | 99 | 6 | 06 | 25 | 3 | 181580 | 1452636 | 25 | 3 | 0 | 560 | 1466236 | 25 | 3 | 0 | 1 | 568 |
| 154 | 9A | 6 | 06 | 25 | 4 | 181650 | 1453196 | 25 | 4 | 0 | 560 | 1466804 | 25 | 4 | 0 | 1 | 568 |
| 155 | 9B | 6 | 06 | 25 | 5 | 181720 | 1453756 | 25 | 5 | 0 | 560 | 1467372 | 25 | 5 | 0 | 1 | 568 |
| 156 | 9C | 6 | 06 | 26 | 0 | 181792 | 1454332 | 26 | 0 | 0 | 576 | 1467956 | 26 | 0 | 0 | 1 | 584 |
| 157 | 9D | 6 | 06 | 26 | 1 | 181864 | 1454908 | 26 | 1 | 0 | 576 | 1468540 | 26 | 1 | 0 | 1 | 584 |
| 158 | 9E | 6 | 06 | 26 | 2 | 181936 | 1455484 | 26 | 2 | 0 | 576 | 1469124 | 26 | 2 | 0 | 1 | 584 |
| 159 | 9F | 6 | 06 | 26 | 3 | 182008 | 1456060 | 26 | 3 | 0 | 576 | 1469708 | 26 | 3 | 0 | 1 | 584 |
| 160 | A0 | 6 | 06 | 26 | 4 | 182080 | 1456636 | 26 | 4 | 0 | 576 | 1470292 | 26 | 4 | 0 | 1 | 584 |
| 161 | A1 | 6 | 06 | 26 | 5 | 182152 | 1457212 | 26 | 5 | 0 | 576 | 1470876 | 26 | 5 | 0 | 1 | 584 |
| 162 | A2 | 6 | 06 | 27 | 0 | 182226 | 1457804 | 27 | 0 | 0 | 592 | 1471476 | 27 | 0 | 0 | 1 | 600 |
| 163 | A3 | 6 | 06 | 27 | 1 | 182300 | 1458396 | 27 | 1 | 0 | 592 | 1472076 | 27 | 1 | 0 | 1 | 600 |
| 164 | A4 | 6 | 06 | 27 | 2 | 182374 | 1458988 | 27 | 2 | 0 | 592 | 1472676 | 27 | 2 | 0 | 1 | 600 |
| 165 | A5 | 6 | 06 | 27 | 3 | 182448 | 1459580 | 27 | 3 | 0 | 592 | 1473276 | 27 | 3 | 0 | 1 | 600 |
| 166 | A6 | 6 | 06 | 27 | 4 | 182522 | 1460172 | 27 | 4 | 0 | 592 | 1473876 | 27 | 4 | 0 | 1 | 600 |
| 167 | A7 | 6 | 06 | 27 | 5 | 182596 | 1460764 | 27 | 5 | 0 | 592 | 1474476 | 27 | 5 | 0 | 1 | 600 |
| 168 | A8 | 6 | 06 | 28 | 0 | 182672 | 1461372 | 28 | 0 | 0 | 608 | 1475092 | 28 | 0 | 0 | 1 | 616 |
| 169 | A9 | 6 | 06 | 28 | 1 | 182748 | 1461980 | 28 | 1 | 0 | 608 | 1475708 | 28 | 1 | 0 | 1 | 616 |
| 170 | AA | 6 | 06 | 28 | 2 | 182824 | 1462588 | 28 | 2 | 0 | 608 | 1476324 | 28 | 2 | 0 | 1 | 616 |
| 171 | AB | 6 | 06 | 28 | 3 | 182900 | 1463196 | 28 | 3 | 0 | 608 | 1476940 | 28 | 3 | 0 | 1 | 616 |
| 172 | AC | 6 | 06 | 28 | 4 | 182976 | 1463804 | 28 | 4 | 0 | 608 | 1477556 | 28 | 4 | 0 | 1 | 616 |
| 173 | AD | 6 | 06 | 28 | 5 | 183052 | 1464412 | 28 | 5 | 0 | 608 | 1478172 | 28 | 5 | 0 | 1 | 616 |
| 174 | AE | 6 | 06 | 29 | 0 | 183130 | 1465036 | 29 | 0 | 0 | 624 | 1478804 | 29 | 0 | 0 | 1 | 632 |
| 175 | AF | 6 | 06 | 29 | 1 | 183208 | 1465660 | 29 | 1 | 0 | 624 | 1479436 | 29 | 1 | 0 | 1 | 632 |
| 176 | B0 | 6 | 06 | 29 | 2 | 183286 | 1466284 | 29 | 2 | 0 | 624 | 1480068 | 29 | 2 | 0 | 1 | 632 |
| 177 | B1 | 6 | 06 | 29 | 3 | 183364 | 1466908 | 29 | 3 | 0 | 624 | 1480700 | 29 | 3 | 0 | 1 | 632 |
| 178 | B2 | 6 | 06 | 29 | 4 | 183442 | 1467532 | 29 | 4 | 0 | 624 | 1481332 | 29 | 4 | 0 | 1 | 632 |
| 179 | B3 | 6 | 06 | 29 | 5 | 183520 | 1468156 | 29 | 5 | 0 | 624 | 1481964 | 29 | 5 | 0 | 1 | 632 |
| 180 | B4 | 6 | 06 | 30 | 0 | 183600 | 1468796 | 30 | 0 | 0 | 640 | 1482612 | 30 | 0 | 0 | 1 | 648 |
| 181 | B5 | 6 | 06 | 30 | 1 | 183680 | 1469436 | 30 | 1 | 0 | 640 | 1483260 | 30 | 1 | 0 | 1 | 648 |
| 182 | B6 | 6 | 06 | 30 | 2 | 183760 | 1470076 | 30 | 2 | 0 | 640 | 1483908 | 30 | 2 | 0 | 1 | 648 |
| 183 | B7 | 6 | 06 | 30 | 3 | 183840 | 1470716 | 30 | 3 | 0 | 640 | 1484556 | 30 | 3 | 0 | 1 | 648 |
| 184 | B8 | 6 | 06 | 30 | 4 | 183920 | 1471356 | 30 | 4 | 0 | 640 | 1485204 | 30 | 4 | 0 | 1 | 648 |
| 185 | B9 | 6 | 06 | 30 | 5 | 184000 | 1471996 | 30 | 5 | 0 | 640 | 1485852 | 30 | 5 | 0 | 1 | 648 |
| 186 | BA | 6 | 06 | 31 | 0 | 184082 | 1472652 | 31 | 0 | 0 | 656 | 1486516 | 31 | 0 | 0 | 1 | 664 |
| 187 | BB | 6 | 06 | 31 | 1 | 184164 | 1473308 | 31 | 1 | 0 | 656 | 1487180 | 31 | 1 | 0 | 1 | 664 |
| 188 | BC | 6 | 06 | 31 | 2 | 184246 | 1473964 | 31 | 2 | 0 | 656 | 1487844 | 31 | 2 | 0 | 1 | 664 |
| 189 | BD | 6 | 06 | 31 | 3 | 184328 | 1474620 | 31 | 3 | 0 | 656 | 1488508 | 31 | 3 | 0 | 1 | 664 |
| 190 | BE | 6 | 06 | 31 | 4 | 184410 | 1475276 | 31 | 4 | 0 | 656 | 1489172 | 31 | 4 | 0 | 1 | 664 |
| 191 | BF | 6 | 06 | 31 | 5 | 184492 | 1475932 | 31 | 5 | 0 | 656 | 1489836 | 31 | 5 | 0 | 1 | 664 |
| 192 | C0 | 6 | 06 | 32 | 0 | 184576 | 1476604 | 32 | 0 | 0 | 672 | 1490516 | 32 | 0 | 0 | 1 | 680 |
| 193 | C1 | 6 | 06 | 32 | 1 | 184660 | 1477276 | 32 | 1 | 0 | 672 | 1491196 | 32 | 1 | 0 | 1 | 680 |
| 194 | C2 | 6 | 06 | 32 | 2 | 184744 | 1477948 | 32 | 2 | 0 | 672 | 1491876 | 32 | 2 | 0 | 1 | 680 |
| 195 | C3 | 6 | 06 | 32 | 3 | 184828 | 1478620 | 32 | 3 | 0 | 672 | 1492556 | 32 | 3 | 0 | 1 | 680 |
| 196 | C4 | 6 | 06 | 32 | 4 | 184912 | 1479292 | 32 | 4 | 0 | 672 | 1493236 | 32 | 4 | 0 | 1 | 680 |
| 197 | C5 | 6 | 06 | 32 | 5 | 184996 | 1479964 | 32 | 5 | 0 | 672 | 1493916 | 32 | 5 | 0 | 1 | 680 |
| 198 | C6 | 6 | 06 | 33 | 0 | 185082 | 1480652 | 33 | 0 | 0 | 688 | 1494612 | 33 | 0 | 0 | 1 | 696 |
| 199 | C7 | 6 | 06 | 33 | 1 | 185168 | 1481340 | 33 | 1 | 0 | 688 | 1495308 | 33 | 1 | 0 | 1 | 696 |
| 200 | C8 | 6 | 06 | 33 | 2 | 185254 | 1482028 | 33 | 2 | 0 | 688 | 1496004 | 33 | 2 | 0 | 1 | 696 |
| 201 | C9 | 6 | 06 | 33 | 3 | 185340 | 1482716 | 33 | 3 | 0 | 688 | 1496700 | 33 | 3 | 0 | 1 | 696 |
| 202 | CA | 6 | 06 | 33 | 4 | 185426 | 1483404 | 33 | 4 | 0 | 688 | 1497396 | 33 | 4 | 0 | 1 | 696 |
| 203 | CB | 6 | 06 | 33 | 5 | 185512 | 1484092 | 33 | 5 | 0 | 688 | 1498092 | 33 | 5 | 0 | 1 | 696 |
| 204 | CC | 6 | 06 | 34 | 0 | 185600 | 1484796 | 34 | 0 | 0 | 704 | 1498804 | 34 | 0 | 0 | 1 | 712 |
| 205 | CD | 6 | 06 | 34 | 1 | 185688 | 1485500 | 34 | 1 | 0 | 704 | 1499516 | 34 | 1 | 0 | 1 | 712 |
| 206 | CE | 6 | 06 | 34 | 2 | 185776 | 1486204 | 34 | 2 | 0 | 704 | 1500228 | 34 | 2 | 0 | 1 | 712 |
| 207 | CF | 6 | 06 | 34 | 3 | 185864 | 1486908 | 34 | 3 | 0 | 704 | 1500940 | 34 | 3 | 0 | 1 | 712 |
| 208 | D0 | 6 | 06 | 34 | 4 | 185952 | 1487612 | 34 | 4 | 0 | 704 | 1501652 | 34 | 4 | 0 | 1 | 712 |
| 209 | D1 | 6 | 06 | 34 | 5 | 186040 | 1488316 | 34 | 5 | 0 | 704 | 1502364 | 34 | 5 | 0 | 1 | 712 |
| 210 | D2 | 6 | 06 | 35 | 0 | 186130 | 1489036 | 35 | 0 | 0 | 720 | 1503092 | 35 | 0 | 0 | 1 | 728 |
| 211 | D3 | 6 | 06 | 35 | 1 | 186220 | 1489756 | 35 | 1 | 0 | 720 | 1503820 | 35 | 1 | 0 | 1 | 728 |

| | | | | | | | | | | | | | | | | | |
|---|---|---|---|---|---|---|---|---|---|---|---|---|---|---|---|---|---|
| 212 | D4 | 6 | 06 | 35 | 2 | 186310 | 1490476 | 35 | 2 | 0 | 720 | 1504548 | 35 | 2 | 0 | 1 | 728 |
| 213 | D5 | 6 | 06 | 35 | 3 | 186400 | 1491196 | 35 | 3 | 0 | 720 | 1505276 | 35 | 3 | 0 | 1 | 728 |
| 214 | D6 | 6 | 06 | 35 | 4 | 186490 | 1491916 | 35 | 4 | 0 | 720 | 1506004 | 35 | 4 | 0 | 1 | 728 |
| 215 | D7 | 6 | 06 | 35 | 5 | 186580 | 1492636 | 35 | 5 | 0 | 720 | 1506732 | 35 | 5 | 0 | 1 | 728 |
| 216 | D8 | 6 | 06 | 36 | 0 | 186672 | 1493372 | 36 | 0 | 0 | 736 | 1507476 | 36 | 0 | 0 | 1 | 744 |
| 217 | D9 | 6 | 06 | 36 | 1 | 186764 | 1494108 | 36 | 1 | 0 | 736 | 1508220 | 36 | 1 | 0 | 1 | 744 |
| 218 | DA | 6 | 06 | 36 | 2 | 186856 | 1494844 | 36 | 2 | 0 | 736 | 1508964 | 36 | 2 | 0 | 1 | 744 |
| 219 | DB | 6 | 06 | 36 | 3 | 186948 | 1495580 | 36 | 3 | 0 | 736 | 1509708 | 36 | 3 | 0 | 1 | 744 |
| 220 | DC | 6 | 06 | 36 | 4 | 187040 | 1496316 | 36 | 4 | 0 | 736 | 1510452 | 36 | 4 | 0 | 1 | 744 |
| 221 | DD | 6 | 06 | 36 | 5 | 187132 | 1497052 | 36 | 5 | 0 | 736 | 1511196 | 36 | 5 | 0 | 1 | 744 |
| 222 | DE | 6 | 06 | 37 | 0 | 187226 | 1497804 | 37 | 0 | 0 | 752 | 1511956 | 37 | 0 | 0 | 1 | 760 |
| 223 | DF | 6 | 06 | 37 | 1 | 187320 | 1498556 | 37 | 1 | 0 | 752 | 1512716 | 37 | 1 | 0 | 1 | 760 |
| 224 | E0 | 6 | 06 | 37 | 2 | 187414 | 1499308 | 37 | 2 | 0 | 752 | 1513476 | 37 | 2 | 0 | 1 | 760 |
| 225 | E1 | 6 | 06 | 37 | 3 | 187508 | 1500060 | 37 | 3 | 0 | 752 | 1514236 | 37 | 3 | 0 | 1 | 760 |
| 226 | E2 | 6 | 06 | 37 | 4 | 187602 | 1500812 | 37 | 4 | 0 | 752 | 1514996 | 37 | 4 | 0 | 1 | 760 |
| 227 | E3 | 6 | 06 | 37 | 5 | 187696 | 1501564 | 37 | 5 | 0 | 752 | 1515756 | 37 | 5 | 0 | 1 | 760 |
| 228 | E4 | 6 | 06 | 38 | 0 | 187792 | 1502332 | 38 | 0 | 0 | 768 | 1516532 | 38 | 0 | 0 | 1 | 776 |
| 229 | E5 | 6 | 06 | 38 | 1 | 187888 | 1503100 | 38 | 1 | 0 | 768 | 1517308 | 38 | 1 | 0 | 1 | 776 |
| 230 | E6 | 6 | 06 | 38 | 2 | 187984 | 1503868 | 38 | 2 | 0 | 768 | 1518084 | 38 | 2 | 0 | 1 | 776 |
| 231 | E7 | 6 | 06 | 38 | 3 | 188080 | 1504636 | 38 | 3 | 0 | 768 | 1518860 | 38 | 3 | 0 | 1 | 776 |
| 232 | E8 | 6 | 06 | 38 | 4 | 188176 | 1505404 | 38 | 4 | 0 | 768 | 1519636 | 38 | 4 | 0 | 1 | 776 |
| 233 | E9 | 6 | 06 | 38 | 5 | 188272 | 1506172 | 38 | 5 | 0 | 768 | 1520412 | 38 | 5 | 0 | 1 | 776 |
| 234 | EA | 6 | 06 | 39 | 0 | 188370 | 1506956 | 39 | 0 | 0 | 784 | 1521204 | 39 | 0 | 0 | 1 | 792 |
| 235 | EB | 6 | 06 | 39 | 1 | 188468 | 1507740 | 39 | 1 | 0 | 784 | 1521996 | 39 | 1 | 0 | 1 | 792 |
| 236 | EC | 6 | 06 | 39 | 2 | 188566 | 1508524 | 39 | 2 | 0 | 784 | 1522788 | 39 | 2 | 0 | 1 | 792 |
| 237 | ED | 6 | 06 | 39 | 3 | 188664 | 1509308 | 39 | 3 | 0 | 784 | 1523580 | 39 | 3 | 0 | 1 | 792 |
| 238 | EE | 6 | 06 | 39 | 4 | 188762 | 1510092 | 39 | 4 | 0 | 784 | 1524372 | 39 | 4 | 0 | 1 | 792 |
| 239 | EF | 6 | 06 | 39 | 5 | 188860 | 1510876 | 39 | 5 | 0 | 784 | 1525164 | 39 | 5 | 0 | 1 | 792 |
| 240 | F0 | 6 | 06 | 40 | 0 | 188960 | 1511676 | 40 | 0 | 0 | 800 | 1525972 | 40 | 0 | 0 | 1 | 808 |
| 241 | F1 | 6 | 06 | 40 | 1 | 189060 | 1512476 | 40 | 1 | 0 | 800 | 1526780 | 40 | 1 | 0 | 1 | 808 |
| 242 | F2 | 6 | 06 | 40 | 2 | 189160 | 1513276 | 40 | 2 | 0 | 800 | 1527588 | 40 | 2 | 0 | 1 | 808 |
| 243 | F3 | 6 | 06 | 40 | 3 | 189260 | 1514076 | 40 | 3 | 0 | 800 | 1528396 | 40 | 3 | 0 | 1 | 808 |
| 244 | F4 | 6 | 06 | 40 | 4 | 189360 | 1514876 | 40 | 4 | 0 | 800 | 1529204 | 40 | 4 | 0 | 1 | 808 |
| 245 | F5 | 6 | 06 | 40 | 5 | 189460 | 1515676 | 40 | 5 | 0 | 800 | 1530012 | 40 | 5 | 0 | 1 | 808 |
| 246 | F6 | 6 | 06 | 41 | 0 | 189562 | 1516492 | 41 | 0 | 0 | 816 | 1530836 | 41 | 0 | 0 | 1 | 824 |
| 247 | F7 | 6 | 06 | 41 | 1 | 189664 | 1517308 | 41 | 1 | 0 | 816 | 1531660 | 41 | 1 | 0 | 1 | 824 |
| 248 | F8 | 6 | 06 | 41 | 2 | 189766 | 1518124 | 41 | 2 | 0 | 816 | 1532484 | 41 | 2 | 0 | 1 | 824 |
| 249 | F9 | 6 | 06 | 41 | 3 | 189868 | 1518940 | 41 | 3 | 0 | 816 | 1533308 | 41 | 3 | 0 | 1 | 824 |
| 250 | FA | 6 | 06 | 41 | 4 | 189970 | 1519756 | 41 | 4 | 0 | 816 | 1534132 | 41 | 4 | 0 | 1 | 824 |
| 251 | FB | 6 | 06 | 41 | 5 | 190072 | 1520572 | 41 | 5 | 0 | 816 | 1534956 | 41 | 5 | 0 | 1 | 824 |
| 252 | FC | 6 | 06 | 42 | 0 | 190176 | 1521404 | 42 | 0 | 0 | 832 | 1535796 | 42 | 0 | 0 | 1 | 840 |
| 253 | FD | 6 | 06 | 42 | 1 | 190280 | 1522236 | 42 | 1 | 0 | 832 | 1536636 | 42 | 1 | 0 | 1 | 840 |
| 254 | FE | 6 | 06 | 42 | 2 | 190384 | 1523068 | 42 | 2 | 0 | 832 | 1537476 | 42 | 2 | 0 | 1 | 840 |
| 255 | FF | 6 | 06 | 42 | 3 | 190488 | 1523900 | 42 | 3 | 0 | 832 | 1538316 | 42 | 3 | 0 | 1 | 840 |
| 0 | 00 | 7 | 07 | 0 | 0 | 190500 | 1523996 | 0 | 0 | 0 | 96 | 1538420 | 0 | 0 | 0 | 1 | 104 |
| 1 | 01 | 7 | 07 | 0 | 1 | 190514 | 1524108 | 0 | 1 | 0 | 112 | 1538540 | 0 | 1 | 0 | 1 | 120 |
| 2 | 02 | 7 | 07 | 0 | 2 | 190528 | 1524220 | 0 | 2 | 0 | 112 | 1538660 | 0 | 2 | 0 | 1 | 120 |
| 3 | 03 | 7 | 07 | 0 | 3 | 190542 | 1524332 | 0 | 3 | 0 | 112 | 1538780 | 0 | 3 | 0 | 1 | 120 |
| 4 | 04 | 7 | 07 | 0 | 4 | 190556 | 1524444 | 0 | 4 | 0 | 112 | 1538900 | 0 | 4 | 0 | 1 | 120 |
| 5 | 05 | 7 | 07 | 0 | 5 | 190570 | 1524556 | 0 | 5 | 0 | 112 | 1539020 | 0 | 5 | 0 | 1 | 120 |
| 6 | 06 | 7 | 07 | 0 | 6 | 190584 | 1524668 | 0 | 6 | 0 | 112 | 1539140 | 0 | 6 | 0 | 1 | 120 |
| 7 | 07 | 7 | 07 | 1 | 0 | 190596 | 1524764 | 1 | 0 | 0 | 96 | 1539244 | 1 | 0 | 0 | 1 | 104 |
| 8 | 08 | 7 | 07 | 1 | 1 | 190613 | 1524900 | 1 | 1 | 0 | 136 | 1539388 | 1 | 1 | 0 | 1 | 144 |
| 9 | 09 | 7 | 07 | 1 | 2 | 190630 | 1525036 | 1 | 2 | 0 | 136 | 1539532 | 1 | 2 | 0 | 1 | 144 |
| 10 | 0A | 7 | 07 | 1 | 3 | 190647 | 1525172 | 1 | 3 | 0 | 136 | 1539676 | 1 | 3 | 0 | 1 | 144 |
| 11 | 0B | 7 | 07 | 1 | 4 | 190664 | 1525308 | 1 | 4 | 0 | 136 | 1539820 | 1 | 4 | 0 | 1 | 144 |
| 12 | 0C | 7 | 07 | 1 | 5 | 190681 | 1525444 | 1 | 5 | 0 | 136 | 1539964 | 1 | 5 | 0 | 1 | 144 |
| 13 | 0D | 7 | 07 | 1 | 6 | 190698 | 1525580 | 1 | 6 | 0 | 136 | 1540108 | 1 | 6 | 0 | 1 | 144 |
| 14 | 0E | 7 | 07 | 2 | 0 | 190717 | 1525732 | 2 | 0 | 0 | 152 | 1540268 | 2 | 0 | 0 | 1 | 160 |
| 15 | 0F | 7 | 07 | 2 | 1 | 190736 | 1525884 | 2 | 1 | 0 | 152 | 1540428 | 2 | 1 | 0 | 1 | 160 |
| 16 | 10 | 7 | 07 | 2 | 2 | 190760 | 1526076 | 2 | 2 | 0 | 192 | 1540628 | 2 | 2 | 0 | 1 | 200 |
| 17 | 11 | 7 | 07 | 2 | 3 | 190784 | 1526268 | 2 | 3 | 0 | 192 | 1540828 | 2 | 3 | 0 | 1 | 200 |
| 18 | 12 | 7 | 07 | 2 | 4 | 190808 | 1526460 | 2 | 4 | 0 | 192 | 1541028 | 2 | 4 | 0 | 1 | 200 |
| 19 | 13 | 7 | 07 | 2 | 5 | 190832 | 1526652 | 2 | 5 | 0 | 192 | 1541228 | 2 | 5 | 0 | 1 | 200 |
| 20 | 14 | 7 | 07 | 2 | 6 | 190856 | 1526844 | 2 | 6 | 0 | 192 | 1541428 | 2 | 6 | 0 | 1 | 200 |
| 21 | 15 | 7 | 07 | 3 | 0 | 190882 | 1527052 | 3 | 0 | 0 | 208 | 1541644 | 3 | 0 | 0 | 1 | 216 |
| 22 | 16 | 7 | 07 | 3 | 1 | 190908 | 1527260 | 3 | 1 | 0 | 208 | 1541860 | 3 | 1 | 0 | 1 | 216 |
| 23 | 17 | 7 | 07 | 3 | 2 | 190934 | 1527468 | 3 | 2 | 0 | 208 | 1542076 | 3 | 2 | 0 | 1 | 216 |
| 24 | 18 | 7 | 07 | 3 | 3 | 190960 | 1527676 | 3 | 3 | 0 | 208 | 1542292 | 3 | 3 | 0 | 1 | 216 |
| 25 | 19 | 7 | 07 | 3 | 4 | 190986 | 1527884 | 3 | 4 | 0 | 208 | 1542508 | 3 | 4 | 0 | 1 | 216 |
| 26 | 1A | 7 | 07 | 3 | 5 | 191012 | 1528092 | 3 | 5 | 0 | 208 | 1542724 | 3 | 5 | 0 | 1 | 216 |
| 27 | 1B | 7 | 07 | 3 | 6 | 191038 | 1528300 | 3 | 6 | 0 | 208 | 1542940 | 3 | 6 | 0 | 1 | 216 |
| 28 | 1C | 7 | 07 | 4 | 0 | 191066 | 1528524 | 4 | 0 | 0 | 224 | 1543172 | 4 | 0 | 0 | 1 | 232 |
| 29 | 1D | 7 | 07 | 4 | 1 | 191094 | 1528748 | 4 | 1 | 0 | 224 | 1543404 | 4 | 1 | 0 | 1 | 232 |
| 30 | 1E | 7 | 07 | 4 | 2 | 191122 | 1528972 | 4 | 2 | 0 | 224 | 1543636 | 4 | 2 | 0 | 1 | 232 |
| 31 | 1F | 7 | 07 | 4 | 3 | 191150 | 1529196 | 4 | 3 | 0 | 224 | 1543868 | 4 | 3 | 0 | 1 | 232 |
| 32 | 20 | 7 | 07 | 4 | 4 | 191178 | 1529420 | 4 | 4 | 0 | 224 | 1544100 | 4 | 4 | 0 | 1 | 232 |
| 33 | 21 | 7 | 07 | 4 | 5 | 191206 | 1529644 | 4 | 5 | 0 | 224 | 1544332 | 4 | 5 | 0 | 1 | 232 |
| 34 | 22 | 7 | 07 | 4 | 6 | 191234 | 1529868 | 4 | 6 | 0 | 224 | 1544564 | 4 | 6 | 0 | 1 | 232 |
| 35 | 23 | 7 | 07 | 5 | 0 | 191264 | 1530108 | 5 | 0 | 0 | 240 | 1544812 | 5 | 0 | 0 | 1 | 248 |
| 36 | 24 | 7 | 07 | 5 | 1 | 191294 | 1530348 | 5 | 1 | 0 | 240 | 1545060 | 5 | 1 | 0 | 1 | 248 |
| 37 | 25 | 7 | 07 | 5 | 2 | 191324 | 1530588 | 5 | 2 | 0 | 240 | 1545308 | 5 | 2 | 0 | 1 | 248 |
| 38 | 26 | 7 | 07 | 5 | 3 | 191354 | 1530828 | 5 | 3 | 0 | 240 | 1545556 | 5 | 3 | 0 | 1 | 248 |
| 39 | 27 | 7 | 07 | 5 | 4 | 191384 | 1531068 | 5 | 4 | 0 | 240 | 1545804 | 5 | 4 | 0 | 1 | 248 |
| 40 | 28 | 7 | 07 | 5 | 5 | 191414 | 1531308 | 5 | 5 | 0 | 240 | 1546052 | 5 | 5 | 0 | 1 | 248 |
| 41 | 29 | 7 | 07 | 5 | 6 | 191444 | 1531548 | 5 | 6 | 0 | 240 | 1546300 | 5 | 6 | 0 | 1 | 248 |
| 42 | 2A | 7 | 07 | 6 | 0 | 191476 | 1531804 | 6 | 0 | 0 | 256 | 1546564 | 6 | 0 | 0 | 1 | 264 |
| 43 | 2B | 7 | 07 | 6 | 1 | 191508 | 1532060 | 6 | 1 | 0 | 256 | 1546828 | 6 | 1 | 0 | 1 | 264 |
| 44 | 2C | 7 | 07 | 6 | 2 | 191540 | 1532316 | 6 | 2 | 0 | 256 | 1547092 | 6 | 2 | 0 | 1 | 264 |
| 45 | 2D | 7 | 07 | 6 | 3 | 191572 | 1532572 | 6 | 3 | 0 | 256 | 1547356 | 6 | 3 | 0 | 1 | 264 |
| 46 | 2E | 7 | 07 | 6 | 4 | 191604 | 1532828 | 6 | 4 | 0 | 256 | 1547620 | 6 | 4 | 0 | 1 | 264 |
| 47 | 2F | 7 | 07 | 6 | 5 | 191636 | 1533084 | 6 | 5 | 0 | 256 | 1547884 | 6 | 5 | 0 | 1 | 264 |
| 48 | 30 | 7 | 07 | 6 | 6 | 191668 | 1533340 | 6 | 6 | 0 | 256 | 1548148 | 6 | 6 | 0 | 1 | 264 |
| 49 | 31 | 7 | 07 | 7 | 0 | 191702 | 1533612 | 7 | 0 | 0 | 272 | 1548428 | 7 | 0 | 0 | 1 | 280 |
| 50 | 32 | 7 | 07 | 7 | 1 | 191736 | 1533884 | 7 | 1 | 0 | 272 | 1548708 | 7 | 1 | 0 | 1 | 280 |
| 51 | 33 | 7 | 07 | 7 | 2 | 191770 | 1534156 | 7 | 2 | 0 | 272 | 1548988 | 7 | 2 | 0 | 1 | 280 |
| 52 | 34 | 7 | 07 | 7 | 3 | 191804 | 1534428 | 7 | 3 | 0 | 272 | 1549268 | 7 | 3 | 0 | 1 | 280 |
| 53 | 35 | 7 | 07 | 7 | 4 | 191838 | 1534700 | 7 | 4 | 0 | 272 | 1549548 | 7 | 4 | 0 | 1 | 280 |
| 54 | 36 | 7 | 07 | 7 | 5 | 191872 | 1534972 | 7 | 5 | 0 | 272 | 1549828 | 7 | 5 | 0 | 1 | 280 |
| 55 | 37 | 7 | 07 | 7 | 6 | 191906 | 1535244 | 7 | 6 | 0 | 272 | 1550108 | 7 | 6 | 0 | 1 | 280 |
| 56 | 38 | 7 | 07 | 8 | 0 | 191942 | 1535532 | 8 | 0 | 0 | 288 | 1550404 | 8 | 0 | 0 | 1 | 296 |
| 57 | 39 | 7 | 07 | 8 | 1 | 191978 | 1535820 | 8 | 1 | 0 | 288 | 1550700 | 8 | 1 | 0 | 1 | 296 |
| 58 | 3A | 7 | 07 | 8 | 2 | 192014 | 1536108 | 8 | 2 | 0 | 288 | 1550996 | 8 | 2 | 0 | 1 | 296 |

| | | | | | | | | | | | | | | | | | |
|---|---|---|---|---|---|---|---|---|---|---|---|---|---|---|---|---|---|
| 59 | 3B | 7 | 07 | 8 | 3 | 192050 | 1536396 | 8 | 3 | 0 | 288 | 1551292 | 8 | 3 | 0 | 1 | 296 |
| 60 | 3C | 7 | 07 | 8 | 4 | 192086 | 1536684 | 8 | 4 | 0 | 288 | 1551588 | 8 | 4 | 0 | 1 | 296 |
| 61 | 3D | 7 | 07 | 8 | 5 | 192122 | 1536972 | 8 | 5 | 0 | 288 | 1551884 | 8 | 5 | 0 | 1 | 296 |
| 62 | 3E | 7 | 07 | 8 | 6 | 192158 | 1537260 | 8 | 6 | 0 | 288 | 1552180 | 8 | 6 | 0 | 1 | 296 |
| 63 | 3F | 7 | 07 | 9 | 0 | 192196 | 1537564 | 9 | 0 | 0 | 304 | 1552492 | 9 | 0 | 0 | 1 | 312 |
| 64 | 40 | 7 | 07 | 9 | 1 | 192234 | 1537868 | 9 | 1 | 0 | 304 | 1552804 | 9 | 1 | 0 | 1 | 312 |
| 65 | 41 | 7 | 07 | 9 | 2 | 192272 | 1538172 | 9 | 2 | 0 | 304 | 1553116 | 9 | 2 | 0 | 1 | 312 |
| 66 | 42 | 7 | 07 | 9 | 3 | 192310 | 1538476 | 9 | 3 | 0 | 304 | 1553428 | 9 | 3 | 0 | 1 | 312 |
| 67 | 43 | 7 | 07 | 9 | 4 | 192348 | 1538780 | 9 | 4 | 0 | 304 | 1553740 | 9 | 4 | 0 | 1 | 312 |
| 68 | 44 | 7 | 07 | 9 | 5 | 192386 | 1539084 | 9 | 5 | 0 | 304 | 1554052 | 9 | 5 | 0 | 1 | 312 |
| 69 | 45 | 7 | 07 | 9 | 6 | 192424 | 1539388 | 9 | 6 | 0 | 304 | 1554364 | 9 | 6 | 0 | 1 | 312 |
| 70 | 46 | 7 | 07 | 10 | 0 | 192464 | 1539708 | 10 | 0 | 0 | 320 | 1554692 | 10 | 0 | 0 | 1 | 328 |
| 71 | 47 | 7 | 07 | 10 | 1 | 192504 | 1540028 | 10 | 1 | 0 | 320 | 1555020 | 10 | 1 | 0 | 1 | 328 |
| 72 | 48 | 7 | 07 | 10 | 2 | 192544 | 1540348 | 10 | 2 | 0 | 320 | 1555348 | 10 | 2 | 0 | 1 | 328 |
| 73 | 49 | 7 | 07 | 10 | 3 | 192584 | 1540668 | 10 | 3 | 0 | 320 | 1555676 | 10 | 3 | 0 | 1 | 328 |
| 74 | 4A | 7 | 07 | 10 | 4 | 192624 | 1540988 | 10 | 4 | 0 | 320 | 1556004 | 10 | 4 | 0 | 1 | 328 |
| 75 | 4B | 7 | 07 | 10 | 5 | 192664 | 1541308 | 10 | 5 | 0 | 320 | 1556332 | 10 | 5 | 0 | 1 | 328 |
| 76 | 4C | 7 | 07 | 10 | 6 | 192704 | 1541628 | 10 | 6 | 0 | 320 | 1556660 | 10 | 6 | 0 | 1 | 328 |
| 77 | 4D | 7 | 07 | 11 | 0 | 192746 | 1541964 | 11 | 0 | 0 | 336 | 1557004 | 11 | 0 | 0 | 1 | 344 |
| 78 | 4E | 7 | 07 | 11 | 1 | 192788 | 1542300 | 11 | 1 | 0 | 336 | 1557348 | 11 | 1 | 0 | 1 | 344 |
| 79 | 4F | 7 | 07 | 11 | 2 | 192830 | 1542636 | 11 | 2 | 0 | 336 | 1557692 | 11 | 2 | 0 | 1 | 344 |
| 80 | 50 | 7 | 07 | 11 | 3 | 192872 | 1542972 | 11 | 3 | 0 | 336 | 1558036 | 11 | 3 | 0 | 1 | 344 |
| 81 | 51 | 7 | 07 | 11 | 4 | 192914 | 1543308 | 11 | 4 | 0 | 336 | 1558380 | 11 | 4 | 0 | 1 | 344 |
| 82 | 52 | 7 | 07 | 11 | 5 | 192956 | 1543644 | 11 | 5 | 0 | 336 | 1558724 | 11 | 5 | 0 | 1 | 344 |
| 83 | 53 | 7 | 07 | 11 | 6 | 192998 | 1543980 | 11 | 6 | 0 | 336 | 1559068 | 11 | 6 | 0 | 1 | 344 |
| 84 | 54 | 7 | 07 | 12 | 0 | 193042 | 1544332 | 12 | 0 | 0 | 352 | 1559428 | 12 | 0 | 0 | 1 | 360 |
| 85 | 55 | 7 | 07 | 12 | 1 | 193086 | 1544684 | 12 | 1 | 0 | 352 | 1559788 | 12 | 1 | 0 | 1 | 360 |
| 86 | 56 | 7 | 07 | 12 | 2 | 193130 | 1545036 | 12 | 2 | 0 | 352 | 1560148 | 12 | 2 | 0 | 1 | 360 |
| 87 | 57 | 7 | 07 | 12 | 3 | 193174 | 1545388 | 12 | 3 | 0 | 352 | 1560508 | 12 | 3 | 0 | 1 | 360 |
| 88 | 58 | 7 | 07 | 12 | 4 | 193218 | 1545740 | 12 | 4 | 0 | 352 | 1560868 | 12 | 4 | 0 | 1 | 360 |
| 89 | 59 | 7 | 07 | 12 | 5 | 193262 | 1546092 | 12 | 5 | 0 | 352 | 1561228 | 12 | 5 | 0 | 1 | 360 |
| 90 | 5A | 7 | 07 | 12 | 6 | 193306 | 1546444 | 12 | 6 | 0 | 352 | 1561588 | 12 | 6 | 0 | 1 | 360 |
| 91 | 5B | 7 | 07 | 13 | 0 | 193352 | 1546812 | 13 | 0 | 0 | 368 | 1561964 | 13 | 0 | 0 | 1 | 376 |
| 92 | 5C | 7 | 07 | 13 | 1 | 193398 | 1547180 | 13 | 1 | 0 | 368 | 1562340 | 13 | 1 | 0 | 1 | 376 |
| 93 | 5D | 7 | 07 | 13 | 2 | 193444 | 1547548 | 13 | 2 | 0 | 368 | 1562716 | 13 | 2 | 0 | 1 | 376 |
| 94 | 5E | 7 | 07 | 13 | 3 | 193490 | 1547916 | 13 | 3 | 0 | 368 | 1563092 | 13 | 3 | 0 | 1 | 376 |
| 95 | 5F | 7 | 07 | 13 | 4 | 193536 | 1548284 | 13 | 4 | 0 | 368 | 1563468 | 13 | 4 | 0 | 1 | 376 |
| 96 | 60 | 7 | 07 | 13 | 5 | 193582 | 1548652 | 13 | 5 | 0 | 368 | 1563844 | 13 | 5 | 0 | 1 | 376 |
| 97 | 61 | 7 | 07 | 13 | 6 | 193628 | 1549020 | 13 | 6 | 0 | 368 | 1564220 | 13 | 6 | 0 | 1 | 376 |
| 98 | 62 | 7 | 07 | 14 | 0 | 193676 | 1549404 | 14 | 0 | 0 | 384 | 1564612 | 14 | 0 | 0 | 1 | 392 |
| 99 | 63 | 7 | 07 | 14 | 1 | 193724 | 1549788 | 14 | 1 | 0 | 384 | 1565004 | 14 | 1 | 0 | 1 | 392 |
| 100 | 64 | 7 | 07 | 14 | 2 | 193772 | 1550172 | 14 | 2 | 0 | 384 | 1565396 | 14 | 2 | 0 | 1 | 392 |
| 101 | 65 | 7 | 07 | 14 | 3 | 193820 | 1550556 | 14 | 3 | 0 | 384 | 1565788 | 14 | 3 | 0 | 1 | 392 |
| 102 | 66 | 7 | 07 | 14 | 4 | 193868 | 1550940 | 14 | 4 | 0 | 384 | 1566180 | 14 | 4 | 0 | 1 | 392 |
| 103 | 67 | 7 | 07 | 14 | 5 | 193916 | 1551324 | 14 | 5 | 0 | 384 | 1566572 | 14 | 5 | 0 | 1 | 392 |
| 104 | 68 | 7 | 07 | 14 | 6 | 193964 | 1551708 | 14 | 6 | 0 | 384 | 1566964 | 14 | 6 | 0 | 1 | 392 |
| 105 | 69 | 7 | 07 | 15 | 0 | 194014 | 1552108 | 15 | 0 | 0 | 400 | 1567372 | 15 | 0 | 0 | 1 | 408 |
| 106 | 6A | 7 | 07 | 15 | 1 | 194064 | 1552508 | 15 | 1 | 0 | 400 | 1567780 | 15 | 1 | 0 | 1 | 408 |
| 107 | 6B | 7 | 07 | 15 | 2 | 194114 | 1552908 | 15 | 2 | 0 | 400 | 1568188 | 15 | 2 | 0 | 1 | 408 |
| 108 | 6C | 7 | 07 | 15 | 3 | 194164 | 1553308 | 15 | 3 | 0 | 400 | 1568596 | 15 | 3 | 0 | 1 | 408 |
| 109 | 6D | 7 | 07 | 15 | 4 | 194214 | 1553708 | 15 | 4 | 0 | 400 | 1569004 | 15 | 4 | 0 | 1 | 408 |
| 110 | 6E | 7 | 07 | 15 | 5 | 194264 | 1554108 | 15 | 5 | 0 | 400 | 1569412 | 15 | 5 | 0 | 1 | 408 |
| 111 | 6F | 7 | 07 | 15 | 6 | 194314 | 1554508 | 15 | 6 | 0 | 400 | 1569820 | 15 | 6 | 0 | 1 | 408 |
| 112 | 70 | 7 | 07 | 16 | 0 | 194366 | 1554924 | 16 | 0 | 0 | 416 | 1570244 | 16 | 0 | 0 | 1 | 424 |
| 113 | 71 | 7 | 07 | 16 | 1 | 194418 | 1555340 | 16 | 1 | 0 | 416 | 1570668 | 16 | 1 | 0 | 1 | 424 |
| 114 | 72 | 7 | 07 | 16 | 2 | 194470 | 1555756 | 16 | 2 | 0 | 416 | 1571092 | 16 | 2 | 0 | 1 | 424 |
| 115 | 73 | 7 | 07 | 16 | 3 | 194522 | 1556172 | 16 | 3 | 0 | 416 | 1571516 | 16 | 3 | 0 | 1 | 424 |
| 116 | 74 | 7 | 07 | 16 | 4 | 194574 | 1556588 | 16 | 4 | 0 | 416 | 1571940 | 16 | 4 | 0 | 1 | 424 |
| 117 | 75 | 7 | 07 | 16 | 5 | 194626 | 1557004 | 16 | 5 | 0 | 416 | 1572364 | 16 | 5 | 0 | 1 | 424 |
| 118 | 76 | 7 | 07 | 16 | 6 | 194678 | 1557420 | 16 | 6 | 0 | 416 | 1572788 | 16 | 6 | 0 | 1 | 424 |
| 119 | 77 | 7 | 07 | 17 | 0 | 194732 | 1557852 | 17 | 0 | 0 | 432 | 1573228 | 17 | 0 | 0 | 1 | 440 |
| 120 | 78 | 7 | 07 | 17 | 1 | 194786 | 1558284 | 17 | 1 | 0 | 432 | 1573668 | 17 | 1 | 0 | 1 | 440 |
| 121 | 79 | 7 | 07 | 17 | 2 | 194840 | 1558716 | 17 | 2 | 0 | 432 | 1574108 | 17 | 2 | 0 | 1 | 440 |
| 122 | 7A | 7 | 07 | 17 | 3 | 194894 | 1559148 | 17 | 3 | 0 | 432 | 1574548 | 17 | 3 | 0 | 1 | 440 |
| 123 | 7B | 7 | 07 | 17 | 4 | 194948 | 1559580 | 17 | 4 | 0 | 432 | 1574988 | 17 | 4 | 0 | 1 | 440 |
| 124 | 7C | 7 | 07 | 17 | 5 | 195002 | 1560012 | 17 | 5 | 0 | 432 | 1575428 | 17 | 5 | 0 | 1 | 440 |
| 125 | 7D | 7 | 07 | 17 | 6 | 195056 | 1560444 | 17 | 6 | 0 | 432 | 1575868 | 17 | 6 | 0 | 1 | 440 |
| 126 | 7E | 7 | 07 | 18 | 0 | 195112 | 1560892 | 18 | 0 | 0 | 448 | 1576324 | 18 | 0 | 0 | 1 | 456 |
| 127 | 7F | 7 | 07 | 18 | 1 | 195168 | 1561340 | 18 | 1 | 0 | 448 | 1576780 | 18 | 1 | 0 | 1 | 456 |
| 128 | 80 | 7 | 07 | 18 | 2 | 195224 | 1561788 | 18 | 2 | 0 | 448 | 1577236 | 18 | 2 | 0 | 1 | 456 |
| 129 | 81 | 7 | 07 | 18 | 3 | 195280 | 1562236 | 18 | 3 | 0 | 448 | 1577692 | 18 | 3 | 0 | 1 | 456 |
| 130 | 82 | 7 | 07 | 18 | 4 | 195336 | 1562684 | 18 | 4 | 0 | 448 | 1578148 | 18 | 4 | 0 | 1 | 456 |
| 131 | 83 | 7 | 07 | 18 | 5 | 195392 | 1563132 | 18 | 5 | 0 | 448 | 1578604 | 18 | 5 | 0 | 1 | 456 |
| 132 | 84 | 7 | 07 | 18 | 6 | 195448 | 1563580 | 18 | 6 | 0 | 448 | 1579060 | 18 | 6 | 0 | 1 | 456 |
| 133 | 85 | 7 | 07 | 19 | 0 | 195506 | 1564044 | 19 | 0 | 0 | 464 | 1579532 | 19 | 0 | 0 | 1 | 472 |
| 134 | 86 | 7 | 07 | 19 | 1 | 195564 | 1564508 | 19 | 1 | 0 | 464 | 1580004 | 19 | 1 | 0 | 1 | 472 |
| 135 | 87 | 7 | 07 | 19 | 2 | 195622 | 1564972 | 19 | 2 | 0 | 464 | 1580476 | 19 | 2 | 0 | 1 | 472 |
| 136 | 88 | 7 | 07 | 19 | 3 | 195680 | 1565436 | 19 | 3 | 0 | 464 | 1580948 | 19 | 3 | 0 | 1 | 472 |
| 137 | 89 | 7 | 07 | 19 | 4 | 195738 | 1565900 | 19 | 4 | 0 | 464 | 1581420 | 19 | 4 | 0 | 1 | 472 |
| 138 | 8A | 7 | 07 | 19 | 5 | 195796 | 1566364 | 19 | 5 | 0 | 464 | 1581892 | 19 | 5 | 0 | 1 | 472 |
| 139 | 8B | 7 | 07 | 19 | 6 | 195854 | 1566828 | 19 | 6 | 0 | 464 | 1582364 | 19 | 6 | 0 | 1 | 472 |
| 140 | 8C | 7 | 07 | 20 | 0 | 195914 | 1567308 | 20 | 0 | 0 | 480 | 1582852 | 20 | 0 | 0 | 1 | 488 |
| 141 | 8D | 7 | 07 | 20 | 1 | 195974 | 1567788 | 20 | 1 | 0 | 480 | 1583340 | 20 | 1 | 0 | 1 | 488 |
| 142 | 8E | 7 | 07 | 20 | 2 | 196034 | 1568268 | 20 | 2 | 0 | 480 | 1583828 | 20 | 2 | 0 | 1 | 488 |
| 143 | 8F | 7 | 07 | 20 | 3 | 196094 | 1568748 | 20 | 3 | 0 | 480 | 1584316 | 20 | 3 | 0 | 1 | 488 |
| 144 | 90 | 7 | 07 | 20 | 4 | 196154 | 1569228 | 20 | 4 | 0 | 480 | 1584804 | 20 | 4 | 0 | 1 | 488 |
| 145 | 91 | 7 | 07 | 20 | 5 | 196214 | 1569708 | 20 | 5 | 0 | 480 | 1585292 | 20 | 5 | 0 | 1 | 488 |
| 146 | 92 | 7 | 07 | 20 | 6 | 196274 | 1570188 | 20 | 6 | 0 | 480 | 1585780 | 20 | 6 | 0 | 1 | 488 |
| 147 | 93 | 7 | 07 | 21 | 0 | 196336 | 1570684 | 21 | 0 | 0 | 496 | 1586284 | 21 | 0 | 0 | 1 | 504 |
| 148 | 94 | 7 | 07 | 21 | 1 | 196398 | 1571180 | 21 | 1 | 0 | 496 | 1586788 | 21 | 1 | 0 | 1 | 504 |
| 149 | 95 | 7 | 07 | 21 | 2 | 196460 | 1571676 | 21 | 2 | 0 | 496 | 1587292 | 21 | 2 | 0 | 1 | 504 |
| 150 | 96 | 7 | 07 | 21 | 3 | 196522 | 1572172 | 21 | 3 | 0 | 496 | 1587796 | 21 | 3 | 0 | 1 | 504 |
| 151 | 97 | 7 | 07 | 21 | 4 | 196584 | 1572668 | 21 | 4 | 0 | 496 | 1588300 | 21 | 4 | 0 | 1 | 504 |
| 152 | 98 | 7 | 07 | 21 | 5 | 196646 | 1573164 | 21 | 5 | 0 | 496 | 1588804 | 21 | 5 | 0 | 1 | 504 |
| 153 | 99 | 7 | 07 | 21 | 6 | 196708 | 1573660 | 21 | 6 | 0 | 496 | 1589308 | 21 | 6 | 0 | 1 | 504 |
| 154 | 9A | 7 | 07 | 22 | 0 | 196772 | 1574172 | 22 | 0 | 0 | 512 | 1589828 | 22 | 0 | 0 | 1 | 520 |
| 155 | 9B | 7 | 07 | 22 | 1 | 196836 | 1574684 | 22 | 1 | 0 | 512 | 1590348 | 22 | 1 | 0 | 1 | 520 |
| 156 | 9C | 7 | 07 | 22 | 2 | 196900 | 1575196 | 22 | 2 | 0 | 512 | 1590868 | 22 | 2 | 0 | 1 | 520 |
| 157 | 9D | 7 | 07 | 22 | 3 | 196964 | 1575708 | 22 | 3 | 0 | 512 | 1591388 | 22 | 3 | 0 | 1 | 520 |
| 158 | 9E | 7 | 07 | 22 | 4 | 197028 | 1576220 | 22 | 4 | 0 | 512 | 1591908 | 22 | 4 | 0 | 1 | 520 |
| 159 | 9F | 7 | 07 | 22 | 5 | 197092 | 1576732 | 22 | 5 | 0 | 512 | 1592428 | 22 | 5 | 0 | 1 | 520 |
| 160 | A0 | 7 | 07 | 22 | 6 | 197156 | 1577244 | 22 | 6 | 0 | 512 | 1592948 | 22 | 6 | 0 | 1 | 520 |
| 161 | A1 | 7 | 07 | 23 | 0 | 197222 | 1577772 | 23 | 0 | 0 | 528 | 1593484 | 23 | 0 | 0 | 1 | 536 |

| | | | | | | | | | | | | | | | | | |
|---|---|---|---|---|---|---|---|---|---|---|---|---|---|---|---|---|---|
| 162 | A2 | 7 | 07 | 23 | 1 | 197288 | 1578300 | 23 | 1 | 0 | 528 | 1594020 | 23 | 1 | 0 | 1 | 536 |
| 163 | A3 | 7 | 07 | 23 | 2 | 197354 | 1578828 | 23 | 2 | 0 | 528 | 1594556 | 23 | 2 | 0 | 1 | 536 |
| 164 | A4 | 7 | 07 | 23 | 3 | 197420 | 1579356 | 23 | 3 | 0 | 528 | 1595092 | 23 | 3 | 0 | 1 | 536 |
| 165 | A5 | 7 | 07 | 23 | 4 | 197486 | 1579884 | 23 | 4 | 0 | 528 | 1595628 | 23 | 4 | 0 | 1 | 536 |
| 166 | A6 | 7 | 07 | 23 | 5 | 197552 | 1580412 | 23 | 5 | 0 | 528 | 1596164 | 23 | 5 | 0 | 1 | 536 |
| 167 | A7 | 7 | 07 | 23 | 6 | 197618 | 1580940 | 23 | 6 | 0 | 528 | 1596700 | 23 | 6 | 0 | 1 | 536 |
| 168 | A8 | 7 | 07 | 24 | 0 | 197686 | 1581484 | 24 | 0 | 0 | 544 | 1597252 | 24 | 0 | 0 | 1 | 552 |
| 169 | A9 | 7 | 07 | 24 | 1 | 197754 | 1582028 | 24 | 1 | 0 | 544 | 1597804 | 24 | 1 | 0 | 1 | 552 |
| 170 | AA | 7 | 07 | 24 | 2 | 197822 | 1582572 | 24 | 2 | 0 | 544 | 1598356 | 24 | 2 | 0 | 1 | 552 |
| 171 | AB | 7 | 07 | 24 | 3 | 197890 | 1583116 | 24 | 3 | 0 | 544 | 1598908 | 24 | 3 | 0 | 1 | 552 |
| 172 | AC | 7 | 07 | 24 | 4 | 197958 | 1583660 | 24 | 4 | 0 | 544 | 1599460 | 24 | 4 | 0 | 1 | 552 |
| 173 | AD | 7 | 07 | 24 | 5 | 198026 | 1584204 | 24 | 5 | 0 | 544 | 1600012 | 24 | 5 | 0 | 1 | 552 |
| 174 | AE | 7 | 07 | 24 | 6 | 198094 | 1584748 | 24 | 6 | 0 | 544 | 1600564 | 24 | 6 | 0 | 1 | 552 |
| 175 | AF | 7 | 07 | 25 | 0 | 198164 | 1585308 | 25 | 0 | 0 | 560 | 1601132 | 25 | 0 | 0 | 1 | 568 |
| 176 | B0 | 7 | 07 | 25 | 1 | 198234 | 1585868 | 25 | 1 | 0 | 560 | 1601700 | 25 | 1 | 0 | 1 | 568 |
| 177 | B1 | 7 | 07 | 25 | 2 | 198304 | 1586428 | 25 | 2 | 0 | 560 | 1602268 | 25 | 2 | 0 | 1 | 568 |
| 178 | B2 | 7 | 07 | 25 | 3 | 198374 | 1586988 | 25 | 3 | 0 | 560 | 1602836 | 25 | 3 | 0 | 1 | 568 |
| 179 | B3 | 7 | 07 | 25 | 4 | 198444 | 1587548 | 25 | 4 | 0 | 560 | 1603404 | 25 | 4 | 0 | 1 | 568 |
| 180 | B4 | 7 | 07 | 25 | 5 | 198514 | 1588108 | 25 | 5 | 0 | 560 | 1603972 | 25 | 5 | 0 | 1 | 568 |
| 181 | B5 | 7 | 07 | 25 | 6 | 198584 | 1588668 | 25 | 6 | 0 | 560 | 1604540 | 25 | 6 | 0 | 1 | 568 |
| 182 | B6 | 7 | 07 | 26 | 0 | 198656 | 1589244 | 26 | 0 | 0 | 576 | 1605124 | 26 | 0 | 0 | 1 | 584 |
| 183 | B7 | 7 | 07 | 26 | 1 | 198728 | 1589820 | 26 | 1 | 0 | 576 | 1605708 | 26 | 1 | 0 | 1 | 584 |
| 184 | B8 | 7 | 07 | 26 | 2 | 198800 | 1590396 | 26 | 2 | 0 | 576 | 1606292 | 26 | 2 | 0 | 1 | 584 |
| 185 | B9 | 7 | 07 | 26 | 3 | 198872 | 1590972 | 26 | 3 | 0 | 576 | 1606876 | 26 | 3 | 0 | 1 | 584 |
| 186 | BA | 7 | 07 | 26 | 4 | 198944 | 1591548 | 26 | 4 | 0 | 576 | 1607460 | 26 | 4 | 0 | 1 | 584 |
| 187 | BB | 7 | 07 | 26 | 5 | 199016 | 1592124 | 26 | 5 | 0 | 576 | 1608044 | 26 | 5 | 0 | 1 | 584 |
| 188 | BC | 7 | 07 | 26 | 6 | 199088 | 1592700 | 26 | 6 | 0 | 576 | 1608628 | 26 | 6 | 0 | 1 | 584 |
| 189 | BD | 7 | 07 | 27 | 0 | 199162 | 1593292 | 27 | 0 | 0 | 592 | 1609228 | 27 | 0 | 0 | 1 | 600 |
| 190 | BE | 7 | 07 | 27 | 1 | 199236 | 1593884 | 27 | 1 | 0 | 592 | 1609828 | 27 | 1 | 0 | 1 | 600 |
| 191 | BF | 7 | 07 | 27 | 2 | 199310 | 1594476 | 27 | 2 | 0 | 592 | 1610428 | 27 | 2 | 0 | 1 | 600 |
| 192 | C0 | 7 | 07 | 27 | 3 | 199384 | 1595068 | 27 | 3 | 0 | 592 | 1611028 | 27 | 3 | 0 | 1 | 600 |
| 193 | C1 | 7 | 07 | 27 | 4 | 199458 | 1595660 | 27 | 4 | 0 | 592 | 1611628 | 27 | 4 | 0 | 1 | 600 |
| 194 | C2 | 7 | 07 | 27 | 5 | 199532 | 1596252 | 27 | 5 | 0 | 592 | 1612228 | 27 | 5 | 0 | 1 | 600 |
| 195 | C3 | 7 | 07 | 27 | 6 | 199606 | 1596844 | 27 | 6 | 0 | 592 | 1612828 | 27 | 6 | 0 | 1 | 600 |
| 196 | C4 | 7 | 07 | 28 | 0 | 199682 | 1597452 | 28 | 0 | 0 | 608 | 1613444 | 28 | 0 | 0 | 1 | 616 |
| 197 | C5 | 7 | 07 | 28 | 1 | 199758 | 1598060 | 28 | 1 | 0 | 608 | 1614060 | 28 | 1 | 0 | 1 | 616 |
| 198 | C6 | 7 | 07 | 28 | 2 | 199834 | 1598668 | 28 | 2 | 0 | 608 | 1614676 | 28 | 2 | 0 | 1 | 616 |
| 199 | C7 | 7 | 07 | 28 | 3 | 199910 | 1599276 | 28 | 3 | 0 | 608 | 1615292 | 28 | 3 | 0 | 1 | 616 |
| 200 | C8 | 7 | 07 | 28 | 4 | 199986 | 1599884 | 28 | 4 | 0 | 608 | 1615908 | 28 | 4 | 0 | 1 | 616 |
| 201 | C9 | 7 | 07 | 28 | 5 | 200062 | 1600492 | 28 | 5 | 0 | 608 | 1616524 | 28 | 5 | 0 | 1 | 616 |
| 202 | CA | 7 | 07 | 28 | 6 | 200138 | 1601100 | 28 | 6 | 0 | 608 | 1617140 | 28 | 6 | 0 | 1 | 616 |
| 203 | CB | 7 | 07 | 29 | 0 | 200216 | 1601724 | 29 | 0 | 0 | 624 | 1617772 | 29 | 0 | 0 | 1 | 632 |
| 204 | CC | 7 | 07 | 29 | 1 | 200294 | 1602348 | 29 | 1 | 0 | 624 | 1618404 | 29 | 1 | 0 | 1 | 632 |
| 205 | CD | 7 | 07 | 29 | 2 | 200372 | 1602972 | 29 | 2 | 0 | 624 | 1619036 | 29 | 2 | 0 | 1 | 632 |
| 206 | CE | 7 | 07 | 29 | 3 | 200450 | 1603596 | 29 | 3 | 0 | 624 | 1619668 | 29 | 3 | 0 | 1 | 632 |
| 207 | CF | 7 | 07 | 29 | 4 | 200528 | 1604220 | 29 | 4 | 0 | 624 | 1620300 | 29 | 4 | 0 | 1 | 632 |
| 208 | D0 | 7 | 07 | 29 | 5 | 200606 | 1604844 | 29 | 5 | 0 | 624 | 1620932 | 29 | 5 | 0 | 1 | 632 |
| 209 | D1 | 7 | 07 | 29 | 6 | 200684 | 1605468 | 29 | 6 | 0 | 624 | 1621564 | 29 | 6 | 0 | 1 | 632 |
| 210 | D2 | 7 | 07 | 30 | 0 | 200764 | 1606108 | 30 | 0 | 0 | 640 | 1622212 | 30 | 0 | 0 | 1 | 648 |
| 211 | D3 | 7 | 07 | 30 | 1 | 200844 | 1606748 | 30 | 1 | 0 | 640 | 1622860 | 30 | 1 | 0 | 1 | 648 |
| 212 | D4 | 7 | 07 | 30 | 2 | 200924 | 1607388 | 30 | 2 | 0 | 640 | 1623508 | 30 | 2 | 0 | 1 | 648 |
| 213 | D5 | 7 | 07 | 30 | 3 | 201004 | 1608028 | 30 | 3 | 0 | 640 | 1624156 | 30 | 3 | 0 | 1 | 648 |
| 214 | D6 | 7 | 07 | 30 | 4 | 201084 | 1608668 | 30 | 4 | 0 | 640 | 1624804 | 30 | 4 | 0 | 1 | 648 |
| 215 | D7 | 7 | 07 | 30 | 5 | 201164 | 1609308 | 30 | 5 | 0 | 640 | 1625452 | 30 | 5 | 0 | 1 | 648 |
| 216 | D8 | 7 | 07 | 30 | 6 | 201244 | 1609948 | 30 | 6 | 0 | 640 | 1626100 | 30 | 6 | 0 | 1 | 648 |
| 217 | D9 | 7 | 07 | 31 | 0 | 201326 | 1610604 | 31 | 0 | 0 | 656 | 1626764 | 31 | 0 | 0 | 1 | 664 |
| 218 | DA | 7 | 07 | 31 | 1 | 201408 | 1611260 | 31 | 1 | 0 | 656 | 1627428 | 31 | 1 | 0 | 1 | 664 |
| 219 | DB | 7 | 07 | 31 | 2 | 201490 | 1611916 | 31 | 2 | 0 | 656 | 1628092 | 31 | 2 | 0 | 1 | 664 |
| 220 | DC | 7 | 07 | 31 | 3 | 201572 | 1612572 | 31 | 3 | 0 | 656 | 1628756 | 31 | 3 | 0 | 1 | 664 |
| 221 | DD | 7 | 07 | 31 | 4 | 201654 | 1613228 | 31 | 4 | 0 | 656 | 1629420 | 31 | 4 | 0 | 1 | 664 |
| 222 | DE | 7 | 07 | 31 | 5 | 201736 | 1613884 | 31 | 5 | 0 | 656 | 1630084 | 31 | 5 | 0 | 1 | 664 |
| 223 | DF | 7 | 07 | 31 | 6 | 201818 | 1614540 | 31 | 6 | 0 | 656 | 1630748 | 31 | 6 | 0 | 1 | 664 |
| 224 | E0 | 7 | 07 | 32 | 0 | 201902 | 1615212 | 32 | 0 | 0 | 672 | 1631428 | 32 | 0 | 0 | 1 | 680 |
| 225 | E1 | 7 | 07 | 32 | 1 | 201986 | 1615884 | 32 | 1 | 0 | 672 | 1632108 | 32 | 1 | 0 | 1 | 680 |
| 226 | E2 | 7 | 07 | 32 | 2 | 202070 | 1616556 | 32 | 2 | 0 | 672 | 1632788 | 32 | 2 | 0 | 1 | 680 |
| 227 | E3 | 7 | 07 | 32 | 3 | 202154 | 1617228 | 32 | 3 | 0 | 672 | 1633468 | 32 | 3 | 0 | 1 | 680 |
| 228 | E4 | 7 | 07 | 32 | 4 | 202238 | 1617900 | 32 | 4 | 0 | 672 | 1634148 | 32 | 4 | 0 | 1 | 680 |
| 229 | E5 | 7 | 07 | 32 | 5 | 202322 | 1618572 | 32 | 5 | 0 | 672 | 1634828 | 32 | 5 | 0 | 1 | 680 |
| 230 | E6 | 7 | 07 | 32 | 6 | 202406 | 1619244 | 32 | 6 | 0 | 672 | 1635508 | 32 | 6 | 0 | 1 | 680 |
| 231 | E7 | 7 | 07 | 33 | 0 | 202492 | 1619932 | 33 | 0 | 0 | 688 | 1636204 | 33 | 0 | 0 | 1 | 696 |
| 232 | E8 | 7 | 07 | 33 | 1 | 202578 | 1620620 | 33 | 1 | 0 | 688 | 1636900 | 33 | 1 | 0 | 1 | 696 |
| 233 | E9 | 7 | 07 | 33 | 2 | 202664 | 1621308 | 33 | 2 | 0 | 688 | 1637596 | 33 | 2 | 0 | 1 | 696 |
| 234 | EA | 7 | 07 | 33 | 3 | 202750 | 1621996 | 33 | 3 | 0 | 688 | 1638292 | 33 | 3 | 0 | 1 | 696 |
| 235 | EB | 7 | 07 | 33 | 4 | 202836 | 1622684 | 33 | 4 | 0 | 688 | 1638988 | 33 | 4 | 0 | 1 | 696 |
| 236 | EC | 7 | 07 | 33 | 5 | 202922 | 1623372 | 33 | 5 | 0 | 688 | 1639684 | 33 | 5 | 0 | 1 | 696 |
| 237 | ED | 7 | 07 | 33 | 6 | 203008 | 1624060 | 33 | 6 | 0 | 688 | 1640380 | 33 | 6 | 0 | 1 | 696 |
| 238 | EE | 7 | 07 | 34 | 0 | 203096 | 1624764 | 34 | 0 | 0 | 704 | 1641092 | 34 | 0 | 0 | 1 | 712 |
| 239 | EF | 7 | 07 | 34 | 1 | 203184 | 1625468 | 34 | 1 | 0 | 704 | 1641804 | 34 | 1 | 0 | 1 | 712 |
| 240 | F0 | 7 | 07 | 34 | 2 | 203272 | 1626172 | 34 | 2 | 0 | 704 | 1642516 | 34 | 2 | 0 | 1 | 712 |
| 241 | F1 | 7 | 07 | 34 | 3 | 203360 | 1626876 | 34 | 3 | 0 | 704 | 1643228 | 34 | 3 | 0 | 1 | 712 |
| 242 | F2 | 7 | 07 | 34 | 4 | 203448 | 1627580 | 34 | 4 | 0 | 704 | 1643940 | 34 | 4 | 0 | 1 | 712 |
| 243 | F3 | 7 | 07 | 34 | 5 | 203536 | 1628284 | 34 | 5 | 0 | 704 | 1644652 | 34 | 5 | 0 | 1 | 712 |
| 244 | F4 | 7 | 07 | 34 | 6 | 203624 | 1628988 | 34 | 6 | 0 | 704 | 1645364 | 34 | 6 | 0 | 1 | 712 |
| 245 | F5 | 7 | 07 | 35 | 0 | 203714 | 1629708 | 35 | 0 | 0 | 720 | 1646092 | 35 | 0 | 0 | 1 | 728 |
| 246 | F6 | 7 | 07 | 35 | 1 | 203804 | 1630428 | 35 | 1 | 0 | 720 | 1646820 | 35 | 1 | 0 | 1 | 728 |
| 247 | F7 | 7 | 07 | 35 | 2 | 203894 | 1631148 | 35 | 2 | 0 | 720 | 1647548 | 35 | 2 | 0 | 1 | 728 |
| 248 | F8 | 7 | 07 | 35 | 3 | 203984 | 1631868 | 35 | 3 | 0 | 720 | 1648276 | 35 | 3 | 0 | 1 | 728 |
| 249 | F9 | 7 | 07 | 35 | 4 | 204074 | 1632588 | 35 | 4 | 0 | 720 | 1649004 | 35 | 4 | 0 | 1 | 728 |
| 250 | FA | 7 | 07 | 35 | 5 | 204164 | 1633308 | 35 | 5 | 0 | 720 | 1649732 | 35 | 5 | 0 | 1 | 728 |
| 251 | FB | 7 | 07 | 35 | 6 | 204254 | 1634028 | 35 | 6 | 0 | 720 | 1650460 | 35 | 6 | 0 | 1 | 728 |
| 252 | FC | 7 | 07 | 36 | 0 | 204346 | 1634764 | 36 | 0 | 0 | 736 | 1651204 | 36 | 0 | 0 | 1 | 744 |
| 253 | FD | 7 | 07 | 36 | 1 | 204438 | 1635500 | 36 | 1 | 0 | 736 | 1651948 | 36 | 1 | 0 | 1 | 744 |
| 254 | FE | 7 | 07 | 36 | 2 | 204530 | 1636236 | 36 | 2 | 0 | 736 | 1652692 | 36 | 2 | 0 | 1 | 744 |
| 255 | FF | 7 | 07 | 36 | 3 | 204622 | 1636972 | 36 | 3 | 0 | 736 | 1653436 | 36 | 3 | 0 | 1 | 744 |
| 0 | 00 | 8 | 08 | 0 | 0 | 204634 | 1637068 | 0 | 0 | 0 | 96 | 1653540 | 0 | 0 | 0 | 1 | 104 |
| 1 | 01 | 8 | 08 | 0 | 1 | 204648 | 1637180 | 0 | 1 | 0 | 112 | 1653660 | 0 | 1 | 0 | 1 | 120 |
| 2 | 02 | 8 | 08 | 0 | 2 | 204662 | 1637292 | 0 | 2 | 0 | 112 | 1653780 | 0 | 2 | 0 | 1 | 120 |
| 3 | 03 | 8 | 08 | 0 | 3 | 204676 | 1637404 | 0 | 3 | 0 | 112 | 1653900 | 0 | 3 | 0 | 1 | 120 |
| 4 | 04 | 8 | 08 | 0 | 4 | 204690 | 1637516 | 0 | 4 | 0 | 112 | 1654020 | 0 | 4 | 0 | 1 | 120 |
| 5 | 05 | 8 | 08 | 0 | 5 | 204704 | 1637628 | 0 | 5 | 0 | 112 | 1654140 | 0 | 5 | 0 | 1 | 120 |
| 6 | 06 | 8 | 08 | 0 | 6 | 204718 | 1637740 | 0 | 6 | 0 | 112 | 1654260 | 0 | 6 | 0 | 1 | 120 |
| 7 | 07 | 8 | 08 | 0 | 7 | 204732 | 1637852 | 0 | 7 | 0 | 112 | 1654380 | 0 | 7 | 0 | 1 | 120 |
| 8 | 08 | 8 | 08 | 1 | 0 | 204744 | 1637948 | 1 | 0 | 0 | 96 | 1654484 | 1 | 0 | 0 | 1 | 104 |

| | | | | | | | | | | | | | | | | | |
|---|---|---|---|---|---|---|---|---|---|---|---|---|---|---|---|---|---|
| 9 | 09 | 8 | 08 | 1 | 1 | 204761 | 1638084 | 1 | 1 | 0 | 136 | 1654628 | 1 | 1 | 0 | 1 | 144 |
| 10 | 0A | 8 | 08 | 1 | 2 | 204778 | 1638220 | 1 | 2 | 0 | 136 | 1654772 | 1 | 2 | 0 | 1 | 144 |
| 11 | 0B | 8 | 08 | 1 | 3 | 204795 | 1638356 | 1 | 3 | 0 | 136 | 1654916 | 1 | 3 | 0 | 1 | 144 |
| 12 | 0C | 8 | 08 | 1 | 4 | 204812 | 1638492 | 1 | 4 | 0 | 136 | 1655060 | 1 | 4 | 0 | 1 | 144 |
| 13 | 0D | 8 | 08 | 1 | 5 | 204829 | 1638628 | 1 | 5 | 0 | 136 | 1655204 | 1 | 5 | 0 | 1 | 144 |
| 14 | 0E | 8 | 08 | 1 | 6 | 204846 | 1638764 | 1 | 6 | 0 | 136 | 1655348 | 1 | 6 | 0 | 1 | 144 |
| 15 | 0F | 8 | 08 | 1 | 7 | 204863 | 1638900 | 1 | 7 | 0 | 136 | 1655492 | 1 | 7 | 0 | 1 | 144 |
| 16 | 10 | 8 | 08 | 2 | 0 | 204882 | 1639052 | 2 | 0 | 0 | 152 | 1655652 | 2 | 0 | 0 | 1 | 160 |
| 17 | 11 | 8 | 08 | 2 | 1 | 204906 | 1639244 | 2 | 1 | 0 | 192 | 1655852 | 2 | 1 | 0 | 1 | 200 |
| 18 | 12 | 8 | 08 | 2 | 2 | 204930 | 1639436 | 2 | 2 | 0 | 192 | 1656052 | 2 | 2 | 0 | 1 | 200 |
| 19 | 13 | 8 | 08 | 2 | 3 | 204954 | 1639628 | 2 | 3 | 0 | 192 | 1656252 | 2 | 3 | 0 | 1 | 200 |
| 20 | 14 | 8 | 08 | 2 | 4 | 204978 | 1639820 | 2 | 4 | 0 | 192 | 1656452 | 2 | 4 | 0 | 1 | 200 |
| 21 | 15 | 8 | 08 | 2 | 5 | 205002 | 1640012 | 2 | 5 | 0 | 192 | 1656652 | 2 | 5 | 0 | 1 | 200 |
| 22 | 16 | 8 | 08 | 2 | 6 | 205026 | 1640204 | 2 | 6 | 0 | 192 | 1656852 | 2 | 6 | 0 | 1 | 200 |
| 23 | 17 | 8 | 08 | 2 | 7 | 205050 | 1640396 | 2 | 7 | 0 | 192 | 1657052 | 2 | 7 | 0 | 1 | 200 |
| 24 | 18 | 8 | 08 | 3 | 0 | 205076 | 1640604 | 3 | 0 | 0 | 208 | 1657268 | 3 | 0 | 0 | 1 | 216 |
| 25 | 19 | 8 | 08 | 3 | 1 | 205102 | 1640812 | 3 | 1 | 0 | 208 | 1657484 | 3 | 1 | 0 | 1 | 216 |
| 26 | 1A | 8 | 08 | 3 | 2 | 205128 | 1641020 | 3 | 2 | 0 | 208 | 1657700 | 3 | 2 | 0 | 1 | 216 |
| 27 | 1B | 8 | 08 | 3 | 3 | 205154 | 1641228 | 3 | 3 | 0 | 208 | 1657916 | 3 | 3 | 0 | 1 | 216 |
| 28 | 1C | 8 | 08 | 3 | 4 | 205180 | 1641436 | 3 | 4 | 0 | 208 | 1658132 | 3 | 4 | 0 | 1 | 216 |
| 29 | 1D | 8 | 08 | 3 | 5 | 205206 | 1641644 | 3 | 5 | 0 | 208 | 1658348 | 3 | 5 | 0 | 1 | 216 |
| 30 | 1E | 8 | 08 | 3 | 6 | 205232 | 1641852 | 3 | 6 | 0 | 208 | 1658564 | 3 | 6 | 0 | 1 | 216 |
| 31 | 1F | 8 | 08 | 3 | 7 | 205258 | 1642060 | 3 | 7 | 0 | 208 | 1658780 | 3 | 7 | 0 | 1 | 216 |
| 32 | 20 | 8 | 08 | 4 | 0 | 205286 | 1642284 | 4 | 0 | 0 | 224 | 1659012 | 4 | 0 | 0 | 1 | 232 |
| 33 | 21 | 8 | 08 | 4 | 1 | 205314 | 1642508 | 4 | 1 | 0 | 224 | 1659244 | 4 | 1 | 0 | 1 | 232 |
| 34 | 22 | 8 | 08 | 4 | 2 | 205342 | 1642732 | 4 | 2 | 0 | 224 | 1659476 | 4 | 2 | 0 | 1 | 232 |
| 35 | 23 | 8 | 08 | 4 | 3 | 205370 | 1642956 | 4 | 3 | 0 | 224 | 1659708 | 4 | 3 | 0 | 1 | 232 |
| 36 | 24 | 8 | 08 | 4 | 4 | 205398 | 1643180 | 4 | 4 | 0 | 224 | 1659940 | 4 | 4 | 0 | 1 | 232 |
| 37 | 25 | 8 | 08 | 4 | 5 | 205426 | 1643404 | 4 | 5 | 0 | 224 | 1660172 | 4 | 5 | 0 | 1 | 232 |
| 38 | 26 | 8 | 08 | 4 | 6 | 205454 | 1643628 | 4 | 6 | 0 | 224 | 1660404 | 4 | 6 | 0 | 1 | 232 |
| 39 | 27 | 8 | 08 | 4 | 7 | 205482 | 1643852 | 4 | 7 | 0 | 224 | 1660636 | 4 | 7 | 0 | 1 | 232 |
| 40 | 28 | 8 | 08 | 5 | 0 | 205512 | 1644092 | 5 | 0 | 0 | 240 | 1660884 | 5 | 0 | 0 | 1 | 248 |
| 41 | 29 | 8 | 08 | 5 | 1 | 205542 | 1644332 | 5 | 1 | 0 | 240 | 1661132 | 5 | 1 | 0 | 1 | 248 |
| 42 | 2A | 8 | 08 | 5 | 2 | 205572 | 1644572 | 5 | 2 | 0 | 240 | 1661380 | 5 | 2 | 0 | 1 | 248 |
| 43 | 2B | 8 | 08 | 5 | 3 | 205602 | 1644812 | 5 | 3 | 0 | 240 | 1661628 | 5 | 3 | 0 | 1 | 248 |
| 44 | 2C | 8 | 08 | 5 | 4 | 205632 | 1645052 | 5 | 4 | 0 | 240 | 1661876 | 5 | 4 | 0 | 1 | 248 |
| 45 | 2D | 8 | 08 | 5 | 5 | 205662 | 1645292 | 5 | 5 | 0 | 240 | 1662124 | 5 | 5 | 0 | 1 | 248 |
| 46 | 2E | 8 | 08 | 5 | 6 | 205692 | 1645532 | 5 | 6 | 0 | 240 | 1662372 | 5 | 6 | 0 | 1 | 248 |
| 47 | 2F | 8 | 08 | 5 | 7 | 205722 | 1645772 | 5 | 7 | 0 | 240 | 1662620 | 5 | 7 | 0 | 1 | 248 |
| 48 | 30 | 8 | 08 | 6 | 0 | 205754 | 1646028 | 6 | 0 | 0 | 256 | 1662884 | 6 | 0 | 0 | 1 | 264 |
| 49 | 31 | 8 | 08 | 6 | 1 | 205786 | 1646284 | 6 | 1 | 0 | 256 | 1663148 | 6 | 1 | 0 | 1 | 264 |
| 50 | 32 | 8 | 08 | 6 | 2 | 205818 | 1646540 | 6 | 2 | 0 | 256 | 1663412 | 6 | 2 | 0 | 1 | 264 |
| 51 | 33 | 8 | 08 | 6 | 3 | 205850 | 1646796 | 6 | 3 | 0 | 256 | 1663676 | 6 | 3 | 0 | 1 | 264 |
| 52 | 34 | 8 | 08 | 6 | 4 | 205882 | 1647052 | 6 | 4 | 0 | 256 | 1663940 | 6 | 4 | 0 | 1 | 264 |
| 53 | 35 | 8 | 08 | 6 | 5 | 205914 | 1647308 | 6 | 5 | 0 | 256 | 1664204 | 6 | 5 | 0 | 1 | 264 |
| 54 | 36 | 8 | 08 | 6 | 6 | 205946 | 1647564 | 6 | 6 | 0 | 256 | 1664468 | 6 | 6 | 0 | 1 | 264 |
| 55 | 37 | 8 | 08 | 6 | 7 | 205978 | 1647820 | 6 | 7 | 0 | 256 | 1664732 | 6 | 7 | 0 | 1 | 264 |
| 56 | 38 | 8 | 08 | 7 | 0 | 206012 | 1648092 | 7 | 0 | 0 | 272 | 1665012 | 7 | 0 | 0 | 1 | 280 |
| 57 | 39 | 8 | 08 | 7 | 1 | 206046 | 1648364 | 7 | 1 | 0 | 272 | 1665292 | 7 | 1 | 0 | 1 | 280 |
| 58 | 3A | 8 | 08 | 7 | 2 | 206080 | 1648636 | 7 | 2 | 0 | 272 | 1665572 | 7 | 2 | 0 | 1 | 280 |
| 59 | 3B | 8 | 08 | 7 | 3 | 206114 | 1648908 | 7 | 3 | 0 | 272 | 1665852 | 7 | 3 | 0 | 1 | 280 |
| 60 | 3C | 8 | 08 | 7 | 4 | 206148 | 1649180 | 7 | 4 | 0 | 272 | 1666132 | 7 | 4 | 0 | 1 | 280 |
| 61 | 3D | 8 | 08 | 7 | 5 | 206182 | 1649452 | 7 | 5 | 0 | 272 | 1666412 | 7 | 5 | 0 | 1 | 280 |
| 62 | 3E | 8 | 08 | 7 | 6 | 206216 | 1649724 | 7 | 6 | 0 | 272 | 1666692 | 7 | 6 | 0 | 1 | 280 |
| 63 | 3F | 8 | 08 | 7 | 7 | 206250 | 1649996 | 7 | 7 | 0 | 272 | 1666972 | 7 | 7 | 0 | 1 | 280 |
| 64 | 40 | 8 | 08 | 8 | 0 | 206286 | 1650284 | 8 | 0 | 0 | 288 | 1667268 | 8 | 0 | 0 | 1 | 296 |
| 65 | 41 | 8 | 08 | 8 | 1 | 206322 | 1650572 | 8 | 1 | 0 | 288 | 1667564 | 8 | 1 | 0 | 1 | 296 |
| 66 | 42 | 8 | 08 | 8 | 2 | 206358 | 1650860 | 8 | 2 | 0 | 288 | 1667860 | 8 | 2 | 0 | 1 | 296 |
| 67 | 43 | 8 | 08 | 8 | 3 | 206394 | 1651148 | 8 | 3 | 0 | 288 | 1668156 | 8 | 3 | 0 | 1 | 296 |
| 68 | 44 | 8 | 08 | 8 | 4 | 206430 | 1651436 | 8 | 4 | 0 | 288 | 1668452 | 8 | 4 | 0 | 1 | 296 |
| 69 | 45 | 8 | 08 | 8 | 5 | 206466 | 1651724 | 8 | 5 | 0 | 288 | 1668748 | 8 | 5 | 0 | 1 | 296 |
| 70 | 46 | 8 | 08 | 8 | 6 | 206502 | 1652012 | 8 | 6 | 0 | 288 | 1669044 | 8 | 6 | 0 | 1 | 296 |
| 71 | 47 | 8 | 08 | 8 | 7 | 206538 | 1652300 | 8 | 7 | 0 | 288 | 1669340 | 8 | 7 | 0 | 1 | 296 |
| 72 | 48 | 8 | 08 | 9 | 0 | 206576 | 1652604 | 9 | 0 | 0 | 304 | 1669652 | 9 | 0 | 0 | 1 | 312 |
| 73 | 49 | 8 | 08 | 9 | 1 | 206614 | 1652908 | 9 | 1 | 0 | 304 | 1669964 | 9 | 1 | 0 | 1 | 312 |
| 74 | 4A | 8 | 08 | 9 | 2 | 206652 | 1653212 | 9 | 2 | 0 | 304 | 1670276 | 9 | 2 | 0 | 1 | 312 |
| 75 | 4B | 8 | 08 | 9 | 3 | 206690 | 1653516 | 9 | 3 | 0 | 304 | 1670588 | 9 | 3 | 0 | 1 | 312 |
| 76 | 4C | 8 | 08 | 9 | 4 | 206728 | 1653820 | 9 | 4 | 0 | 304 | 1670900 | 9 | 4 | 0 | 1 | 312 |
| 77 | 4D | 8 | 08 | 9 | 5 | 206766 | 1654124 | 9 | 5 | 0 | 304 | 1671212 | 9 | 5 | 0 | 1 | 312 |
| 78 | 4E | 8 | 08 | 9 | 6 | 206804 | 1654428 | 9 | 6 | 0 | 304 | 1671524 | 9 | 6 | 0 | 1 | 312 |
| 79 | 4F | 8 | 08 | 9 | 7 | 206842 | 1654732 | 9 | 7 | 0 | 304 | 1671836 | 9 | 7 | 0 | 1 | 312 |
| 80 | 50 | 8 | 08 | 10 | 0 | 206882 | 1655052 | 10 | 0 | 0 | 320 | 1672164 | 10 | 0 | 0 | 1 | 328 |
| 81 | 51 | 8 | 08 | 10 | 1 | 206922 | 1655372 | 10 | 1 | 0 | 320 | 1672492 | 10 | 1 | 0 | 1 | 328 |
| 82 | 52 | 8 | 08 | 10 | 2 | 206962 | 1655692 | 10 | 2 | 0 | 320 | 1672820 | 10 | 2 | 0 | 1 | 328 |
| 83 | 53 | 8 | 08 | 10 | 3 | 207002 | 1656012 | 10 | 3 | 0 | 320 | 1673148 | 10 | 3 | 0 | 1 | 328 |
| 84 | 54 | 8 | 08 | 10 | 4 | 207042 | 1656332 | 10 | 4 | 0 | 320 | 1673476 | 10 | 4 | 0 | 1 | 328 |
| 85 | 55 | 8 | 08 | 10 | 5 | 207082 | 1656652 | 10 | 5 | 0 | 320 | 1673804 | 10 | 5 | 0 | 1 | 328 |
| 86 | 56 | 8 | 08 | 10 | 6 | 207122 | 1656972 | 10 | 6 | 0 | 320 | 1674132 | 10 | 6 | 0 | 1 | 328 |
| 87 | 57 | 8 | 08 | 10 | 7 | 207162 | 1657292 | 10 | 7 | 0 | 320 | 1674460 | 10 | 7 | 0 | 1 | 328 |
| 88 | 58 | 8 | 08 | 11 | 0 | 207204 | 1657628 | 11 | 0 | 0 | 336 | 1674804 | 11 | 0 | 0 | 1 | 344 |
| 89 | 59 | 8 | 08 | 11 | 1 | 207246 | 1657964 | 11 | 1 | 0 | 336 | 1675148 | 11 | 1 | 0 | 1 | 344 |
| 90 | 5A | 8 | 08 | 11 | 2 | 207288 | 1658300 | 11 | 2 | 0 | 336 | 1675492 | 11 | 2 | 0 | 1 | 344 |
| 91 | 5B | 8 | 08 | 11 | 3 | 207330 | 1658636 | 11 | 3 | 0 | 336 | 1675836 | 11 | 3 | 0 | 1 | 344 |
| 92 | 5C | 8 | 08 | 11 | 4 | 207372 | 1658972 | 11 | 4 | 0 | 336 | 1676180 | 11 | 4 | 0 | 1 | 344 |
| 93 | 5D | 8 | 08 | 11 | 5 | 207414 | 1659308 | 11 | 5 | 0 | 336 | 1676524 | 11 | 5 | 0 | 1 | 344 |
| 94 | 5E | 8 | 08 | 11 | 6 | 207456 | 1659644 | 11 | 6 | 0 | 336 | 1676868 | 11 | 6 | 0 | 1 | 344 |
| 95 | 5F | 8 | 08 | 11 | 7 | 207498 | 1659980 | 11 | 7 | 0 | 336 | 1677212 | 11 | 7 | 0 | 1 | 344 |
| 96 | 60 | 8 | 08 | 12 | 0 | 207542 | 1660332 | 12 | 0 | 0 | 352 | 1677572 | 12 | 0 | 0 | 1 | 360 |
| 97 | 61 | 8 | 08 | 12 | 1 | 207586 | 1660684 | 12 | 1 | 0 | 352 | 1677932 | 12 | 1 | 0 | 1 | 360 |
| 98 | 62 | 8 | 08 | 12 | 2 | 207630 | 1661036 | 12 | 2 | 0 | 352 | 1678292 | 12 | 2 | 0 | 1 | 360 |
| 99 | 63 | 8 | 08 | 12 | 3 | 207674 | 1661388 | 12 | 3 | 0 | 352 | 1678652 | 12 | 3 | 0 | 1 | 360 |
| 100 | 64 | 8 | 08 | 12 | 4 | 207718 | 1661740 | 12 | 4 | 0 | 352 | 1679012 | 12 | 4 | 0 | 1 | 360 |
| 101 | 65 | 8 | 08 | 12 | 5 | 207762 | 1662092 | 12 | 5 | 0 | 352 | 1679372 | 12 | 5 | 0 | 1 | 360 |
| 102 | 66 | 8 | 08 | 12 | 6 | 207806 | 1662444 | 12 | 6 | 0 | 352 | 1679732 | 12 | 6 | 0 | 1 | 360 |
| 103 | 67 | 8 | 08 | 12 | 7 | 207850 | 1662796 | 12 | 7 | 0 | 352 | 1680092 | 12 | 7 | 0 | 1 | 360 |
| 104 | 68 | 8 | 08 | 13 | 0 | 207896 | 1663164 | 13 | 0 | 0 | 368 | 1680468 | 13 | 0 | 0 | 1 | 376 |
| 105 | 69 | 8 | 08 | 13 | 1 | 207942 | 1663532 | 13 | 1 | 0 | 368 | 1680844 | 13 | 1 | 0 | 1 | 376 |
| 106 | 6A | 8 | 08 | 13 | 2 | 207988 | 1663900 | 13 | 2 | 0 | 368 | 1681220 | 13 | 2 | 0 | 1 | 376 |
| 107 | 6B | 8 | 08 | 13 | 3 | 208034 | 1664268 | 13 | 3 | 0 | 368 | 1681596 | 13 | 3 | 0 | 1 | 376 |
| 108 | 6C | 8 | 08 | 13 | 4 | 208080 | 1664636 | 13 | 4 | 0 | 368 | 1681972 | 13 | 4 | 0 | 1 | 376 |
| 109 | 6D | 8 | 08 | 13 | 5 | 208126 | 1665004 | 13 | 5 | 0 | 368 | 1682348 | 13 | 5 | 0 | 1 | 376 |
| 110 | 6E | 8 | 08 | 13 | 6 | 208172 | 1665372 | 13 | 6 | 0 | 368 | 1682724 | 13 | 6 | 0 | 1 | 376 |
| 111 | 6F | 8 | 08 | 13 | 7 | 208218 | 1665740 | 13 | 7 | 0 | 368 | 1683100 | 13 | 7 | 0 | 1 | 376 |

| | | | | | | | | | | | | | | | | | |
|---|---|---|---|---|---|---|---|---|---|---|---|---|---|---|---|---|---|
| 112 | 70 | 8 | 08 | 14 | 0 | 208266 | 1666124 | 14 | 0 | 0 | 384 | 1683492 | 14 | 0 | 0 | 1 | 392 |
| 113 | 71 | 8 | 08 | 14 | 1 | 208314 | 1666508 | 14 | 1 | 0 | 384 | 1683884 | 14 | 1 | 0 | 1 | 392 |
| 114 | 72 | 8 | 08 | 14 | 2 | 208362 | 1666892 | 14 | 2 | 0 | 384 | 1684276 | 14 | 2 | 0 | 1 | 392 |
| 115 | 73 | 8 | 08 | 14 | 3 | 208410 | 1667276 | 14 | 3 | 0 | 384 | 1684668 | 14 | 3 | 0 | 1 | 392 |
| 116 | 74 | 8 | 08 | 14 | 4 | 208458 | 1667660 | 14 | 4 | 0 | 384 | 1685060 | 14 | 4 | 0 | 1 | 392 |
| 117 | 75 | 8 | 08 | 14 | 5 | 208506 | 1668044 | 14 | 5 | 0 | 384 | 1685452 | 14 | 5 | 0 | 1 | 392 |
| 118 | 76 | 8 | 08 | 14 | 6 | 208554 | 1668428 | 14 | 6 | 0 | 384 | 1685844 | 14 | 6 | 0 | 1 | 392 |
| 119 | 77 | 8 | 08 | 14 | 7 | 208602 | 1668812 | 14 | 7 | 0 | 384 | 1686236 | 14 | 7 | 0 | 1 | 392 |
| 120 | 78 | 8 | 08 | 15 | 0 | 208652 | 1669212 | 15 | 0 | 0 | 400 | 1686644 | 15 | 0 | 0 | 1 | 408 |
| 121 | 79 | 8 | 08 | 15 | 1 | 208702 | 1669612 | 15 | 1 | 0 | 400 | 1687052 | 15 | 1 | 0 | 1 | 408 |
| 122 | 7A | 8 | 08 | 15 | 2 | 208752 | 1670012 | 15 | 2 | 0 | 400 | 1687460 | 15 | 2 | 0 | 1 | 408 |
| 123 | 7B | 8 | 08 | 15 | 3 | 208802 | 1670412 | 15 | 3 | 0 | 400 | 1687868 | 15 | 3 | 0 | 1 | 408 |
| 124 | 7C | 8 | 08 | 15 | 4 | 208852 | 1670812 | 15 | 4 | 0 | 400 | 1688276 | 15 | 4 | 0 | 1 | 408 |
| 125 | 7D | 8 | 08 | 15 | 5 | 208902 | 1671212 | 15 | 5 | 0 | 400 | 1688684 | 15 | 5 | 0 | 1 | 408 |
| 126 | 7E | 8 | 08 | 15 | 6 | 208952 | 1671612 | 15 | 6 | 0 | 400 | 1689092 | 15 | 6 | 0 | 1 | 408 |
| 127 | 7F | 8 | 08 | 15 | 7 | 209002 | 1672012 | 15 | 7 | 0 | 400 | 1689500 | 15 | 7 | 0 | 1 | 408 |
| 128 | 80 | 8 | 08 | 16 | 0 | 209054 | 1672428 | 16 | 0 | 0 | 416 | 1689924 | 16 | 0 | 0 | 1 | 424 |
| 129 | 81 | 8 | 08 | 16 | 1 | 209106 | 1672844 | 16 | 1 | 0 | 416 | 1690348 | 16 | 1 | 0 | 1 | 424 |
| 130 | 82 | 8 | 08 | 16 | 2 | 209158 | 1673260 | 16 | 2 | 0 | 416 | 1690772 | 16 | 2 | 0 | 1 | 424 |
| 131 | 83 | 8 | 08 | 16 | 3 | 209210 | 1673676 | 16 | 3 | 0 | 416 | 1691196 | 16 | 3 | 0 | 1 | 424 |
| 132 | 84 | 8 | 08 | 16 | 4 | 209262 | 1674092 | 16 | 4 | 0 | 416 | 1691620 | 16 | 4 | 0 | 1 | 424 |
| 133 | 85 | 8 | 08 | 16 | 5 | 209314 | 1674508 | 16 | 5 | 0 | 416 | 1692044 | 16 | 5 | 0 | 1 | 424 |
| 134 | 86 | 8 | 08 | 16 | 6 | 209366 | 1674924 | 16 | 6 | 0 | 416 | 1692468 | 16 | 6 | 0 | 1 | 424 |
| 135 | 87 | 8 | 08 | 16 | 7 | 209418 | 1675340 | 16 | 7 | 0 | 416 | 1692892 | 16 | 7 | 0 | 1 | 424 |
| 136 | 88 | 8 | 08 | 17 | 0 | 209472 | 1675772 | 17 | 0 | 0 | 432 | 1693332 | 17 | 0 | 0 | 1 | 440 |
| 137 | 89 | 8 | 08 | 17 | 1 | 209526 | 1676204 | 17 | 1 | 0 | 432 | 1693772 | 17 | 1 | 0 | 1 | 440 |
| 138 | 8A | 8 | 08 | 17 | 2 | 209580 | 1676636 | 17 | 2 | 0 | 432 | 1694212 | 17 | 2 | 0 | 1 | 440 |
| 139 | 8B | 8 | 08 | 17 | 3 | 209634 | 1677068 | 17 | 3 | 0 | 432 | 1694652 | 17 | 3 | 0 | 1 | 440 |
| 140 | 8C | 8 | 08 | 17 | 4 | 209688 | 1677500 | 17 | 4 | 0 | 432 | 1695092 | 17 | 4 | 0 | 1 | 440 |
| 141 | 8D | 8 | 08 | 17 | 5 | 209742 | 1677932 | 17 | 5 | 0 | 432 | 1695532 | 17 | 5 | 0 | 1 | 440 |
| 142 | 8E | 8 | 08 | 17 | 6 | 209796 | 1678364 | 17 | 6 | 0 | 432 | 1695972 | 17 | 6 | 0 | 1 | 440 |
| 143 | 8F | 8 | 08 | 17 | 7 | 209850 | 1678796 | 17 | 7 | 0 | 432 | 1696412 | 17 | 7 | 0 | 1 | 440 |
| 144 | 90 | 8 | 08 | 18 | 0 | 209906 | 1679244 | 18 | 0 | 0 | 448 | 1696868 | 18 | 0 | 0 | 1 | 456 |
| 145 | 91 | 8 | 08 | 18 | 1 | 209962 | 1679692 | 18 | 1 | 0 | 448 | 1697324 | 18 | 1 | 0 | 1 | 456 |
| 146 | 92 | 8 | 08 | 18 | 2 | 210018 | 1680140 | 18 | 2 | 0 | 448 | 1697780 | 18 | 2 | 0 | 1 | 456 |
| 147 | 93 | 8 | 08 | 18 | 3 | 210074 | 1680588 | 18 | 3 | 0 | 448 | 1698236 | 18 | 3 | 0 | 1 | 456 |
| 148 | 94 | 8 | 08 | 18 | 4 | 210130 | 1681036 | 18 | 4 | 0 | 448 | 1698692 | 18 | 4 | 0 | 1 | 456 |
| 149 | 95 | 8 | 08 | 18 | 5 | 210186 | 1681484 | 18 | 5 | 0 | 448 | 1699148 | 18 | 5 | 0 | 1 | 456 |
| 150 | 96 | 8 | 08 | 18 | 6 | 210242 | 1681932 | 18 | 6 | 0 | 448 | 1699604 | 18 | 6 | 0 | 1 | 456 |
| 151 | 97 | 8 | 08 | 18 | 7 | 210298 | 1682380 | 18 | 7 | 0 | 448 | 1700060 | 18 | 7 | 0 | 1 | 456 |
| 152 | 98 | 8 | 08 | 19 | 0 | 210356 | 1682844 | 19 | 0 | 0 | 464 | 1700532 | 19 | 0 | 0 | 1 | 472 |
| 153 | 99 | 8 | 08 | 19 | 1 | 210414 | 1683308 | 19 | 1 | 0 | 464 | 1701004 | 19 | 1 | 0 | 1 | 472 |
| 154 | 9A | 8 | 08 | 19 | 2 | 210472 | 1683772 | 19 | 2 | 0 | 464 | 1701476 | 19 | 2 | 0 | 1 | 472 |
| 155 | 9B | 8 | 08 | 19 | 3 | 210530 | 1684236 | 19 | 3 | 0 | 464 | 1701948 | 19 | 3 | 0 | 1 | 472 |
| 156 | 9C | 8 | 08 | 19 | 4 | 210588 | 1684700 | 19 | 4 | 0 | 464 | 1702420 | 19 | 4 | 0 | 1 | 472 |
| 157 | 9D | 8 | 08 | 19 | 5 | 210646 | 1685164 | 19 | 5 | 0 | 464 | 1702892 | 19 | 5 | 0 | 1 | 472 |
| 158 | 9E | 8 | 08 | 19 | 6 | 210704 | 1685628 | 19 | 6 | 0 | 464 | 1703364 | 19 | 6 | 0 | 1 | 472 |
| 159 | 9F | 8 | 08 | 19 | 7 | 210762 | 1686092 | 19 | 7 | 0 | 464 | 1703836 | 19 | 7 | 0 | 1 | 472 |
| 160 | A0 | 8 | 08 | 20 | 0 | 210822 | 1686572 | 20 | 0 | 0 | 480 | 1704324 | 20 | 0 | 0 | 1 | 488 |
| 161 | A1 | 8 | 08 | 20 | 1 | 210882 | 1687052 | 20 | 1 | 0 | 480 | 1704812 | 20 | 1 | 0 | 1 | 488 |
| 162 | A2 | 8 | 08 | 20 | 2 | 210942 | 1687532 | 20 | 2 | 0 | 480 | 1705300 | 20 | 2 | 0 | 1 | 488 |
| 163 | A3 | 8 | 08 | 20 | 3 | 211002 | 1688012 | 20 | 3 | 0 | 480 | 1705788 | 20 | 3 | 0 | 1 | 488 |
| 164 | A4 | 8 | 08 | 20 | 4 | 211062 | 1688492 | 20 | 4 | 0 | 480 | 1706276 | 20 | 4 | 0 | 1 | 488 |
| 165 | A5 | 8 | 08 | 20 | 5 | 211122 | 1688972 | 20 | 5 | 0 | 480 | 1706764 | 20 | 5 | 0 | 1 | 488 |
| 166 | A6 | 8 | 08 | 20 | 6 | 211182 | 1689452 | 20 | 6 | 0 | 480 | 1707252 | 20 | 6 | 0 | 1 | 488 |
| 167 | A7 | 8 | 08 | 20 | 7 | 211242 | 1689932 | 20 | 7 | 0 | 480 | 1707740 | 20 | 7 | 0 | 1 | 488 |
| 168 | A8 | 8 | 08 | 21 | 0 | 211304 | 1690428 | 21 | 0 | 0 | 496 | 1708244 | 21 | 0 | 0 | 1 | 504 |
| 169 | A9 | 8 | 08 | 21 | 1 | 211366 | 1690924 | 21 | 1 | 0 | 496 | 1708748 | 21 | 1 | 0 | 1 | 504 |
| 170 | AA | 8 | 08 | 21 | 2 | 211428 | 1691420 | 21 | 2 | 0 | 496 | 1709252 | 21 | 2 | 0 | 1 | 504 |
| 171 | AB | 8 | 08 | 21 | 3 | 211490 | 1691916 | 21 | 3 | 0 | 496 | 1709756 | 21 | 3 | 0 | 1 | 504 |
| 172 | AC | 8 | 08 | 21 | 4 | 211552 | 1692412 | 21 | 4 | 0 | 496 | 1710260 | 21 | 4 | 0 | 1 | 504 |
| 173 | AD | 8 | 08 | 21 | 5 | 211614 | 1692908 | 21 | 5 | 0 | 496 | 1710764 | 21 | 5 | 0 | 1 | 504 |
| 174 | AE | 8 | 08 | 21 | 6 | 211676 | 1693404 | 21 | 6 | 0 | 496 | 1711268 | 21 | 6 | 0 | 1 | 504 |
| 175 | AF | 8 | 08 | 21 | 7 | 211738 | 1693900 | 21 | 7 | 0 | 496 | 1711772 | 21 | 7 | 0 | 1 | 504 |
| 176 | B0 | 8 | 08 | 22 | 0 | 211802 | 1694412 | 22 | 0 | 0 | 512 | 1712292 | 22 | 0 | 0 | 1 | 520 |
| 177 | B1 | 8 | 08 | 22 | 1 | 211866 | 1694924 | 22 | 1 | 0 | 512 | 1712812 | 22 | 1 | 0 | 1 | 520 |
| 178 | B2 | 8 | 08 | 22 | 2 | 211930 | 1695436 | 22 | 2 | 0 | 512 | 1713332 | 22 | 2 | 0 | 1 | 520 |
| 179 | B3 | 8 | 08 | 22 | 3 | 211994 | 1695948 | 22 | 3 | 0 | 512 | 1713852 | 22 | 3 | 0 | 1 | 520 |
| 180 | B4 | 8 | 08 | 22 | 4 | 212058 | 1696460 | 22 | 4 | 0 | 512 | 1714372 | 22 | 4 | 0 | 1 | 520 |
| 181 | B5 | 8 | 08 | 22 | 5 | 212122 | 1696972 | 22 | 5 | 0 | 512 | 1714892 | 22 | 5 | 0 | 1 | 520 |
| 182 | B6 | 8 | 08 | 22 | 6 | 212186 | 1697484 | 22 | 6 | 0 | 512 | 1715412 | 22 | 6 | 0 | 1 | 520 |
| 183 | B7 | 8 | 08 | 22 | 7 | 212250 | 1697996 | 22 | 7 | 0 | 512 | 1715932 | 22 | 7 | 0 | 1 | 520 |
| 184 | B8 | 8 | 08 | 23 | 0 | 212316 | 1698524 | 23 | 0 | 0 | 528 | 1716468 | 23 | 0 | 0 | 1 | 536 |
| 185 | B9 | 8 | 08 | 23 | 1 | 212382 | 1699052 | 23 | 1 | 0 | 528 | 1717004 | 23 | 1 | 0 | 1 | 536 |
| 186 | BA | 8 | 08 | 23 | 2 | 212448 | 1699580 | 23 | 2 | 0 | 528 | 1717540 | 23 | 2 | 0 | 1 | 536 |
| 187 | BB | 8 | 08 | 23 | 3 | 212514 | 1700108 | 23 | 3 | 0 | 528 | 1718076 | 23 | 3 | 0 | 1 | 536 |
| 188 | BC | 8 | 08 | 23 | 4 | 212580 | 1700636 | 23 | 4 | 0 | 528 | 1718612 | 23 | 4 | 0 | 1 | 536 |
| 189 | BD | 8 | 08 | 23 | 5 | 212646 | 1701164 | 23 | 5 | 0 | 528 | 1719148 | 23 | 5 | 0 | 1 | 536 |
| 190 | BE | 8 | 08 | 23 | 6 | 212712 | 1701692 | 23 | 6 | 0 | 528 | 1719684 | 23 | 6 | 0 | 1 | 536 |
| 191 | BF | 8 | 08 | 23 | 7 | 212778 | 1702220 | 23 | 7 | 0 | 528 | 1720220 | 23 | 7 | 0 | 1 | 536 |
| 192 | C0 | 8 | 08 | 24 | 0 | 212846 | 1702764 | 24 | 0 | 0 | 544 | 1720772 | 24 | 0 | 0 | 1 | 552 |
| 193 | C1 | 8 | 08 | 24 | 1 | 212914 | 1703308 | 24 | 1 | 0 | 544 | 1721324 | 24 | 1 | 0 | 1 | 552 |
| 194 | C2 | 8 | 08 | 24 | 2 | 212982 | 1703852 | 24 | 2 | 0 | 544 | 1721876 | 24 | 2 | 0 | 1 | 552 |
| 195 | C3 | 8 | 08 | 24 | 3 | 213050 | 1704396 | 24 | 3 | 0 | 544 | 1722428 | 24 | 3 | 0 | 1 | 552 |
| 196 | C4 | 8 | 08 | 24 | 4 | 213118 | 1704940 | 24 | 4 | 0 | 544 | 1722980 | 24 | 4 | 0 | 1 | 552 |
| 197 | C5 | 8 | 08 | 24 | 5 | 213186 | 1705484 | 24 | 5 | 0 | 544 | 1723532 | 24 | 5 | 0 | 1 | 552 |
| 198 | C6 | 8 | 08 | 24 | 6 | 213254 | 1706028 | 24 | 6 | 0 | 544 | 1724084 | 24 | 6 | 0 | 1 | 552 |
| 199 | C7 | 8 | 08 | 24 | 7 | 213322 | 1706572 | 24 | 7 | 0 | 544 | 1724636 | 24 | 7 | 0 | 1 | 552 |
| 200 | C8 | 8 | 08 | 25 | 0 | 213392 | 1707132 | 25 | 0 | 0 | 560 | 1725204 | 25 | 0 | 0 | 1 | 568 |
| 201 | C9 | 8 | 08 | 25 | 1 | 213462 | 1707692 | 25 | 1 | 0 | 560 | 1725772 | 25 | 1 | 0 | 1 | 568 |
| 202 | CA | 8 | 08 | 25 | 2 | 213532 | 1708252 | 25 | 2 | 0 | 560 | 1726340 | 25 | 2 | 0 | 1 | 568 |
| 203 | CB | 8 | 08 | 25 | 3 | 213602 | 1708812 | 25 | 3 | 0 | 560 | 1726908 | 25 | 3 | 0 | 1 | 568 |
| 204 | CC | 8 | 08 | 25 | 4 | 213672 | 1709372 | 25 | 4 | 0 | 560 | 1727476 | 25 | 4 | 0 | 1 | 568 |
| 205 | CD | 8 | 08 | 25 | 5 | 213742 | 1709932 | 25 | 5 | 0 | 560 | 1728044 | 25 | 5 | 0 | 1 | 568 |
| 206 | CE | 8 | 08 | 25 | 6 | 213812 | 1710492 | 25 | 6 | 0 | 560 | 1728612 | 25 | 6 | 0 | 1 | 568 |
| 207 | CF | 8 | 08 | 25 | 7 | 213882 | 1711052 | 25 | 7 | 0 | 560 | 1729180 | 25 | 7 | 0 | 1 | 568 |
| 208 | D0 | 8 | 08 | 26 | 0 | 213954 | 1711628 | 26 | 0 | 0 | 576 | 1729764 | 26 | 0 | 0 | 1 | 584 |
| 209 | D1 | 8 | 08 | 26 | 1 | 214026 | 1712204 | 26 | 1 | 0 | 576 | 1730348 | 26 | 1 | 0 | 1 | 584 |
| 210 | D2 | 8 | 08 | 26 | 2 | 214098 | 1712780 | 26 | 2 | 0 | 576 | 1730932 | 26 | 2 | 0 | 1 | 584 |
| 211 | D3 | 8 | 08 | 26 | 3 | 214170 | 1713356 | 26 | 3 | 0 | 576 | 1731516 | 26 | 3 | 0 | 1 | 584 |
| 212 | D4 | 8 | 08 | 26 | 4 | 214242 | 1713932 | 26 | 4 | 0 | 576 | 1732100 | 26 | 4 | 0 | 1 | 584 |
| 213 | D5 | 8 | 08 | 26 | 5 | 214314 | 1714508 | 26 | 5 | 0 | 576 | 1732684 | 26 | 5 | 0 | 1 | 584 |
| 214 | D6 | 8 | 08 | 26 | 6 | 214386 | 1715084 | 26 | 6 | 0 | 576 | 1733268 | 26 | 6 | 0 | 1 | 584 |

| | | | | | | | | | | | | | | | | | |
|---|---|---|---|---|---|---|---|---|---|---|---|---|---|---|---|---|---|
| 215 | D7 | 8 | 08 | 26 | 7 | 214458 | 1715660 | 26 | 7 | 0 | 576 | 1733852 | 26 | 7 | 0 | 1 | 584 |
| 216 | D8 | 8 | 08 | 27 | 0 | 214532 | 1716252 | 27 | 0 | 0 | 592 | 1734452 | 27 | 0 | 0 | 1 | 600 |
| 217 | D9 | 8 | 08 | 27 | 1 | 214606 | 1716844 | 27 | 1 | 0 | 592 | 1735052 | 27 | 1 | 0 | 1 | 600 |
| 218 | DA | 8 | 08 | 27 | 2 | 214680 | 1717436 | 27 | 2 | 0 | 592 | 1735652 | 27 | 2 | 0 | 1 | 600 |
| 219 | DB | 8 | 08 | 27 | 3 | 214754 | 1718028 | 27 | 3 | 0 | 592 | 1736252 | 27 | 3 | 0 | 1 | 600 |
| 220 | DC | 8 | 08 | 27 | 4 | 214828 | 1718620 | 27 | 4 | 0 | 592 | 1736852 | 27 | 4 | 0 | 1 | 600 |
| 221 | DD | 8 | 08 | 27 | 5 | 214902 | 1719212 | 27 | 5 | 0 | 592 | 1737452 | 27 | 5 | 0 | 1 | 600 |
| 222 | DE | 8 | 08 | 27 | 6 | 214976 | 1719804 | 27 | 6 | 0 | 592 | 1738052 | 27 | 6 | 0 | 1 | 600 |
| 223 | DF | 8 | 08 | 27 | 7 | 215050 | 1720396 | 27 | 7 | 0 | 592 | 1738652 | 27 | 7 | 0 | 1 | 600 |
| 224 | E0 | 8 | 08 | 28 | 0 | 215126 | 1721004 | 28 | 0 | 0 | 608 | 1739268 | 28 | 0 | 0 | 1 | 616 |
| 225 | E1 | 8 | 08 | 28 | 1 | 215202 | 1721612 | 28 | 1 | 0 | 608 | 1739884 | 28 | 1 | 0 | 1 | 616 |
| 226 | E2 | 8 | 08 | 28 | 2 | 215278 | 1722220 | 28 | 2 | 0 | 608 | 1740500 | 28 | 2 | 0 | 1 | 616 |
| 227 | E3 | 8 | 08 | 28 | 3 | 215354 | 1722828 | 28 | 3 | 0 | 608 | 1741116 | 28 | 3 | 0 | 1 | 616 |
| 228 | E4 | 8 | 08 | 28 | 4 | 215430 | 1723436 | 28 | 4 | 0 | 608 | 1741732 | 28 | 4 | 0 | 1 | 616 |
| 229 | E5 | 8 | 08 | 28 | 5 | 215506 | 1724044 | 28 | 5 | 0 | 608 | 1742348 | 28 | 5 | 0 | 1 | 616 |
| 230 | E6 | 8 | 08 | 28 | 6 | 215582 | 1724652 | 28 | 6 | 0 | 608 | 1742964 | 28 | 6 | 0 | 1 | 616 |
| 231 | E7 | 8 | 08 | 28 | 7 | 215658 | 1725260 | 28 | 7 | 0 | 608 | 1743580 | 28 | 7 | 0 | 1 | 616 |
| 232 | E8 | 8 | 08 | 29 | 0 | 215736 | 1725884 | 29 | 0 | 0 | 624 | 1744212 | 29 | 0 | 0 | 1 | 632 |
| 233 | E9 | 8 | 08 | 29 | 1 | 215814 | 1726508 | 29 | 1 | 0 | 624 | 1744844 | 29 | 1 | 0 | 1 | 632 |
| 234 | EA | 8 | 08 | 29 | 2 | 215892 | 1727132 | 29 | 2 | 0 | 624 | 1745476 | 29 | 2 | 0 | 1 | 632 |
| 235 | EB | 8 | 08 | 29 | 3 | 215970 | 1727756 | 29 | 3 | 0 | 624 | 1746108 | 29 | 3 | 0 | 1 | 632 |
| 236 | EC | 8 | 08 | 29 | 4 | 216048 | 1728380 | 29 | 4 | 0 | 624 | 1746740 | 29 | 4 | 0 | 1 | 632 |
| 237 | ED | 8 | 08 | 29 | 5 | 216126 | 1729004 | 29 | 5 | 0 | 624 | 1747372 | 29 | 5 | 0 | 1 | 632 |
| 238 | EE | 8 | 08 | 29 | 6 | 216204 | 1729628 | 29 | 6 | 0 | 624 | 1748004 | 29 | 6 | 0 | 1 | 632 |
| 239 | EF | 8 | 08 | 29 | 7 | 216282 | 1730252 | 29 | 7 | 0 | 624 | 1748636 | 29 | 7 | 0 | 1 | 632 |
| 240 | F0 | 8 | 08 | 30 | 0 | 216362 | 1730892 | 30 | 0 | 0 | 640 | 1749284 | 30 | 0 | 0 | 1 | 648 |
| 241 | F1 | 8 | 08 | 30 | 1 | 216442 | 1731532 | 30 | 1 | 0 | 640 | 1749932 | 30 | 1 | 0 | 1 | 648 |
| 242 | F2 | 8 | 08 | 30 | 2 | 216522 | 1732172 | 30 | 2 | 0 | 640 | 1750580 | 30 | 2 | 0 | 1 | 648 |
| 243 | F3 | 8 | 08 | 30 | 3 | 216602 | 1732812 | 30 | 3 | 0 | 640 | 1751228 | 30 | 3 | 0 | 1 | 648 |
| 244 | F4 | 8 | 08 | 30 | 4 | 216682 | 1733452 | 30 | 4 | 0 | 640 | 1751876 | 30 | 4 | 0 | 1 | 648 |
| 245 | F5 | 8 | 08 | 30 | 5 | 216762 | 1734092 | 30 | 5 | 0 | 640 | 1752524 | 30 | 5 | 0 | 1 | 648 |
| 246 | F6 | 8 | 08 | 30 | 6 | 216842 | 1734732 | 30 | 6 | 0 | 640 | 1753172 | 30 | 6 | 0 | 1 | 648 |
| 247 | F7 | 8 | 08 | 30 | 7 | 216922 | 1735372 | 30 | 7 | 0 | 640 | 1753820 | 30 | 7 | 0 | 1 | 648 |
| 248 | F8 | 8 | 08 | 31 | 0 | 217004 | 1736028 | 31 | 0 | 0 | 656 | 1754484 | 31 | 0 | 0 | 1 | 664 |
| 249 | F9 | 8 | 08 | 31 | 1 | 217086 | 1736684 | 31 | 1 | 0 | 656 | 1755148 | 31 | 1 | 0 | 1 | 664 |
| 250 | FA | 8 | 08 | 31 | 2 | 217168 | 1737340 | 31 | 2 | 0 | 656 | 1755812 | 31 | 2 | 0 | 1 | 664 |
| 251 | FB | 8 | 08 | 31 | 3 | 217250 | 1737996 | 31 | 3 | 0 | 656 | 1756476 | 31 | 3 | 0 | 1 | 664 |
| 252 | FC | 8 | 08 | 31 | 4 | 217332 | 1738652 | 31 | 4 | 0 | 656 | 1757140 | 31 | 4 | 0 | 1 | 664 |
| 253 | FD | 8 | 08 | 31 | 5 | 217414 | 1739308 | 31 | 5 | 0 | 656 | 1757804 | 31 | 5 | 0 | 1 | 664 |
| 254 | FE | 8 | 08 | 31 | 6 | 217496 | 1739964 | 31 | 6 | 0 | 656 | 1758468 | 31 | 6 | 0 | 1 | 664 |
| 255 | FF | 8 | 08 | 31 | 7 | 217578 | 1740620 | 31 | 7 | 0 | 656 | 1759132 | 31 | 7 | 0 | 1 | 664 |
| 0 | 00 | 9 | 09 | 0 | 0 | 217590 | 1740716 | 0 | 0 | 0 | 96 | 1759236 | 0 | 0 | 0 | 1 | 104 |
| 1 | 01 | 9 | 09 | 0 | 1 | 217604 | 1740828 | 0 | 1 | 0 | 112 | 1759356 | 0 | 1 | 0 | 1 | 120 |
| 2 | 02 | 9 | 09 | 0 | 2 | 217618 | 1740940 | 0 | 2 | 0 | 112 | 1759476 | 0 | 2 | 0 | 1 | 120 |
| 3 | 03 | 9 | 09 | 0 | 3 | 217632 | 1741052 | 0 | 3 | 0 | 112 | 1759596 | 0 | 3 | 0 | 1 | 120 |
| 4 | 04 | 9 | 09 | 0 | 4 | 217646 | 1741164 | 0 | 4 | 0 | 112 | 1759716 | 0 | 4 | 0 | 1 | 120 |
| 5 | 05 | 9 | 09 | 0 | 5 | 217660 | 1741276 | 0 | 5 | 0 | 112 | 1759836 | 0 | 5 | 0 | 1 | 120 |
| 6 | 06 | 9 | 09 | 0 | 6 | 217674 | 1741388 | 0 | 6 | 0 | 112 | 1759956 | 0 | 6 | 0 | 1 | 120 |
| 7 | 07 | 9 | 09 | 0 | 7 | 217688 | 1741500 | 0 | 7 | 0 | 112 | 1760076 | 0 | 7 | 0 | 1 | 120 |
| 8 | 08 | 9 | 09 | 0 | 8 | 217702 | 1741612 | 0 | 8 | 0 | 112 | 1760196 | 0 | 8 | 0 | 1 | 120 |
| 9 | 09 | 9 | 09 | 1 | 0 | 217714 | 1741708 | 1 | 0 | 0 | 96 | 1760300 | 1 | 0 | 0 | 1 | 104 |
| 10 | 0A | 9 | 09 | 1 | 1 | 217731 | 1741844 | 1 | 1 | 0 | 136 | 1760444 | 1 | 1 | 0 | 1 | 144 |
| 11 | 0B | 9 | 09 | 1 | 2 | 217748 | 1741980 | 1 | 2 | 0 | 136 | 1760588 | 1 | 2 | 0 | 1 | 144 |
| 12 | 0C | 9 | 09 | 1 | 3 | 217765 | 1742116 | 1 | 3 | 0 | 136 | 1760732 | 1 | 3 | 0 | 1 | 144 |
| 13 | 0D | 9 | 09 | 1 | 4 | 217782 | 1742252 | 1 | 4 | 0 | 136 | 1760876 | 1 | 4 | 0 | 1 | 144 |
| 14 | 0E | 9 | 09 | 1 | 5 | 217799 | 1742388 | 1 | 5 | 0 | 136 | 1761020 | 1 | 5 | 0 | 1 | 144 |
| 15 | 0F | 9 | 09 | 1 | 6 | 217816 | 1742524 | 1 | 6 | 0 | 136 | 1761164 | 1 | 6 | 0 | 1 | 144 |
| 16 | 10 | 9 | 09 | 1 | 7 | 217837 | 1742692 | 1 | 7 | 0 | 168 | 1761340 | 1 | 7 | 0 | 1 | 176 |
| 17 | 11 | 9 | 09 | 1 | 8 | 217858 | 1742860 | 1 | 8 | 0 | 168 | 1761516 | 1 | 8 | 0 | 1 | 176 |
| 18 | 12 | 9 | 09 | 2 | 0 | 217877 | 1743012 | 2 | 0 | 0 | 152 | 1761676 | 2 | 0 | 0 | 1 | 160 |
| 19 | 13 | 9 | 09 | 2 | 1 | 217901 | 1743204 | 2 | 1 | 0 | 192 | 1761876 | 2 | 1 | 0 | 1 | 200 |
| 20 | 14 | 9 | 09 | 2 | 2 | 217925 | 1743396 | 2 | 2 | 0 | 192 | 1762076 | 2 | 2 | 0 | 1 | 200 |
| 21 | 15 | 9 | 09 | 2 | 3 | 217949 | 1743588 | 2 | 3 | 0 | 192 | 1762276 | 2 | 3 | 0 | 1 | 200 |
| 22 | 16 | 9 | 09 | 2 | 4 | 217973 | 1743780 | 2 | 4 | 0 | 192 | 1762476 | 2 | 4 | 0 | 1 | 200 |
| 23 | 17 | 9 | 09 | 2 | 5 | 217997 | 1743972 | 2 | 5 | 0 | 192 | 1762676 | 2 | 5 | 0 | 1 | 200 |
| 24 | 18 | 9 | 09 | 2 | 6 | 218021 | 1744164 | 2 | 6 | 0 | 192 | 1762876 | 2 | 6 | 0 | 1 | 200 |
| 25 | 19 | 9 | 09 | 2 | 7 | 218045 | 1744356 | 2 | 7 | 0 | 192 | 1763076 | 2 | 7 | 0 | 1 | 200 |
| 26 | 1A | 9 | 09 | 2 | 8 | 218069 | 1744548 | 2 | 8 | 0 | 192 | 1763276 | 2 | 8 | 0 | 1 | 200 |
| 27 | 1B | 9 | 09 | 3 | 0 | 218095 | 1744756 | 3 | 0 | 0 | 208 | 1763492 | 3 | 0 | 0 | 1 | 216 |
| 28 | 1C | 9 | 09 | 3 | 1 | 218121 | 1744964 | 3 | 1 | 0 | 208 | 1763708 | 3 | 1 | 0 | 1 | 216 |
| 29 | 1D | 9 | 09 | 3 | 2 | 218147 | 1745172 | 3 | 2 | 0 | 208 | 1763924 | 3 | 2 | 0 | 1 | 216 |
| 30 | 1E | 9 | 09 | 3 | 3 | 218173 | 1745380 | 3 | 3 | 0 | 208 | 1764140 | 3 | 3 | 0 | 1 | 216 |
| 31 | 1F | 9 | 09 | 3 | 4 | 218199 | 1745588 | 3 | 4 | 0 | 208 | 1764356 | 3 | 4 | 0 | 1 | 216 |
| 32 | 20 | 9 | 09 | 3 | 5 | 218225 | 1745796 | 3 | 5 | 0 | 208 | 1764572 | 3 | 5 | 0 | 1 | 216 |
| 33 | 21 | 9 | 09 | 3 | 6 | 218251 | 1746004 | 3 | 6 | 0 | 208 | 1764788 | 3 | 6 | 0 | 1 | 216 |
| 34 | 22 | 9 | 09 | 3 | 7 | 218277 | 1746212 | 3 | 7 | 0 | 208 | 1765004 | 3 | 7 | 0 | 1 | 216 |
| 35 | 23 | 9 | 09 | 3 | 8 | 218303 | 1746420 | 3 | 8 | 0 | 208 | 1765220 | 3 | 8 | 0 | 1 | 216 |
| 36 | 24 | 9 | 09 | 4 | 0 | 218331 | 1746644 | 4 | 0 | 0 | 224 | 1765452 | 4 | 0 | 0 | 1 | 232 |
| 37 | 25 | 9 | 09 | 4 | 1 | 218359 | 1746868 | 4 | 1 | 0 | 224 | 1765684 | 4 | 1 | 0 | 1 | 232 |
| 38 | 26 | 9 | 09 | 4 | 2 | 218387 | 1747092 | 4 | 2 | 0 | 224 | 1765916 | 4 | 2 | 0 | 1 | 232 |
| 39 | 27 | 9 | 09 | 4 | 3 | 218415 | 1747316 | 4 | 3 | 0 | 224 | 1766148 | 4 | 3 | 0 | 1 | 232 |
| 40 | 28 | 9 | 09 | 4 | 4 | 218443 | 1747540 | 4 | 4 | 0 | 224 | 1766380 | 4 | 4 | 0 | 1 | 232 |
| 41 | 29 | 9 | 09 | 4 | 5 | 218471 | 1747764 | 4 | 5 | 0 | 224 | 1766612 | 4 | 5 | 0 | 1 | 232 |
| 42 | 2A | 9 | 09 | 4 | 6 | 218499 | 1747988 | 4 | 6 | 0 | 224 | 1766844 | 4 | 6 | 0 | 1 | 232 |
| 43 | 2B | 9 | 09 | 4 | 7 | 218527 | 1748212 | 4 | 7 | 0 | 224 | 1767076 | 4 | 7 | 0 | 1 | 232 |
| 44 | 2C | 9 | 09 | 4 | 8 | 218555 | 1748436 | 4 | 8 | 0 | 224 | 1767308 | 4 | 8 | 0 | 1 | 232 |
| 45 | 2D | 9 | 09 | 5 | 0 | 218585 | 1748676 | 5 | 0 | 0 | 240 | 1767556 | 5 | 0 | 0 | 1 | 248 |
| 46 | 2E | 9 | 09 | 5 | 1 | 218615 | 1748916 | 5 | 1 | 0 | 240 | 1767804 | 5 | 1 | 0 | 1 | 248 |
| 47 | 2F | 9 | 09 | 5 | 2 | 218645 | 1749156 | 5 | 2 | 0 | 240 | 1768052 | 5 | 2 | 0 | 1 | 248 |
| 48 | 30 | 9 | 09 | 5 | 3 | 218675 | 1749396 | 5 | 3 | 0 | 240 | 1768300 | 5 | 3 | 0 | 1 | 248 |
| 49 | 31 | 9 | 09 | 5 | 4 | 218705 | 1749636 | 5 | 4 | 0 | 240 | 1768548 | 5 | 4 | 0 | 1 | 248 |
| 50 | 32 | 9 | 09 | 5 | 5 | 218735 | 1749876 | 5 | 5 | 0 | 240 | 1768796 | 5 | 5 | 0 | 1 | 248 |
| 51 | 33 | 9 | 09 | 5 | 6 | 218765 | 1750116 | 5 | 6 | 0 | 240 | 1769044 | 5 | 6 | 0 | 1 | 248 |
| 52 | 34 | 9 | 09 | 5 | 7 | 218795 | 1750356 | 5 | 7 | 0 | 240 | 1769292 | 5 | 7 | 0 | 1 | 248 |
| 53 | 35 | 9 | 09 | 5 | 8 | 218825 | 1750596 | 5 | 8 | 0 | 240 | 1769540 | 5 | 8 | 0 | 1 | 248 |
| 54 | 36 | 9 | 09 | 6 | 0 | 218857 | 1750852 | 6 | 0 | 0 | 256 | 1769804 | 6 | 0 | 0 | 1 | 264 |
| 55 | 37 | 9 | 09 | 6 | 1 | 218889 | 1751108 | 6 | 1 | 0 | 256 | 1770068 | 6 | 1 | 0 | 1 | 264 |
| 56 | 38 | 9 | 09 | 6 | 2 | 218921 | 1751364 | 6 | 2 | 0 | 256 | 1770332 | 6 | 2 | 0 | 1 | 264 |
| 57 | 39 | 9 | 09 | 6 | 3 | 218953 | 1751620 | 6 | 3 | 0 | 256 | 1770596 | 6 | 3 | 0 | 1 | 264 |
| 58 | 3A | 9 | 09 | 6 | 4 | 218985 | 1751876 | 6 | 4 | 0 | 256 | 1770860 | 6 | 4 | 0 | 1 | 264 |
| 59 | 3B | 9 | 09 | 6 | 5 | 219017 | 1752132 | 6 | 5 | 0 | 256 | 1771124 | 6 | 5 | 0 | 1 | 264 |
| 60 | 3C | 9 | 09 | 6 | 6 | 219049 | 1752388 | 6 | 6 | 0 | 256 | 1771388 | 6 | 6 | 0 | 1 | 264 |
| 61 | 3D | 9 | 09 | 6 | 7 | 219081 | 1752644 | 6 | 7 | 0 | 256 | 1771652 | 6 | 7 | 0 | 1 | 264 |

| | | | | | | | | | | | | | | | | | |
|---|---|---|---|---|---|---|---|---|---|---|---|---|---|---|---|---|---|
| 62 | 3E | 9 | 09 | 6 | 8 | 219113 | 1752900 | 6 | 8 | 0 | 256 | 1771916 | 6 | 8 | 0 | 1 | 264 |
| 63 | 3F | 9 | 09 | 7 | 0 | 219147 | 1753172 | 7 | 0 | 0 | 272 | 1772196 | 7 | 0 | 0 | 1 | 280 |
| 64 | 40 | 9 | 09 | 7 | 1 | 219181 | 1753444 | 7 | 1 | 0 | 272 | 1772476 | 7 | 1 | 0 | 1 | 280 |
| 65 | 41 | 9 | 09 | 7 | 2 | 219215 | 1753716 | 7 | 2 | 0 | 272 | 1772756 | 7 | 2 | 0 | 1 | 280 |
| 66 | 42 | 9 | 09 | 7 | 3 | 219249 | 1753988 | 7 | 3 | 0 | 272 | 1773036 | 7 | 3 | 0 | 1 | 280 |
| 67 | 43 | 9 | 09 | 7 | 4 | 219283 | 1754260 | 7 | 4 | 0 | 272 | 1773316 | 7 | 4 | 0 | 1 | 280 |
| 68 | 44 | 9 | 09 | 7 | 5 | 219317 | 1754532 | 7 | 5 | 0 | 272 | 1773596 | 7 | 5 | 0 | 1 | 280 |
| 69 | 45 | 9 | 09 | 7 | 6 | 219351 | 1754804 | 7 | 6 | 0 | 272 | 1773876 | 7 | 6 | 0 | 1 | 280 |
| 70 | 46 | 9 | 09 | 7 | 7 | 219385 | 1755076 | 7 | 7 | 0 | 272 | 1774156 | 7 | 7 | 0 | 1 | 280 |
| 71 | 47 | 9 | 09 | 7 | 8 | 219419 | 1755348 | 7 | 8 | 0 | 272 | 1774436 | 7 | 8 | 0 | 1 | 280 |
| 72 | 48 | 9 | 09 | 8 | 0 | 219455 | 1755636 | 8 | 0 | 0 | 288 | 1774732 | 8 | 0 | 0 | 1 | 296 |
| 73 | 49 | 9 | 09 | 8 | 1 | 219491 | 1755924 | 8 | 1 | 0 | 288 | 1775028 | 8 | 1 | 0 | 1 | 296 |
| 74 | 4A | 9 | 09 | 8 | 2 | 219527 | 1756212 | 8 | 2 | 0 | 288 | 1775324 | 8 | 2 | 0 | 1 | 296 |
| 75 | 4B | 9 | 09 | 8 | 3 | 219563 | 1756500 | 8 | 3 | 0 | 288 | 1775620 | 8 | 3 | 0 | 1 | 296 |
| 76 | 4C | 9 | 09 | 8 | 4 | 219599 | 1756788 | 8 | 4 | 0 | 288 | 1775916 | 8 | 4 | 0 | 1 | 296 |
| 77 | 4D | 9 | 09 | 8 | 5 | 219635 | 1757076 | 8 | 5 | 0 | 288 | 1776212 | 8 | 5 | 0 | 1 | 296 |
| 78 | 4E | 9 | 09 | 8 | 6 | 219671 | 1757364 | 8 | 6 | 0 | 288 | 1776508 | 8 | 6 | 0 | 1 | 296 |
| 79 | 4F | 9 | 09 | 8 | 7 | 219707 | 1757652 | 8 | 7 | 0 | 288 | 1776804 | 8 | 7 | 0 | 1 | 296 |
| 80 | 50 | 9 | 09 | 8 | 8 | 219743 | 1757940 | 8 | 8 | 0 | 288 | 1777100 | 8 | 8 | 0 | 1 | 296 |
| 81 | 51 | 9 | 09 | 9 | 0 | 219781 | 1758244 | 9 | 0 | 0 | 304 | 1777412 | 9 | 0 | 0 | 1 | 312 |
| 82 | 52 | 9 | 09 | 9 | 1 | 219819 | 1758548 | 9 | 1 | 0 | 304 | 1777724 | 9 | 1 | 0 | 1 | 312 |
| 83 | 53 | 9 | 09 | 9 | 2 | 219857 | 1758852 | 9 | 2 | 0 | 304 | 1778036 | 9 | 2 | 0 | 1 | 312 |
| 84 | 54 | 9 | 09 | 9 | 3 | 219895 | 1759156 | 9 | 3 | 0 | 304 | 1778348 | 9 | 3 | 0 | 1 | 312 |
| 85 | 55 | 9 | 09 | 9 | 4 | 219933 | 1759460 | 9 | 4 | 0 | 304 | 1778660 | 9 | 4 | 0 | 1 | 312 |
| 86 | 56 | 9 | 09 | 9 | 5 | 219971 | 1759764 | 9 | 5 | 0 | 304 | 1778972 | 9 | 5 | 0 | 1 | 312 |
| 87 | 57 | 9 | 09 | 9 | 6 | 220009 | 1760068 | 9 | 6 | 0 | 304 | 1779284 | 9 | 6 | 0 | 1 | 312 |
| 88 | 58 | 9 | 09 | 9 | 7 | 220047 | 1760372 | 9 | 7 | 0 | 304 | 1779596 | 9 | 7 | 0 | 1 | 312 |
| 89 | 59 | 9 | 09 | 9 | 8 | 220085 | 1760676 | 9 | 8 | 0 | 304 | 1779908 | 9 | 8 | 0 | 1 | 312 |
| 90 | 5A | 9 | 09 | 10 | 0 | 220125 | 1760996 | 10 | 0 | 0 | 320 | 1780236 | 10 | 0 | 0 | 1 | 328 |
| 91 | 5B | 9 | 09 | 10 | 1 | 220165 | 1761316 | 10 | 1 | 0 | 320 | 1780564 | 10 | 1 | 0 | 1 | 328 |
| 92 | 5C | 9 | 09 | 10 | 2 | 220205 | 1761636 | 10 | 2 | 0 | 320 | 1780892 | 10 | 2 | 0 | 1 | 328 |
| 93 | 5D | 9 | 09 | 10 | 3 | 220245 | 1761956 | 10 | 3 | 0 | 320 | 1781220 | 10 | 3 | 0 | 1 | 328 |
| 94 | 5E | 9 | 09 | 10 | 4 | 220285 | 1762276 | 10 | 4 | 0 | 320 | 1781548 | 10 | 4 | 0 | 1 | 328 |
| 95 | 5F | 9 | 09 | 10 | 5 | 220325 | 1762596 | 10 | 5 | 0 | 320 | 1781876 | 10 | 5 | 0 | 1 | 328 |
| 96 | 60 | 9 | 09 | 10 | 6 | 220365 | 1762916 | 10 | 6 | 0 | 320 | 1782204 | 10 | 6 | 0 | 1 | 328 |
| 97 | 61 | 9 | 09 | 10 | 7 | 220405 | 1763236 | 10 | 7 | 0 | 320 | 1782532 | 10 | 7 | 0 | 1 | 328 |
| 98 | 62 | 9 | 09 | 10 | 8 | 220445 | 1763556 | 10 | 8 | 0 | 320 | 1782860 | 10 | 8 | 0 | 1 | 328 |
| 99 | 63 | 9 | 09 | 11 | 0 | 220487 | 1763892 | 11 | 0 | 0 | 336 | 1783204 | 11 | 0 | 0 | 1 | 344 |
| 100 | 64 | 9 | 09 | 11 | 1 | 220529 | 1764228 | 11 | 1 | 0 | 336 | 1783548 | 11 | 1 | 0 | 1 | 344 |
| 101 | 65 | 9 | 09 | 11 | 2 | 220571 | 1764564 | 11 | 2 | 0 | 336 | 1783892 | 11 | 2 | 0 | 1 | 344 |
| 102 | 66 | 9 | 09 | 11 | 3 | 220613 | 1764900 | 11 | 3 | 0 | 336 | 1784236 | 11 | 3 | 0 | 1 | 344 |
| 103 | 67 | 9 | 09 | 11 | 4 | 220655 | 1765236 | 11 | 4 | 0 | 336 | 1784580 | 11 | 4 | 0 | 1 | 344 |
| 104 | 68 | 9 | 09 | 11 | 5 | 220697 | 1765572 | 11 | 5 | 0 | 336 | 1784924 | 11 | 5 | 0 | 1 | 344 |
| 105 | 69 | 9 | 09 | 11 | 6 | 220739 | 1765908 | 11 | 6 | 0 | 336 | 1785268 | 11 | 6 | 0 | 1 | 344 |
| 106 | 6A | 9 | 09 | 11 | 7 | 220781 | 1766244 | 11 | 7 | 0 | 336 | 1785612 | 11 | 7 | 0 | 1 | 344 |
| 107 | 6B | 9 | 09 | 11 | 8 | 220823 | 1766580 | 11 | 8 | 0 | 336 | 1785956 | 11 | 8 | 0 | 1 | 344 |
| 108 | 6C | 9 | 09 | 12 | 0 | 220867 | 1766932 | 12 | 0 | 0 | 352 | 1786316 | 12 | 0 | 0 | 1 | 360 |
| 109 | 6D | 9 | 09 | 12 | 1 | 220911 | 1767284 | 12 | 1 | 0 | 352 | 1786676 | 12 | 1 | 0 | 1 | 360 |
| 110 | 6E | 9 | 09 | 12 | 2 | 220955 | 1767636 | 12 | 2 | 0 | 352 | 1787036 | 12 | 2 | 0 | 1 | 360 |
| 111 | 6F | 9 | 09 | 12 | 3 | 220999 | 1767988 | 12 | 3 | 0 | 352 | 1787396 | 12 | 3 | 0 | 1 | 360 |
| 112 | 70 | 9 | 09 | 12 | 4 | 221043 | 1768340 | 12 | 4 | 0 | 352 | 1787756 | 12 | 4 | 0 | 1 | 360 |
| 113 | 71 | 9 | 09 | 12 | 5 | 221087 | 1768692 | 12 | 5 | 0 | 352 | 1788116 | 12 | 5 | 0 | 1 | 360 |
| 114 | 72 | 9 | 09 | 12 | 6 | 221131 | 1769044 | 12 | 6 | 0 | 352 | 1788476 | 12 | 6 | 0 | 1 | 360 |
| 115 | 73 | 9 | 09 | 12 | 7 | 221175 | 1769396 | 12 | 7 | 0 | 352 | 1788836 | 12 | 7 | 0 | 1 | 360 |
| 116 | 74 | 9 | 09 | 12 | 8 | 221219 | 1769748 | 12 | 8 | 0 | 352 | 1789196 | 12 | 8 | 0 | 1 | 360 |
| 117 | 75 | 9 | 09 | 13 | 0 | 221265 | 1770116 | 13 | 0 | 0 | 368 | 1789572 | 13 | 0 | 0 | 1 | 376 |
| 118 | 76 | 9 | 09 | 13 | 1 | 221311 | 1770484 | 13 | 1 | 0 | 368 | 1789948 | 13 | 1 | 0 | 1 | 376 |
| 119 | 77 | 9 | 09 | 13 | 2 | 221357 | 1770852 | 13 | 2 | 0 | 368 | 1790324 | 13 | 2 | 0 | 1 | 376 |
| 120 | 78 | 9 | 09 | 13 | 3 | 221403 | 1771220 | 13 | 3 | 0 | 368 | 1790700 | 13 | 3 | 0 | 1 | 376 |
| 121 | 79 | 9 | 09 | 13 | 4 | 221449 | 1771588 | 13 | 4 | 0 | 368 | 1791076 | 13 | 4 | 0 | 1 | 376 |
| 122 | 7A | 9 | 09 | 13 | 5 | 221495 | 1771956 | 13 | 5 | 0 | 368 | 1791452 | 13 | 5 | 0 | 1 | 376 |
| 123 | 7B | 9 | 09 | 13 | 6 | 221541 | 1772324 | 13 | 6 | 0 | 368 | 1791828 | 13 | 6 | 0 | 1 | 376 |
| 124 | 7C | 9 | 09 | 13 | 7 | 221587 | 1772692 | 13 | 7 | 0 | 368 | 1792204 | 13 | 7 | 0 | 1 | 376 |
| 125 | 7D | 9 | 09 | 13 | 8 | 221633 | 1773060 | 13 | 8 | 0 | 368 | 1792580 | 13 | 8 | 0 | 1 | 376 |
| 126 | 7E | 9 | 09 | 14 | 0 | 221681 | 1773444 | 14 | 0 | 0 | 384 | 1792972 | 14 | 0 | 0 | 1 | 392 |
| 127 | 7F | 9 | 09 | 14 | 1 | 221729 | 1773828 | 14 | 1 | 0 | 384 | 1793364 | 14 | 1 | 0 | 1 | 392 |
| 128 | 80 | 9 | 09 | 14 | 2 | 221777 | 1774212 | 14 | 2 | 0 | 384 | 1793756 | 14 | 2 | 0 | 1 | 392 |
| 129 | 81 | 9 | 09 | 14 | 3 | 221825 | 1774596 | 14 | 3 | 0 | 384 | 1794148 | 14 | 3 | 0 | 1 | 392 |
| 130 | 82 | 9 | 09 | 14 | 4 | 221873 | 1774980 | 14 | 4 | 0 | 384 | 1794540 | 14 | 4 | 0 | 1 | 392 |
| 131 | 83 | 9 | 09 | 14 | 5 | 221921 | 1775364 | 14 | 5 | 0 | 384 | 1794932 | 14 | 5 | 0 | 1 | 392 |
| 132 | 84 | 9 | 09 | 14 | 6 | 221969 | 1775748 | 14 | 6 | 0 | 384 | 1795324 | 14 | 6 | 0 | 1 | 392 |
| 133 | 85 | 9 | 09 | 14 | 7 | 222017 | 1776132 | 14 | 7 | 0 | 384 | 1795716 | 14 | 7 | 0 | 1 | 392 |
| 134 | 86 | 9 | 09 | 14 | 8 | 222065 | 1776516 | 14 | 8 | 0 | 384 | 1796108 | 14 | 8 | 0 | 1 | 392 |
| 135 | 87 | 9 | 09 | 15 | 0 | 222115 | 1776916 | 15 | 0 | 0 | 400 | 1796516 | 15 | 0 | 0 | 1 | 408 |
| 136 | 88 | 9 | 09 | 15 | 1 | 222165 | 1777316 | 15 | 1 | 0 | 400 | 1796924 | 15 | 1 | 0 | 1 | 408 |
| 137 | 89 | 9 | 09 | 15 | 2 | 222215 | 1777716 | 15 | 2 | 0 | 400 | 1797332 | 15 | 2 | 0 | 1 | 408 |
| 138 | 8A | 9 | 09 | 15 | 3 | 222265 | 1778116 | 15 | 3 | 0 | 400 | 1797740 | 15 | 3 | 0 | 1 | 408 |
| 139 | 8B | 9 | 09 | 15 | 4 | 222315 | 1778516 | 15 | 4 | 0 | 400 | 1798148 | 15 | 4 | 0 | 1 | 408 |
| 140 | 8C | 9 | 09 | 15 | 5 | 222365 | 1778916 | 15 | 5 | 0 | 400 | 1798556 | 15 | 5 | 0 | 1 | 408 |
| 141 | 8D | 9 | 09 | 15 | 6 | 222415 | 1779316 | 15 | 6 | 0 | 400 | 1798964 | 15 | 6 | 0 | 1 | 408 |
| 142 | 8E | 9 | 09 | 15 | 7 | 222465 | 1779716 | 15 | 7 | 0 | 400 | 1799372 | 15 | 7 | 0 | 1 | 408 |
| 143 | 8F | 9 | 09 | 15 | 8 | 222515 | 1780116 | 15 | 8 | 0 | 400 | 1799780 | 15 | 8 | 0 | 1 | 408 |
| 144 | 90 | 9 | 09 | 16 | 0 | 222567 | 1780532 | 16 | 0 | 0 | 416 | 1800204 | 16 | 0 | 0 | 1 | 424 |
| 145 | 91 | 9 | 09 | 16 | 1 | 222619 | 1780948 | 16 | 1 | 0 | 416 | 1800628 | 16 | 1 | 0 | 1 | 424 |
| 146 | 92 | 9 | 09 | 16 | 2 | 222671 | 1781364 | 16 | 2 | 0 | 416 | 1801052 | 16 | 2 | 0 | 1 | 424 |
| 147 | 93 | 9 | 09 | 16 | 3 | 222723 | 1781780 | 16 | 3 | 0 | 416 | 1801476 | 16 | 3 | 0 | 1 | 424 |
| 148 | 94 | 9 | 09 | 16 | 4 | 222775 | 1782196 | 16 | 4 | 0 | 416 | 1801900 | 16 | 4 | 0 | 1 | 424 |
| 149 | 95 | 9 | 09 | 16 | 5 | 222827 | 1782612 | 16 | 5 | 0 | 416 | 1802324 | 16 | 5 | 0 | 1 | 424 |
| 150 | 96 | 9 | 09 | 16 | 6 | 222879 | 1783028 | 16 | 6 | 0 | 416 | 1802748 | 16 | 6 | 0 | 1 | 424 |
| 151 | 97 | 9 | 09 | 16 | 7 | 222931 | 1783444 | 16 | 7 | 0 | 416 | 1803172 | 16 | 7 | 0 | 1 | 424 |
| 152 | 98 | 9 | 09 | 16 | 8 | 222983 | 1783860 | 16 | 8 | 0 | 416 | 1803596 | 16 | 8 | 0 | 1 | 424 |
| 153 | 99 | 9 | 09 | 17 | 0 | 223037 | 1784292 | 17 | 0 | 0 | 432 | 1804036 | 17 | 0 | 0 | 1 | 440 |
| 154 | 9A | 9 | 09 | 17 | 1 | 223091 | 1784724 | 17 | 1 | 0 | 432 | 1804476 | 17 | 1 | 0 | 1 | 440 |
| 155 | 9B | 9 | 09 | 17 | 2 | 223145 | 1785156 | 17 | 2 | 0 | 432 | 1804916 | 17 | 2 | 0 | 1 | 440 |
| 156 | 9C | 9 | 09 | 17 | 3 | 223199 | 1785588 | 17 | 3 | 0 | 432 | 1805356 | 17 | 3 | 0 | 1 | 440 |
| 157 | 9D | 9 | 09 | 17 | 4 | 223253 | 1786020 | 17 | 4 | 0 | 432 | 1805796 | 17 | 4 | 0 | 1 | 440 |
| 158 | 9E | 9 | 09 | 17 | 5 | 223307 | 1786452 | 17 | 5 | 0 | 432 | 1806236 | 17 | 5 | 0 | 1 | 440 |
| 159 | 9F | 9 | 09 | 17 | 6 | 223361 | 1786884 | 17 | 6 | 0 | 432 | 1806676 | 17 | 6 | 0 | 1 | 440 |
| 160 | A0 | 9 | 09 | 17 | 7 | 223415 | 1787316 | 17 | 7 | 0 | 432 | 1807116 | 17 | 7 | 0 | 1 | 440 |
| 161 | A1 | 9 | 09 | 17 | 8 | 223469 | 1787748 | 17 | 8 | 0 | 432 | 1807556 | 17 | 8 | 0 | 1 | 440 |
| 162 | A2 | 9 | 09 | 18 | 0 | 223525 | 1788196 | 18 | 0 | 0 | 448 | 1808012 | 18 | 0 | 0 | 1 | 456 |
| 163 | A3 | 9 | 09 | 18 | 1 | 223581 | 1788644 | 18 | 1 | 0 | 448 | 1808468 | 18 | 1 | 0 | 1 | 456 |
| 164 | A4 | 9 | 09 | 18 | 2 | 223637 | 1789092 | 18 | 2 | 0 | 448 | 1808924 | 18 | 2 | 0 | 1 | 456 |

| | | | | | | | | | | | | | | | | | |
|---|---|---|---|---|---|---|---|---|---|---|---|---|---|---|---|---|---|
| 165 | A5 | 9 | 09 | 18 | 3 | 223693 | 1789540 | 18 | 3 | 0 | 448 | 1809380 | 18 | 3 | 0 | 1 | 456 |
| 166 | A6 | 9 | 09 | 18 | 4 | 223749 | 1789988 | 18 | 4 | 0 | 448 | 1809836 | 18 | 4 | 0 | 1 | 456 |
| 167 | A7 | 9 | 09 | 18 | 5 | 223805 | 1790436 | 18 | 5 | 0 | 448 | 1810292 | 18 | 5 | 0 | 1 | 456 |
| 168 | A8 | 9 | 09 | 18 | 6 | 223861 | 1790884 | 18 | 6 | 0 | 448 | 1810748 | 18 | 6 | 0 | 1 | 456 |
| 169 | A9 | 9 | 09 | 18 | 7 | 223917 | 1791332 | 18 | 7 | 0 | 448 | 1811204 | 18 | 7 | 0 | 1 | 456 |
| 170 | AA | 9 | 09 | 18 | 8 | 223973 | 1791780 | 18 | 8 | 0 | 448 | 1811660 | 18 | 8 | 0 | 1 | 456 |
| 171 | AB | 9 | 09 | 19 | 0 | 224031 | 1792244 | 19 | 0 | 0 | 464 | 1812132 | 19 | 0 | 0 | 1 | 472 |
| 172 | AC | 9 | 09 | 19 | 1 | 224089 | 1792708 | 19 | 1 | 0 | 464 | 1812604 | 19 | 1 | 0 | 1 | 472 |
| 173 | AD | 9 | 09 | 19 | 2 | 224147 | 1793172 | 19 | 2 | 0 | 464 | 1813076 | 19 | 2 | 0 | 1 | 472 |
| 174 | AE | 9 | 09 | 19 | 3 | 224205 | 1793636 | 19 | 3 | 0 | 464 | 1813548 | 19 | 3 | 0 | 1 | 472 |
| 175 | AF | 9 | 09 | 19 | 4 | 224263 | 1794100 | 19 | 4 | 0 | 464 | 1814020 | 19 | 4 | 0 | 1 | 472 |
| 176 | B0 | 9 | 09 | 19 | 5 | 224321 | 1794564 | 19 | 5 | 0 | 464 | 1814492 | 19 | 5 | 0 | 1 | 472 |
| 177 | B1 | 9 | 09 | 19 | 6 | 224379 | 1795028 | 19 | 6 | 0 | 464 | 1814964 | 19 | 6 | 0 | 1 | 472 |
| 178 | B2 | 9 | 09 | 19 | 7 | 224437 | 1795492 | 19 | 7 | 0 | 464 | 1815436 | 19 | 7 | 0 | 1 | 472 |
| 179 | B3 | 9 | 09 | 19 | 8 | 224495 | 1795956 | 19 | 8 | 0 | 464 | 1815908 | 19 | 8 | 0 | 1 | 472 |
| 180 | B4 | 9 | 09 | 20 | 0 | 224555 | 1796436 | 20 | 0 | 0 | 480 | 1816396 | 20 | 0 | 0 | 1 | 488 |
| 181 | B5 | 9 | 09 | 20 | 1 | 224615 | 1796916 | 20 | 1 | 0 | 480 | 1816884 | 20 | 1 | 0 | 1 | 488 |
| 182 | B6 | 9 | 09 | 20 | 2 | 224675 | 1797396 | 20 | 2 | 0 | 480 | 1817372 | 20 | 2 | 0 | 1 | 488 |
| 183 | B7 | 9 | 09 | 20 | 3 | 224735 | 1797876 | 20 | 3 | 0 | 480 | 1817860 | 20 | 3 | 0 | 1 | 488 |
| 184 | B8 | 9 | 09 | 20 | 4 | 224795 | 1798356 | 20 | 4 | 0 | 480 | 1818348 | 20 | 4 | 0 | 1 | 488 |
| 185 | B9 | 9 | 09 | 20 | 5 | 224855 | 1798836 | 20 | 5 | 0 | 480 | 1818836 | 20 | 5 | 0 | 1 | 488 |
| 186 | BA | 9 | 09 | 20 | 6 | 224915 | 1799316 | 20 | 6 | 0 | 480 | 1819324 | 20 | 6 | 0 | 1 | 488 |
| 187 | BB | 9 | 09 | 20 | 7 | 224975 | 1799796 | 20 | 7 | 0 | 480 | 1819812 | 20 | 7 | 0 | 1 | 488 |
| 188 | BC | 9 | 09 | 20 | 8 | 225035 | 1800276 | 20 | 8 | 0 | 480 | 1820300 | 20 | 8 | 0 | 1 | 488 |
| 189 | BD | 9 | 09 | 21 | 0 | 225097 | 1800772 | 21 | 0 | 0 | 496 | 1820804 | 21 | 0 | 0 | 1 | 504 |
| 190 | BE | 9 | 09 | 21 | 1 | 225159 | 1801268 | 21 | 1 | 0 | 496 | 1821308 | 21 | 1 | 0 | 1 | 504 |
| 191 | BF | 9 | 09 | 21 | 2 | 225221 | 1801764 | 21 | 2 | 0 | 496 | 1821812 | 21 | 2 | 0 | 1 | 504 |
| 192 | C0 | 9 | 09 | 21 | 3 | 225283 | 1802260 | 21 | 3 | 0 | 496 | 1822316 | 21 | 3 | 0 | 1 | 504 |
| 193 | C1 | 9 | 09 | 21 | 4 | 225345 | 1802756 | 21 | 4 | 0 | 496 | 1822820 | 21 | 4 | 0 | 1 | 504 |
| 194 | C2 | 9 | 09 | 21 | 5 | 225407 | 1803252 | 21 | 5 | 0 | 496 | 1823324 | 21 | 5 | 0 | 1 | 504 |
| 195 | C3 | 9 | 09 | 21 | 6 | 225469 | 1803748 | 21 | 6 | 0 | 496 | 1823828 | 21 | 6 | 0 | 1 | 504 |
| 196 | C4 | 9 | 09 | 21 | 7 | 225531 | 1804244 | 21 | 7 | 0 | 496 | 1824332 | 21 | 7 | 0 | 1 | 504 |
| 197 | C5 | 9 | 09 | 21 | 8 | 225593 | 1804740 | 21 | 8 | 0 | 496 | 1824836 | 21 | 8 | 0 | 1 | 504 |
| 198 | C6 | 9 | 09 | 22 | 0 | 225657 | 1805252 | 22 | 0 | 0 | 512 | 1825356 | 22 | 0 | 0 | 1 | 520 |
| 199 | C7 | 9 | 09 | 22 | 1 | 225721 | 1805764 | 22 | 1 | 0 | 512 | 1825876 | 22 | 1 | 0 | 1 | 520 |
| 200 | C8 | 9 | 09 | 22 | 2 | 225785 | 1806276 | 22 | 2 | 0 | 512 | 1826396 | 22 | 2 | 0 | 1 | 520 |
| 201 | C9 | 9 | 09 | 22 | 3 | 225849 | 1806788 | 22 | 3 | 0 | 512 | 1826916 | 22 | 3 | 0 | 1 | 520 |
| 202 | CA | 9 | 09 | 22 | 4 | 225913 | 1807300 | 22 | 4 | 0 | 512 | 1827436 | 22 | 4 | 0 | 1 | 520 |
| 203 | CB | 9 | 09 | 22 | 5 | 225977 | 1807812 | 22 | 5 | 0 | 512 | 1827956 | 22 | 5 | 0 | 1 | 520 |
| 204 | CC | 9 | 09 | 22 | 6 | 226041 | 1808324 | 22 | 6 | 0 | 512 | 1828476 | 22 | 6 | 0 | 1 | 520 |
| 205 | CD | 9 | 09 | 22 | 7 | 226105 | 1808836 | 22 | 7 | 0 | 512 | 1828996 | 22 | 7 | 0 | 1 | 520 |
| 206 | CE | 9 | 09 | 22 | 8 | 226169 | 1809348 | 22 | 8 | 0 | 512 | 1829516 | 22 | 8 | 0 | 1 | 520 |
| 207 | CF | 9 | 09 | 23 | 0 | 226235 | 1809876 | 23 | 0 | 0 | 528 | 1830052 | 23 | 0 | 0 | 1 | 536 |
| 208 | D0 | 9 | 09 | 23 | 1 | 226301 | 1810404 | 23 | 1 | 0 | 528 | 1830588 | 23 | 1 | 0 | 1 | 536 |
| 209 | D1 | 9 | 09 | 23 | 2 | 226367 | 1810932 | 23 | 2 | 0 | 528 | 1831124 | 23 | 2 | 0 | 1 | 536 |
| 210 | D2 | 9 | 09 | 23 | 3 | 226433 | 1811460 | 23 | 3 | 0 | 528 | 1831660 | 23 | 3 | 0 | 1 | 536 |
| 211 | D3 | 9 | 09 | 23 | 4 | 226499 | 1811988 | 23 | 4 | 0 | 528 | 1832196 | 23 | 4 | 0 | 1 | 536 |
| 212 | D4 | 9 | 09 | 23 | 5 | 226565 | 1812516 | 23 | 5 | 0 | 528 | 1832732 | 23 | 5 | 0 | 1 | 536 |
| 213 | D5 | 9 | 09 | 23 | 6 | 226631 | 1813044 | 23 | 6 | 0 | 528 | 1833268 | 23 | 6 | 0 | 1 | 536 |
| 214 | D6 | 9 | 09 | 23 | 7 | 226697 | 1813572 | 23 | 7 | 0 | 528 | 1833804 | 23 | 7 | 0 | 1 | 536 |
| 215 | D7 | 9 | 09 | 23 | 8 | 226763 | 1814100 | 23 | 8 | 0 | 528 | 1834340 | 23 | 8 | 0 | 1 | 536 |
| 216 | D8 | 9 | 09 | 24 | 0 | 226831 | 1814644 | 24 | 0 | 0 | 544 | 1834892 | 24 | 0 | 0 | 1 | 552 |
| 217 | D9 | 9 | 09 | 24 | 1 | 226899 | 1815188 | 24 | 1 | 0 | 544 | 1835444 | 24 | 1 | 0 | 1 | 552 |
| 218 | DA | 9 | 09 | 24 | 2 | 226967 | 1815732 | 24 | 2 | 0 | 544 | 1835996 | 24 | 2 | 0 | 1 | 552 |
| 219 | DB | 9 | 09 | 24 | 3 | 227035 | 1816276 | 24 | 3 | 0 | 544 | 1836548 | 24 | 3 | 0 | 1 | 552 |
| 220 | DC | 9 | 09 | 24 | 4 | 227103 | 1816820 | 24 | 4 | 0 | 544 | 1837100 | 24 | 4 | 0 | 1 | 552 |
| 221 | DD | 9 | 09 | 24 | 5 | 227171 | 1817364 | 24 | 5 | 0 | 544 | 1837652 | 24 | 5 | 0 | 1 | 552 |
| 222 | DE | 9 | 09 | 24 | 6 | 227239 | 1817908 | 24 | 6 | 0 | 544 | 1838204 | 24 | 6 | 0 | 1 | 552 |
| 223 | DF | 9 | 09 | 24 | 7 | 227307 | 1818452 | 24 | 7 | 0 | 544 | 1838756 | 24 | 7 | 0 | 1 | 552 |
| 224 | E0 | 9 | 09 | 24 | 8 | 227375 | 1818996 | 24 | 8 | 0 | 544 | 1839308 | 24 | 8 | 0 | 1 | 552 |
| 225 | E1 | 9 | 09 | 25 | 0 | 227445 | 1819556 | 25 | 0 | 0 | 560 | 1839876 | 25 | 0 | 0 | 1 | 568 |
| 226 | E2 | 9 | 09 | 25 | 1 | 227515 | 1820116 | 25 | 1 | 0 | 560 | 1840444 | 25 | 1 | 0 | 1 | 568 |
| 227 | E3 | 9 | 09 | 25 | 2 | 227585 | 1820676 | 25 | 2 | 0 | 560 | 1841012 | 25 | 2 | 0 | 1 | 568 |
| 228 | E4 | 9 | 09 | 25 | 3 | 227655 | 1821236 | 25 | 3 | 0 | 560 | 1841580 | 25 | 3 | 0 | 1 | 568 |
| 229 | E5 | 9 | 09 | 25 | 4 | 227725 | 1821796 | 25 | 4 | 0 | 560 | 1842148 | 25 | 4 | 0 | 1 | 568 |
| 230 | E6 | 9 | 09 | 25 | 5 | 227795 | 1822356 | 25 | 5 | 0 | 560 | 1842716 | 25 | 5 | 0 | 1 | 568 |
| 231 | E7 | 9 | 09 | 25 | 6 | 227865 | 1822916 | 25 | 6 | 0 | 560 | 1843284 | 25 | 6 | 0 | 1 | 568 |
| 232 | E8 | 9 | 09 | 25 | 7 | 227935 | 1823476 | 25 | 7 | 0 | 560 | 1843852 | 25 | 7 | 0 | 1 | 568 |
| 233 | E9 | 9 | 09 | 25 | 8 | 228005 | 1824036 | 25 | 8 | 0 | 560 | 1844420 | 25 | 8 | 0 | 1 | 568 |
| 234 | EA | 9 | 09 | 26 | 0 | 228077 | 1824612 | 26 | 0 | 0 | 576 | 1845004 | 26 | 0 | 0 | 1 | 584 |
| 235 | EB | 9 | 09 | 26 | 1 | 228149 | 1825188 | 26 | 1 | 0 | 576 | 1845588 | 26 | 1 | 0 | 1 | 584 |
| 236 | EC | 9 | 09 | 26 | 2 | 228221 | 1825764 | 26 | 2 | 0 | 576 | 1846172 | 26 | 2 | 0 | 1 | 584 |
| 237 | ED | 9 | 09 | 26 | 3 | 228293 | 1826340 | 26 | 3 | 0 | 576 | 1846756 | 26 | 3 | 0 | 1 | 584 |
| 238 | EE | 9 | 09 | 26 | 4 | 228365 | 1826916 | 26 | 4 | 0 | 576 | 1847340 | 26 | 4 | 0 | 1 | 584 |
| 239 | EF | 9 | 09 | 26 | 5 | 228437 | 1827492 | 26 | 5 | 0 | 576 | 1847924 | 26 | 5 | 0 | 1 | 584 |
| 240 | F0 | 9 | 09 | 26 | 6 | 228509 | 1828068 | 26 | 6 | 0 | 576 | 1848508 | 26 | 6 | 0 | 1 | 584 |
| 241 | F1 | 9 | 09 | 26 | 7 | 228581 | 1828644 | 26 | 7 | 0 | 576 | 1849092 | 26 | 7 | 0 | 1 | 584 |
| 242 | F2 | 9 | 09 | 26 | 8 | 228653 | 1829220 | 26 | 8 | 0 | 576 | 1849676 | 26 | 8 | 0 | 1 | 584 |
| 243 | F3 | 9 | 09 | 27 | 0 | 228727 | 1829812 | 27 | 0 | 0 | 592 | 1850276 | 27 | 0 | 0 | 1 | 600 |
| 244 | F4 | 9 | 09 | 27 | 1 | 228801 | 1830404 | 27 | 1 | 0 | 592 | 1850876 | 27 | 1 | 0 | 1 | 600 |
| 245 | F5 | 9 | 09 | 27 | 2 | 228875 | 1830996 | 27 | 2 | 0 | 592 | 1851476 | 27 | 2 | 0 | 1 | 600 |
| 246 | F6 | 9 | 09 | 27 | 3 | 228949 | 1831588 | 27 | 3 | 0 | 592 | 1852076 | 27 | 3 | 0 | 1 | 600 |
| 247 | F7 | 9 | 09 | 27 | 4 | 229023 | 1832180 | 27 | 4 | 0 | 592 | 1852676 | 27 | 4 | 0 | 1 | 600 |
| 248 | F8 | 9 | 09 | 27 | 5 | 229097 | 1832772 | 27 | 5 | 0 | 592 | 1853276 | 27 | 5 | 0 | 1 | 600 |
| 249 | F9 | 9 | 09 | 27 | 6 | 229171 | 1833364 | 27 | 6 | 0 | 592 | 1853876 | 27 | 6 | 0 | 1 | 600 |
| 250 | FA | 9 | 09 | 27 | 7 | 229245 | 1833956 | 27 | 7 | 0 | 592 | 1854476 | 27 | 7 | 0 | 1 | 600 |
| 251 | FB | 9 | 09 | 27 | 8 | 229319 | 1834548 | 27 | 8 | 0 | 592 | 1855076 | 27 | 8 | 0 | 1 | 600 |
| 252 | FC | 9 | 09 | 28 | 0 | 229395 | 1835156 | 28 | 0 | 0 | 608 | 1855692 | 28 | 0 | 0 | 1 | 616 |
| 253 | FD | 9 | 09 | 28 | 1 | 229471 | 1835764 | 28 | 1 | 0 | 608 | 1856308 | 28 | 1 | 0 | 1 | 616 |
| 254 | FE | 9 | 09 | 28 | 2 | 229547 | 1836372 | 28 | 2 | 0 | 608 | 1856924 | 28 | 2 | 0 | 1 | 616 |
| 255 | FF | 9 | 09 | 28 | 3 | 229623 | 1836980 | 28 | 3 | 0 | 608 | 1857540 | 28 | 3 | 0 | 1 | 616 |
| 0 | 00 | 10 | 0A | 0 | 0 | 229635 | 1837076 | 0 | 0 | 0 | 96 | 1857644 | 0 | 0 | 0 | 1 | 104 |
| 1 | 01 | 10 | 0A | 0 | 1 | 229649 | 1837188 | 0 | 1 | 0 | 112 | 1857764 | 0 | 1 | 0 | 1 | 120 |
| 2 | 02 | 10 | 0A | 0 | 2 | 229663 | 1837300 | 0 | 2 | 0 | 112 | 1857884 | 0 | 2 | 0 | 1 | 120 |
| 3 | 03 | 10 | 0A | 0 | 3 | 229677 | 1837412 | 0 | 3 | 0 | 112 | 1858004 | 0 | 3 | 0 | 1 | 120 |
| 4 | 04 | 10 | 0A | 0 | 4 | 229691 | 1837524 | 0 | 4 | 0 | 112 | 1858124 | 0 | 4 | 0 | 1 | 120 |
| 5 | 05 | 10 | 0A | 0 | 5 | 229705 | 1837636 | 0 | 5 | 0 | 112 | 1858244 | 0 | 5 | 0 | 1 | 120 |
| 6 | 06 | 10 | 0A | 0 | 6 | 229719 | 1837748 | 0 | 6 | 0 | 112 | 1858364 | 0 | 6 | 0 | 1 | 120 |
| 7 | 07 | 10 | 0A | 0 | 7 | 229733 | 1837860 | 0 | 7 | 0 | 112 | 1858484 | 0 | 7 | 0 | 1 | 120 |
| 8 | 08 | 10 | 0A | 0 | 8 | 229747 | 1837972 | 0 | 8 | 0 | 112 | 1858604 | 0 | 8 | 0 | 1 | 120 |
| 9 | 09 | 10 | 0A | 0 | 9 | 229761 | 1838084 | 0 | 9 | 0 | 112 | 1858724 | 0 | 9 | 0 | 1 | 120 |
| 10 | 0A | 10 | 0A | 1 | 0 | 229773 | 1838180 | 1 | 0 | 0 | 96 | 1858828 | 1 | 0 | 0 | 1 | 104 |
| 11 | 0B | 10 | 0A | 1 | 1 | 229790 | 1838316 | 1 | 1 | 0 | 136 | 1858972 | 1 | 1 | 0 | 1 | 144 |

| | | | | | | | | | | | | | | | | | |
|---|---|---|---|---|---|---|---|---|---|---|---|---|---|---|---|---|---|
| 12 | 0C | 10 | 0A | 1 | 2 | 229807 | 1838452 | 1 | 2 | 0 | 136 | 1859116 | 1 | 2 | 0 | 1 | 144 |
| 13 | 0D | 10 | 0A | 1 | 3 | 229824 | 1838588 | 1 | 3 | 0 | 136 | 1859260 | 1 | 3 | 0 | 1 | 144 |
| 14 | 0E | 10 | 0A | 1 | 4 | 229841 | 1838724 | 1 | 4 | 0 | 136 | 1859404 | 1 | 4 | 0 | 1 | 144 |
| 15 | 0F | 10 | 0A | 1 | 5 | 229858 | 1838860 | 1 | 5 | 0 | 136 | 1859548 | 1 | 5 | 0 | 1 | 144 |
| 16 | 10 | 10 | 0A | 1 | 6 | 229879 | 1839028 | 1 | 6 | 0 | 168 | 1859724 | 1 | 6 | 0 | 1 | 176 |
| 17 | 11 | 10 | 0A | 1 | 7 | 229900 | 1839196 | 1 | 7 | 0 | 168 | 1859900 | 1 | 7 | 0 | 1 | 176 |
| 18 | 12 | 10 | 0A | 1 | 8 | 229921 | 1839364 | 1 | 8 | 0 | 168 | 1860076 | 1 | 8 | 0 | 1 | 176 |
| 19 | 13 | 10 | 0A | 1 | 9 | 229942 | 1839532 | 1 | 9 | 0 | 168 | 1860252 | 1 | 9 | 0 | 1 | 176 |
| 20 | 14 | 10 | 0A | 2 | 0 | 229961 | 1839684 | 2 | 0 | 0 | 152 | 1860412 | 2 | 0 | 0 | 1 | 160 |
| 21 | 15 | 10 | 0A | 2 | 1 | 229985 | 1839876 | 2 | 1 | 0 | 192 | 1860612 | 2 | 1 | 0 | 1 | 200 |
| 22 | 16 | 10 | 0A | 2 | 2 | 230009 | 1840068 | 2 | 2 | 0 | 192 | 1860812 | 2 | 2 | 0 | 1 | 200 |
| 23 | 17 | 10 | 0A | 2 | 3 | 230033 | 1840260 | 2 | 3 | 0 | 192 | 1861012 | 2 | 3 | 0 | 1 | 200 |
| 24 | 18 | 10 | 0A | 2 | 4 | 230057 | 1840452 | 2 | 4 | 0 | 192 | 1861212 | 2 | 4 | 0 | 1 | 200 |
| 25 | 19 | 10 | 0A | 2 | 5 | 230081 | 1840644 | 2 | 5 | 0 | 192 | 1861412 | 2 | 5 | 0 | 1 | 200 |
| 26 | 1A | 10 | 0A | 2 | 6 | 230105 | 1840836 | 2 | 6 | 0 | 192 | 1861612 | 2 | 6 | 0 | 1 | 200 |
| 27 | 1B | 10 | 0A | 2 | 7 | 230129 | 1841028 | 2 | 7 | 0 | 192 | 1861812 | 2 | 7 | 0 | 1 | 200 |
| 28 | 1C | 10 | 0A | 2 | 8 | 230153 | 1841220 | 2 | 8 | 0 | 192 | 1862012 | 2 | 8 | 0 | 1 | 200 |
| 29 | 1D | 10 | 0A | 2 | 9 | 230177 | 1841412 | 2 | 9 | 0 | 192 | 1862212 | 2 | 9 | 0 | 1 | 200 |
| 30 | 1E | 10 | 0A | 3 | 0 | 230203 | 1841620 | 3 | 0 | 0 | 208 | 1862428 | 3 | 0 | 0 | 1 | 216 |
| 31 | 1F | 10 | 0A | 3 | 1 | 230229 | 1841828 | 3 | 1 | 0 | 208 | 1862644 | 3 | 1 | 0 | 1 | 216 |
| 32 | 20 | 10 | 0A | 3 | 2 | 230255 | 1842036 | 3 | 2 | 0 | 208 | 1862860 | 3 | 2 | 0 | 1 | 216 |
| 33 | 21 | 10 | 0A | 3 | 3 | 230281 | 1842244 | 3 | 3 | 0 | 208 | 1863076 | 3 | 3 | 0 | 1 | 216 |
| 34 | 22 | 10 | 0A | 3 | 4 | 230307 | 1842452 | 3 | 4 | 0 | 208 | 1863292 | 3 | 4 | 0 | 1 | 216 |
| 35 | 23 | 10 | 0A | 3 | 5 | 230333 | 1842660 | 3 | 5 | 0 | 208 | 1863508 | 3 | 5 | 0 | 1 | 216 |
| 36 | 24 | 10 | 0A | 3 | 6 | 230359 | 1842868 | 3 | 6 | 0 | 208 | 1863724 | 3 | 6 | 0 | 1 | 216 |
| 37 | 25 | 10 | 0A | 3 | 7 | 230385 | 1843076 | 3 | 7 | 0 | 208 | 1863940 | 3 | 7 | 0 | 1 | 216 |
| 38 | 26 | 10 | 0A | 3 | 8 | 230411 | 1843284 | 3 | 8 | 0 | 208 | 1864156 | 3 | 8 | 0 | 1 | 216 |
| 39 | 27 | 10 | 0A | 3 | 9 | 230437 | 1843492 | 3 | 9 | 0 | 208 | 1864372 | 3 | 9 | 0 | 1 | 216 |
| 40 | 28 | 10 | 0A | 4 | 0 | 230465 | 1843716 | 4 | 0 | 0 | 224 | 1864604 | 4 | 0 | 0 | 1 | 232 |
| 41 | 29 | 10 | 0A | 4 | 1 | 230493 | 1843940 | 4 | 1 | 0 | 224 | 1864836 | 4 | 1 | 0 | 1 | 232 |
| 42 | 2A | 10 | 0A | 4 | 2 | 230521 | 1844164 | 4 | 2 | 0 | 224 | 1865068 | 4 | 2 | 0 | 1 | 232 |
| 43 | 2B | 10 | 0A | 4 | 3 | 230549 | 1844388 | 4 | 3 | 0 | 224 | 1865300 | 4 | 3 | 0 | 1 | 232 |
| 44 | 2C | 10 | 0A | 4 | 4 | 230577 | 1844612 | 4 | 4 | 0 | 224 | 1865532 | 4 | 4 | 0 | 1 | 232 |
| 45 | 2D | 10 | 0A | 4 | 5 | 230605 | 1844836 | 4 | 5 | 0 | 224 | 1865764 | 4 | 5 | 0 | 1 | 232 |
| 46 | 2E | 10 | 0A | 4 | 6 | 230633 | 1845060 | 4 | 6 | 0 | 224 | 1865996 | 4 | 6 | 0 | 1 | 232 |
| 47 | 2F | 10 | 0A | 4 | 7 | 230661 | 1845284 | 4 | 7 | 0 | 224 | 1866228 | 4 | 7 | 0 | 1 | 232 |
| 48 | 30 | 10 | 0A | 4 | 8 | 230689 | 1845508 | 4 | 8 | 0 | 224 | 1866460 | 4 | 8 | 0 | 1 | 232 |
| 49 | 31 | 10 | 0A | 4 | 9 | 230717 | 1845732 | 4 | 9 | 0 | 224 | 1866692 | 4 | 9 | 0 | 1 | 232 |
| 50 | 32 | 10 | 0A | 5 | 0 | 230747 | 1845972 | 5 | 0 | 0 | 240 | 1866940 | 5 | 0 | 0 | 1 | 248 |
| 51 | 33 | 10 | 0A | 5 | 1 | 230777 | 1846212 | 5 | 1 | 0 | 240 | 1867188 | 5 | 1 | 0 | 1 | 248 |
| 52 | 34 | 10 | 0A | 5 | 2 | 230807 | 1846452 | 5 | 2 | 0 | 240 | 1867436 | 5 | 2 | 0 | 1 | 248 |
| 53 | 35 | 10 | 0A | 5 | 3 | 230837 | 1846692 | 5 | 3 | 0 | 240 | 1867684 | 5 | 3 | 0 | 1 | 248 |
| 54 | 36 | 10 | 0A | 5 | 4 | 230867 | 1846932 | 5 | 4 | 0 | 240 | 1867932 | 5 | 4 | 0 | 1 | 248 |
| 55 | 37 | 10 | 0A | 5 | 5 | 230897 | 1847172 | 5 | 5 | 0 | 240 | 1868180 | 5 | 5 | 0 | 1 | 248 |
| 56 | 38 | 10 | 0A | 5 | 6 | 230927 | 1847412 | 5 | 6 | 0 | 240 | 1868428 | 5 | 6 | 0 | 1 | 248 |
| 57 | 39 | 10 | 0A | 5 | 7 | 230957 | 1847652 | 5 | 7 | 0 | 240 | 1868676 | 5 | 7 | 0 | 1 | 248 |
| 58 | 3A | 10 | 0A | 5 | 8 | 230987 | 1847892 | 5 | 8 | 0 | 240 | 1868924 | 5 | 8 | 0 | 1 | 248 |
| 59 | 3B | 10 | 0A | 5 | 9 | 231017 | 1848132 | 5 | 9 | 0 | 240 | 1869172 | 5 | 9 | 0 | 1 | 248 |
| 60 | 3C | 10 | 0A | 6 | 0 | 231049 | 1848388 | 6 | 0 | 0 | 256 | 1869436 | 6 | 0 | 0 | 1 | 264 |
| 61 | 3D | 10 | 0A | 6 | 1 | 231081 | 1848644 | 6 | 1 | 0 | 256 | 1869700 | 6 | 1 | 0 | 1 | 264 |
| 62 | 3E | 10 | 0A | 6 | 2 | 231113 | 1848900 | 6 | 2 | 0 | 256 | 1869964 | 6 | 2 | 0 | 1 | 264 |
| 63 | 3F | 10 | 0A | 6 | 3 | 231145 | 1849156 | 6 | 3 | 0 | 256 | 1870228 | 6 | 3 | 0 | 1 | 264 |
| 64 | 40 | 10 | 0A | 6 | 4 | 231177 | 1849412 | 6 | 4 | 0 | 256 | 1870492 | 6 | 4 | 0 | 1 | 264 |
| 65 | 41 | 10 | 0A | 6 | 5 | 231209 | 1849668 | 6 | 5 | 0 | 256 | 1870756 | 6 | 5 | 0 | 1 | 264 |
| 66 | 42 | 10 | 0A | 6 | 6 | 231241 | 1849924 | 6 | 6 | 0 | 256 | 1871020 | 6 | 6 | 0 | 1 | 264 |
| 67 | 43 | 10 | 0A | 6 | 7 | 231273 | 1850180 | 6 | 7 | 0 | 256 | 1871284 | 6 | 7 | 0 | 1 | 264 |
| 68 | 44 | 10 | 0A | 6 | 8 | 231305 | 1850436 | 6 | 8 | 0 | 256 | 1871548 | 6 | 8 | 0 | 1 | 264 |
| 69 | 45 | 10 | 0A | 6 | 9 | 231337 | 1850692 | 6 | 9 | 0 | 256 | 1871812 | 6 | 9 | 0 | 1 | 264 |
| 70 | 46 | 10 | 0A | 7 | 0 | 231371 | 1850964 | 7 | 0 | 0 | 272 | 1872092 | 7 | 0 | 0 | 1 | 280 |
| 71 | 47 | 10 | 0A | 7 | 1 | 231405 | 1851236 | 7 | 1 | 0 | 272 | 1872372 | 7 | 1 | 0 | 1 | 280 |
| 72 | 48 | 10 | 0A | 7 | 2 | 231439 | 1851508 | 7 | 2 | 0 | 272 | 1872652 | 7 | 2 | 0 | 1 | 280 |
| 73 | 49 | 10 | 0A | 7 | 3 | 231473 | 1851780 | 7 | 3 | 0 | 272 | 1872932 | 7 | 3 | 0 | 1 | 280 |
| 74 | 4A | 10 | 0A | 7 | 4 | 231507 | 1852052 | 7 | 4 | 0 | 272 | 1873212 | 7 | 4 | 0 | 1 | 280 |
| 75 | 4B | 10 | 0A | 7 | 5 | 231541 | 1852324 | 7 | 5 | 0 | 272 | 1873492 | 7 | 5 | 0 | 1 | 280 |
| 76 | 4C | 10 | 0A | 7 | 6 | 231575 | 1852596 | 7 | 6 | 0 | 272 | 1873772 | 7 | 6 | 0 | 1 | 280 |
| 77 | 4D | 10 | 0A | 7 | 7 | 231609 | 1852868 | 7 | 7 | 0 | 272 | 1874052 | 7 | 7 | 0 | 1 | 280 |
| 78 | 4E | 10 | 0A | 7 | 8 | 231643 | 1853140 | 7 | 8 | 0 | 272 | 1874332 | 7 | 8 | 0 | 1 | 280 |
| 79 | 4F | 10 | 0A | 7 | 9 | 231677 | 1853412 | 7 | 9 | 0 | 272 | 1874612 | 7 | 9 | 0 | 1 | 280 |
| 80 | 50 | 10 | 0A | 8 | 0 | 231713 | 1853700 | 8 | 0 | 0 | 288 | 1874908 | 8 | 0 | 0 | 1 | 296 |
| 81 | 51 | 10 | 0A | 8 | 1 | 231749 | 1853988 | 8 | 1 | 0 | 288 | 1875204 | 8 | 1 | 0 | 1 | 296 |
| 82 | 52 | 10 | 0A | 8 | 2 | 231785 | 1854276 | 8 | 2 | 0 | 288 | 1875500 | 8 | 2 | 0 | 1 | 296 |
| 83 | 53 | 10 | 0A | 8 | 3 | 231821 | 1854564 | 8 | 3 | 0 | 288 | 1875796 | 8 | 3 | 0 | 1 | 296 |
| 84 | 54 | 10 | 0A | 8 | 4 | 231857 | 1854852 | 8 | 4 | 0 | 288 | 1876092 | 8 | 4 | 0 | 1 | 296 |
| 85 | 55 | 10 | 0A | 8 | 5 | 231893 | 1855140 | 8 | 5 | 0 | 288 | 1876388 | 8 | 5 | 0 | 1 | 296 |
| 86 | 56 | 10 | 0A | 8 | 6 | 231929 | 1855428 | 8 | 6 | 0 | 288 | 1876684 | 8 | 6 | 0 | 1 | 296 |
| 87 | 57 | 10 | 0A | 8 | 7 | 231965 | 1855716 | 8 | 7 | 0 | 288 | 1876980 | 8 | 7 | 0 | 1 | 296 |
| 88 | 58 | 10 | 0A | 8 | 8 | 232001 | 1856004 | 8 | 8 | 0 | 288 | 1877276 | 8 | 8 | 0 | 1 | 296 |
| 89 | 59 | 10 | 0A | 8 | 9 | 232037 | 1856292 | 8 | 9 | 0 | 288 | 1877572 | 8 | 9 | 0 | 1 | 296 |
| 90 | 5A | 10 | 0A | 9 | 0 | 232075 | 1856596 | 9 | 0 | 0 | 304 | 1877884 | 9 | 0 | 0 | 1 | 312 |
| 91 | 5B | 10 | 0A | 9 | 1 | 232113 | 1856900 | 9 | 1 | 0 | 304 | 1878196 | 9 | 1 | 0 | 1 | 312 |
| 92 | 5C | 10 | 0A | 9 | 2 | 232151 | 1857204 | 9 | 2 | 0 | 304 | 1878508 | 9 | 2 | 0 | 1 | 312 |
| 93 | 5D | 10 | 0A | 9 | 3 | 232189 | 1857508 | 9 | 3 | 0 | 304 | 1878820 | 9 | 3 | 0 | 1 | 312 |
| 94 | 5E | 10 | 0A | 9 | 4 | 232227 | 1857812 | 9 | 4 | 0 | 304 | 1879132 | 9 | 4 | 0 | 1 | 312 |
| 95 | 5F | 10 | 0A | 9 | 5 | 232265 | 1858116 | 9 | 5 | 0 | 304 | 1879444 | 9 | 5 | 0 | 1 | 312 |
| 96 | 60 | 10 | 0A | 9 | 6 | 232303 | 1858420 | 9 | 6 | 0 | 304 | 1879756 | 9 | 6 | 0 | 1 | 312 |
| 97 | 61 | 10 | 0A | 9 | 7 | 232341 | 1858724 | 9 | 7 | 0 | 304 | 1880068 | 9 | 7 | 0 | 1 | 312 |
| 98 | 62 | 10 | 0A | 9 | 8 | 232379 | 1859028 | 9 | 8 | 0 | 304 | 1880380 | 9 | 8 | 0 | 1 | 312 |
| 99 | 63 | 10 | 0A | 9 | 9 | 232417 | 1859332 | 9 | 9 | 0 | 304 | 1880692 | 9 | 9 | 0 | 1 | 312 |
| 100 | 64 | 10 | 0A | 10 | 0 | 232457 | 1859652 | 10 | 0 | 0 | 320 | 1881020 | 10 | 0 | 0 | 1 | 328 |
| 101 | 65 | 10 | 0A | 10 | 1 | 232497 | 1859972 | 10 | 1 | 0 | 320 | 1881348 | 10 | 1 | 0 | 1 | 328 |
| 102 | 66 | 10 | 0A | 10 | 2 | 232537 | 1860292 | 10 | 2 | 0 | 320 | 1881676 | 10 | 2 | 0 | 1 | 328 |
| 103 | 67 | 10 | 0A | 10 | 3 | 232577 | 1860612 | 10 | 3 | 0 | 320 | 1882004 | 10 | 3 | 0 | 1 | 328 |
| 104 | 68 | 10 | 0A | 10 | 4 | 232617 | 1860932 | 10 | 4 | 0 | 320 | 1882332 | 10 | 4 | 0 | 1 | 328 |
| 105 | 69 | 10 | 0A | 10 | 5 | 232657 | 1861252 | 10 | 5 | 0 | 320 | 1882660 | 10 | 5 | 0 | 1 | 328 |
| 106 | 6A | 10 | 0A | 10 | 6 | 232697 | 1861572 | 10 | 6 | 0 | 320 | 1882988 | 10 | 6 | 0 | 1 | 328 |
| 107 | 6B | 10 | 0A | 10 | 7 | 232737 | 1861892 | 10 | 7 | 0 | 320 | 1883316 | 10 | 7 | 0 | 1 | 328 |
| 108 | 6C | 10 | 0A | 10 | 8 | 232777 | 1862212 | 10 | 8 | 0 | 320 | 1883644 | 10 | 8 | 0 | 1 | 328 |
| 109 | 6D | 10 | 0A | 10 | 9 | 232817 | 1862532 | 10 | 9 | 0 | 320 | 1883972 | 10 | 9 | 0 | 1 | 328 |
| 110 | 6E | 10 | 0A | 11 | 0 | 232859 | 1862868 | 11 | 0 | 0 | 336 | 1884316 | 11 | 0 | 0 | 1 | 344 |
| 111 | 6F | 10 | 0A | 11 | 1 | 232901 | 1863204 | 11 | 1 | 0 | 336 | 1884660 | 11 | 1 | 0 | 1 | 344 |
| 112 | 70 | 10 | 0A | 11 | 2 | 232943 | 1863540 | 11 | 2 | 0 | 336 | 1885004 | 11 | 2 | 0 | 1 | 344 |
| 113 | 71 | 10 | 0A | 11 | 3 | 232985 | 1863876 | 11 | 3 | 0 | 336 | 1885348 | 11 | 3 | 0 | 1 | 344 |
| 114 | 72 | 10 | 0A | 11 | 4 | 233027 | 1864212 | 11 | 4 | 0 | 336 | 1885692 | 11 | 4 | 0 | 1 | 344 |

| | | | | | | | | | | | | | | | | | |
|---|---|---|---|---|---|---|---|---|---|---|---|---|---|---|---|---|---|
| 115 | 73 | 10 | 0A | 11 | 5 | 233069 | 1864548 | 11 | 5 | 0 | 336 | 1886036 | 11 | 5 | 0 | 1 | 344 |
| 116 | 74 | 10 | 0A | 11 | 6 | 233111 | 1864884 | 11 | 6 | 0 | 336 | 1886380 | 11 | 6 | 0 | 1 | 344 |
| 117 | 75 | 10 | 0A | 11 | 7 | 233153 | 1865220 | 11 | 7 | 0 | 336 | 1886724 | 11 | 7 | 0 | 1 | 344 |
| 118 | 76 | 10 | 0A | 11 | 8 | 233195 | 1865556 | 11 | 8 | 0 | 336 | 1887068 | 11 | 8 | 0 | 1 | 344 |
| 119 | 77 | 10 | 0A | 11 | 9 | 233237 | 1865892 | 11 | 9 | 0 | 336 | 1887412 | 11 | 9 | 0 | 1 | 344 |
| 120 | 78 | 10 | 0A | 12 | 0 | 233281 | 1866244 | 12 | 0 | 0 | 352 | 1887772 | 12 | 0 | 0 | 1 | 360 |
| 121 | 79 | 10 | 0A | 12 | 1 | 233325 | 1866596 | 12 | 1 | 0 | 352 | 1888132 | 12 | 1 | 0 | 1 | 360 |
| 122 | 7A | 10 | 0A | 12 | 2 | 233369 | 1866948 | 12 | 2 | 0 | 352 | 1888492 | 12 | 2 | 0 | 1 | 360 |
| 123 | 7B | 10 | 0A | 12 | 3 | 233413 | 1867300 | 12 | 3 | 0 | 352 | 1888852 | 12 | 3 | 0 | 1 | 360 |
| 124 | 7C | 10 | 0A | 12 | 4 | 233457 | 1867652 | 12 | 4 | 0 | 352 | 1889212 | 12 | 4 | 0 | 1 | 360 |
| 125 | 7D | 10 | 0A | 12 | 5 | 233501 | 1868004 | 12 | 5 | 0 | 352 | 1889572 | 12 | 5 | 0 | 1 | 360 |
| 126 | 7E | 10 | 0A | 12 | 6 | 233545 | 1868356 | 12 | 6 | 0 | 352 | 1889932 | 12 | 6 | 0 | 1 | 360 |
| 127 | 7F | 10 | 0A | 12 | 7 | 233589 | 1868708 | 12 | 7 | 0 | 352 | 1890292 | 12 | 7 | 0 | 1 | 360 |
| 128 | 80 | 10 | 0A | 12 | 8 | 233633 | 1869060 | 12 | 8 | 0 | 352 | 1890652 | 12 | 8 | 0 | 1 | 360 |
| 129 | 81 | 10 | 0A | 12 | 9 | 233677 | 1869412 | 12 | 9 | 0 | 352 | 1891012 | 12 | 9 | 0 | 1 | 360 |
| 130 | 82 | 10 | 0A | 13 | 0 | 233723 | 1869780 | 13 | 0 | 0 | 368 | 1891388 | 13 | 0 | 0 | 1 | 376 |
| 131 | 83 | 10 | 0A | 13 | 1 | 233769 | 1870148 | 13 | 1 | 0 | 368 | 1891764 | 13 | 1 | 0 | 1 | 376 |
| 132 | 84 | 10 | 0A | 13 | 2 | 233815 | 1870516 | 13 | 2 | 0 | 368 | 1892140 | 13 | 2 | 0 | 1 | 376 |
| 133 | 85 | 10 | 0A | 13 | 3 | 233861 | 1870884 | 13 | 3 | 0 | 368 | 1892516 | 13 | 3 | 0 | 1 | 376 |
| 134 | 86 | 10 | 0A | 13 | 4 | 233907 | 1871252 | 13 | 4 | 0 | 368 | 1892892 | 13 | 4 | 0 | 1 | 376 |
| 135 | 87 | 10 | 0A | 13 | 5 | 233953 | 1871620 | 13 | 5 | 0 | 368 | 1893268 | 13 | 5 | 0 | 1 | 376 |
| 136 | 88 | 10 | 0A | 13 | 6 | 233999 | 1871988 | 13 | 6 | 0 | 368 | 1893644 | 13 | 6 | 0 | 1 | 376 |
| 137 | 89 | 10 | 0A | 13 | 7 | 234045 | 1872356 | 13 | 7 | 0 | 368 | 1894020 | 13 | 7 | 0 | 1 | 376 |
| 138 | 8A | 10 | 0A | 13 | 8 | 234091 | 1872724 | 13 | 8 | 0 | 368 | 1894396 | 13 | 8 | 0 | 1 | 376 |
| 139 | 8B | 10 | 0A | 13 | 9 | 234137 | 1873092 | 13 | 9 | 0 | 368 | 1894772 | 13 | 9 | 0 | 1 | 376 |
| 140 | 8C | 10 | 0A | 14 | 0 | 234185 | 1873476 | 14 | 0 | 0 | 384 | 1895164 | 14 | 0 | 0 | 1 | 392 |
| 141 | 8D | 10 | 0A | 14 | 1 | 234233 | 1873860 | 14 | 1 | 0 | 384 | 1895556 | 14 | 1 | 0 | 1 | 392 |
| 142 | 8E | 10 | 0A | 14 | 2 | 234281 | 1874244 | 14 | 2 | 0 | 384 | 1895948 | 14 | 2 | 0 | 1 | 392 |
| 143 | 8F | 10 | 0A | 14 | 3 | 234329 | 1874628 | 14 | 3 | 0 | 384 | 1896340 | 14 | 3 | 0 | 1 | 392 |
| 144 | 90 | 10 | 0A | 14 | 4 | 234377 | 1875012 | 14 | 4 | 0 | 384 | 1896732 | 14 | 4 | 0 | 1 | 392 |
| 145 | 91 | 10 | 0A | 14 | 5 | 234425 | 1875396 | 14 | 5 | 0 | 384 | 1897124 | 14 | 5 | 0 | 1 | 392 |
| 146 | 92 | 10 | 0A | 14 | 6 | 234473 | 1875780 | 14 | 6 | 0 | 384 | 1897516 | 14 | 6 | 0 | 1 | 392 |
| 147 | 93 | 10 | 0A | 14 | 7 | 234521 | 1876164 | 14 | 7 | 0 | 384 | 1897908 | 14 | 7 | 0 | 1 | 392 |
| 148 | 94 | 10 | 0A | 14 | 8 | 234569 | 1876548 | 14 | 8 | 0 | 384 | 1898300 | 14 | 8 | 0 | 1 | 392 |
| 149 | 95 | 10 | 0A | 14 | 9 | 234617 | 1876932 | 14 | 9 | 0 | 384 | 1898692 | 14 | 9 | 0 | 1 | 392 |
| 150 | 96 | 10 | 0A | 15 | 0 | 234667 | 1877332 | 15 | 0 | 0 | 400 | 1899100 | 15 | 0 | 0 | 1 | 408 |
| 151 | 97 | 10 | 0A | 15 | 1 | 234717 | 1877732 | 15 | 1 | 0 | 400 | 1899508 | 15 | 1 | 0 | 1 | 408 |
| 152 | 98 | 10 | 0A | 15 | 2 | 234767 | 1878132 | 15 | 2 | 0 | 400 | 1899916 | 15 | 2 | 0 | 1 | 408 |
| 153 | 99 | 10 | 0A | 15 | 3 | 234817 | 1878532 | 15 | 3 | 0 | 400 | 1900324 | 15 | 3 | 0 | 1 | 408 |
| 154 | 9A | 10 | 0A | 15 | 4 | 234867 | 1878932 | 15 | 4 | 0 | 400 | 1900732 | 15 | 4 | 0 | 1 | 408 |
| 155 | 9B | 10 | 0A | 15 | 5 | 234917 | 1879332 | 15 | 5 | 0 | 400 | 1901140 | 15 | 5 | 0 | 1 | 408 |
| 156 | 9C | 10 | 0A | 15 | 6 | 234967 | 1879732 | 15 | 6 | 0 | 400 | 1901548 | 15 | 6 | 0 | 1 | 408 |
| 157 | 9D | 10 | 0A | 15 | 7 | 235017 | 1880132 | 15 | 7 | 0 | 400 | 1901956 | 15 | 7 | 0 | 1 | 408 |
| 158 | 9E | 10 | 0A | 15 | 8 | 235067 | 1880532 | 15 | 8 | 0 | 400 | 1902364 | 15 | 8 | 0 | 1 | 408 |
| 159 | 9F | 10 | 0A | 15 | 9 | 235117 | 1880932 | 15 | 9 | 0 | 400 | 1902772 | 15 | 9 | 0 | 1 | 408 |
| 160 | A0 | 10 | 0A | 16 | 0 | 235169 | 1881348 | 16 | 0 | 0 | 416 | 1903196 | 16 | 0 | 0 | 1 | 424 |
| 161 | A1 | 10 | 0A | 16 | 1 | 235221 | 1881764 | 16 | 1 | 0 | 416 | 1903620 | 16 | 1 | 0 | 1 | 424 |
| 162 | A2 | 10 | 0A | 16 | 2 | 235273 | 1882180 | 16 | 2 | 0 | 416 | 1904044 | 16 | 2 | 0 | 1 | 424 |
| 163 | A3 | 10 | 0A | 16 | 3 | 235325 | 1882596 | 16 | 3 | 0 | 416 | 1904468 | 16 | 3 | 0 | 1 | 424 |
| 164 | A4 | 10 | 0A | 16 | 4 | 235377 | 1883012 | 16 | 4 | 0 | 416 | 1904892 | 16 | 4 | 0 | 1 | 424 |
| 165 | A5 | 10 | 0A | 16 | 5 | 235429 | 1883428 | 16 | 5 | 0 | 416 | 1905316 | 16 | 5 | 0 | 1 | 424 |
| 166 | A6 | 10 | 0A | 16 | 6 | 235481 | 1883844 | 16 | 6 | 0 | 416 | 1905740 | 16 | 6 | 0 | 1 | 424 |
| 167 | A7 | 10 | 0A | 16 | 7 | 235533 | 1884260 | 16 | 7 | 0 | 416 | 1906164 | 16 | 7 | 0 | 1 | 424 |
| 168 | A8 | 10 | 0A | 16 | 8 | 235585 | 1884676 | 16 | 8 | 0 | 416 | 1906588 | 16 | 8 | 0 | 1 | 424 |
| 169 | A9 | 10 | 0A | 16 | 9 | 235637 | 1885092 | 16 | 9 | 0 | 416 | 1907012 | 16 | 9 | 0 | 1 | 424 |
| 170 | AA | 10 | 0A | 17 | 0 | 235691 | 1885524 | 17 | 0 | 0 | 432 | 1907452 | 17 | 0 | 0 | 1 | 440 |
| 171 | AB | 10 | 0A | 17 | 1 | 235745 | 1885956 | 17 | 1 | 0 | 432 | 1907892 | 17 | 1 | 0 | 1 | 440 |
| 172 | AC | 10 | 0A | 17 | 2 | 235799 | 1886388 | 17 | 2 | 0 | 432 | 1908332 | 17 | 2 | 0 | 1 | 440 |
| 173 | AD | 10 | 0A | 17 | 3 | 235853 | 1886820 | 17 | 3 | 0 | 432 | 1908772 | 17 | 3 | 0 | 1 | 440 |
| 174 | AE | 10 | 0A | 17 | 4 | 235907 | 1887252 | 17 | 4 | 0 | 432 | 1909212 | 17 | 4 | 0 | 1 | 440 |
| 175 | AF | 10 | 0A | 17 | 5 | 235961 | 1887684 | 17 | 5 | 0 | 432 | 1909652 | 17 | 5 | 0 | 1 | 440 |
| 176 | B0 | 10 | 0A | 17 | 6 | 236015 | 1888116 | 17 | 6 | 0 | 432 | 1910092 | 17 | 6 | 0 | 1 | 440 |
| 177 | B1 | 10 | 0A | 17 | 7 | 236069 | 1888548 | 17 | 7 | 0 | 432 | 1910532 | 17 | 7 | 0 | 1 | 440 |
| 178 | B2 | 10 | 0A | 17 | 8 | 236123 | 1888980 | 17 | 8 | 0 | 432 | 1910972 | 17 | 8 | 0 | 1 | 440 |
| 179 | B3 | 10 | 0A | 17 | 9 | 236177 | 1889412 | 17 | 9 | 0 | 432 | 1911412 | 17 | 9 | 0 | 1 | 440 |
| 180 | B4 | 10 | 0A | 18 | 0 | 236233 | 1889860 | 18 | 0 | 0 | 448 | 1911868 | 18 | 0 | 0 | 1 | 456 |
| 181 | B5 | 10 | 0A | 18 | 1 | 236289 | 1890308 | 18 | 1 | 0 | 448 | 1912324 | 18 | 1 | 0 | 1 | 456 |
| 182 | B6 | 10 | 0A | 18 | 2 | 236345 | 1890756 | 18 | 2 | 0 | 448 | 1912780 | 18 | 2 | 0 | 1 | 456 |
| 183 | B7 | 10 | 0A | 18 | 3 | 236401 | 1891204 | 18 | 3 | 0 | 448 | 1913236 | 18 | 3 | 0 | 1 | 456 |
| 184 | B8 | 10 | 0A | 18 | 4 | 236457 | 1891652 | 18 | 4 | 0 | 448 | 1913692 | 18 | 4 | 0 | 1 | 456 |
| 185 | B9 | 10 | 0A | 18 | 5 | 236513 | 1892100 | 18 | 5 | 0 | 448 | 1914148 | 18 | 5 | 0 | 1 | 456 |
| 186 | BA | 10 | 0A | 18 | 6 | 236569 | 1892548 | 18 | 6 | 0 | 448 | 1914604 | 18 | 6 | 0 | 1 | 456 |
| 187 | BB | 10 | 0A | 18 | 7 | 236625 | 1892996 | 18 | 7 | 0 | 448 | 1915060 | 18 | 7 | 0 | 1 | 456 |
| 188 | BC | 10 | 0A | 18 | 8 | 236681 | 1893444 | 18 | 8 | 0 | 448 | 1915516 | 18 | 8 | 0 | 1 | 456 |
| 189 | BD | 10 | 0A | 18 | 9 | 236737 | 1893892 | 18 | 9 | 0 | 448 | 1915972 | 18 | 9 | 0 | 1 | 456 |
| 190 | BE | 10 | 0A | 19 | 0 | 236795 | 1894356 | 19 | 0 | 0 | 464 | 1916444 | 19 | 0 | 0 | 1 | 472 |
| 191 | BF | 10 | 0A | 19 | 1 | 236853 | 1894820 | 19 | 1 | 0 | 464 | 1916916 | 19 | 1 | 0 | 1 | 472 |
| 192 | C0 | 10 | 0A | 19 | 2 | 236911 | 1895284 | 19 | 2 | 0 | 464 | 1917388 | 19 | 2 | 0 | 1 | 472 |
| 193 | C1 | 10 | 0A | 19 | 3 | 236969 | 1895748 | 19 | 3 | 0 | 464 | 1917860 | 19 | 3 | 0 | 1 | 472 |
| 194 | C2 | 10 | 0A | 19 | 4 | 237027 | 1896212 | 19 | 4 | 0 | 464 | 1918332 | 19 | 4 | 0 | 1 | 472 |
| 195 | C3 | 10 | 0A | 19 | 5 | 237085 | 1896676 | 19 | 5 | 0 | 464 | 1918804 | 19 | 5 | 0 | 1 | 472 |
| 196 | C4 | 10 | 0A | 19 | 6 | 237143 | 1897140 | 19 | 6 | 0 | 464 | 1919276 | 19 | 6 | 0 | 1 | 472 |
| 197 | C5 | 10 | 0A | 19 | 7 | 237201 | 1897604 | 19 | 7 | 0 | 464 | 1919748 | 19 | 7 | 0 | 1 | 472 |
| 198 | C6 | 10 | 0A | 19 | 8 | 237259 | 1898068 | 19 | 8 | 0 | 464 | 1920220 | 19 | 8 | 0 | 1 | 472 |
| 199 | C7 | 10 | 0A | 19 | 9 | 237317 | 1898532 | 19 | 9 | 0 | 464 | 1920692 | 19 | 9 | 0 | 1 | 472 |
| 200 | C8 | 10 | 0A | 20 | 0 | 237377 | 1899012 | 20 | 0 | 0 | 480 | 1921180 | 20 | 0 | 0 | 1 | 488 |
| 201 | C9 | 10 | 0A | 20 | 1 | 237437 | 1899492 | 20 | 1 | 0 | 480 | 1921668 | 20 | 1 | 0 | 1 | 488 |
| 202 | CA | 10 | 0A | 20 | 2 | 237497 | 1899972 | 20 | 2 | 0 | 480 | 1922156 | 20 | 2 | 0 | 1 | 488 |
| 203 | CB | 10 | 0A | 20 | 3 | 237557 | 1900452 | 20 | 3 | 0 | 480 | 1922644 | 20 | 3 | 0 | 1 | 488 |
| 204 | CC | 10 | 0A | 20 | 4 | 237617 | 1900932 | 20 | 4 | 0 | 480 | 1923132 | 20 | 4 | 0 | 1 | 488 |
| 205 | CD | 10 | 0A | 20 | 5 | 237677 | 1901412 | 20 | 5 | 0 | 480 | 1923620 | 20 | 5 | 0 | 1 | 488 |
| 206 | CE | 10 | 0A | 20 | 6 | 237737 | 1901892 | 20 | 6 | 0 | 480 | 1924108 | 20 | 6 | 0 | 1 | 488 |
| 207 | CF | 10 | 0A | 20 | 7 | 237797 | 1902372 | 20 | 7 | 0 | 480 | 1924596 | 20 | 7 | 0 | 1 | 488 |
| 208 | D0 | 10 | 0A | 20 | 8 | 237857 | 1902852 | 20 | 8 | 0 | 480 | 1925084 | 20 | 8 | 0 | 1 | 488 |
| 209 | D1 | 10 | 0A | 20 | 9 | 237917 | 1903332 | 20 | 9 | 0 | 480 | 1925572 | 20 | 9 | 0 | 1 | 488 |
| 210 | D2 | 10 | 0A | 21 | 0 | 237979 | 1903828 | 21 | 0 | 0 | 496 | 1926076 | 21 | 0 | 0 | 1 | 504 |
| 211 | D3 | 10 | 0A | 21 | 1 | 238041 | 1904324 | 21 | 1 | 0 | 496 | 1926580 | 21 | 1 | 0 | 1 | 504 |
| 212 | D4 | 10 | 0A | 21 | 2 | 238103 | 1904820 | 21 | 2 | 0 | 496 | 1927084 | 21 | 2 | 0 | 1 | 504 |
| 213 | D5 | 10 | 0A | 21 | 3 | 238165 | 1905316 | 21 | 3 | 0 | 496 | 1927588 | 21 | 3 | 0 | 1 | 504 |
| 214 | D6 | 10 | 0A | 21 | 4 | 238227 | 1905812 | 21 | 4 | 0 | 496 | 1928092 | 21 | 4 | 0 | 1 | 504 |
| 215 | D7 | 10 | 0A | 21 | 5 | 238289 | 1906308 | 21 | 5 | 0 | 496 | 1928596 | 21 | 5 | 0 | 1 | 504 |
| 216 | D8 | 10 | 0A | 21 | 6 | 238351 | 1906804 | 21 | 6 | 0 | 496 | 1929100 | 21 | 6 | 0 | 1 | 504 |
| 217 | D9 | 10 | 0A | 21 | 7 | 238413 | 1907300 | 21 | 7 | 0 | 496 | 1929604 | 21 | 7 | 0 | 1 | 504 |

| | | | | | | | | | | | | | | | | | |
|---|---|---|---|---|---|---|---|---|---|---|---|---|---|---|---|---|---|
| 218 | DA | 10 | 0A | 21 | 8 | 238475 | 1907796 | 21 | 8 | 0 | 496 | 1930108 | 21 | 8 | 0 | 1 | 504 |
| 219 | DB | 10 | 0A | 21 | 9 | 238537 | 1908292 | 21 | 9 | 0 | 496 | 1930612 | 21 | 9 | 0 | 1 | 504 |
| 220 | DC | 10 | 0A | 22 | 0 | 238601 | 1908804 | 22 | 0 | 0 | 512 | 1931132 | 22 | 0 | 0 | 1 | 520 |
| 221 | DD | 10 | 0A | 22 | 1 | 238665 | 1909316 | 22 | 1 | 0 | 512 | 1931652 | 22 | 1 | 0 | 1 | 520 |
| 222 | DE | 10 | 0A | 22 | 2 | 238729 | 1909828 | 22 | 2 | 0 | 512 | 1932172 | 22 | 2 | 0 | 1 | 520 |
| 223 | DF | 10 | 0A | 22 | 3 | 238793 | 1910340 | 22 | 3 | 0 | 512 | 1932692 | 22 | 3 | 0 | 1 | 520 |
| 224 | E0 | 10 | 0A | 22 | 4 | 238857 | 1910852 | 22 | 4 | 0 | 512 | 1933212 | 22 | 4 | 0 | 1 | 520 |
| 225 | E1 | 10 | 0A | 22 | 5 | 238921 | 1911364 | 22 | 5 | 0 | 512 | 1933732 | 22 | 5 | 0 | 1 | 520 |
| 226 | E2 | 10 | 0A | 22 | 6 | 238985 | 1911876 | 22 | 6 | 0 | 512 | 1934252 | 22 | 6 | 0 | 1 | 520 |
| 227 | E3 | 10 | 0A | 22 | 7 | 239049 | 1912388 | 22 | 7 | 0 | 512 | 1934772 | 22 | 7 | 0 | 1 | 520 |
| 228 | E4 | 10 | 0A | 22 | 8 | 239113 | 1912900 | 22 | 8 | 0 | 512 | 1935292 | 22 | 8 | 0 | 1 | 520 |
| 229 | E5 | 10 | 0A | 22 | 9 | 239177 | 1913412 | 22 | 9 | 0 | 512 | 1935812 | 22 | 9 | 0 | 1 | 520 |
| 230 | E6 | 10 | 0A | 23 | 0 | 239243 | 1913940 | 23 | 0 | 0 | 528 | 1936348 | 23 | 0 | 0 | 1 | 536 |
| 231 | E7 | 10 | 0A | 23 | 1 | 239309 | 1914468 | 23 | 1 | 0 | 528 | 1936884 | 23 | 1 | 0 | 1 | 536 |
| 232 | E8 | 10 | 0A | 23 | 2 | 239375 | 1914996 | 23 | 2 | 0 | 528 | 1937420 | 23 | 2 | 0 | 1 | 536 |
| 233 | E9 | 10 | 0A | 23 | 3 | 239441 | 1915524 | 23 | 3 | 0 | 528 | 1937956 | 23 | 3 | 0 | 1 | 536 |
| 234 | EA | 10 | 0A | 23 | 4 | 239507 | 1916052 | 23 | 4 | 0 | 528 | 1938492 | 23 | 4 | 0 | 1 | 536 |
| 235 | EB | 10 | 0A | 23 | 5 | 239573 | 1916580 | 23 | 5 | 0 | 528 | 1939028 | 23 | 5 | 0 | 1 | 536 |
| 236 | EC | 10 | 0A | 23 | 6 | 239639 | 1917108 | 23 | 6 | 0 | 528 | 1939564 | 23 | 6 | 0 | 1 | 536 |
| 237 | ED | 10 | 0A | 23 | 7 | 239705 | 1917636 | 23 | 7 | 0 | 528 | 1940100 | 23 | 7 | 0 | 1 | 536 |
| 238 | EE | 10 | 0A | 23 | 8 | 239771 | 1918164 | 23 | 8 | 0 | 528 | 1940636 | 23 | 8 | 0 | 1 | 536 |
| 239 | EF | 10 | 0A | 23 | 9 | 239837 | 1918692 | 23 | 9 | 0 | 528 | 1941172 | 23 | 9 | 0 | 1 | 536 |
| 240 | F0 | 10 | 0A | 24 | 0 | 239905 | 1919236 | 24 | 0 | 0 | 544 | 1941724 | 24 | 0 | 0 | 1 | 552 |
| 241 | F1 | 10 | 0A | 24 | 1 | 239973 | 1919780 | 24 | 1 | 0 | 544 | 1942276 | 24 | 1 | 0 | 1 | 552 |
| 242 | F2 | 10 | 0A | 24 | 2 | 240041 | 1920324 | 24 | 2 | 0 | 544 | 1942828 | 24 | 2 | 0 | 1 | 552 |
| 243 | F3 | 10 | 0A | 24 | 3 | 240109 | 1920868 | 24 | 3 | 0 | 544 | 1943380 | 24 | 3 | 0 | 1 | 552 |
| 244 | F4 | 10 | 0A | 24 | 4 | 240177 | 1921412 | 24 | 4 | 0 | 544 | 1943932 | 24 | 4 | 0 | 1 | 552 |
| 245 | F5 | 10 | 0A | 24 | 5 | 240245 | 1921956 | 24 | 5 | 0 | 544 | 1944484 | 24 | 5 | 0 | 1 | 552 |
| 246 | F6 | 10 | 0A | 24 | 6 | 240313 | 1922500 | 24 | 6 | 0 | 544 | 1945036 | 24 | 6 | 0 | 1 | 552 |
| 247 | F7 | 10 | 0A | 24 | 7 | 240381 | 1923044 | 24 | 7 | 0 | 544 | 1945588 | 24 | 7 | 0 | 1 | 552 |
| 248 | F8 | 10 | 0A | 24 | 8 | 240449 | 1923588 | 24 | 8 | 0 | 544 | 1946140 | 24 | 8 | 0 | 1 | 552 |
| 249 | F9 | 10 | 0A | 24 | 9 | 240517 | 1924132 | 24 | 9 | 0 | 544 | 1946692 | 24 | 9 | 0 | 1 | 552 |
| 250 | FA | 10 | 0A | 25 | 0 | 240587 | 1924692 | 25 | 0 | 0 | 560 | 1947260 | 25 | 0 | 0 | 1 | 568 |
| 251 | FB | 10 | 0A | 25 | 1 | 240657 | 1925252 | 25 | 1 | 0 | 560 | 1947828 | 25 | 1 | 0 | 1 | 568 |
| 252 | FC | 10 | 0A | 25 | 2 | 240727 | 1925812 | 25 | 2 | 0 | 560 | 1948396 | 25 | 2 | 0 | 1 | 568 |
| 253 | FD | 10 | 0A | 25 | 3 | 240797 | 1926372 | 25 | 3 | 0 | 560 | 1948964 | 25 | 3 | 0 | 1 | 568 |
| 254 | FE | 10 | 0A | 25 | 4 | 240867 | 1926932 | 25 | 4 | 0 | 560 | 1949532 | 25 | 4 | 0 | 1 | 568 |
| 255 | FF | 10 | 0A | 25 | 5 | 240937 | 1927492 | 25 | 5 | 0 | 560 | 1950100 | 25 | 5 | 0 | 1 | 568 |
| 0 | 00 | 11 | 0B | 0 | 0 | 240949 | 1927588 | 0 | 0 | 0 | 96 | 1950204 | 0 | 0 | 0 | 1 | 104 |
| 1 | 01 | 11 | 0B | 0 | 1 | 240963 | 1927700 | 0 | 1 | 0 | 112 | 1950324 | 0 | 1 | 0 | 1 | 120 |
| 2 | 02 | 11 | 0B | 0 | 2 | 240977 | 1927812 | 0 | 2 | 0 | 112 | 1950444 | 0 | 2 | 0 | 1 | 120 |
| 3 | 03 | 11 | 0B | 0 | 3 | 240991 | 1927924 | 0 | 3 | 0 | 112 | 1950564 | 0 | 3 | 0 | 1 | 120 |
| 4 | 04 | 11 | 0B | 0 | 4 | 241005 | 1928036 | 0 | 4 | 0 | 112 | 1950684 | 0 | 4 | 0 | 1 | 120 |
| 5 | 05 | 11 | 0B | 0 | 5 | 241019 | 1928148 | 0 | 5 | 0 | 112 | 1950804 | 0 | 5 | 0 | 1 | 120 |
| 6 | 06 | 11 | 0B | 0 | 6 | 241033 | 1928260 | 0 | 6 | 0 | 112 | 1950924 | 0 | 6 | 0 | 1 | 120 |
| 7 | 07 | 11 | 0B | 0 | 7 | 241047 | 1928372 | 0 | 7 | 0 | 112 | 1951044 | 0 | 7 | 0 | 1 | 120 |
| 8 | 08 | 11 | 0B | 0 | 8 | 241061 | 1928484 | 0 | 8 | 0 | 112 | 1951164 | 0 | 8 | 0 | 1 | 120 |
| 9 | 09 | 11 | 0B | 0 | 9 | 241075 | 1928596 | 0 | 9 | 0 | 112 | 1951284 | 0 | 9 | 0 | 1 | 120 |
| 10 | 0A | 11 | 0B | 0 | 10 | 241089 | 1928708 | 0 | 10 | 0 | 112 | 1951404 | 0 | 10 | 0 | 1 | 120 |
| 11 | 0B | 11 | 0B | 1 | 0 | 241101 | 1928804 | 1 | 0 | 0 | 96 | 1951508 | 1 | 0 | 0 | 1 | 104 |
| 12 | 0C | 11 | 0B | 1 | 1 | 241118 | 1928940 | 1 | 1 | 0 | 136 | 1951652 | 1 | 1 | 0 | 1 | 144 |
| 13 | 0D | 11 | 0B | 1 | 2 | 241135 | 1929076 | 1 | 2 | 0 | 136 | 1951796 | 1 | 2 | 0 | 1 | 144 |
| 14 | 0E | 11 | 0B | 1 | 3 | 241152 | 1929212 | 1 | 3 | 0 | 136 | 1951940 | 1 | 3 | 0 | 1 | 144 |
| 15 | 0F | 11 | 0B | 1 | 4 | 241169 | 1929348 | 1 | 4 | 0 | 136 | 1952084 | 1 | 4 | 0 | 1 | 144 |
| 16 | 10 | 11 | 0B | 1 | 5 | 241190 | 1929516 | 1 | 5 | 0 | 168 | 1952260 | 1 | 5 | 0 | 1 | 176 |
| 17 | 11 | 11 | 0B | 1 | 6 | 241211 | 1929684 | 1 | 6 | 0 | 168 | 1952436 | 1 | 6 | 0 | 1 | 176 |
| 18 | 12 | 11 | 0B | 1 | 7 | 241232 | 1929852 | 1 | 7 | 0 | 168 | 1952612 | 1 | 7 | 0 | 1 | 176 |
| 19 | 13 | 11 | 0B | 1 | 8 | 241253 | 1930020 | 1 | 8 | 0 | 168 | 1952788 | 1 | 8 | 0 | 1 | 176 |
| 20 | 14 | 11 | 0B | 1 | 9 | 241274 | 1930188 | 1 | 9 | 0 | 168 | 1952964 | 1 | 9 | 0 | 1 | 176 |
| 21 | 15 | 11 | 0B | 1 | 10 | 241295 | 1930356 | 1 | 10 | 0 | 168 | 1953140 | 1 | 10 | 0 | 1 | 176 |
| 22 | 16 | 11 | 0B | 2 | 0 | 241314 | 1930508 | 2 | 0 | 0 | 152 | 1953300 | 2 | 0 | 0 | 1 | 160 |
| 23 | 17 | 11 | 0B | 2 | 1 | 241338 | 1930700 | 2 | 1 | 0 | 192 | 1953500 | 2 | 1 | 0 | 1 | 200 |
| 24 | 18 | 11 | 0B | 2 | 2 | 241362 | 1930892 | 2 | 2 | 0 | 192 | 1953700 | 2 | 2 | 0 | 1 | 200 |
| 25 | 19 | 11 | 0B | 2 | 3 | 241386 | 1931084 | 2 | 3 | 0 | 192 | 1953900 | 2 | 3 | 0 | 1 | 200 |
| 26 | 1A | 11 | 0B | 2 | 4 | 241410 | 1931276 | 2 | 4 | 0 | 192 | 1954100 | 2 | 4 | 0 | 1 | 200 |
| 27 | 1B | 11 | 0B | 2 | 5 | 241434 | 1931468 | 2 | 5 | 0 | 192 | 1954300 | 2 | 5 | 0 | 1 | 200 |
| 28 | 1C | 11 | 0B | 2 | 6 | 241458 | 1931660 | 2 | 6 | 0 | 192 | 1954500 | 2 | 6 | 0 | 1 | 200 |
| 29 | 1D | 11 | 0B | 2 | 7 | 241482 | 1931852 | 2 | 7 | 0 | 192 | 1954700 | 2 | 7 | 0 | 1 | 200 |
| 30 | 1E | 11 | 0B | 2 | 8 | 241506 | 1932044 | 2 | 8 | 0 | 192 | 1954900 | 2 | 8 | 0 | 1 | 200 |
| 31 | 1F | 11 | 0B | 2 | 9 | 241530 | 1932236 | 2 | 9 | 0 | 192 | 1955100 | 2 | 9 | 0 | 1 | 200 |
| 32 | 20 | 11 | 0B | 2 | 10 | 241553 | 1932420 | 2 | 10 | 0 | 184 | 1955292 | 2 | 10 | 0 | 1 | 192 |
| 33 | 21 | 11 | 0B | 3 | 0 | 241574 | 1932588 | 3 | 0 | 0 | 168 | 1955468 | 3 | 0 | 0 | 1 | 176 |
| 34 | 22 | 11 | 0B | 3 | 1 | 241600 | 1932796 | 3 | 1 | 0 | 208 | 1955684 | 3 | 1 | 0 | 1 | 216 |
| 35 | 23 | 11 | 0B | 3 | 2 | 241626 | 1933004 | 3 | 2 | 0 | 208 | 1955900 | 3 | 2 | 0 | 1 | 216 |
| 36 | 24 | 11 | 0B | 3 | 3 | 241652 | 1933212 | 3 | 3 | 0 | 208 | 1956116 | 3 | 3 | 0 | 1 | 216 |
| 37 | 25 | 11 | 0B | 3 | 4 | 241678 | 1933420 | 3 | 4 | 0 | 208 | 1956332 | 3 | 4 | 0 | 1 | 216 |
| 38 | 26 | 11 | 0B | 3 | 5 | 241704 | 1933628 | 3 | 5 | 0 | 208 | 1956548 | 3 | 5 | 0 | 1 | 216 |
| 39 | 27 | 11 | 0B | 3 | 6 | 241730 | 1933836 | 3 | 6 | 0 | 208 | 1956764 | 3 | 6 | 0 | 1 | 216 |
| 40 | 28 | 11 | 0B | 3 | 7 | 241756 | 1934044 | 3 | 7 | 0 | 208 | 1956980 | 3 | 7 | 0 | 1 | 216 |
| 41 | 29 | 11 | 0B | 3 | 8 | 241782 | 1934252 | 3 | 8 | 0 | 208 | 1957196 | 3 | 8 | 0 | 1 | 216 |
| 42 | 2A | 11 | 0B | 3 | 9 | 241808 | 1934460 | 3 | 9 | 0 | 208 | 1957412 | 3 | 9 | 0 | 1 | 216 |
| 43 | 2B | 11 | 0B | 3 | 10 | 241834 | 1934668 | 3 | 10 | 0 | 208 | 1957628 | 3 | 10 | 0 | 1 | 216 |
| 44 | 2C | 11 | 0B | 4 | 0 | 241862 | 1934892 | 4 | 0 | 0 | 224 | 1957860 | 4 | 0 | 0 | 1 | 232 |
| 45 | 2D | 11 | 0B | 4 | 1 | 241890 | 1935116 | 4 | 1 | 0 | 224 | 1958092 | 4 | 1 | 0 | 1 | 232 |
| 46 | 2E | 11 | 0B | 4 | 2 | 241918 | 1935340 | 4 | 2 | 0 | 224 | 1958324 | 4 | 2 | 0 | 1 | 232 |
| 47 | 2F | 11 | 0B | 4 | 3 | 241946 | 1935564 | 4 | 3 | 0 | 224 | 1958556 | 4 | 3 | 0 | 1 | 232 |
| 48 | 30 | 11 | 0B | 4 | 4 | 241974 | 1935788 | 4 | 4 | 0 | 224 | 1958788 | 4 | 4 | 0 | 1 | 232 |
| 49 | 31 | 11 | 0B | 4 | 5 | 242002 | 1936012 | 4 | 5 | 0 | 224 | 1959020 | 4 | 5 | 0 | 1 | 232 |
| 50 | 32 | 11 | 0B | 4 | 6 | 242030 | 1936236 | 4 | 6 | 0 | 224 | 1959252 | 4 | 6 | 0 | 1 | 232 |
| 51 | 33 | 11 | 0B | 4 | 7 | 242058 | 1936460 | 4 | 7 | 0 | 224 | 1959484 | 4 | 7 | 0 | 1 | 232 |
| 52 | 34 | 11 | 0B | 4 | 8 | 242086 | 1936684 | 4 | 8 | 0 | 224 | 1959716 | 4 | 8 | 0 | 1 | 232 |
| 53 | 35 | 11 | 0B | 4 | 9 | 242114 | 1936908 | 4 | 9 | 0 | 224 | 1959948 | 4 | 9 | 0 | 1 | 232 |
| 54 | 36 | 11 | 0B | 4 | 10 | 242142 | 1937132 | 4 | 10 | 0 | 224 | 1960180 | 4 | 10 | 0 | 1 | 232 |
| 55 | 37 | 11 | 0B | 5 | 0 | 242172 | 1937372 | 5 | 0 | 0 | 240 | 1960428 | 5 | 0 | 0 | 1 | 248 |
| 56 | 38 | 11 | 0B | 5 | 1 | 242202 | 1937612 | 5 | 1 | 0 | 240 | 1960676 | 5 | 1 | 0 | 1 | 248 |
| 57 | 39 | 11 | 0B | 5 | 2 | 242232 | 1937852 | 5 | 2 | 0 | 240 | 1960924 | 5 | 2 | 0 | 1 | 248 |
| 58 | 3A | 11 | 0B | 5 | 3 | 242262 | 1938092 | 5 | 3 | 0 | 240 | 1961172 | 5 | 3 | 0 | 1 | 248 |
| 59 | 3B | 11 | 0B | 5 | 4 | 242292 | 1938332 | 5 | 4 | 0 | 240 | 1961420 | 5 | 4 | 0 | 1 | 248 |
| 60 | 3C | 11 | 0B | 5 | 5 | 242322 | 1938572 | 5 | 5 | 0 | 240 | 1961668 | 5 | 5 | 0 | 1 | 248 |
| 61 | 3D | 11 | 0B | 5 | 6 | 242352 | 1938812 | 5 | 6 | 0 | 240 | 1961916 | 5 | 6 | 0 | 1 | 248 |
| 62 | 3E | 11 | 0B | 5 | 7 | 242382 | 1939052 | 5 | 7 | 0 | 240 | 1962164 | 5 | 7 | 0 | 1 | 248 |
| 63 | 3F | 11 | 0B | 5 | 8 | 242412 | 1939292 | 5 | 8 | 0 | 240 | 1962412 | 5 | 8 | 0 | 1 | 248 |
| 64 | 40 | 11 | 0B | 5 | 9 | 242442 | 1939532 | 5 | 9 | 0 | 240 | 1962660 | 5 | 9 | 0 | 1 | 248 |

| | | | | | | | | | | | | | | | | | |
|---|---|---|---|---|---|---|---|---|---|---|---|---|---|---|---|---|---|
| 65 | 41 | 11 | 0B | 5 | 10 | 242472 | 1939772 | 5 | 10 | 0 | 240 | 1962908 | 5 | 10 | 0 | 1 | 248 |
| 66 | 42 | 11 | 0B | 6 | 0 | 242504 | 1940028 | 6 | 0 | 0 | 256 | 1963172 | 6 | 0 | 0 | 1 | 264 |
| 67 | 43 | 11 | 0B | 6 | 1 | 242536 | 1940284 | 6 | 1 | 0 | 256 | 1963436 | 6 | 1 | 0 | 1 | 264 |
| 68 | 44 | 11 | 0B | 6 | 2 | 242568 | 1940540 | 6 | 2 | 0 | 256 | 1963700 | 6 | 2 | 0 | 1 | 264 |
| 69 | 45 | 11 | 0B | 6 | 3 | 242600 | 1940796 | 6 | 3 | 0 | 256 | 1963964 | 6 | 3 | 0 | 1 | 264 |
| 70 | 46 | 11 | 0B | 6 | 4 | 242632 | 1941052 | 6 | 4 | 0 | 256 | 1964228 | 6 | 4 | 0 | 1 | 264 |
| 71 | 47 | 11 | 0B | 6 | 5 | 242664 | 1941308 | 6 | 5 | 0 | 256 | 1964492 | 6 | 5 | 0 | 1 | 264 |
| 72 | 48 | 11 | 0B | 6 | 6 | 242696 | 1941564 | 6 | 6 | 0 | 256 | 1964756 | 6 | 6 | 0 | 1 | 264 |
| 73 | 49 | 11 | 0B | 6 | 7 | 242728 | 1941820 | 6 | 7 | 0 | 256 | 1965020 | 6 | 7 | 0 | 1 | 264 |
| 74 | 4A | 11 | 0B | 6 | 8 | 242760 | 1942076 | 6 | 8 | 0 | 256 | 1965284 | 6 | 8 | 0 | 1 | 264 |
| 75 | 4B | 11 | 0B | 6 | 9 | 242792 | 1942332 | 6 | 9 | 0 | 256 | 1965548 | 6 | 9 | 0 | 1 | 264 |
| 76 | 4C | 11 | 0B | 6 | 10 | 242824 | 1942588 | 6 | 10 | 0 | 256 | 1965812 | 6 | 10 | 0 | 1 | 264 |
| 77 | 4D | 11 | 0B | 7 | 0 | 242858 | 1942860 | 7 | 0 | 0 | 272 | 1966092 | 7 | 0 | 0 | 1 | 280 |
| 78 | 4E | 11 | 0B | 7 | 1 | 242892 | 1943132 | 7 | 1 | 0 | 272 | 1966372 | 7 | 1 | 0 | 1 | 280 |
| 79 | 4F | 11 | 0B | 7 | 2 | 242926 | 1943404 | 7 | 2 | 0 | 272 | 1966652 | 7 | 2 | 0 | 1 | 280 |
| 80 | 50 | 11 | 0B | 7 | 3 | 242960 | 1943676 | 7 | 3 | 0 | 272 | 1966932 | 7 | 3 | 0 | 1 | 280 |
| 81 | 51 | 11 | 0B | 7 | 4 | 242994 | 1943948 | 7 | 4 | 0 | 272 | 1967212 | 7 | 4 | 0 | 1 | 280 |
| 82 | 52 | 11 | 0B | 7 | 5 | 243028 | 1944220 | 7 | 5 | 0 | 272 | 1967492 | 7 | 5 | 0 | 1 | 280 |
| 83 | 53 | 11 | 0B | 7 | 6 | 243062 | 1944492 | 7 | 6 | 0 | 272 | 1967772 | 7 | 6 | 0 | 1 | 280 |
| 84 | 54 | 11 | 0B | 7 | 7 | 243096 | 1944764 | 7 | 7 | 0 | 272 | 1968052 | 7 | 7 | 0 | 1 | 280 |
| 85 | 55 | 11 | 0B | 7 | 8 | 243130 | 1945036 | 7 | 8 | 0 | 272 | 1968332 | 7 | 8 | 0 | 1 | 280 |
| 86 | 56 | 11 | 0B | 7 | 9 | 243164 | 1945308 | 7 | 9 | 0 | 272 | 1968612 | 7 | 9 | 0 | 1 | 280 |
| 87 | 57 | 11 | 0B | 7 | 10 | 243198 | 1945580 | 7 | 10 | 0 | 272 | 1968892 | 7 | 10 | 0 | 1 | 280 |
| 88 | 58 | 11 | 0B | 8 | 0 | 243234 | 1945868 | 8 | 0 | 0 | 288 | 1969188 | 8 | 0 | 0 | 1 | 296 |
| 89 | 59 | 11 | 0B | 8 | 1 | 243270 | 1946156 | 8 | 1 | 0 | 288 | 1969484 | 8 | 1 | 0 | 1 | 296 |
| 90 | 5A | 11 | 0B | 8 | 2 | 243306 | 1946444 | 8 | 2 | 0 | 288 | 1969780 | 8 | 2 | 0 | 1 | 296 |
| 91 | 5B | 11 | 0B | 8 | 3 | 243342 | 1946732 | 8 | 3 | 0 | 288 | 1970076 | 8 | 3 | 0 | 1 | 296 |
| 92 | 5C | 11 | 0B | 8 | 4 | 243378 | 1947020 | 8 | 4 | 0 | 288 | 1970372 | 8 | 4 | 0 | 1 | 296 |
| 93 | 5D | 11 | 0B | 8 | 5 | 243414 | 1947308 | 8 | 5 | 0 | 288 | 1970668 | 8 | 5 | 0 | 1 | 296 |
| 94 | 5E | 11 | 0B | 8 | 6 | 243450 | 1947596 | 8 | 6 | 0 | 288 | 1970964 | 8 | 6 | 0 | 1 | 296 |
| 95 | 5F | 11 | 0B | 8 | 7 | 243486 | 1947884 | 8 | 7 | 0 | 288 | 1971260 | 8 | 7 | 0 | 1 | 296 |
| 96 | 60 | 11 | 0B | 8 | 8 | 243522 | 1948172 | 8 | 8 | 0 | 288 | 1971556 | 8 | 8 | 0 | 1 | 296 |
| 97 | 61 | 11 | 0B | 8 | 9 | 243558 | 1948460 | 8 | 9 | 0 | 288 | 1971852 | 8 | 9 | 0 | 1 | 296 |
| 98 | 62 | 11 | 0B | 8 | 10 | 243594 | 1948748 | 8 | 10 | 0 | 288 | 1972148 | 8 | 10 | 0 | 1 | 296 |
| 99 | 63 | 11 | 0B | 9 | 0 | 243632 | 1949052 | 9 | 0 | 0 | 304 | 1972460 | 9 | 0 | 0 | 1 | 312 |
| 100 | 64 | 11 | 0B | 9 | 1 | 243670 | 1949356 | 9 | 1 | 0 | 304 | 1972772 | 9 | 1 | 0 | 1 | 312 |
| 101 | 65 | 11 | 0B | 9 | 2 | 243708 | 1949660 | 9 | 2 | 0 | 304 | 1973084 | 9 | 2 | 0 | 1 | 312 |
| 102 | 66 | 11 | 0B | 9 | 3 | 243746 | 1949964 | 9 | 3 | 0 | 304 | 1973396 | 9 | 3 | 0 | 1 | 312 |
| 103 | 67 | 11 | 0B | 9 | 4 | 243784 | 1950268 | 9 | 4 | 0 | 304 | 1973708 | 9 | 4 | 0 | 1 | 312 |
| 104 | 68 | 11 | 0B | 9 | 5 | 243822 | 1950572 | 9 | 5 | 0 | 304 | 1974020 | 9 | 5 | 0 | 1 | 312 |
| 105 | 69 | 11 | 0B | 9 | 6 | 243860 | 1950876 | 9 | 6 | 0 | 304 | 1974332 | 9 | 6 | 0 | 1 | 312 |
| 106 | 6A | 11 | 0B | 9 | 7 | 243898 | 1951180 | 9 | 7 | 0 | 304 | 1974644 | 9 | 7 | 0 | 1 | 312 |
| 107 | 6B | 11 | 0B | 9 | 8 | 243936 | 1951484 | 9 | 8 | 0 | 304 | 1974956 | 9 | 8 | 0 | 1 | 312 |
| 108 | 6C | 11 | 0B | 9 | 9 | 243974 | 1951788 | 9 | 9 | 0 | 304 | 1975268 | 9 | 9 | 0 | 1 | 312 |
| 109 | 6D | 11 | 0B | 9 | 10 | 244012 | 1952092 | 9 | 10 | 0 | 304 | 1975580 | 9 | 10 | 0 | 1 | 312 |
| 110 | 6E | 11 | 0B | 10 | 0 | 244052 | 1952412 | 10 | 0 | 0 | 320 | 1975908 | 10 | 0 | 0 | 1 | 328 |
| 111 | 6F | 11 | 0B | 10 | 1 | 244092 | 1952732 | 10 | 1 | 0 | 320 | 1976236 | 10 | 1 | 0 | 1 | 328 |
| 112 | 70 | 11 | 0B | 10 | 2 | 244132 | 1953052 | 10 | 2 | 0 | 320 | 1976564 | 10 | 2 | 0 | 1 | 328 |
| 113 | 71 | 11 | 0B | 10 | 3 | 244172 | 1953372 | 10 | 3 | 0 | 320 | 1976892 | 10 | 3 | 0 | 1 | 328 |
| 114 | 72 | 11 | 0B | 10 | 4 | 244212 | 1953692 | 10 | 4 | 0 | 320 | 1977220 | 10 | 4 | 0 | 1 | 328 |
| 115 | 73 | 11 | 0B | 10 | 5 | 244252 | 1954012 | 10 | 5 | 0 | 320 | 1977548 | 10 | 5 | 0 | 1 | 328 |
| 116 | 74 | 11 | 0B | 10 | 6 | 244292 | 1954332 | 10 | 6 | 0 | 320 | 1977876 | 10 | 6 | 0 | 1 | 328 |
| 117 | 75 | 11 | 0B | 10 | 7 | 244332 | 1954652 | 10 | 7 | 0 | 320 | 1978204 | 10 | 7 | 0 | 1 | 328 |
| 118 | 76 | 11 | 0B | 10 | 8 | 244372 | 1954972 | 10 | 8 | 0 | 320 | 1978532 | 10 | 8 | 0 | 1 | 328 |
| 119 | 77 | 11 | 0B | 10 | 9 | 244412 | 1955292 | 10 | 9 | 0 | 320 | 1978860 | 10 | 9 | 0 | 1 | 328 |
| 120 | 78 | 11 | 0B | 10 | 10 | 244452 | 1955612 | 10 | 10 | 0 | 320 | 1979188 | 10 | 10 | 0 | 1 | 328 |
| 121 | 79 | 11 | 0B | 11 | 0 | 244494 | 1955948 | 11 | 0 | 0 | 336 | 1979532 | 11 | 0 | 0 | 1 | 344 |
| 122 | 7A | 11 | 0B | 11 | 1 | 244536 | 1956284 | 11 | 1 | 0 | 336 | 1979876 | 11 | 1 | 0 | 1 | 344 |
| 123 | 7B | 11 | 0B | 11 | 2 | 244578 | 1956620 | 11 | 2 | 0 | 336 | 1980220 | 11 | 2 | 0 | 1 | 344 |
| 124 | 7C | 11 | 0B | 11 | 3 | 244620 | 1956956 | 11 | 3 | 0 | 336 | 1980564 | 11 | 3 | 0 | 1 | 344 |
| 125 | 7D | 11 | 0B | 11 | 4 | 244662 | 1957292 | 11 | 4 | 0 | 336 | 1980908 | 11 | 4 | 0 | 1 | 344 |
| 126 | 7E | 11 | 0B | 11 | 5 | 244704 | 1957628 | 11 | 5 | 0 | 336 | 1981252 | 11 | 5 | 0 | 1 | 344 |
| 127 | 7F | 11 | 0B | 11 | 6 | 244746 | 1957964 | 11 | 6 | 0 | 336 | 1981596 | 11 | 6 | 0 | 1 | 344 |
| 128 | 80 | 11 | 0B | 11 | 7 | 244788 | 1958300 | 11 | 7 | 0 | 336 | 1981940 | 11 | 7 | 0 | 1 | 344 |
| 129 | 81 | 11 | 0B | 11 | 8 | 244830 | 1958636 | 11 | 8 | 0 | 336 | 1982284 | 11 | 8 | 0 | 1 | 344 |
| 130 | 82 | 11 | 0B | 11 | 9 | 244872 | 1958972 | 11 | 9 | 0 | 336 | 1982628 | 11 | 9 | 0 | 1 | 344 |
| 131 | 83 | 11 | 0B | 11 | 10 | 244914 | 1959308 | 11 | 10 | 0 | 336 | 1982972 | 11 | 10 | 0 | 1 | 344 |
| 132 | 84 | 11 | 0B | 12 | 0 | 244958 | 1959660 | 12 | 0 | 0 | 352 | 1983332 | 12 | 0 | 0 | 1 | 360 |
| 133 | 85 | 11 | 0B | 12 | 1 | 245002 | 1960012 | 12 | 1 | 0 | 352 | 1983692 | 12 | 1 | 0 | 1 | 360 |
| 134 | 86 | 11 | 0B | 12 | 2 | 245046 | 1960364 | 12 | 2 | 0 | 352 | 1984052 | 12 | 2 | 0 | 1 | 360 |
| 135 | 87 | 11 | 0B | 12 | 3 | 245090 | 1960716 | 12 | 3 | 0 | 352 | 1984412 | 12 | 3 | 0 | 1 | 360 |
| 136 | 88 | 11 | 0B | 12 | 4 | 245134 | 1961068 | 12 | 4 | 0 | 352 | 1984772 | 12 | 4 | 0 | 1 | 360 |
| 137 | 89 | 11 | 0B | 12 | 5 | 245178 | 1961420 | 12 | 5 | 0 | 352 | 1985132 | 12 | 5 | 0 | 1 | 360 |
| 138 | 8A | 11 | 0B | 12 | 6 | 245222 | 1961772 | 12 | 6 | 0 | 352 | 1985492 | 12 | 6 | 0 | 1 | 360 |
| 139 | 8B | 11 | 0B | 12 | 7 | 245266 | 1962124 | 12 | 7 | 0 | 352 | 1985852 | 12 | 7 | 0 | 1 | 360 |
| 140 | 8C | 11 | 0B | 12 | 8 | 245310 | 1962476 | 12 | 8 | 0 | 352 | 1986212 | 12 | 8 | 0 | 1 | 360 |
| 141 | 8D | 11 | 0B | 12 | 9 | 245354 | 1962828 | 12 | 9 | 0 | 352 | 1986572 | 12 | 9 | 0 | 1 | 360 |
| 142 | 8E | 11 | 0B | 12 | 10 | 245398 | 1963180 | 12 | 10 | 0 | 352 | 1986932 | 12 | 10 | 0 | 1 | 360 |
| 143 | 8F | 11 | 0B | 13 | 0 | 245444 | 1963548 | 13 | 0 | 0 | 368 | 1987308 | 13 | 0 | 0 | 1 | 376 |
| 144 | 90 | 11 | 0B | 13 | 1 | 245490 | 1963916 | 13 | 1 | 0 | 368 | 1987684 | 13 | 1 | 0 | 1 | 376 |
| 145 | 91 | 11 | 0B | 13 | 2 | 245536 | 1964284 | 13 | 2 | 0 | 368 | 1988060 | 13 | 2 | 0 | 1 | 376 |
| 146 | 92 | 11 | 0B | 13 | 3 | 245582 | 1964652 | 13 | 3 | 0 | 368 | 1988436 | 13 | 3 | 0 | 1 | 376 |
| 147 | 93 | 11 | 0B | 13 | 4 | 245628 | 1965020 | 13 | 4 | 0 | 368 | 1988812 | 13 | 4 | 0 | 1 | 376 |
| 148 | 94 | 11 | 0B | 13 | 5 | 245674 | 1965388 | 13 | 5 | 0 | 368 | 1989188 | 13 | 5 | 0 | 1 | 376 |
| 149 | 95 | 11 | 0B | 13 | 6 | 245720 | 1965756 | 13 | 6 | 0 | 368 | 1989564 | 13 | 6 | 0 | 1 | 376 |
| 150 | 96 | 11 | 0B | 13 | 7 | 245766 | 1966124 | 13 | 7 | 0 | 368 | 1989940 | 13 | 7 | 0 | 1 | 376 |
| 151 | 97 | 11 | 0B | 13 | 8 | 245812 | 1966492 | 13 | 8 | 0 | 368 | 1990316 | 13 | 8 | 0 | 1 | 376 |
| 152 | 98 | 11 | 0B | 13 | 9 | 245858 | 1966860 | 13 | 9 | 0 | 368 | 1990692 | 13 | 9 | 0 | 1 | 376 |
| 153 | 99 | 11 | 0B | 13 | 10 | 245904 | 1967228 | 13 | 10 | 0 | 368 | 1991068 | 13 | 10 | 0 | 1 | 376 |
| 154 | 9A | 11 | 0B | 14 | 0 | 245952 | 1967612 | 14 | 0 | 0 | 384 | 1991460 | 14 | 0 | 0 | 1 | 392 |
| 155 | 9B | 11 | 0B | 14 | 1 | 246000 | 1967996 | 14 | 1 | 0 | 384 | 1991852 | 14 | 1 | 0 | 1 | 392 |
| 156 | 9C | 11 | 0B | 14 | 2 | 246048 | 1968380 | 14 | 2 | 0 | 384 | 1992244 | 14 | 2 | 0 | 1 | 392 |
| 157 | 9D | 11 | 0B | 14 | 3 | 246096 | 1968764 | 14 | 3 | 0 | 384 | 1992636 | 14 | 3 | 0 | 1 | 392 |
| 158 | 9E | 11 | 0B | 14 | 4 | 246144 | 1969148 | 14 | 4 | 0 | 384 | 1993028 | 14 | 4 | 0 | 1 | 392 |
| 159 | 9F | 11 | 0B | 14 | 5 | 246192 | 1969532 | 14 | 5 | 0 | 384 | 1993420 | 14 | 5 | 0 | 1 | 392 |
| 160 | A0 | 11 | 0B | 14 | 6 | 246240 | 1969916 | 14 | 6 | 0 | 384 | 1993812 | 14 | 6 | 0 | 1 | 392 |
| 161 | A1 | 11 | 0B | 14 | 7 | 246288 | 1970300 | 14 | 7 | 0 | 384 | 1994204 | 14 | 7 | 0 | 1 | 392 |
| 162 | A2 | 11 | 0B | 14 | 8 | 246336 | 1970684 | 14 | 8 | 0 | 384 | 1994596 | 14 | 8 | 0 | 1 | 392 |
| 163 | A3 | 11 | 0B | 14 | 9 | 246384 | 1971068 | 14 | 9 | 0 | 384 | 1994988 | 14 | 9 | 0 | 1 | 392 |
| 164 | A4 | 11 | 0B | 14 | 10 | 246432 | 1971452 | 14 | 10 | 0 | 384 | 1995380 | 14 | 10 | 0 | 1 | 392 |
| 165 | A5 | 11 | 0B | 15 | 0 | 246482 | 1971852 | 15 | 0 | 0 | 400 | 1995788 | 15 | 0 | 0 | 1 | 408 |
| 166 | A6 | 11 | 0B | 15 | 1 | 246532 | 1972252 | 15 | 1 | 0 | 400 | 1996196 | 15 | 1 | 0 | 1 | 408 |
| 167 | A7 | 11 | 0B | 15 | 2 | 246582 | 1972652 | 15 | 2 | 0 | 400 | 1996604 | 15 | 2 | 0 | 1 | 408 |

| | | | | | | | | | | | | | | | | | |
|---|---|---|---|---|---|---|---|---|---|---|---|---|---|---|---|---|---|
| 168 | A8 | 11 | 0B | 15 | 3 | 246632 | 1973052 | 15 | 3 | 0 | 400 | 1997012 | 15 | 3 | 0 | 1 | 408 |
| 169 | A9 | 11 | 0B | 15 | 4 | 246682 | 1973452 | 15 | 4 | 0 | 400 | 1997420 | 15 | 4 | 0 | 1 | 408 |
| 170 | AA | 11 | 0B | 15 | 5 | 246732 | 1973852 | 15 | 5 | 0 | 400 | 1997828 | 15 | 5 | 0 | 1 | 408 |
| 171 | AB | 11 | 0B | 15 | 6 | 246782 | 1974252 | 15 | 6 | 0 | 400 | 1998236 | 15 | 6 | 0 | 1 | 408 |
| 172 | AC | 11 | 0B | 15 | 7 | 246832 | 1974652 | 15 | 7 | 0 | 400 | 1998644 | 15 | 7 | 0 | 1 | 408 |
| 173 | AD | 11 | 0B | 15 | 8 | 246882 | 1975052 | 15 | 8 | 0 | 400 | 1999052 | 15 | 8 | 0 | 1 | 408 |
| 174 | AE | 11 | 0B | 15 | 9 | 246932 | 1975452 | 15 | 9 | 0 | 400 | 1999460 | 15 | 9 | 0 | 1 | 408 |
| 175 | AF | 11 | 0B | 15 | 10 | 246982 | 1975852 | 15 | 10 | 0 | 400 | 1999868 | 15 | 10 | 0 | 1 | 408 |
| 176 | B0 | 11 | 0B | 16 | 0 | 247034 | 1976268 | 16 | 0 | 0 | 416 | 2000292 | 16 | 0 | 0 | 1 | 424 |
| 177 | B1 | 11 | 0B | 16 | 1 | 247086 | 1976684 | 16 | 1 | 0 | 416 | 2000716 | 16 | 1 | 0 | 1 | 424 |
| 178 | B2 | 11 | 0B | 16 | 2 | 247138 | 1977100 | 16 | 2 | 0 | 416 | 2001140 | 16 | 2 | 0 | 1 | 424 |
| 179 | B3 | 11 | 0B | 16 | 3 | 247190 | 1977516 | 16 | 3 | 0 | 416 | 2001564 | 16 | 3 | 0 | 1 | 424 |
| 180 | B4 | 11 | 0B | 16 | 4 | 247242 | 1977932 | 16 | 4 | 0 | 416 | 2001988 | 16 | 4 | 0 | 1 | 424 |
| 181 | B5 | 11 | 0B | 16 | 5 | 247294 | 1978348 | 16 | 5 | 0 | 416 | 2002412 | 16 | 5 | 0 | 1 | 424 |
| 182 | B6 | 11 | 0B | 16 | 6 | 247346 | 1978764 | 16 | 6 | 0 | 416 | 2002836 | 16 | 6 | 0 | 1 | 424 |
| 183 | B7 | 11 | 0B | 16 | 7 | 247398 | 1979180 | 16 | 7 | 0 | 416 | 2003260 | 16 | 7 | 0 | 1 | 424 |
| 184 | B8 | 11 | 0B | 16 | 8 | 247450 | 1979596 | 16 | 8 | 0 | 416 | 2003684 | 16 | 8 | 0 | 1 | 424 |
| 185 | B9 | 11 | 0B | 16 | 9 | 247502 | 1980012 | 16 | 9 | 0 | 416 | 2004108 | 16 | 9 | 0 | 1 | 424 |
| 186 | BA | 11 | 0B | 16 | 10 | 247554 | 1980428 | 16 | 10 | 0 | 416 | 2004532 | 16 | 10 | 0 | 1 | 424 |
| 187 | BB | 11 | 0B | 17 | 0 | 247608 | 1980860 | 17 | 0 | 0 | 432 | 2004972 | 17 | 0 | 0 | 1 | 440 |
| 188 | BC | 11 | 0B | 17 | 1 | 247662 | 1981292 | 17 | 1 | 0 | 432 | 2005412 | 17 | 1 | 0 | 1 | 440 |
| 189 | BD | 11 | 0B | 17 | 2 | 247716 | 1981724 | 17 | 2 | 0 | 432 | 2005852 | 17 | 2 | 0 | 1 | 440 |
| 190 | BE | 11 | 0B | 17 | 3 | 247770 | 1982156 | 17 | 3 | 0 | 432 | 2006292 | 17 | 3 | 0 | 1 | 440 |
| 191 | BF | 11 | 0B | 17 | 4 | 247824 | 1982588 | 17 | 4 | 0 | 432 | 2006732 | 17 | 4 | 0 | 1 | 440 |
| 192 | C0 | 11 | 0B | 17 | 5 | 247878 | 1983020 | 17 | 5 | 0 | 432 | 2007172 | 17 | 5 | 0 | 1 | 440 |
| 193 | C1 | 11 | 0B | 17 | 6 | 247932 | 1983452 | 17 | 6 | 0 | 432 | 2007612 | 17 | 6 | 0 | 1 | 440 |
| 194 | C2 | 11 | 0B | 17 | 7 | 247986 | 1983884 | 17 | 7 | 0 | 432 | 2008052 | 17 | 7 | 0 | 1 | 440 |
| 195 | C3 | 11 | 0B | 17 | 8 | 248040 | 1984316 | 17 | 8 | 0 | 432 | 2008492 | 17 | 8 | 0 | 1 | 440 |
| 196 | C4 | 11 | 0B | 17 | 9 | 248094 | 1984748 | 17 | 9 | 0 | 432 | 2008932 | 17 | 9 | 0 | 1 | 440 |
| 197 | C5 | 11 | 0B | 17 | 10 | 248148 | 1985180 | 17 | 10 | 0 | 432 | 2009372 | 17 | 10 | 0 | 1 | 440 |
| 198 | C6 | 11 | 0B | 18 | 0 | 248204 | 1985628 | 18 | 0 | 0 | 448 | 2009828 | 18 | 0 | 0 | 1 | 456 |
| 199 | C7 | 11 | 0B | 18 | 1 | 248260 | 1986076 | 18 | 1 | 0 | 448 | 2010284 | 18 | 1 | 0 | 1 | 456 |
| 200 | C8 | 11 | 0B | 18 | 2 | 248316 | 1986524 | 18 | 2 | 0 | 448 | 2010740 | 18 | 2 | 0 | 1 | 456 |
| 201 | C9 | 11 | 0B | 18 | 3 | 248372 | 1986972 | 18 | 3 | 0 | 448 | 2011196 | 18 | 3 | 0 | 1 | 456 |
| 202 | CA | 11 | 0B | 18 | 4 | 248428 | 1987420 | 18 | 4 | 0 | 448 | 2011652 | 18 | 4 | 0 | 1 | 456 |
| 203 | CB | 11 | 0B | 18 | 5 | 248484 | 1987868 | 18 | 5 | 0 | 448 | 2012108 | 18 | 5 | 0 | 1 | 456 |
| 204 | CC | 11 | 0B | 18 | 6 | 248540 | 1988316 | 18 | 6 | 0 | 448 | 2012564 | 18 | 6 | 0 | 1 | 456 |
| 205 | CD | 11 | 0B | 18 | 7 | 248596 | 1988764 | 18 | 7 | 0 | 448 | 2013020 | 18 | 7 | 0 | 1 | 456 |
| 206 | CE | 11 | 0B | 18 | 8 | 248652 | 1989212 | 18 | 8 | 0 | 448 | 2013476 | 18 | 8 | 0 | 1 | 456 |
| 207 | CF | 11 | 0B | 18 | 9 | 248708 | 1989660 | 18 | 9 | 0 | 448 | 2013932 | 18 | 9 | 0 | 1 | 456 |
| 208 | D0 | 11 | 0B | 18 | 10 | 248764 | 1990108 | 18 | 10 | 0 | 448 | 2014388 | 18 | 10 | 0 | 1 | 456 |
| 209 | D1 | 11 | 0B | 19 | 0 | 248822 | 1990572 | 19 | 0 | 0 | 464 | 2014860 | 19 | 0 | 0 | 1 | 472 |
| 210 | D2 | 11 | 0B | 19 | 1 | 248880 | 1991036 | 19 | 1 | 0 | 464 | 2015332 | 19 | 1 | 0 | 1 | 472 |
| 211 | D3 | 11 | 0B | 19 | 2 | 248938 | 1991500 | 19 | 2 | 0 | 464 | 2015804 | 19 | 2 | 0 | 1 | 472 |
| 212 | D4 | 11 | 0B | 19 | 3 | 248996 | 1991964 | 19 | 3 | 0 | 464 | 2016276 | 19 | 3 | 0 | 1 | 472 |
| 213 | D5 | 11 | 0B | 19 | 4 | 249054 | 1992428 | 19 | 4 | 0 | 464 | 2016748 | 19 | 4 | 0 | 1 | 472 |
| 214 | D6 | 11 | 0B | 19 | 5 | 249112 | 1992892 | 19 | 5 | 0 | 464 | 2017220 | 19 | 5 | 0 | 1 | 472 |
| 215 | D7 | 11 | 0B | 19 | 6 | 249170 | 1993356 | 19 | 6 | 0 | 464 | 2017692 | 19 | 6 | 0 | 1 | 472 |
| 216 | D8 | 11 | 0B | 19 | 7 | 249228 | 1993820 | 19 | 7 | 0 | 464 | 2018164 | 19 | 7 | 0 | 1 | 472 |
| 217 | D9 | 11 | 0B | 19 | 8 | 249286 | 1994284 | 19 | 8 | 0 | 464 | 2018636 | 19 | 8 | 0 | 1 | 472 |
| 218 | DA | 11 | 0B | 19 | 9 | 249344 | 1994748 | 19 | 9 | 0 | 464 | 2019108 | 19 | 9 | 0 | 1 | 472 |
| 219 | DB | 11 | 0B | 19 | 10 | 249402 | 1995212 | 19 | 10 | 0 | 464 | 2019580 | 19 | 10 | 0 | 1 | 472 |
| 220 | DC | 11 | 0B | 20 | 0 | 249462 | 1995692 | 20 | 0 | 0 | 480 | 2020068 | 20 | 0 | 0 | 1 | 488 |
| 221 | DD | 11 | 0B | 20 | 1 | 249522 | 1996172 | 20 | 1 | 0 | 480 | 2020556 | 20 | 1 | 0 | 1 | 488 |
| 222 | DE | 11 | 0B | 20 | 2 | 249582 | 1996652 | 20 | 2 | 0 | 480 | 2021044 | 20 | 2 | 0 | 1 | 488 |
| 223 | DF | 11 | 0B | 20 | 3 | 249642 | 1997132 | 20 | 3 | 0 | 480 | 2021532 | 20 | 3 | 0 | 1 | 488 |
| 224 | E0 | 11 | 0B | 20 | 4 | 249702 | 1997612 | 20 | 4 | 0 | 480 | 2022020 | 20 | 4 | 0 | 1 | 488 |
| 225 | E1 | 11 | 0B | 20 | 5 | 249762 | 1998092 | 20 | 5 | 0 | 480 | 2022508 | 20 | 5 | 0 | 1 | 488 |
| 226 | E2 | 11 | 0B | 20 | 6 | 249822 | 1998572 | 20 | 6 | 0 | 480 | 2022996 | 20 | 6 | 0 | 1 | 488 |
| 227 | E3 | 11 | 0B | 20 | 7 | 249882 | 1999052 | 20 | 7 | 0 | 480 | 2023484 | 20 | 7 | 0 | 1 | 488 |
| 228 | E4 | 11 | 0B | 20 | 8 | 249942 | 1999532 | 20 | 8 | 0 | 480 | 2023972 | 20 | 8 | 0 | 1 | 488 |
| 229 | E5 | 11 | 0B | 20 | 9 | 250002 | 2000012 | 20 | 9 | 0 | 480 | 2024460 | 20 | 9 | 0 | 1 | 488 |
| 230 | E6 | 11 | 0B | 20 | 10 | 250062 | 2000492 | 20 | 10 | 0 | 480 | 2024948 | 20 | 10 | 0 | 1 | 488 |
| 231 | E7 | 11 | 0B | 21 | 0 | 250124 | 2000988 | 21 | 0 | 0 | 496 | 2025452 | 21 | 0 | 0 | 1 | 504 |
| 232 | E8 | 11 | 0B | 21 | 1 | 250186 | 2001484 | 21 | 1 | 0 | 496 | 2025956 | 21 | 1 | 0 | 1 | 504 |
| 233 | E9 | 11 | 0B | 21 | 2 | 250248 | 2001980 | 21 | 2 | 0 | 496 | 2026460 | 21 | 2 | 0 | 1 | 504 |
| 234 | EA | 11 | 0B | 21 | 3 | 250310 | 2002476 | 21 | 3 | 0 | 496 | 2026964 | 21 | 3 | 0 | 1 | 504 |
| 235 | EB | 11 | 0B | 21 | 4 | 250372 | 2002972 | 21 | 4 | 0 | 496 | 2027468 | 21 | 4 | 0 | 1 | 504 |
| 236 | EC | 11 | 0B | 21 | 5 | 250434 | 2003468 | 21 | 5 | 0 | 496 | 2027972 | 21 | 5 | 0 | 1 | 504 |
| 237 | ED | 11 | 0B | 21 | 6 | 250496 | 2003964 | 21 | 6 | 0 | 496 | 2028476 | 21 | 6 | 0 | 1 | 504 |
| 238 | EE | 11 | 0B | 21 | 7 | 250558 | 2004460 | 21 | 7 | 0 | 496 | 2028980 | 21 | 7 | 0 | 1 | 504 |
| 239 | EF | 11 | 0B | 21 | 8 | 250620 | 2004956 | 21 | 8 | 0 | 496 | 2029484 | 21 | 8 | 0 | 1 | 504 |
| 240 | F0 | 11 | 0B | 21 | 9 | 250682 | 2005452 | 21 | 9 | 0 | 496 | 2029988 | 21 | 9 | 0 | 1 | 504 |
| 241 | F1 | 11 | 0B | 21 | 10 | 250744 | 2005948 | 21 | 10 | 0 | 496 | 2030492 | 21 | 10 | 0 | 1 | 504 |
| 242 | F2 | 11 | 0B | 22 | 0 | 250808 | 2006460 | 22 | 0 | 0 | 512 | 2031012 | 22 | 0 | 0 | 1 | 520 |
| 243 | F3 | 11 | 0B | 22 | 1 | 250872 | 2006972 | 22 | 1 | 0 | 512 | 2031532 | 22 | 1 | 0 | 1 | 520 |
| 244 | F4 | 11 | 0B | 22 | 2 | 250936 | 2007484 | 22 | 2 | 0 | 512 | 2032052 | 22 | 2 | 0 | 1 | 520 |
| 245 | F5 | 11 | 0B | 22 | 3 | 251000 | 2007996 | 22 | 3 | 0 | 512 | 2032572 | 22 | 3 | 0 | 1 | 520 |
| 246 | F6 | 11 | 0B | 22 | 4 | 251064 | 2008508 | 22 | 4 | 0 | 512 | 2033092 | 22 | 4 | 0 | 1 | 520 |
| 247 | F7 | 11 | 0B | 22 | 5 | 251128 | 2009020 | 22 | 5 | 0 | 512 | 2033612 | 22 | 5 | 0 | 1 | 520 |
| 248 | F8 | 11 | 0B | 22 | 6 | 251192 | 2009532 | 22 | 6 | 0 | 512 | 2034132 | 22 | 6 | 0 | 1 | 520 |
| 249 | F9 | 11 | 0B | 22 | 7 | 251256 | 2010044 | 22 | 7 | 0 | 512 | 2034652 | 22 | 7 | 0 | 1 | 520 |
| 250 | FA | 11 | 0B | 22 | 8 | 251320 | 2010556 | 22 | 8 | 0 | 512 | 2035172 | 22 | 8 | 0 | 1 | 520 |
| 251 | FB | 11 | 0B | 22 | 9 | 251384 | 2011068 | 22 | 9 | 0 | 512 | 2035692 | 22 | 9 | 0 | 1 | 520 |
| 252 | FC | 11 | 0B | 22 | 10 | 251448 | 2011580 | 22 | 10 | 0 | 512 | 2036212 | 22 | 10 | 0 | 1 | 520 |
| 253 | FD | 11 | 0B | 23 | 0 | 251514 | 2012108 | 23 | 0 | 0 | 528 | 2036748 | 23 | 0 | 0 | 1 | 536 |
| 254 | FE | 11 | 0B | 23 | 1 | 251580 | 2012636 | 23 | 1 | 0 | 528 | 2037284 | 23 | 1 | 0 | 1 | 536 |
| 255 | FF | 11 | 0B | 23 | 2 | 251646 | 2013164 | 23 | 2 | 0 | 528 | 2037820 | 23 | 2 | 0 | 1 | 536 |
| 0 | 00 | 12 | 0C | 0 | 0 | 251658 | 2013260 | 0 | 0 | 0 | 96 | 2037924 | 0 | 0 | 0 | 1 | 104 |
| 1 | 01 | 12 | 0C | 0 | 1 | 251672 | 2013372 | 0 | 1 | 0 | 112 | 2038044 | 0 | 1 | 0 | 1 | 120 |
| 2 | 02 | 12 | 0C | 0 | 2 | 251686 | 2013484 | 0 | 2 | 0 | 112 | 2038164 | 0 | 2 | 0 | 1 | 120 |
| 3 | 03 | 12 | 0C | 0 | 3 | 251700 | 2013596 | 0 | 3 | 0 | 112 | 2038284 | 0 | 3 | 0 | 1 | 120 |
| 4 | 04 | 12 | 0C | 0 | 4 | 251714 | 2013708 | 0 | 4 | 0 | 112 | 2038404 | 0 | 4 | 0 | 1 | 120 |
| 5 | 05 | 12 | 0C | 0 | 5 | 251728 | 2013820 | 0 | 5 | 0 | 112 | 2038524 | 0 | 5 | 0 | 1 | 120 |
| 6 | 06 | 12 | 0C | 0 | 6 | 251742 | 2013932 | 0 | 6 | 0 | 112 | 2038644 | 0 | 6 | 0 | 1 | 120 |
| 7 | 07 | 12 | 0C | 0 | 7 | 251756 | 2014044 | 0 | 7 | 0 | 112 | 2038764 | 0 | 7 | 0 | 1 | 120 |
| 8 | 08 | 12 | 0C | 0 | 8 | 251770 | 2014156 | 0 | 8 | 0 | 112 | 2038884 | 0 | 8 | 0 | 1 | 120 |
| 9 | 09 | 12 | 0C | 0 | 9 | 251784 | 2014268 | 0 | 9 | 0 | 112 | 2039004 | 0 | 9 | 0 | 1 | 120 |
| 10 | 0A | 12 | 0C | 0 | 10 | 251798 | 2014380 | 0 | 10 | 0 | 112 | 2039124 | 0 | 10 | 0 | 1 | 120 |
| 11 | 0B | 12 | 0C | 0 | 11 | 251812 | 2014492 | 0 | 11 | 0 | 112 | 2039244 | 0 | 11 | 0 | 1 | 120 |
| 12 | 0C | 12 | 0C | 1 | 0 | 251824 | 2014588 | 1 | 0 | 0 | 96 | 2039348 | 1 | 0 | 0 | 1 | 104 |
| 13 | 0D | 12 | 0C | 1 | 1 | 251841 | 2014724 | 1 | 1 | 0 | 136 | 2039492 | 1 | 1 | 0 | 1 | 144 |
| 14 | 0E | 12 | 0C | 1 | 2 | 251858 | 2014860 | 1 | 2 | 0 | 136 | 2039636 | 1 | 2 | 0 | 1 | 144 |

| | | | | | | | | | | | | | | | | | |
|---|---|---|---|---|---|---|---|---|---|---|---|---|---|---|---|---|---|
| 15 | 0F | 12 | 0C | 1 | 3 | 251875 | 2014996 | 1 | 3 | 0 | 136 | 2039780 | 1 | 3 | 0 | 1 | 144 |
| 16 | 10 | 12 | 0C | 1 | 4 | 251896 | 2015164 | 1 | 4 | 0 | 168 | 2039956 | 1 | 4 | 0 | 1 | 176 |
| 17 | 11 | 12 | 0C | 1 | 5 | 251917 | 2015332 | 1 | 5 | 0 | 168 | 2040132 | 1 | 5 | 0 | 1 | 176 |
| 18 | 12 | 12 | 0C | 1 | 6 | 251938 | 2015500 | 1 | 6 | 0 | 168 | 2040308 | 1 | 6 | 0 | 1 | 176 |
| 19 | 13 | 12 | 0C | 1 | 7 | 251959 | 2015668 | 1 | 7 | 0 | 168 | 2040484 | 1 | 7 | 0 | 1 | 176 |
| 20 | 14 | 12 | 0C | 1 | 8 | 251980 | 2015836 | 1 | 8 | 0 | 168 | 2040660 | 1 | 8 | 0 | 1 | 176 |
| 21 | 15 | 12 | 0C | 1 | 9 | 252001 | 2016004 | 1 | 9 | 0 | 168 | 2040836 | 1 | 9 | 0 | 1 | 176 |
| 22 | 16 | 12 | 0C | 1 | 10 | 252022 | 2016172 | 1 | 10 | 0 | 168 | 2041012 | 1 | 10 | 0 | 1 | 176 |
| 23 | 17 | 12 | 0C | 1 | 11 | 252043 | 2016340 | 1 | 11 | 0 | 168 | 2041188 | 1 | 11 | 0 | 1 | 176 |
| 24 | 18 | 12 | 0C | 2 | 0 | 252062 | 2016492 | 2 | 0 | 0 | 152 | 2041348 | 2 | 0 | 0 | 1 | 160 |
| 25 | 19 | 12 | 0C | 2 | 1 | 252086 | 2016684 | 2 | 1 | 0 | 192 | 2041548 | 2 | 1 | 0 | 1 | 200 |
| 26 | 1A | 12 | 0C | 2 | 2 | 252110 | 2016876 | 2 | 2 | 0 | 192 | 2041748 | 2 | 2 | 0 | 1 | 200 |
| 27 | 1B | 12 | 0C | 2 | 3 | 252134 | 2017068 | 2 | 3 | 0 | 192 | 2041948 | 2 | 3 | 0 | 1 | 200 |
| 28 | 1C | 12 | 0C | 2 | 4 | 252158 | 2017260 | 2 | 4 | 0 | 192 | 2042148 | 2 | 4 | 0 | 1 | 200 |
| 29 | 1D | 12 | 0C | 2 | 5 | 252182 | 2017452 | 2 | 5 | 0 | 192 | 2042348 | 2 | 5 | 0 | 1 | 200 |
| 30 | 1E | 12 | 0C | 2 | 6 | 252206 | 2017644 | 2 | 6 | 0 | 192 | 2042548 | 2 | 6 | 0 | 1 | 200 |
| 31 | 1F | 12 | 0C | 2 | 7 | 252230 | 2017836 | 2 | 7 | 0 | 192 | 2042748 | 2 | 7 | 0 | 1 | 200 |
| 32 | 20 | 12 | 0C | 2 | 8 | 252253 | 2018020 | 2 | 8 | 0 | 184 | 2042940 | 2 | 8 | 0 | 1 | 192 |
| 33 | 21 | 12 | 0C | 2 | 9 | 252276 | 2018204 | 2 | 9 | 0 | 184 | 2043132 | 2 | 9 | 0 | 1 | 192 |
| 34 | 22 | 12 | 0C | 2 | 10 | 252299 | 2018388 | 2 | 10 | 0 | 184 | 2043324 | 2 | 10 | 0 | 1 | 192 |
| 35 | 23 | 12 | 0C | 2 | 11 | 252322 | 2018572 | 2 | 11 | 0 | 184 | 2043516 | 2 | 11 | 0 | 1 | 192 |
| 36 | 24 | 12 | 0C | 3 | 0 | 252343 | 2018740 | 3 | 0 | 0 | 168 | 2043692 | 3 | 0 | 0 | 1 | 176 |
| 37 | 25 | 12 | 0C | 3 | 1 | 252369 | 2018948 | 3 | 1 | 0 | 208 | 2043908 | 3 | 1 | 0 | 1 | 216 |
| 38 | 26 | 12 | 0C | 3 | 2 | 252395 | 2019156 | 3 | 2 | 0 | 208 | 2044124 | 3 | 2 | 0 | 1 | 216 |
| 39 | 27 | 12 | 0C | 3 | 3 | 252421 | 2019364 | 3 | 3 | 0 | 208 | 2044340 | 3 | 3 | 0 | 1 | 216 |
| 40 | 28 | 12 | 0C | 3 | 4 | 252447 | 2019572 | 3 | 4 | 0 | 208 | 2044556 | 3 | 4 | 0 | 1 | 216 |
| 41 | 29 | 12 | 0C | 3 | 5 | 252473 | 2019780 | 3 | 5 | 0 | 208 | 2044772 | 3 | 5 | 0 | 1 | 216 |
| 42 | 2A | 12 | 0C | 3 | 6 | 252499 | 2019988 | 3 | 6 | 0 | 208 | 2044988 | 3 | 6 | 0 | 1 | 216 |
| 43 | 2B | 12 | 0C | 3 | 7 | 252525 | 2020196 | 3 | 7 | 0 | 208 | 2045204 | 3 | 7 | 0 | 1 | 216 |
| 44 | 2C | 12 | 0C | 3 | 8 | 252551 | 2020404 | 3 | 8 | 0 | 208 | 2045420 | 3 | 8 | 0 | 1 | 216 |
| 45 | 2D | 12 | 0C | 3 | 9 | 252577 | 2020612 | 3 | 9 | 0 | 208 | 2045636 | 3 | 9 | 0 | 1 | 216 |
| 46 | 2E | 12 | 0C | 3 | 10 | 252603 | 2020820 | 3 | 10 | 0 | 208 | 2045852 | 3 | 10 | 0 | 1 | 216 |
| 47 | 2F | 12 | 0C | 3 | 11 | 252629 | 2021028 | 3 | 11 | 0 | 208 | 2046068 | 3 | 11 | 0 | 1 | 216 |
| 48 | 30 | 12 | 0C | 4 | 0 | 252652 | 2021212 | 4 | 0 | 0 | 184 | 2046260 | 4 | 0 | 0 | 1 | 192 |
| 49 | 31 | 12 | 0C | 4 | 1 | 252680 | 2021436 | 4 | 1 | 0 | 224 | 2046492 | 4 | 1 | 0 | 1 | 232 |
| 50 | 32 | 12 | 0C | 4 | 2 | 252708 | 2021660 | 4 | 2 | 0 | 224 | 2046724 | 4 | 2 | 0 | 1 | 232 |
| 51 | 33 | 12 | 0C | 4 | 3 | 252736 | 2021884 | 4 | 3 | 0 | 224 | 2046956 | 4 | 3 | 0 | 1 | 232 |
| 52 | 34 | 12 | 0C | 4 | 4 | 252764 | 2022108 | 4 | 4 | 0 | 224 | 2047188 | 4 | 4 | 0 | 1 | 232 |
| 53 | 35 | 12 | 0C | 4 | 5 | 252792 | 2022332 | 4 | 5 | 0 | 224 | 2047420 | 4 | 5 | 0 | 1 | 232 |
| 54 | 36 | 12 | 0C | 4 | 6 | 252820 | 2022556 | 4 | 6 | 0 | 224 | 2047652 | 4 | 6 | 0 | 1 | 232 |
| 55 | 37 | 12 | 0C | 4 | 7 | 252848 | 2022780 | 4 | 7 | 0 | 224 | 2047884 | 4 | 7 | 0 | 1 | 232 |
| 56 | 38 | 12 | 0C | 4 | 8 | 252876 | 2023004 | 4 | 8 | 0 | 224 | 2048116 | 4 | 8 | 0 | 1 | 232 |
| 57 | 39 | 12 | 0C | 4 | 9 | 252904 | 2023228 | 4 | 9 | 0 | 224 | 2048348 | 4 | 9 | 0 | 1 | 232 |
| 58 | 3A | 12 | 0C | 4 | 10 | 252932 | 2023452 | 4 | 10 | 0 | 224 | 2048580 | 4 | 10 | 0 | 1 | 232 |
| 59 | 3B | 12 | 0C | 4 | 11 | 252960 | 2023676 | 4 | 11 | 0 | 224 | 2048812 | 4 | 11 | 0 | 1 | 232 |
| 60 | 3C | 12 | 0C | 5 | 0 | 252990 | 2023916 | 5 | 0 | 0 | 240 | 2049060 | 5 | 0 | 0 | 1 | 248 |
| 61 | 3D | 12 | 0C | 5 | 1 | 253020 | 2024156 | 5 | 1 | 0 | 240 | 2049308 | 5 | 1 | 0 | 1 | 248 |
| 62 | 3E | 12 | 0C | 5 | 2 | 253050 | 2024396 | 5 | 2 | 0 | 240 | 2049556 | 5 | 2 | 0 | 1 | 248 |
| 63 | 3F | 12 | 0C | 5 | 3 | 253080 | 2024636 | 5 | 3 | 0 | 240 | 2049804 | 5 | 3 | 0 | 1 | 248 |
| 64 | 40 | 12 | 0C | 5 | 4 | 253110 | 2024876 | 5 | 4 | 0 | 240 | 2050052 | 5 | 4 | 0 | 1 | 248 |
| 65 | 41 | 12 | 0C | 5 | 5 | 253140 | 2025116 | 5 | 5 | 0 | 240 | 2050300 | 5 | 5 | 0 | 1 | 248 |
| 66 | 42 | 12 | 0C | 5 | 6 | 253170 | 2025356 | 5 | 6 | 0 | 240 | 2050548 | 5 | 6 | 0 | 1 | 248 |
| 67 | 43 | 12 | 0C | 5 | 7 | 253200 | 2025596 | 5 | 7 | 0 | 240 | 2050796 | 5 | 7 | 0 | 1 | 248 |
| 68 | 44 | 12 | 0C | 5 | 8 | 253230 | 2025836 | 5 | 8 | 0 | 240 | 2051044 | 5 | 8 | 0 | 1 | 248 |
| 69 | 45 | 12 | 0C | 5 | 9 | 253260 | 2026076 | 5 | 9 | 0 | 240 | 2051292 | 5 | 9 | 0 | 1 | 248 |
| 70 | 46 | 12 | 0C | 5 | 10 | 253290 | 2026316 | 5 | 10 | 0 | 240 | 2051540 | 5 | 10 | 0 | 1 | 248 |
| 71 | 47 | 12 | 0C | 5 | 11 | 253320 | 2026556 | 5 | 11 | 0 | 240 | 2051788 | 5 | 11 | 0 | 1 | 248 |
| 72 | 48 | 12 | 0C | 6 | 0 | 253352 | 2026812 | 6 | 0 | 0 | 256 | 2052052 | 6 | 0 | 0 | 1 | 264 |
| 73 | 49 | 12 | 0C | 6 | 1 | 253384 | 2027068 | 6 | 1 | 0 | 256 | 2052316 | 6 | 1 | 0 | 1 | 264 |
| 74 | 4A | 12 | 0C | 6 | 2 | 253416 | 2027324 | 6 | 2 | 0 | 256 | 2052580 | 6 | 2 | 0 | 1 | 264 |
| 75 | 4B | 12 | 0C | 6 | 3 | 253448 | 2027580 | 6 | 3 | 0 | 256 | 2052844 | 6 | 3 | 0 | 1 | 264 |
| 76 | 4C | 12 | 0C | 6 | 4 | 253480 | 2027836 | 6 | 4 | 0 | 256 | 2053108 | 6 | 4 | 0 | 1 | 264 |
| 77 | 4D | 12 | 0C | 6 | 5 | 253512 | 2028092 | 6 | 5 | 0 | 256 | 2053372 | 6 | 5 | 0 | 1 | 264 |
| 78 | 4E | 12 | 0C | 6 | 6 | 253544 | 2028348 | 6 | 6 | 0 | 256 | 2053636 | 6 | 6 | 0 | 1 | 264 |
| 79 | 4F | 12 | 0C | 6 | 7 | 253576 | 2028604 | 6 | 7 | 0 | 256 | 2053900 | 6 | 7 | 0 | 1 | 264 |
| 80 | 50 | 12 | 0C | 6 | 8 | 253608 | 2028860 | 6 | 8 | 0 | 256 | 2054164 | 6 | 8 | 0 | 1 | 264 |
| 81 | 51 | 12 | 0C | 6 | 9 | 253640 | 2029116 | 6 | 9 | 0 | 256 | 2054428 | 6 | 9 | 0 | 1 | 264 |
| 82 | 52 | 12 | 0C | 6 | 10 | 253672 | 2029372 | 6 | 10 | 0 | 256 | 2054692 | 6 | 10 | 0 | 1 | 264 |
| 83 | 53 | 12 | 0C | 6 | 11 | 253704 | 2029628 | 6 | 11 | 0 | 256 | 2054956 | 6 | 11 | 0 | 1 | 264 |
| 84 | 54 | 12 | 0C | 7 | 0 | 253738 | 2029900 | 7 | 0 | 0 | 272 | 2055236 | 7 | 0 | 0 | 1 | 280 |
| 85 | 55 | 12 | 0C | 7 | 1 | 253772 | 2030172 | 7 | 1 | 0 | 272 | 2055516 | 7 | 1 | 0 | 1 | 280 |
| 86 | 56 | 12 | 0C | 7 | 2 | 253806 | 2030444 | 7 | 2 | 0 | 272 | 2055796 | 7 | 2 | 0 | 1 | 280 |
| 87 | 57 | 12 | 0C | 7 | 3 | 253840 | 2030716 | 7 | 3 | 0 | 272 | 2056076 | 7 | 3 | 0 | 1 | 280 |
| 88 | 58 | 12 | 0C | 7 | 4 | 253874 | 2030988 | 7 | 4 | 0 | 272 | 2056356 | 7 | 4 | 0 | 1 | 280 |
| 89 | 59 | 12 | 0C | 7 | 5 | 253908 | 2031260 | 7 | 5 | 0 | 272 | 2056636 | 7 | 5 | 0 | 1 | 280 |
| 90 | 5A | 12 | 0C | 7 | 6 | 253942 | 2031532 | 7 | 6 | 0 | 272 | 2056916 | 7 | 6 | 0 | 1 | 280 |
| 91 | 5B | 12 | 0C | 7 | 7 | 253976 | 2031804 | 7 | 7 | 0 | 272 | 2057196 | 7 | 7 | 0 | 1 | 280 |
| 92 | 5C | 12 | 0C | 7 | 8 | 254010 | 2032076 | 7 | 8 | 0 | 272 | 2057476 | 7 | 8 | 0 | 1 | 280 |
| 93 | 5D | 12 | 0C | 7 | 9 | 254044 | 2032348 | 7 | 9 | 0 | 272 | 2057756 | 7 | 9 | 0 | 1 | 280 |
| 94 | 5E | 12 | 0C | 7 | 10 | 254078 | 2032620 | 7 | 10 | 0 | 272 | 2058036 | 7 | 10 | 0 | 1 | 280 |
| 95 | 5F | 12 | 0C | 7 | 11 | 254112 | 2032892 | 7 | 11 | 0 | 272 | 2058316 | 7 | 11 | 0 | 1 | 280 |
| 96 | 60 | 12 | 0C | 8 | 0 | 254148 | 2033180 | 8 | 0 | 0 | 288 | 2058612 | 8 | 0 | 0 | 1 | 296 |
| 97 | 61 | 12 | 0C | 8 | 1 | 254184 | 2033468 | 8 | 1 | 0 | 288 | 2058908 | 8 | 1 | 0 | 1 | 296 |
| 98 | 62 | 12 | 0C | 8 | 2 | 254220 | 2033756 | 8 | 2 | 0 | 288 | 2059204 | 8 | 2 | 0 | 1 | 296 |
| 99 | 63 | 12 | 0C | 8 | 3 | 254256 | 2034044 | 8 | 3 | 0 | 288 | 2059500 | 8 | 3 | 0 | 1 | 296 |
| 100 | 64 | 12 | 0C | 8 | 4 | 254292 | 2034332 | 8 | 4 | 0 | 288 | 2059796 | 8 | 4 | 0 | 1 | 296 |
| 101 | 65 | 12 | 0C | 8 | 5 | 254328 | 2034620 | 8 | 5 | 0 | 288 | 2060092 | 8 | 5 | 0 | 1 | 296 |
| 102 | 66 | 12 | 0C | 8 | 6 | 254364 | 2034908 | 8 | 6 | 0 | 288 | 2060388 | 8 | 6 | 0 | 1 | 296 |
| 103 | 67 | 12 | 0C | 8 | 7 | 254400 | 2035196 | 8 | 7 | 0 | 288 | 2060684 | 8 | 7 | 0 | 1 | 296 |
| 104 | 68 | 12 | 0C | 8 | 8 | 254436 | 2035484 | 8 | 8 | 0 | 288 | 2060980 | 8 | 8 | 0 | 1 | 296 |
| 105 | 69 | 12 | 0C | 8 | 9 | 254472 | 2035772 | 8 | 9 | 0 | 288 | 2061276 | 8 | 9 | 0 | 1 | 296 |
| 106 | 6A | 12 | 0C | 8 | 10 | 254508 | 2036060 | 8 | 10 | 0 | 288 | 2061572 | 8 | 10 | 0 | 1 | 296 |
| 107 | 6B | 12 | 0C | 8 | 11 | 254544 | 2036348 | 8 | 11 | 0 | 288 | 2061868 | 8 | 11 | 0 | 1 | 296 |
| 108 | 6C | 12 | 0C | 9 | 0 | 254582 | 2036652 | 9 | 0 | 0 | 304 | 2062180 | 9 | 0 | 0 | 1 | 312 |
| 109 | 6D | 12 | 0C | 9 | 1 | 254620 | 2036956 | 9 | 1 | 0 | 304 | 2062492 | 9 | 1 | 0 | 1 | 312 |
| 110 | 6E | 12 | 0C | 9 | 2 | 254658 | 2037260 | 9 | 2 | 0 | 304 | 2062804 | 9 | 2 | 0 | 1 | 312 |
| 111 | 6F | 12 | 0C | 9 | 3 | 254696 | 2037564 | 9 | 3 | 0 | 304 | 2063116 | 9 | 3 | 0 | 1 | 312 |
| 112 | 70 | 12 | 0C | 9 | 4 | 254734 | 2037868 | 9 | 4 | 0 | 304 | 2063428 | 9 | 4 | 0 | 1 | 312 |
| 113 | 71 | 12 | 0C | 9 | 5 | 254772 | 2038172 | 9 | 5 | 0 | 304 | 2063740 | 9 | 5 | 0 | 1 | 312 |
| 114 | 72 | 12 | 0C | 9 | 6 | 254810 | 2038476 | 9 | 6 | 0 | 304 | 2064052 | 9 | 6 | 0 | 1 | 312 |
| 115 | 73 | 12 | 0C | 9 | 7 | 254848 | 2038780 | 9 | 7 | 0 | 304 | 2064364 | 9 | 7 | 0 | 1 | 312 |
| 116 | 74 | 12 | 0C | 9 | 8 | 254886 | 2039084 | 9 | 8 | 0 | 304 | 2064676 | 9 | 8 | 0 | 1 | 312 |
| 117 | 75 | 12 | 0C | 9 | 9 | 254924 | 2039388 | 9 | 9 | 0 | 304 | 2064988 | 9 | 9 | 0 | 1 | 312 |

| | | | | | | | | | | | | | | | | | |
|---|---|---|---|---|---|---|---|---|---|---|---|---|---|---|---|---|---|
| 118 | 76 | 12 | 0C | 9 | 10 | 254962 | 2039692 | 9 | 10 | 0 | 304 | 2065300 | 9 | 10 | 0 | 1 | 312 |
| 119 | 77 | 12 | 0C | 9 | 11 | 255000 | 2039996 | 9 | 11 | 0 | 304 | 2065612 | 9 | 11 | 0 | 1 | 312 |
| 120 | 78 | 12 | 0C | 10 | 0 | 255040 | 2040316 | 10 | 0 | 0 | 320 | 2065940 | 10 | 0 | 0 | 1 | 328 |
| 121 | 79 | 12 | 0C | 10 | 1 | 255080 | 2040636 | 10 | 1 | 0 | 320 | 2066268 | 10 | 1 | 0 | 1 | 328 |
| 122 | 7A | 12 | 0C | 10 | 2 | 255120 | 2040956 | 10 | 2 | 0 | 320 | 2066596 | 10 | 2 | 0 | 1 | 328 |
| 123 | 7B | 12 | 0C | 10 | 3 | 255160 | 2041276 | 10 | 3 | 0 | 320 | 2066924 | 10 | 3 | 0 | 1 | 328 |
| 124 | 7C | 12 | 0C | 10 | 4 | 255200 | 2041596 | 10 | 4 | 0 | 320 | 2067252 | 10 | 4 | 0 | 1 | 328 |
| 125 | 7D | 12 | 0C | 10 | 5 | 255240 | 2041916 | 10 | 5 | 0 | 320 | 2067580 | 10 | 5 | 0 | 1 | 328 |
| 126 | 7E | 12 | 0C | 10 | 6 | 255280 | 2042236 | 10 | 6 | 0 | 320 | 2067908 | 10 | 6 | 0 | 1 | 328 |
| 127 | 7F | 12 | 0C | 10 | 7 | 255320 | 2042556 | 10 | 7 | 0 | 320 | 2068236 | 10 | 7 | 0 | 1 | 328 |
| 128 | 80 | 12 | 0C | 10 | 8 | 255360 | 2042876 | 10 | 8 | 0 | 320 | 2068564 | 10 | 8 | 0 | 1 | 328 |
| 129 | 81 | 12 | 0C | 10 | 9 | 255400 | 2043196 | 10 | 9 | 0 | 320 | 2068892 | 10 | 9 | 0 | 1 | 328 |
| 130 | 82 | 12 | 0C | 10 | 10 | 255440 | 2043516 | 10 | 10 | 0 | 320 | 2069220 | 10 | 10 | 0 | 1 | 328 |
| 131 | 83 | 12 | 0C | 10 | 11 | 255480 | 2043836 | 10 | 11 | 0 | 320 | 2069548 | 10 | 11 | 0 | 1 | 328 |
| 132 | 84 | 12 | 0C | 11 | 0 | 255522 | 2044172 | 11 | 0 | 0 | 336 | 2069892 | 11 | 0 | 0 | 1 | 344 |
| 133 | 85 | 12 | 0C | 11 | 1 | 255564 | 2044508 | 11 | 1 | 0 | 336 | 2070236 | 11 | 1 | 0 | 1 | 344 |
| 134 | 86 | 12 | 0C | 11 | 2 | 255606 | 2044844 | 11 | 2 | 0 | 336 | 2070580 | 11 | 2 | 0 | 1 | 344 |
| 135 | 87 | 12 | 0C | 11 | 3 | 255648 | 2045180 | 11 | 3 | 0 | 336 | 2070924 | 11 | 3 | 0 | 1 | 344 |
| 136 | 88 | 12 | 0C | 11 | 4 | 255690 | 2045516 | 11 | 4 | 0 | 336 | 2071268 | 11 | 4 | 0 | 1 | 344 |
| 137 | 89 | 12 | 0C | 11 | 5 | 255732 | 2045852 | 11 | 5 | 0 | 336 | 2071612 | 11 | 5 | 0 | 1 | 344 |
| 138 | 8A | 12 | 0C | 11 | 6 | 255774 | 2046188 | 11 | 6 | 0 | 336 | 2071956 | 11 | 6 | 0 | 1 | 344 |
| 139 | 8B | 12 | 0C | 11 | 7 | 255816 | 2046524 | 11 | 7 | 0 | 336 | 2072300 | 11 | 7 | 0 | 1 | 344 |
| 140 | 8C | 12 | 0C | 11 | 8 | 255858 | 2046860 | 11 | 8 | 0 | 336 | 2072644 | 11 | 8 | 0 | 1 | 344 |
| 141 | 8D | 12 | 0C | 11 | 9 | 255900 | 2047196 | 11 | 9 | 0 | 336 | 2072988 | 11 | 9 | 0 | 1 | 344 |
| 142 | 8E | 12 | 0C | 11 | 10 | 255942 | 2047532 | 11 | 10 | 0 | 336 | 2073332 | 11 | 10 | 0 | 1 | 344 |
| 143 | 8F | 12 | 0C | 11 | 11 | 255984 | 2047868 | 11 | 11 | 0 | 336 | 2073676 | 11 | 11 | 0 | 1 | 344 |
| 144 | 90 | 12 | 0C | 12 | 0 | 256028 | 2048220 | 12 | 0 | 0 | 352 | 2074036 | 12 | 0 | 0 | 1 | 360 |
| 145 | 91 | 12 | 0C | 12 | 1 | 256072 | 2048572 | 12 | 1 | 0 | 352 | 2074396 | 12 | 1 | 0 | 1 | 360 |
| 146 | 92 | 12 | 0C | 12 | 2 | 256116 | 2048924 | 12 | 2 | 0 | 352 | 2074756 | 12 | 2 | 0 | 1 | 360 |
| 147 | 93 | 12 | 0C | 12 | 3 | 256160 | 2049276 | 12 | 3 | 0 | 352 | 2075116 | 12 | 3 | 0 | 1 | 360 |
| 148 | 94 | 12 | 0C | 12 | 4 | 256204 | 2049628 | 12 | 4 | 0 | 352 | 2075476 | 12 | 4 | 0 | 1 | 360 |
| 149 | 95 | 12 | 0C | 12 | 5 | 256248 | 2049980 | 12 | 5 | 0 | 352 | 2075836 | 12 | 5 | 0 | 1 | 360 |
| 150 | 96 | 12 | 0C | 12 | 6 | 256292 | 2050332 | 12 | 6 | 0 | 352 | 2076196 | 12 | 6 | 0 | 1 | 360 |
| 151 | 97 | 12 | 0C | 12 | 7 | 256336 | 2050684 | 12 | 7 | 0 | 352 | 2076556 | 12 | 7 | 0 | 1 | 360 |
| 152 | 98 | 12 | 0C | 12 | 8 | 256380 | 2051036 | 12 | 8 | 0 | 352 | 2076916 | 12 | 8 | 0 | 1 | 360 |
| 153 | 99 | 12 | 0C | 12 | 9 | 256424 | 2051388 | 12 | 9 | 0 | 352 | 2077276 | 12 | 9 | 0 | 1 | 360 |
| 154 | 9A | 12 | 0C | 12 | 10 | 256468 | 2051740 | 12 | 10 | 0 | 352 | 2077636 | 12 | 10 | 0 | 1 | 360 |
| 155 | 9B | 12 | 0C | 12 | 11 | 256512 | 2052092 | 12 | 11 | 0 | 352 | 2077996 | 12 | 11 | 0 | 1 | 360 |
| 156 | 9C | 12 | 0C | 13 | 0 | 256558 | 2052460 | 13 | 0 | 0 | 368 | 2078372 | 13 | 0 | 0 | 1 | 376 |
| 157 | 9D | 12 | 0C | 13 | 1 | 256604 | 2052828 | 13 | 1 | 0 | 368 | 2078748 | 13 | 1 | 0 | 1 | 376 |
| 158 | 9E | 12 | 0C | 13 | 2 | 256650 | 2053196 | 13 | 2 | 0 | 368 | 2079124 | 13 | 2 | 0 | 1 | 376 |
| 159 | 9F | 12 | 0C | 13 | 3 | 256696 | 2053564 | 13 | 3 | 0 | 368 | 2079500 | 13 | 3 | 0 | 1 | 376 |
| 160 | A0 | 12 | 0C | 13 | 4 | 256742 | 2053932 | 13 | 4 | 0 | 368 | 2079876 | 13 | 4 | 0 | 1 | 376 |
| 161 | A1 | 12 | 0C | 13 | 5 | 256788 | 2054300 | 13 | 5 | 0 | 368 | 2080252 | 13 | 5 | 0 | 1 | 376 |
| 162 | A2 | 12 | 0C | 13 | 6 | 256834 | 2054668 | 13 | 6 | 0 | 368 | 2080628 | 13 | 6 | 0 | 1 | 376 |
| 163 | A3 | 12 | 0C | 13 | 7 | 256880 | 2055036 | 13 | 7 | 0 | 368 | 2081004 | 13 | 7 | 0 | 1 | 376 |
| 164 | A4 | 12 | 0C | 13 | 8 | 256926 | 2055404 | 13 | 8 | 0 | 368 | 2081380 | 13 | 8 | 0 | 1 | 376 |
| 165 | A5 | 12 | 0C | 13 | 9 | 256972 | 2055772 | 13 | 9 | 0 | 368 | 2081756 | 13 | 9 | 0 | 1 | 376 |
| 166 | A6 | 12 | 0C | 13 | 10 | 257018 | 2056140 | 13 | 10 | 0 | 368 | 2082132 | 13 | 10 | 0 | 1 | 376 |
| 167 | A7 | 12 | 0C | 13 | 11 | 257064 | 2056508 | 13 | 11 | 0 | 368 | 2082508 | 13 | 11 | 0 | 1 | 376 |
| 168 | A8 | 12 | 0C | 14 | 0 | 257112 | 2056892 | 14 | 0 | 0 | 384 | 2082900 | 14 | 0 | 0 | 1 | 392 |
| 169 | A9 | 12 | 0C | 14 | 1 | 257160 | 2057276 | 14 | 1 | 0 | 384 | 2083292 | 14 | 1 | 0 | 1 | 392 |
| 170 | AA | 12 | 0C | 14 | 2 | 257208 | 2057660 | 14 | 2 | 0 | 384 | 2083684 | 14 | 2 | 0 | 1 | 392 |
| 171 | AB | 12 | 0C | 14 | 3 | 257256 | 2058044 | 14 | 3 | 0 | 384 | 2084076 | 14 | 3 | 0 | 1 | 392 |
| 172 | AC | 12 | 0C | 14 | 4 | 257304 | 2058428 | 14 | 4 | 0 | 384 | 2084468 | 14 | 4 | 0 | 1 | 392 |
| 173 | AD | 12 | 0C | 14 | 5 | 257352 | 2058812 | 14 | 5 | 0 | 384 | 2084860 | 14 | 5 | 0 | 1 | 392 |
| 174 | AE | 12 | 0C | 14 | 6 | 257400 | 2059196 | 14 | 6 | 0 | 384 | 2085252 | 14 | 6 | 0 | 1 | 392 |
| 175 | AF | 12 | 0C | 14 | 7 | 257448 | 2059580 | 14 | 7 | 0 | 384 | 2085644 | 14 | 7 | 0 | 1 | 392 |
| 176 | B0 | 12 | 0C | 14 | 8 | 257496 | 2059964 | 14 | 8 | 0 | 384 | 2086036 | 14 | 8 | 0 | 1 | 392 |
| 177 | B1 | 12 | 0C | 14 | 9 | 257544 | 2060348 | 14 | 9 | 0 | 384 | 2086428 | 14 | 9 | 0 | 1 | 392 |
| 178 | B2 | 12 | 0C | 14 | 10 | 257592 | 2060732 | 14 | 10 | 0 | 384 | 2086820 | 14 | 10 | 0 | 1 | 392 |
| 179 | B3 | 12 | 0C | 14 | 11 | 257640 | 2061116 | 14 | 11 | 0 | 384 | 2087212 | 14 | 11 | 0 | 1 | 392 |
| 180 | B4 | 12 | 0C | 15 | 0 | 257690 | 2061516 | 15 | 0 | 0 | 400 | 2087620 | 15 | 0 | 0 | 1 | 408 |
| 181 | B5 | 12 | 0C | 15 | 1 | 257740 | 2061916 | 15 | 1 | 0 | 400 | 2088028 | 15 | 1 | 0 | 1 | 408 |
| 182 | B6 | 12 | 0C | 15 | 2 | 257790 | 2062316 | 15 | 2 | 0 | 400 | 2088436 | 15 | 2 | 0 | 1 | 408 |
| 183 | B7 | 12 | 0C | 15 | 3 | 257840 | 2062716 | 15 | 3 | 0 | 400 | 2088844 | 15 | 3 | 0 | 1 | 408 |
| 184 | B8 | 12 | 0C | 15 | 4 | 257890 | 2063116 | 15 | 4 | 0 | 400 | 2089252 | 15 | 4 | 0 | 1 | 408 |
| 185 | B9 | 12 | 0C | 15 | 5 | 257940 | 2063516 | 15 | 5 | 0 | 400 | 2089660 | 15 | 5 | 0 | 1 | 408 |
| 186 | BA | 12 | 0C | 15 | 6 | 257990 | 2063916 | 15 | 6 | 0 | 400 | 2090068 | 15 | 6 | 0 | 1 | 408 |
| 187 | BB | 12 | 0C | 15 | 7 | 258040 | 2064316 | 15 | 7 | 0 | 400 | 2090476 | 15 | 7 | 0 | 1 | 408 |
| 188 | BC | 12 | 0C | 15 | 8 | 258090 | 2064716 | 15 | 8 | 0 | 400 | 2090884 | 15 | 8 | 0 | 1 | 408 |
| 189 | BD | 12 | 0C | 15 | 9 | 258140 | 2065116 | 15 | 9 | 0 | 400 | 2091292 | 15 | 9 | 0 | 1 | 408 |
| 190 | BE | 12 | 0C | 15 | 10 | 258190 | 2065516 | 15 | 10 | 0 | 400 | 2091700 | 15 | 10 | 0 | 1 | 408 |
| 191 | BF | 12 | 0C | 15 | 11 | 258240 | 2065916 | 15 | 11 | 0 | 400 | 2092108 | 15 | 11 | 0 | 1 | 408 |
| 192 | C0 | 12 | 0C | 16 | 0 | 258292 | 2066332 | 16 | 0 | 0 | 416 | 2092532 | 16 | 0 | 0 | 1 | 424 |
| 193 | C1 | 12 | 0C | 16 | 1 | 258344 | 2066748 | 16 | 1 | 0 | 416 | 2092956 | 16 | 1 | 0 | 1 | 424 |
| 194 | C2 | 12 | 0C | 16 | 2 | 258396 | 2067164 | 16 | 2 | 0 | 416 | 2093380 | 16 | 2 | 0 | 1 | 424 |
| 195 | C3 | 12 | 0C | 16 | 3 | 258448 | 2067580 | 16 | 3 | 0 | 416 | 2093804 | 16 | 3 | 0 | 1 | 424 |
| 196 | C4 | 12 | 0C | 16 | 4 | 258500 | 2067996 | 16 | 4 | 0 | 416 | 2094228 | 16 | 4 | 0 | 1 | 424 |
| 197 | C5 | 12 | 0C | 16 | 5 | 258552 | 2068412 | 16 | 5 | 0 | 416 | 2094652 | 16 | 5 | 0 | 1 | 424 |
| 198 | C6 | 12 | 0C | 16 | 6 | 258604 | 2068828 | 16 | 6 | 0 | 416 | 2095076 | 16 | 6 | 0 | 1 | 424 |
| 199 | C7 | 12 | 0C | 16 | 7 | 258656 | 2069244 | 16 | 7 | 0 | 416 | 2095500 | 16 | 7 | 0 | 1 | 424 |
| 200 | C8 | 12 | 0C | 16 | 8 | 258708 | 2069660 | 16 | 8 | 0 | 416 | 2095924 | 16 | 8 | 0 | 1 | 424 |
| 201 | C9 | 12 | 0C | 16 | 9 | 258760 | 2070076 | 16 | 9 | 0 | 416 | 2096348 | 16 | 9 | 0 | 1 | 424 |
| 202 | CA | 12 | 0C | 16 | 10 | 258812 | 2070492 | 16 | 10 | 0 | 416 | 2096772 | 16 | 10 | 0 | 1 | 424 |
| 203 | CB | 12 | 0C | 16 | 11 | 258864 | 2070908 | 16 | 11 | 0 | 416 | 2097196 | 16 | 11 | 0 | 1 | 424 |
| 204 | CC | 12 | 0C | 17 | 0 | 258918 | 2071340 | 17 | 0 | 0 | 432 | 2097636 | 17 | 0 | 0 | 1 | 440 |
| 205 | CD | 12 | 0C | 17 | 1 | 258972 | 2071772 | 17 | 1 | 0 | 432 | 2098076 | 17 | 1 | 0 | 1 | 440 |
| 206 | CE | 12 | 0C | 17 | 2 | 259026 | 2072204 | 17 | 2 | 0 | 432 | 2098516 | 17 | 2 | 0 | 1 | 440 |
| 207 | CF | 12 | 0C | 17 | 3 | 259080 | 2072636 | 17 | 3 | 0 | 432 | 2098956 | 17 | 3 | 0 | 1 | 440 |
| 208 | D0 | 12 | 0C | 17 | 4 | 259134 | 2073068 | 17 | 4 | 0 | 432 | 2099396 | 17 | 4 | 0 | 1 | 440 |
| 209 | D1 | 12 | 0C | 17 | 5 | 259188 | 2073500 | 17 | 5 | 0 | 432 | 2099836 | 17 | 5 | 0 | 1 | 440 |
| 210 | D2 | 12 | 0C | 17 | 6 | 259242 | 2073932 | 17 | 6 | 0 | 432 | 2100276 | 17 | 6 | 0 | 1 | 440 |
| 211 | D3 | 12 | 0C | 17 | 7 | 259296 | 2074364 | 17 | 7 | 0 | 432 | 2100716 | 17 | 7 | 0 | 1 | 440 |
| 212 | D4 | 12 | 0C | 17 | 8 | 259350 | 2074796 | 17 | 8 | 0 | 432 | 2101156 | 17 | 8 | 0 | 1 | 440 |
| 213 | D5 | 12 | 0C | 17 | 9 | 259404 | 2075228 | 17 | 9 | 0 | 432 | 2101596 | 17 | 9 | 0 | 1 | 440 |
| 214 | D6 | 12 | 0C | 17 | 10 | 259458 | 2075660 | 17 | 10 | 0 | 432 | 2102036 | 17 | 10 | 0 | 1 | 440 |
| 215 | D7 | 12 | 0C | 17 | 11 | 259512 | 2076092 | 17 | 11 | 0 | 432 | 2102476 | 17 | 11 | 0 | 1 | 440 |
| 216 | D8 | 12 | 0C | 18 | 0 | 259568 | 2076540 | 18 | 0 | 0 | 448 | 2102932 | 18 | 0 | 0 | 1 | 456 |
| 217 | D9 | 12 | 0C | 18 | 1 | 259624 | 2076988 | 18 | 1 | 0 | 448 | 2103388 | 18 | 1 | 0 | 1 | 456 |
| 218 | DA | 12 | 0C | 18 | 2 | 259680 | 2077436 | 18 | 2 | 0 | 448 | 2103844 | 18 | 2 | 0 | 1 | 456 |
| 219 | DB | 12 | 0C | 18 | 3 | 259736 | 2077884 | 18 | 3 | 0 | 448 | 2104300 | 18 | 3 | 0 | 1 | 456 |
| 220 | DC | 12 | 0C | 18 | 4 | 259792 | 2078332 | 18 | 4 | 0 | 448 | 2104756 | 18 | 4 | 0 | 1 | 456 |

| | | | | | | | | | | | | | | | | | |
|---|---|---|---|---|---|---|---|---|---|---|---|---|---|---|---|---|---|
| 221 | DD | 12 | 0C | 18 | 5 | 259848 | 2078780 | 18 | 5 | 0 | 448 | 2105212 | 18 | 5 | 0 | 1 | 456 |
| 222 | DE | 12 | 0C | 18 | 6 | 259904 | 2079228 | 18 | 6 | 0 | 448 | 2105668 | 18 | 6 | 0 | 1 | 456 |
| 223 | DF | 12 | 0C | 18 | 7 | 259960 | 2079676 | 18 | 7 | 0 | 448 | 2106124 | 18 | 7 | 0 | 1 | 456 |
| 224 | E0 | 12 | 0C | 18 | 8 | 260016 | 2080124 | 18 | 8 | 0 | 448 | 2106580 | 18 | 8 | 0 | 1 | 456 |
| 225 | E1 | 12 | 0C | 18 | 9 | 260072 | 2080572 | 18 | 9 | 0 | 448 | 2107036 | 18 | 9 | 0 | 1 | 456 |
| 226 | E2 | 12 | 0C | 18 | 10 | 260128 | 2081020 | 18 | 10 | 0 | 448 | 2107492 | 18 | 10 | 0 | 1 | 456 |
| 227 | E3 | 12 | 0C | 18 | 11 | 260184 | 2081468 | 18 | 11 | 0 | 448 | 2107948 | 18 | 11 | 0 | 1 | 456 |
| 228 | E4 | 12 | 0C | 19 | 0 | 260242 | 2081932 | 19 | 0 | 0 | 464 | 2108420 | 19 | 0 | 0 | 1 | 472 |
| 229 | E5 | 12 | 0C | 19 | 1 | 260300 | 2082396 | 19 | 1 | 0 | 464 | 2108892 | 19 | 1 | 0 | 1 | 472 |
| 230 | E6 | 12 | 0C | 19 | 2 | 260358 | 2082860 | 19 | 2 | 0 | 464 | 2109364 | 19 | 2 | 0 | 1 | 472 |
| 231 | E7 | 12 | 0C | 19 | 3 | 260416 | 2083324 | 19 | 3 | 0 | 464 | 2109836 | 19 | 3 | 0 | 1 | 472 |
| 232 | E8 | 12 | 0C | 19 | 4 | 260474 | 2083788 | 19 | 4 | 0 | 464 | 2110308 | 19 | 4 | 0 | 1 | 472 |
| 233 | E9 | 12 | 0C | 19 | 5 | 260532 | 2084252 | 19 | 5 | 0 | 464 | 2110780 | 19 | 5 | 0 | 1 | 472 |
| 234 | EA | 12 | 0C | 19 | 6 | 260590 | 2084716 | 19 | 6 | 0 | 464 | 2111252 | 19 | 6 | 0 | 1 | 472 |
| 235 | EB | 12 | 0C | 19 | 7 | 260648 | 2085180 | 19 | 7 | 0 | 464 | 2111724 | 19 | 7 | 0 | 1 | 472 |
| 236 | EC | 12 | 0C | 19 | 8 | 260706 | 2085644 | 19 | 8 | 0 | 464 | 2112196 | 19 | 8 | 0 | 1 | 472 |
| 237 | ED | 12 | 0C | 19 | 9 | 260764 | 2086108 | 19 | 9 | 0 | 464 | 2112668 | 19 | 9 | 0 | 1 | 472 |
| 238 | EE | 12 | 0C | 19 | 10 | 260822 | 2086572 | 19 | 10 | 0 | 464 | 2113140 | 19 | 10 | 0 | 1 | 472 |
| 239 | EF | 12 | 0C | 19 | 11 | 260880 | 2087036 | 19 | 11 | 0 | 464 | 2113612 | 19 | 11 | 0 | 1 | 472 |
| 240 | F0 | 12 | 0C | 20 | 0 | 260940 | 2087516 | 20 | 0 | 0 | 480 | 2114100 | 20 | 0 | 0 | 1 | 488 |
| 241 | F1 | 12 | 0C | 20 | 1 | 261000 | 2087996 | 20 | 1 | 0 | 480 | 2114588 | 20 | 1 | 0 | 1 | 488 |
| 242 | F2 | 12 | 0C | 20 | 2 | 261060 | 2088476 | 20 | 2 | 0 | 480 | 2115076 | 20 | 2 | 0 | 1 | 488 |
| 243 | F3 | 12 | 0C | 20 | 3 | 261120 | 2088956 | 20 | 3 | 0 | 480 | 2115564 | 20 | 3 | 0 | 1 | 488 |
| 244 | F4 | 12 | 0C | 20 | 4 | 261180 | 2089436 | 20 | 4 | 0 | 480 | 2116052 | 20 | 4 | 0 | 1 | 488 |
| 245 | F5 | 12 | 0C | 20 | 5 | 261240 | 2089916 | 20 | 5 | 0 | 480 | 2116540 | 20 | 5 | 0 | 1 | 488 |
| 246 | F6 | 12 | 0C | 20 | 6 | 261300 | 2090396 | 20 | 6 | 0 | 480 | 2117028 | 20 | 6 | 0 | 1 | 488 |
| 247 | F7 | 12 | 0C | 20 | 7 | 261360 | 2090876 | 20 | 7 | 0 | 480 | 2117516 | 20 | 7 | 0 | 1 | 488 |
| 248 | F8 | 12 | 0C | 20 | 8 | 261420 | 2091356 | 20 | 8 | 0 | 480 | 2118004 | 20 | 8 | 0 | 1 | 488 |
| 249 | F9 | 12 | 0C | 20 | 9 | 261480 | 2091836 | 20 | 9 | 0 | 480 | 2118492 | 20 | 9 | 0 | 1 | 488 |
| 250 | FA | 12 | 0C | 20 | 10 | 261540 | 2092316 | 20 | 10 | 0 | 480 | 2118980 | 20 | 10 | 0 | 1 | 488 |
| 251 | FB | 12 | 0C | 20 | 11 | 261600 | 2092796 | 20 | 11 | 0 | 480 | 2119468 | 20 | 11 | 0 | 1 | 488 |
| 252 | FC | 12 | 0C | 21 | 0 | 261662 | 2093292 | 21 | 0 | 0 | 496 | 2119972 | 21 | 0 | 0 | 1 | 504 |
| 253 | FD | 12 | 0C | 21 | 1 | 261724 | 2093788 | 21 | 1 | 0 | 496 | 2120476 | 21 | 1 | 0 | 1 | 504 |
| 254 | FE | 12 | 0C | 21 | 2 | 261786 | 2094284 | 21 | 2 | 0 | 496 | 2120980 | 21 | 2 | 0 | 1 | 504 |
| 255 | FF | 12 | 0C | 21 | 3 | 261848 | 2094780 | 21 | 3 | 0 | 496 | 2121484 | 21 | 3 | 0 | 1 | 504 |
| 0 | 00 | 13 | 0D | 0 | 0 | 261860 | 2094876 | 0 | 0 | 0 | 96 | 2121588 | 0 | 0 | 0 | 1 | 104 |
| 1 | 01 | 13 | 0D | 0 | 1 | 261874 | 2094988 | 0 | 1 | 0 | 112 | 2121708 | 0 | 1 | 0 | 1 | 120 |
| 2 | 02 | 13 | 0D | 0 | 2 | 261888 | 2095100 | 0 | 2 | 0 | 112 | 2121828 | 0 | 2 | 0 | 1 | 120 |
| 3 | 03 | 13 | 0D | 0 | 3 | 261902 | 2095212 | 0 | 3 | 0 | 112 | 2121948 | 0 | 3 | 0 | 1 | 120 |
| 4 | 04 | 13 | 0D | 0 | 4 | 261916 | 2095324 | 0 | 4 | 0 | 112 | 2122068 | 0 | 4 | 0 | 1 | 120 |
| 5 | 05 | 13 | 0D | 0 | 5 | 261930 | 2095436 | 0 | 5 | 0 | 112 | 2122188 | 0 | 5 | 0 | 1 | 120 |
| 6 | 06 | 13 | 0D | 0 | 6 | 261944 | 2095548 | 0 | 6 | 0 | 112 | 2122308 | 0 | 6 | 0 | 1 | 120 |
| 7 | 07 | 13 | 0D | 0 | 7 | 261958 | 2095660 | 0 | 7 | 0 | 112 | 2122428 | 0 | 7 | 0 | 1 | 120 |
| 8 | 08 | 13 | 0D | 0 | 8 | 261972 | 2095772 | 0 | 8 | 0 | 112 | 2122548 | 0 | 8 | 0 | 1 | 120 |
| 9 | 09 | 13 | 0D | 0 | 9 | 261986 | 2095884 | 0 | 9 | 0 | 112 | 2122668 | 0 | 9 | 0 | 1 | 120 |
| 10 | 0A | 13 | 0D | 0 | 10 | 262000 | 2095996 | 0 | 10 | 0 | 112 | 2122788 | 0 | 10 | 0 | 1 | 120 |
| 11 | 0B | 13 | 0D | 0 | 11 | 262014 | 2096108 | 0 | 11 | 0 | 112 | 2122908 | 0 | 11 | 0 | 1 | 120 |
| 12 | 0C | 13 | 0D | 0 | 12 | 262028 | 2096220 | 0 | 12 | 0 | 112 | 2123028 | 0 | 12 | 0 | 1 | 120 |
| 13 | 0D | 13 | 0D | 1 | 0 | 262040 | 2096316 | 1 | 0 | 0 | 96 | 2123132 | 1 | 0 | 0 | 1 | 104 |
| 14 | 0E | 13 | 0D | 1 | 1 | 262057 | 2096452 | 1 | 1 | 0 | 136 | 2123276 | 1 | 1 | 0 | 1 | 144 |
| 15 | 0F | 13 | 0D | 1 | 2 | 262074 | 2096588 | 1 | 2 | 0 | 136 | 2123420 | 1 | 2 | 0 | 1 | 144 |
| 16 | 10 | 13 | 0D | 1 | 3 | 262095 | 2096756 | 1 | 3 | 0 | 168 | 2123596 | 1 | 3 | 0 | 1 | 176 |
| 17 | 11 | 13 | 0D | 1 | 4 | 262116 | 2096924 | 1 | 4 | 0 | 168 | 2123772 | 1 | 4 | 0 | 1 | 176 |
| 18 | 12 | 13 | 0D | 1 | 5 | 262137 | 2097092 | 1 | 5 | 0 | 168 | 2123948 | 1 | 5 | 0 | 1 | 176 |
| 19 | 13 | 13 | 0D | 1 | 6 | 262158 | 2097260 | 1 | 6 | 0 | 168 | 2124124 | 1 | 6 | 0 | 1 | 176 |
| 20 | 14 | 13 | 0D | 1 | 7 | 262179 | 2097428 | 1 | 7 | 0 | 168 | 2124300 | 1 | 7 | 0 | 1 | 176 |
| 21 | 15 | 13 | 0D | 1 | 8 | 262200 | 2097596 | 1 | 8 | 0 | 168 | 2124476 | 1 | 8 | 0 | 1 | 176 |
| 22 | 16 | 13 | 0D | 1 | 9 | 262221 | 2097764 | 1 | 9 | 0 | 168 | 2124652 | 1 | 9 | 0 | 1 | 176 |
| 23 | 17 | 13 | 0D | 1 | 10 | 262242 | 2097932 | 1 | 10 | 0 | 168 | 2124828 | 1 | 10 | 0 | 1 | 176 |
| 24 | 18 | 13 | 0D | 1 | 11 | 262263 | 2098100 | 1 | 11 | 0 | 168 | 2125004 | 1 | 11 | 0 | 1 | 176 |
| 25 | 19 | 13 | 0D | 1 | 12 | 262284 | 2098268 | 1 | 12 | 0 | 168 | 2125180 | 1 | 12 | 0 | 1 | 176 |
| 26 | 1A | 13 | 0D | 2 | 0 | 262303 | 2098420 | 2 | 0 | 0 | 152 | 2125340 | 2 | 0 | 0 | 1 | 160 |
| 27 | 1B | 13 | 0D | 2 | 1 | 262327 | 2098612 | 2 | 1 | 0 | 192 | 2125540 | 2 | 1 | 0 | 1 | 200 |
| 28 | 1C | 13 | 0D | 2 | 2 | 262351 | 2098804 | 2 | 2 | 0 | 192 | 2125740 | 2 | 2 | 0 | 1 | 200 |
| 29 | 1D | 13 | 0D | 2 | 3 | 262375 | 2098996 | 2 | 3 | 0 | 192 | 2125940 | 2 | 3 | 0 | 1 | 200 |
| 30 | 1E | 13 | 0D | 2 | 4 | 262399 | 2099188 | 2 | 4 | 0 | 192 | 2126140 | 2 | 4 | 0 | 1 | 200 |
| 31 | 1F | 13 | 0D | 2 | 5 | 262423 | 2099380 | 2 | 5 | 0 | 192 | 2126340 | 2 | 5 | 0 | 1 | 200 |
| 32 | 20 | 13 | 0D | 2 | 6 | 262446 | 2099564 | 2 | 6 | 0 | 184 | 2126532 | 2 | 6 | 0 | 1 | 192 |
| 33 | 21 | 13 | 0D | 2 | 7 | 262469 | 2099748 | 2 | 7 | 0 | 184 | 2126724 | 2 | 7 | 0 | 1 | 192 |
| 34 | 22 | 13 | 0D | 2 | 8 | 262492 | 2099932 | 2 | 8 | 0 | 184 | 2126916 | 2 | 8 | 0 | 1 | 192 |
| 35 | 23 | 13 | 0D | 2 | 9 | 262515 | 2100116 | 2 | 9 | 0 | 184 | 2127108 | 2 | 9 | 0 | 1 | 192 |
| 36 | 24 | 13 | 0D | 2 | 10 | 262538 | 2100300 | 2 | 10 | 0 | 184 | 2127300 | 2 | 10 | 0 | 1 | 192 |
| 37 | 25 | 13 | 0D | 2 | 11 | 262561 | 2100484 | 2 | 11 | 0 | 184 | 2127492 | 2 | 11 | 0 | 1 | 192 |
| 38 | 26 | 13 | 0D | 2 | 12 | 262584 | 2100668 | 2 | 12 | 0 | 184 | 2127684 | 2 | 12 | 0 | 1 | 192 |
| 39 | 27 | 13 | 0D | 3 | 0 | 262605 | 2100836 | 3 | 0 | 0 | 168 | 2127860 | 3 | 0 | 0 | 1 | 176 |
| 40 | 28 | 13 | 0D | 3 | 1 | 262631 | 2101044 | 3 | 1 | 0 | 208 | 2128076 | 3 | 1 | 0 | 1 | 216 |
| 41 | 29 | 13 | 0D | 3 | 2 | 262657 | 2101252 | 3 | 2 | 0 | 208 | 2128292 | 3 | 2 | 0 | 1 | 216 |
| 42 | 2A | 13 | 0D | 3 | 3 | 262683 | 2101460 | 3 | 3 | 0 | 208 | 2128508 | 3 | 3 | 0 | 1 | 216 |
| 43 | 2B | 13 | 0D | 3 | 4 | 262709 | 2101668 | 3 | 4 | 0 | 208 | 2128724 | 3 | 4 | 0 | 1 | 216 |
| 44 | 2C | 13 | 0D | 3 | 5 | 262735 | 2101876 | 3 | 5 | 0 | 208 | 2128940 | 3 | 5 | 0 | 1 | 216 |
| 45 | 2D | 13 | 0D | 3 | 6 | 262761 | 2102084 | 3 | 6 | 0 | 208 | 2129156 | 3 | 6 | 0 | 1 | 216 |
| 46 | 2E | 13 | 0D | 3 | 7 | 262787 | 2102292 | 3 | 7 | 0 | 208 | 2129372 | 3 | 7 | 0 | 1 | 216 |
| 47 | 2F | 13 | 0D | 3 | 8 | 262813 | 2102500 | 3 | 8 | 0 | 208 | 2129588 | 3 | 8 | 0 | 1 | 216 |
| 48 | 30 | 13 | 0D | 3 | 9 | 262838 | 2102700 | 3 | 9 | 0 | 200 | 2129796 | 3 | 9 | 0 | 1 | 208 |
| 49 | 31 | 13 | 0D | 3 | 10 | 262863 | 2102900 | 3 | 10 | 0 | 200 | 2130004 | 3 | 10 | 0 | 1 | 208 |
| 50 | 32 | 13 | 0D | 3 | 11 | 262888 | 2103100 | 3 | 11 | 0 | 200 | 2130212 | 3 | 11 | 0 | 1 | 208 |
| 51 | 33 | 13 | 0D | 3 | 12 | 262913 | 2103300 | 3 | 12 | 0 | 200 | 2130420 | 3 | 12 | 0 | 1 | 208 |
| 52 | 34 | 13 | 0D | 4 | 0 | 262936 | 2103484 | 4 | 0 | 0 | 184 | 2130612 | 4 | 0 | 0 | 1 | 192 |
| 53 | 35 | 13 | 0D | 4 | 1 | 262964 | 2103708 | 4 | 1 | 0 | 224 | 2130844 | 4 | 1 | 0 | 1 | 232 |
| 54 | 36 | 13 | 0D | 4 | 2 | 262992 | 2103932 | 4 | 2 | 0 | 224 | 2131076 | 4 | 2 | 0 | 1 | 232 |
| 55 | 37 | 13 | 0D | 4 | 3 | 263020 | 2104156 | 4 | 3 | 0 | 224 | 2131308 | 4 | 3 | 0 | 1 | 232 |
| 56 | 38 | 13 | 0D | 4 | 4 | 263048 | 2104380 | 4 | 4 | 0 | 224 | 2131540 | 4 | 4 | 0 | 1 | 232 |
| 57 | 39 | 13 | 0D | 4 | 5 | 263076 | 2104604 | 4 | 5 | 0 | 224 | 2131772 | 4 | 5 | 0 | 1 | 232 |
| 58 | 3A | 13 | 0D | 4 | 6 | 263104 | 2104828 | 4 | 6 | 0 | 224 | 2132004 | 4 | 6 | 0 | 1 | 232 |
| 59 | 3B | 13 | 0D | 4 | 7 | 263132 | 2105052 | 4 | 7 | 0 | 224 | 2132236 | 4 | 7 | 0 | 1 | 232 |
| 60 | 3C | 13 | 0D | 4 | 8 | 263160 | 2105276 | 4 | 8 | 0 | 224 | 2132468 | 4 | 8 | 0 | 1 | 232 |
| 61 | 3D | 13 | 0D | 4 | 9 | 263188 | 2105500 | 4 | 9 | 0 | 224 | 2132700 | 4 | 9 | 0 | 1 | 232 |
| 62 | 3E | 13 | 0D | 4 | 10 | 263216 | 2105724 | 4 | 10 | 0 | 224 | 2132932 | 4 | 10 | 0 | 1 | 232 |
| 63 | 3F | 13 | 0D | 4 | 11 | 263244 | 2105948 | 4 | 11 | 0 | 224 | 2133164 | 4 | 11 | 0 | 1 | 232 |
| 64 | 40 | 13 | 0D | 4 | 12 | 263271 | 2106164 | 4 | 12 | 0 | 216 | 2133388 | 4 | 12 | 0 | 1 | 224 |
| 65 | 41 | 13 | 0D | 5 | 0 | 263296 | 2106364 | 5 | 0 | 0 | 200 | 2133596 | 5 | 0 | 0 | 1 | 208 |
| 66 | 42 | 13 | 0D | 5 | 1 | 263326 | 2106604 | 5 | 1 | 0 | 240 | 2133844 | 5 | 1 | 0 | 1 | 248 |
| 67 | 43 | 13 | 0D | 5 | 2 | 263356 | 2106844 | 5 | 2 | 0 | 240 | 2134092 | 5 | 2 | 0 | 1 | 248 |

| | | | | | | | | | | | | | | | | | |
|---|---|---|---|---|---|---|---|---|---|---|---|---|---|---|---|---|---|
| 68 | 44 | 13 | 0D | 5 | 3 | 263386 | 2107084 | 5 | 3 | 0 | 240 | 2134340 | 5 | 3 | 0 | 1 | 248 |
| 69 | 45 | 13 | 0D | 5 | 4 | 263416 | 2107324 | 5 | 4 | 0 | 240 | 2134588 | 5 | 4 | 0 | 1 | 248 |
| 70 | 46 | 13 | 0D | 5 | 5 | 263446 | 2107564 | 5 | 5 | 0 | 240 | 2134836 | 5 | 5 | 0 | 1 | 248 |
| 71 | 47 | 13 | 0D | 5 | 6 | 263476 | 2107804 | 5 | 6 | 0 | 240 | 2135084 | 5 | 6 | 0 | 1 | 248 |
| 72 | 48 | 13 | 0D | 5 | 7 | 263506 | 2108044 | 5 | 7 | 0 | 240 | 2135332 | 5 | 7 | 0 | 1 | 248 |
| 73 | 49 | 13 | 0D | 5 | 8 | 263536 | 2108284 | 5 | 8 | 0 | 240 | 2135580 | 5 | 8 | 0 | 1 | 248 |
| 74 | 4A | 13 | 0D | 5 | 9 | 263566 | 2108524 | 5 | 9 | 0 | 240 | 2135828 | 5 | 9 | 0 | 1 | 248 |
| 75 | 4B | 13 | 0D | 5 | 10 | 263596 | 2108764 | 5 | 10 | 0 | 240 | 2136076 | 5 | 10 | 0 | 1 | 248 |
| 76 | 4C | 13 | 0D | 5 | 11 | 263626 | 2109004 | 5 | 11 | 0 | 240 | 2136324 | 5 | 11 | 0 | 1 | 248 |
| 77 | 4D | 13 | 0D | 5 | 12 | 263656 | 2109244 | 5 | 12 | 0 | 240 | 2136572 | 5 | 12 | 0 | 1 | 248 |
| 78 | 4E | 13 | 0D | 6 | 0 | 263688 | 2109500 | 6 | 0 | 0 | 256 | 2136836 | 6 | 0 | 0 | 1 | 264 |
| 79 | 4F | 13 | 0D | 6 | 1 | 263720 | 2109756 | 6 | 1 | 0 | 256 | 2137100 | 6 | 1 | 0 | 1 | 264 |
| 80 | 50 | 13 | 0D | 6 | 2 | 263752 | 2110012 | 6 | 2 | 0 | 256 | 2137364 | 6 | 2 | 0 | 1 | 264 |
| 81 | 51 | 13 | 0D | 6 | 3 | 263784 | 2110268 | 6 | 3 | 0 | 256 | 2137628 | 6 | 3 | 0 | 1 | 264 |
| 82 | 52 | 13 | 0D | 6 | 4 | 263816 | 2110524 | 6 | 4 | 0 | 256 | 2137892 | 6 | 4 | 0 | 1 | 264 |
| 83 | 53 | 13 | 0D | 6 | 5 | 263848 | 2110780 | 6 | 5 | 0 | 256 | 2138156 | 6 | 5 | 0 | 1 | 264 |
| 84 | 54 | 13 | 0D | 6 | 6 | 263880 | 2111036 | 6 | 6 | 0 | 256 | 2138420 | 6 | 6 | 0 | 1 | 264 |
| 85 | 55 | 13 | 0D | 6 | 7 | 263912 | 2111292 | 6 | 7 | 0 | 256 | 2138684 | 6 | 7 | 0 | 1 | 264 |
| 86 | 56 | 13 | 0D | 6 | 8 | 263944 | 2111548 | 6 | 8 | 0 | 256 | 2138948 | 6 | 8 | 0 | 1 | 264 |
| 87 | 57 | 13 | 0D | 6 | 9 | 263976 | 2111804 | 6 | 9 | 0 | 256 | 2139212 | 6 | 9 | 0 | 1 | 264 |
| 88 | 58 | 13 | 0D | 6 | 10 | 264008 | 2112060 | 6 | 10 | 0 | 256 | 2139476 | 6 | 10 | 0 | 1 | 264 |
| 89 | 59 | 13 | 0D | 6 | 11 | 264040 | 2112316 | 6 | 11 | 0 | 256 | 2139740 | 6 | 11 | 0 | 1 | 264 |
| 90 | 5A | 13 | 0D | 6 | 12 | 264072 | 2112572 | 6 | 12 | 0 | 256 | 2140004 | 6 | 12 | 0 | 1 | 264 |
| 91 | 5B | 13 | 0D | 7 | 0 | 264106 | 2112844 | 7 | 0 | 0 | 272 | 2140284 | 7 | 0 | 0 | 1 | 280 |
| 92 | 5C | 13 | 0D | 7 | 1 | 264140 | 2113116 | 7 | 1 | 0 | 272 | 2140564 | 7 | 1 | 0 | 1 | 280 |
| 93 | 5D | 13 | 0D | 7 | 2 | 264174 | 2113388 | 7 | 2 | 0 | 272 | 2140844 | 7 | 2 | 0 | 1 | 280 |
| 94 | 5E | 13 | 0D | 7 | 3 | 264208 | 2113660 | 7 | 3 | 0 | 272 | 2141124 | 7 | 3 | 0 | 1 | 280 |
| 95 | 5F | 13 | 0D | 7 | 4 | 264242 | 2113932 | 7 | 4 | 0 | 272 | 2141404 | 7 | 4 | 0 | 1 | 280 |
| 96 | 60 | 13 | 0D | 7 | 5 | 264276 | 2114204 | 7 | 5 | 0 | 272 | 2141684 | 7 | 5 | 0 | 1 | 280 |
| 97 | 61 | 13 | 0D | 7 | 6 | 264310 | 2114476 | 7 | 6 | 0 | 272 | 2141964 | 7 | 6 | 0 | 1 | 280 |
| 98 | 62 | 13 | 0D | 7 | 7 | 264344 | 2114748 | 7 | 7 | 0 | 272 | 2142244 | 7 | 7 | 0 | 1 | 280 |
| 99 | 63 | 13 | 0D | 7 | 8 | 264378 | 2115020 | 7 | 8 | 0 | 272 | 2142524 | 7 | 8 | 0 | 1 | 280 |
| 100 | 64 | 13 | 0D | 7 | 9 | 264412 | 2115292 | 7 | 9 | 0 | 272 | 2142804 | 7 | 9 | 0 | 1 | 280 |
| 101 | 65 | 13 | 0D | 7 | 10 | 264446 | 2115564 | 7 | 10 | 0 | 272 | 2143084 | 7 | 10 | 0 | 1 | 280 |
| 102 | 66 | 13 | 0D | 7 | 11 | 264480 | 2115836 | 7 | 11 | 0 | 272 | 2143364 | 7 | 11 | 0 | 1 | 280 |
| 103 | 67 | 13 | 0D | 7 | 12 | 264514 | 2116108 | 7 | 12 | 0 | 272 | 2143644 | 7 | 12 | 0 | 1 | 280 |
| 104 | 68 | 13 | 0D | 8 | 0 | 264550 | 2116396 | 8 | 0 | 0 | 288 | 2143940 | 8 | 0 | 0 | 1 | 296 |
| 105 | 69 | 13 | 0D | 8 | 1 | 264586 | 2116684 | 8 | 1 | 0 | 288 | 2144236 | 8 | 1 | 0 | 1 | 296 |
| 106 | 6A | 13 | 0D | 8 | 2 | 264622 | 2116972 | 8 | 2 | 0 | 288 | 2144532 | 8 | 2 | 0 | 1 | 296 |
| 107 | 6B | 13 | 0D | 8 | 3 | 264658 | 2117260 | 8 | 3 | 0 | 288 | 2144828 | 8 | 3 | 0 | 1 | 296 |
| 108 | 6C | 13 | 0D | 8 | 4 | 264694 | 2117548 | 8 | 4 | 0 | 288 | 2145124 | 8 | 4 | 0 | 1 | 296 |
| 109 | 6D | 13 | 0D | 8 | 5 | 264730 | 2117836 | 8 | 5 | 0 | 288 | 2145420 | 8 | 5 | 0 | 1 | 296 |
| 110 | 6E | 13 | 0D | 8 | 6 | 264766 | 2118124 | 8 | 6 | 0 | 288 | 2145716 | 8 | 6 | 0 | 1 | 296 |
| 111 | 6F | 13 | 0D | 8 | 7 | 264802 | 2118412 | 8 | 7 | 0 | 288 | 2146012 | 8 | 7 | 0 | 1 | 296 |
| 112 | 70 | 13 | 0D | 8 | 8 | 264838 | 2118700 | 8 | 8 | 0 | 288 | 2146308 | 8 | 8 | 0 | 1 | 296 |
| 113 | 71 | 13 | 0D | 8 | 9 | 264874 | 2118988 | 8 | 9 | 0 | 288 | 2146604 | 8 | 9 | 0 | 1 | 296 |
| 114 | 72 | 13 | 0D | 8 | 10 | 264910 | 2119276 | 8 | 10 | 0 | 288 | 2146900 | 8 | 10 | 0 | 1 | 296 |
| 115 | 73 | 13 | 0D | 8 | 11 | 264946 | 2119564 | 8 | 11 | 0 | 288 | 2147196 | 8 | 11 | 0 | 1 | 296 |
| 116 | 74 | 13 | 0D | 8 | 12 | 264982 | 2119852 | 8 | 12 | 0 | 288 | 2147492 | 8 | 12 | 0 | 1 | 296 |
| 117 | 75 | 13 | 0D | 9 | 0 | 265020 | 2120156 | 9 | 0 | 0 | 304 | 2147804 | 9 | 0 | 0 | 1 | 312 |
| 118 | 76 | 13 | 0D | 9 | 1 | 265058 | 2120460 | 9 | 1 | 0 | 304 | 2148116 | 9 | 1 | 0 | 1 | 312 |
| 119 | 77 | 13 | 0D | 9 | 2 | 265096 | 2120764 | 9 | 2 | 0 | 304 | 2148428 | 9 | 2 | 0 | 1 | 312 |
| 120 | 78 | 13 | 0D | 9 | 3 | 265134 | 2121068 | 9 | 3 | 0 | 304 | 2148740 | 9 | 3 | 0 | 1 | 312 |
| 121 | 79 | 13 | 0D | 9 | 4 | 265172 | 2121372 | 9 | 4 | 0 | 304 | 2149052 | 9 | 4 | 0 | 1 | 312 |
| 122 | 7A | 13 | 0D | 9 | 5 | 265210 | 2121676 | 9 | 5 | 0 | 304 | 2149364 | 9 | 5 | 0 | 1 | 312 |
| 123 | 7B | 13 | 0D | 9 | 6 | 265248 | 2121980 | 9 | 6 | 0 | 304 | 2149676 | 9 | 6 | 0 | 1 | 312 |
| 124 | 7C | 13 | 0D | 9 | 7 | 265286 | 2122284 | 9 | 7 | 0 | 304 | 2149988 | 9 | 7 | 0 | 1 | 312 |
| 125 | 7D | 13 | 0D | 9 | 8 | 265324 | 2122588 | 9 | 8 | 0 | 304 | 2150300 | 9 | 8 | 0 | 1 | 312 |
| 126 | 7E | 13 | 0D | 9 | 9 | 265362 | 2122892 | 9 | 9 | 0 | 304 | 2150612 | 9 | 9 | 0 | 1 | 312 |
| 127 | 7F | 13 | 0D | 9 | 10 | 265400 | 2123196 | 9 | 10 | 0 | 304 | 2150924 | 9 | 10 | 0 | 1 | 312 |
| 128 | 80 | 13 | 0D | 9 | 11 | 265438 | 2123500 | 9 | 11 | 0 | 304 | 2151236 | 9 | 11 | 0 | 1 | 312 |
| 129 | 81 | 13 | 0D | 9 | 12 | 265476 | 2123804 | 9 | 12 | 0 | 304 | 2151548 | 9 | 12 | 0 | 1 | 312 |
| 130 | 82 | 13 | 0D | 10 | 0 | 265516 | 2124124 | 10 | 0 | 0 | 320 | 2151876 | 10 | 0 | 0 | 1 | 328 |
| 131 | 83 | 13 | 0D | 10 | 1 | 265556 | 2124444 | 10 | 1 | 0 | 320 | 2152204 | 10 | 1 | 0 | 1 | 328 |
| 132 | 84 | 13 | 0D | 10 | 2 | 265596 | 2124764 | 10 | 2 | 0 | 320 | 2152532 | 10 | 2 | 0 | 1 | 328 |
| 133 | 85 | 13 | 0D | 10 | 3 | 265636 | 2125084 | 10 | 3 | 0 | 320 | 2152860 | 10 | 3 | 0 | 1 | 328 |
| 134 | 86 | 13 | 0D | 10 | 4 | 265676 | 2125404 | 10 | 4 | 0 | 320 | 2153188 | 10 | 4 | 0 | 1 | 328 |
| 135 | 87 | 13 | 0D | 10 | 5 | 265716 | 2125724 | 10 | 5 | 0 | 320 | 2153516 | 10 | 5 | 0 | 1 | 328 |
| 136 | 88 | 13 | 0D | 10 | 6 | 265756 | 2126044 | 10 | 6 | 0 | 320 | 2153844 | 10 | 6 | 0 | 1 | 328 |
| 137 | 89 | 13 | 0D | 10 | 7 | 265796 | 2126364 | 10 | 7 | 0 | 320 | 2154172 | 10 | 7 | 0 | 1 | 328 |
| 138 | 8A | 13 | 0D | 10 | 8 | 265836 | 2126684 | 10 | 8 | 0 | 320 | 2154500 | 10 | 8 | 0 | 1 | 328 |
| 139 | 8B | 13 | 0D | 10 | 9 | 265876 | 2127004 | 10 | 9 | 0 | 320 | 2154828 | 10 | 9 | 0 | 1 | 328 |
| 140 | 8C | 13 | 0D | 10 | 10 | 265916 | 2127324 | 10 | 10 | 0 | 320 | 2155156 | 10 | 10 | 0 | 1 | 328 |
| 141 | 8D | 13 | 0D | 10 | 11 | 265956 | 2127644 | 10 | 11 | 0 | 320 | 2155484 | 10 | 11 | 0 | 1 | 328 |
| 142 | 8E | 13 | 0D | 10 | 12 | 265996 | 2127964 | 10 | 12 | 0 | 320 | 2155812 | 10 | 12 | 0 | 1 | 328 |
| 143 | 8F | 13 | 0D | 11 | 0 | 266038 | 2128300 | 11 | 0 | 0 | 336 | 2156156 | 11 | 0 | 0 | 1 | 344 |
| 144 | 90 | 13 | 0D | 11 | 1 | 266080 | 2128636 | 11 | 1 | 0 | 336 | 2156500 | 11 | 1 | 0 | 1 | 344 |
| 145 | 91 | 13 | 0D | 11 | 2 | 266122 | 2128972 | 11 | 2 | 0 | 336 | 2156844 | 11 | 2 | 0 | 1 | 344 |
| 146 | 92 | 13 | 0D | 11 | 3 | 266164 | 2129308 | 11 | 3 | 0 | 336 | 2157188 | 11 | 3 | 0 | 1 | 344 |
| 147 | 93 | 13 | 0D | 11 | 4 | 266206 | 2129644 | 11 | 4 | 0 | 336 | 2157532 | 11 | 4 | 0 | 1 | 344 |
| 148 | 94 | 13 | 0D | 11 | 5 | 266248 | 2129980 | 11 | 5 | 0 | 336 | 2157876 | 11 | 5 | 0 | 1 | 344 |
| 149 | 95 | 13 | 0D | 11 | 6 | 266290 | 2130316 | 11 | 6 | 0 | 336 | 2158220 | 11 | 6 | 0 | 1 | 344 |
| 150 | 96 | 13 | 0D | 11 | 7 | 266332 | 2130652 | 11 | 7 | 0 | 336 | 2158564 | 11 | 7 | 0 | 1 | 344 |
| 151 | 97 | 13 | 0D | 11 | 8 | 266374 | 2130988 | 11 | 8 | 0 | 336 | 2158908 | 11 | 8 | 0 | 1 | 344 |
| 152 | 98 | 13 | 0D | 11 | 9 | 266416 | 2131324 | 11 | 9 | 0 | 336 | 2159252 | 11 | 9 | 0 | 1 | 344 |
| 153 | 99 | 13 | 0D | 11 | 10 | 266458 | 2131660 | 11 | 10 | 0 | 336 | 2159596 | 11 | 10 | 0 | 1 | 344 |
| 154 | 9A | 13 | 0D | 11 | 11 | 266500 | 2131996 | 11 | 11 | 0 | 336 | 2159940 | 11 | 11 | 0 | 1 | 344 |
| 155 | 9B | 13 | 0D | 11 | 12 | 266542 | 2132332 | 11 | 12 | 0 | 336 | 2160284 | 11 | 12 | 0 | 1 | 344 |
| 156 | 9C | 13 | 0D | 12 | 0 | 266586 | 2132684 | 12 | 0 | 0 | 352 | 2160644 | 12 | 0 | 0 | 1 | 360 |
| 157 | 9D | 13 | 0D | 12 | 1 | 266630 | 2133036 | 12 | 1 | 0 | 352 | 2161004 | 12 | 1 | 0 | 1 | 360 |
| 158 | 9E | 13 | 0D | 12 | 2 | 266674 | 2133388 | 12 | 2 | 0 | 352 | 2161364 | 12 | 2 | 0 | 1 | 360 |
| 159 | 9F | 13 | 0D | 12 | 3 | 266718 | 2133740 | 12 | 3 | 0 | 352 | 2161724 | 12 | 3 | 0 | 1 | 360 |
| 160 | A0 | 13 | 0D | 12 | 4 | 266762 | 2134092 | 12 | 4 | 0 | 352 | 2162084 | 12 | 4 | 0 | 1 | 360 |
| 161 | A1 | 13 | 0D | 12 | 5 | 266806 | 2134444 | 12 | 5 | 0 | 352 | 2162444 | 12 | 5 | 0 | 1 | 360 |
| 162 | A2 | 13 | 0D | 12 | 6 | 266850 | 2134796 | 12 | 6 | 0 | 352 | 2162804 | 12 | 6 | 0 | 1 | 360 |
| 163 | A3 | 13 | 0D | 12 | 7 | 266894 | 2135148 | 12 | 7 | 0 | 352 | 2163164 | 12 | 7 | 0 | 1 | 360 |
| 164 | A4 | 13 | 0D | 12 | 8 | 266938 | 2135500 | 12 | 8 | 0 | 352 | 2163524 | 12 | 8 | 0 | 1 | 360 |
| 165 | A5 | 13 | 0D | 12 | 9 | 266982 | 2135852 | 12 | 9 | 0 | 352 | 2163884 | 12 | 9 | 0 | 1 | 360 |
| 166 | A6 | 13 | 0D | 12 | 10 | 267026 | 2136204 | 12 | 10 | 0 | 352 | 2164244 | 12 | 10 | 0 | 1 | 360 |
| 167 | A7 | 13 | 0D | 12 | 11 | 267070 | 2136556 | 12 | 11 | 0 | 352 | 2164604 | 12 | 11 | 0 | 1 | 360 |
| 168 | A8 | 13 | 0D | 12 | 12 | 267114 | 2136908 | 12 | 12 | 0 | 352 | 2164964 | 12 | 12 | 0 | 1 | 360 |
| 169 | A9 | 13 | 0D | 13 | 0 | 267160 | 2137276 | 13 | 0 | 0 | 368 | 2165340 | 13 | 0 | 0 | 1 | 376 |
| 170 | AA | 13 | 0D | 13 | 1 | 267206 | 2137644 | 13 | 1 | 0 | 368 | 2165716 | 13 | 1 | 0 | 1 | 376 |

| | | | | | | | | | | | | | | | | | |
|---|---|---|---|---|---|---|---|---|---|---|---|---|---|---|---|---|---|
| 171 | AB | 13 | 0D | 13 | 2 | 267252 | 2138012 | 13 | 2 | 0 | 368 | 2166092 | 13 | 2 | 0 | 1 | 376 |
| 172 | AC | 13 | 0D | 13 | 3 | 267298 | 2138380 | 13 | 3 | 0 | 368 | 2166468 | 13 | 3 | 0 | 1 | 376 |
| 173 | AD | 13 | 0D | 13 | 4 | 267344 | 2138748 | 13 | 4 | 0 | 368 | 2166844 | 13 | 4 | 0 | 1 | 376 |
| 174 | AE | 13 | 0D | 13 | 5 | 267390 | 2139116 | 13 | 5 | 0 | 368 | 2167220 | 13 | 5 | 0 | 1 | 376 |
| 175 | AF | 13 | 0D | 13 | 6 | 267436 | 2139484 | 13 | 6 | 0 | 368 | 2167596 | 13 | 6 | 0 | 1 | 376 |
| 176 | B0 | 13 | 0D | 13 | 7 | 267482 | 2139852 | 13 | 7 | 0 | 368 | 2167972 | 13 | 7 | 0 | 1 | 376 |
| 177 | B1 | 13 | 0D | 13 | 8 | 267528 | 2140220 | 13 | 8 | 0 | 368 | 2168348 | 13 | 8 | 0 | 1 | 376 |
| 178 | B2 | 13 | 0D | 13 | 9 | 267574 | 2140588 | 13 | 9 | 0 | 368 | 2168724 | 13 | 9 | 0 | 1 | 376 |
| 179 | B3 | 13 | 0D | 13 | 10 | 267620 | 2140956 | 13 | 10 | 0 | 368 | 2169100 | 13 | 10 | 0 | 1 | 376 |
| 180 | B4 | 13 | 0D | 13 | 11 | 267666 | 2141324 | 13 | 11 | 0 | 368 | 2169476 | 13 | 11 | 0 | 1 | 376 |
| 181 | B5 | 13 | 0D | 13 | 12 | 267712 | 2141692 | 13 | 12 | 0 | 368 | 2169852 | 13 | 12 | 0 | 1 | 376 |
| 182 | B6 | 13 | 0D | 14 | 0 | 267760 | 2142076 | 14 | 0 | 0 | 384 | 2170244 | 14 | 0 | 0 | 1 | 392 |
| 183 | B7 | 13 | 0D | 14 | 1 | 267808 | 2142460 | 14 | 1 | 0 | 384 | 2170636 | 14 | 1 | 0 | 1 | 392 |
| 184 | B8 | 13 | 0D | 14 | 2 | 267856 | 2142844 | 14 | 2 | 0 | 384 | 2171028 | 14 | 2 | 0 | 1 | 392 |
| 185 | B9 | 13 | 0D | 14 | 3 | 267904 | 2143228 | 14 | 3 | 0 | 384 | 2171420 | 14 | 3 | 0 | 1 | 392 |
| 186 | BA | 13 | 0D | 14 | 4 | 267952 | 2143612 | 14 | 4 | 0 | 384 | 2171812 | 14 | 4 | 0 | 1 | 392 |
| 187 | BB | 13 | 0D | 14 | 5 | 268000 | 2143996 | 14 | 5 | 0 | 384 | 2172204 | 14 | 5 | 0 | 1 | 392 |
| 188 | BC | 13 | 0D | 14 | 6 | 268048 | 2144380 | 14 | 6 | 0 | 384 | 2172596 | 14 | 6 | 0 | 1 | 392 |
| 189 | BD | 13 | 0D | 14 | 7 | 268096 | 2144764 | 14 | 7 | 0 | 384 | 2172988 | 14 | 7 | 0 | 1 | 392 |
| 190 | BE | 13 | 0D | 14 | 8 | 268144 | 2145148 | 14 | 8 | 0 | 384 | 2173380 | 14 | 8 | 0 | 1 | 392 |
| 191 | BF | 13 | 0D | 14 | 9 | 268192 | 2145532 | 14 | 9 | 0 | 384 | 2173772 | 14 | 9 | 0 | 1 | 392 |
| 192 | C0 | 13 | 0D | 14 | 10 | 268240 | 2145916 | 14 | 10 | 0 | 384 | 2174164 | 14 | 10 | 0 | 1 | 392 |
| 193 | C1 | 13 | 0D | 14 | 11 | 268288 | 2146300 | 14 | 11 | 0 | 384 | 2174556 | 14 | 11 | 0 | 1 | 392 |
| 194 | C2 | 13 | 0D | 14 | 12 | 268336 | 2146684 | 14 | 12 | 0 | 384 | 2174948 | 14 | 12 | 0 | 1 | 392 |
| 195 | C3 | 13 | 0D | 15 | 0 | 268386 | 2147084 | 15 | 0 | 0 | 400 | 2175356 | 15 | 0 | 0 | 1 | 408 |
| 196 | C4 | 13 | 0D | 15 | 1 | 268436 | 2147484 | 15 | 1 | 0 | 400 | 2175764 | 15 | 1 | 0 | 1 | 408 |
| 197 | C5 | 13 | 0D | 15 | 2 | 268486 | 2147884 | 15 | 2 | 0 | 400 | 2176172 | 15 | 2 | 0 | 1 | 408 |
| 198 | C6 | 13 | 0D | 15 | 3 | 268536 | 2148284 | 15 | 3 | 0 | 400 | 2176580 | 15 | 3 | 0 | 1 | 408 |
| 199 | C7 | 13 | 0D | 15 | 4 | 268586 | 2148684 | 15 | 4 | 0 | 400 | 2176988 | 15 | 4 | 0 | 1 | 408 |
| 200 | C8 | 13 | 0D | 15 | 5 | 268636 | 2149084 | 15 | 5 | 0 | 400 | 2177396 | 15 | 5 | 0 | 1 | 408 |
| 201 | C9 | 13 | 0D | 15 | 6 | 268686 | 2149484 | 15 | 6 | 0 | 400 | 2177804 | 15 | 6 | 0 | 1 | 408 |
| 202 | CA | 13 | 0D | 15 | 7 | 268736 | 2149884 | 15 | 7 | 0 | 400 | 2178212 | 15 | 7 | 0 | 1 | 408 |
| 203 | CB | 13 | 0D | 15 | 8 | 268786 | 2150284 | 15 | 8 | 0 | 400 | 2178620 | 15 | 8 | 0 | 1 | 408 |
| 204 | CC | 13 | 0D | 15 | 9 | 268836 | 2150684 | 15 | 9 | 0 | 400 | 2179028 | 15 | 9 | 0 | 1 | 408 |
| 205 | CD | 13 | 0D | 15 | 10 | 268886 | 2151084 | 15 | 10 | 0 | 400 | 2179436 | 15 | 10 | 0 | 1 | 408 |
| 206 | CE | 13 | 0D | 15 | 11 | 268936 | 2151484 | 15 | 11 | 0 | 400 | 2179844 | 15 | 11 | 0 | 1 | 408 |
| 207 | CF | 13 | 0D | 15 | 12 | 268986 | 2151884 | 15 | 12 | 0 | 400 | 2180252 | 15 | 12 | 0 | 1 | 408 |
| 208 | D0 | 13 | 0D | 16 | 0 | 269038 | 2152300 | 16 | 0 | 0 | 416 | 2180676 | 16 | 0 | 0 | 1 | 424 |
| 209 | D1 | 13 | 0D | 16 | 1 | 269090 | 2152716 | 16 | 1 | 0 | 416 | 2181100 | 16 | 1 | 0 | 1 | 424 |
| 210 | D2 | 13 | 0D | 16 | 2 | 269142 | 2153132 | 16 | 2 | 0 | 416 | 2181524 | 16 | 2 | 0 | 1 | 424 |
| 211 | D3 | 13 | 0D | 16 | 3 | 269194 | 2153548 | 16 | 3 | 0 | 416 | 2181948 | 16 | 3 | 0 | 1 | 424 |
| 212 | D4 | 13 | 0D | 16 | 4 | 269246 | 2153964 | 16 | 4 | 0 | 416 | 2182372 | 16 | 4 | 0 | 1 | 424 |
| 213 | D5 | 13 | 0D | 16 | 5 | 269298 | 2154380 | 16 | 5 | 0 | 416 | 2182796 | 16 | 5 | 0 | 1 | 424 |
| 214 | D6 | 13 | 0D | 16 | 6 | 269350 | 2154796 | 16 | 6 | 0 | 416 | 2183220 | 16 | 6 | 0 | 1 | 424 |
| 215 | D7 | 13 | 0D | 16 | 7 | 269402 | 2155212 | 16 | 7 | 0 | 416 | 2183644 | 16 | 7 | 0 | 1 | 424 |
| 216 | D8 | 13 | 0D | 16 | 8 | 269454 | 2155628 | 16 | 8 | 0 | 416 | 2184068 | 16 | 8 | 0 | 1 | 424 |
| 217 | D9 | 13 | 0D | 16 | 9 | 269506 | 2156044 | 16 | 9 | 0 | 416 | 2184492 | 16 | 9 | 0 | 1 | 424 |
| 218 | DA | 13 | 0D | 16 | 10 | 269558 | 2156460 | 16 | 10 | 0 | 416 | 2184916 | 16 | 10 | 0 | 1 | 424 |
| 219 | DB | 13 | 0D | 16 | 11 | 269610 | 2156876 | 16 | 11 | 0 | 416 | 2185340 | 16 | 11 | 0 | 1 | 424 |
| 220 | DC | 13 | 0D | 16 | 12 | 269662 | 2157292 | 16 | 12 | 0 | 416 | 2185764 | 16 | 12 | 0 | 1 | 424 |
| 221 | DD | 13 | 0D | 17 | 0 | 269716 | 2157724 | 17 | 0 | 0 | 432 | 2186204 | 17 | 0 | 0 | 1 | 440 |
| 222 | DE | 13 | 0D | 17 | 1 | 269770 | 2158156 | 17 | 1 | 0 | 432 | 2186644 | 17 | 1 | 0 | 1 | 440 |
| 223 | DF | 13 | 0D | 17 | 2 | 269824 | 2158588 | 17 | 2 | 0 | 432 | 2187084 | 17 | 2 | 0 | 1 | 440 |
| 224 | E0 | 13 | 0D | 17 | 3 | 269878 | 2159020 | 17 | 3 | 0 | 432 | 2187524 | 17 | 3 | 0 | 1 | 440 |
| 225 | E1 | 13 | 0D | 17 | 4 | 269932 | 2159452 | 17 | 4 | 0 | 432 | 2187964 | 17 | 4 | 0 | 1 | 440 |
| 226 | E2 | 13 | 0D | 17 | 5 | 269986 | 2159884 | 17 | 5 | 0 | 432 | 2188404 | 17 | 5 | 0 | 1 | 440 |
| 227 | E3 | 13 | 0D | 17 | 6 | 270040 | 2160316 | 17 | 6 | 0 | 432 | 2188844 | 17 | 6 | 0 | 1 | 440 |
| 228 | E4 | 13 | 0D | 17 | 7 | 270094 | 2160748 | 17 | 7 | 0 | 432 | 2189284 | 17 | 7 | 0 | 1 | 440 |
| 229 | E5 | 13 | 0D | 17 | 8 | 270148 | 2161180 | 17 | 8 | 0 | 432 | 2189724 | 17 | 8 | 0 | 1 | 440 |
| 230 | E6 | 13 | 0D | 17 | 9 | 270202 | 2161612 | 17 | 9 | 0 | 432 | 2190164 | 17 | 9 | 0 | 1 | 440 |
| 231 | E7 | 13 | 0D | 17 | 10 | 270256 | 2162044 | 17 | 10 | 0 | 432 | 2190604 | 17 | 10 | 0 | 1 | 440 |
| 232 | E8 | 13 | 0D | 17 | 11 | 270310 | 2162476 | 17 | 11 | 0 | 432 | 2191044 | 17 | 11 | 0 | 1 | 440 |
| 233 | E9 | 13 | 0D | 17 | 12 | 270364 | 2162908 | 17 | 12 | 0 | 432 | 2191484 | 17 | 12 | 0 | 1 | 440 |
| 234 | EA | 13 | 0D | 18 | 0 | 270420 | 2163356 | 18 | 0 | 0 | 448 | 2191940 | 18 | 0 | 0 | 1 | 456 |
| 235 | EB | 13 | 0D | 18 | 1 | 270476 | 2163804 | 18 | 1 | 0 | 448 | 2192396 | 18 | 1 | 0 | 1 | 456 |
| 236 | EC | 13 | 0D | 18 | 2 | 270532 | 2164252 | 18 | 2 | 0 | 448 | 2192852 | 18 | 2 | 0 | 1 | 456 |
| 237 | ED | 13 | 0D | 18 | 3 | 270588 | 2164700 | 18 | 3 | 0 | 448 | 2193308 | 18 | 3 | 0 | 1 | 456 |
| 238 | EE | 13 | 0D | 18 | 4 | 270644 | 2165148 | 18 | 4 | 0 | 448 | 2193764 | 18 | 4 | 0 | 1 | 456 |
| 239 | EF | 13 | 0D | 18 | 5 | 270700 | 2165596 | 18 | 5 | 0 | 448 | 2194220 | 18 | 5 | 0 | 1 | 456 |
| 240 | F0 | 13 | 0D | 18 | 6 | 270756 | 2166044 | 18 | 6 | 0 | 448 | 2194676 | 18 | 6 | 0 | 1 | 456 |
| 241 | F1 | 13 | 0D | 18 | 7 | 270812 | 2166492 | 18 | 7 | 0 | 448 | 2195132 | 18 | 7 | 0 | 1 | 456 |
| 242 | F2 | 13 | 0D | 18 | 8 | 270868 | 2166940 | 18 | 8 | 0 | 448 | 2195588 | 18 | 8 | 0 | 1 | 456 |
| 243 | F3 | 13 | 0D | 18 | 9 | 270924 | 2167388 | 18 | 9 | 0 | 448 | 2196044 | 18 | 9 | 0 | 1 | 456 |
| 244 | F4 | 13 | 0D | 18 | 10 | 270980 | 2167836 | 18 | 10 | 0 | 448 | 2196500 | 18 | 10 | 0 | 1 | 456 |
| 245 | F5 | 13 | 0D | 18 | 11 | 271036 | 2168284 | 18 | 11 | 0 | 448 | 2196956 | 18 | 11 | 0 | 1 | 456 |
| 246 | F6 | 13 | 0D | 18 | 12 | 271092 | 2168732 | 18 | 12 | 0 | 448 | 2197412 | 18 | 12 | 0 | 1 | 456 |
| 247 | F7 | 13 | 0D | 19 | 0 | 271150 | 2169196 | 19 | 0 | 0 | 464 | 2197884 | 19 | 0 | 0 | 1 | 472 |
| 248 | F8 | 13 | 0D | 19 | 1 | 271208 | 2169660 | 19 | 1 | 0 | 464 | 2198356 | 19 | 1 | 0 | 1 | 472 |
| 249 | F9 | 13 | 0D | 19 | 2 | 271266 | 2170124 | 19 | 2 | 0 | 464 | 2198828 | 19 | 2 | 0 | 1 | 472 |
| 250 | FA | 13 | 0D | 19 | 3 | 271324 | 2170588 | 19 | 3 | 0 | 464 | 2199300 | 19 | 3 | 0 | 1 | 472 |
| 251 | FB | 13 | 0D | 19 | 4 | 271382 | 2171052 | 19 | 4 | 0 | 464 | 2199772 | 19 | 4 | 0 | 1 | 472 |
| 252 | FC | 13 | 0D | 19 | 5 | 271440 | 2171516 | 19 | 5 | 0 | 464 | 2200244 | 19 | 5 | 0 | 1 | 472 |
| 253 | FD | 13 | 0D | 19 | 6 | 271498 | 2171980 | 19 | 6 | 0 | 464 | 2200716 | 19 | 6 | 0 | 1 | 472 |
| 254 | FE | 13 | 0D | 19 | 7 | 271556 | 2172444 | 19 | 7 | 0 | 464 | 2201188 | 19 | 7 | 0 | 1 | 472 |
| 255 | FF | 13 | 0D | 19 | 8 | 271614 | 2172908 | 19 | 8 | 0 | 464 | 2201660 | 19 | 8 | 0 | 1 | 472 |
| 0 | 00 | 14 | 0E | 0 | 0 | 271626 | 2173004 | 0 | 0 | 0 | 96 | 2201764 | 0 | 0 | 0 | 1 | 104 |
| 1 | 01 | 14 | 0E | 0 | 1 | 271640 | 2173116 | 0 | 1 | 0 | 112 | 2201884 | 0 | 1 | 0 | 1 | 120 |
| 2 | 02 | 14 | 0E | 0 | 2 | 271654 | 2173228 | 0 | 2 | 0 | 112 | 2202004 | 0 | 2 | 0 | 1 | 120 |
| 3 | 03 | 14 | 0E | 0 | 3 | 271668 | 2173340 | 0 | 3 | 0 | 112 | 2202124 | 0 | 3 | 0 | 1 | 120 |
| 4 | 04 | 14 | 0E | 0 | 4 | 271682 | 2173452 | 0 | 4 | 0 | 112 | 2202244 | 0 | 4 | 0 | 1 | 120 |
| 5 | 05 | 14 | 0E | 0 | 5 | 271696 | 2173564 | 0 | 5 | 0 | 112 | 2202364 | 0 | 5 | 0 | 1 | 120 |
| 6 | 06 | 14 | 0E | 0 | 6 | 271710 | 2173676 | 0 | 6 | 0 | 112 | 2202484 | 0 | 6 | 0 | 1 | 120 |
| 7 | 07 | 14 | 0E | 0 | 7 | 271724 | 2173788 | 0 | 7 | 0 | 112 | 2202604 | 0 | 7 | 0 | 1 | 120 |
| 8 | 08 | 14 | 0E | 0 | 8 | 271738 | 2173900 | 0 | 8 | 0 | 112 | 2202724 | 0 | 8 | 0 | 1 | 120 |
| 9 | 09 | 14 | 0E | 0 | 9 | 271752 | 2174012 | 0 | 9 | 0 | 112 | 2202844 | 0 | 9 | 0 | 1 | 120 |
| 10 | 0A | 14 | 0E | 0 | 10 | 271766 | 2174124 | 0 | 10 | 0 | 112 | 2202964 | 0 | 10 | 0 | 1 | 120 |
| 11 | 0B | 14 | 0E | 0 | 11 | 271780 | 2174236 | 0 | 11 | 0 | 112 | 2203084 | 0 | 11 | 0 | 1 | 120 |
| 12 | 0C | 14 | 0E | 0 | 12 | 271794 | 2174348 | 0 | 12 | 0 | 112 | 2203204 | 0 | 12 | 0 | 1 | 120 |
| 13 | 0D | 14 | 0E | 0 | 13 | 271808 | 2174460 | 0 | 13 | 0 | 112 | 2203324 | 0 | 13 | 0 | 1 | 120 |
| 14 | 0E | 14 | 0E | 1 | 0 | 271820 | 2174556 | 1 | 0 | 0 | 96 | 2203428 | 1 | 0 | 0 | 1 | 104 |
| 15 | 0F | 14 | 0E | 1 | 1 | 271837 | 2174692 | 1 | 1 | 0 | 136 | 2203572 | 1 | 1 | 0 | 1 | 144 |
| 16 | 10 | 14 | 0E | 1 | 2 | 271858 | 2174860 | 1 | 2 | 0 | 168 | 2203748 | 1 | 2 | 0 | 1 | 176 |
| 17 | 11 | 14 | 0E | 1 | 3 | 271879 | 2175028 | 1 | 3 | 0 | 168 | 2203924 | 1 | 3 | 0 | 1 | 176 |

| | | | | | | | | | | | | | | | | | |
|---|---|---|---|---|---|---|---|---|---|---|---|---|---|---|---|---|---|
| 18 | 12 | 14 | 0E | 1 | 4 | 271900 | 2175196 | 1 | 4 | 0 | 168 | 2204100 | 1 | 4 | 0 | 1 | 176 |
| 19 | 13 | 14 | 0E | 1 | 5 | 271921 | 2175364 | 1 | 5 | 0 | 168 | 2204276 | 1 | 5 | 0 | 1 | 176 |
| 20 | 14 | 14 | 0E | 1 | 6 | 271942 | 2175532 | 1 | 6 | 0 | 168 | 2204452 | 1 | 6 | 0 | 1 | 176 |
| 21 | 15 | 14 | 0E | 1 | 7 | 271963 | 2175700 | 1 | 7 | 0 | 168 | 2204628 | 1 | 7 | 0 | 1 | 176 |
| 22 | 16 | 14 | 0E | 1 | 8 | 271984 | 2175868 | 1 | 8 | 0 | 168 | 2204804 | 1 | 8 | 0 | 1 | 176 |
| 23 | 17 | 14 | 0E | 1 | 9 | 272005 | 2176036 | 1 | 9 | 0 | 168 | 2204980 | 1 | 9 | 0 | 1 | 176 |
| 24 | 18 | 14 | 0E | 1 | 10 | 272026 | 2176204 | 1 | 10 | 0 | 168 | 2205156 | 1 | 10 | 0 | 1 | 176 |
| 25 | 19 | 14 | 0E | 1 | 11 | 272047 | 2176372 | 1 | 11 | 0 | 168 | 2205332 | 1 | 11 | 0 | 1 | 176 |
| 26 | 1A | 14 | 0E | 1 | 12 | 272068 | 2176540 | 1 | 12 | 0 | 168 | 2205508 | 1 | 12 | 0 | 1 | 176 |
| 27 | 1B | 14 | 0E | 1 | 13 | 272089 | 2176708 | 1 | 13 | 0 | 168 | 2205684 | 1 | 13 | 0 | 1 | 176 |
| 28 | 1C | 14 | 0E | 2 | 0 | 272108 | 2176860 | 2 | 0 | 0 | 152 | 2205844 | 2 | 0 | 0 | 1 | 160 |
| 29 | 1D | 14 | 0E | 2 | 1 | 272132 | 2177052 | 2 | 1 | 0 | 192 | 2206044 | 2 | 1 | 0 | 1 | 200 |
| 30 | 1E | 14 | 0E | 2 | 2 | 272156 | 2177244 | 2 | 2 | 0 | 192 | 2206244 | 2 | 2 | 0 | 1 | 200 |
| 31 | 1F | 14 | 0E | 2 | 3 | 272180 | 2177436 | 2 | 3 | 0 | 192 | 2206444 | 2 | 3 | 0 | 1 | 200 |
| 32 | 20 | 14 | 0E | 2 | 4 | 272203 | 2177620 | 2 | 4 | 0 | 184 | 2206636 | 2 | 4 | 0 | 1 | 192 |
| 33 | 21 | 14 | 0E | 2 | 5 | 272226 | 2177804 | 2 | 5 | 0 | 184 | 2206828 | 2 | 5 | 0 | 1 | 192 |
| 34 | 22 | 14 | 0E | 2 | 6 | 272249 | 2177988 | 2 | 6 | 0 | 184 | 2207020 | 2 | 6 | 0 | 1 | 192 |
| 35 | 23 | 14 | 0E | 2 | 7 | 272272 | 2178172 | 2 | 7 | 0 | 184 | 2207212 | 2 | 7 | 0 | 1 | 192 |
| 36 | 24 | 14 | 0E | 2 | 8 | 272295 | 2178356 | 2 | 8 | 0 | 184 | 2207404 | 2 | 8 | 0 | 1 | 192 |
| 37 | 25 | 14 | 0E | 2 | 9 | 272318 | 2178540 | 2 | 9 | 0 | 184 | 2207596 | 2 | 9 | 0 | 1 | 192 |
| 38 | 26 | 14 | 0E | 2 | 10 | 272341 | 2178724 | 2 | 10 | 0 | 184 | 2207788 | 2 | 10 | 0 | 1 | 192 |
| 39 | 27 | 14 | 0E | 2 | 11 | 272364 | 2178908 | 2 | 11 | 0 | 184 | 2207980 | 2 | 11 | 0 | 1 | 192 |
| 40 | 28 | 14 | 0E | 2 | 12 | 272387 | 2179092 | 2 | 12 | 0 | 184 | 2208172 | 2 | 12 | 0 | 1 | 192 |
| 41 | 29 | 14 | 0E | 2 | 13 | 272410 | 2179276 | 2 | 13 | 0 | 184 | 2208364 | 2 | 13 | 0 | 1 | 192 |
| 42 | 2A | 14 | 0E | 3 | 0 | 272431 | 2179444 | 3 | 0 | 0 | 168 | 2208540 | 3 | 0 | 0 | 1 | 176 |
| 43 | 2B | 14 | 0E | 3 | 1 | 272457 | 2179652 | 3 | 1 | 0 | 208 | 2208756 | 3 | 1 | 0 | 1 | 216 |
| 44 | 2C | 14 | 0E | 3 | 2 | 272483 | 2179860 | 3 | 2 | 0 | 208 | 2208972 | 3 | 2 | 0 | 1 | 216 |
| 45 | 2D | 14 | 0E | 3 | 3 | 272509 | 2180068 | 3 | 3 | 0 | 208 | 2209188 | 3 | 3 | 0 | 1 | 216 |
| 46 | 2E | 14 | 0E | 3 | 4 | 272535 | 2180276 | 3 | 4 | 0 | 208 | 2209404 | 3 | 4 | 0 | 1 | 216 |
| 47 | 2F | 14 | 0E | 3 | 5 | 272561 | 2180484 | 3 | 5 | 0 | 208 | 2209620 | 3 | 5 | 0 | 1 | 216 |
| 48 | 30 | 14 | 0E | 3 | 6 | 272586 | 2180684 | 3 | 6 | 0 | 200 | 2209828 | 3 | 6 | 0 | 1 | 208 |
| 49 | 31 | 14 | 0E | 3 | 7 | 272611 | 2180884 | 3 | 7 | 0 | 200 | 2210036 | 3 | 7 | 0 | 1 | 208 |
| 50 | 32 | 14 | 0E | 3 | 8 | 272636 | 2181084 | 3 | 8 | 0 | 200 | 2210244 | 3 | 8 | 0 | 1 | 208 |
| 51 | 33 | 14 | 0E | 3 | 9 | 272661 | 2181284 | 3 | 9 | 0 | 200 | 2210452 | 3 | 9 | 0 | 1 | 208 |
| 52 | 34 | 14 | 0E | 3 | 10 | 272686 | 2181484 | 3 | 10 | 0 | 200 | 2210660 | 3 | 10 | 0 | 1 | 208 |
| 53 | 35 | 14 | 0E | 3 | 11 | 272711 | 2181684 | 3 | 11 | 0 | 200 | 2210868 | 3 | 11 | 0 | 1 | 208 |
| 54 | 36 | 14 | 0E | 3 | 12 | 272736 | 2181884 | 3 | 12 | 0 | 200 | 2211076 | 3 | 12 | 0 | 1 | 208 |
| 55 | 37 | 14 | 0E | 3 | 13 | 272761 | 2182084 | 3 | 13 | 0 | 200 | 2211284 | 3 | 13 | 0 | 1 | 208 |
| 56 | 38 | 14 | 0E | 4 | 0 | 272784 | 2182268 | 4 | 0 | 0 | 184 | 2211476 | 4 | 0 | 0 | 1 | 192 |
| 57 | 39 | 14 | 0E | 4 | 1 | 272812 | 2182492 | 4 | 1 | 0 | 224 | 2211708 | 4 | 1 | 0 | 1 | 232 |
| 58 | 3A | 14 | 0E | 4 | 2 | 272840 | 2182716 | 4 | 2 | 0 | 224 | 2211940 | 4 | 2 | 0 | 1 | 232 |
| 59 | 3B | 14 | 0E | 4 | 3 | 272868 | 2182940 | 4 | 3 | 0 | 224 | 2212172 | 4 | 3 | 0 | 1 | 232 |
| 60 | 3C | 14 | 0E | 4 | 4 | 272896 | 2183164 | 4 | 4 | 0 | 224 | 2212404 | 4 | 4 | 0 | 1 | 232 |
| 61 | 3D | 14 | 0E | 4 | 5 | 272924 | 2183388 | 4 | 5 | 0 | 224 | 2212636 | 4 | 5 | 0 | 1 | 232 |
| 62 | 3E | 14 | 0E | 4 | 6 | 272952 | 2183612 | 4 | 6 | 0 | 224 | 2212868 | 4 | 6 | 0 | 1 | 232 |
| 63 | 3F | 14 | 0E | 4 | 7 | 272980 | 2183836 | 4 | 7 | 0 | 224 | 2213100 | 4 | 7 | 0 | 1 | 232 |
| 64 | 40 | 14 | 0E | 4 | 8 | 273007 | 2184052 | 4 | 8 | 0 | 216 | 2213324 | 4 | 8 | 0 | 1 | 224 |
| 65 | 41 | 14 | 0E | 4 | 9 | 273034 | 2184268 | 4 | 9 | 0 | 216 | 2213548 | 4 | 9 | 0 | 1 | 224 |
| 66 | 42 | 14 | 0E | 4 | 10 | 273061 | 2184484 | 4 | 10 | 0 | 216 | 2213772 | 4 | 10 | 0 | 1 | 224 |
| 67 | 43 | 14 | 0E | 4 | 11 | 273088 | 2184700 | 4 | 11 | 0 | 216 | 2213996 | 4 | 11 | 0 | 1 | 224 |
| 68 | 44 | 14 | 0E | 4 | 12 | 273115 | 2184916 | 4 | 12 | 0 | 216 | 2214220 | 4 | 12 | 0 | 1 | 224 |
| 69 | 45 | 14 | 0E | 4 | 13 | 273142 | 2185132 | 4 | 13 | 0 | 216 | 2214444 | 4 | 13 | 0 | 1 | 224 |
| 70 | 46 | 14 | 0E | 5 | 0 | 273167 | 2185332 | 5 | 0 | 0 | 200 | 2214652 | 5 | 0 | 0 | 1 | 208 |
| 71 | 47 | 14 | 0E | 5 | 1 | 273197 | 2185572 | 5 | 1 | 0 | 240 | 2214900 | 5 | 1 | 0 | 1 | 248 |
| 72 | 48 | 14 | 0E | 5 | 2 | 273227 | 2185812 | 5 | 2 | 0 | 240 | 2215148 | 5 | 2 | 0 | 1 | 248 |
| 73 | 49 | 14 | 0E | 5 | 3 | 273257 | 2186052 | 5 | 3 | 0 | 240 | 2215396 | 5 | 3 | 0 | 1 | 248 |
| 74 | 4A | 14 | 0E | 5 | 4 | 273287 | 2186292 | 5 | 4 | 0 | 240 | 2215644 | 5 | 4 | 0 | 1 | 248 |
| 75 | 4B | 14 | 0E | 5 | 5 | 273317 | 2186532 | 5 | 5 | 0 | 240 | 2215892 | 5 | 5 | 0 | 1 | 248 |
| 76 | 4C | 14 | 0E | 5 | 6 | 273347 | 2186772 | 5 | 6 | 0 | 240 | 2216140 | 5 | 6 | 0 | 1 | 248 |
| 77 | 4D | 14 | 0E | 5 | 7 | 273377 | 2187012 | 5 | 7 | 0 | 240 | 2216388 | 5 | 7 | 0 | 1 | 248 |
| 78 | 4E | 14 | 0E | 5 | 8 | 273407 | 2187252 | 5 | 8 | 0 | 240 | 2216636 | 5 | 8 | 0 | 1 | 248 |
| 79 | 4F | 14 | 0E | 5 | 9 | 273437 | 2187492 | 5 | 9 | 0 | 240 | 2216884 | 5 | 9 | 0 | 1 | 248 |
| 80 | 50 | 14 | 0E | 5 | 10 | 273466 | 2187724 | 5 | 10 | 0 | 232 | 2217124 | 5 | 10 | 0 | 1 | 240 |
| 81 | 51 | 14 | 0E | 5 | 11 | 273495 | 2187956 | 5 | 11 | 0 | 232 | 2217364 | 5 | 11 | 0 | 1 | 240 |
| 82 | 52 | 14 | 0E | 5 | 12 | 273524 | 2188188 | 5 | 12 | 0 | 232 | 2217604 | 5 | 12 | 0 | 1 | 240 |
| 83 | 53 | 14 | 0E | 5 | 13 | 273553 | 2188420 | 5 | 13 | 0 | 232 | 2217844 | 5 | 13 | 0 | 1 | 240 |
| 84 | 54 | 14 | 0E | 6 | 0 | 273580 | 2188636 | 6 | 0 | 0 | 216 | 2218068 | 6 | 0 | 0 | 1 | 224 |
| 85 | 55 | 14 | 0E | 6 | 1 | 273612 | 2188892 | 6 | 1 | 0 | 256 | 2218332 | 6 | 1 | 0 | 1 | 264 |
| 86 | 56 | 14 | 0E | 6 | 2 | 273644 | 2189148 | 6 | 2 | 0 | 256 | 2218596 | 6 | 2 | 0 | 1 | 264 |
| 87 | 57 | 14 | 0E | 6 | 3 | 273676 | 2189404 | 6 | 3 | 0 | 256 | 2218860 | 6 | 3 | 0 | 1 | 264 |
| 88 | 58 | 14 | 0E | 6 | 4 | 273708 | 2189660 | 6 | 4 | 0 | 256 | 2219124 | 6 | 4 | 0 | 1 | 264 |
| 89 | 59 | 14 | 0E | 6 | 5 | 273740 | 2189916 | 6 | 5 | 0 | 256 | 2219388 | 6 | 5 | 0 | 1 | 264 |
| 90 | 5A | 14 | 0E | 6 | 6 | 273772 | 2190172 | 6 | 6 | 0 | 256 | 2219652 | 6 | 6 | 0 | 1 | 264 |
| 91 | 5B | 14 | 0E | 6 | 7 | 273804 | 2190428 | 6 | 7 | 0 | 256 | 2219916 | 6 | 7 | 0 | 1 | 264 |
| 92 | 5C | 14 | 0E | 6 | 8 | 273836 | 2190684 | 6 | 8 | 0 | 256 | 2220180 | 6 | 8 | 0 | 1 | 264 |
| 93 | 5D | 14 | 0E | 6 | 9 | 273868 | 2190940 | 6 | 9 | 0 | 256 | 2220444 | 6 | 9 | 0 | 1 | 264 |
| 94 | 5E | 14 | 0E | 6 | 10 | 273900 | 2191196 | 6 | 10 | 0 | 256 | 2220708 | 6 | 10 | 0 | 1 | 264 |
| 95 | 5F | 14 | 0E | 6 | 11 | 273932 | 2191452 | 6 | 11 | 0 | 256 | 2220972 | 6 | 11 | 0 | 1 | 264 |
| 96 | 60 | 14 | 0E | 6 | 12 | 273963 | 2191700 | 6 | 12 | 0 | 248 | 2221228 | 6 | 12 | 0 | 1 | 256 |
| 97 | 61 | 14 | 0E | 6 | 13 | 273994 | 2191948 | 6 | 13 | 0 | 248 | 2221484 | 6 | 13 | 0 | 1 | 256 |
| 98 | 62 | 14 | 0E | 7 | 0 | 274023 | 2192180 | 7 | 0 | 0 | 232 | 2221724 | 7 | 0 | 0 | 1 | 240 |
| 99 | 63 | 14 | 0E | 7 | 1 | 274057 | 2192452 | 7 | 1 | 0 | 272 | 2222004 | 7 | 1 | 0 | 1 | 280 |
| 100 | 64 | 14 | 0E | 7 | 2 | 274091 | 2192724 | 7 | 2 | 0 | 272 | 2222284 | 7 | 2 | 0 | 1 | 280 |
| 101 | 65 | 14 | 0E | 7 | 3 | 274125 | 2192996 | 7 | 3 | 0 | 272 | 2222564 | 7 | 3 | 0 | 1 | 280 |
| 102 | 66 | 14 | 0E | 7 | 4 | 274159 | 2193268 | 7 | 4 | 0 | 272 | 2222844 | 7 | 4 | 0 | 1 | 280 |
| 103 | 67 | 14 | 0E | 7 | 5 | 274193 | 2193540 | 7 | 5 | 0 | 272 | 2223124 | 7 | 5 | 0 | 1 | 280 |
| 104 | 68 | 14 | 0E | 7 | 6 | 274227 | 2193812 | 7 | 6 | 0 | 272 | 2223404 | 7 | 6 | 0 | 1 | 280 |
| 105 | 69 | 14 | 0E | 7 | 7 | 274261 | 2194084 | 7 | 7 | 0 | 272 | 2223684 | 7 | 7 | 0 | 1 | 280 |
| 106 | 6A | 14 | 0E | 7 | 8 | 274295 | 2194356 | 7 | 8 | 0 | 272 | 2223964 | 7 | 8 | 0 | 1 | 280 |
| 107 | 6B | 14 | 0E | 7 | 9 | 274329 | 2194628 | 7 | 9 | 0 | 272 | 2224244 | 7 | 9 | 0 | 1 | 280 |
| 108 | 6C | 14 | 0E | 7 | 10 | 274363 | 2194900 | 7 | 10 | 0 | 272 | 2224524 | 7 | 10 | 0 | 1 | 280 |
| 109 | 6D | 14 | 0E | 7 | 11 | 274397 | 2195172 | 7 | 11 | 0 | 272 | 2224804 | 7 | 11 | 0 | 1 | 280 |
| 110 | 6E | 14 | 0E | 7 | 12 | 274431 | 2195444 | 7 | 12 | 0 | 272 | 2225084 | 7 | 12 | 0 | 1 | 280 |
| 111 | 6F | 14 | 0E | 7 | 13 | 274465 | 2195716 | 7 | 13 | 0 | 272 | 2225364 | 7 | 13 | 0 | 1 | 280 |
| 112 | 70 | 14 | 0E | 8 | 0 | 274496 | 2195964 | 8 | 0 | 0 | 248 | 2225620 | 8 | 0 | 0 | 1 | 256 |
| 113 | 71 | 14 | 0E | 8 | 1 | 274532 | 2196252 | 8 | 1 | 0 | 288 | 2225916 | 8 | 1 | 0 | 1 | 296 |
| 114 | 72 | 14 | 0E | 8 | 2 | 274568 | 2196540 | 8 | 2 | 0 | 288 | 2226212 | 8 | 2 | 0 | 1 | 296 |
| 115 | 73 | 14 | 0E | 8 | 3 | 274604 | 2196828 | 8 | 3 | 0 | 288 | 2226508 | 8 | 3 | 0 | 1 | 296 |
| 116 | 74 | 14 | 0E | 8 | 4 | 274640 | 2197116 | 8 | 4 | 0 | 288 | 2226804 | 8 | 4 | 0 | 1 | 296 |
| 117 | 75 | 14 | 0E | 8 | 5 | 274676 | 2197404 | 8 | 5 | 0 | 288 | 2227100 | 8 | 5 | 0 | 1 | 296 |
| 118 | 76 | 14 | 0E | 8 | 6 | 274712 | 2197692 | 8 | 6 | 0 | 288 | 2227396 | 8 | 6 | 0 | 1 | 296 |
| 119 | 77 | 14 | 0E | 8 | 7 | 274748 | 2197980 | 8 | 7 | 0 | 288 | 2227692 | 8 | 7 | 0 | 1 | 296 |
| 120 | 78 | 14 | 0E | 8 | 8 | 274784 | 2198268 | 8 | 8 | 0 | 288 | 2227988 | 8 | 8 | 0 | 1 | 296 |

| | | | | | | | | | | | | | | | | | |
|---|---|---|---|---|---|---|---|---|---|---|---|---|---|---|---|---|---|
| 121 | 79 | 14 | 0E | 8 | 9 | 274820 | 2198556 | 8 | 9 | 0 | 288 | 2228284 | 8 | 9 | 0 | 1 | 296 |
| 122 | 7A | 14 | 0E | 8 | 10 | 274856 | 2198844 | 8 | 10 | 0 | 288 | 2228580 | 8 | 10 | 0 | 1 | 296 |
| 123 | 7B | 14 | 0E | 8 | 11 | 274892 | 2199132 | 8 | 11 | 0 | 288 | 2228876 | 8 | 11 | 0 | 1 | 296 |
| 124 | 7C | 14 | 0E | 8 | 12 | 274928 | 2199420 | 8 | 12 | 0 | 288 | 2229172 | 8 | 12 | 0 | 1 | 296 |
| 125 | 7D | 14 | 0E | 8 | 13 | 274964 | 2199708 | 8 | 13 | 0 | 288 | 2229468 | 8 | 13 | 0 | 1 | 296 |
| 126 | 7E | 14 | 0E | 9 | 0 | 275002 | 2200012 | 9 | 0 | 0 | 304 | 2229780 | 9 | 0 | 0 | 1 | 312 |
| 127 | 7F | 14 | 0E | 9 | 1 | 275040 | 2200316 | 9 | 1 | 0 | 304 | 2230092 | 9 | 1 | 0 | 1 | 312 |
| 128 | 80 | 14 | 0E | 9 | 2 | 275078 | 2200620 | 9 | 2 | 0 | 304 | 2230404 | 9 | 2 | 0 | 1 | 312 |
| 129 | 81 | 14 | 0E | 9 | 3 | 275116 | 2200924 | 9 | 3 | 0 | 304 | 2230716 | 9 | 3 | 0 | 1 | 312 |
| 130 | 82 | 14 | 0E | 9 | 4 | 275154 | 2201228 | 9 | 4 | 0 | 304 | 2231028 | 9 | 4 | 0 | 1 | 312 |
| 131 | 83 | 14 | 0E | 9 | 5 | 275192 | 2201532 | 9 | 5 | 0 | 304 | 2231340 | 9 | 5 | 0 | 1 | 312 |
| 132 | 84 | 14 | 0E | 9 | 6 | 275230 | 2201836 | 9 | 6 | 0 | 304 | 2231652 | 9 | 6 | 0 | 1 | 312 |
| 133 | 85 | 14 | 0E | 9 | 7 | 275268 | 2202140 | 9 | 7 | 0 | 304 | 2231964 | 9 | 7 | 0 | 1 | 312 |
| 134 | 86 | 14 | 0E | 9 | 8 | 275306 | 2202444 | 9 | 8 | 0 | 304 | 2232276 | 9 | 8 | 0 | 1 | 312 |
| 135 | 87 | 14 | 0E | 9 | 9 | 275344 | 2202748 | 9 | 9 | 0 | 304 | 2232588 | 9 | 9 | 0 | 1 | 312 |
| 136 | 88 | 14 | 0E | 9 | 10 | 275382 | 2203052 | 9 | 10 | 0 | 304 | 2232900 | 9 | 10 | 0 | 1 | 312 |
| 137 | 89 | 14 | 0E | 9 | 11 | 275420 | 2203356 | 9 | 11 | 0 | 304 | 2233212 | 9 | 11 | 0 | 1 | 312 |
| 138 | 8A | 14 | 0E | 9 | 12 | 275458 | 2203660 | 9 | 12 | 0 | 304 | 2233524 | 9 | 12 | 0 | 1 | 312 |
| 139 | 8B | 14 | 0E | 9 | 13 | 275496 | 2203964 | 9 | 13 | 0 | 304 | 2233836 | 9 | 13 | 0 | 1 | 312 |
| 140 | 8C | 14 | 0E | 10 | 0 | 275536 | 2204284 | 10 | 0 | 0 | 320 | 2234164 | 10 | 0 | 0 | 1 | 328 |
| 141 | 8D | 14 | 0E | 10 | 1 | 275576 | 2204604 | 10 | 1 | 0 | 320 | 2234492 | 10 | 1 | 0 | 1 | 328 |
| 142 | 8E | 14 | 0E | 10 | 2 | 275616 | 2204924 | 10 | 2 | 0 | 320 | 2234820 | 10 | 2 | 0 | 1 | 328 |
| 143 | 8F | 14 | 0E | 10 | 3 | 275656 | 2205244 | 10 | 3 | 0 | 320 | 2235148 | 10 | 3 | 0 | 1 | 328 |
| 144 | 90 | 14 | 0E | 10 | 4 | 275696 | 2205564 | 10 | 4 | 0 | 320 | 2235476 | 10 | 4 | 0 | 1 | 328 |
| 145 | 91 | 14 | 0E | 10 | 5 | 275736 | 2205884 | 10 | 5 | 0 | 320 | 2235804 | 10 | 5 | 0 | 1 | 328 |
| 146 | 92 | 14 | 0E | 10 | 6 | 275776 | 2206204 | 10 | 6 | 0 | 320 | 2236132 | 10 | 6 | 0 | 1 | 328 |
| 147 | 93 | 14 | 0E | 10 | 7 | 275816 | 2206524 | 10 | 7 | 0 | 320 | 2236460 | 10 | 7 | 0 | 1 | 328 |
| 148 | 94 | 14 | 0E | 10 | 8 | 275856 | 2206844 | 10 | 8 | 0 | 320 | 2236788 | 10 | 8 | 0 | 1 | 328 |
| 149 | 95 | 14 | 0E | 10 | 9 | 275896 | 2207164 | 10 | 9 | 0 | 320 | 2237116 | 10 | 9 | 0 | 1 | 328 |
| 150 | 96 | 14 | 0E | 10 | 10 | 275936 | 2207484 | 10 | 10 | 0 | 320 | 2237444 | 10 | 10 | 0 | 1 | 328 |
| 151 | 97 | 14 | 0E | 10 | 11 | 275976 | 2207804 | 10 | 11 | 0 | 320 | 2237772 | 10 | 11 | 0 | 1 | 328 |
| 152 | 98 | 14 | 0E | 10 | 12 | 276016 | 2208124 | 10 | 12 | 0 | 320 | 2238100 | 10 | 12 | 0 | 1 | 328 |
| 153 | 99 | 14 | 0E | 10 | 13 | 276056 | 2208444 | 10 | 13 | 0 | 320 | 2238428 | 10 | 13 | 0 | 1 | 328 |
| 154 | 9A | 14 | 0E | 11 | 0 | 276098 | 2208780 | 11 | 0 | 0 | 336 | 2238772 | 11 | 0 | 0 | 1 | 344 |
| 155 | 9B | 14 | 0E | 11 | 1 | 276140 | 2209116 | 11 | 1 | 0 | 336 | 2239116 | 11 | 1 | 0 | 1 | 344 |
| 156 | 9C | 14 | 0E | 11 | 2 | 276182 | 2209452 | 11 | 2 | 0 | 336 | 2239460 | 11 | 2 | 0 | 1 | 344 |
| 157 | 9D | 14 | 0E | 11 | 3 | 276224 | 2209788 | 11 | 3 | 0 | 336 | 2239804 | 11 | 3 | 0 | 1 | 344 |
| 158 | 9E | 14 | 0E | 11 | 4 | 276266 | 2210124 | 11 | 4 | 0 | 336 | 2240148 | 11 | 4 | 0 | 1 | 344 |
| 159 | 9F | 14 | 0E | 11 | 5 | 276308 | 2210460 | 11 | 5 | 0 | 336 | 2240492 | 11 | 5 | 0 | 1 | 344 |
| 160 | A0 | 14 | 0E | 11 | 6 | 276350 | 2210796 | 11 | 6 | 0 | 336 | 2240836 | 11 | 6 | 0 | 1 | 344 |
| 161 | A1 | 14 | 0E | 11 | 7 | 276392 | 2211132 | 11 | 7 | 0 | 336 | 2241180 | 11 | 7 | 0 | 1 | 344 |
| 162 | A2 | 14 | 0E | 11 | 8 | 276434 | 2211468 | 11 | 8 | 0 | 336 | 2241524 | 11 | 8 | 0 | 1 | 344 |
| 163 | A3 | 14 | 0E | 11 | 9 | 276476 | 2211804 | 11 | 9 | 0 | 336 | 2241868 | 11 | 9 | 0 | 1 | 344 |
| 164 | A4 | 14 | 0E | 11 | 10 | 276518 | 2212140 | 11 | 10 | 0 | 336 | 2242212 | 11 | 10 | 0 | 1 | 344 |
| 165 | A5 | 14 | 0E | 11 | 11 | 276560 | 2212476 | 11 | 11 | 0 | 336 | 2242556 | 11 | 11 | 0 | 1 | 344 |
| 166 | A6 | 14 | 0E | 11 | 12 | 276602 | 2212812 | 11 | 12 | 0 | 336 | 2242900 | 11 | 12 | 0 | 1 | 344 |
| 167 | A7 | 14 | 0E | 11 | 13 | 276644 | 2213148 | 11 | 13 | 0 | 336 | 2243244 | 11 | 13 | 0 | 1 | 344 |
| 168 | A8 | 14 | 0E | 12 | 0 | 276688 | 2213500 | 12 | 0 | 0 | 352 | 2243604 | 12 | 0 | 0 | 1 | 360 |
| 169 | A9 | 14 | 0E | 12 | 1 | 276732 | 2213852 | 12 | 1 | 0 | 352 | 2243964 | 12 | 1 | 0 | 1 | 360 |
| 170 | AA | 14 | 0E | 12 | 2 | 276776 | 2214204 | 12 | 2 | 0 | 352 | 2244324 | 12 | 2 | 0 | 1 | 360 |
| 171 | AB | 14 | 0E | 12 | 3 | 276820 | 2214556 | 12 | 3 | 0 | 352 | 2244684 | 12 | 3 | 0 | 1 | 360 |
| 172 | AC | 14 | 0E | 12 | 4 | 276864 | 2214908 | 12 | 4 | 0 | 352 | 2245044 | 12 | 4 | 0 | 1 | 360 |
| 173 | AD | 14 | 0E | 12 | 5 | 276908 | 2215260 | 12 | 5 | 0 | 352 | 2245404 | 12 | 5 | 0 | 1 | 360 |
| 174 | AE | 14 | 0E | 12 | 6 | 276952 | 2215612 | 12 | 6 | 0 | 352 | 2245764 | 12 | 6 | 0 | 1 | 360 |
| 175 | AF | 14 | 0E | 12 | 7 | 276996 | 2215964 | 12 | 7 | 0 | 352 | 2246124 | 12 | 7 | 0 | 1 | 360 |
| 176 | B0 | 14 | 0E | 12 | 8 | 277040 | 2216316 | 12 | 8 | 0 | 352 | 2246484 | 12 | 8 | 0 | 1 | 360 |
| 177 | B1 | 14 | 0E | 12 | 9 | 277084 | 2216668 | 12 | 9 | 0 | 352 | 2246844 | 12 | 9 | 0 | 1 | 360 |
| 178 | B2 | 14 | 0E | 12 | 10 | 277128 | 2217020 | 12 | 10 | 0 | 352 | 2247204 | 12 | 10 | 0 | 1 | 360 |
| 179 | B3 | 14 | 0E | 12 | 11 | 277172 | 2217372 | 12 | 11 | 0 | 352 | 2247564 | 12 | 11 | 0 | 1 | 360 |
| 180 | B4 | 14 | 0E | 12 | 12 | 277216 | 2217724 | 12 | 12 | 0 | 352 | 2247924 | 12 | 12 | 0 | 1 | 360 |
| 181 | B5 | 14 | 0E | 12 | 13 | 277260 | 2218076 | 12 | 13 | 0 | 352 | 2248284 | 12 | 13 | 0 | 1 | 360 |
| 182 | B6 | 14 | 0E | 13 | 0 | 277306 | 2218444 | 13 | 0 | 0 | 368 | 2248660 | 13 | 0 | 0 | 1 | 376 |
| 183 | B7 | 14 | 0E | 13 | 1 | 277352 | 2218812 | 13 | 1 | 0 | 368 | 2249036 | 13 | 1 | 0 | 1 | 376 |
| 184 | B8 | 14 | 0E | 13 | 2 | 277398 | 2219180 | 13 | 2 | 0 | 368 | 2249412 | 13 | 2 | 0 | 1 | 376 |
| 185 | B9 | 14 | 0E | 13 | 3 | 277444 | 2219548 | 13 | 3 | 0 | 368 | 2249788 | 13 | 3 | 0 | 1 | 376 |
| 186 | BA | 14 | 0E | 13 | 4 | 277490 | 2219916 | 13 | 4 | 0 | 368 | 2250164 | 13 | 4 | 0 | 1 | 376 |
| 187 | BB | 14 | 0E | 13 | 5 | 277536 | 2220284 | 13 | 5 | 0 | 368 | 2250540 | 13 | 5 | 0 | 1 | 376 |
| 188 | BC | 14 | 0E | 13 | 6 | 277582 | 2220652 | 13 | 6 | 0 | 368 | 2250916 | 13 | 6 | 0 | 1 | 376 |
| 189 | BD | 14 | 0E | 13 | 7 | 277628 | 2221020 | 13 | 7 | 0 | 368 | 2251292 | 13 | 7 | 0 | 1 | 376 |
| 190 | BE | 14 | 0E | 13 | 8 | 277674 | 2221388 | 13 | 8 | 0 | 368 | 2251668 | 13 | 8 | 0 | 1 | 376 |
| 191 | BF | 14 | 0E | 13 | 9 | 277720 | 2221756 | 13 | 9 | 0 | 368 | 2252044 | 13 | 9 | 0 | 1 | 376 |
| 192 | C0 | 14 | 0E | 13 | 10 | 277766 | 2222124 | 13 | 10 | 0 | 368 | 2252420 | 13 | 10 | 0 | 1 | 376 |
| 193 | C1 | 14 | 0E | 13 | 11 | 277812 | 2222492 | 13 | 11 | 0 | 368 | 2252796 | 13 | 11 | 0 | 1 | 376 |
| 194 | C2 | 14 | 0E | 13 | 12 | 277858 | 2222860 | 13 | 12 | 0 | 368 | 2253172 | 13 | 12 | 0 | 1 | 376 |
| 195 | C3 | 14 | 0E | 13 | 13 | 277904 | 2223228 | 13 | 13 | 0 | 368 | 2253548 | 13 | 13 | 0 | 1 | 376 |
| 196 | C4 | 14 | 0E | 14 | 0 | 277952 | 2223612 | 14 | 0 | 0 | 384 | 2253940 | 14 | 0 | 0 | 1 | 392 |
| 197 | C5 | 14 | 0E | 14 | 1 | 278000 | 2223996 | 14 | 1 | 0 | 384 | 2254332 | 14 | 1 | 0 | 1 | 392 |
| 198 | C6 | 14 | 0E | 14 | 2 | 278048 | 2224380 | 14 | 2 | 0 | 384 | 2254724 | 14 | 2 | 0 | 1 | 392 |
| 199 | C7 | 14 | 0E | 14 | 3 | 278096 | 2224764 | 14 | 3 | 0 | 384 | 2255116 | 14 | 3 | 0 | 1 | 392 |
| 200 | C8 | 14 | 0E | 14 | 4 | 278144 | 2225148 | 14 | 4 | 0 | 384 | 2255508 | 14 | 4 | 0 | 1 | 392 |
| 201 | C9 | 14 | 0E | 14 | 5 | 278192 | 2225532 | 14 | 5 | 0 | 384 | 2255900 | 14 | 5 | 0 | 1 | 392 |
| 202 | CA | 14 | 0E | 14 | 6 | 278240 | 2225916 | 14 | 6 | 0 | 384 | 2256292 | 14 | 6 | 0 | 1 | 392 |
| 203 | CB | 14 | 0E | 14 | 7 | 278288 | 2226300 | 14 | 7 | 0 | 384 | 2256684 | 14 | 7 | 0 | 1 | 392 |
| 204 | CC | 14 | 0E | 14 | 8 | 278336 | 2226684 | 14 | 8 | 0 | 384 | 2257076 | 14 | 8 | 0 | 1 | 392 |
| 205 | CD | 14 | 0E | 14 | 9 | 278384 | 2227068 | 14 | 9 | 0 | 384 | 2257468 | 14 | 9 | 0 | 1 | 392 |
| 206 | CE | 14 | 0E | 14 | 10 | 278432 | 2227452 | 14 | 10 | 0 | 384 | 2257860 | 14 | 10 | 0 | 1 | 392 |
| 207 | CF | 14 | 0E | 14 | 11 | 278480 | 2227836 | 14 | 11 | 0 | 384 | 2258252 | 14 | 11 | 0 | 1 | 392 |
| 208 | D0 | 14 | 0E | 14 | 12 | 278528 | 2228220 | 14 | 12 | 0 | 384 | 2258644 | 14 | 12 | 0 | 1 | 392 |
| 209 | D1 | 14 | 0E | 14 | 13 | 278576 | 2228604 | 14 | 13 | 0 | 384 | 2259036 | 14 | 13 | 0 | 1 | 392 |
| 210 | D2 | 14 | 0E | 15 | 0 | 278626 | 2229004 | 15 | 0 | 0 | 400 | 2259444 | 15 | 0 | 0 | 1 | 408 |
| 211 | D3 | 14 | 0E | 15 | 1 | 278676 | 2229404 | 15 | 1 | 0 | 400 | 2259852 | 15 | 1 | 0 | 1 | 408 |
| 212 | D4 | 14 | 0E | 15 | 2 | 278726 | 2229804 | 15 | 2 | 0 | 400 | 2260260 | 15 | 2 | 0 | 1 | 408 |
| 213 | D5 | 14 | 0E | 15 | 3 | 278776 | 2230204 | 15 | 3 | 0 | 400 | 2260668 | 15 | 3 | 0 | 1 | 408 |
| 214 | D6 | 14 | 0E | 15 | 4 | 278826 | 2230604 | 15 | 4 | 0 | 400 | 2261076 | 15 | 4 | 0 | 1 | 408 |
| 215 | D7 | 14 | 0E | 15 | 5 | 278876 | 2231004 | 15 | 5 | 0 | 400 | 2261484 | 15 | 5 | 0 | 1 | 408 |
| 216 | D8 | 14 | 0E | 15 | 6 | 278926 | 2231404 | 15 | 6 | 0 | 400 | 2261892 | 15 | 6 | 0 | 1 | 408 |
| 217 | D9 | 14 | 0E | 15 | 7 | 278976 | 2231804 | 15 | 7 | 0 | 400 | 2262300 | 15 | 7 | 0 | 1 | 408 |
| 218 | DA | 14 | 0E | 15 | 8 | 279026 | 2232204 | 15 | 8 | 0 | 400 | 2262708 | 15 | 8 | 0 | 1 | 408 |
| 219 | DB | 14 | 0E | 15 | 9 | 279076 | 2232604 | 15 | 9 | 0 | 400 | 2263116 | 15 | 9 | 0 | 1 | 408 |
| 220 | DC | 14 | 0E | 15 | 10 | 279126 | 2233004 | 15 | 10 | 0 | 400 | 2263524 | 15 | 10 | 0 | 1 | 408 |
| 221 | DD | 14 | 0E | 15 | 11 | 279176 | 2233404 | 15 | 11 | 0 | 400 | 2263932 | 15 | 11 | 0 | 1 | 408 |
| 222 | DE | 14 | 0E | 15 | 12 | 279226 | 2233804 | 15 | 12 | 0 | 400 | 2264340 | 15 | 12 | 0 | 1 | 408 |
| 223 | DF | 14 | 0E | 15 | 13 | 279276 | 2234204 | 15 | 13 | 0 | 400 | 2264748 | 15 | 13 | 0 | 1 | 408 |

| | | | | | | | | | | | | | | | | | |
|---|---|---|---|---|---|---|---|---|---|---|---|---|---|---|---|---|---|
| 224 | E0 | 14 | 0E | 16 | 0 | 279328 | 2234620 | 16 | 0 | 0 | 416 | 2265172 | 16 | 0 | 0 | 1 | 424 |
| 225 | E1 | 14 | 0E | 16 | 1 | 279380 | 2235036 | 16 | 1 | 0 | 416 | 2265596 | 16 | 1 | 0 | 1 | 424 |
| 226 | E2 | 14 | 0E | 16 | 2 | 279432 | 2235452 | 16 | 2 | 0 | 416 | 2266020 | 16 | 2 | 0 | 1 | 424 |
| 227 | E3 | 14 | 0E | 16 | 3 | 279484 | 2235868 | 16 | 3 | 0 | 416 | 2266444 | 16 | 3 | 0 | 1 | 424 |
| 228 | E4 | 14 | 0E | 16 | 4 | 279536 | 2236284 | 16 | 4 | 0 | 416 | 2266868 | 16 | 4 | 0 | 1 | 424 |
| 229 | E5 | 14 | 0E | 16 | 5 | 279588 | 2236700 | 16 | 5 | 0 | 416 | 2267292 | 16 | 5 | 0 | 1 | 424 |
| 230 | E6 | 14 | 0E | 16 | 6 | 279640 | 2237116 | 16 | 6 | 0 | 416 | 2267716 | 16 | 6 | 0 | 1 | 424 |
| 231 | E7 | 14 | 0E | 16 | 7 | 279692 | 2237532 | 16 | 7 | 0 | 416 | 2268140 | 16 | 7 | 0 | 1 | 424 |
| 232 | E8 | 14 | 0E | 16 | 8 | 279744 | 2237948 | 16 | 8 | 0 | 416 | 2268564 | 16 | 8 | 0 | 1 | 424 |
| 233 | E9 | 14 | 0E | 16 | 9 | 279796 | 2238364 | 16 | 9 | 0 | 416 | 2268988 | 16 | 9 | 0 | 1 | 424 |
| 234 | EA | 14 | 0E | 16 | 10 | 279848 | 2238780 | 16 | 10 | 0 | 416 | 2269412 | 16 | 10 | 0 | 1 | 424 |
| 235 | EB | 14 | 0E | 16 | 11 | 279900 | 2239196 | 16 | 11 | 0 | 416 | 2269836 | 16 | 11 | 0 | 1 | 424 |
| 236 | EC | 14 | 0E | 16 | 12 | 279952 | 2239612 | 16 | 12 | 0 | 416 | 2270260 | 16 | 12 | 0 | 1 | 424 |
| 237 | ED | 14 | 0E | 16 | 13 | 280004 | 2240028 | 16 | 13 | 0 | 416 | 2270684 | 16 | 13 | 0 | 1 | 424 |
| 238 | EE | 14 | 0E | 17 | 0 | 280058 | 2240460 | 17 | 0 | 0 | 432 | 2271124 | 17 | 0 | 0 | 1 | 440 |
| 239 | EF | 14 | 0E | 17 | 1 | 280112 | 2240892 | 17 | 1 | 0 | 432 | 2271564 | 17 | 1 | 0 | 1 | 440 |
| 240 | F0 | 14 | 0E | 17 | 2 | 280166 | 2241324 | 17 | 2 | 0 | 432 | 2272004 | 17 | 2 | 0 | 1 | 440 |
| 241 | F1 | 14 | 0E | 17 | 3 | 280220 | 2241756 | 17 | 3 | 0 | 432 | 2272444 | 17 | 3 | 0 | 1 | 440 |
| 242 | F2 | 14 | 0E | 17 | 4 | 280274 | 2242188 | 17 | 4 | 0 | 432 | 2272884 | 17 | 4 | 0 | 1 | 440 |
| 243 | F3 | 14 | 0E | 17 | 5 | 280328 | 2242620 | 17 | 5 | 0 | 432 | 2273324 | 17 | 5 | 0 | 1 | 440 |
| 244 | F4 | 14 | 0E | 17 | 6 | 280382 | 2243052 | 17 | 6 | 0 | 432 | 2273764 | 17 | 6 | 0 | 1 | 440 |
| 245 | F5 | 14 | 0E | 17 | 7 | 280436 | 2243484 | 17 | 7 | 0 | 432 | 2274204 | 17 | 7 | 0 | 1 | 440 |
| 246 | F6 | 14 | 0E | 17 | 8 | 280490 | 2243916 | 17 | 8 | 0 | 432 | 2274644 | 17 | 8 | 0 | 1 | 440 |
| 247 | F7 | 14 | 0E | 17 | 9 | 280544 | 2244348 | 17 | 9 | 0 | 432 | 2275084 | 17 | 9 | 0 | 1 | 440 |
| 248 | F8 | 14 | 0E | 17 | 10 | 280598 | 2244780 | 17 | 10 | 0 | 432 | 2275524 | 17 | 10 | 0 | 1 | 440 |
| 249 | F9 | 14 | 0E | 17 | 11 | 280652 | 2245212 | 17 | 11 | 0 | 432 | 2275964 | 17 | 11 | 0 | 1 | 440 |
| 250 | FA | 14 | 0E | 17 | 12 | 280706 | 2245644 | 17 | 12 | 0 | 432 | 2276404 | 17 | 12 | 0 | 1 | 440 |
| 251 | FB | 14 | 0E | 17 | 13 | 280760 | 2246076 | 17 | 13 | 0 | 432 | 2276844 | 17 | 13 | 0 | 1 | 440 |
| 252 | FC | 14 | 0E | 18 | 0 | 280816 | 2246524 | 18 | 0 | 0 | 448 | 2277300 | 18 | 0 | 0 | 1 | 456 |
| 253 | FD | 14 | 0E | 18 | 1 | 280872 | 2246972 | 18 | 1 | 0 | 448 | 2277756 | 18 | 1 | 0 | 1 | 456 |
| 254 | FE | 14 | 0E | 18 | 2 | 280928 | 2247420 | 18 | 2 | 0 | 448 | 2278212 | 18 | 2 | 0 | 1 | 456 |
| 255 | FF | 14 | 0E | 18 | 3 | 280984 | 2247868 | 18 | 3 | 0 | 448 | 2278668 | 18 | 3 | 0 | 1 | 456 |
| 0 | 00 | 15 | 0F | 0 | 0 | 280996 | 2247964 | 0 | 0 | 0 | 96 | 2278772 | 0 | 0 | 0 | 1 | 104 |
| 1 | 01 | 15 | 0F | 0 | 1 | 281010 | 2248076 | 0 | 1 | 0 | 112 | 2278892 | 0 | 1 | 0 | 1 | 120 |
| 2 | 02 | 15 | 0F | 0 | 2 | 281024 | 2248188 | 0 | 2 | 0 | 112 | 2279012 | 0 | 2 | 0 | 1 | 120 |
| 3 | 03 | 15 | 0F | 0 | 3 | 281038 | 2248300 | 0 | 3 | 0 | 112 | 2279132 | 0 | 3 | 0 | 1 | 120 |
| 4 | 04 | 15 | 0F | 0 | 4 | 281052 | 2248412 | 0 | 4 | 0 | 112 | 2279252 | 0 | 4 | 0 | 1 | 120 |
| 5 | 05 | 15 | 0F | 0 | 5 | 281066 | 2248524 | 0 | 5 | 0 | 112 | 2279372 | 0 | 5 | 0 | 1 | 120 |
| 6 | 06 | 15 | 0F | 0 | 6 | 281080 | 2248636 | 0 | 6 | 0 | 112 | 2279492 | 0 | 6 | 0 | 1 | 120 |
| 7 | 07 | 15 | 0F | 0 | 7 | 281094 | 2248748 | 0 | 7 | 0 | 112 | 2279612 | 0 | 7 | 0 | 1 | 120 |
| 8 | 08 | 15 | 0F | 0 | 8 | 281108 | 2248860 | 0 | 8 | 0 | 112 | 2279732 | 0 | 8 | 0 | 1 | 120 |
| 9 | 09 | 15 | 0F | 0 | 9 | 281122 | 2248972 | 0 | 9 | 0 | 112 | 2279852 | 0 | 9 | 0 | 1 | 120 |
| 10 | 0A | 15 | 0F | 0 | 10 | 281136 | 2249084 | 0 | 10 | 0 | 112 | 2279972 | 0 | 10 | 0 | 1 | 120 |
| 11 | 0B | 15 | 0F | 0 | 11 | 281150 | 2249196 | 0 | 11 | 0 | 112 | 2280092 | 0 | 11 | 0 | 1 | 120 |
| 12 | 0C | 15 | 0F | 0 | 12 | 281164 | 2249308 | 0 | 12 | 0 | 112 | 2280212 | 0 | 12 | 0 | 1 | 120 |
| 13 | 0D | 15 | 0F | 0 | 13 | 281178 | 2249420 | 0 | 13 | 0 | 112 | 2280332 | 0 | 13 | 0 | 1 | 120 |
| 14 | 0E | 15 | 0F | 0 | 14 | 281192 | 2249532 | 0 | 14 | 0 | 112 | 2280452 | 0 | 14 | 0 | 1 | 120 |
| 15 | 0F | 15 | 0F | 1 | 0 | 281204 | 2249628 | 1 | 0 | 0 | 96 | 2280556 | 1 | 0 | 0 | 1 | 104 |
| 16 | 10 | 15 | 0F | 1 | 1 | 281225 | 2249796 | 1 | 1 | 0 | 168 | 2280732 | 1 | 1 | 0 | 1 | 176 |
| 17 | 11 | 15 | 0F | 1 | 2 | 281246 | 2249964 | 1 | 2 | 0 | 168 | 2280908 | 1 | 2 | 0 | 1 | 176 |
| 18 | 12 | 15 | 0F | 1 | 3 | 281267 | 2250132 | 1 | 3 | 0 | 168 | 2281084 | 1 | 3 | 0 | 1 | 176 |
| 19 | 13 | 15 | 0F | 1 | 4 | 281288 | 2250300 | 1 | 4 | 0 | 168 | 2281260 | 1 | 4 | 0 | 1 | 176 |
| 20 | 14 | 15 | 0F | 1 | 5 | 281309 | 2250468 | 1 | 5 | 0 | 168 | 2281436 | 1 | 5 | 0 | 1 | 176 |
| 21 | 15 | 15 | 0F | 1 | 6 | 281330 | 2250636 | 1 | 6 | 0 | 168 | 2281612 | 1 | 6 | 0 | 1 | 176 |
| 22 | 16 | 15 | 0F | 1 | 7 | 281351 | 2250804 | 1 | 7 | 0 | 168 | 2281788 | 1 | 7 | 0 | 1 | 176 |
| 23 | 17 | 15 | 0F | 1 | 8 | 281372 | 2250972 | 1 | 8 | 0 | 168 | 2281964 | 1 | 8 | 0 | 1 | 176 |
| 24 | 18 | 15 | 0F | 1 | 9 | 281393 | 2251140 | 1 | 9 | 0 | 168 | 2282140 | 1 | 9 | 0 | 1 | 176 |
| 25 | 19 | 15 | 0F | 1 | 10 | 281414 | 2251308 | 1 | 10 | 0 | 168 | 2282316 | 1 | 10 | 0 | 1 | 176 |
| 26 | 1A | 15 | 0F | 1 | 11 | 281435 | 2251476 | 1 | 11 | 0 | 168 | 2282492 | 1 | 11 | 0 | 1 | 176 |
| 27 | 1B | 15 | 0F | 1 | 12 | 281456 | 2251644 | 1 | 12 | 0 | 168 | 2282668 | 1 | 12 | 0 | 1 | 176 |
| 28 | 1C | 15 | 0F | 1 | 13 | 281477 | 2251812 | 1 | 13 | 0 | 168 | 2282844 | 1 | 13 | 0 | 1 | 176 |
| 29 | 1D | 15 | 0F | 1 | 14 | 281498 | 2251980 | 1 | 14 | 0 | 168 | 2283020 | 1 | 14 | 0 | 1 | 176 |
| 30 | 1E | 15 | 0F | 2 | 0 | 281517 | 2252132 | 2 | 0 | 0 | 152 | 2283180 | 2 | 0 | 0 | 1 | 160 |
| 31 | 1F | 15 | 0F | 2 | 1 | 281541 | 2252324 | 2 | 1 | 0 | 192 | 2283380 | 2 | 1 | 0 | 1 | 200 |
| 32 | 20 | 15 | 0F | 2 | 2 | 281564 | 2252508 | 2 | 2 | 0 | 184 | 2283572 | 2 | 2 | 0 | 1 | 192 |
| 33 | 21 | 15 | 0F | 2 | 3 | 281587 | 2252692 | 2 | 3 | 0 | 184 | 2283764 | 2 | 3 | 0 | 1 | 192 |
| 34 | 22 | 15 | 0F | 2 | 4 | 281610 | 2252876 | 2 | 4 | 0 | 184 | 2283956 | 2 | 4 | 0 | 1 | 192 |
| 35 | 23 | 15 | 0F | 2 | 5 | 281633 | 2253060 | 2 | 5 | 0 | 184 | 2284148 | 2 | 5 | 0 | 1 | 192 |
| 36 | 24 | 15 | 0F | 2 | 6 | 281656 | 2253244 | 2 | 6 | 0 | 184 | 2284340 | 2 | 6 | 0 | 1 | 192 |
| 37 | 25 | 15 | 0F | 2 | 7 | 281679 | 2253428 | 2 | 7 | 0 | 184 | 2284532 | 2 | 7 | 0 | 1 | 192 |
| 38 | 26 | 15 | 0F | 2 | 8 | 281702 | 2253612 | 2 | 8 | 0 | 184 | 2284724 | 2 | 8 | 0 | 1 | 192 |
| 39 | 27 | 15 | 0F | 2 | 9 | 281725 | 2253796 | 2 | 9 | 0 | 184 | 2284916 | 2 | 9 | 0 | 1 | 192 |
| 40 | 28 | 15 | 0F | 2 | 10 | 281748 | 2253980 | 2 | 10 | 0 | 184 | 2285108 | 2 | 10 | 0 | 1 | 192 |
| 41 | 29 | 15 | 0F | 2 | 11 | 281771 | 2254164 | 2 | 11 | 0 | 184 | 2285300 | 2 | 11 | 0 | 1 | 192 |
| 42 | 2A | 15 | 0F | 2 | 12 | 281794 | 2254348 | 2 | 12 | 0 | 184 | 2285492 | 2 | 12 | 0 | 1 | 192 |
| 43 | 2B | 15 | 0F | 2 | 13 | 281817 | 2254532 | 2 | 13 | 0 | 184 | 2285684 | 2 | 13 | 0 | 1 | 192 |
| 44 | 2C | 15 | 0F | 2 | 14 | 281840 | 2254716 | 2 | 14 | 0 | 184 | 2285876 | 2 | 14 | 0 | 1 | 192 |
| 45 | 2D | 15 | 0F | 3 | 0 | 281861 | 2254884 | 3 | 0 | 0 | 168 | 2286052 | 3 | 0 | 0 | 1 | 176 |
| 46 | 2E | 15 | 0F | 3 | 1 | 281887 | 2255092 | 3 | 1 | 0 | 208 | 2286268 | 3 | 1 | 0 | 1 | 216 |
| 47 | 2F | 15 | 0F | 3 | 2 | 281913 | 2255300 | 3 | 2 | 0 | 208 | 2286484 | 3 | 2 | 0 | 1 | 216 |
| 48 | 30 | 15 | 0F | 3 | 3 | 281938 | 2255500 | 3 | 3 | 0 | 200 | 2286692 | 3 | 3 | 0 | 1 | 208 |
| 49 | 31 | 15 | 0F | 3 | 4 | 281963 | 2255700 | 3 | 4 | 0 | 200 | 2286900 | 3 | 4 | 0 | 1 | 208 |
| 50 | 32 | 15 | 0F | 3 | 5 | 281988 | 2255900 | 3 | 5 | 0 | 200 | 2287108 | 3 | 5 | 0 | 1 | 208 |
| 51 | 33 | 15 | 0F | 3 | 6 | 282013 | 2256100 | 3 | 6 | 0 | 200 | 2287316 | 3 | 6 | 0 | 1 | 208 |
| 52 | 34 | 15 | 0F | 3 | 7 | 282038 | 2256300 | 3 | 7 | 0 | 200 | 2287524 | 3 | 7 | 0 | 1 | 208 |
| 53 | 35 | 15 | 0F | 3 | 8 | 282063 | 2256500 | 3 | 8 | 0 | 200 | 2287732 | 3 | 8 | 0 | 1 | 208 |
| 54 | 36 | 15 | 0F | 3 | 9 | 282088 | 2256700 | 3 | 9 | 0 | 200 | 2287940 | 3 | 9 | 0 | 1 | 208 |
| 55 | 37 | 15 | 0F | 3 | 10 | 282113 | 2256900 | 3 | 10 | 0 | 200 | 2288148 | 3 | 10 | 0 | 1 | 208 |
| 56 | 38 | 15 | 0F | 3 | 11 | 282138 | 2257100 | 3 | 11 | 0 | 200 | 2288356 | 3 | 11 | 0 | 1 | 208 |
| 57 | 39 | 15 | 0F | 3 | 12 | 282163 | 2257300 | 3 | 12 | 0 | 200 | 2288564 | 3 | 12 | 0 | 1 | 208 |
| 58 | 3A | 15 | 0F | 3 | 13 | 282188 | 2257500 | 3 | 13 | 0 | 200 | 2288772 | 3 | 13 | 0 | 1 | 208 |
| 59 | 3B | 15 | 0F | 3 | 14 | 282213 | 2257700 | 3 | 14 | 0 | 200 | 2288980 | 3 | 14 | 0 | 1 | 208 |
| 60 | 3C | 15 | 0F | 4 | 0 | 282236 | 2257884 | 4 | 0 | 0 | 184 | 2289172 | 4 | 0 | 0 | 1 | 192 |
| 61 | 3D | 15 | 0F | 4 | 1 | 282264 | 2258108 | 4 | 1 | 0 | 224 | 2289404 | 4 | 1 | 0 | 1 | 232 |
| 62 | 3E | 15 | 0F | 4 | 2 | 282292 | 2258332 | 4 | 2 | 0 | 224 | 2289636 | 4 | 2 | 0 | 1 | 232 |
| 63 | 3F | 15 | 0F | 4 | 3 | 282320 | 2258556 | 4 | 3 | 0 | 224 | 2289868 | 4 | 3 | 0 | 1 | 232 |
| 64 | 40 | 15 | 0F | 4 | 4 | 282347 | 2258772 | 4 | 4 | 0 | 216 | 2290092 | 4 | 4 | 0 | 1 | 224 |
| 65 | 41 | 15 | 0F | 4 | 5 | 282374 | 2258988 | 4 | 5 | 0 | 216 | 2290316 | 4 | 5 | 0 | 1 | 224 |
| 66 | 42 | 15 | 0F | 4 | 6 | 282401 | 2259204 | 4 | 6 | 0 | 216 | 2290540 | 4 | 6 | 0 | 1 | 224 |
| 67 | 43 | 15 | 0F | 4 | 7 | 282428 | 2259420 | 4 | 7 | 0 | 216 | 2290764 | 4 | 7 | 0 | 1 | 224 |
| 68 | 44 | 15 | 0F | 4 | 8 | 282455 | 2259636 | 4 | 8 | 0 | 216 | 2290988 | 4 | 8 | 0 | 1 | 224 |
| 69 | 45 | 15 | 0F | 4 | 9 | 282482 | 2259852 | 4 | 9 | 0 | 216 | 2291212 | 4 | 9 | 0 | 1 | 224 |
| 70 | 46 | 15 | 0F | 4 | 10 | 282509 | 2260068 | 4 | 10 | 0 | 216 | 2291436 | 4 | 10 | 0 | 1 | 224 |

| | | | | | | | | | | | | | | | | | |
|---|---|---|---|---|---|---|---|---|---|---|---|---|---|---|---|---|---|
| 71 | 47 | 15 | 0F | 4 | 11 | 282536 | 2260284 | 4 | 11 | 0 | 216 | 2291660 | 4 | 11 | 0 | 1 | 224 |
| 72 | 48 | 15 | 0F | 4 | 12 | 282563 | 2260500 | 4 | 12 | 0 | 216 | 2291884 | 4 | 12 | 0 | 1 | 224 |
| 73 | 49 | 15 | 0F | 4 | 13 | 282590 | 2260716 | 4 | 13 | 0 | 216 | 2292108 | 4 | 13 | 0 | 1 | 224 |
| 74 | 4A | 15 | 0F | 4 | 14 | 282617 | 2260932 | 4 | 14 | 0 | 216 | 2292332 | 4 | 14 | 0 | 1 | 224 |
| 75 | 4B | 15 | 0F | 5 | 0 | 282642 | 2261132 | 5 | 0 | 0 | 200 | 2292540 | 5 | 0 | 0 | 1 | 208 |
| 76 | 4C | 15 | 0F | 5 | 1 | 282672 | 2261372 | 5 | 1 | 0 | 240 | 2292788 | 5 | 1 | 0 | 1 | 248 |
| 77 | 4D | 15 | 0F | 5 | 2 | 282702 | 2261612 | 5 | 2 | 0 | 240 | 2293036 | 5 | 2 | 0 | 1 | 248 |
| 78 | 4E | 15 | 0F | 5 | 3 | 282732 | 2261852 | 5 | 3 | 0 | 240 | 2293284 | 5 | 3 | 0 | 1 | 248 |
| 79 | 4F | 15 | 0F | 5 | 4 | 282762 | 2262092 | 5 | 4 | 0 | 240 | 2293532 | 5 | 4 | 0 | 1 | 248 |
| 80 | 50 | 15 | 0F | 5 | 5 | 282791 | 2262324 | 5 | 5 | 0 | 232 | 2293772 | 5 | 5 | 0 | 1 | 240 |
| 81 | 51 | 15 | 0F | 5 | 6 | 282820 | 2262556 | 5 | 6 | 0 | 232 | 2294012 | 5 | 6 | 0 | 1 | 240 |
| 82 | 52 | 15 | 0F | 5 | 7 | 282849 | 2262788 | 5 | 7 | 0 | 232 | 2294252 | 5 | 7 | 0 | 1 | 240 |
| 83 | 53 | 15 | 0F | 5 | 8 | 282878 | 2263020 | 5 | 8 | 0 | 232 | 2294492 | 5 | 8 | 0 | 1 | 240 |
| 84 | 54 | 15 | 0F | 5 | 9 | 282907 | 2263252 | 5 | 9 | 0 | 232 | 2294732 | 5 | 9 | 0 | 1 | 240 |
| 85 | 55 | 15 | 0F | 5 | 10 | 282936 | 2263484 | 5 | 10 | 0 | 232 | 2294972 | 5 | 10 | 0 | 1 | 240 |
| 86 | 56 | 15 | 0F | 5 | 11 | 282965 | 2263716 | 5 | 11 | 0 | 232 | 2295212 | 5 | 11 | 0 | 1 | 240 |
| 87 | 57 | 15 | 0F | 5 | 12 | 282994 | 2263948 | 5 | 12 | 0 | 232 | 2295452 | 5 | 12 | 0 | 1 | 240 |
| 88 | 58 | 15 | 0F | 5 | 13 | 283023 | 2264180 | 5 | 13 | 0 | 232 | 2295692 | 5 | 13 | 0 | 1 | 240 |
| 89 | 59 | 15 | 0F | 5 | 14 | 283052 | 2264412 | 5 | 14 | 0 | 232 | 2295932 | 5 | 14 | 0 | 1 | 240 |
| 90 | 5A | 15 | 0F | 6 | 0 | 283079 | 2264628 | 6 | 0 | 0 | 216 | 2296156 | 6 | 0 | 0 | 1 | 224 |
| 91 | 5B | 15 | 0F | 6 | 1 | 283111 | 2264884 | 6 | 1 | 0 | 256 | 2296420 | 6 | 1 | 0 | 1 | 264 |
| 92 | 5C | 15 | 0F | 6 | 2 | 283143 | 2265140 | 6 | 2 | 0 | 256 | 2296684 | 6 | 2 | 0 | 1 | 264 |
| 93 | 5D | 15 | 0F | 6 | 3 | 283175 | 2265396 | 6 | 3 | 0 | 256 | 2296948 | 6 | 3 | 0 | 1 | 264 |
| 94 | 5E | 15 | 0F | 6 | 4 | 283207 | 2265652 | 6 | 4 | 0 | 256 | 2297212 | 6 | 4 | 0 | 1 | 264 |
| 95 | 5F | 15 | 0F | 6 | 5 | 283239 | 2265908 | 6 | 5 | 0 | 256 | 2297476 | 6 | 5 | 0 | 1 | 264 |
| 96 | 60 | 15 | 0F | 6 | 6 | 283270 | 2266156 | 6 | 6 | 0 | 248 | 2297732 | 6 | 6 | 0 | 1 | 256 |
| 97 | 61 | 15 | 0F | 6 | 7 | 283301 | 2266404 | 6 | 7 | 0 | 248 | 2297988 | 6 | 7 | 0 | 1 | 256 |
| 98 | 62 | 15 | 0F | 6 | 8 | 283332 | 2266652 | 6 | 8 | 0 | 248 | 2298244 | 6 | 8 | 0 | 1 | 256 |
| 99 | 63 | 15 | 0F | 6 | 9 | 283363 | 2266900 | 6 | 9 | 0 | 248 | 2298500 | 6 | 9 | 0 | 1 | 256 |
| 100 | 64 | 15 | 0F | 6 | 10 | 283394 | 2267148 | 6 | 10 | 0 | 248 | 2298756 | 6 | 10 | 0 | 1 | 256 |
| 101 | 65 | 15 | 0F | 6 | 11 | 283425 | 2267396 | 6 | 11 | 0 | 248 | 2299012 | 6 | 11 | 0 | 1 | 256 |
| 102 | 66 | 15 | 0F | 6 | 12 | 283456 | 2267644 | 6 | 12 | 0 | 248 | 2299268 | 6 | 12 | 0 | 1 | 256 |
| 103 | 67 | 15 | 0F | 6 | 13 | 283487 | 2267892 | 6 | 13 | 0 | 248 | 2299524 | 6 | 13 | 0 | 1 | 256 |
| 104 | 68 | 15 | 0F | 6 | 14 | 283518 | 2268140 | 6 | 14 | 0 | 248 | 2299780 | 6 | 14 | 0 | 1 | 256 |
| 105 | 69 | 15 | 0F | 7 | 0 | 283547 | 2268372 | 7 | 0 | 0 | 232 | 2300020 | 7 | 0 | 0 | 1 | 240 |
| 106 | 6A | 15 | 0F | 7 | 1 | 283581 | 2268644 | 7 | 1 | 0 | 272 | 2300300 | 7 | 1 | 0 | 1 | 280 |
| 107 | 6B | 15 | 0F | 7 | 2 | 283615 | 2268916 | 7 | 2 | 0 | 272 | 2300580 | 7 | 2 | 0 | 1 | 280 |
| 108 | 6C | 15 | 0F | 7 | 3 | 283649 | 2269188 | 7 | 3 | 0 | 272 | 2300860 | 7 | 3 | 0 | 1 | 280 |
| 109 | 6D | 15 | 0F | 7 | 4 | 283683 | 2269460 | 7 | 4 | 0 | 272 | 2301140 | 7 | 4 | 0 | 1 | 280 |
| 110 | 6E | 15 | 0F | 7 | 5 | 283717 | 2269732 | 7 | 5 | 0 | 272 | 2301420 | 7 | 5 | 0 | 1 | 280 |
| 111 | 6F | 15 | 0F | 7 | 6 | 283751 | 2270004 | 7 | 6 | 0 | 272 | 2301700 | 7 | 6 | 0 | 1 | 280 |
| 112 | 70 | 15 | 0F | 7 | 7 | 283784 | 2270268 | 7 | 7 | 0 | 264 | 2301972 | 7 | 7 | 0 | 1 | 272 |
| 113 | 71 | 15 | 0F | 7 | 8 | 283817 | 2270532 | 7 | 8 | 0 | 264 | 2302244 | 7 | 8 | 0 | 1 | 272 |
| 114 | 72 | 15 | 0F | 7 | 9 | 283850 | 2270796 | 7 | 9 | 0 | 264 | 2302516 | 7 | 9 | 0 | 1 | 272 |
| 115 | 73 | 15 | 0F | 7 | 10 | 283883 | 2271060 | 7 | 10 | 0 | 264 | 2302788 | 7 | 10 | 0 | 1 | 272 |
| 116 | 74 | 15 | 0F | 7 | 11 | 283916 | 2271324 | 7 | 11 | 0 | 264 | 2303060 | 7 | 11 | 0 | 1 | 272 |
| 117 | 75 | 15 | 0F | 7 | 12 | 283949 | 2271588 | 7 | 12 | 0 | 264 | 2303332 | 7 | 12 | 0 | 1 | 272 |
| 118 | 76 | 15 | 0F | 7 | 13 | 283982 | 2271852 | 7 | 13 | 0 | 264 | 2303604 | 7 | 13 | 0 | 1 | 272 |
| 119 | 77 | 15 | 0F | 7 | 14 | 284015 | 2272116 | 7 | 14 | 0 | 264 | 2303876 | 7 | 14 | 0 | 1 | 272 |
| 120 | 78 | 15 | 0F | 8 | 0 | 284046 | 2272364 | 8 | 0 | 0 | 248 | 2304132 | 8 | 0 | 0 | 1 | 256 |
| 121 | 79 | 15 | 0F | 8 | 1 | 284082 | 2272652 | 8 | 1 | 0 | 288 | 2304428 | 8 | 1 | 0 | 1 | 296 |
| 122 | 7A | 15 | 0F | 8 | 2 | 284118 | 2272940 | 8 | 2 | 0 | 288 | 2304724 | 8 | 2 | 0 | 1 | 296 |
| 123 | 7B | 15 | 0F | 8 | 3 | 284154 | 2273228 | 8 | 3 | 0 | 288 | 2305020 | 8 | 3 | 0 | 1 | 296 |
| 124 | 7C | 15 | 0F | 8 | 4 | 284190 | 2273516 | 8 | 4 | 0 | 288 | 2305316 | 8 | 4 | 0 | 1 | 296 |
| 125 | 7D | 15 | 0F | 8 | 5 | 284226 | 2273804 | 8 | 5 | 0 | 288 | 2305612 | 8 | 5 | 0 | 1 | 296 |
| 126 | 7E | 15 | 0F | 8 | 6 | 284262 | 2274092 | 8 | 6 | 0 | 288 | 2305908 | 8 | 6 | 0 | 1 | 296 |
| 127 | 7F | 15 | 0F | 8 | 7 | 284298 | 2274380 | 8 | 7 | 0 | 288 | 2306204 | 8 | 7 | 0 | 1 | 296 |
| 128 | 80 | 15 | 0F | 8 | 8 | 284333 | 2274660 | 8 | 8 | 0 | 280 | 2306492 | 8 | 8 | 0 | 1 | 288 |
| 129 | 81 | 15 | 0F | 8 | 9 | 284368 | 2274940 | 8 | 9 | 0 | 280 | 2306780 | 8 | 9 | 0 | 1 | 288 |
| 130 | 82 | 15 | 0F | 8 | 10 | 284403 | 2275220 | 8 | 10 | 0 | 280 | 2307068 | 8 | 10 | 0 | 1 | 288 |
| 131 | 83 | 15 | 0F | 8 | 11 | 284438 | 2275500 | 8 | 11 | 0 | 280 | 2307356 | 8 | 11 | 0 | 1 | 288 |
| 132 | 84 | 15 | 0F | 8 | 12 | 284473 | 2275780 | 8 | 12 | 0 | 280 | 2307644 | 8 | 12 | 0 | 1 | 288 |
| 133 | 85 | 15 | 0F | 8 | 13 | 284508 | 2276060 | 8 | 13 | 0 | 280 | 2307932 | 8 | 13 | 0 | 1 | 288 |
| 134 | 86 | 15 | 0F | 8 | 14 | 284543 | 2276340 | 8 | 14 | 0 | 280 | 2308220 | 8 | 14 | 0 | 1 | 288 |
| 135 | 87 | 15 | 0F | 9 | 0 | 284576 | 2276604 | 9 | 0 | 0 | 264 | 2308492 | 9 | 0 | 0 | 1 | 272 |
| 136 | 88 | 15 | 0F | 9 | 1 | 284614 | 2276908 | 9 | 1 | 0 | 304 | 2308804 | 9 | 1 | 0 | 1 | 312 |
| 137 | 89 | 15 | 0F | 9 | 2 | 284652 | 2277212 | 9 | 2 | 0 | 304 | 2309116 | 9 | 2 | 0 | 1 | 312 |
| 138 | 8A | 15 | 0F | 9 | 3 | 284690 | 2277516 | 9 | 3 | 0 | 304 | 2309428 | 9 | 3 | 0 | 1 | 312 |
| 139 | 8B | 15 | 0F | 9 | 4 | 284728 | 2277820 | 9 | 4 | 0 | 304 | 2309740 | 9 | 4 | 0 | 1 | 312 |
| 140 | 8C | 15 | 0F | 9 | 5 | 284766 | 2278124 | 9 | 5 | 0 | 304 | 2310052 | 9 | 5 | 0 | 1 | 312 |
| 141 | 8D | 15 | 0F | 9 | 6 | 284804 | 2278428 | 9 | 6 | 0 | 304 | 2310364 | 9 | 6 | 0 | 1 | 312 |
| 142 | 8E | 15 | 0F | 9 | 7 | 284842 | 2278732 | 9 | 7 | 0 | 304 | 2310676 | 9 | 7 | 0 | 1 | 312 |
| 143 | 8F | 15 | 0F | 9 | 8 | 284880 | 2279036 | 9 | 8 | 0 | 304 | 2310988 | 9 | 8 | 0 | 1 | 312 |
| 144 | 90 | 15 | 0F | 9 | 9 | 284917 | 2279332 | 9 | 9 | 0 | 296 | 2311292 | 9 | 9 | 0 | 1 | 304 |
| 145 | 91 | 15 | 0F | 9 | 10 | 284954 | 2279628 | 9 | 10 | 0 | 296 | 2311596 | 9 | 10 | 0 | 1 | 304 |
| 146 | 92 | 15 | 0F | 9 | 11 | 284991 | 2279924 | 9 | 11 | 0 | 296 | 2311900 | 9 | 11 | 0 | 1 | 304 |
| 147 | 93 | 15 | 0F | 9 | 12 | 285028 | 2280220 | 9 | 12 | 0 | 296 | 2312204 | 9 | 12 | 0 | 1 | 304 |
| 148 | 94 | 15 | 0F | 9 | 13 | 285065 | 2280516 | 9 | 13 | 0 | 296 | 2312508 | 9 | 13 | 0 | 1 | 304 |
| 149 | 95 | 15 | 0F | 9 | 14 | 285102 | 2280812 | 9 | 14 | 0 | 296 | 2312812 | 9 | 14 | 0 | 1 | 304 |
| 150 | 96 | 15 | 0F | 10 | 0 | 285137 | 2281092 | 10 | 0 | 0 | 280 | 2313100 | 10 | 0 | 0 | 1 | 288 |
| 151 | 97 | 15 | 0F | 10 | 1 | 285177 | 2281412 | 10 | 1 | 0 | 320 | 2313428 | 10 | 1 | 0 | 1 | 328 |
| 152 | 98 | 15 | 0F | 10 | 2 | 285217 | 2281732 | 10 | 2 | 0 | 320 | 2313756 | 10 | 2 | 0 | 1 | 328 |
| 153 | 99 | 15 | 0F | 10 | 3 | 285257 | 2282052 | 10 | 3 | 0 | 320 | 2314084 | 10 | 3 | 0 | 1 | 328 |
| 154 | 9A | 15 | 0F | 10 | 4 | 285297 | 2282372 | 10 | 4 | 0 | 320 | 2314412 | 10 | 4 | 0 | 1 | 328 |
| 155 | 9B | 15 | 0F | 10 | 5 | 285337 | 2282692 | 10 | 5 | 0 | 320 | 2314740 | 10 | 5 | 0 | 1 | 328 |
| 156 | 9C | 15 | 0F | 10 | 6 | 285377 | 2283012 | 10 | 6 | 0 | 320 | 2315068 | 10 | 6 | 0 | 1 | 328 |
| 157 | 9D | 15 | 0F | 10 | 7 | 285417 | 2283332 | 10 | 7 | 0 | 320 | 2315396 | 10 | 7 | 0 | 1 | 328 |
| 158 | 9E | 15 | 0F | 10 | 8 | 285457 | 2283652 | 10 | 8 | 0 | 320 | 2315724 | 10 | 8 | 0 | 1 | 328 |
| 159 | 9F | 15 | 0F | 10 | 9 | 285497 | 2283972 | 10 | 9 | 0 | 320 | 2316052 | 10 | 9 | 0 | 1 | 328 |
| 160 | A0 | 15 | 0F | 10 | 10 | 285536 | 2284284 | 10 | 10 | 0 | 312 | 2316372 | 10 | 10 | 0 | 1 | 320 |
| 161 | A1 | 15 | 0F | 10 | 11 | 285575 | 2284596 | 10 | 11 | 0 | 312 | 2316692 | 10 | 11 | 0 | 1 | 320 |
| 162 | A2 | 15 | 0F | 10 | 12 | 285614 | 2284908 | 10 | 12 | 0 | 312 | 2317012 | 10 | 12 | 0 | 1 | 320 |
| 163 | A3 | 15 | 0F | 10 | 13 | 285653 | 2285220 | 10 | 13 | 0 | 312 | 2317332 | 10 | 13 | 0 | 1 | 320 |
| 164 | A4 | 15 | 0F | 10 | 14 | 285692 | 2285532 | 10 | 14 | 0 | 312 | 2317652 | 10 | 14 | 0 | 1 | 320 |
| 165 | A5 | 15 | 0F | 11 | 0 | 285729 | 2285828 | 11 | 0 | 0 | 296 | 2317956 | 11 | 0 | 0 | 1 | 304 |
| 166 | A6 | 15 | 0F | 11 | 1 | 285771 | 2286164 | 11 | 1 | 0 | 336 | 2318300 | 11 | 1 | 0 | 1 | 344 |
| 167 | A7 | 15 | 0F | 11 | 2 | 285813 | 2286500 | 11 | 2 | 0 | 336 | 2318644 | 11 | 2 | 0 | 1 | 344 |
| 168 | A8 | 15 | 0F | 11 | 3 | 285855 | 2286836 | 11 | 3 | 0 | 336 | 2318988 | 11 | 3 | 0 | 1 | 344 |
| 169 | A9 | 15 | 0F | 11 | 4 | 285897 | 2287172 | 11 | 4 | 0 | 336 | 2319332 | 11 | 4 | 0 | 1 | 344 |
| 170 | AA | 15 | 0F | 11 | 5 | 285939 | 2287508 | 11 | 5 | 0 | 336 | 2319676 | 11 | 5 | 0 | 1 | 344 |
| 171 | AB | 15 | 0F | 11 | 6 | 285981 | 2287844 | 11 | 6 | 0 | 336 | 2320020 | 11 | 6 | 0 | 1 | 344 |
| 172 | AC | 15 | 0F | 11 | 7 | 286023 | 2288180 | 11 | 7 | 0 | 336 | 2320364 | 11 | 7 | 0 | 1 | 344 |
| 173 | AD | 15 | 0F | 11 | 8 | 286065 | 2288516 | 11 | 8 | 0 | 336 | 2320708 | 11 | 8 | 0 | 1 | 344 |

| | | | | | | | | | | | | | | | | | |
|---|---|---|---|---|---|---|---|---|---|---|---|---|---|---|---|---|---|
| 174 | AE | 15 | 0F | 11 | 9 | 286107 | 2288852 | 11 | 9 | 0 | 336 | 2321052 | 11 | 9 | 0 | 1 | 344 |
| 175 | AF | 15 | 0F | 11 | 10 | 286149 | 2289188 | 11 | 10 | 0 | 336 | 2321396 | 11 | 10 | 0 | 1 | 344 |
| 176 | B0 | 15 | 0F | 11 | 11 | 286190 | 2289516 | 11 | 11 | 0 | 328 | 2321732 | 11 | 11 | 0 | 1 | 336 |
| 177 | B1 | 15 | 0F | 11 | 12 | 286231 | 2289844 | 11 | 12 | 0 | 328 | 2322068 | 11 | 12 | 0 | 1 | 336 |
| 178 | B2 | 15 | 0F | 11 | 13 | 286272 | 2290172 | 11 | 13 | 0 | 328 | 2322404 | 11 | 13 | 0 | 1 | 336 |
| 179 | B3 | 15 | 0F | 11 | 14 | 286313 | 2290500 | 11 | 14 | 0 | 328 | 2322740 | 11 | 14 | 0 | 1 | 336 |
| 180 | B4 | 15 | 0F | 12 | 0 | 286352 | 2290812 | 12 | 0 | 0 | 312 | 2323060 | 12 | 0 | 0 | 1 | 320 |
| 181 | B5 | 15 | 0F | 12 | 1 | 286396 | 2291164 | 12 | 1 | 0 | 352 | 2323420 | 12 | 1 | 0 | 1 | 360 |
| 182 | B6 | 15 | 0F | 12 | 2 | 286440 | 2291516 | 12 | 2 | 0 | 352 | 2323780 | 12 | 2 | 0 | 1 | 360 |
| 183 | B7 | 15 | 0F | 12 | 3 | 286484 | 2291868 | 12 | 3 | 0 | 352 | 2324140 | 12 | 3 | 0 | 1 | 360 |
| 184 | B8 | 15 | 0F | 12 | 4 | 286528 | 2292220 | 12 | 4 | 0 | 352 | 2324500 | 12 | 4 | 0 | 1 | 360 |
| 185 | B9 | 15 | 0F | 12 | 5 | 286572 | 2292572 | 12 | 5 | 0 | 352 | 2324860 | 12 | 5 | 0 | 1 | 360 |
| 186 | BA | 15 | 0F | 12 | 6 | 286616 | 2292924 | 12 | 6 | 0 | 352 | 2325220 | 12 | 6 | 0 | 1 | 360 |
| 187 | BB | 15 | 0F | 12 | 7 | 286660 | 2293276 | 12 | 7 | 0 | 352 | 2325580 | 12 | 7 | 0 | 1 | 360 |
| 188 | BC | 15 | 0F | 12 | 8 | 286704 | 2293628 | 12 | 8 | 0 | 352 | 2325940 | 12 | 8 | 0 | 1 | 360 |
| 189 | BD | 15 | 0F | 12 | 9 | 286748 | 2293980 | 12 | 9 | 0 | 352 | 2326300 | 12 | 9 | 0 | 1 | 360 |
| 190 | BE | 15 | 0F | 12 | 10 | 286792 | 2294332 | 12 | 10 | 0 | 352 | 2326660 | 12 | 10 | 0 | 1 | 360 |
| 191 | BF | 15 | 0F | 12 | 11 | 286836 | 2294684 | 12 | 11 | 0 | 352 | 2327020 | 12 | 11 | 0 | 1 | 360 |
| 192 | C0 | 15 | 0F | 12 | 12 | 286879 | 2295028 | 12 | 12 | 0 | 344 | 2327372 | 12 | 12 | 0 | 1 | 352 |
| 193 | C1 | 15 | 0F | 12 | 13 | 286922 | 2295372 | 12 | 13 | 0 | 344 | 2327724 | 12 | 13 | 0 | 1 | 352 |
| 194 | C2 | 15 | 0F | 12 | 14 | 286965 | 2295716 | 12 | 14 | 0 | 344 | 2328076 | 12 | 14 | 0 | 1 | 352 |
| 195 | C3 | 15 | 0F | 13 | 0 | 287006 | 2296044 | 13 | 0 | 0 | 328 | 2328412 | 13 | 0 | 0 | 1 | 336 |
| 196 | C4 | 15 | 0F | 13 | 1 | 287052 | 2296412 | 13 | 1 | 0 | 368 | 2328788 | 13 | 1 | 0 | 1 | 376 |
| 197 | C5 | 15 | 0F | 13 | 2 | 287098 | 2296780 | 13 | 2 | 0 | 368 | 2329164 | 13 | 2 | 0 | 1 | 376 |
| 198 | C6 | 15 | 0F | 13 | 3 | 287144 | 2297148 | 13 | 3 | 0 | 368 | 2329540 | 13 | 3 | 0 | 1 | 376 |
| 199 | C7 | 15 | 0F | 13 | 4 | 287190 | 2297516 | 13 | 4 | 0 | 368 | 2329916 | 13 | 4 | 0 | 1 | 376 |
| 200 | C8 | 15 | 0F | 13 | 5 | 287236 | 2297884 | 13 | 5 | 0 | 368 | 2330292 | 13 | 5 | 0 | 1 | 376 |
| 201 | C9 | 15 | 0F | 13 | 6 | 287282 | 2298252 | 13 | 6 | 0 | 368 | 2330668 | 13 | 6 | 0 | 1 | 376 |
| 202 | CA | 15 | 0F | 13 | 7 | 287328 | 2298620 | 13 | 7 | 0 | 368 | 2331044 | 13 | 7 | 0 | 1 | 376 |
| 203 | CB | 15 | 0F | 13 | 8 | 287374 | 2298988 | 13 | 8 | 0 | 368 | 2331420 | 13 | 8 | 0 | 1 | 376 |
| 204 | CC | 15 | 0F | 13 | 9 | 287420 | 2299356 | 13 | 9 | 0 | 368 | 2331796 | 13 | 9 | 0 | 1 | 376 |
| 205 | CD | 15 | 0F | 13 | 10 | 287466 | 2299724 | 13 | 10 | 0 | 368 | 2332172 | 13 | 10 | 0 | 1 | 376 |
| 206 | CE | 15 | 0F | 13 | 11 | 287512 | 2300092 | 13 | 11 | 0 | 368 | 2332548 | 13 | 11 | 0 | 1 | 376 |
| 207 | CF | 15 | 0F | 13 | 12 | 287558 | 2300460 | 13 | 12 | 0 | 368 | 2332924 | 13 | 12 | 0 | 1 | 376 |
| 208 | D0 | 15 | 0F | 13 | 13 | 287603 | 2300820 | 13 | 13 | 0 | 360 | 2333292 | 13 | 13 | 0 | 1 | 368 |
| 209 | D1 | 15 | 0F | 13 | 14 | 287648 | 2301180 | 13 | 14 | 0 | 360 | 2333660 | 13 | 14 | 0 | 1 | 368 |
| 210 | D2 | 15 | 0F | 14 | 0 | 287691 | 2301524 | 14 | 0 | 0 | 344 | 2334012 | 14 | 0 | 0 | 1 | 352 |
| 211 | D3 | 15 | 0F | 14 | 1 | 287739 | 2301908 | 14 | 1 | 0 | 384 | 2334404 | 14 | 1 | 0 | 1 | 392 |
| 212 | D4 | 15 | 0F | 14 | 2 | 287787 | 2302292 | 14 | 2 | 0 | 384 | 2334796 | 14 | 2 | 0 | 1 | 392 |
| 213 | D5 | 15 | 0F | 14 | 3 | 287835 | 2302676 | 14 | 3 | 0 | 384 | 2335188 | 14 | 3 | 0 | 1 | 392 |
| 214 | D6 | 15 | 0F | 14 | 4 | 287883 | 2303060 | 14 | 4 | 0 | 384 | 2335580 | 14 | 4 | 0 | 1 | 392 |
| 215 | D7 | 15 | 0F | 14 | 5 | 287931 | 2303444 | 14 | 5 | 0 | 384 | 2335972 | 14 | 5 | 0 | 1 | 392 |
| 216 | D8 | 15 | 0F | 14 | 6 | 287979 | 2303828 | 14 | 6 | 0 | 384 | 2336364 | 14 | 6 | 0 | 1 | 392 |
| 217 | D9 | 15 | 0F | 14 | 7 | 288027 | 2304212 | 14 | 7 | 0 | 384 | 2336756 | 14 | 7 | 0 | 1 | 392 |
| 218 | DA | 15 | 0F | 14 | 8 | 288075 | 2304596 | 14 | 8 | 0 | 384 | 2337148 | 14 | 8 | 0 | 1 | 392 |
| 219 | DB | 15 | 0F | 14 | 9 | 288123 | 2304980 | 14 | 9 | 0 | 384 | 2337540 | 14 | 9 | 0 | 1 | 392 |
| 220 | DC | 15 | 0F | 14 | 10 | 288171 | 2305364 | 14 | 10 | 0 | 384 | 2337932 | 14 | 10 | 0 | 1 | 392 |
| 221 | DD | 15 | 0F | 14 | 11 | 288219 | 2305748 | 14 | 11 | 0 | 384 | 2338324 | 14 | 11 | 0 | 1 | 392 |
| 222 | DE | 15 | 0F | 14 | 12 | 288267 | 2306132 | 14 | 12 | 0 | 384 | 2338716 | 14 | 12 | 0 | 1 | 392 |
| 223 | DF | 15 | 0F | 14 | 13 | 288315 | 2306516 | 14 | 13 | 0 | 384 | 2339108 | 14 | 13 | 0 | 1 | 392 |
| 224 | E0 | 15 | 0F | 14 | 14 | 288362 | 2306892 | 14 | 14 | 0 | 376 | 2339492 | 14 | 14 | 0 | 1 | 384 |
| 225 | E1 | 15 | 0F | 15 | 0 | 288407 | 2307252 | 15 | 0 | 0 | 360 | 2339860 | 15 | 0 | 0 | 1 | 368 |
| 226 | E2 | 15 | 0F | 15 | 1 | 288457 | 2307652 | 15 | 1 | 0 | 400 | 2340268 | 15 | 1 | 0 | 1 | 408 |
| 227 | E3 | 15 | 0F | 15 | 2 | 288507 | 2308052 | 15 | 2 | 0 | 400 | 2340676 | 15 | 2 | 0 | 1 | 408 |
| 228 | E4 | 15 | 0F | 15 | 3 | 288557 | 2308452 | 15 | 3 | 0 | 400 | 2341084 | 15 | 3 | 0 | 1 | 408 |
| 229 | E5 | 15 | 0F | 15 | 4 | 288607 | 2308852 | 15 | 4 | 0 | 400 | 2341492 | 15 | 4 | 0 | 1 | 408 |
| 230 | E6 | 15 | 0F | 15 | 5 | 288657 | 2309252 | 15 | 5 | 0 | 400 | 2341900 | 15 | 5 | 0 | 1 | 408 |
| 231 | E7 | 15 | 0F | 15 | 6 | 288707 | 2309652 | 15 | 6 | 0 | 400 | 2342308 | 15 | 6 | 0 | 1 | 408 |
| 232 | E8 | 15 | 0F | 15 | 7 | 288757 | 2310052 | 15 | 7 | 0 | 400 | 2342716 | 15 | 7 | 0 | 1 | 408 |
| 233 | E9 | 15 | 0F | 15 | 8 | 288807 | 2310452 | 15 | 8 | 0 | 400 | 2343124 | 15 | 8 | 0 | 1 | 408 |
| 234 | EA | 15 | 0F | 15 | 9 | 288857 | 2310852 | 15 | 9 | 0 | 400 | 2343532 | 15 | 9 | 0 | 1 | 408 |
| 235 | EB | 15 | 0F | 15 | 10 | 288907 | 2311252 | 15 | 10 | 0 | 400 | 2343940 | 15 | 10 | 0 | 1 | 408 |
| 236 | EC | 15 | 0F | 15 | 11 | 288957 | 2311652 | 15 | 11 | 0 | 400 | 2344348 | 15 | 11 | 0 | 1 | 408 |
| 237 | ED | 15 | 0F | 15 | 12 | 289007 | 2312052 | 15 | 12 | 0 | 400 | 2344756 | 15 | 12 | 0 | 1 | 408 |
| 238 | EE | 15 | 0F | 15 | 13 | 289057 | 2312452 | 15 | 13 | 0 | 400 | 2345164 | 15 | 13 | 0 | 1 | 408 |
| 239 | EF | 15 | 0F | 15 | 14 | 289107 | 2312852 | 15 | 14 | 0 | 400 | 2345572 | 15 | 14 | 0 | 1 | 408 |
| 240 | F0 | 15 | 0F | 16 | 0 | 289154 | 2313228 | 16 | 0 | 0 | 376 | 2345956 | 16 | 0 | 0 | 1 | 384 |
| 241 | F1 | 15 | 0F | 16 | 1 | 289206 | 2313644 | 16 | 1 | 0 | 416 | 2346380 | 16 | 1 | 0 | 1 | 424 |
| 242 | F2 | 15 | 0F | 16 | 2 | 289258 | 2314060 | 16 | 2 | 0 | 416 | 2346804 | 16 | 2 | 0 | 1 | 424 |
| 243 | F3 | 15 | 0F | 16 | 3 | 289310 | 2314476 | 16 | 3 | 0 | 416 | 2347228 | 16 | 3 | 0 | 1 | 424 |
| 244 | F4 | 15 | 0F | 16 | 4 | 289362 | 2314892 | 16 | 4 | 0 | 416 | 2347652 | 16 | 4 | 0 | 1 | 424 |
| 245 | F5 | 15 | 0F | 16 | 5 | 289414 | 2315308 | 16 | 5 | 0 | 416 | 2348076 | 16 | 5 | 0 | 1 | 424 |
| 246 | F6 | 15 | 0F | 16 | 6 | 289466 | 2315724 | 16 | 6 | 0 | 416 | 2348500 | 16 | 6 | 0 | 1 | 424 |
| 247 | F7 | 15 | 0F | 16 | 7 | 289518 | 2316140 | 16 | 7 | 0 | 416 | 2348924 | 16 | 7 | 0 | 1 | 424 |
| 248 | F8 | 15 | 0F | 16 | 8 | 289570 | 2316556 | 16 | 8 | 0 | 416 | 2349348 | 16 | 8 | 0 | 1 | 424 |
| 249 | F9 | 15 | 0F | 16 | 9 | 289622 | 2316972 | 16 | 9 | 0 | 416 | 2349772 | 16 | 9 | 0 | 1 | 424 |
| 250 | FA | 15 | 0F | 16 | 10 | 289674 | 2317388 | 16 | 10 | 0 | 416 | 2350196 | 16 | 10 | 0 | 1 | 424 |
| 251 | FB | 15 | 0F | 16 | 11 | 289726 | 2317804 | 16 | 11 | 0 | 416 | 2350620 | 16 | 11 | 0 | 1 | 424 |
| 252 | FC | 15 | 0F | 16 | 12 | 289778 | 2318220 | 16 | 12 | 0 | 416 | 2351044 | 16 | 12 | 0 | 1 | 424 |
| 253 | FD | 15 | 0F | 16 | 13 | 289830 | 2318636 | 16 | 13 | 0 | 416 | 2351468 | 16 | 13 | 0 | 1 | 424 |
| 254 | FE | 15 | 0F | 16 | 14 | 289882 | 2319052 | 16 | 14 | 0 | 416 | 2351892 | 16 | 14 | 0 | 1 | 424 |
| 255 | FF | 15 | 0F | 17 | 0 | 289936 | 2319484 | 17 | 0 | 0 | 432 | 2352332 | 17 | 0 | 0 | 1 | 440 |
| 0 | 00 | 16 | 10 | 0 | 0 | 289948 | 2319580 | 0 | 0 | 0 | 96 | 2352436 | 0 | 0 | 0 | 1 | 104 |
| 1 | 01 | 16 | 10 | 0 | 1 | 289962 | 2319692 | 0 | 1 | 0 | 112 | 2352556 | 0 | 1 | 0 | 1 | 120 |
| 2 | 02 | 16 | 10 | 0 | 2 | 289976 | 2319804 | 0 | 2 | 0 | 112 | 2352676 | 0 | 2 | 0 | 1 | 120 |
| 3 | 03 | 16 | 10 | 0 | 3 | 289990 | 2319916 | 0 | 3 | 0 | 112 | 2352796 | 0 | 3 | 0 | 1 | 120 |
| 4 | 04 | 16 | 10 | 0 | 4 | 290004 | 2320028 | 0 | 4 | 0 | 112 | 2352916 | 0 | 4 | 0 | 1 | 120 |
| 5 | 05 | 16 | 10 | 0 | 5 | 290018 | 2320140 | 0 | 5 | 0 | 112 | 2353036 | 0 | 5 | 0 | 1 | 120 |
| 6 | 06 | 16 | 10 | 0 | 6 | 290032 | 2320252 | 0 | 6 | 0 | 112 | 2353156 | 0 | 6 | 0 | 1 | 120 |
| 7 | 07 | 16 | 10 | 0 | 7 | 290046 | 2320364 | 0 | 7 | 0 | 112 | 2353276 | 0 | 7 | 0 | 1 | 120 |
| 8 | 08 | 16 | 10 | 0 | 8 | 290060 | 2320476 | 0 | 8 | 0 | 112 | 2353396 | 0 | 8 | 0 | 1 | 120 |
| 9 | 09 | 16 | 10 | 0 | 9 | 290074 | 2320588 | 0 | 9 | 0 | 112 | 2353516 | 0 | 9 | 0 | 1 | 120 |
| 10 | 0A | 16 | 10 | 0 | 10 | 290088 | 2320700 | 0 | 10 | 0 | 112 | 2353636 | 0 | 10 | 0 | 1 | 120 |
| 11 | 0B | 16 | 10 | 0 | 11 | 290102 | 2320812 | 0 | 11 | 0 | 112 | 2353756 | 0 | 11 | 0 | 1 | 120 |
| 12 | 0C | 16 | 10 | 0 | 12 | 290116 | 2320924 | 0 | 12 | 0 | 112 | 2353876 | 0 | 12 | 0 | 1 | 120 |
| 13 | 0D | 16 | 10 | 0 | 13 | 290130 | 2321036 | 0 | 13 | 0 | 112 | 2353996 | 0 | 13 | 0 | 1 | 120 |
| 14 | 0E | 16 | 10 | 0 | 14 | 290144 | 2321148 | 0 | 14 | 0 | 112 | 2354116 | 0 | 14 | 0 | 1 | 120 |
| 15 | 0F | 16 | 10 | 0 | 15 | 290158 | 2321260 | 0 | 15 | 0 | 112 | 2354236 | 0 | 15 | 0 | 1 | 120 |
| 16 | 10 | 16 | 10 | 1 | 0 | 290177 | 2321412 | 1 | 0 | 0 | 152 | 2354396 | 1 | 0 | 0 | 1 | 160 |
| 17 | 11 | 16 | 10 | 1 | 1 | 290198 | 2321580 | 1 | 1 | 0 | 168 | 2354572 | 1 | 1 | 0 | 1 | 176 |
| 18 | 12 | 16 | 10 | 1 | 2 | 290219 | 2321748 | 1 | 2 | 0 | 168 | 2354748 | 1 | 2 | 0 | 1 | 176 |
| 19 | 13 | 16 | 10 | 1 | 3 | 290240 | 2321916 | 1 | 3 | 0 | 168 | 2354924 | 1 | 3 | 0 | 1 | 176 |
| 20 | 14 | 16 | 10 | 1 | 4 | 290261 | 2322084 | 1 | 4 | 0 | 168 | 2355100 | 1 | 4 | 0 | 1 | 176 |

| | | | | | | | | | | | | | | | | |
|---|---|---|---|---|---|---|---|---|---|---|---|---|---|---|---|---|
| 21 | 15 | 16 | 10 | 1 | 5 | 290282 | 2322252 | 1 | 5 | 0 | 168 | 2355276 | 1 | 5 | 0 | 1 | 176 |
| 22 | 16 | 16 | 10 | 1 | 6 | 290303 | 2322420 | 1 | 6 | 0 | 168 | 2355452 | 1 | 6 | 0 | 1 | 176 |
| 23 | 17 | 16 | 10 | 1 | 7 | 290324 | 2322588 | 1 | 7 | 0 | 168 | 2355628 | 1 | 7 | 0 | 1 | 176 |
| 24 | 18 | 16 | 10 | 1 | 8 | 290345 | 2322756 | 1 | 8 | 0 | 168 | 2355804 | 1 | 8 | 0 | 1 | 176 |
| 25 | 19 | 16 | 10 | 1 | 9 | 290366 | 2322924 | 1 | 9 | 0 | 168 | 2355980 | 1 | 9 | 0 | 1 | 176 |
| 26 | 1A | 16 | 10 | 1 | 10 | 290387 | 2323092 | 1 | 10 | 0 | 168 | 2356156 | 1 | 10 | 0 | 1 | 176 |
| 27 | 1B | 16 | 10 | 1 | 11 | 290408 | 2323260 | 1 | 11 | 0 | 168 | 2356332 | 1 | 11 | 0 | 1 | 176 |
| 28 | 1C | 16 | 10 | 1 | 12 | 290429 | 2323428 | 1 | 12 | 0 | 168 | 2356508 | 1 | 12 | 0 | 1 | 176 |
| 29 | 1D | 16 | 10 | 1 | 13 | 290450 | 2323596 | 1 | 13 | 0 | 168 | 2356684 | 1 | 13 | 0 | 1 | 176 |
| 30 | 1E | 16 | 10 | 1 | 14 | 290471 | 2323764 | 1 | 14 | 0 | 168 | 2356860 | 1 | 14 | 0 | 1 | 176 |
| 31 | 1F | 16 | 10 | 1 | 15 | 290492 | 2323932 | 1 | 15 | 0 | 168 | 2357036 | 1 | 15 | 0 | 1 | 176 |
| 32 | 20 | 16 | 10 | 2 | 0 | 290513 | 2324100 | 2 | 0 | 0 | 168 | 2357212 | 2 | 0 | 0 | 1 | 176 |
| 33 | 21 | 16 | 10 | 2 | 1 | 290536 | 2324284 | 2 | 1 | 0 | 184 | 2357404 | 2 | 1 | 0 | 1 | 192 |
| 34 | 22 | 16 | 10 | 2 | 2 | 290559 | 2324468 | 2 | 2 | 0 | 184 | 2357596 | 2 | 2 | 0 | 1 | 192 |
| 35 | 23 | 16 | 10 | 2 | 3 | 290582 | 2324652 | 2 | 3 | 0 | 184 | 2357788 | 2 | 3 | 0 | 1 | 192 |
| 36 | 24 | 16 | 10 | 2 | 4 | 290605 | 2324836 | 2 | 4 | 0 | 184 | 2357980 | 2 | 4 | 0 | 1 | 192 |
| 37 | 25 | 16 | 10 | 2 | 5 | 290628 | 2325020 | 2 | 5 | 0 | 184 | 2358172 | 2 | 5 | 0 | 1 | 192 |
| 38 | 26 | 16 | 10 | 2 | 6 | 290651 | 2325204 | 2 | 6 | 0 | 184 | 2358364 | 2 | 6 | 0 | 1 | 192 |
| 39 | 27 | 16 | 10 | 2 | 7 | 290674 | 2325388 | 2 | 7 | 0 | 184 | 2358556 | 2 | 7 | 0 | 1 | 192 |
| 40 | 28 | 16 | 10 | 2 | 8 | 290697 | 2325572 | 2 | 8 | 0 | 184 | 2358748 | 2 | 8 | 0 | 1 | 192 |
| 41 | 29 | 16 | 10 | 2 | 9 | 290720 | 2325756 | 2 | 9 | 0 | 184 | 2358940 | 2 | 9 | 0 | 1 | 192 |
| 42 | 2A | 16 | 10 | 2 | 10 | 290743 | 2325940 | 2 | 10 | 0 | 184 | 2359132 | 2 | 10 | 0 | 1 | 192 |
| 43 | 2B | 16 | 10 | 2 | 11 | 290766 | 2326124 | 2 | 11 | 0 | 184 | 2359324 | 2 | 11 | 0 | 1 | 192 |
| 44 | 2C | 16 | 10 | 2 | 12 | 290789 | 2326308 | 2 | 12 | 0 | 184 | 2359516 | 2 | 12 | 0 | 1 | 192 |
| 45 | 2D | 16 | 10 | 2 | 13 | 290812 | 2326492 | 2 | 13 | 0 | 184 | 2359708 | 2 | 13 | 0 | 1 | 192 |
| 46 | 2E | 16 | 10 | 2 | 14 | 290835 | 2326676 | 2 | 14 | 0 | 184 | 2359900 | 2 | 14 | 0 | 1 | 192 |
| 47 | 2F | 16 | 10 | 2 | 15 | 290858 | 2326860 | 2 | 15 | 0 | 184 | 2360092 | 2 | 15 | 0 | 1 | 192 |
| 48 | 30 | 16 | 10 | 3 | 0 | 290881 | 2327044 | 3 | 0 | 0 | 184 | 2360284 | 3 | 0 | 0 | 1 | 192 |
| 49 | 31 | 16 | 10 | 3 | 1 | 290906 | 2327244 | 3 | 1 | 0 | 200 | 2360492 | 3 | 1 | 0 | 1 | 208 |
| 50 | 32 | 16 | 10 | 3 | 2 | 290931 | 2327444 | 3 | 2 | 0 | 200 | 2360700 | 3 | 2 | 0 | 1 | 208 |
| 51 | 33 | 16 | 10 | 3 | 3 | 290956 | 2327644 | 3 | 3 | 0 | 200 | 2360908 | 3 | 3 | 0 | 1 | 208 |
| 52 | 34 | 16 | 10 | 3 | 4 | 290981 | 2327844 | 3 | 4 | 0 | 200 | 2361116 | 3 | 4 | 0 | 1 | 208 |
| 53 | 35 | 16 | 10 | 3 | 5 | 291006 | 2328044 | 3 | 5 | 0 | 200 | 2361324 | 3 | 5 | 0 | 1 | 208 |
| 54 | 36 | 16 | 10 | 3 | 6 | 291031 | 2328244 | 3 | 6 | 0 | 200 | 2361532 | 3 | 6 | 0 | 1 | 208 |
| 55 | 37 | 16 | 10 | 3 | 7 | 291056 | 2328444 | 3 | 7 | 0 | 200 | 2361740 | 3 | 7 | 0 | 1 | 208 |
| 56 | 38 | 16 | 10 | 3 | 8 | 291081 | 2328644 | 3 | 8 | 0 | 200 | 2361948 | 3 | 8 | 0 | 1 | 208 |
| 57 | 39 | 16 | 10 | 3 | 9 | 291106 | 2328844 | 3 | 9 | 0 | 200 | 2362156 | 3 | 9 | 0 | 1 | 208 |
| 58 | 3A | 16 | 10 | 3 | 10 | 291131 | 2329044 | 3 | 10 | 0 | 200 | 2362364 | 3 | 10 | 0 | 1 | 208 |
| 59 | 3B | 16 | 10 | 3 | 11 | 291156 | 2329244 | 3 | 11 | 0 | 200 | 2362572 | 3 | 11 | 0 | 1 | 208 |
| 60 | 3C | 16 | 10 | 3 | 12 | 291181 | 2329444 | 3 | 12 | 0 | 200 | 2362780 | 3 | 12 | 0 | 1 | 208 |
| 61 | 3D | 16 | 10 | 3 | 13 | 291206 | 2329644 | 3 | 13 | 0 | 200 | 2362988 | 3 | 13 | 0 | 1 | 208 |
| 62 | 3E | 16 | 10 | 3 | 14 | 291231 | 2329844 | 3 | 14 | 0 | 200 | 2363196 | 3 | 14 | 0 | 1 | 208 |
| 63 | 3F | 16 | 10 | 3 | 15 | 291256 | 2330044 | 3 | 15 | 0 | 200 | 2363404 | 3 | 15 | 0 | 1 | 208 |
| 64 | 40 | 16 | 10 | 4 | 0 | 291281 | 2330244 | 4 | 0 | 0 | 200 | 2363612 | 4 | 0 | 0 | 1 | 208 |
| 65 | 41 | 16 | 10 | 4 | 1 | 291308 | 2330460 | 4 | 1 | 0 | 216 | 2363836 | 4 | 1 | 0 | 1 | 224 |
| 66 | 42 | 16 | 10 | 4 | 2 | 291335 | 2330676 | 4 | 2 | 0 | 216 | 2364060 | 4 | 2 | 0 | 1 | 224 |
| 67 | 43 | 16 | 10 | 4 | 3 | 291362 | 2330892 | 4 | 3 | 0 | 216 | 2364284 | 4 | 3 | 0 | 1 | 224 |
| 68 | 44 | 16 | 10 | 4 | 4 | 291389 | 2331108 | 4 | 4 | 0 | 216 | 2364508 | 4 | 4 | 0 | 1 | 224 |
| 69 | 45 | 16 | 10 | 4 | 5 | 291416 | 2331324 | 4 | 5 | 0 | 216 | 2364732 | 4 | 5 | 0 | 1 | 224 |
| 70 | 46 | 16 | 10 | 4 | 6 | 291443 | 2331540 | 4 | 6 | 0 | 216 | 2364956 | 4 | 6 | 0 | 1 | 224 |
| 71 | 47 | 16 | 10 | 4 | 7 | 291470 | 2331756 | 4 | 7 | 0 | 216 | 2365180 | 4 | 7 | 0 | 1 | 224 |
| 72 | 48 | 16 | 10 | 4 | 8 | 291497 | 2331972 | 4 | 8 | 0 | 216 | 2365404 | 4 | 8 | 0 | 1 | 224 |
| 73 | 49 | 16 | 10 | 4 | 9 | 291524 | 2332188 | 4 | 9 | 0 | 216 | 2365628 | 4 | 9 | 0 | 1 | 224 |
| 74 | 4A | 16 | 10 | 4 | 10 | 291551 | 2332404 | 4 | 10 | 0 | 216 | 2365852 | 4 | 10 | 0 | 1 | 224 |
| 75 | 4B | 16 | 10 | 4 | 11 | 291578 | 2332620 | 4 | 11 | 0 | 216 | 2366076 | 4 | 11 | 0 | 1 | 224 |
| 76 | 4C | 16 | 10 | 4 | 12 | 291605 | 2332836 | 4 | 12 | 0 | 216 | 2366300 | 4 | 12 | 0 | 1 | 224 |
| 77 | 4D | 16 | 10 | 4 | 13 | 291632 | 2333052 | 4 | 13 | 0 | 216 | 2366524 | 4 | 13 | 0 | 1 | 224 |
| 78 | 4E | 16 | 10 | 4 | 14 | 291659 | 2333268 | 4 | 14 | 0 | 216 | 2366748 | 4 | 14 | 0 | 1 | 224 |
| 79 | 4F | 16 | 10 | 4 | 15 | 291686 | 2333484 | 4 | 15 | 0 | 216 | 2366972 | 4 | 15 | 0 | 1 | 224 |
| 80 | 50 | 16 | 10 | 5 | 0 | 291713 | 2333700 | 5 | 0 | 0 | 216 | 2367196 | 5 | 0 | 0 | 1 | 224 |
| 81 | 51 | 16 | 10 | 5 | 1 | 291742 | 2333932 | 5 | 1 | 0 | 232 | 2367436 | 5 | 1 | 0 | 1 | 240 |
| 82 | 52 | 16 | 10 | 5 | 2 | 291771 | 2334164 | 5 | 2 | 0 | 232 | 2367676 | 5 | 2 | 0 | 1 | 240 |
| 83 | 53 | 16 | 10 | 5 | 3 | 291800 | 2334396 | 5 | 3 | 0 | 232 | 2367916 | 5 | 3 | 0 | 1 | 240 |
| 84 | 54 | 16 | 10 | 5 | 4 | 291829 | 2334628 | 5 | 4 | 0 | 232 | 2368156 | 5 | 4 | 0 | 1 | 240 |
| 85 | 55 | 16 | 10 | 5 | 5 | 291858 | 2334860 | 5 | 5 | 0 | 232 | 2368396 | 5 | 5 | 0 | 1 | 240 |
| 86 | 56 | 16 | 10 | 5 | 6 | 291887 | 2335092 | 5 | 6 | 0 | 232 | 2368636 | 5 | 6 | 0 | 1 | 240 |
| 87 | 57 | 16 | 10 | 5 | 7 | 291916 | 2335324 | 5 | 7 | 0 | 232 | 2368876 | 5 | 7 | 0 | 1 | 240 |
| 88 | 58 | 16 | 10 | 5 | 8 | 291945 | 2335556 | 5 | 8 | 0 | 232 | 2369116 | 5 | 8 | 0 | 1 | 240 |
| 89 | 59 | 16 | 10 | 5 | 9 | 291974 | 2335788 | 5 | 9 | 0 | 232 | 2369356 | 5 | 9 | 0 | 1 | 240 |
| 90 | 5A | 16 | 10 | 5 | 10 | 292003 | 2336020 | 5 | 10 | 0 | 232 | 2369596 | 5 | 10 | 0 | 1 | 240 |
| 91 | 5B | 16 | 10 | 5 | 11 | 292032 | 2336252 | 5 | 11 | 0 | 232 | 2369836 | 5 | 11 | 0 | 1 | 240 |
| 92 | 5C | 16 | 10 | 5 | 12 | 292061 | 2336484 | 5 | 12 | 0 | 232 | 2370076 | 5 | 12 | 0 | 1 | 240 |
| 93 | 5D | 16 | 10 | 5 | 13 | 292090 | 2336716 | 5 | 13 | 0 | 232 | 2370316 | 5 | 13 | 0 | 1 | 240 |
| 94 | 5E | 16 | 10 | 5 | 14 | 292119 | 2336948 | 5 | 14 | 0 | 232 | 2370556 | 5 | 14 | 0 | 1 | 240 |
| 95 | 5F | 16 | 10 | 5 | 15 | 292148 | 2337180 | 5 | 15 | 0 | 232 | 2370796 | 5 | 15 | 0 | 1 | 240 |
| 96 | 60 | 16 | 10 | 6 | 0 | 292177 | 2337412 | 6 | 0 | 0 | 232 | 2371036 | 6 | 0 | 0 | 1 | 240 |
| 97 | 61 | 16 | 10 | 6 | 1 | 292208 | 2337660 | 6 | 1 | 0 | 248 | 2371292 | 6 | 1 | 0 | 1 | 256 |
| 98 | 62 | 16 | 10 | 6 | 2 | 292239 | 2337908 | 6 | 2 | 0 | 248 | 2371548 | 6 | 2 | 0 | 1 | 256 |
| 99 | 63 | 16 | 10 | 6 | 3 | 292270 | 2338156 | 6 | 3 | 0 | 248 | 2371804 | 6 | 3 | 0 | 1 | 256 |
| 100 | 64 | 16 | 10 | 6 | 4 | 292301 | 2338404 | 6 | 4 | 0 | 248 | 2372060 | 6 | 4 | 0 | 1 | 256 |
| 101 | 65 | 16 | 10 | 6 | 5 | 292332 | 2338652 | 6 | 5 | 0 | 248 | 2372316 | 6 | 5 | 0 | 1 | 256 |
| 102 | 66 | 16 | 10 | 6 | 6 | 292363 | 2338900 | 6 | 6 | 0 | 248 | 2372572 | 6 | 6 | 0 | 1 | 256 |
| 103 | 67 | 16 | 10 | 6 | 7 | 292394 | 2339148 | 6 | 7 | 0 | 248 | 2372828 | 6 | 7 | 0 | 1 | 256 |
| 104 | 68 | 16 | 10 | 6 | 8 | 292425 | 2339396 | 6 | 8 | 0 | 248 | 2373084 | 6 | 8 | 0 | 1 | 256 |
| 105 | 69 | 16 | 10 | 6 | 9 | 292456 | 2339644 | 6 | 9 | 0 | 248 | 2373340 | 6 | 9 | 0 | 1 | 256 |
| 106 | 6A | 16 | 10 | 6 | 10 | 292487 | 2339892 | 6 | 10 | 0 | 248 | 2373596 | 6 | 10 | 0 | 1 | 256 |
| 107 | 6B | 16 | 10 | 6 | 11 | 292518 | 2340140 | 6 | 11 | 0 | 248 | 2373852 | 6 | 11 | 0 | 1 | 256 |
| 108 | 6C | 16 | 10 | 6 | 12 | 292549 | 2340388 | 6 | 12 | 0 | 248 | 2374108 | 6 | 12 | 0 | 1 | 256 |
| 109 | 6D | 16 | 10 | 6 | 13 | 292580 | 2340636 | 6 | 13 | 0 | 248 | 2374364 | 6 | 13 | 0 | 1 | 256 |
| 110 | 6E | 16 | 10 | 6 | 14 | 292611 | 2340884 | 6 | 14 | 0 | 248 | 2374620 | 6 | 14 | 0 | 1 | 256 |
| 111 | 6F | 16 | 10 | 6 | 15 | 292642 | 2341132 | 6 | 15 | 0 | 248 | 2374876 | 6 | 15 | 0 | 1 | 256 |
| 112 | 70 | 16 | 10 | 7 | 0 | 292673 | 2341380 | 7 | 0 | 0 | 248 | 2375132 | 7 | 0 | 0 | 1 | 256 |
| 113 | 71 | 16 | 10 | 7 | 1 | 292706 | 2341644 | 7 | 1 | 0 | 264 | 2375404 | 7 | 1 | 0 | 1 | 272 |
| 114 | 72 | 16 | 10 | 7 | 2 | 292739 | 2341908 | 7 | 2 | 0 | 264 | 2375676 | 7 | 2 | 0 | 1 | 272 |
| 115 | 73 | 16 | 10 | 7 | 3 | 292772 | 2342172 | 7 | 3 | 0 | 264 | 2375948 | 7 | 3 | 0 | 1 | 272 |
| 116 | 74 | 16 | 10 | 7 | 4 | 292805 | 2342436 | 7 | 4 | 0 | 264 | 2376220 | 7 | 4 | 0 | 1 | 272 |
| 117 | 75 | 16 | 10 | 7 | 5 | 292838 | 2342700 | 7 | 5 | 0 | 264 | 2376492 | 7 | 5 | 0 | 1 | 272 |
| 118 | 76 | 16 | 10 | 7 | 6 | 292871 | 2342964 | 7 | 6 | 0 | 264 | 2376764 | 7 | 6 | 0 | 1 | 272 |
| 119 | 77 | 16 | 10 | 7 | 7 | 292904 | 2343228 | 7 | 7 | 0 | 264 | 2377036 | 7 | 7 | 0 | 1 | 272 |
| 120 | 78 | 16 | 10 | 7 | 8 | 292937 | 2343492 | 7 | 8 | 0 | 264 | 2377308 | 7 | 8 | 0 | 1 | 272 |
| 121 | 79 | 16 | 10 | 7 | 9 | 292970 | 2343756 | 7 | 9 | 0 | 264 | 2377580 | 7 | 9 | 0 | 1 | 272 |
| 122 | 7A | 16 | 10 | 7 | 10 | 293003 | 2344020 | 7 | 10 | 0 | 264 | 2377852 | 7 | 10 | 0 | 1 | 272 |
| 123 | 7B | 16 | 10 | 7 | 11 | 293036 | 2344284 | 7 | 11 | 0 | 264 | 2378124 | 7 | 11 | 0 | 1 | 272 |

| | | | | | | | | | | | | | | | | | |
|---|---|---|---|---|---|---|---|---|---|---|---|---|---|---|---|---|---|
| 124 | 7C | 16 | 10 | 7 | 12 | 293069 | 2344548 | 7 | 12 | 0 | 264 | 2378396 | 7 | 12 | 0 | 1 | 272 |
| 125 | 7D | 16 | 10 | 7 | 13 | 293102 | 2344812 | 7 | 13 | 0 | 264 | 2378668 | 7 | 13 | 0 | 1 | 272 |
| 126 | 7E | 16 | 10 | 7 | 14 | 293135 | 2345076 | 7 | 14 | 0 | 264 | 2378940 | 7 | 14 | 0 | 1 | 272 |
| 127 | 7F | 16 | 10 | 7 | 15 | 293168 | 2345340 | 7 | 15 | 0 | 264 | 2379212 | 7 | 15 | 0 | 1 | 272 |
| 128 | 80 | 16 | 10 | 8 | 0 | 293201 | 2345604 | 8 | 0 | 0 | 264 | 2379484 | 8 | 0 | 0 | 1 | 272 |
| 129 | 81 | 16 | 10 | 8 | 1 | 293236 | 2345884 | 8 | 1 | 0 | 280 | 2379772 | 8 | 1 | 0 | 1 | 288 |
| 130 | 82 | 16 | 10 | 8 | 2 | 293271 | 2346164 | 8 | 2 | 0 | 280 | 2380060 | 8 | 2 | 0 | 1 | 288 |
| 131 | 83 | 16 | 10 | 8 | 3 | 293306 | 2346444 | 8 | 3 | 0 | 280 | 2380348 | 8 | 3 | 0 | 1 | 288 |
| 132 | 84 | 16 | 10 | 8 | 4 | 293341 | 2346724 | 8 | 4 | 0 | 280 | 2380636 | 8 | 4 | 0 | 1 | 288 |
| 133 | 85 | 16 | 10 | 8 | 5 | 293376 | 2347004 | 8 | 5 | 0 | 280 | 2380924 | 8 | 5 | 0 | 1 | 288 |
| 134 | 86 | 16 | 10 | 8 | 6 | 293411 | 2347284 | 8 | 6 | 0 | 280 | 2381212 | 8 | 6 | 0 | 1 | 288 |
| 135 | 87 | 16 | 10 | 8 | 7 | 293446 | 2347564 | 8 | 7 | 0 | 280 | 2381500 | 8 | 7 | 0 | 1 | 288 |
| 136 | 88 | 16 | 10 | 8 | 8 | 293481 | 2347844 | 8 | 8 | 0 | 280 | 2381788 | 8 | 8 | 0 | 1 | 288 |
| 137 | 89 | 16 | 10 | 8 | 9 | 293516 | 2348124 | 8 | 9 | 0 | 280 | 2382076 | 8 | 9 | 0 | 1 | 288 |
| 138 | 8A | 16 | 10 | 8 | 10 | 293551 | 2348404 | 8 | 10 | 0 | 280 | 2382364 | 8 | 10 | 0 | 1 | 288 |
| 139 | 8B | 16 | 10 | 8 | 11 | 293586 | 2348684 | 8 | 11 | 0 | 280 | 2382652 | 8 | 11 | 0 | 1 | 288 |
| 140 | 8C | 16 | 10 | 8 | 12 | 293621 | 2348964 | 8 | 12 | 0 | 280 | 2382940 | 8 | 12 | 0 | 1 | 288 |
| 141 | 8D | 16 | 10 | 8 | 13 | 293656 | 2349244 | 8 | 13 | 0 | 280 | 2383228 | 8 | 13 | 0 | 1 | 288 |
| 142 | 8E | 16 | 10 | 8 | 14 | 293691 | 2349524 | 8 | 14 | 0 | 280 | 2383516 | 8 | 14 | 0 | 1 | 288 |
| 143 | 8F | 16 | 10 | 8 | 15 | 293726 | 2349804 | 8 | 15 | 0 | 280 | 2383804 | 8 | 15 | 0 | 1 | 288 |
| 144 | 90 | 16 | 10 | 9 | 0 | 293761 | 2350084 | 9 | 0 | 0 | 280 | 2384092 | 9 | 0 | 0 | 1 | 288 |
| 145 | 91 | 16 | 10 | 9 | 1 | 293798 | 2350380 | 9 | 1 | 0 | 296 | 2384396 | 9 | 1 | 0 | 1 | 304 |
| 146 | 92 | 16 | 10 | 9 | 2 | 293835 | 2350676 | 9 | 2 | 0 | 296 | 2384700 | 9 | 2 | 0 | 1 | 304 |
| 147 | 93 | 16 | 10 | 9 | 3 | 293872 | 2350972 | 9 | 3 | 0 | 296 | 2385004 | 9 | 3 | 0 | 1 | 304 |
| 148 | 94 | 16 | 10 | 9 | 4 | 293909 | 2351268 | 9 | 4 | 0 | 296 | 2385308 | 9 | 4 | 0 | 1 | 304 |
| 149 | 95 | 16 | 10 | 9 | 5 | 293946 | 2351564 | 9 | 5 | 0 | 296 | 2385612 | 9 | 5 | 0 | 1 | 304 |
| 150 | 96 | 16 | 10 | 9 | 6 | 293983 | 2351860 | 9 | 6 | 0 | 296 | 2385916 | 9 | 6 | 0 | 1 | 304 |
| 151 | 97 | 16 | 10 | 9 | 7 | 294020 | 2352156 | 9 | 7 | 0 | 296 | 2386220 | 9 | 7 | 0 | 1 | 304 |
| 152 | 98 | 16 | 10 | 9 | 8 | 294057 | 2352452 | 9 | 8 | 0 | 296 | 2386524 | 9 | 8 | 0 | 1 | 304 |
| 153 | 99 | 16 | 10 | 9 | 9 | 294094 | 2352748 | 9 | 9 | 0 | 296 | 2386828 | 9 | 9 | 0 | 1 | 304 |
| 154 | 9A | 16 | 10 | 9 | 10 | 294131 | 2353044 | 9 | 10 | 0 | 296 | 2387132 | 9 | 10 | 0 | 1 | 304 |
| 155 | 9B | 16 | 10 | 9 | 11 | 294168 | 2353340 | 9 | 11 | 0 | 296 | 2387436 | 9 | 11 | 0 | 1 | 304 |
| 156 | 9C | 16 | 10 | 9 | 12 | 294205 | 2353636 | 9 | 12 | 0 | 296 | 2387740 | 9 | 12 | 0 | 1 | 304 |
| 157 | 9D | 16 | 10 | 9 | 13 | 294242 | 2353932 | 9 | 13 | 0 | 296 | 2388044 | 9 | 13 | 0 | 1 | 304 |
| 158 | 9E | 16 | 10 | 9 | 14 | 294279 | 2354228 | 9 | 14 | 0 | 296 | 2388348 | 9 | 14 | 0 | 1 | 304 |
| 159 | 9F | 16 | 10 | 9 | 15 | 294316 | 2354524 | 9 | 15 | 0 | 296 | 2388652 | 9 | 15 | 0 | 1 | 304 |
| 160 | A0 | 16 | 10 | 10 | 0 | 294353 | 2354820 | 10 | 0 | 0 | 296 | 2388956 | 10 | 0 | 0 | 1 | 304 |
| 161 | A1 | 16 | 10 | 10 | 1 | 294392 | 2355132 | 10 | 1 | 0 | 312 | 2389276 | 10 | 1 | 0 | 1 | 320 |
| 162 | A2 | 16 | 10 | 10 | 2 | 294431 | 2355444 | 10 | 2 | 0 | 312 | 2389596 | 10 | 2 | 0 | 1 | 320 |
| 163 | A3 | 16 | 10 | 10 | 3 | 294470 | 2355756 | 10 | 3 | 0 | 312 | 2389916 | 10 | 3 | 0 | 1 | 320 |
| 164 | A4 | 16 | 10 | 10 | 4 | 294509 | 2356068 | 10 | 4 | 0 | 312 | 2390236 | 10 | 4 | 0 | 1 | 320 |
| 165 | A5 | 16 | 10 | 10 | 5 | 294548 | 2356380 | 10 | 5 | 0 | 312 | 2390556 | 10 | 5 | 0 | 1 | 320 |
| 166 | A6 | 16 | 10 | 10 | 6 | 294587 | 2356692 | 10 | 6 | 0 | 312 | 2390876 | 10 | 6 | 0 | 1 | 320 |
| 167 | A7 | 16 | 10 | 10 | 7 | 294626 | 2357004 | 10 | 7 | 0 | 312 | 2391196 | 10 | 7 | 0 | 1 | 320 |
| 168 | A8 | 16 | 10 | 10 | 8 | 294665 | 2357316 | 10 | 8 | 0 | 312 | 2391516 | 10 | 8 | 0 | 1 | 320 |
| 169 | A9 | 16 | 10 | 10 | 9 | 294704 | 2357628 | 10 | 9 | 0 | 312 | 2391836 | 10 | 9 | 0 | 1 | 320 |
| 170 | AA | 16 | 10 | 10 | 10 | 294743 | 2357940 | 10 | 10 | 0 | 312 | 2392156 | 10 | 10 | 0 | 1 | 320 |
| 171 | AB | 16 | 10 | 10 | 11 | 294782 | 2358252 | 10 | 11 | 0 | 312 | 2392476 | 10 | 11 | 0 | 1 | 320 |
| 172 | AC | 16 | 10 | 10 | 12 | 294821 | 2358564 | 10 | 12 | 0 | 312 | 2392796 | 10 | 12 | 0 | 1 | 320 |
| 173 | AD | 16 | 10 | 10 | 13 | 294860 | 2358876 | 10 | 13 | 0 | 312 | 2393116 | 10 | 13 | 0 | 1 | 320 |
| 174 | AE | 16 | 10 | 10 | 14 | 294899 | 2359188 | 10 | 14 | 0 | 312 | 2393436 | 10 | 14 | 0 | 1 | 320 |
| 175 | AF | 16 | 10 | 10 | 15 | 294938 | 2359500 | 10 | 15 | 0 | 312 | 2393756 | 10 | 15 | 0 | 1 | 320 |
| 176 | B0 | 16 | 10 | 11 | 0 | 294977 | 2359812 | 11 | 0 | 0 | 312 | 2394076 | 11 | 0 | 0 | 1 | 320 |
| 177 | B1 | 16 | 10 | 11 | 1 | 295018 | 2360140 | 11 | 1 | 0 | 328 | 2394412 | 11 | 1 | 0 | 1 | 336 |
| 178 | B2 | 16 | 10 | 11 | 2 | 295059 | 2360468 | 11 | 2 | 0 | 328 | 2394748 | 11 | 2 | 0 | 1 | 336 |
| 179 | B3 | 16 | 10 | 11 | 3 | 295100 | 2360796 | 11 | 3 | 0 | 328 | 2395084 | 11 | 3 | 0 | 1 | 336 |
| 180 | B4 | 16 | 10 | 11 | 4 | 295141 | 2361124 | 11 | 4 | 0 | 328 | 2395420 | 11 | 4 | 0 | 1 | 336 |
| 181 | B5 | 16 | 10 | 11 | 5 | 295182 | 2361452 | 11 | 5 | 0 | 328 | 2395756 | 11 | 5 | 0 | 1 | 336 |
| 182 | B6 | 16 | 10 | 11 | 6 | 295223 | 2361780 | 11 | 6 | 0 | 328 | 2396092 | 11 | 6 | 0 | 1 | 336 |
| 183 | B7 | 16 | 10 | 11 | 7 | 295264 | 2362108 | 11 | 7 | 0 | 328 | 2396428 | 11 | 7 | 0 | 1 | 336 |
| 184 | B8 | 16 | 10 | 11 | 8 | 295305 | 2362436 | 11 | 8 | 0 | 328 | 2396764 | 11 | 8 | 0 | 1 | 336 |
| 185 | B9 | 16 | 10 | 11 | 9 | 295346 | 2362764 | 11 | 9 | 0 | 328 | 2397100 | 11 | 9 | 0 | 1 | 336 |
| 186 | BA | 16 | 10 | 11 | 10 | 295387 | 2363092 | 11 | 10 | 0 | 328 | 2397436 | 11 | 10 | 0 | 1 | 336 |
| 187 | BB | 16 | 10 | 11 | 11 | 295428 | 2363420 | 11 | 11 | 0 | 328 | 2397772 | 11 | 11 | 0 | 1 | 336 |
| 188 | BC | 16 | 10 | 11 | 12 | 295469 | 2363748 | 11 | 12 | 0 | 328 | 2398108 | 11 | 12 | 0 | 1 | 336 |
| 189 | BD | 16 | 10 | 11 | 13 | 295510 | 2364076 | 11 | 13 | 0 | 328 | 2398444 | 11 | 13 | 0 | 1 | 336 |
| 190 | BE | 16 | 10 | 11 | 14 | 295551 | 2364404 | 11 | 14 | 0 | 328 | 2398780 | 11 | 14 | 0 | 1 | 336 |
| 191 | BF | 16 | 10 | 11 | 15 | 295592 | 2364732 | 11 | 15 | 0 | 328 | 2399116 | 11 | 15 | 0 | 1 | 336 |
| 192 | C0 | 16 | 10 | 12 | 0 | 295633 | 2365060 | 12 | 0 | 0 | 328 | 2399452 | 12 | 0 | 0 | 1 | 336 |
| 193 | C1 | 16 | 10 | 12 | 1 | 295676 | 2365404 | 12 | 1 | 0 | 344 | 2399804 | 12 | 1 | 0 | 1 | 352 |
| 194 | C2 | 16 | 10 | 12 | 2 | 295719 | 2365748 | 12 | 2 | 0 | 344 | 2400156 | 12 | 2 | 0 | 1 | 352 |
| 195 | C3 | 16 | 10 | 12 | 3 | 295762 | 2366092 | 12 | 3 | 0 | 344 | 2400508 | 12 | 3 | 0 | 1 | 352 |
| 196 | C4 | 16 | 10 | 12 | 4 | 295805 | 2366436 | 12 | 4 | 0 | 344 | 2400860 | 12 | 4 | 0 | 1 | 352 |
| 197 | C5 | 16 | 10 | 12 | 5 | 295848 | 2366780 | 12 | 5 | 0 | 344 | 2401212 | 12 | 5 | 0 | 1 | 352 |
| 198 | C6 | 16 | 10 | 12 | 6 | 295891 | 2367124 | 12 | 6 | 0 | 344 | 2401564 | 12 | 6 | 0 | 1 | 352 |
| 199 | C7 | 16 | 10 | 12 | 7 | 295934 | 2367468 | 12 | 7 | 0 | 344 | 2401916 | 12 | 7 | 0 | 1 | 352 |
| 200 | C8 | 16 | 10 | 12 | 8 | 295977 | 2367812 | 12 | 8 | 0 | 344 | 2402268 | 12 | 8 | 0 | 1 | 352 |
| 201 | C9 | 16 | 10 | 12 | 9 | 296020 | 2368156 | 12 | 9 | 0 | 344 | 2402620 | 12 | 9 | 0 | 1 | 352 |
| 202 | CA | 16 | 10 | 12 | 10 | 296063 | 2368500 | 12 | 10 | 0 | 344 | 2402972 | 12 | 10 | 0 | 1 | 352 |
| 203 | CB | 16 | 10 | 12 | 11 | 296106 | 2368844 | 12 | 11 | 0 | 344 | 2403324 | 12 | 11 | 0 | 1 | 352 |
| 204 | CC | 16 | 10 | 12 | 12 | 296149 | 2369188 | 12 | 12 | 0 | 344 | 2403676 | 12 | 12 | 0 | 1 | 352 |
| 205 | CD | 16 | 10 | 12 | 13 | 296192 | 2369532 | 12 | 13 | 0 | 344 | 2404028 | 12 | 13 | 0 | 1 | 352 |
| 206 | CE | 16 | 10 | 12 | 14 | 296235 | 2369876 | 12 | 14 | 0 | 344 | 2404380 | 12 | 14 | 0 | 1 | 352 |
| 207 | CF | 16 | 10 | 12 | 15 | 296278 | 2370220 | 12 | 15 | 0 | 344 | 2404732 | 12 | 15 | 0 | 1 | 352 |
| 208 | D0 | 16 | 10 | 13 | 0 | 296321 | 2370564 | 13 | 0 | 0 | 344 | 2405084 | 13 | 0 | 0 | 1 | 352 |
| 209 | D1 | 16 | 10 | 13 | 1 | 296366 | 2370924 | 13 | 1 | 0 | 360 | 2405452 | 13 | 1 | 0 | 1 | 368 |
| 210 | D2 | 16 | 10 | 13 | 2 | 296411 | 2371284 | 13 | 2 | 0 | 360 | 2405820 | 13 | 2 | 0 | 1 | 368 |
| 211 | D3 | 16 | 10 | 13 | 3 | 296456 | 2371644 | 13 | 3 | 0 | 360 | 2406188 | 13 | 3 | 0 | 1 | 368 |
| 212 | D4 | 16 | 10 | 13 | 4 | 296501 | 2372004 | 13 | 4 | 0 | 360 | 2406556 | 13 | 4 | 0 | 1 | 368 |
| 213 | D5 | 16 | 10 | 13 | 5 | 296546 | 2372364 | 13 | 5 | 0 | 360 | 2406924 | 13 | 5 | 0 | 1 | 368 |
| 214 | D6 | 16 | 10 | 13 | 6 | 296591 | 2372724 | 13 | 6 | 0 | 360 | 2407292 | 13 | 6 | 0 | 1 | 368 |
| 215 | D7 | 16 | 10 | 13 | 7 | 296636 | 2373084 | 13 | 7 | 0 | 360 | 2407660 | 13 | 7 | 0 | 1 | 368 |
| 216 | D8 | 16 | 10 | 13 | 8 | 296681 | 2373444 | 13 | 8 | 0 | 360 | 2408028 | 13 | 8 | 0 | 1 | 368 |
| 217 | D9 | 16 | 10 | 13 | 9 | 296726 | 2373804 | 13 | 9 | 0 | 360 | 2408396 | 13 | 9 | 0 | 1 | 368 |
| 218 | DA | 16 | 10 | 13 | 10 | 296771 | 2374164 | 13 | 10 | 0 | 360 | 2408764 | 13 | 10 | 0 | 1 | 368 |
| 219 | DB | 16 | 10 | 13 | 11 | 296816 | 2374524 | 13 | 11 | 0 | 360 | 2409132 | 13 | 11 | 0 | 1 | 368 |
| 220 | DC | 16 | 10 | 13 | 12 | 296861 | 2374884 | 13 | 12 | 0 | 360 | 2409500 | 13 | 12 | 0 | 1 | 368 |
| 221 | DD | 16 | 10 | 13 | 13 | 296906 | 2375244 | 13 | 13 | 0 | 360 | 2409868 | 13 | 13 | 0 | 1 | 368 |
| 222 | DE | 16 | 10 | 13 | 14 | 296951 | 2375604 | 13 | 14 | 0 | 360 | 2410236 | 13 | 14 | 0 | 1 | 368 |
| 223 | DF | 16 | 10 | 13 | 15 | 296996 | 2375964 | 13 | 15 | 0 | 360 | 2410604 | 13 | 15 | 0 | 1 | 368 |
| 224 | E0 | 16 | 10 | 14 | 0 | 297041 | 2376324 | 14 | 0 | 0 | 360 | 2410972 | 14 | 0 | 0 | 1 | 368 |
| 225 | E1 | 16 | 10 | 14 | 1 | 297088 | 2376700 | 14 | 1 | 0 | 376 | 2411356 | 14 | 1 | 0 | 1 | 384 |
| 226 | E2 | 16 | 10 | 14 | 2 | 297135 | 2377076 | 14 | 2 | 0 | 376 | 2411740 | 14 | 2 | 0 | 1 | 384 |

| | | | | | | | | | | | | | | | | | |
|---|---|---|---|---|---|---|---|---|---|---|---|---|---|---|---|---|---|
| 227 | E3 | 16 | 10 | 14 | 3 | 297182 | 2377452 | 14 | 3 | 0 | 376 | 2412124 | 14 | 3 | 0 | 1 | 384 |
| 228 | E4 | 16 | 10 | 14 | 4 | 297229 | 2377828 | 14 | 4 | 0 | 376 | 2412508 | 14 | 4 | 0 | 1 | 384 |
| 229 | E5 | 16 | 10 | 14 | 5 | 297276 | 2378204 | 14 | 5 | 0 | 376 | 2412892 | 14 | 5 | 0 | 1 | 384 |
| 230 | E6 | 16 | 10 | 14 | 6 | 297323 | 2378580 | 14 | 6 | 0 | 376 | 2413276 | 14 | 6 | 0 | 1 | 384 |
| 231 | E7 | 16 | 10 | 14 | 7 | 297370 | 2378956 | 14 | 7 | 0 | 376 | 2413660 | 14 | 7 | 0 | 1 | 384 |
| 232 | E8 | 16 | 10 | 14 | 8 | 297417 | 2379332 | 14 | 8 | 0 | 376 | 2414044 | 14 | 8 | 0 | 1 | 384 |
| 233 | E9 | 16 | 10 | 14 | 9 | 297464 | 2379708 | 14 | 9 | 0 | 376 | 2414428 | 14 | 9 | 0 | 1 | 384 |
| 234 | EA | 16 | 10 | 14 | 10 | 297511 | 2380084 | 14 | 10 | 0 | 376 | 2414812 | 14 | 10 | 0 | 1 | 384 |
| 235 | EB | 16 | 10 | 14 | 11 | 297558 | 2380460 | 14 | 11 | 0 | 376 | 2415196 | 14 | 11 | 0 | 1 | 384 |
| 236 | EC | 16 | 10 | 14 | 12 | 297605 | 2380836 | 14 | 12 | 0 | 376 | 2415580 | 14 | 12 | 0 | 1 | 384 |
| 237 | ED | 16 | 10 | 14 | 13 | 297652 | 2381212 | 14 | 13 | 0 | 376 | 2415964 | 14 | 13 | 0 | 1 | 384 |
| 238 | EE | 16 | 10 | 14 | 14 | 297699 | 2381588 | 14 | 14 | 0 | 376 | 2416348 | 14 | 14 | 0 | 1 | 384 |
| 239 | EF | 16 | 10 | 14 | 15 | 297746 | 2381964 | 14 | 15 | 0 | 376 | 2416732 | 14 | 15 | 0 | 1 | 384 |
| 240 | F0 | 16 | 10 | 15 | 0 | 297793 | 2382340 | 15 | 0 | 0 | 376 | 2417116 | 15 | 0 | 0 | 1 | 384 |
| 241 | F1 | 16 | 10 | 15 | 1 | 297842 | 2382732 | 15 | 1 | 0 | 392 | 2417516 | 15 | 1 | 0 | 1 | 400 |
| 242 | F2 | 16 | 10 | 15 | 2 | 297891 | 2383124 | 15 | 2 | 0 | 392 | 2417916 | 15 | 2 | 0 | 1 | 400 |
| 243 | F3 | 16 | 10 | 15 | 3 | 297940 | 2383516 | 15 | 3 | 0 | 392 | 2418316 | 15 | 3 | 0 | 1 | 400 |
| 244 | F4 | 16 | 10 | 15 | 4 | 297989 | 2383908 | 15 | 4 | 0 | 392 | 2418716 | 15 | 4 | 0 | 1 | 400 |
| 245 | F5 | 16 | 10 | 15 | 5 | 298038 | 2384300 | 15 | 5 | 0 | 392 | 2419116 | 15 | 5 | 0 | 1 | 400 |
| 246 | F6 | 16 | 10 | 15 | 6 | 298087 | 2384692 | 15 | 6 | 0 | 392 | 2419516 | 15 | 6 | 0 | 1 | 400 |
| 247 | F7 | 16 | 10 | 15 | 7 | 298136 | 2385084 | 15 | 7 | 0 | 392 | 2419916 | 15 | 7 | 0 | 1 | 400 |
| 248 | F8 | 16 | 10 | 15 | 8 | 298185 | 2385476 | 15 | 8 | 0 | 392 | 2420316 | 15 | 8 | 0 | 1 | 400 |
| 249 | F9 | 16 | 10 | 15 | 9 | 298234 | 2385868 | 15 | 9 | 0 | 392 | 2420716 | 15 | 9 | 0 | 1 | 400 |
| 250 | FA | 16 | 10 | 15 | 10 | 298283 | 2386260 | 15 | 10 | 0 | 392 | 2421116 | 15 | 10 | 0 | 1 | 400 |
| 251 | FB | 16 | 10 | 15 | 11 | 298332 | 2386652 | 15 | 11 | 0 | 392 | 2421516 | 15 | 11 | 0 | 1 | 400 |
| 252 | FC | 16 | 10 | 15 | 12 | 298381 | 2387044 | 15 | 12 | 0 | 392 | 2421916 | 15 | 12 | 0 | 1 | 400 |
| 253 | FD | 16 | 10 | 15 | 13 | 298430 | 2387436 | 15 | 13 | 0 | 392 | 2422316 | 15 | 13 | 0 | 1 | 400 |
| 254 | FE | 16 | 10 | 15 | 14 | 298479 | 2387828 | 15 | 14 | 0 | 392 | 2422716 | 15 | 14 | 0 | 1 | 400 |
| 255 | FF | 16 | 10 | 15 | 15 | 298528 | 2388220 | 15 | 15 | 0 | 392 | 2423116 | 15 | 15 | 0 | 1 | 400 |
| 0 | 00 | 17 | 11 | 0 | 0 | 298540 | 2388316 | 0 | 0 | 0 | 96 | 2423220 | 0 | 0 | 0 | 1 | 104 |
| 1 | 01 | 17 | 11 | 0 | 1 | 298554 | 2388428 | 0 | 1 | 0 | 112 | 2423340 | 0 | 1 | 0 | 1 | 120 |
| 2 | 02 | 17 | 11 | 0 | 2 | 298568 | 2388540 | 0 | 2 | 0 | 112 | 2423460 | 0 | 2 | 0 | 1 | 120 |
| 3 | 03 | 17 | 11 | 0 | 3 | 298582 | 2388652 | 0 | 3 | 0 | 112 | 2423580 | 0 | 3 | 0 | 1 | 120 |
| 4 | 04 | 17 | 11 | 0 | 4 | 298596 | 2388764 | 0 | 4 | 0 | 112 | 2423700 | 0 | 4 | 0 | 1 | 120 |
| 5 | 05 | 17 | 11 | 0 | 5 | 298610 | 2388876 | 0 | 5 | 0 | 112 | 2423820 | 0 | 5 | 0 | 1 | 120 |
| 6 | 06 | 17 | 11 | 0 | 6 | 298624 | 2388988 | 0 | 6 | 0 | 112 | 2423940 | 0 | 6 | 0 | 1 | 120 |
| 7 | 07 | 17 | 11 | 0 | 7 | 298638 | 2389100 | 0 | 7 | 0 | 112 | 2424060 | 0 | 7 | 0 | 1 | 120 |
| 8 | 08 | 17 | 11 | 0 | 8 | 298652 | 2389212 | 0 | 8 | 0 | 112 | 2424180 | 0 | 8 | 0 | 1 | 120 |
| 9 | 09 | 17 | 11 | 0 | 9 | 298666 | 2389324 | 0 | 9 | 0 | 112 | 2424300 | 0 | 9 | 0 | 1 | 120 |
| 10 | 0A | 17 | 11 | 0 | 10 | 298680 | 2389436 | 0 | 10 | 0 | 112 | 2424420 | 0 | 10 | 0 | 1 | 120 |
| 11 | 0B | 17 | 11 | 0 | 11 | 298694 | 2389548 | 0 | 11 | 0 | 112 | 2424540 | 0 | 11 | 0 | 1 | 120 |
| 12 | 0C | 17 | 11 | 0 | 12 | 298708 | 2389660 | 0 | 12 | 0 | 112 | 2424660 | 0 | 12 | 0 | 1 | 120 |
| 13 | 0D | 17 | 11 | 0 | 13 | 298722 | 2389772 | 0 | 13 | 0 | 112 | 2424780 | 0 | 13 | 0 | 1 | 120 |
| 14 | 0E | 17 | 11 | 0 | 14 | 298736 | 2389884 | 0 | 14 | 0 | 112 | 2424900 | 0 | 14 | 0 | 1 | 120 |
| 15 | 0F | 17 | 11 | 0 | 15 | 298750 | 2389996 | 0 | 15 | 0 | 112 | 2425020 | 0 | 15 | 0 | 1 | 120 |
| 16 | 10 | 17 | 11 | 0 | 16 | 298764 | 2390108 | 0 | 16 | 0 | 112 | 2425140 | 0 | 16 | 0 | 1 | 120 |
| 17 | 11 | 17 | 11 | 1 | 0 | 298776 | 2390204 | 1 | 0 | 0 | 96 | 2425244 | 1 | 0 | 0 | 1 | 104 |
| 18 | 12 | 17 | 11 | 1 | 1 | 298793 | 2390340 | 1 | 1 | 0 | 136 | 2425388 | 1 | 1 | 0 | 1 | 144 |
| 19 | 13 | 17 | 11 | 1 | 2 | 298810 | 2390476 | 1 | 2 | 0 | 136 | 2425532 | 1 | 2 | 0 | 1 | 144 |
| 20 | 14 | 17 | 11 | 1 | 3 | 298827 | 2390612 | 1 | 3 | 0 | 136 | 2425676 | 1 | 3 | 0 | 1 | 144 |
| 21 | 15 | 17 | 11 | 1 | 4 | 298844 | 2390748 | 1 | 4 | 0 | 136 | 2425820 | 1 | 4 | 0 | 1 | 144 |
| 22 | 16 | 17 | 11 | 1 | 5 | 298861 | 2390884 | 1 | 5 | 0 | 136 | 2425964 | 1 | 5 | 0 | 1 | 144 |
| 23 | 17 | 17 | 11 | 1 | 6 | 298878 | 2391020 | 1 | 6 | 0 | 136 | 2426108 | 1 | 6 | 0 | 1 | 144 |
| 24 | 18 | 17 | 11 | 1 | 7 | 298895 | 2391156 | 1 | 7 | 0 | 136 | 2426252 | 1 | 7 | 0 | 1 | 144 |
| 25 | 19 | 17 | 11 | 1 | 8 | 298912 | 2391292 | 1 | 8 | 0 | 136 | 2426396 | 1 | 8 | 0 | 1 | 144 |
| 26 | 1A | 17 | 11 | 1 | 9 | 298929 | 2391428 | 1 | 9 | 0 | 136 | 2426540 | 1 | 9 | 0 | 1 | 144 |
| 27 | 1B | 17 | 11 | 1 | 10 | 298946 | 2391564 | 1 | 10 | 0 | 136 | 2426684 | 1 | 10 | 0 | 1 | 144 |
| 28 | 1C | 17 | 11 | 1 | 11 | 298963 | 2391700 | 1 | 11 | 0 | 136 | 2426828 | 1 | 11 | 0 | 1 | 144 |
| 29 | 1D | 17 | 11 | 1 | 12 | 298980 | 2391836 | 1 | 12 | 0 | 136 | 2426972 | 1 | 12 | 0 | 1 | 144 |
| 30 | 1E | 17 | 11 | 1 | 13 | 298997 | 2391972 | 1 | 13 | 0 | 136 | 2427116 | 1 | 13 | 0 | 1 | 144 |
| 31 | 1F | 17 | 11 | 1 | 14 | 299014 | 2392108 | 1 | 14 | 0 | 136 | 2427260 | 1 | 14 | 0 | 1 | 144 |
| 32 | 20 | 17 | 11 | 1 | 15 | 299035 | 2392276 | 1 | 15 | 0 | 168 | 2427436 | 1 | 15 | 0 | 1 | 176 |
| 33 | 21 | 17 | 11 | 1 | 16 | 299056 | 2392444 | 1 | 16 | 0 | 168 | 2427612 | 1 | 16 | 0 | 1 | 176 |
| 34 | 22 | 17 | 11 | 2 | 0 | 299075 | 2392596 | 2 | 0 | 0 | 152 | 2427772 | 2 | 0 | 0 | 1 | 160 |
| 35 | 23 | 17 | 11 | 2 | 1 | 299099 | 2392788 | 2 | 1 | 0 | 192 | 2427972 | 2 | 1 | 0 | 1 | 200 |
| 36 | 24 | 17 | 11 | 2 | 2 | 299123 | 2392980 | 2 | 2 | 0 | 192 | 2428172 | 2 | 2 | 0 | 1 | 200 |
| 37 | 25 | 17 | 11 | 2 | 3 | 299147 | 2393172 | 2 | 3 | 0 | 192 | 2428372 | 2 | 3 | 0 | 1 | 200 |
| 38 | 26 | 17 | 11 | 2 | 4 | 299171 | 2393364 | 2 | 4 | 0 | 192 | 2428572 | 2 | 4 | 0 | 1 | 200 |
| 39 | 27 | 17 | 11 | 2 | 5 | 299195 | 2393556 | 2 | 5 | 0 | 192 | 2428772 | 2 | 5 | 0 | 1 | 200 |
| 40 | 28 | 17 | 11 | 2 | 6 | 299219 | 2393748 | 2 | 6 | 0 | 192 | 2428972 | 2 | 6 | 0 | 1 | 200 |
| 41 | 29 | 17 | 11 | 2 | 7 | 299243 | 2393940 | 2 | 7 | 0 | 192 | 2429172 | 2 | 7 | 0 | 1 | 200 |
| 42 | 2A | 17 | 11 | 2 | 8 | 299267 | 2394132 | 2 | 8 | 0 | 192 | 2429372 | 2 | 8 | 0 | 1 | 200 |
| 43 | 2B | 17 | 11 | 2 | 9 | 299291 | 2394324 | 2 | 9 | 0 | 192 | 2429572 | 2 | 9 | 0 | 1 | 200 |
| 44 | 2C | 17 | 11 | 2 | 10 | 299315 | 2394516 | 2 | 10 | 0 | 192 | 2429772 | 2 | 10 | 0 | 1 | 200 |
| 45 | 2D | 17 | 11 | 2 | 11 | 299339 | 2394708 | 2 | 11 | 0 | 192 | 2429972 | 2 | 11 | 0 | 1 | 200 |
| 46 | 2E | 17 | 11 | 2 | 12 | 299363 | 2394900 | 2 | 12 | 0 | 192 | 2430172 | 2 | 12 | 0 | 1 | 200 |
| 47 | 2F | 17 | 11 | 2 | 13 | 299387 | 2395092 | 2 | 13 | 0 | 192 | 2430372 | 2 | 13 | 0 | 1 | 200 |
| 48 | 30 | 17 | 11 | 2 | 14 | 299413 | 2395300 | 2 | 14 | 0 | 208 | 2430588 | 2 | 14 | 0 | 1 | 216 |
| 49 | 31 | 17 | 11 | 2 | 15 | 299439 | 2395508 | 2 | 15 | 0 | 208 | 2430804 | 2 | 15 | 0 | 1 | 216 |
| 50 | 32 | 17 | 11 | 2 | 16 | 299465 | 2395716 | 2 | 16 | 0 | 208 | 2431020 | 2 | 16 | 0 | 1 | 216 |
| 51 | 33 | 17 | 11 | 3 | 0 | 299493 | 2395940 | 3 | 0 | 0 | 224 | 2431252 | 3 | 0 | 0 | 1 | 232 |
| 52 | 34 | 17 | 11 | 3 | 1 | 299521 | 2396164 | 3 | 1 | 0 | 224 | 2431484 | 3 | 1 | 0 | 1 | 232 |
| 53 | 35 | 17 | 11 | 3 | 2 | 299549 | 2396388 | 3 | 2 | 0 | 224 | 2431716 | 3 | 2 | 0 | 1 | 232 |
| 54 | 36 | 17 | 11 | 3 | 3 | 299577 | 2396612 | 3 | 3 | 0 | 224 | 2431948 | 3 | 3 | 0 | 1 | 232 |
| 55 | 37 | 17 | 11 | 3 | 4 | 299605 | 2396836 | 3 | 4 | 0 | 224 | 2432180 | 3 | 4 | 0 | 1 | 232 |
| 56 | 38 | 17 | 11 | 3 | 5 | 299633 | 2397060 | 3 | 5 | 0 | 224 | 2432412 | 3 | 5 | 0 | 1 | 232 |
| 57 | 39 | 17 | 11 | 3 | 6 | 299661 | 2397284 | 3 | 6 | 0 | 224 | 2432644 | 3 | 6 | 0 | 1 | 232 |
| 58 | 3A | 17 | 11 | 3 | 7 | 299689 | 2397508 | 3 | 7 | 0 | 224 | 2432876 | 3 | 7 | 0 | 1 | 232 |
| 59 | 3B | 17 | 11 | 3 | 8 | 299717 | 2397732 | 3 | 8 | 0 | 224 | 2433108 | 3 | 8 | 0 | 1 | 232 |
| 60 | 3C | 17 | 11 | 3 | 9 | 299745 | 2397956 | 3 | 9 | 0 | 224 | 2433340 | 3 | 9 | 0 | 1 | 232 |
| 61 | 3D | 17 | 11 | 3 | 10 | 299773 | 2398180 | 3 | 10 | 0 | 224 | 2433572 | 3 | 10 | 0 | 1 | 232 |
| 62 | 3E | 17 | 11 | 3 | 11 | 299801 | 2398404 | 3 | 11 | 0 | 224 | 2433804 | 3 | 11 | 0 | 1 | 232 |
| 63 | 3F | 17 | 11 | 3 | 12 | 299829 | 2398628 | 3 | 12 | 0 | 224 | 2434036 | 3 | 12 | 0 | 1 | 232 |
| 64 | 40 | 17 | 11 | 3 | 13 | 299855 | 2398836 | 3 | 13 | 0 | 208 | 2434252 | 3 | 13 | 0 | 1 | 216 |
| 65 | 41 | 17 | 11 | 3 | 14 | 299881 | 2399044 | 3 | 14 | 0 | 208 | 2434468 | 3 | 14 | 0 | 1 | 216 |
| 66 | 42 | 17 | 11 | 3 | 15 | 299907 | 2399252 | 3 | 15 | 0 | 208 | 2434684 | 3 | 15 | 0 | 1 | 216 |
| 67 | 43 | 17 | 11 | 3 | 16 | 299933 | 2399460 | 3 | 16 | 0 | 208 | 2434900 | 3 | 16 | 0 | 1 | 216 |
| 68 | 44 | 17 | 11 | 4 | 0 | 299961 | 2399684 | 4 | 0 | 0 | 224 | 2435132 | 4 | 0 | 0 | 1 | 232 |
| 69 | 45 | 17 | 11 | 4 | 1 | 299989 | 2399908 | 4 | 1 | 0 | 224 | 2435364 | 4 | 1 | 0 | 1 | 232 |
| 70 | 46 | 17 | 11 | 4 | 2 | 300017 | 2400132 | 4 | 2 | 0 | 224 | 2435596 | 4 | 2 | 0 | 1 | 232 |
| 71 | 47 | 17 | 11 | 4 | 3 | 300045 | 2400356 | 4 | 3 | 0 | 224 | 2435828 | 4 | 3 | 0 | 1 | 232 |
| 72 | 48 | 17 | 11 | 4 | 4 | 300073 | 2400580 | 4 | 4 | 0 | 224 | 2436060 | 4 | 4 | 0 | 1 | 232 |
| 73 | 49 | 17 | 11 | 4 | 5 | 300101 | 2400804 | 4 | 5 | 0 | 224 | 2436292 | 4 | 5 | 0 | 1 | 232 |

| | | | | | | | | | | | | | | | | | |
|---|---|---|---|---|---|---|---|---|---|---|---|---|---|---|---|---|---|
| 74 | 4A | 17 | 11 | 4 | 6 | 300129 | 2401028 | 4 | 6 | 0 | 224 | 2436524 | 4 | 6 | 0 | 1 | 232 |
| 75 | 4B | 17 | 11 | 4 | 7 | 300157 | 2401252 | 4 | 7 | 0 | 224 | 2436756 | 4 | 7 | 0 | 1 | 232 |
| 76 | 4C | 17 | 11 | 4 | 8 | 300185 | 2401476 | 4 | 8 | 0 | 224 | 2436988 | 4 | 8 | 0 | 1 | 232 |
| 77 | 4D | 17 | 11 | 4 | 9 | 300213 | 2401700 | 4 | 9 | 0 | 224 | 2437220 | 4 | 9 | 0 | 1 | 232 |
| 78 | 4E | 17 | 11 | 4 | 10 | 300241 | 2401924 | 4 | 10 | 0 | 224 | 2437452 | 4 | 10 | 0 | 1 | 232 |
| 79 | 4F | 17 | 11 | 4 | 11 | 300269 | 2402148 | 4 | 11 | 0 | 224 | 2437684 | 4 | 11 | 0 | 1 | 232 |
| 80 | 50 | 17 | 11 | 4 | 12 | 300299 | 2402388 | 4 | 12 | 0 | 240 | 2437932 | 4 | 12 | 0 | 1 | 248 |
| 81 | 51 | 17 | 11 | 4 | 13 | 300329 | 2402628 | 4 | 13 | 0 | 240 | 2438180 | 4 | 13 | 0 | 1 | 248 |
| 82 | 52 | 17 | 11 | 4 | 14 | 300359 | 2402868 | 4 | 14 | 0 | 240 | 2438428 | 4 | 14 | 0 | 1 | 248 |
| 83 | 53 | 17 | 11 | 4 | 15 | 300389 | 2403108 | 4 | 15 | 0 | 240 | 2438676 | 4 | 15 | 0 | 1 | 248 |
| 84 | 54 | 17 | 11 | 4 | 16 | 300419 | 2403348 | 4 | 16 | 0 | 240 | 2438924 | 4 | 16 | 0 | 1 | 248 |
| 85 | 55 | 17 | 11 | 5 | 0 | 300451 | 2403604 | 5 | 0 | 0 | 256 | 2439188 | 5 | 0 | 0 | 1 | 264 |
| 86 | 56 | 17 | 11 | 5 | 1 | 300483 | 2403860 | 5 | 1 | 0 | 256 | 2439452 | 5 | 1 | 0 | 1 | 264 |
| 87 | 57 | 17 | 11 | 5 | 2 | 300515 | 2404116 | 5 | 2 | 0 | 256 | 2439716 | 5 | 2 | 0 | 1 | 264 |
| 88 | 58 | 17 | 11 | 5 | 3 | 300547 | 2404372 | 5 | 3 | 0 | 256 | 2439980 | 5 | 3 | 0 | 1 | 264 |
| 89 | 59 | 17 | 11 | 5 | 4 | 300579 | 2404628 | 5 | 4 | 0 | 256 | 2440244 | 5 | 4 | 0 | 1 | 264 |
| 90 | 5A | 17 | 11 | 5 | 5 | 300611 | 2404884 | 5 | 5 | 0 | 256 | 2440508 | 5 | 5 | 0 | 1 | 264 |
| 91 | 5B | 17 | 11 | 5 | 6 | 300643 | 2405140 | 5 | 6 | 0 | 256 | 2440772 | 5 | 6 | 0 | 1 | 264 |
| 92 | 5C | 17 | 11 | 5 | 7 | 300675 | 2405396 | 5 | 7 | 0 | 256 | 2441036 | 5 | 7 | 0 | 1 | 264 |
| 93 | 5D | 17 | 11 | 5 | 8 | 300707 | 2405652 | 5 | 8 | 0 | 256 | 2441300 | 5 | 8 | 0 | 1 | 264 |
| 94 | 5E | 17 | 11 | 5 | 9 | 300739 | 2405908 | 5 | 9 | 0 | 256 | 2441564 | 5 | 9 | 0 | 1 | 264 |
| 95 | 5F | 17 | 11 | 5 | 10 | 300771 | 2406164 | 5 | 10 | 0 | 256 | 2441828 | 5 | 10 | 0 | 1 | 264 |
| 96 | 60 | 17 | 11 | 5 | 11 | 300801 | 2406404 | 5 | 11 | 0 | 240 | 2442076 | 5 | 11 | 0 | 1 | 248 |
| 97 | 61 | 17 | 11 | 5 | 12 | 300831 | 2406644 | 5 | 12 | 0 | 240 | 2442324 | 5 | 12 | 0 | 1 | 248 |
| 98 | 62 | 17 | 11 | 5 | 13 | 300861 | 2406884 | 5 | 13 | 0 | 240 | 2442572 | 5 | 13 | 0 | 1 | 248 |
| 99 | 63 | 17 | 11 | 5 | 14 | 300891 | 2407124 | 5 | 14 | 0 | 240 | 2442820 | 5 | 14 | 0 | 1 | 248 |
| 100 | 64 | 17 | 11 | 5 | 15 | 300921 | 2407364 | 5 | 15 | 0 | 240 | 2443068 | 5 | 15 | 0 | 1 | 248 |
| 101 | 65 | 17 | 11 | 5 | 16 | 300951 | 2407604 | 5 | 16 | 0 | 240 | 2443316 | 5 | 16 | 0 | 1 | 248 |
| 102 | 66 | 17 | 11 | 6 | 0 | 300983 | 2407860 | 6 | 0 | 0 | 256 | 2443580 | 6 | 0 | 0 | 1 | 264 |
| 103 | 67 | 17 | 11 | 6 | 1 | 301015 | 2408116 | 6 | 1 | 0 | 256 | 2443844 | 6 | 1 | 0 | 1 | 264 |
| 104 | 68 | 17 | 11 | 6 | 2 | 301047 | 2408372 | 6 | 2 | 0 | 256 | 2444108 | 6 | 2 | 0 | 1 | 264 |
| 105 | 69 | 17 | 11 | 6 | 3 | 301079 | 2408628 | 6 | 3 | 0 | 256 | 2444372 | 6 | 3 | 0 | 1 | 264 |
| 106 | 6A | 17 | 11 | 6 | 4 | 301111 | 2408884 | 6 | 4 | 0 | 256 | 2444636 | 6 | 4 | 0 | 1 | 264 |
| 107 | 6B | 17 | 11 | 6 | 5 | 301143 | 2409140 | 6 | 5 | 0 | 256 | 2444900 | 6 | 5 | 0 | 1 | 264 |
| 108 | 6C | 17 | 11 | 6 | 6 | 301175 | 2409396 | 6 | 6 | 0 | 256 | 2445164 | 6 | 6 | 0 | 1 | 264 |
| 109 | 6D | 17 | 11 | 6 | 7 | 301207 | 2409652 | 6 | 7 | 0 | 256 | 2445428 | 6 | 7 | 0 | 1 | 264 |
| 110 | 6E | 17 | 11 | 6 | 8 | 301239 | 2409908 | 6 | 8 | 0 | 256 | 2445692 | 6 | 8 | 0 | 1 | 264 |
| 111 | 6F | 17 | 11 | 6 | 9 | 301271 | 2410164 | 6 | 9 | 0 | 256 | 2445956 | 6 | 9 | 0 | 1 | 264 |
| 112 | 70 | 17 | 11 | 6 | 10 | 301305 | 2410436 | 6 | 10 | 0 | 272 | 2446236 | 6 | 10 | 0 | 1 | 280 |
| 113 | 71 | 17 | 11 | 6 | 11 | 301339 | 2410708 | 6 | 11 | 0 | 272 | 2446516 | 6 | 11 | 0 | 1 | 280 |
| 114 | 72 | 17 | 11 | 6 | 12 | 301373 | 2410980 | 6 | 12 | 0 | 272 | 2446796 | 6 | 12 | 0 | 1 | 280 |
| 115 | 73 | 17 | 11 | 6 | 13 | 301407 | 2411252 | 6 | 13 | 0 | 272 | 2447076 | 6 | 13 | 0 | 1 | 280 |
| 116 | 74 | 17 | 11 | 6 | 14 | 301441 | 2411524 | 6 | 14 | 0 | 272 | 2447356 | 6 | 14 | 0 | 1 | 280 |
| 117 | 75 | 17 | 11 | 6 | 15 | 301475 | 2411796 | 6 | 15 | 0 | 272 | 2447636 | 6 | 15 | 0 | 1 | 280 |
| 118 | 76 | 17 | 11 | 6 | 16 | 301509 | 2412068 | 6 | 16 | 0 | 272 | 2447916 | 6 | 16 | 0 | 1 | 280 |
| 119 | 77 | 17 | 11 | 7 | 0 | 301545 | 2412356 | 7 | 0 | 0 | 288 | 2448212 | 7 | 0 | 0 | 1 | 296 |
| 120 | 78 | 17 | 11 | 7 | 1 | 301581 | 2412644 | 7 | 1 | 0 | 288 | 2448508 | 7 | 1 | 0 | 1 | 296 |
| 121 | 79 | 17 | 11 | 7 | 2 | 301617 | 2412932 | 7 | 2 | 0 | 288 | 2448804 | 7 | 2 | 0 | 1 | 296 |
| 122 | 7A | 17 | 11 | 7 | 3 | 301653 | 2413220 | 7 | 3 | 0 | 288 | 2449100 | 7 | 3 | 0 | 1 | 296 |
| 123 | 7B | 17 | 11 | 7 | 4 | 301689 | 2413508 | 7 | 4 | 0 | 288 | 2449396 | 7 | 4 | 0 | 1 | 296 |
| 124 | 7C | 17 | 11 | 7 | 5 | 301725 | 2413796 | 7 | 5 | 0 | 288 | 2449692 | 7 | 5 | 0 | 1 | 296 |
| 125 | 7D | 17 | 11 | 7 | 6 | 301761 | 2414084 | 7 | 6 | 0 | 288 | 2449988 | 7 | 6 | 0 | 1 | 296 |
| 126 | 7E | 17 | 11 | 7 | 7 | 301797 | 2414372 | 7 | 7 | 0 | 288 | 2450284 | 7 | 7 | 0 | 1 | 296 |
| 127 | 7F | 17 | 11 | 7 | 8 | 301833 | 2414660 | 7 | 8 | 0 | 288 | 2450580 | 7 | 8 | 0 | 1 | 296 |
| 128 | 80 | 17 | 11 | 7 | 9 | 301867 | 2414932 | 7 | 9 | 0 | 272 | 2450860 | 7 | 9 | 0 | 1 | 280 |
| 129 | 81 | 17 | 11 | 7 | 10 | 301901 | 2415204 | 7 | 10 | 0 | 272 | 2451140 | 7 | 10 | 0 | 1 | 280 |
| 130 | 82 | 17 | 11 | 7 | 11 | 301935 | 2415476 | 7 | 11 | 0 | 272 | 2451420 | 7 | 11 | 0 | 1 | 280 |
| 131 | 83 | 17 | 11 | 7 | 12 | 301969 | 2415748 | 7 | 12 | 0 | 272 | 2451700 | 7 | 12 | 0 | 1 | 280 |
| 132 | 84 | 17 | 11 | 7 | 13 | 302003 | 2416020 | 7 | 13 | 0 | 272 | 2451980 | 7 | 13 | 0 | 1 | 280 |
| 133 | 85 | 17 | 11 | 7 | 14 | 302037 | 2416292 | 7 | 14 | 0 | 272 | 2452260 | 7 | 14 | 0 | 1 | 280 |
| 134 | 86 | 17 | 11 | 7 | 15 | 302071 | 2416564 | 7 | 15 | 0 | 272 | 2452540 | 7 | 15 | 0 | 1 | 280 |
| 135 | 87 | 17 | 11 | 7 | 16 | 302105 | 2416836 | 7 | 16 | 0 | 272 | 2452820 | 7 | 16 | 0 | 1 | 280 |
| 136 | 88 | 17 | 11 | 8 | 0 | 302141 | 2417124 | 8 | 0 | 0 | 288 | 2453116 | 8 | 0 | 0 | 1 | 296 |
| 137 | 89 | 17 | 11 | 8 | 1 | 302177 | 2417412 | 8 | 1 | 0 | 288 | 2453412 | 8 | 1 | 0 | 1 | 296 |
| 138 | 8A | 17 | 11 | 8 | 2 | 302213 | 2417700 | 8 | 2 | 0 | 288 | 2453708 | 8 | 2 | 0 | 1 | 296 |
| 139 | 8B | 17 | 11 | 8 | 3 | 302249 | 2417988 | 8 | 3 | 0 | 288 | 2454004 | 8 | 3 | 0 | 1 | 296 |
| 140 | 8C | 17 | 11 | 8 | 4 | 302285 | 2418276 | 8 | 4 | 0 | 288 | 2454300 | 8 | 4 | 0 | 1 | 296 |
| 141 | 8D | 17 | 11 | 8 | 5 | 302321 | 2418564 | 8 | 5 | 0 | 288 | 2454596 | 8 | 5 | 0 | 1 | 296 |
| 142 | 8E | 17 | 11 | 8 | 6 | 302357 | 2418852 | 8 | 6 | 0 | 288 | 2454892 | 8 | 6 | 0 | 1 | 296 |
| 143 | 8F | 17 | 11 | 8 | 7 | 302393 | 2419140 | 8 | 7 | 0 | 288 | 2455188 | 8 | 7 | 0 | 1 | 296 |
| 144 | 90 | 17 | 11 | 8 | 8 | 302431 | 2419444 | 8 | 8 | 0 | 304 | 2455500 | 8 | 8 | 0 | 1 | 312 |
| 145 | 91 | 17 | 11 | 8 | 9 | 302469 | 2419748 | 8 | 9 | 0 | 304 | 2455812 | 8 | 9 | 0 | 1 | 312 |
| 146 | 92 | 17 | 11 | 8 | 10 | 302507 | 2420052 | 8 | 10 | 0 | 304 | 2456124 | 8 | 10 | 0 | 1 | 312 |
| 147 | 93 | 17 | 11 | 8 | 11 | 302545 | 2420356 | 8 | 11 | 0 | 304 | 2456436 | 8 | 11 | 0 | 1 | 312 |
| 148 | 94 | 17 | 11 | 8 | 12 | 302583 | 2420660 | 8 | 12 | 0 | 304 | 2456748 | 8 | 12 | 0 | 1 | 312 |
| 149 | 95 | 17 | 11 | 8 | 13 | 302621 | 2420964 | 8 | 13 | 0 | 304 | 2457060 | 8 | 13 | 0 | 1 | 312 |
| 150 | 96 | 17 | 11 | 8 | 14 | 302659 | 2421268 | 8 | 14 | 0 | 304 | 2457372 | 8 | 14 | 0 | 1 | 312 |
| 151 | 97 | 17 | 11 | 8 | 15 | 302697 | 2421572 | 8 | 15 | 0 | 304 | 2457684 | 8 | 15 | 0 | 1 | 312 |
| 152 | 98 | 17 | 11 | 8 | 16 | 302735 | 2421876 | 8 | 16 | 0 | 304 | 2457996 | 8 | 16 | 0 | 1 | 312 |
| 153 | 99 | 17 | 11 | 9 | 0 | 302775 | 2422196 | 9 | 0 | 0 | 320 | 2458324 | 9 | 0 | 0 | 1 | 328 |
| 154 | 9A | 17 | 11 | 9 | 1 | 302815 | 2422516 | 9 | 1 | 0 | 320 | 2458652 | 9 | 1 | 0 | 1 | 328 |
| 155 | 9B | 17 | 11 | 9 | 2 | 302855 | 2422836 | 9 | 2 | 0 | 320 | 2458980 | 9 | 2 | 0 | 1 | 328 |
| 156 | 9C | 17 | 11 | 9 | 3 | 302895 | 2423156 | 9 | 3 | 0 | 320 | 2459308 | 9 | 3 | 0 | 1 | 328 |
| 157 | 9D | 17 | 11 | 9 | 4 | 302935 | 2423476 | 9 | 4 | 0 | 320 | 2459636 | 9 | 4 | 0 | 1 | 328 |
| 158 | 9E | 17 | 11 | 9 | 5 | 302975 | 2423796 | 9 | 5 | 0 | 320 | 2459964 | 9 | 5 | 0 | 1 | 328 |
| 159 | 9F | 17 | 11 | 9 | 6 | 303015 | 2424116 | 9 | 6 | 0 | 320 | 2460292 | 9 | 6 | 0 | 1 | 328 |
| 160 | A0 | 17 | 11 | 9 | 7 | 303053 | 2424420 | 9 | 7 | 0 | 304 | 2460604 | 9 | 7 | 0 | 1 | 312 |
| 161 | A1 | 17 | 11 | 9 | 8 | 303091 | 2424724 | 9 | 8 | 0 | 304 | 2460916 | 9 | 8 | 0 | 1 | 312 |
| 162 | A2 | 17 | 11 | 9 | 9 | 303129 | 2425028 | 9 | 9 | 0 | 304 | 2461228 | 9 | 9 | 0 | 1 | 312 |
| 163 | A3 | 17 | 11 | 9 | 10 | 303167 | 2425332 | 9 | 10 | 0 | 304 | 2461540 | 9 | 10 | 0 | 1 | 312 |
| 164 | A4 | 17 | 11 | 9 | 11 | 303205 | 2425636 | 9 | 11 | 0 | 304 | 2461852 | 9 | 11 | 0 | 1 | 312 |
| 165 | A5 | 17 | 11 | 9 | 12 | 303243 | 2425940 | 9 | 12 | 0 | 304 | 2462164 | 9 | 12 | 0 | 1 | 312 |
| 166 | A6 | 17 | 11 | 9 | 13 | 303281 | 2426244 | 9 | 13 | 0 | 304 | 2462476 | 9 | 13 | 0 | 1 | 312 |
| 167 | A7 | 17 | 11 | 9 | 14 | 303319 | 2426548 | 9 | 14 | 0 | 304 | 2462788 | 9 | 14 | 0 | 1 | 312 |
| 168 | A8 | 17 | 11 | 9 | 15 | 303357 | 2426852 | 9 | 15 | 0 | 304 | 2463100 | 9 | 15 | 0 | 1 | 312 |
| 169 | A9 | 17 | 11 | 9 | 16 | 303395 | 2427156 | 9 | 16 | 0 | 304 | 2463412 | 9 | 16 | 0 | 1 | 312 |
| 170 | AA | 17 | 11 | 10 | 0 | 303435 | 2427476 | 10 | 0 | 0 | 320 | 2463740 | 10 | 0 | 0 | 1 | 328 |
| 171 | AB | 17 | 11 | 10 | 1 | 303475 | 2427796 | 10 | 1 | 0 | 320 | 2464068 | 10 | 1 | 0 | 1 | 328 |
| 172 | AC | 17 | 11 | 10 | 2 | 303515 | 2428116 | 10 | 2 | 0 | 320 | 2464396 | 10 | 2 | 0 | 1 | 328 |
| 173 | AD | 17 | 11 | 10 | 3 | 303555 | 2428436 | 10 | 3 | 0 | 320 | 2464724 | 10 | 3 | 0 | 1 | 328 |
| 174 | AE | 17 | 11 | 10 | 4 | 303595 | 2428756 | 10 | 4 | 0 | 320 | 2465052 | 10 | 4 | 0 | 1 | 328 |
| 175 | AF | 17 | 11 | 10 | 5 | 303635 | 2429076 | 10 | 5 | 0 | 320 | 2465380 | 10 | 5 | 0 | 1 | 328 |
| 176 | B0 | 17 | 11 | 10 | 6 | 303677 | 2429412 | 10 | 6 | 0 | 336 | 2465724 | 10 | 6 | 0 | 1 | 344 |

| | | | | | | | | | | | | | | | | | |
|---|---|---|---|---|---|---|---|---|---|---|---|---|---|---|---|---|---|
| 177 | B1 | 17 | 11 | 10 | 7 | 303719 | 2429748 | 10 | 7 | 0 | 336 | 2466068 | 10 | 7 | 0 | 1 | 344 |
| 178 | B2 | 17 | 11 | 10 | 8 | 303761 | 2430084 | 10 | 8 | 0 | 336 | 2466412 | 10 | 8 | 0 | 1 | 344 |
| 179 | B3 | 17 | 11 | 10 | 9 | 303803 | 2430420 | 10 | 9 | 0 | 336 | 2466756 | 10 | 9 | 0 | 1 | 344 |
| 180 | B4 | 17 | 11 | 10 | 10 | 303845 | 2430756 | 10 | 10 | 0 | 336 | 2467100 | 10 | 10 | 0 | 1 | 344 |
| 181 | B5 | 17 | 11 | 10 | 11 | 303887 | 2431092 | 10 | 11 | 0 | 336 | 2467444 | 10 | 11 | 0 | 1 | 344 |
| 182 | B6 | 17 | 11 | 10 | 12 | 303929 | 2431428 | 10 | 12 | 0 | 336 | 2467788 | 10 | 12 | 0 | 1 | 344 |
| 183 | B7 | 17 | 11 | 10 | 13 | 303971 | 2431764 | 10 | 13 | 0 | 336 | 2468132 | 10 | 13 | 0 | 1 | 344 |
| 184 | B8 | 17 | 11 | 10 | 14 | 304013 | 2432100 | 10 | 14 | 0 | 336 | 2468476 | 10 | 14 | 0 | 1 | 344 |
| 185 | B9 | 17 | 11 | 10 | 15 | 304055 | 2432436 | 10 | 15 | 0 | 336 | 2468820 | 10 | 15 | 0 | 1 | 344 |
| 186 | BA | 17 | 11 | 10 | 16 | 304097 | 2432772 | 10 | 16 | 0 | 336 | 2469164 | 10 | 16 | 0 | 1 | 344 |
| 187 | BB | 17 | 11 | 11 | 0 | 304141 | 2433124 | 11 | 0 | 0 | 352 | 2469524 | 11 | 0 | 0 | 1 | 360 |
| 188 | BC | 17 | 11 | 11 | 1 | 304185 | 2433476 | 11 | 1 | 0 | 352 | 2469884 | 11 | 1 | 0 | 1 | 360 |
| 189 | BD | 17 | 11 | 11 | 2 | 304229 | 2433828 | 11 | 2 | 0 | 352 | 2470244 | 11 | 2 | 0 | 1 | 360 |
| 190 | BE | 17 | 11 | 11 | 3 | 304273 | 2434180 | 11 | 3 | 0 | 352 | 2470604 | 11 | 3 | 0 | 1 | 360 |
| 191 | BF | 17 | 11 | 11 | 4 | 304317 | 2434532 | 11 | 4 | 0 | 352 | 2470964 | 11 | 4 | 0 | 1 | 360 |
| 192 | C0 | 17 | 11 | 11 | 5 | 304359 | 2434868 | 11 | 5 | 0 | 336 | 2471308 | 11 | 5 | 0 | 1 | 344 |
| 193 | C1 | 17 | 11 | 11 | 6 | 304401 | 2435204 | 11 | 6 | 0 | 336 | 2471652 | 11 | 6 | 0 | 1 | 344 |
| 194 | C2 | 17 | 11 | 11 | 7 | 304443 | 2435540 | 11 | 7 | 0 | 336 | 2471996 | 11 | 7 | 0 | 1 | 344 |
| 195 | C3 | 17 | 11 | 11 | 8 | 304485 | 2435876 | 11 | 8 | 0 | 336 | 2472340 | 11 | 8 | 0 | 1 | 344 |
| 196 | C4 | 17 | 11 | 11 | 9 | 304527 | 2436212 | 11 | 9 | 0 | 336 | 2472684 | 11 | 9 | 0 | 1 | 344 |
| 197 | C5 | 17 | 11 | 11 | 10 | 304569 | 2436548 | 11 | 10 | 0 | 336 | 2473028 | 11 | 10 | 0 | 1 | 344 |
| 198 | C6 | 17 | 11 | 11 | 11 | 304611 | 2436884 | 11 | 11 | 0 | 336 | 2473372 | 11 | 11 | 0 | 1 | 344 |
| 199 | C7 | 17 | 11 | 11 | 12 | 304653 | 2437220 | 11 | 12 | 0 | 336 | 2473716 | 11 | 12 | 0 | 1 | 344 |
| 200 | C8 | 17 | 11 | 11 | 13 | 304695 | 2437556 | 11 | 13 | 0 | 336 | 2474060 | 11 | 13 | 0 | 1 | 344 |
| 201 | C9 | 17 | 11 | 11 | 14 | 304737 | 2437892 | 11 | 14 | 0 | 336 | 2474404 | 11 | 14 | 0 | 1 | 344 |
| 202 | CA | 17 | 11 | 11 | 15 | 304779 | 2438228 | 11 | 15 | 0 | 336 | 2474748 | 11 | 15 | 0 | 1 | 344 |
| 203 | CB | 17 | 11 | 11 | 16 | 304821 | 2438564 | 11 | 16 | 0 | 336 | 2475092 | 11 | 16 | 0 | 1 | 344 |
| 204 | CC | 17 | 11 | 12 | 0 | 304865 | 2438916 | 12 | 0 | 0 | 352 | 2475452 | 12 | 0 | 0 | 1 | 360 |
| 205 | CD | 17 | 11 | 12 | 1 | 304909 | 2439268 | 12 | 1 | 0 | 352 | 2475812 | 12 | 1 | 0 | 1 | 360 |
| 206 | CE | 17 | 11 | 12 | 2 | 304953 | 2439620 | 12 | 2 | 0 | 352 | 2476172 | 12 | 2 | 0 | 1 | 360 |
| 207 | CF | 17 | 11 | 12 | 3 | 304997 | 2439972 | 12 | 3 | 0 | 352 | 2476532 | 12 | 3 | 0 | 1 | 360 |
| 208 | D0 | 17 | 11 | 12 | 4 | 305043 | 2440340 | 12 | 4 | 0 | 368 | 2476908 | 12 | 4 | 0 | 1 | 376 |
| 209 | D1 | 17 | 11 | 12 | 5 | 305089 | 2440708 | 12 | 5 | 0 | 368 | 2477284 | 12 | 5 | 0 | 1 | 376 |
| 210 | D2 | 17 | 11 | 12 | 6 | 305135 | 2441076 | 12 | 6 | 0 | 368 | 2477660 | 12 | 6 | 0 | 1 | 376 |
| 211 | D3 | 17 | 11 | 12 | 7 | 305181 | 2441444 | 12 | 7 | 0 | 368 | 2478036 | 12 | 7 | 0 | 1 | 376 |
| 212 | D4 | 17 | 11 | 12 | 8 | 305227 | 2441812 | 12 | 8 | 0 | 368 | 2478412 | 12 | 8 | 0 | 1 | 376 |
| 213 | D5 | 17 | 11 | 12 | 9 | 305273 | 2442180 | 12 | 9 | 0 | 368 | 2478788 | 12 | 9 | 0 | 1 | 376 |
| 214 | D6 | 17 | 11 | 12 | 10 | 305319 | 2442548 | 12 | 10 | 0 | 368 | 2479164 | 12 | 10 | 0 | 1 | 376 |
| 215 | D7 | 17 | 11 | 12 | 11 | 305365 | 2442916 | 12 | 11 | 0 | 368 | 2479540 | 12 | 11 | 0 | 1 | 376 |
| 216 | D8 | 17 | 11 | 12 | 12 | 305411 | 2443284 | 12 | 12 | 0 | 368 | 2479916 | 12 | 12 | 0 | 1 | 376 |
| 217 | D9 | 17 | 11 | 12 | 13 | 305457 | 2443652 | 12 | 13 | 0 | 368 | 2480292 | 12 | 13 | 0 | 1 | 376 |
| 218 | DA | 17 | 11 | 12 | 14 | 305503 | 2444020 | 12 | 14 | 0 | 368 | 2480668 | 12 | 14 | 0 | 1 | 376 |
| 219 | DB | 17 | 11 | 12 | 15 | 305549 | 2444388 | 12 | 15 | 0 | 368 | 2481044 | 12 | 15 | 0 | 1 | 376 |
| 220 | DC | 17 | 11 | 12 | 16 | 305595 | 2444756 | 12 | 16 | 0 | 368 | 2481420 | 12 | 16 | 0 | 1 | 376 |
| 221 | DD | 17 | 11 | 13 | 0 | 305643 | 2445140 | 13 | 0 | 0 | 384 | 2481812 | 13 | 0 | 0 | 1 | 392 |
| 222 | DE | 17 | 11 | 13 | 1 | 305691 | 2445524 | 13 | 1 | 0 | 384 | 2482204 | 13 | 1 | 0 | 1 | 392 |
| 223 | DF | 17 | 11 | 13 | 2 | 305739 | 2445908 | 13 | 2 | 0 | 384 | 2482596 | 13 | 2 | 0 | 1 | 392 |
| 224 | E0 | 17 | 11 | 13 | 3 | 305785 | 2446276 | 13 | 3 | 0 | 368 | 2482972 | 13 | 3 | 0 | 1 | 376 |
| 225 | E1 | 17 | 11 | 13 | 4 | 305831 | 2446644 | 13 | 4 | 0 | 368 | 2483348 | 13 | 4 | 0 | 1 | 376 |
| 226 | E2 | 17 | 11 | 13 | 5 | 305877 | 2447012 | 13 | 5 | 0 | 368 | 2483724 | 13 | 5 | 0 | 1 | 376 |
| 227 | E3 | 17 | 11 | 13 | 6 | 305923 | 2447380 | 13 | 6 | 0 | 368 | 2484100 | 13 | 6 | 0 | 1 | 376 |
| 228 | E4 | 17 | 11 | 13 | 7 | 305969 | 2447748 | 13 | 7 | 0 | 368 | 2484476 | 13 | 7 | 0 | 1 | 376 |
| 229 | E5 | 17 | 11 | 13 | 8 | 306015 | 2448116 | 13 | 8 | 0 | 368 | 2484852 | 13 | 8 | 0 | 1 | 376 |
| 230 | E6 | 17 | 11 | 13 | 9 | 306061 | 2448484 | 13 | 9 | 0 | 368 | 2485228 | 13 | 9 | 0 | 1 | 376 |
| 231 | E7 | 17 | 11 | 13 | 10 | 306107 | 2448852 | 13 | 10 | 0 | 368 | 2485604 | 13 | 10 | 0 | 1 | 376 |
| 232 | E8 | 17 | 11 | 13 | 11 | 306153 | 2449220 | 13 | 11 | 0 | 368 | 2485980 | 13 | 11 | 0 | 1 | 376 |
| 233 | E9 | 17 | 11 | 13 | 12 | 306199 | 2449588 | 13 | 12 | 0 | 368 | 2486356 | 13 | 12 | 0 | 1 | 376 |
| 234 | EA | 17 | 11 | 13 | 13 | 306245 | 2449956 | 13 | 13 | 0 | 368 | 2486732 | 13 | 13 | 0 | 1 | 376 |
| 235 | EB | 17 | 11 | 13 | 14 | 306291 | 2450324 | 13 | 14 | 0 | 368 | 2487108 | 13 | 14 | 0 | 1 | 376 |
| 236 | EC | 17 | 11 | 13 | 15 | 306337 | 2450692 | 13 | 15 | 0 | 368 | 2487484 | 13 | 15 | 0 | 1 | 376 |
| 237 | ED | 17 | 11 | 13 | 16 | 306383 | 2451060 | 13 | 16 | 0 | 368 | 2487860 | 13 | 16 | 0 | 1 | 376 |
| 238 | EE | 17 | 11 | 14 | 0 | 306431 | 2451444 | 14 | 0 | 0 | 384 | 2488252 | 14 | 0 | 0 | 1 | 392 |
| 239 | EF | 17 | 11 | 14 | 1 | 306479 | 2451828 | 14 | 1 | 0 | 384 | 2488644 | 14 | 1 | 0 | 1 | 392 |
| 240 | F0 | 17 | 11 | 14 | 2 | 306529 | 2452228 | 14 | 2 | 0 | 400 | 2489052 | 14 | 2 | 0 | 1 | 408 |
| 241 | F1 | 17 | 11 | 14 | 3 | 306579 | 2452628 | 14 | 3 | 0 | 400 | 2489460 | 14 | 3 | 0 | 1 | 408 |
| 242 | F2 | 17 | 11 | 14 | 4 | 306629 | 2453028 | 14 | 4 | 0 | 400 | 2489868 | 14 | 4 | 0 | 1 | 408 |
| 243 | F3 | 17 | 11 | 14 | 5 | 306679 | 2453428 | 14 | 5 | 0 | 400 | 2490276 | 14 | 5 | 0 | 1 | 408 |
| 244 | F4 | 17 | 11 | 14 | 6 | 306729 | 2453828 | 14 | 6 | 0 | 400 | 2490684 | 14 | 6 | 0 | 1 | 408 |
| 245 | F5 | 17 | 11 | 14 | 7 | 306779 | 2454228 | 14 | 7 | 0 | 400 | 2491092 | 14 | 7 | 0 | 1 | 408 |
| 246 | F6 | 17 | 11 | 14 | 8 | 306829 | 2454628 | 14 | 8 | 0 | 400 | 2491500 | 14 | 8 | 0 | 1 | 408 |
| 247 | F7 | 17 | 11 | 14 | 9 | 306879 | 2455028 | 14 | 9 | 0 | 400 | 2491908 | 14 | 9 | 0 | 1 | 408 |
| 248 | F8 | 17 | 11 | 14 | 10 | 306929 | 2455428 | 14 | 10 | 0 | 400 | 2492316 | 14 | 10 | 0 | 1 | 408 |
| 249 | F9 | 17 | 11 | 14 | 11 | 306979 | 2455828 | 14 | 11 | 0 | 400 | 2492724 | 14 | 11 | 0 | 1 | 408 |
| 250 | FA | 17 | 11 | 14 | 12 | 307029 | 2456228 | 14 | 12 | 0 | 400 | 2493132 | 14 | 12 | 0 | 1 | 408 |
| 251 | FB | 17 | 11 | 14 | 13 | 307079 | 2456628 | 14 | 13 | 0 | 400 | 2493540 | 14 | 13 | 0 | 1 | 408 |
| 252 | FC | 17 | 11 | 14 | 14 | 307129 | 2457028 | 14 | 14 | 0 | 400 | 2493948 | 14 | 14 | 0 | 1 | 408 |
| 253 | FD | 17 | 11 | 14 | 15 | 307179 | 2457428 | 14 | 15 | 0 | 400 | 2494356 | 14 | 15 | 0 | 1 | 408 |
| 254 | FE | 17 | 11 | 14 | 16 | 307229 | 2457828 | 14 | 16 | 0 | 400 | 2494764 | 14 | 16 | 0 | 1 | 408 |
| 255 | FF | 17 | 11 | 15 | 0 | 307281 | 2458244 | 15 | 0 | 0 | 416 | 2495188 | 15 | 0 | 0 | 1 | 424 |
| 0 | 00 | 18 | 12 | 0 | 0 | 307293 | 2458340 | 0 | 0 | 0 | 96 | 2495292 | 0 | 0 | 0 | 1 | 104 |
| 1 | 01 | 18 | 12 | 0 | 1 | 307307 | 2458452 | 0 | 1 | 0 | 112 | 2495412 | 0 | 1 | 0 | 1 | 120 |
| 2 | 02 | 18 | 12 | 0 | 2 | 307321 | 2458564 | 0 | 2 | 0 | 112 | 2495532 | 0 | 2 | 0 | 1 | 120 |
| 3 | 03 | 18 | 12 | 0 | 3 | 307335 | 2458676 | 0 | 3 | 0 | 112 | 2495652 | 0 | 3 | 0 | 1 | 120 |
| 4 | 04 | 18 | 12 | 0 | 4 | 307349 | 2458788 | 0 | 4 | 0 | 112 | 2495772 | 0 | 4 | 0 | 1 | 120 |
| 5 | 05 | 18 | 12 | 0 | 5 | 307363 | 2458900 | 0 | 5 | 0 | 112 | 2495892 | 0 | 5 | 0 | 1 | 120 |
| 6 | 06 | 18 | 12 | 0 | 6 | 307377 | 2459012 | 0 | 6 | 0 | 112 | 2496012 | 0 | 6 | 0 | 1 | 120 |
| 7 | 07 | 18 | 12 | 0 | 7 | 307391 | 2459124 | 0 | 7 | 0 | 112 | 2496132 | 0 | 7 | 0 | 1 | 120 |
| 8 | 08 | 18 | 12 | 0 | 8 | 307405 | 2459236 | 0 | 8 | 0 | 112 | 2496252 | 0 | 8 | 0 | 1 | 120 |
| 9 | 09 | 18 | 12 | 0 | 9 | 307419 | 2459348 | 0 | 9 | 0 | 112 | 2496372 | 0 | 9 | 0 | 1 | 120 |
| 10 | 0A | 18 | 12 | 0 | 10 | 307433 | 2459460 | 0 | 10 | 0 | 112 | 2496492 | 0 | 10 | 0 | 1 | 120 |
| 11 | 0B | 18 | 12 | 0 | 11 | 307447 | 2459572 | 0 | 11 | 0 | 112 | 2496612 | 0 | 11 | 0 | 1 | 120 |
| 12 | 0C | 18 | 12 | 0 | 12 | 307461 | 2459684 | 0 | 12 | 0 | 112 | 2496732 | 0 | 12 | 0 | 1 | 120 |
| 13 | 0D | 18 | 12 | 0 | 13 | 307475 | 2459796 | 0 | 13 | 0 | 112 | 2496852 | 0 | 13 | 0 | 1 | 120 |
| 14 | 0E | 18 | 12 | 0 | 14 | 307489 | 2459908 | 0 | 14 | 0 | 112 | 2496972 | 0 | 14 | 0 | 1 | 120 |
| 15 | 0F | 18 | 12 | 0 | 15 | 307503 | 2460020 | 0 | 15 | 0 | 112 | 2497092 | 0 | 15 | 0 | 1 | 120 |
| 16 | 10 | 18 | 12 | 0 | 16 | 307517 | 2460132 | 0 | 16 | 0 | 112 | 2497212 | 0 | 16 | 0 | 1 | 120 |
| 17 | 11 | 18 | 12 | 0 | 17 | 307531 | 2460244 | 0 | 17 | 0 | 112 | 2497332 | 0 | 17 | 0 | 1 | 120 |
| 18 | 12 | 18 | 12 | 1 | 0 | 307543 | 2460340 | 1 | 0 | 0 | 96 | 2497436 | 1 | 0 | 0 | 1 | 104 |
| 19 | 13 | 18 | 12 | 1 | 1 | 307560 | 2460476 | 1 | 1 | 0 | 136 | 2497580 | 1 | 1 | 0 | 1 | 144 |
| 20 | 14 | 18 | 12 | 1 | 2 | 307577 | 2460612 | 1 | 2 | 0 | 136 | 2497724 | 1 | 2 | 0 | 1 | 144 |
| 21 | 15 | 18 | 12 | 1 | 3 | 307594 | 2460748 | 1 | 3 | 0 | 136 | 2497868 | 1 | 3 | 0 | 1 | 144 |
| 22 | 16 | 18 | 12 | 1 | 4 | 307611 | 2460884 | 1 | 4 | 0 | 136 | 2498012 | 1 | 4 | 0 | 1 | 144 |
| 23 | 17 | 18 | 12 | 1 | 5 | 307628 | 2461020 | 1 | 5 | 0 | 136 | 2498156 | 1 | 5 | 0 | 1 | 144 |

| | | | | | | | | | | | | | | | | | |
|---|---|---|---|---|---|---|---|---|---|---|---|---|---|---|---|---|---|
| 24 | 18 | 18 | 12 | 1 | 6 | 307645 | 2461156 | 1 | 6 | 0 | 136 | 2498300 | 1 | 6 | 0 | 1 | 144 |
| 25 | 19 | 18 | 12 | 1 | 7 | 307662 | 2461292 | 1 | 7 | 0 | 136 | 2498444 | 1 | 7 | 0 | 1 | 144 |
| 26 | 1A | 18 | 12 | 1 | 8 | 307679 | 2461428 | 1 | 8 | 0 | 136 | 2498588 | 1 | 8 | 0 | 1 | 144 |
| 27 | 1B | 18 | 12 | 1 | 9 | 307696 | 2461564 | 1 | 9 | 0 | 136 | 2498732 | 1 | 9 | 0 | 1 | 144 |
| 28 | 1C | 18 | 12 | 1 | 10 | 307713 | 2461700 | 1 | 10 | 0 | 136 | 2498876 | 1 | 10 | 0 | 1 | 144 |
| 29 | 1D | 18 | 12 | 1 | 11 | 307730 | 2461836 | 1 | 11 | 0 | 136 | 2499020 | 1 | 11 | 0 | 1 | 144 |
| 30 | 1E | 18 | 12 | 1 | 12 | 307747 | 2461972 | 1 | 12 | 0 | 136 | 2499164 | 1 | 12 | 0 | 1 | 144 |
| 31 | 1F | 18 | 12 | 1 | 13 | 307764 | 2462108 | 1 | 13 | 0 | 136 | 2499308 | 1 | 13 | 0 | 1 | 144 |
| 32 | 20 | 18 | 12 | 1 | 14 | 307785 | 2462276 | 1 | 14 | 0 | 168 | 2499484 | 1 | 14 | 0 | 1 | 176 |
| 33 | 21 | 18 | 12 | 1 | 15 | 307806 | 2462444 | 1 | 15 | 0 | 168 | 2499660 | 1 | 15 | 0 | 1 | 176 |
| 34 | 22 | 18 | 12 | 1 | 16 | 307827 | 2462612 | 1 | 16 | 0 | 168 | 2499836 | 1 | 16 | 0 | 1 | 176 |
| 35 | 23 | 18 | 12 | 1 | 17 | 307848 | 2462780 | 1 | 17 | 0 | 168 | 2500012 | 1 | 17 | 0 | 1 | 176 |
| 36 | 24 | 18 | 12 | 2 | 0 | 307867 | 2462932 | 2 | 0 | 0 | 152 | 2500172 | 2 | 0 | 0 | 1 | 160 |
| 37 | 25 | 18 | 12 | 2 | 1 | 307891 | 2463124 | 2 | 1 | 0 | 192 | 2500372 | 2 | 1 | 0 | 1 | 200 |
| 38 | 26 | 18 | 12 | 2 | 2 | 307915 | 2463316 | 2 | 2 | 0 | 192 | 2500572 | 2 | 2 | 0 | 1 | 200 |
| 39 | 27 | 18 | 12 | 2 | 3 | 307939 | 2463508 | 2 | 3 | 0 | 192 | 2500772 | 2 | 3 | 0 | 1 | 200 |
| 40 | 28 | 18 | 12 | 2 | 4 | 307963 | 2463700 | 2 | 4 | 0 | 192 | 2500972 | 2 | 4 | 0 | 1 | 200 |
| 41 | 29 | 18 | 12 | 2 | 5 | 307987 | 2463892 | 2 | 5 | 0 | 192 | 2501172 | 2 | 5 | 0 | 1 | 200 |
| 42 | 2A | 18 | 12 | 2 | 6 | 308011 | 2464084 | 2 | 6 | 0 | 192 | 2501372 | 2 | 6 | 0 | 1 | 200 |
| 43 | 2B | 18 | 12 | 2 | 7 | 308035 | 2464276 | 2 | 7 | 0 | 192 | 2501572 | 2 | 7 | 0 | 1 | 200 |
| 44 | 2C | 18 | 12 | 2 | 8 | 308059 | 2464468 | 2 | 8 | 0 | 192 | 2501772 | 2 | 8 | 0 | 1 | 200 |
| 45 | 2D | 18 | 12 | 2 | 9 | 308083 | 2464660 | 2 | 9 | 0 | 192 | 2501972 | 2 | 9 | 0 | 1 | 200 |
| 46 | 2E | 18 | 12 | 2 | 10 | 308107 | 2464852 | 2 | 10 | 0 | 192 | 2502172 | 2 | 10 | 0 | 1 | 200 |
| 47 | 2F | 18 | 12 | 2 | 11 | 308131 | 2465044 | 2 | 11 | 0 | 192 | 2502372 | 2 | 11 | 0 | 1 | 200 |
| 48 | 30 | 18 | 12 | 2 | 12 | 308157 | 2465252 | 2 | 12 | 0 | 208 | 2502588 | 2 | 12 | 0 | 1 | 216 |
| 49 | 31 | 18 | 12 | 2 | 13 | 308183 | 2465460 | 2 | 13 | 0 | 208 | 2502804 | 2 | 13 | 0 | 1 | 216 |
| 50 | 32 | 18 | 12 | 2 | 14 | 308209 | 2465668 | 2 | 14 | 0 | 208 | 2503020 | 2 | 14 | 0 | 1 | 216 |
| 51 | 33 | 18 | 12 | 2 | 15 | 308235 | 2465876 | 2 | 15 | 0 | 208 | 2503236 | 2 | 15 | 0 | 1 | 216 |
| 52 | 34 | 18 | 12 | 2 | 16 | 308261 | 2466084 | 2 | 16 | 0 | 208 | 2503452 | 2 | 16 | 0 | 1 | 216 |
| 53 | 35 | 18 | 12 | 2 | 17 | 308287 | 2466292 | 2 | 17 | 0 | 208 | 2503668 | 2 | 17 | 0 | 1 | 216 |
| 54 | 36 | 18 | 12 | 3 | 0 | 308315 | 2466516 | 3 | 0 | 0 | 224 | 2503900 | 3 | 0 | 0 | 1 | 232 |
| 55 | 37 | 18 | 12 | 3 | 1 | 308343 | 2466740 | 3 | 1 | 0 | 224 | 2504132 | 3 | 1 | 0 | 1 | 232 |
| 56 | 38 | 18 | 12 | 3 | 2 | 308371 | 2466964 | 3 | 2 | 0 | 224 | 2504364 | 3 | 2 | 0 | 1 | 232 |
| 57 | 39 | 18 | 12 | 3 | 3 | 308399 | 2467188 | 3 | 3 | 0 | 224 | 2504596 | 3 | 3 | 0 | 1 | 232 |
| 58 | 3A | 18 | 12 | 3 | 4 | 308427 | 2467412 | 3 | 4 | 0 | 224 | 2504828 | 3 | 4 | 0 | 1 | 232 |
| 59 | 3B | 18 | 12 | 3 | 5 | 308455 | 2467636 | 3 | 5 | 0 | 224 | 2505060 | 3 | 5 | 0 | 1 | 232 |
| 60 | 3C | 18 | 12 | 3 | 6 | 308483 | 2467860 | 3 | 6 | 0 | 224 | 2505292 | 3 | 6 | 0 | 1 | 232 |
| 61 | 3D | 18 | 12 | 3 | 7 | 308511 | 2468084 | 3 | 7 | 0 | 224 | 2505524 | 3 | 7 | 0 | 1 | 232 |
| 62 | 3E | 18 | 12 | 3 | 8 | 308539 | 2468308 | 3 | 8 | 0 | 224 | 2505756 | 3 | 8 | 0 | 1 | 232 |
| 63 | 3F | 18 | 12 | 3 | 9 | 308567 | 2468532 | 3 | 9 | 0 | 224 | 2505988 | 3 | 9 | 0 | 1 | 232 |
| 64 | 40 | 18 | 12 | 3 | 10 | 308593 | 2468740 | 3 | 10 | 0 | 208 | 2506204 | 3 | 10 | 0 | 1 | 216 |
| 65 | 41 | 18 | 12 | 3 | 11 | 308619 | 2468948 | 3 | 11 | 0 | 208 | 2506420 | 3 | 11 | 0 | 1 | 216 |
| 66 | 42 | 18 | 12 | 3 | 12 | 308645 | 2469156 | 3 | 12 | 0 | 208 | 2506636 | 3 | 12 | 0 | 1 | 216 |
| 67 | 43 | 18 | 12 | 3 | 13 | 308671 | 2469364 | 3 | 13 | 0 | 208 | 2506852 | 3 | 13 | 0 | 1 | 216 |
| 68 | 44 | 18 | 12 | 3 | 14 | 308697 | 2469572 | 3 | 14 | 0 | 208 | 2507068 | 3 | 14 | 0 | 1 | 216 |
| 69 | 45 | 18 | 12 | 3 | 15 | 308723 | 2469780 | 3 | 15 | 0 | 208 | 2507284 | 3 | 15 | 0 | 1 | 216 |
| 70 | 46 | 18 | 12 | 3 | 16 | 308749 | 2469988 | 3 | 16 | 0 | 208 | 2507500 | 3 | 16 | 0 | 1 | 216 |
| 71 | 47 | 18 | 12 | 3 | 17 | 308775 | 2470196 | 3 | 17 | 0 | 208 | 2507716 | 3 | 17 | 0 | 1 | 216 |
| 72 | 48 | 18 | 12 | 4 | 0 | 308803 | 2470420 | 4 | 0 | 0 | 224 | 2507948 | 4 | 0 | 0 | 1 | 232 |
| 73 | 49 | 18 | 12 | 4 | 1 | 308831 | 2470644 | 4 | 1 | 0 | 224 | 2508180 | 4 | 1 | 0 | 1 | 232 |
| 74 | 4A | 18 | 12 | 4 | 2 | 308859 | 2470868 | 4 | 2 | 0 | 224 | 2508412 | 4 | 2 | 0 | 1 | 232 |
| 75 | 4B | 18 | 12 | 4 | 3 | 308887 | 2471092 | 4 | 3 | 0 | 224 | 2508644 | 4 | 3 | 0 | 1 | 232 |
| 76 | 4C | 18 | 12 | 4 | 4 | 308915 | 2471316 | 4 | 4 | 0 | 224 | 2508876 | 4 | 4 | 0 | 1 | 232 |
| 77 | 4D | 18 | 12 | 4 | 5 | 308943 | 2471540 | 4 | 5 | 0 | 224 | 2509108 | 4 | 5 | 0 | 1 | 232 |
| 78 | 4E | 18 | 12 | 4 | 6 | 308971 | 2471764 | 4 | 6 | 0 | 224 | 2509340 | 4 | 6 | 0 | 1 | 232 |
| 79 | 4F | 18 | 12 | 4 | 7 | 308999 | 2471988 | 4 | 7 | 0 | 224 | 2509572 | 4 | 7 | 0 | 1 | 232 |
| 80 | 50 | 18 | 12 | 4 | 8 | 309029 | 2472228 | 4 | 8 | 0 | 240 | 2509820 | 4 | 8 | 0 | 1 | 248 |
| 81 | 51 | 18 | 12 | 4 | 9 | 309059 | 2472468 | 4 | 9 | 0 | 240 | 2510068 | 4 | 9 | 0 | 1 | 248 |
| 82 | 52 | 18 | 12 | 4 | 10 | 309089 | 2472708 | 4 | 10 | 0 | 240 | 2510316 | 4 | 10 | 0 | 1 | 248 |
| 83 | 53 | 18 | 12 | 4 | 11 | 309119 | 2472948 | 4 | 11 | 0 | 240 | 2510564 | 4 | 11 | 0 | 1 | 248 |
| 84 | 54 | 18 | 12 | 4 | 12 | 309149 | 2473188 | 4 | 12 | 0 | 240 | 2510812 | 4 | 12 | 0 | 1 | 248 |
| 85 | 55 | 18 | 12 | 4 | 13 | 309179 | 2473428 | 4 | 13 | 0 | 240 | 2511060 | 4 | 13 | 0 | 1 | 248 |
| 86 | 56 | 18 | 12 | 4 | 14 | 309209 | 2473668 | 4 | 14 | 0 | 240 | 2511308 | 4 | 14 | 0 | 1 | 248 |
| 87 | 57 | 18 | 12 | 4 | 15 | 309239 | 2473908 | 4 | 15 | 0 | 240 | 2511556 | 4 | 15 | 0 | 1 | 248 |
| 88 | 58 | 18 | 12 | 4 | 16 | 309269 | 2474148 | 4 | 16 | 0 | 240 | 2511804 | 4 | 16 | 0 | 1 | 248 |
| 89 | 59 | 18 | 12 | 4 | 17 | 309299 | 2474388 | 4 | 17 | 0 | 240 | 2512052 | 4 | 17 | 0 | 1 | 248 |
| 90 | 5A | 18 | 12 | 5 | 0 | 309331 | 2474644 | 5 | 0 | 0 | 256 | 2512316 | 5 | 0 | 0 | 1 | 264 |
| 91 | 5B | 18 | 12 | 5 | 1 | 309363 | 2474900 | 5 | 1 | 0 | 256 | 2512580 | 5 | 1 | 0 | 1 | 264 |
| 92 | 5C | 18 | 12 | 5 | 2 | 309395 | 2475156 | 5 | 2 | 0 | 256 | 2512844 | 5 | 2 | 0 | 1 | 264 |
| 93 | 5D | 18 | 12 | 5 | 3 | 309427 | 2475412 | 5 | 3 | 0 | 256 | 2513108 | 5 | 3 | 0 | 1 | 264 |
| 94 | 5E | 18 | 12 | 5 | 4 | 309459 | 2475668 | 5 | 4 | 0 | 256 | 2513372 | 5 | 4 | 0 | 1 | 264 |
| 95 | 5F | 18 | 12 | 5 | 5 | 309491 | 2475924 | 5 | 5 | 0 | 256 | 2513636 | 5 | 5 | 0 | 1 | 264 |
| 96 | 60 | 18 | 12 | 5 | 6 | 309521 | 2476164 | 5 | 6 | 0 | 240 | 2513884 | 5 | 6 | 0 | 1 | 248 |
| 97 | 61 | 18 | 12 | 5 | 7 | 309551 | 2476404 | 5 | 7 | 0 | 240 | 2514132 | 5 | 7 | 0 | 1 | 248 |
| 98 | 62 | 18 | 12 | 5 | 8 | 309581 | 2476644 | 5 | 8 | 0 | 240 | 2514380 | 5 | 8 | 0 | 1 | 248 |
| 99 | 63 | 18 | 12 | 5 | 9 | 309611 | 2476884 | 5 | 9 | 0 | 240 | 2514628 | 5 | 9 | 0 | 1 | 248 |
| 100 | 64 | 18 | 12 | 5 | 10 | 309641 | 2477124 | 5 | 10 | 0 | 240 | 2514876 | 5 | 10 | 0 | 1 | 248 |
| 101 | 65 | 18 | 12 | 5 | 11 | 309671 | 2477364 | 5 | 11 | 0 | 240 | 2515124 | 5 | 11 | 0 | 1 | 248 |
| 102 | 66 | 18 | 12 | 5 | 12 | 309701 | 2477604 | 5 | 12 | 0 | 240 | 2515372 | 5 | 12 | 0 | 1 | 248 |
| 103 | 67 | 18 | 12 | 5 | 13 | 309731 | 2477844 | 5 | 13 | 0 | 240 | 2515620 | 5 | 13 | 0 | 1 | 248 |
| 104 | 68 | 18 | 12 | 5 | 14 | 309761 | 2478084 | 5 | 14 | 0 | 240 | 2515868 | 5 | 14 | 0 | 1 | 248 |
| 105 | 69 | 18 | 12 | 5 | 15 | 309791 | 2478324 | 5 | 15 | 0 | 240 | 2516116 | 5 | 15 | 0 | 1 | 248 |
| 106 | 6A | 18 | 12 | 5 | 16 | 309821 | 2478564 | 5 | 16 | 0 | 240 | 2516364 | 5 | 16 | 0 | 1 | 248 |
| 107 | 6B | 18 | 12 | 5 | 17 | 309851 | 2478804 | 5 | 17 | 0 | 240 | 2516612 | 5 | 17 | 0 | 1 | 248 |
| 108 | 6C | 18 | 12 | 6 | 0 | 309883 | 2479060 | 6 | 0 | 0 | 256 | 2516876 | 6 | 0 | 0 | 1 | 264 |
| 109 | 6D | 18 | 12 | 6 | 1 | 309915 | 2479316 | 6 | 1 | 0 | 256 | 2517140 | 6 | 1 | 0 | 1 | 264 |
| 110 | 6E | 18 | 12 | 6 | 2 | 309947 | 2479572 | 6 | 2 | 0 | 256 | 2517404 | 6 | 2 | 0 | 1 | 264 |
| 111 | 6F | 18 | 12 | 6 | 3 | 309979 | 2479828 | 6 | 3 | 0 | 256 | 2517668 | 6 | 3 | 0 | 1 | 264 |
| 112 | 70 | 18 | 12 | 6 | 4 | 310013 | 2480100 | 6 | 4 | 0 | 272 | 2517948 | 6 | 4 | 0 | 1 | 280 |
| 113 | 71 | 18 | 12 | 6 | 5 | 310047 | 2480372 | 6 | 5 | 0 | 272 | 2518228 | 6 | 5 | 0 | 1 | 280 |
| 114 | 72 | 18 | 12 | 6 | 6 | 310081 | 2480644 | 6 | 6 | 0 | 272 | 2518508 | 6 | 6 | 0 | 1 | 280 |
| 115 | 73 | 18 | 12 | 6 | 7 | 310115 | 2480916 | 6 | 7 | 0 | 272 | 2518788 | 6 | 7 | 0 | 1 | 280 |
| 116 | 74 | 18 | 12 | 6 | 8 | 310149 | 2481188 | 6 | 8 | 0 | 272 | 2519068 | 6 | 8 | 0 | 1 | 280 |
| 117 | 75 | 18 | 12 | 6 | 9 | 310183 | 2481460 | 6 | 9 | 0 | 272 | 2519348 | 6 | 9 | 0 | 1 | 280 |
| 118 | 76 | 18 | 12 | 6 | 10 | 310217 | 2481732 | 6 | 10 | 0 | 272 | 2519628 | 6 | 10 | 0 | 1 | 280 |
| 119 | 77 | 18 | 12 | 6 | 11 | 310251 | 2482004 | 6 | 11 | 0 | 272 | 2519908 | 6 | 11 | 0 | 1 | 280 |
| 120 | 78 | 18 | 12 | 6 | 12 | 310285 | 2482276 | 6 | 12 | 0 | 272 | 2520188 | 6 | 12 | 0 | 1 | 280 |
| 121 | 79 | 18 | 12 | 6 | 13 | 310319 | 2482548 | 6 | 13 | 0 | 272 | 2520468 | 6 | 13 | 0 | 1 | 280 |
| 122 | 7A | 18 | 12 | 6 | 14 | 310353 | 2482820 | 6 | 14 | 0 | 272 | 2520748 | 6 | 14 | 0 | 1 | 280 |
| 123 | 7B | 18 | 12 | 6 | 15 | 310387 | 2483092 | 6 | 15 | 0 | 272 | 2521028 | 6 | 15 | 0 | 1 | 280 |
| 124 | 7C | 18 | 12 | 6 | 16 | 310421 | 2483364 | 6 | 16 | 0 | 272 | 2521308 | 6 | 16 | 0 | 1 | 280 |
| 125 | 7D | 18 | 12 | 6 | 17 | 310455 | 2483636 | 6 | 17 | 0 | 272 | 2521588 | 6 | 17 | 0 | 1 | 280 |
| 126 | 7E | 18 | 12 | 7 | 0 | 310491 | 2483924 | 7 | 0 | 0 | 288 | 2521884 | 7 | 0 | 0 | 1 | 296 |

| | | | | | | | | | | | | | | | | | |
|---|---|---|---|---|---|---|---|---|---|---|---|---|---|---|---|---|---|
| 127 | 7F | 18 | 12 | 7 | 1 | 310527 | 2484212 | 7 | 1 | 0 | 288 | 2522180 | 7 | 1 | 0 | 1 | 296 |
| 128 | 80 | 18 | 12 | 7 | 2 | 310561 | 2484484 | 7 | 2 | 0 | 272 | 2522460 | 7 | 2 | 0 | 1 | 280 |
| 129 | 81 | 18 | 12 | 7 | 3 | 310595 | 2484756 | 7 | 3 | 0 | 272 | 2522740 | 7 | 3 | 0 | 1 | 280 |
| 130 | 82 | 18 | 12 | 7 | 4 | 310629 | 2485028 | 7 | 4 | 0 | 272 | 2523020 | 7 | 4 | 0 | 1 | 280 |
| 131 | 83 | 18 | 12 | 7 | 5 | 310663 | 2485300 | 7 | 5 | 0 | 272 | 2523300 | 7 | 5 | 0 | 1 | 280 |
| 132 | 84 | 18 | 12 | 7 | 6 | 310697 | 2485572 | 7 | 6 | 0 | 272 | 2523580 | 7 | 6 | 0 | 1 | 280 |
| 133 | 85 | 18 | 12 | 7 | 7 | 310731 | 2485844 | 7 | 7 | 0 | 272 | 2523860 | 7 | 7 | 0 | 1 | 280 |
| 134 | 86 | 18 | 12 | 7 | 8 | 310765 | 2486116 | 7 | 8 | 0 | 272 | 2524140 | 7 | 8 | 0 | 1 | 280 |
| 135 | 87 | 18 | 12 | 7 | 9 | 310799 | 2486388 | 7 | 9 | 0 | 272 | 2524420 | 7 | 9 | 0 | 1 | 280 |
| 136 | 88 | 18 | 12 | 7 | 10 | 310833 | 2486660 | 7 | 10 | 0 | 272 | 2524700 | 7 | 10 | 0 | 1 | 280 |
| 137 | 89 | 18 | 12 | 7 | 11 | 310867 | 2486932 | 7 | 11 | 0 | 272 | 2524980 | 7 | 11 | 0 | 1 | 280 |
| 138 | 8A | 18 | 12 | 7 | 12 | 310901 | 2487204 | 7 | 12 | 0 | 272 | 2525260 | 7 | 12 | 0 | 1 | 280 |
| 139 | 8B | 18 | 12 | 7 | 13 | 310935 | 2487476 | 7 | 13 | 0 | 272 | 2525540 | 7 | 13 | 0 | 1 | 280 |
| 140 | 8C | 18 | 12 | 7 | 14 | 310969 | 2487748 | 7 | 14 | 0 | 272 | 2525820 | 7 | 14 | 0 | 1 | 280 |
| 141 | 8D | 18 | 12 | 7 | 15 | 311003 | 2488020 | 7 | 15 | 0 | 272 | 2526100 | 7 | 15 | 0 | 1 | 280 |
| 142 | 8E | 18 | 12 | 7 | 16 | 311037 | 2488292 | 7 | 16 | 0 | 272 | 2526380 | 7 | 16 | 0 | 1 | 280 |
| 143 | 8F | 18 | 12 | 7 | 17 | 311071 | 2488564 | 7 | 17 | 0 | 272 | 2526660 | 7 | 17 | 0 | 1 | 280 |
| 144 | 90 | 18 | 12 | 8 | 0 | 311109 | 2488868 | 8 | 0 | 0 | 304 | 2526972 | 8 | 0 | 0 | 1 | 312 |
| 145 | 91 | 18 | 12 | 8 | 1 | 311147 | 2489172 | 8 | 1 | 0 | 304 | 2527284 | 8 | 1 | 0 | 1 | 312 |
| 146 | 92 | 18 | 12 | 8 | 2 | 311185 | 2489476 | 8 | 2 | 0 | 304 | 2527596 | 8 | 2 | 0 | 1 | 312 |
| 147 | 93 | 18 | 12 | 8 | 3 | 311223 | 2489780 | 8 | 3 | 0 | 304 | 2527908 | 8 | 3 | 0 | 1 | 312 |
| 148 | 94 | 18 | 12 | 8 | 4 | 311261 | 2490084 | 8 | 4 | 0 | 304 | 2528220 | 8 | 4 | 0 | 1 | 312 |
| 149 | 95 | 18 | 12 | 8 | 5 | 311299 | 2490388 | 8 | 5 | 0 | 304 | 2528532 | 8 | 5 | 0 | 1 | 312 |
| 150 | 96 | 18 | 12 | 8 | 6 | 311337 | 2490692 | 8 | 6 | 0 | 304 | 2528844 | 8 | 6 | 0 | 1 | 312 |
| 151 | 97 | 18 | 12 | 8 | 7 | 311375 | 2490996 | 8 | 7 | 0 | 304 | 2529156 | 8 | 7 | 0 | 1 | 312 |
| 152 | 98 | 18 | 12 | 8 | 8 | 311413 | 2491300 | 8 | 8 | 0 | 304 | 2529468 | 8 | 8 | 0 | 1 | 312 |
| 153 | 99 | 18 | 12 | 8 | 9 | 311451 | 2491604 | 8 | 9 | 0 | 304 | 2529780 | 8 | 9 | 0 | 1 | 312 |
| 154 | 9A | 18 | 12 | 8 | 10 | 311489 | 2491908 | 8 | 10 | 0 | 304 | 2530092 | 8 | 10 | 0 | 1 | 312 |
| 155 | 9B | 18 | 12 | 8 | 11 | 311527 | 2492212 | 8 | 11 | 0 | 304 | 2530404 | 8 | 11 | 0 | 1 | 312 |
| 156 | 9C | 18 | 12 | 8 | 12 | 311565 | 2492516 | 8 | 12 | 0 | 304 | 2530716 | 8 | 12 | 0 | 1 | 312 |
| 157 | 9D | 18 | 12 | 8 | 13 | 311603 | 2492820 | 8 | 13 | 0 | 304 | 2531028 | 8 | 13 | 0 | 1 | 312 |
| 158 | 9E | 18 | 12 | 8 | 14 | 311641 | 2493124 | 8 | 14 | 0 | 304 | 2531340 | 8 | 14 | 0 | 1 | 312 |
| 159 | 9F | 18 | 12 | 8 | 15 | 311679 | 2493428 | 8 | 15 | 0 | 304 | 2531652 | 8 | 15 | 0 | 1 | 312 |
| 160 | A0 | 18 | 12 | 8 | 16 | 311715 | 2493716 | 8 | 16 | 0 | 288 | 2531948 | 8 | 16 | 0 | 1 | 296 |
| 161 | A1 | 18 | 12 | 8 | 17 | 311751 | 2494004 | 8 | 17 | 0 | 288 | 2532244 | 8 | 17 | 0 | 1 | 296 |
| 162 | A2 | 18 | 12 | 9 | 0 | 311789 | 2494308 | 9 | 0 | 0 | 304 | 2532556 | 9 | 0 | 0 | 1 | 312 |
| 163 | A3 | 18 | 12 | 9 | 1 | 311827 | 2494612 | 9 | 1 | 0 | 304 | 2532868 | 9 | 1 | 0 | 1 | 312 |
| 164 | A4 | 18 | 12 | 9 | 2 | 311865 | 2494916 | 9 | 2 | 0 | 304 | 2533180 | 9 | 2 | 0 | 1 | 312 |
| 165 | A5 | 18 | 12 | 9 | 3 | 311903 | 2495220 | 9 | 3 | 0 | 304 | 2533492 | 9 | 3 | 0 | 1 | 312 |
| 166 | A6 | 18 | 12 | 9 | 4 | 311941 | 2495524 | 9 | 4 | 0 | 304 | 2533804 | 9 | 4 | 0 | 1 | 312 |
| 167 | A7 | 18 | 12 | 9 | 5 | 311979 | 2495828 | 9 | 5 | 0 | 304 | 2534116 | 9 | 5 | 0 | 1 | 312 |
| 168 | A8 | 18 | 12 | 9 | 6 | 312017 | 2496132 | 9 | 6 | 0 | 304 | 2534428 | 9 | 6 | 0 | 1 | 312 |
| 169 | A9 | 18 | 12 | 9 | 7 | 312055 | 2496436 | 9 | 7 | 0 | 304 | 2534740 | 9 | 7 | 0 | 1 | 312 |
| 170 | AA | 18 | 12 | 9 | 8 | 312093 | 2496740 | 9 | 8 | 0 | 304 | 2535052 | 9 | 8 | 0 | 1 | 312 |
| 171 | AB | 18 | 12 | 9 | 9 | 312131 | 2497044 | 9 | 9 | 0 | 304 | 2535364 | 9 | 9 | 0 | 1 | 312 |
| 172 | AC | 18 | 12 | 9 | 10 | 312169 | 2497348 | 9 | 10 | 0 | 304 | 2535676 | 9 | 10 | 0 | 1 | 312 |
| 173 | AD | 18 | 12 | 9 | 11 | 312207 | 2497652 | 9 | 11 | 0 | 304 | 2535988 | 9 | 11 | 0 | 1 | 312 |
| 174 | AE | 18 | 12 | 9 | 12 | 312245 | 2497956 | 9 | 12 | 0 | 304 | 2536300 | 9 | 12 | 0 | 1 | 312 |
| 175 | AF | 18 | 12 | 9 | 13 | 312283 | 2498260 | 9 | 13 | 0 | 304 | 2536612 | 9 | 13 | 0 | 1 | 312 |
| 176 | B0 | 18 | 12 | 9 | 14 | 312323 | 2498580 | 9 | 14 | 0 | 320 | 2536940 | 9 | 14 | 0 | 1 | 328 |
| 177 | B1 | 18 | 12 | 9 | 15 | 312363 | 2498900 | 9 | 15 | 0 | 320 | 2537268 | 9 | 15 | 0 | 1 | 328 |
| 178 | B2 | 18 | 12 | 9 | 16 | 312403 | 2499220 | 9 | 16 | 0 | 320 | 2537596 | 9 | 16 | 0 | 1 | 328 |
| 179 | B3 | 18 | 12 | 9 | 17 | 312443 | 2499540 | 9 | 17 | 0 | 320 | 2537924 | 9 | 17 | 0 | 1 | 328 |
| 180 | B4 | 18 | 12 | 10 | 0 | 312485 | 2499876 | 10 | 0 | 0 | 336 | 2538268 | 10 | 0 | 0 | 1 | 344 |
| 181 | B5 | 18 | 12 | 10 | 1 | 312527 | 2500212 | 10 | 1 | 0 | 336 | 2538612 | 10 | 1 | 0 | 1 | 344 |
| 182 | B6 | 18 | 12 | 10 | 2 | 312569 | 2500548 | 10 | 2 | 0 | 336 | 2538956 | 10 | 2 | 0 | 1 | 344 |
| 183 | B7 | 18 | 12 | 10 | 3 | 312611 | 2500884 | 10 | 3 | 0 | 336 | 2539300 | 10 | 3 | 0 | 1 | 344 |
| 184 | B8 | 18 | 12 | 10 | 4 | 312653 | 2501220 | 10 | 4 | 0 | 336 | 2539644 | 10 | 4 | 0 | 1 | 344 |
| 185 | B9 | 18 | 12 | 10 | 5 | 312695 | 2501556 | 10 | 5 | 0 | 336 | 2539988 | 10 | 5 | 0 | 1 | 344 |
| 186 | BA | 18 | 12 | 10 | 6 | 312737 | 2501892 | 10 | 6 | 0 | 336 | 2540332 | 10 | 6 | 0 | 1 | 344 |
| 187 | BB | 18 | 12 | 10 | 7 | 312779 | 2502228 | 10 | 7 | 0 | 336 | 2540676 | 10 | 7 | 0 | 1 | 344 |
| 188 | BC | 18 | 12 | 10 | 8 | 312821 | 2502564 | 10 | 8 | 0 | 336 | 2541020 | 10 | 8 | 0 | 1 | 344 |
| 189 | BD | 18 | 12 | 10 | 9 | 312863 | 2502900 | 10 | 9 | 0 | 336 | 2541364 | 10 | 9 | 0 | 1 | 344 |
| 190 | BE | 18 | 12 | 10 | 10 | 312905 | 2503236 | 10 | 10 | 0 | 336 | 2541708 | 10 | 10 | 0 | 1 | 344 |
| 191 | BF | 18 | 12 | 10 | 11 | 312947 | 2503572 | 10 | 11 | 0 | 336 | 2542052 | 10 | 11 | 0 | 1 | 344 |
| 192 | C0 | 18 | 12 | 10 | 12 | 312987 | 2503892 | 10 | 12 | 0 | 320 | 2542380 | 10 | 12 | 0 | 1 | 328 |
| 193 | C1 | 18 | 12 | 10 | 13 | 313027 | 2504212 | 10 | 13 | 0 | 320 | 2542708 | 10 | 13 | 0 | 1 | 328 |
| 194 | C2 | 18 | 12 | 10 | 14 | 313067 | 2504532 | 10 | 14 | 0 | 320 | 2543036 | 10 | 14 | 0 | 1 | 328 |
| 195 | C3 | 18 | 12 | 10 | 15 | 313107 | 2504852 | 10 | 15 | 0 | 320 | 2543364 | 10 | 15 | 0 | 1 | 328 |
| 196 | C4 | 18 | 12 | 10 | 16 | 313147 | 2505172 | 10 | 16 | 0 | 320 | 2543692 | 10 | 16 | 0 | 1 | 328 |
| 197 | C5 | 18 | 12 | 10 | 17 | 313187 | 2505492 | 10 | 17 | 0 | 320 | 2544020 | 10 | 17 | 0 | 1 | 328 |
| 198 | C6 | 18 | 12 | 11 | 0 | 313229 | 2505828 | 11 | 0 | 0 | 336 | 2544364 | 11 | 0 | 0 | 1 | 344 |
| 199 | C7 | 18 | 12 | 11 | 1 | 313271 | 2506164 | 11 | 1 | 0 | 336 | 2544708 | 11 | 1 | 0 | 1 | 344 |
| 200 | C8 | 18 | 12 | 11 | 2 | 313313 | 2506500 | 11 | 2 | 0 | 336 | 2545052 | 11 | 2 | 0 | 1 | 344 |
| 201 | C9 | 18 | 12 | 11 | 3 | 313355 | 2506836 | 11 | 3 | 0 | 336 | 2545396 | 11 | 3 | 0 | 1 | 344 |
| 202 | CA | 18 | 12 | 11 | 4 | 313397 | 2507172 | 11 | 4 | 0 | 336 | 2545740 | 11 | 4 | 0 | 1 | 344 |
| 203 | CB | 18 | 12 | 11 | 5 | 313439 | 2507508 | 11 | 5 | 0 | 336 | 2546084 | 11 | 5 | 0 | 1 | 344 |
| 204 | CC | 18 | 12 | 11 | 6 | 313481 | 2507844 | 11 | 6 | 0 | 336 | 2546428 | 11 | 6 | 0 | 1 | 344 |
| 205 | CD | 18 | 12 | 11 | 7 | 313523 | 2508180 | 11 | 7 | 0 | 336 | 2546772 | 11 | 7 | 0 | 1 | 344 |
| 206 | CE | 18 | 12 | 11 | 8 | 313565 | 2508516 | 11 | 8 | 0 | 336 | 2547116 | 11 | 8 | 0 | 1 | 344 |
| 207 | CF | 18 | 12 | 11 | 9 | 313607 | 2508852 | 11 | 9 | 0 | 336 | 2547460 | 11 | 9 | 0 | 1 | 344 |
| 208 | D0 | 18 | 12 | 11 | 10 | 313651 | 2509204 | 11 | 10 | 0 | 352 | 2547820 | 11 | 10 | 0 | 1 | 360 |
| 209 | D1 | 18 | 12 | 11 | 11 | 313695 | 2509556 | 11 | 11 | 0 | 352 | 2548180 | 11 | 11 | 0 | 1 | 360 |
| 210 | D2 | 18 | 12 | 11 | 12 | 313739 | 2509908 | 11 | 12 | 0 | 352 | 2548540 | 11 | 12 | 0 | 1 | 360 |
| 211 | D3 | 18 | 12 | 11 | 13 | 313783 | 2510260 | 11 | 13 | 0 | 352 | 2548900 | 11 | 13 | 0 | 1 | 360 |
| 212 | D4 | 18 | 12 | 11 | 14 | 313827 | 2510612 | 11 | 14 | 0 | 352 | 2549260 | 11 | 14 | 0 | 1 | 360 |
| 213 | D5 | 18 | 12 | 11 | 15 | 313871 | 2510964 | 11 | 15 | 0 | 352 | 2549620 | 11 | 15 | 0 | 1 | 360 |
| 214 | D6 | 18 | 12 | 11 | 16 | 313915 | 2511316 | 11 | 16 | 0 | 352 | 2549980 | 11 | 16 | 0 | 1 | 360 |
| 215 | D7 | 18 | 12 | 11 | 17 | 313959 | 2511668 | 11 | 17 | 0 | 352 | 2550340 | 11 | 17 | 0 | 1 | 360 |
| 216 | D8 | 18 | 12 | 12 | 0 | 314005 | 2512036 | 12 | 0 | 0 | 368 | 2550716 | 12 | 0 | 0 | 1 | 376 |
| 217 | D9 | 18 | 12 | 12 | 1 | 314051 | 2512404 | 12 | 1 | 0 | 368 | 2551092 | 12 | 1 | 0 | 1 | 376 |
| 218 | DA | 18 | 12 | 12 | 2 | 314097 | 2512772 | 12 | 2 | 0 | 368 | 2551468 | 12 | 2 | 0 | 1 | 376 |
| 219 | DB | 18 | 12 | 12 | 3 | 314143 | 2513140 | 12 | 3 | 0 | 368 | 2551844 | 12 | 3 | 0 | 1 | 376 |
| 220 | DC | 18 | 12 | 12 | 4 | 314189 | 2513508 | 12 | 4 | 0 | 368 | 2552220 | 12 | 4 | 0 | 1 | 376 |
| 221 | DD | 18 | 12 | 12 | 5 | 314235 | 2513876 | 12 | 5 | 0 | 368 | 2552596 | 12 | 5 | 0 | 1 | 376 |
| 222 | DE | 18 | 12 | 12 | 6 | 314281 | 2514244 | 12 | 6 | 0 | 368 | 2552972 | 12 | 6 | 0 | 1 | 376 |
| 223 | DF | 18 | 12 | 12 | 7 | 314327 | 2514612 | 12 | 7 | 0 | 368 | 2553348 | 12 | 7 | 0 | 1 | 376 |
| 224 | E0 | 18 | 12 | 12 | 8 | 314371 | 2514964 | 12 | 8 | 0 | 352 | 2553708 | 12 | 8 | 0 | 1 | 360 |
| 225 | E1 | 18 | 12 | 12 | 9 | 314415 | 2515316 | 12 | 9 | 0 | 352 | 2554068 | 12 | 9 | 0 | 1 | 360 |
| 226 | E2 | 18 | 12 | 12 | 10 | 314459 | 2515668 | 12 | 10 | 0 | 352 | 2554428 | 12 | 10 | 0 | 1 | 360 |
| 227 | E3 | 18 | 12 | 12 | 11 | 314503 | 2516020 | 12 | 11 | 0 | 352 | 2554788 | 12 | 11 | 0 | 1 | 360 |
| 228 | E4 | 18 | 12 | 12 | 12 | 314547 | 2516372 | 12 | 12 | 0 | 352 | 2555148 | 12 | 12 | 0 | 1 | 360 |
| 229 | E5 | 18 | 12 | 12 | 13 | 314591 | 2516724 | 12 | 13 | 0 | 352 | 2555508 | 12 | 13 | 0 | 1 | 360 |

| | | | | | | | | | | | | | | | | | |
|---|---|---|---|---|---|---|---|---|---|---|---|---|---|---|---|---|---|
| 230 | E6 | 18 | 12 | 12 | 14 | 314635 | 2517076 | 12 | 14 | 0 | 352 | 2555868 | 12 | 14 | 0 | 1 | 360 |
| 231 | E7 | 18 | 12 | 12 | 15 | 314679 | 2517428 | 12 | 15 | 0 | 352 | 2556228 | 12 | 15 | 0 | 1 | 360 |
| 232 | E8 | 18 | 12 | 12 | 16 | 314723 | 2517780 | 12 | 16 | 0 | 352 | 2556588 | 12 | 16 | 0 | 1 | 360 |
| 233 | E9 | 18 | 12 | 12 | 17 | 314767 | 2518132 | 12 | 17 | 0 | 352 | 2556948 | 12 | 17 | 0 | 1 | 360 |
| 234 | EA | 18 | 12 | 13 | 0 | 314813 | 2518500 | 13 | 0 | 0 | 368 | 2557324 | 13 | 0 | 0 | 1 | 376 |
| 235 | EB | 18 | 12 | 13 | 1 | 314859 | 2518868 | 13 | 1 | 0 | 368 | 2557700 | 13 | 1 | 0 | 1 | 376 |
| 236 | EC | 18 | 12 | 13 | 2 | 314905 | 2519236 | 13 | 2 | 0 | 368 | 2558076 | 13 | 2 | 0 | 1 | 376 |
| 237 | ED | 18 | 12 | 13 | 3 | 314951 | 2519604 | 13 | 3 | 0 | 368 | 2558452 | 13 | 3 | 0 | 1 | 376 |
| 238 | EE | 18 | 12 | 13 | 4 | 314997 | 2519972 | 13 | 4 | 0 | 368 | 2558828 | 13 | 4 | 0 | 1 | 376 |
| 239 | EF | 18 | 12 | 13 | 5 | 315043 | 2520340 | 13 | 5 | 0 | 368 | 2559204 | 13 | 5 | 0 | 1 | 376 |
| 240 | F0 | 18 | 12 | 13 | 6 | 315091 | 2520724 | 13 | 6 | 0 | 384 | 2559596 | 13 | 6 | 0 | 1 | 392 |
| 241 | F1 | 18 | 12 | 13 | 7 | 315139 | 2521108 | 13 | 7 | 0 | 384 | 2559988 | 13 | 7 | 0 | 1 | 392 |
| 242 | F2 | 18 | 12 | 13 | 8 | 315187 | 2521492 | 13 | 8 | 0 | 384 | 2560380 | 13 | 8 | 0 | 1 | 392 |
| 243 | F3 | 18 | 12 | 13 | 9 | 315235 | 2521876 | 13 | 9 | 0 | 384 | 2560772 | 13 | 9 | 0 | 1 | 392 |
| 244 | F4 | 18 | 12 | 13 | 10 | 315283 | 2522260 | 13 | 10 | 0 | 384 | 2561164 | 13 | 10 | 0 | 1 | 392 |
| 245 | F5 | 18 | 12 | 13 | 11 | 315331 | 2522644 | 13 | 11 | 0 | 384 | 2561556 | 13 | 11 | 0 | 1 | 392 |
| 246 | F6 | 18 | 12 | 13 | 12 | 315379 | 2523028 | 13 | 12 | 0 | 384 | 2561948 | 13 | 12 | 0 | 1 | 392 |
| 247 | F7 | 18 | 12 | 13 | 13 | 315427 | 2523412 | 13 | 13 | 0 | 384 | 2562340 | 13 | 13 | 0 | 1 | 392 |
| 248 | F8 | 18 | 12 | 13 | 14 | 315475 | 2523796 | 13 | 14 | 0 | 384 | 2562732 | 13 | 14 | 0 | 1 | 392 |
| 249 | F9 | 18 | 12 | 13 | 15 | 315523 | 2524180 | 13 | 15 | 0 | 384 | 2563124 | 13 | 15 | 0 | 1 | 392 |
| 250 | FA | 18 | 12 | 13 | 16 | 315571 | 2524564 | 13 | 16 | 0 | 384 | 2563516 | 13 | 16 | 0 | 1 | 392 |
| 251 | FB | 18 | 12 | 13 | 17 | 315619 | 2524948 | 13 | 17 | 0 | 384 | 2563908 | 13 | 17 | 0 | 1 | 392 |
| 252 | FC | 18 | 12 | 14 | 0 | 315669 | 2525348 | 14 | 0 | 0 | 400 | 2564316 | 14 | 0 | 0 | 1 | 408 |
| 253 | FD | 18 | 12 | 14 | 1 | 315719 | 2525748 | 14 | 1 | 0 | 400 | 2564724 | 14 | 1 | 0 | 1 | 408 |
| 254 | FE | 18 | 12 | 14 | 2 | 315769 | 2526148 | 14 | 2 | 0 | 400 | 2565132 | 14 | 2 | 0 | 1 | 408 |
| 255 | FF | 18 | 12 | 14 | 3 | 315819 | 2526548 | 14 | 3 | 0 | 400 | 2565540 | 14 | 3 | 0 | 1 | 408 |
| 0 | 00 | 19 | 13 | 0 | 0 | 315831 | 2526644 | 0 | 0 | 0 | 96 | 2565644 | 0 | 0 | 0 | 1 | 104 |
| 1 | 01 | 19 | 13 | 0 | 1 | 315845 | 2526756 | 0 | 1 | 0 | 112 | 2565764 | 0 | 1 | 0 | 1 | 120 |
| 2 | 02 | 19 | 13 | 0 | 2 | 315859 | 2526868 | 0 | 2 | 0 | 112 | 2565884 | 0 | 2 | 0 | 1 | 120 |
| 3 | 03 | 19 | 13 | 0 | 3 | 315873 | 2526980 | 0 | 3 | 0 | 112 | 2566004 | 0 | 3 | 0 | 1 | 120 |
| 4 | 04 | 19 | 13 | 0 | 4 | 315887 | 2527092 | 0 | 4 | 0 | 112 | 2566124 | 0 | 4 | 0 | 1 | 120 |
| 5 | 05 | 19 | 13 | 0 | 5 | 315901 | 2527204 | 0 | 5 | 0 | 112 | 2566244 | 0 | 5 | 0 | 1 | 120 |
| 6 | 06 | 19 | 13 | 0 | 6 | 315915 | 2527316 | 0 | 6 | 0 | 112 | 2566364 | 0 | 6 | 0 | 1 | 120 |
| 7 | 07 | 19 | 13 | 0 | 7 | 315929 | 2527428 | 0 | 7 | 0 | 112 | 2566484 | 0 | 7 | 0 | 1 | 120 |
| 8 | 08 | 19 | 13 | 0 | 8 | 315943 | 2527540 | 0 | 8 | 0 | 112 | 2566604 | 0 | 8 | 0 | 1 | 120 |
| 9 | 09 | 19 | 13 | 0 | 9 | 315957 | 2527652 | 0 | 9 | 0 | 112 | 2566724 | 0 | 9 | 0 | 1 | 120 |
| 10 | 0A | 19 | 13 | 0 | 10 | 315971 | 2527764 | 0 | 10 | 0 | 112 | 2566844 | 0 | 10 | 0 | 1 | 120 |
| 11 | 0B | 19 | 13 | 0 | 11 | 315985 | 2527876 | 0 | 11 | 0 | 112 | 2566964 | 0 | 11 | 0 | 1 | 120 |
| 12 | 0C | 19 | 13 | 0 | 12 | 315999 | 2527988 | 0 | 12 | 0 | 112 | 2567084 | 0 | 12 | 0 | 1 | 120 |
| 13 | 0D | 19 | 13 | 0 | 13 | 316013 | 2528100 | 0 | 13 | 0 | 112 | 2567204 | 0 | 13 | 0 | 1 | 120 |
| 14 | 0E | 19 | 13 | 0 | 14 | 316027 | 2528212 | 0 | 14 | 0 | 112 | 2567324 | 0 | 14 | 0 | 1 | 120 |
| 15 | 0F | 19 | 13 | 0 | 15 | 316041 | 2528324 | 0 | 15 | 0 | 112 | 2567444 | 0 | 15 | 0 | 1 | 120 |
| 16 | 10 | 19 | 13 | 0 | 16 | 316055 | 2528436 | 0 | 16 | 0 | 112 | 2567564 | 0 | 16 | 0 | 1 | 120 |
| 17 | 11 | 19 | 13 | 0 | 17 | 316069 | 2528548 | 0 | 17 | 0 | 112 | 2567684 | 0 | 17 | 0 | 1 | 120 |
| 18 | 12 | 19 | 13 | 0 | 18 | 316083 | 2528660 | 0 | 18 | 0 | 112 | 2567804 | 0 | 18 | 0 | 1 | 120 |
| 19 | 13 | 19 | 13 | 1 | 0 | 316095 | 2528756 | 1 | 0 | 0 | 96 | 2567908 | 1 | 0 | 0 | 1 | 104 |
| 20 | 14 | 19 | 13 | 1 | 1 | 316112 | 2528892 | 1 | 1 | 0 | 136 | 2568052 | 1 | 1 | 0 | 1 | 144 |
| 21 | 15 | 19 | 13 | 1 | 2 | 316129 | 2529028 | 1 | 2 | 0 | 136 | 2568196 | 1 | 2 | 0 | 1 | 144 |
| 22 | 16 | 19 | 13 | 1 | 3 | 316146 | 2529164 | 1 | 3 | 0 | 136 | 2568340 | 1 | 3 | 0 | 1 | 144 |
| 23 | 17 | 19 | 13 | 1 | 4 | 316163 | 2529300 | 1 | 4 | 0 | 136 | 2568484 | 1 | 4 | 0 | 1 | 144 |
| 24 | 18 | 19 | 13 | 1 | 5 | 316180 | 2529436 | 1 | 5 | 0 | 136 | 2568628 | 1 | 5 | 0 | 1 | 144 |
| 25 | 19 | 19 | 13 | 1 | 6 | 316197 | 2529572 | 1 | 6 | 0 | 136 | 2568772 | 1 | 6 | 0 | 1 | 144 |
| 26 | 1A | 19 | 13 | 1 | 7 | 316214 | 2529708 | 1 | 7 | 0 | 136 | 2568916 | 1 | 7 | 0 | 1 | 144 |
| 27 | 1B | 19 | 13 | 1 | 8 | 316231 | 2529844 | 1 | 8 | 0 | 136 | 2569060 | 1 | 8 | 0 | 1 | 144 |
| 28 | 1C | 19 | 13 | 1 | 9 | 316248 | 2529980 | 1 | 9 | 0 | 136 | 2569204 | 1 | 9 | 0 | 1 | 144 |
| 29 | 1D | 19 | 13 | 1 | 10 | 316265 | 2530116 | 1 | 10 | 0 | 136 | 2569348 | 1 | 10 | 0 | 1 | 144 |
| 30 | 1E | 19 | 13 | 1 | 11 | 316282 | 2530252 | 1 | 11 | 0 | 136 | 2569492 | 1 | 11 | 0 | 1 | 144 |
| 31 | 1F | 19 | 13 | 1 | 12 | 316299 | 2530388 | 1 | 12 | 0 | 136 | 2569636 | 1 | 12 | 0 | 1 | 144 |
| 32 | 20 | 19 | 13 | 1 | 13 | 316320 | 2530556 | 1 | 13 | 0 | 168 | 2569812 | 1 | 13 | 0 | 1 | 176 |
| 33 | 21 | 19 | 13 | 1 | 14 | 316341 | 2530724 | 1 | 14 | 0 | 168 | 2569988 | 1 | 14 | 0 | 1 | 176 |
| 34 | 22 | 19 | 13 | 1 | 15 | 316362 | 2530892 | 1 | 15 | 0 | 168 | 2570164 | 1 | 15 | 0 | 1 | 176 |
| 35 | 23 | 19 | 13 | 1 | 16 | 316383 | 2531060 | 1 | 16 | 0 | 168 | 2570340 | 1 | 16 | 0 | 1 | 176 |
| 36 | 24 | 19 | 13 | 1 | 17 | 316404 | 2531228 | 1 | 17 | 0 | 168 | 2570516 | 1 | 17 | 0 | 1 | 176 |
| 37 | 25 | 19 | 13 | 1 | 18 | 316425 | 2531396 | 1 | 18 | 0 | 168 | 2570692 | 1 | 18 | 0 | 1 | 176 |
| 38 | 26 | 19 | 13 | 2 | 0 | 316444 | 2531548 | 2 | 0 | 0 | 152 | 2570852 | 2 | 0 | 0 | 1 | 160 |
| 39 | 27 | 19 | 13 | 2 | 1 | 316468 | 2531740 | 2 | 1 | 0 | 192 | 2571052 | 2 | 1 | 0 | 1 | 200 |
| 40 | 28 | 19 | 13 | 2 | 2 | 316492 | 2531932 | 2 | 2 | 0 | 192 | 2571252 | 2 | 2 | 0 | 1 | 200 |
| 41 | 29 | 19 | 13 | 2 | 3 | 316516 | 2532124 | 2 | 3 | 0 | 192 | 2571452 | 2 | 3 | 0 | 1 | 200 |
| 42 | 2A | 19 | 13 | 2 | 4 | 316540 | 2532316 | 2 | 4 | 0 | 192 | 2571652 | 2 | 4 | 0 | 1 | 200 |
| 43 | 2B | 19 | 13 | 2 | 5 | 316564 | 2532508 | 2 | 5 | 0 | 192 | 2571852 | 2 | 5 | 0 | 1 | 200 |
| 44 | 2C | 19 | 13 | 2 | 6 | 316588 | 2532700 | 2 | 6 | 0 | 192 | 2572052 | 2 | 6 | 0 | 1 | 200 |
| 45 | 2D | 19 | 13 | 2 | 7 | 316612 | 2532892 | 2 | 7 | 0 | 192 | 2572252 | 2 | 7 | 0 | 1 | 200 |
| 46 | 2E | 19 | 13 | 2 | 8 | 316636 | 2533084 | 2 | 8 | 0 | 192 | 2572452 | 2 | 8 | 0 | 1 | 200 |
| 47 | 2F | 19 | 13 | 2 | 9 | 316660 | 2533276 | 2 | 9 | 0 | 192 | 2572652 | 2 | 9 | 0 | 1 | 200 |
| 48 | 30 | 19 | 13 | 2 | 10 | 316686 | 2533484 | 2 | 10 | 0 | 208 | 2572868 | 2 | 10 | 0 | 1 | 216 |
| 49 | 31 | 19 | 13 | 2 | 11 | 316712 | 2533692 | 2 | 11 | 0 | 208 | 2573084 | 2 | 11 | 0 | 1 | 216 |
| 50 | 32 | 19 | 13 | 2 | 12 | 316738 | 2533900 | 2 | 12 | 0 | 208 | 2573300 | 2 | 12 | 0 | 1 | 216 |
| 51 | 33 | 19 | 13 | 2 | 13 | 316764 | 2534108 | 2 | 13 | 0 | 208 | 2573516 | 2 | 13 | 0 | 1 | 216 |
| 52 | 34 | 19 | 13 | 2 | 14 | 316790 | 2534316 | 2 | 14 | 0 | 208 | 2573732 | 2 | 14 | 0 | 1 | 216 |
| 53 | 35 | 19 | 13 | 2 | 15 | 316816 | 2534524 | 2 | 15 | 0 | 208 | 2573948 | 2 | 15 | 0 | 1 | 216 |
| 54 | 36 | 19 | 13 | 2 | 16 | 316842 | 2534732 | 2 | 16 | 0 | 208 | 2574164 | 2 | 16 | 0 | 1 | 216 |
| 55 | 37 | 19 | 13 | 2 | 17 | 316868 | 2534940 | 2 | 17 | 0 | 208 | 2574380 | 2 | 17 | 0 | 1 | 216 |
| 56 | 38 | 19 | 13 | 2 | 18 | 316894 | 2535148 | 2 | 18 | 0 | 208 | 2574596 | 2 | 18 | 0 | 1 | 216 |
| 57 | 39 | 19 | 13 | 3 | 0 | 316922 | 2535372 | 3 | 0 | 0 | 224 | 2574828 | 3 | 0 | 0 | 1 | 232 |
| 58 | 3A | 19 | 13 | 3 | 1 | 316950 | 2535596 | 3 | 1 | 0 | 224 | 2575060 | 3 | 1 | 0 | 1 | 232 |
| 59 | 3B | 19 | 13 | 3 | 2 | 316978 | 2535820 | 3 | 2 | 0 | 224 | 2575292 | 3 | 2 | 0 | 1 | 232 |
| 60 | 3C | 19 | 13 | 3 | 3 | 317006 | 2536044 | 3 | 3 | 0 | 224 | 2575524 | 3 | 3 | 0 | 1 | 232 |
| 61 | 3D | 19 | 13 | 3 | 4 | 317034 | 2536268 | 3 | 4 | 0 | 224 | 2575756 | 3 | 4 | 0 | 1 | 232 |
| 62 | 3E | 19 | 13 | 3 | 5 | 317062 | 2536492 | 3 | 5 | 0 | 224 | 2575988 | 3 | 5 | 0 | 1 | 232 |
| 63 | 3F | 19 | 13 | 3 | 6 | 317090 | 2536716 | 3 | 6 | 0 | 224 | 2576220 | 3 | 6 | 0 | 1 | 232 |
| 64 | 40 | 19 | 13 | 3 | 7 | 317116 | 2536924 | 3 | 7 | 0 | 208 | 2576436 | 3 | 7 | 0 | 1 | 216 |
| 65 | 41 | 19 | 13 | 3 | 8 | 317142 | 2537132 | 3 | 8 | 0 | 208 | 2576652 | 3 | 8 | 0 | 1 | 216 |
| 66 | 42 | 19 | 13 | 3 | 9 | 317168 | 2537340 | 3 | 9 | 0 | 208 | 2576868 | 3 | 9 | 0 | 1 | 216 |
| 67 | 43 | 19 | 13 | 3 | 10 | 317194 | 2537548 | 3 | 10 | 0 | 208 | 2577084 | 3 | 10 | 0 | 1 | 216 |
| 68 | 44 | 19 | 13 | 3 | 11 | 317220 | 2537756 | 3 | 11 | 0 | 208 | 2577300 | 3 | 11 | 0 | 1 | 216 |
| 69 | 45 | 19 | 13 | 3 | 12 | 317246 | 2537964 | 3 | 12 | 0 | 208 | 2577516 | 3 | 12 | 0 | 1 | 216 |
| 70 | 46 | 19 | 13 | 3 | 13 | 317272 | 2538172 | 3 | 13 | 0 | 208 | 2577732 | 3 | 13 | 0 | 1 | 216 |
| 71 | 47 | 19 | 13 | 3 | 14 | 317298 | 2538380 | 3 | 14 | 0 | 208 | 2577948 | 3 | 14 | 0 | 1 | 216 |
| 72 | 48 | 19 | 13 | 3 | 15 | 317324 | 2538588 | 3 | 15 | 0 | 208 | 2578164 | 3 | 15 | 0 | 1 | 216 |
| 73 | 49 | 19 | 13 | 3 | 16 | 317350 | 2538796 | 3 | 16 | 0 | 208 | 2578380 | 3 | 16 | 0 | 1 | 216 |
| 74 | 4A | 19 | 13 | 3 | 17 | 317376 | 2539004 | 3 | 17 | 0 | 208 | 2578596 | 3 | 17 | 0 | 1 | 216 |
| 75 | 4B | 19 | 13 | 3 | 18 | 317402 | 2539212 | 3 | 18 | 0 | 208 | 2578812 | 3 | 18 | 0 | 1 | 216 |
| 76 | 4C | 19 | 13 | 4 | 0 | 317430 | 2539436 | 4 | 0 | 0 | 224 | 2579044 | 4 | 0 | 0 | 1 | 232 |

| | | | | | | | | | | | | | | | | | |
|---|---|---|---|---|---|---|---|---|---|---|---|---|---|---|---|---|---|
| 77 | 4D | 19 | 13 | 4 | 1 | 317458 | 2539660 | 4 | 1 | 0 | 224 | 2579276 | 4 | 1 | 0 | 1 | 232 |
| 78 | 4E | 19 | 13 | 4 | 2 | 317486 | 2539884 | 4 | 2 | 0 | 224 | 2579508 | 4 | 2 | 0 | 1 | 232 |
| 79 | 4F | 19 | 13 | 4 | 3 | 317514 | 2540108 | 4 | 3 | 0 | 224 | 2579740 | 4 | 3 | 0 | 1 | 232 |
| 80 | 50 | 19 | 13 | 4 | 4 | 317544 | 2540348 | 4 | 4 | 0 | 240 | 2579988 | 4 | 4 | 0 | 1 | 248 |
| 81 | 51 | 19 | 13 | 4 | 5 | 317574 | 2540588 | 4 | 5 | 0 | 240 | 2580236 | 4 | 5 | 0 | 1 | 248 |
| 82 | 52 | 19 | 13 | 4 | 6 | 317604 | 2540828 | 4 | 6 | 0 | 240 | 2580484 | 4 | 6 | 0 | 1 | 248 |
| 83 | 53 | 19 | 13 | 4 | 7 | 317634 | 2541068 | 4 | 7 | 0 | 240 | 2580732 | 4 | 7 | 0 | 1 | 248 |
| 84 | 54 | 19 | 13 | 4 | 8 | 317664 | 2541308 | 4 | 8 | 0 | 240 | 2580980 | 4 | 8 | 0 | 1 | 248 |
| 85 | 55 | 19 | 13 | 4 | 9 | 317694 | 2541548 | 4 | 9 | 0 | 240 | 2581228 | 4 | 9 | 0 | 1 | 248 |
| 86 | 56 | 19 | 13 | 4 | 10 | 317724 | 2541788 | 4 | 10 | 0 | 240 | 2581476 | 4 | 10 | 0 | 1 | 248 |
| 87 | 57 | 19 | 13 | 4 | 11 | 317754 | 2542028 | 4 | 11 | 0 | 240 | 2581724 | 4 | 11 | 0 | 1 | 248 |
| 88 | 58 | 19 | 13 | 4 | 12 | 317784 | 2542268 | 4 | 12 | 0 | 240 | 2581972 | 4 | 12 | 0 | 1 | 248 |
| 89 | 59 | 19 | 13 | 4 | 13 | 317814 | 2542508 | 4 | 13 | 0 | 240 | 2582220 | 4 | 13 | 0 | 1 | 248 |
| 90 | 5A | 19 | 13 | 4 | 14 | 317844 | 2542748 | 4 | 14 | 0 | 240 | 2582468 | 4 | 14 | 0 | 1 | 248 |
| 91 | 5B | 19 | 13 | 4 | 15 | 317874 | 2542988 | 4 | 15 | 0 | 240 | 2582716 | 4 | 15 | 0 | 1 | 248 |
| 92 | 5C | 19 | 13 | 4 | 16 | 317904 | 2543228 | 4 | 16 | 0 | 240 | 2582964 | 4 | 16 | 0 | 1 | 248 |
| 93 | 5D | 19 | 13 | 4 | 17 | 317934 | 2543468 | 4 | 17 | 0 | 240 | 2583212 | 4 | 17 | 0 | 1 | 248 |
| 94 | 5E | 19 | 13 | 4 | 18 | 317964 | 2543708 | 4 | 18 | 0 | 240 | 2583460 | 4 | 18 | 0 | 1 | 248 |
| 95 | 5F | 19 | 13 | 5 | 0 | 317996 | 2543964 | 5 | 0 | 0 | 256 | 2583724 | 5 | 0 | 0 | 1 | 264 |
| 96 | 60 | 19 | 13 | 5 | 1 | 318026 | 2544204 | 5 | 1 | 0 | 240 | 2583972 | 5 | 1 | 0 | 1 | 248 |
| 97 | 61 | 19 | 13 | 5 | 2 | 318056 | 2544444 | 5 | 2 | 0 | 240 | 2584220 | 5 | 2 | 0 | 1 | 248 |
| 98 | 62 | 19 | 13 | 5 | 3 | 318086 | 2544684 | 5 | 3 | 0 | 240 | 2584468 | 5 | 3 | 0 | 1 | 248 |
| 99 | 63 | 19 | 13 | 5 | 4 | 318116 | 2544924 | 5 | 4 | 0 | 240 | 2584716 | 5 | 4 | 0 | 1 | 248 |
| 100 | 64 | 19 | 13 | 5 | 5 | 318146 | 2545164 | 5 | 5 | 0 | 240 | 2584964 | 5 | 5 | 0 | 1 | 248 |
| 101 | 65 | 19 | 13 | 5 | 6 | 318176 | 2545404 | 5 | 6 | 0 | 240 | 2585212 | 5 | 6 | 0 | 1 | 248 |
| 102 | 66 | 19 | 13 | 5 | 7 | 318206 | 2545644 | 5 | 7 | 0 | 240 | 2585460 | 5 | 7 | 0 | 1 | 248 |
| 103 | 67 | 19 | 13 | 5 | 8 | 318236 | 2545884 | 5 | 8 | 0 | 240 | 2585708 | 5 | 8 | 0 | 1 | 248 |
| 104 | 68 | 19 | 13 | 5 | 9 | 318266 | 2546124 | 5 | 9 | 0 | 240 | 2585956 | 5 | 9 | 0 | 1 | 248 |
| 105 | 69 | 19 | 13 | 5 | 10 | 318296 | 2546364 | 5 | 10 | 0 | 240 | 2586204 | 5 | 10 | 0 | 1 | 248 |
| 106 | 6A | 19 | 13 | 5 | 11 | 318326 | 2546604 | 5 | 11 | 0 | 240 | 2586452 | 5 | 11 | 0 | 1 | 248 |
| 107 | 6B | 19 | 13 | 5 | 12 | 318356 | 2546844 | 5 | 12 | 0 | 240 | 2586700 | 5 | 12 | 0 | 1 | 248 |
| 108 | 6C | 19 | 13 | 5 | 13 | 318386 | 2547084 | 5 | 13 | 0 | 240 | 2586948 | 5 | 13 | 0 | 1 | 248 |
| 109 | 6D | 19 | 13 | 5 | 14 | 318416 | 2547324 | 5 | 14 | 0 | 240 | 2587196 | 5 | 14 | 0 | 1 | 248 |
| 110 | 6E | 19 | 13 | 5 | 15 | 318446 | 2547564 | 5 | 15 | 0 | 240 | 2587444 | 5 | 15 | 0 | 1 | 248 |
| 111 | 6F | 19 | 13 | 5 | 16 | 318476 | 2547804 | 5 | 16 | 0 | 240 | 2587692 | 5 | 16 | 0 | 1 | 248 |
| 112 | 70 | 19 | 13 | 5 | 17 | 318508 | 2548060 | 5 | 17 | 0 | 256 | 2587956 | 5 | 17 | 0 | 1 | 264 |
| 113 | 71 | 19 | 13 | 5 | 18 | 318540 | 2548316 | 5 | 18 | 0 | 256 | 2588220 | 5 | 18 | 0 | 1 | 264 |
| 114 | 72 | 19 | 13 | 6 | 0 | 318574 | 2548588 | 6 | 0 | 0 | 272 | 2588500 | 6 | 0 | 0 | 1 | 280 |
| 115 | 73 | 19 | 13 | 6 | 1 | 318608 | 2548860 | 6 | 1 | 0 | 272 | 2588780 | 6 | 1 | 0 | 1 | 280 |
| 116 | 74 | 19 | 13 | 6 | 2 | 318642 | 2549132 | 6 | 2 | 0 | 272 | 2589060 | 6 | 2 | 0 | 1 | 280 |
| 117 | 75 | 19 | 13 | 6 | 3 | 318676 | 2549404 | 6 | 3 | 0 | 272 | 2589340 | 6 | 3 | 0 | 1 | 280 |
| 118 | 76 | 19 | 13 | 6 | 4 | 318710 | 2549676 | 6 | 4 | 0 | 272 | 2589620 | 6 | 4 | 0 | 1 | 280 |
| 119 | 77 | 19 | 13 | 6 | 5 | 318744 | 2549948 | 6 | 5 | 0 | 272 | 2589900 | 6 | 5 | 0 | 1 | 280 |
| 120 | 78 | 19 | 13 | 6 | 6 | 318778 | 2550220 | 6 | 6 | 0 | 272 | 2590180 | 6 | 6 | 0 | 1 | 280 |
| 121 | 79 | 19 | 13 | 6 | 7 | 318812 | 2550492 | 6 | 7 | 0 | 272 | 2590460 | 6 | 7 | 0 | 1 | 280 |
| 122 | 7A | 19 | 13 | 6 | 8 | 318846 | 2550764 | 6 | 8 | 0 | 272 | 2590740 | 6 | 8 | 0 | 1 | 280 |
| 123 | 7B | 19 | 13 | 6 | 9 | 318880 | 2551036 | 6 | 9 | 0 | 272 | 2591020 | 6 | 9 | 0 | 1 | 280 |
| 124 | 7C | 19 | 13 | 6 | 10 | 318914 | 2551308 | 6 | 10 | 0 | 272 | 2591300 | 6 | 10 | 0 | 1 | 280 |
| 125 | 7D | 19 | 13 | 6 | 11 | 318948 | 2551580 | 6 | 11 | 0 | 272 | 2591580 | 6 | 11 | 0 | 1 | 280 |
| 126 | 7E | 19 | 13 | 6 | 12 | 318982 | 2551852 | 6 | 12 | 0 | 272 | 2591860 | 6 | 12 | 0 | 1 | 280 |
| 127 | 7F | 19 | 13 | 6 | 13 | 319016 | 2552124 | 6 | 13 | 0 | 272 | 2592140 | 6 | 13 | 0 | 1 | 280 |
| 128 | 80 | 19 | 13 | 6 | 14 | 319048 | 2552380 | 6 | 14 | 0 | 256 | 2592404 | 6 | 14 | 0 | 1 | 264 |
| 129 | 81 | 19 | 13 | 6 | 15 | 319080 | 2552636 | 6 | 15 | 0 | 256 | 2592668 | 6 | 15 | 0 | 1 | 264 |
| 130 | 82 | 19 | 13 | 6 | 16 | 319112 | 2552892 | 6 | 16 | 0 | 256 | 2592932 | 6 | 16 | 0 | 1 | 264 |
| 131 | 83 | 19 | 13 | 6 | 17 | 319144 | 2553148 | 6 | 17 | 0 | 256 | 2593196 | 6 | 17 | 0 | 1 | 264 |
| 132 | 84 | 19 | 13 | 6 | 18 | 319176 | 2553404 | 6 | 18 | 0 | 256 | 2593460 | 6 | 18 | 0 | 1 | 264 |
| 133 | 85 | 19 | 13 | 7 | 0 | 319210 | 2553676 | 7 | 0 | 0 | 272 | 2593740 | 7 | 0 | 0 | 1 | 280 |
| 134 | 86 | 19 | 13 | 7 | 1 | 319244 | 2553948 | 7 | 1 | 0 | 272 | 2594020 | 7 | 1 | 0 | 1 | 280 |
| 135 | 87 | 19 | 13 | 7 | 2 | 319278 | 2554220 | 7 | 2 | 0 | 272 | 2594300 | 7 | 2 | 0 | 1 | 280 |
| 136 | 88 | 19 | 13 | 7 | 3 | 319312 | 2554492 | 7 | 3 | 0 | 272 | 2594580 | 7 | 3 | 0 | 1 | 280 |
| 137 | 89 | 19 | 13 | 7 | 4 | 319346 | 2554764 | 7 | 4 | 0 | 272 | 2594860 | 7 | 4 | 0 | 1 | 280 |
| 138 | 8A | 19 | 13 | 7 | 5 | 319380 | 2555036 | 7 | 5 | 0 | 272 | 2595140 | 7 | 5 | 0 | 1 | 280 |
| 139 | 8B | 19 | 13 | 7 | 6 | 319414 | 2555308 | 7 | 6 | 0 | 272 | 2595420 | 7 | 6 | 0 | 1 | 280 |
| 140 | 8C | 19 | 13 | 7 | 7 | 319448 | 2555580 | 7 | 7 | 0 | 272 | 2595700 | 7 | 7 | 0 | 1 | 280 |
| 141 | 8D | 19 | 13 | 7 | 8 | 319482 | 2555852 | 7 | 8 | 0 | 272 | 2595980 | 7 | 8 | 0 | 1 | 280 |
| 142 | 8E | 19 | 13 | 7 | 9 | 319516 | 2556124 | 7 | 9 | 0 | 272 | 2596260 | 7 | 9 | 0 | 1 | 280 |
| 143 | 8F | 19 | 13 | 7 | 10 | 319550 | 2556396 | 7 | 10 | 0 | 272 | 2596540 | 7 | 10 | 0 | 1 | 280 |
| 144 | 90 | 19 | 13 | 7 | 11 | 319586 | 2556684 | 7 | 11 | 0 | 288 | 2596836 | 7 | 11 | 0 | 1 | 296 |
| 145 | 91 | 19 | 13 | 7 | 12 | 319622 | 2556972 | 7 | 12 | 0 | 288 | 2597132 | 7 | 12 | 0 | 1 | 296 |
| 146 | 92 | 19 | 13 | 7 | 13 | 319658 | 2557260 | 7 | 13 | 0 | 288 | 2597428 | 7 | 13 | 0 | 1 | 296 |
| 147 | 93 | 19 | 13 | 7 | 14 | 319694 | 2557548 | 7 | 14 | 0 | 288 | 2597724 | 7 | 14 | 0 | 1 | 296 |
| 148 | 94 | 19 | 13 | 7 | 15 | 319730 | 2557836 | 7 | 15 | 0 | 288 | 2598020 | 7 | 15 | 0 | 1 | 296 |
| 149 | 95 | 19 | 13 | 7 | 16 | 319766 | 2558124 | 7 | 16 | 0 | 288 | 2598316 | 7 | 16 | 0 | 1 | 296 |
| 150 | 96 | 19 | 13 | 7 | 17 | 319802 | 2558412 | 7 | 17 | 0 | 288 | 2598612 | 7 | 17 | 0 | 1 | 296 |
| 151 | 97 | 19 | 13 | 7 | 18 | 319838 | 2558700 | 7 | 18 | 0 | 288 | 2598908 | 7 | 18 | 0 | 1 | 296 |
| 152 | 98 | 19 | 13 | 8 | 0 | 319876 | 2559004 | 8 | 0 | 0 | 304 | 2599220 | 8 | 0 | 0 | 1 | 312 |
| 153 | 99 | 19 | 13 | 8 | 1 | 319914 | 2559308 | 8 | 1 | 0 | 304 | 2599532 | 8 | 1 | 0 | 1 | 312 |
| 154 | 9A | 19 | 13 | 8 | 2 | 319952 | 2559612 | 8 | 2 | 0 | 304 | 2599844 | 8 | 2 | 0 | 1 | 312 |
| 155 | 9B | 19 | 13 | 8 | 3 | 319990 | 2559916 | 8 | 3 | 0 | 304 | 2600156 | 8 | 3 | 0 | 1 | 312 |
| 156 | 9C | 19 | 13 | 8 | 4 | 320028 | 2560220 | 8 | 4 | 0 | 304 | 2600468 | 8 | 4 | 0 | 1 | 312 |
| 157 | 9D | 19 | 13 | 8 | 5 | 320066 | 2560524 | 8 | 5 | 0 | 304 | 2600780 | 8 | 5 | 0 | 1 | 312 |
| 158 | 9E | 19 | 13 | 8 | 6 | 320104 | 2560828 | 8 | 6 | 0 | 304 | 2601092 | 8 | 6 | 0 | 1 | 312 |
| 159 | 9F | 19 | 13 | 8 | 7 | 320142 | 2561132 | 8 | 7 | 0 | 304 | 2601404 | 8 | 7 | 0 | 1 | 312 |
| 160 | A0 | 19 | 13 | 8 | 8 | 320178 | 2561420 | 8 | 8 | 0 | 288 | 2601700 | 8 | 8 | 0 | 1 | 296 |
| 161 | A1 | 19 | 13 | 8 | 9 | 320214 | 2561708 | 8 | 9 | 0 | 288 | 2601996 | 8 | 9 | 0 | 1 | 296 |
| 162 | A2 | 19 | 13 | 8 | 10 | 320250 | 2561996 | 8 | 10 | 0 | 288 | 2602292 | 8 | 10 | 0 | 1 | 296 |
| 163 | A3 | 19 | 13 | 8 | 11 | 320286 | 2562284 | 8 | 11 | 0 | 288 | 2602588 | 8 | 11 | 0 | 1 | 296 |
| 164 | A4 | 19 | 13 | 8 | 12 | 320322 | 2562572 | 8 | 12 | 0 | 288 | 2602884 | 8 | 12 | 0 | 1 | 296 |
| 165 | A5 | 19 | 13 | 8 | 13 | 320358 | 2562860 | 8 | 13 | 0 | 288 | 2603180 | 8 | 13 | 0 | 1 | 296 |
| 166 | A6 | 19 | 13 | 8 | 14 | 320394 | 2563148 | 8 | 14 | 0 | 288 | 2603476 | 8 | 14 | 0 | 1 | 296 |
| 167 | A7 | 19 | 13 | 8 | 15 | 320430 | 2563436 | 8 | 15 | 0 | 288 | 2603772 | 8 | 15 | 0 | 1 | 296 |
| 168 | A8 | 19 | 13 | 8 | 16 | 320466 | 2563724 | 8 | 16 | 0 | 288 | 2604068 | 8 | 16 | 0 | 1 | 296 |
| 169 | A9 | 19 | 13 | 8 | 17 | 320502 | 2564012 | 8 | 17 | 0 | 288 | 2604364 | 8 | 17 | 0 | 1 | 296 |
| 170 | AA | 19 | 13 | 8 | 18 | 320538 | 2564300 | 8 | 18 | 0 | 288 | 2604660 | 8 | 18 | 0 | 1 | 296 |
| 171 | AB | 19 | 13 | 9 | 0 | 320576 | 2564604 | 9 | 0 | 0 | 304 | 2604972 | 9 | 0 | 0 | 1 | 312 |
| 172 | AC | 19 | 13 | 9 | 1 | 320614 | 2564908 | 9 | 1 | 0 | 304 | 2605284 | 9 | 1 | 0 | 1 | 312 |
| 173 | AD | 19 | 13 | 9 | 2 | 320652 | 2565212 | 9 | 2 | 0 | 304 | 2605596 | 9 | 2 | 0 | 1 | 312 |
| 174 | AE | 19 | 13 | 9 | 3 | 320690 | 2565516 | 9 | 3 | 0 | 304 | 2605908 | 9 | 3 | 0 | 1 | 312 |
| 175 | AF | 19 | 13 | 9 | 4 | 320728 | 2565820 | 9 | 4 | 0 | 304 | 2606220 | 9 | 4 | 0 | 1 | 312 |
| 176 | B0 | 19 | 13 | 9 | 5 | 320768 | 2566140 | 9 | 5 | 0 | 320 | 2606548 | 9 | 5 | 0 | 1 | 328 |
| 177 | B1 | 19 | 13 | 9 | 6 | 320808 | 2566460 | 9 | 6 | 0 | 320 | 2606876 | 9 | 6 | 0 | 1 | 328 |
| 178 | B2 | 19 | 13 | 9 | 7 | 320848 | 2566780 | 9 | 7 | 0 | 320 | 2607204 | 9 | 7 | 0 | 1 | 328 |
| 179 | B3 | 19 | 13 | 9 | 8 | 320888 | 2567100 | 9 | 8 | 0 | 320 | 2607532 | 9 | 8 | 0 | 1 | 328 |

| | | | | | | | | | | | | | | | | | |
|---|---|---|---|---|---|---|---|---|---|---|---|---|---|---|---|---|---|
| 180 | B4 | 19 | 13 | 9 | 9 | 320928 | 2567420 | 9 | 9 | 0 | 320 | 2607860 | 9 | 9 | 0 | 1 | 328 |
| 181 | B5 | 19 | 13 | 9 | 10 | 320968 | 2567740 | 9 | 10 | 0 | 320 | 2608188 | 9 | 10 | 0 | 1 | 328 |
| 182 | B6 | 19 | 13 | 9 | 11 | 321008 | 2568060 | 9 | 11 | 0 | 320 | 2608516 | 9 | 11 | 0 | 1 | 328 |
| 183 | B7 | 19 | 13 | 9 | 12 | 321048 | 2568380 | 9 | 12 | 0 | 320 | 2608844 | 9 | 12 | 0 | 1 | 328 |
| 184 | B8 | 19 | 13 | 9 | 13 | 321088 | 2568700 | 9 | 13 | 0 | 320 | 2609172 | 9 | 13 | 0 | 1 | 328 |
| 185 | B9 | 19 | 13 | 9 | 14 | 321128 | 2569020 | 9 | 14 | 0 | 320 | 2609500 | 9 | 14 | 0 | 1 | 328 |
| 186 | BA | 19 | 13 | 9 | 15 | 321168 | 2569340 | 9 | 15 | 0 | 320 | 2609828 | 9 | 15 | 0 | 1 | 328 |
| 187 | BB | 19 | 13 | 9 | 16 | 321208 | 2569660 | 9 | 16 | 0 | 320 | 2610156 | 9 | 16 | 0 | 1 | 328 |
| 188 | BC | 19 | 13 | 9 | 17 | 321248 | 2569980 | 9 | 17 | 0 | 320 | 2610484 | 9 | 17 | 0 | 1 | 328 |
| 189 | BD | 19 | 13 | 9 | 18 | 321288 | 2570300 | 9 | 18 | 0 | 320 | 2610812 | 9 | 18 | 0 | 1 | 328 |
| 190 | BE | 19 | 13 | 10 | 0 | 321330 | 2570636 | 10 | 0 | 0 | 336 | 2611156 | 10 | 0 | 0 | 1 | 344 |
| 191 | BF | 19 | 13 | 10 | 1 | 321372 | 2570972 | 10 | 1 | 0 | 336 | 2611500 | 10 | 1 | 0 | 1 | 344 |
| 192 | C0 | 19 | 13 | 10 | 2 | 321412 | 2571292 | 10 | 2 | 0 | 320 | 2611828 | 10 | 2 | 0 | 1 | 328 |
| 193 | C1 | 19 | 13 | 10 | 3 | 321452 | 2571612 | 10 | 3 | 0 | 320 | 2612156 | 10 | 3 | 0 | 1 | 328 |
| 194 | C2 | 19 | 13 | 10 | 4 | 321492 | 2571932 | 10 | 4 | 0 | 320 | 2612484 | 10 | 4 | 0 | 1 | 328 |
| 195 | C3 | 19 | 13 | 10 | 5 | 321532 | 2572252 | 10 | 5 | 0 | 320 | 2612812 | 10 | 5 | 0 | 1 | 328 |
| 196 | C4 | 19 | 13 | 10 | 6 | 321572 | 2572572 | 10 | 6 | 0 | 320 | 2613140 | 10 | 6 | 0 | 1 | 328 |
| 197 | C5 | 19 | 13 | 10 | 7 | 321612 | 2572892 | 10 | 7 | 0 | 320 | 2613468 | 10 | 7 | 0 | 1 | 328 |
| 198 | C6 | 19 | 13 | 10 | 8 | 321652 | 2573212 | 10 | 8 | 0 | 320 | 2613796 | 10 | 8 | 0 | 1 | 328 |
| 199 | C7 | 19 | 13 | 10 | 9 | 321692 | 2573532 | 10 | 9 | 0 | 320 | 2614124 | 10 | 9 | 0 | 1 | 328 |
| 200 | C8 | 19 | 13 | 10 | 10 | 321732 | 2573852 | 10 | 10 | 0 | 320 | 2614452 | 10 | 10 | 0 | 1 | 328 |
| 201 | C9 | 19 | 13 | 10 | 11 | 321772 | 2574172 | 10 | 11 | 0 | 320 | 2614780 | 10 | 11 | 0 | 1 | 328 |
| 202 | CA | 19 | 13 | 10 | 12 | 321812 | 2574492 | 10 | 12 | 0 | 320 | 2615108 | 10 | 12 | 0 | 1 | 328 |
| 203 | CB | 19 | 13 | 10 | 13 | 321852 | 2574812 | 10 | 13 | 0 | 320 | 2615436 | 10 | 13 | 0 | 1 | 328 |
| 204 | CC | 19 | 13 | 10 | 14 | 321892 | 2575132 | 10 | 14 | 0 | 320 | 2615764 | 10 | 14 | 0 | 1 | 328 |
| 205 | CD | 19 | 13 | 10 | 15 | 321932 | 2575452 | 10 | 15 | 0 | 320 | 2616092 | 10 | 15 | 0 | 1 | 328 |
| 206 | CE | 19 | 13 | 10 | 16 | 321972 | 2575772 | 10 | 16 | 0 | 320 | 2616420 | 10 | 16 | 0 | 1 | 328 |
| 207 | CF | 19 | 13 | 10 | 17 | 322012 | 2576092 | 10 | 17 | 0 | 320 | 2616748 | 10 | 17 | 0 | 1 | 328 |
| 208 | D0 | 19 | 13 | 10 | 18 | 322054 | 2576428 | 10 | 18 | 0 | 336 | 2617092 | 10 | 18 | 0 | 1 | 344 |
| 209 | D1 | 19 | 13 | 11 | 0 | 322098 | 2576780 | 11 | 0 | 0 | 352 | 2617452 | 11 | 0 | 0 | 1 | 360 |
| 210 | D2 | 19 | 13 | 11 | 1 | 322142 | 2577132 | 11 | 1 | 0 | 352 | 2617812 | 11 | 1 | 0 | 1 | 360 |
| 211 | D3 | 19 | 13 | 11 | 2 | 322186 | 2577484 | 11 | 2 | 0 | 352 | 2618172 | 11 | 2 | 0 | 1 | 360 |
| 212 | D4 | 19 | 13 | 11 | 3 | 322230 | 2577836 | 11 | 3 | 0 | 352 | 2618532 | 11 | 3 | 0 | 1 | 360 |
| 213 | D5 | 19 | 13 | 11 | 4 | 322274 | 2578188 | 11 | 4 | 0 | 352 | 2618892 | 11 | 4 | 0 | 1 | 360 |
| 214 | D6 | 19 | 13 | 11 | 5 | 322318 | 2578540 | 11 | 5 | 0 | 352 | 2619252 | 11 | 5 | 0 | 1 | 360 |
| 215 | D7 | 19 | 13 | 11 | 6 | 322362 | 2578892 | 11 | 6 | 0 | 352 | 2619612 | 11 | 6 | 0 | 1 | 360 |
| 216 | D8 | 19 | 13 | 11 | 7 | 322406 | 2579244 | 11 | 7 | 0 | 352 | 2619972 | 11 | 7 | 0 | 1 | 360 |
| 217 | D9 | 19 | 13 | 11 | 8 | 322450 | 2579596 | 11 | 8 | 0 | 352 | 2620332 | 11 | 8 | 0 | 1 | 360 |
| 218 | DA | 19 | 13 | 11 | 9 | 322494 | 2579948 | 11 | 9 | 0 | 352 | 2620692 | 11 | 9 | 0 | 1 | 360 |
| 219 | DB | 19 | 13 | 11 | 10 | 322538 | 2580300 | 11 | 10 | 0 | 352 | 2621052 | 11 | 10 | 0 | 1 | 360 |
| 220 | DC | 19 | 13 | 11 | 11 | 322582 | 2580652 | 11 | 11 | 0 | 352 | 2621412 | 11 | 11 | 0 | 1 | 360 |
| 221 | DD | 19 | 13 | 11 | 12 | 322626 | 2581004 | 11 | 12 | 0 | 352 | 2621772 | 11 | 12 | 0 | 1 | 360 |
| 222 | DE | 19 | 13 | 11 | 13 | 322670 | 2581356 | 11 | 13 | 0 | 352 | 2622132 | 11 | 13 | 0 | 1 | 360 |
| 223 | DF | 19 | 13 | 11 | 14 | 322714 | 2581708 | 11 | 14 | 0 | 352 | 2622492 | 11 | 14 | 0 | 1 | 360 |
| 224 | E0 | 19 | 13 | 11 | 15 | 322756 | 2582044 | 11 | 15 | 0 | 336 | 2622836 | 11 | 15 | 0 | 1 | 344 |
| 225 | E1 | 19 | 13 | 11 | 16 | 322798 | 2582380 | 11 | 16 | 0 | 336 | 2623180 | 11 | 16 | 0 | 1 | 344 |
| 226 | E2 | 19 | 13 | 11 | 17 | 322840 | 2582716 | 11 | 17 | 0 | 336 | 2623524 | 11 | 17 | 0 | 1 | 344 |
| 227 | E3 | 19 | 13 | 11 | 18 | 322882 | 2583052 | 11 | 18 | 0 | 336 | 2623868 | 11 | 18 | 0 | 1 | 344 |
| 228 | E4 | 19 | 13 | 12 | 0 | 322926 | 2583404 | 12 | 0 | 0 | 352 | 2624228 | 12 | 0 | 0 | 1 | 360 |
| 229 | E5 | 19 | 13 | 12 | 1 | 322970 | 2583756 | 12 | 1 | 0 | 352 | 2624588 | 12 | 1 | 0 | 1 | 360 |
| 230 | E6 | 19 | 13 | 12 | 2 | 323014 | 2584108 | 12 | 2 | 0 | 352 | 2624948 | 12 | 2 | 0 | 1 | 360 |
| 231 | E7 | 19 | 13 | 12 | 3 | 323058 | 2584460 | 12 | 3 | 0 | 352 | 2625308 | 12 | 3 | 0 | 1 | 360 |
| 232 | E8 | 19 | 13 | 12 | 4 | 323102 | 2584812 | 12 | 4 | 0 | 352 | 2625668 | 12 | 4 | 0 | 1 | 360 |
| 233 | E9 | 19 | 13 | 12 | 5 | 323146 | 2585164 | 12 | 5 | 0 | 352 | 2626028 | 12 | 5 | 0 | 1 | 360 |
| 234 | EA | 19 | 13 | 12 | 6 | 323190 | 2585516 | 12 | 6 | 0 | 352 | 2626388 | 12 | 6 | 0 | 1 | 360 |
| 235 | EB | 19 | 13 | 12 | 7 | 323234 | 2585868 | 12 | 7 | 0 | 352 | 2626748 | 12 | 7 | 0 | 1 | 360 |
| 236 | EC | 19 | 13 | 12 | 8 | 323278 | 2586220 | 12 | 8 | 0 | 352 | 2627108 | 12 | 8 | 0 | 1 | 360 |
| 237 | ED | 19 | 13 | 12 | 9 | 323322 | 2586572 | 12 | 9 | 0 | 352 | 2627468 | 12 | 9 | 0 | 1 | 360 |
| 238 | EE | 19 | 13 | 12 | 10 | 323366 | 2586924 | 12 | 10 | 0 | 352 | 2627828 | 12 | 10 | 0 | 1 | 360 |
| 239 | EF | 19 | 13 | 12 | 11 | 323410 | 2587276 | 12 | 11 | 0 | 352 | 2628188 | 12 | 11 | 0 | 1 | 360 |
| 240 | F0 | 19 | 13 | 12 | 12 | 323456 | 2587644 | 12 | 12 | 0 | 368 | 2628564 | 12 | 12 | 0 | 1 | 376 |
| 241 | F1 | 19 | 13 | 12 | 13 | 323502 | 2588012 | 12 | 13 | 0 | 368 | 2628940 | 12 | 13 | 0 | 1 | 376 |
| 242 | F2 | 19 | 13 | 12 | 14 | 323548 | 2588380 | 12 | 14 | 0 | 368 | 2629316 | 12 | 14 | 0 | 1 | 376 |
| 243 | F3 | 19 | 13 | 12 | 15 | 323594 | 2588748 | 12 | 15 | 0 | 368 | 2629692 | 12 | 15 | 0 | 1 | 376 |
| 244 | F4 | 19 | 13 | 12 | 16 | 323640 | 2589116 | 12 | 16 | 0 | 368 | 2630068 | 12 | 16 | 0 | 1 | 376 |
| 245 | F5 | 19 | 13 | 12 | 17 | 323686 | 2589484 | 12 | 17 | 0 | 368 | 2630444 | 12 | 17 | 0 | 1 | 376 |
| 246 | F6 | 19 | 13 | 12 | 18 | 323732 | 2589852 | 12 | 18 | 0 | 368 | 2630820 | 12 | 18 | 0 | 1 | 376 |
| 247 | F7 | 19 | 13 | 13 | 0 | 323780 | 2590236 | 13 | 0 | 0 | 384 | 2631212 | 13 | 0 | 0 | 1 | 392 |
| 248 | F8 | 19 | 13 | 13 | 1 | 323828 | 2590620 | 13 | 1 | 0 | 384 | 2631604 | 13 | 1 | 0 | 1 | 392 |
| 249 | F9 | 19 | 13 | 13 | 2 | 323876 | 2591004 | 13 | 2 | 0 | 384 | 2631996 | 13 | 2 | 0 | 1 | 392 |
| 250 | FA | 19 | 13 | 13 | 3 | 323924 | 2591388 | 13 | 3 | 0 | 384 | 2632388 | 13 | 3 | 0 | 1 | 392 |
| 251 | FB | 19 | 13 | 13 | 4 | 323972 | 2591772 | 13 | 4 | 0 | 384 | 2632780 | 13 | 4 | 0 | 1 | 392 |
| 252 | FC | 19 | 13 | 13 | 5 | 324020 | 2592156 | 13 | 5 | 0 | 384 | 2633172 | 13 | 5 | 0 | 1 | 392 |
| 253 | FD | 19 | 13 | 13 | 6 | 324068 | 2592540 | 13 | 6 | 0 | 384 | 2633564 | 13 | 6 | 0 | 1 | 392 |
| 254 | FE | 19 | 13 | 13 | 7 | 324116 | 2592924 | 13 | 7 | 0 | 384 | 2633956 | 13 | 7 | 0 | 1 | 392 |
| 255 | FF | 19 | 13 | 13 | 8 | 324164 | 2593308 | 13 | 8 | 0 | 384 | 2634348 | 13 | 8 | 0 | 1 | 392 |
| 0 | 00 | 20 | 14 | 0 | 0 | 324176 | 2593404 | 0 | 0 | 0 | 96 | 2634452 | 0 | 0 | 0 | 1 | 104 |
| 1 | 01 | 20 | 14 | 0 | 1 | 324190 | 2593516 | 0 | 1 | 0 | 112 | 2634572 | 0 | 1 | 0 | 1 | 120 |
| 2 | 02 | 20 | 14 | 0 | 2 | 324204 | 2593628 | 0 | 2 | 0 | 112 | 2634692 | 0 | 2 | 0 | 1 | 120 |
| 3 | 03 | 20 | 14 | 0 | 3 | 324218 | 2593740 | 0 | 3 | 0 | 112 | 2634812 | 0 | 3 | 0 | 1 | 120 |
| 4 | 04 | 20 | 14 | 0 | 4 | 324232 | 2593852 | 0 | 4 | 0 | 112 | 2634932 | 0 | 4 | 0 | 1 | 120 |
| 5 | 05 | 20 | 14 | 0 | 5 | 324246 | 2593964 | 0 | 5 | 0 | 112 | 2635052 | 0 | 5 | 0 | 1 | 120 |
| 6 | 06 | 20 | 14 | 0 | 6 | 324260 | 2594076 | 0 | 6 | 0 | 112 | 2635172 | 0 | 6 | 0 | 1 | 120 |
| 7 | 07 | 20 | 14 | 0 | 7 | 324274 | 2594188 | 0 | 7 | 0 | 112 | 2635292 | 0 | 7 | 0 | 1 | 120 |
| 8 | 08 | 20 | 14 | 0 | 8 | 324288 | 2594300 | 0 | 8 | 0 | 112 | 2635412 | 0 | 8 | 0 | 1 | 120 |
| 9 | 09 | 20 | 14 | 0 | 9 | 324302 | 2594412 | 0 | 9 | 0 | 112 | 2635532 | 0 | 9 | 0 | 1 | 120 |
| 10 | 0A | 20 | 14 | 0 | 10 | 324316 | 2594524 | 0 | 10 | 0 | 112 | 2635652 | 0 | 10 | 0 | 1 | 120 |
| 11 | 0B | 20 | 14 | 0 | 11 | 324330 | 2594636 | 0 | 11 | 0 | 112 | 2635772 | 0 | 11 | 0 | 1 | 120 |
| 12 | 0C | 20 | 14 | 0 | 12 | 324344 | 2594748 | 0 | 12 | 0 | 112 | 2635892 | 0 | 12 | 0 | 1 | 120 |
| 13 | 0D | 20 | 14 | 0 | 13 | 324358 | 2594860 | 0 | 13 | 0 | 112 | 2636012 | 0 | 13 | 0 | 1 | 120 |
| 14 | 0E | 20 | 14 | 0 | 14 | 324372 | 2594972 | 0 | 14 | 0 | 112 | 2636132 | 0 | 14 | 0 | 1 | 120 |
| 15 | 0F | 20 | 14 | 0 | 15 | 324386 | 2595084 | 0 | 15 | 0 | 112 | 2636252 | 0 | 15 | 0 | 1 | 120 |
| 16 | 10 | 20 | 14 | 0 | 16 | 324400 | 2595196 | 0 | 16 | 0 | 112 | 2636372 | 0 | 16 | 0 | 1 | 120 |
| 17 | 11 | 20 | 14 | 0 | 17 | 324414 | 2595308 | 0 | 17 | 0 | 112 | 2636492 | 0 | 17 | 0 | 1 | 120 |
| 18 | 12 | 20 | 14 | 0 | 18 | 324428 | 2595420 | 0 | 18 | 0 | 112 | 2636612 | 0 | 18 | 0 | 1 | 120 |
| 19 | 13 | 20 | 14 | 0 | 19 | 324442 | 2595532 | 0 | 19 | 0 | 112 | 2636732 | 0 | 19 | 0 | 1 | 120 |
| 20 | 14 | 20 | 14 | 1 | 0 | 324454 | 2595628 | 1 | 0 | 0 | 96 | 2636836 | 1 | 0 | 0 | 1 | 104 |
| 21 | 15 | 20 | 14 | 1 | 1 | 324471 | 2595764 | 1 | 1 | 0 | 136 | 2636980 | 1 | 1 | 0 | 1 | 144 |
| 22 | 16 | 20 | 14 | 1 | 2 | 324488 | 2595900 | 1 | 2 | 0 | 136 | 2637124 | 1 | 2 | 0 | 1 | 144 |
| 23 | 17 | 20 | 14 | 1 | 3 | 324505 | 2596036 | 1 | 3 | 0 | 136 | 2637268 | 1 | 3 | 0 | 1 | 144 |
| 24 | 18 | 20 | 14 | 1 | 4 | 324522 | 2596172 | 1 | 4 | 0 | 136 | 2637412 | 1 | 4 | 0 | 1 | 144 |
| 25 | 19 | 20 | 14 | 1 | 5 | 324539 | 2596308 | 1 | 5 | 0 | 136 | 2637556 | 1 | 5 | 0 | 1 | 144 |
| 26 | 1A | 20 | 14 | 1 | 6 | 324556 | 2596444 | 1 | 6 | 0 | 136 | 2637700 | 1 | 6 | 0 | 1 | 144 |

| | | | | | | | | | | | | | | | | | |
|---|---|---|---|---|---|---|---|---|---|---|---|---|---|---|---|---|---|
| 27 | 1B | 20 | 14 | 1 | 7 | 324573 | 2596580 | 1 | 7 | 0 | 136 | 2637844 | 1 | 7 | 0 | 1 | 144 |
| 28 | 1C | 20 | 14 | 1 | 8 | 324590 | 2596716 | 1 | 8 | 0 | 136 | 2637988 | 1 | 8 | 0 | 1 | 144 |
| 29 | 1D | 20 | 14 | 1 | 9 | 324607 | 2596852 | 1 | 9 | 0 | 136 | 2638132 | 1 | 9 | 0 | 1 | 144 |
| 30 | 1E | 20 | 14 | 1 | 10 | 324624 | 2596988 | 1 | 10 | 0 | 136 | 2638276 | 1 | 10 | 0 | 1 | 144 |
| 31 | 1F | 20 | 14 | 1 | 11 | 324641 | 2597124 | 1 | 11 | 0 | 136 | 2638420 | 1 | 11 | 0 | 1 | 144 |
| 32 | 20 | 20 | 14 | 1 | 12 | 324662 | 2597292 | 1 | 12 | 0 | 168 | 2638596 | 1 | 12 | 0 | 1 | 176 |
| 33 | 21 | 20 | 14 | 1 | 13 | 324683 | 2597460 | 1 | 13 | 0 | 168 | 2638772 | 1 | 13 | 0 | 1 | 176 |
| 34 | 22 | 20 | 14 | 1 | 14 | 324704 | 2597628 | 1 | 14 | 0 | 168 | 2638948 | 1 | 14 | 0 | 1 | 176 |
| 35 | 23 | 20 | 14 | 1 | 15 | 324725 | 2597796 | 1 | 15 | 0 | 168 | 2639124 | 1 | 15 | 0 | 1 | 176 |
| 36 | 24 | 20 | 14 | 1 | 16 | 324746 | 2597964 | 1 | 16 | 0 | 168 | 2639300 | 1 | 16 | 0 | 1 | 176 |
| 37 | 25 | 20 | 14 | 1 | 17 | 324767 | 2598132 | 1 | 17 | 0 | 168 | 2639476 | 1 | 17 | 0 | 1 | 176 |
| 38 | 26 | 20 | 14 | 1 | 18 | 324788 | 2598300 | 1 | 18 | 0 | 168 | 2639652 | 1 | 18 | 0 | 1 | 176 |
| 39 | 27 | 20 | 14 | 1 | 19 | 324809 | 2598468 | 1 | 19 | 0 | 168 | 2639828 | 1 | 19 | 0 | 1 | 176 |
| 40 | 28 | 20 | 14 | 2 | 0 | 324828 | 2598620 | 2 | 0 | 0 | 152 | 2639988 | 2 | 0 | 0 | 1 | 160 |
| 41 | 29 | 20 | 14 | 2 | 1 | 324852 | 2598812 | 2 | 1 | 0 | 192 | 2640188 | 2 | 1 | 0 | 1 | 200 |
| 42 | 2A | 20 | 14 | 2 | 2 | 324876 | 2599004 | 2 | 2 | 0 | 192 | 2640388 | 2 | 2 | 0 | 1 | 200 |
| 43 | 2B | 20 | 14 | 2 | 3 | 324900 | 2599196 | 2 | 3 | 0 | 192 | 2640588 | 2 | 3 | 0 | 1 | 200 |
| 44 | 2C | 20 | 14 | 2 | 4 | 324924 | 2599388 | 2 | 4 | 0 | 192 | 2640788 | 2 | 4 | 0 | 1 | 200 |
| 45 | 2D | 20 | 14 | 2 | 5 | 324948 | 2599580 | 2 | 5 | 0 | 192 | 2640988 | 2 | 5 | 0 | 1 | 200 |
| 46 | 2E | 20 | 14 | 2 | 6 | 324972 | 2599772 | 2 | 6 | 0 | 192 | 2641188 | 2 | 6 | 0 | 1 | 200 |
| 47 | 2F | 20 | 14 | 2 | 7 | 324996 | 2599964 | 2 | 7 | 0 | 192 | 2641388 | 2 | 7 | 0 | 1 | 200 |
| 48 | 30 | 20 | 14 | 2 | 8 | 325022 | 2600172 | 2 | 8 | 0 | 208 | 2641604 | 2 | 8 | 0 | 1 | 216 |
| 49 | 31 | 20 | 14 | 2 | 9 | 325048 | 2600380 | 2 | 9 | 0 | 208 | 2641820 | 2 | 9 | 0 | 1 | 216 |
| 50 | 32 | 20 | 14 | 2 | 10 | 325074 | 2600588 | 2 | 10 | 0 | 208 | 2642036 | 2 | 10 | 0 | 1 | 216 |
| 51 | 33 | 20 | 14 | 2 | 11 | 325100 | 2600796 | 2 | 11 | 0 | 208 | 2642252 | 2 | 11 | 0 | 1 | 216 |
| 52 | 34 | 20 | 14 | 2 | 12 | 325126 | 2601004 | 2 | 12 | 0 | 208 | 2642468 | 2 | 12 | 0 | 1 | 216 |
| 53 | 35 | 20 | 14 | 2 | 13 | 325152 | 2601212 | 2 | 13 | 0 | 208 | 2642684 | 2 | 13 | 0 | 1 | 216 |
| 54 | 36 | 20 | 14 | 2 | 14 | 325178 | 2601420 | 2 | 14 | 0 | 208 | 2642900 | 2 | 14 | 0 | 1 | 216 |
| 55 | 37 | 20 | 14 | 2 | 15 | 325204 | 2601628 | 2 | 15 | 0 | 208 | 2643116 | 2 | 15 | 0 | 1 | 216 |
| 56 | 38 | 20 | 14 | 2 | 16 | 325230 | 2601836 | 2 | 16 | 0 | 208 | 2643332 | 2 | 16 | 0 | 1 | 216 |
| 57 | 39 | 20 | 14 | 2 | 17 | 325256 | 2602044 | 2 | 17 | 0 | 208 | 2643548 | 2 | 17 | 0 | 1 | 216 |
| 58 | 3A | 20 | 14 | 2 | 18 | 325282 | 2602252 | 2 | 18 | 0 | 208 | 2643764 | 2 | 18 | 0 | 1 | 216 |
| 59 | 3B | 20 | 14 | 2 | 19 | 325308 | 2602460 | 2 | 19 | 0 | 208 | 2643980 | 2 | 19 | 0 | 1 | 216 |
| 60 | 3C | 20 | 14 | 3 | 0 | 325336 | 2602684 | 3 | 0 | 0 | 224 | 2644212 | 3 | 0 | 0 | 1 | 232 |
| 61 | 3D | 20 | 14 | 3 | 1 | 325364 | 2602908 | 3 | 1 | 0 | 224 | 2644444 | 3 | 1 | 0 | 1 | 232 |
| 62 | 3E | 20 | 14 | 3 | 2 | 325392 | 2603132 | 3 | 2 | 0 | 224 | 2644676 | 3 | 2 | 0 | 1 | 232 |
| 63 | 3F | 20 | 14 | 3 | 3 | 325420 | 2603356 | 3 | 3 | 0 | 224 | 2644908 | 3 | 3 | 0 | 1 | 232 |
| 64 | 40 | 20 | 14 | 3 | 4 | 325446 | 2603564 | 3 | 4 | 0 | 208 | 2645124 | 3 | 4 | 0 | 1 | 216 |
| 65 | 41 | 20 | 14 | 3 | 5 | 325472 | 2603772 | 3 | 5 | 0 | 208 | 2645340 | 3 | 5 | 0 | 1 | 216 |
| 66 | 42 | 20 | 14 | 3 | 6 | 325498 | 2603980 | 3 | 6 | 0 | 208 | 2645556 | 3 | 6 | 0 | 1 | 216 |
| 67 | 43 | 20 | 14 | 3 | 7 | 325524 | 2604188 | 3 | 7 | 0 | 208 | 2645772 | 3 | 7 | 0 | 1 | 216 |
| 68 | 44 | 20 | 14 | 3 | 8 | 325550 | 2604396 | 3 | 8 | 0 | 208 | 2645988 | 3 | 8 | 0 | 1 | 216 |
| 69 | 45 | 20 | 14 | 3 | 9 | 325576 | 2604604 | 3 | 9 | 0 | 208 | 2646204 | 3 | 9 | 0 | 1 | 216 |
| 70 | 46 | 20 | 14 | 3 | 10 | 325602 | 2604812 | 3 | 10 | 0 | 208 | 2646420 | 3 | 10 | 0 | 1 | 216 |
| 71 | 47 | 20 | 14 | 3 | 11 | 325628 | 2605020 | 3 | 11 | 0 | 208 | 2646636 | 3 | 11 | 0 | 1 | 216 |
| 72 | 48 | 20 | 14 | 3 | 12 | 325654 | 2605228 | 3 | 12 | 0 | 208 | 2646852 | 3 | 12 | 0 | 1 | 216 |
| 73 | 49 | 20 | 14 | 3 | 13 | 325680 | 2605436 | 3 | 13 | 0 | 208 | 2647068 | 3 | 13 | 0 | 1 | 216 |
| 74 | 4A | 20 | 14 | 3 | 14 | 325706 | 2605644 | 3 | 14 | 0 | 208 | 2647284 | 3 | 14 | 0 | 1 | 216 |
| 75 | 4B | 20 | 14 | 3 | 15 | 325732 | 2605852 | 3 | 15 | 0 | 208 | 2647500 | 3 | 15 | 0 | 1 | 216 |
| 76 | 4C | 20 | 14 | 3 | 16 | 325758 | 2606060 | 3 | 16 | 0 | 208 | 2647716 | 3 | 16 | 0 | 1 | 216 |
| 77 | 4D | 20 | 14 | 3 | 17 | 325784 | 2606268 | 3 | 17 | 0 | 208 | 2647932 | 3 | 17 | 0 | 1 | 216 |
| 78 | 4E | 20 | 14 | 3 | 18 | 325810 | 2606476 | 3 | 18 | 0 | 208 | 2648148 | 3 | 18 | 0 | 1 | 216 |
| 79 | 4F | 20 | 14 | 3 | 19 | 325836 | 2606684 | 3 | 19 | 0 | 208 | 2648364 | 3 | 19 | 0 | 1 | 216 |
| 80 | 50 | 20 | 14 | 4 | 0 | 325866 | 2606924 | 4 | 0 | 0 | 240 | 2648612 | 4 | 0 | 0 | 1 | 248 |
| 81 | 51 | 20 | 14 | 4 | 1 | 325896 | 2607164 | 4 | 1 | 0 | 240 | 2648860 | 4 | 1 | 0 | 1 | 248 |
| 82 | 52 | 20 | 14 | 4 | 2 | 325926 | 2607404 | 4 | 2 | 0 | 240 | 2649108 | 4 | 2 | 0 | 1 | 248 |
| 83 | 53 | 20 | 14 | 4 | 3 | 325956 | 2607644 | 4 | 3 | 0 | 240 | 2649356 | 4 | 3 | 0 | 1 | 248 |
| 84 | 54 | 20 | 14 | 4 | 4 | 325986 | 2607884 | 4 | 4 | 0 | 240 | 2649604 | 4 | 4 | 0 | 1 | 248 |
| 85 | 55 | 20 | 14 | 4 | 5 | 326016 | 2608124 | 4 | 5 | 0 | 240 | 2649852 | 4 | 5 | 0 | 1 | 248 |
| 86 | 56 | 20 | 14 | 4 | 6 | 326046 | 2608364 | 4 | 6 | 0 | 240 | 2650100 | 4 | 6 | 0 | 1 | 248 |
| 87 | 57 | 20 | 14 | 4 | 7 | 326076 | 2608604 | 4 | 7 | 0 | 240 | 2650348 | 4 | 7 | 0 | 1 | 248 |
| 88 | 58 | 20 | 14 | 4 | 8 | 326106 | 2608844 | 4 | 8 | 0 | 240 | 2650596 | 4 | 8 | 0 | 1 | 248 |
| 89 | 59 | 20 | 14 | 4 | 9 | 326136 | 2609084 | 4 | 9 | 0 | 240 | 2650844 | 4 | 9 | 0 | 1 | 248 |
| 90 | 5A | 20 | 14 | 4 | 10 | 326166 | 2609324 | 4 | 10 | 0 | 240 | 2651092 | 4 | 10 | 0 | 1 | 248 |
| 91 | 5B | 20 | 14 | 4 | 11 | 326196 | 2609564 | 4 | 11 | 0 | 240 | 2651340 | 4 | 11 | 0 | 1 | 248 |
| 92 | 5C | 20 | 14 | 4 | 12 | 326226 | 2609804 | 4 | 12 | 0 | 240 | 2651588 | 4 | 12 | 0 | 1 | 248 |
| 93 | 5D | 20 | 14 | 4 | 13 | 326256 | 2610044 | 4 | 13 | 0 | 240 | 2651836 | 4 | 13 | 0 | 1 | 248 |
| 94 | 5E | 20 | 14 | 4 | 14 | 326286 | 2610284 | 4 | 14 | 0 | 240 | 2652084 | 4 | 14 | 0 | 1 | 248 |
| 95 | 5F | 20 | 14 | 4 | 15 | 326316 | 2610524 | 4 | 15 | 0 | 240 | 2652332 | 4 | 15 | 0 | 1 | 248 |
| 96 | 60 | 20 | 14 | 4 | 16 | 326344 | 2610748 | 4 | 16 | 0 | 224 | 2652564 | 4 | 16 | 0 | 1 | 232 |
| 97 | 61 | 20 | 14 | 4 | 17 | 326372 | 2610972 | 4 | 17 | 0 | 224 | 2652796 | 4 | 17 | 0 | 1 | 232 |
| 98 | 62 | 20 | 14 | 4 | 18 | 326400 | 2611196 | 4 | 18 | 0 | 224 | 2653028 | 4 | 18 | 0 | 1 | 232 |
| 99 | 63 | 20 | 14 | 4 | 19 | 326428 | 2611420 | 4 | 19 | 0 | 224 | 2653260 | 4 | 19 | 0 | 1 | 232 |
| 100 | 64 | 20 | 14 | 5 | 0 | 326458 | 2611660 | 5 | 0 | 0 | 240 | 2653508 | 5 | 0 | 0 | 1 | 248 |
| 101 | 65 | 20 | 14 | 5 | 1 | 326488 | 2611900 | 5 | 1 | 0 | 240 | 2653756 | 5 | 1 | 0 | 1 | 248 |
| 102 | 66 | 20 | 14 | 5 | 2 | 326518 | 2612140 | 5 | 2 | 0 | 240 | 2654004 | 5 | 2 | 0 | 1 | 248 |
| 103 | 67 | 20 | 14 | 5 | 3 | 326548 | 2612380 | 5 | 3 | 0 | 240 | 2654252 | 5 | 3 | 0 | 1 | 248 |
| 104 | 68 | 20 | 14 | 5 | 4 | 326578 | 2612620 | 5 | 4 | 0 | 240 | 2654500 | 5 | 4 | 0 | 1 | 248 |
| 105 | 69 | 20 | 14 | 5 | 5 | 326608 | 2612860 | 5 | 5 | 0 | 240 | 2654748 | 5 | 5 | 0 | 1 | 248 |
| 106 | 6A | 20 | 14 | 5 | 6 | 326638 | 2613100 | 5 | 6 | 0 | 240 | 2654996 | 5 | 6 | 0 | 1 | 248 |
| 107 | 6B | 20 | 14 | 5 | 7 | 326668 | 2613340 | 5 | 7 | 0 | 240 | 2655244 | 5 | 7 | 0 | 1 | 248 |
| 108 | 6C | 20 | 14 | 5 | 8 | 326698 | 2613580 | 5 | 8 | 0 | 240 | 2655492 | 5 | 8 | 0 | 1 | 248 |
| 109 | 6D | 20 | 14 | 5 | 9 | 326728 | 2613820 | 5 | 9 | 0 | 240 | 2655740 | 5 | 9 | 0 | 1 | 248 |
| 110 | 6E | 20 | 14 | 5 | 10 | 326758 | 2614060 | 5 | 10 | 0 | 240 | 2655988 | 5 | 10 | 0 | 1 | 248 |
| 111 | 6F | 20 | 14 | 5 | 11 | 326788 | 2614300 | 5 | 11 | 0 | 240 | 2656236 | 5 | 11 | 0 | 1 | 248 |
| 112 | 70 | 20 | 14 | 5 | 12 | 326820 | 2614556 | 5 | 12 | 0 | 256 | 2656500 | 5 | 12 | 0 | 1 | 264 |
| 113 | 71 | 20 | 14 | 5 | 13 | 326852 | 2614812 | 5 | 13 | 0 | 256 | 2656764 | 5 | 13 | 0 | 1 | 264 |
| 114 | 72 | 20 | 14 | 5 | 14 | 326884 | 2615068 | 5 | 14 | 0 | 256 | 2657028 | 5 | 14 | 0 | 1 | 264 |
| 115 | 73 | 20 | 14 | 5 | 15 | 326916 | 2615324 | 5 | 15 | 0 | 256 | 2657292 | 5 | 15 | 0 | 1 | 264 |
| 116 | 74 | 20 | 14 | 5 | 16 | 326948 | 2615580 | 5 | 16 | 0 | 256 | 2657556 | 5 | 16 | 0 | 1 | 264 |
| 117 | 75 | 20 | 14 | 5 | 17 | 326980 | 2615836 | 5 | 17 | 0 | 256 | 2657820 | 5 | 17 | 0 | 1 | 264 |
| 118 | 76 | 20 | 14 | 5 | 18 | 327012 | 2616092 | 5 | 18 | 0 | 256 | 2658084 | 5 | 18 | 0 | 1 | 264 |
| 119 | 77 | 20 | 14 | 5 | 19 | 327044 | 2616348 | 5 | 19 | 0 | 256 | 2658348 | 5 | 19 | 0 | 1 | 264 |
| 120 | 78 | 20 | 14 | 6 | 0 | 327078 | 2616620 | 6 | 0 | 0 | 272 | 2658628 | 6 | 0 | 0 | 1 | 280 |
| 121 | 79 | 20 | 14 | 6 | 1 | 327112 | 2616892 | 6 | 1 | 0 | 272 | 2658908 | 6 | 1 | 0 | 1 | 280 |
| 122 | 7A | 20 | 14 | 6 | 2 | 327146 | 2617164 | 6 | 2 | 0 | 272 | 2659188 | 6 | 2 | 0 | 1 | 280 |
| 123 | 7B | 20 | 14 | 6 | 3 | 327180 | 2617436 | 6 | 3 | 0 | 272 | 2659468 | 6 | 3 | 0 | 1 | 280 |
| 124 | 7C | 20 | 14 | 6 | 4 | 327214 | 2617708 | 6 | 4 | 0 | 272 | 2659748 | 6 | 4 | 0 | 1 | 280 |
| 125 | 7D | 20 | 14 | 6 | 5 | 327248 | 2617980 | 6 | 5 | 0 | 272 | 2660028 | 6 | 5 | 0 | 1 | 280 |
| 126 | 7E | 20 | 14 | 6 | 6 | 327282 | 2618252 | 6 | 6 | 0 | 272 | 2660308 | 6 | 6 | 0 | 1 | 280 |
| 127 | 7F | 20 | 14 | 6 | 7 | 327316 | 2618524 | 6 | 7 | 0 | 272 | 2660588 | 6 | 7 | 0 | 1 | 280 |
| 128 | 80 | 20 | 14 | 6 | 8 | 327348 | 2618780 | 6 | 8 | 0 | 256 | 2660852 | 6 | 8 | 0 | 1 | 264 |
| 129 | 81 | 20 | 14 | 6 | 9 | 327380 | 2619036 | 6 | 9 | 0 | 256 | 2661116 | 6 | 9 | 0 | 1 | 264 |

| | | | | | | | | | | | | | | | | | |
|---|---|---|---|---|---|---|---|---|---|---|---|---|---|---|---|---|---|
| 130 | 82 | 20 | 14 | 6 | 10 | 327412 | 2619292 | 6 | 10 | 0 | 256 | 2661380 | 6 | 10 | 0 | 1 | 264 |
| 131 | 83 | 20 | 14 | 6 | 11 | 327444 | 2619548 | 6 | 11 | 0 | 256 | 2661644 | 6 | 11 | 0 | 1 | 264 |
| 132 | 84 | 20 | 14 | 6 | 12 | 327476 | 2619804 | 6 | 12 | 0 | 256 | 2661908 | 6 | 12 | 0 | 1 | 264 |
| 133 | 85 | 20 | 14 | 6 | 13 | 327508 | 2620060 | 6 | 13 | 0 | 256 | 2662172 | 6 | 13 | 0 | 1 | 264 |
| 134 | 86 | 20 | 14 | 6 | 14 | 327540 | 2620316 | 6 | 14 | 0 | 256 | 2662436 | 6 | 14 | 0 | 1 | 264 |
| 135 | 87 | 20 | 14 | 6 | 15 | 327572 | 2620572 | 6 | 15 | 0 | 256 | 2662700 | 6 | 15 | 0 | 1 | 264 |
| 136 | 88 | 20 | 14 | 6 | 16 | 327604 | 2620828 | 6 | 16 | 0 | 256 | 2662964 | 6 | 16 | 0 | 1 | 264 |
| 137 | 89 | 20 | 14 | 6 | 17 | 327636 | 2621084 | 6 | 17 | 0 | 256 | 2663228 | 6 | 17 | 0 | 1 | 264 |
| 138 | 8A | 20 | 14 | 6 | 18 | 327668 | 2621340 | 6 | 18 | 0 | 256 | 2663492 | 6 | 18 | 0 | 1 | 264 |
| 139 | 8B | 20 | 14 | 6 | 19 | 327700 | 2621596 | 6 | 19 | 0 | 256 | 2663756 | 6 | 19 | 0 | 1 | 264 |
| 140 | 8C | 20 | 14 | 7 | 0 | 327734 | 2621868 | 7 | 0 | 0 | 272 | 2664036 | 7 | 0 | 0 | 1 | 280 |
| 141 | 8D | 20 | 14 | 7 | 1 | 327768 | 2622140 | 7 | 1 | 0 | 272 | 2664316 | 7 | 1 | 0 | 1 | 280 |
| 142 | 8E | 20 | 14 | 7 | 2 | 327802 | 2622412 | 7 | 2 | 0 | 272 | 2664596 | 7 | 2 | 0 | 1 | 280 |
| 143 | 8F | 20 | 14 | 7 | 3 | 327836 | 2622684 | 7 | 3 | 0 | 272 | 2664876 | 7 | 3 | 0 | 1 | 280 |
| 144 | 90 | 20 | 14 | 7 | 4 | 327872 | 2622972 | 7 | 4 | 0 | 288 | 2665172 | 7 | 4 | 0 | 1 | 296 |
| 145 | 91 | 20 | 14 | 7 | 5 | 327908 | 2623260 | 7 | 5 | 0 | 288 | 2665468 | 7 | 5 | 0 | 1 | 296 |
| 146 | 92 | 20 | 14 | 7 | 6 | 327944 | 2623548 | 7 | 6 | 0 | 288 | 2665764 | 7 | 6 | 0 | 1 | 296 |
| 147 | 93 | 20 | 14 | 7 | 7 | 327980 | 2623836 | 7 | 7 | 0 | 288 | 2666060 | 7 | 7 | 0 | 1 | 296 |
| 148 | 94 | 20 | 14 | 7 | 8 | 328016 | 2624124 | 7 | 8 | 0 | 288 | 2666356 | 7 | 8 | 0 | 1 | 296 |
| 149 | 95 | 20 | 14 | 7 | 9 | 328052 | 2624412 | 7 | 9 | 0 | 288 | 2666652 | 7 | 9 | 0 | 1 | 296 |
| 150 | 96 | 20 | 14 | 7 | 10 | 328088 | 2624700 | 7 | 10 | 0 | 288 | 2666948 | 7 | 10 | 0 | 1 | 296 |
| 151 | 97 | 20 | 14 | 7 | 11 | 328124 | 2624988 | 7 | 11 | 0 | 288 | 2667244 | 7 | 11 | 0 | 1 | 296 |
| 152 | 98 | 20 | 14 | 7 | 12 | 328160 | 2625276 | 7 | 12 | 0 | 288 | 2667540 | 7 | 12 | 0 | 1 | 296 |
| 153 | 99 | 20 | 14 | 7 | 13 | 328196 | 2625564 | 7 | 13 | 0 | 288 | 2667836 | 7 | 13 | 0 | 1 | 296 |
| 154 | 9A | 20 | 14 | 7 | 14 | 328232 | 2625852 | 7 | 14 | 0 | 288 | 2668132 | 7 | 14 | 0 | 1 | 296 |
| 155 | 9B | 20 | 14 | 7 | 15 | 328268 | 2626140 | 7 | 15 | 0 | 288 | 2668428 | 7 | 15 | 0 | 1 | 296 |
| 156 | 9C | 20 | 14 | 7 | 16 | 328304 | 2626428 | 7 | 16 | 0 | 288 | 2668724 | 7 | 16 | 0 | 1 | 296 |
| 157 | 9D | 20 | 14 | 7 | 17 | 328340 | 2626716 | 7 | 17 | 0 | 288 | 2669020 | 7 | 17 | 0 | 1 | 296 |
| 158 | 9E | 20 | 14 | 7 | 18 | 328376 | 2627004 | 7 | 18 | 0 | 288 | 2669316 | 7 | 18 | 0 | 1 | 296 |
| 159 | 9F | 20 | 14 | 7 | 19 | 328412 | 2627292 | 7 | 19 | 0 | 288 | 2669612 | 7 | 19 | 0 | 1 | 296 |
| 160 | A0 | 20 | 14 | 8 | 0 | 328448 | 2627580 | 8 | 0 | 0 | 288 | 2669908 | 8 | 0 | 0 | 1 | 296 |
| 161 | A1 | 20 | 14 | 8 | 1 | 328484 | 2627868 | 8 | 1 | 0 | 288 | 2670204 | 8 | 1 | 0 | 1 | 296 |
| 162 | A2 | 20 | 14 | 8 | 2 | 328520 | 2628156 | 8 | 2 | 0 | 288 | 2670500 | 8 | 2 | 0 | 1 | 296 |
| 163 | A3 | 20 | 14 | 8 | 3 | 328556 | 2628444 | 8 | 3 | 0 | 288 | 2670796 | 8 | 3 | 0 | 1 | 296 |
| 164 | A4 | 20 | 14 | 8 | 4 | 328592 | 2628732 | 8 | 4 | 0 | 288 | 2671092 | 8 | 4 | 0 | 1 | 296 |
| 165 | A5 | 20 | 14 | 8 | 5 | 328628 | 2629020 | 8 | 5 | 0 | 288 | 2671388 | 8 | 5 | 0 | 1 | 296 |
| 166 | A6 | 20 | 14 | 8 | 6 | 328664 | 2629308 | 8 | 6 | 0 | 288 | 2671684 | 8 | 6 | 0 | 1 | 296 |
| 167 | A7 | 20 | 14 | 8 | 7 | 328700 | 2629596 | 8 | 7 | 0 | 288 | 2671980 | 8 | 7 | 0 | 1 | 296 |
| 168 | A8 | 20 | 14 | 8 | 8 | 328736 | 2629884 | 8 | 8 | 0 | 288 | 2672276 | 8 | 8 | 0 | 1 | 296 |
| 169 | A9 | 20 | 14 | 8 | 9 | 328772 | 2630172 | 8 | 9 | 0 | 288 | 2672572 | 8 | 9 | 0 | 1 | 296 |
| 170 | AA | 20 | 14 | 8 | 10 | 328808 | 2630460 | 8 | 10 | 0 | 288 | 2672868 | 8 | 10 | 0 | 1 | 296 |
| 171 | AB | 20 | 14 | 8 | 11 | 328844 | 2630748 | 8 | 11 | 0 | 288 | 2673164 | 8 | 11 | 0 | 1 | 296 |
| 172 | AC | 20 | 14 | 8 | 12 | 328880 | 2631036 | 8 | 12 | 0 | 288 | 2673460 | 8 | 12 | 0 | 1 | 296 |
| 173 | AD | 20 | 14 | 8 | 13 | 328916 | 2631324 | 8 | 13 | 0 | 288 | 2673756 | 8 | 13 | 0 | 1 | 296 |
| 174 | AE | 20 | 14 | 8 | 14 | 328952 | 2631612 | 8 | 14 | 0 | 288 | 2674052 | 8 | 14 | 0 | 1 | 296 |
| 175 | AF | 20 | 14 | 8 | 15 | 328988 | 2631900 | 8 | 15 | 0 | 288 | 2674348 | 8 | 15 | 0 | 1 | 296 |
| 176 | B0 | 20 | 14 | 8 | 16 | 329026 | 2632204 | 8 | 16 | 0 | 304 | 2674660 | 8 | 16 | 0 | 1 | 312 |
| 177 | B1 | 20 | 14 | 8 | 17 | 329064 | 2632508 | 8 | 17 | 0 | 304 | 2674972 | 8 | 17 | 0 | 1 | 312 |
| 178 | B2 | 20 | 14 | 8 | 18 | 329102 | 2632812 | 8 | 18 | 0 | 304 | 2675284 | 8 | 18 | 0 | 1 | 312 |
| 179 | B3 | 20 | 14 | 8 | 19 | 329140 | 2633116 | 8 | 19 | 0 | 304 | 2675596 | 8 | 19 | 0 | 1 | 312 |
| 180 | B4 | 20 | 14 | 9 | 0 | 329180 | 2633436 | 9 | 0 | 0 | 320 | 2675924 | 9 | 0 | 0 | 1 | 328 |
| 181 | B5 | 20 | 14 | 9 | 1 | 329220 | 2633756 | 9 | 1 | 0 | 320 | 2676252 | 9 | 1 | 0 | 1 | 328 |
| 182 | B6 | 20 | 14 | 9 | 2 | 329260 | 2634076 | 9 | 2 | 0 | 320 | 2676580 | 9 | 2 | 0 | 1 | 328 |
| 183 | B7 | 20 | 14 | 9 | 3 | 329300 | 2634396 | 9 | 3 | 0 | 320 | 2676908 | 9 | 3 | 0 | 1 | 328 |
| 184 | B8 | 20 | 14 | 9 | 4 | 329340 | 2634716 | 9 | 4 | 0 | 320 | 2677236 | 9 | 4 | 0 | 1 | 328 |
| 185 | B9 | 20 | 14 | 9 | 5 | 329380 | 2635036 | 9 | 5 | 0 | 320 | 2677564 | 9 | 5 | 0 | 1 | 328 |
| 186 | BA | 20 | 14 | 9 | 6 | 329420 | 2635356 | 9 | 6 | 0 | 320 | 2677892 | 9 | 6 | 0 | 1 | 328 |
| 187 | BB | 20 | 14 | 9 | 7 | 329460 | 2635676 | 9 | 7 | 0 | 320 | 2678220 | 9 | 7 | 0 | 1 | 328 |
| 188 | BC | 20 | 14 | 9 | 8 | 329500 | 2635996 | 9 | 8 | 0 | 320 | 2678548 | 9 | 8 | 0 | 1 | 328 |
| 189 | BD | 20 | 14 | 9 | 9 | 329540 | 2636316 | 9 | 9 | 0 | 320 | 2678876 | 9 | 9 | 0 | 1 | 328 |
| 190 | BE | 20 | 14 | 9 | 10 | 329580 | 2636636 | 9 | 10 | 0 | 320 | 2679204 | 9 | 10 | 0 | 1 | 328 |
| 191 | BF | 20 | 14 | 9 | 11 | 329620 | 2636956 | 9 | 11 | 0 | 320 | 2679532 | 9 | 11 | 0 | 1 | 328 |
| 192 | C0 | 20 | 14 | 9 | 12 | 329658 | 2637260 | 9 | 12 | 0 | 304 | 2679844 | 9 | 12 | 0 | 1 | 312 |
| 193 | C1 | 20 | 14 | 9 | 13 | 329696 | 2637564 | 9 | 13 | 0 | 304 | 2680156 | 9 | 13 | 0 | 1 | 312 |
| 194 | C2 | 20 | 14 | 9 | 14 | 329734 | 2637868 | 9 | 14 | 0 | 304 | 2680468 | 9 | 14 | 0 | 1 | 312 |
| 195 | C3 | 20 | 14 | 9 | 15 | 329772 | 2638172 | 9 | 15 | 0 | 304 | 2680780 | 9 | 15 | 0 | 1 | 312 |
| 196 | C4 | 20 | 14 | 9 | 16 | 329810 | 2638476 | 9 | 16 | 0 | 304 | 2681092 | 9 | 16 | 0 | 1 | 312 |
| 197 | C5 | 20 | 14 | 9 | 17 | 329848 | 2638780 | 9 | 17 | 0 | 304 | 2681404 | 9 | 17 | 0 | 1 | 312 |
| 198 | C6 | 20 | 14 | 9 | 18 | 329886 | 2639084 | 9 | 18 | 0 | 304 | 2681716 | 9 | 18 | 0 | 1 | 312 |
| 199 | C7 | 20 | 14 | 9 | 19 | 329924 | 2639388 | 9 | 19 | 0 | 304 | 2682028 | 9 | 19 | 0 | 1 | 312 |
| 200 | C8 | 20 | 14 | 10 | 0 | 329964 | 2639708 | 10 | 0 | 0 | 320 | 2682356 | 10 | 0 | 0 | 1 | 328 |
| 201 | C9 | 20 | 14 | 10 | 1 | 330004 | 2640028 | 10 | 1 | 0 | 320 | 2682684 | 10 | 1 | 0 | 1 | 328 |
| 202 | CA | 20 | 14 | 10 | 2 | 330044 | 2640348 | 10 | 2 | 0 | 320 | 2683012 | 10 | 2 | 0 | 1 | 328 |
| 203 | CB | 20 | 14 | 10 | 3 | 330084 | 2640668 | 10 | 3 | 0 | 320 | 2683340 | 10 | 3 | 0 | 1 | 328 |
| 204 | CC | 20 | 14 | 10 | 4 | 330124 | 2640988 | 10 | 4 | 0 | 320 | 2683668 | 10 | 4 | 0 | 1 | 328 |
| 205 | CD | 20 | 14 | 10 | 5 | 330164 | 2641308 | 10 | 5 | 0 | 320 | 2683996 | 10 | 5 | 0 | 1 | 328 |
| 206 | CE | 20 | 14 | 10 | 6 | 330204 | 2641628 | 10 | 6 | 0 | 320 | 2684324 | 10 | 6 | 0 | 1 | 328 |
| 207 | CF | 20 | 14 | 10 | 7 | 330244 | 2641948 | 10 | 7 | 0 | 320 | 2684652 | 10 | 7 | 0 | 1 | 328 |
| 208 | D0 | 20 | 14 | 10 | 8 | 330286 | 2642284 | 10 | 8 | 0 | 336 | 2684996 | 10 | 8 | 0 | 1 | 344 |
| 209 | D1 | 20 | 14 | 10 | 9 | 330328 | 2642620 | 10 | 9 | 0 | 336 | 2685340 | 10 | 9 | 0 | 1 | 344 |
| 210 | D2 | 20 | 14 | 10 | 10 | 330370 | 2642956 | 10 | 10 | 0 | 336 | 2685684 | 10 | 10 | 0 | 1 | 344 |
| 211 | D3 | 20 | 14 | 10 | 11 | 330412 | 2643292 | 10 | 11 | 0 | 336 | 2686028 | 10 | 11 | 0 | 1 | 344 |
| 212 | D4 | 20 | 14 | 10 | 12 | 330454 | 2643628 | 10 | 12 | 0 | 336 | 2686372 | 10 | 12 | 0 | 1 | 344 |
| 213 | D5 | 20 | 14 | 10 | 13 | 330496 | 2643964 | 10 | 13 | 0 | 336 | 2686716 | 10 | 13 | 0 | 1 | 344 |
| 214 | D6 | 20 | 14 | 10 | 14 | 330538 | 2644300 | 10 | 14 | 0 | 336 | 2687060 | 10 | 14 | 0 | 1 | 344 |
| 215 | D7 | 20 | 14 | 10 | 15 | 330580 | 2644636 | 10 | 15 | 0 | 336 | 2687404 | 10 | 15 | 0 | 1 | 344 |
| 216 | D8 | 20 | 14 | 10 | 16 | 330622 | 2644972 | 10 | 16 | 0 | 336 | 2687748 | 10 | 16 | 0 | 1 | 344 |
| 217 | D9 | 20 | 14 | 10 | 17 | 330664 | 2645308 | 10 | 17 | 0 | 336 | 2688092 | 10 | 17 | 0 | 1 | 344 |
| 218 | DA | 20 | 14 | 10 | 18 | 330706 | 2645644 | 10 | 18 | 0 | 336 | 2688436 | 10 | 18 | 0 | 1 | 344 |
| 219 | DB | 20 | 14 | 10 | 19 | 330748 | 2645980 | 10 | 19 | 0 | 336 | 2688780 | 10 | 19 | 0 | 1 | 344 |
| 220 | DC | 20 | 14 | 11 | 0 | 330792 | 2646332 | 11 | 0 | 0 | 352 | 2689140 | 11 | 0 | 0 | 1 | 360 |
| 221 | DD | 20 | 14 | 11 | 1 | 330836 | 2646684 | 11 | 1 | 0 | 352 | 2689500 | 11 | 1 | 0 | 1 | 360 |
| 222 | DE | 20 | 14 | 11 | 2 | 330880 | 2647036 | 11 | 2 | 0 | 352 | 2689860 | 11 | 2 | 0 | 1 | 360 |
| 223 | DF | 20 | 14 | 11 | 3 | 330924 | 2647388 | 11 | 3 | 0 | 352 | 2690220 | 11 | 3 | 0 | 1 | 360 |
| 224 | E0 | 20 | 14 | 11 | 4 | 330966 | 2647724 | 11 | 4 | 0 | 336 | 2690564 | 11 | 4 | 0 | 1 | 344 |
| 225 | E1 | 20 | 14 | 11 | 5 | 331008 | 2648060 | 11 | 5 | 0 | 336 | 2690908 | 11 | 5 | 0 | 1 | 344 |
| 226 | E2 | 20 | 14 | 11 | 6 | 331050 | 2648396 | 11 | 6 | 0 | 336 | 2691252 | 11 | 6 | 0 | 1 | 344 |
| 227 | E3 | 20 | 14 | 11 | 7 | 331092 | 2648732 | 11 | 7 | 0 | 336 | 2691596 | 11 | 7 | 0 | 1 | 344 |
| 228 | E4 | 20 | 14 | 11 | 8 | 331134 | 2649068 | 11 | 8 | 0 | 336 | 2691940 | 11 | 8 | 0 | 1 | 344 |
| 229 | E5 | 20 | 14 | 11 | 9 | 331176 | 2649404 | 11 | 9 | 0 | 336 | 2692284 | 11 | 9 | 0 | 1 | 344 |
| 230 | E6 | 20 | 14 | 11 | 10 | 331218 | 2649740 | 11 | 10 | 0 | 336 | 2692628 | 11 | 10 | 0 | 1 | 344 |
| 231 | E7 | 20 | 14 | 11 | 11 | 331260 | 2650076 | 11 | 11 | 0 | 336 | 2692972 | 11 | 11 | 0 | 1 | 344 |
| 232 | E8 | 20 | 14 | 11 | 12 | 331302 | 2650412 | 11 | 12 | 0 | 336 | 2693316 | 11 | 12 | 0 | 1 | 344 |

| | | | | | | | | | | | | | | | | | |
|---|---|---|---|---|---|---|---|---|---|---|---|---|---|---|---|---|---|
| 233 | E9 | 20 | 14 | 11 | 13 | 331344 | 2650748 | 11 | 13 | 0 | 336 | 2693660 | 11 | 13 | 0 | 1 | 344 |
| 234 | EA | 20 | 14 | 11 | 14 | 331386 | 2651084 | 11 | 14 | 0 | 336 | 2694004 | 11 | 14 | 0 | 1 | 344 |
| 235 | EB | 20 | 14 | 11 | 15 | 331428 | 2651420 | 11 | 15 | 0 | 336 | 2694348 | 11 | 15 | 0 | 1 | 344 |
| 236 | EC | 20 | 14 | 11 | 16 | 331470 | 2651756 | 11 | 16 | 0 | 336 | 2694692 | 11 | 16 | 0 | 1 | 344 |
| 237 | ED | 20 | 14 | 11 | 17 | 331512 | 2652092 | 11 | 17 | 0 | 336 | 2695036 | 11 | 17 | 0 | 1 | 344 |
| 238 | EE | 20 | 14 | 11 | 18 | 331554 | 2652428 | 11 | 18 | 0 | 336 | 2695380 | 11 | 18 | 0 | 1 | 344 |
| 239 | EF | 20 | 14 | 11 | 19 | 331596 | 2652764 | 11 | 19 | 0 | 336 | 2695724 | 11 | 19 | 0 | 1 | 344 |
| 240 | F0 | 20 | 14 | 12 | 0 | 331642 | 2653132 | 12 | 0 | 0 | 368 | 2696100 | 12 | 0 | 0 | 1 | 376 |
| 241 | F1 | 20 | 14 | 12 | 1 | 331688 | 2653500 | 12 | 1 | 0 | 368 | 2696476 | 12 | 1 | 0 | 1 | 376 |
| 242 | F2 | 20 | 14 | 12 | 2 | 331734 | 2653868 | 12 | 2 | 0 | 368 | 2696852 | 12 | 2 | 0 | 1 | 376 |
| 243 | F3 | 20 | 14 | 12 | 3 | 331780 | 2654236 | 12 | 3 | 0 | 368 | 2697228 | 12 | 3 | 0 | 1 | 376 |
| 244 | F4 | 20 | 14 | 12 | 4 | 331826 | 2654604 | 12 | 4 | 0 | 368 | 2697604 | 12 | 4 | 0 | 1 | 376 |
| 245 | F5 | 20 | 14 | 12 | 5 | 331872 | 2654972 | 12 | 5 | 0 | 368 | 2697980 | 12 | 5 | 0 | 1 | 376 |
| 246 | F6 | 20 | 14 | 12 | 6 | 331918 | 2655340 | 12 | 6 | 0 | 368 | 2698356 | 12 | 6 | 0 | 1 | 376 |
| 247 | F7 | 20 | 14 | 12 | 7 | 331964 | 2655708 | 12 | 7 | 0 | 368 | 2698732 | 12 | 7 | 0 | 1 | 376 |
| 248 | F8 | 20 | 14 | 12 | 8 | 332010 | 2656076 | 12 | 8 | 0 | 368 | 2699108 | 12 | 8 | 0 | 1 | 376 |
| 249 | F9 | 20 | 14 | 12 | 9 | 332056 | 2656444 | 12 | 9 | 0 | 368 | 2699484 | 12 | 9 | 0 | 1 | 376 |
| 250 | FA | 20 | 14 | 12 | 10 | 332102 | 2656812 | 12 | 10 | 0 | 368 | 2699860 | 12 | 10 | 0 | 1 | 376 |
| 251 | FB | 20 | 14 | 12 | 11 | 332148 | 2657180 | 12 | 11 | 0 | 368 | 2700236 | 12 | 11 | 0 | 1 | 376 |
| 252 | FC | 20 | 14 | 12 | 12 | 332194 | 2657548 | 12 | 12 | 0 | 368 | 2700612 | 12 | 12 | 0 | 1 | 376 |
| 253 | FD | 20 | 14 | 12 | 13 | 332240 | 2657916 | 12 | 13 | 0 | 368 | 2700988 | 12 | 13 | 0 | 1 | 376 |
| 254 | FE | 20 | 14 | 12 | 14 | 332286 | 2658284 | 12 | 14 | 0 | 368 | 2701364 | 12 | 14 | 0 | 1 | 376 |
| 255 | FF | 20 | 14 | 12 | 15 | 332332 | 2658652 | 12 | 15 | 0 | 368 | 2701740 | 12 | 15 | 0 | 1 | 376 |
| 0 | 00 | 21 | 15 | 0 | 0 | 332344 | 2658748 | 0 | 0 | 0 | 96 | 2701844 | 0 | 0 | 0 | 1 | 104 |
| 1 | 01 | 21 | 15 | 0 | 1 | 332358 | 2658860 | 0 | 1 | 0 | 112 | 2701964 | 0 | 1 | 0 | 1 | 120 |
| 2 | 02 | 21 | 15 | 0 | 2 | 332372 | 2658972 | 0 | 2 | 0 | 112 | 2702084 | 0 | 2 | 0 | 1 | 120 |
| 3 | 03 | 21 | 15 | 0 | 3 | 332386 | 2659084 | 0 | 3 | 0 | 112 | 2702204 | 0 | 3 | 0 | 1 | 120 |
| 4 | 04 | 21 | 15 | 0 | 4 | 332400 | 2659196 | 0 | 4 | 0 | 112 | 2702324 | 0 | 4 | 0 | 1 | 120 |
| 5 | 05 | 21 | 15 | 0 | 5 | 332414 | 2659308 | 0 | 5 | 0 | 112 | 2702444 | 0 | 5 | 0 | 1 | 120 |
| 6 | 06 | 21 | 15 | 0 | 6 | 332428 | 2659420 | 0 | 6 | 0 | 112 | 2702564 | 0 | 6 | 0 | 1 | 120 |
| 7 | 07 | 21 | 15 | 0 | 7 | 332442 | 2659532 | 0 | 7 | 0 | 112 | 2702684 | 0 | 7 | 0 | 1 | 120 |
| 8 | 08 | 21 | 15 | 0 | 8 | 332456 | 2659644 | 0 | 8 | 0 | 112 | 2702804 | 0 | 8 | 0 | 1 | 120 |
| 9 | 09 | 21 | 15 | 0 | 9 | 332470 | 2659756 | 0 | 9 | 0 | 112 | 2702924 | 0 | 9 | 0 | 1 | 120 |
| 10 | 0A | 21 | 15 | 0 | 10 | 332484 | 2659868 | 0 | 10 | 0 | 112 | 2703044 | 0 | 10 | 0 | 1 | 120 |
| 11 | 0B | 21 | 15 | 0 | 11 | 332498 | 2659980 | 0 | 11 | 0 | 112 | 2703164 | 0 | 11 | 0 | 1 | 120 |
| 12 | 0C | 21 | 15 | 0 | 12 | 332512 | 2660092 | 0 | 12 | 0 | 112 | 2703284 | 0 | 12 | 0 | 1 | 120 |
| 13 | 0D | 21 | 15 | 0 | 13 | 332526 | 2660204 | 0 | 13 | 0 | 112 | 2703404 | 0 | 13 | 0 | 1 | 120 |
| 14 | 0E | 21 | 15 | 0 | 14 | 332540 | 2660316 | 0 | 14 | 0 | 112 | 2703524 | 0 | 14 | 0 | 1 | 120 |
| 15 | 0F | 21 | 15 | 0 | 15 | 332554 | 2660428 | 0 | 15 | 0 | 112 | 2703644 | 0 | 15 | 0 | 1 | 120 |
| 16 | 10 | 21 | 15 | 0 | 16 | 332568 | 2660540 | 0 | 16 | 0 | 112 | 2703764 | 0 | 16 | 0 | 1 | 120 |
| 17 | 11 | 21 | 15 | 0 | 17 | 332582 | 2660652 | 0 | 17 | 0 | 112 | 2703884 | 0 | 17 | 0 | 1 | 120 |
| 18 | 12 | 21 | 15 | 0 | 18 | 332596 | 2660764 | 0 | 18 | 0 | 112 | 2704004 | 0 | 18 | 0 | 1 | 120 |
| 19 | 13 | 21 | 15 | 0 | 19 | 332610 | 2660876 | 0 | 19 | 0 | 112 | 2704124 | 0 | 19 | 0 | 1 | 120 |
| 20 | 14 | 21 | 15 | 0 | 20 | 332624 | 2660988 | 0 | 20 | 0 | 112 | 2704244 | 0 | 20 | 0 | 1 | 120 |
| 21 | 15 | 21 | 15 | 1 | 0 | 332636 | 2661084 | 1 | 0 | 0 | 96 | 2704348 | 1 | 0 | 0 | 1 | 104 |
| 22 | 16 | 21 | 15 | 1 | 1 | 332653 | 2661220 | 1 | 1 | 0 | 136 | 2704492 | 1 | 1 | 0 | 1 | 144 |
| 23 | 17 | 21 | 15 | 1 | 2 | 332670 | 2661356 | 1 | 2 | 0 | 136 | 2704636 | 1 | 2 | 0 | 1 | 144 |
| 24 | 18 | 21 | 15 | 1 | 3 | 332687 | 2661492 | 1 | 3 | 0 | 136 | 2704780 | 1 | 3 | 0 | 1 | 144 |
| 25 | 19 | 21 | 15 | 1 | 4 | 332704 | 2661628 | 1 | 4 | 0 | 136 | 2704924 | 1 | 4 | 0 | 1 | 144 |
| 26 | 1A | 21 | 15 | 1 | 5 | 332721 | 2661764 | 1 | 5 | 0 | 136 | 2705068 | 1 | 5 | 0 | 1 | 144 |
| 27 | 1B | 21 | 15 | 1 | 6 | 332738 | 2661900 | 1 | 6 | 0 | 136 | 2705212 | 1 | 6 | 0 | 1 | 144 |
| 28 | 1C | 21 | 15 | 1 | 7 | 332755 | 2662036 | 1 | 7 | 0 | 136 | 2705356 | 1 | 7 | 0 | 1 | 144 |
| 29 | 1D | 21 | 15 | 1 | 8 | 332772 | 2662172 | 1 | 8 | 0 | 136 | 2705500 | 1 | 8 | 0 | 1 | 144 |
| 30 | 1E | 21 | 15 | 1 | 9 | 332789 | 2662308 | 1 | 9 | 0 | 136 | 2705644 | 1 | 9 | 0 | 1 | 144 |
| 31 | 1F | 21 | 15 | 1 | 10 | 332806 | 2662444 | 1 | 10 | 0 | 136 | 2705788 | 1 | 10 | 0 | 1 | 144 |
| 32 | 20 | 21 | 15 | 1 | 11 | 332827 | 2662612 | 1 | 11 | 0 | 168 | 2705964 | 1 | 11 | 0 | 1 | 176 |
| 33 | 21 | 21 | 15 | 1 | 12 | 332848 | 2662780 | 1 | 12 | 0 | 168 | 2706140 | 1 | 12 | 0 | 1 | 176 |
| 34 | 22 | 21 | 15 | 1 | 13 | 332869 | 2662948 | 1 | 13 | 0 | 168 | 2706316 | 1 | 13 | 0 | 1 | 176 |
| 35 | 23 | 21 | 15 | 1 | 14 | 332890 | 2663116 | 1 | 14 | 0 | 168 | 2706492 | 1 | 14 | 0 | 1 | 176 |
| 36 | 24 | 21 | 15 | 1 | 15 | 332911 | 2663284 | 1 | 15 | 0 | 168 | 2706668 | 1 | 15 | 0 | 1 | 176 |
| 37 | 25 | 21 | 15 | 1 | 16 | 332932 | 2663452 | 1 | 16 | 0 | 168 | 2706844 | 1 | 16 | 0 | 1 | 176 |
| 38 | 26 | 21 | 15 | 1 | 17 | 332953 | 2663620 | 1 | 17 | 0 | 168 | 2707020 | 1 | 17 | 0 | 1 | 176 |
| 39 | 27 | 21 | 15 | 1 | 18 | 332974 | 2663788 | 1 | 18 | 0 | 168 | 2707196 | 1 | 18 | 0 | 1 | 176 |
| 40 | 28 | 21 | 15 | 1 | 19 | 332995 | 2663956 | 1 | 19 | 0 | 168 | 2707372 | 1 | 19 | 0 | 1 | 176 |
| 41 | 29 | 21 | 15 | 1 | 20 | 333016 | 2664124 | 1 | 20 | 0 | 168 | 2707548 | 1 | 20 | 0 | 1 | 176 |
| 42 | 2A | 21 | 15 | 2 | 0 | 333035 | 2664276 | 2 | 0 | 0 | 152 | 2707708 | 2 | 0 | 0 | 1 | 160 |
| 43 | 2B | 21 | 15 | 2 | 1 | 333059 | 2664468 | 2 | 1 | 0 | 192 | 2707908 | 2 | 1 | 0 | 1 | 200 |
| 44 | 2C | 21 | 15 | 2 | 2 | 333083 | 2664660 | 2 | 2 | 0 | 192 | 2708108 | 2 | 2 | 0 | 1 | 200 |
| 45 | 2D | 21 | 15 | 2 | 3 | 333107 | 2664852 | 2 | 3 | 0 | 192 | 2708308 | 2 | 3 | 0 | 1 | 200 |
| 46 | 2E | 21 | 15 | 2 | 4 | 333131 | 2665044 | 2 | 4 | 0 | 192 | 2708508 | 2 | 4 | 0 | 1 | 200 |
| 47 | 2F | 21 | 15 | 2 | 5 | 333155 | 2665236 | 2 | 5 | 0 | 192 | 2708708 | 2 | 5 | 0 | 1 | 200 |
| 48 | 30 | 21 | 15 | 2 | 6 | 333181 | 2665444 | 2 | 6 | 0 | 208 | 2708924 | 2 | 6 | 0 | 1 | 216 |
| 49 | 31 | 21 | 15 | 2 | 7 | 333207 | 2665652 | 2 | 7 | 0 | 208 | 2709140 | 2 | 7 | 0 | 1 | 216 |
| 50 | 32 | 21 | 15 | 2 | 8 | 333233 | 2665860 | 2 | 8 | 0 | 208 | 2709356 | 2 | 8 | 0 | 1 | 216 |
| 51 | 33 | 21 | 15 | 2 | 9 | 333259 | 2666068 | 2 | 9 | 0 | 208 | 2709572 | 2 | 9 | 0 | 1 | 216 |
| 52 | 34 | 21 | 15 | 2 | 10 | 333285 | 2666276 | 2 | 10 | 0 | 208 | 2709788 | 2 | 10 | 0 | 1 | 216 |
| 53 | 35 | 21 | 15 | 2 | 11 | 333311 | 2666484 | 2 | 11 | 0 | 208 | 2710004 | 2 | 11 | 0 | 1 | 216 |
| 54 | 36 | 21 | 15 | 2 | 12 | 333337 | 2666692 | 2 | 12 | 0 | 208 | 2710220 | 2 | 12 | 0 | 1 | 216 |
| 55 | 37 | 21 | 15 | 2 | 13 | 333363 | 2666900 | 2 | 13 | 0 | 208 | 2710436 | 2 | 13 | 0 | 1 | 216 |
| 56 | 38 | 21 | 15 | 2 | 14 | 333389 | 2667108 | 2 | 14 | 0 | 208 | 2710652 | 2 | 14 | 0 | 1 | 216 |
| 57 | 39 | 21 | 15 | 2 | 15 | 333415 | 2667316 | 2 | 15 | 0 | 208 | 2710868 | 2 | 15 | 0 | 1 | 216 |
| 58 | 3A | 21 | 15 | 2 | 16 | 333441 | 2667524 | 2 | 16 | 0 | 208 | 2711084 | 2 | 16 | 0 | 1 | 216 |
| 59 | 3B | 21 | 15 | 2 | 17 | 333467 | 2667732 | 2 | 17 | 0 | 208 | 2711300 | 2 | 17 | 0 | 1 | 216 |
| 60 | 3C | 21 | 15 | 2 | 18 | 333493 | 2667940 | 2 | 18 | 0 | 208 | 2711516 | 2 | 18 | 0 | 1 | 216 |
| 61 | 3D | 21 | 15 | 2 | 19 | 333519 | 2668148 | 2 | 19 | 0 | 208 | 2711732 | 2 | 19 | 0 | 1 | 216 |
| 62 | 3E | 21 | 15 | 2 | 20 | 333545 | 2668356 | 2 | 20 | 0 | 208 | 2711948 | 2 | 20 | 0 | 1 | 216 |
| 63 | 3F | 21 | 15 | 3 | 0 | 333573 | 2668580 | 3 | 0 | 0 | 224 | 2712180 | 3 | 0 | 0 | 1 | 232 |
| 64 | 40 | 21 | 15 | 3 | 1 | 333599 | 2668788 | 3 | 1 | 0 | 208 | 2712396 | 3 | 1 | 0 | 1 | 216 |
| 65 | 41 | 21 | 15 | 3 | 2 | 333625 | 2668996 | 3 | 2 | 0 | 208 | 2712612 | 3 | 2 | 0 | 1 | 216 |
| 66 | 42 | 21 | 15 | 3 | 3 | 333651 | 2669204 | 3 | 3 | 0 | 208 | 2712828 | 3 | 3 | 0 | 1 | 216 |
| 67 | 43 | 21 | 15 | 3 | 4 | 333677 | 2669412 | 3 | 4 | 0 | 208 | 2713044 | 3 | 4 | 0 | 1 | 216 |
| 68 | 44 | 21 | 15 | 3 | 5 | 333703 | 2669620 | 3 | 5 | 0 | 208 | 2713260 | 3 | 5 | 0 | 1 | 216 |
| 69 | 45 | 21 | 15 | 3 | 6 | 333729 | 2669828 | 3 | 6 | 0 | 208 | 2713476 | 3 | 6 | 0 | 1 | 216 |
| 70 | 46 | 21 | 15 | 3 | 7 | 333755 | 2670036 | 3 | 7 | 0 | 208 | 2713692 | 3 | 7 | 0 | 1 | 216 |
| 71 | 47 | 21 | 15 | 3 | 8 | 333781 | 2670244 | 3 | 8 | 0 | 208 | 2713908 | 3 | 8 | 0 | 1 | 216 |
| 72 | 48 | 21 | 15 | 3 | 9 | 333807 | 2670452 | 3 | 9 | 0 | 208 | 2714124 | 3 | 9 | 0 | 1 | 216 |
| 73 | 49 | 21 | 15 | 3 | 10 | 333833 | 2670660 | 3 | 10 | 0 | 208 | 2714340 | 3 | 10 | 0 | 1 | 216 |
| 74 | 4A | 21 | 15 | 3 | 11 | 333859 | 2670868 | 3 | 11 | 0 | 208 | 2714556 | 3 | 11 | 0 | 1 | 216 |
| 75 | 4B | 21 | 15 | 3 | 12 | 333885 | 2671076 | 3 | 12 | 0 | 208 | 2714772 | 3 | 12 | 0 | 1 | 216 |
| 76 | 4C | 21 | 15 | 3 | 13 | 333911 | 2671284 | 3 | 13 | 0 | 208 | 2714988 | 3 | 13 | 0 | 1 | 216 |
| 77 | 4D | 21 | 15 | 3 | 14 | 333937 | 2671492 | 3 | 14 | 0 | 208 | 2715204 | 3 | 14 | 0 | 1 | 216 |
| 78 | 4E | 21 | 15 | 3 | 15 | 333963 | 2671700 | 3 | 15 | 0 | 208 | 2715420 | 3 | 15 | 0 | 1 | 216 |
| 79 | 4F | 21 | 15 | 3 | 16 | 333989 | 2671908 | 3 | 16 | 0 | 208 | 2715636 | 3 | 16 | 0 | 1 | 216 |

| | | | | | | | | | | | | | | | | | |
|---|---|---|---|---|---|---|---|---|---|---|---|---|---|---|---|---|---|
| 80 | 50 | 21 | 15 | 3 | 17 | 334017 | 2672132 | 3 | 17 | 0 | 224 | 2715868 | 3 | 17 | 0 | 1 | 232 |
| 81 | 51 | 21 | 15 | 3 | 18 | 334045 | 2672356 | 3 | 18 | 0 | 224 | 2716100 | 3 | 18 | 0 | 1 | 232 |
| 82 | 52 | 21 | 15 | 3 | 19 | 334073 | 2672580 | 3 | 19 | 0 | 224 | 2716332 | 3 | 19 | 0 | 1 | 232 |
| 83 | 53 | 21 | 15 | 3 | 20 | 334101 | 2672804 | 3 | 20 | 0 | 224 | 2716564 | 3 | 20 | 0 | 1 | 232 |
| 84 | 54 | 21 | 15 | 4 | 0 | 334131 | 2673044 | 4 | 0 | 0 | 240 | 2716812 | 4 | 0 | 0 | 1 | 248 |
| 85 | 55 | 21 | 15 | 4 | 1 | 334161 | 2673284 | 4 | 1 | 0 | 240 | 2717060 | 4 | 1 | 0 | 1 | 248 |
| 86 | 56 | 21 | 15 | 4 | 2 | 334191 | 2673524 | 4 | 2 | 0 | 240 | 2717308 | 4 | 2 | 0 | 1 | 248 |
| 87 | 57 | 21 | 15 | 4 | 3 | 334221 | 2673764 | 4 | 3 | 0 | 240 | 2717556 | 4 | 3 | 0 | 1 | 248 |
| 88 | 58 | 21 | 15 | 4 | 4 | 334251 | 2674004 | 4 | 4 | 0 | 240 | 2717804 | 4 | 4 | 0 | 1 | 248 |
| 89 | 59 | 21 | 15 | 4 | 5 | 334281 | 2674244 | 4 | 5 | 0 | 240 | 2718052 | 4 | 5 | 0 | 1 | 248 |
| 90 | 5A | 21 | 15 | 4 | 6 | 334311 | 2674484 | 4 | 6 | 0 | 240 | 2718300 | 4 | 6 | 0 | 1 | 248 |
| 91 | 5B | 21 | 15 | 4 | 7 | 334341 | 2674724 | 4 | 7 | 0 | 240 | 2718548 | 4 | 7 | 0 | 1 | 248 |
| 92 | 5C | 21 | 15 | 4 | 8 | 334371 | 2674964 | 4 | 8 | 0 | 240 | 2718796 | 4 | 8 | 0 | 1 | 248 |
| 93 | 5D | 21 | 15 | 4 | 9 | 334401 | 2675204 | 4 | 9 | 0 | 240 | 2719044 | 4 | 9 | 0 | 1 | 248 |
| 94 | 5E | 21 | 15 | 4 | 10 | 334431 | 2675444 | 4 | 10 | 0 | 240 | 2719292 | 4 | 10 | 0 | 1 | 248 |
| 95 | 5F | 21 | 15 | 4 | 11 | 334461 | 2675684 | 4 | 11 | 0 | 240 | 2719540 | 4 | 11 | 0 | 1 | 248 |
| 96 | 60 | 21 | 15 | 4 | 12 | 334489 | 2675908 | 4 | 12 | 0 | 224 | 2719772 | 4 | 12 | 0 | 1 | 232 |
| 97 | 61 | 21 | 15 | 4 | 13 | 334517 | 2676132 | 4 | 13 | 0 | 224 | 2720004 | 4 | 13 | 0 | 1 | 232 |
| 98 | 62 | 21 | 15 | 4 | 14 | 334545 | 2676356 | 4 | 14 | 0 | 224 | 2720236 | 4 | 14 | 0 | 1 | 232 |
| 99 | 63 | 21 | 15 | 4 | 15 | 334573 | 2676580 | 4 | 15 | 0 | 224 | 2720468 | 4 | 15 | 0 | 1 | 232 |
| 100 | 64 | 21 | 15 | 4 | 16 | 334601 | 2676804 | 4 | 16 | 0 | 224 | 2720700 | 4 | 16 | 0 | 1 | 232 |
| 101 | 65 | 21 | 15 | 4 | 17 | 334629 | 2677028 | 4 | 17 | 0 | 224 | 2720932 | 4 | 17 | 0 | 1 | 232 |
| 102 | 66 | 21 | 15 | 4 | 18 | 334657 | 2677252 | 4 | 18 | 0 | 224 | 2721164 | 4 | 18 | 0 | 1 | 232 |
| 103 | 67 | 21 | 15 | 4 | 19 | 334685 | 2677476 | 4 | 19 | 0 | 224 | 2721396 | 4 | 19 | 0 | 1 | 232 |
| 104 | 68 | 21 | 15 | 4 | 20 | 334713 | 2677700 | 4 | 20 | 0 | 224 | 2721628 | 4 | 20 | 0 | 1 | 232 |
| 105 | 69 | 21 | 15 | 5 | 0 | 334743 | 2677940 | 5 | 0 | 0 | 240 | 2721876 | 5 | 0 | 0 | 1 | 248 |
| 106 | 6A | 21 | 15 | 5 | 1 | 334773 | 2678180 | 5 | 1 | 0 | 240 | 2722124 | 5 | 1 | 0 | 1 | 248 |
| 107 | 6B | 21 | 15 | 5 | 2 | 334803 | 2678420 | 5 | 2 | 0 | 240 | 2722372 | 5 | 2 | 0 | 1 | 248 |
| 108 | 6C | 21 | 15 | 5 | 3 | 334833 | 2678660 | 5 | 3 | 0 | 240 | 2722620 | 5 | 3 | 0 | 1 | 248 |
| 109 | 6D | 21 | 15 | 5 | 4 | 334863 | 2678900 | 5 | 4 | 0 | 240 | 2722868 | 5 | 4 | 0 | 1 | 248 |
| 110 | 6E | 21 | 15 | 5 | 5 | 334893 | 2679140 | 5 | 5 | 0 | 240 | 2723116 | 5 | 5 | 0 | 1 | 248 |
| 111 | 6F | 21 | 15 | 5 | 6 | 334923 | 2679380 | 5 | 6 | 0 | 240 | 2723364 | 5 | 6 | 0 | 1 | 248 |
| 112 | 70 | 21 | 15 | 5 | 7 | 334955 | 2679636 | 5 | 7 | 0 | 256 | 2723628 | 5 | 7 | 0 | 1 | 264 |
| 113 | 71 | 21 | 15 | 5 | 8 | 334987 | 2679892 | 5 | 8 | 0 | 256 | 2723892 | 5 | 8 | 0 | 1 | 264 |
| 114 | 72 | 21 | 15 | 5 | 9 | 335019 | 2680148 | 5 | 9 | 0 | 256 | 2724156 | 5 | 9 | 0 | 1 | 264 |
| 115 | 73 | 21 | 15 | 5 | 10 | 335051 | 2680404 | 5 | 10 | 0 | 256 | 2724420 | 5 | 10 | 0 | 1 | 264 |
| 116 | 74 | 21 | 15 | 5 | 11 | 335083 | 2680660 | 5 | 11 | 0 | 256 | 2724684 | 5 | 11 | 0 | 1 | 264 |
| 117 | 75 | 21 | 15 | 5 | 12 | 335115 | 2680916 | 5 | 12 | 0 | 256 | 2724948 | 5 | 12 | 0 | 1 | 264 |
| 118 | 76 | 21 | 15 | 5 | 13 | 335147 | 2681172 | 5 | 13 | 0 | 256 | 2725212 | 5 | 13 | 0 | 1 | 264 |
| 119 | 77 | 21 | 15 | 5 | 14 | 335179 | 2681428 | 5 | 14 | 0 | 256 | 2725476 | 5 | 14 | 0 | 1 | 264 |
| 120 | 78 | 21 | 15 | 5 | 15 | 335211 | 2681684 | 5 | 15 | 0 | 256 | 2725740 | 5 | 15 | 0 | 1 | 264 |
| 121 | 79 | 21 | 15 | 5 | 16 | 335243 | 2681940 | 5 | 16 | 0 | 256 | 2726004 | 5 | 16 | 0 | 1 | 264 |
| 122 | 7A | 21 | 15 | 5 | 17 | 335275 | 2682196 | 5 | 17 | 0 | 256 | 2726268 | 5 | 17 | 0 | 1 | 264 |
| 123 | 7B | 21 | 15 | 5 | 18 | 335307 | 2682452 | 5 | 18 | 0 | 256 | 2726532 | 5 | 18 | 0 | 1 | 264 |
| 124 | 7C | 21 | 15 | 5 | 19 | 335339 | 2682708 | 5 | 19 | 0 | 256 | 2726796 | 5 | 19 | 0 | 1 | 264 |
| 125 | 7D | 21 | 15 | 5 | 20 | 335371 | 2682964 | 5 | 20 | 0 | 256 | 2727060 | 5 | 20 | 0 | 1 | 264 |
| 126 | 7E | 21 | 15 | 6 | 0 | 335405 | 2683236 | 6 | 0 | 0 | 272 | 2727340 | 6 | 0 | 0 | 1 | 280 |
| 127 | 7F | 21 | 15 | 6 | 1 | 335439 | 2683508 | 6 | 1 | 0 | 272 | 2727620 | 6 | 1 | 0 | 1 | 280 |
| 128 | 80 | 21 | 15 | 6 | 2 | 335471 | 2683764 | 6 | 2 | 0 | 256 | 2727884 | 6 | 2 | 0 | 1 | 264 |
| 129 | 81 | 21 | 15 | 6 | 3 | 335503 | 2684020 | 6 | 3 | 0 | 256 | 2728148 | 6 | 3 | 0 | 1 | 264 |
| 130 | 82 | 21 | 15 | 6 | 4 | 335535 | 2684276 | 6 | 4 | 0 | 256 | 2728412 | 6 | 4 | 0 | 1 | 264 |
| 131 | 83 | 21 | 15 | 6 | 5 | 335567 | 2684532 | 6 | 5 | 0 | 256 | 2728676 | 6 | 5 | 0 | 1 | 264 |
| 132 | 84 | 21 | 15 | 6 | 6 | 335599 | 2684788 | 6 | 6 | 0 | 256 | 2728940 | 6 | 6 | 0 | 1 | 264 |
| 133 | 85 | 21 | 15 | 6 | 7 | 335631 | 2685044 | 6 | 7 | 0 | 256 | 2729204 | 6 | 7 | 0 | 1 | 264 |
| 134 | 86 | 21 | 15 | 6 | 8 | 335663 | 2685300 | 6 | 8 | 0 | 256 | 2729468 | 6 | 8 | 0 | 1 | 264 |
| 135 | 87 | 21 | 15 | 6 | 9 | 335695 | 2685556 | 6 | 9 | 0 | 256 | 2729732 | 6 | 9 | 0 | 1 | 264 |
| 136 | 88 | 21 | 15 | 6 | 10 | 335727 | 2685812 | 6 | 10 | 0 | 256 | 2729996 | 6 | 10 | 0 | 1 | 264 |
| 137 | 89 | 21 | 15 | 6 | 11 | 335759 | 2686068 | 6 | 11 | 0 | 256 | 2730260 | 6 | 11 | 0 | 1 | 264 |
| 138 | 8A | 21 | 15 | 6 | 12 | 335791 | 2686324 | 6 | 12 | 0 | 256 | 2730524 | 6 | 12 | 0 | 1 | 264 |
| 139 | 8B | 21 | 15 | 6 | 13 | 335823 | 2686580 | 6 | 13 | 0 | 256 | 2730788 | 6 | 13 | 0 | 1 | 264 |
| 140 | 8C | 21 | 15 | 6 | 14 | 335855 | 2686836 | 6 | 14 | 0 | 256 | 2731052 | 6 | 14 | 0 | 1 | 264 |
| 141 | 8D | 21 | 15 | 6 | 15 | 335887 | 2687092 | 6 | 15 | 0 | 256 | 2731316 | 6 | 15 | 0 | 1 | 264 |
| 142 | 8E | 21 | 15 | 6 | 16 | 335919 | 2687348 | 6 | 16 | 0 | 256 | 2731580 | 6 | 16 | 0 | 1 | 264 |
| 143 | 8F | 21 | 15 | 6 | 17 | 335951 | 2687604 | 6 | 17 | 0 | 256 | 2731844 | 6 | 17 | 0 | 1 | 264 |
| 144 | 90 | 21 | 15 | 6 | 18 | 335985 | 2687876 | 6 | 18 | 0 | 272 | 2732124 | 6 | 18 | 0 | 1 | 280 |
| 145 | 91 | 21 | 15 | 6 | 19 | 336019 | 2688148 | 6 | 19 | 0 | 272 | 2732404 | 6 | 19 | 0 | 1 | 280 |
| 146 | 92 | 21 | 15 | 6 | 20 | 336053 | 2688420 | 6 | 20 | 0 | 272 | 2732684 | 6 | 20 | 0 | 1 | 280 |
| 147 | 93 | 21 | 15 | 7 | 0 | 336089 | 2688708 | 7 | 0 | 0 | 288 | 2732980 | 7 | 0 | 0 | 1 | 296 |
| 148 | 94 | 21 | 15 | 7 | 1 | 336125 | 2688996 | 7 | 1 | 0 | 288 | 2733276 | 7 | 1 | 0 | 1 | 296 |
| 149 | 95 | 21 | 15 | 7 | 2 | 336161 | 2689284 | 7 | 2 | 0 | 288 | 2733572 | 7 | 2 | 0 | 1 | 296 |
| 150 | 96 | 21 | 15 | 7 | 3 | 336197 | 2689572 | 7 | 3 | 0 | 288 | 2733868 | 7 | 3 | 0 | 1 | 296 |
| 151 | 97 | 21 | 15 | 7 | 4 | 336233 | 2689860 | 7 | 4 | 0 | 288 | 2734164 | 7 | 4 | 0 | 1 | 296 |
| 152 | 98 | 21 | 15 | 7 | 5 | 336269 | 2690148 | 7 | 5 | 0 | 288 | 2734460 | 7 | 5 | 0 | 1 | 296 |
| 153 | 99 | 21 | 15 | 7 | 6 | 336305 | 2690436 | 7 | 6 | 0 | 288 | 2734756 | 7 | 6 | 0 | 1 | 296 |
| 154 | 9A | 21 | 15 | 7 | 7 | 336341 | 2690724 | 7 | 7 | 0 | 288 | 2735052 | 7 | 7 | 0 | 1 | 296 |
| 155 | 9B | 21 | 15 | 7 | 8 | 336377 | 2691012 | 7 | 8 | 0 | 288 | 2735348 | 7 | 8 | 0 | 1 | 296 |
| 156 | 9C | 21 | 15 | 7 | 9 | 336413 | 2691300 | 7 | 9 | 0 | 288 | 2735644 | 7 | 9 | 0 | 1 | 296 |
| 157 | 9D | 21 | 15 | 7 | 10 | 336449 | 2691588 | 7 | 10 | 0 | 288 | 2735940 | 7 | 10 | 0 | 1 | 296 |
| 158 | 9E | 21 | 15 | 7 | 11 | 336485 | 2691876 | 7 | 11 | 0 | 288 | 2736236 | 7 | 11 | 0 | 1 | 296 |
| 159 | 9F | 21 | 15 | 7 | 12 | 336521 | 2692164 | 7 | 12 | 0 | 288 | 2736532 | 7 | 12 | 0 | 1 | 296 |
| 160 | A0 | 21 | 15 | 7 | 13 | 336555 | 2692436 | 7 | 13 | 0 | 272 | 2736812 | 7 | 13 | 0 | 1 | 280 |
| 161 | A1 | 21 | 15 | 7 | 14 | 336589 | 2692708 | 7 | 14 | 0 | 272 | 2737092 | 7 | 14 | 0 | 1 | 280 |
| 162 | A2 | 21 | 15 | 7 | 15 | 336623 | 2692980 | 7 | 15 | 0 | 272 | 2737372 | 7 | 15 | 0 | 1 | 280 |
| 163 | A3 | 21 | 15 | 7 | 16 | 336657 | 2693252 | 7 | 16 | 0 | 272 | 2737652 | 7 | 16 | 0 | 1 | 280 |
| 164 | A4 | 21 | 15 | 7 | 17 | 336691 | 2693524 | 7 | 17 | 0 | 272 | 2737932 | 7 | 17 | 0 | 1 | 280 |
| 165 | A5 | 21 | 15 | 7 | 18 | 336725 | 2693796 | 7 | 18 | 0 | 272 | 2738212 | 7 | 18 | 0 | 1 | 280 |
| 166 | A6 | 21 | 15 | 7 | 19 | 336759 | 2694068 | 7 | 19 | 0 | 272 | 2738492 | 7 | 19 | 0 | 1 | 280 |
| 167 | A7 | 21 | 15 | 7 | 20 | 336793 | 2694340 | 7 | 20 | 0 | 272 | 2738772 | 7 | 20 | 0 | 1 | 280 |
| 168 | A8 | 21 | 15 | 8 | 0 | 336829 | 2694628 | 8 | 0 | 0 | 288 | 2739068 | 8 | 0 | 0 | 1 | 296 |
| 169 | A9 | 21 | 15 | 8 | 1 | 336865 | 2694916 | 8 | 1 | 0 | 288 | 2739364 | 8 | 1 | 0 | 1 | 296 |
| 170 | AA | 21 | 15 | 8 | 2 | 336901 | 2695204 | 8 | 2 | 0 | 288 | 2739660 | 8 | 2 | 0 | 1 | 296 |
| 171 | AB | 21 | 15 | 8 | 3 | 336937 | 2695492 | 8 | 3 | 0 | 288 | 2739956 | 8 | 3 | 0 | 1 | 296 |
| 172 | AC | 21 | 15 | 8 | 4 | 336973 | 2695780 | 8 | 4 | 0 | 288 | 2740252 | 8 | 4 | 0 | 1 | 296 |
| 173 | AD | 21 | 15 | 8 | 5 | 337009 | 2696068 | 8 | 5 | 0 | 288 | 2740548 | 8 | 5 | 0 | 1 | 296 |
| 174 | AE | 21 | 15 | 8 | 6 | 337045 | 2696356 | 8 | 6 | 0 | 288 | 2740844 | 8 | 6 | 0 | 1 | 296 |
| 175 | AF | 21 | 15 | 8 | 7 | 337081 | 2696644 | 8 | 7 | 0 | 288 | 2741140 | 8 | 7 | 0 | 1 | 296 |
| 176 | B0 | 21 | 15 | 8 | 8 | 337119 | 2696948 | 8 | 8 | 0 | 304 | 2741452 | 8 | 8 | 0 | 1 | 312 |
| 177 | B1 | 21 | 15 | 8 | 9 | 337157 | 2697252 | 8 | 9 | 0 | 304 | 2741764 | 8 | 9 | 0 | 1 | 312 |
| 178 | B2 | 21 | 15 | 8 | 10 | 337195 | 2697556 | 8 | 10 | 0 | 304 | 2742076 | 8 | 10 | 0 | 1 | 312 |
| 179 | B3 | 21 | 15 | 8 | 11 | 337233 | 2697860 | 8 | 11 | 0 | 304 | 2742388 | 8 | 11 | 0 | 1 | 312 |
| 180 | B4 | 21 | 15 | 8 | 12 | 337271 | 2698164 | 8 | 12 | 0 | 304 | 2742700 | 8 | 12 | 0 | 1 | 312 |
| 181 | B5 | 21 | 15 | 8 | 13 | 337309 | 2698468 | 8 | 13 | 0 | 304 | 2743012 | 8 | 13 | 0 | 1 | 312 |
| 182 | B6 | 21 | 15 | 8 | 14 | 337347 | 2698772 | 8 | 14 | 0 | 304 | 2743324 | 8 | 14 | 0 | 1 | 312 |

| | | | | | | | | | | | | | | | | | |
|---|---|---|---|---|---|---|---|---|---|---|---|---|---|---|---|---|---|
| 183 | B7 | 21 | 15 | 8 | 15 | 337385 | 2699076 | 8 | 15 | 0 | 304 | 2743636 | 8 | 15 | 0 | 1 | 312 |
| 184 | B8 | 21 | 15 | 8 | 16 | 337423 | 2699380 | 8 | 16 | 0 | 304 | 2743948 | 8 | 16 | 0 | 1 | 312 |
| 185 | B9 | 21 | 15 | 8 | 17 | 337461 | 2699684 | 8 | 17 | 0 | 304 | 2744260 | 8 | 17 | 0 | 1 | 312 |
| 186 | BA | 21 | 15 | 8 | 18 | 337499 | 2699988 | 8 | 18 | 0 | 304 | 2744572 | 8 | 18 | 0 | 1 | 312 |
| 187 | BB | 21 | 15 | 8 | 19 | 337537 | 2700292 | 8 | 19 | 0 | 304 | 2744884 | 8 | 19 | 0 | 1 | 312 |
| 188 | BC | 21 | 15 | 8 | 20 | 337575 | 2700596 | 8 | 20 | 0 | 304 | 2745196 | 8 | 20 | 0 | 1 | 312 |
| 189 | BD | 21 | 15 | 9 | 0 | 337615 | 2700916 | 9 | 0 | 0 | 320 | 2745524 | 9 | 0 | 0 | 1 | 328 |
| 190 | BE | 21 | 15 | 9 | 1 | 337655 | 2701236 | 9 | 1 | 0 | 320 | 2745852 | 9 | 1 | 0 | 1 | 328 |
| 191 | BF | 21 | 15 | 9 | 2 | 337695 | 2701556 | 9 | 2 | 0 | 320 | 2746180 | 9 | 2 | 0 | 1 | 328 |
| 192 | C0 | 21 | 15 | 9 | 3 | 337733 | 2701860 | 9 | 3 | 0 | 304 | 2746492 | 9 | 3 | 0 | 1 | 312 |
| 193 | C1 | 21 | 15 | 9 | 4 | 337771 | 2702164 | 9 | 4 | 0 | 304 | 2746804 | 9 | 4 | 0 | 1 | 312 |
| 194 | C2 | 21 | 15 | 9 | 5 | 337809 | 2702468 | 9 | 5 | 0 | 304 | 2747116 | 9 | 5 | 0 | 1 | 312 |
| 195 | C3 | 21 | 15 | 9 | 6 | 337847 | 2702772 | 9 | 6 | 0 | 304 | 2747428 | 9 | 6 | 0 | 1 | 312 |
| 196 | C4 | 21 | 15 | 9 | 7 | 337885 | 2703076 | 9 | 7 | 0 | 304 | 2747740 | 9 | 7 | 0 | 1 | 312 |
| 197 | C5 | 21 | 15 | 9 | 8 | 337923 | 2703380 | 9 | 8 | 0 | 304 | 2748052 | 9 | 8 | 0 | 1 | 312 |
| 198 | C6 | 21 | 15 | 9 | 9 | 337961 | 2703684 | 9 | 9 | 0 | 304 | 2748364 | 9 | 9 | 0 | 1 | 312 |
| 199 | C7 | 21 | 15 | 9 | 10 | 337999 | 2703988 | 9 | 10 | 0 | 304 | 2748676 | 9 | 10 | 0 | 1 | 312 |
| 200 | C8 | 21 | 15 | 9 | 11 | 338037 | 2704292 | 9 | 11 | 0 | 304 | 2748988 | 9 | 11 | 0 | 1 | 312 |
| 201 | C9 | 21 | 15 | 9 | 12 | 338075 | 2704596 | 9 | 12 | 0 | 304 | 2749300 | 9 | 12 | 0 | 1 | 312 |
| 202 | CA | 21 | 15 | 9 | 13 | 338113 | 2704900 | 9 | 13 | 0 | 304 | 2749612 | 9 | 13 | 0 | 1 | 312 |
| 203 | CB | 21 | 15 | 9 | 14 | 338151 | 2705204 | 9 | 14 | 0 | 304 | 2749924 | 9 | 14 | 0 | 1 | 312 |
| 204 | CC | 21 | 15 | 9 | 15 | 338189 | 2705508 | 9 | 15 | 0 | 304 | 2750236 | 9 | 15 | 0 | 1 | 312 |
| 205 | CD | 21 | 15 | 9 | 16 | 338227 | 2705812 | 9 | 16 | 0 | 304 | 2750548 | 9 | 16 | 0 | 1 | 312 |
| 206 | CE | 21 | 15 | 9 | 17 | 338265 | 2706116 | 9 | 17 | 0 | 304 | 2750860 | 9 | 17 | 0 | 1 | 312 |
| 207 | CF | 21 | 15 | 9 | 18 | 338303 | 2706420 | 9 | 18 | 0 | 304 | 2751172 | 9 | 18 | 0 | 1 | 312 |
| 208 | D0 | 21 | 15 | 9 | 19 | 338343 | 2706740 | 9 | 19 | 0 | 320 | 2751500 | 9 | 19 | 0 | 1 | 328 |
| 209 | D1 | 21 | 15 | 9 | 20 | 338383 | 2707060 | 9 | 20 | 0 | 320 | 2751828 | 9 | 20 | 0 | 1 | 328 |
| 210 | D2 | 21 | 15 | 10 | 0 | 338425 | 2707396 | 10 | 0 | 0 | 336 | 2752172 | 10 | 0 | 0 | 1 | 344 |
| 211 | D3 | 21 | 15 | 10 | 1 | 338467 | 2707732 | 10 | 1 | 0 | 336 | 2752516 | 10 | 1 | 0 | 1 | 344 |
| 212 | D4 | 21 | 15 | 10 | 2 | 338509 | 2708068 | 10 | 2 | 0 | 336 | 2752860 | 10 | 2 | 0 | 1 | 344 |
| 213 | D5 | 21 | 15 | 10 | 3 | 338551 | 2708404 | 10 | 3 | 0 | 336 | 2753204 | 10 | 3 | 0 | 1 | 344 |
| 214 | D6 | 21 | 15 | 10 | 4 | 338593 | 2708740 | 10 | 4 | 0 | 336 | 2753548 | 10 | 4 | 0 | 1 | 344 |
| 215 | D7 | 21 | 15 | 10 | 5 | 338635 | 2709076 | 10 | 5 | 0 | 336 | 2753892 | 10 | 5 | 0 | 1 | 344 |
| 216 | D8 | 21 | 15 | 10 | 6 | 338677 | 2709412 | 10 | 6 | 0 | 336 | 2754236 | 10 | 6 | 0 | 1 | 344 |
| 217 | D9 | 21 | 15 | 10 | 7 | 338719 | 2709748 | 10 | 7 | 0 | 336 | 2754580 | 10 | 7 | 0 | 1 | 344 |
| 218 | DA | 21 | 15 | 10 | 8 | 338761 | 2710084 | 10 | 8 | 0 | 336 | 2754924 | 10 | 8 | 0 | 1 | 344 |
| 219 | DB | 21 | 15 | 10 | 9 | 338803 | 2710420 | 10 | 9 | 0 | 336 | 2755268 | 10 | 9 | 0 | 1 | 344 |
| 220 | DC | 21 | 15 | 10 | 10 | 338845 | 2710756 | 10 | 10 | 0 | 336 | 2755612 | 10 | 10 | 0 | 1 | 344 |
| 221 | DD | 21 | 15 | 10 | 11 | 338887 | 2711092 | 10 | 11 | 0 | 336 | 2755956 | 10 | 11 | 0 | 1 | 344 |
| 222 | DE | 21 | 15 | 10 | 12 | 338929 | 2711428 | 10 | 12 | 0 | 336 | 2756300 | 10 | 12 | 0 | 1 | 344 |
| 223 | DF | 21 | 15 | 10 | 13 | 338971 | 2711764 | 10 | 13 | 0 | 336 | 2756644 | 10 | 13 | 0 | 1 | 344 |
| 224 | E0 | 21 | 15 | 10 | 14 | 339011 | 2712084 | 10 | 14 | 0 | 320 | 2756972 | 10 | 14 | 0 | 1 | 328 |
| 225 | E1 | 21 | 15 | 10 | 15 | 339051 | 2712404 | 10 | 15 | 0 | 320 | 2757300 | 10 | 15 | 0 | 1 | 328 |
| 226 | E2 | 21 | 15 | 10 | 16 | 339091 | 2712724 | 10 | 16 | 0 | 320 | 2757628 | 10 | 16 | 0 | 1 | 328 |
| 227 | E3 | 21 | 15 | 10 | 17 | 339131 | 2713044 | 10 | 17 | 0 | 320 | 2757956 | 10 | 17 | 0 | 1 | 328 |
| 228 | E4 | 21 | 15 | 10 | 18 | 339171 | 2713364 | 10 | 18 | 0 | 320 | 2758284 | 10 | 18 | 0 | 1 | 328 |
| 229 | E5 | 21 | 15 | 10 | 19 | 339211 | 2713684 | 10 | 19 | 0 | 320 | 2758612 | 10 | 19 | 0 | 1 | 328 |
| 230 | E6 | 21 | 15 | 10 | 20 | 339251 | 2714004 | 10 | 20 | 0 | 320 | 2758940 | 10 | 20 | 0 | 1 | 328 |
| 231 | E7 | 21 | 15 | 11 | 0 | 339293 | 2714340 | 11 | 0 | 0 | 336 | 2759284 | 11 | 0 | 0 | 1 | 344 |
| 232 | E8 | 21 | 15 | 11 | 1 | 339335 | 2714676 | 11 | 1 | 0 | 336 | 2759628 | 11 | 1 | 0 | 1 | 344 |
| 233 | E9 | 21 | 15 | 11 | 2 | 339377 | 2715012 | 11 | 2 | 0 | 336 | 2759972 | 11 | 2 | 0 | 1 | 344 |
| 234 | EA | 21 | 15 | 11 | 3 | 339419 | 2715348 | 11 | 3 | 0 | 336 | 2760316 | 11 | 3 | 0 | 1 | 344 |
| 235 | EB | 21 | 15 | 11 | 4 | 339461 | 2715684 | 11 | 4 | 0 | 336 | 2760660 | 11 | 4 | 0 | 1 | 344 |
| 236 | EC | 21 | 15 | 11 | 5 | 339503 | 2716020 | 11 | 5 | 0 | 336 | 2761004 | 11 | 5 | 0 | 1 | 344 |
| 237 | ED | 21 | 15 | 11 | 6 | 339545 | 2716356 | 11 | 6 | 0 | 336 | 2761348 | 11 | 6 | 0 | 1 | 344 |
| 238 | EE | 21 | 15 | 11 | 7 | 339587 | 2716692 | 11 | 7 | 0 | 336 | 2761692 | 11 | 7 | 0 | 1 | 344 |
| 239 | EF | 21 | 15 | 11 | 8 | 339629 | 2717028 | 11 | 8 | 0 | 336 | 2762036 | 11 | 8 | 0 | 1 | 344 |
| 240 | F0 | 21 | 15 | 11 | 9 | 339673 | 2717380 | 11 | 9 | 0 | 352 | 2762396 | 11 | 9 | 0 | 1 | 360 |
| 241 | F1 | 21 | 15 | 11 | 10 | 339717 | 2717732 | 11 | 10 | 0 | 352 | 2762756 | 11 | 10 | 0 | 1 | 360 |
| 242 | F2 | 21 | 15 | 11 | 11 | 339761 | 2718084 | 11 | 11 | 0 | 352 | 2763116 | 11 | 11 | 0 | 1 | 360 |
| 243 | F3 | 21 | 15 | 11 | 12 | 339805 | 2718436 | 11 | 12 | 0 | 352 | 2763476 | 11 | 12 | 0 | 1 | 360 |
| 244 | F4 | 21 | 15 | 11 | 13 | 339849 | 2718788 | 11 | 13 | 0 | 352 | 2763836 | 11 | 13 | 0 | 1 | 360 |
| 245 | F5 | 21 | 15 | 11 | 14 | 339893 | 2719140 | 11 | 14 | 0 | 352 | 2764196 | 11 | 14 | 0 | 1 | 360 |
| 246 | F6 | 21 | 15 | 11 | 15 | 339937 | 2719492 | 11 | 15 | 0 | 352 | 2764556 | 11 | 15 | 0 | 1 | 360 |
| 247 | F7 | 21 | 15 | 11 | 16 | 339981 | 2719844 | 11 | 16 | 0 | 352 | 2764916 | 11 | 16 | 0 | 1 | 360 |
| 248 | F8 | 21 | 15 | 11 | 17 | 340025 | 2720196 | 11 | 17 | 0 | 352 | 2765276 | 11 | 17 | 0 | 1 | 360 |
| 249 | F9 | 21 | 15 | 11 | 18 | 340069 | 2720548 | 11 | 18 | 0 | 352 | 2765636 | 11 | 18 | 0 | 1 | 360 |
| 250 | FA | 21 | 15 | 11 | 19 | 340113 | 2720900 | 11 | 19 | 0 | 352 | 2765996 | 11 | 19 | 0 | 1 | 360 |
| 251 | FB | 21 | 15 | 11 | 20 | 340157 | 2721252 | 11 | 20 | 0 | 352 | 2766356 | 11 | 20 | 0 | 1 | 360 |
| 252 | FC | 21 | 15 | 12 | 0 | 340203 | 2721620 | 12 | 0 | 0 | 368 | 2766732 | 12 | 0 | 0 | 1 | 376 |
| 253 | FD | 21 | 15 | 12 | 1 | 340249 | 2721988 | 12 | 1 | 0 | 368 | 2767108 | 12 | 1 | 0 | 1 | 376 |
| 254 | FE | 21 | 15 | 12 | 2 | 340295 | 2722356 | 12 | 2 | 0 | 368 | 2767484 | 12 | 2 | 0 | 1 | 376 |
| 255 | FF | 21 | 15 | 12 | 3 | 340341 | 2722724 | 12 | 3 | 0 | 368 | 2767860 | 12 | 3 | 0 | 1 | 376 |
| 0 | 00 | 22 | 16 | 0 | 0 | 340353 | 2722820 | 0 | 0 | 0 | 96 | 2767964 | 0 | 0 | 0 | 1 | 104 |
| 1 | 01 | 22 | 16 | 0 | 1 | 340367 | 2722932 | 0 | 1 | 0 | 112 | 2768084 | 0 | 1 | 0 | 1 | 120 |
| 2 | 02 | 22 | 16 | 0 | 2 | 340381 | 2723044 | 0 | 2 | 0 | 112 | 2768204 | 0 | 2 | 0 | 1 | 120 |
| 3 | 03 | 22 | 16 | 0 | 3 | 340395 | 2723156 | 0 | 3 | 0 | 112 | 2768324 | 0 | 3 | 0 | 1 | 120 |
| 4 | 04 | 22 | 16 | 0 | 4 | 340409 | 2723268 | 0 | 4 | 0 | 112 | 2768444 | 0 | 4 | 0 | 1 | 120 |
| 5 | 05 | 22 | 16 | 0 | 5 | 340423 | 2723380 | 0 | 5 | 0 | 112 | 2768564 | 0 | 5 | 0 | 1 | 120 |
| 6 | 06 | 22 | 16 | 0 | 6 | 340437 | 2723492 | 0 | 6 | 0 | 112 | 2768684 | 0 | 6 | 0 | 1 | 120 |
| 7 | 07 | 22 | 16 | 0 | 7 | 340451 | 2723604 | 0 | 7 | 0 | 112 | 2768804 | 0 | 7 | 0 | 1 | 120 |
| 8 | 08 | 22 | 16 | 0 | 8 | 340465 | 2723716 | 0 | 8 | 0 | 112 | 2768924 | 0 | 8 | 0 | 1 | 120 |
| 9 | 09 | 22 | 16 | 0 | 9 | 340479 | 2723828 | 0 | 9 | 0 | 112 | 2769044 | 0 | 9 | 0 | 1 | 120 |
| 10 | 0A | 22 | 16 | 0 | 10 | 340493 | 2723940 | 0 | 10 | 0 | 112 | 2769164 | 0 | 10 | 0 | 1 | 120 |
| 11 | 0B | 22 | 16 | 0 | 11 | 340507 | 2724052 | 0 | 11 | 0 | 112 | 2769284 | 0 | 11 | 0 | 1 | 120 |
| 12 | 0C | 22 | 16 | 0 | 12 | 340521 | 2724164 | 0 | 12 | 0 | 112 | 2769404 | 0 | 12 | 0 | 1 | 120 |
| 13 | 0D | 22 | 16 | 0 | 13 | 340535 | 2724276 | 0 | 13 | 0 | 112 | 2769524 | 0 | 13 | 0 | 1 | 120 |
| 14 | 0E | 22 | 16 | 0 | 14 | 340549 | 2724388 | 0 | 14 | 0 | 112 | 2769644 | 0 | 14 | 0 | 1 | 120 |
| 15 | 0F | 22 | 16 | 0 | 15 | 340563 | 2724500 | 0 | 15 | 0 | 112 | 2769764 | 0 | 15 | 0 | 1 | 120 |
| 16 | 10 | 22 | 16 | 0 | 16 | 340577 | 2724612 | 0 | 16 | 0 | 112 | 2769884 | 0 | 16 | 0 | 1 | 120 |
| 17 | 11 | 22 | 16 | 0 | 17 | 340591 | 2724724 | 0 | 17 | 0 | 112 | 2770004 | 0 | 17 | 0 | 1 | 120 |
| 18 | 12 | 22 | 16 | 0 | 18 | 340605 | 2724836 | 0 | 18 | 0 | 112 | 2770124 | 0 | 18 | 0 | 1 | 120 |
| 19 | 13 | 22 | 16 | 0 | 19 | 340619 | 2724948 | 0 | 19 | 0 | 112 | 2770244 | 0 | 19 | 0 | 1 | 120 |
| 20 | 14 | 22 | 16 | 0 | 20 | 340633 | 2725060 | 0 | 20 | 0 | 112 | 2770364 | 0 | 20 | 0 | 1 | 120 |
| 21 | 15 | 22 | 16 | 0 | 21 | 340647 | 2725172 | 0 | 21 | 0 | 112 | 2770484 | 0 | 21 | 0 | 1 | 120 |
| 22 | 16 | 22 | 16 | 1 | 0 | 340659 | 2725268 | 1 | 0 | 0 | 96 | 2770588 | 1 | 0 | 0 | 1 | 104 |
| 23 | 17 | 22 | 16 | 1 | 1 | 340676 | 2725404 | 1 | 1 | 0 | 136 | 2770732 | 1 | 1 | 0 | 1 | 144 |
| 24 | 18 | 22 | 16 | 1 | 2 | 340693 | 2725540 | 1 | 2 | 0 | 136 | 2770876 | 1 | 2 | 0 | 1 | 144 |
| 25 | 19 | 22 | 16 | 1 | 3 | 340710 | 2725676 | 1 | 3 | 0 | 136 | 2771020 | 1 | 3 | 0 | 1 | 144 |
| 26 | 1A | 22 | 16 | 1 | 4 | 340727 | 2725812 | 1 | 4 | 0 | 136 | 2771164 | 1 | 4 | 0 | 1 | 144 |
| 27 | 1B | 22 | 16 | 1 | 5 | 340744 | 2725948 | 1 | 5 | 0 | 136 | 2771308 | 1 | 5 | 0 | 1 | 144 |
| 28 | 1C | 22 | 16 | 1 | 6 | 340761 | 2726084 | 1 | 6 | 0 | 136 | 2771452 | 1 | 6 | 0 | 1 | 144 |
| 29 | 1D | 22 | 16 | 1 | 7 | 340778 | 2726220 | 1 | 7 | 0 | 136 | 2771596 | 1 | 7 | 0 | 1 | 144 |

| | | | | | | | | | | | | | | | | | |
|---|---|---|---|---|---|---|---|---|---|---|---|---|---|---|---|---|---|
| 30 | 1E | 22 | 16 | 1 | 8 | 340795 | 2726356 | 1 | 8 | 0 | 136 | 2771740 | 1 | 8 | 0 | 1 | 144 |
| 31 | 1F | 22 | 16 | 1 | 9 | 340812 | 2726492 | 1 | 9 | 0 | 136 | 2771884 | 1 | 9 | 0 | 1 | 144 |
| 32 | 20 | 22 | 16 | 1 | 10 | 340833 | 2726660 | 1 | 10 | 0 | 168 | 2772060 | 1 | 10 | 0 | 1 | 176 |
| 33 | 21 | 22 | 16 | 1 | 11 | 340854 | 2726828 | 1 | 11 | 0 | 168 | 2772236 | 1 | 11 | 0 | 1 | 176 |
| 34 | 22 | 22 | 16 | 1 | 12 | 340875 | 2726996 | 1 | 12 | 0 | 168 | 2772412 | 1 | 12 | 0 | 1 | 176 |
| 35 | 23 | 22 | 16 | 1 | 13 | 340896 | 2727164 | 1 | 13 | 0 | 168 | 2772588 | 1 | 13 | 0 | 1 | 176 |
| 36 | 24 | 22 | 16 | 1 | 14 | 340917 | 2727332 | 1 | 14 | 0 | 168 | 2772764 | 1 | 14 | 0 | 1 | 176 |
| 37 | 25 | 22 | 16 | 1 | 15 | 340938 | 2727500 | 1 | 15 | 0 | 168 | 2772940 | 1 | 15 | 0 | 1 | 176 |
| 38 | 26 | 22 | 16 | 1 | 16 | 340959 | 2727668 | 1 | 16 | 0 | 168 | 2773116 | 1 | 16 | 0 | 1 | 176 |
| 39 | 27 | 22 | 16 | 1 | 17 | 340980 | 2727836 | 1 | 17 | 0 | 168 | 2773292 | 1 | 17 | 0 | 1 | 176 |
| 40 | 28 | 22 | 16 | 1 | 18 | 341001 | 2728004 | 1 | 18 | 0 | 168 | 2773468 | 1 | 18 | 0 | 1 | 176 |
| 41 | 29 | 22 | 16 | 1 | 19 | 341022 | 2728172 | 1 | 19 | 0 | 168 | 2773644 | 1 | 19 | 0 | 1 | 176 |
| 42 | 2A | 22 | 16 | 1 | 20 | 341043 | 2728340 | 1 | 20 | 0 | 168 | 2773820 | 1 | 20 | 0 | 1 | 176 |
| 43 | 2B | 22 | 16 | 1 | 21 | 341064 | 2728508 | 1 | 21 | 0 | 168 | 2773996 | 1 | 21 | 0 | 1 | 176 |
| 44 | 2C | 22 | 16 | 2 | 0 | 341083 | 2728660 | 2 | 0 | 0 | 152 | 2774156 | 2 | 0 | 0 | 1 | 160 |
| 45 | 2D | 22 | 16 | 2 | 1 | 341107 | 2728852 | 2 | 1 | 0 | 192 | 2774356 | 2 | 1 | 0 | 1 | 200 |
| 46 | 2E | 22 | 16 | 2 | 2 | 341131 | 2729044 | 2 | 2 | 0 | 192 | 2774556 | 2 | 2 | 0 | 1 | 200 |
| 47 | 2F | 22 | 16 | 2 | 3 | 341155 | 2729236 | 2 | 3 | 0 | 192 | 2774756 | 2 | 3 | 0 | 1 | 200 |
| 48 | 30 | 22 | 16 | 2 | 4 | 341181 | 2729444 | 2 | 4 | 0 | 208 | 2774972 | 2 | 4 | 0 | 1 | 216 |
| 49 | 31 | 22 | 16 | 2 | 5 | 341207 | 2729652 | 2 | 5 | 0 | 208 | 2775188 | 2 | 5 | 0 | 1 | 216 |
| 50 | 32 | 22 | 16 | 2 | 6 | 341233 | 2729860 | 2 | 6 | 0 | 208 | 2775404 | 2 | 6 | 0 | 1 | 216 |
| 51 | 33 | 22 | 16 | 2 | 7 | 341259 | 2730068 | 2 | 7 | 0 | 208 | 2775620 | 2 | 7 | 0 | 1 | 216 |
| 52 | 34 | 22 | 16 | 2 | 8 | 341285 | 2730276 | 2 | 8 | 0 | 208 | 2775836 | 2 | 8 | 0 | 1 | 216 |
| 53 | 35 | 22 | 16 | 2 | 9 | 341311 | 2730484 | 2 | 9 | 0 | 208 | 2776052 | 2 | 9 | 0 | 1 | 216 |
| 54 | 36 | 22 | 16 | 2 | 10 | 341337 | 2730692 | 2 | 10 | 0 | 208 | 2776268 | 2 | 10 | 0 | 1 | 216 |
| 55 | 37 | 22 | 16 | 2 | 11 | 341363 | 2730900 | 2 | 11 | 0 | 208 | 2776484 | 2 | 11 | 0 | 1 | 216 |
| 56 | 38 | 22 | 16 | 2 | 12 | 341389 | 2731108 | 2 | 12 | 0 | 208 | 2776700 | 2 | 12 | 0 | 1 | 216 |
| 57 | 39 | 22 | 16 | 2 | 13 | 341415 | 2731316 | 2 | 13 | 0 | 208 | 2776916 | 2 | 13 | 0 | 1 | 216 |
| 58 | 3A | 22 | 16 | 2 | 14 | 341441 | 2731524 | 2 | 14 | 0 | 208 | 2777132 | 2 | 14 | 0 | 1 | 216 |
| 59 | 3B | 22 | 16 | 2 | 15 | 341467 | 2731732 | 2 | 15 | 0 | 208 | 2777348 | 2 | 15 | 0 | 1 | 216 |
| 60 | 3C | 22 | 16 | 2 | 16 | 341493 | 2731940 | 2 | 16 | 0 | 208 | 2777564 | 2 | 16 | 0 | 1 | 216 |
| 61 | 3D | 22 | 16 | 2 | 17 | 341519 | 2732148 | 2 | 17 | 0 | 208 | 2777780 | 2 | 17 | 0 | 1 | 216 |
| 62 | 3E | 22 | 16 | 2 | 18 | 341545 | 2732356 | 2 | 18 | 0 | 208 | 2777996 | 2 | 18 | 0 | 1 | 216 |
| 63 | 3F | 22 | 16 | 2 | 19 | 341571 | 2732564 | 2 | 19 | 0 | 208 | 2778212 | 2 | 19 | 0 | 1 | 216 |
| 64 | 40 | 22 | 16 | 2 | 20 | 341594 | 2732748 | 2 | 20 | 0 | 184 | 2778404 | 2 | 20 | 0 | 1 | 192 |
| 65 | 41 | 22 | 16 | 2 | 21 | 341617 | 2732932 | 2 | 21 | 0 | 184 | 2778596 | 2 | 21 | 0 | 1 | 192 |
| 66 | 42 | 22 | 16 | 3 | 0 | 341638 | 2733100 | 3 | 0 | 0 | 168 | 2778772 | 3 | 0 | 0 | 1 | 176 |
| 67 | 43 | 22 | 16 | 3 | 1 | 341664 | 2733308 | 3 | 1 | 0 | 208 | 2778988 | 3 | 1 | 0 | 1 | 216 |
| 68 | 44 | 22 | 16 | 3 | 2 | 341690 | 2733516 | 3 | 2 | 0 | 208 | 2779204 | 3 | 2 | 0 | 1 | 216 |
| 69 | 45 | 22 | 16 | 3 | 3 | 341716 | 2733724 | 3 | 3 | 0 | 208 | 2779420 | 3 | 3 | 0 | 1 | 216 |
| 70 | 46 | 22 | 16 | 3 | 4 | 341742 | 2733932 | 3 | 4 | 0 | 208 | 2779636 | 3 | 4 | 0 | 1 | 216 |
| 71 | 47 | 22 | 16 | 3 | 5 | 341768 | 2734140 | 3 | 5 | 0 | 208 | 2779852 | 3 | 5 | 0 | 1 | 216 |
| 72 | 48 | 22 | 16 | 3 | 6 | 341794 | 2734348 | 3 | 6 | 0 | 208 | 2780068 | 3 | 6 | 0 | 1 | 216 |
| 73 | 49 | 22 | 16 | 3 | 7 | 341820 | 2734556 | 3 | 7 | 0 | 208 | 2780284 | 3 | 7 | 0 | 1 | 216 |
| 74 | 4A | 22 | 16 | 3 | 8 | 341846 | 2734764 | 3 | 8 | 0 | 208 | 2780500 | 3 | 8 | 0 | 1 | 216 |
| 75 | 4B | 22 | 16 | 3 | 9 | 341872 | 2734972 | 3 | 9 | 0 | 208 | 2780716 | 3 | 9 | 0 | 1 | 216 |
| 76 | 4C | 22 | 16 | 3 | 10 | 341898 | 2735180 | 3 | 10 | 0 | 208 | 2780932 | 3 | 10 | 0 | 1 | 216 |
| 77 | 4D | 22 | 16 | 3 | 11 | 341924 | 2735388 | 3 | 11 | 0 | 208 | 2781148 | 3 | 11 | 0 | 1 | 216 |
| 78 | 4E | 22 | 16 | 3 | 12 | 341950 | 2735596 | 3 | 12 | 0 | 208 | 2781364 | 3 | 12 | 0 | 1 | 216 |
| 79 | 4F | 22 | 16 | 3 | 13 | 341976 | 2735804 | 3 | 13 | 0 | 208 | 2781580 | 3 | 13 | 0 | 1 | 216 |
| 80 | 50 | 22 | 16 | 3 | 14 | 342004 | 2736028 | 3 | 14 | 0 | 224 | 2781812 | 3 | 14 | 0 | 1 | 232 |
| 81 | 51 | 22 | 16 | 3 | 15 | 342032 | 2736252 | 3 | 15 | 0 | 224 | 2782044 | 3 | 15 | 0 | 1 | 232 |
| 82 | 52 | 22 | 16 | 3 | 16 | 342060 | 2736476 | 3 | 16 | 0 | 224 | 2782276 | 3 | 16 | 0 | 1 | 232 |
| 83 | 53 | 22 | 16 | 3 | 17 | 342088 | 2736700 | 3 | 17 | 0 | 224 | 2782508 | 3 | 17 | 0 | 1 | 232 |
| 84 | 54 | 22 | 16 | 3 | 18 | 342116 | 2736924 | 3 | 18 | 0 | 224 | 2782740 | 3 | 18 | 0 | 1 | 232 |
| 85 | 55 | 22 | 16 | 3 | 19 | 342144 | 2737148 | 3 | 19 | 0 | 224 | 2782972 | 3 | 19 | 0 | 1 | 232 |
| 86 | 56 | 22 | 16 | 3 | 20 | 342172 | 2737372 | 3 | 20 | 0 | 224 | 2783204 | 3 | 20 | 0 | 1 | 232 |
| 87 | 57 | 22 | 16 | 3 | 21 | 342200 | 2737596 | 3 | 21 | 0 | 224 | 2783436 | 3 | 21 | 0 | 1 | 232 |
| 88 | 58 | 22 | 16 | 4 | 0 | 342230 | 2737836 | 4 | 0 | 0 | 240 | 2783684 | 4 | 0 | 0 | 1 | 248 |
| 89 | 59 | 22 | 16 | 4 | 1 | 342260 | 2738076 | 4 | 1 | 0 | 240 | 2783932 | 4 | 1 | 0 | 1 | 248 |
| 90 | 5A | 22 | 16 | 4 | 2 | 342290 | 2738316 | 4 | 2 | 0 | 240 | 2784180 | 4 | 2 | 0 | 1 | 248 |
| 91 | 5B | 22 | 16 | 4 | 3 | 342320 | 2738556 | 4 | 3 | 0 | 240 | 2784428 | 4 | 3 | 0 | 1 | 248 |
| 92 | 5C | 22 | 16 | 4 | 4 | 342350 | 2738796 | 4 | 4 | 0 | 240 | 2784676 | 4 | 4 | 0 | 1 | 248 |
| 93 | 5D | 22 | 16 | 4 | 5 | 342380 | 2739036 | 4 | 5 | 0 | 240 | 2784924 | 4 | 5 | 0 | 1 | 248 |
| 94 | 5E | 22 | 16 | 4 | 6 | 342410 | 2739276 | 4 | 6 | 0 | 240 | 2785172 | 4 | 6 | 0 | 1 | 248 |
| 95 | 5F | 22 | 16 | 4 | 7 | 342440 | 2739516 | 4 | 7 | 0 | 240 | 2785420 | 4 | 7 | 0 | 1 | 248 |
| 96 | 60 | 22 | 16 | 4 | 8 | 342468 | 2739740 | 4 | 8 | 0 | 224 | 2785652 | 4 | 8 | 0 | 1 | 232 |
| 97 | 61 | 22 | 16 | 4 | 9 | 342496 | 2739964 | 4 | 9 | 0 | 224 | 2785884 | 4 | 9 | 0 | 1 | 232 |
| 98 | 62 | 22 | 16 | 4 | 10 | 342524 | 2740188 | 4 | 10 | 0 | 224 | 2786116 | 4 | 10 | 0 | 1 | 232 |
| 99 | 63 | 22 | 16 | 4 | 11 | 342552 | 2740412 | 4 | 11 | 0 | 224 | 2786348 | 4 | 11 | 0 | 1 | 232 |
| 100 | 64 | 22 | 16 | 4 | 12 | 342580 | 2740636 | 4 | 12 | 0 | 224 | 2786580 | 4 | 12 | 0 | 1 | 232 |
| 101 | 65 | 22 | 16 | 4 | 13 | 342608 | 2740860 | 4 | 13 | 0 | 224 | 2786812 | 4 | 13 | 0 | 1 | 232 |
| 102 | 66 | 22 | 16 | 4 | 14 | 342636 | 2741084 | 4 | 14 | 0 | 224 | 2787044 | 4 | 14 | 0 | 1 | 232 |
| 103 | 67 | 22 | 16 | 4 | 15 | 342664 | 2741308 | 4 | 15 | 0 | 224 | 2787276 | 4 | 15 | 0 | 1 | 232 |
| 104 | 68 | 22 | 16 | 4 | 16 | 342692 | 2741532 | 4 | 16 | 0 | 224 | 2787508 | 4 | 16 | 0 | 1 | 232 |
| 105 | 69 | 22 | 16 | 4 | 17 | 342720 | 2741756 | 4 | 17 | 0 | 224 | 2787740 | 4 | 17 | 0 | 1 | 232 |
| 106 | 6A | 22 | 16 | 4 | 18 | 342748 | 2741980 | 4 | 18 | 0 | 224 | 2787972 | 4 | 18 | 0 | 1 | 232 |
| 107 | 6B | 22 | 16 | 4 | 19 | 342776 | 2742204 | 4 | 19 | 0 | 224 | 2788204 | 4 | 19 | 0 | 1 | 232 |
| 108 | 6C | 22 | 16 | 4 | 20 | 342804 | 2742428 | 4 | 20 | 0 | 224 | 2788436 | 4 | 20 | 0 | 1 | 232 |
| 109 | 6D | 22 | 16 | 4 | 21 | 342832 | 2742652 | 4 | 21 | 0 | 224 | 2788668 | 4 | 21 | 0 | 1 | 232 |
| 110 | 6E | 22 | 16 | 5 | 0 | 342862 | 2742892 | 5 | 0 | 0 | 240 | 2788916 | 5 | 0 | 0 | 1 | 248 |
| 111 | 6F | 22 | 16 | 5 | 1 | 342892 | 2743132 | 5 | 1 | 0 | 240 | 2789164 | 5 | 1 | 0 | 1 | 248 |
| 112 | 70 | 22 | 16 | 5 | 2 | 342924 | 2743388 | 5 | 2 | 0 | 256 | 2789428 | 5 | 2 | 0 | 1 | 264 |
| 113 | 71 | 22 | 16 | 5 | 3 | 342956 | 2743644 | 5 | 3 | 0 | 256 | 2789692 | 5 | 3 | 0 | 1 | 264 |
| 114 | 72 | 22 | 16 | 5 | 4 | 342988 | 2743900 | 5 | 4 | 0 | 256 | 2789956 | 5 | 4 | 0 | 1 | 264 |
| 115 | 73 | 22 | 16 | 5 | 5 | 343020 | 2744156 | 5 | 5 | 0 | 256 | 2790220 | 5 | 5 | 0 | 1 | 264 |
| 116 | 74 | 22 | 16 | 5 | 6 | 343052 | 2744412 | 5 | 6 | 0 | 256 | 2790484 | 5 | 6 | 0 | 1 | 264 |
| 117 | 75 | 22 | 16 | 5 | 7 | 343084 | 2744668 | 5 | 7 | 0 | 256 | 2790748 | 5 | 7 | 0 | 1 | 264 |
| 118 | 76 | 22 | 16 | 5 | 8 | 343116 | 2744924 | 5 | 8 | 0 | 256 | 2791012 | 5 | 8 | 0 | 1 | 264 |
| 119 | 77 | 22 | 16 | 5 | 9 | 343148 | 2745180 | 5 | 9 | 0 | 256 | 2791276 | 5 | 9 | 0 | 1 | 264 |
| 120 | 78 | 22 | 16 | 5 | 10 | 343180 | 2745436 | 5 | 10 | 0 | 256 | 2791540 | 5 | 10 | 0 | 1 | 264 |
| 121 | 79 | 22 | 16 | 5 | 11 | 343212 | 2745692 | 5 | 11 | 0 | 256 | 2791804 | 5 | 11 | 0 | 1 | 264 |
| 122 | 7A | 22 | 16 | 5 | 12 | 343244 | 2745948 | 5 | 12 | 0 | 256 | 2792068 | 5 | 12 | 0 | 1 | 264 |
| 123 | 7B | 22 | 16 | 5 | 13 | 343276 | 2746204 | 5 | 13 | 0 | 256 | 2792332 | 5 | 13 | 0 | 1 | 264 |
| 124 | 7C | 22 | 16 | 5 | 14 | 343308 | 2746460 | 5 | 14 | 0 | 256 | 2792596 | 5 | 14 | 0 | 1 | 264 |
| 125 | 7D | 22 | 16 | 5 | 15 | 343340 | 2746716 | 5 | 15 | 0 | 256 | 2792860 | 5 | 15 | 0 | 1 | 264 |
| 126 | 7E | 22 | 16 | 5 | 16 | 343372 | 2746972 | 5 | 16 | 0 | 256 | 2793124 | 5 | 16 | 0 | 1 | 264 |
| 127 | 7F | 22 | 16 | 5 | 17 | 343404 | 2747228 | 5 | 17 | 0 | 256 | 2793388 | 5 | 17 | 0 | 1 | 264 |
| 128 | 80 | 22 | 16 | 5 | 18 | 343434 | 2747468 | 5 | 18 | 0 | 240 | 2793636 | 5 | 18 | 0 | 1 | 248 |
| 129 | 81 | 22 | 16 | 5 | 19 | 343464 | 2747708 | 5 | 19 | 0 | 240 | 2793884 | 5 | 19 | 0 | 1 | 248 |
| 130 | 82 | 22 | 16 | 5 | 20 | 343494 | 2747948 | 5 | 20 | 0 | 240 | 2794132 | 5 | 20 | 0 | 1 | 248 |
| 131 | 83 | 22 | 16 | 5 | 21 | 343524 | 2748188 | 5 | 21 | 0 | 240 | 2794380 | 5 | 21 | 0 | 1 | 248 |
| 132 | 84 | 22 | 16 | 6 | 0 | 343556 | 2748444 | 6 | 0 | 0 | 256 | 2794644 | 6 | 0 | 0 | 1 | 264 |

| | | | | | | | | | | | | | | | | | |
|---|---|---|---|---|---|---|---|---|---|---|---|---|---|---|---|---|---|
| 133 | 85 | 22 | 16 | 6 | 1 | 343588 | 2748700 | 6 | 1 | 0 | 256 | 2794908 | 6 | 1 | 0 | 1 | 264 |
| 134 | 86 | 22 | 16 | 6 | 2 | 343620 | 2748956 | 6 | 2 | 0 | 256 | 2795172 | 6 | 2 | 0 | 1 | 264 |
| 135 | 87 | 22 | 16 | 6 | 3 | 343652 | 2749212 | 6 | 3 | 0 | 256 | 2795436 | 6 | 3 | 0 | 1 | 264 |
| 136 | 88 | 22 | 16 | 6 | 4 | 343684 | 2749468 | 6 | 4 | 0 | 256 | 2795700 | 6 | 4 | 0 | 1 | 264 |
| 137 | 89 | 22 | 16 | 6 | 5 | 343716 | 2749724 | 6 | 5 | 0 | 256 | 2795964 | 6 | 5 | 0 | 1 | 264 |
| 138 | 8A | 22 | 16 | 6 | 6 | 343748 | 2749980 | 6 | 6 | 0 | 256 | 2796228 | 6 | 6 | 0 | 1 | 264 |
| 139 | 8B | 22 | 16 | 6 | 7 | 343780 | 2750236 | 6 | 7 | 0 | 256 | 2796492 | 6 | 7 | 0 | 1 | 264 |
| 140 | 8C | 22 | 16 | 6 | 8 | 343812 | 2750492 | 6 | 8 | 0 | 256 | 2796756 | 6 | 8 | 0 | 1 | 264 |
| 141 | 8D | 22 | 16 | 6 | 9 | 343844 | 2750748 | 6 | 9 | 0 | 256 | 2797020 | 6 | 9 | 0 | 1 | 264 |
| 142 | 8E | 22 | 16 | 6 | 10 | 343876 | 2751004 | 6 | 10 | 0 | 256 | 2797284 | 6 | 10 | 0 | 1 | 264 |
| 143 | 8F | 22 | 16 | 6 | 11 | 343908 | 2751260 | 6 | 11 | 0 | 256 | 2797548 | 6 | 11 | 0 | 1 | 264 |
| 144 | 90 | 22 | 16 | 6 | 12 | 343942 | 2751532 | 6 | 12 | 0 | 272 | 2797828 | 6 | 12 | 0 | 1 | 280 |
| 145 | 91 | 22 | 16 | 6 | 13 | 343976 | 2751804 | 6 | 13 | 0 | 272 | 2798108 | 6 | 13 | 0 | 1 | 280 |
| 146 | 92 | 22 | 16 | 6 | 14 | 344010 | 2752076 | 6 | 14 | 0 | 272 | 2798388 | 6 | 14 | 0 | 1 | 280 |
| 147 | 93 | 22 | 16 | 6 | 15 | 344044 | 2752348 | 6 | 15 | 0 | 272 | 2798668 | 6 | 15 | 0 | 1 | 280 |
| 148 | 94 | 22 | 16 | 6 | 16 | 344078 | 2752620 | 6 | 16 | 0 | 272 | 2798948 | 6 | 16 | 0 | 1 | 280 |
| 149 | 95 | 22 | 16 | 6 | 17 | 344112 | 2752892 | 6 | 17 | 0 | 272 | 2799228 | 6 | 17 | 0 | 1 | 280 |
| 150 | 96 | 22 | 16 | 6 | 18 | 344146 | 2753164 | 6 | 18 | 0 | 272 | 2799508 | 6 | 18 | 0 | 1 | 280 |
| 151 | 97 | 22 | 16 | 6 | 19 | 344180 | 2753436 | 6 | 19 | 0 | 272 | 2799788 | 6 | 19 | 0 | 1 | 280 |
| 152 | 98 | 22 | 16 | 6 | 20 | 344214 | 2753708 | 6 | 20 | 0 | 272 | 2800068 | 6 | 20 | 0 | 1 | 280 |
| 153 | 99 | 22 | 16 | 6 | 21 | 344248 | 2753980 | 6 | 21 | 0 | 272 | 2800348 | 6 | 21 | 0 | 1 | 280 |
| 154 | 9A | 22 | 16 | 7 | 0 | 344284 | 2754268 | 7 | 0 | 0 | 288 | 2800644 | 7 | 0 | 0 | 1 | 296 |
| 155 | 9B | 22 | 16 | 7 | 1 | 344320 | 2754556 | 7 | 1 | 0 | 288 | 2800940 | 7 | 1 | 0 | 1 | 296 |
| 156 | 9C | 22 | 16 | 7 | 2 | 344356 | 2754844 | 7 | 2 | 0 | 288 | 2801236 | 7 | 2 | 0 | 1 | 296 |
| 157 | 9D | 22 | 16 | 7 | 3 | 344392 | 2755132 | 7 | 3 | 0 | 288 | 2801532 | 7 | 3 | 0 | 1 | 296 |
| 158 | 9E | 22 | 16 | 7 | 4 | 344428 | 2755420 | 7 | 4 | 0 | 288 | 2801828 | 7 | 4 | 0 | 1 | 296 |
| 159 | 9F | 22 | 16 | 7 | 5 | 344464 | 2755708 | 7 | 5 | 0 | 288 | 2802124 | 7 | 5 | 0 | 1 | 296 |
| 160 | A0 | 22 | 16 | 7 | 6 | 344498 | 2755980 | 7 | 6 | 0 | 272 | 2802404 | 7 | 6 | 0 | 1 | 280 |
| 161 | A1 | 22 | 16 | 7 | 7 | 344532 | 2756252 | 7 | 7 | 0 | 272 | 2802684 | 7 | 7 | 0 | 1 | 280 |
| 162 | A2 | 22 | 16 | 7 | 8 | 344566 | 2756524 | 7 | 8 | 0 | 272 | 2802964 | 7 | 8 | 0 | 1 | 280 |
| 163 | A3 | 22 | 16 | 7 | 9 | 344600 | 2756796 | 7 | 9 | 0 | 272 | 2803244 | 7 | 9 | 0 | 1 | 280 |
| 164 | A4 | 22 | 16 | 7 | 10 | 344634 | 2757068 | 7 | 10 | 0 | 272 | 2803524 | 7 | 10 | 0 | 1 | 280 |
| 165 | A5 | 22 | 16 | 7 | 11 | 344668 | 2757340 | 7 | 11 | 0 | 272 | 2803804 | 7 | 11 | 0 | 1 | 280 |
| 166 | A6 | 22 | 16 | 7 | 12 | 344702 | 2757612 | 7 | 12 | 0 | 272 | 2804084 | 7 | 12 | 0 | 1 | 280 |
| 167 | A7 | 22 | 16 | 7 | 13 | 344736 | 2757884 | 7 | 13 | 0 | 272 | 2804364 | 7 | 13 | 0 | 1 | 280 |
| 168 | A8 | 22 | 16 | 7 | 14 | 344770 | 2758156 | 7 | 14 | 0 | 272 | 2804644 | 7 | 14 | 0 | 1 | 280 |
| 169 | A9 | 22 | 16 | 7 | 15 | 344804 | 2758428 | 7 | 15 | 0 | 272 | 2804924 | 7 | 15 | 0 | 1 | 280 |
| 170 | AA | 22 | 16 | 7 | 16 | 344838 | 2758700 | 7 | 16 | 0 | 272 | 2805204 | 7 | 16 | 0 | 1 | 280 |
| 171 | AB | 22 | 16 | 7 | 17 | 344872 | 2758972 | 7 | 17 | 0 | 272 | 2805484 | 7 | 17 | 0 | 1 | 280 |
| 172 | AC | 22 | 16 | 7 | 18 | 344906 | 2759244 | 7 | 18 | 0 | 272 | 2805764 | 7 | 18 | 0 | 1 | 280 |
| 173 | AD | 22 | 16 | 7 | 19 | 344940 | 2759516 | 7 | 19 | 0 | 272 | 2806044 | 7 | 19 | 0 | 1 | 280 |
| 174 | AE | 22 | 16 | 7 | 20 | 344974 | 2759788 | 7 | 20 | 0 | 272 | 2806324 | 7 | 20 | 0 | 1 | 280 |
| 175 | AF | 22 | 16 | 7 | 21 | 345008 | 2760060 | 7 | 21 | 0 | 272 | 2806604 | 7 | 21 | 0 | 1 | 280 |
| 176 | B0 | 22 | 16 | 8 | 0 | 345046 | 2760364 | 8 | 0 | 0 | 304 | 2806916 | 8 | 0 | 0 | 1 | 312 |
| 177 | B1 | 22 | 16 | 8 | 1 | 345084 | 2760668 | 8 | 1 | 0 | 304 | 2807228 | 8 | 1 | 0 | 1 | 312 |
| 178 | B2 | 22 | 16 | 8 | 2 | 345122 | 2760972 | 8 | 2 | 0 | 304 | 2807540 | 8 | 2 | 0 | 1 | 312 |
| 179 | B3 | 22 | 16 | 8 | 3 | 345160 | 2761276 | 8 | 3 | 0 | 304 | 2807852 | 8 | 3 | 0 | 1 | 312 |
| 180 | B4 | 22 | 16 | 8 | 4 | 345198 | 2761580 | 8 | 4 | 0 | 304 | 2808164 | 8 | 4 | 0 | 1 | 312 |
| 181 | B5 | 22 | 16 | 8 | 5 | 345236 | 2761884 | 8 | 5 | 0 | 304 | 2808476 | 8 | 5 | 0 | 1 | 312 |
| 182 | B6 | 22 | 16 | 8 | 6 | 345274 | 2762188 | 8 | 6 | 0 | 304 | 2808788 | 8 | 6 | 0 | 1 | 312 |
| 183 | B7 | 22 | 16 | 8 | 7 | 345312 | 2762492 | 8 | 7 | 0 | 304 | 2809100 | 8 | 7 | 0 | 1 | 312 |
| 184 | B8 | 22 | 16 | 8 | 8 | 345350 | 2762796 | 8 | 8 | 0 | 304 | 2809412 | 8 | 8 | 0 | 1 | 312 |
| 185 | B9 | 22 | 16 | 8 | 9 | 345388 | 2763100 | 8 | 9 | 0 | 304 | 2809724 | 8 | 9 | 0 | 1 | 312 |
| 186 | BA | 22 | 16 | 8 | 10 | 345426 | 2763404 | 8 | 10 | 0 | 304 | 2810036 | 8 | 10 | 0 | 1 | 312 |
| 187 | BB | 22 | 16 | 8 | 11 | 345464 | 2763708 | 8 | 11 | 0 | 304 | 2810348 | 8 | 11 | 0 | 1 | 312 |
| 188 | BC | 22 | 16 | 8 | 12 | 345502 | 2764012 | 8 | 12 | 0 | 304 | 2810660 | 8 | 12 | 0 | 1 | 312 |
| 189 | BD | 22 | 16 | 8 | 13 | 345540 | 2764316 | 8 | 13 | 0 | 304 | 2810972 | 8 | 13 | 0 | 1 | 312 |
| 190 | BE | 22 | 16 | 8 | 14 | 345578 | 2764620 | 8 | 14 | 0 | 304 | 2811284 | 8 | 14 | 0 | 1 | 312 |
| 191 | BF | 22 | 16 | 8 | 15 | 345616 | 2764924 | 8 | 15 | 0 | 304 | 2811596 | 8 | 15 | 0 | 1 | 312 |
| 192 | C0 | 22 | 16 | 8 | 16 | 345652 | 2765212 | 8 | 16 | 0 | 288 | 2811892 | 8 | 16 | 0 | 1 | 296 |
| 193 | C1 | 22 | 16 | 8 | 17 | 345688 | 2765500 | 8 | 17 | 0 | 288 | 2812188 | 8 | 17 | 0 | 1 | 296 |
| 194 | C2 | 22 | 16 | 8 | 18 | 345724 | 2765788 | 8 | 18 | 0 | 288 | 2812484 | 8 | 18 | 0 | 1 | 296 |
| 195 | C3 | 22 | 16 | 8 | 19 | 345760 | 2766076 | 8 | 19 | 0 | 288 | 2812780 | 8 | 19 | 0 | 1 | 296 |
| 196 | C4 | 22 | 16 | 8 | 20 | 345796 | 2766364 | 8 | 20 | 0 | 288 | 2813076 | 8 | 20 | 0 | 1 | 296 |
| 197 | C5 | 22 | 16 | 8 | 21 | 345832 | 2766652 | 8 | 21 | 0 | 288 | 2813372 | 8 | 21 | 0 | 1 | 296 |
| 198 | C6 | 22 | 16 | 9 | 0 | 345870 | 2766956 | 9 | 0 | 0 | 304 | 2813684 | 9 | 0 | 0 | 1 | 312 |
| 199 | C7 | 22 | 16 | 9 | 1 | 345908 | 2767260 | 9 | 1 | 0 | 304 | 2813996 | 9 | 1 | 0 | 1 | 312 |
| 200 | C8 | 22 | 16 | 9 | 2 | 345946 | 2767564 | 9 | 2 | 0 | 304 | 2814308 | 9 | 2 | 0 | 1 | 312 |
| 201 | C9 | 22 | 16 | 9 | 3 | 345984 | 2767868 | 9 | 3 | 0 | 304 | 2814620 | 9 | 3 | 0 | 1 | 312 |
| 202 | CA | 22 | 16 | 9 | 4 | 346022 | 2768172 | 9 | 4 | 0 | 304 | 2814932 | 9 | 4 | 0 | 1 | 312 |
| 203 | CB | 22 | 16 | 9 | 5 | 346060 | 2768476 | 9 | 5 | 0 | 304 | 2815244 | 9 | 5 | 0 | 1 | 312 |
| 204 | CC | 22 | 16 | 9 | 6 | 346098 | 2768780 | 9 | 6 | 0 | 304 | 2815556 | 9 | 6 | 0 | 1 | 312 |
| 205 | CD | 22 | 16 | 9 | 7 | 346136 | 2769084 | 9 | 7 | 0 | 304 | 2815868 | 9 | 7 | 0 | 1 | 312 |
| 206 | CE | 22 | 16 | 9 | 8 | 346174 | 2769388 | 9 | 8 | 0 | 304 | 2816180 | 9 | 8 | 0 | 1 | 312 |
| 207 | CF | 22 | 16 | 9 | 9 | 346212 | 2769692 | 9 | 9 | 0 | 304 | 2816492 | 9 | 9 | 0 | 1 | 312 |
| 208 | D0 | 22 | 16 | 9 | 10 | 346252 | 2770012 | 9 | 10 | 0 | 320 | 2816820 | 9 | 10 | 0 | 1 | 328 |
| 209 | D1 | 22 | 16 | 9 | 11 | 346292 | 2770332 | 9 | 11 | 0 | 320 | 2817148 | 9 | 11 | 0 | 1 | 328 |
| 210 | D2 | 22 | 16 | 9 | 12 | 346332 | 2770652 | 9 | 12 | 0 | 320 | 2817476 | 9 | 12 | 0 | 1 | 328 |
| 211 | D3 | 22 | 16 | 9 | 13 | 346372 | 2770972 | 9 | 13 | 0 | 320 | 2817804 | 9 | 13 | 0 | 1 | 328 |
| 212 | D4 | 22 | 16 | 9 | 14 | 346412 | 2771292 | 9 | 14 | 0 | 320 | 2818132 | 9 | 14 | 0 | 1 | 328 |
| 213 | D5 | 22 | 16 | 9 | 15 | 346452 | 2771612 | 9 | 15 | 0 | 320 | 2818460 | 9 | 15 | 0 | 1 | 328 |
| 214 | D6 | 22 | 16 | 9 | 16 | 346492 | 2771932 | 9 | 16 | 0 | 320 | 2818788 | 9 | 16 | 0 | 1 | 328 |
| 215 | D7 | 22 | 16 | 9 | 17 | 346532 | 2772252 | 9 | 17 | 0 | 320 | 2819116 | 9 | 17 | 0 | 1 | 328 |
| 216 | D8 | 22 | 16 | 9 | 18 | 346572 | 2772572 | 9 | 18 | 0 | 320 | 2819444 | 9 | 18 | 0 | 1 | 328 |
| 217 | D9 | 22 | 16 | 9 | 19 | 346612 | 2772892 | 9 | 19 | 0 | 320 | 2819772 | 9 | 19 | 0 | 1 | 328 |
| 218 | DA | 22 | 16 | 9 | 20 | 346652 | 2773212 | 9 | 20 | 0 | 320 | 2820100 | 9 | 20 | 0 | 1 | 328 |
| 219 | DB | 22 | 16 | 9 | 21 | 346692 | 2773532 | 9 | 21 | 0 | 320 | 2820428 | 9 | 21 | 0 | 1 | 328 |
| 220 | DC | 22 | 16 | 10 | 0 | 346734 | 2773868 | 10 | 0 | 0 | 336 | 2820772 | 10 | 0 | 0 | 1 | 344 |
| 221 | DD | 22 | 16 | 10 | 1 | 346776 | 2774204 | 10 | 1 | 0 | 336 | 2821116 | 10 | 1 | 0 | 1 | 344 |
| 222 | DE | 22 | 16 | 10 | 2 | 346818 | 2774540 | 10 | 2 | 0 | 336 | 2821460 | 10 | 2 | 0 | 1 | 344 |
| 223 | DF | 22 | 16 | 10 | 3 | 346860 | 2774876 | 10 | 3 | 0 | 336 | 2821804 | 10 | 3 | 0 | 1 | 344 |
| 224 | E0 | 22 | 16 | 10 | 4 | 346900 | 2775196 | 10 | 4 | 0 | 320 | 2822132 | 10 | 4 | 0 | 1 | 328 |
| 225 | E1 | 22 | 16 | 10 | 5 | 346940 | 2775516 | 10 | 5 | 0 | 320 | 2822460 | 10 | 5 | 0 | 1 | 328 |
| 226 | E2 | 22 | 16 | 10 | 6 | 346980 | 2775836 | 10 | 6 | 0 | 320 | 2822788 | 10 | 6 | 0 | 1 | 328 |
| 227 | E3 | 22 | 16 | 10 | 7 | 347020 | 2776156 | 10 | 7 | 0 | 320 | 2823116 | 10 | 7 | 0 | 1 | 328 |
| 228 | E4 | 22 | 16 | 10 | 8 | 347060 | 2776476 | 10 | 8 | 0 | 320 | 2823444 | 10 | 8 | 0 | 1 | 328 |
| 229 | E5 | 22 | 16 | 10 | 9 | 347100 | 2776796 | 10 | 9 | 0 | 320 | 2823772 | 10 | 9 | 0 | 1 | 328 |
| 230 | E6 | 22 | 16 | 10 | 10 | 347140 | 2777116 | 10 | 10 | 0 | 320 | 2824100 | 10 | 10 | 0 | 1 | 328 |
| 231 | E7 | 22 | 16 | 10 | 11 | 347180 | 2777436 | 10 | 11 | 0 | 320 | 2824428 | 10 | 11 | 0 | 1 | 328 |
| 232 | E8 | 22 | 16 | 10 | 12 | 347220 | 2777756 | 10 | 12 | 0 | 320 | 2824756 | 10 | 12 | 0 | 1 | 328 |
| 233 | E9 | 22 | 16 | 10 | 13 | 347260 | 2778076 | 10 | 13 | 0 | 320 | 2825084 | 10 | 13 | 0 | 1 | 328 |
| 234 | EA | 22 | 16 | 10 | 14 | 347300 | 2778396 | 10 | 14 | 0 | 320 | 2825412 | 10 | 14 | 0 | 1 | 328 |
| 235 | EB | 22 | 16 | 10 | 15 | 347340 | 2778716 | 10 | 15 | 0 | 320 | 2825740 | 10 | 15 | 0 | 1 | 328 |

| | | | | | | | | | | | | | | | | | |
|---|---|---|---|---|---|---|---|---|---|---|---|---|---|---|---|---|---|
| 236 | EC | 22 | 16 | 10 | 16 | 347380 | 2779036 | 10 | 16 | 0 | 320 | 2826068 | 10 | 16 | 0 | 1 | 328 |
| 237 | ED | 22 | 16 | 10 | 17 | 347420 | 2779356 | 10 | 17 | 0 | 320 | 2826396 | 10 | 17 | 0 | 1 | 328 |
| 238 | EE | 22 | 16 | 10 | 18 | 347460 | 2779676 | 10 | 18 | 0 | 320 | 2826724 | 10 | 18 | 0 | 1 | 328 |
| 239 | EF | 22 | 16 | 10 | 19 | 347500 | 2779996 | 10 | 19 | 0 | 320 | 2827052 | 10 | 19 | 0 | 1 | 328 |
| 240 | F0 | 22 | 16 | 10 | 20 | 347542 | 2780332 | 10 | 20 | 0 | 336 | 2827396 | 10 | 20 | 0 | 1 | 344 |
| 241 | F1 | 22 | 16 | 10 | 21 | 347584 | 2780668 | 10 | 21 | 0 | 336 | 2827740 | 10 | 21 | 0 | 1 | 344 |
| 242 | F2 | 22 | 16 | 11 | 0 | 347628 | 2781020 | 11 | 0 | 0 | 352 | 2828100 | 11 | 0 | 0 | 1 | 360 |
| 243 | F3 | 22 | 16 | 11 | 1 | 347672 | 2781372 | 11 | 1 | 0 | 352 | 2828460 | 11 | 1 | 0 | 1 | 360 |
| 244 | F4 | 22 | 16 | 11 | 2 | 347716 | 2781724 | 11 | 2 | 0 | 352 | 2828820 | 11 | 2 | 0 | 1 | 360 |
| 245 | F5 | 22 | 16 | 11 | 3 | 347760 | 2782076 | 11 | 3 | 0 | 352 | 2829180 | 11 | 3 | 0 | 1 | 360 |
| 246 | F6 | 22 | 16 | 11 | 4 | 347804 | 2782428 | 11 | 4 | 0 | 352 | 2829540 | 11 | 4 | 0 | 1 | 360 |
| 247 | F7 | 22 | 16 | 11 | 5 | 347848 | 2782780 | 11 | 5 | 0 | 352 | 2829900 | 11 | 5 | 0 | 1 | 360 |
| 248 | F8 | 22 | 16 | 11 | 6 | 347892 | 2783132 | 11 | 6 | 0 | 352 | 2830260 | 11 | 6 | 0 | 1 | 360 |
| 249 | F9 | 22 | 16 | 11 | 7 | 347936 | 2783484 | 11 | 7 | 0 | 352 | 2830620 | 11 | 7 | 0 | 1 | 360 |
| 250 | FA | 22 | 16 | 11 | 8 | 347980 | 2783836 | 11 | 8 | 0 | 352 | 2830980 | 11 | 8 | 0 | 1 | 360 |
| 251 | FB | 22 | 16 | 11 | 9 | 348024 | 2784188 | 11 | 9 | 0 | 352 | 2831340 | 11 | 9 | 0 | 1 | 360 |
| 252 | FC | 22 | 16 | 11 | 10 | 348068 | 2784540 | 11 | 10 | 0 | 352 | 2831700 | 11 | 10 | 0 | 1 | 360 |
| 253 | FD | 22 | 16 | 11 | 11 | 348112 | 2784892 | 11 | 11 | 0 | 352 | 2832060 | 11 | 11 | 0 | 1 | 360 |
| 254 | FE | 22 | 16 | 11 | 12 | 348156 | 2785244 | 11 | 12 | 0 | 352 | 2832420 | 11 | 12 | 0 | 1 | 360 |
| 255 | FF | 22 | 16 | 11 | 13 | 348200 | 2785596 | 11 | 13 | 0 | 352 | 2832780 | 11 | 13 | 0 | 1 | 360 |
| 0 | 00 | 23 | 17 | 0 | 0 | 348212 | 2785692 | 0 | 0 | 0 | 96 | 2832884 | 0 | 0 | 0 | 1 | 104 |
| 1 | 01 | 23 | 17 | 0 | 1 | 348226 | 2785804 | 0 | 1 | 0 | 112 | 2833004 | 0 | 1 | 0 | 1 | 120 |
| 2 | 02 | 23 | 17 | 0 | 2 | 348240 | 2785916 | 0 | 2 | 0 | 112 | 2833124 | 0 | 2 | 0 | 1 | 120 |
| 3 | 03 | 23 | 17 | 0 | 3 | 348254 | 2786028 | 0 | 3 | 0 | 112 | 2833244 | 0 | 3 | 0 | 1 | 120 |
| 4 | 04 | 23 | 17 | 0 | 4 | 348268 | 2786140 | 0 | 4 | 0 | 112 | 2833364 | 0 | 4 | 0 | 1 | 120 |
| 5 | 05 | 23 | 17 | 0 | 5 | 348282 | 2786252 | 0 | 5 | 0 | 112 | 2833484 | 0 | 5 | 0 | 1 | 120 |
| 6 | 06 | 23 | 17 | 0 | 6 | 348296 | 2786364 | 0 | 6 | 0 | 112 | 2833604 | 0 | 6 | 0 | 1 | 120 |
| 7 | 07 | 23 | 17 | 0 | 7 | 348310 | 2786476 | 0 | 7 | 0 | 112 | 2833724 | 0 | 7 | 0 | 1 | 120 |
| 8 | 08 | 23 | 17 | 0 | 8 | 348324 | 2786588 | 0 | 8 | 0 | 112 | 2833844 | 0 | 8 | 0 | 1 | 120 |
| 9 | 09 | 23 | 17 | 0 | 9 | 348338 | 2786700 | 0 | 9 | 0 | 112 | 2833964 | 0 | 9 | 0 | 1 | 120 |
| 10 | 0A | 23 | 17 | 0 | 10 | 348352 | 2786812 | 0 | 10 | 0 | 112 | 2834084 | 0 | 10 | 0 | 1 | 120 |
| 11 | 0B | 23 | 17 | 0 | 11 | 348366 | 2786924 | 0 | 11 | 0 | 112 | 2834204 | 0 | 11 | 0 | 1 | 120 |
| 12 | 0C | 23 | 17 | 0 | 12 | 348380 | 2787036 | 0 | 12 | 0 | 112 | 2834324 | 0 | 12 | 0 | 1 | 120 |
| 13 | 0D | 23 | 17 | 0 | 13 | 348394 | 2787148 | 0 | 13 | 0 | 112 | 2834444 | 0 | 13 | 0 | 1 | 120 |
| 14 | 0E | 23 | 17 | 0 | 14 | 348408 | 2787260 | 0 | 14 | 0 | 112 | 2834564 | 0 | 14 | 0 | 1 | 120 |
| 15 | 0F | 23 | 17 | 0 | 15 | 348422 | 2787372 | 0 | 15 | 0 | 112 | 2834684 | 0 | 15 | 0 | 1 | 120 |
| 16 | 10 | 23 | 17 | 0 | 16 | 348436 | 2787484 | 0 | 16 | 0 | 112 | 2834804 | 0 | 16 | 0 | 1 | 120 |
| 17 | 11 | 23 | 17 | 0 | 17 | 348450 | 2787596 | 0 | 17 | 0 | 112 | 2834924 | 0 | 17 | 0 | 1 | 120 |
| 18 | 12 | 23 | 17 | 0 | 18 | 348464 | 2787708 | 0 | 18 | 0 | 112 | 2835044 | 0 | 18 | 0 | 1 | 120 |
| 19 | 13 | 23 | 17 | 0 | 19 | 348478 | 2787820 | 0 | 19 | 0 | 112 | 2835164 | 0 | 19 | 0 | 1 | 120 |
| 20 | 14 | 23 | 17 | 0 | 20 | 348492 | 2787932 | 0 | 20 | 0 | 112 | 2835284 | 0 | 20 | 0 | 1 | 120 |
| 21 | 15 | 23 | 17 | 0 | 21 | 348506 | 2788044 | 0 | 21 | 0 | 112 | 2835404 | 0 | 21 | 0 | 1 | 120 |
| 22 | 16 | 23 | 17 | 0 | 22 | 348520 | 2788156 | 0 | 22 | 0 | 112 | 2835524 | 0 | 22 | 0 | 1 | 120 |
| 23 | 17 | 23 | 17 | 1 | 0 | 348532 | 2788252 | 1 | 0 | 0 | 96 | 2835628 | 1 | 0 | 0 | 1 | 104 |
| 24 | 18 | 23 | 17 | 1 | 1 | 348549 | 2788388 | 1 | 1 | 0 | 136 | 2835772 | 1 | 1 | 0 | 1 | 144 |
| 25 | 19 | 23 | 17 | 1 | 2 | 348566 | 2788524 | 1 | 2 | 0 | 136 | 2835916 | 1 | 2 | 0 | 1 | 144 |
| 26 | 1A | 23 | 17 | 1 | 3 | 348583 | 2788660 | 1 | 3 | 0 | 136 | 2836060 | 1 | 3 | 0 | 1 | 144 |
| 27 | 1B | 23 | 17 | 1 | 4 | 348600 | 2788796 | 1 | 4 | 0 | 136 | 2836204 | 1 | 4 | 0 | 1 | 144 |
| 28 | 1C | 23 | 17 | 1 | 5 | 348617 | 2788932 | 1 | 5 | 0 | 136 | 2836348 | 1 | 5 | 0 | 1 | 144 |
| 29 | 1D | 23 | 17 | 1 | 6 | 348634 | 2789068 | 1 | 6 | 0 | 136 | 2836492 | 1 | 6 | 0 | 1 | 144 |
| 30 | 1E | 23 | 17 | 1 | 7 | 348651 | 2789204 | 1 | 7 | 0 | 136 | 2836636 | 1 | 7 | 0 | 1 | 144 |
| 31 | 1F | 23 | 17 | 1 | 8 | 348668 | 2789340 | 1 | 8 | 0 | 136 | 2836780 | 1 | 8 | 0 | 1 | 144 |
| 32 | 20 | 23 | 17 | 1 | 9 | 348689 | 2789508 | 1 | 9 | 0 | 168 | 2836956 | 1 | 9 | 0 | 1 | 176 |
| 33 | 21 | 23 | 17 | 1 | 10 | 348710 | 2789676 | 1 | 10 | 0 | 168 | 2837132 | 1 | 10 | 0 | 1 | 176 |
| 34 | 22 | 23 | 17 | 1 | 11 | 348731 | 2789844 | 1 | 11 | 0 | 168 | 2837308 | 1 | 11 | 0 | 1 | 176 |
| 35 | 23 | 23 | 17 | 1 | 12 | 348752 | 2790012 | 1 | 12 | 0 | 168 | 2837484 | 1 | 12 | 0 | 1 | 176 |
| 36 | 24 | 23 | 17 | 1 | 13 | 348773 | 2790180 | 1 | 13 | 0 | 168 | 2837660 | 1 | 13 | 0 | 1 | 176 |
| 37 | 25 | 23 | 17 | 1 | 14 | 348794 | 2790348 | 1 | 14 | 0 | 168 | 2837836 | 1 | 14 | 0 | 1 | 176 |
| 38 | 26 | 23 | 17 | 1 | 15 | 348815 | 2790516 | 1 | 15 | 0 | 168 | 2838012 | 1 | 15 | 0 | 1 | 176 |
| 39 | 27 | 23 | 17 | 1 | 16 | 348836 | 2790684 | 1 | 16 | 0 | 168 | 2838188 | 1 | 16 | 0 | 1 | 176 |
| 40 | 28 | 23 | 17 | 1 | 17 | 348857 | 2790852 | 1 | 17 | 0 | 168 | 2838364 | 1 | 17 | 0 | 1 | 176 |
| 41 | 29 | 23 | 17 | 1 | 18 | 348878 | 2791020 | 1 | 18 | 0 | 168 | 2838540 | 1 | 18 | 0 | 1 | 176 |
| 42 | 2A | 23 | 17 | 1 | 19 | 348899 | 2791188 | 1 | 19 | 0 | 168 | 2838716 | 1 | 19 | 0 | 1 | 176 |
| 43 | 2B | 23 | 17 | 1 | 20 | 348920 | 2791356 | 1 | 20 | 0 | 168 | 2838892 | 1 | 20 | 0 | 1 | 176 |
| 44 | 2C | 23 | 17 | 1 | 21 | 348941 | 2791524 | 1 | 21 | 0 | 168 | 2839068 | 1 | 21 | 0 | 1 | 176 |
| 45 | 2D | 23 | 17 | 1 | 22 | 348962 | 2791692 | 1 | 22 | 0 | 168 | 2839244 | 1 | 22 | 0 | 1 | 176 |
| 46 | 2E | 23 | 17 | 2 | 0 | 348981 | 2791844 | 2 | 0 | 0 | 152 | 2839404 | 2 | 0 | 0 | 1 | 160 |
| 47 | 2F | 23 | 17 | 2 | 1 | 349005 | 2792036 | 2 | 1 | 0 | 192 | 2839604 | 2 | 1 | 0 | 1 | 200 |
| 48 | 30 | 23 | 17 | 2 | 2 | 349031 | 2792244 | 2 | 2 | 0 | 208 | 2839820 | 2 | 2 | 0 | 1 | 216 |
| 49 | 31 | 23 | 17 | 2 | 3 | 349057 | 2792452 | 2 | 3 | 0 | 208 | 2840036 | 2 | 3 | 0 | 1 | 216 |
| 50 | 32 | 23 | 17 | 2 | 4 | 349083 | 2792660 | 2 | 4 | 0 | 208 | 2840252 | 2 | 4 | 0 | 1 | 216 |
| 51 | 33 | 23 | 17 | 2 | 5 | 349109 | 2792868 | 2 | 5 | 0 | 208 | 2840468 | 2 | 5 | 0 | 1 | 216 |
| 52 | 34 | 23 | 17 | 2 | 6 | 349135 | 2793076 | 2 | 6 | 0 | 208 | 2840684 | 2 | 6 | 0 | 1 | 216 |
| 53 | 35 | 23 | 17 | 2 | 7 | 349161 | 2793284 | 2 | 7 | 0 | 208 | 2840900 | 2 | 7 | 0 | 1 | 216 |
| 54 | 36 | 23 | 17 | 2 | 8 | 349187 | 2793492 | 2 | 8 | 0 | 208 | 2841116 | 2 | 8 | 0 | 1 | 216 |
| 55 | 37 | 23 | 17 | 2 | 9 | 349213 | 2793700 | 2 | 9 | 0 | 208 | 2841332 | 2 | 9 | 0 | 1 | 216 |
| 56 | 38 | 23 | 17 | 2 | 10 | 349239 | 2793908 | 2 | 10 | 0 | 208 | 2841548 | 2 | 10 | 0 | 1 | 216 |
| 57 | 39 | 23 | 17 | 2 | 11 | 349265 | 2794116 | 2 | 11 | 0 | 208 | 2841764 | 2 | 11 | 0 | 1 | 216 |
| 58 | 3A | 23 | 17 | 2 | 12 | 349291 | 2794324 | 2 | 12 | 0 | 208 | 2841980 | 2 | 12 | 0 | 1 | 216 |
| 59 | 3B | 23 | 17 | 2 | 13 | 349317 | 2794532 | 2 | 13 | 0 | 208 | 2842196 | 2 | 13 | 0 | 1 | 216 |
| 60 | 3C | 23 | 17 | 2 | 14 | 349343 | 2794740 | 2 | 14 | 0 | 208 | 2842412 | 2 | 14 | 0 | 1 | 216 |
| 61 | 3D | 23 | 17 | 2 | 15 | 349369 | 2794948 | 2 | 15 | 0 | 208 | 2842628 | 2 | 15 | 0 | 1 | 216 |
| 62 | 3E | 23 | 17 | 2 | 16 | 349395 | 2795156 | 2 | 16 | 0 | 208 | 2842844 | 2 | 16 | 0 | 1 | 216 |
| 63 | 3F | 23 | 17 | 2 | 17 | 349421 | 2795364 | 2 | 17 | 0 | 208 | 2843060 | 2 | 17 | 0 | 1 | 216 |
| 64 | 40 | 23 | 17 | 2 | 18 | 349444 | 2795548 | 2 | 18 | 0 | 184 | 2843252 | 2 | 18 | 0 | 1 | 192 |
| 65 | 41 | 23 | 17 | 2 | 19 | 349467 | 2795732 | 2 | 19 | 0 | 184 | 2843444 | 2 | 19 | 0 | 1 | 192 |
| 66 | 42 | 23 | 17 | 2 | 20 | 349490 | 2795916 | 2 | 20 | 0 | 184 | 2843636 | 2 | 20 | 0 | 1 | 192 |
| 67 | 43 | 23 | 17 | 2 | 21 | 349513 | 2796100 | 2 | 21 | 0 | 184 | 2843828 | 2 | 21 | 0 | 1 | 192 |
| 68 | 44 | 23 | 17 | 2 | 22 | 349536 | 2796284 | 2 | 22 | 0 | 184 | 2844020 | 2 | 22 | 0 | 1 | 192 |
| 69 | 45 | 23 | 17 | 3 | 0 | 349557 | 2796452 | 3 | 0 | 0 | 168 | 2844196 | 3 | 0 | 0 | 1 | 176 |
| 70 | 46 | 23 | 17 | 3 | 1 | 349583 | 2796660 | 3 | 1 | 0 | 208 | 2844412 | 3 | 1 | 0 | 1 | 216 |
| 71 | 47 | 23 | 17 | 3 | 2 | 349609 | 2796868 | 3 | 2 | 0 | 208 | 2844628 | 3 | 2 | 0 | 1 | 216 |
| 72 | 48 | 23 | 17 | 3 | 3 | 349635 | 2797076 | 3 | 3 | 0 | 208 | 2844844 | 3 | 3 | 0 | 1 | 216 |
| 73 | 49 | 23 | 17 | 3 | 4 | 349661 | 2797284 | 3 | 4 | 0 | 208 | 2845060 | 3 | 4 | 0 | 1 | 216 |
| 74 | 4A | 23 | 17 | 3 | 5 | 349687 | 2797492 | 3 | 5 | 0 | 208 | 2845276 | 3 | 5 | 0 | 1 | 216 |
| 75 | 4B | 23 | 17 | 3 | 6 | 349713 | 2797700 | 3 | 6 | 0 | 208 | 2845492 | 3 | 6 | 0 | 1 | 216 |
| 76 | 4C | 23 | 17 | 3 | 7 | 349739 | 2797908 | 3 | 7 | 0 | 208 | 2845708 | 3 | 7 | 0 | 1 | 216 |
| 77 | 4D | 23 | 17 | 3 | 8 | 349765 | 2798116 | 3 | 8 | 0 | 208 | 2845924 | 3 | 8 | 0 | 1 | 216 |
| 78 | 4E | 23 | 17 | 3 | 9 | 349791 | 2798324 | 3 | 9 | 0 | 208 | 2846140 | 3 | 9 | 0 | 1 | 216 |
| 79 | 4F | 23 | 17 | 3 | 10 | 349817 | 2798532 | 3 | 10 | 0 | 208 | 2846356 | 3 | 10 | 0 | 1 | 216 |
| 80 | 50 | 23 | 17 | 3 | 11 | 349845 | 2798756 | 3 | 11 | 0 | 224 | 2846588 | 3 | 11 | 0 | 1 | 232 |
| 81 | 51 | 23 | 17 | 3 | 12 | 349873 | 2798980 | 3 | 12 | 0 | 224 | 2846820 | 3 | 12 | 0 | 1 | 232 |
| 82 | 52 | 23 | 17 | 3 | 13 | 349901 | 2799204 | 3 | 13 | 0 | 224 | 2847052 | 3 | 13 | 0 | 1 | 232 |

| | | | | | | | | | | | | | | | | | |
|---|---|---|---|---|---|---|---|---|---|---|---|---|---|---|---|---|---|
| 83 | 53 | 23 | 17 | 3 | 14 | 349929 | 2799428 | 3 | 14 | 0 | 224 | 2847284 | 3 | 14 | 0 | 1 | 232 |
| 84 | 54 | 23 | 17 | 3 | 15 | 349957 | 2799652 | 3 | 15 | 0 | 224 | 2847516 | 3 | 15 | 0 | 1 | 232 |
| 85 | 55 | 23 | 17 | 3 | 16 | 349985 | 2799876 | 3 | 16 | 0 | 224 | 2847748 | 3 | 16 | 0 | 1 | 232 |
| 86 | 56 | 23 | 17 | 3 | 17 | 350013 | 2800100 | 3 | 17 | 0 | 224 | 2847980 | 3 | 17 | 0 | 1 | 232 |
| 87 | 57 | 23 | 17 | 3 | 18 | 350041 | 2800324 | 3 | 18 | 0 | 224 | 2848212 | 3 | 18 | 0 | 1 | 232 |
| 88 | 58 | 23 | 17 | 3 | 19 | 350069 | 2800548 | 3 | 19 | 0 | 224 | 2848444 | 3 | 19 | 0 | 1 | 232 |
| 89 | 59 | 23 | 17 | 3 | 20 | 350097 | 2800772 | 3 | 20 | 0 | 224 | 2848676 | 3 | 20 | 0 | 1 | 232 |
| 90 | 5A | 23 | 17 | 3 | 21 | 350125 | 2800996 | 3 | 21 | 0 | 224 | 2848908 | 3 | 21 | 0 | 1 | 232 |
| 91 | 5B | 23 | 17 | 3 | 22 | 350153 | 2801220 | 3 | 22 | 0 | 224 | 2849140 | 3 | 22 | 0 | 1 | 232 |
| 92 | 5C | 23 | 17 | 4 | 0 | 350183 | 2801460 | 4 | 0 | 0 | 240 | 2849388 | 4 | 0 | 0 | 1 | 248 |
| 93 | 5D | 23 | 17 | 4 | 1 | 350213 | 2801700 | 4 | 1 | 0 | 240 | 2849636 | 4 | 1 | 0 | 1 | 248 |
| 94 | 5E | 23 | 17 | 4 | 2 | 350243 | 2801940 | 4 | 2 | 0 | 240 | 2849884 | 4 | 2 | 0 | 1 | 248 |
| 95 | 5F | 23 | 17 | 4 | 3 | 350273 | 2802180 | 4 | 3 | 0 | 240 | 2850132 | 4 | 3 | 0 | 1 | 248 |
| 96 | 60 | 23 | 17 | 4 | 4 | 350301 | 2802404 | 4 | 4 | 0 | 224 | 2850364 | 4 | 4 | 0 | 1 | 232 |
| 97 | 61 | 23 | 17 | 4 | 5 | 350329 | 2802628 | 4 | 5 | 0 | 224 | 2850596 | 4 | 5 | 0 | 1 | 232 |
| 98 | 62 | 23 | 17 | 4 | 6 | 350357 | 2802852 | 4 | 6 | 0 | 224 | 2850828 | 4 | 6 | 0 | 1 | 232 |
| 99 | 63 | 23 | 17 | 4 | 7 | 350385 | 2803076 | 4 | 7 | 0 | 224 | 2851060 | 4 | 7 | 0 | 1 | 232 |
| 100 | 64 | 23 | 17 | 4 | 8 | 350413 | 2803300 | 4 | 8 | 0 | 224 | 2851292 | 4 | 8 | 0 | 1 | 232 |
| 101 | 65 | 23 | 17 | 4 | 9 | 350441 | 2803524 | 4 | 9 | 0 | 224 | 2851524 | 4 | 9 | 0 | 1 | 232 |
| 102 | 66 | 23 | 17 | 4 | 10 | 350469 | 2803748 | 4 | 10 | 0 | 224 | 2851756 | 4 | 10 | 0 | 1 | 232 |
| 103 | 67 | 23 | 17 | 4 | 11 | 350497 | 2803972 | 4 | 11 | 0 | 224 | 2851988 | 4 | 11 | 0 | 1 | 232 |
| 104 | 68 | 23 | 17 | 4 | 12 | 350525 | 2804196 | 4 | 12 | 0 | 224 | 2852220 | 4 | 12 | 0 | 1 | 232 |
| 105 | 69 | 23 | 17 | 4 | 13 | 350553 | 2804420 | 4 | 13 | 0 | 224 | 2852452 | 4 | 13 | 0 | 1 | 232 |
| 106 | 6A | 23 | 17 | 4 | 14 | 350581 | 2804644 | 4 | 14 | 0 | 224 | 2852684 | 4 | 14 | 0 | 1 | 232 |
| 107 | 6B | 23 | 17 | 4 | 15 | 350609 | 2804868 | 4 | 15 | 0 | 224 | 2852916 | 4 | 15 | 0 | 1 | 232 |
| 108 | 6C | 23 | 17 | 4 | 16 | 350637 | 2805092 | 4 | 16 | 0 | 224 | 2853148 | 4 | 16 | 0 | 1 | 232 |
| 109 | 6D | 23 | 17 | 4 | 17 | 350665 | 2805316 | 4 | 17 | 0 | 224 | 2853380 | 4 | 17 | 0 | 1 | 232 |
| 110 | 6E | 23 | 17 | 4 | 18 | 350693 | 2805540 | 4 | 18 | 0 | 224 | 2853612 | 4 | 18 | 0 | 1 | 232 |
| 111 | 6F | 23 | 17 | 4 | 19 | 350721 | 2805764 | 4 | 19 | 0 | 224 | 2853844 | 4 | 19 | 0 | 1 | 232 |
| 112 | 70 | 23 | 17 | 4 | 20 | 350751 | 2806004 | 4 | 20 | 0 | 240 | 2854092 | 4 | 20 | 0 | 1 | 248 |
| 113 | 71 | 23 | 17 | 4 | 21 | 350781 | 2806244 | 4 | 21 | 0 | 240 | 2854340 | 4 | 21 | 0 | 1 | 248 |
| 114 | 72 | 23 | 17 | 4 | 22 | 350811 | 2806484 | 4 | 22 | 0 | 240 | 2854588 | 4 | 22 | 0 | 1 | 248 |
| 115 | 73 | 23 | 17 | 5 | 0 | 350843 | 2806740 | 5 | 0 | 0 | 256 | 2854852 | 5 | 0 | 0 | 1 | 264 |
| 116 | 74 | 23 | 17 | 5 | 1 | 350875 | 2806996 | 5 | 1 | 0 | 256 | 2855116 | 5 | 1 | 0 | 1 | 264 |
| 117 | 75 | 23 | 17 | 5 | 2 | 350907 | 2807252 | 5 | 2 | 0 | 256 | 2855380 | 5 | 2 | 0 | 1 | 264 |
| 118 | 76 | 23 | 17 | 5 | 3 | 350939 | 2807508 | 5 | 3 | 0 | 256 | 2855644 | 5 | 3 | 0 | 1 | 264 |
| 119 | 77 | 23 | 17 | 5 | 4 | 350971 | 2807764 | 5 | 4 | 0 | 256 | 2855908 | 5 | 4 | 0 | 1 | 264 |
| 120 | 78 | 23 | 17 | 5 | 5 | 351003 | 2808020 | 5 | 5 | 0 | 256 | 2856172 | 5 | 5 | 0 | 1 | 264 |
| 121 | 79 | 23 | 17 | 5 | 6 | 351035 | 2808276 | 5 | 6 | 0 | 256 | 2856436 | 5 | 6 | 0 | 1 | 264 |
| 122 | 7A | 23 | 17 | 5 | 7 | 351067 | 2808532 | 5 | 7 | 0 | 256 | 2856700 | 5 | 7 | 0 | 1 | 264 |
| 123 | 7B | 23 | 17 | 5 | 8 | 351099 | 2808788 | 5 | 8 | 0 | 256 | 2856964 | 5 | 8 | 0 | 1 | 264 |
| 124 | 7C | 23 | 17 | 5 | 9 | 351131 | 2809044 | 5 | 9 | 0 | 256 | 2857228 | 5 | 9 | 0 | 1 | 264 |
| 125 | 7D | 23 | 17 | 5 | 10 | 351163 | 2809300 | 5 | 10 | 0 | 256 | 2857492 | 5 | 10 | 0 | 1 | 264 |
| 126 | 7E | 23 | 17 | 5 | 11 | 351195 | 2809556 | 5 | 11 | 0 | 256 | 2857756 | 5 | 11 | 0 | 1 | 264 |
| 127 | 7F | 23 | 17 | 5 | 12 | 351227 | 2809812 | 5 | 12 | 0 | 256 | 2858020 | 5 | 12 | 0 | 1 | 264 |
| 128 | 80 | 23 | 17 | 5 | 13 | 351257 | 2810052 | 5 | 13 | 0 | 240 | 2858268 | 5 | 13 | 0 | 1 | 248 |
| 129 | 81 | 23 | 17 | 5 | 14 | 351287 | 2810292 | 5 | 14 | 0 | 240 | 2858516 | 5 | 14 | 0 | 1 | 248 |
| 130 | 82 | 23 | 17 | 5 | 15 | 351317 | 2810532 | 5 | 15 | 0 | 240 | 2858764 | 5 | 15 | 0 | 1 | 248 |
| 131 | 83 | 23 | 17 | 5 | 16 | 351347 | 2810772 | 5 | 16 | 0 | 240 | 2859012 | 5 | 16 | 0 | 1 | 248 |
| 132 | 84 | 23 | 17 | 5 | 17 | 351377 | 2811012 | 5 | 17 | 0 | 240 | 2859260 | 5 | 17 | 0 | 1 | 248 |
| 133 | 85 | 23 | 17 | 5 | 18 | 351407 | 2811252 | 5 | 18 | 0 | 240 | 2859508 | 5 | 18 | 0 | 1 | 248 |
| 134 | 86 | 23 | 17 | 5 | 19 | 351437 | 2811492 | 5 | 19 | 0 | 240 | 2859756 | 5 | 19 | 0 | 1 | 248 |
| 135 | 87 | 23 | 17 | 5 | 20 | 351467 | 2811732 | 5 | 20 | 0 | 240 | 2860004 | 5 | 20 | 0 | 1 | 248 |
| 136 | 88 | 23 | 17 | 5 | 21 | 351497 | 2811972 | 5 | 21 | 0 | 240 | 2860252 | 5 | 21 | 0 | 1 | 248 |
| 137 | 89 | 23 | 17 | 5 | 22 | 351527 | 2812212 | 5 | 22 | 0 | 240 | 2860500 | 5 | 22 | 0 | 1 | 248 |
| 138 | 8A | 23 | 17 | 6 | 0 | 351559 | 2812468 | 6 | 0 | 0 | 256 | 2860764 | 6 | 0 | 0 | 1 | 264 |
| 139 | 8B | 23 | 17 | 6 | 1 | 351591 | 2812724 | 6 | 1 | 0 | 256 | 2861028 | 6 | 1 | 0 | 1 | 264 |
| 140 | 8C | 23 | 17 | 6 | 2 | 351623 | 2812980 | 6 | 2 | 0 | 256 | 2861292 | 6 | 2 | 0 | 1 | 264 |
| 141 | 8D | 23 | 17 | 6 | 3 | 351655 | 2813236 | 6 | 3 | 0 | 256 | 2861556 | 6 | 3 | 0 | 1 | 264 |
| 142 | 8E | 23 | 17 | 6 | 4 | 351687 | 2813492 | 6 | 4 | 0 | 256 | 2861820 | 6 | 4 | 0 | 1 | 264 |
| 143 | 8F | 23 | 17 | 6 | 5 | 351719 | 2813748 | 6 | 5 | 0 | 256 | 2862084 | 6 | 5 | 0 | 1 | 264 |
| 144 | 90 | 23 | 17 | 6 | 6 | 351753 | 2814020 | 6 | 6 | 0 | 272 | 2862364 | 6 | 6 | 0 | 1 | 280 |
| 145 | 91 | 23 | 17 | 6 | 7 | 351787 | 2814292 | 6 | 7 | 0 | 272 | 2862644 | 6 | 7 | 0 | 1 | 280 |
| 146 | 92 | 23 | 17 | 6 | 8 | 351821 | 2814564 | 6 | 8 | 0 | 272 | 2862924 | 6 | 8 | 0 | 1 | 280 |
| 147 | 93 | 23 | 17 | 6 | 9 | 351855 | 2814836 | 6 | 9 | 0 | 272 | 2863204 | 6 | 9 | 0 | 1 | 280 |
| 148 | 94 | 23 | 17 | 6 | 10 | 351889 | 2815108 | 6 | 10 | 0 | 272 | 2863484 | 6 | 10 | 0 | 1 | 280 |
| 149 | 95 | 23 | 17 | 6 | 11 | 351923 | 2815380 | 6 | 11 | 0 | 272 | 2863764 | 6 | 11 | 0 | 1 | 280 |
| 150 | 96 | 23 | 17 | 6 | 12 | 351957 | 2815652 | 6 | 12 | 0 | 272 | 2864044 | 6 | 12 | 0 | 1 | 280 |
| 151 | 97 | 23 | 17 | 6 | 13 | 351991 | 2815924 | 6 | 13 | 0 | 272 | 2864324 | 6 | 13 | 0 | 1 | 280 |
| 152 | 98 | 23 | 17 | 6 | 14 | 352025 | 2816196 | 6 | 14 | 0 | 272 | 2864604 | 6 | 14 | 0 | 1 | 280 |
| 153 | 99 | 23 | 17 | 6 | 15 | 352059 | 2816468 | 6 | 15 | 0 | 272 | 2864884 | 6 | 15 | 0 | 1 | 280 |
| 154 | 9A | 23 | 17 | 6 | 16 | 352093 | 2816740 | 6 | 16 | 0 | 272 | 2865164 | 6 | 16 | 0 | 1 | 280 |
| 155 | 9B | 23 | 17 | 6 | 17 | 352127 | 2817012 | 6 | 17 | 0 | 272 | 2865444 | 6 | 17 | 0 | 1 | 280 |
| 156 | 9C | 23 | 17 | 6 | 18 | 352161 | 2817284 | 6 | 18 | 0 | 272 | 2865724 | 6 | 18 | 0 | 1 | 280 |
| 157 | 9D | 23 | 17 | 6 | 19 | 352195 | 2817556 | 6 | 19 | 0 | 272 | 2866004 | 6 | 19 | 0 | 1 | 280 |
| 158 | 9E | 23 | 17 | 6 | 20 | 352229 | 2817828 | 6 | 20 | 0 | 272 | 2866284 | 6 | 20 | 0 | 1 | 280 |
| 159 | 9F | 23 | 17 | 6 | 21 | 352263 | 2818100 | 6 | 21 | 0 | 272 | 2866564 | 6 | 21 | 0 | 1 | 280 |
| 160 | A0 | 23 | 17 | 6 | 22 | 352295 | 2818356 | 6 | 22 | 0 | 256 | 2866828 | 6 | 22 | 0 | 1 | 264 |
| 161 | A1 | 23 | 17 | 7 | 0 | 352329 | 2818628 | 7 | 0 | 0 | 272 | 2867108 | 7 | 0 | 0 | 1 | 280 |
| 162 | A2 | 23 | 17 | 7 | 1 | 352363 | 2818900 | 7 | 1 | 0 | 272 | 2867388 | 7 | 1 | 0 | 1 | 280 |
| 163 | A3 | 23 | 17 | 7 | 2 | 352397 | 2819172 | 7 | 2 | 0 | 272 | 2867668 | 7 | 2 | 0 | 1 | 280 |
| 164 | A4 | 23 | 17 | 7 | 3 | 352431 | 2819444 | 7 | 3 | 0 | 272 | 2867948 | 7 | 3 | 0 | 1 | 280 |
| 165 | A5 | 23 | 17 | 7 | 4 | 352465 | 2819716 | 7 | 4 | 0 | 272 | 2868228 | 7 | 4 | 0 | 1 | 280 |
| 166 | A6 | 23 | 17 | 7 | 5 | 352499 | 2819988 | 7 | 5 | 0 | 272 | 2868508 | 7 | 5 | 0 | 1 | 280 |
| 167 | A7 | 23 | 17 | 7 | 6 | 352533 | 2820260 | 7 | 6 | 0 | 272 | 2868788 | 7 | 6 | 0 | 1 | 280 |
| 168 | A8 | 23 | 17 | 7 | 7 | 352567 | 2820532 | 7 | 7 | 0 | 272 | 2869068 | 7 | 7 | 0 | 1 | 280 |
| 169 | A9 | 23 | 17 | 7 | 8 | 352601 | 2820804 | 7 | 8 | 0 | 272 | 2869348 | 7 | 8 | 0 | 1 | 280 |
| 170 | AA | 23 | 17 | 7 | 9 | 352635 | 2821076 | 7 | 9 | 0 | 272 | 2869628 | 7 | 9 | 0 | 1 | 280 |
| 171 | AB | 23 | 17 | 7 | 10 | 352669 | 2821348 | 7 | 10 | 0 | 272 | 2869908 | 7 | 10 | 0 | 1 | 280 |
| 172 | AC | 23 | 17 | 7 | 11 | 352703 | 2821620 | 7 | 11 | 0 | 272 | 2870188 | 7 | 11 | 0 | 1 | 280 |
| 173 | AD | 23 | 17 | 7 | 12 | 352737 | 2821892 | 7 | 12 | 0 | 272 | 2870468 | 7 | 12 | 0 | 1 | 280 |
| 174 | AE | 23 | 17 | 7 | 13 | 352771 | 2822164 | 7 | 13 | 0 | 272 | 2870748 | 7 | 13 | 0 | 1 | 280 |
| 175 | AF | 23 | 17 | 7 | 14 | 352805 | 2822436 | 7 | 14 | 0 | 272 | 2871028 | 7 | 14 | 0 | 1 | 280 |
| 176 | B0 | 23 | 17 | 7 | 15 | 352841 | 2822724 | 7 | 15 | 0 | 288 | 2871324 | 7 | 15 | 0 | 1 | 296 |
| 177 | B1 | 23 | 17 | 7 | 16 | 352877 | 2823012 | 7 | 16 | 0 | 288 | 2871620 | 7 | 16 | 0 | 1 | 296 |
| 178 | B2 | 23 | 17 | 7 | 17 | 352913 | 2823300 | 7 | 17 | 0 | 288 | 2871916 | 7 | 17 | 0 | 1 | 296 |
| 179 | B3 | 23 | 17 | 7 | 18 | 352949 | 2823588 | 7 | 18 | 0 | 288 | 2872212 | 7 | 18 | 0 | 1 | 296 |
| 180 | B4 | 23 | 17 | 7 | 19 | 352985 | 2823876 | 7 | 19 | 0 | 288 | 2872508 | 7 | 19 | 0 | 1 | 296 |
| 181 | B5 | 23 | 17 | 7 | 20 | 353021 | 2824164 | 7 | 20 | 0 | 288 | 2872804 | 7 | 20 | 0 | 1 | 296 |
| 182 | B6 | 23 | 17 | 7 | 21 | 353057 | 2824452 | 7 | 21 | 0 | 288 | 2873100 | 7 | 21 | 0 | 1 | 296 |
| 183 | B7 | 23 | 17 | 7 | 22 | 353093 | 2824740 | 7 | 22 | 0 | 288 | 2873396 | 7 | 22 | 0 | 1 | 296 |
| 184 | B8 | 23 | 17 | 8 | 0 | 353131 | 2825044 | 8 | 0 | 0 | 304 | 2873708 | 8 | 0 | 0 | 1 | 312 |
| 185 | B9 | 23 | 17 | 8 | 1 | 353169 | 2825348 | 8 | 1 | 0 | 304 | 2874020 | 8 | 1 | 0 | 1 | 312 |

| | | | | | | | | | | | | | | | | | |
|---|---|---|---|---|---|---|---|---|---|---|---|---|---|---|---|---|---|
| 186 | BA | 23 | 17 | 8 | 2 | 353207 | 2825652 | 8 | 2 | 0 | 304 | 2874332 | 8 | 2 | 0 | 1 | 312 |
| 187 | BB | 23 | 17 | 8 | 3 | 353245 | 2825956 | 8 | 3 | 0 | 304 | 2874644 | 8 | 3 | 0 | 1 | 312 |
| 188 | BC | 23 | 17 | 8 | 4 | 353283 | 2826260 | 8 | 4 | 0 | 304 | 2874956 | 8 | 4 | 0 | 1 | 312 |
| 189 | BD | 23 | 17 | 8 | 5 | 353321 | 2826564 | 8 | 5 | 0 | 304 | 2875268 | 8 | 5 | 0 | 1 | 312 |
| 190 | BE | 23 | 17 | 8 | 6 | 353359 | 2826868 | 8 | 6 | 0 | 304 | 2875580 | 8 | 6 | 0 | 1 | 312 |
| 191 | BF | 23 | 17 | 8 | 7 | 353397 | 2827172 | 8 | 7 | 0 | 304 | 2875892 | 8 | 7 | 0 | 1 | 312 |
| 192 | C0 | 23 | 17 | 8 | 8 | 353433 | 2827460 | 8 | 8 | 0 | 288 | 2876188 | 8 | 8 | 0 | 1 | 296 |
| 193 | C1 | 23 | 17 | 8 | 9 | 353469 | 2827748 | 8 | 9 | 0 | 288 | 2876484 | 8 | 9 | 0 | 1 | 296 |
| 194 | C2 | 23 | 17 | 8 | 10 | 353505 | 2828036 | 8 | 10 | 0 | 288 | 2876780 | 8 | 10 | 0 | 1 | 296 |
| 195 | C3 | 23 | 17 | 8 | 11 | 353541 | 2828324 | 8 | 11 | 0 | 288 | 2877076 | 8 | 11 | 0 | 1 | 296 |
| 196 | C4 | 23 | 17 | 8 | 12 | 353577 | 2828612 | 8 | 12 | 0 | 288 | 2877372 | 8 | 12 | 0 | 1 | 296 |
| 197 | C5 | 23 | 17 | 8 | 13 | 353613 | 2828900 | 8 | 13 | 0 | 288 | 2877668 | 8 | 13 | 0 | 1 | 296 |
| 198 | C6 | 23 | 17 | 8 | 14 | 353649 | 2829188 | 8 | 14 | 0 | 288 | 2877964 | 8 | 14 | 0 | 1 | 296 |
| 199 | C7 | 23 | 17 | 8 | 15 | 353685 | 2829476 | 8 | 15 | 0 | 288 | 2878260 | 8 | 15 | 0 | 1 | 296 |
| 200 | C8 | 23 | 17 | 8 | 16 | 353721 | 2829764 | 8 | 16 | 0 | 288 | 2878556 | 8 | 16 | 0 | 1 | 296 |
| 201 | C9 | 23 | 17 | 8 | 17 | 353757 | 2830052 | 8 | 17 | 0 | 288 | 2878852 | 8 | 17 | 0 | 1 | 296 |
| 202 | CA | 23 | 17 | 8 | 18 | 353793 | 2830340 | 8 | 18 | 0 | 288 | 2879148 | 8 | 18 | 0 | 1 | 296 |
| 203 | CB | 23 | 17 | 8 | 19 | 353829 | 2830628 | 8 | 19 | 0 | 288 | 2879444 | 8 | 19 | 0 | 1 | 296 |
| 204 | CC | 23 | 17 | 8 | 20 | 353865 | 2830916 | 8 | 20 | 0 | 288 | 2879740 | 8 | 20 | 0 | 1 | 296 |
| 205 | CD | 23 | 17 | 8 | 21 | 353901 | 2831204 | 8 | 21 | 0 | 288 | 2880036 | 8 | 21 | 0 | 1 | 296 |
| 206 | CE | 23 | 17 | 8 | 22 | 353937 | 2831492 | 8 | 22 | 0 | 288 | 2880332 | 8 | 22 | 0 | 1 | 296 |
| 207 | CF | 23 | 17 | 9 | 0 | 353975 | 2831796 | 9 | 0 | 0 | 304 | 2880644 | 9 | 0 | 0 | 1 | 312 |
| 208 | D0 | 23 | 17 | 9 | 1 | 354015 | 2832116 | 9 | 1 | 0 | 320 | 2880972 | 9 | 1 | 0 | 1 | 328 |
| 209 | D1 | 23 | 17 | 9 | 2 | 354055 | 2832436 | 9 | 2 | 0 | 320 | 2881300 | 9 | 2 | 0 | 1 | 328 |
| 210 | D2 | 23 | 17 | 9 | 3 | 354095 | 2832756 | 9 | 3 | 0 | 320 | 2881628 | 9 | 3 | 0 | 1 | 328 |
| 211 | D3 | 23 | 17 | 9 | 4 | 354135 | 2833076 | 9 | 4 | 0 | 320 | 2881956 | 9 | 4 | 0 | 1 | 328 |
| 212 | D4 | 23 | 17 | 9 | 5 | 354175 | 2833396 | 9 | 5 | 0 | 320 | 2882284 | 9 | 5 | 0 | 1 | 328 |
| 213 | D5 | 23 | 17 | 9 | 6 | 354215 | 2833716 | 9 | 6 | 0 | 320 | 2882612 | 9 | 6 | 0 | 1 | 328 |
| 214 | D6 | 23 | 17 | 9 | 7 | 354255 | 2834036 | 9 | 7 | 0 | 320 | 2882940 | 9 | 7 | 0 | 1 | 328 |
| 215 | D7 | 23 | 17 | 9 | 8 | 354295 | 2834356 | 9 | 8 | 0 | 320 | 2883268 | 9 | 8 | 0 | 1 | 328 |
| 216 | D8 | 23 | 17 | 9 | 9 | 354335 | 2834676 | 9 | 9 | 0 | 320 | 2883596 | 9 | 9 | 0 | 1 | 328 |
| 217 | D9 | 23 | 17 | 9 | 10 | 354375 | 2834996 | 9 | 10 | 0 | 320 | 2883924 | 9 | 10 | 0 | 1 | 328 |
| 218 | DA | 23 | 17 | 9 | 11 | 354415 | 2835316 | 9 | 11 | 0 | 320 | 2884252 | 9 | 11 | 0 | 1 | 328 |
| 219 | DB | 23 | 17 | 9 | 12 | 354455 | 2835636 | 9 | 12 | 0 | 320 | 2884580 | 9 | 12 | 0 | 1 | 328 |
| 220 | DC | 23 | 17 | 9 | 13 | 354495 | 2835956 | 9 | 13 | 0 | 320 | 2884908 | 9 | 13 | 0 | 1 | 328 |
| 221 | DD | 23 | 17 | 9 | 14 | 354535 | 2836276 | 9 | 14 | 0 | 320 | 2885236 | 9 | 14 | 0 | 1 | 328 |
| 222 | DE | 23 | 17 | 9 | 15 | 354575 | 2836596 | 9 | 15 | 0 | 320 | 2885564 | 9 | 15 | 0 | 1 | 328 |
| 223 | DF | 23 | 17 | 9 | 16 | 354615 | 2836916 | 9 | 16 | 0 | 320 | 2885892 | 9 | 16 | 0 | 1 | 328 |
| 224 | E0 | 23 | 17 | 9 | 17 | 354653 | 2837220 | 9 | 17 | 0 | 304 | 2886204 | 9 | 17 | 0 | 1 | 312 |
| 225 | E1 | 23 | 17 | 9 | 18 | 354691 | 2837524 | 9 | 18 | 0 | 304 | 2886516 | 9 | 18 | 0 | 1 | 312 |
| 226 | E2 | 23 | 17 | 9 | 19 | 354729 | 2837828 | 9 | 19 | 0 | 304 | 2886828 | 9 | 19 | 0 | 1 | 312 |
| 227 | E3 | 23 | 17 | 9 | 20 | 354767 | 2838132 | 9 | 20 | 0 | 304 | 2887140 | 9 | 20 | 0 | 1 | 312 |
| 228 | E4 | 23 | 17 | 9 | 21 | 354805 | 2838436 | 9 | 21 | 0 | 304 | 2887452 | 9 | 21 | 0 | 1 | 312 |
| 229 | E5 | 23 | 17 | 9 | 22 | 354843 | 2838740 | 9 | 22 | 0 | 304 | 2887764 | 9 | 22 | 0 | 1 | 312 |
| 230 | E6 | 23 | 17 | 10 | 0 | 354883 | 2839060 | 10 | 0 | 0 | 320 | 2888092 | 10 | 0 | 0 | 1 | 328 |
| 231 | E7 | 23 | 17 | 10 | 1 | 354923 | 2839380 | 10 | 1 | 0 | 320 | 2888420 | 10 | 1 | 0 | 1 | 328 |
| 232 | E8 | 23 | 17 | 10 | 2 | 354963 | 2839700 | 10 | 2 | 0 | 320 | 2888748 | 10 | 2 | 0 | 1 | 328 |
| 233 | E9 | 23 | 17 | 10 | 3 | 355003 | 2840020 | 10 | 3 | 0 | 320 | 2889076 | 10 | 3 | 0 | 1 | 328 |
| 234 | EA | 23 | 17 | 10 | 4 | 355043 | 2840340 | 10 | 4 | 0 | 320 | 2889404 | 10 | 4 | 0 | 1 | 328 |
| 235 | EB | 23 | 17 | 10 | 5 | 355083 | 2840660 | 10 | 5 | 0 | 320 | 2889732 | 10 | 5 | 0 | 1 | 328 |
| 236 | EC | 23 | 17 | 10 | 6 | 355123 | 2840980 | 10 | 6 | 0 | 320 | 2890060 | 10 | 6 | 0 | 1 | 328 |
| 237 | ED | 23 | 17 | 10 | 7 | 355163 | 2841300 | 10 | 7 | 0 | 320 | 2890388 | 10 | 7 | 0 | 1 | 328 |
| 238 | EE | 23 | 17 | 10 | 8 | 355203 | 2841620 | 10 | 8 | 0 | 320 | 2890716 | 10 | 8 | 0 | 1 | 328 |
| 239 | EF | 23 | 17 | 10 | 9 | 355243 | 2841940 | 10 | 9 | 0 | 320 | 2891044 | 10 | 9 | 0 | 1 | 328 |
| 240 | F0 | 23 | 17 | 10 | 10 | 355285 | 2842276 | 10 | 10 | 0 | 336 | 2891388 | 10 | 10 | 0 | 1 | 344 |
| 241 | F1 | 23 | 17 | 10 | 11 | 355327 | 2842612 | 10 | 11 | 0 | 336 | 2891732 | 10 | 11 | 0 | 1 | 344 |
| 242 | F2 | 23 | 17 | 10 | 12 | 355369 | 2842948 | 10 | 12 | 0 | 336 | 2892076 | 10 | 12 | 0 | 1 | 344 |
| 243 | F3 | 23 | 17 | 10 | 13 | 355411 | 2843284 | 10 | 13 | 0 | 336 | 2892420 | 10 | 13 | 0 | 1 | 344 |
| 244 | F4 | 23 | 17 | 10 | 14 | 355453 | 2843620 | 10 | 14 | 0 | 336 | 2892764 | 10 | 14 | 0 | 1 | 344 |
| 245 | F5 | 23 | 17 | 10 | 15 | 355495 | 2843956 | 10 | 15 | 0 | 336 | 2893108 | 10 | 15 | 0 | 1 | 344 |
| 246 | F6 | 23 | 17 | 10 | 16 | 355537 | 2844292 | 10 | 16 | 0 | 336 | 2893452 | 10 | 16 | 0 | 1 | 344 |
| 247 | F7 | 23 | 17 | 10 | 17 | 355579 | 2844628 | 10 | 17 | 0 | 336 | 2893796 | 10 | 17 | 0 | 1 | 344 |
| 248 | F8 | 23 | 17 | 10 | 18 | 355621 | 2844964 | 10 | 18 | 0 | 336 | 2894140 | 10 | 18 | 0 | 1 | 344 |
| 249 | F9 | 23 | 17 | 10 | 19 | 355663 | 2845300 | 10 | 19 | 0 | 336 | 2894484 | 10 | 19 | 0 | 1 | 344 |
| 250 | FA | 23 | 17 | 10 | 20 | 355705 | 2845636 | 10 | 20 | 0 | 336 | 2894828 | 10 | 20 | 0 | 1 | 344 |
| 251 | FB | 23 | 17 | 10 | 21 | 355747 | 2845972 | 10 | 21 | 0 | 336 | 2895172 | 10 | 21 | 0 | 1 | 344 |
| 252 | FC | 23 | 17 | 10 | 22 | 355789 | 2846308 | 10 | 22 | 0 | 336 | 2895516 | 10 | 22 | 0 | 1 | 344 |
| 253 | FD | 23 | 17 | 11 | 0 | 355833 | 2846660 | 11 | 0 | 0 | 352 | 2895876 | 11 | 0 | 0 | 1 | 360 |
| 254 | FE | 23 | 17 | 11 | 1 | 355877 | 2847012 | 11 | 1 | 0 | 352 | 2896236 | 11 | 1 | 0 | 1 | 360 |
| 255 | FF | 23 | 17 | 11 | 2 | 355921 | 2847364 | 11 | 2 | 0 | 352 | 2896596 | 11 | 2 | 0 | 1 | 360 |
| 0 | 00 | 24 | 18 | 0 | 0 | 355933 | 2847460 | 0 | 0 | 0 | 96 | 2896700 | 0 | 0 | 0 | 1 | 104 |
| 1 | 01 | 24 | 18 | 0 | 1 | 355947 | 2847572 | 0 | 1 | 0 | 112 | 2896820 | 0 | 1 | 0 | 1 | 120 |
| 2 | 02 | 24 | 18 | 0 | 2 | 355961 | 2847684 | 0 | 2 | 0 | 112 | 2896940 | 0 | 2 | 0 | 1 | 120 |
| 3 | 03 | 24 | 18 | 0 | 3 | 355975 | 2847796 | 0 | 3 | 0 | 112 | 2897060 | 0 | 3 | 0 | 1 | 120 |
| 4 | 04 | 24 | 18 | 0 | 4 | 355989 | 2847908 | 0 | 4 | 0 | 112 | 2897180 | 0 | 4 | 0 | 1 | 120 |
| 5 | 05 | 24 | 18 | 0 | 5 | 356003 | 2848020 | 0 | 5 | 0 | 112 | 2897300 | 0 | 5 | 0 | 1 | 120 |
| 6 | 06 | 24 | 18 | 0 | 6 | 356017 | 2848132 | 0 | 6 | 0 | 112 | 2897420 | 0 | 6 | 0 | 1 | 120 |
| 7 | 07 | 24 | 18 | 0 | 7 | 356031 | 2848244 | 0 | 7 | 0 | 112 | 2897540 | 0 | 7 | 0 | 1 | 120 |
| 8 | 08 | 24 | 18 | 0 | 8 | 356045 | 2848356 | 0 | 8 | 0 | 112 | 2897660 | 0 | 8 | 0 | 1 | 120 |
| 9 | 09 | 24 | 18 | 0 | 9 | 356059 | 2848468 | 0 | 9 | 0 | 112 | 2897780 | 0 | 9 | 0 | 1 | 120 |
| 10 | 0A | 24 | 18 | 0 | 10 | 356073 | 2848580 | 0 | 10 | 0 | 112 | 2897900 | 0 | 10 | 0 | 1 | 120 |
| 11 | 0B | 24 | 18 | 0 | 11 | 356087 | 2848692 | 0 | 11 | 0 | 112 | 2898020 | 0 | 11 | 0 | 1 | 120 |
| 12 | 0C | 24 | 18 | 0 | 12 | 356101 | 2848804 | 0 | 12 | 0 | 112 | 2898140 | 0 | 12 | 0 | 1 | 120 |
| 13 | 0D | 24 | 18 | 0 | 13 | 356115 | 2848916 | 0 | 13 | 0 | 112 | 2898260 | 0 | 13 | 0 | 1 | 120 |
| 14 | 0E | 24 | 18 | 0 | 14 | 356129 | 2849028 | 0 | 14 | 0 | 112 | 2898380 | 0 | 14 | 0 | 1 | 120 |
| 15 | 0F | 24 | 18 | 0 | 15 | 356143 | 2849140 | 0 | 15 | 0 | 112 | 2898500 | 0 | 15 | 0 | 1 | 120 |
| 16 | 10 | 24 | 18 | 0 | 16 | 356157 | 2849252 | 0 | 16 | 0 | 112 | 2898620 | 0 | 16 | 0 | 1 | 120 |
| 17 | 11 | 24 | 18 | 0 | 17 | 356171 | 2849364 | 0 | 17 | 0 | 112 | 2898740 | 0 | 17 | 0 | 1 | 120 |
| 18 | 12 | 24 | 18 | 0 | 18 | 356185 | 2849476 | 0 | 18 | 0 | 112 | 2898860 | 0 | 18 | 0 | 1 | 120 |
| 19 | 13 | 24 | 18 | 0 | 19 | 356199 | 2849588 | 0 | 19 | 0 | 112 | 2898980 | 0 | 19 | 0 | 1 | 120 |
| 20 | 14 | 24 | 18 | 0 | 20 | 356213 | 2849700 | 0 | 20 | 0 | 112 | 2899100 | 0 | 20 | 0 | 1 | 120 |
| 21 | 15 | 24 | 18 | 0 | 21 | 356227 | 2849812 | 0 | 21 | 0 | 112 | 2899220 | 0 | 21 | 0 | 1 | 120 |
| 22 | 16 | 24 | 18 | 0 | 22 | 356241 | 2849924 | 0 | 22 | 0 | 112 | 2899340 | 0 | 22 | 0 | 1 | 120 |
| 23 | 17 | 24 | 18 | 0 | 23 | 356255 | 2850036 | 0 | 23 | 0 | 112 | 2899460 | 0 | 23 | 0 | 1 | 120 |
| 24 | 18 | 24 | 18 | 1 | 0 | 356267 | 2850132 | 1 | 0 | 0 | 96 | 2899564 | 1 | 0 | 0 | 1 | 104 |
| 25 | 19 | 24 | 18 | 1 | 1 | 356284 | 2850268 | 1 | 1 | 0 | 136 | 2899708 | 1 | 1 | 0 | 1 | 144 |
| 26 | 1A | 24 | 18 | 1 | 2 | 356301 | 2850404 | 1 | 2 | 0 | 136 | 2899852 | 1 | 2 | 0 | 1 | 144 |
| 27 | 1B | 24 | 18 | 1 | 3 | 356318 | 2850540 | 1 | 3 | 0 | 136 | 2899996 | 1 | 3 | 0 | 1 | 144 |
| 28 | 1C | 24 | 18 | 1 | 4 | 356335 | 2850676 | 1 | 4 | 0 | 136 | 2900140 | 1 | 4 | 0 | 1 | 144 |
| 29 | 1D | 24 | 18 | 1 | 5 | 356352 | 2850812 | 1 | 5 | 0 | 136 | 2900284 | 1 | 5 | 0 | 1 | 144 |
| 30 | 1E | 24 | 18 | 1 | 6 | 356369 | 2850948 | 1 | 6 | 0 | 136 | 2900428 | 1 | 6 | 0 | 1 | 144 |
| 31 | 1F | 24 | 18 | 1 | 7 | 356386 | 2851084 | 1 | 7 | 0 | 136 | 2900572 | 1 | 7 | 0 | 1 | 144 |
| 32 | 20 | 24 | 18 | 1 | 8 | 356407 | 2851252 | 1 | 8 | 0 | 168 | 2900748 | 1 | 8 | 0 | 1 | 176 |

| | | | | | | | | | | | | | | | | | |
|---|---|---|---|---|---|---|---|---|---|---|---|---|---|---|---|---|---|
| 33 | 21 | 24 | 18 | 1 | 9 | 356428 | 2851420 | 1 | 9 | 0 | 168 | 2900924 | 1 | 9 | 0 | 1 | 176 |
| 34 | 22 | 24 | 18 | 1 | 10 | 356449 | 2851588 | 1 | 10 | 0 | 168 | 2901100 | 1 | 10 | 0 | 1 | 176 |
| 35 | 23 | 24 | 18 | 1 | 11 | 356470 | 2851756 | 1 | 11 | 0 | 168 | 2901276 | 1 | 11 | 0 | 1 | 176 |
| 36 | 24 | 24 | 18 | 1 | 12 | 356491 | 2851924 | 1 | 12 | 0 | 168 | 2901452 | 1 | 12 | 0 | 1 | 176 |
| 37 | 25 | 24 | 18 | 1 | 13 | 356512 | 2852092 | 1 | 13 | 0 | 168 | 2901628 | 1 | 13 | 0 | 1 | 176 |
| 38 | 26 | 24 | 18 | 1 | 14 | 356533 | 2852260 | 1 | 14 | 0 | 168 | 2901804 | 1 | 14 | 0 | 1 | 176 |
| 39 | 27 | 24 | 18 | 1 | 15 | 356554 | 2852428 | 1 | 15 | 0 | 168 | 2901980 | 1 | 15 | 0 | 1 | 176 |
| 40 | 28 | 24 | 18 | 1 | 16 | 356575 | 2852596 | 1 | 16 | 0 | 168 | 2902156 | 1 | 16 | 0 | 1 | 176 |
| 41 | 29 | 24 | 18 | 1 | 17 | 356596 | 2852764 | 1 | 17 | 0 | 168 | 2902332 | 1 | 17 | 0 | 1 | 176 |
| 42 | 2A | 24 | 18 | 1 | 18 | 356617 | 2852932 | 1 | 18 | 0 | 168 | 2902508 | 1 | 18 | 0 | 1 | 176 |
| 43 | 2B | 24 | 18 | 1 | 19 | 356638 | 2853100 | 1 | 19 | 0 | 168 | 2902684 | 1 | 19 | 0 | 1 | 176 |
| 44 | 2C | 24 | 18 | 1 | 20 | 356659 | 2853268 | 1 | 20 | 0 | 168 | 2902860 | 1 | 20 | 0 | 1 | 176 |
| 45 | 2D | 24 | 18 | 1 | 21 | 356680 | 2853436 | 1 | 21 | 0 | 168 | 2903036 | 1 | 21 | 0 | 1 | 176 |
| 46 | 2E | 24 | 18 | 1 | 22 | 356701 | 2853604 | 1 | 22 | 0 | 168 | 2903212 | 1 | 22 | 0 | 1 | 176 |
| 47 | 2F | 24 | 18 | 1 | 23 | 356722 | 2853772 | 1 | 23 | 0 | 168 | 2903388 | 1 | 23 | 0 | 1 | 176 |
| 48 | 30 | 24 | 18 | 2 | 0 | 356743 | 2853940 | 2 | 0 | 0 | 168 | 2903564 | 2 | 0 | 0 | 1 | 176 |
| 49 | 31 | 24 | 18 | 2 | 1 | 356769 | 2854148 | 2 | 1 | 0 | 208 | 2903780 | 2 | 1 | 0 | 1 | 216 |
| 50 | 32 | 24 | 18 | 2 | 2 | 356795 | 2854356 | 2 | 2 | 0 | 208 | 2903996 | 2 | 2 | 0 | 1 | 216 |
| 51 | 33 | 24 | 18 | 2 | 3 | 356821 | 2854564 | 2 | 3 | 0 | 208 | 2904212 | 2 | 3 | 0 | 1 | 216 |
| 52 | 34 | 24 | 18 | 2 | 4 | 356847 | 2854772 | 2 | 4 | 0 | 208 | 2904428 | 2 | 4 | 0 | 1 | 216 |
| 53 | 35 | 24 | 18 | 2 | 5 | 356873 | 2854980 | 2 | 5 | 0 | 208 | 2904644 | 2 | 5 | 0 | 1 | 216 |
| 54 | 36 | 24 | 18 | 2 | 6 | 356899 | 2855188 | 2 | 6 | 0 | 208 | 2904860 | 2 | 6 | 0 | 1 | 216 |
| 55 | 37 | 24 | 18 | 2 | 7 | 356925 | 2855396 | 2 | 7 | 0 | 208 | 2905076 | 2 | 7 | 0 | 1 | 216 |
| 56 | 38 | 24 | 18 | 2 | 8 | 356951 | 2855604 | 2 | 8 | 0 | 208 | 2905292 | 2 | 8 | 0 | 1 | 216 |
| 57 | 39 | 24 | 18 | 2 | 9 | 356977 | 2855812 | 2 | 9 | 0 | 208 | 2905508 | 2 | 9 | 0 | 1 | 216 |
| 58 | 3A | 24 | 18 | 2 | 10 | 357003 | 2856020 | 2 | 10 | 0 | 208 | 2905724 | 2 | 10 | 0 | 1 | 216 |
| 59 | 3B | 24 | 18 | 2 | 11 | 357029 | 2856228 | 2 | 11 | 0 | 208 | 2905940 | 2 | 11 | 0 | 1 | 216 |
| 60 | 3C | 24 | 18 | 2 | 12 | 357055 | 2856436 | 2 | 12 | 0 | 208 | 2906156 | 2 | 12 | 0 | 1 | 216 |
| 61 | 3D | 24 | 18 | 2 | 13 | 357081 | 2856644 | 2 | 13 | 0 | 208 | 2906372 | 2 | 13 | 0 | 1 | 216 |
| 62 | 3E | 24 | 18 | 2 | 14 | 357107 | 2856852 | 2 | 14 | 0 | 208 | 2906588 | 2 | 14 | 0 | 1 | 216 |
| 63 | 3F | 24 | 18 | 2 | 15 | 357133 | 2857060 | 2 | 15 | 0 | 208 | 2906804 | 2 | 15 | 0 | 1 | 216 |
| 64 | 40 | 24 | 18 | 2 | 16 | 357156 | 2857244 | 2 | 16 | 0 | 184 | 2906996 | 2 | 16 | 0 | 1 | 192 |
| 65 | 41 | 24 | 18 | 2 | 17 | 357179 | 2857428 | 2 | 17 | 0 | 184 | 2907188 | 2 | 17 | 0 | 1 | 192 |
| 66 | 42 | 24 | 18 | 2 | 18 | 357202 | 2857612 | 2 | 18 | 0 | 184 | 2907380 | 2 | 18 | 0 | 1 | 192 |
| 67 | 43 | 24 | 18 | 2 | 19 | 357225 | 2857796 | 2 | 19 | 0 | 184 | 2907572 | 2 | 19 | 0 | 1 | 192 |
| 68 | 44 | 24 | 18 | 2 | 20 | 357248 | 2857980 | 2 | 20 | 0 | 184 | 2907764 | 2 | 20 | 0 | 1 | 192 |
| 69 | 45 | 24 | 18 | 2 | 21 | 357271 | 2858164 | 2 | 21 | 0 | 184 | 2907956 | 2 | 21 | 0 | 1 | 192 |
| 70 | 46 | 24 | 18 | 2 | 22 | 357294 | 2858348 | 2 | 22 | 0 | 184 | 2908148 | 2 | 22 | 0 | 1 | 192 |
| 71 | 47 | 24 | 18 | 2 | 23 | 357317 | 2858532 | 2 | 23 | 0 | 184 | 2908340 | 2 | 23 | 0 | 1 | 192 |
| 72 | 48 | 24 | 18 | 3 | 0 | 357338 | 2858700 | 3 | 0 | 0 | 168 | 2908516 | 3 | 0 | 0 | 1 | 176 |
| 73 | 49 | 24 | 18 | 3 | 1 | 357364 | 2858908 | 3 | 1 | 0 | 208 | 2908732 | 3 | 1 | 0 | 1 | 216 |
| 74 | 4A | 24 | 18 | 3 | 2 | 357390 | 2859116 | 3 | 2 | 0 | 208 | 2908948 | 3 | 2 | 0 | 1 | 216 |
| 75 | 4B | 24 | 18 | 3 | 3 | 357416 | 2859324 | 3 | 3 | 0 | 208 | 2909164 | 3 | 3 | 0 | 1 | 216 |
| 76 | 4C | 24 | 18 | 3 | 4 | 357442 | 2859532 | 3 | 4 | 0 | 208 | 2909380 | 3 | 4 | 0 | 1 | 216 |
| 77 | 4D | 24 | 18 | 3 | 5 | 357468 | 2859740 | 3 | 5 | 0 | 208 | 2909596 | 3 | 5 | 0 | 1 | 216 |
| 78 | 4E | 24 | 18 | 3 | 6 | 357494 | 2859948 | 3 | 6 | 0 | 208 | 2909812 | 3 | 6 | 0 | 1 | 216 |
| 79 | 4F | 24 | 18 | 3 | 7 | 357520 | 2860156 | 3 | 7 | 0 | 208 | 2910028 | 3 | 7 | 0 | 1 | 216 |
| 80 | 50 | 24 | 18 | 3 | 8 | 357548 | 2860380 | 3 | 8 | 0 | 224 | 2910260 | 3 | 8 | 0 | 1 | 232 |
| 81 | 51 | 24 | 18 | 3 | 9 | 357576 | 2860604 | 3 | 9 | 0 | 224 | 2910492 | 3 | 9 | 0 | 1 | 232 |
| 82 | 52 | 24 | 18 | 3 | 10 | 357604 | 2860828 | 3 | 10 | 0 | 224 | 2910724 | 3 | 10 | 0 | 1 | 232 |
| 83 | 53 | 24 | 18 | 3 | 11 | 357632 | 2861052 | 3 | 11 | 0 | 224 | 2910956 | 3 | 11 | 0 | 1 | 232 |
| 84 | 54 | 24 | 18 | 3 | 12 | 357660 | 2861276 | 3 | 12 | 0 | 224 | 2911188 | 3 | 12 | 0 | 1 | 232 |
| 85 | 55 | 24 | 18 | 3 | 13 | 357688 | 2861500 | 3 | 13 | 0 | 224 | 2911420 | 3 | 13 | 0 | 1 | 232 |
| 86 | 56 | 24 | 18 | 3 | 14 | 357716 | 2861724 | 3 | 14 | 0 | 224 | 2911652 | 3 | 14 | 0 | 1 | 232 |
| 87 | 57 | 24 | 18 | 3 | 15 | 357744 | 2861948 | 3 | 15 | 0 | 224 | 2911884 | 3 | 15 | 0 | 1 | 232 |
| 88 | 58 | 24 | 18 | 3 | 16 | 357772 | 2862172 | 3 | 16 | 0 | 224 | 2912116 | 3 | 16 | 0 | 1 | 232 |
| 89 | 59 | 24 | 18 | 3 | 17 | 357800 | 2862396 | 3 | 17 | 0 | 224 | 2912348 | 3 | 17 | 0 | 1 | 232 |
| 90 | 5A | 24 | 18 | 3 | 18 | 357828 | 2862620 | 3 | 18 | 0 | 224 | 2912580 | 3 | 18 | 0 | 1 | 232 |
| 91 | 5B | 24 | 18 | 3 | 19 | 357856 | 2862844 | 3 | 19 | 0 | 224 | 2912812 | 3 | 19 | 0 | 1 | 232 |
| 92 | 5C | 24 | 18 | 3 | 20 | 357884 | 2863068 | 3 | 20 | 0 | 224 | 2913044 | 3 | 20 | 0 | 1 | 232 |
| 93 | 5D | 24 | 18 | 3 | 21 | 357912 | 2863292 | 3 | 21 | 0 | 224 | 2913276 | 3 | 21 | 0 | 1 | 232 |
| 94 | 5E | 24 | 18 | 3 | 22 | 357940 | 2863516 | 3 | 22 | 0 | 224 | 2913508 | 3 | 22 | 0 | 1 | 232 |
| 95 | 5F | 24 | 18 | 3 | 23 | 357968 | 2863740 | 3 | 23 | 0 | 224 | 2913740 | 3 | 23 | 0 | 1 | 232 |
| 96 | 60 | 24 | 18 | 4 | 0 | 357991 | 2863924 | 4 | 0 | 0 | 184 | 2913932 | 4 | 0 | 0 | 1 | 192 |
| 97 | 61 | 24 | 18 | 4 | 1 | 358019 | 2864148 | 4 | 1 | 0 | 224 | 2914164 | 4 | 1 | 0 | 1 | 232 |
| 98 | 62 | 24 | 18 | 4 | 2 | 358047 | 2864372 | 4 | 2 | 0 | 224 | 2914396 | 4 | 2 | 0 | 1 | 232 |
| 99 | 63 | 24 | 18 | 4 | 3 | 358075 | 2864596 | 4 | 3 | 0 | 224 | 2914628 | 4 | 3 | 0 | 1 | 232 |
| 100 | 64 | 24 | 18 | 4 | 4 | 358103 | 2864820 | 4 | 4 | 0 | 224 | 2914860 | 4 | 4 | 0 | 1 | 232 |
| 101 | 65 | 24 | 18 | 4 | 5 | 358131 | 2865044 | 4 | 5 | 0 | 224 | 2915092 | 4 | 5 | 0 | 1 | 232 |
| 102 | 66 | 24 | 18 | 4 | 6 | 358159 | 2865268 | 4 | 6 | 0 | 224 | 2915324 | 4 | 6 | 0 | 1 | 232 |
| 103 | 67 | 24 | 18 | 4 | 7 | 358187 | 2865492 | 4 | 7 | 0 | 224 | 2915556 | 4 | 7 | 0 | 1 | 232 |
| 104 | 68 | 24 | 18 | 4 | 8 | 358215 | 2865716 | 4 | 8 | 0 | 224 | 2915788 | 4 | 8 | 0 | 1 | 232 |
| 105 | 69 | 24 | 18 | 4 | 9 | 358243 | 2865940 | 4 | 9 | 0 | 224 | 2916020 | 4 | 9 | 0 | 1 | 232 |
| 106 | 6A | 24 | 18 | 4 | 10 | 358271 | 2866164 | 4 | 10 | 0 | 224 | 2916252 | 4 | 10 | 0 | 1 | 232 |
| 107 | 6B | 24 | 18 | 4 | 11 | 358299 | 2866388 | 4 | 11 | 0 | 224 | 2916484 | 4 | 11 | 0 | 1 | 232 |
| 108 | 6C | 24 | 18 | 4 | 12 | 358327 | 2866612 | 4 | 12 | 0 | 224 | 2916716 | 4 | 12 | 0 | 1 | 232 |
| 109 | 6D | 24 | 18 | 4 | 13 | 358355 | 2866836 | 4 | 13 | 0 | 224 | 2916948 | 4 | 13 | 0 | 1 | 232 |
| 110 | 6E | 24 | 18 | 4 | 14 | 358383 | 2867060 | 4 | 14 | 0 | 224 | 2917180 | 4 | 14 | 0 | 1 | 232 |
| 111 | 6F | 24 | 18 | 4 | 15 | 358411 | 2867284 | 4 | 15 | 0 | 224 | 2917412 | 4 | 15 | 0 | 1 | 232 |
| 112 | 70 | 24 | 18 | 4 | 16 | 358441 | 2867524 | 4 | 16 | 0 | 240 | 2917660 | 4 | 16 | 0 | 1 | 248 |
| 113 | 71 | 24 | 18 | 4 | 17 | 358471 | 2867764 | 4 | 17 | 0 | 240 | 2917908 | 4 | 17 | 0 | 1 | 248 |
| 114 | 72 | 24 | 18 | 4 | 18 | 358501 | 2868004 | 4 | 18 | 0 | 240 | 2918156 | 4 | 18 | 0 | 1 | 248 |
| 115 | 73 | 24 | 18 | 4 | 19 | 358531 | 2868244 | 4 | 19 | 0 | 240 | 2918404 | 4 | 19 | 0 | 1 | 248 |
| 116 | 74 | 24 | 18 | 4 | 20 | 358561 | 2868484 | 4 | 20 | 0 | 240 | 2918652 | 4 | 20 | 0 | 1 | 248 |
| 117 | 75 | 24 | 18 | 4 | 21 | 358591 | 2868724 | 4 | 21 | 0 | 240 | 2918900 | 4 | 21 | 0 | 1 | 248 |
| 118 | 76 | 24 | 18 | 4 | 22 | 358621 | 2868964 | 4 | 22 | 0 | 240 | 2919148 | 4 | 22 | 0 | 1 | 248 |
| 119 | 77 | 24 | 18 | 4 | 23 | 358651 | 2869204 | 4 | 23 | 0 | 240 | 2919396 | 4 | 23 | 0 | 1 | 248 |
| 120 | 78 | 24 | 18 | 5 | 0 | 358683 | 2869460 | 5 | 0 | 0 | 256 | 2919660 | 5 | 0 | 0 | 1 | 264 |
| 121 | 79 | 24 | 18 | 5 | 1 | 358715 | 2869716 | 5 | 1 | 0 | 256 | 2919924 | 5 | 1 | 0 | 1 | 264 |
| 122 | 7A | 24 | 18 | 5 | 2 | 358747 | 2869972 | 5 | 2 | 0 | 256 | 2920188 | 5 | 2 | 0 | 1 | 264 |
| 123 | 7B | 24 | 18 | 5 | 3 | 358779 | 2870228 | 5 | 3 | 0 | 256 | 2920452 | 5 | 3 | 0 | 1 | 264 |
| 124 | 7C | 24 | 18 | 5 | 4 | 358811 | 2870484 | 5 | 4 | 0 | 256 | 2920716 | 5 | 4 | 0 | 1 | 264 |
| 125 | 7D | 24 | 18 | 5 | 5 | 358843 | 2870740 | 5 | 5 | 0 | 256 | 2920980 | 5 | 5 | 0 | 1 | 264 |
| 126 | 7E | 24 | 18 | 5 | 6 | 358875 | 2870996 | 5 | 6 | 0 | 256 | 2921244 | 5 | 6 | 0 | 1 | 264 |
| 127 | 7F | 24 | 18 | 5 | 7 | 358907 | 2871252 | 5 | 7 | 0 | 256 | 2921508 | 5 | 7 | 0 | 1 | 264 |
| 128 | 80 | 24 | 18 | 5 | 8 | 358937 | 2871492 | 5 | 8 | 0 | 240 | 2921756 | 5 | 8 | 0 | 1 | 248 |
| 129 | 81 | 24 | 18 | 5 | 9 | 358967 | 2871732 | 5 | 9 | 0 | 240 | 2922004 | 5 | 9 | 0 | 1 | 248 |
| 130 | 82 | 24 | 18 | 5 | 10 | 358997 | 2871972 | 5 | 10 | 0 | 240 | 2922252 | 5 | 10 | 0 | 1 | 248 |
| 131 | 83 | 24 | 18 | 5 | 11 | 359027 | 2872212 | 5 | 11 | 0 | 240 | 2922500 | 5 | 11 | 0 | 1 | 248 |
| 132 | 84 | 24 | 18 | 5 | 12 | 359057 | 2872452 | 5 | 12 | 0 | 240 | 2922748 | 5 | 12 | 0 | 1 | 248 |
| 133 | 85 | 24 | 18 | 5 | 13 | 359087 | 2872692 | 5 | 13 | 0 | 240 | 2922996 | 5 | 13 | 0 | 1 | 248 |
| 134 | 86 | 24 | 18 | 5 | 14 | 359117 | 2872932 | 5 | 14 | 0 | 240 | 2923244 | 5 | 14 | 0 | 1 | 248 |
| 135 | 87 | 24 | 18 | 5 | 15 | 359147 | 2873172 | 5 | 15 | 0 | 240 | 2923492 | 5 | 15 | 0 | 1 | 248 |

| | | | | | | | | | | | | | | | | | |
|---|---|---|---|---|---|---|---|---|---|---|---|---|---|---|---|---|---|
| 136 | 88 | 24 | 18 | 5 | 16 | 359177 | 2873412 | 5 | 16 | 0 | 240 | 2923740 | 5 | 16 | 0 | 1 | 248 |
| 137 | 89 | 24 | 18 | 5 | 17 | 359207 | 2873652 | 5 | 17 | 0 | 240 | 2923988 | 5 | 17 | 0 | 1 | 248 |
| 138 | 8A | 24 | 18 | 5 | 18 | 359237 | 2873892 | 5 | 18 | 0 | 240 | 2924236 | 5 | 18 | 0 | 1 | 248 |
| 139 | 8B | 24 | 18 | 5 | 19 | 359267 | 2874132 | 5 | 19 | 0 | 240 | 2924484 | 5 | 19 | 0 | 1 | 248 |
| 140 | 8C | 24 | 18 | 5 | 20 | 359297 | 2874372 | 5 | 20 | 0 | 240 | 2924732 | 5 | 20 | 0 | 1 | 248 |
| 141 | 8D | 24 | 18 | 5 | 21 | 359327 | 2874612 | 5 | 21 | 0 | 240 | 2924980 | 5 | 21 | 0 | 1 | 248 |
| 142 | 8E | 24 | 18 | 5 | 22 | 359357 | 2874852 | 5 | 22 | 0 | 240 | 2925228 | 5 | 22 | 0 | 1 | 248 |
| 143 | 8F | 24 | 18 | 5 | 23 | 359387 | 2875092 | 5 | 23 | 0 | 240 | 2925476 | 5 | 23 | 0 | 1 | 248 |
| 144 | 90 | 24 | 18 | 6 | 0 | 359421 | 2875364 | 6 | 0 | 0 | 272 | 2925756 | 6 | 0 | 0 | 1 | 280 |
| 145 | 91 | 24 | 18 | 6 | 1 | 359455 | 2875636 | 6 | 1 | 0 | 272 | 2926036 | 6 | 1 | 0 | 1 | 280 |
| 146 | 92 | 24 | 18 | 6 | 2 | 359489 | 2875908 | 6 | 2 | 0 | 272 | 2926316 | 6 | 2 | 0 | 1 | 280 |
| 147 | 93 | 24 | 18 | 6 | 3 | 359523 | 2876180 | 6 | 3 | 0 | 272 | 2926596 | 6 | 3 | 0 | 1 | 280 |
| 148 | 94 | 24 | 18 | 6 | 4 | 359557 | 2876452 | 6 | 4 | 0 | 272 | 2926876 | 6 | 4 | 0 | 1 | 280 |
| 149 | 95 | 24 | 18 | 6 | 5 | 359591 | 2876724 | 6 | 5 | 0 | 272 | 2927156 | 6 | 5 | 0 | 1 | 280 |
| 150 | 96 | 24 | 18 | 6 | 6 | 359625 | 2876996 | 6 | 6 | 0 | 272 | 2927436 | 6 | 6 | 0 | 1 | 280 |
| 151 | 97 | 24 | 18 | 6 | 7 | 359659 | 2877268 | 6 | 7 | 0 | 272 | 2927716 | 6 | 7 | 0 | 1 | 280 |
| 152 | 98 | 24 | 18 | 6 | 8 | 359693 | 2877540 | 6 | 8 | 0 | 272 | 2927996 | 6 | 8 | 0 | 1 | 280 |
| 153 | 99 | 24 | 18 | 6 | 9 | 359727 | 2877812 | 6 | 9 | 0 | 272 | 2928276 | 6 | 9 | 0 | 1 | 280 |
| 154 | 9A | 24 | 18 | 6 | 10 | 359761 | 2878084 | 6 | 10 | 0 | 272 | 2928556 | 6 | 10 | 0 | 1 | 280 |
| 155 | 9B | 24 | 18 | 6 | 11 | 359795 | 2878356 | 6 | 11 | 0 | 272 | 2928836 | 6 | 11 | 0 | 1 | 280 |
| 156 | 9C | 24 | 18 | 6 | 12 | 359829 | 2878628 | 6 | 12 | 0 | 272 | 2929116 | 6 | 12 | 0 | 1 | 280 |
| 157 | 9D | 24 | 18 | 6 | 13 | 359863 | 2878900 | 6 | 13 | 0 | 272 | 2929396 | 6 | 13 | 0 | 1 | 280 |
| 158 | 9E | 24 | 18 | 6 | 14 | 359897 | 2879172 | 6 | 14 | 0 | 272 | 2929676 | 6 | 14 | 0 | 1 | 280 |
| 159 | 9F | 24 | 18 | 6 | 15 | 359931 | 2879444 | 6 | 15 | 0 | 272 | 2929956 | 6 | 15 | 0 | 1 | 280 |
| 160 | A0 | 24 | 18 | 6 | 16 | 359963 | 2879700 | 6 | 16 | 0 | 256 | 2930220 | 6 | 16 | 0 | 1 | 264 |
| 161 | A1 | 24 | 18 | 6 | 17 | 359995 | 2879956 | 6 | 17 | 0 | 256 | 2930484 | 6 | 17 | 0 | 1 | 264 |
| 162 | A2 | 24 | 18 | 6 | 18 | 360027 | 2880212 | 6 | 18 | 0 | 256 | 2930748 | 6 | 18 | 0 | 1 | 264 |
| 163 | A3 | 24 | 18 | 6 | 19 | 360059 | 2880468 | 6 | 19 | 0 | 256 | 2931012 | 6 | 19 | 0 | 1 | 264 |
| 164 | A4 | 24 | 18 | 6 | 20 | 360091 | 2880724 | 6 | 20 | 0 | 256 | 2931276 | 6 | 20 | 0 | 1 | 264 |
| 165 | A5 | 24 | 18 | 6 | 21 | 360123 | 2880980 | 6 | 21 | 0 | 256 | 2931540 | 6 | 21 | 0 | 1 | 264 |
| 166 | A6 | 24 | 18 | 6 | 22 | 360155 | 2881236 | 6 | 22 | 0 | 256 | 2931804 | 6 | 22 | 0 | 1 | 264 |
| 167 | A7 | 24 | 18 | 6 | 23 | 360187 | 2881492 | 6 | 23 | 0 | 256 | 2932068 | 6 | 23 | 0 | 1 | 264 |
| 168 | A8 | 24 | 18 | 7 | 0 | 360221 | 2881764 | 7 | 0 | 0 | 272 | 2932348 | 7 | 0 | 0 | 1 | 280 |
| 169 | A9 | 24 | 18 | 7 | 1 | 360255 | 2882036 | 7 | 1 | 0 | 272 | 2932628 | 7 | 1 | 0 | 1 | 280 |
| 170 | AA | 24 | 18 | 7 | 2 | 360289 | 2882308 | 7 | 2 | 0 | 272 | 2932908 | 7 | 2 | 0 | 1 | 280 |
| 171 | AB | 24 | 18 | 7 | 3 | 360323 | 2882580 | 7 | 3 | 0 | 272 | 2933188 | 7 | 3 | 0 | 1 | 280 |
| 172 | AC | 24 | 18 | 7 | 4 | 360357 | 2882852 | 7 | 4 | 0 | 272 | 2933468 | 7 | 4 | 0 | 1 | 280 |
| 173 | AD | 24 | 18 | 7 | 5 | 360391 | 2883124 | 7 | 5 | 0 | 272 | 2933748 | 7 | 5 | 0 | 1 | 280 |
| 174 | AE | 24 | 18 | 7 | 6 | 360425 | 2883396 | 7 | 6 | 0 | 272 | 2934028 | 7 | 6 | 0 | 1 | 280 |
| 175 | AF | 24 | 18 | 7 | 7 | 360459 | 2883668 | 7 | 7 | 0 | 272 | 2934308 | 7 | 7 | 0 | 1 | 280 |
| 176 | B0 | 24 | 18 | 7 | 8 | 360495 | 2883956 | 7 | 8 | 0 | 288 | 2934604 | 7 | 8 | 0 | 1 | 296 |
| 177 | B1 | 24 | 18 | 7 | 9 | 360531 | 2884244 | 7 | 9 | 0 | 288 | 2934900 | 7 | 9 | 0 | 1 | 296 |
| 178 | B2 | 24 | 18 | 7 | 10 | 360567 | 2884532 | 7 | 10 | 0 | 288 | 2935196 | 7 | 10 | 0 | 1 | 296 |
| 179 | B3 | 24 | 18 | 7 | 11 | 360603 | 2884820 | 7 | 11 | 0 | 288 | 2935492 | 7 | 11 | 0 | 1 | 296 |
| 180 | B4 | 24 | 18 | 7 | 12 | 360639 | 2885108 | 7 | 12 | 0 | 288 | 2935788 | 7 | 12 | 0 | 1 | 296 |
| 181 | B5 | 24 | 18 | 7 | 13 | 360675 | 2885396 | 7 | 13 | 0 | 288 | 2936084 | 7 | 13 | 0 | 1 | 296 |
| 182 | B6 | 24 | 18 | 7 | 14 | 360711 | 2885684 | 7 | 14 | 0 | 288 | 2936380 | 7 | 14 | 0 | 1 | 296 |
| 183 | B7 | 24 | 18 | 7 | 15 | 360747 | 2885972 | 7 | 15 | 0 | 288 | 2936676 | 7 | 15 | 0 | 1 | 296 |
| 184 | B8 | 24 | 18 | 7 | 16 | 360783 | 2886260 | 7 | 16 | 0 | 288 | 2936972 | 7 | 16 | 0 | 1 | 296 |
| 185 | B9 | 24 | 18 | 7 | 17 | 360819 | 2886548 | 7 | 17 | 0 | 288 | 2937268 | 7 | 17 | 0 | 1 | 296 |
| 186 | BA | 24 | 18 | 7 | 18 | 360855 | 2886836 | 7 | 18 | 0 | 288 | 2937564 | 7 | 18 | 0 | 1 | 296 |
| 187 | BB | 24 | 18 | 7 | 19 | 360891 | 2887124 | 7 | 19 | 0 | 288 | 2937860 | 7 | 19 | 0 | 1 | 296 |
| 188 | BC | 24 | 18 | 7 | 20 | 360927 | 2887412 | 7 | 20 | 0 | 288 | 2938156 | 7 | 20 | 0 | 1 | 296 |
| 189 | BD | 24 | 18 | 7 | 21 | 360963 | 2887700 | 7 | 21 | 0 | 288 | 2938452 | 7 | 21 | 0 | 1 | 296 |
| 190 | BE | 24 | 18 | 7 | 22 | 360999 | 2887988 | 7 | 22 | 0 | 288 | 2938748 | 7 | 22 | 0 | 1 | 296 |
| 191 | BF | 24 | 18 | 7 | 23 | 361035 | 2888276 | 7 | 23 | 0 | 288 | 2939044 | 7 | 23 | 0 | 1 | 296 |
| 192 | C0 | 24 | 18 | 8 | 0 | 361071 | 2888564 | 8 | 0 | 0 | 288 | 2939340 | 8 | 0 | 0 | 1 | 296 |
| 193 | C1 | 24 | 18 | 8 | 1 | 361107 | 2888852 | 8 | 1 | 0 | 288 | 2939636 | 8 | 1 | 0 | 1 | 296 |
| 194 | C2 | 24 | 18 | 8 | 2 | 361143 | 2889140 | 8 | 2 | 0 | 288 | 2939932 | 8 | 2 | 0 | 1 | 296 |
| 195 | C3 | 24 | 18 | 8 | 3 | 361179 | 2889428 | 8 | 3 | 0 | 288 | 2940228 | 8 | 3 | 0 | 1 | 296 |
| 196 | C4 | 24 | 18 | 8 | 4 | 361215 | 2889716 | 8 | 4 | 0 | 288 | 2940524 | 8 | 4 | 0 | 1 | 296 |
| 197 | C5 | 24 | 18 | 8 | 5 | 361251 | 2890004 | 8 | 5 | 0 | 288 | 2940820 | 8 | 5 | 0 | 1 | 296 |
| 198 | C6 | 24 | 18 | 8 | 6 | 361287 | 2890292 | 8 | 6 | 0 | 288 | 2941116 | 8 | 6 | 0 | 1 | 296 |
| 199 | C7 | 24 | 18 | 8 | 7 | 361323 | 2890580 | 8 | 7 | 0 | 288 | 2941412 | 8 | 7 | 0 | 1 | 296 |
| 200 | C8 | 24 | 18 | 8 | 8 | 361359 | 2890868 | 8 | 8 | 0 | 288 | 2941708 | 8 | 8 | 0 | 1 | 296 |
| 201 | C9 | 24 | 18 | 8 | 9 | 361395 | 2891156 | 8 | 9 | 0 | 288 | 2942004 | 8 | 9 | 0 | 1 | 296 |
| 202 | CA | 24 | 18 | 8 | 10 | 361431 | 2891444 | 8 | 10 | 0 | 288 | 2942300 | 8 | 10 | 0 | 1 | 296 |
| 203 | CB | 24 | 18 | 8 | 11 | 361467 | 2891732 | 8 | 11 | 0 | 288 | 2942596 | 8 | 11 | 0 | 1 | 296 |
| 204 | CC | 24 | 18 | 8 | 12 | 361503 | 2892020 | 8 | 12 | 0 | 288 | 2942892 | 8 | 12 | 0 | 1 | 296 |
| 205 | CD | 24 | 18 | 8 | 13 | 361539 | 2892308 | 8 | 13 | 0 | 288 | 2943188 | 8 | 13 | 0 | 1 | 296 |
| 206 | CE | 24 | 18 | 8 | 14 | 361575 | 2892596 | 8 | 14 | 0 | 288 | 2943484 | 8 | 14 | 0 | 1 | 296 |
| 207 | CF | 24 | 18 | 8 | 15 | 361611 | 2892884 | 8 | 15 | 0 | 288 | 2943780 | 8 | 15 | 0 | 1 | 296 |
| 208 | D0 | 24 | 18 | 8 | 16 | 361649 | 2893188 | 8 | 16 | 0 | 304 | 2944092 | 8 | 16 | 0 | 1 | 312 |
| 209 | D1 | 24 | 18 | 8 | 17 | 361687 | 2893492 | 8 | 17 | 0 | 304 | 2944404 | 8 | 17 | 0 | 1 | 312 |
| 210 | D2 | 24 | 18 | 8 | 18 | 361725 | 2893796 | 8 | 18 | 0 | 304 | 2944716 | 8 | 18 | 0 | 1 | 312 |
| 211 | D3 | 24 | 18 | 8 | 19 | 361763 | 2894100 | 8 | 19 | 0 | 304 | 2945028 | 8 | 19 | 0 | 1 | 312 |
| 212 | D4 | 24 | 18 | 8 | 20 | 361801 | 2894404 | 8 | 20 | 0 | 304 | 2945340 | 8 | 20 | 0 | 1 | 312 |
| 213 | D5 | 24 | 18 | 8 | 21 | 361839 | 2894708 | 8 | 21 | 0 | 304 | 2945652 | 8 | 21 | 0 | 1 | 312 |
| 214 | D6 | 24 | 18 | 8 | 22 | 361877 | 2895012 | 8 | 22 | 0 | 304 | 2945964 | 8 | 22 | 0 | 1 | 312 |
| 215 | D7 | 24 | 18 | 8 | 23 | 361915 | 2895316 | 8 | 23 | 0 | 304 | 2946276 | 8 | 23 | 0 | 1 | 312 |
| 216 | D8 | 24 | 18 | 9 | 0 | 361955 | 2895636 | 9 | 0 | 0 | 320 | 2946604 | 9 | 0 | 0 | 1 | 328 |
| 217 | D9 | 24 | 18 | 9 | 1 | 361995 | 2895956 | 9 | 1 | 0 | 320 | 2946932 | 9 | 1 | 0 | 1 | 328 |
| 218 | DA | 24 | 18 | 9 | 2 | 362035 | 2896276 | 9 | 2 | 0 | 320 | 2947260 | 9 | 2 | 0 | 1 | 328 |
| 219 | DB | 24 | 18 | 9 | 3 | 362075 | 2896596 | 9 | 3 | 0 | 320 | 2947588 | 9 | 3 | 0 | 1 | 328 |
| 220 | DC | 24 | 18 | 9 | 4 | 362115 | 2896916 | 9 | 4 | 0 | 320 | 2947916 | 9 | 4 | 0 | 1 | 328 |
| 221 | DD | 24 | 18 | 9 | 5 | 362155 | 2897236 | 9 | 5 | 0 | 320 | 2948244 | 9 | 5 | 0 | 1 | 328 |
| 222 | DE | 24 | 18 | 9 | 6 | 362195 | 2897556 | 9 | 6 | 0 | 320 | 2948572 | 9 | 6 | 0 | 1 | 328 |
| 223 | DF | 24 | 18 | 9 | 7 | 362235 | 2897876 | 9 | 7 | 0 | 320 | 2948900 | 9 | 7 | 0 | 1 | 328 |
| 224 | E0 | 24 | 18 | 9 | 8 | 362273 | 2898180 | 9 | 8 | 0 | 304 | 2949212 | 9 | 8 | 0 | 1 | 312 |
| 225 | E1 | 24 | 18 | 9 | 9 | 362311 | 2898484 | 9 | 9 | 0 | 304 | 2949524 | 9 | 9 | 0 | 1 | 312 |
| 226 | E2 | 24 | 18 | 9 | 10 | 362349 | 2898788 | 9 | 10 | 0 | 304 | 2949836 | 9 | 10 | 0 | 1 | 312 |
| 227 | E3 | 24 | 18 | 9 | 11 | 362387 | 2899092 | 9 | 11 | 0 | 304 | 2950148 | 9 | 11 | 0 | 1 | 312 |
| 228 | E4 | 24 | 18 | 9 | 12 | 362425 | 2899396 | 9 | 12 | 0 | 304 | 2950460 | 9 | 12 | 0 | 1 | 312 |
| 229 | E5 | 24 | 18 | 9 | 13 | 362463 | 2899700 | 9 | 13 | 0 | 304 | 2950772 | 9 | 13 | 0 | 1 | 312 |
| 230 | E6 | 24 | 18 | 9 | 14 | 362501 | 2900004 | 9 | 14 | 0 | 304 | 2951084 | 9 | 14 | 0 | 1 | 312 |
| 231 | E7 | 24 | 18 | 9 | 15 | 362539 | 2900308 | 9 | 15 | 0 | 304 | 2951396 | 9 | 15 | 0 | 1 | 312 |
| 232 | E8 | 24 | 18 | 9 | 16 | 362577 | 2900612 | 9 | 16 | 0 | 304 | 2951708 | 9 | 16 | 0 | 1 | 312 |
| 233 | E9 | 24 | 18 | 9 | 17 | 362615 | 2900916 | 9 | 17 | 0 | 304 | 2952020 | 9 | 17 | 0 | 1 | 312 |
| 234 | EA | 24 | 18 | 9 | 18 | 362653 | 2901220 | 9 | 18 | 0 | 304 | 2952332 | 9 | 18 | 0 | 1 | 312 |
| 235 | EB | 24 | 18 | 9 | 19 | 362691 | 2901524 | 9 | 19 | 0 | 304 | 2952644 | 9 | 19 | 0 | 1 | 312 |
| 236 | EC | 24 | 18 | 9 | 20 | 362729 | 2901828 | 9 | 20 | 0 | 304 | 2952956 | 9 | 20 | 0 | 1 | 312 |
| 237 | ED | 24 | 18 | 9 | 21 | 362767 | 2902132 | 9 | 21 | 0 | 304 | 2953268 | 9 | 21 | 0 | 1 | 312 |
| 238 | EE | 24 | 18 | 9 | 22 | 362805 | 2902436 | 9 | 22 | 0 | 304 | 2953580 | 9 | 22 | 0 | 1 | 312 |

| | | | | | | | | | | | | | | | | | |
|---|---|---|---|---|---|---|---|---|---|---|---|---|---|---|---|---|---|
| 239 | EF | 24 | 18 | 9 | 23 | 362843 | 2902740 | 9 | 23 | 0 | 304 | 2953892 | 9 | 23 | 0 | 1 | 312 |
| 240 | F0 | 24 | 18 | 10 | 0 | 362885 | 2903076 | 10 | 0 | 0 | 336 | 2954236 | 10 | 0 | 0 | 1 | 344 |
| 241 | F1 | 24 | 18 | 10 | 1 | 362927 | 2903412 | 10 | 1 | 0 | 336 | 2954580 | 10 | 1 | 0 | 1 | 344 |
| 242 | F2 | 24 | 18 | 10 | 2 | 362969 | 2903748 | 10 | 2 | 0 | 336 | 2954924 | 10 | 2 | 0 | 1 | 344 |
| 243 | F3 | 24 | 18 | 10 | 3 | 363011 | 2904084 | 10 | 3 | 0 | 336 | 2955268 | 10 | 3 | 0 | 1 | 344 |
| 244 | F4 | 24 | 18 | 10 | 4 | 363053 | 2904420 | 10 | 4 | 0 | 336 | 2955612 | 10 | 4 | 0 | 1 | 344 |
| 245 | F5 | 24 | 18 | 10 | 5 | 363095 | 2904756 | 10 | 5 | 0 | 336 | 2955956 | 10 | 5 | 0 | 1 | 344 |
| 246 | F6 | 24 | 18 | 10 | 6 | 363137 | 2905092 | 10 | 6 | 0 | 336 | 2956300 | 10 | 6 | 0 | 1 | 344 |
| 247 | F7 | 24 | 18 | 10 | 7 | 363179 | 2905428 | 10 | 7 | 0 | 336 | 2956644 | 10 | 7 | 0 | 1 | 344 |
| 248 | F8 | 24 | 18 | 10 | 8 | 363221 | 2905764 | 10 | 8 | 0 | 336 | 2956988 | 10 | 8 | 0 | 1 | 344 |
| 249 | F9 | 24 | 18 | 10 | 9 | 363263 | 2906100 | 10 | 9 | 0 | 336 | 2957332 | 10 | 9 | 0 | 1 | 344 |
| 250 | FA | 24 | 18 | 10 | 10 | 363305 | 2906436 | 10 | 10 | 0 | 336 | 2957676 | 10 | 10 | 0 | 1 | 344 |
| 251 | FB | 24 | 18 | 10 | 11 | 363347 | 2906772 | 10 | 11 | 0 | 336 | 2958020 | 10 | 11 | 0 | 1 | 344 |
| 252 | FC | 24 | 18 | 10 | 12 | 363389 | 2907108 | 10 | 12 | 0 | 336 | 2958364 | 10 | 12 | 0 | 1 | 344 |
| 253 | FD | 24 | 18 | 10 | 13 | 363431 | 2907444 | 10 | 13 | 0 | 336 | 2958708 | 10 | 13 | 0 | 1 | 344 |
| 254 | FE | 24 | 18 | 10 | 14 | 363473 | 2907780 | 10 | 14 | 0 | 336 | 2959052 | 10 | 14 | 0 | 1 | 344 |
| 255 | FF | 24 | 18 | 10 | 15 | 363515 | 2908116 | 10 | 15 | 0 | 336 | 2959396 | 10 | 15 | 0 | 1 | 344 |
| 0 | 00 | 25 | 19 | 0 | 0 | 363527 | 2908212 | 0 | 0 | 0 | 96 | 2959500 | 0 | 0 | 0 | 1 | 104 |
| 1 | 01 | 25 | 19 | 0 | 1 | 363541 | 2908324 | 0 | 1 | 0 | 112 | 2959620 | 0 | 1 | 0 | 1 | 120 |
| 2 | 02 | 25 | 19 | 0 | 2 | 363555 | 2908436 | 0 | 2 | 0 | 112 | 2959740 | 0 | 2 | 0 | 1 | 120 |
| 3 | 03 | 25 | 19 | 0 | 3 | 363569 | 2908548 | 0 | 3 | 0 | 112 | 2959860 | 0 | 3 | 0 | 1 | 120 |
| 4 | 04 | 25 | 19 | 0 | 4 | 363583 | 2908660 | 0 | 4 | 0 | 112 | 2959980 | 0 | 4 | 0 | 1 | 120 |
| 5 | 05 | 25 | 19 | 0 | 5 | 363597 | 2908772 | 0 | 5 | 0 | 112 | 2960100 | 0 | 5 | 0 | 1 | 120 |
| 6 | 06 | 25 | 19 | 0 | 6 | 363611 | 2908884 | 0 | 6 | 0 | 112 | 2960220 | 0 | 6 | 0 | 1 | 120 |
| 7 | 07 | 25 | 19 | 0 | 7 | 363625 | 2908996 | 0 | 7 | 0 | 112 | 2960340 | 0 | 7 | 0 | 1 | 120 |
| 8 | 08 | 25 | 19 | 0 | 8 | 363639 | 2909108 | 0 | 8 | 0 | 112 | 2960460 | 0 | 8 | 0 | 1 | 120 |
| 9 | 09 | 25 | 19 | 0 | 9 | 363653 | 2909220 | 0 | 9 | 0 | 112 | 2960580 | 0 | 9 | 0 | 1 | 120 |
| 10 | 0A | 25 | 19 | 0 | 10 | 363667 | 2909332 | 0 | 10 | 0 | 112 | 2960700 | 0 | 10 | 0 | 1 | 120 |
| 11 | 0B | 25 | 19 | 0 | 11 | 363681 | 2909444 | 0 | 11 | 0 | 112 | 2960820 | 0 | 11 | 0 | 1 | 120 |
| 12 | 0C | 25 | 19 | 0 | 12 | 363695 | 2909556 | 0 | 12 | 0 | 112 | 2960940 | 0 | 12 | 0 | 1 | 120 |
| 13 | 0D | 25 | 19 | 0 | 13 | 363709 | 2909668 | 0 | 13 | 0 | 112 | 2961060 | 0 | 13 | 0 | 1 | 120 |
| 14 | 0E | 25 | 19 | 0 | 14 | 363723 | 2909780 | 0 | 14 | 0 | 112 | 2961180 | 0 | 14 | 0 | 1 | 120 |
| 15 | 0F | 25 | 19 | 0 | 15 | 363737 | 2909892 | 0 | 15 | 0 | 112 | 2961300 | 0 | 15 | 0 | 1 | 120 |
| 16 | 10 | 25 | 19 | 0 | 16 | 363751 | 2910004 | 0 | 16 | 0 | 112 | 2961420 | 0 | 16 | 0 | 1 | 120 |
| 17 | 11 | 25 | 19 | 0 | 17 | 363765 | 2910116 | 0 | 17 | 0 | 112 | 2961540 | 0 | 17 | 0 | 1 | 120 |
| 18 | 12 | 25 | 19 | 0 | 18 | 363779 | 2910228 | 0 | 18 | 0 | 112 | 2961660 | 0 | 18 | 0 | 1 | 120 |
| 19 | 13 | 25 | 19 | 0 | 19 | 363793 | 2910340 | 0 | 19 | 0 | 112 | 2961780 | 0 | 19 | 0 | 1 | 120 |
| 20 | 14 | 25 | 19 | 0 | 20 | 363807 | 2910452 | 0 | 20 | 0 | 112 | 2961900 | 0 | 20 | 0 | 1 | 120 |
| 21 | 15 | 25 | 19 | 0 | 21 | 363821 | 2910564 | 0 | 21 | 0 | 112 | 2962020 | 0 | 21 | 0 | 1 | 120 |
| 22 | 16 | 25 | 19 | 0 | 22 | 363835 | 2910676 | 0 | 22 | 0 | 112 | 2962140 | 0 | 22 | 0 | 1 | 120 |
| 23 | 17 | 25 | 19 | 0 | 23 | 363849 | 2910788 | 0 | 23 | 0 | 112 | 2962260 | 0 | 23 | 0 | 1 | 120 |
| 24 | 18 | 25 | 19 | 0 | 24 | 363863 | 2910900 | 0 | 24 | 0 | 112 | 2962380 | 0 | 24 | 0 | 1 | 120 |
| 25 | 19 | 25 | 19 | 1 | 0 | 363875 | 2910996 | 1 | 0 | 0 | 96 | 2962484 | 1 | 0 | 0 | 1 | 104 |
| 26 | 1A | 25 | 19 | 1 | 1 | 363892 | 2911132 | 1 | 1 | 0 | 136 | 2962628 | 1 | 1 | 0 | 1 | 144 |
| 27 | 1B | 25 | 19 | 1 | 2 | 363909 | 2911268 | 1 | 2 | 0 | 136 | 2962772 | 1 | 2 | 0 | 1 | 144 |
| 28 | 1C | 25 | 19 | 1 | 3 | 363926 | 2911404 | 1 | 3 | 0 | 136 | 2962916 | 1 | 3 | 0 | 1 | 144 |
| 29 | 1D | 25 | 19 | 1 | 4 | 363943 | 2911540 | 1 | 4 | 0 | 136 | 2963060 | 1 | 4 | 0 | 1 | 144 |
| 30 | 1E | 25 | 19 | 1 | 5 | 363960 | 2911676 | 1 | 5 | 0 | 136 | 2963204 | 1 | 5 | 0 | 1 | 144 |
| 31 | 1F | 25 | 19 | 1 | 6 | 363977 | 2911812 | 1 | 6 | 0 | 136 | 2963348 | 1 | 6 | 0 | 1 | 144 |
| 32 | 20 | 25 | 19 | 1 | 7 | 363998 | 2911980 | 1 | 7 | 0 | 168 | 2963524 | 1 | 7 | 0 | 1 | 176 |
| 33 | 21 | 25 | 19 | 1 | 8 | 364019 | 2912148 | 1 | 8 | 0 | 168 | 2963700 | 1 | 8 | 0 | 1 | 176 |
| 34 | 22 | 25 | 19 | 1 | 9 | 364040 | 2912316 | 1 | 9 | 0 | 168 | 2963876 | 1 | 9 | 0 | 1 | 176 |
| 35 | 23 | 25 | 19 | 1 | 10 | 364061 | 2912484 | 1 | 10 | 0 | 168 | 2964052 | 1 | 10 | 0 | 1 | 176 |
| 36 | 24 | 25 | 19 | 1 | 11 | 364082 | 2912652 | 1 | 11 | 0 | 168 | 2964228 | 1 | 11 | 0 | 1 | 176 |
| 37 | 25 | 25 | 19 | 1 | 12 | 364103 | 2912820 | 1 | 12 | 0 | 168 | 2964404 | 1 | 12 | 0 | 1 | 176 |
| 38 | 26 | 25 | 19 | 1 | 13 | 364124 | 2912988 | 1 | 13 | 0 | 168 | 2964580 | 1 | 13 | 0 | 1 | 176 |
| 39 | 27 | 25 | 19 | 1 | 14 | 364145 | 2913156 | 1 | 14 | 0 | 168 | 2964756 | 1 | 14 | 0 | 1 | 176 |
| 40 | 28 | 25 | 19 | 1 | 15 | 364166 | 2913324 | 1 | 15 | 0 | 168 | 2964932 | 1 | 15 | 0 | 1 | 176 |
| 41 | 29 | 25 | 19 | 1 | 16 | 364187 | 2913492 | 1 | 16 | 0 | 168 | 2965108 | 1 | 16 | 0 | 1 | 176 |
| 42 | 2A | 25 | 19 | 1 | 17 | 364208 | 2913660 | 1 | 17 | 0 | 168 | 2965284 | 1 | 17 | 0 | 1 | 176 |
| 43 | 2B | 25 | 19 | 1 | 18 | 364229 | 2913828 | 1 | 18 | 0 | 168 | 2965460 | 1 | 18 | 0 | 1 | 176 |
| 44 | 2C | 25 | 19 | 1 | 19 | 364250 | 2913996 | 1 | 19 | 0 | 168 | 2965636 | 1 | 19 | 0 | 1 | 176 |
| 45 | 2D | 25 | 19 | 1 | 20 | 364271 | 2914164 | 1 | 20 | 0 | 168 | 2965812 | 1 | 20 | 0 | 1 | 176 |
| 46 | 2E | 25 | 19 | 1 | 21 | 364292 | 2914332 | 1 | 21 | 0 | 168 | 2965988 | 1 | 21 | 0 | 1 | 176 |
| 47 | 2F | 25 | 19 | 1 | 22 | 364313 | 2914500 | 1 | 22 | 0 | 168 | 2966164 | 1 | 22 | 0 | 1 | 176 |
| 48 | 30 | 25 | 19 | 1 | 23 | 364336 | 2914684 | 1 | 23 | 0 | 184 | 2966356 | 1 | 23 | 0 | 1 | 192 |
| 49 | 31 | 25 | 19 | 1 | 24 | 364359 | 2914868 | 1 | 24 | 0 | 184 | 2966548 | 1 | 24 | 0 | 1 | 192 |
| 50 | 32 | 25 | 19 | 2 | 0 | 364380 | 2915036 | 2 | 0 | 0 | 168 | 2966724 | 2 | 0 | 0 | 1 | 176 |
| 51 | 33 | 25 | 19 | 2 | 1 | 364406 | 2915244 | 2 | 1 | 0 | 208 | 2966940 | 2 | 1 | 0 | 1 | 216 |
| 52 | 34 | 25 | 19 | 2 | 2 | 364432 | 2915452 | 2 | 2 | 0 | 208 | 2967156 | 2 | 2 | 0 | 1 | 216 |
| 53 | 35 | 25 | 19 | 2 | 3 | 364458 | 2915660 | 2 | 3 | 0 | 208 | 2967372 | 2 | 3 | 0 | 1 | 216 |
| 54 | 36 | 25 | 19 | 2 | 4 | 364484 | 2915868 | 2 | 4 | 0 | 208 | 2967588 | 2 | 4 | 0 | 1 | 216 |
| 55 | 37 | 25 | 19 | 2 | 5 | 364510 | 2916076 | 2 | 5 | 0 | 208 | 2967804 | 2 | 5 | 0 | 1 | 216 |
| 56 | 38 | 25 | 19 | 2 | 6 | 364536 | 2916284 | 2 | 6 | 0 | 208 | 2968020 | 2 | 6 | 0 | 1 | 216 |
| 57 | 39 | 25 | 19 | 2 | 7 | 364562 | 2916492 | 2 | 7 | 0 | 208 | 2968236 | 2 | 7 | 0 | 1 | 216 |
| 58 | 3A | 25 | 19 | 2 | 8 | 364588 | 2916700 | 2 | 8 | 0 | 208 | 2968452 | 2 | 8 | 0 | 1 | 216 |
| 59 | 3B | 25 | 19 | 2 | 9 | 364614 | 2916908 | 2 | 9 | 0 | 208 | 2968668 | 2 | 9 | 0 | 1 | 216 |
| 60 | 3C | 25 | 19 | 2 | 10 | 364640 | 2917116 | 2 | 10 | 0 | 208 | 2968884 | 2 | 10 | 0 | 1 | 216 |
| 61 | 3D | 25 | 19 | 2 | 11 | 364666 | 2917324 | 2 | 11 | 0 | 208 | 2969100 | 2 | 11 | 0 | 1 | 216 |
| 62 | 3E | 25 | 19 | 2 | 12 | 364692 | 2917532 | 2 | 12 | 0 | 208 | 2969316 | 2 | 12 | 0 | 1 | 216 |
| 63 | 3F | 25 | 19 | 2 | 13 | 364718 | 2917740 | 2 | 13 | 0 | 208 | 2969532 | 2 | 13 | 0 | 1 | 216 |
| 64 | 40 | 25 | 19 | 2 | 14 | 364741 | 2917924 | 2 | 14 | 0 | 184 | 2969724 | 2 | 14 | 0 | 1 | 192 |
| 65 | 41 | 25 | 19 | 2 | 15 | 364764 | 2918108 | 2 | 15 | 0 | 184 | 2969916 | 2 | 15 | 0 | 1 | 192 |
| 66 | 42 | 25 | 19 | 2 | 16 | 364787 | 2918292 | 2 | 16 | 0 | 184 | 2970108 | 2 | 16 | 0 | 1 | 192 |
| 67 | 43 | 25 | 19 | 2 | 17 | 364810 | 2918476 | 2 | 17 | 0 | 184 | 2970300 | 2 | 17 | 0 | 1 | 192 |
| 68 | 44 | 25 | 19 | 2 | 18 | 364833 | 2918660 | 2 | 18 | 0 | 184 | 2970492 | 2 | 18 | 0 | 1 | 192 |
| 69 | 45 | 25 | 19 | 2 | 19 | 364856 | 2918844 | 2 | 19 | 0 | 184 | 2970684 | 2 | 19 | 0 | 1 | 192 |
| 70 | 46 | 25 | 19 | 2 | 20 | 364879 | 2919028 | 2 | 20 | 0 | 184 | 2970876 | 2 | 20 | 0 | 1 | 192 |
| 71 | 47 | 25 | 19 | 2 | 21 | 364902 | 2919212 | 2 | 21 | 0 | 184 | 2971068 | 2 | 21 | 0 | 1 | 192 |
| 72 | 48 | 25 | 19 | 2 | 22 | 364925 | 2919396 | 2 | 22 | 0 | 184 | 2971260 | 2 | 22 | 0 | 1 | 192 |
| 73 | 49 | 25 | 19 | 2 | 23 | 364948 | 2919580 | 2 | 23 | 0 | 184 | 2971452 | 2 | 23 | 0 | 1 | 192 |
| 74 | 4A | 25 | 19 | 2 | 24 | 364971 | 2919764 | 2 | 24 | 0 | 184 | 2971644 | 2 | 24 | 0 | 1 | 192 |
| 75 | 4B | 25 | 19 | 3 | 0 | 364992 | 2919932 | 3 | 0 | 0 | 168 | 2971820 | 3 | 0 | 0 | 1 | 176 |
| 76 | 4C | 25 | 19 | 3 | 1 | 365018 | 2920140 | 3 | 1 | 0 | 208 | 2972036 | 3 | 1 | 0 | 1 | 216 |
| 77 | 4D | 25 | 19 | 3 | 2 | 365044 | 2920348 | 3 | 2 | 0 | 208 | 2972252 | 3 | 2 | 0 | 1 | 216 |
| 78 | 4E | 25 | 19 | 3 | 3 | 365070 | 2920556 | 3 | 3 | 0 | 208 | 2972468 | 3 | 3 | 0 | 1 | 216 |
| 79 | 4F | 25 | 19 | 3 | 4 | 365096 | 2920764 | 3 | 4 | 0 | 208 | 2972684 | 3 | 4 | 0 | 1 | 216 |
| 80 | 50 | 25 | 19 | 3 | 5 | 365124 | 2920988 | 3 | 5 | 0 | 224 | 2972916 | 3 | 5 | 0 | 1 | 232 |
| 81 | 51 | 25 | 19 | 3 | 6 | 365152 | 2921212 | 3 | 6 | 0 | 224 | 2973148 | 3 | 6 | 0 | 1 | 232 |
| 82 | 52 | 25 | 19 | 3 | 7 | 365180 | 2921436 | 3 | 7 | 0 | 224 | 2973380 | 3 | 7 | 0 | 1 | 232 |
| 83 | 53 | 25 | 19 | 3 | 8 | 365208 | 2921660 | 3 | 8 | 0 | 224 | 2973612 | 3 | 8 | 0 | 1 | 232 |
| 84 | 54 | 25 | 19 | 3 | 9 | 365236 | 2921884 | 3 | 9 | 0 | 224 | 2973844 | 3 | 9 | 0 | 1 | 232 |
| 85 | 55 | 25 | 19 | 3 | 10 | 365264 | 2922108 | 3 | 10 | 0 | 224 | 2974076 | 3 | 10 | 0 | 1 | 232 |

| | | | | | | | | | | | | | | | | | |
|---|---|---|---|---|---|---|---|---|---|---|---|---|---|---|---|---|---|
| 86 | 56 | 25 | 19 | 3 | 11 | 365292 | 2922332 | 3 | 11 | 0 | 224 | 2974308 | 3 | 11 | 0 | 1 | 232 |
| 87 | 57 | 25 | 19 | 3 | 12 | 365320 | 2922556 | 3 | 12 | 0 | 224 | 2974540 | 3 | 12 | 0 | 1 | 232 |
| 88 | 58 | 25 | 19 | 3 | 13 | 365348 | 2922780 | 3 | 13 | 0 | 224 | 2974772 | 3 | 13 | 0 | 1 | 232 |
| 89 | 59 | 25 | 19 | 3 | 14 | 365376 | 2923004 | 3 | 14 | 0 | 224 | 2975004 | 3 | 14 | 0 | 1 | 232 |
| 90 | 5A | 25 | 19 | 3 | 15 | 365404 | 2923228 | 3 | 15 | 0 | 224 | 2975236 | 3 | 15 | 0 | 1 | 232 |
| 91 | 5B | 25 | 19 | 3 | 16 | 365432 | 2923452 | 3 | 16 | 0 | 224 | 2975468 | 3 | 16 | 0 | 1 | 232 |
| 92 | 5C | 25 | 19 | 3 | 17 | 365460 | 2923676 | 3 | 17 | 0 | 224 | 2975700 | 3 | 17 | 0 | 1 | 232 |
| 93 | 5D | 25 | 19 | 3 | 18 | 365488 | 2923900 | 3 | 18 | 0 | 224 | 2975932 | 3 | 18 | 0 | 1 | 232 |
| 94 | 5E | 25 | 19 | 3 | 19 | 365516 | 2924124 | 3 | 19 | 0 | 224 | 2976164 | 3 | 19 | 0 | 1 | 232 |
| 95 | 5F | 25 | 19 | 3 | 20 | 365544 | 2924348 | 3 | 20 | 0 | 224 | 2976396 | 3 | 20 | 0 | 1 | 232 |
| 96 | 60 | 25 | 19 | 3 | 21 | 365569 | 2924548 | 3 | 21 | 0 | 200 | 2976604 | 3 | 21 | 0 | 1 | 208 |
| 97 | 61 | 25 | 19 | 3 | 22 | 365594 | 2924748 | 3 | 22 | 0 | 200 | 2976812 | 3 | 22 | 0 | 1 | 208 |
| 98 | 62 | 25 | 19 | 3 | 23 | 365619 | 2924948 | 3 | 23 | 0 | 200 | 2977020 | 3 | 23 | 0 | 1 | 208 |
| 99 | 63 | 25 | 19 | 3 | 24 | 365644 | 2925148 | 3 | 24 | 0 | 200 | 2977228 | 3 | 24 | 0 | 1 | 208 |
| 100 | 64 | 25 | 19 | 4 | 0 | 365667 | 2925332 | 4 | 0 | 0 | 184 | 2977420 | 4 | 0 | 0 | 1 | 192 |
| 101 | 65 | 25 | 19 | 4 | 1 | 365695 | 2925556 | 4 | 1 | 0 | 224 | 2977652 | 4 | 1 | 0 | 1 | 232 |
| 102 | 66 | 25 | 19 | 4 | 2 | 365723 | 2925780 | 4 | 2 | 0 | 224 | 2977884 | 4 | 2 | 0 | 1 | 232 |
| 103 | 67 | 25 | 19 | 4 | 3 | 365751 | 2926004 | 4 | 3 | 0 | 224 | 2978116 | 4 | 3 | 0 | 1 | 232 |
| 104 | 68 | 25 | 19 | 4 | 4 | 365779 | 2926228 | 4 | 4 | 0 | 224 | 2978348 | 4 | 4 | 0 | 1 | 232 |
| 105 | 69 | 25 | 19 | 4 | 5 | 365807 | 2926452 | 4 | 5 | 0 | 224 | 2978580 | 4 | 5 | 0 | 1 | 232 |
| 106 | 6A | 25 | 19 | 4 | 6 | 365835 | 2926676 | 4 | 6 | 0 | 224 | 2978812 | 4 | 6 | 0 | 1 | 232 |
| 107 | 6B | 25 | 19 | 4 | 7 | 365863 | 2926900 | 4 | 7 | 0 | 224 | 2979044 | 4 | 7 | 0 | 1 | 232 |
| 108 | 6C | 25 | 19 | 4 | 8 | 365891 | 2927124 | 4 | 8 | 0 | 224 | 2979276 | 4 | 8 | 0 | 1 | 232 |
| 109 | 6D | 25 | 19 | 4 | 9 | 365919 | 2927348 | 4 | 9 | 0 | 224 | 2979508 | 4 | 9 | 0 | 1 | 232 |
| 110 | 6E | 25 | 19 | 4 | 10 | 365947 | 2927572 | 4 | 10 | 0 | 224 | 2979740 | 4 | 10 | 0 | 1 | 232 |
| 111 | 6F | 25 | 19 | 4 | 11 | 365975 | 2927796 | 4 | 11 | 0 | 224 | 2979972 | 4 | 11 | 0 | 1 | 232 |
| 112 | 70 | 25 | 19 | 4 | 12 | 366005 | 2928036 | 4 | 12 | 0 | 240 | 2980220 | 4 | 12 | 0 | 1 | 248 |
| 113 | 71 | 25 | 19 | 4 | 13 | 366035 | 2928276 | 4 | 13 | 0 | 240 | 2980468 | 4 | 13 | 0 | 1 | 248 |
| 114 | 72 | 25 | 19 | 4 | 14 | 366065 | 2928516 | 4 | 14 | 0 | 240 | 2980716 | 4 | 14 | 0 | 1 | 248 |
| 115 | 73 | 25 | 19 | 4 | 15 | 366095 | 2928756 | 4 | 15 | 0 | 240 | 2980964 | 4 | 15 | 0 | 1 | 248 |
| 116 | 74 | 25 | 19 | 4 | 16 | 366125 | 2928996 | 4 | 16 | 0 | 240 | 2981212 | 4 | 16 | 0 | 1 | 248 |
| 117 | 75 | 25 | 19 | 4 | 17 | 366155 | 2929236 | 4 | 17 | 0 | 240 | 2981460 | 4 | 17 | 0 | 1 | 248 |
| 118 | 76 | 25 | 19 | 4 | 18 | 366185 | 2929476 | 4 | 18 | 0 | 240 | 2981708 | 4 | 18 | 0 | 1 | 248 |
| 119 | 77 | 25 | 19 | 4 | 19 | 366215 | 2929716 | 4 | 19 | 0 | 240 | 2981956 | 4 | 19 | 0 | 1 | 248 |
| 120 | 78 | 25 | 19 | 4 | 20 | 366245 | 2929956 | 4 | 20 | 0 | 240 | 2982204 | 4 | 20 | 0 | 1 | 248 |
| 121 | 79 | 25 | 19 | 4 | 21 | 366275 | 2930196 | 4 | 21 | 0 | 240 | 2982452 | 4 | 21 | 0 | 1 | 248 |
| 122 | 7A | 25 | 19 | 4 | 22 | 366305 | 2930436 | 4 | 22 | 0 | 240 | 2982700 | 4 | 22 | 0 | 1 | 248 |
| 123 | 7B | 25 | 19 | 4 | 23 | 366335 | 2930676 | 4 | 23 | 0 | 240 | 2982948 | 4 | 23 | 0 | 1 | 248 |
| 124 | 7C | 25 | 19 | 4 | 24 | 366365 | 2930916 | 4 | 24 | 0 | 240 | 2983196 | 4 | 24 | 0 | 1 | 248 |
| 125 | 7D | 25 | 19 | 5 | 0 | 366397 | 2931172 | 5 | 0 | 0 | 256 | 2983460 | 5 | 0 | 0 | 1 | 264 |
| 126 | 7E | 25 | 19 | 5 | 1 | 366429 | 2931428 | 5 | 1 | 0 | 256 | 2983724 | 5 | 1 | 0 | 1 | 264 |
| 127 | 7F | 25 | 19 | 5 | 2 | 366461 | 2931684 | 5 | 2 | 0 | 256 | 2983988 | 5 | 2 | 0 | 1 | 264 |
| 128 | 80 | 25 | 19 | 5 | 3 | 366491 | 2931924 | 5 | 3 | 0 | 240 | 2984236 | 5 | 3 | 0 | 1 | 248 |
| 129 | 81 | 25 | 19 | 5 | 4 | 366521 | 2932164 | 5 | 4 | 0 | 240 | 2984484 | 5 | 4 | 0 | 1 | 248 |
| 130 | 82 | 25 | 19 | 5 | 5 | 366551 | 2932404 | 5 | 5 | 0 | 240 | 2984732 | 5 | 5 | 0 | 1 | 248 |
| 131 | 83 | 25 | 19 | 5 | 6 | 366581 | 2932644 | 5 | 6 | 0 | 240 | 2984980 | 5 | 6 | 0 | 1 | 248 |
| 132 | 84 | 25 | 19 | 5 | 7 | 366611 | 2932884 | 5 | 7 | 0 | 240 | 2985228 | 5 | 7 | 0 | 1 | 248 |
| 133 | 85 | 25 | 19 | 5 | 8 | 366641 | 2933124 | 5 | 8 | 0 | 240 | 2985476 | 5 | 8 | 0 | 1 | 248 |
| 134 | 86 | 25 | 19 | 5 | 9 | 366671 | 2933364 | 5 | 9 | 0 | 240 | 2985724 | 5 | 9 | 0 | 1 | 248 |
| 135 | 87 | 25 | 19 | 5 | 10 | 366701 | 2933604 | 5 | 10 | 0 | 240 | 2985972 | 5 | 10 | 0 | 1 | 248 |
| 136 | 88 | 25 | 19 | 5 | 11 | 366731 | 2933844 | 5 | 11 | 0 | 240 | 2986220 | 5 | 11 | 0 | 1 | 248 |
| 137 | 89 | 25 | 19 | 5 | 12 | 366761 | 2934084 | 5 | 12 | 0 | 240 | 2986468 | 5 | 12 | 0 | 1 | 248 |
| 138 | 8A | 25 | 19 | 5 | 13 | 366791 | 2934324 | 5 | 13 | 0 | 240 | 2986716 | 5 | 13 | 0 | 1 | 248 |
| 139 | 8B | 25 | 19 | 5 | 14 | 366821 | 2934564 | 5 | 14 | 0 | 240 | 2986964 | 5 | 14 | 0 | 1 | 248 |
| 140 | 8C | 25 | 19 | 5 | 15 | 366851 | 2934804 | 5 | 15 | 0 | 240 | 2987212 | 5 | 15 | 0 | 1 | 248 |
| 141 | 8D | 25 | 19 | 5 | 16 | 366881 | 2935044 | 5 | 16 | 0 | 240 | 2987460 | 5 | 16 | 0 | 1 | 248 |
| 142 | 8E | 25 | 19 | 5 | 17 | 366911 | 2935284 | 5 | 17 | 0 | 240 | 2987708 | 5 | 17 | 0 | 1 | 248 |
| 143 | 8F | 25 | 19 | 5 | 18 | 366941 | 2935524 | 5 | 18 | 0 | 240 | 2987956 | 5 | 18 | 0 | 1 | 248 |
| 144 | 90 | 25 | 19 | 5 | 19 | 366973 | 2935780 | 5 | 19 | 0 | 256 | 2988220 | 5 | 19 | 0 | 1 | 264 |
| 145 | 91 | 25 | 19 | 5 | 20 | 367005 | 2936036 | 5 | 20 | 0 | 256 | 2988484 | 5 | 20 | 0 | 1 | 264 |
| 146 | 92 | 25 | 19 | 5 | 21 | 367037 | 2936292 | 5 | 21 | 0 | 256 | 2988748 | 5 | 21 | 0 | 1 | 264 |
| 147 | 93 | 25 | 19 | 5 | 22 | 367069 | 2936548 | 5 | 22 | 0 | 256 | 2989012 | 5 | 22 | 0 | 1 | 264 |
| 148 | 94 | 25 | 19 | 5 | 23 | 367101 | 2936804 | 5 | 23 | 0 | 256 | 2989276 | 5 | 23 | 0 | 1 | 264 |
| 149 | 95 | 25 | 19 | 5 | 24 | 367133 | 2937060 | 5 | 24 | 0 | 256 | 2989540 | 5 | 24 | 0 | 1 | 264 |
| 150 | 96 | 25 | 19 | 6 | 0 | 367167 | 2937332 | 6 | 0 | 0 | 272 | 2989820 | 6 | 0 | 0 | 1 | 280 |
| 151 | 97 | 25 | 19 | 6 | 1 | 367201 | 2937604 | 6 | 1 | 0 | 272 | 2990100 | 6 | 1 | 0 | 1 | 280 |
| 152 | 98 | 25 | 19 | 6 | 2 | 367235 | 2937876 | 6 | 2 | 0 | 272 | 2990380 | 6 | 2 | 0 | 1 | 280 |
| 153 | 99 | 25 | 19 | 6 | 3 | 367269 | 2938148 | 6 | 3 | 0 | 272 | 2990660 | 6 | 3 | 0 | 1 | 280 |
| 154 | 9A | 25 | 19 | 6 | 4 | 367303 | 2938420 | 6 | 4 | 0 | 272 | 2990940 | 6 | 4 | 0 | 1 | 280 |
| 155 | 9B | 25 | 19 | 6 | 5 | 367337 | 2938692 | 6 | 5 | 0 | 272 | 2991220 | 6 | 5 | 0 | 1 | 280 |
| 156 | 9C | 25 | 19 | 6 | 6 | 367371 | 2938964 | 6 | 6 | 0 | 272 | 2991500 | 6 | 6 | 0 | 1 | 280 |
| 157 | 9D | 25 | 19 | 6 | 7 | 367405 | 2939236 | 6 | 7 | 0 | 272 | 2991780 | 6 | 7 | 0 | 1 | 280 |
| 158 | 9E | 25 | 19 | 6 | 8 | 367439 | 2939508 | 6 | 8 | 0 | 272 | 2992060 | 6 | 8 | 0 | 1 | 280 |
| 159 | 9F | 25 | 19 | 6 | 9 | 367473 | 2939780 | 6 | 9 | 0 | 272 | 2992340 | 6 | 9 | 0 | 1 | 280 |
| 160 | A0 | 25 | 19 | 6 | 10 | 367505 | 2940036 | 6 | 10 | 0 | 256 | 2992604 | 6 | 10 | 0 | 1 | 264 |
| 161 | A1 | 25 | 19 | 6 | 11 | 367537 | 2940292 | 6 | 11 | 0 | 256 | 2992868 | 6 | 11 | 0 | 1 | 264 |
| 162 | A2 | 25 | 19 | 6 | 12 | 367569 | 2940548 | 6 | 12 | 0 | 256 | 2993132 | 6 | 12 | 0 | 1 | 264 |
| 163 | A3 | 25 | 19 | 6 | 13 | 367601 | 2940804 | 6 | 13 | 0 | 256 | 2993396 | 6 | 13 | 0 | 1 | 264 |
| 164 | A4 | 25 | 19 | 6 | 14 | 367633 | 2941060 | 6 | 14 | 0 | 256 | 2993660 | 6 | 14 | 0 | 1 | 264 |
| 165 | A5 | 25 | 19 | 6 | 15 | 367665 | 2941316 | 6 | 15 | 0 | 256 | 2993924 | 6 | 15 | 0 | 1 | 264 |
| 166 | A6 | 25 | 19 | 6 | 16 | 367697 | 2941572 | 6 | 16 | 0 | 256 | 2994188 | 6 | 16 | 0 | 1 | 264 |
| 167 | A7 | 25 | 19 | 6 | 17 | 367729 | 2941828 | 6 | 17 | 0 | 256 | 2994452 | 6 | 17 | 0 | 1 | 264 |
| 168 | A8 | 25 | 19 | 6 | 18 | 367761 | 2942084 | 6 | 18 | 0 | 256 | 2994716 | 6 | 18 | 0 | 1 | 264 |
| 169 | A9 | 25 | 19 | 6 | 19 | 367793 | 2942340 | 6 | 19 | 0 | 256 | 2994980 | 6 | 19 | 0 | 1 | 264 |
| 170 | AA | 25 | 19 | 6 | 20 | 367825 | 2942596 | 6 | 20 | 0 | 256 | 2995244 | 6 | 20 | 0 | 1 | 264 |
| 171 | AB | 25 | 19 | 6 | 21 | 367857 | 2942852 | 6 | 21 | 0 | 256 | 2995508 | 6 | 21 | 0 | 1 | 264 |
| 172 | AC | 25 | 19 | 6 | 22 | 367889 | 2943108 | 6 | 22 | 0 | 256 | 2995772 | 6 | 22 | 0 | 1 | 264 |
| 173 | AD | 25 | 19 | 6 | 23 | 367921 | 2943364 | 6 | 23 | 0 | 256 | 2996036 | 6 | 23 | 0 | 1 | 264 |
| 174 | AE | 25 | 19 | 6 | 24 | 367953 | 2943620 | 6 | 24 | 0 | 256 | 2996300 | 6 | 24 | 0 | 1 | 264 |
| 175 | AF | 25 | 19 | 7 | 0 | 367987 | 2943892 | 7 | 0 | 0 | 272 | 2996580 | 7 | 0 | 0 | 1 | 280 |
| 176 | B0 | 25 | 19 | 7 | 1 | 368023 | 2944180 | 7 | 1 | 0 | 288 | 2996876 | 7 | 1 | 0 | 1 | 296 |
| 177 | B1 | 25 | 19 | 7 | 2 | 368059 | 2944468 | 7 | 2 | 0 | 288 | 2997172 | 7 | 2 | 0 | 1 | 296 |
| 178 | B2 | 25 | 19 | 7 | 3 | 368095 | 2944756 | 7 | 3 | 0 | 288 | 2997468 | 7 | 3 | 0 | 1 | 296 |
| 179 | B3 | 25 | 19 | 7 | 4 | 368131 | 2945044 | 7 | 4 | 0 | 288 | 2997764 | 7 | 4 | 0 | 1 | 296 |
| 180 | B4 | 25 | 19 | 7 | 5 | 368167 | 2945332 | 7 | 5 | 0 | 288 | 2998060 | 7 | 5 | 0 | 1 | 296 |
| 181 | B5 | 25 | 19 | 7 | 6 | 368203 | 2945620 | 7 | 6 | 0 | 288 | 2998356 | 7 | 6 | 0 | 1 | 296 |
| 182 | B6 | 25 | 19 | 7 | 7 | 368239 | 2945908 | 7 | 7 | 0 | 288 | 2998652 | 7 | 7 | 0 | 1 | 296 |
| 183 | B7 | 25 | 19 | 7 | 8 | 368275 | 2946196 | 7 | 8 | 0 | 288 | 2998948 | 7 | 8 | 0 | 1 | 296 |
| 184 | B8 | 25 | 19 | 7 | 9 | 368311 | 2946484 | 7 | 9 | 0 | 288 | 2999244 | 7 | 9 | 0 | 1 | 296 |
| 185 | B9 | 25 | 19 | 7 | 10 | 368347 | 2946772 | 7 | 10 | 0 | 288 | 2999540 | 7 | 10 | 0 | 1 | 296 |
| 186 | BA | 25 | 19 | 7 | 11 | 368383 | 2947060 | 7 | 11 | 0 | 288 | 2999836 | 7 | 11 | 0 | 1 | 296 |
| 187 | BB | 25 | 19 | 7 | 12 | 368419 | 2947348 | 7 | 12 | 0 | 288 | 3000132 | 7 | 12 | 0 | 1 | 296 |
| 188 | BC | 25 | 19 | 7 | 13 | 368455 | 2947636 | 7 | 13 | 0 | 288 | 3000428 | 7 | 13 | 0 | 1 | 296 |

| | | | | | | | | | | | | | | | | | |
|---|---|---|---|---|---|---|---|---|---|---|---|---|---|---|---|---|---|
| 189 | BD | 25 | 19 | 7 | 14 | 368491 | 2947924 | 7 | 14 | 0 | 288 | 3000724 | 7 | 14 | 0 | 1 | 296 |
| 190 | BE | 25 | 19 | 7 | 15 | 368527 | 2948212 | 7 | 15 | 0 | 288 | 3001020 | 7 | 15 | 0 | 1 | 296 |
| 191 | BF | 25 | 19 | 7 | 16 | 368563 | 2948500 | 7 | 16 | 0 | 288 | 3001316 | 7 | 16 | 0 | 1 | 296 |
| 192 | C0 | 25 | 19 | 7 | 17 | 368597 | 2948772 | 7 | 17 | 0 | 272 | 3001596 | 7 | 17 | 0 | 1 | 280 |
| 193 | C1 | 25 | 19 | 7 | 18 | 368631 | 2949044 | 7 | 18 | 0 | 272 | 3001876 | 7 | 18 | 0 | 1 | 280 |
| 194 | C2 | 25 | 19 | 7 | 19 | 368665 | 2949316 | 7 | 19 | 0 | 272 | 3002156 | 7 | 19 | 0 | 1 | 280 |
| 195 | C3 | 25 | 19 | 7 | 20 | 368699 | 2949588 | 7 | 20 | 0 | 272 | 3002436 | 7 | 20 | 0 | 1 | 280 |
| 196 | C4 | 25 | 19 | 7 | 21 | 368733 | 2949860 | 7 | 21 | 0 | 272 | 3002716 | 7 | 21 | 0 | 1 | 280 |
| 197 | C5 | 25 | 19 | 7 | 22 | 368767 | 2950132 | 7 | 22 | 0 | 272 | 3002996 | 7 | 22 | 0 | 1 | 280 |
| 198 | C6 | 25 | 19 | 7 | 23 | 368801 | 2950404 | 7 | 23 | 0 | 272 | 3003276 | 7 | 23 | 0 | 1 | 280 |
| 199 | C7 | 25 | 19 | 7 | 24 | 368835 | 2950676 | 7 | 24 | 0 | 272 | 3003556 | 7 | 24 | 0 | 1 | 280 |
| 200 | C8 | 25 | 19 | 8 | 0 | 368871 | 2950964 | 8 | 0 | 0 | 288 | 3003852 | 8 | 0 | 0 | 1 | 296 |
| 201 | C9 | 25 | 19 | 8 | 1 | 368907 | 2951252 | 8 | 1 | 0 | 288 | 3004148 | 8 | 1 | 0 | 1 | 296 |
| 202 | CA | 25 | 19 | 8 | 2 | 368943 | 2951540 | 8 | 2 | 0 | 288 | 3004444 | 8 | 2 | 0 | 1 | 296 |
| 203 | CB | 25 | 19 | 8 | 3 | 368979 | 2951828 | 8 | 3 | 0 | 288 | 3004740 | 8 | 3 | 0 | 1 | 296 |
| 204 | CC | 25 | 19 | 8 | 4 | 369015 | 2952116 | 8 | 4 | 0 | 288 | 3005036 | 8 | 4 | 0 | 1 | 296 |
| 205 | CD | 25 | 19 | 8 | 5 | 369051 | 2952404 | 8 | 5 | 0 | 288 | 3005332 | 8 | 5 | 0 | 1 | 296 |
| 206 | CE | 25 | 19 | 8 | 6 | 369087 | 2952692 | 8 | 6 | 0 | 288 | 3005628 | 8 | 6 | 0 | 1 | 296 |
| 207 | CF | 25 | 19 | 8 | 7 | 369123 | 2952980 | 8 | 7 | 0 | 288 | 3005924 | 8 | 7 | 0 | 1 | 296 |
| 208 | D0 | 25 | 19 | 8 | 8 | 369161 | 2953284 | 8 | 8 | 0 | 304 | 3006236 | 8 | 8 | 0 | 1 | 312 |
| 209 | D1 | 25 | 19 | 8 | 9 | 369199 | 2953588 | 8 | 9 | 0 | 304 | 3006548 | 8 | 9 | 0 | 1 | 312 |
| 210 | D2 | 25 | 19 | 8 | 10 | 369237 | 2953892 | 8 | 10 | 0 | 304 | 3006860 | 8 | 10 | 0 | 1 | 312 |
| 211 | D3 | 25 | 19 | 8 | 11 | 369275 | 2954196 | 8 | 11 | 0 | 304 | 3007172 | 8 | 11 | 0 | 1 | 312 |
| 212 | D4 | 25 | 19 | 8 | 12 | 369313 | 2954500 | 8 | 12 | 0 | 304 | 3007484 | 8 | 12 | 0 | 1 | 312 |
| 213 | D5 | 25 | 19 | 8 | 13 | 369351 | 2954804 | 8 | 13 | 0 | 304 | 3007796 | 8 | 13 | 0 | 1 | 312 |
| 214 | D6 | 25 | 19 | 8 | 14 | 369389 | 2955108 | 8 | 14 | 0 | 304 | 3008108 | 8 | 14 | 0 | 1 | 312 |
| 215 | D7 | 25 | 19 | 8 | 15 | 369427 | 2955412 | 8 | 15 | 0 | 304 | 3008420 | 8 | 15 | 0 | 1 | 312 |
| 216 | D8 | 25 | 19 | 8 | 16 | 369465 | 2955716 | 8 | 16 | 0 | 304 | 3008732 | 8 | 16 | 0 | 1 | 312 |
| 217 | D9 | 25 | 19 | 8 | 17 | 369503 | 2956020 | 8 | 17 | 0 | 304 | 3009044 | 8 | 17 | 0 | 1 | 312 |
| 218 | DA | 25 | 19 | 8 | 18 | 369541 | 2956324 | 8 | 18 | 0 | 304 | 3009356 | 8 | 18 | 0 | 1 | 312 |
| 219 | DB | 25 | 19 | 8 | 19 | 369579 | 2956628 | 8 | 19 | 0 | 304 | 3009668 | 8 | 19 | 0 | 1 | 312 |
| 220 | DC | 25 | 19 | 8 | 20 | 369617 | 2956932 | 8 | 20 | 0 | 304 | 3009980 | 8 | 20 | 0 | 1 | 312 |
| 221 | DD | 25 | 19 | 8 | 21 | 369655 | 2957236 | 8 | 21 | 0 | 304 | 3010292 | 8 | 21 | 0 | 1 | 312 |
| 222 | DE | 25 | 19 | 8 | 22 | 369693 | 2957540 | 8 | 22 | 0 | 304 | 3010604 | 8 | 22 | 0 | 1 | 312 |
| 223 | DF | 25 | 19 | 8 | 23 | 369731 | 2957844 | 8 | 23 | 0 | 304 | 3010916 | 8 | 23 | 0 | 1 | 312 |
| 224 | E0 | 25 | 19 | 8 | 24 | 369767 | 2958132 | 8 | 24 | 0 | 288 | 3011212 | 8 | 24 | 0 | 1 | 296 |
| 225 | E1 | 25 | 19 | 9 | 0 | 369805 | 2958436 | 9 | 0 | 0 | 304 | 3011524 | 9 | 0 | 0 | 1 | 312 |
| 226 | E2 | 25 | 19 | 9 | 1 | 369843 | 2958740 | 9 | 1 | 0 | 304 | 3011836 | 9 | 1 | 0 | 1 | 312 |
| 227 | E3 | 25 | 19 | 9 | 2 | 369881 | 2959044 | 9 | 2 | 0 | 304 | 3012148 | 9 | 2 | 0 | 1 | 312 |
| 228 | E4 | 25 | 19 | 9 | 3 | 369919 | 2959348 | 9 | 3 | 0 | 304 | 3012460 | 9 | 3 | 0 | 1 | 312 |
| 229 | E5 | 25 | 19 | 9 | 4 | 369957 | 2959652 | 9 | 4 | 0 | 304 | 3012772 | 9 | 4 | 0 | 1 | 312 |
| 230 | E6 | 25 | 19 | 9 | 5 | 369995 | 2959956 | 9 | 5 | 0 | 304 | 3013084 | 9 | 5 | 0 | 1 | 312 |
| 231 | E7 | 25 | 19 | 9 | 6 | 370033 | 2960260 | 9 | 6 | 0 | 304 | 3013396 | 9 | 6 | 0 | 1 | 312 |
| 232 | E8 | 25 | 19 | 9 | 7 | 370071 | 2960564 | 9 | 7 | 0 | 304 | 3013708 | 9 | 7 | 0 | 1 | 312 |
| 233 | E9 | 25 | 19 | 9 | 8 | 370109 | 2960868 | 9 | 8 | 0 | 304 | 3014020 | 9 | 8 | 0 | 1 | 312 |
| 234 | EA | 25 | 19 | 9 | 9 | 370147 | 2961172 | 9 | 9 | 0 | 304 | 3014332 | 9 | 9 | 0 | 1 | 312 |
| 235 | EB | 25 | 19 | 9 | 10 | 370185 | 2961476 | 9 | 10 | 0 | 304 | 3014644 | 9 | 10 | 0 | 1 | 312 |
| 236 | EC | 25 | 19 | 9 | 11 | 370223 | 2961780 | 9 | 11 | 0 | 304 | 3014956 | 9 | 11 | 0 | 1 | 312 |
| 237 | ED | 25 | 19 | 9 | 12 | 370261 | 2962084 | 9 | 12 | 0 | 304 | 3015268 | 9 | 12 | 0 | 1 | 312 |
| 238 | EE | 25 | 19 | 9 | 13 | 370299 | 2962388 | 9 | 13 | 0 | 304 | 3015580 | 9 | 13 | 0 | 1 | 312 |
| 239 | EF | 25 | 19 | 9 | 14 | 370337 | 2962692 | 9 | 14 | 0 | 304 | 3015892 | 9 | 14 | 0 | 1 | 312 |
| 240 | F0 | 25 | 19 | 9 | 15 | 370377 | 2963012 | 9 | 15 | 0 | 320 | 3016220 | 9 | 15 | 0 | 1 | 328 |
| 241 | F1 | 25 | 19 | 9 | 16 | 370417 | 2963332 | 9 | 16 | 0 | 320 | 3016548 | 9 | 16 | 0 | 1 | 328 |
| 242 | F2 | 25 | 19 | 9 | 17 | 370457 | 2963652 | 9 | 17 | 0 | 320 | 3016876 | 9 | 17 | 0 | 1 | 328 |
| 243 | F3 | 25 | 19 | 9 | 18 | 370497 | 2963972 | 9 | 18 | 0 | 320 | 3017204 | 9 | 18 | 0 | 1 | 328 |
| 244 | F4 | 25 | 19 | 9 | 19 | 370537 | 2964292 | 9 | 19 | 0 | 320 | 3017532 | 9 | 19 | 0 | 1 | 328 |
| 245 | F5 | 25 | 19 | 9 | 20 | 370577 | 2964612 | 9 | 20 | 0 | 320 | 3017860 | 9 | 20 | 0 | 1 | 328 |
| 246 | F6 | 25 | 19 | 9 | 21 | 370617 | 2964932 | 9 | 21 | 0 | 320 | 3018188 | 9 | 21 | 0 | 1 | 328 |
| 247 | F7 | 25 | 19 | 9 | 22 | 370657 | 2965252 | 9 | 22 | 0 | 320 | 3018516 | 9 | 22 | 0 | 1 | 328 |
| 248 | F8 | 25 | 19 | 9 | 23 | 370697 | 2965572 | 9 | 23 | 0 | 320 | 3018844 | 9 | 23 | 0 | 1 | 328 |
| 249 | F9 | 25 | 19 | 9 | 24 | 370737 | 2965892 | 9 | 24 | 0 | 320 | 3019172 | 9 | 24 | 0 | 1 | 328 |
| 250 | FA | 25 | 19 | 10 | 0 | 370779 | 2966228 | 10 | 0 | 0 | 336 | 3019516 | 10 | 0 | 0 | 1 | 344 |
| 251 | FB | 25 | 19 | 10 | 1 | 370821 | 2966564 | 10 | 1 | 0 | 336 | 3019860 | 10 | 1 | 0 | 1 | 344 |
| 252 | FC | 25 | 19 | 10 | 2 | 370863 | 2966900 | 10 | 2 | 0 | 336 | 3020204 | 10 | 2 | 0 | 1 | 344 |
| 253 | FD | 25 | 19 | 10 | 3 | 370905 | 2967236 | 10 | 3 | 0 | 336 | 3020548 | 10 | 3 | 0 | 1 | 344 |
| 254 | FE | 25 | 19 | 10 | 4 | 370947 | 2967572 | 10 | 4 | 0 | 336 | 3020892 | 10 | 4 | 0 | 1 | 344 |
| 255 | FF | 25 | 19 | 10 | 5 | 370989 | 2967908 | 10 | 5 | 0 | 336 | 3021236 | 10 | 5 | 0 | 1 | 344 |
| 0 | 00 | 26 | 1A | 0 | 0 | 371001 | 2968004 | 0 | 0 | 0 | 96 | 3021340 | 0 | 0 | 0 | 1 | 104 |
| 1 | 01 | 26 | 1A | 0 | 1 | 371015 | 2968116 | 0 | 1 | 0 | 112 | 3021460 | 0 | 1 | 0 | 1 | 120 |
| 2 | 02 | 26 | 1A | 0 | 2 | 371029 | 2968228 | 0 | 2 | 0 | 112 | 3021580 | 0 | 2 | 0 | 1 | 120 |
| 3 | 03 | 26 | 1A | 0 | 3 | 371043 | 2968340 | 0 | 3 | 0 | 112 | 3021700 | 0 | 3 | 0 | 1 | 120 |
| 4 | 04 | 26 | 1A | 0 | 4 | 371057 | 2968452 | 0 | 4 | 0 | 112 | 3021820 | 0 | 4 | 0 | 1 | 120 |
| 5 | 05 | 26 | 1A | 0 | 5 | 371071 | 2968564 | 0 | 5 | 0 | 112 | 3021940 | 0 | 5 | 0 | 1 | 120 |
| 6 | 06 | 26 | 1A | 0 | 6 | 371085 | 2968676 | 0 | 6 | 0 | 112 | 3022060 | 0 | 6 | 0 | 1 | 120 |
| 7 | 07 | 26 | 1A | 0 | 7 | 371099 | 2968788 | 0 | 7 | 0 | 112 | 3022180 | 0 | 7 | 0 | 1 | 120 |
| 8 | 08 | 26 | 1A | 0 | 8 | 371113 | 2968900 | 0 | 8 | 0 | 112 | 3022300 | 0 | 8 | 0 | 1 | 120 |
| 9 | 09 | 26 | 1A | 0 | 9 | 371127 | 2969012 | 0 | 9 | 0 | 112 | 3022420 | 0 | 9 | 0 | 1 | 120 |
| 10 | 0A | 26 | 1A | 0 | 10 | 371141 | 2969124 | 0 | 10 | 0 | 112 | 3022540 | 0 | 10 | 0 | 1 | 120 |
| 11 | 0B | 26 | 1A | 0 | 11 | 371155 | 2969236 | 0 | 11 | 0 | 112 | 3022660 | 0 | 11 | 0 | 1 | 120 |
| 12 | 0C | 26 | 1A | 0 | 12 | 371169 | 2969348 | 0 | 12 | 0 | 112 | 3022780 | 0 | 12 | 0 | 1 | 120 |
| 13 | 0D | 26 | 1A | 0 | 13 | 371183 | 2969460 | 0 | 13 | 0 | 112 | 3022900 | 0 | 13 | 0 | 1 | 120 |
| 14 | 0E | 26 | 1A | 0 | 14 | 371197 | 2969572 | 0 | 14 | 0 | 112 | 3023020 | 0 | 14 | 0 | 1 | 120 |
| 15 | 0F | 26 | 1A | 0 | 15 | 371211 | 2969684 | 0 | 15 | 0 | 112 | 3023140 | 0 | 15 | 0 | 1 | 120 |
| 16 | 10 | 26 | 1A | 0 | 16 | 371225 | 2969796 | 0 | 16 | 0 | 112 | 3023260 | 0 | 16 | 0 | 1 | 120 |
| 17 | 11 | 26 | 1A | 0 | 17 | 371239 | 2969908 | 0 | 17 | 0 | 112 | 3023380 | 0 | 17 | 0 | 1 | 120 |
| 18 | 12 | 26 | 1A | 0 | 18 | 371253 | 2970020 | 0 | 18 | 0 | 112 | 3023500 | 0 | 18 | 0 | 1 | 120 |
| 19 | 13 | 26 | 1A | 0 | 19 | 371267 | 2970132 | 0 | 19 | 0 | 112 | 3023620 | 0 | 19 | 0 | 1 | 120 |
| 20 | 14 | 26 | 1A | 0 | 20 | 371281 | 2970244 | 0 | 20 | 0 | 112 | 3023740 | 0 | 20 | 0 | 1 | 120 |
| 21 | 15 | 26 | 1A | 0 | 21 | 371295 | 2970356 | 0 | 21 | 0 | 112 | 3023860 | 0 | 21 | 0 | 1 | 120 |
| 22 | 16 | 26 | 1A | 0 | 22 | 371309 | 2970468 | 0 | 22 | 0 | 112 | 3023980 | 0 | 22 | 0 | 1 | 120 |
| 23 | 17 | 26 | 1A | 0 | 23 | 371323 | 2970580 | 0 | 23 | 0 | 112 | 3024100 | 0 | 23 | 0 | 1 | 120 |
| 24 | 18 | 26 | 1A | 0 | 24 | 371337 | 2970692 | 0 | 24 | 0 | 112 | 3024220 | 0 | 24 | 0 | 1 | 120 |
| 25 | 19 | 26 | 1A | 0 | 25 | 371351 | 2970804 | 0 | 25 | 0 | 112 | 3024340 | 0 | 25 | 0 | 1 | 120 |
| 26 | 1A | 26 | 1A | 1 | 0 | 371363 | 2970900 | 1 | 0 | 0 | 96 | 3024444 | 1 | 0 | 0 | 1 | 104 |
| 27 | 1B | 26 | 1A | 1 | 1 | 371380 | 2971036 | 1 | 1 | 0 | 136 | 3024588 | 1 | 1 | 0 | 1 | 144 |
| 28 | 1C | 26 | 1A | 1 | 2 | 371397 | 2971172 | 1 | 2 | 0 | 136 | 3024732 | 1 | 2 | 0 | 1 | 144 |
| 29 | 1D | 26 | 1A | 1 | 3 | 371414 | 2971308 | 1 | 3 | 0 | 136 | 3024876 | 1 | 3 | 0 | 1 | 144 |
| 30 | 1E | 26 | 1A | 1 | 4 | 371431 | 2971444 | 1 | 4 | 0 | 136 | 3025020 | 1 | 4 | 0 | 1 | 144 |
| 31 | 1F | 26 | 1A | 1 | 5 | 371448 | 2971580 | 1 | 5 | 0 | 136 | 3025164 | 1 | 5 | 0 | 1 | 144 |
| 32 | 20 | 26 | 1A | 1 | 6 | 371469 | 2971748 | 1 | 6 | 0 | 168 | 3025340 | 1 | 6 | 0 | 1 | 176 |
| 33 | 21 | 26 | 1A | 1 | 7 | 371490 | 2971916 | 1 | 7 | 0 | 168 | 3025516 | 1 | 7 | 0 | 1 | 176 |
| 34 | 22 | 26 | 1A | 1 | 8 | 371511 | 2972084 | 1 | 8 | 0 | 168 | 3025692 | 1 | 8 | 0 | 1 | 176 |
| 35 | 23 | 26 | 1A | 1 | 9 | 371532 | 2972252 | 1 | 9 | 0 | 168 | 3025868 | 1 | 9 | 0 | 1 | 176 |

| | | | | | | | | | | | | | | | | | |
|---|---|---|---|---|---|---|---|---|---|---|---|---|---|---|---|---|---|
| 36 | 24 | 26 | 1A | 1 | 10 | 371553 | 2972420 | 1 | 10 | 0 | 168 | 3026044 | 1 | 10 | 0 | 1 | 176 |
| 37 | 25 | 26 | 1A | 1 | 11 | 371574 | 2972588 | 1 | 11 | 0 | 168 | 3026220 | 1 | 11 | 0 | 1 | 176 |
| 38 | 26 | 26 | 1A | 1 | 12 | 371595 | 2972756 | 1 | 12 | 0 | 168 | 3026396 | 1 | 12 | 0 | 1 | 176 |
| 39 | 27 | 26 | 1A | 1 | 13 | 371616 | 2972924 | 1 | 13 | 0 | 168 | 3026572 | 1 | 13 | 0 | 1 | 176 |
| 40 | 28 | 26 | 1A | 1 | 14 | 371637 | 2973092 | 1 | 14 | 0 | 168 | 3026748 | 1 | 14 | 0 | 1 | 176 |
| 41 | 29 | 26 | 1A | 1 | 15 | 371658 | 2973260 | 1 | 15 | 0 | 168 | 3026924 | 1 | 15 | 0 | 1 | 176 |
| 42 | 2A | 26 | 1A | 1 | 16 | 371679 | 2973428 | 1 | 16 | 0 | 168 | 3027100 | 1 | 16 | 0 | 1 | 176 |
| 43 | 2B | 26 | 1A | 1 | 17 | 371700 | 2973596 | 1 | 17 | 0 | 168 | 3027276 | 1 | 17 | 0 | 1 | 176 |
| 44 | 2C | 26 | 1A | 1 | 18 | 371721 | 2973764 | 1 | 18 | 0 | 168 | 3027452 | 1 | 18 | 0 | 1 | 176 |
| 45 | 2D | 26 | 1A | 1 | 19 | 371742 | 2973932 | 1 | 19 | 0 | 168 | 3027628 | 1 | 19 | 0 | 1 | 176 |
| 46 | 2E | 26 | 1A | 1 | 20 | 371763 | 2974100 | 1 | 20 | 0 | 168 | 3027804 | 1 | 20 | 0 | 1 | 176 |
| 47 | 2F | 26 | 1A | 1 | 21 | 371784 | 2974268 | 1 | 21 | 0 | 168 | 3027980 | 1 | 21 | 0 | 1 | 176 |
| 48 | 30 | 26 | 1A | 1 | 22 | 371807 | 2974452 | 1 | 22 | 0 | 184 | 3028172 | 1 | 22 | 0 | 1 | 192 |
| 49 | 31 | 26 | 1A | 1 | 23 | 371830 | 2974636 | 1 | 23 | 0 | 184 | 3028364 | 1 | 23 | 0 | 1 | 192 |
| 50 | 32 | 26 | 1A | 1 | 24 | 371853 | 2974820 | 1 | 24 | 0 | 184 | 3028556 | 1 | 24 | 0 | 1 | 192 |
| 51 | 33 | 26 | 1A | 1 | 25 | 371876 | 2975004 | 1 | 25 | 0 | 184 | 3028748 | 1 | 25 | 0 | 1 | 192 |
| 52 | 34 | 26 | 1A | 2 | 0 | 371897 | 2975172 | 2 | 0 | 0 | 168 | 3028924 | 2 | 0 | 0 | 1 | 176 |
| 53 | 35 | 26 | 1A | 2 | 1 | 371923 | 2975380 | 2 | 1 | 0 | 208 | 3029140 | 2 | 1 | 0 | 1 | 216 |
| 54 | 36 | 26 | 1A | 2 | 2 | 371949 | 2975588 | 2 | 2 | 0 | 208 | 3029356 | 2 | 2 | 0 | 1 | 216 |
| 55 | 37 | 26 | 1A | 2 | 3 | 371975 | 2975796 | 2 | 3 | 0 | 208 | 3029572 | 2 | 3 | 0 | 1 | 216 |
| 56 | 38 | 26 | 1A | 2 | 4 | 372001 | 2976004 | 2 | 4 | 0 | 208 | 3029788 | 2 | 4 | 0 | 1 | 216 |
| 57 | 39 | 26 | 1A | 2 | 5 | 372027 | 2976212 | 2 | 5 | 0 | 208 | 3030004 | 2 | 5 | 0 | 1 | 216 |
| 58 | 3A | 26 | 1A | 2 | 6 | 372053 | 2976420 | 2 | 6 | 0 | 208 | 3030220 | 2 | 6 | 0 | 1 | 216 |
| 59 | 3B | 26 | 1A | 2 | 7 | 372079 | 2976628 | 2 | 7 | 0 | 208 | 3030436 | 2 | 7 | 0 | 1 | 216 |
| 60 | 3C | 26 | 1A | 2 | 8 | 372105 | 2976836 | 2 | 8 | 0 | 208 | 3030652 | 2 | 8 | 0 | 1 | 216 |
| 61 | 3D | 26 | 1A | 2 | 9 | 372131 | 2977044 | 2 | 9 | 0 | 208 | 3030868 | 2 | 9 | 0 | 1 | 216 |
| 62 | 3E | 26 | 1A | 2 | 10 | 372157 | 2977252 | 2 | 10 | 0 | 208 | 3031084 | 2 | 10 | 0 | 1 | 216 |
| 63 | 3F | 26 | 1A | 2 | 11 | 372183 | 2977460 | 2 | 11 | 0 | 208 | 3031300 | 2 | 11 | 0 | 1 | 216 |
| 64 | 40 | 26 | 1A | 2 | 12 | 372206 | 2977644 | 2 | 12 | 0 | 184 | 3031492 | 2 | 12 | 0 | 1 | 192 |
| 65 | 41 | 26 | 1A | 2 | 13 | 372229 | 2977828 | 2 | 13 | 0 | 184 | 3031684 | 2 | 13 | 0 | 1 | 192 |
| 66 | 42 | 26 | 1A | 2 | 14 | 372252 | 2978012 | 2 | 14 | 0 | 184 | 3031876 | 2 | 14 | 0 | 1 | 192 |
| 67 | 43 | 26 | 1A | 2 | 15 | 372275 | 2978196 | 2 | 15 | 0 | 184 | 3032068 | 2 | 15 | 0 | 1 | 192 |
| 68 | 44 | 26 | 1A | 2 | 16 | 372298 | 2978380 | 2 | 16 | 0 | 184 | 3032260 | 2 | 16 | 0 | 1 | 192 |
| 69 | 45 | 26 | 1A | 2 | 17 | 372321 | 2978564 | 2 | 17 | 0 | 184 | 3032452 | 2 | 17 | 0 | 1 | 192 |
| 70 | 46 | 26 | 1A | 2 | 18 | 372344 | 2978748 | 2 | 18 | 0 | 184 | 3032644 | 2 | 18 | 0 | 1 | 192 |
| 71 | 47 | 26 | 1A | 2 | 19 | 372367 | 2978932 | 2 | 19 | 0 | 184 | 3032836 | 2 | 19 | 0 | 1 | 192 |
| 72 | 48 | 26 | 1A | 2 | 20 | 372390 | 2979116 | 2 | 20 | 0 | 184 | 3033028 | 2 | 20 | 0 | 1 | 192 |
| 73 | 49 | 26 | 1A | 2 | 21 | 372413 | 2979300 | 2 | 21 | 0 | 184 | 3033220 | 2 | 21 | 0 | 1 | 192 |
| 74 | 4A | 26 | 1A | 2 | 22 | 372436 | 2979484 | 2 | 22 | 0 | 184 | 3033412 | 2 | 22 | 0 | 1 | 192 |
| 75 | 4B | 26 | 1A | 2 | 23 | 372459 | 2979668 | 2 | 23 | 0 | 184 | 3033604 | 2 | 23 | 0 | 1 | 192 |
| 76 | 4C | 26 | 1A | 2 | 24 | 372482 | 2979852 | 2 | 24 | 0 | 184 | 3033796 | 2 | 24 | 0 | 1 | 192 |
| 77 | 4D | 26 | 1A | 2 | 25 | 372505 | 2980036 | 2 | 25 | 0 | 184 | 3033988 | 2 | 25 | 0 | 1 | 192 |
| 78 | 4E | 26 | 1A | 3 | 0 | 372526 | 2980204 | 3 | 0 | 0 | 168 | 3034164 | 3 | 0 | 0 | 1 | 176 |
| 79 | 4F | 26 | 1A | 3 | 1 | 372552 | 2980412 | 3 | 1 | 0 | 208 | 3034380 | 3 | 1 | 0 | 1 | 216 |
| 80 | 50 | 26 | 1A | 3 | 2 | 372580 | 2980636 | 3 | 2 | 0 | 224 | 3034612 | 3 | 2 | 0 | 1 | 232 |
| 81 | 51 | 26 | 1A | 3 | 3 | 372608 | 2980860 | 3 | 3 | 0 | 224 | 3034844 | 3 | 3 | 0 | 1 | 232 |
| 82 | 52 | 26 | 1A | 3 | 4 | 372636 | 2981084 | 3 | 4 | 0 | 224 | 3035076 | 3 | 4 | 0 | 1 | 232 |
| 83 | 53 | 26 | 1A | 3 | 5 | 372664 | 2981308 | 3 | 5 | 0 | 224 | 3035308 | 3 | 5 | 0 | 1 | 232 |
| 84 | 54 | 26 | 1A | 3 | 6 | 372692 | 2981532 | 3 | 6 | 0 | 224 | 3035540 | 3 | 6 | 0 | 1 | 232 |
| 85 | 55 | 26 | 1A | 3 | 7 | 372720 | 2981756 | 3 | 7 | 0 | 224 | 3035772 | 3 | 7 | 0 | 1 | 232 |
| 86 | 56 | 26 | 1A | 3 | 8 | 372748 | 2981980 | 3 | 8 | 0 | 224 | 3036004 | 3 | 8 | 0 | 1 | 232 |
| 87 | 57 | 26 | 1A | 3 | 9 | 372776 | 2982204 | 3 | 9 | 0 | 224 | 3036236 | 3 | 9 | 0 | 1 | 232 |
| 88 | 58 | 26 | 1A | 3 | 10 | 372804 | 2982428 | 3 | 10 | 0 | 224 | 3036468 | 3 | 10 | 0 | 1 | 232 |
| 89 | 59 | 26 | 1A | 3 | 11 | 372832 | 2982652 | 3 | 11 | 0 | 224 | 3036700 | 3 | 11 | 0 | 1 | 232 |
| 90 | 5A | 26 | 1A | 3 | 12 | 372860 | 2982876 | 3 | 12 | 0 | 224 | 3036932 | 3 | 12 | 0 | 1 | 232 |
| 91 | 5B | 26 | 1A | 3 | 13 | 372888 | 2983100 | 3 | 13 | 0 | 224 | 3037164 | 3 | 13 | 0 | 1 | 232 |
| 92 | 5C | 26 | 1A | 3 | 14 | 372916 | 2983324 | 3 | 14 | 0 | 224 | 3037396 | 3 | 14 | 0 | 1 | 232 |
| 93 | 5D | 26 | 1A | 3 | 15 | 372944 | 2983548 | 3 | 15 | 0 | 224 | 3037628 | 3 | 15 | 0 | 1 | 232 |
| 94 | 5E | 26 | 1A | 3 | 16 | 372972 | 2983772 | 3 | 16 | 0 | 224 | 3037860 | 3 | 16 | 0 | 1 | 232 |
| 95 | 5F | 26 | 1A | 3 | 17 | 373000 | 2983996 | 3 | 17 | 0 | 224 | 3038092 | 3 | 17 | 0 | 1 | 232 |
| 96 | 60 | 26 | 1A | 3 | 18 | 373025 | 2984196 | 3 | 18 | 0 | 200 | 3038300 | 3 | 18 | 0 | 1 | 208 |
| 97 | 61 | 26 | 1A | 3 | 19 | 373050 | 2984396 | 3 | 19 | 0 | 200 | 3038508 | 3 | 19 | 0 | 1 | 208 |
| 98 | 62 | 26 | 1A | 3 | 20 | 373075 | 2984596 | 3 | 20 | 0 | 200 | 3038716 | 3 | 20 | 0 | 1 | 208 |
| 99 | 63 | 26 | 1A | 3 | 21 | 373100 | 2984796 | 3 | 21 | 0 | 200 | 3038924 | 3 | 21 | 0 | 1 | 208 |
| 100 | 64 | 26 | 1A | 3 | 22 | 373125 | 2984996 | 3 | 22 | 0 | 200 | 3039132 | 3 | 22 | 0 | 1 | 208 |
| 101 | 65 | 26 | 1A | 3 | 23 | 373150 | 2985196 | 3 | 23 | 0 | 200 | 3039340 | 3 | 23 | 0 | 1 | 208 |
| 102 | 66 | 26 | 1A | 3 | 24 | 373175 | 2985396 | 3 | 24 | 0 | 200 | 3039548 | 3 | 24 | 0 | 1 | 208 |
| 103 | 67 | 26 | 1A | 3 | 25 | 373200 | 2985596 | 3 | 25 | 0 | 200 | 3039756 | 3 | 25 | 0 | 1 | 208 |
| 104 | 68 | 26 | 1A | 4 | 0 | 373223 | 2985780 | 4 | 0 | 0 | 184 | 3039948 | 4 | 0 | 0 | 1 | 192 |
| 105 | 69 | 26 | 1A | 4 | 1 | 373251 | 2986004 | 4 | 1 | 0 | 224 | 3040180 | 4 | 1 | 0 | 1 | 232 |
| 106 | 6A | 26 | 1A | 4 | 2 | 373279 | 2986228 | 4 | 2 | 0 | 224 | 3040412 | 4 | 2 | 0 | 1 | 232 |
| 107 | 6B | 26 | 1A | 4 | 3 | 373307 | 2986452 | 4 | 3 | 0 | 224 | 3040644 | 4 | 3 | 0 | 1 | 232 |
| 108 | 6C | 26 | 1A | 4 | 4 | 373335 | 2986676 | 4 | 4 | 0 | 224 | 3040876 | 4 | 4 | 0 | 1 | 232 |
| 109 | 6D | 26 | 1A | 4 | 5 | 373363 | 2986900 | 4 | 5 | 0 | 224 | 3041108 | 4 | 5 | 0 | 1 | 232 |
| 110 | 6E | 26 | 1A | 4 | 6 | 373391 | 2987124 | 4 | 6 | 0 | 224 | 3041340 | 4 | 6 | 0 | 1 | 232 |
| 111 | 6F | 26 | 1A | 4 | 7 | 373419 | 2987348 | 4 | 7 | 0 | 224 | 3041572 | 4 | 7 | 0 | 1 | 232 |
| 112 | 70 | 26 | 1A | 4 | 8 | 373449 | 2987588 | 4 | 8 | 0 | 240 | 3041820 | 4 | 8 | 0 | 1 | 248 |
| 113 | 71 | 26 | 1A | 4 | 9 | 373479 | 2987828 | 4 | 9 | 0 | 240 | 3042068 | 4 | 9 | 0 | 1 | 248 |
| 114 | 72 | 26 | 1A | 4 | 10 | 373509 | 2988068 | 4 | 10 | 0 | 240 | 3042316 | 4 | 10 | 0 | 1 | 248 |
| 115 | 73 | 26 | 1A | 4 | 11 | 373539 | 2988308 | 4 | 11 | 0 | 240 | 3042564 | 4 | 11 | 0 | 1 | 248 |
| 116 | 74 | 26 | 1A | 4 | 12 | 373569 | 2988548 | 4 | 12 | 0 | 240 | 3042812 | 4 | 12 | 0 | 1 | 248 |
| 117 | 75 | 26 | 1A | 4 | 13 | 373599 | 2988788 | 4 | 13 | 0 | 240 | 3043060 | 4 | 13 | 0 | 1 | 248 |
| 118 | 76 | 26 | 1A | 4 | 14 | 373629 | 2989028 | 4 | 14 | 0 | 240 | 3043308 | 4 | 14 | 0 | 1 | 248 |
| 119 | 77 | 26 | 1A | 4 | 15 | 373659 | 2989268 | 4 | 15 | 0 | 240 | 3043556 | 4 | 15 | 0 | 1 | 248 |
| 120 | 78 | 26 | 1A | 4 | 16 | 373689 | 2989508 | 4 | 16 | 0 | 240 | 3043804 | 4 | 16 | 0 | 1 | 248 |
| 121 | 79 | 26 | 1A | 4 | 17 | 373719 | 2989748 | 4 | 17 | 0 | 240 | 3044052 | 4 | 17 | 0 | 1 | 248 |
| 122 | 7A | 26 | 1A | 4 | 18 | 373749 | 2989988 | 4 | 18 | 0 | 240 | 3044300 | 4 | 18 | 0 | 1 | 248 |
| 123 | 7B | 26 | 1A | 4 | 19 | 373779 | 2990228 | 4 | 19 | 0 | 240 | 3044548 | 4 | 19 | 0 | 1 | 248 |
| 124 | 7C | 26 | 1A | 4 | 20 | 373809 | 2990468 | 4 | 20 | 0 | 240 | 3044796 | 4 | 20 | 0 | 1 | 248 |
| 125 | 7D | 26 | 1A | 4 | 21 | 373839 | 2990708 | 4 | 21 | 0 | 240 | 3045044 | 4 | 21 | 0 | 1 | 248 |
| 126 | 7E | 26 | 1A | 4 | 22 | 373869 | 2990948 | 4 | 22 | 0 | 240 | 3045292 | 4 | 22 | 0 | 1 | 248 |
| 127 | 7F | 26 | 1A | 4 | 23 | 373899 | 2991188 | 4 | 23 | 0 | 240 | 3045540 | 4 | 23 | 0 | 1 | 248 |
| 128 | 80 | 26 | 1A | 4 | 24 | 373926 | 2991404 | 4 | 24 | 0 | 216 | 3045764 | 4 | 24 | 0 | 1 | 224 |
| 129 | 81 | 26 | 1A | 4 | 25 | 373953 | 2991620 | 4 | 25 | 0 | 216 | 3045988 | 4 | 25 | 0 | 1 | 224 |
| 130 | 82 | 26 | 1A | 5 | 0 | 373978 | 2991820 | 5 | 0 | 0 | 200 | 3046196 | 5 | 0 | 0 | 1 | 208 |
| 131 | 83 | 26 | 1A | 5 | 1 | 374008 | 2992060 | 5 | 1 | 0 | 240 | 3046444 | 5 | 1 | 0 | 1 | 248 |
| 132 | 84 | 26 | 1A | 5 | 2 | 374038 | 2992300 | 5 | 2 | 0 | 240 | 3046692 | 5 | 2 | 0 | 1 | 248 |
| 133 | 85 | 26 | 1A | 5 | 3 | 374068 | 2992540 | 5 | 3 | 0 | 240 | 3046940 | 5 | 3 | 0 | 1 | 248 |
| 134 | 86 | 26 | 1A | 5 | 4 | 374098 | 2992780 | 5 | 4 | 0 | 240 | 3047188 | 5 | 4 | 0 | 1 | 248 |
| 135 | 87 | 26 | 1A | 5 | 5 | 374128 | 2993020 | 5 | 5 | 0 | 240 | 3047436 | 5 | 5 | 0 | 1 | 248 |
| 136 | 88 | 26 | 1A | 5 | 6 | 374158 | 2993260 | 5 | 6 | 0 | 240 | 3047684 | 5 | 6 | 0 | 1 | 248 |
| 137 | 89 | 26 | 1A | 5 | 7 | 374188 | 2993500 | 5 | 7 | 0 | 240 | 3047932 | 5 | 7 | 0 | 1 | 248 |
| 138 | 8A | 26 | 1A | 5 | 8 | 374218 | 2993740 | 5 | 8 | 0 | 240 | 3048180 | 5 | 8 | 0 | 1 | 248 |

| | | | | | | | | | | | | | | | | | |
|---|---|---|---|---|---|---|---|---|---|---|---|---|---|---|---|---|---|
| 139 | 8B | 26 | 1A | 5 | 9 | 374248 | 2993980 | 5 | 9 | 0 | 240 | 3048428 | 5 | 9 | 0 | 1 | 248 |
| 140 | 8C | 26 | 1A | 5 | 10 | 374278 | 2994220 | 5 | 10 | 0 | 240 | 3048676 | 5 | 10 | 0 | 1 | 248 |
| 141 | 8D | 26 | 1A | 5 | 11 | 374308 | 2994460 | 5 | 11 | 0 | 240 | 3048924 | 5 | 11 | 0 | 1 | 248 |
| 142 | 8E | 26 | 1A | 5 | 12 | 374338 | 2994700 | 5 | 12 | 0 | 240 | 3049172 | 5 | 12 | 0 | 1 | 248 |
| 143 | 8F | 26 | 1A | 5 | 13 | 374368 | 2994940 | 5 | 13 | 0 | 240 | 3049420 | 5 | 13 | 0 | 1 | 248 |
| 144 | 90 | 26 | 1A | 5 | 14 | 374400 | 2995196 | 5 | 14 | 0 | 256 | 3049684 | 5 | 14 | 0 | 1 | 264 |
| 145 | 91 | 26 | 1A | 5 | 15 | 374432 | 2995452 | 5 | 15 | 0 | 256 | 3049948 | 5 | 15 | 0 | 1 | 264 |
| 146 | 92 | 26 | 1A | 5 | 16 | 374464 | 2995708 | 5 | 16 | 0 | 256 | 3050212 | 5 | 16 | 0 | 1 | 264 |
| 147 | 93 | 26 | 1A | 5 | 17 | 374496 | 2995964 | 5 | 17 | 0 | 256 | 3050476 | 5 | 17 | 0 | 1 | 264 |
| 148 | 94 | 26 | 1A | 5 | 18 | 374528 | 2996220 | 5 | 18 | 0 | 256 | 3050740 | 5 | 18 | 0 | 1 | 264 |
| 149 | 95 | 26 | 1A | 5 | 19 | 374560 | 2996476 | 5 | 19 | 0 | 256 | 3051004 | 5 | 19 | 0 | 1 | 264 |
| 150 | 96 | 26 | 1A | 5 | 20 | 374592 | 2996732 | 5 | 20 | 0 | 256 | 3051268 | 5 | 20 | 0 | 1 | 264 |
| 151 | 97 | 26 | 1A | 5 | 21 | 374624 | 2996988 | 5 | 21 | 0 | 256 | 3051532 | 5 | 21 | 0 | 1 | 264 |
| 152 | 98 | 26 | 1A | 5 | 22 | 374656 | 2997244 | 5 | 22 | 0 | 256 | 3051796 | 5 | 22 | 0 | 1 | 264 |
| 153 | 99 | 26 | 1A | 5 | 23 | 374688 | 2997500 | 5 | 23 | 0 | 256 | 3052060 | 5 | 23 | 0 | 1 | 264 |
| 154 | 9A | 26 | 1A | 5 | 24 | 374720 | 2997756 | 5 | 24 | 0 | 256 | 3052324 | 5 | 24 | 0 | 1 | 264 |
| 155 | 9B | 26 | 1A | 5 | 25 | 374752 | 2998012 | 5 | 25 | 0 | 256 | 3052588 | 5 | 25 | 0 | 1 | 264 |
| 156 | 9C | 26 | 1A | 6 | 0 | 374786 | 2998284 | 6 | 0 | 0 | 272 | 3052868 | 6 | 0 | 0 | 1 | 280 |
| 157 | 9D | 26 | 1A | 6 | 1 | 374820 | 2998556 | 6 | 1 | 0 | 272 | 3053148 | 6 | 1 | 0 | 1 | 280 |
| 158 | 9E | 26 | 1A | 6 | 2 | 374854 | 2998828 | 6 | 2 | 0 | 272 | 3053428 | 6 | 2 | 0 | 1 | 280 |
| 159 | 9F | 26 | 1A | 6 | 3 | 374888 | 2999100 | 6 | 3 | 0 | 272 | 3053708 | 6 | 3 | 0 | 1 | 280 |
| 160 | A0 | 26 | 1A | 6 | 4 | 374920 | 2999356 | 6 | 4 | 0 | 256 | 3053972 | 6 | 4 | 0 | 1 | 264 |
| 161 | A1 | 26 | 1A | 6 | 5 | 374952 | 2999612 | 6 | 5 | 0 | 256 | 3054236 | 6 | 5 | 0 | 1 | 264 |
| 162 | A2 | 26 | 1A | 6 | 6 | 374984 | 2999868 | 6 | 6 | 0 | 256 | 3054500 | 6 | 6 | 0 | 1 | 264 |
| 163 | A3 | 26 | 1A | 6 | 7 | 375016 | 3000124 | 6 | 7 | 0 | 256 | 3054764 | 6 | 7 | 0 | 1 | 264 |
| 164 | A4 | 26 | 1A | 6 | 8 | 375048 | 3000380 | 6 | 8 | 0 | 256 | 3055028 | 6 | 8 | 0 | 1 | 264 |
| 165 | A5 | 26 | 1A | 6 | 9 | 375080 | 3000636 | 6 | 9 | 0 | 256 | 3055292 | 6 | 9 | 0 | 1 | 264 |
| 166 | A6 | 26 | 1A | 6 | 10 | 375112 | 3000892 | 6 | 10 | 0 | 256 | 3055556 | 6 | 10 | 0 | 1 | 264 |
| 167 | A7 | 26 | 1A | 6 | 11 | 375144 | 3001148 | 6 | 11 | 0 | 256 | 3055820 | 6 | 11 | 0 | 1 | 264 |
| 168 | A8 | 26 | 1A | 6 | 12 | 375176 | 3001404 | 6 | 12 | 0 | 256 | 3056084 | 6 | 12 | 0 | 1 | 264 |
| 169 | A9 | 26 | 1A | 6 | 13 | 375208 | 3001660 | 6 | 13 | 0 | 256 | 3056348 | 6 | 13 | 0 | 1 | 264 |
| 170 | AA | 26 | 1A | 6 | 14 | 375240 | 3001916 | 6 | 14 | 0 | 256 | 3056612 | 6 | 14 | 0 | 1 | 264 |
| 171 | AB | 26 | 1A | 6 | 15 | 375272 | 3002172 | 6 | 15 | 0 | 256 | 3056876 | 6 | 15 | 0 | 1 | 264 |
| 172 | AC | 26 | 1A | 6 | 16 | 375304 | 3002428 | 6 | 16 | 0 | 256 | 3057140 | 6 | 16 | 0 | 1 | 264 |
| 173 | AD | 26 | 1A | 6 | 17 | 375336 | 3002684 | 6 | 17 | 0 | 256 | 3057404 | 6 | 17 | 0 | 1 | 264 |
| 174 | AE | 26 | 1A | 6 | 18 | 375368 | 3002940 | 6 | 18 | 0 | 256 | 3057668 | 6 | 18 | 0 | 1 | 264 |
| 175 | AF | 26 | 1A | 6 | 19 | 375400 | 3003196 | 6 | 19 | 0 | 256 | 3057932 | 6 | 19 | 0 | 1 | 264 |
| 176 | B0 | 26 | 1A | 6 | 20 | 375434 | 3003468 | 6 | 20 | 0 | 272 | 3058212 | 6 | 20 | 0 | 1 | 280 |
| 177 | B1 | 26 | 1A | 6 | 21 | 375468 | 3003740 | 6 | 21 | 0 | 272 | 3058492 | 6 | 21 | 0 | 1 | 280 |
| 178 | B2 | 26 | 1A | 6 | 22 | 375502 | 3004012 | 6 | 22 | 0 | 272 | 3058772 | 6 | 22 | 0 | 1 | 280 |
| 179 | B3 | 26 | 1A | 6 | 23 | 375536 | 3004284 | 6 | 23 | 0 | 272 | 3059052 | 6 | 23 | 0 | 1 | 280 |
| 180 | B4 | 26 | 1A | 6 | 24 | 375570 | 3004556 | 6 | 24 | 0 | 272 | 3059332 | 6 | 24 | 0 | 1 | 280 |
| 181 | B5 | 26 | 1A | 6 | 25 | 375604 | 3004828 | 6 | 25 | 0 | 272 | 3059612 | 6 | 25 | 0 | 1 | 280 |
| 182 | B6 | 26 | 1A | 7 | 0 | 375640 | 3005116 | 7 | 0 | 0 | 288 | 3059908 | 7 | 0 | 0 | 1 | 296 |
| 183 | B7 | 26 | 1A | 7 | 1 | 375676 | 3005404 | 7 | 1 | 0 | 288 | 3060204 | 7 | 1 | 0 | 1 | 296 |
| 184 | B8 | 26 | 1A | 7 | 2 | 375712 | 3005692 | 7 | 2 | 0 | 288 | 3060500 | 7 | 2 | 0 | 1 | 296 |
| 185 | B9 | 26 | 1A | 7 | 3 | 375748 | 3005980 | 7 | 3 | 0 | 288 | 3060796 | 7 | 3 | 0 | 1 | 296 |
| 186 | BA | 26 | 1A | 7 | 4 | 375784 | 3006268 | 7 | 4 | 0 | 288 | 3061092 | 7 | 4 | 0 | 1 | 296 |
| 187 | BB | 26 | 1A | 7 | 5 | 375820 | 3006556 | 7 | 5 | 0 | 288 | 3061388 | 7 | 5 | 0 | 1 | 296 |
| 188 | BC | 26 | 1A | 7 | 6 | 375856 | 3006844 | 7 | 6 | 0 | 288 | 3061684 | 7 | 6 | 0 | 1 | 296 |
| 189 | BD | 26 | 1A | 7 | 7 | 375892 | 3007132 | 7 | 7 | 0 | 288 | 3061980 | 7 | 7 | 0 | 1 | 296 |
| 190 | BE | 26 | 1A | 7 | 8 | 375928 | 3007420 | 7 | 8 | 0 | 288 | 3062276 | 7 | 8 | 0 | 1 | 296 |
| 191 | BF | 26 | 1A | 7 | 9 | 375964 | 3007708 | 7 | 9 | 0 | 288 | 3062572 | 7 | 9 | 0 | 1 | 296 |
| 192 | C0 | 26 | 1A | 7 | 10 | 375998 | 3007980 | 7 | 10 | 0 | 272 | 3062852 | 7 | 10 | 0 | 1 | 280 |
| 193 | C1 | 26 | 1A | 7 | 11 | 376032 | 3008252 | 7 | 11 | 0 | 272 | 3063132 | 7 | 11 | 0 | 1 | 280 |
| 194 | C2 | 26 | 1A | 7 | 12 | 376066 | 3008524 | 7 | 12 | 0 | 272 | 3063412 | 7 | 12 | 0 | 1 | 280 |
| 195 | C3 | 26 | 1A | 7 | 13 | 376100 | 3008796 | 7 | 13 | 0 | 272 | 3063692 | 7 | 13 | 0 | 1 | 280 |
| 196 | C4 | 26 | 1A | 7 | 14 | 376134 | 3009068 | 7 | 14 | 0 | 272 | 3063972 | 7 | 14 | 0 | 1 | 280 |
| 197 | C5 | 26 | 1A | 7 | 15 | 376168 | 3009340 | 7 | 15 | 0 | 272 | 3064252 | 7 | 15 | 0 | 1 | 280 |
| 198 | C6 | 26 | 1A | 7 | 16 | 376202 | 3009612 | 7 | 16 | 0 | 272 | 3064532 | 7 | 16 | 0 | 1 | 280 |
| 199 | C7 | 26 | 1A | 7 | 17 | 376236 | 3009884 | 7 | 17 | 0 | 272 | 3064812 | 7 | 17 | 0 | 1 | 280 |
| 200 | C8 | 26 | 1A | 7 | 18 | 376270 | 3010156 | 7 | 18 | 0 | 272 | 3065092 | 7 | 18 | 0 | 1 | 280 |
| 201 | C9 | 26 | 1A | 7 | 19 | 376304 | 3010428 | 7 | 19 | 0 | 272 | 3065372 | 7 | 19 | 0 | 1 | 280 |
| 202 | CA | 26 | 1A | 7 | 20 | 376338 | 3010700 | 7 | 20 | 0 | 272 | 3065652 | 7 | 20 | 0 | 1 | 280 |
| 203 | CB | 26 | 1A | 7 | 21 | 376372 | 3010972 | 7 | 21 | 0 | 272 | 3065932 | 7 | 21 | 0 | 1 | 280 |
| 204 | CC | 26 | 1A | 7 | 22 | 376406 | 3011244 | 7 | 22 | 0 | 272 | 3066212 | 7 | 22 | 0 | 1 | 280 |
| 205 | CD | 26 | 1A | 7 | 23 | 376440 | 3011516 | 7 | 23 | 0 | 272 | 3066492 | 7 | 23 | 0 | 1 | 280 |
| 206 | CE | 26 | 1A | 7 | 24 | 376474 | 3011788 | 7 | 24 | 0 | 272 | 3066772 | 7 | 24 | 0 | 1 | 280 |
| 207 | CF | 26 | 1A | 7 | 25 | 376508 | 3012060 | 7 | 25 | 0 | 272 | 3067052 | 7 | 25 | 0 | 1 | 280 |
| 208 | D0 | 26 | 1A | 8 | 0 | 376546 | 3012364 | 8 | 0 | 0 | 304 | 3067364 | 8 | 0 | 0 | 1 | 312 |
| 209 | D1 | 26 | 1A | 8 | 1 | 376584 | 3012668 | 8 | 1 | 0 | 304 | 3067676 | 8 | 1 | 0 | 1 | 312 |
| 210 | D2 | 26 | 1A | 8 | 2 | 376622 | 3012972 | 8 | 2 | 0 | 304 | 3067988 | 8 | 2 | 0 | 1 | 312 |
| 211 | D3 | 26 | 1A | 8 | 3 | 376660 | 3013276 | 8 | 3 | 0 | 304 | 3068300 | 8 | 3 | 0 | 1 | 312 |
| 212 | D4 | 26 | 1A | 8 | 4 | 376698 | 3013580 | 8 | 4 | 0 | 304 | 3068612 | 8 | 4 | 0 | 1 | 312 |
| 213 | D5 | 26 | 1A | 8 | 5 | 376736 | 3013884 | 8 | 5 | 0 | 304 | 3068924 | 8 | 5 | 0 | 1 | 312 |
| 214 | D6 | 26 | 1A | 8 | 6 | 376774 | 3014188 | 8 | 6 | 0 | 304 | 3069236 | 8 | 6 | 0 | 1 | 312 |
| 215 | D7 | 26 | 1A | 8 | 7 | 376812 | 3014492 | 8 | 7 | 0 | 304 | 3069548 | 8 | 7 | 0 | 1 | 312 |
| 216 | D8 | 26 | 1A | 8 | 8 | 376850 | 3014796 | 8 | 8 | 0 | 304 | 3069860 | 8 | 8 | 0 | 1 | 312 |
| 217 | D9 | 26 | 1A | 8 | 9 | 376888 | 3015100 | 8 | 9 | 0 | 304 | 3070172 | 8 | 9 | 0 | 1 | 312 |
| 218 | DA | 26 | 1A | 8 | 10 | 376926 | 3015404 | 8 | 10 | 0 | 304 | 3070484 | 8 | 10 | 0 | 1 | 312 |
| 219 | DB | 26 | 1A | 8 | 11 | 376964 | 3015708 | 8 | 11 | 0 | 304 | 3070796 | 8 | 11 | 0 | 1 | 312 |
| 220 | DC | 26 | 1A | 8 | 12 | 377002 | 3016012 | 8 | 12 | 0 | 304 | 3071108 | 8 | 12 | 0 | 1 | 312 |
| 221 | DD | 26 | 1A | 8 | 13 | 377040 | 3016316 | 8 | 13 | 0 | 304 | 3071420 | 8 | 13 | 0 | 1 | 312 |
| 222 | DE | 26 | 1A | 8 | 14 | 377078 | 3016620 | 8 | 14 | 0 | 304 | 3071732 | 8 | 14 | 0 | 1 | 312 |
| 223 | DF | 26 | 1A | 8 | 15 | 377116 | 3016924 | 8 | 15 | 0 | 304 | 3072044 | 8 | 15 | 0 | 1 | 312 |
| 224 | E0 | 26 | 1A | 8 | 16 | 377152 | 3017212 | 8 | 16 | 0 | 288 | 3072340 | 8 | 16 | 0 | 1 | 296 |
| 225 | E1 | 26 | 1A | 8 | 17 | 377188 | 3017500 | 8 | 17 | 0 | 288 | 3072636 | 8 | 17 | 0 | 1 | 296 |
| 226 | E2 | 26 | 1A | 8 | 18 | 377224 | 3017788 | 8 | 18 | 0 | 288 | 3072932 | 8 | 18 | 0 | 1 | 296 |
| 227 | E3 | 26 | 1A | 8 | 19 | 377260 | 3018076 | 8 | 19 | 0 | 288 | 3073228 | 8 | 19 | 0 | 1 | 296 |
| 228 | E4 | 26 | 1A | 8 | 20 | 377296 | 3018364 | 8 | 20 | 0 | 288 | 3073524 | 8 | 20 | 0 | 1 | 296 |
| 229 | E5 | 26 | 1A | 8 | 21 | 377332 | 3018652 | 8 | 21 | 0 | 288 | 3073820 | 8 | 21 | 0 | 1 | 296 |
| 230 | E6 | 26 | 1A | 8 | 22 | 377368 | 3018940 | 8 | 22 | 0 | 288 | 3074116 | 8 | 22 | 0 | 1 | 296 |
| 231 | E7 | 26 | 1A | 8 | 23 | 377404 | 3019228 | 8 | 23 | 0 | 288 | 3074412 | 8 | 23 | 0 | 1 | 296 |
| 232 | E8 | 26 | 1A | 8 | 24 | 377440 | 3019516 | 8 | 24 | 0 | 288 | 3074708 | 8 | 24 | 0 | 1 | 296 |
| 233 | E9 | 26 | 1A | 8 | 25 | 377476 | 3019804 | 8 | 25 | 0 | 288 | 3075004 | 8 | 25 | 0 | 1 | 296 |
| 234 | EA | 26 | 1A | 9 | 0 | 377514 | 3020108 | 9 | 0 | 0 | 304 | 3075316 | 9 | 0 | 0 | 1 | 312 |
| 235 | EB | 26 | 1A | 9 | 1 | 377552 | 3020412 | 9 | 1 | 0 | 304 | 3075628 | 9 | 1 | 0 | 1 | 312 |
| 236 | EC | 26 | 1A | 9 | 2 | 377590 | 3020716 | 9 | 2 | 0 | 304 | 3075940 | 9 | 2 | 0 | 1 | 312 |
| 237 | ED | 26 | 1A | 9 | 3 | 377628 | 3021020 | 9 | 3 | 0 | 304 | 3076252 | 9 | 3 | 0 | 1 | 312 |
| 238 | EE | 26 | 1A | 9 | 4 | 377666 | 3021324 | 9 | 4 | 0 | 304 | 3076564 | 9 | 4 | 0 | 1 | 312 |
| 239 | EF | 26 | 1A | 9 | 5 | 377704 | 3021628 | 9 | 5 | 0 | 304 | 3076876 | 9 | 5 | 0 | 1 | 312 |
| 240 | F0 | 26 | 1A | 9 | 6 | 377744 | 3021948 | 9 | 6 | 0 | 320 | 3077204 | 9 | 6 | 0 | 1 | 328 |
| 241 | F1 | 26 | 1A | 9 | 7 | 377784 | 3022268 | 9 | 7 | 0 | 320 | 3077532 | 9 | 7 | 0 | 1 | 328 |

| | | | | | | | | | | | | | | | | | |
|---|---|---|---|---|---|---|---|---|---|---|---|---|---|---|---|---|---|
| 242 | F2 | 26 | 1A | 9 | 8 | 377824 | 3022588 | 9 | 8 | 0 | 320 | 3077860 | 9 | 8 | 0 | 1 | 328 |
| 243 | F3 | 26 | 1A | 9 | 9 | 377864 | 3022908 | 9 | 9 | 0 | 320 | 3078188 | 9 | 9 | 0 | 1 | 328 |
| 244 | F4 | 26 | 1A | 9 | 10 | 377904 | 3023228 | 9 | 10 | 0 | 320 | 3078516 | 9 | 10 | 0 | 1 | 328 |
| 245 | F5 | 26 | 1A | 9 | 11 | 377944 | 3023548 | 9 | 11 | 0 | 320 | 3078844 | 9 | 11 | 0 | 1 | 328 |
| 246 | F6 | 26 | 1A | 9 | 12 | 377984 | 3023868 | 9 | 12 | 0 | 320 | 3079172 | 9 | 12 | 0 | 1 | 328 |
| 247 | F7 | 26 | 1A | 9 | 13 | 378024 | 3024188 | 9 | 13 | 0 | 320 | 3079500 | 9 | 13 | 0 | 1 | 328 |
| 248 | F8 | 26 | 1A | 9 | 14 | 378064 | 3024508 | 9 | 14 | 0 | 320 | 3079828 | 9 | 14 | 0 | 1 | 328 |
| 249 | F9 | 26 | 1A | 9 | 15 | 378104 | 3024828 | 9 | 15 | 0 | 320 | 3080156 | 9 | 15 | 0 | 1 | 328 |
| 250 | FA | 26 | 1A | 9 | 16 | 378144 | 3025148 | 9 | 16 | 0 | 320 | 3080484 | 9 | 16 | 0 | 1 | 328 |
| 251 | FB | 26 | 1A | 9 | 17 | 378184 | 3025468 | 9 | 17 | 0 | 320 | 3080812 | 9 | 17 | 0 | 1 | 328 |
| 252 | FC | 26 | 1A | 9 | 18 | 378224 | 3025788 | 9 | 18 | 0 | 320 | 3081140 | 9 | 18 | 0 | 1 | 328 |
| 253 | FD | 26 | 1A | 9 | 19 | 378264 | 3026108 | 9 | 19 | 0 | 320 | 3081468 | 9 | 19 | 0 | 1 | 328 |
| 254 | FE | 26 | 1A | 9 | 20 | 378304 | 3026428 | 9 | 20 | 0 | 320 | 3081796 | 9 | 20 | 0 | 1 | 328 |
| 255 | FF | 26 | 1A | 9 | 21 | 378344 | 3026748 | 9 | 21 | 0 | 320 | 3082124 | 9 | 21 | 0 | 1 | 328 |
| 0 | 00 | 27 | 1B | 0 | 0 | 378356 | 3026844 | 0 | 0 | 0 | 96 | 3082228 | 0 | 0 | 0 | 1 | 104 |
| 1 | 01 | 27 | 1B | 0 | 1 | 378370 | 3026956 | 0 | 1 | 0 | 112 | 3082348 | 0 | 1 | 0 | 1 | 120 |
| 2 | 02 | 27 | 1B | 0 | 2 | 378384 | 3027068 | 0 | 2 | 0 | 112 | 3082468 | 0 | 2 | 0 | 1 | 120 |
| 3 | 03 | 27 | 1B | 0 | 3 | 378398 | 3027180 | 0 | 3 | 0 | 112 | 3082588 | 0 | 3 | 0 | 1 | 120 |
| 4 | 04 | 27 | 1B | 0 | 4 | 378412 | 3027292 | 0 | 4 | 0 | 112 | 3082708 | 0 | 4 | 0 | 1 | 120 |
| 5 | 05 | 27 | 1B | 0 | 5 | 378426 | 3027404 | 0 | 5 | 0 | 112 | 3082828 | 0 | 5 | 0 | 1 | 120 |
| 6 | 06 | 27 | 1B | 0 | 6 | 378440 | 3027516 | 0 | 6 | 0 | 112 | 3082948 | 0 | 6 | 0 | 1 | 120 |
| 7 | 07 | 27 | 1B | 0 | 7 | 378454 | 3027628 | 0 | 7 | 0 | 112 | 3083068 | 0 | 7 | 0 | 1 | 120 |
| 8 | 08 | 27 | 1B | 0 | 8 | 378468 | 3027740 | 0 | 8 | 0 | 112 | 3083188 | 0 | 8 | 0 | 1 | 120 |
| 9 | 09 | 27 | 1B | 0 | 9 | 378482 | 3027852 | 0 | 9 | 0 | 112 | 3083308 | 0 | 9 | 0 | 1 | 120 |
| 10 | 0A | 27 | 1B | 0 | 10 | 378496 | 3027964 | 0 | 10 | 0 | 112 | 3083428 | 0 | 10 | 0 | 1 | 120 |
| 11 | 0B | 27 | 1B | 0 | 11 | 378510 | 3028076 | 0 | 11 | 0 | 112 | 3083548 | 0 | 11 | 0 | 1 | 120 |
| 12 | 0C | 27 | 1B | 0 | 12 | 378524 | 3028188 | 0 | 12 | 0 | 112 | 3083668 | 0 | 12 | 0 | 1 | 120 |
| 13 | 0D | 27 | 1B | 0 | 13 | 378538 | 3028300 | 0 | 13 | 0 | 112 | 3083788 | 0 | 13 | 0 | 1 | 120 |
| 14 | 0E | 27 | 1B | 0 | 14 | 378552 | 3028412 | 0 | 14 | 0 | 112 | 3083908 | 0 | 14 | 0 | 1 | 120 |
| 15 | 0F | 27 | 1B | 0 | 15 | 378566 | 3028524 | 0 | 15 | 0 | 112 | 3084028 | 0 | 15 | 0 | 1 | 120 |
| 16 | 10 | 27 | 1B | 0 | 16 | 378580 | 3028636 | 0 | 16 | 0 | 112 | 3084148 | 0 | 16 | 0 | 1 | 120 |
| 17 | 11 | 27 | 1B | 0 | 17 | 378594 | 3028748 | 0 | 17 | 0 | 112 | 3084268 | 0 | 17 | 0 | 1 | 120 |
| 18 | 12 | 27 | 1B | 0 | 18 | 378608 | 3028860 | 0 | 18 | 0 | 112 | 3084388 | 0 | 18 | 0 | 1 | 120 |
| 19 | 13 | 27 | 1B | 0 | 19 | 378622 | 3028972 | 0 | 19 | 0 | 112 | 3084508 | 0 | 19 | 0 | 1 | 120 |
| 20 | 14 | 27 | 1B | 0 | 20 | 378636 | 3029084 | 0 | 20 | 0 | 112 | 3084628 | 0 | 20 | 0 | 1 | 120 |
| 21 | 15 | 27 | 1B | 0 | 21 | 378650 | 3029196 | 0 | 21 | 0 | 112 | 3084748 | 0 | 21 | 0 | 1 | 120 |
| 22 | 16 | 27 | 1B | 0 | 22 | 378664 | 3029308 | 0 | 22 | 0 | 112 | 3084868 | 0 | 22 | 0 | 1 | 120 |
| 23 | 17 | 27 | 1B | 0 | 23 | 378678 | 3029420 | 0 | 23 | 0 | 112 | 3084988 | 0 | 23 | 0 | 1 | 120 |
| 24 | 18 | 27 | 1B | 0 | 24 | 378692 | 3029532 | 0 | 24 | 0 | 112 | 3085108 | 0 | 24 | 0 | 1 | 120 |
| 25 | 19 | 27 | 1B | 0 | 25 | 378706 | 3029644 | 0 | 25 | 0 | 112 | 3085228 | 0 | 25 | 0 | 1 | 120 |
| 26 | 1A | 27 | 1B | 0 | 26 | 378720 | 3029756 | 0 | 26 | 0 | 112 | 3085348 | 0 | 26 | 0 | 1 | 120 |
| 27 | 1B | 27 | 1B | 1 | 0 | 378732 | 3029852 | 1 | 0 | 0 | 96 | 3085452 | 1 | 0 | 0 | 1 | 104 |
| 28 | 1C | 27 | 1B | 1 | 1 | 378749 | 3029988 | 1 | 1 | 0 | 136 | 3085596 | 1 | 1 | 0 | 1 | 144 |
| 29 | 1D | 27 | 1B | 1 | 2 | 378766 | 3030124 | 1 | 2 | 0 | 136 | 3085740 | 1 | 2 | 0 | 1 | 144 |
| 30 | 1E | 27 | 1B | 1 | 3 | 378783 | 3030260 | 1 | 3 | 0 | 136 | 3085884 | 1 | 3 | 0 | 1 | 144 |
| 31 | 1F | 27 | 1B | 1 | 4 | 378800 | 3030396 | 1 | 4 | 0 | 136 | 3086028 | 1 | 4 | 0 | 1 | 144 |
| 32 | 20 | 27 | 1B | 1 | 5 | 378821 | 3030564 | 1 | 5 | 0 | 168 | 3086204 | 1 | 5 | 0 | 1 | 176 |
| 33 | 21 | 27 | 1B | 1 | 6 | 378842 | 3030732 | 1 | 6 | 0 | 168 | 3086380 | 1 | 6 | 0 | 1 | 176 |
| 34 | 22 | 27 | 1B | 1 | 7 | 378863 | 3030900 | 1 | 7 | 0 | 168 | 3086556 | 1 | 7 | 0 | 1 | 176 |
| 35 | 23 | 27 | 1B | 1 | 8 | 378884 | 3031068 | 1 | 8 | 0 | 168 | 3086732 | 1 | 8 | 0 | 1 | 176 |
| 36 | 24 | 27 | 1B | 1 | 9 | 378905 | 3031236 | 1 | 9 | 0 | 168 | 3086908 | 1 | 9 | 0 | 1 | 176 |
| 37 | 25 | 27 | 1B | 1 | 10 | 378926 | 3031404 | 1 | 10 | 0 | 168 | 3087084 | 1 | 10 | 0 | 1 | 176 |
| 38 | 26 | 27 | 1B | 1 | 11 | 378947 | 3031572 | 1 | 11 | 0 | 168 | 3087260 | 1 | 11 | 0 | 1 | 176 |
| 39 | 27 | 27 | 1B | 1 | 12 | 378968 | 3031740 | 1 | 12 | 0 | 168 | 3087436 | 1 | 12 | 0 | 1 | 176 |
| 40 | 28 | 27 | 1B | 1 | 13 | 378989 | 3031908 | 1 | 13 | 0 | 168 | 3087612 | 1 | 13 | 0 | 1 | 176 |
| 41 | 29 | 27 | 1B | 1 | 14 | 379010 | 3032076 | 1 | 14 | 0 | 168 | 3087788 | 1 | 14 | 0 | 1 | 176 |
| 42 | 2A | 27 | 1B | 1 | 15 | 379031 | 3032244 | 1 | 15 | 0 | 168 | 3087964 | 1 | 15 | 0 | 1 | 176 |
| 43 | 2B | 27 | 1B | 1 | 16 | 379052 | 3032412 | 1 | 16 | 0 | 168 | 3088140 | 1 | 16 | 0 | 1 | 176 |
| 44 | 2C | 27 | 1B | 1 | 17 | 379073 | 3032580 | 1 | 17 | 0 | 168 | 3088316 | 1 | 17 | 0 | 1 | 176 |
| 45 | 2D | 27 | 1B | 1 | 18 | 379094 | 3032748 | 1 | 18 | 0 | 168 | 3088492 | 1 | 18 | 0 | 1 | 176 |
| 46 | 2E | 27 | 1B | 1 | 19 | 379115 | 3032916 | 1 | 19 | 0 | 168 | 3088668 | 1 | 19 | 0 | 1 | 176 |
| 47 | 2F | 27 | 1B | 1 | 20 | 379136 | 3033084 | 1 | 20 | 0 | 168 | 3088844 | 1 | 20 | 0 | 1 | 176 |
| 48 | 30 | 27 | 1B | 1 | 21 | 379159 | 3033268 | 1 | 21 | 0 | 184 | 3089036 | 1 | 21 | 0 | 1 | 192 |
| 49 | 31 | 27 | 1B | 1 | 22 | 379182 | 3033452 | 1 | 22 | 0 | 184 | 3089228 | 1 | 22 | 0 | 1 | 192 |
| 50 | 32 | 27 | 1B | 1 | 23 | 379205 | 3033636 | 1 | 23 | 0 | 184 | 3089420 | 1 | 23 | 0 | 1 | 192 |
| 51 | 33 | 27 | 1B | 1 | 24 | 379228 | 3033820 | 1 | 24 | 0 | 184 | 3089612 | 1 | 24 | 0 | 1 | 192 |
| 52 | 34 | 27 | 1B | 1 | 25 | 379251 | 3034004 | 1 | 25 | 0 | 184 | 3089804 | 1 | 25 | 0 | 1 | 192 |
| 53 | 35 | 27 | 1B | 1 | 26 | 379274 | 3034188 | 1 | 26 | 0 | 184 | 3089996 | 1 | 26 | 0 | 1 | 192 |
| 54 | 36 | 27 | 1B | 2 | 0 | 379295 | 3034356 | 2 | 0 | 0 | 168 | 3090172 | 2 | 0 | 0 | 1 | 176 |
| 55 | 37 | 27 | 1B | 2 | 1 | 379321 | 3034564 | 2 | 1 | 0 | 208 | 3090388 | 2 | 1 | 0 | 1 | 216 |
| 56 | 38 | 27 | 1B | 2 | 2 | 379347 | 3034772 | 2 | 2 | 0 | 208 | 3090604 | 2 | 2 | 0 | 1 | 216 |
| 57 | 39 | 27 | 1B | 2 | 3 | 379373 | 3034980 | 2 | 3 | 0 | 208 | 3090820 | 2 | 3 | 0 | 1 | 216 |
| 58 | 3A | 27 | 1B | 2 | 4 | 379399 | 3035188 | 2 | 4 | 0 | 208 | 3091036 | 2 | 4 | 0 | 1 | 216 |
| 59 | 3B | 27 | 1B | 2 | 5 | 379425 | 3035396 | 2 | 5 | 0 | 208 | 3091252 | 2 | 5 | 0 | 1 | 216 |
| 60 | 3C | 27 | 1B | 2 | 6 | 379451 | 3035604 | 2 | 6 | 0 | 208 | 3091468 | 2 | 6 | 0 | 1 | 216 |
| 61 | 3D | 27 | 1B | 2 | 7 | 379477 | 3035812 | 2 | 7 | 0 | 208 | 3091684 | 2 | 7 | 0 | 1 | 216 |
| 62 | 3E | 27 | 1B | 2 | 8 | 379503 | 3036020 | 2 | 8 | 0 | 208 | 3091900 | 2 | 8 | 0 | 1 | 216 |
| 63 | 3F | 27 | 1B | 2 | 9 | 379529 | 3036228 | 2 | 9 | 0 | 208 | 3092116 | 2 | 9 | 0 | 1 | 216 |
| 64 | 40 | 27 | 1B | 2 | 10 | 379552 | 3036412 | 2 | 10 | 0 | 184 | 3092308 | 2 | 10 | 0 | 1 | 192 |
| 65 | 41 | 27 | 1B | 2 | 11 | 379575 | 3036596 | 2 | 11 | 0 | 184 | 3092500 | 2 | 11 | 0 | 1 | 192 |
| 66 | 42 | 27 | 1B | 2 | 12 | 379598 | 3036780 | 2 | 12 | 0 | 184 | 3092692 | 2 | 12 | 0 | 1 | 192 |
| 67 | 43 | 27 | 1B | 2 | 13 | 379621 | 3036964 | 2 | 13 | 0 | 184 | 3092884 | 2 | 13 | 0 | 1 | 192 |
| 68 | 44 | 27 | 1B | 2 | 14 | 379644 | 3037148 | 2 | 14 | 0 | 184 | 3093076 | 2 | 14 | 0 | 1 | 192 |
| 69 | 45 | 27 | 1B | 2 | 15 | 379667 | 3037332 | 2 | 15 | 0 | 184 | 3093268 | 2 | 15 | 0 | 1 | 192 |
| 70 | 46 | 27 | 1B | 2 | 16 | 379690 | 3037516 | 2 | 16 | 0 | 184 | 3093460 | 2 | 16 | 0 | 1 | 192 |
| 71 | 47 | 27 | 1B | 2 | 17 | 379713 | 3037700 | 2 | 17 | 0 | 184 | 3093652 | 2 | 17 | 0 | 1 | 192 |
| 72 | 48 | 27 | 1B | 2 | 18 | 379736 | 3037884 | 2 | 18 | 0 | 184 | 3093844 | 2 | 18 | 0 | 1 | 192 |
| 73 | 49 | 27 | 1B | 2 | 19 | 379759 | 3038068 | 2 | 19 | 0 | 184 | 3094036 | 2 | 19 | 0 | 1 | 192 |
| 74 | 4A | 27 | 1B | 2 | 20 | 379782 | 3038252 | 2 | 20 | 0 | 184 | 3094228 | 2 | 20 | 0 | 1 | 192 |
| 75 | 4B | 27 | 1B | 2 | 21 | 379805 | 3038436 | 2 | 21 | 0 | 184 | 3094420 | 2 | 21 | 0 | 1 | 192 |
| 76 | 4C | 27 | 1B | 2 | 22 | 379828 | 3038620 | 2 | 22 | 0 | 184 | 3094612 | 2 | 22 | 0 | 1 | 192 |
| 77 | 4D | 27 | 1B | 2 | 23 | 379851 | 3038804 | 2 | 23 | 0 | 184 | 3094804 | 2 | 23 | 0 | 1 | 192 |
| 78 | 4E | 27 | 1B | 2 | 24 | 379874 | 3038988 | 2 | 24 | 0 | 184 | 3094996 | 2 | 24 | 0 | 1 | 192 |
| 79 | 4F | 27 | 1B | 2 | 25 | 379897 | 3039172 | 2 | 25 | 0 | 184 | 3095188 | 2 | 25 | 0 | 1 | 192 |
| 80 | 50 | 27 | 1B | 2 | 26 | 379922 | 3039372 | 2 | 26 | 0 | 200 | 3095396 | 2 | 26 | 0 | 1 | 208 |
| 81 | 51 | 27 | 1B | 3 | 0 | 379945 | 3039556 | 3 | 0 | 0 | 184 | 3095588 | 3 | 0 | 0 | 1 | 192 |
| 82 | 52 | 27 | 1B | 3 | 1 | 379973 | 3039780 | 3 | 1 | 0 | 224 | 3095820 | 3 | 1 | 0 | 1 | 232 |
| 83 | 53 | 27 | 1B | 3 | 2 | 380001 | 3040004 | 3 | 2 | 0 | 224 | 3096052 | 3 | 2 | 0 | 1 | 232 |
| 84 | 54 | 27 | 1B | 3 | 3 | 380029 | 3040228 | 3 | 3 | 0 | 224 | 3096284 | 3 | 3 | 0 | 1 | 232 |
| 85 | 55 | 27 | 1B | 3 | 4 | 380057 | 3040452 | 3 | 4 | 0 | 224 | 3096516 | 3 | 4 | 0 | 1 | 232 |
| 86 | 56 | 27 | 1B | 3 | 5 | 380085 | 3040676 | 3 | 5 | 0 | 224 | 3096748 | 3 | 5 | 0 | 1 | 232 |
| 87 | 57 | 27 | 1B | 3 | 6 | 380113 | 3040900 | 3 | 6 | 0 | 224 | 3096980 | 3 | 6 | 0 | 1 | 232 |
| 88 | 58 | 27 | 1B | 3 | 7 | 380141 | 3041124 | 3 | 7 | 0 | 224 | 3097212 | 3 | 7 | 0 | 1 | 232 |

| | | | | | | | | | | | | | | | | | |
|---|---|---|---|---|---|---|---|---|---|---|---|---|---|---|---|---|---|
| 89 | 59 | 27 | 1B | 3 | 8 | 380169 | 3041348 | 3 | 8 | 0 | 224 | 3097444 | 3 | 8 | 0 | 1 | 232 |
| 90 | 5A | 27 | 1B | 3 | 9 | 380197 | 3041572 | 3 | 9 | 0 | 224 | 3097676 | 3 | 9 | 0 | 1 | 232 |
| 91 | 5B | 27 | 1B | 3 | 10 | 380225 | 3041796 | 3 | 10 | 0 | 224 | 3097908 | 3 | 10 | 0 | 1 | 232 |
| 92 | 5C | 27 | 1B | 3 | 11 | 380253 | 3042020 | 3 | 11 | 0 | 224 | 3098140 | 3 | 11 | 0 | 1 | 232 |
| 93 | 5D | 27 | 1B | 3 | 12 | 380281 | 3042244 | 3 | 12 | 0 | 224 | 3098372 | 3 | 12 | 0 | 1 | 232 |
| 94 | 5E | 27 | 1B | 3 | 13 | 380309 | 3042468 | 3 | 13 | 0 | 224 | 3098604 | 3 | 13 | 0 | 1 | 232 |
| 95 | 5F | 27 | 1B | 3 | 14 | 380337 | 3042692 | 3 | 14 | 0 | 224 | 3098836 | 3 | 14 | 0 | 1 | 232 |
| 96 | 60 | 27 | 1B | 3 | 15 | 380362 | 3042892 | 3 | 15 | 0 | 200 | 3099044 | 3 | 15 | 0 | 1 | 208 |
| 97 | 61 | 27 | 1B | 3 | 16 | 380387 | 3043092 | 3 | 16 | 0 | 200 | 3099252 | 3 | 16 | 0 | 1 | 208 |
| 98 | 62 | 27 | 1B | 3 | 17 | 380412 | 3043292 | 3 | 17 | 0 | 200 | 3099460 | 3 | 17 | 0 | 1 | 208 |
| 99 | 63 | 27 | 1B | 3 | 18 | 380437 | 3043492 | 3 | 18 | 0 | 200 | 3099668 | 3 | 18 | 0 | 1 | 208 |
| 100 | 64 | 27 | 1B | 3 | 19 | 380462 | 3043692 | 3 | 19 | 0 | 200 | 3099876 | 3 | 19 | 0 | 1 | 208 |
| 101 | 65 | 27 | 1B | 3 | 20 | 380487 | 3043892 | 3 | 20 | 0 | 200 | 3100084 | 3 | 20 | 0 | 1 | 208 |
| 102 | 66 | 27 | 1B | 3 | 21 | 380512 | 3044092 | 3 | 21 | 0 | 200 | 3100292 | 3 | 21 | 0 | 1 | 208 |
| 103 | 67 | 27 | 1B | 3 | 22 | 380537 | 3044292 | 3 | 22 | 0 | 200 | 3100500 | 3 | 22 | 0 | 1 | 208 |
| 104 | 68 | 27 | 1B | 3 | 23 | 380562 | 3044492 | 3 | 23 | 0 | 200 | 3100708 | 3 | 23 | 0 | 1 | 208 |
| 105 | 69 | 27 | 1B | 3 | 24 | 380587 | 3044692 | 3 | 24 | 0 | 200 | 3100916 | 3 | 24 | 0 | 1 | 208 |
| 106 | 6A | 27 | 1B | 3 | 25 | 380612 | 3044892 | 3 | 25 | 0 | 200 | 3101124 | 3 | 25 | 0 | 1 | 208 |
| 107 | 6B | 27 | 1B | 3 | 26 | 380637 | 3045092 | 3 | 26 | 0 | 200 | 3101332 | 3 | 26 | 0 | 1 | 208 |
| 108 | 6C | 27 | 1B | 4 | 0 | 380660 | 3045276 | 4 | 0 | 0 | 184 | 3101524 | 4 | 0 | 0 | 1 | 192 |
| 109 | 6D | 27 | 1B | 4 | 1 | 380688 | 3045500 | 4 | 1 | 0 | 224 | 3101756 | 4 | 1 | 0 | 1 | 232 |
| 110 | 6E | 27 | 1B | 4 | 2 | 380716 | 3045724 | 4 | 2 | 0 | 224 | 3101988 | 4 | 2 | 0 | 1 | 232 |
| 111 | 6F | 27 | 1B | 4 | 3 | 380744 | 3045948 | 4 | 3 | 0 | 224 | 3102220 | 4 | 3 | 0 | 1 | 232 |
| 112 | 70 | 27 | 1B | 4 | 4 | 380774 | 3046188 | 4 | 4 | 0 | 240 | 3102468 | 4 | 4 | 0 | 1 | 248 |
| 113 | 71 | 27 | 1B | 4 | 5 | 380804 | 3046428 | 4 | 5 | 0 | 240 | 3102716 | 4 | 5 | 0 | 1 | 248 |
| 114 | 72 | 27 | 1B | 4 | 6 | 380834 | 3046668 | 4 | 6 | 0 | 240 | 3102964 | 4 | 6 | 0 | 1 | 248 |
| 115 | 73 | 27 | 1B | 4 | 7 | 380864 | 3046908 | 4 | 7 | 0 | 240 | 3103212 | 4 | 7 | 0 | 1 | 248 |
| 116 | 74 | 27 | 1B | 4 | 8 | 380894 | 3047148 | 4 | 8 | 0 | 240 | 3103460 | 4 | 8 | 0 | 1 | 248 |
| 117 | 75 | 27 | 1B | 4 | 9 | 380924 | 3047388 | 4 | 9 | 0 | 240 | 3103708 | 4 | 9 | 0 | 1 | 248 |
| 118 | 76 | 27 | 1B | 4 | 10 | 380954 | 3047628 | 4 | 10 | 0 | 240 | 3103956 | 4 | 10 | 0 | 1 | 248 |
| 119 | 77 | 27 | 1B | 4 | 11 | 380984 | 3047868 | 4 | 11 | 0 | 240 | 3104204 | 4 | 11 | 0 | 1 | 248 |
| 120 | 78 | 27 | 1B | 4 | 12 | 381014 | 3048108 | 4 | 12 | 0 | 240 | 3104452 | 4 | 12 | 0 | 1 | 248 |
| 121 | 79 | 27 | 1B | 4 | 13 | 381044 | 3048348 | 4 | 13 | 0 | 240 | 3104700 | 4 | 13 | 0 | 1 | 248 |
| 122 | 7A | 27 | 1B | 4 | 14 | 381074 | 3048588 | 4 | 14 | 0 | 240 | 3104948 | 4 | 14 | 0 | 1 | 248 |
| 123 | 7B | 27 | 1B | 4 | 15 | 381104 | 3048828 | 4 | 15 | 0 | 240 | 3105196 | 4 | 15 | 0 | 1 | 248 |
| 124 | 7C | 27 | 1B | 4 | 16 | 381134 | 3049068 | 4 | 16 | 0 | 240 | 3105444 | 4 | 16 | 0 | 1 | 248 |
| 125 | 7D | 27 | 1B | 4 | 17 | 381164 | 3049308 | 4 | 17 | 0 | 240 | 3105692 | 4 | 17 | 0 | 1 | 248 |
| 126 | 7E | 27 | 1B | 4 | 18 | 381194 | 3049548 | 4 | 18 | 0 | 240 | 3105940 | 4 | 18 | 0 | 1 | 248 |
| 127 | 7F | 27 | 1B | 4 | 19 | 381224 | 3049788 | 4 | 19 | 0 | 240 | 3106188 | 4 | 19 | 0 | 1 | 248 |
| 128 | 80 | 27 | 1B | 4 | 20 | 381251 | 3050004 | 4 | 20 | 0 | 216 | 3106412 | 4 | 20 | 0 | 1 | 224 |
| 129 | 81 | 27 | 1B | 4 | 21 | 381278 | 3050220 | 4 | 21 | 0 | 216 | 3106636 | 4 | 21 | 0 | 1 | 224 |
| 130 | 82 | 27 | 1B | 4 | 22 | 381305 | 3050436 | 4 | 22 | 0 | 216 | 3106860 | 4 | 22 | 0 | 1 | 224 |
| 131 | 83 | 27 | 1B | 4 | 23 | 381332 | 3050652 | 4 | 23 | 0 | 216 | 3107084 | 4 | 23 | 0 | 1 | 224 |
| 132 | 84 | 27 | 1B | 4 | 24 | 381359 | 3050868 | 4 | 24 | 0 | 216 | 3107308 | 4 | 24 | 0 | 1 | 224 |
| 133 | 85 | 27 | 1B | 4 | 25 | 381386 | 3051084 | 4 | 25 | 0 | 216 | 3107532 | 4 | 25 | 0 | 1 | 224 |
| 134 | 86 | 27 | 1B | 4 | 26 | 381413 | 3051300 | 4 | 26 | 0 | 216 | 3107756 | 4 | 26 | 0 | 1 | 224 |
| 135 | 87 | 27 | 1B | 5 | 0 | 381438 | 3051500 | 5 | 0 | 0 | 200 | 3107964 | 5 | 0 | 0 | 1 | 208 |
| 136 | 88 | 27 | 1B | 5 | 1 | 381468 | 3051740 | 5 | 1 | 0 | 240 | 3108212 | 5 | 1 | 0 | 1 | 248 |
| 137 | 89 | 27 | 1B | 5 | 2 | 381498 | 3051980 | 5 | 2 | 0 | 240 | 3108460 | 5 | 2 | 0 | 1 | 248 |
| 138 | 8A | 27 | 1B | 5 | 3 | 381528 | 3052220 | 5 | 3 | 0 | 240 | 3108708 | 5 | 3 | 0 | 1 | 248 |
| 139 | 8B | 27 | 1B | 5 | 4 | 381558 | 3052460 | 5 | 4 | 0 | 240 | 3108956 | 5 | 4 | 0 | 1 | 248 |
| 140 | 8C | 27 | 1B | 5 | 5 | 381588 | 3052700 | 5 | 5 | 0 | 240 | 3109204 | 5 | 5 | 0 | 1 | 248 |
| 141 | 8D | 27 | 1B | 5 | 6 | 381618 | 3052940 | 5 | 6 | 0 | 240 | 3109452 | 5 | 6 | 0 | 1 | 248 |
| 142 | 8E | 27 | 1B | 5 | 7 | 381648 | 3053180 | 5 | 7 | 0 | 240 | 3109700 | 5 | 7 | 0 | 1 | 248 |
| 143 | 8F | 27 | 1B | 5 | 8 | 381678 | 3053420 | 5 | 8 | 0 | 240 | 3109948 | 5 | 8 | 0 | 1 | 248 |
| 144 | 90 | 27 | 1B | 5 | 9 | 381710 | 3053676 | 5 | 9 | 0 | 256 | 3110212 | 5 | 9 | 0 | 1 | 264 |
| 145 | 91 | 27 | 1B | 5 | 10 | 381742 | 3053932 | 5 | 10 | 0 | 256 | 3110476 | 5 | 10 | 0 | 1 | 264 |
| 146 | 92 | 27 | 1B | 5 | 11 | 381774 | 3054188 | 5 | 11 | 0 | 256 | 3110740 | 5 | 11 | 0 | 1 | 264 |
| 147 | 93 | 27 | 1B | 5 | 12 | 381806 | 3054444 | 5 | 12 | 0 | 256 | 3111004 | 5 | 12 | 0 | 1 | 264 |
| 148 | 94 | 27 | 1B | 5 | 13 | 381838 | 3054700 | 5 | 13 | 0 | 256 | 3111268 | 5 | 13 | 0 | 1 | 264 |
| 149 | 95 | 27 | 1B | 5 | 14 | 381870 | 3054956 | 5 | 14 | 0 | 256 | 3111532 | 5 | 14 | 0 | 1 | 264 |
| 150 | 96 | 27 | 1B | 5 | 15 | 381902 | 3055212 | 5 | 15 | 0 | 256 | 3111796 | 5 | 15 | 0 | 1 | 264 |
| 151 | 97 | 27 | 1B | 5 | 16 | 381934 | 3055468 | 5 | 16 | 0 | 256 | 3112060 | 5 | 16 | 0 | 1 | 264 |
| 152 | 98 | 27 | 1B | 5 | 17 | 381966 | 3055724 | 5 | 17 | 0 | 256 | 3112324 | 5 | 17 | 0 | 1 | 264 |
| 153 | 99 | 27 | 1B | 5 | 18 | 381998 | 3055980 | 5 | 18 | 0 | 256 | 3112588 | 5 | 18 | 0 | 1 | 264 |
| 154 | 9A | 27 | 1B | 5 | 19 | 382030 | 3056236 | 5 | 19 | 0 | 256 | 3112852 | 5 | 19 | 0 | 1 | 264 |
| 155 | 9B | 27 | 1B | 5 | 20 | 382062 | 3056492 | 5 | 20 | 0 | 256 | 3113116 | 5 | 20 | 0 | 1 | 264 |
| 156 | 9C | 27 | 1B | 5 | 21 | 382094 | 3056748 | 5 | 21 | 0 | 256 | 3113380 | 5 | 21 | 0 | 1 | 264 |
| 157 | 9D | 27 | 1B | 5 | 22 | 382126 | 3057004 | 5 | 22 | 0 | 256 | 3113644 | 5 | 22 | 0 | 1 | 264 |
| 158 | 9E | 27 | 1B | 5 | 23 | 382158 | 3057260 | 5 | 23 | 0 | 256 | 3113908 | 5 | 23 | 0 | 1 | 264 |
| 159 | 9F | 27 | 1B | 5 | 24 | 382190 | 3057516 | 5 | 24 | 0 | 256 | 3114172 | 5 | 24 | 0 | 1 | 264 |
| 160 | A0 | 27 | 1B | 5 | 25 | 382219 | 3057748 | 5 | 25 | 0 | 232 | 3114412 | 5 | 25 | 0 | 1 | 240 |
| 161 | A1 | 27 | 1B | 5 | 26 | 382248 | 3057980 | 5 | 26 | 0 | 232 | 3114652 | 5 | 26 | 0 | 1 | 240 |
| 162 | A2 | 27 | 1B | 6 | 0 | 382275 | 3058196 | 6 | 0 | 0 | 216 | 3114876 | 6 | 0 | 0 | 1 | 224 |
| 163 | A3 | 27 | 1B | 6 | 1 | 382307 | 3058452 | 6 | 1 | 0 | 256 | 3115140 | 6 | 1 | 0 | 1 | 264 |
| 164 | A4 | 27 | 1B | 6 | 2 | 382339 | 3058708 | 6 | 2 | 0 | 256 | 3115404 | 6 | 2 | 0 | 1 | 264 |
| 165 | A5 | 27 | 1B | 6 | 3 | 382371 | 3058964 | 6 | 3 | 0 | 256 | 3115668 | 6 | 3 | 0 | 1 | 264 |
| 166 | A6 | 27 | 1B | 6 | 4 | 382403 | 3059220 | 6 | 4 | 0 | 256 | 3115932 | 6 | 4 | 0 | 1 | 264 |
| 167 | A7 | 27 | 1B | 6 | 5 | 382435 | 3059476 | 6 | 5 | 0 | 256 | 3116196 | 6 | 5 | 0 | 1 | 264 |
| 168 | A8 | 27 | 1B | 6 | 6 | 382467 | 3059732 | 6 | 6 | 0 | 256 | 3116460 | 6 | 6 | 0 | 1 | 264 |
| 169 | A9 | 27 | 1B | 6 | 7 | 382499 | 3059988 | 6 | 7 | 0 | 256 | 3116724 | 6 | 7 | 0 | 1 | 264 |
| 170 | AA | 27 | 1B | 6 | 8 | 382531 | 3060244 | 6 | 8 | 0 | 256 | 3116988 | 6 | 8 | 0 | 1 | 264 |
| 171 | AB | 27 | 1B | 6 | 9 | 382563 | 3060500 | 6 | 9 | 0 | 256 | 3117252 | 6 | 9 | 0 | 1 | 264 |
| 172 | AC | 27 | 1B | 6 | 10 | 382595 | 3060756 | 6 | 10 | 0 | 256 | 3117516 | 6 | 10 | 0 | 1 | 264 |
| 173 | AD | 27 | 1B | 6 | 11 | 382627 | 3061012 | 6 | 11 | 0 | 256 | 3117780 | 6 | 11 | 0 | 1 | 264 |
| 174 | AE | 27 | 1B | 6 | 12 | 382659 | 3061268 | 6 | 12 | 0 | 256 | 3118044 | 6 | 12 | 0 | 1 | 264 |
| 175 | AF | 27 | 1B | 6 | 13 | 382691 | 3061524 | 6 | 13 | 0 | 256 | 3118308 | 6 | 13 | 0 | 1 | 264 |
| 176 | B0 | 27 | 1B | 6 | 14 | 382725 | 3061796 | 6 | 14 | 0 | 272 | 3118588 | 6 | 14 | 0 | 1 | 280 |
| 177 | B1 | 27 | 1B | 6 | 15 | 382759 | 3062068 | 6 | 15 | 0 | 272 | 3118868 | 6 | 15 | 0 | 1 | 280 |
| 178 | B2 | 27 | 1B | 6 | 16 | 382793 | 3062340 | 6 | 16 | 0 | 272 | 3119148 | 6 | 16 | 0 | 1 | 280 |
| 179 | B3 | 27 | 1B | 6 | 17 | 382827 | 3062612 | 6 | 17 | 0 | 272 | 3119428 | 6 | 17 | 0 | 1 | 280 |
| 180 | B4 | 27 | 1B | 6 | 18 | 382861 | 3062884 | 6 | 18 | 0 | 272 | 3119708 | 6 | 18 | 0 | 1 | 280 |
| 181 | B5 | 27 | 1B | 6 | 19 | 382895 | 3063156 | 6 | 19 | 0 | 272 | 3119988 | 6 | 19 | 0 | 1 | 280 |
| 182 | B6 | 27 | 1B | 6 | 20 | 382929 | 3063428 | 6 | 20 | 0 | 272 | 3120268 | 6 | 20 | 0 | 1 | 280 |
| 183 | B7 | 27 | 1B | 6 | 21 | 382963 | 3063700 | 6 | 21 | 0 | 272 | 3120548 | 6 | 21 | 0 | 1 | 280 |
| 184 | B8 | 27 | 1B | 6 | 22 | 382997 | 3063972 | 6 | 22 | 0 | 272 | 3120828 | 6 | 22 | 0 | 1 | 280 |
| 185 | B9 | 27 | 1B | 6 | 23 | 383031 | 3064244 | 6 | 23 | 0 | 272 | 3121108 | 6 | 23 | 0 | 1 | 280 |
| 186 | BA | 27 | 1B | 6 | 24 | 383065 | 3064516 | 6 | 24 | 0 | 272 | 3121388 | 6 | 24 | 0 | 1 | 280 |
| 187 | BB | 27 | 1B | 6 | 25 | 383099 | 3064788 | 6 | 25 | 0 | 272 | 3121668 | 6 | 25 | 0 | 1 | 280 |
| 188 | BC | 27 | 1B | 6 | 26 | 383133 | 3065060 | 6 | 26 | 0 | 272 | 3121948 | 6 | 26 | 0 | 1 | 280 |
| 189 | BD | 27 | 1B | 7 | 0 | 383169 | 3065348 | 7 | 0 | 0 | 288 | 3122244 | 7 | 0 | 0 | 1 | 296 |
| 190 | BE | 27 | 1B | 7 | 1 | 383205 | 3065636 | 7 | 1 | 0 | 288 | 3122540 | 7 | 1 | 0 | 1 | 296 |
| 191 | BF | 27 | 1B | 7 | 2 | 383241 | 3065924 | 7 | 2 | 0 | 288 | 3122836 | 7 | 2 | 0 | 1 | 296 |

| | | | | | | | | | | | | | | | | | |
|---|---|---|---|---|---|---|---|---|---|---|---|---|---|---|---|---|---|
| 192 | C0 | 27 | 1B | 7 | 3 | 383275 | 3066196 | 7 | 3 | 0 | 272 | 3123116 | 7 | 3 | 0 | 1 | 280 |
| 193 | C1 | 27 | 1B | 7 | 4 | 383309 | 3066468 | 7 | 4 | 0 | 272 | 3123396 | 7 | 4 | 0 | 1 | 280 |
| 194 | C2 | 27 | 1B | 7 | 5 | 383343 | 3066740 | 7 | 5 | 0 | 272 | 3123676 | 7 | 5 | 0 | 1 | 280 |
| 195 | C3 | 27 | 1B | 7 | 6 | 383377 | 3067012 | 7 | 6 | 0 | 272 | 3123956 | 7 | 6 | 0 | 1 | 280 |
| 196 | C4 | 27 | 1B | 7 | 7 | 383411 | 3067284 | 7 | 7 | 0 | 272 | 3124236 | 7 | 7 | 0 | 1 | 280 |
| 197 | C5 | 27 | 1B | 7 | 8 | 383445 | 3067556 | 7 | 8 | 0 | 272 | 3124516 | 7 | 8 | 0 | 1 | 280 |
| 198 | C6 | 27 | 1B | 7 | 9 | 383479 | 3067828 | 7 | 9 | 0 | 272 | 3124796 | 7 | 9 | 0 | 1 | 280 |
| 199 | C7 | 27 | 1B | 7 | 10 | 383513 | 3068100 | 7 | 10 | 0 | 272 | 3125076 | 7 | 10 | 0 | 1 | 280 |
| 200 | C8 | 27 | 1B | 7 | 11 | 383547 | 3068372 | 7 | 11 | 0 | 272 | 3125356 | 7 | 11 | 0 | 1 | 280 |
| 201 | C9 | 27 | 1B | 7 | 12 | 383581 | 3068644 | 7 | 12 | 0 | 272 | 3125636 | 7 | 12 | 0 | 1 | 280 |
| 202 | CA | 27 | 1B | 7 | 13 | 383615 | 3068916 | 7 | 13 | 0 | 272 | 3125916 | 7 | 13 | 0 | 1 | 280 |
| 203 | CB | 27 | 1B | 7 | 14 | 383649 | 3069188 | 7 | 14 | 0 | 272 | 3126196 | 7 | 14 | 0 | 1 | 280 |
| 204 | CC | 27 | 1B | 7 | 15 | 383683 | 3069460 | 7 | 15 | 0 | 272 | 3126476 | 7 | 15 | 0 | 1 | 280 |
| 205 | CD | 27 | 1B | 7 | 16 | 383717 | 3069732 | 7 | 16 | 0 | 272 | 3126756 | 7 | 16 | 0 | 1 | 280 |
| 206 | CE | 27 | 1B | 7 | 17 | 383751 | 3070004 | 7 | 17 | 0 | 272 | 3127036 | 7 | 17 | 0 | 1 | 280 |
| 207 | CF | 27 | 1B | 7 | 18 | 383785 | 3070276 | 7 | 18 | 0 | 272 | 3127316 | 7 | 18 | 0 | 1 | 280 |
| 208 | D0 | 27 | 1B | 7 | 19 | 383821 | 3070564 | 7 | 19 | 0 | 288 | 3127612 | 7 | 19 | 0 | 1 | 296 |
| 209 | D1 | 27 | 1B | 7 | 20 | 383857 | 3070852 | 7 | 20 | 0 | 288 | 3127908 | 7 | 20 | 0 | 1 | 296 |
| 210 | D2 | 27 | 1B | 7 | 21 | 383893 | 3071140 | 7 | 21 | 0 | 288 | 3128204 | 7 | 21 | 0 | 1 | 296 |
| 211 | D3 | 27 | 1B | 7 | 22 | 383929 | 3071428 | 7 | 22 | 0 | 288 | 3128500 | 7 | 22 | 0 | 1 | 296 |
| 212 | D4 | 27 | 1B | 7 | 23 | 383965 | 3071716 | 7 | 23 | 0 | 288 | 3128796 | 7 | 23 | 0 | 1 | 296 |
| 213 | D5 | 27 | 1B | 7 | 24 | 384001 | 3072004 | 7 | 24 | 0 | 288 | 3129092 | 7 | 24 | 0 | 1 | 296 |
| 214 | D6 | 27 | 1B | 7 | 25 | 384037 | 3072292 | 7 | 25 | 0 | 288 | 3129388 | 7 | 25 | 0 | 1 | 296 |
| 215 | D7 | 27 | 1B | 7 | 26 | 384073 | 3072580 | 7 | 26 | 0 | 288 | 3129684 | 7 | 26 | 0 | 1 | 296 |
| 216 | D8 | 27 | 1B | 8 | 0 | 384111 | 3072884 | 8 | 0 | 0 | 304 | 3129996 | 8 | 0 | 0 | 1 | 312 |
| 217 | D9 | 27 | 1B | 8 | 1 | 384149 | 3073188 | 8 | 1 | 0 | 304 | 3130308 | 8 | 1 | 0 | 1 | 312 |
| 218 | DA | 27 | 1B | 8 | 2 | 384187 | 3073492 | 8 | 2 | 0 | 304 | 3130620 | 8 | 2 | 0 | 1 | 312 |
| 219 | DB | 27 | 1B | 8 | 3 | 384225 | 3073796 | 8 | 3 | 0 | 304 | 3130932 | 8 | 3 | 0 | 1 | 312 |
| 220 | DC | 27 | 1B | 8 | 4 | 384263 | 3074100 | 8 | 4 | 0 | 304 | 3131244 | 8 | 4 | 0 | 1 | 312 |
| 221 | DD | 27 | 1B | 8 | 5 | 384301 | 3074404 | 8 | 5 | 0 | 304 | 3131556 | 8 | 5 | 0 | 1 | 312 |
| 222 | DE | 27 | 1B | 8 | 6 | 384339 | 3074708 | 8 | 6 | 0 | 304 | 3131868 | 8 | 6 | 0 | 1 | 312 |
| 223 | DF | 27 | 1B | 8 | 7 | 384377 | 3075012 | 8 | 7 | 0 | 304 | 3132180 | 8 | 7 | 0 | 1 | 312 |
| 224 | E0 | 27 | 1B | 8 | 8 | 384413 | 3075300 | 8 | 8 | 0 | 288 | 3132476 | 8 | 8 | 0 | 1 | 296 |
| 225 | E1 | 27 | 1B | 8 | 9 | 384449 | 3075588 | 8 | 9 | 0 | 288 | 3132772 | 8 | 9 | 0 | 1 | 296 |
| 226 | E2 | 27 | 1B | 8 | 10 | 384485 | 3075876 | 8 | 10 | 0 | 288 | 3133068 | 8 | 10 | 0 | 1 | 296 |
| 227 | E3 | 27 | 1B | 8 | 11 | 384521 | 3076164 | 8 | 11 | 0 | 288 | 3133364 | 8 | 11 | 0 | 1 | 296 |
| 228 | E4 | 27 | 1B | 8 | 12 | 384557 | 3076452 | 8 | 12 | 0 | 288 | 3133660 | 8 | 12 | 0 | 1 | 296 |
| 229 | E5 | 27 | 1B | 8 | 13 | 384593 | 3076740 | 8 | 13 | 0 | 288 | 3133956 | 8 | 13 | 0 | 1 | 296 |
| 230 | E6 | 27 | 1B | 8 | 14 | 384629 | 3077028 | 8 | 14 | 0 | 288 | 3134252 | 8 | 14 | 0 | 1 | 296 |
| 231 | E7 | 27 | 1B | 8 | 15 | 384665 | 3077316 | 8 | 15 | 0 | 288 | 3134548 | 8 | 15 | 0 | 1 | 296 |
| 232 | E8 | 27 | 1B | 8 | 16 | 384701 | 3077604 | 8 | 16 | 0 | 288 | 3134844 | 8 | 16 | 0 | 1 | 296 |
| 233 | E9 | 27 | 1B | 8 | 17 | 384737 | 3077892 | 8 | 17 | 0 | 288 | 3135140 | 8 | 17 | 0 | 1 | 296 |
| 234 | EA | 27 | 1B | 8 | 18 | 384773 | 3078180 | 8 | 18 | 0 | 288 | 3135436 | 8 | 18 | 0 | 1 | 296 |
| 235 | EB | 27 | 1B | 8 | 19 | 384809 | 3078468 | 8 | 19 | 0 | 288 | 3135732 | 8 | 19 | 0 | 1 | 296 |
| 236 | EC | 27 | 1B | 8 | 20 | 384845 | 3078756 | 8 | 20 | 0 | 288 | 3136028 | 8 | 20 | 0 | 1 | 296 |
| 237 | ED | 27 | 1B | 8 | 21 | 384881 | 3079044 | 8 | 21 | 0 | 288 | 3136324 | 8 | 21 | 0 | 1 | 296 |
| 238 | EE | 27 | 1B | 8 | 22 | 384917 | 3079332 | 8 | 22 | 0 | 288 | 3136620 | 8 | 22 | 0 | 1 | 296 |
| 239 | EF | 27 | 1B | 8 | 23 | 384953 | 3079620 | 8 | 23 | 0 | 288 | 3136916 | 8 | 23 | 0 | 1 | 296 |
| 240 | F0 | 27 | 1B | 8 | 24 | 384991 | 3079924 | 8 | 24 | 0 | 304 | 3137228 | 8 | 24 | 0 | 1 | 312 |
| 241 | F1 | 27 | 1B | 8 | 25 | 385029 | 3080228 | 8 | 25 | 0 | 304 | 3137540 | 8 | 25 | 0 | 1 | 312 |
| 242 | F2 | 27 | 1B | 8 | 26 | 385067 | 3080532 | 8 | 26 | 0 | 304 | 3137852 | 8 | 26 | 0 | 1 | 312 |
| 243 | F3 | 27 | 1B | 9 | 0 | 385107 | 3080852 | 9 | 0 | 0 | 320 | 3138180 | 9 | 0 | 0 | 1 | 328 |
| 244 | F4 | 27 | 1B | 9 | 1 | 385147 | 3081172 | 9 | 1 | 0 | 320 | 3138508 | 9 | 1 | 0 | 1 | 328 |
| 245 | F5 | 27 | 1B | 9 | 2 | 385187 | 3081492 | 9 | 2 | 0 | 320 | 3138836 | 9 | 2 | 0 | 1 | 328 |
| 246 | F6 | 27 | 1B | 9 | 3 | 385227 | 3081812 | 9 | 3 | 0 | 320 | 3139164 | 9 | 3 | 0 | 1 | 328 |
| 247 | F7 | 27 | 1B | 9 | 4 | 385267 | 3082132 | 9 | 4 | 0 | 320 | 3139492 | 9 | 4 | 0 | 1 | 328 |
| 248 | F8 | 27 | 1B | 9 | 5 | 385307 | 3082452 | 9 | 5 | 0 | 320 | 3139820 | 9 | 5 | 0 | 1 | 328 |
| 249 | F9 | 27 | 1B | 9 | 6 | 385347 | 3082772 | 9 | 6 | 0 | 320 | 3140148 | 9 | 6 | 0 | 1 | 328 |
| 250 | FA | 27 | 1B | 9 | 7 | 385387 | 3083092 | 9 | 7 | 0 | 320 | 3140476 | 9 | 7 | 0 | 1 | 328 |
| 251 | FB | 27 | 1B | 9 | 8 | 385427 | 3083412 | 9 | 8 | 0 | 320 | 3140804 | 9 | 8 | 0 | 1 | 328 |
| 252 | FC | 27 | 1B | 9 | 9 | 385467 | 3083732 | 9 | 9 | 0 | 320 | 3141132 | 9 | 9 | 0 | 1 | 328 |
| 253 | FD | 27 | 1B | 9 | 10 | 385507 | 3084052 | 9 | 10 | 0 | 320 | 3141460 | 9 | 10 | 0 | 1 | 328 |
| 254 | FE | 27 | 1B | 9 | 11 | 385547 | 3084372 | 9 | 11 | 0 | 320 | 3141788 | 9 | 11 | 0 | 1 | 328 |
| 255 | FF | 27 | 1B | 9 | 12 | 385587 | 3084692 | 9 | 12 | 0 | 320 | 3142116 | 9 | 12 | 0 | 1 | 328 |
| 0 | 00 | 28 | 1C | 0 | 0 | 385599 | 3084788 | 0 | 0 | 0 | 96 | 3142220 | 0 | 0 | 0 | 1 | 104 |
| 1 | 01 | 28 | 1C | 0 | 1 | 385613 | 3084900 | 0 | 1 | 0 | 112 | 3142340 | 0 | 1 | 0 | 1 | 120 |
| 2 | 02 | 28 | 1C | 0 | 2 | 385627 | 3085012 | 0 | 2 | 0 | 112 | 3142460 | 0 | 2 | 0 | 1 | 120 |
| 3 | 03 | 28 | 1C | 0 | 3 | 385641 | 3085124 | 0 | 3 | 0 | 112 | 3142580 | 0 | 3 | 0 | 1 | 120 |
| 4 | 04 | 28 | 1C | 0 | 4 | 385655 | 3085236 | 0 | 4 | 0 | 112 | 3142700 | 0 | 4 | 0 | 1 | 120 |
| 5 | 05 | 28 | 1C | 0 | 5 | 385669 | 3085348 | 0 | 5 | 0 | 112 | 3142820 | 0 | 5 | 0 | 1 | 120 |
| 6 | 06 | 28 | 1C | 0 | 6 | 385683 | 3085460 | 0 | 6 | 0 | 112 | 3142940 | 0 | 6 | 0 | 1 | 120 |
| 7 | 07 | 28 | 1C | 0 | 7 | 385697 | 3085572 | 0 | 7 | 0 | 112 | 3143060 | 0 | 7 | 0 | 1 | 120 |
| 8 | 08 | 28 | 1C | 0 | 8 | 385711 | 3085684 | 0 | 8 | 0 | 112 | 3143180 | 0 | 8 | 0 | 1 | 120 |
| 9 | 09 | 28 | 1C | 0 | 9 | 385725 | 3085796 | 0 | 9 | 0 | 112 | 3143300 | 0 | 9 | 0 | 1 | 120 |
| 10 | 0A | 28 | 1C | 0 | 10 | 385739 | 3085908 | 0 | 10 | 0 | 112 | 3143420 | 0 | 10 | 0 | 1 | 120 |
| 11 | 0B | 28 | 1C | 0 | 11 | 385753 | 3086020 | 0 | 11 | 0 | 112 | 3143540 | 0 | 11 | 0 | 1 | 120 |
| 12 | 0C | 28 | 1C | 0 | 12 | 385767 | 3086132 | 0 | 12 | 0 | 112 | 3143660 | 0 | 12 | 0 | 1 | 120 |
| 13 | 0D | 28 | 1C | 0 | 13 | 385781 | 3086244 | 0 | 13 | 0 | 112 | 3143780 | 0 | 13 | 0 | 1 | 120 |
| 14 | 0E | 28 | 1C | 0 | 14 | 385795 | 3086356 | 0 | 14 | 0 | 112 | 3143900 | 0 | 14 | 0 | 1 | 120 |
| 15 | 0F | 28 | 1C | 0 | 15 | 385809 | 3086468 | 0 | 15 | 0 | 112 | 3144020 | 0 | 15 | 0 | 1 | 120 |
| 16 | 10 | 28 | 1C | 0 | 16 | 385823 | 3086580 | 0 | 16 | 0 | 112 | 3144140 | 0 | 16 | 0 | 1 | 120 |
| 17 | 11 | 28 | 1C | 0 | 17 | 385837 | 3086692 | 0 | 17 | 0 | 112 | 3144260 | 0 | 17 | 0 | 1 | 120 |
| 18 | 12 | 28 | 1C | 0 | 18 | 385851 | 3086804 | 0 | 18 | 0 | 112 | 3144380 | 0 | 18 | 0 | 1 | 120 |
| 19 | 13 | 28 | 1C | 0 | 19 | 385865 | 3086916 | 0 | 19 | 0 | 112 | 3144500 | 0 | 19 | 0 | 1 | 120 |
| 20 | 14 | 28 | 1C | 0 | 20 | 385879 | 3087028 | 0 | 20 | 0 | 112 | 3144620 | 0 | 20 | 0 | 1 | 120 |
| 21 | 15 | 28 | 1C | 0 | 21 | 385893 | 3087140 | 0 | 21 | 0 | 112 | 3144740 | 0 | 21 | 0 | 1 | 120 |
| 22 | 16 | 28 | 1C | 0 | 22 | 385907 | 3087252 | 0 | 22 | 0 | 112 | 3144860 | 0 | 22 | 0 | 1 | 120 |
| 23 | 17 | 28 | 1C | 0 | 23 | 385921 | 3087364 | 0 | 23 | 0 | 112 | 3144980 | 0 | 23 | 0 | 1 | 120 |
| 24 | 18 | 28 | 1C | 0 | 24 | 385935 | 3087476 | 0 | 24 | 0 | 112 | 3145100 | 0 | 24 | 0 | 1 | 120 |
| 25 | 19 | 28 | 1C | 0 | 25 | 385949 | 3087588 | 0 | 25 | 0 | 112 | 3145220 | 0 | 25 | 0 | 1 | 120 |
| 26 | 1A | 28 | 1C | 0 | 26 | 385963 | 3087700 | 0 | 26 | 0 | 112 | 3145340 | 0 | 26 | 0 | 1 | 120 |
| 27 | 1B | 28 | 1C | 0 | 27 | 385977 | 3087812 | 0 | 27 | 0 | 112 | 3145460 | 0 | 27 | 0 | 1 | 120 |
| 28 | 1C | 28 | 1C | 1 | 0 | 385989 | 3087908 | 1 | 0 | 0 | 96 | 3145564 | 1 | 0 | 0 | 1 | 104 |
| 29 | 1D | 28 | 1C | 1 | 1 | 386006 | 3088044 | 1 | 1 | 0 | 136 | 3145708 | 1 | 1 | 0 | 1 | 144 |
| 30 | 1E | 28 | 1C | 1 | 2 | 386023 | 3088180 | 1 | 2 | 0 | 136 | 3145852 | 1 | 2 | 0 | 1 | 144 |
| 31 | 1F | 28 | 1C | 1 | 3 | 386040 | 3088316 | 1 | 3 | 0 | 136 | 3145996 | 1 | 3 | 0 | 1 | 144 |
| 32 | 20 | 28 | 1C | 1 | 4 | 386061 | 3088484 | 1 | 4 | 0 | 168 | 3146172 | 1 | 4 | 0 | 1 | 176 |
| 33 | 21 | 28 | 1C | 1 | 5 | 386082 | 3088652 | 1 | 5 | 0 | 168 | 3146348 | 1 | 5 | 0 | 1 | 176 |
| 34 | 22 | 28 | 1C | 1 | 6 | 386103 | 3088820 | 1 | 6 | 0 | 168 | 3146524 | 1 | 6 | 0 | 1 | 176 |
| 35 | 23 | 28 | 1C | 1 | 7 | 386124 | 3088988 | 1 | 7 | 0 | 168 | 3146700 | 1 | 7 | 0 | 1 | 176 |
| 36 | 24 | 28 | 1C | 1 | 8 | 386145 | 3089156 | 1 | 8 | 0 | 168 | 3146876 | 1 | 8 | 0 | 1 | 176 |
| 37 | 25 | 28 | 1C | 1 | 9 | 386166 | 3089324 | 1 | 9 | 0 | 168 | 3147052 | 1 | 9 | 0 | 1 | 176 |
| 38 | 26 | 28 | 1C | 1 | 10 | 386187 | 3089492 | 1 | 10 | 0 | 168 | 3147228 | 1 | 10 | 0 | 1 | 176 |

| | | | | | | | | | | | | | | | | | |
|---|---|---|---|---|---|---|---|---|---|---|---|---|---|---|---|---|---|
| 39 | 27 | 28 | 1C | 1 | 11 | 386208 | 3089660 | 1 | 11 | 0 | 168 | 3147404 | 1 | 11 | 0 | 1 | 176 |
| 40 | 28 | 28 | 1C | 1 | 12 | 386229 | 3089828 | 1 | 12 | 0 | 168 | 3147580 | 1 | 12 | 0 | 1 | 176 |
| 41 | 29 | 28 | 1C | 1 | 13 | 386250 | 3089996 | 1 | 13 | 0 | 168 | 3147756 | 1 | 13 | 0 | 1 | 176 |
| 42 | 2A | 28 | 1C | 1 | 14 | 386271 | 3090164 | 1 | 14 | 0 | 168 | 3147932 | 1 | 14 | 0 | 1 | 176 |
| 43 | 2B | 28 | 1C | 1 | 15 | 386292 | 3090332 | 1 | 15 | 0 | 168 | 3148108 | 1 | 15 | 0 | 1 | 176 |
| 44 | 2C | 28 | 1C | 1 | 16 | 386313 | 3090500 | 1 | 16 | 0 | 168 | 3148284 | 1 | 16 | 0 | 1 | 176 |
| 45 | 2D | 28 | 1C | 1 | 17 | 386334 | 3090668 | 1 | 17 | 0 | 168 | 3148460 | 1 | 17 | 0 | 1 | 176 |
| 46 | 2E | 28 | 1C | 1 | 18 | 386355 | 3090836 | 1 | 18 | 0 | 168 | 3148636 | 1 | 18 | 0 | 1 | 176 |
| 47 | 2F | 28 | 1C | 1 | 19 | 386376 | 3091004 | 1 | 19 | 0 | 168 | 3148812 | 1 | 19 | 0 | 1 | 176 |
| 48 | 30 | 28 | 1C | 1 | 20 | 386399 | 3091188 | 1 | 20 | 0 | 184 | 3149004 | 1 | 20 | 0 | 1 | 192 |
| 49 | 31 | 28 | 1C | 1 | 21 | 386422 | 3091372 | 1 | 21 | 0 | 184 | 3149196 | 1 | 21 | 0 | 1 | 192 |
| 50 | 32 | 28 | 1C | 1 | 22 | 386445 | 3091556 | 1 | 22 | 0 | 184 | 3149388 | 1 | 22 | 0 | 1 | 192 |
| 51 | 33 | 28 | 1C | 1 | 23 | 386468 | 3091740 | 1 | 23 | 0 | 184 | 3149580 | 1 | 23 | 0 | 1 | 192 |
| 52 | 34 | 28 | 1C | 1 | 24 | 386491 | 3091924 | 1 | 24 | 0 | 184 | 3149772 | 1 | 24 | 0 | 1 | 192 |
| 53 | 35 | 28 | 1C | 1 | 25 | 386514 | 3092108 | 1 | 25 | 0 | 184 | 3149964 | 1 | 25 | 0 | 1 | 192 |
| 54 | 36 | 28 | 1C | 1 | 26 | 386537 | 3092292 | 1 | 26 | 0 | 184 | 3150156 | 1 | 26 | 0 | 1 | 192 |
| 55 | 37 | 28 | 1C | 1 | 27 | 386560 | 3092476 | 1 | 27 | 0 | 184 | 3150348 | 1 | 27 | 0 | 1 | 192 |
| 56 | 38 | 28 | 1C | 2 | 0 | 386581 | 3092644 | 2 | 0 | 0 | 168 | 3150524 | 2 | 0 | 0 | 1 | 176 |
| 57 | 39 | 28 | 1C | 2 | 1 | 386607 | 3092852 | 2 | 1 | 0 | 208 | 3150740 | 2 | 1 | 0 | 1 | 216 |
| 58 | 3A | 28 | 1C | 2 | 2 | 386633 | 3093060 | 2 | 2 | 0 | 208 | 3150956 | 2 | 2 | 0 | 1 | 216 |
| 59 | 3B | 28 | 1C | 2 | 3 | 386659 | 3093268 | 2 | 3 | 0 | 208 | 3151172 | 2 | 3 | 0 | 1 | 216 |
| 60 | 3C | 28 | 1C | 2 | 4 | 386685 | 3093476 | 2 | 4 | 0 | 208 | 3151388 | 2 | 4 | 0 | 1 | 216 |
| 61 | 3D | 28 | 1C | 2 | 5 | 386711 | 3093684 | 2 | 5 | 0 | 208 | 3151604 | 2 | 5 | 0 | 1 | 216 |
| 62 | 3E | 28 | 1C | 2 | 6 | 386737 | 3093892 | 2 | 6 | 0 | 208 | 3151820 | 2 | 6 | 0 | 1 | 216 |
| 63 | 3F | 28 | 1C | 2 | 7 | 386763 | 3094100 | 2 | 7 | 0 | 208 | 3152036 | 2 | 7 | 0 | 1 | 216 |
| 64 | 40 | 28 | 1C | 2 | 8 | 386786 | 3094284 | 2 | 8 | 0 | 184 | 3152228 | 2 | 8 | 0 | 1 | 192 |
| 65 | 41 | 28 | 1C | 2 | 9 | 386809 | 3094468 | 2 | 9 | 0 | 184 | 3152420 | 2 | 9 | 0 | 1 | 192 |
| 66 | 42 | 28 | 1C | 2 | 10 | 386832 | 3094652 | 2 | 10 | 0 | 184 | 3152612 | 2 | 10 | 0 | 1 | 192 |
| 67 | 43 | 28 | 1C | 2 | 11 | 386855 | 3094836 | 2 | 11 | 0 | 184 | 3152804 | 2 | 11 | 0 | 1 | 192 |
| 68 | 44 | 28 | 1C | 2 | 12 | 386878 | 3095020 | 2 | 12 | 0 | 184 | 3152996 | 2 | 12 | 0 | 1 | 192 |
| 69 | 45 | 28 | 1C | 2 | 13 | 386901 | 3095204 | 2 | 13 | 0 | 184 | 3153188 | 2 | 13 | 0 | 1 | 192 |
| 70 | 46 | 28 | 1C | 2 | 14 | 386924 | 3095388 | 2 | 14 | 0 | 184 | 3153380 | 2 | 14 | 0 | 1 | 192 |
| 71 | 47 | 28 | 1C | 2 | 15 | 386947 | 3095572 | 2 | 15 | 0 | 184 | 3153572 | 2 | 15 | 0 | 1 | 192 |
| 72 | 48 | 28 | 1C | 2 | 16 | 386970 | 3095756 | 2 | 16 | 0 | 184 | 3153764 | 2 | 16 | 0 | 1 | 192 |
| 73 | 49 | 28 | 1C | 2 | 17 | 386993 | 3095940 | 2 | 17 | 0 | 184 | 3153956 | 2 | 17 | 0 | 1 | 192 |
| 74 | 4A | 28 | 1C | 2 | 18 | 387016 | 3096124 | 2 | 18 | 0 | 184 | 3154148 | 2 | 18 | 0 | 1 | 192 |
| 75 | 4B | 28 | 1C | 2 | 19 | 387039 | 3096308 | 2 | 19 | 0 | 184 | 3154340 | 2 | 19 | 0 | 1 | 192 |
| 76 | 4C | 28 | 1C | 2 | 20 | 387062 | 3096492 | 2 | 20 | 0 | 184 | 3154532 | 2 | 20 | 0 | 1 | 192 |
| 77 | 4D | 28 | 1C | 2 | 21 | 387085 | 3096676 | 2 | 21 | 0 | 184 | 3154724 | 2 | 21 | 0 | 1 | 192 |
| 78 | 4E | 28 | 1C | 2 | 22 | 387108 | 3096860 | 2 | 22 | 0 | 184 | 3154916 | 2 | 22 | 0 | 1 | 192 |
| 79 | 4F | 28 | 1C | 2 | 23 | 387131 | 3097044 | 2 | 23 | 0 | 184 | 3155108 | 2 | 23 | 0 | 1 | 192 |
| 80 | 50 | 28 | 1C | 2 | 24 | 387156 | 3097244 | 2 | 24 | 0 | 200 | 3155316 | 2 | 24 | 0 | 1 | 208 |
| 81 | 51 | 28 | 1C | 2 | 25 | 387181 | 3097444 | 2 | 25 | 0 | 200 | 3155524 | 2 | 25 | 0 | 1 | 208 |
| 82 | 52 | 28 | 1C | 2 | 26 | 387206 | 3097644 | 2 | 26 | 0 | 200 | 3155732 | 2 | 26 | 0 | 1 | 208 |
| 83 | 53 | 28 | 1C | 2 | 27 | 387231 | 3097844 | 2 | 27 | 0 | 200 | 3155940 | 2 | 27 | 0 | 1 | 208 |
| 84 | 54 | 28 | 1C | 3 | 0 | 387254 | 3098028 | 3 | 0 | 0 | 184 | 3156132 | 3 | 0 | 0 | 1 | 192 |
| 85 | 55 | 28 | 1C | 3 | 1 | 387282 | 3098252 | 3 | 1 | 0 | 224 | 3156364 | 3 | 1 | 0 | 1 | 232 |
| 86 | 56 | 28 | 1C | 3 | 2 | 387310 | 3098476 | 3 | 2 | 0 | 224 | 3156596 | 3 | 2 | 0 | 1 | 232 |
| 87 | 57 | 28 | 1C | 3 | 3 | 387338 | 3098700 | 3 | 3 | 0 | 224 | 3156828 | 3 | 3 | 0 | 1 | 232 |
| 88 | 58 | 28 | 1C | 3 | 4 | 387366 | 3098924 | 3 | 4 | 0 | 224 | 3157060 | 3 | 4 | 0 | 1 | 232 |
| 89 | 59 | 28 | 1C | 3 | 5 | 387394 | 3099148 | 3 | 5 | 0 | 224 | 3157292 | 3 | 5 | 0 | 1 | 232 |
| 90 | 5A | 28 | 1C | 3 | 6 | 387422 | 3099372 | 3 | 6 | 0 | 224 | 3157524 | 3 | 6 | 0 | 1 | 232 |
| 91 | 5B | 28 | 1C | 3 | 7 | 387450 | 3099596 | 3 | 7 | 0 | 224 | 3157756 | 3 | 7 | 0 | 1 | 232 |
| 92 | 5C | 28 | 1C | 3 | 8 | 387478 | 3099820 | 3 | 8 | 0 | 224 | 3157988 | 3 | 8 | 0 | 1 | 232 |
| 93 | 5D | 28 | 1C | 3 | 9 | 387506 | 3100044 | 3 | 9 | 0 | 224 | 3158220 | 3 | 9 | 0 | 1 | 232 |
| 94 | 5E | 28 | 1C | 3 | 10 | 387534 | 3100268 | 3 | 10 | 0 | 224 | 3158452 | 3 | 10 | 0 | 1 | 232 |
| 95 | 5F | 28 | 1C | 3 | 11 | 387562 | 3100492 | 3 | 11 | 0 | 224 | 3158684 | 3 | 11 | 0 | 1 | 232 |
| 96 | 60 | 28 | 1C | 3 | 12 | 387587 | 3100692 | 3 | 12 | 0 | 200 | 3158892 | 3 | 12 | 0 | 1 | 208 |
| 97 | 61 | 28 | 1C | 3 | 13 | 387612 | 3100892 | 3 | 13 | 0 | 200 | 3159100 | 3 | 13 | 0 | 1 | 208 |
| 98 | 62 | 28 | 1C | 3 | 14 | 387637 | 3101092 | 3 | 14 | 0 | 200 | 3159308 | 3 | 14 | 0 | 1 | 208 |
| 99 | 63 | 28 | 1C | 3 | 15 | 387662 | 3101292 | 3 | 15 | 0 | 200 | 3159516 | 3 | 15 | 0 | 1 | 208 |
| 100 | 64 | 28 | 1C | 3 | 16 | 387687 | 3101492 | 3 | 16 | 0 | 200 | 3159724 | 3 | 16 | 0 | 1 | 208 |
| 101 | 65 | 28 | 1C | 3 | 17 | 387712 | 3101692 | 3 | 17 | 0 | 200 | 3159932 | 3 | 17 | 0 | 1 | 208 |
| 102 | 66 | 28 | 1C | 3 | 18 | 387737 | 3101892 | 3 | 18 | 0 | 200 | 3160140 | 3 | 18 | 0 | 1 | 208 |
| 103 | 67 | 28 | 1C | 3 | 19 | 387762 | 3102092 | 3 | 19 | 0 | 200 | 3160348 | 3 | 19 | 0 | 1 | 208 |
| 104 | 68 | 28 | 1C | 3 | 20 | 387787 | 3102292 | 3 | 20 | 0 | 200 | 3160556 | 3 | 20 | 0 | 1 | 208 |
| 105 | 69 | 28 | 1C | 3 | 21 | 387812 | 3102492 | 3 | 21 | 0 | 200 | 3160764 | 3 | 21 | 0 | 1 | 208 |
| 106 | 6A | 28 | 1C | 3 | 22 | 387837 | 3102692 | 3 | 22 | 0 | 200 | 3160972 | 3 | 22 | 0 | 1 | 208 |
| 107 | 6B | 28 | 1C | 3 | 23 | 387862 | 3102892 | 3 | 23 | 0 | 200 | 3161180 | 3 | 23 | 0 | 1 | 208 |
| 108 | 6C | 28 | 1C | 3 | 24 | 387887 | 3103092 | 3 | 24 | 0 | 200 | 3161388 | 3 | 24 | 0 | 1 | 208 |
| 109 | 6D | 28 | 1C | 3 | 25 | 387912 | 3103292 | 3 | 25 | 0 | 200 | 3161596 | 3 | 25 | 0 | 1 | 208 |
| 110 | 6E | 28 | 1C | 3 | 26 | 387937 | 3103492 | 3 | 26 | 0 | 200 | 3161804 | 3 | 26 | 0 | 1 | 208 |
| 111 | 6F | 28 | 1C | 3 | 27 | 387962 | 3103692 | 3 | 27 | 0 | 200 | 3162012 | 3 | 27 | 0 | 1 | 208 |
| 112 | 70 | 28 | 1C | 4 | 0 | 387987 | 3103892 | 4 | 0 | 0 | 200 | 3162220 | 4 | 0 | 0 | 1 | 208 |
| 113 | 71 | 28 | 1C | 4 | 1 | 388017 | 3104132 | 4 | 1 | 0 | 240 | 3162468 | 4 | 1 | 0 | 1 | 248 |
| 114 | 72 | 28 | 1C | 4 | 2 | 388047 | 3104372 | 4 | 2 | 0 | 240 | 3162716 | 4 | 2 | 0 | 1 | 248 |
| 115 | 73 | 28 | 1C | 4 | 3 | 388077 | 3104612 | 4 | 3 | 0 | 240 | 3162964 | 4 | 3 | 0 | 1 | 248 |
| 116 | 74 | 28 | 1C | 4 | 4 | 388107 | 3104852 | 4 | 4 | 0 | 240 | 3163212 | 4 | 4 | 0 | 1 | 248 |
| 117 | 75 | 28 | 1C | 4 | 5 | 388137 | 3105092 | 4 | 5 | 0 | 240 | 3163460 | 4 | 5 | 0 | 1 | 248 |
| 118 | 76 | 28 | 1C | 4 | 6 | 388167 | 3105332 | 4 | 6 | 0 | 240 | 3163708 | 4 | 6 | 0 | 1 | 248 |
| 119 | 77 | 28 | 1C | 4 | 7 | 388197 | 3105572 | 4 | 7 | 0 | 240 | 3163956 | 4 | 7 | 0 | 1 | 248 |
| 120 | 78 | 28 | 1C | 4 | 8 | 388227 | 3105812 | 4 | 8 | 0 | 240 | 3164204 | 4 | 8 | 0 | 1 | 248 |
| 121 | 79 | 28 | 1C | 4 | 9 | 388257 | 3106052 | 4 | 9 | 0 | 240 | 3164452 | 4 | 9 | 0 | 1 | 248 |
| 122 | 7A | 28 | 1C | 4 | 10 | 388287 | 3106292 | 4 | 10 | 0 | 240 | 3164700 | 4 | 10 | 0 | 1 | 248 |
| 123 | 7B | 28 | 1C | 4 | 11 | 388317 | 3106532 | 4 | 11 | 0 | 240 | 3164948 | 4 | 11 | 0 | 1 | 248 |
| 124 | 7C | 28 | 1C | 4 | 12 | 388347 | 3106772 | 4 | 12 | 0 | 240 | 3165196 | 4 | 12 | 0 | 1 | 248 |
| 125 | 7D | 28 | 1C | 4 | 13 | 388377 | 3107012 | 4 | 13 | 0 | 240 | 3165444 | 4 | 13 | 0 | 1 | 248 |
| 126 | 7E | 28 | 1C | 4 | 14 | 388407 | 3107252 | 4 | 14 | 0 | 240 | 3165692 | 4 | 14 | 0 | 1 | 248 |
| 127 | 7F | 28 | 1C | 4 | 15 | 388437 | 3107492 | 4 | 15 | 0 | 240 | 3165940 | 4 | 15 | 0 | 1 | 248 |
| 128 | 80 | 28 | 1C | 4 | 16 | 388464 | 3107708 | 4 | 16 | 0 | 216 | 3166164 | 4 | 16 | 0 | 1 | 224 |
| 129 | 81 | 28 | 1C | 4 | 17 | 388491 | 3107924 | 4 | 17 | 0 | 216 | 3166388 | 4 | 17 | 0 | 1 | 224 |
| 130 | 82 | 28 | 1C | 4 | 18 | 388518 | 3108140 | 4 | 18 | 0 | 216 | 3166612 | 4 | 18 | 0 | 1 | 224 |
| 131 | 83 | 28 | 1C | 4 | 19 | 388545 | 3108356 | 4 | 19 | 0 | 216 | 3166836 | 4 | 19 | 0 | 1 | 224 |
| 132 | 84 | 28 | 1C | 4 | 20 | 388572 | 3108572 | 4 | 20 | 0 | 216 | 3167060 | 4 | 20 | 0 | 1 | 224 |
| 133 | 85 | 28 | 1C | 4 | 21 | 388599 | 3108788 | 4 | 21 | 0 | 216 | 3167284 | 4 | 21 | 0 | 1 | 224 |
| 134 | 86 | 28 | 1C | 4 | 22 | 388626 | 3109004 | 4 | 22 | 0 | 216 | 3167508 | 4 | 22 | 0 | 1 | 224 |
| 135 | 87 | 28 | 1C | 4 | 23 | 388653 | 3109220 | 4 | 23 | 0 | 216 | 3167732 | 4 | 23 | 0 | 1 | 224 |
| 136 | 88 | 28 | 1C | 4 | 24 | 388680 | 3109436 | 4 | 24 | 0 | 216 | 3167956 | 4 | 24 | 0 | 1 | 224 |
| 137 | 89 | 28 | 1C | 4 | 25 | 388707 | 3109652 | 4 | 25 | 0 | 216 | 3168180 | 4 | 25 | 0 | 1 | 224 |
| 138 | 8A | 28 | 1C | 4 | 26 | 388734 | 3109868 | 4 | 26 | 0 | 216 | 3168404 | 4 | 26 | 0 | 1 | 224 |
| 139 | 8B | 28 | 1C | 4 | 27 | 388761 | 3110084 | 4 | 27 | 0 | 216 | 3168628 | 4 | 27 | 0 | 1 | 224 |
| 140 | 8C | 28 | 1C | 5 | 0 | 388786 | 3110284 | 5 | 0 | 0 | 200 | 3168836 | 5 | 0 | 0 | 1 | 208 |
| 141 | 8D | 28 | 1C | 5 | 1 | 388816 | 3110524 | 5 | 1 | 0 | 240 | 3169084 | 5 | 1 | 0 | 1 | 248 |

| | | | | | | | | | | | | | | | | | |
|---|---|---|---|---|---|---|---|---|---|---|---|---|---|---|---|---|---|
| 142 | 8E | 28 | 1C | 5 | 2 | 388846 | 3110764 | 5 | 2 | 0 | 240 | 3169332 | 5 | 2 | 0 | 1 | 248 |
| 143 | 8F | 28 | 1C | 5 | 3 | 388876 | 3111004 | 5 | 3 | 0 | 240 | 3169580 | 5 | 3 | 0 | 1 | 248 |
| 144 | 90 | 28 | 1C | 5 | 4 | 388908 | 3111260 | 5 | 4 | 0 | 256 | 3169844 | 5 | 4 | 0 | 1 | 264 |
| 145 | 91 | 28 | 1C | 5 | 5 | 388940 | 3111516 | 5 | 5 | 0 | 256 | 3170108 | 5 | 5 | 0 | 1 | 264 |
| 146 | 92 | 28 | 1C | 5 | 6 | 388972 | 3111772 | 5 | 6 | 0 | 256 | 3170372 | 5 | 6 | 0 | 1 | 264 |
| 147 | 93 | 28 | 1C | 5 | 7 | 389004 | 3112028 | 5 | 7 | 0 | 256 | 3170636 | 5 | 7 | 0 | 1 | 264 |
| 148 | 94 | 28 | 1C | 5 | 8 | 389036 | 3112284 | 5 | 8 | 0 | 256 | 3170900 | 5 | 8 | 0 | 1 | 264 |
| 149 | 95 | 28 | 1C | 5 | 9 | 389068 | 3112540 | 5 | 9 | 0 | 256 | 3171164 | 5 | 9 | 0 | 1 | 264 |
| 150 | 96 | 28 | 1C | 5 | 10 | 389100 | 3112796 | 5 | 10 | 0 | 256 | 3171428 | 5 | 10 | 0 | 1 | 264 |
| 151 | 97 | 28 | 1C | 5 | 11 | 389132 | 3113052 | 5 | 11 | 0 | 256 | 3171692 | 5 | 11 | 0 | 1 | 264 |
| 152 | 98 | 28 | 1C | 5 | 12 | 389164 | 3113308 | 5 | 12 | 0 | 256 | 3171956 | 5 | 12 | 0 | 1 | 264 |
| 153 | 99 | 28 | 1C | 5 | 13 | 389196 | 3113564 | 5 | 13 | 0 | 256 | 3172220 | 5 | 13 | 0 | 1 | 264 |
| 154 | 9A | 28 | 1C | 5 | 14 | 389228 | 3113820 | 5 | 14 | 0 | 256 | 3172484 | 5 | 14 | 0 | 1 | 264 |
| 155 | 9B | 28 | 1C | 5 | 15 | 389260 | 3114076 | 5 | 15 | 0 | 256 | 3172748 | 5 | 15 | 0 | 1 | 264 |
| 156 | 9C | 28 | 1C | 5 | 16 | 389292 | 3114332 | 5 | 16 | 0 | 256 | 3173012 | 5 | 16 | 0 | 1 | 264 |
| 157 | 9D | 28 | 1C | 5 | 17 | 389324 | 3114588 | 5 | 17 | 0 | 256 | 3173276 | 5 | 17 | 0 | 1 | 264 |
| 158 | 9E | 28 | 1C | 5 | 18 | 389356 | 3114844 | 5 | 18 | 0 | 256 | 3173540 | 5 | 18 | 0 | 1 | 264 |
| 159 | 9F | 28 | 1C | 5 | 19 | 389388 | 3115100 | 5 | 19 | 0 | 256 | 3173804 | 5 | 19 | 0 | 1 | 264 |
| 160 | A0 | 28 | 1C | 5 | 20 | 389417 | 3115332 | 5 | 20 | 0 | 232 | 3174044 | 5 | 20 | 0 | 1 | 240 |
| 161 | A1 | 28 | 1C | 5 | 21 | 389446 | 3115564 | 5 | 21 | 0 | 232 | 3174284 | 5 | 21 | 0 | 1 | 240 |
| 162 | A2 | 28 | 1C | 5 | 22 | 389475 | 3115796 | 5 | 22 | 0 | 232 | 3174524 | 5 | 22 | 0 | 1 | 240 |
| 163 | A3 | 28 | 1C | 5 | 23 | 389504 | 3116028 | 5 | 23 | 0 | 232 | 3174764 | 5 | 23 | 0 | 1 | 240 |
| 164 | A4 | 28 | 1C | 5 | 24 | 389533 | 3116260 | 5 | 24 | 0 | 232 | 3175004 | 5 | 24 | 0 | 1 | 240 |
| 165 | A5 | 28 | 1C | 5 | 25 | 389562 | 3116492 | 5 | 25 | 0 | 232 | 3175244 | 5 | 25 | 0 | 1 | 240 |
| 166 | A6 | 28 | 1C | 5 | 26 | 389591 | 3116724 | 5 | 26 | 0 | 232 | 3175484 | 5 | 26 | 0 | 1 | 240 |
| 167 | A7 | 28 | 1C | 5 | 27 | 389620 | 3116956 | 5 | 27 | 0 | 232 | 3175724 | 5 | 27 | 0 | 1 | 240 |
| 168 | A8 | 28 | 1C | 6 | 0 | 389647 | 3117172 | 6 | 0 | 0 | 216 | 3175948 | 6 | 0 | 0 | 1 | 224 |
| 169 | A9 | 28 | 1C | 6 | 1 | 389679 | 3117428 | 6 | 1 | 0 | 256 | 3176212 | 6 | 1 | 0 | 1 | 264 |
| 170 | AA | 28 | 1C | 6 | 2 | 389711 | 3117684 | 6 | 2 | 0 | 256 | 3176476 | 6 | 2 | 0 | 1 | 264 |
| 171 | AB | 28 | 1C | 6 | 3 | 389743 | 3117940 | 6 | 3 | 0 | 256 | 3176740 | 6 | 3 | 0 | 1 | 264 |
| 172 | AC | 28 | 1C | 6 | 4 | 389775 | 3118196 | 6 | 4 | 0 | 256 | 3177004 | 6 | 4 | 0 | 1 | 264 |
| 173 | AD | 28 | 1C | 6 | 5 | 389807 | 3118452 | 6 | 5 | 0 | 256 | 3177268 | 6 | 5 | 0 | 1 | 264 |
| 174 | AE | 28 | 1C | 6 | 6 | 389839 | 3118708 | 6 | 6 | 0 | 256 | 3177532 | 6 | 6 | 0 | 1 | 264 |
| 175 | AF | 28 | 1C | 6 | 7 | 389871 | 3118964 | 6 | 7 | 0 | 256 | 3177796 | 6 | 7 | 0 | 1 | 264 |
| 176 | B0 | 28 | 1C | 6 | 8 | 389905 | 3119236 | 6 | 8 | 0 | 272 | 3178076 | 6 | 8 | 0 | 1 | 280 |
| 177 | B1 | 28 | 1C | 6 | 9 | 389939 | 3119508 | 6 | 9 | 0 | 272 | 3178356 | 6 | 9 | 0 | 1 | 280 |
| 178 | B2 | 28 | 1C | 6 | 10 | 389973 | 3119780 | 6 | 10 | 0 | 272 | 3178636 | 6 | 10 | 0 | 1 | 280 |
| 179 | B3 | 28 | 1C | 6 | 11 | 390007 | 3120052 | 6 | 11 | 0 | 272 | 3178916 | 6 | 11 | 0 | 1 | 280 |
| 180 | B4 | 28 | 1C | 6 | 12 | 390041 | 3120324 | 6 | 12 | 0 | 272 | 3179196 | 6 | 12 | 0 | 1 | 280 |
| 181 | B5 | 28 | 1C | 6 | 13 | 390075 | 3120596 | 6 | 13 | 0 | 272 | 3179476 | 6 | 13 | 0 | 1 | 280 |
| 182 | B6 | 28 | 1C | 6 | 14 | 390109 | 3120868 | 6 | 14 | 0 | 272 | 3179756 | 6 | 14 | 0 | 1 | 280 |
| 183 | B7 | 28 | 1C | 6 | 15 | 390143 | 3121140 | 6 | 15 | 0 | 272 | 3180036 | 6 | 15 | 0 | 1 | 280 |
| 184 | B8 | 28 | 1C | 6 | 16 | 390177 | 3121412 | 6 | 16 | 0 | 272 | 3180316 | 6 | 16 | 0 | 1 | 280 |
| 185 | B9 | 28 | 1C | 6 | 17 | 390211 | 3121684 | 6 | 17 | 0 | 272 | 3180596 | 6 | 17 | 0 | 1 | 280 |
| 186 | BA | 28 | 1C | 6 | 18 | 390245 | 3121956 | 6 | 18 | 0 | 272 | 3180876 | 6 | 18 | 0 | 1 | 280 |
| 187 | BB | 28 | 1C | 6 | 19 | 390279 | 3122228 | 6 | 19 | 0 | 272 | 3181156 | 6 | 19 | 0 | 1 | 280 |
| 188 | BC | 28 | 1C | 6 | 20 | 390313 | 3122500 | 6 | 20 | 0 | 272 | 3181436 | 6 | 20 | 0 | 1 | 280 |
| 189 | BD | 28 | 1C | 6 | 21 | 390347 | 3122772 | 6 | 21 | 0 | 272 | 3181716 | 6 | 21 | 0 | 1 | 280 |
| 190 | BE | 28 | 1C | 6 | 22 | 390381 | 3123044 | 6 | 22 | 0 | 272 | 3181996 | 6 | 22 | 0 | 1 | 280 |
| 191 | BF | 28 | 1C | 6 | 23 | 390415 | 3123316 | 6 | 23 | 0 | 272 | 3182276 | 6 | 23 | 0 | 1 | 280 |
| 192 | C0 | 28 | 1C | 6 | 24 | 390446 | 3123564 | 6 | 24 | 0 | 248 | 3182532 | 6 | 24 | 0 | 1 | 256 |
| 193 | C1 | 28 | 1C | 6 | 25 | 390477 | 3123812 | 6 | 25 | 0 | 248 | 3182788 | 6 | 25 | 0 | 1 | 256 |
| 194 | C2 | 28 | 1C | 6 | 26 | 390508 | 3124060 | 6 | 26 | 0 | 248 | 3183044 | 6 | 26 | 0 | 1 | 256 |
| 195 | C3 | 28 | 1C | 6 | 27 | 390539 | 3124308 | 6 | 27 | 0 | 248 | 3183300 | 6 | 27 | 0 | 1 | 256 |
| 196 | C4 | 28 | 1C | 7 | 0 | 390568 | 3124540 | 7 | 0 | 0 | 232 | 3183540 | 7 | 0 | 0 | 1 | 240 |
| 197 | C5 | 28 | 1C | 7 | 1 | 390602 | 3124812 | 7 | 1 | 0 | 272 | 3183820 | 7 | 1 | 0 | 1 | 280 |
| 198 | C6 | 28 | 1C | 7 | 2 | 390636 | 3125084 | 7 | 2 | 0 | 272 | 3184100 | 7 | 2 | 0 | 1 | 280 |
| 199 | C7 | 28 | 1C | 7 | 3 | 390670 | 3125356 | 7 | 3 | 0 | 272 | 3184380 | 7 | 3 | 0 | 1 | 280 |
| 200 | C8 | 28 | 1C | 7 | 4 | 390704 | 3125628 | 7 | 4 | 0 | 272 | 3184660 | 7 | 4 | 0 | 1 | 280 |
| 201 | C9 | 28 | 1C | 7 | 5 | 390738 | 3125900 | 7 | 5 | 0 | 272 | 3184940 | 7 | 5 | 0 | 1 | 280 |
| 202 | CA | 28 | 1C | 7 | 6 | 390772 | 3126172 | 7 | 6 | 0 | 272 | 3185220 | 7 | 6 | 0 | 1 | 280 |
| 203 | CB | 28 | 1C | 7 | 7 | 390806 | 3126444 | 7 | 7 | 0 | 272 | 3185500 | 7 | 7 | 0 | 1 | 280 |
| 204 | CC | 28 | 1C | 7 | 8 | 390840 | 3126716 | 7 | 8 | 0 | 272 | 3185780 | 7 | 8 | 0 | 1 | 280 |
| 205 | CD | 28 | 1C | 7 | 9 | 390874 | 3126988 | 7 | 9 | 0 | 272 | 3186060 | 7 | 9 | 0 | 1 | 280 |
| 206 | CE | 28 | 1C | 7 | 10 | 390908 | 3127260 | 7 | 10 | 0 | 272 | 3186340 | 7 | 10 | 0 | 1 | 280 |
| 207 | CF | 28 | 1C | 7 | 11 | 390942 | 3127532 | 7 | 11 | 0 | 272 | 3186620 | 7 | 11 | 0 | 1 | 280 |
| 208 | D0 | 28 | 1C | 7 | 12 | 390978 | 3127820 | 7 | 12 | 0 | 288 | 3186916 | 7 | 12 | 0 | 1 | 296 |
| 209 | D1 | 28 | 1C | 7 | 13 | 391014 | 3128108 | 7 | 13 | 0 | 288 | 3187212 | 7 | 13 | 0 | 1 | 296 |
| 210 | D2 | 28 | 1C | 7 | 14 | 391050 | 3128396 | 7 | 14 | 0 | 288 | 3187508 | 7 | 14 | 0 | 1 | 296 |
| 211 | D3 | 28 | 1C | 7 | 15 | 391086 | 3128684 | 7 | 15 | 0 | 288 | 3187804 | 7 | 15 | 0 | 1 | 296 |
| 212 | D4 | 28 | 1C | 7 | 16 | 391122 | 3128972 | 7 | 16 | 0 | 288 | 3188100 | 7 | 16 | 0 | 1 | 296 |
| 213 | D5 | 28 | 1C | 7 | 17 | 391158 | 3129260 | 7 | 17 | 0 | 288 | 3188396 | 7 | 17 | 0 | 1 | 296 |
| 214 | D6 | 28 | 1C | 7 | 18 | 391194 | 3129548 | 7 | 18 | 0 | 288 | 3188692 | 7 | 18 | 0 | 1 | 296 |
| 215 | D7 | 28 | 1C | 7 | 19 | 391230 | 3129836 | 7 | 19 | 0 | 288 | 3188988 | 7 | 19 | 0 | 1 | 296 |
| 216 | D8 | 28 | 1C | 7 | 20 | 391266 | 3130124 | 7 | 20 | 0 | 288 | 3189284 | 7 | 20 | 0 | 1 | 296 |
| 217 | D9 | 28 | 1C | 7 | 21 | 391302 | 3130412 | 7 | 21 | 0 | 288 | 3189580 | 7 | 21 | 0 | 1 | 296 |
| 218 | DA | 28 | 1C | 7 | 22 | 391338 | 3130700 | 7 | 22 | 0 | 288 | 3189876 | 7 | 22 | 0 | 1 | 296 |
| 219 | DB | 28 | 1C | 7 | 23 | 391374 | 3130988 | 7 | 23 | 0 | 288 | 3190172 | 7 | 23 | 0 | 1 | 296 |
| 220 | DC | 28 | 1C | 7 | 24 | 391410 | 3131276 | 7 | 24 | 0 | 288 | 3190468 | 7 | 24 | 0 | 1 | 296 |
| 221 | DD | 28 | 1C | 7 | 25 | 391446 | 3131564 | 7 | 25 | 0 | 288 | 3190764 | 7 | 25 | 0 | 1 | 296 |
| 222 | DE | 28 | 1C | 7 | 26 | 391482 | 3131852 | 7 | 26 | 0 | 288 | 3191060 | 7 | 26 | 0 | 1 | 296 |
| 223 | DF | 28 | 1C | 7 | 27 | 391518 | 3132140 | 7 | 27 | 0 | 288 | 3191356 | 7 | 27 | 0 | 1 | 296 |
| 224 | E0 | 28 | 1C | 8 | 0 | 391549 | 3132388 | 8 | 0 | 0 | 248 | 3191612 | 8 | 0 | 0 | 1 | 256 |
| 225 | E1 | 28 | 1C | 8 | 1 | 391585 | 3132676 | 8 | 1 | 0 | 288 | 3191908 | 8 | 1 | 0 | 1 | 296 |
| 226 | E2 | 28 | 1C | 8 | 2 | 391621 | 3132964 | 8 | 2 | 0 | 288 | 3192204 | 8 | 2 | 0 | 1 | 296 |
| 227 | E3 | 28 | 1C | 8 | 3 | 391657 | 3133252 | 8 | 3 | 0 | 288 | 3192500 | 8 | 3 | 0 | 1 | 296 |
| 228 | E4 | 28 | 1C | 8 | 4 | 391693 | 3133540 | 8 | 4 | 0 | 288 | 3192796 | 8 | 4 | 0 | 1 | 296 |
| 229 | E5 | 28 | 1C | 8 | 5 | 391729 | 3133828 | 8 | 5 | 0 | 288 | 3193092 | 8 | 5 | 0 | 1 | 296 |
| 230 | E6 | 28 | 1C | 8 | 6 | 391765 | 3134116 | 8 | 6 | 0 | 288 | 3193388 | 8 | 6 | 0 | 1 | 296 |
| 231 | E7 | 28 | 1C | 8 | 7 | 391801 | 3134404 | 8 | 7 | 0 | 288 | 3193684 | 8 | 7 | 0 | 1 | 296 |
| 232 | E8 | 28 | 1C | 8 | 8 | 391837 | 3134692 | 8 | 8 | 0 | 288 | 3193980 | 8 | 8 | 0 | 1 | 296 |
| 233 | E9 | 28 | 1C | 8 | 9 | 391873 | 3134980 | 8 | 9 | 0 | 288 | 3194276 | 8 | 9 | 0 | 1 | 296 |
| 234 | EA | 28 | 1C | 8 | 10 | 391909 | 3135268 | 8 | 10 | 0 | 288 | 3194572 | 8 | 10 | 0 | 1 | 296 |
| 235 | EB | 28 | 1C | 8 | 11 | 391945 | 3135556 | 8 | 11 | 0 | 288 | 3194868 | 8 | 11 | 0 | 1 | 296 |
| 236 | EC | 28 | 1C | 8 | 12 | 391981 | 3135844 | 8 | 12 | 0 | 288 | 3195164 | 8 | 12 | 0 | 1 | 296 |
| 237 | ED | 28 | 1C | 8 | 13 | 392017 | 3136132 | 8 | 13 | 0 | 288 | 3195460 | 8 | 13 | 0 | 1 | 296 |
| 238 | EE | 28 | 1C | 8 | 14 | 392053 | 3136420 | 8 | 14 | 0 | 288 | 3195756 | 8 | 14 | 0 | 1 | 296 |
| 239 | EF | 28 | 1C | 8 | 15 | 392089 | 3136708 | 8 | 15 | 0 | 288 | 3196052 | 8 | 15 | 0 | 1 | 296 |
| 240 | F0 | 28 | 1C | 8 | 16 | 392127 | 3137012 | 8 | 16 | 0 | 304 | 3196364 | 8 | 16 | 0 | 1 | 312 |
| 241 | F1 | 28 | 1C | 8 | 17 | 392165 | 3137316 | 8 | 17 | 0 | 304 | 3196676 | 8 | 17 | 0 | 1 | 312 |
| 242 | F2 | 28 | 1C | 8 | 18 | 392203 | 3137620 | 8 | 18 | 0 | 304 | 3196988 | 8 | 18 | 0 | 1 | 312 |
| 243 | F3 | 28 | 1C | 8 | 19 | 392241 | 3137924 | 8 | 19 | 0 | 304 | 3197300 | 8 | 19 | 0 | 1 | 312 |
| 244 | F4 | 28 | 1C | 8 | 20 | 392279 | 3138228 | 8 | 20 | 0 | 304 | 3197612 | 8 | 20 | 0 | 1 | 312 |

| | | | | | | | | | | | | | | | | | |
|---|---|---|---|---|---|---|---|---|---|---|---|---|---|---|---|---|---|
| 245 | F5 | 28 | 1C | 8 | 21 | 392317 | 3138532 | 8 | 21 | 0 | 304 | 3197924 | 8 | 21 | 0 | 1 | 312 |
| 246 | F6 | 28 | 1C | 8 | 22 | 392355 | 3138836 | 8 | 22 | 0 | 304 | 3198236 | 8 | 22 | 0 | 1 | 312 |
| 247 | F7 | 28 | 1C | 8 | 23 | 392393 | 3139140 | 8 | 23 | 0 | 304 | 3198548 | 8 | 23 | 0 | 1 | 312 |
| 248 | F8 | 28 | 1C | 8 | 24 | 392431 | 3139444 | 8 | 24 | 0 | 304 | 3198860 | 8 | 24 | 0 | 1 | 312 |
| 249 | F9 | 28 | 1C | 8 | 25 | 392469 | 3139748 | 8 | 25 | 0 | 304 | 3199172 | 8 | 25 | 0 | 1 | 312 |
| 250 | FA | 28 | 1C | 8 | 26 | 392507 | 3140052 | 8 | 26 | 0 | 304 | 3199484 | 8 | 26 | 0 | 1 | 312 |
| 251 | FB | 28 | 1C | 8 | 27 | 392545 | 3140356 | 8 | 27 | 0 | 304 | 3199796 | 8 | 27 | 0 | 1 | 312 |
| 252 | FC | 28 | 1C | 9 | 0 | 392585 | 3140676 | 9 | 0 | 0 | 320 | 3200124 | 9 | 0 | 0 | 1 | 328 |
| 253 | FD | 28 | 1C | 9 | 1 | 392625 | 3140996 | 9 | 1 | 0 | 320 | 3200452 | 9 | 1 | 0 | 1 | 328 |
| 254 | FE | 28 | 1C | 9 | 2 | 392665 | 3141316 | 9 | 2 | 0 | 320 | 3200780 | 9 | 2 | 0 | 1 | 328 |
| 255 | FF | 28 | 1C | 9 | 3 | 392705 | 3141636 | 9 | 3 | 0 | 320 | 3201108 | 9 | 3 | 0 | 1 | 328 |
| 0 | 00 | 29 | 1D | 0 | 0 | 392717 | 3141732 | 0 | 0 | 0 | 96 | 3201212 | 0 | 0 | 0 | 1 | 104 |
| 1 | 01 | 29 | 1D | 0 | 1 | 392731 | 3141844 | 0 | 1 | 0 | 112 | 3201332 | 0 | 1 | 0 | 1 | 120 |
| 2 | 02 | 29 | 1D | 0 | 2 | 392745 | 3141956 | 0 | 2 | 0 | 112 | 3201452 | 0 | 2 | 0 | 1 | 120 |
| 3 | 03 | 29 | 1D | 0 | 3 | 392759 | 3142068 | 0 | 3 | 0 | 112 | 3201572 | 0 | 3 | 0 | 1 | 120 |
| 4 | 04 | 29 | 1D | 0 | 4 | 392773 | 3142180 | 0 | 4 | 0 | 112 | 3201692 | 0 | 4 | 0 | 1 | 120 |
| 5 | 05 | 29 | 1D | 0 | 5 | 392787 | 3142292 | 0 | 5 | 0 | 112 | 3201812 | 0 | 5 | 0 | 1 | 120 |
| 6 | 06 | 29 | 1D | 0 | 6 | 392801 | 3142404 | 0 | 6 | 0 | 112 | 3201932 | 0 | 6 | 0 | 1 | 120 |
| 7 | 07 | 29 | 1D | 0 | 7 | 392815 | 3142516 | 0 | 7 | 0 | 112 | 3202052 | 0 | 7 | 0 | 1 | 120 |
| 8 | 08 | 29 | 1D | 0 | 8 | 392829 | 3142628 | 0 | 8 | 0 | 112 | 3202172 | 0 | 8 | 0 | 1 | 120 |
| 9 | 09 | 29 | 1D | 0 | 9 | 392843 | 3142740 | 0 | 9 | 0 | 112 | 3202292 | 0 | 9 | 0 | 1 | 120 |
| 10 | 0A | 29 | 1D | 0 | 10 | 392857 | 3142852 | 0 | 10 | 0 | 112 | 3202412 | 0 | 10 | 0 | 1 | 120 |
| 11 | 0B | 29 | 1D | 0 | 11 | 392871 | 3142964 | 0 | 11 | 0 | 112 | 3202532 | 0 | 11 | 0 | 1 | 120 |
| 12 | 0C | 29 | 1D | 0 | 12 | 392885 | 3143076 | 0 | 12 | 0 | 112 | 3202652 | 0 | 12 | 0 | 1 | 120 |
| 13 | 0D | 29 | 1D | 0 | 13 | 392899 | 3143188 | 0 | 13 | 0 | 112 | 3202772 | 0 | 13 | 0 | 1 | 120 |
| 14 | 0E | 29 | 1D | 0 | 14 | 392913 | 3143300 | 0 | 14 | 0 | 112 | 3202892 | 0 | 14 | 0 | 1 | 120 |
| 15 | 0F | 29 | 1D | 0 | 15 | 392927 | 3143412 | 0 | 15 | 0 | 112 | 3203012 | 0 | 15 | 0 | 1 | 120 |
| 16 | 10 | 29 | 1D | 0 | 16 | 392941 | 3143524 | 0 | 16 | 0 | 112 | 3203132 | 0 | 16 | 0 | 1 | 120 |
| 17 | 11 | 29 | 1D | 0 | 17 | 392955 | 3143636 | 0 | 17 | 0 | 112 | 3203252 | 0 | 17 | 0 | 1 | 120 |
| 18 | 12 | 29 | 1D | 0 | 18 | 392969 | 3143748 | 0 | 18 | 0 | 112 | 3203372 | 0 | 18 | 0 | 1 | 120 |
| 19 | 13 | 29 | 1D | 0 | 19 | 392983 | 3143860 | 0 | 19 | 0 | 112 | 3203492 | 0 | 19 | 0 | 1 | 120 |
| 20 | 14 | 29 | 1D | 0 | 20 | 392997 | 3143972 | 0 | 20 | 0 | 112 | 3203612 | 0 | 20 | 0 | 1 | 120 |
| 21 | 15 | 29 | 1D | 0 | 21 | 393011 | 3144084 | 0 | 21 | 0 | 112 | 3203732 | 0 | 21 | 0 | 1 | 120 |
| 22 | 16 | 29 | 1D | 0 | 22 | 393025 | 3144196 | 0 | 22 | 0 | 112 | 3203852 | 0 | 22 | 0 | 1 | 120 |
| 23 | 17 | 29 | 1D | 0 | 23 | 393039 | 3144308 | 0 | 23 | 0 | 112 | 3203972 | 0 | 23 | 0 | 1 | 120 |
| 24 | 18 | 29 | 1D | 0 | 24 | 393053 | 3144420 | 0 | 24 | 0 | 112 | 3204092 | 0 | 24 | 0 | 1 | 120 |
| 25 | 19 | 29 | 1D | 0 | 25 | 393067 | 3144532 | 0 | 25 | 0 | 112 | 3204212 | 0 | 25 | 0 | 1 | 120 |
| 26 | 1A | 29 | 1D | 0 | 26 | 393081 | 3144644 | 0 | 26 | 0 | 112 | 3204332 | 0 | 26 | 0 | 1 | 120 |
| 27 | 1B | 29 | 1D | 0 | 27 | 393095 | 3144756 | 0 | 27 | 0 | 112 | 3204452 | 0 | 27 | 0 | 1 | 120 |
| 28 | 1C | 29 | 1D | 0 | 28 | 393109 | 3144868 | 0 | 28 | 0 | 112 | 3204572 | 0 | 28 | 0 | 1 | 120 |
| 29 | 1D | 29 | 1D | 1 | 0 | 393121 | 3144964 | 1 | 0 | 0 | 96 | 3204676 | 1 | 0 | 0 | 1 | 104 |
| 30 | 1E | 29 | 1D | 1 | 1 | 393138 | 3145100 | 1 | 1 | 0 | 136 | 3204820 | 1 | 1 | 0 | 1 | 144 |
| 31 | 1F | 29 | 1D | 1 | 2 | 393155 | 3145236 | 1 | 2 | 0 | 136 | 3204964 | 1 | 2 | 0 | 1 | 144 |
| 32 | 20 | 29 | 1D | 1 | 3 | 393176 | 3145404 | 1 | 3 | 0 | 168 | 3205140 | 1 | 3 | 0 | 1 | 176 |
| 33 | 21 | 29 | 1D | 1 | 4 | 393197 | 3145572 | 1 | 4 | 0 | 168 | 3205316 | 1 | 4 | 0 | 1 | 176 |
| 34 | 22 | 29 | 1D | 1 | 5 | 393218 | 3145740 | 1 | 5 | 0 | 168 | 3205492 | 1 | 5 | 0 | 1 | 176 |
| 35 | 23 | 29 | 1D | 1 | 6 | 393239 | 3145908 | 1 | 6 | 0 | 168 | 3205668 | 1 | 6 | 0 | 1 | 176 |
| 36 | 24 | 29 | 1D | 1 | 7 | 393260 | 3146076 | 1 | 7 | 0 | 168 | 3205844 | 1 | 7 | 0 | 1 | 176 |
| 37 | 25 | 29 | 1D | 1 | 8 | 393281 | 3146244 | 1 | 8 | 0 | 168 | 3206020 | 1 | 8 | 0 | 1 | 176 |
| 38 | 26 | 29 | 1D | 1 | 9 | 393302 | 3146412 | 1 | 9 | 0 | 168 | 3206196 | 1 | 9 | 0 | 1 | 176 |
| 39 | 27 | 29 | 1D | 1 | 10 | 393323 | 3146580 | 1 | 10 | 0 | 168 | 3206372 | 1 | 10 | 0 | 1 | 176 |
| 40 | 28 | 29 | 1D | 1 | 11 | 393344 | 3146748 | 1 | 11 | 0 | 168 | 3206548 | 1 | 11 | 0 | 1 | 176 |
| 41 | 29 | 29 | 1D | 1 | 12 | 393365 | 3146916 | 1 | 12 | 0 | 168 | 3206724 | 1 | 12 | 0 | 1 | 176 |
| 42 | 2A | 29 | 1D | 1 | 13 | 393386 | 3147084 | 1 | 13 | 0 | 168 | 3206900 | 1 | 13 | 0 | 1 | 176 |
| 43 | 2B | 29 | 1D | 1 | 14 | 393407 | 3147252 | 1 | 14 | 0 | 168 | 3207076 | 1 | 14 | 0 | 1 | 176 |
| 44 | 2C | 29 | 1D | 1 | 15 | 393428 | 3147420 | 1 | 15 | 0 | 168 | 3207252 | 1 | 15 | 0 | 1 | 176 |
| 45 | 2D | 29 | 1D | 1 | 16 | 393449 | 3147588 | 1 | 16 | 0 | 168 | 3207428 | 1 | 16 | 0 | 1 | 176 |
| 46 | 2E | 29 | 1D | 1 | 17 | 393470 | 3147756 | 1 | 17 | 0 | 168 | 3207604 | 1 | 17 | 0 | 1 | 176 |
| 47 | 2F | 29 | 1D | 1 | 18 | 393491 | 3147924 | 1 | 18 | 0 | 168 | 3207780 | 1 | 18 | 0 | 1 | 176 |
| 48 | 30 | 29 | 1D | 1 | 19 | 393514 | 3148108 | 1 | 19 | 0 | 184 | 3207972 | 1 | 19 | 0 | 1 | 192 |
| 49 | 31 | 29 | 1D | 1 | 20 | 393537 | 3148292 | 1 | 20 | 0 | 184 | 3208164 | 1 | 20 | 0 | 1 | 192 |
| 50 | 32 | 29 | 1D | 1 | 21 | 393560 | 3148476 | 1 | 21 | 0 | 184 | 3208356 | 1 | 21 | 0 | 1 | 192 |
| 51 | 33 | 29 | 1D | 1 | 22 | 393583 | 3148660 | 1 | 22 | 0 | 184 | 3208548 | 1 | 22 | 0 | 1 | 192 |
| 52 | 34 | 29 | 1D | 1 | 23 | 393606 | 3148844 | 1 | 23 | 0 | 184 | 3208740 | 1 | 23 | 0 | 1 | 192 |
| 53 | 35 | 29 | 1D | 1 | 24 | 393629 | 3149028 | 1 | 24 | 0 | 184 | 3208932 | 1 | 24 | 0 | 1 | 192 |
| 54 | 36 | 29 | 1D | 1 | 25 | 393652 | 3149212 | 1 | 25 | 0 | 184 | 3209124 | 1 | 25 | 0 | 1 | 192 |
| 55 | 37 | 29 | 1D | 1 | 26 | 393675 | 3149396 | 1 | 26 | 0 | 184 | 3209316 | 1 | 26 | 0 | 1 | 192 |
| 56 | 38 | 29 | 1D | 1 | 27 | 393698 | 3149580 | 1 | 27 | 0 | 184 | 3209508 | 1 | 27 | 0 | 1 | 192 |
| 57 | 39 | 29 | 1D | 1 | 28 | 393721 | 3149764 | 1 | 28 | 0 | 184 | 3209700 | 1 | 28 | 0 | 1 | 192 |
| 58 | 3A | 29 | 1D | 2 | 0 | 393742 | 3149932 | 2 | 0 | 0 | 168 | 3209876 | 2 | 0 | 0 | 1 | 176 |
| 59 | 3B | 29 | 1D | 2 | 1 | 393768 | 3150140 | 2 | 1 | 0 | 208 | 3210092 | 2 | 1 | 0 | 1 | 216 |
| 60 | 3C | 29 | 1D | 2 | 2 | 393794 | 3150348 | 2 | 2 | 0 | 208 | 3210308 | 2 | 2 | 0 | 1 | 216 |
| 61 | 3D | 29 | 1D | 2 | 3 | 393820 | 3150556 | 2 | 3 | 0 | 208 | 3210524 | 2 | 3 | 0 | 1 | 216 |
| 62 | 3E | 29 | 1D | 2 | 4 | 393846 | 3150764 | 2 | 4 | 0 | 208 | 3210740 | 2 | 4 | 0 | 1 | 216 |
| 63 | 3F | 29 | 1D | 2 | 5 | 393872 | 3150972 | 2 | 5 | 0 | 208 | 3210956 | 2 | 5 | 0 | 1 | 216 |
| 64 | 40 | 29 | 1D | 2 | 6 | 393895 | 3151156 | 2 | 6 | 0 | 184 | 3211148 | 2 | 6 | 0 | 1 | 192 |
| 65 | 41 | 29 | 1D | 2 | 7 | 393918 | 3151340 | 2 | 7 | 0 | 184 | 3211340 | 2 | 7 | 0 | 1 | 192 |
| 66 | 42 | 29 | 1D | 2 | 8 | 393941 | 3151524 | 2 | 8 | 0 | 184 | 3211532 | 2 | 8 | 0 | 1 | 192 |
| 67 | 43 | 29 | 1D | 2 | 9 | 393964 | 3151708 | 2 | 9 | 0 | 184 | 3211724 | 2 | 9 | 0 | 1 | 192 |
| 68 | 44 | 29 | 1D | 2 | 10 | 393987 | 3151892 | 2 | 10 | 0 | 184 | 3211916 | 2 | 10 | 0 | 1 | 192 |
| 69 | 45 | 29 | 1D | 2 | 11 | 394010 | 3152076 | 2 | 11 | 0 | 184 | 3212108 | 2 | 11 | 0 | 1 | 192 |
| 70 | 46 | 29 | 1D | 2 | 12 | 394033 | 3152260 | 2 | 12 | 0 | 184 | 3212300 | 2 | 12 | 0 | 1 | 192 |
| 71 | 47 | 29 | 1D | 2 | 13 | 394056 | 3152444 | 2 | 13 | 0 | 184 | 3212492 | 2 | 13 | 0 | 1 | 192 |
| 72 | 48 | 29 | 1D | 2 | 14 | 394079 | 3152628 | 2 | 14 | 0 | 184 | 3212684 | 2 | 14 | 0 | 1 | 192 |
| 73 | 49 | 29 | 1D | 2 | 15 | 394102 | 3152812 | 2 | 15 | 0 | 184 | 3212876 | 2 | 15 | 0 | 1 | 192 |
| 74 | 4A | 29 | 1D | 2 | 16 | 394125 | 3152996 | 2 | 16 | 0 | 184 | 3213068 | 2 | 16 | 0 | 1 | 192 |
| 75 | 4B | 29 | 1D | 2 | 17 | 394148 | 3153180 | 2 | 17 | 0 | 184 | 3213260 | 2 | 17 | 0 | 1 | 192 |
| 76 | 4C | 29 | 1D | 2 | 18 | 394171 | 3153364 | 2 | 18 | 0 | 184 | 3213452 | 2 | 18 | 0 | 1 | 192 |
| 77 | 4D | 29 | 1D | 2 | 19 | 394194 | 3153548 | 2 | 19 | 0 | 184 | 3213644 | 2 | 19 | 0 | 1 | 192 |
| 78 | 4E | 29 | 1D | 2 | 20 | 394217 | 3153732 | 2 | 20 | 0 | 184 | 3213836 | 2 | 20 | 0 | 1 | 192 |
| 79 | 4F | 29 | 1D | 2 | 21 | 394240 | 3153916 | 2 | 21 | 0 | 184 | 3214028 | 2 | 21 | 0 | 1 | 192 |
| 80 | 50 | 29 | 1D | 2 | 22 | 394265 | 3154116 | 2 | 22 | 0 | 200 | 3214236 | 2 | 22 | 0 | 1 | 208 |
| 81 | 51 | 29 | 1D | 2 | 23 | 394290 | 3154316 | 2 | 23 | 0 | 200 | 3214444 | 2 | 23 | 0 | 1 | 208 |
| 82 | 52 | 29 | 1D | 2 | 24 | 394315 | 3154516 | 2 | 24 | 0 | 200 | 3214652 | 2 | 24 | 0 | 1 | 208 |
| 83 | 53 | 29 | 1D | 2 | 25 | 394340 | 3154716 | 2 | 25 | 0 | 200 | 3214860 | 2 | 25 | 0 | 1 | 208 |
| 84 | 54 | 29 | 1D | 2 | 26 | 394365 | 3154916 | 2 | 26 | 0 | 200 | 3215068 | 2 | 26 | 0 | 1 | 208 |
| 85 | 55 | 29 | 1D | 2 | 27 | 394390 | 3155116 | 2 | 27 | 0 | 200 | 3215276 | 2 | 27 | 0 | 1 | 208 |
| 86 | 56 | 29 | 1D | 2 | 28 | 394415 | 3155316 | 2 | 28 | 0 | 200 | 3215484 | 2 | 28 | 0 | 1 | 208 |
| 87 | 57 | 29 | 1D | 3 | 0 | 394438 | 3155500 | 3 | 0 | 0 | 184 | 3215676 | 3 | 0 | 0 | 1 | 192 |
| 88 | 58 | 29 | 1D | 3 | 1 | 394466 | 3155724 | 3 | 1 | 0 | 224 | 3215908 | 3 | 1 | 0 | 1 | 232 |
| 89 | 59 | 29 | 1D | 3 | 2 | 394494 | 3155948 | 3 | 2 | 0 | 224 | 3216140 | 3 | 2 | 0 | 1 | 232 |
| 90 | 5A | 29 | 1D | 3 | 3 | 394522 | 3156172 | 3 | 3 | 0 | 224 | 3216372 | 3 | 3 | 0 | 1 | 232 |
| 91 | 5B | 29 | 1D | 3 | 4 | 394550 | 3156396 | 3 | 4 | 0 | 224 | 3216604 | 3 | 4 | 0 | 1 | 232 |

| | | | | | | | | | | | | | | | | | |
|---|---|---|---|---|---|---|---|---|---|---|---|---|---|---|---|---|---|
| 92 | 5C | 29 | 1D | 3 | 5 | 394578 | 3156620 | 3 | 5 | 0 | 224 | 3216836 | 3 | 5 | 0 | 1 | 232 |
| 93 | 5D | 29 | 1D | 3 | 6 | 394606 | 3156844 | 3 | 6 | 0 | 224 | 3217068 | 3 | 6 | 0 | 1 | 232 |
| 94 | 5E | 29 | 1D | 3 | 7 | 394634 | 3157068 | 3 | 7 | 0 | 224 | 3217300 | 3 | 7 | 0 | 1 | 232 |
| 95 | 5F | 29 | 1D | 3 | 8 | 394662 | 3157292 | 3 | 8 | 0 | 224 | 3217532 | 3 | 8 | 0 | 1 | 232 |
| 96 | 60 | 29 | 1D | 3 | 9 | 394687 | 3157492 | 3 | 9 | 0 | 200 | 3217740 | 3 | 9 | 0 | 1 | 208 |
| 97 | 61 | 29 | 1D | 3 | 10 | 394712 | 3157692 | 3 | 10 | 0 | 200 | 3217948 | 3 | 10 | 0 | 1 | 208 |
| 98 | 62 | 29 | 1D | 3 | 11 | 394737 | 3157892 | 3 | 11 | 0 | 200 | 3218156 | 3 | 11 | 0 | 1 | 208 |
| 99 | 63 | 29 | 1D | 3 | 12 | 394762 | 3158092 | 3 | 12 | 0 | 200 | 3218364 | 3 | 12 | 0 | 1 | 208 |
| 100 | 64 | 29 | 1D | 3 | 13 | 394787 | 3158292 | 3 | 13 | 0 | 200 | 3218572 | 3 | 13 | 0 | 1 | 208 |
| 101 | 65 | 29 | 1D | 3 | 14 | 394812 | 3158492 | 3 | 14 | 0 | 200 | 3218780 | 3 | 14 | 0 | 1 | 208 |
| 102 | 66 | 29 | 1D | 3 | 15 | 394837 | 3158692 | 3 | 15 | 0 | 200 | 3218988 | 3 | 15 | 0 | 1 | 208 |
| 103 | 67 | 29 | 1D | 3 | 16 | 394862 | 3158892 | 3 | 16 | 0 | 200 | 3219196 | 3 | 16 | 0 | 1 | 208 |
| 104 | 68 | 29 | 1D | 3 | 17 | 394887 | 3159092 | 3 | 17 | 0 | 200 | 3219404 | 3 | 17 | 0 | 1 | 208 |
| 105 | 69 | 29 | 1D | 3 | 18 | 394912 | 3159292 | 3 | 18 | 0 | 200 | 3219612 | 3 | 18 | 0 | 1 | 208 |
| 106 | 6A | 29 | 1D | 3 | 19 | 394937 | 3159492 | 3 | 19 | 0 | 200 | 3219820 | 3 | 19 | 0 | 1 | 208 |
| 107 | 6B | 29 | 1D | 3 | 20 | 394962 | 3159692 | 3 | 20 | 0 | 200 | 3220028 | 3 | 20 | 0 | 1 | 208 |
| 108 | 6C | 29 | 1D | 3 | 21 | 394987 | 3159892 | 3 | 21 | 0 | 200 | 3220236 | 3 | 21 | 0 | 1 | 208 |
| 109 | 6D | 29 | 1D | 3 | 22 | 395012 | 3160092 | 3 | 22 | 0 | 200 | 3220444 | 3 | 22 | 0 | 1 | 208 |
| 110 | 6E | 29 | 1D | 3 | 23 | 395037 | 3160292 | 3 | 23 | 0 | 200 | 3220652 | 3 | 23 | 0 | 1 | 208 |
| 111 | 6F | 29 | 1D | 3 | 24 | 395062 | 3160492 | 3 | 24 | 0 | 200 | 3220860 | 3 | 24 | 0 | 1 | 208 |
| 112 | 70 | 29 | 1D | 3 | 25 | 395089 | 3160708 | 3 | 25 | 0 | 216 | 3221084 | 3 | 25 | 0 | 1 | 224 |
| 113 | 71 | 29 | 1D | 3 | 26 | 395116 | 3160924 | 3 | 26 | 0 | 216 | 3221308 | 3 | 26 | 0 | 1 | 224 |
| 114 | 72 | 29 | 1D | 3 | 27 | 395143 | 3161140 | 3 | 27 | 0 | 216 | 3221532 | 3 | 27 | 0 | 1 | 224 |
| 115 | 73 | 29 | 1D | 3 | 28 | 395170 | 3161356 | 3 | 28 | 0 | 216 | 3221756 | 3 | 28 | 0 | 1 | 224 |
| 116 | 74 | 29 | 1D | 4 | 0 | 395195 | 3161556 | 4 | 0 | 0 | 200 | 3221964 | 4 | 0 | 0 | 1 | 208 |
| 117 | 75 | 29 | 1D | 4 | 1 | 395225 | 3161796 | 4 | 1 | 0 | 240 | 3222212 | 4 | 1 | 0 | 1 | 248 |
| 118 | 76 | 29 | 1D | 4 | 2 | 395255 | 3162036 | 4 | 2 | 0 | 240 | 3222460 | 4 | 2 | 0 | 1 | 248 |
| 119 | 77 | 29 | 1D | 4 | 3 | 395285 | 3162276 | 4 | 3 | 0 | 240 | 3222708 | 4 | 3 | 0 | 1 | 248 |
| 120 | 78 | 29 | 1D | 4 | 4 | 395315 | 3162516 | 4 | 4 | 0 | 240 | 3222956 | 4 | 4 | 0 | 1 | 248 |
| 121 | 79 | 29 | 1D | 4 | 5 | 395345 | 3162756 | 4 | 5 | 0 | 240 | 3223204 | 4 | 5 | 0 | 1 | 248 |
| 122 | 7A | 29 | 1D | 4 | 6 | 395375 | 3162996 | 4 | 6 | 0 | 240 | 3223452 | 4 | 6 | 0 | 1 | 248 |
| 123 | 7B | 29 | 1D | 4 | 7 | 395405 | 3163236 | 4 | 7 | 0 | 240 | 3223700 | 4 | 7 | 0 | 1 | 248 |
| 124 | 7C | 29 | 1D | 4 | 8 | 395435 | 3163476 | 4 | 8 | 0 | 240 | 3223948 | 4 | 8 | 0 | 1 | 248 |
| 125 | 7D | 29 | 1D | 4 | 9 | 395465 | 3163716 | 4 | 9 | 0 | 240 | 3224196 | 4 | 9 | 0 | 1 | 248 |
| 126 | 7E | 29 | 1D | 4 | 10 | 395495 | 3163956 | 4 | 10 | 0 | 240 | 3224444 | 4 | 10 | 0 | 1 | 248 |
| 127 | 7F | 29 | 1D | 4 | 11 | 395525 | 3164196 | 4 | 11 | 0 | 240 | 3224692 | 4 | 11 | 0 | 1 | 248 |
| 128 | 80 | 29 | 1D | 4 | 12 | 395552 | 3164412 | 4 | 12 | 0 | 216 | 3224916 | 4 | 12 | 0 | 1 | 224 |
| 129 | 81 | 29 | 1D | 4 | 13 | 395579 | 3164628 | 4 | 13 | 0 | 216 | 3225140 | 4 | 13 | 0 | 1 | 224 |
| 130 | 82 | 29 | 1D | 4 | 14 | 395606 | 3164844 | 4 | 14 | 0 | 216 | 3225364 | 4 | 14 | 0 | 1 | 224 |
| 131 | 83 | 29 | 1D | 4 | 15 | 395633 | 3165060 | 4 | 15 | 0 | 216 | 3225588 | 4 | 15 | 0 | 1 | 224 |
| 132 | 84 | 29 | 1D | 4 | 16 | 395660 | 3165276 | 4 | 16 | 0 | 216 | 3225812 | 4 | 16 | 0 | 1 | 224 |
| 133 | 85 | 29 | 1D | 4 | 17 | 395687 | 3165492 | 4 | 17 | 0 | 216 | 3226036 | 4 | 17 | 0 | 1 | 224 |
| 134 | 86 | 29 | 1D | 4 | 18 | 395714 | 3165708 | 4 | 18 | 0 | 216 | 3226260 | 4 | 18 | 0 | 1 | 224 |
| 135 | 87 | 29 | 1D | 4 | 19 | 395741 | 3165924 | 4 | 19 | 0 | 216 | 3226484 | 4 | 19 | 0 | 1 | 224 |
| 136 | 88 | 29 | 1D | 4 | 20 | 395768 | 3166140 | 4 | 20 | 0 | 216 | 3226708 | 4 | 20 | 0 | 1 | 224 |
| 137 | 89 | 29 | 1D | 4 | 21 | 395795 | 3166356 | 4 | 21 | 0 | 216 | 3226932 | 4 | 21 | 0 | 1 | 224 |
| 138 | 8A | 29 | 1D | 4 | 22 | 395822 | 3166572 | 4 | 22 | 0 | 216 | 3227156 | 4 | 22 | 0 | 1 | 224 |
| 139 | 8B | 29 | 1D | 4 | 23 | 395849 | 3166788 | 4 | 23 | 0 | 216 | 3227380 | 4 | 23 | 0 | 1 | 224 |
| 140 | 8C | 29 | 1D | 4 | 24 | 395876 | 3167004 | 4 | 24 | 0 | 216 | 3227604 | 4 | 24 | 0 | 1 | 224 |
| 141 | 8D | 29 | 1D | 4 | 25 | 395903 | 3167220 | 4 | 25 | 0 | 216 | 3227828 | 4 | 25 | 0 | 1 | 224 |
| 142 | 8E | 29 | 1D | 4 | 26 | 395930 | 3167436 | 4 | 26 | 0 | 216 | 3228052 | 4 | 26 | 0 | 1 | 224 |
| 143 | 8F | 29 | 1D | 4 | 27 | 395957 | 3167652 | 4 | 27 | 0 | 216 | 3228276 | 4 | 27 | 0 | 1 | 224 |
| 144 | 90 | 29 | 1D | 4 | 28 | 395986 | 3167884 | 4 | 28 | 0 | 232 | 3228516 | 4 | 28 | 0 | 1 | 240 |
| 145 | 91 | 29 | 1D | 5 | 0 | 396013 | 3168100 | 5 | 0 | 0 | 216 | 3228740 | 5 | 0 | 0 | 1 | 224 |
| 146 | 92 | 29 | 1D | 5 | 1 | 396045 | 3168356 | 5 | 1 | 0 | 256 | 3229004 | 5 | 1 | 0 | 1 | 264 |
| 147 | 93 | 29 | 1D | 5 | 2 | 396077 | 3168612 | 5 | 2 | 0 | 256 | 3229268 | 5 | 2 | 0 | 1 | 264 |
| 148 | 94 | 29 | 1D | 5 | 3 | 396109 | 3168868 | 5 | 3 | 0 | 256 | 3229532 | 5 | 3 | 0 | 1 | 264 |
| 149 | 95 | 29 | 1D | 5 | 4 | 396141 | 3169124 | 5 | 4 | 0 | 256 | 3229796 | 5 | 4 | 0 | 1 | 264 |
| 150 | 96 | 29 | 1D | 5 | 5 | 396173 | 3169380 | 5 | 5 | 0 | 256 | 3230060 | 5 | 5 | 0 | 1 | 264 |
| 151 | 97 | 29 | 1D | 5 | 6 | 396205 | 3169636 | 5 | 6 | 0 | 256 | 3230324 | 5 | 6 | 0 | 1 | 264 |
| 152 | 98 | 29 | 1D | 5 | 7 | 396237 | 3169892 | 5 | 7 | 0 | 256 | 3230588 | 5 | 7 | 0 | 1 | 264 |
| 153 | 99 | 29 | 1D | 5 | 8 | 396269 | 3170148 | 5 | 8 | 0 | 256 | 3230852 | 5 | 8 | 0 | 1 | 264 |
| 154 | 9A | 29 | 1D | 5 | 9 | 396301 | 3170404 | 5 | 9 | 0 | 256 | 3231116 | 5 | 9 | 0 | 1 | 264 |
| 155 | 9B | 29 | 1D | 5 | 10 | 396333 | 3170660 | 5 | 10 | 0 | 256 | 3231380 | 5 | 10 | 0 | 1 | 264 |
| 156 | 9C | 29 | 1D | 5 | 11 | 396365 | 3170916 | 5 | 11 | 0 | 256 | 3231644 | 5 | 11 | 0 | 1 | 264 |
| 157 | 9D | 29 | 1D | 5 | 12 | 396397 | 3171172 | 5 | 12 | 0 | 256 | 3231908 | 5 | 12 | 0 | 1 | 264 |
| 158 | 9E | 29 | 1D | 5 | 13 | 396429 | 3171428 | 5 | 13 | 0 | 256 | 3232172 | 5 | 13 | 0 | 1 | 264 |
| 159 | 9F | 29 | 1D | 5 | 14 | 396461 | 3171684 | 5 | 14 | 0 | 256 | 3232436 | 5 | 14 | 0 | 1 | 264 |
| 160 | A0 | 29 | 1D | 5 | 15 | 396490 | 3171916 | 5 | 15 | 0 | 232 | 3232676 | 5 | 15 | 0 | 1 | 240 |
| 161 | A1 | 29 | 1D | 5 | 16 | 396519 | 3172148 | 5 | 16 | 0 | 232 | 3232916 | 5 | 16 | 0 | 1 | 240 |
| 162 | A2 | 29 | 1D | 5 | 17 | 396548 | 3172380 | 5 | 17 | 0 | 232 | 3233156 | 5 | 17 | 0 | 1 | 240 |
| 163 | A3 | 29 | 1D | 5 | 18 | 396577 | 3172612 | 5 | 18 | 0 | 232 | 3233396 | 5 | 18 | 0 | 1 | 240 |
| 164 | A4 | 29 | 1D | 5 | 19 | 396606 | 3172844 | 5 | 19 | 0 | 232 | 3233636 | 5 | 19 | 0 | 1 | 240 |
| 165 | A5 | 29 | 1D | 5 | 20 | 396635 | 3173076 | 5 | 20 | 0 | 232 | 3233876 | 5 | 20 | 0 | 1 | 240 |
| 166 | A6 | 29 | 1D | 5 | 21 | 396664 | 3173308 | 5 | 21 | 0 | 232 | 3234116 | 5 | 21 | 0 | 1 | 240 |
| 167 | A7 | 29 | 1D | 5 | 22 | 396693 | 3173540 | 5 | 22 | 0 | 232 | 3234356 | 5 | 22 | 0 | 1 | 240 |
| 168 | A8 | 29 | 1D | 5 | 23 | 396722 | 3173772 | 5 | 23 | 0 | 232 | 3234596 | 5 | 23 | 0 | 1 | 240 |
| 169 | A9 | 29 | 1D | 5 | 24 | 396751 | 3174004 | 5 | 24 | 0 | 232 | 3234836 | 5 | 24 | 0 | 1 | 240 |
| 170 | AA | 29 | 1D | 5 | 25 | 396780 | 3174236 | 5 | 25 | 0 | 232 | 3235076 | 5 | 25 | 0 | 1 | 240 |
| 171 | AB | 29 | 1D | 5 | 26 | 396809 | 3174468 | 5 | 26 | 0 | 232 | 3235316 | 5 | 26 | 0 | 1 | 240 |
| 172 | AC | 29 | 1D | 5 | 27 | 396838 | 3174700 | 5 | 27 | 0 | 232 | 3235556 | 5 | 27 | 0 | 1 | 240 |
| 173 | AD | 29 | 1D | 5 | 28 | 396867 | 3174932 | 5 | 28 | 0 | 232 | 3235796 | 5 | 28 | 0 | 1 | 240 |
| 174 | AE | 29 | 1D | 6 | 0 | 396894 | 3175148 | 6 | 0 | 0 | 216 | 3236020 | 6 | 0 | 0 | 1 | 224 |
| 175 | AF | 29 | 1D | 6 | 1 | 396926 | 3175404 | 6 | 1 | 0 | 256 | 3236284 | 6 | 1 | 0 | 1 | 264 |
| 176 | B0 | 29 | 1D | 6 | 2 | 396960 | 3175676 | 6 | 2 | 0 | 272 | 3236564 | 6 | 2 | 0 | 1 | 280 |
| 177 | B1 | 29 | 1D | 6 | 3 | 396994 | 3175948 | 6 | 3 | 0 | 272 | 3236844 | 6 | 3 | 0 | 1 | 280 |
| 178 | B2 | 29 | 1D | 6 | 4 | 397028 | 3176220 | 6 | 4 | 0 | 272 | 3237124 | 6 | 4 | 0 | 1 | 280 |
| 179 | B3 | 29 | 1D | 6 | 5 | 397062 | 3176492 | 6 | 5 | 0 | 272 | 3237404 | 6 | 5 | 0 | 1 | 280 |
| 180 | B4 | 29 | 1D | 6 | 6 | 397096 | 3176764 | 6 | 6 | 0 | 272 | 3237684 | 6 | 6 | 0 | 1 | 280 |
| 181 | B5 | 29 | 1D | 6 | 7 | 397130 | 3177036 | 6 | 7 | 0 | 272 | 3237964 | 6 | 7 | 0 | 1 | 280 |
| 182 | B6 | 29 | 1D | 6 | 8 | 397164 | 3177308 | 6 | 8 | 0 | 272 | 3238244 | 6 | 8 | 0 | 1 | 280 |
| 183 | B7 | 29 | 1D | 6 | 9 | 397198 | 3177580 | 6 | 9 | 0 | 272 | 3238524 | 6 | 9 | 0 | 1 | 280 |
| 184 | B8 | 29 | 1D | 6 | 10 | 397232 | 3177852 | 6 | 10 | 0 | 272 | 3238804 | 6 | 10 | 0 | 1 | 280 |
| 185 | B9 | 29 | 1D | 6 | 11 | 397266 | 3178124 | 6 | 11 | 0 | 272 | 3239084 | 6 | 11 | 0 | 1 | 280 |
| 186 | BA | 29 | 1D | 6 | 12 | 397300 | 3178396 | 6 | 12 | 0 | 272 | 3239364 | 6 | 12 | 0 | 1 | 280 |
| 187 | BB | 29 | 1D | 6 | 13 | 397334 | 3178668 | 6 | 13 | 0 | 272 | 3239644 | 6 | 13 | 0 | 1 | 280 |
| 188 | BC | 29 | 1D | 6 | 14 | 397368 | 3178940 | 6 | 14 | 0 | 272 | 3239924 | 6 | 14 | 0 | 1 | 280 |
| 189 | BD | 29 | 1D | 6 | 15 | 397402 | 3179212 | 6 | 15 | 0 | 272 | 3240204 | 6 | 15 | 0 | 1 | 280 |
| 190 | BE | 29 | 1D | 6 | 16 | 397436 | 3179484 | 6 | 16 | 0 | 272 | 3240484 | 6 | 16 | 0 | 1 | 280 |
| 191 | BF | 29 | 1D | 6 | 17 | 397470 | 3179756 | 6 | 17 | 0 | 272 | 3240764 | 6 | 17 | 0 | 1 | 280 |
| 192 | C0 | 29 | 1D | 6 | 18 | 397501 | 3180004 | 6 | 18 | 0 | 248 | 3241020 | 6 | 18 | 0 | 1 | 256 |
| 193 | C1 | 29 | 1D | 6 | 19 | 397532 | 3180252 | 6 | 19 | 0 | 248 | 3241276 | 6 | 19 | 0 | 1 | 256 |
| 194 | C2 | 29 | 1D | 6 | 20 | 397563 | 3180500 | 6 | 20 | 0 | 248 | 3241532 | 6 | 20 | 0 | 1 | 256 |

| | | | | | | | | | | | | | | | | | |
|---|---|---|---|---|---|---|---|---|---|---|---|---|---|---|---|---|---|
| 195 | C3 | 29 | 1D | 6 | 21 | 397594 | 3180748 | 6 | 21 | 0 | 248 | 3241788 | 6 | 21 | 0 | 1 | 256 |
| 196 | C4 | 29 | 1D | 6 | 22 | 397625 | 3180996 | 6 | 22 | 0 | 248 | 3242044 | 6 | 22 | 0 | 1 | 256 |
| 197 | C5 | 29 | 1D | 6 | 23 | 397656 | 3181244 | 6 | 23 | 0 | 248 | 3242300 | 6 | 23 | 0 | 1 | 256 |
| 198 | C6 | 29 | 1D | 6 | 24 | 397687 | 3181492 | 6 | 24 | 0 | 248 | 3242556 | 6 | 24 | 0 | 1 | 256 |
| 199 | C7 | 29 | 1D | 6 | 25 | 397718 | 3181740 | 6 | 25 | 0 | 248 | 3242812 | 6 | 25 | 0 | 1 | 256 |
| 200 | C8 | 29 | 1D | 6 | 26 | 397749 | 3181988 | 6 | 26 | 0 | 248 | 3243068 | 6 | 26 | 0 | 1 | 256 |
| 201 | C9 | 29 | 1D | 6 | 27 | 397780 | 3182236 | 6 | 27 | 0 | 248 | 3243324 | 6 | 27 | 0 | 1 | 256 |
| 202 | CA | 29 | 1D | 6 | 28 | 397811 | 3182484 | 6 | 28 | 0 | 248 | 3243580 | 6 | 28 | 0 | 1 | 256 |
| 203 | CB | 29 | 1D | 7 | 0 | 397840 | 3182716 | 7 | 0 | 0 | 232 | 3243820 | 7 | 0 | 0 | 1 | 240 |
| 204 | CC | 29 | 1D | 7 | 1 | 397874 | 3182988 | 7 | 1 | 0 | 272 | 3244100 | 7 | 1 | 0 | 1 | 280 |
| 205 | CD | 29 | 1D | 7 | 2 | 397908 | 3183260 | 7 | 2 | 0 | 272 | 3244380 | 7 | 2 | 0 | 1 | 280 |
| 206 | CE | 29 | 1D | 7 | 3 | 397942 | 3183532 | 7 | 3 | 0 | 272 | 3244660 | 7 | 3 | 0 | 1 | 280 |
| 207 | CF | 29 | 1D | 7 | 4 | 397976 | 3183804 | 7 | 4 | 0 | 272 | 3244940 | 7 | 4 | 0 | 1 | 280 |
| 208 | D0 | 29 | 1D | 7 | 5 | 398012 | 3184092 | 7 | 5 | 0 | 288 | 3245236 | 7 | 5 | 0 | 1 | 296 |
| 209 | D1 | 29 | 1D | 7 | 6 | 398048 | 3184380 | 7 | 6 | 0 | 288 | 3245532 | 7 | 6 | 0 | 1 | 296 |
| 210 | D2 | 29 | 1D | 7 | 7 | 398084 | 3184668 | 7 | 7 | 0 | 288 | 3245828 | 7 | 7 | 0 | 1 | 296 |
| 211 | D3 | 29 | 1D | 7 | 8 | 398120 | 3184956 | 7 | 8 | 0 | 288 | 3246124 | 7 | 8 | 0 | 1 | 296 |
| 212 | D4 | 29 | 1D | 7 | 9 | 398156 | 3185244 | 7 | 9 | 0 | 288 | 3246420 | 7 | 9 | 0 | 1 | 296 |
| 213 | D5 | 29 | 1D | 7 | 10 | 398192 | 3185532 | 7 | 10 | 0 | 288 | 3246716 | 7 | 10 | 0 | 1 | 296 |
| 214 | D6 | 29 | 1D | 7 | 11 | 398228 | 3185820 | 7 | 11 | 0 | 288 | 3247012 | 7 | 11 | 0 | 1 | 296 |
| 215 | D7 | 29 | 1D | 7 | 12 | 398264 | 3186108 | 7 | 12 | 0 | 288 | 3247308 | 7 | 12 | 0 | 1 | 296 |
| 216 | D8 | 29 | 1D | 7 | 13 | 398300 | 3186396 | 7 | 13 | 0 | 288 | 3247604 | 7 | 13 | 0 | 1 | 296 |
| 217 | D9 | 29 | 1D | 7 | 14 | 398336 | 3186684 | 7 | 14 | 0 | 288 | 3247900 | 7 | 14 | 0 | 1 | 296 |
| 218 | DA | 29 | 1D | 7 | 15 | 398372 | 3186972 | 7 | 15 | 0 | 288 | 3248196 | 7 | 15 | 0 | 1 | 296 |
| 219 | DB | 29 | 1D | 7 | 16 | 398408 | 3187260 | 7 | 16 | 0 | 288 | 3248492 | 7 | 16 | 0 | 1 | 296 |
| 220 | DC | 29 | 1D | 7 | 17 | 398444 | 3187548 | 7 | 17 | 0 | 288 | 3248788 | 7 | 17 | 0 | 1 | 296 |
| 221 | DD | 29 | 1D | 7 | 18 | 398480 | 3187836 | 7 | 18 | 0 | 288 | 3249084 | 7 | 18 | 0 | 1 | 296 |
| 222 | DE | 29 | 1D | 7 | 19 | 398516 | 3188124 | 7 | 19 | 0 | 288 | 3249380 | 7 | 19 | 0 | 1 | 296 |
| 223 | DF | 29 | 1D | 7 | 20 | 398552 | 3188412 | 7 | 20 | 0 | 288 | 3249676 | 7 | 20 | 0 | 1 | 296 |
| 224 | E0 | 29 | 1D | 7 | 21 | 398585 | 3188676 | 7 | 21 | 0 | 264 | 3249948 | 7 | 21 | 0 | 1 | 272 |
| 225 | E1 | 29 | 1D | 7 | 22 | 398618 | 3188940 | 7 | 22 | 0 | 264 | 3250220 | 7 | 22 | 0 | 1 | 272 |
| 226 | E2 | 29 | 1D | 7 | 23 | 398651 | 3189204 | 7 | 23 | 0 | 264 | 3250492 | 7 | 23 | 0 | 1 | 272 |
| 227 | E3 | 29 | 1D | 7 | 24 | 398684 | 3189468 | 7 | 24 | 0 | 264 | 3250764 | 7 | 24 | 0 | 1 | 272 |
| 228 | E4 | 29 | 1D | 7 | 25 | 398717 | 3189732 | 7 | 25 | 0 | 264 | 3251036 | 7 | 25 | 0 | 1 | 272 |
| 229 | E5 | 29 | 1D | 7 | 26 | 398750 | 3189996 | 7 | 26 | 0 | 264 | 3251308 | 7 | 26 | 0 | 1 | 272 |
| 230 | E6 | 29 | 1D | 7 | 27 | 398783 | 3190260 | 7 | 27 | 0 | 264 | 3251580 | 7 | 27 | 0 | 1 | 272 |
| 231 | E7 | 29 | 1D | 7 | 28 | 398816 | 3190524 | 7 | 28 | 0 | 264 | 3251852 | 7 | 28 | 0 | 1 | 272 |
| 232 | E8 | 29 | 1D | 8 | 0 | 398847 | 3190772 | 8 | 0 | 0 | 248 | 3252108 | 8 | 0 | 0 | 1 | 256 |
| 233 | E9 | 29 | 1D | 8 | 1 | 398883 | 3191060 | 8 | 1 | 0 | 288 | 3252404 | 8 | 1 | 0 | 1 | 296 |
| 234 | EA | 29 | 1D | 8 | 2 | 398919 | 3191348 | 8 | 2 | 0 | 288 | 3252700 | 8 | 2 | 0 | 1 | 296 |
| 235 | EB | 29 | 1D | 8 | 3 | 398955 | 3191636 | 8 | 3 | 0 | 288 | 3252996 | 8 | 3 | 0 | 1 | 296 |
| 236 | EC | 29 | 1D | 8 | 4 | 398991 | 3191924 | 8 | 4 | 0 | 288 | 3253292 | 8 | 4 | 0 | 1 | 296 |
| 237 | ED | 29 | 1D | 8 | 5 | 399027 | 3192212 | 8 | 5 | 0 | 288 | 3253588 | 8 | 5 | 0 | 1 | 296 |
| 238 | EE | 29 | 1D | 8 | 6 | 399063 | 3192500 | 8 | 6 | 0 | 288 | 3253884 | 8 | 6 | 0 | 1 | 296 |
| 239 | EF | 29 | 1D | 8 | 7 | 399099 | 3192788 | 8 | 7 | 0 | 288 | 3254180 | 8 | 7 | 0 | 1 | 296 |
| 240 | F0 | 29 | 1D | 8 | 8 | 399137 | 3193092 | 8 | 8 | 0 | 304 | 3254492 | 8 | 8 | 0 | 1 | 312 |
| 241 | F1 | 29 | 1D | 8 | 9 | 399175 | 3193396 | 8 | 9 | 0 | 304 | 3254804 | 8 | 9 | 0 | 1 | 312 |
| 242 | F2 | 29 | 1D | 8 | 10 | 399213 | 3193700 | 8 | 10 | 0 | 304 | 3255116 | 8 | 10 | 0 | 1 | 312 |
| 243 | F3 | 29 | 1D | 8 | 11 | 399251 | 3194004 | 8 | 11 | 0 | 304 | 3255428 | 8 | 11 | 0 | 1 | 312 |
| 244 | F4 | 29 | 1D | 8 | 12 | 399289 | 3194308 | 8 | 12 | 0 | 304 | 3255740 | 8 | 12 | 0 | 1 | 312 |
| 245 | F5 | 29 | 1D | 8 | 13 | 399327 | 3194612 | 8 | 13 | 0 | 304 | 3256052 | 8 | 13 | 0 | 1 | 312 |
| 246 | F6 | 29 | 1D | 8 | 14 | 399365 | 3194916 | 8 | 14 | 0 | 304 | 3256364 | 8 | 14 | 0 | 1 | 312 |
| 247 | F7 | 29 | 1D | 8 | 15 | 399403 | 3195220 | 8 | 15 | 0 | 304 | 3256676 | 8 | 15 | 0 | 1 | 312 |
| 248 | F8 | 29 | 1D | 8 | 16 | 399441 | 3195524 | 8 | 16 | 0 | 304 | 3256988 | 8 | 16 | 0 | 1 | 312 |
| 249 | F9 | 29 | 1D | 8 | 17 | 399479 | 3195828 | 8 | 17 | 0 | 304 | 3257300 | 8 | 17 | 0 | 1 | 312 |
| 250 | FA | 29 | 1D | 8 | 18 | 399517 | 3196132 | 8 | 18 | 0 | 304 | 3257612 | 8 | 18 | 0 | 1 | 312 |
| 251 | FB | 29 | 1D | 8 | 19 | 399555 | 3196436 | 8 | 19 | 0 | 304 | 3257924 | 8 | 19 | 0 | 1 | 312 |
| 252 | FC | 29 | 1D | 8 | 20 | 399593 | 3196740 | 8 | 20 | 0 | 304 | 3258236 | 8 | 20 | 0 | 1 | 312 |
| 253 | FD | 29 | 1D | 8 | 21 | 399631 | 3197044 | 8 | 21 | 0 | 304 | 3258548 | 8 | 21 | 0 | 1 | 312 |
| 254 | FE | 29 | 1D | 8 | 22 | 399669 | 3197348 | 8 | 22 | 0 | 304 | 3258860 | 8 | 22 | 0 | 1 | 312 |
| 255 | FF | 29 | 1D | 8 | 23 | 399707 | 3197652 | 8 | 23 | 0 | 304 | 3259172 | 8 | 23 | 0 | 1 | 312 |
| 0 | 00 | 30 | 1E | 0 | 0 | 399719 | 3197748 | 0 | 0 | 0 | 96 | 3259276 | 0 | 0 | 0 | 1 | 104 |
| 1 | 01 | 30 | 1E | 0 | 1 | 399733 | 3197860 | 0 | 1 | 0 | 112 | 3259396 | 0 | 1 | 0 | 1 | 120 |
| 2 | 02 | 30 | 1E | 0 | 2 | 399747 | 3197972 | 0 | 2 | 0 | 112 | 3259516 | 0 | 2 | 0 | 1 | 120 |
| 3 | 03 | 30 | 1E | 0 | 3 | 399761 | 3198084 | 0 | 3 | 0 | 112 | 3259636 | 0 | 3 | 0 | 1 | 120 |
| 4 | 04 | 30 | 1E | 0 | 4 | 399775 | 3198196 | 0 | 4 | 0 | 112 | 3259756 | 0 | 4 | 0 | 1 | 120 |
| 5 | 05 | 30 | 1E | 0 | 5 | 399789 | 3198308 | 0 | 5 | 0 | 112 | 3259876 | 0 | 5 | 0 | 1 | 120 |
| 6 | 06 | 30 | 1E | 0 | 6 | 399803 | 3198420 | 0 | 6 | 0 | 112 | 3259996 | 0 | 6 | 0 | 1 | 120 |
| 7 | 07 | 30 | 1E | 0 | 7 | 399817 | 3198532 | 0 | 7 | 0 | 112 | 3260116 | 0 | 7 | 0 | 1 | 120 |
| 8 | 08 | 30 | 1E | 0 | 8 | 399831 | 3198644 | 0 | 8 | 0 | 112 | 3260236 | 0 | 8 | 0 | 1 | 120 |
| 9 | 09 | 30 | 1E | 0 | 9 | 399845 | 3198756 | 0 | 9 | 0 | 112 | 3260356 | 0 | 9 | 0 | 1 | 120 |
| 10 | 0A | 30 | 1E | 0 | 10 | 399859 | 3198868 | 0 | 10 | 0 | 112 | 3260476 | 0 | 10 | 0 | 1 | 120 |
| 11 | 0B | 30 | 1E | 0 | 11 | 399873 | 3198980 | 0 | 11 | 0 | 112 | 3260596 | 0 | 11 | 0 | 1 | 120 |
| 12 | 0C | 30 | 1E | 0 | 12 | 399887 | 3199092 | 0 | 12 | 0 | 112 | 3260716 | 0 | 12 | 0 | 1 | 120 |
| 13 | 0D | 30 | 1E | 0 | 13 | 399901 | 3199204 | 0 | 13 | 0 | 112 | 3260836 | 0 | 13 | 0 | 1 | 120 |
| 14 | 0E | 30 | 1E | 0 | 14 | 399915 | 3199316 | 0 | 14 | 0 | 112 | 3260956 | 0 | 14 | 0 | 1 | 120 |
| 15 | 0F | 30 | 1E | 0 | 15 | 399929 | 3199428 | 0 | 15 | 0 | 112 | 3261076 | 0 | 15 | 0 | 1 | 120 |
| 16 | 10 | 30 | 1E | 0 | 16 | 399943 | 3199540 | 0 | 16 | 0 | 112 | 3261196 | 0 | 16 | 0 | 1 | 120 |
| 17 | 11 | 30 | 1E | 0 | 17 | 399957 | 3199652 | 0 | 17 | 0 | 112 | 3261316 | 0 | 17 | 0 | 1 | 120 |
| 18 | 12 | 30 | 1E | 0 | 18 | 399971 | 3199764 | 0 | 18 | 0 | 112 | 3261436 | 0 | 18 | 0 | 1 | 120 |
| 19 | 13 | 30 | 1E | 0 | 19 | 399985 | 3199876 | 0 | 19 | 0 | 112 | 3261556 | 0 | 19 | 0 | 1 | 120 |
| 20 | 14 | 30 | 1E | 0 | 20 | 399999 | 3199988 | 0 | 20 | 0 | 112 | 3261676 | 0 | 20 | 0 | 1 | 120 |
| 21 | 15 | 30 | 1E | 0 | 21 | 400013 | 3200100 | 0 | 21 | 0 | 112 | 3261796 | 0 | 21 | 0 | 1 | 120 |
| 22 | 16 | 30 | 1E | 0 | 22 | 400027 | 3200212 | 0 | 22 | 0 | 112 | 3261916 | 0 | 22 | 0 | 1 | 120 |
| 23 | 17 | 30 | 1E | 0 | 23 | 400041 | 3200324 | 0 | 23 | 0 | 112 | 3262036 | 0 | 23 | 0 | 1 | 120 |
| 24 | 18 | 30 | 1E | 0 | 24 | 400055 | 3200436 | 0 | 24 | 0 | 112 | 3262156 | 0 | 24 | 0 | 1 | 120 |
| 25 | 19 | 30 | 1E | 0 | 25 | 400069 | 3200548 | 0 | 25 | 0 | 112 | 3262276 | 0 | 25 | 0 | 1 | 120 |
| 26 | 1A | 30 | 1E | 0 | 26 | 400083 | 3200660 | 0 | 26 | 0 | 112 | 3262396 | 0 | 26 | 0 | 1 | 120 |
| 27 | 1B | 30 | 1E | 0 | 27 | 400097 | 3200772 | 0 | 27 | 0 | 112 | 3262516 | 0 | 27 | 0 | 1 | 120 |
| 28 | 1C | 30 | 1E | 0 | 28 | 400111 | 3200884 | 0 | 28 | 0 | 112 | 3262636 | 0 | 28 | 0 | 1 | 120 |
| 29 | 1D | 30 | 1E | 0 | 29 | 400125 | 3200996 | 0 | 29 | 0 | 112 | 3262756 | 0 | 29 | 0 | 1 | 120 |
| 30 | 1E | 30 | 1E | 1 | 0 | 400137 | 3201092 | 1 | 0 | 0 | 96 | 3262860 | 1 | 0 | 0 | 1 | 104 |
| 31 | 1F | 30 | 1E | 1 | 1 | 400154 | 3201228 | 1 | 1 | 0 | 136 | 3263004 | 1 | 1 | 0 | 1 | 144 |
| 32 | 20 | 30 | 1E | 1 | 2 | 400175 | 3201396 | 1 | 2 | 0 | 168 | 3263180 | 1 | 2 | 0 | 1 | 176 |
| 33 | 21 | 30 | 1E | 1 | 3 | 400196 | 3201564 | 1 | 3 | 0 | 168 | 3263356 | 1 | 3 | 0 | 1 | 176 |
| 34 | 22 | 30 | 1E | 1 | 4 | 400217 | 3201732 | 1 | 4 | 0 | 168 | 3263532 | 1 | 4 | 0 | 1 | 176 |
| 35 | 23 | 30 | 1E | 1 | 5 | 400238 | 3201900 | 1 | 5 | 0 | 168 | 3263708 | 1 | 5 | 0 | 1 | 176 |
| 36 | 24 | 30 | 1E | 1 | 6 | 400259 | 3202068 | 1 | 6 | 0 | 168 | 3263884 | 1 | 6 | 0 | 1 | 176 |
| 37 | 25 | 30 | 1E | 1 | 7 | 400280 | 3202236 | 1 | 7 | 0 | 168 | 3264060 | 1 | 7 | 0 | 1 | 176 |
| 38 | 26 | 30 | 1E | 1 | 8 | 400301 | 3202404 | 1 | 8 | 0 | 168 | 3264236 | 1 | 8 | 0 | 1 | 176 |
| 39 | 27 | 30 | 1E | 1 | 9 | 400322 | 3202572 | 1 | 9 | 0 | 168 | 3264412 | 1 | 9 | 0 | 1 | 176 |
| 40 | 28 | 30 | 1E | 1 | 10 | 400343 | 3202740 | 1 | 10 | 0 | 168 | 3264588 | 1 | 10 | 0 | 1 | 176 |
| 41 | 29 | 30 | 1E | 1 | 11 | 400364 | 3202908 | 1 | 11 | 0 | 168 | 3264764 | 1 | 11 | 0 | 1 | 176 |

| | | | | | | | | | | | | | | | | | |
|---|---|---|---|---|---|---|---|---|---|---|---|---|---|---|---|---|---|
| 42 | 2A | 30 | 1E | 1 | 12 | 400385 | 3203076 | 1 | 12 | 0 | 168 | 3264940 | 1 | 12 | 0 | 1 | 176 |
| 43 | 2B | 30 | 1E | 1 | 13 | 400406 | 3203244 | 1 | 13 | 0 | 168 | 3265116 | 1 | 13 | 0 | 1 | 176 |
| 44 | 2C | 30 | 1E | 1 | 14 | 400427 | 3203412 | 1 | 14 | 0 | 168 | 3265292 | 1 | 14 | 0 | 1 | 176 |
| 45 | 2D | 30 | 1E | 1 | 15 | 400448 | 3203580 | 1 | 15 | 0 | 168 | 3265468 | 1 | 15 | 0 | 1 | 176 |
| 46 | 2E | 30 | 1E | 1 | 16 | 400469 | 3203748 | 1 | 16 | 0 | 168 | 3265644 | 1 | 16 | 0 | 1 | 176 |
| 47 | 2F | 30 | 1E | 1 | 17 | 400490 | 3203916 | 1 | 17 | 0 | 168 | 3265820 | 1 | 17 | 0 | 1 | 176 |
| 48 | 30 | 30 | 1E | 1 | 18 | 400513 | 3204100 | 1 | 18 | 0 | 184 | 3266012 | 1 | 18 | 0 | 1 | 192 |
| 49 | 31 | 30 | 1E | 1 | 19 | 400536 | 3204284 | 1 | 19 | 0 | 184 | 3266204 | 1 | 19 | 0 | 1 | 192 |
| 50 | 32 | 30 | 1E | 1 | 20 | 400559 | 3204468 | 1 | 20 | 0 | 184 | 3266396 | 1 | 20 | 0 | 1 | 192 |
| 51 | 33 | 30 | 1E | 1 | 21 | 400582 | 3204652 | 1 | 21 | 0 | 184 | 3266588 | 1 | 21 | 0 | 1 | 192 |
| 52 | 34 | 30 | 1E | 1 | 22 | 400605 | 3204836 | 1 | 22 | 0 | 184 | 3266780 | 1 | 22 | 0 | 1 | 192 |
| 53 | 35 | 30 | 1E | 1 | 23 | 400628 | 3205020 | 1 | 23 | 0 | 184 | 3266972 | 1 | 23 | 0 | 1 | 192 |
| 54 | 36 | 30 | 1E | 1 | 24 | 400651 | 3205204 | 1 | 24 | 0 | 184 | 3267164 | 1 | 24 | 0 | 1 | 192 |
| 55 | 37 | 30 | 1E | 1 | 25 | 400674 | 3205388 | 1 | 25 | 0 | 184 | 3267356 | 1 | 25 | 0 | 1 | 192 |
| 56 | 38 | 30 | 1E | 1 | 26 | 400697 | 3205572 | 1 | 26 | 0 | 184 | 3267548 | 1 | 26 | 0 | 1 | 192 |
| 57 | 39 | 30 | 1E | 1 | 27 | 400720 | 3205756 | 1 | 27 | 0 | 184 | 3267740 | 1 | 27 | 0 | 1 | 192 |
| 58 | 3A | 30 | 1E | 1 | 28 | 400743 | 3205940 | 1 | 28 | 0 | 184 | 3267932 | 1 | 28 | 0 | 1 | 192 |
| 59 | 3B | 30 | 1E | 1 | 29 | 400766 | 3206124 | 1 | 29 | 0 | 184 | 3268124 | 1 | 29 | 0 | 1 | 192 |
| 60 | 3C | 30 | 1E | 2 | 0 | 400787 | 3206292 | 2 | 0 | 0 | 168 | 3268300 | 2 | 0 | 0 | 1 | 176 |
| 61 | 3D | 30 | 1E | 2 | 1 | 400813 | 3206500 | 2 | 1 | 0 | 208 | 3268516 | 2 | 1 | 0 | 1 | 216 |
| 62 | 3E | 30 | 1E | 2 | 2 | 400839 | 3206708 | 2 | 2 | 0 | 208 | 3268732 | 2 | 2 | 0 | 1 | 216 |
| 63 | 3F | 30 | 1E | 2 | 3 | 400865 | 3206916 | 2 | 3 | 0 | 208 | 3268948 | 2 | 3 | 0 | 1 | 216 |
| 64 | 40 | 30 | 1E | 2 | 4 | 400888 | 3207100 | 2 | 4 | 0 | 184 | 3269140 | 2 | 4 | 0 | 1 | 192 |
| 65 | 41 | 30 | 1E | 2 | 5 | 400911 | 3207284 | 2 | 5 | 0 | 184 | 3269332 | 2 | 5 | 0 | 1 | 192 |
| 66 | 42 | 30 | 1E | 2 | 6 | 400934 | 3207468 | 2 | 6 | 0 | 184 | 3269524 | 2 | 6 | 0 | 1 | 192 |
| 67 | 43 | 30 | 1E | 2 | 7 | 400957 | 3207652 | 2 | 7 | 0 | 184 | 3269716 | 2 | 7 | 0 | 1 | 192 |
| 68 | 44 | 30 | 1E | 2 | 8 | 400980 | 3207836 | 2 | 8 | 0 | 184 | 3269908 | 2 | 8 | 0 | 1 | 192 |
| 69 | 45 | 30 | 1E | 2 | 9 | 401003 | 3208020 | 2 | 9 | 0 | 184 | 3270100 | 2 | 9 | 0 | 1 | 192 |
| 70 | 46 | 30 | 1E | 2 | 10 | 401026 | 3208204 | 2 | 10 | 0 | 184 | 3270292 | 2 | 10 | 0 | 1 | 192 |
| 71 | 47 | 30 | 1E | 2 | 11 | 401049 | 3208388 | 2 | 11 | 0 | 184 | 3270484 | 2 | 11 | 0 | 1 | 192 |
| 72 | 48 | 30 | 1E | 2 | 12 | 401072 | 3208572 | 2 | 12 | 0 | 184 | 3270676 | 2 | 12 | 0 | 1 | 192 |
| 73 | 49 | 30 | 1E | 2 | 13 | 401095 | 3208756 | 2 | 13 | 0 | 184 | 3270868 | 2 | 13 | 0 | 1 | 192 |
| 74 | 4A | 30 | 1E | 2 | 14 | 401118 | 3208940 | 2 | 14 | 0 | 184 | 3271060 | 2 | 14 | 0 | 1 | 192 |
| 75 | 4B | 30 | 1E | 2 | 15 | 401141 | 3209124 | 2 | 15 | 0 | 184 | 3271252 | 2 | 15 | 0 | 1 | 192 |
| 76 | 4C | 30 | 1E | 2 | 16 | 401164 | 3209308 | 2 | 16 | 0 | 184 | 3271444 | 2 | 16 | 0 | 1 | 192 |
| 77 | 4D | 30 | 1E | 2 | 17 | 401187 | 3209492 | 2 | 17 | 0 | 184 | 3271636 | 2 | 17 | 0 | 1 | 192 |
| 78 | 4E | 30 | 1E | 2 | 18 | 401210 | 3209676 | 2 | 18 | 0 | 184 | 3271828 | 2 | 18 | 0 | 1 | 192 |
| 79 | 4F | 30 | 1E | 2 | 19 | 401233 | 3209860 | 2 | 19 | 0 | 184 | 3272020 | 2 | 19 | 0 | 1 | 192 |
| 80 | 50 | 30 | 1E | 2 | 20 | 401258 | 3210060 | 2 | 20 | 0 | 200 | 3272228 | 2 | 20 | 0 | 1 | 208 |
| 81 | 51 | 30 | 1E | 2 | 21 | 401283 | 3210260 | 2 | 21 | 0 | 200 | 3272436 | 2 | 21 | 0 | 1 | 208 |
| 82 | 52 | 30 | 1E | 2 | 22 | 401308 | 3210460 | 2 | 22 | 0 | 200 | 3272644 | 2 | 22 | 0 | 1 | 208 |
| 83 | 53 | 30 | 1E | 2 | 23 | 401333 | 3210660 | 2 | 23 | 0 | 200 | 3272852 | 2 | 23 | 0 | 1 | 208 |
| 84 | 54 | 30 | 1E | 2 | 24 | 401358 | 3210860 | 2 | 24 | 0 | 200 | 3273060 | 2 | 24 | 0 | 1 | 208 |
| 85 | 55 | 30 | 1E | 2 | 25 | 401383 | 3211060 | 2 | 25 | 0 | 200 | 3273268 | 2 | 25 | 0 | 1 | 208 |
| 86 | 56 | 30 | 1E | 2 | 26 | 401408 | 3211260 | 2 | 26 | 0 | 200 | 3273476 | 2 | 26 | 0 | 1 | 208 |
| 87 | 57 | 30 | 1E | 2 | 27 | 401433 | 3211460 | 2 | 27 | 0 | 200 | 3273684 | 2 | 27 | 0 | 1 | 208 |
| 88 | 58 | 30 | 1E | 2 | 28 | 401458 | 3211660 | 2 | 28 | 0 | 200 | 3273892 | 2 | 28 | 0 | 1 | 208 |
| 89 | 59 | 30 | 1E | 2 | 29 | 401483 | 3211860 | 2 | 29 | 0 | 200 | 3274100 | 2 | 29 | 0 | 1 | 208 |
| 90 | 5A | 30 | 1E | 3 | 0 | 401506 | 3212044 | 3 | 0 | 0 | 184 | 3274292 | 3 | 0 | 0 | 1 | 192 |
| 91 | 5B | 30 | 1E | 3 | 1 | 401534 | 3212268 | 3 | 1 | 0 | 224 | 3274524 | 3 | 1 | 0 | 1 | 232 |
| 92 | 5C | 30 | 1E | 3 | 2 | 401562 | 3212492 | 3 | 2 | 0 | 224 | 3274756 | 3 | 2 | 0 | 1 | 232 |
| 93 | 5D | 30 | 1E | 3 | 3 | 401590 | 3212716 | 3 | 3 | 0 | 224 | 3274988 | 3 | 3 | 0 | 1 | 232 |
| 94 | 5E | 30 | 1E | 3 | 4 | 401618 | 3212940 | 3 | 4 | 0 | 224 | 3275220 | 3 | 4 | 0 | 1 | 232 |
| 95 | 5F | 30 | 1E | 3 | 5 | 401646 | 3213164 | 3 | 5 | 0 | 224 | 3275452 | 3 | 5 | 0 | 1 | 232 |
| 96 | 60 | 30 | 1E | 3 | 6 | 401671 | 3213364 | 3 | 6 | 0 | 200 | 3275660 | 3 | 6 | 0 | 1 | 208 |
| 97 | 61 | 30 | 1E | 3 | 7 | 401696 | 3213564 | 3 | 7 | 0 | 200 | 3275868 | 3 | 7 | 0 | 1 | 208 |
| 98 | 62 | 30 | 1E | 3 | 8 | 401721 | 3213764 | 3 | 8 | 0 | 200 | 3276076 | 3 | 8 | 0 | 1 | 208 |
| 99 | 63 | 30 | 1E | 3 | 9 | 401746 | 3213964 | 3 | 9 | 0 | 200 | 3276284 | 3 | 9 | 0 | 1 | 208 |
| 100 | 64 | 30 | 1E | 3 | 10 | 401771 | 3214164 | 3 | 10 | 0 | 200 | 3276492 | 3 | 10 | 0 | 1 | 208 |
| 101 | 65 | 30 | 1E | 3 | 11 | 401796 | 3214364 | 3 | 11 | 0 | 200 | 3276700 | 3 | 11 | 0 | 1 | 208 |
| 102 | 66 | 30 | 1E | 3 | 12 | 401821 | 3214564 | 3 | 12 | 0 | 200 | 3276908 | 3 | 12 | 0 | 1 | 208 |
| 103 | 67 | 30 | 1E | 3 | 13 | 401846 | 3214764 | 3 | 13 | 0 | 200 | 3277116 | 3 | 13 | 0 | 1 | 208 |
| 104 | 68 | 30 | 1E | 3 | 14 | 401871 | 3214964 | 3 | 14 | 0 | 200 | 3277324 | 3 | 14 | 0 | 1 | 208 |
| 105 | 69 | 30 | 1E | 3 | 15 | 401896 | 3215164 | 3 | 15 | 0 | 200 | 3277532 | 3 | 15 | 0 | 1 | 208 |
| 106 | 6A | 30 | 1E | 3 | 16 | 401921 | 3215364 | 3 | 16 | 0 | 200 | 3277740 | 3 | 16 | 0 | 1 | 208 |
| 107 | 6B | 30 | 1E | 3 | 17 | 401946 | 3215564 | 3 | 17 | 0 | 200 | 3277948 | 3 | 17 | 0 | 1 | 208 |
| 108 | 6C | 30 | 1E | 3 | 18 | 401971 | 3215764 | 3 | 18 | 0 | 200 | 3278156 | 3 | 18 | 0 | 1 | 208 |
| 109 | 6D | 30 | 1E | 3 | 19 | 401996 | 3215964 | 3 | 19 | 0 | 200 | 3278364 | 3 | 19 | 0 | 1 | 208 |
| 110 | 6E | 30 | 1E | 3 | 20 | 402021 | 3216164 | 3 | 20 | 0 | 200 | 3278572 | 3 | 20 | 0 | 1 | 208 |
| 111 | 6F | 30 | 1E | 3 | 21 | 402046 | 3216364 | 3 | 21 | 0 | 200 | 3278780 | 3 | 21 | 0 | 1 | 208 |
| 112 | 70 | 30 | 1E | 3 | 22 | 402073 | 3216580 | 3 | 22 | 0 | 216 | 3279004 | 3 | 22 | 0 | 1 | 224 |
| 113 | 71 | 30 | 1E | 3 | 23 | 402100 | 3216796 | 3 | 23 | 0 | 216 | 3279228 | 3 | 23 | 0 | 1 | 224 |
| 114 | 72 | 30 | 1E | 3 | 24 | 402127 | 3217012 | 3 | 24 | 0 | 216 | 3279452 | 3 | 24 | 0 | 1 | 224 |
| 115 | 73 | 30 | 1E | 3 | 25 | 402154 | 3217228 | 3 | 25 | 0 | 216 | 3279676 | 3 | 25 | 0 | 1 | 224 |
| 116 | 74 | 30 | 1E | 3 | 26 | 402181 | 3217444 | 3 | 26 | 0 | 216 | 3279900 | 3 | 26 | 0 | 1 | 224 |
| 117 | 75 | 30 | 1E | 3 | 27 | 402208 | 3217660 | 3 | 27 | 0 | 216 | 3280124 | 3 | 27 | 0 | 1 | 224 |
| 118 | 76 | 30 | 1E | 3 | 28 | 402235 | 3217876 | 3 | 28 | 0 | 216 | 3280348 | 3 | 28 | 0 | 1 | 224 |
| 119 | 77 | 30 | 1E | 3 | 29 | 402262 | 3218092 | 3 | 29 | 0 | 216 | 3280572 | 3 | 29 | 0 | 1 | 224 |
| 120 | 78 | 30 | 1E | 4 | 0 | 402287 | 3218292 | 4 | 0 | 0 | 200 | 3280780 | 4 | 0 | 0 | 1 | 208 |
| 121 | 79 | 30 | 1E | 4 | 1 | 402317 | 3218532 | 4 | 1 | 0 | 240 | 3281028 | 4 | 1 | 0 | 1 | 248 |
| 122 | 7A | 30 | 1E | 4 | 2 | 402347 | 3218772 | 4 | 2 | 0 | 240 | 3281276 | 4 | 2 | 0 | 1 | 248 |
| 123 | 7B | 30 | 1E | 4 | 3 | 402377 | 3219012 | 4 | 3 | 0 | 240 | 3281524 | 4 | 3 | 0 | 1 | 248 |
| 124 | 7C | 30 | 1E | 4 | 4 | 402407 | 3219252 | 4 | 4 | 0 | 240 | 3281772 | 4 | 4 | 0 | 1 | 248 |
| 125 | 7D | 30 | 1E | 4 | 5 | 402437 | 3219492 | 4 | 5 | 0 | 240 | 3282020 | 4 | 5 | 0 | 1 | 248 |
| 126 | 7E | 30 | 1E | 4 | 6 | 402467 | 3219732 | 4 | 6 | 0 | 240 | 3282268 | 4 | 6 | 0 | 1 | 248 |
| 127 | 7F | 30 | 1E | 4 | 7 | 402497 | 3219972 | 4 | 7 | 0 | 240 | 3282516 | 4 | 7 | 0 | 1 | 248 |
| 128 | 80 | 30 | 1E | 4 | 8 | 402524 | 3220188 | 4 | 8 | 0 | 216 | 3282740 | 4 | 8 | 0 | 1 | 224 |
| 129 | 81 | 30 | 1E | 4 | 9 | 402551 | 3220404 | 4 | 9 | 0 | 216 | 3282964 | 4 | 9 | 0 | 1 | 224 |
| 130 | 82 | 30 | 1E | 4 | 10 | 402578 | 3220620 | 4 | 10 | 0 | 216 | 3283188 | 4 | 10 | 0 | 1 | 224 |
| 131 | 83 | 30 | 1E | 4 | 11 | 402605 | 3220836 | 4 | 11 | 0 | 216 | 3283412 | 4 | 11 | 0 | 1 | 224 |
| 132 | 84 | 30 | 1E | 4 | 12 | 402632 | 3221052 | 4 | 12 | 0 | 216 | 3283636 | 4 | 12 | 0 | 1 | 224 |
| 133 | 85 | 30 | 1E | 4 | 13 | 402659 | 3221268 | 4 | 13 | 0 | 216 | 3283860 | 4 | 13 | 0 | 1 | 224 |
| 134 | 86 | 30 | 1E | 4 | 14 | 402686 | 3221484 | 4 | 14 | 0 | 216 | 3284084 | 4 | 14 | 0 | 1 | 224 |
| 135 | 87 | 30 | 1E | 4 | 15 | 402713 | 3221700 | 4 | 15 | 0 | 216 | 3284308 | 4 | 15 | 0 | 1 | 224 |
| 136 | 88 | 30 | 1E | 4 | 16 | 402740 | 3221916 | 4 | 16 | 0 | 216 | 3284532 | 4 | 16 | 0 | 1 | 224 |
| 137 | 89 | 30 | 1E | 4 | 17 | 402767 | 3222132 | 4 | 17 | 0 | 216 | 3284756 | 4 | 17 | 0 | 1 | 224 |
| 138 | 8A | 30 | 1E | 4 | 18 | 402794 | 3222348 | 4 | 18 | 0 | 216 | 3284980 | 4 | 18 | 0 | 1 | 224 |
| 139 | 8B | 30 | 1E | 4 | 19 | 402821 | 3222564 | 4 | 19 | 0 | 216 | 3285204 | 4 | 19 | 0 | 1 | 224 |
| 140 | 8C | 30 | 1E | 4 | 20 | 402848 | 3222780 | 4 | 20 | 0 | 216 | 3285428 | 4 | 20 | 0 | 1 | 224 |
| 141 | 8D | 30 | 1E | 4 | 21 | 402875 | 3222996 | 4 | 21 | 0 | 216 | 3285652 | 4 | 21 | 0 | 1 | 224 |
| 142 | 8E | 30 | 1E | 4 | 22 | 402902 | 3223212 | 4 | 22 | 0 | 216 | 3285876 | 4 | 22 | 0 | 1 | 224 |
| 143 | 8F | 30 | 1E | 4 | 23 | 402929 | 3223428 | 4 | 23 | 0 | 216 | 3286100 | 4 | 23 | 0 | 1 | 224 |
| 144 | 90 | 30 | 1E | 4 | 24 | 402958 | 3223660 | 4 | 24 | 0 | 232 | 3286340 | 4 | 24 | 0 | 1 | 240 |

| | | | | | | | | | | | | | | | | | |
|---|---|---|---|---|---|---|---|---|---|---|---|---|---|---|---|---|---|
| 145 | 91 | 30 | 1E | 4 | 25 | 402987 | 3223892 | 4 | 25 | 0 | 232 | 3286580 | 4 | 25 | 0 | 1 | 240 |
| 146 | 92 | 30 | 1E | 4 | 26 | 403016 | 3224124 | 4 | 26 | 0 | 232 | 3286820 | 4 | 26 | 0 | 1 | 240 |
| 147 | 93 | 30 | 1E | 4 | 27 | 403045 | 3224356 | 4 | 27 | 0 | 232 | 3287060 | 4 | 27 | 0 | 1 | 240 |
| 148 | 94 | 30 | 1E | 4 | 28 | 403074 | 3224588 | 4 | 28 | 0 | 232 | 3287300 | 4 | 28 | 0 | 1 | 240 |
| 149 | 95 | 30 | 1E | 4 | 29 | 403103 | 3224820 | 4 | 29 | 0 | 232 | 3287540 | 4 | 29 | 0 | 1 | 240 |
| 150 | 96 | 30 | 1E | 5 | 0 | 403130 | 3225036 | 5 | 0 | 0 | 216 | 3287764 | 5 | 0 | 0 | 1 | 224 |
| 151 | 97 | 30 | 1E | 5 | 1 | 403162 | 3225292 | 5 | 1 | 0 | 256 | 3288028 | 5 | 1 | 0 | 1 | 264 |
| 152 | 98 | 30 | 1E | 5 | 2 | 403194 | 3225548 | 5 | 2 | 0 | 256 | 3288292 | 5 | 2 | 0 | 1 | 264 |
| 153 | 99 | 30 | 1E | 5 | 3 | 403226 | 3225804 | 5 | 3 | 0 | 256 | 3288556 | 5 | 3 | 0 | 1 | 264 |
| 154 | 9A | 30 | 1E | 5 | 4 | 403258 | 3226060 | 5 | 4 | 0 | 256 | 3288820 | 5 | 4 | 0 | 1 | 264 |
| 155 | 9B | 30 | 1E | 5 | 5 | 403290 | 3226316 | 5 | 5 | 0 | 256 | 3289084 | 5 | 5 | 0 | 1 | 264 |
| 156 | 9C | 30 | 1E | 5 | 6 | 403322 | 3226572 | 5 | 6 | 0 | 256 | 3289348 | 5 | 6 | 0 | 1 | 264 |
| 157 | 9D | 30 | 1E | 5 | 7 | 403354 | 3226828 | 5 | 7 | 0 | 256 | 3289612 | 5 | 7 | 0 | 1 | 264 |
| 158 | 9E | 30 | 1E | 5 | 8 | 403386 | 3227084 | 5 | 8 | 0 | 256 | 3289876 | 5 | 8 | 0 | 1 | 264 |
| 159 | 9F | 30 | 1E | 5 | 9 | 403418 | 3227340 | 5 | 9 | 0 | 256 | 3290140 | 5 | 9 | 0 | 1 | 264 |
| 160 | A0 | 30 | 1E | 5 | 10 | 403447 | 3227572 | 5 | 10 | 0 | 232 | 3290380 | 5 | 10 | 0 | 1 | 240 |
| 161 | A1 | 30 | 1E | 5 | 11 | 403476 | 3227804 | 5 | 11 | 0 | 232 | 3290620 | 5 | 11 | 0 | 1 | 240 |
| 162 | A2 | 30 | 1E | 5 | 12 | 403505 | 3228036 | 5 | 12 | 0 | 232 | 3290860 | 5 | 12 | 0 | 1 | 240 |
| 163 | A3 | 30 | 1E | 5 | 13 | 403534 | 3228268 | 5 | 13 | 0 | 232 | 3291100 | 5 | 13 | 0 | 1 | 240 |
| 164 | A4 | 30 | 1E | 5 | 14 | 403563 | 3228500 | 5 | 14 | 0 | 232 | 3291340 | 5 | 14 | 0 | 1 | 240 |
| 165 | A5 | 30 | 1E | 5 | 15 | 403592 | 3228732 | 5 | 15 | 0 | 232 | 3291580 | 5 | 15 | 0 | 1 | 240 |
| 166 | A6 | 30 | 1E | 5 | 16 | 403621 | 3228964 | 5 | 16 | 0 | 232 | 3291820 | 5 | 16 | 0 | 1 | 240 |
| 167 | A7 | 30 | 1E | 5 | 17 | 403650 | 3229196 | 5 | 17 | 0 | 232 | 3292060 | 5 | 17 | 0 | 1 | 240 |
| 168 | A8 | 30 | 1E | 5 | 18 | 403679 | 3229428 | 5 | 18 | 0 | 232 | 3292300 | 5 | 18 | 0 | 1 | 240 |
| 169 | A9 | 30 | 1E | 5 | 19 | 403708 | 3229660 | 5 | 19 | 0 | 232 | 3292540 | 5 | 19 | 0 | 1 | 240 |
| 170 | AA | 30 | 1E | 5 | 20 | 403737 | 3229892 | 5 | 20 | 0 | 232 | 3292780 | 5 | 20 | 0 | 1 | 240 |
| 171 | AB | 30 | 1E | 5 | 21 | 403766 | 3230124 | 5 | 21 | 0 | 232 | 3293020 | 5 | 21 | 0 | 1 | 240 |
| 172 | AC | 30 | 1E | 5 | 22 | 403795 | 3230356 | 5 | 22 | 0 | 232 | 3293260 | 5 | 22 | 0 | 1 | 240 |
| 173 | AD | 30 | 1E | 5 | 23 | 403824 | 3230588 | 5 | 23 | 0 | 232 | 3293500 | 5 | 23 | 0 | 1 | 240 |
| 174 | AE | 30 | 1E | 5 | 24 | 403853 | 3230820 | 5 | 24 | 0 | 232 | 3293740 | 5 | 24 | 0 | 1 | 240 |
| 175 | AF | 30 | 1E | 5 | 25 | 403882 | 3231052 | 5 | 25 | 0 | 232 | 3293980 | 5 | 25 | 0 | 1 | 240 |
| 176 | B0 | 30 | 1E | 5 | 26 | 403913 | 3231300 | 5 | 26 | 0 | 248 | 3294236 | 5 | 26 | 0 | 1 | 256 |
| 177 | B1 | 30 | 1E | 5 | 27 | 403944 | 3231548 | 5 | 27 | 0 | 248 | 3294492 | 5 | 27 | 0 | 1 | 256 |
| 178 | B2 | 30 | 1E | 5 | 28 | 403975 | 3231796 | 5 | 28 | 0 | 248 | 3294748 | 5 | 28 | 0 | 1 | 256 |
| 179 | B3 | 30 | 1E | 5 | 29 | 404006 | 3232044 | 5 | 29 | 0 | 248 | 3295004 | 5 | 29 | 0 | 1 | 256 |
| 180 | B4 | 30 | 1E | 6 | 0 | 404035 | 3232276 | 6 | 0 | 0 | 232 | 3295244 | 6 | 0 | 0 | 1 | 240 |
| 181 | B5 | 30 | 1E | 6 | 1 | 404069 | 3232548 | 6 | 1 | 0 | 272 | 3295524 | 6 | 1 | 0 | 1 | 280 |
| 182 | B6 | 30 | 1E | 6 | 2 | 404103 | 3232820 | 6 | 2 | 0 | 272 | 3295804 | 6 | 2 | 0 | 1 | 280 |
| 183 | B7 | 30 | 1E | 6 | 3 | 404137 | 3233092 | 6 | 3 | 0 | 272 | 3296084 | 6 | 3 | 0 | 1 | 280 |
| 184 | B8 | 30 | 1E | 6 | 4 | 404171 | 3233364 | 6 | 4 | 0 | 272 | 3296364 | 6 | 4 | 0 | 1 | 280 |
| 185 | B9 | 30 | 1E | 6 | 5 | 404205 | 3233636 | 6 | 5 | 0 | 272 | 3296644 | 6 | 5 | 0 | 1 | 280 |
| 186 | BA | 30 | 1E | 6 | 6 | 404239 | 3233908 | 6 | 6 | 0 | 272 | 3296924 | 6 | 6 | 0 | 1 | 280 |
| 187 | BB | 30 | 1E | 6 | 7 | 404273 | 3234180 | 6 | 7 | 0 | 272 | 3297204 | 6 | 7 | 0 | 1 | 280 |
| 188 | BC | 30 | 1E | 6 | 8 | 404307 | 3234452 | 6 | 8 | 0 | 272 | 3297484 | 6 | 8 | 0 | 1 | 280 |
| 189 | BD | 30 | 1E | 6 | 9 | 404341 | 3234724 | 6 | 9 | 0 | 272 | 3297764 | 6 | 9 | 0 | 1 | 280 |
| 190 | BE | 30 | 1E | 6 | 10 | 404375 | 3234996 | 6 | 10 | 0 | 272 | 3298044 | 6 | 10 | 0 | 1 | 280 |
| 191 | BF | 30 | 1E | 6 | 11 | 404409 | 3235268 | 6 | 11 | 0 | 272 | 3298324 | 6 | 11 | 0 | 1 | 280 |
| 192 | C0 | 30 | 1E | 6 | 12 | 404440 | 3235516 | 6 | 12 | 0 | 248 | 3298580 | 6 | 12 | 0 | 1 | 256 |
| 193 | C1 | 30 | 1E | 6 | 13 | 404471 | 3235764 | 6 | 13 | 0 | 248 | 3298836 | 6 | 13 | 0 | 1 | 256 |
| 194 | C2 | 30 | 1E | 6 | 14 | 404502 | 3236012 | 6 | 14 | 0 | 248 | 3299092 | 6 | 14 | 0 | 1 | 256 |
| 195 | C3 | 30 | 1E | 6 | 15 | 404533 | 3236260 | 6 | 15 | 0 | 248 | 3299348 | 6 | 15 | 0 | 1 | 256 |
| 196 | C4 | 30 | 1E | 6 | 16 | 404564 | 3236508 | 6 | 16 | 0 | 248 | 3299604 | 6 | 16 | 0 | 1 | 256 |
| 197 | C5 | 30 | 1E | 6 | 17 | 404595 | 3236756 | 6 | 17 | 0 | 248 | 3299860 | 6 | 17 | 0 | 1 | 256 |
| 198 | C6 | 30 | 1E | 6 | 18 | 404626 | 3237004 | 6 | 18 | 0 | 248 | 3300116 | 6 | 18 | 0 | 1 | 256 |
| 199 | C7 | 30 | 1E | 6 | 19 | 404657 | 3237252 | 6 | 19 | 0 | 248 | 3300372 | 6 | 19 | 0 | 1 | 256 |
| 200 | C8 | 30 | 1E | 6 | 20 | 404688 | 3237500 | 6 | 20 | 0 | 248 | 3300628 | 6 | 20 | 0 | 1 | 256 |
| 201 | C9 | 30 | 1E | 6 | 21 | 404719 | 3237748 | 6 | 21 | 0 | 248 | 3300884 | 6 | 21 | 0 | 1 | 256 |
| 202 | CA | 30 | 1E | 6 | 22 | 404750 | 3237996 | 6 | 22 | 0 | 248 | 3301140 | 6 | 22 | 0 | 1 | 256 |
| 203 | CB | 30 | 1E | 6 | 23 | 404781 | 3238244 | 6 | 23 | 0 | 248 | 3301396 | 6 | 23 | 0 | 1 | 256 |
| 204 | CC | 30 | 1E | 6 | 24 | 404812 | 3238492 | 6 | 24 | 0 | 248 | 3301652 | 6 | 24 | 0 | 1 | 256 |
| 205 | CD | 30 | 1E | 6 | 25 | 404843 | 3238740 | 6 | 25 | 0 | 248 | 3301908 | 6 | 25 | 0 | 1 | 256 |
| 206 | CE | 30 | 1E | 6 | 26 | 404874 | 3238988 | 6 | 26 | 0 | 248 | 3302164 | 6 | 26 | 0 | 1 | 256 |
| 207 | CF | 30 | 1E | 6 | 27 | 404905 | 3239236 | 6 | 27 | 0 | 248 | 3302420 | 6 | 27 | 0 | 1 | 256 |
| 208 | D0 | 30 | 1E | 6 | 28 | 404938 | 3239500 | 6 | 28 | 0 | 264 | 3302692 | 6 | 28 | 0 | 1 | 272 |
| 209 | D1 | 30 | 1E | 6 | 29 | 404971 | 3239764 | 6 | 29 | 0 | 264 | 3302964 | 6 | 29 | 0 | 1 | 272 |
| 210 | D2 | 30 | 1E | 7 | 0 | 405002 | 3240012 | 7 | 0 | 0 | 248 | 3303220 | 7 | 0 | 0 | 1 | 256 |
| 211 | D3 | 30 | 1E | 7 | 1 | 405038 | 3240300 | 7 | 1 | 0 | 288 | 3303516 | 7 | 1 | 0 | 1 | 296 |
| 212 | D4 | 30 | 1E | 7 | 2 | 405074 | 3240588 | 7 | 2 | 0 | 288 | 3303812 | 7 | 2 | 0 | 1 | 296 |
| 213 | D5 | 30 | 1E | 7 | 3 | 405110 | 3240876 | 7 | 3 | 0 | 288 | 3304108 | 7 | 3 | 0 | 1 | 296 |
| 214 | D6 | 30 | 1E | 7 | 4 | 405146 | 3241164 | 7 | 4 | 0 | 288 | 3304404 | 7 | 4 | 0 | 1 | 296 |
| 215 | D7 | 30 | 1E | 7 | 5 | 405182 | 3241452 | 7 | 5 | 0 | 288 | 3304700 | 7 | 5 | 0 | 1 | 296 |
| 216 | D8 | 30 | 1E | 7 | 6 | 405218 | 3241740 | 7 | 6 | 0 | 288 | 3304996 | 7 | 6 | 0 | 1 | 296 |
| 217 | D9 | 30 | 1E | 7 | 7 | 405254 | 3242028 | 7 | 7 | 0 | 288 | 3305292 | 7 | 7 | 0 | 1 | 296 |
| 218 | DA | 30 | 1E | 7 | 8 | 405290 | 3242316 | 7 | 8 | 0 | 288 | 3305588 | 7 | 8 | 0 | 1 | 296 |
| 219 | DB | 30 | 1E | 7 | 9 | 405326 | 3242604 | 7 | 9 | 0 | 288 | 3305884 | 7 | 9 | 0 | 1 | 296 |
| 220 | DC | 30 | 1E | 7 | 10 | 405362 | 3242892 | 7 | 10 | 0 | 288 | 3306180 | 7 | 10 | 0 | 1 | 296 |
| 221 | DD | 30 | 1E | 7 | 11 | 405398 | 3243180 | 7 | 11 | 0 | 288 | 3306476 | 7 | 11 | 0 | 1 | 296 |
| 222 | DE | 30 | 1E | 7 | 12 | 405434 | 3243468 | 7 | 12 | 0 | 288 | 3306772 | 7 | 12 | 0 | 1 | 296 |
| 223 | DF | 30 | 1E | 7 | 13 | 405470 | 3243756 | 7 | 13 | 0 | 288 | 3307068 | 7 | 13 | 0 | 1 | 296 |
| 224 | E0 | 30 | 1E | 7 | 14 | 405503 | 3244020 | 7 | 14 | 0 | 264 | 3307340 | 7 | 14 | 0 | 1 | 272 |
| 225 | E1 | 30 | 1E | 7 | 15 | 405536 | 3244284 | 7 | 15 | 0 | 264 | 3307612 | 7 | 15 | 0 | 1 | 272 |
| 226 | E2 | 30 | 1E | 7 | 16 | 405569 | 3244548 | 7 | 16 | 0 | 264 | 3307884 | 7 | 16 | 0 | 1 | 272 |
| 227 | E3 | 30 | 1E | 7 | 17 | 405602 | 3244812 | 7 | 17 | 0 | 264 | 3308156 | 7 | 17 | 0 | 1 | 272 |
| 228 | E4 | 30 | 1E | 7 | 18 | 405635 | 3245076 | 7 | 18 | 0 | 264 | 3308428 | 7 | 18 | 0 | 1 | 272 |
| 229 | E5 | 30 | 1E | 7 | 19 | 405668 | 3245340 | 7 | 19 | 0 | 264 | 3308700 | 7 | 19 | 0 | 1 | 272 |
| 230 | E6 | 30 | 1E | 7 | 20 | 405701 | 3245604 | 7 | 20 | 0 | 264 | 3308972 | 7 | 20 | 0 | 1 | 272 |
| 231 | E7 | 30 | 1E | 7 | 21 | 405734 | 3245868 | 7 | 21 | 0 | 264 | 3309244 | 7 | 21 | 0 | 1 | 272 |
| 232 | E8 | 30 | 1E | 7 | 22 | 405767 | 3246132 | 7 | 22 | 0 | 264 | 3309516 | 7 | 22 | 0 | 1 | 272 |
| 233 | E9 | 30 | 1E | 7 | 23 | 405800 | 3246396 | 7 | 23 | 0 | 264 | 3309788 | 7 | 23 | 0 | 1 | 272 |
| 234 | EA | 30 | 1E | 7 | 24 | 405833 | 3246660 | 7 | 24 | 0 | 264 | 3310060 | 7 | 24 | 0 | 1 | 272 |
| 235 | EB | 30 | 1E | 7 | 25 | 405866 | 3246924 | 7 | 25 | 0 | 264 | 3310332 | 7 | 25 | 0 | 1 | 272 |
| 236 | EC | 30 | 1E | 7 | 26 | 405899 | 3247188 | 7 | 26 | 0 | 264 | 3310604 | 7 | 26 | 0 | 1 | 272 |
| 237 | ED | 30 | 1E | 7 | 27 | 405932 | 3247452 | 7 | 27 | 0 | 264 | 3310876 | 7 | 27 | 0 | 1 | 272 |
| 238 | EE | 30 | 1E | 7 | 28 | 405965 | 3247716 | 7 | 28 | 0 | 264 | 3311148 | 7 | 28 | 0 | 1 | 272 |
| 239 | EF | 30 | 1E | 7 | 29 | 405998 | 3247980 | 7 | 29 | 0 | 264 | 3311420 | 7 | 29 | 0 | 1 | 272 |
| 240 | F0 | 30 | 1E | 8 | 0 | 406031 | 3248244 | 8 | 0 | 0 | 264 | 3311692 | 8 | 0 | 0 | 1 | 272 |
| 241 | F1 | 30 | 1E | 8 | 1 | 406069 | 3248548 | 8 | 1 | 0 | 304 | 3312004 | 8 | 1 | 0 | 1 | 312 |
| 242 | F2 | 30 | 1E | 8 | 2 | 406107 | 3248852 | 8 | 2 | 0 | 304 | 3312316 | 8 | 2 | 0 | 1 | 312 |
| 243 | F3 | 30 | 1E | 8 | 3 | 406145 | 3249156 | 8 | 3 | 0 | 304 | 3312628 | 8 | 3 | 0 | 1 | 312 |
| 244 | F4 | 30 | 1E | 8 | 4 | 406183 | 3249460 | 8 | 4 | 0 | 304 | 3312940 | 8 | 4 | 0 | 1 | 312 |
| 245 | F5 | 30 | 1E | 8 | 5 | 406221 | 3249764 | 8 | 5 | 0 | 304 | 3313252 | 8 | 5 | 0 | 1 | 312 |
| 246 | F6 | 30 | 1E | 8 | 6 | 406259 | 3250068 | 8 | 6 | 0 | 304 | 3313564 | 8 | 6 | 0 | 1 | 312 |
| 247 | F7 | 30 | 1E | 8 | 7 | 406297 | 3250372 | 8 | 7 | 0 | 304 | 3313876 | 8 | 7 | 0 | 1 | 312 |

| | | | | | | | | | | | | | | | | | |
|---|---|---|---|---|---|---|---|---|---|---|---|---|---|---|---|---|---|
| 248 | F8 | 30 | 1E | 8 | 8 | 406335 | 3250676 | 8 | 8 | 0 | 304 | 3314188 | 8 | 8 | 0 | 1 | 312 |
| 249 | F9 | 30 | 1E | 8 | 9 | 406373 | 3250980 | 8 | 9 | 0 | 304 | 3314500 | 8 | 9 | 0 | 1 | 312 |
| 250 | FA | 30 | 1E | 8 | 10 | 406411 | 3251284 | 8 | 10 | 0 | 304 | 3314812 | 8 | 10 | 0 | 1 | 312 |
| 251 | FB | 30 | 1E | 8 | 11 | 406449 | 3251588 | 8 | 11 | 0 | 304 | 3315124 | 8 | 11 | 0 | 1 | 312 |
| 252 | FC | 30 | 1E | 8 | 12 | 406487 | 3251892 | 8 | 12 | 0 | 304 | 3315436 | 8 | 12 | 0 | 1 | 312 |
| 253 | FD | 30 | 1E | 8 | 13 | 406525 | 3252196 | 8 | 13 | 0 | 304 | 3315748 | 8 | 13 | 0 | 1 | 312 |
| 254 | FE | 30 | 1E | 8 | 14 | 406563 | 3252500 | 8 | 14 | 0 | 304 | 3316060 | 8 | 14 | 0 | 1 | 312 |
| 255 | FF | 30 | 1E | 8 | 15 | 406601 | 3252804 | 8 | 15 | 0 | 304 | 3316372 | 8 | 15 | 0 | 1 | 312 |
| 0 | 00 | 31 | 1F | 0 | 0 | 406613 | 3252900 | 0 | 0 | 0 | 96 | 3316476 | 0 | 0 | 0 | 1 | 104 |
| 1 | 01 | 31 | 1F | 0 | 1 | 406627 | 3253012 | 0 | 1 | 0 | 112 | 3316596 | 0 | 1 | 0 | 1 | 120 |
| 2 | 02 | 31 | 1F | 0 | 2 | 406641 | 3253124 | 0 | 2 | 0 | 112 | 3316716 | 0 | 2 | 0 | 1 | 120 |
| 3 | 03 | 31 | 1F | 0 | 3 | 406655 | 3253236 | 0 | 3 | 0 | 112 | 3316836 | 0 | 3 | 0 | 1 | 120 |
| 4 | 04 | 31 | 1F | 0 | 4 | 406669 | 3253348 | 0 | 4 | 0 | 112 | 3316956 | 0 | 4 | 0 | 1 | 120 |
| 5 | 05 | 31 | 1F | 0 | 5 | 406683 | 3253460 | 0 | 5 | 0 | 112 | 3317076 | 0 | 5 | 0 | 1 | 120 |
| 6 | 06 | 31 | 1F | 0 | 6 | 406697 | 3253572 | 0 | 6 | 0 | 112 | 3317196 | 0 | 6 | 0 | 1 | 120 |
| 7 | 07 | 31 | 1F | 0 | 7 | 406711 | 3253684 | 0 | 7 | 0 | 112 | 3317316 | 0 | 7 | 0 | 1 | 120 |
| 8 | 08 | 31 | 1F | 0 | 8 | 406725 | 3253796 | 0 | 8 | 0 | 112 | 3317436 | 0 | 8 | 0 | 1 | 120 |
| 9 | 09 | 31 | 1F | 0 | 9 | 406739 | 3253908 | 0 | 9 | 0 | 112 | 3317556 | 0 | 9 | 0 | 1 | 120 |
| 10 | 0A | 31 | 1F | 0 | 10 | 406753 | 3254020 | 0 | 10 | 0 | 112 | 3317676 | 0 | 10 | 0 | 1 | 120 |
| 11 | 0B | 31 | 1F | 0 | 11 | 406767 | 3254132 | 0 | 11 | 0 | 112 | 3317796 | 0 | 11 | 0 | 1 | 120 |
| 12 | 0C | 31 | 1F | 0 | 12 | 406781 | 3254244 | 0 | 12 | 0 | 112 | 3317916 | 0 | 12 | 0 | 1 | 120 |
| 13 | 0D | 31 | 1F | 0 | 13 | 406795 | 3254356 | 0 | 13 | 0 | 112 | 3318036 | 0 | 13 | 0 | 1 | 120 |
| 14 | 0E | 31 | 1F | 0 | 14 | 406809 | 3254468 | 0 | 14 | 0 | 112 | 3318156 | 0 | 14 | 0 | 1 | 120 |
| 15 | 0F | 31 | 1F | 0 | 15 | 406823 | 3254580 | 0 | 15 | 0 | 112 | 3318276 | 0 | 15 | 0 | 1 | 120 |
| 16 | 10 | 31 | 1F | 0 | 16 | 406837 | 3254692 | 0 | 16 | 0 | 112 | 3318396 | 0 | 16 | 0 | 1 | 120 |
| 17 | 11 | 31 | 1F | 0 | 17 | 406851 | 3254804 | 0 | 17 | 0 | 112 | 3318516 | 0 | 17 | 0 | 1 | 120 |
| 18 | 12 | 31 | 1F | 0 | 18 | 406865 | 3254916 | 0 | 18 | 0 | 112 | 3318636 | 0 | 18 | 0 | 1 | 120 |
| 19 | 13 | 31 | 1F | 0 | 19 | 406879 | 3255028 | 0 | 19 | 0 | 112 | 3318756 | 0 | 19 | 0 | 1 | 120 |
| 20 | 14 | 31 | 1F | 0 | 20 | 406893 | 3255140 | 0 | 20 | 0 | 112 | 3318876 | 0 | 20 | 0 | 1 | 120 |
| 21 | 15 | 31 | 1F | 0 | 21 | 406907 | 3255252 | 0 | 21 | 0 | 112 | 3318996 | 0 | 21 | 0 | 1 | 120 |
| 22 | 16 | 31 | 1F | 0 | 22 | 406921 | 3255364 | 0 | 22 | 0 | 112 | 3319116 | 0 | 22 | 0 | 1 | 120 |
| 23 | 17 | 31 | 1F | 0 | 23 | 406935 | 3255476 | 0 | 23 | 0 | 112 | 3319236 | 0 | 23 | 0 | 1 | 120 |
| 24 | 18 | 31 | 1F | 0 | 24 | 406949 | 3255588 | 0 | 24 | 0 | 112 | 3319356 | 0 | 24 | 0 | 1 | 120 |
| 25 | 19 | 31 | 1F | 0 | 25 | 406963 | 3255700 | 0 | 25 | 0 | 112 | 3319476 | 0 | 25 | 0 | 1 | 120 |
| 26 | 1A | 31 | 1F | 0 | 26 | 406977 | 3255812 | 0 | 26 | 0 | 112 | 3319596 | 0 | 26 | 0 | 1 | 120 |
| 27 | 1B | 31 | 1F | 0 | 27 | 406991 | 3255924 | 0 | 27 | 0 | 112 | 3319716 | 0 | 27 | 0 | 1 | 120 |
| 28 | 1C | 31 | 1F | 0 | 28 | 407005 | 3256036 | 0 | 28 | 0 | 112 | 3319836 | 0 | 28 | 0 | 1 | 120 |
| 29 | 1D | 31 | 1F | 0 | 29 | 407019 | 3256148 | 0 | 29 | 0 | 112 | 3319956 | 0 | 29 | 0 | 1 | 120 |
| 30 | 1E | 31 | 1F | 0 | 30 | 407033 | 3256260 | 0 | 30 | 0 | 112 | 3320076 | 0 | 30 | 0 | 1 | 120 |
| 31 | 1F | 31 | 1F | 1 | 0 | 407045 | 3256356 | 1 | 0 | 0 | 96 | 3320180 | 1 | 0 | 0 | 1 | 104 |
| 32 | 20 | 31 | 1F | 1 | 1 | 407066 | 3256524 | 1 | 1 | 0 | 168 | 3320356 | 1 | 1 | 0 | 1 | 176 |
| 33 | 21 | 31 | 1F | 1 | 2 | 407087 | 3256692 | 1 | 2 | 0 | 168 | 3320532 | 1 | 2 | 0 | 1 | 176 |
| 34 | 22 | 31 | 1F | 1 | 3 | 407108 | 3256860 | 1 | 3 | 0 | 168 | 3320708 | 1 | 3 | 0 | 1 | 176 |
| 35 | 23 | 31 | 1F | 1 | 4 | 407129 | 3257028 | 1 | 4 | 0 | 168 | 3320884 | 1 | 4 | 0 | 1 | 176 |
| 36 | 24 | 31 | 1F | 1 | 5 | 407150 | 3257196 | 1 | 5 | 0 | 168 | 3321060 | 1 | 5 | 0 | 1 | 176 |
| 37 | 25 | 31 | 1F | 1 | 6 | 407171 | 3257364 | 1 | 6 | 0 | 168 | 3321236 | 1 | 6 | 0 | 1 | 176 |
| 38 | 26 | 31 | 1F | 1 | 7 | 407192 | 3257532 | 1 | 7 | 0 | 168 | 3321412 | 1 | 7 | 0 | 1 | 176 |
| 39 | 27 | 31 | 1F | 1 | 8 | 407213 | 3257700 | 1 | 8 | 0 | 168 | 3321588 | 1 | 8 | 0 | 1 | 176 |
| 40 | 28 | 31 | 1F | 1 | 9 | 407234 | 3257868 | 1 | 9 | 0 | 168 | 3321764 | 1 | 9 | 0 | 1 | 176 |
| 41 | 29 | 31 | 1F | 1 | 10 | 407255 | 3258036 | 1 | 10 | 0 | 168 | 3321940 | 1 | 10 | 0 | 1 | 176 |
| 42 | 2A | 31 | 1F | 1 | 11 | 407276 | 3258204 | 1 | 11 | 0 | 168 | 3322116 | 1 | 11 | 0 | 1 | 176 |
| 43 | 2B | 31 | 1F | 1 | 12 | 407297 | 3258372 | 1 | 12 | 0 | 168 | 3322292 | 1 | 12 | 0 | 1 | 176 |
| 44 | 2C | 31 | 1F | 1 | 13 | 407318 | 3258540 | 1 | 13 | 0 | 168 | 3322468 | 1 | 13 | 0 | 1 | 176 |
| 45 | 2D | 31 | 1F | 1 | 14 | 407339 | 3258708 | 1 | 14 | 0 | 168 | 3322644 | 1 | 14 | 0 | 1 | 176 |
| 46 | 2E | 31 | 1F | 1 | 15 | 407360 | 3258876 | 1 | 15 | 0 | 168 | 3322820 | 1 | 15 | 0 | 1 | 176 |
| 47 | 2F | 31 | 1F | 1 | 16 | 407381 | 3259044 | 1 | 16 | 0 | 168 | 3322996 | 1 | 16 | 0 | 1 | 176 |
| 48 | 30 | 31 | 1F | 1 | 17 | 407404 | 3259228 | 1 | 17 | 0 | 184 | 3323188 | 1 | 17 | 0 | 1 | 192 |
| 49 | 31 | 31 | 1F | 1 | 18 | 407427 | 3259412 | 1 | 18 | 0 | 184 | 3323380 | 1 | 18 | 0 | 1 | 192 |
| 50 | 32 | 31 | 1F | 1 | 19 | 407450 | 3259596 | 1 | 19 | 0 | 184 | 3323572 | 1 | 19 | 0 | 1 | 192 |
| 51 | 33 | 31 | 1F | 1 | 20 | 407473 | 3259780 | 1 | 20 | 0 | 184 | 3323764 | 1 | 20 | 0 | 1 | 192 |
| 52 | 34 | 31 | 1F | 1 | 21 | 407496 | 3259964 | 1 | 21 | 0 | 184 | 3323956 | 1 | 21 | 0 | 1 | 192 |
| 53 | 35 | 31 | 1F | 1 | 22 | 407519 | 3260148 | 1 | 22 | 0 | 184 | 3324148 | 1 | 22 | 0 | 1 | 192 |
| 54 | 36 | 31 | 1F | 1 | 23 | 407542 | 3260332 | 1 | 23 | 0 | 184 | 3324340 | 1 | 23 | 0 | 1 | 192 |
| 55 | 37 | 31 | 1F | 1 | 24 | 407565 | 3260516 | 1 | 24 | 0 | 184 | 3324532 | 1 | 24 | 0 | 1 | 192 |
| 56 | 38 | 31 | 1F | 1 | 25 | 407588 | 3260700 | 1 | 25 | 0 | 184 | 3324724 | 1 | 25 | 0 | 1 | 192 |
| 57 | 39 | 31 | 1F | 1 | 26 | 407611 | 3260884 | 1 | 26 | 0 | 184 | 3324916 | 1 | 26 | 0 | 1 | 192 |
| 58 | 3A | 31 | 1F | 1 | 27 | 407634 | 3261068 | 1 | 27 | 0 | 184 | 3325108 | 1 | 27 | 0 | 1 | 192 |
| 59 | 3B | 31 | 1F | 1 | 28 | 407657 | 3261252 | 1 | 28 | 0 | 184 | 3325300 | 1 | 28 | 0 | 1 | 192 |
| 60 | 3C | 31 | 1F | 1 | 29 | 407680 | 3261436 | 1 | 29 | 0 | 184 | 3325492 | 1 | 29 | 0 | 1 | 192 |
| 61 | 3D | 31 | 1F | 1 | 30 | 407703 | 3261620 | 1 | 30 | 0 | 184 | 3325684 | 1 | 30 | 0 | 1 | 192 |
| 62 | 3E | 31 | 1F | 2 | 0 | 407724 | 3261788 | 2 | 0 | 0 | 168 | 3325860 | 2 | 0 | 0 | 1 | 176 |
| 63 | 3F | 31 | 1F | 2 | 1 | 407750 | 3261996 | 2 | 1 | 0 | 208 | 3326076 | 2 | 1 | 0 | 1 | 216 |
| 64 | 40 | 31 | 1F | 2 | 2 | 407773 | 3262180 | 2 | 2 | 0 | 184 | 3326268 | 2 | 2 | 0 | 1 | 192 |
| 65 | 41 | 31 | 1F | 2 | 3 | 407796 | 3262364 | 2 | 3 | 0 | 184 | 3326460 | 2 | 3 | 0 | 1 | 192 |
| 66 | 42 | 31 | 1F | 2 | 4 | 407819 | 3262548 | 2 | 4 | 0 | 184 | 3326652 | 2 | 4 | 0 | 1 | 192 |
| 67 | 43 | 31 | 1F | 2 | 5 | 407842 | 3262732 | 2 | 5 | 0 | 184 | 3326844 | 2 | 5 | 0 | 1 | 192 |
| 68 | 44 | 31 | 1F | 2 | 6 | 407865 | 3262916 | 2 | 6 | 0 | 184 | 3327036 | 2 | 6 | 0 | 1 | 192 |
| 69 | 45 | 31 | 1F | 2 | 7 | 407888 | 3263100 | 2 | 7 | 0 | 184 | 3327228 | 2 | 7 | 0 | 1 | 192 |
| 70 | 46 | 31 | 1F | 2 | 8 | 407911 | 3263284 | 2 | 8 | 0 | 184 | 3327420 | 2 | 8 | 0 | 1 | 192 |
| 71 | 47 | 31 | 1F | 2 | 9 | 407934 | 3263468 | 2 | 9 | 0 | 184 | 3327612 | 2 | 9 | 0 | 1 | 192 |
| 72 | 48 | 31 | 1F | 2 | 10 | 407957 | 3263652 | 2 | 10 | 0 | 184 | 3327804 | 2 | 10 | 0 | 1 | 192 |
| 73 | 49 | 31 | 1F | 2 | 11 | 407980 | 3263836 | 2 | 11 | 0 | 184 | 3327996 | 2 | 11 | 0 | 1 | 192 |
| 74 | 4A | 31 | 1F | 2 | 12 | 408003 | 3264020 | 2 | 12 | 0 | 184 | 3328188 | 2 | 12 | 0 | 1 | 192 |
| 75 | 4B | 31 | 1F | 2 | 13 | 408026 | 3264204 | 2 | 13 | 0 | 184 | 3328380 | 2 | 13 | 0 | 1 | 192 |
| 76 | 4C | 31 | 1F | 2 | 14 | 408049 | 3264388 | 2 | 14 | 0 | 184 | 3328572 | 2 | 14 | 0 | 1 | 192 |
| 77 | 4D | 31 | 1F | 2 | 15 | 408072 | 3264572 | 2 | 15 | 0 | 184 | 3328764 | 2 | 15 | 0 | 1 | 192 |
| 78 | 4E | 31 | 1F | 2 | 16 | 408095 | 3264756 | 2 | 16 | 0 | 184 | 3328956 | 2 | 16 | 0 | 1 | 192 |
| 79 | 4F | 31 | 1F | 2 | 17 | 408118 | 3264940 | 2 | 17 | 0 | 184 | 3329148 | 2 | 17 | 0 | 1 | 192 |
| 80 | 50 | 31 | 1F | 2 | 18 | 408143 | 3265140 | 2 | 18 | 0 | 200 | 3329356 | 2 | 18 | 0 | 1 | 208 |
| 81 | 51 | 31 | 1F | 2 | 19 | 408168 | 3265340 | 2 | 19 | 0 | 200 | 3329564 | 2 | 19 | 0 | 1 | 208 |
| 82 | 52 | 31 | 1F | 2 | 20 | 408193 | 3265540 | 2 | 20 | 0 | 200 | 3329772 | 2 | 20 | 0 | 1 | 208 |
| 83 | 53 | 31 | 1F | 2 | 21 | 408218 | 3265740 | 2 | 21 | 0 | 200 | 3329980 | 2 | 21 | 0 | 1 | 208 |
| 84 | 54 | 31 | 1F | 2 | 22 | 408243 | 3265940 | 2 | 22 | 0 | 200 | 3330188 | 2 | 22 | 0 | 1 | 208 |
| 85 | 55 | 31 | 1F | 2 | 23 | 408268 | 3266140 | 2 | 23 | 0 | 200 | 3330396 | 2 | 23 | 0 | 1 | 208 |
| 86 | 56 | 31 | 1F | 2 | 24 | 408293 | 3266340 | 2 | 24 | 0 | 200 | 3330604 | 2 | 24 | 0 | 1 | 208 |
| 87 | 57 | 31 | 1F | 2 | 25 | 408318 | 3266540 | 2 | 25 | 0 | 200 | 3330812 | 2 | 25 | 0 | 1 | 208 |
| 88 | 58 | 31 | 1F | 2 | 26 | 408343 | 3266740 | 2 | 26 | 0 | 200 | 3331020 | 2 | 26 | 0 | 1 | 208 |
| 89 | 59 | 31 | 1F | 2 | 27 | 408368 | 3266940 | 2 | 27 | 0 | 200 | 3331228 | 2 | 27 | 0 | 1 | 208 |
| 90 | 5A | 31 | 1F | 2 | 28 | 408393 | 3267140 | 2 | 28 | 0 | 200 | 3331436 | 2 | 28 | 0 | 1 | 208 |
| 91 | 5B | 31 | 1F | 2 | 29 | 408418 | 3267340 | 2 | 29 | 0 | 200 | 3331644 | 2 | 29 | 0 | 1 | 208 |
| 92 | 5C | 31 | 1F | 2 | 30 | 408443 | 3267540 | 2 | 30 | 0 | 200 | 3331852 | 2 | 30 | 0 | 1 | 208 |
| 93 | 5D | 31 | 1F | 3 | 0 | 408466 | 3267724 | 3 | 0 | 0 | 184 | 3332044 | 3 | 0 | 0 | 1 | 192 |
| 94 | 5E | 31 | 1F | 3 | 1 | 408494 | 3267948 | 3 | 1 | 0 | 224 | 3332276 | 3 | 1 | 0 | 1 | 232 |

| | | | | | | | | | | | | | | | | | |
|---|---|---|---|---|---|---|---|---|---|---|---|---|---|---|---|---|---|
| 95 | 5F | 31 | 1F | 3 | 2 | 408522 | 3268172 | 3 | 2 | 0 | 224 | 3332508 | 3 | 2 | 0 | 1 | 232 |
| 96 | 60 | 31 | 1F | 3 | 3 | 408547 | 3268372 | 3 | 3 | 0 | 200 | 3332716 | 3 | 3 | 0 | 1 | 208 |
| 97 | 61 | 31 | 1F | 3 | 4 | 408572 | 3268572 | 3 | 4 | 0 | 200 | 3332924 | 3 | 4 | 0 | 1 | 208 |
| 98 | 62 | 31 | 1F | 3 | 5 | 408597 | 3268772 | 3 | 5 | 0 | 200 | 3333132 | 3 | 5 | 0 | 1 | 208 |
| 99 | 63 | 31 | 1F | 3 | 6 | 408622 | 3268972 | 3 | 6 | 0 | 200 | 3333340 | 3 | 6 | 0 | 1 | 208 |
| 100 | 64 | 31 | 1F | 3 | 7 | 408647 | 3269172 | 3 | 7 | 0 | 200 | 3333548 | 3 | 7 | 0 | 1 | 208 |
| 101 | 65 | 31 | 1F | 3 | 8 | 408672 | 3269372 | 3 | 8 | 0 | 200 | 3333756 | 3 | 8 | 0 | 1 | 208 |
| 102 | 66 | 31 | 1F | 3 | 9 | 408697 | 3269572 | 3 | 9 | 0 | 200 | 3333964 | 3 | 9 | 0 | 1 | 208 |
| 103 | 67 | 31 | 1F | 3 | 10 | 408722 | 3269772 | 3 | 10 | 0 | 200 | 3334172 | 3 | 10 | 0 | 1 | 208 |
| 104 | 68 | 31 | 1F | 3 | 11 | 408747 | 3269972 | 3 | 11 | 0 | 200 | 3334380 | 3 | 11 | 0 | 1 | 208 |
| 105 | 69 | 31 | 1F | 3 | 12 | 408772 | 3270172 | 3 | 12 | 0 | 200 | 3334588 | 3 | 12 | 0 | 1 | 208 |
| 106 | 6A | 31 | 1F | 3 | 13 | 408797 | 3270372 | 3 | 13 | 0 | 200 | 3334796 | 3 | 13 | 0 | 1 | 208 |
| 107 | 6B | 31 | 1F | 3 | 14 | 408822 | 3270572 | 3 | 14 | 0 | 200 | 3335004 | 3 | 14 | 0 | 1 | 208 |
| 108 | 6C | 31 | 1F | 3 | 15 | 408847 | 3270772 | 3 | 15 | 0 | 200 | 3335212 | 3 | 15 | 0 | 1 | 208 |
| 109 | 6D | 31 | 1F | 3 | 16 | 408872 | 3270972 | 3 | 16 | 0 | 200 | 3335420 | 3 | 16 | 0 | 1 | 208 |
| 110 | 6E | 31 | 1F | 3 | 17 | 408897 | 3271172 | 3 | 17 | 0 | 200 | 3335628 | 3 | 17 | 0 | 1 | 208 |
| 111 | 6F | 31 | 1F | 3 | 18 | 408922 | 3271372 | 3 | 18 | 0 | 200 | 3335836 | 3 | 18 | 0 | 1 | 208 |
| 112 | 70 | 31 | 1F | 3 | 19 | 408949 | 3271588 | 3 | 19 | 0 | 216 | 3336060 | 3 | 19 | 0 | 1 | 224 |
| 113 | 71 | 31 | 1F | 3 | 20 | 408976 | 3271804 | 3 | 20 | 0 | 216 | 3336284 | 3 | 20 | 0 | 1 | 224 |
| 114 | 72 | 31 | 1F | 3 | 21 | 409003 | 3272020 | 3 | 21 | 0 | 216 | 3336508 | 3 | 21 | 0 | 1 | 224 |
| 115 | 73 | 31 | 1F | 3 | 22 | 409030 | 3272236 | 3 | 22 | 0 | 216 | 3336732 | 3 | 22 | 0 | 1 | 224 |
| 116 | 74 | 31 | 1F | 3 | 23 | 409057 | 3272452 | 3 | 23 | 0 | 216 | 3336956 | 3 | 23 | 0 | 1 | 224 |
| 117 | 75 | 31 | 1F | 3 | 24 | 409084 | 3272668 | 3 | 24 | 0 | 216 | 3337180 | 3 | 24 | 0 | 1 | 224 |
| 118 | 76 | 31 | 1F | 3 | 25 | 409111 | 3272884 | 3 | 25 | 0 | 216 | 3337404 | 3 | 25 | 0 | 1 | 224 |
| 119 | 77 | 31 | 1F | 3 | 26 | 409138 | 3273100 | 3 | 26 | 0 | 216 | 3337628 | 3 | 26 | 0 | 1 | 224 |
| 120 | 78 | 31 | 1F | 3 | 27 | 409165 | 3273316 | 3 | 27 | 0 | 216 | 3337852 | 3 | 27 | 0 | 1 | 224 |
| 121 | 79 | 31 | 1F | 3 | 28 | 409192 | 3273532 | 3 | 28 | 0 | 216 | 3338076 | 3 | 28 | 0 | 1 | 224 |
| 122 | 7A | 31 | 1F | 3 | 29 | 409219 | 3273748 | 3 | 29 | 0 | 216 | 3338300 | 3 | 29 | 0 | 1 | 224 |
| 123 | 7B | 31 | 1F | 3 | 30 | 409246 | 3273964 | 3 | 30 | 0 | 216 | 3338524 | 3 | 30 | 0 | 1 | 224 |
| 124 | 7C | 31 | 1F | 4 | 0 | 409271 | 3274164 | 4 | 0 | 0 | 200 | 3338732 | 4 | 0 | 0 | 1 | 208 |
| 125 | 7D | 31 | 1F | 4 | 1 | 409301 | 3274404 | 4 | 1 | 0 | 240 | 3338980 | 4 | 1 | 0 | 1 | 248 |
| 126 | 7E | 31 | 1F | 4 | 2 | 409331 | 3274644 | 4 | 2 | 0 | 240 | 3339228 | 4 | 2 | 0 | 1 | 248 |
| 127 | 7F | 31 | 1F | 4 | 3 | 409361 | 3274884 | 4 | 3 | 0 | 240 | 3339476 | 4 | 3 | 0 | 1 | 248 |
| 128 | 80 | 31 | 1F | 4 | 4 | 409388 | 3275100 | 4 | 4 | 0 | 216 | 3339700 | 4 | 4 | 0 | 1 | 224 |
| 129 | 81 | 31 | 1F | 4 | 5 | 409415 | 3275316 | 4 | 5 | 0 | 216 | 3339924 | 4 | 5 | 0 | 1 | 224 |
| 130 | 82 | 31 | 1F | 4 | 6 | 409442 | 3275532 | 4 | 6 | 0 | 216 | 3340148 | 4 | 6 | 0 | 1 | 224 |
| 131 | 83 | 31 | 1F | 4 | 7 | 409469 | 3275748 | 4 | 7 | 0 | 216 | 3340372 | 4 | 7 | 0 | 1 | 224 |
| 132 | 84 | 31 | 1F | 4 | 8 | 409496 | 3275964 | 4 | 8 | 0 | 216 | 3340596 | 4 | 8 | 0 | 1 | 224 |
| 133 | 85 | 31 | 1F | 4 | 9 | 409523 | 3276180 | 4 | 9 | 0 | 216 | 3340820 | 4 | 9 | 0 | 1 | 224 |
| 134 | 86 | 31 | 1F | 4 | 10 | 409550 | 3276396 | 4 | 10 | 0 | 216 | 3341044 | 4 | 10 | 0 | 1 | 224 |
| 135 | 87 | 31 | 1F | 4 | 11 | 409577 | 3276612 | 4 | 11 | 0 | 216 | 3341268 | 4 | 11 | 0 | 1 | 224 |
| 136 | 88 | 31 | 1F | 4 | 12 | 409604 | 3276828 | 4 | 12 | 0 | 216 | 3341492 | 4 | 12 | 0 | 1 | 224 |
| 137 | 89 | 31 | 1F | 4 | 13 | 409631 | 3277044 | 4 | 13 | 0 | 216 | 3341716 | 4 | 13 | 0 | 1 | 224 |
| 138 | 8A | 31 | 1F | 4 | 14 | 409658 | 3277260 | 4 | 14 | 0 | 216 | 3341940 | 4 | 14 | 0 | 1 | 224 |
| 139 | 8B | 31 | 1F | 4 | 15 | 409685 | 3277476 | 4 | 15 | 0 | 216 | 3342164 | 4 | 15 | 0 | 1 | 224 |
| 140 | 8C | 31 | 1F | 4 | 16 | 409712 | 3277692 | 4 | 16 | 0 | 216 | 3342388 | 4 | 16 | 0 | 1 | 224 |
| 141 | 8D | 31 | 1F | 4 | 17 | 409739 | 3277908 | 4 | 17 | 0 | 216 | 3342612 | 4 | 17 | 0 | 1 | 224 |
| 142 | 8E | 31 | 1F | 4 | 18 | 409766 | 3278124 | 4 | 18 | 0 | 216 | 3342836 | 4 | 18 | 0 | 1 | 224 |
| 143 | 8F | 31 | 1F | 4 | 19 | 409793 | 3278340 | 4 | 19 | 0 | 216 | 3343060 | 4 | 19 | 0 | 1 | 224 |
| 144 | 90 | 31 | 1F | 4 | 20 | 409822 | 3278572 | 4 | 20 | 0 | 232 | 3343300 | 4 | 20 | 0 | 1 | 240 |
| 145 | 91 | 31 | 1F | 4 | 21 | 409851 | 3278804 | 4 | 21 | 0 | 232 | 3343540 | 4 | 21 | 0 | 1 | 240 |
| 146 | 92 | 31 | 1F | 4 | 22 | 409880 | 3279036 | 4 | 22 | 0 | 232 | 3343780 | 4 | 22 | 0 | 1 | 240 |
| 147 | 93 | 31 | 1F | 4 | 23 | 409909 | 3279268 | 4 | 23 | 0 | 232 | 3344020 | 4 | 23 | 0 | 1 | 240 |
| 148 | 94 | 31 | 1F | 4 | 24 | 409938 | 3279500 | 4 | 24 | 0 | 232 | 3344260 | 4 | 24 | 0 | 1 | 240 |
| 149 | 95 | 31 | 1F | 4 | 25 | 409967 | 3279732 | 4 | 25 | 0 | 232 | 3344500 | 4 | 25 | 0 | 1 | 240 |
| 150 | 96 | 31 | 1F | 4 | 26 | 409996 | 3279964 | 4 | 26 | 0 | 232 | 3344740 | 4 | 26 | 0 | 1 | 240 |
| 151 | 97 | 31 | 1F | 4 | 27 | 410025 | 3280196 | 4 | 27 | 0 | 232 | 3344980 | 4 | 27 | 0 | 1 | 240 |
| 152 | 98 | 31 | 1F | 4 | 28 | 410054 | 3280428 | 4 | 28 | 0 | 232 | 3345220 | 4 | 28 | 0 | 1 | 240 |
| 153 | 99 | 31 | 1F | 4 | 29 | 410083 | 3280660 | 4 | 29 | 0 | 232 | 3345460 | 4 | 29 | 0 | 1 | 240 |
| 154 | 9A | 31 | 1F | 4 | 30 | 410112 | 3280892 | 4 | 30 | 0 | 232 | 3345700 | 4 | 30 | 0 | 1 | 240 |
| 155 | 9B | 31 | 1F | 5 | 0 | 410139 | 3281108 | 5 | 0 | 0 | 216 | 3345924 | 5 | 0 | 0 | 1 | 224 |
| 156 | 9C | 31 | 1F | 5 | 1 | 410171 | 3281364 | 5 | 1 | 0 | 256 | 3346188 | 5 | 1 | 0 | 1 | 264 |
| 157 | 9D | 31 | 1F | 5 | 2 | 410203 | 3281620 | 5 | 2 | 0 | 256 | 3346452 | 5 | 2 | 0 | 1 | 264 |
| 158 | 9E | 31 | 1F | 5 | 3 | 410235 | 3281876 | 5 | 3 | 0 | 256 | 3346716 | 5 | 3 | 0 | 1 | 264 |
| 159 | 9F | 31 | 1F | 5 | 4 | 410267 | 3282132 | 5 | 4 | 0 | 256 | 3346980 | 5 | 4 | 0 | 1 | 264 |
| 160 | A0 | 31 | 1F | 5 | 5 | 410296 | 3282364 | 5 | 5 | 0 | 232 | 3347220 | 5 | 5 | 0 | 1 | 240 |
| 161 | A1 | 31 | 1F | 5 | 6 | 410325 | 3282596 | 5 | 6 | 0 | 232 | 3347460 | 5 | 6 | 0 | 1 | 240 |
| 162 | A2 | 31 | 1F | 5 | 7 | 410354 | 3282828 | 5 | 7 | 0 | 232 | 3347700 | 5 | 7 | 0 | 1 | 240 |
| 163 | A3 | 31 | 1F | 5 | 8 | 410383 | 3283060 | 5 | 8 | 0 | 232 | 3347940 | 5 | 8 | 0 | 1 | 240 |
| 164 | A4 | 31 | 1F | 5 | 9 | 410412 | 3283292 | 5 | 9 | 0 | 232 | 3348180 | 5 | 9 | 0 | 1 | 240 |
| 165 | A5 | 31 | 1F | 5 | 10 | 410441 | 3283524 | 5 | 10 | 0 | 232 | 3348420 | 5 | 10 | 0 | 1 | 240 |
| 166 | A6 | 31 | 1F | 5 | 11 | 410470 | 3283756 | 5 | 11 | 0 | 232 | 3348660 | 5 | 11 | 0 | 1 | 240 |
| 167 | A7 | 31 | 1F | 5 | 12 | 410499 | 3283988 | 5 | 12 | 0 | 232 | 3348900 | 5 | 12 | 0 | 1 | 240 |
| 168 | A8 | 31 | 1F | 5 | 13 | 410528 | 3284220 | 5 | 13 | 0 | 232 | 3349140 | 5 | 13 | 0 | 1 | 240 |
| 169 | A9 | 31 | 1F | 5 | 14 | 410557 | 3284452 | 5 | 14 | 0 | 232 | 3349380 | 5 | 14 | 0 | 1 | 240 |
| 170 | AA | 31 | 1F | 5 | 15 | 410586 | 3284684 | 5 | 15 | 0 | 232 | 3349620 | 5 | 15 | 0 | 1 | 240 |
| 171 | AB | 31 | 1F | 5 | 16 | 410615 | 3284916 | 5 | 16 | 0 | 232 | 3349860 | 5 | 16 | 0 | 1 | 240 |
| 172 | AC | 31 | 1F | 5 | 17 | 410644 | 3285148 | 5 | 17 | 0 | 232 | 3350100 | 5 | 17 | 0 | 1 | 240 |
| 173 | AD | 31 | 1F | 5 | 18 | 410673 | 3285380 | 5 | 18 | 0 | 232 | 3350340 | 5 | 18 | 0 | 1 | 240 |
| 174 | AE | 31 | 1F | 5 | 19 | 410702 | 3285612 | 5 | 19 | 0 | 232 | 3350580 | 5 | 19 | 0 | 1 | 240 |
| 175 | AF | 31 | 1F | 5 | 20 | 410731 | 3285844 | 5 | 20 | 0 | 232 | 3350820 | 5 | 20 | 0 | 1 | 240 |
| 176 | B0 | 31 | 1F | 5 | 21 | 410762 | 3286092 | 5 | 21 | 0 | 248 | 3351076 | 5 | 21 | 0 | 1 | 256 |
| 177 | B1 | 31 | 1F | 5 | 22 | 410793 | 3286340 | 5 | 22 | 0 | 248 | 3351332 | 5 | 22 | 0 | 1 | 256 |
| 178 | B2 | 31 | 1F | 5 | 23 | 410824 | 3286588 | 5 | 23 | 0 | 248 | 3351588 | 5 | 23 | 0 | 1 | 256 |
| 179 | B3 | 31 | 1F | 5 | 24 | 410855 | 3286836 | 5 | 24 | 0 | 248 | 3351844 | 5 | 24 | 0 | 1 | 256 |
| 180 | B4 | 31 | 1F | 5 | 25 | 410886 | 3287084 | 5 | 25 | 0 | 248 | 3352100 | 5 | 25 | 0 | 1 | 256 |
| 181 | B5 | 31 | 1F | 5 | 26 | 410917 | 3287332 | 5 | 26 | 0 | 248 | 3352356 | 5 | 26 | 0 | 1 | 256 |
| 182 | B6 | 31 | 1F | 5 | 27 | 410948 | 3287580 | 5 | 27 | 0 | 248 | 3352612 | 5 | 27 | 0 | 1 | 256 |
| 183 | B7 | 31 | 1F | 5 | 28 | 410979 | 3287828 | 5 | 28 | 0 | 248 | 3352868 | 5 | 28 | 0 | 1 | 256 |
| 184 | B8 | 31 | 1F | 5 | 29 | 411010 | 3288076 | 5 | 29 | 0 | 248 | 3353124 | 5 | 29 | 0 | 1 | 256 |
| 185 | B9 | 31 | 1F | 5 | 30 | 411041 | 3288324 | 5 | 30 | 0 | 248 | 3353380 | 5 | 30 | 0 | 1 | 256 |
| 186 | BA | 31 | 1F | 6 | 0 | 411070 | 3288556 | 6 | 0 | 0 | 232 | 3353620 | 6 | 0 | 0 | 1 | 240 |
| 187 | BB | 31 | 1F | 6 | 1 | 411104 | 3288828 | 6 | 1 | 0 | 272 | 3353900 | 6 | 1 | 0 | 1 | 280 |
| 188 | BC | 31 | 1F | 6 | 2 | 411138 | 3289100 | 6 | 2 | 0 | 272 | 3354180 | 6 | 2 | 0 | 1 | 280 |
| 189 | BD | 31 | 1F | 6 | 3 | 411172 | 3289372 | 6 | 3 | 0 | 272 | 3354460 | 6 | 3 | 0 | 1 | 280 |
| 190 | BE | 31 | 1F | 6 | 4 | 411206 | 3289644 | 6 | 4 | 0 | 272 | 3354740 | 6 | 4 | 0 | 1 | 280 |
| 191 | BF | 31 | 1F | 6 | 5 | 411240 | 3289916 | 6 | 5 | 0 | 272 | 3355020 | 6 | 5 | 0 | 1 | 280 |
| 192 | C0 | 31 | 1F | 6 | 6 | 411271 | 3290164 | 6 | 6 | 0 | 248 | 3355276 | 6 | 6 | 0 | 1 | 256 |
| 193 | C1 | 31 | 1F | 6 | 7 | 411302 | 3290412 | 6 | 7 | 0 | 248 | 3355532 | 6 | 7 | 0 | 1 | 256 |
| 194 | C2 | 31 | 1F | 6 | 8 | 411333 | 3290660 | 6 | 8 | 0 | 248 | 3355788 | 6 | 8 | 0 | 1 | 256 |
| 195 | C3 | 31 | 1F | 6 | 9 | 411364 | 3290908 | 6 | 9 | 0 | 248 | 3356044 | 6 | 9 | 0 | 1 | 256 |
| 196 | C4 | 31 | 1F | 6 | 10 | 411395 | 3291156 | 6 | 10 | 0 | 248 | 3356300 | 6 | 10 | 0 | 1 | 256 |
| 197 | C5 | 31 | 1F | 6 | 11 | 411426 | 3291404 | 6 | 11 | 0 | 248 | 3356556 | 6 | 11 | 0 | 1 | 256 |

| | | | | | | | | | | | | | | | | |
|---|---|---|---|---|---|---|---|---|---|---|---|---|---|---|---|---|
| 198 | C6 | 31 | 1F | 6 | 12 | 411457 | 3291652 | 6 | 12 | 0 | 248 | 3356812 | 6 | 12 | 0 | 1 | 256 |
| 199 | C7 | 31 | 1F | 6 | 13 | 411488 | 3291900 | 6 | 13 | 0 | 248 | 3357068 | 6 | 13 | 0 | 1 | 256 |
| 200 | C8 | 31 | 1F | 6 | 14 | 411519 | 3292148 | 6 | 14 | 0 | 248 | 3357324 | 6 | 14 | 0 | 1 | 256 |
| 201 | C9 | 31 | 1F | 6 | 15 | 411550 | 3292396 | 6 | 15 | 0 | 248 | 3357580 | 6 | 15 | 0 | 1 | 256 |
| 202 | CA | 31 | 1F | 6 | 16 | 411581 | 3292644 | 6 | 16 | 0 | 248 | 3357836 | 6 | 16 | 0 | 1 | 256 |
| 203 | CB | 31 | 1F | 6 | 17 | 411612 | 3292892 | 6 | 17 | 0 | 248 | 3358092 | 6 | 17 | 0 | 1 | 256 |
| 204 | CC | 31 | 1F | 6 | 18 | 411643 | 3293140 | 6 | 18 | 0 | 248 | 3358348 | 6 | 18 | 0 | 1 | 256 |
| 205 | CD | 31 | 1F | 6 | 19 | 411674 | 3293388 | 6 | 19 | 0 | 248 | 3358604 | 6 | 19 | 0 | 1 | 256 |
| 206 | CE | 31 | 1F | 6 | 20 | 411705 | 3293636 | 6 | 20 | 0 | 248 | 3358860 | 6 | 20 | 0 | 1 | 256 |
| 207 | CF | 31 | 1F | 6 | 21 | 411736 | 3293884 | 6 | 21 | 0 | 248 | 3359116 | 6 | 21 | 0 | 1 | 256 |
| 208 | D0 | 31 | 1F | 6 | 22 | 411769 | 3294148 | 6 | 22 | 0 | 264 | 3359388 | 6 | 22 | 0 | 1 | 272 |
| 209 | D1 | 31 | 1F | 6 | 23 | 411802 | 3294412 | 6 | 23 | 0 | 264 | 3359660 | 6 | 23 | 0 | 1 | 272 |
| 210 | D2 | 31 | 1F | 6 | 24 | 411835 | 3294676 | 6 | 24 | 0 | 264 | 3359932 | 6 | 24 | 0 | 1 | 272 |
| 211 | D3 | 31 | 1F | 6 | 25 | 411868 | 3294940 | 6 | 25 | 0 | 264 | 3360204 | 6 | 25 | 0 | 1 | 272 |
| 212 | D4 | 31 | 1F | 6 | 26 | 411901 | 3295204 | 6 | 26 | 0 | 264 | 3360476 | 6 | 26 | 0 | 1 | 272 |
| 213 | D5 | 31 | 1F | 6 | 27 | 411934 | 3295468 | 6 | 27 | 0 | 264 | 3360748 | 6 | 27 | 0 | 1 | 272 |
| 214 | D6 | 31 | 1F | 6 | 28 | 411967 | 3295732 | 6 | 28 | 0 | 264 | 3361020 | 6 | 28 | 0 | 1 | 272 |
| 215 | D7 | 31 | 1F | 6 | 29 | 412000 | 3295996 | 6 | 29 | 0 | 264 | 3361292 | 6 | 29 | 0 | 1 | 272 |
| 216 | D8 | 31 | 1F | 6 | 30 | 412033 | 3296260 | 6 | 30 | 0 | 264 | 3361564 | 6 | 30 | 0 | 1 | 272 |
| 217 | D9 | 31 | 1F | 7 | 0 | 412064 | 3296508 | 7 | 0 | 0 | 248 | 3361820 | 7 | 0 | 0 | 1 | 256 |
| 218 | DA | 31 | 1F | 7 | 1 | 412100 | 3296796 | 7 | 1 | 0 | 288 | 3362116 | 7 | 1 | 0 | 1 | 296 |
| 219 | DB | 31 | 1F | 7 | 2 | 412136 | 3297084 | 7 | 2 | 0 | 288 | 3362412 | 7 | 2 | 0 | 1 | 296 |
| 220 | DC | 31 | 1F | 7 | 3 | 412172 | 3297372 | 7 | 3 | 0 | 288 | 3362708 | 7 | 3 | 0 | 1 | 296 |
| 221 | DD | 31 | 1F | 7 | 4 | 412208 | 3297660 | 7 | 4 | 0 | 288 | 3363004 | 7 | 4 | 0 | 1 | 296 |
| 222 | DE | 31 | 1F | 7 | 5 | 412244 | 3297948 | 7 | 5 | 0 | 288 | 3363300 | 7 | 5 | 0 | 1 | 296 |
| 223 | DF | 31 | 1F | 7 | 6 | 412280 | 3298236 | 7 | 6 | 0 | 288 | 3363596 | 7 | 6 | 0 | 1 | 296 |
| 224 | E0 | 31 | 1F | 7 | 7 | 412313 | 3298500 | 7 | 7 | 0 | 264 | 3363868 | 7 | 7 | 0 | 1 | 272 |
| 225 | E1 | 31 | 1F | 7 | 8 | 412346 | 3298764 | 7 | 8 | 0 | 264 | 3364140 | 7 | 8 | 0 | 1 | 272 |
| 226 | E2 | 31 | 1F | 7 | 9 | 412379 | 3299028 | 7 | 9 | 0 | 264 | 3364412 | 7 | 9 | 0 | 1 | 272 |
| 227 | E3 | 31 | 1F | 7 | 10 | 412412 | 3299292 | 7 | 10 | 0 | 264 | 3364684 | 7 | 10 | 0 | 1 | 272 |
| 228 | E4 | 31 | 1F | 7 | 11 | 412445 | 3299556 | 7 | 11 | 0 | 264 | 3364956 | 7 | 11 | 0 | 1 | 272 |
| 229 | E5 | 31 | 1F | 7 | 12 | 412478 | 3299820 | 7 | 12 | 0 | 264 | 3365228 | 7 | 12 | 0 | 1 | 272 |
| 230 | E6 | 31 | 1F | 7 | 13 | 412511 | 3300084 | 7 | 13 | 0 | 264 | 3365500 | 7 | 13 | 0 | 1 | 272 |
| 231 | E7 | 31 | 1F | 7 | 14 | 412544 | 3300348 | 7 | 14 | 0 | 264 | 3365772 | 7 | 14 | 0 | 1 | 272 |
| 232 | E8 | 31 | 1F | 7 | 15 | 412577 | 3300612 | 7 | 15 | 0 | 264 | 3366044 | 7 | 15 | 0 | 1 | 272 |
| 233 | E9 | 31 | 1F | 7 | 16 | 412610 | 3300876 | 7 | 16 | 0 | 264 | 3366316 | 7 | 16 | 0 | 1 | 272 |
| 234 | EA | 31 | 1F | 7 | 17 | 412643 | 3301140 | 7 | 17 | 0 | 264 | 3366588 | 7 | 17 | 0 | 1 | 272 |
| 235 | EB | 31 | 1F | 7 | 18 | 412676 | 3301404 | 7 | 18 | 0 | 264 | 3366860 | 7 | 18 | 0 | 1 | 272 |
| 236 | EC | 31 | 1F | 7 | 19 | 412709 | 3301668 | 7 | 19 | 0 | 264 | 3367132 | 7 | 19 | 0 | 1 | 272 |
| 237 | ED | 31 | 1F | 7 | 20 | 412742 | 3301932 | 7 | 20 | 0 | 264 | 3367404 | 7 | 20 | 0 | 1 | 272 |
| 238 | EE | 31 | 1F | 7 | 21 | 412775 | 3302196 | 7 | 21 | 0 | 264 | 3367676 | 7 | 21 | 0 | 1 | 272 |
| 239 | EF | 31 | 1F | 7 | 22 | 412808 | 3302460 | 7 | 22 | 0 | 264 | 3367948 | 7 | 22 | 0 | 1 | 272 |
| 240 | F0 | 31 | 1F | 7 | 23 | 412843 | 3302740 | 7 | 23 | 0 | 280 | 3368236 | 7 | 23 | 0 | 1 | 288 |
| 241 | F1 | 31 | 1F | 7 | 24 | 412878 | 3303020 | 7 | 24 | 0 | 280 | 3368524 | 7 | 24 | 0 | 1 | 288 |
| 242 | F2 | 31 | 1F | 7 | 25 | 412913 | 3303300 | 7 | 25 | 0 | 280 | 3368812 | 7 | 25 | 0 | 1 | 288 |
| 243 | F3 | 31 | 1F | 7 | 26 | 412948 | 3303580 | 7 | 26 | 0 | 280 | 3369100 | 7 | 26 | 0 | 1 | 288 |
| 244 | F4 | 31 | 1F | 7 | 27 | 412983 | 3303860 | 7 | 27 | 0 | 280 | 3369388 | 7 | 27 | 0 | 1 | 288 |
| 245 | F5 | 31 | 1F | 7 | 28 | 413018 | 3304140 | 7 | 28 | 0 | 280 | 3369676 | 7 | 28 | 0 | 1 | 288 |
| 246 | F6 | 31 | 1F | 7 | 29 | 413053 | 3304420 | 7 | 29 | 0 | 280 | 3369964 | 7 | 29 | 0 | 1 | 288 |
| 247 | F7 | 31 | 1F | 7 | 30 | 413088 | 3304700 | 7 | 30 | 0 | 280 | 3370252 | 7 | 30 | 0 | 1 | 288 |
| 248 | F8 | 31 | 1F | 8 | 0 | 413121 | 3304964 | 8 | 0 | 0 | 264 | 3370524 | 8 | 0 | 0 | 1 | 272 |
| 249 | F9 | 31 | 1F | 8 | 1 | 413159 | 3305268 | 8 | 1 | 0 | 304 | 3370836 | 8 | 1 | 0 | 1 | 312 |
| 250 | FA | 31 | 1F | 8 | 2 | 413197 | 3305572 | 8 | 2 | 0 | 304 | 3371148 | 8 | 2 | 0 | 1 | 312 |
| 251 | FB | 31 | 1F | 8 | 3 | 413235 | 3305876 | 8 | 3 | 0 | 304 | 3371460 | 8 | 3 | 0 | 1 | 312 |
| 252 | FC | 31 | 1F | 8 | 4 | 413273 | 3306180 | 8 | 4 | 0 | 304 | 3371772 | 8 | 4 | 0 | 1 | 312 |
| 253 | FD | 31 | 1F | 8 | 5 | 413311 | 3306484 | 8 | 5 | 0 | 304 | 3372084 | 8 | 5 | 0 | 1 | 312 |
| 254 | FE | 31 | 1F | 8 | 6 | 413349 | 3306788 | 8 | 6 | 0 | 304 | 3372396 | 8 | 6 | 0 | 1 | 312 |
| 255 | FF | 31 | 1F | 8 | 7 | 413387 | 3307092 | 8 | 7 | 0 | 304 | 3372708 | 8 | 7 | 0 | 1 | 312 |
| 0 | 00 | 32 | 20 | 0 | 0 | 413399 | 3307188 | 0 | 0 | 0 | 96 | 3372812 | 0 | 0 | 0 | 1 | 104 |
| 1 | 01 | 32 | 20 | 0 | 1 | 413413 | 3307300 | 0 | 1 | 0 | 112 | 3372932 | 0 | 1 | 0 | 1 | 120 |
| 2 | 02 | 32 | 20 | 0 | 2 | 413427 | 3307412 | 0 | 2 | 0 | 112 | 3373052 | 0 | 2 | 0 | 1 | 120 |
| 3 | 03 | 32 | 20 | 0 | 3 | 413441 | 3307524 | 0 | 3 | 0 | 112 | 3373172 | 0 | 3 | 0 | 1 | 120 |
| 4 | 04 | 32 | 20 | 0 | 4 | 413455 | 3307636 | 0 | 4 | 0 | 112 | 3373292 | 0 | 4 | 0 | 1 | 120 |
| 5 | 05 | 32 | 20 | 0 | 5 | 413469 | 3307748 | 0 | 5 | 0 | 112 | 3373412 | 0 | 5 | 0 | 1 | 120 |
| 6 | 06 | 32 | 20 | 0 | 6 | 413483 | 3307860 | 0 | 6 | 0 | 112 | 3373532 | 0 | 6 | 0 | 1 | 120 |
| 7 | 07 | 32 | 20 | 0 | 7 | 413497 | 3307972 | 0 | 7 | 0 | 112 | 3373652 | 0 | 7 | 0 | 1 | 120 |
| 8 | 08 | 32 | 20 | 0 | 8 | 413511 | 3308084 | 0 | 8 | 0 | 112 | 3373772 | 0 | 8 | 0 | 1 | 120 |
| 9 | 09 | 32 | 20 | 0 | 9 | 413525 | 3308196 | 0 | 9 | 0 | 112 | 3373892 | 0 | 9 | 0 | 1 | 120 |
| 10 | 0A | 32 | 20 | 0 | 10 | 413539 | 3308308 | 0 | 10 | 0 | 112 | 3374012 | 0 | 10 | 0 | 1 | 120 |
| 11 | 0B | 32 | 20 | 0 | 11 | 413553 | 3308420 | 0 | 11 | 0 | 112 | 3374132 | 0 | 11 | 0 | 1 | 120 |
| 12 | 0C | 32 | 20 | 0 | 12 | 413567 | 3308532 | 0 | 12 | 0 | 112 | 3374252 | 0 | 12 | 0 | 1 | 120 |
| 13 | 0D | 32 | 20 | 0 | 13 | 413581 | 3308644 | 0 | 13 | 0 | 112 | 3374372 | 0 | 13 | 0 | 1 | 120 |
| 14 | 0E | 32 | 20 | 0 | 14 | 413595 | 3308756 | 0 | 14 | 0 | 112 | 3374492 | 0 | 14 | 0 | 1 | 120 |
| 15 | 0F | 32 | 20 | 0 | 15 | 413609 | 3308868 | 0 | 15 | 0 | 112 | 3374612 | 0 | 15 | 0 | 1 | 120 |
| 16 | 10 | 32 | 20 | 0 | 16 | 413623 | 3308980 | 0 | 16 | 0 | 112 | 3374732 | 0 | 16 | 0 | 1 | 120 |
| 17 | 11 | 32 | 20 | 0 | 17 | 413637 | 3309092 | 0 | 17 | 0 | 112 | 3374852 | 0 | 17 | 0 | 1 | 120 |
| 18 | 12 | 32 | 20 | 0 | 18 | 413651 | 3309204 | 0 | 18 | 0 | 112 | 3374972 | 0 | 18 | 0 | 1 | 120 |
| 19 | 13 | 32 | 20 | 0 | 19 | 413665 | 3309316 | 0 | 19 | 0 | 112 | 3375092 | 0 | 19 | 0 | 1 | 120 |
| 20 | 14 | 32 | 20 | 0 | 20 | 413679 | 3309428 | 0 | 20 | 0 | 112 | 3375212 | 0 | 20 | 0 | 1 | 120 |
| 21 | 15 | 32 | 20 | 0 | 21 | 413693 | 3309540 | 0 | 21 | 0 | 112 | 3375332 | 0 | 21 | 0 | 1 | 120 |
| 22 | 16 | 32 | 20 | 0 | 22 | 413707 | 3309652 | 0 | 22 | 0 | 112 | 3375452 | 0 | 22 | 0 | 1 | 120 |
| 23 | 17 | 32 | 20 | 0 | 23 | 413721 | 3309764 | 0 | 23 | 0 | 112 | 3375572 | 0 | 23 | 0 | 1 | 120 |
| 24 | 18 | 32 | 20 | 0 | 24 | 413735 | 3309876 | 0 | 24 | 0 | 112 | 3375692 | 0 | 24 | 0 | 1 | 120 |
| 25 | 19 | 32 | 20 | 0 | 25 | 413749 | 3309988 | 0 | 25 | 0 | 112 | 3375812 | 0 | 25 | 0 | 1 | 120 |
| 26 | 1A | 32 | 20 | 0 | 26 | 413763 | 3310100 | 0 | 26 | 0 | 112 | 3375932 | 0 | 26 | 0 | 1 | 120 |
| 27 | 1B | 32 | 20 | 0 | 27 | 413777 | 3310212 | 0 | 27 | 0 | 112 | 3376052 | 0 | 27 | 0 | 1 | 120 |
| 28 | 1C | 32 | 20 | 0 | 28 | 413791 | 3310324 | 0 | 28 | 0 | 112 | 3376172 | 0 | 28 | 0 | 1 | 120 |
| 29 | 1D | 32 | 20 | 0 | 29 | 413805 | 3310436 | 0 | 29 | 0 | 112 | 3376292 | 0 | 29 | 0 | 1 | 120 |
| 30 | 1E | 32 | 20 | 0 | 30 | 413819 | 3310548 | 0 | 30 | 0 | 112 | 3376412 | 0 | 30 | 0 | 1 | 120 |
| 31 | 1F | 32 | 20 | 0 | 31 | 413833 | 3310660 | 0 | 31 | 0 | 112 | 3376532 | 0 | 31 | 0 | 1 | 120 |
| 32 | 20 | 32 | 20 | 1 | 0 | 413852 | 3310812 | 1 | 0 | 0 | 152 | 3376692 | 1 | 0 | 0 | 1 | 160 |
| 33 | 21 | 32 | 20 | 1 | 1 | 413873 | 3310980 | 1 | 1 | 0 | 168 | 3376868 | 1 | 1 | 0 | 1 | 176 |
| 34 | 22 | 32 | 20 | 1 | 2 | 413894 | 3311148 | 1 | 2 | 0 | 168 | 3377044 | 1 | 2 | 0 | 1 | 176 |
| 35 | 23 | 32 | 20 | 1 | 3 | 413915 | 3311316 | 1 | 3 | 0 | 168 | 3377220 | 1 | 3 | 0 | 1 | 176 |
| 36 | 24 | 32 | 20 | 1 | 4 | 413936 | 3311484 | 1 | 4 | 0 | 168 | 3377396 | 1 | 4 | 0 | 1 | 176 |
| 37 | 25 | 32 | 20 | 1 | 5 | 413957 | 3311652 | 1 | 5 | 0 | 168 | 3377572 | 1 | 5 | 0 | 1 | 176 |
| 38 | 26 | 32 | 20 | 1 | 6 | 413978 | 3311820 | 1 | 6 | 0 | 168 | 3377748 | 1 | 6 | 0 | 1 | 176 |
| 39 | 27 | 32 | 20 | 1 | 7 | 413999 | 3311988 | 1 | 7 | 0 | 168 | 3377924 | 1 | 7 | 0 | 1 | 176 |
| 40 | 28 | 32 | 20 | 1 | 8 | 414020 | 3312156 | 1 | 8 | 0 | 168 | 3378100 | 1 | 8 | 0 | 1 | 176 |
| 41 | 29 | 32 | 20 | 1 | 9 | 414041 | 3312324 | 1 | 9 | 0 | 168 | 3378276 | 1 | 9 | 0 | 1 | 176 |
| 42 | 2A | 32 | 20 | 1 | 10 | 414062 | 3312492 | 1 | 10 | 0 | 168 | 3378452 | 1 | 10 | 0 | 1 | 176 |
| 43 | 2B | 32 | 20 | 1 | 11 | 414083 | 3312660 | 1 | 11 | 0 | 168 | 3378628 | 1 | 11 | 0 | 1 | 176 |
| 44 | 2C | 32 | 20 | 1 | 12 | 414104 | 3312828 | 1 | 12 | 0 | 168 | 3378804 | 1 | 12 | 0 | 1 | 176 |

| | | | | | | | | | | | | | | | | | |
|---|---|---|---|---|---|---|---|---|---|---|---|---|---|---|---|---|---|
| 45 | 2D | 32 | 20 | 1 | 13 | 414125 | 3312996 | 1 | 13 | 0 | 168 | 3378980 | 1 | 13 | 0 | 1 | 176 |
| 46 | 2E | 32 | 20 | 1 | 14 | 414146 | 3313164 | 1 | 14 | 0 | 168 | 3379156 | 1 | 14 | 0 | 1 | 176 |
| 47 | 2F | 32 | 20 | 1 | 15 | 414167 | 3313332 | 1 | 15 | 0 | 168 | 3379332 | 1 | 15 | 0 | 1 | 176 |
| 48 | 30 | 32 | 20 | 1 | 16 | 414190 | 3313516 | 1 | 16 | 0 | 184 | 3379524 | 1 | 16 | 0 | 1 | 192 |
| 49 | 31 | 32 | 20 | 1 | 17 | 414213 | 3313700 | 1 | 17 | 0 | 184 | 3379716 | 1 | 17 | 0 | 1 | 192 |
| 50 | 32 | 32 | 20 | 1 | 18 | 414236 | 3313884 | 1 | 18 | 0 | 184 | 3379908 | 1 | 18 | 0 | 1 | 192 |
| 51 | 33 | 32 | 20 | 1 | 19 | 414259 | 3314068 | 1 | 19 | 0 | 184 | 3380100 | 1 | 19 | 0 | 1 | 192 |
| 52 | 34 | 32 | 20 | 1 | 20 | 414282 | 3314252 | 1 | 20 | 0 | 184 | 3380292 | 1 | 20 | 0 | 1 | 192 |
| 53 | 35 | 32 | 20 | 1 | 21 | 414305 | 3314436 | 1 | 21 | 0 | 184 | 3380484 | 1 | 21 | 0 | 1 | 192 |
| 54 | 36 | 32 | 20 | 1 | 22 | 414328 | 3314620 | 1 | 22 | 0 | 184 | 3380676 | 1 | 22 | 0 | 1 | 192 |
| 55 | 37 | 32 | 20 | 1 | 23 | 414351 | 3314804 | 1 | 23 | 0 | 184 | 3380868 | 1 | 23 | 0 | 1 | 192 |
| 56 | 38 | 32 | 20 | 1 | 24 | 414374 | 3314988 | 1 | 24 | 0 | 184 | 3381060 | 1 | 24 | 0 | 1 | 192 |
| 57 | 39 | 32 | 20 | 1 | 25 | 414397 | 3315172 | 1 | 25 | 0 | 184 | 3381252 | 1 | 25 | 0 | 1 | 192 |
| 58 | 3A | 32 | 20 | 1 | 26 | 414420 | 3315356 | 1 | 26 | 0 | 184 | 3381444 | 1 | 26 | 0 | 1 | 192 |
| 59 | 3B | 32 | 20 | 1 | 27 | 414443 | 3315540 | 1 | 27 | 0 | 184 | 3381636 | 1 | 27 | 0 | 1 | 192 |
| 60 | 3C | 32 | 20 | 1 | 28 | 414466 | 3315724 | 1 | 28 | 0 | 184 | 3381828 | 1 | 28 | 0 | 1 | 192 |
| 61 | 3D | 32 | 20 | 1 | 29 | 414489 | 3315908 | 1 | 29 | 0 | 184 | 3382020 | 1 | 29 | 0 | 1 | 192 |
| 62 | 3E | 32 | 20 | 1 | 30 | 414512 | 3316092 | 1 | 30 | 0 | 184 | 3382212 | 1 | 30 | 0 | 1 | 192 |
| 63 | 3F | 32 | 20 | 1 | 31 | 414535 | 3316276 | 1 | 31 | 0 | 184 | 3382404 | 1 | 31 | 0 | 1 | 192 |
| 64 | 40 | 32 | 20 | 2 | 0 | 414556 | 3316444 | 2 | 0 | 0 | 168 | 3382580 | 2 | 0 | 0 | 1 | 176 |
| 65 | 41 | 32 | 20 | 2 | 1 | 414579 | 3316628 | 2 | 1 | 0 | 184 | 3382772 | 2 | 1 | 0 | 1 | 192 |
| 66 | 42 | 32 | 20 | 2 | 2 | 414602 | 3316812 | 2 | 2 | 0 | 184 | 3382964 | 2 | 2 | 0 | 1 | 192 |
| 67 | 43 | 32 | 20 | 2 | 3 | 414625 | 3316996 | 2 | 3 | 0 | 184 | 3383156 | 2 | 3 | 0 | 1 | 192 |
| 68 | 44 | 32 | 20 | 2 | 4 | 414648 | 3317180 | 2 | 4 | 0 | 184 | 3383348 | 2 | 4 | 0 | 1 | 192 |
| 69 | 45 | 32 | 20 | 2 | 5 | 414671 | 3317364 | 2 | 5 | 0 | 184 | 3383540 | 2 | 5 | 0 | 1 | 192 |
| 70 | 46 | 32 | 20 | 2 | 6 | 414694 | 3317548 | 2 | 6 | 0 | 184 | 3383732 | 2 | 6 | 0 | 1 | 192 |
| 71 | 47 | 32 | 20 | 2 | 7 | 414717 | 3317732 | 2 | 7 | 0 | 184 | 3383924 | 2 | 7 | 0 | 1 | 192 |
| 72 | 48 | 32 | 20 | 2 | 8 | 414740 | 3317916 | 2 | 8 | 0 | 184 | 3384116 | 2 | 8 | 0 | 1 | 192 |
| 73 | 49 | 32 | 20 | 2 | 9 | 414763 | 3318100 | 2 | 9 | 0 | 184 | 3384308 | 2 | 9 | 0 | 1 | 192 |
| 74 | 4A | 32 | 20 | 2 | 10 | 414786 | 3318284 | 2 | 10 | 0 | 184 | 3384500 | 2 | 10 | 0 | 1 | 192 |
| 75 | 4B | 32 | 20 | 2 | 11 | 414809 | 3318468 | 2 | 11 | 0 | 184 | 3384692 | 2 | 11 | 0 | 1 | 192 |
| 76 | 4C | 32 | 20 | 2 | 12 | 414832 | 3318652 | 2 | 12 | 0 | 184 | 3384884 | 2 | 12 | 0 | 1 | 192 |
| 77 | 4D | 32 | 20 | 2 | 13 | 414855 | 3318836 | 2 | 13 | 0 | 184 | 3385076 | 2 | 13 | 0 | 1 | 192 |
| 78 | 4E | 32 | 20 | 2 | 14 | 414878 | 3319020 | 2 | 14 | 0 | 184 | 3385268 | 2 | 14 | 0 | 1 | 192 |
| 79 | 4F | 32 | 20 | 2 | 15 | 414901 | 3319204 | 2 | 15 | 0 | 184 | 3385460 | 2 | 15 | 0 | 1 | 192 |
| 80 | 50 | 32 | 20 | 2 | 16 | 414926 | 3319404 | 2 | 16 | 0 | 200 | 3385668 | 2 | 16 | 0 | 1 | 208 |
| 81 | 51 | 32 | 20 | 2 | 17 | 414951 | 3319604 | 2 | 17 | 0 | 200 | 3385876 | 2 | 17 | 0 | 1 | 208 |
| 82 | 52 | 32 | 20 | 2 | 18 | 414976 | 3319804 | 2 | 18 | 0 | 200 | 3386084 | 2 | 18 | 0 | 1 | 208 |
| 83 | 53 | 32 | 20 | 2 | 19 | 415001 | 3320004 | 2 | 19 | 0 | 200 | 3386292 | 2 | 19 | 0 | 1 | 208 |
| 84 | 54 | 32 | 20 | 2 | 20 | 415026 | 3320204 | 2 | 20 | 0 | 200 | 3386500 | 2 | 20 | 0 | 1 | 208 |
| 85 | 55 | 32 | 20 | 2 | 21 | 415051 | 3320404 | 2 | 21 | 0 | 200 | 3386708 | 2 | 21 | 0 | 1 | 208 |
| 86 | 56 | 32 | 20 | 2 | 22 | 415076 | 3320604 | 2 | 22 | 0 | 200 | 3386916 | 2 | 22 | 0 | 1 | 208 |
| 87 | 57 | 32 | 20 | 2 | 23 | 415101 | 3320804 | 2 | 23 | 0 | 200 | 3387124 | 2 | 23 | 0 | 1 | 208 |
| 88 | 58 | 32 | 20 | 2 | 24 | 415126 | 3321004 | 2 | 24 | 0 | 200 | 3387332 | 2 | 24 | 0 | 1 | 208 |
| 89 | 59 | 32 | 20 | 2 | 25 | 415151 | 3321204 | 2 | 25 | 0 | 200 | 3387540 | 2 | 25 | 0 | 1 | 208 |
| 90 | 5A | 32 | 20 | 2 | 26 | 415176 | 3321404 | 2 | 26 | 0 | 200 | 3387748 | 2 | 26 | 0 | 1 | 208 |
| 91 | 5B | 32 | 20 | 2 | 27 | 415201 | 3321604 | 2 | 27 | 0 | 200 | 3387956 | 2 | 27 | 0 | 1 | 208 |
| 92 | 5C | 32 | 20 | 2 | 28 | 415226 | 3321804 | 2 | 28 | 0 | 200 | 3388164 | 2 | 28 | 0 | 1 | 208 |
| 93 | 5D | 32 | 20 | 2 | 29 | 415251 | 3322004 | 2 | 29 | 0 | 200 | 3388372 | 2 | 29 | 0 | 1 | 208 |
| 94 | 5E | 32 | 20 | 2 | 30 | 415276 | 3322204 | 2 | 30 | 0 | 200 | 3388580 | 2 | 30 | 0 | 1 | 208 |
| 95 | 5F | 32 | 20 | 2 | 31 | 415301 | 3322404 | 2 | 31 | 0 | 200 | 3388788 | 2 | 31 | 0 | 1 | 208 |
| 96 | 60 | 32 | 20 | 3 | 0 | 415324 | 3322588 | 3 | 0 | 0 | 184 | 3388980 | 3 | 0 | 0 | 1 | 192 |
| 97 | 61 | 32 | 20 | 3 | 1 | 415349 | 3322788 | 3 | 1 | 0 | 200 | 3389188 | 3 | 1 | 0 | 1 | 208 |
| 98 | 62 | 32 | 20 | 3 | 2 | 415374 | 3322988 | 3 | 2 | 0 | 200 | 3389396 | 3 | 2 | 0 | 1 | 208 |
| 99 | 63 | 32 | 20 | 3 | 3 | 415399 | 3323188 | 3 | 3 | 0 | 200 | 3389604 | 3 | 3 | 0 | 1 | 208 |
| 100 | 64 | 32 | 20 | 3 | 4 | 415424 | 3323388 | 3 | 4 | 0 | 200 | 3389812 | 3 | 4 | 0 | 1 | 208 |
| 101 | 65 | 32 | 20 | 3 | 5 | 415449 | 3323588 | 3 | 5 | 0 | 200 | 3390020 | 3 | 5 | 0 | 1 | 208 |
| 102 | 66 | 32 | 20 | 3 | 6 | 415474 | 3323788 | 3 | 6 | 0 | 200 | 3390228 | 3 | 6 | 0 | 1 | 208 |
| 103 | 67 | 32 | 20 | 3 | 7 | 415499 | 3323988 | 3 | 7 | 0 | 200 | 3390436 | 3 | 7 | 0 | 1 | 208 |
| 104 | 68 | 32 | 20 | 3 | 8 | 415524 | 3324188 | 3 | 8 | 0 | 200 | 3390644 | 3 | 8 | 0 | 1 | 208 |
| 105 | 69 | 32 | 20 | 3 | 9 | 415549 | 3324388 | 3 | 9 | 0 | 200 | 3390852 | 3 | 9 | 0 | 1 | 208 |
| 106 | 6A | 32 | 20 | 3 | 10 | 415574 | 3324588 | 3 | 10 | 0 | 200 | 3391060 | 3 | 10 | 0 | 1 | 208 |
| 107 | 6B | 32 | 20 | 3 | 11 | 415599 | 3324788 | 3 | 11 | 0 | 200 | 3391268 | 3 | 11 | 0 | 1 | 208 |
| 108 | 6C | 32 | 20 | 3 | 12 | 415624 | 3324988 | 3 | 12 | 0 | 200 | 3391476 | 3 | 12 | 0 | 1 | 208 |
| 109 | 6D | 32 | 20 | 3 | 13 | 415649 | 3325188 | 3 | 13 | 0 | 200 | 3391684 | 3 | 13 | 0 | 1 | 208 |
| 110 | 6E | 32 | 20 | 3 | 14 | 415674 | 3325388 | 3 | 14 | 0 | 200 | 3391892 | 3 | 14 | 0 | 1 | 208 |
| 111 | 6F | 32 | 20 | 3 | 15 | 415699 | 3325588 | 3 | 15 | 0 | 200 | 3392100 | 3 | 15 | 0 | 1 | 208 |
| 112 | 70 | 32 | 20 | 3 | 16 | 415726 | 3325804 | 3 | 16 | 0 | 216 | 3392324 | 3 | 16 | 0 | 1 | 224 |
| 113 | 71 | 32 | 20 | 3 | 17 | 415753 | 3326020 | 3 | 17 | 0 | 216 | 3392548 | 3 | 17 | 0 | 1 | 224 |
| 114 | 72 | 32 | 20 | 3 | 18 | 415780 | 3326236 | 3 | 18 | 0 | 216 | 3392772 | 3 | 18 | 0 | 1 | 224 |
| 115 | 73 | 32 | 20 | 3 | 19 | 415807 | 3326452 | 3 | 19 | 0 | 216 | 3392996 | 3 | 19 | 0 | 1 | 224 |
| 116 | 74 | 32 | 20 | 3 | 20 | 415834 | 3326668 | 3 | 20 | 0 | 216 | 3393220 | 3 | 20 | 0 | 1 | 224 |
| 117 | 75 | 32 | 20 | 3 | 21 | 415861 | 3326884 | 3 | 21 | 0 | 216 | 3393444 | 3 | 21 | 0 | 1 | 224 |
| 118 | 76 | 32 | 20 | 3 | 22 | 415888 | 3327100 | 3 | 22 | 0 | 216 | 3393668 | 3 | 22 | 0 | 1 | 224 |
| 119 | 77 | 32 | 20 | 3 | 23 | 415915 | 3327316 | 3 | 23 | 0 | 216 | 3393892 | 3 | 23 | 0 | 1 | 224 |
| 120 | 78 | 32 | 20 | 3 | 24 | 415942 | 3327532 | 3 | 24 | 0 | 216 | 3394116 | 3 | 24 | 0 | 1 | 224 |
| 121 | 79 | 32 | 20 | 3 | 25 | 415969 | 3327748 | 3 | 25 | 0 | 216 | 3394340 | 3 | 25 | 0 | 1 | 224 |
| 122 | 7A | 32 | 20 | 3 | 26 | 415996 | 3327964 | 3 | 26 | 0 | 216 | 3394564 | 3 | 26 | 0 | 1 | 224 |
| 123 | 7B | 32 | 20 | 3 | 27 | 416023 | 3328180 | 3 | 27 | 0 | 216 | 3394788 | 3 | 27 | 0 | 1 | 224 |
| 124 | 7C | 32 | 20 | 3 | 28 | 416050 | 3328396 | 3 | 28 | 0 | 216 | 3395012 | 3 | 28 | 0 | 1 | 224 |
| 125 | 7D | 32 | 20 | 3 | 29 | 416077 | 3328612 | 3 | 29 | 0 | 216 | 3395236 | 3 | 29 | 0 | 1 | 224 |
| 126 | 7E | 32 | 20 | 3 | 30 | 416104 | 3328828 | 3 | 30 | 0 | 216 | 3395460 | 3 | 30 | 0 | 1 | 224 |
| 127 | 7F | 32 | 20 | 3 | 31 | 416131 | 3329044 | 3 | 31 | 0 | 216 | 3395684 | 3 | 31 | 0 | 1 | 224 |
| 128 | 80 | 32 | 20 | 4 | 0 | 416156 | 3329244 | 4 | 0 | 0 | 200 | 3395892 | 4 | 0 | 0 | 1 | 208 |
| 129 | 81 | 32 | 20 | 4 | 1 | 416183 | 3329460 | 4 | 1 | 0 | 216 | 3396116 | 4 | 1 | 0 | 1 | 224 |
| 130 | 82 | 32 | 20 | 4 | 2 | 416210 | 3329676 | 4 | 2 | 0 | 216 | 3396340 | 4 | 2 | 0 | 1 | 224 |
| 131 | 83 | 32 | 20 | 4 | 3 | 416237 | 3329892 | 4 | 3 | 0 | 216 | 3396564 | 4 | 3 | 0 | 1 | 224 |
| 132 | 84 | 32 | 20 | 4 | 4 | 416264 | 3330108 | 4 | 4 | 0 | 216 | 3396788 | 4 | 4 | 0 | 1 | 224 |
| 133 | 85 | 32 | 20 | 4 | 5 | 416291 | 3330324 | 4 | 5 | 0 | 216 | 3397012 | 4 | 5 | 0 | 1 | 224 |
| 134 | 86 | 32 | 20 | 4 | 6 | 416318 | 3330540 | 4 | 6 | 0 | 216 | 3397236 | 4 | 6 | 0 | 1 | 224 |
| 135 | 87 | 32 | 20 | 4 | 7 | 416345 | 3330756 | 4 | 7 | 0 | 216 | 3397460 | 4 | 7 | 0 | 1 | 224 |
| 136 | 88 | 32 | 20 | 4 | 8 | 416372 | 3330972 | 4 | 8 | 0 | 216 | 3397684 | 4 | 8 | 0 | 1 | 224 |
| 137 | 89 | 32 | 20 | 4 | 9 | 416399 | 3331188 | 4 | 9 | 0 | 216 | 3397908 | 4 | 9 | 0 | 1 | 224 |
| 138 | 8A | 32 | 20 | 4 | 10 | 416426 | 3331404 | 4 | 10 | 0 | 216 | 3398132 | 4 | 10 | 0 | 1 | 224 |
| 139 | 8B | 32 | 20 | 4 | 11 | 416453 | 3331620 | 4 | 11 | 0 | 216 | 3398356 | 4 | 11 | 0 | 1 | 224 |
| 140 | 8C | 32 | 20 | 4 | 12 | 416480 | 3331836 | 4 | 12 | 0 | 216 | 3398580 | 4 | 12 | 0 | 1 | 224 |
| 141 | 8D | 32 | 20 | 4 | 13 | 416507 | 3332052 | 4 | 13 | 0 | 216 | 3398804 | 4 | 13 | 0 | 1 | 224 |
| 142 | 8E | 32 | 20 | 4 | 14 | 416534 | 3332268 | 4 | 14 | 0 | 216 | 3399028 | 4 | 14 | 0 | 1 | 224 |
| 143 | 8F | 32 | 20 | 4 | 15 | 416561 | 3332484 | 4 | 15 | 0 | 216 | 3399252 | 4 | 15 | 0 | 1 | 224 |
| 144 | 90 | 32 | 20 | 4 | 16 | 416590 | 3332716 | 4 | 16 | 0 | 232 | 3399492 | 4 | 16 | 0 | 1 | 240 |
| 145 | 91 | 32 | 20 | 4 | 17 | 416619 | 3332948 | 4 | 17 | 0 | 232 | 3399732 | 4 | 17 | 0 | 1 | 240 |
| 146 | 92 | 32 | 20 | 4 | 18 | 416648 | 3333180 | 4 | 18 | 0 | 232 | 3399972 | 4 | 18 | 0 | 1 | 240 |
| 147 | 93 | 32 | 20 | 4 | 19 | 416677 | 3333412 | 4 | 19 | 0 | 232 | 3400212 | 4 | 19 | 0 | 1 | 240 |

| | | | | | | | | | | | | | | | | | |
|---|---|---|---|---|---|---|---|---|---|---|---|---|---|---|---|---|---|
| 148 | 94 | 32 | 20 | 4 | 20 | 416706 | 3333644 | 4 | 20 | 0 | 232 | 3400452 | 4 | 20 | 0 | 1 | 240 |
| 149 | 95 | 32 | 20 | 4 | 21 | 416735 | 3333876 | 4 | 21 | 0 | 232 | 3400692 | 4 | 21 | 0 | 1 | 240 |
| 150 | 96 | 32 | 20 | 4 | 22 | 416764 | 3334108 | 4 | 22 | 0 | 232 | 3400932 | 4 | 22 | 0 | 1 | 240 |
| 151 | 97 | 32 | 20 | 4 | 23 | 416793 | 3334340 | 4 | 23 | 0 | 232 | 3401172 | 4 | 23 | 0 | 1 | 240 |
| 152 | 98 | 32 | 20 | 4 | 24 | 416822 | 3334572 | 4 | 24 | 0 | 232 | 3401412 | 4 | 24 | 0 | 1 | 240 |
| 153 | 99 | 32 | 20 | 4 | 25 | 416851 | 3334804 | 4 | 25 | 0 | 232 | 3401652 | 4 | 25 | 0 | 1 | 240 |
| 154 | 9A | 32 | 20 | 4 | 26 | 416880 | 3335036 | 4 | 26 | 0 | 232 | 3401892 | 4 | 26 | 0 | 1 | 240 |
| 155 | 9B | 32 | 20 | 4 | 27 | 416909 | 3335268 | 4 | 27 | 0 | 232 | 3402132 | 4 | 27 | 0 | 1 | 240 |
| 156 | 9C | 32 | 20 | 4 | 28 | 416938 | 3335500 | 4 | 28 | 0 | 232 | 3402372 | 4 | 28 | 0 | 1 | 240 |
| 157 | 9D | 32 | 20 | 4 | 29 | 416967 | 3335732 | 4 | 29 | 0 | 232 | 3402612 | 4 | 29 | 0 | 1 | 240 |
| 158 | 9E | 32 | 20 | 4 | 30 | 416996 | 3335964 | 4 | 30 | 0 | 232 | 3402852 | 4 | 30 | 0 | 1 | 240 |
| 159 | 9F | 32 | 20 | 4 | 31 | 417025 | 3336196 | 4 | 31 | 0 | 232 | 3403092 | 4 | 31 | 0 | 1 | 240 |
| 160 | A0 | 32 | 20 | 5 | 0 | 417052 | 3336412 | 5 | 0 | 0 | 216 | 3403316 | 5 | 0 | 0 | 1 | 224 |
| 161 | A1 | 32 | 20 | 5 | 1 | 417081 | 3336644 | 5 | 1 | 0 | 232 | 3403556 | 5 | 1 | 0 | 1 | 240 |
| 162 | A2 | 32 | 20 | 5 | 2 | 417110 | 3336876 | 5 | 2 | 0 | 232 | 3403796 | 5 | 2 | 0 | 1 | 240 |
| 163 | A3 | 32 | 20 | 5 | 3 | 417139 | 3337108 | 5 | 3 | 0 | 232 | 3404036 | 5 | 3 | 0 | 1 | 240 |
| 164 | A4 | 32 | 20 | 5 | 4 | 417168 | 3337340 | 5 | 4 | 0 | 232 | 3404276 | 5 | 4 | 0 | 1 | 240 |
| 165 | A5 | 32 | 20 | 5 | 5 | 417197 | 3337572 | 5 | 5 | 0 | 232 | 3404516 | 5 | 5 | 0 | 1 | 240 |
| 166 | A6 | 32 | 20 | 5 | 6 | 417226 | 3337804 | 5 | 6 | 0 | 232 | 3404756 | 5 | 6 | 0 | 1 | 240 |
| 167 | A7 | 32 | 20 | 5 | 7 | 417255 | 3338036 | 5 | 7 | 0 | 232 | 3404996 | 5 | 7 | 0 | 1 | 240 |
| 168 | A8 | 32 | 20 | 5 | 8 | 417284 | 3338268 | 5 | 8 | 0 | 232 | 3405236 | 5 | 8 | 0 | 1 | 240 |
| 169 | A9 | 32 | 20 | 5 | 9 | 417313 | 3338500 | 5 | 9 | 0 | 232 | 3405476 | 5 | 9 | 0 | 1 | 240 |
| 170 | AA | 32 | 20 | 5 | 10 | 417342 | 3338732 | 5 | 10 | 0 | 232 | 3405716 | 5 | 10 | 0 | 1 | 240 |
| 171 | AB | 32 | 20 | 5 | 11 | 417371 | 3338964 | 5 | 11 | 0 | 232 | 3405956 | 5 | 11 | 0 | 1 | 240 |
| 172 | AC | 32 | 20 | 5 | 12 | 417400 | 3339196 | 5 | 12 | 0 | 232 | 3406196 | 5 | 12 | 0 | 1 | 240 |
| 173 | AD | 32 | 20 | 5 | 13 | 417429 | 3339428 | 5 | 13 | 0 | 232 | 3406436 | 5 | 13 | 0 | 1 | 240 |
| 174 | AE | 32 | 20 | 5 | 14 | 417458 | 3339660 | 5 | 14 | 0 | 232 | 3406676 | 5 | 14 | 0 | 1 | 240 |
| 175 | AF | 32 | 20 | 5 | 15 | 417487 | 3339892 | 5 | 15 | 0 | 232 | 3406916 | 5 | 15 | 0 | 1 | 240 |
| 176 | B0 | 32 | 20 | 5 | 16 | 417518 | 3340140 | 5 | 16 | 0 | 248 | 3407172 | 5 | 16 | 0 | 1 | 256 |
| 177 | B1 | 32 | 20 | 5 | 17 | 417549 | 3340388 | 5 | 17 | 0 | 248 | 3407428 | 5 | 17 | 0 | 1 | 256 |
| 178 | B2 | 32 | 20 | 5 | 18 | 417580 | 3340636 | 5 | 18 | 0 | 248 | 3407684 | 5 | 18 | 0 | 1 | 256 |
| 179 | B3 | 32 | 20 | 5 | 19 | 417611 | 3340884 | 5 | 19 | 0 | 248 | 3407940 | 5 | 19 | 0 | 1 | 256 |
| 180 | B4 | 32 | 20 | 5 | 20 | 417642 | 3341132 | 5 | 20 | 0 | 248 | 3408196 | 5 | 20 | 0 | 1 | 256 |
| 181 | B5 | 32 | 20 | 5 | 21 | 417673 | 3341380 | 5 | 21 | 0 | 248 | 3408452 | 5 | 21 | 0 | 1 | 256 |
| 182 | B6 | 32 | 20 | 5 | 22 | 417704 | 3341628 | 5 | 22 | 0 | 248 | 3408708 | 5 | 22 | 0 | 1 | 256 |
| 183 | B7 | 32 | 20 | 5 | 23 | 417735 | 3341876 | 5 | 23 | 0 | 248 | 3408964 | 5 | 23 | 0 | 1 | 256 |
| 184 | B8 | 32 | 20 | 5 | 24 | 417766 | 3342124 | 5 | 24 | 0 | 248 | 3409220 | 5 | 24 | 0 | 1 | 256 |
| 185 | B9 | 32 | 20 | 5 | 25 | 417797 | 3342372 | 5 | 25 | 0 | 248 | 3409476 | 5 | 25 | 0 | 1 | 256 |
| 186 | BA | 32 | 20 | 5 | 26 | 417828 | 3342620 | 5 | 26 | 0 | 248 | 3409732 | 5 | 26 | 0 | 1 | 256 |
| 187 | BB | 32 | 20 | 5 | 27 | 417859 | 3342868 | 5 | 27 | 0 | 248 | 3409988 | 5 | 27 | 0 | 1 | 256 |
| 188 | BC | 32 | 20 | 5 | 28 | 417890 | 3343116 | 5 | 28 | 0 | 248 | 3410244 | 5 | 28 | 0 | 1 | 256 |
| 189 | BD | 32 | 20 | 5 | 29 | 417921 | 3343364 | 5 | 29 | 0 | 248 | 3410500 | 5 | 29 | 0 | 1 | 256 |
| 190 | BE | 32 | 20 | 5 | 30 | 417952 | 3343612 | 5 | 30 | 0 | 248 | 3410756 | 5 | 30 | 0 | 1 | 256 |
| 191 | BF | 32 | 20 | 5 | 31 | 417983 | 3343860 | 5 | 31 | 0 | 248 | 3411012 | 5 | 31 | 0 | 1 | 256 |
| 192 | C0 | 32 | 20 | 6 | 0 | 418012 | 3344092 | 6 | 0 | 0 | 232 | 3411252 | 6 | 0 | 0 | 1 | 240 |
| 193 | C1 | 32 | 20 | 6 | 1 | 418043 | 3344340 | 6 | 1 | 0 | 248 | 3411508 | 6 | 1 | 0 | 1 | 256 |
| 194 | C2 | 32 | 20 | 6 | 2 | 418074 | 3344588 | 6 | 2 | 0 | 248 | 3411764 | 6 | 2 | 0 | 1 | 256 |
| 195 | C3 | 32 | 20 | 6 | 3 | 418105 | 3344836 | 6 | 3 | 0 | 248 | 3412020 | 6 | 3 | 0 | 1 | 256 |
| 196 | C4 | 32 | 20 | 6 | 4 | 418136 | 3345084 | 6 | 4 | 0 | 248 | 3412276 | 6 | 4 | 0 | 1 | 256 |
| 197 | C5 | 32 | 20 | 6 | 5 | 418167 | 3345332 | 6 | 5 | 0 | 248 | 3412532 | 6 | 5 | 0 | 1 | 256 |
| 198 | C6 | 32 | 20 | 6 | 6 | 418198 | 3345580 | 6 | 6 | 0 | 248 | 3412788 | 6 | 6 | 0 | 1 | 256 |
| 199 | C7 | 32 | 20 | 6 | 7 | 418229 | 3345828 | 6 | 7 | 0 | 248 | 3413044 | 6 | 7 | 0 | 1 | 256 |
| 200 | C8 | 32 | 20 | 6 | 8 | 418260 | 3346076 | 6 | 8 | 0 | 248 | 3413300 | 6 | 8 | 0 | 1 | 256 |
| 201 | C9 | 32 | 20 | 6 | 9 | 418291 | 3346324 | 6 | 9 | 0 | 248 | 3413556 | 6 | 9 | 0 | 1 | 256 |
| 202 | CA | 32 | 20 | 6 | 10 | 418322 | 3346572 | 6 | 10 | 0 | 248 | 3413812 | 6 | 10 | 0 | 1 | 256 |
| 203 | CB | 32 | 20 | 6 | 11 | 418353 | 3346820 | 6 | 11 | 0 | 248 | 3414068 | 6 | 11 | 0 | 1 | 256 |
| 204 | CC | 32 | 20 | 6 | 12 | 418384 | 3347068 | 6 | 12 | 0 | 248 | 3414324 | 6 | 12 | 0 | 1 | 256 |
| 205 | CD | 32 | 20 | 6 | 13 | 418415 | 3347316 | 6 | 13 | 0 | 248 | 3414580 | 6 | 13 | 0 | 1 | 256 |
| 206 | CE | 32 | 20 | 6 | 14 | 418446 | 3347564 | 6 | 14 | 0 | 248 | 3414836 | 6 | 14 | 0 | 1 | 256 |
| 207 | CF | 32 | 20 | 6 | 15 | 418477 | 3347812 | 6 | 15 | 0 | 248 | 3415092 | 6 | 15 | 0 | 1 | 256 |
| 208 | D0 | 32 | 20 | 6 | 16 | 418510 | 3348076 | 6 | 16 | 0 | 264 | 3415364 | 6 | 16 | 0 | 1 | 272 |
| 209 | D1 | 32 | 20 | 6 | 17 | 418543 | 3348340 | 6 | 17 | 0 | 264 | 3415636 | 6 | 17 | 0 | 1 | 272 |
| 210 | D2 | 32 | 20 | 6 | 18 | 418576 | 3348604 | 6 | 18 | 0 | 264 | 3415908 | 6 | 18 | 0 | 1 | 272 |
| 211 | D3 | 32 | 20 | 6 | 19 | 418609 | 3348868 | 6 | 19 | 0 | 264 | 3416180 | 6 | 19 | 0 | 1 | 272 |
| 212 | D4 | 32 | 20 | 6 | 20 | 418642 | 3349132 | 6 | 20 | 0 | 264 | 3416452 | 6 | 20 | 0 | 1 | 272 |
| 213 | D5 | 32 | 20 | 6 | 21 | 418675 | 3349396 | 6 | 21 | 0 | 264 | 3416724 | 6 | 21 | 0 | 1 | 272 |
| 214 | D6 | 32 | 20 | 6 | 22 | 418708 | 3349660 | 6 | 22 | 0 | 264 | 3416996 | 6 | 22 | 0 | 1 | 272 |
| 215 | D7 | 32 | 20 | 6 | 23 | 418741 | 3349924 | 6 | 23 | 0 | 264 | 3417268 | 6 | 23 | 0 | 1 | 272 |
| 216 | D8 | 32 | 20 | 6 | 24 | 418774 | 3350188 | 6 | 24 | 0 | 264 | 3417540 | 6 | 24 | 0 | 1 | 272 |
| 217 | D9 | 32 | 20 | 6 | 25 | 418807 | 3350452 | 6 | 25 | 0 | 264 | 3417812 | 6 | 25 | 0 | 1 | 272 |
| 218 | DA | 32 | 20 | 6 | 26 | 418840 | 3350716 | 6 | 26 | 0 | 264 | 3418084 | 6 | 26 | 0 | 1 | 272 |
| 219 | DB | 32 | 20 | 6 | 27 | 418873 | 3350980 | 6 | 27 | 0 | 264 | 3418356 | 6 | 27 | 0 | 1 | 272 |
| 220 | DC | 32 | 20 | 6 | 28 | 418906 | 3351244 | 6 | 28 | 0 | 264 | 3418628 | 6 | 28 | 0 | 1 | 272 |
| 221 | DD | 32 | 20 | 6 | 29 | 418939 | 3351508 | 6 | 29 | 0 | 264 | 3418900 | 6 | 29 | 0 | 1 | 272 |
| 222 | DE | 32 | 20 | 6 | 30 | 418972 | 3351772 | 6 | 30 | 0 | 264 | 3419172 | 6 | 30 | 0 | 1 | 272 |
| 223 | DF | 32 | 20 | 6 | 31 | 419005 | 3352036 | 6 | 31 | 0 | 264 | 3419444 | 6 | 31 | 0 | 1 | 272 |
| 224 | E0 | 32 | 20 | 7 | 0 | 419036 | 3352284 | 7 | 0 | 0 | 248 | 3419700 | 7 | 0 | 0 | 1 | 256 |
| 225 | E1 | 32 | 20 | 7 | 1 | 419069 | 3352548 | 7 | 1 | 0 | 264 | 3419972 | 7 | 1 | 0 | 1 | 272 |
| 226 | E2 | 32 | 20 | 7 | 2 | 419102 | 3352812 | 7 | 2 | 0 | 264 | 3420244 | 7 | 2 | 0 | 1 | 272 |
| 227 | E3 | 32 | 20 | 7 | 3 | 419135 | 3353076 | 7 | 3 | 0 | 264 | 3420516 | 7 | 3 | 0 | 1 | 272 |
| 228 | E4 | 32 | 20 | 7 | 4 | 419168 | 3353340 | 7 | 4 | 0 | 264 | 3420788 | 7 | 4 | 0 | 1 | 272 |
| 229 | E5 | 32 | 20 | 7 | 5 | 419201 | 3353604 | 7 | 5 | 0 | 264 | 3421060 | 7 | 5 | 0 | 1 | 272 |
| 230 | E6 | 32 | 20 | 7 | 6 | 419234 | 3353868 | 7 | 6 | 0 | 264 | 3421332 | 7 | 6 | 0 | 1 | 272 |
| 231 | E7 | 32 | 20 | 7 | 7 | 419267 | 3354132 | 7 | 7 | 0 | 264 | 3421604 | 7 | 7 | 0 | 1 | 272 |
| 232 | E8 | 32 | 20 | 7 | 8 | 419300 | 3354396 | 7 | 8 | 0 | 264 | 3421876 | 7 | 8 | 0 | 1 | 272 |
| 233 | E9 | 32 | 20 | 7 | 9 | 419333 | 3354660 | 7 | 9 | 0 | 264 | 3422148 | 7 | 9 | 0 | 1 | 272 |
| 234 | EA | 32 | 20 | 7 | 10 | 419366 | 3354924 | 7 | 10 | 0 | 264 | 3422420 | 7 | 10 | 0 | 1 | 272 |
| 235 | EB | 32 | 20 | 7 | 11 | 419399 | 3355188 | 7 | 11 | 0 | 264 | 3422692 | 7 | 11 | 0 | 1 | 272 |
| 236 | EC | 32 | 20 | 7 | 12 | 419432 | 3355452 | 7 | 12 | 0 | 264 | 3422964 | 7 | 12 | 0 | 1 | 272 |
| 237 | ED | 32 | 20 | 7 | 13 | 419465 | 3355716 | 7 | 13 | 0 | 264 | 3423236 | 7 | 13 | 0 | 1 | 272 |
| 238 | EE | 32 | 20 | 7 | 14 | 419498 | 3355980 | 7 | 14 | 0 | 264 | 3423508 | 7 | 14 | 0 | 1 | 272 |
| 239 | EF | 32 | 20 | 7 | 15 | 419531 | 3356244 | 7 | 15 | 0 | 264 | 3423780 | 7 | 15 | 0 | 1 | 272 |
| 240 | F0 | 32 | 20 | 7 | 16 | 419566 | 3356524 | 7 | 16 | 0 | 280 | 3424068 | 7 | 16 | 0 | 1 | 288 |
| 241 | F1 | 32 | 20 | 7 | 17 | 419601 | 3356804 | 7 | 17 | 0 | 280 | 3424356 | 7 | 17 | 0 | 1 | 288 |
| 242 | F2 | 32 | 20 | 7 | 18 | 419636 | 3357084 | 7 | 18 | 0 | 280 | 3424644 | 7 | 18 | 0 | 1 | 288 |
| 243 | F3 | 32 | 20 | 7 | 19 | 419671 | 3357364 | 7 | 19 | 0 | 280 | 3424932 | 7 | 19 | 0 | 1 | 288 |
| 244 | F4 | 32 | 20 | 7 | 20 | 419706 | 3357644 | 7 | 20 | 0 | 280 | 3425220 | 7 | 20 | 0 | 1 | 288 |
| 245 | F5 | 32 | 20 | 7 | 21 | 419741 | 3357924 | 7 | 21 | 0 | 280 | 3425508 | 7 | 21 | 0 | 1 | 288 |
| 246 | F6 | 32 | 20 | 7 | 22 | 419776 | 3358204 | 7 | 22 | 0 | 280 | 3425796 | 7 | 22 | 0 | 1 | 288 |
| 247 | F7 | 32 | 20 | 7 | 23 | 419811 | 3358484 | 7 | 23 | 0 | 280 | 3426084 | 7 | 23 | 0 | 1 | 288 |
| 248 | F8 | 32 | 20 | 7 | 24 | 419846 | 3358764 | 7 | 24 | 0 | 280 | 3426372 | 7 | 24 | 0 | 1 | 288 |
| 249 | F9 | 32 | 20 | 7 | 25 | 419881 | 3359044 | 7 | 25 | 0 | 280 | 3426660 | 7 | 25 | 0 | 1 | 288 |
| 250 | FA | 32 | 20 | 7 | 26 | 419916 | 3359324 | 7 | 26 | 0 | 280 | 3426948 | 7 | 26 | 0 | 1 | 288 |

| | | | | | | | | | | | | | | | | | |
|---|---|---|---|---|---|---|---|---|---|---|---|---|---|---|---|---|---|
| 251 | FB | 32 | 20 | 7 | 27 | 419951 | 3359604 | 7 | 27 | 0 | 280 | 3427236 | 7 | 27 | 0 | 1 | 288 |
| 252 | FC | 32 | 20 | 7 | 28 | 419986 | 3359884 | 7 | 28 | 0 | 280 | 3427524 | 7 | 28 | 0 | 1 | 288 |
| 253 | FD | 32 | 20 | 7 | 29 | 420021 | 3360164 | 7 | 29 | 0 | 280 | 3427812 | 7 | 29 | 0 | 1 | 288 |
| 254 | FE | 32 | 20 | 7 | 30 | 420056 | 3360444 | 7 | 30 | 0 | 280 | 3428100 | 7 | 30 | 0 | 1 | 288 |
| 255 | FF | 32 | 20 | 7 | 31 | 420091 | 3360724 | 7 | 31 | 0 | 280 | 3428388 | 7 | 31 | 0 | 1 | 288 |
| 0 | 00 | 33 | 21 | 0 | 0 | 420103 | 3360820 | 0 | 0 | 0 | 96 | 3428492 | 0 | 0 | 0 | 1 | 104 |
| 1 | 01 | 33 | 21 | 0 | 1 | 420117 | 3360932 | 0 | 1 | 0 | 112 | 3428612 | 0 | 1 | 0 | 1 | 120 |
| 2 | 02 | 33 | 21 | 0 | 2 | 420131 | 3361044 | 0 | 2 | 0 | 112 | 3428732 | 0 | 2 | 0 | 1 | 120 |
| 3 | 03 | 33 | 21 | 0 | 3 | 420145 | 3361156 | 0 | 3 | 0 | 112 | 3428852 | 0 | 3 | 0 | 1 | 120 |
| 4 | 04 | 33 | 21 | 0 | 4 | 420159 | 3361268 | 0 | 4 | 0 | 112 | 3428972 | 0 | 4 | 0 | 1 | 120 |
| 5 | 05 | 33 | 21 | 0 | 5 | 420173 | 3361380 | 0 | 5 | 0 | 112 | 3429092 | 0 | 5 | 0 | 1 | 120 |
| 6 | 06 | 33 | 21 | 0 | 6 | 420187 | 3361492 | 0 | 6 | 0 | 112 | 3429212 | 0 | 6 | 0 | 1 | 120 |
| 7 | 07 | 33 | 21 | 0 | 7 | 420201 | 3361604 | 0 | 7 | 0 | 112 | 3429332 | 0 | 7 | 0 | 1 | 120 |
| 8 | 08 | 33 | 21 | 0 | 8 | 420215 | 3361716 | 0 | 8 | 0 | 112 | 3429452 | 0 | 8 | 0 | 1 | 120 |
| 9 | 09 | 33 | 21 | 0 | 9 | 420229 | 3361828 | 0 | 9 | 0 | 112 | 3429572 | 0 | 9 | 0 | 1 | 120 |
| 10 | 0A | 33 | 21 | 0 | 10 | 420243 | 3361940 | 0 | 10 | 0 | 112 | 3429692 | 0 | 10 | 0 | 1 | 120 |
| 11 | 0B | 33 | 21 | 0 | 11 | 420257 | 3362052 | 0 | 11 | 0 | 112 | 3429812 | 0 | 11 | 0 | 1 | 120 |
| 12 | 0C | 33 | 21 | 0 | 12 | 420271 | 3362164 | 0 | 12 | 0 | 112 | 3429932 | 0 | 12 | 0 | 1 | 120 |
| 13 | 0D | 33 | 21 | 0 | 13 | 420285 | 3362276 | 0 | 13 | 0 | 112 | 3430052 | 0 | 13 | 0 | 1 | 120 |
| 14 | 0E | 33 | 21 | 0 | 14 | 420299 | 3362388 | 0 | 14 | 0 | 112 | 3430172 | 0 | 14 | 0 | 1 | 120 |
| 15 | 0F | 33 | 21 | 0 | 15 | 420313 | 3362500 | 0 | 15 | 0 | 112 | 3430292 | 0 | 15 | 0 | 1 | 120 |
| 16 | 10 | 33 | 21 | 0 | 16 | 420327 | 3362612 | 0 | 16 | 0 | 112 | 3430412 | 0 | 16 | 0 | 1 | 120 |
| 17 | 11 | 33 | 21 | 0 | 17 | 420341 | 3362724 | 0 | 17 | 0 | 112 | 3430532 | 0 | 17 | 0 | 1 | 120 |
| 18 | 12 | 33 | 21 | 0 | 18 | 420355 | 3362836 | 0 | 18 | 0 | 112 | 3430652 | 0 | 18 | 0 | 1 | 120 |
| 19 | 13 | 33 | 21 | 0 | 19 | 420369 | 3362948 | 0 | 19 | 0 | 112 | 3430772 | 0 | 19 | 0 | 1 | 120 |
| 20 | 14 | 33 | 21 | 0 | 20 | 420383 | 3363060 | 0 | 20 | 0 | 112 | 3430892 | 0 | 20 | 0 | 1 | 120 |
| 21 | 15 | 33 | 21 | 0 | 21 | 420397 | 3363172 | 0 | 21 | 0 | 112 | 3431012 | 0 | 21 | 0 | 1 | 120 |
| 22 | 16 | 33 | 21 | 0 | 22 | 420411 | 3363284 | 0 | 22 | 0 | 112 | 3431132 | 0 | 22 | 0 | 1 | 120 |
| 23 | 17 | 33 | 21 | 0 | 23 | 420425 | 3363396 | 0 | 23 | 0 | 112 | 3431252 | 0 | 23 | 0 | 1 | 120 |
| 24 | 18 | 33 | 21 | 0 | 24 | 420439 | 3363508 | 0 | 24 | 0 | 112 | 3431372 | 0 | 24 | 0 | 1 | 120 |
| 25 | 19 | 33 | 21 | 0 | 25 | 420453 | 3363620 | 0 | 25 | 0 | 112 | 3431492 | 0 | 25 | 0 | 1 | 120 |
| 26 | 1A | 33 | 21 | 0 | 26 | 420467 | 3363732 | 0 | 26 | 0 | 112 | 3431612 | 0 | 26 | 0 | 1 | 120 |
| 27 | 1B | 33 | 21 | 0 | 27 | 420481 | 3363844 | 0 | 27 | 0 | 112 | 3431732 | 0 | 27 | 0 | 1 | 120 |
| 28 | 1C | 33 | 21 | 0 | 28 | 420495 | 3363956 | 0 | 28 | 0 | 112 | 3431852 | 0 | 28 | 0 | 1 | 120 |
| 29 | 1D | 33 | 21 | 0 | 29 | 420509 | 3364068 | 0 | 29 | 0 | 112 | 3431972 | 0 | 29 | 0 | 1 | 120 |
| 30 | 1E | 33 | 21 | 0 | 30 | 420523 | 3364180 | 0 | 30 | 0 | 112 | 3432092 | 0 | 30 | 0 | 1 | 120 |
| 31 | 1F | 33 | 21 | 0 | 31 | 420537 | 3364292 | 0 | 31 | 0 | 112 | 3432212 | 0 | 31 | 0 | 1 | 120 |
| 32 | 20 | 33 | 21 | 0 | 32 | 420551 | 3364404 | 0 | 32 | 0 | 112 | 3432332 | 0 | 32 | 0 | 1 | 120 |
| 33 | 21 | 33 | 21 | 1 | 0 | 420563 | 3364500 | 1 | 0 | 0 | 96 | 3432436 | 1 | 0 | 0 | 1 | 104 |
| 34 | 22 | 33 | 21 | 1 | 1 | 420580 | 3364636 | 1 | 1 | 0 | 136 | 3432580 | 1 | 1 | 0 | 1 | 144 |
| 35 | 23 | 33 | 21 | 1 | 2 | 420597 | 3364772 | 1 | 2 | 0 | 136 | 3432724 | 1 | 2 | 0 | 1 | 144 |
| 36 | 24 | 33 | 21 | 1 | 3 | 420614 | 3364908 | 1 | 3 | 0 | 136 | 3432868 | 1 | 3 | 0 | 1 | 144 |
| 37 | 25 | 33 | 21 | 1 | 4 | 420631 | 3365044 | 1 | 4 | 0 | 136 | 3433012 | 1 | 4 | 0 | 1 | 144 |
| 38 | 26 | 33 | 21 | 1 | 5 | 420648 | 3365180 | 1 | 5 | 0 | 136 | 3433156 | 1 | 5 | 0 | 1 | 144 |
| 39 | 27 | 33 | 21 | 1 | 6 | 420665 | 3365316 | 1 | 6 | 0 | 136 | 3433300 | 1 | 6 | 0 | 1 | 144 |
| 40 | 28 | 33 | 21 | 1 | 7 | 420682 | 3365452 | 1 | 7 | 0 | 136 | 3433444 | 1 | 7 | 0 | 1 | 144 |
| 41 | 29 | 33 | 21 | 1 | 8 | 420699 | 3365588 | 1 | 8 | 0 | 136 | 3433588 | 1 | 8 | 0 | 1 | 144 |
| 42 | 2A | 33 | 21 | 1 | 9 | 420716 | 3365724 | 1 | 9 | 0 | 136 | 3433732 | 1 | 9 | 0 | 1 | 144 |
| 43 | 2B | 33 | 21 | 1 | 10 | 420733 | 3365860 | 1 | 10 | 0 | 136 | 3433876 | 1 | 10 | 0 | 1 | 144 |
| 44 | 2C | 33 | 21 | 1 | 11 | 420750 | 3365996 | 1 | 11 | 0 | 136 | 3434020 | 1 | 11 | 0 | 1 | 144 |
| 45 | 2D | 33 | 21 | 1 | 12 | 420767 | 3366132 | 1 | 12 | 0 | 136 | 3434164 | 1 | 12 | 0 | 1 | 144 |
| 46 | 2E | 33 | 21 | 1 | 13 | 420784 | 3366268 | 1 | 13 | 0 | 136 | 3434308 | 1 | 13 | 0 | 1 | 144 |
| 47 | 2F | 33 | 21 | 1 | 14 | 420801 | 3366404 | 1 | 14 | 0 | 136 | 3434452 | 1 | 14 | 0 | 1 | 144 |
| 48 | 30 | 33 | 21 | 1 | 15 | 420822 | 3366572 | 1 | 15 | 0 | 168 | 3434628 | 1 | 15 | 0 | 1 | 176 |
| 49 | 31 | 33 | 21 | 1 | 16 | 420843 | 3366740 | 1 | 16 | 0 | 168 | 3434804 | 1 | 16 | 0 | 1 | 176 |
| 50 | 32 | 33 | 21 | 1 | 17 | 420864 | 3366908 | 1 | 17 | 0 | 168 | 3434980 | 1 | 17 | 0 | 1 | 176 |
| 51 | 33 | 33 | 21 | 1 | 18 | 420885 | 3367076 | 1 | 18 | 0 | 168 | 3435156 | 1 | 18 | 0 | 1 | 176 |
| 52 | 34 | 33 | 21 | 1 | 19 | 420906 | 3367244 | 1 | 19 | 0 | 168 | 3435332 | 1 | 19 | 0 | 1 | 176 |
| 53 | 35 | 33 | 21 | 1 | 20 | 420927 | 3367412 | 1 | 20 | 0 | 168 | 3435508 | 1 | 20 | 0 | 1 | 176 |
| 54 | 36 | 33 | 21 | 1 | 21 | 420948 | 3367580 | 1 | 21 | 0 | 168 | 3435684 | 1 | 21 | 0 | 1 | 176 |
| 55 | 37 | 33 | 21 | 1 | 22 | 420969 | 3367748 | 1 | 22 | 0 | 168 | 3435860 | 1 | 22 | 0 | 1 | 176 |
| 56 | 38 | 33 | 21 | 1 | 23 | 420990 | 3367916 | 1 | 23 | 0 | 168 | 3436036 | 1 | 23 | 0 | 1 | 176 |
| 57 | 39 | 33 | 21 | 1 | 24 | 421011 | 3368084 | 1 | 24 | 0 | 168 | 3436212 | 1 | 24 | 0 | 1 | 176 |
| 58 | 3A | 33 | 21 | 1 | 25 | 421032 | 3368252 | 1 | 25 | 0 | 168 | 3436388 | 1 | 25 | 0 | 1 | 176 |
| 59 | 3B | 33 | 21 | 1 | 26 | 421053 | 3368420 | 1 | 26 | 0 | 168 | 3436564 | 1 | 26 | 0 | 1 | 176 |
| 60 | 3C | 33 | 21 | 1 | 27 | 421074 | 3368588 | 1 | 27 | 0 | 168 | 3436740 | 1 | 27 | 0 | 1 | 176 |
| 61 | 3D | 33 | 21 | 1 | 28 | 421095 | 3368756 | 1 | 28 | 0 | 168 | 3436916 | 1 | 28 | 0 | 1 | 176 |
| 62 | 3E | 33 | 21 | 1 | 29 | 421116 | 3368924 | 1 | 29 | 0 | 168 | 3437092 | 1 | 29 | 0 | 1 | 176 |
| 63 | 3F | 33 | 21 | 1 | 30 | 421137 | 3369092 | 1 | 30 | 0 | 168 | 3437268 | 1 | 30 | 0 | 1 | 176 |
| 64 | 40 | 33 | 21 | 1 | 31 | 421160 | 3369276 | 1 | 31 | 0 | 184 | 3437460 | 1 | 31 | 0 | 1 | 192 |
| 65 | 41 | 33 | 21 | 1 | 32 | 421183 | 3369460 | 1 | 32 | 0 | 184 | 3437652 | 1 | 32 | 0 | 1 | 192 |
| 66 | 42 | 33 | 21 | 2 | 0 | 421204 | 3369628 | 2 | 0 | 0 | 168 | 3437828 | 2 | 0 | 0 | 1 | 176 |
| 67 | 43 | 33 | 21 | 2 | 1 | 421230 | 3369836 | 2 | 1 | 0 | 208 | 3438044 | 2 | 1 | 0 | 1 | 216 |
| 68 | 44 | 33 | 21 | 2 | 2 | 421256 | 3370044 | 2 | 2 | 0 | 208 | 3438260 | 2 | 2 | 0 | 1 | 216 |
| 69 | 45 | 33 | 21 | 2 | 3 | 421282 | 3370252 | 2 | 3 | 0 | 208 | 3438476 | 2 | 3 | 0 | 1 | 216 |
| 70 | 46 | 33 | 21 | 2 | 4 | 421308 | 3370460 | 2 | 4 | 0 | 208 | 3438692 | 2 | 4 | 0 | 1 | 216 |
| 71 | 47 | 33 | 21 | 2 | 5 | 421334 | 3370668 | 2 | 5 | 0 | 208 | 3438908 | 2 | 5 | 0 | 1 | 216 |
| 72 | 48 | 33 | 21 | 2 | 6 | 421360 | 3370876 | 2 | 6 | 0 | 208 | 3439124 | 2 | 6 | 0 | 1 | 216 |
| 73 | 49 | 33 | 21 | 2 | 7 | 421386 | 3371084 | 2 | 7 | 0 | 208 | 3439340 | 2 | 7 | 0 | 1 | 216 |
| 74 | 4A | 33 | 21 | 2 | 8 | 421412 | 3371292 | 2 | 8 | 0 | 208 | 3439556 | 2 | 8 | 0 | 1 | 216 |
| 75 | 4B | 33 | 21 | 2 | 9 | 421438 | 3371500 | 2 | 9 | 0 | 208 | 3439772 | 2 | 9 | 0 | 1 | 216 |
| 76 | 4C | 33 | 21 | 2 | 10 | 421464 | 3371708 | 2 | 10 | 0 | 208 | 3439988 | 2 | 10 | 0 | 1 | 216 |
| 77 | 4D | 33 | 21 | 2 | 11 | 421490 | 3371916 | 2 | 11 | 0 | 208 | 3440204 | 2 | 11 | 0 | 1 | 216 |
| 78 | 4E | 33 | 21 | 2 | 12 | 421516 | 3372124 | 2 | 12 | 0 | 208 | 3440420 | 2 | 12 | 0 | 1 | 216 |
| 79 | 4F | 33 | 21 | 2 | 13 | 421542 | 3372332 | 2 | 13 | 0 | 208 | 3440636 | 2 | 13 | 0 | 1 | 216 |
| 80 | 50 | 33 | 21 | 2 | 14 | 421568 | 3372540 | 2 | 14 | 0 | 208 | 3440852 | 2 | 14 | 0 | 1 | 216 |
| 81 | 51 | 33 | 21 | 2 | 15 | 421594 | 3372748 | 2 | 15 | 0 | 208 | 3441068 | 2 | 15 | 0 | 1 | 216 |
| 82 | 52 | 33 | 21 | 2 | 16 | 421620 | 3372956 | 2 | 16 | 0 | 208 | 3441284 | 2 | 16 | 0 | 1 | 216 |
| 83 | 53 | 33 | 21 | 2 | 17 | 421646 | 3373164 | 2 | 17 | 0 | 208 | 3441500 | 2 | 17 | 0 | 1 | 216 |
| 84 | 54 | 33 | 21 | 2 | 18 | 421672 | 3373372 | 2 | 18 | 0 | 208 | 3441716 | 2 | 18 | 0 | 1 | 216 |
| 85 | 55 | 33 | 21 | 2 | 19 | 421698 | 3373580 | 2 | 19 | 0 | 208 | 3441932 | 2 | 19 | 0 | 1 | 216 |
| 86 | 56 | 33 | 21 | 2 | 20 | 421724 | 3373788 | 2 | 20 | 0 | 208 | 3442148 | 2 | 20 | 0 | 1 | 216 |
| 87 | 57 | 33 | 21 | 2 | 21 | 421750 | 3373996 | 2 | 21 | 0 | 208 | 3442364 | 2 | 21 | 0 | 1 | 216 |
| 88 | 58 | 33 | 21 | 2 | 22 | 421776 | 3374204 | 2 | 22 | 0 | 208 | 3442580 | 2 | 22 | 0 | 1 | 216 |
| 89 | 59 | 33 | 21 | 2 | 23 | 421802 | 3374412 | 2 | 23 | 0 | 208 | 3442796 | 2 | 23 | 0 | 1 | 216 |
| 90 | 5A | 33 | 21 | 2 | 24 | 421828 | 3374620 | 2 | 24 | 0 | 208 | 3443012 | 2 | 24 | 0 | 1 | 216 |
| 91 | 5B | 33 | 21 | 2 | 25 | 421854 | 3374828 | 2 | 25 | 0 | 208 | 3443228 | 2 | 25 | 0 | 1 | 216 |
| 92 | 5C | 33 | 21 | 2 | 26 | 421880 | 3375036 | 2 | 26 | 0 | 208 | 3443444 | 2 | 26 | 0 | 1 | 216 |
| 93 | 5D | 33 | 21 | 2 | 27 | 421906 | 3375244 | 2 | 27 | 0 | 208 | 3443660 | 2 | 27 | 0 | 1 | 216 |
| 94 | 5E | 33 | 21 | 2 | 28 | 421932 | 3375452 | 2 | 28 | 0 | 208 | 3443876 | 2 | 28 | 0 | 1 | 216 |
| 95 | 5F | 33 | 21 | 2 | 29 | 421958 | 3375660 | 2 | 29 | 0 | 208 | 3444092 | 2 | 29 | 0 | 1 | 216 |
| 96 | 60 | 33 | 21 | 2 | 30 | 421981 | 3375844 | 2 | 30 | 0 | 184 | 3444284 | 2 | 30 | 0 | 1 | 192 |
| 97 | 61 | 33 | 21 | 2 | 31 | 422004 | 3376028 | 2 | 31 | 0 | 184 | 3444476 | 2 | 31 | 0 | 1 | 192 |

| | | | | | | | | | | | | | | | | | |
|---|---|---|---|---|---|---|---|---|---|---|---|---|---|---|---|---|---|
| 98 | 62 | 33 | 21 | 2 | 32 | 422027 | 3376212 | 2 | 32 | 0 | 184 | 3444668 | 2 | 32 | 0 | 1 | 192 |
| 99 | 63 | 33 | 21 | 3 | 0 | 422048 | 3376380 | 3 | 0 | 0 | 168 | 3444844 | 3 | 0 | 0 | 1 | 176 |
| 100 | 64 | 33 | 21 | 3 | 1 | 422074 | 3376588 | 3 | 1 | 0 | 208 | 3445060 | 3 | 1 | 0 | 1 | 216 |
| 101 | 65 | 33 | 21 | 3 | 2 | 422100 | 3376796 | 3 | 2 | 0 | 208 | 3445276 | 3 | 2 | 0 | 1 | 216 |
| 102 | 66 | 33 | 21 | 3 | 3 | 422126 | 3377004 | 3 | 3 | 0 | 208 | 3445492 | 3 | 3 | 0 | 1 | 216 |
| 103 | 67 | 33 | 21 | 3 | 4 | 422152 | 3377212 | 3 | 4 | 0 | 208 | 3445708 | 3 | 4 | 0 | 1 | 216 |
| 104 | 68 | 33 | 21 | 3 | 5 | 422178 | 3377420 | 3 | 5 | 0 | 208 | 3445924 | 3 | 5 | 0 | 1 | 216 |
| 105 | 69 | 33 | 21 | 3 | 6 | 422204 | 3377628 | 3 | 6 | 0 | 208 | 3446140 | 3 | 6 | 0 | 1 | 216 |
| 106 | 6A | 33 | 21 | 3 | 7 | 422230 | 3377836 | 3 | 7 | 0 | 208 | 3446356 | 3 | 7 | 0 | 1 | 216 |
| 107 | 6B | 33 | 21 | 3 | 8 | 422256 | 3378044 | 3 | 8 | 0 | 208 | 3446572 | 3 | 8 | 0 | 1 | 216 |
| 108 | 6C | 33 | 21 | 3 | 9 | 422282 | 3378252 | 3 | 9 | 0 | 208 | 3446788 | 3 | 9 | 0 | 1 | 216 |
| 109 | 6D | 33 | 21 | 3 | 10 | 422308 | 3378460 | 3 | 10 | 0 | 208 | 3447004 | 3 | 10 | 0 | 1 | 216 |
| 110 | 6E | 33 | 21 | 3 | 11 | 422334 | 3378668 | 3 | 11 | 0 | 208 | 3447220 | 3 | 11 | 0 | 1 | 216 |
| 111 | 6F | 33 | 21 | 3 | 12 | 422360 | 3378876 | 3 | 12 | 0 | 208 | 3447436 | 3 | 12 | 0 | 1 | 216 |
| 112 | 70 | 33 | 21 | 3 | 13 | 422388 | 3379100 | 3 | 13 | 0 | 224 | 3447668 | 3 | 13 | 0 | 1 | 232 |
| 113 | 71 | 33 | 21 | 3 | 14 | 422416 | 3379324 | 3 | 14 | 0 | 224 | 3447900 | 3 | 14 | 0 | 1 | 232 |
| 114 | 72 | 33 | 21 | 3 | 15 | 422444 | 3379548 | 3 | 15 | 0 | 224 | 3448132 | 3 | 15 | 0 | 1 | 232 |
| 115 | 73 | 33 | 21 | 3 | 16 | 422472 | 3379772 | 3 | 16 | 0 | 224 | 3448364 | 3 | 16 | 0 | 1 | 232 |
| 116 | 74 | 33 | 21 | 3 | 17 | 422500 | 3379996 | 3 | 17 | 0 | 224 | 3448596 | 3 | 17 | 0 | 1 | 232 |
| 117 | 75 | 33 | 21 | 3 | 18 | 422528 | 3380220 | 3 | 18 | 0 | 224 | 3448828 | 3 | 18 | 0 | 1 | 232 |
| 118 | 76 | 33 | 21 | 3 | 19 | 422556 | 3380444 | 3 | 19 | 0 | 224 | 3449060 | 3 | 19 | 0 | 1 | 232 |
| 119 | 77 | 33 | 21 | 3 | 20 | 422584 | 3380668 | 3 | 20 | 0 | 224 | 3449292 | 3 | 20 | 0 | 1 | 232 |
| 120 | 78 | 33 | 21 | 3 | 21 | 422612 | 3380892 | 3 | 21 | 0 | 224 | 3449524 | 3 | 21 | 0 | 1 | 232 |
| 121 | 79 | 33 | 21 | 3 | 22 | 422640 | 3381116 | 3 | 22 | 0 | 224 | 3449756 | 3 | 22 | 0 | 1 | 232 |
| 122 | 7A | 33 | 21 | 3 | 23 | 422668 | 3381340 | 3 | 23 | 0 | 224 | 3449988 | 3 | 23 | 0 | 1 | 232 |
| 123 | 7B | 33 | 21 | 3 | 24 | 422696 | 3381564 | 3 | 24 | 0 | 224 | 3450220 | 3 | 24 | 0 | 1 | 232 |
| 124 | 7C | 33 | 21 | 3 | 25 | 422724 | 3381788 | 3 | 25 | 0 | 224 | 3450452 | 3 | 25 | 0 | 1 | 232 |
| 125 | 7D | 33 | 21 | 3 | 26 | 422752 | 3382012 | 3 | 26 | 0 | 224 | 3450684 | 3 | 26 | 0 | 1 | 232 |
| 126 | 7E | 33 | 21 | 3 | 27 | 422780 | 3382236 | 3 | 27 | 0 | 224 | 3450916 | 3 | 27 | 0 | 1 | 232 |
| 127 | 7F | 33 | 21 | 3 | 28 | 422808 | 3382460 | 3 | 28 | 0 | 224 | 3451148 | 3 | 28 | 0 | 1 | 232 |
| 128 | 80 | 33 | 21 | 3 | 29 | 422836 | 3382684 | 3 | 29 | 0 | 224 | 3451380 | 3 | 29 | 0 | 1 | 232 |
| 129 | 81 | 33 | 21 | 3 | 30 | 422864 | 3382908 | 3 | 30 | 0 | 224 | 3451612 | 3 | 30 | 0 | 1 | 232 |
| 130 | 82 | 33 | 21 | 3 | 31 | 422892 | 3383132 | 3 | 31 | 0 | 224 | 3451844 | 3 | 31 | 0 | 1 | 232 |
| 131 | 83 | 33 | 21 | 3 | 32 | 422920 | 3383356 | 3 | 32 | 0 | 224 | 3452076 | 3 | 32 | 0 | 1 | 232 |
| 132 | 84 | 33 | 21 | 4 | 0 | 422950 | 3383596 | 4 | 0 | 0 | 240 | 3452324 | 4 | 0 | 0 | 1 | 248 |
| 133 | 85 | 33 | 21 | 4 | 1 | 422980 | 3383836 | 4 | 1 | 0 | 240 | 3452572 | 4 | 1 | 0 | 1 | 248 |
| 134 | 86 | 33 | 21 | 4 | 2 | 423010 | 3384076 | 4 | 2 | 0 | 240 | 3452820 | 4 | 2 | 0 | 1 | 248 |
| 135 | 87 | 33 | 21 | 4 | 3 | 423040 | 3384316 | 4 | 3 | 0 | 240 | 3453068 | 4 | 3 | 0 | 1 | 248 |
| 136 | 88 | 33 | 21 | 4 | 4 | 423070 | 3384556 | 4 | 4 | 0 | 240 | 3453316 | 4 | 4 | 0 | 1 | 248 |
| 137 | 89 | 33 | 21 | 4 | 5 | 423100 | 3384796 | 4 | 5 | 0 | 240 | 3453564 | 4 | 5 | 0 | 1 | 248 |
| 138 | 8A | 33 | 21 | 4 | 6 | 423130 | 3385036 | 4 | 6 | 0 | 240 | 3453812 | 4 | 6 | 0 | 1 | 248 |
| 139 | 8B | 33 | 21 | 4 | 7 | 423160 | 3385276 | 4 | 7 | 0 | 240 | 3454060 | 4 | 7 | 0 | 1 | 248 |
| 140 | 8C | 33 | 21 | 4 | 8 | 423190 | 3385516 | 4 | 8 | 0 | 240 | 3454308 | 4 | 8 | 0 | 1 | 248 |
| 141 | 8D | 33 | 21 | 4 | 9 | 423220 | 3385756 | 4 | 9 | 0 | 240 | 3454556 | 4 | 9 | 0 | 1 | 248 |
| 142 | 8E | 33 | 21 | 4 | 10 | 423250 | 3385996 | 4 | 10 | 0 | 240 | 3454804 | 4 | 10 | 0 | 1 | 248 |
| 143 | 8F | 33 | 21 | 4 | 11 | 423280 | 3386236 | 4 | 11 | 0 | 240 | 3455052 | 4 | 11 | 0 | 1 | 248 |
| 144 | 90 | 33 | 21 | 4 | 12 | 423308 | 3386460 | 4 | 12 | 0 | 224 | 3455284 | 4 | 12 | 0 | 1 | 232 |
| 145 | 91 | 33 | 21 | 4 | 13 | 423336 | 3386684 | 4 | 13 | 0 | 224 | 3455516 | 4 | 13 | 0 | 1 | 232 |
| 146 | 92 | 33 | 21 | 4 | 14 | 423364 | 3386908 | 4 | 14 | 0 | 224 | 3455748 | 4 | 14 | 0 | 1 | 232 |
| 147 | 93 | 33 | 21 | 4 | 15 | 423392 | 3387132 | 4 | 15 | 0 | 224 | 3455980 | 4 | 15 | 0 | 1 | 232 |
| 148 | 94 | 33 | 21 | 4 | 16 | 423420 | 3387356 | 4 | 16 | 0 | 224 | 3456212 | 4 | 16 | 0 | 1 | 232 |
| 149 | 95 | 33 | 21 | 4 | 17 | 423448 | 3387580 | 4 | 17 | 0 | 224 | 3456444 | 4 | 17 | 0 | 1 | 232 |
| 150 | 96 | 33 | 21 | 4 | 18 | 423476 | 3387804 | 4 | 18 | 0 | 224 | 3456676 | 4 | 18 | 0 | 1 | 232 |
| 151 | 97 | 33 | 21 | 4 | 19 | 423504 | 3388028 | 4 | 19 | 0 | 224 | 3456908 | 4 | 19 | 0 | 1 | 232 |
| 152 | 98 | 33 | 21 | 4 | 20 | 423532 | 3388252 | 4 | 20 | 0 | 224 | 3457140 | 4 | 20 | 0 | 1 | 232 |
| 153 | 99 | 33 | 21 | 4 | 21 | 423560 | 3388476 | 4 | 21 | 0 | 224 | 3457372 | 4 | 21 | 0 | 1 | 232 |
| 154 | 9A | 33 | 21 | 4 | 22 | 423588 | 3388700 | 4 | 22 | 0 | 224 | 3457604 | 4 | 22 | 0 | 1 | 232 |
| 155 | 9B | 33 | 21 | 4 | 23 | 423616 | 3388924 | 4 | 23 | 0 | 224 | 3457836 | 4 | 23 | 0 | 1 | 232 |
| 156 | 9C | 33 | 21 | 4 | 24 | 423644 | 3389148 | 4 | 24 | 0 | 224 | 3458068 | 4 | 24 | 0 | 1 | 232 |
| 157 | 9D | 33 | 21 | 4 | 25 | 423672 | 3389372 | 4 | 25 | 0 | 224 | 3458300 | 4 | 25 | 0 | 1 | 232 |
| 158 | 9E | 33 | 21 | 4 | 26 | 423700 | 3389596 | 4 | 26 | 0 | 224 | 3458532 | 4 | 26 | 0 | 1 | 232 |
| 159 | 9F | 33 | 21 | 4 | 27 | 423728 | 3389820 | 4 | 27 | 0 | 224 | 3458764 | 4 | 27 | 0 | 1 | 232 |
| 160 | A0 | 33 | 21 | 4 | 28 | 423758 | 3390060 | 4 | 28 | 0 | 240 | 3459012 | 4 | 28 | 0 | 1 | 248 |
| 161 | A1 | 33 | 21 | 4 | 29 | 423788 | 3390300 | 4 | 29 | 0 | 240 | 3459260 | 4 | 29 | 0 | 1 | 248 |
| 162 | A2 | 33 | 21 | 4 | 30 | 423818 | 3390540 | 4 | 30 | 0 | 240 | 3459508 | 4 | 30 | 0 | 1 | 248 |
| 163 | A3 | 33 | 21 | 4 | 31 | 423848 | 3390780 | 4 | 31 | 0 | 240 | 3459756 | 4 | 31 | 0 | 1 | 248 |
| 164 | A4 | 33 | 21 | 4 | 32 | 423878 | 3391020 | 4 | 32 | 0 | 240 | 3460004 | 4 | 32 | 0 | 1 | 248 |
| 165 | A5 | 33 | 21 | 5 | 0 | 423910 | 3391276 | 5 | 0 | 0 | 256 | 3460268 | 5 | 0 | 0 | 1 | 264 |
| 166 | A6 | 33 | 21 | 5 | 1 | 423942 | 3391532 | 5 | 1 | 0 | 256 | 3460532 | 5 | 1 | 0 | 1 | 264 |
| 167 | A7 | 33 | 21 | 5 | 2 | 423974 | 3391788 | 5 | 2 | 0 | 256 | 3460796 | 5 | 2 | 0 | 1 | 264 |
| 168 | A8 | 33 | 21 | 5 | 3 | 424006 | 3392044 | 5 | 3 | 0 | 256 | 3461060 | 5 | 3 | 0 | 1 | 264 |
| 169 | A9 | 33 | 21 | 5 | 4 | 424038 | 3392300 | 5 | 4 | 0 | 256 | 3461324 | 5 | 4 | 0 | 1 | 264 |
| 170 | AA | 33 | 21 | 5 | 5 | 424070 | 3392556 | 5 | 5 | 0 | 256 | 3461588 | 5 | 5 | 0 | 1 | 264 |
| 171 | AB | 33 | 21 | 5 | 6 | 424102 | 3392812 | 5 | 6 | 0 | 256 | 3461852 | 5 | 6 | 0 | 1 | 264 |
| 172 | AC | 33 | 21 | 5 | 7 | 424134 | 3393068 | 5 | 7 | 0 | 256 | 3462116 | 5 | 7 | 0 | 1 | 264 |
| 173 | AD | 33 | 21 | 5 | 8 | 424166 | 3393324 | 5 | 8 | 0 | 256 | 3462380 | 5 | 8 | 0 | 1 | 264 |
| 174 | AE | 33 | 21 | 5 | 9 | 424198 | 3393580 | 5 | 9 | 0 | 256 | 3462644 | 5 | 9 | 0 | 1 | 264 |
| 175 | AF | 33 | 21 | 5 | 10 | 424230 | 3393836 | 5 | 10 | 0 | 256 | 3462908 | 5 | 10 | 0 | 1 | 264 |
| 176 | B0 | 33 | 21 | 5 | 11 | 424262 | 3394092 | 5 | 11 | 0 | 256 | 3463172 | 5 | 11 | 0 | 1 | 264 |
| 177 | B1 | 33 | 21 | 5 | 12 | 424294 | 3394348 | 5 | 12 | 0 | 256 | 3463436 | 5 | 12 | 0 | 1 | 264 |
| 178 | B2 | 33 | 21 | 5 | 13 | 424326 | 3394604 | 5 | 13 | 0 | 256 | 3463700 | 5 | 13 | 0 | 1 | 264 |
| 179 | B3 | 33 | 21 | 5 | 14 | 424358 | 3394860 | 5 | 14 | 0 | 256 | 3463964 | 5 | 14 | 0 | 1 | 264 |
| 180 | B4 | 33 | 21 | 5 | 15 | 424390 | 3395116 | 5 | 15 | 0 | 256 | 3464228 | 5 | 15 | 0 | 1 | 264 |
| 181 | B5 | 33 | 21 | 5 | 16 | 424422 | 3395372 | 5 | 16 | 0 | 256 | 3464492 | 5 | 16 | 0 | 1 | 264 |
| 182 | B6 | 33 | 21 | 5 | 17 | 424454 | 3395628 | 5 | 17 | 0 | 256 | 3464756 | 5 | 17 | 0 | 1 | 264 |
| 183 | B7 | 33 | 21 | 5 | 18 | 424486 | 3395884 | 5 | 18 | 0 | 256 | 3465020 | 5 | 18 | 0 | 1 | 264 |
| 184 | B8 | 33 | 21 | 5 | 19 | 424518 | 3396140 | 5 | 19 | 0 | 256 | 3465284 | 5 | 19 | 0 | 1 | 264 |
| 185 | B9 | 33 | 21 | 5 | 20 | 424550 | 3396396 | 5 | 20 | 0 | 256 | 3465548 | 5 | 20 | 0 | 1 | 264 |
| 186 | BA | 33 | 21 | 5 | 21 | 424582 | 3396652 | 5 | 21 | 0 | 256 | 3465812 | 5 | 21 | 0 | 1 | 264 |
| 187 | BB | 33 | 21 | 5 | 22 | 424614 | 3396908 | 5 | 22 | 0 | 256 | 3466076 | 5 | 22 | 0 | 1 | 264 |
| 188 | BC | 33 | 21 | 5 | 23 | 424646 | 3397164 | 5 | 23 | 0 | 256 | 3466340 | 5 | 23 | 0 | 1 | 264 |
| 189 | BD | 33 | 21 | 5 | 24 | 424678 | 3397420 | 5 | 24 | 0 | 256 | 3466604 | 5 | 24 | 0 | 1 | 264 |
| 190 | BE | 33 | 21 | 5 | 25 | 424710 | 3397676 | 5 | 25 | 0 | 256 | 3466868 | 5 | 25 | 0 | 1 | 264 |
| 191 | BF | 33 | 21 | 5 | 26 | 424742 | 3397932 | 5 | 26 | 0 | 256 | 3467132 | 5 | 26 | 0 | 1 | 264 |
| 192 | C0 | 33 | 21 | 5 | 27 | 424772 | 3398172 | 5 | 27 | 0 | 240 | 3467380 | 5 | 27 | 0 | 1 | 248 |
| 193 | C1 | 33 | 21 | 5 | 28 | 424802 | 3398412 | 5 | 28 | 0 | 240 | 3467628 | 5 | 28 | 0 | 1 | 248 |
| 194 | C2 | 33 | 21 | 5 | 29 | 424832 | 3398652 | 5 | 29 | 0 | 240 | 3467876 | 5 | 29 | 0 | 1 | 248 |
| 195 | C3 | 33 | 21 | 5 | 30 | 424862 | 3398892 | 5 | 30 | 0 | 240 | 3468124 | 5 | 30 | 0 | 1 | 248 |
| 196 | C4 | 33 | 21 | 5 | 31 | 424892 | 3399132 | 5 | 31 | 0 | 240 | 3468372 | 5 | 31 | 0 | 1 | 248 |
| 197 | C5 | 33 | 21 | 5 | 32 | 424922 | 3399372 | 5 | 32 | 0 | 240 | 3468620 | 5 | 32 | 0 | 1 | 248 |
| 198 | C6 | 33 | 21 | 6 | 0 | 424954 | 3399628 | 6 | 0 | 0 | 256 | 3468884 | 6 | 0 | 0 | 1 | 264 |
| 199 | C7 | 33 | 21 | 6 | 1 | 424986 | 3399884 | 6 | 1 | 0 | 256 | 3469148 | 6 | 1 | 0 | 1 | 264 |
| 200 | C8 | 33 | 21 | 6 | 2 | 425018 | 3400140 | 6 | 2 | 0 | 256 | 3469412 | 6 | 2 | 0 | 1 | 264 |

| | | | | | | | | | | | | | | | | | |
|---|---|---|---|---|---|---|---|---|---|---|---|---|---|---|---|---|---|
| 201 | C9 | 33 | 21 | 6 | 3 | 425050 | 3400396 | 6 | 3 | 0 | 256 | 3469676 | 6 | 3 | 0 | 1 | 264 |
| 202 | CA | 33 | 21 | 6 | 4 | 425082 | 3400652 | 6 | 4 | 0 | 256 | 3469940 | 6 | 4 | 0 | 1 | 264 |
| 203 | CB | 33 | 21 | 6 | 5 | 425114 | 3400908 | 6 | 5 | 0 | 256 | 3470204 | 6 | 5 | 0 | 1 | 264 |
| 204 | CC | 33 | 21 | 6 | 6 | 425146 | 3401164 | 6 | 6 | 0 | 256 | 3470468 | 6 | 6 | 0 | 1 | 264 |
| 205 | CD | 33 | 21 | 6 | 7 | 425178 | 3401420 | 6 | 7 | 0 | 256 | 3470732 | 6 | 7 | 0 | 1 | 264 |
| 206 | CE | 33 | 21 | 6 | 8 | 425210 | 3401676 | 6 | 8 | 0 | 256 | 3470996 | 6 | 8 | 0 | 1 | 264 |
| 207 | CF | 33 | 21 | 6 | 9 | 425242 | 3401932 | 6 | 9 | 0 | 256 | 3471260 | 6 | 9 | 0 | 1 | 264 |
| 208 | D0 | 33 | 21 | 6 | 10 | 425276 | 3402204 | 6 | 10 | 0 | 272 | 3471540 | 6 | 10 | 0 | 1 | 280 |
| 209 | D1 | 33 | 21 | 6 | 11 | 425310 | 3402476 | 6 | 11 | 0 | 272 | 3471820 | 6 | 11 | 0 | 1 | 280 |
| 210 | D2 | 33 | 21 | 6 | 12 | 425344 | 3402748 | 6 | 12 | 0 | 272 | 3472100 | 6 | 12 | 0 | 1 | 280 |
| 211 | D3 | 33 | 21 | 6 | 13 | 425378 | 3403020 | 6 | 13 | 0 | 272 | 3472380 | 6 | 13 | 0 | 1 | 280 |
| 212 | D4 | 33 | 21 | 6 | 14 | 425412 | 3403292 | 6 | 14 | 0 | 272 | 3472660 | 6 | 14 | 0 | 1 | 280 |
| 213 | D5 | 33 | 21 | 6 | 15 | 425446 | 3403564 | 6 | 15 | 0 | 272 | 3472940 | 6 | 15 | 0 | 1 | 280 |
| 214 | D6 | 33 | 21 | 6 | 16 | 425480 | 3403836 | 6 | 16 | 0 | 272 | 3473220 | 6 | 16 | 0 | 1 | 280 |
| 215 | D7 | 33 | 21 | 6 | 17 | 425514 | 3404108 | 6 | 17 | 0 | 272 | 3473500 | 6 | 17 | 0 | 1 | 280 |
| 216 | D8 | 33 | 21 | 6 | 18 | 425548 | 3404380 | 6 | 18 | 0 | 272 | 3473780 | 6 | 18 | 0 | 1 | 280 |
| 217 | D9 | 33 | 21 | 6 | 19 | 425582 | 3404652 | 6 | 19 | 0 | 272 | 3474060 | 6 | 19 | 0 | 1 | 280 |
| 218 | DA | 33 | 21 | 6 | 20 | 425616 | 3404924 | 6 | 20 | 0 | 272 | 3474340 | 6 | 20 | 0 | 1 | 280 |
| 219 | DB | 33 | 21 | 6 | 21 | 425650 | 3405196 | 6 | 21 | 0 | 272 | 3474620 | 6 | 21 | 0 | 1 | 280 |
| 220 | DC | 33 | 21 | 6 | 22 | 425684 | 3405468 | 6 | 22 | 0 | 272 | 3474900 | 6 | 22 | 0 | 1 | 280 |
| 221 | DD | 33 | 21 | 6 | 23 | 425718 | 3405740 | 6 | 23 | 0 | 272 | 3475180 | 6 | 23 | 0 | 1 | 280 |
| 222 | DE | 33 | 21 | 6 | 24 | 425752 | 3406012 | 6 | 24 | 0 | 272 | 3475460 | 6 | 24 | 0 | 1 | 280 |
| 223 | DF | 33 | 21 | 6 | 25 | 425786 | 3406284 | 6 | 25 | 0 | 272 | 3475740 | 6 | 25 | 0 | 1 | 280 |
| 224 | E0 | 33 | 21 | 6 | 26 | 425820 | 3406556 | 6 | 26 | 0 | 272 | 3476020 | 6 | 26 | 0 | 1 | 280 |
| 225 | E1 | 33 | 21 | 6 | 27 | 425854 | 3406828 | 6 | 27 | 0 | 272 | 3476300 | 6 | 27 | 0 | 1 | 280 |
| 226 | E2 | 33 | 21 | 6 | 28 | 425888 | 3407100 | 6 | 28 | 0 | 272 | 3476580 | 6 | 28 | 0 | 1 | 280 |
| 227 | E3 | 33 | 21 | 6 | 29 | 425922 | 3407372 | 6 | 29 | 0 | 272 | 3476860 | 6 | 29 | 0 | 1 | 280 |
| 228 | E4 | 33 | 21 | 6 | 30 | 425956 | 3407644 | 6 | 30 | 0 | 272 | 3477140 | 6 | 30 | 0 | 1 | 280 |
| 229 | E5 | 33 | 21 | 6 | 31 | 425990 | 3407916 | 6 | 31 | 0 | 272 | 3477420 | 6 | 31 | 0 | 1 | 280 |
| 230 | E6 | 33 | 21 | 6 | 32 | 426024 | 3408188 | 6 | 32 | 0 | 272 | 3477700 | 6 | 32 | 0 | 1 | 280 |
| 231 | E7 | 33 | 21 | 7 | 0 | 426060 | 3408476 | 7 | 0 | 0 | 288 | 3477996 | 7 | 0 | 0 | 1 | 296 |
| 232 | E8 | 33 | 21 | 7 | 1 | 426096 | 3408764 | 7 | 1 | 0 | 288 | 3478292 | 7 | 1 | 0 | 1 | 296 |
| 233 | E9 | 33 | 21 | 7 | 2 | 426132 | 3409052 | 7 | 2 | 0 | 288 | 3478588 | 7 | 2 | 0 | 1 | 296 |
| 234 | EA | 33 | 21 | 7 | 3 | 426168 | 3409340 | 7 | 3 | 0 | 288 | 3478884 | 7 | 3 | 0 | 1 | 296 |
| 235 | EB | 33 | 21 | 7 | 4 | 426204 | 3409628 | 7 | 4 | 0 | 288 | 3479180 | 7 | 4 | 0 | 1 | 296 |
| 236 | EC | 33 | 21 | 7 | 5 | 426240 | 3409916 | 7 | 5 | 0 | 288 | 3479476 | 7 | 5 | 0 | 1 | 296 |
| 237 | ED | 33 | 21 | 7 | 6 | 426276 | 3410204 | 7 | 6 | 0 | 288 | 3479772 | 7 | 6 | 0 | 1 | 296 |
| 238 | EE | 33 | 21 | 7 | 7 | 426312 | 3410492 | 7 | 7 | 0 | 288 | 3480068 | 7 | 7 | 0 | 1 | 296 |
| 239 | EF | 33 | 21 | 7 | 8 | 426348 | 3410780 | 7 | 8 | 0 | 288 | 3480364 | 7 | 8 | 0 | 1 | 296 |
| 240 | F0 | 33 | 21 | 7 | 9 | 426382 | 3411052 | 7 | 9 | 0 | 272 | 3480644 | 7 | 9 | 0 | 1 | 280 |
| 241 | F1 | 33 | 21 | 7 | 10 | 426416 | 3411324 | 7 | 10 | 0 | 272 | 3480924 | 7 | 10 | 0 | 1 | 280 |
| 242 | F2 | 33 | 21 | 7 | 11 | 426450 | 3411596 | 7 | 11 | 0 | 272 | 3481204 | 7 | 11 | 0 | 1 | 280 |
| 243 | F3 | 33 | 21 | 7 | 12 | 426484 | 3411868 | 7 | 12 | 0 | 272 | 3481484 | 7 | 12 | 0 | 1 | 280 |
| 244 | F4 | 33 | 21 | 7 | 13 | 426518 | 3412140 | 7 | 13 | 0 | 272 | 3481764 | 7 | 13 | 0 | 1 | 280 |
| 245 | F5 | 33 | 21 | 7 | 14 | 426552 | 3412412 | 7 | 14 | 0 | 272 | 3482044 | 7 | 14 | 0 | 1 | 280 |
| 246 | F6 | 33 | 21 | 7 | 15 | 426586 | 3412684 | 7 | 15 | 0 | 272 | 3482324 | 7 | 15 | 0 | 1 | 280 |
| 247 | F7 | 33 | 21 | 7 | 16 | 426620 | 3412956 | 7 | 16 | 0 | 272 | 3482604 | 7 | 16 | 0 | 1 | 280 |
| 248 | F8 | 33 | 21 | 7 | 17 | 426654 | 3413228 | 7 | 17 | 0 | 272 | 3482884 | 7 | 17 | 0 | 1 | 280 |
| 249 | F9 | 33 | 21 | 7 | 18 | 426688 | 3413500 | 7 | 18 | 0 | 272 | 3483164 | 7 | 18 | 0 | 1 | 280 |
| 250 | FA | 33 | 21 | 7 | 19 | 426722 | 3413772 | 7 | 19 | 0 | 272 | 3483444 | 7 | 19 | 0 | 1 | 280 |
| 251 | FB | 33 | 21 | 7 | 20 | 426756 | 3414044 | 7 | 20 | 0 | 272 | 3483724 | 7 | 20 | 0 | 1 | 280 |
| 252 | FC | 33 | 21 | 7 | 21 | 426790 | 3414316 | 7 | 21 | 0 | 272 | 3484004 | 7 | 21 | 0 | 1 | 280 |
| 253 | FD | 33 | 21 | 7 | 22 | 426824 | 3414588 | 7 | 22 | 0 | 272 | 3484284 | 7 | 22 | 0 | 1 | 280 |
| 254 | FE | 33 | 21 | 7 | 23 | 426858 | 3414860 | 7 | 23 | 0 | 272 | 3484564 | 7 | 23 | 0 | 1 | 280 |
| 255 | FF | 33 | 21 | 7 | 24 | 426892 | 3415132 | 7 | 24 | 0 | 272 | 3484844 | 7 | 24 | 0 | 1 | 280 |
| 0 | 00 | 34 | 22 | 0 | 0 | 426904 | 3415228 | 0 | 0 | 0 | 96 | 3484948 | 0 | 0 | 0 | 1 | 104 |
| 1 | 01 | 34 | 22 | 0 | 1 | 426918 | 3415340 | 0 | 1 | 0 | 112 | 3485068 | 0 | 1 | 0 | 1 | 120 |
| 2 | 02 | 34 | 22 | 0 | 2 | 426932 | 3415452 | 0 | 2 | 0 | 112 | 3485188 | 0 | 2 | 0 | 1 | 120 |
| 3 | 03 | 34 | 22 | 0 | 3 | 426946 | 3415564 | 0 | 3 | 0 | 112 | 3485308 | 0 | 3 | 0 | 1 | 120 |
| 4 | 04 | 34 | 22 | 0 | 4 | 426960 | 3415676 | 0 | 4 | 0 | 112 | 3485428 | 0 | 4 | 0 | 1 | 120 |
| 5 | 05 | 34 | 22 | 0 | 5 | 426974 | 3415788 | 0 | 5 | 0 | 112 | 3485548 | 0 | 5 | 0 | 1 | 120 |
| 6 | 06 | 34 | 22 | 0 | 6 | 426988 | 3415900 | 0 | 6 | 0 | 112 | 3485668 | 0 | 6 | 0 | 1 | 120 |
| 7 | 07 | 34 | 22 | 0 | 7 | 427002 | 3416012 | 0 | 7 | 0 | 112 | 3485788 | 0 | 7 | 0 | 1 | 120 |
| 8 | 08 | 34 | 22 | 0 | 8 | 427016 | 3416124 | 0 | 8 | 0 | 112 | 3485908 | 0 | 8 | 0 | 1 | 120 |
| 9 | 09 | 34 | 22 | 0 | 9 | 427030 | 3416236 | 0 | 9 | 0 | 112 | 3486028 | 0 | 9 | 0 | 1 | 120 |
| 10 | 0A | 34 | 22 | 0 | 10 | 427044 | 3416348 | 0 | 10 | 0 | 112 | 3486148 | 0 | 10 | 0 | 1 | 120 |
| 11 | 0B | 34 | 22 | 0 | 11 | 427058 | 3416460 | 0 | 11 | 0 | 112 | 3486268 | 0 | 11 | 0 | 1 | 120 |
| 12 | 0C | 34 | 22 | 0 | 12 | 427072 | 3416572 | 0 | 12 | 0 | 112 | 3486388 | 0 | 12 | 0 | 1 | 120 |
| 13 | 0D | 34 | 22 | 0 | 13 | 427086 | 3416684 | 0 | 13 | 0 | 112 | 3486508 | 0 | 13 | 0 | 1 | 120 |
| 14 | 0E | 34 | 22 | 0 | 14 | 427100 | 3416796 | 0 | 14 | 0 | 112 | 3486628 | 0 | 14 | 0 | 1 | 120 |
| 15 | 0F | 34 | 22 | 0 | 15 | 427114 | 3416908 | 0 | 15 | 0 | 112 | 3486748 | 0 | 15 | 0 | 1 | 120 |
| 16 | 10 | 34 | 22 | 0 | 16 | 427128 | 3417020 | 0 | 16 | 0 | 112 | 3486868 | 0 | 16 | 0 | 1 | 120 |
| 17 | 11 | 34 | 22 | 0 | 17 | 427142 | 3417132 | 0 | 17 | 0 | 112 | 3486988 | 0 | 17 | 0 | 1 | 120 |
| 18 | 12 | 34 | 22 | 0 | 18 | 427156 | 3417244 | 0 | 18 | 0 | 112 | 3487108 | 0 | 18 | 0 | 1 | 120 |
| 19 | 13 | 34 | 22 | 0 | 19 | 427170 | 3417356 | 0 | 19 | 0 | 112 | 3487228 | 0 | 19 | 0 | 1 | 120 |
| 20 | 14 | 34 | 22 | 0 | 20 | 427184 | 3417468 | 0 | 20 | 0 | 112 | 3487348 | 0 | 20 | 0 | 1 | 120 |
| 21 | 15 | 34 | 22 | 0 | 21 | 427198 | 3417580 | 0 | 21 | 0 | 112 | 3487468 | 0 | 21 | 0 | 1 | 120 |
| 22 | 16 | 34 | 22 | 0 | 22 | 427212 | 3417692 | 0 | 22 | 0 | 112 | 3487588 | 0 | 22 | 0 | 1 | 120 |
| 23 | 17 | 34 | 22 | 0 | 23 | 427226 | 3417804 | 0 | 23 | 0 | 112 | 3487708 | 0 | 23 | 0 | 1 | 120 |
| 24 | 18 | 34 | 22 | 0 | 24 | 427240 | 3417916 | 0 | 24 | 0 | 112 | 3487828 | 0 | 24 | 0 | 1 | 120 |
| 25 | 19 | 34 | 22 | 0 | 25 | 427254 | 3418028 | 0 | 25 | 0 | 112 | 3487948 | 0 | 25 | 0 | 1 | 120 |
| 26 | 1A | 34 | 22 | 0 | 26 | 427268 | 3418140 | 0 | 26 | 0 | 112 | 3488068 | 0 | 26 | 0 | 1 | 120 |
| 27 | 1B | 34 | 22 | 0 | 27 | 427282 | 3418252 | 0 | 27 | 0 | 112 | 3488188 | 0 | 27 | 0 | 1 | 120 |
| 28 | 1C | 34 | 22 | 0 | 28 | 427296 | 3418364 | 0 | 28 | 0 | 112 | 3488308 | 0 | 28 | 0 | 1 | 120 |
| 29 | 1D | 34 | 22 | 0 | 29 | 427310 | 3418476 | 0 | 29 | 0 | 112 | 3488428 | 0 | 29 | 0 | 1 | 120 |
| 30 | 1E | 34 | 22 | 0 | 30 | 427324 | 3418588 | 0 | 30 | 0 | 112 | 3488548 | 0 | 30 | 0 | 1 | 120 |
| 31 | 1F | 34 | 22 | 0 | 31 | 427338 | 3418700 | 0 | 31 | 0 | 112 | 3488668 | 0 | 31 | 0 | 1 | 120 |
| 32 | 20 | 34 | 22 | 0 | 32 | 427352 | 3418812 | 0 | 32 | 0 | 112 | 3488788 | 0 | 32 | 0 | 1 | 120 |
| 33 | 21 | 34 | 22 | 0 | 33 | 427366 | 3418924 | 0 | 33 | 0 | 112 | 3488908 | 0 | 33 | 0 | 1 | 120 |
| 34 | 22 | 34 | 22 | 1 | 0 | 427378 | 3419020 | 1 | 0 | 0 | 96 | 3489012 | 1 | 0 | 0 | 1 | 104 |
| 35 | 23 | 34 | 22 | 1 | 1 | 427395 | 3419156 | 1 | 1 | 0 | 136 | 3489156 | 1 | 1 | 0 | 1 | 144 |
| 36 | 24 | 34 | 22 | 1 | 2 | 427412 | 3419292 | 1 | 2 | 0 | 136 | 3489300 | 1 | 2 | 0 | 1 | 144 |
| 37 | 25 | 34 | 22 | 1 | 3 | 427429 | 3419428 | 1 | 3 | 0 | 136 | 3489444 | 1 | 3 | 0 | 1 | 144 |
| 38 | 26 | 34 | 22 | 1 | 4 | 427446 | 3419564 | 1 | 4 | 0 | 136 | 3489588 | 1 | 4 | 0 | 1 | 144 |
| 39 | 27 | 34 | 22 | 1 | 5 | 427463 | 3419700 | 1 | 5 | 0 | 136 | 3489732 | 1 | 5 | 0 | 1 | 144 |
| 40 | 28 | 34 | 22 | 1 | 6 | 427480 | 3419836 | 1 | 6 | 0 | 136 | 3489876 | 1 | 6 | 0 | 1 | 144 |
| 41 | 29 | 34 | 22 | 1 | 7 | 427497 | 3419972 | 1 | 7 | 0 | 136 | 3490020 | 1 | 7 | 0 | 1 | 144 |
| 42 | 2A | 34 | 22 | 1 | 8 | 427514 | 3420108 | 1 | 8 | 0 | 136 | 3490164 | 1 | 8 | 0 | 1 | 144 |
| 43 | 2B | 34 | 22 | 1 | 9 | 427531 | 3420244 | 1 | 9 | 0 | 136 | 3490308 | 1 | 9 | 0 | 1 | 144 |
| 44 | 2C | 34 | 22 | 1 | 10 | 427548 | 3420380 | 1 | 10 | 0 | 136 | 3490452 | 1 | 10 | 0 | 1 | 144 |
| 45 | 2D | 34 | 22 | 1 | 11 | 427565 | 3420516 | 1 | 11 | 0 | 136 | 3490596 | 1 | 11 | 0 | 1 | 144 |
| 46 | 2E | 34 | 22 | 1 | 12 | 427582 | 3420652 | 1 | 12 | 0 | 136 | 3490740 | 1 | 12 | 0 | 1 | 144 |
| 47 | 2F | 34 | 22 | 1 | 13 | 427599 | 3420788 | 1 | 13 | 0 | 136 | 3490884 | 1 | 13 | 0 | 1 | 144 |

| | | | | | | | | | | | | | | | | | |
|---|---|---|---|---|---|---|---|---|---|---|---|---|---|---|---|---|---|
| 48 | 30 | 34 | 22 | 1 | 14 | 427620 | 3420956 | 1 | 14 | 0 | 168 | 3491060 | 1 | 14 | 0 | 1 | 176 |
| 49 | 31 | 34 | 22 | 1 | 15 | 427641 | 3421124 | 1 | 15 | 0 | 168 | 3491236 | 1 | 15 | 0 | 1 | 176 |
| 50 | 32 | 34 | 22 | 1 | 16 | 427662 | 3421292 | 1 | 16 | 0 | 168 | 3491412 | 1 | 16 | 0 | 1 | 176 |
| 51 | 33 | 34 | 22 | 1 | 17 | 427683 | 3421460 | 1 | 17 | 0 | 168 | 3491588 | 1 | 17 | 0 | 1 | 176 |
| 52 | 34 | 34 | 22 | 1 | 18 | 427704 | 3421628 | 1 | 18 | 0 | 168 | 3491764 | 1 | 18 | 0 | 1 | 176 |
| 53 | 35 | 34 | 22 | 1 | 19 | 427725 | 3421796 | 1 | 19 | 0 | 168 | 3491940 | 1 | 19 | 0 | 1 | 176 |
| 54 | 36 | 34 | 22 | 1 | 20 | 427746 | 3421964 | 1 | 20 | 0 | 168 | 3492116 | 1 | 20 | 0 | 1 | 176 |
| 55 | 37 | 34 | 22 | 1 | 21 | 427767 | 3422132 | 1 | 21 | 0 | 168 | 3492292 | 1 | 21 | 0 | 1 | 176 |
| 56 | 38 | 34 | 22 | 1 | 22 | 427788 | 3422300 | 1 | 22 | 0 | 168 | 3492468 | 1 | 22 | 0 | 1 | 176 |
| 57 | 39 | 34 | 22 | 1 | 23 | 427809 | 3422468 | 1 | 23 | 0 | 168 | 3492644 | 1 | 23 | 0 | 1 | 176 |
| 58 | 3A | 34 | 22 | 1 | 24 | 427830 | 3422636 | 1 | 24 | 0 | 168 | 3492820 | 1 | 24 | 0 | 1 | 176 |
| 59 | 3B | 34 | 22 | 1 | 25 | 427851 | 3422804 | 1 | 25 | 0 | 168 | 3492996 | 1 | 25 | 0 | 1 | 176 |
| 60 | 3C | 34 | 22 | 1 | 26 | 427872 | 3422972 | 1 | 26 | 0 | 168 | 3493172 | 1 | 26 | 0 | 1 | 176 |
| 61 | 3D | 34 | 22 | 1 | 27 | 427893 | 3423140 | 1 | 27 | 0 | 168 | 3493348 | 1 | 27 | 0 | 1 | 176 |
| 62 | 3E | 34 | 22 | 1 | 28 | 427914 | 3423308 | 1 | 28 | 0 | 168 | 3493524 | 1 | 28 | 0 | 1 | 176 |
| 63 | 3F | 34 | 22 | 1 | 29 | 427935 | 3423476 | 1 | 29 | 0 | 168 | 3493700 | 1 | 29 | 0 | 1 | 176 |
| 64 | 40 | 34 | 22 | 1 | 30 | 427958 | 3423660 | 1 | 30 | 0 | 184 | 3493892 | 1 | 30 | 0 | 1 | 192 |
| 65 | 41 | 34 | 22 | 1 | 31 | 427981 | 3423844 | 1 | 31 | 0 | 184 | 3494084 | 1 | 31 | 0 | 1 | 192 |
| 66 | 42 | 34 | 22 | 1 | 32 | 428004 | 3424028 | 1 | 32 | 0 | 184 | 3494276 | 1 | 32 | 0 | 1 | 192 |
| 67 | 43 | 34 | 22 | 1 | 33 | 428027 | 3424212 | 1 | 33 | 0 | 184 | 3494468 | 1 | 33 | 0 | 1 | 192 |
| 68 | 44 | 34 | 22 | 2 | 0 | 428048 | 3424380 | 2 | 0 | 0 | 168 | 3494644 | 2 | 0 | 0 | 1 | 176 |
| 69 | 45 | 34 | 22 | 2 | 1 | 428074 | 3424588 | 2 | 1 | 0 | 208 | 3494860 | 2 | 1 | 0 | 1 | 216 |
| 70 | 46 | 34 | 22 | 2 | 2 | 428100 | 3424796 | 2 | 2 | 0 | 208 | 3495076 | 2 | 2 | 0 | 1 | 216 |
| 71 | 47 | 34 | 22 | 2 | 3 | 428126 | 3425004 | 2 | 3 | 0 | 208 | 3495292 | 2 | 3 | 0 | 1 | 216 |
| 72 | 48 | 34 | 22 | 2 | 4 | 428152 | 3425212 | 2 | 4 | 0 | 208 | 3495508 | 2 | 4 | 0 | 1 | 216 |
| 73 | 49 | 34 | 22 | 2 | 5 | 428178 | 3425420 | 2 | 5 | 0 | 208 | 3495724 | 2 | 5 | 0 | 1 | 216 |
| 74 | 4A | 34 | 22 | 2 | 6 | 428204 | 3425628 | 2 | 6 | 0 | 208 | 3495940 | 2 | 6 | 0 | 1 | 216 |
| 75 | 4B | 34 | 22 | 2 | 7 | 428230 | 3425836 | 2 | 7 | 0 | 208 | 3496156 | 2 | 7 | 0 | 1 | 216 |
| 76 | 4C | 34 | 22 | 2 | 8 | 428256 | 3426044 | 2 | 8 | 0 | 208 | 3496372 | 2 | 8 | 0 | 1 | 216 |
| 77 | 4D | 34 | 22 | 2 | 9 | 428282 | 3426252 | 2 | 9 | 0 | 208 | 3496588 | 2 | 9 | 0 | 1 | 216 |
| 78 | 4E | 34 | 22 | 2 | 10 | 428308 | 3426460 | 2 | 10 | 0 | 208 | 3496804 | 2 | 10 | 0 | 1 | 216 |
| 79 | 4F | 34 | 22 | 2 | 11 | 428334 | 3426668 | 2 | 11 | 0 | 208 | 3497020 | 2 | 11 | 0 | 1 | 216 |
| 80 | 50 | 34 | 22 | 2 | 12 | 428360 | 3426876 | 2 | 12 | 0 | 208 | 3497236 | 2 | 12 | 0 | 1 | 216 |
| 81 | 51 | 34 | 22 | 2 | 13 | 428386 | 3427084 | 2 | 13 | 0 | 208 | 3497452 | 2 | 13 | 0 | 1 | 216 |
| 82 | 52 | 34 | 22 | 2 | 14 | 428412 | 3427292 | 2 | 14 | 0 | 208 | 3497668 | 2 | 14 | 0 | 1 | 216 |
| 83 | 53 | 34 | 22 | 2 | 15 | 428438 | 3427500 | 2 | 15 | 0 | 208 | 3497884 | 2 | 15 | 0 | 1 | 216 |
| 84 | 54 | 34 | 22 | 2 | 16 | 428464 | 3427708 | 2 | 16 | 0 | 208 | 3498100 | 2 | 16 | 0 | 1 | 216 |
| 85 | 55 | 34 | 22 | 2 | 17 | 428490 | 3427916 | 2 | 17 | 0 | 208 | 3498316 | 2 | 17 | 0 | 1 | 216 |
| 86 | 56 | 34 | 22 | 2 | 18 | 428516 | 3428124 | 2 | 18 | 0 | 208 | 3498532 | 2 | 18 | 0 | 1 | 216 |
| 87 | 57 | 34 | 22 | 2 | 19 | 428542 | 3428332 | 2 | 19 | 0 | 208 | 3498748 | 2 | 19 | 0 | 1 | 216 |
| 88 | 58 | 34 | 22 | 2 | 20 | 428568 | 3428540 | 2 | 20 | 0 | 208 | 3498964 | 2 | 20 | 0 | 1 | 216 |
| 89 | 59 | 34 | 22 | 2 | 21 | 428594 | 3428748 | 2 | 21 | 0 | 208 | 3499180 | 2 | 21 | 0 | 1 | 216 |
| 90 | 5A | 34 | 22 | 2 | 22 | 428620 | 3428956 | 2 | 22 | 0 | 208 | 3499396 | 2 | 22 | 0 | 1 | 216 |
| 91 | 5B | 34 | 22 | 2 | 23 | 428646 | 3429164 | 2 | 23 | 0 | 208 | 3499612 | 2 | 23 | 0 | 1 | 216 |
| 92 | 5C | 34 | 22 | 2 | 24 | 428672 | 3429372 | 2 | 24 | 0 | 208 | 3499828 | 2 | 24 | 0 | 1 | 216 |
| 93 | 5D | 34 | 22 | 2 | 25 | 428698 | 3429580 | 2 | 25 | 0 | 208 | 3500044 | 2 | 25 | 0 | 1 | 216 |
| 94 | 5E | 34 | 22 | 2 | 26 | 428724 | 3429788 | 2 | 26 | 0 | 208 | 3500260 | 2 | 26 | 0 | 1 | 216 |
| 95 | 5F | 34 | 22 | 2 | 27 | 428750 | 3429996 | 2 | 27 | 0 | 208 | 3500476 | 2 | 27 | 0 | 1 | 216 |
| 96 | 60 | 34 | 22 | 2 | 28 | 428773 | 3430180 | 2 | 28 | 0 | 184 | 3500668 | 2 | 28 | 0 | 1 | 192 |
| 97 | 61 | 34 | 22 | 2 | 29 | 428796 | 3430364 | 2 | 29 | 0 | 184 | 3500860 | 2 | 29 | 0 | 1 | 192 |
| 98 | 62 | 34 | 22 | 2 | 30 | 428819 | 3430548 | 2 | 30 | 0 | 184 | 3501052 | 2 | 30 | 0 | 1 | 192 |
| 99 | 63 | 34 | 22 | 2 | 31 | 428842 | 3430732 | 2 | 31 | 0 | 184 | 3501244 | 2 | 31 | 0 | 1 | 192 |
| 100 | 64 | 34 | 22 | 2 | 32 | 428865 | 3430916 | 2 | 32 | 0 | 184 | 3501436 | 2 | 32 | 0 | 1 | 192 |
| 101 | 65 | 34 | 22 | 2 | 33 | 428888 | 3431100 | 2 | 33 | 0 | 184 | 3501628 | 2 | 33 | 0 | 1 | 192 |
| 102 | 66 | 34 | 22 | 3 | 0 | 428909 | 3431268 | 3 | 0 | 0 | 168 | 3501804 | 3 | 0 | 0 | 1 | 176 |
| 103 | 67 | 34 | 22 | 3 | 1 | 428935 | 3431476 | 3 | 1 | 0 | 208 | 3502020 | 3 | 1 | 0 | 1 | 216 |
| 104 | 68 | 34 | 22 | 3 | 2 | 428961 | 3431684 | 3 | 2 | 0 | 208 | 3502236 | 3 | 2 | 0 | 1 | 216 |
| 105 | 69 | 34 | 22 | 3 | 3 | 428987 | 3431892 | 3 | 3 | 0 | 208 | 3502452 | 3 | 3 | 0 | 1 | 216 |
| 106 | 6A | 34 | 22 | 3 | 4 | 429013 | 3432100 | 3 | 4 | 0 | 208 | 3502668 | 3 | 4 | 0 | 1 | 216 |
| 107 | 6B | 34 | 22 | 3 | 5 | 429039 | 3432308 | 3 | 5 | 0 | 208 | 3502884 | 3 | 5 | 0 | 1 | 216 |
| 108 | 6C | 34 | 22 | 3 | 6 | 429065 | 3432516 | 3 | 6 | 0 | 208 | 3503100 | 3 | 6 | 0 | 1 | 216 |
| 109 | 6D | 34 | 22 | 3 | 7 | 429091 | 3432724 | 3 | 7 | 0 | 208 | 3503316 | 3 | 7 | 0 | 1 | 216 |
| 110 | 6E | 34 | 22 | 3 | 8 | 429117 | 3432932 | 3 | 8 | 0 | 208 | 3503532 | 3 | 8 | 0 | 1 | 216 |
| 111 | 6F | 34 | 22 | 3 | 9 | 429143 | 3433140 | 3 | 9 | 0 | 208 | 3503748 | 3 | 9 | 0 | 1 | 216 |
| 112 | 70 | 34 | 22 | 3 | 10 | 429171 | 3433364 | 3 | 10 | 0 | 224 | 3503980 | 3 | 10 | 0 | 1 | 232 |
| 113 | 71 | 34 | 22 | 3 | 11 | 429199 | 3433588 | 3 | 11 | 0 | 224 | 3504212 | 3 | 11 | 0 | 1 | 232 |
| 114 | 72 | 34 | 22 | 3 | 12 | 429227 | 3433812 | 3 | 12 | 0 | 224 | 3504444 | 3 | 12 | 0 | 1 | 232 |
| 115 | 73 | 34 | 22 | 3 | 13 | 429255 | 3434036 | 3 | 13 | 0 | 224 | 3504676 | 3 | 13 | 0 | 1 | 232 |
| 116 | 74 | 34 | 22 | 3 | 14 | 429283 | 3434260 | 3 | 14 | 0 | 224 | 3504908 | 3 | 14 | 0 | 1 | 232 |
| 117 | 75 | 34 | 22 | 3 | 15 | 429311 | 3434484 | 3 | 15 | 0 | 224 | 3505140 | 3 | 15 | 0 | 1 | 232 |
| 118 | 76 | 34 | 22 | 3 | 16 | 429339 | 3434708 | 3 | 16 | 0 | 224 | 3505372 | 3 | 16 | 0 | 1 | 232 |
| 119 | 77 | 34 | 22 | 3 | 17 | 429367 | 3434932 | 3 | 17 | 0 | 224 | 3505604 | 3 | 17 | 0 | 1 | 232 |
| 120 | 78 | 34 | 22 | 3 | 18 | 429395 | 3435156 | 3 | 18 | 0 | 224 | 3505836 | 3 | 18 | 0 | 1 | 232 |
| 121 | 79 | 34 | 22 | 3 | 19 | 429423 | 3435380 | 3 | 19 | 0 | 224 | 3506068 | 3 | 19 | 0 | 1 | 232 |
| 122 | 7A | 34 | 22 | 3 | 20 | 429451 | 3435604 | 3 | 20 | 0 | 224 | 3506300 | 3 | 20 | 0 | 1 | 232 |
| 123 | 7B | 34 | 22 | 3 | 21 | 429479 | 3435828 | 3 | 21 | 0 | 224 | 3506532 | 3 | 21 | 0 | 1 | 232 |
| 124 | 7C | 34 | 22 | 3 | 22 | 429507 | 3436052 | 3 | 22 | 0 | 224 | 3506764 | 3 | 22 | 0 | 1 | 232 |
| 125 | 7D | 34 | 22 | 3 | 23 | 429535 | 3436276 | 3 | 23 | 0 | 224 | 3506996 | 3 | 23 | 0 | 1 | 232 |
| 126 | 7E | 34 | 22 | 3 | 24 | 429563 | 3436500 | 3 | 24 | 0 | 224 | 3507228 | 3 | 24 | 0 | 1 | 232 |
| 127 | 7F | 34 | 22 | 3 | 25 | 429591 | 3436724 | 3 | 25 | 0 | 224 | 3507460 | 3 | 25 | 0 | 1 | 232 |
| 128 | 80 | 34 | 22 | 3 | 26 | 429619 | 3436948 | 3 | 26 | 0 | 224 | 3507692 | 3 | 26 | 0 | 1 | 232 |
| 129 | 81 | 34 | 22 | 3 | 27 | 429647 | 3437172 | 3 | 27 | 0 | 224 | 3507924 | 3 | 27 | 0 | 1 | 232 |
| 130 | 82 | 34 | 22 | 3 | 28 | 429675 | 3437396 | 3 | 28 | 0 | 224 | 3508156 | 3 | 28 | 0 | 1 | 232 |
| 131 | 83 | 34 | 22 | 3 | 29 | 429703 | 3437620 | 3 | 29 | 0 | 224 | 3508388 | 3 | 29 | 0 | 1 | 232 |
| 132 | 84 | 34 | 22 | 3 | 30 | 429731 | 3437844 | 3 | 30 | 0 | 224 | 3508620 | 3 | 30 | 0 | 1 | 232 |
| 133 | 85 | 34 | 22 | 3 | 31 | 429759 | 3438068 | 3 | 31 | 0 | 224 | 3508852 | 3 | 31 | 0 | 1 | 232 |
| 134 | 86 | 34 | 22 | 3 | 32 | 429787 | 3438292 | 3 | 32 | 0 | 224 | 3509084 | 3 | 32 | 0 | 1 | 232 |
| 135 | 87 | 34 | 22 | 3 | 33 | 429815 | 3438516 | 3 | 33 | 0 | 224 | 3509316 | 3 | 33 | 0 | 1 | 232 |
| 136 | 88 | 34 | 22 | 4 | 0 | 429845 | 3438756 | 4 | 0 | 0 | 240 | 3509564 | 4 | 0 | 0 | 1 | 248 |
| 137 | 89 | 34 | 22 | 4 | 1 | 429875 | 3438996 | 4 | 1 | 0 | 240 | 3509812 | 4 | 1 | 0 | 1 | 248 |
| 138 | 8A | 34 | 22 | 4 | 2 | 429905 | 3439236 | 4 | 2 | 0 | 240 | 3510060 | 4 | 2 | 0 | 1 | 248 |
| 139 | 8B | 34 | 22 | 4 | 3 | 429935 | 3439476 | 4 | 3 | 0 | 240 | 3510308 | 4 | 3 | 0 | 1 | 248 |
| 140 | 8C | 34 | 22 | 4 | 4 | 429965 | 3439716 | 4 | 4 | 0 | 240 | 3510556 | 4 | 4 | 0 | 1 | 248 |
| 141 | 8D | 34 | 22 | 4 | 5 | 429995 | 3439956 | 4 | 5 | 0 | 240 | 3510804 | 4 | 5 | 0 | 1 | 248 |
| 142 | 8E | 34 | 22 | 4 | 6 | 430025 | 3440196 | 4 | 6 | 0 | 240 | 3511052 | 4 | 6 | 0 | 1 | 248 |
| 143 | 8F | 34 | 22 | 4 | 7 | 430055 | 3440436 | 4 | 7 | 0 | 240 | 3511300 | 4 | 7 | 0 | 1 | 248 |
| 144 | 90 | 34 | 22 | 4 | 8 | 430083 | 3440660 | 4 | 8 | 0 | 224 | 3511532 | 4 | 8 | 0 | 1 | 232 |
| 145 | 91 | 34 | 22 | 4 | 9 | 430111 | 3440884 | 4 | 9 | 0 | 224 | 3511764 | 4 | 9 | 0 | 1 | 232 |
| 146 | 92 | 34 | 22 | 4 | 10 | 430139 | 3441108 | 4 | 10 | 0 | 224 | 3511996 | 4 | 10 | 0 | 1 | 232 |
| 147 | 93 | 34 | 22 | 4 | 11 | 430167 | 3441332 | 4 | 11 | 0 | 224 | 3512228 | 4 | 11 | 0 | 1 | 232 |
| 148 | 94 | 34 | 22 | 4 | 12 | 430195 | 3441556 | 4 | 12 | 0 | 224 | 3512460 | 4 | 12 | 0 | 1 | 232 |
| 149 | 95 | 34 | 22 | 4 | 13 | 430223 | 3441780 | 4 | 13 | 0 | 224 | 3512692 | 4 | 13 | 0 | 1 | 232 |
| 150 | 96 | 34 | 22 | 4 | 14 | 430251 | 3442004 | 4 | 14 | 0 | 224 | 3512924 | 4 | 14 | 0 | 1 | 232 |

| | | | | | | | | | | | | | | | | | |
|---|---|---|---|---|---|---|---|---|---|---|---|---|---|---|---|---|---|
| 151 | 97 | 34 | 22 | 4 | 15 | 430279 | 3442228 | 4 | 15 | 0 | 224 | 3513156 | 4 | 15 | 0 | 1 | 232 |
| 152 | 98 | 34 | 22 | 4 | 16 | 430307 | 3442452 | 4 | 16 | 0 | 224 | 3513388 | 4 | 16 | 0 | 1 | 232 |
| 153 | 99 | 34 | 22 | 4 | 17 | 430335 | 3442676 | 4 | 17 | 0 | 224 | 3513620 | 4 | 17 | 0 | 1 | 232 |
| 154 | 9A | 34 | 22 | 4 | 18 | 430363 | 3442900 | 4 | 18 | 0 | 224 | 3513852 | 4 | 18 | 0 | 1 | 232 |
| 155 | 9B | 34 | 22 | 4 | 19 | 430391 | 3443124 | 4 | 19 | 0 | 224 | 3514084 | 4 | 19 | 0 | 1 | 232 |
| 156 | 9C | 34 | 22 | 4 | 20 | 430419 | 3443348 | 4 | 20 | 0 | 224 | 3514316 | 4 | 20 | 0 | 1 | 232 |
| 157 | 9D | 34 | 22 | 4 | 21 | 430447 | 3443572 | 4 | 21 | 0 | 224 | 3514548 | 4 | 21 | 0 | 1 | 232 |
| 158 | 9E | 34 | 22 | 4 | 22 | 430475 | 3443796 | 4 | 22 | 0 | 224 | 3514780 | 4 | 22 | 0 | 1 | 232 |
| 159 | 9F | 34 | 22 | 4 | 23 | 430503 | 3444020 | 4 | 23 | 0 | 224 | 3515012 | 4 | 23 | 0 | 1 | 232 |
| 160 | A0 | 34 | 22 | 4 | 24 | 430533 | 3444260 | 4 | 24 | 0 | 240 | 3515260 | 4 | 24 | 0 | 1 | 248 |
| 161 | A1 | 34 | 22 | 4 | 25 | 430563 | 3444500 | 4 | 25 | 0 | 240 | 3515508 | 4 | 25 | 0 | 1 | 248 |
| 162 | A2 | 34 | 22 | 4 | 26 | 430593 | 3444740 | 4 | 26 | 0 | 240 | 3515756 | 4 | 26 | 0 | 1 | 248 |
| 163 | A3 | 34 | 22 | 4 | 27 | 430623 | 3444980 | 4 | 27 | 0 | 240 | 3516004 | 4 | 27 | 0 | 1 | 248 |
| 164 | A4 | 34 | 22 | 4 | 28 | 430653 | 3445220 | 4 | 28 | 0 | 240 | 3516252 | 4 | 28 | 0 | 1 | 248 |
| 165 | A5 | 34 | 22 | 4 | 29 | 430683 | 3445460 | 4 | 29 | 0 | 240 | 3516500 | 4 | 29 | 0 | 1 | 248 |
| 166 | A6 | 34 | 22 | 4 | 30 | 430713 | 3445700 | 4 | 30 | 0 | 240 | 3516748 | 4 | 30 | 0 | 1 | 248 |
| 167 | A7 | 34 | 22 | 4 | 31 | 430743 | 3445940 | 4 | 31 | 0 | 240 | 3516996 | 4 | 31 | 0 | 1 | 248 |
| 168 | A8 | 34 | 22 | 4 | 32 | 430773 | 3446180 | 4 | 32 | 0 | 240 | 3517244 | 4 | 32 | 0 | 1 | 248 |
| 169 | A9 | 34 | 22 | 4 | 33 | 430803 | 3446420 | 4 | 33 | 0 | 240 | 3517492 | 4 | 33 | 0 | 1 | 248 |
| 170 | AA | 34 | 22 | 5 | 0 | 430835 | 3446676 | 5 | 0 | 0 | 256 | 3517756 | 5 | 0 | 0 | 1 | 264 |
| 171 | AB | 34 | 22 | 5 | 1 | 430867 | 3446932 | 5 | 1 | 0 | 256 | 3518020 | 5 | 1 | 0 | 1 | 264 |
| 172 | AC | 34 | 22 | 5 | 2 | 430899 | 3447188 | 5 | 2 | 0 | 256 | 3518284 | 5 | 2 | 0 | 1 | 264 |
| 173 | AD | 34 | 22 | 5 | 3 | 430931 | 3447444 | 5 | 3 | 0 | 256 | 3518548 | 5 | 3 | 0 | 1 | 264 |
| 174 | AE | 34 | 22 | 5 | 4 | 430963 | 3447700 | 5 | 4 | 0 | 256 | 3518812 | 5 | 4 | 0 | 1 | 264 |
| 175 | AF | 34 | 22 | 5 | 5 | 430995 | 3447956 | 5 | 5 | 0 | 256 | 3519076 | 5 | 5 | 0 | 1 | 264 |
| 176 | B0 | 34 | 22 | 5 | 6 | 431027 | 3448212 | 5 | 6 | 0 | 256 | 3519340 | 5 | 6 | 0 | 1 | 264 |
| 177 | B1 | 34 | 22 | 5 | 7 | 431059 | 3448468 | 5 | 7 | 0 | 256 | 3519604 | 5 | 7 | 0 | 1 | 264 |
| 178 | B2 | 34 | 22 | 5 | 8 | 431091 | 3448724 | 5 | 8 | 0 | 256 | 3519868 | 5 | 8 | 0 | 1 | 264 |
| 179 | B3 | 34 | 22 | 5 | 9 | 431123 | 3448980 | 5 | 9 | 0 | 256 | 3520132 | 5 | 9 | 0 | 1 | 264 |
| 180 | B4 | 34 | 22 | 5 | 10 | 431155 | 3449236 | 5 | 10 | 0 | 256 | 3520396 | 5 | 10 | 0 | 1 | 264 |
| 181 | B5 | 34 | 22 | 5 | 11 | 431187 | 3449492 | 5 | 11 | 0 | 256 | 3520660 | 5 | 11 | 0 | 1 | 264 |
| 182 | B6 | 34 | 22 | 5 | 12 | 431219 | 3449748 | 5 | 12 | 0 | 256 | 3520924 | 5 | 12 | 0 | 1 | 264 |
| 183 | B7 | 34 | 22 | 5 | 13 | 431251 | 3450004 | 5 | 13 | 0 | 256 | 3521188 | 5 | 13 | 0 | 1 | 264 |
| 184 | B8 | 34 | 22 | 5 | 14 | 431283 | 3450260 | 5 | 14 | 0 | 256 | 3521452 | 5 | 14 | 0 | 1 | 264 |
| 185 | B9 | 34 | 22 | 5 | 15 | 431315 | 3450516 | 5 | 15 | 0 | 256 | 3521716 | 5 | 15 | 0 | 1 | 264 |
| 186 | BA | 34 | 22 | 5 | 16 | 431347 | 3450772 | 5 | 16 | 0 | 256 | 3521980 | 5 | 16 | 0 | 1 | 264 |
| 187 | BB | 34 | 22 | 5 | 17 | 431379 | 3451028 | 5 | 17 | 0 | 256 | 3522244 | 5 | 17 | 0 | 1 | 264 |
| 188 | BC | 34 | 22 | 5 | 18 | 431411 | 3451284 | 5 | 18 | 0 | 256 | 3522508 | 5 | 18 | 0 | 1 | 264 |
| 189 | BD | 34 | 22 | 5 | 19 | 431443 | 3451540 | 5 | 19 | 0 | 256 | 3522772 | 5 | 19 | 0 | 1 | 264 |
| 190 | BE | 34 | 22 | 5 | 20 | 431475 | 3451796 | 5 | 20 | 0 | 256 | 3523036 | 5 | 20 | 0 | 1 | 264 |
| 191 | BF | 34 | 22 | 5 | 21 | 431507 | 3452052 | 5 | 21 | 0 | 256 | 3523300 | 5 | 21 | 0 | 1 | 264 |
| 192 | C0 | 34 | 22 | 5 | 22 | 431537 | 3452292 | 5 | 22 | 0 | 240 | 3523548 | 5 | 22 | 0 | 1 | 248 |
| 193 | C1 | 34 | 22 | 5 | 23 | 431567 | 3452532 | 5 | 23 | 0 | 240 | 3523796 | 5 | 23 | 0 | 1 | 248 |
| 194 | C2 | 34 | 22 | 5 | 24 | 431597 | 3452772 | 5 | 24 | 0 | 240 | 3524044 | 5 | 24 | 0 | 1 | 248 |
| 195 | C3 | 34 | 22 | 5 | 25 | 431627 | 3453012 | 5 | 25 | 0 | 240 | 3524292 | 5 | 25 | 0 | 1 | 248 |
| 196 | C4 | 34 | 22 | 5 | 26 | 431657 | 3453252 | 5 | 26 | 0 | 240 | 3524540 | 5 | 26 | 0 | 1 | 248 |
| 197 | C5 | 34 | 22 | 5 | 27 | 431687 | 3453492 | 5 | 27 | 0 | 240 | 3524788 | 5 | 27 | 0 | 1 | 248 |
| 198 | C6 | 34 | 22 | 5 | 28 | 431717 | 3453732 | 5 | 28 | 0 | 240 | 3525036 | 5 | 28 | 0 | 1 | 248 |
| 199 | C7 | 34 | 22 | 5 | 29 | 431747 | 3453972 | 5 | 29 | 0 | 240 | 3525284 | 5 | 29 | 0 | 1 | 248 |
| 200 | C8 | 34 | 22 | 5 | 30 | 431777 | 3454212 | 5 | 30 | 0 | 240 | 3525532 | 5 | 30 | 0 | 1 | 248 |
| 201 | C9 | 34 | 22 | 5 | 31 | 431807 | 3454452 | 5 | 31 | 0 | 240 | 3525780 | 5 | 31 | 0 | 1 | 248 |
| 202 | CA | 34 | 22 | 5 | 32 | 431837 | 3454692 | 5 | 32 | 0 | 240 | 3526028 | 5 | 32 | 0 | 1 | 248 |
| 203 | CB | 34 | 22 | 5 | 33 | 431867 | 3454932 | 5 | 33 | 0 | 240 | 3526276 | 5 | 33 | 0 | 1 | 248 |
| 204 | CC | 34 | 22 | 6 | 0 | 431899 | 3455188 | 6 | 0 | 0 | 256 | 3526540 | 6 | 0 | 0 | 1 | 264 |
| 205 | CD | 34 | 22 | 6 | 1 | 431931 | 3455444 | 6 | 1 | 0 | 256 | 3526804 | 6 | 1 | 0 | 1 | 264 |
| 206 | CE | 34 | 22 | 6 | 2 | 431963 | 3455700 | 6 | 2 | 0 | 256 | 3527068 | 6 | 2 | 0 | 1 | 264 |
| 207 | CF | 34 | 22 | 6 | 3 | 431995 | 3455956 | 6 | 3 | 0 | 256 | 3527332 | 6 | 3 | 0 | 1 | 264 |
| 208 | D0 | 34 | 22 | 6 | 4 | 432029 | 3456228 | 6 | 4 | 0 | 272 | 3527612 | 6 | 4 | 0 | 1 | 280 |
| 209 | D1 | 34 | 22 | 6 | 5 | 432063 | 3456500 | 6 | 5 | 0 | 272 | 3527892 | 6 | 5 | 0 | 1 | 280 |
| 210 | D2 | 34 | 22 | 6 | 6 | 432097 | 3456772 | 6 | 6 | 0 | 272 | 3528172 | 6 | 6 | 0 | 1 | 280 |
| 211 | D3 | 34 | 22 | 6 | 7 | 432131 | 3457044 | 6 | 7 | 0 | 272 | 3528452 | 6 | 7 | 0 | 1 | 280 |
| 212 | D4 | 34 | 22 | 6 | 8 | 432165 | 3457316 | 6 | 8 | 0 | 272 | 3528732 | 6 | 8 | 0 | 1 | 280 |
| 213 | D5 | 34 | 22 | 6 | 9 | 432199 | 3457588 | 6 | 9 | 0 | 272 | 3529012 | 6 | 9 | 0 | 1 | 280 |
| 214 | D6 | 34 | 22 | 6 | 10 | 432233 | 3457860 | 6 | 10 | 0 | 272 | 3529292 | 6 | 10 | 0 | 1 | 280 |
| 215 | D7 | 34 | 22 | 6 | 11 | 432267 | 3458132 | 6 | 11 | 0 | 272 | 3529572 | 6 | 11 | 0 | 1 | 280 |
| 216 | D8 | 34 | 22 | 6 | 12 | 432301 | 3458404 | 6 | 12 | 0 | 272 | 3529852 | 6 | 12 | 0 | 1 | 280 |
| 217 | D9 | 34 | 22 | 6 | 13 | 432335 | 3458676 | 6 | 13 | 0 | 272 | 3530132 | 6 | 13 | 0 | 1 | 280 |
| 218 | DA | 34 | 22 | 6 | 14 | 432369 | 3458948 | 6 | 14 | 0 | 272 | 3530412 | 6 | 14 | 0 | 1 | 280 |
| 219 | DB | 34 | 22 | 6 | 15 | 432403 | 3459220 | 6 | 15 | 0 | 272 | 3530692 | 6 | 15 | 0 | 1 | 280 |
| 220 | DC | 34 | 22 | 6 | 16 | 432437 | 3459492 | 6 | 16 | 0 | 272 | 3530972 | 6 | 16 | 0 | 1 | 280 |
| 221 | DD | 34 | 22 | 6 | 17 | 432471 | 3459764 | 6 | 17 | 0 | 272 | 3531252 | 6 | 17 | 0 | 1 | 280 |
| 222 | DE | 34 | 22 | 6 | 18 | 432505 | 3460036 | 6 | 18 | 0 | 272 | 3531532 | 6 | 18 | 0 | 1 | 280 |
| 223 | DF | 34 | 22 | 6 | 19 | 432539 | 3460308 | 6 | 19 | 0 | 272 | 3531812 | 6 | 19 | 0 | 1 | 280 |
| 224 | E0 | 34 | 22 | 6 | 20 | 432573 | 3460580 | 6 | 20 | 0 | 272 | 3532092 | 6 | 20 | 0 | 1 | 280 |
| 225 | E1 | 34 | 22 | 6 | 21 | 432607 | 3460852 | 6 | 21 | 0 | 272 | 3532372 | 6 | 21 | 0 | 1 | 280 |
| 226 | E2 | 34 | 22 | 6 | 22 | 432641 | 3461124 | 6 | 22 | 0 | 272 | 3532652 | 6 | 22 | 0 | 1 | 280 |
| 227 | E3 | 34 | 22 | 6 | 23 | 432675 | 3461396 | 6 | 23 | 0 | 272 | 3532932 | 6 | 23 | 0 | 1 | 280 |
| 228 | E4 | 34 | 22 | 6 | 24 | 432709 | 3461668 | 6 | 24 | 0 | 272 | 3533212 | 6 | 24 | 0 | 1 | 280 |
| 229 | E5 | 34 | 22 | 6 | 25 | 432743 | 3461940 | 6 | 25 | 0 | 272 | 3533492 | 6 | 25 | 0 | 1 | 280 |
| 230 | E6 | 34 | 22 | 6 | 26 | 432777 | 3462212 | 6 | 26 | 0 | 272 | 3533772 | 6 | 26 | 0 | 1 | 280 |
| 231 | E7 | 34 | 22 | 6 | 27 | 432811 | 3462484 | 6 | 27 | 0 | 272 | 3534052 | 6 | 27 | 0 | 1 | 280 |
| 232 | E8 | 34 | 22 | 6 | 28 | 432845 | 3462756 | 6 | 28 | 0 | 272 | 3534332 | 6 | 28 | 0 | 1 | 280 |
| 233 | E9 | 34 | 22 | 6 | 29 | 432879 | 3463028 | 6 | 29 | 0 | 272 | 3534612 | 6 | 29 | 0 | 1 | 280 |
| 234 | EA | 34 | 22 | 6 | 30 | 432913 | 3463300 | 6 | 30 | 0 | 272 | 3534892 | 6 | 30 | 0 | 1 | 280 |
| 235 | EB | 34 | 22 | 6 | 31 | 432947 | 3463572 | 6 | 31 | 0 | 272 | 3535172 | 6 | 31 | 0 | 1 | 280 |
| 236 | EC | 34 | 22 | 6 | 32 | 432981 | 3463844 | 6 | 32 | 0 | 272 | 3535452 | 6 | 32 | 0 | 1 | 280 |
| 237 | ED | 34 | 22 | 6 | 33 | 433015 | 3464116 | 6 | 33 | 0 | 272 | 3535732 | 6 | 33 | 0 | 1 | 280 |
| 238 | EE | 34 | 22 | 7 | 0 | 433051 | 3464404 | 7 | 0 | 0 | 288 | 3536028 | 7 | 0 | 0 | 1 | 296 |
| 239 | EF | 34 | 22 | 7 | 1 | 433087 | 3464692 | 7 | 1 | 0 | 288 | 3536324 | 7 | 1 | 0 | 1 | 296 |
| 240 | F0 | 34 | 22 | 7 | 2 | 433121 | 3464964 | 7 | 2 | 0 | 272 | 3536604 | 7 | 2 | 0 | 1 | 280 |
| 241 | F1 | 34 | 22 | 7 | 3 | 433155 | 3465236 | 7 | 3 | 0 | 272 | 3536884 | 7 | 3 | 0 | 1 | 280 |
| 242 | F2 | 34 | 22 | 7 | 4 | 433189 | 3465508 | 7 | 4 | 0 | 272 | 3537164 | 7 | 4 | 0 | 1 | 280 |
| 243 | F3 | 34 | 22 | 7 | 5 | 433223 | 3465780 | 7 | 5 | 0 | 272 | 3537444 | 7 | 5 | 0 | 1 | 280 |
| 244 | F4 | 34 | 22 | 7 | 6 | 433257 | 3466052 | 7 | 6 | 0 | 272 | 3537724 | 7 | 6 | 0 | 1 | 280 |
| 245 | F5 | 34 | 22 | 7 | 7 | 433291 | 3466324 | 7 | 7 | 0 | 272 | 3538004 | 7 | 7 | 0 | 1 | 280 |
| 246 | F6 | 34 | 22 | 7 | 8 | 433325 | 3466596 | 7 | 8 | 0 | 272 | 3538284 | 7 | 8 | 0 | 1 | 280 |
| 247 | F7 | 34 | 22 | 7 | 9 | 433359 | 3466868 | 7 | 9 | 0 | 272 | 3538564 | 7 | 9 | 0 | 1 | 280 |
| 248 | F8 | 34 | 22 | 7 | 10 | 433393 | 3467140 | 7 | 10 | 0 | 272 | 3538844 | 7 | 10 | 0 | 1 | 280 |
| 249 | F9 | 34 | 22 | 7 | 11 | 433427 | 3467412 | 7 | 11 | 0 | 272 | 3539124 | 7 | 11 | 0 | 1 | 280 |
| 250 | FA | 34 | 22 | 7 | 12 | 433461 | 3467684 | 7 | 12 | 0 | 272 | 3539404 | 7 | 12 | 0 | 1 | 280 |
| 251 | FB | 34 | 22 | 7 | 13 | 433495 | 3467956 | 7 | 13 | 0 | 272 | 3539684 | 7 | 13 | 0 | 1 | 280 |
| 252 | FC | 34 | 22 | 7 | 14 | 433529 | 3468228 | 7 | 14 | 0 | 272 | 3539964 | 7 | 14 | 0 | 1 | 280 |
| 253 | FD | 34 | 22 | 7 | 15 | 433563 | 3468500 | 7 | 15 | 0 | 272 | 3540244 | 7 | 15 | 0 | 1 | 280 |

| | | | | | | | | | | | | | | | | |
|---|---|---|---|---|---|---|---|---|---|---|---|---|---|---|---|---|
| 254 | FE | 34 | 22 | 7 | 16 | 433597 | 3468772 | 7 | 16 | 0 | 272 | 3540524 | 7 | 16 | 0 | 1 | 280 |
| 255 | FF | 34 | 22 | 7 | 17 | 433631 | 3469044 | 7 | 17 | 0 | 272 | 3540804 | 7 | 17 | 0 | 1 | 280 |
| 0 | 00 | 35 | 23 | 0 | 0 | 433643 | 3469140 | 0 | 0 | 0 | 96 | 3540908 | 0 | 0 | 0 | 1 | 104 |
| 1 | 01 | 35 | 23 | 0 | 1 | 433657 | 3469252 | 0 | 1 | 0 | 112 | 3541028 | 0 | 1 | 0 | 1 | 120 |
| 2 | 02 | 35 | 23 | 0 | 2 | 433671 | 3469364 | 0 | 2 | 0 | 112 | 3541148 | 0 | 2 | 0 | 1 | 120 |
| 3 | 03 | 35 | 23 | 0 | 3 | 433685 | 3469476 | 0 | 3 | 0 | 112 | 3541268 | 0 | 3 | 0 | 1 | 120 |
| 4 | 04 | 35 | 23 | 0 | 4 | 433699 | 3469588 | 0 | 4 | 0 | 112 | 3541388 | 0 | 4 | 0 | 1 | 120 |
| 5 | 05 | 35 | 23 | 0 | 5 | 433713 | 3469700 | 0 | 5 | 0 | 112 | 3541508 | 0 | 5 | 0 | 1 | 120 |
| 6 | 06 | 35 | 23 | 0 | 6 | 433727 | 3469812 | 0 | 6 | 0 | 112 | 3541628 | 0 | 6 | 0 | 1 | 120 |
| 7 | 07 | 35 | 23 | 0 | 7 | 433741 | 3469924 | 0 | 7 | 0 | 112 | 3541748 | 0 | 7 | 0 | 1 | 120 |
| 8 | 08 | 35 | 23 | 0 | 8 | 433755 | 3470036 | 0 | 8 | 0 | 112 | 3541868 | 0 | 8 | 0 | 1 | 120 |
| 9 | 09 | 35 | 23 | 0 | 9 | 433769 | 3470148 | 0 | 9 | 0 | 112 | 3541988 | 0 | 9 | 0 | 1 | 120 |
| 10 | 0A | 35 | 23 | 0 | 10 | 433783 | 3470260 | 0 | 10 | 0 | 112 | 3542108 | 0 | 10 | 0 | 1 | 120 |
| 11 | 0B | 35 | 23 | 0 | 11 | 433797 | 3470372 | 0 | 11 | 0 | 112 | 3542228 | 0 | 11 | 0 | 1 | 120 |
| 12 | 0C | 35 | 23 | 0 | 12 | 433811 | 3470484 | 0 | 12 | 0 | 112 | 3542348 | 0 | 12 | 0 | 1 | 120 |
| 13 | 0D | 35 | 23 | 0 | 13 | 433825 | 3470596 | 0 | 13 | 0 | 112 | 3542468 | 0 | 13 | 0 | 1 | 120 |
| 14 | 0E | 35 | 23 | 0 | 14 | 433839 | 3470708 | 0 | 14 | 0 | 112 | 3542588 | 0 | 14 | 0 | 1 | 120 |
| 15 | 0F | 35 | 23 | 0 | 15 | 433853 | 3470820 | 0 | 15 | 0 | 112 | 3542708 | 0 | 15 | 0 | 1 | 120 |
| 16 | 10 | 35 | 23 | 0 | 16 | 433867 | 3470932 | 0 | 16 | 0 | 112 | 3542828 | 0 | 16 | 0 | 1 | 120 |
| 17 | 11 | 35 | 23 | 0 | 17 | 433881 | 3471044 | 0 | 17 | 0 | 112 | 3542948 | 0 | 17 | 0 | 1 | 120 |
| 18 | 12 | 35 | 23 | 0 | 18 | 433895 | 3471156 | 0 | 18 | 0 | 112 | 3543068 | 0 | 18 | 0 | 1 | 120 |
| 19 | 13 | 35 | 23 | 0 | 19 | 433909 | 3471268 | 0 | 19 | 0 | 112 | 3543188 | 0 | 19 | 0 | 1 | 120 |
| 20 | 14 | 35 | 23 | 0 | 20 | 433923 | 3471380 | 0 | 20 | 0 | 112 | 3543308 | 0 | 20 | 0 | 1 | 120 |
| 21 | 15 | 35 | 23 | 0 | 21 | 433937 | 3471492 | 0 | 21 | 0 | 112 | 3543428 | 0 | 21 | 0 | 1 | 120 |
| 22 | 16 | 35 | 23 | 0 | 22 | 433951 | 3471604 | 0 | 22 | 0 | 112 | 3543548 | 0 | 22 | 0 | 1 | 120 |
| 23 | 17 | 35 | 23 | 0 | 23 | 433965 | 3471716 | 0 | 23 | 0 | 112 | 3543668 | 0 | 23 | 0 | 1 | 120 |
| 24 | 18 | 35 | 23 | 0 | 24 | 433979 | 3471828 | 0 | 24 | 0 | 112 | 3543788 | 0 | 24 | 0 | 1 | 120 |
| 25 | 19 | 35 | 23 | 0 | 25 | 433993 | 3471940 | 0 | 25 | 0 | 112 | 3543908 | 0 | 25 | 0 | 1 | 120 |
| 26 | 1A | 35 | 23 | 0 | 26 | 434007 | 3472052 | 0 | 26 | 0 | 112 | 3544028 | 0 | 26 | 0 | 1 | 120 |
| 27 | 1B | 35 | 23 | 0 | 27 | 434021 | 3472164 | 0 | 27 | 0 | 112 | 3544148 | 0 | 27 | 0 | 1 | 120 |
| 28 | 1C | 35 | 23 | 0 | 28 | 434035 | 3472276 | 0 | 28 | 0 | 112 | 3544268 | 0 | 28 | 0 | 1 | 120 |
| 29 | 1D | 35 | 23 | 0 | 29 | 434049 | 3472388 | 0 | 29 | 0 | 112 | 3544388 | 0 | 29 | 0 | 1 | 120 |
| 30 | 1E | 35 | 23 | 0 | 30 | 434063 | 3472500 | 0 | 30 | 0 | 112 | 3544508 | 0 | 30 | 0 | 1 | 120 |
| 31 | 1F | 35 | 23 | 0 | 31 | 434077 | 3472612 | 0 | 31 | 0 | 112 | 3544628 | 0 | 31 | 0 | 1 | 120 |
| 32 | 20 | 35 | 23 | 0 | 32 | 434091 | 3472724 | 0 | 32 | 0 | 112 | 3544748 | 0 | 32 | 0 | 1 | 120 |
| 33 | 21 | 35 | 23 | 0 | 33 | 434105 | 3472836 | 0 | 33 | 0 | 112 | 3544868 | 0 | 33 | 0 | 1 | 120 |
| 34 | 22 | 35 | 23 | 0 | 34 | 434119 | 3472948 | 0 | 34 | 0 | 112 | 3544988 | 0 | 34 | 0 | 1 | 120 |
| 35 | 23 | 35 | 23 | 1 | 0 | 434131 | 3473044 | 1 | 0 | 0 | 96 | 3545092 | 1 | 0 | 0 | 1 | 104 |
| 36 | 24 | 35 | 23 | 1 | 1 | 434148 | 3473180 | 1 | 1 | 0 | 136 | 3545236 | 1 | 1 | 0 | 1 | 144 |
| 37 | 25 | 35 | 23 | 1 | 2 | 434165 | 3473316 | 1 | 2 | 0 | 136 | 3545380 | 1 | 2 | 0 | 1 | 144 |
| 38 | 26 | 35 | 23 | 1 | 3 | 434182 | 3473452 | 1 | 3 | 0 | 136 | 3545524 | 1 | 3 | 0 | 1 | 144 |
| 39 | 27 | 35 | 23 | 1 | 4 | 434199 | 3473588 | 1 | 4 | 0 | 136 | 3545668 | 1 | 4 | 0 | 1 | 144 |
| 40 | 28 | 35 | 23 | 1 | 5 | 434216 | 3473724 | 1 | 5 | 0 | 136 | 3545812 | 1 | 5 | 0 | 1 | 144 |
| 41 | 29 | 35 | 23 | 1 | 6 | 434233 | 3473860 | 1 | 6 | 0 | 136 | 3545956 | 1 | 6 | 0 | 1 | 144 |
| 42 | 2A | 35 | 23 | 1 | 7 | 434250 | 3473996 | 1 | 7 | 0 | 136 | 3546100 | 1 | 7 | 0 | 1 | 144 |
| 43 | 2B | 35 | 23 | 1 | 8 | 434267 | 3474132 | 1 | 8 | 0 | 136 | 3546244 | 1 | 8 | 0 | 1 | 144 |
| 44 | 2C | 35 | 23 | 1 | 9 | 434284 | 3474268 | 1 | 9 | 0 | 136 | 3546388 | 1 | 9 | 0 | 1 | 144 |
| 45 | 2D | 35 | 23 | 1 | 10 | 434301 | 3474404 | 1 | 10 | 0 | 136 | 3546532 | 1 | 10 | 0 | 1 | 144 |
| 46 | 2E | 35 | 23 | 1 | 11 | 434318 | 3474540 | 1 | 11 | 0 | 136 | 3546676 | 1 | 11 | 0 | 1 | 144 |
| 47 | 2F | 35 | 23 | 1 | 12 | 434335 | 3474676 | 1 | 12 | 0 | 136 | 3546820 | 1 | 12 | 0 | 1 | 144 |
| 48 | 30 | 35 | 23 | 1 | 13 | 434356 | 3474844 | 1 | 13 | 0 | 168 | 3546996 | 1 | 13 | 0 | 1 | 176 |
| 49 | 31 | 35 | 23 | 1 | 14 | 434377 | 3475012 | 1 | 14 | 0 | 168 | 3547172 | 1 | 14 | 0 | 1 | 176 |
| 50 | 32 | 35 | 23 | 1 | 15 | 434398 | 3475180 | 1 | 15 | 0 | 168 | 3547348 | 1 | 15 | 0 | 1 | 176 |
| 51 | 33 | 35 | 23 | 1 | 16 | 434419 | 3475348 | 1 | 16 | 0 | 168 | 3547524 | 1 | 16 | 0 | 1 | 176 |
| 52 | 34 | 35 | 23 | 1 | 17 | 434440 | 3475516 | 1 | 17 | 0 | 168 | 3547700 | 1 | 17 | 0 | 1 | 176 |
| 53 | 35 | 35 | 23 | 1 | 18 | 434461 | 3475684 | 1 | 18 | 0 | 168 | 3547876 | 1 | 18 | 0 | 1 | 176 |
| 54 | 36 | 35 | 23 | 1 | 19 | 434482 | 3475852 | 1 | 19 | 0 | 168 | 3548052 | 1 | 19 | 0 | 1 | 176 |
| 55 | 37 | 35 | 23 | 1 | 20 | 434503 | 3476020 | 1 | 20 | 0 | 168 | 3548228 | 1 | 20 | 0 | 1 | 176 |
| 56 | 38 | 35 | 23 | 1 | 21 | 434524 | 3476188 | 1 | 21 | 0 | 168 | 3548404 | 1 | 21 | 0 | 1 | 176 |
| 57 | 39 | 35 | 23 | 1 | 22 | 434545 | 3476356 | 1 | 22 | 0 | 168 | 3548580 | 1 | 22 | 0 | 1 | 176 |
| 58 | 3A | 35 | 23 | 1 | 23 | 434566 | 3476524 | 1 | 23 | 0 | 168 | 3548756 | 1 | 23 | 0 | 1 | 176 |
| 59 | 3B | 35 | 23 | 1 | 24 | 434587 | 3476692 | 1 | 24 | 0 | 168 | 3548932 | 1 | 24 | 0 | 1 | 176 |
| 60 | 3C | 35 | 23 | 1 | 25 | 434608 | 3476860 | 1 | 25 | 0 | 168 | 3549108 | 1 | 25 | 0 | 1 | 176 |
| 61 | 3D | 35 | 23 | 1 | 26 | 434629 | 3477028 | 1 | 26 | 0 | 168 | 3549284 | 1 | 26 | 0 | 1 | 176 |
| 62 | 3E | 35 | 23 | 1 | 27 | 434650 | 3477196 | 1 | 27 | 0 | 168 | 3549460 | 1 | 27 | 0 | 1 | 176 |
| 63 | 3F | 35 | 23 | 1 | 28 | 434671 | 3477364 | 1 | 28 | 0 | 168 | 3549636 | 1 | 28 | 0 | 1 | 176 |
| 64 | 40 | 35 | 23 | 1 | 29 | 434694 | 3477548 | 1 | 29 | 0 | 184 | 3549828 | 1 | 29 | 0 | 1 | 192 |
| 65 | 41 | 35 | 23 | 1 | 30 | 434717 | 3477732 | 1 | 30 | 0 | 184 | 3550020 | 1 | 30 | 0 | 1 | 192 |
| 66 | 42 | 35 | 23 | 1 | 31 | 434740 | 3477916 | 1 | 31 | 0 | 184 | 3550212 | 1 | 31 | 0 | 1 | 192 |
| 67 | 43 | 35 | 23 | 1 | 32 | 434763 | 3478100 | 1 | 32 | 0 | 184 | 3550404 | 1 | 32 | 0 | 1 | 192 |
| 68 | 44 | 35 | 23 | 1 | 33 | 434786 | 3478284 | 1 | 33 | 0 | 184 | 3550596 | 1 | 33 | 0 | 1 | 192 |
| 69 | 45 | 35 | 23 | 1 | 34 | 434809 | 3478468 | 1 | 34 | 0 | 184 | 3550788 | 1 | 34 | 0 | 1 | 192 |
| 70 | 46 | 35 | 23 | 2 | 0 | 434830 | 3478636 | 2 | 0 | 0 | 168 | 3550964 | 2 | 0 | 0 | 1 | 176 |
| 71 | 47 | 35 | 23 | 2 | 1 | 434856 | 3478844 | 2 | 1 | 0 | 208 | 3551180 | 2 | 1 | 0 | 1 | 216 |
| 72 | 48 | 35 | 23 | 2 | 2 | 434882 | 3479052 | 2 | 2 | 0 | 208 | 3551396 | 2 | 2 | 0 | 1 | 216 |
| 73 | 49 | 35 | 23 | 2 | 3 | 434908 | 3479260 | 2 | 3 | 0 | 208 | 3551612 | 2 | 3 | 0 | 1 | 216 |
| 74 | 4A | 35 | 23 | 2 | 4 | 434934 | 3479468 | 2 | 4 | 0 | 208 | 3551828 | 2 | 4 | 0 | 1 | 216 |
| 75 | 4B | 35 | 23 | 2 | 5 | 434960 | 3479676 | 2 | 5 | 0 | 208 | 3552044 | 2 | 5 | 0 | 1 | 216 |
| 76 | 4C | 35 | 23 | 2 | 6 | 434986 | 3479884 | 2 | 6 | 0 | 208 | 3552260 | 2 | 6 | 0 | 1 | 216 |
| 77 | 4D | 35 | 23 | 2 | 7 | 435012 | 3480092 | 2 | 7 | 0 | 208 | 3552476 | 2 | 7 | 0 | 1 | 216 |
| 78 | 4E | 35 | 23 | 2 | 8 | 435038 | 3480300 | 2 | 8 | 0 | 208 | 3552692 | 2 | 8 | 0 | 1 | 216 |
| 79 | 4F | 35 | 23 | 2 | 9 | 435064 | 3480508 | 2 | 9 | 0 | 208 | 3552908 | 2 | 9 | 0 | 1 | 216 |
| 80 | 50 | 35 | 23 | 2 | 10 | 435090 | 3480716 | 2 | 10 | 0 | 208 | 3553124 | 2 | 10 | 0 | 1 | 216 |
| 81 | 51 | 35 | 23 | 2 | 11 | 435116 | 3480924 | 2 | 11 | 0 | 208 | 3553340 | 2 | 11 | 0 | 1 | 216 |
| 82 | 52 | 35 | 23 | 2 | 12 | 435142 | 3481132 | 2 | 12 | 0 | 208 | 3553556 | 2 | 12 | 0 | 1 | 216 |
| 83 | 53 | 35 | 23 | 2 | 13 | 435168 | 3481340 | 2 | 13 | 0 | 208 | 3553772 | 2 | 13 | 0 | 1 | 216 |
| 84 | 54 | 35 | 23 | 2 | 14 | 435194 | 3481548 | 2 | 14 | 0 | 208 | 3553988 | 2 | 14 | 0 | 1 | 216 |
| 85 | 55 | 35 | 23 | 2 | 15 | 435220 | 3481756 | 2 | 15 | 0 | 208 | 3554204 | 2 | 15 | 0 | 1 | 216 |
| 86 | 56 | 35 | 23 | 2 | 16 | 435246 | 3481964 | 2 | 16 | 0 | 208 | 3554420 | 2 | 16 | 0 | 1 | 216 |
| 87 | 57 | 35 | 23 | 2 | 17 | 435272 | 3482172 | 2 | 17 | 0 | 208 | 3554636 | 2 | 17 | 0 | 1 | 216 |
| 88 | 58 | 35 | 23 | 2 | 18 | 435298 | 3482380 | 2 | 18 | 0 | 208 | 3554852 | 2 | 18 | 0 | 1 | 216 |
| 89 | 59 | 35 | 23 | 2 | 19 | 435324 | 3482588 | 2 | 19 | 0 | 208 | 3555068 | 2 | 19 | 0 | 1 | 216 |
| 90 | 5A | 35 | 23 | 2 | 20 | 435350 | 3482796 | 2 | 20 | 0 | 208 | 3555284 | 2 | 20 | 0 | 1 | 216 |
| 91 | 5B | 35 | 23 | 2 | 21 | 435376 | 3483004 | 2 | 21 | 0 | 208 | 3555500 | 2 | 21 | 0 | 1 | 216 |
| 92 | 5C | 35 | 23 | 2 | 22 | 435402 | 3483212 | 2 | 22 | 0 | 208 | 3555716 | 2 | 22 | 0 | 1 | 216 |
| 93 | 5D | 35 | 23 | 2 | 23 | 435428 | 3483420 | 2 | 23 | 0 | 208 | 3555932 | 2 | 23 | 0 | 1 | 216 |
| 94 | 5E | 35 | 23 | 2 | 24 | 435454 | 3483628 | 2 | 24 | 0 | 208 | 3556148 | 2 | 24 | 0 | 1 | 216 |
| 95 | 5F | 35 | 23 | 2 | 25 | 435480 | 3483836 | 2 | 25 | 0 | 208 | 3556364 | 2 | 25 | 0 | 1 | 216 |
| 96 | 60 | 35 | 23 | 2 | 26 | 435503 | 3484020 | 2 | 26 | 0 | 184 | 3556556 | 2 | 26 | 0 | 1 | 192 |
| 97 | 61 | 35 | 23 | 2 | 27 | 435526 | 3484204 | 2 | 27 | 0 | 184 | 3556748 | 2 | 27 | 0 | 1 | 192 |
| 98 | 62 | 35 | 23 | 2 | 28 | 435549 | 3484388 | 2 | 28 | 0 | 184 | 3556940 | 2 | 28 | 0 | 1 | 192 |
| 99 | 63 | 35 | 23 | 2 | 29 | 435572 | 3484572 | 2 | 29 | 0 | 184 | 3557132 | 2 | 29 | 0 | 1 | 192 |
| 100 | 64 | 35 | 23 | 2 | 30 | 435595 | 3484756 | 2 | 30 | 0 | 184 | 3557324 | 2 | 30 | 0 | 1 | 192 |

| | | | | | | | | | | | | | | | | | |
|---|---|---|---|---|---|---|---|---|---|---|---|---|---|---|---|---|---|
| 101 | 65 | 35 | 23 | 2 | 31 | 435618 | 3484940 | 2 | 31 | 0 | 184 | 3557516 | 2 | 31 | 0 | 1 | 192 |
| 102 | 66 | 35 | 23 | 2 | 32 | 435641 | 3485124 | 2 | 32 | 0 | 184 | 3557708 | 2 | 32 | 0 | 1 | 192 |
| 103 | 67 | 35 | 23 | 2 | 33 | 435664 | 3485308 | 2 | 33 | 0 | 184 | 3557900 | 2 | 33 | 0 | 1 | 192 |
| 104 | 68 | 35 | 23 | 2 | 34 | 435687 | 3485492 | 2 | 34 | 0 | 184 | 3558092 | 2 | 34 | 0 | 1 | 192 |
| 105 | 69 | 35 | 23 | 3 | 0 | 435708 | 3485660 | 3 | 0 | 0 | 168 | 3558268 | 3 | 0 | 0 | 1 | 176 |
| 106 | 6A | 35 | 23 | 3 | 1 | 435734 | 3485868 | 3 | 1 | 0 | 208 | 3558484 | 3 | 1 | 0 | 1 | 216 |
| 107 | 6B | 35 | 23 | 3 | 2 | 435760 | 3486076 | 3 | 2 | 0 | 208 | 3558700 | 3 | 2 | 0 | 1 | 216 |
| 108 | 6C | 35 | 23 | 3 | 3 | 435786 | 3486284 | 3 | 3 | 0 | 208 | 3558916 | 3 | 3 | 0 | 1 | 216 |
| 109 | 6D | 35 | 23 | 3 | 4 | 435812 | 3486492 | 3 | 4 | 0 | 208 | 3559132 | 3 | 4 | 0 | 1 | 216 |
| 110 | 6E | 35 | 23 | 3 | 5 | 435838 | 3486700 | 3 | 5 | 0 | 208 | 3559348 | 3 | 5 | 0 | 1 | 216 |
| 111 | 6F | 35 | 23 | 3 | 6 | 435864 | 3486908 | 3 | 6 | 0 | 208 | 3559564 | 3 | 6 | 0 | 1 | 216 |
| 112 | 70 | 35 | 23 | 3 | 7 | 435892 | 3487132 | 3 | 7 | 0 | 224 | 3559796 | 3 | 7 | 0 | 1 | 232 |
| 113 | 71 | 35 | 23 | 3 | 8 | 435920 | 3487356 | 3 | 8 | 0 | 224 | 3560028 | 3 | 8 | 0 | 1 | 232 |
| 114 | 72 | 35 | 23 | 3 | 9 | 435948 | 3487580 | 3 | 9 | 0 | 224 | 3560260 | 3 | 9 | 0 | 1 | 232 |
| 115 | 73 | 35 | 23 | 3 | 10 | 435976 | 3487804 | 3 | 10 | 0 | 224 | 3560492 | 3 | 10 | 0 | 1 | 232 |
| 116 | 74 | 35 | 23 | 3 | 11 | 436004 | 3488028 | 3 | 11 | 0 | 224 | 3560724 | 3 | 11 | 0 | 1 | 232 |
| 117 | 75 | 35 | 23 | 3 | 12 | 436032 | 3488252 | 3 | 12 | 0 | 224 | 3560956 | 3 | 12 | 0 | 1 | 232 |
| 118 | 76 | 35 | 23 | 3 | 13 | 436060 | 3488476 | 3 | 13 | 0 | 224 | 3561188 | 3 | 13 | 0 | 1 | 232 |
| 119 | 77 | 35 | 23 | 3 | 14 | 436088 | 3488700 | 3 | 14 | 0 | 224 | 3561420 | 3 | 14 | 0 | 1 | 232 |
| 120 | 78 | 35 | 23 | 3 | 15 | 436116 | 3488924 | 3 | 15 | 0 | 224 | 3561652 | 3 | 15 | 0 | 1 | 232 |
| 121 | 79 | 35 | 23 | 3 | 16 | 436144 | 3489148 | 3 | 16 | 0 | 224 | 3561884 | 3 | 16 | 0 | 1 | 232 |
| 122 | 7A | 35 | 23 | 3 | 17 | 436172 | 3489372 | 3 | 17 | 0 | 224 | 3562116 | 3 | 17 | 0 | 1 | 232 |
| 123 | 7B | 35 | 23 | 3 | 18 | 436200 | 3489596 | 3 | 18 | 0 | 224 | 3562348 | 3 | 18 | 0 | 1 | 232 |
| 124 | 7C | 35 | 23 | 3 | 19 | 436228 | 3489820 | 3 | 19 | 0 | 224 | 3562580 | 3 | 19 | 0 | 1 | 232 |
| 125 | 7D | 35 | 23 | 3 | 20 | 436256 | 3490044 | 3 | 20 | 0 | 224 | 3562812 | 3 | 20 | 0 | 1 | 232 |
| 126 | 7E | 35 | 23 | 3 | 21 | 436284 | 3490268 | 3 | 21 | 0 | 224 | 3563044 | 3 | 21 | 0 | 1 | 232 |
| 127 | 7F | 35 | 23 | 3 | 22 | 436312 | 3490492 | 3 | 22 | 0 | 224 | 3563276 | 3 | 22 | 0 | 1 | 232 |
| 128 | 80 | 35 | 23 | 3 | 23 | 436340 | 3490716 | 3 | 23 | 0 | 224 | 3563508 | 3 | 23 | 0 | 1 | 232 |
| 129 | 81 | 35 | 23 | 3 | 24 | 436368 | 3490940 | 3 | 24 | 0 | 224 | 3563740 | 3 | 24 | 0 | 1 | 232 |
| 130 | 82 | 35 | 23 | 3 | 25 | 436396 | 3491164 | 3 | 25 | 0 | 224 | 3563972 | 3 | 25 | 0 | 1 | 232 |
| 131 | 83 | 35 | 23 | 3 | 26 | 436424 | 3491388 | 3 | 26 | 0 | 224 | 3564204 | 3 | 26 | 0 | 1 | 232 |
| 132 | 84 | 35 | 23 | 3 | 27 | 436452 | 3491612 | 3 | 27 | 0 | 224 | 3564436 | 3 | 27 | 0 | 1 | 232 |
| 133 | 85 | 35 | 23 | 3 | 28 | 436480 | 3491836 | 3 | 28 | 0 | 224 | 3564668 | 3 | 28 | 0 | 1 | 232 |
| 134 | 86 | 35 | 23 | 3 | 29 | 436508 | 3492060 | 3 | 29 | 0 | 224 | 3564900 | 3 | 29 | 0 | 1 | 232 |
| 135 | 87 | 35 | 23 | 3 | 30 | 436536 | 3492284 | 3 | 30 | 0 | 224 | 3565132 | 3 | 30 | 0 | 1 | 232 |
| 136 | 88 | 35 | 23 | 3 | 31 | 436564 | 3492508 | 3 | 31 | 0 | 224 | 3565364 | 3 | 31 | 0 | 1 | 232 |
| 137 | 89 | 35 | 23 | 3 | 32 | 436592 | 3492732 | 3 | 32 | 0 | 224 | 3565596 | 3 | 32 | 0 | 1 | 232 |
| 138 | 8A | 35 | 23 | 3 | 33 | 436620 | 3492956 | 3 | 33 | 0 | 224 | 3565828 | 3 | 33 | 0 | 1 | 232 |
| 139 | 8B | 35 | 23 | 3 | 34 | 436648 | 3493180 | 3 | 34 | 0 | 224 | 3566060 | 3 | 34 | 0 | 1 | 232 |
| 140 | 8C | 35 | 23 | 4 | 0 | 436678 | 3493420 | 4 | 0 | 0 | 240 | 3566308 | 4 | 0 | 0 | 1 | 248 |
| 141 | 8D | 35 | 23 | 4 | 1 | 436708 | 3493660 | 4 | 1 | 0 | 240 | 3566556 | 4 | 1 | 0 | 1 | 248 |
| 142 | 8E | 35 | 23 | 4 | 2 | 436738 | 3493900 | 4 | 2 | 0 | 240 | 3566804 | 4 | 2 | 0 | 1 | 248 |
| 143 | 8F | 35 | 23 | 4 | 3 | 436768 | 3494140 | 4 | 3 | 0 | 240 | 3567052 | 4 | 3 | 0 | 1 | 248 |
| 144 | 90 | 35 | 23 | 4 | 4 | 436796 | 3494364 | 4 | 4 | 0 | 224 | 3567284 | 4 | 4 | 0 | 1 | 232 |
| 145 | 91 | 35 | 23 | 4 | 5 | 436824 | 3494588 | 4 | 5 | 0 | 224 | 3567516 | 4 | 5 | 0 | 1 | 232 |
| 146 | 92 | 35 | 23 | 4 | 6 | 436852 | 3494812 | 4 | 6 | 0 | 224 | 3567748 | 4 | 6 | 0 | 1 | 232 |
| 147 | 93 | 35 | 23 | 4 | 7 | 436880 | 3495036 | 4 | 7 | 0 | 224 | 3567980 | 4 | 7 | 0 | 1 | 232 |
| 148 | 94 | 35 | 23 | 4 | 8 | 436908 | 3495260 | 4 | 8 | 0 | 224 | 3568212 | 4 | 8 | 0 | 1 | 232 |
| 149 | 95 | 35 | 23 | 4 | 9 | 436936 | 3495484 | 4 | 9 | 0 | 224 | 3568444 | 4 | 9 | 0 | 1 | 232 |
| 150 | 96 | 35 | 23 | 4 | 10 | 436964 | 3495708 | 4 | 10 | 0 | 224 | 3568676 | 4 | 10 | 0 | 1 | 232 |
| 151 | 97 | 35 | 23 | 4 | 11 | 436992 | 3495932 | 4 | 11 | 0 | 224 | 3568908 | 4 | 11 | 0 | 1 | 232 |
| 152 | 98 | 35 | 23 | 4 | 12 | 437020 | 3496156 | 4 | 12 | 0 | 224 | 3569140 | 4 | 12 | 0 | 1 | 232 |
| 153 | 99 | 35 | 23 | 4 | 13 | 437048 | 3496380 | 4 | 13 | 0 | 224 | 3569372 | 4 | 13 | 0 | 1 | 232 |
| 154 | 9A | 35 | 23 | 4 | 14 | 437076 | 3496604 | 4 | 14 | 0 | 224 | 3569604 | 4 | 14 | 0 | 1 | 232 |
| 155 | 9B | 35 | 23 | 4 | 15 | 437104 | 3496828 | 4 | 15 | 0 | 224 | 3569836 | 4 | 15 | 0 | 1 | 232 |
| 156 | 9C | 35 | 23 | 4 | 16 | 437132 | 3497052 | 4 | 16 | 0 | 224 | 3570068 | 4 | 16 | 0 | 1 | 232 |
| 157 | 9D | 35 | 23 | 4 | 17 | 437160 | 3497276 | 4 | 17 | 0 | 224 | 3570300 | 4 | 17 | 0 | 1 | 232 |
| 158 | 9E | 35 | 23 | 4 | 18 | 437188 | 3497500 | 4 | 18 | 0 | 224 | 3570532 | 4 | 18 | 0 | 1 | 232 |
| 159 | 9F | 35 | 23 | 4 | 19 | 437216 | 3497724 | 4 | 19 | 0 | 224 | 3570764 | 4 | 19 | 0 | 1 | 232 |
| 160 | A0 | 35 | 23 | 4 | 20 | 437246 | 3497964 | 4 | 20 | 0 | 240 | 3571012 | 4 | 20 | 0 | 1 | 248 |
| 161 | A1 | 35 | 23 | 4 | 21 | 437276 | 3498204 | 4 | 21 | 0 | 240 | 3571260 | 4 | 21 | 0 | 1 | 248 |
| 162 | A2 | 35 | 23 | 4 | 22 | 437306 | 3498444 | 4 | 22 | 0 | 240 | 3571508 | 4 | 22 | 0 | 1 | 248 |
| 163 | A3 | 35 | 23 | 4 | 23 | 437336 | 3498684 | 4 | 23 | 0 | 240 | 3571756 | 4 | 23 | 0 | 1 | 248 |
| 164 | A4 | 35 | 23 | 4 | 24 | 437366 | 3498924 | 4 | 24 | 0 | 240 | 3572004 | 4 | 24 | 0 | 1 | 248 |
| 165 | A5 | 35 | 23 | 4 | 25 | 437396 | 3499164 | 4 | 25 | 0 | 240 | 3572252 | 4 | 25 | 0 | 1 | 248 |
| 166 | A6 | 35 | 23 | 4 | 26 | 437426 | 3499404 | 4 | 26 | 0 | 240 | 3572500 | 4 | 26 | 0 | 1 | 248 |
| 167 | A7 | 35 | 23 | 4 | 27 | 437456 | 3499644 | 4 | 27 | 0 | 240 | 3572748 | 4 | 27 | 0 | 1 | 248 |
| 168 | A8 | 35 | 23 | 4 | 28 | 437486 | 3499884 | 4 | 28 | 0 | 240 | 3572996 | 4 | 28 | 0 | 1 | 248 |
| 169 | A9 | 35 | 23 | 4 | 29 | 437516 | 3500124 | 4 | 29 | 0 | 240 | 3573244 | 4 | 29 | 0 | 1 | 248 |
| 170 | AA | 35 | 23 | 4 | 30 | 437546 | 3500364 | 4 | 30 | 0 | 240 | 3573492 | 4 | 30 | 0 | 1 | 248 |
| 171 | AB | 35 | 23 | 4 | 31 | 437576 | 3500604 | 4 | 31 | 0 | 240 | 3573740 | 4 | 31 | 0 | 1 | 248 |
| 172 | AC | 35 | 23 | 4 | 32 | 437606 | 3500844 | 4 | 32 | 0 | 240 | 3573988 | 4 | 32 | 0 | 1 | 248 |
| 173 | AD | 35 | 23 | 4 | 33 | 437636 | 3501084 | 4 | 33 | 0 | 240 | 3574236 | 4 | 33 | 0 | 1 | 248 |
| 174 | AE | 35 | 23 | 4 | 34 | 437666 | 3501324 | 4 | 34 | 0 | 240 | 3574484 | 4 | 34 | 0 | 1 | 248 |
| 175 | AF | 35 | 23 | 5 | 0 | 437698 | 3501580 | 5 | 0 | 0 | 256 | 3574748 | 5 | 0 | 0 | 1 | 264 |
| 176 | B0 | 35 | 23 | 5 | 1 | 437730 | 3501836 | 5 | 1 | 0 | 256 | 3575012 | 5 | 1 | 0 | 1 | 264 |
| 177 | B1 | 35 | 23 | 5 | 2 | 437762 | 3502092 | 5 | 2 | 0 | 256 | 3575276 | 5 | 2 | 0 | 1 | 264 |
| 178 | B2 | 35 | 23 | 5 | 3 | 437794 | 3502348 | 5 | 3 | 0 | 256 | 3575540 | 5 | 3 | 0 | 1 | 264 |
| 179 | B3 | 35 | 23 | 5 | 4 | 437826 | 3502604 | 5 | 4 | 0 | 256 | 3575804 | 5 | 4 | 0 | 1 | 264 |
| 180 | B4 | 35 | 23 | 5 | 5 | 437858 | 3502860 | 5 | 5 | 0 | 256 | 3576068 | 5 | 5 | 0 | 1 | 264 |
| 181 | B5 | 35 | 23 | 5 | 6 | 437890 | 3503116 | 5 | 6 | 0 | 256 | 3576332 | 5 | 6 | 0 | 1 | 264 |
| 182 | B6 | 35 | 23 | 5 | 7 | 437922 | 3503372 | 5 | 7 | 0 | 256 | 3576596 | 5 | 7 | 0 | 1 | 264 |
| 183 | B7 | 35 | 23 | 5 | 8 | 437954 | 3503628 | 5 | 8 | 0 | 256 | 3576860 | 5 | 8 | 0 | 1 | 264 |
| 184 | B8 | 35 | 23 | 5 | 9 | 437986 | 3503884 | 5 | 9 | 0 | 256 | 3577124 | 5 | 9 | 0 | 1 | 264 |
| 185 | B9 | 35 | 23 | 5 | 10 | 438018 | 3504140 | 5 | 10 | 0 | 256 | 3577388 | 5 | 10 | 0 | 1 | 264 |
| 186 | BA | 35 | 23 | 5 | 11 | 438050 | 3504396 | 5 | 11 | 0 | 256 | 3577652 | 5 | 11 | 0 | 1 | 264 |
| 187 | BB | 35 | 23 | 5 | 12 | 438082 | 3504652 | 5 | 12 | 0 | 256 | 3577916 | 5 | 12 | 0 | 1 | 264 |
| 188 | BC | 35 | 23 | 5 | 13 | 438114 | 3504908 | 5 | 13 | 0 | 256 | 3578180 | 5 | 13 | 0 | 1 | 264 |
| 189 | BD | 35 | 23 | 5 | 14 | 438146 | 3505164 | 5 | 14 | 0 | 256 | 3578444 | 5 | 14 | 0 | 1 | 264 |
| 190 | BE | 35 | 23 | 5 | 15 | 438178 | 3505420 | 5 | 15 | 0 | 256 | 3578708 | 5 | 15 | 0 | 1 | 264 |
| 191 | BF | 35 | 23 | 5 | 16 | 438210 | 3505676 | 5 | 16 | 0 | 256 | 3578972 | 5 | 16 | 0 | 1 | 264 |
| 192 | C0 | 35 | 23 | 5 | 17 | 438240 | 3505916 | 5 | 17 | 0 | 240 | 3579220 | 5 | 17 | 0 | 1 | 248 |
| 193 | C1 | 35 | 23 | 5 | 18 | 438270 | 3506156 | 5 | 18 | 0 | 240 | 3579468 | 5 | 18 | 0 | 1 | 248 |
| 194 | C2 | 35 | 23 | 5 | 19 | 438300 | 3506396 | 5 | 19 | 0 | 240 | 3579716 | 5 | 19 | 0 | 1 | 248 |
| 195 | C3 | 35 | 23 | 5 | 20 | 438330 | 3506636 | 5 | 20 | 0 | 240 | 3579964 | 5 | 20 | 0 | 1 | 248 |
| 196 | C4 | 35 | 23 | 5 | 21 | 438360 | 3506876 | 5 | 21 | 0 | 240 | 3580212 | 5 | 21 | 0 | 1 | 248 |
| 197 | C5 | 35 | 23 | 5 | 22 | 438390 | 3507116 | 5 | 22 | 0 | 240 | 3580460 | 5 | 22 | 0 | 1 | 248 |
| 198 | C6 | 35 | 23 | 5 | 23 | 438420 | 3507356 | 5 | 23 | 0 | 240 | 3580708 | 5 | 23 | 0 | 1 | 248 |
| 199 | C7 | 35 | 23 | 5 | 24 | 438450 | 3507596 | 5 | 24 | 0 | 240 | 3580956 | 5 | 24 | 0 | 1 | 248 |
| 200 | C8 | 35 | 23 | 5 | 25 | 438480 | 3507836 | 5 | 25 | 0 | 240 | 3581204 | 5 | 25 | 0 | 1 | 248 |
| 201 | C9 | 35 | 23 | 5 | 26 | 438510 | 3508076 | 5 | 26 | 0 | 240 | 3581452 | 5 | 26 | 0 | 1 | 248 |
| 202 | CA | 35 | 23 | 5 | 27 | 438540 | 3508316 | 5 | 27 | 0 | 240 | 3581700 | 5 | 27 | 0 | 1 | 248 |
| 203 | CB | 35 | 23 | 5 | 28 | 438570 | 3508556 | 5 | 28 | 0 | 240 | 3581948 | 5 | 28 | 0 | 1 | 248 |

| | | | | | | | | | | | | | | | | | |
|---|---|---|---|---|---|---|---|---|---|---|---|---|---|---|---|---|---|
| 204 | CC | 35 | 23 | 5 | 29 | 438600 | 3508796 | 5 | 29 | 0 | 240 | 3582196 | 5 | 29 | 0 | 1 | 248 |
| 205 | CD | 35 | 23 | 5 | 30 | 438630 | 3509036 | 5 | 30 | 0 | 240 | 3582444 | 5 | 30 | 0 | 1 | 248 |
| 206 | CE | 35 | 23 | 5 | 31 | 438660 | 3509276 | 5 | 31 | 0 | 240 | 3582692 | 5 | 31 | 0 | 1 | 248 |
| 207 | CF | 35 | 23 | 5 | 32 | 438690 | 3509516 | 5 | 32 | 0 | 240 | 3582940 | 5 | 32 | 0 | 1 | 248 |
| 208 | D0 | 35 | 23 | 5 | 33 | 438722 | 3509772 | 5 | 33 | 0 | 256 | 3583204 | 5 | 33 | 0 | 1 | 264 |
| 209 | D1 | 35 | 23 | 5 | 34 | 438754 | 3510028 | 5 | 34 | 0 | 256 | 3583468 | 5 | 34 | 0 | 1 | 264 |
| 210 | D2 | 35 | 23 | 6 | 0 | 438788 | 3510300 | 6 | 0 | 0 | 272 | 3583748 | 6 | 0 | 0 | 1 | 280 |
| 211 | D3 | 35 | 23 | 6 | 1 | 438822 | 3510572 | 6 | 1 | 0 | 272 | 3584028 | 6 | 1 | 0 | 1 | 280 |
| 212 | D4 | 35 | 23 | 6 | 2 | 438856 | 3510844 | 6 | 2 | 0 | 272 | 3584308 | 6 | 2 | 0 | 1 | 280 |
| 213 | D5 | 35 | 23 | 6 | 3 | 438890 | 3511116 | 6 | 3 | 0 | 272 | 3584588 | 6 | 3 | 0 | 1 | 280 |
| 214 | D6 | 35 | 23 | 6 | 4 | 438924 | 3511388 | 6 | 4 | 0 | 272 | 3584868 | 6 | 4 | 0 | 1 | 280 |
| 215 | D7 | 35 | 23 | 6 | 5 | 438958 | 3511660 | 6 | 5 | 0 | 272 | 3585148 | 6 | 5 | 0 | 1 | 280 |
| 216 | D8 | 35 | 23 | 6 | 6 | 438992 | 3511932 | 6 | 6 | 0 | 272 | 3585428 | 6 | 6 | 0 | 1 | 280 |
| 217 | D9 | 35 | 23 | 6 | 7 | 439026 | 3512204 | 6 | 7 | 0 | 272 | 3585708 | 6 | 7 | 0 | 1 | 280 |
| 218 | DA | 35 | 23 | 6 | 8 | 439060 | 3512476 | 6 | 8 | 0 | 272 | 3585988 | 6 | 8 | 0 | 1 | 280 |
| 219 | DB | 35 | 23 | 6 | 9 | 439094 | 3512748 | 6 | 9 | 0 | 272 | 3586268 | 6 | 9 | 0 | 1 | 280 |
| 220 | DC | 35 | 23 | 6 | 10 | 439128 | 3513020 | 6 | 10 | 0 | 272 | 3586548 | 6 | 10 | 0 | 1 | 280 |
| 221 | DD | 35 | 23 | 6 | 11 | 439162 | 3513292 | 6 | 11 | 0 | 272 | 3586828 | 6 | 11 | 0 | 1 | 280 |
| 222 | DE | 35 | 23 | 6 | 12 | 439196 | 3513564 | 6 | 12 | 0 | 272 | 3587108 | 6 | 12 | 0 | 1 | 280 |
| 223 | DF | 35 | 23 | 6 | 13 | 439230 | 3513836 | 6 | 13 | 0 | 272 | 3587388 | 6 | 13 | 0 | 1 | 280 |
| 224 | E0 | 35 | 23 | 6 | 14 | 439264 | 3514108 | 6 | 14 | 0 | 272 | 3587668 | 6 | 14 | 0 | 1 | 280 |
| 225 | E1 | 35 | 23 | 6 | 15 | 439298 | 3514380 | 6 | 15 | 0 | 272 | 3587948 | 6 | 15 | 0 | 1 | 280 |
| 226 | E2 | 35 | 23 | 6 | 16 | 439332 | 3514652 | 6 | 16 | 0 | 272 | 3588228 | 6 | 16 | 0 | 1 | 280 |
| 227 | E3 | 35 | 23 | 6 | 17 | 439366 | 3514924 | 6 | 17 | 0 | 272 | 3588508 | 6 | 17 | 0 | 1 | 280 |
| 228 | E4 | 35 | 23 | 6 | 18 | 439400 | 3515196 | 6 | 18 | 0 | 272 | 3588788 | 6 | 18 | 0 | 1 | 280 |
| 229 | E5 | 35 | 23 | 6 | 19 | 439434 | 3515468 | 6 | 19 | 0 | 272 | 3589068 | 6 | 19 | 0 | 1 | 280 |
| 230 | E6 | 35 | 23 | 6 | 20 | 439468 | 3515740 | 6 | 20 | 0 | 272 | 3589348 | 6 | 20 | 0 | 1 | 280 |
| 231 | E7 | 35 | 23 | 6 | 21 | 439502 | 3516012 | 6 | 21 | 0 | 272 | 3589628 | 6 | 21 | 0 | 1 | 280 |
| 232 | E8 | 35 | 23 | 6 | 22 | 439536 | 3516284 | 6 | 22 | 0 | 272 | 3589908 | 6 | 22 | 0 | 1 | 280 |
| 233 | E9 | 35 | 23 | 6 | 23 | 439570 | 3516556 | 6 | 23 | 0 | 272 | 3590188 | 6 | 23 | 0 | 1 | 280 |
| 234 | EA | 35 | 23 | 6 | 24 | 439604 | 3516828 | 6 | 24 | 0 | 272 | 3590468 | 6 | 24 | 0 | 1 | 280 |
| 235 | EB | 35 | 23 | 6 | 25 | 439638 | 3517100 | 6 | 25 | 0 | 272 | 3590748 | 6 | 25 | 0 | 1 | 280 |
| 236 | EC | 35 | 23 | 6 | 26 | 439672 | 3517372 | 6 | 26 | 0 | 272 | 3591028 | 6 | 26 | 0 | 1 | 280 |
| 237 | ED | 35 | 23 | 6 | 27 | 439706 | 3517644 | 6 | 27 | 0 | 272 | 3591308 | 6 | 27 | 0 | 1 | 280 |
| 238 | EE | 35 | 23 | 6 | 28 | 439740 | 3517916 | 6 | 28 | 0 | 272 | 3591588 | 6 | 28 | 0 | 1 | 280 |
| 239 | EF | 35 | 23 | 6 | 29 | 439774 | 3518188 | 6 | 29 | 0 | 272 | 3591868 | 6 | 29 | 0 | 1 | 280 |
| 240 | F0 | 35 | 23 | 6 | 30 | 439806 | 3518444 | 6 | 30 | 0 | 256 | 3592132 | 6 | 30 | 0 | 1 | 264 |
| 241 | F1 | 35 | 23 | 6 | 31 | 439838 | 3518700 | 6 | 31 | 0 | 256 | 3592396 | 6 | 31 | 0 | 1 | 264 |
| 242 | F2 | 35 | 23 | 6 | 32 | 439870 | 3518956 | 6 | 32 | 0 | 256 | 3592660 | 6 | 32 | 0 | 1 | 264 |
| 243 | F3 | 35 | 23 | 6 | 33 | 439902 | 3519212 | 6 | 33 | 0 | 256 | 3592924 | 6 | 33 | 0 | 1 | 264 |
| 244 | F4 | 35 | 23 | 6 | 34 | 439934 | 3519468 | 6 | 34 | 0 | 256 | 3593188 | 6 | 34 | 0 | 1 | 264 |
| 245 | F5 | 35 | 23 | 7 | 0 | 439968 | 3519740 | 7 | 0 | 0 | 272 | 3593468 | 7 | 0 | 0 | 1 | 280 |
| 246 | F6 | 35 | 23 | 7 | 1 | 440002 | 3520012 | 7 | 1 | 0 | 272 | 3593748 | 7 | 1 | 0 | 1 | 280 |
| 247 | F7 | 35 | 23 | 7 | 2 | 440036 | 3520284 | 7 | 2 | 0 | 272 | 3594028 | 7 | 2 | 0 | 1 | 280 |
| 248 | F8 | 35 | 23 | 7 | 3 | 440070 | 3520556 | 7 | 3 | 0 | 272 | 3594308 | 7 | 3 | 0 | 1 | 280 |
| 249 | F9 | 35 | 23 | 7 | 4 | 440104 | 3520828 | 7 | 4 | 0 | 272 | 3594588 | 7 | 4 | 0 | 1 | 280 |
| 250 | FA | 35 | 23 | 7 | 5 | 440138 | 3521100 | 7 | 5 | 0 | 272 | 3594868 | 7 | 5 | 0 | 1 | 280 |
| 251 | FB | 35 | 23 | 7 | 6 | 440172 | 3521372 | 7 | 6 | 0 | 272 | 3595148 | 7 | 6 | 0 | 1 | 280 |
| 252 | FC | 35 | 23 | 7 | 7 | 440206 | 3521644 | 7 | 7 | 0 | 272 | 3595428 | 7 | 7 | 0 | 1 | 280 |
| 253 | FD | 35 | 23 | 7 | 8 | 440240 | 3521916 | 7 | 8 | 0 | 272 | 3595708 | 7 | 8 | 0 | 1 | 280 |
| 254 | FE | 35 | 23 | 7 | 9 | 440274 | 3522188 | 7 | 9 | 0 | 272 | 3595988 | 7 | 9 | 0 | 1 | 280 |
| 255 | FF | 35 | 23 | 7 | 10 | 440308 | 3522460 | 7 | 10 | 0 | 272 | 3596268 | 7 | 10 | 0 | 1 | 280 |
| 0 | 00 | 36 | 24 | 0 | 0 | 440320 | 3522556 | 0 | 0 | 0 | 96 | 3596372 | 0 | 0 | 0 | 1 | 104 |
| 1 | 01 | 36 | 24 | 0 | 1 | 440334 | 3522668 | 0 | 1 | 0 | 112 | 3596492 | 0 | 1 | 0 | 1 | 120 |
| 2 | 02 | 36 | 24 | 0 | 2 | 440348 | 3522780 | 0 | 2 | 0 | 112 | 3596612 | 0 | 2 | 0 | 1 | 120 |
| 3 | 03 | 36 | 24 | 0 | 3 | 440362 | 3522892 | 0 | 3 | 0 | 112 | 3596732 | 0 | 3 | 0 | 1 | 120 |
| 4 | 04 | 36 | 24 | 0 | 4 | 440376 | 3523004 | 0 | 4 | 0 | 112 | 3596852 | 0 | 4 | 0 | 1 | 120 |
| 5 | 05 | 36 | 24 | 0 | 5 | 440390 | 3523116 | 0 | 5 | 0 | 112 | 3596972 | 0 | 5 | 0 | 1 | 120 |
| 6 | 06 | 36 | 24 | 0 | 6 | 440404 | 3523228 | 0 | 6 | 0 | 112 | 3597092 | 0 | 6 | 0 | 1 | 120 |
| 7 | 07 | 36 | 24 | 0 | 7 | 440418 | 3523340 | 0 | 7 | 0 | 112 | 3597212 | 0 | 7 | 0 | 1 | 120 |
| 8 | 08 | 36 | 24 | 0 | 8 | 440432 | 3523452 | 0 | 8 | 0 | 112 | 3597332 | 0 | 8 | 0 | 1 | 120 |
| 9 | 09 | 36 | 24 | 0 | 9 | 440446 | 3523564 | 0 | 9 | 0 | 112 | 3597452 | 0 | 9 | 0 | 1 | 120 |
| 10 | 0A | 36 | 24 | 0 | 10 | 440460 | 3523676 | 0 | 10 | 0 | 112 | 3597572 | 0 | 10 | 0 | 1 | 120 |
| 11 | 0B | 36 | 24 | 0 | 11 | 440474 | 3523788 | 0 | 11 | 0 | 112 | 3597692 | 0 | 11 | 0 | 1 | 120 |
| 12 | 0C | 36 | 24 | 0 | 12 | 440488 | 3523900 | 0 | 12 | 0 | 112 | 3597812 | 0 | 12 | 0 | 1 | 120 |
| 13 | 0D | 36 | 24 | 0 | 13 | 440502 | 3524012 | 0 | 13 | 0 | 112 | 3597932 | 0 | 13 | 0 | 1 | 120 |
| 14 | 0E | 36 | 24 | 0 | 14 | 440516 | 3524124 | 0 | 14 | 0 | 112 | 3598052 | 0 | 14 | 0 | 1 | 120 |
| 15 | 0F | 36 | 24 | 0 | 15 | 440530 | 3524236 | 0 | 15 | 0 | 112 | 3598172 | 0 | 15 | 0 | 1 | 120 |
| 16 | 10 | 36 | 24 | 0 | 16 | 440544 | 3524348 | 0 | 16 | 0 | 112 | 3598292 | 0 | 16 | 0 | 1 | 120 |
| 17 | 11 | 36 | 24 | 0 | 17 | 440558 | 3524460 | 0 | 17 | 0 | 112 | 3598412 | 0 | 17 | 0 | 1 | 120 |
| 18 | 12 | 36 | 24 | 0 | 18 | 440572 | 3524572 | 0 | 18 | 0 | 112 | 3598532 | 0 | 18 | 0 | 1 | 120 |
| 19 | 13 | 36 | 24 | 0 | 19 | 440586 | 3524684 | 0 | 19 | 0 | 112 | 3598652 | 0 | 19 | 0 | 1 | 120 |
| 20 | 14 | 36 | 24 | 0 | 20 | 440600 | 3524796 | 0 | 20 | 0 | 112 | 3598772 | 0 | 20 | 0 | 1 | 120 |
| 21 | 15 | 36 | 24 | 0 | 21 | 440614 | 3524908 | 0 | 21 | 0 | 112 | 3598892 | 0 | 21 | 0 | 1 | 120 |
| 22 | 16 | 36 | 24 | 0 | 22 | 440628 | 3525020 | 0 | 22 | 0 | 112 | 3599012 | 0 | 22 | 0 | 1 | 120 |
| 23 | 17 | 36 | 24 | 0 | 23 | 440642 | 3525132 | 0 | 23 | 0 | 112 | 3599132 | 0 | 23 | 0 | 1 | 120 |
| 24 | 18 | 36 | 24 | 0 | 24 | 440656 | 3525244 | 0 | 24 | 0 | 112 | 3599252 | 0 | 24 | 0 | 1 | 120 |
| 25 | 19 | 36 | 24 | 0 | 25 | 440670 | 3525356 | 0 | 25 | 0 | 112 | 3599372 | 0 | 25 | 0 | 1 | 120 |
| 26 | 1A | 36 | 24 | 0 | 26 | 440684 | 3525468 | 0 | 26 | 0 | 112 | 3599492 | 0 | 26 | 0 | 1 | 120 |
| 27 | 1B | 36 | 24 | 0 | 27 | 440698 | 3525580 | 0 | 27 | 0 | 112 | 3599612 | 0 | 27 | 0 | 1 | 120 |
| 28 | 1C | 36 | 24 | 0 | 28 | 440712 | 3525692 | 0 | 28 | 0 | 112 | 3599732 | 0 | 28 | 0 | 1 | 120 |
| 29 | 1D | 36 | 24 | 0 | 29 | 440726 | 3525804 | 0 | 29 | 0 | 112 | 3599852 | 0 | 29 | 0 | 1 | 120 |
| 30 | 1E | 36 | 24 | 0 | 30 | 440740 | 3525916 | 0 | 30 | 0 | 112 | 3599972 | 0 | 30 | 0 | 1 | 120 |
| 31 | 1F | 36 | 24 | 0 | 31 | 440754 | 3526028 | 0 | 31 | 0 | 112 | 3600092 | 0 | 31 | 0 | 1 | 120 |
| 32 | 20 | 36 | 24 | 0 | 32 | 440768 | 3526140 | 0 | 32 | 0 | 112 | 3600212 | 0 | 32 | 0 | 1 | 120 |
| 33 | 21 | 36 | 24 | 0 | 33 | 440782 | 3526252 | 0 | 33 | 0 | 112 | 3600332 | 0 | 33 | 0 | 1 | 120 |
| 34 | 22 | 36 | 24 | 0 | 34 | 440796 | 3526364 | 0 | 34 | 0 | 112 | 3600452 | 0 | 34 | 0 | 1 | 120 |
| 35 | 23 | 36 | 24 | 0 | 35 | 440810 | 3526476 | 0 | 35 | 0 | 112 | 3600572 | 0 | 35 | 0 | 1 | 120 |
| 36 | 24 | 36 | 24 | 1 | 0 | 440822 | 3526572 | 1 | 0 | 0 | 96 | 3600676 | 1 | 0 | 0 | 1 | 104 |
| 37 | 25 | 36 | 24 | 1 | 1 | 440839 | 3526708 | 1 | 1 | 0 | 136 | 3600820 | 1 | 1 | 0 | 1 | 144 |
| 38 | 26 | 36 | 24 | 1 | 2 | 440856 | 3526844 | 1 | 2 | 0 | 136 | 3600964 | 1 | 2 | 0 | 1 | 144 |
| 39 | 27 | 36 | 24 | 1 | 3 | 440873 | 3526980 | 1 | 3 | 0 | 136 | 3601108 | 1 | 3 | 0 | 1 | 144 |
| 40 | 28 | 36 | 24 | 1 | 4 | 440890 | 3527116 | 1 | 4 | 0 | 136 | 3601252 | 1 | 4 | 0 | 1 | 144 |
| 41 | 29 | 36 | 24 | 1 | 5 | 440907 | 3527252 | 1 | 5 | 0 | 136 | 3601396 | 1 | 5 | 0 | 1 | 144 |
| 42 | 2A | 36 | 24 | 1 | 6 | 440924 | 3527388 | 1 | 6 | 0 | 136 | 3601540 | 1 | 6 | 0 | 1 | 144 |
| 43 | 2B | 36 | 24 | 1 | 7 | 440941 | 3527524 | 1 | 7 | 0 | 136 | 3601684 | 1 | 7 | 0 | 1 | 144 |
| 44 | 2C | 36 | 24 | 1 | 8 | 440958 | 3527660 | 1 | 8 | 0 | 136 | 3601828 | 1 | 8 | 0 | 1 | 144 |
| 45 | 2D | 36 | 24 | 1 | 9 | 440975 | 3527796 | 1 | 9 | 0 | 136 | 3601972 | 1 | 9 | 0 | 1 | 144 |
| 46 | 2E | 36 | 24 | 1 | 10 | 440992 | 3527932 | 1 | 10 | 0 | 136 | 3602116 | 1 | 10 | 0 | 1 | 144 |
| 47 | 2F | 36 | 24 | 1 | 11 | 441009 | 3528068 | 1 | 11 | 0 | 136 | 3602260 | 1 | 11 | 0 | 1 | 144 |
| 48 | 30 | 36 | 24 | 1 | 12 | 441030 | 3528236 | 1 | 12 | 0 | 168 | 3602436 | 1 | 12 | 0 | 1 | 176 |
| 49 | 31 | 36 | 24 | 1 | 13 | 441051 | 3528404 | 1 | 13 | 0 | 168 | 3602612 | 1 | 13 | 0 | 1 | 176 |
| 50 | 32 | 36 | 24 | 1 | 14 | 441072 | 3528572 | 1 | 14 | 0 | 168 | 3602788 | 1 | 14 | 0 | 1 | 176 |

| | | | | | | | | | | | | | | | | | |
|---|---|---|---|---|---|---|---|---|---|---|---|---|---|---|---|---|---|
| 51 | 33 | 36 | 24 | 1 | 15 | 441093 | 3528740 | 1 | 15 | 0 | 168 | 3602964 | 1 | 15 | 0 | 1 | 176 |
| 52 | 34 | 36 | 24 | 1 | 16 | 441114 | 3528908 | 1 | 16 | 0 | 168 | 3603140 | 1 | 16 | 0 | 1 | 176 |
| 53 | 35 | 36 | 24 | 1 | 17 | 441135 | 3529076 | 1 | 17 | 0 | 168 | 3603316 | 1 | 17 | 0 | 1 | 176 |
| 54 | 36 | 36 | 24 | 1 | 18 | 441156 | 3529244 | 1 | 18 | 0 | 168 | 3603492 | 1 | 18 | 0 | 1 | 176 |
| 55 | 37 | 36 | 24 | 1 | 19 | 441177 | 3529412 | 1 | 19 | 0 | 168 | 3603668 | 1 | 19 | 0 | 1 | 176 |
| 56 | 38 | 36 | 24 | 1 | 20 | 441198 | 3529580 | 1 | 20 | 0 | 168 | 3603844 | 1 | 20 | 0 | 1 | 176 |
| 57 | 39 | 36 | 24 | 1 | 21 | 441219 | 3529748 | 1 | 21 | 0 | 168 | 3604020 | 1 | 21 | 0 | 1 | 176 |
| 58 | 3A | 36 | 24 | 1 | 22 | 441240 | 3529916 | 1 | 22 | 0 | 168 | 3604196 | 1 | 22 | 0 | 1 | 176 |
| 59 | 3B | 36 | 24 | 1 | 23 | 441261 | 3530084 | 1 | 23 | 0 | 168 | 3604372 | 1 | 23 | 0 | 1 | 176 |
| 60 | 3C | 36 | 24 | 1 | 24 | 441282 | 3530252 | 1 | 24 | 0 | 168 | 3604548 | 1 | 24 | 0 | 1 | 176 |
| 61 | 3D | 36 | 24 | 1 | 25 | 441303 | 3530420 | 1 | 25 | 0 | 168 | 3604724 | 1 | 25 | 0 | 1 | 176 |
| 62 | 3E | 36 | 24 | 1 | 26 | 441324 | 3530588 | 1 | 26 | 0 | 168 | 3604900 | 1 | 26 | 0 | 1 | 176 |
| 63 | 3F | 36 | 24 | 1 | 27 | 441345 | 3530756 | 1 | 27 | 0 | 168 | 3605076 | 1 | 27 | 0 | 1 | 176 |
| 64 | 40 | 36 | 24 | 1 | 28 | 441368 | 3530940 | 1 | 28 | 0 | 184 | 3605268 | 1 | 28 | 0 | 1 | 192 |
| 65 | 41 | 36 | 24 | 1 | 29 | 441391 | 3531124 | 1 | 29 | 0 | 184 | 3605460 | 1 | 29 | 0 | 1 | 192 |
| 66 | 42 | 36 | 24 | 1 | 30 | 441414 | 3531308 | 1 | 30 | 0 | 184 | 3605652 | 1 | 30 | 0 | 1 | 192 |
| 67 | 43 | 36 | 24 | 1 | 31 | 441437 | 3531492 | 1 | 31 | 0 | 184 | 3605844 | 1 | 31 | 0 | 1 | 192 |
| 68 | 44 | 36 | 24 | 1 | 32 | 441460 | 3531676 | 1 | 32 | 0 | 184 | 3606036 | 1 | 32 | 0 | 1 | 192 |
| 69 | 45 | 36 | 24 | 1 | 33 | 441483 | 3531860 | 1 | 33 | 0 | 184 | 3606228 | 1 | 33 | 0 | 1 | 192 |
| 70 | 46 | 36 | 24 | 1 | 34 | 441506 | 3532044 | 1 | 34 | 0 | 184 | 3606420 | 1 | 34 | 0 | 1 | 192 |
| 71 | 47 | 36 | 24 | 1 | 35 | 441529 | 3532228 | 1 | 35 | 0 | 184 | 3606612 | 1 | 35 | 0 | 1 | 192 |
| 72 | 48 | 36 | 24 | 2 | 0 | 441550 | 3532396 | 2 | 0 | 0 | 168 | 3606788 | 2 | 0 | 0 | 1 | 176 |
| 73 | 49 | 36 | 24 | 2 | 1 | 441576 | 3532604 | 2 | 1 | 0 | 208 | 3607004 | 2 | 1 | 0 | 1 | 216 |
| 74 | 4A | 36 | 24 | 2 | 2 | 441602 | 3532812 | 2 | 2 | 0 | 208 | 3607220 | 2 | 2 | 0 | 1 | 216 |
| 75 | 4B | 36 | 24 | 2 | 3 | 441628 | 3533020 | 2 | 3 | 0 | 208 | 3607436 | 2 | 3 | 0 | 1 | 216 |
| 76 | 4C | 36 | 24 | 2 | 4 | 441654 | 3533228 | 2 | 4 | 0 | 208 | 3607652 | 2 | 4 | 0 | 1 | 216 |
| 77 | 4D | 36 | 24 | 2 | 5 | 441680 | 3533436 | 2 | 5 | 0 | 208 | 3607868 | 2 | 5 | 0 | 1 | 216 |
| 78 | 4E | 36 | 24 | 2 | 6 | 441706 | 3533644 | 2 | 6 | 0 | 208 | 3608084 | 2 | 6 | 0 | 1 | 216 |
| 79 | 4F | 36 | 24 | 2 | 7 | 441732 | 3533852 | 2 | 7 | 0 | 208 | 3608300 | 2 | 7 | 0 | 1 | 216 |
| 80 | 50 | 36 | 24 | 2 | 8 | 441758 | 3534060 | 2 | 8 | 0 | 208 | 3608516 | 2 | 8 | 0 | 1 | 216 |
| 81 | 51 | 36 | 24 | 2 | 9 | 441784 | 3534268 | 2 | 9 | 0 | 208 | 3608732 | 2 | 9 | 0 | 1 | 216 |
| 82 | 52 | 36 | 24 | 2 | 10 | 441810 | 3534476 | 2 | 10 | 0 | 208 | 3608948 | 2 | 10 | 0 | 1 | 216 |
| 83 | 53 | 36 | 24 | 2 | 11 | 441836 | 3534684 | 2 | 11 | 0 | 208 | 3609164 | 2 | 11 | 0 | 1 | 216 |
| 84 | 54 | 36 | 24 | 2 | 12 | 441862 | 3534892 | 2 | 12 | 0 | 208 | 3609380 | 2 | 12 | 0 | 1 | 216 |
| 85 | 55 | 36 | 24 | 2 | 13 | 441888 | 3535100 | 2 | 13 | 0 | 208 | 3609596 | 2 | 13 | 0 | 1 | 216 |
| 86 | 56 | 36 | 24 | 2 | 14 | 441914 | 3535308 | 2 | 14 | 0 | 208 | 3609812 | 2 | 14 | 0 | 1 | 216 |
| 87 | 57 | 36 | 24 | 2 | 15 | 441940 | 3535516 | 2 | 15 | 0 | 208 | 3610028 | 2 | 15 | 0 | 1 | 216 |
| 88 | 58 | 36 | 24 | 2 | 16 | 441966 | 3535724 | 2 | 16 | 0 | 208 | 3610244 | 2 | 16 | 0 | 1 | 216 |
| 89 | 59 | 36 | 24 | 2 | 17 | 441992 | 3535932 | 2 | 17 | 0 | 208 | 3610460 | 2 | 17 | 0 | 1 | 216 |
| 90 | 5A | 36 | 24 | 2 | 18 | 442018 | 3536140 | 2 | 18 | 0 | 208 | 3610676 | 2 | 18 | 0 | 1 | 216 |
| 91 | 5B | 36 | 24 | 2 | 19 | 442044 | 3536348 | 2 | 19 | 0 | 208 | 3610892 | 2 | 19 | 0 | 1 | 216 |
| 92 | 5C | 36 | 24 | 2 | 20 | 442070 | 3536556 | 2 | 20 | 0 | 208 | 3611108 | 2 | 20 | 0 | 1 | 216 |
| 93 | 5D | 36 | 24 | 2 | 21 | 442096 | 3536764 | 2 | 21 | 0 | 208 | 3611324 | 2 | 21 | 0 | 1 | 216 |
| 94 | 5E | 36 | 24 | 2 | 22 | 442122 | 3536972 | 2 | 22 | 0 | 208 | 3611540 | 2 | 22 | 0 | 1 | 216 |
| 95 | 5F | 36 | 24 | 2 | 23 | 442148 | 3537180 | 2 | 23 | 0 | 208 | 3611756 | 2 | 23 | 0 | 1 | 216 |
| 96 | 60 | 36 | 24 | 2 | 24 | 442171 | 3537364 | 2 | 24 | 0 | 184 | 3611948 | 2 | 24 | 0 | 1 | 192 |
| 97 | 61 | 36 | 24 | 2 | 25 | 442194 | 3537548 | 2 | 25 | 0 | 184 | 3612140 | 2 | 25 | 0 | 1 | 192 |
| 98 | 62 | 36 | 24 | 2 | 26 | 442217 | 3537732 | 2 | 26 | 0 | 184 | 3612332 | 2 | 26 | 0 | 1 | 192 |
| 99 | 63 | 36 | 24 | 2 | 27 | 442240 | 3537916 | 2 | 27 | 0 | 184 | 3612524 | 2 | 27 | 0 | 1 | 192 |
| 100 | 64 | 36 | 24 | 2 | 28 | 442263 | 3538100 | 2 | 28 | 0 | 184 | 3612716 | 2 | 28 | 0 | 1 | 192 |
| 101 | 65 | 36 | 24 | 2 | 29 | 442286 | 3538284 | 2 | 29 | 0 | 184 | 3612908 | 2 | 29 | 0 | 1 | 192 |
| 102 | 66 | 36 | 24 | 2 | 30 | 442309 | 3538468 | 2 | 30 | 0 | 184 | 3613100 | 2 | 30 | 0 | 1 | 192 |
| 103 | 67 | 36 | 24 | 2 | 31 | 442332 | 3538652 | 2 | 31 | 0 | 184 | 3613292 | 2 | 31 | 0 | 1 | 192 |
| 104 | 68 | 36 | 24 | 2 | 32 | 442355 | 3538836 | 2 | 32 | 0 | 184 | 3613484 | 2 | 32 | 0 | 1 | 192 |
| 105 | 69 | 36 | 24 | 2 | 33 | 442378 | 3539020 | 2 | 33 | 0 | 184 | 3613676 | 2 | 33 | 0 | 1 | 192 |
| 106 | 6A | 36 | 24 | 2 | 34 | 442401 | 3539204 | 2 | 34 | 0 | 184 | 3613868 | 2 | 34 | 0 | 1 | 192 |
| 107 | 6B | 36 | 24 | 2 | 35 | 442424 | 3539388 | 2 | 35 | 0 | 184 | 3614060 | 2 | 35 | 0 | 1 | 192 |
| 108 | 6C | 36 | 24 | 3 | 0 | 442445 | 3539556 | 3 | 0 | 0 | 168 | 3614236 | 3 | 0 | 0 | 1 | 176 |
| 109 | 6D | 36 | 24 | 3 | 1 | 442471 | 3539764 | 3 | 1 | 0 | 208 | 3614452 | 3 | 1 | 0 | 1 | 216 |
| 110 | 6E | 36 | 24 | 3 | 2 | 442497 | 3539972 | 3 | 2 | 0 | 208 | 3614668 | 3 | 2 | 0 | 1 | 216 |
| 111 | 6F | 36 | 24 | 3 | 3 | 442523 | 3540180 | 3 | 3 | 0 | 208 | 3614884 | 3 | 3 | 0 | 1 | 216 |
| 112 | 70 | 36 | 24 | 3 | 4 | 442551 | 3540404 | 3 | 4 | 0 | 224 | 3615116 | 3 | 4 | 0 | 1 | 232 |
| 113 | 71 | 36 | 24 | 3 | 5 | 442579 | 3540628 | 3 | 5 | 0 | 224 | 3615348 | 3 | 5 | 0 | 1 | 232 |
| 114 | 72 | 36 | 24 | 3 | 6 | 442607 | 3540852 | 3 | 6 | 0 | 224 | 3615580 | 3 | 6 | 0 | 1 | 232 |
| 115 | 73 | 36 | 24 | 3 | 7 | 442635 | 3541076 | 3 | 7 | 0 | 224 | 3615812 | 3 | 7 | 0 | 1 | 232 |
| 116 | 74 | 36 | 24 | 3 | 8 | 442663 | 3541300 | 3 | 8 | 0 | 224 | 3616044 | 3 | 8 | 0 | 1 | 232 |
| 117 | 75 | 36 | 24 | 3 | 9 | 442691 | 3541524 | 3 | 9 | 0 | 224 | 3616276 | 3 | 9 | 0 | 1 | 232 |
| 118 | 76 | 36 | 24 | 3 | 10 | 442719 | 3541748 | 3 | 10 | 0 | 224 | 3616508 | 3 | 10 | 0 | 1 | 232 |
| 119 | 77 | 36 | 24 | 3 | 11 | 442747 | 3541972 | 3 | 11 | 0 | 224 | 3616740 | 3 | 11 | 0 | 1 | 232 |
| 120 | 78 | 36 | 24 | 3 | 12 | 442775 | 3542196 | 3 | 12 | 0 | 224 | 3616972 | 3 | 12 | 0 | 1 | 232 |
| 121 | 79 | 36 | 24 | 3 | 13 | 442803 | 3542420 | 3 | 13 | 0 | 224 | 3617204 | 3 | 13 | 0 | 1 | 232 |
| 122 | 7A | 36 | 24 | 3 | 14 | 442831 | 3542644 | 3 | 14 | 0 | 224 | 3617436 | 3 | 14 | 0 | 1 | 232 |
| 123 | 7B | 36 | 24 | 3 | 15 | 442859 | 3542868 | 3 | 15 | 0 | 224 | 3617668 | 3 | 15 | 0 | 1 | 232 |
| 124 | 7C | 36 | 24 | 3 | 16 | 442887 | 3543092 | 3 | 16 | 0 | 224 | 3617900 | 3 | 16 | 0 | 1 | 232 |
| 125 | 7D | 36 | 24 | 3 | 17 | 442915 | 3543316 | 3 | 17 | 0 | 224 | 3618132 | 3 | 17 | 0 | 1 | 232 |
| 126 | 7E | 36 | 24 | 3 | 18 | 442943 | 3543540 | 3 | 18 | 0 | 224 | 3618364 | 3 | 18 | 0 | 1 | 232 |
| 127 | 7F | 36 | 24 | 3 | 19 | 442971 | 3543764 | 3 | 19 | 0 | 224 | 3618596 | 3 | 19 | 0 | 1 | 232 |
| 128 | 80 | 36 | 24 | 3 | 20 | 442999 | 3543988 | 3 | 20 | 0 | 224 | 3618828 | 3 | 20 | 0 | 1 | 232 |
| 129 | 81 | 36 | 24 | 3 | 21 | 443027 | 3544212 | 3 | 21 | 0 | 224 | 3619060 | 3 | 21 | 0 | 1 | 232 |
| 130 | 82 | 36 | 24 | 3 | 22 | 443055 | 3544436 | 3 | 22 | 0 | 224 | 3619292 | 3 | 22 | 0 | 1 | 232 |
| 131 | 83 | 36 | 24 | 3 | 23 | 443083 | 3544660 | 3 | 23 | 0 | 224 | 3619524 | 3 | 23 | 0 | 1 | 232 |
| 132 | 84 | 36 | 24 | 3 | 24 | 443111 | 3544884 | 3 | 24 | 0 | 224 | 3619756 | 3 | 24 | 0 | 1 | 232 |
| 133 | 85 | 36 | 24 | 3 | 25 | 443139 | 3545108 | 3 | 25 | 0 | 224 | 3619988 | 3 | 25 | 0 | 1 | 232 |
| 134 | 86 | 36 | 24 | 3 | 26 | 443167 | 3545332 | 3 | 26 | 0 | 224 | 3620220 | 3 | 26 | 0 | 1 | 232 |
| 135 | 87 | 36 | 24 | 3 | 27 | 443195 | 3545556 | 3 | 27 | 0 | 224 | 3620452 | 3 | 27 | 0 | 1 | 232 |
| 136 | 88 | 36 | 24 | 3 | 28 | 443223 | 3545780 | 3 | 28 | 0 | 224 | 3620684 | 3 | 28 | 0 | 1 | 232 |
| 137 | 89 | 36 | 24 | 3 | 29 | 443251 | 3546004 | 3 | 29 | 0 | 224 | 3620916 | 3 | 29 | 0 | 1 | 232 |
| 138 | 8A | 36 | 24 | 3 | 30 | 443279 | 3546228 | 3 | 30 | 0 | 224 | 3621148 | 3 | 30 | 0 | 1 | 232 |
| 139 | 8B | 36 | 24 | 3 | 31 | 443307 | 3546452 | 3 | 31 | 0 | 224 | 3621380 | 3 | 31 | 0 | 1 | 232 |
| 140 | 8C | 36 | 24 | 3 | 32 | 443335 | 3546676 | 3 | 32 | 0 | 224 | 3621612 | 3 | 32 | 0 | 1 | 232 |
| 141 | 8D | 36 | 24 | 3 | 33 | 443363 | 3546900 | 3 | 33 | 0 | 224 | 3621844 | 3 | 33 | 0 | 1 | 232 |
| 142 | 8E | 36 | 24 | 3 | 34 | 443391 | 3547124 | 3 | 34 | 0 | 224 | 3622076 | 3 | 34 | 0 | 1 | 232 |
| 143 | 8F | 36 | 24 | 3 | 35 | 443419 | 3547348 | 3 | 35 | 0 | 224 | 3622308 | 3 | 35 | 0 | 1 | 232 |
| 144 | 90 | 36 | 24 | 4 | 0 | 443442 | 3547532 | 4 | 0 | 0 | 184 | 3622500 | 4 | 0 | 0 | 1 | 192 |
| 145 | 91 | 36 | 24 | 4 | 1 | 443470 | 3547756 | 4 | 1 | 0 | 224 | 3622732 | 4 | 1 | 0 | 1 | 232 |
| 146 | 92 | 36 | 24 | 4 | 2 | 443498 | 3547980 | 4 | 2 | 0 | 224 | 3622964 | 4 | 2 | 0 | 1 | 232 |
| 147 | 93 | 36 | 24 | 4 | 3 | 443526 | 3548204 | 4 | 3 | 0 | 224 | 3623196 | 4 | 3 | 0 | 1 | 232 |
| 148 | 94 | 36 | 24 | 4 | 4 | 443554 | 3548428 | 4 | 4 | 0 | 224 | 3623428 | 4 | 4 | 0 | 1 | 232 |
| 149 | 95 | 36 | 24 | 4 | 5 | 443582 | 3548652 | 4 | 5 | 0 | 224 | 3623660 | 4 | 5 | 0 | 1 | 232 |
| 150 | 96 | 36 | 24 | 4 | 6 | 443610 | 3548876 | 4 | 6 | 0 | 224 | 3623892 | 4 | 6 | 0 | 1 | 232 |
| 151 | 97 | 36 | 24 | 4 | 7 | 443638 | 3549100 | 4 | 7 | 0 | 224 | 3624124 | 4 | 7 | 0 | 1 | 232 |
| 152 | 98 | 36 | 24 | 4 | 8 | 443666 | 3549324 | 4 | 8 | 0 | 224 | 3624356 | 4 | 8 | 0 | 1 | 232 |
| 153 | 99 | 36 | 24 | 4 | 9 | 443694 | 3549548 | 4 | 9 | 0 | 224 | 3624588 | 4 | 9 | 0 | 1 | 232 |

| | | | | | | | | | | | | | | | | | |
|---|---|---|---|---|---|---|---|---|---|---|---|---|---|---|---|---|---|
| 154 | 9A | 36 | 24 | 4 | 10 | 443722 | 3549772 | 4 | 10 | 0 | 224 | 3624820 | 4 | 10 | 0 | 1 | 232 |
| 155 | 9B | 36 | 24 | 4 | 11 | 443750 | 3549996 | 4 | 11 | 0 | 224 | 3625052 | 4 | 11 | 0 | 1 | 232 |
| 156 | 9C | 36 | 24 | 4 | 12 | 443778 | 3550220 | 4 | 12 | 0 | 224 | 3625284 | 4 | 12 | 0 | 1 | 232 |
| 157 | 9D | 36 | 24 | 4 | 13 | 443806 | 3550444 | 4 | 13 | 0 | 224 | 3625516 | 4 | 13 | 0 | 1 | 232 |
| 158 | 9E | 36 | 24 | 4 | 14 | 443834 | 3550668 | 4 | 14 | 0 | 224 | 3625748 | 4 | 14 | 0 | 1 | 232 |
| 159 | 9F | 36 | 24 | 4 | 15 | 443862 | 3550892 | 4 | 15 | 0 | 224 | 3625980 | 4 | 15 | 0 | 1 | 232 |
| 160 | A0 | 36 | 24 | 4 | 16 | 443892 | 3551132 | 4 | 16 | 0 | 240 | 3626228 | 4 | 16 | 0 | 1 | 248 |
| 161 | A1 | 36 | 24 | 4 | 17 | 443922 | 3551372 | 4 | 17 | 0 | 240 | 3626476 | 4 | 17 | 0 | 1 | 248 |
| 162 | A2 | 36 | 24 | 4 | 18 | 443952 | 3551612 | 4 | 18 | 0 | 240 | 3626724 | 4 | 18 | 0 | 1 | 248 |
| 163 | A3 | 36 | 24 | 4 | 19 | 443982 | 3551852 | 4 | 19 | 0 | 240 | 3626972 | 4 | 19 | 0 | 1 | 248 |
| 164 | A4 | 36 | 24 | 4 | 20 | 444012 | 3552092 | 4 | 20 | 0 | 240 | 3627220 | 4 | 20 | 0 | 1 | 248 |
| 165 | A5 | 36 | 24 | 4 | 21 | 444042 | 3552332 | 4 | 21 | 0 | 240 | 3627468 | 4 | 21 | 0 | 1 | 248 |
| 166 | A6 | 36 | 24 | 4 | 22 | 444072 | 3552572 | 4 | 22 | 0 | 240 | 3627716 | 4 | 22 | 0 | 1 | 248 |
| 167 | A7 | 36 | 24 | 4 | 23 | 444102 | 3552812 | 4 | 23 | 0 | 240 | 3627964 | 4 | 23 | 0 | 1 | 248 |
| 168 | A8 | 36 | 24 | 4 | 24 | 444132 | 3553052 | 4 | 24 | 0 | 240 | 3628212 | 4 | 24 | 0 | 1 | 248 |
| 169 | A9 | 36 | 24 | 4 | 25 | 444162 | 3553292 | 4 | 25 | 0 | 240 | 3628460 | 4 | 25 | 0 | 1 | 248 |
| 170 | AA | 36 | 24 | 4 | 26 | 444192 | 3553532 | 4 | 26 | 0 | 240 | 3628708 | 4 | 26 | 0 | 1 | 248 |
| 171 | AB | 36 | 24 | 4 | 27 | 444222 | 3553772 | 4 | 27 | 0 | 240 | 3628956 | 4 | 27 | 0 | 1 | 248 |
| 172 | AC | 36 | 24 | 4 | 28 | 444252 | 3554012 | 4 | 28 | 0 | 240 | 3629204 | 4 | 28 | 0 | 1 | 248 |
| 173 | AD | 36 | 24 | 4 | 29 | 444282 | 3554252 | 4 | 29 | 0 | 240 | 3629452 | 4 | 29 | 0 | 1 | 248 |
| 174 | AE | 36 | 24 | 4 | 30 | 444312 | 3554492 | 4 | 30 | 0 | 240 | 3629700 | 4 | 30 | 0 | 1 | 248 |
| 175 | AF | 36 | 24 | 4 | 31 | 444342 | 3554732 | 4 | 31 | 0 | 240 | 3629948 | 4 | 31 | 0 | 1 | 248 |
| 176 | B0 | 36 | 24 | 4 | 32 | 444372 | 3554972 | 4 | 32 | 0 | 240 | 3630196 | 4 | 32 | 0 | 1 | 248 |
| 177 | B1 | 36 | 24 | 4 | 33 | 444402 | 3555212 | 4 | 33 | 0 | 240 | 3630444 | 4 | 33 | 0 | 1 | 248 |
| 178 | B2 | 36 | 24 | 4 | 34 | 444432 | 3555452 | 4 | 34 | 0 | 240 | 3630692 | 4 | 34 | 0 | 1 | 248 |
| 179 | B3 | 36 | 24 | 4 | 35 | 444462 | 3555692 | 4 | 35 | 0 | 240 | 3630940 | 4 | 35 | 0 | 1 | 248 |
| 180 | B4 | 36 | 24 | 5 | 0 | 444494 | 3555948 | 5 | 0 | 0 | 256 | 3631204 | 5 | 0 | 0 | 1 | 264 |
| 181 | B5 | 36 | 24 | 5 | 1 | 444526 | 3556204 | 5 | 1 | 0 | 256 | 3631468 | 5 | 1 | 0 | 1 | 264 |
| 182 | B6 | 36 | 24 | 5 | 2 | 444558 | 3556460 | 5 | 2 | 0 | 256 | 3631732 | 5 | 2 | 0 | 1 | 264 |
| 183 | B7 | 36 | 24 | 5 | 3 | 444590 | 3556716 | 5 | 3 | 0 | 256 | 3631996 | 5 | 3 | 0 | 1 | 264 |
| 184 | B8 | 36 | 24 | 5 | 4 | 444622 | 3556972 | 5 | 4 | 0 | 256 | 3632260 | 5 | 4 | 0 | 1 | 264 |
| 185 | B9 | 36 | 24 | 5 | 5 | 444654 | 3557228 | 5 | 5 | 0 | 256 | 3632524 | 5 | 5 | 0 | 1 | 264 |
| 186 | BA | 36 | 24 | 5 | 6 | 444686 | 3557484 | 5 | 6 | 0 | 256 | 3632788 | 5 | 6 | 0 | 1 | 264 |
| 187 | BB | 36 | 24 | 5 | 7 | 444718 | 3557740 | 5 | 7 | 0 | 256 | 3633052 | 5 | 7 | 0 | 1 | 264 |
| 188 | BC | 36 | 24 | 5 | 8 | 444750 | 3557996 | 5 | 8 | 0 | 256 | 3633316 | 5 | 8 | 0 | 1 | 264 |
| 189 | BD | 36 | 24 | 5 | 9 | 444782 | 3558252 | 5 | 9 | 0 | 256 | 3633580 | 5 | 9 | 0 | 1 | 264 |
| 190 | BE | 36 | 24 | 5 | 10 | 444814 | 3558508 | 5 | 10 | 0 | 256 | 3633844 | 5 | 10 | 0 | 1 | 264 |
| 191 | BF | 36 | 24 | 5 | 11 | 444846 | 3558764 | 5 | 11 | 0 | 256 | 3634108 | 5 | 11 | 0 | 1 | 264 |
| 192 | C0 | 36 | 24 | 5 | 12 | 444876 | 3559004 | 5 | 12 | 0 | 240 | 3634356 | 5 | 12 | 0 | 1 | 248 |
| 193 | C1 | 36 | 24 | 5 | 13 | 444906 | 3559244 | 5 | 13 | 0 | 240 | 3634604 | 5 | 13 | 0 | 1 | 248 |
| 194 | C2 | 36 | 24 | 5 | 14 | 444936 | 3559484 | 5 | 14 | 0 | 240 | 3634852 | 5 | 14 | 0 | 1 | 248 |
| 195 | C3 | 36 | 24 | 5 | 15 | 444966 | 3559724 | 5 | 15 | 0 | 240 | 3635100 | 5 | 15 | 0 | 1 | 248 |
| 196 | C4 | 36 | 24 | 5 | 16 | 444996 | 3559964 | 5 | 16 | 0 | 240 | 3635348 | 5 | 16 | 0 | 1 | 248 |
| 197 | C5 | 36 | 24 | 5 | 17 | 445026 | 3560204 | 5 | 17 | 0 | 240 | 3635596 | 5 | 17 | 0 | 1 | 248 |
| 198 | C6 | 36 | 24 | 5 | 18 | 445056 | 3560444 | 5 | 18 | 0 | 240 | 3635844 | 5 | 18 | 0 | 1 | 248 |
| 199 | C7 | 36 | 24 | 5 | 19 | 445086 | 3560684 | 5 | 19 | 0 | 240 | 3636092 | 5 | 19 | 0 | 1 | 248 |
| 200 | C8 | 36 | 24 | 5 | 20 | 445116 | 3560924 | 5 | 20 | 0 | 240 | 3636340 | 5 | 20 | 0 | 1 | 248 |
| 201 | C9 | 36 | 24 | 5 | 21 | 445146 | 3561164 | 5 | 21 | 0 | 240 | 3636588 | 5 | 21 | 0 | 1 | 248 |
| 202 | CA | 36 | 24 | 5 | 22 | 445176 | 3561404 | 5 | 22 | 0 | 240 | 3636836 | 5 | 22 | 0 | 1 | 248 |
| 203 | CB | 36 | 24 | 5 | 23 | 445206 | 3561644 | 5 | 23 | 0 | 240 | 3637084 | 5 | 23 | 0 | 1 | 248 |
| 204 | CC | 36 | 24 | 5 | 24 | 445236 | 3561884 | 5 | 24 | 0 | 240 | 3637332 | 5 | 24 | 0 | 1 | 248 |
| 205 | CD | 36 | 24 | 5 | 25 | 445266 | 3562124 | 5 | 25 | 0 | 240 | 3637580 | 5 | 25 | 0 | 1 | 248 |
| 206 | CE | 36 | 24 | 5 | 26 | 445296 | 3562364 | 5 | 26 | 0 | 240 | 3637828 | 5 | 26 | 0 | 1 | 248 |
| 207 | CF | 36 | 24 | 5 | 27 | 445326 | 3562604 | 5 | 27 | 0 | 240 | 3638076 | 5 | 27 | 0 | 1 | 248 |
| 208 | D0 | 36 | 24 | 5 | 28 | 445358 | 3562860 | 5 | 28 | 0 | 256 | 3638340 | 5 | 28 | 0 | 1 | 264 |
| 209 | D1 | 36 | 24 | 5 | 29 | 445390 | 3563116 | 5 | 29 | 0 | 256 | 3638604 | 5 | 29 | 0 | 1 | 264 |
| 210 | D2 | 36 | 24 | 5 | 30 | 445422 | 3563372 | 5 | 30 | 0 | 256 | 3638868 | 5 | 30 | 0 | 1 | 264 |
| 211 | D3 | 36 | 24 | 5 | 31 | 445454 | 3563628 | 5 | 31 | 0 | 256 | 3639132 | 5 | 31 | 0 | 1 | 264 |
| 212 | D4 | 36 | 24 | 5 | 32 | 445486 | 3563884 | 5 | 32 | 0 | 256 | 3639396 | 5 | 32 | 0 | 1 | 264 |
| 213 | D5 | 36 | 24 | 5 | 33 | 445518 | 3564140 | 5 | 33 | 0 | 256 | 3639660 | 5 | 33 | 0 | 1 | 264 |
| 214 | D6 | 36 | 24 | 5 | 34 | 445550 | 3564396 | 5 | 34 | 0 | 256 | 3639924 | 5 | 34 | 0 | 1 | 264 |
| 215 | D7 | 36 | 24 | 5 | 35 | 445582 | 3564652 | 5 | 35 | 0 | 256 | 3640188 | 5 | 35 | 0 | 1 | 264 |
| 216 | D8 | 36 | 24 | 6 | 0 | 445616 | 3564924 | 6 | 0 | 0 | 272 | 3640468 | 6 | 0 | 0 | 1 | 280 |
| 217 | D9 | 36 | 24 | 6 | 1 | 445650 | 3565196 | 6 | 1 | 0 | 272 | 3640748 | 6 | 1 | 0 | 1 | 280 |
| 218 | DA | 36 | 24 | 6 | 2 | 445684 | 3565468 | 6 | 2 | 0 | 272 | 3641028 | 6 | 2 | 0 | 1 | 280 |
| 219 | DB | 36 | 24 | 6 | 3 | 445718 | 3565740 | 6 | 3 | 0 | 272 | 3641308 | 6 | 3 | 0 | 1 | 280 |
| 220 | DC | 36 | 24 | 6 | 4 | 445752 | 3566012 | 6 | 4 | 0 | 272 | 3641588 | 6 | 4 | 0 | 1 | 280 |
| 221 | DD | 36 | 24 | 6 | 5 | 445786 | 3566284 | 6 | 5 | 0 | 272 | 3641868 | 6 | 5 | 0 | 1 | 280 |
| 222 | DE | 36 | 24 | 6 | 6 | 445820 | 3566556 | 6 | 6 | 0 | 272 | 3642148 | 6 | 6 | 0 | 1 | 280 |
| 223 | DF | 36 | 24 | 6 | 7 | 445854 | 3566828 | 6 | 7 | 0 | 272 | 3642428 | 6 | 7 | 0 | 1 | 280 |
| 224 | E0 | 36 | 24 | 6 | 8 | 445888 | 3567100 | 6 | 8 | 0 | 272 | 3642708 | 6 | 8 | 0 | 1 | 280 |
| 225 | E1 | 36 | 24 | 6 | 9 | 445922 | 3567372 | 6 | 9 | 0 | 272 | 3642988 | 6 | 9 | 0 | 1 | 280 |
| 226 | E2 | 36 | 24 | 6 | 10 | 445956 | 3567644 | 6 | 10 | 0 | 272 | 3643268 | 6 | 10 | 0 | 1 | 280 |
| 227 | E3 | 36 | 24 | 6 | 11 | 445990 | 3567916 | 6 | 11 | 0 | 272 | 3643548 | 6 | 11 | 0 | 1 | 280 |
| 228 | E4 | 36 | 24 | 6 | 12 | 446024 | 3568188 | 6 | 12 | 0 | 272 | 3643828 | 6 | 12 | 0 | 1 | 280 |
| 229 | E5 | 36 | 24 | 6 | 13 | 446058 | 3568460 | 6 | 13 | 0 | 272 | 3644108 | 6 | 13 | 0 | 1 | 280 |
| 230 | E6 | 36 | 24 | 6 | 14 | 446092 | 3568732 | 6 | 14 | 0 | 272 | 3644388 | 6 | 14 | 0 | 1 | 280 |
| 231 | E7 | 36 | 24 | 6 | 15 | 446126 | 3569004 | 6 | 15 | 0 | 272 | 3644668 | 6 | 15 | 0 | 1 | 280 |
| 232 | E8 | 36 | 24 | 6 | 16 | 446160 | 3569276 | 6 | 16 | 0 | 272 | 3644948 | 6 | 16 | 0 | 1 | 280 |
| 233 | E9 | 36 | 24 | 6 | 17 | 446194 | 3569548 | 6 | 17 | 0 | 272 | 3645228 | 6 | 17 | 0 | 1 | 280 |
| 234 | EA | 36 | 24 | 6 | 18 | 446228 | 3569820 | 6 | 18 | 0 | 272 | 3645508 | 6 | 18 | 0 | 1 | 280 |
| 235 | EB | 36 | 24 | 6 | 19 | 446262 | 3570092 | 6 | 19 | 0 | 272 | 3645788 | 6 | 19 | 0 | 1 | 280 |
| 236 | EC | 36 | 24 | 6 | 20 | 446296 | 3570364 | 6 | 20 | 0 | 272 | 3646068 | 6 | 20 | 0 | 1 | 280 |
| 237 | ED | 36 | 24 | 6 | 21 | 446330 | 3570636 | 6 | 21 | 0 | 272 | 3646348 | 6 | 21 | 0 | 1 | 280 |
| 238 | EE | 36 | 24 | 6 | 22 | 446364 | 3570908 | 6 | 22 | 0 | 272 | 3646628 | 6 | 22 | 0 | 1 | 280 |
| 239 | EF | 36 | 24 | 6 | 23 | 446398 | 3571180 | 6 | 23 | 0 | 272 | 3646908 | 6 | 23 | 0 | 1 | 280 |
| 240 | F0 | 36 | 24 | 6 | 24 | 446430 | 3571436 | 6 | 24 | 0 | 256 | 3647172 | 6 | 24 | 0 | 1 | 264 |
| 241 | F1 | 36 | 24 | 6 | 25 | 446462 | 3571692 | 6 | 25 | 0 | 256 | 3647436 | 6 | 25 | 0 | 1 | 264 |
| 242 | F2 | 36 | 24 | 6 | 26 | 446494 | 3571948 | 6 | 26 | 0 | 256 | 3647700 | 6 | 26 | 0 | 1 | 264 |
| 243 | F3 | 36 | 24 | 6 | 27 | 446526 | 3572204 | 6 | 27 | 0 | 256 | 3647964 | 6 | 27 | 0 | 1 | 264 |
| 244 | F4 | 36 | 24 | 6 | 28 | 446558 | 3572460 | 6 | 28 | 0 | 256 | 3648228 | 6 | 28 | 0 | 1 | 264 |
| 245 | F5 | 36 | 24 | 6 | 29 | 446590 | 3572716 | 6 | 29 | 0 | 256 | 3648492 | 6 | 29 | 0 | 1 | 264 |
| 246 | F6 | 36 | 24 | 6 | 30 | 446622 | 3572972 | 6 | 30 | 0 | 256 | 3648756 | 6 | 30 | 0 | 1 | 264 |
| 247 | F7 | 36 | 24 | 6 | 31 | 446654 | 3573228 | 6 | 31 | 0 | 256 | 3649020 | 6 | 31 | 0 | 1 | 264 |
| 248 | F8 | 36 | 24 | 6 | 32 | 446686 | 3573484 | 6 | 32 | 0 | 256 | 3649284 | 6 | 32 | 0 | 1 | 264 |
| 249 | F9 | 36 | 24 | 6 | 33 | 446718 | 3573740 | 6 | 33 | 0 | 256 | 3649548 | 6 | 33 | 0 | 1 | 264 |
| 250 | FA | 36 | 24 | 6 | 34 | 446750 | 3573996 | 6 | 34 | 0 | 256 | 3649812 | 6 | 34 | 0 | 1 | 264 |
| 251 | FB | 36 | 24 | 6 | 35 | 446782 | 3574252 | 6 | 35 | 0 | 256 | 3650076 | 6 | 35 | 0 | 1 | 264 |
| 252 | FC | 36 | 24 | 7 | 0 | 446816 | 3574524 | 7 | 0 | 0 | 272 | 3650356 | 7 | 0 | 0 | 1 | 280 |
| 253 | FD | 36 | 24 | 7 | 1 | 446850 | 3574796 | 7 | 1 | 0 | 272 | 3650636 | 7 | 1 | 0 | 1 | 280 |
| 254 | FE | 36 | 24 | 7 | 2 | 446884 | 3575068 | 7 | 2 | 0 | 272 | 3650916 | 7 | 2 | 0 | 1 | 280 |
| 255 | FF | 36 | 24 | 7 | 3 | 446918 | 3575340 | 7 | 3 | 0 | 272 | 3651196 | 7 | 3 | 0 | 1 | 280 |
| 0 | 00 | 37 | 25 | 0 | 0 | 446930 | 3575436 | 0 | 0 | 0 | 96 | 3651300 | 0 | 0 | 0 | 1 | 104 |

| | | | | | | | | | | | | | | | | | |
|---|---|---|---|---|---|---|---|---|---|---|---|---|---|---|---|---|---|
| 1 | 01 | 37 | 25 | 0 | 1 | 446944 | 3575548 | 0 | 1 | 0 | 112 | 3651420 | 0 | 1 | 0 | 1 | 120 |
| 2 | 02 | 37 | 25 | 0 | 2 | 446958 | 3575660 | 0 | 2 | 0 | 112 | 3651540 | 0 | 2 | 0 | 1 | 120 |
| 3 | 03 | 37 | 25 | 0 | 3 | 446972 | 3575772 | 0 | 3 | 0 | 112 | 3651660 | 0 | 3 | 0 | 1 | 120 |
| 4 | 04 | 37 | 25 | 0 | 4 | 446986 | 3575884 | 0 | 4 | 0 | 112 | 3651780 | 0 | 4 | 0 | 1 | 120 |
| 5 | 05 | 37 | 25 | 0 | 5 | 447000 | 3575996 | 0 | 5 | 0 | 112 | 3651900 | 0 | 5 | 0 | 1 | 120 |
| 6 | 06 | 37 | 25 | 0 | 6 | 447014 | 3576108 | 0 | 6 | 0 | 112 | 3652020 | 0 | 6 | 0 | 1 | 120 |
| 7 | 07 | 37 | 25 | 0 | 7 | 447028 | 3576220 | 0 | 7 | 0 | 112 | 3652140 | 0 | 7 | 0 | 1 | 120 |
| 8 | 08 | 37 | 25 | 0 | 8 | 447042 | 3576332 | 0 | 8 | 0 | 112 | 3652260 | 0 | 8 | 0 | 1 | 120 |
| 9 | 09 | 37 | 25 | 0 | 9 | 447056 | 3576444 | 0 | 9 | 0 | 112 | 3652380 | 0 | 9 | 0 | 1 | 120 |
| 10 | 0A | 37 | 25 | 0 | 10 | 447070 | 3576556 | 0 | 10 | 0 | 112 | 3652500 | 0 | 10 | 0 | 1 | 120 |
| 11 | 0B | 37 | 25 | 0 | 11 | 447084 | 3576668 | 0 | 11 | 0 | 112 | 3652620 | 0 | 11 | 0 | 1 | 120 |
| 12 | 0C | 37 | 25 | 0 | 12 | 447098 | 3576780 | 0 | 12 | 0 | 112 | 3652740 | 0 | 12 | 0 | 1 | 120 |
| 13 | 0D | 37 | 25 | 0 | 13 | 447112 | 3576892 | 0 | 13 | 0 | 112 | 3652860 | 0 | 13 | 0 | 1 | 120 |
| 14 | 0E | 37 | 25 | 0 | 14 | 447126 | 3577004 | 0 | 14 | 0 | 112 | 3652980 | 0 | 14 | 0 | 1 | 120 |
| 15 | 0F | 37 | 25 | 0 | 15 | 447140 | 3577116 | 0 | 15 | 0 | 112 | 3653100 | 0 | 15 | 0 | 1 | 120 |
| 16 | 10 | 37 | 25 | 0 | 16 | 447154 | 3577228 | 0 | 16 | 0 | 112 | 3653220 | 0 | 16 | 0 | 1 | 120 |
| 17 | 11 | 37 | 25 | 0 | 17 | 447168 | 3577340 | 0 | 17 | 0 | 112 | 3653340 | 0 | 17 | 0 | 1 | 120 |
| 18 | 12 | 37 | 25 | 0 | 18 | 447182 | 3577452 | 0 | 18 | 0 | 112 | 3653460 | 0 | 18 | 0 | 1 | 120 |
| 19 | 13 | 37 | 25 | 0 | 19 | 447196 | 3577564 | 0 | 19 | 0 | 112 | 3653580 | 0 | 19 | 0 | 1 | 120 |
| 20 | 14 | 37 | 25 | 0 | 20 | 447210 | 3577676 | 0 | 20 | 0 | 112 | 3653700 | 0 | 20 | 0 | 1 | 120 |
| 21 | 15 | 37 | 25 | 0 | 21 | 447224 | 3577788 | 0 | 21 | 0 | 112 | 3653820 | 0 | 21 | 0 | 1 | 120 |
| 22 | 16 | 37 | 25 | 0 | 22 | 447238 | 3577900 | 0 | 22 | 0 | 112 | 3653940 | 0 | 22 | 0 | 1 | 120 |
| 23 | 17 | 37 | 25 | 0 | 23 | 447252 | 3578012 | 0 | 23 | 0 | 112 | 3654060 | 0 | 23 | 0 | 1 | 120 |
| 24 | 18 | 37 | 25 | 0 | 24 | 447266 | 3578124 | 0 | 24 | 0 | 112 | 3654180 | 0 | 24 | 0 | 1 | 120 |
| 25 | 19 | 37 | 25 | 0 | 25 | 447280 | 3578236 | 0 | 25 | 0 | 112 | 3654300 | 0 | 25 | 0 | 1 | 120 |
| 26 | 1A | 37 | 25 | 0 | 26 | 447294 | 3578348 | 0 | 26 | 0 | 112 | 3654420 | 0 | 26 | 0 | 1 | 120 |
| 27 | 1B | 37 | 25 | 0 | 27 | 447308 | 3578460 | 0 | 27 | 0 | 112 | 3654540 | 0 | 27 | 0 | 1 | 120 |
| 28 | 1C | 37 | 25 | 0 | 28 | 447322 | 3578572 | 0 | 28 | 0 | 112 | 3654660 | 0 | 28 | 0 | 1 | 120 |
| 29 | 1D | 37 | 25 | 0 | 29 | 447336 | 3578684 | 0 | 29 | 0 | 112 | 3654780 | 0 | 29 | 0 | 1 | 120 |
| 30 | 1E | 37 | 25 | 0 | 30 | 447350 | 3578796 | 0 | 30 | 0 | 112 | 3654900 | 0 | 30 | 0 | 1 | 120 |
| 31 | 1F | 37 | 25 | 0 | 31 | 447364 | 3578908 | 0 | 31 | 0 | 112 | 3655020 | 0 | 31 | 0 | 1 | 120 |
| 32 | 20 | 37 | 25 | 0 | 32 | 447378 | 3579020 | 0 | 32 | 0 | 112 | 3655140 | 0 | 32 | 0 | 1 | 120 |
| 33 | 21 | 37 | 25 | 0 | 33 | 447392 | 3579132 | 0 | 33 | 0 | 112 | 3655260 | 0 | 33 | 0 | 1 | 120 |
| 34 | 22 | 37 | 25 | 0 | 34 | 447406 | 3579244 | 0 | 34 | 0 | 112 | 3655380 | 0 | 34 | 0 | 1 | 120 |
| 35 | 23 | 37 | 25 | 0 | 35 | 447420 | 3579356 | 0 | 35 | 0 | 112 | 3655500 | 0 | 35 | 0 | 1 | 120 |
| 36 | 24 | 37 | 25 | 0 | 36 | 447434 | 3579468 | 0 | 36 | 0 | 112 | 3655620 | 0 | 36 | 0 | 1 | 120 |
| 37 | 25 | 37 | 25 | 1 | 0 | 447446 | 3579564 | 1 | 0 | 0 | 96 | 3655724 | 1 | 0 | 0 | 1 | 104 |
| 38 | 26 | 37 | 25 | 1 | 1 | 447463 | 3579700 | 1 | 1 | 0 | 136 | 3655868 | 1 | 1 | 0 | 1 | 144 |
| 39 | 27 | 37 | 25 | 1 | 2 | 447480 | 3579836 | 1 | 2 | 0 | 136 | 3656012 | 1 | 2 | 0 | 1 | 144 |
| 40 | 28 | 37 | 25 | 1 | 3 | 447497 | 3579972 | 1 | 3 | 0 | 136 | 3656156 | 1 | 3 | 0 | 1 | 144 |
| 41 | 29 | 37 | 25 | 1 | 4 | 447514 | 3580108 | 1 | 4 | 0 | 136 | 3656300 | 1 | 4 | 0 | 1 | 144 |
| 42 | 2A | 37 | 25 | 1 | 5 | 447531 | 3580244 | 1 | 5 | 0 | 136 | 3656444 | 1 | 5 | 0 | 1 | 144 |
| 43 | 2B | 37 | 25 | 1 | 6 | 447548 | 3580380 | 1 | 6 | 0 | 136 | 3656588 | 1 | 6 | 0 | 1 | 144 |
| 44 | 2C | 37 | 25 | 1 | 7 | 447565 | 3580516 | 1 | 7 | 0 | 136 | 3656732 | 1 | 7 | 0 | 1 | 144 |
| 45 | 2D | 37 | 25 | 1 | 8 | 447582 | 3580652 | 1 | 8 | 0 | 136 | 3656876 | 1 | 8 | 0 | 1 | 144 |
| 46 | 2E | 37 | 25 | 1 | 9 | 447599 | 3580788 | 1 | 9 | 0 | 136 | 3657020 | 1 | 9 | 0 | 1 | 144 |
| 47 | 2F | 37 | 25 | 1 | 10 | 447616 | 3580924 | 1 | 10 | 0 | 136 | 3657164 | 1 | 10 | 0 | 1 | 144 |
| 48 | 30 | 37 | 25 | 1 | 11 | 447637 | 3581092 | 1 | 11 | 0 | 168 | 3657340 | 1 | 11 | 0 | 1 | 176 |
| 49 | 31 | 37 | 25 | 1 | 12 | 447658 | 3581260 | 1 | 12 | 0 | 168 | 3657516 | 1 | 12 | 0 | 1 | 176 |
| 50 | 32 | 37 | 25 | 1 | 13 | 447679 | 3581428 | 1 | 13 | 0 | 168 | 3657692 | 1 | 13 | 0 | 1 | 176 |
| 51 | 33 | 37 | 25 | 1 | 14 | 447700 | 3581596 | 1 | 14 | 0 | 168 | 3657868 | 1 | 14 | 0 | 1 | 176 |
| 52 | 34 | 37 | 25 | 1 | 15 | 447721 | 3581764 | 1 | 15 | 0 | 168 | 3658044 | 1 | 15 | 0 | 1 | 176 |
| 53 | 35 | 37 | 25 | 1 | 16 | 447742 | 3581932 | 1 | 16 | 0 | 168 | 3658220 | 1 | 16 | 0 | 1 | 176 |
| 54 | 36 | 37 | 25 | 1 | 17 | 447763 | 3582100 | 1 | 17 | 0 | 168 | 3658396 | 1 | 17 | 0 | 1 | 176 |
| 55 | 37 | 37 | 25 | 1 | 18 | 447784 | 3582268 | 1 | 18 | 0 | 168 | 3658572 | 1 | 18 | 0 | 1 | 176 |
| 56 | 38 | 37 | 25 | 1 | 19 | 447805 | 3582436 | 1 | 19 | 0 | 168 | 3658748 | 1 | 19 | 0 | 1 | 176 |
| 57 | 39 | 37 | 25 | 1 | 20 | 447826 | 3582604 | 1 | 20 | 0 | 168 | 3658924 | 1 | 20 | 0 | 1 | 176 |
| 58 | 3A | 37 | 25 | 1 | 21 | 447847 | 3582772 | 1 | 21 | 0 | 168 | 3659100 | 1 | 21 | 0 | 1 | 176 |
| 59 | 3B | 37 | 25 | 1 | 22 | 447868 | 3582940 | 1 | 22 | 0 | 168 | 3659276 | 1 | 22 | 0 | 1 | 176 |
| 60 | 3C | 37 | 25 | 1 | 23 | 447889 | 3583108 | 1 | 23 | 0 | 168 | 3659452 | 1 | 23 | 0 | 1 | 176 |
| 61 | 3D | 37 | 25 | 1 | 24 | 447910 | 3583276 | 1 | 24 | 0 | 168 | 3659628 | 1 | 24 | 0 | 1 | 176 |
| 62 | 3E | 37 | 25 | 1 | 25 | 447931 | 3583444 | 1 | 25 | 0 | 168 | 3659804 | 1 | 25 | 0 | 1 | 176 |
| 63 | 3F | 37 | 25 | 1 | 26 | 447952 | 3583612 | 1 | 26 | 0 | 168 | 3659980 | 1 | 26 | 0 | 1 | 176 |
| 64 | 40 | 37 | 25 | 1 | 27 | 447975 | 3583796 | 1 | 27 | 0 | 184 | 3660172 | 1 | 27 | 0 | 1 | 192 |
| 65 | 41 | 37 | 25 | 1 | 28 | 447998 | 3583980 | 1 | 28 | 0 | 184 | 3660364 | 1 | 28 | 0 | 1 | 192 |
| 66 | 42 | 37 | 25 | 1 | 29 | 448021 | 3584164 | 1 | 29 | 0 | 184 | 3660556 | 1 | 29 | 0 | 1 | 192 |
| 67 | 43 | 37 | 25 | 1 | 30 | 448044 | 3584348 | 1 | 30 | 0 | 184 | 3660748 | 1 | 30 | 0 | 1 | 192 |
| 68 | 44 | 37 | 25 | 1 | 31 | 448067 | 3584532 | 1 | 31 | 0 | 184 | 3660940 | 1 | 31 | 0 | 1 | 192 |
| 69 | 45 | 37 | 25 | 1 | 32 | 448090 | 3584716 | 1 | 32 | 0 | 184 | 3661132 | 1 | 32 | 0 | 1 | 192 |
| 70 | 46 | 37 | 25 | 1 | 33 | 448113 | 3584900 | 1 | 33 | 0 | 184 | 3661324 | 1 | 33 | 0 | 1 | 192 |
| 71 | 47 | 37 | 25 | 1 | 34 | 448136 | 3585084 | 1 | 34 | 0 | 184 | 3661516 | 1 | 34 | 0 | 1 | 192 |
| 72 | 48 | 37 | 25 | 1 | 35 | 448159 | 3585268 | 1 | 35 | 0 | 184 | 3661708 | 1 | 35 | 0 | 1 | 192 |
| 73 | 49 | 37 | 25 | 1 | 36 | 448182 | 3585452 | 1 | 36 | 0 | 184 | 3661900 | 1 | 36 | 0 | 1 | 192 |
| 74 | 4A | 37 | 25 | 2 | 0 | 448203 | 3585620 | 2 | 0 | 0 | 168 | 3662076 | 2 | 0 | 0 | 1 | 176 |
| 75 | 4B | 37 | 25 | 2 | 1 | 448229 | 3585828 | 2 | 1 | 0 | 208 | 3662292 | 2 | 1 | 0 | 1 | 216 |
| 76 | 4C | 37 | 25 | 2 | 2 | 448255 | 3586036 | 2 | 2 | 0 | 208 | 3662508 | 2 | 2 | 0 | 1 | 216 |
| 77 | 4D | 37 | 25 | 2 | 3 | 448281 | 3586244 | 2 | 3 | 0 | 208 | 3662724 | 2 | 3 | 0 | 1 | 216 |
| 78 | 4E | 37 | 25 | 2 | 4 | 448307 | 3586452 | 2 | 4 | 0 | 208 | 3662940 | 2 | 4 | 0 | 1 | 216 |
| 79 | 4F | 37 | 25 | 2 | 5 | 448333 | 3586660 | 2 | 5 | 0 | 208 | 3663156 | 2 | 5 | 0 | 1 | 216 |
| 80 | 50 | 37 | 25 | 2 | 6 | 448359 | 3586868 | 2 | 6 | 0 | 208 | 3663372 | 2 | 6 | 0 | 1 | 216 |
| 81 | 51 | 37 | 25 | 2 | 7 | 448385 | 3587076 | 2 | 7 | 0 | 208 | 3663588 | 2 | 7 | 0 | 1 | 216 |
| 82 | 52 | 37 | 25 | 2 | 8 | 448411 | 3587284 | 2 | 8 | 0 | 208 | 3663804 | 2 | 8 | 0 | 1 | 216 |
| 83 | 53 | 37 | 25 | 2 | 9 | 448437 | 3587492 | 2 | 9 | 0 | 208 | 3664020 | 2 | 9 | 0 | 1 | 216 |
| 84 | 54 | 37 | 25 | 2 | 10 | 448463 | 3587700 | 2 | 10 | 0 | 208 | 3664236 | 2 | 10 | 0 | 1 | 216 |
| 85 | 55 | 37 | 25 | 2 | 11 | 448489 | 3587908 | 2 | 11 | 0 | 208 | 3664452 | 2 | 11 | 0 | 1 | 216 |
| 86 | 56 | 37 | 25 | 2 | 12 | 448515 | 3588116 | 2 | 12 | 0 | 208 | 3664668 | 2 | 12 | 0 | 1 | 216 |
| 87 | 57 | 37 | 25 | 2 | 13 | 448541 | 3588324 | 2 | 13 | 0 | 208 | 3664884 | 2 | 13 | 0 | 1 | 216 |
| 88 | 58 | 37 | 25 | 2 | 14 | 448567 | 3588532 | 2 | 14 | 0 | 208 | 3665100 | 2 | 14 | 0 | 1 | 216 |
| 89 | 59 | 37 | 25 | 2 | 15 | 448593 | 3588740 | 2 | 15 | 0 | 208 | 3665316 | 2 | 15 | 0 | 1 | 216 |
| 90 | 5A | 37 | 25 | 2 | 16 | 448619 | 3588948 | 2 | 16 | 0 | 208 | 3665532 | 2 | 16 | 0 | 1 | 216 |
| 91 | 5B | 37 | 25 | 2 | 17 | 448645 | 3589156 | 2 | 17 | 0 | 208 | 3665748 | 2 | 17 | 0 | 1 | 216 |
| 92 | 5C | 37 | 25 | 2 | 18 | 448671 | 3589364 | 2 | 18 | 0 | 208 | 3665964 | 2 | 18 | 0 | 1 | 216 |
| 93 | 5D | 37 | 25 | 2 | 19 | 448697 | 3589572 | 2 | 19 | 0 | 208 | 3666180 | 2 | 19 | 0 | 1 | 216 |
| 94 | 5E | 37 | 25 | 2 | 20 | 448723 | 3589780 | 2 | 20 | 0 | 208 | 3666396 | 2 | 20 | 0 | 1 | 216 |
| 95 | 5F | 37 | 25 | 2 | 21 | 448749 | 3589988 | 2 | 21 | 0 | 208 | 3666612 | 2 | 21 | 0 | 1 | 216 |
| 96 | 60 | 37 | 25 | 2 | 22 | 448772 | 3590172 | 2 | 22 | 0 | 184 | 3666804 | 2 | 22 | 0 | 1 | 192 |
| 97 | 61 | 37 | 25 | 2 | 23 | 448795 | 3590356 | 2 | 23 | 0 | 184 | 3666996 | 2 | 23 | 0 | 1 | 192 |
| 98 | 62 | 37 | 25 | 2 | 24 | 448818 | 3590540 | 2 | 24 | 0 | 184 | 3667188 | 2 | 24 | 0 | 1 | 192 |
| 99 | 63 | 37 | 25 | 2 | 25 | 448841 | 3590724 | 2 | 25 | 0 | 184 | 3667380 | 2 | 25 | 0 | 1 | 192 |
| 100 | 64 | 37 | 25 | 2 | 26 | 448864 | 3590908 | 2 | 26 | 0 | 184 | 3667572 | 2 | 26 | 0 | 1 | 192 |
| 101 | 65 | 37 | 25 | 2 | 27 | 448887 | 3591092 | 2 | 27 | 0 | 184 | 3667764 | 2 | 27 | 0 | 1 | 192 |
| 102 | 66 | 37 | 25 | 2 | 28 | 448910 | 3591276 | 2 | 28 | 0 | 184 | 3667956 | 2 | 28 | 0 | 1 | 192 |
| 103 | 67 | 37 | 25 | 2 | 29 | 448933 | 3591460 | 2 | 29 | 0 | 184 | 3668148 | 2 | 29 | 0 | 1 | 192 |

| | | | | | | | | | | | | | | | | | |
|---|---|---|---|---|---|---|---|---|---|---|---|---|---|---|---|---|---|
| 104 | 68 | 37 | 25 | 2 | 30 | 448956 | 3591644 | 2 | 30 | 0 | 184 | 3668340 | 2 | 30 | 0 | 1 | 192 |
| 105 | 69 | 37 | 25 | 2 | 31 | 448979 | 3591828 | 2 | 31 | 0 | 184 | 3668532 | 2 | 31 | 0 | 1 | 192 |
| 106 | 6A | 37 | 25 | 2 | 32 | 449002 | 3592012 | 2 | 32 | 0 | 184 | 3668724 | 2 | 32 | 0 | 1 | 192 |
| 107 | 6B | 37 | 25 | 2 | 33 | 449025 | 3592196 | 2 | 33 | 0 | 184 | 3668916 | 2 | 33 | 0 | 1 | 192 |
| 108 | 6C | 37 | 25 | 2 | 34 | 449048 | 3592380 | 2 | 34 | 0 | 184 | 3669108 | 2 | 34 | 0 | 1 | 192 |
| 109 | 6D | 37 | 25 | 2 | 35 | 449071 | 3592564 | 2 | 35 | 0 | 184 | 3669300 | 2 | 35 | 0 | 1 | 192 |
| 110 | 6E | 37 | 25 | 2 | 36 | 449094 | 3592748 | 2 | 36 | 0 | 184 | 3669492 | 2 | 36 | 0 | 1 | 192 |
| 111 | 6F | 37 | 25 | 3 | 0 | 449115 | 3592916 | 3 | 0 | 0 | 168 | 3669668 | 3 | 0 | 0 | 1 | 176 |
| 112 | 70 | 37 | 25 | 3 | 1 | 449143 | 3593140 | 3 | 1 | 0 | 224 | 3669900 | 3 | 1 | 0 | 1 | 232 |
| 113 | 71 | 37 | 25 | 3 | 2 | 449171 | 3593364 | 3 | 2 | 0 | 224 | 3670132 | 3 | 2 | 0 | 1 | 232 |
| 114 | 72 | 37 | 25 | 3 | 3 | 449199 | 3593588 | 3 | 3 | 0 | 224 | 3670364 | 3 | 3 | 0 | 1 | 232 |
| 115 | 73 | 37 | 25 | 3 | 4 | 449227 | 3593812 | 3 | 4 | 0 | 224 | 3670596 | 3 | 4 | 0 | 1 | 232 |
| 116 | 74 | 37 | 25 | 3 | 5 | 449255 | 3594036 | 3 | 5 | 0 | 224 | 3670828 | 3 | 5 | 0 | 1 | 232 |
| 117 | 75 | 37 | 25 | 3 | 6 | 449283 | 3594260 | 3 | 6 | 0 | 224 | 3671060 | 3 | 6 | 0 | 1 | 232 |
| 118 | 76 | 37 | 25 | 3 | 7 | 449311 | 3594484 | 3 | 7 | 0 | 224 | 3671292 | 3 | 7 | 0 | 1 | 232 |
| 119 | 77 | 37 | 25 | 3 | 8 | 449339 | 3594708 | 3 | 8 | 0 | 224 | 3671524 | 3 | 8 | 0 | 1 | 232 |
| 120 | 78 | 37 | 25 | 3 | 9 | 449367 | 3594932 | 3 | 9 | 0 | 224 | 3671756 | 3 | 9 | 0 | 1 | 232 |
| 121 | 79 | 37 | 25 | 3 | 10 | 449395 | 3595156 | 3 | 10 | 0 | 224 | 3671988 | 3 | 10 | 0 | 1 | 232 |
| 122 | 7A | 37 | 25 | 3 | 11 | 449423 | 3595380 | 3 | 11 | 0 | 224 | 3672220 | 3 | 11 | 0 | 1 | 232 |
| 123 | 7B | 37 | 25 | 3 | 12 | 449451 | 3595604 | 3 | 12 | 0 | 224 | 3672452 | 3 | 12 | 0 | 1 | 232 |
| 124 | 7C | 37 | 25 | 3 | 13 | 449479 | 3595828 | 3 | 13 | 0 | 224 | 3672684 | 3 | 13 | 0 | 1 | 232 |
| 125 | 7D | 37 | 25 | 3 | 14 | 449507 | 3596052 | 3 | 14 | 0 | 224 | 3672916 | 3 | 14 | 0 | 1 | 232 |
| 126 | 7E | 37 | 25 | 3 | 15 | 449535 | 3596276 | 3 | 15 | 0 | 224 | 3673148 | 3 | 15 | 0 | 1 | 232 |
| 127 | 7F | 37 | 25 | 3 | 16 | 449563 | 3596500 | 3 | 16 | 0 | 224 | 3673380 | 3 | 16 | 0 | 1 | 232 |
| 128 | 80 | 37 | 25 | 3 | 17 | 449591 | 3596724 | 3 | 17 | 0 | 224 | 3673612 | 3 | 17 | 0 | 1 | 232 |
| 129 | 81 | 37 | 25 | 3 | 18 | 449619 | 3596948 | 3 | 18 | 0 | 224 | 3673844 | 3 | 18 | 0 | 1 | 232 |
| 130 | 82 | 37 | 25 | 3 | 19 | 449647 | 3597172 | 3 | 19 | 0 | 224 | 3674076 | 3 | 19 | 0 | 1 | 232 |
| 131 | 83 | 37 | 25 | 3 | 20 | 449675 | 3597396 | 3 | 20 | 0 | 224 | 3674308 | 3 | 20 | 0 | 1 | 232 |
| 132 | 84 | 37 | 25 | 3 | 21 | 449703 | 3597620 | 3 | 21 | 0 | 224 | 3674540 | 3 | 21 | 0 | 1 | 232 |
| 133 | 85 | 37 | 25 | 3 | 22 | 449731 | 3597844 | 3 | 22 | 0 | 224 | 3674772 | 3 | 22 | 0 | 1 | 232 |
| 134 | 86 | 37 | 25 | 3 | 23 | 449759 | 3598068 | 3 | 23 | 0 | 224 | 3675004 | 3 | 23 | 0 | 1 | 232 |
| 135 | 87 | 37 | 25 | 3 | 24 | 449787 | 3598292 | 3 | 24 | 0 | 224 | 3675236 | 3 | 24 | 0 | 1 | 232 |
| 136 | 88 | 37 | 25 | 3 | 25 | 449815 | 3598516 | 3 | 25 | 0 | 224 | 3675468 | 3 | 25 | 0 | 1 | 232 |
| 137 | 89 | 37 | 25 | 3 | 26 | 449843 | 3598740 | 3 | 26 | 0 | 224 | 3675700 | 3 | 26 | 0 | 1 | 232 |
| 138 | 8A | 37 | 25 | 3 | 27 | 449871 | 3598964 | 3 | 27 | 0 | 224 | 3675932 | 3 | 27 | 0 | 1 | 232 |
| 139 | 8B | 37 | 25 | 3 | 28 | 449899 | 3599188 | 3 | 28 | 0 | 224 | 3676164 | 3 | 28 | 0 | 1 | 232 |
| 140 | 8C | 37 | 25 | 3 | 29 | 449927 | 3599412 | 3 | 29 | 0 | 224 | 3676396 | 3 | 29 | 0 | 1 | 232 |
| 141 | 8D | 37 | 25 | 3 | 30 | 449955 | 3599636 | 3 | 30 | 0 | 224 | 3676628 | 3 | 30 | 0 | 1 | 232 |
| 142 | 8E | 37 | 25 | 3 | 31 | 449983 | 3599860 | 3 | 31 | 0 | 224 | 3676860 | 3 | 31 | 0 | 1 | 232 |
| 143 | 8F | 37 | 25 | 3 | 32 | 450011 | 3600084 | 3 | 32 | 0 | 224 | 3677092 | 3 | 32 | 0 | 1 | 232 |
| 144 | 90 | 37 | 25 | 3 | 33 | 450036 | 3600284 | 3 | 33 | 0 | 200 | 3677300 | 3 | 33 | 0 | 1 | 208 |
| 145 | 91 | 37 | 25 | 3 | 34 | 450061 | 3600484 | 3 | 34 | 0 | 200 | 3677508 | 3 | 34 | 0 | 1 | 208 |
| 146 | 92 | 37 | 25 | 3 | 35 | 450086 | 3600684 | 3 | 35 | 0 | 200 | 3677716 | 3 | 35 | 0 | 1 | 208 |
| 147 | 93 | 37 | 25 | 3 | 36 | 450111 | 3600884 | 3 | 36 | 0 | 200 | 3677924 | 3 | 36 | 0 | 1 | 208 |
| 148 | 94 | 37 | 25 | 4 | 0 | 450134 | 3601068 | 4 | 0 | 0 | 184 | 3678116 | 4 | 0 | 0 | 1 | 192 |
| 149 | 95 | 37 | 25 | 4 | 1 | 450162 | 3601292 | 4 | 1 | 0 | 224 | 3678348 | 4 | 1 | 0 | 1 | 232 |
| 150 | 96 | 37 | 25 | 4 | 2 | 450190 | 3601516 | 4 | 2 | 0 | 224 | 3678580 | 4 | 2 | 0 | 1 | 232 |
| 151 | 97 | 37 | 25 | 4 | 3 | 450218 | 3601740 | 4 | 3 | 0 | 224 | 3678812 | 4 | 3 | 0 | 1 | 232 |
| 152 | 98 | 37 | 25 | 4 | 4 | 450246 | 3601964 | 4 | 4 | 0 | 224 | 3679044 | 4 | 4 | 0 | 1 | 232 |
| 153 | 99 | 37 | 25 | 4 | 5 | 450274 | 3602188 | 4 | 5 | 0 | 224 | 3679276 | 4 | 5 | 0 | 1 | 232 |
| 154 | 9A | 37 | 25 | 4 | 6 | 450302 | 3602412 | 4 | 6 | 0 | 224 | 3679508 | 4 | 6 | 0 | 1 | 232 |
| 155 | 9B | 37 | 25 | 4 | 7 | 450330 | 3602636 | 4 | 7 | 0 | 224 | 3679740 | 4 | 7 | 0 | 1 | 232 |
| 156 | 9C | 37 | 25 | 4 | 8 | 450358 | 3602860 | 4 | 8 | 0 | 224 | 3679972 | 4 | 8 | 0 | 1 | 232 |
| 157 | 9D | 37 | 25 | 4 | 9 | 450386 | 3603084 | 4 | 9 | 0 | 224 | 3680204 | 4 | 9 | 0 | 1 | 232 |
| 158 | 9E | 37 | 25 | 4 | 10 | 450414 | 3603308 | 4 | 10 | 0 | 224 | 3680436 | 4 | 10 | 0 | 1 | 232 |
| 159 | 9F | 37 | 25 | 4 | 11 | 450442 | 3603532 | 4 | 11 | 0 | 224 | 3680668 | 4 | 11 | 0 | 1 | 232 |
| 160 | A0 | 37 | 25 | 4 | 12 | 450472 | 3603772 | 4 | 12 | 0 | 240 | 3680916 | 4 | 12 | 0 | 1 | 248 |
| 161 | A1 | 37 | 25 | 4 | 13 | 450502 | 3604012 | 4 | 13 | 0 | 240 | 3681164 | 4 | 13 | 0 | 1 | 248 |
| 162 | A2 | 37 | 25 | 4 | 14 | 450532 | 3604252 | 4 | 14 | 0 | 240 | 3681412 | 4 | 14 | 0 | 1 | 248 |
| 163 | A3 | 37 | 25 | 4 | 15 | 450562 | 3604492 | 4 | 15 | 0 | 240 | 3681660 | 4 | 15 | 0 | 1 | 248 |
| 164 | A4 | 37 | 25 | 4 | 16 | 450592 | 3604732 | 4 | 16 | 0 | 240 | 3681908 | 4 | 16 | 0 | 1 | 248 |
| 165 | A5 | 37 | 25 | 4 | 17 | 450622 | 3604972 | 4 | 17 | 0 | 240 | 3682156 | 4 | 17 | 0 | 1 | 248 |
| 166 | A6 | 37 | 25 | 4 | 18 | 450652 | 3605212 | 4 | 18 | 0 | 240 | 3682404 | 4 | 18 | 0 | 1 | 248 |
| 167 | A7 | 37 | 25 | 4 | 19 | 450682 | 3605452 | 4 | 19 | 0 | 240 | 3682652 | 4 | 19 | 0 | 1 | 248 |
| 168 | A8 | 37 | 25 | 4 | 20 | 450712 | 3605692 | 4 | 20 | 0 | 240 | 3682900 | 4 | 20 | 0 | 1 | 248 |
| 169 | A9 | 37 | 25 | 4 | 21 | 450742 | 3605932 | 4 | 21 | 0 | 240 | 3683148 | 4 | 21 | 0 | 1 | 248 |
| 170 | AA | 37 | 25 | 4 | 22 | 450772 | 3606172 | 4 | 22 | 0 | 240 | 3683396 | 4 | 22 | 0 | 1 | 248 |
| 171 | AB | 37 | 25 | 4 | 23 | 450802 | 3606412 | 4 | 23 | 0 | 240 | 3683644 | 4 | 23 | 0 | 1 | 248 |
| 172 | AC | 37 | 25 | 4 | 24 | 450832 | 3606652 | 4 | 24 | 0 | 240 | 3683892 | 4 | 24 | 0 | 1 | 248 |
| 173 | AD | 37 | 25 | 4 | 25 | 450862 | 3606892 | 4 | 25 | 0 | 240 | 3684140 | 4 | 25 | 0 | 1 | 248 |
| 174 | AE | 37 | 25 | 4 | 26 | 450892 | 3607132 | 4 | 26 | 0 | 240 | 3684388 | 4 | 26 | 0 | 1 | 248 |
| 175 | AF | 37 | 25 | 4 | 27 | 450922 | 3607372 | 4 | 27 | 0 | 240 | 3684636 | 4 | 27 | 0 | 1 | 248 |
| 176 | B0 | 37 | 25 | 4 | 28 | 450952 | 3607612 | 4 | 28 | 0 | 240 | 3684884 | 4 | 28 | 0 | 1 | 248 |
| 177 | B1 | 37 | 25 | 4 | 29 | 450982 | 3607852 | 4 | 29 | 0 | 240 | 3685132 | 4 | 29 | 0 | 1 | 248 |
| 178 | B2 | 37 | 25 | 4 | 30 | 451012 | 3608092 | 4 | 30 | 0 | 240 | 3685380 | 4 | 30 | 0 | 1 | 248 |
| 179 | B3 | 37 | 25 | 4 | 31 | 451042 | 3608332 | 4 | 31 | 0 | 240 | 3685628 | 4 | 31 | 0 | 1 | 248 |
| 180 | B4 | 37 | 25 | 4 | 32 | 451072 | 3608572 | 4 | 32 | 0 | 240 | 3685876 | 4 | 32 | 0 | 1 | 248 |
| 181 | B5 | 37 | 25 | 4 | 33 | 451102 | 3608812 | 4 | 33 | 0 | 240 | 3686124 | 4 | 33 | 0 | 1 | 248 |
| 182 | B6 | 37 | 25 | 4 | 34 | 451132 | 3609052 | 4 | 34 | 0 | 240 | 3686372 | 4 | 34 | 0 | 1 | 248 |
| 183 | B7 | 37 | 25 | 4 | 35 | 451162 | 3609292 | 4 | 35 | 0 | 240 | 3686620 | 4 | 35 | 0 | 1 | 248 |
| 184 | B8 | 37 | 25 | 4 | 36 | 451192 | 3609532 | 4 | 36 | 0 | 240 | 3686868 | 4 | 36 | 0 | 1 | 248 |
| 185 | B9 | 37 | 25 | 5 | 0 | 451224 | 3609788 | 5 | 0 | 0 | 256 | 3687132 | 5 | 0 | 0 | 1 | 264 |
| 186 | BA | 37 | 25 | 5 | 1 | 451256 | 3610044 | 5 | 1 | 0 | 256 | 3687396 | 5 | 1 | 0 | 1 | 264 |
| 187 | BB | 37 | 25 | 5 | 2 | 451288 | 3610300 | 5 | 2 | 0 | 256 | 3687660 | 5 | 2 | 0 | 1 | 264 |
| 188 | BC | 37 | 25 | 5 | 3 | 451320 | 3610556 | 5 | 3 | 0 | 256 | 3687924 | 5 | 3 | 0 | 1 | 264 |
| 189 | BD | 37 | 25 | 5 | 4 | 451352 | 3610812 | 5 | 4 | 0 | 256 | 3688188 | 5 | 4 | 0 | 1 | 264 |
| 190 | BE | 37 | 25 | 5 | 5 | 451384 | 3611068 | 5 | 5 | 0 | 256 | 3688452 | 5 | 5 | 0 | 1 | 264 |
| 191 | BF | 37 | 25 | 5 | 6 | 451416 | 3611324 | 5 | 6 | 0 | 256 | 3688716 | 5 | 6 | 0 | 1 | 264 |
| 192 | C0 | 37 | 25 | 5 | 7 | 451446 | 3611564 | 5 | 7 | 0 | 240 | 3688964 | 5 | 7 | 0 | 1 | 248 |
| 193 | C1 | 37 | 25 | 5 | 8 | 451476 | 3611804 | 5 | 8 | 0 | 240 | 3689212 | 5 | 8 | 0 | 1 | 248 |
| 194 | C2 | 37 | 25 | 5 | 9 | 451506 | 3612044 | 5 | 9 | 0 | 240 | 3689460 | 5 | 9 | 0 | 1 | 248 |
| 195 | C3 | 37 | 25 | 5 | 10 | 451536 | 3612284 | 5 | 10 | 0 | 240 | 3689708 | 5 | 10 | 0 | 1 | 248 |
| 196 | C4 | 37 | 25 | 5 | 11 | 451566 | 3612524 | 5 | 11 | 0 | 240 | 3689956 | 5 | 11 | 0 | 1 | 248 |
| 197 | C5 | 37 | 25 | 5 | 12 | 451596 | 3612764 | 5 | 12 | 0 | 240 | 3690204 | 5 | 12 | 0 | 1 | 248 |
| 198 | C6 | 37 | 25 | 5 | 13 | 451626 | 3613004 | 5 | 13 | 0 | 240 | 3690452 | 5 | 13 | 0 | 1 | 248 |
| 199 | C7 | 37 | 25 | 5 | 14 | 451656 | 3613244 | 5 | 14 | 0 | 240 | 3690700 | 5 | 14 | 0 | 1 | 248 |
| 200 | C8 | 37 | 25 | 5 | 15 | 451686 | 3613484 | 5 | 15 | 0 | 240 | 3690948 | 5 | 15 | 0 | 1 | 248 |
| 201 | C9 | 37 | 25 | 5 | 16 | 451716 | 3613724 | 5 | 16 | 0 | 240 | 3691196 | 5 | 16 | 0 | 1 | 248 |
| 202 | CA | 37 | 25 | 5 | 17 | 451746 | 3613964 | 5 | 17 | 0 | 240 | 3691444 | 5 | 17 | 0 | 1 | 248 |
| 203 | CB | 37 | 25 | 5 | 18 | 451776 | 3614204 | 5 | 18 | 0 | 240 | 3691692 | 5 | 18 | 0 | 1 | 248 |
| 204 | CC | 37 | 25 | 5 | 19 | 451806 | 3614444 | 5 | 19 | 0 | 240 | 3691940 | 5 | 19 | 0 | 1 | 248 |
| 205 | CD | 37 | 25 | 5 | 20 | 451836 | 3614684 | 5 | 20 | 0 | 240 | 3692188 | 5 | 20 | 0 | 1 | 248 |
| 206 | CE | 37 | 25 | 5 | 21 | 451866 | 3614924 | 5 | 21 | 0 | 240 | 3692436 | 5 | 21 | 0 | 1 | 248 |

| | | | | | | | | | | | | | | | | | |
|---|---|---|---|---|---|---|---|---|---|---|---|---|---|---|---|---|---|
| 207 | CF | 37 | 25 | 5 | 22 | 451896 | 3615164 | 5 | 22 | 0 | 240 | 3692684 | 5 | 22 | 0 | 1 | 248 |
| 208 | D0 | 37 | 25 | 5 | 23 | 451928 | 3615420 | 5 | 23 | 0 | 256 | 3692948 | 5 | 23 | 0 | 1 | 264 |
| 209 | D1 | 37 | 25 | 5 | 24 | 451960 | 3615676 | 5 | 24 | 0 | 256 | 3693212 | 5 | 24 | 0 | 1 | 264 |
| 210 | D2 | 37 | 25 | 5 | 25 | 451992 | 3615932 | 5 | 25 | 0 | 256 | 3693476 | 5 | 25 | 0 | 1 | 264 |
| 211 | D3 | 37 | 25 | 5 | 26 | 452024 | 3616188 | 5 | 26 | 0 | 256 | 3693740 | 5 | 26 | 0 | 1 | 264 |
| 212 | D4 | 37 | 25 | 5 | 27 | 452056 | 3616444 | 5 | 27 | 0 | 256 | 3694004 | 5 | 27 | 0 | 1 | 264 |
| 213 | D5 | 37 | 25 | 5 | 28 | 452088 | 3616700 | 5 | 28 | 0 | 256 | 3694268 | 5 | 28 | 0 | 1 | 264 |
| 214 | D6 | 37 | 25 | 5 | 29 | 452120 | 3616956 | 5 | 29 | 0 | 256 | 3694532 | 5 | 29 | 0 | 1 | 264 |
| 215 | D7 | 37 | 25 | 5 | 30 | 452152 | 3617212 | 5 | 30 | 0 | 256 | 3694796 | 5 | 30 | 0 | 1 | 264 |
| 216 | D8 | 37 | 25 | 5 | 31 | 452184 | 3617468 | 5 | 31 | 0 | 256 | 3695060 | 5 | 31 | 0 | 1 | 264 |
| 217 | D9 | 37 | 25 | 5 | 32 | 452216 | 3617724 | 5 | 32 | 0 | 256 | 3695324 | 5 | 32 | 0 | 1 | 264 |
| 218 | DA | 37 | 25 | 5 | 33 | 452248 | 3617980 | 5 | 33 | 0 | 256 | 3695588 | 5 | 33 | 0 | 1 | 264 |
| 219 | DB | 37 | 25 | 5 | 34 | 452280 | 3618236 | 5 | 34 | 0 | 256 | 3695852 | 5 | 34 | 0 | 1 | 264 |
| 220 | DC | 37 | 25 | 5 | 35 | 452312 | 3618492 | 5 | 35 | 0 | 256 | 3696116 | 5 | 35 | 0 | 1 | 264 |
| 221 | DD | 37 | 25 | 5 | 36 | 452344 | 3618748 | 5 | 36 | 0 | 256 | 3696380 | 5 | 36 | 0 | 1 | 264 |
| 222 | DE | 37 | 25 | 6 | 0 | 452378 | 3619020 | 6 | 0 | 0 | 272 | 3696660 | 6 | 0 | 0 | 1 | 280 |
| 223 | DF | 37 | 25 | 6 | 1 | 452412 | 3619292 | 6 | 1 | 0 | 272 | 3696940 | 6 | 1 | 0 | 1 | 280 |
| 224 | E0 | 37 | 25 | 6 | 2 | 452446 | 3619564 | 6 | 2 | 0 | 272 | 3697220 | 6 | 2 | 0 | 1 | 280 |
| 225 | E1 | 37 | 25 | 6 | 3 | 452480 | 3619836 | 6 | 3 | 0 | 272 | 3697500 | 6 | 3 | 0 | 1 | 280 |
| 226 | E2 | 37 | 25 | 6 | 4 | 452514 | 3620108 | 6 | 4 | 0 | 272 | 3697780 | 6 | 4 | 0 | 1 | 280 |
| 227 | E3 | 37 | 25 | 6 | 5 | 452548 | 3620380 | 6 | 5 | 0 | 272 | 3698060 | 6 | 5 | 0 | 1 | 280 |
| 228 | E4 | 37 | 25 | 6 | 6 | 452582 | 3620652 | 6 | 6 | 0 | 272 | 3698340 | 6 | 6 | 0 | 1 | 280 |
| 229 | E5 | 37 | 25 | 6 | 7 | 452616 | 3620924 | 6 | 7 | 0 | 272 | 3698620 | 6 | 7 | 0 | 1 | 280 |
| 230 | E6 | 37 | 25 | 6 | 8 | 452650 | 3621196 | 6 | 8 | 0 | 272 | 3698900 | 6 | 8 | 0 | 1 | 280 |
| 231 | E7 | 37 | 25 | 6 | 9 | 452684 | 3621468 | 6 | 9 | 0 | 272 | 3699180 | 6 | 9 | 0 | 1 | 280 |
| 232 | E8 | 37 | 25 | 6 | 10 | 452718 | 3621740 | 6 | 10 | 0 | 272 | 3699460 | 6 | 10 | 0 | 1 | 280 |
| 233 | E9 | 37 | 25 | 6 | 11 | 452752 | 3622012 | 6 | 11 | 0 | 272 | 3699740 | 6 | 11 | 0 | 1 | 280 |
| 234 | EA | 37 | 25 | 6 | 12 | 452786 | 3622284 | 6 | 12 | 0 | 272 | 3700020 | 6 | 12 | 0 | 1 | 280 |
| 235 | EB | 37 | 25 | 6 | 13 | 452820 | 3622556 | 6 | 13 | 0 | 272 | 3700300 | 6 | 13 | 0 | 1 | 280 |
| 236 | EC | 37 | 25 | 6 | 14 | 452854 | 3622828 | 6 | 14 | 0 | 272 | 3700580 | 6 | 14 | 0 | 1 | 280 |
| 237 | ED | 37 | 25 | 6 | 15 | 452888 | 3623100 | 6 | 15 | 0 | 272 | 3700860 | 6 | 15 | 0 | 1 | 280 |
| 238 | EE | 37 | 25 | 6 | 16 | 452922 | 3623372 | 6 | 16 | 0 | 272 | 3701140 | 6 | 16 | 0 | 1 | 280 |
| 239 | EF | 37 | 25 | 6 | 17 | 452956 | 3623644 | 6 | 17 | 0 | 272 | 3701420 | 6 | 17 | 0 | 1 | 280 |
| 240 | F0 | 37 | 25 | 6 | 18 | 452988 | 3623900 | 6 | 18 | 0 | 256 | 3701684 | 6 | 18 | 0 | 1 | 264 |
| 241 | F1 | 37 | 25 | 6 | 19 | 453020 | 3624156 | 6 | 19 | 0 | 256 | 3701948 | 6 | 19 | 0 | 1 | 264 |
| 242 | F2 | 37 | 25 | 6 | 20 | 453052 | 3624412 | 6 | 20 | 0 | 256 | 3702212 | 6 | 20 | 0 | 1 | 264 |
| 243 | F3 | 37 | 25 | 6 | 21 | 453084 | 3624668 | 6 | 21 | 0 | 256 | 3702476 | 6 | 21 | 0 | 1 | 264 |
| 244 | F4 | 37 | 25 | 6 | 22 | 453116 | 3624924 | 6 | 22 | 0 | 256 | 3702740 | 6 | 22 | 0 | 1 | 264 |
| 245 | F5 | 37 | 25 | 6 | 23 | 453148 | 3625180 | 6 | 23 | 0 | 256 | 3703004 | 6 | 23 | 0 | 1 | 264 |
| 246 | F6 | 37 | 25 | 6 | 24 | 453180 | 3625436 | 6 | 24 | 0 | 256 | 3703268 | 6 | 24 | 0 | 1 | 264 |
| 247 | F7 | 37 | 25 | 6 | 25 | 453212 | 3625692 | 6 | 25 | 0 | 256 | 3703532 | 6 | 25 | 0 | 1 | 264 |
| 248 | F8 | 37 | 25 | 6 | 26 | 453244 | 3625948 | 6 | 26 | 0 | 256 | 3703796 | 6 | 26 | 0 | 1 | 264 |
| 249 | F9 | 37 | 25 | 6 | 27 | 453276 | 3626204 | 6 | 27 | 0 | 256 | 3704060 | 6 | 27 | 0 | 1 | 264 |
| 250 | FA | 37 | 25 | 6 | 28 | 453308 | 3626460 | 6 | 28 | 0 | 256 | 3704324 | 6 | 28 | 0 | 1 | 264 |
| 251 | FB | 37 | 25 | 6 | 29 | 453340 | 3626716 | 6 | 29 | 0 | 256 | 3704588 | 6 | 29 | 0 | 1 | 264 |
| 252 | FC | 37 | 25 | 6 | 30 | 453372 | 3626972 | 6 | 30 | 0 | 256 | 3704852 | 6 | 30 | 0 | 1 | 264 |
| 253 | FD | 37 | 25 | 6 | 31 | 453404 | 3627228 | 6 | 31 | 0 | 256 | 3705116 | 6 | 31 | 0 | 1 | 264 |
| 254 | FE | 37 | 25 | 6 | 32 | 453436 | 3627484 | 6 | 32 | 0 | 256 | 3705380 | 6 | 32 | 0 | 1 | 264 |
| 255 | FF | 37 | 25 | 6 | 33 | 453468 | 3627740 | 6 | 33 | 0 | 256 | 3705644 | 6 | 33 | 0 | 1 | 264 |
| 0 | 00 | 38 | 26 | 0 | 0 | 453480 | 3627836 | 0 | 0 | 0 | 96 | 3705748 | 0 | 0 | 0 | 1 | 104 |
| 1 | 01 | 38 | 26 | 0 | 1 | 453494 | 3627948 | 0 | 1 | 0 | 112 | 3705868 | 0 | 1 | 0 | 1 | 120 |
| 2 | 02 | 38 | 26 | 0 | 2 | 453508 | 3628060 | 0 | 2 | 0 | 112 | 3705988 | 0 | 2 | 0 | 1 | 120 |
| 3 | 03 | 38 | 26 | 0 | 3 | 453522 | 3628172 | 0 | 3 | 0 | 112 | 3706108 | 0 | 3 | 0 | 1 | 120 |
| 4 | 04 | 38 | 26 | 0 | 4 | 453536 | 3628284 | 0 | 4 | 0 | 112 | 3706228 | 0 | 4 | 0 | 1 | 120 |
| 5 | 05 | 38 | 26 | 0 | 5 | 453550 | 3628396 | 0 | 5 | 0 | 112 | 3706348 | 0 | 5 | 0 | 1 | 120 |
| 6 | 06 | 38 | 26 | 0 | 6 | 453564 | 3628508 | 0 | 6 | 0 | 112 | 3706468 | 0 | 6 | 0 | 1 | 120 |
| 7 | 07 | 38 | 26 | 0 | 7 | 453578 | 3628620 | 0 | 7 | 0 | 112 | 3706588 | 0 | 7 | 0 | 1 | 120 |
| 8 | 08 | 38 | 26 | 0 | 8 | 453592 | 3628732 | 0 | 8 | 0 | 112 | 3706708 | 0 | 8 | 0 | 1 | 120 |
| 9 | 09 | 38 | 26 | 0 | 9 | 453606 | 3628844 | 0 | 9 | 0 | 112 | 3706828 | 0 | 9 | 0 | 1 | 120 |
| 10 | 0A | 38 | 26 | 0 | 10 | 453620 | 3628956 | 0 | 10 | 0 | 112 | 3706948 | 0 | 10 | 0 | 1 | 120 |
| 11 | 0B | 38 | 26 | 0 | 11 | 453634 | 3629068 | 0 | 11 | 0 | 112 | 3707068 | 0 | 11 | 0 | 1 | 120 |
| 12 | 0C | 38 | 26 | 0 | 12 | 453648 | 3629180 | 0 | 12 | 0 | 112 | 3707188 | 0 | 12 | 0 | 1 | 120 |
| 13 | 0D | 38 | 26 | 0 | 13 | 453662 | 3629292 | 0 | 13 | 0 | 112 | 3707308 | 0 | 13 | 0 | 1 | 120 |
| 14 | 0E | 38 | 26 | 0 | 14 | 453676 | 3629404 | 0 | 14 | 0 | 112 | 3707428 | 0 | 14 | 0 | 1 | 120 |
| 15 | 0F | 38 | 26 | 0 | 15 | 453690 | 3629516 | 0 | 15 | 0 | 112 | 3707548 | 0 | 15 | 0 | 1 | 120 |
| 16 | 10 | 38 | 26 | 0 | 16 | 453704 | 3629628 | 0 | 16 | 0 | 112 | 3707668 | 0 | 16 | 0 | 1 | 120 |
| 17 | 11 | 38 | 26 | 0 | 17 | 453718 | 3629740 | 0 | 17 | 0 | 112 | 3707788 | 0 | 17 | 0 | 1 | 120 |
| 18 | 12 | 38 | 26 | 0 | 18 | 453732 | 3629852 | 0 | 18 | 0 | 112 | 3707908 | 0 | 18 | 0 | 1 | 120 |
| 19 | 13 | 38 | 26 | 0 | 19 | 453746 | 3629964 | 0 | 19 | 0 | 112 | 3708028 | 0 | 19 | 0 | 1 | 120 |
| 20 | 14 | 38 | 26 | 0 | 20 | 453760 | 3630076 | 0 | 20 | 0 | 112 | 3708148 | 0 | 20 | 0 | 1 | 120 |
| 21 | 15 | 38 | 26 | 0 | 21 | 453774 | 3630188 | 0 | 21 | 0 | 112 | 3708268 | 0 | 21 | 0 | 1 | 120 |
| 22 | 16 | 38 | 26 | 0 | 22 | 453788 | 3630300 | 0 | 22 | 0 | 112 | 3708388 | 0 | 22 | 0 | 1 | 120 |
| 23 | 17 | 38 | 26 | 0 | 23 | 453802 | 3630412 | 0 | 23 | 0 | 112 | 3708508 | 0 | 23 | 0 | 1 | 120 |
| 24 | 18 | 38 | 26 | 0 | 24 | 453816 | 3630524 | 0 | 24 | 0 | 112 | 3708628 | 0 | 24 | 0 | 1 | 120 |
| 25 | 19 | 38 | 26 | 0 | 25 | 453830 | 3630636 | 0 | 25 | 0 | 112 | 3708748 | 0 | 25 | 0 | 1 | 120 |
| 26 | 1A | 38 | 26 | 0 | 26 | 453844 | 3630748 | 0 | 26 | 0 | 112 | 3708868 | 0 | 26 | 0 | 1 | 120 |
| 27 | 1B | 38 | 26 | 0 | 27 | 453858 | 3630860 | 0 | 27 | 0 | 112 | 3708988 | 0 | 27 | 0 | 1 | 120 |
| 28 | 1C | 38 | 26 | 0 | 28 | 453872 | 3630972 | 0 | 28 | 0 | 112 | 3709108 | 0 | 28 | 0 | 1 | 120 |
| 29 | 1D | 38 | 26 | 0 | 29 | 453886 | 3631084 | 0 | 29 | 0 | 112 | 3709228 | 0 | 29 | 0 | 1 | 120 |
| 30 | 1E | 38 | 26 | 0 | 30 | 453900 | 3631196 | 0 | 30 | 0 | 112 | 3709348 | 0 | 30 | 0 | 1 | 120 |
| 31 | 1F | 38 | 26 | 0 | 31 | 453914 | 3631308 | 0 | 31 | 0 | 112 | 3709468 | 0 | 31 | 0 | 1 | 120 |
| 32 | 20 | 38 | 26 | 0 | 32 | 453928 | 3631420 | 0 | 32 | 0 | 112 | 3709588 | 0 | 32 | 0 | 1 | 120 |
| 33 | 21 | 38 | 26 | 0 | 33 | 453942 | 3631532 | 0 | 33 | 0 | 112 | 3709708 | 0 | 33 | 0 | 1 | 120 |
| 34 | 22 | 38 | 26 | 0 | 34 | 453956 | 3631644 | 0 | 34 | 0 | 112 | 3709828 | 0 | 34 | 0 | 1 | 120 |
| 35 | 23 | 38 | 26 | 0 | 35 | 453970 | 3631756 | 0 | 35 | 0 | 112 | 3709948 | 0 | 35 | 0 | 1 | 120 |
| 36 | 24 | 38 | 26 | 0 | 36 | 453984 | 3631868 | 0 | 36 | 0 | 112 | 3710068 | 0 | 36 | 0 | 1 | 120 |
| 37 | 25 | 38 | 26 | 0 | 37 | 453998 | 3631980 | 0 | 37 | 0 | 112 | 3710188 | 0 | 37 | 0 | 1 | 120 |
| 38 | 26 | 38 | 26 | 1 | 0 | 454010 | 3632076 | 1 | 0 | 0 | 96 | 3710292 | 1 | 0 | 0 | 1 | 104 |
| 39 | 27 | 38 | 26 | 1 | 1 | 454027 | 3632212 | 1 | 1 | 0 | 136 | 3710436 | 1 | 1 | 0 | 1 | 144 |
| 40 | 28 | 38 | 26 | 1 | 2 | 454044 | 3632348 | 1 | 2 | 0 | 136 | 3710580 | 1 | 2 | 0 | 1 | 144 |
| 41 | 29 | 38 | 26 | 1 | 3 | 454061 | 3632484 | 1 | 3 | 0 | 136 | 3710724 | 1 | 3 | 0 | 1 | 144 |
| 42 | 2A | 38 | 26 | 1 | 4 | 454078 | 3632620 | 1 | 4 | 0 | 136 | 3710868 | 1 | 4 | 0 | 1 | 144 |
| 43 | 2B | 38 | 26 | 1 | 5 | 454095 | 3632756 | 1 | 5 | 0 | 136 | 3711012 | 1 | 5 | 0 | 1 | 144 |
| 44 | 2C | 38 | 26 | 1 | 6 | 454112 | 3632892 | 1 | 6 | 0 | 136 | 3711156 | 1 | 6 | 0 | 1 | 144 |
| 45 | 2D | 38 | 26 | 1 | 7 | 454129 | 3633028 | 1 | 7 | 0 | 136 | 3711300 | 1 | 7 | 0 | 1 | 144 |
| 46 | 2E | 38 | 26 | 1 | 8 | 454146 | 3633164 | 1 | 8 | 0 | 136 | 3711444 | 1 | 8 | 0 | 1 | 144 |
| 47 | 2F | 38 | 26 | 1 | 9 | 454163 | 3633300 | 1 | 9 | 0 | 136 | 3711588 | 1 | 9 | 0 | 1 | 144 |
| 48 | 30 | 38 | 26 | 1 | 10 | 454184 | 3633468 | 1 | 10 | 0 | 168 | 3711764 | 1 | 10 | 0 | 1 | 176 |
| 49 | 31 | 38 | 26 | 1 | 11 | 454205 | 3633636 | 1 | 11 | 0 | 168 | 3711940 | 1 | 11 | 0 | 1 | 176 |
| 50 | 32 | 38 | 26 | 1 | 12 | 454226 | 3633804 | 1 | 12 | 0 | 168 | 3712116 | 1 | 12 | 0 | 1 | 176 |
| 51 | 33 | 38 | 26 | 1 | 13 | 454247 | 3633972 | 1 | 13 | 0 | 168 | 3712292 | 1 | 13 | 0 | 1 | 176 |
| 52 | 34 | 38 | 26 | 1 | 14 | 454268 | 3634140 | 1 | 14 | 0 | 168 | 3712468 | 1 | 14 | 0 | 1 | 176 |
| 53 | 35 | 38 | 26 | 1 | 15 | 454289 | 3634308 | 1 | 15 | 0 | 168 | 3712644 | 1 | 15 | 0 | 1 | 176 |

| | | | | | | | | | | | | | | | | | |
|---|---|---|---|---|---|---|---|---|---|---|---|---|---|---|---|---|---|
| 54 | 36 | 38 | 26 | 1 | 16 | 454310 | 3634476 | 1 | 16 | 0 | 168 | 3712820 | 1 | 16 | 0 | 1 | 176 |
| 55 | 37 | 38 | 26 | 1 | 17 | 454331 | 3634644 | 1 | 17 | 0 | 168 | 3712996 | 1 | 17 | 0 | 1 | 176 |
| 56 | 38 | 38 | 26 | 1 | 18 | 454352 | 3634812 | 1 | 18 | 0 | 168 | 3713172 | 1 | 18 | 0 | 1 | 176 |
| 57 | 39 | 38 | 26 | 1 | 19 | 454373 | 3634980 | 1 | 19 | 0 | 168 | 3713348 | 1 | 19 | 0 | 1 | 176 |
| 58 | 3A | 38 | 26 | 1 | 20 | 454394 | 3635148 | 1 | 20 | 0 | 168 | 3713524 | 1 | 20 | 0 | 1 | 176 |
| 59 | 3B | 38 | 26 | 1 | 21 | 454415 | 3635316 | 1 | 21 | 0 | 168 | 3713700 | 1 | 21 | 0 | 1 | 176 |
| 60 | 3C | 38 | 26 | 1 | 22 | 454436 | 3635484 | 1 | 22 | 0 | 168 | 3713876 | 1 | 22 | 0 | 1 | 176 |
| 61 | 3D | 38 | 26 | 1 | 23 | 454457 | 3635652 | 1 | 23 | 0 | 168 | 3714052 | 1 | 23 | 0 | 1 | 176 |
| 62 | 3E | 38 | 26 | 1 | 24 | 454478 | 3635820 | 1 | 24 | 0 | 168 | 3714228 | 1 | 24 | 0 | 1 | 176 |
| 63 | 3F | 38 | 26 | 1 | 25 | 454499 | 3635988 | 1 | 25 | 0 | 168 | 3714404 | 1 | 25 | 0 | 1 | 176 |
| 64 | 40 | 38 | 26 | 1 | 26 | 454522 | 3636172 | 1 | 26 | 0 | 184 | 3714596 | 1 | 26 | 0 | 1 | 192 |
| 65 | 41 | 38 | 26 | 1 | 27 | 454545 | 3636356 | 1 | 27 | 0 | 184 | 3714788 | 1 | 27 | 0 | 1 | 192 |
| 66 | 42 | 38 | 26 | 1 | 28 | 454568 | 3636540 | 1 | 28 | 0 | 184 | 3714980 | 1 | 28 | 0 | 1 | 192 |
| 67 | 43 | 38 | 26 | 1 | 29 | 454591 | 3636724 | 1 | 29 | 0 | 184 | 3715172 | 1 | 29 | 0 | 1 | 192 |
| 68 | 44 | 38 | 26 | 1 | 30 | 454614 | 3636908 | 1 | 30 | 0 | 184 | 3715364 | 1 | 30 | 0 | 1 | 192 |
| 69 | 45 | 38 | 26 | 1 | 31 | 454637 | 3637092 | 1 | 31 | 0 | 184 | 3715556 | 1 | 31 | 0 | 1 | 192 |
| 70 | 46 | 38 | 26 | 1 | 32 | 454660 | 3637276 | 1 | 32 | 0 | 184 | 3715748 | 1 | 32 | 0 | 1 | 192 |
| 71 | 47 | 38 | 26 | 1 | 33 | 454683 | 3637460 | 1 | 33 | 0 | 184 | 3715940 | 1 | 33 | 0 | 1 | 192 |
| 72 | 48 | 38 | 26 | 1 | 34 | 454706 | 3637644 | 1 | 34 | 0 | 184 | 3716132 | 1 | 34 | 0 | 1 | 192 |
| 73 | 49 | 38 | 26 | 1 | 35 | 454729 | 3637828 | 1 | 35 | 0 | 184 | 3716324 | 1 | 35 | 0 | 1 | 192 |
| 74 | 4A | 38 | 26 | 1 | 36 | 454752 | 3638012 | 1 | 36 | 0 | 184 | 3716516 | 1 | 36 | 0 | 1 | 192 |
| 75 | 4B | 38 | 26 | 1 | 37 | 454775 | 3638196 | 1 | 37 | 0 | 184 | 3716708 | 1 | 37 | 0 | 1 | 192 |
| 76 | 4C | 38 | 26 | 2 | 0 | 454796 | 3638364 | 2 | 0 | 0 | 168 | 3716884 | 2 | 0 | 0 | 1 | 176 |
| 77 | 4D | 38 | 26 | 2 | 1 | 454822 | 3638572 | 2 | 1 | 0 | 208 | 3717100 | 2 | 1 | 0 | 1 | 216 |
| 78 | 4E | 38 | 26 | 2 | 2 | 454848 | 3638780 | 2 | 2 | 0 | 208 | 3717316 | 2 | 2 | 0 | 1 | 216 |
| 79 | 4F | 38 | 26 | 2 | 3 | 454874 | 3638988 | 2 | 3 | 0 | 208 | 3717532 | 2 | 3 | 0 | 1 | 216 |
| 80 | 50 | 38 | 26 | 2 | 4 | 454900 | 3639196 | 2 | 4 | 0 | 208 | 3717748 | 2 | 4 | 0 | 1 | 216 |
| 81 | 51 | 38 | 26 | 2 | 5 | 454926 | 3639404 | 2 | 5 | 0 | 208 | 3717964 | 2 | 5 | 0 | 1 | 216 |
| 82 | 52 | 38 | 26 | 2 | 6 | 454952 | 3639612 | 2 | 6 | 0 | 208 | 3718180 | 2 | 6 | 0 | 1 | 216 |
| 83 | 53 | 38 | 26 | 2 | 7 | 454978 | 3639820 | 2 | 7 | 0 | 208 | 3718396 | 2 | 7 | 0 | 1 | 216 |
| 84 | 54 | 38 | 26 | 2 | 8 | 455004 | 3640028 | 2 | 8 | 0 | 208 | 3718612 | 2 | 8 | 0 | 1 | 216 |
| 85 | 55 | 38 | 26 | 2 | 9 | 455030 | 3640236 | 2 | 9 | 0 | 208 | 3718828 | 2 | 9 | 0 | 1 | 216 |
| 86 | 56 | 38 | 26 | 2 | 10 | 455056 | 3640444 | 2 | 10 | 0 | 208 | 3719044 | 2 | 10 | 0 | 1 | 216 |
| 87 | 57 | 38 | 26 | 2 | 11 | 455082 | 3640652 | 2 | 11 | 0 | 208 | 3719260 | 2 | 11 | 0 | 1 | 216 |
| 88 | 58 | 38 | 26 | 2 | 12 | 455108 | 3640860 | 2 | 12 | 0 | 208 | 3719476 | 2 | 12 | 0 | 1 | 216 |
| 89 | 59 | 38 | 26 | 2 | 13 | 455134 | 3641068 | 2 | 13 | 0 | 208 | 3719692 | 2 | 13 | 0 | 1 | 216 |
| 90 | 5A | 38 | 26 | 2 | 14 | 455160 | 3641276 | 2 | 14 | 0 | 208 | 3719908 | 2 | 14 | 0 | 1 | 216 |
| 91 | 5B | 38 | 26 | 2 | 15 | 455186 | 3641484 | 2 | 15 | 0 | 208 | 3720124 | 2 | 15 | 0 | 1 | 216 |
| 92 | 5C | 38 | 26 | 2 | 16 | 455212 | 3641692 | 2 | 16 | 0 | 208 | 3720340 | 2 | 16 | 0 | 1 | 216 |
| 93 | 5D | 38 | 26 | 2 | 17 | 455238 | 3641900 | 2 | 17 | 0 | 208 | 3720556 | 2 | 17 | 0 | 1 | 216 |
| 94 | 5E | 38 | 26 | 2 | 18 | 455264 | 3642108 | 2 | 18 | 0 | 208 | 3720772 | 2 | 18 | 0 | 1 | 216 |
| 95 | 5F | 38 | 26 | 2 | 19 | 455290 | 3642316 | 2 | 19 | 0 | 208 | 3720988 | 2 | 19 | 0 | 1 | 216 |
| 96 | 60 | 38 | 26 | 2 | 20 | 455313 | 3642500 | 2 | 20 | 0 | 184 | 3721180 | 2 | 20 | 0 | 1 | 192 |
| 97 | 61 | 38 | 26 | 2 | 21 | 455336 | 3642684 | 2 | 21 | 0 | 184 | 3721372 | 2 | 21 | 0 | 1 | 192 |
| 98 | 62 | 38 | 26 | 2 | 22 | 455359 | 3642868 | 2 | 22 | 0 | 184 | 3721564 | 2 | 22 | 0 | 1 | 192 |
| 99 | 63 | 38 | 26 | 2 | 23 | 455382 | 3643052 | 2 | 23 | 0 | 184 | 3721756 | 2 | 23 | 0 | 1 | 192 |
| 100 | 64 | 38 | 26 | 2 | 24 | 455405 | 3643236 | 2 | 24 | 0 | 184 | 3721948 | 2 | 24 | 0 | 1 | 192 |
| 101 | 65 | 38 | 26 | 2 | 25 | 455428 | 3643420 | 2 | 25 | 0 | 184 | 3722140 | 2 | 25 | 0 | 1 | 192 |
| 102 | 66 | 38 | 26 | 2 | 26 | 455451 | 3643604 | 2 | 26 | 0 | 184 | 3722332 | 2 | 26 | 0 | 1 | 192 |
| 103 | 67 | 38 | 26 | 2 | 27 | 455474 | 3643788 | 2 | 27 | 0 | 184 | 3722524 | 2 | 27 | 0 | 1 | 192 |
| 104 | 68 | 38 | 26 | 2 | 28 | 455497 | 3643972 | 2 | 28 | 0 | 184 | 3722716 | 2 | 28 | 0 | 1 | 192 |
| 105 | 69 | 38 | 26 | 2 | 29 | 455520 | 3644156 | 2 | 29 | 0 | 184 | 3722908 | 2 | 29 | 0 | 1 | 192 |
| 106 | 6A | 38 | 26 | 2 | 30 | 455543 | 3644340 | 2 | 30 | 0 | 184 | 3723100 | 2 | 30 | 0 | 1 | 192 |
| 107 | 6B | 38 | 26 | 2 | 31 | 455566 | 3644524 | 2 | 31 | 0 | 184 | 3723292 | 2 | 31 | 0 | 1 | 192 |
| 108 | 6C | 38 | 26 | 2 | 32 | 455589 | 3644708 | 2 | 32 | 0 | 184 | 3723484 | 2 | 32 | 0 | 1 | 192 |
| 109 | 6D | 38 | 26 | 2 | 33 | 455612 | 3644892 | 2 | 33 | 0 | 184 | 3723676 | 2 | 33 | 0 | 1 | 192 |
| 110 | 6E | 38 | 26 | 2 | 34 | 455635 | 3645076 | 2 | 34 | 0 | 184 | 3723868 | 2 | 34 | 0 | 1 | 192 |
| 111 | 6F | 38 | 26 | 2 | 35 | 455658 | 3645260 | 2 | 35 | 0 | 184 | 3724060 | 2 | 35 | 0 | 1 | 192 |
| 112 | 70 | 38 | 26 | 2 | 36 | 455683 | 3645460 | 2 | 36 | 0 | 200 | 3724268 | 2 | 36 | 0 | 1 | 208 |
| 113 | 71 | 38 | 26 | 2 | 37 | 455708 | 3645660 | 2 | 37 | 0 | 200 | 3724476 | 2 | 37 | 0 | 1 | 208 |
| 114 | 72 | 38 | 26 | 3 | 0 | 455731 | 3645844 | 3 | 0 | 0 | 184 | 3724668 | 3 | 0 | 0 | 1 | 192 |
| 115 | 73 | 38 | 26 | 3 | 1 | 455759 | 3646068 | 3 | 1 | 0 | 224 | 3724900 | 3 | 1 | 0 | 1 | 232 |
| 116 | 74 | 38 | 26 | 3 | 2 | 455787 | 3646292 | 3 | 2 | 0 | 224 | 3725132 | 3 | 2 | 0 | 1 | 232 |
| 117 | 75 | 38 | 26 | 3 | 3 | 455815 | 3646516 | 3 | 3 | 0 | 224 | 3725364 | 3 | 3 | 0 | 1 | 232 |
| 118 | 76 | 38 | 26 | 3 | 4 | 455843 | 3646740 | 3 | 4 | 0 | 224 | 3725596 | 3 | 4 | 0 | 1 | 232 |
| 119 | 77 | 38 | 26 | 3 | 5 | 455871 | 3646964 | 3 | 5 | 0 | 224 | 3725828 | 3 | 5 | 0 | 1 | 232 |
| 120 | 78 | 38 | 26 | 3 | 6 | 455899 | 3647188 | 3 | 6 | 0 | 224 | 3726060 | 3 | 6 | 0 | 1 | 232 |
| 121 | 79 | 38 | 26 | 3 | 7 | 455927 | 3647412 | 3 | 7 | 0 | 224 | 3726292 | 3 | 7 | 0 | 1 | 232 |
| 122 | 7A | 38 | 26 | 3 | 8 | 455955 | 3647636 | 3 | 8 | 0 | 224 | 3726524 | 3 | 8 | 0 | 1 | 232 |
| 123 | 7B | 38 | 26 | 3 | 9 | 455983 | 3647860 | 3 | 9 | 0 | 224 | 3726756 | 3 | 9 | 0 | 1 | 232 |
| 124 | 7C | 38 | 26 | 3 | 10 | 456011 | 3648084 | 3 | 10 | 0 | 224 | 3726988 | 3 | 10 | 0 | 1 | 232 |
| 125 | 7D | 38 | 26 | 3 | 11 | 456039 | 3648308 | 3 | 11 | 0 | 224 | 3727220 | 3 | 11 | 0 | 1 | 232 |
| 126 | 7E | 38 | 26 | 3 | 12 | 456067 | 3648532 | 3 | 12 | 0 | 224 | 3727452 | 3 | 12 | 0 | 1 | 232 |
| 127 | 7F | 38 | 26 | 3 | 13 | 456095 | 3648756 | 3 | 13 | 0 | 224 | 3727684 | 3 | 13 | 0 | 1 | 232 |
| 128 | 80 | 38 | 26 | 3 | 14 | 456123 | 3648980 | 3 | 14 | 0 | 224 | 3727916 | 3 | 14 | 0 | 1 | 232 |
| 129 | 81 | 38 | 26 | 3 | 15 | 456151 | 3649204 | 3 | 15 | 0 | 224 | 3728148 | 3 | 15 | 0 | 1 | 232 |
| 130 | 82 | 38 | 26 | 3 | 16 | 456179 | 3649428 | 3 | 16 | 0 | 224 | 3728380 | 3 | 16 | 0 | 1 | 232 |
| 131 | 83 | 38 | 26 | 3 | 17 | 456207 | 3649652 | 3 | 17 | 0 | 224 | 3728612 | 3 | 17 | 0 | 1 | 232 |
| 132 | 84 | 38 | 26 | 3 | 18 | 456235 | 3649876 | 3 | 18 | 0 | 224 | 3728844 | 3 | 18 | 0 | 1 | 232 |
| 133 | 85 | 38 | 26 | 3 | 19 | 456263 | 3650100 | 3 | 19 | 0 | 224 | 3729076 | 3 | 19 | 0 | 1 | 232 |
| 134 | 86 | 38 | 26 | 3 | 20 | 456291 | 3650324 | 3 | 20 | 0 | 224 | 3729308 | 3 | 20 | 0 | 1 | 232 |
| 135 | 87 | 38 | 26 | 3 | 21 | 456319 | 3650548 | 3 | 21 | 0 | 224 | 3729540 | 3 | 21 | 0 | 1 | 232 |
| 136 | 88 | 38 | 26 | 3 | 22 | 456347 | 3650772 | 3 | 22 | 0 | 224 | 3729772 | 3 | 22 | 0 | 1 | 232 |
| 137 | 89 | 38 | 26 | 3 | 23 | 456375 | 3650996 | 3 | 23 | 0 | 224 | 3730004 | 3 | 23 | 0 | 1 | 232 |
| 138 | 8A | 38 | 26 | 3 | 24 | 456403 | 3651220 | 3 | 24 | 0 | 224 | 3730236 | 3 | 24 | 0 | 1 | 232 |
| 139 | 8B | 38 | 26 | 3 | 25 | 456431 | 3651444 | 3 | 25 | 0 | 224 | 3730468 | 3 | 25 | 0 | 1 | 232 |
| 140 | 8C | 38 | 26 | 3 | 26 | 456459 | 3651668 | 3 | 26 | 0 | 224 | 3730700 | 3 | 26 | 0 | 1 | 232 |
| 141 | 8D | 38 | 26 | 3 | 27 | 456487 | 3651892 | 3 | 27 | 0 | 224 | 3730932 | 3 | 27 | 0 | 1 | 232 |
| 142 | 8E | 38 | 26 | 3 | 28 | 456515 | 3652116 | 3 | 28 | 0 | 224 | 3731164 | 3 | 28 | 0 | 1 | 232 |
| 143 | 8F | 38 | 26 | 3 | 29 | 456543 | 3652340 | 3 | 29 | 0 | 224 | 3731396 | 3 | 29 | 0 | 1 | 232 |
| 144 | 90 | 38 | 26 | 3 | 30 | 456568 | 3652540 | 3 | 30 | 0 | 200 | 3731604 | 3 | 30 | 0 | 1 | 208 |
| 145 | 91 | 38 | 26 | 3 | 31 | 456593 | 3652740 | 3 | 31 | 0 | 200 | 3731812 | 3 | 31 | 0 | 1 | 208 |
| 146 | 92 | 38 | 26 | 3 | 32 | 456618 | 3652940 | 3 | 32 | 0 | 200 | 3732020 | 3 | 32 | 0 | 1 | 208 |
| 147 | 93 | 38 | 26 | 3 | 33 | 456643 | 3653140 | 3 | 33 | 0 | 200 | 3732228 | 3 | 33 | 0 | 1 | 208 |
| 148 | 94 | 38 | 26 | 3 | 34 | 456668 | 3653340 | 3 | 34 | 0 | 200 | 3732436 | 3 | 34 | 0 | 1 | 208 |
| 149 | 95 | 38 | 26 | 3 | 35 | 456693 | 3653540 | 3 | 35 | 0 | 200 | 3732644 | 3 | 35 | 0 | 1 | 208 |
| 150 | 96 | 38 | 26 | 3 | 36 | 456718 | 3653740 | 3 | 36 | 0 | 200 | 3732852 | 3 | 36 | 0 | 1 | 208 |
| 151 | 97 | 38 | 26 | 3 | 37 | 456743 | 3653940 | 3 | 37 | 0 | 200 | 3733060 | 3 | 37 | 0 | 1 | 208 |
| 152 | 98 | 38 | 26 | 4 | 0 | 456766 | 3654124 | 4 | 0 | 0 | 184 | 3733252 | 4 | 0 | 0 | 1 | 192 |
| 153 | 99 | 38 | 26 | 4 | 1 | 456794 | 3654348 | 4 | 1 | 0 | 224 | 3733484 | 4 | 1 | 0 | 1 | 232 |
| 154 | 9A | 38 | 26 | 4 | 2 | 456822 | 3654572 | 4 | 2 | 0 | 224 | 3733716 | 4 | 2 | 0 | 1 | 232 |
| 155 | 9B | 38 | 26 | 4 | 3 | 456850 | 3654796 | 4 | 3 | 0 | 224 | 3733948 | 4 | 3 | 0 | 1 | 232 |
| 156 | 9C | 38 | 26 | 4 | 4 | 456878 | 3655020 | 4 | 4 | 0 | 224 | 3734180 | 4 | 4 | 0 | 1 | 232 |

| | | | | | | | | | | | | | | | | | |
|---|---|---|---|---|---|---|---|---|---|---|---|---|---|---|---|---|---|
| 157 | 9D | 38 | 26 | 4 | 5 | 456906 | 3655244 | 4 | 5 | 0 | 224 | 3734412 | 4 | 5 | 0 | 1 | 232 |
| 158 | 9E | 38 | 26 | 4 | 6 | 456934 | 3655468 | 4 | 6 | 0 | 224 | 3734644 | 4 | 6 | 0 | 1 | 232 |
| 159 | 9F | 38 | 26 | 4 | 7 | 456962 | 3655692 | 4 | 7 | 0 | 224 | 3734876 | 4 | 7 | 0 | 1 | 232 |
| 160 | A0 | 38 | 26 | 4 | 8 | 456992 | 3655932 | 4 | 8 | 0 | 240 | 3735124 | 4 | 8 | 0 | 1 | 248 |
| 161 | A1 | 38 | 26 | 4 | 9 | 457022 | 3656172 | 4 | 9 | 0 | 240 | 3735372 | 4 | 9 | 0 | 1 | 248 |
| 162 | A2 | 38 | 26 | 4 | 10 | 457052 | 3656412 | 4 | 10 | 0 | 240 | 3735620 | 4 | 10 | 0 | 1 | 248 |
| 163 | A3 | 38 | 26 | 4 | 11 | 457082 | 3656652 | 4 | 11 | 0 | 240 | 3735868 | 4 | 11 | 0 | 1 | 248 |
| 164 | A4 | 38 | 26 | 4 | 12 | 457112 | 3656892 | 4 | 12 | 0 | 240 | 3736116 | 4 | 12 | 0 | 1 | 248 |
| 165 | A5 | 38 | 26 | 4 | 13 | 457142 | 3657132 | 4 | 13 | 0 | 240 | 3736364 | 4 | 13 | 0 | 1 | 248 |
| 166 | A6 | 38 | 26 | 4 | 14 | 457172 | 3657372 | 4 | 14 | 0 | 240 | 3736612 | 4 | 14 | 0 | 1 | 248 |
| 167 | A7 | 38 | 26 | 4 | 15 | 457202 | 3657612 | 4 | 15 | 0 | 240 | 3736860 | 4 | 15 | 0 | 1 | 248 |
| 168 | A8 | 38 | 26 | 4 | 16 | 457232 | 3657852 | 4 | 16 | 0 | 240 | 3737108 | 4 | 16 | 0 | 1 | 248 |
| 169 | A9 | 38 | 26 | 4 | 17 | 457262 | 3658092 | 4 | 17 | 0 | 240 | 3737356 | 4 | 17 | 0 | 1 | 248 |
| 170 | AA | 38 | 26 | 4 | 18 | 457292 | 3658332 | 4 | 18 | 0 | 240 | 3737604 | 4 | 18 | 0 | 1 | 248 |
| 171 | AB | 38 | 26 | 4 | 19 | 457322 | 3658572 | 4 | 19 | 0 | 240 | 3737852 | 4 | 19 | 0 | 1 | 248 |
| 172 | AC | 38 | 26 | 4 | 20 | 457352 | 3658812 | 4 | 20 | 0 | 240 | 3738100 | 4 | 20 | 0 | 1 | 248 |
| 173 | AD | 38 | 26 | 4 | 21 | 457382 | 3659052 | 4 | 21 | 0 | 240 | 3738348 | 4 | 21 | 0 | 1 | 248 |
| 174 | AE | 38 | 26 | 4 | 22 | 457412 | 3659292 | 4 | 22 | 0 | 240 | 3738596 | 4 | 22 | 0 | 1 | 248 |
| 175 | AF | 38 | 26 | 4 | 23 | 457442 | 3659532 | 4 | 23 | 0 | 240 | 3738844 | 4 | 23 | 0 | 1 | 248 |
| 176 | B0 | 38 | 26 | 4 | 24 | 457472 | 3659772 | 4 | 24 | 0 | 240 | 3739092 | 4 | 24 | 0 | 1 | 248 |
| 177 | B1 | 38 | 26 | 4 | 25 | 457502 | 3660012 | 4 | 25 | 0 | 240 | 3739340 | 4 | 25 | 0 | 1 | 248 |
| 178 | B2 | 38 | 26 | 4 | 26 | 457532 | 3660252 | 4 | 26 | 0 | 240 | 3739588 | 4 | 26 | 0 | 1 | 248 |
| 179 | B3 | 38 | 26 | 4 | 27 | 457562 | 3660492 | 4 | 27 | 0 | 240 | 3739836 | 4 | 27 | 0 | 1 | 248 |
| 180 | B4 | 38 | 26 | 4 | 28 | 457592 | 3660732 | 4 | 28 | 0 | 240 | 3740084 | 4 | 28 | 0 | 1 | 248 |
| 181 | B5 | 38 | 26 | 4 | 29 | 457622 | 3660972 | 4 | 29 | 0 | 240 | 3740332 | 4 | 29 | 0 | 1 | 248 |
| 182 | B6 | 38 | 26 | 4 | 30 | 457652 | 3661212 | 4 | 30 | 0 | 240 | 3740580 | 4 | 30 | 0 | 1 | 248 |
| 183 | B7 | 38 | 26 | 4 | 31 | 457682 | 3661452 | 4 | 31 | 0 | 240 | 3740828 | 4 | 31 | 0 | 1 | 248 |
| 184 | B8 | 38 | 26 | 4 | 32 | 457712 | 3661692 | 4 | 32 | 0 | 240 | 3741076 | 4 | 32 | 0 | 1 | 248 |
| 185 | B9 | 38 | 26 | 4 | 33 | 457742 | 3661932 | 4 | 33 | 0 | 240 | 3741324 | 4 | 33 | 0 | 1 | 248 |
| 186 | BA | 38 | 26 | 4 | 34 | 457772 | 3662172 | 4 | 34 | 0 | 240 | 3741572 | 4 | 34 | 0 | 1 | 248 |
| 187 | BB | 38 | 26 | 4 | 35 | 457802 | 3662412 | 4 | 35 | 0 | 240 | 3741820 | 4 | 35 | 0 | 1 | 248 |
| 188 | BC | 38 | 26 | 4 | 36 | 457832 | 3662652 | 4 | 36 | 0 | 240 | 3742068 | 4 | 36 | 0 | 1 | 248 |
| 189 | BD | 38 | 26 | 4 | 37 | 457862 | 3662892 | 4 | 37 | 0 | 240 | 3742316 | 4 | 37 | 0 | 1 | 248 |
| 190 | BE | 38 | 26 | 5 | 0 | 457894 | 3663148 | 5 | 0 | 0 | 256 | 3742580 | 5 | 0 | 0 | 1 | 264 |
| 191 | BF | 38 | 26 | 5 | 1 | 457926 | 3663404 | 5 | 1 | 0 | 256 | 3742844 | 5 | 1 | 0 | 1 | 264 |
| 192 | C0 | 38 | 26 | 5 | 2 | 457956 | 3663644 | 5 | 2 | 0 | 240 | 3743092 | 5 | 2 | 0 | 1 | 248 |
| 193 | C1 | 38 | 26 | 5 | 3 | 457986 | 3663884 | 5 | 3 | 0 | 240 | 3743340 | 5 | 3 | 0 | 1 | 248 |
| 194 | C2 | 38 | 26 | 5 | 4 | 458016 | 3664124 | 5 | 4 | 0 | 240 | 3743588 | 5 | 4 | 0 | 1 | 248 |
| 195 | C3 | 38 | 26 | 5 | 5 | 458046 | 3664364 | 5 | 5 | 0 | 240 | 3743836 | 5 | 5 | 0 | 1 | 248 |
| 196 | C4 | 38 | 26 | 5 | 6 | 458076 | 3664604 | 5 | 6 | 0 | 240 | 3744084 | 5 | 6 | 0 | 1 | 248 |
| 197 | C5 | 38 | 26 | 5 | 7 | 458106 | 3664844 | 5 | 7 | 0 | 240 | 3744332 | 5 | 7 | 0 | 1 | 248 |
| 198 | C6 | 38 | 26 | 5 | 8 | 458136 | 3665084 | 5 | 8 | 0 | 240 | 3744580 | 5 | 8 | 0 | 1 | 248 |
| 199 | C7 | 38 | 26 | 5 | 9 | 458166 | 3665324 | 5 | 9 | 0 | 240 | 3744828 | 5 | 9 | 0 | 1 | 248 |
| 200 | C8 | 38 | 26 | 5 | 10 | 458196 | 3665564 | 5 | 10 | 0 | 240 | 3745076 | 5 | 10 | 0 | 1 | 248 |
| 201 | C9 | 38 | 26 | 5 | 11 | 458226 | 3665804 | 5 | 11 | 0 | 240 | 3745324 | 5 | 11 | 0 | 1 | 248 |
| 202 | CA | 38 | 26 | 5 | 12 | 458256 | 3666044 | 5 | 12 | 0 | 240 | 3745572 | 5 | 12 | 0 | 1 | 248 |
| 203 | CB | 38 | 26 | 5 | 13 | 458286 | 3666284 | 5 | 13 | 0 | 240 | 3745820 | 5 | 13 | 0 | 1 | 248 |
| 204 | CC | 38 | 26 | 5 | 14 | 458316 | 3666524 | 5 | 14 | 0 | 240 | 3746068 | 5 | 14 | 0 | 1 | 248 |
| 205 | CD | 38 | 26 | 5 | 15 | 458346 | 3666764 | 5 | 15 | 0 | 240 | 3746316 | 5 | 15 | 0 | 1 | 248 |
| 206 | CE | 38 | 26 | 5 | 16 | 458376 | 3667004 | 5 | 16 | 0 | 240 | 3746564 | 5 | 16 | 0 | 1 | 248 |
| 207 | CF | 38 | 26 | 5 | 17 | 458406 | 3667244 | 5 | 17 | 0 | 240 | 3746812 | 5 | 17 | 0 | 1 | 248 |
| 208 | D0 | 38 | 26 | 5 | 18 | 458438 | 3667500 | 5 | 18 | 0 | 256 | 3747076 | 5 | 18 | 0 | 1 | 264 |
| 209 | D1 | 38 | 26 | 5 | 19 | 458470 | 3667756 | 5 | 19 | 0 | 256 | 3747340 | 5 | 19 | 0 | 1 | 264 |
| 210 | D2 | 38 | 26 | 5 | 20 | 458502 | 3668012 | 5 | 20 | 0 | 256 | 3747604 | 5 | 20 | 0 | 1 | 264 |
| 211 | D3 | 38 | 26 | 5 | 21 | 458534 | 3668268 | 5 | 21 | 0 | 256 | 3747868 | 5 | 21 | 0 | 1 | 264 |
| 212 | D4 | 38 | 26 | 5 | 22 | 458566 | 3668524 | 5 | 22 | 0 | 256 | 3748132 | 5 | 22 | 0 | 1 | 264 |
| 213 | D5 | 38 | 26 | 5 | 23 | 458598 | 3668780 | 5 | 23 | 0 | 256 | 3748396 | 5 | 23 | 0 | 1 | 264 |
| 214 | D6 | 38 | 26 | 5 | 24 | 458630 | 3669036 | 5 | 24 | 0 | 256 | 3748660 | 5 | 24 | 0 | 1 | 264 |
| 215 | D7 | 38 | 26 | 5 | 25 | 458662 | 3669292 | 5 | 25 | 0 | 256 | 3748924 | 5 | 25 | 0 | 1 | 264 |
| 216 | D8 | 38 | 26 | 5 | 26 | 458694 | 3669548 | 5 | 26 | 0 | 256 | 3749188 | 5 | 26 | 0 | 1 | 264 |
| 217 | D9 | 38 | 26 | 5 | 27 | 458726 | 3669804 | 5 | 27 | 0 | 256 | 3749452 | 5 | 27 | 0 | 1 | 264 |
| 218 | DA | 38 | 26 | 5 | 28 | 458758 | 3670060 | 5 | 28 | 0 | 256 | 3749716 | 5 | 28 | 0 | 1 | 264 |
| 219 | DB | 38 | 26 | 5 | 29 | 458790 | 3670316 | 5 | 29 | 0 | 256 | 3749980 | 5 | 29 | 0 | 1 | 264 |
| 220 | DC | 38 | 26 | 5 | 30 | 458822 | 3670572 | 5 | 30 | 0 | 256 | 3750244 | 5 | 30 | 0 | 1 | 264 |
| 221 | DD | 38 | 26 | 5 | 31 | 458854 | 3670828 | 5 | 31 | 0 | 256 | 3750508 | 5 | 31 | 0 | 1 | 264 |
| 222 | DE | 38 | 26 | 5 | 32 | 458886 | 3671084 | 5 | 32 | 0 | 256 | 3750772 | 5 | 32 | 0 | 1 | 264 |
| 223 | DF | 38 | 26 | 5 | 33 | 458918 | 3671340 | 5 | 33 | 0 | 256 | 3751036 | 5 | 33 | 0 | 1 | 264 |
| 224 | E0 | 38 | 26 | 5 | 34 | 458950 | 3671596 | 5 | 34 | 0 | 256 | 3751300 | 5 | 34 | 0 | 1 | 264 |
| 225 | E1 | 38 | 26 | 5 | 35 | 458982 | 3671852 | 5 | 35 | 0 | 256 | 3751564 | 5 | 35 | 0 | 1 | 264 |
| 226 | E2 | 38 | 26 | 5 | 36 | 459014 | 3672108 | 5 | 36 | 0 | 256 | 3751828 | 5 | 36 | 0 | 1 | 264 |
| 227 | E3 | 38 | 26 | 5 | 37 | 459046 | 3672364 | 5 | 37 | 0 | 256 | 3752092 | 5 | 37 | 0 | 1 | 264 |
| 228 | E4 | 38 | 26 | 6 | 0 | 459080 | 3672636 | 6 | 0 | 0 | 272 | 3752372 | 6 | 0 | 0 | 1 | 280 |
| 229 | E5 | 38 | 26 | 6 | 1 | 459114 | 3672908 | 6 | 1 | 0 | 272 | 3752652 | 6 | 1 | 0 | 1 | 280 |
| 230 | E6 | 38 | 26 | 6 | 2 | 459148 | 3673180 | 6 | 2 | 0 | 272 | 3752932 | 6 | 2 | 0 | 1 | 280 |
| 231 | E7 | 38 | 26 | 6 | 3 | 459182 | 3673452 | 6 | 3 | 0 | 272 | 3753212 | 6 | 3 | 0 | 1 | 280 |
| 232 | E8 | 38 | 26 | 6 | 4 | 459216 | 3673724 | 6 | 4 | 0 | 272 | 3753492 | 6 | 4 | 0 | 1 | 280 |
| 233 | E9 | 38 | 26 | 6 | 5 | 459250 | 3673996 | 6 | 5 | 0 | 272 | 3753772 | 6 | 5 | 0 | 1 | 280 |
| 234 | EA | 38 | 26 | 6 | 6 | 459284 | 3674268 | 6 | 6 | 0 | 272 | 3754052 | 6 | 6 | 0 | 1 | 280 |
| 235 | EB | 38 | 26 | 6 | 7 | 459318 | 3674540 | 6 | 7 | 0 | 272 | 3754332 | 6 | 7 | 0 | 1 | 280 |
| 236 | EC | 38 | 26 | 6 | 8 | 459352 | 3674812 | 6 | 8 | 0 | 272 | 3754612 | 6 | 8 | 0 | 1 | 280 |
| 237 | ED | 38 | 26 | 6 | 9 | 459386 | 3675084 | 6 | 9 | 0 | 272 | 3754892 | 6 | 9 | 0 | 1 | 280 |
| 238 | EE | 38 | 26 | 6 | 10 | 459420 | 3675356 | 6 | 10 | 0 | 272 | 3755172 | 6 | 10 | 0 | 1 | 280 |
| 239 | EF | 38 | 26 | 6 | 11 | 459454 | 3675628 | 6 | 11 | 0 | 272 | 3755452 | 6 | 11 | 0 | 1 | 280 |
| 240 | F0 | 38 | 26 | 6 | 12 | 459486 | 3675884 | 6 | 12 | 0 | 256 | 3755716 | 6 | 12 | 0 | 1 | 264 |
| 241 | F1 | 38 | 26 | 6 | 13 | 459518 | 3676140 | 6 | 13 | 0 | 256 | 3755980 | 6 | 13 | 0 | 1 | 264 |
| 242 | F2 | 38 | 26 | 6 | 14 | 459550 | 3676396 | 6 | 14 | 0 | 256 | 3756244 | 6 | 14 | 0 | 1 | 264 |
| 243 | F3 | 38 | 26 | 6 | 15 | 459582 | 3676652 | 6 | 15 | 0 | 256 | 3756508 | 6 | 15 | 0 | 1 | 264 |
| 244 | F4 | 38 | 26 | 6 | 16 | 459614 | 3676908 | 6 | 16 | 0 | 256 | 3756772 | 6 | 16 | 0 | 1 | 264 |
| 245 | F5 | 38 | 26 | 6 | 17 | 459646 | 3677164 | 6 | 17 | 0 | 256 | 3757036 | 6 | 17 | 0 | 1 | 264 |
| 246 | F6 | 38 | 26 | 6 | 18 | 459678 | 3677420 | 6 | 18 | 0 | 256 | 3757300 | 6 | 18 | 0 | 1 | 264 |
| 247 | F7 | 38 | 26 | 6 | 19 | 459710 | 3677676 | 6 | 19 | 0 | 256 | 3757564 | 6 | 19 | 0 | 1 | 264 |
| 248 | F8 | 38 | 26 | 6 | 20 | 459742 | 3677932 | 6 | 20 | 0 | 256 | 3757828 | 6 | 20 | 0 | 1 | 264 |
| 249 | F9 | 38 | 26 | 6 | 21 | 459774 | 3678188 | 6 | 21 | 0 | 256 | 3758092 | 6 | 21 | 0 | 1 | 264 |
| 250 | FA | 38 | 26 | 6 | 22 | 459806 | 3678444 | 6 | 22 | 0 | 256 | 3758356 | 6 | 22 | 0 | 1 | 264 |
| 251 | FB | 38 | 26 | 6 | 23 | 459838 | 3678700 | 6 | 23 | 0 | 256 | 3758620 | 6 | 23 | 0 | 1 | 264 |
| 252 | FC | 38 | 26 | 6 | 24 | 459870 | 3678956 | 6 | 24 | 0 | 256 | 3758884 | 6 | 24 | 0 | 1 | 264 |
| 253 | FD | 38 | 26 | 6 | 25 | 459902 | 3679212 | 6 | 25 | 0 | 256 | 3759148 | 6 | 25 | 0 | 1 | 264 |
| 254 | FE | 38 | 26 | 6 | 26 | 459934 | 3679468 | 6 | 26 | 0 | 256 | 3759412 | 6 | 26 | 0 | 1 | 264 |
| 255 | FF | 38 | 26 | 6 | 27 | 459966 | 3679724 | 6 | 27 | 0 | 256 | 3759676 | 6 | 27 | 0 | 1 | 264 |
| 0 | 00 | 39 | 27 | 0 | 0 | 459978 | 3679820 | 0 | 0 | 0 | 96 | 3759780 | 0 | 0 | 0 | 1 | 104 |
| 1 | 01 | 39 | 27 | 0 | 1 | 459992 | 3679932 | 0 | 1 | 0 | 112 | 3759900 | 0 | 1 | 0 | 1 | 120 |
| 2 | 02 | 39 | 27 | 0 | 2 | 460006 | 3680044 | 0 | 2 | 0 | 112 | 3760020 | 0 | 2 | 0 | 1 | 120 |
| 3 | 03 | 39 | 27 | 0 | 3 | 460020 | 3680156 | 0 | 3 | 0 | 112 | 3760140 | 0 | 3 | 0 | 1 | 120 |

| | | | | | | | | | | | | | | | | | |
|---|---|---|---|---|---|---|---|---|---|---|---|---|---|---|---|---|---|
| 4 | 04 | 39 | 27 | 0 | 4 | 460034 | 3680268 | 0 | 4 | 0 | 112 | 3760260 | 0 | 4 | 0 | 1 | 120 |
| 5 | 05 | 39 | 27 | 0 | 5 | 460048 | 3680380 | 0 | 5 | 0 | 112 | 3760380 | 0 | 5 | 0 | 1 | 120 |
| 6 | 06 | 39 | 27 | 0 | 6 | 460062 | 3680492 | 0 | 6 | 0 | 112 | 3760500 | 0 | 6 | 0 | 1 | 120 |
| 7 | 07 | 39 | 27 | 0 | 7 | 460076 | 3680604 | 0 | 7 | 0 | 112 | 3760620 | 0 | 7 | 0 | 1 | 120 |
| 8 | 08 | 39 | 27 | 0 | 8 | 460090 | 3680716 | 0 | 8 | 0 | 112 | 3760740 | 0 | 8 | 0 | 1 | 120 |
| 9 | 09 | 39 | 27 | 0 | 9 | 460104 | 3680828 | 0 | 9 | 0 | 112 | 3760860 | 0 | 9 | 0 | 1 | 120 |
| 10 | 0A | 39 | 27 | 0 | 10 | 460118 | 3680940 | 0 | 10 | 0 | 112 | 3760980 | 0 | 10 | 0 | 1 | 120 |
| 11 | 0B | 39 | 27 | 0 | 11 | 460132 | 3681052 | 0 | 11 | 0 | 112 | 3761100 | 0 | 11 | 0 | 1 | 120 |
| 12 | 0C | 39 | 27 | 0 | 12 | 460146 | 3681164 | 0 | 12 | 0 | 112 | 3761220 | 0 | 12 | 0 | 1 | 120 |
| 13 | 0D | 39 | 27 | 0 | 13 | 460160 | 3681276 | 0 | 13 | 0 | 112 | 3761340 | 0 | 13 | 0 | 1 | 120 |
| 14 | 0E | 39 | 27 | 0 | 14 | 460174 | 3681388 | 0 | 14 | 0 | 112 | 3761460 | 0 | 14 | 0 | 1 | 120 |
| 15 | 0F | 39 | 27 | 0 | 15 | 460188 | 3681500 | 0 | 15 | 0 | 112 | 3761580 | 0 | 15 | 0 | 1 | 120 |
| 16 | 10 | 39 | 27 | 0 | 16 | 460202 | 3681612 | 0 | 16 | 0 | 112 | 3761700 | 0 | 16 | 0 | 1 | 120 |
| 17 | 11 | 39 | 27 | 0 | 17 | 460216 | 3681724 | 0 | 17 | 0 | 112 | 3761820 | 0 | 17 | 0 | 1 | 120 |
| 18 | 12 | 39 | 27 | 0 | 18 | 460230 | 3681836 | 0 | 18 | 0 | 112 | 3761940 | 0 | 18 | 0 | 1 | 120 |
| 19 | 13 | 39 | 27 | 0 | 19 | 460244 | 3681948 | 0 | 19 | 0 | 112 | 3762060 | 0 | 19 | 0 | 1 | 120 |
| 20 | 14 | 39 | 27 | 0 | 20 | 460258 | 3682060 | 0 | 20 | 0 | 112 | 3762180 | 0 | 20 | 0 | 1 | 120 |
| 21 | 15 | 39 | 27 | 0 | 21 | 460272 | 3682172 | 0 | 21 | 0 | 112 | 3762300 | 0 | 21 | 0 | 1 | 120 |
| 22 | 16 | 39 | 27 | 0 | 22 | 460286 | 3682284 | 0 | 22 | 0 | 112 | 3762420 | 0 | 22 | 0 | 1 | 120 |
| 23 | 17 | 39 | 27 | 0 | 23 | 460300 | 3682396 | 0 | 23 | 0 | 112 | 3762540 | 0 | 23 | 0 | 1 | 120 |
| 24 | 18 | 39 | 27 | 0 | 24 | 460314 | 3682508 | 0 | 24 | 0 | 112 | 3762660 | 0 | 24 | 0 | 1 | 120 |
| 25 | 19 | 39 | 27 | 0 | 25 | 460328 | 3682620 | 0 | 25 | 0 | 112 | 3762780 | 0 | 25 | 0 | 1 | 120 |
| 26 | 1A | 39 | 27 | 0 | 26 | 460342 | 3682732 | 0 | 26 | 0 | 112 | 3762900 | 0 | 26 | 0 | 1 | 120 |
| 27 | 1B | 39 | 27 | 0 | 27 | 460356 | 3682844 | 0 | 27 | 0 | 112 | 3763020 | 0 | 27 | 0 | 1 | 120 |
| 28 | 1C | 39 | 27 | 0 | 28 | 460370 | 3682956 | 0 | 28 | 0 | 112 | 3763140 | 0 | 28 | 0 | 1 | 120 |
| 29 | 1D | 39 | 27 | 0 | 29 | 460384 | 3683068 | 0 | 29 | 0 | 112 | 3763260 | 0 | 29 | 0 | 1 | 120 |
| 30 | 1E | 39 | 27 | 0 | 30 | 460398 | 3683180 | 0 | 30 | 0 | 112 | 3763380 | 0 | 30 | 0 | 1 | 120 |
| 31 | 1F | 39 | 27 | 0 | 31 | 460412 | 3683292 | 0 | 31 | 0 | 112 | 3763500 | 0 | 31 | 0 | 1 | 120 |
| 32 | 20 | 39 | 27 | 0 | 32 | 460426 | 3683404 | 0 | 32 | 0 | 112 | 3763620 | 0 | 32 | 0 | 1 | 120 |
| 33 | 21 | 39 | 27 | 0 | 33 | 460440 | 3683516 | 0 | 33 | 0 | 112 | 3763740 | 0 | 33 | 0 | 1 | 120 |
| 34 | 22 | 39 | 27 | 0 | 34 | 460454 | 3683628 | 0 | 34 | 0 | 112 | 3763860 | 0 | 34 | 0 | 1 | 120 |
| 35 | 23 | 39 | 27 | 0 | 35 | 460468 | 3683740 | 0 | 35 | 0 | 112 | 3763980 | 0 | 35 | 0 | 1 | 120 |
| 36 | 24 | 39 | 27 | 0 | 36 | 460482 | 3683852 | 0 | 36 | 0 | 112 | 3764100 | 0 | 36 | 0 | 1 | 120 |
| 37 | 25 | 39 | 27 | 0 | 37 | 460496 | 3683964 | 0 | 37 | 0 | 112 | 3764220 | 0 | 37 | 0 | 1 | 120 |
| 38 | 26 | 39 | 27 | 0 | 38 | 460510 | 3684076 | 0 | 38 | 0 | 112 | 3764340 | 0 | 38 | 0 | 1 | 120 |
| 39 | 27 | 39 | 27 | 1 | 0 | 460522 | 3684172 | 1 | 0 | 0 | 96 | 3764444 | 1 | 0 | 0 | 1 | 104 |
| 40 | 28 | 39 | 27 | 1 | 1 | 460539 | 3684308 | 1 | 1 | 0 | 136 | 3764588 | 1 | 1 | 0 | 1 | 144 |
| 41 | 29 | 39 | 27 | 1 | 2 | 460556 | 3684444 | 1 | 2 | 0 | 136 | 3764732 | 1 | 2 | 0 | 1 | 144 |
| 42 | 2A | 39 | 27 | 1 | 3 | 460573 | 3684580 | 1 | 3 | 0 | 136 | 3764876 | 1 | 3 | 0 | 1 | 144 |
| 43 | 2B | 39 | 27 | 1 | 4 | 460590 | 3684716 | 1 | 4 | 0 | 136 | 3765020 | 1 | 4 | 0 | 1 | 144 |
| 44 | 2C | 39 | 27 | 1 | 5 | 460607 | 3684852 | 1 | 5 | 0 | 136 | 3765164 | 1 | 5 | 0 | 1 | 144 |
| 45 | 2D | 39 | 27 | 1 | 6 | 460624 | 3684988 | 1 | 6 | 0 | 136 | 3765308 | 1 | 6 | 0 | 1 | 144 |
| 46 | 2E | 39 | 27 | 1 | 7 | 460641 | 3685124 | 1 | 7 | 0 | 136 | 3765452 | 1 | 7 | 0 | 1 | 144 |
| 47 | 2F | 39 | 27 | 1 | 8 | 460658 | 3685260 | 1 | 8 | 0 | 136 | 3765596 | 1 | 8 | 0 | 1 | 144 |
| 48 | 30 | 39 | 27 | 1 | 9 | 460679 | 3685428 | 1 | 9 | 0 | 168 | 3765772 | 1 | 9 | 0 | 1 | 176 |
| 49 | 31 | 39 | 27 | 1 | 10 | 460700 | 3685596 | 1 | 10 | 0 | 168 | 3765948 | 1 | 10 | 0 | 1 | 176 |
| 50 | 32 | 39 | 27 | 1 | 11 | 460721 | 3685764 | 1 | 11 | 0 | 168 | 3766124 | 1 | 11 | 0 | 1 | 176 |
| 51 | 33 | 39 | 27 | 1 | 12 | 460742 | 3685932 | 1 | 12 | 0 | 168 | 3766300 | 1 | 12 | 0 | 1 | 176 |
| 52 | 34 | 39 | 27 | 1 | 13 | 460763 | 3686100 | 1 | 13 | 0 | 168 | 3766476 | 1 | 13 | 0 | 1 | 176 |
| 53 | 35 | 39 | 27 | 1 | 14 | 460784 | 3686268 | 1 | 14 | 0 | 168 | 3766652 | 1 | 14 | 0 | 1 | 176 |
| 54 | 36 | 39 | 27 | 1 | 15 | 460805 | 3686436 | 1 | 15 | 0 | 168 | 3766828 | 1 | 15 | 0 | 1 | 176 |
| 55 | 37 | 39 | 27 | 1 | 16 | 460826 | 3686604 | 1 | 16 | 0 | 168 | 3767004 | 1 | 16 | 0 | 1 | 176 |
| 56 | 38 | 39 | 27 | 1 | 17 | 460847 | 3686772 | 1 | 17 | 0 | 168 | 3767180 | 1 | 17 | 0 | 1 | 176 |
| 57 | 39 | 39 | 27 | 1 | 18 | 460868 | 3686940 | 1 | 18 | 0 | 168 | 3767356 | 1 | 18 | 0 | 1 | 176 |
| 58 | 3A | 39 | 27 | 1 | 19 | 460889 | 3687108 | 1 | 19 | 0 | 168 | 3767532 | 1 | 19 | 0 | 1 | 176 |
| 59 | 3B | 39 | 27 | 1 | 20 | 460910 | 3687276 | 1 | 20 | 0 | 168 | 3767708 | 1 | 20 | 0 | 1 | 176 |
| 60 | 3C | 39 | 27 | 1 | 21 | 460931 | 3687444 | 1 | 21 | 0 | 168 | 3767884 | 1 | 21 | 0 | 1 | 176 |
| 61 | 3D | 39 | 27 | 1 | 22 | 460952 | 3687612 | 1 | 22 | 0 | 168 | 3768060 | 1 | 22 | 0 | 1 | 176 |
| 62 | 3E | 39 | 27 | 1 | 23 | 460973 | 3687780 | 1 | 23 | 0 | 168 | 3768236 | 1 | 23 | 0 | 1 | 176 |
| 63 | 3F | 39 | 27 | 1 | 24 | 460994 | 3687948 | 1 | 24 | 0 | 168 | 3768412 | 1 | 24 | 0 | 1 | 176 |
| 64 | 40 | 39 | 27 | 1 | 25 | 461017 | 3688132 | 1 | 25 | 0 | 184 | 3768604 | 1 | 25 | 0 | 1 | 192 |
| 65 | 41 | 39 | 27 | 1 | 26 | 461040 | 3688316 | 1 | 26 | 0 | 184 | 3768796 | 1 | 26 | 0 | 1 | 192 |
| 66 | 42 | 39 | 27 | 1 | 27 | 461063 | 3688500 | 1 | 27 | 0 | 184 | 3768988 | 1 | 27 | 0 | 1 | 192 |
| 67 | 43 | 39 | 27 | 1 | 28 | 461086 | 3688684 | 1 | 28 | 0 | 184 | 3769180 | 1 | 28 | 0 | 1 | 192 |
| 68 | 44 | 39 | 27 | 1 | 29 | 461109 | 3688868 | 1 | 29 | 0 | 184 | 3769372 | 1 | 29 | 0 | 1 | 192 |
| 69 | 45 | 39 | 27 | 1 | 30 | 461132 | 3689052 | 1 | 30 | 0 | 184 | 3769564 | 1 | 30 | 0 | 1 | 192 |
| 70 | 46 | 39 | 27 | 1 | 31 | 461155 | 3689236 | 1 | 31 | 0 | 184 | 3769756 | 1 | 31 | 0 | 1 | 192 |
| 71 | 47 | 39 | 27 | 1 | 32 | 461178 | 3689420 | 1 | 32 | 0 | 184 | 3769948 | 1 | 32 | 0 | 1 | 192 |
| 72 | 48 | 39 | 27 | 1 | 33 | 461201 | 3689604 | 1 | 33 | 0 | 184 | 3770140 | 1 | 33 | 0 | 1 | 192 |
| 73 | 49 | 39 | 27 | 1 | 34 | 461224 | 3689788 | 1 | 34 | 0 | 184 | 3770332 | 1 | 34 | 0 | 1 | 192 |
| 74 | 4A | 39 | 27 | 1 | 35 | 461247 | 3689972 | 1 | 35 | 0 | 184 | 3770524 | 1 | 35 | 0 | 1 | 192 |
| 75 | 4B | 39 | 27 | 1 | 36 | 461270 | 3690156 | 1 | 36 | 0 | 184 | 3770716 | 1 | 36 | 0 | 1 | 192 |
| 76 | 4C | 39 | 27 | 1 | 37 | 461293 | 3690340 | 1 | 37 | 0 | 184 | 3770908 | 1 | 37 | 0 | 1 | 192 |
| 77 | 4D | 39 | 27 | 1 | 38 | 461316 | 3690524 | 1 | 38 | 0 | 184 | 3771100 | 1 | 38 | 0 | 1 | 192 |
| 78 | 4E | 39 | 27 | 2 | 0 | 461337 | 3690692 | 2 | 0 | 0 | 168 | 3771276 | 2 | 0 | 0 | 1 | 176 |
| 79 | 4F | 39 | 27 | 2 | 1 | 461363 | 3690900 | 2 | 1 | 0 | 208 | 3771492 | 2 | 1 | 0 | 1 | 216 |
| 80 | 50 | 39 | 27 | 2 | 2 | 461389 | 3691108 | 2 | 2 | 0 | 208 | 3771708 | 2 | 2 | 0 | 1 | 216 |
| 81 | 51 | 39 | 27 | 2 | 3 | 461415 | 3691316 | 2 | 3 | 0 | 208 | 3771924 | 2 | 3 | 0 | 1 | 216 |
| 82 | 52 | 39 | 27 | 2 | 4 | 461441 | 3691524 | 2 | 4 | 0 | 208 | 3772140 | 2 | 4 | 0 | 1 | 216 |
| 83 | 53 | 39 | 27 | 2 | 5 | 461467 | 3691732 | 2 | 5 | 0 | 208 | 3772356 | 2 | 5 | 0 | 1 | 216 |
| 84 | 54 | 39 | 27 | 2 | 6 | 461493 | 3691940 | 2 | 6 | 0 | 208 | 3772572 | 2 | 6 | 0 | 1 | 216 |
| 85 | 55 | 39 | 27 | 2 | 7 | 461519 | 3692148 | 2 | 7 | 0 | 208 | 3772788 | 2 | 7 | 0 | 1 | 216 |
| 86 | 56 | 39 | 27 | 2 | 8 | 461545 | 3692356 | 2 | 8 | 0 | 208 | 3773004 | 2 | 8 | 0 | 1 | 216 |
| 87 | 57 | 39 | 27 | 2 | 9 | 461571 | 3692564 | 2 | 9 | 0 | 208 | 3773220 | 2 | 9 | 0 | 1 | 216 |
| 88 | 58 | 39 | 27 | 2 | 10 | 461597 | 3692772 | 2 | 10 | 0 | 208 | 3773436 | 2 | 10 | 0 | 1 | 216 |
| 89 | 59 | 39 | 27 | 2 | 11 | 461623 | 3692980 | 2 | 11 | 0 | 208 | 3773652 | 2 | 11 | 0 | 1 | 216 |
| 90 | 5A | 39 | 27 | 2 | 12 | 461649 | 3693188 | 2 | 12 | 0 | 208 | 3773868 | 2 | 12 | 0 | 1 | 216 |
| 91 | 5B | 39 | 27 | 2 | 13 | 461675 | 3693396 | 2 | 13 | 0 | 208 | 3774084 | 2 | 13 | 0 | 1 | 216 |
| 92 | 5C | 39 | 27 | 2 | 14 | 461701 | 3693604 | 2 | 14 | 0 | 208 | 3774300 | 2 | 14 | 0 | 1 | 216 |
| 93 | 5D | 39 | 27 | 2 | 15 | 461727 | 3693812 | 2 | 15 | 0 | 208 | 3774516 | 2 | 15 | 0 | 1 | 216 |
| 94 | 5E | 39 | 27 | 2 | 16 | 461753 | 3694020 | 2 | 16 | 0 | 208 | 3774732 | 2 | 16 | 0 | 1 | 216 |
| 95 | 5F | 39 | 27 | 2 | 17 | 461779 | 3694228 | 2 | 17 | 0 | 208 | 3774948 | 2 | 17 | 0 | 1 | 216 |
| 96 | 60 | 39 | 27 | 2 | 18 | 461802 | 3694412 | 2 | 18 | 0 | 184 | 3775140 | 2 | 18 | 0 | 1 | 192 |
| 97 | 61 | 39 | 27 | 2 | 19 | 461825 | 3694596 | 2 | 19 | 0 | 184 | 3775332 | 2 | 19 | 0 | 1 | 192 |
| 98 | 62 | 39 | 27 | 2 | 20 | 461848 | 3694780 | 2 | 20 | 0 | 184 | 3775524 | 2 | 20 | 0 | 1 | 192 |
| 99 | 63 | 39 | 27 | 2 | 21 | 461871 | 3694964 | 2 | 21 | 0 | 184 | 3775716 | 2 | 21 | 0 | 1 | 192 |
| 100 | 64 | 39 | 27 | 2 | 22 | 461894 | 3695148 | 2 | 22 | 0 | 184 | 3775908 | 2 | 22 | 0 | 1 | 192 |
| 101 | 65 | 39 | 27 | 2 | 23 | 461917 | 3695332 | 2 | 23 | 0 | 184 | 3776100 | 2 | 23 | 0 | 1 | 192 |
| 102 | 66 | 39 | 27 | 2 | 24 | 461940 | 3695516 | 2 | 24 | 0 | 184 | 3776292 | 2 | 24 | 0 | 1 | 192 |
| 103 | 67 | 39 | 27 | 2 | 25 | 461963 | 3695700 | 2 | 25 | 0 | 184 | 3776484 | 2 | 25 | 0 | 1 | 192 |
| 104 | 68 | 39 | 27 | 2 | 26 | 461986 | 3695884 | 2 | 26 | 0 | 184 | 3776676 | 2 | 26 | 0 | 1 | 192 |
| 105 | 69 | 39 | 27 | 2 | 27 | 462009 | 3696068 | 2 | 27 | 0 | 184 | 3776868 | 2 | 27 | 0 | 1 | 192 |
| 106 | 6A | 39 | 27 | 2 | 28 | 462032 | 3696252 | 2 | 28 | 0 | 184 | 3777060 | 2 | 28 | 0 | 1 | 192 |

| | | | | | | | | | | | | | | | | | |
|---|---|---|---|---|---|---|---|---|---|---|---|---|---|---|---|---|---|
| 107 | 6B | 39 | 27 | 2 | 29 | 462055 | 3696436 | 2 | 29 | 0 | 184 | 3777252 | 2 | 29 | 0 | 1 | 192 |
| 108 | 6C | 39 | 27 | 2 | 30 | 462078 | 3696620 | 2 | 30 | 0 | 184 | 3777444 | 2 | 30 | 0 | 1 | 192 |
| 109 | 6D | 39 | 27 | 2 | 31 | 462101 | 3696804 | 2 | 31 | 0 | 184 | 3777636 | 2 | 31 | 0 | 1 | 192 |
| 110 | 6E | 39 | 27 | 2 | 32 | 462124 | 3696988 | 2 | 32 | 0 | 184 | 3777828 | 2 | 32 | 0 | 1 | 192 |
| 111 | 6F | 39 | 27 | 2 | 33 | 462147 | 3697172 | 2 | 33 | 0 | 184 | 3778020 | 2 | 33 | 0 | 1 | 192 |
| 112 | 70 | 39 | 27 | 2 | 34 | 462172 | 3697372 | 2 | 34 | 0 | 200 | 3778228 | 2 | 34 | 0 | 1 | 208 |
| 113 | 71 | 39 | 27 | 2 | 35 | 462197 | 3697572 | 2 | 35 | 0 | 200 | 3778436 | 2 | 35 | 0 | 1 | 208 |
| 114 | 72 | 39 | 27 | 2 | 36 | 462222 | 3697772 | 2 | 36 | 0 | 200 | 3778644 | 2 | 36 | 0 | 1 | 208 |
| 115 | 73 | 39 | 27 | 2 | 37 | 462247 | 3697972 | 2 | 37 | 0 | 200 | 3778852 | 2 | 37 | 0 | 1 | 208 |
| 116 | 74 | 39 | 27 | 2 | 38 | 462272 | 3698172 | 2 | 38 | 0 | 200 | 3779060 | 2 | 38 | 0 | 1 | 208 |
| 117 | 75 | 39 | 27 | 3 | 0 | 462295 | 3698356 | 3 | 0 | 0 | 184 | 3779252 | 3 | 0 | 0 | 1 | 192 |
| 118 | 76 | 39 | 27 | 3 | 1 | 462323 | 3698580 | 3 | 1 | 0 | 224 | 3779484 | 3 | 1 | 0 | 1 | 232 |
| 119 | 77 | 39 | 27 | 3 | 2 | 462351 | 3698804 | 3 | 2 | 0 | 224 | 3779716 | 3 | 2 | 0 | 1 | 232 |
| 120 | 78 | 39 | 27 | 3 | 3 | 462379 | 3699028 | 3 | 3 | 0 | 224 | 3779948 | 3 | 3 | 0 | 1 | 232 |
| 121 | 79 | 39 | 27 | 3 | 4 | 462407 | 3699252 | 3 | 4 | 0 | 224 | 3780180 | 3 | 4 | 0 | 1 | 232 |
| 122 | 7A | 39 | 27 | 3 | 5 | 462435 | 3699476 | 3 | 5 | 0 | 224 | 3780412 | 3 | 5 | 0 | 1 | 232 |
| 123 | 7B | 39 | 27 | 3 | 6 | 462463 | 3699700 | 3 | 6 | 0 | 224 | 3780644 | 3 | 6 | 0 | 1 | 232 |
| 124 | 7C | 39 | 27 | 3 | 7 | 462491 | 3699924 | 3 | 7 | 0 | 224 | 3780876 | 3 | 7 | 0 | 1 | 232 |
| 125 | 7D | 39 | 27 | 3 | 8 | 462519 | 3700148 | 3 | 8 | 0 | 224 | 3781108 | 3 | 8 | 0 | 1 | 232 |
| 126 | 7E | 39 | 27 | 3 | 9 | 462547 | 3700372 | 3 | 9 | 0 | 224 | 3781340 | 3 | 9 | 0 | 1 | 232 |
| 127 | 7F | 39 | 27 | 3 | 10 | 462575 | 3700596 | 3 | 10 | 0 | 224 | 3781572 | 3 | 10 | 0 | 1 | 232 |
| 128 | 80 | 39 | 27 | 3 | 11 | 462603 | 3700820 | 3 | 11 | 0 | 224 | 3781804 | 3 | 11 | 0 | 1 | 232 |
| 129 | 81 | 39 | 27 | 3 | 12 | 462631 | 3701044 | 3 | 12 | 0 | 224 | 3782036 | 3 | 12 | 0 | 1 | 232 |
| 130 | 82 | 39 | 27 | 3 | 13 | 462659 | 3701268 | 3 | 13 | 0 | 224 | 3782268 | 3 | 13 | 0 | 1 | 232 |
| 131 | 83 | 39 | 27 | 3 | 14 | 462687 | 3701492 | 3 | 14 | 0 | 224 | 3782500 | 3 | 14 | 0 | 1 | 232 |
| 132 | 84 | 39 | 27 | 3 | 15 | 462715 | 3701716 | 3 | 15 | 0 | 224 | 3782732 | 3 | 15 | 0 | 1 | 232 |
| 133 | 85 | 39 | 27 | 3 | 16 | 462743 | 3701940 | 3 | 16 | 0 | 224 | 3782964 | 3 | 16 | 0 | 1 | 232 |
| 134 | 86 | 39 | 27 | 3 | 17 | 462771 | 3702164 | 3 | 17 | 0 | 224 | 3783196 | 3 | 17 | 0 | 1 | 232 |
| 135 | 87 | 39 | 27 | 3 | 18 | 462799 | 3702388 | 3 | 18 | 0 | 224 | 3783428 | 3 | 18 | 0 | 1 | 232 |
| 136 | 88 | 39 | 27 | 3 | 19 | 462827 | 3702612 | 3 | 19 | 0 | 224 | 3783660 | 3 | 19 | 0 | 1 | 232 |
| 137 | 89 | 39 | 27 | 3 | 20 | 462855 | 3702836 | 3 | 20 | 0 | 224 | 3783892 | 3 | 20 | 0 | 1 | 232 |
| 138 | 8A | 39 | 27 | 3 | 21 | 462883 | 3703060 | 3 | 21 | 0 | 224 | 3784124 | 3 | 21 | 0 | 1 | 232 |
| 139 | 8B | 39 | 27 | 3 | 22 | 462911 | 3703284 | 3 | 22 | 0 | 224 | 3784356 | 3 | 22 | 0 | 1 | 232 |
| 140 | 8C | 39 | 27 | 3 | 23 | 462939 | 3703508 | 3 | 23 | 0 | 224 | 3784588 | 3 | 23 | 0 | 1 | 232 |
| 141 | 8D | 39 | 27 | 3 | 24 | 462967 | 3703732 | 3 | 24 | 0 | 224 | 3784820 | 3 | 24 | 0 | 1 | 232 |
| 142 | 8E | 39 | 27 | 3 | 25 | 462995 | 3703956 | 3 | 25 | 0 | 224 | 3785052 | 3 | 25 | 0 | 1 | 232 |
| 143 | 8F | 39 | 27 | 3 | 26 | 463023 | 3704180 | 3 | 26 | 0 | 224 | 3785284 | 3 | 26 | 0 | 1 | 232 |
| 144 | 90 | 39 | 27 | 3 | 27 | 463048 | 3704380 | 3 | 27 | 0 | 200 | 3785492 | 3 | 27 | 0 | 1 | 208 |
| 145 | 91 | 39 | 27 | 3 | 28 | 463073 | 3704580 | 3 | 28 | 0 | 200 | 3785700 | 3 | 28 | 0 | 1 | 208 |
| 146 | 92 | 39 | 27 | 3 | 29 | 463098 | 3704780 | 3 | 29 | 0 | 200 | 3785908 | 3 | 29 | 0 | 1 | 208 |
| 147 | 93 | 39 | 27 | 3 | 30 | 463123 | 3704980 | 3 | 30 | 0 | 200 | 3786116 | 3 | 30 | 0 | 1 | 208 |
| 148 | 94 | 39 | 27 | 3 | 31 | 463148 | 3705180 | 3 | 31 | 0 | 200 | 3786324 | 3 | 31 | 0 | 1 | 208 |
| 149 | 95 | 39 | 27 | 3 | 32 | 463173 | 3705380 | 3 | 32 | 0 | 200 | 3786532 | 3 | 32 | 0 | 1 | 208 |
| 150 | 96 | 39 | 27 | 3 | 33 | 463198 | 3705580 | 3 | 33 | 0 | 200 | 3786740 | 3 | 33 | 0 | 1 | 208 |
| 151 | 97 | 39 | 27 | 3 | 34 | 463223 | 3705780 | 3 | 34 | 0 | 200 | 3786948 | 3 | 34 | 0 | 1 | 208 |
| 152 | 98 | 39 | 27 | 3 | 35 | 463248 | 3705980 | 3 | 35 | 0 | 200 | 3787156 | 3 | 35 | 0 | 1 | 208 |
| 153 | 99 | 39 | 27 | 3 | 36 | 463273 | 3706180 | 3 | 36 | 0 | 200 | 3787364 | 3 | 36 | 0 | 1 | 208 |
| 154 | 9A | 39 | 27 | 3 | 37 | 463298 | 3706380 | 3 | 37 | 0 | 200 | 3787572 | 3 | 37 | 0 | 1 | 208 |
| 155 | 9B | 39 | 27 | 3 | 38 | 463323 | 3706580 | 3 | 38 | 0 | 200 | 3787780 | 3 | 38 | 0 | 1 | 208 |
| 156 | 9C | 39 | 27 | 4 | 0 | 463346 | 3706764 | 4 | 0 | 0 | 184 | 3787972 | 4 | 0 | 0 | 1 | 192 |
| 157 | 9D | 39 | 27 | 4 | 1 | 463374 | 3706988 | 4 | 1 | 0 | 224 | 3788204 | 4 | 1 | 0 | 1 | 232 |
| 158 | 9E | 39 | 27 | 4 | 2 | 463402 | 3707212 | 4 | 2 | 0 | 224 | 3788436 | 4 | 2 | 0 | 1 | 232 |
| 159 | 9F | 39 | 27 | 4 | 3 | 463430 | 3707436 | 4 | 3 | 0 | 224 | 3788668 | 4 | 3 | 0 | 1 | 232 |
| 160 | A0 | 39 | 27 | 4 | 4 | 463460 | 3707676 | 4 | 4 | 0 | 240 | 3788916 | 4 | 4 | 0 | 1 | 248 |
| 161 | A1 | 39 | 27 | 4 | 5 | 463490 | 3707916 | 4 | 5 | 0 | 240 | 3789164 | 4 | 5 | 0 | 1 | 248 |
| 162 | A2 | 39 | 27 | 4 | 6 | 463520 | 3708156 | 4 | 6 | 0 | 240 | 3789412 | 4 | 6 | 0 | 1 | 248 |
| 163 | A3 | 39 | 27 | 4 | 7 | 463550 | 3708396 | 4 | 7 | 0 | 240 | 3789660 | 4 | 7 | 0 | 1 | 248 |
| 164 | A4 | 39 | 27 | 4 | 8 | 463580 | 3708636 | 4 | 8 | 0 | 240 | 3789908 | 4 | 8 | 0 | 1 | 248 |
| 165 | A5 | 39 | 27 | 4 | 9 | 463610 | 3708876 | 4 | 9 | 0 | 240 | 3790156 | 4 | 9 | 0 | 1 | 248 |
| 166 | A6 | 39 | 27 | 4 | 10 | 463640 | 3709116 | 4 | 10 | 0 | 240 | 3790404 | 4 | 10 | 0 | 1 | 248 |
| 167 | A7 | 39 | 27 | 4 | 11 | 463670 | 3709356 | 4 | 11 | 0 | 240 | 3790652 | 4 | 11 | 0 | 1 | 248 |
| 168 | A8 | 39 | 27 | 4 | 12 | 463700 | 3709596 | 4 | 12 | 0 | 240 | 3790900 | 4 | 12 | 0 | 1 | 248 |
| 169 | A9 | 39 | 27 | 4 | 13 | 463730 | 3709836 | 4 | 13 | 0 | 240 | 3791148 | 4 | 13 | 0 | 1 | 248 |
| 170 | AA | 39 | 27 | 4 | 14 | 463760 | 3710076 | 4 | 14 | 0 | 240 | 3791396 | 4 | 14 | 0 | 1 | 248 |
| 171 | AB | 39 | 27 | 4 | 15 | 463790 | 3710316 | 4 | 15 | 0 | 240 | 3791644 | 4 | 15 | 0 | 1 | 248 |
| 172 | AC | 39 | 27 | 4 | 16 | 463820 | 3710556 | 4 | 16 | 0 | 240 | 3791892 | 4 | 16 | 0 | 1 | 248 |
| 173 | AD | 39 | 27 | 4 | 17 | 463850 | 3710796 | 4 | 17 | 0 | 240 | 3792140 | 4 | 17 | 0 | 1 | 248 |
| 174 | AE | 39 | 27 | 4 | 18 | 463880 | 3711036 | 4 | 18 | 0 | 240 | 3792388 | 4 | 18 | 0 | 1 | 248 |
| 175 | AF | 39 | 27 | 4 | 19 | 463910 | 3711276 | 4 | 19 | 0 | 240 | 3792636 | 4 | 19 | 0 | 1 | 248 |
| 176 | B0 | 39 | 27 | 4 | 20 | 463940 | 3711516 | 4 | 20 | 0 | 240 | 3792884 | 4 | 20 | 0 | 1 | 248 |
| 177 | B1 | 39 | 27 | 4 | 21 | 463970 | 3711756 | 4 | 21 | 0 | 240 | 3793132 | 4 | 21 | 0 | 1 | 248 |
| 178 | B2 | 39 | 27 | 4 | 22 | 464000 | 3711996 | 4 | 22 | 0 | 240 | 3793380 | 4 | 22 | 0 | 1 | 248 |
| 179 | B3 | 39 | 27 | 4 | 23 | 464030 | 3712236 | 4 | 23 | 0 | 240 | 3793628 | 4 | 23 | 0 | 1 | 248 |
| 180 | B4 | 39 | 27 | 4 | 24 | 464060 | 3712476 | 4 | 24 | 0 | 240 | 3793876 | 4 | 24 | 0 | 1 | 248 |
| 181 | B5 | 39 | 27 | 4 | 25 | 464090 | 3712716 | 4 | 25 | 0 | 240 | 3794124 | 4 | 25 | 0 | 1 | 248 |
| 182 | B6 | 39 | 27 | 4 | 26 | 464120 | 3712956 | 4 | 26 | 0 | 240 | 3794372 | 4 | 26 | 0 | 1 | 248 |
| 183 | B7 | 39 | 27 | 4 | 27 | 464150 | 3713196 | 4 | 27 | 0 | 240 | 3794620 | 4 | 27 | 0 | 1 | 248 |
| 184 | B8 | 39 | 27 | 4 | 28 | 464180 | 3713436 | 4 | 28 | 0 | 240 | 3794868 | 4 | 28 | 0 | 1 | 248 |
| 185 | B9 | 39 | 27 | 4 | 29 | 464210 | 3713676 | 4 | 29 | 0 | 240 | 3795116 | 4 | 29 | 0 | 1 | 248 |
| 186 | BA | 39 | 27 | 4 | 30 | 464240 | 3713916 | 4 | 30 | 0 | 240 | 3795364 | 4 | 30 | 0 | 1 | 248 |
| 187 | BB | 39 | 27 | 4 | 31 | 464270 | 3714156 | 4 | 31 | 0 | 240 | 3795612 | 4 | 31 | 0 | 1 | 248 |
| 188 | BC | 39 | 27 | 4 | 32 | 464300 | 3714396 | 4 | 32 | 0 | 240 | 3795860 | 4 | 32 | 0 | 1 | 248 |
| 189 | BD | 39 | 27 | 4 | 33 | 464330 | 3714636 | 4 | 33 | 0 | 240 | 3796108 | 4 | 33 | 0 | 1 | 248 |
| 190 | BE | 39 | 27 | 4 | 34 | 464360 | 3714876 | 4 | 34 | 0 | 240 | 3796356 | 4 | 34 | 0 | 1 | 248 |
| 191 | BF | 39 | 27 | 4 | 35 | 464390 | 3715116 | 4 | 35 | 0 | 240 | 3796604 | 4 | 35 | 0 | 1 | 248 |
| 192 | C0 | 39 | 27 | 4 | 36 | 464417 | 3715332 | 4 | 36 | 0 | 216 | 3796828 | 4 | 36 | 0 | 1 | 224 |
| 193 | C1 | 39 | 27 | 4 | 37 | 464444 | 3715548 | 4 | 37 | 0 | 216 | 3797052 | 4 | 37 | 0 | 1 | 224 |
| 194 | C2 | 39 | 27 | 4 | 38 | 464471 | 3715764 | 4 | 38 | 0 | 216 | 3797276 | 4 | 38 | 0 | 1 | 224 |
| 195 | C3 | 39 | 27 | 5 | 0 | 464496 | 3715964 | 5 | 0 | 0 | 200 | 3797484 | 5 | 0 | 0 | 1 | 208 |
| 196 | C4 | 39 | 27 | 5 | 1 | 464526 | 3716204 | 5 | 1 | 0 | 240 | 3797732 | 5 | 1 | 0 | 1 | 248 |
| 197 | C5 | 39 | 27 | 5 | 2 | 464556 | 3716444 | 5 | 2 | 0 | 240 | 3797980 | 5 | 2 | 0 | 1 | 248 |
| 198 | C6 | 39 | 27 | 5 | 3 | 464586 | 3716684 | 5 | 3 | 0 | 240 | 3798228 | 5 | 3 | 0 | 1 | 248 |
| 199 | C7 | 39 | 27 | 5 | 4 | 464616 | 3716924 | 5 | 4 | 0 | 240 | 3798476 | 5 | 4 | 0 | 1 | 248 |
| 200 | C8 | 39 | 27 | 5 | 5 | 464646 | 3717164 | 5 | 5 | 0 | 240 | 3798724 | 5 | 5 | 0 | 1 | 248 |
| 201 | C9 | 39 | 27 | 5 | 6 | 464676 | 3717404 | 5 | 6 | 0 | 240 | 3798972 | 5 | 6 | 0 | 1 | 248 |
| 202 | CA | 39 | 27 | 5 | 7 | 464706 | 3717644 | 5 | 7 | 0 | 240 | 3799220 | 5 | 7 | 0 | 1 | 248 |
| 203 | CB | 39 | 27 | 5 | 8 | 464736 | 3717884 | 5 | 8 | 0 | 240 | 3799468 | 5 | 8 | 0 | 1 | 248 |
| 204 | CC | 39 | 27 | 5 | 9 | 464766 | 3718124 | 5 | 9 | 0 | 240 | 3799716 | 5 | 9 | 0 | 1 | 248 |
| 205 | CD | 39 | 27 | 5 | 10 | 464796 | 3718364 | 5 | 10 | 0 | 240 | 3799964 | 5 | 10 | 0 | 1 | 248 |
| 206 | CE | 39 | 27 | 5 | 11 | 464826 | 3718604 | 5 | 11 | 0 | 240 | 3800212 | 5 | 11 | 0 | 1 | 248 |
| 207 | CF | 39 | 27 | 5 | 12 | 464856 | 3718844 | 5 | 12 | 0 | 240 | 3800460 | 5 | 12 | 0 | 1 | 248 |
| 208 | D0 | 39 | 27 | 5 | 13 | 464888 | 3719100 | 5 | 13 | 0 | 256 | 3800724 | 5 | 13 | 0 | 1 | 264 |
| 209 | D1 | 39 | 27 | 5 | 14 | 464920 | 3719356 | 5 | 14 | 0 | 256 | 3800988 | 5 | 14 | 0 | 1 | 264 |

| | | | | | | | | | | | | | | | | | |
|---|---|---|---|---|---|---|---|---|---|---|---|---|---|---|---|---|---|
| 210 | D2 | 39 | 27 | 5 | 15 | 464952 | 3719612 | 5 | 15 | 0 | 256 | 3801252 | 5 | 15 | 0 | 1 | 264 |
| 211 | D3 | 39 | 27 | 5 | 16 | 464984 | 3719868 | 5 | 16 | 0 | 256 | 3801516 | 5 | 16 | 0 | 1 | 264 |
| 212 | D4 | 39 | 27 | 5 | 17 | 465016 | 3720124 | 5 | 17 | 0 | 256 | 3801780 | 5 | 17 | 0 | 1 | 264 |
| 213 | D5 | 39 | 27 | 5 | 18 | 465048 | 3720380 | 5 | 18 | 0 | 256 | 3802044 | 5 | 18 | 0 | 1 | 264 |
| 214 | D6 | 39 | 27 | 5 | 19 | 465080 | 3720636 | 5 | 19 | 0 | 256 | 3802308 | 5 | 19 | 0 | 1 | 264 |
| 215 | D7 | 39 | 27 | 5 | 20 | 465112 | 3720892 | 5 | 20 | 0 | 256 | 3802572 | 5 | 20 | 0 | 1 | 264 |
| 216 | D8 | 39 | 27 | 5 | 21 | 465144 | 3721148 | 5 | 21 | 0 | 256 | 3802836 | 5 | 21 | 0 | 1 | 264 |
| 217 | D9 | 39 | 27 | 5 | 22 | 465176 | 3721404 | 5 | 22 | 0 | 256 | 3803100 | 5 | 22 | 0 | 1 | 264 |
| 218 | DA | 39 | 27 | 5 | 23 | 465208 | 3721660 | 5 | 23 | 0 | 256 | 3803364 | 5 | 23 | 0 | 1 | 264 |
| 219 | DB | 39 | 27 | 5 | 24 | 465240 | 3721916 | 5 | 24 | 0 | 256 | 3803628 | 5 | 24 | 0 | 1 | 264 |
| 220 | DC | 39 | 27 | 5 | 25 | 465272 | 3722172 | 5 | 25 | 0 | 256 | 3803892 | 5 | 25 | 0 | 1 | 264 |
| 221 | DD | 39 | 27 | 5 | 26 | 465304 | 3722428 | 5 | 26 | 0 | 256 | 3804156 | 5 | 26 | 0 | 1 | 264 |
| 222 | DE | 39 | 27 | 5 | 27 | 465336 | 3722684 | 5 | 27 | 0 | 256 | 3804420 | 5 | 27 | 0 | 1 | 264 |
| 223 | DF | 39 | 27 | 5 | 28 | 465368 | 3722940 | 5 | 28 | 0 | 256 | 3804684 | 5 | 28 | 0 | 1 | 264 |
| 224 | E0 | 39 | 27 | 5 | 29 | 465400 | 3723196 | 5 | 29 | 0 | 256 | 3804948 | 5 | 29 | 0 | 1 | 264 |
| 225 | E1 | 39 | 27 | 5 | 30 | 465432 | 3723452 | 5 | 30 | 0 | 256 | 3805212 | 5 | 30 | 0 | 1 | 264 |
| 226 | E2 | 39 | 27 | 5 | 31 | 465464 | 3723708 | 5 | 31 | 0 | 256 | 3805476 | 5 | 31 | 0 | 1 | 264 |
| 227 | E3 | 39 | 27 | 5 | 32 | 465496 | 3723964 | 5 | 32 | 0 | 256 | 3805740 | 5 | 32 | 0 | 1 | 264 |
| 228 | E4 | 39 | 27 | 5 | 33 | 465528 | 3724220 | 5 | 33 | 0 | 256 | 3806004 | 5 | 33 | 0 | 1 | 264 |
| 229 | E5 | 39 | 27 | 5 | 34 | 465560 | 3724476 | 5 | 34 | 0 | 256 | 3806268 | 5 | 34 | 0 | 1 | 264 |
| 230 | E6 | 39 | 27 | 5 | 35 | 465592 | 3724732 | 5 | 35 | 0 | 256 | 3806532 | 5 | 35 | 0 | 1 | 264 |
| 231 | E7 | 39 | 27 | 5 | 36 | 465624 | 3724988 | 5 | 36 | 0 | 256 | 3806796 | 5 | 36 | 0 | 1 | 264 |
| 232 | E8 | 39 | 27 | 5 | 37 | 465656 | 3725244 | 5 | 37 | 0 | 256 | 3807060 | 5 | 37 | 0 | 1 | 264 |
| 233 | E9 | 39 | 27 | 5 | 38 | 465688 | 3725500 | 5 | 38 | 0 | 256 | 3807324 | 5 | 38 | 0 | 1 | 264 |
| 234 | EA | 39 | 27 | 6 | 0 | 465722 | 3725772 | 6 | 0 | 0 | 272 | 3807604 | 6 | 0 | 0 | 1 | 280 |
| 235 | EB | 39 | 27 | 6 | 1 | 465756 | 3726044 | 6 | 1 | 0 | 272 | 3807884 | 6 | 1 | 0 | 1 | 280 |
| 236 | EC | 39 | 27 | 6 | 2 | 465790 | 3726316 | 6 | 2 | 0 | 272 | 3808164 | 6 | 2 | 0 | 1 | 280 |
| 237 | ED | 39 | 27 | 6 | 3 | 465824 | 3726588 | 6 | 3 | 0 | 272 | 3808444 | 6 | 3 | 0 | 1 | 280 |
| 238 | EE | 39 | 27 | 6 | 4 | 465858 | 3726860 | 6 | 4 | 0 | 272 | 3808724 | 6 | 4 | 0 | 1 | 280 |
| 239 | EF | 39 | 27 | 6 | 5 | 465892 | 3727132 | 6 | 5 | 0 | 272 | 3809004 | 6 | 5 | 0 | 1 | 280 |
| 240 | F0 | 39 | 27 | 6 | 6 | 465924 | 3727388 | 6 | 6 | 0 | 256 | 3809268 | 6 | 6 | 0 | 1 | 264 |
| 241 | F1 | 39 | 27 | 6 | 7 | 465956 | 3727644 | 6 | 7 | 0 | 256 | 3809532 | 6 | 7 | 0 | 1 | 264 |
| 242 | F2 | 39 | 27 | 6 | 8 | 465988 | 3727900 | 6 | 8 | 0 | 256 | 3809796 | 6 | 8 | 0 | 1 | 264 |
| 243 | F3 | 39 | 27 | 6 | 9 | 466020 | 3728156 | 6 | 9 | 0 | 256 | 3810060 | 6 | 9 | 0 | 1 | 264 |
| 244 | F4 | 39 | 27 | 6 | 10 | 466052 | 3728412 | 6 | 10 | 0 | 256 | 3810324 | 6 | 10 | 0 | 1 | 264 |
| 245 | F5 | 39 | 27 | 6 | 11 | 466084 | 3728668 | 6 | 11 | 0 | 256 | 3810588 | 6 | 11 | 0 | 1 | 264 |
| 246 | F6 | 39 | 27 | 6 | 12 | 466116 | 3728924 | 6 | 12 | 0 | 256 | 3810852 | 6 | 12 | 0 | 1 | 264 |
| 247 | F7 | 39 | 27 | 6 | 13 | 466148 | 3729180 | 6 | 13 | 0 | 256 | 3811116 | 6 | 13 | 0 | 1 | 264 |
| 248 | F8 | 39 | 27 | 6 | 14 | 466180 | 3729436 | 6 | 14 | 0 | 256 | 3811380 | 6 | 14 | 0 | 1 | 264 |
| 249 | F9 | 39 | 27 | 6 | 15 | 466212 | 3729692 | 6 | 15 | 0 | 256 | 3811644 | 6 | 15 | 0 | 1 | 264 |
| 250 | FA | 39 | 27 | 6 | 16 | 466244 | 3729948 | 6 | 16 | 0 | 256 | 3811908 | 6 | 16 | 0 | 1 | 264 |
| 251 | FB | 39 | 27 | 6 | 17 | 466276 | 3730204 | 6 | 17 | 0 | 256 | 3812172 | 6 | 17 | 0 | 1 | 264 |
| 252 | FC | 39 | 27 | 6 | 18 | 466308 | 3730460 | 6 | 18 | 0 | 256 | 3812436 | 6 | 18 | 0 | 1 | 264 |
| 253 | FD | 39 | 27 | 6 | 19 | 466340 | 3730716 | 6 | 19 | 0 | 256 | 3812700 | 6 | 19 | 0 | 1 | 264 |
| 254 | FE | 39 | 27 | 6 | 20 | 466372 | 3730972 | 6 | 20 | 0 | 256 | 3812964 | 6 | 20 | 0 | 1 | 264 |
| 255 | FF | 39 | 27 | 6 | 21 | 466404 | 3731228 | 6 | 21 | 0 | 256 | 3813228 | 6 | 21 | 0 | 1 | 264 |
| 0 | 00 | 40 | 28 | 0 | 0 | 466416 | 3731324 | 0 | 0 | 0 | 96 | 3813332 | 0 | 0 | 0 | 1 | 104 |
| 1 | 01 | 40 | 28 | 0 | 1 | 466430 | 3731436 | 0 | 1 | 0 | 112 | 3813452 | 0 | 1 | 0 | 1 | 120 |
| 2 | 02 | 40 | 28 | 0 | 2 | 466444 | 3731548 | 0 | 2 | 0 | 112 | 3813572 | 0 | 2 | 0 | 1 | 120 |
| 3 | 03 | 40 | 28 | 0 | 3 | 466458 | 3731660 | 0 | 3 | 0 | 112 | 3813692 | 0 | 3 | 0 | 1 | 120 |
| 4 | 04 | 40 | 28 | 0 | 4 | 466472 | 3731772 | 0 | 4 | 0 | 112 | 3813812 | 0 | 4 | 0 | 1 | 120 |
| 5 | 05 | 40 | 28 | 0 | 5 | 466486 | 3731884 | 0 | 5 | 0 | 112 | 3813932 | 0 | 5 | 0 | 1 | 120 |
| 6 | 06 | 40 | 28 | 0 | 6 | 466500 | 3731996 | 0 | 6 | 0 | 112 | 3814052 | 0 | 6 | 0 | 1 | 120 |
| 7 | 07 | 40 | 28 | 0 | 7 | 466514 | 3732108 | 0 | 7 | 0 | 112 | 3814172 | 0 | 7 | 0 | 1 | 120 |
| 8 | 08 | 40 | 28 | 0 | 8 | 466528 | 3732220 | 0 | 8 | 0 | 112 | 3814292 | 0 | 8 | 0 | 1 | 120 |
| 9 | 09 | 40 | 28 | 0 | 9 | 466542 | 3732332 | 0 | 9 | 0 | 112 | 3814412 | 0 | 9 | 0 | 1 | 120 |
| 10 | 0A | 40 | 28 | 0 | 10 | 466556 | 3732444 | 0 | 10 | 0 | 112 | 3814532 | 0 | 10 | 0 | 1 | 120 |
| 11 | 0B | 40 | 28 | 0 | 11 | 466570 | 3732556 | 0 | 11 | 0 | 112 | 3814652 | 0 | 11 | 0 | 1 | 120 |
| 12 | 0C | 40 | 28 | 0 | 12 | 466584 | 3732668 | 0 | 12 | 0 | 112 | 3814772 | 0 | 12 | 0 | 1 | 120 |
| 13 | 0D | 40 | 28 | 0 | 13 | 466598 | 3732780 | 0 | 13 | 0 | 112 | 3814892 | 0 | 13 | 0 | 1 | 120 |
| 14 | 0E | 40 | 28 | 0 | 14 | 466612 | 3732892 | 0 | 14 | 0 | 112 | 3815012 | 0 | 14 | 0 | 1 | 120 |
| 15 | 0F | 40 | 28 | 0 | 15 | 466626 | 3733004 | 0 | 15 | 0 | 112 | 3815132 | 0 | 15 | 0 | 1 | 120 |
| 16 | 10 | 40 | 28 | 0 | 16 | 466640 | 3733116 | 0 | 16 | 0 | 112 | 3815252 | 0 | 16 | 0 | 1 | 120 |
| 17 | 11 | 40 | 28 | 0 | 17 | 466654 | 3733228 | 0 | 17 | 0 | 112 | 3815372 | 0 | 17 | 0 | 1 | 120 |
| 18 | 12 | 40 | 28 | 0 | 18 | 466668 | 3733340 | 0 | 18 | 0 | 112 | 3815492 | 0 | 18 | 0 | 1 | 120 |
| 19 | 13 | 40 | 28 | 0 | 19 | 466682 | 3733452 | 0 | 19 | 0 | 112 | 3815612 | 0 | 19 | 0 | 1 | 120 |
| 20 | 14 | 40 | 28 | 0 | 20 | 466696 | 3733564 | 0 | 20 | 0 | 112 | 3815732 | 0 | 20 | 0 | 1 | 120 |
| 21 | 15 | 40 | 28 | 0 | 21 | 466710 | 3733676 | 0 | 21 | 0 | 112 | 3815852 | 0 | 21 | 0 | 1 | 120 |
| 22 | 16 | 40 | 28 | 0 | 22 | 466724 | 3733788 | 0 | 22 | 0 | 112 | 3815972 | 0 | 22 | 0 | 1 | 120 |
| 23 | 17 | 40 | 28 | 0 | 23 | 466738 | 3733900 | 0 | 23 | 0 | 112 | 3816092 | 0 | 23 | 0 | 1 | 120 |
| 24 | 18 | 40 | 28 | 0 | 24 | 466752 | 3734012 | 0 | 24 | 0 | 112 | 3816212 | 0 | 24 | 0 | 1 | 120 |
| 25 | 19 | 40 | 28 | 0 | 25 | 466766 | 3734124 | 0 | 25 | 0 | 112 | 3816332 | 0 | 25 | 0 | 1 | 120 |
| 26 | 1A | 40 | 28 | 0 | 26 | 466780 | 3734236 | 0 | 26 | 0 | 112 | 3816452 | 0 | 26 | 0 | 1 | 120 |
| 27 | 1B | 40 | 28 | 0 | 27 | 466794 | 3734348 | 0 | 27 | 0 | 112 | 3816572 | 0 | 27 | 0 | 1 | 120 |
| 28 | 1C | 40 | 28 | 0 | 28 | 466808 | 3734460 | 0 | 28 | 0 | 112 | 3816692 | 0 | 28 | 0 | 1 | 120 |
| 29 | 1D | 40 | 28 | 0 | 29 | 466822 | 3734572 | 0 | 29 | 0 | 112 | 3816812 | 0 | 29 | 0 | 1 | 120 |
| 30 | 1E | 40 | 28 | 0 | 30 | 466836 | 3734684 | 0 | 30 | 0 | 112 | 3816932 | 0 | 30 | 0 | 1 | 120 |
| 31 | 1F | 40 | 28 | 0 | 31 | 466850 | 3734796 | 0 | 31 | 0 | 112 | 3817052 | 0 | 31 | 0 | 1 | 120 |
| 32 | 20 | 40 | 28 | 0 | 32 | 466864 | 3734908 | 0 | 32 | 0 | 112 | 3817172 | 0 | 32 | 0 | 1 | 120 |
| 33 | 21 | 40 | 28 | 0 | 33 | 466878 | 3735020 | 0 | 33 | 0 | 112 | 3817292 | 0 | 33 | 0 | 1 | 120 |
| 34 | 22 | 40 | 28 | 0 | 34 | 466892 | 3735132 | 0 | 34 | 0 | 112 | 3817412 | 0 | 34 | 0 | 1 | 120 |
| 35 | 23 | 40 | 28 | 0 | 35 | 466906 | 3735244 | 0 | 35 | 0 | 112 | 3817532 | 0 | 35 | 0 | 1 | 120 |
| 36 | 24 | 40 | 28 | 0 | 36 | 466920 | 3735356 | 0 | 36 | 0 | 112 | 3817652 | 0 | 36 | 0 | 1 | 120 |
| 37 | 25 | 40 | 28 | 0 | 37 | 466934 | 3735468 | 0 | 37 | 0 | 112 | 3817772 | 0 | 37 | 0 | 1 | 120 |
| 38 | 26 | 40 | 28 | 0 | 38 | 466948 | 3735580 | 0 | 38 | 0 | 112 | 3817892 | 0 | 38 | 0 | 1 | 120 |
| 39 | 27 | 40 | 28 | 0 | 39 | 466962 | 3735692 | 0 | 39 | 0 | 112 | 3818012 | 0 | 39 | 0 | 1 | 120 |
| 40 | 28 | 40 | 28 | 1 | 0 | 466974 | 3735788 | 1 | 0 | 0 | 96 | 3818116 | 1 | 0 | 0 | 1 | 104 |
| 41 | 29 | 40 | 28 | 1 | 1 | 466991 | 3735924 | 1 | 1 | 0 | 136 | 3818260 | 1 | 1 | 0 | 1 | 144 |
| 42 | 2A | 40 | 28 | 1 | 2 | 467008 | 3736060 | 1 | 2 | 0 | 136 | 3818404 | 1 | 2 | 0 | 1 | 144 |
| 43 | 2B | 40 | 28 | 1 | 3 | 467025 | 3736196 | 1 | 3 | 0 | 136 | 3818548 | 1 | 3 | 0 | 1 | 144 |
| 44 | 2C | 40 | 28 | 1 | 4 | 467042 | 3736332 | 1 | 4 | 0 | 136 | 3818692 | 1 | 4 | 0 | 1 | 144 |
| 45 | 2D | 40 | 28 | 1 | 5 | 467059 | 3736468 | 1 | 5 | 0 | 136 | 3818836 | 1 | 5 | 0 | 1 | 144 |
| 46 | 2E | 40 | 28 | 1 | 6 | 467076 | 3736604 | 1 | 6 | 0 | 136 | 3818980 | 1 | 6 | 0 | 1 | 144 |
| 47 | 2F | 40 | 28 | 1 | 7 | 467093 | 3736740 | 1 | 7 | 0 | 136 | 3819124 | 1 | 7 | 0 | 1 | 144 |
| 48 | 30 | 40 | 28 | 1 | 8 | 467114 | 3736908 | 1 | 8 | 0 | 168 | 3819300 | 1 | 8 | 0 | 1 | 176 |
| 49 | 31 | 40 | 28 | 1 | 9 | 467135 | 3737076 | 1 | 9 | 0 | 168 | 3819476 | 1 | 9 | 0 | 1 | 176 |
| 50 | 32 | 40 | 28 | 1 | 10 | 467156 | 3737244 | 1 | 10 | 0 | 168 | 3819652 | 1 | 10 | 0 | 1 | 176 |
| 51 | 33 | 40 | 28 | 1 | 11 | 467177 | 3737412 | 1 | 11 | 0 | 168 | 3819828 | 1 | 11 | 0 | 1 | 176 |
| 52 | 34 | 40 | 28 | 1 | 12 | 467198 | 3737580 | 1 | 12 | 0 | 168 | 3820004 | 1 | 12 | 0 | 1 | 176 |
| 53 | 35 | 40 | 28 | 1 | 13 | 467219 | 3737748 | 1 | 13 | 0 | 168 | 3820180 | 1 | 13 | 0 | 1 | 176 |
| 54 | 36 | 40 | 28 | 1 | 14 | 467240 | 3737916 | 1 | 14 | 0 | 168 | 3820356 | 1 | 14 | 0 | 1 | 176 |
| 55 | 37 | 40 | 28 | 1 | 15 | 467261 | 3738084 | 1 | 15 | 0 | 168 | 3820532 | 1 | 15 | 0 | 1 | 176 |
| 56 | 38 | 40 | 28 | 1 | 16 | 467282 | 3738252 | 1 | 16 | 0 | 168 | 3820708 | 1 | 16 | 0 | 1 | 176 |

| | | | | | | | | | | | | | | | | | |
|---|---|---|---|---|---|---|---|---|---|---|---|---|---|---|---|---|---|
| 57 | 39 | 40 | 28 | 1 | 17 | 467303 | 3738420 | 1 | 17 | 0 | 168 | 3820884 | 1 | 17 | 0 | 1 | 176 |
| 58 | 3A | 40 | 28 | 1 | 18 | 467324 | 3738588 | 1 | 18 | 0 | 168 | 3821060 | 1 | 18 | 0 | 1 | 176 |
| 59 | 3B | 40 | 28 | 1 | 19 | 467345 | 3738756 | 1 | 19 | 0 | 168 | 3821236 | 1 | 19 | 0 | 1 | 176 |
| 60 | 3C | 40 | 28 | 1 | 20 | 467366 | 3738924 | 1 | 20 | 0 | 168 | 3821412 | 1 | 20 | 0 | 1 | 176 |
| 61 | 3D | 40 | 28 | 1 | 21 | 467387 | 3739092 | 1 | 21 | 0 | 168 | 3821588 | 1 | 21 | 0 | 1 | 176 |
| 62 | 3E | 40 | 28 | 1 | 22 | 467408 | 3739260 | 1 | 22 | 0 | 168 | 3821764 | 1 | 22 | 0 | 1 | 176 |
| 63 | 3F | 40 | 28 | 1 | 23 | 467429 | 3739428 | 1 | 23 | 0 | 168 | 3821940 | 1 | 23 | 0 | 1 | 176 |
| 64 | 40 | 40 | 28 | 1 | 24 | 467452 | 3739612 | 1 | 24 | 0 | 184 | 3822132 | 1 | 24 | 0 | 1 | 192 |
| 65 | 41 | 40 | 28 | 1 | 25 | 467475 | 3739796 | 1 | 25 | 0 | 184 | 3822324 | 1 | 25 | 0 | 1 | 192 |
| 66 | 42 | 40 | 28 | 1 | 26 | 467498 | 3739980 | 1 | 26 | 0 | 184 | 3822516 | 1 | 26 | 0 | 1 | 192 |
| 67 | 43 | 40 | 28 | 1 | 27 | 467521 | 3740164 | 1 | 27 | 0 | 184 | 3822708 | 1 | 27 | 0 | 1 | 192 |
| 68 | 44 | 40 | 28 | 1 | 28 | 467544 | 3740348 | 1 | 28 | 0 | 184 | 3822900 | 1 | 28 | 0 | 1 | 192 |
| 69 | 45 | 40 | 28 | 1 | 29 | 467567 | 3740532 | 1 | 29 | 0 | 184 | 3823092 | 1 | 29 | 0 | 1 | 192 |
| 70 | 46 | 40 | 28 | 1 | 30 | 467590 | 3740716 | 1 | 30 | 0 | 184 | 3823284 | 1 | 30 | 0 | 1 | 192 |
| 71 | 47 | 40 | 28 | 1 | 31 | 467613 | 3740900 | 1 | 31 | 0 | 184 | 3823476 | 1 | 31 | 0 | 1 | 192 |
| 72 | 48 | 40 | 28 | 1 | 32 | 467636 | 3741084 | 1 | 32 | 0 | 184 | 3823668 | 1 | 32 | 0 | 1 | 192 |
| 73 | 49 | 40 | 28 | 1 | 33 | 467659 | 3741268 | 1 | 33 | 0 | 184 | 3823860 | 1 | 33 | 0 | 1 | 192 |
| 74 | 4A | 40 | 28 | 1 | 34 | 467682 | 3741452 | 1 | 34 | 0 | 184 | 3824052 | 1 | 34 | 0 | 1 | 192 |
| 75 | 4B | 40 | 28 | 1 | 35 | 467705 | 3741636 | 1 | 35 | 0 | 184 | 3824244 | 1 | 35 | 0 | 1 | 192 |
| 76 | 4C | 40 | 28 | 1 | 36 | 467728 | 3741820 | 1 | 36 | 0 | 184 | 3824436 | 1 | 36 | 0 | 1 | 192 |
| 77 | 4D | 40 | 28 | 1 | 37 | 467751 | 3742004 | 1 | 37 | 0 | 184 | 3824628 | 1 | 37 | 0 | 1 | 192 |
| 78 | 4E | 40 | 28 | 1 | 38 | 467774 | 3742188 | 1 | 38 | 0 | 184 | 3824820 | 1 | 38 | 0 | 1 | 192 |
| 79 | 4F | 40 | 28 | 1 | 39 | 467797 | 3742372 | 1 | 39 | 0 | 184 | 3825012 | 1 | 39 | 0 | 1 | 192 |
| 80 | 50 | 40 | 28 | 2 | 0 | 467818 | 3742540 | 2 | 0 | 0 | 168 | 3825188 | 2 | 0 | 0 | 1 | 176 |
| 81 | 51 | 40 | 28 | 2 | 1 | 467844 | 3742748 | 2 | 1 | 0 | 208 | 3825404 | 2 | 1 | 0 | 1 | 216 |
| 82 | 52 | 40 | 28 | 2 | 2 | 467870 | 3742956 | 2 | 2 | 0 | 208 | 3825620 | 2 | 2 | 0 | 1 | 216 |
| 83 | 53 | 40 | 28 | 2 | 3 | 467896 | 3743164 | 2 | 3 | 0 | 208 | 3825836 | 2 | 3 | 0 | 1 | 216 |
| 84 | 54 | 40 | 28 | 2 | 4 | 467922 | 3743372 | 2 | 4 | 0 | 208 | 3826052 | 2 | 4 | 0 | 1 | 216 |
| 85 | 55 | 40 | 28 | 2 | 5 | 467948 | 3743580 | 2 | 5 | 0 | 208 | 3826268 | 2 | 5 | 0 | 1 | 216 |
| 86 | 56 | 40 | 28 | 2 | 6 | 467974 | 3743788 | 2 | 6 | 0 | 208 | 3826484 | 2 | 6 | 0 | 1 | 216 |
| 87 | 57 | 40 | 28 | 2 | 7 | 468000 | 3743996 | 2 | 7 | 0 | 208 | 3826700 | 2 | 7 | 0 | 1 | 216 |
| 88 | 58 | 40 | 28 | 2 | 8 | 468026 | 3744204 | 2 | 8 | 0 | 208 | 3826916 | 2 | 8 | 0 | 1 | 216 |
| 89 | 59 | 40 | 28 | 2 | 9 | 468052 | 3744412 | 2 | 9 | 0 | 208 | 3827132 | 2 | 9 | 0 | 1 | 216 |
| 90 | 5A | 40 | 28 | 2 | 10 | 468078 | 3744620 | 2 | 10 | 0 | 208 | 3827348 | 2 | 10 | 0 | 1 | 216 |
| 91 | 5B | 40 | 28 | 2 | 11 | 468104 | 3744828 | 2 | 11 | 0 | 208 | 3827564 | 2 | 11 | 0 | 1 | 216 |
| 92 | 5C | 40 | 28 | 2 | 12 | 468130 | 3745036 | 2 | 12 | 0 | 208 | 3827780 | 2 | 12 | 0 | 1 | 216 |
| 93 | 5D | 40 | 28 | 2 | 13 | 468156 | 3745244 | 2 | 13 | 0 | 208 | 3827996 | 2 | 13 | 0 | 1 | 216 |
| 94 | 5E | 40 | 28 | 2 | 14 | 468182 | 3745452 | 2 | 14 | 0 | 208 | 3828212 | 2 | 14 | 0 | 1 | 216 |
| 95 | 5F | 40 | 28 | 2 | 15 | 468208 | 3745660 | 2 | 15 | 0 | 208 | 3828428 | 2 | 15 | 0 | 1 | 216 |
| 96 | 60 | 40 | 28 | 2 | 16 | 468231 | 3745844 | 2 | 16 | 0 | 184 | 3828620 | 2 | 16 | 0 | 1 | 192 |
| 97 | 61 | 40 | 28 | 2 | 17 | 468254 | 3746028 | 2 | 17 | 0 | 184 | 3828812 | 2 | 17 | 0 | 1 | 192 |
| 98 | 62 | 40 | 28 | 2 | 18 | 468277 | 3746212 | 2 | 18 | 0 | 184 | 3829004 | 2 | 18 | 0 | 1 | 192 |
| 99 | 63 | 40 | 28 | 2 | 19 | 468300 | 3746396 | 2 | 19 | 0 | 184 | 3829196 | 2 | 19 | 0 | 1 | 192 |
| 100 | 64 | 40 | 28 | 2 | 20 | 468323 | 3746580 | 2 | 20 | 0 | 184 | 3829388 | 2 | 20 | 0 | 1 | 192 |
| 101 | 65 | 40 | 28 | 2 | 21 | 468346 | 3746764 | 2 | 21 | 0 | 184 | 3829580 | 2 | 21 | 0 | 1 | 192 |
| 102 | 66 | 40 | 28 | 2 | 22 | 468369 | 3746948 | 2 | 22 | 0 | 184 | 3829772 | 2 | 22 | 0 | 1 | 192 |
| 103 | 67 | 40 | 28 | 2 | 23 | 468392 | 3747132 | 2 | 23 | 0 | 184 | 3829964 | 2 | 23 | 0 | 1 | 192 |
| 104 | 68 | 40 | 28 | 2 | 24 | 468415 | 3747316 | 2 | 24 | 0 | 184 | 3830156 | 2 | 24 | 0 | 1 | 192 |
| 105 | 69 | 40 | 28 | 2 | 25 | 468438 | 3747500 | 2 | 25 | 0 | 184 | 3830348 | 2 | 25 | 0 | 1 | 192 |
| 106 | 6A | 40 | 28 | 2 | 26 | 468461 | 3747684 | 2 | 26 | 0 | 184 | 3830540 | 2 | 26 | 0 | 1 | 192 |
| 107 | 6B | 40 | 28 | 2 | 27 | 468484 | 3747868 | 2 | 27 | 0 | 184 | 3830732 | 2 | 27 | 0 | 1 | 192 |
| 108 | 6C | 40 | 28 | 2 | 28 | 468507 | 3748052 | 2 | 28 | 0 | 184 | 3830924 | 2 | 28 | 0 | 1 | 192 |
| 109 | 6D | 40 | 28 | 2 | 29 | 468530 | 3748236 | 2 | 29 | 0 | 184 | 3831116 | 2 | 29 | 0 | 1 | 192 |
| 110 | 6E | 40 | 28 | 2 | 30 | 468553 | 3748420 | 2 | 30 | 0 | 184 | 3831308 | 2 | 30 | 0 | 1 | 192 |
| 111 | 6F | 40 | 28 | 2 | 31 | 468576 | 3748604 | 2 | 31 | 0 | 184 | 3831500 | 2 | 31 | 0 | 1 | 192 |
| 112 | 70 | 40 | 28 | 2 | 32 | 468601 | 3748804 | 2 | 32 | 0 | 200 | 3831708 | 2 | 32 | 0 | 1 | 208 |
| 113 | 71 | 40 | 28 | 2 | 33 | 468626 | 3749004 | 2 | 33 | 0 | 200 | 3831916 | 2 | 33 | 0 | 1 | 208 |
| 114 | 72 | 40 | 28 | 2 | 34 | 468651 | 3749204 | 2 | 34 | 0 | 200 | 3832124 | 2 | 34 | 0 | 1 | 208 |
| 115 | 73 | 40 | 28 | 2 | 35 | 468676 | 3749404 | 2 | 35 | 0 | 200 | 3832332 | 2 | 35 | 0 | 1 | 208 |
| 116 | 74 | 40 | 28 | 2 | 36 | 468701 | 3749604 | 2 | 36 | 0 | 200 | 3832540 | 2 | 36 | 0 | 1 | 208 |
| 117 | 75 | 40 | 28 | 2 | 37 | 468726 | 3749804 | 2 | 37 | 0 | 200 | 3832748 | 2 | 37 | 0 | 1 | 208 |
| 118 | 76 | 40 | 28 | 2 | 38 | 468751 | 3750004 | 2 | 38 | 0 | 200 | 3832956 | 2 | 38 | 0 | 1 | 208 |
| 119 | 77 | 40 | 28 | 2 | 39 | 468776 | 3750204 | 2 | 39 | 0 | 200 | 3833164 | 2 | 39 | 0 | 1 | 208 |
| 120 | 78 | 40 | 28 | 3 | 0 | 468799 | 3750388 | 3 | 0 | 0 | 184 | 3833356 | 3 | 0 | 0 | 1 | 192 |
| 121 | 79 | 40 | 28 | 3 | 1 | 468827 | 3750612 | 3 | 1 | 0 | 224 | 3833588 | 3 | 1 | 0 | 1 | 232 |
| 122 | 7A | 40 | 28 | 3 | 2 | 468855 | 3750836 | 3 | 2 | 0 | 224 | 3833820 | 3 | 2 | 0 | 1 | 232 |
| 123 | 7B | 40 | 28 | 3 | 3 | 468883 | 3751060 | 3 | 3 | 0 | 224 | 3834052 | 3 | 3 | 0 | 1 | 232 |
| 124 | 7C | 40 | 28 | 3 | 4 | 468911 | 3751284 | 3 | 4 | 0 | 224 | 3834284 | 3 | 4 | 0 | 1 | 232 |
| 125 | 7D | 40 | 28 | 3 | 5 | 468939 | 3751508 | 3 | 5 | 0 | 224 | 3834516 | 3 | 5 | 0 | 1 | 232 |
| 126 | 7E | 40 | 28 | 3 | 6 | 468967 | 3751732 | 3 | 6 | 0 | 224 | 3834748 | 3 | 6 | 0 | 1 | 232 |
| 127 | 7F | 40 | 28 | 3 | 7 | 468995 | 3751956 | 3 | 7 | 0 | 224 | 3834980 | 3 | 7 | 0 | 1 | 232 |
| 128 | 80 | 40 | 28 | 3 | 8 | 469023 | 3752180 | 3 | 8 | 0 | 224 | 3835212 | 3 | 8 | 0 | 1 | 232 |
| 129 | 81 | 40 | 28 | 3 | 9 | 469051 | 3752404 | 3 | 9 | 0 | 224 | 3835444 | 3 | 9 | 0 | 1 | 232 |
| 130 | 82 | 40 | 28 | 3 | 10 | 469079 | 3752628 | 3 | 10 | 0 | 224 | 3835676 | 3 | 10 | 0 | 1 | 232 |
| 131 | 83 | 40 | 28 | 3 | 11 | 469107 | 3752852 | 3 | 11 | 0 | 224 | 3835908 | 3 | 11 | 0 | 1 | 232 |
| 132 | 84 | 40 | 28 | 3 | 12 | 469135 | 3753076 | 3 | 12 | 0 | 224 | 3836140 | 3 | 12 | 0 | 1 | 232 |
| 133 | 85 | 40 | 28 | 3 | 13 | 469163 | 3753300 | 3 | 13 | 0 | 224 | 3836372 | 3 | 13 | 0 | 1 | 232 |
| 134 | 86 | 40 | 28 | 3 | 14 | 469191 | 3753524 | 3 | 14 | 0 | 224 | 3836604 | 3 | 14 | 0 | 1 | 232 |
| 135 | 87 | 40 | 28 | 3 | 15 | 469219 | 3753748 | 3 | 15 | 0 | 224 | 3836836 | 3 | 15 | 0 | 1 | 232 |
| 136 | 88 | 40 | 28 | 3 | 16 | 469247 | 3753972 | 3 | 16 | 0 | 224 | 3837068 | 3 | 16 | 0 | 1 | 232 |
| 137 | 89 | 40 | 28 | 3 | 17 | 469275 | 3754196 | 3 | 17 | 0 | 224 | 3837300 | 3 | 17 | 0 | 1 | 232 |
| 138 | 8A | 40 | 28 | 3 | 18 | 469303 | 3754420 | 3 | 18 | 0 | 224 | 3837532 | 3 | 18 | 0 | 1 | 232 |
| 139 | 8B | 40 | 28 | 3 | 19 | 469331 | 3754644 | 3 | 19 | 0 | 224 | 3837764 | 3 | 19 | 0 | 1 | 232 |
| 140 | 8C | 40 | 28 | 3 | 20 | 469359 | 3754868 | 3 | 20 | 0 | 224 | 3837996 | 3 | 20 | 0 | 1 | 232 |
| 141 | 8D | 40 | 28 | 3 | 21 | 469387 | 3755092 | 3 | 21 | 0 | 224 | 3838228 | 3 | 21 | 0 | 1 | 232 |
| 142 | 8E | 40 | 28 | 3 | 22 | 469415 | 3755316 | 3 | 22 | 0 | 224 | 3838460 | 3 | 22 | 0 | 1 | 232 |
| 143 | 8F | 40 | 28 | 3 | 23 | 469443 | 3755540 | 3 | 23 | 0 | 224 | 3838692 | 3 | 23 | 0 | 1 | 232 |
| 144 | 90 | 40 | 28 | 3 | 24 | 469468 | 3755740 | 3 | 24 | 0 | 200 | 3838900 | 3 | 24 | 0 | 1 | 208 |
| 145 | 91 | 40 | 28 | 3 | 25 | 469493 | 3755940 | 3 | 25 | 0 | 200 | 3839108 | 3 | 25 | 0 | 1 | 208 |
| 146 | 92 | 40 | 28 | 3 | 26 | 469518 | 3756140 | 3 | 26 | 0 | 200 | 3839316 | 3 | 26 | 0 | 1 | 208 |
| 147 | 93 | 40 | 28 | 3 | 27 | 469543 | 3756340 | 3 | 27 | 0 | 200 | 3839524 | 3 | 27 | 0 | 1 | 208 |
| 148 | 94 | 40 | 28 | 3 | 28 | 469568 | 3756540 | 3 | 28 | 0 | 200 | 3839732 | 3 | 28 | 0 | 1 | 208 |
| 149 | 95 | 40 | 28 | 3 | 29 | 469593 | 3756740 | 3 | 29 | 0 | 200 | 3839940 | 3 | 29 | 0 | 1 | 208 |
| 150 | 96 | 40 | 28 | 3 | 30 | 469618 | 3756940 | 3 | 30 | 0 | 200 | 3840148 | 3 | 30 | 0 | 1 | 208 |
| 151 | 97 | 40 | 28 | 3 | 31 | 469643 | 3757140 | 3 | 31 | 0 | 200 | 3840356 | 3 | 31 | 0 | 1 | 208 |
| 152 | 98 | 40 | 28 | 3 | 32 | 469668 | 3757340 | 3 | 32 | 0 | 200 | 3840564 | 3 | 32 | 0 | 1 | 208 |
| 153 | 99 | 40 | 28 | 3 | 33 | 469693 | 3757540 | 3 | 33 | 0 | 200 | 3840772 | 3 | 33 | 0 | 1 | 208 |
| 154 | 9A | 40 | 28 | 3 | 34 | 469718 | 3757740 | 3 | 34 | 0 | 200 | 3840980 | 3 | 34 | 0 | 1 | 208 |
| 155 | 9B | 40 | 28 | 3 | 35 | 469743 | 3757940 | 3 | 35 | 0 | 200 | 3841188 | 3 | 35 | 0 | 1 | 208 |
| 156 | 9C | 40 | 28 | 3 | 36 | 469768 | 3758140 | 3 | 36 | 0 | 200 | 3841396 | 3 | 36 | 0 | 1 | 208 |
| 157 | 9D | 40 | 28 | 3 | 37 | 469793 | 3758340 | 3 | 37 | 0 | 200 | 3841604 | 3 | 37 | 0 | 1 | 208 |
| 158 | 9E | 40 | 28 | 3 | 38 | 469818 | 3758540 | 3 | 38 | 0 | 200 | 3841812 | 3 | 38 | 0 | 1 | 208 |
| 159 | 9F | 40 | 28 | 3 | 39 | 469843 | 3758740 | 3 | 39 | 0 | 200 | 3842020 | 3 | 39 | 0 | 1 | 208 |

| | | | | | | | | | | | | | | | | | |
|---|---|---|---|---|---|---|---|---|---|---|---|---|---|---|---|---|---|
| 160 | A0 | 40 | 28 | 4 | 0 | 469868 | 3758940 | 4 | 0 | 0 | 200 | 3842228 | 4 | 0 | 0 | 1 | 208 |
| 161 | A1 | 40 | 28 | 4 | 1 | 469898 | 3759180 | 4 | 1 | 0 | 240 | 3842476 | 4 | 1 | 0 | 1 | 248 |
| 162 | A2 | 40 | 28 | 4 | 2 | 469928 | 3759420 | 4 | 2 | 0 | 240 | 3842724 | 4 | 2 | 0 | 1 | 248 |
| 163 | A3 | 40 | 28 | 4 | 3 | 469958 | 3759660 | 4 | 3 | 0 | 240 | 3842972 | 4 | 3 | 0 | 1 | 248 |
| 164 | A4 | 40 | 28 | 4 | 4 | 469988 | 3759900 | 4 | 4 | 0 | 240 | 3843220 | 4 | 4 | 0 | 1 | 248 |
| 165 | A5 | 40 | 28 | 4 | 5 | 470018 | 3760140 | 4 | 5 | 0 | 240 | 3843468 | 4 | 5 | 0 | 1 | 248 |
| 166 | A6 | 40 | 28 | 4 | 6 | 470048 | 3760380 | 4 | 6 | 0 | 240 | 3843716 | 4 | 6 | 0 | 1 | 248 |
| 167 | A7 | 40 | 28 | 4 | 7 | 470078 | 3760620 | 4 | 7 | 0 | 240 | 3843964 | 4 | 7 | 0 | 1 | 248 |
| 168 | A8 | 40 | 28 | 4 | 8 | 470108 | 3760860 | 4 | 8 | 0 | 240 | 3844212 | 4 | 8 | 0 | 1 | 248 |
| 169 | A9 | 40 | 28 | 4 | 9 | 470138 | 3761100 | 4 | 9 | 0 | 240 | 3844460 | 4 | 9 | 0 | 1 | 248 |
| 170 | AA | 40 | 28 | 4 | 10 | 470168 | 3761340 | 4 | 10 | 0 | 240 | 3844708 | 4 | 10 | 0 | 1 | 248 |
| 171 | AB | 40 | 28 | 4 | 11 | 470198 | 3761580 | 4 | 11 | 0 | 240 | 3844956 | 4 | 11 | 0 | 1 | 248 |
| 172 | AC | 40 | 28 | 4 | 12 | 470228 | 3761820 | 4 | 12 | 0 | 240 | 3845204 | 4 | 12 | 0 | 1 | 248 |
| 173 | AD | 40 | 28 | 4 | 13 | 470258 | 3762060 | 4 | 13 | 0 | 240 | 3845452 | 4 | 13 | 0 | 1 | 248 |
| 174 | AE | 40 | 28 | 4 | 14 | 470288 | 3762300 | 4 | 14 | 0 | 240 | 3845700 | 4 | 14 | 0 | 1 | 248 |
| 175 | AF | 40 | 28 | 4 | 15 | 470318 | 3762540 | 4 | 15 | 0 | 240 | 3845948 | 4 | 15 | 0 | 1 | 248 |
| 176 | B0 | 40 | 28 | 4 | 16 | 470348 | 3762780 | 4 | 16 | 0 | 240 | 3846196 | 4 | 16 | 0 | 1 | 248 |
| 177 | B1 | 40 | 28 | 4 | 17 | 470378 | 3763020 | 4 | 17 | 0 | 240 | 3846444 | 4 | 17 | 0 | 1 | 248 |
| 178 | B2 | 40 | 28 | 4 | 18 | 470408 | 3763260 | 4 | 18 | 0 | 240 | 3846692 | 4 | 18 | 0 | 1 | 248 |
| 179 | B3 | 40 | 28 | 4 | 19 | 470438 | 3763500 | 4 | 19 | 0 | 240 | 3846940 | 4 | 19 | 0 | 1 | 248 |
| 180 | B4 | 40 | 28 | 4 | 20 | 470468 | 3763740 | 4 | 20 | 0 | 240 | 3847188 | 4 | 20 | 0 | 1 | 248 |
| 181 | B5 | 40 | 28 | 4 | 21 | 470498 | 3763980 | 4 | 21 | 0 | 240 | 3847436 | 4 | 21 | 0 | 1 | 248 |
| 182 | B6 | 40 | 28 | 4 | 22 | 470528 | 3764220 | 4 | 22 | 0 | 240 | 3847684 | 4 | 22 | 0 | 1 | 248 |
| 183 | B7 | 40 | 28 | 4 | 23 | 470558 | 3764460 | 4 | 23 | 0 | 240 | 3847932 | 4 | 23 | 0 | 1 | 248 |
| 184 | B8 | 40 | 28 | 4 | 24 | 470588 | 3764700 | 4 | 24 | 0 | 240 | 3848180 | 4 | 24 | 0 | 1 | 248 |
| 185 | B9 | 40 | 28 | 4 | 25 | 470618 | 3764940 | 4 | 25 | 0 | 240 | 3848428 | 4 | 25 | 0 | 1 | 248 |
| 186 | BA | 40 | 28 | 4 | 26 | 470648 | 3765180 | 4 | 26 | 0 | 240 | 3848676 | 4 | 26 | 0 | 1 | 248 |
| 187 | BB | 40 | 28 | 4 | 27 | 470678 | 3765420 | 4 | 27 | 0 | 240 | 3848924 | 4 | 27 | 0 | 1 | 248 |
| 188 | BC | 40 | 28 | 4 | 28 | 470708 | 3765660 | 4 | 28 | 0 | 240 | 3849172 | 4 | 28 | 0 | 1 | 248 |
| 189 | BD | 40 | 28 | 4 | 29 | 470738 | 3765900 | 4 | 29 | 0 | 240 | 3849420 | 4 | 29 | 0 | 1 | 248 |
| 190 | BE | 40 | 28 | 4 | 30 | 470768 | 3766140 | 4 | 30 | 0 | 240 | 3849668 | 4 | 30 | 0 | 1 | 248 |
| 191 | BF | 40 | 28 | 4 | 31 | 470798 | 3766380 | 4 | 31 | 0 | 240 | 3849916 | 4 | 31 | 0 | 1 | 248 |
| 192 | C0 | 40 | 28 | 4 | 32 | 470825 | 3766596 | 4 | 32 | 0 | 216 | 3850140 | 4 | 32 | 0 | 1 | 224 |
| 193 | C1 | 40 | 28 | 4 | 33 | 470852 | 3766812 | 4 | 33 | 0 | 216 | 3850364 | 4 | 33 | 0 | 1 | 224 |
| 194 | C2 | 40 | 28 | 4 | 34 | 470879 | 3767028 | 4 | 34 | 0 | 216 | 3850588 | 4 | 34 | 0 | 1 | 224 |
| 195 | C3 | 40 | 28 | 4 | 35 | 470906 | 3767244 | 4 | 35 | 0 | 216 | 3850812 | 4 | 35 | 0 | 1 | 224 |
| 196 | C4 | 40 | 28 | 4 | 36 | 470933 | 3767460 | 4 | 36 | 0 | 216 | 3851036 | 4 | 36 | 0 | 1 | 224 |
| 197 | C5 | 40 | 28 | 4 | 37 | 470960 | 3767676 | 4 | 37 | 0 | 216 | 3851260 | 4 | 37 | 0 | 1 | 224 |
| 198 | C6 | 40 | 28 | 4 | 38 | 470987 | 3767892 | 4 | 38 | 0 | 216 | 3851484 | 4 | 38 | 0 | 1 | 224 |
| 199 | C7 | 40 | 28 | 4 | 39 | 471014 | 3768108 | 4 | 39 | 0 | 216 | 3851708 | 4 | 39 | 0 | 1 | 224 |
| 200 | C8 | 40 | 28 | 5 | 0 | 471039 | 3768308 | 5 | 0 | 0 | 200 | 3851916 | 5 | 0 | 0 | 1 | 208 |
| 201 | C9 | 40 | 28 | 5 | 1 | 471069 | 3768548 | 5 | 1 | 0 | 240 | 3852164 | 5 | 1 | 0 | 1 | 248 |
| 202 | CA | 40 | 28 | 5 | 2 | 471099 | 3768788 | 5 | 2 | 0 | 240 | 3852412 | 5 | 2 | 0 | 1 | 248 |
| 203 | CB | 40 | 28 | 5 | 3 | 471129 | 3769028 | 5 | 3 | 0 | 240 | 3852660 | 5 | 3 | 0 | 1 | 248 |
| 204 | CC | 40 | 28 | 5 | 4 | 471159 | 3769268 | 5 | 4 | 0 | 240 | 3852908 | 5 | 4 | 0 | 1 | 248 |
| 205 | CD | 40 | 28 | 5 | 5 | 471189 | 3769508 | 5 | 5 | 0 | 240 | 3853156 | 5 | 5 | 0 | 1 | 248 |
| 206 | CE | 40 | 28 | 5 | 6 | 471219 | 3769748 | 5 | 6 | 0 | 240 | 3853404 | 5 | 6 | 0 | 1 | 248 |
| 207 | CF | 40 | 28 | 5 | 7 | 471249 | 3769988 | 5 | 7 | 0 | 240 | 3853652 | 5 | 7 | 0 | 1 | 248 |
| 208 | D0 | 40 | 28 | 5 | 8 | 471281 | 3770244 | 5 | 8 | 0 | 256 | 3853916 | 5 | 8 | 0 | 1 | 264 |
| 209 | D1 | 40 | 28 | 5 | 9 | 471313 | 3770500 | 5 | 9 | 0 | 256 | 3854180 | 5 | 9 | 0 | 1 | 264 |
| 210 | D2 | 40 | 28 | 5 | 10 | 471345 | 3770756 | 5 | 10 | 0 | 256 | 3854444 | 5 | 10 | 0 | 1 | 264 |
| 211 | D3 | 40 | 28 | 5 | 11 | 471377 | 3771012 | 5 | 11 | 0 | 256 | 3854708 | 5 | 11 | 0 | 1 | 264 |
| 212 | D4 | 40 | 28 | 5 | 12 | 471409 | 3771268 | 5 | 12 | 0 | 256 | 3854972 | 5 | 12 | 0 | 1 | 264 |
| 213 | D5 | 40 | 28 | 5 | 13 | 471441 | 3771524 | 5 | 13 | 0 | 256 | 3855236 | 5 | 13 | 0 | 1 | 264 |
| 214 | D6 | 40 | 28 | 5 | 14 | 471473 | 3771780 | 5 | 14 | 0 | 256 | 3855500 | 5 | 14 | 0 | 1 | 264 |
| 215 | D7 | 40 | 28 | 5 | 15 | 471505 | 3772036 | 5 | 15 | 0 | 256 | 3855764 | 5 | 15 | 0 | 1 | 264 |
| 216 | D8 | 40 | 28 | 5 | 16 | 471537 | 3772292 | 5 | 16 | 0 | 256 | 3856028 | 5 | 16 | 0 | 1 | 264 |
| 217 | D9 | 40 | 28 | 5 | 17 | 471569 | 3772548 | 5 | 17 | 0 | 256 | 3856292 | 5 | 17 | 0 | 1 | 264 |
| 218 | DA | 40 | 28 | 5 | 18 | 471601 | 3772804 | 5 | 18 | 0 | 256 | 3856556 | 5 | 18 | 0 | 1 | 264 |
| 219 | DB | 40 | 28 | 5 | 19 | 471633 | 3773060 | 5 | 19 | 0 | 256 | 3856820 | 5 | 19 | 0 | 1 | 264 |
| 220 | DC | 40 | 28 | 5 | 20 | 471665 | 3773316 | 5 | 20 | 0 | 256 | 3857084 | 5 | 20 | 0 | 1 | 264 |
| 221 | DD | 40 | 28 | 5 | 21 | 471697 | 3773572 | 5 | 21 | 0 | 256 | 3857348 | 5 | 21 | 0 | 1 | 264 |
| 222 | DE | 40 | 28 | 5 | 22 | 471729 | 3773828 | 5 | 22 | 0 | 256 | 3857612 | 5 | 22 | 0 | 1 | 264 |
| 223 | DF | 40 | 28 | 5 | 23 | 471761 | 3774084 | 5 | 23 | 0 | 256 | 3857876 | 5 | 23 | 0 | 1 | 264 |
| 224 | E0 | 40 | 28 | 5 | 24 | 471793 | 3774340 | 5 | 24 | 0 | 256 | 3858140 | 5 | 24 | 0 | 1 | 264 |
| 225 | E1 | 40 | 28 | 5 | 25 | 471825 | 3774596 | 5 | 25 | 0 | 256 | 3858404 | 5 | 25 | 0 | 1 | 264 |
| 226 | E2 | 40 | 28 | 5 | 26 | 471857 | 3774852 | 5 | 26 | 0 | 256 | 3858668 | 5 | 26 | 0 | 1 | 264 |
| 227 | E3 | 40 | 28 | 5 | 27 | 471889 | 3775108 | 5 | 27 | 0 | 256 | 3858932 | 5 | 27 | 0 | 1 | 264 |
| 228 | E4 | 40 | 28 | 5 | 28 | 471921 | 3775364 | 5 | 28 | 0 | 256 | 3859196 | 5 | 28 | 0 | 1 | 264 |
| 229 | E5 | 40 | 28 | 5 | 29 | 471953 | 3775620 | 5 | 29 | 0 | 256 | 3859460 | 5 | 29 | 0 | 1 | 264 |
| 230 | E6 | 40 | 28 | 5 | 30 | 471985 | 3775876 | 5 | 30 | 0 | 256 | 3859724 | 5 | 30 | 0 | 1 | 264 |
| 231 | E7 | 40 | 28 | 5 | 31 | 472017 | 3776132 | 5 | 31 | 0 | 256 | 3859988 | 5 | 31 | 0 | 1 | 264 |
| 232 | E8 | 40 | 28 | 5 | 32 | 472049 | 3776388 | 5 | 32 | 0 | 256 | 3860252 | 5 | 32 | 0 | 1 | 264 |
| 233 | E9 | 40 | 28 | 5 | 33 | 472081 | 3776644 | 5 | 33 | 0 | 256 | 3860516 | 5 | 33 | 0 | 1 | 264 |
| 234 | EA | 40 | 28 | 5 | 34 | 472113 | 3776900 | 5 | 34 | 0 | 256 | 3860780 | 5 | 34 | 0 | 1 | 264 |
| 235 | EB | 40 | 28 | 5 | 35 | 472145 | 3777156 | 5 | 35 | 0 | 256 | 3861044 | 5 | 35 | 0 | 1 | 264 |
| 236 | EC | 40 | 28 | 5 | 36 | 472177 | 3777412 | 5 | 36 | 0 | 256 | 3861308 | 5 | 36 | 0 | 1 | 264 |
| 237 | ED | 40 | 28 | 5 | 37 | 472209 | 3777668 | 5 | 37 | 0 | 256 | 3861572 | 5 | 37 | 0 | 1 | 264 |
| 238 | EE | 40 | 28 | 5 | 38 | 472241 | 3777924 | 5 | 38 | 0 | 256 | 3861836 | 5 | 38 | 0 | 1 | 264 |
| 239 | EF | 40 | 28 | 5 | 39 | 472273 | 3778180 | 5 | 39 | 0 | 256 | 3862100 | 5 | 39 | 0 | 1 | 264 |
| 240 | F0 | 40 | 28 | 6 | 0 | 472300 | 3778396 | 6 | 0 | 0 | 216 | 3862324 | 6 | 0 | 0 | 1 | 224 |
| 241 | F1 | 40 | 28 | 6 | 1 | 472332 | 3778652 | 6 | 1 | 0 | 256 | 3862588 | 6 | 1 | 0 | 1 | 264 |
| 242 | F2 | 40 | 28 | 6 | 2 | 472364 | 3778908 | 6 | 2 | 0 | 256 | 3862852 | 6 | 2 | 0 | 1 | 264 |
| 243 | F3 | 40 | 28 | 6 | 3 | 472396 | 3779164 | 6 | 3 | 0 | 256 | 3863116 | 6 | 3 | 0 | 1 | 264 |
| 244 | F4 | 40 | 28 | 6 | 4 | 472428 | 3779420 | 6 | 4 | 0 | 256 | 3863380 | 6 | 4 | 0 | 1 | 264 |
| 245 | F5 | 40 | 28 | 6 | 5 | 472460 | 3779676 | 6 | 5 | 0 | 256 | 3863644 | 6 | 5 | 0 | 1 | 264 |
| 246 | F6 | 40 | 28 | 6 | 6 | 472492 | 3779932 | 6 | 6 | 0 | 256 | 3863908 | 6 | 6 | 0 | 1 | 264 |
| 247 | F7 | 40 | 28 | 6 | 7 | 472524 | 3780188 | 6 | 7 | 0 | 256 | 3864172 | 6 | 7 | 0 | 1 | 264 |
| 248 | F8 | 40 | 28 | 6 | 8 | 472556 | 3780444 | 6 | 8 | 0 | 256 | 3864436 | 6 | 8 | 0 | 1 | 264 |
| 249 | F9 | 40 | 28 | 6 | 9 | 472588 | 3780700 | 6 | 9 | 0 | 256 | 3864700 | 6 | 9 | 0 | 1 | 264 |
| 250 | FA | 40 | 28 | 6 | 10 | 472620 | 3780956 | 6 | 10 | 0 | 256 | 3864964 | 6 | 10 | 0 | 1 | 264 |
| 251 | FB | 40 | 28 | 6 | 11 | 472652 | 3781212 | 6 | 11 | 0 | 256 | 3865228 | 6 | 11 | 0 | 1 | 264 |
| 252 | FC | 40 | 28 | 6 | 12 | 472684 | 3781468 | 6 | 12 | 0 | 256 | 3865492 | 6 | 12 | 0 | 1 | 264 |
| 253 | FD | 40 | 28 | 6 | 13 | 472716 | 3781724 | 6 | 13 | 0 | 256 | 3865756 | 6 | 13 | 0 | 1 | 264 |
| 254 | FE | 40 | 28 | 6 | 14 | 472748 | 3781980 | 6 | 14 | 0 | 256 | 3866020 | 6 | 14 | 0 | 1 | 264 |
| 255 | FF | 40 | 28 | 6 | 15 | 472780 | 3782236 | 6 | 15 | 0 | 256 | 3866284 | 6 | 15 | 0 | 1 | 264 |
| 0 | 00 | 41 | 29 | 0 | 0 | 472792 | 3782332 | 0 | 0 | 0 | 96 | 3866388 | 0 | 0 | 0 | 1 | 104 |
| 1 | 01 | 41 | 29 | 0 | 1 | 472806 | 3782444 | 0 | 1 | 0 | 112 | 3866508 | 0 | 1 | 0 | 1 | 120 |
| 2 | 02 | 41 | 29 | 0 | 2 | 472820 | 3782556 | 0 | 2 | 0 | 112 | 3866628 | 0 | 2 | 0 | 1 | 120 |
| 3 | 03 | 41 | 29 | 0 | 3 | 472834 | 3782668 | 0 | 3 | 0 | 112 | 3866748 | 0 | 3 | 0 | 1 | 120 |
| 4 | 04 | 41 | 29 | 0 | 4 | 472848 | 3782780 | 0 | 4 | 0 | 112 | 3866868 | 0 | 4 | 0 | 1 | 120 |
| 5 | 05 | 41 | 29 | 0 | 5 | 472862 | 3782892 | 0 | 5 | 0 | 112 | 3866988 | 0 | 5 | 0 | 1 | 120 |
| 6 | 06 | 41 | 29 | 0 | 6 | 472876 | 3783004 | 0 | 6 | 0 | 112 | 3867108 | 0 | 6 | 0 | 1 | 120 |

| | | | | | | | | | | | | | | | | | | |
|---|---|---|---|---|---|---|---|---|---|---|---|---|---|---|---|---|---|---|
| 7 | 07 | 41 | 29 | 0 | 7 | 472890 | 3783116 | 0 | 7 | 0 | 112 | 3867228 | 0 | 7 | 0 | 1 | 120 |
| 8 | 08 | 41 | 29 | 0 | 8 | 472904 | 3783228 | 0 | 8 | 0 | 112 | 3867348 | 0 | 8 | 0 | 1 | 120 |
| 9 | 09 | 41 | 29 | 0 | 9 | 472918 | 3783340 | 0 | 9 | 0 | 112 | 3867468 | 0 | 9 | 0 | 1 | 120 |
| 10 | 0A | 41 | 29 | 0 | 10 | 472932 | 3783452 | 0 | 10 | 0 | 112 | 3867588 | 0 | 10 | 0 | 1 | 120 |
| 11 | 0B | 41 | 29 | 0 | 11 | 472946 | 3783564 | 0 | 11 | 0 | 112 | 3867708 | 0 | 11 | 0 | 1 | 120 |
| 12 | 0C | 41 | 29 | 0 | 12 | 472960 | 3783676 | 0 | 12 | 0 | 112 | 3867828 | 0 | 12 | 0 | 1 | 120 |
| 13 | 0D | 41 | 29 | 0 | 13 | 472974 | 3783788 | 0 | 13 | 0 | 112 | 3867948 | 0 | 13 | 0 | 1 | 120 |
| 14 | 0E | 41 | 29 | 0 | 14 | 472988 | 3783900 | 0 | 14 | 0 | 112 | 3868068 | 0 | 14 | 0 | 1 | 120 |
| 15 | 0F | 41 | 29 | 0 | 15 | 473002 | 3784012 | 0 | 15 | 0 | 112 | 3868188 | 0 | 15 | 0 | 1 | 120 |
| 16 | 10 | 41 | 29 | 0 | 16 | 473016 | 3784124 | 0 | 16 | 0 | 112 | 3868308 | 0 | 16 | 0 | 1 | 120 |
| 17 | 11 | 41 | 29 | 0 | 17 | 473030 | 3784236 | 0 | 17 | 0 | 112 | 3868428 | 0 | 17 | 0 | 1 | 120 |
| 18 | 12 | 41 | 29 | 0 | 18 | 473044 | 3784348 | 0 | 18 | 0 | 112 | 3868548 | 0 | 18 | 0 | 1 | 120 |
| 19 | 13 | 41 | 29 | 0 | 19 | 473058 | 3784460 | 0 | 19 | 0 | 112 | 3868668 | 0 | 19 | 0 | 1 | 120 |
| 20 | 14 | 41 | 29 | 0 | 20 | 473072 | 3784572 | 0 | 20 | 0 | 112 | 3868788 | 0 | 20 | 0 | 1 | 120 |
| 21 | 15 | 41 | 29 | 0 | 21 | 473086 | 3784684 | 0 | 21 | 0 | 112 | 3868908 | 0 | 21 | 0 | 1 | 120 |
| 22 | 16 | 41 | 29 | 0 | 22 | 473100 | 3784796 | 0 | 22 | 0 | 112 | 3869028 | 0 | 22 | 0 | 1 | 120 |
| 23 | 17 | 41 | 29 | 0 | 23 | 473114 | 3784908 | 0 | 23 | 0 | 112 | 3869148 | 0 | 23 | 0 | 1 | 120 |
| 24 | 18 | 41 | 29 | 0 | 24 | 473128 | 3785020 | 0 | 24 | 0 | 112 | 3869268 | 0 | 24 | 0 | 1 | 120 |
| 25 | 19 | 41 | 29 | 0 | 25 | 473142 | 3785132 | 0 | 25 | 0 | 112 | 3869388 | 0 | 25 | 0 | 1 | 120 |
| 26 | 1A | 41 | 29 | 0 | 26 | 473156 | 3785244 | 0 | 26 | 0 | 112 | 3869508 | 0 | 26 | 0 | 1 | 120 |
| 27 | 1B | 41 | 29 | 0 | 27 | 473170 | 3785356 | 0 | 27 | 0 | 112 | 3869628 | 0 | 27 | 0 | 1 | 120 |
| 28 | 1C | 41 | 29 | 0 | 28 | 473184 | 3785468 | 0 | 28 | 0 | 112 | 3869748 | 0 | 28 | 0 | 1 | 120 |
| 29 | 1D | 41 | 29 | 0 | 29 | 473198 | 3785580 | 0 | 29 | 0 | 112 | 3869868 | 0 | 29 | 0 | 1 | 120 |
| 30 | 1E | 41 | 29 | 0 | 30 | 473212 | 3785692 | 0 | 30 | 0 | 112 | 3869988 | 0 | 30 | 0 | 1 | 120 |
| 31 | 1F | 41 | 29 | 0 | 31 | 473226 | 3785804 | 0 | 31 | 0 | 112 | 3870108 | 0 | 31 | 0 | 1 | 120 |
| 32 | 20 | 41 | 29 | 0 | 32 | 473240 | 3785916 | 0 | 32 | 0 | 112 | 3870228 | 0 | 32 | 0 | 1 | 120 |
| 33 | 21 | 41 | 29 | 0 | 33 | 473254 | 3786028 | 0 | 33 | 0 | 112 | 3870348 | 0 | 33 | 0 | 1 | 120 |
| 34 | 22 | 41 | 29 | 0 | 34 | 473268 | 3786140 | 0 | 34 | 0 | 112 | 3870468 | 0 | 34 | 0 | 1 | 120 |
| 35 | 23 | 41 | 29 | 0 | 35 | 473282 | 3786252 | 0 | 35 | 0 | 112 | 3870588 | 0 | 35 | 0 | 1 | 120 |
| 36 | 24 | 41 | 29 | 0 | 36 | 473296 | 3786364 | 0 | 36 | 0 | 112 | 3870708 | 0 | 36 | 0 | 1 | 120 |
| 37 | 25 | 41 | 29 | 0 | 37 | 473310 | 3786476 | 0 | 37 | 0 | 112 | 3870828 | 0 | 37 | 0 | 1 | 120 |
| 38 | 26 | 41 | 29 | 0 | 38 | 473324 | 3786588 | 0 | 38 | 0 | 112 | 3870948 | 0 | 38 | 0 | 1 | 120 |
| 39 | 27 | 41 | 29 | 0 | 39 | 473338 | 3786700 | 0 | 39 | 0 | 112 | 3871068 | 0 | 39 | 0 | 1 | 120 |
| 40 | 28 | 41 | 29 | 0 | 40 | 473352 | 3786812 | 0 | 40 | 0 | 112 | 3871188 | 0 | 40 | 0 | 1 | 120 |
| 41 | 29 | 41 | 29 | 1 | 0 | 473364 | 3786908 | 1 | 0 | 0 | 96 | 3871292 | 1 | 0 | 0 | 1 | 104 |
| 42 | 2A | 41 | 29 | 1 | 1 | 473381 | 3787044 | 1 | 1 | 0 | 136 | 3871436 | 1 | 1 | 0 | 1 | 144 |
| 43 | 2B | 41 | 29 | 1 | 2 | 473398 | 3787180 | 1 | 2 | 0 | 136 | 3871580 | 1 | 2 | 0 | 1 | 144 |
| 44 | 2C | 41 | 29 | 1 | 3 | 473415 | 3787316 | 1 | 3 | 0 | 136 | 3871724 | 1 | 3 | 0 | 1 | 144 |
| 45 | 2D | 41 | 29 | 1 | 4 | 473432 | 3787452 | 1 | 4 | 0 | 136 | 3871868 | 1 | 4 | 0 | 1 | 144 |
| 46 | 2E | 41 | 29 | 1 | 5 | 473449 | 3787588 | 1 | 5 | 0 | 136 | 3872012 | 1 | 5 | 0 | 1 | 144 |
| 47 | 2F | 41 | 29 | 1 | 6 | 473466 | 3787724 | 1 | 6 | 0 | 136 | 3872156 | 1 | 6 | 0 | 1 | 144 |
| 48 | 30 | 41 | 29 | 1 | 7 | 473487 | 3787892 | 1 | 7 | 0 | 168 | 3872332 | 1 | 7 | 0 | 1 | 176 |
| 49 | 31 | 41 | 29 | 1 | 8 | 473508 | 3788060 | 1 | 8 | 0 | 168 | 3872508 | 1 | 8 | 0 | 1 | 176 |
| 50 | 32 | 41 | 29 | 1 | 9 | 473529 | 3788228 | 1 | 9 | 0 | 168 | 3872684 | 1 | 9 | 0 | 1 | 176 |
| 51 | 33 | 41 | 29 | 1 | 10 | 473550 | 3788396 | 1 | 10 | 0 | 168 | 3872860 | 1 | 10 | 0 | 1 | 176 |
| 52 | 34 | 41 | 29 | 1 | 11 | 473571 | 3788564 | 1 | 11 | 0 | 168 | 3873036 | 1 | 11 | 0 | 1 | 176 |
| 53 | 35 | 41 | 29 | 1 | 12 | 473592 | 3788732 | 1 | 12 | 0 | 168 | 3873212 | 1 | 12 | 0 | 1 | 176 |
| 54 | 36 | 41 | 29 | 1 | 13 | 473613 | 3788900 | 1 | 13 | 0 | 168 | 3873388 | 1 | 13 | 0 | 1 | 176 |
| 55 | 37 | 41 | 29 | 1 | 14 | 473634 | 3789068 | 1 | 14 | 0 | 168 | 3873564 | 1 | 14 | 0 | 1 | 176 |
| 56 | 38 | 41 | 29 | 1 | 15 | 473655 | 3789236 | 1 | 15 | 0 | 168 | 3873740 | 1 | 15 | 0 | 1 | 176 |
| 57 | 39 | 41 | 29 | 1 | 16 | 473676 | 3789404 | 1 | 16 | 0 | 168 | 3873916 | 1 | 16 | 0 | 1 | 176 |
| 58 | 3A | 41 | 29 | 1 | 17 | 473697 | 3789572 | 1 | 17 | 0 | 168 | 3874092 | 1 | 17 | 0 | 1 | 176 |
| 59 | 3B | 41 | 29 | 1 | 18 | 473718 | 3789740 | 1 | 18 | 0 | 168 | 3874268 | 1 | 18 | 0 | 1 | 176 |
| 60 | 3C | 41 | 29 | 1 | 19 | 473739 | 3789908 | 1 | 19 | 0 | 168 | 3874444 | 1 | 19 | 0 | 1 | 176 |
| 61 | 3D | 41 | 29 | 1 | 20 | 473760 | 3790076 | 1 | 20 | 0 | 168 | 3874620 | 1 | 20 | 0 | 1 | 176 |
| 62 | 3E | 41 | 29 | 1 | 21 | 473781 | 3790244 | 1 | 21 | 0 | 168 | 3874796 | 1 | 21 | 0 | 1 | 176 |
| 63 | 3F | 41 | 29 | 1 | 22 | 473802 | 3790412 | 1 | 22 | 0 | 168 | 3874972 | 1 | 22 | 0 | 1 | 176 |
| 64 | 40 | 41 | 29 | 1 | 23 | 473825 | 3790596 | 1 | 23 | 0 | 184 | 3875164 | 1 | 23 | 0 | 1 | 192 |
| 65 | 41 | 41 | 29 | 1 | 24 | 473848 | 3790780 | 1 | 24 | 0 | 184 | 3875356 | 1 | 24 | 0 | 1 | 192 |
| 66 | 42 | 41 | 29 | 1 | 25 | 473871 | 3790964 | 1 | 25 | 0 | 184 | 3875548 | 1 | 25 | 0 | 1 | 192 |
| 67 | 43 | 41 | 29 | 1 | 26 | 473894 | 3791148 | 1 | 26 | 0 | 184 | 3875740 | 1 | 26 | 0 | 1 | 192 |
| 68 | 44 | 41 | 29 | 1 | 27 | 473917 | 3791332 | 1 | 27 | 0 | 184 | 3875932 | 1 | 27 | 0 | 1 | 192 |
| 69 | 45 | 41 | 29 | 1 | 28 | 473940 | 3791516 | 1 | 28 | 0 | 184 | 3876124 | 1 | 28 | 0 | 1 | 192 |
| 70 | 46 | 41 | 29 | 1 | 29 | 473963 | 3791700 | 1 | 29 | 0 | 184 | 3876316 | 1 | 29 | 0 | 1 | 192 |
| 71 | 47 | 41 | 29 | 1 | 30 | 473986 | 3791884 | 1 | 30 | 0 | 184 | 3876508 | 1 | 30 | 0 | 1 | 192 |
| 72 | 48 | 41 | 29 | 1 | 31 | 474009 | 3792068 | 1 | 31 | 0 | 184 | 3876700 | 1 | 31 | 0 | 1 | 192 |
| 73 | 49 | 41 | 29 | 1 | 32 | 474032 | 3792252 | 1 | 32 | 0 | 184 | 3876892 | 1 | 32 | 0 | 1 | 192 |
| 74 | 4A | 41 | 29 | 1 | 33 | 474055 | 3792436 | 1 | 33 | 0 | 184 | 3877084 | 1 | 33 | 0 | 1 | 192 |
| 75 | 4B | 41 | 29 | 1 | 34 | 474078 | 3792620 | 1 | 34 | 0 | 184 | 3877276 | 1 | 34 | 0 | 1 | 192 |
| 76 | 4C | 41 | 29 | 1 | 35 | 474101 | 3792804 | 1 | 35 | 0 | 184 | 3877468 | 1 | 35 | 0 | 1 | 192 |
| 77 | 4D | 41 | 29 | 1 | 36 | 474124 | 3792988 | 1 | 36 | 0 | 184 | 3877660 | 1 | 36 | 0 | 1 | 192 |
| 78 | 4E | 41 | 29 | 1 | 37 | 474147 | 3793172 | 1 | 37 | 0 | 184 | 3877852 | 1 | 37 | 0 | 1 | 192 |
| 79 | 4F | 41 | 29 | 1 | 38 | 474170 | 3793356 | 1 | 38 | 0 | 184 | 3878044 | 1 | 38 | 0 | 1 | 192 |
| 80 | 50 | 41 | 29 | 1 | 39 | 474193 | 3793540 | 1 | 39 | 0 | 184 | 3878236 | 1 | 39 | 0 | 1 | 192 |
| 81 | 51 | 41 | 29 | 1 | 40 | 474216 | 3793724 | 1 | 40 | 0 | 184 | 3878428 | 1 | 40 | 0 | 1 | 192 |
| 82 | 52 | 41 | 29 | 2 | 0 | 474237 | 3793892 | 2 | 0 | 0 | 168 | 3878604 | 2 | 0 | 0 | 1 | 176 |
| 83 | 53 | 41 | 29 | 2 | 1 | 474263 | 3794100 | 2 | 1 | 0 | 208 | 3878820 | 2 | 1 | 0 | 1 | 216 |
| 84 | 54 | 41 | 29 | 2 | 2 | 474289 | 3794308 | 2 | 2 | 0 | 208 | 3879036 | 2 | 2 | 0 | 1 | 216 |
| 85 | 55 | 41 | 29 | 2 | 3 | 474315 | 3794516 | 2 | 3 | 0 | 208 | 3879252 | 2 | 3 | 0 | 1 | 216 |
| 86 | 56 | 41 | 29 | 2 | 4 | 474341 | 3794724 | 2 | 4 | 0 | 208 | 3879468 | 2 | 4 | 0 | 1 | 216 |
| 87 | 57 | 41 | 29 | 2 | 5 | 474367 | 3794932 | 2 | 5 | 0 | 208 | 3879684 | 2 | 5 | 0 | 1 | 216 |
| 88 | 58 | 41 | 29 | 2 | 6 | 474393 | 3795140 | 2 | 6 | 0 | 208 | 3879900 | 2 | 6 | 0 | 1 | 216 |
| 89 | 59 | 41 | 29 | 2 | 7 | 474419 | 3795348 | 2 | 7 | 0 | 208 | 3880116 | 2 | 7 | 0 | 1 | 216 |
| 90 | 5A | 41 | 29 | 2 | 8 | 474445 | 3795556 | 2 | 8 | 0 | 208 | 3880332 | 2 | 8 | 0 | 1 | 216 |
| 91 | 5B | 41 | 29 | 2 | 9 | 474471 | 3795764 | 2 | 9 | 0 | 208 | 3880548 | 2 | 9 | 0 | 1 | 216 |
| 92 | 5C | 41 | 29 | 2 | 10 | 474497 | 3795972 | 2 | 10 | 0 | 208 | 3880764 | 2 | 10 | 0 | 1 | 216 |
| 93 | 5D | 41 | 29 | 2 | 11 | 474523 | 3796180 | 2 | 11 | 0 | 208 | 3880980 | 2 | 11 | 0 | 1 | 216 |
| 94 | 5E | 41 | 29 | 2 | 12 | 474549 | 3796388 | 2 | 12 | 0 | 208 | 3881196 | 2 | 12 | 0 | 1 | 216 |
| 95 | 5F | 41 | 29 | 2 | 13 | 474575 | 3796596 | 2 | 13 | 0 | 208 | 3881412 | 2 | 13 | 0 | 1 | 216 |
| 96 | 60 | 41 | 29 | 2 | 14 | 474598 | 3796780 | 2 | 14 | 0 | 184 | 3881604 | 2 | 14 | 0 | 1 | 192 |
| 97 | 61 | 41 | 29 | 2 | 15 | 474621 | 3796964 | 2 | 15 | 0 | 184 | 3881796 | 2 | 15 | 0 | 1 | 192 |
| 98 | 62 | 41 | 29 | 2 | 16 | 474644 | 3797148 | 2 | 16 | 0 | 184 | 3881988 | 2 | 16 | 0 | 1 | 192 |
| 99 | 63 | 41 | 29 | 2 | 17 | 474667 | 3797332 | 2 | 17 | 0 | 184 | 3882180 | 2 | 17 | 0 | 1 | 192 |
| 100 | 64 | 41 | 29 | 2 | 18 | 474690 | 3797516 | 2 | 18 | 0 | 184 | 3882372 | 2 | 18 | 0 | 1 | 192 |
| 101 | 65 | 41 | 29 | 2 | 19 | 474713 | 3797700 | 2 | 19 | 0 | 184 | 3882564 | 2 | 19 | 0 | 1 | 192 |
| 102 | 66 | 41 | 29 | 2 | 20 | 474736 | 3797884 | 2 | 20 | 0 | 184 | 3882756 | 2 | 20 | 0 | 1 | 192 |
| 103 | 67 | 41 | 29 | 2 | 21 | 474759 | 3798068 | 2 | 21 | 0 | 184 | 3882948 | 2 | 21 | 0 | 1 | 192 |
| 104 | 68 | 41 | 29 | 2 | 22 | 474782 | 3798252 | 2 | 22 | 0 | 184 | 3883140 | 2 | 22 | 0 | 1 | 192 |
| 105 | 69 | 41 | 29 | 2 | 23 | 474805 | 3798436 | 2 | 23 | 0 | 184 | 3883332 | 2 | 23 | 0 | 1 | 192 |
| 106 | 6A | 41 | 29 | 2 | 24 | 474828 | 3798620 | 2 | 24 | 0 | 184 | 3883524 | 2 | 24 | 0 | 1 | 192 |
| 107 | 6B | 41 | 29 | 2 | 25 | 474851 | 3798804 | 2 | 25 | 0 | 184 | 3883716 | 2 | 25 | 0 | 1 | 192 |
| 108 | 6C | 41 | 29 | 2 | 26 | 474874 | 3798988 | 2 | 26 | 0 | 184 | 3883908 | 2 | 26 | 0 | 1 | 192 |
| 109 | 6D | 41 | 29 | 2 | 27 | 474897 | 3799172 | 2 | 27 | 0 | 184 | 3884100 | 2 | 27 | 0 | 1 | 192 |

| | | | | | | | | | | | | | | | | | |
|---|---|---|---|---|---|---|---|---|---|---|---|---|---|---|---|---|---|
| 110 | 6E | 41 | 29 | 2 | 28 | 474920 | 3799356 | 2 | 28 | 0 | 184 | 3884292 | 2 | 28 | 0 | 1 | 192 |
| 111 | 6F | 41 | 29 | 2 | 29 | 474943 | 3799540 | 2 | 29 | 0 | 184 | 3884484 | 2 | 29 | 0 | 1 | 192 |
| 112 | 70 | 41 | 29 | 2 | 30 | 474968 | 3799740 | 2 | 30 | 0 | 200 | 3884692 | 2 | 30 | 0 | 1 | 208 |
| 113 | 71 | 41 | 29 | 2 | 31 | 474993 | 3799940 | 2 | 31 | 0 | 200 | 3884900 | 2 | 31 | 0 | 1 | 208 |
| 114 | 72 | 41 | 29 | 2 | 32 | 475018 | 3800140 | 2 | 32 | 0 | 200 | 3885108 | 2 | 32 | 0 | 1 | 208 |
| 115 | 73 | 41 | 29 | 2 | 33 | 475043 | 3800340 | 2 | 33 | 0 | 200 | 3885316 | 2 | 33 | 0 | 1 | 208 |
| 116 | 74 | 41 | 29 | 2 | 34 | 475068 | 3800540 | 2 | 34 | 0 | 200 | 3885524 | 2 | 34 | 0 | 1 | 208 |
| 117 | 75 | 41 | 29 | 2 | 35 | 475093 | 3800740 | 2 | 35 | 0 | 200 | 3885732 | 2 | 35 | 0 | 1 | 208 |
| 118 | 76 | 41 | 29 | 2 | 36 | 475118 | 3800940 | 2 | 36 | 0 | 200 | 3885940 | 2 | 36 | 0 | 1 | 208 |
| 119 | 77 | 41 | 29 | 2 | 37 | 475143 | 3801140 | 2 | 37 | 0 | 200 | 3886148 | 2 | 37 | 0 | 1 | 208 |
| 120 | 78 | 41 | 29 | 2 | 38 | 475168 | 3801340 | 2 | 38 | 0 | 200 | 3886356 | 2 | 38 | 0 | 1 | 208 |
| 121 | 79 | 41 | 29 | 2 | 39 | 475193 | 3801540 | 2 | 39 | 0 | 200 | 3886564 | 2 | 39 | 0 | 1 | 208 |
| 122 | 7A | 41 | 29 | 2 | 40 | 475218 | 3801740 | 2 | 40 | 0 | 200 | 3886772 | 2 | 40 | 0 | 1 | 208 |
| 123 | 7B | 41 | 29 | 3 | 0 | 475241 | 3801924 | 3 | 0 | 0 | 184 | 3886964 | 3 | 0 | 0 | 1 | 192 |
| 124 | 7C | 41 | 29 | 3 | 1 | 475269 | 3802148 | 3 | 1 | 0 | 224 | 3887196 | 3 | 1 | 0 | 1 | 232 |
| 125 | 7D | 41 | 29 | 3 | 2 | 475297 | 3802372 | 3 | 2 | 0 | 224 | 3887428 | 3 | 2 | 0 | 1 | 232 |
| 126 | 7E | 41 | 29 | 3 | 3 | 475325 | 3802596 | 3 | 3 | 0 | 224 | 3887660 | 3 | 3 | 0 | 1 | 232 |
| 127 | 7F | 41 | 29 | 3 | 4 | 475353 | 3802820 | 3 | 4 | 0 | 224 | 3887892 | 3 | 4 | 0 | 1 | 232 |
| 128 | 80 | 41 | 29 | 3 | 5 | 475381 | 3803044 | 3 | 5 | 0 | 224 | 3888124 | 3 | 5 | 0 | 1 | 232 |
| 129 | 81 | 41 | 29 | 3 | 6 | 475409 | 3803268 | 3 | 6 | 0 | 224 | 3888356 | 3 | 6 | 0 | 1 | 232 |
| 130 | 82 | 41 | 29 | 3 | 7 | 475437 | 3803492 | 3 | 7 | 0 | 224 | 3888588 | 3 | 7 | 0 | 1 | 232 |
| 131 | 83 | 41 | 29 | 3 | 8 | 475465 | 3803716 | 3 | 8 | 0 | 224 | 3888820 | 3 | 8 | 0 | 1 | 232 |
| 132 | 84 | 41 | 29 | 3 | 9 | 475493 | 3803940 | 3 | 9 | 0 | 224 | 3889052 | 3 | 9 | 0 | 1 | 232 |
| 133 | 85 | 41 | 29 | 3 | 10 | 475521 | 3804164 | 3 | 10 | 0 | 224 | 3889284 | 3 | 10 | 0 | 1 | 232 |
| 134 | 86 | 41 | 29 | 3 | 11 | 475549 | 3804388 | 3 | 11 | 0 | 224 | 3889516 | 3 | 11 | 0 | 1 | 232 |
| 135 | 87 | 41 | 29 | 3 | 12 | 475577 | 3804612 | 3 | 12 | 0 | 224 | 3889748 | 3 | 12 | 0 | 1 | 232 |
| 136 | 88 | 41 | 29 | 3 | 13 | 475605 | 3804836 | 3 | 13 | 0 | 224 | 3889980 | 3 | 13 | 0 | 1 | 232 |
| 137 | 89 | 41 | 29 | 3 | 14 | 475633 | 3805060 | 3 | 14 | 0 | 224 | 3890212 | 3 | 14 | 0 | 1 | 232 |
| 138 | 8A | 41 | 29 | 3 | 15 | 475661 | 3805284 | 3 | 15 | 0 | 224 | 3890444 | 3 | 15 | 0 | 1 | 232 |
| 139 | 8B | 41 | 29 | 3 | 16 | 475689 | 3805508 | 3 | 16 | 0 | 224 | 3890676 | 3 | 16 | 0 | 1 | 232 |
| 140 | 8C | 41 | 29 | 3 | 17 | 475717 | 3805732 | 3 | 17 | 0 | 224 | 3890908 | 3 | 17 | 0 | 1 | 232 |
| 141 | 8D | 41 | 29 | 3 | 18 | 475745 | 3805956 | 3 | 18 | 0 | 224 | 3891140 | 3 | 18 | 0 | 1 | 232 |
| 142 | 8E | 41 | 29 | 3 | 19 | 475773 | 3806180 | 3 | 19 | 0 | 224 | 3891372 | 3 | 19 | 0 | 1 | 232 |
| 143 | 8F | 41 | 29 | 3 | 20 | 475801 | 3806404 | 3 | 20 | 0 | 224 | 3891604 | 3 | 20 | 0 | 1 | 232 |
| 144 | 90 | 41 | 29 | 3 | 21 | 475826 | 3806604 | 3 | 21 | 0 | 200 | 3891812 | 3 | 21 | 0 | 1 | 208 |
| 145 | 91 | 41 | 29 | 3 | 22 | 475851 | 3806804 | 3 | 22 | 0 | 200 | 3892020 | 3 | 22 | 0 | 1 | 208 |
| 146 | 92 | 41 | 29 | 3 | 23 | 475876 | 3807004 | 3 | 23 | 0 | 200 | 3892228 | 3 | 23 | 0 | 1 | 208 |
| 147 | 93 | 41 | 29 | 3 | 24 | 475901 | 3807204 | 3 | 24 | 0 | 200 | 3892436 | 3 | 24 | 0 | 1 | 208 |
| 148 | 94 | 41 | 29 | 3 | 25 | 475926 | 3807404 | 3 | 25 | 0 | 200 | 3892644 | 3 | 25 | 0 | 1 | 208 |
| 149 | 95 | 41 | 29 | 3 | 26 | 475951 | 3807604 | 3 | 26 | 0 | 200 | 3892852 | 3 | 26 | 0 | 1 | 208 |
| 150 | 96 | 41 | 29 | 3 | 27 | 475976 | 3807804 | 3 | 27 | 0 | 200 | 3893060 | 3 | 27 | 0 | 1 | 208 |
| 151 | 97 | 41 | 29 | 3 | 28 | 476001 | 3808004 | 3 | 28 | 0 | 200 | 3893268 | 3 | 28 | 0 | 1 | 208 |
| 152 | 98 | 41 | 29 | 3 | 29 | 476026 | 3808204 | 3 | 29 | 0 | 200 | 3893476 | 3 | 29 | 0 | 1 | 208 |
| 153 | 99 | 41 | 29 | 3 | 30 | 476051 | 3808404 | 3 | 30 | 0 | 200 | 3893684 | 3 | 30 | 0 | 1 | 208 |
| 154 | 9A | 41 | 29 | 3 | 31 | 476076 | 3808604 | 3 | 31 | 0 | 200 | 3893892 | 3 | 31 | 0 | 1 | 208 |
| 155 | 9B | 41 | 29 | 3 | 32 | 476101 | 3808804 | 3 | 32 | 0 | 200 | 3894100 | 3 | 32 | 0 | 1 | 208 |
| 156 | 9C | 41 | 29 | 3 | 33 | 476126 | 3809004 | 3 | 33 | 0 | 200 | 3894308 | 3 | 33 | 0 | 1 | 208 |
| 157 | 9D | 41 | 29 | 3 | 34 | 476151 | 3809204 | 3 | 34 | 0 | 200 | 3894516 | 3 | 34 | 0 | 1 | 208 |
| 158 | 9E | 41 | 29 | 3 | 35 | 476176 | 3809404 | 3 | 35 | 0 | 200 | 3894724 | 3 | 35 | 0 | 1 | 208 |
| 159 | 9F | 41 | 29 | 3 | 36 | 476201 | 3809604 | 3 | 36 | 0 | 200 | 3894932 | 3 | 36 | 0 | 1 | 208 |
| 160 | A0 | 41 | 29 | 3 | 37 | 476228 | 3809820 | 3 | 37 | 0 | 216 | 3895156 | 3 | 37 | 0 | 1 | 224 |
| 161 | A1 | 41 | 29 | 3 | 38 | 476255 | 3810036 | 3 | 38 | 0 | 216 | 3895380 | 3 | 38 | 0 | 1 | 224 |
| 162 | A2 | 41 | 29 | 3 | 39 | 476282 | 3810252 | 3 | 39 | 0 | 216 | 3895604 | 3 | 39 | 0 | 1 | 224 |
| 163 | A3 | 41 | 29 | 3 | 40 | 476309 | 3810468 | 3 | 40 | 0 | 216 | 3895828 | 3 | 40 | 0 | 1 | 224 |
| 164 | A4 | 41 | 29 | 4 | 0 | 476334 | 3810668 | 4 | 0 | 0 | 200 | 3896036 | 4 | 0 | 0 | 1 | 208 |
| 165 | A5 | 41 | 29 | 4 | 1 | 476364 | 3810908 | 4 | 1 | 0 | 240 | 3896284 | 4 | 1 | 0 | 1 | 248 |
| 166 | A6 | 41 | 29 | 4 | 2 | 476394 | 3811148 | 4 | 2 | 0 | 240 | 3896532 | 4 | 2 | 0 | 1 | 248 |
| 167 | A7 | 41 | 29 | 4 | 3 | 476424 | 3811388 | 4 | 3 | 0 | 240 | 3896780 | 4 | 3 | 0 | 1 | 248 |
| 168 | A8 | 41 | 29 | 4 | 4 | 476454 | 3811628 | 4 | 4 | 0 | 240 | 3897028 | 4 | 4 | 0 | 1 | 248 |
| 169 | A9 | 41 | 29 | 4 | 5 | 476484 | 3811868 | 4 | 5 | 0 | 240 | 3897276 | 4 | 5 | 0 | 1 | 248 |
| 170 | AA | 41 | 29 | 4 | 6 | 476514 | 3812108 | 4 | 6 | 0 | 240 | 3897524 | 4 | 6 | 0 | 1 | 248 |
| 171 | AB | 41 | 29 | 4 | 7 | 476544 | 3812348 | 4 | 7 | 0 | 240 | 3897772 | 4 | 7 | 0 | 1 | 248 |
| 172 | AC | 41 | 29 | 4 | 8 | 476574 | 3812588 | 4 | 8 | 0 | 240 | 3898020 | 4 | 8 | 0 | 1 | 248 |
| 173 | AD | 41 | 29 | 4 | 9 | 476604 | 3812828 | 4 | 9 | 0 | 240 | 3898268 | 4 | 9 | 0 | 1 | 248 |
| 174 | AE | 41 | 29 | 4 | 10 | 476634 | 3813068 | 4 | 10 | 0 | 240 | 3898516 | 4 | 10 | 0 | 1 | 248 |
| 175 | AF | 41 | 29 | 4 | 11 | 476664 | 3813308 | 4 | 11 | 0 | 240 | 3898764 | 4 | 11 | 0 | 1 | 248 |
| 176 | B0 | 41 | 29 | 4 | 12 | 476694 | 3813548 | 4 | 12 | 0 | 240 | 3899012 | 4 | 12 | 0 | 1 | 248 |
| 177 | B1 | 41 | 29 | 4 | 13 | 476724 | 3813788 | 4 | 13 | 0 | 240 | 3899260 | 4 | 13 | 0 | 1 | 248 |
| 178 | B2 | 41 | 29 | 4 | 14 | 476754 | 3814028 | 4 | 14 | 0 | 240 | 3899508 | 4 | 14 | 0 | 1 | 248 |
| 179 | B3 | 41 | 29 | 4 | 15 | 476784 | 3814268 | 4 | 15 | 0 | 240 | 3899756 | 4 | 15 | 0 | 1 | 248 |
| 180 | B4 | 41 | 29 | 4 | 16 | 476814 | 3814508 | 4 | 16 | 0 | 240 | 3900004 | 4 | 16 | 0 | 1 | 248 |
| 181 | B5 | 41 | 29 | 4 | 17 | 476844 | 3814748 | 4 | 17 | 0 | 240 | 3900252 | 4 | 17 | 0 | 1 | 248 |
| 182 | B6 | 41 | 29 | 4 | 18 | 476874 | 3814988 | 4 | 18 | 0 | 240 | 3900500 | 4 | 18 | 0 | 1 | 248 |
| 183 | B7 | 41 | 29 | 4 | 19 | 476904 | 3815228 | 4 | 19 | 0 | 240 | 3900748 | 4 | 19 | 0 | 1 | 248 |
| 184 | B8 | 41 | 29 | 4 | 20 | 476934 | 3815468 | 4 | 20 | 0 | 240 | 3900996 | 4 | 20 | 0 | 1 | 248 |
| 185 | B9 | 41 | 29 | 4 | 21 | 476964 | 3815708 | 4 | 21 | 0 | 240 | 3901244 | 4 | 21 | 0 | 1 | 248 |
| 186 | BA | 41 | 29 | 4 | 22 | 476994 | 3815948 | 4 | 22 | 0 | 240 | 3901492 | 4 | 22 | 0 | 1 | 248 |
| 187 | BB | 41 | 29 | 4 | 23 | 477024 | 3816188 | 4 | 23 | 0 | 240 | 3901740 | 4 | 23 | 0 | 1 | 248 |
| 188 | BC | 41 | 29 | 4 | 24 | 477054 | 3816428 | 4 | 24 | 0 | 240 | 3901988 | 4 | 24 | 0 | 1 | 248 |
| 189 | BD | 41 | 29 | 4 | 25 | 477084 | 3816668 | 4 | 25 | 0 | 240 | 3902236 | 4 | 25 | 0 | 1 | 248 |
| 190 | BE | 41 | 29 | 4 | 26 | 477114 | 3816908 | 4 | 26 | 0 | 240 | 3902484 | 4 | 26 | 0 | 1 | 248 |
| 191 | BF | 41 | 29 | 4 | 27 | 477144 | 3817148 | 4 | 27 | 0 | 240 | 3902732 | 4 | 27 | 0 | 1 | 248 |
| 192 | C0 | 41 | 29 | 4 | 28 | 477171 | 3817364 | 4 | 28 | 0 | 216 | 3902956 | 4 | 28 | 0 | 1 | 224 |
| 193 | C1 | 41 | 29 | 4 | 29 | 477198 | 3817580 | 4 | 29 | 0 | 216 | 3903180 | 4 | 29 | 0 | 1 | 224 |
| 194 | C2 | 41 | 29 | 4 | 30 | 477225 | 3817796 | 4 | 30 | 0 | 216 | 3903404 | 4 | 30 | 0 | 1 | 224 |
| 195 | C3 | 41 | 29 | 4 | 31 | 477252 | 3818012 | 4 | 31 | 0 | 216 | 3903628 | 4 | 31 | 0 | 1 | 224 |
| 196 | C4 | 41 | 29 | 4 | 32 | 477279 | 3818228 | 4 | 32 | 0 | 216 | 3903852 | 4 | 32 | 0 | 1 | 224 |
| 197 | C5 | 41 | 29 | 4 | 33 | 477306 | 3818444 | 4 | 33 | 0 | 216 | 3904076 | 4 | 33 | 0 | 1 | 224 |
| 198 | C6 | 41 | 29 | 4 | 34 | 477333 | 3818660 | 4 | 34 | 0 | 216 | 3904300 | 4 | 34 | 0 | 1 | 224 |
| 199 | C7 | 41 | 29 | 4 | 35 | 477360 | 3818876 | 4 | 35 | 0 | 216 | 3904524 | 4 | 35 | 0 | 1 | 224 |
| 200 | C8 | 41 | 29 | 4 | 36 | 477387 | 3819092 | 4 | 36 | 0 | 216 | 3904748 | 4 | 36 | 0 | 1 | 224 |
| 201 | C9 | 41 | 29 | 4 | 37 | 477414 | 3819308 | 4 | 37 | 0 | 216 | 3904972 | 4 | 37 | 0 | 1 | 224 |
| 202 | CA | 41 | 29 | 4 | 38 | 477441 | 3819524 | 4 | 38 | 0 | 216 | 3905196 | 4 | 38 | 0 | 1 | 224 |
| 203 | CB | 41 | 29 | 4 | 39 | 477468 | 3819740 | 4 | 39 | 0 | 216 | 3905420 | 4 | 39 | 0 | 1 | 224 |
| 204 | CC | 41 | 29 | 4 | 40 | 477495 | 3819956 | 4 | 40 | 0 | 216 | 3905644 | 4 | 40 | 0 | 1 | 224 |
| 205 | CD | 41 | 29 | 5 | 0 | 477520 | 3820156 | 5 | 0 | 0 | 200 | 3905852 | 5 | 0 | 0 | 1 | 208 |
| 206 | CE | 41 | 29 | 5 | 1 | 477550 | 3820396 | 5 | 1 | 0 | 240 | 3906100 | 5 | 1 | 0 | 1 | 248 |
| 207 | CF | 41 | 29 | 5 | 2 | 477580 | 3820636 | 5 | 2 | 0 | 240 | 3906348 | 5 | 2 | 0 | 1 | 248 |
| 208 | D0 | 41 | 29 | 5 | 3 | 477612 | 3820892 | 5 | 3 | 0 | 256 | 3906612 | 5 | 3 | 0 | 1 | 264 |
| 209 | D1 | 41 | 29 | 5 | 4 | 477644 | 3821148 | 5 | 4 | 0 | 256 | 3906876 | 5 | 4 | 0 | 1 | 264 |
| 210 | D2 | 41 | 29 | 5 | 5 | 477676 | 3821404 | 5 | 5 | 0 | 256 | 3907140 | 5 | 5 | 0 | 1 | 264 |
| 211 | D3 | 41 | 29 | 5 | 6 | 477708 | 3821660 | 5 | 6 | 0 | 256 | 3907404 | 5 | 6 | 0 | 1 | 264 |
| 212 | D4 | 41 | 29 | 5 | 7 | 477740 | 3821916 | 5 | 7 | 0 | 256 | 3907668 | 5 | 7 | 0 | 1 | 264 |

| | | | | | | | | | | | | | | | | | |
|---|---|---|---|---|---|---|---|---|---|---|---|---|---|---|---|---|---|
| 213 | D5 | 41 | 29 | 5 | 8 | 477772 | 3822172 | 5 | 8 | 0 | 256 | 3907932 | 5 | 8 | 0 | 1 | 264 |
| 214 | D6 | 41 | 29 | 5 | 9 | 477804 | 3822428 | 5 | 9 | 0 | 256 | 3908196 | 5 | 9 | 0 | 1 | 264 |
| 215 | D7 | 41 | 29 | 5 | 10 | 477836 | 3822684 | 5 | 10 | 0 | 256 | 3908460 | 5 | 10 | 0 | 1 | 264 |
| 216 | D8 | 41 | 29 | 5 | 11 | 477868 | 3822940 | 5 | 11 | 0 | 256 | 3908724 | 5 | 11 | 0 | 1 | 264 |
| 217 | D9 | 41 | 29 | 5 | 12 | 477900 | 3823196 | 5 | 12 | 0 | 256 | 3908988 | 5 | 12 | 0 | 1 | 264 |
| 218 | DA | 41 | 29 | 5 | 13 | 477932 | 3823452 | 5 | 13 | 0 | 256 | 3909252 | 5 | 13 | 0 | 1 | 264 |
| 219 | DB | 41 | 29 | 5 | 14 | 477964 | 3823708 | 5 | 14 | 0 | 256 | 3909516 | 5 | 14 | 0 | 1 | 264 |
| 220 | DC | 41 | 29 | 5 | 15 | 477996 | 3823964 | 5 | 15 | 0 | 256 | 3909780 | 5 | 15 | 0 | 1 | 264 |
| 221 | DD | 41 | 29 | 5 | 16 | 478028 | 3824220 | 5 | 16 | 0 | 256 | 3910044 | 5 | 16 | 0 | 1 | 264 |
| 222 | DE | 41 | 29 | 5 | 17 | 478060 | 3824476 | 5 | 17 | 0 | 256 | 3910308 | 5 | 17 | 0 | 1 | 264 |
| 223 | DF | 41 | 29 | 5 | 18 | 478092 | 3824732 | 5 | 18 | 0 | 256 | 3910572 | 5 | 18 | 0 | 1 | 264 |
| 224 | E0 | 41 | 29 | 5 | 19 | 478124 | 3824988 | 5 | 19 | 0 | 256 | 3910836 | 5 | 19 | 0 | 1 | 264 |
| 225 | E1 | 41 | 29 | 5 | 20 | 478156 | 3825244 | 5 | 20 | 0 | 256 | 3911100 | 5 | 20 | 0 | 1 | 264 |
| 226 | E2 | 41 | 29 | 5 | 21 | 478188 | 3825500 | 5 | 21 | 0 | 256 | 3911364 | 5 | 21 | 0 | 1 | 264 |
| 227 | E3 | 41 | 29 | 5 | 22 | 478220 | 3825756 | 5 | 22 | 0 | 256 | 3911628 | 5 | 22 | 0 | 1 | 264 |
| 228 | E4 | 41 | 29 | 5 | 23 | 478252 | 3826012 | 5 | 23 | 0 | 256 | 3911892 | 5 | 23 | 0 | 1 | 264 |
| 229 | E5 | 41 | 29 | 5 | 24 | 478284 | 3826268 | 5 | 24 | 0 | 256 | 3912156 | 5 | 24 | 0 | 1 | 264 |
| 230 | E6 | 41 | 29 | 5 | 25 | 478316 | 3826524 | 5 | 25 | 0 | 256 | 3912420 | 5 | 25 | 0 | 1 | 264 |
| 231 | E7 | 41 | 29 | 5 | 26 | 478348 | 3826780 | 5 | 26 | 0 | 256 | 3912684 | 5 | 26 | 0 | 1 | 264 |
| 232 | E8 | 41 | 29 | 5 | 27 | 478380 | 3827036 | 5 | 27 | 0 | 256 | 3912948 | 5 | 27 | 0 | 1 | 264 |
| 233 | E9 | 41 | 29 | 5 | 28 | 478412 | 3827292 | 5 | 28 | 0 | 256 | 3913212 | 5 | 28 | 0 | 1 | 264 |
| 234 | EA | 41 | 29 | 5 | 29 | 478444 | 3827548 | 5 | 29 | 0 | 256 | 3913476 | 5 | 29 | 0 | 1 | 264 |
| 235 | EB | 41 | 29 | 5 | 30 | 478476 | 3827804 | 5 | 30 | 0 | 256 | 3913740 | 5 | 30 | 0 | 1 | 264 |
| 236 | EC | 41 | 29 | 5 | 31 | 478508 | 3828060 | 5 | 31 | 0 | 256 | 3914004 | 5 | 31 | 0 | 1 | 264 |
| 237 | ED | 41 | 29 | 5 | 32 | 478540 | 3828316 | 5 | 32 | 0 | 256 | 3914268 | 5 | 32 | 0 | 1 | 264 |
| 238 | EE | 41 | 29 | 5 | 33 | 478572 | 3828572 | 5 | 33 | 0 | 256 | 3914532 | 5 | 33 | 0 | 1 | 264 |
| 239 | EF | 41 | 29 | 5 | 34 | 478604 | 3828828 | 5 | 34 | 0 | 256 | 3914796 | 5 | 34 | 0 | 1 | 264 |
| 240 | F0 | 41 | 29 | 5 | 35 | 478633 | 3829060 | 5 | 35 | 0 | 232 | 3915036 | 5 | 35 | 0 | 1 | 240 |
| 241 | F1 | 41 | 29 | 5 | 36 | 478662 | 3829292 | 5 | 36 | 0 | 232 | 3915276 | 5 | 36 | 0 | 1 | 240 |
| 242 | F2 | 41 | 29 | 5 | 37 | 478691 | 3829524 | 5 | 37 | 0 | 232 | 3915516 | 5 | 37 | 0 | 1 | 240 |
| 243 | F3 | 41 | 29 | 5 | 38 | 478720 | 3829756 | 5 | 38 | 0 | 232 | 3915756 | 5 | 38 | 0 | 1 | 240 |
| 244 | F4 | 41 | 29 | 5 | 39 | 478749 | 3829988 | 5 | 39 | 0 | 232 | 3915996 | 5 | 39 | 0 | 1 | 240 |
| 245 | F5 | 41 | 29 | 5 | 40 | 478778 | 3830220 | 5 | 40 | 0 | 232 | 3916236 | 5 | 40 | 0 | 1 | 240 |
| 246 | F6 | 41 | 29 | 6 | 0 | 478805 | 3830436 | 6 | 0 | 0 | 216 | 3916460 | 6 | 0 | 0 | 1 | 224 |
| 247 | F7 | 41 | 29 | 6 | 1 | 478837 | 3830692 | 6 | 1 | 0 | 256 | 3916724 | 6 | 1 | 0 | 1 | 264 |
| 248 | F8 | 41 | 29 | 6 | 2 | 478869 | 3830948 | 6 | 2 | 0 | 256 | 3916988 | 6 | 2 | 0 | 1 | 264 |
| 249 | F9 | 41 | 29 | 6 | 3 | 478901 | 3831204 | 6 | 3 | 0 | 256 | 3917252 | 6 | 3 | 0 | 1 | 264 |
| 250 | FA | 41 | 29 | 6 | 4 | 478933 | 3831460 | 6 | 4 | 0 | 256 | 3917516 | 6 | 4 | 0 | 1 | 264 |
| 251 | FB | 41 | 29 | 6 | 5 | 478965 | 3831716 | 6 | 5 | 0 | 256 | 3917780 | 6 | 5 | 0 | 1 | 264 |
| 252 | FC | 41 | 29 | 6 | 6 | 478997 | 3831972 | 6 | 6 | 0 | 256 | 3918044 | 6 | 6 | 0 | 1 | 264 |
| 253 | FD | 41 | 29 | 6 | 7 | 479029 | 3832228 | 6 | 7 | 0 | 256 | 3918308 | 6 | 7 | 0 | 1 | 264 |
| 254 | FE | 41 | 29 | 6 | 8 | 479061 | 3832484 | 6 | 8 | 0 | 256 | 3918572 | 6 | 8 | 0 | 1 | 264 |
| 255 | FF | 41 | 29 | 6 | 9 | 479093 | 3832740 | 6 | 9 | 0 | 256 | 3918836 | 6 | 9 | 0 | 1 | 264 |
| 0 | 00 | 42 | 2A | 0 | 0 | 479105 | 3832836 | 0 | 0 | 0 | 96 | 3918940 | 0 | 0 | 0 | 1 | 104 |
| 1 | 01 | 42 | 2A | 0 | 1 | 479119 | 3832948 | 0 | 1 | 0 | 112 | 3919060 | 0 | 1 | 0 | 1 | 120 |
| 2 | 02 | 42 | 2A | 0 | 2 | 479133 | 3833060 | 0 | 2 | 0 | 112 | 3919180 | 0 | 2 | 0 | 1 | 120 |
| 3 | 03 | 42 | 2A | 0 | 3 | 479147 | 3833172 | 0 | 3 | 0 | 112 | 3919300 | 0 | 3 | 0 | 1 | 120 |
| 4 | 04 | 42 | 2A | 0 | 4 | 479161 | 3833284 | 0 | 4 | 0 | 112 | 3919420 | 0 | 4 | 0 | 1 | 120 |
| 5 | 05 | 42 | 2A | 0 | 5 | 479175 | 3833396 | 0 | 5 | 0 | 112 | 3919540 | 0 | 5 | 0 | 1 | 120 |
| 6 | 06 | 42 | 2A | 0 | 6 | 479189 | 3833508 | 0 | 6 | 0 | 112 | 3919660 | 0 | 6 | 0 | 1 | 120 |
| 7 | 07 | 42 | 2A | 0 | 7 | 479203 | 3833620 | 0 | 7 | 0 | 112 | 3919780 | 0 | 7 | 0 | 1 | 120 |
| 8 | 08 | 42 | 2A | 0 | 8 | 479217 | 3833732 | 0 | 8 | 0 | 112 | 3919900 | 0 | 8 | 0 | 1 | 120 |
| 9 | 09 | 42 | 2A | 0 | 9 | 479231 | 3833844 | 0 | 9 | 0 | 112 | 3920020 | 0 | 9 | 0 | 1 | 120 |
| 10 | 0A | 42 | 2A | 0 | 10 | 479245 | 3833956 | 0 | 10 | 0 | 112 | 3920140 | 0 | 10 | 0 | 1 | 120 |
| 11 | 0B | 42 | 2A | 0 | 11 | 479259 | 3834068 | 0 | 11 | 0 | 112 | 3920260 | 0 | 11 | 0 | 1 | 120 |
| 12 | 0C | 42 | 2A | 0 | 12 | 479273 | 3834180 | 0 | 12 | 0 | 112 | 3920380 | 0 | 12 | 0 | 1 | 120 |
| 13 | 0D | 42 | 2A | 0 | 13 | 479287 | 3834292 | 0 | 13 | 0 | 112 | 3920500 | 0 | 13 | 0 | 1 | 120 |
| 14 | 0E | 42 | 2A | 0 | 14 | 479301 | 3834404 | 0 | 14 | 0 | 112 | 3920620 | 0 | 14 | 0 | 1 | 120 |
| 15 | 0F | 42 | 2A | 0 | 15 | 479315 | 3834516 | 0 | 15 | 0 | 112 | 3920740 | 0 | 15 | 0 | 1 | 120 |
| 16 | 10 | 42 | 2A | 0 | 16 | 479329 | 3834628 | 0 | 16 | 0 | 112 | 3920860 | 0 | 16 | 0 | 1 | 120 |
| 17 | 11 | 42 | 2A | 0 | 17 | 479343 | 3834740 | 0 | 17 | 0 | 112 | 3920980 | 0 | 17 | 0 | 1 | 120 |
| 18 | 12 | 42 | 2A | 0 | 18 | 479357 | 3834852 | 0 | 18 | 0 | 112 | 3921100 | 0 | 18 | 0 | 1 | 120 |
| 19 | 13 | 42 | 2A | 0 | 19 | 479371 | 3834964 | 0 | 19 | 0 | 112 | 3921220 | 0 | 19 | 0 | 1 | 120 |
| 20 | 14 | 42 | 2A | 0 | 20 | 479385 | 3835076 | 0 | 20 | 0 | 112 | 3921340 | 0 | 20 | 0 | 1 | 120 |
| 21 | 15 | 42 | 2A | 0 | 21 | 479399 | 3835188 | 0 | 21 | 0 | 112 | 3921460 | 0 | 21 | 0 | 1 | 120 |
| 22 | 16 | 42 | 2A | 0 | 22 | 479413 | 3835300 | 0 | 22 | 0 | 112 | 3921580 | 0 | 22 | 0 | 1 | 120 |
| 23 | 17 | 42 | 2A | 0 | 23 | 479427 | 3835412 | 0 | 23 | 0 | 112 | 3921700 | 0 | 23 | 0 | 1 | 120 |
| 24 | 18 | 42 | 2A | 0 | 24 | 479441 | 3835524 | 0 | 24 | 0 | 112 | 3921820 | 0 | 24 | 0 | 1 | 120 |
| 25 | 19 | 42 | 2A | 0 | 25 | 479455 | 3835636 | 0 | 25 | 0 | 112 | 3921940 | 0 | 25 | 0 | 1 | 120 |
| 26 | 1A | 42 | 2A | 0 | 26 | 479469 | 3835748 | 0 | 26 | 0 | 112 | 3922060 | 0 | 26 | 0 | 1 | 120 |
| 27 | 1B | 42 | 2A | 0 | 27 | 479483 | 3835860 | 0 | 27 | 0 | 112 | 3922180 | 0 | 27 | 0 | 1 | 120 |
| 28 | 1C | 42 | 2A | 0 | 28 | 479497 | 3835972 | 0 | 28 | 0 | 112 | 3922300 | 0 | 28 | 0 | 1 | 120 |
| 29 | 1D | 42 | 2A | 0 | 29 | 479511 | 3836084 | 0 | 29 | 0 | 112 | 3922420 | 0 | 29 | 0 | 1 | 120 |
| 30 | 1E | 42 | 2A | 0 | 30 | 479525 | 3836196 | 0 | 30 | 0 | 112 | 3922540 | 0 | 30 | 0 | 1 | 120 |
| 31 | 1F | 42 | 2A | 0 | 31 | 479539 | 3836308 | 0 | 31 | 0 | 112 | 3922660 | 0 | 31 | 0 | 1 | 120 |
| 32 | 20 | 42 | 2A | 0 | 32 | 479553 | 3836420 | 0 | 32 | 0 | 112 | 3922780 | 0 | 32 | 0 | 1 | 120 |
| 33 | 21 | 42 | 2A | 0 | 33 | 479567 | 3836532 | 0 | 33 | 0 | 112 | 3922900 | 0 | 33 | 0 | 1 | 120 |
| 34 | 22 | 42 | 2A | 0 | 34 | 479581 | 3836644 | 0 | 34 | 0 | 112 | 3923020 | 0 | 34 | 0 | 1 | 120 |
| 35 | 23 | 42 | 2A | 0 | 35 | 479595 | 3836756 | 0 | 35 | 0 | 112 | 3923140 | 0 | 35 | 0 | 1 | 120 |
| 36 | 24 | 42 | 2A | 0 | 36 | 479609 | 3836868 | 0 | 36 | 0 | 112 | 3923260 | 0 | 36 | 0 | 1 | 120 |
| 37 | 25 | 42 | 2A | 0 | 37 | 479623 | 3836980 | 0 | 37 | 0 | 112 | 3923380 | 0 | 37 | 0 | 1 | 120 |
| 38 | 26 | 42 | 2A | 0 | 38 | 479637 | 3837092 | 0 | 38 | 0 | 112 | 3923500 | 0 | 38 | 0 | 1 | 120 |
| 39 | 27 | 42 | 2A | 0 | 39 | 479651 | 3837204 | 0 | 39 | 0 | 112 | 3923620 | 0 | 39 | 0 | 1 | 120 |
| 40 | 28 | 42 | 2A | 0 | 40 | 479665 | 3837316 | 0 | 40 | 0 | 112 | 3923740 | 0 | 40 | 0 | 1 | 120 |
| 41 | 29 | 42 | 2A | 0 | 41 | 479679 | 3837428 | 0 | 41 | 0 | 112 | 3923860 | 0 | 41 | 0 | 1 | 120 |
| 42 | 2A | 42 | 2A | 1 | 0 | 479691 | 3837524 | 1 | 0 | 0 | 96 | 3923964 | 1 | 0 | 0 | 1 | 104 |
| 43 | 2B | 42 | 2A | 1 | 1 | 479708 | 3837660 | 1 | 1 | 0 | 136 | 3924108 | 1 | 1 | 0 | 1 | 144 |
| 44 | 2C | 42 | 2A | 1 | 2 | 479725 | 3837796 | 1 | 2 | 0 | 136 | 3924252 | 1 | 2 | 0 | 1 | 144 |
| 45 | 2D | 42 | 2A | 1 | 3 | 479742 | 3837932 | 1 | 3 | 0 | 136 | 3924396 | 1 | 3 | 0 | 1 | 144 |
| 46 | 2E | 42 | 2A | 1 | 4 | 479759 | 3838068 | 1 | 4 | 0 | 136 | 3924540 | 1 | 4 | 0 | 1 | 144 |
| 47 | 2F | 42 | 2A | 1 | 5 | 479776 | 3838204 | 1 | 5 | 0 | 136 | 3924684 | 1 | 5 | 0 | 1 | 144 |
| 48 | 30 | 42 | 2A | 1 | 6 | 479797 | 3838372 | 1 | 6 | 0 | 168 | 3924860 | 1 | 6 | 0 | 1 | 176 |
| 49 | 31 | 42 | 2A | 1 | 7 | 479818 | 3838540 | 1 | 7 | 0 | 168 | 3925036 | 1 | 7 | 0 | 1 | 176 |
| 50 | 32 | 42 | 2A | 1 | 8 | 479839 | 3838708 | 1 | 8 | 0 | 168 | 3925212 | 1 | 8 | 0 | 1 | 176 |
| 51 | 33 | 42 | 2A | 1 | 9 | 479860 | 3838876 | 1 | 9 | 0 | 168 | 3925388 | 1 | 9 | 0 | 1 | 176 |
| 52 | 34 | 42 | 2A | 1 | 10 | 479881 | 3839044 | 1 | 10 | 0 | 168 | 3925564 | 1 | 10 | 0 | 1 | 176 |
| 53 | 35 | 42 | 2A | 1 | 11 | 479902 | 3839212 | 1 | 11 | 0 | 168 | 3925740 | 1 | 11 | 0 | 1 | 176 |
| 54 | 36 | 42 | 2A | 1 | 12 | 479923 | 3839380 | 1 | 12 | 0 | 168 | 3925916 | 1 | 12 | 0 | 1 | 176 |
| 55 | 37 | 42 | 2A | 1 | 13 | 479944 | 3839548 | 1 | 13 | 0 | 168 | 3926092 | 1 | 13 | 0 | 1 | 176 |
| 56 | 38 | 42 | 2A | 1 | 14 | 479965 | 3839716 | 1 | 14 | 0 | 168 | 3926268 | 1 | 14 | 0 | 1 | 176 |
| 57 | 39 | 42 | 2A | 1 | 15 | 479986 | 3839884 | 1 | 15 | 0 | 168 | 3926444 | 1 | 15 | 0 | 1 | 176 |
| 58 | 3A | 42 | 2A | 1 | 16 | 480007 | 3840052 | 1 | 16 | 0 | 168 | 3926620 | 1 | 16 | 0 | 1 | 176 |
| 59 | 3B | 42 | 2A | 1 | 17 | 480028 | 3840220 | 1 | 17 | 0 | 168 | 3926796 | 1 | 17 | 0 | 1 | 176 |

| | | | | | | | | | | | | | | | | | |
|---|---|---|---|---|---|---|---|---|---|---|---|---|---|---|---|---|---|
| 60 | 3C | 42 | 2A | 1 | 18 | 480049 | 3840388 | 1 | 18 | 0 | 168 | 3926972 | 1 | 18 | 0 | 1 | 176 |
| 61 | 3D | 42 | 2A | 1 | 19 | 480070 | 3840556 | 1 | 19 | 0 | 168 | 3927148 | 1 | 19 | 0 | 1 | 176 |
| 62 | 3E | 42 | 2A | 1 | 20 | 480091 | 3840724 | 1 | 20 | 0 | 168 | 3927324 | 1 | 20 | 0 | 1 | 176 |
| 63 | 3F | 42 | 2A | 1 | 21 | 480112 | 3840892 | 1 | 21 | 0 | 168 | 3927500 | 1 | 21 | 0 | 1 | 176 |
| 64 | 40 | 42 | 2A | 1 | 22 | 480135 | 3841076 | 1 | 22 | 0 | 184 | 3927692 | 1 | 22 | 0 | 1 | 192 |
| 65 | 41 | 42 | 2A | 1 | 23 | 480158 | 3841260 | 1 | 23 | 0 | 184 | 3927884 | 1 | 23 | 0 | 1 | 192 |
| 66 | 42 | 42 | 2A | 1 | 24 | 480181 | 3841444 | 1 | 24 | 0 | 184 | 3928076 | 1 | 24 | 0 | 1 | 192 |
| 67 | 43 | 42 | 2A | 1 | 25 | 480204 | 3841628 | 1 | 25 | 0 | 184 | 3928268 | 1 | 25 | 0 | 1 | 192 |
| 68 | 44 | 42 | 2A | 1 | 26 | 480227 | 3841812 | 1 | 26 | 0 | 184 | 3928460 | 1 | 26 | 0 | 1 | 192 |
| 69 | 45 | 42 | 2A | 1 | 27 | 480250 | 3841996 | 1 | 27 | 0 | 184 | 3928652 | 1 | 27 | 0 | 1 | 192 |
| 70 | 46 | 42 | 2A | 1 | 28 | 480273 | 3842180 | 1 | 28 | 0 | 184 | 3928844 | 1 | 28 | 0 | 1 | 192 |
| 71 | 47 | 42 | 2A | 1 | 29 | 480296 | 3842364 | 1 | 29 | 0 | 184 | 3929036 | 1 | 29 | 0 | 1 | 192 |
| 72 | 48 | 42 | 2A | 1 | 30 | 480319 | 3842548 | 1 | 30 | 0 | 184 | 3929228 | 1 | 30 | 0 | 1 | 192 |
| 73 | 49 | 42 | 2A | 1 | 31 | 480342 | 3842732 | 1 | 31 | 0 | 184 | 3929420 | 1 | 31 | 0 | 1 | 192 |
| 74 | 4A | 42 | 2A | 1 | 32 | 480365 | 3842916 | 1 | 32 | 0 | 184 | 3929612 | 1 | 32 | 0 | 1 | 192 |
| 75 | 4B | 42 | 2A | 1 | 33 | 480388 | 3843100 | 1 | 33 | 0 | 184 | 3929804 | 1 | 33 | 0 | 1 | 192 |
| 76 | 4C | 42 | 2A | 1 | 34 | 480411 | 3843284 | 1 | 34 | 0 | 184 | 3929996 | 1 | 34 | 0 | 1 | 192 |
| 77 | 4D | 42 | 2A | 1 | 35 | 480434 | 3843468 | 1 | 35 | 0 | 184 | 3930188 | 1 | 35 | 0 | 1 | 192 |
| 78 | 4E | 42 | 2A | 1 | 36 | 480457 | 3843652 | 1 | 36 | 0 | 184 | 3930380 | 1 | 36 | 0 | 1 | 192 |
| 79 | 4F | 42 | 2A | 1 | 37 | 480480 | 3843836 | 1 | 37 | 0 | 184 | 3930572 | 1 | 37 | 0 | 1 | 192 |
| 80 | 50 | 42 | 2A | 1 | 38 | 480503 | 3844020 | 1 | 38 | 0 | 184 | 3930764 | 1 | 38 | 0 | 1 | 192 |
| 81 | 51 | 42 | 2A | 1 | 39 | 480526 | 3844204 | 1 | 39 | 0 | 184 | 3930956 | 1 | 39 | 0 | 1 | 192 |
| 82 | 52 | 42 | 2A | 1 | 40 | 480549 | 3844388 | 1 | 40 | 0 | 184 | 3931148 | 1 | 40 | 0 | 1 | 192 |
| 83 | 53 | 42 | 2A | 1 | 41 | 480572 | 3844572 | 1 | 41 | 0 | 184 | 3931340 | 1 | 41 | 0 | 1 | 192 |
| 84 | 54 | 42 | 2A | 2 | 0 | 480593 | 3844740 | 2 | 0 | 0 | 168 | 3931516 | 2 | 0 | 0 | 1 | 176 |
| 85 | 55 | 42 | 2A | 2 | 1 | 480619 | 3844948 | 2 | 1 | 0 | 208 | 3931732 | 2 | 1 | 0 | 1 | 216 |
| 86 | 56 | 42 | 2A | 2 | 2 | 480645 | 3845156 | 2 | 2 | 0 | 208 | 3931948 | 2 | 2 | 0 | 1 | 216 |
| 87 | 57 | 42 | 2A | 2 | 3 | 480671 | 3845364 | 2 | 3 | 0 | 208 | 3932164 | 2 | 3 | 0 | 1 | 216 |
| 88 | 58 | 42 | 2A | 2 | 4 | 480697 | 3845572 | 2 | 4 | 0 | 208 | 3932380 | 2 | 4 | 0 | 1 | 216 |
| 89 | 59 | 42 | 2A | 2 | 5 | 480723 | 3845780 | 2 | 5 | 0 | 208 | 3932596 | 2 | 5 | 0 | 1 | 216 |
| 90 | 5A | 42 | 2A | 2 | 6 | 480749 | 3845988 | 2 | 6 | 0 | 208 | 3932812 | 2 | 6 | 0 | 1 | 216 |
| 91 | 5B | 42 | 2A | 2 | 7 | 480775 | 3846196 | 2 | 7 | 0 | 208 | 3933028 | 2 | 7 | 0 | 1 | 216 |
| 92 | 5C | 42 | 2A | 2 | 8 | 480801 | 3846404 | 2 | 8 | 0 | 208 | 3933244 | 2 | 8 | 0 | 1 | 216 |
| 93 | 5D | 42 | 2A | 2 | 9 | 480827 | 3846612 | 2 | 9 | 0 | 208 | 3933460 | 2 | 9 | 0 | 1 | 216 |
| 94 | 5E | 42 | 2A | 2 | 10 | 480853 | 3846820 | 2 | 10 | 0 | 208 | 3933676 | 2 | 10 | 0 | 1 | 216 |
| 95 | 5F | 42 | 2A | 2 | 11 | 480879 | 3847028 | 2 | 11 | 0 | 208 | 3933892 | 2 | 11 | 0 | 1 | 216 |
| 96 | 60 | 42 | 2A | 2 | 12 | 480902 | 3847212 | 2 | 12 | 0 | 184 | 3934084 | 2 | 12 | 0 | 1 | 192 |
| 97 | 61 | 42 | 2A | 2 | 13 | 480925 | 3847396 | 2 | 13 | 0 | 184 | 3934276 | 2 | 13 | 0 | 1 | 192 |
| 98 | 62 | 42 | 2A | 2 | 14 | 480948 | 3847580 | 2 | 14 | 0 | 184 | 3934468 | 2 | 14 | 0 | 1 | 192 |
| 99 | 63 | 42 | 2A | 2 | 15 | 480971 | 3847764 | 2 | 15 | 0 | 184 | 3934660 | 2 | 15 | 0 | 1 | 192 |
| 100 | 64 | 42 | 2A | 2 | 16 | 480994 | 3847948 | 2 | 16 | 0 | 184 | 3934852 | 2 | 16 | 0 | 1 | 192 |
| 101 | 65 | 42 | 2A | 2 | 17 | 481017 | 3848132 | 2 | 17 | 0 | 184 | 3935044 | 2 | 17 | 0 | 1 | 192 |
| 102 | 66 | 42 | 2A | 2 | 18 | 481040 | 3848316 | 2 | 18 | 0 | 184 | 3935236 | 2 | 18 | 0 | 1 | 192 |
| 103 | 67 | 42 | 2A | 2 | 19 | 481063 | 3848500 | 2 | 19 | 0 | 184 | 3935428 | 2 | 19 | 0 | 1 | 192 |
| 104 | 68 | 42 | 2A | 2 | 20 | 481086 | 3848684 | 2 | 20 | 0 | 184 | 3935620 | 2 | 20 | 0 | 1 | 192 |
| 105 | 69 | 42 | 2A | 2 | 21 | 481109 | 3848868 | 2 | 21 | 0 | 184 | 3935812 | 2 | 21 | 0 | 1 | 192 |
| 106 | 6A | 42 | 2A | 2 | 22 | 481132 | 3849052 | 2 | 22 | 0 | 184 | 3936004 | 2 | 22 | 0 | 1 | 192 |
| 107 | 6B | 42 | 2A | 2 | 23 | 481155 | 3849236 | 2 | 23 | 0 | 184 | 3936196 | 2 | 23 | 0 | 1 | 192 |
| 108 | 6C | 42 | 2A | 2 | 24 | 481178 | 3849420 | 2 | 24 | 0 | 184 | 3936388 | 2 | 24 | 0 | 1 | 192 |
| 109 | 6D | 42 | 2A | 2 | 25 | 481201 | 3849604 | 2 | 25 | 0 | 184 | 3936580 | 2 | 25 | 0 | 1 | 192 |
| 110 | 6E | 42 | 2A | 2 | 26 | 481224 | 3849788 | 2 | 26 | 0 | 184 | 3936772 | 2 | 26 | 0 | 1 | 192 |
| 111 | 6F | 42 | 2A | 2 | 27 | 481247 | 3849972 | 2 | 27 | 0 | 184 | 3936964 | 2 | 27 | 0 | 1 | 192 |
| 112 | 70 | 42 | 2A | 2 | 28 | 481272 | 3850172 | 2 | 28 | 0 | 200 | 3937172 | 2 | 28 | 0 | 1 | 208 |
| 113 | 71 | 42 | 2A | 2 | 29 | 481297 | 3850372 | 2 | 29 | 0 | 200 | 3937380 | 2 | 29 | 0 | 1 | 208 |
| 114 | 72 | 42 | 2A | 2 | 30 | 481322 | 3850572 | 2 | 30 | 0 | 200 | 3937588 | 2 | 30 | 0 | 1 | 208 |
| 115 | 73 | 42 | 2A | 2 | 31 | 481347 | 3850772 | 2 | 31 | 0 | 200 | 3937796 | 2 | 31 | 0 | 1 | 208 |
| 116 | 74 | 42 | 2A | 2 | 32 | 481372 | 3850972 | 2 | 32 | 0 | 200 | 3938004 | 2 | 32 | 0 | 1 | 208 |
| 117 | 75 | 42 | 2A | 2 | 33 | 481397 | 3851172 | 2 | 33 | 0 | 200 | 3938212 | 2 | 33 | 0 | 1 | 208 |
| 118 | 76 | 42 | 2A | 2 | 34 | 481422 | 3851372 | 2 | 34 | 0 | 200 | 3938420 | 2 | 34 | 0 | 1 | 208 |
| 119 | 77 | 42 | 2A | 2 | 35 | 481447 | 3851572 | 2 | 35 | 0 | 200 | 3938628 | 2 | 35 | 0 | 1 | 208 |
| 120 | 78 | 42 | 2A | 2 | 36 | 481472 | 3851772 | 2 | 36 | 0 | 200 | 3938836 | 2 | 36 | 0 | 1 | 208 |
| 121 | 79 | 42 | 2A | 2 | 37 | 481497 | 3851972 | 2 | 37 | 0 | 200 | 3939044 | 2 | 37 | 0 | 1 | 208 |
| 122 | 7A | 42 | 2A | 2 | 38 | 481522 | 3852172 | 2 | 38 | 0 | 200 | 3939252 | 2 | 38 | 0 | 1 | 208 |
| 123 | 7B | 42 | 2A | 2 | 39 | 481547 | 3852372 | 2 | 39 | 0 | 200 | 3939460 | 2 | 39 | 0 | 1 | 208 |
| 124 | 7C | 42 | 2A | 2 | 40 | 481572 | 3852572 | 2 | 40 | 0 | 200 | 3939668 | 2 | 40 | 0 | 1 | 208 |
| 125 | 7D | 42 | 2A | 2 | 41 | 481597 | 3852772 | 2 | 41 | 0 | 200 | 3939876 | 2 | 41 | 0 | 1 | 208 |
| 126 | 7E | 42 | 2A | 3 | 0 | 481620 | 3852956 | 3 | 0 | 0 | 184 | 3940068 | 3 | 0 | 0 | 1 | 192 |
| 127 | 7F | 42 | 2A | 3 | 1 | 481648 | 3853180 | 3 | 1 | 0 | 224 | 3940300 | 3 | 1 | 0 | 1 | 232 |
| 128 | 80 | 42 | 2A | 3 | 2 | 481676 | 3853404 | 3 | 2 | 0 | 224 | 3940532 | 3 | 2 | 0 | 1 | 232 |
| 129 | 81 | 42 | 2A | 3 | 3 | 481704 | 3853628 | 3 | 3 | 0 | 224 | 3940764 | 3 | 3 | 0 | 1 | 232 |
| 130 | 82 | 42 | 2A | 3 | 4 | 481732 | 3853852 | 3 | 4 | 0 | 224 | 3940996 | 3 | 4 | 0 | 1 | 232 |
| 131 | 83 | 42 | 2A | 3 | 5 | 481760 | 3854076 | 3 | 5 | 0 | 224 | 3941228 | 3 | 5 | 0 | 1 | 232 |
| 132 | 84 | 42 | 2A | 3 | 6 | 481788 | 3854300 | 3 | 6 | 0 | 224 | 3941460 | 3 | 6 | 0 | 1 | 232 |
| 133 | 85 | 42 | 2A | 3 | 7 | 481816 | 3854524 | 3 | 7 | 0 | 224 | 3941692 | 3 | 7 | 0 | 1 | 232 |
| 134 | 86 | 42 | 2A | 3 | 8 | 481844 | 3854748 | 3 | 8 | 0 | 224 | 3941924 | 3 | 8 | 0 | 1 | 232 |
| 135 | 87 | 42 | 2A | 3 | 9 | 481872 | 3854972 | 3 | 9 | 0 | 224 | 3942156 | 3 | 9 | 0 | 1 | 232 |
| 136 | 88 | 42 | 2A | 3 | 10 | 481900 | 3855196 | 3 | 10 | 0 | 224 | 3942388 | 3 | 10 | 0 | 1 | 232 |
| 137 | 89 | 42 | 2A | 3 | 11 | 481928 | 3855420 | 3 | 11 | 0 | 224 | 3942620 | 3 | 11 | 0 | 1 | 232 |
| 138 | 8A | 42 | 2A | 3 | 12 | 481956 | 3855644 | 3 | 12 | 0 | 224 | 3942852 | 3 | 12 | 0 | 1 | 232 |
| 139 | 8B | 42 | 2A | 3 | 13 | 481984 | 3855868 | 3 | 13 | 0 | 224 | 3943084 | 3 | 13 | 0 | 1 | 232 |
| 140 | 8C | 42 | 2A | 3 | 14 | 482012 | 3856092 | 3 | 14 | 0 | 224 | 3943316 | 3 | 14 | 0 | 1 | 232 |
| 141 | 8D | 42 | 2A | 3 | 15 | 482040 | 3856316 | 3 | 15 | 0 | 224 | 3943548 | 3 | 15 | 0 | 1 | 232 |
| 142 | 8E | 42 | 2A | 3 | 16 | 482068 | 3856540 | 3 | 16 | 0 | 224 | 3943780 | 3 | 16 | 0 | 1 | 232 |
| 143 | 8F | 42 | 2A | 3 | 17 | 482096 | 3856764 | 3 | 17 | 0 | 224 | 3944012 | 3 | 17 | 0 | 1 | 232 |
| 144 | 90 | 42 | 2A | 3 | 18 | 482121 | 3856964 | 3 | 18 | 0 | 200 | 3944220 | 3 | 18 | 0 | 1 | 208 |
| 145 | 91 | 42 | 2A | 3 | 19 | 482146 | 3857164 | 3 | 19 | 0 | 200 | 3944428 | 3 | 19 | 0 | 1 | 208 |
| 146 | 92 | 42 | 2A | 3 | 20 | 482171 | 3857364 | 3 | 20 | 0 | 200 | 3944636 | 3 | 20 | 0 | 1 | 208 |
| 147 | 93 | 42 | 2A | 3 | 21 | 482196 | 3857564 | 3 | 21 | 0 | 200 | 3944844 | 3 | 21 | 0 | 1 | 208 |
| 148 | 94 | 42 | 2A | 3 | 22 | 482221 | 3857764 | 3 | 22 | 0 | 200 | 3945052 | 3 | 22 | 0 | 1 | 208 |
| 149 | 95 | 42 | 2A | 3 | 23 | 482246 | 3857964 | 3 | 23 | 0 | 200 | 3945260 | 3 | 23 | 0 | 1 | 208 |
| 150 | 96 | 42 | 2A | 3 | 24 | 482271 | 3858164 | 3 | 24 | 0 | 200 | 3945468 | 3 | 24 | 0 | 1 | 208 |
| 151 | 97 | 42 | 2A | 3 | 25 | 482296 | 3858364 | 3 | 25 | 0 | 200 | 3945676 | 3 | 25 | 0 | 1 | 208 |
| 152 | 98 | 42 | 2A | 3 | 26 | 482321 | 3858564 | 3 | 26 | 0 | 200 | 3945884 | 3 | 26 | 0 | 1 | 208 |
| 153 | 99 | 42 | 2A | 3 | 27 | 482346 | 3858764 | 3 | 27 | 0 | 200 | 3946092 | 3 | 27 | 0 | 1 | 208 |
| 154 | 9A | 42 | 2A | 3 | 28 | 482371 | 3858964 | 3 | 28 | 0 | 200 | 3946300 | 3 | 28 | 0 | 1 | 208 |
| 155 | 9B | 42 | 2A | 3 | 29 | 482396 | 3859164 | 3 | 29 | 0 | 200 | 3946508 | 3 | 29 | 0 | 1 | 208 |
| 156 | 9C | 42 | 2A | 3 | 30 | 482421 | 3859364 | 3 | 30 | 0 | 200 | 3946716 | 3 | 30 | 0 | 1 | 208 |
| 157 | 9D | 42 | 2A | 3 | 31 | 482446 | 3859564 | 3 | 31 | 0 | 200 | 3946924 | 3 | 31 | 0 | 1 | 208 |
| 158 | 9E | 42 | 2A | 3 | 32 | 482471 | 3859764 | 3 | 32 | 0 | 200 | 3947132 | 3 | 32 | 0 | 1 | 208 |
| 159 | 9F | 42 | 2A | 3 | 33 | 482496 | 3859964 | 3 | 33 | 0 | 200 | 3947340 | 3 | 33 | 0 | 1 | 208 |
| 160 | A0 | 42 | 2A | 3 | 34 | 482523 | 3860180 | 3 | 34 | 0 | 216 | 3947564 | 3 | 34 | 0 | 1 | 224 |
| 161 | A1 | 42 | 2A | 3 | 35 | 482550 | 3860396 | 3 | 35 | 0 | 216 | 3947788 | 3 | 35 | 0 | 1 | 224 |
| 162 | A2 | 42 | 2A | 3 | 36 | 482577 | 3860612 | 3 | 36 | 0 | 216 | 3948012 | 3 | 36 | 0 | 1 | 224 |

| | | | | | | | | | | | | | | | | | |
|---|---|---|---|---|---|---|---|---|---|---|---|---|---|---|---|---|---|
| 163 | A3 | 42 | 2A | 3 | 37 | 482604 | 3860828 | 3 | 37 | 0 | 216 | 3948236 | 3 | 37 | 0 | 1 | 224 |
| 164 | A4 | 42 | 2A | 3 | 38 | 482631 | 3861044 | 3 | 38 | 0 | 216 | 3948460 | 3 | 38 | 0 | 1 | 224 |
| 165 | A5 | 42 | 2A | 3 | 39 | 482658 | 3861260 | 3 | 39 | 0 | 216 | 3948684 | 3 | 39 | 0 | 1 | 224 |
| 166 | A6 | 42 | 2A | 3 | 40 | 482685 | 3861476 | 3 | 40 | 0 | 216 | 3948908 | 3 | 40 | 0 | 1 | 224 |
| 167 | A7 | 42 | 2A | 3 | 41 | 482712 | 3861692 | 3 | 41 | 0 | 216 | 3949132 | 3 | 41 | 0 | 1 | 224 |
| 168 | A8 | 42 | 2A | 4 | 0 | 482737 | 3861892 | 4 | 0 | 0 | 200 | 3949340 | 4 | 0 | 0 | 1 | 208 |
| 169 | A9 | 42 | 2A | 4 | 1 | 482767 | 3862132 | 4 | 1 | 0 | 240 | 3949588 | 4 | 1 | 0 | 1 | 248 |
| 170 | AA | 42 | 2A | 4 | 2 | 482797 | 3862372 | 4 | 2 | 0 | 240 | 3949836 | 4 | 2 | 0 | 1 | 248 |
| 171 | AB | 42 | 2A | 4 | 3 | 482827 | 3862612 | 4 | 3 | 0 | 240 | 3950084 | 4 | 3 | 0 | 1 | 248 |
| 172 | AC | 42 | 2A | 4 | 4 | 482857 | 3862852 | 4 | 4 | 0 | 240 | 3950332 | 4 | 4 | 0 | 1 | 248 |
| 173 | AD | 42 | 2A | 4 | 5 | 482887 | 3863092 | 4 | 5 | 0 | 240 | 3950580 | 4 | 5 | 0 | 1 | 248 |
| 174 | AE | 42 | 2A | 4 | 6 | 482917 | 3863332 | 4 | 6 | 0 | 240 | 3950828 | 4 | 6 | 0 | 1 | 248 |
| 175 | AF | 42 | 2A | 4 | 7 | 482947 | 3863572 | 4 | 7 | 0 | 240 | 3951076 | 4 | 7 | 0 | 1 | 248 |
| 176 | B0 | 42 | 2A | 4 | 8 | 482977 | 3863812 | 4 | 8 | 0 | 240 | 3951324 | 4 | 8 | 0 | 1 | 248 |
| 177 | B1 | 42 | 2A | 4 | 9 | 483007 | 3864052 | 4 | 9 | 0 | 240 | 3951572 | 4 | 9 | 0 | 1 | 248 |
| 178 | B2 | 42 | 2A | 4 | 10 | 483037 | 3864292 | 4 | 10 | 0 | 240 | 3951820 | 4 | 10 | 0 | 1 | 248 |
| 179 | B3 | 42 | 2A | 4 | 11 | 483067 | 3864532 | 4 | 11 | 0 | 240 | 3952068 | 4 | 11 | 0 | 1 | 248 |
| 180 | B4 | 42 | 2A | 4 | 12 | 483097 | 3864772 | 4 | 12 | 0 | 240 | 3952316 | 4 | 12 | 0 | 1 | 248 |
| 181 | B5 | 42 | 2A | 4 | 13 | 483127 | 3865012 | 4 | 13 | 0 | 240 | 3952564 | 4 | 13 | 0 | 1 | 248 |
| 182 | B6 | 42 | 2A | 4 | 14 | 483157 | 3865252 | 4 | 14 | 0 | 240 | 3952812 | 4 | 14 | 0 | 1 | 248 |
| 183 | B7 | 42 | 2A | 4 | 15 | 483187 | 3865492 | 4 | 15 | 0 | 240 | 3953060 | 4 | 15 | 0 | 1 | 248 |
| 184 | B8 | 42 | 2A | 4 | 16 | 483217 | 3865732 | 4 | 16 | 0 | 240 | 3953308 | 4 | 16 | 0 | 1 | 248 |
| 185 | B9 | 42 | 2A | 4 | 17 | 483247 | 3865972 | 4 | 17 | 0 | 240 | 3953556 | 4 | 17 | 0 | 1 | 248 |
| 186 | BA | 42 | 2A | 4 | 18 | 483277 | 3866212 | 4 | 18 | 0 | 240 | 3953804 | 4 | 18 | 0 | 1 | 248 |
| 187 | BB | 42 | 2A | 4 | 19 | 483307 | 3866452 | 4 | 19 | 0 | 240 | 3954052 | 4 | 19 | 0 | 1 | 248 |
| 188 | BC | 42 | 2A | 4 | 20 | 483337 | 3866692 | 4 | 20 | 0 | 240 | 3954300 | 4 | 20 | 0 | 1 | 248 |
| 189 | BD | 42 | 2A | 4 | 21 | 483367 | 3866932 | 4 | 21 | 0 | 240 | 3954548 | 4 | 21 | 0 | 1 | 248 |
| 190 | BE | 42 | 2A | 4 | 22 | 483397 | 3867172 | 4 | 22 | 0 | 240 | 3954796 | 4 | 22 | 0 | 1 | 248 |
| 191 | BF | 42 | 2A | 4 | 23 | 483427 | 3867412 | 4 | 23 | 0 | 240 | 3955044 | 4 | 23 | 0 | 1 | 248 |
| 192 | C0 | 42 | 2A | 4 | 24 | 483454 | 3867628 | 4 | 24 | 0 | 216 | 3955268 | 4 | 24 | 0 | 1 | 224 |
| 193 | C1 | 42 | 2A | 4 | 25 | 483481 | 3867844 | 4 | 25 | 0 | 216 | 3955492 | 4 | 25 | 0 | 1 | 224 |
| 194 | C2 | 42 | 2A | 4 | 26 | 483508 | 3868060 | 4 | 26 | 0 | 216 | 3955716 | 4 | 26 | 0 | 1 | 224 |
| 195 | C3 | 42 | 2A | 4 | 27 | 483535 | 3868276 | 4 | 27 | 0 | 216 | 3955940 | 4 | 27 | 0 | 1 | 224 |
| 196 | C4 | 42 | 2A | 4 | 28 | 483562 | 3868492 | 4 | 28 | 0 | 216 | 3956164 | 4 | 28 | 0 | 1 | 224 |
| 197 | C5 | 42 | 2A | 4 | 29 | 483589 | 3868708 | 4 | 29 | 0 | 216 | 3956388 | 4 | 29 | 0 | 1 | 224 |
| 198 | C6 | 42 | 2A | 4 | 30 | 483616 | 3868924 | 4 | 30 | 0 | 216 | 3956612 | 4 | 30 | 0 | 1 | 224 |
| 199 | C7 | 42 | 2A | 4 | 31 | 483643 | 3869140 | 4 | 31 | 0 | 216 | 3956836 | 4 | 31 | 0 | 1 | 224 |
| 200 | C8 | 42 | 2A | 4 | 32 | 483670 | 3869356 | 4 | 32 | 0 | 216 | 3957060 | 4 | 32 | 0 | 1 | 224 |
| 201 | C9 | 42 | 2A | 4 | 33 | 483697 | 3869572 | 4 | 33 | 0 | 216 | 3957284 | 4 | 33 | 0 | 1 | 224 |
| 202 | CA | 42 | 2A | 4 | 34 | 483724 | 3869788 | 4 | 34 | 0 | 216 | 3957508 | 4 | 34 | 0 | 1 | 224 |
| 203 | CB | 42 | 2A | 4 | 35 | 483751 | 3870004 | 4 | 35 | 0 | 216 | 3957732 | 4 | 35 | 0 | 1 | 224 |
| 204 | CC | 42 | 2A | 4 | 36 | 483778 | 3870220 | 4 | 36 | 0 | 216 | 3957956 | 4 | 36 | 0 | 1 | 224 |
| 205 | CD | 42 | 2A | 4 | 37 | 483805 | 3870436 | 4 | 37 | 0 | 216 | 3958180 | 4 | 37 | 0 | 1 | 224 |
| 206 | CE | 42 | 2A | 4 | 38 | 483832 | 3870652 | 4 | 38 | 0 | 216 | 3958404 | 4 | 38 | 0 | 1 | 224 |
| 207 | CF | 42 | 2A | 4 | 39 | 483859 | 3870868 | 4 | 39 | 0 | 216 | 3958628 | 4 | 39 | 0 | 1 | 224 |
| 208 | D0 | 42 | 2A | 4 | 40 | 483888 | 3871100 | 4 | 40 | 0 | 232 | 3958868 | 4 | 40 | 0 | 1 | 240 |
| 209 | D1 | 42 | 2A | 4 | 41 | 483917 | 3871332 | 4 | 41 | 0 | 232 | 3959108 | 4 | 41 | 0 | 1 | 240 |
| 210 | D2 | 42 | 2A | 5 | 0 | 483944 | 3871548 | 5 | 0 | 0 | 216 | 3959332 | 5 | 0 | 0 | 1 | 224 |
| 211 | D3 | 42 | 2A | 5 | 1 | 483976 | 3871804 | 5 | 1 | 0 | 256 | 3959596 | 5 | 1 | 0 | 1 | 264 |
| 212 | D4 | 42 | 2A | 5 | 2 | 484008 | 3872060 | 5 | 2 | 0 | 256 | 3959860 | 5 | 2 | 0 | 1 | 264 |
| 213 | D5 | 42 | 2A | 5 | 3 | 484040 | 3872316 | 5 | 3 | 0 | 256 | 3960124 | 5 | 3 | 0 | 1 | 264 |
| 214 | D6 | 42 | 2A | 5 | 4 | 484072 | 3872572 | 5 | 4 | 0 | 256 | 3960388 | 5 | 4 | 0 | 1 | 264 |
| 215 | D7 | 42 | 2A | 5 | 5 | 484104 | 3872828 | 5 | 5 | 0 | 256 | 3960652 | 5 | 5 | 0 | 1 | 264 |
| 216 | D8 | 42 | 2A | 5 | 6 | 484136 | 3873084 | 5 | 6 | 0 | 256 | 3960916 | 5 | 6 | 0 | 1 | 264 |
| 217 | D9 | 42 | 2A | 5 | 7 | 484168 | 3873340 | 5 | 7 | 0 | 256 | 3961180 | 5 | 7 | 0 | 1 | 264 |
| 218 | DA | 42 | 2A | 5 | 8 | 484200 | 3873596 | 5 | 8 | 0 | 256 | 3961444 | 5 | 8 | 0 | 1 | 264 |
| 219 | DB | 42 | 2A | 5 | 9 | 484232 | 3873852 | 5 | 9 | 0 | 256 | 3961708 | 5 | 9 | 0 | 1 | 264 |
| 220 | DC | 42 | 2A | 5 | 10 | 484264 | 3874108 | 5 | 10 | 0 | 256 | 3961972 | 5 | 10 | 0 | 1 | 264 |
| 221 | DD | 42 | 2A | 5 | 11 | 484296 | 3874364 | 5 | 11 | 0 | 256 | 3962236 | 5 | 11 | 0 | 1 | 264 |
| 222 | DE | 42 | 2A | 5 | 12 | 484328 | 3874620 | 5 | 12 | 0 | 256 | 3962500 | 5 | 12 | 0 | 1 | 264 |
| 223 | DF | 42 | 2A | 5 | 13 | 484360 | 3874876 | 5 | 13 | 0 | 256 | 3962764 | 5 | 13 | 0 | 1 | 264 |
| 224 | E0 | 42 | 2A | 5 | 14 | 484392 | 3875132 | 5 | 14 | 0 | 256 | 3963028 | 5 | 14 | 0 | 1 | 264 |
| 225 | E1 | 42 | 2A | 5 | 15 | 484424 | 3875388 | 5 | 15 | 0 | 256 | 3963292 | 5 | 15 | 0 | 1 | 264 |
| 226 | E2 | 42 | 2A | 5 | 16 | 484456 | 3875644 | 5 | 16 | 0 | 256 | 3963556 | 5 | 16 | 0 | 1 | 264 |
| 227 | E3 | 42 | 2A | 5 | 17 | 484488 | 3875900 | 5 | 17 | 0 | 256 | 3963820 | 5 | 17 | 0 | 1 | 264 |
| 228 | E4 | 42 | 2A | 5 | 18 | 484520 | 3876156 | 5 | 18 | 0 | 256 | 3964084 | 5 | 18 | 0 | 1 | 264 |
| 229 | E5 | 42 | 2A | 5 | 19 | 484552 | 3876412 | 5 | 19 | 0 | 256 | 3964348 | 5 | 19 | 0 | 1 | 264 |
| 230 | E6 | 42 | 2A | 5 | 20 | 484584 | 3876668 | 5 | 20 | 0 | 256 | 3964612 | 5 | 20 | 0 | 1 | 264 |
| 231 | E7 | 42 | 2A | 5 | 21 | 484616 | 3876924 | 5 | 21 | 0 | 256 | 3964876 | 5 | 21 | 0 | 1 | 264 |
| 232 | E8 | 42 | 2A | 5 | 22 | 484648 | 3877180 | 5 | 22 | 0 | 256 | 3965140 | 5 | 22 | 0 | 1 | 264 |
| 233 | E9 | 42 | 2A | 5 | 23 | 484680 | 3877436 | 5 | 23 | 0 | 256 | 3965404 | 5 | 23 | 0 | 1 | 264 |
| 234 | EA | 42 | 2A | 5 | 24 | 484712 | 3877692 | 5 | 24 | 0 | 256 | 3965668 | 5 | 24 | 0 | 1 | 264 |
| 235 | EB | 42 | 2A | 5 | 25 | 484744 | 3877948 | 5 | 25 | 0 | 256 | 3965932 | 5 | 25 | 0 | 1 | 264 |
| 236 | EC | 42 | 2A | 5 | 26 | 484776 | 3878204 | 5 | 26 | 0 | 256 | 3966196 | 5 | 26 | 0 | 1 | 264 |
| 237 | ED | 42 | 2A | 5 | 27 | 484808 | 3878460 | 5 | 27 | 0 | 256 | 3966460 | 5 | 27 | 0 | 1 | 264 |
| 238 | EE | 42 | 2A | 5 | 28 | 484840 | 3878716 | 5 | 28 | 0 | 256 | 3966724 | 5 | 28 | 0 | 1 | 264 |
| 239 | EF | 42 | 2A | 5 | 29 | 484872 | 3878972 | 5 | 29 | 0 | 256 | 3966988 | 5 | 29 | 0 | 1 | 264 |
| 240 | F0 | 42 | 2A | 5 | 30 | 484901 | 3879204 | 5 | 30 | 0 | 232 | 3967228 | 5 | 30 | 0 | 1 | 240 |
| 241 | F1 | 42 | 2A | 5 | 31 | 484930 | 3879436 | 5 | 31 | 0 | 232 | 3967468 | 5 | 31 | 0 | 1 | 240 |
| 242 | F2 | 42 | 2A | 5 | 32 | 484959 | 3879668 | 5 | 32 | 0 | 232 | 3967708 | 5 | 32 | 0 | 1 | 240 |
| 243 | F3 | 42 | 2A | 5 | 33 | 484988 | 3879900 | 5 | 33 | 0 | 232 | 3967948 | 5 | 33 | 0 | 1 | 240 |
| 244 | F4 | 42 | 2A | 5 | 34 | 485017 | 3880132 | 5 | 34 | 0 | 232 | 3968188 | 5 | 34 | 0 | 1 | 240 |
| 245 | F5 | 42 | 2A | 5 | 35 | 485046 | 3880364 | 5 | 35 | 0 | 232 | 3968428 | 5 | 35 | 0 | 1 | 240 |
| 246 | F6 | 42 | 2A | 5 | 36 | 485075 | 3880596 | 5 | 36 | 0 | 232 | 3968668 | 5 | 36 | 0 | 1 | 240 |
| 247 | F7 | 42 | 2A | 5 | 37 | 485104 | 3880828 | 5 | 37 | 0 | 232 | 3968908 | 5 | 37 | 0 | 1 | 240 |
| 248 | F8 | 42 | 2A | 5 | 38 | 485133 | 3881060 | 5 | 38 | 0 | 232 | 3969148 | 5 | 38 | 0 | 1 | 240 |
| 249 | F9 | 42 | 2A | 5 | 39 | 485162 | 3881292 | 5 | 39 | 0 | 232 | 3969388 | 5 | 39 | 0 | 1 | 240 |
| 250 | FA | 42 | 2A | 5 | 40 | 485191 | 3881524 | 5 | 40 | 0 | 232 | 3969628 | 5 | 40 | 0 | 1 | 240 |
| 251 | FB | 42 | 2A | 5 | 41 | 485220 | 3881756 | 5 | 41 | 0 | 232 | 3969868 | 5 | 41 | 0 | 1 | 240 |
| 252 | FC | 42 | 2A | 6 | 0 | 485247 | 3881972 | 6 | 0 | 0 | 216 | 3970092 | 6 | 0 | 0 | 1 | 224 |
| 253 | FD | 42 | 2A | 6 | 1 | 485279 | 3882228 | 6 | 1 | 0 | 256 | 3970356 | 6 | 1 | 0 | 1 | 264 |
| 254 | FE | 42 | 2A | 6 | 2 | 485311 | 3882484 | 6 | 2 | 0 | 256 | 3970620 | 6 | 2 | 0 | 1 | 264 |
| 255 | FF | 42 | 2A | 6 | 3 | 485343 | 3882740 | 6 | 3 | 0 | 256 | 3970884 | 6 | 3 | 0 | 1 | 264 |
| 0 | 00 | 43 | 2B | 0 | 0 | 485355 | 3882836 | 0 | 0 | 0 | 96 | 3970988 | 0 | 0 | 0 | 1 | 104 |
| 1 | 01 | 43 | 2B | 0 | 1 | 485369 | 3882948 | 0 | 1 | 0 | 112 | 3971108 | 0 | 1 | 0 | 1 | 120 |
| 2 | 02 | 43 | 2B | 0 | 2 | 485383 | 3883060 | 0 | 2 | 0 | 112 | 3971228 | 0 | 2 | 0 | 1 | 120 |
| 3 | 03 | 43 | 2B | 0 | 3 | 485397 | 3883172 | 0 | 3 | 0 | 112 | 3971348 | 0 | 3 | 0 | 1 | 120 |
| 4 | 04 | 43 | 2B | 0 | 4 | 485411 | 3883284 | 0 | 4 | 0 | 112 | 3971468 | 0 | 4 | 0 | 1 | 120 |
| 5 | 05 | 43 | 2B | 0 | 5 | 485425 | 3883396 | 0 | 5 | 0 | 112 | 3971588 | 0 | 5 | 0 | 1 | 120 |
| 6 | 06 | 43 | 2B | 0 | 6 | 485439 | 3883508 | 0 | 6 | 0 | 112 | 3971708 | 0 | 6 | 0 | 1 | 120 |
| 7 | 07 | 43 | 2B | 0 | 7 | 485453 | 3883620 | 0 | 7 | 0 | 112 | 3971828 | 0 | 7 | 0 | 1 | 120 |
| 8 | 08 | 43 | 2B | 0 | 8 | 485467 | 3883732 | 0 | 8 | 0 | 112 | 3971948 | 0 | 8 | 0 | 1 | 120 |
| 9 | 09 | 43 | 2B | 0 | 9 | 485481 | 3883844 | 0 | 9 | 0 | 112 | 3972068 | 0 | 9 | 0 | 1 | 120 |

| | | | | | | | | | | | | | | | | | |
|---|---|---|---|---|---|---|---|---|---|---|---|---|---|---|---|---|---|
| 10 | 0A | 43 | 2B | 0 | 10 | 485495 | 3883956 | 0 | 10 | 0 | 112 | 3972188 | 0 | 10 | 0 | 1 | 120 |
| 11 | 0B | 43 | 2B | 0 | 11 | 485509 | 3884068 | 0 | 11 | 0 | 112 | 3972308 | 0 | 11 | 0 | 1 | 120 |
| 12 | 0C | 43 | 2B | 0 | 12 | 485523 | 3884180 | 0 | 12 | 0 | 112 | 3972428 | 0 | 12 | 0 | 1 | 120 |
| 13 | 0D | 43 | 2B | 0 | 13 | 485537 | 3884292 | 0 | 13 | 0 | 112 | 3972548 | 0 | 13 | 0 | 1 | 120 |
| 14 | 0E | 43 | 2B | 0 | 14 | 485551 | 3884404 | 0 | 14 | 0 | 112 | 3972668 | 0 | 14 | 0 | 1 | 120 |
| 15 | 0F | 43 | 2B | 0 | 15 | 485565 | 3884516 | 0 | 15 | 0 | 112 | 3972788 | 0 | 15 | 0 | 1 | 120 |
| 16 | 10 | 43 | 2B | 0 | 16 | 485579 | 3884628 | 0 | 16 | 0 | 112 | 3972908 | 0 | 16 | 0 | 1 | 120 |
| 17 | 11 | 43 | 2B | 0 | 17 | 485593 | 3884740 | 0 | 17 | 0 | 112 | 3973028 | 0 | 17 | 0 | 1 | 120 |
| 18 | 12 | 43 | 2B | 0 | 18 | 485607 | 3884852 | 0 | 18 | 0 | 112 | 3973148 | 0 | 18 | 0 | 1 | 120 |
| 19 | 13 | 43 | 2B | 0 | 19 | 485621 | 3884964 | 0 | 19 | 0 | 112 | 3973268 | 0 | 19 | 0 | 1 | 120 |
| 20 | 14 | 43 | 2B | 0 | 20 | 485635 | 3885076 | 0 | 20 | 0 | 112 | 3973388 | 0 | 20 | 0 | 1 | 120 |
| 21 | 15 | 43 | 2B | 0 | 21 | 485649 | 3885188 | 0 | 21 | 0 | 112 | 3973508 | 0 | 21 | 0 | 1 | 120 |
| 22 | 16 | 43 | 2B | 0 | 22 | 485663 | 3885300 | 0 | 22 | 0 | 112 | 3973628 | 0 | 22 | 0 | 1 | 120 |
| 23 | 17 | 43 | 2B | 0 | 23 | 485677 | 3885412 | 0 | 23 | 0 | 112 | 3973748 | 0 | 23 | 0 | 1 | 120 |
| 24 | 18 | 43 | 2B | 0 | 24 | 485691 | 3885524 | 0 | 24 | 0 | 112 | 3973868 | 0 | 24 | 0 | 1 | 120 |
| 25 | 19 | 43 | 2B | 0 | 25 | 485705 | 3885636 | 0 | 25 | 0 | 112 | 3973988 | 0 | 25 | 0 | 1 | 120 |
| 26 | 1A | 43 | 2B | 0 | 26 | 485719 | 3885748 | 0 | 26 | 0 | 112 | 3974108 | 0 | 26 | 0 | 1 | 120 |
| 27 | 1B | 43 | 2B | 0 | 27 | 485733 | 3885860 | 0 | 27 | 0 | 112 | 3974228 | 0 | 27 | 0 | 1 | 120 |
| 28 | 1C | 43 | 2B | 0 | 28 | 485747 | 3885972 | 0 | 28 | 0 | 112 | 3974348 | 0 | 28 | 0 | 1 | 120 |
| 29 | 1D | 43 | 2B | 0 | 29 | 485761 | 3886084 | 0 | 29 | 0 | 112 | 3974468 | 0 | 29 | 0 | 1 | 120 |
| 30 | 1E | 43 | 2B | 0 | 30 | 485775 | 3886196 | 0 | 30 | 0 | 112 | 3974588 | 0 | 30 | 0 | 1 | 120 |
| 31 | 1F | 43 | 2B | 0 | 31 | 485789 | 3886308 | 0 | 31 | 0 | 112 | 3974708 | 0 | 31 | 0 | 1 | 120 |
| 32 | 20 | 43 | 2B | 0 | 32 | 485803 | 3886420 | 0 | 32 | 0 | 112 | 3974828 | 0 | 32 | 0 | 1 | 120 |
| 33 | 21 | 43 | 2B | 0 | 33 | 485817 | 3886532 | 0 | 33 | 0 | 112 | 3974948 | 0 | 33 | 0 | 1 | 120 |
| 34 | 22 | 43 | 2B | 0 | 34 | 485831 | 3886644 | 0 | 34 | 0 | 112 | 3975068 | 0 | 34 | 0 | 1 | 120 |
| 35 | 23 | 43 | 2B | 0 | 35 | 485845 | 3886756 | 0 | 35 | 0 | 112 | 3975188 | 0 | 35 | 0 | 1 | 120 |
| 36 | 24 | 43 | 2B | 0 | 36 | 485859 | 3886868 | 0 | 36 | 0 | 112 | 3975308 | 0 | 36 | 0 | 1 | 120 |
| 37 | 25 | 43 | 2B | 0 | 37 | 485873 | 3886980 | 0 | 37 | 0 | 112 | 3975428 | 0 | 37 | 0 | 1 | 120 |
| 38 | 26 | 43 | 2B | 0 | 38 | 485887 | 3887092 | 0 | 38 | 0 | 112 | 3975548 | 0 | 38 | 0 | 1 | 120 |
| 39 | 27 | 43 | 2B | 0 | 39 | 485901 | 3887204 | 0 | 39 | 0 | 112 | 3975668 | 0 | 39 | 0 | 1 | 120 |
| 40 | 28 | 43 | 2B | 0 | 40 | 485915 | 3887316 | 0 | 40 | 0 | 112 | 3975788 | 0 | 40 | 0 | 1 | 120 |
| 41 | 29 | 43 | 2B | 0 | 41 | 485929 | 3887428 | 0 | 41 | 0 | 112 | 3975908 | 0 | 41 | 0 | 1 | 120 |
| 42 | 2A | 43 | 2B | 0 | 42 | 485943 | 3887540 | 0 | 42 | 0 | 112 | 3976028 | 0 | 42 | 0 | 1 | 120 |
| 43 | 2B | 43 | 2B | 1 | 0 | 485955 | 3887636 | 1 | 0 | 0 | 96 | 3976132 | 1 | 0 | 0 | 1 | 104 |
| 44 | 2C | 43 | 2B | 1 | 1 | 485972 | 3887772 | 1 | 1 | 0 | 136 | 3976276 | 1 | 1 | 0 | 1 | 144 |
| 45 | 2D | 43 | 2B | 1 | 2 | 485989 | 3887908 | 1 | 2 | 0 | 136 | 3976420 | 1 | 2 | 0 | 1 | 144 |
| 46 | 2E | 43 | 2B | 1 | 3 | 486006 | 3888044 | 1 | 3 | 0 | 136 | 3976564 | 1 | 3 | 0 | 1 | 144 |
| 47 | 2F | 43 | 2B | 1 | 4 | 486023 | 3888180 | 1 | 4 | 0 | 136 | 3976708 | 1 | 4 | 0 | 1 | 144 |
| 48 | 30 | 43 | 2B | 1 | 5 | 486044 | 3888348 | 1 | 5 | 0 | 168 | 3976884 | 1 | 5 | 0 | 1 | 176 |
| 49 | 31 | 43 | 2B | 1 | 6 | 486065 | 3888516 | 1 | 6 | 0 | 168 | 3977060 | 1 | 6 | 0 | 1 | 176 |
| 50 | 32 | 43 | 2B | 1 | 7 | 486086 | 3888684 | 1 | 7 | 0 | 168 | 3977236 | 1 | 7 | 0 | 1 | 176 |
| 51 | 33 | 43 | 2B | 1 | 8 | 486107 | 3888852 | 1 | 8 | 0 | 168 | 3977412 | 1 | 8 | 0 | 1 | 176 |
| 52 | 34 | 43 | 2B | 1 | 9 | 486128 | 3889020 | 1 | 9 | 0 | 168 | 3977588 | 1 | 9 | 0 | 1 | 176 |
| 53 | 35 | 43 | 2B | 1 | 10 | 486149 | 3889188 | 1 | 10 | 0 | 168 | 3977764 | 1 | 10 | 0 | 1 | 176 |
| 54 | 36 | 43 | 2B | 1 | 11 | 486170 | 3889356 | 1 | 11 | 0 | 168 | 3977940 | 1 | 11 | 0 | 1 | 176 |
| 55 | 37 | 43 | 2B | 1 | 12 | 486191 | 3889524 | 1 | 12 | 0 | 168 | 3978116 | 1 | 12 | 0 | 1 | 176 |
| 56 | 38 | 43 | 2B | 1 | 13 | 486212 | 3889692 | 1 | 13 | 0 | 168 | 3978292 | 1 | 13 | 0 | 1 | 176 |
| 57 | 39 | 43 | 2B | 1 | 14 | 486233 | 3889860 | 1 | 14 | 0 | 168 | 3978468 | 1 | 14 | 0 | 1 | 176 |
| 58 | 3A | 43 | 2B | 1 | 15 | 486254 | 3890028 | 1 | 15 | 0 | 168 | 3978644 | 1 | 15 | 0 | 1 | 176 |
| 59 | 3B | 43 | 2B | 1 | 16 | 486275 | 3890196 | 1 | 16 | 0 | 168 | 3978820 | 1 | 16 | 0 | 1 | 176 |
| 60 | 3C | 43 | 2B | 1 | 17 | 486296 | 3890364 | 1 | 17 | 0 | 168 | 3978996 | 1 | 17 | 0 | 1 | 176 |
| 61 | 3D | 43 | 2B | 1 | 18 | 486317 | 3890532 | 1 | 18 | 0 | 168 | 3979172 | 1 | 18 | 0 | 1 | 176 |
| 62 | 3E | 43 | 2B | 1 | 19 | 486338 | 3890700 | 1 | 19 | 0 | 168 | 3979348 | 1 | 19 | 0 | 1 | 176 |
| 63 | 3F | 43 | 2B | 1 | 20 | 486359 | 3890868 | 1 | 20 | 0 | 168 | 3979524 | 1 | 20 | 0 | 1 | 176 |
| 64 | 40 | 43 | 2B | 1 | 21 | 486382 | 3891052 | 1 | 21 | 0 | 184 | 3979716 | 1 | 21 | 0 | 1 | 192 |
| 65 | 41 | 43 | 2B | 1 | 22 | 486405 | 3891236 | 1 | 22 | 0 | 184 | 3979908 | 1 | 22 | 0 | 1 | 192 |
| 66 | 42 | 43 | 2B | 1 | 23 | 486428 | 3891420 | 1 | 23 | 0 | 184 | 3980100 | 1 | 23 | 0 | 1 | 192 |
| 67 | 43 | 43 | 2B | 1 | 24 | 486451 | 3891604 | 1 | 24 | 0 | 184 | 3980292 | 1 | 24 | 0 | 1 | 192 |
| 68 | 44 | 43 | 2B | 1 | 25 | 486474 | 3891788 | 1 | 25 | 0 | 184 | 3980484 | 1 | 25 | 0 | 1 | 192 |
| 69 | 45 | 43 | 2B | 1 | 26 | 486497 | 3891972 | 1 | 26 | 0 | 184 | 3980676 | 1 | 26 | 0 | 1 | 192 |
| 70 | 46 | 43 | 2B | 1 | 27 | 486520 | 3892156 | 1 | 27 | 0 | 184 | 3980868 | 1 | 27 | 0 | 1 | 192 |
| 71 | 47 | 43 | 2B | 1 | 28 | 486543 | 3892340 | 1 | 28 | 0 | 184 | 3981060 | 1 | 28 | 0 | 1 | 192 |
| 72 | 48 | 43 | 2B | 1 | 29 | 486566 | 3892524 | 1 | 29 | 0 | 184 | 3981252 | 1 | 29 | 0 | 1 | 192 |
| 73 | 49 | 43 | 2B | 1 | 30 | 486589 | 3892708 | 1 | 30 | 0 | 184 | 3981444 | 1 | 30 | 0 | 1 | 192 |
| 74 | 4A | 43 | 2B | 1 | 31 | 486612 | 3892892 | 1 | 31 | 0 | 184 | 3981636 | 1 | 31 | 0 | 1 | 192 |
| 75 | 4B | 43 | 2B | 1 | 32 | 486635 | 3893076 | 1 | 32 | 0 | 184 | 3981828 | 1 | 32 | 0 | 1 | 192 |
| 76 | 4C | 43 | 2B | 1 | 33 | 486658 | 3893260 | 1 | 33 | 0 | 184 | 3982020 | 1 | 33 | 0 | 1 | 192 |
| 77 | 4D | 43 | 2B | 1 | 34 | 486681 | 3893444 | 1 | 34 | 0 | 184 | 3982212 | 1 | 34 | 0 | 1 | 192 |
| 78 | 4E | 43 | 2B | 1 | 35 | 486704 | 3893628 | 1 | 35 | 0 | 184 | 3982404 | 1 | 35 | 0 | 1 | 192 |
| 79 | 4F | 43 | 2B | 1 | 36 | 486727 | 3893812 | 1 | 36 | 0 | 184 | 3982596 | 1 | 36 | 0 | 1 | 192 |
| 80 | 50 | 43 | 2B | 1 | 37 | 486750 | 3893996 | 1 | 37 | 0 | 184 | 3982788 | 1 | 37 | 0 | 1 | 192 |
| 81 | 51 | 43 | 2B | 1 | 38 | 486773 | 3894180 | 1 | 38 | 0 | 184 | 3982980 | 1 | 38 | 0 | 1 | 192 |
| 82 | 52 | 43 | 2B | 1 | 39 | 486796 | 3894364 | 1 | 39 | 0 | 184 | 3983172 | 1 | 39 | 0 | 1 | 192 |
| 83 | 53 | 43 | 2B | 1 | 40 | 486819 | 3894548 | 1 | 40 | 0 | 184 | 3983364 | 1 | 40 | 0 | 1 | 192 |
| 84 | 54 | 43 | 2B | 1 | 41 | 486842 | 3894732 | 1 | 41 | 0 | 184 | 3983556 | 1 | 41 | 0 | 1 | 192 |
| 85 | 55 | 43 | 2B | 1 | 42 | 486865 | 3894916 | 1 | 42 | 0 | 184 | 3983748 | 1 | 42 | 0 | 1 | 192 |
| 86 | 56 | 43 | 2B | 2 | 0 | 486886 | 3895084 | 2 | 0 | 0 | 168 | 3983924 | 2 | 0 | 0 | 1 | 176 |
| 87 | 57 | 43 | 2B | 2 | 1 | 486912 | 3895292 | 2 | 1 | 0 | 208 | 3984140 | 2 | 1 | 0 | 1 | 216 |
| 88 | 58 | 43 | 2B | 2 | 2 | 486938 | 3895500 | 2 | 2 | 0 | 208 | 3984356 | 2 | 2 | 0 | 1 | 216 |
| 89 | 59 | 43 | 2B | 2 | 3 | 486964 | 3895708 | 2 | 3 | 0 | 208 | 3984572 | 2 | 3 | 0 | 1 | 216 |
| 90 | 5A | 43 | 2B | 2 | 4 | 486990 | 3895916 | 2 | 4 | 0 | 208 | 3984788 | 2 | 4 | 0 | 1 | 216 |
| 91 | 5B | 43 | 2B | 2 | 5 | 487016 | 3896124 | 2 | 5 | 0 | 208 | 3985004 | 2 | 5 | 0 | 1 | 216 |
| 92 | 5C | 43 | 2B | 2 | 6 | 487042 | 3896332 | 2 | 6 | 0 | 208 | 3985220 | 2 | 6 | 0 | 1 | 216 |
| 93 | 5D | 43 | 2B | 2 | 7 | 487068 | 3896540 | 2 | 7 | 0 | 208 | 3985436 | 2 | 7 | 0 | 1 | 216 |
| 94 | 5E | 43 | 2B | 2 | 8 | 487094 | 3896748 | 2 | 8 | 0 | 208 | 3985652 | 2 | 8 | 0 | 1 | 216 |
| 95 | 5F | 43 | 2B | 2 | 9 | 487120 | 3896956 | 2 | 9 | 0 | 208 | 3985868 | 2 | 9 | 0 | 1 | 216 |
| 96 | 60 | 43 | 2B | 2 | 10 | 487143 | 3897140 | 2 | 10 | 0 | 184 | 3986060 | 2 | 10 | 0 | 1 | 192 |
| 97 | 61 | 43 | 2B | 2 | 11 | 487166 | 3897324 | 2 | 11 | 0 | 184 | 3986252 | 2 | 11 | 0 | 1 | 192 |
| 98 | 62 | 43 | 2B | 2 | 12 | 487189 | 3897508 | 2 | 12 | 0 | 184 | 3986444 | 2 | 12 | 0 | 1 | 192 |
| 99 | 63 | 43 | 2B | 2 | 13 | 487212 | 3897692 | 2 | 13 | 0 | 184 | 3986636 | 2 | 13 | 0 | 1 | 192 |
| 100 | 64 | 43 | 2B | 2 | 14 | 487235 | 3897876 | 2 | 14 | 0 | 184 | 3986828 | 2 | 14 | 0 | 1 | 192 |
| 101 | 65 | 43 | 2B | 2 | 15 | 487258 | 3898060 | 2 | 15 | 0 | 184 | 3987020 | 2 | 15 | 0 | 1 | 192 |
| 102 | 66 | 43 | 2B | 2 | 16 | 487281 | 3898244 | 2 | 16 | 0 | 184 | 3987212 | 2 | 16 | 0 | 1 | 192 |
| 103 | 67 | 43 | 2B | 2 | 17 | 487304 | 3898428 | 2 | 17 | 0 | 184 | 3987404 | 2 | 17 | 0 | 1 | 192 |
| 104 | 68 | 43 | 2B | 2 | 18 | 487327 | 3898612 | 2 | 18 | 0 | 184 | 3987596 | 2 | 18 | 0 | 1 | 192 |
| 105 | 69 | 43 | 2B | 2 | 19 | 487350 | 3898796 | 2 | 19 | 0 | 184 | 3987788 | 2 | 19 | 0 | 1 | 192 |
| 106 | 6A | 43 | 2B | 2 | 20 | 487373 | 3898980 | 2 | 20 | 0 | 184 | 3987980 | 2 | 20 | 0 | 1 | 192 |
| 107 | 6B | 43 | 2B | 2 | 21 | 487396 | 3899164 | 2 | 21 | 0 | 184 | 3988172 | 2 | 21 | 0 | 1 | 192 |
| 108 | 6C | 43 | 2B | 2 | 22 | 487419 | 3899348 | 2 | 22 | 0 | 184 | 3988364 | 2 | 22 | 0 | 1 | 192 |
| 109 | 6D | 43 | 2B | 2 | 23 | 487442 | 3899532 | 2 | 23 | 0 | 184 | 3988556 | 2 | 23 | 0 | 1 | 192 |
| 110 | 6E | 43 | 2B | 2 | 24 | 487465 | 3899716 | 2 | 24 | 0 | 184 | 3988748 | 2 | 24 | 0 | 1 | 192 |
| 111 | 6F | 43 | 2B | 2 | 25 | 487488 | 3899900 | 2 | 25 | 0 | 184 | 3988940 | 2 | 25 | 0 | 1 | 192 |
| 112 | 70 | 43 | 2B | 2 | 26 | 487513 | 3900100 | 2 | 26 | 0 | 200 | 3989148 | 2 | 26 | 0 | 1 | 208 |

| | | | | | | | | | | | | | | | | | |
|---|---|---|---|---|---|---|---|---|---|---|---|---|---|---|---|---|---|
| 113 | 71 | 43 | 2B | 2 | 27 | 487538 | 3900300 | 2 | 27 | 0 | 200 | 3989356 | 2 | 27 | 0 | 1 | 208 |
| 114 | 72 | 43 | 2B | 2 | 28 | 487563 | 3900500 | 2 | 28 | 0 | 200 | 3989564 | 2 | 28 | 0 | 1 | 208 |
| 115 | 73 | 43 | 2B | 2 | 29 | 487588 | 3900700 | 2 | 29 | 0 | 200 | 3989772 | 2 | 29 | 0 | 1 | 208 |
| 116 | 74 | 43 | 2B | 2 | 30 | 487613 | 3900900 | 2 | 30 | 0 | 200 | 3989980 | 2 | 30 | 0 | 1 | 208 |
| 117 | 75 | 43 | 2B | 2 | 31 | 487638 | 3901100 | 2 | 31 | 0 | 200 | 3990188 | 2 | 31 | 0 | 1 | 208 |
| 118 | 76 | 43 | 2B | 2 | 32 | 487663 | 3901300 | 2 | 32 | 0 | 200 | 3990396 | 2 | 32 | 0 | 1 | 208 |
| 119 | 77 | 43 | 2B | 2 | 33 | 487688 | 3901500 | 2 | 33 | 0 | 200 | 3990604 | 2 | 33 | 0 | 1 | 208 |
| 120 | 78 | 43 | 2B | 2 | 34 | 487713 | 3901700 | 2 | 34 | 0 | 200 | 3990812 | 2 | 34 | 0 | 1 | 208 |
| 121 | 79 | 43 | 2B | 2 | 35 | 487738 | 3901900 | 2 | 35 | 0 | 200 | 3991020 | 2 | 35 | 0 | 1 | 208 |
| 122 | 7A | 43 | 2B | 2 | 36 | 487763 | 3902100 | 2 | 36 | 0 | 200 | 3991228 | 2 | 36 | 0 | 1 | 208 |
| 123 | 7B | 43 | 2B | 2 | 37 | 487788 | 3902300 | 2 | 37 | 0 | 200 | 3991436 | 2 | 37 | 0 | 1 | 208 |
| 124 | 7C | 43 | 2B | 2 | 38 | 487813 | 3902500 | 2 | 38 | 0 | 200 | 3991644 | 2 | 38 | 0 | 1 | 208 |
| 125 | 7D | 43 | 2B | 2 | 39 | 487838 | 3902700 | 2 | 39 | 0 | 200 | 3991852 | 2 | 39 | 0 | 1 | 208 |
| 126 | 7E | 43 | 2B | 2 | 40 | 487863 | 3902900 | 2 | 40 | 0 | 200 | 3992060 | 2 | 40 | 0 | 1 | 208 |
| 127 | 7F | 43 | 2B | 2 | 41 | 487888 | 3903100 | 2 | 41 | 0 | 200 | 3992268 | 2 | 41 | 0 | 1 | 208 |
| 128 | 80 | 43 | 2B | 2 | 42 | 487913 | 3903300 | 2 | 42 | 0 | 200 | 3992476 | 2 | 42 | 0 | 1 | 208 |
| 129 | 81 | 43 | 2B | 3 | 0 | 487936 | 3903484 | 3 | 0 | 0 | 184 | 3992668 | 3 | 0 | 0 | 1 | 192 |
| 130 | 82 | 43 | 2B | 3 | 1 | 487964 | 3903708 | 3 | 1 | 0 | 224 | 3992900 | 3 | 1 | 0 | 1 | 232 |
| 131 | 83 | 43 | 2B | 3 | 2 | 487992 | 3903932 | 3 | 2 | 0 | 224 | 3993132 | 3 | 2 | 0 | 1 | 232 |
| 132 | 84 | 43 | 2B | 3 | 3 | 488020 | 3904156 | 3 | 3 | 0 | 224 | 3993364 | 3 | 3 | 0 | 1 | 232 |
| 133 | 85 | 43 | 2B | 3 | 4 | 488048 | 3904380 | 3 | 4 | 0 | 224 | 3993596 | 3 | 4 | 0 | 1 | 232 |
| 134 | 86 | 43 | 2B | 3 | 5 | 488076 | 3904604 | 3 | 5 | 0 | 224 | 3993828 | 3 | 5 | 0 | 1 | 232 |
| 135 | 87 | 43 | 2B | 3 | 6 | 488104 | 3904828 | 3 | 6 | 0 | 224 | 3994060 | 3 | 6 | 0 | 1 | 232 |
| 136 | 88 | 43 | 2B | 3 | 7 | 488132 | 3905052 | 3 | 7 | 0 | 224 | 3994292 | 3 | 7 | 0 | 1 | 232 |
| 137 | 89 | 43 | 2B | 3 | 8 | 488160 | 3905276 | 3 | 8 | 0 | 224 | 3994524 | 3 | 8 | 0 | 1 | 232 |
| 138 | 8A | 43 | 2B | 3 | 9 | 488188 | 3905500 | 3 | 9 | 0 | 224 | 3994756 | 3 | 9 | 0 | 1 | 232 |
| 139 | 8B | 43 | 2B | 3 | 10 | 488216 | 3905724 | 3 | 10 | 0 | 224 | 3994988 | 3 | 10 | 0 | 1 | 232 |
| 140 | 8C | 43 | 2B | 3 | 11 | 488244 | 3905948 | 3 | 11 | 0 | 224 | 3995220 | 3 | 11 | 0 | 1 | 232 |
| 141 | 8D | 43 | 2B | 3 | 12 | 488272 | 3906172 | 3 | 12 | 0 | 224 | 3995452 | 3 | 12 | 0 | 1 | 232 |
| 142 | 8E | 43 | 2B | 3 | 13 | 488300 | 3906396 | 3 | 13 | 0 | 224 | 3995684 | 3 | 13 | 0 | 1 | 232 |
| 143 | 8F | 43 | 2B | 3 | 14 | 488328 | 3906620 | 3 | 14 | 0 | 224 | 3995916 | 3 | 14 | 0 | 1 | 232 |
| 144 | 90 | 43 | 2B | 3 | 15 | 488353 | 3906820 | 3 | 15 | 0 | 200 | 3996124 | 3 | 15 | 0 | 1 | 208 |
| 145 | 91 | 43 | 2B | 3 | 16 | 488378 | 3907020 | 3 | 16 | 0 | 200 | 3996332 | 3 | 16 | 0 | 1 | 208 |
| 146 | 92 | 43 | 2B | 3 | 17 | 488403 | 3907220 | 3 | 17 | 0 | 200 | 3996540 | 3 | 17 | 0 | 1 | 208 |
| 147 | 93 | 43 | 2B | 3 | 18 | 488428 | 3907420 | 3 | 18 | 0 | 200 | 3996748 | 3 | 18 | 0 | 1 | 208 |
| 148 | 94 | 43 | 2B | 3 | 19 | 488453 | 3907620 | 3 | 19 | 0 | 200 | 3996956 | 3 | 19 | 0 | 1 | 208 |
| 149 | 95 | 43 | 2B | 3 | 20 | 488478 | 3907820 | 3 | 20 | 0 | 200 | 3997164 | 3 | 20 | 0 | 1 | 208 |
| 150 | 96 | 43 | 2B | 3 | 21 | 488503 | 3908020 | 3 | 21 | 0 | 200 | 3997372 | 3 | 21 | 0 | 1 | 208 |
| 151 | 97 | 43 | 2B | 3 | 22 | 488528 | 3908220 | 3 | 22 | 0 | 200 | 3997580 | 3 | 22 | 0 | 1 | 208 |
| 152 | 98 | 43 | 2B | 3 | 23 | 488553 | 3908420 | 3 | 23 | 0 | 200 | 3997788 | 3 | 23 | 0 | 1 | 208 |
| 153 | 99 | 43 | 2B | 3 | 24 | 488578 | 3908620 | 3 | 24 | 0 | 200 | 3997996 | 3 | 24 | 0 | 1 | 208 |
| 154 | 9A | 43 | 2B | 3 | 25 | 488603 | 3908820 | 3 | 25 | 0 | 200 | 3998204 | 3 | 25 | 0 | 1 | 208 |
| 155 | 9B | 43 | 2B | 3 | 26 | 488628 | 3909020 | 3 | 26 | 0 | 200 | 3998412 | 3 | 26 | 0 | 1 | 208 |
| 156 | 9C | 43 | 2B | 3 | 27 | 488653 | 3909220 | 3 | 27 | 0 | 200 | 3998620 | 3 | 27 | 0 | 1 | 208 |
| 157 | 9D | 43 | 2B | 3 | 28 | 488678 | 3909420 | 3 | 28 | 0 | 200 | 3998828 | 3 | 28 | 0 | 1 | 208 |
| 158 | 9E | 43 | 2B | 3 | 29 | 488703 | 3909620 | 3 | 29 | 0 | 200 | 3999036 | 3 | 29 | 0 | 1 | 208 |
| 159 | 9F | 43 | 2B | 3 | 30 | 488728 | 3909820 | 3 | 30 | 0 | 200 | 3999244 | 3 | 30 | 0 | 1 | 208 |
| 160 | A0 | 43 | 2B | 3 | 31 | 488755 | 3910036 | 3 | 31 | 0 | 216 | 3999468 | 3 | 31 | 0 | 1 | 224 |
| 161 | A1 | 43 | 2B | 3 | 32 | 488782 | 3910252 | 3 | 32 | 0 | 216 | 3999692 | 3 | 32 | 0 | 1 | 224 |
| 162 | A2 | 43 | 2B | 3 | 33 | 488809 | 3910468 | 3 | 33 | 0 | 216 | 3999916 | 3 | 33 | 0 | 1 | 224 |
| 163 | A3 | 43 | 2B | 3 | 34 | 488836 | 3910684 | 3 | 34 | 0 | 216 | 4000140 | 3 | 34 | 0 | 1 | 224 |
| 164 | A4 | 43 | 2B | 3 | 35 | 488863 | 3910900 | 3 | 35 | 0 | 216 | 4000364 | 3 | 35 | 0 | 1 | 224 |
| 165 | A5 | 43 | 2B | 3 | 36 | 488890 | 3911116 | 3 | 36 | 0 | 216 | 4000588 | 3 | 36 | 0 | 1 | 224 |
| 166 | A6 | 43 | 2B | 3 | 37 | 488917 | 3911332 | 3 | 37 | 0 | 216 | 4000812 | 3 | 37 | 0 | 1 | 224 |
| 167 | A7 | 43 | 2B | 3 | 38 | 488944 | 3911548 | 3 | 38 | 0 | 216 | 4001036 | 3 | 38 | 0 | 1 | 224 |
| 168 | A8 | 43 | 2B | 3 | 39 | 488971 | 3911764 | 3 | 39 | 0 | 216 | 4001260 | 3 | 39 | 0 | 1 | 224 |
| 169 | A9 | 43 | 2B | 3 | 40 | 488998 | 3911980 | 3 | 40 | 0 | 216 | 4001484 | 3 | 40 | 0 | 1 | 224 |
| 170 | AA | 43 | 2B | 3 | 41 | 489025 | 3912196 | 3 | 41 | 0 | 216 | 4001708 | 3 | 41 | 0 | 1 | 224 |
| 171 | AB | 43 | 2B | 3 | 42 | 489052 | 3912412 | 3 | 42 | 0 | 216 | 4001932 | 3 | 42 | 0 | 1 | 224 |
| 172 | AC | 43 | 2B | 4 | 0 | 489077 | 3912612 | 4 | 0 | 0 | 200 | 4002140 | 4 | 0 | 0 | 1 | 208 |
| 173 | AD | 43 | 2B | 4 | 1 | 489107 | 3912852 | 4 | 1 | 0 | 240 | 4002388 | 4 | 1 | 0 | 1 | 248 |
| 174 | AE | 43 | 2B | 4 | 2 | 489137 | 3913092 | 4 | 2 | 0 | 240 | 4002636 | 4 | 2 | 0 | 1 | 248 |
| 175 | AF | 43 | 2B | 4 | 3 | 489167 | 3913332 | 4 | 3 | 0 | 240 | 4002884 | 4 | 3 | 0 | 1 | 248 |
| 176 | B0 | 43 | 2B | 4 | 4 | 489197 | 3913572 | 4 | 4 | 0 | 240 | 4003132 | 4 | 4 | 0 | 1 | 248 |
| 177 | B1 | 43 | 2B | 4 | 5 | 489227 | 3913812 | 4 | 5 | 0 | 240 | 4003380 | 4 | 5 | 0 | 1 | 248 |
| 178 | B2 | 43 | 2B | 4 | 6 | 489257 | 3914052 | 4 | 6 | 0 | 240 | 4003628 | 4 | 6 | 0 | 1 | 248 |
| 179 | B3 | 43 | 2B | 4 | 7 | 489287 | 3914292 | 4 | 7 | 0 | 240 | 4003876 | 4 | 7 | 0 | 1 | 248 |
| 180 | B4 | 43 | 2B | 4 | 8 | 489317 | 3914532 | 4 | 8 | 0 | 240 | 4004124 | 4 | 8 | 0 | 1 | 248 |
| 181 | B5 | 43 | 2B | 4 | 9 | 489347 | 3914772 | 4 | 9 | 0 | 240 | 4004372 | 4 | 9 | 0 | 1 | 248 |
| 182 | B6 | 43 | 2B | 4 | 10 | 489377 | 3915012 | 4 | 10 | 0 | 240 | 4004620 | 4 | 10 | 0 | 1 | 248 |
| 183 | B7 | 43 | 2B | 4 | 11 | 489407 | 3915252 | 4 | 11 | 0 | 240 | 4004868 | 4 | 11 | 0 | 1 | 248 |
| 184 | B8 | 43 | 2B | 4 | 12 | 489437 | 3915492 | 4 | 12 | 0 | 240 | 4005116 | 4 | 12 | 0 | 1 | 248 |
| 185 | B9 | 43 | 2B | 4 | 13 | 489467 | 3915732 | 4 | 13 | 0 | 240 | 4005364 | 4 | 13 | 0 | 1 | 248 |
| 186 | BA | 43 | 2B | 4 | 14 | 489497 | 3915972 | 4 | 14 | 0 | 240 | 4005612 | 4 | 14 | 0 | 1 | 248 |
| 187 | BB | 43 | 2B | 4 | 15 | 489527 | 3916212 | 4 | 15 | 0 | 240 | 4005860 | 4 | 15 | 0 | 1 | 248 |
| 188 | BC | 43 | 2B | 4 | 16 | 489557 | 3916452 | 4 | 16 | 0 | 240 | 4006108 | 4 | 16 | 0 | 1 | 248 |
| 189 | BD | 43 | 2B | 4 | 17 | 489587 | 3916692 | 4 | 17 | 0 | 240 | 4006356 | 4 | 17 | 0 | 1 | 248 |
| 190 | BE | 43 | 2B | 4 | 18 | 489617 | 3916932 | 4 | 18 | 0 | 240 | 4006604 | 4 | 18 | 0 | 1 | 248 |
| 191 | BF | 43 | 2B | 4 | 19 | 489647 | 3917172 | 4 | 19 | 0 | 240 | 4006852 | 4 | 19 | 0 | 1 | 248 |
| 192 | C0 | 43 | 2B | 4 | 20 | 489674 | 3917388 | 4 | 20 | 0 | 216 | 4007076 | 4 | 20 | 0 | 1 | 224 |
| 193 | C1 | 43 | 2B | 4 | 21 | 489701 | 3917604 | 4 | 21 | 0 | 216 | 4007300 | 4 | 21 | 0 | 1 | 224 |
| 194 | C2 | 43 | 2B | 4 | 22 | 489728 | 3917820 | 4 | 22 | 0 | 216 | 4007524 | 4 | 22 | 0 | 1 | 224 |
| 195 | C3 | 43 | 2B | 4 | 23 | 489755 | 3918036 | 4 | 23 | 0 | 216 | 4007748 | 4 | 23 | 0 | 1 | 224 |
| 196 | C4 | 43 | 2B | 4 | 24 | 489782 | 3918252 | 4 | 24 | 0 | 216 | 4007972 | 4 | 24 | 0 | 1 | 224 |
| 197 | C5 | 43 | 2B | 4 | 25 | 489809 | 3918468 | 4 | 25 | 0 | 216 | 4008196 | 4 | 25 | 0 | 1 | 224 |
| 198 | C6 | 43 | 2B | 4 | 26 | 489836 | 3918684 | 4 | 26 | 0 | 216 | 4008420 | 4 | 26 | 0 | 1 | 224 |
| 199 | C7 | 43 | 2B | 4 | 27 | 489863 | 3918900 | 4 | 27 | 0 | 216 | 4008644 | 4 | 27 | 0 | 1 | 224 |
| 200 | C8 | 43 | 2B | 4 | 28 | 489890 | 3919116 | 4 | 28 | 0 | 216 | 4008868 | 4 | 28 | 0 | 1 | 224 |
| 201 | C9 | 43 | 2B | 4 | 29 | 489917 | 3919332 | 4 | 29 | 0 | 216 | 4009092 | 4 | 29 | 0 | 1 | 224 |
| 202 | CA | 43 | 2B | 4 | 30 | 489944 | 3919548 | 4 | 30 | 0 | 216 | 4009316 | 4 | 30 | 0 | 1 | 224 |
| 203 | CB | 43 | 2B | 4 | 31 | 489971 | 3919764 | 4 | 31 | 0 | 216 | 4009540 | 4 | 31 | 0 | 1 | 224 |
| 204 | CC | 43 | 2B | 4 | 32 | 489998 | 3919980 | 4 | 32 | 0 | 216 | 4009764 | 4 | 32 | 0 | 1 | 224 |
| 205 | CD | 43 | 2B | 4 | 33 | 490025 | 3920196 | 4 | 33 | 0 | 216 | 4009988 | 4 | 33 | 0 | 1 | 224 |
| 206 | CE | 43 | 2B | 4 | 34 | 490052 | 3920412 | 4 | 34 | 0 | 216 | 4010212 | 4 | 34 | 0 | 1 | 224 |
| 207 | CF | 43 | 2B | 4 | 35 | 490079 | 3920628 | 4 | 35 | 0 | 216 | 4010436 | 4 | 35 | 0 | 1 | 224 |
| 208 | D0 | 43 | 2B | 4 | 36 | 490108 | 3920860 | 4 | 36 | 0 | 232 | 4010676 | 4 | 36 | 0 | 1 | 240 |
| 209 | D1 | 43 | 2B | 4 | 37 | 490137 | 3921092 | 4 | 37 | 0 | 232 | 4010916 | 4 | 37 | 0 | 1 | 240 |
| 210 | D2 | 43 | 2B | 4 | 38 | 490166 | 3921324 | 4 | 38 | 0 | 232 | 4011156 | 4 | 38 | 0 | 1 | 240 |
| 211 | D3 | 43 | 2B | 4 | 39 | 490195 | 3921556 | 4 | 39 | 0 | 232 | 4011396 | 4 | 39 | 0 | 1 | 240 |
| 212 | D4 | 43 | 2B | 4 | 40 | 490224 | 3921788 | 4 | 40 | 0 | 232 | 4011636 | 4 | 40 | 0 | 1 | 240 |
| 213 | D5 | 43 | 2B | 4 | 41 | 490253 | 3922020 | 4 | 41 | 0 | 232 | 4011876 | 4 | 41 | 0 | 1 | 240 |
| 214 | D6 | 43 | 2B | 4 | 42 | 490282 | 3922252 | 4 | 42 | 0 | 232 | 4012116 | 4 | 42 | 0 | 1 | 240 |
| 215 | D7 | 43 | 2B | 5 | 0 | 490309 | 3922468 | 5 | 0 | 0 | 216 | 4012340 | 5 | 0 | 0 | 1 | 224 |

| | | | | | | | | | | | | | | | | | |
|---|---|---|---|---|---|---|---|---|---|---|---|---|---|---|---|---|---|
| 216 | D8 | 43 | 2B | 5 | 1 | 490341 | 3922724 | 5 | 1 | 0 | 256 | 4012604 | 5 | 1 | 0 | 1 | 264 |
| 217 | D9 | 43 | 2B | 5 | 2 | 490373 | 3922980 | 5 | 2 | 0 | 256 | 4012868 | 5 | 2 | 0 | 1 | 264 |
| 218 | DA | 43 | 2B | 5 | 3 | 490405 | 3923236 | 5 | 3 | 0 | 256 | 4013132 | 5 | 3 | 0 | 1 | 264 |
| 219 | DB | 43 | 2B | 5 | 4 | 490437 | 3923492 | 5 | 4 | 0 | 256 | 4013396 | 5 | 4 | 0 | 1 | 264 |
| 220 | DC | 43 | 2B | 5 | 5 | 490469 | 3923748 | 5 | 5 | 0 | 256 | 4013660 | 5 | 5 | 0 | 1 | 264 |
| 221 | DD | 43 | 2B | 5 | 6 | 490501 | 3924004 | 5 | 6 | 0 | 256 | 4013924 | 5 | 6 | 0 | 1 | 264 |
| 222 | DE | 43 | 2B | 5 | 7 | 490533 | 3924260 | 5 | 7 | 0 | 256 | 4014188 | 5 | 7 | 0 | 1 | 264 |
| 223 | DF | 43 | 2B | 5 | 8 | 490565 | 3924516 | 5 | 8 | 0 | 256 | 4014452 | 5 | 8 | 0 | 1 | 264 |
| 224 | E0 | 43 | 2B | 5 | 9 | 490597 | 3924772 | 5 | 9 | 0 | 256 | 4014716 | 5 | 9 | 0 | 1 | 264 |
| 225 | E1 | 43 | 2B | 5 | 10 | 490629 | 3925028 | 5 | 10 | 0 | 256 | 4014980 | 5 | 10 | 0 | 1 | 264 |
| 226 | E2 | 43 | 2B | 5 | 11 | 490661 | 3925284 | 5 | 11 | 0 | 256 | 4015244 | 5 | 11 | 0 | 1 | 264 |
| 227 | E3 | 43 | 2B | 5 | 12 | 490693 | 3925540 | 5 | 12 | 0 | 256 | 4015508 | 5 | 12 | 0 | 1 | 264 |
| 228 | E4 | 43 | 2B | 5 | 13 | 490725 | 3925796 | 5 | 13 | 0 | 256 | 4015772 | 5 | 13 | 0 | 1 | 264 |
| 229 | E5 | 43 | 2B | 5 | 14 | 490757 | 3926052 | 5 | 14 | 0 | 256 | 4016036 | 5 | 14 | 0 | 1 | 264 |
| 230 | E6 | 43 | 2B | 5 | 15 | 490789 | 3926308 | 5 | 15 | 0 | 256 | 4016300 | 5 | 15 | 0 | 1 | 264 |
| 231 | E7 | 43 | 2B | 5 | 16 | 490821 | 3926564 | 5 | 16 | 0 | 256 | 4016564 | 5 | 16 | 0 | 1 | 264 |
| 232 | E8 | 43 | 2B | 5 | 17 | 490853 | 3926820 | 5 | 17 | 0 | 256 | 4016828 | 5 | 17 | 0 | 1 | 264 |
| 233 | E9 | 43 | 2B | 5 | 18 | 490885 | 3927076 | 5 | 18 | 0 | 256 | 4017092 | 5 | 18 | 0 | 1 | 264 |
| 234 | EA | 43 | 2B | 5 | 19 | 490917 | 3927332 | 5 | 19 | 0 | 256 | 4017356 | 5 | 19 | 0 | 1 | 264 |
| 235 | EB | 43 | 2B | 5 | 20 | 490949 | 3927588 | 5 | 20 | 0 | 256 | 4017620 | 5 | 20 | 0 | 1 | 264 |
| 236 | EC | 43 | 2B | 5 | 21 | 490981 | 3927844 | 5 | 21 | 0 | 256 | 4017884 | 5 | 21 | 0 | 1 | 264 |
| 237 | ED | 43 | 2B | 5 | 22 | 491013 | 3928100 | 5 | 22 | 0 | 256 | 4018148 | 5 | 22 | 0 | 1 | 264 |
| 238 | EE | 43 | 2B | 5 | 23 | 491045 | 3928356 | 5 | 23 | 0 | 256 | 4018412 | 5 | 23 | 0 | 1 | 264 |
| 239 | EF | 43 | 2B | 5 | 24 | 491077 | 3928612 | 5 | 24 | 0 | 256 | 4018676 | 5 | 24 | 0 | 1 | 264 |
| 240 | F0 | 43 | 2B | 5 | 25 | 491106 | 3928844 | 5 | 25 | 0 | 232 | 4018916 | 5 | 25 | 0 | 1 | 240 |
| 241 | F1 | 43 | 2B | 5 | 26 | 491135 | 3929076 | 5 | 26 | 0 | 232 | 4019156 | 5 | 26 | 0 | 1 | 240 |
| 242 | F2 | 43 | 2B | 5 | 27 | 491164 | 3929308 | 5 | 27 | 0 | 232 | 4019396 | 5 | 27 | 0 | 1 | 240 |
| 243 | F3 | 43 | 2B | 5 | 28 | 491193 | 3929540 | 5 | 28 | 0 | 232 | 4019636 | 5 | 28 | 0 | 1 | 240 |
| 244 | F4 | 43 | 2B | 5 | 29 | 491222 | 3929772 | 5 | 29 | 0 | 232 | 4019876 | 5 | 29 | 0 | 1 | 240 |
| 245 | F5 | 43 | 2B | 5 | 30 | 491251 | 3930004 | 5 | 30 | 0 | 232 | 4020116 | 5 | 30 | 0 | 1 | 240 |
| 246 | F6 | 43 | 2B | 5 | 31 | 491280 | 3930236 | 5 | 31 | 0 | 232 | 4020356 | 5 | 31 | 0 | 1 | 240 |
| 247 | F7 | 43 | 2B | 5 | 32 | 491309 | 3930468 | 5 | 32 | 0 | 232 | 4020596 | 5 | 32 | 0 | 1 | 240 |
| 248 | F8 | 43 | 2B | 5 | 33 | 491338 | 3930700 | 5 | 33 | 0 | 232 | 4020836 | 5 | 33 | 0 | 1 | 240 |
| 249 | F9 | 43 | 2B | 5 | 34 | 491367 | 3930932 | 5 | 34 | 0 | 232 | 4021076 | 5 | 34 | 0 | 1 | 240 |
| 250 | FA | 43 | 2B | 5 | 35 | 491396 | 3931164 | 5 | 35 | 0 | 232 | 4021316 | 5 | 35 | 0 | 1 | 240 |
| 251 | FB | 43 | 2B | 5 | 36 | 491425 | 3931396 | 5 | 36 | 0 | 232 | 4021556 | 5 | 36 | 0 | 1 | 240 |
| 252 | FC | 43 | 2B | 5 | 37 | 491454 | 3931628 | 5 | 37 | 0 | 232 | 4021796 | 5 | 37 | 0 | 1 | 240 |
| 253 | FD | 43 | 2B | 5 | 38 | 491483 | 3931860 | 5 | 38 | 0 | 232 | 4022036 | 5 | 38 | 0 | 1 | 240 |
| 254 | FE | 43 | 2B | 5 | 39 | 491512 | 3932092 | 5 | 39 | 0 | 232 | 4022276 | 5 | 39 | 0 | 1 | 240 |
| 255 | FF | 43 | 2B | 5 | 40 | 491541 | 3932324 | 5 | 40 | 0 | 232 | 4022516 | 5 | 40 | 0 | 1 | 240 |
| 0 | 00 | 44 | 2C | 0 | 0 | 491553 | 3932420 | 0 | 0 | 0 | 96 | 4022620 | 0 | 0 | 0 | 1 | 104 |
| 1 | 01 | 44 | 2C | 0 | 1 | 491567 | 3932532 | 0 | 1 | 0 | 112 | 4022740 | 0 | 1 | 0 | 1 | 120 |
| 2 | 02 | 44 | 2C | 0 | 2 | 491581 | 3932644 | 0 | 2 | 0 | 112 | 4022860 | 0 | 2 | 0 | 1 | 120 |
| 3 | 03 | 44 | 2C | 0 | 3 | 491595 | 3932756 | 0 | 3 | 0 | 112 | 4022980 | 0 | 3 | 0 | 1 | 120 |
| 4 | 04 | 44 | 2C | 0 | 4 | 491609 | 3932868 | 0 | 4 | 0 | 112 | 4023100 | 0 | 4 | 0 | 1 | 120 |
| 5 | 05 | 44 | 2C | 0 | 5 | 491623 | 3932980 | 0 | 5 | 0 | 112 | 4023220 | 0 | 5 | 0 | 1 | 120 |
| 6 | 06 | 44 | 2C | 0 | 6 | 491637 | 3933092 | 0 | 6 | 0 | 112 | 4023340 | 0 | 6 | 0 | 1 | 120 |
| 7 | 07 | 44 | 2C | 0 | 7 | 491651 | 3933204 | 0 | 7 | 0 | 112 | 4023460 | 0 | 7 | 0 | 1 | 120 |
| 8 | 08 | 44 | 2C | 0 | 8 | 491665 | 3933316 | 0 | 8 | 0 | 112 | 4023580 | 0 | 8 | 0 | 1 | 120 |
| 9 | 09 | 44 | 2C | 0 | 9 | 491679 | 3933428 | 0 | 9 | 0 | 112 | 4023700 | 0 | 9 | 0 | 1 | 120 |
| 10 | 0A | 44 | 2C | 0 | 10 | 491693 | 3933540 | 0 | 10 | 0 | 112 | 4023820 | 0 | 10 | 0 | 1 | 120 |
| 11 | 0B | 44 | 2C | 0 | 11 | 491707 | 3933652 | 0 | 11 | 0 | 112 | 4023940 | 0 | 11 | 0 | 1 | 120 |
| 12 | 0C | 44 | 2C | 0 | 12 | 491721 | 3933764 | 0 | 12 | 0 | 112 | 4024060 | 0 | 12 | 0 | 1 | 120 |
| 13 | 0D | 44 | 2C | 0 | 13 | 491735 | 3933876 | 0 | 13 | 0 | 112 | 4024180 | 0 | 13 | 0 | 1 | 120 |
| 14 | 0E | 44 | 2C | 0 | 14 | 491749 | 3933988 | 0 | 14 | 0 | 112 | 4024300 | 0 | 14 | 0 | 1 | 120 |
| 15 | 0F | 44 | 2C | 0 | 15 | 491763 | 3934100 | 0 | 15 | 0 | 112 | 4024420 | 0 | 15 | 0 | 1 | 120 |
| 16 | 10 | 44 | 2C | 0 | 16 | 491777 | 3934212 | 0 | 16 | 0 | 112 | 4024540 | 0 | 16 | 0 | 1 | 120 |
| 17 | 11 | 44 | 2C | 0 | 17 | 491791 | 3934324 | 0 | 17 | 0 | 112 | 4024660 | 0 | 17 | 0 | 1 | 120 |
| 18 | 12 | 44 | 2C | 0 | 18 | 491805 | 3934436 | 0 | 18 | 0 | 112 | 4024780 | 0 | 18 | 0 | 1 | 120 |
| 19 | 13 | 44 | 2C | 0 | 19 | 491819 | 3934548 | 0 | 19 | 0 | 112 | 4024900 | 0 | 19 | 0 | 1 | 120 |
| 20 | 14 | 44 | 2C | 0 | 20 | 491833 | 3934660 | 0 | 20 | 0 | 112 | 4025020 | 0 | 20 | 0 | 1 | 120 |
| 21 | 15 | 44 | 2C | 0 | 21 | 491847 | 3934772 | 0 | 21 | 0 | 112 | 4025140 | 0 | 21 | 0 | 1 | 120 |
| 22 | 16 | 44 | 2C | 0 | 22 | 491861 | 3934884 | 0 | 22 | 0 | 112 | 4025260 | 0 | 22 | 0 | 1 | 120 |
| 23 | 17 | 44 | 2C | 0 | 23 | 491875 | 3934996 | 0 | 23 | 0 | 112 | 4025380 | 0 | 23 | 0 | 1 | 120 |
| 24 | 18 | 44 | 2C | 0 | 24 | 491889 | 3935108 | 0 | 24 | 0 | 112 | 4025500 | 0 | 24 | 0 | 1 | 120 |
| 25 | 19 | 44 | 2C | 0 | 25 | 491903 | 3935220 | 0 | 25 | 0 | 112 | 4025620 | 0 | 25 | 0 | 1 | 120 |
| 26 | 1A | 44 | 2C | 0 | 26 | 491917 | 3935332 | 0 | 26 | 0 | 112 | 4025740 | 0 | 26 | 0 | 1 | 120 |
| 27 | 1B | 44 | 2C | 0 | 27 | 491931 | 3935444 | 0 | 27 | 0 | 112 | 4025860 | 0 | 27 | 0 | 1 | 120 |
| 28 | 1C | 44 | 2C | 0 | 28 | 491945 | 3935556 | 0 | 28 | 0 | 112 | 4025980 | 0 | 28 | 0 | 1 | 120 |
| 29 | 1D | 44 | 2C | 0 | 29 | 491959 | 3935668 | 0 | 29 | 0 | 112 | 4026100 | 0 | 29 | 0 | 1 | 120 |
| 30 | 1E | 44 | 2C | 0 | 30 | 491973 | 3935780 | 0 | 30 | 0 | 112 | 4026220 | 0 | 30 | 0 | 1 | 120 |
| 31 | 1F | 44 | 2C | 0 | 31 | 491987 | 3935892 | 0 | 31 | 0 | 112 | 4026340 | 0 | 31 | 0 | 1 | 120 |
| 32 | 20 | 44 | 2C | 0 | 32 | 492001 | 3936004 | 0 | 32 | 0 | 112 | 4026460 | 0 | 32 | 0 | 1 | 120 |
| 33 | 21 | 44 | 2C | 0 | 33 | 492015 | 3936116 | 0 | 33 | 0 | 112 | 4026580 | 0 | 33 | 0 | 1 | 120 |
| 34 | 22 | 44 | 2C | 0 | 34 | 492029 | 3936228 | 0 | 34 | 0 | 112 | 4026700 | 0 | 34 | 0 | 1 | 120 |
| 35 | 23 | 44 | 2C | 0 | 35 | 492043 | 3936340 | 0 | 35 | 0 | 112 | 4026820 | 0 | 35 | 0 | 1 | 120 |
| 36 | 24 | 44 | 2C | 0 | 36 | 492057 | 3936452 | 0 | 36 | 0 | 112 | 4026940 | 0 | 36 | 0 | 1 | 120 |
| 37 | 25 | 44 | 2C | 0 | 37 | 492071 | 3936564 | 0 | 37 | 0 | 112 | 4027060 | 0 | 37 | 0 | 1 | 120 |
| 38 | 26 | 44 | 2C | 0 | 38 | 492085 | 3936676 | 0 | 38 | 0 | 112 | 4027180 | 0 | 38 | 0 | 1 | 120 |
| 39 | 27 | 44 | 2C | 0 | 39 | 492099 | 3936788 | 0 | 39 | 0 | 112 | 4027300 | 0 | 39 | 0 | 1 | 120 |
| 40 | 28 | 44 | 2C | 0 | 40 | 492113 | 3936900 | 0 | 40 | 0 | 112 | 4027420 | 0 | 40 | 0 | 1 | 120 |
| 41 | 29 | 44 | 2C | 0 | 41 | 492127 | 3937012 | 0 | 41 | 0 | 112 | 4027540 | 0 | 41 | 0 | 1 | 120 |
| 42 | 2A | 44 | 2C | 0 | 42 | 492141 | 3937124 | 0 | 42 | 0 | 112 | 4027660 | 0 | 42 | 0 | 1 | 120 |
| 43 | 2B | 44 | 2C | 0 | 43 | 492155 | 3937236 | 0 | 43 | 0 | 112 | 4027780 | 0 | 43 | 0 | 1 | 120 |
| 44 | 2C | 44 | 2C | 1 | 0 | 492167 | 3937332 | 1 | 0 | 0 | 96 | 4027884 | 1 | 0 | 0 | 1 | 104 |
| 45 | 2D | 44 | 2C | 1 | 1 | 492184 | 3937468 | 1 | 1 | 0 | 136 | 4028028 | 1 | 1 | 0 | 1 | 144 |
| 46 | 2E | 44 | 2C | 1 | 2 | 492201 | 3937604 | 1 | 2 | 0 | 136 | 4028172 | 1 | 2 | 0 | 1 | 144 |
| 47 | 2F | 44 | 2C | 1 | 3 | 492218 | 3937740 | 1 | 3 | 0 | 136 | 4028316 | 1 | 3 | 0 | 1 | 144 |
| 48 | 30 | 44 | 2C | 1 | 4 | 492239 | 3937908 | 1 | 4 | 0 | 168 | 4028492 | 1 | 4 | 0 | 1 | 176 |
| 49 | 31 | 44 | 2C | 1 | 5 | 492260 | 3938076 | 1 | 5 | 0 | 168 | 4028668 | 1 | 5 | 0 | 1 | 176 |
| 50 | 32 | 44 | 2C | 1 | 6 | 492281 | 3938244 | 1 | 6 | 0 | 168 | 4028844 | 1 | 6 | 0 | 1 | 176 |
| 51 | 33 | 44 | 2C | 1 | 7 | 492302 | 3938412 | 1 | 7 | 0 | 168 | 4029020 | 1 | 7 | 0 | 1 | 176 |
| 52 | 34 | 44 | 2C | 1 | 8 | 492323 | 3938580 | 1 | 8 | 0 | 168 | 4029196 | 1 | 8 | 0 | 1 | 176 |
| 53 | 35 | 44 | 2C | 1 | 9 | 492344 | 3938748 | 1 | 9 | 0 | 168 | 4029372 | 1 | 9 | 0 | 1 | 176 |
| 54 | 36 | 44 | 2C | 1 | 10 | 492365 | 3938916 | 1 | 10 | 0 | 168 | 4029548 | 1 | 10 | 0 | 1 | 176 |
| 55 | 37 | 44 | 2C | 1 | 11 | 492386 | 3939084 | 1 | 11 | 0 | 168 | 4029724 | 1 | 11 | 0 | 1 | 176 |
| 56 | 38 | 44 | 2C | 1 | 12 | 492407 | 3939252 | 1 | 12 | 0 | 168 | 4029900 | 1 | 12 | 0 | 1 | 176 |
| 57 | 39 | 44 | 2C | 1 | 13 | 492428 | 3939420 | 1 | 13 | 0 | 168 | 4030076 | 1 | 13 | 0 | 1 | 176 |
| 58 | 3A | 44 | 2C | 1 | 14 | 492449 | 3939588 | 1 | 14 | 0 | 168 | 4030252 | 1 | 14 | 0 | 1 | 176 |
| 59 | 3B | 44 | 2C | 1 | 15 | 492470 | 3939756 | 1 | 15 | 0 | 168 | 4030428 | 1 | 15 | 0 | 1 | 176 |
| 60 | 3C | 44 | 2C | 1 | 16 | 492491 | 3939924 | 1 | 16 | 0 | 168 | 4030604 | 1 | 16 | 0 | 1 | 176 |
| 61 | 3D | 44 | 2C | 1 | 17 | 492512 | 3940092 | 1 | 17 | 0 | 168 | 4030780 | 1 | 17 | 0 | 1 | 176 |
| 62 | 3E | 44 | 2C | 1 | 18 | 492533 | 3940260 | 1 | 18 | 0 | 168 | 4030956 | 1 | 18 | 0 | 1 | 176 |

| | | | | | | | | | | | | | | | | | |
|---|---|---|---|---|---|---|---|---|---|---|---|---|---|---|---|---|---|
| 63 | 3F | 44 | 2C | 1 | 19 | 492554 | 3940428 | 1 | 19 | 0 | 168 | 4031132 | 1 | 19 | 0 | 1 | 176 |
| 64 | 40 | 44 | 2C | 1 | 20 | 492577 | 3940612 | 1 | 20 | 0 | 184 | 4031324 | 1 | 20 | 0 | 1 | 192 |
| 65 | 41 | 44 | 2C | 1 | 21 | 492600 | 3940796 | 1 | 21 | 0 | 184 | 4031516 | 1 | 21 | 0 | 1 | 192 |
| 66 | 42 | 44 | 2C | 1 | 22 | 492623 | 3940980 | 1 | 22 | 0 | 184 | 4031708 | 1 | 22 | 0 | 1 | 192 |
| 67 | 43 | 44 | 2C | 1 | 23 | 492646 | 3941164 | 1 | 23 | 0 | 184 | 4031900 | 1 | 23 | 0 | 1 | 192 |
| 68 | 44 | 44 | 2C | 1 | 24 | 492669 | 3941348 | 1 | 24 | 0 | 184 | 4032092 | 1 | 24 | 0 | 1 | 192 |
| 69 | 45 | 44 | 2C | 1 | 25 | 492692 | 3941532 | 1 | 25 | 0 | 184 | 4032284 | 1 | 25 | 0 | 1 | 192 |
| 70 | 46 | 44 | 2C | 1 | 26 | 492715 | 3941716 | 1 | 26 | 0 | 184 | 4032476 | 1 | 26 | 0 | 1 | 192 |
| 71 | 47 | 44 | 2C | 1 | 27 | 492738 | 3941900 | 1 | 27 | 0 | 184 | 4032668 | 1 | 27 | 0 | 1 | 192 |
| 72 | 48 | 44 | 2C | 1 | 28 | 492761 | 3942084 | 1 | 28 | 0 | 184 | 4032860 | 1 | 28 | 0 | 1 | 192 |
| 73 | 49 | 44 | 2C | 1 | 29 | 492784 | 3942268 | 1 | 29 | 0 | 184 | 4033052 | 1 | 29 | 0 | 1 | 192 |
| 74 | 4A | 44 | 2C | 1 | 30 | 492807 | 3942452 | 1 | 30 | 0 | 184 | 4033244 | 1 | 30 | 0 | 1 | 192 |
| 75 | 4B | 44 | 2C | 1 | 31 | 492830 | 3942636 | 1 | 31 | 0 | 184 | 4033436 | 1 | 31 | 0 | 1 | 192 |
| 76 | 4C | 44 | 2C | 1 | 32 | 492853 | 3942820 | 1 | 32 | 0 | 184 | 4033628 | 1 | 32 | 0 | 1 | 192 |
| 77 | 4D | 44 | 2C | 1 | 33 | 492876 | 3943004 | 1 | 33 | 0 | 184 | 4033820 | 1 | 33 | 0 | 1 | 192 |
| 78 | 4E | 44 | 2C | 1 | 34 | 492899 | 3943188 | 1 | 34 | 0 | 184 | 4034012 | 1 | 34 | 0 | 1 | 192 |
| 79 | 4F | 44 | 2C | 1 | 35 | 492922 | 3943372 | 1 | 35 | 0 | 184 | 4034204 | 1 | 35 | 0 | 1 | 192 |
| 80 | 50 | 44 | 2C | 1 | 36 | 492945 | 3943556 | 1 | 36 | 0 | 184 | 4034396 | 1 | 36 | 0 | 1 | 192 |
| 81 | 51 | 44 | 2C | 1 | 37 | 492968 | 3943740 | 1 | 37 | 0 | 184 | 4034588 | 1 | 37 | 0 | 1 | 192 |
| 82 | 52 | 44 | 2C | 1 | 38 | 492991 | 3943924 | 1 | 38 | 0 | 184 | 4034780 | 1 | 38 | 0 | 1 | 192 |
| 83 | 53 | 44 | 2C | 1 | 39 | 493014 | 3944108 | 1 | 39 | 0 | 184 | 4034972 | 1 | 39 | 0 | 1 | 192 |
| 84 | 54 | 44 | 2C | 1 | 40 | 493037 | 3944292 | 1 | 40 | 0 | 184 | 4035164 | 1 | 40 | 0 | 1 | 192 |
| 85 | 55 | 44 | 2C | 1 | 41 | 493060 | 3944476 | 1 | 41 | 0 | 184 | 4035356 | 1 | 41 | 0 | 1 | 192 |
| 86 | 56 | 44 | 2C | 1 | 42 | 493083 | 3944660 | 1 | 42 | 0 | 184 | 4035548 | 1 | 42 | 0 | 1 | 192 |
| 87 | 57 | 44 | 2C | 1 | 43 | 493106 | 3944844 | 1 | 43 | 0 | 184 | 4035740 | 1 | 43 | 0 | 1 | 192 |
| 88 | 58 | 44 | 2C | 2 | 0 | 493127 | 3945012 | 2 | 0 | 0 | 168 | 4035916 | 2 | 0 | 0 | 1 | 176 |
| 89 | 59 | 44 | 2C | 2 | 1 | 493153 | 3945220 | 2 | 1 | 0 | 208 | 4036132 | 2 | 1 | 0 | 1 | 216 |
| 90 | 5A | 44 | 2C | 2 | 2 | 493179 | 3945428 | 2 | 2 | 0 | 208 | 4036348 | 2 | 2 | 0 | 1 | 216 |
| 91 | 5B | 44 | 2C | 2 | 3 | 493205 | 3945636 | 2 | 3 | 0 | 208 | 4036564 | 2 | 3 | 0 | 1 | 216 |
| 92 | 5C | 44 | 2C | 2 | 4 | 493231 | 3945844 | 2 | 4 | 0 | 208 | 4036780 | 2 | 4 | 0 | 1 | 216 |
| 93 | 5D | 44 | 2C | 2 | 5 | 493257 | 3946052 | 2 | 5 | 0 | 208 | 4036996 | 2 | 5 | 0 | 1 | 216 |
| 94 | 5E | 44 | 2C | 2 | 6 | 493283 | 3946260 | 2 | 6 | 0 | 208 | 4037212 | 2 | 6 | 0 | 1 | 216 |
| 95 | 5F | 44 | 2C | 2 | 7 | 493309 | 3946468 | 2 | 7 | 0 | 208 | 4037428 | 2 | 7 | 0 | 1 | 216 |
| 96 | 60 | 44 | 2C | 2 | 8 | 493332 | 3946652 | 2 | 8 | 0 | 184 | 4037620 | 2 | 8 | 0 | 1 | 192 |
| 97 | 61 | 44 | 2C | 2 | 9 | 493355 | 3946836 | 2 | 9 | 0 | 184 | 4037812 | 2 | 9 | 0 | 1 | 192 |
| 98 | 62 | 44 | 2C | 2 | 10 | 493378 | 3947020 | 2 | 10 | 0 | 184 | 4038004 | 2 | 10 | 0 | 1 | 192 |
| 99 | 63 | 44 | 2C | 2 | 11 | 493401 | 3947204 | 2 | 11 | 0 | 184 | 4038196 | 2 | 11 | 0 | 1 | 192 |
| 100 | 64 | 44 | 2C | 2 | 12 | 493424 | 3947388 | 2 | 12 | 0 | 184 | 4038388 | 2 | 12 | 0 | 1 | 192 |
| 101 | 65 | 44 | 2C | 2 | 13 | 493447 | 3947572 | 2 | 13 | 0 | 184 | 4038580 | 2 | 13 | 0 | 1 | 192 |
| 102 | 66 | 44 | 2C | 2 | 14 | 493470 | 3947756 | 2 | 14 | 0 | 184 | 4038772 | 2 | 14 | 0 | 1 | 192 |
| 103 | 67 | 44 | 2C | 2 | 15 | 493493 | 3947940 | 2 | 15 | 0 | 184 | 4038964 | 2 | 15 | 0 | 1 | 192 |
| 104 | 68 | 44 | 2C | 2 | 16 | 493516 | 3948124 | 2 | 16 | 0 | 184 | 4039156 | 2 | 16 | 0 | 1 | 192 |
| 105 | 69 | 44 | 2C | 2 | 17 | 493539 | 3948308 | 2 | 17 | 0 | 184 | 4039348 | 2 | 17 | 0 | 1 | 192 |
| 106 | 6A | 44 | 2C | 2 | 18 | 493562 | 3948492 | 2 | 18 | 0 | 184 | 4039540 | 2 | 18 | 0 | 1 | 192 |
| 107 | 6B | 44 | 2C | 2 | 19 | 493585 | 3948676 | 2 | 19 | 0 | 184 | 4039732 | 2 | 19 | 0 | 1 | 192 |
| 108 | 6C | 44 | 2C | 2 | 20 | 493608 | 3948860 | 2 | 20 | 0 | 184 | 4039924 | 2 | 20 | 0 | 1 | 192 |
| 109 | 6D | 44 | 2C | 2 | 21 | 493631 | 3949044 | 2 | 21 | 0 | 184 | 4040116 | 2 | 21 | 0 | 1 | 192 |
| 110 | 6E | 44 | 2C | 2 | 22 | 493654 | 3949228 | 2 | 22 | 0 | 184 | 4040308 | 2 | 22 | 0 | 1 | 192 |
| 111 | 6F | 44 | 2C | 2 | 23 | 493677 | 3949412 | 2 | 23 | 0 | 184 | 4040500 | 2 | 23 | 0 | 1 | 192 |
| 112 | 70 | 44 | 2C | 2 | 24 | 493702 | 3949612 | 2 | 24 | 0 | 200 | 4040708 | 2 | 24 | 0 | 1 | 208 |
| 113 | 71 | 44 | 2C | 2 | 25 | 493727 | 3949812 | 2 | 25 | 0 | 200 | 4040916 | 2 | 25 | 0 | 1 | 208 |
| 114 | 72 | 44 | 2C | 2 | 26 | 493752 | 3950012 | 2 | 26 | 0 | 200 | 4041124 | 2 | 26 | 0 | 1 | 208 |
| 115 | 73 | 44 | 2C | 2 | 27 | 493777 | 3950212 | 2 | 27 | 0 | 200 | 4041332 | 2 | 27 | 0 | 1 | 208 |
| 116 | 74 | 44 | 2C | 2 | 28 | 493802 | 3950412 | 2 | 28 | 0 | 200 | 4041540 | 2 | 28 | 0 | 1 | 208 |
| 117 | 75 | 44 | 2C | 2 | 29 | 493827 | 3950612 | 2 | 29 | 0 | 200 | 4041748 | 2 | 29 | 0 | 1 | 208 |
| 118 | 76 | 44 | 2C | 2 | 30 | 493852 | 3950812 | 2 | 30 | 0 | 200 | 4041956 | 2 | 30 | 0 | 1 | 208 |
| 119 | 77 | 44 | 2C | 2 | 31 | 493877 | 3951012 | 2 | 31 | 0 | 200 | 4042164 | 2 | 31 | 0 | 1 | 208 |
| 120 | 78 | 44 | 2C | 2 | 32 | 493902 | 3951212 | 2 | 32 | 0 | 200 | 4042372 | 2 | 32 | 0 | 1 | 208 |
| 121 | 79 | 44 | 2C | 2 | 33 | 493927 | 3951412 | 2 | 33 | 0 | 200 | 4042580 | 2 | 33 | 0 | 1 | 208 |
| 122 | 7A | 44 | 2C | 2 | 34 | 493952 | 3951612 | 2 | 34 | 0 | 200 | 4042788 | 2 | 34 | 0 | 1 | 208 |
| 123 | 7B | 44 | 2C | 2 | 35 | 493977 | 3951812 | 2 | 35 | 0 | 200 | 4042996 | 2 | 35 | 0 | 1 | 208 |
| 124 | 7C | 44 | 2C | 2 | 36 | 494002 | 3952012 | 2 | 36 | 0 | 200 | 4043204 | 2 | 36 | 0 | 1 | 208 |
| 125 | 7D | 44 | 2C | 2 | 37 | 494027 | 3952212 | 2 | 37 | 0 | 200 | 4043412 | 2 | 37 | 0 | 1 | 208 |
| 126 | 7E | 44 | 2C | 2 | 38 | 494052 | 3952412 | 2 | 38 | 0 | 200 | 4043620 | 2 | 38 | 0 | 1 | 208 |
| 127 | 7F | 44 | 2C | 2 | 39 | 494077 | 3952612 | 2 | 39 | 0 | 200 | 4043828 | 2 | 39 | 0 | 1 | 208 |
| 128 | 80 | 44 | 2C | 2 | 40 | 494102 | 3952812 | 2 | 40 | 0 | 200 | 4044036 | 2 | 40 | 0 | 1 | 208 |
| 129 | 81 | 44 | 2C | 2 | 41 | 494127 | 3953012 | 2 | 41 | 0 | 200 | 4044244 | 2 | 41 | 0 | 1 | 208 |
| 130 | 82 | 44 | 2C | 2 | 42 | 494152 | 3953212 | 2 | 42 | 0 | 200 | 4044452 | 2 | 42 | 0 | 1 | 208 |
| 131 | 83 | 44 | 2C | 2 | 43 | 494177 | 3953412 | 2 | 43 | 0 | 200 | 4044660 | 2 | 43 | 0 | 1 | 208 |
| 132 | 84 | 44 | 2C | 3 | 0 | 494200 | 3953596 | 3 | 0 | 0 | 184 | 4044852 | 3 | 0 | 0 | 1 | 192 |
| 133 | 85 | 44 | 2C | 3 | 1 | 494228 | 3953820 | 3 | 1 | 0 | 224 | 4045084 | 3 | 1 | 0 | 1 | 232 |
| 134 | 86 | 44 | 2C | 3 | 2 | 494256 | 3954044 | 3 | 2 | 0 | 224 | 4045316 | 3 | 2 | 0 | 1 | 232 |
| 135 | 87 | 44 | 2C | 3 | 3 | 494284 | 3954268 | 3 | 3 | 0 | 224 | 4045548 | 3 | 3 | 0 | 1 | 232 |
| 136 | 88 | 44 | 2C | 3 | 4 | 494312 | 3954492 | 3 | 4 | 0 | 224 | 4045780 | 3 | 4 | 0 | 1 | 232 |
| 137 | 89 | 44 | 2C | 3 | 5 | 494340 | 3954716 | 3 | 5 | 0 | 224 | 4046012 | 3 | 5 | 0 | 1 | 232 |
| 138 | 8A | 44 | 2C | 3 | 6 | 494368 | 3954940 | 3 | 6 | 0 | 224 | 4046244 | 3 | 6 | 0 | 1 | 232 |
| 139 | 8B | 44 | 2C | 3 | 7 | 494396 | 3955164 | 3 | 7 | 0 | 224 | 4046476 | 3 | 7 | 0 | 1 | 232 |
| 140 | 8C | 44 | 2C | 3 | 8 | 494424 | 3955388 | 3 | 8 | 0 | 224 | 4046708 | 3 | 8 | 0 | 1 | 232 |
| 141 | 8D | 44 | 2C | 3 | 9 | 494452 | 3955612 | 3 | 9 | 0 | 224 | 4046940 | 3 | 9 | 0 | 1 | 232 |
| 142 | 8E | 44 | 2C | 3 | 10 | 494480 | 3955836 | 3 | 10 | 0 | 224 | 4047172 | 3 | 10 | 0 | 1 | 232 |
| 143 | 8F | 44 | 2C | 3 | 11 | 494508 | 3956060 | 3 | 11 | 0 | 224 | 4047404 | 3 | 11 | 0 | 1 | 232 |
| 144 | 90 | 44 | 2C | 3 | 12 | 494533 | 3956260 | 3 | 12 | 0 | 200 | 4047612 | 3 | 12 | 0 | 1 | 208 |
| 145 | 91 | 44 | 2C | 3 | 13 | 494558 | 3956460 | 3 | 13 | 0 | 200 | 4047820 | 3 | 13 | 0 | 1 | 208 |
| 146 | 92 | 44 | 2C | 3 | 14 | 494583 | 3956660 | 3 | 14 | 0 | 200 | 4048028 | 3 | 14 | 0 | 1 | 208 |
| 147 | 93 | 44 | 2C | 3 | 15 | 494608 | 3956860 | 3 | 15 | 0 | 200 | 4048236 | 3 | 15 | 0 | 1 | 208 |
| 148 | 94 | 44 | 2C | 3 | 16 | 494633 | 3957060 | 3 | 16 | 0 | 200 | 4048444 | 3 | 16 | 0 | 1 | 208 |
| 149 | 95 | 44 | 2C | 3 | 17 | 494658 | 3957260 | 3 | 17 | 0 | 200 | 4048652 | 3 | 17 | 0 | 1 | 208 |
| 150 | 96 | 44 | 2C | 3 | 18 | 494683 | 3957460 | 3 | 18 | 0 | 200 | 4048860 | 3 | 18 | 0 | 1 | 208 |
| 151 | 97 | 44 | 2C | 3 | 19 | 494708 | 3957660 | 3 | 19 | 0 | 200 | 4049068 | 3 | 19 | 0 | 1 | 208 |
| 152 | 98 | 44 | 2C | 3 | 20 | 494733 | 3957860 | 3 | 20 | 0 | 200 | 4049276 | 3 | 20 | 0 | 1 | 208 |
| 153 | 99 | 44 | 2C | 3 | 21 | 494758 | 3958060 | 3 | 21 | 0 | 200 | 4049484 | 3 | 21 | 0 | 1 | 208 |
| 154 | 9A | 44 | 2C | 3 | 22 | 494783 | 3958260 | 3 | 22 | 0 | 200 | 4049692 | 3 | 22 | 0 | 1 | 208 |
| 155 | 9B | 44 | 2C | 3 | 23 | 494808 | 3958460 | 3 | 23 | 0 | 200 | 4049900 | 3 | 23 | 0 | 1 | 208 |
| 156 | 9C | 44 | 2C | 3 | 24 | 494833 | 3958660 | 3 | 24 | 0 | 200 | 4050108 | 3 | 24 | 0 | 1 | 208 |
| 157 | 9D | 44 | 2C | 3 | 25 | 494858 | 3958860 | 3 | 25 | 0 | 200 | 4050316 | 3 | 25 | 0 | 1 | 208 |
| 158 | 9E | 44 | 2C | 3 | 26 | 494883 | 3959060 | 3 | 26 | 0 | 200 | 4050524 | 3 | 26 | 0 | 1 | 208 |
| 159 | 9F | 44 | 2C | 3 | 27 | 494908 | 3959260 | 3 | 27 | 0 | 200 | 4050732 | 3 | 27 | 0 | 1 | 208 |
| 160 | A0 | 44 | 2C | 3 | 28 | 494935 | 3959476 | 3 | 28 | 0 | 216 | 4050956 | 3 | 28 | 0 | 1 | 224 |
| 161 | A1 | 44 | 2C | 3 | 29 | 494962 | 3959692 | 3 | 29 | 0 | 216 | 4051180 | 3 | 29 | 0 | 1 | 224 |
| 162 | A2 | 44 | 2C | 3 | 30 | 494989 | 3959908 | 3 | 30 | 0 | 216 | 4051404 | 3 | 30 | 0 | 1 | 224 |
| 163 | A3 | 44 | 2C | 3 | 31 | 495016 | 3960124 | 3 | 31 | 0 | 216 | 4051628 | 3 | 31 | 0 | 1 | 224 |
| 164 | A4 | 44 | 2C | 3 | 32 | 495043 | 3960340 | 3 | 32 | 0 | 216 | 4051852 | 3 | 32 | 0 | 1 | 224 |
| 165 | A5 | 44 | 2C | 3 | 33 | 495070 | 3960556 | 3 | 33 | 0 | 216 | 4052076 | 3 | 33 | 0 | 1 | 224 |

| | | | | | | | | | | | | | | | | | |
|---|---|---|---|---|---|---|---|---|---|---|---|---|---|---|---|---|---|
| 166 | A6 | 44 | 2C | 3 | 34 | 495097 | 3960772 | 3 | 34 | 0 | 216 | 4052300 | 3 | 34 | 0 | 1 | 224 |
| 167 | A7 | 44 | 2C | 3 | 35 | 495124 | 3960988 | 3 | 35 | 0 | 216 | 4052524 | 3 | 35 | 0 | 1 | 224 |
| 168 | A8 | 44 | 2C | 3 | 36 | 495151 | 3961204 | 3 | 36 | 0 | 216 | 4052748 | 3 | 36 | 0 | 1 | 224 |
| 169 | A9 | 44 | 2C | 3 | 37 | 495178 | 3961420 | 3 | 37 | 0 | 216 | 4052972 | 3 | 37 | 0 | 1 | 224 |
| 170 | AA | 44 | 2C | 3 | 38 | 495205 | 3961636 | 3 | 38 | 0 | 216 | 4053196 | 3 | 38 | 0 | 1 | 224 |
| 171 | AB | 44 | 2C | 3 | 39 | 495232 | 3961852 | 3 | 39 | 0 | 216 | 4053420 | 3 | 39 | 0 | 1 | 224 |
| 172 | AC | 44 | 2C | 3 | 40 | 495259 | 3962068 | 3 | 40 | 0 | 216 | 4053644 | 3 | 40 | 0 | 1 | 224 |
| 173 | AD | 44 | 2C | 3 | 41 | 495286 | 3962284 | 3 | 41 | 0 | 216 | 4053868 | 3 | 41 | 0 | 1 | 224 |
| 174 | AE | 44 | 2C | 3 | 42 | 495313 | 3962500 | 3 | 42 | 0 | 216 | 4054092 | 3 | 42 | 0 | 1 | 224 |
| 175 | AF | 44 | 2C | 3 | 43 | 495340 | 3962716 | 3 | 43 | 0 | 216 | 4054316 | 3 | 43 | 0 | 1 | 224 |
| 176 | B0 | 44 | 2C | 4 | 0 | 495365 | 3962916 | 4 | 0 | 0 | 200 | 4054524 | 4 | 0 | 0 | 1 | 208 |
| 177 | B1 | 44 | 2C | 4 | 1 | 495395 | 3963156 | 4 | 1 | 0 | 240 | 4054772 | 4 | 1 | 0 | 1 | 248 |
| 178 | B2 | 44 | 2C | 4 | 2 | 495425 | 3963396 | 4 | 2 | 0 | 240 | 4055020 | 4 | 2 | 0 | 1 | 248 |
| 179 | B3 | 44 | 2C | 4 | 3 | 495455 | 3963636 | 4 | 3 | 0 | 240 | 4055268 | 4 | 3 | 0 | 1 | 248 |
| 180 | B4 | 44 | 2C | 4 | 4 | 495485 | 3963876 | 4 | 4 | 0 | 240 | 4055516 | 4 | 4 | 0 | 1 | 248 |
| 181 | B5 | 44 | 2C | 4 | 5 | 495515 | 3964116 | 4 | 5 | 0 | 240 | 4055764 | 4 | 5 | 0 | 1 | 248 |
| 182 | B6 | 44 | 2C | 4 | 6 | 495545 | 3964356 | 4 | 6 | 0 | 240 | 4056012 | 4 | 6 | 0 | 1 | 248 |
| 183 | B7 | 44 | 2C | 4 | 7 | 495575 | 3964596 | 4 | 7 | 0 | 240 | 4056260 | 4 | 7 | 0 | 1 | 248 |
| 184 | B8 | 44 | 2C | 4 | 8 | 495605 | 3964836 | 4 | 8 | 0 | 240 | 4056508 | 4 | 8 | 0 | 1 | 248 |
| 185 | B9 | 44 | 2C | 4 | 9 | 495635 | 3965076 | 4 | 9 | 0 | 240 | 4056756 | 4 | 9 | 0 | 1 | 248 |
| 186 | BA | 44 | 2C | 4 | 10 | 495665 | 3965316 | 4 | 10 | 0 | 240 | 4057004 | 4 | 10 | 0 | 1 | 248 |
| 187 | BB | 44 | 2C | 4 | 11 | 495695 | 3965556 | 4 | 11 | 0 | 240 | 4057252 | 4 | 11 | 0 | 1 | 248 |
| 188 | BC | 44 | 2C | 4 | 12 | 495725 | 3965796 | 4 | 12 | 0 | 240 | 4057500 | 4 | 12 | 0 | 1 | 248 |
| 189 | BD | 44 | 2C | 4 | 13 | 495755 | 3966036 | 4 | 13 | 0 | 240 | 4057748 | 4 | 13 | 0 | 1 | 248 |
| 190 | BE | 44 | 2C | 4 | 14 | 495785 | 3966276 | 4 | 14 | 0 | 240 | 4057996 | 4 | 14 | 0 | 1 | 248 |
| 191 | BF | 44 | 2C | 4 | 15 | 495815 | 3966516 | 4 | 15 | 0 | 240 | 4058244 | 4 | 15 | 0 | 1 | 248 |
| 192 | C0 | 44 | 2C | 4 | 16 | 495842 | 3966732 | 4 | 16 | 0 | 216 | 4058468 | 4 | 16 | 0 | 1 | 224 |
| 193 | C1 | 44 | 2C | 4 | 17 | 495869 | 3966948 | 4 | 17 | 0 | 216 | 4058692 | 4 | 17 | 0 | 1 | 224 |
| 194 | C2 | 44 | 2C | 4 | 18 | 495896 | 3967164 | 4 | 18 | 0 | 216 | 4058916 | 4 | 18 | 0 | 1 | 224 |
| 195 | C3 | 44 | 2C | 4 | 19 | 495923 | 3967380 | 4 | 19 | 0 | 216 | 4059140 | 4 | 19 | 0 | 1 | 224 |
| 196 | C4 | 44 | 2C | 4 | 20 | 495950 | 3967596 | 4 | 20 | 0 | 216 | 4059364 | 4 | 20 | 0 | 1 | 224 |
| 197 | C5 | 44 | 2C | 4 | 21 | 495977 | 3967812 | 4 | 21 | 0 | 216 | 4059588 | 4 | 21 | 0 | 1 | 224 |
| 198 | C6 | 44 | 2C | 4 | 22 | 496004 | 3968028 | 4 | 22 | 0 | 216 | 4059812 | 4 | 22 | 0 | 1 | 224 |
| 199 | C7 | 44 | 2C | 4 | 23 | 496031 | 3968244 | 4 | 23 | 0 | 216 | 4060036 | 4 | 23 | 0 | 1 | 224 |
| 200 | C8 | 44 | 2C | 4 | 24 | 496058 | 3968460 | 4 | 24 | 0 | 216 | 4060260 | 4 | 24 | 0 | 1 | 224 |
| 201 | C9 | 44 | 2C | 4 | 25 | 496085 | 3968676 | 4 | 25 | 0 | 216 | 4060484 | 4 | 25 | 0 | 1 | 224 |
| 202 | CA | 44 | 2C | 4 | 26 | 496112 | 3968892 | 4 | 26 | 0 | 216 | 4060708 | 4 | 26 | 0 | 1 | 224 |
| 203 | CB | 44 | 2C | 4 | 27 | 496139 | 3969108 | 4 | 27 | 0 | 216 | 4060932 | 4 | 27 | 0 | 1 | 224 |
| 204 | CC | 44 | 2C | 4 | 28 | 496166 | 3969324 | 4 | 28 | 0 | 216 | 4061156 | 4 | 28 | 0 | 1 | 224 |
| 205 | CD | 44 | 2C | 4 | 29 | 496193 | 3969540 | 4 | 29 | 0 | 216 | 4061380 | 4 | 29 | 0 | 1 | 224 |
| 206 | CE | 44 | 2C | 4 | 30 | 496220 | 3969756 | 4 | 30 | 0 | 216 | 4061604 | 4 | 30 | 0 | 1 | 224 |
| 207 | CF | 44 | 2C | 4 | 31 | 496247 | 3969972 | 4 | 31 | 0 | 216 | 4061828 | 4 | 31 | 0 | 1 | 224 |
| 208 | D0 | 44 | 2C | 4 | 32 | 496276 | 3970204 | 4 | 32 | 0 | 232 | 4062068 | 4 | 32 | 0 | 1 | 240 |
| 209 | D1 | 44 | 2C | 4 | 33 | 496305 | 3970436 | 4 | 33 | 0 | 232 | 4062308 | 4 | 33 | 0 | 1 | 240 |
| 210 | D2 | 44 | 2C | 4 | 34 | 496334 | 3970668 | 4 | 34 | 0 | 232 | 4062548 | 4 | 34 | 0 | 1 | 240 |
| 211 | D3 | 44 | 2C | 4 | 35 | 496363 | 3970900 | 4 | 35 | 0 | 232 | 4062788 | 4 | 35 | 0 | 1 | 240 |
| 212 | D4 | 44 | 2C | 4 | 36 | 496392 | 3971132 | 4 | 36 | 0 | 232 | 4063028 | 4 | 36 | 0 | 1 | 240 |
| 213 | D5 | 44 | 2C | 4 | 37 | 496421 | 3971364 | 4 | 37 | 0 | 232 | 4063268 | 4 | 37 | 0 | 1 | 240 |
| 214 | D6 | 44 | 2C | 4 | 38 | 496450 | 3971596 | 4 | 38 | 0 | 232 | 4063508 | 4 | 38 | 0 | 1 | 240 |
| 215 | D7 | 44 | 2C | 4 | 39 | 496479 | 3971828 | 4 | 39 | 0 | 232 | 4063748 | 4 | 39 | 0 | 1 | 240 |
| 216 | D8 | 44 | 2C | 4 | 40 | 496508 | 3972060 | 4 | 40 | 0 | 232 | 4063988 | 4 | 40 | 0 | 1 | 240 |
| 217 | D9 | 44 | 2C | 4 | 41 | 496537 | 3972292 | 4 | 41 | 0 | 232 | 4064228 | 4 | 41 | 0 | 1 | 240 |
| 218 | DA | 44 | 2C | 4 | 42 | 496566 | 3972524 | 4 | 42 | 0 | 232 | 4064468 | 4 | 42 | 0 | 1 | 240 |
| 219 | DB | 44 | 2C | 4 | 43 | 496595 | 3972756 | 4 | 43 | 0 | 232 | 4064708 | 4 | 43 | 0 | 1 | 240 |
| 220 | DC | 44 | 2C | 5 | 0 | 496622 | 3972972 | 5 | 0 | 0 | 216 | 4064932 | 5 | 0 | 0 | 1 | 224 |
| 221 | DD | 44 | 2C | 5 | 1 | 496654 | 3973228 | 5 | 1 | 0 | 256 | 4065196 | 5 | 1 | 0 | 1 | 264 |
| 222 | DE | 44 | 2C | 5 | 2 | 496686 | 3973484 | 5 | 2 | 0 | 256 | 4065460 | 5 | 2 | 0 | 1 | 264 |
| 223 | DF | 44 | 2C | 5 | 3 | 496718 | 3973740 | 5 | 3 | 0 | 256 | 4065724 | 5 | 3 | 0 | 1 | 264 |
| 224 | E0 | 44 | 2C | 5 | 4 | 496750 | 3973996 | 5 | 4 | 0 | 256 | 4065988 | 5 | 4 | 0 | 1 | 264 |
| 225 | E1 | 44 | 2C | 5 | 5 | 496782 | 3974252 | 5 | 5 | 0 | 256 | 4066252 | 5 | 5 | 0 | 1 | 264 |
| 226 | E2 | 44 | 2C | 5 | 6 | 496814 | 3974508 | 5 | 6 | 0 | 256 | 4066516 | 5 | 6 | 0 | 1 | 264 |
| 227 | E3 | 44 | 2C | 5 | 7 | 496846 | 3974764 | 5 | 7 | 0 | 256 | 4066780 | 5 | 7 | 0 | 1 | 264 |
| 228 | E4 | 44 | 2C | 5 | 8 | 496878 | 3975020 | 5 | 8 | 0 | 256 | 4067044 | 5 | 8 | 0 | 1 | 264 |
| 229 | E5 | 44 | 2C | 5 | 9 | 496910 | 3975276 | 5 | 9 | 0 | 256 | 4067308 | 5 | 9 | 0 | 1 | 264 |
| 230 | E6 | 44 | 2C | 5 | 10 | 496942 | 3975532 | 5 | 10 | 0 | 256 | 4067572 | 5 | 10 | 0 | 1 | 264 |
| 231 | E7 | 44 | 2C | 5 | 11 | 496974 | 3975788 | 5 | 11 | 0 | 256 | 4067836 | 5 | 11 | 0 | 1 | 264 |
| 232 | E8 | 44 | 2C | 5 | 12 | 497006 | 3976044 | 5 | 12 | 0 | 256 | 4068100 | 5 | 12 | 0 | 1 | 264 |
| 233 | E9 | 44 | 2C | 5 | 13 | 497038 | 3976300 | 5 | 13 | 0 | 256 | 4068364 | 5 | 13 | 0 | 1 | 264 |
| 234 | EA | 44 | 2C | 5 | 14 | 497070 | 3976556 | 5 | 14 | 0 | 256 | 4068628 | 5 | 14 | 0 | 1 | 264 |
| 235 | EB | 44 | 2C | 5 | 15 | 497102 | 3976812 | 5 | 15 | 0 | 256 | 4068892 | 5 | 15 | 0 | 1 | 264 |
| 236 | EC | 44 | 2C | 5 | 16 | 497134 | 3977068 | 5 | 16 | 0 | 256 | 4069156 | 5 | 16 | 0 | 1 | 264 |
| 237 | ED | 44 | 2C | 5 | 17 | 497166 | 3977324 | 5 | 17 | 0 | 256 | 4069420 | 5 | 17 | 0 | 1 | 264 |
| 238 | EE | 44 | 2C | 5 | 18 | 497198 | 3977580 | 5 | 18 | 0 | 256 | 4069684 | 5 | 18 | 0 | 1 | 264 |
| 239 | EF | 44 | 2C | 5 | 19 | 497230 | 3977836 | 5 | 19 | 0 | 256 | 4069948 | 5 | 19 | 0 | 1 | 264 |
| 240 | F0 | 44 | 2C | 5 | 20 | 497259 | 3978068 | 5 | 20 | 0 | 232 | 4070188 | 5 | 20 | 0 | 1 | 240 |
| 241 | F1 | 44 | 2C | 5 | 21 | 497288 | 3978300 | 5 | 21 | 0 | 232 | 4070428 | 5 | 21 | 0 | 1 | 240 |
| 242 | F2 | 44 | 2C | 5 | 22 | 497317 | 3978532 | 5 | 22 | 0 | 232 | 4070668 | 5 | 22 | 0 | 1 | 240 |
| 243 | F3 | 44 | 2C | 5 | 23 | 497346 | 3978764 | 5 | 23 | 0 | 232 | 4070908 | 5 | 23 | 0 | 1 | 240 |
| 244 | F4 | 44 | 2C | 5 | 24 | 497375 | 3978996 | 5 | 24 | 0 | 232 | 4071148 | 5 | 24 | 0 | 1 | 240 |
| 245 | F5 | 44 | 2C | 5 | 25 | 497404 | 3979228 | 5 | 25 | 0 | 232 | 4071388 | 5 | 25 | 0 | 1 | 240 |
| 246 | F6 | 44 | 2C | 5 | 26 | 497433 | 3979460 | 5 | 26 | 0 | 232 | 4071628 | 5 | 26 | 0 | 1 | 240 |
| 247 | F7 | 44 | 2C | 5 | 27 | 497462 | 3979692 | 5 | 27 | 0 | 232 | 4071868 | 5 | 27 | 0 | 1 | 240 |
| 248 | F8 | 44 | 2C | 5 | 28 | 497491 | 3979924 | 5 | 28 | 0 | 232 | 4072108 | 5 | 28 | 0 | 1 | 240 |
| 249 | F9 | 44 | 2C | 5 | 29 | 497520 | 3980156 | 5 | 29 | 0 | 232 | 4072348 | 5 | 29 | 0 | 1 | 240 |
| 250 | FA | 44 | 2C | 5 | 30 | 497549 | 3980388 | 5 | 30 | 0 | 232 | 4072588 | 5 | 30 | 0 | 1 | 240 |
| 251 | FB | 44 | 2C | 5 | 31 | 497578 | 3980620 | 5 | 31 | 0 | 232 | 4072828 | 5 | 31 | 0 | 1 | 240 |
| 252 | FC | 44 | 2C | 5 | 32 | 497607 | 3980852 | 5 | 32 | 0 | 232 | 4073068 | 5 | 32 | 0 | 1 | 240 |
| 253 | FD | 44 | 2C | 5 | 33 | 497636 | 3981084 | 5 | 33 | 0 | 232 | 4073308 | 5 | 33 | 0 | 1 | 240 |
| 254 | FE | 44 | 2C | 5 | 34 | 497665 | 3981316 | 5 | 34 | 0 | 232 | 4073548 | 5 | 34 | 0 | 1 | 240 |
| 255 | FF | 44 | 2C | 5 | 35 | 497694 | 3981548 | 5 | 35 | 0 | 232 | 4073788 | 5 | 35 | 0 | 1 | 240 |
| 0 | 00 | 45 | 2D | 0 | 0 | 497706 | 3981644 | 0 | 0 | 0 | 96 | 4073892 | 0 | 0 | 0 | 1 | 104 |
| 1 | 01 | 45 | 2D | 0 | 1 | 497720 | 3981756 | 0 | 1 | 0 | 112 | 4074012 | 0 | 1 | 0 | 1 | 120 |
| 2 | 02 | 45 | 2D | 0 | 2 | 497734 | 3981868 | 0 | 2 | 0 | 112 | 4074132 | 0 | 2 | 0 | 1 | 120 |
| 3 | 03 | 45 | 2D | 0 | 3 | 497748 | 3981980 | 0 | 3 | 0 | 112 | 4074252 | 0 | 3 | 0 | 1 | 120 |
| 4 | 04 | 45 | 2D | 0 | 4 | 497762 | 3982092 | 0 | 4 | 0 | 112 | 4074372 | 0 | 4 | 0 | 1 | 120 |
| 5 | 05 | 45 | 2D | 0 | 5 | 497776 | 3982204 | 0 | 5 | 0 | 112 | 4074492 | 0 | 5 | 0 | 1 | 120 |
| 6 | 06 | 45 | 2D | 0 | 6 | 497790 | 3982316 | 0 | 6 | 0 | 112 | 4074612 | 0 | 6 | 0 | 1 | 120 |
| 7 | 07 | 45 | 2D | 0 | 7 | 497804 | 3982428 | 0 | 7 | 0 | 112 | 4074732 | 0 | 7 | 0 | 1 | 120 |
| 8 | 08 | 45 | 2D | 0 | 8 | 497818 | 3982540 | 0 | 8 | 0 | 112 | 4074852 | 0 | 8 | 0 | 1 | 120 |
| 9 | 09 | 45 | 2D | 0 | 9 | 497832 | 3982652 | 0 | 9 | 0 | 112 | 4074972 | 0 | 9 | 0 | 1 | 120 |
| 10 | 0A | 45 | 2D | 0 | 10 | 497846 | 3982764 | 0 | 10 | 0 | 112 | 4075092 | 0 | 10 | 0 | 1 | 120 |
| 11 | 0B | 45 | 2D | 0 | 11 | 497860 | 3982876 | 0 | 11 | 0 | 112 | 4075212 | 0 | 11 | 0 | 1 | 120 |
| 12 | 0C | 45 | 2D | 0 | 12 | 497874 | 3982988 | 0 | 12 | 0 | 112 | 4075332 | 0 | 12 | 0 | 1 | 120 |

| | | | | | | | | | | | | | | | | | |
|---|---|---|---|---|---|---|---|---|---|---|---|---|---|---|---|---|---|
| 13 | 0D | 45 | 2D | 0 | 13 | 497888 | 3983100 | 0 | 13 | 0 | 112 | 4075452 | 0 | 13 | 0 | 1 | 120 |
| 14 | 0E | 45 | 2D | 0 | 14 | 497902 | 3983212 | 0 | 14 | 0 | 112 | 4075572 | 0 | 14 | 0 | 1 | 120 |
| 15 | 0F | 45 | 2D | 0 | 15 | 497916 | 3983324 | 0 | 15 | 0 | 112 | 4075692 | 0 | 15 | 0 | 1 | 120 |
| 16 | 10 | 45 | 2D | 0 | 16 | 497930 | 3983436 | 0 | 16 | 0 | 112 | 4075812 | 0 | 16 | 0 | 1 | 120 |
| 17 | 11 | 45 | 2D | 0 | 17 | 497944 | 3983548 | 0 | 17 | 0 | 112 | 4075932 | 0 | 17 | 0 | 1 | 120 |
| 18 | 12 | 45 | 2D | 0 | 18 | 497958 | 3983660 | 0 | 18 | 0 | 112 | 4076052 | 0 | 18 | 0 | 1 | 120 |
| 19 | 13 | 45 | 2D | 0 | 19 | 497972 | 3983772 | 0 | 19 | 0 | 112 | 4076172 | 0 | 19 | 0 | 1 | 120 |
| 20 | 14 | 45 | 2D | 0 | 20 | 497986 | 3983884 | 0 | 20 | 0 | 112 | 4076292 | 0 | 20 | 0 | 1 | 120 |
| 21 | 15 | 45 | 2D | 0 | 21 | 498000 | 3983996 | 0 | 21 | 0 | 112 | 4076412 | 0 | 21 | 0 | 1 | 120 |
| 22 | 16 | 45 | 2D | 0 | 22 | 498014 | 3984108 | 0 | 22 | 0 | 112 | 4076532 | 0 | 22 | 0 | 1 | 120 |
| 23 | 17 | 45 | 2D | 0 | 23 | 498028 | 3984220 | 0 | 23 | 0 | 112 | 4076652 | 0 | 23 | 0 | 1 | 120 |
| 24 | 18 | 45 | 2D | 0 | 24 | 498042 | 3984332 | 0 | 24 | 0 | 112 | 4076772 | 0 | 24 | 0 | 1 | 120 |
| 25 | 19 | 45 | 2D | 0 | 25 | 498056 | 3984444 | 0 | 25 | 0 | 112 | 4076892 | 0 | 25 | 0 | 1 | 120 |
| 26 | 1A | 45 | 2D | 0 | 26 | 498070 | 3984556 | 0 | 26 | 0 | 112 | 4077012 | 0 | 26 | 0 | 1 | 120 |
| 27 | 1B | 45 | 2D | 0 | 27 | 498084 | 3984668 | 0 | 27 | 0 | 112 | 4077132 | 0 | 27 | 0 | 1 | 120 |
| 28 | 1C | 45 | 2D | 0 | 28 | 498098 | 3984780 | 0 | 28 | 0 | 112 | 4077252 | 0 | 28 | 0 | 1 | 120 |
| 29 | 1D | 45 | 2D | 0 | 29 | 498112 | 3984892 | 0 | 29 | 0 | 112 | 4077372 | 0 | 29 | 0 | 1 | 120 |
| 30 | 1E | 45 | 2D | 0 | 30 | 498126 | 3985004 | 0 | 30 | 0 | 112 | 4077492 | 0 | 30 | 0 | 1 | 120 |
| 31 | 1F | 45 | 2D | 0 | 31 | 498140 | 3985116 | 0 | 31 | 0 | 112 | 4077612 | 0 | 31 | 0 | 1 | 120 |
| 32 | 20 | 45 | 2D | 0 | 32 | 498154 | 3985228 | 0 | 32 | 0 | 112 | 4077732 | 0 | 32 | 0 | 1 | 120 |
| 33 | 21 | 45 | 2D | 0 | 33 | 498168 | 3985340 | 0 | 33 | 0 | 112 | 4077852 | 0 | 33 | 0 | 1 | 120 |
| 34 | 22 | 45 | 2D | 0 | 34 | 498182 | 3985452 | 0 | 34 | 0 | 112 | 4077972 | 0 | 34 | 0 | 1 | 120 |
| 35 | 23 | 45 | 2D | 0 | 35 | 498196 | 3985564 | 0 | 35 | 0 | 112 | 4078092 | 0 | 35 | 0 | 1 | 120 |
| 36 | 24 | 45 | 2D | 0 | 36 | 498210 | 3985676 | 0 | 36 | 0 | 112 | 4078212 | 0 | 36 | 0 | 1 | 120 |
| 37 | 25 | 45 | 2D | 0 | 37 | 498224 | 3985788 | 0 | 37 | 0 | 112 | 4078332 | 0 | 37 | 0 | 1 | 120 |
| 38 | 26 | 45 | 2D | 0 | 38 | 498238 | 3985900 | 0 | 38 | 0 | 112 | 4078452 | 0 | 38 | 0 | 1 | 120 |
| 39 | 27 | 45 | 2D | 0 | 39 | 498252 | 3986012 | 0 | 39 | 0 | 112 | 4078572 | 0 | 39 | 0 | 1 | 120 |
| 40 | 28 | 45 | 2D | 0 | 40 | 498266 | 3986124 | 0 | 40 | 0 | 112 | 4078692 | 0 | 40 | 0 | 1 | 120 |
| 41 | 29 | 45 | 2D | 0 | 41 | 498280 | 3986236 | 0 | 41 | 0 | 112 | 4078812 | 0 | 41 | 0 | 1 | 120 |
| 42 | 2A | 45 | 2D | 0 | 42 | 498294 | 3986348 | 0 | 42 | 0 | 112 | 4078932 | 0 | 42 | 0 | 1 | 120 |
| 43 | 2B | 45 | 2D | 0 | 43 | 498308 | 3986460 | 0 | 43 | 0 | 112 | 4079052 | 0 | 43 | 0 | 1 | 120 |
| 44 | 2C | 45 | 2D | 0 | 44 | 498322 | 3986572 | 0 | 44 | 0 | 112 | 4079172 | 0 | 44 | 0 | 1 | 120 |
| 45 | 2D | 45 | 2D | 1 | 0 | 498334 | 3986668 | 1 | 0 | 0 | 96 | 4079276 | 1 | 0 | 0 | 1 | 104 |
| 46 | 2E | 45 | 2D | 1 | 1 | 498351 | 3986804 | 1 | 1 | 0 | 136 | 4079420 | 1 | 1 | 0 | 1 | 144 |
| 47 | 2F | 45 | 2D | 1 | 2 | 498368 | 3986940 | 1 | 2 | 0 | 136 | 4079564 | 1 | 2 | 0 | 1 | 144 |
| 48 | 30 | 45 | 2D | 1 | 3 | 498389 | 3987108 | 1 | 3 | 0 | 168 | 4079740 | 1 | 3 | 0 | 1 | 176 |
| 49 | 31 | 45 | 2D | 1 | 4 | 498410 | 3987276 | 1 | 4 | 0 | 168 | 4079916 | 1 | 4 | 0 | 1 | 176 |
| 50 | 32 | 45 | 2D | 1 | 5 | 498431 | 3987444 | 1 | 5 | 0 | 168 | 4080092 | 1 | 5 | 0 | 1 | 176 |
| 51 | 33 | 45 | 2D | 1 | 6 | 498452 | 3987612 | 1 | 6 | 0 | 168 | 4080268 | 1 | 6 | 0 | 1 | 176 |
| 52 | 34 | 45 | 2D | 1 | 7 | 498473 | 3987780 | 1 | 7 | 0 | 168 | 4080444 | 1 | 7 | 0 | 1 | 176 |
| 53 | 35 | 45 | 2D | 1 | 8 | 498494 | 3987948 | 1 | 8 | 0 | 168 | 4080620 | 1 | 8 | 0 | 1 | 176 |
| 54 | 36 | 45 | 2D | 1 | 9 | 498515 | 3988116 | 1 | 9 | 0 | 168 | 4080796 | 1 | 9 | 0 | 1 | 176 |
| 55 | 37 | 45 | 2D | 1 | 10 | 498536 | 3988284 | 1 | 10 | 0 | 168 | 4080972 | 1 | 10 | 0 | 1 | 176 |
| 56 | 38 | 45 | 2D | 1 | 11 | 498557 | 3988452 | 1 | 11 | 0 | 168 | 4081148 | 1 | 11 | 0 | 1 | 176 |
| 57 | 39 | 45 | 2D | 1 | 12 | 498578 | 3988620 | 1 | 12 | 0 | 168 | 4081324 | 1 | 12 | 0 | 1 | 176 |
| 58 | 3A | 45 | 2D | 1 | 13 | 498599 | 3988788 | 1 | 13 | 0 | 168 | 4081500 | 1 | 13 | 0 | 1 | 176 |
| 59 | 3B | 45 | 2D | 1 | 14 | 498620 | 3988956 | 1 | 14 | 0 | 168 | 4081676 | 1 | 14 | 0 | 1 | 176 |
| 60 | 3C | 45 | 2D | 1 | 15 | 498641 | 3989124 | 1 | 15 | 0 | 168 | 4081852 | 1 | 15 | 0 | 1 | 176 |
| 61 | 3D | 45 | 2D | 1 | 16 | 498662 | 3989292 | 1 | 16 | 0 | 168 | 4082028 | 1 | 16 | 0 | 1 | 176 |
| 62 | 3E | 45 | 2D | 1 | 17 | 498683 | 3989460 | 1 | 17 | 0 | 168 | 4082204 | 1 | 17 | 0 | 1 | 176 |
| 63 | 3F | 45 | 2D | 1 | 18 | 498704 | 3989628 | 1 | 18 | 0 | 168 | 4082380 | 1 | 18 | 0 | 1 | 176 |
| 64 | 40 | 45 | 2D | 1 | 19 | 498727 | 3989812 | 1 | 19 | 0 | 184 | 4082572 | 1 | 19 | 0 | 1 | 192 |
| 65 | 41 | 45 | 2D | 1 | 20 | 498750 | 3989996 | 1 | 20 | 0 | 184 | 4082764 | 1 | 20 | 0 | 1 | 192 |
| 66 | 42 | 45 | 2D | 1 | 21 | 498773 | 3990180 | 1 | 21 | 0 | 184 | 4082956 | 1 | 21 | 0 | 1 | 192 |
| 67 | 43 | 45 | 2D | 1 | 22 | 498796 | 3990364 | 1 | 22 | 0 | 184 | 4083148 | 1 | 22 | 0 | 1 | 192 |
| 68 | 44 | 45 | 2D | 1 | 23 | 498819 | 3990548 | 1 | 23 | 0 | 184 | 4083340 | 1 | 23 | 0 | 1 | 192 |
| 69 | 45 | 45 | 2D | 1 | 24 | 498842 | 3990732 | 1 | 24 | 0 | 184 | 4083532 | 1 | 24 | 0 | 1 | 192 |
| 70 | 46 | 45 | 2D | 1 | 25 | 498865 | 3990916 | 1 | 25 | 0 | 184 | 4083724 | 1 | 25 | 0 | 1 | 192 |
| 71 | 47 | 45 | 2D | 1 | 26 | 498888 | 3991100 | 1 | 26 | 0 | 184 | 4083916 | 1 | 26 | 0 | 1 | 192 |
| 72 | 48 | 45 | 2D | 1 | 27 | 498911 | 3991284 | 1 | 27 | 0 | 184 | 4084108 | 1 | 27 | 0 | 1 | 192 |
| 73 | 49 | 45 | 2D | 1 | 28 | 498934 | 3991468 | 1 | 28 | 0 | 184 | 4084300 | 1 | 28 | 0 | 1 | 192 |
| 74 | 4A | 45 | 2D | 1 | 29 | 498957 | 3991652 | 1 | 29 | 0 | 184 | 4084492 | 1 | 29 | 0 | 1 | 192 |
| 75 | 4B | 45 | 2D | 1 | 30 | 498980 | 3991836 | 1 | 30 | 0 | 184 | 4084684 | 1 | 30 | 0 | 1 | 192 |
| 76 | 4C | 45 | 2D | 1 | 31 | 499003 | 3992020 | 1 | 31 | 0 | 184 | 4084876 | 1 | 31 | 0 | 1 | 192 |
| 77 | 4D | 45 | 2D | 1 | 32 | 499026 | 3992204 | 1 | 32 | 0 | 184 | 4085068 | 1 | 32 | 0 | 1 | 192 |
| 78 | 4E | 45 | 2D | 1 | 33 | 499049 | 3992388 | 1 | 33 | 0 | 184 | 4085260 | 1 | 33 | 0 | 1 | 192 |
| 79 | 4F | 45 | 2D | 1 | 34 | 499072 | 3992572 | 1 | 34 | 0 | 184 | 4085452 | 1 | 34 | 0 | 1 | 192 |
| 80 | 50 | 45 | 2D | 1 | 35 | 499095 | 3992756 | 1 | 35 | 0 | 184 | 4085644 | 1 | 35 | 0 | 1 | 192 |
| 81 | 51 | 45 | 2D | 1 | 36 | 499118 | 3992940 | 1 | 36 | 0 | 184 | 4085836 | 1 | 36 | 0 | 1 | 192 |
| 82 | 52 | 45 | 2D | 1 | 37 | 499141 | 3993124 | 1 | 37 | 0 | 184 | 4086028 | 1 | 37 | 0 | 1 | 192 |
| 83 | 53 | 45 | 2D | 1 | 38 | 499164 | 3993308 | 1 | 38 | 0 | 184 | 4086220 | 1 | 38 | 0 | 1 | 192 |
| 84 | 54 | 45 | 2D | 1 | 39 | 499187 | 3993492 | 1 | 39 | 0 | 184 | 4086412 | 1 | 39 | 0 | 1 | 192 |
| 85 | 55 | 45 | 2D | 1 | 40 | 499210 | 3993676 | 1 | 40 | 0 | 184 | 4086604 | 1 | 40 | 0 | 1 | 192 |
| 86 | 56 | 45 | 2D | 1 | 41 | 499233 | 3993860 | 1 | 41 | 0 | 184 | 4086796 | 1 | 41 | 0 | 1 | 192 |
| 87 | 57 | 45 | 2D | 1 | 42 | 499256 | 3994044 | 1 | 42 | 0 | 184 | 4086988 | 1 | 42 | 0 | 1 | 192 |
| 88 | 58 | 45 | 2D | 1 | 43 | 499279 | 3994228 | 1 | 43 | 0 | 184 | 4087180 | 1 | 43 | 0 | 1 | 192 |
| 89 | 59 | 45 | 2D | 1 | 44 | 499302 | 3994412 | 1 | 44 | 0 | 184 | 4087372 | 1 | 44 | 0 | 1 | 192 |
| 90 | 5A | 45 | 2D | 2 | 0 | 499323 | 3994580 | 2 | 0 | 0 | 168 | 4087548 | 2 | 0 | 0 | 1 | 176 |
| 91 | 5B | 45 | 2D | 2 | 1 | 499349 | 3994788 | 2 | 1 | 0 | 208 | 4087764 | 2 | 1 | 0 | 1 | 216 |
| 92 | 5C | 45 | 2D | 2 | 2 | 499375 | 3994996 | 2 | 2 | 0 | 208 | 4087980 | 2 | 2 | 0 | 1 | 216 |
| 93 | 5D | 45 | 2D | 2 | 3 | 499401 | 3995204 | 2 | 3 | 0 | 208 | 4088196 | 2 | 3 | 0 | 1 | 216 |
| 94 | 5E | 45 | 2D | 2 | 4 | 499427 | 3995412 | 2 | 4 | 0 | 208 | 4088412 | 2 | 4 | 0 | 1 | 216 |
| 95 | 5F | 45 | 2D | 2 | 5 | 499453 | 3995620 | 2 | 5 | 0 | 208 | 4088628 | 2 | 5 | 0 | 1 | 216 |
| 96 | 60 | 45 | 2D | 2 | 6 | 499476 | 3995804 | 2 | 6 | 0 | 184 | 4088820 | 2 | 6 | 0 | 1 | 192 |
| 97 | 61 | 45 | 2D | 2 | 7 | 499499 | 3995988 | 2 | 7 | 0 | 184 | 4089012 | 2 | 7 | 0 | 1 | 192 |
| 98 | 62 | 45 | 2D | 2 | 8 | 499522 | 3996172 | 2 | 8 | 0 | 184 | 4089204 | 2 | 8 | 0 | 1 | 192 |
| 99 | 63 | 45 | 2D | 2 | 9 | 499545 | 3996356 | 2 | 9 | 0 | 184 | 4089396 | 2 | 9 | 0 | 1 | 192 |
| 100 | 64 | 45 | 2D | 2 | 10 | 499568 | 3996540 | 2 | 10 | 0 | 184 | 4089588 | 2 | 10 | 0 | 1 | 192 |
| 101 | 65 | 45 | 2D | 2 | 11 | 499591 | 3996724 | 2 | 11 | 0 | 184 | 4089780 | 2 | 11 | 0 | 1 | 192 |
| 102 | 66 | 45 | 2D | 2 | 12 | 499614 | 3996908 | 2 | 12 | 0 | 184 | 4089972 | 2 | 12 | 0 | 1 | 192 |
| 103 | 67 | 45 | 2D | 2 | 13 | 499637 | 3997092 | 2 | 13 | 0 | 184 | 4090164 | 2 | 13 | 0 | 1 | 192 |
| 104 | 68 | 45 | 2D | 2 | 14 | 499660 | 3997276 | 2 | 14 | 0 | 184 | 4090356 | 2 | 14 | 0 | 1 | 192 |
| 105 | 69 | 45 | 2D | 2 | 15 | 499683 | 3997460 | 2 | 15 | 0 | 184 | 4090548 | 2 | 15 | 0 | 1 | 192 |
| 106 | 6A | 45 | 2D | 2 | 16 | 499706 | 3997644 | 2 | 16 | 0 | 184 | 4090740 | 2 | 16 | 0 | 1 | 192 |
| 107 | 6B | 45 | 2D | 2 | 17 | 499729 | 3997828 | 2 | 17 | 0 | 184 | 4090932 | 2 | 17 | 0 | 1 | 192 |
| 108 | 6C | 45 | 2D | 2 | 18 | 499752 | 3998012 | 2 | 18 | 0 | 184 | 4091124 | 2 | 18 | 0 | 1 | 192 |
| 109 | 6D | 45 | 2D | 2 | 19 | 499775 | 3998196 | 2 | 19 | 0 | 184 | 4091316 | 2 | 19 | 0 | 1 | 192 |
| 110 | 6E | 45 | 2D | 2 | 20 | 499798 | 3998380 | 2 | 20 | 0 | 184 | 4091508 | 2 | 20 | 0 | 1 | 192 |
| 111 | 6F | 45 | 2D | 2 | 21 | 499821 | 3998564 | 2 | 21 | 0 | 184 | 4091700 | 2 | 21 | 0 | 1 | 192 |
| 112 | 70 | 45 | 2D | 2 | 22 | 499846 | 3998764 | 2 | 22 | 0 | 200 | 4091908 | 2 | 22 | 0 | 1 | 208 |
| 113 | 71 | 45 | 2D | 2 | 23 | 499871 | 3998964 | 2 | 23 | 0 | 200 | 4092116 | 2 | 23 | 0 | 1 | 208 |
| 114 | 72 | 45 | 2D | 2 | 24 | 499896 | 3999164 | 2 | 24 | 0 | 200 | 4092324 | 2 | 24 | 0 | 1 | 208 |
| 115 | 73 | 45 | 2D | 2 | 25 | 499921 | 3999364 | 2 | 25 | 0 | 200 | 4092532 | 2 | 25 | 0 | 1 | 208 |

| | | | | | | | | | | | | | | | | | |
|---|---|---|---|---|---|---|---|---|---|---|---|---|---|---|---|---|---|
| 116 | 74 | 45 | 2D | 2 | 26 | 499946 | 3999564 | 2 | 26 | 0 | 200 | 4092740 | 2 | 26 | 0 | 1 | 208 |
| 117 | 75 | 45 | 2D | 2 | 27 | 499971 | 3999764 | 2 | 27 | 0 | 200 | 4092948 | 2 | 27 | 0 | 1 | 208 |
| 118 | 76 | 45 | 2D | 2 | 28 | 499996 | 3999964 | 2 | 28 | 0 | 200 | 4093156 | 2 | 28 | 0 | 1 | 208 |
| 119 | 77 | 45 | 2D | 2 | 29 | 500021 | 4000164 | 2 | 29 | 0 | 200 | 4093364 | 2 | 29 | 0 | 1 | 208 |
| 120 | 78 | 45 | 2D | 2 | 30 | 500046 | 4000364 | 2 | 30 | 0 | 200 | 4093572 | 2 | 30 | 0 | 1 | 208 |
| 121 | 79 | 45 | 2D | 2 | 31 | 500071 | 4000564 | 2 | 31 | 0 | 200 | 4093780 | 2 | 31 | 0 | 1 | 208 |
| 122 | 7A | 45 | 2D | 2 | 32 | 500096 | 4000764 | 2 | 32 | 0 | 200 | 4093988 | 2 | 32 | 0 | 1 | 208 |
| 123 | 7B | 45 | 2D | 2 | 33 | 500121 | 4000964 | 2 | 33 | 0 | 200 | 4094196 | 2 | 33 | 0 | 1 | 208 |
| 124 | 7C | 45 | 2D | 2 | 34 | 500146 | 4001164 | 2 | 34 | 0 | 200 | 4094404 | 2 | 34 | 0 | 1 | 208 |
| 125 | 7D | 45 | 2D | 2 | 35 | 500171 | 4001364 | 2 | 35 | 0 | 200 | 4094612 | 2 | 35 | 0 | 1 | 208 |
| 126 | 7E | 45 | 2D | 2 | 36 | 500196 | 4001564 | 2 | 36 | 0 | 200 | 4094820 | 2 | 36 | 0 | 1 | 208 |
| 127 | 7F | 45 | 2D | 2 | 37 | 500221 | 4001764 | 2 | 37 | 0 | 200 | 4095028 | 2 | 37 | 0 | 1 | 208 |
| 128 | 80 | 45 | 2D | 2 | 38 | 500246 | 4001964 | 2 | 38 | 0 | 200 | 4095236 | 2 | 38 | 0 | 1 | 208 |
| 129 | 81 | 45 | 2D | 2 | 39 | 500271 | 4002164 | 2 | 39 | 0 | 200 | 4095444 | 2 | 39 | 0 | 1 | 208 |
| 130 | 82 | 45 | 2D | 2 | 40 | 500296 | 4002364 | 2 | 40 | 0 | 200 | 4095652 | 2 | 40 | 0 | 1 | 208 |
| 131 | 83 | 45 | 2D | 2 | 41 | 500321 | 4002564 | 2 | 41 | 0 | 200 | 4095860 | 2 | 41 | 0 | 1 | 208 |
| 132 | 84 | 45 | 2D | 2 | 42 | 500346 | 4002764 | 2 | 42 | 0 | 200 | 4096068 | 2 | 42 | 0 | 1 | 208 |
| 133 | 85 | 45 | 2D | 2 | 43 | 500371 | 4002964 | 2 | 43 | 0 | 200 | 4096276 | 2 | 43 | 0 | 1 | 208 |
| 134 | 86 | 45 | 2D | 2 | 44 | 500396 | 4003164 | 2 | 44 | 0 | 200 | 4096484 | 2 | 44 | 0 | 1 | 208 |
| 135 | 87 | 45 | 2D | 3 | 0 | 500419 | 4003348 | 3 | 0 | 0 | 184 | 4096676 | 3 | 0 | 0 | 1 | 192 |
| 136 | 88 | 45 | 2D | 3 | 1 | 500447 | 4003572 | 3 | 1 | 0 | 224 | 4096908 | 3 | 1 | 0 | 1 | 232 |
| 137 | 89 | 45 | 2D | 3 | 2 | 500475 | 4003796 | 3 | 2 | 0 | 224 | 4097140 | 3 | 2 | 0 | 1 | 232 |
| 138 | 8A | 45 | 2D | 3 | 3 | 500503 | 4004020 | 3 | 3 | 0 | 224 | 4097372 | 3 | 3 | 0 | 1 | 232 |
| 139 | 8B | 45 | 2D | 3 | 4 | 500531 | 4004244 | 3 | 4 | 0 | 224 | 4097604 | 3 | 4 | 0 | 1 | 232 |
| 140 | 8C | 45 | 2D | 3 | 5 | 500559 | 4004468 | 3 | 5 | 0 | 224 | 4097836 | 3 | 5 | 0 | 1 | 232 |
| 141 | 8D | 45 | 2D | 3 | 6 | 500587 | 4004692 | 3 | 6 | 0 | 224 | 4098068 | 3 | 6 | 0 | 1 | 232 |
| 142 | 8E | 45 | 2D | 3 | 7 | 500615 | 4004916 | 3 | 7 | 0 | 224 | 4098300 | 3 | 7 | 0 | 1 | 232 |
| 143 | 8F | 45 | 2D | 3 | 8 | 500643 | 4005140 | 3 | 8 | 0 | 224 | 4098532 | 3 | 8 | 0 | 1 | 232 |
| 144 | 90 | 45 | 2D | 3 | 9 | 500668 | 4005340 | 3 | 9 | 0 | 200 | 4098740 | 3 | 9 | 0 | 1 | 208 |
| 145 | 91 | 45 | 2D | 3 | 10 | 500693 | 4005540 | 3 | 10 | 0 | 200 | 4098948 | 3 | 10 | 0 | 1 | 208 |
| 146 | 92 | 45 | 2D | 3 | 11 | 500718 | 4005740 | 3 | 11 | 0 | 200 | 4099156 | 3 | 11 | 0 | 1 | 208 |
| 147 | 93 | 45 | 2D | 3 | 12 | 500743 | 4005940 | 3 | 12 | 0 | 200 | 4099364 | 3 | 12 | 0 | 1 | 208 |
| 148 | 94 | 45 | 2D | 3 | 13 | 500768 | 4006140 | 3 | 13 | 0 | 200 | 4099572 | 3 | 13 | 0 | 1 | 208 |
| 149 | 95 | 45 | 2D | 3 | 14 | 500793 | 4006340 | 3 | 14 | 0 | 200 | 4099780 | 3 | 14 | 0 | 1 | 208 |
| 150 | 96 | 45 | 2D | 3 | 15 | 500818 | 4006540 | 3 | 15 | 0 | 200 | 4099988 | 3 | 15 | 0 | 1 | 208 |
| 151 | 97 | 45 | 2D | 3 | 16 | 500843 | 4006740 | 3 | 16 | 0 | 200 | 4100196 | 3 | 16 | 0 | 1 | 208 |
| 152 | 98 | 45 | 2D | 3 | 17 | 500868 | 4006940 | 3 | 17 | 0 | 200 | 4100404 | 3 | 17 | 0 | 1 | 208 |
| 153 | 99 | 45 | 2D | 3 | 18 | 500893 | 4007140 | 3 | 18 | 0 | 200 | 4100612 | 3 | 18 | 0 | 1 | 208 |
| 154 | 9A | 45 | 2D | 3 | 19 | 500918 | 4007340 | 3 | 19 | 0 | 200 | 4100820 | 3 | 19 | 0 | 1 | 208 |
| 155 | 9B | 45 | 2D | 3 | 20 | 500943 | 4007540 | 3 | 20 | 0 | 200 | 4101028 | 3 | 20 | 0 | 1 | 208 |
| 156 | 9C | 45 | 2D | 3 | 21 | 500968 | 4007740 | 3 | 21 | 0 | 200 | 4101236 | 3 | 21 | 0 | 1 | 208 |
| 157 | 9D | 45 | 2D | 3 | 22 | 500993 | 4007940 | 3 | 22 | 0 | 200 | 4101444 | 3 | 22 | 0 | 1 | 208 |
| 158 | 9E | 45 | 2D | 3 | 23 | 501018 | 4008140 | 3 | 23 | 0 | 200 | 4101652 | 3 | 23 | 0 | 1 | 208 |
| 159 | 9F | 45 | 2D | 3 | 24 | 501043 | 4008340 | 3 | 24 | 0 | 200 | 4101860 | 3 | 24 | 0 | 1 | 208 |
| 160 | A0 | 45 | 2D | 3 | 25 | 501070 | 4008556 | 3 | 25 | 0 | 216 | 4102084 | 3 | 25 | 0 | 1 | 224 |
| 161 | A1 | 45 | 2D | 3 | 26 | 501097 | 4008772 | 3 | 26 | 0 | 216 | 4102308 | 3 | 26 | 0 | 1 | 224 |
| 162 | A2 | 45 | 2D | 3 | 27 | 501124 | 4008988 | 3 | 27 | 0 | 216 | 4102532 | 3 | 27 | 0 | 1 | 224 |
| 163 | A3 | 45 | 2D | 3 | 28 | 501151 | 4009204 | 3 | 28 | 0 | 216 | 4102756 | 3 | 28 | 0 | 1 | 224 |
| 164 | A4 | 45 | 2D | 3 | 29 | 501178 | 4009420 | 3 | 29 | 0 | 216 | 4102980 | 3 | 29 | 0 | 1 | 224 |
| 165 | A5 | 45 | 2D | 3 | 30 | 501205 | 4009636 | 3 | 30 | 0 | 216 | 4103204 | 3 | 30 | 0 | 1 | 224 |
| 166 | A6 | 45 | 2D | 3 | 31 | 501232 | 4009852 | 3 | 31 | 0 | 216 | 4103428 | 3 | 31 | 0 | 1 | 224 |
| 167 | A7 | 45 | 2D | 3 | 32 | 501259 | 4010068 | 3 | 32 | 0 | 216 | 4103652 | 3 | 32 | 0 | 1 | 224 |
| 168 | A8 | 45 | 2D | 3 | 33 | 501286 | 4010284 | 3 | 33 | 0 | 216 | 4103876 | 3 | 33 | 0 | 1 | 224 |
| 169 | A9 | 45 | 2D | 3 | 34 | 501313 | 4010500 | 3 | 34 | 0 | 216 | 4104100 | 3 | 34 | 0 | 1 | 224 |
| 170 | AA | 45 | 2D | 3 | 35 | 501340 | 4010716 | 3 | 35 | 0 | 216 | 4104324 | 3 | 35 | 0 | 1 | 224 |
| 171 | AB | 45 | 2D | 3 | 36 | 501367 | 4010932 | 3 | 36 | 0 | 216 | 4104548 | 3 | 36 | 0 | 1 | 224 |
| 172 | AC | 45 | 2D | 3 | 37 | 501394 | 4011148 | 3 | 37 | 0 | 216 | 4104772 | 3 | 37 | 0 | 1 | 224 |
| 173 | AD | 45 | 2D | 3 | 38 | 501421 | 4011364 | 3 | 38 | 0 | 216 | 4104996 | 3 | 38 | 0 | 1 | 224 |
| 174 | AE | 45 | 2D | 3 | 39 | 501448 | 4011580 | 3 | 39 | 0 | 216 | 4105220 | 3 | 39 | 0 | 1 | 224 |
| 175 | AF | 45 | 2D | 3 | 40 | 501475 | 4011796 | 3 | 40 | 0 | 216 | 4105444 | 3 | 40 | 0 | 1 | 224 |
| 176 | B0 | 45 | 2D | 3 | 41 | 501502 | 4012012 | 3 | 41 | 0 | 216 | 4105668 | 3 | 41 | 0 | 1 | 224 |
| 177 | B1 | 45 | 2D | 3 | 42 | 501529 | 4012228 | 3 | 42 | 0 | 216 | 4105892 | 3 | 42 | 0 | 1 | 224 |
| 178 | B2 | 45 | 2D | 3 | 43 | 501556 | 4012444 | 3 | 43 | 0 | 216 | 4106116 | 3 | 43 | 0 | 1 | 224 |
| 179 | B3 | 45 | 2D | 3 | 44 | 501583 | 4012660 | 3 | 44 | 0 | 216 | 4106340 | 3 | 44 | 0 | 1 | 224 |
| 180 | B4 | 45 | 2D | 4 | 0 | 501608 | 4012860 | 4 | 0 | 0 | 200 | 4106548 | 4 | 0 | 0 | 1 | 208 |
| 181 | B5 | 45 | 2D | 4 | 1 | 501638 | 4013100 | 4 | 1 | 0 | 240 | 4106796 | 4 | 1 | 0 | 1 | 248 |
| 182 | B6 | 45 | 2D | 4 | 2 | 501668 | 4013340 | 4 | 2 | 0 | 240 | 4107044 | 4 | 2 | 0 | 1 | 248 |
| 183 | B7 | 45 | 2D | 4 | 3 | 501698 | 4013580 | 4 | 3 | 0 | 240 | 4107292 | 4 | 3 | 0 | 1 | 248 |
| 184 | B8 | 45 | 2D | 4 | 4 | 501728 | 4013820 | 4 | 4 | 0 | 240 | 4107540 | 4 | 4 | 0 | 1 | 248 |
| 185 | B9 | 45 | 2D | 4 | 5 | 501758 | 4014060 | 4 | 5 | 0 | 240 | 4107788 | 4 | 5 | 0 | 1 | 248 |
| 186 | BA | 45 | 2D | 4 | 6 | 501788 | 4014300 | 4 | 6 | 0 | 240 | 4108036 | 4 | 6 | 0 | 1 | 248 |
| 187 | BB | 45 | 2D | 4 | 7 | 501818 | 4014540 | 4 | 7 | 0 | 240 | 4108284 | 4 | 7 | 0 | 1 | 248 |
| 188 | BC | 45 | 2D | 4 | 8 | 501848 | 4014780 | 4 | 8 | 0 | 240 | 4108532 | 4 | 8 | 0 | 1 | 248 |
| 189 | BD | 45 | 2D | 4 | 9 | 501878 | 4015020 | 4 | 9 | 0 | 240 | 4108780 | 4 | 9 | 0 | 1 | 248 |
| 190 | BE | 45 | 2D | 4 | 10 | 501908 | 4015260 | 4 | 10 | 0 | 240 | 4109028 | 4 | 10 | 0 | 1 | 248 |
| 191 | BF | 45 | 2D | 4 | 11 | 501938 | 4015500 | 4 | 11 | 0 | 240 | 4109276 | 4 | 11 | 0 | 1 | 248 |
| 192 | C0 | 45 | 2D | 4 | 12 | 501965 | 4015716 | 4 | 12 | 0 | 216 | 4109500 | 4 | 12 | 0 | 1 | 224 |
| 193 | C1 | 45 | 2D | 4 | 13 | 501992 | 4015932 | 4 | 13 | 0 | 216 | 4109724 | 4 | 13 | 0 | 1 | 224 |
| 194 | C2 | 45 | 2D | 4 | 14 | 502019 | 4016148 | 4 | 14 | 0 | 216 | 4109948 | 4 | 14 | 0 | 1 | 224 |
| 195 | C3 | 45 | 2D | 4 | 15 | 502046 | 4016364 | 4 | 15 | 0 | 216 | 4110172 | 4 | 15 | 0 | 1 | 224 |
| 196 | C4 | 45 | 2D | 4 | 16 | 502073 | 4016580 | 4 | 16 | 0 | 216 | 4110396 | 4 | 16 | 0 | 1 | 224 |
| 197 | C5 | 45 | 2D | 4 | 17 | 502100 | 4016796 | 4 | 17 | 0 | 216 | 4110620 | 4 | 17 | 0 | 1 | 224 |
| 198 | C6 | 45 | 2D | 4 | 18 | 502127 | 4017012 | 4 | 18 | 0 | 216 | 4110844 | 4 | 18 | 0 | 1 | 224 |
| 199 | C7 | 45 | 2D | 4 | 19 | 502154 | 4017228 | 4 | 19 | 0 | 216 | 4111068 | 4 | 19 | 0 | 1 | 224 |
| 200 | C8 | 45 | 2D | 4 | 20 | 502181 | 4017444 | 4 | 20 | 0 | 216 | 4111292 | 4 | 20 | 0 | 1 | 224 |
| 201 | C9 | 45 | 2D | 4 | 21 | 502208 | 4017660 | 4 | 21 | 0 | 216 | 4111516 | 4 | 21 | 0 | 1 | 224 |
| 202 | CA | 45 | 2D | 4 | 22 | 502235 | 4017876 | 4 | 22 | 0 | 216 | 4111740 | 4 | 22 | 0 | 1 | 224 |
| 203 | CB | 45 | 2D | 4 | 23 | 502262 | 4018092 | 4 | 23 | 0 | 216 | 4111964 | 4 | 23 | 0 | 1 | 224 |
| 204 | CC | 45 | 2D | 4 | 24 | 502289 | 4018308 | 4 | 24 | 0 | 216 | 4112188 | 4 | 24 | 0 | 1 | 224 |
| 205 | CD | 45 | 2D | 4 | 25 | 502316 | 4018524 | 4 | 25 | 0 | 216 | 4112412 | 4 | 25 | 0 | 1 | 224 |
| 206 | CE | 45 | 2D | 4 | 26 | 502343 | 4018740 | 4 | 26 | 0 | 216 | 4112636 | 4 | 26 | 0 | 1 | 224 |
| 207 | CF | 45 | 2D | 4 | 27 | 502370 | 4018956 | 4 | 27 | 0 | 216 | 4112860 | 4 | 27 | 0 | 1 | 224 |
| 208 | D0 | 45 | 2D | 4 | 28 | 502399 | 4019188 | 4 | 28 | 0 | 232 | 4113100 | 4 | 28 | 0 | 1 | 240 |
| 209 | D1 | 45 | 2D | 4 | 29 | 502428 | 4019420 | 4 | 29 | 0 | 232 | 4113340 | 4 | 29 | 0 | 1 | 240 |
| 210 | D2 | 45 | 2D | 4 | 30 | 502457 | 4019652 | 4 | 30 | 0 | 232 | 4113580 | 4 | 30 | 0 | 1 | 240 |
| 211 | D3 | 45 | 2D | 4 | 31 | 502486 | 4019884 | 4 | 31 | 0 | 232 | 4113820 | 4 | 31 | 0 | 1 | 240 |
| 212 | D4 | 45 | 2D | 4 | 32 | 502515 | 4020116 | 4 | 32 | 0 | 232 | 4114060 | 4 | 32 | 0 | 1 | 240 |
| 213 | D5 | 45 | 2D | 4 | 33 | 502544 | 4020348 | 4 | 33 | 0 | 232 | 4114300 | 4 | 33 | 0 | 1 | 240 |
| 214 | D6 | 45 | 2D | 4 | 34 | 502573 | 4020580 | 4 | 34 | 0 | 232 | 4114540 | 4 | 34 | 0 | 1 | 240 |
| 215 | D7 | 45 | 2D | 4 | 35 | 502602 | 4020812 | 4 | 35 | 0 | 232 | 4114780 | 4 | 35 | 0 | 1 | 240 |
| 216 | D8 | 45 | 2D | 4 | 36 | 502631 | 4021044 | 4 | 36 | 0 | 232 | 4115020 | 4 | 36 | 0 | 1 | 240 |
| 217 | D9 | 45 | 2D | 4 | 37 | 502660 | 4021276 | 4 | 37 | 0 | 232 | 4115260 | 4 | 37 | 0 | 1 | 240 |
| 218 | DA | 45 | 2D | 4 | 38 | 502689 | 4021508 | 4 | 38 | 0 | 232 | 4115500 | 4 | 38 | 0 | 1 | 240 |

| | | | | | | | | | | | | | | | | | |
|---|---|---|---|---|---|---|---|---|---|---|---|---|---|---|---|---|---|
| 219 | DB | 45 | 2D | 4 | 39 | 502718 | 4021740 | 4 | 39 | 0 | 232 | 4115740 | 4 | 39 | 0 | 1 | 240 |
| 220 | DC | 45 | 2D | 4 | 40 | 502747 | 4021972 | 4 | 40 | 0 | 232 | 4115980 | 4 | 40 | 0 | 1 | 240 |
| 221 | DD | 45 | 2D | 4 | 41 | 502776 | 4022204 | 4 | 41 | 0 | 232 | 4116220 | 4 | 41 | 0 | 1 | 240 |
| 222 | DE | 45 | 2D | 4 | 42 | 502805 | 4022436 | 4 | 42 | 0 | 232 | 4116460 | 4 | 42 | 0 | 1 | 240 |
| 223 | DF | 45 | 2D | 4 | 43 | 502834 | 4022668 | 4 | 43 | 0 | 232 | 4116700 | 4 | 43 | 0 | 1 | 240 |
| 224 | E0 | 45 | 2D | 4 | 44 | 502863 | 4022900 | 4 | 44 | 0 | 232 | 4116940 | 4 | 44 | 0 | 1 | 240 |
| 225 | E1 | 45 | 2D | 5 | 0 | 502890 | 4023116 | 5 | 0 | 0 | 216 | 4117164 | 5 | 0 | 0 | 1 | 224 |
| 226 | E2 | 45 | 2D | 5 | 1 | 502922 | 4023372 | 5 | 1 | 0 | 256 | 4117428 | 5 | 1 | 0 | 1 | 264 |
| 227 | E3 | 45 | 2D | 5 | 2 | 502954 | 4023628 | 5 | 2 | 0 | 256 | 4117692 | 5 | 2 | 0 | 1 | 264 |
| 228 | E4 | 45 | 2D | 5 | 3 | 502986 | 4023884 | 5 | 3 | 0 | 256 | 4117956 | 5 | 3 | 0 | 1 | 264 |
| 229 | E5 | 45 | 2D | 5 | 4 | 503018 | 4024140 | 5 | 4 | 0 | 256 | 4118220 | 5 | 4 | 0 | 1 | 264 |
| 230 | E6 | 45 | 2D | 5 | 5 | 503050 | 4024396 | 5 | 5 | 0 | 256 | 4118484 | 5 | 5 | 0 | 1 | 264 |
| 231 | E7 | 45 | 2D | 5 | 6 | 503082 | 4024652 | 5 | 6 | 0 | 256 | 4118748 | 5 | 6 | 0 | 1 | 264 |
| 232 | E8 | 45 | 2D | 5 | 7 | 503114 | 4024908 | 5 | 7 | 0 | 256 | 4119012 | 5 | 7 | 0 | 1 | 264 |
| 233 | E9 | 45 | 2D | 5 | 8 | 503146 | 4025164 | 5 | 8 | 0 | 256 | 4119276 | 5 | 8 | 0 | 1 | 264 |
| 234 | EA | 45 | 2D | 5 | 9 | 503178 | 4025420 | 5 | 9 | 0 | 256 | 4119540 | 5 | 9 | 0 | 1 | 264 |
| 235 | EB | 45 | 2D | 5 | 10 | 503210 | 4025676 | 5 | 10 | 0 | 256 | 4119804 | 5 | 10 | 0 | 1 | 264 |
| 236 | EC | 45 | 2D | 5 | 11 | 503242 | 4025932 | 5 | 11 | 0 | 256 | 4120068 | 5 | 11 | 0 | 1 | 264 |
| 237 | ED | 45 | 2D | 5 | 12 | 503274 | 4026188 | 5 | 12 | 0 | 256 | 4120332 | 5 | 12 | 0 | 1 | 264 |
| 238 | EE | 45 | 2D | 5 | 13 | 503306 | 4026444 | 5 | 13 | 0 | 256 | 4120596 | 5 | 13 | 0 | 1 | 264 |
| 239 | EF | 45 | 2D | 5 | 14 | 503338 | 4026700 | 5 | 14 | 0 | 256 | 4120860 | 5 | 14 | 0 | 1 | 264 |
| 240 | F0 | 45 | 2D | 5 | 15 | 503367 | 4026932 | 5 | 15 | 0 | 232 | 4121100 | 5 | 15 | 0 | 1 | 240 |
| 241 | F1 | 45 | 2D | 5 | 16 | 503396 | 4027164 | 5 | 16 | 0 | 232 | 4121340 | 5 | 16 | 0 | 1 | 240 |
| 242 | F2 | 45 | 2D | 5 | 17 | 503425 | 4027396 | 5 | 17 | 0 | 232 | 4121580 | 5 | 17 | 0 | 1 | 240 |
| 243 | F3 | 45 | 2D | 5 | 18 | 503454 | 4027628 | 5 | 18 | 0 | 232 | 4121820 | 5 | 18 | 0 | 1 | 240 |
| 244 | F4 | 45 | 2D | 5 | 19 | 503483 | 4027860 | 5 | 19 | 0 | 232 | 4122060 | 5 | 19 | 0 | 1 | 240 |
| 245 | F5 | 45 | 2D | 5 | 20 | 503512 | 4028092 | 5 | 20 | 0 | 232 | 4122300 | 5 | 20 | 0 | 1 | 240 |
| 246 | F6 | 45 | 2D | 5 | 21 | 503541 | 4028324 | 5 | 21 | 0 | 232 | 4122540 | 5 | 21 | 0 | 1 | 240 |
| 247 | F7 | 45 | 2D | 5 | 22 | 503570 | 4028556 | 5 | 22 | 0 | 232 | 4122780 | 5 | 22 | 0 | 1 | 240 |
| 248 | F8 | 45 | 2D | 5 | 23 | 503599 | 4028788 | 5 | 23 | 0 | 232 | 4123020 | 5 | 23 | 0 | 1 | 240 |
| 249 | F9 | 45 | 2D | 5 | 24 | 503628 | 4029020 | 5 | 24 | 0 | 232 | 4123260 | 5 | 24 | 0 | 1 | 240 |
| 250 | FA | 45 | 2D | 5 | 25 | 503657 | 4029252 | 5 | 25 | 0 | 232 | 4123500 | 5 | 25 | 0 | 1 | 240 |
| 251 | FB | 45 | 2D | 5 | 26 | 503686 | 4029484 | 5 | 26 | 0 | 232 | 4123740 | 5 | 26 | 0 | 1 | 240 |
| 252 | FC | 45 | 2D | 5 | 27 | 503715 | 4029716 | 5 | 27 | 0 | 232 | 4123980 | 5 | 27 | 0 | 1 | 240 |
| 253 | FD | 45 | 2D | 5 | 28 | 503744 | 4029948 | 5 | 28 | 0 | 232 | 4124220 | 5 | 28 | 0 | 1 | 240 |
| 254 | FE | 45 | 2D | 5 | 29 | 503773 | 4030180 | 5 | 29 | 0 | 232 | 4124460 | 5 | 29 | 0 | 1 | 240 |
| 255 | FF | 45 | 2D | 5 | 30 | 503802 | 4030412 | 5 | 30 | 0 | 232 | 4124700 | 5 | 30 | 0 | 1 | 240 |
| 0 | 00 | 46 | 2E | 0 | 0 | 503814 | 4030508 | 0 | 0 | 0 | 96 | 4124804 | 0 | 0 | 0 | 1 | 104 |
| 1 | 01 | 46 | 2E | 0 | 1 | 503828 | 4030620 | 0 | 1 | 0 | 112 | 4124924 | 0 | 1 | 0 | 1 | 120 |
| 2 | 02 | 46 | 2E | 0 | 2 | 503842 | 4030732 | 0 | 2 | 0 | 112 | 4125044 | 0 | 2 | 0 | 1 | 120 |
| 3 | 03 | 46 | 2E | 0 | 3 | 503856 | 4030844 | 0 | 3 | 0 | 112 | 4125164 | 0 | 3 | 0 | 1 | 120 |
| 4 | 04 | 46 | 2E | 0 | 4 | 503870 | 4030956 | 0 | 4 | 0 | 112 | 4125284 | 0 | 4 | 0 | 1 | 120 |
| 5 | 05 | 46 | 2E | 0 | 5 | 503884 | 4031068 | 0 | 5 | 0 | 112 | 4125404 | 0 | 5 | 0 | 1 | 120 |
| 6 | 06 | 46 | 2E | 0 | 6 | 503898 | 4031180 | 0 | 6 | 0 | 112 | 4125524 | 0 | 6 | 0 | 1 | 120 |
| 7 | 07 | 46 | 2E | 0 | 7 | 503912 | 4031292 | 0 | 7 | 0 | 112 | 4125644 | 0 | 7 | 0 | 1 | 120 |
| 8 | 08 | 46 | 2E | 0 | 8 | 503926 | 4031404 | 0 | 8 | 0 | 112 | 4125764 | 0 | 8 | 0 | 1 | 120 |
| 9 | 09 | 46 | 2E | 0 | 9 | 503940 | 4031516 | 0 | 9 | 0 | 112 | 4125884 | 0 | 9 | 0 | 1 | 120 |
| 10 | 0A | 46 | 2E | 0 | 10 | 503954 | 4031628 | 0 | 10 | 0 | 112 | 4126004 | 0 | 10 | 0 | 1 | 120 |
| 11 | 0B | 46 | 2E | 0 | 11 | 503968 | 4031740 | 0 | 11 | 0 | 112 | 4126124 | 0 | 11 | 0 | 1 | 120 |
| 12 | 0C | 46 | 2E | 0 | 12 | 503982 | 4031852 | 0 | 12 | 0 | 112 | 4126244 | 0 | 12 | 0 | 1 | 120 |
| 13 | 0D | 46 | 2E | 0 | 13 | 503996 | 4031964 | 0 | 13 | 0 | 112 | 4126364 | 0 | 13 | 0 | 1 | 120 |
| 14 | 0E | 46 | 2E | 0 | 14 | 504010 | 4032076 | 0 | 14 | 0 | 112 | 4126484 | 0 | 14 | 0 | 1 | 120 |
| 15 | 0F | 46 | 2E | 0 | 15 | 504024 | 4032188 | 0 | 15 | 0 | 112 | 4126604 | 0 | 15 | 0 | 1 | 120 |
| 16 | 10 | 46 | 2E | 0 | 16 | 504038 | 4032300 | 0 | 16 | 0 | 112 | 4126724 | 0 | 16 | 0 | 1 | 120 |
| 17 | 11 | 46 | 2E | 0 | 17 | 504052 | 4032412 | 0 | 17 | 0 | 112 | 4126844 | 0 | 17 | 0 | 1 | 120 |
| 18 | 12 | 46 | 2E | 0 | 18 | 504066 | 4032524 | 0 | 18 | 0 | 112 | 4126964 | 0 | 18 | 0 | 1 | 120 |
| 19 | 13 | 46 | 2E | 0 | 19 | 504080 | 4032636 | 0 | 19 | 0 | 112 | 4127084 | 0 | 19 | 0 | 1 | 120 |
| 20 | 14 | 46 | 2E | 0 | 20 | 504094 | 4032748 | 0 | 20 | 0 | 112 | 4127204 | 0 | 20 | 0 | 1 | 120 |
| 21 | 15 | 46 | 2E | 0 | 21 | 504108 | 4032860 | 0 | 21 | 0 | 112 | 4127324 | 0 | 21 | 0 | 1 | 120 |
| 22 | 16 | 46 | 2E | 0 | 22 | 504122 | 4032972 | 0 | 22 | 0 | 112 | 4127444 | 0 | 22 | 0 | 1 | 120 |
| 23 | 17 | 46 | 2E | 0 | 23 | 504136 | 4033084 | 0 | 23 | 0 | 112 | 4127564 | 0 | 23 | 0 | 1 | 120 |
| 24 | 18 | 46 | 2E | 0 | 24 | 504150 | 4033196 | 0 | 24 | 0 | 112 | 4127684 | 0 | 24 | 0 | 1 | 120 |
| 25 | 19 | 46 | 2E | 0 | 25 | 504164 | 4033308 | 0 | 25 | 0 | 112 | 4127804 | 0 | 25 | 0 | 1 | 120 |
| 26 | 1A | 46 | 2E | 0 | 26 | 504178 | 4033420 | 0 | 26 | 0 | 112 | 4127924 | 0 | 26 | 0 | 1 | 120 |
| 27 | 1B | 46 | 2E | 0 | 27 | 504192 | 4033532 | 0 | 27 | 0 | 112 | 4128044 | 0 | 27 | 0 | 1 | 120 |
| 28 | 1C | 46 | 2E | 0 | 28 | 504206 | 4033644 | 0 | 28 | 0 | 112 | 4128164 | 0 | 28 | 0 | 1 | 120 |
| 29 | 1D | 46 | 2E | 0 | 29 | 504220 | 4033756 | 0 | 29 | 0 | 112 | 4128284 | 0 | 29 | 0 | 1 | 120 |
| 30 | 1E | 46 | 2E | 0 | 30 | 504234 | 4033868 | 0 | 30 | 0 | 112 | 4128404 | 0 | 30 | 0 | 1 | 120 |
| 31 | 1F | 46 | 2E | 0 | 31 | 504248 | 4033980 | 0 | 31 | 0 | 112 | 4128524 | 0 | 31 | 0 | 1 | 120 |
| 32 | 20 | 46 | 2E | 0 | 32 | 504262 | 4034092 | 0 | 32 | 0 | 112 | 4128644 | 0 | 32 | 0 | 1 | 120 |
| 33 | 21 | 46 | 2E | 0 | 33 | 504276 | 4034204 | 0 | 33 | 0 | 112 | 4128764 | 0 | 33 | 0 | 1 | 120 |
| 34 | 22 | 46 | 2E | 0 | 34 | 504290 | 4034316 | 0 | 34 | 0 | 112 | 4128884 | 0 | 34 | 0 | 1 | 120 |
| 35 | 23 | 46 | 2E | 0 | 35 | 504304 | 4034428 | 0 | 35 | 0 | 112 | 4129004 | 0 | 35 | 0 | 1 | 120 |
| 36 | 24 | 46 | 2E | 0 | 36 | 504318 | 4034540 | 0 | 36 | 0 | 112 | 4129124 | 0 | 36 | 0 | 1 | 120 |
| 37 | 25 | 46 | 2E | 0 | 37 | 504332 | 4034652 | 0 | 37 | 0 | 112 | 4129244 | 0 | 37 | 0 | 1 | 120 |
| 38 | 26 | 46 | 2E | 0 | 38 | 504346 | 4034764 | 0 | 38 | 0 | 112 | 4129364 | 0 | 38 | 0 | 1 | 120 |
| 39 | 27 | 46 | 2E | 0 | 39 | 504360 | 4034876 | 0 | 39 | 0 | 112 | 4129484 | 0 | 39 | 0 | 1 | 120 |
| 40 | 28 | 46 | 2E | 0 | 40 | 504374 | 4034988 | 0 | 40 | 0 | 112 | 4129604 | 0 | 40 | 0 | 1 | 120 |
| 41 | 29 | 46 | 2E | 0 | 41 | 504388 | 4035100 | 0 | 41 | 0 | 112 | 4129724 | 0 | 41 | 0 | 1 | 120 |
| 42 | 2A | 46 | 2E | 0 | 42 | 504402 | 4035212 | 0 | 42 | 0 | 112 | 4129844 | 0 | 42 | 0 | 1 | 120 |
| 43 | 2B | 46 | 2E | 0 | 43 | 504416 | 4035324 | 0 | 43 | 0 | 112 | 4129964 | 0 | 43 | 0 | 1 | 120 |
| 44 | 2C | 46 | 2E | 0 | 44 | 504430 | 4035436 | 0 | 44 | 0 | 112 | 4130084 | 0 | 44 | 0 | 1 | 120 |
| 45 | 2D | 46 | 2E | 0 | 45 | 504444 | 4035548 | 0 | 45 | 0 | 112 | 4130204 | 0 | 45 | 0 | 1 | 120 |
| 46 | 2E | 46 | 2E | 1 | 0 | 504456 | 4035644 | 1 | 0 | 0 | 96 | 4130308 | 1 | 0 | 0 | 1 | 104 |
| 47 | 2F | 46 | 2E | 1 | 1 | 504473 | 4035780 | 1 | 1 | 0 | 136 | 4130452 | 1 | 1 | 0 | 1 | 144 |
| 48 | 30 | 46 | 2E | 1 | 2 | 504494 | 4035948 | 1 | 2 | 0 | 168 | 4130628 | 1 | 2 | 0 | 1 | 176 |
| 49 | 31 | 46 | 2E | 1 | 3 | 504515 | 4036116 | 1 | 3 | 0 | 168 | 4130804 | 1 | 3 | 0 | 1 | 176 |
| 50 | 32 | 46 | 2E | 1 | 4 | 504536 | 4036284 | 1 | 4 | 0 | 168 | 4130980 | 1 | 4 | 0 | 1 | 176 |
| 51 | 33 | 46 | 2E | 1 | 5 | 504557 | 4036452 | 1 | 5 | 0 | 168 | 4131156 | 1 | 5 | 0 | 1 | 176 |
| 52 | 34 | 46 | 2E | 1 | 6 | 504578 | 4036620 | 1 | 6 | 0 | 168 | 4131332 | 1 | 6 | 0 | 1 | 176 |
| 53 | 35 | 46 | 2E | 1 | 7 | 504599 | 4036788 | 1 | 7 | 0 | 168 | 4131508 | 1 | 7 | 0 | 1 | 176 |
| 54 | 36 | 46 | 2E | 1 | 8 | 504620 | 4036956 | 1 | 8 | 0 | 168 | 4131684 | 1 | 8 | 0 | 1 | 176 |
| 55 | 37 | 46 | 2E | 1 | 9 | 504641 | 4037124 | 1 | 9 | 0 | 168 | 4131860 | 1 | 9 | 0 | 1 | 176 |
| 56 | 38 | 46 | 2E | 1 | 10 | 504662 | 4037292 | 1 | 10 | 0 | 168 | 4132036 | 1 | 10 | 0 | 1 | 176 |
| 57 | 39 | 46 | 2E | 1 | 11 | 504683 | 4037460 | 1 | 11 | 0 | 168 | 4132212 | 1 | 11 | 0 | 1 | 176 |
| 58 | 3A | 46 | 2E | 1 | 12 | 504704 | 4037628 | 1 | 12 | 0 | 168 | 4132388 | 1 | 12 | 0 | 1 | 176 |
| 59 | 3B | 46 | 2E | 1 | 13 | 504725 | 4037796 | 1 | 13 | 0 | 168 | 4132564 | 1 | 13 | 0 | 1 | 176 |
| 60 | 3C | 46 | 2E | 1 | 14 | 504746 | 4037964 | 1 | 14 | 0 | 168 | 4132740 | 1 | 14 | 0 | 1 | 176 |
| 61 | 3D | 46 | 2E | 1 | 15 | 504767 | 4038132 | 1 | 15 | 0 | 168 | 4132916 | 1 | 15 | 0 | 1 | 176 |
| 62 | 3E | 46 | 2E | 1 | 16 | 504788 | 4038300 | 1 | 16 | 0 | 168 | 4133092 | 1 | 16 | 0 | 1 | 176 |
| 63 | 3F | 46 | 2E | 1 | 17 | 504809 | 4038468 | 1 | 17 | 0 | 168 | 4133268 | 1 | 17 | 0 | 1 | 176 |
| 64 | 40 | 46 | 2E | 1 | 18 | 504832 | 4038652 | 1 | 18 | 0 | 184 | 4133460 | 1 | 18 | 0 | 1 | 192 |
| 65 | 41 | 46 | 2E | 1 | 19 | 504855 | 4038836 | 1 | 19 | 0 | 184 | 4133652 | 1 | 19 | 0 | 1 | 192 |

| | | | | | | | | | | | | | | | | | |
|---|---|---|---|---|---|---|---|---|---|---|---|---|---|---|---|---|---|
| 66 | 42 | 46 | 2E | 1 | 20 | 504878 | 4039020 | 1 | 20 | 0 | 184 | 4133844 | 1 | 20 | 0 | 1 | 192 |
| 67 | 43 | 46 | 2E | 1 | 21 | 504901 | 4039204 | 1 | 21 | 0 | 184 | 4134036 | 1 | 21 | 0 | 1 | 192 |
| 68 | 44 | 46 | 2E | 1 | 22 | 504924 | 4039388 | 1 | 22 | 0 | 184 | 4134228 | 1 | 22 | 0 | 1 | 192 |
| 69 | 45 | 46 | 2E | 1 | 23 | 504947 | 4039572 | 1 | 23 | 0 | 184 | 4134420 | 1 | 23 | 0 | 1 | 192 |
| 70 | 46 | 46 | 2E | 1 | 24 | 504970 | 4039756 | 1 | 24 | 0 | 184 | 4134612 | 1 | 24 | 0 | 1 | 192 |
| 71 | 47 | 46 | 2E | 1 | 25 | 504993 | 4039940 | 1 | 25 | 0 | 184 | 4134804 | 1 | 25 | 0 | 1 | 192 |
| 72 | 48 | 46 | 2E | 1 | 26 | 505016 | 4040124 | 1 | 26 | 0 | 184 | 4134996 | 1 | 26 | 0 | 1 | 192 |
| 73 | 49 | 46 | 2E | 1 | 27 | 505039 | 4040308 | 1 | 27 | 0 | 184 | 4135188 | 1 | 27 | 0 | 1 | 192 |
| 74 | 4A | 46 | 2E | 1 | 28 | 505062 | 4040492 | 1 | 28 | 0 | 184 | 4135380 | 1 | 28 | 0 | 1 | 192 |
| 75 | 4B | 46 | 2E | 1 | 29 | 505085 | 4040676 | 1 | 29 | 0 | 184 | 4135572 | 1 | 29 | 0 | 1 | 192 |
| 76 | 4C | 46 | 2E | 1 | 30 | 505108 | 4040860 | 1 | 30 | 0 | 184 | 4135764 | 1 | 30 | 0 | 1 | 192 |
| 77 | 4D | 46 | 2E | 1 | 31 | 505131 | 4041044 | 1 | 31 | 0 | 184 | 4135956 | 1 | 31 | 0 | 1 | 192 |
| 78 | 4E | 46 | 2E | 1 | 32 | 505154 | 4041228 | 1 | 32 | 0 | 184 | 4136148 | 1 | 32 | 0 | 1 | 192 |
| 79 | 4F | 46 | 2E | 1 | 33 | 505177 | 4041412 | 1 | 33 | 0 | 184 | 4136340 | 1 | 33 | 0 | 1 | 192 |
| 80 | 50 | 46 | 2E | 1 | 34 | 505200 | 4041596 | 1 | 34 | 0 | 184 | 4136532 | 1 | 34 | 0 | 1 | 192 |
| 81 | 51 | 46 | 2E | 1 | 35 | 505223 | 4041780 | 1 | 35 | 0 | 184 | 4136724 | 1 | 35 | 0 | 1 | 192 |
| 82 | 52 | 46 | 2E | 1 | 36 | 505246 | 4041964 | 1 | 36 | 0 | 184 | 4136916 | 1 | 36 | 0 | 1 | 192 |
| 83 | 53 | 46 | 2E | 1 | 37 | 505269 | 4042148 | 1 | 37 | 0 | 184 | 4137108 | 1 | 37 | 0 | 1 | 192 |
| 84 | 54 | 46 | 2E | 1 | 38 | 505292 | 4042332 | 1 | 38 | 0 | 184 | 4137300 | 1 | 38 | 0 | 1 | 192 |
| 85 | 55 | 46 | 2E | 1 | 39 | 505315 | 4042516 | 1 | 39 | 0 | 184 | 4137492 | 1 | 39 | 0 | 1 | 192 |
| 86 | 56 | 46 | 2E | 1 | 40 | 505338 | 4042700 | 1 | 40 | 0 | 184 | 4137684 | 1 | 40 | 0 | 1 | 192 |
| 87 | 57 | 46 | 2E | 1 | 41 | 505361 | 4042884 | 1 | 41 | 0 | 184 | 4137876 | 1 | 41 | 0 | 1 | 192 |
| 88 | 58 | 46 | 2E | 1 | 42 | 505384 | 4043068 | 1 | 42 | 0 | 184 | 4138068 | 1 | 42 | 0 | 1 | 192 |
| 89 | 59 | 46 | 2E | 1 | 43 | 505407 | 4043252 | 1 | 43 | 0 | 184 | 4138260 | 1 | 43 | 0 | 1 | 192 |
| 90 | 5A | 46 | 2E | 1 | 44 | 505430 | 4043436 | 1 | 44 | 0 | 184 | 4138452 | 1 | 44 | 0 | 1 | 192 |
| 91 | 5B | 46 | 2E | 1 | 45 | 505453 | 4043620 | 1 | 45 | 0 | 184 | 4138644 | 1 | 45 | 0 | 1 | 192 |
| 92 | 5C | 46 | 2E | 2 | 0 | 505474 | 4043788 | 2 | 0 | 0 | 168 | 4138820 | 2 | 0 | 0 | 1 | 176 |
| 93 | 5D | 46 | 2E | 2 | 1 | 505500 | 4043996 | 2 | 1 | 0 | 208 | 4139036 | 2 | 1 | 0 | 1 | 216 |
| 94 | 5E | 46 | 2E | 2 | 2 | 505526 | 4044204 | 2 | 2 | 0 | 208 | 4139252 | 2 | 2 | 0 | 1 | 216 |
| 95 | 5F | 46 | 2E | 2 | 3 | 505552 | 4044412 | 2 | 3 | 0 | 208 | 4139468 | 2 | 3 | 0 | 1 | 216 |
| 96 | 60 | 46 | 2E | 2 | 4 | 505575 | 4044596 | 2 | 4 | 0 | 184 | 4139660 | 2 | 4 | 0 | 1 | 192 |
| 97 | 61 | 46 | 2E | 2 | 5 | 505598 | 4044780 | 2 | 5 | 0 | 184 | 4139852 | 2 | 5 | 0 | 1 | 192 |
| 98 | 62 | 46 | 2E | 2 | 6 | 505621 | 4044964 | 2 | 6 | 0 | 184 | 4140044 | 2 | 6 | 0 | 1 | 192 |
| 99 | 63 | 46 | 2E | 2 | 7 | 505644 | 4045148 | 2 | 7 | 0 | 184 | 4140236 | 2 | 7 | 0 | 1 | 192 |
| 100 | 64 | 46 | 2E | 2 | 8 | 505667 | 4045332 | 2 | 8 | 0 | 184 | 4140428 | 2 | 8 | 0 | 1 | 192 |
| 101 | 65 | 46 | 2E | 2 | 9 | 505690 | 4045516 | 2 | 9 | 0 | 184 | 4140620 | 2 | 9 | 0 | 1 | 192 |
| 102 | 66 | 46 | 2E | 2 | 10 | 505713 | 4045700 | 2 | 10 | 0 | 184 | 4140812 | 2 | 10 | 0 | 1 | 192 |
| 103 | 67 | 46 | 2E | 2 | 11 | 505736 | 4045884 | 2 | 11 | 0 | 184 | 4141004 | 2 | 11 | 0 | 1 | 192 |
| 104 | 68 | 46 | 2E | 2 | 12 | 505759 | 4046068 | 2 | 12 | 0 | 184 | 4141196 | 2 | 12 | 0 | 1 | 192 |
| 105 | 69 | 46 | 2E | 2 | 13 | 505782 | 4046252 | 2 | 13 | 0 | 184 | 4141388 | 2 | 13 | 0 | 1 | 192 |
| 106 | 6A | 46 | 2E | 2 | 14 | 505805 | 4046436 | 2 | 14 | 0 | 184 | 4141580 | 2 | 14 | 0 | 1 | 192 |
| 107 | 6B | 46 | 2E | 2 | 15 | 505828 | 4046620 | 2 | 15 | 0 | 184 | 4141772 | 2 | 15 | 0 | 1 | 192 |
| 108 | 6C | 46 | 2E | 2 | 16 | 505851 | 4046804 | 2 | 16 | 0 | 184 | 4141964 | 2 | 16 | 0 | 1 | 192 |
| 109 | 6D | 46 | 2E | 2 | 17 | 505874 | 4046988 | 2 | 17 | 0 | 184 | 4142156 | 2 | 17 | 0 | 1 | 192 |
| 110 | 6E | 46 | 2E | 2 | 18 | 505897 | 4047172 | 2 | 18 | 0 | 184 | 4142348 | 2 | 18 | 0 | 1 | 192 |
| 111 | 6F | 46 | 2E | 2 | 19 | 505920 | 4047356 | 2 | 19 | 0 | 184 | 4142540 | 2 | 19 | 0 | 1 | 192 |
| 112 | 70 | 46 | 2E | 2 | 20 | 505945 | 4047556 | 2 | 20 | 0 | 200 | 4142748 | 2 | 20 | 0 | 1 | 208 |
| 113 | 71 | 46 | 2E | 2 | 21 | 505970 | 4047756 | 2 | 21 | 0 | 200 | 4142956 | 2 | 21 | 0 | 1 | 208 |
| 114 | 72 | 46 | 2E | 2 | 22 | 505995 | 4047956 | 2 | 22 | 0 | 200 | 4143164 | 2 | 22 | 0 | 1 | 208 |
| 115 | 73 | 46 | 2E | 2 | 23 | 506020 | 4048156 | 2 | 23 | 0 | 200 | 4143372 | 2 | 23 | 0 | 1 | 208 |
| 116 | 74 | 46 | 2E | 2 | 24 | 506045 | 4048356 | 2 | 24 | 0 | 200 | 4143580 | 2 | 24 | 0 | 1 | 208 |
| 117 | 75 | 46 | 2E | 2 | 25 | 506070 | 4048556 | 2 | 25 | 0 | 200 | 4143788 | 2 | 25 | 0 | 1 | 208 |
| 118 | 76 | 46 | 2E | 2 | 26 | 506095 | 4048756 | 2 | 26 | 0 | 200 | 4143996 | 2 | 26 | 0 | 1 | 208 |
| 119 | 77 | 46 | 2E | 2 | 27 | 506120 | 4048956 | 2 | 27 | 0 | 200 | 4144204 | 2 | 27 | 0 | 1 | 208 |
| 120 | 78 | 46 | 2E | 2 | 28 | 506145 | 4049156 | 2 | 28 | 0 | 200 | 4144412 | 2 | 28 | 0 | 1 | 208 |
| 121 | 79 | 46 | 2E | 2 | 29 | 506170 | 4049356 | 2 | 29 | 0 | 200 | 4144620 | 2 | 29 | 0 | 1 | 208 |
| 122 | 7A | 46 | 2E | 2 | 30 | 506195 | 4049556 | 2 | 30 | 0 | 200 | 4144828 | 2 | 30 | 0 | 1 | 208 |
| 123 | 7B | 46 | 2E | 2 | 31 | 506220 | 4049756 | 2 | 31 | 0 | 200 | 4145036 | 2 | 31 | 0 | 1 | 208 |
| 124 | 7C | 46 | 2E | 2 | 32 | 506245 | 4049956 | 2 | 32 | 0 | 200 | 4145244 | 2 | 32 | 0 | 1 | 208 |
| 125 | 7D | 46 | 2E | 2 | 33 | 506270 | 4050156 | 2 | 33 | 0 | 200 | 4145452 | 2 | 33 | 0 | 1 | 208 |
| 126 | 7E | 46 | 2E | 2 | 34 | 506295 | 4050356 | 2 | 34 | 0 | 200 | 4145660 | 2 | 34 | 0 | 1 | 208 |
| 127 | 7F | 46 | 2E | 2 | 35 | 506320 | 4050556 | 2 | 35 | 0 | 200 | 4145868 | 2 | 35 | 0 | 1 | 208 |
| 128 | 80 | 46 | 2E | 2 | 36 | 506345 | 4050756 | 2 | 36 | 0 | 200 | 4146076 | 2 | 36 | 0 | 1 | 208 |
| 129 | 81 | 46 | 2E | 2 | 37 | 506370 | 4050956 | 2 | 37 | 0 | 200 | 4146284 | 2 | 37 | 0 | 1 | 208 |
| 130 | 82 | 46 | 2E | 2 | 38 | 506395 | 4051156 | 2 | 38 | 0 | 200 | 4146492 | 2 | 38 | 0 | 1 | 208 |
| 131 | 83 | 46 | 2E | 2 | 39 | 506420 | 4051356 | 2 | 39 | 0 | 200 | 4146700 | 2 | 39 | 0 | 1 | 208 |
| 132 | 84 | 46 | 2E | 2 | 40 | 506445 | 4051556 | 2 | 40 | 0 | 200 | 4146908 | 2 | 40 | 0 | 1 | 208 |
| 133 | 85 | 46 | 2E | 2 | 41 | 506470 | 4051756 | 2 | 41 | 0 | 200 | 4147116 | 2 | 41 | 0 | 1 | 208 |
| 134 | 86 | 46 | 2E | 2 | 42 | 506495 | 4051956 | 2 | 42 | 0 | 200 | 4147324 | 2 | 42 | 0 | 1 | 208 |
| 135 | 87 | 46 | 2E | 2 | 43 | 506520 | 4052156 | 2 | 43 | 0 | 200 | 4147532 | 2 | 43 | 0 | 1 | 208 |
| 136 | 88 | 46 | 2E | 2 | 44 | 506545 | 4052356 | 2 | 44 | 0 | 200 | 4147740 | 2 | 44 | 0 | 1 | 208 |
| 137 | 89 | 46 | 2E | 2 | 45 | 506570 | 4052556 | 2 | 45 | 0 | 200 | 4147948 | 2 | 45 | 0 | 1 | 208 |
| 138 | 8A | 46 | 2E | 3 | 0 | 506593 | 4052740 | 3 | 0 | 0 | 184 | 4148140 | 3 | 0 | 0 | 1 | 192 |
| 139 | 8B | 46 | 2E | 3 | 1 | 506621 | 4052964 | 3 | 1 | 0 | 224 | 4148372 | 3 | 1 | 0 | 1 | 232 |
| 140 | 8C | 46 | 2E | 3 | 2 | 506649 | 4053188 | 3 | 2 | 0 | 224 | 4148604 | 3 | 2 | 0 | 1 | 232 |
| 141 | 8D | 46 | 2E | 3 | 3 | 506677 | 4053412 | 3 | 3 | 0 | 224 | 4148836 | 3 | 3 | 0 | 1 | 232 |
| 142 | 8E | 46 | 2E | 3 | 4 | 506705 | 4053636 | 3 | 4 | 0 | 224 | 4149068 | 3 | 4 | 0 | 1 | 232 |
| 143 | 8F | 46 | 2E | 3 | 5 | 506733 | 4053860 | 3 | 5 | 0 | 224 | 4149300 | 3 | 5 | 0 | 1 | 232 |
| 144 | 90 | 46 | 2E | 3 | 6 | 506758 | 4054060 | 3 | 6 | 0 | 200 | 4149508 | 3 | 6 | 0 | 1 | 208 |
| 145 | 91 | 46 | 2E | 3 | 7 | 506783 | 4054260 | 3 | 7 | 0 | 200 | 4149716 | 3 | 7 | 0 | 1 | 208 |
| 146 | 92 | 46 | 2E | 3 | 8 | 506808 | 4054460 | 3 | 8 | 0 | 200 | 4149924 | 3 | 8 | 0 | 1 | 208 |
| 147 | 93 | 46 | 2E | 3 | 9 | 506833 | 4054660 | 3 | 9 | 0 | 200 | 4150132 | 3 | 9 | 0 | 1 | 208 |
| 148 | 94 | 46 | 2E | 3 | 10 | 506858 | 4054860 | 3 | 10 | 0 | 200 | 4150340 | 3 | 10 | 0 | 1 | 208 |
| 149 | 95 | 46 | 2E | 3 | 11 | 506883 | 4055060 | 3 | 11 | 0 | 200 | 4150548 | 3 | 11 | 0 | 1 | 208 |
| 150 | 96 | 46 | 2E | 3 | 12 | 506908 | 4055260 | 3 | 12 | 0 | 200 | 4150756 | 3 | 12 | 0 | 1 | 208 |
| 151 | 97 | 46 | 2E | 3 | 13 | 506933 | 4055460 | 3 | 13 | 0 | 200 | 4150964 | 3 | 13 | 0 | 1 | 208 |
| 152 | 98 | 46 | 2E | 3 | 14 | 506958 | 4055660 | 3 | 14 | 0 | 200 | 4151172 | 3 | 14 | 0 | 1 | 208 |
| 153 | 99 | 46 | 2E | 3 | 15 | 506983 | 4055860 | 3 | 15 | 0 | 200 | 4151380 | 3 | 15 | 0 | 1 | 208 |
| 154 | 9A | 46 | 2E | 3 | 16 | 507008 | 4056060 | 3 | 16 | 0 | 200 | 4151588 | 3 | 16 | 0 | 1 | 208 |
| 155 | 9B | 46 | 2E | 3 | 17 | 507033 | 4056260 | 3 | 17 | 0 | 200 | 4151796 | 3 | 17 | 0 | 1 | 208 |
| 156 | 9C | 46 | 2E | 3 | 18 | 507058 | 4056460 | 3 | 18 | 0 | 200 | 4152004 | 3 | 18 | 0 | 1 | 208 |
| 157 | 9D | 46 | 2E | 3 | 19 | 507083 | 4056660 | 3 | 19 | 0 | 200 | 4152212 | 3 | 19 | 0 | 1 | 208 |
| 158 | 9E | 46 | 2E | 3 | 20 | 507108 | 4056860 | 3 | 20 | 0 | 200 | 4152420 | 3 | 20 | 0 | 1 | 208 |
| 159 | 9F | 46 | 2E | 3 | 21 | 507133 | 4057060 | 3 | 21 | 0 | 200 | 4152628 | 3 | 21 | 0 | 1 | 208 |
| 160 | A0 | 46 | 2E | 3 | 22 | 507160 | 4057276 | 3 | 22 | 0 | 216 | 4152852 | 3 | 22 | 0 | 1 | 224 |
| 161 | A1 | 46 | 2E | 3 | 23 | 507187 | 4057492 | 3 | 23 | 0 | 216 | 4153076 | 3 | 23 | 0 | 1 | 224 |
| 162 | A2 | 46 | 2E | 3 | 24 | 507214 | 4057708 | 3 | 24 | 0 | 216 | 4153300 | 3 | 24 | 0 | 1 | 224 |
| 163 | A3 | 46 | 2E | 3 | 25 | 507241 | 4057924 | 3 | 25 | 0 | 216 | 4153524 | 3 | 25 | 0 | 1 | 224 |
| 164 | A4 | 46 | 2E | 3 | 26 | 507268 | 4058140 | 3 | 26 | 0 | 216 | 4153748 | 3 | 26 | 0 | 1 | 224 |
| 165 | A5 | 46 | 2E | 3 | 27 | 507295 | 4058356 | 3 | 27 | 0 | 216 | 4153972 | 3 | 27 | 0 | 1 | 224 |
| 166 | A6 | 46 | 2E | 3 | 28 | 507322 | 4058572 | 3 | 28 | 0 | 216 | 4154196 | 3 | 28 | 0 | 1 | 224 |
| 167 | A7 | 46 | 2E | 3 | 29 | 507349 | 4058788 | 3 | 29 | 0 | 216 | 4154420 | 3 | 29 | 0 | 1 | 224 |
| 168 | A8 | 46 | 2E | 3 | 30 | 507376 | 4059004 | 3 | 30 | 0 | 216 | 4154644 | 3 | 30 | 0 | 1 | 224 |

| | | | | | | | | | | | | | | | | | |
|---|---|---|---|---|---|---|---|---|---|---|---|---|---|---|---|---|---|
| 169 | A9 | 46 | 2E | 3 | 31 | 507403 | 4059220 | 3 | 31 | 0 | 216 | 4154868 | 3 | 31 | 0 | 1 | 224 |
| 170 | AA | 46 | 2E | 3 | 32 | 507430 | 4059436 | 3 | 32 | 0 | 216 | 4155092 | 3 | 32 | 0 | 1 | 224 |
| 171 | AB | 46 | 2E | 3 | 33 | 507457 | 4059652 | 3 | 33 | 0 | 216 | 4155316 | 3 | 33 | 0 | 1 | 224 |
| 172 | AC | 46 | 2E | 3 | 34 | 507484 | 4059868 | 3 | 34 | 0 | 216 | 4155540 | 3 | 34 | 0 | 1 | 224 |
| 173 | AD | 46 | 2E | 3 | 35 | 507511 | 4060084 | 3 | 35 | 0 | 216 | 4155764 | 3 | 35 | 0 | 1 | 224 |
| 174 | AE | 46 | 2E | 3 | 36 | 507538 | 4060300 | 3 | 36 | 0 | 216 | 4155988 | 3 | 36 | 0 | 1 | 224 |
| 175 | AF | 46 | 2E | 3 | 37 | 507565 | 4060516 | 3 | 37 | 0 | 216 | 4156212 | 3 | 37 | 0 | 1 | 224 |
| 176 | B0 | 46 | 2E | 3 | 38 | 507592 | 4060732 | 3 | 38 | 0 | 216 | 4156436 | 3 | 38 | 0 | 1 | 224 |
| 177 | B1 | 46 | 2E | 3 | 39 | 507619 | 4060948 | 3 | 39 | 0 | 216 | 4156660 | 3 | 39 | 0 | 1 | 224 |
| 178 | B2 | 46 | 2E | 3 | 40 | 507646 | 4061164 | 3 | 40 | 0 | 216 | 4156884 | 3 | 40 | 0 | 1 | 224 |
| 179 | B3 | 46 | 2E | 3 | 41 | 507673 | 4061380 | 3 | 41 | 0 | 216 | 4157108 | 3 | 41 | 0 | 1 | 224 |
| 180 | B4 | 46 | 2E | 3 | 42 | 507700 | 4061596 | 3 | 42 | 0 | 216 | 4157332 | 3 | 42 | 0 | 1 | 224 |
| 181 | B5 | 46 | 2E | 3 | 43 | 507727 | 4061812 | 3 | 43 | 0 | 216 | 4157556 | 3 | 43 | 0 | 1 | 224 |
| 182 | B6 | 46 | 2E | 3 | 44 | 507754 | 4062028 | 3 | 44 | 0 | 216 | 4157780 | 3 | 44 | 0 | 1 | 224 |
| 183 | B7 | 46 | 2E | 3 | 45 | 507781 | 4062244 | 3 | 45 | 0 | 216 | 4158004 | 3 | 45 | 0 | 1 | 224 |
| 184 | B8 | 46 | 2E | 4 | 0 | 507806 | 4062444 | 4 | 0 | 0 | 200 | 4158212 | 4 | 0 | 0 | 1 | 208 |
| 185 | B9 | 46 | 2E | 4 | 1 | 507836 | 4062684 | 4 | 1 | 0 | 240 | 4158460 | 4 | 1 | 0 | 1 | 248 |
| 186 | BA | 46 | 2E | 4 | 2 | 507866 | 4062924 | 4 | 2 | 0 | 240 | 4158708 | 4 | 2 | 0 | 1 | 248 |
| 187 | BB | 46 | 2E | 4 | 3 | 507896 | 4063164 | 4 | 3 | 0 | 240 | 4158956 | 4 | 3 | 0 | 1 | 248 |
| 188 | BC | 46 | 2E | 4 | 4 | 507926 | 4063404 | 4 | 4 | 0 | 240 | 4159204 | 4 | 4 | 0 | 1 | 248 |
| 189 | BD | 46 | 2E | 4 | 5 | 507956 | 4063644 | 4 | 5 | 0 | 240 | 4159452 | 4 | 5 | 0 | 1 | 248 |
| 190 | BE | 46 | 2E | 4 | 6 | 507986 | 4063884 | 4 | 6 | 0 | 240 | 4159700 | 4 | 6 | 0 | 1 | 248 |
| 191 | BF | 46 | 2E | 4 | 7 | 508016 | 4064124 | 4 | 7 | 0 | 240 | 4159948 | 4 | 7 | 0 | 1 | 248 |
| 192 | C0 | 46 | 2E | 4 | 8 | 508043 | 4064340 | 4 | 8 | 0 | 216 | 4160172 | 4 | 8 | 0 | 1 | 224 |
| 193 | C1 | 46 | 2E | 4 | 9 | 508070 | 4064556 | 4 | 9 | 0 | 216 | 4160396 | 4 | 9 | 0 | 1 | 224 |
| 194 | C2 | 46 | 2E | 4 | 10 | 508097 | 4064772 | 4 | 10 | 0 | 216 | 4160620 | 4 | 10 | 0 | 1 | 224 |
| 195 | C3 | 46 | 2E | 4 | 11 | 508124 | 4064988 | 4 | 11 | 0 | 216 | 4160844 | 4 | 11 | 0 | 1 | 224 |
| 196 | C4 | 46 | 2E | 4 | 12 | 508151 | 4065204 | 4 | 12 | 0 | 216 | 4161068 | 4 | 12 | 0 | 1 | 224 |
| 197 | C5 | 46 | 2E | 4 | 13 | 508178 | 4065420 | 4 | 13 | 0 | 216 | 4161292 | 4 | 13 | 0 | 1 | 224 |
| 198 | C6 | 46 | 2E | 4 | 14 | 508205 | 4065636 | 4 | 14 | 0 | 216 | 4161516 | 4 | 14 | 0 | 1 | 224 |
| 199 | C7 | 46 | 2E | 4 | 15 | 508232 | 4065852 | 4 | 15 | 0 | 216 | 4161740 | 4 | 15 | 0 | 1 | 224 |
| 200 | C8 | 46 | 2E | 4 | 16 | 508259 | 4066068 | 4 | 16 | 0 | 216 | 4161964 | 4 | 16 | 0 | 1 | 224 |
| 201 | C9 | 46 | 2E | 4 | 17 | 508286 | 4066284 | 4 | 17 | 0 | 216 | 4162188 | 4 | 17 | 0 | 1 | 224 |
| 202 | CA | 46 | 2E | 4 | 18 | 508313 | 4066500 | 4 | 18 | 0 | 216 | 4162412 | 4 | 18 | 0 | 1 | 224 |
| 203 | CB | 46 | 2E | 4 | 19 | 508340 | 4066716 | 4 | 19 | 0 | 216 | 4162636 | 4 | 19 | 0 | 1 | 224 |
| 204 | CC | 46 | 2E | 4 | 20 | 508367 | 4066932 | 4 | 20 | 0 | 216 | 4162860 | 4 | 20 | 0 | 1 | 224 |
| 205 | CD | 46 | 2E | 4 | 21 | 508394 | 4067148 | 4 | 21 | 0 | 216 | 4163084 | 4 | 21 | 0 | 1 | 224 |
| 206 | CE | 46 | 2E | 4 | 22 | 508421 | 4067364 | 4 | 22 | 0 | 216 | 4163308 | 4 | 22 | 0 | 1 | 224 |
| 207 | CF | 46 | 2E | 4 | 23 | 508448 | 4067580 | 4 | 23 | 0 | 216 | 4163532 | 4 | 23 | 0 | 1 | 224 |
| 208 | D0 | 46 | 2E | 4 | 24 | 508477 | 4067812 | 4 | 24 | 0 | 232 | 4163772 | 4 | 24 | 0 | 1 | 240 |
| 209 | D1 | 46 | 2E | 4 | 25 | 508506 | 4068044 | 4 | 25 | 0 | 232 | 4164012 | 4 | 25 | 0 | 1 | 240 |
| 210 | D2 | 46 | 2E | 4 | 26 | 508535 | 4068276 | 4 | 26 | 0 | 232 | 4164252 | 4 | 26 | 0 | 1 | 240 |
| 211 | D3 | 46 | 2E | 4 | 27 | 508564 | 4068508 | 4 | 27 | 0 | 232 | 4164492 | 4 | 27 | 0 | 1 | 240 |
| 212 | D4 | 46 | 2E | 4 | 28 | 508593 | 4068740 | 4 | 28 | 0 | 232 | 4164732 | 4 | 28 | 0 | 1 | 240 |
| 213 | D5 | 46 | 2E | 4 | 29 | 508622 | 4068972 | 4 | 29 | 0 | 232 | 4164972 | 4 | 29 | 0 | 1 | 240 |
| 214 | D6 | 46 | 2E | 4 | 30 | 508651 | 4069204 | 4 | 30 | 0 | 232 | 4165212 | 4 | 30 | 0 | 1 | 240 |
| 215 | D7 | 46 | 2E | 4 | 31 | 508680 | 4069436 | 4 | 31 | 0 | 232 | 4165452 | 4 | 31 | 0 | 1 | 240 |
| 216 | D8 | 46 | 2E | 4 | 32 | 508709 | 4069668 | 4 | 32 | 0 | 232 | 4165692 | 4 | 32 | 0 | 1 | 240 |
| 217 | D9 | 46 | 2E | 4 | 33 | 508738 | 4069900 | 4 | 33 | 0 | 232 | 4165932 | 4 | 33 | 0 | 1 | 240 |
| 218 | DA | 46 | 2E | 4 | 34 | 508767 | 4070132 | 4 | 34 | 0 | 232 | 4166172 | 4 | 34 | 0 | 1 | 240 |
| 219 | DB | 46 | 2E | 4 | 35 | 508796 | 4070364 | 4 | 35 | 0 | 232 | 4166412 | 4 | 35 | 0 | 1 | 240 |
| 220 | DC | 46 | 2E | 4 | 36 | 508825 | 4070596 | 4 | 36 | 0 | 232 | 4166652 | 4 | 36 | 0 | 1 | 240 |
| 221 | DD | 46 | 2E | 4 | 37 | 508854 | 4070828 | 4 | 37 | 0 | 232 | 4166892 | 4 | 37 | 0 | 1 | 240 |
| 222 | DE | 46 | 2E | 4 | 38 | 508883 | 4071060 | 4 | 38 | 0 | 232 | 4167132 | 4 | 38 | 0 | 1 | 240 |
| 223 | DF | 46 | 2E | 4 | 39 | 508912 | 4071292 | 4 | 39 | 0 | 232 | 4167372 | 4 | 39 | 0 | 1 | 240 |
| 224 | E0 | 46 | 2E | 4 | 40 | 508941 | 4071524 | 4 | 40 | 0 | 232 | 4167612 | 4 | 40 | 0 | 1 | 240 |
| 225 | E1 | 46 | 2E | 4 | 41 | 508970 | 4071756 | 4 | 41 | 0 | 232 | 4167852 | 4 | 41 | 0 | 1 | 240 |
| 226 | E2 | 46 | 2E | 4 | 42 | 508999 | 4071988 | 4 | 42 | 0 | 232 | 4168092 | 4 | 42 | 0 | 1 | 240 |
| 227 | E3 | 46 | 2E | 4 | 43 | 509028 | 4072220 | 4 | 43 | 0 | 232 | 4168332 | 4 | 43 | 0 | 1 | 240 |
| 228 | E4 | 46 | 2E | 4 | 44 | 509057 | 4072452 | 4 | 44 | 0 | 232 | 4168572 | 4 | 44 | 0 | 1 | 240 |
| 229 | E5 | 46 | 2E | 4 | 45 | 509086 | 4072684 | 4 | 45 | 0 | 232 | 4168812 | 4 | 45 | 0 | 1 | 240 |
| 230 | E6 | 46 | 2E | 5 | 0 | 509113 | 4072900 | 5 | 0 | 0 | 216 | 4169036 | 5 | 0 | 0 | 1 | 224 |
| 231 | E7 | 46 | 2E | 5 | 1 | 509145 | 4073156 | 5 | 1 | 0 | 256 | 4169300 | 5 | 1 | 0 | 1 | 264 |
| 232 | E8 | 46 | 2E | 5 | 2 | 509177 | 4073412 | 5 | 2 | 0 | 256 | 4169564 | 5 | 2 | 0 | 1 | 264 |
| 233 | E9 | 46 | 2E | 5 | 3 | 509209 | 4073668 | 5 | 3 | 0 | 256 | 4169828 | 5 | 3 | 0 | 1 | 264 |
| 234 | EA | 46 | 2E | 5 | 4 | 509241 | 4073924 | 5 | 4 | 0 | 256 | 4170092 | 5 | 4 | 0 | 1 | 264 |
| 235 | EB | 46 | 2E | 5 | 5 | 509273 | 4074180 | 5 | 5 | 0 | 256 | 4170356 | 5 | 5 | 0 | 1 | 264 |
| 236 | EC | 46 | 2E | 5 | 6 | 509305 | 4074436 | 5 | 6 | 0 | 256 | 4170620 | 5 | 6 | 0 | 1 | 264 |
| 237 | ED | 46 | 2E | 5 | 7 | 509337 | 4074692 | 5 | 7 | 0 | 256 | 4170884 | 5 | 7 | 0 | 1 | 264 |
| 238 | EE | 46 | 2E | 5 | 8 | 509369 | 4074948 | 5 | 8 | 0 | 256 | 4171148 | 5 | 8 | 0 | 1 | 264 |
| 239 | EF | 46 | 2E | 5 | 9 | 509401 | 4075204 | 5 | 9 | 0 | 256 | 4171412 | 5 | 9 | 0 | 1 | 264 |
| 240 | F0 | 46 | 2E | 5 | 10 | 509430 | 4075436 | 5 | 10 | 0 | 232 | 4171652 | 5 | 10 | 0 | 1 | 240 |
| 241 | F1 | 46 | 2E | 5 | 11 | 509459 | 4075668 | 5 | 11 | 0 | 232 | 4171892 | 5 | 11 | 0 | 1 | 240 |
| 242 | F2 | 46 | 2E | 5 | 12 | 509488 | 4075900 | 5 | 12 | 0 | 232 | 4172132 | 5 | 12 | 0 | 1 | 240 |
| 243 | F3 | 46 | 2E | 5 | 13 | 509517 | 4076132 | 5 | 13 | 0 | 232 | 4172372 | 5 | 13 | 0 | 1 | 240 |
| 244 | F4 | 46 | 2E | 5 | 14 | 509546 | 4076364 | 5 | 14 | 0 | 232 | 4172612 | 5 | 14 | 0 | 1 | 240 |
| 245 | F5 | 46 | 2E | 5 | 15 | 509575 | 4076596 | 5 | 15 | 0 | 232 | 4172852 | 5 | 15 | 0 | 1 | 240 |
| 246 | F6 | 46 | 2E | 5 | 16 | 509604 | 4076828 | 5 | 16 | 0 | 232 | 4173092 | 5 | 16 | 0 | 1 | 240 |
| 247 | F7 | 46 | 2E | 5 | 17 | 509633 | 4077060 | 5 | 17 | 0 | 232 | 4173332 | 5 | 17 | 0 | 1 | 240 |
| 248 | F8 | 46 | 2E | 5 | 18 | 509662 | 4077292 | 5 | 18 | 0 | 232 | 4173572 | 5 | 18 | 0 | 1 | 240 |
| 249 | F9 | 46 | 2E | 5 | 19 | 509691 | 4077524 | 5 | 19 | 0 | 232 | 4173812 | 5 | 19 | 0 | 1 | 240 |
| 250 | FA | 46 | 2E | 5 | 20 | 509720 | 4077756 | 5 | 20 | 0 | 232 | 4174052 | 5 | 20 | 0 | 1 | 240 |
| 251 | FB | 46 | 2E | 5 | 21 | 509749 | 4077988 | 5 | 21 | 0 | 232 | 4174292 | 5 | 21 | 0 | 1 | 240 |
| 252 | FC | 46 | 2E | 5 | 22 | 509778 | 4078220 | 5 | 22 | 0 | 232 | 4174532 | 5 | 22 | 0 | 1 | 240 |
| 253 | FD | 46 | 2E | 5 | 23 | 509807 | 4078452 | 5 | 23 | 0 | 232 | 4174772 | 5 | 23 | 0 | 1 | 240 |
| 254 | FE | 46 | 2E | 5 | 24 | 509836 | 4078684 | 5 | 24 | 0 | 232 | 4175012 | 5 | 24 | 0 | 1 | 240 |
| 255 | FF | 46 | 2E | 5 | 25 | 509865 | 4078916 | 5 | 25 | 0 | 232 | 4175252 | 5 | 25 | 0 | 1 | 240 |
| 0 | 00 | 47 | 2F | 0 | 0 | 509877 | 4079012 | 0 | 0 | 0 | 96 | 4175356 | 0 | 0 | 0 | 1 | 104 |
| 1 | 01 | 47 | 2F | 0 | 1 | 509891 | 4079124 | 0 | 1 | 0 | 112 | 4175476 | 0 | 1 | 0 | 1 | 120 |
| 2 | 02 | 47 | 2F | 0 | 2 | 509905 | 4079236 | 0 | 2 | 0 | 112 | 4175596 | 0 | 2 | 0 | 1 | 120 |
| 3 | 03 | 47 | 2F | 0 | 3 | 509919 | 4079348 | 0 | 3 | 0 | 112 | 4175716 | 0 | 3 | 0 | 1 | 120 |
| 4 | 04 | 47 | 2F | 0 | 4 | 509933 | 4079460 | 0 | 4 | 0 | 112 | 4175836 | 0 | 4 | 0 | 1 | 120 |
| 5 | 05 | 47 | 2F | 0 | 5 | 509947 | 4079572 | 0 | 5 | 0 | 112 | 4175956 | 0 | 5 | 0 | 1 | 120 |
| 6 | 06 | 47 | 2F | 0 | 6 | 509961 | 4079684 | 0 | 6 | 0 | 112 | 4176076 | 0 | 6 | 0 | 1 | 120 |
| 7 | 07 | 47 | 2F | 0 | 7 | 509975 | 4079796 | 0 | 7 | 0 | 112 | 4176196 | 0 | 7 | 0 | 1 | 120 |
| 8 | 08 | 47 | 2F | 0 | 8 | 509989 | 4079908 | 0 | 8 | 0 | 112 | 4176316 | 0 | 8 | 0 | 1 | 120 |
| 9 | 09 | 47 | 2F | 0 | 9 | 510003 | 4080020 | 0 | 9 | 0 | 112 | 4176436 | 0 | 9 | 0 | 1 | 120 |
| 10 | 0A | 47 | 2F | 0 | 10 | 510017 | 4080132 | 0 | 10 | 0 | 112 | 4176556 | 0 | 10 | 0 | 1 | 120 |
| 11 | 0B | 47 | 2F | 0 | 11 | 510031 | 4080244 | 0 | 11 | 0 | 112 | 4176676 | 0 | 11 | 0 | 1 | 120 |
| 12 | 0C | 47 | 2F | 0 | 12 | 510045 | 4080356 | 0 | 12 | 0 | 112 | 4176796 | 0 | 12 | 0 | 1 | 120 |
| 13 | 0D | 47 | 2F | 0 | 13 | 510059 | 4080468 | 0 | 13 | 0 | 112 | 4176916 | 0 | 13 | 0 | 1 | 120 |
| 14 | 0E | 47 | 2F | 0 | 14 | 510073 | 4080580 | 0 | 14 | 0 | 112 | 4177036 | 0 | 14 | 0 | 1 | 120 |
| 15 | 0F | 47 | 2F | 0 | 15 | 510087 | 4080692 | 0 | 15 | 0 | 112 | 4177156 | 0 | 15 | 0 | 1 | 120 |

| | | | | | | | | | | | | | | | | |
|---|---|---|---|---|---|---|---|---|---|---|---|---|---|---|---|---|
| 16 | 10 | 47 | 2F | 0 | 16 | 510101 | 4080804 | 0 | 16 | 0 | 112 | 4177276 | 0 | 16 | 0 | 1 | 120 |
| 17 | 11 | 47 | 2F | 0 | 17 | 510115 | 4080916 | 0 | 17 | 0 | 112 | 4177396 | 0 | 17 | 0 | 1 | 120 |
| 18 | 12 | 47 | 2F | 0 | 18 | 510129 | 4081028 | 0 | 18 | 0 | 112 | 4177516 | 0 | 18 | 0 | 1 | 120 |
| 19 | 13 | 47 | 2F | 0 | 19 | 510143 | 4081140 | 0 | 19 | 0 | 112 | 4177636 | 0 | 19 | 0 | 1 | 120 |
| 20 | 14 | 47 | 2F | 0 | 20 | 510157 | 4081252 | 0 | 20 | 0 | 112 | 4177756 | 0 | 20 | 0 | 1 | 120 |
| 21 | 15 | 47 | 2F | 0 | 21 | 510171 | 4081364 | 0 | 21 | 0 | 112 | 4177876 | 0 | 21 | 0 | 1 | 120 |
| 22 | 16 | 47 | 2F | 0 | 22 | 510185 | 4081476 | 0 | 22 | 0 | 112 | 4177996 | 0 | 22 | 0 | 1 | 120 |
| 23 | 17 | 47 | 2F | 0 | 23 | 510199 | 4081588 | 0 | 23 | 0 | 112 | 4178116 | 0 | 23 | 0 | 1 | 120 |
| 24 | 18 | 47 | 2F | 0 | 24 | 510213 | 4081700 | 0 | 24 | 0 | 112 | 4178236 | 0 | 24 | 0 | 1 | 120 |
| 25 | 19 | 47 | 2F | 0 | 25 | 510227 | 4081812 | 0 | 25 | 0 | 112 | 4178356 | 0 | 25 | 0 | 1 | 120 |
| 26 | 1A | 47 | 2F | 0 | 26 | 510241 | 4081924 | 0 | 26 | 0 | 112 | 4178476 | 0 | 26 | 0 | 1 | 120 |
| 27 | 1B | 47 | 2F | 0 | 27 | 510255 | 4082036 | 0 | 27 | 0 | 112 | 4178596 | 0 | 27 | 0 | 1 | 120 |
| 28 | 1C | 47 | 2F | 0 | 28 | 510269 | 4082148 | 0 | 28 | 0 | 112 | 4178716 | 0 | 28 | 0 | 1 | 120 |
| 29 | 1D | 47 | 2F | 0 | 29 | 510283 | 4082260 | 0 | 29 | 0 | 112 | 4178836 | 0 | 29 | 0 | 1 | 120 |
| 30 | 1E | 47 | 2F | 0 | 30 | 510297 | 4082372 | 0 | 30 | 0 | 112 | 4178956 | 0 | 30 | 0 | 1 | 120 |
| 31 | 1F | 47 | 2F | 0 | 31 | 510311 | 4082484 | 0 | 31 | 0 | 112 | 4179076 | 0 | 31 | 0 | 1 | 120 |
| 32 | 20 | 47 | 2F | 0 | 32 | 510325 | 4082596 | 0 | 32 | 0 | 112 | 4179196 | 0 | 32 | 0 | 1 | 120 |
| 33 | 21 | 47 | 2F | 0 | 33 | 510339 | 4082708 | 0 | 33 | 0 | 112 | 4179316 | 0 | 33 | 0 | 1 | 120 |
| 34 | 22 | 47 | 2F | 0 | 34 | 510353 | 4082820 | 0 | 34 | 0 | 112 | 4179436 | 0 | 34 | 0 | 1 | 120 |
| 35 | 23 | 47 | 2F | 0 | 35 | 510367 | 4082932 | 0 | 35 | 0 | 112 | 4179556 | 0 | 35 | 0 | 1 | 120 |
| 36 | 24 | 47 | 2F | 0 | 36 | 510381 | 4083044 | 0 | 36 | 0 | 112 | 4179676 | 0 | 36 | 0 | 1 | 120 |
| 37 | 25 | 47 | 2F | 0 | 37 | 510395 | 4083156 | 0 | 37 | 0 | 112 | 4179796 | 0 | 37 | 0 | 1 | 120 |
| 38 | 26 | 47 | 2F | 0 | 38 | 510409 | 4083268 | 0 | 38 | 0 | 112 | 4179916 | 0 | 38 | 0 | 1 | 120 |
| 39 | 27 | 47 | 2F | 0 | 39 | 510423 | 4083380 | 0 | 39 | 0 | 112 | 4180036 | 0 | 39 | 0 | 1 | 120 |
| 40 | 28 | 47 | 2F | 0 | 40 | 510437 | 4083492 | 0 | 40 | 0 | 112 | 4180156 | 0 | 40 | 0 | 1 | 120 |
| 41 | 29 | 47 | 2F | 0 | 41 | 510451 | 4083604 | 0 | 41 | 0 | 112 | 4180276 | 0 | 41 | 0 | 1 | 120 |
| 42 | 2A | 47 | 2F | 0 | 42 | 510465 | 4083716 | 0 | 42 | 0 | 112 | 4180396 | 0 | 42 | 0 | 1 | 120 |
| 43 | 2B | 47 | 2F | 0 | 43 | 510479 | 4083828 | 0 | 43 | 0 | 112 | 4180516 | 0 | 43 | 0 | 1 | 120 |
| 44 | 2C | 47 | 2F | 0 | 44 | 510493 | 4083940 | 0 | 44 | 0 | 112 | 4180636 | 0 | 44 | 0 | 1 | 120 |
| 45 | 2D | 47 | 2F | 0 | 45 | 510507 | 4084052 | 0 | 45 | 0 | 112 | 4180756 | 0 | 45 | 0 | 1 | 120 |
| 46 | 2E | 47 | 2F | 0 | 46 | 510521 | 4084164 | 0 | 46 | 0 | 112 | 4180876 | 0 | 46 | 0 | 1 | 120 |
| 47 | 2F | 47 | 2F | 1 | 0 | 510533 | 4084260 | 1 | 0 | 0 | 96 | 4180980 | 1 | 0 | 0 | 1 | 104 |
| 48 | 30 | 47 | 2F | 1 | 1 | 510554 | 4084428 | 1 | 1 | 0 | 168 | 4181156 | 1 | 1 | 0 | 1 | 176 |
| 49 | 31 | 47 | 2F | 1 | 2 | 510575 | 4084596 | 1 | 2 | 0 | 168 | 4181332 | 1 | 2 | 0 | 1 | 176 |
| 50 | 32 | 47 | 2F | 1 | 3 | 510596 | 4084764 | 1 | 3 | 0 | 168 | 4181508 | 1 | 3 | 0 | 1 | 176 |
| 51 | 33 | 47 | 2F | 1 | 4 | 510617 | 4084932 | 1 | 4 | 0 | 168 | 4181684 | 1 | 4 | 0 | 1 | 176 |
| 52 | 34 | 47 | 2F | 1 | 5 | 510638 | 4085100 | 1 | 5 | 0 | 168 | 4181860 | 1 | 5 | 0 | 1 | 176 |
| 53 | 35 | 47 | 2F | 1 | 6 | 510659 | 4085268 | 1 | 6 | 0 | 168 | 4182036 | 1 | 6 | 0 | 1 | 176 |
| 54 | 36 | 47 | 2F | 1 | 7 | 510680 | 4085436 | 1 | 7 | 0 | 168 | 4182212 | 1 | 7 | 0 | 1 | 176 |
| 55 | 37 | 47 | 2F | 1 | 8 | 510701 | 4085604 | 1 | 8 | 0 | 168 | 4182388 | 1 | 8 | 0 | 1 | 176 |
| 56 | 38 | 47 | 2F | 1 | 9 | 510722 | 4085772 | 1 | 9 | 0 | 168 | 4182564 | 1 | 9 | 0 | 1 | 176 |
| 57 | 39 | 47 | 2F | 1 | 10 | 510743 | 4085940 | 1 | 10 | 0 | 168 | 4182740 | 1 | 10 | 0 | 1 | 176 |
| 58 | 3A | 47 | 2F | 1 | 11 | 510764 | 4086108 | 1 | 11 | 0 | 168 | 4182916 | 1 | 11 | 0 | 1 | 176 |
| 59 | 3B | 47 | 2F | 1 | 12 | 510785 | 4086276 | 1 | 12 | 0 | 168 | 4183092 | 1 | 12 | 0 | 1 | 176 |
| 60 | 3C | 47 | 2F | 1 | 13 | 510806 | 4086444 | 1 | 13 | 0 | 168 | 4183268 | 1 | 13 | 0 | 1 | 176 |
| 61 | 3D | 47 | 2F | 1 | 14 | 510827 | 4086612 | 1 | 14 | 0 | 168 | 4183444 | 1 | 14 | 0 | 1 | 176 |
| 62 | 3E | 47 | 2F | 1 | 15 | 510848 | 4086780 | 1 | 15 | 0 | 168 | 4183620 | 1 | 15 | 0 | 1 | 176 |
| 63 | 3F | 47 | 2F | 1 | 16 | 510869 | 4086948 | 1 | 16 | 0 | 168 | 4183796 | 1 | 16 | 0 | 1 | 176 |
| 64 | 40 | 47 | 2F | 1 | 17 | 510892 | 4087132 | 1 | 17 | 0 | 184 | 4183988 | 1 | 17 | 0 | 1 | 192 |
| 65 | 41 | 47 | 2F | 1 | 18 | 510915 | 4087316 | 1 | 18 | 0 | 184 | 4184180 | 1 | 18 | 0 | 1 | 192 |
| 66 | 42 | 47 | 2F | 1 | 19 | 510938 | 4087500 | 1 | 19 | 0 | 184 | 4184372 | 1 | 19 | 0 | 1 | 192 |
| 67 | 43 | 47 | 2F | 1 | 20 | 510961 | 4087684 | 1 | 20 | 0 | 184 | 4184564 | 1 | 20 | 0 | 1 | 192 |
| 68 | 44 | 47 | 2F | 1 | 21 | 510984 | 4087868 | 1 | 21 | 0 | 184 | 4184756 | 1 | 21 | 0 | 1 | 192 |
| 69 | 45 | 47 | 2F | 1 | 22 | 511007 | 4088052 | 1 | 22 | 0 | 184 | 4184948 | 1 | 22 | 0 | 1 | 192 |
| 70 | 46 | 47 | 2F | 1 | 23 | 511030 | 4088236 | 1 | 23 | 0 | 184 | 4185140 | 1 | 23 | 0 | 1 | 192 |
| 71 | 47 | 47 | 2F | 1 | 24 | 511053 | 4088420 | 1 | 24 | 0 | 184 | 4185332 | 1 | 24 | 0 | 1 | 192 |
| 72 | 48 | 47 | 2F | 1 | 25 | 511076 | 4088604 | 1 | 25 | 0 | 184 | 4185524 | 1 | 25 | 0 | 1 | 192 |
| 73 | 49 | 47 | 2F | 1 | 26 | 511099 | 4088788 | 1 | 26 | 0 | 184 | 4185716 | 1 | 26 | 0 | 1 | 192 |
| 74 | 4A | 47 | 2F | 1 | 27 | 511122 | 4088972 | 1 | 27 | 0 | 184 | 4185908 | 1 | 27 | 0 | 1 | 192 |
| 75 | 4B | 47 | 2F | 1 | 28 | 511145 | 4089156 | 1 | 28 | 0 | 184 | 4186100 | 1 | 28 | 0 | 1 | 192 |
| 76 | 4C | 47 | 2F | 1 | 29 | 511168 | 4089340 | 1 | 29 | 0 | 184 | 4186292 | 1 | 29 | 0 | 1 | 192 |
| 77 | 4D | 47 | 2F | 1 | 30 | 511191 | 4089524 | 1 | 30 | 0 | 184 | 4186484 | 1 | 30 | 0 | 1 | 192 |
| 78 | 4E | 47 | 2F | 1 | 31 | 511214 | 4089708 | 1 | 31 | 0 | 184 | 4186676 | 1 | 31 | 0 | 1 | 192 |
| 79 | 4F | 47 | 2F | 1 | 32 | 511237 | 4089892 | 1 | 32 | 0 | 184 | 4186868 | 1 | 32 | 0 | 1 | 192 |
| 80 | 50 | 47 | 2F | 1 | 33 | 511260 | 4090076 | 1 | 33 | 0 | 184 | 4187060 | 1 | 33 | 0 | 1 | 192 |
| 81 | 51 | 47 | 2F | 1 | 34 | 511283 | 4090260 | 1 | 34 | 0 | 184 | 4187252 | 1 | 34 | 0 | 1 | 192 |
| 82 | 52 | 47 | 2F | 1 | 35 | 511306 | 4090444 | 1 | 35 | 0 | 184 | 4187444 | 1 | 35 | 0 | 1 | 192 |
| 83 | 53 | 47 | 2F | 1 | 36 | 511329 | 4090628 | 1 | 36 | 0 | 184 | 4187636 | 1 | 36 | 0 | 1 | 192 |
| 84 | 54 | 47 | 2F | 1 | 37 | 511352 | 4090812 | 1 | 37 | 0 | 184 | 4187828 | 1 | 37 | 0 | 1 | 192 |
| 85 | 55 | 47 | 2F | 1 | 38 | 511375 | 4090996 | 1 | 38 | 0 | 184 | 4188020 | 1 | 38 | 0 | 1 | 192 |
| 86 | 56 | 47 | 2F | 1 | 39 | 511398 | 4091180 | 1 | 39 | 0 | 184 | 4188212 | 1 | 39 | 0 | 1 | 192 |
| 87 | 57 | 47 | 2F | 1 | 40 | 511421 | 4091364 | 1 | 40 | 0 | 184 | 4188404 | 1 | 40 | 0 | 1 | 192 |
| 88 | 58 | 47 | 2F | 1 | 41 | 511444 | 4091548 | 1 | 41 | 0 | 184 | 4188596 | 1 | 41 | 0 | 1 | 192 |
| 89 | 59 | 47 | 2F | 1 | 42 | 511467 | 4091732 | 1 | 42 | 0 | 184 | 4188788 | 1 | 42 | 0 | 1 | 192 |
| 90 | 5A | 47 | 2F | 1 | 43 | 511490 | 4091916 | 1 | 43 | 0 | 184 | 4188980 | 1 | 43 | 0 | 1 | 192 |
| 91 | 5B | 47 | 2F | 1 | 44 | 511513 | 4092100 | 1 | 44 | 0 | 184 | 4189172 | 1 | 44 | 0 | 1 | 192 |
| 92 | 5C | 47 | 2F | 1 | 45 | 511536 | 4092284 | 1 | 45 | 0 | 184 | 4189364 | 1 | 45 | 0 | 1 | 192 |
| 93 | 5D | 47 | 2F | 1 | 46 | 511559 | 4092468 | 1 | 46 | 0 | 184 | 4189556 | 1 | 46 | 0 | 1 | 192 |
| 94 | 5E | 47 | 2F | 2 | 0 | 511580 | 4092636 | 2 | 0 | 0 | 168 | 4189732 | 2 | 0 | 0 | 1 | 176 |
| 95 | 5F | 47 | 2F | 2 | 1 | 511606 | 4092844 | 2 | 1 | 0 | 208 | 4189948 | 2 | 1 | 0 | 1 | 216 |
| 96 | 60 | 47 | 2F | 2 | 2 | 511629 | 4093028 | 2 | 2 | 0 | 184 | 4190140 | 2 | 2 | 0 | 1 | 192 |
| 97 | 61 | 47 | 2F | 2 | 3 | 511652 | 4093212 | 2 | 3 | 0 | 184 | 4190332 | 2 | 3 | 0 | 1 | 192 |
| 98 | 62 | 47 | 2F | 2 | 4 | 511675 | 4093396 | 2 | 4 | 0 | 184 | 4190524 | 2 | 4 | 0 | 1 | 192 |
| 99 | 63 | 47 | 2F | 2 | 5 | 511698 | 4093580 | 2 | 5 | 0 | 184 | 4190716 | 2 | 5 | 0 | 1 | 192 |
| 100 | 64 | 47 | 2F | 2 | 6 | 511721 | 4093764 | 2 | 6 | 0 | 184 | 4190908 | 2 | 6 | 0 | 1 | 192 |
| 101 | 65 | 47 | 2F | 2 | 7 | 511744 | 4093948 | 2 | 7 | 0 | 184 | 4191100 | 2 | 7 | 0 | 1 | 192 |
| 102 | 66 | 47 | 2F | 2 | 8 | 511767 | 4094132 | 2 | 8 | 0 | 184 | 4191292 | 2 | 8 | 0 | 1 | 192 |
| 103 | 67 | 47 | 2F | 2 | 9 | 511790 | 4094316 | 2 | 9 | 0 | 184 | 4191484 | 2 | 9 | 0 | 1 | 192 |
| 104 | 68 | 47 | 2F | 2 | 10 | 511813 | 4094500 | 2 | 10 | 0 | 184 | 4191676 | 2 | 10 | 0 | 1 | 192 |
| 105 | 69 | 47 | 2F | 2 | 11 | 511836 | 4094684 | 2 | 11 | 0 | 184 | 4191868 | 2 | 11 | 0 | 1 | 192 |
| 106 | 6A | 47 | 2F | 2 | 12 | 511859 | 4094868 | 2 | 12 | 0 | 184 | 4192060 | 2 | 12 | 0 | 1 | 192 |
| 107 | 6B | 47 | 2F | 2 | 13 | 511882 | 4095052 | 2 | 13 | 0 | 184 | 4192252 | 2 | 13 | 0 | 1 | 192 |
| 108 | 6C | 47 | 2F | 2 | 14 | 511905 | 4095236 | 2 | 14 | 0 | 184 | 4192444 | 2 | 14 | 0 | 1 | 192 |
| 109 | 6D | 47 | 2F | 2 | 15 | 511928 | 4095420 | 2 | 15 | 0 | 184 | 4192636 | 2 | 15 | 0 | 1 | 192 |
| 110 | 6E | 47 | 2F | 2 | 16 | 511951 | 4095604 | 2 | 16 | 0 | 184 | 4192828 | 2 | 16 | 0 | 1 | 192 |
| 111 | 6F | 47 | 2F | 2 | 17 | 511974 | 4095788 | 2 | 17 | 0 | 184 | 4193020 | 2 | 17 | 0 | 1 | 192 |
| 112 | 70 | 47 | 2F | 2 | 18 | 511999 | 4095988 | 2 | 18 | 0 | 200 | 4193228 | 2 | 18 | 0 | 1 | 208 |
| 113 | 71 | 47 | 2F | 2 | 19 | 512024 | 4096188 | 2 | 19 | 0 | 200 | 4193436 | 2 | 19 | 0 | 1 | 208 |
| 114 | 72 | 47 | 2F | 2 | 20 | 512049 | 4096388 | 2 | 20 | 0 | 200 | 4193644 | 2 | 20 | 0 | 1 | 208 |
| 115 | 73 | 47 | 2F | 2 | 21 | 512074 | 4096588 | 2 | 21 | 0 | 200 | 4193852 | 2 | 21 | 0 | 1 | 208 |
| 116 | 74 | 47 | 2F | 2 | 22 | 512099 | 4096788 | 2 | 22 | 0 | 200 | 4194060 | 2 | 22 | 0 | 1 | 208 |
| 117 | 75 | 47 | 2F | 2 | 23 | 512124 | 4096988 | 2 | 23 | 0 | 200 | 4194268 | 2 | 23 | 0 | 1 | 208 |
| 118 | 76 | 47 | 2F | 2 | 24 | 512149 | 4097188 | 2 | 24 | 0 | 200 | 4194476 | 2 | 24 | 0 | 1 | 208 |

| | | | | | | | | | | | | | | | | | |
|---|---|---|---|---|---|---|---|---|---|---|---|---|---|---|---|---|---|
| 119 | 77 | 47 | 2F | 2 | 25 | 512174 | 4097388 | 2 | 25 | 0 | 200 | 4194684 | 2 | 25 | 0 | 1 | 208 |
| 120 | 78 | 47 | 2F | 2 | 26 | 512199 | 4097588 | 2 | 26 | 0 | 200 | 4194892 | 2 | 26 | 0 | 1 | 208 |
| 121 | 79 | 47 | 2F | 2 | 27 | 512224 | 4097788 | 2 | 27 | 0 | 200 | 4195100 | 2 | 27 | 0 | 1 | 208 |
| 122 | 7A | 47 | 2F | 2 | 28 | 512249 | 4097988 | 2 | 28 | 0 | 200 | 4195308 | 2 | 28 | 0 | 1 | 208 |
| 123 | 7B | 47 | 2F | 2 | 29 | 512274 | 4098188 | 2 | 29 | 0 | 200 | 4195516 | 2 | 29 | 0 | 1 | 208 |
| 124 | 7C | 47 | 2F | 2 | 30 | 512299 | 4098388 | 2 | 30 | 0 | 200 | 4195724 | 2 | 30 | 0 | 1 | 208 |
| 125 | 7D | 47 | 2F | 2 | 31 | 512324 | 4098588 | 2 | 31 | 0 | 200 | 4195932 | 2 | 31 | 0 | 1 | 208 |
| 126 | 7E | 47 | 2F | 2 | 32 | 512349 | 4098788 | 2 | 32 | 0 | 200 | 4196140 | 2 | 32 | 0 | 1 | 208 |
| 127 | 7F | 47 | 2F | 2 | 33 | 512374 | 4098988 | 2 | 33 | 0 | 200 | 4196348 | 2 | 33 | 0 | 1 | 208 |
| 128 | 80 | 47 | 2F | 2 | 34 | 512399 | 4099188 | 2 | 34 | 0 | 200 | 4196556 | 2 | 34 | 0 | 1 | 208 |
| 129 | 81 | 47 | 2F | 2 | 35 | 512424 | 4099388 | 2 | 35 | 0 | 200 | 4196764 | 2 | 35 | 0 | 1 | 208 |
| 130 | 82 | 47 | 2F | 2 | 36 | 512449 | 4099588 | 2 | 36 | 0 | 200 | 4196972 | 2 | 36 | 0 | 1 | 208 |
| 131 | 83 | 47 | 2F | 2 | 37 | 512474 | 4099788 | 2 | 37 | 0 | 200 | 4197180 | 2 | 37 | 0 | 1 | 208 |
| 132 | 84 | 47 | 2F | 2 | 38 | 512499 | 4099988 | 2 | 38 | 0 | 200 | 4197388 | 2 | 38 | 0 | 1 | 208 |
| 133 | 85 | 47 | 2F | 2 | 39 | 512524 | 4100188 | 2 | 39 | 0 | 200 | 4197596 | 2 | 39 | 0 | 1 | 208 |
| 134 | 86 | 47 | 2F | 2 | 40 | 512549 | 4100388 | 2 | 40 | 0 | 200 | 4197804 | 2 | 40 | 0 | 1 | 208 |
| 135 | 87 | 47 | 2F | 2 | 41 | 512574 | 4100588 | 2 | 41 | 0 | 200 | 4198012 | 2 | 41 | 0 | 1 | 208 |
| 136 | 88 | 47 | 2F | 2 | 42 | 512599 | 4100788 | 2 | 42 | 0 | 200 | 4198220 | 2 | 42 | 0 | 1 | 208 |
| 137 | 89 | 47 | 2F | 2 | 43 | 512624 | 4100988 | 2 | 43 | 0 | 200 | 4198428 | 2 | 43 | 0 | 1 | 208 |
| 138 | 8A | 47 | 2F | 2 | 44 | 512649 | 4101188 | 2 | 44 | 0 | 200 | 4198636 | 2 | 44 | 0 | 1 | 208 |
| 139 | 8B | 47 | 2F | 2 | 45 | 512674 | 4101388 | 2 | 45 | 0 | 200 | 4198844 | 2 | 45 | 0 | 1 | 208 |
| 140 | 8C | 47 | 2F | 2 | 46 | 512699 | 4101588 | 2 | 46 | 0 | 200 | 4199052 | 2 | 46 | 0 | 1 | 208 |
| 141 | 8D | 47 | 2F | 3 | 0 | 512722 | 4101772 | 3 | 0 | 0 | 184 | 4199244 | 3 | 0 | 0 | 1 | 192 |
| 142 | 8E | 47 | 2F | 3 | 1 | 512750 | 4101996 | 3 | 1 | 0 | 224 | 4199476 | 3 | 1 | 0 | 1 | 232 |
| 143 | 8F | 47 | 2F | 3 | 2 | 512778 | 4102220 | 3 | 2 | 0 | 224 | 4199708 | 3 | 2 | 0 | 1 | 232 |
| 144 | 90 | 47 | 2F | 3 | 3 | 512803 | 4102420 | 3 | 3 | 0 | 200 | 4199916 | 3 | 3 | 0 | 1 | 208 |
| 145 | 91 | 47 | 2F | 3 | 4 | 512828 | 4102620 | 3 | 4 | 0 | 200 | 4200124 | 3 | 4 | 0 | 1 | 208 |
| 146 | 92 | 47 | 2F | 3 | 5 | 512853 | 4102820 | 3 | 5 | 0 | 200 | 4200332 | 3 | 5 | 0 | 1 | 208 |
| 147 | 93 | 47 | 2F | 3 | 6 | 512878 | 4103020 | 3 | 6 | 0 | 200 | 4200540 | 3 | 6 | 0 | 1 | 208 |
| 148 | 94 | 47 | 2F | 3 | 7 | 512903 | 4103220 | 3 | 7 | 0 | 200 | 4200748 | 3 | 7 | 0 | 1 | 208 |
| 149 | 95 | 47 | 2F | 3 | 8 | 512928 | 4103420 | 3 | 8 | 0 | 200 | 4200956 | 3 | 8 | 0 | 1 | 208 |
| 150 | 96 | 47 | 2F | 3 | 9 | 512953 | 4103620 | 3 | 9 | 0 | 200 | 4201164 | 3 | 9 | 0 | 1 | 208 |
| 151 | 97 | 47 | 2F | 3 | 10 | 512978 | 4103820 | 3 | 10 | 0 | 200 | 4201372 | 3 | 10 | 0 | 1 | 208 |
| 152 | 98 | 47 | 2F | 3 | 11 | 513003 | 4104020 | 3 | 11 | 0 | 200 | 4201580 | 3 | 11 | 0 | 1 | 208 |
| 153 | 99 | 47 | 2F | 3 | 12 | 513028 | 4104220 | 3 | 12 | 0 | 200 | 4201788 | 3 | 12 | 0 | 1 | 208 |
| 154 | 9A | 47 | 2F | 3 | 13 | 513053 | 4104420 | 3 | 13 | 0 | 200 | 4201996 | 3 | 13 | 0 | 1 | 208 |
| 155 | 9B | 47 | 2F | 3 | 14 | 513078 | 4104620 | 3 | 14 | 0 | 200 | 4202204 | 3 | 14 | 0 | 1 | 208 |
| 156 | 9C | 47 | 2F | 3 | 15 | 513103 | 4104820 | 3 | 15 | 0 | 200 | 4202412 | 3 | 15 | 0 | 1 | 208 |
| 157 | 9D | 47 | 2F | 3 | 16 | 513128 | 4105020 | 3 | 16 | 0 | 200 | 4202620 | 3 | 16 | 0 | 1 | 208 |
| 158 | 9E | 47 | 2F | 3 | 17 | 513153 | 4105220 | 3 | 17 | 0 | 200 | 4202828 | 3 | 17 | 0 | 1 | 208 |
| 159 | 9F | 47 | 2F | 3 | 18 | 513178 | 4105420 | 3 | 18 | 0 | 200 | 4203036 | 3 | 18 | 0 | 1 | 208 |
| 160 | A0 | 47 | 2F | 3 | 19 | 513205 | 4105636 | 3 | 19 | 0 | 216 | 4203260 | 3 | 19 | 0 | 1 | 224 |
| 161 | A1 | 47 | 2F | 3 | 20 | 513232 | 4105852 | 3 | 20 | 0 | 216 | 4203484 | 3 | 20 | 0 | 1 | 224 |
| 162 | A2 | 47 | 2F | 3 | 21 | 513259 | 4106068 | 3 | 21 | 0 | 216 | 4203708 | 3 | 21 | 0 | 1 | 224 |
| 163 | A3 | 47 | 2F | 3 | 22 | 513286 | 4106284 | 3 | 22 | 0 | 216 | 4203932 | 3 | 22 | 0 | 1 | 224 |
| 164 | A4 | 47 | 2F | 3 | 23 | 513313 | 4106500 | 3 | 23 | 0 | 216 | 4204156 | 3 | 23 | 0 | 1 | 224 |
| 165 | A5 | 47 | 2F | 3 | 24 | 513340 | 4106716 | 3 | 24 | 0 | 216 | 4204380 | 3 | 24 | 0 | 1 | 224 |
| 166 | A6 | 47 | 2F | 3 | 25 | 513367 | 4106932 | 3 | 25 | 0 | 216 | 4204604 | 3 | 25 | 0 | 1 | 224 |
| 167 | A7 | 47 | 2F | 3 | 26 | 513394 | 4107148 | 3 | 26 | 0 | 216 | 4204828 | 3 | 26 | 0 | 1 | 224 |
| 168 | A8 | 47 | 2F | 3 | 27 | 513421 | 4107364 | 3 | 27 | 0 | 216 | 4205052 | 3 | 27 | 0 | 1 | 224 |
| 169 | A9 | 47 | 2F | 3 | 28 | 513448 | 4107580 | 3 | 28 | 0 | 216 | 4205276 | 3 | 28 | 0 | 1 | 224 |
| 170 | AA | 47 | 2F | 3 | 29 | 513475 | 4107796 | 3 | 29 | 0 | 216 | 4205500 | 3 | 29 | 0 | 1 | 224 |
| 171 | AB | 47 | 2F | 3 | 30 | 513502 | 4108012 | 3 | 30 | 0 | 216 | 4205724 | 3 | 30 | 0 | 1 | 224 |
| 172 | AC | 47 | 2F | 3 | 31 | 513529 | 4108228 | 3 | 31 | 0 | 216 | 4205948 | 3 | 31 | 0 | 1 | 224 |
| 173 | AD | 47 | 2F | 3 | 32 | 513556 | 4108444 | 3 | 32 | 0 | 216 | 4206172 | 3 | 32 | 0 | 1 | 224 |
| 174 | AE | 47 | 2F | 3 | 33 | 513583 | 4108660 | 3 | 33 | 0 | 216 | 4206396 | 3 | 33 | 0 | 1 | 224 |
| 175 | AF | 47 | 2F | 3 | 34 | 513610 | 4108876 | 3 | 34 | 0 | 216 | 4206620 | 3 | 34 | 0 | 1 | 224 |
| 176 | B0 | 47 | 2F | 3 | 35 | 513637 | 4109092 | 3 | 35 | 0 | 216 | 4206844 | 3 | 35 | 0 | 1 | 224 |
| 177 | B1 | 47 | 2F | 3 | 36 | 513664 | 4109308 | 3 | 36 | 0 | 216 | 4207068 | 3 | 36 | 0 | 1 | 224 |
| 178 | B2 | 47 | 2F | 3 | 37 | 513691 | 4109524 | 3 | 37 | 0 | 216 | 4207292 | 3 | 37 | 0 | 1 | 224 |
| 179 | B3 | 47 | 2F | 3 | 38 | 513718 | 4109740 | 3 | 38 | 0 | 216 | 4207516 | 3 | 38 | 0 | 1 | 224 |
| 180 | B4 | 47 | 2F | 3 | 39 | 513745 | 4109956 | 3 | 39 | 0 | 216 | 4207740 | 3 | 39 | 0 | 1 | 224 |
| 181 | B5 | 47 | 2F | 3 | 40 | 513772 | 4110172 | 3 | 40 | 0 | 216 | 4207964 | 3 | 40 | 0 | 1 | 224 |
| 182 | B6 | 47 | 2F | 3 | 41 | 513799 | 4110388 | 3 | 41 | 0 | 216 | 4208188 | 3 | 41 | 0 | 1 | 224 |
| 183 | B7 | 47 | 2F | 3 | 42 | 513826 | 4110604 | 3 | 42 | 0 | 216 | 4208412 | 3 | 42 | 0 | 1 | 224 |
| 184 | B8 | 47 | 2F | 3 | 43 | 513853 | 4110820 | 3 | 43 | 0 | 216 | 4208636 | 3 | 43 | 0 | 1 | 224 |
| 185 | B9 | 47 | 2F | 3 | 44 | 513880 | 4111036 | 3 | 44 | 0 | 216 | 4208860 | 3 | 44 | 0 | 1 | 224 |
| 186 | BA | 47 | 2F | 3 | 45 | 513907 | 4111252 | 3 | 45 | 0 | 216 | 4209084 | 3 | 45 | 0 | 1 | 224 |
| 187 | BB | 47 | 2F | 3 | 46 | 513934 | 4111468 | 3 | 46 | 0 | 216 | 4209308 | 3 | 46 | 0 | 1 | 224 |
| 188 | BC | 47 | 2F | 4 | 0 | 513959 | 4111668 | 4 | 0 | 0 | 200 | 4209516 | 4 | 0 | 0 | 1 | 208 |
| 189 | BD | 47 | 2F | 4 | 1 | 513989 | 4111908 | 4 | 1 | 0 | 240 | 4209764 | 4 | 1 | 0 | 1 | 248 |
| 190 | BE | 47 | 2F | 4 | 2 | 514019 | 4112148 | 4 | 2 | 0 | 240 | 4210012 | 4 | 2 | 0 | 1 | 248 |
| 191 | BF | 47 | 2F | 4 | 3 | 514049 | 4112388 | 4 | 3 | 0 | 240 | 4210260 | 4 | 3 | 0 | 1 | 248 |
| 192 | C0 | 47 | 2F | 4 | 4 | 514076 | 4112604 | 4 | 4 | 0 | 216 | 4210484 | 4 | 4 | 0 | 1 | 224 |
| 193 | C1 | 47 | 2F | 4 | 5 | 514103 | 4112820 | 4 | 5 | 0 | 216 | 4210708 | 4 | 5 | 0 | 1 | 224 |
| 194 | C2 | 47 | 2F | 4 | 6 | 514130 | 4113036 | 4 | 6 | 0 | 216 | 4210932 | 4 | 6 | 0 | 1 | 224 |
| 195 | C3 | 47 | 2F | 4 | 7 | 514157 | 4113252 | 4 | 7 | 0 | 216 | 4211156 | 4 | 7 | 0 | 1 | 224 |
| 196 | C4 | 47 | 2F | 4 | 8 | 514184 | 4113468 | 4 | 8 | 0 | 216 | 4211380 | 4 | 8 | 0 | 1 | 224 |
| 197 | C5 | 47 | 2F | 4 | 9 | 514211 | 4113684 | 4 | 9 | 0 | 216 | 4211604 | 4 | 9 | 0 | 1 | 224 |
| 198 | C6 | 47 | 2F | 4 | 10 | 514238 | 4113900 | 4 | 10 | 0 | 216 | 4211828 | 4 | 10 | 0 | 1 | 224 |
| 199 | C7 | 47 | 2F | 4 | 11 | 514265 | 4114116 | 4 | 11 | 0 | 216 | 4212052 | 4 | 11 | 0 | 1 | 224 |
| 200 | C8 | 47 | 2F | 4 | 12 | 514292 | 4114332 | 4 | 12 | 0 | 216 | 4212276 | 4 | 12 | 0 | 1 | 224 |
| 201 | C9 | 47 | 2F | 4 | 13 | 514319 | 4114548 | 4 | 13 | 0 | 216 | 4212500 | 4 | 13 | 0 | 1 | 224 |
| 202 | CA | 47 | 2F | 4 | 14 | 514346 | 4114764 | 4 | 14 | 0 | 216 | 4212724 | 4 | 14 | 0 | 1 | 224 |
| 203 | CB | 47 | 2F | 4 | 15 | 514373 | 4114980 | 4 | 15 | 0 | 216 | 4212948 | 4 | 15 | 0 | 1 | 224 |
| 204 | CC | 47 | 2F | 4 | 16 | 514400 | 4115196 | 4 | 16 | 0 | 216 | 4213172 | 4 | 16 | 0 | 1 | 224 |
| 205 | CD | 47 | 2F | 4 | 17 | 514427 | 4115412 | 4 | 17 | 0 | 216 | 4213396 | 4 | 17 | 0 | 1 | 224 |
| 206 | CE | 47 | 2F | 4 | 18 | 514454 | 4115628 | 4 | 18 | 0 | 216 | 4213620 | 4 | 18 | 0 | 1 | 224 |
| 207 | CF | 47 | 2F | 4 | 19 | 514481 | 4115844 | 4 | 19 | 0 | 216 | 4213844 | 4 | 19 | 0 | 1 | 224 |
| 208 | D0 | 47 | 2F | 4 | 20 | 514510 | 4116076 | 4 | 20 | 0 | 232 | 4214084 | 4 | 20 | 0 | 1 | 240 |
| 209 | D1 | 47 | 2F | 4 | 21 | 514539 | 4116308 | 4 | 21 | 0 | 232 | 4214324 | 4 | 21 | 0 | 1 | 240 |
| 210 | D2 | 47 | 2F | 4 | 22 | 514568 | 4116540 | 4 | 22 | 0 | 232 | 4214564 | 4 | 22 | 0 | 1 | 240 |
| 211 | D3 | 47 | 2F | 4 | 23 | 514597 | 4116772 | 4 | 23 | 0 | 232 | 4214804 | 4 | 23 | 0 | 1 | 240 |
| 212 | D4 | 47 | 2F | 4 | 24 | 514626 | 4117004 | 4 | 24 | 0 | 232 | 4215044 | 4 | 24 | 0 | 1 | 240 |
| 213 | D5 | 47 | 2F | 4 | 25 | 514655 | 4117236 | 4 | 25 | 0 | 232 | 4215284 | 4 | 25 | 0 | 1 | 240 |
| 214 | D6 | 47 | 2F | 4 | 26 | 514684 | 4117468 | 4 | 26 | 0 | 232 | 4215524 | 4 | 26 | 0 | 1 | 240 |
| 215 | D7 | 47 | 2F | 4 | 27 | 514713 | 4117700 | 4 | 27 | 0 | 232 | 4215764 | 4 | 27 | 0 | 1 | 240 |
| 216 | D8 | 47 | 2F | 4 | 28 | 514742 | 4117932 | 4 | 28 | 0 | 232 | 4216004 | 4 | 28 | 0 | 1 | 240 |
| 217 | D9 | 47 | 2F | 4 | 29 | 514771 | 4118164 | 4 | 29 | 0 | 232 | 4216244 | 4 | 29 | 0 | 1 | 240 |
| 218 | DA | 47 | 2F | 4 | 30 | 514800 | 4118396 | 4 | 30 | 0 | 232 | 4216484 | 4 | 30 | 0 | 1 | 240 |
| 219 | DB | 47 | 2F | 4 | 31 | 514829 | 4118628 | 4 | 31 | 0 | 232 | 4216724 | 4 | 31 | 0 | 1 | 240 |
| 220 | DC | 47 | 2F | 4 | 32 | 514858 | 4118860 | 4 | 32 | 0 | 232 | 4216964 | 4 | 32 | 0 | 1 | 240 |
| 221 | DD | 47 | 2F | 4 | 33 | 514887 | 4119092 | 4 | 33 | 0 | 232 | 4217204 | 4 | 33 | 0 | 1 | 240 |

| | | | | | | | | | | | | | | | | | |
|---|---|---|---|---|---|---|---|---|---|---|---|---|---|---|---|---|---|
| 222 | DE | 47 | 2F | 4 | 34 | 514916 | 4119324 | 4 | 34 | 0 | 232 | 4217444 | 4 | 34 | 0 | 1 | 240 |
| 223 | DF | 47 | 2F | 4 | 35 | 514945 | 4119556 | 4 | 35 | 0 | 232 | 4217684 | 4 | 35 | 0 | 1 | 240 |
| 224 | E0 | 47 | 2F | 4 | 36 | 514974 | 4119788 | 4 | 36 | 0 | 232 | 4217924 | 4 | 36 | 0 | 1 | 240 |
| 225 | E1 | 47 | 2F | 4 | 37 | 515003 | 4120020 | 4 | 37 | 0 | 232 | 4218164 | 4 | 37 | 0 | 1 | 240 |
| 226 | E2 | 47 | 2F | 4 | 38 | 515032 | 4120252 | 4 | 38 | 0 | 232 | 4218404 | 4 | 38 | 0 | 1 | 240 |
| 227 | E3 | 47 | 2F | 4 | 39 | 515061 | 4120484 | 4 | 39 | 0 | 232 | 4218644 | 4 | 39 | 0 | 1 | 240 |
| 228 | E4 | 47 | 2F | 4 | 40 | 515090 | 4120716 | 4 | 40 | 0 | 232 | 4218884 | 4 | 40 | 0 | 1 | 240 |
| 229 | E5 | 47 | 2F | 4 | 41 | 515119 | 4120948 | 4 | 41 | 0 | 232 | 4219124 | 4 | 41 | 0 | 1 | 240 |
| 230 | E6 | 47 | 2F | 4 | 42 | 515148 | 4121180 | 4 | 42 | 0 | 232 | 4219364 | 4 | 42 | 0 | 1 | 240 |
| 231 | E7 | 47 | 2F | 4 | 43 | 515177 | 4121412 | 4 | 43 | 0 | 232 | 4219604 | 4 | 43 | 0 | 1 | 240 |
| 232 | E8 | 47 | 2F | 4 | 44 | 515206 | 4121644 | 4 | 44 | 0 | 232 | 4219844 | 4 | 44 | 0 | 1 | 240 |
| 233 | E9 | 47 | 2F | 4 | 45 | 515235 | 4121876 | 4 | 45 | 0 | 232 | 4220084 | 4 | 45 | 0 | 1 | 240 |
| 234 | EA | 47 | 2F | 4 | 46 | 515264 | 4122108 | 4 | 46 | 0 | 232 | 4220324 | 4 | 46 | 0 | 1 | 240 |
| 235 | EB | 47 | 2F | 5 | 0 | 515291 | 4122324 | 5 | 0 | 0 | 216 | 4220548 | 5 | 0 | 0 | 1 | 224 |
| 236 | EC | 47 | 2F | 5 | 1 | 515323 | 4122580 | 5 | 1 | 0 | 256 | 4220812 | 5 | 1 | 0 | 1 | 264 |
| 237 | ED | 47 | 2F | 5 | 2 | 515355 | 4122836 | 5 | 2 | 0 | 256 | 4221076 | 5 | 2 | 0 | 1 | 264 |
| 238 | EE | 47 | 2F | 5 | 3 | 515387 | 4123092 | 5 | 3 | 0 | 256 | 4221340 | 5 | 3 | 0 | 1 | 264 |
| 239 | EF | 47 | 2F | 5 | 4 | 515419 | 4123348 | 5 | 4 | 0 | 256 | 4221604 | 5 | 4 | 0 | 1 | 264 |
| 240 | F0 | 47 | 2F | 5 | 5 | 515448 | 4123580 | 5 | 5 | 0 | 232 | 4221844 | 5 | 5 | 0 | 1 | 240 |
| 241 | F1 | 47 | 2F | 5 | 6 | 515477 | 4123812 | 5 | 6 | 0 | 232 | 4222084 | 5 | 6 | 0 | 1 | 240 |
| 242 | F2 | 47 | 2F | 5 | 7 | 515506 | 4124044 | 5 | 7 | 0 | 232 | 4222324 | 5 | 7 | 0 | 1 | 240 |
| 243 | F3 | 47 | 2F | 5 | 8 | 515535 | 4124276 | 5 | 8 | 0 | 232 | 4222564 | 5 | 8 | 0 | 1 | 240 |
| 244 | F4 | 47 | 2F | 5 | 9 | 515564 | 4124508 | 5 | 9 | 0 | 232 | 4222804 | 5 | 9 | 0 | 1 | 240 |
| 245 | F5 | 47 | 2F | 5 | 10 | 515593 | 4124740 | 5 | 10 | 0 | 232 | 4223044 | 5 | 10 | 0 | 1 | 240 |
| 246 | F6 | 47 | 2F | 5 | 11 | 515622 | 4124972 | 5 | 11 | 0 | 232 | 4223284 | 5 | 11 | 0 | 1 | 240 |
| 247 | F7 | 47 | 2F | 5 | 12 | 515651 | 4125204 | 5 | 12 | 0 | 232 | 4223524 | 5 | 12 | 0 | 1 | 240 |
| 248 | F8 | 47 | 2F | 5 | 13 | 515680 | 4125436 | 5 | 13 | 0 | 232 | 4223764 | 5 | 13 | 0 | 1 | 240 |
| 249 | F9 | 47 | 2F | 5 | 14 | 515709 | 4125668 | 5 | 14 | 0 | 232 | 4224004 | 5 | 14 | 0 | 1 | 240 |
| 250 | FA | 47 | 2F | 5 | 15 | 515738 | 4125900 | 5 | 15 | 0 | 232 | 4224244 | 5 | 15 | 0 | 1 | 240 |
| 251 | FB | 47 | 2F | 5 | 16 | 515767 | 4126132 | 5 | 16 | 0 | 232 | 4224484 | 5 | 16 | 0 | 1 | 240 |
| 252 | FC | 47 | 2F | 5 | 17 | 515796 | 4126364 | 5 | 17 | 0 | 232 | 4224724 | 5 | 17 | 0 | 1 | 240 |
| 253 | FD | 47 | 2F | 5 | 18 | 515825 | 4126596 | 5 | 18 | 0 | 232 | 4224964 | 5 | 18 | 0 | 1 | 240 |
| 254 | FE | 47 | 2F | 5 | 19 | 515854 | 4126828 | 5 | 19 | 0 | 232 | 4225204 | 5 | 19 | 0 | 1 | 240 |
| 255 | FF | 47 | 2F | 5 | 20 | 515883 | 4127060 | 5 | 20 | 0 | 232 | 4225444 | 5 | 20 | 0 | 1 | 240 |
| 0 | 00 | 48 | 30 | 0 | 0 | 515895 | 4127156 | 0 | 0 | 0 | 96 | 4225548 | 0 | 0 | 0 | 1 | 104 |
| 1 | 01 | 48 | 30 | 0 | 1 | 515909 | 4127268 | 0 | 1 | 0 | 112 | 4225668 | 0 | 1 | 0 | 1 | 120 |
| 2 | 02 | 48 | 30 | 0 | 2 | 515923 | 4127380 | 0 | 2 | 0 | 112 | 4225788 | 0 | 2 | 0 | 1 | 120 |
| 3 | 03 | 48 | 30 | 0 | 3 | 515937 | 4127492 | 0 | 3 | 0 | 112 | 4225908 | 0 | 3 | 0 | 1 | 120 |
| 4 | 04 | 48 | 30 | 0 | 4 | 515951 | 4127604 | 0 | 4 | 0 | 112 | 4226028 | 0 | 4 | 0 | 1 | 120 |
| 5 | 05 | 48 | 30 | 0 | 5 | 515965 | 4127716 | 0 | 5 | 0 | 112 | 4226148 | 0 | 5 | 0 | 1 | 120 |
| 6 | 06 | 48 | 30 | 0 | 6 | 515979 | 4127828 | 0 | 6 | 0 | 112 | 4226268 | 0 | 6 | 0 | 1 | 120 |
| 7 | 07 | 48 | 30 | 0 | 7 | 515993 | 4127940 | 0 | 7 | 0 | 112 | 4226388 | 0 | 7 | 0 | 1 | 120 |
| 8 | 08 | 48 | 30 | 0 | 8 | 516007 | 4128052 | 0 | 8 | 0 | 112 | 4226508 | 0 | 8 | 0 | 1 | 120 |
| 9 | 09 | 48 | 30 | 0 | 9 | 516021 | 4128164 | 0 | 9 | 0 | 112 | 4226628 | 0 | 9 | 0 | 1 | 120 |
| 10 | 0A | 48 | 30 | 0 | 10 | 516035 | 4128276 | 0 | 10 | 0 | 112 | 4226748 | 0 | 10 | 0 | 1 | 120 |
| 11 | 0B | 48 | 30 | 0 | 11 | 516049 | 4128388 | 0 | 11 | 0 | 112 | 4226868 | 0 | 11 | 0 | 1 | 120 |
| 12 | 0C | 48 | 30 | 0 | 12 | 516063 | 4128500 | 0 | 12 | 0 | 112 | 4226988 | 0 | 12 | 0 | 1 | 120 |
| 13 | 0D | 48 | 30 | 0 | 13 | 516077 | 4128612 | 0 | 13 | 0 | 112 | 4227108 | 0 | 13 | 0 | 1 | 120 |
| 14 | 0E | 48 | 30 | 0 | 14 | 516091 | 4128724 | 0 | 14 | 0 | 112 | 4227228 | 0 | 14 | 0 | 1 | 120 |
| 15 | 0F | 48 | 30 | 0 | 15 | 516105 | 4128836 | 0 | 15 | 0 | 112 | 4227348 | 0 | 15 | 0 | 1 | 120 |
| 16 | 10 | 48 | 30 | 0 | 16 | 516119 | 4128948 | 0 | 16 | 0 | 112 | 4227468 | 0 | 16 | 0 | 1 | 120 |
| 17 | 11 | 48 | 30 | 0 | 17 | 516133 | 4129060 | 0 | 17 | 0 | 112 | 4227588 | 0 | 17 | 0 | 1 | 120 |
| 18 | 12 | 48 | 30 | 0 | 18 | 516147 | 4129172 | 0 | 18 | 0 | 112 | 4227708 | 0 | 18 | 0 | 1 | 120 |
| 19 | 13 | 48 | 30 | 0 | 19 | 516161 | 4129284 | 0 | 19 | 0 | 112 | 4227828 | 0 | 19 | 0 | 1 | 120 |
| 20 | 14 | 48 | 30 | 0 | 20 | 516175 | 4129396 | 0 | 20 | 0 | 112 | 4227948 | 0 | 20 | 0 | 1 | 120 |
| 21 | 15 | 48 | 30 | 0 | 21 | 516189 | 4129508 | 0 | 21 | 0 | 112 | 4228068 | 0 | 21 | 0 | 1 | 120 |
| 22 | 16 | 48 | 30 | 0 | 22 | 516203 | 4129620 | 0 | 22 | 0 | 112 | 4228188 | 0 | 22 | 0 | 1 | 120 |
| 23 | 17 | 48 | 30 | 0 | 23 | 516217 | 4129732 | 0 | 23 | 0 | 112 | 4228308 | 0 | 23 | 0 | 1 | 120 |
| 24 | 18 | 48 | 30 | 0 | 24 | 516231 | 4129844 | 0 | 24 | 0 | 112 | 4228428 | 0 | 24 | 0 | 1 | 120 |
| 25 | 19 | 48 | 30 | 0 | 25 | 516245 | 4129956 | 0 | 25 | 0 | 112 | 4228548 | 0 | 25 | 0 | 1 | 120 |
| 26 | 1A | 48 | 30 | 0 | 26 | 516259 | 4130068 | 0 | 26 | 0 | 112 | 4228668 | 0 | 26 | 0 | 1 | 120 |
| 27 | 1B | 48 | 30 | 0 | 27 | 516273 | 4130180 | 0 | 27 | 0 | 112 | 4228788 | 0 | 27 | 0 | 1 | 120 |
| 28 | 1C | 48 | 30 | 0 | 28 | 516287 | 4130292 | 0 | 28 | 0 | 112 | 4228908 | 0 | 28 | 0 | 1 | 120 |
| 29 | 1D | 48 | 30 | 0 | 29 | 516301 | 4130404 | 0 | 29 | 0 | 112 | 4229028 | 0 | 29 | 0 | 1 | 120 |
| 30 | 1E | 48 | 30 | 0 | 30 | 516315 | 4130516 | 0 | 30 | 0 | 112 | 4229148 | 0 | 30 | 0 | 1 | 120 |
| 31 | 1F | 48 | 30 | 0 | 31 | 516329 | 4130628 | 0 | 31 | 0 | 112 | 4229268 | 0 | 31 | 0 | 1 | 120 |
| 32 | 20 | 48 | 30 | 0 | 32 | 516343 | 4130740 | 0 | 32 | 0 | 112 | 4229388 | 0 | 32 | 0 | 1 | 120 |
| 33 | 21 | 48 | 30 | 0 | 33 | 516357 | 4130852 | 0 | 33 | 0 | 112 | 4229508 | 0 | 33 | 0 | 1 | 120 |
| 34 | 22 | 48 | 30 | 0 | 34 | 516371 | 4130964 | 0 | 34 | 0 | 112 | 4229628 | 0 | 34 | 0 | 1 | 120 |
| 35 | 23 | 48 | 30 | 0 | 35 | 516385 | 4131076 | 0 | 35 | 0 | 112 | 4229748 | 0 | 35 | 0 | 1 | 120 |
| 36 | 24 | 48 | 30 | 0 | 36 | 516399 | 4131188 | 0 | 36 | 0 | 112 | 4229868 | 0 | 36 | 0 | 1 | 120 |
| 37 | 25 | 48 | 30 | 0 | 37 | 516413 | 4131300 | 0 | 37 | 0 | 112 | 4229988 | 0 | 37 | 0 | 1 | 120 |
| 38 | 26 | 48 | 30 | 0 | 38 | 516427 | 4131412 | 0 | 38 | 0 | 112 | 4230108 | 0 | 38 | 0 | 1 | 120 |
| 39 | 27 | 48 | 30 | 0 | 39 | 516441 | 4131524 | 0 | 39 | 0 | 112 | 4230228 | 0 | 39 | 0 | 1 | 120 |
| 40 | 28 | 48 | 30 | 0 | 40 | 516455 | 4131636 | 0 | 40 | 0 | 112 | 4230348 | 0 | 40 | 0 | 1 | 120 |
| 41 | 29 | 48 | 30 | 0 | 41 | 516469 | 4131748 | 0 | 41 | 0 | 112 | 4230468 | 0 | 41 | 0 | 1 | 120 |
| 42 | 2A | 48 | 30 | 0 | 42 | 516483 | 4131860 | 0 | 42 | 0 | 112 | 4230588 | 0 | 42 | 0 | 1 | 120 |
| 43 | 2B | 48 | 30 | 0 | 43 | 516497 | 4131972 | 0 | 43 | 0 | 112 | 4230708 | 0 | 43 | 0 | 1 | 120 |
| 44 | 2C | 48 | 30 | 0 | 44 | 516511 | 4132084 | 0 | 44 | 0 | 112 | 4230828 | 0 | 44 | 0 | 1 | 120 |
| 45 | 2D | 48 | 30 | 0 | 45 | 516525 | 4132196 | 0 | 45 | 0 | 112 | 4230948 | 0 | 45 | 0 | 1 | 120 |
| 46 | 2E | 48 | 30 | 0 | 46 | 516539 | 4132308 | 0 | 46 | 0 | 112 | 4231068 | 0 | 46 | 0 | 1 | 120 |
| 47 | 2F | 48 | 30 | 0 | 47 | 516553 | 4132420 | 0 | 47 | 0 | 112 | 4231188 | 0 | 47 | 0 | 1 | 120 |
| 48 | 30 | 48 | 30 | 1 | 0 | 516572 | 4132572 | 1 | 0 | 0 | 152 | 4231348 | 1 | 0 | 0 | 1 | 160 |
| 49 | 31 | 48 | 30 | 1 | 1 | 516593 | 4132740 | 1 | 1 | 0 | 168 | 4231524 | 1 | 1 | 0 | 1 | 176 |
| 50 | 32 | 48 | 30 | 1 | 2 | 516614 | 4132908 | 1 | 2 | 0 | 168 | 4231700 | 1 | 2 | 0 | 1 | 176 |
| 51 | 33 | 48 | 30 | 1 | 3 | 516635 | 4133076 | 1 | 3 | 0 | 168 | 4231876 | 1 | 3 | 0 | 1 | 176 |
| 52 | 34 | 48 | 30 | 1 | 4 | 516656 | 4133244 | 1 | 4 | 0 | 168 | 4232052 | 1 | 4 | 0 | 1 | 176 |
| 53 | 35 | 48 | 30 | 1 | 5 | 516677 | 4133412 | 1 | 5 | 0 | 168 | 4232228 | 1 | 5 | 0 | 1 | 176 |
| 54 | 36 | 48 | 30 | 1 | 6 | 516698 | 4133580 | 1 | 6 | 0 | 168 | 4232404 | 1 | 6 | 0 | 1 | 176 |
| 55 | 37 | 48 | 30 | 1 | 7 | 516719 | 4133748 | 1 | 7 | 0 | 168 | 4232580 | 1 | 7 | 0 | 1 | 176 |
| 56 | 38 | 48 | 30 | 1 | 8 | 516740 | 4133916 | 1 | 8 | 0 | 168 | 4232756 | 1 | 8 | 0 | 1 | 176 |
| 57 | 39 | 48 | 30 | 1 | 9 | 516761 | 4134084 | 1 | 9 | 0 | 168 | 4232932 | 1 | 9 | 0 | 1 | 176 |
| 58 | 3A | 48 | 30 | 1 | 10 | 516782 | 4134252 | 1 | 10 | 0 | 168 | 4233108 | 1 | 10 | 0 | 1 | 176 |
| 59 | 3B | 48 | 30 | 1 | 11 | 516803 | 4134420 | 1 | 11 | 0 | 168 | 4233284 | 1 | 11 | 0 | 1 | 176 |
| 60 | 3C | 48 | 30 | 1 | 12 | 516824 | 4134588 | 1 | 12 | 0 | 168 | 4233460 | 1 | 12 | 0 | 1 | 176 |
| 61 | 3D | 48 | 30 | 1 | 13 | 516845 | 4134756 | 1 | 13 | 0 | 168 | 4233636 | 1 | 13 | 0 | 1 | 176 |
| 62 | 3E | 48 | 30 | 1 | 14 | 516866 | 4134924 | 1 | 14 | 0 | 168 | 4233812 | 1 | 14 | 0 | 1 | 176 |
| 63 | 3F | 48 | 30 | 1 | 15 | 516887 | 4135092 | 1 | 15 | 0 | 168 | 4233988 | 1 | 15 | 0 | 1 | 176 |
| 64 | 40 | 48 | 30 | 1 | 16 | 516910 | 4135276 | 1 | 16 | 0 | 184 | 4234180 | 1 | 16 | 0 | 1 | 192 |
| 65 | 41 | 48 | 30 | 1 | 17 | 516933 | 4135460 | 1 | 17 | 0 | 184 | 4234372 | 1 | 17 | 0 | 1 | 192 |
| 66 | 42 | 48 | 30 | 1 | 18 | 516956 | 4135644 | 1 | 18 | 0 | 184 | 4234564 | 1 | 18 | 0 | 1 | 192 |
| 67 | 43 | 48 | 30 | 1 | 19 | 516979 | 4135828 | 1 | 19 | 0 | 184 | 4234756 | 1 | 19 | 0 | 1 | 192 |
| 68 | 44 | 48 | 30 | 1 | 20 | 517002 | 4136012 | 1 | 20 | 0 | 184 | 4234948 | 1 | 20 | 0 | 1 | 192 |

| | | | | | | | | | | | | | | | | | |
|---|---|---|---|---|---|---|---|---|---|---|---|---|---|---|---|---|---|
| 69 | 45 | 48 | 30 | 1 | 21 | 517025 | 4136196 | 1 | 21 | 0 | 184 | 4235140 | 1 | 21 | 0 | 1 | 192 |
| 70 | 46 | 48 | 30 | 1 | 22 | 517048 | 4136380 | 1 | 22 | 0 | 184 | 4235332 | 1 | 22 | 0 | 1 | 192 |
| 71 | 47 | 48 | 30 | 1 | 23 | 517071 | 4136564 | 1 | 23 | 0 | 184 | 4235524 | 1 | 23 | 0 | 1 | 192 |
| 72 | 48 | 48 | 30 | 1 | 24 | 517094 | 4136748 | 1 | 24 | 0 | 184 | 4235716 | 1 | 24 | 0 | 1 | 192 |
| 73 | 49 | 48 | 30 | 1 | 25 | 517117 | 4136932 | 1 | 25 | 0 | 184 | 4235908 | 1 | 25 | 0 | 1 | 192 |
| 74 | 4A | 48 | 30 | 1 | 26 | 517140 | 4137116 | 1 | 26 | 0 | 184 | 4236100 | 1 | 26 | 0 | 1 | 192 |
| 75 | 4B | 48 | 30 | 1 | 27 | 517163 | 4137300 | 1 | 27 | 0 | 184 | 4236292 | 1 | 27 | 0 | 1 | 192 |
| 76 | 4C | 48 | 30 | 1 | 28 | 517186 | 4137484 | 1 | 28 | 0 | 184 | 4236484 | 1 | 28 | 0 | 1 | 192 |
| 77 | 4D | 48 | 30 | 1 | 29 | 517209 | 4137668 | 1 | 29 | 0 | 184 | 4236676 | 1 | 29 | 0 | 1 | 192 |
| 78 | 4E | 48 | 30 | 1 | 30 | 517232 | 4137852 | 1 | 30 | 0 | 184 | 4236868 | 1 | 30 | 0 | 1 | 192 |
| 79 | 4F | 48 | 30 | 1 | 31 | 517255 | 4138036 | 1 | 31 | 0 | 184 | 4237060 | 1 | 31 | 0 | 1 | 192 |
| 80 | 50 | 48 | 30 | 1 | 32 | 517278 | 4138220 | 1 | 32 | 0 | 184 | 4237252 | 1 | 32 | 0 | 1 | 192 |
| 81 | 51 | 48 | 30 | 1 | 33 | 517301 | 4138404 | 1 | 33 | 0 | 184 | 4237444 | 1 | 33 | 0 | 1 | 192 |
| 82 | 52 | 48 | 30 | 1 | 34 | 517324 | 4138588 | 1 | 34 | 0 | 184 | 4237636 | 1 | 34 | 0 | 1 | 192 |
| 83 | 53 | 48 | 30 | 1 | 35 | 517347 | 4138772 | 1 | 35 | 0 | 184 | 4237828 | 1 | 35 | 0 | 1 | 192 |
| 84 | 54 | 48 | 30 | 1 | 36 | 517370 | 4138956 | 1 | 36 | 0 | 184 | 4238020 | 1 | 36 | 0 | 1 | 192 |
| 85 | 55 | 48 | 30 | 1 | 37 | 517393 | 4139140 | 1 | 37 | 0 | 184 | 4238212 | 1 | 37 | 0 | 1 | 192 |
| 86 | 56 | 48 | 30 | 1 | 38 | 517416 | 4139324 | 1 | 38 | 0 | 184 | 4238404 | 1 | 38 | 0 | 1 | 192 |
| 87 | 57 | 48 | 30 | 1 | 39 | 517439 | 4139508 | 1 | 39 | 0 | 184 | 4238596 | 1 | 39 | 0 | 1 | 192 |
| 88 | 58 | 48 | 30 | 1 | 40 | 517462 | 4139692 | 1 | 40 | 0 | 184 | 4238788 | 1 | 40 | 0 | 1 | 192 |
| 89 | 59 | 48 | 30 | 1 | 41 | 517485 | 4139876 | 1 | 41 | 0 | 184 | 4238980 | 1 | 41 | 0 | 1 | 192 |
| 90 | 5A | 48 | 30 | 1 | 42 | 517508 | 4140060 | 1 | 42 | 0 | 184 | 4239172 | 1 | 42 | 0 | 1 | 192 |
| 91 | 5B | 48 | 30 | 1 | 43 | 517531 | 4140244 | 1 | 43 | 0 | 184 | 4239364 | 1 | 43 | 0 | 1 | 192 |
| 92 | 5C | 48 | 30 | 1 | 44 | 517554 | 4140428 | 1 | 44 | 0 | 184 | 4239556 | 1 | 44 | 0 | 1 | 192 |
| 93 | 5D | 48 | 30 | 1 | 45 | 517577 | 4140612 | 1 | 45 | 0 | 184 | 4239748 | 1 | 45 | 0 | 1 | 192 |
| 94 | 5E | 48 | 30 | 1 | 46 | 517600 | 4140796 | 1 | 46 | 0 | 184 | 4239940 | 1 | 46 | 0 | 1 | 192 |
| 95 | 5F | 48 | 30 | 1 | 47 | 517623 | 4140980 | 1 | 47 | 0 | 184 | 4240132 | 1 | 47 | 0 | 1 | 192 |
| 96 | 60 | 48 | 30 | 2 | 0 | 517644 | 4141148 | 2 | 0 | 0 | 168 | 4240308 | 2 | 0 | 0 | 1 | 176 |
| 97 | 61 | 48 | 30 | 2 | 1 | 517667 | 4141332 | 2 | 1 | 0 | 184 | 4240500 | 2 | 1 | 0 | 1 | 192 |
| 98 | 62 | 48 | 30 | 2 | 2 | 517690 | 4141516 | 2 | 2 | 0 | 184 | 4240692 | 2 | 2 | 0 | 1 | 192 |
| 99 | 63 | 48 | 30 | 2 | 3 | 517713 | 4141700 | 2 | 3 | 0 | 184 | 4240884 | 2 | 3 | 0 | 1 | 192 |
| 100 | 64 | 48 | 30 | 2 | 4 | 517736 | 4141884 | 2 | 4 | 0 | 184 | 4241076 | 2 | 4 | 0 | 1 | 192 |
| 101 | 65 | 48 | 30 | 2 | 5 | 517759 | 4142068 | 2 | 5 | 0 | 184 | 4241268 | 2 | 5 | 0 | 1 | 192 |
| 102 | 66 | 48 | 30 | 2 | 6 | 517782 | 4142252 | 2 | 6 | 0 | 184 | 4241460 | 2 | 6 | 0 | 1 | 192 |
| 103 | 67 | 48 | 30 | 2 | 7 | 517805 | 4142436 | 2 | 7 | 0 | 184 | 4241652 | 2 | 7 | 0 | 1 | 192 |
| 104 | 68 | 48 | 30 | 2 | 8 | 517828 | 4142620 | 2 | 8 | 0 | 184 | 4241844 | 2 | 8 | 0 | 1 | 192 |
| 105 | 69 | 48 | 30 | 2 | 9 | 517851 | 4142804 | 2 | 9 | 0 | 184 | 4242036 | 2 | 9 | 0 | 1 | 192 |
| 106 | 6A | 48 | 30 | 2 | 10 | 517874 | 4142988 | 2 | 10 | 0 | 184 | 4242228 | 2 | 10 | 0 | 1 | 192 |
| 107 | 6B | 48 | 30 | 2 | 11 | 517897 | 4143172 | 2 | 11 | 0 | 184 | 4242420 | 2 | 11 | 0 | 1 | 192 |
| 108 | 6C | 48 | 30 | 2 | 12 | 517920 | 4143356 | 2 | 12 | 0 | 184 | 4242612 | 2 | 12 | 0 | 1 | 192 |
| 109 | 6D | 48 | 30 | 2 | 13 | 517943 | 4143540 | 2 | 13 | 0 | 184 | 4242804 | 2 | 13 | 0 | 1 | 192 |
| 110 | 6E | 48 | 30 | 2 | 14 | 517966 | 4143724 | 2 | 14 | 0 | 184 | 4242996 | 2 | 14 | 0 | 1 | 192 |
| 111 | 6F | 48 | 30 | 2 | 15 | 517989 | 4143908 | 2 | 15 | 0 | 184 | 4243188 | 2 | 15 | 0 | 1 | 192 |
| 112 | 70 | 48 | 30 | 2 | 16 | 518014 | 4144108 | 2 | 16 | 0 | 200 | 4243396 | 2 | 16 | 0 | 1 | 208 |
| 113 | 71 | 48 | 30 | 2 | 17 | 518039 | 4144308 | 2 | 17 | 0 | 200 | 4243604 | 2 | 17 | 0 | 1 | 208 |
| 114 | 72 | 48 | 30 | 2 | 18 | 518064 | 4144508 | 2 | 18 | 0 | 200 | 4243812 | 2 | 18 | 0 | 1 | 208 |
| 115 | 73 | 48 | 30 | 2 | 19 | 518089 | 4144708 | 2 | 19 | 0 | 200 | 4244020 | 2 | 19 | 0 | 1 | 208 |
| 116 | 74 | 48 | 30 | 2 | 20 | 518114 | 4144908 | 2 | 20 | 0 | 200 | 4244228 | 2 | 20 | 0 | 1 | 208 |
| 117 | 75 | 48 | 30 | 2 | 21 | 518139 | 4145108 | 2 | 21 | 0 | 200 | 4244436 | 2 | 21 | 0 | 1 | 208 |
| 118 | 76 | 48 | 30 | 2 | 22 | 518164 | 4145308 | 2 | 22 | 0 | 200 | 4244644 | 2 | 22 | 0 | 1 | 208 |
| 119 | 77 | 48 | 30 | 2 | 23 | 518189 | 4145508 | 2 | 23 | 0 | 200 | 4244852 | 2 | 23 | 0 | 1 | 208 |
| 120 | 78 | 48 | 30 | 2 | 24 | 518214 | 4145708 | 2 | 24 | 0 | 200 | 4245060 | 2 | 24 | 0 | 1 | 208 |
| 121 | 79 | 48 | 30 | 2 | 25 | 518239 | 4145908 | 2 | 25 | 0 | 200 | 4245268 | 2 | 25 | 0 | 1 | 208 |
| 122 | 7A | 48 | 30 | 2 | 26 | 518264 | 4146108 | 2 | 26 | 0 | 200 | 4245476 | 2 | 26 | 0 | 1 | 208 |
| 123 | 7B | 48 | 30 | 2 | 27 | 518289 | 4146308 | 2 | 27 | 0 | 200 | 4245684 | 2 | 27 | 0 | 1 | 208 |
| 124 | 7C | 48 | 30 | 2 | 28 | 518314 | 4146508 | 2 | 28 | 0 | 200 | 4245892 | 2 | 28 | 0 | 1 | 208 |
| 125 | 7D | 48 | 30 | 2 | 29 | 518339 | 4146708 | 2 | 29 | 0 | 200 | 4246100 | 2 | 29 | 0 | 1 | 208 |
| 126 | 7E | 48 | 30 | 2 | 30 | 518364 | 4146908 | 2 | 30 | 0 | 200 | 4246308 | 2 | 30 | 0 | 1 | 208 |
| 127 | 7F | 48 | 30 | 2 | 31 | 518389 | 4147108 | 2 | 31 | 0 | 200 | 4246516 | 2 | 31 | 0 | 1 | 208 |
| 128 | 80 | 48 | 30 | 2 | 32 | 518414 | 4147308 | 2 | 32 | 0 | 200 | 4246724 | 2 | 32 | 0 | 1 | 208 |
| 129 | 81 | 48 | 30 | 2 | 33 | 518439 | 4147508 | 2 | 33 | 0 | 200 | 4246932 | 2 | 33 | 0 | 1 | 208 |
| 130 | 82 | 48 | 30 | 2 | 34 | 518464 | 4147708 | 2 | 34 | 0 | 200 | 4247140 | 2 | 34 | 0 | 1 | 208 |
| 131 | 83 | 48 | 30 | 2 | 35 | 518489 | 4147908 | 2 | 35 | 0 | 200 | 4247348 | 2 | 35 | 0 | 1 | 208 |
| 132 | 84 | 48 | 30 | 2 | 36 | 518514 | 4148108 | 2 | 36 | 0 | 200 | 4247556 | 2 | 36 | 0 | 1 | 208 |
| 133 | 85 | 48 | 30 | 2 | 37 | 518539 | 4148308 | 2 | 37 | 0 | 200 | 4247764 | 2 | 37 | 0 | 1 | 208 |
| 134 | 86 | 48 | 30 | 2 | 38 | 518564 | 4148508 | 2 | 38 | 0 | 200 | 4247972 | 2 | 38 | 0 | 1 | 208 |
| 135 | 87 | 48 | 30 | 2 | 39 | 518589 | 4148708 | 2 | 39 | 0 | 200 | 4248180 | 2 | 39 | 0 | 1 | 208 |
| 136 | 88 | 48 | 30 | 2 | 40 | 518614 | 4148908 | 2 | 40 | 0 | 200 | 4248388 | 2 | 40 | 0 | 1 | 208 |
| 137 | 89 | 48 | 30 | 2 | 41 | 518639 | 4149108 | 2 | 41 | 0 | 200 | 4248596 | 2 | 41 | 0 | 1 | 208 |
| 138 | 8A | 48 | 30 | 2 | 42 | 518664 | 4149308 | 2 | 42 | 0 | 200 | 4248804 | 2 | 42 | 0 | 1 | 208 |
| 139 | 8B | 48 | 30 | 2 | 43 | 518689 | 4149508 | 2 | 43 | 0 | 200 | 4249012 | 2 | 43 | 0 | 1 | 208 |
| 140 | 8C | 48 | 30 | 2 | 44 | 518714 | 4149708 | 2 | 44 | 0 | 200 | 4249220 | 2 | 44 | 0 | 1 | 208 |
| 141 | 8D | 48 | 30 | 2 | 45 | 518739 | 4149908 | 2 | 45 | 0 | 200 | 4249428 | 2 | 45 | 0 | 1 | 208 |
| 142 | 8E | 48 | 30 | 2 | 46 | 518764 | 4150108 | 2 | 46 | 0 | 200 | 4249636 | 2 | 46 | 0 | 1 | 208 |
| 143 | 8F | 48 | 30 | 2 | 47 | 518789 | 4150308 | 2 | 47 | 0 | 200 | 4249844 | 2 | 47 | 0 | 1 | 208 |
| 144 | 90 | 48 | 30 | 3 | 0 | 518812 | 4150492 | 3 | 0 | 0 | 184 | 4250036 | 3 | 0 | 0 | 1 | 192 |
| 145 | 91 | 48 | 30 | 3 | 1 | 518837 | 4150692 | 3 | 1 | 0 | 200 | 4250244 | 3 | 1 | 0 | 1 | 208 |
| 146 | 92 | 48 | 30 | 3 | 2 | 518862 | 4150892 | 3 | 2 | 0 | 200 | 4250452 | 3 | 2 | 0 | 1 | 208 |
| 147 | 93 | 48 | 30 | 3 | 3 | 518887 | 4151092 | 3 | 3 | 0 | 200 | 4250660 | 3 | 3 | 0 | 1 | 208 |
| 148 | 94 | 48 | 30 | 3 | 4 | 518912 | 4151292 | 3 | 4 | 0 | 200 | 4250868 | 3 | 4 | 0 | 1 | 208 |
| 149 | 95 | 48 | 30 | 3 | 5 | 518937 | 4151492 | 3 | 5 | 0 | 200 | 4251076 | 3 | 5 | 0 | 1 | 208 |
| 150 | 96 | 48 | 30 | 3 | 6 | 518962 | 4151692 | 3 | 6 | 0 | 200 | 4251284 | 3 | 6 | 0 | 1 | 208 |
| 151 | 97 | 48 | 30 | 3 | 7 | 518987 | 4151892 | 3 | 7 | 0 | 200 | 4251492 | 3 | 7 | 0 | 1 | 208 |
| 152 | 98 | 48 | 30 | 3 | 8 | 519012 | 4152092 | 3 | 8 | 0 | 200 | 4251700 | 3 | 8 | 0 | 1 | 208 |
| 153 | 99 | 48 | 30 | 3 | 9 | 519037 | 4152292 | 3 | 9 | 0 | 200 | 4251908 | 3 | 9 | 0 | 1 | 208 |
| 154 | 9A | 48 | 30 | 3 | 10 | 519062 | 4152492 | 3 | 10 | 0 | 200 | 4252116 | 3 | 10 | 0 | 1 | 208 |
| 155 | 9B | 48 | 30 | 3 | 11 | 519087 | 4152692 | 3 | 11 | 0 | 200 | 4252324 | 3 | 11 | 0 | 1 | 208 |
| 156 | 9C | 48 | 30 | 3 | 12 | 519112 | 4152892 | 3 | 12 | 0 | 200 | 4252532 | 3 | 12 | 0 | 1 | 208 |
| 157 | 9D | 48 | 30 | 3 | 13 | 519137 | 4153092 | 3 | 13 | 0 | 200 | 4252740 | 3 | 13 | 0 | 1 | 208 |
| 158 | 9E | 48 | 30 | 3 | 14 | 519162 | 4153292 | 3 | 14 | 0 | 200 | 4252948 | 3 | 14 | 0 | 1 | 208 |
| 159 | 9F | 48 | 30 | 3 | 15 | 519187 | 4153492 | 3 | 15 | 0 | 200 | 4253156 | 3 | 15 | 0 | 1 | 208 |
| 160 | A0 | 48 | 30 | 3 | 16 | 519214 | 4153708 | 3 | 16 | 0 | 216 | 4253380 | 3 | 16 | 0 | 1 | 224 |
| 161 | A1 | 48 | 30 | 3 | 17 | 519241 | 4153924 | 3 | 17 | 0 | 216 | 4253604 | 3 | 17 | 0 | 1 | 224 |
| 162 | A2 | 48 | 30 | 3 | 18 | 519268 | 4154140 | 3 | 18 | 0 | 216 | 4253828 | 3 | 18 | 0 | 1 | 224 |
| 163 | A3 | 48 | 30 | 3 | 19 | 519295 | 4154356 | 3 | 19 | 0 | 216 | 4254052 | 3 | 19 | 0 | 1 | 224 |
| 164 | A4 | 48 | 30 | 3 | 20 | 519322 | 4154572 | 3 | 20 | 0 | 216 | 4254276 | 3 | 20 | 0 | 1 | 224 |
| 165 | A5 | 48 | 30 | 3 | 21 | 519349 | 4154788 | 3 | 21 | 0 | 216 | 4254500 | 3 | 21 | 0 | 1 | 224 |
| 166 | A6 | 48 | 30 | 3 | 22 | 519376 | 4155004 | 3 | 22 | 0 | 216 | 4254724 | 3 | 22 | 0 | 1 | 224 |
| 167 | A7 | 48 | 30 | 3 | 23 | 519403 | 4155220 | 3 | 23 | 0 | 216 | 4254948 | 3 | 23 | 0 | 1 | 224 |
| 168 | A8 | 48 | 30 | 3 | 24 | 519430 | 4155436 | 3 | 24 | 0 | 216 | 4255172 | 3 | 24 | 0 | 1 | 224 |
| 169 | A9 | 48 | 30 | 3 | 25 | 519457 | 4155652 | 3 | 25 | 0 | 216 | 4255396 | 3 | 25 | 0 | 1 | 224 |
| 170 | AA | 48 | 30 | 3 | 26 | 519484 | 4155868 | 3 | 26 | 0 | 216 | 4255620 | 3 | 26 | 0 | 1 | 224 |
| 171 | AB | 48 | 30 | 3 | 27 | 519511 | 4156084 | 3 | 27 | 0 | 216 | 4255844 | 3 | 27 | 0 | 1 | 224 |

| | | | | | | | | | | | | | | | | | |
|---|---|---|---|---|---|---|---|---|---|---|---|---|---|---|---|---|---|
| 172 | AC | 48 | 30 | 3 | 28 | 519538 | 4156300 | 3 | 28 | 0 | 216 | 4256068 | 3 | 28 | 0 | 1 | 224 |
| 173 | AD | 48 | 30 | 3 | 29 | 519565 | 4156516 | 3 | 29 | 0 | 216 | 4256292 | 3 | 29 | 0 | 1 | 224 |
| 174 | AE | 48 | 30 | 3 | 30 | 519592 | 4156732 | 3 | 30 | 0 | 216 | 4256516 | 3 | 30 | 0 | 1 | 224 |
| 175 | AF | 48 | 30 | 3 | 31 | 519619 | 4156948 | 3 | 31 | 0 | 216 | 4256740 | 3 | 31 | 0 | 1 | 224 |
| 176 | B0 | 48 | 30 | 3 | 32 | 519646 | 4157164 | 3 | 32 | 0 | 216 | 4256964 | 3 | 32 | 0 | 1 | 224 |
| 177 | B1 | 48 | 30 | 3 | 33 | 519673 | 4157380 | 3 | 33 | 0 | 216 | 4257188 | 3 | 33 | 0 | 1 | 224 |
| 178 | B2 | 48 | 30 | 3 | 34 | 519700 | 4157596 | 3 | 34 | 0 | 216 | 4257412 | 3 | 34 | 0 | 1 | 224 |
| 179 | B3 | 48 | 30 | 3 | 35 | 519727 | 4157812 | 3 | 35 | 0 | 216 | 4257636 | 3 | 35 | 0 | 1 | 224 |
| 180 | B4 | 48 | 30 | 3 | 36 | 519754 | 4158028 | 3 | 36 | 0 | 216 | 4257860 | 3 | 36 | 0 | 1 | 224 |
| 181 | B5 | 48 | 30 | 3 | 37 | 519781 | 4158244 | 3 | 37 | 0 | 216 | 4258084 | 3 | 37 | 0 | 1 | 224 |
| 182 | B6 | 48 | 30 | 3 | 38 | 519808 | 4158460 | 3 | 38 | 0 | 216 | 4258308 | 3 | 38 | 0 | 1 | 224 |
| 183 | B7 | 48 | 30 | 3 | 39 | 519835 | 4158676 | 3 | 39 | 0 | 216 | 4258532 | 3 | 39 | 0 | 1 | 224 |
| 184 | B8 | 48 | 30 | 3 | 40 | 519862 | 4158892 | 3 | 40 | 0 | 216 | 4258756 | 3 | 40 | 0 | 1 | 224 |
| 185 | B9 | 48 | 30 | 3 | 41 | 519889 | 4159108 | 3 | 41 | 0 | 216 | 4258980 | 3 | 41 | 0 | 1 | 224 |
| 186 | BA | 48 | 30 | 3 | 42 | 519916 | 4159324 | 3 | 42 | 0 | 216 | 4259204 | 3 | 42 | 0 | 1 | 224 |
| 187 | BB | 48 | 30 | 3 | 43 | 519943 | 4159540 | 3 | 43 | 0 | 216 | 4259428 | 3 | 43 | 0 | 1 | 224 |
| 188 | BC | 48 | 30 | 3 | 44 | 519970 | 4159756 | 3 | 44 | 0 | 216 | 4259652 | 3 | 44 | 0 | 1 | 224 |
| 189 | BD | 48 | 30 | 3 | 45 | 519997 | 4159972 | 3 | 45 | 0 | 216 | 4259876 | 3 | 45 | 0 | 1 | 224 |
| 190 | BE | 48 | 30 | 3 | 46 | 520024 | 4160188 | 3 | 46 | 0 | 216 | 4260100 | 3 | 46 | 0 | 1 | 224 |
| 191 | BF | 48 | 30 | 3 | 47 | 520051 | 4160404 | 3 | 47 | 0 | 216 | 4260324 | 3 | 47 | 0 | 1 | 224 |
| 192 | C0 | 48 | 30 | 4 | 0 | 520076 | 4160604 | 4 | 0 | 0 | 200 | 4260532 | 4 | 0 | 0 | 1 | 208 |
| 193 | C1 | 48 | 30 | 4 | 1 | 520103 | 4160820 | 4 | 1 | 0 | 216 | 4260756 | 4 | 1 | 0 | 1 | 224 |
| 194 | C2 | 48 | 30 | 4 | 2 | 520130 | 4161036 | 4 | 2 | 0 | 216 | 4260980 | 4 | 2 | 0 | 1 | 224 |
| 195 | C3 | 48 | 30 | 4 | 3 | 520157 | 4161252 | 4 | 3 | 0 | 216 | 4261204 | 4 | 3 | 0 | 1 | 224 |
| 196 | C4 | 48 | 30 | 4 | 4 | 520184 | 4161468 | 4 | 4 | 0 | 216 | 4261428 | 4 | 4 | 0 | 1 | 224 |
| 197 | C5 | 48 | 30 | 4 | 5 | 520211 | 4161684 | 4 | 5 | 0 | 216 | 4261652 | 4 | 5 | 0 | 1 | 224 |
| 198 | C6 | 48 | 30 | 4 | 6 | 520238 | 4161900 | 4 | 6 | 0 | 216 | 4261876 | 4 | 6 | 0 | 1 | 224 |
| 199 | C7 | 48 | 30 | 4 | 7 | 520265 | 4162116 | 4 | 7 | 0 | 216 | 4262100 | 4 | 7 | 0 | 1 | 224 |
| 200 | C8 | 48 | 30 | 4 | 8 | 520292 | 4162332 | 4 | 8 | 0 | 216 | 4262324 | 4 | 8 | 0 | 1 | 224 |
| 201 | C9 | 48 | 30 | 4 | 9 | 520319 | 4162548 | 4 | 9 | 0 | 216 | 4262548 | 4 | 9 | 0 | 1 | 224 |
| 202 | CA | 48 | 30 | 4 | 10 | 520346 | 4162764 | 4 | 10 | 0 | 216 | 4262772 | 4 | 10 | 0 | 1 | 224 |
| 203 | CB | 48 | 30 | 4 | 11 | 520373 | 4162980 | 4 | 11 | 0 | 216 | 4262996 | 4 | 11 | 0 | 1 | 224 |
| 204 | CC | 48 | 30 | 4 | 12 | 520400 | 4163196 | 4 | 12 | 0 | 216 | 4263220 | 4 | 12 | 0 | 1 | 224 |
| 205 | CD | 48 | 30 | 4 | 13 | 520427 | 4163412 | 4 | 13 | 0 | 216 | 4263444 | 4 | 13 | 0 | 1 | 224 |
| 206 | CE | 48 | 30 | 4 | 14 | 520454 | 4163628 | 4 | 14 | 0 | 216 | 4263668 | 4 | 14 | 0 | 1 | 224 |
| 207 | CF | 48 | 30 | 4 | 15 | 520481 | 4163844 | 4 | 15 | 0 | 216 | 4263892 | 4 | 15 | 0 | 1 | 224 |
| 208 | D0 | 48 | 30 | 4 | 16 | 520510 | 4164076 | 4 | 16 | 0 | 232 | 4264132 | 4 | 16 | 0 | 1 | 240 |
| 209 | D1 | 48 | 30 | 4 | 17 | 520539 | 4164308 | 4 | 17 | 0 | 232 | 4264372 | 4 | 17 | 0 | 1 | 240 |
| 210 | D2 | 48 | 30 | 4 | 18 | 520568 | 4164540 | 4 | 18 | 0 | 232 | 4264612 | 4 | 18 | 0 | 1 | 240 |
| 211 | D3 | 48 | 30 | 4 | 19 | 520597 | 4164772 | 4 | 19 | 0 | 232 | 4264852 | 4 | 19 | 0 | 1 | 240 |
| 212 | D4 | 48 | 30 | 4 | 20 | 520626 | 4165004 | 4 | 20 | 0 | 232 | 4265092 | 4 | 20 | 0 | 1 | 240 |
| 213 | D5 | 48 | 30 | 4 | 21 | 520655 | 4165236 | 4 | 21 | 0 | 232 | 4265332 | 4 | 21 | 0 | 1 | 240 |
| 214 | D6 | 48 | 30 | 4 | 22 | 520684 | 4165468 | 4 | 22 | 0 | 232 | 4265572 | 4 | 22 | 0 | 1 | 240 |
| 215 | D7 | 48 | 30 | 4 | 23 | 520713 | 4165700 | 4 | 23 | 0 | 232 | 4265812 | 4 | 23 | 0 | 1 | 240 |
| 216 | D8 | 48 | 30 | 4 | 24 | 520742 | 4165932 | 4 | 24 | 0 | 232 | 4266052 | 4 | 24 | 0 | 1 | 240 |
| 217 | D9 | 48 | 30 | 4 | 25 | 520771 | 4166164 | 4 | 25 | 0 | 232 | 4266292 | 4 | 25 | 0 | 1 | 240 |
| 218 | DA | 48 | 30 | 4 | 26 | 520800 | 4166396 | 4 | 26 | 0 | 232 | 4266532 | 4 | 26 | 0 | 1 | 240 |
| 219 | DB | 48 | 30 | 4 | 27 | 520829 | 4166628 | 4 | 27 | 0 | 232 | 4266772 | 4 | 27 | 0 | 1 | 240 |
| 220 | DC | 48 | 30 | 4 | 28 | 520858 | 4166860 | 4 | 28 | 0 | 232 | 4267012 | 4 | 28 | 0 | 1 | 240 |
| 221 | DD | 48 | 30 | 4 | 29 | 520887 | 4167092 | 4 | 29 | 0 | 232 | 4267252 | 4 | 29 | 0 | 1 | 240 |
| 222 | DE | 48 | 30 | 4 | 30 | 520916 | 4167324 | 4 | 30 | 0 | 232 | 4267492 | 4 | 30 | 0 | 1 | 240 |
| 223 | DF | 48 | 30 | 4 | 31 | 520945 | 4167556 | 4 | 31 | 0 | 232 | 4267732 | 4 | 31 | 0 | 1 | 240 |
| 224 | E0 | 48 | 30 | 4 | 32 | 520974 | 4167788 | 4 | 32 | 0 | 232 | 4267972 | 4 | 32 | 0 | 1 | 240 |
| 225 | E1 | 48 | 30 | 4 | 33 | 521003 | 4168020 | 4 | 33 | 0 | 232 | 4268212 | 4 | 33 | 0 | 1 | 240 |
| 226 | E2 | 48 | 30 | 4 | 34 | 521032 | 4168252 | 4 | 34 | 0 | 232 | 4268452 | 4 | 34 | 0 | 1 | 240 |
| 227 | E3 | 48 | 30 | 4 | 35 | 521061 | 4168484 | 4 | 35 | 0 | 232 | 4268692 | 4 | 35 | 0 | 1 | 240 |
| 228 | E4 | 48 | 30 | 4 | 36 | 521090 | 4168716 | 4 | 36 | 0 | 232 | 4268932 | 4 | 36 | 0 | 1 | 240 |
| 229 | E5 | 48 | 30 | 4 | 37 | 521119 | 4168948 | 4 | 37 | 0 | 232 | 4269172 | 4 | 37 | 0 | 1 | 240 |
| 230 | E6 | 48 | 30 | 4 | 38 | 521148 | 4169180 | 4 | 38 | 0 | 232 | 4269412 | 4 | 38 | 0 | 1 | 240 |
| 231 | E7 | 48 | 30 | 4 | 39 | 521177 | 4169412 | 4 | 39 | 0 | 232 | 4269652 | 4 | 39 | 0 | 1 | 240 |
| 232 | E8 | 48 | 30 | 4 | 40 | 521206 | 4169644 | 4 | 40 | 0 | 232 | 4269892 | 4 | 40 | 0 | 1 | 240 |
| 233 | E9 | 48 | 30 | 4 | 41 | 521235 | 4169876 | 4 | 41 | 0 | 232 | 4270132 | 4 | 41 | 0 | 1 | 240 |
| 234 | EA | 48 | 30 | 4 | 42 | 521264 | 4170108 | 4 | 42 | 0 | 232 | 4270372 | 4 | 42 | 0 | 1 | 240 |
| 235 | EB | 48 | 30 | 4 | 43 | 521293 | 4170340 | 4 | 43 | 0 | 232 | 4270612 | 4 | 43 | 0 | 1 | 240 |
| 236 | EC | 48 | 30 | 4 | 44 | 521322 | 4170572 | 4 | 44 | 0 | 232 | 4270852 | 4 | 44 | 0 | 1 | 240 |
| 237 | ED | 48 | 30 | 4 | 45 | 521351 | 4170804 | 4 | 45 | 0 | 232 | 4271092 | 4 | 45 | 0 | 1 | 240 |
| 238 | EE | 48 | 30 | 4 | 46 | 521380 | 4171036 | 4 | 46 | 0 | 232 | 4271332 | 4 | 46 | 0 | 1 | 240 |
| 239 | EF | 48 | 30 | 4 | 47 | 521409 | 4171268 | 4 | 47 | 0 | 232 | 4271572 | 4 | 47 | 0 | 1 | 240 |
| 240 | F0 | 48 | 30 | 5 | 0 | 521436 | 4171484 | 5 | 0 | 0 | 216 | 4271796 | 5 | 0 | 0 | 1 | 224 |
| 241 | F1 | 48 | 30 | 5 | 1 | 521465 | 4171716 | 5 | 1 | 0 | 232 | 4272036 | 5 | 1 | 0 | 1 | 240 |
| 242 | F2 | 48 | 30 | 5 | 2 | 521494 | 4171948 | 5 | 2 | 0 | 232 | 4272276 | 5 | 2 | 0 | 1 | 240 |
| 243 | F3 | 48 | 30 | 5 | 3 | 521523 | 4172180 | 5 | 3 | 0 | 232 | 4272516 | 5 | 3 | 0 | 1 | 240 |
| 244 | F4 | 48 | 30 | 5 | 4 | 521552 | 4172412 | 5 | 4 | 0 | 232 | 4272756 | 5 | 4 | 0 | 1 | 240 |
| 245 | F5 | 48 | 30 | 5 | 5 | 521581 | 4172644 | 5 | 5 | 0 | 232 | 4272996 | 5 | 5 | 0 | 1 | 240 |
| 246 | F6 | 48 | 30 | 5 | 6 | 521610 | 4172876 | 5 | 6 | 0 | 232 | 4273236 | 5 | 6 | 0 | 1 | 240 |
| 247 | F7 | 48 | 30 | 5 | 7 | 521639 | 4173108 | 5 | 7 | 0 | 232 | 4273476 | 5 | 7 | 0 | 1 | 240 |
| 248 | F8 | 48 | 30 | 5 | 8 | 521668 | 4173340 | 5 | 8 | 0 | 232 | 4273716 | 5 | 8 | 0 | 1 | 240 |
| 249 | F9 | 48 | 30 | 5 | 9 | 521697 | 4173572 | 5 | 9 | 0 | 232 | 4273956 | 5 | 9 | 0 | 1 | 240 |
| 250 | FA | 48 | 30 | 5 | 10 | 521726 | 4173804 | 5 | 10 | 0 | 232 | 4274196 | 5 | 10 | 0 | 1 | 240 |
| 251 | FB | 48 | 30 | 5 | 11 | 521755 | 4174036 | 5 | 11 | 0 | 232 | 4274436 | 5 | 11 | 0 | 1 | 240 |
| 252 | FC | 48 | 30 | 5 | 12 | 521784 | 4174268 | 5 | 12 | 0 | 232 | 4274676 | 5 | 12 | 0 | 1 | 240 |
| 253 | FD | 48 | 30 | 5 | 13 | 521813 | 4174500 | 5 | 13 | 0 | 232 | 4274916 | 5 | 13 | 0 | 1 | 240 |
| 254 | FE | 48 | 30 | 5 | 14 | 521842 | 4174732 | 5 | 14 | 0 | 232 | 4275156 | 5 | 14 | 0 | 1 | 240 |
| 255 | FF | 48 | 30 | 5 | 15 | 521871 | 4174964 | 5 | 15 | 0 | 232 | 4275396 | 5 | 15 | 0 | 1 | 240 |
| 0 | 00 | 49 | 31 | 0 | 0 | 521883 | 4175060 | 0 | 0 | 0 | 96 | 4275500 | 0 | 0 | 0 | 1 | 104 |
| 1 | 01 | 49 | 31 | 0 | 1 | 521897 | 4175172 | 0 | 1 | 0 | 112 | 4275620 | 0 | 1 | 0 | 1 | 120 |
| 2 | 02 | 49 | 31 | 0 | 2 | 521911 | 4175284 | 0 | 2 | 0 | 112 | 4275740 | 0 | 2 | 0 | 1 | 120 |
| 3 | 03 | 49 | 31 | 0 | 3 | 521925 | 4175396 | 0 | 3 | 0 | 112 | 4275860 | 0 | 3 | 0 | 1 | 120 |
| 4 | 04 | 49 | 31 | 0 | 4 | 521939 | 4175508 | 0 | 4 | 0 | 112 | 4275980 | 0 | 4 | 0 | 1 | 120 |
| 5 | 05 | 49 | 31 | 0 | 5 | 521953 | 4175620 | 0 | 5 | 0 | 112 | 4276100 | 0 | 5 | 0 | 1 | 120 |
| 6 | 06 | 49 | 31 | 0 | 6 | 521967 | 4175732 | 0 | 6 | 0 | 112 | 4276220 | 0 | 6 | 0 | 1 | 120 |
| 7 | 07 | 49 | 31 | 0 | 7 | 521981 | 4175844 | 0 | 7 | 0 | 112 | 4276340 | 0 | 7 | 0 | 1 | 120 |
| 8 | 08 | 49 | 31 | 0 | 8 | 521995 | 4175956 | 0 | 8 | 0 | 112 | 4276460 | 0 | 8 | 0 | 1 | 120 |
| 9 | 09 | 49 | 31 | 0 | 9 | 522009 | 4176068 | 0 | 9 | 0 | 112 | 4276580 | 0 | 9 | 0 | 1 | 120 |
| 10 | 0A | 49 | 31 | 0 | 10 | 522023 | 4176180 | 0 | 10 | 0 | 112 | 4276700 | 0 | 10 | 0 | 1 | 120 |
| 11 | 0B | 49 | 31 | 0 | 11 | 522037 | 4176292 | 0 | 11 | 0 | 112 | 4276820 | 0 | 11 | 0 | 1 | 120 |
| 12 | 0C | 49 | 31 | 0 | 12 | 522051 | 4176404 | 0 | 12 | 0 | 112 | 4276940 | 0 | 12 | 0 | 1 | 120 |
| 13 | 0D | 49 | 31 | 0 | 13 | 522065 | 4176516 | 0 | 13 | 0 | 112 | 4277060 | 0 | 13 | 0 | 1 | 120 |
| 14 | 0E | 49 | 31 | 0 | 14 | 522079 | 4176628 | 0 | 14 | 0 | 112 | 4277180 | 0 | 14 | 0 | 1 | 120 |
| 15 | 0F | 49 | 31 | 0 | 15 | 522093 | 4176740 | 0 | 15 | 0 | 112 | 4277300 | 0 | 15 | 0 | 1 | 120 |
| 16 | 10 | 49 | 31 | 0 | 16 | 522107 | 4176852 | 0 | 16 | 0 | 112 | 4277420 | 0 | 16 | 0 | 1 | 120 |
| 17 | 11 | 49 | 31 | 0 | 17 | 522121 | 4176964 | 0 | 17 | 0 | 112 | 4277540 | 0 | 17 | 0 | 1 | 120 |
| 18 | 12 | 49 | 31 | 0 | 18 | 522135 | 4177076 | 0 | 18 | 0 | 112 | 4277660 | 0 | 18 | 0 | 1 | 120 |

| | | | | | | | | | | | | | | | | | |
|---|---|---|---|---|---|---|---|---|---|---|---|---|---|---|---|---|---|
| 19 | 13 | 49 | 31 | 0 | 19 | 522149 | 4177188 | 0 | 19 | 0 | 112 | 4277780 | 0 | 19 | 0 | 1 | 120 |
| 20 | 14 | 49 | 31 | 0 | 20 | 522163 | 4177300 | 0 | 20 | 0 | 112 | 4277900 | 0 | 20 | 0 | 1 | 120 |
| 21 | 15 | 49 | 31 | 0 | 21 | 522177 | 4177412 | 0 | 21 | 0 | 112 | 4278020 | 0 | 21 | 0 | 1 | 120 |
| 22 | 16 | 49 | 31 | 0 | 22 | 522191 | 4177524 | 0 | 22 | 0 | 112 | 4278140 | 0 | 22 | 0 | 1 | 120 |
| 23 | 17 | 49 | 31 | 0 | 23 | 522205 | 4177636 | 0 | 23 | 0 | 112 | 4278260 | 0 | 23 | 0 | 1 | 120 |
| 24 | 18 | 49 | 31 | 0 | 24 | 522219 | 4177748 | 0 | 24 | 0 | 112 | 4278380 | 0 | 24 | 0 | 1 | 120 |
| 25 | 19 | 49 | 31 | 0 | 25 | 522233 | 4177860 | 0 | 25 | 0 | 112 | 4278500 | 0 | 25 | 0 | 1 | 120 |
| 26 | 1A | 49 | 31 | 0 | 26 | 522247 | 4177972 | 0 | 26 | 0 | 112 | 4278620 | 0 | 26 | 0 | 1 | 120 |
| 27 | 1B | 49 | 31 | 0 | 27 | 522261 | 4178084 | 0 | 27 | 0 | 112 | 4278740 | 0 | 27 | 0 | 1 | 120 |
| 28 | 1C | 49 | 31 | 0 | 28 | 522275 | 4178196 | 0 | 28 | 0 | 112 | 4278860 | 0 | 28 | 0 | 1 | 120 |
| 29 | 1D | 49 | 31 | 0 | 29 | 522289 | 4178308 | 0 | 29 | 0 | 112 | 4278980 | 0 | 29 | 0 | 1 | 120 |
| 30 | 1E | 49 | 31 | 0 | 30 | 522303 | 4178420 | 0 | 30 | 0 | 112 | 4279100 | 0 | 30 | 0 | 1 | 120 |
| 31 | 1F | 49 | 31 | 0 | 31 | 522317 | 4178532 | 0 | 31 | 0 | 112 | 4279220 | 0 | 31 | 0 | 1 | 120 |
| 32 | 20 | 49 | 31 | 0 | 32 | 522331 | 4178644 | 0 | 32 | 0 | 112 | 4279340 | 0 | 32 | 0 | 1 | 120 |
| 33 | 21 | 49 | 31 | 0 | 33 | 522345 | 4178756 | 0 | 33 | 0 | 112 | 4279460 | 0 | 33 | 0 | 1 | 120 |
| 34 | 22 | 49 | 31 | 0 | 34 | 522359 | 4178868 | 0 | 34 | 0 | 112 | 4279580 | 0 | 34 | 0 | 1 | 120 |
| 35 | 23 | 49 | 31 | 0 | 35 | 522373 | 4178980 | 0 | 35 | 0 | 112 | 4279700 | 0 | 35 | 0 | 1 | 120 |
| 36 | 24 | 49 | 31 | 0 | 36 | 522387 | 4179092 | 0 | 36 | 0 | 112 | 4279820 | 0 | 36 | 0 | 1 | 120 |
| 37 | 25 | 49 | 31 | 0 | 37 | 522401 | 4179204 | 0 | 37 | 0 | 112 | 4279940 | 0 | 37 | 0 | 1 | 120 |
| 38 | 26 | 49 | 31 | 0 | 38 | 522415 | 4179316 | 0 | 38 | 0 | 112 | 4280060 | 0 | 38 | 0 | 1 | 120 |
| 39 | 27 | 49 | 31 | 0 | 39 | 522429 | 4179428 | 0 | 39 | 0 | 112 | 4280180 | 0 | 39 | 0 | 1 | 120 |
| 40 | 28 | 49 | 31 | 0 | 40 | 522443 | 4179540 | 0 | 40 | 0 | 112 | 4280300 | 0 | 40 | 0 | 1 | 120 |
| 41 | 29 | 49 | 31 | 0 | 41 | 522457 | 4179652 | 0 | 41 | 0 | 112 | 4280420 | 0 | 41 | 0 | 1 | 120 |
| 42 | 2A | 49 | 31 | 0 | 42 | 522471 | 4179764 | 0 | 42 | 0 | 112 | 4280540 | 0 | 42 | 0 | 1 | 120 |
| 43 | 2B | 49 | 31 | 0 | 43 | 522485 | 4179876 | 0 | 43 | 0 | 112 | 4280660 | 0 | 43 | 0 | 1 | 120 |
| 44 | 2C | 49 | 31 | 0 | 44 | 522499 | 4179988 | 0 | 44 | 0 | 112 | 4280780 | 0 | 44 | 0 | 1 | 120 |
| 45 | 2D | 49 | 31 | 0 | 45 | 522513 | 4180100 | 0 | 45 | 0 | 112 | 4280900 | 0 | 45 | 0 | 1 | 120 |
| 46 | 2E | 49 | 31 | 0 | 46 | 522527 | 4180212 | 0 | 46 | 0 | 112 | 4281020 | 0 | 46 | 0 | 1 | 120 |
| 47 | 2F | 49 | 31 | 0 | 47 | 522541 | 4180324 | 0 | 47 | 0 | 112 | 4281140 | 0 | 47 | 0 | 1 | 120 |
| 48 | 30 | 49 | 31 | 0 | 48 | 522555 | 4180436 | 0 | 48 | 0 | 112 | 4281260 | 0 | 48 | 0 | 1 | 120 |
| 49 | 31 | 49 | 31 | 1 | 0 | 522567 | 4180532 | 1 | 0 | 0 | 96 | 4281364 | 1 | 0 | 0 | 1 | 104 |
| 50 | 32 | 49 | 31 | 1 | 1 | 522584 | 4180668 | 1 | 1 | 0 | 136 | 4281508 | 1 | 1 | 0 | 1 | 144 |
| 51 | 33 | 49 | 31 | 1 | 2 | 522601 | 4180804 | 1 | 2 | 0 | 136 | 4281652 | 1 | 2 | 0 | 1 | 144 |
| 52 | 34 | 49 | 31 | 1 | 3 | 522618 | 4180940 | 1 | 3 | 0 | 136 | 4281796 | 1 | 3 | 0 | 1 | 144 |
| 53 | 35 | 49 | 31 | 1 | 4 | 522635 | 4181076 | 1 | 4 | 0 | 136 | 4281940 | 1 | 4 | 0 | 1 | 144 |
| 54 | 36 | 49 | 31 | 1 | 5 | 522652 | 4181212 | 1 | 5 | 0 | 136 | 4282084 | 1 | 5 | 0 | 1 | 144 |
| 55 | 37 | 49 | 31 | 1 | 6 | 522669 | 4181348 | 1 | 6 | 0 | 136 | 4282228 | 1 | 6 | 0 | 1 | 144 |
| 56 | 38 | 49 | 31 | 1 | 7 | 522686 | 4181484 | 1 | 7 | 0 | 136 | 4282372 | 1 | 7 | 0 | 1 | 144 |
| 57 | 39 | 49 | 31 | 1 | 8 | 522703 | 4181620 | 1 | 8 | 0 | 136 | 4282516 | 1 | 8 | 0 | 1 | 144 |
| 58 | 3A | 49 | 31 | 1 | 9 | 522720 | 4181756 | 1 | 9 | 0 | 136 | 4282660 | 1 | 9 | 0 | 1 | 144 |
| 59 | 3B | 49 | 31 | 1 | 10 | 522737 | 4181892 | 1 | 10 | 0 | 136 | 4282804 | 1 | 10 | 0 | 1 | 144 |
| 60 | 3C | 49 | 31 | 1 | 11 | 522754 | 4182028 | 1 | 11 | 0 | 136 | 4282948 | 1 | 11 | 0 | 1 | 144 |
| 61 | 3D | 49 | 31 | 1 | 12 | 522771 | 4182164 | 1 | 12 | 0 | 136 | 4283092 | 1 | 12 | 0 | 1 | 144 |
| 62 | 3E | 49 | 31 | 1 | 13 | 522788 | 4182300 | 1 | 13 | 0 | 136 | 4283236 | 1 | 13 | 0 | 1 | 144 |
| 63 | 3F | 49 | 31 | 1 | 14 | 522805 | 4182436 | 1 | 14 | 0 | 136 | 4283380 | 1 | 14 | 0 | 1 | 144 |
| 64 | 40 | 49 | 31 | 1 | 15 | 522826 | 4182604 | 1 | 15 | 0 | 168 | 4283556 | 1 | 15 | 0 | 1 | 176 |
| 65 | 41 | 49 | 31 | 1 | 16 | 522847 | 4182772 | 1 | 16 | 0 | 168 | 4283732 | 1 | 16 | 0 | 1 | 176 |
| 66 | 42 | 49 | 31 | 1 | 17 | 522868 | 4182940 | 1 | 17 | 0 | 168 | 4283908 | 1 | 17 | 0 | 1 | 176 |
| 67 | 43 | 49 | 31 | 1 | 18 | 522889 | 4183108 | 1 | 18 | 0 | 168 | 4284084 | 1 | 18 | 0 | 1 | 176 |
| 68 | 44 | 49 | 31 | 1 | 19 | 522910 | 4183276 | 1 | 19 | 0 | 168 | 4284260 | 1 | 19 | 0 | 1 | 176 |
| 69 | 45 | 49 | 31 | 1 | 20 | 522931 | 4183444 | 1 | 20 | 0 | 168 | 4284436 | 1 | 20 | 0 | 1 | 176 |
| 70 | 46 | 49 | 31 | 1 | 21 | 522952 | 4183612 | 1 | 21 | 0 | 168 | 4284612 | 1 | 21 | 0 | 1 | 176 |
| 71 | 47 | 49 | 31 | 1 | 22 | 522973 | 4183780 | 1 | 22 | 0 | 168 | 4284788 | 1 | 22 | 0 | 1 | 176 |
| 72 | 48 | 49 | 31 | 1 | 23 | 522994 | 4183948 | 1 | 23 | 0 | 168 | 4284964 | 1 | 23 | 0 | 1 | 176 |
| 73 | 49 | 49 | 31 | 1 | 24 | 523015 | 4184116 | 1 | 24 | 0 | 168 | 4285140 | 1 | 24 | 0 | 1 | 176 |
| 74 | 4A | 49 | 31 | 1 | 25 | 523036 | 4184284 | 1 | 25 | 0 | 168 | 4285316 | 1 | 25 | 0 | 1 | 176 |
| 75 | 4B | 49 | 31 | 1 | 26 | 523057 | 4184452 | 1 | 26 | 0 | 168 | 4285492 | 1 | 26 | 0 | 1 | 176 |
| 76 | 4C | 49 | 31 | 1 | 27 | 523078 | 4184620 | 1 | 27 | 0 | 168 | 4285668 | 1 | 27 | 0 | 1 | 176 |
| 77 | 4D | 49 | 31 | 1 | 28 | 523099 | 4184788 | 1 | 28 | 0 | 168 | 4285844 | 1 | 28 | 0 | 1 | 176 |
| 78 | 4E | 49 | 31 | 1 | 29 | 523120 | 4184956 | 1 | 29 | 0 | 168 | 4286020 | 1 | 29 | 0 | 1 | 176 |
| 79 | 4F | 49 | 31 | 1 | 30 | 523141 | 4185124 | 1 | 30 | 0 | 168 | 4286196 | 1 | 30 | 0 | 1 | 176 |
| 80 | 50 | 49 | 31 | 1 | 31 | 523164 | 4185308 | 1 | 31 | 0 | 184 | 4286388 | 1 | 31 | 0 | 1 | 192 |
| 81 | 51 | 49 | 31 | 1 | 32 | 523187 | 4185492 | 1 | 32 | 0 | 184 | 4286580 | 1 | 32 | 0 | 1 | 192 |
| 82 | 52 | 49 | 31 | 1 | 33 | 523210 | 4185676 | 1 | 33 | 0 | 184 | 4286772 | 1 | 33 | 0 | 1 | 192 |
| 83 | 53 | 49 | 31 | 1 | 34 | 523233 | 4185860 | 1 | 34 | 0 | 184 | 4286964 | 1 | 34 | 0 | 1 | 192 |
| 84 | 54 | 49 | 31 | 1 | 35 | 523256 | 4186044 | 1 | 35 | 0 | 184 | 4287156 | 1 | 35 | 0 | 1 | 192 |
| 85 | 55 | 49 | 31 | 1 | 36 | 523279 | 4186228 | 1 | 36 | 0 | 184 | 4287348 | 1 | 36 | 0 | 1 | 192 |
| 86 | 56 | 49 | 31 | 1 | 37 | 523302 | 4186412 | 1 | 37 | 0 | 184 | 4287540 | 1 | 37 | 0 | 1 | 192 |
| 87 | 57 | 49 | 31 | 1 | 38 | 523325 | 4186596 | 1 | 38 | 0 | 184 | 4287732 | 1 | 38 | 0 | 1 | 192 |
| 88 | 58 | 49 | 31 | 1 | 39 | 523348 | 4186780 | 1 | 39 | 0 | 184 | 4287924 | 1 | 39 | 0 | 1 | 192 |
| 89 | 59 | 49 | 31 | 1 | 40 | 523371 | 4186964 | 1 | 40 | 0 | 184 | 4288116 | 1 | 40 | 0 | 1 | 192 |
| 90 | 5A | 49 | 31 | 1 | 41 | 523394 | 4187148 | 1 | 41 | 0 | 184 | 4288308 | 1 | 41 | 0 | 1 | 192 |
| 91 | 5B | 49 | 31 | 1 | 42 | 523417 | 4187332 | 1 | 42 | 0 | 184 | 4288500 | 1 | 42 | 0 | 1 | 192 |
| 92 | 5C | 49 | 31 | 1 | 43 | 523440 | 4187516 | 1 | 43 | 0 | 184 | 4288692 | 1 | 43 | 0 | 1 | 192 |
| 93 | 5D | 49 | 31 | 1 | 44 | 523463 | 4187700 | 1 | 44 | 0 | 184 | 4288884 | 1 | 44 | 0 | 1 | 192 |
| 94 | 5E | 49 | 31 | 1 | 45 | 523486 | 4187884 | 1 | 45 | 0 | 184 | 4289076 | 1 | 45 | 0 | 1 | 192 |
| 95 | 5F | 49 | 31 | 1 | 46 | 523509 | 4188068 | 1 | 46 | 0 | 184 | 4289268 | 1 | 46 | 0 | 1 | 192 |
| 96 | 60 | 49 | 31 | 1 | 47 | 523532 | 4188252 | 1 | 47 | 0 | 184 | 4289460 | 1 | 47 | 0 | 1 | 192 |
| 97 | 61 | 49 | 31 | 1 | 48 | 523555 | 4188436 | 1 | 48 | 0 | 184 | 4289652 | 1 | 48 | 0 | 1 | 192 |
| 98 | 62 | 49 | 31 | 2 | 0 | 523576 | 4188604 | 2 | 0 | 0 | 168 | 4289828 | 2 | 0 | 0 | 1 | 176 |
| 99 | 63 | 49 | 31 | 2 | 1 | 523602 | 4188812 | 2 | 1 | 0 | 208 | 4290044 | 2 | 1 | 0 | 1 | 216 |
| 100 | 64 | 49 | 31 | 2 | 2 | 523628 | 4189020 | 2 | 2 | 0 | 208 | 4290260 | 2 | 2 | 0 | 1 | 216 |
| 101 | 65 | 49 | 31 | 2 | 3 | 523654 | 4189228 | 2 | 3 | 0 | 208 | 4290476 | 2 | 3 | 0 | 1 | 216 |
| 102 | 66 | 49 | 31 | 2 | 4 | 523680 | 4189436 | 2 | 4 | 0 | 208 | 4290692 | 2 | 4 | 0 | 1 | 216 |
| 103 | 67 | 49 | 31 | 2 | 5 | 523706 | 4189644 | 2 | 5 | 0 | 208 | 4290908 | 2 | 5 | 0 | 1 | 216 |
| 104 | 68 | 49 | 31 | 2 | 6 | 523732 | 4189852 | 2 | 6 | 0 | 208 | 4291124 | 2 | 6 | 0 | 1 | 216 |
| 105 | 69 | 49 | 31 | 2 | 7 | 523758 | 4190060 | 2 | 7 | 0 | 208 | 4291340 | 2 | 7 | 0 | 1 | 216 |
| 106 | 6A | 49 | 31 | 2 | 8 | 523784 | 4190268 | 2 | 8 | 0 | 208 | 4291556 | 2 | 8 | 0 | 1 | 216 |
| 107 | 6B | 49 | 31 | 2 | 9 | 523810 | 4190476 | 2 | 9 | 0 | 208 | 4291772 | 2 | 9 | 0 | 1 | 216 |
| 108 | 6C | 49 | 31 | 2 | 10 | 523836 | 4190684 | 2 | 10 | 0 | 208 | 4291988 | 2 | 10 | 0 | 1 | 216 |
| 109 | 6D | 49 | 31 | 2 | 11 | 523862 | 4190892 | 2 | 11 | 0 | 208 | 4292204 | 2 | 11 | 0 | 1 | 216 |
| 110 | 6E | 49 | 31 | 2 | 12 | 523888 | 4191100 | 2 | 12 | 0 | 208 | 4292420 | 2 | 12 | 0 | 1 | 216 |
| 111 | 6F | 49 | 31 | 2 | 13 | 523914 | 4191308 | 2 | 13 | 0 | 208 | 4292636 | 2 | 13 | 0 | 1 | 216 |
| 112 | 70 | 49 | 31 | 2 | 14 | 523940 | 4191516 | 2 | 14 | 0 | 208 | 4292852 | 2 | 14 | 0 | 1 | 216 |
| 113 | 71 | 49 | 31 | 2 | 15 | 523966 | 4191724 | 2 | 15 | 0 | 208 | 4293068 | 2 | 15 | 0 | 1 | 216 |
| 114 | 72 | 49 | 31 | 2 | 16 | 523992 | 4191932 | 2 | 16 | 0 | 208 | 4293284 | 2 | 16 | 0 | 1 | 216 |
| 115 | 73 | 49 | 31 | 2 | 17 | 524018 | 4192140 | 2 | 17 | 0 | 208 | 4293500 | 2 | 17 | 0 | 1 | 216 |
| 116 | 74 | 49 | 31 | 2 | 18 | 524044 | 4192348 | 2 | 18 | 0 | 208 | 4293716 | 2 | 18 | 0 | 1 | 216 |
| 117 | 75 | 49 | 31 | 2 | 19 | 524070 | 4192556 | 2 | 19 | 0 | 208 | 4293932 | 2 | 19 | 0 | 1 | 216 |
| 118 | 76 | 49 | 31 | 2 | 20 | 524096 | 4192764 | 2 | 20 | 0 | 208 | 4294148 | 2 | 20 | 0 | 1 | 216 |
| 119 | 77 | 49 | 31 | 2 | 21 | 524122 | 4192972 | 2 | 21 | 0 | 208 | 4294364 | 2 | 21 | 0 | 1 | 216 |
| 120 | 78 | 49 | 31 | 2 | 22 | 524148 | 4193180 | 2 | 22 | 0 | 208 | 4294580 | 2 | 22 | 0 | 1 | 216 |
| 121 | 79 | 49 | 31 | 2 | 23 | 524174 | 4193388 | 2 | 23 | 0 | 208 | 4294796 | 2 | 23 | 0 | 1 | 216 |

| | | | | | | | | | | | | | | | | | |
|---|---|---|---|---|---|---|---|---|---|---|---|---|---|---|---|---|---|
| 122 | 7A | 49 | 31 | 2 | 24 | 524200 | 4193596 | 2 | 24 | 0 | 208 | 4295012 | 2 | 24 | 0 | 1 | 216 |
| 123 | 7B | 49 | 31 | 2 | 25 | 524226 | 4193804 | 2 | 25 | 0 | 208 | 4295228 | 2 | 25 | 0 | 1 | 216 |
| 124 | 7C | 49 | 31 | 2 | 26 | 524252 | 4194012 | 2 | 26 | 0 | 208 | 4295444 | 2 | 26 | 0 | 1 | 216 |
| 125 | 7D | 49 | 31 | 2 | 27 | 524278 | 4194220 | 2 | 27 | 0 | 208 | 4295660 | 2 | 27 | 0 | 1 | 216 |
| 126 | 7E | 49 | 31 | 2 | 28 | 524304 | 4194428 | 2 | 28 | 0 | 208 | 4295876 | 2 | 28 | 0 | 1 | 216 |
| 127 | 7F | 49 | 31 | 2 | 29 | 524330 | 4194636 | 2 | 29 | 0 | 208 | 4296092 | 2 | 29 | 0 | 1 | 216 |
| 128 | 80 | 49 | 31 | 2 | 30 | 524353 | 4194820 | 2 | 30 | 0 | 184 | 4296284 | 2 | 30 | 0 | 1 | 192 |
| 129 | 81 | 49 | 31 | 2 | 31 | 524376 | 4195004 | 2 | 31 | 0 | 184 | 4296476 | 2 | 31 | 0 | 1 | 192 |
| 130 | 82 | 49 | 31 | 2 | 32 | 524399 | 4195188 | 2 | 32 | 0 | 184 | 4296668 | 2 | 32 | 0 | 1 | 192 |
| 131 | 83 | 49 | 31 | 2 | 33 | 524422 | 4195372 | 2 | 33 | 0 | 184 | 4296860 | 2 | 33 | 0 | 1 | 192 |
| 132 | 84 | 49 | 31 | 2 | 34 | 524445 | 4195556 | 2 | 34 | 0 | 184 | 4297052 | 2 | 34 | 0 | 1 | 192 |
| 133 | 85 | 49 | 31 | 2 | 35 | 524468 | 4195740 | 2 | 35 | 0 | 184 | 4297244 | 2 | 35 | 0 | 1 | 192 |
| 134 | 86 | 49 | 31 | 2 | 36 | 524491 | 4195924 | 2 | 36 | 0 | 184 | 4297436 | 2 | 36 | 0 | 1 | 192 |
| 135 | 87 | 49 | 31 | 2 | 37 | 524514 | 4196108 | 2 | 37 | 0 | 184 | 4297628 | 2 | 37 | 0 | 1 | 192 |
| 136 | 88 | 49 | 31 | 2 | 38 | 524537 | 4196292 | 2 | 38 | 0 | 184 | 4297820 | 2 | 38 | 0 | 1 | 192 |
| 137 | 89 | 49 | 31 | 2 | 39 | 524560 | 4196476 | 2 | 39 | 0 | 184 | 4298012 | 2 | 39 | 0 | 1 | 192 |
| 138 | 8A | 49 | 31 | 2 | 40 | 524583 | 4196660 | 2 | 40 | 0 | 184 | 4298204 | 2 | 40 | 0 | 1 | 192 |
| 139 | 8B | 49 | 31 | 2 | 41 | 524606 | 4196844 | 2 | 41 | 0 | 184 | 4298396 | 2 | 41 | 0 | 1 | 192 |
| 140 | 8C | 49 | 31 | 2 | 42 | 524629 | 4197028 | 2 | 42 | 0 | 184 | 4298588 | 2 | 42 | 0 | 1 | 192 |
| 141 | 8D | 49 | 31 | 2 | 43 | 524652 | 4197212 | 2 | 43 | 0 | 184 | 4298780 | 2 | 43 | 0 | 1 | 192 |
| 142 | 8E | 49 | 31 | 2 | 44 | 524675 | 4197396 | 2 | 44 | 0 | 184 | 4298972 | 2 | 44 | 0 | 1 | 192 |
| 143 | 8F | 49 | 31 | 2 | 45 | 524698 | 4197580 | 2 | 45 | 0 | 184 | 4299164 | 2 | 45 | 0 | 1 | 192 |
| 144 | 90 | 49 | 31 | 2 | 46 | 524723 | 4197780 | 2 | 46 | 0 | 200 | 4299372 | 2 | 46 | 0 | 1 | 208 |
| 145 | 91 | 49 | 31 | 2 | 47 | 524748 | 4197980 | 2 | 47 | 0 | 200 | 4299580 | 2 | 47 | 0 | 1 | 208 |
| 146 | 92 | 49 | 31 | 2 | 48 | 524773 | 4198180 | 2 | 48 | 0 | 200 | 4299788 | 2 | 48 | 0 | 1 | 208 |
| 147 | 93 | 49 | 31 | 3 | 0 | 524796 | 4198364 | 3 | 0 | 0 | 184 | 4299980 | 3 | 0 | 0 | 1 | 192 |
| 148 | 94 | 49 | 31 | 3 | 1 | 524824 | 4198588 | 3 | 1 | 0 | 224 | 4300212 | 3 | 1 | 0 | 1 | 232 |
| 149 | 95 | 49 | 31 | 3 | 2 | 524852 | 4198812 | 3 | 2 | 0 | 224 | 4300444 | 3 | 2 | 0 | 1 | 232 |
| 150 | 96 | 49 | 31 | 3 | 3 | 524880 | 4199036 | 3 | 3 | 0 | 224 | 4300676 | 3 | 3 | 0 | 1 | 232 |
| 151 | 97 | 49 | 31 | 3 | 4 | 524908 | 4199260 | 3 | 4 | 0 | 224 | 4300908 | 3 | 4 | 0 | 1 | 232 |
| 152 | 98 | 49 | 31 | 3 | 5 | 524936 | 4199484 | 3 | 5 | 0 | 224 | 4301140 | 3 | 5 | 0 | 1 | 232 |
| 153 | 99 | 49 | 31 | 3 | 6 | 524964 | 4199708 | 3 | 6 | 0 | 224 | 4301372 | 3 | 6 | 0 | 1 | 232 |
| 154 | 9A | 49 | 31 | 3 | 7 | 524992 | 4199932 | 3 | 7 | 0 | 224 | 4301604 | 3 | 7 | 0 | 1 | 232 |
| 155 | 9B | 49 | 31 | 3 | 8 | 525020 | 4200156 | 3 | 8 | 0 | 224 | 4301836 | 3 | 8 | 0 | 1 | 232 |
| 156 | 9C | 49 | 31 | 3 | 9 | 525048 | 4200380 | 3 | 9 | 0 | 224 | 4302068 | 3 | 9 | 0 | 1 | 232 |
| 157 | 9D | 49 | 31 | 3 | 10 | 525076 | 4200604 | 3 | 10 | 0 | 224 | 4302300 | 3 | 10 | 0 | 1 | 232 |
| 158 | 9E | 49 | 31 | 3 | 11 | 525104 | 4200828 | 3 | 11 | 0 | 224 | 4302532 | 3 | 11 | 0 | 1 | 232 |
| 159 | 9F | 49 | 31 | 3 | 12 | 525132 | 4201052 | 3 | 12 | 0 | 224 | 4302764 | 3 | 12 | 0 | 1 | 232 |
| 160 | A0 | 49 | 31 | 3 | 13 | 525160 | 4201276 | 3 | 13 | 0 | 224 | 4302996 | 3 | 13 | 0 | 1 | 232 |
| 161 | A1 | 49 | 31 | 3 | 14 | 525188 | 4201500 | 3 | 14 | 0 | 224 | 4303228 | 3 | 14 | 0 | 1 | 232 |
| 162 | A2 | 49 | 31 | 3 | 15 | 525216 | 4201724 | 3 | 15 | 0 | 224 | 4303460 | 3 | 15 | 0 | 1 | 232 |
| 163 | A3 | 49 | 31 | 3 | 16 | 525244 | 4201948 | 3 | 16 | 0 | 224 | 4303692 | 3 | 16 | 0 | 1 | 232 |
| 164 | A4 | 49 | 31 | 3 | 17 | 525272 | 4202172 | 3 | 17 | 0 | 224 | 4303924 | 3 | 17 | 0 | 1 | 232 |
| 165 | A5 | 49 | 31 | 3 | 18 | 525300 | 4202396 | 3 | 18 | 0 | 224 | 4304156 | 3 | 18 | 0 | 1 | 232 |
| 166 | A6 | 49 | 31 | 3 | 19 | 525328 | 4202620 | 3 | 19 | 0 | 224 | 4304388 | 3 | 19 | 0 | 1 | 232 |
| 167 | A7 | 49 | 31 | 3 | 20 | 525356 | 4202844 | 3 | 20 | 0 | 224 | 4304620 | 3 | 20 | 0 | 1 | 232 |
| 168 | A8 | 49 | 31 | 3 | 21 | 525384 | 4203068 | 3 | 21 | 0 | 224 | 4304852 | 3 | 21 | 0 | 1 | 232 |
| 169 | A9 | 49 | 31 | 3 | 22 | 525412 | 4203292 | 3 | 22 | 0 | 224 | 4305084 | 3 | 22 | 0 | 1 | 232 |
| 170 | AA | 49 | 31 | 3 | 23 | 525440 | 4203516 | 3 | 23 | 0 | 224 | 4305316 | 3 | 23 | 0 | 1 | 232 |
| 171 | AB | 49 | 31 | 3 | 24 | 525468 | 4203740 | 3 | 24 | 0 | 224 | 4305548 | 3 | 24 | 0 | 1 | 232 |
| 172 | AC | 49 | 31 | 3 | 25 | 525496 | 4203964 | 3 | 25 | 0 | 224 | 4305780 | 3 | 25 | 0 | 1 | 232 |
| 173 | AD | 49 | 31 | 3 | 26 | 525524 | 4204188 | 3 | 26 | 0 | 224 | 4306012 | 3 | 26 | 0 | 1 | 232 |
| 174 | AE | 49 | 31 | 3 | 27 | 525552 | 4204412 | 3 | 27 | 0 | 224 | 4306244 | 3 | 27 | 0 | 1 | 232 |
| 175 | AF | 49 | 31 | 3 | 28 | 525580 | 4204636 | 3 | 28 | 0 | 224 | 4306476 | 3 | 28 | 0 | 1 | 232 |
| 176 | B0 | 49 | 31 | 3 | 29 | 525608 | 4204860 | 3 | 29 | 0 | 224 | 4306708 | 3 | 29 | 0 | 1 | 232 |
| 177 | B1 | 49 | 31 | 3 | 30 | 525636 | 4205084 | 3 | 30 | 0 | 224 | 4306940 | 3 | 30 | 0 | 1 | 232 |
| 178 | B2 | 49 | 31 | 3 | 31 | 525664 | 4205308 | 3 | 31 | 0 | 224 | 4307172 | 3 | 31 | 0 | 1 | 232 |
| 179 | B3 | 49 | 31 | 3 | 32 | 525692 | 4205532 | 3 | 32 | 0 | 224 | 4307404 | 3 | 32 | 0 | 1 | 232 |
| 180 | B4 | 49 | 31 | 3 | 33 | 525720 | 4205756 | 3 | 33 | 0 | 224 | 4307636 | 3 | 33 | 0 | 1 | 232 |
| 181 | B5 | 49 | 31 | 3 | 34 | 525748 | 4205980 | 3 | 34 | 0 | 224 | 4307868 | 3 | 34 | 0 | 1 | 232 |
| 182 | B6 | 49 | 31 | 3 | 35 | 525776 | 4206204 | 3 | 35 | 0 | 224 | 4308100 | 3 | 35 | 0 | 1 | 232 |
| 183 | B7 | 49 | 31 | 3 | 36 | 525804 | 4206428 | 3 | 36 | 0 | 224 | 4308332 | 3 | 36 | 0 | 1 | 232 |
| 184 | B8 | 49 | 31 | 3 | 37 | 525832 | 4206652 | 3 | 37 | 0 | 224 | 4308564 | 3 | 37 | 0 | 1 | 232 |
| 185 | B9 | 49 | 31 | 3 | 38 | 525860 | 4206876 | 3 | 38 | 0 | 224 | 4308796 | 3 | 38 | 0 | 1 | 232 |
| 186 | BA | 49 | 31 | 3 | 39 | 525888 | 4207100 | 3 | 39 | 0 | 224 | 4309028 | 3 | 39 | 0 | 1 | 232 |
| 187 | BB | 49 | 31 | 3 | 40 | 525916 | 4207324 | 3 | 40 | 0 | 224 | 4309260 | 3 | 40 | 0 | 1 | 232 |
| 188 | BC | 49 | 31 | 3 | 41 | 525944 | 4207548 | 3 | 41 | 0 | 224 | 4309492 | 3 | 41 | 0 | 1 | 232 |
| 189 | BD | 49 | 31 | 3 | 42 | 525972 | 4207772 | 3 | 42 | 0 | 224 | 4309724 | 3 | 42 | 0 | 1 | 232 |
| 190 | BE | 49 | 31 | 3 | 43 | 526000 | 4207996 | 3 | 43 | 0 | 224 | 4309956 | 3 | 43 | 0 | 1 | 232 |
| 191 | BF | 49 | 31 | 3 | 44 | 526028 | 4208220 | 3 | 44 | 0 | 224 | 4310188 | 3 | 44 | 0 | 1 | 232 |
| 192 | C0 | 49 | 31 | 3 | 45 | 526053 | 4208420 | 3 | 45 | 0 | 200 | 4310396 | 3 | 45 | 0 | 1 | 208 |
| 193 | C1 | 49 | 31 | 3 | 46 | 526078 | 4208620 | 3 | 46 | 0 | 200 | 4310604 | 3 | 46 | 0 | 1 | 208 |
| 194 | C2 | 49 | 31 | 3 | 47 | 526103 | 4208820 | 3 | 47 | 0 | 200 | 4310812 | 3 | 47 | 0 | 1 | 208 |
| 195 | C3 | 49 | 31 | 3 | 48 | 526128 | 4209020 | 3 | 48 | 0 | 200 | 4311020 | 3 | 48 | 0 | 1 | 208 |
| 196 | C4 | 49 | 31 | 4 | 0 | 526151 | 4209204 | 4 | 0 | 0 | 184 | 4311212 | 4 | 0 | 0 | 1 | 192 |
| 197 | C5 | 49 | 31 | 4 | 1 | 526179 | 4209428 | 4 | 1 | 0 | 224 | 4311444 | 4 | 1 | 0 | 1 | 232 |
| 198 | C6 | 49 | 31 | 4 | 2 | 526207 | 4209652 | 4 | 2 | 0 | 224 | 4311676 | 4 | 2 | 0 | 1 | 232 |
| 199 | C7 | 49 | 31 | 4 | 3 | 526235 | 4209876 | 4 | 3 | 0 | 224 | 4311908 | 4 | 3 | 0 | 1 | 232 |
| 200 | C8 | 49 | 31 | 4 | 4 | 526263 | 4210100 | 4 | 4 | 0 | 224 | 4312140 | 4 | 4 | 0 | 1 | 232 |
| 201 | C9 | 49 | 31 | 4 | 5 | 526291 | 4210324 | 4 | 5 | 0 | 224 | 4312372 | 4 | 5 | 0 | 1 | 232 |
| 202 | CA | 49 | 31 | 4 | 6 | 526319 | 4210548 | 4 | 6 | 0 | 224 | 4312604 | 4 | 6 | 0 | 1 | 232 |
| 203 | CB | 49 | 31 | 4 | 7 | 526347 | 4210772 | 4 | 7 | 0 | 224 | 4312836 | 4 | 7 | 0 | 1 | 232 |
| 204 | CC | 49 | 31 | 4 | 8 | 526375 | 4210996 | 4 | 8 | 0 | 224 | 4313068 | 4 | 8 | 0 | 1 | 232 |
| 205 | CD | 49 | 31 | 4 | 9 | 526403 | 4211220 | 4 | 9 | 0 | 224 | 4313300 | 4 | 9 | 0 | 1 | 232 |
| 206 | CE | 49 | 31 | 4 | 10 | 526431 | 4211444 | 4 | 10 | 0 | 224 | 4313532 | 4 | 10 | 0 | 1 | 232 |
| 207 | CF | 49 | 31 | 4 | 11 | 526459 | 4211668 | 4 | 11 | 0 | 224 | 4313764 | 4 | 11 | 0 | 1 | 232 |
| 208 | D0 | 49 | 31 | 4 | 12 | 526489 | 4211908 | 4 | 12 | 0 | 240 | 4314012 | 4 | 12 | 0 | 1 | 248 |
| 209 | D1 | 49 | 31 | 4 | 13 | 526519 | 4212148 | 4 | 13 | 0 | 240 | 4314260 | 4 | 13 | 0 | 1 | 248 |
| 210 | D2 | 49 | 31 | 4 | 14 | 526549 | 4212388 | 4 | 14 | 0 | 240 | 4314508 | 4 | 14 | 0 | 1 | 248 |
| 211 | D3 | 49 | 31 | 4 | 15 | 526579 | 4212628 | 4 | 15 | 0 | 240 | 4314756 | 4 | 15 | 0 | 1 | 248 |
| 212 | D4 | 49 | 31 | 4 | 16 | 526609 | 4212868 | 4 | 16 | 0 | 240 | 4315004 | 4 | 16 | 0 | 1 | 248 |
| 213 | D5 | 49 | 31 | 4 | 17 | 526639 | 4213108 | 4 | 17 | 0 | 240 | 4315252 | 4 | 17 | 0 | 1 | 248 |
| 214 | D6 | 49 | 31 | 4 | 18 | 526669 | 4213348 | 4 | 18 | 0 | 240 | 4315500 | 4 | 18 | 0 | 1 | 248 |
| 215 | D7 | 49 | 31 | 4 | 19 | 526699 | 4213588 | 4 | 19 | 0 | 240 | 4315748 | 4 | 19 | 0 | 1 | 248 |
| 216 | D8 | 49 | 31 | 4 | 20 | 526729 | 4213828 | 4 | 20 | 0 | 240 | 4315996 | 4 | 20 | 0 | 1 | 248 |
| 217 | D9 | 49 | 31 | 4 | 21 | 526759 | 4214068 | 4 | 21 | 0 | 240 | 4316244 | 4 | 21 | 0 | 1 | 248 |
| 218 | DA | 49 | 31 | 4 | 22 | 526789 | 4214308 | 4 | 22 | 0 | 240 | 4316492 | 4 | 22 | 0 | 1 | 248 |
| 219 | DB | 49 | 31 | 4 | 23 | 526819 | 4214548 | 4 | 23 | 0 | 240 | 4316740 | 4 | 23 | 0 | 1 | 248 |
| 220 | DC | 49 | 31 | 4 | 24 | 526849 | 4214788 | 4 | 24 | 0 | 240 | 4316988 | 4 | 24 | 0 | 1 | 248 |
| 221 | DD | 49 | 31 | 4 | 25 | 526879 | 4215028 | 4 | 25 | 0 | 240 | 4317236 | 4 | 25 | 0 | 1 | 248 |
| 222 | DE | 49 | 31 | 4 | 26 | 526909 | 4215268 | 4 | 26 | 0 | 240 | 4317484 | 4 | 26 | 0 | 1 | 248 |
| 223 | DF | 49 | 31 | 4 | 27 | 526939 | 4215508 | 4 | 27 | 0 | 240 | 4317732 | 4 | 27 | 0 | 1 | 248 |
| 224 | E0 | 49 | 31 | 4 | 28 | 526969 | 4215748 | 4 | 28 | 0 | 240 | 4317980 | 4 | 28 | 0 | 1 | 248 |

| | | | | | | | | | | | | | | | | | |
|---|---|---|---|---|---|---|---|---|---|---|---|---|---|---|---|---|---|
| 225 | E1 | 49 | 31 | 4 | 29 | 526999 | 4215988 | 4 | 29 | 0 | 240 | 4318228 | 4 | 29 | 0 | 1 | 248 |
| 226 | E2 | 49 | 31 | 4 | 30 | 527029 | 4216228 | 4 | 30 | 0 | 240 | 4318476 | 4 | 30 | 0 | 1 | 248 |
| 227 | E3 | 49 | 31 | 4 | 31 | 527059 | 4216468 | 4 | 31 | 0 | 240 | 4318724 | 4 | 31 | 0 | 1 | 248 |
| 228 | E4 | 49 | 31 | 4 | 32 | 527089 | 4216708 | 4 | 32 | 0 | 240 | 4318972 | 4 | 32 | 0 | 1 | 248 |
| 229 | E5 | 49 | 31 | 4 | 33 | 527119 | 4216948 | 4 | 33 | 0 | 240 | 4319220 | 4 | 33 | 0 | 1 | 248 |
| 230 | E6 | 49 | 31 | 4 | 34 | 527149 | 4217188 | 4 | 34 | 0 | 240 | 4319468 | 4 | 34 | 0 | 1 | 248 |
| 231 | E7 | 49 | 31 | 4 | 35 | 527179 | 4217428 | 4 | 35 | 0 | 240 | 4319716 | 4 | 35 | 0 | 1 | 248 |
| 232 | E8 | 49 | 31 | 4 | 36 | 527209 | 4217668 | 4 | 36 | 0 | 240 | 4319964 | 4 | 36 | 0 | 1 | 248 |
| 233 | E9 | 49 | 31 | 4 | 37 | 527239 | 4217908 | 4 | 37 | 0 | 240 | 4320212 | 4 | 37 | 0 | 1 | 248 |
| 234 | EA | 49 | 31 | 4 | 38 | 527269 | 4218148 | 4 | 38 | 0 | 240 | 4320460 | 4 | 38 | 0 | 1 | 248 |
| 235 | EB | 49 | 31 | 4 | 39 | 527299 | 4218388 | 4 | 39 | 0 | 240 | 4320708 | 4 | 39 | 0 | 1 | 248 |
| 236 | EC | 49 | 31 | 4 | 40 | 527329 | 4218628 | 4 | 40 | 0 | 240 | 4320956 | 4 | 40 | 0 | 1 | 248 |
| 237 | ED | 49 | 31 | 4 | 41 | 527359 | 4218868 | 4 | 41 | 0 | 240 | 4321204 | 4 | 41 | 0 | 1 | 248 |
| 238 | EE | 49 | 31 | 4 | 42 | 527389 | 4219108 | 4 | 42 | 0 | 240 | 4321452 | 4 | 42 | 0 | 1 | 248 |
| 239 | EF | 49 | 31 | 4 | 43 | 527419 | 4219348 | 4 | 43 | 0 | 240 | 4321700 | 4 | 43 | 0 | 1 | 248 |
| 240 | F0 | 49 | 31 | 4 | 44 | 527449 | 4219588 | 4 | 44 | 0 | 240 | 4321948 | 4 | 44 | 0 | 1 | 248 |
| 241 | F1 | 49 | 31 | 4 | 45 | 527479 | 4219828 | 4 | 45 | 0 | 240 | 4322196 | 4 | 45 | 0 | 1 | 248 |
| 242 | F2 | 49 | 31 | 4 | 46 | 527509 | 4220068 | 4 | 46 | 0 | 240 | 4322444 | 4 | 46 | 0 | 1 | 248 |
| 243 | F3 | 49 | 31 | 4 | 47 | 527539 | 4220308 | 4 | 47 | 0 | 240 | 4322692 | 4 | 47 | 0 | 1 | 248 |
| 244 | F4 | 49 | 31 | 4 | 48 | 527569 | 4220548 | 4 | 48 | 0 | 240 | 4322940 | 4 | 48 | 0 | 1 | 248 |
| 245 | F5 | 49 | 31 | 5 | 0 | 527601 | 4220804 | 5 | 0 | 0 | 256 | 4323204 | 5 | 0 | 0 | 1 | 264 |
| 246 | F6 | 49 | 31 | 5 | 1 | 527633 | 4221060 | 5 | 1 | 0 | 256 | 4323468 | 5 | 1 | 0 | 1 | 264 |
| 247 | F7 | 49 | 31 | 5 | 2 | 527665 | 4221316 | 5 | 2 | 0 | 256 | 4323732 | 5 | 2 | 0 | 1 | 264 |
| 248 | F8 | 49 | 31 | 5 | 3 | 527697 | 4221572 | 5 | 3 | 0 | 256 | 4323996 | 5 | 3 | 0 | 1 | 264 |
| 249 | F9 | 49 | 31 | 5 | 4 | 527729 | 4221828 | 5 | 4 | 0 | 256 | 4324260 | 5 | 4 | 0 | 1 | 264 |
| 250 | FA | 49 | 31 | 5 | 5 | 527761 | 4222084 | 5 | 5 | 0 | 256 | 4324524 | 5 | 5 | 0 | 1 | 264 |
| 251 | FB | 49 | 31 | 5 | 6 | 527793 | 4222340 | 5 | 6 | 0 | 256 | 4324788 | 5 | 6 | 0 | 1 | 264 |
| 252 | FC | 49 | 31 | 5 | 7 | 527825 | 4222596 | 5 | 7 | 0 | 256 | 4325052 | 5 | 7 | 0 | 1 | 264 |
| 253 | FD | 49 | 31 | 5 | 8 | 527857 | 4222852 | 5 | 8 | 0 | 256 | 4325316 | 5 | 8 | 0 | 1 | 264 |
| 254 | FE | 49 | 31 | 5 | 9 | 527889 | 4223108 | 5 | 9 | 0 | 256 | 4325580 | 5 | 9 | 0 | 1 | 264 |
| 255 | FF | 49 | 31 | 5 | 10 | 527921 | 4223364 | 5 | 10 | 0 | 256 | 4325844 | 5 | 10 | 0 | 1 | 264 |
| 0 | 00 | 50 | 32 | 0 | 0 | 527933 | 4223460 | 0 | 0 | 0 | 96 | 4325948 | 0 | 0 | 0 | 1 | 104 |
| 1 | 01 | 50 | 32 | 0 | 1 | 527947 | 4223572 | 0 | 1 | 0 | 112 | 4326068 | 0 | 1 | 0 | 1 | 120 |
| 2 | 02 | 50 | 32 | 0 | 2 | 527961 | 4223684 | 0 | 2 | 0 | 112 | 4326188 | 0 | 2 | 0 | 1 | 120 |
| 3 | 03 | 50 | 32 | 0 | 3 | 527975 | 4223796 | 0 | 3 | 0 | 112 | 4326308 | 0 | 3 | 0 | 1 | 120 |
| 4 | 04 | 50 | 32 | 0 | 4 | 527989 | 4223908 | 0 | 4 | 0 | 112 | 4326428 | 0 | 4 | 0 | 1 | 120 |
| 5 | 05 | 50 | 32 | 0 | 5 | 528003 | 4224020 | 0 | 5 | 0 | 112 | 4326548 | 0 | 5 | 0 | 1 | 120 |
| 6 | 06 | 50 | 32 | 0 | 6 | 528017 | 4224132 | 0 | 6 | 0 | 112 | 4326668 | 0 | 6 | 0 | 1 | 120 |
| 7 | 07 | 50 | 32 | 0 | 7 | 528031 | 4224244 | 0 | 7 | 0 | 112 | 4326788 | 0 | 7 | 0 | 1 | 120 |
| 8 | 08 | 50 | 32 | 0 | 8 | 528045 | 4224356 | 0 | 8 | 0 | 112 | 4326908 | 0 | 8 | 0 | 1 | 120 |
| 9 | 09 | 50 | 32 | 0 | 9 | 528059 | 4224468 | 0 | 9 | 0 | 112 | 4327028 | 0 | 9 | 0 | 1 | 120 |
| 10 | 0A | 50 | 32 | 0 | 10 | 528073 | 4224580 | 0 | 10 | 0 | 112 | 4327148 | 0 | 10 | 0 | 1 | 120 |
| 11 | 0B | 50 | 32 | 0 | 11 | 528087 | 4224692 | 0 | 11 | 0 | 112 | 4327268 | 0 | 11 | 0 | 1 | 120 |
| 12 | 0C | 50 | 32 | 0 | 12 | 528101 | 4224804 | 0 | 12 | 0 | 112 | 4327388 | 0 | 12 | 0 | 1 | 120 |
| 13 | 0D | 50 | 32 | 0 | 13 | 528115 | 4224916 | 0 | 13 | 0 | 112 | 4327508 | 0 | 13 | 0 | 1 | 120 |
| 14 | 0E | 50 | 32 | 0 | 14 | 528129 | 4225028 | 0 | 14 | 0 | 112 | 4327628 | 0 | 14 | 0 | 1 | 120 |
| 15 | 0F | 50 | 32 | 0 | 15 | 528143 | 4225140 | 0 | 15 | 0 | 112 | 4327748 | 0 | 15 | 0 | 1 | 120 |
| 16 | 10 | 50 | 32 | 0 | 16 | 528157 | 4225252 | 0 | 16 | 0 | 112 | 4327868 | 0 | 16 | 0 | 1 | 120 |
| 17 | 11 | 50 | 32 | 0 | 17 | 528171 | 4225364 | 0 | 17 | 0 | 112 | 4327988 | 0 | 17 | 0 | 1 | 120 |
| 18 | 12 | 50 | 32 | 0 | 18 | 528185 | 4225476 | 0 | 18 | 0 | 112 | 4328108 | 0 | 18 | 0 | 1 | 120 |
| 19 | 13 | 50 | 32 | 0 | 19 | 528199 | 4225588 | 0 | 19 | 0 | 112 | 4328228 | 0 | 19 | 0 | 1 | 120 |
| 20 | 14 | 50 | 32 | 0 | 20 | 528213 | 4225700 | 0 | 20 | 0 | 112 | 4328348 | 0 | 20 | 0 | 1 | 120 |
| 21 | 15 | 50 | 32 | 0 | 21 | 528227 | 4225812 | 0 | 21 | 0 | 112 | 4328468 | 0 | 21 | 0 | 1 | 120 |
| 22 | 16 | 50 | 32 | 0 | 22 | 528241 | 4225924 | 0 | 22 | 0 | 112 | 4328588 | 0 | 22 | 0 | 1 | 120 |
| 23 | 17 | 50 | 32 | 0 | 23 | 528255 | 4226036 | 0 | 23 | 0 | 112 | 4328708 | 0 | 23 | 0 | 1 | 120 |
| 24 | 18 | 50 | 32 | 0 | 24 | 528269 | 4226148 | 0 | 24 | 0 | 112 | 4328828 | 0 | 24 | 0 | 1 | 120 |
| 25 | 19 | 50 | 32 | 0 | 25 | 528283 | 4226260 | 0 | 25 | 0 | 112 | 4328948 | 0 | 25 | 0 | 1 | 120 |
| 26 | 1A | 50 | 32 | 0 | 26 | 528297 | 4226372 | 0 | 26 | 0 | 112 | 4329068 | 0 | 26 | 0 | 1 | 120 |
| 27 | 1B | 50 | 32 | 0 | 27 | 528311 | 4226484 | 0 | 27 | 0 | 112 | 4329188 | 0 | 27 | 0 | 1 | 120 |
| 28 | 1C | 50 | 32 | 0 | 28 | 528325 | 4226596 | 0 | 28 | 0 | 112 | 4329308 | 0 | 28 | 0 | 1 | 120 |
| 29 | 1D | 50 | 32 | 0 | 29 | 528339 | 4226708 | 0 | 29 | 0 | 112 | 4329428 | 0 | 29 | 0 | 1 | 120 |
| 30 | 1E | 50 | 32 | 0 | 30 | 528353 | 4226820 | 0 | 30 | 0 | 112 | 4329548 | 0 | 30 | 0 | 1 | 120 |
| 31 | 1F | 50 | 32 | 0 | 31 | 528367 | 4226932 | 0 | 31 | 0 | 112 | 4329668 | 0 | 31 | 0 | 1 | 120 |
| 32 | 20 | 50 | 32 | 0 | 32 | 528381 | 4227044 | 0 | 32 | 0 | 112 | 4329788 | 0 | 32 | 0 | 1 | 120 |
| 33 | 21 | 50 | 32 | 0 | 33 | 528395 | 4227156 | 0 | 33 | 0 | 112 | 4329908 | 0 | 33 | 0 | 1 | 120 |
| 34 | 22 | 50 | 32 | 0 | 34 | 528409 | 4227268 | 0 | 34 | 0 | 112 | 4330028 | 0 | 34 | 0 | 1 | 120 |
| 35 | 23 | 50 | 32 | 0 | 35 | 528423 | 4227380 | 0 | 35 | 0 | 112 | 4330148 | 0 | 35 | 0 | 1 | 120 |
| 36 | 24 | 50 | 32 | 0 | 36 | 528437 | 4227492 | 0 | 36 | 0 | 112 | 4330268 | 0 | 36 | 0 | 1 | 120 |
| 37 | 25 | 50 | 32 | 0 | 37 | 528451 | 4227604 | 0 | 37 | 0 | 112 | 4330388 | 0 | 37 | 0 | 1 | 120 |
| 38 | 26 | 50 | 32 | 0 | 38 | 528465 | 4227716 | 0 | 38 | 0 | 112 | 4330508 | 0 | 38 | 0 | 1 | 120 |
| 39 | 27 | 50 | 32 | 0 | 39 | 528479 | 4227828 | 0 | 39 | 0 | 112 | 4330628 | 0 | 39 | 0 | 1 | 120 |
| 40 | 28 | 50 | 32 | 0 | 40 | 528493 | 4227940 | 0 | 40 | 0 | 112 | 4330748 | 0 | 40 | 0 | 1 | 120 |
| 41 | 29 | 50 | 32 | 0 | 41 | 528507 | 4228052 | 0 | 41 | 0 | 112 | 4330868 | 0 | 41 | 0 | 1 | 120 |
| 42 | 2A | 50 | 32 | 0 | 42 | 528521 | 4228164 | 0 | 42 | 0 | 112 | 4330988 | 0 | 42 | 0 | 1 | 120 |
| 43 | 2B | 50 | 32 | 0 | 43 | 528535 | 4228276 | 0 | 43 | 0 | 112 | 4331108 | 0 | 43 | 0 | 1 | 120 |
| 44 | 2C | 50 | 32 | 0 | 44 | 528549 | 4228388 | 0 | 44 | 0 | 112 | 4331228 | 0 | 44 | 0 | 1 | 120 |
| 45 | 2D | 50 | 32 | 0 | 45 | 528563 | 4228500 | 0 | 45 | 0 | 112 | 4331348 | 0 | 45 | 0 | 1 | 120 |
| 46 | 2E | 50 | 32 | 0 | 46 | 528577 | 4228612 | 0 | 46 | 0 | 112 | 4331468 | 0 | 46 | 0 | 1 | 120 |
| 47 | 2F | 50 | 32 | 0 | 47 | 528591 | 4228724 | 0 | 47 | 0 | 112 | 4331588 | 0 | 47 | 0 | 1 | 120 |
| 48 | 30 | 50 | 32 | 0 | 48 | 528605 | 4228836 | 0 | 48 | 0 | 112 | 4331708 | 0 | 48 | 0 | 1 | 120 |
| 49 | 31 | 50 | 32 | 0 | 49 | 528619 | 4228948 | 0 | 49 | 0 | 112 | 4331828 | 0 | 49 | 0 | 1 | 120 |
| 50 | 32 | 50 | 32 | 1 | 0 | 528631 | 4229044 | 1 | 0 | 0 | 96 | 4331932 | 1 | 0 | 0 | 1 | 104 |
| 51 | 33 | 50 | 32 | 1 | 1 | 528648 | 4229180 | 1 | 1 | 0 | 136 | 4332076 | 1 | 1 | 0 | 1 | 144 |
| 52 | 34 | 50 | 32 | 1 | 2 | 528665 | 4229316 | 1 | 2 | 0 | 136 | 4332220 | 1 | 2 | 0 | 1 | 144 |
| 53 | 35 | 50 | 32 | 1 | 3 | 528682 | 4229452 | 1 | 3 | 0 | 136 | 4332364 | 1 | 3 | 0 | 1 | 144 |
| 54 | 36 | 50 | 32 | 1 | 4 | 528699 | 4229588 | 1 | 4 | 0 | 136 | 4332508 | 1 | 4 | 0 | 1 | 144 |
| 55 | 37 | 50 | 32 | 1 | 5 | 528716 | 4229724 | 1 | 5 | 0 | 136 | 4332652 | 1 | 5 | 0 | 1 | 144 |
| 56 | 38 | 50 | 32 | 1 | 6 | 528733 | 4229860 | 1 | 6 | 0 | 136 | 4332796 | 1 | 6 | 0 | 1 | 144 |
| 57 | 39 | 50 | 32 | 1 | 7 | 528750 | 4229996 | 1 | 7 | 0 | 136 | 4332940 | 1 | 7 | 0 | 1 | 144 |
| 58 | 3A | 50 | 32 | 1 | 8 | 528767 | 4230132 | 1 | 8 | 0 | 136 | 4333084 | 1 | 8 | 0 | 1 | 144 |
| 59 | 3B | 50 | 32 | 1 | 9 | 528784 | 4230268 | 1 | 9 | 0 | 136 | 4333228 | 1 | 9 | 0 | 1 | 144 |
| 60 | 3C | 50 | 32 | 1 | 10 | 528801 | 4230404 | 1 | 10 | 0 | 136 | 4333372 | 1 | 10 | 0 | 1 | 144 |
| 61 | 3D | 50 | 32 | 1 | 11 | 528818 | 4230540 | 1 | 11 | 0 | 136 | 4333516 | 1 | 11 | 0 | 1 | 144 |
| 62 | 3E | 50 | 32 | 1 | 12 | 528835 | 4230676 | 1 | 12 | 0 | 136 | 4333660 | 1 | 12 | 0 | 1 | 144 |
| 63 | 3F | 50 | 32 | 1 | 13 | 528852 | 4230812 | 1 | 13 | 0 | 136 | 4333804 | 1 | 13 | 0 | 1 | 144 |
| 64 | 40 | 50 | 32 | 1 | 14 | 528873 | 4230980 | 1 | 14 | 0 | 168 | 4333980 | 1 | 14 | 0 | 1 | 176 |
| 65 | 41 | 50 | 32 | 1 | 15 | 528894 | 4231148 | 1 | 15 | 0 | 168 | 4334156 | 1 | 15 | 0 | 1 | 176 |
| 66 | 42 | 50 | 32 | 1 | 16 | 528915 | 4231316 | 1 | 16 | 0 | 168 | 4334332 | 1 | 16 | 0 | 1 | 176 |
| 67 | 43 | 50 | 32 | 1 | 17 | 528936 | 4231484 | 1 | 17 | 0 | 168 | 4334508 | 1 | 17 | 0 | 1 | 176 |
| 68 | 44 | 50 | 32 | 1 | 18 | 528957 | 4231652 | 1 | 18 | 0 | 168 | 4334684 | 1 | 18 | 0 | 1 | 176 |
| 69 | 45 | 50 | 32 | 1 | 19 | 528978 | 4231820 | 1 | 19 | 0 | 168 | 4334860 | 1 | 19 | 0 | 1 | 176 |
| 70 | 46 | 50 | 32 | 1 | 20 | 528999 | 4231988 | 1 | 20 | 0 | 168 | 4335036 | 1 | 20 | 0 | 1 | 176 |
| 71 | 47 | 50 | 32 | 1 | 21 | 529020 | 4232156 | 1 | 21 | 0 | 168 | 4335212 | 1 | 21 | 0 | 1 | 176 |

| | | | | | | | | | | | | | | | | | |
|---|---|---|---|---|---|---|---|---|---|---|---|---|---|---|---|---|---|
| 72 | 48 | 50 | 32 | 1 | 22 | 529041 | 4232324 | 1 | 22 | 0 | 168 | 4335388 | 1 | 22 | 0 | 1 | 176 |
| 73 | 49 | 50 | 32 | 1 | 23 | 529062 | 4232492 | 1 | 23 | 0 | 168 | 4335564 | 1 | 23 | 0 | 1 | 176 |
| 74 | 4A | 50 | 32 | 1 | 24 | 529083 | 4232660 | 1 | 24 | 0 | 168 | 4335740 | 1 | 24 | 0 | 1 | 176 |
| 75 | 4B | 50 | 32 | 1 | 25 | 529104 | 4232828 | 1 | 25 | 0 | 168 | 4335916 | 1 | 25 | 0 | 1 | 176 |
| 76 | 4C | 50 | 32 | 1 | 26 | 529125 | 4232996 | 1 | 26 | 0 | 168 | 4336092 | 1 | 26 | 0 | 1 | 176 |
| 77 | 4D | 50 | 32 | 1 | 27 | 529146 | 4233164 | 1 | 27 | 0 | 168 | 4336268 | 1 | 27 | 0 | 1 | 176 |
| 78 | 4E | 50 | 32 | 1 | 28 | 529167 | 4233332 | 1 | 28 | 0 | 168 | 4336444 | 1 | 28 | 0 | 1 | 176 |
| 79 | 4F | 50 | 32 | 1 | 29 | 529188 | 4233500 | 1 | 29 | 0 | 168 | 4336620 | 1 | 29 | 0 | 1 | 176 |
| 80 | 50 | 50 | 32 | 1 | 30 | 529211 | 4233684 | 1 | 30 | 0 | 184 | 4336812 | 1 | 30 | 0 | 1 | 192 |
| 81 | 51 | 50 | 32 | 1 | 31 | 529234 | 4233868 | 1 | 31 | 0 | 184 | 4337004 | 1 | 31 | 0 | 1 | 192 |
| 82 | 52 | 50 | 32 | 1 | 32 | 529257 | 4234052 | 1 | 32 | 0 | 184 | 4337196 | 1 | 32 | 0 | 1 | 192 |
| 83 | 53 | 50 | 32 | 1 | 33 | 529280 | 4234236 | 1 | 33 | 0 | 184 | 4337388 | 1 | 33 | 0 | 1 | 192 |
| 84 | 54 | 50 | 32 | 1 | 34 | 529303 | 4234420 | 1 | 34 | 0 | 184 | 4337580 | 1 | 34 | 0 | 1 | 192 |
| 85 | 55 | 50 | 32 | 1 | 35 | 529326 | 4234604 | 1 | 35 | 0 | 184 | 4337772 | 1 | 35 | 0 | 1 | 192 |
| 86 | 56 | 50 | 32 | 1 | 36 | 529349 | 4234788 | 1 | 36 | 0 | 184 | 4337964 | 1 | 36 | 0 | 1 | 192 |
| 87 | 57 | 50 | 32 | 1 | 37 | 529372 | 4234972 | 1 | 37 | 0 | 184 | 4338156 | 1 | 37 | 0 | 1 | 192 |
| 88 | 58 | 50 | 32 | 1 | 38 | 529395 | 4235156 | 1 | 38 | 0 | 184 | 4338348 | 1 | 38 | 0 | 1 | 192 |
| 89 | 59 | 50 | 32 | 1 | 39 | 529418 | 4235340 | 1 | 39 | 0 | 184 | 4338540 | 1 | 39 | 0 | 1 | 192 |
| 90 | 5A | 50 | 32 | 1 | 40 | 529441 | 4235524 | 1 | 40 | 0 | 184 | 4338732 | 1 | 40 | 0 | 1 | 192 |
| 91 | 5B | 50 | 32 | 1 | 41 | 529464 | 4235708 | 1 | 41 | 0 | 184 | 4338924 | 1 | 41 | 0 | 1 | 192 |
| 92 | 5C | 50 | 32 | 1 | 42 | 529487 | 4235892 | 1 | 42 | 0 | 184 | 4339116 | 1 | 42 | 0 | 1 | 192 |
| 93 | 5D | 50 | 32 | 1 | 43 | 529510 | 4236076 | 1 | 43 | 0 | 184 | 4339308 | 1 | 43 | 0 | 1 | 192 |
| 94 | 5E | 50 | 32 | 1 | 44 | 529533 | 4236260 | 1 | 44 | 0 | 184 | 4339500 | 1 | 44 | 0 | 1 | 192 |
| 95 | 5F | 50 | 32 | 1 | 45 | 529556 | 4236444 | 1 | 45 | 0 | 184 | 4339692 | 1 | 45 | 0 | 1 | 192 |
| 96 | 60 | 50 | 32 | 1 | 46 | 529579 | 4236628 | 1 | 46 | 0 | 184 | 4339884 | 1 | 46 | 0 | 1 | 192 |
| 97 | 61 | 50 | 32 | 1 | 47 | 529602 | 4236812 | 1 | 47 | 0 | 184 | 4340076 | 1 | 47 | 0 | 1 | 192 |
| 98 | 62 | 50 | 32 | 1 | 48 | 529625 | 4236996 | 1 | 48 | 0 | 184 | 4340268 | 1 | 48 | 0 | 1 | 192 |
| 99 | 63 | 50 | 32 | 1 | 49 | 529648 | 4237180 | 1 | 49 | 0 | 184 | 4340460 | 1 | 49 | 0 | 1 | 192 |
| 100 | 64 | 50 | 32 | 2 | 0 | 529669 | 4237348 | 2 | 0 | 0 | 168 | 4340636 | 2 | 0 | 0 | 1 | 176 |
| 101 | 65 | 50 | 32 | 2 | 1 | 529695 | 4237556 | 2 | 1 | 0 | 208 | 4340852 | 2 | 1 | 0 | 1 | 216 |
| 102 | 66 | 50 | 32 | 2 | 2 | 529721 | 4237764 | 2 | 2 | 0 | 208 | 4341068 | 2 | 2 | 0 | 1 | 216 |
| 103 | 67 | 50 | 32 | 2 | 3 | 529747 | 4237972 | 2 | 3 | 0 | 208 | 4341284 | 2 | 3 | 0 | 1 | 216 |
| 104 | 68 | 50 | 32 | 2 | 4 | 529773 | 4238180 | 2 | 4 | 0 | 208 | 4341500 | 2 | 4 | 0 | 1 | 216 |
| 105 | 69 | 50 | 32 | 2 | 5 | 529799 | 4238388 | 2 | 5 | 0 | 208 | 4341716 | 2 | 5 | 0 | 1 | 216 |
| 106 | 6A | 50 | 32 | 2 | 6 | 529825 | 4238596 | 2 | 6 | 0 | 208 | 4341932 | 2 | 6 | 0 | 1 | 216 |
| 107 | 6B | 50 | 32 | 2 | 7 | 529851 | 4238804 | 2 | 7 | 0 | 208 | 4342148 | 2 | 7 | 0 | 1 | 216 |
| 108 | 6C | 50 | 32 | 2 | 8 | 529877 | 4239012 | 2 | 8 | 0 | 208 | 4342364 | 2 | 8 | 0 | 1 | 216 |
| 109 | 6D | 50 | 32 | 2 | 9 | 529903 | 4239220 | 2 | 9 | 0 | 208 | 4342580 | 2 | 9 | 0 | 1 | 216 |
| 110 | 6E | 50 | 32 | 2 | 10 | 529929 | 4239428 | 2 | 10 | 0 | 208 | 4342796 | 2 | 10 | 0 | 1 | 216 |
| 111 | 6F | 50 | 32 | 2 | 11 | 529955 | 4239636 | 2 | 11 | 0 | 208 | 4343012 | 2 | 11 | 0 | 1 | 216 |
| 112 | 70 | 50 | 32 | 2 | 12 | 529981 | 4239844 | 2 | 12 | 0 | 208 | 4343228 | 2 | 12 | 0 | 1 | 216 |
| 113 | 71 | 50 | 32 | 2 | 13 | 530007 | 4240052 | 2 | 13 | 0 | 208 | 4343444 | 2 | 13 | 0 | 1 | 216 |
| 114 | 72 | 50 | 32 | 2 | 14 | 530033 | 4240260 | 2 | 14 | 0 | 208 | 4343660 | 2 | 14 | 0 | 1 | 216 |
| 115 | 73 | 50 | 32 | 2 | 15 | 530059 | 4240468 | 2 | 15 | 0 | 208 | 4343876 | 2 | 15 | 0 | 1 | 216 |
| 116 | 74 | 50 | 32 | 2 | 16 | 530085 | 4240676 | 2 | 16 | 0 | 208 | 4344092 | 2 | 16 | 0 | 1 | 216 |
| 117 | 75 | 50 | 32 | 2 | 17 | 530111 | 4240884 | 2 | 17 | 0 | 208 | 4344308 | 2 | 17 | 0 | 1 | 216 |
| 118 | 76 | 50 | 32 | 2 | 18 | 530137 | 4241092 | 2 | 18 | 0 | 208 | 4344524 | 2 | 18 | 0 | 1 | 216 |
| 119 | 77 | 50 | 32 | 2 | 19 | 530163 | 4241300 | 2 | 19 | 0 | 208 | 4344740 | 2 | 19 | 0 | 1 | 216 |
| 120 | 78 | 50 | 32 | 2 | 20 | 530189 | 4241508 | 2 | 20 | 0 | 208 | 4344956 | 2 | 20 | 0 | 1 | 216 |
| 121 | 79 | 50 | 32 | 2 | 21 | 530215 | 4241716 | 2 | 21 | 0 | 208 | 4345172 | 2 | 21 | 0 | 1 | 216 |
| 122 | 7A | 50 | 32 | 2 | 22 | 530241 | 4241924 | 2 | 22 | 0 | 208 | 4345388 | 2 | 22 | 0 | 1 | 216 |
| 123 | 7B | 50 | 32 | 2 | 23 | 530267 | 4242132 | 2 | 23 | 0 | 208 | 4345604 | 2 | 23 | 0 | 1 | 216 |
| 124 | 7C | 50 | 32 | 2 | 24 | 530293 | 4242340 | 2 | 24 | 0 | 208 | 4345820 | 2 | 24 | 0 | 1 | 216 |
| 125 | 7D | 50 | 32 | 2 | 25 | 530319 | 4242548 | 2 | 25 | 0 | 208 | 4346036 | 2 | 25 | 0 | 1 | 216 |
| 126 | 7E | 50 | 32 | 2 | 26 | 530345 | 4242756 | 2 | 26 | 0 | 208 | 4346252 | 2 | 26 | 0 | 1 | 216 |
| 127 | 7F | 50 | 32 | 2 | 27 | 530371 | 4242964 | 2 | 27 | 0 | 208 | 4346468 | 2 | 27 | 0 | 1 | 216 |
| 128 | 80 | 50 | 32 | 2 | 28 | 530394 | 4243148 | 2 | 28 | 0 | 184 | 4346660 | 2 | 28 | 0 | 1 | 192 |
| 129 | 81 | 50 | 32 | 2 | 29 | 530417 | 4243332 | 2 | 29 | 0 | 184 | 4346852 | 2 | 29 | 0 | 1 | 192 |
| 130 | 82 | 50 | 32 | 2 | 30 | 530440 | 4243516 | 2 | 30 | 0 | 184 | 4347044 | 2 | 30 | 0 | 1 | 192 |
| 131 | 83 | 50 | 32 | 2 | 31 | 530463 | 4243700 | 2 | 31 | 0 | 184 | 4347236 | 2 | 31 | 0 | 1 | 192 |
| 132 | 84 | 50 | 32 | 2 | 32 | 530486 | 4243884 | 2 | 32 | 0 | 184 | 4347428 | 2 | 32 | 0 | 1 | 192 |
| 133 | 85 | 50 | 32 | 2 | 33 | 530509 | 4244068 | 2 | 33 | 0 | 184 | 4347620 | 2 | 33 | 0 | 1 | 192 |
| 134 | 86 | 50 | 32 | 2 | 34 | 530532 | 4244252 | 2 | 34 | 0 | 184 | 4347812 | 2 | 34 | 0 | 1 | 192 |
| 135 | 87 | 50 | 32 | 2 | 35 | 530555 | 4244436 | 2 | 35 | 0 | 184 | 4348004 | 2 | 35 | 0 | 1 | 192 |
| 136 | 88 | 50 | 32 | 2 | 36 | 530578 | 4244620 | 2 | 36 | 0 | 184 | 4348196 | 2 | 36 | 0 | 1 | 192 |
| 137 | 89 | 50 | 32 | 2 | 37 | 530601 | 4244804 | 2 | 37 | 0 | 184 | 4348388 | 2 | 37 | 0 | 1 | 192 |
| 138 | 8A | 50 | 32 | 2 | 38 | 530624 | 4244988 | 2 | 38 | 0 | 184 | 4348580 | 2 | 38 | 0 | 1 | 192 |
| 139 | 8B | 50 | 32 | 2 | 39 | 530647 | 4245172 | 2 | 39 | 0 | 184 | 4348772 | 2 | 39 | 0 | 1 | 192 |
| 140 | 8C | 50 | 32 | 2 | 40 | 530670 | 4245356 | 2 | 40 | 0 | 184 | 4348964 | 2 | 40 | 0 | 1 | 192 |
| 141 | 8D | 50 | 32 | 2 | 41 | 530693 | 4245540 | 2 | 41 | 0 | 184 | 4349156 | 2 | 41 | 0 | 1 | 192 |
| 142 | 8E | 50 | 32 | 2 | 42 | 530716 | 4245724 | 2 | 42 | 0 | 184 | 4349348 | 2 | 42 | 0 | 1 | 192 |
| 143 | 8F | 50 | 32 | 2 | 43 | 530739 | 4245908 | 2 | 43 | 0 | 184 | 4349540 | 2 | 43 | 0 | 1 | 192 |
| 144 | 90 | 50 | 32 | 2 | 44 | 530764 | 4246108 | 2 | 44 | 0 | 200 | 4349748 | 2 | 44 | 0 | 1 | 208 |
| 145 | 91 | 50 | 32 | 2 | 45 | 530789 | 4246308 | 2 | 45 | 0 | 200 | 4349956 | 2 | 45 | 0 | 1 | 208 |
| 146 | 92 | 50 | 32 | 2 | 46 | 530814 | 4246508 | 2 | 46 | 0 | 200 | 4350164 | 2 | 46 | 0 | 1 | 208 |
| 147 | 93 | 50 | 32 | 2 | 47 | 530839 | 4246708 | 2 | 47 | 0 | 200 | 4350372 | 2 | 47 | 0 | 1 | 208 |
| 148 | 94 | 50 | 32 | 2 | 48 | 530864 | 4246908 | 2 | 48 | 0 | 200 | 4350580 | 2 | 48 | 0 | 1 | 208 |
| 149 | 95 | 50 | 32 | 2 | 49 | 530889 | 4247108 | 2 | 49 | 0 | 200 | 4350788 | 2 | 49 | 0 | 1 | 208 |
| 150 | 96 | 50 | 32 | 3 | 0 | 530912 | 4247292 | 3 | 0 | 0 | 184 | 4350980 | 3 | 0 | 0 | 1 | 192 |
| 151 | 97 | 50 | 32 | 3 | 1 | 530940 | 4247516 | 3 | 1 | 0 | 224 | 4351212 | 3 | 1 | 0 | 1 | 232 |
| 152 | 98 | 50 | 32 | 3 | 2 | 530968 | 4247740 | 3 | 2 | 0 | 224 | 4351444 | 3 | 2 | 0 | 1 | 232 |
| 153 | 99 | 50 | 32 | 3 | 3 | 530996 | 4247964 | 3 | 3 | 0 | 224 | 4351676 | 3 | 3 | 0 | 1 | 232 |
| 154 | 9A | 50 | 32 | 3 | 4 | 531024 | 4248188 | 3 | 4 | 0 | 224 | 4351908 | 3 | 4 | 0 | 1 | 232 |
| 155 | 9B | 50 | 32 | 3 | 5 | 531052 | 4248412 | 3 | 5 | 0 | 224 | 4352140 | 3 | 5 | 0 | 1 | 232 |
| 156 | 9C | 50 | 32 | 3 | 6 | 531080 | 4248636 | 3 | 6 | 0 | 224 | 4352372 | 3 | 6 | 0 | 1 | 232 |
| 157 | 9D | 50 | 32 | 3 | 7 | 531108 | 4248860 | 3 | 7 | 0 | 224 | 4352604 | 3 | 7 | 0 | 1 | 232 |
| 158 | 9E | 50 | 32 | 3 | 8 | 531136 | 4249084 | 3 | 8 | 0 | 224 | 4352836 | 3 | 8 | 0 | 1 | 232 |
| 159 | 9F | 50 | 32 | 3 | 9 | 531164 | 4249308 | 3 | 9 | 0 | 224 | 4353068 | 3 | 9 | 0 | 1 | 232 |
| 160 | A0 | 50 | 32 | 3 | 10 | 531192 | 4249532 | 3 | 10 | 0 | 224 | 4353300 | 3 | 10 | 0 | 1 | 232 |
| 161 | A1 | 50 | 32 | 3 | 11 | 531220 | 4249756 | 3 | 11 | 0 | 224 | 4353532 | 3 | 11 | 0 | 1 | 232 |
| 162 | A2 | 50 | 32 | 3 | 12 | 531248 | 4249980 | 3 | 12 | 0 | 224 | 4353764 | 3 | 12 | 0 | 1 | 232 |
| 163 | A3 | 50 | 32 | 3 | 13 | 531276 | 4250204 | 3 | 13 | 0 | 224 | 4353996 | 3 | 13 | 0 | 1 | 232 |
| 164 | A4 | 50 | 32 | 3 | 14 | 531304 | 4250428 | 3 | 14 | 0 | 224 | 4354228 | 3 | 14 | 0 | 1 | 232 |
| 165 | A5 | 50 | 32 | 3 | 15 | 531332 | 4250652 | 3 | 15 | 0 | 224 | 4354460 | 3 | 15 | 0 | 1 | 232 |
| 166 | A6 | 50 | 32 | 3 | 16 | 531360 | 4250876 | 3 | 16 | 0 | 224 | 4354692 | 3 | 16 | 0 | 1 | 232 |
| 167 | A7 | 50 | 32 | 3 | 17 | 531388 | 4251100 | 3 | 17 | 0 | 224 | 4354924 | 3 | 17 | 0 | 1 | 232 |
| 168 | A8 | 50 | 32 | 3 | 18 | 531416 | 4251324 | 3 | 18 | 0 | 224 | 4355156 | 3 | 18 | 0 | 1 | 232 |
| 169 | A9 | 50 | 32 | 3 | 19 | 531444 | 4251548 | 3 | 19 | 0 | 224 | 4355388 | 3 | 19 | 0 | 1 | 232 |
| 170 | AA | 50 | 32 | 3 | 20 | 531472 | 4251772 | 3 | 20 | 0 | 224 | 4355620 | 3 | 20 | 0 | 1 | 232 |
| 171 | AB | 50 | 32 | 3 | 21 | 531500 | 4251996 | 3 | 21 | 0 | 224 | 4355852 | 3 | 21 | 0 | 1 | 232 |
| 172 | AC | 50 | 32 | 3 | 22 | 531528 | 4252220 | 3 | 22 | 0 | 224 | 4356084 | 3 | 22 | 0 | 1 | 232 |
| 173 | AD | 50 | 32 | 3 | 23 | 531556 | 4252444 | 3 | 23 | 0 | 224 | 4356316 | 3 | 23 | 0 | 1 | 232 |
| 174 | AE | 50 | 32 | 3 | 24 | 531584 | 4252668 | 3 | 24 | 0 | 224 | 4356548 | 3 | 24 | 0 | 1 | 232 |

| | | | | | | | | | | | | | | | | | |
|---|---|---|---|---|---|---|---|---|---|---|---|---|---|---|---|---|---|
| 175 | AF | 50 | 32 | 3 | 25 | 531612 | 4252892 | 3 | 25 | 0 | 224 | 4356780 | 3 | 25 | 0 | 1 | 232 |
| 176 | B0 | 50 | 32 | 3 | 26 | 531640 | 4253116 | 3 | 26 | 0 | 224 | 4357012 | 3 | 26 | 0 | 1 | 232 |
| 177 | B1 | 50 | 32 | 3 | 27 | 531668 | 4253340 | 3 | 27 | 0 | 224 | 4357244 | 3 | 27 | 0 | 1 | 232 |
| 178 | B2 | 50 | 32 | 3 | 28 | 531696 | 4253564 | 3 | 28 | 0 | 224 | 4357476 | 3 | 28 | 0 | 1 | 232 |
| 179 | B3 | 50 | 32 | 3 | 29 | 531724 | 4253788 | 3 | 29 | 0 | 224 | 4357708 | 3 | 29 | 0 | 1 | 232 |
| 180 | B4 | 50 | 32 | 3 | 30 | 531752 | 4254012 | 3 | 30 | 0 | 224 | 4357940 | 3 | 30 | 0 | 1 | 232 |
| 181 | B5 | 50 | 32 | 3 | 31 | 531780 | 4254236 | 3 | 31 | 0 | 224 | 4358172 | 3 | 31 | 0 | 1 | 232 |
| 182 | B6 | 50 | 32 | 3 | 32 | 531808 | 4254460 | 3 | 32 | 0 | 224 | 4358404 | 3 | 32 | 0 | 1 | 232 |
| 183 | B7 | 50 | 32 | 3 | 33 | 531836 | 4254684 | 3 | 33 | 0 | 224 | 4358636 | 3 | 33 | 0 | 1 | 232 |
| 184 | B8 | 50 | 32 | 3 | 34 | 531864 | 4254908 | 3 | 34 | 0 | 224 | 4358868 | 3 | 34 | 0 | 1 | 232 |
| 185 | B9 | 50 | 32 | 3 | 35 | 531892 | 4255132 | 3 | 35 | 0 | 224 | 4359100 | 3 | 35 | 0 | 1 | 232 |
| 186 | BA | 50 | 32 | 3 | 36 | 531920 | 4255356 | 3 | 36 | 0 | 224 | 4359332 | 3 | 36 | 0 | 1 | 232 |
| 187 | BB | 50 | 32 | 3 | 37 | 531948 | 4255580 | 3 | 37 | 0 | 224 | 4359564 | 3 | 37 | 0 | 1 | 232 |
| 188 | BC | 50 | 32 | 3 | 38 | 531976 | 4255804 | 3 | 38 | 0 | 224 | 4359796 | 3 | 38 | 0 | 1 | 232 |
| 189 | BD | 50 | 32 | 3 | 39 | 532004 | 4256028 | 3 | 39 | 0 | 224 | 4360028 | 3 | 39 | 0 | 1 | 232 |
| 190 | BE | 50 | 32 | 3 | 40 | 532032 | 4256252 | 3 | 40 | 0 | 224 | 4360260 | 3 | 40 | 0 | 1 | 232 |
| 191 | BF | 50 | 32 | 3 | 41 | 532060 | 4256476 | 3 | 41 | 0 | 224 | 4360492 | 3 | 41 | 0 | 1 | 232 |
| 192 | C0 | 50 | 32 | 3 | 42 | 532085 | 4256676 | 3 | 42 | 0 | 200 | 4360700 | 3 | 42 | 0 | 1 | 208 |
| 193 | C1 | 50 | 32 | 3 | 43 | 532110 | 4256876 | 3 | 43 | 0 | 200 | 4360908 | 3 | 43 | 0 | 1 | 208 |
| 194 | C2 | 50 | 32 | 3 | 44 | 532135 | 4257076 | 3 | 44 | 0 | 200 | 4361116 | 3 | 44 | 0 | 1 | 208 |
| 195 | C3 | 50 | 32 | 3 | 45 | 532160 | 4257276 | 3 | 45 | 0 | 200 | 4361324 | 3 | 45 | 0 | 1 | 208 |
| 196 | C4 | 50 | 32 | 3 | 46 | 532185 | 4257476 | 3 | 46 | 0 | 200 | 4361532 | 3 | 46 | 0 | 1 | 208 |
| 197 | C5 | 50 | 32 | 3 | 47 | 532210 | 4257676 | 3 | 47 | 0 | 200 | 4361740 | 3 | 47 | 0 | 1 | 208 |
| 198 | C6 | 50 | 32 | 3 | 48 | 532235 | 4257876 | 3 | 48 | 0 | 200 | 4361948 | 3 | 48 | 0 | 1 | 208 |
| 199 | C7 | 50 | 32 | 3 | 49 | 532260 | 4258076 | 3 | 49 | 0 | 200 | 4362156 | 3 | 49 | 0 | 1 | 208 |
| 200 | C8 | 50 | 32 | 4 | 0 | 532283 | 4258260 | 4 | 0 | 0 | 184 | 4362348 | 4 | 0 | 0 | 1 | 192 |
| 201 | C9 | 50 | 32 | 4 | 1 | 532311 | 4258484 | 4 | 1 | 0 | 224 | 4362580 | 4 | 1 | 0 | 1 | 232 |
| 202 | CA | 50 | 32 | 4 | 2 | 532339 | 4258708 | 4 | 2 | 0 | 224 | 4362812 | 4 | 2 | 0 | 1 | 232 |
| 203 | CB | 50 | 32 | 4 | 3 | 532367 | 4258932 | 4 | 3 | 0 | 224 | 4363044 | 4 | 3 | 0 | 1 | 232 |
| 204 | CC | 50 | 32 | 4 | 4 | 532395 | 4259156 | 4 | 4 | 0 | 224 | 4363276 | 4 | 4 | 0 | 1 | 232 |
| 205 | CD | 50 | 32 | 4 | 5 | 532423 | 4259380 | 4 | 5 | 0 | 224 | 4363508 | 4 | 5 | 0 | 1 | 232 |
| 206 | CE | 50 | 32 | 4 | 6 | 532451 | 4259604 | 4 | 6 | 0 | 224 | 4363740 | 4 | 6 | 0 | 1 | 232 |
| 207 | CF | 50 | 32 | 4 | 7 | 532479 | 4259828 | 4 | 7 | 0 | 224 | 4363972 | 4 | 7 | 0 | 1 | 232 |
| 208 | D0 | 50 | 32 | 4 | 8 | 532509 | 4260068 | 4 | 8 | 0 | 240 | 4364220 | 4 | 8 | 0 | 1 | 248 |
| 209 | D1 | 50 | 32 | 4 | 9 | 532539 | 4260308 | 4 | 9 | 0 | 240 | 4364468 | 4 | 9 | 0 | 1 | 248 |
| 210 | D2 | 50 | 32 | 4 | 10 | 532569 | 4260548 | 4 | 10 | 0 | 240 | 4364716 | 4 | 10 | 0 | 1 | 248 |
| 211 | D3 | 50 | 32 | 4 | 11 | 532599 | 4260788 | 4 | 11 | 0 | 240 | 4364964 | 4 | 11 | 0 | 1 | 248 |
| 212 | D4 | 50 | 32 | 4 | 12 | 532629 | 4261028 | 4 | 12 | 0 | 240 | 4365212 | 4 | 12 | 0 | 1 | 248 |
| 213 | D5 | 50 | 32 | 4 | 13 | 532659 | 4261268 | 4 | 13 | 0 | 240 | 4365460 | 4 | 13 | 0 | 1 | 248 |
| 214 | D6 | 50 | 32 | 4 | 14 | 532689 | 4261508 | 4 | 14 | 0 | 240 | 4365708 | 4 | 14 | 0 | 1 | 248 |
| 215 | D7 | 50 | 32 | 4 | 15 | 532719 | 4261748 | 4 | 15 | 0 | 240 | 4365956 | 4 | 15 | 0 | 1 | 248 |
| 216 | D8 | 50 | 32 | 4 | 16 | 532749 | 4261988 | 4 | 16 | 0 | 240 | 4366204 | 4 | 16 | 0 | 1 | 248 |
| 217 | D9 | 50 | 32 | 4 | 17 | 532779 | 4262228 | 4 | 17 | 0 | 240 | 4366452 | 4 | 17 | 0 | 1 | 248 |
| 218 | DA | 50 | 32 | 4 | 18 | 532809 | 4262468 | 4 | 18 | 0 | 240 | 4366700 | 4 | 18 | 0 | 1 | 248 |
| 219 | DB | 50 | 32 | 4 | 19 | 532839 | 4262708 | 4 | 19 | 0 | 240 | 4366948 | 4 | 19 | 0 | 1 | 248 |
| 220 | DC | 50 | 32 | 4 | 20 | 532869 | 4262948 | 4 | 20 | 0 | 240 | 4367196 | 4 | 20 | 0 | 1 | 248 |
| 221 | DD | 50 | 32 | 4 | 21 | 532899 | 4263188 | 4 | 21 | 0 | 240 | 4367444 | 4 | 21 | 0 | 1 | 248 |
| 222 | DE | 50 | 32 | 4 | 22 | 532929 | 4263428 | 4 | 22 | 0 | 240 | 4367692 | 4 | 22 | 0 | 1 | 248 |
| 223 | DF | 50 | 32 | 4 | 23 | 532959 | 4263668 | 4 | 23 | 0 | 240 | 4367940 | 4 | 23 | 0 | 1 | 248 |
| 224 | E0 | 50 | 32 | 4 | 24 | 532989 | 4263908 | 4 | 24 | 0 | 240 | 4368188 | 4 | 24 | 0 | 1 | 248 |
| 225 | E1 | 50 | 32 | 4 | 25 | 533019 | 4264148 | 4 | 25 | 0 | 240 | 4368436 | 4 | 25 | 0 | 1 | 248 |
| 226 | E2 | 50 | 32 | 4 | 26 | 533049 | 4264388 | 4 | 26 | 0 | 240 | 4368684 | 4 | 26 | 0 | 1 | 248 |
| 227 | E3 | 50 | 32 | 4 | 27 | 533079 | 4264628 | 4 | 27 | 0 | 240 | 4368932 | 4 | 27 | 0 | 1 | 248 |
| 228 | E4 | 50 | 32 | 4 | 28 | 533109 | 4264868 | 4 | 28 | 0 | 240 | 4369180 | 4 | 28 | 0 | 1 | 248 |
| 229 | E5 | 50 | 32 | 4 | 29 | 533139 | 4265108 | 4 | 29 | 0 | 240 | 4369428 | 4 | 29 | 0 | 1 | 248 |
| 230 | E6 | 50 | 32 | 4 | 30 | 533169 | 4265348 | 4 | 30 | 0 | 240 | 4369676 | 4 | 30 | 0 | 1 | 248 |
| 231 | E7 | 50 | 32 | 4 | 31 | 533199 | 4265588 | 4 | 31 | 0 | 240 | 4369924 | 4 | 31 | 0 | 1 | 248 |
| 232 | E8 | 50 | 32 | 4 | 32 | 533229 | 4265828 | 4 | 32 | 0 | 240 | 4370172 | 4 | 32 | 0 | 1 | 248 |
| 233 | E9 | 50 | 32 | 4 | 33 | 533259 | 4266068 | 4 | 33 | 0 | 240 | 4370420 | 4 | 33 | 0 | 1 | 248 |
| 234 | EA | 50 | 32 | 4 | 34 | 533289 | 4266308 | 4 | 34 | 0 | 240 | 4370668 | 4 | 34 | 0 | 1 | 248 |
| 235 | EB | 50 | 32 | 4 | 35 | 533319 | 4266548 | 4 | 35 | 0 | 240 | 4370916 | 4 | 35 | 0 | 1 | 248 |
| 236 | EC | 50 | 32 | 4 | 36 | 533349 | 4266788 | 4 | 36 | 0 | 240 | 4371164 | 4 | 36 | 0 | 1 | 248 |
| 237 | ED | 50 | 32 | 4 | 37 | 533379 | 4267028 | 4 | 37 | 0 | 240 | 4371412 | 4 | 37 | 0 | 1 | 248 |
| 238 | EE | 50 | 32 | 4 | 38 | 533409 | 4267268 | 4 | 38 | 0 | 240 | 4371660 | 4 | 38 | 0 | 1 | 248 |
| 239 | EF | 50 | 32 | 4 | 39 | 533439 | 4267508 | 4 | 39 | 0 | 240 | 4371908 | 4 | 39 | 0 | 1 | 248 |
| 240 | F0 | 50 | 32 | 4 | 40 | 533469 | 4267748 | 4 | 40 | 0 | 240 | 4372156 | 4 | 40 | 0 | 1 | 248 |
| 241 | F1 | 50 | 32 | 4 | 41 | 533499 | 4267988 | 4 | 41 | 0 | 240 | 4372404 | 4 | 41 | 0 | 1 | 248 |
| 242 | F2 | 50 | 32 | 4 | 42 | 533529 | 4268228 | 4 | 42 | 0 | 240 | 4372652 | 4 | 42 | 0 | 1 | 248 |
| 243 | F3 | 50 | 32 | 4 | 43 | 533559 | 4268468 | 4 | 43 | 0 | 240 | 4372900 | 4 | 43 | 0 | 1 | 248 |
| 244 | F4 | 50 | 32 | 4 | 44 | 533589 | 4268708 | 4 | 44 | 0 | 240 | 4373148 | 4 | 44 | 0 | 1 | 248 |
| 245 | F5 | 50 | 32 | 4 | 45 | 533619 | 4268948 | 4 | 45 | 0 | 240 | 4373396 | 4 | 45 | 0 | 1 | 248 |
| 246 | F6 | 50 | 32 | 4 | 46 | 533649 | 4269188 | 4 | 46 | 0 | 240 | 4373644 | 4 | 46 | 0 | 1 | 248 |
| 247 | F7 | 50 | 32 | 4 | 47 | 533679 | 4269428 | 4 | 47 | 0 | 240 | 4373892 | 4 | 47 | 0 | 1 | 248 |
| 248 | F8 | 50 | 32 | 4 | 48 | 533709 | 4269668 | 4 | 48 | 0 | 240 | 4374140 | 4 | 48 | 0 | 1 | 248 |
| 249 | F9 | 50 | 32 | 4 | 49 | 533739 | 4269908 | 4 | 49 | 0 | 240 | 4374388 | 4 | 49 | 0 | 1 | 248 |
| 250 | FA | 50 | 32 | 5 | 0 | 533771 | 4270164 | 5 | 0 | 0 | 256 | 4374652 | 5 | 0 | 0 | 1 | 264 |
| 251 | FB | 50 | 32 | 5 | 1 | 533803 | 4270420 | 5 | 1 | 0 | 256 | 4374916 | 5 | 1 | 0 | 1 | 264 |
| 252 | FC | 50 | 32 | 5 | 2 | 533835 | 4270676 | 5 | 2 | 0 | 256 | 4375180 | 5 | 2 | 0 | 1 | 264 |
| 253 | FD | 50 | 32 | 5 | 3 | 533867 | 4270932 | 5 | 3 | 0 | 256 | 4375444 | 5 | 3 | 0 | 1 | 264 |
| 254 | FE | 50 | 32 | 5 | 4 | 533899 | 4271188 | 5 | 4 | 0 | 256 | 4375708 | 5 | 4 | 0 | 1 | 264 |
| 255 | FF | 50 | 32 | 5 | 5 | 533931 | 4271444 | 5 | 5 | 0 | 256 | 4375972 | 5 | 5 | 0 | 1 | 264 |
| 0 | 00 | 51 | 33 | 0 | 0 | 533943 | 4271540 | 0 | 0 | 0 | 96 | 4376076 | 0 | 0 | 0 | 1 | 104 |
| 1 | 01 | 51 | 33 | 0 | 1 | 533957 | 4271652 | 0 | 1 | 0 | 112 | 4376196 | 0 | 1 | 0 | 1 | 120 |
| 2 | 02 | 51 | 33 | 0 | 2 | 533971 | 4271764 | 0 | 2 | 0 | 112 | 4376316 | 0 | 2 | 0 | 1 | 120 |
| 3 | 03 | 51 | 33 | 0 | 3 | 533985 | 4271876 | 0 | 3 | 0 | 112 | 4376436 | 0 | 3 | 0 | 1 | 120 |
| 4 | 04 | 51 | 33 | 0 | 4 | 533999 | 4271988 | 0 | 4 | 0 | 112 | 4376556 | 0 | 4 | 0 | 1 | 120 |
| 5 | 05 | 51 | 33 | 0 | 5 | 534013 | 4272100 | 0 | 5 | 0 | 112 | 4376676 | 0 | 5 | 0 | 1 | 120 |
| 6 | 06 | 51 | 33 | 0 | 6 | 534027 | 4272212 | 0 | 6 | 0 | 112 | 4376796 | 0 | 6 | 0 | 1 | 120 |
| 7 | 07 | 51 | 33 | 0 | 7 | 534041 | 4272324 | 0 | 7 | 0 | 112 | 4376916 | 0 | 7 | 0 | 1 | 120 |
| 8 | 08 | 51 | 33 | 0 | 8 | 534055 | 4272436 | 0 | 8 | 0 | 112 | 4377036 | 0 | 8 | 0 | 1 | 120 |
| 9 | 09 | 51 | 33 | 0 | 9 | 534069 | 4272548 | 0 | 9 | 0 | 112 | 4377156 | 0 | 9 | 0 | 1 | 120 |
| 10 | 0A | 51 | 33 | 0 | 10 | 534083 | 4272660 | 0 | 10 | 0 | 112 | 4377276 | 0 | 10 | 0 | 1 | 120 |
| 11 | 0B | 51 | 33 | 0 | 11 | 534097 | 4272772 | 0 | 11 | 0 | 112 | 4377396 | 0 | 11 | 0 | 1 | 120 |
| 12 | 0C | 51 | 33 | 0 | 12 | 534111 | 4272884 | 0 | 12 | 0 | 112 | 4377516 | 0 | 12 | 0 | 1 | 120 |
| 13 | 0D | 51 | 33 | 0 | 13 | 534125 | 4272996 | 0 | 13 | 0 | 112 | 4377636 | 0 | 13 | 0 | 1 | 120 |
| 14 | 0E | 51 | 33 | 0 | 14 | 534139 | 4273108 | 0 | 14 | 0 | 112 | 4377756 | 0 | 14 | 0 | 1 | 120 |
| 15 | 0F | 51 | 33 | 0 | 15 | 534153 | 4273220 | 0 | 15 | 0 | 112 | 4377876 | 0 | 15 | 0 | 1 | 120 |
| 16 | 10 | 51 | 33 | 0 | 16 | 534167 | 4273332 | 0 | 16 | 0 | 112 | 4377996 | 0 | 16 | 0 | 1 | 120 |
| 17 | 11 | 51 | 33 | 0 | 17 | 534181 | 4273444 | 0 | 17 | 0 | 112 | 4378116 | 0 | 17 | 0 | 1 | 120 |
| 18 | 12 | 51 | 33 | 0 | 18 | 534195 | 4273556 | 0 | 18 | 0 | 112 | 4378236 | 0 | 18 | 0 | 1 | 120 |
| 19 | 13 | 51 | 33 | 0 | 19 | 534209 | 4273668 | 0 | 19 | 0 | 112 | 4378356 | 0 | 19 | 0 | 1 | 120 |
| 20 | 14 | 51 | 33 | 0 | 20 | 534223 | 4273780 | 0 | 20 | 0 | 112 | 4378476 | 0 | 20 | 0 | 1 | 120 |
| 21 | 15 | 51 | 33 | 0 | 21 | 534237 | 4273892 | 0 | 21 | 0 | 112 | 4378596 | 0 | 21 | 0 | 1 | 120 |

| | | | | | | | | | | | | | | | | | |
|---|---|---|---|---|---|---|---|---|---|---|---|---|---|---|---|---|---|
| 22 | 16 | 51 | 33 | 0 | 22 | 534251 | 4274004 | 0 | 22 | 0 | 112 | 4378716 | 0 | 22 | 0 | 1 | 120 |
| 23 | 17 | 51 | 33 | 0 | 23 | 534265 | 4274116 | 0 | 23 | 0 | 112 | 4378836 | 0 | 23 | 0 | 1 | 120 |
| 24 | 18 | 51 | 33 | 0 | 24 | 534279 | 4274228 | 0 | 24 | 0 | 112 | 4378956 | 0 | 24 | 0 | 1 | 120 |
| 25 | 19 | 51 | 33 | 0 | 25 | 534293 | 4274340 | 0 | 25 | 0 | 112 | 4379076 | 0 | 25 | 0 | 1 | 120 |
| 26 | 1A | 51 | 33 | 0 | 26 | 534307 | 4274452 | 0 | 26 | 0 | 112 | 4379196 | 0 | 26 | 0 | 1 | 120 |
| 27 | 1B | 51 | 33 | 0 | 27 | 534321 | 4274564 | 0 | 27 | 0 | 112 | 4379316 | 0 | 27 | 0 | 1 | 120 |
| 28 | 1C | 51 | 33 | 0 | 28 | 534335 | 4274676 | 0 | 28 | 0 | 112 | 4379436 | 0 | 28 | 0 | 1 | 120 |
| 29 | 1D | 51 | 33 | 0 | 29 | 534349 | 4274788 | 0 | 29 | 0 | 112 | 4379556 | 0 | 29 | 0 | 1 | 120 |
| 30 | 1E | 51 | 33 | 0 | 30 | 534363 | 4274900 | 0 | 30 | 0 | 112 | 4379676 | 0 | 30 | 0 | 1 | 120 |
| 31 | 1F | 51 | 33 | 0 | 31 | 534377 | 4275012 | 0 | 31 | 0 | 112 | 4379796 | 0 | 31 | 0 | 1 | 120 |
| 32 | 20 | 51 | 33 | 0 | 32 | 534391 | 4275124 | 0 | 32 | 0 | 112 | 4379916 | 0 | 32 | 0 | 1 | 120 |
| 33 | 21 | 51 | 33 | 0 | 33 | 534405 | 4275236 | 0 | 33 | 0 | 112 | 4380036 | 0 | 33 | 0 | 1 | 120 |
| 34 | 22 | 51 | 33 | 0 | 34 | 534419 | 4275348 | 0 | 34 | 0 | 112 | 4380156 | 0 | 34 | 0 | 1 | 120 |
| 35 | 23 | 51 | 33 | 0 | 35 | 534433 | 4275460 | 0 | 35 | 0 | 112 | 4380276 | 0 | 35 | 0 | 1 | 120 |
| 36 | 24 | 51 | 33 | 0 | 36 | 534447 | 4275572 | 0 | 36 | 0 | 112 | 4380396 | 0 | 36 | 0 | 1 | 120 |
| 37 | 25 | 51 | 33 | 0 | 37 | 534461 | 4275684 | 0 | 37 | 0 | 112 | 4380516 | 0 | 37 | 0 | 1 | 120 |
| 38 | 26 | 51 | 33 | 0 | 38 | 534475 | 4275796 | 0 | 38 | 0 | 112 | 4380636 | 0 | 38 | 0 | 1 | 120 |
| 39 | 27 | 51 | 33 | 0 | 39 | 534489 | 4275908 | 0 | 39 | 0 | 112 | 4380756 | 0 | 39 | 0 | 1 | 120 |
| 40 | 28 | 51 | 33 | 0 | 40 | 534503 | 4276020 | 0 | 40 | 0 | 112 | 4380876 | 0 | 40 | 0 | 1 | 120 |
| 41 | 29 | 51 | 33 | 0 | 41 | 534517 | 4276132 | 0 | 41 | 0 | 112 | 4380996 | 0 | 41 | 0 | 1 | 120 |
| 42 | 2A | 51 | 33 | 0 | 42 | 534531 | 4276244 | 0 | 42 | 0 | 112 | 4381116 | 0 | 42 | 0 | 1 | 120 |
| 43 | 2B | 51 | 33 | 0 | 43 | 534545 | 4276356 | 0 | 43 | 0 | 112 | 4381236 | 0 | 43 | 0 | 1 | 120 |
| 44 | 2C | 51 | 33 | 0 | 44 | 534559 | 4276468 | 0 | 44 | 0 | 112 | 4381356 | 0 | 44 | 0 | 1 | 120 |
| 45 | 2D | 51 | 33 | 0 | 45 | 534573 | 4276580 | 0 | 45 | 0 | 112 | 4381476 | 0 | 45 | 0 | 1 | 120 |
| 46 | 2E | 51 | 33 | 0 | 46 | 534587 | 4276692 | 0 | 46 | 0 | 112 | 4381596 | 0 | 46 | 0 | 1 | 120 |
| 47 | 2F | 51 | 33 | 0 | 47 | 534601 | 4276804 | 0 | 47 | 0 | 112 | 4381716 | 0 | 47 | 0 | 1 | 120 |
| 48 | 30 | 51 | 33 | 0 | 48 | 534615 | 4276916 | 0 | 48 | 0 | 112 | 4381836 | 0 | 48 | 0 | 1 | 120 |
| 49 | 31 | 51 | 33 | 0 | 49 | 534629 | 4277028 | 0 | 49 | 0 | 112 | 4381956 | 0 | 49 | 0 | 1 | 120 |
| 50 | 32 | 51 | 33 | 0 | 50 | 534643 | 4277140 | 0 | 50 | 0 | 112 | 4382076 | 0 | 50 | 0 | 1 | 120 |
| 51 | 33 | 51 | 33 | 1 | 0 | 534655 | 4277236 | 1 | 0 | 0 | 96 | 4382180 | 1 | 0 | 0 | 1 | 104 |
| 52 | 34 | 51 | 33 | 1 | 1 | 534672 | 4277372 | 1 | 1 | 0 | 136 | 4382324 | 1 | 1 | 0 | 1 | 144 |
| 53 | 35 | 51 | 33 | 1 | 2 | 534689 | 4277508 | 1 | 2 | 0 | 136 | 4382468 | 1 | 2 | 0 | 1 | 144 |
| 54 | 36 | 51 | 33 | 1 | 3 | 534706 | 4277644 | 1 | 3 | 0 | 136 | 4382612 | 1 | 3 | 0 | 1 | 144 |
| 55 | 37 | 51 | 33 | 1 | 4 | 534723 | 4277780 | 1 | 4 | 0 | 136 | 4382756 | 1 | 4 | 0 | 1 | 144 |
| 56 | 38 | 51 | 33 | 1 | 5 | 534740 | 4277916 | 1 | 5 | 0 | 136 | 4382900 | 1 | 5 | 0 | 1 | 144 |
| 57 | 39 | 51 | 33 | 1 | 6 | 534757 | 4278052 | 1 | 6 | 0 | 136 | 4383044 | 1 | 6 | 0 | 1 | 144 |
| 58 | 3A | 51 | 33 | 1 | 7 | 534774 | 4278188 | 1 | 7 | 0 | 136 | 4383188 | 1 | 7 | 0 | 1 | 144 |
| 59 | 3B | 51 | 33 | 1 | 8 | 534791 | 4278324 | 1 | 8 | 0 | 136 | 4383332 | 1 | 8 | 0 | 1 | 144 |
| 60 | 3C | 51 | 33 | 1 | 9 | 534808 | 4278460 | 1 | 9 | 0 | 136 | 4383476 | 1 | 9 | 0 | 1 | 144 |
| 61 | 3D | 51 | 33 | 1 | 10 | 534825 | 4278596 | 1 | 10 | 0 | 136 | 4383620 | 1 | 10 | 0 | 1 | 144 |
| 62 | 3E | 51 | 33 | 1 | 11 | 534842 | 4278732 | 1 | 11 | 0 | 136 | 4383764 | 1 | 11 | 0 | 1 | 144 |
| 63 | 3F | 51 | 33 | 1 | 12 | 534859 | 4278868 | 1 | 12 | 0 | 136 | 4383908 | 1 | 12 | 0 | 1 | 144 |
| 64 | 40 | 51 | 33 | 1 | 13 | 534880 | 4279036 | 1 | 13 | 0 | 168 | 4384084 | 1 | 13 | 0 | 1 | 176 |
| 65 | 41 | 51 | 33 | 1 | 14 | 534901 | 4279204 | 1 | 14 | 0 | 168 | 4384260 | 1 | 14 | 0 | 1 | 176 |
| 66 | 42 | 51 | 33 | 1 | 15 | 534922 | 4279372 | 1 | 15 | 0 | 168 | 4384436 | 1 | 15 | 0 | 1 | 176 |
| 67 | 43 | 51 | 33 | 1 | 16 | 534943 | 4279540 | 1 | 16 | 0 | 168 | 4384612 | 1 | 16 | 0 | 1 | 176 |
| 68 | 44 | 51 | 33 | 1 | 17 | 534964 | 4279708 | 1 | 17 | 0 | 168 | 4384788 | 1 | 17 | 0 | 1 | 176 |
| 69 | 45 | 51 | 33 | 1 | 18 | 534985 | 4279876 | 1 | 18 | 0 | 168 | 4384964 | 1 | 18 | 0 | 1 | 176 |
| 70 | 46 | 51 | 33 | 1 | 19 | 535006 | 4280044 | 1 | 19 | 0 | 168 | 4385140 | 1 | 19 | 0 | 1 | 176 |
| 71 | 47 | 51 | 33 | 1 | 20 | 535027 | 4280212 | 1 | 20 | 0 | 168 | 4385316 | 1 | 20 | 0 | 1 | 176 |
| 72 | 48 | 51 | 33 | 1 | 21 | 535048 | 4280380 | 1 | 21 | 0 | 168 | 4385492 | 1 | 21 | 0 | 1 | 176 |
| 73 | 49 | 51 | 33 | 1 | 22 | 535069 | 4280548 | 1 | 22 | 0 | 168 | 4385668 | 1 | 22 | 0 | 1 | 176 |
| 74 | 4A | 51 | 33 | 1 | 23 | 535090 | 4280716 | 1 | 23 | 0 | 168 | 4385844 | 1 | 23 | 0 | 1 | 176 |
| 75 | 4B | 51 | 33 | 1 | 24 | 535111 | 4280884 | 1 | 24 | 0 | 168 | 4386020 | 1 | 24 | 0 | 1 | 176 |
| 76 | 4C | 51 | 33 | 1 | 25 | 535132 | 4281052 | 1 | 25 | 0 | 168 | 4386196 | 1 | 25 | 0 | 1 | 176 |
| 77 | 4D | 51 | 33 | 1 | 26 | 535153 | 4281220 | 1 | 26 | 0 | 168 | 4386372 | 1 | 26 | 0 | 1 | 176 |
| 78 | 4E | 51 | 33 | 1 | 27 | 535174 | 4281388 | 1 | 27 | 0 | 168 | 4386548 | 1 | 27 | 0 | 1 | 176 |
| 79 | 4F | 51 | 33 | 1 | 28 | 535195 | 4281556 | 1 | 28 | 0 | 168 | 4386724 | 1 | 28 | 0 | 1 | 176 |
| 80 | 50 | 51 | 33 | 1 | 29 | 535218 | 4281740 | 1 | 29 | 0 | 184 | 4386916 | 1 | 29 | 0 | 1 | 192 |
| 81 | 51 | 51 | 33 | 1 | 30 | 535241 | 4281924 | 1 | 30 | 0 | 184 | 4387108 | 1 | 30 | 0 | 1 | 192 |
| 82 | 52 | 51 | 33 | 1 | 31 | 535264 | 4282108 | 1 | 31 | 0 | 184 | 4387300 | 1 | 31 | 0 | 1 | 192 |
| 83 | 53 | 51 | 33 | 1 | 32 | 535287 | 4282292 | 1 | 32 | 0 | 184 | 4387492 | 1 | 32 | 0 | 1 | 192 |
| 84 | 54 | 51 | 33 | 1 | 33 | 535310 | 4282476 | 1 | 33 | 0 | 184 | 4387684 | 1 | 33 | 0 | 1 | 192 |
| 85 | 55 | 51 | 33 | 1 | 34 | 535333 | 4282660 | 1 | 34 | 0 | 184 | 4387876 | 1 | 34 | 0 | 1 | 192 |
| 86 | 56 | 51 | 33 | 1 | 35 | 535356 | 4282844 | 1 | 35 | 0 | 184 | 4388068 | 1 | 35 | 0 | 1 | 192 |
| 87 | 57 | 51 | 33 | 1 | 36 | 535379 | 4283028 | 1 | 36 | 0 | 184 | 4388260 | 1 | 36 | 0 | 1 | 192 |
| 88 | 58 | 51 | 33 | 1 | 37 | 535402 | 4283212 | 1 | 37 | 0 | 184 | 4388452 | 1 | 37 | 0 | 1 | 192 |
| 89 | 59 | 51 | 33 | 1 | 38 | 535425 | 4283396 | 1 | 38 | 0 | 184 | 4388644 | 1 | 38 | 0 | 1 | 192 |
| 90 | 5A | 51 | 33 | 1 | 39 | 535448 | 4283580 | 1 | 39 | 0 | 184 | 4388836 | 1 | 39 | 0 | 1 | 192 |
| 91 | 5B | 51 | 33 | 1 | 40 | 535471 | 4283764 | 1 | 40 | 0 | 184 | 4389028 | 1 | 40 | 0 | 1 | 192 |
| 92 | 5C | 51 | 33 | 1 | 41 | 535494 | 4283948 | 1 | 41 | 0 | 184 | 4389220 | 1 | 41 | 0 | 1 | 192 |
| 93 | 5D | 51 | 33 | 1 | 42 | 535517 | 4284132 | 1 | 42 | 0 | 184 | 4389412 | 1 | 42 | 0 | 1 | 192 |
| 94 | 5E | 51 | 33 | 1 | 43 | 535540 | 4284316 | 1 | 43 | 0 | 184 | 4389604 | 1 | 43 | 0 | 1 | 192 |
| 95 | 5F | 51 | 33 | 1 | 44 | 535563 | 4284500 | 1 | 44 | 0 | 184 | 4389796 | 1 | 44 | 0 | 1 | 192 |
| 96 | 60 | 51 | 33 | 1 | 45 | 535586 | 4284684 | 1 | 45 | 0 | 184 | 4389988 | 1 | 45 | 0 | 1 | 192 |
| 97 | 61 | 51 | 33 | 1 | 46 | 535609 | 4284868 | 1 | 46 | 0 | 184 | 4390180 | 1 | 46 | 0 | 1 | 192 |
| 98 | 62 | 51 | 33 | 1 | 47 | 535632 | 4285052 | 1 | 47 | 0 | 184 | 4390372 | 1 | 47 | 0 | 1 | 192 |
| 99 | 63 | 51 | 33 | 1 | 48 | 535655 | 4285236 | 1 | 48 | 0 | 184 | 4390564 | 1 | 48 | 0 | 1 | 192 |
| 100 | 64 | 51 | 33 | 1 | 49 | 535678 | 4285420 | 1 | 49 | 0 | 184 | 4390756 | 1 | 49 | 0 | 1 | 192 |
| 101 | 65 | 51 | 33 | 1 | 50 | 535701 | 4285604 | 1 | 50 | 0 | 184 | 4390948 | 1 | 50 | 0 | 1 | 192 |
| 102 | 66 | 51 | 33 | 2 | 0 | 535722 | 4285772 | 2 | 0 | 0 | 168 | 4391124 | 2 | 0 | 0 | 1 | 176 |
| 103 | 67 | 51 | 33 | 2 | 1 | 535748 | 4285980 | 2 | 1 | 0 | 208 | 4391340 | 2 | 1 | 0 | 1 | 216 |
| 104 | 68 | 51 | 33 | 2 | 2 | 535774 | 4286188 | 2 | 2 | 0 | 208 | 4391556 | 2 | 2 | 0 | 1 | 216 |
| 105 | 69 | 51 | 33 | 2 | 3 | 535800 | 4286396 | 2 | 3 | 0 | 208 | 4391772 | 2 | 3 | 0 | 1 | 216 |
| 106 | 6A | 51 | 33 | 2 | 4 | 535826 | 4286604 | 2 | 4 | 0 | 208 | 4391988 | 2 | 4 | 0 | 1 | 216 |
| 107 | 6B | 51 | 33 | 2 | 5 | 535852 | 4286812 | 2 | 5 | 0 | 208 | 4392204 | 2 | 5 | 0 | 1 | 216 |
| 108 | 6C | 51 | 33 | 2 | 6 | 535878 | 4287020 | 2 | 6 | 0 | 208 | 4392420 | 2 | 6 | 0 | 1 | 216 |
| 109 | 6D | 51 | 33 | 2 | 7 | 535904 | 4287228 | 2 | 7 | 0 | 208 | 4392636 | 2 | 7 | 0 | 1 | 216 |
| 110 | 6E | 51 | 33 | 2 | 8 | 535930 | 4287436 | 2 | 8 | 0 | 208 | 4392852 | 2 | 8 | 0 | 1 | 216 |
| 111 | 6F | 51 | 33 | 2 | 9 | 535956 | 4287644 | 2 | 9 | 0 | 208 | 4393068 | 2 | 9 | 0 | 1 | 216 |
| 112 | 70 | 51 | 33 | 2 | 10 | 535982 | 4287852 | 2 | 10 | 0 | 208 | 4393284 | 2 | 10 | 0 | 1 | 216 |
| 113 | 71 | 51 | 33 | 2 | 11 | 536008 | 4288060 | 2 | 11 | 0 | 208 | 4393500 | 2 | 11 | 0 | 1 | 216 |
| 114 | 72 | 51 | 33 | 2 | 12 | 536034 | 4288268 | 2 | 12 | 0 | 208 | 4393716 | 2 | 12 | 0 | 1 | 216 |
| 115 | 73 | 51 | 33 | 2 | 13 | 536060 | 4288476 | 2 | 13 | 0 | 208 | 4393932 | 2 | 13 | 0 | 1 | 216 |
| 116 | 74 | 51 | 33 | 2 | 14 | 536086 | 4288684 | 2 | 14 | 0 | 208 | 4394148 | 2 | 14 | 0 | 1 | 216 |
| 117 | 75 | 51 | 33 | 2 | 15 | 536112 | 4288892 | 2 | 15 | 0 | 208 | 4394364 | 2 | 15 | 0 | 1 | 216 |
| 118 | 76 | 51 | 33 | 2 | 16 | 536138 | 4289100 | 2 | 16 | 0 | 208 | 4394580 | 2 | 16 | 0 | 1 | 216 |
| 119 | 77 | 51 | 33 | 2 | 17 | 536164 | 4289308 | 2 | 17 | 0 | 208 | 4394796 | 2 | 17 | 0 | 1 | 216 |
| 120 | 78 | 51 | 33 | 2 | 18 | 536190 | 4289516 | 2 | 18 | 0 | 208 | 4395012 | 2 | 18 | 0 | 1 | 216 |
| 121 | 79 | 51 | 33 | 2 | 19 | 536216 | 4289724 | 2 | 19 | 0 | 208 | 4395228 | 2 | 19 | 0 | 1 | 216 |
| 122 | 7A | 51 | 33 | 2 | 20 | 536242 | 4289932 | 2 | 20 | 0 | 208 | 4395444 | 2 | 20 | 0 | 1 | 216 |
| 123 | 7B | 51 | 33 | 2 | 21 | 536268 | 4290140 | 2 | 21 | 0 | 208 | 4395660 | 2 | 21 | 0 | 1 | 216 |
| 124 | 7C | 51 | 33 | 2 | 22 | 536294 | 4290348 | 2 | 22 | 0 | 208 | 4395876 | 2 | 22 | 0 | 1 | 216 |

| | | | | | | | | | | | | | | | | | |
|---|---|---|---|---|---|---|---|---|---|---|---|---|---|---|---|---|---|
| 125 | 7D | 51 | 33 | 2 | 23 | 536320 | 4290556 | 2 | 23 | 0 | 208 | 4396092 | 2 | 23 | 0 | 1 | 216 |
| 126 | 7E | 51 | 33 | 2 | 24 | 536346 | 4290764 | 2 | 24 | 0 | 208 | 4396308 | 2 | 24 | 0 | 1 | 216 |
| 127 | 7F | 51 | 33 | 2 | 25 | 536372 | 4290972 | 2 | 25 | 0 | 208 | 4396524 | 2 | 25 | 0 | 1 | 216 |
| 128 | 80 | 51 | 33 | 2 | 26 | 536395 | 4291156 | 2 | 26 | 0 | 184 | 4396716 | 2 | 26 | 0 | 1 | 192 |
| 129 | 81 | 51 | 33 | 2 | 27 | 536418 | 4291340 | 2 | 27 | 0 | 184 | 4396908 | 2 | 27 | 0 | 1 | 192 |
| 130 | 82 | 51 | 33 | 2 | 28 | 536441 | 4291524 | 2 | 28 | 0 | 184 | 4397100 | 2 | 28 | 0 | 1 | 192 |
| 131 | 83 | 51 | 33 | 2 | 29 | 536464 | 4291708 | 2 | 29 | 0 | 184 | 4397292 | 2 | 29 | 0 | 1 | 192 |
| 132 | 84 | 51 | 33 | 2 | 30 | 536487 | 4291892 | 2 | 30 | 0 | 184 | 4397484 | 2 | 30 | 0 | 1 | 192 |
| 133 | 85 | 51 | 33 | 2 | 31 | 536510 | 4292076 | 2 | 31 | 0 | 184 | 4397676 | 2 | 31 | 0 | 1 | 192 |
| 134 | 86 | 51 | 33 | 2 | 32 | 536533 | 4292260 | 2 | 32 | 0 | 184 | 4397868 | 2 | 32 | 0 | 1 | 192 |
| 135 | 87 | 51 | 33 | 2 | 33 | 536556 | 4292444 | 2 | 33 | 0 | 184 | 4398060 | 2 | 33 | 0 | 1 | 192 |
| 136 | 88 | 51 | 33 | 2 | 34 | 536579 | 4292628 | 2 | 34 | 0 | 184 | 4398252 | 2 | 34 | 0 | 1 | 192 |
| 137 | 89 | 51 | 33 | 2 | 35 | 536602 | 4292812 | 2 | 35 | 0 | 184 | 4398444 | 2 | 35 | 0 | 1 | 192 |
| 138 | 8A | 51 | 33 | 2 | 36 | 536625 | 4292996 | 2 | 36 | 0 | 184 | 4398636 | 2 | 36 | 0 | 1 | 192 |
| 139 | 8B | 51 | 33 | 2 | 37 | 536648 | 4293180 | 2 | 37 | 0 | 184 | 4398828 | 2 | 37 | 0 | 1 | 192 |
| 140 | 8C | 51 | 33 | 2 | 38 | 536671 | 4293364 | 2 | 38 | 0 | 184 | 4399020 | 2 | 38 | 0 | 1 | 192 |
| 141 | 8D | 51 | 33 | 2 | 39 | 536694 | 4293548 | 2 | 39 | 0 | 184 | 4399212 | 2 | 39 | 0 | 1 | 192 |
| 142 | 8E | 51 | 33 | 2 | 40 | 536717 | 4293732 | 2 | 40 | 0 | 184 | 4399404 | 2 | 40 | 0 | 1 | 192 |
| 143 | 8F | 51 | 33 | 2 | 41 | 536740 | 4293916 | 2 | 41 | 0 | 184 | 4399596 | 2 | 41 | 0 | 1 | 192 |
| 144 | 90 | 51 | 33 | 2 | 42 | 536765 | 4294116 | 2 | 42 | 0 | 200 | 4399804 | 2 | 42 | 0 | 1 | 208 |
| 145 | 91 | 51 | 33 | 2 | 43 | 536790 | 4294316 | 2 | 43 | 0 | 200 | 4400012 | 2 | 43 | 0 | 1 | 208 |
| 146 | 92 | 51 | 33 | 2 | 44 | 536815 | 4294516 | 2 | 44 | 0 | 200 | 4400220 | 2 | 44 | 0 | 1 | 208 |
| 147 | 93 | 51 | 33 | 2 | 45 | 536840 | 4294716 | 2 | 45 | 0 | 200 | 4400428 | 2 | 45 | 0 | 1 | 208 |
| 148 | 94 | 51 | 33 | 2 | 46 | 536865 | 4294916 | 2 | 46 | 0 | 200 | 4400636 | 2 | 46 | 0 | 1 | 208 |
| 149 | 95 | 51 | 33 | 2 | 47 | 536890 | 4295116 | 2 | 47 | 0 | 200 | 4400844 | 2 | 47 | 0 | 1 | 208 |
| 150 | 96 | 51 | 33 | 2 | 48 | 536915 | 4295316 | 2 | 48 | 0 | 200 | 4401052 | 2 | 48 | 0 | 1 | 208 |
| 151 | 97 | 51 | 33 | 2 | 49 | 536940 | 4295516 | 2 | 49 | 0 | 200 | 4401260 | 2 | 49 | 0 | 1 | 208 |
| 152 | 98 | 51 | 33 | 2 | 50 | 536965 | 4295716 | 2 | 50 | 0 | 200 | 4401468 | 2 | 50 | 0 | 1 | 208 |
| 153 | 99 | 51 | 33 | 3 | 0 | 536988 | 4295900 | 3 | 0 | 0 | 184 | 4401660 | 3 | 0 | 0 | 1 | 192 |
| 154 | 9A | 51 | 33 | 3 | 1 | 537016 | 4296124 | 3 | 1 | 0 | 224 | 4401892 | 3 | 1 | 0 | 1 | 232 |
| 155 | 9B | 51 | 33 | 3 | 2 | 537044 | 4296348 | 3 | 2 | 0 | 224 | 4402124 | 3 | 2 | 0 | 1 | 232 |
| 156 | 9C | 51 | 33 | 3 | 3 | 537072 | 4296572 | 3 | 3 | 0 | 224 | 4402356 | 3 | 3 | 0 | 1 | 232 |
| 157 | 9D | 51 | 33 | 3 | 4 | 537100 | 4296796 | 3 | 4 | 0 | 224 | 4402588 | 3 | 4 | 0 | 1 | 232 |
| 158 | 9E | 51 | 33 | 3 | 5 | 537128 | 4297020 | 3 | 5 | 0 | 224 | 4402820 | 3 | 5 | 0 | 1 | 232 |
| 159 | 9F | 51 | 33 | 3 | 6 | 537156 | 4297244 | 3 | 6 | 0 | 224 | 4403052 | 3 | 6 | 0 | 1 | 232 |
| 160 | A0 | 51 | 33 | 3 | 7 | 537184 | 4297468 | 3 | 7 | 0 | 224 | 4403284 | 3 | 7 | 0 | 1 | 232 |
| 161 | A1 | 51 | 33 | 3 | 8 | 537212 | 4297692 | 3 | 8 | 0 | 224 | 4403516 | 3 | 8 | 0 | 1 | 232 |
| 162 | A2 | 51 | 33 | 3 | 9 | 537240 | 4297916 | 3 | 9 | 0 | 224 | 4403748 | 3 | 9 | 0 | 1 | 232 |
| 163 | A3 | 51 | 33 | 3 | 10 | 537268 | 4298140 | 3 | 10 | 0 | 224 | 4403980 | 3 | 10 | 0 | 1 | 232 |
| 164 | A4 | 51 | 33 | 3 | 11 | 537296 | 4298364 | 3 | 11 | 0 | 224 | 4404212 | 3 | 11 | 0 | 1 | 232 |
| 165 | A5 | 51 | 33 | 3 | 12 | 537324 | 4298588 | 3 | 12 | 0 | 224 | 4404444 | 3 | 12 | 0 | 1 | 232 |
| 166 | A6 | 51 | 33 | 3 | 13 | 537352 | 4298812 | 3 | 13 | 0 | 224 | 4404676 | 3 | 13 | 0 | 1 | 232 |
| 167 | A7 | 51 | 33 | 3 | 14 | 537380 | 4299036 | 3 | 14 | 0 | 224 | 4404908 | 3 | 14 | 0 | 1 | 232 |
| 168 | A8 | 51 | 33 | 3 | 15 | 537408 | 4299260 | 3 | 15 | 0 | 224 | 4405140 | 3 | 15 | 0 | 1 | 232 |
| 169 | A9 | 51 | 33 | 3 | 16 | 537436 | 4299484 | 3 | 16 | 0 | 224 | 4405372 | 3 | 16 | 0 | 1 | 232 |
| 170 | AA | 51 | 33 | 3 | 17 | 537464 | 4299708 | 3 | 17 | 0 | 224 | 4405604 | 3 | 17 | 0 | 1 | 232 |
| 171 | AB | 51 | 33 | 3 | 18 | 537492 | 4299932 | 3 | 18 | 0 | 224 | 4405836 | 3 | 18 | 0 | 1 | 232 |
| 172 | AC | 51 | 33 | 3 | 19 | 537520 | 4300156 | 3 | 19 | 0 | 224 | 4406068 | 3 | 19 | 0 | 1 | 232 |
| 173 | AD | 51 | 33 | 3 | 20 | 537548 | 4300380 | 3 | 20 | 0 | 224 | 4406300 | 3 | 20 | 0 | 1 | 232 |
| 174 | AE | 51 | 33 | 3 | 21 | 537576 | 4300604 | 3 | 21 | 0 | 224 | 4406532 | 3 | 21 | 0 | 1 | 232 |
| 175 | AF | 51 | 33 | 3 | 22 | 537604 | 4300828 | 3 | 22 | 0 | 224 | 4406764 | 3 | 22 | 0 | 1 | 232 |
| 176 | B0 | 51 | 33 | 3 | 23 | 537632 | 4301052 | 3 | 23 | 0 | 224 | 4406996 | 3 | 23 | 0 | 1 | 232 |
| 177 | B1 | 51 | 33 | 3 | 24 | 537660 | 4301276 | 3 | 24 | 0 | 224 | 4407228 | 3 | 24 | 0 | 1 | 232 |
| 178 | B2 | 51 | 33 | 3 | 25 | 537688 | 4301500 | 3 | 25 | 0 | 224 | 4407460 | 3 | 25 | 0 | 1 | 232 |
| 179 | B3 | 51 | 33 | 3 | 26 | 537716 | 4301724 | 3 | 26 | 0 | 224 | 4407692 | 3 | 26 | 0 | 1 | 232 |
| 180 | B4 | 51 | 33 | 3 | 27 | 537744 | 4301948 | 3 | 27 | 0 | 224 | 4407924 | 3 | 27 | 0 | 1 | 232 |
| 181 | B5 | 51 | 33 | 3 | 28 | 537772 | 4302172 | 3 | 28 | 0 | 224 | 4408156 | 3 | 28 | 0 | 1 | 232 |
| 182 | B6 | 51 | 33 | 3 | 29 | 537800 | 4302396 | 3 | 29 | 0 | 224 | 4408388 | 3 | 29 | 0 | 1 | 232 |
| 183 | B7 | 51 | 33 | 3 | 30 | 537828 | 4302620 | 3 | 30 | 0 | 224 | 4408620 | 3 | 30 | 0 | 1 | 232 |
| 184 | B8 | 51 | 33 | 3 | 31 | 537856 | 4302844 | 3 | 31 | 0 | 224 | 4408852 | 3 | 31 | 0 | 1 | 232 |
| 185 | B9 | 51 | 33 | 3 | 32 | 537884 | 4303068 | 3 | 32 | 0 | 224 | 4409084 | 3 | 32 | 0 | 1 | 232 |
| 186 | BA | 51 | 33 | 3 | 33 | 537912 | 4303292 | 3 | 33 | 0 | 224 | 4409316 | 3 | 33 | 0 | 1 | 232 |
| 187 | BB | 51 | 33 | 3 | 34 | 537940 | 4303516 | 3 | 34 | 0 | 224 | 4409548 | 3 | 34 | 0 | 1 | 232 |
| 188 | BC | 51 | 33 | 3 | 35 | 537968 | 4303740 | 3 | 35 | 0 | 224 | 4409780 | 3 | 35 | 0 | 1 | 232 |
| 189 | BD | 51 | 33 | 3 | 36 | 537996 | 4303964 | 3 | 36 | 0 | 224 | 4410012 | 3 | 36 | 0 | 1 | 232 |
| 190 | BE | 51 | 33 | 3 | 37 | 538024 | 4304188 | 3 | 37 | 0 | 224 | 4410244 | 3 | 37 | 0 | 1 | 232 |
| 191 | BF | 51 | 33 | 3 | 38 | 538052 | 4304412 | 3 | 38 | 0 | 224 | 4410476 | 3 | 38 | 0 | 1 | 232 |
| 192 | C0 | 51 | 33 | 3 | 39 | 538077 | 4304612 | 3 | 39 | 0 | 200 | 4410684 | 3 | 39 | 0 | 1 | 208 |
| 193 | C1 | 51 | 33 | 3 | 40 | 538102 | 4304812 | 3 | 40 | 0 | 200 | 4410892 | 3 | 40 | 0 | 1 | 208 |
| 194 | C2 | 51 | 33 | 3 | 41 | 538127 | 4305012 | 3 | 41 | 0 | 200 | 4411100 | 3 | 41 | 0 | 1 | 208 |
| 195 | C3 | 51 | 33 | 3 | 42 | 538152 | 4305212 | 3 | 42 | 0 | 200 | 4411308 | 3 | 42 | 0 | 1 | 208 |
| 196 | C4 | 51 | 33 | 3 | 43 | 538177 | 4305412 | 3 | 43 | 0 | 200 | 4411516 | 3 | 43 | 0 | 1 | 208 |
| 197 | C5 | 51 | 33 | 3 | 44 | 538202 | 4305612 | 3 | 44 | 0 | 200 | 4411724 | 3 | 44 | 0 | 1 | 208 |
| 198 | C6 | 51 | 33 | 3 | 45 | 538227 | 4305812 | 3 | 45 | 0 | 200 | 4411932 | 3 | 45 | 0 | 1 | 208 |
| 199 | C7 | 51 | 33 | 3 | 46 | 538252 | 4306012 | 3 | 46 | 0 | 200 | 4412140 | 3 | 46 | 0 | 1 | 208 |
| 200 | C8 | 51 | 33 | 3 | 47 | 538277 | 4306212 | 3 | 47 | 0 | 200 | 4412348 | 3 | 47 | 0 | 1 | 208 |
| 201 | C9 | 51 | 33 | 3 | 48 | 538302 | 4306412 | 3 | 48 | 0 | 200 | 4412556 | 3 | 48 | 0 | 1 | 208 |
| 202 | CA | 51 | 33 | 3 | 49 | 538327 | 4306612 | 3 | 49 | 0 | 200 | 4412764 | 3 | 49 | 0 | 1 | 208 |
| 203 | CB | 51 | 33 | 3 | 50 | 538352 | 4306812 | 3 | 50 | 0 | 200 | 4412972 | 3 | 50 | 0 | 1 | 208 |
| 204 | CC | 51 | 33 | 4 | 0 | 538375 | 4306996 | 4 | 0 | 0 | 184 | 4413164 | 4 | 0 | 0 | 1 | 192 |
| 205 | CD | 51 | 33 | 4 | 1 | 538403 | 4307220 | 4 | 1 | 0 | 224 | 4413396 | 4 | 1 | 0 | 1 | 232 |
| 206 | CE | 51 | 33 | 4 | 2 | 538431 | 4307444 | 4 | 2 | 0 | 224 | 4413628 | 4 | 2 | 0 | 1 | 232 |
| 207 | CF | 51 | 33 | 4 | 3 | 538459 | 4307668 | 4 | 3 | 0 | 224 | 4413860 | 4 | 3 | 0 | 1 | 232 |
| 208 | D0 | 51 | 33 | 4 | 4 | 538489 | 4307908 | 4 | 4 | 0 | 240 | 4414108 | 4 | 4 | 0 | 1 | 248 |
| 209 | D1 | 51 | 33 | 4 | 5 | 538519 | 4308148 | 4 | 5 | 0 | 240 | 4414356 | 4 | 5 | 0 | 1 | 248 |
| 210 | D2 | 51 | 33 | 4 | 6 | 538549 | 4308388 | 4 | 6 | 0 | 240 | 4414604 | 4 | 6 | 0 | 1 | 248 |
| 211 | D3 | 51 | 33 | 4 | 7 | 538579 | 4308628 | 4 | 7 | 0 | 240 | 4414852 | 4 | 7 | 0 | 1 | 248 |
| 212 | D4 | 51 | 33 | 4 | 8 | 538609 | 4308868 | 4 | 8 | 0 | 240 | 4415100 | 4 | 8 | 0 | 1 | 248 |
| 213 | D5 | 51 | 33 | 4 | 9 | 538639 | 4309108 | 4 | 9 | 0 | 240 | 4415348 | 4 | 9 | 0 | 1 | 248 |
| 214 | D6 | 51 | 33 | 4 | 10 | 538669 | 4309348 | 4 | 10 | 0 | 240 | 4415596 | 4 | 10 | 0 | 1 | 248 |
| 215 | D7 | 51 | 33 | 4 | 11 | 538699 | 4309588 | 4 | 11 | 0 | 240 | 4415844 | 4 | 11 | 0 | 1 | 248 |
| 216 | D8 | 51 | 33 | 4 | 12 | 538729 | 4309828 | 4 | 12 | 0 | 240 | 4416092 | 4 | 12 | 0 | 1 | 248 |
| 217 | D9 | 51 | 33 | 4 | 13 | 538759 | 4310068 | 4 | 13 | 0 | 240 | 4416340 | 4 | 13 | 0 | 1 | 248 |
| 218 | DA | 51 | 33 | 4 | 14 | 538789 | 4310308 | 4 | 14 | 0 | 240 | 4416588 | 4 | 14 | 0 | 1 | 248 |
| 219 | DB | 51 | 33 | 4 | 15 | 538819 | 4310548 | 4 | 15 | 0 | 240 | 4416836 | 4 | 15 | 0 | 1 | 248 |
| 220 | DC | 51 | 33 | 4 | 16 | 538849 | 4310788 | 4 | 16 | 0 | 240 | 4417084 | 4 | 16 | 0 | 1 | 248 |
| 221 | DD | 51 | 33 | 4 | 17 | 538879 | 4311028 | 4 | 17 | 0 | 240 | 4417332 | 4 | 17 | 0 | 1 | 248 |
| 222 | DE | 51 | 33 | 4 | 18 | 538909 | 4311268 | 4 | 18 | 0 | 240 | 4417580 | 4 | 18 | 0 | 1 | 248 |
| 223 | DF | 51 | 33 | 4 | 19 | 538939 | 4311508 | 4 | 19 | 0 | 240 | 4417828 | 4 | 19 | 0 | 1 | 248 |
| 224 | E0 | 51 | 33 | 4 | 20 | 538969 | 4311748 | 4 | 20 | 0 | 240 | 4418076 | 4 | 20 | 0 | 1 | 248 |
| 225 | E1 | 51 | 33 | 4 | 21 | 538999 | 4311988 | 4 | 21 | 0 | 240 | 4418324 | 4 | 21 | 0 | 1 | 248 |
| 226 | E2 | 51 | 33 | 4 | 22 | 539029 | 4312228 | 4 | 22 | 0 | 240 | 4418572 | 4 | 22 | 0 | 1 | 248 |
| 227 | E3 | 51 | 33 | 4 | 23 | 539059 | 4312468 | 4 | 23 | 0 | 240 | 4418820 | 4 | 23 | 0 | 1 | 248 |

| | | | | | | | | | | | | | | | | | |
|---|---|---|---|---|---|---|---|---|---|---|---|---|---|---|---|---|---|
| 228 | E4 | 51 | 33 | 4 | 24 | 539089 | 4312708 | 4 | 24 | 0 | 240 | 4419068 | 4 | 24 | 0 | 1 | 248 |
| 229 | E5 | 51 | 33 | 4 | 25 | 539119 | 4312948 | 4 | 25 | 0 | 240 | 4419316 | 4 | 25 | 0 | 1 | 248 |
| 230 | E6 | 51 | 33 | 4 | 26 | 539149 | 4313188 | 4 | 26 | 0 | 240 | 4419564 | 4 | 26 | 0 | 1 | 248 |
| 231 | E7 | 51 | 33 | 4 | 27 | 539179 | 4313428 | 4 | 27 | 0 | 240 | 4419812 | 4 | 27 | 0 | 1 | 248 |
| 232 | E8 | 51 | 33 | 4 | 28 | 539209 | 4313668 | 4 | 28 | 0 | 240 | 4420060 | 4 | 28 | 0 | 1 | 248 |
| 233 | E9 | 51 | 33 | 4 | 29 | 539239 | 4313908 | 4 | 29 | 0 | 240 | 4420308 | 4 | 29 | 0 | 1 | 248 |
| 234 | EA | 51 | 33 | 4 | 30 | 539269 | 4314148 | 4 | 30 | 0 | 240 | 4420556 | 4 | 30 | 0 | 1 | 248 |
| 235 | EB | 51 | 33 | 4 | 31 | 539299 | 4314388 | 4 | 31 | 0 | 240 | 4420804 | 4 | 31 | 0 | 1 | 248 |
| 236 | EC | 51 | 33 | 4 | 32 | 539329 | 4314628 | 4 | 32 | 0 | 240 | 4421052 | 4 | 32 | 0 | 1 | 248 |
| 237 | ED | 51 | 33 | 4 | 33 | 539359 | 4314868 | 4 | 33 | 0 | 240 | 4421300 | 4 | 33 | 0 | 1 | 248 |
| 238 | EE | 51 | 33 | 4 | 34 | 539389 | 4315108 | 4 | 34 | 0 | 240 | 4421548 | 4 | 34 | 0 | 1 | 248 |
| 239 | EF | 51 | 33 | 4 | 35 | 539419 | 4315348 | 4 | 35 | 0 | 240 | 4421796 | 4 | 35 | 0 | 1 | 248 |
| 240 | F0 | 51 | 33 | 4 | 36 | 539449 | 4315588 | 4 | 36 | 0 | 240 | 4422044 | 4 | 36 | 0 | 1 | 248 |
| 241 | F1 | 51 | 33 | 4 | 37 | 539479 | 4315828 | 4 | 37 | 0 | 240 | 4422292 | 4 | 37 | 0 | 1 | 248 |
| 242 | F2 | 51 | 33 | 4 | 38 | 539509 | 4316068 | 4 | 38 | 0 | 240 | 4422540 | 4 | 38 | 0 | 1 | 248 |
| 243 | F3 | 51 | 33 | 4 | 39 | 539539 | 4316308 | 4 | 39 | 0 | 240 | 4422788 | 4 | 39 | 0 | 1 | 248 |
| 244 | F4 | 51 | 33 | 4 | 40 | 539569 | 4316548 | 4 | 40 | 0 | 240 | 4423036 | 4 | 40 | 0 | 1 | 248 |
| 245 | F5 | 51 | 33 | 4 | 41 | 539599 | 4316788 | 4 | 41 | 0 | 240 | 4423284 | 4 | 41 | 0 | 1 | 248 |
| 246 | F6 | 51 | 33 | 4 | 42 | 539629 | 4317028 | 4 | 42 | 0 | 240 | 4423532 | 4 | 42 | 0 | 1 | 248 |
| 247 | F7 | 51 | 33 | 4 | 43 | 539659 | 4317268 | 4 | 43 | 0 | 240 | 4423780 | 4 | 43 | 0 | 1 | 248 |
| 248 | F8 | 51 | 33 | 4 | 44 | 539689 | 4317508 | 4 | 44 | 0 | 240 | 4424028 | 4 | 44 | 0 | 1 | 248 |
| 249 | F9 | 51 | 33 | 4 | 45 | 539719 | 4317748 | 4 | 45 | 0 | 240 | 4424276 | 4 | 45 | 0 | 1 | 248 |
| 250 | FA | 51 | 33 | 4 | 46 | 539749 | 4317988 | 4 | 46 | 0 | 240 | 4424524 | 4 | 46 | 0 | 1 | 248 |
| 251 | FB | 51 | 33 | 4 | 47 | 539779 | 4318228 | 4 | 47 | 0 | 240 | 4424772 | 4 | 47 | 0 | 1 | 248 |
| 252 | FC | 51 | 33 | 4 | 48 | 539809 | 4318468 | 4 | 48 | 0 | 240 | 4425020 | 4 | 48 | 0 | 1 | 248 |
| 253 | FD | 51 | 33 | 4 | 49 | 539839 | 4318708 | 4 | 49 | 0 | 240 | 4425268 | 4 | 49 | 0 | 1 | 248 |
| 254 | FE | 51 | 33 | 4 | 50 | 539869 | 4318948 | 4 | 50 | 0 | 240 | 4425516 | 4 | 50 | 0 | 1 | 248 |
| 255 | FF | 51 | 33 | 5 | 0 | 539901 | 4319204 | 5 | 0 | 0 | 256 | 4425780 | 5 | 0 | 0 | 1 | 264 |
| 0 | 00 | 52 | 34 | 0 | 0 | 539913 | 4319300 | 0 | 0 | 0 | 96 | 4425884 | 0 | 0 | 0 | 1 | 104 |
| 1 | 01 | 52 | 34 | 0 | 1 | 539927 | 4319412 | 0 | 1 | 0 | 112 | 4426004 | 0 | 1 | 0 | 1 | 120 |
| 2 | 02 | 52 | 34 | 0 | 2 | 539941 | 4319524 | 0 | 2 | 0 | 112 | 4426124 | 0 | 2 | 0 | 1 | 120 |
| 3 | 03 | 52 | 34 | 0 | 3 | 539955 | 4319636 | 0 | 3 | 0 | 112 | 4426244 | 0 | 3 | 0 | 1 | 120 |
| 4 | 04 | 52 | 34 | 0 | 4 | 539969 | 4319748 | 0 | 4 | 0 | 112 | 4426364 | 0 | 4 | 0 | 1 | 120 |
| 5 | 05 | 52 | 34 | 0 | 5 | 539983 | 4319860 | 0 | 5 | 0 | 112 | 4426484 | 0 | 5 | 0 | 1 | 120 |
| 6 | 06 | 52 | 34 | 0 | 6 | 539997 | 4319972 | 0 | 6 | 0 | 112 | 4426604 | 0 | 6 | 0 | 1 | 120 |
| 7 | 07 | 52 | 34 | 0 | 7 | 540011 | 4320084 | 0 | 7 | 0 | 112 | 4426724 | 0 | 7 | 0 | 1 | 120 |
| 8 | 08 | 52 | 34 | 0 | 8 | 540025 | 4320196 | 0 | 8 | 0 | 112 | 4426844 | 0 | 8 | 0 | 1 | 120 |
| 9 | 09 | 52 | 34 | 0 | 9 | 540039 | 4320308 | 0 | 9 | 0 | 112 | 4426964 | 0 | 9 | 0 | 1 | 120 |
| 10 | 0A | 52 | 34 | 0 | 10 | 540053 | 4320420 | 0 | 10 | 0 | 112 | 4427084 | 0 | 10 | 0 | 1 | 120 |
| 11 | 0B | 52 | 34 | 0 | 11 | 540067 | 4320532 | 0 | 11 | 0 | 112 | 4427204 | 0 | 11 | 0 | 1 | 120 |
| 12 | 0C | 52 | 34 | 0 | 12 | 540081 | 4320644 | 0 | 12 | 0 | 112 | 4427324 | 0 | 12 | 0 | 1 | 120 |
| 13 | 0D | 52 | 34 | 0 | 13 | 540095 | 4320756 | 0 | 13 | 0 | 112 | 4427444 | 0 | 13 | 0 | 1 | 120 |
| 14 | 0E | 52 | 34 | 0 | 14 | 540109 | 4320868 | 0 | 14 | 0 | 112 | 4427564 | 0 | 14 | 0 | 1 | 120 |
| 15 | 0F | 52 | 34 | 0 | 15 | 540123 | 4320980 | 0 | 15 | 0 | 112 | 4427684 | 0 | 15 | 0 | 1 | 120 |
| 16 | 10 | 52 | 34 | 0 | 16 | 540137 | 4321092 | 0 | 16 | 0 | 112 | 4427804 | 0 | 16 | 0 | 1 | 120 |
| 17 | 11 | 52 | 34 | 0 | 17 | 540151 | 4321204 | 0 | 17 | 0 | 112 | 4427924 | 0 | 17 | 0 | 1 | 120 |
| 18 | 12 | 52 | 34 | 0 | 18 | 540165 | 4321316 | 0 | 18 | 0 | 112 | 4428044 | 0 | 18 | 0 | 1 | 120 |
| 19 | 13 | 52 | 34 | 0 | 19 | 540179 | 4321428 | 0 | 19 | 0 | 112 | 4428164 | 0 | 19 | 0 | 1 | 120 |
| 20 | 14 | 52 | 34 | 0 | 20 | 540193 | 4321540 | 0 | 20 | 0 | 112 | 4428284 | 0 | 20 | 0 | 1 | 120 |
| 21 | 15 | 52 | 34 | 0 | 21 | 540207 | 4321652 | 0 | 21 | 0 | 112 | 4428404 | 0 | 21 | 0 | 1 | 120 |
| 22 | 16 | 52 | 34 | 0 | 22 | 540221 | 4321764 | 0 | 22 | 0 | 112 | 4428524 | 0 | 22 | 0 | 1 | 120 |
| 23 | 17 | 52 | 34 | 0 | 23 | 540235 | 4321876 | 0 | 23 | 0 | 112 | 4428644 | 0 | 23 | 0 | 1 | 120 |
| 24 | 18 | 52 | 34 | 0 | 24 | 540249 | 4321988 | 0 | 24 | 0 | 112 | 4428764 | 0 | 24 | 0 | 1 | 120 |
| 25 | 19 | 52 | 34 | 0 | 25 | 540263 | 4322100 | 0 | 25 | 0 | 112 | 4428884 | 0 | 25 | 0 | 1 | 120 |
| 26 | 1A | 52 | 34 | 0 | 26 | 540277 | 4322212 | 0 | 26 | 0 | 112 | 4429004 | 0 | 26 | 0 | 1 | 120 |
| 27 | 1B | 52 | 34 | 0 | 27 | 540291 | 4322324 | 0 | 27 | 0 | 112 | 4429124 | 0 | 27 | 0 | 1 | 120 |
| 28 | 1C | 52 | 34 | 0 | 28 | 540305 | 4322436 | 0 | 28 | 0 | 112 | 4429244 | 0 | 28 | 0 | 1 | 120 |
| 29 | 1D | 52 | 34 | 0 | 29 | 540319 | 4322548 | 0 | 29 | 0 | 112 | 4429364 | 0 | 29 | 0 | 1 | 120 |
| 30 | 1E | 52 | 34 | 0 | 30 | 540333 | 4322660 | 0 | 30 | 0 | 112 | 4429484 | 0 | 30 | 0 | 1 | 120 |
| 31 | 1F | 52 | 34 | 0 | 31 | 540347 | 4322772 | 0 | 31 | 0 | 112 | 4429604 | 0 | 31 | 0 | 1 | 120 |
| 32 | 20 | 52 | 34 | 0 | 32 | 540361 | 4322884 | 0 | 32 | 0 | 112 | 4429724 | 0 | 32 | 0 | 1 | 120 |
| 33 | 21 | 52 | 34 | 0 | 33 | 540375 | 4322996 | 0 | 33 | 0 | 112 | 4429844 | 0 | 33 | 0 | 1 | 120 |
| 34 | 22 | 52 | 34 | 0 | 34 | 540389 | 4323108 | 0 | 34 | 0 | 112 | 4429964 | 0 | 34 | 0 | 1 | 120 |
| 35 | 23 | 52 | 34 | 0 | 35 | 540403 | 4323220 | 0 | 35 | 0 | 112 | 4430084 | 0 | 35 | 0 | 1 | 120 |
| 36 | 24 | 52 | 34 | 0 | 36 | 540417 | 4323332 | 0 | 36 | 0 | 112 | 4430204 | 0 | 36 | 0 | 1 | 120 |
| 37 | 25 | 52 | 34 | 0 | 37 | 540431 | 4323444 | 0 | 37 | 0 | 112 | 4430324 | 0 | 37 | 0 | 1 | 120 |
| 38 | 26 | 52 | 34 | 0 | 38 | 540445 | 4323556 | 0 | 38 | 0 | 112 | 4430444 | 0 | 38 | 0 | 1 | 120 |
| 39 | 27 | 52 | 34 | 0 | 39 | 540459 | 4323668 | 0 | 39 | 0 | 112 | 4430564 | 0 | 39 | 0 | 1 | 120 |
| 40 | 28 | 52 | 34 | 0 | 40 | 540473 | 4323780 | 0 | 40 | 0 | 112 | 4430684 | 0 | 40 | 0 | 1 | 120 |
| 41 | 29 | 52 | 34 | 0 | 41 | 540487 | 4323892 | 0 | 41 | 0 | 112 | 4430804 | 0 | 41 | 0 | 1 | 120 |
| 42 | 2A | 52 | 34 | 0 | 42 | 540501 | 4324004 | 0 | 42 | 0 | 112 | 4430924 | 0 | 42 | 0 | 1 | 120 |
| 43 | 2B | 52 | 34 | 0 | 43 | 540515 | 4324116 | 0 | 43 | 0 | 112 | 4431044 | 0 | 43 | 0 | 1 | 120 |
| 44 | 2C | 52 | 34 | 0 | 44 | 540529 | 4324228 | 0 | 44 | 0 | 112 | 4431164 | 0 | 44 | 0 | 1 | 120 |
| 45 | 2D | 52 | 34 | 0 | 45 | 540543 | 4324340 | 0 | 45 | 0 | 112 | 4431284 | 0 | 45 | 0 | 1 | 120 |
| 46 | 2E | 52 | 34 | 0 | 46 | 540557 | 4324452 | 0 | 46 | 0 | 112 | 4431404 | 0 | 46 | 0 | 1 | 120 |
| 47 | 2F | 52 | 34 | 0 | 47 | 540571 | 4324564 | 0 | 47 | 0 | 112 | 4431524 | 0 | 47 | 0 | 1 | 120 |
| 48 | 30 | 52 | 34 | 0 | 48 | 540585 | 4324676 | 0 | 48 | 0 | 112 | 4431644 | 0 | 48 | 0 | 1 | 120 |
| 49 | 31 | 52 | 34 | 0 | 49 | 540599 | 4324788 | 0 | 49 | 0 | 112 | 4431764 | 0 | 49 | 0 | 1 | 120 |
| 50 | 32 | 52 | 34 | 0 | 50 | 540613 | 4324900 | 0 | 50 | 0 | 112 | 4431884 | 0 | 50 | 0 | 1 | 120 |
| 51 | 33 | 52 | 34 | 0 | 51 | 540627 | 4325012 | 0 | 51 | 0 | 112 | 4432004 | 0 | 51 | 0 | 1 | 120 |
| 52 | 34 | 52 | 34 | 1 | 0 | 540639 | 4325108 | 1 | 0 | 0 | 96 | 4432108 | 1 | 0 | 0 | 1 | 104 |
| 53 | 35 | 52 | 34 | 1 | 1 | 540656 | 4325244 | 1 | 1 | 0 | 136 | 4432252 | 1 | 1 | 0 | 1 | 144 |
| 54 | 36 | 52 | 34 | 1 | 2 | 540673 | 4325380 | 1 | 2 | 0 | 136 | 4432396 | 1 | 2 | 0 | 1 | 144 |
| 55 | 37 | 52 | 34 | 1 | 3 | 540690 | 4325516 | 1 | 3 | 0 | 136 | 4432540 | 1 | 3 | 0 | 1 | 144 |
| 56 | 38 | 52 | 34 | 1 | 4 | 540707 | 4325652 | 1 | 4 | 0 | 136 | 4432684 | 1 | 4 | 0 | 1 | 144 |
| 57 | 39 | 52 | 34 | 1 | 5 | 540724 | 4325788 | 1 | 5 | 0 | 136 | 4432828 | 1 | 5 | 0 | 1 | 144 |
| 58 | 3A | 52 | 34 | 1 | 6 | 540741 | 4325924 | 1 | 6 | 0 | 136 | 4432972 | 1 | 6 | 0 | 1 | 144 |
| 59 | 3B | 52 | 34 | 1 | 7 | 540758 | 4326060 | 1 | 7 | 0 | 136 | 4433116 | 1 | 7 | 0 | 1 | 144 |
| 60 | 3C | 52 | 34 | 1 | 8 | 540775 | 4326196 | 1 | 8 | 0 | 136 | 4433260 | 1 | 8 | 0 | 1 | 144 |
| 61 | 3D | 52 | 34 | 1 | 9 | 540792 | 4326332 | 1 | 9 | 0 | 136 | 4433404 | 1 | 9 | 0 | 1 | 144 |
| 62 | 3E | 52 | 34 | 1 | 10 | 540809 | 4326468 | 1 | 10 | 0 | 136 | 4433548 | 1 | 10 | 0 | 1 | 144 |
| 63 | 3F | 52 | 34 | 1 | 11 | 540826 | 4326604 | 1 | 11 | 0 | 136 | 4433692 | 1 | 11 | 0 | 1 | 144 |
| 64 | 40 | 52 | 34 | 1 | 12 | 540847 | 4326772 | 1 | 12 | 0 | 168 | 4433868 | 1 | 12 | 0 | 1 | 176 |
| 65 | 41 | 52 | 34 | 1 | 13 | 540868 | 4326940 | 1 | 13 | 0 | 168 | 4434044 | 1 | 13 | 0 | 1 | 176 |
| 66 | 42 | 52 | 34 | 1 | 14 | 540889 | 4327108 | 1 | 14 | 0 | 168 | 4434220 | 1 | 14 | 0 | 1 | 176 |
| 67 | 43 | 52 | 34 | 1 | 15 | 540910 | 4327276 | 1 | 15 | 0 | 168 | 4434396 | 1 | 15 | 0 | 1 | 176 |
| 68 | 44 | 52 | 34 | 1 | 16 | 540931 | 4327444 | 1 | 16 | 0 | 168 | 4434572 | 1 | 16 | 0 | 1 | 176 |
| 69 | 45 | 52 | 34 | 1 | 17 | 540952 | 4327612 | 1 | 17 | 0 | 168 | 4434748 | 1 | 17 | 0 | 1 | 176 |
| 70 | 46 | 52 | 34 | 1 | 18 | 540973 | 4327780 | 1 | 18 | 0 | 168 | 4434924 | 1 | 18 | 0 | 1 | 176 |
| 71 | 47 | 52 | 34 | 1 | 19 | 540994 | 4327948 | 1 | 19 | 0 | 168 | 4435100 | 1 | 19 | 0 | 1 | 176 |
| 72 | 48 | 52 | 34 | 1 | 20 | 541015 | 4328116 | 1 | 20 | 0 | 168 | 4435276 | 1 | 20 | 0 | 1 | 176 |
| 73 | 49 | 52 | 34 | 1 | 21 | 541036 | 4328284 | 1 | 21 | 0 | 168 | 4435452 | 1 | 21 | 0 | 1 | 176 |
| 74 | 4A | 52 | 34 | 1 | 22 | 541057 | 4328452 | 1 | 22 | 0 | 168 | 4435628 | 1 | 22 | 0 | 1 | 176 |

| | | | | | | | | | | | | | | | | | |
|---|---|---|---|---|---|---|---|---|---|---|---|---|---|---|---|---|---|
| 75 | 4B | 52 | 34 | 1 | 23 | 541078 | 4328620 | 1 | 23 | 0 | 168 | 4435804 | 1 | 23 | 0 | 1 | 176 |
| 76 | 4C | 52 | 34 | 1 | 24 | 541099 | 4328788 | 1 | 24 | 0 | 168 | 4435980 | 1 | 24 | 0 | 1 | 176 |
| 77 | 4D | 52 | 34 | 1 | 25 | 541120 | 4328956 | 1 | 25 | 0 | 168 | 4436156 | 1 | 25 | 0 | 1 | 176 |
| 78 | 4E | 52 | 34 | 1 | 26 | 541141 | 4329124 | 1 | 26 | 0 | 168 | 4436332 | 1 | 26 | 0 | 1 | 176 |
| 79 | 4F | 52 | 34 | 1 | 27 | 541162 | 4329292 | 1 | 27 | 0 | 168 | 4436508 | 1 | 27 | 0 | 1 | 176 |
| 80 | 50 | 52 | 34 | 1 | 28 | 541185 | 4329476 | 1 | 28 | 0 | 184 | 4436700 | 1 | 28 | 0 | 1 | 192 |
| 81 | 51 | 52 | 34 | 1 | 29 | 541208 | 4329660 | 1 | 29 | 0 | 184 | 4436892 | 1 | 29 | 0 | 1 | 192 |
| 82 | 52 | 52 | 34 | 1 | 30 | 541231 | 4329844 | 1 | 30 | 0 | 184 | 4437084 | 1 | 30 | 0 | 1 | 192 |
| 83 | 53 | 52 | 34 | 1 | 31 | 541254 | 4330028 | 1 | 31 | 0 | 184 | 4437276 | 1 | 31 | 0 | 1 | 192 |
| 84 | 54 | 52 | 34 | 1 | 32 | 541277 | 4330212 | 1 | 32 | 0 | 184 | 4437468 | 1 | 32 | 0 | 1 | 192 |
| 85 | 55 | 52 | 34 | 1 | 33 | 541300 | 4330396 | 1 | 33 | 0 | 184 | 4437660 | 1 | 33 | 0 | 1 | 192 |
| 86 | 56 | 52 | 34 | 1 | 34 | 541323 | 4330580 | 1 | 34 | 0 | 184 | 4437852 | 1 | 34 | 0 | 1 | 192 |
| 87 | 57 | 52 | 34 | 1 | 35 | 541346 | 4330764 | 1 | 35 | 0 | 184 | 4438044 | 1 | 35 | 0 | 1 | 192 |
| 88 | 58 | 52 | 34 | 1 | 36 | 541369 | 4330948 | 1 | 36 | 0 | 184 | 4438236 | 1 | 36 | 0 | 1 | 192 |
| 89 | 59 | 52 | 34 | 1 | 37 | 541392 | 4331132 | 1 | 37 | 0 | 184 | 4438428 | 1 | 37 | 0 | 1 | 192 |
| 90 | 5A | 52 | 34 | 1 | 38 | 541415 | 4331316 | 1 | 38 | 0 | 184 | 4438620 | 1 | 38 | 0 | 1 | 192 |
| 91 | 5B | 52 | 34 | 1 | 39 | 541438 | 4331500 | 1 | 39 | 0 | 184 | 4438812 | 1 | 39 | 0 | 1 | 192 |
| 92 | 5C | 52 | 34 | 1 | 40 | 541461 | 4331684 | 1 | 40 | 0 | 184 | 4439004 | 1 | 40 | 0 | 1 | 192 |
| 93 | 5D | 52 | 34 | 1 | 41 | 541484 | 4331868 | 1 | 41 | 0 | 184 | 4439196 | 1 | 41 | 0 | 1 | 192 |
| 94 | 5E | 52 | 34 | 1 | 42 | 541507 | 4332052 | 1 | 42 | 0 | 184 | 4439388 | 1 | 42 | 0 | 1 | 192 |
| 95 | 5F | 52 | 34 | 1 | 43 | 541530 | 4332236 | 1 | 43 | 0 | 184 | 4439580 | 1 | 43 | 0 | 1 | 192 |
| 96 | 60 | 52 | 34 | 1 | 44 | 541553 | 4332420 | 1 | 44 | 0 | 184 | 4439772 | 1 | 44 | 0 | 1 | 192 |
| 97 | 61 | 52 | 34 | 1 | 45 | 541576 | 4332604 | 1 | 45 | 0 | 184 | 4439964 | 1 | 45 | 0 | 1 | 192 |
| 98 | 62 | 52 | 34 | 1 | 46 | 541599 | 4332788 | 1 | 46 | 0 | 184 | 4440156 | 1 | 46 | 0 | 1 | 192 |
| 99 | 63 | 52 | 34 | 1 | 47 | 541622 | 4332972 | 1 | 47 | 0 | 184 | 4440348 | 1 | 47 | 0 | 1 | 192 |
| 100 | 64 | 52 | 34 | 1 | 48 | 541645 | 4333156 | 1 | 48 | 0 | 184 | 4440540 | 1 | 48 | 0 | 1 | 192 |
| 101 | 65 | 52 | 34 | 1 | 49 | 541668 | 4333340 | 1 | 49 | 0 | 184 | 4440732 | 1 | 49 | 0 | 1 | 192 |
| 102 | 66 | 52 | 34 | 1 | 50 | 541691 | 4333524 | 1 | 50 | 0 | 184 | 4440924 | 1 | 50 | 0 | 1 | 192 |
| 103 | 67 | 52 | 34 | 1 | 51 | 541714 | 4333708 | 1 | 51 | 0 | 184 | 4441116 | 1 | 51 | 0 | 1 | 192 |
| 104 | 68 | 52 | 34 | 2 | 0 | 541735 | 4333876 | 2 | 0 | 0 | 168 | 4441292 | 2 | 0 | 0 | 1 | 176 |
| 105 | 69 | 52 | 34 | 2 | 1 | 541761 | 4334084 | 2 | 1 | 0 | 208 | 4441508 | 2 | 1 | 0 | 1 | 216 |
| 106 | 6A | 52 | 34 | 2 | 2 | 541787 | 4334292 | 2 | 2 | 0 | 208 | 4441724 | 2 | 2 | 0 | 1 | 216 |
| 107 | 6B | 52 | 34 | 2 | 3 | 541813 | 4334500 | 2 | 3 | 0 | 208 | 4441940 | 2 | 3 | 0 | 1 | 216 |
| 108 | 6C | 52 | 34 | 2 | 4 | 541839 | 4334708 | 2 | 4 | 0 | 208 | 4442156 | 2 | 4 | 0 | 1 | 216 |
| 109 | 6D | 52 | 34 | 2 | 5 | 541865 | 4334916 | 2 | 5 | 0 | 208 | 4442372 | 2 | 5 | 0 | 1 | 216 |
| 110 | 6E | 52 | 34 | 2 | 6 | 541891 | 4335124 | 2 | 6 | 0 | 208 | 4442588 | 2 | 6 | 0 | 1 | 216 |
| 111 | 6F | 52 | 34 | 2 | 7 | 541917 | 4335332 | 2 | 7 | 0 | 208 | 4442804 | 2 | 7 | 0 | 1 | 216 |
| 112 | 70 | 52 | 34 | 2 | 8 | 541943 | 4335540 | 2 | 8 | 0 | 208 | 4443020 | 2 | 8 | 0 | 1 | 216 |
| 113 | 71 | 52 | 34 | 2 | 9 | 541969 | 4335748 | 2 | 9 | 0 | 208 | 4443236 | 2 | 9 | 0 | 1 | 216 |
| 114 | 72 | 52 | 34 | 2 | 10 | 541995 | 4335956 | 2 | 10 | 0 | 208 | 4443452 | 2 | 10 | 0 | 1 | 216 |
| 115 | 73 | 52 | 34 | 2 | 11 | 542021 | 4336164 | 2 | 11 | 0 | 208 | 4443668 | 2 | 11 | 0 | 1 | 216 |
| 116 | 74 | 52 | 34 | 2 | 12 | 542047 | 4336372 | 2 | 12 | 0 | 208 | 4443884 | 2 | 12 | 0 | 1 | 216 |
| 117 | 75 | 52 | 34 | 2 | 13 | 542073 | 4336580 | 2 | 13 | 0 | 208 | 4444100 | 2 | 13 | 0 | 1 | 216 |
| 118 | 76 | 52 | 34 | 2 | 14 | 542099 | 4336788 | 2 | 14 | 0 | 208 | 4444316 | 2 | 14 | 0 | 1 | 216 |
| 119 | 77 | 52 | 34 | 2 | 15 | 542125 | 4336996 | 2 | 15 | 0 | 208 | 4444532 | 2 | 15 | 0 | 1 | 216 |
| 120 | 78 | 52 | 34 | 2 | 16 | 542151 | 4337204 | 2 | 16 | 0 | 208 | 4444748 | 2 | 16 | 0 | 1 | 216 |
| 121 | 79 | 52 | 34 | 2 | 17 | 542177 | 4337412 | 2 | 17 | 0 | 208 | 4444964 | 2 | 17 | 0 | 1 | 216 |
| 122 | 7A | 52 | 34 | 2 | 18 | 542203 | 4337620 | 2 | 18 | 0 | 208 | 4445180 | 2 | 18 | 0 | 1 | 216 |
| 123 | 7B | 52 | 34 | 2 | 19 | 542229 | 4337828 | 2 | 19 | 0 | 208 | 4445396 | 2 | 19 | 0 | 1 | 216 |
| 124 | 7C | 52 | 34 | 2 | 20 | 542255 | 4338036 | 2 | 20 | 0 | 208 | 4445612 | 2 | 20 | 0 | 1 | 216 |
| 125 | 7D | 52 | 34 | 2 | 21 | 542281 | 4338244 | 2 | 21 | 0 | 208 | 4445828 | 2 | 21 | 0 | 1 | 216 |
| 126 | 7E | 52 | 34 | 2 | 22 | 542307 | 4338452 | 2 | 22 | 0 | 208 | 4446044 | 2 | 22 | 0 | 1 | 216 |
| 127 | 7F | 52 | 34 | 2 | 23 | 542333 | 4338660 | 2 | 23 | 0 | 208 | 4446260 | 2 | 23 | 0 | 1 | 216 |
| 128 | 80 | 52 | 34 | 2 | 24 | 542356 | 4338844 | 2 | 24 | 0 | 184 | 4446452 | 2 | 24 | 0 | 1 | 192 |
| 129 | 81 | 52 | 34 | 2 | 25 | 542379 | 4339028 | 2 | 25 | 0 | 184 | 4446644 | 2 | 25 | 0 | 1 | 192 |
| 130 | 82 | 52 | 34 | 2 | 26 | 542402 | 4339212 | 2 | 26 | 0 | 184 | 4446836 | 2 | 26 | 0 | 1 | 192 |
| 131 | 83 | 52 | 34 | 2 | 27 | 542425 | 4339396 | 2 | 27 | 0 | 184 | 4447028 | 2 | 27 | 0 | 1 | 192 |
| 132 | 84 | 52 | 34 | 2 | 28 | 542448 | 4339580 | 2 | 28 | 0 | 184 | 4447220 | 2 | 28 | 0 | 1 | 192 |
| 133 | 85 | 52 | 34 | 2 | 29 | 542471 | 4339764 | 2 | 29 | 0 | 184 | 4447412 | 2 | 29 | 0 | 1 | 192 |
| 134 | 86 | 52 | 34 | 2 | 30 | 542494 | 4339948 | 2 | 30 | 0 | 184 | 4447604 | 2 | 30 | 0 | 1 | 192 |
| 135 | 87 | 52 | 34 | 2 | 31 | 542517 | 4340132 | 2 | 31 | 0 | 184 | 4447796 | 2 | 31 | 0 | 1 | 192 |
| 136 | 88 | 52 | 34 | 2 | 32 | 542540 | 4340316 | 2 | 32 | 0 | 184 | 4447988 | 2 | 32 | 0 | 1 | 192 |
| 137 | 89 | 52 | 34 | 2 | 33 | 542563 | 4340500 | 2 | 33 | 0 | 184 | 4448180 | 2 | 33 | 0 | 1 | 192 |
| 138 | 8A | 52 | 34 | 2 | 34 | 542586 | 4340684 | 2 | 34 | 0 | 184 | 4448372 | 2 | 34 | 0 | 1 | 192 |
| 139 | 8B | 52 | 34 | 2 | 35 | 542609 | 4340868 | 2 | 35 | 0 | 184 | 4448564 | 2 | 35 | 0 | 1 | 192 |
| 140 | 8C | 52 | 34 | 2 | 36 | 542632 | 4341052 | 2 | 36 | 0 | 184 | 4448756 | 2 | 36 | 0 | 1 | 192 |
| 141 | 8D | 52 | 34 | 2 | 37 | 542655 | 4341236 | 2 | 37 | 0 | 184 | 4448948 | 2 | 37 | 0 | 1 | 192 |
| 142 | 8E | 52 | 34 | 2 | 38 | 542678 | 4341420 | 2 | 38 | 0 | 184 | 4449140 | 2 | 38 | 0 | 1 | 192 |
| 143 | 8F | 52 | 34 | 2 | 39 | 542701 | 4341604 | 2 | 39 | 0 | 184 | 4449332 | 2 | 39 | 0 | 1 | 192 |
| 144 | 90 | 52 | 34 | 2 | 40 | 542726 | 4341804 | 2 | 40 | 0 | 200 | 4449540 | 2 | 40 | 0 | 1 | 208 |
| 145 | 91 | 52 | 34 | 2 | 41 | 542751 | 4342004 | 2 | 41 | 0 | 200 | 4449748 | 2 | 41 | 0 | 1 | 208 |
| 146 | 92 | 52 | 34 | 2 | 42 | 542776 | 4342204 | 2 | 42 | 0 | 200 | 4449956 | 2 | 42 | 0 | 1 | 208 |
| 147 | 93 | 52 | 34 | 2 | 43 | 542801 | 4342404 | 2 | 43 | 0 | 200 | 4450164 | 2 | 43 | 0 | 1 | 208 |
| 148 | 94 | 52 | 34 | 2 | 44 | 542826 | 4342604 | 2 | 44 | 0 | 200 | 4450372 | 2 | 44 | 0 | 1 | 208 |
| 149 | 95 | 52 | 34 | 2 | 45 | 542851 | 4342804 | 2 | 45 | 0 | 200 | 4450580 | 2 | 45 | 0 | 1 | 208 |
| 150 | 96 | 52 | 34 | 2 | 46 | 542876 | 4343004 | 2 | 46 | 0 | 200 | 4450788 | 2 | 46 | 0 | 1 | 208 |
| 151 | 97 | 52 | 34 | 2 | 47 | 542901 | 4343204 | 2 | 47 | 0 | 200 | 4450996 | 2 | 47 | 0 | 1 | 208 |
| 152 | 98 | 52 | 34 | 2 | 48 | 542926 | 4343404 | 2 | 48 | 0 | 200 | 4451204 | 2 | 48 | 0 | 1 | 208 |
| 153 | 99 | 52 | 34 | 2 | 49 | 542951 | 4343604 | 2 | 49 | 0 | 200 | 4451412 | 2 | 49 | 0 | 1 | 208 |
| 154 | 9A | 52 | 34 | 2 | 50 | 542976 | 4343804 | 2 | 50 | 0 | 200 | 4451620 | 2 | 50 | 0 | 1 | 208 |
| 155 | 9B | 52 | 34 | 2 | 51 | 543001 | 4344004 | 2 | 51 | 0 | 200 | 4451828 | 2 | 51 | 0 | 1 | 208 |
| 156 | 9C | 52 | 34 | 3 | 0 | 543024 | 4344188 | 3 | 0 | 0 | 184 | 4452020 | 3 | 0 | 0 | 1 | 192 |
| 157 | 9D | 52 | 34 | 3 | 1 | 543052 | 4344412 | 3 | 1 | 0 | 224 | 4452252 | 3 | 1 | 0 | 1 | 232 |
| 158 | 9E | 52 | 34 | 3 | 2 | 543080 | 4344636 | 3 | 2 | 0 | 224 | 4452484 | 3 | 2 | 0 | 1 | 232 |
| 159 | 9F | 52 | 34 | 3 | 3 | 543108 | 4344860 | 3 | 3 | 0 | 224 | 4452716 | 3 | 3 | 0 | 1 | 232 |
| 160 | A0 | 52 | 34 | 3 | 4 | 543136 | 4345084 | 3 | 4 | 0 | 224 | 4452948 | 3 | 4 | 0 | 1 | 232 |
| 161 | A1 | 52 | 34 | 3 | 5 | 543164 | 4345308 | 3 | 5 | 0 | 224 | 4453180 | 3 | 5 | 0 | 1 | 232 |
| 162 | A2 | 52 | 34 | 3 | 6 | 543192 | 4345532 | 3 | 6 | 0 | 224 | 4453412 | 3 | 6 | 0 | 1 | 232 |
| 163 | A3 | 52 | 34 | 3 | 7 | 543220 | 4345756 | 3 | 7 | 0 | 224 | 4453644 | 3 | 7 | 0 | 1 | 232 |
| 164 | A4 | 52 | 34 | 3 | 8 | 543248 | 4345980 | 3 | 8 | 0 | 224 | 4453876 | 3 | 8 | 0 | 1 | 232 |
| 165 | A5 | 52 | 34 | 3 | 9 | 543276 | 4346204 | 3 | 9 | 0 | 224 | 4454108 | 3 | 9 | 0 | 1 | 232 |
| 166 | A6 | 52 | 34 | 3 | 10 | 543304 | 4346428 | 3 | 10 | 0 | 224 | 4454340 | 3 | 10 | 0 | 1 | 232 |
| 167 | A7 | 52 | 34 | 3 | 11 | 543332 | 4346652 | 3 | 11 | 0 | 224 | 4454572 | 3 | 11 | 0 | 1 | 232 |
| 168 | A8 | 52 | 34 | 3 | 12 | 543360 | 4346876 | 3 | 12 | 0 | 224 | 4454804 | 3 | 12 | 0 | 1 | 232 |
| 169 | A9 | 52 | 34 | 3 | 13 | 543388 | 4347100 | 3 | 13 | 0 | 224 | 4455036 | 3 | 13 | 0 | 1 | 232 |
| 170 | AA | 52 | 34 | 3 | 14 | 543416 | 4347324 | 3 | 14 | 0 | 224 | 4455268 | 3 | 14 | 0 | 1 | 232 |
| 171 | AB | 52 | 34 | 3 | 15 | 543444 | 4347548 | 3 | 15 | 0 | 224 | 4455500 | 3 | 15 | 0 | 1 | 232 |
| 172 | AC | 52 | 34 | 3 | 16 | 543472 | 4347772 | 3 | 16 | 0 | 224 | 4455732 | 3 | 16 | 0 | 1 | 232 |
| 173 | AD | 52 | 34 | 3 | 17 | 543500 | 4347996 | 3 | 17 | 0 | 224 | 4455964 | 3 | 17 | 0 | 1 | 232 |
| 174 | AE | 52 | 34 | 3 | 18 | 543528 | 4348220 | 3 | 18 | 0 | 224 | 4456196 | 3 | 18 | 0 | 1 | 232 |
| 175 | AF | 52 | 34 | 3 | 19 | 543556 | 4348444 | 3 | 19 | 0 | 224 | 4456428 | 3 | 19 | 0 | 1 | 232 |
| 176 | B0 | 52 | 34 | 3 | 20 | 543584 | 4348668 | 3 | 20 | 0 | 224 | 4456660 | 3 | 20 | 0 | 1 | 232 |
| 177 | B1 | 52 | 34 | 3 | 21 | 543612 | 4348892 | 3 | 21 | 0 | 224 | 4456892 | 3 | 21 | 0 | 1 | 232 |

| | | | | | | | | | | | | | | | | |
|---|---|---|---|---|---|---|---|---|---|---|---|---|---|---|---|---|
| 178 | B2 | 52 | 34 | 3 | 22 | 543640 | 4349116 | 3 | 22 | 0 | 224 | 4457124 | 3 | 22 | 0 | 1 | 232 |
| 179 | B3 | 52 | 34 | 3 | 23 | 543668 | 4349340 | 3 | 23 | 0 | 224 | 4457356 | 3 | 23 | 0 | 1 | 232 |
| 180 | B4 | 52 | 34 | 3 | 24 | 543696 | 4349564 | 3 | 24 | 0 | 224 | 4457588 | 3 | 24 | 0 | 1 | 232 |
| 181 | B5 | 52 | 34 | 3 | 25 | 543724 | 4349788 | 3 | 25 | 0 | 224 | 4457820 | 3 | 25 | 0 | 1 | 232 |
| 182 | B6 | 52 | 34 | 3 | 26 | 543752 | 4350012 | 3 | 26 | 0 | 224 | 4458052 | 3 | 26 | 0 | 1 | 232 |
| 183 | B7 | 52 | 34 | 3 | 27 | 543780 | 4350236 | 3 | 27 | 0 | 224 | 4458284 | 3 | 27 | 0 | 1 | 232 |
| 184 | B8 | 52 | 34 | 3 | 28 | 543808 | 4350460 | 3 | 28 | 0 | 224 | 4458516 | 3 | 28 | 0 | 1 | 232 |
| 185 | B9 | 52 | 34 | 3 | 29 | 543836 | 4350684 | 3 | 29 | 0 | 224 | 4458748 | 3 | 29 | 0 | 1 | 232 |
| 186 | BA | 52 | 34 | 3 | 30 | 543864 | 4350908 | 3 | 30 | 0 | 224 | 4458980 | 3 | 30 | 0 | 1 | 232 |
| 187 | BB | 52 | 34 | 3 | 31 | 543892 | 4351132 | 3 | 31 | 0 | 224 | 4459212 | 3 | 31 | 0 | 1 | 232 |
| 188 | BC | 52 | 34 | 3 | 32 | 543920 | 4351356 | 3 | 32 | 0 | 224 | 4459444 | 3 | 32 | 0 | 1 | 232 |
| 189 | BD | 52 | 34 | 3 | 33 | 543948 | 4351580 | 3 | 33 | 0 | 224 | 4459676 | 3 | 33 | 0 | 1 | 232 |
| 190 | BE | 52 | 34 | 3 | 34 | 543976 | 4351804 | 3 | 34 | 0 | 224 | 4459908 | 3 | 34 | 0 | 1 | 232 |
| 191 | BF | 52 | 34 | 3 | 35 | 544004 | 4352028 | 3 | 35 | 0 | 224 | 4460140 | 3 | 35 | 0 | 1 | 232 |
| 192 | C0 | 52 | 34 | 3 | 36 | 544029 | 4352228 | 3 | 36 | 0 | 200 | 4460348 | 3 | 36 | 0 | 1 | 208 |
| 193 | C1 | 52 | 34 | 3 | 37 | 544054 | 4352428 | 3 | 37 | 0 | 200 | 4460556 | 3 | 37 | 0 | 1 | 208 |
| 194 | C2 | 52 | 34 | 3 | 38 | 544079 | 4352628 | 3 | 38 | 0 | 200 | 4460764 | 3 | 38 | 0 | 1 | 208 |
| 195 | C3 | 52 | 34 | 3 | 39 | 544104 | 4352828 | 3 | 39 | 0 | 200 | 4460972 | 3 | 39 | 0 | 1 | 208 |
| 196 | C4 | 52 | 34 | 3 | 40 | 544129 | 4353028 | 3 | 40 | 0 | 200 | 4461180 | 3 | 40 | 0 | 1 | 208 |
| 197 | C5 | 52 | 34 | 3 | 41 | 544154 | 4353228 | 3 | 41 | 0 | 200 | 4461388 | 3 | 41 | 0 | 1 | 208 |
| 198 | C6 | 52 | 34 | 3 | 42 | 544179 | 4353428 | 3 | 42 | 0 | 200 | 4461596 | 3 | 42 | 0 | 1 | 208 |
| 199 | C7 | 52 | 34 | 3 | 43 | 544204 | 4353628 | 3 | 43 | 0 | 200 | 4461804 | 3 | 43 | 0 | 1 | 208 |
| 200 | C8 | 52 | 34 | 3 | 44 | 544229 | 4353828 | 3 | 44 | 0 | 200 | 4462012 | 3 | 44 | 0 | 1 | 208 |
| 201 | C9 | 52 | 34 | 3 | 45 | 544254 | 4354028 | 3 | 45 | 0 | 200 | 4462220 | 3 | 45 | 0 | 1 | 208 |
| 202 | CA | 52 | 34 | 3 | 46 | 544279 | 4354228 | 3 | 46 | 0 | 200 | 4462428 | 3 | 46 | 0 | 1 | 208 |
| 203 | CB | 52 | 34 | 3 | 47 | 544304 | 4354428 | 3 | 47 | 0 | 200 | 4462636 | 3 | 47 | 0 | 1 | 208 |
| 204 | CC | 52 | 34 | 3 | 48 | 544329 | 4354628 | 3 | 48 | 0 | 200 | 4462844 | 3 | 48 | 0 | 1 | 208 |
| 205 | CD | 52 | 34 | 3 | 49 | 544354 | 4354828 | 3 | 49 | 0 | 200 | 4463052 | 3 | 49 | 0 | 1 | 208 |
| 206 | CE | 52 | 34 | 3 | 50 | 544379 | 4355028 | 3 | 50 | 0 | 200 | 4463260 | 3 | 50 | 0 | 1 | 208 |
| 207 | CF | 52 | 34 | 3 | 51 | 544404 | 4355228 | 3 | 51 | 0 | 200 | 4463468 | 3 | 51 | 0 | 1 | 208 |
| 208 | D0 | 52 | 34 | 4 | 0 | 544429 | 4355428 | 4 | 0 | 0 | 200 | 4463676 | 4 | 0 | 0 | 1 | 208 |
| 209 | D1 | 52 | 34 | 4 | 1 | 544459 | 4355668 | 4 | 1 | 0 | 240 | 4463924 | 4 | 1 | 0 | 1 | 248 |
| 210 | D2 | 52 | 34 | 4 | 2 | 544489 | 4355908 | 4 | 2 | 0 | 240 | 4464172 | 4 | 2 | 0 | 1 | 248 |
| 211 | D3 | 52 | 34 | 4 | 3 | 544519 | 4356148 | 4 | 3 | 0 | 240 | 4464420 | 4 | 3 | 0 | 1 | 248 |
| 212 | D4 | 52 | 34 | 4 | 4 | 544549 | 4356388 | 4 | 4 | 0 | 240 | 4464668 | 4 | 4 | 0 | 1 | 248 |
| 213 | D5 | 52 | 34 | 4 | 5 | 544579 | 4356628 | 4 | 5 | 0 | 240 | 4464916 | 4 | 5 | 0 | 1 | 248 |
| 214 | D6 | 52 | 34 | 4 | 6 | 544609 | 4356868 | 4 | 6 | 0 | 240 | 4465164 | 4 | 6 | 0 | 1 | 248 |
| 215 | D7 | 52 | 34 | 4 | 7 | 544639 | 4357108 | 4 | 7 | 0 | 240 | 4465412 | 4 | 7 | 0 | 1 | 248 |
| 216 | D8 | 52 | 34 | 4 | 8 | 544669 | 4357348 | 4 | 8 | 0 | 240 | 4465660 | 4 | 8 | 0 | 1 | 248 |
| 217 | D9 | 52 | 34 | 4 | 9 | 544699 | 4357588 | 4 | 9 | 0 | 240 | 4465908 | 4 | 9 | 0 | 1 | 248 |
| 218 | DA | 52 | 34 | 4 | 10 | 544729 | 4357828 | 4 | 10 | 0 | 240 | 4466156 | 4 | 10 | 0 | 1 | 248 |
| 219 | DB | 52 | 34 | 4 | 11 | 544759 | 4358068 | 4 | 11 | 0 | 240 | 4466404 | 4 | 11 | 0 | 1 | 248 |
| 220 | DC | 52 | 34 | 4 | 12 | 544789 | 4358308 | 4 | 12 | 0 | 240 | 4466652 | 4 | 12 | 0 | 1 | 248 |
| 221 | DD | 52 | 34 | 4 | 13 | 544819 | 4358548 | 4 | 13 | 0 | 240 | 4466900 | 4 | 13 | 0 | 1 | 248 |
| 222 | DE | 52 | 34 | 4 | 14 | 544849 | 4358788 | 4 | 14 | 0 | 240 | 4467148 | 4 | 14 | 0 | 1 | 248 |
| 223 | DF | 52 | 34 | 4 | 15 | 544879 | 4359028 | 4 | 15 | 0 | 240 | 4467396 | 4 | 15 | 0 | 1 | 248 |
| 224 | E0 | 52 | 34 | 4 | 16 | 544909 | 4359268 | 4 | 16 | 0 | 240 | 4467644 | 4 | 16 | 0 | 1 | 248 |
| 225 | E1 | 52 | 34 | 4 | 17 | 544939 | 4359508 | 4 | 17 | 0 | 240 | 4467892 | 4 | 17 | 0 | 1 | 248 |
| 226 | E2 | 52 | 34 | 4 | 18 | 544969 | 4359748 | 4 | 18 | 0 | 240 | 4468140 | 4 | 18 | 0 | 1 | 248 |
| 227 | E3 | 52 | 34 | 4 | 19 | 544999 | 4359988 | 4 | 19 | 0 | 240 | 4468388 | 4 | 19 | 0 | 1 | 248 |
| 228 | E4 | 52 | 34 | 4 | 20 | 545029 | 4360228 | 4 | 20 | 0 | 240 | 4468636 | 4 | 20 | 0 | 1 | 248 |
| 229 | E5 | 52 | 34 | 4 | 21 | 545059 | 4360468 | 4 | 21 | 0 | 240 | 4468884 | 4 | 21 | 0 | 1 | 248 |
| 230 | E6 | 52 | 34 | 4 | 22 | 545089 | 4360708 | 4 | 22 | 0 | 240 | 4469132 | 4 | 22 | 0 | 1 | 248 |
| 231 | E7 | 52 | 34 | 4 | 23 | 545119 | 4360948 | 4 | 23 | 0 | 240 | 4469380 | 4 | 23 | 0 | 1 | 248 |
| 232 | E8 | 52 | 34 | 4 | 24 | 545149 | 4361188 | 4 | 24 | 0 | 240 | 4469628 | 4 | 24 | 0 | 1 | 248 |
| 233 | E9 | 52 | 34 | 4 | 25 | 545179 | 4361428 | 4 | 25 | 0 | 240 | 4469876 | 4 | 25 | 0 | 1 | 248 |
| 234 | EA | 52 | 34 | 4 | 26 | 545209 | 4361668 | 4 | 26 | 0 | 240 | 4470124 | 4 | 26 | 0 | 1 | 248 |
| 235 | EB | 52 | 34 | 4 | 27 | 545239 | 4361908 | 4 | 27 | 0 | 240 | 4470372 | 4 | 27 | 0 | 1 | 248 |
| 236 | EC | 52 | 34 | 4 | 28 | 545269 | 4362148 | 4 | 28 | 0 | 240 | 4470620 | 4 | 28 | 0 | 1 | 248 |
| 237 | ED | 52 | 34 | 4 | 29 | 545299 | 4362388 | 4 | 29 | 0 | 240 | 4470868 | 4 | 29 | 0 | 1 | 248 |
| 238 | EE | 52 | 34 | 4 | 30 | 545329 | 4362628 | 4 | 30 | 0 | 240 | 4471116 | 4 | 30 | 0 | 1 | 248 |
| 239 | EF | 52 | 34 | 4 | 31 | 545359 | 4362868 | 4 | 31 | 0 | 240 | 4471364 | 4 | 31 | 0 | 1 | 248 |
| 240 | F0 | 52 | 34 | 4 | 32 | 545389 | 4363108 | 4 | 32 | 0 | 240 | 4471612 | 4 | 32 | 0 | 1 | 248 |
| 241 | F1 | 52 | 34 | 4 | 33 | 545419 | 4363348 | 4 | 33 | 0 | 240 | 4471860 | 4 | 33 | 0 | 1 | 248 |
| 242 | F2 | 52 | 34 | 4 | 34 | 545449 | 4363588 | 4 | 34 | 0 | 240 | 4472108 | 4 | 34 | 0 | 1 | 248 |
| 243 | F3 | 52 | 34 | 4 | 35 | 545479 | 4363828 | 4 | 35 | 0 | 240 | 4472356 | 4 | 35 | 0 | 1 | 248 |
| 244 | F4 | 52 | 34 | 4 | 36 | 545509 | 4364068 | 4 | 36 | 0 | 240 | 4472604 | 4 | 36 | 0 | 1 | 248 |
| 245 | F5 | 52 | 34 | 4 | 37 | 545539 | 4364308 | 4 | 37 | 0 | 240 | 4472852 | 4 | 37 | 0 | 1 | 248 |
| 246 | F6 | 52 | 34 | 4 | 38 | 545569 | 4364548 | 4 | 38 | 0 | 240 | 4473100 | 4 | 38 | 0 | 1 | 248 |
| 247 | F7 | 52 | 34 | 4 | 39 | 545599 | 4364788 | 4 | 39 | 0 | 240 | 4473348 | 4 | 39 | 0 | 1 | 248 |
| 248 | F8 | 52 | 34 | 4 | 40 | 545629 | 4365028 | 4 | 40 | 0 | 240 | 4473596 | 4 | 40 | 0 | 1 | 248 |
| 249 | F9 | 52 | 34 | 4 | 41 | 545659 | 4365268 | 4 | 41 | 0 | 240 | 4473844 | 4 | 41 | 0 | 1 | 248 |
| 250 | FA | 52 | 34 | 4 | 42 | 545689 | 4365508 | 4 | 42 | 0 | 240 | 4474092 | 4 | 42 | 0 | 1 | 248 |
| 251 | FB | 52 | 34 | 4 | 43 | 545719 | 4365748 | 4 | 43 | 0 | 240 | 4474340 | 4 | 43 | 0 | 1 | 248 |
| 252 | FC | 52 | 34 | 4 | 44 | 545749 | 4365988 | 4 | 44 | 0 | 240 | 4474588 | 4 | 44 | 0 | 1 | 248 |
| 253 | FD | 52 | 34 | 4 | 45 | 545779 | 4366228 | 4 | 45 | 0 | 240 | 4474836 | 4 | 45 | 0 | 1 | 248 |
| 254 | FE | 52 | 34 | 4 | 46 | 545809 | 4366468 | 4 | 46 | 0 | 240 | 4475084 | 4 | 46 | 0 | 1 | 248 |
| 255 | FF | 52 | 34 | 4 | 47 | 545839 | 4366708 | 4 | 47 | 0 | 240 | 4475332 | 4 | 47 | 0 | 1 | 248 |
| 0 | 00 | 53 | 35 | 0 | 0 | 545851 | 4366804 | 0 | 0 | 0 | 96 | 4475436 | 0 | 0 | 0 | 1 | 104 |
| 1 | 01 | 53 | 35 | 0 | 1 | 545865 | 4366916 | 0 | 1 | 0 | 112 | 4475556 | 0 | 1 | 0 | 1 | 120 |
| 2 | 02 | 53 | 35 | 0 | 2 | 545879 | 4367028 | 0 | 2 | 0 | 112 | 4475676 | 0 | 2 | 0 | 1 | 120 |
| 3 | 03 | 53 | 35 | 0 | 3 | 545893 | 4367140 | 0 | 3 | 0 | 112 | 4475796 | 0 | 3 | 0 | 1 | 120 |
| 4 | 04 | 53 | 35 | 0 | 4 | 545907 | 4367252 | 0 | 4 | 0 | 112 | 4475916 | 0 | 4 | 0 | 1 | 120 |
| 5 | 05 | 53 | 35 | 0 | 5 | 545921 | 4367364 | 0 | 5 | 0 | 112 | 4476036 | 0 | 5 | 0 | 1 | 120 |
| 6 | 06 | 53 | 35 | 0 | 6 | 545935 | 4367476 | 0 | 6 | 0 | 112 | 4476156 | 0 | 6 | 0 | 1 | 120 |
| 7 | 07 | 53 | 35 | 0 | 7 | 545949 | 4367588 | 0 | 7 | 0 | 112 | 4476276 | 0 | 7 | 0 | 1 | 120 |
| 8 | 08 | 53 | 35 | 0 | 8 | 545963 | 4367700 | 0 | 8 | 0 | 112 | 4476396 | 0 | 8 | 0 | 1 | 120 |
| 9 | 09 | 53 | 35 | 0 | 9 | 545977 | 4367812 | 0 | 9 | 0 | 112 | 4476516 | 0 | 9 | 0 | 1 | 120 |
| 10 | 0A | 53 | 35 | 0 | 10 | 545991 | 4367924 | 0 | 10 | 0 | 112 | 4476636 | 0 | 10 | 0 | 1 | 120 |
| 11 | 0B | 53 | 35 | 0 | 11 | 546005 | 4368036 | 0 | 11 | 0 | 112 | 4476756 | 0 | 11 | 0 | 1 | 120 |
| 12 | 0C | 53 | 35 | 0 | 12 | 546019 | 4368148 | 0 | 12 | 0 | 112 | 4476876 | 0 | 12 | 0 | 1 | 120 |
| 13 | 0D | 53 | 35 | 0 | 13 | 546033 | 4368260 | 0 | 13 | 0 | 112 | 4476996 | 0 | 13 | 0 | 1 | 120 |
| 14 | 0E | 53 | 35 | 0 | 14 | 546047 | 4368372 | 0 | 14 | 0 | 112 | 4477116 | 0 | 14 | 0 | 1 | 120 |
| 15 | 0F | 53 | 35 | 0 | 15 | 546061 | 4368484 | 0 | 15 | 0 | 112 | 4477236 | 0 | 15 | 0 | 1 | 120 |
| 16 | 10 | 53 | 35 | 0 | 16 | 546075 | 4368596 | 0 | 16 | 0 | 112 | 4477356 | 0 | 16 | 0 | 1 | 120 |
| 17 | 11 | 53 | 35 | 0 | 17 | 546089 | 4368708 | 0 | 17 | 0 | 112 | 4477476 | 0 | 17 | 0 | 1 | 120 |
| 18 | 12 | 53 | 35 | 0 | 18 | 546103 | 4368820 | 0 | 18 | 0 | 112 | 4477596 | 0 | 18 | 0 | 1 | 120 |
| 19 | 13 | 53 | 35 | 0 | 19 | 546117 | 4368932 | 0 | 19 | 0 | 112 | 4477716 | 0 | 19 | 0 | 1 | 120 |
| 20 | 14 | 53 | 35 | 0 | 20 | 546131 | 4369044 | 0 | 20 | 0 | 112 | 4477836 | 0 | 20 | 0 | 1 | 120 |
| 21 | 15 | 53 | 35 | 0 | 21 | 546145 | 4369156 | 0 | 21 | 0 | 112 | 4477956 | 0 | 21 | 0 | 1 | 120 |
| 22 | 16 | 53 | 35 | 0 | 22 | 546159 | 4369268 | 0 | 22 | 0 | 112 | 4478076 | 0 | 22 | 0 | 1 | 120 |
| 23 | 17 | 53 | 35 | 0 | 23 | 546173 | 4369380 | 0 | 23 | 0 | 112 | 4478196 | 0 | 23 | 0 | 1 | 120 |
| 24 | 18 | 53 | 35 | 0 | 24 | 546187 | 4369492 | 0 | 24 | 0 | 112 | 4478316 | 0 | 24 | 0 | 1 | 120 |

| | | | | | | | | | | | | | | | | | |
|---|---|---|---|---|---|---|---|---|---|---|---|---|---|---|---|---|---|
| 25 | 19 | 53 | 35 | 0 | 25 | 546201 | 4369604 | 0 | 25 | 0 | 112 | 4478436 | 0 | 25 | 0 | 1 | 120 |
| 26 | 1A | 53 | 35 | 0 | 26 | 546215 | 4369716 | 0 | 26 | 0 | 112 | 4478556 | 0 | 26 | 0 | 1 | 120 |
| 27 | 1B | 53 | 35 | 0 | 27 | 546229 | 4369828 | 0 | 27 | 0 | 112 | 4478676 | 0 | 27 | 0 | 1 | 120 |
| 28 | 1C | 53 | 35 | 0 | 28 | 546243 | 4369940 | 0 | 28 | 0 | 112 | 4478796 | 0 | 28 | 0 | 1 | 120 |
| 29 | 1D | 53 | 35 | 0 | 29 | 546257 | 4370052 | 0 | 29 | 0 | 112 | 4478916 | 0 | 29 | 0 | 1 | 120 |
| 30 | 1E | 53 | 35 | 0 | 30 | 546271 | 4370164 | 0 | 30 | 0 | 112 | 4479036 | 0 | 30 | 0 | 1 | 120 |
| 31 | 1F | 53 | 35 | 0 | 31 | 546285 | 4370276 | 0 | 31 | 0 | 112 | 4479156 | 0 | 31 | 0 | 1 | 120 |
| 32 | 20 | 53 | 35 | 0 | 32 | 546299 | 4370388 | 0 | 32 | 0 | 112 | 4479276 | 0 | 32 | 0 | 1 | 120 |
| 33 | 21 | 53 | 35 | 0 | 33 | 546313 | 4370500 | 0 | 33 | 0 | 112 | 4479396 | 0 | 33 | 0 | 1 | 120 |
| 34 | 22 | 53 | 35 | 0 | 34 | 546327 | 4370612 | 0 | 34 | 0 | 112 | 4479516 | 0 | 34 | 0 | 1 | 120 |
| 35 | 23 | 53 | 35 | 0 | 35 | 546341 | 4370724 | 0 | 35 | 0 | 112 | 4479636 | 0 | 35 | 0 | 1 | 120 |
| 36 | 24 | 53 | 35 | 0 | 36 | 546355 | 4370836 | 0 | 36 | 0 | 112 | 4479756 | 0 | 36 | 0 | 1 | 120 |
| 37 | 25 | 53 | 35 | 0 | 37 | 546369 | 4370948 | 0 | 37 | 0 | 112 | 4479876 | 0 | 37 | 0 | 1 | 120 |
| 38 | 26 | 53 | 35 | 0 | 38 | 546383 | 4371060 | 0 | 38 | 0 | 112 | 4479996 | 0 | 38 | 0 | 1 | 120 |
| 39 | 27 | 53 | 35 | 0 | 39 | 546397 | 4371172 | 0 | 39 | 0 | 112 | 4480116 | 0 | 39 | 0 | 1 | 120 |
| 40 | 28 | 53 | 35 | 0 | 40 | 546411 | 4371284 | 0 | 40 | 0 | 112 | 4480236 | 0 | 40 | 0 | 1 | 120 |
| 41 | 29 | 53 | 35 | 0 | 41 | 546425 | 4371396 | 0 | 41 | 0 | 112 | 4480356 | 0 | 41 | 0 | 1 | 120 |
| 42 | 2A | 53 | 35 | 0 | 42 | 546439 | 4371508 | 0 | 42 | 0 | 112 | 4480476 | 0 | 42 | 0 | 1 | 120 |
| 43 | 2B | 53 | 35 | 0 | 43 | 546453 | 4371620 | 0 | 43 | 0 | 112 | 4480596 | 0 | 43 | 0 | 1 | 120 |
| 44 | 2C | 53 | 35 | 0 | 44 | 546467 | 4371732 | 0 | 44 | 0 | 112 | 4480716 | 0 | 44 | 0 | 1 | 120 |
| 45 | 2D | 53 | 35 | 0 | 45 | 546481 | 4371844 | 0 | 45 | 0 | 112 | 4480836 | 0 | 45 | 0 | 1 | 120 |
| 46 | 2E | 53 | 35 | 0 | 46 | 546495 | 4371956 | 0 | 46 | 0 | 112 | 4480956 | 0 | 46 | 0 | 1 | 120 |
| 47 | 2F | 53 | 35 | 0 | 47 | 546509 | 4372068 | 0 | 47 | 0 | 112 | 4481076 | 0 | 47 | 0 | 1 | 120 |
| 48 | 30 | 53 | 35 | 0 | 48 | 546523 | 4372180 | 0 | 48 | 0 | 112 | 4481196 | 0 | 48 | 0 | 1 | 120 |
| 49 | 31 | 53 | 35 | 0 | 49 | 546537 | 4372292 | 0 | 49 | 0 | 112 | 4481316 | 0 | 49 | 0 | 1 | 120 |
| 50 | 32 | 53 | 35 | 0 | 50 | 546551 | 4372404 | 0 | 50 | 0 | 112 | 4481436 | 0 | 50 | 0 | 1 | 120 |
| 51 | 33 | 53 | 35 | 0 | 51 | 546565 | 4372516 | 0 | 51 | 0 | 112 | 4481556 | 0 | 51 | 0 | 1 | 120 |
| 52 | 34 | 53 | 35 | 0 | 52 | 546579 | 4372628 | 0 | 52 | 0 | 112 | 4481676 | 0 | 52 | 0 | 1 | 120 |
| 53 | 35 | 53 | 35 | 1 | 0 | 546591 | 4372724 | 1 | 0 | 0 | 96 | 4481780 | 1 | 0 | 0 | 1 | 104 |
| 54 | 36 | 53 | 35 | 1 | 1 | 546608 | 4372860 | 1 | 1 | 0 | 136 | 4481924 | 1 | 1 | 0 | 1 | 144 |
| 55 | 37 | 53 | 35 | 1 | 2 | 546625 | 4372996 | 1 | 2 | 0 | 136 | 4482068 | 1 | 2 | 0 | 1 | 144 |
| 56 | 38 | 53 | 35 | 1 | 3 | 546642 | 4373132 | 1 | 3 | 0 | 136 | 4482212 | 1 | 3 | 0 | 1 | 144 |
| 57 | 39 | 53 | 35 | 1 | 4 | 546659 | 4373268 | 1 | 4 | 0 | 136 | 4482356 | 1 | 4 | 0 | 1 | 144 |
| 58 | 3A | 53 | 35 | 1 | 5 | 546676 | 4373404 | 1 | 5 | 0 | 136 | 4482500 | 1 | 5 | 0 | 1 | 144 |
| 59 | 3B | 53 | 35 | 1 | 6 | 546693 | 4373540 | 1 | 6 | 0 | 136 | 4482644 | 1 | 6 | 0 | 1 | 144 |
| 60 | 3C | 53 | 35 | 1 | 7 | 546710 | 4373676 | 1 | 7 | 0 | 136 | 4482788 | 1 | 7 | 0 | 1 | 144 |
| 61 | 3D | 53 | 35 | 1 | 8 | 546727 | 4373812 | 1 | 8 | 0 | 136 | 4482932 | 1 | 8 | 0 | 1 | 144 |
| 62 | 3E | 53 | 35 | 1 | 9 | 546744 | 4373948 | 1 | 9 | 0 | 136 | 4483076 | 1 | 9 | 0 | 1 | 144 |
| 63 | 3F | 53 | 35 | 1 | 10 | 546761 | 4374084 | 1 | 10 | 0 | 136 | 4483220 | 1 | 10 | 0 | 1 | 144 |
| 64 | 40 | 53 | 35 | 1 | 11 | 546782 | 4374252 | 1 | 11 | 0 | 168 | 4483396 | 1 | 11 | 0 | 1 | 176 |
| 65 | 41 | 53 | 35 | 1 | 12 | 546803 | 4374420 | 1 | 12 | 0 | 168 | 4483572 | 1 | 12 | 0 | 1 | 176 |
| 66 | 42 | 53 | 35 | 1 | 13 | 546824 | 4374588 | 1 | 13 | 0 | 168 | 4483748 | 1 | 13 | 0 | 1 | 176 |
| 67 | 43 | 53 | 35 | 1 | 14 | 546845 | 4374756 | 1 | 14 | 0 | 168 | 4483924 | 1 | 14 | 0 | 1 | 176 |
| 68 | 44 | 53 | 35 | 1 | 15 | 546866 | 4374924 | 1 | 15 | 0 | 168 | 4484100 | 1 | 15 | 0 | 1 | 176 |
| 69 | 45 | 53 | 35 | 1 | 16 | 546887 | 4375092 | 1 | 16 | 0 | 168 | 4484276 | 1 | 16 | 0 | 1 | 176 |
| 70 | 46 | 53 | 35 | 1 | 17 | 546908 | 4375260 | 1 | 17 | 0 | 168 | 4484452 | 1 | 17 | 0 | 1 | 176 |
| 71 | 47 | 53 | 35 | 1 | 18 | 546929 | 4375428 | 1 | 18 | 0 | 168 | 4484628 | 1 | 18 | 0 | 1 | 176 |
| 72 | 48 | 53 | 35 | 1 | 19 | 546950 | 4375596 | 1 | 19 | 0 | 168 | 4484804 | 1 | 19 | 0 | 1 | 176 |
| 73 | 49 | 53 | 35 | 1 | 20 | 546971 | 4375764 | 1 | 20 | 0 | 168 | 4484980 | 1 | 20 | 0 | 1 | 176 |
| 74 | 4A | 53 | 35 | 1 | 21 | 546992 | 4375932 | 1 | 21 | 0 | 168 | 4485156 | 1 | 21 | 0 | 1 | 176 |
| 75 | 4B | 53 | 35 | 1 | 22 | 547013 | 4376100 | 1 | 22 | 0 | 168 | 4485332 | 1 | 22 | 0 | 1 | 176 |
| 76 | 4C | 53 | 35 | 1 | 23 | 547034 | 4376268 | 1 | 23 | 0 | 168 | 4485508 | 1 | 23 | 0 | 1 | 176 |
| 77 | 4D | 53 | 35 | 1 | 24 | 547055 | 4376436 | 1 | 24 | 0 | 168 | 4485684 | 1 | 24 | 0 | 1 | 176 |
| 78 | 4E | 53 | 35 | 1 | 25 | 547076 | 4376604 | 1 | 25 | 0 | 168 | 4485860 | 1 | 25 | 0 | 1 | 176 |
| 79 | 4F | 53 | 35 | 1 | 26 | 547097 | 4376772 | 1 | 26 | 0 | 168 | 4486036 | 1 | 26 | 0 | 1 | 176 |
| 80 | 50 | 53 | 35 | 1 | 27 | 547120 | 4376956 | 1 | 27 | 0 | 184 | 4486228 | 1 | 27 | 0 | 1 | 192 |
| 81 | 51 | 53 | 35 | 1 | 28 | 547143 | 4377140 | 1 | 28 | 0 | 184 | 4486420 | 1 | 28 | 0 | 1 | 192 |
| 82 | 52 | 53 | 35 | 1 | 29 | 547166 | 4377324 | 1 | 29 | 0 | 184 | 4486612 | 1 | 29 | 0 | 1 | 192 |
| 83 | 53 | 53 | 35 | 1 | 30 | 547189 | 4377508 | 1 | 30 | 0 | 184 | 4486804 | 1 | 30 | 0 | 1 | 192 |
| 84 | 54 | 53 | 35 | 1 | 31 | 547212 | 4377692 | 1 | 31 | 0 | 184 | 4486996 | 1 | 31 | 0 | 1 | 192 |
| 85 | 55 | 53 | 35 | 1 | 32 | 547235 | 4377876 | 1 | 32 | 0 | 184 | 4487188 | 1 | 32 | 0 | 1 | 192 |
| 86 | 56 | 53 | 35 | 1 | 33 | 547258 | 4378060 | 1 | 33 | 0 | 184 | 4487380 | 1 | 33 | 0 | 1 | 192 |
| 87 | 57 | 53 | 35 | 1 | 34 | 547281 | 4378244 | 1 | 34 | 0 | 184 | 4487572 | 1 | 34 | 0 | 1 | 192 |
| 88 | 58 | 53 | 35 | 1 | 35 | 547304 | 4378428 | 1 | 35 | 0 | 184 | 4487764 | 1 | 35 | 0 | 1 | 192 |
| 89 | 59 | 53 | 35 | 1 | 36 | 547327 | 4378612 | 1 | 36 | 0 | 184 | 4487956 | 1 | 36 | 0 | 1 | 192 |
| 90 | 5A | 53 | 35 | 1 | 37 | 547350 | 4378796 | 1 | 37 | 0 | 184 | 4488148 | 1 | 37 | 0 | 1 | 192 |
| 91 | 5B | 53 | 35 | 1 | 38 | 547373 | 4378980 | 1 | 38 | 0 | 184 | 4488340 | 1 | 38 | 0 | 1 | 192 |
| 92 | 5C | 53 | 35 | 1 | 39 | 547396 | 4379164 | 1 | 39 | 0 | 184 | 4488532 | 1 | 39 | 0 | 1 | 192 |
| 93 | 5D | 53 | 35 | 1 | 40 | 547419 | 4379348 | 1 | 40 | 0 | 184 | 4488724 | 1 | 40 | 0 | 1 | 192 |
| 94 | 5E | 53 | 35 | 1 | 41 | 547442 | 4379532 | 1 | 41 | 0 | 184 | 4488916 | 1 | 41 | 0 | 1 | 192 |
| 95 | 5F | 53 | 35 | 1 | 42 | 547465 | 4379716 | 1 | 42 | 0 | 184 | 4489108 | 1 | 42 | 0 | 1 | 192 |
| 96 | 60 | 53 | 35 | 1 | 43 | 547488 | 4379900 | 1 | 43 | 0 | 184 | 4489300 | 1 | 43 | 0 | 1 | 192 |
| 97 | 61 | 53 | 35 | 1 | 44 | 547511 | 4380084 | 1 | 44 | 0 | 184 | 4489492 | 1 | 44 | 0 | 1 | 192 |
| 98 | 62 | 53 | 35 | 1 | 45 | 547534 | 4380268 | 1 | 45 | 0 | 184 | 4489684 | 1 | 45 | 0 | 1 | 192 |
| 99 | 63 | 53 | 35 | 1 | 46 | 547557 | 4380452 | 1 | 46 | 0 | 184 | 4489876 | 1 | 46 | 0 | 1 | 192 |
| 100 | 64 | 53 | 35 | 1 | 47 | 547580 | 4380636 | 1 | 47 | 0 | 184 | 4490068 | 1 | 47 | 0 | 1 | 192 |
| 101 | 65 | 53 | 35 | 1 | 48 | 547603 | 4380820 | 1 | 48 | 0 | 184 | 4490260 | 1 | 48 | 0 | 1 | 192 |
| 102 | 66 | 53 | 35 | 1 | 49 | 547626 | 4381004 | 1 | 49 | 0 | 184 | 4490452 | 1 | 49 | 0 | 1 | 192 |
| 103 | 67 | 53 | 35 | 1 | 50 | 547649 | 4381188 | 1 | 50 | 0 | 184 | 4490644 | 1 | 50 | 0 | 1 | 192 |
| 104 | 68 | 53 | 35 | 1 | 51 | 547672 | 4381372 | 1 | 51 | 0 | 184 | 4490836 | 1 | 51 | 0 | 1 | 192 |
| 105 | 69 | 53 | 35 | 1 | 52 | 547695 | 4381556 | 1 | 52 | 0 | 184 | 4491028 | 1 | 52 | 0 | 1 | 192 |
| 106 | 6A | 53 | 35 | 2 | 0 | 547716 | 4381724 | 2 | 0 | 0 | 168 | 4491204 | 2 | 0 | 0 | 1 | 176 |
| 107 | 6B | 53 | 35 | 2 | 1 | 547742 | 4381932 | 2 | 1 | 0 | 208 | 4491420 | 2 | 1 | 0 | 1 | 216 |
| 108 | 6C | 53 | 35 | 2 | 2 | 547768 | 4382140 | 2 | 2 | 0 | 208 | 4491636 | 2 | 2 | 0 | 1 | 216 |
| 109 | 6D | 53 | 35 | 2 | 3 | 547794 | 4382348 | 2 | 3 | 0 | 208 | 4491852 | 2 | 3 | 0 | 1 | 216 |
| 110 | 6E | 53 | 35 | 2 | 4 | 547820 | 4382556 | 2 | 4 | 0 | 208 | 4492068 | 2 | 4 | 0 | 1 | 216 |
| 111 | 6F | 53 | 35 | 2 | 5 | 547846 | 4382764 | 2 | 5 | 0 | 208 | 4492284 | 2 | 5 | 0 | 1 | 216 |
| 112 | 70 | 53 | 35 | 2 | 6 | 547872 | 4382972 | 2 | 6 | 0 | 208 | 4492500 | 2 | 6 | 0 | 1 | 216 |
| 113 | 71 | 53 | 35 | 2 | 7 | 547898 | 4383180 | 2 | 7 | 0 | 208 | 4492716 | 2 | 7 | 0 | 1 | 216 |
| 114 | 72 | 53 | 35 | 2 | 8 | 547924 | 4383388 | 2 | 8 | 0 | 208 | 4492932 | 2 | 8 | 0 | 1 | 216 |
| 115 | 73 | 53 | 35 | 2 | 9 | 547950 | 4383596 | 2 | 9 | 0 | 208 | 4493148 | 2 | 9 | 0 | 1 | 216 |
| 116 | 74 | 53 | 35 | 2 | 10 | 547976 | 4383804 | 2 | 10 | 0 | 208 | 4493364 | 2 | 10 | 0 | 1 | 216 |
| 117 | 75 | 53 | 35 | 2 | 11 | 548002 | 4384012 | 2 | 11 | 0 | 208 | 4493580 | 2 | 11 | 0 | 1 | 216 |
| 118 | 76 | 53 | 35 | 2 | 12 | 548028 | 4384220 | 2 | 12 | 0 | 208 | 4493796 | 2 | 12 | 0 | 1 | 216 |
| 119 | 77 | 53 | 35 | 2 | 13 | 548054 | 4384428 | 2 | 13 | 0 | 208 | 4494012 | 2 | 13 | 0 | 1 | 216 |
| 120 | 78 | 53 | 35 | 2 | 14 | 548080 | 4384636 | 2 | 14 | 0 | 208 | 4494228 | 2 | 14 | 0 | 1 | 216 |
| 121 | 79 | 53 | 35 | 2 | 15 | 548106 | 4384844 | 2 | 15 | 0 | 208 | 4494444 | 2 | 15 | 0 | 1 | 216 |
| 122 | 7A | 53 | 35 | 2 | 16 | 548132 | 4385052 | 2 | 16 | 0 | 208 | 4494660 | 2 | 16 | 0 | 1 | 216 |
| 123 | 7B | 53 | 35 | 2 | 17 | 548158 | 4385260 | 2 | 17 | 0 | 208 | 4494876 | 2 | 17 | 0 | 1 | 216 |
| 124 | 7C | 53 | 35 | 2 | 18 | 548184 | 4385468 | 2 | 18 | 0 | 208 | 4495092 | 2 | 18 | 0 | 1 | 216 |
| 125 | 7D | 53 | 35 | 2 | 19 | 548210 | 4385676 | 2 | 19 | 0 | 208 | 4495308 | 2 | 19 | 0 | 1 | 216 |
| 126 | 7E | 53 | 35 | 2 | 20 | 548236 | 4385884 | 2 | 20 | 0 | 208 | 4495524 | 2 | 20 | 0 | 1 | 216 |
| 127 | 7F | 53 | 35 | 2 | 21 | 548262 | 4386092 | 2 | 21 | 0 | 208 | 4495740 | 2 | 21 | 0 | 1 | 216 |

| | | | | | | | | | | | | | | | | | |
|---|---|---|---|---|---|---|---|---|---|---|---|---|---|---|---|---|---|
| 128 | 80 | 53 | 35 | 2 | 22 | 548285 | 4386276 | 2 | 22 | 0 | 184 | 4495932 | 2 | 22 | 0 | 1 | 192 |
| 129 | 81 | 53 | 35 | 2 | 23 | 548308 | 4386460 | 2 | 23 | 0 | 184 | 4496124 | 2 | 23 | 0 | 1 | 192 |
| 130 | 82 | 53 | 35 | 2 | 24 | 548331 | 4386644 | 2 | 24 | 0 | 184 | 4496316 | 2 | 24 | 0 | 1 | 192 |
| 131 | 83 | 53 | 35 | 2 | 25 | 548354 | 4386828 | 2 | 25 | 0 | 184 | 4496508 | 2 | 25 | 0 | 1 | 192 |
| 132 | 84 | 53 | 35 | 2 | 26 | 548377 | 4387012 | 2 | 26 | 0 | 184 | 4496700 | 2 | 26 | 0 | 1 | 192 |
| 133 | 85 | 53 | 35 | 2 | 27 | 548400 | 4387196 | 2 | 27 | 0 | 184 | 4496892 | 2 | 27 | 0 | 1 | 192 |
| 134 | 86 | 53 | 35 | 2 | 28 | 548423 | 4387380 | 2 | 28 | 0 | 184 | 4497084 | 2 | 28 | 0 | 1 | 192 |
| 135 | 87 | 53 | 35 | 2 | 29 | 548446 | 4387564 | 2 | 29 | 0 | 184 | 4497276 | 2 | 29 | 0 | 1 | 192 |
| 136 | 88 | 53 | 35 | 2 | 30 | 548469 | 4387748 | 2 | 30 | 0 | 184 | 4497468 | 2 | 30 | 0 | 1 | 192 |
| 137 | 89 | 53 | 35 | 2 | 31 | 548492 | 4387932 | 2 | 31 | 0 | 184 | 4497660 | 2 | 31 | 0 | 1 | 192 |
| 138 | 8A | 53 | 35 | 2 | 32 | 548515 | 4388116 | 2 | 32 | 0 | 184 | 4497852 | 2 | 32 | 0 | 1 | 192 |
| 139 | 8B | 53 | 35 | 2 | 33 | 548538 | 4388300 | 2 | 33 | 0 | 184 | 4498044 | 2 | 33 | 0 | 1 | 192 |
| 140 | 8C | 53 | 35 | 2 | 34 | 548561 | 4388484 | 2 | 34 | 0 | 184 | 4498236 | 2 | 34 | 0 | 1 | 192 |
| 141 | 8D | 53 | 35 | 2 | 35 | 548584 | 4388668 | 2 | 35 | 0 | 184 | 4498428 | 2 | 35 | 0 | 1 | 192 |
| 142 | 8E | 53 | 35 | 2 | 36 | 548607 | 4388852 | 2 | 36 | 0 | 184 | 4498620 | 2 | 36 | 0 | 1 | 192 |
| 143 | 8F | 53 | 35 | 2 | 37 | 548630 | 4389036 | 2 | 37 | 0 | 184 | 4498812 | 2 | 37 | 0 | 1 | 192 |
| 144 | 90 | 53 | 35 | 2 | 38 | 548655 | 4389236 | 2 | 38 | 0 | 200 | 4499020 | 2 | 38 | 0 | 1 | 208 |
| 145 | 91 | 53 | 35 | 2 | 39 | 548680 | 4389436 | 2 | 39 | 0 | 200 | 4499228 | 2 | 39 | 0 | 1 | 208 |
| 146 | 92 | 53 | 35 | 2 | 40 | 548705 | 4389636 | 2 | 40 | 0 | 200 | 4499436 | 2 | 40 | 0 | 1 | 208 |
| 147 | 93 | 53 | 35 | 2 | 41 | 548730 | 4389836 | 2 | 41 | 0 | 200 | 4499644 | 2 | 41 | 0 | 1 | 208 |
| 148 | 94 | 53 | 35 | 2 | 42 | 548755 | 4390036 | 2 | 42 | 0 | 200 | 4499852 | 2 | 42 | 0 | 1 | 208 |
| 149 | 95 | 53 | 35 | 2 | 43 | 548780 | 4390236 | 2 | 43 | 0 | 200 | 4500060 | 2 | 43 | 0 | 1 | 208 |
| 150 | 96 | 53 | 35 | 2 | 44 | 548805 | 4390436 | 2 | 44 | 0 | 200 | 4500268 | 2 | 44 | 0 | 1 | 208 |
| 151 | 97 | 53 | 35 | 2 | 45 | 548830 | 4390636 | 2 | 45 | 0 | 200 | 4500476 | 2 | 45 | 0 | 1 | 208 |
| 152 | 98 | 53 | 35 | 2 | 46 | 548855 | 4390836 | 2 | 46 | 0 | 200 | 4500684 | 2 | 46 | 0 | 1 | 208 |
| 153 | 99 | 53 | 35 | 2 | 47 | 548880 | 4391036 | 2 | 47 | 0 | 200 | 4500892 | 2 | 47 | 0 | 1 | 208 |
| 154 | 9A | 53 | 35 | 2 | 48 | 548905 | 4391236 | 2 | 48 | 0 | 200 | 4501100 | 2 | 48 | 0 | 1 | 208 |
| 155 | 9B | 53 | 35 | 2 | 49 | 548930 | 4391436 | 2 | 49 | 0 | 200 | 4501308 | 2 | 49 | 0 | 1 | 208 |
| 156 | 9C | 53 | 35 | 2 | 50 | 548955 | 4391636 | 2 | 50 | 0 | 200 | 4501516 | 2 | 50 | 0 | 1 | 208 |
| 157 | 9D | 53 | 35 | 2 | 51 | 548980 | 4391836 | 2 | 51 | 0 | 200 | 4501724 | 2 | 51 | 0 | 1 | 208 |
| 158 | 9E | 53 | 35 | 2 | 52 | 549005 | 4392036 | 2 | 52 | 0 | 200 | 4501932 | 2 | 52 | 0 | 1 | 208 |
| 159 | 9F | 53 | 35 | 3 | 0 | 549028 | 4392220 | 3 | 0 | 0 | 184 | 4502124 | 3 | 0 | 0 | 1 | 192 |
| 160 | A0 | 53 | 35 | 3 | 1 | 549056 | 4392444 | 3 | 1 | 0 | 224 | 4502356 | 3 | 1 | 0 | 1 | 232 |
| 161 | A1 | 53 | 35 | 3 | 2 | 549084 | 4392668 | 3 | 2 | 0 | 224 | 4502588 | 3 | 2 | 0 | 1 | 232 |
| 162 | A2 | 53 | 35 | 3 | 3 | 549112 | 4392892 | 3 | 3 | 0 | 224 | 4502820 | 3 | 3 | 0 | 1 | 232 |
| 163 | A3 | 53 | 35 | 3 | 4 | 549140 | 4393116 | 3 | 4 | 0 | 224 | 4503052 | 3 | 4 | 0 | 1 | 232 |
| 164 | A4 | 53 | 35 | 3 | 5 | 549168 | 4393340 | 3 | 5 | 0 | 224 | 4503284 | 3 | 5 | 0 | 1 | 232 |
| 165 | A5 | 53 | 35 | 3 | 6 | 549196 | 4393564 | 3 | 6 | 0 | 224 | 4503516 | 3 | 6 | 0 | 1 | 232 |
| 166 | A6 | 53 | 35 | 3 | 7 | 549224 | 4393788 | 3 | 7 | 0 | 224 | 4503748 | 3 | 7 | 0 | 1 | 232 |
| 167 | A7 | 53 | 35 | 3 | 8 | 549252 | 4394012 | 3 | 8 | 0 | 224 | 4503980 | 3 | 8 | 0 | 1 | 232 |
| 168 | A8 | 53 | 35 | 3 | 9 | 549280 | 4394236 | 3 | 9 | 0 | 224 | 4504212 | 3 | 9 | 0 | 1 | 232 |
| 169 | A9 | 53 | 35 | 3 | 10 | 549308 | 4394460 | 3 | 10 | 0 | 224 | 4504444 | 3 | 10 | 0 | 1 | 232 |
| 170 | AA | 53 | 35 | 3 | 11 | 549336 | 4394684 | 3 | 11 | 0 | 224 | 4504676 | 3 | 11 | 0 | 1 | 232 |
| 171 | AB | 53 | 35 | 3 | 12 | 549364 | 4394908 | 3 | 12 | 0 | 224 | 4504908 | 3 | 12 | 0 | 1 | 232 |
| 172 | AC | 53 | 35 | 3 | 13 | 549392 | 4395132 | 3 | 13 | 0 | 224 | 4505140 | 3 | 13 | 0 | 1 | 232 |
| 173 | AD | 53 | 35 | 3 | 14 | 549420 | 4395356 | 3 | 14 | 0 | 224 | 4505372 | 3 | 14 | 0 | 1 | 232 |
| 174 | AE | 53 | 35 | 3 | 15 | 549448 | 4395580 | 3 | 15 | 0 | 224 | 4505604 | 3 | 15 | 0 | 1 | 232 |
| 175 | AF | 53 | 35 | 3 | 16 | 549476 | 4395804 | 3 | 16 | 0 | 224 | 4505836 | 3 | 16 | 0 | 1 | 232 |
| 176 | B0 | 53 | 35 | 3 | 17 | 549504 | 4396028 | 3 | 17 | 0 | 224 | 4506068 | 3 | 17 | 0 | 1 | 232 |
| 177 | B1 | 53 | 35 | 3 | 18 | 549532 | 4396252 | 3 | 18 | 0 | 224 | 4506300 | 3 | 18 | 0 | 1 | 232 |
| 178 | B2 | 53 | 35 | 3 | 19 | 549560 | 4396476 | 3 | 19 | 0 | 224 | 4506532 | 3 | 19 | 0 | 1 | 232 |
| 179 | B3 | 53 | 35 | 3 | 20 | 549588 | 4396700 | 3 | 20 | 0 | 224 | 4506764 | 3 | 20 | 0 | 1 | 232 |
| 180 | B4 | 53 | 35 | 3 | 21 | 549616 | 4396924 | 3 | 21 | 0 | 224 | 4506996 | 3 | 21 | 0 | 1 | 232 |
| 181 | B5 | 53 | 35 | 3 | 22 | 549644 | 4397148 | 3 | 22 | 0 | 224 | 4507228 | 3 | 22 | 0 | 1 | 232 |
| 182 | B6 | 53 | 35 | 3 | 23 | 549672 | 4397372 | 3 | 23 | 0 | 224 | 4507460 | 3 | 23 | 0 | 1 | 232 |
| 183 | B7 | 53 | 35 | 3 | 24 | 549700 | 4397596 | 3 | 24 | 0 | 224 | 4507692 | 3 | 24 | 0 | 1 | 232 |
| 184 | B8 | 53 | 35 | 3 | 25 | 549728 | 4397820 | 3 | 25 | 0 | 224 | 4507924 | 3 | 25 | 0 | 1 | 232 |
| 185 | B9 | 53 | 35 | 3 | 26 | 549756 | 4398044 | 3 | 26 | 0 | 224 | 4508156 | 3 | 26 | 0 | 1 | 232 |
| 186 | BA | 53 | 35 | 3 | 27 | 549784 | 4398268 | 3 | 27 | 0 | 224 | 4508388 | 3 | 27 | 0 | 1 | 232 |
| 187 | BB | 53 | 35 | 3 | 28 | 549812 | 4398492 | 3 | 28 | 0 | 224 | 4508620 | 3 | 28 | 0 | 1 | 232 |
| 188 | BC | 53 | 35 | 3 | 29 | 549840 | 4398716 | 3 | 29 | 0 | 224 | 4508852 | 3 | 29 | 0 | 1 | 232 |
| 189 | BD | 53 | 35 | 3 | 30 | 549868 | 4398940 | 3 | 30 | 0 | 224 | 4509084 | 3 | 30 | 0 | 1 | 232 |
| 190 | BE | 53 | 35 | 3 | 31 | 549896 | 4399164 | 3 | 31 | 0 | 224 | 4509316 | 3 | 31 | 0 | 1 | 232 |
| 191 | BF | 53 | 35 | 3 | 32 | 549924 | 4399388 | 3 | 32 | 0 | 224 | 4509548 | 3 | 32 | 0 | 1 | 232 |
| 192 | C0 | 53 | 35 | 3 | 33 | 549949 | 4399588 | 3 | 33 | 0 | 200 | 4509756 | 3 | 33 | 0 | 1 | 208 |
| 193 | C1 | 53 | 35 | 3 | 34 | 549974 | 4399788 | 3 | 34 | 0 | 200 | 4509964 | 3 | 34 | 0 | 1 | 208 |
| 194 | C2 | 53 | 35 | 3 | 35 | 549999 | 4399988 | 3 | 35 | 0 | 200 | 4510172 | 3 | 35 | 0 | 1 | 208 |
| 195 | C3 | 53 | 35 | 3 | 36 | 550024 | 4400188 | 3 | 36 | 0 | 200 | 4510380 | 3 | 36 | 0 | 1 | 208 |
| 196 | C4 | 53 | 35 | 3 | 37 | 550049 | 4400388 | 3 | 37 | 0 | 200 | 4510588 | 3 | 37 | 0 | 1 | 208 |
| 197 | C5 | 53 | 35 | 3 | 38 | 550074 | 4400588 | 3 | 38 | 0 | 200 | 4510796 | 3 | 38 | 0 | 1 | 208 |
| 198 | C6 | 53 | 35 | 3 | 39 | 550099 | 4400788 | 3 | 39 | 0 | 200 | 4511004 | 3 | 39 | 0 | 1 | 208 |
| 199 | C7 | 53 | 35 | 3 | 40 | 550124 | 4400988 | 3 | 40 | 0 | 200 | 4511212 | 3 | 40 | 0 | 1 | 208 |
| 200 | C8 | 53 | 35 | 3 | 41 | 550149 | 4401188 | 3 | 41 | 0 | 200 | 4511420 | 3 | 41 | 0 | 1 | 208 |
| 201 | C9 | 53 | 35 | 3 | 42 | 550174 | 4401388 | 3 | 42 | 0 | 200 | 4511628 | 3 | 42 | 0 | 1 | 208 |
| 202 | CA | 53 | 35 | 3 | 43 | 550199 | 4401588 | 3 | 43 | 0 | 200 | 4511836 | 3 | 43 | 0 | 1 | 208 |
| 203 | CB | 53 | 35 | 3 | 44 | 550224 | 4401788 | 3 | 44 | 0 | 200 | 4512044 | 3 | 44 | 0 | 1 | 208 |
| 204 | CC | 53 | 35 | 3 | 45 | 550249 | 4401988 | 3 | 45 | 0 | 200 | 4512252 | 3 | 45 | 0 | 1 | 208 |
| 205 | CD | 53 | 35 | 3 | 46 | 550274 | 4402188 | 3 | 46 | 0 | 200 | 4512460 | 3 | 46 | 0 | 1 | 208 |
| 206 | CE | 53 | 35 | 3 | 47 | 550299 | 4402388 | 3 | 47 | 0 | 200 | 4512668 | 3 | 47 | 0 | 1 | 208 |
| 207 | CF | 53 | 35 | 3 | 48 | 550324 | 4402588 | 3 | 48 | 0 | 200 | 4512876 | 3 | 48 | 0 | 1 | 208 |
| 208 | D0 | 53 | 35 | 3 | 49 | 550351 | 4402804 | 3 | 49 | 0 | 216 | 4513100 | 3 | 49 | 0 | 1 | 224 |
| 209 | D1 | 53 | 35 | 3 | 50 | 550378 | 4403020 | 3 | 50 | 0 | 216 | 4513324 | 3 | 50 | 0 | 1 | 224 |
| 210 | D2 | 53 | 35 | 3 | 51 | 550405 | 4403236 | 3 | 51 | 0 | 216 | 4513548 | 3 | 51 | 0 | 1 | 224 |
| 211 | D3 | 53 | 35 | 3 | 52 | 550432 | 4403452 | 3 | 52 | 0 | 216 | 4513772 | 3 | 52 | 0 | 1 | 224 |
| 212 | D4 | 53 | 35 | 4 | 0 | 550457 | 4403652 | 4 | 0 | 0 | 200 | 4513980 | 4 | 0 | 0 | 1 | 208 |
| 213 | D5 | 53 | 35 | 4 | 1 | 550487 | 4403892 | 4 | 1 | 0 | 240 | 4514228 | 4 | 1 | 0 | 1 | 248 |
| 214 | D6 | 53 | 35 | 4 | 2 | 550517 | 4404132 | 4 | 2 | 0 | 240 | 4514476 | 4 | 2 | 0 | 1 | 248 |
| 215 | D7 | 53 | 35 | 4 | 3 | 550547 | 4404372 | 4 | 3 | 0 | 240 | 4514724 | 4 | 3 | 0 | 1 | 248 |
| 216 | D8 | 53 | 35 | 4 | 4 | 550577 | 4404612 | 4 | 4 | 0 | 240 | 4514972 | 4 | 4 | 0 | 1 | 248 |
| 217 | D9 | 53 | 35 | 4 | 5 | 550607 | 4404852 | 4 | 5 | 0 | 240 | 4515220 | 4 | 5 | 0 | 1 | 248 |
| 218 | DA | 53 | 35 | 4 | 6 | 550637 | 4405092 | 4 | 6 | 0 | 240 | 4515468 | 4 | 6 | 0 | 1 | 248 |
| 219 | DB | 53 | 35 | 4 | 7 | 550667 | 4405332 | 4 | 7 | 0 | 240 | 4515716 | 4 | 7 | 0 | 1 | 248 |
| 220 | DC | 53 | 35 | 4 | 8 | 550697 | 4405572 | 4 | 8 | 0 | 240 | 4515964 | 4 | 8 | 0 | 1 | 248 |
| 221 | DD | 53 | 35 | 4 | 9 | 550727 | 4405812 | 4 | 9 | 0 | 240 | 4516212 | 4 | 9 | 0 | 1 | 248 |
| 222 | DE | 53 | 35 | 4 | 10 | 550757 | 4406052 | 4 | 10 | 0 | 240 | 4516460 | 4 | 10 | 0 | 1 | 248 |
| 223 | DF | 53 | 35 | 4 | 11 | 550787 | 4406292 | 4 | 11 | 0 | 240 | 4516708 | 4 | 11 | 0 | 1 | 248 |
| 224 | E0 | 53 | 35 | 4 | 12 | 550817 | 4406532 | 4 | 12 | 0 | 240 | 4516956 | 4 | 12 | 0 | 1 | 248 |
| 225 | E1 | 53 | 35 | 4 | 13 | 550847 | 4406772 | 4 | 13 | 0 | 240 | 4517204 | 4 | 13 | 0 | 1 | 248 |
| 226 | E2 | 53 | 35 | 4 | 14 | 550877 | 4407012 | 4 | 14 | 0 | 240 | 4517452 | 4 | 14 | 0 | 1 | 248 |
| 227 | E3 | 53 | 35 | 4 | 15 | 550907 | 4407252 | 4 | 15 | 0 | 240 | 4517700 | 4 | 15 | 0 | 1 | 248 |
| 228 | E4 | 53 | 35 | 4 | 16 | 550937 | 4407492 | 4 | 16 | 0 | 240 | 4517948 | 4 | 16 | 0 | 1 | 248 |
| 229 | E5 | 53 | 35 | 4 | 17 | 550967 | 4407732 | 4 | 17 | 0 | 240 | 4518196 | 4 | 17 | 0 | 1 | 248 |
| 230 | E6 | 53 | 35 | 4 | 18 | 550997 | 4407972 | 4 | 18 | 0 | 240 | 4518444 | 4 | 18 | 0 | 1 | 248 |

| | | | | | | | | | | | | | | | | | |
|---|---|---|---|---|---|---|---|---|---|---|---|---|---|---|---|---|---|
| 231 | E7 | 53 | 35 | 4 | 19 | 551027 | 4408212 | 4 | 19 | 0 | 240 | 4518692 | 4 | 19 | 0 | 1 | 248 |
| 232 | E8 | 53 | 35 | 4 | 20 | 551057 | 4408452 | 4 | 20 | 0 | 240 | 4518940 | 4 | 20 | 0 | 1 | 248 |
| 233 | E9 | 53 | 35 | 4 | 21 | 551087 | 4408692 | 4 | 21 | 0 | 240 | 4519188 | 4 | 21 | 0 | 1 | 248 |
| 234 | EA | 53 | 35 | 4 | 22 | 551117 | 4408932 | 4 | 22 | 0 | 240 | 4519436 | 4 | 22 | 0 | 1 | 248 |
| 235 | EB | 53 | 35 | 4 | 23 | 551147 | 4409172 | 4 | 23 | 0 | 240 | 4519684 | 4 | 23 | 0 | 1 | 248 |
| 236 | EC | 53 | 35 | 4 | 24 | 551177 | 4409412 | 4 | 24 | 0 | 240 | 4519932 | 4 | 24 | 0 | 1 | 248 |
| 237 | ED | 53 | 35 | 4 | 25 | 551207 | 4409652 | 4 | 25 | 0 | 240 | 4520180 | 4 | 25 | 0 | 1 | 248 |
| 238 | EE | 53 | 35 | 4 | 26 | 551237 | 4409892 | 4 | 26 | 0 | 240 | 4520428 | 4 | 26 | 0 | 1 | 248 |
| 239 | EF | 53 | 35 | 4 | 27 | 551267 | 4410132 | 4 | 27 | 0 | 240 | 4520676 | 4 | 27 | 0 | 1 | 248 |
| 240 | F0 | 53 | 35 | 4 | 28 | 551297 | 4410372 | 4 | 28 | 0 | 240 | 4520924 | 4 | 28 | 0 | 1 | 248 |
| 241 | F1 | 53 | 35 | 4 | 29 | 551327 | 4410612 | 4 | 29 | 0 | 240 | 4521172 | 4 | 29 | 0 | 1 | 248 |
| 242 | F2 | 53 | 35 | 4 | 30 | 551357 | 4410852 | 4 | 30 | 0 | 240 | 4521420 | 4 | 30 | 0 | 1 | 248 |
| 243 | F3 | 53 | 35 | 4 | 31 | 551387 | 4411092 | 4 | 31 | 0 | 240 | 4521668 | 4 | 31 | 0 | 1 | 248 |
| 244 | F4 | 53 | 35 | 4 | 32 | 551417 | 4411332 | 4 | 32 | 0 | 240 | 4521916 | 4 | 32 | 0 | 1 | 248 |
| 245 | F5 | 53 | 35 | 4 | 33 | 551447 | 4411572 | 4 | 33 | 0 | 240 | 4522164 | 4 | 33 | 0 | 1 | 248 |
| 246 | F6 | 53 | 35 | 4 | 34 | 551477 | 4411812 | 4 | 34 | 0 | 240 | 4522412 | 4 | 34 | 0 | 1 | 248 |
| 247 | F7 | 53 | 35 | 4 | 35 | 551507 | 4412052 | 4 | 35 | 0 | 240 | 4522660 | 4 | 35 | 0 | 1 | 248 |
| 248 | F8 | 53 | 35 | 4 | 36 | 551537 | 4412292 | 4 | 36 | 0 | 240 | 4522908 | 4 | 36 | 0 | 1 | 248 |
| 249 | F9 | 53 | 35 | 4 | 37 | 551567 | 4412532 | 4 | 37 | 0 | 240 | 4523156 | 4 | 37 | 0 | 1 | 248 |
| 250 | FA | 53 | 35 | 4 | 38 | 551597 | 4412772 | 4 | 38 | 0 | 240 | 4523404 | 4 | 38 | 0 | 1 | 248 |
| 251 | FB | 53 | 35 | 4 | 39 | 551627 | 4413012 | 4 | 39 | 0 | 240 | 4523652 | 4 | 39 | 0 | 1 | 248 |
| 252 | FC | 53 | 35 | 4 | 40 | 551657 | 4413252 | 4 | 40 | 0 | 240 | 4523900 | 4 | 40 | 0 | 1 | 248 |
| 253 | FD | 53 | 35 | 4 | 41 | 551687 | 4413492 | 4 | 41 | 0 | 240 | 4524148 | 4 | 41 | 0 | 1 | 248 |
| 254 | FE | 53 | 35 | 4 | 42 | 551717 | 4413732 | 4 | 42 | 0 | 240 | 4524396 | 4 | 42 | 0 | 1 | 248 |
| 255 | FF | 53 | 35 | 4 | 43 | 551747 | 4413972 | 4 | 43 | 0 | 240 | 4524644 | 4 | 43 | 0 | 1 | 248 |
| 0 | 00 | 54 | 36 | 0 | 0 | 551759 | 4414068 | 0 | 0 | 0 | 96 | 4524748 | 0 | 0 | 0 | 1 | 104 |
| 1 | 01 | 54 | 36 | 0 | 1 | 551773 | 4414180 | 0 | 1 | 0 | 112 | 4524868 | 0 | 1 | 0 | 1 | 120 |
| 2 | 02 | 54 | 36 | 0 | 2 | 551787 | 4414292 | 0 | 2 | 0 | 112 | 4524988 | 0 | 2 | 0 | 1 | 120 |
| 3 | 03 | 54 | 36 | 0 | 3 | 551801 | 4414404 | 0 | 3 | 0 | 112 | 4525108 | 0 | 3 | 0 | 1 | 120 |
| 4 | 04 | 54 | 36 | 0 | 4 | 551815 | 4414516 | 0 | 4 | 0 | 112 | 4525228 | 0 | 4 | 0 | 1 | 120 |
| 5 | 05 | 54 | 36 | 0 | 5 | 551829 | 4414628 | 0 | 5 | 0 | 112 | 4525348 | 0 | 5 | 0 | 1 | 120 |
| 6 | 06 | 54 | 36 | 0 | 6 | 551843 | 4414740 | 0 | 6 | 0 | 112 | 4525468 | 0 | 6 | 0 | 1 | 120 |
| 7 | 07 | 54 | 36 | 0 | 7 | 551857 | 4414852 | 0 | 7 | 0 | 112 | 4525588 | 0 | 7 | 0 | 1 | 120 |
| 8 | 08 | 54 | 36 | 0 | 8 | 551871 | 4414964 | 0 | 8 | 0 | 112 | 4525708 | 0 | 8 | 0 | 1 | 120 |
| 9 | 09 | 54 | 36 | 0 | 9 | 551885 | 4415076 | 0 | 9 | 0 | 112 | 4525828 | 0 | 9 | 0 | 1 | 120 |
| 10 | 0A | 54 | 36 | 0 | 10 | 551899 | 4415188 | 0 | 10 | 0 | 112 | 4525948 | 0 | 10 | 0 | 1 | 120 |
| 11 | 0B | 54 | 36 | 0 | 11 | 551913 | 4415300 | 0 | 11 | 0 | 112 | 4526068 | 0 | 11 | 0 | 1 | 120 |
| 12 | 0C | 54 | 36 | 0 | 12 | 551927 | 4415412 | 0 | 12 | 0 | 112 | 4526188 | 0 | 12 | 0 | 1 | 120 |
| 13 | 0D | 54 | 36 | 0 | 13 | 551941 | 4415524 | 0 | 13 | 0 | 112 | 4526308 | 0 | 13 | 0 | 1 | 120 |
| 14 | 0E | 54 | 36 | 0 | 14 | 551955 | 4415636 | 0 | 14 | 0 | 112 | 4526428 | 0 | 14 | 0 | 1 | 120 |
| 15 | 0F | 54 | 36 | 0 | 15 | 551969 | 4415748 | 0 | 15 | 0 | 112 | 4526548 | 0 | 15 | 0 | 1 | 120 |
| 16 | 10 | 54 | 36 | 0 | 16 | 551983 | 4415860 | 0 | 16 | 0 | 112 | 4526668 | 0 | 16 | 0 | 1 | 120 |
| 17 | 11 | 54 | 36 | 0 | 17 | 551997 | 4415972 | 0 | 17 | 0 | 112 | 4526788 | 0 | 17 | 0 | 1 | 120 |
| 18 | 12 | 54 | 36 | 0 | 18 | 552011 | 4416084 | 0 | 18 | 0 | 112 | 4526908 | 0 | 18 | 0 | 1 | 120 |
| 19 | 13 | 54 | 36 | 0 | 19 | 552025 | 4416196 | 0 | 19 | 0 | 112 | 4527028 | 0 | 19 | 0 | 1 | 120 |
| 20 | 14 | 54 | 36 | 0 | 20 | 552039 | 4416308 | 0 | 20 | 0 | 112 | 4527148 | 0 | 20 | 0 | 1 | 120 |
| 21 | 15 | 54 | 36 | 0 | 21 | 552053 | 4416420 | 0 | 21 | 0 | 112 | 4527268 | 0 | 21 | 0 | 1 | 120 |
| 22 | 16 | 54 | 36 | 0 | 22 | 552067 | 4416532 | 0 | 22 | 0 | 112 | 4527388 | 0 | 22 | 0 | 1 | 120 |
| 23 | 17 | 54 | 36 | 0 | 23 | 552081 | 4416644 | 0 | 23 | 0 | 112 | 4527508 | 0 | 23 | 0 | 1 | 120 |
| 24 | 18 | 54 | 36 | 0 | 24 | 552095 | 4416756 | 0 | 24 | 0 | 112 | 4527628 | 0 | 24 | 0 | 1 | 120 |
| 25 | 19 | 54 | 36 | 0 | 25 | 552109 | 4416868 | 0 | 25 | 0 | 112 | 4527748 | 0 | 25 | 0 | 1 | 120 |
| 26 | 1A | 54 | 36 | 0 | 26 | 552123 | 4416980 | 0 | 26 | 0 | 112 | 4527868 | 0 | 26 | 0 | 1 | 120 |
| 27 | 1B | 54 | 36 | 0 | 27 | 552137 | 4417092 | 0 | 27 | 0 | 112 | 4527988 | 0 | 27 | 0 | 1 | 120 |
| 28 | 1C | 54 | 36 | 0 | 28 | 552151 | 4417204 | 0 | 28 | 0 | 112 | 4528108 | 0 | 28 | 0 | 1 | 120 |
| 29 | 1D | 54 | 36 | 0 | 29 | 552165 | 4417316 | 0 | 29 | 0 | 112 | 4528228 | 0 | 29 | 0 | 1 | 120 |
| 30 | 1E | 54 | 36 | 0 | 30 | 552179 | 4417428 | 0 | 30 | 0 | 112 | 4528348 | 0 | 30 | 0 | 1 | 120 |
| 31 | 1F | 54 | 36 | 0 | 31 | 552193 | 4417540 | 0 | 31 | 0 | 112 | 4528468 | 0 | 31 | 0 | 1 | 120 |
| 32 | 20 | 54 | 36 | 0 | 32 | 552207 | 4417652 | 0 | 32 | 0 | 112 | 4528588 | 0 | 32 | 0 | 1 | 120 |
| 33 | 21 | 54 | 36 | 0 | 33 | 552221 | 4417764 | 0 | 33 | 0 | 112 | 4528708 | 0 | 33 | 0 | 1 | 120 |
| 34 | 22 | 54 | 36 | 0 | 34 | 552235 | 4417876 | 0 | 34 | 0 | 112 | 4528828 | 0 | 34 | 0 | 1 | 120 |
| 35 | 23 | 54 | 36 | 0 | 35 | 552249 | 4417988 | 0 | 35 | 0 | 112 | 4528948 | 0 | 35 | 0 | 1 | 120 |
| 36 | 24 | 54 | 36 | 0 | 36 | 552263 | 4418100 | 0 | 36 | 0 | 112 | 4529068 | 0 | 36 | 0 | 1 | 120 |
| 37 | 25 | 54 | 36 | 0 | 37 | 552277 | 4418212 | 0 | 37 | 0 | 112 | 4529188 | 0 | 37 | 0 | 1 | 120 |
| 38 | 26 | 54 | 36 | 0 | 38 | 552291 | 4418324 | 0 | 38 | 0 | 112 | 4529308 | 0 | 38 | 0 | 1 | 120 |
| 39 | 27 | 54 | 36 | 0 | 39 | 552305 | 4418436 | 0 | 39 | 0 | 112 | 4529428 | 0 | 39 | 0 | 1 | 120 |
| 40 | 28 | 54 | 36 | 0 | 40 | 552319 | 4418548 | 0 | 40 | 0 | 112 | 4529548 | 0 | 40 | 0 | 1 | 120 |
| 41 | 29 | 54 | 36 | 0 | 41 | 552333 | 4418660 | 0 | 41 | 0 | 112 | 4529668 | 0 | 41 | 0 | 1 | 120 |
| 42 | 2A | 54 | 36 | 0 | 42 | 552347 | 4418772 | 0 | 42 | 0 | 112 | 4529788 | 0 | 42 | 0 | 1 | 120 |
| 43 | 2B | 54 | 36 | 0 | 43 | 552361 | 4418884 | 0 | 43 | 0 | 112 | 4529908 | 0 | 43 | 0 | 1 | 120 |
| 44 | 2C | 54 | 36 | 0 | 44 | 552375 | 4418996 | 0 | 44 | 0 | 112 | 4530028 | 0 | 44 | 0 | 1 | 120 |
| 45 | 2D | 54 | 36 | 0 | 45 | 552389 | 4419108 | 0 | 45 | 0 | 112 | 4530148 | 0 | 45 | 0 | 1 | 120 |
| 46 | 2E | 54 | 36 | 0 | 46 | 552403 | 4419220 | 0 | 46 | 0 | 112 | 4530268 | 0 | 46 | 0 | 1 | 120 |
| 47 | 2F | 54 | 36 | 0 | 47 | 552417 | 4419332 | 0 | 47 | 0 | 112 | 4530388 | 0 | 47 | 0 | 1 | 120 |
| 48 | 30 | 54 | 36 | 0 | 48 | 552431 | 4419444 | 0 | 48 | 0 | 112 | 4530508 | 0 | 48 | 0 | 1 | 120 |
| 49 | 31 | 54 | 36 | 0 | 49 | 552445 | 4419556 | 0 | 49 | 0 | 112 | 4530628 | 0 | 49 | 0 | 1 | 120 |
| 50 | 32 | 54 | 36 | 0 | 50 | 552459 | 4419668 | 0 | 50 | 0 | 112 | 4530748 | 0 | 50 | 0 | 1 | 120 |
| 51 | 33 | 54 | 36 | 0 | 51 | 552473 | 4419780 | 0 | 51 | 0 | 112 | 4530868 | 0 | 51 | 0 | 1 | 120 |
| 52 | 34 | 54 | 36 | 0 | 52 | 552487 | 4419892 | 0 | 52 | 0 | 112 | 4530988 | 0 | 52 | 0 | 1 | 120 |
| 53 | 35 | 54 | 36 | 0 | 53 | 552501 | 4420004 | 0 | 53 | 0 | 112 | 4531108 | 0 | 53 | 0 | 1 | 120 |
| 54 | 36 | 54 | 36 | 1 | 0 | 552513 | 4420100 | 1 | 0 | 0 | 96 | 4531212 | 1 | 0 | 0 | 1 | 104 |
| 55 | 37 | 54 | 36 | 1 | 1 | 552530 | 4420236 | 1 | 1 | 0 | 136 | 4531356 | 1 | 1 | 0 | 1 | 144 |
| 56 | 38 | 54 | 36 | 1 | 2 | 552547 | 4420372 | 1 | 2 | 0 | 136 | 4531500 | 1 | 2 | 0 | 1 | 144 |
| 57 | 39 | 54 | 36 | 1 | 3 | 552564 | 4420508 | 1 | 3 | 0 | 136 | 4531644 | 1 | 3 | 0 | 1 | 144 |
| 58 | 3A | 54 | 36 | 1 | 4 | 552581 | 4420644 | 1 | 4 | 0 | 136 | 4531788 | 1 | 4 | 0 | 1 | 144 |
| 59 | 3B | 54 | 36 | 1 | 5 | 552598 | 4420780 | 1 | 5 | 0 | 136 | 4531932 | 1 | 5 | 0 | 1 | 144 |
| 60 | 3C | 54 | 36 | 1 | 6 | 552615 | 4420916 | 1 | 6 | 0 | 136 | 4532076 | 1 | 6 | 0 | 1 | 144 |
| 61 | 3D | 54 | 36 | 1 | 7 | 552632 | 4421052 | 1 | 7 | 0 | 136 | 4532220 | 1 | 7 | 0 | 1 | 144 |
| 62 | 3E | 54 | 36 | 1 | 8 | 552649 | 4421188 | 1 | 8 | 0 | 136 | 4532364 | 1 | 8 | 0 | 1 | 144 |
| 63 | 3F | 54 | 36 | 1 | 9 | 552666 | 4421324 | 1 | 9 | 0 | 136 | 4532508 | 1 | 9 | 0 | 1 | 144 |
| 64 | 40 | 54 | 36 | 1 | 10 | 552687 | 4421492 | 1 | 10 | 0 | 168 | 4532684 | 1 | 10 | 0 | 1 | 176 |
| 65 | 41 | 54 | 36 | 1 | 11 | 552708 | 4421660 | 1 | 11 | 0 | 168 | 4532860 | 1 | 11 | 0 | 1 | 176 |
| 66 | 42 | 54 | 36 | 1 | 12 | 552729 | 4421828 | 1 | 12 | 0 | 168 | 4533036 | 1 | 12 | 0 | 1 | 176 |
| 67 | 43 | 54 | 36 | 1 | 13 | 552750 | 4421996 | 1 | 13 | 0 | 168 | 4533212 | 1 | 13 | 0 | 1 | 176 |
| 68 | 44 | 54 | 36 | 1 | 14 | 552771 | 4422164 | 1 | 14 | 0 | 168 | 4533388 | 1 | 14 | 0 | 1 | 176 |
| 69 | 45 | 54 | 36 | 1 | 15 | 552792 | 4422332 | 1 | 15 | 0 | 168 | 4533564 | 1 | 15 | 0 | 1 | 176 |
| 70 | 46 | 54 | 36 | 1 | 16 | 552813 | 4422500 | 1 | 16 | 0 | 168 | 4533740 | 1 | 16 | 0 | 1 | 176 |
| 71 | 47 | 54 | 36 | 1 | 17 | 552834 | 4422668 | 1 | 17 | 0 | 168 | 4533916 | 1 | 17 | 0 | 1 | 176 |
| 72 | 48 | 54 | 36 | 1 | 18 | 552855 | 4422836 | 1 | 18 | 0 | 168 | 4534092 | 1 | 18 | 0 | 1 | 176 |
| 73 | 49 | 54 | 36 | 1 | 19 | 552876 | 4423004 | 1 | 19 | 0 | 168 | 4534268 | 1 | 19 | 0 | 1 | 176 |
| 74 | 4A | 54 | 36 | 1 | 20 | 552897 | 4423172 | 1 | 20 | 0 | 168 | 4534444 | 1 | 20 | 0 | 1 | 176 |
| 75 | 4B | 54 | 36 | 1 | 21 | 552918 | 4423340 | 1 | 21 | 0 | 168 | 4534620 | 1 | 21 | 0 | 1 | 176 |
| 76 | 4C | 54 | 36 | 1 | 22 | 552939 | 4423508 | 1 | 22 | 0 | 168 | 4534796 | 1 | 22 | 0 | 1 | 176 |
| 77 | 4D | 54 | 36 | 1 | 23 | 552960 | 4423676 | 1 | 23 | 0 | 168 | 4534972 | 1 | 23 | 0 | 1 | 176 |

| | | | | | | | | | | | | | | | | | |
|---|---|---|---|---|---|---|---|---|---|---|---|---|---|---|---|---|---|
| 78 | 4E | 54 | 36 | 1 | 24 | 552981 | 4423844 | 1 | 24 | 0 | 168 | 4535148 | 1 | 24 | 0 | 1 | 176 |
| 79 | 4F | 54 | 36 | 1 | 25 | 553002 | 4424012 | 1 | 25 | 0 | 168 | 4535324 | 1 | 25 | 0 | 1 | 176 |
| 80 | 50 | 54 | 36 | 1 | 26 | 553025 | 4424196 | 1 | 26 | 0 | 184 | 4535516 | 1 | 26 | 0 | 1 | 192 |
| 81 | 51 | 54 | 36 | 1 | 27 | 553048 | 4424380 | 1 | 27 | 0 | 184 | 4535708 | 1 | 27 | 0 | 1 | 192 |
| 82 | 52 | 54 | 36 | 1 | 28 | 553071 | 4424564 | 1 | 28 | 0 | 184 | 4535900 | 1 | 28 | 0 | 1 | 192 |
| 83 | 53 | 54 | 36 | 1 | 29 | 553094 | 4424748 | 1 | 29 | 0 | 184 | 4536092 | 1 | 29 | 0 | 1 | 192 |
| 84 | 54 | 54 | 36 | 1 | 30 | 553117 | 4424932 | 1 | 30 | 0 | 184 | 4536284 | 1 | 30 | 0 | 1 | 192 |
| 85 | 55 | 54 | 36 | 1 | 31 | 553140 | 4425116 | 1 | 31 | 0 | 184 | 4536476 | 1 | 31 | 0 | 1 | 192 |
| 86 | 56 | 54 | 36 | 1 | 32 | 553163 | 4425300 | 1 | 32 | 0 | 184 | 4536668 | 1 | 32 | 0 | 1 | 192 |
| 87 | 57 | 54 | 36 | 1 | 33 | 553186 | 4425484 | 1 | 33 | 0 | 184 | 4536860 | 1 | 33 | 0 | 1 | 192 |
| 88 | 58 | 54 | 36 | 1 | 34 | 553209 | 4425668 | 1 | 34 | 0 | 184 | 4537052 | 1 | 34 | 0 | 1 | 192 |
| 89 | 59 | 54 | 36 | 1 | 35 | 553232 | 4425852 | 1 | 35 | 0 | 184 | 4537244 | 1 | 35 | 0 | 1 | 192 |
| 90 | 5A | 54 | 36 | 1 | 36 | 553255 | 4426036 | 1 | 36 | 0 | 184 | 4537436 | 1 | 36 | 0 | 1 | 192 |
| 91 | 5B | 54 | 36 | 1 | 37 | 553278 | 4426220 | 1 | 37 | 0 | 184 | 4537628 | 1 | 37 | 0 | 1 | 192 |
| 92 | 5C | 54 | 36 | 1 | 38 | 553301 | 4426404 | 1 | 38 | 0 | 184 | 4537820 | 1 | 38 | 0 | 1 | 192 |
| 93 | 5D | 54 | 36 | 1 | 39 | 553324 | 4426588 | 1 | 39 | 0 | 184 | 4538012 | 1 | 39 | 0 | 1 | 192 |
| 94 | 5E | 54 | 36 | 1 | 40 | 553347 | 4426772 | 1 | 40 | 0 | 184 | 4538204 | 1 | 40 | 0 | 1 | 192 |
| 95 | 5F | 54 | 36 | 1 | 41 | 553370 | 4426956 | 1 | 41 | 0 | 184 | 4538396 | 1 | 41 | 0 | 1 | 192 |
| 96 | 60 | 54 | 36 | 1 | 42 | 553393 | 4427140 | 1 | 42 | 0 | 184 | 4538588 | 1 | 42 | 0 | 1 | 192 |
| 97 | 61 | 54 | 36 | 1 | 43 | 553416 | 4427324 | 1 | 43 | 0 | 184 | 4538780 | 1 | 43 | 0 | 1 | 192 |
| 98 | 62 | 54 | 36 | 1 | 44 | 553439 | 4427508 | 1 | 44 | 0 | 184 | 4538972 | 1 | 44 | 0 | 1 | 192 |
| 99 | 63 | 54 | 36 | 1 | 45 | 553462 | 4427692 | 1 | 45 | 0 | 184 | 4539164 | 1 | 45 | 0 | 1 | 192 |
| 100 | 64 | 54 | 36 | 1 | 46 | 553485 | 4427876 | 1 | 46 | 0 | 184 | 4539356 | 1 | 46 | 0 | 1 | 192 |
| 101 | 65 | 54 | 36 | 1 | 47 | 553508 | 4428060 | 1 | 47 | 0 | 184 | 4539548 | 1 | 47 | 0 | 1 | 192 |
| 102 | 66 | 54 | 36 | 1 | 48 | 553531 | 4428244 | 1 | 48 | 0 | 184 | 4539740 | 1 | 48 | 0 | 1 | 192 |
| 103 | 67 | 54 | 36 | 1 | 49 | 553554 | 4428428 | 1 | 49 | 0 | 184 | 4539932 | 1 | 49 | 0 | 1 | 192 |
| 104 | 68 | 54 | 36 | 1 | 50 | 553577 | 4428612 | 1 | 50 | 0 | 184 | 4540124 | 1 | 50 | 0 | 1 | 192 |
| 105 | 69 | 54 | 36 | 1 | 51 | 553600 | 4428796 | 1 | 51 | 0 | 184 | 4540316 | 1 | 51 | 0 | 1 | 192 |
| 106 | 6A | 54 | 36 | 1 | 52 | 553623 | 4428980 | 1 | 52 | 0 | 184 | 4540508 | 1 | 52 | 0 | 1 | 192 |
| 107 | 6B | 54 | 36 | 1 | 53 | 553646 | 4429164 | 1 | 53 | 0 | 184 | 4540700 | 1 | 53 | 0 | 1 | 192 |
| 108 | 6C | 54 | 36 | 2 | 0 | 553667 | 4429332 | 2 | 0 | 0 | 168 | 4540876 | 2 | 0 | 0 | 1 | 176 |
| 109 | 6D | 54 | 36 | 2 | 1 | 553693 | 4429540 | 2 | 1 | 0 | 208 | 4541092 | 2 | 1 | 0 | 1 | 216 |
| 110 | 6E | 54 | 36 | 2 | 2 | 553719 | 4429748 | 2 | 2 | 0 | 208 | 4541308 | 2 | 2 | 0 | 1 | 216 |
| 111 | 6F | 54 | 36 | 2 | 3 | 553745 | 4429956 | 2 | 3 | 0 | 208 | 4541524 | 2 | 3 | 0 | 1 | 216 |
| 112 | 70 | 54 | 36 | 2 | 4 | 553771 | 4430164 | 2 | 4 | 0 | 208 | 4541740 | 2 | 4 | 0 | 1 | 216 |
| 113 | 71 | 54 | 36 | 2 | 5 | 553797 | 4430372 | 2 | 5 | 0 | 208 | 4541956 | 2 | 5 | 0 | 1 | 216 |
| 114 | 72 | 54 | 36 | 2 | 6 | 553823 | 4430580 | 2 | 6 | 0 | 208 | 4542172 | 2 | 6 | 0 | 1 | 216 |
| 115 | 73 | 54 | 36 | 2 | 7 | 553849 | 4430788 | 2 | 7 | 0 | 208 | 4542388 | 2 | 7 | 0 | 1 | 216 |
| 116 | 74 | 54 | 36 | 2 | 8 | 553875 | 4430996 | 2 | 8 | 0 | 208 | 4542604 | 2 | 8 | 0 | 1 | 216 |
| 117 | 75 | 54 | 36 | 2 | 9 | 553901 | 4431204 | 2 | 9 | 0 | 208 | 4542820 | 2 | 9 | 0 | 1 | 216 |
| 118 | 76 | 54 | 36 | 2 | 10 | 553927 | 4431412 | 2 | 10 | 0 | 208 | 4543036 | 2 | 10 | 0 | 1 | 216 |
| 119 | 77 | 54 | 36 | 2 | 11 | 553953 | 4431620 | 2 | 11 | 0 | 208 | 4543252 | 2 | 11 | 0 | 1 | 216 |
| 120 | 78 | 54 | 36 | 2 | 12 | 553979 | 4431828 | 2 | 12 | 0 | 208 | 4543468 | 2 | 12 | 0 | 1 | 216 |
| 121 | 79 | 54 | 36 | 2 | 13 | 554005 | 4432036 | 2 | 13 | 0 | 208 | 4543684 | 2 | 13 | 0 | 1 | 216 |
| 122 | 7A | 54 | 36 | 2 | 14 | 554031 | 4432244 | 2 | 14 | 0 | 208 | 4543900 | 2 | 14 | 0 | 1 | 216 |
| 123 | 7B | 54 | 36 | 2 | 15 | 554057 | 4432452 | 2 | 15 | 0 | 208 | 4544116 | 2 | 15 | 0 | 1 | 216 |
| 124 | 7C | 54 | 36 | 2 | 16 | 554083 | 4432660 | 2 | 16 | 0 | 208 | 4544332 | 2 | 16 | 0 | 1 | 216 |
| 125 | 7D | 54 | 36 | 2 | 17 | 554109 | 4432868 | 2 | 17 | 0 | 208 | 4544548 | 2 | 17 | 0 | 1 | 216 |
| 126 | 7E | 54 | 36 | 2 | 18 | 554135 | 4433076 | 2 | 18 | 0 | 208 | 4544764 | 2 | 18 | 0 | 1 | 216 |
| 127 | 7F | 54 | 36 | 2 | 19 | 554161 | 4433284 | 2 | 19 | 0 | 208 | 4544980 | 2 | 19 | 0 | 1 | 216 |
| 128 | 80 | 54 | 36 | 2 | 20 | 554184 | 4433468 | 2 | 20 | 0 | 184 | 4545172 | 2 | 20 | 0 | 1 | 192 |
| 129 | 81 | 54 | 36 | 2 | 21 | 554207 | 4433652 | 2 | 21 | 0 | 184 | 4545364 | 2 | 21 | 0 | 1 | 192 |
| 130 | 82 | 54 | 36 | 2 | 22 | 554230 | 4433836 | 2 | 22 | 0 | 184 | 4545556 | 2 | 22 | 0 | 1 | 192 |
| 131 | 83 | 54 | 36 | 2 | 23 | 554253 | 4434020 | 2 | 23 | 0 | 184 | 4545748 | 2 | 23 | 0 | 1 | 192 |
| 132 | 84 | 54 | 36 | 2 | 24 | 554276 | 4434204 | 2 | 24 | 0 | 184 | 4545940 | 2 | 24 | 0 | 1 | 192 |
| 133 | 85 | 54 | 36 | 2 | 25 | 554299 | 4434388 | 2 | 25 | 0 | 184 | 4546132 | 2 | 25 | 0 | 1 | 192 |
| 134 | 86 | 54 | 36 | 2 | 26 | 554322 | 4434572 | 2 | 26 | 0 | 184 | 4546324 | 2 | 26 | 0 | 1 | 192 |
| 135 | 87 | 54 | 36 | 2 | 27 | 554345 | 4434756 | 2 | 27 | 0 | 184 | 4546516 | 2 | 27 | 0 | 1 | 192 |
| 136 | 88 | 54 | 36 | 2 | 28 | 554368 | 4434940 | 2 | 28 | 0 | 184 | 4546708 | 2 | 28 | 0 | 1 | 192 |
| 137 | 89 | 54 | 36 | 2 | 29 | 554391 | 4435124 | 2 | 29 | 0 | 184 | 4546900 | 2 | 29 | 0 | 1 | 192 |
| 138 | 8A | 54 | 36 | 2 | 30 | 554414 | 4435308 | 2 | 30 | 0 | 184 | 4547092 | 2 | 30 | 0 | 1 | 192 |
| 139 | 8B | 54 | 36 | 2 | 31 | 554437 | 4435492 | 2 | 31 | 0 | 184 | 4547284 | 2 | 31 | 0 | 1 | 192 |
| 140 | 8C | 54 | 36 | 2 | 32 | 554460 | 4435676 | 2 | 32 | 0 | 184 | 4547476 | 2 | 32 | 0 | 1 | 192 |
| 141 | 8D | 54 | 36 | 2 | 33 | 554483 | 4435860 | 2 | 33 | 0 | 184 | 4547668 | 2 | 33 | 0 | 1 | 192 |
| 142 | 8E | 54 | 36 | 2 | 34 | 554506 | 4436044 | 2 | 34 | 0 | 184 | 4547860 | 2 | 34 | 0 | 1 | 192 |
| 143 | 8F | 54 | 36 | 2 | 35 | 554529 | 4436228 | 2 | 35 | 0 | 184 | 4548052 | 2 | 35 | 0 | 1 | 192 |
| 144 | 90 | 54 | 36 | 2 | 36 | 554554 | 4436428 | 2 | 36 | 0 | 200 | 4548260 | 2 | 36 | 0 | 1 | 208 |
| 145 | 91 | 54 | 36 | 2 | 37 | 554579 | 4436628 | 2 | 37 | 0 | 200 | 4548468 | 2 | 37 | 0 | 1 | 208 |
| 146 | 92 | 54 | 36 | 2 | 38 | 554604 | 4436828 | 2 | 38 | 0 | 200 | 4548676 | 2 | 38 | 0 | 1 | 208 |
| 147 | 93 | 54 | 36 | 2 | 39 | 554629 | 4437028 | 2 | 39 | 0 | 200 | 4548884 | 2 | 39 | 0 | 1 | 208 |
| 148 | 94 | 54 | 36 | 2 | 40 | 554654 | 4437228 | 2 | 40 | 0 | 200 | 4549092 | 2 | 40 | 0 | 1 | 208 |
| 149 | 95 | 54 | 36 | 2 | 41 | 554679 | 4437428 | 2 | 41 | 0 | 200 | 4549300 | 2 | 41 | 0 | 1 | 208 |
| 150 | 96 | 54 | 36 | 2 | 42 | 554704 | 4437628 | 2 | 42 | 0 | 200 | 4549508 | 2 | 42 | 0 | 1 | 208 |
| 151 | 97 | 54 | 36 | 2 | 43 | 554729 | 4437828 | 2 | 43 | 0 | 200 | 4549716 | 2 | 43 | 0 | 1 | 208 |
| 152 | 98 | 54 | 36 | 2 | 44 | 554754 | 4438028 | 2 | 44 | 0 | 200 | 4549924 | 2 | 44 | 0 | 1 | 208 |
| 153 | 99 | 54 | 36 | 2 | 45 | 554779 | 4438228 | 2 | 45 | 0 | 200 | 4550132 | 2 | 45 | 0 | 1 | 208 |
| 154 | 9A | 54 | 36 | 2 | 46 | 554804 | 4438428 | 2 | 46 | 0 | 200 | 4550340 | 2 | 46 | 0 | 1 | 208 |
| 155 | 9B | 54 | 36 | 2 | 47 | 554829 | 4438628 | 2 | 47 | 0 | 200 | 4550548 | 2 | 47 | 0 | 1 | 208 |
| 156 | 9C | 54 | 36 | 2 | 48 | 554854 | 4438828 | 2 | 48 | 0 | 200 | 4550756 | 2 | 48 | 0 | 1 | 208 |
| 157 | 9D | 54 | 36 | 2 | 49 | 554879 | 4439028 | 2 | 49 | 0 | 200 | 4550964 | 2 | 49 | 0 | 1 | 208 |
| 158 | 9E | 54 | 36 | 2 | 50 | 554904 | 4439228 | 2 | 50 | 0 | 200 | 4551172 | 2 | 50 | 0 | 1 | 208 |
| 159 | 9F | 54 | 36 | 2 | 51 | 554929 | 4439428 | 2 | 51 | 0 | 200 | 4551380 | 2 | 51 | 0 | 1 | 208 |
| 160 | A0 | 54 | 36 | 2 | 52 | 554954 | 4439628 | 2 | 52 | 0 | 200 | 4551588 | 2 | 52 | 0 | 1 | 208 |
| 161 | A1 | 54 | 36 | 2 | 53 | 554979 | 4439828 | 2 | 53 | 0 | 200 | 4551796 | 2 | 53 | 0 | 1 | 208 |
| 162 | A2 | 54 | 36 | 3 | 0 | 555002 | 4440012 | 3 | 0 | 0 | 184 | 4551988 | 3 | 0 | 0 | 1 | 192 |
| 163 | A3 | 54 | 36 | 3 | 1 | 555030 | 4440236 | 3 | 1 | 0 | 224 | 4552220 | 3 | 1 | 0 | 1 | 232 |
| 164 | A4 | 54 | 36 | 3 | 2 | 555058 | 4440460 | 3 | 2 | 0 | 224 | 4552452 | 3 | 2 | 0 | 1 | 232 |
| 165 | A5 | 54 | 36 | 3 | 3 | 555086 | 4440684 | 3 | 3 | 0 | 224 | 4552684 | 3 | 3 | 0 | 1 | 232 |
| 166 | A6 | 54 | 36 | 3 | 4 | 555114 | 4440908 | 3 | 4 | 0 | 224 | 4552916 | 3 | 4 | 0 | 1 | 232 |
| 167 | A7 | 54 | 36 | 3 | 5 | 555142 | 4441132 | 3 | 5 | 0 | 224 | 4553148 | 3 | 5 | 0 | 1 | 232 |
| 168 | A8 | 54 | 36 | 3 | 6 | 555170 | 4441356 | 3 | 6 | 0 | 224 | 4553380 | 3 | 6 | 0 | 1 | 232 |
| 169 | A9 | 54 | 36 | 3 | 7 | 555198 | 4441580 | 3 | 7 | 0 | 224 | 4553612 | 3 | 7 | 0 | 1 | 232 |
| 170 | AA | 54 | 36 | 3 | 8 | 555226 | 4441804 | 3 | 8 | 0 | 224 | 4553844 | 3 | 8 | 0 | 1 | 232 |
| 171 | AB | 54 | 36 | 3 | 9 | 555254 | 4442028 | 3 | 9 | 0 | 224 | 4554076 | 3 | 9 | 0 | 1 | 232 |
| 172 | AC | 54 | 36 | 3 | 10 | 555282 | 4442252 | 3 | 10 | 0 | 224 | 4554308 | 3 | 10 | 0 | 1 | 232 |
| 173 | AD | 54 | 36 | 3 | 11 | 555310 | 4442476 | 3 | 11 | 0 | 224 | 4554540 | 3 | 11 | 0 | 1 | 232 |
| 174 | AE | 54 | 36 | 3 | 12 | 555338 | 4442700 | 3 | 12 | 0 | 224 | 4554772 | 3 | 12 | 0 | 1 | 232 |
| 175 | AF | 54 | 36 | 3 | 13 | 555366 | 4442924 | 3 | 13 | 0 | 224 | 4555004 | 3 | 13 | 0 | 1 | 232 |
| 176 | B0 | 54 | 36 | 3 | 14 | 555394 | 4443148 | 3 | 14 | 0 | 224 | 4555236 | 3 | 14 | 0 | 1 | 232 |
| 177 | B1 | 54 | 36 | 3 | 15 | 555422 | 4443372 | 3 | 15 | 0 | 224 | 4555468 | 3 | 15 | 0 | 1 | 232 |
| 178 | B2 | 54 | 36 | 3 | 16 | 555450 | 4443596 | 3 | 16 | 0 | 224 | 4555700 | 3 | 16 | 0 | 1 | 232 |
| 179 | B3 | 54 | 36 | 3 | 17 | 555478 | 4443820 | 3 | 17 | 0 | 224 | 4555932 | 3 | 17 | 0 | 1 | 232 |
| 180 | B4 | 54 | 36 | 3 | 18 | 555506 | 4444044 | 3 | 18 | 0 | 224 | 4556164 | 3 | 18 | 0 | 1 | 232 |

| | | | | | | | | | | | | | | | | | |
|---|---|---|---|---|---|---|---|---|---|---|---|---|---|---|---|---|---|
| 181 | B5 | 54 | 36 | 3 | 19 | 555534 | 4444268 | 3 | 19 | 0 | 224 | 4556396 | 3 | 19 | 0 | 1 | 232 |
| 182 | B6 | 54 | 36 | 3 | 20 | 555562 | 4444492 | 3 | 20 | 0 | 224 | 4556628 | 3 | 20 | 0 | 1 | 232 |
| 183 | B7 | 54 | 36 | 3 | 21 | 555590 | 4444716 | 3 | 21 | 0 | 224 | 4556860 | 3 | 21 | 0 | 1 | 232 |
| 184 | B8 | 54 | 36 | 3 | 22 | 555618 | 4444940 | 3 | 22 | 0 | 224 | 4557092 | 3 | 22 | 0 | 1 | 232 |
| 185 | B9 | 54 | 36 | 3 | 23 | 555646 | 4445164 | 3 | 23 | 0 | 224 | 4557324 | 3 | 23 | 0 | 1 | 232 |
| 186 | BA | 54 | 36 | 3 | 24 | 555674 | 4445388 | 3 | 24 | 0 | 224 | 4557556 | 3 | 24 | 0 | 1 | 232 |
| 187 | BB | 54 | 36 | 3 | 25 | 555702 | 4445612 | 3 | 25 | 0 | 224 | 4557788 | 3 | 25 | 0 | 1 | 232 |
| 188 | BC | 54 | 36 | 3 | 26 | 555730 | 4445836 | 3 | 26 | 0 | 224 | 4558020 | 3 | 26 | 0 | 1 | 232 |
| 189 | BD | 54 | 36 | 3 | 27 | 555758 | 4446060 | 3 | 27 | 0 | 224 | 4558252 | 3 | 27 | 0 | 1 | 232 |
| 190 | BE | 54 | 36 | 3 | 28 | 555786 | 4446284 | 3 | 28 | 0 | 224 | 4558484 | 3 | 28 | 0 | 1 | 232 |
| 191 | BF | 54 | 36 | 3 | 29 | 555814 | 4446508 | 3 | 29 | 0 | 224 | 4558716 | 3 | 29 | 0 | 1 | 232 |
| 192 | C0 | 54 | 36 | 3 | 30 | 555839 | 4446708 | 3 | 30 | 0 | 200 | 4558924 | 3 | 30 | 0 | 1 | 208 |
| 193 | C1 | 54 | 36 | 3 | 31 | 555864 | 4446908 | 3 | 31 | 0 | 200 | 4559132 | 3 | 31 | 0 | 1 | 208 |
| 194 | C2 | 54 | 36 | 3 | 32 | 555889 | 4447108 | 3 | 32 | 0 | 200 | 4559340 | 3 | 32 | 0 | 1 | 208 |
| 195 | C3 | 54 | 36 | 3 | 33 | 555914 | 4447308 | 3 | 33 | 0 | 200 | 4559548 | 3 | 33 | 0 | 1 | 208 |
| 196 | C4 | 54 | 36 | 3 | 34 | 555939 | 4447508 | 3 | 34 | 0 | 200 | 4559756 | 3 | 34 | 0 | 1 | 208 |
| 197 | C5 | 54 | 36 | 3 | 35 | 555964 | 4447708 | 3 | 35 | 0 | 200 | 4559964 | 3 | 35 | 0 | 1 | 208 |
| 198 | C6 | 54 | 36 | 3 | 36 | 555989 | 4447908 | 3 | 36 | 0 | 200 | 4560172 | 3 | 36 | 0 | 1 | 208 |
| 199 | C7 | 54 | 36 | 3 | 37 | 556014 | 4448108 | 3 | 37 | 0 | 200 | 4560380 | 3 | 37 | 0 | 1 | 208 |
| 200 | C8 | 54 | 36 | 3 | 38 | 556039 | 4448308 | 3 | 38 | 0 | 200 | 4560588 | 3 | 38 | 0 | 1 | 208 |
| 201 | C9 | 54 | 36 | 3 | 39 | 556064 | 4448508 | 3 | 39 | 0 | 200 | 4560796 | 3 | 39 | 0 | 1 | 208 |
| 202 | CA | 54 | 36 | 3 | 40 | 556089 | 4448708 | 3 | 40 | 0 | 200 | 4561004 | 3 | 40 | 0 | 1 | 208 |
| 203 | CB | 54 | 36 | 3 | 41 | 556114 | 4448908 | 3 | 41 | 0 | 200 | 4561212 | 3 | 41 | 0 | 1 | 208 |
| 204 | CC | 54 | 36 | 3 | 42 | 556139 | 4449108 | 3 | 42 | 0 | 200 | 4561420 | 3 | 42 | 0 | 1 | 208 |
| 205 | CD | 54 | 36 | 3 | 43 | 556164 | 4449308 | 3 | 43 | 0 | 200 | 4561628 | 3 | 43 | 0 | 1 | 208 |
| 206 | CE | 54 | 36 | 3 | 44 | 556189 | 4449508 | 3 | 44 | 0 | 200 | 4561836 | 3 | 44 | 0 | 1 | 208 |
| 207 | CF | 54 | 36 | 3 | 45 | 556214 | 4449708 | 3 | 45 | 0 | 200 | 4562044 | 3 | 45 | 0 | 1 | 208 |
| 208 | D0 | 54 | 36 | 3 | 46 | 556241 | 4449924 | 3 | 46 | 0 | 216 | 4562268 | 3 | 46 | 0 | 1 | 224 |
| 209 | D1 | 54 | 36 | 3 | 47 | 556268 | 4450140 | 3 | 47 | 0 | 216 | 4562492 | 3 | 47 | 0 | 1 | 224 |
| 210 | D2 | 54 | 36 | 3 | 48 | 556295 | 4450356 | 3 | 48 | 0 | 216 | 4562716 | 3 | 48 | 0 | 1 | 224 |
| 211 | D3 | 54 | 36 | 3 | 49 | 556322 | 4450572 | 3 | 49 | 0 | 216 | 4562940 | 3 | 49 | 0 | 1 | 224 |
| 212 | D4 | 54 | 36 | 3 | 50 | 556349 | 4450788 | 3 | 50 | 0 | 216 | 4563164 | 3 | 50 | 0 | 1 | 224 |
| 213 | D5 | 54 | 36 | 3 | 51 | 556376 | 4451004 | 3 | 51 | 0 | 216 | 4563388 | 3 | 51 | 0 | 1 | 224 |
| 214 | D6 | 54 | 36 | 3 | 52 | 556403 | 4451220 | 3 | 52 | 0 | 216 | 4563612 | 3 | 52 | 0 | 1 | 224 |
| 215 | D7 | 54 | 36 | 3 | 53 | 556430 | 4451436 | 3 | 53 | 0 | 216 | 4563836 | 3 | 53 | 0 | 1 | 224 |
| 216 | D8 | 54 | 36 | 4 | 0 | 556455 | 4451636 | 4 | 0 | 0 | 200 | 4564044 | 4 | 0 | 0 | 1 | 208 |
| 217 | D9 | 54 | 36 | 4 | 1 | 556485 | 4451876 | 4 | 1 | 0 | 240 | 4564292 | 4 | 1 | 0 | 1 | 248 |
| 218 | DA | 54 | 36 | 4 | 2 | 556515 | 4452116 | 4 | 2 | 0 | 240 | 4564540 | 4 | 2 | 0 | 1 | 248 |
| 219 | DB | 54 | 36 | 4 | 3 | 556545 | 4452356 | 4 | 3 | 0 | 240 | 4564788 | 4 | 3 | 0 | 1 | 248 |
| 220 | DC | 54 | 36 | 4 | 4 | 556575 | 4452596 | 4 | 4 | 0 | 240 | 4565036 | 4 | 4 | 0 | 1 | 248 |
| 221 | DD | 54 | 36 | 4 | 5 | 556605 | 4452836 | 4 | 5 | 0 | 240 | 4565284 | 4 | 5 | 0 | 1 | 248 |
| 222 | DE | 54 | 36 | 4 | 6 | 556635 | 4453076 | 4 | 6 | 0 | 240 | 4565532 | 4 | 6 | 0 | 1 | 248 |
| 223 | DF | 54 | 36 | 4 | 7 | 556665 | 4453316 | 4 | 7 | 0 | 240 | 4565780 | 4 | 7 | 0 | 1 | 248 |
| 224 | E0 | 54 | 36 | 4 | 8 | 556695 | 4453556 | 4 | 8 | 0 | 240 | 4566028 | 4 | 8 | 0 | 1 | 248 |
| 225 | E1 | 54 | 36 | 4 | 9 | 556725 | 4453796 | 4 | 9 | 0 | 240 | 4566276 | 4 | 9 | 0 | 1 | 248 |
| 226 | E2 | 54 | 36 | 4 | 10 | 556755 | 4454036 | 4 | 10 | 0 | 240 | 4566524 | 4 | 10 | 0 | 1 | 248 |
| 227 | E3 | 54 | 36 | 4 | 11 | 556785 | 4454276 | 4 | 11 | 0 | 240 | 4566772 | 4 | 11 | 0 | 1 | 248 |
| 228 | E4 | 54 | 36 | 4 | 12 | 556815 | 4454516 | 4 | 12 | 0 | 240 | 4567020 | 4 | 12 | 0 | 1 | 248 |
| 229 | E5 | 54 | 36 | 4 | 13 | 556845 | 4454756 | 4 | 13 | 0 | 240 | 4567268 | 4 | 13 | 0 | 1 | 248 |
| 230 | E6 | 54 | 36 | 4 | 14 | 556875 | 4454996 | 4 | 14 | 0 | 240 | 4567516 | 4 | 14 | 0 | 1 | 248 |
| 231 | E7 | 54 | 36 | 4 | 15 | 556905 | 4455236 | 4 | 15 | 0 | 240 | 4567764 | 4 | 15 | 0 | 1 | 248 |
| 232 | E8 | 54 | 36 | 4 | 16 | 556935 | 4455476 | 4 | 16 | 0 | 240 | 4568012 | 4 | 16 | 0 | 1 | 248 |
| 233 | E9 | 54 | 36 | 4 | 17 | 556965 | 4455716 | 4 | 17 | 0 | 240 | 4568260 | 4 | 17 | 0 | 1 | 248 |
| 234 | EA | 54 | 36 | 4 | 18 | 556995 | 4455956 | 4 | 18 | 0 | 240 | 4568508 | 4 | 18 | 0 | 1 | 248 |
| 235 | EB | 54 | 36 | 4 | 19 | 557025 | 4456196 | 4 | 19 | 0 | 240 | 4568756 | 4 | 19 | 0 | 1 | 248 |
| 236 | EC | 54 | 36 | 4 | 20 | 557055 | 4456436 | 4 | 20 | 0 | 240 | 4569004 | 4 | 20 | 0 | 1 | 248 |
| 237 | ED | 54 | 36 | 4 | 21 | 557085 | 4456676 | 4 | 21 | 0 | 240 | 4569252 | 4 | 21 | 0 | 1 | 248 |
| 238 | EE | 54 | 36 | 4 | 22 | 557115 | 4456916 | 4 | 22 | 0 | 240 | 4569500 | 4 | 22 | 0 | 1 | 248 |
| 239 | EF | 54 | 36 | 4 | 23 | 557145 | 4457156 | 4 | 23 | 0 | 240 | 4569748 | 4 | 23 | 0 | 1 | 248 |
| 240 | F0 | 54 | 36 | 4 | 24 | 557175 | 4457396 | 4 | 24 | 0 | 240 | 4569996 | 4 | 24 | 0 | 1 | 248 |
| 241 | F1 | 54 | 36 | 4 | 25 | 557205 | 4457636 | 4 | 25 | 0 | 240 | 4570244 | 4 | 25 | 0 | 1 | 248 |
| 242 | F2 | 54 | 36 | 4 | 26 | 557235 | 4457876 | 4 | 26 | 0 | 240 | 4570492 | 4 | 26 | 0 | 1 | 248 |
| 243 | F3 | 54 | 36 | 4 | 27 | 557265 | 4458116 | 4 | 27 | 0 | 240 | 4570740 | 4 | 27 | 0 | 1 | 248 |
| 244 | F4 | 54 | 36 | 4 | 28 | 557295 | 4458356 | 4 | 28 | 0 | 240 | 4570988 | 4 | 28 | 0 | 1 | 248 |
| 245 | F5 | 54 | 36 | 4 | 29 | 557325 | 4458596 | 4 | 29 | 0 | 240 | 4571236 | 4 | 29 | 0 | 1 | 248 |
| 246 | F6 | 54 | 36 | 4 | 30 | 557355 | 4458836 | 4 | 30 | 0 | 240 | 4571484 | 4 | 30 | 0 | 1 | 248 |
| 247 | F7 | 54 | 36 | 4 | 31 | 557385 | 4459076 | 4 | 31 | 0 | 240 | 4571732 | 4 | 31 | 0 | 1 | 248 |
| 248 | F8 | 54 | 36 | 4 | 32 | 557415 | 4459316 | 4 | 32 | 0 | 240 | 4571980 | 4 | 32 | 0 | 1 | 248 |
| 249 | F9 | 54 | 36 | 4 | 33 | 557445 | 4459556 | 4 | 33 | 0 | 240 | 4572228 | 4 | 33 | 0 | 1 | 248 |
| 250 | FA | 54 | 36 | 4 | 34 | 557475 | 4459796 | 4 | 34 | 0 | 240 | 4572476 | 4 | 34 | 0 | 1 | 248 |
| 251 | FB | 54 | 36 | 4 | 35 | 557505 | 4460036 | 4 | 35 | 0 | 240 | 4572724 | 4 | 35 | 0 | 1 | 248 |
| 252 | FC | 54 | 36 | 4 | 36 | 557535 | 4460276 | 4 | 36 | 0 | 240 | 4572972 | 4 | 36 | 0 | 1 | 248 |
| 253 | FD | 54 | 36 | 4 | 37 | 557565 | 4460516 | 4 | 37 | 0 | 240 | 4573220 | 4 | 37 | 0 | 1 | 248 |
| 254 | FE | 54 | 36 | 4 | 38 | 557595 | 4460756 | 4 | 38 | 0 | 240 | 4573468 | 4 | 38 | 0 | 1 | 248 |
| 255 | FF | 54 | 36 | 4 | 39 | 557625 | 4460996 | 4 | 39 | 0 | 240 | 4573716 | 4 | 39 | 0 | 1 | 248 |
| 0 | 00 | 55 | 37 | 0 | 0 | 557637 | 4461092 | 0 | 0 | 0 | 96 | 4573820 | 0 | 0 | 0 | 1 | 104 |
| 1 | 01 | 55 | 37 | 0 | 1 | 557651 | 4461204 | 0 | 1 | 0 | 112 | 4573940 | 0 | 1 | 0 | 1 | 120 |
| 2 | 02 | 55 | 37 | 0 | 2 | 557665 | 4461316 | 0 | 2 | 0 | 112 | 4574060 | 0 | 2 | 0 | 1 | 120 |
| 3 | 03 | 55 | 37 | 0 | 3 | 557679 | 4461428 | 0 | 3 | 0 | 112 | 4574180 | 0 | 3 | 0 | 1 | 120 |
| 4 | 04 | 55 | 37 | 0 | 4 | 557693 | 4461540 | 0 | 4 | 0 | 112 | 4574300 | 0 | 4 | 0 | 1 | 120 |
| 5 | 05 | 55 | 37 | 0 | 5 | 557707 | 4461652 | 0 | 5 | 0 | 112 | 4574420 | 0 | 5 | 0 | 1 | 120 |
| 6 | 06 | 55 | 37 | 0 | 6 | 557721 | 4461764 | 0 | 6 | 0 | 112 | 4574540 | 0 | 6 | 0 | 1 | 120 |
| 7 | 07 | 55 | 37 | 0 | 7 | 557735 | 4461876 | 0 | 7 | 0 | 112 | 4574660 | 0 | 7 | 0 | 1 | 120 |
| 8 | 08 | 55 | 37 | 0 | 8 | 557749 | 4461988 | 0 | 8 | 0 | 112 | 4574780 | 0 | 8 | 0 | 1 | 120 |
| 9 | 09 | 55 | 37 | 0 | 9 | 557763 | 4462100 | 0 | 9 | 0 | 112 | 4574900 | 0 | 9 | 0 | 1 | 120 |
| 10 | 0A | 55 | 37 | 0 | 10 | 557777 | 4462212 | 0 | 10 | 0 | 112 | 4575020 | 0 | 10 | 0 | 1 | 120 |
| 11 | 0B | 55 | 37 | 0 | 11 | 557791 | 4462324 | 0 | 11 | 0 | 112 | 4575140 | 0 | 11 | 0 | 1 | 120 |
| 12 | 0C | 55 | 37 | 0 | 12 | 557805 | 4462436 | 0 | 12 | 0 | 112 | 4575260 | 0 | 12 | 0 | 1 | 120 |
| 13 | 0D | 55 | 37 | 0 | 13 | 557819 | 4462548 | 0 | 13 | 0 | 112 | 4575380 | 0 | 13 | 0 | 1 | 120 |
| 14 | 0E | 55 | 37 | 0 | 14 | 557833 | 4462660 | 0 | 14 | 0 | 112 | 4575500 | 0 | 14 | 0 | 1 | 120 |
| 15 | 0F | 55 | 37 | 0 | 15 | 557847 | 4462772 | 0 | 15 | 0 | 112 | 4575620 | 0 | 15 | 0 | 1 | 120 |
| 16 | 10 | 55 | 37 | 0 | 16 | 557861 | 4462884 | 0 | 16 | 0 | 112 | 4575740 | 0 | 16 | 0 | 1 | 120 |
| 17 | 11 | 55 | 37 | 0 | 17 | 557875 | 4462996 | 0 | 17 | 0 | 112 | 4575860 | 0 | 17 | 0 | 1 | 120 |
| 18 | 12 | 55 | 37 | 0 | 18 | 557889 | 4463108 | 0 | 18 | 0 | 112 | 4575980 | 0 | 18 | 0 | 1 | 120 |
| 19 | 13 | 55 | 37 | 0 | 19 | 557903 | 4463220 | 0 | 19 | 0 | 112 | 4576100 | 0 | 19 | 0 | 1 | 120 |
| 20 | 14 | 55 | 37 | 0 | 20 | 557917 | 4463332 | 0 | 20 | 0 | 112 | 4576220 | 0 | 20 | 0 | 1 | 120 |
| 21 | 15 | 55 | 37 | 0 | 21 | 557931 | 4463444 | 0 | 21 | 0 | 112 | 4576340 | 0 | 21 | 0 | 1 | 120 |
| 22 | 16 | 55 | 37 | 0 | 22 | 557945 | 4463556 | 0 | 22 | 0 | 112 | 4576460 | 0 | 22 | 0 | 1 | 120 |
| 23 | 17 | 55 | 37 | 0 | 23 | 557959 | 4463668 | 0 | 23 | 0 | 112 | 4576580 | 0 | 23 | 0 | 1 | 120 |
| 24 | 18 | 55 | 37 | 0 | 24 | 557973 | 4463780 | 0 | 24 | 0 | 112 | 4576700 | 0 | 24 | 0 | 1 | 120 |
| 25 | 19 | 55 | 37 | 0 | 25 | 557987 | 4463892 | 0 | 25 | 0 | 112 | 4576820 | 0 | 25 | 0 | 1 | 120 |
| 26 | 1A | 55 | 37 | 0 | 26 | 558001 | 4464004 | 0 | 26 | 0 | 112 | 4576940 | 0 | 26 | 0 | 1 | 120 |
| 27 | 1B | 55 | 37 | 0 | 27 | 558015 | 4464116 | 0 | 27 | 0 | 112 | 4577060 | 0 | 27 | 0 | 1 | 120 |

| | | | | | | | | | | | | | | | | | |
|---|---|---|---|---|---|---|---|---|---|---|---|---|---|---|---|---|---|
| 28 | 1C | 55 | 37 | 0 | 28 | 558029 | 4464228 | 0 | 28 | 0 | 112 | 4577180 | 0 | 28 | 0 | 1 | 120 |
| 29 | 1D | 55 | 37 | 0 | 29 | 558043 | 4464340 | 0 | 29 | 0 | 112 | 4577300 | 0 | 29 | 0 | 1 | 120 |
| 30 | 1E | 55 | 37 | 0 | 30 | 558057 | 4464452 | 0 | 30 | 0 | 112 | 4577420 | 0 | 30 | 0 | 1 | 120 |
| 31 | 1F | 55 | 37 | 0 | 31 | 558071 | 4464564 | 0 | 31 | 0 | 112 | 4577540 | 0 | 31 | 0 | 1 | 120 |
| 32 | 20 | 55 | 37 | 0 | 32 | 558085 | 4464676 | 0 | 32 | 0 | 112 | 4577660 | 0 | 32 | 0 | 1 | 120 |
| 33 | 21 | 55 | 37 | 0 | 33 | 558099 | 4464788 | 0 | 33 | 0 | 112 | 4577780 | 0 | 33 | 0 | 1 | 120 |
| 34 | 22 | 55 | 37 | 0 | 34 | 558113 | 4464900 | 0 | 34 | 0 | 112 | 4577900 | 0 | 34 | 0 | 1 | 120 |
| 35 | 23 | 55 | 37 | 0 | 35 | 558127 | 4465012 | 0 | 35 | 0 | 112 | 4578020 | 0 | 35 | 0 | 1 | 120 |
| 36 | 24 | 55 | 37 | 0 | 36 | 558141 | 4465124 | 0 | 36 | 0 | 112 | 4578140 | 0 | 36 | 0 | 1 | 120 |
| 37 | 25 | 55 | 37 | 0 | 37 | 558155 | 4465236 | 0 | 37 | 0 | 112 | 4578260 | 0 | 37 | 0 | 1 | 120 |
| 38 | 26 | 55 | 37 | 0 | 38 | 558169 | 4465348 | 0 | 38 | 0 | 112 | 4578380 | 0 | 38 | 0 | 1 | 120 |
| 39 | 27 | 55 | 37 | 0 | 39 | 558183 | 4465460 | 0 | 39 | 0 | 112 | 4578500 | 0 | 39 | 0 | 1 | 120 |
| 40 | 28 | 55 | 37 | 0 | 40 | 558197 | 4465572 | 0 | 40 | 0 | 112 | 4578620 | 0 | 40 | 0 | 1 | 120 |
| 41 | 29 | 55 | 37 | 0 | 41 | 558211 | 4465684 | 0 | 41 | 0 | 112 | 4578740 | 0 | 41 | 0 | 1 | 120 |
| 42 | 2A | 55 | 37 | 0 | 42 | 558225 | 4465796 | 0 | 42 | 0 | 112 | 4578860 | 0 | 42 | 0 | 1 | 120 |
| 43 | 2B | 55 | 37 | 0 | 43 | 558239 | 4465908 | 0 | 43 | 0 | 112 | 4578980 | 0 | 43 | 0 | 1 | 120 |
| 44 | 2C | 55 | 37 | 0 | 44 | 558253 | 4466020 | 0 | 44 | 0 | 112 | 4579100 | 0 | 44 | 0 | 1 | 120 |
| 45 | 2D | 55 | 37 | 0 | 45 | 558267 | 4466132 | 0 | 45 | 0 | 112 | 4579220 | 0 | 45 | 0 | 1 | 120 |
| 46 | 2E | 55 | 37 | 0 | 46 | 558281 | 4466244 | 0 | 46 | 0 | 112 | 4579340 | 0 | 46 | 0 | 1 | 120 |
| 47 | 2F | 55 | 37 | 0 | 47 | 558295 | 4466356 | 0 | 47 | 0 | 112 | 4579460 | 0 | 47 | 0 | 1 | 120 |
| 48 | 30 | 55 | 37 | 0 | 48 | 558309 | 4466468 | 0 | 48 | 0 | 112 | 4579580 | 0 | 48 | 0 | 1 | 120 |
| 49 | 31 | 55 | 37 | 0 | 49 | 558323 | 4466580 | 0 | 49 | 0 | 112 | 4579700 | 0 | 49 | 0 | 1 | 120 |
| 50 | 32 | 55 | 37 | 0 | 50 | 558337 | 4466692 | 0 | 50 | 0 | 112 | 4579820 | 0 | 50 | 0 | 1 | 120 |
| 51 | 33 | 55 | 37 | 0 | 51 | 558351 | 4466804 | 0 | 51 | 0 | 112 | 4579940 | 0 | 51 | 0 | 1 | 120 |
| 52 | 34 | 55 | 37 | 0 | 52 | 558365 | 4466916 | 0 | 52 | 0 | 112 | 4580060 | 0 | 52 | 0 | 1 | 120 |
| 53 | 35 | 55 | 37 | 0 | 53 | 558379 | 4467028 | 0 | 53 | 0 | 112 | 4580180 | 0 | 53 | 0 | 1 | 120 |
| 54 | 36 | 55 | 37 | 0 | 54 | 558393 | 4467140 | 0 | 54 | 0 | 112 | 4580300 | 0 | 54 | 0 | 1 | 120 |
| 55 | 37 | 55 | 37 | 1 | 0 | 558405 | 4467236 | 1 | 0 | 0 | 96 | 4580404 | 1 | 0 | 0 | 1 | 104 |
| 56 | 38 | 55 | 37 | 1 | 1 | 558422 | 4467372 | 1 | 1 | 0 | 136 | 4580548 | 1 | 1 | 0 | 1 | 144 |
| 57 | 39 | 55 | 37 | 1 | 2 | 558439 | 4467508 | 1 | 2 | 0 | 136 | 4580692 | 1 | 2 | 0 | 1 | 144 |
| 58 | 3A | 55 | 37 | 1 | 3 | 558456 | 4467644 | 1 | 3 | 0 | 136 | 4580836 | 1 | 3 | 0 | 1 | 144 |
| 59 | 3B | 55 | 37 | 1 | 4 | 558473 | 4467780 | 1 | 4 | 0 | 136 | 4580980 | 1 | 4 | 0 | 1 | 144 |
| 60 | 3C | 55 | 37 | 1 | 5 | 558490 | 4467916 | 1 | 5 | 0 | 136 | 4581124 | 1 | 5 | 0 | 1 | 144 |
| 61 | 3D | 55 | 37 | 1 | 6 | 558507 | 4468052 | 1 | 6 | 0 | 136 | 4581268 | 1 | 6 | 0 | 1 | 144 |
| 62 | 3E | 55 | 37 | 1 | 7 | 558524 | 4468188 | 1 | 7 | 0 | 136 | 4581412 | 1 | 7 | 0 | 1 | 144 |
| 63 | 3F | 55 | 37 | 1 | 8 | 558541 | 4468324 | 1 | 8 | 0 | 136 | 4581556 | 1 | 8 | 0 | 1 | 144 |
| 64 | 40 | 55 | 37 | 1 | 9 | 558562 | 4468492 | 1 | 9 | 0 | 168 | 4581732 | 1 | 9 | 0 | 1 | 176 |
| 65 | 41 | 55 | 37 | 1 | 10 | 558583 | 4468660 | 1 | 10 | 0 | 168 | 4581908 | 1 | 10 | 0 | 1 | 176 |
| 66 | 42 | 55 | 37 | 1 | 11 | 558604 | 4468828 | 1 | 11 | 0 | 168 | 4582084 | 1 | 11 | 0 | 1 | 176 |
| 67 | 43 | 55 | 37 | 1 | 12 | 558625 | 4468996 | 1 | 12 | 0 | 168 | 4582260 | 1 | 12 | 0 | 1 | 176 |
| 68 | 44 | 55 | 37 | 1 | 13 | 558646 | 4469164 | 1 | 13 | 0 | 168 | 4582436 | 1 | 13 | 0 | 1 | 176 |
| 69 | 45 | 55 | 37 | 1 | 14 | 558667 | 4469332 | 1 | 14 | 0 | 168 | 4582612 | 1 | 14 | 0 | 1 | 176 |
| 70 | 46 | 55 | 37 | 1 | 15 | 558688 | 4469500 | 1 | 15 | 0 | 168 | 4582788 | 1 | 15 | 0 | 1 | 176 |
| 71 | 47 | 55 | 37 | 1 | 16 | 558709 | 4469668 | 1 | 16 | 0 | 168 | 4582964 | 1 | 16 | 0 | 1 | 176 |
| 72 | 48 | 55 | 37 | 1 | 17 | 558730 | 4469836 | 1 | 17 | 0 | 168 | 4583140 | 1 | 17 | 0 | 1 | 176 |
| 73 | 49 | 55 | 37 | 1 | 18 | 558751 | 4470004 | 1 | 18 | 0 | 168 | 4583316 | 1 | 18 | 0 | 1 | 176 |
| 74 | 4A | 55 | 37 | 1 | 19 | 558772 | 4470172 | 1 | 19 | 0 | 168 | 4583492 | 1 | 19 | 0 | 1 | 176 |
| 75 | 4B | 55 | 37 | 1 | 20 | 558793 | 4470340 | 1 | 20 | 0 | 168 | 4583668 | 1 | 20 | 0 | 1 | 176 |
| 76 | 4C | 55 | 37 | 1 | 21 | 558814 | 4470508 | 1 | 21 | 0 | 168 | 4583844 | 1 | 21 | 0 | 1 | 176 |
| 77 | 4D | 55 | 37 | 1 | 22 | 558835 | 4470676 | 1 | 22 | 0 | 168 | 4584020 | 1 | 22 | 0 | 1 | 176 |
| 78 | 4E | 55 | 37 | 1 | 23 | 558856 | 4470844 | 1 | 23 | 0 | 168 | 4584196 | 1 | 23 | 0 | 1 | 176 |
| 79 | 4F | 55 | 37 | 1 | 24 | 558877 | 4471012 | 1 | 24 | 0 | 168 | 4584372 | 1 | 24 | 0 | 1 | 176 |
| 80 | 50 | 55 | 37 | 1 | 25 | 558900 | 4471196 | 1 | 25 | 0 | 184 | 4584564 | 1 | 25 | 0 | 1 | 192 |
| 81 | 51 | 55 | 37 | 1 | 26 | 558923 | 4471380 | 1 | 26 | 0 | 184 | 4584756 | 1 | 26 | 0 | 1 | 192 |
| 82 | 52 | 55 | 37 | 1 | 27 | 558946 | 4471564 | 1 | 27 | 0 | 184 | 4584948 | 1 | 27 | 0 | 1 | 192 |
| 83 | 53 | 55 | 37 | 1 | 28 | 558969 | 4471748 | 1 | 28 | 0 | 184 | 4585140 | 1 | 28 | 0 | 1 | 192 |
| 84 | 54 | 55 | 37 | 1 | 29 | 558992 | 4471932 | 1 | 29 | 0 | 184 | 4585332 | 1 | 29 | 0 | 1 | 192 |
| 85 | 55 | 55 | 37 | 1 | 30 | 559015 | 4472116 | 1 | 30 | 0 | 184 | 4585524 | 1 | 30 | 0 | 1 | 192 |
| 86 | 56 | 55 | 37 | 1 | 31 | 559038 | 4472300 | 1 | 31 | 0 | 184 | 4585716 | 1 | 31 | 0 | 1 | 192 |
| 87 | 57 | 55 | 37 | 1 | 32 | 559061 | 4472484 | 1 | 32 | 0 | 184 | 4585908 | 1 | 32 | 0 | 1 | 192 |
| 88 | 58 | 55 | 37 | 1 | 33 | 559084 | 4472668 | 1 | 33 | 0 | 184 | 4586100 | 1 | 33 | 0 | 1 | 192 |
| 89 | 59 | 55 | 37 | 1 | 34 | 559107 | 4472852 | 1 | 34 | 0 | 184 | 4586292 | 1 | 34 | 0 | 1 | 192 |
| 90 | 5A | 55 | 37 | 1 | 35 | 559130 | 4473036 | 1 | 35 | 0 | 184 | 4586484 | 1 | 35 | 0 | 1 | 192 |
| 91 | 5B | 55 | 37 | 1 | 36 | 559153 | 4473220 | 1 | 36 | 0 | 184 | 4586676 | 1 | 36 | 0 | 1 | 192 |
| 92 | 5C | 55 | 37 | 1 | 37 | 559176 | 4473404 | 1 | 37 | 0 | 184 | 4586868 | 1 | 37 | 0 | 1 | 192 |
| 93 | 5D | 55 | 37 | 1 | 38 | 559199 | 4473588 | 1 | 38 | 0 | 184 | 4587060 | 1 | 38 | 0 | 1 | 192 |
| 94 | 5E | 55 | 37 | 1 | 39 | 559222 | 4473772 | 1 | 39 | 0 | 184 | 4587252 | 1 | 39 | 0 | 1 | 192 |
| 95 | 5F | 55 | 37 | 1 | 40 | 559245 | 4473956 | 1 | 40 | 0 | 184 | 4587444 | 1 | 40 | 0 | 1 | 192 |
| 96 | 60 | 55 | 37 | 1 | 41 | 559268 | 4474140 | 1 | 41 | 0 | 184 | 4587636 | 1 | 41 | 0 | 1 | 192 |
| 97 | 61 | 55 | 37 | 1 | 42 | 559291 | 4474324 | 1 | 42 | 0 | 184 | 4587828 | 1 | 42 | 0 | 1 | 192 |
| 98 | 62 | 55 | 37 | 1 | 43 | 559314 | 4474508 | 1 | 43 | 0 | 184 | 4588020 | 1 | 43 | 0 | 1 | 192 |
| 99 | 63 | 55 | 37 | 1 | 44 | 559337 | 4474692 | 1 | 44 | 0 | 184 | 4588212 | 1 | 44 | 0 | 1 | 192 |
| 100 | 64 | 55 | 37 | 1 | 45 | 559360 | 4474876 | 1 | 45 | 0 | 184 | 4588404 | 1 | 45 | 0 | 1 | 192 |
| 101 | 65 | 55 | 37 | 1 | 46 | 559383 | 4475060 | 1 | 46 | 0 | 184 | 4588596 | 1 | 46 | 0 | 1 | 192 |
| 102 | 66 | 55 | 37 | 1 | 47 | 559406 | 4475244 | 1 | 47 | 0 | 184 | 4588788 | 1 | 47 | 0 | 1 | 192 |
| 103 | 67 | 55 | 37 | 1 | 48 | 559429 | 4475428 | 1 | 48 | 0 | 184 | 4588980 | 1 | 48 | 0 | 1 | 192 |
| 104 | 68 | 55 | 37 | 1 | 49 | 559452 | 4475612 | 1 | 49 | 0 | 184 | 4589172 | 1 | 49 | 0 | 1 | 192 |
| 105 | 69 | 55 | 37 | 1 | 50 | 559475 | 4475796 | 1 | 50 | 0 | 184 | 4589364 | 1 | 50 | 0 | 1 | 192 |
| 106 | 6A | 55 | 37 | 1 | 51 | 559498 | 4475980 | 1 | 51 | 0 | 184 | 4589556 | 1 | 51 | 0 | 1 | 192 |
| 107 | 6B | 55 | 37 | 1 | 52 | 559521 | 4476164 | 1 | 52 | 0 | 184 | 4589748 | 1 | 52 | 0 | 1 | 192 |
| 108 | 6C | 55 | 37 | 1 | 53 | 559544 | 4476348 | 1 | 53 | 0 | 184 | 4589940 | 1 | 53 | 0 | 1 | 192 |
| 109 | 6D | 55 | 37 | 1 | 54 | 559567 | 4476532 | 1 | 54 | 0 | 184 | 4590132 | 1 | 54 | 0 | 1 | 192 |
| 110 | 6E | 55 | 37 | 2 | 0 | 559588 | 4476700 | 2 | 0 | 0 | 168 | 4590308 | 2 | 0 | 0 | 1 | 176 |
| 111 | 6F | 55 | 37 | 2 | 1 | 559614 | 4476908 | 2 | 1 | 0 | 208 | 4590524 | 2 | 1 | 0 | 1 | 216 |
| 112 | 70 | 55 | 37 | 2 | 2 | 559640 | 4477116 | 2 | 2 | 0 | 208 | 4590740 | 2 | 2 | 0 | 1 | 216 |
| 113 | 71 | 55 | 37 | 2 | 3 | 559666 | 4477324 | 2 | 3 | 0 | 208 | 4590956 | 2 | 3 | 0 | 1 | 216 |
| 114 | 72 | 55 | 37 | 2 | 4 | 559692 | 4477532 | 2 | 4 | 0 | 208 | 4591172 | 2 | 4 | 0 | 1 | 216 |
| 115 | 73 | 55 | 37 | 2 | 5 | 559718 | 4477740 | 2 | 5 | 0 | 208 | 4591388 | 2 | 5 | 0 | 1 | 216 |
| 116 | 74 | 55 | 37 | 2 | 6 | 559744 | 4477948 | 2 | 6 | 0 | 208 | 4591604 | 2 | 6 | 0 | 1 | 216 |
| 117 | 75 | 55 | 37 | 2 | 7 | 559770 | 4478156 | 2 | 7 | 0 | 208 | 4591820 | 2 | 7 | 0 | 1 | 216 |
| 118 | 76 | 55 | 37 | 2 | 8 | 559796 | 4478364 | 2 | 8 | 0 | 208 | 4592036 | 2 | 8 | 0 | 1 | 216 |
| 119 | 77 | 55 | 37 | 2 | 9 | 559822 | 4478572 | 2 | 9 | 0 | 208 | 4592252 | 2 | 9 | 0 | 1 | 216 |
| 120 | 78 | 55 | 37 | 2 | 10 | 559848 | 4478780 | 2 | 10 | 0 | 208 | 4592468 | 2 | 10 | 0 | 1 | 216 |
| 121 | 79 | 55 | 37 | 2 | 11 | 559874 | 4478988 | 2 | 11 | 0 | 208 | 4592684 | 2 | 11 | 0 | 1 | 216 |
| 122 | 7A | 55 | 37 | 2 | 12 | 559900 | 4479196 | 2 | 12 | 0 | 208 | 4592900 | 2 | 12 | 0 | 1 | 216 |
| 123 | 7B | 55 | 37 | 2 | 13 | 559926 | 4479404 | 2 | 13 | 0 | 208 | 4593116 | 2 | 13 | 0 | 1 | 216 |
| 124 | 7C | 55 | 37 | 2 | 14 | 559952 | 4479612 | 2 | 14 | 0 | 208 | 4593332 | 2 | 14 | 0 | 1 | 216 |
| 125 | 7D | 55 | 37 | 2 | 15 | 559978 | 4479820 | 2 | 15 | 0 | 208 | 4593548 | 2 | 15 | 0 | 1 | 216 |
| 126 | 7E | 55 | 37 | 2 | 16 | 560004 | 4480028 | 2 | 16 | 0 | 208 | 4593764 | 2 | 16 | 0 | 1 | 216 |
| 127 | 7F | 55 | 37 | 2 | 17 | 560030 | 4480236 | 2 | 17 | 0 | 208 | 4593980 | 2 | 17 | 0 | 1 | 216 |
| 128 | 80 | 55 | 37 | 2 | 18 | 560053 | 4480420 | 2 | 18 | 0 | 184 | 4594172 | 2 | 18 | 0 | 1 | 192 |
| 129 | 81 | 55 | 37 | 2 | 19 | 560076 | 4480604 | 2 | 19 | 0 | 184 | 4594364 | 2 | 19 | 0 | 1 | 192 |
| 130 | 82 | 55 | 37 | 2 | 20 | 560099 | 4480788 | 2 | 20 | 0 | 184 | 4594556 | 2 | 20 | 0 | 1 | 192 |

| | | | | | | | | | | | | | | | | | |
|---|---|---|---|---|---|---|---|---|---|---|---|---|---|---|---|---|---|
| 131 | 83 | 55 | 37 | 2 | 21 | 560122 | 4480972 | 2 | 21 | 0 | 184 | 4594748 | 2 | 21 | 0 | 1 | 192 |
| 132 | 84 | 55 | 37 | 2 | 22 | 560145 | 4481156 | 2 | 22 | 0 | 184 | 4594940 | 2 | 22 | 0 | 1 | 192 |
| 133 | 85 | 55 | 37 | 2 | 23 | 560168 | 4481340 | 2 | 23 | 0 | 184 | 4595132 | 2 | 23 | 0 | 1 | 192 |
| 134 | 86 | 55 | 37 | 2 | 24 | 560191 | 4481524 | 2 | 24 | 0 | 184 | 4595324 | 2 | 24 | 0 | 1 | 192 |
| 135 | 87 | 55 | 37 | 2 | 25 | 560214 | 4481708 | 2 | 25 | 0 | 184 | 4595516 | 2 | 25 | 0 | 1 | 192 |
| 136 | 88 | 55 | 37 | 2 | 26 | 560237 | 4481892 | 2 | 26 | 0 | 184 | 4595708 | 2 | 26 | 0 | 1 | 192 |
| 137 | 89 | 55 | 37 | 2 | 27 | 560260 | 4482076 | 2 | 27 | 0 | 184 | 4595900 | 2 | 27 | 0 | 1 | 192 |
| 138 | 8A | 55 | 37 | 2 | 28 | 560283 | 4482260 | 2 | 28 | 0 | 184 | 4596092 | 2 | 28 | 0 | 1 | 192 |
| 139 | 8B | 55 | 37 | 2 | 29 | 560306 | 4482444 | 2 | 29 | 0 | 184 | 4596284 | 2 | 29 | 0 | 1 | 192 |
| 140 | 8C | 55 | 37 | 2 | 30 | 560329 | 4482628 | 2 | 30 | 0 | 184 | 4596476 | 2 | 30 | 0 | 1 | 192 |
| 141 | 8D | 55 | 37 | 2 | 31 | 560352 | 4482812 | 2 | 31 | 0 | 184 | 4596668 | 2 | 31 | 0 | 1 | 192 |
| 142 | 8E | 55 | 37 | 2 | 32 | 560375 | 4482996 | 2 | 32 | 0 | 184 | 4596860 | 2 | 32 | 0 | 1 | 192 |
| 143 | 8F | 55 | 37 | 2 | 33 | 560398 | 4483180 | 2 | 33 | 0 | 184 | 4597052 | 2 | 33 | 0 | 1 | 192 |
| 144 | 90 | 55 | 37 | 2 | 34 | 560423 | 4483380 | 2 | 34 | 0 | 200 | 4597260 | 2 | 34 | 0 | 1 | 208 |
| 145 | 91 | 55 | 37 | 2 | 35 | 560448 | 4483580 | 2 | 35 | 0 | 200 | 4597468 | 2 | 35 | 0 | 1 | 208 |
| 146 | 92 | 55 | 37 | 2 | 36 | 560473 | 4483780 | 2 | 36 | 0 | 200 | 4597676 | 2 | 36 | 0 | 1 | 208 |
| 147 | 93 | 55 | 37 | 2 | 37 | 560498 | 4483980 | 2 | 37 | 0 | 200 | 4597884 | 2 | 37 | 0 | 1 | 208 |
| 148 | 94 | 55 | 37 | 2 | 38 | 560523 | 4484180 | 2 | 38 | 0 | 200 | 4598092 | 2 | 38 | 0 | 1 | 208 |
| 149 | 95 | 55 | 37 | 2 | 39 | 560548 | 4484380 | 2 | 39 | 0 | 200 | 4598300 | 2 | 39 | 0 | 1 | 208 |
| 150 | 96 | 55 | 37 | 2 | 40 | 560573 | 4484580 | 2 | 40 | 0 | 200 | 4598508 | 2 | 40 | 0 | 1 | 208 |
| 151 | 97 | 55 | 37 | 2 | 41 | 560598 | 4484780 | 2 | 41 | 0 | 200 | 4598716 | 2 | 41 | 0 | 1 | 208 |
| 152 | 98 | 55 | 37 | 2 | 42 | 560623 | 4484980 | 2 | 42 | 0 | 200 | 4598924 | 2 | 42 | 0 | 1 | 208 |
| 153 | 99 | 55 | 37 | 2 | 43 | 560648 | 4485180 | 2 | 43 | 0 | 200 | 4599132 | 2 | 43 | 0 | 1 | 208 |
| 154 | 9A | 55 | 37 | 2 | 44 | 560673 | 4485380 | 2 | 44 | 0 | 200 | 4599340 | 2 | 44 | 0 | 1 | 208 |
| 155 | 9B | 55 | 37 | 2 | 45 | 560698 | 4485580 | 2 | 45 | 0 | 200 | 4599548 | 2 | 45 | 0 | 1 | 208 |
| 156 | 9C | 55 | 37 | 2 | 46 | 560723 | 4485780 | 2 | 46 | 0 | 200 | 4599756 | 2 | 46 | 0 | 1 | 208 |
| 157 | 9D | 55 | 37 | 2 | 47 | 560748 | 4485980 | 2 | 47 | 0 | 200 | 4599964 | 2 | 47 | 0 | 1 | 208 |
| 158 | 9E | 55 | 37 | 2 | 48 | 560773 | 4486180 | 2 | 48 | 0 | 200 | 4600172 | 2 | 48 | 0 | 1 | 208 |
| 159 | 9F | 55 | 37 | 2 | 49 | 560798 | 4486380 | 2 | 49 | 0 | 200 | 4600380 | 2 | 49 | 0 | 1 | 208 |
| 160 | A0 | 55 | 37 | 2 | 50 | 560823 | 4486580 | 2 | 50 | 0 | 200 | 4600588 | 2 | 50 | 0 | 1 | 208 |
| 161 | A1 | 55 | 37 | 2 | 51 | 560848 | 4486780 | 2 | 51 | 0 | 200 | 4600796 | 2 | 51 | 0 | 1 | 208 |
| 162 | A2 | 55 | 37 | 2 | 52 | 560873 | 4486980 | 2 | 52 | 0 | 200 | 4601004 | 2 | 52 | 0 | 1 | 208 |
| 163 | A3 | 55 | 37 | 2 | 53 | 560898 | 4487180 | 2 | 53 | 0 | 200 | 4601212 | 2 | 53 | 0 | 1 | 208 |
| 164 | A4 | 55 | 37 | 2 | 54 | 560923 | 4487380 | 2 | 54 | 0 | 200 | 4601420 | 2 | 54 | 0 | 1 | 208 |
| 165 | A5 | 55 | 37 | 3 | 0 | 560946 | 4487564 | 3 | 0 | 0 | 184 | 4601612 | 3 | 0 | 0 | 1 | 192 |
| 166 | A6 | 55 | 37 | 3 | 1 | 560974 | 4487788 | 3 | 1 | 0 | 224 | 4601844 | 3 | 1 | 0 | 1 | 232 |
| 167 | A7 | 55 | 37 | 3 | 2 | 561002 | 4488012 | 3 | 2 | 0 | 224 | 4602076 | 3 | 2 | 0 | 1 | 232 |
| 168 | A8 | 55 | 37 | 3 | 3 | 561030 | 4488236 | 3 | 3 | 0 | 224 | 4602308 | 3 | 3 | 0 | 1 | 232 |
| 169 | A9 | 55 | 37 | 3 | 4 | 561058 | 4488460 | 3 | 4 | 0 | 224 | 4602540 | 3 | 4 | 0 | 1 | 232 |
| 170 | AA | 55 | 37 | 3 | 5 | 561086 | 4488684 | 3 | 5 | 0 | 224 | 4602772 | 3 | 5 | 0 | 1 | 232 |
| 171 | AB | 55 | 37 | 3 | 6 | 561114 | 4488908 | 3 | 6 | 0 | 224 | 4603004 | 3 | 6 | 0 | 1 | 232 |
| 172 | AC | 55 | 37 | 3 | 7 | 561142 | 4489132 | 3 | 7 | 0 | 224 | 4603236 | 3 | 7 | 0 | 1 | 232 |
| 173 | AD | 55 | 37 | 3 | 8 | 561170 | 4489356 | 3 | 8 | 0 | 224 | 4603468 | 3 | 8 | 0 | 1 | 232 |
| 174 | AE | 55 | 37 | 3 | 9 | 561198 | 4489580 | 3 | 9 | 0 | 224 | 4603700 | 3 | 9 | 0 | 1 | 232 |
| 175 | AF | 55 | 37 | 3 | 10 | 561226 | 4489804 | 3 | 10 | 0 | 224 | 4603932 | 3 | 10 | 0 | 1 | 232 |
| 176 | B0 | 55 | 37 | 3 | 11 | 561254 | 4490028 | 3 | 11 | 0 | 224 | 4604164 | 3 | 11 | 0 | 1 | 232 |
| 177 | B1 | 55 | 37 | 3 | 12 | 561282 | 4490252 | 3 | 12 | 0 | 224 | 4604396 | 3 | 12 | 0 | 1 | 232 |
| 178 | B2 | 55 | 37 | 3 | 13 | 561310 | 4490476 | 3 | 13 | 0 | 224 | 4604628 | 3 | 13 | 0 | 1 | 232 |
| 179 | B3 | 55 | 37 | 3 | 14 | 561338 | 4490700 | 3 | 14 | 0 | 224 | 4604860 | 3 | 14 | 0 | 1 | 232 |
| 180 | B4 | 55 | 37 | 3 | 15 | 561366 | 4490924 | 3 | 15 | 0 | 224 | 4605092 | 3 | 15 | 0 | 1 | 232 |
| 181 | B5 | 55 | 37 | 3 | 16 | 561394 | 4491148 | 3 | 16 | 0 | 224 | 4605324 | 3 | 16 | 0 | 1 | 232 |
| 182 | B6 | 55 | 37 | 3 | 17 | 561422 | 4491372 | 3 | 17 | 0 | 224 | 4605556 | 3 | 17 | 0 | 1 | 232 |
| 183 | B7 | 55 | 37 | 3 | 18 | 561450 | 4491596 | 3 | 18 | 0 | 224 | 4605788 | 3 | 18 | 0 | 1 | 232 |
| 184 | B8 | 55 | 37 | 3 | 19 | 561478 | 4491820 | 3 | 19 | 0 | 224 | 4606020 | 3 | 19 | 0 | 1 | 232 |
| 185 | B9 | 55 | 37 | 3 | 20 | 561506 | 4492044 | 3 | 20 | 0 | 224 | 4606252 | 3 | 20 | 0 | 1 | 232 |
| 186 | BA | 55 | 37 | 3 | 21 | 561534 | 4492268 | 3 | 21 | 0 | 224 | 4606484 | 3 | 21 | 0 | 1 | 232 |
| 187 | BB | 55 | 37 | 3 | 22 | 561562 | 4492492 | 3 | 22 | 0 | 224 | 4606716 | 3 | 22 | 0 | 1 | 232 |
| 188 | BC | 55 | 37 | 3 | 23 | 561590 | 4492716 | 3 | 23 | 0 | 224 | 4606948 | 3 | 23 | 0 | 1 | 232 |
| 189 | BD | 55 | 37 | 3 | 24 | 561618 | 4492940 | 3 | 24 | 0 | 224 | 4607180 | 3 | 24 | 0 | 1 | 232 |
| 190 | BE | 55 | 37 | 3 | 25 | 561646 | 4493164 | 3 | 25 | 0 | 224 | 4607412 | 3 | 25 | 0 | 1 | 232 |
| 191 | BF | 55 | 37 | 3 | 26 | 561674 | 4493388 | 3 | 26 | 0 | 224 | 4607644 | 3 | 26 | 0 | 1 | 232 |
| 192 | C0 | 55 | 37 | 3 | 27 | 561699 | 4493588 | 3 | 27 | 0 | 200 | 4607852 | 3 | 27 | 0 | 1 | 208 |
| 193 | C1 | 55 | 37 | 3 | 28 | 561724 | 4493788 | 3 | 28 | 0 | 200 | 4608060 | 3 | 28 | 0 | 1 | 208 |
| 194 | C2 | 55 | 37 | 3 | 29 | 561749 | 4493988 | 3 | 29 | 0 | 200 | 4608268 | 3 | 29 | 0 | 1 | 208 |
| 195 | C3 | 55 | 37 | 3 | 30 | 561774 | 4494188 | 3 | 30 | 0 | 200 | 4608476 | 3 | 30 | 0 | 1 | 208 |
| 196 | C4 | 55 | 37 | 3 | 31 | 561799 | 4494388 | 3 | 31 | 0 | 200 | 4608684 | 3 | 31 | 0 | 1 | 208 |
| 197 | C5 | 55 | 37 | 3 | 32 | 561824 | 4494588 | 3 | 32 | 0 | 200 | 4608892 | 3 | 32 | 0 | 1 | 208 |
| 198 | C6 | 55 | 37 | 3 | 33 | 561849 | 4494788 | 3 | 33 | 0 | 200 | 4609100 | 3 | 33 | 0 | 1 | 208 |
| 199 | C7 | 55 | 37 | 3 | 34 | 561874 | 4494988 | 3 | 34 | 0 | 200 | 4609308 | 3 | 34 | 0 | 1 | 208 |
| 200 | C8 | 55 | 37 | 3 | 35 | 561899 | 4495188 | 3 | 35 | 0 | 200 | 4609516 | 3 | 35 | 0 | 1 | 208 |
| 201 | C9 | 55 | 37 | 3 | 36 | 561924 | 4495388 | 3 | 36 | 0 | 200 | 4609724 | 3 | 36 | 0 | 1 | 208 |
| 202 | CA | 55 | 37 | 3 | 37 | 561949 | 4495588 | 3 | 37 | 0 | 200 | 4609932 | 3 | 37 | 0 | 1 | 208 |
| 203 | CB | 55 | 37 | 3 | 38 | 561974 | 4495788 | 3 | 38 | 0 | 200 | 4610140 | 3 | 38 | 0 | 1 | 208 |
| 204 | CC | 55 | 37 | 3 | 39 | 561999 | 4495988 | 3 | 39 | 0 | 200 | 4610348 | 3 | 39 | 0 | 1 | 208 |
| 205 | CD | 55 | 37 | 3 | 40 | 562024 | 4496188 | 3 | 40 | 0 | 200 | 4610556 | 3 | 40 | 0 | 1 | 208 |
| 206 | CE | 55 | 37 | 3 | 41 | 562049 | 4496388 | 3 | 41 | 0 | 200 | 4610764 | 3 | 41 | 0 | 1 | 208 |
| 207 | CF | 55 | 37 | 3 | 42 | 562074 | 4496588 | 3 | 42 | 0 | 200 | 4610972 | 3 | 42 | 0 | 1 | 208 |
| 208 | D0 | 55 | 37 | 3 | 43 | 562101 | 4496804 | 3 | 43 | 0 | 216 | 4611196 | 3 | 43 | 0 | 1 | 224 |
| 209 | D1 | 55 | 37 | 3 | 44 | 562128 | 4497020 | 3 | 44 | 0 | 216 | 4611420 | 3 | 44 | 0 | 1 | 224 |
| 210 | D2 | 55 | 37 | 3 | 45 | 562155 | 4497236 | 3 | 45 | 0 | 216 | 4611644 | 3 | 45 | 0 | 1 | 224 |
| 211 | D3 | 55 | 37 | 3 | 46 | 562182 | 4497452 | 3 | 46 | 0 | 216 | 4611868 | 3 | 46 | 0 | 1 | 224 |
| 212 | D4 | 55 | 37 | 3 | 47 | 562209 | 4497668 | 3 | 47 | 0 | 216 | 4612092 | 3 | 47 | 0 | 1 | 224 |
| 213 | D5 | 55 | 37 | 3 | 48 | 562236 | 4497884 | 3 | 48 | 0 | 216 | 4612316 | 3 | 48 | 0 | 1 | 224 |
| 214 | D6 | 55 | 37 | 3 | 49 | 562263 | 4498100 | 3 | 49 | 0 | 216 | 4612540 | 3 | 49 | 0 | 1 | 224 |
| 215 | D7 | 55 | 37 | 3 | 50 | 562290 | 4498316 | 3 | 50 | 0 | 216 | 4612764 | 3 | 50 | 0 | 1 | 224 |
| 216 | D8 | 55 | 37 | 3 | 51 | 562317 | 4498532 | 3 | 51 | 0 | 216 | 4612988 | 3 | 51 | 0 | 1 | 224 |
| 217 | D9 | 55 | 37 | 3 | 52 | 562344 | 4498748 | 3 | 52 | 0 | 216 | 4613212 | 3 | 52 | 0 | 1 | 224 |
| 218 | DA | 55 | 37 | 3 | 53 | 562371 | 4498964 | 3 | 53 | 0 | 216 | 4613436 | 3 | 53 | 0 | 1 | 224 |
| 219 | DB | 55 | 37 | 3 | 54 | 562398 | 4499180 | 3 | 54 | 0 | 216 | 4613660 | 3 | 54 | 0 | 1 | 224 |
| 220 | DC | 55 | 37 | 4 | 0 | 562423 | 4499380 | 4 | 0 | 0 | 200 | 4613868 | 4 | 0 | 0 | 1 | 208 |
| 221 | DD | 55 | 37 | 4 | 1 | 562453 | 4499620 | 4 | 1 | 0 | 240 | 4614116 | 4 | 1 | 0 | 1 | 248 |
| 222 | DE | 55 | 37 | 4 | 2 | 562483 | 4499860 | 4 | 2 | 0 | 240 | 4614364 | 4 | 2 | 0 | 1 | 248 |
| 223 | DF | 55 | 37 | 4 | 3 | 562513 | 4500100 | 4 | 3 | 0 | 240 | 4614612 | 4 | 3 | 0 | 1 | 248 |
| 224 | E0 | 55 | 37 | 4 | 4 | 562543 | 4500340 | 4 | 4 | 0 | 240 | 4614860 | 4 | 4 | 0 | 1 | 248 |
| 225 | E1 | 55 | 37 | 4 | 5 | 562573 | 4500580 | 4 | 5 | 0 | 240 | 4615108 | 4 | 5 | 0 | 1 | 248 |
| 226 | E2 | 55 | 37 | 4 | 6 | 562603 | 4500820 | 4 | 6 | 0 | 240 | 4615356 | 4 | 6 | 0 | 1 | 248 |
| 227 | E3 | 55 | 37 | 4 | 7 | 562633 | 4501060 | 4 | 7 | 0 | 240 | 4615604 | 4 | 7 | 0 | 1 | 248 |
| 228 | E4 | 55 | 37 | 4 | 8 | 562663 | 4501300 | 4 | 8 | 0 | 240 | 4615852 | 4 | 8 | 0 | 1 | 248 |
| 229 | E5 | 55 | 37 | 4 | 9 | 562693 | 4501540 | 4 | 9 | 0 | 240 | 4616100 | 4 | 9 | 0 | 1 | 248 |
| 230 | E6 | 55 | 37 | 4 | 10 | 562723 | 4501780 | 4 | 10 | 0 | 240 | 4616348 | 4 | 10 | 0 | 1 | 248 |
| 231 | E7 | 55 | 37 | 4 | 11 | 562753 | 4502020 | 4 | 11 | 0 | 240 | 4616596 | 4 | 11 | 0 | 1 | 248 |
| 232 | E8 | 55 | 37 | 4 | 12 | 562783 | 4502260 | 4 | 12 | 0 | 240 | 4616844 | 4 | 12 | 0 | 1 | 248 |
| 233 | E9 | 55 | 37 | 4 | 13 | 562813 | 4502500 | 4 | 13 | 0 | 240 | 4617092 | 4 | 13 | 0 | 1 | 248 |

| | | | | | | | | | | | | | | | | | |
|---|---|---|---|---|---|---|---|---|---|---|---|---|---|---|---|---|---|
| 234 | EA | 55 | 37 | 4 | 14 | 562843 | 4502740 | 4 | 14 | 0 | 240 | 4617340 | 4 | 14 | 0 | 1 | 248 |
| 235 | EB | 55 | 37 | 4 | 15 | 562873 | 4502980 | 4 | 15 | 0 | 240 | 4617588 | 4 | 15 | 0 | 1 | 248 |
| 236 | EC | 55 | 37 | 4 | 16 | 562903 | 4503220 | 4 | 16 | 0 | 240 | 4617836 | 4 | 16 | 0 | 1 | 248 |
| 237 | ED | 55 | 37 | 4 | 17 | 562933 | 4503460 | 4 | 17 | 0 | 240 | 4618084 | 4 | 17 | 0 | 1 | 248 |
| 238 | EE | 55 | 37 | 4 | 18 | 562963 | 4503700 | 4 | 18 | 0 | 240 | 4618332 | 4 | 18 | 0 | 1 | 248 |
| 239 | EF | 55 | 37 | 4 | 19 | 562993 | 4503940 | 4 | 19 | 0 | 240 | 4618580 | 4 | 19 | 0 | 1 | 248 |
| 240 | F0 | 55 | 37 | 4 | 20 | 563023 | 4504180 | 4 | 20 | 0 | 240 | 4618828 | 4 | 20 | 0 | 1 | 248 |
| 241 | F1 | 55 | 37 | 4 | 21 | 563053 | 4504420 | 4 | 21 | 0 | 240 | 4619076 | 4 | 21 | 0 | 1 | 248 |
| 242 | F2 | 55 | 37 | 4 | 22 | 563083 | 4504660 | 4 | 22 | 0 | 240 | 4619324 | 4 | 22 | 0 | 1 | 248 |
| 243 | F3 | 55 | 37 | 4 | 23 | 563113 | 4504900 | 4 | 23 | 0 | 240 | 4619572 | 4 | 23 | 0 | 1 | 248 |
| 244 | F4 | 55 | 37 | 4 | 24 | 563143 | 4505140 | 4 | 24 | 0 | 240 | 4619820 | 4 | 24 | 0 | 1 | 248 |
| 245 | F5 | 55 | 37 | 4 | 25 | 563173 | 4505380 | 4 | 25 | 0 | 240 | 4620068 | 4 | 25 | 0 | 1 | 248 |
| 246 | F6 | 55 | 37 | 4 | 26 | 563203 | 4505620 | 4 | 26 | 0 | 240 | 4620316 | 4 | 26 | 0 | 1 | 248 |
| 247 | F7 | 55 | 37 | 4 | 27 | 563233 | 4505860 | 4 | 27 | 0 | 240 | 4620564 | 4 | 27 | 0 | 1 | 248 |
| 248 | F8 | 55 | 37 | 4 | 28 | 563263 | 4506100 | 4 | 28 | 0 | 240 | 4620812 | 4 | 28 | 0 | 1 | 248 |
| 249 | F9 | 55 | 37 | 4 | 29 | 563293 | 4506340 | 4 | 29 | 0 | 240 | 4621060 | 4 | 29 | 0 | 1 | 248 |
| 250 | FA | 55 | 37 | 4 | 30 | 563323 | 4506580 | 4 | 30 | 0 | 240 | 4621308 | 4 | 30 | 0 | 1 | 248 |
| 251 | FB | 55 | 37 | 4 | 31 | 563353 | 4506820 | 4 | 31 | 0 | 240 | 4621556 | 4 | 31 | 0 | 1 | 248 |
| 252 | FC | 55 | 37 | 4 | 32 | 563383 | 4507060 | 4 | 32 | 0 | 240 | 4621804 | 4 | 32 | 0 | 1 | 248 |
| 253 | FD | 55 | 37 | 4 | 33 | 563413 | 4507300 | 4 | 33 | 0 | 240 | 4622052 | 4 | 33 | 0 | 1 | 248 |
| 254 | FE | 55 | 37 | 4 | 34 | 563443 | 4507540 | 4 | 34 | 0 | 240 | 4622300 | 4 | 34 | 0 | 1 | 248 |
| 255 | FF | 55 | 37 | 4 | 35 | 563473 | 4507780 | 4 | 35 | 0 | 240 | 4622548 | 4 | 35 | 0 | 1 | 248 |
| 0 | 00 | 56 | 38 | 0 | 0 | 563485 | 4507876 | 0 | 0 | 0 | 96 | 4622652 | 0 | 0 | 0 | 1 | 104 |
| 1 | 01 | 56 | 38 | 0 | 1 | 563499 | 4507988 | 0 | 1 | 0 | 112 | 4622772 | 0 | 1 | 0 | 1 | 120 |
| 2 | 02 | 56 | 38 | 0 | 2 | 563513 | 4508100 | 0 | 2 | 0 | 112 | 4622892 | 0 | 2 | 0 | 1 | 120 |
| 3 | 03 | 56 | 38 | 0 | 3 | 563527 | 4508212 | 0 | 3 | 0 | 112 | 4623012 | 0 | 3 | 0 | 1 | 120 |
| 4 | 04 | 56 | 38 | 0 | 4 | 563541 | 4508324 | 0 | 4 | 0 | 112 | 4623132 | 0 | 4 | 0 | 1 | 120 |
| 5 | 05 | 56 | 38 | 0 | 5 | 563555 | 4508436 | 0 | 5 | 0 | 112 | 4623252 | 0 | 5 | 0 | 1 | 120 |
| 6 | 06 | 56 | 38 | 0 | 6 | 563569 | 4508548 | 0 | 6 | 0 | 112 | 4623372 | 0 | 6 | 0 | 1 | 120 |
| 7 | 07 | 56 | 38 | 0 | 7 | 563583 | 4508660 | 0 | 7 | 0 | 112 | 4623492 | 0 | 7 | 0 | 1 | 120 |
| 8 | 08 | 56 | 38 | 0 | 8 | 563597 | 4508772 | 0 | 8 | 0 | 112 | 4623612 | 0 | 8 | 0 | 1 | 120 |
| 9 | 09 | 56 | 38 | 0 | 9 | 563611 | 4508884 | 0 | 9 | 0 | 112 | 4623732 | 0 | 9 | 0 | 1 | 120 |
| 10 | 0A | 56 | 38 | 0 | 10 | 563625 | 4508996 | 0 | 10 | 0 | 112 | 4623852 | 0 | 10 | 0 | 1 | 120 |
| 11 | 0B | 56 | 38 | 0 | 11 | 563639 | 4509108 | 0 | 11 | 0 | 112 | 4623972 | 0 | 11 | 0 | 1 | 120 |
| 12 | 0C | 56 | 38 | 0 | 12 | 563653 | 4509220 | 0 | 12 | 0 | 112 | 4624092 | 0 | 12 | 0 | 1 | 120 |
| 13 | 0D | 56 | 38 | 0 | 13 | 563667 | 4509332 | 0 | 13 | 0 | 112 | 4624212 | 0 | 13 | 0 | 1 | 120 |
| 14 | 0E | 56 | 38 | 0 | 14 | 563681 | 4509444 | 0 | 14 | 0 | 112 | 4624332 | 0 | 14 | 0 | 1 | 120 |
| 15 | 0F | 56 | 38 | 0 | 15 | 563695 | 4509556 | 0 | 15 | 0 | 112 | 4624452 | 0 | 15 | 0 | 1 | 120 |
| 16 | 10 | 56 | 38 | 0 | 16 | 563709 | 4509668 | 0 | 16 | 0 | 112 | 4624572 | 0 | 16 | 0 | 1 | 120 |
| 17 | 11 | 56 | 38 | 0 | 17 | 563723 | 4509780 | 0 | 17 | 0 | 112 | 4624692 | 0 | 17 | 0 | 1 | 120 |
| 18 | 12 | 56 | 38 | 0 | 18 | 563737 | 4509892 | 0 | 18 | 0 | 112 | 4624812 | 0 | 18 | 0 | 1 | 120 |
| 19 | 13 | 56 | 38 | 0 | 19 | 563751 | 4510004 | 0 | 19 | 0 | 112 | 4624932 | 0 | 19 | 0 | 1 | 120 |
| 20 | 14 | 56 | 38 | 0 | 20 | 563765 | 4510116 | 0 | 20 | 0 | 112 | 4625052 | 0 | 20 | 0 | 1 | 120 |
| 21 | 15 | 56 | 38 | 0 | 21 | 563779 | 4510228 | 0 | 21 | 0 | 112 | 4625172 | 0 | 21 | 0 | 1 | 120 |
| 22 | 16 | 56 | 38 | 0 | 22 | 563793 | 4510340 | 0 | 22 | 0 | 112 | 4625292 | 0 | 22 | 0 | 1 | 120 |
| 23 | 17 | 56 | 38 | 0 | 23 | 563807 | 4510452 | 0 | 23 | 0 | 112 | 4625412 | 0 | 23 | 0 | 1 | 120 |
| 24 | 18 | 56 | 38 | 0 | 24 | 563821 | 4510564 | 0 | 24 | 0 | 112 | 4625532 | 0 | 24 | 0 | 1 | 120 |
| 25 | 19 | 56 | 38 | 0 | 25 | 563835 | 4510676 | 0 | 25 | 0 | 112 | 4625652 | 0 | 25 | 0 | 1 | 120 |
| 26 | 1A | 56 | 38 | 0 | 26 | 563849 | 4510788 | 0 | 26 | 0 | 112 | 4625772 | 0 | 26 | 0 | 1 | 120 |
| 27 | 1B | 56 | 38 | 0 | 27 | 563863 | 4510900 | 0 | 27 | 0 | 112 | 4625892 | 0 | 27 | 0 | 1 | 120 |
| 28 | 1C | 56 | 38 | 0 | 28 | 563877 | 4511012 | 0 | 28 | 0 | 112 | 4626012 | 0 | 28 | 0 | 1 | 120 |
| 29 | 1D | 56 | 38 | 0 | 29 | 563891 | 4511124 | 0 | 29 | 0 | 112 | 4626132 | 0 | 29 | 0 | 1 | 120 |
| 30 | 1E | 56 | 38 | 0 | 30 | 563905 | 4511236 | 0 | 30 | 0 | 112 | 4626252 | 0 | 30 | 0 | 1 | 120 |
| 31 | 1F | 56 | 38 | 0 | 31 | 563919 | 4511348 | 0 | 31 | 0 | 112 | 4626372 | 0 | 31 | 0 | 1 | 120 |
| 32 | 20 | 56 | 38 | 0 | 32 | 563933 | 4511460 | 0 | 32 | 0 | 112 | 4626492 | 0 | 32 | 0 | 1 | 120 |
| 33 | 21 | 56 | 38 | 0 | 33 | 563947 | 4511572 | 0 | 33 | 0 | 112 | 4626612 | 0 | 33 | 0 | 1 | 120 |
| 34 | 22 | 56 | 38 | 0 | 34 | 563961 | 4511684 | 0 | 34 | 0 | 112 | 4626732 | 0 | 34 | 0 | 1 | 120 |
| 35 | 23 | 56 | 38 | 0 | 35 | 563975 | 4511796 | 0 | 35 | 0 | 112 | 4626852 | 0 | 35 | 0 | 1 | 120 |
| 36 | 24 | 56 | 38 | 0 | 36 | 563989 | 4511908 | 0 | 36 | 0 | 112 | 4626972 | 0 | 36 | 0 | 1 | 120 |
| 37 | 25 | 56 | 38 | 0 | 37 | 564003 | 4512020 | 0 | 37 | 0 | 112 | 4627092 | 0 | 37 | 0 | 1 | 120 |
| 38 | 26 | 56 | 38 | 0 | 38 | 564017 | 4512132 | 0 | 38 | 0 | 112 | 4627212 | 0 | 38 | 0 | 1 | 120 |
| 39 | 27 | 56 | 38 | 0 | 39 | 564031 | 4512244 | 0 | 39 | 0 | 112 | 4627332 | 0 | 39 | 0 | 1 | 120 |
| 40 | 28 | 56 | 38 | 0 | 40 | 564045 | 4512356 | 0 | 40 | 0 | 112 | 4627452 | 0 | 40 | 0 | 1 | 120 |
| 41 | 29 | 56 | 38 | 0 | 41 | 564059 | 4512468 | 0 | 41 | 0 | 112 | 4627572 | 0 | 41 | 0 | 1 | 120 |
| 42 | 2A | 56 | 38 | 0 | 42 | 564073 | 4512580 | 0 | 42 | 0 | 112 | 4627692 | 0 | 42 | 0 | 1 | 120 |
| 43 | 2B | 56 | 38 | 0 | 43 | 564087 | 4512692 | 0 | 43 | 0 | 112 | 4627812 | 0 | 43 | 0 | 1 | 120 |
| 44 | 2C | 56 | 38 | 0 | 44 | 564101 | 4512804 | 0 | 44 | 0 | 112 | 4627932 | 0 | 44 | 0 | 1 | 120 |
| 45 | 2D | 56 | 38 | 0 | 45 | 564115 | 4512916 | 0 | 45 | 0 | 112 | 4628052 | 0 | 45 | 0 | 1 | 120 |
| 46 | 2E | 56 | 38 | 0 | 46 | 564129 | 4513028 | 0 | 46 | 0 | 112 | 4628172 | 0 | 46 | 0 | 1 | 120 |
| 47 | 2F | 56 | 38 | 0 | 47 | 564143 | 4513140 | 0 | 47 | 0 | 112 | 4628292 | 0 | 47 | 0 | 1 | 120 |
| 48 | 30 | 56 | 38 | 0 | 48 | 564157 | 4513252 | 0 | 48 | 0 | 112 | 4628412 | 0 | 48 | 0 | 1 | 120 |
| 49 | 31 | 56 | 38 | 0 | 49 | 564171 | 4513364 | 0 | 49 | 0 | 112 | 4628532 | 0 | 49 | 0 | 1 | 120 |
| 50 | 32 | 56 | 38 | 0 | 50 | 564185 | 4513476 | 0 | 50 | 0 | 112 | 4628652 | 0 | 50 | 0 | 1 | 120 |
| 51 | 33 | 56 | 38 | 0 | 51 | 564199 | 4513588 | 0 | 51 | 0 | 112 | 4628772 | 0 | 51 | 0 | 1 | 120 |
| 52 | 34 | 56 | 38 | 0 | 52 | 564213 | 4513700 | 0 | 52 | 0 | 112 | 4628892 | 0 | 52 | 0 | 1 | 120 |
| 53 | 35 | 56 | 38 | 0 | 53 | 564227 | 4513812 | 0 | 53 | 0 | 112 | 4629012 | 0 | 53 | 0 | 1 | 120 |
| 54 | 36 | 56 | 38 | 0 | 54 | 564241 | 4513924 | 0 | 54 | 0 | 112 | 4629132 | 0 | 54 | 0 | 1 | 120 |
| 55 | 37 | 56 | 38 | 0 | 55 | 564255 | 4514036 | 0 | 55 | 0 | 112 | 4629252 | 0 | 55 | 0 | 1 | 120 |
| 56 | 38 | 56 | 38 | 1 | 0 | 564267 | 4514132 | 1 | 0 | 0 | 96 | 4629356 | 1 | 0 | 0 | 1 | 104 |
| 57 | 39 | 56 | 38 | 1 | 1 | 564284 | 4514268 | 1 | 1 | 0 | 136 | 4629500 | 1 | 1 | 0 | 1 | 144 |
| 58 | 3A | 56 | 38 | 1 | 2 | 564301 | 4514404 | 1 | 2 | 0 | 136 | 4629644 | 1 | 2 | 0 | 1 | 144 |
| 59 | 3B | 56 | 38 | 1 | 3 | 564318 | 4514540 | 1 | 3 | 0 | 136 | 4629788 | 1 | 3 | 0 | 1 | 144 |
| 60 | 3C | 56 | 38 | 1 | 4 | 564335 | 4514676 | 1 | 4 | 0 | 136 | 4629932 | 1 | 4 | 0 | 1 | 144 |
| 61 | 3D | 56 | 38 | 1 | 5 | 564352 | 4514812 | 1 | 5 | 0 | 136 | 4630076 | 1 | 5 | 0 | 1 | 144 |
| 62 | 3E | 56 | 38 | 1 | 6 | 564369 | 4514948 | 1 | 6 | 0 | 136 | 4630220 | 1 | 6 | 0 | 1 | 144 |
| 63 | 3F | 56 | 38 | 1 | 7 | 564386 | 4515084 | 1 | 7 | 0 | 136 | 4630364 | 1 | 7 | 0 | 1 | 144 |
| 64 | 40 | 56 | 38 | 1 | 8 | 564407 | 4515252 | 1 | 8 | 0 | 168 | 4630540 | 1 | 8 | 0 | 1 | 176 |
| 65 | 41 | 56 | 38 | 1 | 9 | 564428 | 4515420 | 1 | 9 | 0 | 168 | 4630716 | 1 | 9 | 0 | 1 | 176 |
| 66 | 42 | 56 | 38 | 1 | 10 | 564449 | 4515588 | 1 | 10 | 0 | 168 | 4630892 | 1 | 10 | 0 | 1 | 176 |
| 67 | 43 | 56 | 38 | 1 | 11 | 564470 | 4515756 | 1 | 11 | 0 | 168 | 4631068 | 1 | 11 | 0 | 1 | 176 |
| 68 | 44 | 56 | 38 | 1 | 12 | 564491 | 4515924 | 1 | 12 | 0 | 168 | 4631244 | 1 | 12 | 0 | 1 | 176 |
| 69 | 45 | 56 | 38 | 1 | 13 | 564512 | 4516092 | 1 | 13 | 0 | 168 | 4631420 | 1 | 13 | 0 | 1 | 176 |
| 70 | 46 | 56 | 38 | 1 | 14 | 564533 | 4516260 | 1 | 14 | 0 | 168 | 4631596 | 1 | 14 | 0 | 1 | 176 |
| 71 | 47 | 56 | 38 | 1 | 15 | 564554 | 4516428 | 1 | 15 | 0 | 168 | 4631772 | 1 | 15 | 0 | 1 | 176 |
| 72 | 48 | 56 | 38 | 1 | 16 | 564575 | 4516596 | 1 | 16 | 0 | 168 | 4631948 | 1 | 16 | 0 | 1 | 176 |
| 73 | 49 | 56 | 38 | 1 | 17 | 564596 | 4516764 | 1 | 17 | 0 | 168 | 4632124 | 1 | 17 | 0 | 1 | 176 |
| 74 | 4A | 56 | 38 | 1 | 18 | 564617 | 4516932 | 1 | 18 | 0 | 168 | 4632300 | 1 | 18 | 0 | 1 | 176 |
| 75 | 4B | 56 | 38 | 1 | 19 | 564638 | 4517100 | 1 | 19 | 0 | 168 | 4632476 | 1 | 19 | 0 | 1 | 176 |
| 76 | 4C | 56 | 38 | 1 | 20 | 564659 | 4517268 | 1 | 20 | 0 | 168 | 4632652 | 1 | 20 | 0 | 1 | 176 |
| 77 | 4D | 56 | 38 | 1 | 21 | 564680 | 4517436 | 1 | 21 | 0 | 168 | 4632828 | 1 | 21 | 0 | 1 | 176 |
| 78 | 4E | 56 | 38 | 1 | 22 | 564701 | 4517604 | 1 | 22 | 0 | 168 | 4633004 | 1 | 22 | 0 | 1 | 176 |
| 79 | 4F | 56 | 38 | 1 | 23 | 564722 | 4517772 | 1 | 23 | 0 | 168 | 4633180 | 1 | 23 | 0 | 1 | 176 |
| 80 | 50 | 56 | 38 | 1 | 24 | 564745 | 4517956 | 1 | 24 | 0 | 184 | 4633372 | 1 | 24 | 0 | 1 | 192 |

| | | | | | | | | | | | | | | | | | |
|---|---|---|---|---|---|---|---|---|---|---|---|---|---|---|---|---|---|
| 81 | 51 | 56 | 38 | 1 | 25 | 564768 | 4518140 | 1 | 25 | 0 | 184 | 4633564 | 1 | 25 | 0 | 1 | 192 |
| 82 | 52 | 56 | 38 | 1 | 26 | 564791 | 4518324 | 1 | 26 | 0 | 184 | 4633756 | 1 | 26 | 0 | 1 | 192 |
| 83 | 53 | 56 | 38 | 1 | 27 | 564814 | 4518508 | 1 | 27 | 0 | 184 | 4633948 | 1 | 27 | 0 | 1 | 192 |
| 84 | 54 | 56 | 38 | 1 | 28 | 564837 | 4518692 | 1 | 28 | 0 | 184 | 4634140 | 1 | 28 | 0 | 1 | 192 |
| 85 | 55 | 56 | 38 | 1 | 29 | 564860 | 4518876 | 1 | 29 | 0 | 184 | 4634332 | 1 | 29 | 0 | 1 | 192 |
| 86 | 56 | 56 | 38 | 1 | 30 | 564883 | 4519060 | 1 | 30 | 0 | 184 | 4634524 | 1 | 30 | 0 | 1 | 192 |
| 87 | 57 | 56 | 38 | 1 | 31 | 564906 | 4519244 | 1 | 31 | 0 | 184 | 4634716 | 1 | 31 | 0 | 1 | 192 |
| 88 | 58 | 56 | 38 | 1 | 32 | 564929 | 4519428 | 1 | 32 | 0 | 184 | 4634908 | 1 | 32 | 0 | 1 | 192 |
| 89 | 59 | 56 | 38 | 1 | 33 | 564952 | 4519612 | 1 | 33 | 0 | 184 | 4635100 | 1 | 33 | 0 | 1 | 192 |
| 90 | 5A | 56 | 38 | 1 | 34 | 564975 | 4519796 | 1 | 34 | 0 | 184 | 4635292 | 1 | 34 | 0 | 1 | 192 |
| 91 | 5B | 56 | 38 | 1 | 35 | 564998 | 4519980 | 1 | 35 | 0 | 184 | 4635484 | 1 | 35 | 0 | 1 | 192 |
| 92 | 5C | 56 | 38 | 1 | 36 | 565021 | 4520164 | 1 | 36 | 0 | 184 | 4635676 | 1 | 36 | 0 | 1 | 192 |
| 93 | 5D | 56 | 38 | 1 | 37 | 565044 | 4520348 | 1 | 37 | 0 | 184 | 4635868 | 1 | 37 | 0 | 1 | 192 |
| 94 | 5E | 56 | 38 | 1 | 38 | 565067 | 4520532 | 1 | 38 | 0 | 184 | 4636060 | 1 | 38 | 0 | 1 | 192 |
| 95 | 5F | 56 | 38 | 1 | 39 | 565090 | 4520716 | 1 | 39 | 0 | 184 | 4636252 | 1 | 39 | 0 | 1 | 192 |
| 96 | 60 | 56 | 38 | 1 | 40 | 565113 | 4520900 | 1 | 40 | 0 | 184 | 4636444 | 1 | 40 | 0 | 1 | 192 |
| 97 | 61 | 56 | 38 | 1 | 41 | 565136 | 4521084 | 1 | 41 | 0 | 184 | 4636636 | 1 | 41 | 0 | 1 | 192 |
| 98 | 62 | 56 | 38 | 1 | 42 | 565159 | 4521268 | 1 | 42 | 0 | 184 | 4636828 | 1 | 42 | 0 | 1 | 192 |
| 99 | 63 | 56 | 38 | 1 | 43 | 565182 | 4521452 | 1 | 43 | 0 | 184 | 4637020 | 1 | 43 | 0 | 1 | 192 |
| 100 | 64 | 56 | 38 | 1 | 44 | 565205 | 4521636 | 1 | 44 | 0 | 184 | 4637212 | 1 | 44 | 0 | 1 | 192 |
| 101 | 65 | 56 | 38 | 1 | 45 | 565228 | 4521820 | 1 | 45 | 0 | 184 | 4637404 | 1 | 45 | 0 | 1 | 192 |
| 102 | 66 | 56 | 38 | 1 | 46 | 565251 | 4522004 | 1 | 46 | 0 | 184 | 4637596 | 1 | 46 | 0 | 1 | 192 |
| 103 | 67 | 56 | 38 | 1 | 47 | 565274 | 4522188 | 1 | 47 | 0 | 184 | 4637788 | 1 | 47 | 0 | 1 | 192 |
| 104 | 68 | 56 | 38 | 1 | 48 | 565297 | 4522372 | 1 | 48 | 0 | 184 | 4637980 | 1 | 48 | 0 | 1 | 192 |
| 105 | 69 | 56 | 38 | 1 | 49 | 565320 | 4522556 | 1 | 49 | 0 | 184 | 4638172 | 1 | 49 | 0 | 1 | 192 |
| 106 | 6A | 56 | 38 | 1 | 50 | 565343 | 4522740 | 1 | 50 | 0 | 184 | 4638364 | 1 | 50 | 0 | 1 | 192 |
| 107 | 6B | 56 | 38 | 1 | 51 | 565366 | 4522924 | 1 | 51 | 0 | 184 | 4638556 | 1 | 51 | 0 | 1 | 192 |
| 108 | 6C | 56 | 38 | 1 | 52 | 565389 | 4523108 | 1 | 52 | 0 | 184 | 4638748 | 1 | 52 | 0 | 1 | 192 |
| 109 | 6D | 56 | 38 | 1 | 53 | 565412 | 4523292 | 1 | 53 | 0 | 184 | 4638940 | 1 | 53 | 0 | 1 | 192 |
| 110 | 6E | 56 | 38 | 1 | 54 | 565435 | 4523476 | 1 | 54 | 0 | 184 | 4639132 | 1 | 54 | 0 | 1 | 192 |
| 111 | 6F | 56 | 38 | 1 | 55 | 565458 | 4523660 | 1 | 55 | 0 | 184 | 4639324 | 1 | 55 | 0 | 1 | 192 |
| 112 | 70 | 56 | 38 | 2 | 0 | 565479 | 4523828 | 2 | 0 | 0 | 168 | 4639500 | 2 | 0 | 0 | 1 | 176 |
| 113 | 71 | 56 | 38 | 2 | 1 | 565505 | 4524036 | 2 | 1 | 0 | 208 | 4639716 | 2 | 1 | 0 | 1 | 216 |
| 114 | 72 | 56 | 38 | 2 | 2 | 565531 | 4524244 | 2 | 2 | 0 | 208 | 4639932 | 2 | 2 | 0 | 1 | 216 |
| 115 | 73 | 56 | 38 | 2 | 3 | 565557 | 4524452 | 2 | 3 | 0 | 208 | 4640148 | 2 | 3 | 0 | 1 | 216 |
| 116 | 74 | 56 | 38 | 2 | 4 | 565583 | 4524660 | 2 | 4 | 0 | 208 | 4640364 | 2 | 4 | 0 | 1 | 216 |
| 117 | 75 | 56 | 38 | 2 | 5 | 565609 | 4524868 | 2 | 5 | 0 | 208 | 4640580 | 2 | 5 | 0 | 1 | 216 |
| 118 | 76 | 56 | 38 | 2 | 6 | 565635 | 4525076 | 2 | 6 | 0 | 208 | 4640796 | 2 | 6 | 0 | 1 | 216 |
| 119 | 77 | 56 | 38 | 2 | 7 | 565661 | 4525284 | 2 | 7 | 0 | 208 | 4641012 | 2 | 7 | 0 | 1 | 216 |
| 120 | 78 | 56 | 38 | 2 | 8 | 565687 | 4525492 | 2 | 8 | 0 | 208 | 4641228 | 2 | 8 | 0 | 1 | 216 |
| 121 | 79 | 56 | 38 | 2 | 9 | 565713 | 4525700 | 2 | 9 | 0 | 208 | 4641444 | 2 | 9 | 0 | 1 | 216 |
| 122 | 7A | 56 | 38 | 2 | 10 | 565739 | 4525908 | 2 | 10 | 0 | 208 | 4641660 | 2 | 10 | 0 | 1 | 216 |
| 123 | 7B | 56 | 38 | 2 | 11 | 565765 | 4526116 | 2 | 11 | 0 | 208 | 4641876 | 2 | 11 | 0 | 1 | 216 |
| 124 | 7C | 56 | 38 | 2 | 12 | 565791 | 4526324 | 2 | 12 | 0 | 208 | 4642092 | 2 | 12 | 0 | 1 | 216 |
| 125 | 7D | 56 | 38 | 2 | 13 | 565817 | 4526532 | 2 | 13 | 0 | 208 | 4642308 | 2 | 13 | 0 | 1 | 216 |
| 126 | 7E | 56 | 38 | 2 | 14 | 565843 | 4526740 | 2 | 14 | 0 | 208 | 4642524 | 2 | 14 | 0 | 1 | 216 |
| 127 | 7F | 56 | 38 | 2 | 15 | 565869 | 4526948 | 2 | 15 | 0 | 208 | 4642740 | 2 | 15 | 0 | 1 | 216 |
| 128 | 80 | 56 | 38 | 2 | 16 | 565892 | 4527132 | 2 | 16 | 0 | 184 | 4642932 | 2 | 16 | 0 | 1 | 192 |
| 129 | 81 | 56 | 38 | 2 | 17 | 565915 | 4527316 | 2 | 17 | 0 | 184 | 4643124 | 2 | 17 | 0 | 1 | 192 |
| 130 | 82 | 56 | 38 | 2 | 18 | 565938 | 4527500 | 2 | 18 | 0 | 184 | 4643316 | 2 | 18 | 0 | 1 | 192 |
| 131 | 83 | 56 | 38 | 2 | 19 | 565961 | 4527684 | 2 | 19 | 0 | 184 | 4643508 | 2 | 19 | 0 | 1 | 192 |
| 132 | 84 | 56 | 38 | 2 | 20 | 565984 | 4527868 | 2 | 20 | 0 | 184 | 4643700 | 2 | 20 | 0 | 1 | 192 |
| 133 | 85 | 56 | 38 | 2 | 21 | 566007 | 4528052 | 2 | 21 | 0 | 184 | 4643892 | 2 | 21 | 0 | 1 | 192 |
| 134 | 86 | 56 | 38 | 2 | 22 | 566030 | 4528236 | 2 | 22 | 0 | 184 | 4644084 | 2 | 22 | 0 | 1 | 192 |
| 135 | 87 | 56 | 38 | 2 | 23 | 566053 | 4528420 | 2 | 23 | 0 | 184 | 4644276 | 2 | 23 | 0 | 1 | 192 |
| 136 | 88 | 56 | 38 | 2 | 24 | 566076 | 4528604 | 2 | 24 | 0 | 184 | 4644468 | 2 | 24 | 0 | 1 | 192 |
| 137 | 89 | 56 | 38 | 2 | 25 | 566099 | 4528788 | 2 | 25 | 0 | 184 | 4644660 | 2 | 25 | 0 | 1 | 192 |
| 138 | 8A | 56 | 38 | 2 | 26 | 566122 | 4528972 | 2 | 26 | 0 | 184 | 4644852 | 2 | 26 | 0 | 1 | 192 |
| 139 | 8B | 56 | 38 | 2 | 27 | 566145 | 4529156 | 2 | 27 | 0 | 184 | 4645044 | 2 | 27 | 0 | 1 | 192 |
| 140 | 8C | 56 | 38 | 2 | 28 | 566168 | 4529340 | 2 | 28 | 0 | 184 | 4645236 | 2 | 28 | 0 | 1 | 192 |
| 141 | 8D | 56 | 38 | 2 | 29 | 566191 | 4529524 | 2 | 29 | 0 | 184 | 4645428 | 2 | 29 | 0 | 1 | 192 |
| 142 | 8E | 56 | 38 | 2 | 30 | 566214 | 4529708 | 2 | 30 | 0 | 184 | 4645620 | 2 | 30 | 0 | 1 | 192 |
| 143 | 8F | 56 | 38 | 2 | 31 | 566237 | 4529892 | 2 | 31 | 0 | 184 | 4645812 | 2 | 31 | 0 | 1 | 192 |
| 144 | 90 | 56 | 38 | 2 | 32 | 566262 | 4530092 | 2 | 32 | 0 | 200 | 4646020 | 2 | 32 | 0 | 1 | 208 |
| 145 | 91 | 56 | 38 | 2 | 33 | 566287 | 4530292 | 2 | 33 | 0 | 200 | 4646228 | 2 | 33 | 0 | 1 | 208 |
| 146 | 92 | 56 | 38 | 2 | 34 | 566312 | 4530492 | 2 | 34 | 0 | 200 | 4646436 | 2 | 34 | 0 | 1 | 208 |
| 147 | 93 | 56 | 38 | 2 | 35 | 566337 | 4530692 | 2 | 35 | 0 | 200 | 4646644 | 2 | 35 | 0 | 1 | 208 |
| 148 | 94 | 56 | 38 | 2 | 36 | 566362 | 4530892 | 2 | 36 | 0 | 200 | 4646852 | 2 | 36 | 0 | 1 | 208 |
| 149 | 95 | 56 | 38 | 2 | 37 | 566387 | 4531092 | 2 | 37 | 0 | 200 | 4647060 | 2 | 37 | 0 | 1 | 208 |
| 150 | 96 | 56 | 38 | 2 | 38 | 566412 | 4531292 | 2 | 38 | 0 | 200 | 4647268 | 2 | 38 | 0 | 1 | 208 |
| 151 | 97 | 56 | 38 | 2 | 39 | 566437 | 4531492 | 2 | 39 | 0 | 200 | 4647476 | 2 | 39 | 0 | 1 | 208 |
| 152 | 98 | 56 | 38 | 2 | 40 | 566462 | 4531692 | 2 | 40 | 0 | 200 | 4647684 | 2 | 40 | 0 | 1 | 208 |
| 153 | 99 | 56 | 38 | 2 | 41 | 566487 | 4531892 | 2 | 41 | 0 | 200 | 4647892 | 2 | 41 | 0 | 1 | 208 |
| 154 | 9A | 56 | 38 | 2 | 42 | 566512 | 4532092 | 2 | 42 | 0 | 200 | 4648100 | 2 | 42 | 0 | 1 | 208 |
| 155 | 9B | 56 | 38 | 2 | 43 | 566537 | 4532292 | 2 | 43 | 0 | 200 | 4648308 | 2 | 43 | 0 | 1 | 208 |
| 156 | 9C | 56 | 38 | 2 | 44 | 566562 | 4532492 | 2 | 44 | 0 | 200 | 4648516 | 2 | 44 | 0 | 1 | 208 |
| 157 | 9D | 56 | 38 | 2 | 45 | 566587 | 4532692 | 2 | 45 | 0 | 200 | 4648724 | 2 | 45 | 0 | 1 | 208 |
| 158 | 9E | 56 | 38 | 2 | 46 | 566612 | 4532892 | 2 | 46 | 0 | 200 | 4648932 | 2 | 46 | 0 | 1 | 208 |
| 159 | 9F | 56 | 38 | 2 | 47 | 566637 | 4533092 | 2 | 47 | 0 | 200 | 4649140 | 2 | 47 | 0 | 1 | 208 |
| 160 | A0 | 56 | 38 | 2 | 48 | 566662 | 4533292 | 2 | 48 | 0 | 200 | 4649348 | 2 | 48 | 0 | 1 | 208 |
| 161 | A1 | 56 | 38 | 2 | 49 | 566687 | 4533492 | 2 | 49 | 0 | 200 | 4649556 | 2 | 49 | 0 | 1 | 208 |
| 162 | A2 | 56 | 38 | 2 | 50 | 566712 | 4533692 | 2 | 50 | 0 | 200 | 4649764 | 2 | 50 | 0 | 1 | 208 |
| 163 | A3 | 56 | 38 | 2 | 51 | 566737 | 4533892 | 2 | 51 | 0 | 200 | 4649972 | 2 | 51 | 0 | 1 | 208 |
| 164 | A4 | 56 | 38 | 2 | 52 | 566762 | 4534092 | 2 | 52 | 0 | 200 | 4650180 | 2 | 52 | 0 | 1 | 208 |
| 165 | A5 | 56 | 38 | 2 | 53 | 566787 | 4534292 | 2 | 53 | 0 | 200 | 4650388 | 2 | 53 | 0 | 1 | 208 |
| 166 | A6 | 56 | 38 | 2 | 54 | 566812 | 4534492 | 2 | 54 | 0 | 200 | 4650596 | 2 | 54 | 0 | 1 | 208 |
| 167 | A7 | 56 | 38 | 2 | 55 | 566837 | 4534692 | 2 | 55 | 0 | 200 | 4650804 | 2 | 55 | 0 | 1 | 208 |
| 168 | A8 | 56 | 38 | 3 | 0 | 566860 | 4534876 | 3 | 0 | 0 | 184 | 4650996 | 3 | 0 | 0 | 1 | 192 |
| 169 | A9 | 56 | 38 | 3 | 1 | 566888 | 4535100 | 3 | 1 | 0 | 224 | 4651228 | 3 | 1 | 0 | 1 | 232 |
| 170 | AA | 56 | 38 | 3 | 2 | 566916 | 4535324 | 3 | 2 | 0 | 224 | 4651460 | 3 | 2 | 0 | 1 | 232 |
| 171 | AB | 56 | 38 | 3 | 3 | 566944 | 4535548 | 3 | 3 | 0 | 224 | 4651692 | 3 | 3 | 0 | 1 | 232 |
| 172 | AC | 56 | 38 | 3 | 4 | 566972 | 4535772 | 3 | 4 | 0 | 224 | 4651924 | 3 | 4 | 0 | 1 | 232 |
| 173 | AD | 56 | 38 | 3 | 5 | 567000 | 4535996 | 3 | 5 | 0 | 224 | 4652156 | 3 | 5 | 0 | 1 | 232 |
| 174 | AE | 56 | 38 | 3 | 6 | 567028 | 4536220 | 3 | 6 | 0 | 224 | 4652388 | 3 | 6 | 0 | 1 | 232 |
| 175 | AF | 56 | 38 | 3 | 7 | 567056 | 4536444 | 3 | 7 | 0 | 224 | 4652620 | 3 | 7 | 0 | 1 | 232 |
| 176 | B0 | 56 | 38 | 3 | 8 | 567084 | 4536668 | 3 | 8 | 0 | 224 | 4652852 | 3 | 8 | 0 | 1 | 232 |
| 177 | B1 | 56 | 38 | 3 | 9 | 567112 | 4536892 | 3 | 9 | 0 | 224 | 4653084 | 3 | 9 | 0 | 1 | 232 |
| 178 | B2 | 56 | 38 | 3 | 10 | 567140 | 4537116 | 3 | 10 | 0 | 224 | 4653316 | 3 | 10 | 0 | 1 | 232 |
| 179 | B3 | 56 | 38 | 3 | 11 | 567168 | 4537340 | 3 | 11 | 0 | 224 | 4653548 | 3 | 11 | 0 | 1 | 232 |
| 180 | B4 | 56 | 38 | 3 | 12 | 567196 | 4537564 | 3 | 12 | 0 | 224 | 4653780 | 3 | 12 | 0 | 1 | 232 |
| 181 | B5 | 56 | 38 | 3 | 13 | 567224 | 4537788 | 3 | 13 | 0 | 224 | 4654012 | 3 | 13 | 0 | 1 | 232 |
| 182 | B6 | 56 | 38 | 3 | 14 | 567252 | 4538012 | 3 | 14 | 0 | 224 | 4654244 | 3 | 14 | 0 | 1 | 232 |
| 183 | B7 | 56 | 38 | 3 | 15 | 567280 | 4538236 | 3 | 15 | 0 | 224 | 4654476 | 3 | 15 | 0 | 1 | 232 |

| | | | | | | | | | | | | | | | | | |
|---|---|---|---|---|---|---|---|---|---|---|---|---|---|---|---|---|---|
| 184 | B8 | 56 | 38 | 3 | 16 | 567308 | 4538460 | 3 | 16 | 0 | 224 | 4654708 | 3 | 16 | 0 | 1 | 232 |
| 185 | B9 | 56 | 38 | 3 | 17 | 567336 | 4538684 | 3 | 17 | 0 | 224 | 4654940 | 3 | 17 | 0 | 1 | 232 |
| 186 | BA | 56 | 38 | 3 | 18 | 567364 | 4538908 | 3 | 18 | 0 | 224 | 4655172 | 3 | 18 | 0 | 1 | 232 |
| 187 | BB | 56 | 38 | 3 | 19 | 567392 | 4539132 | 3 | 19 | 0 | 224 | 4655404 | 3 | 19 | 0 | 1 | 232 |
| 188 | BC | 56 | 38 | 3 | 20 | 567420 | 4539356 | 3 | 20 | 0 | 224 | 4655636 | 3 | 20 | 0 | 1 | 232 |
| 189 | BD | 56 | 38 | 3 | 21 | 567448 | 4539580 | 3 | 21 | 0 | 224 | 4655868 | 3 | 21 | 0 | 1 | 232 |
| 190 | BE | 56 | 38 | 3 | 22 | 567476 | 4539804 | 3 | 22 | 0 | 224 | 4656100 | 3 | 22 | 0 | 1 | 232 |
| 191 | BF | 56 | 38 | 3 | 23 | 567504 | 4540028 | 3 | 23 | 0 | 224 | 4656332 | 3 | 23 | 0 | 1 | 232 |
| 192 | C0 | 56 | 38 | 3 | 24 | 567529 | 4540228 | 3 | 24 | 0 | 200 | 4656540 | 3 | 24 | 0 | 1 | 208 |
| 193 | C1 | 56 | 38 | 3 | 25 | 567554 | 4540428 | 3 | 25 | 0 | 200 | 4656748 | 3 | 25 | 0 | 1 | 208 |
| 194 | C2 | 56 | 38 | 3 | 26 | 567579 | 4540628 | 3 | 26 | 0 | 200 | 4656956 | 3 | 26 | 0 | 1 | 208 |
| 195 | C3 | 56 | 38 | 3 | 27 | 567604 | 4540828 | 3 | 27 | 0 | 200 | 4657164 | 3 | 27 | 0 | 1 | 208 |
| 196 | C4 | 56 | 38 | 3 | 28 | 567629 | 4541028 | 3 | 28 | 0 | 200 | 4657372 | 3 | 28 | 0 | 1 | 208 |
| 197 | C5 | 56 | 38 | 3 | 29 | 567654 | 4541228 | 3 | 29 | 0 | 200 | 4657580 | 3 | 29 | 0 | 1 | 208 |
| 198 | C6 | 56 | 38 | 3 | 30 | 567679 | 4541428 | 3 | 30 | 0 | 200 | 4657788 | 3 | 30 | 0 | 1 | 208 |
| 199 | C7 | 56 | 38 | 3 | 31 | 567704 | 4541628 | 3 | 31 | 0 | 200 | 4657996 | 3 | 31 | 0 | 1 | 208 |
| 200 | C8 | 56 | 38 | 3 | 32 | 567729 | 4541828 | 3 | 32 | 0 | 200 | 4658204 | 3 | 32 | 0 | 1 | 208 |
| 201 | C9 | 56 | 38 | 3 | 33 | 567754 | 4542028 | 3 | 33 | 0 | 200 | 4658412 | 3 | 33 | 0 | 1 | 208 |
| 202 | CA | 56 | 38 | 3 | 34 | 567779 | 4542228 | 3 | 34 | 0 | 200 | 4658620 | 3 | 34 | 0 | 1 | 208 |
| 203 | CB | 56 | 38 | 3 | 35 | 567804 | 4542428 | 3 | 35 | 0 | 200 | 4658828 | 3 | 35 | 0 | 1 | 208 |
| 204 | CC | 56 | 38 | 3 | 36 | 567829 | 4542628 | 3 | 36 | 0 | 200 | 4659036 | 3 | 36 | 0 | 1 | 208 |
| 205 | CD | 56 | 38 | 3 | 37 | 567854 | 4542828 | 3 | 37 | 0 | 200 | 4659244 | 3 | 37 | 0 | 1 | 208 |
| 206 | CE | 56 | 38 | 3 | 38 | 567879 | 4543028 | 3 | 38 | 0 | 200 | 4659452 | 3 | 38 | 0 | 1 | 208 |
| 207 | CF | 56 | 38 | 3 | 39 | 567904 | 4543228 | 3 | 39 | 0 | 200 | 4659660 | 3 | 39 | 0 | 1 | 208 |
| 208 | D0 | 56 | 38 | 3 | 40 | 567931 | 4543444 | 3 | 40 | 0 | 216 | 4659884 | 3 | 40 | 0 | 1 | 224 |
| 209 | D1 | 56 | 38 | 3 | 41 | 567958 | 4543660 | 3 | 41 | 0 | 216 | 4660108 | 3 | 41 | 0 | 1 | 224 |
| 210 | D2 | 56 | 38 | 3 | 42 | 567985 | 4543876 | 3 | 42 | 0 | 216 | 4660332 | 3 | 42 | 0 | 1 | 224 |
| 211 | D3 | 56 | 38 | 3 | 43 | 568012 | 4544092 | 3 | 43 | 0 | 216 | 4660556 | 3 | 43 | 0 | 1 | 224 |
| 212 | D4 | 56 | 38 | 3 | 44 | 568039 | 4544308 | 3 | 44 | 0 | 216 | 4660780 | 3 | 44 | 0 | 1 | 224 |
| 213 | D5 | 56 | 38 | 3 | 45 | 568066 | 4544524 | 3 | 45 | 0 | 216 | 4661004 | 3 | 45 | 0 | 1 | 224 |
| 214 | D6 | 56 | 38 | 3 | 46 | 568093 | 4544740 | 3 | 46 | 0 | 216 | 4661228 | 3 | 46 | 0 | 1 | 224 |
| 215 | D7 | 56 | 38 | 3 | 47 | 568120 | 4544956 | 3 | 47 | 0 | 216 | 4661452 | 3 | 47 | 0 | 1 | 224 |
| 216 | D8 | 56 | 38 | 3 | 48 | 568147 | 4545172 | 3 | 48 | 0 | 216 | 4661676 | 3 | 48 | 0 | 1 | 224 |
| 217 | D9 | 56 | 38 | 3 | 49 | 568174 | 4545388 | 3 | 49 | 0 | 216 | 4661900 | 3 | 49 | 0 | 1 | 224 |
| 218 | DA | 56 | 38 | 3 | 50 | 568201 | 4545604 | 3 | 50 | 0 | 216 | 4662124 | 3 | 50 | 0 | 1 | 224 |
| 219 | DB | 56 | 38 | 3 | 51 | 568228 | 4545820 | 3 | 51 | 0 | 216 | 4662348 | 3 | 51 | 0 | 1 | 224 |
| 220 | DC | 56 | 38 | 3 | 52 | 568255 | 4546036 | 3 | 52 | 0 | 216 | 4662572 | 3 | 52 | 0 | 1 | 224 |
| 221 | DD | 56 | 38 | 3 | 53 | 568282 | 4546252 | 3 | 53 | 0 | 216 | 4662796 | 3 | 53 | 0 | 1 | 224 |
| 222 | DE | 56 | 38 | 3 | 54 | 568309 | 4546468 | 3 | 54 | 0 | 216 | 4663020 | 3 | 54 | 0 | 1 | 224 |
| 223 | DF | 56 | 38 | 3 | 55 | 568336 | 4546684 | 3 | 55 | 0 | 216 | 4663244 | 3 | 55 | 0 | 1 | 224 |
| 224 | E0 | 56 | 38 | 4 | 0 | 568361 | 4546884 | 4 | 0 | 0 | 200 | 4663452 | 4 | 0 | 0 | 1 | 208 |
| 225 | E1 | 56 | 38 | 4 | 1 | 568391 | 4547124 | 4 | 1 | 0 | 240 | 4663700 | 4 | 1 | 0 | 1 | 248 |
| 226 | E2 | 56 | 38 | 4 | 2 | 568421 | 4547364 | 4 | 2 | 0 | 240 | 4663948 | 4 | 2 | 0 | 1 | 248 |
| 227 | E3 | 56 | 38 | 4 | 3 | 568451 | 4547604 | 4 | 3 | 0 | 240 | 4664196 | 4 | 3 | 0 | 1 | 248 |
| 228 | E4 | 56 | 38 | 4 | 4 | 568481 | 4547844 | 4 | 4 | 0 | 240 | 4664444 | 4 | 4 | 0 | 1 | 248 |
| 229 | E5 | 56 | 38 | 4 | 5 | 568511 | 4548084 | 4 | 5 | 0 | 240 | 4664692 | 4 | 5 | 0 | 1 | 248 |
| 230 | E6 | 56 | 38 | 4 | 6 | 568541 | 4548324 | 4 | 6 | 0 | 240 | 4664940 | 4 | 6 | 0 | 1 | 248 |
| 231 | E7 | 56 | 38 | 4 | 7 | 568571 | 4548564 | 4 | 7 | 0 | 240 | 4665188 | 4 | 7 | 0 | 1 | 248 |
| 232 | E8 | 56 | 38 | 4 | 8 | 568601 | 4548804 | 4 | 8 | 0 | 240 | 4665436 | 4 | 8 | 0 | 1 | 248 |
| 233 | E9 | 56 | 38 | 4 | 9 | 568631 | 4549044 | 4 | 9 | 0 | 240 | 4665684 | 4 | 9 | 0 | 1 | 248 |
| 234 | EA | 56 | 38 | 4 | 10 | 568661 | 4549284 | 4 | 10 | 0 | 240 | 4665932 | 4 | 10 | 0 | 1 | 248 |
| 235 | EB | 56 | 38 | 4 | 11 | 568691 | 4549524 | 4 | 11 | 0 | 240 | 4666180 | 4 | 11 | 0 | 1 | 248 |
| 236 | EC | 56 | 38 | 4 | 12 | 568721 | 4549764 | 4 | 12 | 0 | 240 | 4666428 | 4 | 12 | 0 | 1 | 248 |
| 237 | ED | 56 | 38 | 4 | 13 | 568751 | 4550004 | 4 | 13 | 0 | 240 | 4666676 | 4 | 13 | 0 | 1 | 248 |
| 238 | EE | 56 | 38 | 4 | 14 | 568781 | 4550244 | 4 | 14 | 0 | 240 | 4666924 | 4 | 14 | 0 | 1 | 248 |
| 239 | EF | 56 | 38 | 4 | 15 | 568811 | 4550484 | 4 | 15 | 0 | 240 | 4667172 | 4 | 15 | 0 | 1 | 248 |
| 240 | F0 | 56 | 38 | 4 | 16 | 568841 | 4550724 | 4 | 16 | 0 | 240 | 4667420 | 4 | 16 | 0 | 1 | 248 |
| 241 | F1 | 56 | 38 | 4 | 17 | 568871 | 4550964 | 4 | 17 | 0 | 240 | 4667668 | 4 | 17 | 0 | 1 | 248 |
| 242 | F2 | 56 | 38 | 4 | 18 | 568901 | 4551204 | 4 | 18 | 0 | 240 | 4667916 | 4 | 18 | 0 | 1 | 248 |
| 243 | F3 | 56 | 38 | 4 | 19 | 568931 | 4551444 | 4 | 19 | 0 | 240 | 4668164 | 4 | 19 | 0 | 1 | 248 |
| 244 | F4 | 56 | 38 | 4 | 20 | 568961 | 4551684 | 4 | 20 | 0 | 240 | 4668412 | 4 | 20 | 0 | 1 | 248 |
| 245 | F5 | 56 | 38 | 4 | 21 | 568991 | 4551924 | 4 | 21 | 0 | 240 | 4668660 | 4 | 21 | 0 | 1 | 248 |
| 246 | F6 | 56 | 38 | 4 | 22 | 569021 | 4552164 | 4 | 22 | 0 | 240 | 4668908 | 4 | 22 | 0 | 1 | 248 |
| 247 | F7 | 56 | 38 | 4 | 23 | 569051 | 4552404 | 4 | 23 | 0 | 240 | 4669156 | 4 | 23 | 0 | 1 | 248 |
| 248 | F8 | 56 | 38 | 4 | 24 | 569081 | 4552644 | 4 | 24 | 0 | 240 | 4669404 | 4 | 24 | 0 | 1 | 248 |
| 249 | F9 | 56 | 38 | 4 | 25 | 569111 | 4552884 | 4 | 25 | 0 | 240 | 4669652 | 4 | 25 | 0 | 1 | 248 |
| 250 | FA | 56 | 38 | 4 | 26 | 569141 | 4553124 | 4 | 26 | 0 | 240 | 4669900 | 4 | 26 | 0 | 1 | 248 |
| 251 | FB | 56 | 38 | 4 | 27 | 569171 | 4553364 | 4 | 27 | 0 | 240 | 4670148 | 4 | 27 | 0 | 1 | 248 |
| 252 | FC | 56 | 38 | 4 | 28 | 569201 | 4553604 | 4 | 28 | 0 | 240 | 4670396 | 4 | 28 | 0 | 1 | 248 |
| 253 | FD | 56 | 38 | 4 | 29 | 569231 | 4553844 | 4 | 29 | 0 | 240 | 4670644 | 4 | 29 | 0 | 1 | 248 |
| 254 | FE | 56 | 38 | 4 | 30 | 569261 | 4554084 | 4 | 30 | 0 | 240 | 4670892 | 4 | 30 | 0 | 1 | 248 |
| 255 | FF | 56 | 38 | 4 | 31 | 569291 | 4554324 | 4 | 31 | 0 | 240 | 4671140 | 4 | 31 | 0 | 1 | 248 |
| 0 | 00 | 57 | 39 | 0 | 0 | 569303 | 4554420 | 0 | 0 | 0 | 96 | 4671244 | 0 | 0 | 0 | 1 | 104 |
| 1 | 01 | 57 | 39 | 0 | 1 | 569317 | 4554532 | 0 | 1 | 0 | 112 | 4671364 | 0 | 1 | 0 | 1 | 120 |
| 2 | 02 | 57 | 39 | 0 | 2 | 569331 | 4554644 | 0 | 2 | 0 | 112 | 4671484 | 0 | 2 | 0 | 1 | 120 |
| 3 | 03 | 57 | 39 | 0 | 3 | 569345 | 4554756 | 0 | 3 | 0 | 112 | 4671604 | 0 | 3 | 0 | 1 | 120 |
| 4 | 04 | 57 | 39 | 0 | 4 | 569359 | 4554868 | 0 | 4 | 0 | 112 | 4671724 | 0 | 4 | 0 | 1 | 120 |
| 5 | 05 | 57 | 39 | 0 | 5 | 569373 | 4554980 | 0 | 5 | 0 | 112 | 4671844 | 0 | 5 | 0 | 1 | 120 |
| 6 | 06 | 57 | 39 | 0 | 6 | 569387 | 4555092 | 0 | 6 | 0 | 112 | 4671964 | 0 | 6 | 0 | 1 | 120 |
| 7 | 07 | 57 | 39 | 0 | 7 | 569401 | 4555204 | 0 | 7 | 0 | 112 | 4672084 | 0 | 7 | 0 | 1 | 120 |
| 8 | 08 | 57 | 39 | 0 | 8 | 569415 | 4555316 | 0 | 8 | 0 | 112 | 4672204 | 0 | 8 | 0 | 1 | 120 |
| 9 | 09 | 57 | 39 | 0 | 9 | 569429 | 4555428 | 0 | 9 | 0 | 112 | 4672324 | 0 | 9 | 0 | 1 | 120 |
| 10 | 0A | 57 | 39 | 0 | 10 | 569443 | 4555540 | 0 | 10 | 0 | 112 | 4672444 | 0 | 10 | 0 | 1 | 120 |
| 11 | 0B | 57 | 39 | 0 | 11 | 569457 | 4555652 | 0 | 11 | 0 | 112 | 4672564 | 0 | 11 | 0 | 1 | 120 |
| 12 | 0C | 57 | 39 | 0 | 12 | 569471 | 4555764 | 0 | 12 | 0 | 112 | 4672684 | 0 | 12 | 0 | 1 | 120 |
| 13 | 0D | 57 | 39 | 0 | 13 | 569485 | 4555876 | 0 | 13 | 0 | 112 | 4672804 | 0 | 13 | 0 | 1 | 120 |
| 14 | 0E | 57 | 39 | 0 | 14 | 569499 | 4555988 | 0 | 14 | 0 | 112 | 4672924 | 0 | 14 | 0 | 1 | 120 |
| 15 | 0F | 57 | 39 | 0 | 15 | 569513 | 4556100 | 0 | 15 | 0 | 112 | 4673044 | 0 | 15 | 0 | 1 | 120 |
| 16 | 10 | 57 | 39 | 0 | 16 | 569527 | 4556212 | 0 | 16 | 0 | 112 | 4673164 | 0 | 16 | 0 | 1 | 120 |
| 17 | 11 | 57 | 39 | 0 | 17 | 569541 | 4556324 | 0 | 17 | 0 | 112 | 4673284 | 0 | 17 | 0 | 1 | 120 |
| 18 | 12 | 57 | 39 | 0 | 18 | 569555 | 4556436 | 0 | 18 | 0 | 112 | 4673404 | 0 | 18 | 0 | 1 | 120 |
| 19 | 13 | 57 | 39 | 0 | 19 | 569569 | 4556548 | 0 | 19 | 0 | 112 | 4673524 | 0 | 19 | 0 | 1 | 120 |
| 20 | 14 | 57 | 39 | 0 | 20 | 569583 | 4556660 | 0 | 20 | 0 | 112 | 4673644 | 0 | 20 | 0 | 1 | 120 |
| 21 | 15 | 57 | 39 | 0 | 21 | 569597 | 4556772 | 0 | 21 | 0 | 112 | 4673764 | 0 | 21 | 0 | 1 | 120 |
| 22 | 16 | 57 | 39 | 0 | 22 | 569611 | 4556884 | 0 | 22 | 0 | 112 | 4673884 | 0 | 22 | 0 | 1 | 120 |
| 23 | 17 | 57 | 39 | 0 | 23 | 569625 | 4556996 | 0 | 23 | 0 | 112 | 4674004 | 0 | 23 | 0 | 1 | 120 |
| 24 | 18 | 57 | 39 | 0 | 24 | 569639 | 4557108 | 0 | 24 | 0 | 112 | 4674124 | 0 | 24 | 0 | 1 | 120 |
| 25 | 19 | 57 | 39 | 0 | 25 | 569653 | 4557220 | 0 | 25 | 0 | 112 | 4674244 | 0 | 25 | 0 | 1 | 120 |
| 26 | 1A | 57 | 39 | 0 | 26 | 569667 | 4557332 | 0 | 26 | 0 | 112 | 4674364 | 0 | 26 | 0 | 1 | 120 |
| 27 | 1B | 57 | 39 | 0 | 27 | 569681 | 4557444 | 0 | 27 | 0 | 112 | 4674484 | 0 | 27 | 0 | 1 | 120 |
| 28 | 1C | 57 | 39 | 0 | 28 | 569695 | 4557556 | 0 | 28 | 0 | 112 | 4674604 | 0 | 28 | 0 | 1 | 120 |
| 29 | 1D | 57 | 39 | 0 | 29 | 569709 | 4557668 | 0 | 29 | 0 | 112 | 4674724 | 0 | 29 | 0 | 1 | 120 |
| 30 | 1E | 57 | 39 | 0 | 30 | 569723 | 4557780 | 0 | 30 | 0 | 112 | 4674844 | 0 | 30 | 0 | 1 | 120 |

| | | | | | | | | | | | | | | | | | |
|---|---|---|---|---|---|---|---|---|---|---|---|---|---|---|---|---|---|
| 31 | 1F | 57 | 39 | 0 | 31 | 569737 | 4557892 | 0 | 31 | 0 | 112 | 4674964 | 0 | 31 | 0 | 1 | 120 |
| 32 | 20 | 57 | 39 | 0 | 32 | 569751 | 4558004 | 0 | 32 | 0 | 112 | 4675084 | 0 | 32 | 0 | 1 | 120 |
| 33 | 21 | 57 | 39 | 0 | 33 | 569765 | 4558116 | 0 | 33 | 0 | 112 | 4675204 | 0 | 33 | 0 | 1 | 120 |
| 34 | 22 | 57 | 39 | 0 | 34 | 569779 | 4558228 | 0 | 34 | 0 | 112 | 4675324 | 0 | 34 | 0 | 1 | 120 |
| 35 | 23 | 57 | 39 | 0 | 35 | 569793 | 4558340 | 0 | 35 | 0 | 112 | 4675444 | 0 | 35 | 0 | 1 | 120 |
| 36 | 24 | 57 | 39 | 0 | 36 | 569807 | 4558452 | 0 | 36 | 0 | 112 | 4675564 | 0 | 36 | 0 | 1 | 120 |
| 37 | 25 | 57 | 39 | 0 | 37 | 569821 | 4558564 | 0 | 37 | 0 | 112 | 4675684 | 0 | 37 | 0 | 1 | 120 |
| 38 | 26 | 57 | 39 | 0 | 38 | 569835 | 4558676 | 0 | 38 | 0 | 112 | 4675804 | 0 | 38 | 0 | 1 | 120 |
| 39 | 27 | 57 | 39 | 0 | 39 | 569849 | 4558788 | 0 | 39 | 0 | 112 | 4675924 | 0 | 39 | 0 | 1 | 120 |
| 40 | 28 | 57 | 39 | 0 | 40 | 569863 | 4558900 | 0 | 40 | 0 | 112 | 4676044 | 0 | 40 | 0 | 1 | 120 |
| 41 | 29 | 57 | 39 | 0 | 41 | 569877 | 4559012 | 0 | 41 | 0 | 112 | 4676164 | 0 | 41 | 0 | 1 | 120 |
| 42 | 2A | 57 | 39 | 0 | 42 | 569891 | 4559124 | 0 | 42 | 0 | 112 | 4676284 | 0 | 42 | 0 | 1 | 120 |
| 43 | 2B | 57 | 39 | 0 | 43 | 569905 | 4559236 | 0 | 43 | 0 | 112 | 4676404 | 0 | 43 | 0 | 1 | 120 |
| 44 | 2C | 57 | 39 | 0 | 44 | 569919 | 4559348 | 0 | 44 | 0 | 112 | 4676524 | 0 | 44 | 0 | 1 | 120 |
| 45 | 2D | 57 | 39 | 0 | 45 | 569933 | 4559460 | 0 | 45 | 0 | 112 | 4676644 | 0 | 45 | 0 | 1 | 120 |
| 46 | 2E | 57 | 39 | 0 | 46 | 569947 | 4559572 | 0 | 46 | 0 | 112 | 4676764 | 0 | 46 | 0 | 1 | 120 |
| 47 | 2F | 57 | 39 | 0 | 47 | 569961 | 4559684 | 0 | 47 | 0 | 112 | 4676884 | 0 | 47 | 0 | 1 | 120 |
| 48 | 30 | 57 | 39 | 0 | 48 | 569975 | 4559796 | 0 | 48 | 0 | 112 | 4677004 | 0 | 48 | 0 | 1 | 120 |
| 49 | 31 | 57 | 39 | 0 | 49 | 569989 | 4559908 | 0 | 49 | 0 | 112 | 4677124 | 0 | 49 | 0 | 1 | 120 |
| 50 | 32 | 57 | 39 | 0 | 50 | 570003 | 4560020 | 0 | 50 | 0 | 112 | 4677244 | 0 | 50 | 0 | 1 | 120 |
| 51 | 33 | 57 | 39 | 0 | 51 | 570017 | 4560132 | 0 | 51 | 0 | 112 | 4677364 | 0 | 51 | 0 | 1 | 120 |
| 52 | 34 | 57 | 39 | 0 | 52 | 570031 | 4560244 | 0 | 52 | 0 | 112 | 4677484 | 0 | 52 | 0 | 1 | 120 |
| 53 | 35 | 57 | 39 | 0 | 53 | 570045 | 4560356 | 0 | 53 | 0 | 112 | 4677604 | 0 | 53 | 0 | 1 | 120 |
| 54 | 36 | 57 | 39 | 0 | 54 | 570059 | 4560468 | 0 | 54 | 0 | 112 | 4677724 | 0 | 54 | 0 | 1 | 120 |
| 55 | 37 | 57 | 39 | 0 | 55 | 570073 | 4560580 | 0 | 55 | 0 | 112 | 4677844 | 0 | 55 | 0 | 1 | 120 |
| 56 | 38 | 57 | 39 | 0 | 56 | 570087 | 4560692 | 0 | 56 | 0 | 112 | 4677964 | 0 | 56 | 0 | 1 | 120 |
| 57 | 39 | 57 | 39 | 1 | 0 | 570099 | 4560788 | 1 | 0 | 0 | 96 | 4678068 | 1 | 0 | 0 | 1 | 104 |
| 58 | 3A | 57 | 39 | 1 | 1 | 570116 | 4560924 | 1 | 1 | 0 | 136 | 4678212 | 1 | 1 | 0 | 1 | 144 |
| 59 | 3B | 57 | 39 | 1 | 2 | 570133 | 4561060 | 1 | 2 | 0 | 136 | 4678356 | 1 | 2 | 0 | 1 | 144 |
| 60 | 3C | 57 | 39 | 1 | 3 | 570150 | 4561196 | 1 | 3 | 0 | 136 | 4678500 | 1 | 3 | 0 | 1 | 144 |
| 61 | 3D | 57 | 39 | 1 | 4 | 570167 | 4561332 | 1 | 4 | 0 | 136 | 4678644 | 1 | 4 | 0 | 1 | 144 |
| 62 | 3E | 57 | 39 | 1 | 5 | 570184 | 4561468 | 1 | 5 | 0 | 136 | 4678788 | 1 | 5 | 0 | 1 | 144 |
| 63 | 3F | 57 | 39 | 1 | 6 | 570201 | 4561604 | 1 | 6 | 0 | 136 | 4678932 | 1 | 6 | 0 | 1 | 144 |
| 64 | 40 | 57 | 39 | 1 | 7 | 570222 | 4561772 | 1 | 7 | 0 | 168 | 4679108 | 1 | 7 | 0 | 1 | 176 |
| 65 | 41 | 57 | 39 | 1 | 8 | 570243 | 4561940 | 1 | 8 | 0 | 168 | 4679284 | 1 | 8 | 0 | 1 | 176 |
| 66 | 42 | 57 | 39 | 1 | 9 | 570264 | 4562108 | 1 | 9 | 0 | 168 | 4679460 | 1 | 9 | 0 | 1 | 176 |
| 67 | 43 | 57 | 39 | 1 | 10 | 570285 | 4562276 | 1 | 10 | 0 | 168 | 4679636 | 1 | 10 | 0 | 1 | 176 |
| 68 | 44 | 57 | 39 | 1 | 11 | 570306 | 4562444 | 1 | 11 | 0 | 168 | 4679812 | 1 | 11 | 0 | 1 | 176 |
| 69 | 45 | 57 | 39 | 1 | 12 | 570327 | 4562612 | 1 | 12 | 0 | 168 | 4679988 | 1 | 12 | 0 | 1 | 176 |
| 70 | 46 | 57 | 39 | 1 | 13 | 570348 | 4562780 | 1 | 13 | 0 | 168 | 4680164 | 1 | 13 | 0 | 1 | 176 |
| 71 | 47 | 57 | 39 | 1 | 14 | 570369 | 4562948 | 1 | 14 | 0 | 168 | 4680340 | 1 | 14 | 0 | 1 | 176 |
| 72 | 48 | 57 | 39 | 1 | 15 | 570390 | 4563116 | 1 | 15 | 0 | 168 | 4680516 | 1 | 15 | 0 | 1 | 176 |
| 73 | 49 | 57 | 39 | 1 | 16 | 570411 | 4563284 | 1 | 16 | 0 | 168 | 4680692 | 1 | 16 | 0 | 1 | 176 |
| 74 | 4A | 57 | 39 | 1 | 17 | 570432 | 4563452 | 1 | 17 | 0 | 168 | 4680868 | 1 | 17 | 0 | 1 | 176 |
| 75 | 4B | 57 | 39 | 1 | 18 | 570453 | 4563620 | 1 | 18 | 0 | 168 | 4681044 | 1 | 18 | 0 | 1 | 176 |
| 76 | 4C | 57 | 39 | 1 | 19 | 570474 | 4563788 | 1 | 19 | 0 | 168 | 4681220 | 1 | 19 | 0 | 1 | 176 |
| 77 | 4D | 57 | 39 | 1 | 20 | 570495 | 4563956 | 1 | 20 | 0 | 168 | 4681396 | 1 | 20 | 0 | 1 | 176 |
| 78 | 4E | 57 | 39 | 1 | 21 | 570516 | 4564124 | 1 | 21 | 0 | 168 | 4681572 | 1 | 21 | 0 | 1 | 176 |
| 79 | 4F | 57 | 39 | 1 | 22 | 570537 | 4564292 | 1 | 22 | 0 | 168 | 4681748 | 1 | 22 | 0 | 1 | 176 |
| 80 | 50 | 57 | 39 | 1 | 23 | 570560 | 4564476 | 1 | 23 | 0 | 184 | 4681940 | 1 | 23 | 0 | 1 | 192 |
| 81 | 51 | 57 | 39 | 1 | 24 | 570583 | 4564660 | 1 | 24 | 0 | 184 | 4682132 | 1 | 24 | 0 | 1 | 192 |
| 82 | 52 | 57 | 39 | 1 | 25 | 570606 | 4564844 | 1 | 25 | 0 | 184 | 4682324 | 1 | 25 | 0 | 1 | 192 |
| 83 | 53 | 57 | 39 | 1 | 26 | 570629 | 4565028 | 1 | 26 | 0 | 184 | 4682516 | 1 | 26 | 0 | 1 | 192 |
| 84 | 54 | 57 | 39 | 1 | 27 | 570652 | 4565212 | 1 | 27 | 0 | 184 | 4682708 | 1 | 27 | 0 | 1 | 192 |
| 85 | 55 | 57 | 39 | 1 | 28 | 570675 | 4565396 | 1 | 28 | 0 | 184 | 4682900 | 1 | 28 | 0 | 1 | 192 |
| 86 | 56 | 57 | 39 | 1 | 29 | 570698 | 4565580 | 1 | 29 | 0 | 184 | 4683092 | 1 | 29 | 0 | 1 | 192 |
| 87 | 57 | 57 | 39 | 1 | 30 | 570721 | 4565764 | 1 | 30 | 0 | 184 | 4683284 | 1 | 30 | 0 | 1 | 192 |
| 88 | 58 | 57 | 39 | 1 | 31 | 570744 | 4565948 | 1 | 31 | 0 | 184 | 4683476 | 1 | 31 | 0 | 1 | 192 |
| 89 | 59 | 57 | 39 | 1 | 32 | 570767 | 4566132 | 1 | 32 | 0 | 184 | 4683668 | 1 | 32 | 0 | 1 | 192 |
| 90 | 5A | 57 | 39 | 1 | 33 | 570790 | 4566316 | 1 | 33 | 0 | 184 | 4683860 | 1 | 33 | 0 | 1 | 192 |
| 91 | 5B | 57 | 39 | 1 | 34 | 570813 | 4566500 | 1 | 34 | 0 | 184 | 4684052 | 1 | 34 | 0 | 1 | 192 |
| 92 | 5C | 57 | 39 | 1 | 35 | 570836 | 4566684 | 1 | 35 | 0 | 184 | 4684244 | 1 | 35 | 0 | 1 | 192 |
| 93 | 5D | 57 | 39 | 1 | 36 | 570859 | 4566868 | 1 | 36 | 0 | 184 | 4684436 | 1 | 36 | 0 | 1 | 192 |
| 94 | 5E | 57 | 39 | 1 | 37 | 570882 | 4567052 | 1 | 37 | 0 | 184 | 4684628 | 1 | 37 | 0 | 1 | 192 |
| 95 | 5F | 57 | 39 | 1 | 38 | 570905 | 4567236 | 1 | 38 | 0 | 184 | 4684820 | 1 | 38 | 0 | 1 | 192 |
| 96 | 60 | 57 | 39 | 1 | 39 | 570928 | 4567420 | 1 | 39 | 0 | 184 | 4685012 | 1 | 39 | 0 | 1 | 192 |
| 97 | 61 | 57 | 39 | 1 | 40 | 570951 | 4567604 | 1 | 40 | 0 | 184 | 4685204 | 1 | 40 | 0 | 1 | 192 |
| 98 | 62 | 57 | 39 | 1 | 41 | 570974 | 4567788 | 1 | 41 | 0 | 184 | 4685396 | 1 | 41 | 0 | 1 | 192 |
| 99 | 63 | 57 | 39 | 1 | 42 | 570997 | 4567972 | 1 | 42 | 0 | 184 | 4685588 | 1 | 42 | 0 | 1 | 192 |
| 100 | 64 | 57 | 39 | 1 | 43 | 571020 | 4568156 | 1 | 43 | 0 | 184 | 4685780 | 1 | 43 | 0 | 1 | 192 |
| 101 | 65 | 57 | 39 | 1 | 44 | 571043 | 4568340 | 1 | 44 | 0 | 184 | 4685972 | 1 | 44 | 0 | 1 | 192 |
| 102 | 66 | 57 | 39 | 1 | 45 | 571066 | 4568524 | 1 | 45 | 0 | 184 | 4686164 | 1 | 45 | 0 | 1 | 192 |
| 103 | 67 | 57 | 39 | 1 | 46 | 571089 | 4568708 | 1 | 46 | 0 | 184 | 4686356 | 1 | 46 | 0 | 1 | 192 |
| 104 | 68 | 57 | 39 | 1 | 47 | 571112 | 4568892 | 1 | 47 | 0 | 184 | 4686548 | 1 | 47 | 0 | 1 | 192 |
| 105 | 69 | 57 | 39 | 1 | 48 | 571135 | 4569076 | 1 | 48 | 0 | 184 | 4686740 | 1 | 48 | 0 | 1 | 192 |
| 106 | 6A | 57 | 39 | 1 | 49 | 571158 | 4569260 | 1 | 49 | 0 | 184 | 4686932 | 1 | 49 | 0 | 1 | 192 |
| 107 | 6B | 57 | 39 | 1 | 50 | 571181 | 4569444 | 1 | 50 | 0 | 184 | 4687124 | 1 | 50 | 0 | 1 | 192 |
| 108 | 6C | 57 | 39 | 1 | 51 | 571204 | 4569628 | 1 | 51 | 0 | 184 | 4687316 | 1 | 51 | 0 | 1 | 192 |
| 109 | 6D | 57 | 39 | 1 | 52 | 571227 | 4569812 | 1 | 52 | 0 | 184 | 4687508 | 1 | 52 | 0 | 1 | 192 |
| 110 | 6E | 57 | 39 | 1 | 53 | 571250 | 4569996 | 1 | 53 | 0 | 184 | 4687700 | 1 | 53 | 0 | 1 | 192 |
| 111 | 6F | 57 | 39 | 1 | 54 | 571273 | 4570180 | 1 | 54 | 0 | 184 | 4687892 | 1 | 54 | 0 | 1 | 192 |
| 112 | 70 | 57 | 39 | 1 | 55 | 571296 | 4570364 | 1 | 55 | 0 | 184 | 4688084 | 1 | 55 | 0 | 1 | 192 |
| 113 | 71 | 57 | 39 | 1 | 56 | 571319 | 4570548 | 1 | 56 | 0 | 184 | 4688276 | 1 | 56 | 0 | 1 | 192 |
| 114 | 72 | 57 | 39 | 2 | 0 | 571340 | 4570716 | 2 | 0 | 0 | 168 | 4688452 | 2 | 0 | 0 | 1 | 176 |
| 115 | 73 | 57 | 39 | 2 | 1 | 571366 | 4570924 | 2 | 1 | 0 | 208 | 4688668 | 2 | 1 | 0 | 1 | 216 |
| 116 | 74 | 57 | 39 | 2 | 2 | 571392 | 4571132 | 2 | 2 | 0 | 208 | 4688884 | 2 | 2 | 0 | 1 | 216 |
| 117 | 75 | 57 | 39 | 2 | 3 | 571418 | 4571340 | 2 | 3 | 0 | 208 | 4689100 | 2 | 3 | 0 | 1 | 216 |
| 118 | 76 | 57 | 39 | 2 | 4 | 571444 | 4571548 | 2 | 4 | 0 | 208 | 4689316 | 2 | 4 | 0 | 1 | 216 |
| 119 | 77 | 57 | 39 | 2 | 5 | 571470 | 4571756 | 2 | 5 | 0 | 208 | 4689532 | 2 | 5 | 0 | 1 | 216 |
| 120 | 78 | 57 | 39 | 2 | 6 | 571496 | 4571964 | 2 | 6 | 0 | 208 | 4689748 | 2 | 6 | 0 | 1 | 216 |
| 121 | 79 | 57 | 39 | 2 | 7 | 571522 | 4572172 | 2 | 7 | 0 | 208 | 4689964 | 2 | 7 | 0 | 1 | 216 |
| 122 | 7A | 57 | 39 | 2 | 8 | 571548 | 4572380 | 2 | 8 | 0 | 208 | 4690180 | 2 | 8 | 0 | 1 | 216 |
| 123 | 7B | 57 | 39 | 2 | 9 | 571574 | 4572588 | 2 | 9 | 0 | 208 | 4690396 | 2 | 9 | 0 | 1 | 216 |
| 124 | 7C | 57 | 39 | 2 | 10 | 571600 | 4572796 | 2 | 10 | 0 | 208 | 4690612 | 2 | 10 | 0 | 1 | 216 |
| 125 | 7D | 57 | 39 | 2 | 11 | 571626 | 4573004 | 2 | 11 | 0 | 208 | 4690828 | 2 | 11 | 0 | 1 | 216 |
| 126 | 7E | 57 | 39 | 2 | 12 | 571652 | 4573212 | 2 | 12 | 0 | 208 | 4691044 | 2 | 12 | 0 | 1 | 216 |
| 127 | 7F | 57 | 39 | 2 | 13 | 571678 | 4573420 | 2 | 13 | 0 | 208 | 4691260 | 2 | 13 | 0 | 1 | 216 |
| 128 | 80 | 57 | 39 | 2 | 14 | 571701 | 4573604 | 2 | 14 | 0 | 184 | 4691452 | 2 | 14 | 0 | 1 | 192 |
| 129 | 81 | 57 | 39 | 2 | 15 | 571724 | 4573788 | 2 | 15 | 0 | 184 | 4691644 | 2 | 15 | 0 | 1 | 192 |
| 130 | 82 | 57 | 39 | 2 | 16 | 571747 | 4573972 | 2 | 16 | 0 | 184 | 4691836 | 2 | 16 | 0 | 1 | 192 |
| 131 | 83 | 57 | 39 | 2 | 17 | 571770 | 4574156 | 2 | 17 | 0 | 184 | 4692028 | 2 | 17 | 0 | 1 | 192 |
| 132 | 84 | 57 | 39 | 2 | 18 | 571793 | 4574340 | 2 | 18 | 0 | 184 | 4692220 | 2 | 18 | 0 | 1 | 192 |
| 133 | 85 | 57 | 39 | 2 | 19 | 571816 | 4574524 | 2 | 19 | 0 | 184 | 4692412 | 2 | 19 | 0 | 1 | 192 |

| | | | | | | | | | | | | | | | | | |
|---|---|---|---|---|---|---|---|---|---|---|---|---|---|---|---|---|---|
| 134 | 86 | 57 | 39 | 2 | 20 | 571839 | 4574708 | 2 | 20 | 0 | 184 | 4692604 | 2 | 20 | 0 | 1 | 192 |
| 135 | 87 | 57 | 39 | 2 | 21 | 571862 | 4574892 | 2 | 21 | 0 | 184 | 4692796 | 2 | 21 | 0 | 1 | 192 |
| 136 | 88 | 57 | 39 | 2 | 22 | 571885 | 4575076 | 2 | 22 | 0 | 184 | 4692988 | 2 | 22 | 0 | 1 | 192 |
| 137 | 89 | 57 | 39 | 2 | 23 | 571908 | 4575260 | 2 | 23 | 0 | 184 | 4693180 | 2 | 23 | 0 | 1 | 192 |
| 138 | 8A | 57 | 39 | 2 | 24 | 571931 | 4575444 | 2 | 24 | 0 | 184 | 4693372 | 2 | 24 | 0 | 1 | 192 |
| 139 | 8B | 57 | 39 | 2 | 25 | 571954 | 4575628 | 2 | 25 | 0 | 184 | 4693564 | 2 | 25 | 0 | 1 | 192 |
| 140 | 8C | 57 | 39 | 2 | 26 | 571977 | 4575812 | 2 | 26 | 0 | 184 | 4693756 | 2 | 26 | 0 | 1 | 192 |
| 141 | 8D | 57 | 39 | 2 | 27 | 572000 | 4575996 | 2 | 27 | 0 | 184 | 4693948 | 2 | 27 | 0 | 1 | 192 |
| 142 | 8E | 57 | 39 | 2 | 28 | 572023 | 4576180 | 2 | 28 | 0 | 184 | 4694140 | 2 | 28 | 0 | 1 | 192 |
| 143 | 8F | 57 | 39 | 2 | 29 | 572046 | 4576364 | 2 | 29 | 0 | 184 | 4694332 | 2 | 29 | 0 | 1 | 192 |
| 144 | 90 | 57 | 39 | 2 | 30 | 572071 | 4576564 | 2 | 30 | 0 | 200 | 4694540 | 2 | 30 | 0 | 1 | 208 |
| 145 | 91 | 57 | 39 | 2 | 31 | 572096 | 4576764 | 2 | 31 | 0 | 200 | 4694748 | 2 | 31 | 0 | 1 | 208 |
| 146 | 92 | 57 | 39 | 2 | 32 | 572121 | 4576964 | 2 | 32 | 0 | 200 | 4694956 | 2 | 32 | 0 | 1 | 208 |
| 147 | 93 | 57 | 39 | 2 | 33 | 572146 | 4577164 | 2 | 33 | 0 | 200 | 4695164 | 2 | 33 | 0 | 1 | 208 |
| 148 | 94 | 57 | 39 | 2 | 34 | 572171 | 4577364 | 2 | 34 | 0 | 200 | 4695372 | 2 | 34 | 0 | 1 | 208 |
| 149 | 95 | 57 | 39 | 2 | 35 | 572196 | 4577564 | 2 | 35 | 0 | 200 | 4695580 | 2 | 35 | 0 | 1 | 208 |
| 150 | 96 | 57 | 39 | 2 | 36 | 572221 | 4577764 | 2 | 36 | 0 | 200 | 4695788 | 2 | 36 | 0 | 1 | 208 |
| 151 | 97 | 57 | 39 | 2 | 37 | 572246 | 4577964 | 2 | 37 | 0 | 200 | 4695996 | 2 | 37 | 0 | 1 | 208 |
| 152 | 98 | 57 | 39 | 2 | 38 | 572271 | 4578164 | 2 | 38 | 0 | 200 | 4696204 | 2 | 38 | 0 | 1 | 208 |
| 153 | 99 | 57 | 39 | 2 | 39 | 572296 | 4578364 | 2 | 39 | 0 | 200 | 4696412 | 2 | 39 | 0 | 1 | 208 |
| 154 | 9A | 57 | 39 | 2 | 40 | 572321 | 4578564 | 2 | 40 | 0 | 200 | 4696620 | 2 | 40 | 0 | 1 | 208 |
| 155 | 9B | 57 | 39 | 2 | 41 | 572346 | 4578764 | 2 | 41 | 0 | 200 | 4696828 | 2 | 41 | 0 | 1 | 208 |
| 156 | 9C | 57 | 39 | 2 | 42 | 572371 | 4578964 | 2 | 42 | 0 | 200 | 4697036 | 2 | 42 | 0 | 1 | 208 |
| 157 | 9D | 57 | 39 | 2 | 43 | 572396 | 4579164 | 2 | 43 | 0 | 200 | 4697244 | 2 | 43 | 0 | 1 | 208 |
| 158 | 9E | 57 | 39 | 2 | 44 | 572421 | 4579364 | 2 | 44 | 0 | 200 | 4697452 | 2 | 44 | 0 | 1 | 208 |
| 159 | 9F | 57 | 39 | 2 | 45 | 572446 | 4579564 | 2 | 45 | 0 | 200 | 4697660 | 2 | 45 | 0 | 1 | 208 |
| 160 | A0 | 57 | 39 | 2 | 46 | 572471 | 4579764 | 2 | 46 | 0 | 200 | 4697868 | 2 | 46 | 0 | 1 | 208 |
| 161 | A1 | 57 | 39 | 2 | 47 | 572496 | 4579964 | 2 | 47 | 0 | 200 | 4698076 | 2 | 47 | 0 | 1 | 208 |
| 162 | A2 | 57 | 39 | 2 | 48 | 572521 | 4580164 | 2 | 48 | 0 | 200 | 4698284 | 2 | 48 | 0 | 1 | 208 |
| 163 | A3 | 57 | 39 | 2 | 49 | 572546 | 4580364 | 2 | 49 | 0 | 200 | 4698492 | 2 | 49 | 0 | 1 | 208 |
| 164 | A4 | 57 | 39 | 2 | 50 | 572571 | 4580564 | 2 | 50 | 0 | 200 | 4698700 | 2 | 50 | 0 | 1 | 208 |
| 165 | A5 | 57 | 39 | 2 | 51 | 572596 | 4580764 | 2 | 51 | 0 | 200 | 4698908 | 2 | 51 | 0 | 1 | 208 |
| 166 | A6 | 57 | 39 | 2 | 52 | 572621 | 4580964 | 2 | 52 | 0 | 200 | 4699116 | 2 | 52 | 0 | 1 | 208 |
| 167 | A7 | 57 | 39 | 2 | 53 | 572646 | 4581164 | 2 | 53 | 0 | 200 | 4699324 | 2 | 53 | 0 | 1 | 208 |
| 168 | A8 | 57 | 39 | 2 | 54 | 572671 | 4581364 | 2 | 54 | 0 | 200 | 4699532 | 2 | 54 | 0 | 1 | 208 |
| 169 | A9 | 57 | 39 | 2 | 55 | 572696 | 4581564 | 2 | 55 | 0 | 200 | 4699740 | 2 | 55 | 0 | 1 | 208 |
| 170 | AA | 57 | 39 | 2 | 56 | 572721 | 4581764 | 2 | 56 | 0 | 200 | 4699948 | 2 | 56 | 0 | 1 | 208 |
| 171 | AB | 57 | 39 | 3 | 0 | 572744 | 4581948 | 3 | 0 | 0 | 184 | 4700140 | 3 | 0 | 0 | 1 | 192 |
| 172 | AC | 57 | 39 | 3 | 1 | 572772 | 4582172 | 3 | 1 | 0 | 224 | 4700372 | 3 | 1 | 0 | 1 | 232 |
| 173 | AD | 57 | 39 | 3 | 2 | 572800 | 4582396 | 3 | 2 | 0 | 224 | 4700604 | 3 | 2 | 0 | 1 | 232 |
| 174 | AE | 57 | 39 | 3 | 3 | 572828 | 4582620 | 3 | 3 | 0 | 224 | 4700836 | 3 | 3 | 0 | 1 | 232 |
| 175 | AF | 57 | 39 | 3 | 4 | 572856 | 4582844 | 3 | 4 | 0 | 224 | 4701068 | 3 | 4 | 0 | 1 | 232 |
| 176 | B0 | 57 | 39 | 3 | 5 | 572884 | 4583068 | 3 | 5 | 0 | 224 | 4701300 | 3 | 5 | 0 | 1 | 232 |
| 177 | B1 | 57 | 39 | 3 | 6 | 572912 | 4583292 | 3 | 6 | 0 | 224 | 4701532 | 3 | 6 | 0 | 1 | 232 |
| 178 | B2 | 57 | 39 | 3 | 7 | 572940 | 4583516 | 3 | 7 | 0 | 224 | 4701764 | 3 | 7 | 0 | 1 | 232 |
| 179 | B3 | 57 | 39 | 3 | 8 | 572968 | 4583740 | 3 | 8 | 0 | 224 | 4701996 | 3 | 8 | 0 | 1 | 232 |
| 180 | B4 | 57 | 39 | 3 | 9 | 572996 | 4583964 | 3 | 9 | 0 | 224 | 4702228 | 3 | 9 | 0 | 1 | 232 |
| 181 | B5 | 57 | 39 | 3 | 10 | 573024 | 4584188 | 3 | 10 | 0 | 224 | 4702460 | 3 | 10 | 0 | 1 | 232 |
| 182 | B6 | 57 | 39 | 3 | 11 | 573052 | 4584412 | 3 | 11 | 0 | 224 | 4702692 | 3 | 11 | 0 | 1 | 232 |
| 183 | B7 | 57 | 39 | 3 | 12 | 573080 | 4584636 | 3 | 12 | 0 | 224 | 4702924 | 3 | 12 | 0 | 1 | 232 |
| 184 | B8 | 57 | 39 | 3 | 13 | 573108 | 4584860 | 3 | 13 | 0 | 224 | 4703156 | 3 | 13 | 0 | 1 | 232 |
| 185 | B9 | 57 | 39 | 3 | 14 | 573136 | 4585084 | 3 | 14 | 0 | 224 | 4703388 | 3 | 14 | 0 | 1 | 232 |
| 186 | BA | 57 | 39 | 3 | 15 | 573164 | 4585308 | 3 | 15 | 0 | 224 | 4703620 | 3 | 15 | 0 | 1 | 232 |
| 187 | BB | 57 | 39 | 3 | 16 | 573192 | 4585532 | 3 | 16 | 0 | 224 | 4703852 | 3 | 16 | 0 | 1 | 232 |
| 188 | BC | 57 | 39 | 3 | 17 | 573220 | 4585756 | 3 | 17 | 0 | 224 | 4704084 | 3 | 17 | 0 | 1 | 232 |
| 189 | BD | 57 | 39 | 3 | 18 | 573248 | 4585980 | 3 | 18 | 0 | 224 | 4704316 | 3 | 18 | 0 | 1 | 232 |
| 190 | BE | 57 | 39 | 3 | 19 | 573276 | 4586204 | 3 | 19 | 0 | 224 | 4704548 | 3 | 19 | 0 | 1 | 232 |
| 191 | BF | 57 | 39 | 3 | 20 | 573304 | 4586428 | 3 | 20 | 0 | 224 | 4704780 | 3 | 20 | 0 | 1 | 232 |
| 192 | C0 | 57 | 39 | 3 | 21 | 573329 | 4586628 | 3 | 21 | 0 | 200 | 4704988 | 3 | 21 | 0 | 1 | 208 |
| 193 | C1 | 57 | 39 | 3 | 22 | 573354 | 4586828 | 3 | 22 | 0 | 200 | 4705196 | 3 | 22 | 0 | 1 | 208 |
| 194 | C2 | 57 | 39 | 3 | 23 | 573379 | 4587028 | 3 | 23 | 0 | 200 | 4705404 | 3 | 23 | 0 | 1 | 208 |
| 195 | C3 | 57 | 39 | 3 | 24 | 573404 | 4587228 | 3 | 24 | 0 | 200 | 4705612 | 3 | 24 | 0 | 1 | 208 |
| 196 | C4 | 57 | 39 | 3 | 25 | 573429 | 4587428 | 3 | 25 | 0 | 200 | 4705820 | 3 | 25 | 0 | 1 | 208 |
| 197 | C5 | 57 | 39 | 3 | 26 | 573454 | 4587628 | 3 | 26 | 0 | 200 | 4706028 | 3 | 26 | 0 | 1 | 208 |
| 198 | C6 | 57 | 39 | 3 | 27 | 573479 | 4587828 | 3 | 27 | 0 | 200 | 4706236 | 3 | 27 | 0 | 1 | 208 |
| 199 | C7 | 57 | 39 | 3 | 28 | 573504 | 4588028 | 3 | 28 | 0 | 200 | 4706444 | 3 | 28 | 0 | 1 | 208 |
| 200 | C8 | 57 | 39 | 3 | 29 | 573529 | 4588228 | 3 | 29 | 0 | 200 | 4706652 | 3 | 29 | 0 | 1 | 208 |
| 201 | C9 | 57 | 39 | 3 | 30 | 573554 | 4588428 | 3 | 30 | 0 | 200 | 4706860 | 3 | 30 | 0 | 1 | 208 |
| 202 | CA | 57 | 39 | 3 | 31 | 573579 | 4588628 | 3 | 31 | 0 | 200 | 4707068 | 3 | 31 | 0 | 1 | 208 |
| 203 | CB | 57 | 39 | 3 | 32 | 573604 | 4588828 | 3 | 32 | 0 | 200 | 4707276 | 3 | 32 | 0 | 1 | 208 |
| 204 | CC | 57 | 39 | 3 | 33 | 573629 | 4589028 | 3 | 33 | 0 | 200 | 4707484 | 3 | 33 | 0 | 1 | 208 |
| 205 | CD | 57 | 39 | 3 | 34 | 573654 | 4589228 | 3 | 34 | 0 | 200 | 4707692 | 3 | 34 | 0 | 1 | 208 |
| 206 | CE | 57 | 39 | 3 | 35 | 573679 | 4589428 | 3 | 35 | 0 | 200 | 4707900 | 3 | 35 | 0 | 1 | 208 |
| 207 | CF | 57 | 39 | 3 | 36 | 573704 | 4589628 | 3 | 36 | 0 | 200 | 4708108 | 3 | 36 | 0 | 1 | 208 |
| 208 | D0 | 57 | 39 | 3 | 37 | 573731 | 4589844 | 3 | 37 | 0 | 216 | 4708332 | 3 | 37 | 0 | 1 | 224 |
| 209 | D1 | 57 | 39 | 3 | 38 | 573758 | 4590060 | 3 | 38 | 0 | 216 | 4708556 | 3 | 38 | 0 | 1 | 224 |
| 210 | D2 | 57 | 39 | 3 | 39 | 573785 | 4590276 | 3 | 39 | 0 | 216 | 4708780 | 3 | 39 | 0 | 1 | 224 |
| 211 | D3 | 57 | 39 | 3 | 40 | 573812 | 4590492 | 3 | 40 | 0 | 216 | 4709004 | 3 | 40 | 0 | 1 | 224 |
| 212 | D4 | 57 | 39 | 3 | 41 | 573839 | 4590708 | 3 | 41 | 0 | 216 | 4709228 | 3 | 41 | 0 | 1 | 224 |
| 213 | D5 | 57 | 39 | 3 | 42 | 573866 | 4590924 | 3 | 42 | 0 | 216 | 4709452 | 3 | 42 | 0 | 1 | 224 |
| 214 | D6 | 57 | 39 | 3 | 43 | 573893 | 4591140 | 3 | 43 | 0 | 216 | 4709676 | 3 | 43 | 0 | 1 | 224 |
| 215 | D7 | 57 | 39 | 3 | 44 | 573920 | 4591356 | 3 | 44 | 0 | 216 | 4709900 | 3 | 44 | 0 | 1 | 224 |
| 216 | D8 | 57 | 39 | 3 | 45 | 573947 | 4591572 | 3 | 45 | 0 | 216 | 4710124 | 3 | 45 | 0 | 1 | 224 |
| 217 | D9 | 57 | 39 | 3 | 46 | 573974 | 4591788 | 3 | 46 | 0 | 216 | 4710348 | 3 | 46 | 0 | 1 | 224 |
| 218 | DA | 57 | 39 | 3 | 47 | 574001 | 4592004 | 3 | 47 | 0 | 216 | 4710572 | 3 | 47 | 0 | 1 | 224 |
| 219 | DB | 57 | 39 | 3 | 48 | 574028 | 4592220 | 3 | 48 | 0 | 216 | 4710796 | 3 | 48 | 0 | 1 | 224 |
| 220 | DC | 57 | 39 | 3 | 49 | 574055 | 4592436 | 3 | 49 | 0 | 216 | 4711020 | 3 | 49 | 0 | 1 | 224 |
| 221 | DD | 57 | 39 | 3 | 50 | 574082 | 4592652 | 3 | 50 | 0 | 216 | 4711244 | 3 | 50 | 0 | 1 | 224 |
| 222 | DE | 57 | 39 | 3 | 51 | 574109 | 4592868 | 3 | 51 | 0 | 216 | 4711468 | 3 | 51 | 0 | 1 | 224 |
| 223 | DF | 57 | 39 | 3 | 52 | 574136 | 4593084 | 3 | 52 | 0 | 216 | 4711692 | 3 | 52 | 0 | 1 | 224 |
| 224 | E0 | 57 | 39 | 3 | 53 | 574163 | 4593300 | 3 | 53 | 0 | 216 | 4711916 | 3 | 53 | 0 | 1 | 224 |
| 225 | E1 | 57 | 39 | 3 | 54 | 574190 | 4593516 | 3 | 54 | 0 | 216 | 4712140 | 3 | 54 | 0 | 1 | 224 |
| 226 | E2 | 57 | 39 | 3 | 55 | 574217 | 4593732 | 3 | 55 | 0 | 216 | 4712364 | 3 | 55 | 0 | 1 | 224 |
| 227 | E3 | 57 | 39 | 3 | 56 | 574244 | 4593948 | 3 | 56 | 0 | 216 | 4712588 | 3 | 56 | 0 | 1 | 224 |
| 228 | E4 | 57 | 39 | 4 | 0 | 574269 | 4594148 | 4 | 0 | 0 | 200 | 4712796 | 4 | 0 | 0 | 1 | 208 |
| 229 | E5 | 57 | 39 | 4 | 1 | 574299 | 4594388 | 4 | 1 | 0 | 240 | 4713044 | 4 | 1 | 0 | 1 | 248 |
| 230 | E6 | 57 | 39 | 4 | 2 | 574329 | 4594628 | 4 | 2 | 0 | 240 | 4713292 | 4 | 2 | 0 | 1 | 248 |
| 231 | E7 | 57 | 39 | 4 | 3 | 574359 | 4594868 | 4 | 3 | 0 | 240 | 4713540 | 4 | 3 | 0 | 1 | 248 |
| 232 | E8 | 57 | 39 | 4 | 4 | 574389 | 4595108 | 4 | 4 | 0 | 240 | 4713788 | 4 | 4 | 0 | 1 | 248 |
| 233 | E9 | 57 | 39 | 4 | 5 | 574419 | 4595348 | 4 | 5 | 0 | 240 | 4714036 | 4 | 5 | 0 | 1 | 248 |
| 234 | EA | 57 | 39 | 4 | 6 | 574449 | 4595588 | 4 | 6 | 0 | 240 | 4714284 | 4 | 6 | 0 | 1 | 248 |
| 235 | EB | 57 | 39 | 4 | 7 | 574479 | 4595828 | 4 | 7 | 0 | 240 | 4714532 | 4 | 7 | 0 | 1 | 248 |
| 236 | EC | 57 | 39 | 4 | 8 | 574509 | 4596068 | 4 | 8 | 0 | 240 | 4714780 | 4 | 8 | 0 | 1 | 248 |

| | | | | | | | | | | | | | | | | |
|---|---|---|---|---|---|---|---|---|---|---|---|---|---|---|---|---|
| 237 | ED | 57 | 39 | 4 | 9 | 574539 | 4596308 | 4 | 9 | 0 | 240 | 4715028 | 4 | 9 | 0 | 1 | 248 |
| 238 | EE | 57 | 39 | 4 | 10 | 574569 | 4596548 | 4 | 10 | 0 | 240 | 4715276 | 4 | 10 | 0 | 1 | 248 |
| 239 | EF | 57 | 39 | 4 | 11 | 574599 | 4596788 | 4 | 11 | 0 | 240 | 4715524 | 4 | 11 | 0 | 1 | 248 |
| 240 | F0 | 57 | 39 | 4 | 12 | 574629 | 4597028 | 4 | 12 | 0 | 240 | 4715772 | 4 | 12 | 0 | 1 | 248 |
| 241 | F1 | 57 | 39 | 4 | 13 | 574659 | 4597268 | 4 | 13 | 0 | 240 | 4716020 | 4 | 13 | 0 | 1 | 248 |
| 242 | F2 | 57 | 39 | 4 | 14 | 574689 | 4597508 | 4 | 14 | 0 | 240 | 4716268 | 4 | 14 | 0 | 1 | 248 |
| 243 | F3 | 57 | 39 | 4 | 15 | 574719 | 4597748 | 4 | 15 | 0 | 240 | 4716516 | 4 | 15 | 0 | 1 | 248 |
| 244 | F4 | 57 | 39 | 4 | 16 | 574749 | 4597988 | 4 | 16 | 0 | 240 | 4716764 | 4 | 16 | 0 | 1 | 248 |
| 245 | F5 | 57 | 39 | 4 | 17 | 574779 | 4598228 | 4 | 17 | 0 | 240 | 4717012 | 4 | 17 | 0 | 1 | 248 |
| 246 | F6 | 57 | 39 | 4 | 18 | 574809 | 4598468 | 4 | 18 | 0 | 240 | 4717260 | 4 | 18 | 0 | 1 | 248 |
| 247 | F7 | 57 | 39 | 4 | 19 | 574839 | 4598708 | 4 | 19 | 0 | 240 | 4717508 | 4 | 19 | 0 | 1 | 248 |
| 248 | F8 | 57 | 39 | 4 | 20 | 574869 | 4598948 | 4 | 20 | 0 | 240 | 4717756 | 4 | 20 | 0 | 1 | 248 |
| 249 | F9 | 57 | 39 | 4 | 21 | 574899 | 4599188 | 4 | 21 | 0 | 240 | 4718004 | 4 | 21 | 0 | 1 | 248 |
| 250 | FA | 57 | 39 | 4 | 22 | 574929 | 4599428 | 4 | 22 | 0 | 240 | 4718252 | 4 | 22 | 0 | 1 | 248 |
| 251 | FB | 57 | 39 | 4 | 23 | 574959 | 4599668 | 4 | 23 | 0 | 240 | 4718500 | 4 | 23 | 0 | 1 | 248 |
| 252 | FC | 57 | 39 | 4 | 24 | 574989 | 4599908 | 4 | 24 | 0 | 240 | 4718748 | 4 | 24 | 0 | 1 | 248 |
| 253 | FD | 57 | 39 | 4 | 25 | 575019 | 4600148 | 4 | 25 | 0 | 240 | 4718996 | 4 | 25 | 0 | 1 | 248 |
| 254 | FE | 57 | 39 | 4 | 26 | 575049 | 4600388 | 4 | 26 | 0 | 240 | 4719244 | 4 | 26 | 0 | 1 | 248 |
| 255 | FF | 57 | 39 | 4 | 27 | 575079 | 4600628 | 4 | 27 | 0 | 240 | 4719492 | 4 | 27 | 0 | 1 | 248 |
| 0 | 00 | 58 | 3A | 0 | 0 | 575091 | 4600724 | 0 | 0 | 0 | 96 | 4719596 | 0 | 0 | 0 | 1 | 104 |
| 1 | 01 | 58 | 3A | 0 | 1 | 575105 | 4600836 | 0 | 1 | 0 | 112 | 4719716 | 0 | 1 | 0 | 1 | 120 |
| 2 | 02 | 58 | 3A | 0 | 2 | 575119 | 4600948 | 0 | 2 | 0 | 112 | 4719836 | 0 | 2 | 0 | 1 | 120 |
| 3 | 03 | 58 | 3A | 0 | 3 | 575133 | 4601060 | 0 | 3 | 0 | 112 | 4719956 | 0 | 3 | 0 | 1 | 120 |
| 4 | 04 | 58 | 3A | 0 | 4 | 575147 | 4601172 | 0 | 4 | 0 | 112 | 4720076 | 0 | 4 | 0 | 1 | 120 |
| 5 | 05 | 58 | 3A | 0 | 5 | 575161 | 4601284 | 0 | 5 | 0 | 112 | 4720196 | 0 | 5 | 0 | 1 | 120 |
| 6 | 06 | 58 | 3A | 0 | 6 | 575175 | 4601396 | 0 | 6 | 0 | 112 | 4720316 | 0 | 6 | 0 | 1 | 120 |
| 7 | 07 | 58 | 3A | 0 | 7 | 575189 | 4601508 | 0 | 7 | 0 | 112 | 4720436 | 0 | 7 | 0 | 1 | 120 |
| 8 | 08 | 58 | 3A | 0 | 8 | 575203 | 4601620 | 0 | 8 | 0 | 112 | 4720556 | 0 | 8 | 0 | 1 | 120 |
| 9 | 09 | 58 | 3A | 0 | 9 | 575217 | 4601732 | 0 | 9 | 0 | 112 | 4720676 | 0 | 9 | 0 | 1 | 120 |
| 10 | 0A | 58 | 3A | 0 | 10 | 575231 | 4601844 | 0 | 10 | 0 | 112 | 4720796 | 0 | 10 | 0 | 1 | 120 |
| 11 | 0B | 58 | 3A | 0 | 11 | 575245 | 4601956 | 0 | 11 | 0 | 112 | 4720916 | 0 | 11 | 0 | 1 | 120 |
| 12 | 0C | 58 | 3A | 0 | 12 | 575259 | 4602068 | 0 | 12 | 0 | 112 | 4721036 | 0 | 12 | 0 | 1 | 120 |
| 13 | 0D | 58 | 3A | 0 | 13 | 575273 | 4602180 | 0 | 13 | 0 | 112 | 4721156 | 0 | 13 | 0 | 1 | 120 |
| 14 | 0E | 58 | 3A | 0 | 14 | 575287 | 4602292 | 0 | 14 | 0 | 112 | 4721276 | 0 | 14 | 0 | 1 | 120 |
| 15 | 0F | 58 | 3A | 0 | 15 | 575301 | 4602404 | 0 | 15 | 0 | 112 | 4721396 | 0 | 15 | 0 | 1 | 120 |
| 16 | 10 | 58 | 3A | 0 | 16 | 575315 | 4602516 | 0 | 16 | 0 | 112 | 4721516 | 0 | 16 | 0 | 1 | 120 |
| 17 | 11 | 58 | 3A | 0 | 17 | 575329 | 4602628 | 0 | 17 | 0 | 112 | 4721636 | 0 | 17 | 0 | 1 | 120 |
| 18 | 12 | 58 | 3A | 0 | 18 | 575343 | 4602740 | 0 | 18 | 0 | 112 | 4721756 | 0 | 18 | 0 | 1 | 120 |
| 19 | 13 | 58 | 3A | 0 | 19 | 575357 | 4602852 | 0 | 19 | 0 | 112 | 4721876 | 0 | 19 | 0 | 1 | 120 |
| 20 | 14 | 58 | 3A | 0 | 20 | 575371 | 4602964 | 0 | 20 | 0 | 112 | 4721996 | 0 | 20 | 0 | 1 | 120 |
| 21 | 15 | 58 | 3A | 0 | 21 | 575385 | 4603076 | 0 | 21 | 0 | 112 | 4722116 | 0 | 21 | 0 | 1 | 120 |
| 22 | 16 | 58 | 3A | 0 | 22 | 575399 | 4603188 | 0 | 22 | 0 | 112 | 4722236 | 0 | 22 | 0 | 1 | 120 |
| 23 | 17 | 58 | 3A | 0 | 23 | 575413 | 4603300 | 0 | 23 | 0 | 112 | 4722356 | 0 | 23 | 0 | 1 | 120 |
| 24 | 18 | 58 | 3A | 0 | 24 | 575427 | 4603412 | 0 | 24 | 0 | 112 | 4722476 | 0 | 24 | 0 | 1 | 120 |
| 25 | 19 | 58 | 3A | 0 | 25 | 575441 | 4603524 | 0 | 25 | 0 | 112 | 4722596 | 0 | 25 | 0 | 1 | 120 |
| 26 | 1A | 58 | 3A | 0 | 26 | 575455 | 4603636 | 0 | 26 | 0 | 112 | 4722716 | 0 | 26 | 0 | 1 | 120 |
| 27 | 1B | 58 | 3A | 0 | 27 | 575469 | 4603748 | 0 | 27 | 0 | 112 | 4722836 | 0 | 27 | 0 | 1 | 120 |
| 28 | 1C | 58 | 3A | 0 | 28 | 575483 | 4603860 | 0 | 28 | 0 | 112 | 4722956 | 0 | 28 | 0 | 1 | 120 |
| 29 | 1D | 58 | 3A | 0 | 29 | 575497 | 4603972 | 0 | 29 | 0 | 112 | 4723076 | 0 | 29 | 0 | 1 | 120 |
| 30 | 1E | 58 | 3A | 0 | 30 | 575511 | 4604084 | 0 | 30 | 0 | 112 | 4723196 | 0 | 30 | 0 | 1 | 120 |
| 31 | 1F | 58 | 3A | 0 | 31 | 575525 | 4604196 | 0 | 31 | 0 | 112 | 4723316 | 0 | 31 | 0 | 1 | 120 |
| 32 | 20 | 58 | 3A | 0 | 32 | 575539 | 4604308 | 0 | 32 | 0 | 112 | 4723436 | 0 | 32 | 0 | 1 | 120 |
| 33 | 21 | 58 | 3A | 0 | 33 | 575553 | 4604420 | 0 | 33 | 0 | 112 | 4723556 | 0 | 33 | 0 | 1 | 120 |
| 34 | 22 | 58 | 3A | 0 | 34 | 575567 | 4604532 | 0 | 34 | 0 | 112 | 4723676 | 0 | 34 | 0 | 1 | 120 |
| 35 | 23 | 58 | 3A | 0 | 35 | 575581 | 4604644 | 0 | 35 | 0 | 112 | 4723796 | 0 | 35 | 0 | 1 | 120 |
| 36 | 24 | 58 | 3A | 0 | 36 | 575595 | 4604756 | 0 | 36 | 0 | 112 | 4723916 | 0 | 36 | 0 | 1 | 120 |
| 37 | 25 | 58 | 3A | 0 | 37 | 575609 | 4604868 | 0 | 37 | 0 | 112 | 4724036 | 0 | 37 | 0 | 1 | 120 |
| 38 | 26 | 58 | 3A | 0 | 38 | 575623 | 4604980 | 0 | 38 | 0 | 112 | 4724156 | 0 | 38 | 0 | 1 | 120 |
| 39 | 27 | 58 | 3A | 0 | 39 | 575637 | 4605092 | 0 | 39 | 0 | 112 | 4724276 | 0 | 39 | 0 | 1 | 120 |
| 40 | 28 | 58 | 3A | 0 | 40 | 575651 | 4605204 | 0 | 40 | 0 | 112 | 4724396 | 0 | 40 | 0 | 1 | 120 |
| 41 | 29 | 58 | 3A | 0 | 41 | 575665 | 4605316 | 0 | 41 | 0 | 112 | 4724516 | 0 | 41 | 0 | 1 | 120 |
| 42 | 2A | 58 | 3A | 0 | 42 | 575679 | 4605428 | 0 | 42 | 0 | 112 | 4724636 | 0 | 42 | 0 | 1 | 120 |
| 43 | 2B | 58 | 3A | 0 | 43 | 575693 | 4605540 | 0 | 43 | 0 | 112 | 4724756 | 0 | 43 | 0 | 1 | 120 |
| 44 | 2C | 58 | 3A | 0 | 44 | 575707 | 4605652 | 0 | 44 | 0 | 112 | 4724876 | 0 | 44 | 0 | 1 | 120 |
| 45 | 2D | 58 | 3A | 0 | 45 | 575721 | 4605764 | 0 | 45 | 0 | 112 | 4724996 | 0 | 45 | 0 | 1 | 120 |
| 46 | 2E | 58 | 3A | 0 | 46 | 575735 | 4605876 | 0 | 46 | 0 | 112 | 4725116 | 0 | 46 | 0 | 1 | 120 |
| 47 | 2F | 58 | 3A | 0 | 47 | 575749 | 4605988 | 0 | 47 | 0 | 112 | 4725236 | 0 | 47 | 0 | 1 | 120 |
| 48 | 30 | 58 | 3A | 0 | 48 | 575763 | 4606100 | 0 | 48 | 0 | 112 | 4725356 | 0 | 48 | 0 | 1 | 120 |
| 49 | 31 | 58 | 3A | 0 | 49 | 575777 | 4606212 | 0 | 49 | 0 | 112 | 4725476 | 0 | 49 | 0 | 1 | 120 |
| 50 | 32 | 58 | 3A | 0 | 50 | 575791 | 4606324 | 0 | 50 | 0 | 112 | 4725596 | 0 | 50 | 0 | 1 | 120 |
| 51 | 33 | 58 | 3A | 0 | 51 | 575805 | 4606436 | 0 | 51 | 0 | 112 | 4725716 | 0 | 51 | 0 | 1 | 120 |
| 52 | 34 | 58 | 3A | 0 | 52 | 575819 | 4606548 | 0 | 52 | 0 | 112 | 4725836 | 0 | 52 | 0 | 1 | 120 |
| 53 | 35 | 58 | 3A | 0 | 53 | 575833 | 4606660 | 0 | 53 | 0 | 112 | 4725956 | 0 | 53 | 0 | 1 | 120 |
| 54 | 36 | 58 | 3A | 0 | 54 | 575847 | 4606772 | 0 | 54 | 0 | 112 | 4726076 | 0 | 54 | 0 | 1 | 120 |
| 55 | 37 | 58 | 3A | 0 | 55 | 575861 | 4606884 | 0 | 55 | 0 | 112 | 4726196 | 0 | 55 | 0 | 1 | 120 |
| 56 | 38 | 58 | 3A | 0 | 56 | 575875 | 4606996 | 0 | 56 | 0 | 112 | 4726316 | 0 | 56 | 0 | 1 | 120 |
| 57 | 39 | 58 | 3A | 0 | 57 | 575889 | 4607108 | 0 | 57 | 0 | 112 | 4726436 | 0 | 57 | 0 | 1 | 120 |
| 58 | 3A | 58 | 3A | 1 | 0 | 575901 | 4607204 | 1 | 0 | 0 | 96 | 4726540 | 1 | 0 | 0 | 1 | 104 |
| 59 | 3B | 58 | 3A | 1 | 1 | 575918 | 4607340 | 1 | 1 | 0 | 136 | 4726684 | 1 | 1 | 0 | 1 | 144 |
| 60 | 3C | 58 | 3A | 1 | 2 | 575935 | 4607476 | 1 | 2 | 0 | 136 | 4726828 | 1 | 2 | 0 | 1 | 144 |
| 61 | 3D | 58 | 3A | 1 | 3 | 575952 | 4607612 | 1 | 3 | 0 | 136 | 4726972 | 1 | 3 | 0 | 1 | 144 |
| 62 | 3E | 58 | 3A | 1 | 4 | 575969 | 4607748 | 1 | 4 | 0 | 136 | 4727116 | 1 | 4 | 0 | 1 | 144 |
| 63 | 3F | 58 | 3A | 1 | 5 | 575986 | 4607884 | 1 | 5 | 0 | 136 | 4727260 | 1 | 5 | 0 | 1 | 144 |
| 64 | 40 | 58 | 3A | 1 | 6 | 576007 | 4608052 | 1 | 6 | 0 | 168 | 4727436 | 1 | 6 | 0 | 1 | 176 |
| 65 | 41 | 58 | 3A | 1 | 7 | 576028 | 4608220 | 1 | 7 | 0 | 168 | 4727612 | 1 | 7 | 0 | 1 | 176 |
| 66 | 42 | 58 | 3A | 1 | 8 | 576049 | 4608388 | 1 | 8 | 0 | 168 | 4727788 | 1 | 8 | 0 | 1 | 176 |
| 67 | 43 | 58 | 3A | 1 | 9 | 576070 | 4608556 | 1 | 9 | 0 | 168 | 4727964 | 1 | 9 | 0 | 1 | 176 |
| 68 | 44 | 58 | 3A | 1 | 10 | 576091 | 4608724 | 1 | 10 | 0 | 168 | 4728140 | 1 | 10 | 0 | 1 | 176 |
| 69 | 45 | 58 | 3A | 1 | 11 | 576112 | 4608892 | 1 | 11 | 0 | 168 | 4728316 | 1 | 11 | 0 | 1 | 176 |
| 70 | 46 | 58 | 3A | 1 | 12 | 576133 | 4609060 | 1 | 12 | 0 | 168 | 4728492 | 1 | 12 | 0 | 1 | 176 |
| 71 | 47 | 58 | 3A | 1 | 13 | 576154 | 4609228 | 1 | 13 | 0 | 168 | 4728668 | 1 | 13 | 0 | 1 | 176 |
| 72 | 48 | 58 | 3A | 1 | 14 | 576175 | 4609396 | 1 | 14 | 0 | 168 | 4728844 | 1 | 14 | 0 | 1 | 176 |
| 73 | 49 | 58 | 3A | 1 | 15 | 576196 | 4609564 | 1 | 15 | 0 | 168 | 4729020 | 1 | 15 | 0 | 1 | 176 |
| 74 | 4A | 58 | 3A | 1 | 16 | 576217 | 4609732 | 1 | 16 | 0 | 168 | 4729196 | 1 | 16 | 0 | 1 | 176 |
| 75 | 4B | 58 | 3A | 1 | 17 | 576238 | 4609900 | 1 | 17 | 0 | 168 | 4729372 | 1 | 17 | 0 | 1 | 176 |
| 76 | 4C | 58 | 3A | 1 | 18 | 576259 | 4610068 | 1 | 18 | 0 | 168 | 4729548 | 1 | 18 | 0 | 1 | 176 |
| 77 | 4D | 58 | 3A | 1 | 19 | 576280 | 4610236 | 1 | 19 | 0 | 168 | 4729724 | 1 | 19 | 0 | 1 | 176 |
| 78 | 4E | 58 | 3A | 1 | 20 | 576301 | 4610404 | 1 | 20 | 0 | 168 | 4729900 | 1 | 20 | 0 | 1 | 176 |
| 79 | 4F | 58 | 3A | 1 | 21 | 576322 | 4610572 | 1 | 21 | 0 | 168 | 4730076 | 1 | 21 | 0 | 1 | 176 |
| 80 | 50 | 58 | 3A | 1 | 22 | 576345 | 4610756 | 1 | 22 | 0 | 184 | 4730268 | 1 | 22 | 0 | 1 | 192 |
| 81 | 51 | 58 | 3A | 1 | 23 | 576368 | 4610940 | 1 | 23 | 0 | 184 | 4730460 | 1 | 23 | 0 | 1 | 192 |
| 82 | 52 | 58 | 3A | 1 | 24 | 576391 | 4611124 | 1 | 24 | 0 | 184 | 4730652 | 1 | 24 | 0 | 1 | 192 |
| 83 | 53 | 58 | 3A | 1 | 25 | 576414 | 4611308 | 1 | 25 | 0 | 184 | 4730844 | 1 | 25 | 0 | 1 | 192 |

| | | | | | | | | | | | | | | | | | |
|---|---|---|---|---|---|---|---|---|---|---|---|---|---|---|---|---|---|
| 84 | 54 | 58 | 3A | 1 | 26 | 576437 | 4611492 | 1 | 26 | 0 | 184 | 4731036 | 1 | 26 | 0 | 1 | 192 |
| 85 | 55 | 58 | 3A | 1 | 27 | 576460 | 4611676 | 1 | 27 | 0 | 184 | 4731228 | 1 | 27 | 0 | 1 | 192 |
| 86 | 56 | 58 | 3A | 1 | 28 | 576483 | 4611860 | 1 | 28 | 0 | 184 | 4731420 | 1 | 28 | 0 | 1 | 192 |
| 87 | 57 | 58 | 3A | 1 | 29 | 576506 | 4612044 | 1 | 29 | 0 | 184 | 4731612 | 1 | 29 | 0 | 1 | 192 |
| 88 | 58 | 58 | 3A | 1 | 30 | 576529 | 4612228 | 1 | 30 | 0 | 184 | 4731804 | 1 | 30 | 0 | 1 | 192 |
| 89 | 59 | 58 | 3A | 1 | 31 | 576552 | 4612412 | 1 | 31 | 0 | 184 | 4731996 | 1 | 31 | 0 | 1 | 192 |
| 90 | 5A | 58 | 3A | 1 | 32 | 576575 | 4612596 | 1 | 32 | 0 | 184 | 4732188 | 1 | 32 | 0 | 1 | 192 |
| 91 | 5B | 58 | 3A | 1 | 33 | 576598 | 4612780 | 1 | 33 | 0 | 184 | 4732380 | 1 | 33 | 0 | 1 | 192 |
| 92 | 5C | 58 | 3A | 1 | 34 | 576621 | 4612964 | 1 | 34 | 0 | 184 | 4732572 | 1 | 34 | 0 | 1 | 192 |
| 93 | 5D | 58 | 3A | 1 | 35 | 576644 | 4613148 | 1 | 35 | 0 | 184 | 4732764 | 1 | 35 | 0 | 1 | 192 |
| 94 | 5E | 58 | 3A | 1 | 36 | 576667 | 4613332 | 1 | 36 | 0 | 184 | 4732956 | 1 | 36 | 0 | 1 | 192 |
| 95 | 5F | 58 | 3A | 1 | 37 | 576690 | 4613516 | 1 | 37 | 0 | 184 | 4733148 | 1 | 37 | 0 | 1 | 192 |
| 96 | 60 | 58 | 3A | 1 | 38 | 576713 | 4613700 | 1 | 38 | 0 | 184 | 4733340 | 1 | 38 | 0 | 1 | 192 |
| 97 | 61 | 58 | 3A | 1 | 39 | 576736 | 4613884 | 1 | 39 | 0 | 184 | 4733532 | 1 | 39 | 0 | 1 | 192 |
| 98 | 62 | 58 | 3A | 1 | 40 | 576759 | 4614068 | 1 | 40 | 0 | 184 | 4733724 | 1 | 40 | 0 | 1 | 192 |
| 99 | 63 | 58 | 3A | 1 | 41 | 576782 | 4614252 | 1 | 41 | 0 | 184 | 4733916 | 1 | 41 | 0 | 1 | 192 |
| 100 | 64 | 58 | 3A | 1 | 42 | 576805 | 4614436 | 1 | 42 | 0 | 184 | 4734108 | 1 | 42 | 0 | 1 | 192 |
| 101 | 65 | 58 | 3A | 1 | 43 | 576828 | 4614620 | 1 | 43 | 0 | 184 | 4734300 | 1 | 43 | 0 | 1 | 192 |
| 102 | 66 | 58 | 3A | 1 | 44 | 576851 | 4614804 | 1 | 44 | 0 | 184 | 4734492 | 1 | 44 | 0 | 1 | 192 |
| 103 | 67 | 58 | 3A | 1 | 45 | 576874 | 4614988 | 1 | 45 | 0 | 184 | 4734684 | 1 | 45 | 0 | 1 | 192 |
| 104 | 68 | 58 | 3A | 1 | 46 | 576897 | 4615172 | 1 | 46 | 0 | 184 | 4734876 | 1 | 46 | 0 | 1 | 192 |
| 105 | 69 | 58 | 3A | 1 | 47 | 576920 | 4615356 | 1 | 47 | 0 | 184 | 4735068 | 1 | 47 | 0 | 1 | 192 |
| 106 | 6A | 58 | 3A | 1 | 48 | 576943 | 4615540 | 1 | 48 | 0 | 184 | 4735260 | 1 | 48 | 0 | 1 | 192 |
| 107 | 6B | 58 | 3A | 1 | 49 | 576966 | 4615724 | 1 | 49 | 0 | 184 | 4735452 | 1 | 49 | 0 | 1 | 192 |
| 108 | 6C | 58 | 3A | 1 | 50 | 576989 | 4615908 | 1 | 50 | 0 | 184 | 4735644 | 1 | 50 | 0 | 1 | 192 |
| 109 | 6D | 58 | 3A | 1 | 51 | 577012 | 4616092 | 1 | 51 | 0 | 184 | 4735836 | 1 | 51 | 0 | 1 | 192 |
| 110 | 6E | 58 | 3A | 1 | 52 | 577035 | 4616276 | 1 | 52 | 0 | 184 | 4736028 | 1 | 52 | 0 | 1 | 192 |
| 111 | 6F | 58 | 3A | 1 | 53 | 577058 | 4616460 | 1 | 53 | 0 | 184 | 4736220 | 1 | 53 | 0 | 1 | 192 |
| 112 | 70 | 58 | 3A | 1 | 54 | 577081 | 4616644 | 1 | 54 | 0 | 184 | 4736412 | 1 | 54 | 0 | 1 | 192 |
| 113 | 71 | 58 | 3A | 1 | 55 | 577104 | 4616828 | 1 | 55 | 0 | 184 | 4736604 | 1 | 55 | 0 | 1 | 192 |
| 114 | 72 | 58 | 3A | 1 | 56 | 577127 | 4617012 | 1 | 56 | 0 | 184 | 4736796 | 1 | 56 | 0 | 1 | 192 |
| 115 | 73 | 58 | 3A | 1 | 57 | 577150 | 4617196 | 1 | 57 | 0 | 184 | 4736988 | 1 | 57 | 0 | 1 | 192 |
| 116 | 74 | 58 | 3A | 2 | 0 | 577171 | 4617364 | 2 | 0 | 0 | 168 | 4737164 | 2 | 0 | 0 | 1 | 176 |
| 117 | 75 | 58 | 3A | 2 | 1 | 577197 | 4617572 | 2 | 1 | 0 | 208 | 4737380 | 2 | 1 | 0 | 1 | 216 |
| 118 | 76 | 58 | 3A | 2 | 2 | 577223 | 4617780 | 2 | 2 | 0 | 208 | 4737596 | 2 | 2 | 0 | 1 | 216 |
| 119 | 77 | 58 | 3A | 2 | 3 | 577249 | 4617988 | 2 | 3 | 0 | 208 | 4737812 | 2 | 3 | 0 | 1 | 216 |
| 120 | 78 | 58 | 3A | 2 | 4 | 577275 | 4618196 | 2 | 4 | 0 | 208 | 4738028 | 2 | 4 | 0 | 1 | 216 |
| 121 | 79 | 58 | 3A | 2 | 5 | 577301 | 4618404 | 2 | 5 | 0 | 208 | 4738244 | 2 | 5 | 0 | 1 | 216 |
| 122 | 7A | 58 | 3A | 2 | 6 | 577327 | 4618612 | 2 | 6 | 0 | 208 | 4738460 | 2 | 6 | 0 | 1 | 216 |
| 123 | 7B | 58 | 3A | 2 | 7 | 577353 | 4618820 | 2 | 7 | 0 | 208 | 4738676 | 2 | 7 | 0 | 1 | 216 |
| 124 | 7C | 58 | 3A | 2 | 8 | 577379 | 4619028 | 2 | 8 | 0 | 208 | 4738892 | 2 | 8 | 0 | 1 | 216 |
| 125 | 7D | 58 | 3A | 2 | 9 | 577405 | 4619236 | 2 | 9 | 0 | 208 | 4739108 | 2 | 9 | 0 | 1 | 216 |
| 126 | 7E | 58 | 3A | 2 | 10 | 577431 | 4619444 | 2 | 10 | 0 | 208 | 4739324 | 2 | 10 | 0 | 1 | 216 |
| 127 | 7F | 58 | 3A | 2 | 11 | 577457 | 4619652 | 2 | 11 | 0 | 208 | 4739540 | 2 | 11 | 0 | 1 | 216 |
| 128 | 80 | 58 | 3A | 2 | 12 | 577480 | 4619836 | 2 | 12 | 0 | 184 | 4739732 | 2 | 12 | 0 | 1 | 192 |
| 129 | 81 | 58 | 3A | 2 | 13 | 577503 | 4620020 | 2 | 13 | 0 | 184 | 4739924 | 2 | 13 | 0 | 1 | 192 |
| 130 | 82 | 58 | 3A | 2 | 14 | 577526 | 4620204 | 2 | 14 | 0 | 184 | 4740116 | 2 | 14 | 0 | 1 | 192 |
| 131 | 83 | 58 | 3A | 2 | 15 | 577549 | 4620388 | 2 | 15 | 0 | 184 | 4740308 | 2 | 15 | 0 | 1 | 192 |
| 132 | 84 | 58 | 3A | 2 | 16 | 577572 | 4620572 | 2 | 16 | 0 | 184 | 4740500 | 2 | 16 | 0 | 1 | 192 |
| 133 | 85 | 58 | 3A | 2 | 17 | 577595 | 4620756 | 2 | 17 | 0 | 184 | 4740692 | 2 | 17 | 0 | 1 | 192 |
| 134 | 86 | 58 | 3A | 2 | 18 | 577618 | 4620940 | 2 | 18 | 0 | 184 | 4740884 | 2 | 18 | 0 | 1 | 192 |
| 135 | 87 | 58 | 3A | 2 | 19 | 577641 | 4621124 | 2 | 19 | 0 | 184 | 4741076 | 2 | 19 | 0 | 1 | 192 |
| 136 | 88 | 58 | 3A | 2 | 20 | 577664 | 4621308 | 2 | 20 | 0 | 184 | 4741268 | 2 | 20 | 0 | 1 | 192 |
| 137 | 89 | 58 | 3A | 2 | 21 | 577687 | 4621492 | 2 | 21 | 0 | 184 | 4741460 | 2 | 21 | 0 | 1 | 192 |
| 138 | 8A | 58 | 3A | 2 | 22 | 577710 | 4621676 | 2 | 22 | 0 | 184 | 4741652 | 2 | 22 | 0 | 1 | 192 |
| 139 | 8B | 58 | 3A | 2 | 23 | 577733 | 4621860 | 2 | 23 | 0 | 184 | 4741844 | 2 | 23 | 0 | 1 | 192 |
| 140 | 8C | 58 | 3A | 2 | 24 | 577756 | 4622044 | 2 | 24 | 0 | 184 | 4742036 | 2 | 24 | 0 | 1 | 192 |
| 141 | 8D | 58 | 3A | 2 | 25 | 577779 | 4622228 | 2 | 25 | 0 | 184 | 4742228 | 2 | 25 | 0 | 1 | 192 |
| 142 | 8E | 58 | 3A | 2 | 26 | 577802 | 4622412 | 2 | 26 | 0 | 184 | 4742420 | 2 | 26 | 0 | 1 | 192 |
| 143 | 8F | 58 | 3A | 2 | 27 | 577825 | 4622596 | 2 | 27 | 0 | 184 | 4742612 | 2 | 27 | 0 | 1 | 192 |
| 144 | 90 | 58 | 3A | 2 | 28 | 577850 | 4622796 | 2 | 28 | 0 | 200 | 4742820 | 2 | 28 | 0 | 1 | 208 |
| 145 | 91 | 58 | 3A | 2 | 29 | 577875 | 4622996 | 2 | 29 | 0 | 200 | 4743028 | 2 | 29 | 0 | 1 | 208 |
| 146 | 92 | 58 | 3A | 2 | 30 | 577900 | 4623196 | 2 | 30 | 0 | 200 | 4743236 | 2 | 30 | 0 | 1 | 208 |
| 147 | 93 | 58 | 3A | 2 | 31 | 577925 | 4623396 | 2 | 31 | 0 | 200 | 4743444 | 2 | 31 | 0 | 1 | 208 |
| 148 | 94 | 58 | 3A | 2 | 32 | 577950 | 4623596 | 2 | 32 | 0 | 200 | 4743652 | 2 | 32 | 0 | 1 | 208 |
| 149 | 95 | 58 | 3A | 2 | 33 | 577975 | 4623796 | 2 | 33 | 0 | 200 | 4743860 | 2 | 33 | 0 | 1 | 208 |
| 150 | 96 | 58 | 3A | 2 | 34 | 578000 | 4623996 | 2 | 34 | 0 | 200 | 4744068 | 2 | 34 | 0 | 1 | 208 |
| 151 | 97 | 58 | 3A | 2 | 35 | 578025 | 4624196 | 2 | 35 | 0 | 200 | 4744276 | 2 | 35 | 0 | 1 | 208 |
| 152 | 98 | 58 | 3A | 2 | 36 | 578050 | 4624396 | 2 | 36 | 0 | 200 | 4744484 | 2 | 36 | 0 | 1 | 208 |
| 153 | 99 | 58 | 3A | 2 | 37 | 578075 | 4624596 | 2 | 37 | 0 | 200 | 4744692 | 2 | 37 | 0 | 1 | 208 |
| 154 | 9A | 58 | 3A | 2 | 38 | 578100 | 4624796 | 2 | 38 | 0 | 200 | 4744900 | 2 | 38 | 0 | 1 | 208 |
| 155 | 9B | 58 | 3A | 2 | 39 | 578125 | 4624996 | 2 | 39 | 0 | 200 | 4745108 | 2 | 39 | 0 | 1 | 208 |
| 156 | 9C | 58 | 3A | 2 | 40 | 578150 | 4625196 | 2 | 40 | 0 | 200 | 4745316 | 2 | 40 | 0 | 1 | 208 |
| 157 | 9D | 58 | 3A | 2 | 41 | 578175 | 4625396 | 2 | 41 | 0 | 200 | 4745524 | 2 | 41 | 0 | 1 | 208 |
| 158 | 9E | 58 | 3A | 2 | 42 | 578200 | 4625596 | 2 | 42 | 0 | 200 | 4745732 | 2 | 42 | 0 | 1 | 208 |
| 159 | 9F | 58 | 3A | 2 | 43 | 578225 | 4625796 | 2 | 43 | 0 | 200 | 4745940 | 2 | 43 | 0 | 1 | 208 |
| 160 | A0 | 58 | 3A | 2 | 44 | 578250 | 4625996 | 2 | 44 | 0 | 200 | 4746148 | 2 | 44 | 0 | 1 | 208 |
| 161 | A1 | 58 | 3A | 2 | 45 | 578275 | 4626196 | 2 | 45 | 0 | 200 | 4746356 | 2 | 45 | 0 | 1 | 208 |
| 162 | A2 | 58 | 3A | 2 | 46 | 578300 | 4626396 | 2 | 46 | 0 | 200 | 4746564 | 2 | 46 | 0 | 1 | 208 |
| 163 | A3 | 58 | 3A | 2 | 47 | 578325 | 4626596 | 2 | 47 | 0 | 200 | 4746772 | 2 | 47 | 0 | 1 | 208 |
| 164 | A4 | 58 | 3A | 2 | 48 | 578350 | 4626796 | 2 | 48 | 0 | 200 | 4746980 | 2 | 48 | 0 | 1 | 208 |
| 165 | A5 | 58 | 3A | 2 | 49 | 578375 | 4626996 | 2 | 49 | 0 | 200 | 4747188 | 2 | 49 | 0 | 1 | 208 |
| 166 | A6 | 58 | 3A | 2 | 50 | 578400 | 4627196 | 2 | 50 | 0 | 200 | 4747396 | 2 | 50 | 0 | 1 | 208 |
| 167 | A7 | 58 | 3A | 2 | 51 | 578425 | 4627396 | 2 | 51 | 0 | 200 | 4747604 | 2 | 51 | 0 | 1 | 208 |
| 168 | A8 | 58 | 3A | 2 | 52 | 578450 | 4627596 | 2 | 52 | 0 | 200 | 4747812 | 2 | 52 | 0 | 1 | 208 |
| 169 | A9 | 58 | 3A | 2 | 53 | 578475 | 4627796 | 2 | 53 | 0 | 200 | 4748020 | 2 | 53 | 0 | 1 | 208 |
| 170 | AA | 58 | 3A | 2 | 54 | 578500 | 4627996 | 2 | 54 | 0 | 200 | 4748228 | 2 | 54 | 0 | 1 | 208 |
| 171 | AB | 58 | 3A | 2 | 55 | 578525 | 4628196 | 2 | 55 | 0 | 200 | 4748436 | 2 | 55 | 0 | 1 | 208 |
| 172 | AC | 58 | 3A | 2 | 56 | 578550 | 4628396 | 2 | 56 | 0 | 200 | 4748644 | 2 | 56 | 0 | 1 | 208 |
| 173 | AD | 58 | 3A | 2 | 57 | 578575 | 4628596 | 2 | 57 | 0 | 200 | 4748852 | 2 | 57 | 0 | 1 | 208 |
| 174 | AE | 58 | 3A | 3 | 0 | 578598 | 4628780 | 3 | 0 | 0 | 184 | 4749044 | 3 | 0 | 0 | 1 | 192 |
| 175 | AF | 58 | 3A | 3 | 1 | 578626 | 4629004 | 3 | 1 | 0 | 224 | 4749276 | 3 | 1 | 0 | 1 | 232 |
| 176 | B0 | 58 | 3A | 3 | 2 | 578654 | 4629228 | 3 | 2 | 0 | 224 | 4749508 | 3 | 2 | 0 | 1 | 232 |
| 177 | B1 | 58 | 3A | 3 | 3 | 578682 | 4629452 | 3 | 3 | 0 | 224 | 4749740 | 3 | 3 | 0 | 1 | 232 |
| 178 | B2 | 58 | 3A | 3 | 4 | 578710 | 4629676 | 3 | 4 | 0 | 224 | 4749972 | 3 | 4 | 0 | 1 | 232 |
| 179 | B3 | 58 | 3A | 3 | 5 | 578738 | 4629900 | 3 | 5 | 0 | 224 | 4750204 | 3 | 5 | 0 | 1 | 232 |
| 180 | B4 | 58 | 3A | 3 | 6 | 578766 | 4630124 | 3 | 6 | 0 | 224 | 4750436 | 3 | 6 | 0 | 1 | 232 |
| 181 | B5 | 58 | 3A | 3 | 7 | 578794 | 4630348 | 3 | 7 | 0 | 224 | 4750668 | 3 | 7 | 0 | 1 | 232 |
| 182 | B6 | 58 | 3A | 3 | 8 | 578822 | 4630572 | 3 | 8 | 0 | 224 | 4750900 | 3 | 8 | 0 | 1 | 232 |
| 183 | B7 | 58 | 3A | 3 | 9 | 578850 | 4630796 | 3 | 9 | 0 | 224 | 4751132 | 3 | 9 | 0 | 1 | 232 |
| 184 | B8 | 58 | 3A | 3 | 10 | 578878 | 4631020 | 3 | 10 | 0 | 224 | 4751364 | 3 | 10 | 0 | 1 | 232 |
| 185 | B9 | 58 | 3A | 3 | 11 | 578906 | 4631244 | 3 | 11 | 0 | 224 | 4751596 | 3 | 11 | 0 | 1 | 232 |
| 186 | BA | 58 | 3A | 3 | 12 | 578934 | 4631468 | 3 | 12 | 0 | 224 | 4751828 | 3 | 12 | 0 | 1 | 232 |

| | | | | | | | | | | | | | | | | | |
|---|---|---|---|---|---|---|---|---|---|---|---|---|---|---|---|---|---|
| 187 | BB | 58 | 3A | 3 | 13 | 578962 | 4631692 | 3 | 13 | 0 | 224 | 4752060 | 3 | 13 | 0 | 1 | 232 |
| 188 | BC | 58 | 3A | 3 | 14 | 578990 | 4631916 | 3 | 14 | 0 | 224 | 4752292 | 3 | 14 | 0 | 1 | 232 |
| 189 | BD | 58 | 3A | 3 | 15 | 579018 | 4632140 | 3 | 15 | 0 | 224 | 4752524 | 3 | 15 | 0 | 1 | 232 |
| 190 | BE | 58 | 3A | 3 | 16 | 579046 | 4632364 | 3 | 16 | 0 | 224 | 4752756 | 3 | 16 | 0 | 1 | 232 |
| 191 | BF | 58 | 3A | 3 | 17 | 579074 | 4632588 | 3 | 17 | 0 | 224 | 4752988 | 3 | 17 | 0 | 1 | 232 |
| 192 | C0 | 58 | 3A | 3 | 18 | 579099 | 4632788 | 3 | 18 | 0 | 200 | 4753196 | 3 | 18 | 0 | 1 | 208 |
| 193 | C1 | 58 | 3A | 3 | 19 | 579124 | 4632988 | 3 | 19 | 0 | 200 | 4753404 | 3 | 19 | 0 | 1 | 208 |
| 194 | C2 | 58 | 3A | 3 | 20 | 579149 | 4633188 | 3 | 20 | 0 | 200 | 4753612 | 3 | 20 | 0 | 1 | 208 |
| 195 | C3 | 58 | 3A | 3 | 21 | 579174 | 4633388 | 3 | 21 | 0 | 200 | 4753820 | 3 | 21 | 0 | 1 | 208 |
| 196 | C4 | 58 | 3A | 3 | 22 | 579199 | 4633588 | 3 | 22 | 0 | 200 | 4754028 | 3 | 22 | 0 | 1 | 208 |
| 197 | C5 | 58 | 3A | 3 | 23 | 579224 | 4633788 | 3 | 23 | 0 | 200 | 4754236 | 3 | 23 | 0 | 1 | 208 |
| 198 | C6 | 58 | 3A | 3 | 24 | 579249 | 4633988 | 3 | 24 | 0 | 200 | 4754444 | 3 | 24 | 0 | 1 | 208 |
| 199 | C7 | 58 | 3A | 3 | 25 | 579274 | 4634188 | 3 | 25 | 0 | 200 | 4754652 | 3 | 25 | 0 | 1 | 208 |
| 200 | C8 | 58 | 3A | 3 | 26 | 579299 | 4634388 | 3 | 26 | 0 | 200 | 4754860 | 3 | 26 | 0 | 1 | 208 |
| 201 | C9 | 58 | 3A | 3 | 27 | 579324 | 4634588 | 3 | 27 | 0 | 200 | 4755068 | 3 | 27 | 0 | 1 | 208 |
| 202 | CA | 58 | 3A | 3 | 28 | 579349 | 4634788 | 3 | 28 | 0 | 200 | 4755276 | 3 | 28 | 0 | 1 | 208 |
| 203 | CB | 58 | 3A | 3 | 29 | 579374 | 4634988 | 3 | 29 | 0 | 200 | 4755484 | 3 | 29 | 0 | 1 | 208 |
| 204 | CC | 58 | 3A | 3 | 30 | 579399 | 4635188 | 3 | 30 | 0 | 200 | 4755692 | 3 | 30 | 0 | 1 | 208 |
| 205 | CD | 58 | 3A | 3 | 31 | 579424 | 4635388 | 3 | 31 | 0 | 200 | 4755900 | 3 | 31 | 0 | 1 | 208 |
| 206 | CE | 58 | 3A | 3 | 32 | 579449 | 4635588 | 3 | 32 | 0 | 200 | 4756108 | 3 | 32 | 0 | 1 | 208 |
| 207 | CF | 58 | 3A | 3 | 33 | 579474 | 4635788 | 3 | 33 | 0 | 200 | 4756316 | 3 | 33 | 0 | 1 | 208 |
| 208 | D0 | 58 | 3A | 3 | 34 | 579501 | 4636004 | 3 | 34 | 0 | 216 | 4756540 | 3 | 34 | 0 | 1 | 224 |
| 209 | D1 | 58 | 3A | 3 | 35 | 579528 | 4636220 | 3 | 35 | 0 | 216 | 4756764 | 3 | 35 | 0 | 1 | 224 |
| 210 | D2 | 58 | 3A | 3 | 36 | 579555 | 4636436 | 3 | 36 | 0 | 216 | 4756988 | 3 | 36 | 0 | 1 | 224 |
| 211 | D3 | 58 | 3A | 3 | 37 | 579582 | 4636652 | 3 | 37 | 0 | 216 | 4757212 | 3 | 37 | 0 | 1 | 224 |
| 212 | D4 | 58 | 3A | 3 | 38 | 579609 | 4636868 | 3 | 38 | 0 | 216 | 4757436 | 3 | 38 | 0 | 1 | 224 |
| 213 | D5 | 58 | 3A | 3 | 39 | 579636 | 4637084 | 3 | 39 | 0 | 216 | 4757660 | 3 | 39 | 0 | 1 | 224 |
| 214 | D6 | 58 | 3A | 3 | 40 | 579663 | 4637300 | 3 | 40 | 0 | 216 | 4757884 | 3 | 40 | 0 | 1 | 224 |
| 215 | D7 | 58 | 3A | 3 | 41 | 579690 | 4637516 | 3 | 41 | 0 | 216 | 4758108 | 3 | 41 | 0 | 1 | 224 |
| 216 | D8 | 58 | 3A | 3 | 42 | 579717 | 4637732 | 3 | 42 | 0 | 216 | 4758332 | 3 | 42 | 0 | 1 | 224 |
| 217 | D9 | 58 | 3A | 3 | 43 | 579744 | 4637948 | 3 | 43 | 0 | 216 | 4758556 | 3 | 43 | 0 | 1 | 224 |
| 218 | DA | 58 | 3A | 3 | 44 | 579771 | 4638164 | 3 | 44 | 0 | 216 | 4758780 | 3 | 44 | 0 | 1 | 224 |
| 219 | DB | 58 | 3A | 3 | 45 | 579798 | 4638380 | 3 | 45 | 0 | 216 | 4759004 | 3 | 45 | 0 | 1 | 224 |
| 220 | DC | 58 | 3A | 3 | 46 | 579825 | 4638596 | 3 | 46 | 0 | 216 | 4759228 | 3 | 46 | 0 | 1 | 224 |
| 221 | DD | 58 | 3A | 3 | 47 | 579852 | 4638812 | 3 | 47 | 0 | 216 | 4759452 | 3 | 47 | 0 | 1 | 224 |
| 222 | DE | 58 | 3A | 3 | 48 | 579879 | 4639028 | 3 | 48 | 0 | 216 | 4759676 | 3 | 48 | 0 | 1 | 224 |
| 223 | DF | 58 | 3A | 3 | 49 | 579906 | 4639244 | 3 | 49 | 0 | 216 | 4759900 | 3 | 49 | 0 | 1 | 224 |
| 224 | E0 | 58 | 3A | 3 | 50 | 579933 | 4639460 | 3 | 50 | 0 | 216 | 4760124 | 3 | 50 | 0 | 1 | 224 |
| 225 | E1 | 58 | 3A | 3 | 51 | 579960 | 4639676 | 3 | 51 | 0 | 216 | 4760348 | 3 | 51 | 0 | 1 | 224 |
| 226 | E2 | 58 | 3A | 3 | 52 | 579987 | 4639892 | 3 | 52 | 0 | 216 | 4760572 | 3 | 52 | 0 | 1 | 224 |
| 227 | E3 | 58 | 3A | 3 | 53 | 580014 | 4640108 | 3 | 53 | 0 | 216 | 4760796 | 3 | 53 | 0 | 1 | 224 |
| 228 | E4 | 58 | 3A | 3 | 54 | 580041 | 4640324 | 3 | 54 | 0 | 216 | 4761020 | 3 | 54 | 0 | 1 | 224 |
| 229 | E5 | 58 | 3A | 3 | 55 | 580068 | 4640540 | 3 | 55 | 0 | 216 | 4761244 | 3 | 55 | 0 | 1 | 224 |
| 230 | E6 | 58 | 3A | 3 | 56 | 580095 | 4640756 | 3 | 56 | 0 | 216 | 4761468 | 3 | 56 | 0 | 1 | 224 |
| 231 | E7 | 58 | 3A | 3 | 57 | 580122 | 4640972 | 3 | 57 | 0 | 216 | 4761692 | 3 | 57 | 0 | 1 | 224 |
| 232 | E8 | 58 | 3A | 4 | 0 | 580147 | 4641172 | 4 | 0 | 0 | 200 | 4761900 | 4 | 0 | 0 | 1 | 208 |
| 233 | E9 | 58 | 3A | 4 | 1 | 580177 | 4641412 | 4 | 1 | 0 | 240 | 4762148 | 4 | 1 | 0 | 1 | 248 |
| 234 | EA | 58 | 3A | 4 | 2 | 580207 | 4641652 | 4 | 2 | 0 | 240 | 4762396 | 4 | 2 | 0 | 1 | 248 |
| 235 | EB | 58 | 3A | 4 | 3 | 580237 | 4641892 | 4 | 3 | 0 | 240 | 4762644 | 4 | 3 | 0 | 1 | 248 |
| 236 | EC | 58 | 3A | 4 | 4 | 580267 | 4642132 | 4 | 4 | 0 | 240 | 4762892 | 4 | 4 | 0 | 1 | 248 |
| 237 | ED | 58 | 3A | 4 | 5 | 580297 | 4642372 | 4 | 5 | 0 | 240 | 4763140 | 4 | 5 | 0 | 1 | 248 |
| 238 | EE | 58 | 3A | 4 | 6 | 580327 | 4642612 | 4 | 6 | 0 | 240 | 4763388 | 4 | 6 | 0 | 1 | 248 |
| 239 | EF | 58 | 3A | 4 | 7 | 580357 | 4642852 | 4 | 7 | 0 | 240 | 4763636 | 4 | 7 | 0 | 1 | 248 |
| 240 | F0 | 58 | 3A | 4 | 8 | 580387 | 4643092 | 4 | 8 | 0 | 240 | 4763884 | 4 | 8 | 0 | 1 | 248 |
| 241 | F1 | 58 | 3A | 4 | 9 | 580417 | 4643332 | 4 | 9 | 0 | 240 | 4764132 | 4 | 9 | 0 | 1 | 248 |
| 242 | F2 | 58 | 3A | 4 | 10 | 580447 | 4643572 | 4 | 10 | 0 | 240 | 4764380 | 4 | 10 | 0 | 1 | 248 |
| 243 | F3 | 58 | 3A | 4 | 11 | 580477 | 4643812 | 4 | 11 | 0 | 240 | 4764628 | 4 | 11 | 0 | 1 | 248 |
| 244 | F4 | 58 | 3A | 4 | 12 | 580507 | 4644052 | 4 | 12 | 0 | 240 | 4764876 | 4 | 12 | 0 | 1 | 248 |
| 245 | F5 | 58 | 3A | 4 | 13 | 580537 | 4644292 | 4 | 13 | 0 | 240 | 4765124 | 4 | 13 | 0 | 1 | 248 |
| 246 | F6 | 58 | 3A | 4 | 14 | 580567 | 4644532 | 4 | 14 | 0 | 240 | 4765372 | 4 | 14 | 0 | 1 | 248 |
| 247 | F7 | 58 | 3A | 4 | 15 | 580597 | 4644772 | 4 | 15 | 0 | 240 | 4765620 | 4 | 15 | 0 | 1 | 248 |
| 248 | F8 | 58 | 3A | 4 | 16 | 580627 | 4645012 | 4 | 16 | 0 | 240 | 4765868 | 4 | 16 | 0 | 1 | 248 |
| 249 | F9 | 58 | 3A | 4 | 17 | 580657 | 4645252 | 4 | 17 | 0 | 240 | 4766116 | 4 | 17 | 0 | 1 | 248 |
| 250 | FA | 58 | 3A | 4 | 18 | 580687 | 4645492 | 4 | 18 | 0 | 240 | 4766364 | 4 | 18 | 0 | 1 | 248 |
| 251 | FB | 58 | 3A | 4 | 19 | 580717 | 4645732 | 4 | 19 | 0 | 240 | 4766612 | 4 | 19 | 0 | 1 | 248 |
| 252 | FC | 58 | 3A | 4 | 20 | 580747 | 4645972 | 4 | 20 | 0 | 240 | 4766860 | 4 | 20 | 0 | 1 | 248 |
| 253 | FD | 58 | 3A | 4 | 21 | 580777 | 4646212 | 4 | 21 | 0 | 240 | 4767108 | 4 | 21 | 0 | 1 | 248 |
| 254 | FE | 58 | 3A | 4 | 22 | 580807 | 4646452 | 4 | 22 | 0 | 240 | 4767356 | 4 | 22 | 0 | 1 | 248 |
| 255 | FF | 58 | 3A | 4 | 23 | 580837 | 4646692 | 4 | 23 | 0 | 240 | 4767604 | 4 | 23 | 0 | 1 | 248 |
| 0 | 00 | 59 | 3B | 0 | 0 | 580849 | 4646788 | 0 | 0 | 0 | 96 | 4767708 | 0 | 0 | 0 | 1 | 104 |
| 1 | 01 | 59 | 3B | 0 | 1 | 580863 | 4646900 | 0 | 1 | 0 | 112 | 4767828 | 0 | 1 | 0 | 1 | 120 |
| 2 | 02 | 59 | 3B | 0 | 2 | 580877 | 4647012 | 0 | 2 | 0 | 112 | 4767948 | 0 | 2 | 0 | 1 | 120 |
| 3 | 03 | 59 | 3B | 0 | 3 | 580891 | 4647124 | 0 | 3 | 0 | 112 | 4768068 | 0 | 3 | 0 | 1 | 120 |
| 4 | 04 | 59 | 3B | 0 | 4 | 580905 | 4647236 | 0 | 4 | 0 | 112 | 4768188 | 0 | 4 | 0 | 1 | 120 |
| 5 | 05 | 59 | 3B | 0 | 5 | 580919 | 4647348 | 0 | 5 | 0 | 112 | 4768308 | 0 | 5 | 0 | 1 | 120 |
| 6 | 06 | 59 | 3B | 0 | 6 | 580933 | 4647460 | 0 | 6 | 0 | 112 | 4768428 | 0 | 6 | 0 | 1 | 120 |
| 7 | 07 | 59 | 3B | 0 | 7 | 580947 | 4647572 | 0 | 7 | 0 | 112 | 4768548 | 0 | 7 | 0 | 1 | 120 |
| 8 | 08 | 59 | 3B | 0 | 8 | 580961 | 4647684 | 0 | 8 | 0 | 112 | 4768668 | 0 | 8 | 0 | 1 | 120 |
| 9 | 09 | 59 | 3B | 0 | 9 | 580975 | 4647796 | 0 | 9 | 0 | 112 | 4768788 | 0 | 9 | 0 | 1 | 120 |
| 10 | 0A | 59 | 3B | 0 | 10 | 580989 | 4647908 | 0 | 10 | 0 | 112 | 4768908 | 0 | 10 | 0 | 1 | 120 |
| 11 | 0B | 59 | 3B | 0 | 11 | 581003 | 4648020 | 0 | 11 | 0 | 112 | 4769028 | 0 | 11 | 0 | 1 | 120 |
| 12 | 0C | 59 | 3B | 0 | 12 | 581017 | 4648132 | 0 | 12 | 0 | 112 | 4769148 | 0 | 12 | 0 | 1 | 120 |
| 13 | 0D | 59 | 3B | 0 | 13 | 581031 | 4648244 | 0 | 13 | 0 | 112 | 4769268 | 0 | 13 | 0 | 1 | 120 |
| 14 | 0E | 59 | 3B | 0 | 14 | 581045 | 4648356 | 0 | 14 | 0 | 112 | 4769388 | 0 | 14 | 0 | 1 | 120 |
| 15 | 0F | 59 | 3B | 0 | 15 | 581059 | 4648468 | 0 | 15 | 0 | 112 | 4769508 | 0 | 15 | 0 | 1 | 120 |
| 16 | 10 | 59 | 3B | 0 | 16 | 581073 | 4648580 | 0 | 16 | 0 | 112 | 4769628 | 0 | 16 | 0 | 1 | 120 |
| 17 | 11 | 59 | 3B | 0 | 17 | 581087 | 4648692 | 0 | 17 | 0 | 112 | 4769748 | 0 | 17 | 0 | 1 | 120 |
| 18 | 12 | 59 | 3B | 0 | 18 | 581101 | 4648804 | 0 | 18 | 0 | 112 | 4769868 | 0 | 18 | 0 | 1 | 120 |
| 19 | 13 | 59 | 3B | 0 | 19 | 581115 | 4648916 | 0 | 19 | 0 | 112 | 4769988 | 0 | 19 | 0 | 1 | 120 |
| 20 | 14 | 59 | 3B | 0 | 20 | 581129 | 4649028 | 0 | 20 | 0 | 112 | 4770108 | 0 | 20 | 0 | 1 | 120 |
| 21 | 15 | 59 | 3B | 0 | 21 | 581143 | 4649140 | 0 | 21 | 0 | 112 | 4770228 | 0 | 21 | 0 | 1 | 120 |
| 22 | 16 | 59 | 3B | 0 | 22 | 581157 | 4649252 | 0 | 22 | 0 | 112 | 4770348 | 0 | 22 | 0 | 1 | 120 |
| 23 | 17 | 59 | 3B | 0 | 23 | 581171 | 4649364 | 0 | 23 | 0 | 112 | 4770468 | 0 | 23 | 0 | 1 | 120 |
| 24 | 18 | 59 | 3B | 0 | 24 | 581185 | 4649476 | 0 | 24 | 0 | 112 | 4770588 | 0 | 24 | 0 | 1 | 120 |
| 25 | 19 | 59 | 3B | 0 | 25 | 581199 | 4649588 | 0 | 25 | 0 | 112 | 4770708 | 0 | 25 | 0 | 1 | 120 |
| 26 | 1A | 59 | 3B | 0 | 26 | 581213 | 4649700 | 0 | 26 | 0 | 112 | 4770828 | 0 | 26 | 0 | 1 | 120 |
| 27 | 1B | 59 | 3B | 0 | 27 | 581227 | 4649812 | 0 | 27 | 0 | 112 | 4770948 | 0 | 27 | 0 | 1 | 120 |
| 28 | 1C | 59 | 3B | 0 | 28 | 581241 | 4649924 | 0 | 28 | 0 | 112 | 4771068 | 0 | 28 | 0 | 1 | 120 |
| 29 | 1D | 59 | 3B | 0 | 29 | 581255 | 4650036 | 0 | 29 | 0 | 112 | 4771188 | 0 | 29 | 0 | 1 | 120 |
| 30 | 1E | 59 | 3B | 0 | 30 | 581269 | 4650148 | 0 | 30 | 0 | 112 | 4771308 | 0 | 30 | 0 | 1 | 120 |
| 31 | 1F | 59 | 3B | 0 | 31 | 581283 | 4650260 | 0 | 31 | 0 | 112 | 4771428 | 0 | 31 | 0 | 1 | 120 |
| 32 | 20 | 59 | 3B | 0 | 32 | 581297 | 4650372 | 0 | 32 | 0 | 112 | 4771548 | 0 | 32 | 0 | 1 | 120 |
| 33 | 21 | 59 | 3B | 0 | 33 | 581311 | 4650484 | 0 | 33 | 0 | 112 | 4771668 | 0 | 33 | 0 | 1 | 120 |

| | | | | | | | | | | | | | | | | | |
|---|---|---|---|---|---|---|---|---|---|---|---|---|---|---|---|---|---|
| 34 | 22 | 59 | 3B | 0 | 34 | 581325 | 4650596 | 0 | 34 | 0 | 112 | 4771788 | 0 | 34 | 0 | 1 | 120 |
| 35 | 23 | 59 | 3B | 0 | 35 | 581339 | 4650708 | 0 | 35 | 0 | 112 | 4771908 | 0 | 35 | 0 | 1 | 120 |
| 36 | 24 | 59 | 3B | 0 | 36 | 581353 | 4650820 | 0 | 36 | 0 | 112 | 4772028 | 0 | 36 | 0 | 1 | 120 |
| 37 | 25 | 59 | 3B | 0 | 37 | 581367 | 4650932 | 0 | 37 | 0 | 112 | 4772148 | 0 | 37 | 0 | 1 | 120 |
| 38 | 26 | 59 | 3B | 0 | 38 | 581381 | 4651044 | 0 | 38 | 0 | 112 | 4772268 | 0 | 38 | 0 | 1 | 120 |
| 39 | 27 | 59 | 3B | 0 | 39 | 581395 | 4651156 | 0 | 39 | 0 | 112 | 4772388 | 0 | 39 | 0 | 1 | 120 |
| 40 | 28 | 59 | 3B | 0 | 40 | 581409 | 4651268 | 0 | 40 | 0 | 112 | 4772508 | 0 | 40 | 0 | 1 | 120 |
| 41 | 29 | 59 | 3B | 0 | 41 | 581423 | 4651380 | 0 | 41 | 0 | 112 | 4772628 | 0 | 41 | 0 | 1 | 120 |
| 42 | 2A | 59 | 3B | 0 | 42 | 581437 | 4651492 | 0 | 42 | 0 | 112 | 4772748 | 0 | 42 | 0 | 1 | 120 |
| 43 | 2B | 59 | 3B | 0 | 43 | 581451 | 4651604 | 0 | 43 | 0 | 112 | 4772868 | 0 | 43 | 0 | 1 | 120 |
| 44 | 2C | 59 | 3B | 0 | 44 | 581465 | 4651716 | 0 | 44 | 0 | 112 | 4772988 | 0 | 44 | 0 | 1 | 120 |
| 45 | 2D | 59 | 3B | 0 | 45 | 581479 | 4651828 | 0 | 45 | 0 | 112 | 4773108 | 0 | 45 | 0 | 1 | 120 |
| 46 | 2E | 59 | 3B | 0 | 46 | 581493 | 4651940 | 0 | 46 | 0 | 112 | 4773228 | 0 | 46 | 0 | 1 | 120 |
| 47 | 2F | 59 | 3B | 0 | 47 | 581507 | 4652052 | 0 | 47 | 0 | 112 | 4773348 | 0 | 47 | 0 | 1 | 120 |
| 48 | 30 | 59 | 3B | 0 | 48 | 581521 | 4652164 | 0 | 48 | 0 | 112 | 4773468 | 0 | 48 | 0 | 1 | 120 |
| 49 | 31 | 59 | 3B | 0 | 49 | 581535 | 4652276 | 0 | 49 | 0 | 112 | 4773588 | 0 | 49 | 0 | 1 | 120 |
| 50 | 32 | 59 | 3B | 0 | 50 | 581549 | 4652388 | 0 | 50 | 0 | 112 | 4773708 | 0 | 50 | 0 | 1 | 120 |
| 51 | 33 | 59 | 3B | 0 | 51 | 581563 | 4652500 | 0 | 51 | 0 | 112 | 4773828 | 0 | 51 | 0 | 1 | 120 |
| 52 | 34 | 59 | 3B | 0 | 52 | 581577 | 4652612 | 0 | 52 | 0 | 112 | 4773948 | 0 | 52 | 0 | 1 | 120 |
| 53 | 35 | 59 | 3B | 0 | 53 | 581591 | 4652724 | 0 | 53 | 0 | 112 | 4774068 | 0 | 53 | 0 | 1 | 120 |
| 54 | 36 | 59 | 3B | 0 | 54 | 581605 | 4652836 | 0 | 54 | 0 | 112 | 4774188 | 0 | 54 | 0 | 1 | 120 |
| 55 | 37 | 59 | 3B | 0 | 55 | 581619 | 4652948 | 0 | 55 | 0 | 112 | 4774308 | 0 | 55 | 0 | 1 | 120 |
| 56 | 38 | 59 | 3B | 0 | 56 | 581633 | 4653060 | 0 | 56 | 0 | 112 | 4774428 | 0 | 56 | 0 | 1 | 120 |
| 57 | 39 | 59 | 3B | 0 | 57 | 581647 | 4653172 | 0 | 57 | 0 | 112 | 4774548 | 0 | 57 | 0 | 1 | 120 |
| 58 | 3A | 59 | 3B | 0 | 58 | 581661 | 4653284 | 0 | 58 | 0 | 112 | 4774668 | 0 | 58 | 0 | 1 | 120 |
| 59 | 3B | 59 | 3B | 1 | 0 | 581673 | 4653380 | 1 | 0 | 0 | 96 | 4774772 | 1 | 0 | 0 | 1 | 104 |
| 60 | 3C | 59 | 3B | 1 | 1 | 581690 | 4653516 | 1 | 1 | 0 | 136 | 4774916 | 1 | 1 | 0 | 1 | 144 |
| 61 | 3D | 59 | 3B | 1 | 2 | 581707 | 4653652 | 1 | 2 | 0 | 136 | 4775060 | 1 | 2 | 0 | 1 | 144 |
| 62 | 3E | 59 | 3B | 1 | 3 | 581724 | 4653788 | 1 | 3 | 0 | 136 | 4775204 | 1 | 3 | 0 | 1 | 144 |
| 63 | 3F | 59 | 3B | 1 | 4 | 581741 | 4653924 | 1 | 4 | 0 | 136 | 4775348 | 1 | 4 | 0 | 1 | 144 |
| 64 | 40 | 59 | 3B | 1 | 5 | 581762 | 4654092 | 1 | 5 | 0 | 168 | 4775524 | 1 | 5 | 0 | 1 | 176 |
| 65 | 41 | 59 | 3B | 1 | 6 | 581783 | 4654260 | 1 | 6 | 0 | 168 | 4775700 | 1 | 6 | 0 | 1 | 176 |
| 66 | 42 | 59 | 3B | 1 | 7 | 581804 | 4654428 | 1 | 7 | 0 | 168 | 4775876 | 1 | 7 | 0 | 1 | 176 |
| 67 | 43 | 59 | 3B | 1 | 8 | 581825 | 4654596 | 1 | 8 | 0 | 168 | 4776052 | 1 | 8 | 0 | 1 | 176 |
| 68 | 44 | 59 | 3B | 1 | 9 | 581846 | 4654764 | 1 | 9 | 0 | 168 | 4776228 | 1 | 9 | 0 | 1 | 176 |
| 69 | 45 | 59 | 3B | 1 | 10 | 581867 | 4654932 | 1 | 10 | 0 | 168 | 4776404 | 1 | 10 | 0 | 1 | 176 |
| 70 | 46 | 59 | 3B | 1 | 11 | 581888 | 4655100 | 1 | 11 | 0 | 168 | 4776580 | 1 | 11 | 0 | 1 | 176 |
| 71 | 47 | 59 | 3B | 1 | 12 | 581909 | 4655268 | 1 | 12 | 0 | 168 | 4776756 | 1 | 12 | 0 | 1 | 176 |
| 72 | 48 | 59 | 3B | 1 | 13 | 581930 | 4655436 | 1 | 13 | 0 | 168 | 4776932 | 1 | 13 | 0 | 1 | 176 |
| 73 | 49 | 59 | 3B | 1 | 14 | 581951 | 4655604 | 1 | 14 | 0 | 168 | 4777108 | 1 | 14 | 0 | 1 | 176 |
| 74 | 4A | 59 | 3B | 1 | 15 | 581972 | 4655772 | 1 | 15 | 0 | 168 | 4777284 | 1 | 15 | 0 | 1 | 176 |
| 75 | 4B | 59 | 3B | 1 | 16 | 581993 | 4655940 | 1 | 16 | 0 | 168 | 4777460 | 1 | 16 | 0 | 1 | 176 |
| 76 | 4C | 59 | 3B | 1 | 17 | 582014 | 4656108 | 1 | 17 | 0 | 168 | 4777636 | 1 | 17 | 0 | 1 | 176 |
| 77 | 4D | 59 | 3B | 1 | 18 | 582035 | 4656276 | 1 | 18 | 0 | 168 | 4777812 | 1 | 18 | 0 | 1 | 176 |
| 78 | 4E | 59 | 3B | 1 | 19 | 582056 | 4656444 | 1 | 19 | 0 | 168 | 4777988 | 1 | 19 | 0 | 1 | 176 |
| 79 | 4F | 59 | 3B | 1 | 20 | 582077 | 4656612 | 1 | 20 | 0 | 168 | 4778164 | 1 | 20 | 0 | 1 | 176 |
| 80 | 50 | 59 | 3B | 1 | 21 | 582100 | 4656796 | 1 | 21 | 0 | 184 | 4778356 | 1 | 21 | 0 | 1 | 192 |
| 81 | 51 | 59 | 3B | 1 | 22 | 582123 | 4656980 | 1 | 22 | 0 | 184 | 4778548 | 1 | 22 | 0 | 1 | 192 |
| 82 | 52 | 59 | 3B | 1 | 23 | 582146 | 4657164 | 1 | 23 | 0 | 184 | 4778740 | 1 | 23 | 0 | 1 | 192 |
| 83 | 53 | 59 | 3B | 1 | 24 | 582169 | 4657348 | 1 | 24 | 0 | 184 | 4778932 | 1 | 24 | 0 | 1 | 192 |
| 84 | 54 | 59 | 3B | 1 | 25 | 582192 | 4657532 | 1 | 25 | 0 | 184 | 4779124 | 1 | 25 | 0 | 1 | 192 |
| 85 | 55 | 59 | 3B | 1 | 26 | 582215 | 4657716 | 1 | 26 | 0 | 184 | 4779316 | 1 | 26 | 0 | 1 | 192 |
| 86 | 56 | 59 | 3B | 1 | 27 | 582238 | 4657900 | 1 | 27 | 0 | 184 | 4779508 | 1 | 27 | 0 | 1 | 192 |
| 87 | 57 | 59 | 3B | 1 | 28 | 582261 | 4658084 | 1 | 28 | 0 | 184 | 4779700 | 1 | 28 | 0 | 1 | 192 |
| 88 | 58 | 59 | 3B | 1 | 29 | 582284 | 4658268 | 1 | 29 | 0 | 184 | 4779892 | 1 | 29 | 0 | 1 | 192 |
| 89 | 59 | 59 | 3B | 1 | 30 | 582307 | 4658452 | 1 | 30 | 0 | 184 | 4780084 | 1 | 30 | 0 | 1 | 192 |
| 90 | 5A | 59 | 3B | 1 | 31 | 582330 | 4658636 | 1 | 31 | 0 | 184 | 4780276 | 1 | 31 | 0 | 1 | 192 |
| 91 | 5B | 59 | 3B | 1 | 32 | 582353 | 4658820 | 1 | 32 | 0 | 184 | 4780468 | 1 | 32 | 0 | 1 | 192 |
| 92 | 5C | 59 | 3B | 1 | 33 | 582376 | 4659004 | 1 | 33 | 0 | 184 | 4780660 | 1 | 33 | 0 | 1 | 192 |
| 93 | 5D | 59 | 3B | 1 | 34 | 582399 | 4659188 | 1 | 34 | 0 | 184 | 4780852 | 1 | 34 | 0 | 1 | 192 |
| 94 | 5E | 59 | 3B | 1 | 35 | 582422 | 4659372 | 1 | 35 | 0 | 184 | 4781044 | 1 | 35 | 0 | 1 | 192 |
| 95 | 5F | 59 | 3B | 1 | 36 | 582445 | 4659556 | 1 | 36 | 0 | 184 | 4781236 | 1 | 36 | 0 | 1 | 192 |
| 96 | 60 | 59 | 3B | 1 | 37 | 582468 | 4659740 | 1 | 37 | 0 | 184 | 4781428 | 1 | 37 | 0 | 1 | 192 |
| 97 | 61 | 59 | 3B | 1 | 38 | 582491 | 4659924 | 1 | 38 | 0 | 184 | 4781620 | 1 | 38 | 0 | 1 | 192 |
| 98 | 62 | 59 | 3B | 1 | 39 | 582514 | 4660108 | 1 | 39 | 0 | 184 | 4781812 | 1 | 39 | 0 | 1 | 192 |
| 99 | 63 | 59 | 3B | 1 | 40 | 582537 | 4660292 | 1 | 40 | 0 | 184 | 4782004 | 1 | 40 | 0 | 1 | 192 |
| 100 | 64 | 59 | 3B | 1 | 41 | 582560 | 4660476 | 1 | 41 | 0 | 184 | 4782196 | 1 | 41 | 0 | 1 | 192 |
| 101 | 65 | 59 | 3B | 1 | 42 | 582583 | 4660660 | 1 | 42 | 0 | 184 | 4782388 | 1 | 42 | 0 | 1 | 192 |
| 102 | 66 | 59 | 3B | 1 | 43 | 582606 | 4660844 | 1 | 43 | 0 | 184 | 4782580 | 1 | 43 | 0 | 1 | 192 |
| 103 | 67 | 59 | 3B | 1 | 44 | 582629 | 4661028 | 1 | 44 | 0 | 184 | 4782772 | 1 | 44 | 0 | 1 | 192 |
| 104 | 68 | 59 | 3B | 1 | 45 | 582652 | 4661212 | 1 | 45 | 0 | 184 | 4782964 | 1 | 45 | 0 | 1 | 192 |
| 105 | 69 | 59 | 3B | 1 | 46 | 582675 | 4661396 | 1 | 46 | 0 | 184 | 4783156 | 1 | 46 | 0 | 1 | 192 |
| 106 | 6A | 59 | 3B | 1 | 47 | 582698 | 4661580 | 1 | 47 | 0 | 184 | 4783348 | 1 | 47 | 0 | 1 | 192 |
| 107 | 6B | 59 | 3B | 1 | 48 | 582721 | 4661764 | 1 | 48 | 0 | 184 | 4783540 | 1 | 48 | 0 | 1 | 192 |
| 108 | 6C | 59 | 3B | 1 | 49 | 582744 | 4661948 | 1 | 49 | 0 | 184 | 4783732 | 1 | 49 | 0 | 1 | 192 |
| 109 | 6D | 59 | 3B | 1 | 50 | 582767 | 4662132 | 1 | 50 | 0 | 184 | 4783924 | 1 | 50 | 0 | 1 | 192 |
| 110 | 6E | 59 | 3B | 1 | 51 | 582790 | 4662316 | 1 | 51 | 0 | 184 | 4784116 | 1 | 51 | 0 | 1 | 192 |
| 111 | 6F | 59 | 3B | 1 | 52 | 582813 | 4662500 | 1 | 52 | 0 | 184 | 4784308 | 1 | 52 | 0 | 1 | 192 |
| 112 | 70 | 59 | 3B | 1 | 53 | 582836 | 4662684 | 1 | 53 | 0 | 184 | 4784500 | 1 | 53 | 0 | 1 | 192 |
| 113 | 71 | 59 | 3B | 1 | 54 | 582859 | 4662868 | 1 | 54 | 0 | 184 | 4784692 | 1 | 54 | 0 | 1 | 192 |
| 114 | 72 | 59 | 3B | 1 | 55 | 582882 | 4663052 | 1 | 55 | 0 | 184 | 4784884 | 1 | 55 | 0 | 1 | 192 |
| 115 | 73 | 59 | 3B | 1 | 56 | 582905 | 4663236 | 1 | 56 | 0 | 184 | 4785076 | 1 | 56 | 0 | 1 | 192 |
| 116 | 74 | 59 | 3B | 1 | 57 | 582928 | 4663420 | 1 | 57 | 0 | 184 | 4785268 | 1 | 57 | 0 | 1 | 192 |
| 117 | 75 | 59 | 3B | 1 | 58 | 582951 | 4663604 | 1 | 58 | 0 | 184 | 4785460 | 1 | 58 | 0 | 1 | 192 |
| 118 | 76 | 59 | 3B | 2 | 0 | 582972 | 4663772 | 2 | 0 | 0 | 168 | 4785636 | 2 | 0 | 0 | 1 | 176 |
| 119 | 77 | 59 | 3B | 2 | 1 | 582998 | 4663980 | 2 | 1 | 0 | 208 | 4785852 | 2 | 1 | 0 | 1 | 216 |
| 120 | 78 | 59 | 3B | 2 | 2 | 583024 | 4664188 | 2 | 2 | 0 | 208 | 4786068 | 2 | 2 | 0 | 1 | 216 |
| 121 | 79 | 59 | 3B | 2 | 3 | 583050 | 4664396 | 2 | 3 | 0 | 208 | 4786284 | 2 | 3 | 0 | 1 | 216 |
| 122 | 7A | 59 | 3B | 2 | 4 | 583076 | 4664604 | 2 | 4 | 0 | 208 | 4786500 | 2 | 4 | 0 | 1 | 216 |
| 123 | 7B | 59 | 3B | 2 | 5 | 583102 | 4664812 | 2 | 5 | 0 | 208 | 4786716 | 2 | 5 | 0 | 1 | 216 |
| 124 | 7C | 59 | 3B | 2 | 6 | 583128 | 4665020 | 2 | 6 | 0 | 208 | 4786932 | 2 | 6 | 0 | 1 | 216 |
| 125 | 7D | 59 | 3B | 2 | 7 | 583154 | 4665228 | 2 | 7 | 0 | 208 | 4787148 | 2 | 7 | 0 | 1 | 216 |
| 126 | 7E | 59 | 3B | 2 | 8 | 583180 | 4665436 | 2 | 8 | 0 | 208 | 4787364 | 2 | 8 | 0 | 1 | 216 |
| 127 | 7F | 59 | 3B | 2 | 9 | 583206 | 4665644 | 2 | 9 | 0 | 208 | 4787580 | 2 | 9 | 0 | 1 | 216 |
| 128 | 80 | 59 | 3B | 2 | 10 | 583229 | 4665828 | 2 | 10 | 0 | 184 | 4787772 | 2 | 10 | 0 | 1 | 192 |
| 129 | 81 | 59 | 3B | 2 | 11 | 583252 | 4666012 | 2 | 11 | 0 | 184 | 4787964 | 2 | 11 | 0 | 1 | 192 |
| 130 | 82 | 59 | 3B | 2 | 12 | 583275 | 4666196 | 2 | 12 | 0 | 184 | 4788156 | 2 | 12 | 0 | 1 | 192 |
| 131 | 83 | 59 | 3B | 2 | 13 | 583298 | 4666380 | 2 | 13 | 0 | 184 | 4788348 | 2 | 13 | 0 | 1 | 192 |
| 132 | 84 | 59 | 3B | 2 | 14 | 583321 | 4666564 | 2 | 14 | 0 | 184 | 4788540 | 2 | 14 | 0 | 1 | 192 |
| 133 | 85 | 59 | 3B | 2 | 15 | 583344 | 4666748 | 2 | 15 | 0 | 184 | 4788732 | 2 | 15 | 0 | 1 | 192 |
| 134 | 86 | 59 | 3B | 2 | 16 | 583367 | 4666932 | 2 | 16 | 0 | 184 | 4788924 | 2 | 16 | 0 | 1 | 192 |
| 135 | 87 | 59 | 3B | 2 | 17 | 583390 | 4667116 | 2 | 17 | 0 | 184 | 4789116 | 2 | 17 | 0 | 1 | 192 |
| 136 | 88 | 59 | 3B | 2 | 18 | 583413 | 4667300 | 2 | 18 | 0 | 184 | 4789308 | 2 | 18 | 0 | 1 | 192 |

| | | | | | | | | | | | | | | | | | |
|---|---|---|---|---|---|---|---|---|---|---|---|---|---|---|---|---|---|
| 137 | 89 | 59 | 3B | 2 | 19 | 583436 | 4667484 | 2 | 19 | 0 | 184 | 4789500 | 2 | 19 | 0 | 1 | 192 |
| 138 | 8A | 59 | 3B | 2 | 20 | 583459 | 4667668 | 2 | 20 | 0 | 184 | 4789692 | 2 | 20 | 0 | 1 | 192 |
| 139 | 8B | 59 | 3B | 2 | 21 | 583482 | 4667852 | 2 | 21 | 0 | 184 | 4789884 | 2 | 21 | 0 | 1 | 192 |
| 140 | 8C | 59 | 3B | 2 | 22 | 583505 | 4668036 | 2 | 22 | 0 | 184 | 4790076 | 2 | 22 | 0 | 1 | 192 |
| 141 | 8D | 59 | 3B | 2 | 23 | 583528 | 4668220 | 2 | 23 | 0 | 184 | 4790268 | 2 | 23 | 0 | 1 | 192 |
| 142 | 8E | 59 | 3B | 2 | 24 | 583551 | 4668404 | 2 | 24 | 0 | 184 | 4790460 | 2 | 24 | 0 | 1 | 192 |
| 143 | 8F | 59 | 3B | 2 | 25 | 583574 | 4668588 | 2 | 25 | 0 | 184 | 4790652 | 2 | 25 | 0 | 1 | 192 |
| 144 | 90 | 59 | 3B | 2 | 26 | 583599 | 4668788 | 2 | 26 | 0 | 200 | 4790860 | 2 | 26 | 0 | 1 | 208 |
| 145 | 91 | 59 | 3B | 2 | 27 | 583624 | 4668988 | 2 | 27 | 0 | 200 | 4791068 | 2 | 27 | 0 | 1 | 208 |
| 146 | 92 | 59 | 3B | 2 | 28 | 583649 | 4669188 | 2 | 28 | 0 | 200 | 4791276 | 2 | 28 | 0 | 1 | 208 |
| 147 | 93 | 59 | 3B | 2 | 29 | 583674 | 4669388 | 2 | 29 | 0 | 200 | 4791484 | 2 | 29 | 0 | 1 | 208 |
| 148 | 94 | 59 | 3B | 2 | 30 | 583699 | 4669588 | 2 | 30 | 0 | 200 | 4791692 | 2 | 30 | 0 | 1 | 208 |
| 149 | 95 | 59 | 3B | 2 | 31 | 583724 | 4669788 | 2 | 31 | 0 | 200 | 4791900 | 2 | 31 | 0 | 1 | 208 |
| 150 | 96 | 59 | 3B | 2 | 32 | 583749 | 4669988 | 2 | 32 | 0 | 200 | 4792108 | 2 | 32 | 0 | 1 | 208 |
| 151 | 97 | 59 | 3B | 2 | 33 | 583774 | 4670188 | 2 | 33 | 0 | 200 | 4792316 | 2 | 33 | 0 | 1 | 208 |
| 152 | 98 | 59 | 3B | 2 | 34 | 583799 | 4670388 | 2 | 34 | 0 | 200 | 4792524 | 2 | 34 | 0 | 1 | 208 |
| 153 | 99 | 59 | 3B | 2 | 35 | 583824 | 4670588 | 2 | 35 | 0 | 200 | 4792732 | 2 | 35 | 0 | 1 | 208 |
| 154 | 9A | 59 | 3B | 2 | 36 | 583849 | 4670788 | 2 | 36 | 0 | 200 | 4792940 | 2 | 36 | 0 | 1 | 208 |
| 155 | 9B | 59 | 3B | 2 | 37 | 583874 | 4670988 | 2 | 37 | 0 | 200 | 4793148 | 2 | 37 | 0 | 1 | 208 |
| 156 | 9C | 59 | 3B | 2 | 38 | 583899 | 4671188 | 2 | 38 | 0 | 200 | 4793356 | 2 | 38 | 0 | 1 | 208 |
| 157 | 9D | 59 | 3B | 2 | 39 | 583924 | 4671388 | 2 | 39 | 0 | 200 | 4793564 | 2 | 39 | 0 | 1 | 208 |
| 158 | 9E | 59 | 3B | 2 | 40 | 583949 | 4671588 | 2 | 40 | 0 | 200 | 4793772 | 2 | 40 | 0 | 1 | 208 |
| 159 | 9F | 59 | 3B | 2 | 41 | 583974 | 4671788 | 2 | 41 | 0 | 200 | 4793980 | 2 | 41 | 0 | 1 | 208 |
| 160 | A0 | 59 | 3B | 2 | 42 | 583999 | 4671988 | 2 | 42 | 0 | 200 | 4794188 | 2 | 42 | 0 | 1 | 208 |
| 161 | A1 | 59 | 3B | 2 | 43 | 584024 | 4672188 | 2 | 43 | 0 | 200 | 4794396 | 2 | 43 | 0 | 1 | 208 |
| 162 | A2 | 59 | 3B | 2 | 44 | 584049 | 4672388 | 2 | 44 | 0 | 200 | 4794604 | 2 | 44 | 0 | 1 | 208 |
| 163 | A3 | 59 | 3B | 2 | 45 | 584074 | 4672588 | 2 | 45 | 0 | 200 | 4794812 | 2 | 45 | 0 | 1 | 208 |
| 164 | A4 | 59 | 3B | 2 | 46 | 584099 | 4672788 | 2 | 46 | 0 | 200 | 4795020 | 2 | 46 | 0 | 1 | 208 |
| 165 | A5 | 59 | 3B | 2 | 47 | 584124 | 4672988 | 2 | 47 | 0 | 200 | 4795228 | 2 | 47 | 0 | 1 | 208 |
| 166 | A6 | 59 | 3B | 2 | 48 | 584149 | 4673188 | 2 | 48 | 0 | 200 | 4795436 | 2 | 48 | 0 | 1 | 208 |
| 167 | A7 | 59 | 3B | 2 | 49 | 584174 | 4673388 | 2 | 49 | 0 | 200 | 4795644 | 2 | 49 | 0 | 1 | 208 |
| 168 | A8 | 59 | 3B | 2 | 50 | 584199 | 4673588 | 2 | 50 | 0 | 200 | 4795852 | 2 | 50 | 0 | 1 | 208 |
| 169 | A9 | 59 | 3B | 2 | 51 | 584224 | 4673788 | 2 | 51 | 0 | 200 | 4796060 | 2 | 51 | 0 | 1 | 208 |
| 170 | AA | 59 | 3B | 2 | 52 | 584249 | 4673988 | 2 | 52 | 0 | 200 | 4796268 | 2 | 52 | 0 | 1 | 208 |
| 171 | AB | 59 | 3B | 2 | 53 | 584274 | 4674188 | 2 | 53 | 0 | 200 | 4796476 | 2 | 53 | 0 | 1 | 208 |
| 172 | AC | 59 | 3B | 2 | 54 | 584299 | 4674388 | 2 | 54 | 0 | 200 | 4796684 | 2 | 54 | 0 | 1 | 208 |
| 173 | AD | 59 | 3B | 2 | 55 | 584324 | 4674588 | 2 | 55 | 0 | 200 | 4796892 | 2 | 55 | 0 | 1 | 208 |
| 174 | AE | 59 | 3B | 2 | 56 | 584349 | 4674788 | 2 | 56 | 0 | 200 | 4797100 | 2 | 56 | 0 | 1 | 208 |
| 175 | AF | 59 | 3B | 2 | 57 | 584374 | 4674988 | 2 | 57 | 0 | 200 | 4797308 | 2 | 57 | 0 | 1 | 208 |
| 176 | B0 | 59 | 3B | 2 | 58 | 584399 | 4675188 | 2 | 58 | 0 | 200 | 4797516 | 2 | 58 | 0 | 1 | 208 |
| 177 | B1 | 59 | 3B | 3 | 0 | 584422 | 4675372 | 3 | 0 | 0 | 184 | 4797708 | 3 | 0 | 0 | 1 | 192 |
| 178 | B2 | 59 | 3B | 3 | 1 | 584450 | 4675596 | 3 | 1 | 0 | 224 | 4797940 | 3 | 1 | 0 | 1 | 232 |
| 179 | B3 | 59 | 3B | 3 | 2 | 584478 | 4675820 | 3 | 2 | 0 | 224 | 4798172 | 3 | 2 | 0 | 1 | 232 |
| 180 | B4 | 59 | 3B | 3 | 3 | 584506 | 4676044 | 3 | 3 | 0 | 224 | 4798404 | 3 | 3 | 0 | 1 | 232 |
| 181 | B5 | 59 | 3B | 3 | 4 | 584534 | 4676268 | 3 | 4 | 0 | 224 | 4798636 | 3 | 4 | 0 | 1 | 232 |
| 182 | B6 | 59 | 3B | 3 | 5 | 584562 | 4676492 | 3 | 5 | 0 | 224 | 4798868 | 3 | 5 | 0 | 1 | 232 |
| 183 | B7 | 59 | 3B | 3 | 6 | 584590 | 4676716 | 3 | 6 | 0 | 224 | 4799100 | 3 | 6 | 0 | 1 | 232 |
| 184 | B8 | 59 | 3B | 3 | 7 | 584618 | 4676940 | 3 | 7 | 0 | 224 | 4799332 | 3 | 7 | 0 | 1 | 232 |
| 185 | B9 | 59 | 3B | 3 | 8 | 584646 | 4677164 | 3 | 8 | 0 | 224 | 4799564 | 3 | 8 | 0 | 1 | 232 |
| 186 | BA | 59 | 3B | 3 | 9 | 584674 | 4677388 | 3 | 9 | 0 | 224 | 4799796 | 3 | 9 | 0 | 1 | 232 |
| 187 | BB | 59 | 3B | 3 | 10 | 584702 | 4677612 | 3 | 10 | 0 | 224 | 4800028 | 3 | 10 | 0 | 1 | 232 |
| 188 | BC | 59 | 3B | 3 | 11 | 584730 | 4677836 | 3 | 11 | 0 | 224 | 4800260 | 3 | 11 | 0 | 1 | 232 |
| 189 | BD | 59 | 3B | 3 | 12 | 584758 | 4678060 | 3 | 12 | 0 | 224 | 4800492 | 3 | 12 | 0 | 1 | 232 |
| 190 | BE | 59 | 3B | 3 | 13 | 584786 | 4678284 | 3 | 13 | 0 | 224 | 4800724 | 3 | 13 | 0 | 1 | 232 |
| 191 | BF | 59 | 3B | 3 | 14 | 584814 | 4678508 | 3 | 14 | 0 | 224 | 4800956 | 3 | 14 | 0 | 1 | 232 |
| 192 | C0 | 59 | 3B | 3 | 15 | 584839 | 4678708 | 3 | 15 | 0 | 200 | 4801164 | 3 | 15 | 0 | 1 | 208 |
| 193 | C1 | 59 | 3B | 3 | 16 | 584864 | 4678908 | 3 | 16 | 0 | 200 | 4801372 | 3 | 16 | 0 | 1 | 208 |
| 194 | C2 | 59 | 3B | 3 | 17 | 584889 | 4679108 | 3 | 17 | 0 | 200 | 4801580 | 3 | 17 | 0 | 1 | 208 |
| 195 | C3 | 59 | 3B | 3 | 18 | 584914 | 4679308 | 3 | 18 | 0 | 200 | 4801788 | 3 | 18 | 0 | 1 | 208 |
| 196 | C4 | 59 | 3B | 3 | 19 | 584939 | 4679508 | 3 | 19 | 0 | 200 | 4801996 | 3 | 19 | 0 | 1 | 208 |
| 197 | C5 | 59 | 3B | 3 | 20 | 584964 | 4679708 | 3 | 20 | 0 | 200 | 4802204 | 3 | 20 | 0 | 1 | 208 |
| 198 | C6 | 59 | 3B | 3 | 21 | 584989 | 4679908 | 3 | 21 | 0 | 200 | 4802412 | 3 | 21 | 0 | 1 | 208 |
| 199 | C7 | 59 | 3B | 3 | 22 | 585014 | 4680108 | 3 | 22 | 0 | 200 | 4802620 | 3 | 22 | 0 | 1 | 208 |
| 200 | C8 | 59 | 3B | 3 | 23 | 585039 | 4680308 | 3 | 23 | 0 | 200 | 4802828 | 3 | 23 | 0 | 1 | 208 |
| 201 | C9 | 59 | 3B | 3 | 24 | 585064 | 4680508 | 3 | 24 | 0 | 200 | 4803036 | 3 | 24 | 0 | 1 | 208 |
| 202 | CA | 59 | 3B | 3 | 25 | 585089 | 4680708 | 3 | 25 | 0 | 200 | 4803244 | 3 | 25 | 0 | 1 | 208 |
| 203 | CB | 59 | 3B | 3 | 26 | 585114 | 4680908 | 3 | 26 | 0 | 200 | 4803452 | 3 | 26 | 0 | 1 | 208 |
| 204 | CC | 59 | 3B | 3 | 27 | 585139 | 4681108 | 3 | 27 | 0 | 200 | 4803660 | 3 | 27 | 0 | 1 | 208 |
| 205 | CD | 59 | 3B | 3 | 28 | 585164 | 4681308 | 3 | 28 | 0 | 200 | 4803868 | 3 | 28 | 0 | 1 | 208 |
| 206 | CE | 59 | 3B | 3 | 29 | 585189 | 4681508 | 3 | 29 | 0 | 200 | 4804076 | 3 | 29 | 0 | 1 | 208 |
| 207 | CF | 59 | 3B | 3 | 30 | 585214 | 4681708 | 3 | 30 | 0 | 200 | 4804284 | 3 | 30 | 0 | 1 | 208 |
| 208 | D0 | 59 | 3B | 3 | 31 | 585241 | 4681924 | 3 | 31 | 0 | 216 | 4804508 | 3 | 31 | 0 | 1 | 224 |
| 209 | D1 | 59 | 3B | 3 | 32 | 585268 | 4682140 | 3 | 32 | 0 | 216 | 4804732 | 3 | 32 | 0 | 1 | 224 |
| 210 | D2 | 59 | 3B | 3 | 33 | 585295 | 4682356 | 3 | 33 | 0 | 216 | 4804956 | 3 | 33 | 0 | 1 | 224 |
| 211 | D3 | 59 | 3B | 3 | 34 | 585322 | 4682572 | 3 | 34 | 0 | 216 | 4805180 | 3 | 34 | 0 | 1 | 224 |
| 212 | D4 | 59 | 3B | 3 | 35 | 585349 | 4682788 | 3 | 35 | 0 | 216 | 4805404 | 3 | 35 | 0 | 1 | 224 |
| 213 | D5 | 59 | 3B | 3 | 36 | 585376 | 4683004 | 3 | 36 | 0 | 216 | 4805628 | 3 | 36 | 0 | 1 | 224 |
| 214 | D6 | 59 | 3B | 3 | 37 | 585403 | 4683220 | 3 | 37 | 0 | 216 | 4805852 | 3 | 37 | 0 | 1 | 224 |
| 215 | D7 | 59 | 3B | 3 | 38 | 585430 | 4683436 | 3 | 38 | 0 | 216 | 4806076 | 3 | 38 | 0 | 1 | 224 |
| 216 | D8 | 59 | 3B | 3 | 39 | 585457 | 4683652 | 3 | 39 | 0 | 216 | 4806300 | 3 | 39 | 0 | 1 | 224 |
| 217 | D9 | 59 | 3B | 3 | 40 | 585484 | 4683868 | 3 | 40 | 0 | 216 | 4806524 | 3 | 40 | 0 | 1 | 224 |
| 218 | DA | 59 | 3B | 3 | 41 | 585511 | 4684084 | 3 | 41 | 0 | 216 | 4806748 | 3 | 41 | 0 | 1 | 224 |
| 219 | DB | 59 | 3B | 3 | 42 | 585538 | 4684300 | 3 | 42 | 0 | 216 | 4806972 | 3 | 42 | 0 | 1 | 224 |
| 220 | DC | 59 | 3B | 3 | 43 | 585565 | 4684516 | 3 | 43 | 0 | 216 | 4807196 | 3 | 43 | 0 | 1 | 224 |
| 221 | DD | 59 | 3B | 3 | 44 | 585592 | 4684732 | 3 | 44 | 0 | 216 | 4807420 | 3 | 44 | 0 | 1 | 224 |
| 222 | DE | 59 | 3B | 3 | 45 | 585619 | 4684948 | 3 | 45 | 0 | 216 | 4807644 | 3 | 45 | 0 | 1 | 224 |
| 223 | DF | 59 | 3B | 3 | 46 | 585646 | 4685164 | 3 | 46 | 0 | 216 | 4807868 | 3 | 46 | 0 | 1 | 224 |
| 224 | E0 | 59 | 3B | 3 | 47 | 585673 | 4685380 | 3 | 47 | 0 | 216 | 4808092 | 3 | 47 | 0 | 1 | 224 |
| 225 | E1 | 59 | 3B | 3 | 48 | 585700 | 4685596 | 3 | 48 | 0 | 216 | 4808316 | 3 | 48 | 0 | 1 | 224 |
| 226 | E2 | 59 | 3B | 3 | 49 | 585727 | 4685812 | 3 | 49 | 0 | 216 | 4808540 | 3 | 49 | 0 | 1 | 224 |
| 227 | E3 | 59 | 3B | 3 | 50 | 585754 | 4686028 | 3 | 50 | 0 | 216 | 4808764 | 3 | 50 | 0 | 1 | 224 |
| 228 | E4 | 59 | 3B | 3 | 51 | 585781 | 4686244 | 3 | 51 | 0 | 216 | 4808988 | 3 | 51 | 0 | 1 | 224 |
| 229 | E5 | 59 | 3B | 3 | 52 | 585808 | 4686460 | 3 | 52 | 0 | 216 | 4809212 | 3 | 52 | 0 | 1 | 224 |
| 230 | E6 | 59 | 3B | 3 | 53 | 585835 | 4686676 | 3 | 53 | 0 | 216 | 4809436 | 3 | 53 | 0 | 1 | 224 |
| 231 | E7 | 59 | 3B | 3 | 54 | 585862 | 4686892 | 3 | 54 | 0 | 216 | 4809660 | 3 | 54 | 0 | 1 | 224 |
| 232 | E8 | 59 | 3B | 3 | 55 | 585889 | 4687108 | 3 | 55 | 0 | 216 | 4809884 | 3 | 55 | 0 | 1 | 224 |
| 233 | E9 | 59 | 3B | 3 | 56 | 585916 | 4687324 | 3 | 56 | 0 | 216 | 4810108 | 3 | 56 | 0 | 1 | 224 |
| 234 | EA | 59 | 3B | 3 | 57 | 585943 | 4687540 | 3 | 57 | 0 | 216 | 4810332 | 3 | 57 | 0 | 1 | 224 |
| 235 | EB | 59 | 3B | 3 | 58 | 585970 | 4687756 | 3 | 58 | 0 | 216 | 4810556 | 3 | 58 | 0 | 1 | 224 |
| 236 | EC | 59 | 3B | 4 | 0 | 585995 | 4687956 | 4 | 0 | 0 | 200 | 4810764 | 4 | 0 | 0 | 1 | 208 |
| 237 | ED | 59 | 3B | 4 | 1 | 586025 | 4688196 | 4 | 1 | 0 | 240 | 4811012 | 4 | 1 | 0 | 1 | 248 |
| 238 | EE | 59 | 3B | 4 | 2 | 586055 | 4688436 | 4 | 2 | 0 | 240 | 4811260 | 4 | 2 | 0 | 1 | 248 |
| 239 | EF | 59 | 3B | 4 | 3 | 586085 | 4688676 | 4 | 3 | 0 | 240 | 4811508 | 4 | 3 | 0 | 1 | 248 |

| | | | | | | | | | | | | | | | | | |
|---|---|---|---|---|---|---|---|---|---|---|---|---|---|---|---|---|---|
| 240 | F0 | 59 | 3B | 4 | 4 | 586115 | 4688916 | 4 | 4 | 0 | 240 | 4811756 | 4 | 4 | 0 | 1 | 248 |
| 241 | F1 | 59 | 3B | 4 | 5 | 586145 | 4689156 | 4 | 5 | 0 | 240 | 4812004 | 4 | 5 | 0 | 1 | 248 |
| 242 | F2 | 59 | 3B | 4 | 6 | 586175 | 4689396 | 4 | 6 | 0 | 240 | 4812252 | 4 | 6 | 0 | 1 | 248 |
| 243 | F3 | 59 | 3B | 4 | 7 | 586205 | 4689636 | 4 | 7 | 0 | 240 | 4812500 | 4 | 7 | 0 | 1 | 248 |
| 244 | F4 | 59 | 3B | 4 | 8 | 586235 | 4689876 | 4 | 8 | 0 | 240 | 4812748 | 4 | 8 | 0 | 1 | 248 |
| 245 | F5 | 59 | 3B | 4 | 9 | 586265 | 4690116 | 4 | 9 | 0 | 240 | 4812996 | 4 | 9 | 0 | 1 | 248 |
| 246 | F6 | 59 | 3B | 4 | 10 | 586295 | 4690356 | 4 | 10 | 0 | 240 | 4813244 | 4 | 10 | 0 | 1 | 248 |
| 247 | F7 | 59 | 3B | 4 | 11 | 586325 | 4690596 | 4 | 11 | 0 | 240 | 4813492 | 4 | 11 | 0 | 1 | 248 |
| 248 | F8 | 59 | 3B | 4 | 12 | 586355 | 4690836 | 4 | 12 | 0 | 240 | 4813740 | 4 | 12 | 0 | 1 | 248 |
| 249 | F9 | 59 | 3B | 4 | 13 | 586385 | 4691076 | 4 | 13 | 0 | 240 | 4813988 | 4 | 13 | 0 | 1 | 248 |
| 250 | FA | 59 | 3B | 4 | 14 | 586415 | 4691316 | 4 | 14 | 0 | 240 | 4814236 | 4 | 14 | 0 | 1 | 248 |
| 251 | FB | 59 | 3B | 4 | 15 | 586445 | 4691556 | 4 | 15 | 0 | 240 | 4814484 | 4 | 15 | 0 | 1 | 248 |
| 252 | FC | 59 | 3B | 4 | 16 | 586475 | 4691796 | 4 | 16 | 0 | 240 | 4814732 | 4 | 16 | 0 | 1 | 248 |
| 253 | FD | 59 | 3B | 4 | 17 | 586505 | 4692036 | 4 | 17 | 0 | 240 | 4814980 | 4 | 17 | 0 | 1 | 248 |
| 254 | FE | 59 | 3B | 4 | 18 | 586535 | 4692276 | 4 | 18 | 0 | 240 | 4815228 | 4 | 18 | 0 | 1 | 248 |
| 255 | FF | 59 | 3B | 4 | 19 | 586565 | 4692516 | 4 | 19 | 0 | 240 | 4815476 | 4 | 19 | 0 | 1 | 248 |
| 0 | 00 | 60 | 3C | 0 | 0 | 586577 | 4692612 | 0 | 0 | 0 | 96 | 4815580 | 0 | 0 | 0 | 1 | 104 |
| 1 | 01 | 60 | 3C | 0 | 1 | 586591 | 4692724 | 0 | 1 | 0 | 112 | 4815700 | 0 | 1 | 0 | 1 | 120 |
| 2 | 02 | 60 | 3C | 0 | 2 | 586605 | 4692836 | 0 | 2 | 0 | 112 | 4815820 | 0 | 2 | 0 | 1 | 120 |
| 3 | 03 | 60 | 3C | 0 | 3 | 586619 | 4692948 | 0 | 3 | 0 | 112 | 4815940 | 0 | 3 | 0 | 1 | 120 |
| 4 | 04 | 60 | 3C | 0 | 4 | 586633 | 4693060 | 0 | 4 | 0 | 112 | 4816060 | 0 | 4 | 0 | 1 | 120 |
| 5 | 05 | 60 | 3C | 0 | 5 | 586647 | 4693172 | 0 | 5 | 0 | 112 | 4816180 | 0 | 5 | 0 | 1 | 120 |
| 6 | 06 | 60 | 3C | 0 | 6 | 586661 | 4693284 | 0 | 6 | 0 | 112 | 4816300 | 0 | 6 | 0 | 1 | 120 |
| 7 | 07 | 60 | 3C | 0 | 7 | 586675 | 4693396 | 0 | 7 | 0 | 112 | 4816420 | 0 | 7 | 0 | 1 | 120 |
| 8 | 08 | 60 | 3C | 0 | 8 | 586689 | 4693508 | 0 | 8 | 0 | 112 | 4816540 | 0 | 8 | 0 | 1 | 120 |
| 9 | 09 | 60 | 3C | 0 | 9 | 586703 | 4693620 | 0 | 9 | 0 | 112 | 4816660 | 0 | 9 | 0 | 1 | 120 |
| 10 | 0A | 60 | 3C | 0 | 10 | 586717 | 4693732 | 0 | 10 | 0 | 112 | 4816780 | 0 | 10 | 0 | 1 | 120 |
| 11 | 0B | 60 | 3C | 0 | 11 | 586731 | 4693844 | 0 | 11 | 0 | 112 | 4816900 | 0 | 11 | 0 | 1 | 120 |
| 12 | 0C | 60 | 3C | 0 | 12 | 586745 | 4693956 | 0 | 12 | 0 | 112 | 4817020 | 0 | 12 | 0 | 1 | 120 |
| 13 | 0D | 60 | 3C | 0 | 13 | 586759 | 4694068 | 0 | 13 | 0 | 112 | 4817140 | 0 | 13 | 0 | 1 | 120 |
| 14 | 0E | 60 | 3C | 0 | 14 | 586773 | 4694180 | 0 | 14 | 0 | 112 | 4817260 | 0 | 14 | 0 | 1 | 120 |
| 15 | 0F | 60 | 3C | 0 | 15 | 586787 | 4694292 | 0 | 15 | 0 | 112 | 4817380 | 0 | 15 | 0 | 1 | 120 |
| 16 | 10 | 60 | 3C | 0 | 16 | 586801 | 4694404 | 0 | 16 | 0 | 112 | 4817500 | 0 | 16 | 0 | 1 | 120 |
| 17 | 11 | 60 | 3C | 0 | 17 | 586815 | 4694516 | 0 | 17 | 0 | 112 | 4817620 | 0 | 17 | 0 | 1 | 120 |
| 18 | 12 | 60 | 3C | 0 | 18 | 586829 | 4694628 | 0 | 18 | 0 | 112 | 4817740 | 0 | 18 | 0 | 1 | 120 |
| 19 | 13 | 60 | 3C | 0 | 19 | 586843 | 4694740 | 0 | 19 | 0 | 112 | 4817860 | 0 | 19 | 0 | 1 | 120 |
| 20 | 14 | 60 | 3C | 0 | 20 | 586857 | 4694852 | 0 | 20 | 0 | 112 | 4817980 | 0 | 20 | 0 | 1 | 120 |
| 21 | 15 | 60 | 3C | 0 | 21 | 586871 | 4694964 | 0 | 21 | 0 | 112 | 4818100 | 0 | 21 | 0 | 1 | 120 |
| 22 | 16 | 60 | 3C | 0 | 22 | 586885 | 4695076 | 0 | 22 | 0 | 112 | 4818220 | 0 | 22 | 0 | 1 | 120 |
| 23 | 17 | 60 | 3C | 0 | 23 | 586899 | 4695188 | 0 | 23 | 0 | 112 | 4818340 | 0 | 23 | 0 | 1 | 120 |
| 24 | 18 | 60 | 3C | 0 | 24 | 586913 | 4695300 | 0 | 24 | 0 | 112 | 4818460 | 0 | 24 | 0 | 1 | 120 |
| 25 | 19 | 60 | 3C | 0 | 25 | 586927 | 4695412 | 0 | 25 | 0 | 112 | 4818580 | 0 | 25 | 0 | 1 | 120 |
| 26 | 1A | 60 | 3C | 0 | 26 | 586941 | 4695524 | 0 | 26 | 0 | 112 | 4818700 | 0 | 26 | 0 | 1 | 120 |
| 27 | 1B | 60 | 3C | 0 | 27 | 586955 | 4695636 | 0 | 27 | 0 | 112 | 4818820 | 0 | 27 | 0 | 1 | 120 |
| 28 | 1C | 60 | 3C | 0 | 28 | 586969 | 4695748 | 0 | 28 | 0 | 112 | 4818940 | 0 | 28 | 0 | 1 | 120 |
| 29 | 1D | 60 | 3C | 0 | 29 | 586983 | 4695860 | 0 | 29 | 0 | 112 | 4819060 | 0 | 29 | 0 | 1 | 120 |
| 30 | 1E | 60 | 3C | 0 | 30 | 586997 | 4695972 | 0 | 30 | 0 | 112 | 4819180 | 0 | 30 | 0 | 1 | 120 |
| 31 | 1F | 60 | 3C | 0 | 31 | 587011 | 4696084 | 0 | 31 | 0 | 112 | 4819300 | 0 | 31 | 0 | 1 | 120 |
| 32 | 20 | 60 | 3C | 0 | 32 | 587025 | 4696196 | 0 | 32 | 0 | 112 | 4819420 | 0 | 32 | 0 | 1 | 120 |
| 33 | 21 | 60 | 3C | 0 | 33 | 587039 | 4696308 | 0 | 33 | 0 | 112 | 4819540 | 0 | 33 | 0 | 1 | 120 |
| 34 | 22 | 60 | 3C | 0 | 34 | 587053 | 4696420 | 0 | 34 | 0 | 112 | 4819660 | 0 | 34 | 0 | 1 | 120 |
| 35 | 23 | 60 | 3C | 0 | 35 | 587067 | 4696532 | 0 | 35 | 0 | 112 | 4819780 | 0 | 35 | 0 | 1 | 120 |
| 36 | 24 | 60 | 3C | 0 | 36 | 587081 | 4696644 | 0 | 36 | 0 | 112 | 4819900 | 0 | 36 | 0 | 1 | 120 |
| 37 | 25 | 60 | 3C | 0 | 37 | 587095 | 4696756 | 0 | 37 | 0 | 112 | 4820020 | 0 | 37 | 0 | 1 | 120 |
| 38 | 26 | 60 | 3C | 0 | 38 | 587109 | 4696868 | 0 | 38 | 0 | 112 | 4820140 | 0 | 38 | 0 | 1 | 120 |
| 39 | 27 | 60 | 3C | 0 | 39 | 587123 | 4696980 | 0 | 39 | 0 | 112 | 4820260 | 0 | 39 | 0 | 1 | 120 |
| 40 | 28 | 60 | 3C | 0 | 40 | 587137 | 4697092 | 0 | 40 | 0 | 112 | 4820380 | 0 | 40 | 0 | 1 | 120 |
| 41 | 29 | 60 | 3C | 0 | 41 | 587151 | 4697204 | 0 | 41 | 0 | 112 | 4820500 | 0 | 41 | 0 | 1 | 120 |
| 42 | 2A | 60 | 3C | 0 | 42 | 587165 | 4697316 | 0 | 42 | 0 | 112 | 4820620 | 0 | 42 | 0 | 1 | 120 |
| 43 | 2B | 60 | 3C | 0 | 43 | 587179 | 4697428 | 0 | 43 | 0 | 112 | 4820740 | 0 | 43 | 0 | 1 | 120 |
| 44 | 2C | 60 | 3C | 0 | 44 | 587193 | 4697540 | 0 | 44 | 0 | 112 | 4820860 | 0 | 44 | 0 | 1 | 120 |
| 45 | 2D | 60 | 3C | 0 | 45 | 587207 | 4697652 | 0 | 45 | 0 | 112 | 4820980 | 0 | 45 | 0 | 1 | 120 |
| 46 | 2E | 60 | 3C | 0 | 46 | 587221 | 4697764 | 0 | 46 | 0 | 112 | 4821100 | 0 | 46 | 0 | 1 | 120 |
| 47 | 2F | 60 | 3C | 0 | 47 | 587235 | 4697876 | 0 | 47 | 0 | 112 | 4821220 | 0 | 47 | 0 | 1 | 120 |
| 48 | 30 | 60 | 3C | 0 | 48 | 587249 | 4697988 | 0 | 48 | 0 | 112 | 4821340 | 0 | 48 | 0 | 1 | 120 |
| 49 | 31 | 60 | 3C | 0 | 49 | 587263 | 4698100 | 0 | 49 | 0 | 112 | 4821460 | 0 | 49 | 0 | 1 | 120 |
| 50 | 32 | 60 | 3C | 0 | 50 | 587277 | 4698212 | 0 | 50 | 0 | 112 | 4821580 | 0 | 50 | 0 | 1 | 120 |
| 51 | 33 | 60 | 3C | 0 | 51 | 587291 | 4698324 | 0 | 51 | 0 | 112 | 4821700 | 0 | 51 | 0 | 1 | 120 |
| 52 | 34 | 60 | 3C | 0 | 52 | 587305 | 4698436 | 0 | 52 | 0 | 112 | 4821820 | 0 | 52 | 0 | 1 | 120 |
| 53 | 35 | 60 | 3C | 0 | 53 | 587319 | 4698548 | 0 | 53 | 0 | 112 | 4821940 | 0 | 53 | 0 | 1 | 120 |
| 54 | 36 | 60 | 3C | 0 | 54 | 587333 | 4698660 | 0 | 54 | 0 | 112 | 4822060 | 0 | 54 | 0 | 1 | 120 |
| 55 | 37 | 60 | 3C | 0 | 55 | 587347 | 4698772 | 0 | 55 | 0 | 112 | 4822180 | 0 | 55 | 0 | 1 | 120 |
| 56 | 38 | 60 | 3C | 0 | 56 | 587361 | 4698884 | 0 | 56 | 0 | 112 | 4822300 | 0 | 56 | 0 | 1 | 120 |
| 57 | 39 | 60 | 3C | 0 | 57 | 587375 | 4698996 | 0 | 57 | 0 | 112 | 4822420 | 0 | 57 | 0 | 1 | 120 |
| 58 | 3A | 60 | 3C | 0 | 58 | 587389 | 4699108 | 0 | 58 | 0 | 112 | 4822540 | 0 | 58 | 0 | 1 | 120 |
| 59 | 3B | 60 | 3C | 0 | 59 | 587403 | 4699220 | 0 | 59 | 0 | 112 | 4822660 | 0 | 59 | 0 | 1 | 120 |
| 60 | 3C | 60 | 3C | 1 | 0 | 587415 | 4699316 | 1 | 0 | 0 | 96 | 4822764 | 1 | 0 | 0 | 1 | 104 |
| 61 | 3D | 60 | 3C | 1 | 1 | 587432 | 4699452 | 1 | 1 | 0 | 136 | 4822908 | 1 | 1 | 0 | 1 | 144 |
| 62 | 3E | 60 | 3C | 1 | 2 | 587449 | 4699588 | 1 | 2 | 0 | 136 | 4823052 | 1 | 2 | 0 | 1 | 144 |
| 63 | 3F | 60 | 3C | 1 | 3 | 587466 | 4699724 | 1 | 3 | 0 | 136 | 4823196 | 1 | 3 | 0 | 1 | 144 |
| 64 | 40 | 60 | 3C | 1 | 4 | 587487 | 4699892 | 1 | 4 | 0 | 168 | 4823372 | 1 | 4 | 0 | 1 | 176 |
| 65 | 41 | 60 | 3C | 1 | 5 | 587508 | 4700060 | 1 | 5 | 0 | 168 | 4823548 | 1 | 5 | 0 | 1 | 176 |
| 66 | 42 | 60 | 3C | 1 | 6 | 587529 | 4700228 | 1 | 6 | 0 | 168 | 4823724 | 1 | 6 | 0 | 1 | 176 |
| 67 | 43 | 60 | 3C | 1 | 7 | 587550 | 4700396 | 1 | 7 | 0 | 168 | 4823900 | 1 | 7 | 0 | 1 | 176 |
| 68 | 44 | 60 | 3C | 1 | 8 | 587571 | 4700564 | 1 | 8 | 0 | 168 | 4824076 | 1 | 8 | 0 | 1 | 176 |
| 69 | 45 | 60 | 3C | 1 | 9 | 587592 | 4700732 | 1 | 9 | 0 | 168 | 4824252 | 1 | 9 | 0 | 1 | 176 |
| 70 | 46 | 60 | 3C | 1 | 10 | 587613 | 4700900 | 1 | 10 | 0 | 168 | 4824428 | 1 | 10 | 0 | 1 | 176 |
| 71 | 47 | 60 | 3C | 1 | 11 | 587634 | 4701068 | 1 | 11 | 0 | 168 | 4824604 | 1 | 11 | 0 | 1 | 176 |
| 72 | 48 | 60 | 3C | 1 | 12 | 587655 | 4701236 | 1 | 12 | 0 | 168 | 4824780 | 1 | 12 | 0 | 1 | 176 |
| 73 | 49 | 60 | 3C | 1 | 13 | 587676 | 4701404 | 1 | 13 | 0 | 168 | 4824956 | 1 | 13 | 0 | 1 | 176 |
| 74 | 4A | 60 | 3C | 1 | 14 | 587697 | 4701572 | 1 | 14 | 0 | 168 | 4825132 | 1 | 14 | 0 | 1 | 176 |
| 75 | 4B | 60 | 3C | 1 | 15 | 587718 | 4701740 | 1 | 15 | 0 | 168 | 4825308 | 1 | 15 | 0 | 1 | 176 |
| 76 | 4C | 60 | 3C | 1 | 16 | 587739 | 4701908 | 1 | 16 | 0 | 168 | 4825484 | 1 | 16 | 0 | 1 | 176 |
| 77 | 4D | 60 | 3C | 1 | 17 | 587760 | 4702076 | 1 | 17 | 0 | 168 | 4825660 | 1 | 17 | 0 | 1 | 176 |
| 78 | 4E | 60 | 3C | 1 | 18 | 587781 | 4702244 | 1 | 18 | 0 | 168 | 4825836 | 1 | 18 | 0 | 1 | 176 |
| 79 | 4F | 60 | 3C | 1 | 19 | 587802 | 4702412 | 1 | 19 | 0 | 168 | 4826012 | 1 | 19 | 0 | 1 | 176 |
| 80 | 50 | 60 | 3C | 1 | 20 | 587825 | 4702596 | 1 | 20 | 0 | 184 | 4826204 | 1 | 20 | 0 | 1 | 192 |
| 81 | 51 | 60 | 3C | 1 | 21 | 587848 | 4702780 | 1 | 21 | 0 | 184 | 4826396 | 1 | 21 | 0 | 1 | 192 |
| 82 | 52 | 60 | 3C | 1 | 22 | 587871 | 4702964 | 1 | 22 | 0 | 184 | 4826588 | 1 | 22 | 0 | 1 | 192 |
| 83 | 53 | 60 | 3C | 1 | 23 | 587894 | 4703148 | 1 | 23 | 0 | 184 | 4826780 | 1 | 23 | 0 | 1 | 192 |
| 84 | 54 | 60 | 3C | 1 | 24 | 587917 | 4703332 | 1 | 24 | 0 | 184 | 4826972 | 1 | 24 | 0 | 1 | 192 |
| 85 | 55 | 60 | 3C | 1 | 25 | 587940 | 4703516 | 1 | 25 | 0 | 184 | 4827164 | 1 | 25 | 0 | 1 | 192 |
| 86 | 56 | 60 | 3C | 1 | 26 | 587963 | 4703700 | 1 | 26 | 0 | 184 | 4827356 | 1 | 26 | 0 | 1 | 192 |

| | | | | | | | | | | | | | | | | |
|---|---|---|---|---|---|---|---|---|---|---|---|---|---|---|---|---|
| 87 | 57 | 60 | 3C | 1 | 27 | 587986 | 4703884 | 1 | 27 | 0 | 184 | 4827548 | 1 | 27 | 0 | 1 | 192 |
| 88 | 58 | 60 | 3C | 1 | 28 | 588009 | 4704068 | 1 | 28 | 0 | 184 | 4827740 | 1 | 28 | 0 | 1 | 192 |
| 89 | 59 | 60 | 3C | 1 | 29 | 588032 | 4704252 | 1 | 29 | 0 | 184 | 4827932 | 1 | 29 | 0 | 1 | 192 |
| 90 | 5A | 60 | 3C | 1 | 30 | 588055 | 4704436 | 1 | 30 | 0 | 184 | 4828124 | 1 | 30 | 0 | 1 | 192 |
| 91 | 5B | 60 | 3C | 1 | 31 | 588078 | 4704620 | 1 | 31 | 0 | 184 | 4828316 | 1 | 31 | 0 | 1 | 192 |
| 92 | 5C | 60 | 3C | 1 | 32 | 588101 | 4704804 | 1 | 32 | 0 | 184 | 4828508 | 1 | 32 | 0 | 1 | 192 |
| 93 | 5D | 60 | 3C | 1 | 33 | 588124 | 4704988 | 1 | 33 | 0 | 184 | 4828700 | 1 | 33 | 0 | 1 | 192 |
| 94 | 5E | 60 | 3C | 1 | 34 | 588147 | 4705172 | 1 | 34 | 0 | 184 | 4828892 | 1 | 34 | 0 | 1 | 192 |
| 95 | 5F | 60 | 3C | 1 | 35 | 588170 | 4705356 | 1 | 35 | 0 | 184 | 4829084 | 1 | 35 | 0 | 1 | 192 |
| 96 | 60 | 60 | 3C | 1 | 36 | 588193 | 4705540 | 1 | 36 | 0 | 184 | 4829276 | 1 | 36 | 0 | 1 | 192 |
| 97 | 61 | 60 | 3C | 1 | 37 | 588216 | 4705724 | 1 | 37 | 0 | 184 | 4829468 | 1 | 37 | 0 | 1 | 192 |
| 98 | 62 | 60 | 3C | 1 | 38 | 588239 | 4705908 | 1 | 38 | 0 | 184 | 4829660 | 1 | 38 | 0 | 1 | 192 |
| 99 | 63 | 60 | 3C | 1 | 39 | 588262 | 4706092 | 1 | 39 | 0 | 184 | 4829852 | 1 | 39 | 0 | 1 | 192 |
| 100 | 64 | 60 | 3C | 1 | 40 | 588285 | 4706276 | 1 | 40 | 0 | 184 | 4830044 | 1 | 40 | 0 | 1 | 192 |
| 101 | 65 | 60 | 3C | 1 | 41 | 588308 | 4706460 | 1 | 41 | 0 | 184 | 4830236 | 1 | 41 | 0 | 1 | 192 |
| 102 | 66 | 60 | 3C | 1 | 42 | 588331 | 4706644 | 1 | 42 | 0 | 184 | 4830428 | 1 | 42 | 0 | 1 | 192 |
| 103 | 67 | 60 | 3C | 1 | 43 | 588354 | 4706828 | 1 | 43 | 0 | 184 | 4830620 | 1 | 43 | 0 | 1 | 192 |
| 104 | 68 | 60 | 3C | 1 | 44 | 588377 | 4707012 | 1 | 44 | 0 | 184 | 4830812 | 1 | 44 | 0 | 1 | 192 |
| 105 | 69 | 60 | 3C | 1 | 45 | 588400 | 4707196 | 1 | 45 | 0 | 184 | 4831004 | 1 | 45 | 0 | 1 | 192 |
| 106 | 6A | 60 | 3C | 1 | 46 | 588423 | 4707380 | 1 | 46 | 0 | 184 | 4831196 | 1 | 46 | 0 | 1 | 192 |
| 107 | 6B | 60 | 3C | 1 | 47 | 588446 | 4707564 | 1 | 47 | 0 | 184 | 4831388 | 1 | 47 | 0 | 1 | 192 |
| 108 | 6C | 60 | 3C | 1 | 48 | 588469 | 4707748 | 1 | 48 | 0 | 184 | 4831580 | 1 | 48 | 0 | 1 | 192 |
| 109 | 6D | 60 | 3C | 1 | 49 | 588492 | 4707932 | 1 | 49 | 0 | 184 | 4831772 | 1 | 49 | 0 | 1 | 192 |
| 110 | 6E | 60 | 3C | 1 | 50 | 588515 | 4708116 | 1 | 50 | 0 | 184 | 4831964 | 1 | 50 | 0 | 1 | 192 |
| 111 | 6F | 60 | 3C | 1 | 51 | 588538 | 4708300 | 1 | 51 | 0 | 184 | 4832156 | 1 | 51 | 0 | 1 | 192 |
| 112 | 70 | 60 | 3C | 1 | 52 | 588561 | 4708484 | 1 | 52 | 0 | 184 | 4832348 | 1 | 52 | 0 | 1 | 192 |
| 113 | 71 | 60 | 3C | 1 | 53 | 588584 | 4708668 | 1 | 53 | 0 | 184 | 4832540 | 1 | 53 | 0 | 1 | 192 |
| 114 | 72 | 60 | 3C | 1 | 54 | 588607 | 4708852 | 1 | 54 | 0 | 184 | 4832732 | 1 | 54 | 0 | 1 | 192 |
| 115 | 73 | 60 | 3C | 1 | 55 | 588630 | 4709036 | 1 | 55 | 0 | 184 | 4832924 | 1 | 55 | 0 | 1 | 192 |
| 116 | 74 | 60 | 3C | 1 | 56 | 588653 | 4709220 | 1 | 56 | 0 | 184 | 4833116 | 1 | 56 | 0 | 1 | 192 |
| 117 | 75 | 60 | 3C | 1 | 57 | 588676 | 4709404 | 1 | 57 | 0 | 184 | 4833308 | 1 | 57 | 0 | 1 | 192 |
| 118 | 76 | 60 | 3C | 1 | 58 | 588699 | 4709588 | 1 | 58 | 0 | 184 | 4833500 | 1 | 58 | 0 | 1 | 192 |
| 119 | 77 | 60 | 3C | 1 | 59 | 588722 | 4709772 | 1 | 59 | 0 | 184 | 4833692 | 1 | 59 | 0 | 1 | 192 |
| 120 | 78 | 60 | 3C | 2 | 0 | 588743 | 4709940 | 2 | 0 | 0 | 168 | 4833868 | 2 | 0 | 0 | 1 | 176 |
| 121 | 79 | 60 | 3C | 2 | 1 | 588769 | 4710148 | 2 | 1 | 0 | 208 | 4834084 | 2 | 1 | 0 | 1 | 216 |
| 122 | 7A | 60 | 3C | 2 | 2 | 588795 | 4710356 | 2 | 2 | 0 | 208 | 4834300 | 2 | 2 | 0 | 1 | 216 |
| 123 | 7B | 60 | 3C | 2 | 3 | 588821 | 4710564 | 2 | 3 | 0 | 208 | 4834516 | 2 | 3 | 0 | 1 | 216 |
| 124 | 7C | 60 | 3C | 2 | 4 | 588847 | 4710772 | 2 | 4 | 0 | 208 | 4834732 | 2 | 4 | 0 | 1 | 216 |
| 125 | 7D | 60 | 3C | 2 | 5 | 588873 | 4710980 | 2 | 5 | 0 | 208 | 4834948 | 2 | 5 | 0 | 1 | 216 |
| 126 | 7E | 60 | 3C | 2 | 6 | 588899 | 4711188 | 2 | 6 | 0 | 208 | 4835164 | 2 | 6 | 0 | 1 | 216 |
| 127 | 7F | 60 | 3C | 2 | 7 | 588925 | 4711396 | 2 | 7 | 0 | 208 | 4835380 | 2 | 7 | 0 | 1 | 216 |
| 128 | 80 | 60 | 3C | 2 | 8 | 588948 | 4711580 | 2 | 8 | 0 | 184 | 4835572 | 2 | 8 | 0 | 1 | 192 |
| 129 | 81 | 60 | 3C | 2 | 9 | 588971 | 4711764 | 2 | 9 | 0 | 184 | 4835764 | 2 | 9 | 0 | 1 | 192 |
| 130 | 82 | 60 | 3C | 2 | 10 | 588994 | 4711948 | 2 | 10 | 0 | 184 | 4835956 | 2 | 10 | 0 | 1 | 192 |
| 131 | 83 | 60 | 3C | 2 | 11 | 589017 | 4712132 | 2 | 11 | 0 | 184 | 4836148 | 2 | 11 | 0 | 1 | 192 |
| 132 | 84 | 60 | 3C | 2 | 12 | 589040 | 4712316 | 2 | 12 | 0 | 184 | 4836340 | 2 | 12 | 0 | 1 | 192 |
| 133 | 85 | 60 | 3C | 2 | 13 | 589063 | 4712500 | 2 | 13 | 0 | 184 | 4836532 | 2 | 13 | 0 | 1 | 192 |
| 134 | 86 | 60 | 3C | 2 | 14 | 589086 | 4712684 | 2 | 14 | 0 | 184 | 4836724 | 2 | 14 | 0 | 1 | 192 |
| 135 | 87 | 60 | 3C | 2 | 15 | 589109 | 4712868 | 2 | 15 | 0 | 184 | 4836916 | 2 | 15 | 0 | 1 | 192 |
| 136 | 88 | 60 | 3C | 2 | 16 | 589132 | 4713052 | 2 | 16 | 0 | 184 | 4837108 | 2 | 16 | 0 | 1 | 192 |
| 137 | 89 | 60 | 3C | 2 | 17 | 589155 | 4713236 | 2 | 17 | 0 | 184 | 4837300 | 2 | 17 | 0 | 1 | 192 |
| 138 | 8A | 60 | 3C | 2 | 18 | 589178 | 4713420 | 2 | 18 | 0 | 184 | 4837492 | 2 | 18 | 0 | 1 | 192 |
| 139 | 8B | 60 | 3C | 2 | 19 | 589201 | 4713604 | 2 | 19 | 0 | 184 | 4837684 | 2 | 19 | 0 | 1 | 192 |
| 140 | 8C | 60 | 3C | 2 | 20 | 589224 | 4713788 | 2 | 20 | 0 | 184 | 4837876 | 2 | 20 | 0 | 1 | 192 |
| 141 | 8D | 60 | 3C | 2 | 21 | 589247 | 4713972 | 2 | 21 | 0 | 184 | 4838068 | 2 | 21 | 0 | 1 | 192 |
| 142 | 8E | 60 | 3C | 2 | 22 | 589270 | 4714156 | 2 | 22 | 0 | 184 | 4838260 | 2 | 22 | 0 | 1 | 192 |
| 143 | 8F | 60 | 3C | 2 | 23 | 589293 | 4714340 | 2 | 23 | 0 | 184 | 4838452 | 2 | 23 | 0 | 1 | 192 |
| 144 | 90 | 60 | 3C | 2 | 24 | 589318 | 4714540 | 2 | 24 | 0 | 200 | 4838660 | 2 | 24 | 0 | 1 | 208 |
| 145 | 91 | 60 | 3C | 2 | 25 | 589343 | 4714740 | 2 | 25 | 0 | 200 | 4838868 | 2 | 25 | 0 | 1 | 208 |
| 146 | 92 | 60 | 3C | 2 | 26 | 589368 | 4714940 | 2 | 26 | 0 | 200 | 4839076 | 2 | 26 | 0 | 1 | 208 |
| 147 | 93 | 60 | 3C | 2 | 27 | 589393 | 4715140 | 2 | 27 | 0 | 200 | 4839284 | 2 | 27 | 0 | 1 | 208 |
| 148 | 94 | 60 | 3C | 2 | 28 | 589418 | 4715340 | 2 | 28 | 0 | 200 | 4839492 | 2 | 28 | 0 | 1 | 208 |
| 149 | 95 | 60 | 3C | 2 | 29 | 589443 | 4715540 | 2 | 29 | 0 | 200 | 4839700 | 2 | 29 | 0 | 1 | 208 |
| 150 | 96 | 60 | 3C | 2 | 30 | 589468 | 4715740 | 2 | 30 | 0 | 200 | 4839908 | 2 | 30 | 0 | 1 | 208 |
| 151 | 97 | 60 | 3C | 2 | 31 | 589493 | 4715940 | 2 | 31 | 0 | 200 | 4840116 | 2 | 31 | 0 | 1 | 208 |
| 152 | 98 | 60 | 3C | 2 | 32 | 589518 | 4716140 | 2 | 32 | 0 | 200 | 4840324 | 2 | 32 | 0 | 1 | 208 |
| 153 | 99 | 60 | 3C | 2 | 33 | 589543 | 4716340 | 2 | 33 | 0 | 200 | 4840532 | 2 | 33 | 0 | 1 | 208 |
| 154 | 9A | 60 | 3C | 2 | 34 | 589568 | 4716540 | 2 | 34 | 0 | 200 | 4840740 | 2 | 34 | 0 | 1 | 208 |
| 155 | 9B | 60 | 3C | 2 | 35 | 589593 | 4716740 | 2 | 35 | 0 | 200 | 4840948 | 2 | 35 | 0 | 1 | 208 |
| 156 | 9C | 60 | 3C | 2 | 36 | 589618 | 4716940 | 2 | 36 | 0 | 200 | 4841156 | 2 | 36 | 0 | 1 | 208 |
| 157 | 9D | 60 | 3C | 2 | 37 | 589643 | 4717140 | 2 | 37 | 0 | 200 | 4841364 | 2 | 37 | 0 | 1 | 208 |
| 158 | 9E | 60 | 3C | 2 | 38 | 589668 | 4717340 | 2 | 38 | 0 | 200 | 4841572 | 2 | 38 | 0 | 1 | 208 |
| 159 | 9F | 60 | 3C | 2 | 39 | 589693 | 4717540 | 2 | 39 | 0 | 200 | 4841780 | 2 | 39 | 0 | 1 | 208 |
| 160 | A0 | 60 | 3C | 2 | 40 | 589718 | 4717740 | 2 | 40 | 0 | 200 | 4841988 | 2 | 40 | 0 | 1 | 208 |
| 161 | A1 | 60 | 3C | 2 | 41 | 589743 | 4717940 | 2 | 41 | 0 | 200 | 4842196 | 2 | 41 | 0 | 1 | 208 |
| 162 | A2 | 60 | 3C | 2 | 42 | 589768 | 4718140 | 2 | 42 | 0 | 200 | 4842404 | 2 | 42 | 0 | 1 | 208 |
| 163 | A3 | 60 | 3C | 2 | 43 | 589793 | 4718340 | 2 | 43 | 0 | 200 | 4842612 | 2 | 43 | 0 | 1 | 208 |
| 164 | A4 | 60 | 3C | 2 | 44 | 589818 | 4718540 | 2 | 44 | 0 | 200 | 4842820 | 2 | 44 | 0 | 1 | 208 |
| 165 | A5 | 60 | 3C | 2 | 45 | 589843 | 4718740 | 2 | 45 | 0 | 200 | 4843028 | 2 | 45 | 0 | 1 | 208 |
| 166 | A6 | 60 | 3C | 2 | 46 | 589868 | 4718940 | 2 | 46 | 0 | 200 | 4843236 | 2 | 46 | 0 | 1 | 208 |
| 167 | A7 | 60 | 3C | 2 | 47 | 589893 | 4719140 | 2 | 47 | 0 | 200 | 4843444 | 2 | 47 | 0 | 1 | 208 |
| 168 | A8 | 60 | 3C | 2 | 48 | 589918 | 4719340 | 2 | 48 | 0 | 200 | 4843652 | 2 | 48 | 0 | 1 | 208 |
| 169 | A9 | 60 | 3C | 2 | 49 | 589943 | 4719540 | 2 | 49 | 0 | 200 | 4843860 | 2 | 49 | 0 | 1 | 208 |
| 170 | AA | 60 | 3C | 2 | 50 | 589968 | 4719740 | 2 | 50 | 0 | 200 | 4844068 | 2 | 50 | 0 | 1 | 208 |
| 171 | AB | 60 | 3C | 2 | 51 | 589993 | 4719940 | 2 | 51 | 0 | 200 | 4844276 | 2 | 51 | 0 | 1 | 208 |
| 172 | AC | 60 | 3C | 2 | 52 | 590018 | 4720140 | 2 | 52 | 0 | 200 | 4844484 | 2 | 52 | 0 | 1 | 208 |
| 173 | AD | 60 | 3C | 2 | 53 | 590043 | 4720340 | 2 | 53 | 0 | 200 | 4844692 | 2 | 53 | 0 | 1 | 208 |
| 174 | AE | 60 | 3C | 2 | 54 | 590068 | 4720540 | 2 | 54 | 0 | 200 | 4844900 | 2 | 54 | 0 | 1 | 208 |
| 175 | AF | 60 | 3C | 2 | 55 | 590093 | 4720740 | 2 | 55 | 0 | 200 | 4845108 | 2 | 55 | 0 | 1 | 208 |
| 176 | B0 | 60 | 3C | 2 | 56 | 590118 | 4720940 | 2 | 56 | 0 | 200 | 4845316 | 2 | 56 | 0 | 1 | 208 |
| 177 | B1 | 60 | 3C | 2 | 57 | 590143 | 4721140 | 2 | 57 | 0 | 200 | 4845524 | 2 | 57 | 0 | 1 | 208 |
| 178 | B2 | 60 | 3C | 2 | 58 | 590168 | 4721340 | 2 | 58 | 0 | 200 | 4845732 | 2 | 58 | 0 | 1 | 208 |
| 179 | B3 | 60 | 3C | 2 | 59 | 590193 | 4721540 | 2 | 59 | 0 | 200 | 4845940 | 2 | 59 | 0 | 1 | 208 |
| 180 | B4 | 60 | 3C | 3 | 0 | 590216 | 4721724 | 3 | 0 | 0 | 184 | 4846132 | 3 | 0 | 0 | 1 | 192 |
| 181 | B5 | 60 | 3C | 3 | 1 | 590244 | 4721948 | 3 | 1 | 0 | 224 | 4846364 | 3 | 1 | 0 | 1 | 232 |
| 182 | B6 | 60 | 3C | 3 | 2 | 590272 | 4722172 | 3 | 2 | 0 | 224 | 4846596 | 3 | 2 | 0 | 1 | 232 |
| 183 | B7 | 60 | 3C | 3 | 3 | 590300 | 4722396 | 3 | 3 | 0 | 224 | 4846828 | 3 | 3 | 0 | 1 | 232 |
| 184 | B8 | 60 | 3C | 3 | 4 | 590328 | 4722620 | 3 | 4 | 0 | 224 | 4847060 | 3 | 4 | 0 | 1 | 232 |
| 185 | B9 | 60 | 3C | 3 | 5 | 590356 | 4722844 | 3 | 5 | 0 | 224 | 4847292 | 3 | 5 | 0 | 1 | 232 |
| 186 | BA | 60 | 3C | 3 | 6 | 590384 | 4723068 | 3 | 6 | 0 | 224 | 4847524 | 3 | 6 | 0 | 1 | 232 |
| 187 | BB | 60 | 3C | 3 | 7 | 590412 | 4723292 | 3 | 7 | 0 | 224 | 4847756 | 3 | 7 | 0 | 1 | 232 |
| 188 | BC | 60 | 3C | 3 | 8 | 590440 | 4723516 | 3 | 8 | 0 | 224 | 4847988 | 3 | 8 | 0 | 1 | 232 |
| 189 | BD | 60 | 3C | 3 | 9 | 590468 | 4723740 | 3 | 9 | 0 | 224 | 4848220 | 3 | 9 | 0 | 1 | 232 |

| | | | | | | | | | | | | | | | | | |
|---|---|---|---|---|---|---|---|---|---|---|---|---|---|---|---|---|---|
| 190 | BE | 60 | 3C | 3 | 10 | 590496 | 4723964 | 3 | 10 | 0 | 224 | 4848452 | 3 | 10 | 0 | 1 | 232 |
| 191 | BF | 60 | 3C | 3 | 11 | 590524 | 4724188 | 3 | 11 | 0 | 224 | 4848684 | 3 | 11 | 0 | 1 | 232 |
| 192 | C0 | 60 | 3C | 3 | 12 | 590549 | 4724388 | 3 | 12 | 0 | 200 | 4848892 | 3 | 12 | 0 | 1 | 208 |
| 193 | C1 | 60 | 3C | 3 | 13 | 590574 | 4724588 | 3 | 13 | 0 | 200 | 4849100 | 3 | 13 | 0 | 1 | 208 |
| 194 | C2 | 60 | 3C | 3 | 14 | 590599 | 4724788 | 3 | 14 | 0 | 200 | 4849308 | 3 | 14 | 0 | 1 | 208 |
| 195 | C3 | 60 | 3C | 3 | 15 | 590624 | 4724988 | 3 | 15 | 0 | 200 | 4849516 | 3 | 15 | 0 | 1 | 208 |
| 196 | C4 | 60 | 3C | 3 | 16 | 590649 | 4725188 | 3 | 16 | 0 | 200 | 4849724 | 3 | 16 | 0 | 1 | 208 |
| 197 | C5 | 60 | 3C | 3 | 17 | 590674 | 4725388 | 3 | 17 | 0 | 200 | 4849932 | 3 | 17 | 0 | 1 | 208 |
| 198 | C6 | 60 | 3C | 3 | 18 | 590699 | 4725588 | 3 | 18 | 0 | 200 | 4850140 | 3 | 18 | 0 | 1 | 208 |
| 199 | C7 | 60 | 3C | 3 | 19 | 590724 | 4725788 | 3 | 19 | 0 | 200 | 4850348 | 3 | 19 | 0 | 1 | 208 |
| 200 | C8 | 60 | 3C | 3 | 20 | 590749 | 4725988 | 3 | 20 | 0 | 200 | 4850556 | 3 | 20 | 0 | 1 | 208 |
| 201 | C9 | 60 | 3C | 3 | 21 | 590774 | 4726188 | 3 | 21 | 0 | 200 | 4850764 | 3 | 21 | 0 | 1 | 208 |
| 202 | CA | 60 | 3C | 3 | 22 | 590799 | 4726388 | 3 | 22 | 0 | 200 | 4850972 | 3 | 22 | 0 | 1 | 208 |
| 203 | CB | 60 | 3C | 3 | 23 | 590824 | 4726588 | 3 | 23 | 0 | 200 | 4851180 | 3 | 23 | 0 | 1 | 208 |
| 204 | CC | 60 | 3C | 3 | 24 | 590849 | 4726788 | 3 | 24 | 0 | 200 | 4851388 | 3 | 24 | 0 | 1 | 208 |
| 205 | CD | 60 | 3C | 3 | 25 | 590874 | 4726988 | 3 | 25 | 0 | 200 | 4851596 | 3 | 25 | 0 | 1 | 208 |
| 206 | CE | 60 | 3C | 3 | 26 | 590899 | 4727188 | 3 | 26 | 0 | 200 | 4851804 | 3 | 26 | 0 | 1 | 208 |
| 207 | CF | 60 | 3C | 3 | 27 | 590924 | 4727388 | 3 | 27 | 0 | 200 | 4852012 | 3 | 27 | 0 | 1 | 208 |
| 208 | D0 | 60 | 3C | 3 | 28 | 590951 | 4727604 | 3 | 28 | 0 | 216 | 4852236 | 3 | 28 | 0 | 1 | 224 |
| 209 | D1 | 60 | 3C | 3 | 29 | 590978 | 4727820 | 3 | 29 | 0 | 216 | 4852460 | 3 | 29 | 0 | 1 | 224 |
| 210 | D2 | 60 | 3C | 3 | 30 | 591005 | 4728036 | 3 | 30 | 0 | 216 | 4852684 | 3 | 30 | 0 | 1 | 224 |
| 211 | D3 | 60 | 3C | 3 | 31 | 591032 | 4728252 | 3 | 31 | 0 | 216 | 4852908 | 3 | 31 | 0 | 1 | 224 |
| 212 | D4 | 60 | 3C | 3 | 32 | 591059 | 4728468 | 3 | 32 | 0 | 216 | 4853132 | 3 | 32 | 0 | 1 | 224 |
| 213 | D5 | 60 | 3C | 3 | 33 | 591086 | 4728684 | 3 | 33 | 0 | 216 | 4853356 | 3 | 33 | 0 | 1 | 224 |
| 214 | D6 | 60 | 3C | 3 | 34 | 591113 | 4728900 | 3 | 34 | 0 | 216 | 4853580 | 3 | 34 | 0 | 1 | 224 |
| 215 | D7 | 60 | 3C | 3 | 35 | 591140 | 4729116 | 3 | 35 | 0 | 216 | 4853804 | 3 | 35 | 0 | 1 | 224 |
| 216 | D8 | 60 | 3C | 3 | 36 | 591167 | 4729332 | 3 | 36 | 0 | 216 | 4854028 | 3 | 36 | 0 | 1 | 224 |
| 217 | D9 | 60 | 3C | 3 | 37 | 591194 | 4729548 | 3 | 37 | 0 | 216 | 4854252 | 3 | 37 | 0 | 1 | 224 |
| 218 | DA | 60 | 3C | 3 | 38 | 591221 | 4729764 | 3 | 38 | 0 | 216 | 4854476 | 3 | 38 | 0 | 1 | 224 |
| 219 | DB | 60 | 3C | 3 | 39 | 591248 | 4729980 | 3 | 39 | 0 | 216 | 4854700 | 3 | 39 | 0 | 1 | 224 |
| 220 | DC | 60 | 3C | 3 | 40 | 591275 | 4730196 | 3 | 40 | 0 | 216 | 4854924 | 3 | 40 | 0 | 1 | 224 |
| 221 | DD | 60 | 3C | 3 | 41 | 591302 | 4730412 | 3 | 41 | 0 | 216 | 4855148 | 3 | 41 | 0 | 1 | 224 |
| 222 | DE | 60 | 3C | 3 | 42 | 591329 | 4730628 | 3 | 42 | 0 | 216 | 4855372 | 3 | 42 | 0 | 1 | 224 |
| 223 | DF | 60 | 3C | 3 | 43 | 591356 | 4730844 | 3 | 43 | 0 | 216 | 4855596 | 3 | 43 | 0 | 1 | 224 |
| 224 | E0 | 60 | 3C | 3 | 44 | 591383 | 4731060 | 3 | 44 | 0 | 216 | 4855820 | 3 | 44 | 0 | 1 | 224 |
| 225 | E1 | 60 | 3C | 3 | 45 | 591410 | 4731276 | 3 | 45 | 0 | 216 | 4856044 | 3 | 45 | 0 | 1 | 224 |
| 226 | E2 | 60 | 3C | 3 | 46 | 591437 | 4731492 | 3 | 46 | 0 | 216 | 4856268 | 3 | 46 | 0 | 1 | 224 |
| 227 | E3 | 60 | 3C | 3 | 47 | 591464 | 4731708 | 3 | 47 | 0 | 216 | 4856492 | 3 | 47 | 0 | 1 | 224 |
| 228 | E4 | 60 | 3C | 3 | 48 | 591491 | 4731924 | 3 | 48 | 0 | 216 | 4856716 | 3 | 48 | 0 | 1 | 224 |
| 229 | E5 | 60 | 3C | 3 | 49 | 591518 | 4732140 | 3 | 49 | 0 | 216 | 4856940 | 3 | 49 | 0 | 1 | 224 |
| 230 | E6 | 60 | 3C | 3 | 50 | 591545 | 4732356 | 3 | 50 | 0 | 216 | 4857164 | 3 | 50 | 0 | 1 | 224 |
| 231 | E7 | 60 | 3C | 3 | 51 | 591572 | 4732572 | 3 | 51 | 0 | 216 | 4857388 | 3 | 51 | 0 | 1 | 224 |
| 232 | E8 | 60 | 3C | 3 | 52 | 591599 | 4732788 | 3 | 52 | 0 | 216 | 4857612 | 3 | 52 | 0 | 1 | 224 |
| 233 | E9 | 60 | 3C | 3 | 53 | 591626 | 4733004 | 3 | 53 | 0 | 216 | 4857836 | 3 | 53 | 0 | 1 | 224 |
| 234 | EA | 60 | 3C | 3 | 54 | 591653 | 4733220 | 3 | 54 | 0 | 216 | 4858060 | 3 | 54 | 0 | 1 | 224 |
| 235 | EB | 60 | 3C | 3 | 55 | 591680 | 4733436 | 3 | 55 | 0 | 216 | 4858284 | 3 | 55 | 0 | 1 | 224 |
| 236 | EC | 60 | 3C | 3 | 56 | 591707 | 4733652 | 3 | 56 | 0 | 216 | 4858508 | 3 | 56 | 0 | 1 | 224 |
| 237 | ED | 60 | 3C | 3 | 57 | 591734 | 4733868 | 3 | 57 | 0 | 216 | 4858732 | 3 | 57 | 0 | 1 | 224 |
| 238 | EE | 60 | 3C | 3 | 58 | 591761 | 4734084 | 3 | 58 | 0 | 216 | 4858956 | 3 | 58 | 0 | 1 | 224 |
| 239 | EF | 60 | 3C | 3 | 59 | 591788 | 4734300 | 3 | 59 | 0 | 216 | 4859180 | 3 | 59 | 0 | 1 | 224 |
| 240 | F0 | 60 | 3C | 4 | 0 | 591813 | 4734500 | 4 | 0 | 0 | 200 | 4859388 | 4 | 0 | 0 | 1 | 208 |
| 241 | F1 | 60 | 3C | 4 | 1 | 591843 | 4734740 | 4 | 1 | 0 | 240 | 4859636 | 4 | 1 | 0 | 1 | 248 |
| 242 | F2 | 60 | 3C | 4 | 2 | 591873 | 4734980 | 4 | 2 | 0 | 240 | 4859884 | 4 | 2 | 0 | 1 | 248 |
| 243 | F3 | 60 | 3C | 4 | 3 | 591903 | 4735220 | 4 | 3 | 0 | 240 | 4860132 | 4 | 3 | 0 | 1 | 248 |
| 244 | F4 | 60 | 3C | 4 | 4 | 591933 | 4735460 | 4 | 4 | 0 | 240 | 4860380 | 4 | 4 | 0 | 1 | 248 |
| 245 | F5 | 60 | 3C | 4 | 5 | 591963 | 4735700 | 4 | 5 | 0 | 240 | 4860628 | 4 | 5 | 0 | 1 | 248 |
| 246 | F6 | 60 | 3C | 4 | 6 | 591993 | 4735940 | 4 | 6 | 0 | 240 | 4860876 | 4 | 6 | 0 | 1 | 248 |
| 247 | F7 | 60 | 3C | 4 | 7 | 592023 | 4736180 | 4 | 7 | 0 | 240 | 4861124 | 4 | 7 | 0 | 1 | 248 |
| 248 | F8 | 60 | 3C | 4 | 8 | 592053 | 4736420 | 4 | 8 | 0 | 240 | 4861372 | 4 | 8 | 0 | 1 | 248 |
| 249 | F9 | 60 | 3C | 4 | 9 | 592083 | 4736660 | 4 | 9 | 0 | 240 | 4861620 | 4 | 9 | 0 | 1 | 248 |
| 250 | FA | 60 | 3C | 4 | 10 | 592113 | 4736900 | 4 | 10 | 0 | 240 | 4861868 | 4 | 10 | 0 | 1 | 248 |
| 251 | FB | 60 | 3C | 4 | 11 | 592143 | 4737140 | 4 | 11 | 0 | 240 | 4862116 | 4 | 11 | 0 | 1 | 248 |
| 252 | FC | 60 | 3C | 4 | 12 | 592173 | 4737380 | 4 | 12 | 0 | 240 | 4862364 | 4 | 12 | 0 | 1 | 248 |
| 253 | FD | 60 | 3C | 4 | 13 | 592203 | 4737620 | 4 | 13 | 0 | 240 | 4862612 | 4 | 13 | 0 | 1 | 248 |
| 254 | FE | 60 | 3C | 4 | 14 | 592233 | 4737860 | 4 | 14 | 0 | 240 | 4862860 | 4 | 14 | 0 | 1 | 248 |
| 255 | FF | 60 | 3C | 4 | 15 | 592263 | 4738100 | 4 | 15 | 0 | 240 | 4863108 | 4 | 15 | 0 | 1 | 248 |
| 0 | 00 | 61 | 3D | 0 | 0 | 592275 | 4738196 | 0 | 0 | 0 | 96 | 4863212 | 0 | 0 | 0 | 1 | 104 |
| 1 | 01 | 61 | 3D | 0 | 1 | 592289 | 4738308 | 0 | 1 | 0 | 112 | 4863332 | 0 | 1 | 0 | 1 | 120 |
| 2 | 02 | 61 | 3D | 0 | 2 | 592303 | 4738420 | 0 | 2 | 0 | 112 | 4863452 | 0 | 2 | 0 | 1 | 120 |
| 3 | 03 | 61 | 3D | 0 | 3 | 592317 | 4738532 | 0 | 3 | 0 | 112 | 4863572 | 0 | 3 | 0 | 1 | 120 |
| 4 | 04 | 61 | 3D | 0 | 4 | 592331 | 4738644 | 0 | 4 | 0 | 112 | 4863692 | 0 | 4 | 0 | 1 | 120 |
| 5 | 05 | 61 | 3D | 0 | 5 | 592345 | 4738756 | 0 | 5 | 0 | 112 | 4863812 | 0 | 5 | 0 | 1 | 120 |
| 6 | 06 | 61 | 3D | 0 | 6 | 592359 | 4738868 | 0 | 6 | 0 | 112 | 4863932 | 0 | 6 | 0 | 1 | 120 |
| 7 | 07 | 61 | 3D | 0 | 7 | 592373 | 4738980 | 0 | 7 | 0 | 112 | 4864052 | 0 | 7 | 0 | 1 | 120 |
| 8 | 08 | 61 | 3D | 0 | 8 | 592387 | 4739092 | 0 | 8 | 0 | 112 | 4864172 | 0 | 8 | 0 | 1 | 120 |
| 9 | 09 | 61 | 3D | 0 | 9 | 592401 | 4739204 | 0 | 9 | 0 | 112 | 4864292 | 0 | 9 | 0 | 1 | 120 |
| 10 | 0A | 61 | 3D | 0 | 10 | 592415 | 4739316 | 0 | 10 | 0 | 112 | 4864412 | 0 | 10 | 0 | 1 | 120 |
| 11 | 0B | 61 | 3D | 0 | 11 | 592429 | 4739428 | 0 | 11 | 0 | 112 | 4864532 | 0 | 11 | 0 | 1 | 120 |
| 12 | 0C | 61 | 3D | 0 | 12 | 592443 | 4739540 | 0 | 12 | 0 | 112 | 4864652 | 0 | 12 | 0 | 1 | 120 |
| 13 | 0D | 61 | 3D | 0 | 13 | 592457 | 4739652 | 0 | 13 | 0 | 112 | 4864772 | 0 | 13 | 0 | 1 | 120 |
| 14 | 0E | 61 | 3D | 0 | 14 | 592471 | 4739764 | 0 | 14 | 0 | 112 | 4864892 | 0 | 14 | 0 | 1 | 120 |
| 15 | 0F | 61 | 3D | 0 | 15 | 592485 | 4739876 | 0 | 15 | 0 | 112 | 4865012 | 0 | 15 | 0 | 1 | 120 |
| 16 | 10 | 61 | 3D | 0 | 16 | 592499 | 4739988 | 0 | 16 | 0 | 112 | 4865132 | 0 | 16 | 0 | 1 | 120 |
| 17 | 11 | 61 | 3D | 0 | 17 | 592513 | 4740100 | 0 | 17 | 0 | 112 | 4865252 | 0 | 17 | 0 | 1 | 120 |
| 18 | 12 | 61 | 3D | 0 | 18 | 592527 | 4740212 | 0 | 18 | 0 | 112 | 4865372 | 0 | 18 | 0 | 1 | 120 |
| 19 | 13 | 61 | 3D | 0 | 19 | 592541 | 4740324 | 0 | 19 | 0 | 112 | 4865492 | 0 | 19 | 0 | 1 | 120 |
| 20 | 14 | 61 | 3D | 0 | 20 | 592555 | 4740436 | 0 | 20 | 0 | 112 | 4865612 | 0 | 20 | 0 | 1 | 120 |
| 21 | 15 | 61 | 3D | 0 | 21 | 592569 | 4740548 | 0 | 21 | 0 | 112 | 4865732 | 0 | 21 | 0 | 1 | 120 |
| 22 | 16 | 61 | 3D | 0 | 22 | 592583 | 4740660 | 0 | 22 | 0 | 112 | 4865852 | 0 | 22 | 0 | 1 | 120 |
| 23 | 17 | 61 | 3D | 0 | 23 | 592597 | 4740772 | 0 | 23 | 0 | 112 | 4865972 | 0 | 23 | 0 | 1 | 120 |
| 24 | 18 | 61 | 3D | 0 | 24 | 592611 | 4740884 | 0 | 24 | 0 | 112 | 4866092 | 0 | 24 | 0 | 1 | 120 |
| 25 | 19 | 61 | 3D | 0 | 25 | 592625 | 4740996 | 0 | 25 | 0 | 112 | 4866212 | 0 | 25 | 0 | 1 | 120 |
| 26 | 1A | 61 | 3D | 0 | 26 | 592639 | 4741108 | 0 | 26 | 0 | 112 | 4866332 | 0 | 26 | 0 | 1 | 120 |
| 27 | 1B | 61 | 3D | 0 | 27 | 592653 | 4741220 | 0 | 27 | 0 | 112 | 4866452 | 0 | 27 | 0 | 1 | 120 |
| 28 | 1C | 61 | 3D | 0 | 28 | 592667 | 4741332 | 0 | 28 | 0 | 112 | 4866572 | 0 | 28 | 0 | 1 | 120 |
| 29 | 1D | 61 | 3D | 0 | 29 | 592681 | 4741444 | 0 | 29 | 0 | 112 | 4866692 | 0 | 29 | 0 | 1 | 120 |
| 30 | 1E | 61 | 3D | 0 | 30 | 592695 | 4741556 | 0 | 30 | 0 | 112 | 4866812 | 0 | 30 | 0 | 1 | 120 |
| 31 | 1F | 61 | 3D | 0 | 31 | 592709 | 4741668 | 0 | 31 | 0 | 112 | 4866932 | 0 | 31 | 0 | 1 | 120 |
| 32 | 20 | 61 | 3D | 0 | 32 | 592723 | 4741780 | 0 | 32 | 0 | 112 | 4867052 | 0 | 32 | 0 | 1 | 120 |
| 33 | 21 | 61 | 3D | 0 | 33 | 592737 | 4741892 | 0 | 33 | 0 | 112 | 4867172 | 0 | 33 | 0 | 1 | 120 |
| 34 | 22 | 61 | 3D | 0 | 34 | 592751 | 4742004 | 0 | 34 | 0 | 112 | 4867292 | 0 | 34 | 0 | 1 | 120 |
| 35 | 23 | 61 | 3D | 0 | 35 | 592765 | 4742116 | 0 | 35 | 0 | 112 | 4867412 | 0 | 35 | 0 | 1 | 120 |
| 36 | 24 | 61 | 3D | 0 | 36 | 592779 | 4742228 | 0 | 36 | 0 | 112 | 4867532 | 0 | 36 | 0 | 1 | 120 |

| | | | | | | | | | | | | | | | | | |
|---|---|---|---|---|---|---|---|---|---|---|---|---|---|---|---|---|---|
| 37 | 25 | 61 | 3D | 0 | 37 | 592793 | 4742340 | 0 | 37 | 0 | 112 | 4867652 | 0 | 37 | 0 | 1 | 120 |
| 38 | 26 | 61 | 3D | 0 | 38 | 592807 | 4742452 | 0 | 38 | 0 | 112 | 4867772 | 0 | 38 | 0 | 1 | 120 |
| 39 | 27 | 61 | 3D | 0 | 39 | 592821 | 4742564 | 0 | 39 | 0 | 112 | 4867892 | 0 | 39 | 0 | 1 | 120 |
| 40 | 28 | 61 | 3D | 0 | 40 | 592835 | 4742676 | 0 | 40 | 0 | 112 | 4868012 | 0 | 40 | 0 | 1 | 120 |
| 41 | 29 | 61 | 3D | 0 | 41 | 592849 | 4742788 | 0 | 41 | 0 | 112 | 4868132 | 0 | 41 | 0 | 1 | 120 |
| 42 | 2A | 61 | 3D | 0 | 42 | 592863 | 4742900 | 0 | 42 | 0 | 112 | 4868252 | 0 | 42 | 0 | 1 | 120 |
| 43 | 2B | 61 | 3D | 0 | 43 | 592877 | 4743012 | 0 | 43 | 0 | 112 | 4868372 | 0 | 43 | 0 | 1 | 120 |
| 44 | 2C | 61 | 3D | 0 | 44 | 592891 | 4743124 | 0 | 44 | 0 | 112 | 4868492 | 0 | 44 | 0 | 1 | 120 |
| 45 | 2D | 61 | 3D | 0 | 45 | 592905 | 4743236 | 0 | 45 | 0 | 112 | 4868612 | 0 | 45 | 0 | 1 | 120 |
| 46 | 2E | 61 | 3D | 0 | 46 | 592919 | 4743348 | 0 | 46 | 0 | 112 | 4868732 | 0 | 46 | 0 | 1 | 120 |
| 47 | 2F | 61 | 3D | 0 | 47 | 592933 | 4743460 | 0 | 47 | 0 | 112 | 4868852 | 0 | 47 | 0 | 1 | 120 |
| 48 | 30 | 61 | 3D | 0 | 48 | 592947 | 4743572 | 0 | 48 | 0 | 112 | 4868972 | 0 | 48 | 0 | 1 | 120 |
| 49 | 31 | 61 | 3D | 0 | 49 | 592961 | 4743684 | 0 | 49 | 0 | 112 | 4869092 | 0 | 49 | 0 | 1 | 120 |
| 50 | 32 | 61 | 3D | 0 | 50 | 592975 | 4743796 | 0 | 50 | 0 | 112 | 4869212 | 0 | 50 | 0 | 1 | 120 |
| 51 | 33 | 61 | 3D | 0 | 51 | 592989 | 4743908 | 0 | 51 | 0 | 112 | 4869332 | 0 | 51 | 0 | 1 | 120 |
| 52 | 34 | 61 | 3D | 0 | 52 | 593003 | 4744020 | 0 | 52 | 0 | 112 | 4869452 | 0 | 52 | 0 | 1 | 120 |
| 53 | 35 | 61 | 3D | 0 | 53 | 593017 | 4744132 | 0 | 53 | 0 | 112 | 4869572 | 0 | 53 | 0 | 1 | 120 |
| 54 | 36 | 61 | 3D | 0 | 54 | 593031 | 4744244 | 0 | 54 | 0 | 112 | 4869692 | 0 | 54 | 0 | 1 | 120 |
| 55 | 37 | 61 | 3D | 0 | 55 | 593045 | 4744356 | 0 | 55 | 0 | 112 | 4869812 | 0 | 55 | 0 | 1 | 120 |
| 56 | 38 | 61 | 3D | 0 | 56 | 593059 | 4744468 | 0 | 56 | 0 | 112 | 4869932 | 0 | 56 | 0 | 1 | 120 |
| 57 | 39 | 61 | 3D | 0 | 57 | 593073 | 4744580 | 0 | 57 | 0 | 112 | 4870052 | 0 | 57 | 0 | 1 | 120 |
| 58 | 3A | 61 | 3D | 0 | 58 | 593087 | 4744692 | 0 | 58 | 0 | 112 | 4870172 | 0 | 58 | 0 | 1 | 120 |
| 59 | 3B | 61 | 3D | 0 | 59 | 593101 | 4744804 | 0 | 59 | 0 | 112 | 4870292 | 0 | 59 | 0 | 1 | 120 |
| 60 | 3C | 61 | 3D | 0 | 60 | 593115 | 4744916 | 0 | 60 | 0 | 112 | 4870412 | 0 | 60 | 0 | 1 | 120 |
| 61 | 3D | 61 | 3D | 1 | 0 | 593127 | 4745012 | 1 | 0 | 0 | 96 | 4870516 | 1 | 0 | 0 | 1 | 104 |
| 62 | 3E | 61 | 3D | 1 | 1 | 593144 | 4745148 | 1 | 1 | 0 | 136 | 4870660 | 1 | 1 | 0 | 1 | 144 |
| 63 | 3F | 61 | 3D | 1 | 2 | 593161 | 4745284 | 1 | 2 | 0 | 136 | 4870804 | 1 | 2 | 0 | 1 | 144 |
| 64 | 40 | 61 | 3D | 1 | 3 | 593182 | 4745452 | 1 | 3 | 0 | 168 | 4870980 | 1 | 3 | 0 | 1 | 176 |
| 65 | 41 | 61 | 3D | 1 | 4 | 593203 | 4745620 | 1 | 4 | 0 | 168 | 4871156 | 1 | 4 | 0 | 1 | 176 |
| 66 | 42 | 61 | 3D | 1 | 5 | 593224 | 4745788 | 1 | 5 | 0 | 168 | 4871332 | 1 | 5 | 0 | 1 | 176 |
| 67 | 43 | 61 | 3D | 1 | 6 | 593245 | 4745956 | 1 | 6 | 0 | 168 | 4871508 | 1 | 6 | 0 | 1 | 176 |
| 68 | 44 | 61 | 3D | 1 | 7 | 593266 | 4746124 | 1 | 7 | 0 | 168 | 4871684 | 1 | 7 | 0 | 1 | 176 |
| 69 | 45 | 61 | 3D | 1 | 8 | 593287 | 4746292 | 1 | 8 | 0 | 168 | 4871860 | 1 | 8 | 0 | 1 | 176 |
| 70 | 46 | 61 | 3D | 1 | 9 | 593308 | 4746460 | 1 | 9 | 0 | 168 | 4872036 | 1 | 9 | 0 | 1 | 176 |
| 71 | 47 | 61 | 3D | 1 | 10 | 593329 | 4746628 | 1 | 10 | 0 | 168 | 4872212 | 1 | 10 | 0 | 1 | 176 |
| 72 | 48 | 61 | 3D | 1 | 11 | 593350 | 4746796 | 1 | 11 | 0 | 168 | 4872388 | 1 | 11 | 0 | 1 | 176 |
| 73 | 49 | 61 | 3D | 1 | 12 | 593371 | 4746964 | 1 | 12 | 0 | 168 | 4872564 | 1 | 12 | 0 | 1 | 176 |
| 74 | 4A | 61 | 3D | 1 | 13 | 593392 | 4747132 | 1 | 13 | 0 | 168 | 4872740 | 1 | 13 | 0 | 1 | 176 |
| 75 | 4B | 61 | 3D | 1 | 14 | 593413 | 4747300 | 1 | 14 | 0 | 168 | 4872916 | 1 | 14 | 0 | 1 | 176 |
| 76 | 4C | 61 | 3D | 1 | 15 | 593434 | 4747468 | 1 | 15 | 0 | 168 | 4873092 | 1 | 15 | 0 | 1 | 176 |
| 77 | 4D | 61 | 3D | 1 | 16 | 593455 | 4747636 | 1 | 16 | 0 | 168 | 4873268 | 1 | 16 | 0 | 1 | 176 |
| 78 | 4E | 61 | 3D | 1 | 17 | 593476 | 4747804 | 1 | 17 | 0 | 168 | 4873444 | 1 | 17 | 0 | 1 | 176 |
| 79 | 4F | 61 | 3D | 1 | 18 | 593497 | 4747972 | 1 | 18 | 0 | 168 | 4873620 | 1 | 18 | 0 | 1 | 176 |
| 80 | 50 | 61 | 3D | 1 | 19 | 593520 | 4748156 | 1 | 19 | 0 | 184 | 4873812 | 1 | 19 | 0 | 1 | 192 |
| 81 | 51 | 61 | 3D | 1 | 20 | 593543 | 4748340 | 1 | 20 | 0 | 184 | 4874004 | 1 | 20 | 0 | 1 | 192 |
| 82 | 52 | 61 | 3D | 1 | 21 | 593566 | 4748524 | 1 | 21 | 0 | 184 | 4874196 | 1 | 21 | 0 | 1 | 192 |
| 83 | 53 | 61 | 3D | 1 | 22 | 593589 | 4748708 | 1 | 22 | 0 | 184 | 4874388 | 1 | 22 | 0 | 1 | 192 |
| 84 | 54 | 61 | 3D | 1 | 23 | 593612 | 4748892 | 1 | 23 | 0 | 184 | 4874580 | 1 | 23 | 0 | 1 | 192 |
| 85 | 55 | 61 | 3D | 1 | 24 | 593635 | 4749076 | 1 | 24 | 0 | 184 | 4874772 | 1 | 24 | 0 | 1 | 192 |
| 86 | 56 | 61 | 3D | 1 | 25 | 593658 | 4749260 | 1 | 25 | 0 | 184 | 4874964 | 1 | 25 | 0 | 1 | 192 |
| 87 | 57 | 61 | 3D | 1 | 26 | 593681 | 4749444 | 1 | 26 | 0 | 184 | 4875156 | 1 | 26 | 0 | 1 | 192 |
| 88 | 58 | 61 | 3D | 1 | 27 | 593704 | 4749628 | 1 | 27 | 0 | 184 | 4875348 | 1 | 27 | 0 | 1 | 192 |
| 89 | 59 | 61 | 3D | 1 | 28 | 593727 | 4749812 | 1 | 28 | 0 | 184 | 4875540 | 1 | 28 | 0 | 1 | 192 |
| 90 | 5A | 61 | 3D | 1 | 29 | 593750 | 4749996 | 1 | 29 | 0 | 184 | 4875732 | 1 | 29 | 0 | 1 | 192 |
| 91 | 5B | 61 | 3D | 1 | 30 | 593773 | 4750180 | 1 | 30 | 0 | 184 | 4875924 | 1 | 30 | 0 | 1 | 192 |
| 92 | 5C | 61 | 3D | 1 | 31 | 593796 | 4750364 | 1 | 31 | 0 | 184 | 4876116 | 1 | 31 | 0 | 1 | 192 |
| 93 | 5D | 61 | 3D | 1 | 32 | 593819 | 4750548 | 1 | 32 | 0 | 184 | 4876308 | 1 | 32 | 0 | 1 | 192 |
| 94 | 5E | 61 | 3D | 1 | 33 | 593842 | 4750732 | 1 | 33 | 0 | 184 | 4876500 | 1 | 33 | 0 | 1 | 192 |
| 95 | 5F | 61 | 3D | 1 | 34 | 593865 | 4750916 | 1 | 34 | 0 | 184 | 4876692 | 1 | 34 | 0 | 1 | 192 |
| 96 | 60 | 61 | 3D | 1 | 35 | 593888 | 4751100 | 1 | 35 | 0 | 184 | 4876884 | 1 | 35 | 0 | 1 | 192 |
| 97 | 61 | 61 | 3D | 1 | 36 | 593911 | 4751284 | 1 | 36 | 0 | 184 | 4877076 | 1 | 36 | 0 | 1 | 192 |
| 98 | 62 | 61 | 3D | 1 | 37 | 593934 | 4751468 | 1 | 37 | 0 | 184 | 4877268 | 1 | 37 | 0 | 1 | 192 |
| 99 | 63 | 61 | 3D | 1 | 38 | 593957 | 4751652 | 1 | 38 | 0 | 184 | 4877460 | 1 | 38 | 0 | 1 | 192 |
| 100 | 64 | 61 | 3D | 1 | 39 | 593980 | 4751836 | 1 | 39 | 0 | 184 | 4877652 | 1 | 39 | 0 | 1 | 192 |
| 101 | 65 | 61 | 3D | 1 | 40 | 594003 | 4752020 | 1 | 40 | 0 | 184 | 4877844 | 1 | 40 | 0 | 1 | 192 |
| 102 | 66 | 61 | 3D | 1 | 41 | 594026 | 4752204 | 1 | 41 | 0 | 184 | 4878036 | 1 | 41 | 0 | 1 | 192 |
| 103 | 67 | 61 | 3D | 1 | 42 | 594049 | 4752388 | 1 | 42 | 0 | 184 | 4878228 | 1 | 42 | 0 | 1 | 192 |
| 104 | 68 | 61 | 3D | 1 | 43 | 594072 | 4752572 | 1 | 43 | 0 | 184 | 4878420 | 1 | 43 | 0 | 1 | 192 |
| 105 | 69 | 61 | 3D | 1 | 44 | 594095 | 4752756 | 1 | 44 | 0 | 184 | 4878612 | 1 | 44 | 0 | 1 | 192 |
| 106 | 6A | 61 | 3D | 1 | 45 | 594118 | 4752940 | 1 | 45 | 0 | 184 | 4878804 | 1 | 45 | 0 | 1 | 192 |
| 107 | 6B | 61 | 3D | 1 | 46 | 594141 | 4753124 | 1 | 46 | 0 | 184 | 4878996 | 1 | 46 | 0 | 1 | 192 |
| 108 | 6C | 61 | 3D | 1 | 47 | 594164 | 4753308 | 1 | 47 | 0 | 184 | 4879188 | 1 | 47 | 0 | 1 | 192 |
| 109 | 6D | 61 | 3D | 1 | 48 | 594187 | 4753492 | 1 | 48 | 0 | 184 | 4879380 | 1 | 48 | 0 | 1 | 192 |
| 110 | 6E | 61 | 3D | 1 | 49 | 594210 | 4753676 | 1 | 49 | 0 | 184 | 4879572 | 1 | 49 | 0 | 1 | 192 |
| 111 | 6F | 61 | 3D | 1 | 50 | 594233 | 4753860 | 1 | 50 | 0 | 184 | 4879764 | 1 | 50 | 0 | 1 | 192 |
| 112 | 70 | 61 | 3D | 1 | 51 | 594256 | 4754044 | 1 | 51 | 0 | 184 | 4879956 | 1 | 51 | 0 | 1 | 192 |
| 113 | 71 | 61 | 3D | 1 | 52 | 594279 | 4754228 | 1 | 52 | 0 | 184 | 4880148 | 1 | 52 | 0 | 1 | 192 |
| 114 | 72 | 61 | 3D | 1 | 53 | 594302 | 4754412 | 1 | 53 | 0 | 184 | 4880340 | 1 | 53 | 0 | 1 | 192 |
| 115 | 73 | 61 | 3D | 1 | 54 | 594325 | 4754596 | 1 | 54 | 0 | 184 | 4880532 | 1 | 54 | 0 | 1 | 192 |
| 116 | 74 | 61 | 3D | 1 | 55 | 594348 | 4754780 | 1 | 55 | 0 | 184 | 4880724 | 1 | 55 | 0 | 1 | 192 |
| 117 | 75 | 61 | 3D | 1 | 56 | 594371 | 4754964 | 1 | 56 | 0 | 184 | 4880916 | 1 | 56 | 0 | 1 | 192 |
| 118 | 76 | 61 | 3D | 1 | 57 | 594394 | 4755148 | 1 | 57 | 0 | 184 | 4881108 | 1 | 57 | 0 | 1 | 192 |
| 119 | 77 | 61 | 3D | 1 | 58 | 594417 | 4755332 | 1 | 58 | 0 | 184 | 4881300 | 1 | 58 | 0 | 1 | 192 |
| 120 | 78 | 61 | 3D | 1 | 59 | 594440 | 4755516 | 1 | 59 | 0 | 184 | 4881492 | 1 | 59 | 0 | 1 | 192 |
| 121 | 79 | 61 | 3D | 1 | 60 | 594463 | 4755700 | 1 | 60 | 0 | 184 | 4881684 | 1 | 60 | 0 | 1 | 192 |
| 122 | 7A | 61 | 3D | 2 | 0 | 594484 | 4755868 | 2 | 0 | 0 | 168 | 4881860 | 2 | 0 | 0 | 1 | 176 |
| 123 | 7B | 61 | 3D | 2 | 1 | 594510 | 4756076 | 2 | 1 | 0 | 208 | 4882076 | 2 | 1 | 0 | 1 | 216 |
| 124 | 7C | 61 | 3D | 2 | 2 | 594536 | 4756284 | 2 | 2 | 0 | 208 | 4882292 | 2 | 2 | 0 | 1 | 216 |
| 125 | 7D | 61 | 3D | 2 | 3 | 594562 | 4756492 | 2 | 3 | 0 | 208 | 4882508 | 2 | 3 | 0 | 1 | 216 |
| 126 | 7E | 61 | 3D | 2 | 4 | 594588 | 4756700 | 2 | 4 | 0 | 208 | 4882724 | 2 | 4 | 0 | 1 | 216 |
| 127 | 7F | 61 | 3D | 2 | 5 | 594614 | 4756908 | 2 | 5 | 0 | 208 | 4882940 | 2 | 5 | 0 | 1 | 216 |
| 128 | 80 | 61 | 3D | 2 | 6 | 594637 | 4757092 | 2 | 6 | 0 | 184 | 4883132 | 2 | 6 | 0 | 1 | 192 |
| 129 | 81 | 61 | 3D | 2 | 7 | 594660 | 4757276 | 2 | 7 | 0 | 184 | 4883324 | 2 | 7 | 0 | 1 | 192 |
| 130 | 82 | 61 | 3D | 2 | 8 | 594683 | 4757460 | 2 | 8 | 0 | 184 | 4883516 | 2 | 8 | 0 | 1 | 192 |
| 131 | 83 | 61 | 3D | 2 | 9 | 594706 | 4757644 | 2 | 9 | 0 | 184 | 4883708 | 2 | 9 | 0 | 1 | 192 |
| 132 | 84 | 61 | 3D | 2 | 10 | 594729 | 4757828 | 2 | 10 | 0 | 184 | 4883900 | 2 | 10 | 0 | 1 | 192 |
| 133 | 85 | 61 | 3D | 2 | 11 | 594752 | 4758012 | 2 | 11 | 0 | 184 | 4884092 | 2 | 11 | 0 | 1 | 192 |
| 134 | 86 | 61 | 3D | 2 | 12 | 594775 | 4758196 | 2 | 12 | 0 | 184 | 4884284 | 2 | 12 | 0 | 1 | 192 |
| 135 | 87 | 61 | 3D | 2 | 13 | 594798 | 4758380 | 2 | 13 | 0 | 184 | 4884476 | 2 | 13 | 0 | 1 | 192 |
| 136 | 88 | 61 | 3D | 2 | 14 | 594821 | 4758564 | 2 | 14 | 0 | 184 | 4884668 | 2 | 14 | 0 | 1 | 192 |
| 137 | 89 | 61 | 3D | 2 | 15 | 594844 | 4758748 | 2 | 15 | 0 | 184 | 4884860 | 2 | 15 | 0 | 1 | 192 |
| 138 | 8A | 61 | 3D | 2 | 16 | 594867 | 4758932 | 2 | 16 | 0 | 184 | 4885052 | 2 | 16 | 0 | 1 | 192 |
| 139 | 8B | 61 | 3D | 2 | 17 | 594890 | 4759116 | 2 | 17 | 0 | 184 | 4885244 | 2 | 17 | 0 | 1 | 192 |

| | | | | | | | | | | | | | | | | | |
|---|---|---|---|---|---|---|---|---|---|---|---|---|---|---|---|---|---|
| 140 | 8C | 61 | 3D | 2 | 18 | 594913 | 4759300 | 2 | 18 | 0 | 184 | 4885436 | 2 | 18 | 0 | 1 | 192 |
| 141 | 8D | 61 | 3D | 2 | 19 | 594936 | 4759484 | 2 | 19 | 0 | 184 | 4885628 | 2 | 19 | 0 | 1 | 192 |
| 142 | 8E | 61 | 3D | 2 | 20 | 594959 | 4759668 | 2 | 20 | 0 | 184 | 4885820 | 2 | 20 | 0 | 1 | 192 |
| 143 | 8F | 61 | 3D | 2 | 21 | 594982 | 4759852 | 2 | 21 | 0 | 184 | 4886012 | 2 | 21 | 0 | 1 | 192 |
| 144 | 90 | 61 | 3D | 2 | 22 | 595007 | 4760052 | 2 | 22 | 0 | 200 | 4886220 | 2 | 22 | 0 | 1 | 208 |
| 145 | 91 | 61 | 3D | 2 | 23 | 595032 | 4760252 | 2 | 23 | 0 | 200 | 4886428 | 2 | 23 | 0 | 1 | 208 |
| 146 | 92 | 61 | 3D | 2 | 24 | 595057 | 4760452 | 2 | 24 | 0 | 200 | 4886636 | 2 | 24 | 0 | 1 | 208 |
| 147 | 93 | 61 | 3D | 2 | 25 | 595082 | 4760652 | 2 | 25 | 0 | 200 | 4886844 | 2 | 25 | 0 | 1 | 208 |
| 148 | 94 | 61 | 3D | 2 | 26 | 595107 | 4760852 | 2 | 26 | 0 | 200 | 4887052 | 2 | 26 | 0 | 1 | 208 |
| 149 | 95 | 61 | 3D | 2 | 27 | 595132 | 4761052 | 2 | 27 | 0 | 200 | 4887260 | 2 | 27 | 0 | 1 | 208 |
| 150 | 96 | 61 | 3D | 2 | 28 | 595157 | 4761252 | 2 | 28 | 0 | 200 | 4887468 | 2 | 28 | 0 | 1 | 208 |
| 151 | 97 | 61 | 3D | 2 | 29 | 595182 | 4761452 | 2 | 29 | 0 | 200 | 4887676 | 2 | 29 | 0 | 1 | 208 |
| 152 | 98 | 61 | 3D | 2 | 30 | 595207 | 4761652 | 2 | 30 | 0 | 200 | 4887884 | 2 | 30 | 0 | 1 | 208 |
| 153 | 99 | 61 | 3D | 2 | 31 | 595232 | 4761852 | 2 | 31 | 0 | 200 | 4888092 | 2 | 31 | 0 | 1 | 208 |
| 154 | 9A | 61 | 3D | 2 | 32 | 595257 | 4762052 | 2 | 32 | 0 | 200 | 4888300 | 2 | 32 | 0 | 1 | 208 |
| 155 | 9B | 61 | 3D | 2 | 33 | 595282 | 4762252 | 2 | 33 | 0 | 200 | 4888508 | 2 | 33 | 0 | 1 | 208 |
| 156 | 9C | 61 | 3D | 2 | 34 | 595307 | 4762452 | 2 | 34 | 0 | 200 | 4888716 | 2 | 34 | 0 | 1 | 208 |
| 157 | 9D | 61 | 3D | 2 | 35 | 595332 | 4762652 | 2 | 35 | 0 | 200 | 4888924 | 2 | 35 | 0 | 1 | 208 |
| 158 | 9E | 61 | 3D | 2 | 36 | 595357 | 4762852 | 2 | 36 | 0 | 200 | 4889132 | 2 | 36 | 0 | 1 | 208 |
| 159 | 9F | 61 | 3D | 2 | 37 | 595382 | 4763052 | 2 | 37 | 0 | 200 | 4889340 | 2 | 37 | 0 | 1 | 208 |
| 160 | A0 | 61 | 3D | 2 | 38 | 595407 | 4763252 | 2 | 38 | 0 | 200 | 4889548 | 2 | 38 | 0 | 1 | 208 |
| 161 | A1 | 61 | 3D | 2 | 39 | 595432 | 4763452 | 2 | 39 | 0 | 200 | 4889756 | 2 | 39 | 0 | 1 | 208 |
| 162 | A2 | 61 | 3D | 2 | 40 | 595457 | 4763652 | 2 | 40 | 0 | 200 | 4889964 | 2 | 40 | 0 | 1 | 208 |
| 163 | A3 | 61 | 3D | 2 | 41 | 595482 | 4763852 | 2 | 41 | 0 | 200 | 4890172 | 2 | 41 | 0 | 1 | 208 |
| 164 | A4 | 61 | 3D | 2 | 42 | 595507 | 4764052 | 2 | 42 | 0 | 200 | 4890380 | 2 | 42 | 0 | 1 | 208 |
| 165 | A5 | 61 | 3D | 2 | 43 | 595532 | 4764252 | 2 | 43 | 0 | 200 | 4890588 | 2 | 43 | 0 | 1 | 208 |
| 166 | A6 | 61 | 3D | 2 | 44 | 595557 | 4764452 | 2 | 44 | 0 | 200 | 4890796 | 2 | 44 | 0 | 1 | 208 |
| 167 | A7 | 61 | 3D | 2 | 45 | 595582 | 4764652 | 2 | 45 | 0 | 200 | 4891004 | 2 | 45 | 0 | 1 | 208 |
| 168 | A8 | 61 | 3D | 2 | 46 | 595607 | 4764852 | 2 | 46 | 0 | 200 | 4891212 | 2 | 46 | 0 | 1 | 208 |
| 169 | A9 | 61 | 3D | 2 | 47 | 595632 | 4765052 | 2 | 47 | 0 | 200 | 4891420 | 2 | 47 | 0 | 1 | 208 |
| 170 | AA | 61 | 3D | 2 | 48 | 595657 | 4765252 | 2 | 48 | 0 | 200 | 4891628 | 2 | 48 | 0 | 1 | 208 |
| 171 | AB | 61 | 3D | 2 | 49 | 595682 | 4765452 | 2 | 49 | 0 | 200 | 4891836 | 2 | 49 | 0 | 1 | 208 |
| 172 | AC | 61 | 3D | 2 | 50 | 595707 | 4765652 | 2 | 50 | 0 | 200 | 4892044 | 2 | 50 | 0 | 1 | 208 |
| 173 | AD | 61 | 3D | 2 | 51 | 595732 | 4765852 | 2 | 51 | 0 | 200 | 4892252 | 2 | 51 | 0 | 1 | 208 |
| 174 | AE | 61 | 3D | 2 | 52 | 595757 | 4766052 | 2 | 52 | 0 | 200 | 4892460 | 2 | 52 | 0 | 1 | 208 |
| 175 | AF | 61 | 3D | 2 | 53 | 595782 | 4766252 | 2 | 53 | 0 | 200 | 4892668 | 2 | 53 | 0 | 1 | 208 |
| 176 | B0 | 61 | 3D | 2 | 54 | 595807 | 4766452 | 2 | 54 | 0 | 200 | 4892876 | 2 | 54 | 0 | 1 | 208 |
| 177 | B1 | 61 | 3D | 2 | 55 | 595832 | 4766652 | 2 | 55 | 0 | 200 | 4893084 | 2 | 55 | 0 | 1 | 208 |
| 178 | B2 | 61 | 3D | 2 | 56 | 595857 | 4766852 | 2 | 56 | 0 | 200 | 4893292 | 2 | 56 | 0 | 1 | 208 |
| 179 | B3 | 61 | 3D | 2 | 57 | 595882 | 4767052 | 2 | 57 | 0 | 200 | 4893500 | 2 | 57 | 0 | 1 | 208 |
| 180 | B4 | 61 | 3D | 2 | 58 | 595907 | 4767252 | 2 | 58 | 0 | 200 | 4893708 | 2 | 58 | 0 | 1 | 208 |
| 181 | B5 | 61 | 3D | 2 | 59 | 595932 | 4767452 | 2 | 59 | 0 | 200 | 4893916 | 2 | 59 | 0 | 1 | 208 |
| 182 | B6 | 61 | 3D | 2 | 60 | 595957 | 4767652 | 2 | 60 | 0 | 200 | 4894124 | 2 | 60 | 0 | 1 | 208 |
| 183 | B7 | 61 | 3D | 3 | 0 | 595980 | 4767836 | 3 | 0 | 0 | 184 | 4894316 | 3 | 0 | 0 | 1 | 192 |
| 184 | B8 | 61 | 3D | 3 | 1 | 596008 | 4768060 | 3 | 1 | 0 | 224 | 4894548 | 3 | 1 | 0 | 1 | 232 |
| 185 | B9 | 61 | 3D | 3 | 2 | 596036 | 4768284 | 3 | 2 | 0 | 224 | 4894780 | 3 | 2 | 0 | 1 | 232 |
| 186 | BA | 61 | 3D | 3 | 3 | 596064 | 4768508 | 3 | 3 | 0 | 224 | 4895012 | 3 | 3 | 0 | 1 | 232 |
| 187 | BB | 61 | 3D | 3 | 4 | 596092 | 4768732 | 3 | 4 | 0 | 224 | 4895244 | 3 | 4 | 0 | 1 | 232 |
| 188 | BC | 61 | 3D | 3 | 5 | 596120 | 4768956 | 3 | 5 | 0 | 224 | 4895476 | 3 | 5 | 0 | 1 | 232 |
| 189 | BD | 61 | 3D | 3 | 6 | 596148 | 4769180 | 3 | 6 | 0 | 224 | 4895708 | 3 | 6 | 0 | 1 | 232 |
| 190 | BE | 61 | 3D | 3 | 7 | 596176 | 4769404 | 3 | 7 | 0 | 224 | 4895940 | 3 | 7 | 0 | 1 | 232 |
| 191 | BF | 61 | 3D | 3 | 8 | 596204 | 4769628 | 3 | 8 | 0 | 224 | 4896172 | 3 | 8 | 0 | 1 | 232 |
| 192 | C0 | 61 | 3D | 3 | 9 | 596229 | 4769828 | 3 | 9 | 0 | 200 | 4896380 | 3 | 9 | 0 | 1 | 208 |
| 193 | C1 | 61 | 3D | 3 | 10 | 596254 | 4770028 | 3 | 10 | 0 | 200 | 4896588 | 3 | 10 | 0 | 1 | 208 |
| 194 | C2 | 61 | 3D | 3 | 11 | 596279 | 4770228 | 3 | 11 | 0 | 200 | 4896796 | 3 | 11 | 0 | 1 | 208 |
| 195 | C3 | 61 | 3D | 3 | 12 | 596304 | 4770428 | 3 | 12 | 0 | 200 | 4897004 | 3 | 12 | 0 | 1 | 208 |
| 196 | C4 | 61 | 3D | 3 | 13 | 596329 | 4770628 | 3 | 13 | 0 | 200 | 4897212 | 3 | 13 | 0 | 1 | 208 |
| 197 | C5 | 61 | 3D | 3 | 14 | 596354 | 4770828 | 3 | 14 | 0 | 200 | 4897420 | 3 | 14 | 0 | 1 | 208 |
| 198 | C6 | 61 | 3D | 3 | 15 | 596379 | 4771028 | 3 | 15 | 0 | 200 | 4897628 | 3 | 15 | 0 | 1 | 208 |
| 199 | C7 | 61 | 3D | 3 | 16 | 596404 | 4771228 | 3 | 16 | 0 | 200 | 4897836 | 3 | 16 | 0 | 1 | 208 |
| 200 | C8 | 61 | 3D | 3 | 17 | 596429 | 4771428 | 3 | 17 | 0 | 200 | 4898044 | 3 | 17 | 0 | 1 | 208 |
| 201 | C9 | 61 | 3D | 3 | 18 | 596454 | 4771628 | 3 | 18 | 0 | 200 | 4898252 | 3 | 18 | 0 | 1 | 208 |
| 202 | CA | 61 | 3D | 3 | 19 | 596479 | 4771828 | 3 | 19 | 0 | 200 | 4898460 | 3 | 19 | 0 | 1 | 208 |
| 203 | CB | 61 | 3D | 3 | 20 | 596504 | 4772028 | 3 | 20 | 0 | 200 | 4898668 | 3 | 20 | 0 | 1 | 208 |
| 204 | CC | 61 | 3D | 3 | 21 | 596529 | 4772228 | 3 | 21 | 0 | 200 | 4898876 | 3 | 21 | 0 | 1 | 208 |
| 205 | CD | 61 | 3D | 3 | 22 | 596554 | 4772428 | 3 | 22 | 0 | 200 | 4899084 | 3 | 22 | 0 | 1 | 208 |
| 206 | CE | 61 | 3D | 3 | 23 | 596579 | 4772628 | 3 | 23 | 0 | 200 | 4899292 | 3 | 23 | 0 | 1 | 208 |
| 207 | CF | 61 | 3D | 3 | 24 | 596604 | 4772828 | 3 | 24 | 0 | 200 | 4899500 | 3 | 24 | 0 | 1 | 208 |
| 208 | D0 | 61 | 3D | 3 | 25 | 596631 | 4773044 | 3 | 25 | 0 | 216 | 4899724 | 3 | 25 | 0 | 1 | 224 |
| 209 | D1 | 61 | 3D | 3 | 26 | 596658 | 4773260 | 3 | 26 | 0 | 216 | 4899948 | 3 | 26 | 0 | 1 | 224 |
| 210 | D2 | 61 | 3D | 3 | 27 | 596685 | 4773476 | 3 | 27 | 0 | 216 | 4900172 | 3 | 27 | 0 | 1 | 224 |
| 211 | D3 | 61 | 3D | 3 | 28 | 596712 | 4773692 | 3 | 28 | 0 | 216 | 4900396 | 3 | 28 | 0 | 1 | 224 |
| 212 | D4 | 61 | 3D | 3 | 29 | 596739 | 4773908 | 3 | 29 | 0 | 216 | 4900620 | 3 | 29 | 0 | 1 | 224 |
| 213 | D5 | 61 | 3D | 3 | 30 | 596766 | 4774124 | 3 | 30 | 0 | 216 | 4900844 | 3 | 30 | 0 | 1 | 224 |
| 214 | D6 | 61 | 3D | 3 | 31 | 596793 | 4774340 | 3 | 31 | 0 | 216 | 4901068 | 3 | 31 | 0 | 1 | 224 |
| 215 | D7 | 61 | 3D | 3 | 32 | 596820 | 4774556 | 3 | 32 | 0 | 216 | 4901292 | 3 | 32 | 0 | 1 | 224 |
| 216 | D8 | 61 | 3D | 3 | 33 | 596847 | 4774772 | 3 | 33 | 0 | 216 | 4901516 | 3 | 33 | 0 | 1 | 224 |
| 217 | D9 | 61 | 3D | 3 | 34 | 596874 | 4774988 | 3 | 34 | 0 | 216 | 4901740 | 3 | 34 | 0 | 1 | 224 |
| 218 | DA | 61 | 3D | 3 | 35 | 596901 | 4775204 | 3 | 35 | 0 | 216 | 4901964 | 3 | 35 | 0 | 1 | 224 |
| 219 | DB | 61 | 3D | 3 | 36 | 596928 | 4775420 | 3 | 36 | 0 | 216 | 4902188 | 3 | 36 | 0 | 1 | 224 |
| 220 | DC | 61 | 3D | 3 | 37 | 596955 | 4775636 | 3 | 37 | 0 | 216 | 4902412 | 3 | 37 | 0 | 1 | 224 |
| 221 | DD | 61 | 3D | 3 | 38 | 596982 | 4775852 | 3 | 38 | 0 | 216 | 4902636 | 3 | 38 | 0 | 1 | 224 |
| 222 | DE | 61 | 3D | 3 | 39 | 597009 | 4776068 | 3 | 39 | 0 | 216 | 4902860 | 3 | 39 | 0 | 1 | 224 |
| 223 | DF | 61 | 3D | 3 | 40 | 597036 | 4776284 | 3 | 40 | 0 | 216 | 4903084 | 3 | 40 | 0 | 1 | 224 |
| 224 | E0 | 61 | 3D | 3 | 41 | 597063 | 4776500 | 3 | 41 | 0 | 216 | 4903308 | 3 | 41 | 0 | 1 | 224 |
| 225 | E1 | 61 | 3D | 3 | 42 | 597090 | 4776716 | 3 | 42 | 0 | 216 | 4903532 | 3 | 42 | 0 | 1 | 224 |
| 226 | E2 | 61 | 3D | 3 | 43 | 597117 | 4776932 | 3 | 43 | 0 | 216 | 4903756 | 3 | 43 | 0 | 1 | 224 |
| 227 | E3 | 61 | 3D | 3 | 44 | 597144 | 4777148 | 3 | 44 | 0 | 216 | 4903980 | 3 | 44 | 0 | 1 | 224 |
| 228 | E4 | 61 | 3D | 3 | 45 | 597171 | 4777364 | 3 | 45 | 0 | 216 | 4904204 | 3 | 45 | 0 | 1 | 224 |
| 229 | E5 | 61 | 3D | 3 | 46 | 597198 | 4777580 | 3 | 46 | 0 | 216 | 4904428 | 3 | 46 | 0 | 1 | 224 |
| 230 | E6 | 61 | 3D | 3 | 47 | 597225 | 4777796 | 3 | 47 | 0 | 216 | 4904652 | 3 | 47 | 0 | 1 | 224 |
| 231 | E7 | 61 | 3D | 3 | 48 | 597252 | 4778012 | 3 | 48 | 0 | 216 | 4904876 | 3 | 48 | 0 | 1 | 224 |
| 232 | E8 | 61 | 3D | 3 | 49 | 597279 | 4778228 | 3 | 49 | 0 | 216 | 4905100 | 3 | 49 | 0 | 1 | 224 |
| 233 | E9 | 61 | 3D | 3 | 50 | 597306 | 4778444 | 3 | 50 | 0 | 216 | 4905324 | 3 | 50 | 0 | 1 | 224 |
| 234 | EA | 61 | 3D | 3 | 51 | 597333 | 4778660 | 3 | 51 | 0 | 216 | 4905548 | 3 | 51 | 0 | 1 | 224 |
| 235 | EB | 61 | 3D | 3 | 52 | 597360 | 4778876 | 3 | 52 | 0 | 216 | 4905772 | 3 | 52 | 0 | 1 | 224 |
| 236 | EC | 61 | 3D | 3 | 53 | 597387 | 4779092 | 3 | 53 | 0 | 216 | 4905996 | 3 | 53 | 0 | 1 | 224 |
| 237 | ED | 61 | 3D | 3 | 54 | 597414 | 4779308 | 3 | 54 | 0 | 216 | 4906220 | 3 | 54 | 0 | 1 | 224 |
| 238 | EE | 61 | 3D | 3 | 55 | 597441 | 4779524 | 3 | 55 | 0 | 216 | 4906444 | 3 | 55 | 0 | 1 | 224 |
| 239 | EF | 61 | 3D | 3 | 56 | 597468 | 4779740 | 3 | 56 | 0 | 216 | 4906668 | 3 | 56 | 0 | 1 | 224 |
| 240 | F0 | 61 | 3D | 3 | 57 | 597495 | 4779956 | 3 | 57 | 0 | 216 | 4906892 | 3 | 57 | 0 | 1 | 224 |
| 241 | F1 | 61 | 3D | 3 | 58 | 597522 | 4780172 | 3 | 58 | 0 | 216 | 4907116 | 3 | 58 | 0 | 1 | 224 |
| 242 | F2 | 61 | 3D | 3 | 59 | 597549 | 4780388 | 3 | 59 | 0 | 216 | 4907340 | 3 | 59 | 0 | 1 | 224 |

| | | | | | | | | | | | | | | | | | |
|---|---|---|---|---|---|---|---|---|---|---|---|---|---|---|---|---|---|
| 243 | F3 | 61 | 3D | 3 | 60 | 597576 | 4780604 | 3 | 60 | 0 | 216 | 4907564 | 3 | 60 | 0 | 1 | 224 |
| 244 | F4 | 61 | 3D | 4 | 0 | 597601 | 4780804 | 4 | 0 | 0 | 200 | 4907772 | 4 | 0 | 0 | 1 | 208 |
| 245 | F5 | 61 | 3D | 4 | 1 | 597631 | 4781044 | 4 | 1 | 0 | 240 | 4908020 | 4 | 1 | 0 | 1 | 248 |
| 246 | F6 | 61 | 3D | 4 | 2 | 597661 | 4781284 | 4 | 2 | 0 | 240 | 4908268 | 4 | 2 | 0 | 1 | 248 |
| 247 | F7 | 61 | 3D | 4 | 3 | 597691 | 4781524 | 4 | 3 | 0 | 240 | 4908516 | 4 | 3 | 0 | 1 | 248 |
| 248 | F8 | 61 | 3D | 4 | 4 | 597721 | 4781764 | 4 | 4 | 0 | 240 | 4908764 | 4 | 4 | 0 | 1 | 248 |
| 249 | F9 | 61 | 3D | 4 | 5 | 597751 | 4782004 | 4 | 5 | 0 | 240 | 4909012 | 4 | 5 | 0 | 1 | 248 |
| 250 | FA | 61 | 3D | 4 | 6 | 597781 | 4782244 | 4 | 6 | 0 | 240 | 4909260 | 4 | 6 | 0 | 1 | 248 |
| 251 | FB | 61 | 3D | 4 | 7 | 597811 | 4782484 | 4 | 7 | 0 | 240 | 4909508 | 4 | 7 | 0 | 1 | 248 |
| 252 | FC | 61 | 3D | 4 | 8 | 597841 | 4782724 | 4 | 8 | 0 | 240 | 4909756 | 4 | 8 | 0 | 1 | 248 |
| 253 | FD | 61 | 3D | 4 | 9 | 597871 | 4782964 | 4 | 9 | 0 | 240 | 4910004 | 4 | 9 | 0 | 1 | 248 |
| 254 | FE | 61 | 3D | 4 | 10 | 597901 | 4783204 | 4 | 10 | 0 | 240 | 4910252 | 4 | 10 | 0 | 1 | 248 |
| 255 | FF | 61 | 3D | 4 | 11 | 597931 | 4783444 | 4 | 11 | 0 | 240 | 4910500 | 4 | 11 | 0 | 1 | 248 |
| 0 | 00 | 62 | 3E | 0 | 0 | 597943 | 4783540 | 0 | 0 | 0 | 96 | 4910604 | 0 | 0 | 0 | 1 | 104 |
| 1 | 01 | 62 | 3E | 0 | 1 | 597957 | 4783652 | 0 | 1 | 0 | 112 | 4910724 | 0 | 1 | 0 | 1 | 120 |
| 2 | 02 | 62 | 3E | 0 | 2 | 597971 | 4783764 | 0 | 2 | 0 | 112 | 4910844 | 0 | 2 | 0 | 1 | 120 |
| 3 | 03 | 62 | 3E | 0 | 3 | 597985 | 4783876 | 0 | 3 | 0 | 112 | 4910964 | 0 | 3 | 0 | 1 | 120 |
| 4 | 04 | 62 | 3E | 0 | 4 | 597999 | 4783988 | 0 | 4 | 0 | 112 | 4911084 | 0 | 4 | 0 | 1 | 120 |
| 5 | 05 | 62 | 3E | 0 | 5 | 598013 | 4784100 | 0 | 5 | 0 | 112 | 4911204 | 0 | 5 | 0 | 1 | 120 |
| 6 | 06 | 62 | 3E | 0 | 6 | 598027 | 4784212 | 0 | 6 | 0 | 112 | 4911324 | 0 | 6 | 0 | 1 | 120 |
| 7 | 07 | 62 | 3E | 0 | 7 | 598041 | 4784324 | 0 | 7 | 0 | 112 | 4911444 | 0 | 7 | 0 | 1 | 120 |
| 8 | 08 | 62 | 3E | 0 | 8 | 598055 | 4784436 | 0 | 8 | 0 | 112 | 4911564 | 0 | 8 | 0 | 1 | 120 |
| 9 | 09 | 62 | 3E | 0 | 9 | 598069 | 4784548 | 0 | 9 | 0 | 112 | 4911684 | 0 | 9 | 0 | 1 | 120 |
| 10 | 0A | 62 | 3E | 0 | 10 | 598083 | 4784660 | 0 | 10 | 0 | 112 | 4911804 | 0 | 10 | 0 | 1 | 120 |
| 11 | 0B | 62 | 3E | 0 | 11 | 598097 | 4784772 | 0 | 11 | 0 | 112 | 4911924 | 0 | 11 | 0 | 1 | 120 |
| 12 | 0C | 62 | 3E | 0 | 12 | 598111 | 4784884 | 0 | 12 | 0 | 112 | 4912044 | 0 | 12 | 0 | 1 | 120 |
| 13 | 0D | 62 | 3E | 0 | 13 | 598125 | 4784996 | 0 | 13 | 0 | 112 | 4912164 | 0 | 13 | 0 | 1 | 120 |
| 14 | 0E | 62 | 3E | 0 | 14 | 598139 | 4785108 | 0 | 14 | 0 | 112 | 4912284 | 0 | 14 | 0 | 1 | 120 |
| 15 | 0F | 62 | 3E | 0 | 15 | 598153 | 4785220 | 0 | 15 | 0 | 112 | 4912404 | 0 | 15 | 0 | 1 | 120 |
| 16 | 10 | 62 | 3E | 0 | 16 | 598167 | 4785332 | 0 | 16 | 0 | 112 | 4912524 | 0 | 16 | 0 | 1 | 120 |
| 17 | 11 | 62 | 3E | 0 | 17 | 598181 | 4785444 | 0 | 17 | 0 | 112 | 4912644 | 0 | 17 | 0 | 1 | 120 |
| 18 | 12 | 62 | 3E | 0 | 18 | 598195 | 4785556 | 0 | 18 | 0 | 112 | 4912764 | 0 | 18 | 0 | 1 | 120 |
| 19 | 13 | 62 | 3E | 0 | 19 | 598209 | 4785668 | 0 | 19 | 0 | 112 | 4912884 | 0 | 19 | 0 | 1 | 120 |
| 20 | 14 | 62 | 3E | 0 | 20 | 598223 | 4785780 | 0 | 20 | 0 | 112 | 4913004 | 0 | 20 | 0 | 1 | 120 |
| 21 | 15 | 62 | 3E | 0 | 21 | 598237 | 4785892 | 0 | 21 | 0 | 112 | 4913124 | 0 | 21 | 0 | 1 | 120 |
| 22 | 16 | 62 | 3E | 0 | 22 | 598251 | 4786004 | 0 | 22 | 0 | 112 | 4913244 | 0 | 22 | 0 | 1 | 120 |
| 23 | 17 | 62 | 3E | 0 | 23 | 598265 | 4786116 | 0 | 23 | 0 | 112 | 4913364 | 0 | 23 | 0 | 1 | 120 |
| 24 | 18 | 62 | 3E | 0 | 24 | 598279 | 4786228 | 0 | 24 | 0 | 112 | 4913484 | 0 | 24 | 0 | 1 | 120 |
| 25 | 19 | 62 | 3E | 0 | 25 | 598293 | 4786340 | 0 | 25 | 0 | 112 | 4913604 | 0 | 25 | 0 | 1 | 120 |
| 26 | 1A | 62 | 3E | 0 | 26 | 598307 | 4786452 | 0 | 26 | 0 | 112 | 4913724 | 0 | 26 | 0 | 1 | 120 |
| 27 | 1B | 62 | 3E | 0 | 27 | 598321 | 4786564 | 0 | 27 | 0 | 112 | 4913844 | 0 | 27 | 0 | 1 | 120 |
| 28 | 1C | 62 | 3E | 0 | 28 | 598335 | 4786676 | 0 | 28 | 0 | 112 | 4913964 | 0 | 28 | 0 | 1 | 120 |
| 29 | 1D | 62 | 3E | 0 | 29 | 598349 | 4786788 | 0 | 29 | 0 | 112 | 4914084 | 0 | 29 | 0 | 1 | 120 |
| 30 | 1E | 62 | 3E | 0 | 30 | 598363 | 4786900 | 0 | 30 | 0 | 112 | 4914204 | 0 | 30 | 0 | 1 | 120 |
| 31 | 1F | 62 | 3E | 0 | 31 | 598377 | 4787012 | 0 | 31 | 0 | 112 | 4914324 | 0 | 31 | 0 | 1 | 120 |
| 32 | 20 | 62 | 3E | 0 | 32 | 598391 | 4787124 | 0 | 32 | 0 | 112 | 4914444 | 0 | 32 | 0 | 1 | 120 |
| 33 | 21 | 62 | 3E | 0 | 33 | 598405 | 4787236 | 0 | 33 | 0 | 112 | 4914564 | 0 | 33 | 0 | 1 | 120 |
| 34 | 22 | 62 | 3E | 0 | 34 | 598419 | 4787348 | 0 | 34 | 0 | 112 | 4914684 | 0 | 34 | 0 | 1 | 120 |
| 35 | 23 | 62 | 3E | 0 | 35 | 598433 | 4787460 | 0 | 35 | 0 | 112 | 4914804 | 0 | 35 | 0 | 1 | 120 |
| 36 | 24 | 62 | 3E | 0 | 36 | 598447 | 4787572 | 0 | 36 | 0 | 112 | 4914924 | 0 | 36 | 0 | 1 | 120 |
| 37 | 25 | 62 | 3E | 0 | 37 | 598461 | 4787684 | 0 | 37 | 0 | 112 | 4915044 | 0 | 37 | 0 | 1 | 120 |
| 38 | 26 | 62 | 3E | 0 | 38 | 598475 | 4787796 | 0 | 38 | 0 | 112 | 4915164 | 0 | 38 | 0 | 1 | 120 |
| 39 | 27 | 62 | 3E | 0 | 39 | 598489 | 4787908 | 0 | 39 | 0 | 112 | 4915284 | 0 | 39 | 0 | 1 | 120 |
| 40 | 28 | 62 | 3E | 0 | 40 | 598503 | 4788020 | 0 | 40 | 0 | 112 | 4915404 | 0 | 40 | 0 | 1 | 120 |
| 41 | 29 | 62 | 3E | 0 | 41 | 598517 | 4788132 | 0 | 41 | 0 | 112 | 4915524 | 0 | 41 | 0 | 1 | 120 |
| 42 | 2A | 62 | 3E | 0 | 42 | 598531 | 4788244 | 0 | 42 | 0 | 112 | 4915644 | 0 | 42 | 0 | 1 | 120 |
| 43 | 2B | 62 | 3E | 0 | 43 | 598545 | 4788356 | 0 | 43 | 0 | 112 | 4915764 | 0 | 43 | 0 | 1 | 120 |
| 44 | 2C | 62 | 3E | 0 | 44 | 598559 | 4788468 | 0 | 44 | 0 | 112 | 4915884 | 0 | 44 | 0 | 1 | 120 |
| 45 | 2D | 62 | 3E | 0 | 45 | 598573 | 4788580 | 0 | 45 | 0 | 112 | 4916004 | 0 | 45 | 0 | 1 | 120 |
| 46 | 2E | 62 | 3E | 0 | 46 | 598587 | 4788692 | 0 | 46 | 0 | 112 | 4916124 | 0 | 46 | 0 | 1 | 120 |
| 47 | 2F | 62 | 3E | 0 | 47 | 598601 | 4788804 | 0 | 47 | 0 | 112 | 4916244 | 0 | 47 | 0 | 1 | 120 |
| 48 | 30 | 62 | 3E | 0 | 48 | 598615 | 4788916 | 0 | 48 | 0 | 112 | 4916364 | 0 | 48 | 0 | 1 | 120 |
| 49 | 31 | 62 | 3E | 0 | 49 | 598629 | 4789028 | 0 | 49 | 0 | 112 | 4916484 | 0 | 49 | 0 | 1 | 120 |
| 50 | 32 | 62 | 3E | 0 | 50 | 598643 | 4789140 | 0 | 50 | 0 | 112 | 4916604 | 0 | 50 | 0 | 1 | 120 |
| 51 | 33 | 62 | 3E | 0 | 51 | 598657 | 4789252 | 0 | 51 | 0 | 112 | 4916724 | 0 | 51 | 0 | 1 | 120 |
| 52 | 34 | 62 | 3E | 0 | 52 | 598671 | 4789364 | 0 | 52 | 0 | 112 | 4916844 | 0 | 52 | 0 | 1 | 120 |
| 53 | 35 | 62 | 3E | 0 | 53 | 598685 | 4789476 | 0 | 53 | 0 | 112 | 4916964 | 0 | 53 | 0 | 1 | 120 |
| 54 | 36 | 62 | 3E | 0 | 54 | 598699 | 4789588 | 0 | 54 | 0 | 112 | 4917084 | 0 | 54 | 0 | 1 | 120 |
| 55 | 37 | 62 | 3E | 0 | 55 | 598713 | 4789700 | 0 | 55 | 0 | 112 | 4917204 | 0 | 55 | 0 | 1 | 120 |
| 56 | 38 | 62 | 3E | 0 | 56 | 598727 | 4789812 | 0 | 56 | 0 | 112 | 4917324 | 0 | 56 | 0 | 1 | 120 |
| 57 | 39 | 62 | 3E | 0 | 57 | 598741 | 4789924 | 0 | 57 | 0 | 112 | 4917444 | 0 | 57 | 0 | 1 | 120 |
| 58 | 3A | 62 | 3E | 0 | 58 | 598755 | 4790036 | 0 | 58 | 0 | 112 | 4917564 | 0 | 58 | 0 | 1 | 120 |
| 59 | 3B | 62 | 3E | 0 | 59 | 598769 | 4790148 | 0 | 59 | 0 | 112 | 4917684 | 0 | 59 | 0 | 1 | 120 |
| 60 | 3C | 62 | 3E | 0 | 60 | 598783 | 4790260 | 0 | 60 | 0 | 112 | 4917804 | 0 | 60 | 0 | 1 | 120 |
| 61 | 3D | 62 | 3E | 0 | 61 | 598797 | 4790372 | 0 | 61 | 0 | 112 | 4917924 | 0 | 61 | 0 | 1 | 120 |
| 62 | 3E | 62 | 3E | 1 | 0 | 598809 | 4790468 | 1 | 0 | 0 | 96 | 4918028 | 1 | 0 | 0 | 1 | 104 |
| 63 | 3F | 62 | 3E | 1 | 1 | 598826 | 4790604 | 1 | 1 | 0 | 136 | 4918172 | 1 | 1 | 0 | 1 | 144 |
| 64 | 40 | 62 | 3E | 1 | 2 | 598847 | 4790772 | 1 | 2 | 0 | 168 | 4918348 | 1 | 2 | 0 | 1 | 176 |
| 65 | 41 | 62 | 3E | 1 | 3 | 598868 | 4790940 | 1 | 3 | 0 | 168 | 4918524 | 1 | 3 | 0 | 1 | 176 |
| 66 | 42 | 62 | 3E | 1 | 4 | 598889 | 4791108 | 1 | 4 | 0 | 168 | 4918700 | 1 | 4 | 0 | 1 | 176 |
| 67 | 43 | 62 | 3E | 1 | 5 | 598910 | 4791276 | 1 | 5 | 0 | 168 | 4918876 | 1 | 5 | 0 | 1 | 176 |
| 68 | 44 | 62 | 3E | 1 | 6 | 598931 | 4791444 | 1 | 6 | 0 | 168 | 4919052 | 1 | 6 | 0 | 1 | 176 |
| 69 | 45 | 62 | 3E | 1 | 7 | 598952 | 4791612 | 1 | 7 | 0 | 168 | 4919228 | 1 | 7 | 0 | 1 | 176 |
| 70 | 46 | 62 | 3E | 1 | 8 | 598973 | 4791780 | 1 | 8 | 0 | 168 | 4919404 | 1 | 8 | 0 | 1 | 176 |
| 71 | 47 | 62 | 3E | 1 | 9 | 598994 | 4791948 | 1 | 9 | 0 | 168 | 4919580 | 1 | 9 | 0 | 1 | 176 |
| 72 | 48 | 62 | 3E | 1 | 10 | 599015 | 4792116 | 1 | 10 | 0 | 168 | 4919756 | 1 | 10 | 0 | 1 | 176 |
| 73 | 49 | 62 | 3E | 1 | 11 | 599036 | 4792284 | 1 | 11 | 0 | 168 | 4919932 | 1 | 11 | 0 | 1 | 176 |
| 74 | 4A | 62 | 3E | 1 | 12 | 599057 | 4792452 | 1 | 12 | 0 | 168 | 4920108 | 1 | 12 | 0 | 1 | 176 |
| 75 | 4B | 62 | 3E | 1 | 13 | 599078 | 4792620 | 1 | 13 | 0 | 168 | 4920284 | 1 | 13 | 0 | 1 | 176 |
| 76 | 4C | 62 | 3E | 1 | 14 | 599099 | 4792788 | 1 | 14 | 0 | 168 | 4920460 | 1 | 14 | 0 | 1 | 176 |
| 77 | 4D | 62 | 3E | 1 | 15 | 599120 | 4792956 | 1 | 15 | 0 | 168 | 4920636 | 1 | 15 | 0 | 1 | 176 |
| 78 | 4E | 62 | 3E | 1 | 16 | 599141 | 4793124 | 1 | 16 | 0 | 168 | 4920812 | 1 | 16 | 0 | 1 | 176 |
| 79 | 4F | 62 | 3E | 1 | 17 | 599162 | 4793292 | 1 | 17 | 0 | 168 | 4920988 | 1 | 17 | 0 | 1 | 176 |
| 80 | 50 | 62 | 3E | 1 | 18 | 599185 | 4793476 | 1 | 18 | 0 | 184 | 4921180 | 1 | 18 | 0 | 1 | 192 |
| 81 | 51 | 62 | 3E | 1 | 19 | 599208 | 4793660 | 1 | 19 | 0 | 184 | 4921372 | 1 | 19 | 0 | 1 | 192 |
| 82 | 52 | 62 | 3E | 1 | 20 | 599231 | 4793844 | 1 | 20 | 0 | 184 | 4921564 | 1 | 20 | 0 | 1 | 192 |
| 83 | 53 | 62 | 3E | 1 | 21 | 599254 | 4794028 | 1 | 21 | 0 | 184 | 4921756 | 1 | 21 | 0 | 1 | 192 |
| 84 | 54 | 62 | 3E | 1 | 22 | 599277 | 4794212 | 1 | 22 | 0 | 184 | 4921948 | 1 | 22 | 0 | 1 | 192 |
| 85 | 55 | 62 | 3E | 1 | 23 | 599300 | 4794396 | 1 | 23 | 0 | 184 | 4922140 | 1 | 23 | 0 | 1 | 192 |
| 86 | 56 | 62 | 3E | 1 | 24 | 599323 | 4794580 | 1 | 24 | 0 | 184 | 4922332 | 1 | 24 | 0 | 1 | 192 |
| 87 | 57 | 62 | 3E | 1 | 25 | 599346 | 4794764 | 1 | 25 | 0 | 184 | 4922524 | 1 | 25 | 0 | 1 | 192 |
| 88 | 58 | 62 | 3E | 1 | 26 | 599369 | 4794948 | 1 | 26 | 0 | 184 | 4922716 | 1 | 26 | 0 | 1 | 192 |
| 89 | 59 | 62 | 3E | 1 | 27 | 599392 | 4795132 | 1 | 27 | 0 | 184 | 4922908 | 1 | 27 | 0 | 1 | 192 |

| | | | | | | | | | | | | | | | | | |
|---|---|---|---|---|---|---|---|---|---|---|---|---|---|---|---|---|---|
| 90 | 5A | 62 | 3E | 1 | 28 | 599415 | 4795316 | 1 | 28 | 0 | 184 | 4923100 | 1 | 28 | 0 | 1 | 192 |
| 91 | 5B | 62 | 3E | 1 | 29 | 599438 | 4795500 | 1 | 29 | 0 | 184 | 4923292 | 1 | 29 | 0 | 1 | 192 |
| 92 | 5C | 62 | 3E | 1 | 30 | 599461 | 4795684 | 1 | 30 | 0 | 184 | 4923484 | 1 | 30 | 0 | 1 | 192 |
| 93 | 5D | 62 | 3E | 1 | 31 | 599484 | 4795868 | 1 | 31 | 0 | 184 | 4923676 | 1 | 31 | 0 | 1 | 192 |
| 94 | 5E | 62 | 3E | 1 | 32 | 599507 | 4796052 | 1 | 32 | 0 | 184 | 4923868 | 1 | 32 | 0 | 1 | 192 |
| 95 | 5F | 62 | 3E | 1 | 33 | 599530 | 4796236 | 1 | 33 | 0 | 184 | 4924060 | 1 | 33 | 0 | 1 | 192 |
| 96 | 60 | 62 | 3E | 1 | 34 | 599553 | 4796420 | 1 | 34 | 0 | 184 | 4924252 | 1 | 34 | 0 | 1 | 192 |
| 97 | 61 | 62 | 3E | 1 | 35 | 599576 | 4796604 | 1 | 35 | 0 | 184 | 4924444 | 1 | 35 | 0 | 1 | 192 |
| 98 | 62 | 62 | 3E | 1 | 36 | 599599 | 4796788 | 1 | 36 | 0 | 184 | 4924636 | 1 | 36 | 0 | 1 | 192 |
| 99 | 63 | 62 | 3E | 1 | 37 | 599622 | 4796972 | 1 | 37 | 0 | 184 | 4924828 | 1 | 37 | 0 | 1 | 192 |
| 100 | 64 | 62 | 3E | 1 | 38 | 599645 | 4797156 | 1 | 38 | 0 | 184 | 4925020 | 1 | 38 | 0 | 1 | 192 |
| 101 | 65 | 62 | 3E | 1 | 39 | 599668 | 4797340 | 1 | 39 | 0 | 184 | 4925212 | 1 | 39 | 0 | 1 | 192 |
| 102 | 66 | 62 | 3E | 1 | 40 | 599691 | 4797524 | 1 | 40 | 0 | 184 | 4925404 | 1 | 40 | 0 | 1 | 192 |
| 103 | 67 | 62 | 3E | 1 | 41 | 599714 | 4797708 | 1 | 41 | 0 | 184 | 4925596 | 1 | 41 | 0 | 1 | 192 |
| 104 | 68 | 62 | 3E | 1 | 42 | 599737 | 4797892 | 1 | 42 | 0 | 184 | 4925788 | 1 | 42 | 0 | 1 | 192 |
| 105 | 69 | 62 | 3E | 1 | 43 | 599760 | 4798076 | 1 | 43 | 0 | 184 | 4925980 | 1 | 43 | 0 | 1 | 192 |
| 106 | 6A | 62 | 3E | 1 | 44 | 599783 | 4798260 | 1 | 44 | 0 | 184 | 4926172 | 1 | 44 | 0 | 1 | 192 |
| 107 | 6B | 62 | 3E | 1 | 45 | 599806 | 4798444 | 1 | 45 | 0 | 184 | 4926364 | 1 | 45 | 0 | 1 | 192 |
| 108 | 6C | 62 | 3E | 1 | 46 | 599829 | 4798628 | 1 | 46 | 0 | 184 | 4926556 | 1 | 46 | 0 | 1 | 192 |
| 109 | 6D | 62 | 3E | 1 | 47 | 599852 | 4798812 | 1 | 47 | 0 | 184 | 4926748 | 1 | 47 | 0 | 1 | 192 |
| 110 | 6E | 62 | 3E | 1 | 48 | 599875 | 4798996 | 1 | 48 | 0 | 184 | 4926940 | 1 | 48 | 0 | 1 | 192 |
| 111 | 6F | 62 | 3E | 1 | 49 | 599898 | 4799180 | 1 | 49 | 0 | 184 | 4927132 | 1 | 49 | 0 | 1 | 192 |
| 112 | 70 | 62 | 3E | 1 | 50 | 599921 | 4799364 | 1 | 50 | 0 | 184 | 4927324 | 1 | 50 | 0 | 1 | 192 |
| 113 | 71 | 62 | 3E | 1 | 51 | 599944 | 4799548 | 1 | 51 | 0 | 184 | 4927516 | 1 | 51 | 0 | 1 | 192 |
| 114 | 72 | 62 | 3E | 1 | 52 | 599967 | 4799732 | 1 | 52 | 0 | 184 | 4927708 | 1 | 52 | 0 | 1 | 192 |
| 115 | 73 | 62 | 3E | 1 | 53 | 599990 | 4799916 | 1 | 53 | 0 | 184 | 4927900 | 1 | 53 | 0 | 1 | 192 |
| 116 | 74 | 62 | 3E | 1 | 54 | 600013 | 4800100 | 1 | 54 | 0 | 184 | 4928092 | 1 | 54 | 0 | 1 | 192 |
| 117 | 75 | 62 | 3E | 1 | 55 | 600036 | 4800284 | 1 | 55 | 0 | 184 | 4928284 | 1 | 55 | 0 | 1 | 192 |
| 118 | 76 | 62 | 3E | 1 | 56 | 600059 | 4800468 | 1 | 56 | 0 | 184 | 4928476 | 1 | 56 | 0 | 1 | 192 |
| 119 | 77 | 62 | 3E | 1 | 57 | 600082 | 4800652 | 1 | 57 | 0 | 184 | 4928668 | 1 | 57 | 0 | 1 | 192 |
| 120 | 78 | 62 | 3E | 1 | 58 | 600105 | 4800836 | 1 | 58 | 0 | 184 | 4928860 | 1 | 58 | 0 | 1 | 192 |
| 121 | 79 | 62 | 3E | 1 | 59 | 600128 | 4801020 | 1 | 59 | 0 | 184 | 4929052 | 1 | 59 | 0 | 1 | 192 |
| 122 | 7A | 62 | 3E | 1 | 60 | 600151 | 4801204 | 1 | 60 | 0 | 184 | 4929244 | 1 | 60 | 0 | 1 | 192 |
| 123 | 7B | 62 | 3E | 1 | 61 | 600174 | 4801388 | 1 | 61 | 0 | 184 | 4929436 | 1 | 61 | 0 | 1 | 192 |
| 124 | 7C | 62 | 3E | 2 | 0 | 600195 | 4801556 | 2 | 0 | 0 | 168 | 4929612 | 2 | 0 | 0 | 1 | 176 |
| 125 | 7D | 62 | 3E | 2 | 1 | 600221 | 4801764 | 2 | 1 | 0 | 208 | 4929828 | 2 | 1 | 0 | 1 | 216 |
| 126 | 7E | 62 | 3E | 2 | 2 | 600247 | 4801972 | 2 | 2 | 0 | 208 | 4930044 | 2 | 2 | 0 | 1 | 216 |
| 127 | 7F | 62 | 3E | 2 | 3 | 600273 | 4802180 | 2 | 3 | 0 | 208 | 4930260 | 2 | 3 | 0 | 1 | 216 |
| 128 | 80 | 62 | 3E | 2 | 4 | 600296 | 4802364 | 2 | 4 | 0 | 184 | 4930452 | 2 | 4 | 0 | 1 | 192 |
| 129 | 81 | 62 | 3E | 2 | 5 | 600319 | 4802548 | 2 | 5 | 0 | 184 | 4930644 | 2 | 5 | 0 | 1 | 192 |
| 130 | 82 | 62 | 3E | 2 | 6 | 600342 | 4802732 | 2 | 6 | 0 | 184 | 4930836 | 2 | 6 | 0 | 1 | 192 |
| 131 | 83 | 62 | 3E | 2 | 7 | 600365 | 4802916 | 2 | 7 | 0 | 184 | 4931028 | 2 | 7 | 0 | 1 | 192 |
| 132 | 84 | 62 | 3E | 2 | 8 | 600388 | 4803100 | 2 | 8 | 0 | 184 | 4931220 | 2 | 8 | 0 | 1 | 192 |
| 133 | 85 | 62 | 3E | 2 | 9 | 600411 | 4803284 | 2 | 9 | 0 | 184 | 4931412 | 2 | 9 | 0 | 1 | 192 |
| 134 | 86 | 62 | 3E | 2 | 10 | 600434 | 4803468 | 2 | 10 | 0 | 184 | 4931604 | 2 | 10 | 0 | 1 | 192 |
| 135 | 87 | 62 | 3E | 2 | 11 | 600457 | 4803652 | 2 | 11 | 0 | 184 | 4931796 | 2 | 11 | 0 | 1 | 192 |
| 136 | 88 | 62 | 3E | 2 | 12 | 600480 | 4803836 | 2 | 12 | 0 | 184 | 4931988 | 2 | 12 | 0 | 1 | 192 |
| 137 | 89 | 62 | 3E | 2 | 13 | 600503 | 4804020 | 2 | 13 | 0 | 184 | 4932180 | 2 | 13 | 0 | 1 | 192 |
| 138 | 8A | 62 | 3E | 2 | 14 | 600526 | 4804204 | 2 | 14 | 0 | 184 | 4932372 | 2 | 14 | 0 | 1 | 192 |
| 139 | 8B | 62 | 3E | 2 | 15 | 600549 | 4804388 | 2 | 15 | 0 | 184 | 4932564 | 2 | 15 | 0 | 1 | 192 |
| 140 | 8C | 62 | 3E | 2 | 16 | 600572 | 4804572 | 2 | 16 | 0 | 184 | 4932756 | 2 | 16 | 0 | 1 | 192 |
| 141 | 8D | 62 | 3E | 2 | 17 | 600595 | 4804756 | 2 | 17 | 0 | 184 | 4932948 | 2 | 17 | 0 | 1 | 192 |
| 142 | 8E | 62 | 3E | 2 | 18 | 600618 | 4804940 | 2 | 18 | 0 | 184 | 4933140 | 2 | 18 | 0 | 1 | 192 |
| 143 | 8F | 62 | 3E | 2 | 19 | 600641 | 4805124 | 2 | 19 | 0 | 184 | 4933332 | 2 | 19 | 0 | 1 | 192 |
| 144 | 90 | 62 | 3E | 2 | 20 | 600666 | 4805324 | 2 | 20 | 0 | 200 | 4933540 | 2 | 20 | 0 | 1 | 208 |
| 145 | 91 | 62 | 3E | 2 | 21 | 600691 | 4805524 | 2 | 21 | 0 | 200 | 4933748 | 2 | 21 | 0 | 1 | 208 |
| 146 | 92 | 62 | 3E | 2 | 22 | 600716 | 4805724 | 2 | 22 | 0 | 200 | 4933956 | 2 | 22 | 0 | 1 | 208 |
| 147 | 93 | 62 | 3E | 2 | 23 | 600741 | 4805924 | 2 | 23 | 0 | 200 | 4934164 | 2 | 23 | 0 | 1 | 208 |
| 148 | 94 | 62 | 3E | 2 | 24 | 600766 | 4806124 | 2 | 24 | 0 | 200 | 4934372 | 2 | 24 | 0 | 1 | 208 |
| 149 | 95 | 62 | 3E | 2 | 25 | 600791 | 4806324 | 2 | 25 | 0 | 200 | 4934580 | 2 | 25 | 0 | 1 | 208 |
| 150 | 96 | 62 | 3E | 2 | 26 | 600816 | 4806524 | 2 | 26 | 0 | 200 | 4934788 | 2 | 26 | 0 | 1 | 208 |
| 151 | 97 | 62 | 3E | 2 | 27 | 600841 | 4806724 | 2 | 27 | 0 | 200 | 4934996 | 2 | 27 | 0 | 1 | 208 |
| 152 | 98 | 62 | 3E | 2 | 28 | 600866 | 4806924 | 2 | 28 | 0 | 200 | 4935204 | 2 | 28 | 0 | 1 | 208 |
| 153 | 99 | 62 | 3E | 2 | 29 | 600891 | 4807124 | 2 | 29 | 0 | 200 | 4935412 | 2 | 29 | 0 | 1 | 208 |
| 154 | 9A | 62 | 3E | 2 | 30 | 600916 | 4807324 | 2 | 30 | 0 | 200 | 4935620 | 2 | 30 | 0 | 1 | 208 |
| 155 | 9B | 62 | 3E | 2 | 31 | 600941 | 4807524 | 2 | 31 | 0 | 200 | 4935828 | 2 | 31 | 0 | 1 | 208 |
| 156 | 9C | 62 | 3E | 2 | 32 | 600966 | 4807724 | 2 | 32 | 0 | 200 | 4936036 | 2 | 32 | 0 | 1 | 208 |
| 157 | 9D | 62 | 3E | 2 | 33 | 600991 | 4807924 | 2 | 33 | 0 | 200 | 4936244 | 2 | 33 | 0 | 1 | 208 |
| 158 | 9E | 62 | 3E | 2 | 34 | 601016 | 4808124 | 2 | 34 | 0 | 200 | 4936452 | 2 | 34 | 0 | 1 | 208 |
| 159 | 9F | 62 | 3E | 2 | 35 | 601041 | 4808324 | 2 | 35 | 0 | 200 | 4936660 | 2 | 35 | 0 | 1 | 208 |
| 160 | A0 | 62 | 3E | 2 | 36 | 601066 | 4808524 | 2 | 36 | 0 | 200 | 4936868 | 2 | 36 | 0 | 1 | 208 |
| 161 | A1 | 62 | 3E | 2 | 37 | 601091 | 4808724 | 2 | 37 | 0 | 200 | 4937076 | 2 | 37 | 0 | 1 | 208 |
| 162 | A2 | 62 | 3E | 2 | 38 | 601116 | 4808924 | 2 | 38 | 0 | 200 | 4937284 | 2 | 38 | 0 | 1 | 208 |
| 163 | A3 | 62 | 3E | 2 | 39 | 601141 | 4809124 | 2 | 39 | 0 | 200 | 4937492 | 2 | 39 | 0 | 1 | 208 |
| 164 | A4 | 62 | 3E | 2 | 40 | 601166 | 4809324 | 2 | 40 | 0 | 200 | 4937700 | 2 | 40 | 0 | 1 | 208 |
| 165 | A5 | 62 | 3E | 2 | 41 | 601191 | 4809524 | 2 | 41 | 0 | 200 | 4937908 | 2 | 41 | 0 | 1 | 208 |
| 166 | A6 | 62 | 3E | 2 | 42 | 601216 | 4809724 | 2 | 42 | 0 | 200 | 4938116 | 2 | 42 | 0 | 1 | 208 |
| 167 | A7 | 62 | 3E | 2 | 43 | 601241 | 4809924 | 2 | 43 | 0 | 200 | 4938324 | 2 | 43 | 0 | 1 | 208 |
| 168 | A8 | 62 | 3E | 2 | 44 | 601266 | 4810124 | 2 | 44 | 0 | 200 | 4938532 | 2 | 44 | 0 | 1 | 208 |
| 169 | A9 | 62 | 3E | 2 | 45 | 601291 | 4810324 | 2 | 45 | 0 | 200 | 4938740 | 2 | 45 | 0 | 1 | 208 |
| 170 | AA | 62 | 3E | 2 | 46 | 601316 | 4810524 | 2 | 46 | 0 | 200 | 4938948 | 2 | 46 | 0 | 1 | 208 |
| 171 | AB | 62 | 3E | 2 | 47 | 601341 | 4810724 | 2 | 47 | 0 | 200 | 4939156 | 2 | 47 | 0 | 1 | 208 |
| 172 | AC | 62 | 3E | 2 | 48 | 601366 | 4810924 | 2 | 48 | 0 | 200 | 4939364 | 2 | 48 | 0 | 1 | 208 |
| 173 | AD | 62 | 3E | 2 | 49 | 601391 | 4811124 | 2 | 49 | 0 | 200 | 4939572 | 2 | 49 | 0 | 1 | 208 |
| 174 | AE | 62 | 3E | 2 | 50 | 601416 | 4811324 | 2 | 50 | 0 | 200 | 4939780 | 2 | 50 | 0 | 1 | 208 |
| 175 | AF | 62 | 3E | 2 | 51 | 601441 | 4811524 | 2 | 51 | 0 | 200 | 4939988 | 2 | 51 | 0 | 1 | 208 |
| 176 | B0 | 62 | 3E | 2 | 52 | 601466 | 4811724 | 2 | 52 | 0 | 200 | 4940196 | 2 | 52 | 0 | 1 | 208 |
| 177 | B1 | 62 | 3E | 2 | 53 | 601491 | 4811924 | 2 | 53 | 0 | 200 | 4940404 | 2 | 53 | 0 | 1 | 208 |
| 178 | B2 | 62 | 3E | 2 | 54 | 601516 | 4812124 | 2 | 54 | 0 | 200 | 4940612 | 2 | 54 | 0 | 1 | 208 |
| 179 | B3 | 62 | 3E | 2 | 55 | 601541 | 4812324 | 2 | 55 | 0 | 200 | 4940820 | 2 | 55 | 0 | 1 | 208 |
| 180 | B4 | 62 | 3E | 2 | 56 | 601566 | 4812524 | 2 | 56 | 0 | 200 | 4941028 | 2 | 56 | 0 | 1 | 208 |
| 181 | B5 | 62 | 3E | 2 | 57 | 601591 | 4812724 | 2 | 57 | 0 | 200 | 4941236 | 2 | 57 | 0 | 1 | 208 |
| 182 | B6 | 62 | 3E | 2 | 58 | 601616 | 4812924 | 2 | 58 | 0 | 200 | 4941444 | 2 | 58 | 0 | 1 | 208 |
| 183 | B7 | 62 | 3E | 2 | 59 | 601641 | 4813124 | 2 | 59 | 0 | 200 | 4941652 | 2 | 59 | 0 | 1 | 208 |
| 184 | B8 | 62 | 3E | 2 | 60 | 601666 | 4813324 | 2 | 60 | 0 | 200 | 4941860 | 2 | 60 | 0 | 1 | 208 |
| 185 | B9 | 62 | 3E | 2 | 61 | 601691 | 4813524 | 2 | 61 | 0 | 200 | 4942068 | 2 | 61 | 0 | 1 | 208 |
| 186 | BA | 62 | 3E | 3 | 0 | 601714 | 4813708 | 3 | 0 | 0 | 184 | 4942260 | 3 | 0 | 0 | 1 | 192 |
| 187 | BB | 62 | 3E | 3 | 1 | 601742 | 4813932 | 3 | 1 | 0 | 224 | 4942492 | 3 | 1 | 0 | 1 | 232 |
| 188 | BC | 62 | 3E | 3 | 2 | 601770 | 4814156 | 3 | 2 | 0 | 224 | 4942724 | 3 | 2 | 0 | 1 | 232 |
| 189 | BD | 62 | 3E | 3 | 3 | 601798 | 4814380 | 3 | 3 | 0 | 224 | 4942956 | 3 | 3 | 0 | 1 | 232 |
| 190 | BE | 62 | 3E | 3 | 4 | 601826 | 4814604 | 3 | 4 | 0 | 224 | 4943188 | 3 | 4 | 0 | 1 | 232 |
| 191 | BF | 62 | 3E | 3 | 5 | 601854 | 4814828 | 3 | 5 | 0 | 224 | 4943420 | 3 | 5 | 0 | 1 | 232 |
| 192 | C0 | 62 | 3E | 3 | 6 | 601879 | 4815028 | 3 | 6 | 0 | 200 | 4943628 | 3 | 6 | 0 | 1 | 208 |

| | | | | | | | | | | | | | | | | | | |
|---|---|---|---|---|---|---|---|---|---|---|---|---|---|---|---|---|---|---|
| 193 | C1 | 62 | 3E | 3 | 7 | 601904 | 4815228 | 3 | 7 | 0 | 200 | 4943836 | 3 | 7 | 0 | 1 | 208 |
| 194 | C2 | 62 | 3E | 3 | 8 | 601929 | 4815428 | 3 | 8 | 0 | 200 | 4944044 | 3 | 8 | 0 | 1 | 208 |
| 195 | C3 | 62 | 3E | 3 | 9 | 601954 | 4815628 | 3 | 9 | 0 | 200 | 4944252 | 3 | 9 | 0 | 1 | 208 |
| 196 | C4 | 62 | 3E | 3 | 10 | 601979 | 4815828 | 3 | 10 | 0 | 200 | 4944460 | 3 | 10 | 0 | 1 | 208 |
| 197 | C5 | 62 | 3E | 3 | 11 | 602004 | 4816028 | 3 | 11 | 0 | 200 | 4944668 | 3 | 11 | 0 | 1 | 208 |
| 198 | C6 | 62 | 3E | 3 | 12 | 602029 | 4816228 | 3 | 12 | 0 | 200 | 4944876 | 3 | 12 | 0 | 1 | 208 |
| 199 | C7 | 62 | 3E | 3 | 13 | 602054 | 4816428 | 3 | 13 | 0 | 200 | 4945084 | 3 | 13 | 0 | 1 | 208 |
| 200 | C8 | 62 | 3E | 3 | 14 | 602079 | 4816628 | 3 | 14 | 0 | 200 | 4945292 | 3 | 14 | 0 | 1 | 208 |
| 201 | C9 | 62 | 3E | 3 | 15 | 602104 | 4816828 | 3 | 15 | 0 | 200 | 4945500 | 3 | 15 | 0 | 1 | 208 |
| 202 | CA | 62 | 3E | 3 | 16 | 602129 | 4817028 | 3 | 16 | 0 | 200 | 4945708 | 3 | 16 | 0 | 1 | 208 |
| 203 | CB | 62 | 3E | 3 | 17 | 602154 | 4817228 | 3 | 17 | 0 | 200 | 4945916 | 3 | 17 | 0 | 1 | 208 |
| 204 | CC | 62 | 3E | 3 | 18 | 602179 | 4817428 | 3 | 18 | 0 | 200 | 4946124 | 3 | 18 | 0 | 1 | 208 |
| 205 | CD | 62 | 3E | 3 | 19 | 602204 | 4817628 | 3 | 19 | 0 | 200 | 4946332 | 3 | 19 | 0 | 1 | 208 |
| 206 | CE | 62 | 3E | 3 | 20 | 602229 | 4817828 | 3 | 20 | 0 | 200 | 4946540 | 3 | 20 | 0 | 1 | 208 |
| 207 | CF | 62 | 3E | 3 | 21 | 602254 | 4818028 | 3 | 21 | 0 | 200 | 4946748 | 3 | 21 | 0 | 1 | 208 |
| 208 | D0 | 62 | 3E | 3 | 22 | 602281 | 4818244 | 3 | 22 | 0 | 216 | 4946972 | 3 | 22 | 0 | 1 | 224 |
| 209 | D1 | 62 | 3E | 3 | 23 | 602308 | 4818460 | 3 | 23 | 0 | 216 | 4947196 | 3 | 23 | 0 | 1 | 224 |
| 210 | D2 | 62 | 3E | 3 | 24 | 602335 | 4818676 | 3 | 24 | 0 | 216 | 4947420 | 3 | 24 | 0 | 1 | 224 |
| 211 | D3 | 62 | 3E | 3 | 25 | 602362 | 4818892 | 3 | 25 | 0 | 216 | 4947644 | 3 | 25 | 0 | 1 | 224 |
| 212 | D4 | 62 | 3E | 3 | 26 | 602389 | 4819108 | 3 | 26 | 0 | 216 | 4947868 | 3 | 26 | 0 | 1 | 224 |
| 213 | D5 | 62 | 3E | 3 | 27 | 602416 | 4819324 | 3 | 27 | 0 | 216 | 4948092 | 3 | 27 | 0 | 1 | 224 |
| 214 | D6 | 62 | 3E | 3 | 28 | 602443 | 4819540 | 3 | 28 | 0 | 216 | 4948316 | 3 | 28 | 0 | 1 | 224 |
| 215 | D7 | 62 | 3E | 3 | 29 | 602470 | 4819756 | 3 | 29 | 0 | 216 | 4948540 | 3 | 29 | 0 | 1 | 224 |
| 216 | D8 | 62 | 3E | 3 | 30 | 602497 | 4819972 | 3 | 30 | 0 | 216 | 4948764 | 3 | 30 | 0 | 1 | 224 |
| 217 | D9 | 62 | 3E | 3 | 31 | 602524 | 4820188 | 3 | 31 | 0 | 216 | 4948988 | 3 | 31 | 0 | 1 | 224 |
| 218 | DA | 62 | 3E | 3 | 32 | 602551 | 4820404 | 3 | 32 | 0 | 216 | 4949212 | 3 | 32 | 0 | 1 | 224 |
| 219 | DB | 62 | 3E | 3 | 33 | 602578 | 4820620 | 3 | 33 | 0 | 216 | 4949436 | 3 | 33 | 0 | 1 | 224 |
| 220 | DC | 62 | 3E | 3 | 34 | 602605 | 4820836 | 3 | 34 | 0 | 216 | 4949660 | 3 | 34 | 0 | 1 | 224 |
| 221 | DD | 62 | 3E | 3 | 35 | 602632 | 4821052 | 3 | 35 | 0 | 216 | 4949884 | 3 | 35 | 0 | 1 | 224 |
| 222 | DE | 62 | 3E | 3 | 36 | 602659 | 4821268 | 3 | 36 | 0 | 216 | 4950108 | 3 | 36 | 0 | 1 | 224 |
| 223 | DF | 62 | 3E | 3 | 37 | 602686 | 4821484 | 3 | 37 | 0 | 216 | 4950332 | 3 | 37 | 0 | 1 | 224 |
| 224 | E0 | 62 | 3E | 3 | 38 | 602713 | 4821700 | 3 | 38 | 0 | 216 | 4950556 | 3 | 38 | 0 | 1 | 224 |
| 225 | E1 | 62 | 3E | 3 | 39 | 602740 | 4821916 | 3 | 39 | 0 | 216 | 4950780 | 3 | 39 | 0 | 1 | 224 |
| 226 | E2 | 62 | 3E | 3 | 40 | 602767 | 4822132 | 3 | 40 | 0 | 216 | 4951004 | 3 | 40 | 0 | 1 | 224 |
| 227 | E3 | 62 | 3E | 3 | 41 | 602794 | 4822348 | 3 | 41 | 0 | 216 | 4951228 | 3 | 41 | 0 | 1 | 224 |
| 228 | E4 | 62 | 3E | 3 | 42 | 602821 | 4822564 | 3 | 42 | 0 | 216 | 4951452 | 3 | 42 | 0 | 1 | 224 |
| 229 | E5 | 62 | 3E | 3 | 43 | 602848 | 4822780 | 3 | 43 | 0 | 216 | 4951676 | 3 | 43 | 0 | 1 | 224 |
| 230 | E6 | 62 | 3E | 3 | 44 | 602875 | 4822996 | 3 | 44 | 0 | 216 | 4951900 | 3 | 44 | 0 | 1 | 224 |
| 231 | E7 | 62 | 3E | 3 | 45 | 602902 | 4823212 | 3 | 45 | 0 | 216 | 4952124 | 3 | 45 | 0 | 1 | 224 |
| 232 | E8 | 62 | 3E | 3 | 46 | 602929 | 4823428 | 3 | 46 | 0 | 216 | 4952348 | 3 | 46 | 0 | 1 | 224 |
| 233 | E9 | 62 | 3E | 3 | 47 | 602956 | 4823644 | 3 | 47 | 0 | 216 | 4952572 | 3 | 47 | 0 | 1 | 224 |
| 234 | EA | 62 | 3E | 3 | 48 | 602983 | 4823860 | 3 | 48 | 0 | 216 | 4952796 | 3 | 48 | 0 | 1 | 224 |
| 235 | EB | 62 | 3E | 3 | 49 | 603010 | 4824076 | 3 | 49 | 0 | 216 | 4953020 | 3 | 49 | 0 | 1 | 224 |
| 236 | EC | 62 | 3E | 3 | 50 | 603037 | 4824292 | 3 | 50 | 0 | 216 | 4953244 | 3 | 50 | 0 | 1 | 224 |
| 237 | ED | 62 | 3E | 3 | 51 | 603064 | 4824508 | 3 | 51 | 0 | 216 | 4953468 | 3 | 51 | 0 | 1 | 224 |
| 238 | EE | 62 | 3E | 3 | 52 | 603091 | 4824724 | 3 | 52 | 0 | 216 | 4953692 | 3 | 52 | 0 | 1 | 224 |
| 239 | EF | 62 | 3E | 3 | 53 | 603118 | 4824940 | 3 | 53 | 0 | 216 | 4953916 | 3 | 53 | 0 | 1 | 224 |
| 240 | F0 | 62 | 3E | 3 | 54 | 603145 | 4825156 | 3 | 54 | 0 | 216 | 4954140 | 3 | 54 | 0 | 1 | 224 |
| 241 | F1 | 62 | 3E | 3 | 55 | 603172 | 4825372 | 3 | 55 | 0 | 216 | 4954364 | 3 | 55 | 0 | 1 | 224 |
| 242 | F2 | 62 | 3E | 3 | 56 | 603199 | 4825588 | 3 | 56 | 0 | 216 | 4954588 | 3 | 56 | 0 | 1 | 224 |
| 243 | F3 | 62 | 3E | 3 | 57 | 603226 | 4825804 | 3 | 57 | 0 | 216 | 4954812 | 3 | 57 | 0 | 1 | 224 |
| 244 | F4 | 62 | 3E | 3 | 58 | 603253 | 4826020 | 3 | 58 | 0 | 216 | 4955036 | 3 | 58 | 0 | 1 | 224 |
| 245 | F5 | 62 | 3E | 3 | 59 | 603280 | 4826236 | 3 | 59 | 0 | 216 | 4955260 | 3 | 59 | 0 | 1 | 224 |
| 246 | F6 | 62 | 3E | 3 | 60 | 603307 | 4826452 | 3 | 60 | 0 | 216 | 4955484 | 3 | 60 | 0 | 1 | 224 |
| 247 | F7 | 62 | 3E | 3 | 61 | 603334 | 4826668 | 3 | 61 | 0 | 216 | 4955708 | 3 | 61 | 0 | 1 | 224 |
| 248 | F8 | 62 | 3E | 4 | 0 | 603359 | 4826868 | 4 | 0 | 0 | 200 | 4955916 | 4 | 0 | 0 | 1 | 208 |
| 249 | F9 | 62 | 3E | 4 | 1 | 603389 | 4827108 | 4 | 1 | 0 | 240 | 4956164 | 4 | 1 | 0 | 1 | 248 |
| 250 | FA | 62 | 3E | 4 | 2 | 603419 | 4827348 | 4 | 2 | 0 | 240 | 4956412 | 4 | 2 | 0 | 1 | 248 |
| 251 | FB | 62 | 3E | 4 | 3 | 603449 | 4827588 | 4 | 3 | 0 | 240 | 4956660 | 4 | 3 | 0 | 1 | 248 |
| 252 | FC | 62 | 3E | 4 | 4 | 603479 | 4827828 | 4 | 4 | 0 | 240 | 4956908 | 4 | 4 | 0 | 1 | 248 |
| 253 | FD | 62 | 3E | 4 | 5 | 603509 | 4828068 | 4 | 5 | 0 | 240 | 4957156 | 4 | 5 | 0 | 1 | 248 |
| 254 | FE | 62 | 3E | 4 | 6 | 603539 | 4828308 | 4 | 6 | 0 | 240 | 4957404 | 4 | 6 | 0 | 1 | 248 |
| 255 | FF | 62 | 3E | 4 | 7 | 603569 | 4828548 | 4 | 7 | 0 | 240 | 4957652 | 4 | 7 | 0 | 1 | 248 |
| 0 | 00 | 63 | 3F | 0 | 0 | 603581 | 4828644 | 0 | 0 | 0 | 96 | 4957756 | 0 | 0 | 0 | 1 | 104 |
| 1 | 01 | 63 | 3F | 0 | 1 | 603595 | 4828756 | 0 | 1 | 0 | 112 | 4957876 | 0 | 1 | 0 | 1 | 120 |
| 2 | 02 | 63 | 3F | 0 | 2 | 603609 | 4828868 | 0 | 2 | 0 | 112 | 4957996 | 0 | 2 | 0 | 1 | 120 |
| 3 | 03 | 63 | 3F | 0 | 3 | 603623 | 4828980 | 0 | 3 | 0 | 112 | 4958116 | 0 | 3 | 0 | 1 | 120 |
| 4 | 04 | 63 | 3F | 0 | 4 | 603637 | 4829092 | 0 | 4 | 0 | 112 | 4958236 | 0 | 4 | 0 | 1 | 120 |
| 5 | 05 | 63 | 3F | 0 | 5 | 603651 | 4829204 | 0 | 5 | 0 | 112 | 4958356 | 0 | 5 | 0 | 1 | 120 |
| 6 | 06 | 63 | 3F | 0 | 6 | 603665 | 4829316 | 0 | 6 | 0 | 112 | 4958476 | 0 | 6 | 0 | 1 | 120 |
| 7 | 07 | 63 | 3F | 0 | 7 | 603679 | 4829428 | 0 | 7 | 0 | 112 | 4958596 | 0 | 7 | 0 | 1 | 120 |
| 8 | 08 | 63 | 3F | 0 | 8 | 603693 | 4829540 | 0 | 8 | 0 | 112 | 4958716 | 0 | 8 | 0 | 1 | 120 |
| 9 | 09 | 63 | 3F | 0 | 9 | 603707 | 4829652 | 0 | 9 | 0 | 112 | 4958836 | 0 | 9 | 0 | 1 | 120 |
| 10 | 0A | 63 | 3F | 0 | 10 | 603721 | 4829764 | 0 | 10 | 0 | 112 | 4958956 | 0 | 10 | 0 | 1 | 120 |
| 11 | 0B | 63 | 3F | 0 | 11 | 603735 | 4829876 | 0 | 11 | 0 | 112 | 4959076 | 0 | 11 | 0 | 1 | 120 |
| 12 | 0C | 63 | 3F | 0 | 12 | 603749 | 4829988 | 0 | 12 | 0 | 112 | 4959196 | 0 | 12 | 0 | 1 | 120 |
| 13 | 0D | 63 | 3F | 0 | 13 | 603763 | 4830100 | 0 | 13 | 0 | 112 | 4959316 | 0 | 13 | 0 | 1 | 120 |
| 14 | 0E | 63 | 3F | 0 | 14 | 603777 | 4830212 | 0 | 14 | 0 | 112 | 4959436 | 0 | 14 | 0 | 1 | 120 |
| 15 | 0F | 63 | 3F | 0 | 15 | 603791 | 4830324 | 0 | 15 | 0 | 112 | 4959556 | 0 | 15 | 0 | 1 | 120 |
| 16 | 10 | 63 | 3F | 0 | 16 | 603805 | 4830436 | 0 | 16 | 0 | 112 | 4959676 | 0 | 16 | 0 | 1 | 120 |
| 17 | 11 | 63 | 3F | 0 | 17 | 603819 | 4830548 | 0 | 17 | 0 | 112 | 4959796 | 0 | 17 | 0 | 1 | 120 |
| 18 | 12 | 63 | 3F | 0 | 18 | 603833 | 4830660 | 0 | 18 | 0 | 112 | 4959916 | 0 | 18 | 0 | 1 | 120 |
| 19 | 13 | 63 | 3F | 0 | 19 | 603847 | 4830772 | 0 | 19 | 0 | 112 | 4960036 | 0 | 19 | 0 | 1 | 120 |
| 20 | 14 | 63 | 3F | 0 | 20 | 603861 | 4830884 | 0 | 20 | 0 | 112 | 4960156 | 0 | 20 | 0 | 1 | 120 |
| 21 | 15 | 63 | 3F | 0 | 21 | 603875 | 4830996 | 0 | 21 | 0 | 112 | 4960276 | 0 | 21 | 0 | 1 | 120 |
| 22 | 16 | 63 | 3F | 0 | 22 | 603889 | 4831108 | 0 | 22 | 0 | 112 | 4960396 | 0 | 22 | 0 | 1 | 120 |
| 23 | 17 | 63 | 3F | 0 | 23 | 603903 | 4831220 | 0 | 23 | 0 | 112 | 4960516 | 0 | 23 | 0 | 1 | 120 |
| 24 | 18 | 63 | 3F | 0 | 24 | 603917 | 4831332 | 0 | 24 | 0 | 112 | 4960636 | 0 | 24 | 0 | 1 | 120 |
| 25 | 19 | 63 | 3F | 0 | 25 | 603931 | 4831444 | 0 | 25 | 0 | 112 | 4960756 | 0 | 25 | 0 | 1 | 120 |
| 26 | 1A | 63 | 3F | 0 | 26 | 603945 | 4831556 | 0 | 26 | 0 | 112 | 4960876 | 0 | 26 | 0 | 1 | 120 |
| 27 | 1B | 63 | 3F | 0 | 27 | 603959 | 4831668 | 0 | 27 | 0 | 112 | 4960996 | 0 | 27 | 0 | 1 | 120 |
| 28 | 1C | 63 | 3F | 0 | 28 | 603973 | 4831780 | 0 | 28 | 0 | 112 | 4961116 | 0 | 28 | 0 | 1 | 120 |
| 29 | 1D | 63 | 3F | 0 | 29 | 603987 | 4831892 | 0 | 29 | 0 | 112 | 4961236 | 0 | 29 | 0 | 1 | 120 |
| 30 | 1E | 63 | 3F | 0 | 30 | 604001 | 4832004 | 0 | 30 | 0 | 112 | 4961356 | 0 | 30 | 0 | 1 | 120 |
| 31 | 1F | 63 | 3F | 0 | 31 | 604015 | 4832116 | 0 | 31 | 0 | 112 | 4961476 | 0 | 31 | 0 | 1 | 120 |
| 32 | 20 | 63 | 3F | 0 | 32 | 604029 | 4832228 | 0 | 32 | 0 | 112 | 4961596 | 0 | 32 | 0 | 1 | 120 |
| 33 | 21 | 63 | 3F | 0 | 33 | 604043 | 4832340 | 0 | 33 | 0 | 112 | 4961716 | 0 | 33 | 0 | 1 | 120 |
| 34 | 22 | 63 | 3F | 0 | 34 | 604057 | 4832452 | 0 | 34 | 0 | 112 | 4961836 | 0 | 34 | 0 | 1 | 120 |
| 35 | 23 | 63 | 3F | 0 | 35 | 604071 | 4832564 | 0 | 35 | 0 | 112 | 4961956 | 0 | 35 | 0 | 1 | 120 |
| 36 | 24 | 63 | 3F | 0 | 36 | 604085 | 4832676 | 0 | 36 | 0 | 112 | 4962076 | 0 | 36 | 0 | 1 | 120 |
| 37 | 25 | 63 | 3F | 0 | 37 | 604099 | 4832788 | 0 | 37 | 0 | 112 | 4962196 | 0 | 37 | 0 | 1 | 120 |
| 38 | 26 | 63 | 3F | 0 | 38 | 604113 | 4832900 | 0 | 38 | 0 | 112 | 4962316 | 0 | 38 | 0 | 1 | 120 |
| 39 | 27 | 63 | 3F | 0 | 39 | 604127 | 4833012 | 0 | 39 | 0 | 112 | 4962436 | 0 | 39 | 0 | 1 | 120 |

| | | | | | | | | | | | | | | | | | |
|---|---|---|---|---|---|---|---|---|---|---|---|---|---|---|---|---|---|
| 40 | 28 | 63 | 3F | 0 | 40 | 604141 | 4833124 | 0 | 40 | 0 | 112 | 4962556 | 0 | 40 | 0 | 1 | 120 |
| 41 | 29 | 63 | 3F | 0 | 41 | 604155 | 4833236 | 0 | 41 | 0 | 112 | 4962676 | 0 | 41 | 0 | 1 | 120 |
| 42 | 2A | 63 | 3F | 0 | 42 | 604169 | 4833348 | 0 | 42 | 0 | 112 | 4962796 | 0 | 42 | 0 | 1 | 120 |
| 43 | 2B | 63 | 3F | 0 | 43 | 604183 | 4833460 | 0 | 43 | 0 | 112 | 4962916 | 0 | 43 | 0 | 1 | 120 |
| 44 | 2C | 63 | 3F | 0 | 44 | 604197 | 4833572 | 0 | 44 | 0 | 112 | 4963036 | 0 | 44 | 0 | 1 | 120 |
| 45 | 2D | 63 | 3F | 0 | 45 | 604211 | 4833684 | 0 | 45 | 0 | 112 | 4963156 | 0 | 45 | 0 | 1 | 120 |
| 46 | 2E | 63 | 3F | 0 | 46 | 604225 | 4833796 | 0 | 46 | 0 | 112 | 4963276 | 0 | 46 | 0 | 1 | 120 |
| 47 | 2F | 63 | 3F | 0 | 47 | 604239 | 4833908 | 0 | 47 | 0 | 112 | 4963396 | 0 | 47 | 0 | 1 | 120 |
| 48 | 30 | 63 | 3F | 0 | 48 | 604253 | 4834020 | 0 | 48 | 0 | 112 | 4963516 | 0 | 48 | 0 | 1 | 120 |
| 49 | 31 | 63 | 3F | 0 | 49 | 604267 | 4834132 | 0 | 49 | 0 | 112 | 4963636 | 0 | 49 | 0 | 1 | 120 |
| 50 | 32 | 63 | 3F | 0 | 50 | 604281 | 4834244 | 0 | 50 | 0 | 112 | 4963756 | 0 | 50 | 0 | 1 | 120 |
| 51 | 33 | 63 | 3F | 0 | 51 | 604295 | 4834356 | 0 | 51 | 0 | 112 | 4963876 | 0 | 51 | 0 | 1 | 120 |
| 52 | 34 | 63 | 3F | 0 | 52 | 604309 | 4834468 | 0 | 52 | 0 | 112 | 4963996 | 0 | 52 | 0 | 1 | 120 |
| 53 | 35 | 63 | 3F | 0 | 53 | 604323 | 4834580 | 0 | 53 | 0 | 112 | 4964116 | 0 | 53 | 0 | 1 | 120 |
| 54 | 36 | 63 | 3F | 0 | 54 | 604337 | 4834692 | 0 | 54 | 0 | 112 | 4964236 | 0 | 54 | 0 | 1 | 120 |
| 55 | 37 | 63 | 3F | 0 | 55 | 604351 | 4834804 | 0 | 55 | 0 | 112 | 4964356 | 0 | 55 | 0 | 1 | 120 |
| 56 | 38 | 63 | 3F | 0 | 56 | 604365 | 4834916 | 0 | 56 | 0 | 112 | 4964476 | 0 | 56 | 0 | 1 | 120 |
| 57 | 39 | 63 | 3F | 0 | 57 | 604379 | 4835028 | 0 | 57 | 0 | 112 | 4964596 | 0 | 57 | 0 | 1 | 120 |
| 58 | 3A | 63 | 3F | 0 | 58 | 604393 | 4835140 | 0 | 58 | 0 | 112 | 4964716 | 0 | 58 | 0 | 1 | 120 |
| 59 | 3B | 63 | 3F | 0 | 59 | 604407 | 4835252 | 0 | 59 | 0 | 112 | 4964836 | 0 | 59 | 0 | 1 | 120 |
| 60 | 3C | 63 | 3F | 0 | 60 | 604421 | 4835364 | 0 | 60 | 0 | 112 | 4964956 | 0 | 60 | 0 | 1 | 120 |
| 61 | 3D | 63 | 3F | 0 | 61 | 604435 | 4835476 | 0 | 61 | 0 | 112 | 4965076 | 0 | 61 | 0 | 1 | 120 |
| 62 | 3E | 63 | 3F | 0 | 62 | 604449 | 4835588 | 0 | 62 | 0 | 112 | 4965196 | 0 | 62 | 0 | 1 | 120 |
| 63 | 3F | 63 | 3F | 1 | 0 | 604461 | 4835684 | 1 | 0 | 0 | 96 | 4965300 | 1 | 0 | 0 | 1 | 104 |
| 64 | 40 | 63 | 3F | 1 | 1 | 604482 | 4835852 | 1 | 1 | 0 | 168 | 4965476 | 1 | 1 | 0 | 1 | 176 |
| 65 | 41 | 63 | 3F | 1 | 2 | 604503 | 4836020 | 1 | 2 | 0 | 168 | 4965652 | 1 | 2 | 0 | 1 | 176 |
| 66 | 42 | 63 | 3F | 1 | 3 | 604524 | 4836188 | 1 | 3 | 0 | 168 | 4965828 | 1 | 3 | 0 | 1 | 176 |
| 67 | 43 | 63 | 3F | 1 | 4 | 604545 | 4836356 | 1 | 4 | 0 | 168 | 4966004 | 1 | 4 | 0 | 1 | 176 |
| 68 | 44 | 63 | 3F | 1 | 5 | 604566 | 4836524 | 1 | 5 | 0 | 168 | 4966180 | 1 | 5 | 0 | 1 | 176 |
| 69 | 45 | 63 | 3F | 1 | 6 | 604587 | 4836692 | 1 | 6 | 0 | 168 | 4966356 | 1 | 6 | 0 | 1 | 176 |
| 70 | 46 | 63 | 3F | 1 | 7 | 604608 | 4836860 | 1 | 7 | 0 | 168 | 4966532 | 1 | 7 | 0 | 1 | 176 |
| 71 | 47 | 63 | 3F | 1 | 8 | 604629 | 4837028 | 1 | 8 | 0 | 168 | 4966708 | 1 | 8 | 0 | 1 | 176 |
| 72 | 48 | 63 | 3F | 1 | 9 | 604650 | 4837196 | 1 | 9 | 0 | 168 | 4966884 | 1 | 9 | 0 | 1 | 176 |
| 73 | 49 | 63 | 3F | 1 | 10 | 604671 | 4837364 | 1 | 10 | 0 | 168 | 4967060 | 1 | 10 | 0 | 1 | 176 |
| 74 | 4A | 63 | 3F | 1 | 11 | 604692 | 4837532 | 1 | 11 | 0 | 168 | 4967236 | 1 | 11 | 0 | 1 | 176 |
| 75 | 4B | 63 | 3F | 1 | 12 | 604713 | 4837700 | 1 | 12 | 0 | 168 | 4967412 | 1 | 12 | 0 | 1 | 176 |
| 76 | 4C | 63 | 3F | 1 | 13 | 604734 | 4837868 | 1 | 13 | 0 | 168 | 4967588 | 1 | 13 | 0 | 1 | 176 |
| 77 | 4D | 63 | 3F | 1 | 14 | 604755 | 4838036 | 1 | 14 | 0 | 168 | 4967764 | 1 | 14 | 0 | 1 | 176 |
| 78 | 4E | 63 | 3F | 1 | 15 | 604776 | 4838204 | 1 | 15 | 0 | 168 | 4967940 | 1 | 15 | 0 | 1 | 176 |
| 79 | 4F | 63 | 3F | 1 | 16 | 604797 | 4838372 | 1 | 16 | 0 | 168 | 4968116 | 1 | 16 | 0 | 1 | 176 |
| 80 | 50 | 63 | 3F | 1 | 17 | 604820 | 4838556 | 1 | 17 | 0 | 184 | 4968308 | 1 | 17 | 0 | 1 | 192 |
| 81 | 51 | 63 | 3F | 1 | 18 | 604843 | 4838740 | 1 | 18 | 0 | 184 | 4968500 | 1 | 18 | 0 | 1 | 192 |
| 82 | 52 | 63 | 3F | 1 | 19 | 604866 | 4838924 | 1 | 19 | 0 | 184 | 4968692 | 1 | 19 | 0 | 1 | 192 |
| 83 | 53 | 63 | 3F | 1 | 20 | 604889 | 4839108 | 1 | 20 | 0 | 184 | 4968884 | 1 | 20 | 0 | 1 | 192 |
| 84 | 54 | 63 | 3F | 1 | 21 | 604912 | 4839292 | 1 | 21 | 0 | 184 | 4969076 | 1 | 21 | 0 | 1 | 192 |
| 85 | 55 | 63 | 3F | 1 | 22 | 604935 | 4839476 | 1 | 22 | 0 | 184 | 4969268 | 1 | 22 | 0 | 1 | 192 |
| 86 | 56 | 63 | 3F | 1 | 23 | 604958 | 4839660 | 1 | 23 | 0 | 184 | 4969460 | 1 | 23 | 0 | 1 | 192 |
| 87 | 57 | 63 | 3F | 1 | 24 | 604981 | 4839844 | 1 | 24 | 0 | 184 | 4969652 | 1 | 24 | 0 | 1 | 192 |
| 88 | 58 | 63 | 3F | 1 | 25 | 605004 | 4840028 | 1 | 25 | 0 | 184 | 4969844 | 1 | 25 | 0 | 1 | 192 |
| 89 | 59 | 63 | 3F | 1 | 26 | 605027 | 4840212 | 1 | 26 | 0 | 184 | 4970036 | 1 | 26 | 0 | 1 | 192 |
| 90 | 5A | 63 | 3F | 1 | 27 | 605050 | 4840396 | 1 | 27 | 0 | 184 | 4970228 | 1 | 27 | 0 | 1 | 192 |
| 91 | 5B | 63 | 3F | 1 | 28 | 605073 | 4840580 | 1 | 28 | 0 | 184 | 4970420 | 1 | 28 | 0 | 1 | 192 |
| 92 | 5C | 63 | 3F | 1 | 29 | 605096 | 4840764 | 1 | 29 | 0 | 184 | 4970612 | 1 | 29 | 0 | 1 | 192 |
| 93 | 5D | 63 | 3F | 1 | 30 | 605119 | 4840948 | 1 | 30 | 0 | 184 | 4970804 | 1 | 30 | 0 | 1 | 192 |
| 94 | 5E | 63 | 3F | 1 | 31 | 605142 | 4841132 | 1 | 31 | 0 | 184 | 4970996 | 1 | 31 | 0 | 1 | 192 |
| 95 | 5F | 63 | 3F | 1 | 32 | 605165 | 4841316 | 1 | 32 | 0 | 184 | 4971188 | 1 | 32 | 0 | 1 | 192 |
| 96 | 60 | 63 | 3F | 1 | 33 | 605188 | 4841500 | 1 | 33 | 0 | 184 | 4971380 | 1 | 33 | 0 | 1 | 192 |
| 97 | 61 | 63 | 3F | 1 | 34 | 605211 | 4841684 | 1 | 34 | 0 | 184 | 4971572 | 1 | 34 | 0 | 1 | 192 |
| 98 | 62 | 63 | 3F | 1 | 35 | 605234 | 4841868 | 1 | 35 | 0 | 184 | 4971764 | 1 | 35 | 0 | 1 | 192 |
| 99 | 63 | 63 | 3F | 1 | 36 | 605257 | 4842052 | 1 | 36 | 0 | 184 | 4971956 | 1 | 36 | 0 | 1 | 192 |
| 100 | 64 | 63 | 3F | 1 | 37 | 605280 | 4842236 | 1 | 37 | 0 | 184 | 4972148 | 1 | 37 | 0 | 1 | 192 |
| 101 | 65 | 63 | 3F | 1 | 38 | 605303 | 4842420 | 1 | 38 | 0 | 184 | 4972340 | 1 | 38 | 0 | 1 | 192 |
| 102 | 66 | 63 | 3F | 1 | 39 | 605326 | 4842604 | 1 | 39 | 0 | 184 | 4972532 | 1 | 39 | 0 | 1 | 192 |
| 103 | 67 | 63 | 3F | 1 | 40 | 605349 | 4842788 | 1 | 40 | 0 | 184 | 4972724 | 1 | 40 | 0 | 1 | 192 |
| 104 | 68 | 63 | 3F | 1 | 41 | 605372 | 4842972 | 1 | 41 | 0 | 184 | 4972916 | 1 | 41 | 0 | 1 | 192 |
| 105 | 69 | 63 | 3F | 1 | 42 | 605395 | 4843156 | 1 | 42 | 0 | 184 | 4973108 | 1 | 42 | 0 | 1 | 192 |
| 106 | 6A | 63 | 3F | 1 | 43 | 605418 | 4843340 | 1 | 43 | 0 | 184 | 4973300 | 1 | 43 | 0 | 1 | 192 |
| 107 | 6B | 63 | 3F | 1 | 44 | 605441 | 4843524 | 1 | 44 | 0 | 184 | 4973492 | 1 | 44 | 0 | 1 | 192 |
| 108 | 6C | 63 | 3F | 1 | 45 | 605464 | 4843708 | 1 | 45 | 0 | 184 | 4973684 | 1 | 45 | 0 | 1 | 192 |
| 109 | 6D | 63 | 3F | 1 | 46 | 605487 | 4843892 | 1 | 46 | 0 | 184 | 4973876 | 1 | 46 | 0 | 1 | 192 |
| 110 | 6E | 63 | 3F | 1 | 47 | 605510 | 4844076 | 1 | 47 | 0 | 184 | 4974068 | 1 | 47 | 0 | 1 | 192 |
| 111 | 6F | 63 | 3F | 1 | 48 | 605533 | 4844260 | 1 | 48 | 0 | 184 | 4974260 | 1 | 48 | 0 | 1 | 192 |
| 112 | 70 | 63 | 3F | 1 | 49 | 605556 | 4844444 | 1 | 49 | 0 | 184 | 4974452 | 1 | 49 | 0 | 1 | 192 |
| 113 | 71 | 63 | 3F | 1 | 50 | 605579 | 4844628 | 1 | 50 | 0 | 184 | 4974644 | 1 | 50 | 0 | 1 | 192 |
| 114 | 72 | 63 | 3F | 1 | 51 | 605602 | 4844812 | 1 | 51 | 0 | 184 | 4974836 | 1 | 51 | 0 | 1 | 192 |
| 115 | 73 | 63 | 3F | 1 | 52 | 605625 | 4844996 | 1 | 52 | 0 | 184 | 4975028 | 1 | 52 | 0 | 1 | 192 |
| 116 | 74 | 63 | 3F | 1 | 53 | 605648 | 4845180 | 1 | 53 | 0 | 184 | 4975220 | 1 | 53 | 0 | 1 | 192 |
| 117 | 75 | 63 | 3F | 1 | 54 | 605671 | 4845364 | 1 | 54 | 0 | 184 | 4975412 | 1 | 54 | 0 | 1 | 192 |
| 118 | 76 | 63 | 3F | 1 | 55 | 605694 | 4845548 | 1 | 55 | 0 | 184 | 4975604 | 1 | 55 | 0 | 1 | 192 |
| 119 | 77 | 63 | 3F | 1 | 56 | 605717 | 4845732 | 1 | 56 | 0 | 184 | 4975796 | 1 | 56 | 0 | 1 | 192 |
| 120 | 78 | 63 | 3F | 1 | 57 | 605740 | 4845916 | 1 | 57 | 0 | 184 | 4975988 | 1 | 57 | 0 | 1 | 192 |
| 121 | 79 | 63 | 3F | 1 | 58 | 605763 | 4846100 | 1 | 58 | 0 | 184 | 4976180 | 1 | 58 | 0 | 1 | 192 |
| 122 | 7A | 63 | 3F | 1 | 59 | 605786 | 4846284 | 1 | 59 | 0 | 184 | 4976372 | 1 | 59 | 0 | 1 | 192 |
| 123 | 7B | 63 | 3F | 1 | 60 | 605809 | 4846468 | 1 | 60 | 0 | 184 | 4976564 | 1 | 60 | 0 | 1 | 192 |
| 124 | 7C | 63 | 3F | 1 | 61 | 605832 | 4846652 | 1 | 61 | 0 | 184 | 4976756 | 1 | 61 | 0 | 1 | 192 |
| 125 | 7D | 63 | 3F | 1 | 62 | 605855 | 4846836 | 1 | 62 | 0 | 184 | 4976948 | 1 | 62 | 0 | 1 | 192 |
| 126 | 7E | 63 | 3F | 2 | 0 | 605876 | 4847004 | 2 | 0 | 0 | 168 | 4977124 | 2 | 0 | 0 | 1 | 176 |
| 127 | 7F | 63 | 3F | 2 | 1 | 605902 | 4847212 | 2 | 1 | 0 | 208 | 4977340 | 2 | 1 | 0 | 1 | 216 |
| 128 | 80 | 63 | 3F | 2 | 2 | 605925 | 4847396 | 2 | 2 | 0 | 184 | 4977532 | 2 | 2 | 0 | 1 | 192 |
| 129 | 81 | 63 | 3F | 2 | 3 | 605948 | 4847580 | 2 | 3 | 0 | 184 | 4977724 | 2 | 3 | 0 | 1 | 192 |
| 130 | 82 | 63 | 3F | 2 | 4 | 605971 | 4847764 | 2 | 4 | 0 | 184 | 4977916 | 2 | 4 | 0 | 1 | 192 |
| 131 | 83 | 63 | 3F | 2 | 5 | 605994 | 4847948 | 2 | 5 | 0 | 184 | 4978108 | 2 | 5 | 0 | 1 | 192 |
| 132 | 84 | 63 | 3F | 2 | 6 | 606017 | 4848132 | 2 | 6 | 0 | 184 | 4978300 | 2 | 6 | 0 | 1 | 192 |
| 133 | 85 | 63 | 3F | 2 | 7 | 606040 | 4848316 | 2 | 7 | 0 | 184 | 4978492 | 2 | 7 | 0 | 1 | 192 |
| 134 | 86 | 63 | 3F | 2 | 8 | 606063 | 4848500 | 2 | 8 | 0 | 184 | 4978684 | 2 | 8 | 0 | 1 | 192 |
| 135 | 87 | 63 | 3F | 2 | 9 | 606086 | 4848684 | 2 | 9 | 0 | 184 | 4978876 | 2 | 9 | 0 | 1 | 192 |
| 136 | 88 | 63 | 3F | 2 | 10 | 606109 | 4848868 | 2 | 10 | 0 | 184 | 4979068 | 2 | 10 | 0 | 1 | 192 |
| 137 | 89 | 63 | 3F | 2 | 11 | 606132 | 4849052 | 2 | 11 | 0 | 184 | 4979260 | 2 | 11 | 0 | 1 | 192 |
| 138 | 8A | 63 | 3F | 2 | 12 | 606155 | 4849236 | 2 | 12 | 0 | 184 | 4979452 | 2 | 12 | 0 | 1 | 192 |
| 139 | 8B | 63 | 3F | 2 | 13 | 606178 | 4849420 | 2 | 13 | 0 | 184 | 4979644 | 2 | 13 | 0 | 1 | 192 |
| 140 | 8C | 63 | 3F | 2 | 14 | 606201 | 4849604 | 2 | 14 | 0 | 184 | 4979836 | 2 | 14 | 0 | 1 | 192 |
| 141 | 8D | 63 | 3F | 2 | 15 | 606224 | 4849788 | 2 | 15 | 0 | 184 | 4980028 | 2 | 15 | 0 | 1 | 192 |
| 142 | 8E | 63 | 3F | 2 | 16 | 606247 | 4849972 | 2 | 16 | 0 | 184 | 4980220 | 2 | 16 | 0 | 1 | 192 |

| | | | | | | | | | | | | | | | | | |
|---|---|---|---|---|---|---|---|---|---|---|---|---|---|---|---|---|---|
| 143 | 8F | 63 | 3F | 2 | 17 | 606270 | 4850156 | 2 | 17 | 0 | 184 | 4980412 | 2 | 17 | 0 | 1 | 192 |
| 144 | 90 | 63 | 3F | 2 | 18 | 606295 | 4850356 | 2 | 18 | 0 | 200 | 4980620 | 2 | 18 | 0 | 1 | 208 |
| 145 | 91 | 63 | 3F | 2 | 19 | 606320 | 4850556 | 2 | 19 | 0 | 200 | 4980828 | 2 | 19 | 0 | 1 | 208 |
| 146 | 92 | 63 | 3F | 2 | 20 | 606345 | 4850756 | 2 | 20 | 0 | 200 | 4981036 | 2 | 20 | 0 | 1 | 208 |
| 147 | 93 | 63 | 3F | 2 | 21 | 606370 | 4850956 | 2 | 21 | 0 | 200 | 4981244 | 2 | 21 | 0 | 1 | 208 |
| 148 | 94 | 63 | 3F | 2 | 22 | 606395 | 4851156 | 2 | 22 | 0 | 200 | 4981452 | 2 | 22 | 0 | 1 | 208 |
| 149 | 95 | 63 | 3F | 2 | 23 | 606420 | 4851356 | 2 | 23 | 0 | 200 | 4981660 | 2 | 23 | 0 | 1 | 208 |
| 150 | 96 | 63 | 3F | 2 | 24 | 606445 | 4851556 | 2 | 24 | 0 | 200 | 4981868 | 2 | 24 | 0 | 1 | 208 |
| 151 | 97 | 63 | 3F | 2 | 25 | 606470 | 4851756 | 2 | 25 | 0 | 200 | 4982076 | 2 | 25 | 0 | 1 | 208 |
| 152 | 98 | 63 | 3F | 2 | 26 | 606495 | 4851956 | 2 | 26 | 0 | 200 | 4982284 | 2 | 26 | 0 | 1 | 208 |
| 153 | 99 | 63 | 3F | 2 | 27 | 606520 | 4852156 | 2 | 27 | 0 | 200 | 4982492 | 2 | 27 | 0 | 1 | 208 |
| 154 | 9A | 63 | 3F | 2 | 28 | 606545 | 4852356 | 2 | 28 | 0 | 200 | 4982700 | 2 | 28 | 0 | 1 | 208 |
| 155 | 9B | 63 | 3F | 2 | 29 | 606570 | 4852556 | 2 | 29 | 0 | 200 | 4982908 | 2 | 29 | 0 | 1 | 208 |
| 156 | 9C | 63 | 3F | 2 | 30 | 606595 | 4852756 | 2 | 30 | 0 | 200 | 4983116 | 2 | 30 | 0 | 1 | 208 |
| 157 | 9D | 63 | 3F | 2 | 31 | 606620 | 4852956 | 2 | 31 | 0 | 200 | 4983324 | 2 | 31 | 0 | 1 | 208 |
| 158 | 9E | 63 | 3F | 2 | 32 | 606645 | 4853156 | 2 | 32 | 0 | 200 | 4983532 | 2 | 32 | 0 | 1 | 208 |
| 159 | 9F | 63 | 3F | 2 | 33 | 606670 | 4853356 | 2 | 33 | 0 | 200 | 4983740 | 2 | 33 | 0 | 1 | 208 |
| 160 | A0 | 63 | 3F | 2 | 34 | 606695 | 4853556 | 2 | 34 | 0 | 200 | 4983948 | 2 | 34 | 0 | 1 | 208 |
| 161 | A1 | 63 | 3F | 2 | 35 | 606720 | 4853756 | 2 | 35 | 0 | 200 | 4984156 | 2 | 35 | 0 | 1 | 208 |
| 162 | A2 | 63 | 3F | 2 | 36 | 606745 | 4853956 | 2 | 36 | 0 | 200 | 4984364 | 2 | 36 | 0 | 1 | 208 |
| 163 | A3 | 63 | 3F | 2 | 37 | 606770 | 4854156 | 2 | 37 | 0 | 200 | 4984572 | 2 | 37 | 0 | 1 | 208 |
| 164 | A4 | 63 | 3F | 2 | 38 | 606795 | 4854356 | 2 | 38 | 0 | 200 | 4984780 | 2 | 38 | 0 | 1 | 208 |
| 165 | A5 | 63 | 3F | 2 | 39 | 606820 | 4854556 | 2 | 39 | 0 | 200 | 4984988 | 2 | 39 | 0 | 1 | 208 |
| 166 | A6 | 63 | 3F | 2 | 40 | 606845 | 4854756 | 2 | 40 | 0 | 200 | 4985196 | 2 | 40 | 0 | 1 | 208 |
| 167 | A7 | 63 | 3F | 2 | 41 | 606870 | 4854956 | 2 | 41 | 0 | 200 | 4985404 | 2 | 41 | 0 | 1 | 208 |
| 168 | A8 | 63 | 3F | 2 | 42 | 606895 | 4855156 | 2 | 42 | 0 | 200 | 4985612 | 2 | 42 | 0 | 1 | 208 |
| 169 | A9 | 63 | 3F | 2 | 43 | 606920 | 4855356 | 2 | 43 | 0 | 200 | 4985820 | 2 | 43 | 0 | 1 | 208 |
| 170 | AA | 63 | 3F | 2 | 44 | 606945 | 4855556 | 2 | 44 | 0 | 200 | 4986028 | 2 | 44 | 0 | 1 | 208 |
| 171 | AB | 63 | 3F | 2 | 45 | 606970 | 4855756 | 2 | 45 | 0 | 200 | 4986236 | 2 | 45 | 0 | 1 | 208 |
| 172 | AC | 63 | 3F | 2 | 46 | 606995 | 4855956 | 2 | 46 | 0 | 200 | 4986444 | 2 | 46 | 0 | 1 | 208 |
| 173 | AD | 63 | 3F | 2 | 47 | 607020 | 4856156 | 2 | 47 | 0 | 200 | 4986652 | 2 | 47 | 0 | 1 | 208 |
| 174 | AE | 63 | 3F | 2 | 48 | 607045 | 4856356 | 2 | 48 | 0 | 200 | 4986860 | 2 | 48 | 0 | 1 | 208 |
| 175 | AF | 63 | 3F | 2 | 49 | 607070 | 4856556 | 2 | 49 | 0 | 200 | 4987068 | 2 | 49 | 0 | 1 | 208 |
| 176 | B0 | 63 | 3F | 2 | 50 | 607095 | 4856756 | 2 | 50 | 0 | 200 | 4987276 | 2 | 50 | 0 | 1 | 208 |
| 177 | B1 | 63 | 3F | 2 | 51 | 607120 | 4856956 | 2 | 51 | 0 | 200 | 4987484 | 2 | 51 | 0 | 1 | 208 |
| 178 | B2 | 63 | 3F | 2 | 52 | 607145 | 4857156 | 2 | 52 | 0 | 200 | 4987692 | 2 | 52 | 0 | 1 | 208 |
| 179 | B3 | 63 | 3F | 2 | 53 | 607170 | 4857356 | 2 | 53 | 0 | 200 | 4987900 | 2 | 53 | 0 | 1 | 208 |
| 180 | B4 | 63 | 3F | 2 | 54 | 607195 | 4857556 | 2 | 54 | 0 | 200 | 4988108 | 2 | 54 | 0 | 1 | 208 |
| 181 | B5 | 63 | 3F | 2 | 55 | 607220 | 4857756 | 2 | 55 | 0 | 200 | 4988316 | 2 | 55 | 0 | 1 | 208 |
| 182 | B6 | 63 | 3F | 2 | 56 | 607245 | 4857956 | 2 | 56 | 0 | 200 | 4988524 | 2 | 56 | 0 | 1 | 208 |
| 183 | B7 | 63 | 3F | 2 | 57 | 607270 | 4858156 | 2 | 57 | 0 | 200 | 4988732 | 2 | 57 | 0 | 1 | 208 |
| 184 | B8 | 63 | 3F | 2 | 58 | 607295 | 4858356 | 2 | 58 | 0 | 200 | 4988940 | 2 | 58 | 0 | 1 | 208 |
| 185 | B9 | 63 | 3F | 2 | 59 | 607320 | 4858556 | 2 | 59 | 0 | 200 | 4989148 | 2 | 59 | 0 | 1 | 208 |
| 186 | BA | 63 | 3F | 2 | 60 | 607345 | 4858756 | 2 | 60 | 0 | 200 | 4989356 | 2 | 60 | 0 | 1 | 208 |
| 187 | BB | 63 | 3F | 2 | 61 | 607370 | 4858956 | 2 | 61 | 0 | 200 | 4989564 | 2 | 61 | 0 | 1 | 208 |
| 188 | BC | 63 | 3F | 2 | 62 | 607395 | 4859156 | 2 | 62 | 0 | 200 | 4989772 | 2 | 62 | 0 | 1 | 208 |
| 189 | BD | 63 | 3F | 3 | 0 | 607418 | 4859340 | 3 | 0 | 0 | 184 | 4989964 | 3 | 0 | 0 | 1 | 192 |
| 190 | BE | 63 | 3F | 3 | 1 | 607446 | 4859564 | 3 | 1 | 0 | 224 | 4990196 | 3 | 1 | 0 | 1 | 232 |
| 191 | BF | 63 | 3F | 3 | 2 | 607474 | 4859788 | 3 | 2 | 0 | 224 | 4990428 | 3 | 2 | 0 | 1 | 232 |
| 192 | C0 | 63 | 3F | 3 | 3 | 607499 | 4859988 | 3 | 3 | 0 | 200 | 4990636 | 3 | 3 | 0 | 1 | 208 |
| 193 | C1 | 63 | 3F | 3 | 4 | 607524 | 4860188 | 3 | 4 | 0 | 200 | 4990844 | 3 | 4 | 0 | 1 | 208 |
| 194 | C2 | 63 | 3F | 3 | 5 | 607549 | 4860388 | 3 | 5 | 0 | 200 | 4991052 | 3 | 5 | 0 | 1 | 208 |
| 195 | C3 | 63 | 3F | 3 | 6 | 607574 | 4860588 | 3 | 6 | 0 | 200 | 4991260 | 3 | 6 | 0 | 1 | 208 |
| 196 | C4 | 63 | 3F | 3 | 7 | 607599 | 4860788 | 3 | 7 | 0 | 200 | 4991468 | 3 | 7 | 0 | 1 | 208 |
| 197 | C5 | 63 | 3F | 3 | 8 | 607624 | 4860988 | 3 | 8 | 0 | 200 | 4991676 | 3 | 8 | 0 | 1 | 208 |
| 198 | C6 | 63 | 3F | 3 | 9 | 607649 | 4861188 | 3 | 9 | 0 | 200 | 4991884 | 3 | 9 | 0 | 1 | 208 |
| 199 | C7 | 63 | 3F | 3 | 10 | 607674 | 4861388 | 3 | 10 | 0 | 200 | 4992092 | 3 | 10 | 0 | 1 | 208 |
| 200 | C8 | 63 | 3F | 3 | 11 | 607699 | 4861588 | 3 | 11 | 0 | 200 | 4992300 | 3 | 11 | 0 | 1 | 208 |
| 201 | C9 | 63 | 3F | 3 | 12 | 607724 | 4861788 | 3 | 12 | 0 | 200 | 4992508 | 3 | 12 | 0 | 1 | 208 |
| 202 | CA | 63 | 3F | 3 | 13 | 607749 | 4861988 | 3 | 13 | 0 | 200 | 4992716 | 3 | 13 | 0 | 1 | 208 |
| 203 | CB | 63 | 3F | 3 | 14 | 607774 | 4862188 | 3 | 14 | 0 | 200 | 4992924 | 3 | 14 | 0 | 1 | 208 |
| 204 | CC | 63 | 3F | 3 | 15 | 607799 | 4862388 | 3 | 15 | 0 | 200 | 4993132 | 3 | 15 | 0 | 1 | 208 |
| 205 | CD | 63 | 3F | 3 | 16 | 607824 | 4862588 | 3 | 16 | 0 | 200 | 4993340 | 3 | 16 | 0 | 1 | 208 |
| 206 | CE | 63 | 3F | 3 | 17 | 607849 | 4862788 | 3 | 17 | 0 | 200 | 4993548 | 3 | 17 | 0 | 1 | 208 |
| 207 | CF | 63 | 3F | 3 | 18 | 607874 | 4862988 | 3 | 18 | 0 | 200 | 4993756 | 3 | 18 | 0 | 1 | 208 |
| 208 | D0 | 63 | 3F | 3 | 19 | 607901 | 4863204 | 3 | 19 | 0 | 216 | 4993980 | 3 | 19 | 0 | 1 | 224 |
| 209 | D1 | 63 | 3F | 3 | 20 | 607928 | 4863420 | 3 | 20 | 0 | 216 | 4994204 | 3 | 20 | 0 | 1 | 224 |
| 210 | D2 | 63 | 3F | 3 | 21 | 607955 | 4863636 | 3 | 21 | 0 | 216 | 4994428 | 3 | 21 | 0 | 1 | 224 |
| 211 | D3 | 63 | 3F | 3 | 22 | 607982 | 4863852 | 3 | 22 | 0 | 216 | 4994652 | 3 | 22 | 0 | 1 | 224 |
| 212 | D4 | 63 | 3F | 3 | 23 | 608009 | 4864068 | 3 | 23 | 0 | 216 | 4994876 | 3 | 23 | 0 | 1 | 224 |
| 213 | D5 | 63 | 3F | 3 | 24 | 608036 | 4864284 | 3 | 24 | 0 | 216 | 4995100 | 3 | 24 | 0 | 1 | 224 |
| 214 | D6 | 63 | 3F | 3 | 25 | 608063 | 4864500 | 3 | 25 | 0 | 216 | 4995324 | 3 | 25 | 0 | 1 | 224 |
| 215 | D7 | 63 | 3F | 3 | 26 | 608090 | 4864716 | 3 | 26 | 0 | 216 | 4995548 | 3 | 26 | 0 | 1 | 224 |
| 216 | D8 | 63 | 3F | 3 | 27 | 608117 | 4864932 | 3 | 27 | 0 | 216 | 4995772 | 3 | 27 | 0 | 1 | 224 |
| 217 | D9 | 63 | 3F | 3 | 28 | 608144 | 4865148 | 3 | 28 | 0 | 216 | 4995996 | 3 | 28 | 0 | 1 | 224 |
| 218 | DA | 63 | 3F | 3 | 29 | 608171 | 4865364 | 3 | 29 | 0 | 216 | 4996220 | 3 | 29 | 0 | 1 | 224 |
| 219 | DB | 63 | 3F | 3 | 30 | 608198 | 4865580 | 3 | 30 | 0 | 216 | 4996444 | 3 | 30 | 0 | 1 | 224 |
| 220 | DC | 63 | 3F | 3 | 31 | 608225 | 4865796 | 3 | 31 | 0 | 216 | 4996668 | 3 | 31 | 0 | 1 | 224 |
| 221 | DD | 63 | 3F | 3 | 32 | 608252 | 4866012 | 3 | 32 | 0 | 216 | 4996892 | 3 | 32 | 0 | 1 | 224 |
| 222 | DE | 63 | 3F | 3 | 33 | 608279 | 4866228 | 3 | 33 | 0 | 216 | 4997116 | 3 | 33 | 0 | 1 | 224 |
| 223 | DF | 63 | 3F | 3 | 34 | 608306 | 4866444 | 3 | 34 | 0 | 216 | 4997340 | 3 | 34 | 0 | 1 | 224 |
| 224 | E0 | 63 | 3F | 3 | 35 | 608333 | 4866660 | 3 | 35 | 0 | 216 | 4997564 | 3 | 35 | 0 | 1 | 224 |
| 225 | E1 | 63 | 3F | 3 | 36 | 608360 | 4866876 | 3 | 36 | 0 | 216 | 4997788 | 3 | 36 | 0 | 1 | 224 |
| 226 | E2 | 63 | 3F | 3 | 37 | 608387 | 4867092 | 3 | 37 | 0 | 216 | 4998012 | 3 | 37 | 0 | 1 | 224 |
| 227 | E3 | 63 | 3F | 3 | 38 | 608414 | 4867308 | 3 | 38 | 0 | 216 | 4998236 | 3 | 38 | 0 | 1 | 224 |
| 228 | E4 | 63 | 3F | 3 | 39 | 608441 | 4867524 | 3 | 39 | 0 | 216 | 4998460 | 3 | 39 | 0 | 1 | 224 |
| 229 | E5 | 63 | 3F | 3 | 40 | 608468 | 4867740 | 3 | 40 | 0 | 216 | 4998684 | 3 | 40 | 0 | 1 | 224 |
| 230 | E6 | 63 | 3F | 3 | 41 | 608495 | 4867956 | 3 | 41 | 0 | 216 | 4998908 | 3 | 41 | 0 | 1 | 224 |
| 231 | E7 | 63 | 3F | 3 | 42 | 608522 | 4868172 | 3 | 42 | 0 | 216 | 4999132 | 3 | 42 | 0 | 1 | 224 |
| 232 | E8 | 63 | 3F | 3 | 43 | 608549 | 4868388 | 3 | 43 | 0 | 216 | 4999356 | 3 | 43 | 0 | 1 | 224 |
| 233 | E9 | 63 | 3F | 3 | 44 | 608576 | 4868604 | 3 | 44 | 0 | 216 | 4999580 | 3 | 44 | 0 | 1 | 224 |
| 234 | EA | 63 | 3F | 3 | 45 | 608603 | 4868820 | 3 | 45 | 0 | 216 | 4999804 | 3 | 45 | 0 | 1 | 224 |
| 235 | EB | 63 | 3F | 3 | 46 | 608630 | 4869036 | 3 | 46 | 0 | 216 | 5000028 | 3 | 46 | 0 | 1 | 224 |
| 236 | EC | 63 | 3F | 3 | 47 | 608657 | 4869252 | 3 | 47 | 0 | 216 | 5000252 | 3 | 47 | 0 | 1 | 224 |
| 237 | ED | 63 | 3F | 3 | 48 | 608684 | 4869468 | 3 | 48 | 0 | 216 | 5000476 | 3 | 48 | 0 | 1 | 224 |
| 238 | EE | 63 | 3F | 3 | 49 | 608711 | 4869684 | 3 | 49 | 0 | 216 | 5000700 | 3 | 49 | 0 | 1 | 224 |
| 239 | EF | 63 | 3F | 3 | 50 | 608738 | 4869900 | 3 | 50 | 0 | 216 | 5000924 | 3 | 50 | 0 | 1 | 224 |
| 240 | F0 | 63 | 3F | 3 | 51 | 608765 | 4870116 | 3 | 51 | 0 | 216 | 5001148 | 3 | 51 | 0 | 1 | 224 |
| 241 | F1 | 63 | 3F | 3 | 52 | 608792 | 4870332 | 3 | 52 | 0 | 216 | 5001372 | 3 | 52 | 0 | 1 | 224 |
| 242 | F2 | 63 | 3F | 3 | 53 | 608819 | 4870548 | 3 | 53 | 0 | 216 | 5001596 | 3 | 53 | 0 | 1 | 224 |
| 243 | F3 | 63 | 3F | 3 | 54 | 608846 | 4870764 | 3 | 54 | 0 | 216 | 5001820 | 3 | 54 | 0 | 1 | 224 |
| 244 | F4 | 63 | 3F | 3 | 55 | 608873 | 4870980 | 3 | 55 | 0 | 216 | 5002044 | 3 | 55 | 0 | 1 | 224 |
| 245 | F5 | 63 | 3F | 3 | 56 | 608900 | 4871196 | 3 | 56 | 0 | 216 | 5002268 | 3 | 56 | 0 | 1 | 224 |

| | | | | | | | | | | | | | | | | |
|---|---|---|---|---|---|---|---|---|---|---|---|---|---|---|---|---|
| 246 | F6 | 63 | 3F | 3 | 57 | 608927 | 4871412 | 3 | 57 | 0 | 216 | 5002492 | 3 | 57 | 0 | 1 | 224 |
| 247 | F7 | 63 | 3F | 3 | 58 | 608954 | 4871628 | 3 | 58 | 0 | 216 | 5002716 | 3 | 58 | 0 | 1 | 224 |
| 248 | F8 | 63 | 3F | 3 | 59 | 608981 | 4871844 | 3 | 59 | 0 | 216 | 5002940 | 3 | 59 | 0 | 1 | 224 |
| 249 | F9 | 63 | 3F | 3 | 60 | 609008 | 4872060 | 3 | 60 | 0 | 216 | 5003164 | 3 | 60 | 0 | 1 | 224 |
| 250 | FA | 63 | 3F | 3 | 61 | 609035 | 4872276 | 3 | 61 | 0 | 216 | 5003388 | 3 | 61 | 0 | 1 | 224 |
| 251 | FB | 63 | 3F | 3 | 62 | 609062 | 4872492 | 3 | 62 | 0 | 216 | 5003612 | 3 | 62 | 0 | 1 | 224 |
| 252 | FC | 63 | 3F | 4 | 0 | 609087 | 4872692 | 4 | 0 | 0 | 200 | 5003820 | 4 | 0 | 0 | 1 | 208 |
| 253 | FD | 63 | 3F | 4 | 1 | 609117 | 4872932 | 4 | 1 | 0 | 240 | 5004068 | 4 | 1 | 0 | 1 | 248 |
| 254 | FE | 63 | 3F | 4 | 2 | 609147 | 4873172 | 4 | 2 | 0 | 240 | 5004316 | 4 | 2 | 0 | 1 | 248 |
| 255 | FF | 63 | 3F | 4 | 3 | 609177 | 4873412 | 4 | 3 | 0 | 240 | 5004564 | 4 | 3 | 0 | 1 | 248 |
| 0 | 00 | 64 | 40 | 0 | 0 | 609189 | 4873508 | 0 | 0 | 0 | 96 | 5004668 | 0 | 0 | 0 | 1 | 104 |
| 1 | 01 | 64 | 40 | 0 | 1 | 609203 | 4873620 | 0 | 1 | 0 | 112 | 5004788 | 0 | 1 | 0 | 1 | 120 |
| 2 | 02 | 64 | 40 | 0 | 2 | 609217 | 4873732 | 0 | 2 | 0 | 112 | 5004908 | 0 | 2 | 0 | 1 | 120 |
| 3 | 03 | 64 | 40 | 0 | 3 | 609231 | 4873844 | 0 | 3 | 0 | 112 | 5005028 | 0 | 3 | 0 | 1 | 120 |
| 4 | 04 | 64 | 40 | 0 | 4 | 609245 | 4873956 | 0 | 4 | 0 | 112 | 5005148 | 0 | 4 | 0 | 1 | 120 |
| 5 | 05 | 64 | 40 | 0 | 5 | 609259 | 4874068 | 0 | 5 | 0 | 112 | 5005268 | 0 | 5 | 0 | 1 | 120 |
| 6 | 06 | 64 | 40 | 0 | 6 | 609273 | 4874180 | 0 | 6 | 0 | 112 | 5005388 | 0 | 6 | 0 | 1 | 120 |
| 7 | 07 | 64 | 40 | 0 | 7 | 609287 | 4874292 | 0 | 7 | 0 | 112 | 5005508 | 0 | 7 | 0 | 1 | 120 |
| 8 | 08 | 64 | 40 | 0 | 8 | 609301 | 4874404 | 0 | 8 | 0 | 112 | 5005628 | 0 | 8 | 0 | 1 | 120 |
| 9 | 09 | 64 | 40 | 0 | 9 | 609315 | 4874516 | 0 | 9 | 0 | 112 | 5005748 | 0 | 9 | 0 | 1 | 120 |
| 10 | 0A | 64 | 40 | 0 | 10 | 609329 | 4874628 | 0 | 10 | 0 | 112 | 5005868 | 0 | 10 | 0 | 1 | 120 |
| 11 | 0B | 64 | 40 | 0 | 11 | 609343 | 4874740 | 0 | 11 | 0 | 112 | 5005988 | 0 | 11 | 0 | 1 | 120 |
| 12 | 0C | 64 | 40 | 0 | 12 | 609357 | 4874852 | 0 | 12 | 0 | 112 | 5006108 | 0 | 12 | 0 | 1 | 120 |
| 13 | 0D | 64 | 40 | 0 | 13 | 609371 | 4874964 | 0 | 13 | 0 | 112 | 5006228 | 0 | 13 | 0 | 1 | 120 |
| 14 | 0E | 64 | 40 | 0 | 14 | 609385 | 4875076 | 0 | 14 | 0 | 112 | 5006348 | 0 | 14 | 0 | 1 | 120 |
| 15 | 0F | 64 | 40 | 0 | 15 | 609399 | 4875188 | 0 | 15 | 0 | 112 | 5006468 | 0 | 15 | 0 | 1 | 120 |
| 16 | 10 | 64 | 40 | 0 | 16 | 609413 | 4875300 | 0 | 16 | 0 | 112 | 5006588 | 0 | 16 | 0 | 1 | 120 |
| 17 | 11 | 64 | 40 | 0 | 17 | 609427 | 4875412 | 0 | 17 | 0 | 112 | 5006708 | 0 | 17 | 0 | 1 | 120 |
| 18 | 12 | 64 | 40 | 0 | 18 | 609441 | 4875524 | 0 | 18 | 0 | 112 | 5006828 | 0 | 18 | 0 | 1 | 120 |
| 19 | 13 | 64 | 40 | 0 | 19 | 609455 | 4875636 | 0 | 19 | 0 | 112 | 5006948 | 0 | 19 | 0 | 1 | 120 |
| 20 | 14 | 64 | 40 | 0 | 20 | 609469 | 4875748 | 0 | 20 | 0 | 112 | 5007068 | 0 | 20 | 0 | 1 | 120 |
| 21 | 15 | 64 | 40 | 0 | 21 | 609483 | 4875860 | 0 | 21 | 0 | 112 | 5007188 | 0 | 21 | 0 | 1 | 120 |
| 22 | 16 | 64 | 40 | 0 | 22 | 609497 | 4875972 | 0 | 22 | 0 | 112 | 5007308 | 0 | 22 | 0 | 1 | 120 |
| 23 | 17 | 64 | 40 | 0 | 23 | 609511 | 4876084 | 0 | 23 | 0 | 112 | 5007428 | 0 | 23 | 0 | 1 | 120 |
| 24 | 18 | 64 | 40 | 0 | 24 | 609525 | 4876196 | 0 | 24 | 0 | 112 | 5007548 | 0 | 24 | 0 | 1 | 120 |
| 25 | 19 | 64 | 40 | 0 | 25 | 609539 | 4876308 | 0 | 25 | 0 | 112 | 5007668 | 0 | 25 | 0 | 1 | 120 |
| 26 | 1A | 64 | 40 | 0 | 26 | 609553 | 4876420 | 0 | 26 | 0 | 112 | 5007788 | 0 | 26 | 0 | 1 | 120 |
| 27 | 1B | 64 | 40 | 0 | 27 | 609567 | 4876532 | 0 | 27 | 0 | 112 | 5007908 | 0 | 27 | 0 | 1 | 120 |
| 28 | 1C | 64 | 40 | 0 | 28 | 609581 | 4876644 | 0 | 28 | 0 | 112 | 5008028 | 0 | 28 | 0 | 1 | 120 |
| 29 | 1D | 64 | 40 | 0 | 29 | 609595 | 4876756 | 0 | 29 | 0 | 112 | 5008148 | 0 | 29 | 0 | 1 | 120 |
| 30 | 1E | 64 | 40 | 0 | 30 | 609609 | 4876868 | 0 | 30 | 0 | 112 | 5008268 | 0 | 30 | 0 | 1 | 120 |
| 31 | 1F | 64 | 40 | 0 | 31 | 609623 | 4876980 | 0 | 31 | 0 | 112 | 5008388 | 0 | 31 | 0 | 1 | 120 |
| 32 | 20 | 64 | 40 | 0 | 32 | 609637 | 4877092 | 0 | 32 | 0 | 112 | 5008508 | 0 | 32 | 0 | 1 | 120 |
| 33 | 21 | 64 | 40 | 0 | 33 | 609651 | 4877204 | 0 | 33 | 0 | 112 | 5008628 | 0 | 33 | 0 | 1 | 120 |
| 34 | 22 | 64 | 40 | 0 | 34 | 609665 | 4877316 | 0 | 34 | 0 | 112 | 5008748 | 0 | 34 | 0 | 1 | 120 |
| 35 | 23 | 64 | 40 | 0 | 35 | 609679 | 4877428 | 0 | 35 | 0 | 112 | 5008868 | 0 | 35 | 0 | 1 | 120 |
| 36 | 24 | 64 | 40 | 0 | 36 | 609693 | 4877540 | 0 | 36 | 0 | 112 | 5008988 | 0 | 36 | 0 | 1 | 120 |
| 37 | 25 | 64 | 40 | 0 | 37 | 609707 | 4877652 | 0 | 37 | 0 | 112 | 5009108 | 0 | 37 | 0 | 1 | 120 |
| 38 | 26 | 64 | 40 | 0 | 38 | 609721 | 4877764 | 0 | 38 | 0 | 112 | 5009228 | 0 | 38 | 0 | 1 | 120 |
| 39 | 27 | 64 | 40 | 0 | 39 | 609735 | 4877876 | 0 | 39 | 0 | 112 | 5009348 | 0 | 39 | 0 | 1 | 120 |
| 40 | 28 | 64 | 40 | 0 | 40 | 609749 | 4877988 | 0 | 40 | 0 | 112 | 5009468 | 0 | 40 | 0 | 1 | 120 |
| 41 | 29 | 64 | 40 | 0 | 41 | 609763 | 4878100 | 0 | 41 | 0 | 112 | 5009588 | 0 | 41 | 0 | 1 | 120 |
| 42 | 2A | 64 | 40 | 0 | 42 | 609777 | 4878212 | 0 | 42 | 0 | 112 | 5009708 | 0 | 42 | 0 | 1 | 120 |
| 43 | 2B | 64 | 40 | 0 | 43 | 609791 | 4878324 | 0 | 43 | 0 | 112 | 5009828 | 0 | 43 | 0 | 1 | 120 |
| 44 | 2C | 64 | 40 | 0 | 44 | 609805 | 4878436 | 0 | 44 | 0 | 112 | 5009948 | 0 | 44 | 0 | 1 | 120 |
| 45 | 2D | 64 | 40 | 0 | 45 | 609819 | 4878548 | 0 | 45 | 0 | 112 | 5010068 | 0 | 45 | 0 | 1 | 120 |
| 46 | 2E | 64 | 40 | 0 | 46 | 609833 | 4878660 | 0 | 46 | 0 | 112 | 5010188 | 0 | 46 | 0 | 1 | 120 |
| 47 | 2F | 64 | 40 | 0 | 47 | 609847 | 4878772 | 0 | 47 | 0 | 112 | 5010308 | 0 | 47 | 0 | 1 | 120 |
| 48 | 30 | 64 | 40 | 0 | 48 | 609861 | 4878884 | 0 | 48 | 0 | 112 | 5010428 | 0 | 48 | 0 | 1 | 120 |
| 49 | 31 | 64 | 40 | 0 | 49 | 609875 | 4878996 | 0 | 49 | 0 | 112 | 5010548 | 0 | 49 | 0 | 1 | 120 |
| 50 | 32 | 64 | 40 | 0 | 50 | 609889 | 4879108 | 0 | 50 | 0 | 112 | 5010668 | 0 | 50 | 0 | 1 | 120 |
| 51 | 33 | 64 | 40 | 0 | 51 | 609903 | 4879220 | 0 | 51 | 0 | 112 | 5010788 | 0 | 51 | 0 | 1 | 120 |
| 52 | 34 | 64 | 40 | 0 | 52 | 609917 | 4879332 | 0 | 52 | 0 | 112 | 5010908 | 0 | 52 | 0 | 1 | 120 |
| 53 | 35 | 64 | 40 | 0 | 53 | 609931 | 4879444 | 0 | 53 | 0 | 112 | 5011028 | 0 | 53 | 0 | 1 | 120 |
| 54 | 36 | 64 | 40 | 0 | 54 | 609945 | 4879556 | 0 | 54 | 0 | 112 | 5011148 | 0 | 54 | 0 | 1 | 120 |
| 55 | 37 | 64 | 40 | 0 | 55 | 609959 | 4879668 | 0 | 55 | 0 | 112 | 5011268 | 0 | 55 | 0 | 1 | 120 |
| 56 | 38 | 64 | 40 | 0 | 56 | 609973 | 4879780 | 0 | 56 | 0 | 112 | 5011388 | 0 | 56 | 0 | 1 | 120 |
| 57 | 39 | 64 | 40 | 0 | 57 | 609987 | 4879892 | 0 | 57 | 0 | 112 | 5011508 | 0 | 57 | 0 | 1 | 120 |
| 58 | 3A | 64 | 40 | 0 | 58 | 610001 | 4880004 | 0 | 58 | 0 | 112 | 5011628 | 0 | 58 | 0 | 1 | 120 |
| 59 | 3B | 64 | 40 | 0 | 59 | 610015 | 4880116 | 0 | 59 | 0 | 112 | 5011748 | 0 | 59 | 0 | 1 | 120 |
| 60 | 3C | 64 | 40 | 0 | 60 | 610029 | 4880228 | 0 | 60 | 0 | 112 | 5011868 | 0 | 60 | 0 | 1 | 120 |
| 61 | 3D | 64 | 40 | 0 | 61 | 610043 | 4880340 | 0 | 61 | 0 | 112 | 5011988 | 0 | 61 | 0 | 1 | 120 |
| 62 | 3E | 64 | 40 | 0 | 62 | 610057 | 4880452 | 0 | 62 | 0 | 112 | 5012108 | 0 | 62 | 0 | 1 | 120 |
| 63 | 3F | 64 | 40 | 0 | 63 | 610071 | 4880564 | 0 | 63 | 0 | 112 | 5012228 | 0 | 63 | 0 | 1 | 120 |
| 64 | 40 | 64 | 40 | 1 | 0 | 610090 | 4880716 | 1 | 0 | 0 | 152 | 5012388 | 1 | 0 | 0 | 1 | 160 |
| 65 | 41 | 64 | 40 | 1 | 1 | 610111 | 4880884 | 1 | 1 | 0 | 168 | 5012564 | 1 | 1 | 0 | 1 | 176 |
| 66 | 42 | 64 | 40 | 1 | 2 | 610132 | 4881052 | 1 | 2 | 0 | 168 | 5012740 | 1 | 2 | 0 | 1 | 176 |
| 67 | 43 | 64 | 40 | 1 | 3 | 610153 | 4881220 | 1 | 3 | 0 | 168 | 5012916 | 1 | 3 | 0 | 1 | 176 |
| 68 | 44 | 64 | 40 | 1 | 4 | 610174 | 4881388 | 1 | 4 | 0 | 168 | 5013092 | 1 | 4 | 0 | 1 | 176 |
| 69 | 45 | 64 | 40 | 1 | 5 | 610195 | 4881556 | 1 | 5 | 0 | 168 | 5013268 | 1 | 5 | 0 | 1 | 176 |
| 70 | 46 | 64 | 40 | 1 | 6 | 610216 | 4881724 | 1 | 6 | 0 | 168 | 5013444 | 1 | 6 | 0 | 1 | 176 |
| 71 | 47 | 64 | 40 | 1 | 7 | 610237 | 4881892 | 1 | 7 | 0 | 168 | 5013620 | 1 | 7 | 0 | 1 | 176 |
| 72 | 48 | 64 | 40 | 1 | 8 | 610258 | 4882060 | 1 | 8 | 0 | 168 | 5013796 | 1 | 8 | 0 | 1 | 176 |
| 73 | 49 | 64 | 40 | 1 | 9 | 610279 | 4882228 | 1 | 9 | 0 | 168 | 5013972 | 1 | 9 | 0 | 1 | 176 |
| 74 | 4A | 64 | 40 | 1 | 10 | 610300 | 4882396 | 1 | 10 | 0 | 168 | 5014148 | 1 | 10 | 0 | 1 | 176 |
| 75 | 4B | 64 | 40 | 1 | 11 | 610321 | 4882564 | 1 | 11 | 0 | 168 | 5014324 | 1 | 11 | 0 | 1 | 176 |
| 76 | 4C | 64 | 40 | 1 | 12 | 610342 | 4882732 | 1 | 12 | 0 | 168 | 5014500 | 1 | 12 | 0 | 1 | 176 |
| 77 | 4D | 64 | 40 | 1 | 13 | 610363 | 4882900 | 1 | 13 | 0 | 168 | 5014676 | 1 | 13 | 0 | 1 | 176 |
| 78 | 4E | 64 | 40 | 1 | 14 | 610384 | 4883068 | 1 | 14 | 0 | 168 | 5014852 | 1 | 14 | 0 | 1 | 176 |
| 79 | 4F | 64 | 40 | 1 | 15 | 610405 | 4883236 | 1 | 15 | 0 | 168 | 5015028 | 1 | 15 | 0 | 1 | 176 |
| 80 | 50 | 64 | 40 | 1 | 16 | 610428 | 4883420 | 1 | 16 | 0 | 184 | 5015220 | 1 | 16 | 0 | 1 | 192 |
| 81 | 51 | 64 | 40 | 1 | 17 | 610451 | 4883604 | 1 | 17 | 0 | 184 | 5015412 | 1 | 17 | 0 | 1 | 192 |
| 82 | 52 | 64 | 40 | 1 | 18 | 610474 | 4883788 | 1 | 18 | 0 | 184 | 5015604 | 1 | 18 | 0 | 1 | 192 |
| 83 | 53 | 64 | 40 | 1 | 19 | 610497 | 4883972 | 1 | 19 | 0 | 184 | 5015796 | 1 | 19 | 0 | 1 | 192 |
| 84 | 54 | 64 | 40 | 1 | 20 | 610520 | 4884156 | 1 | 20 | 0 | 184 | 5015988 | 1 | 20 | 0 | 1 | 192 |
| 85 | 55 | 64 | 40 | 1 | 21 | 610543 | 4884340 | 1 | 21 | 0 | 184 | 5016180 | 1 | 21 | 0 | 1 | 192 |
| 86 | 56 | 64 | 40 | 1 | 22 | 610566 | 4884524 | 1 | 22 | 0 | 184 | 5016372 | 1 | 22 | 0 | 1 | 192 |
| 87 | 57 | 64 | 40 | 1 | 23 | 610589 | 4884708 | 1 | 23 | 0 | 184 | 5016564 | 1 | 23 | 0 | 1 | 192 |
| 88 | 58 | 64 | 40 | 1 | 24 | 610612 | 4884892 | 1 | 24 | 0 | 184 | 5016756 | 1 | 24 | 0 | 1 | 192 |
| 89 | 59 | 64 | 40 | 1 | 25 | 610635 | 4885076 | 1 | 25 | 0 | 184 | 5016948 | 1 | 25 | 0 | 1 | 192 |
| 90 | 5A | 64 | 40 | 1 | 26 | 610658 | 4885260 | 1 | 26 | 0 | 184 | 5017140 | 1 | 26 | 0 | 1 | 192 |
| 91 | 5B | 64 | 40 | 1 | 27 | 610681 | 4885444 | 1 | 27 | 0 | 184 | 5017332 | 1 | 27 | 0 | 1 | 192 |
| 92 | 5C | 64 | 40 | 1 | 28 | 610704 | 4885628 | 1 | 28 | 0 | 184 | 5017524 | 1 | 28 | 0 | 1 | 192 |

| | | | | | | | | | | | | | | | | | |
|---|---|---|---|---|---|---|---|---|---|---|---|---|---|---|---|---|---|
| 93 | 5D | 64 | 40 | 1 | 29 | 610727 | 4885812 | 1 | 29 | 0 | 184 | 5017716 | 1 | 29 | 0 | 1 | 192 |
| 94 | 5E | 64 | 40 | 1 | 30 | 610750 | 4885996 | 1 | 30 | 0 | 184 | 5017908 | 1 | 30 | 0 | 1 | 192 |
| 95 | 5F | 64 | 40 | 1 | 31 | 610773 | 4886180 | 1 | 31 | 0 | 184 | 5018100 | 1 | 31 | 0 | 1 | 192 |
| 96 | 60 | 64 | 40 | 1 | 32 | 610796 | 4886364 | 1 | 32 | 0 | 184 | 5018292 | 1 | 32 | 0 | 1 | 192 |
| 97 | 61 | 64 | 40 | 1 | 33 | 610819 | 4886548 | 1 | 33 | 0 | 184 | 5018484 | 1 | 33 | 0 | 1 | 192 |
| 98 | 62 | 64 | 40 | 1 | 34 | 610842 | 4886732 | 1 | 34 | 0 | 184 | 5018676 | 1 | 34 | 0 | 1 | 192 |
| 99 | 63 | 64 | 40 | 1 | 35 | 610865 | 4886916 | 1 | 35 | 0 | 184 | 5018868 | 1 | 35 | 0 | 1 | 192 |
| 100 | 64 | 64 | 40 | 1 | 36 | 610888 | 4887100 | 1 | 36 | 0 | 184 | 5019060 | 1 | 36 | 0 | 1 | 192 |
| 101 | 65 | 64 | 40 | 1 | 37 | 610911 | 4887284 | 1 | 37 | 0 | 184 | 5019252 | 1 | 37 | 0 | 1 | 192 |
| 102 | 66 | 64 | 40 | 1 | 38 | 610934 | 4887468 | 1 | 38 | 0 | 184 | 5019444 | 1 | 38 | 0 | 1 | 192 |
| 103 | 67 | 64 | 40 | 1 | 39 | 610957 | 4887652 | 1 | 39 | 0 | 184 | 5019636 | 1 | 39 | 0 | 1 | 192 |
| 104 | 68 | 64 | 40 | 1 | 40 | 610980 | 4887836 | 1 | 40 | 0 | 184 | 5019828 | 1 | 40 | 0 | 1 | 192 |
| 105 | 69 | 64 | 40 | 1 | 41 | 611003 | 4888020 | 1 | 41 | 0 | 184 | 5020020 | 1 | 41 | 0 | 1 | 192 |
| 106 | 6A | 64 | 40 | 1 | 42 | 611026 | 4888204 | 1 | 42 | 0 | 184 | 5020212 | 1 | 42 | 0 | 1 | 192 |
| 107 | 6B | 64 | 40 | 1 | 43 | 611049 | 4888388 | 1 | 43 | 0 | 184 | 5020404 | 1 | 43 | 0 | 1 | 192 |
| 108 | 6C | 64 | 40 | 1 | 44 | 611072 | 4888572 | 1 | 44 | 0 | 184 | 5020596 | 1 | 44 | 0 | 1 | 192 |
| 109 | 6D | 64 | 40 | 1 | 45 | 611095 | 4888756 | 1 | 45 | 0 | 184 | 5020788 | 1 | 45 | 0 | 1 | 192 |
| 110 | 6E | 64 | 40 | 1 | 46 | 611118 | 4888940 | 1 | 46 | 0 | 184 | 5020980 | 1 | 46 | 0 | 1 | 192 |
| 111 | 6F | 64 | 40 | 1 | 47 | 611141 | 4889124 | 1 | 47 | 0 | 184 | 5021172 | 1 | 47 | 0 | 1 | 192 |
| 112 | 70 | 64 | 40 | 1 | 48 | 611164 | 4889308 | 1 | 48 | 0 | 184 | 5021364 | 1 | 48 | 0 | 1 | 192 |
| 113 | 71 | 64 | 40 | 1 | 49 | 611187 | 4889492 | 1 | 49 | 0 | 184 | 5021556 | 1 | 49 | 0 | 1 | 192 |
| 114 | 72 | 64 | 40 | 1 | 50 | 611210 | 4889676 | 1 | 50 | 0 | 184 | 5021748 | 1 | 50 | 0 | 1 | 192 |
| 115 | 73 | 64 | 40 | 1 | 51 | 611233 | 4889860 | 1 | 51 | 0 | 184 | 5021940 | 1 | 51 | 0 | 1 | 192 |
| 116 | 74 | 64 | 40 | 1 | 52 | 611256 | 4890044 | 1 | 52 | 0 | 184 | 5022132 | 1 | 52 | 0 | 1 | 192 |
| 117 | 75 | 64 | 40 | 1 | 53 | 611279 | 4890228 | 1 | 53 | 0 | 184 | 5022324 | 1 | 53 | 0 | 1 | 192 |
| 118 | 76 | 64 | 40 | 1 | 54 | 611302 | 4890412 | 1 | 54 | 0 | 184 | 5022516 | 1 | 54 | 0 | 1 | 192 |
| 119 | 77 | 64 | 40 | 1 | 55 | 611325 | 4890596 | 1 | 55 | 0 | 184 | 5022708 | 1 | 55 | 0 | 1 | 192 |
| 120 | 78 | 64 | 40 | 1 | 56 | 611348 | 4890780 | 1 | 56 | 0 | 184 | 5022900 | 1 | 56 | 0 | 1 | 192 |
| 121 | 79 | 64 | 40 | 1 | 57 | 611371 | 4890964 | 1 | 57 | 0 | 184 | 5023092 | 1 | 57 | 0 | 1 | 192 |
| 122 | 7A | 64 | 40 | 1 | 58 | 611394 | 4891148 | 1 | 58 | 0 | 184 | 5023284 | 1 | 58 | 0 | 1 | 192 |
| 123 | 7B | 64 | 40 | 1 | 59 | 611417 | 4891332 | 1 | 59 | 0 | 184 | 5023476 | 1 | 59 | 0 | 1 | 192 |
| 124 | 7C | 64 | 40 | 1 | 60 | 611440 | 4891516 | 1 | 60 | 0 | 184 | 5023668 | 1 | 60 | 0 | 1 | 192 |
| 125 | 7D | 64 | 40 | 1 | 61 | 611463 | 4891700 | 1 | 61 | 0 | 184 | 5023860 | 1 | 61 | 0 | 1 | 192 |
| 126 | 7E | 64 | 40 | 1 | 62 | 611486 | 4891884 | 1 | 62 | 0 | 184 | 5024052 | 1 | 62 | 0 | 1 | 192 |
| 127 | 7F | 64 | 40 | 1 | 63 | 611509 | 4892068 | 1 | 63 | 0 | 184 | 5024244 | 1 | 63 | 0 | 1 | 192 |
| 128 | 80 | 64 | 40 | 2 | 0 | 611530 | 4892236 | 2 | 0 | 0 | 168 | 5024420 | 2 | 0 | 0 | 1 | 176 |
| 129 | 81 | 64 | 40 | 2 | 1 | 611553 | 4892420 | 2 | 1 | 0 | 184 | 5024612 | 2 | 1 | 0 | 1 | 192 |
| 130 | 82 | 64 | 40 | 2 | 2 | 611576 | 4892604 | 2 | 2 | 0 | 184 | 5024804 | 2 | 2 | 0 | 1 | 192 |
| 131 | 83 | 64 | 40 | 2 | 3 | 611599 | 4892788 | 2 | 3 | 0 | 184 | 5024996 | 2 | 3 | 0 | 1 | 192 |
| 132 | 84 | 64 | 40 | 2 | 4 | 611622 | 4892972 | 2 | 4 | 0 | 184 | 5025188 | 2 | 4 | 0 | 1 | 192 |
| 133 | 85 | 64 | 40 | 2 | 5 | 611645 | 4893156 | 2 | 5 | 0 | 184 | 5025380 | 2 | 5 | 0 | 1 | 192 |
| 134 | 86 | 64 | 40 | 2 | 6 | 611668 | 4893340 | 2 | 6 | 0 | 184 | 5025572 | 2 | 6 | 0 | 1 | 192 |
| 135 | 87 | 64 | 40 | 2 | 7 | 611691 | 4893524 | 2 | 7 | 0 | 184 | 5025764 | 2 | 7 | 0 | 1 | 192 |
| 136 | 88 | 64 | 40 | 2 | 8 | 611714 | 4893708 | 2 | 8 | 0 | 184 | 5025956 | 2 | 8 | 0 | 1 | 192 |
| 137 | 89 | 64 | 40 | 2 | 9 | 611737 | 4893892 | 2 | 9 | 0 | 184 | 5026148 | 2 | 9 | 0 | 1 | 192 |
| 138 | 8A | 64 | 40 | 2 | 10 | 611760 | 4894076 | 2 | 10 | 0 | 184 | 5026340 | 2 | 10 | 0 | 1 | 192 |
| 139 | 8B | 64 | 40 | 2 | 11 | 611783 | 4894260 | 2 | 11 | 0 | 184 | 5026532 | 2 | 11 | 0 | 1 | 192 |
| 140 | 8C | 64 | 40 | 2 | 12 | 611806 | 4894444 | 2 | 12 | 0 | 184 | 5026724 | 2 | 12 | 0 | 1 | 192 |
| 141 | 8D | 64 | 40 | 2 | 13 | 611829 | 4894628 | 2 | 13 | 0 | 184 | 5026916 | 2 | 13 | 0 | 1 | 192 |
| 142 | 8E | 64 | 40 | 2 | 14 | 611852 | 4894812 | 2 | 14 | 0 | 184 | 5027108 | 2 | 14 | 0 | 1 | 192 |
| 143 | 8F | 64 | 40 | 2 | 15 | 611875 | 4894996 | 2 | 15 | 0 | 184 | 5027300 | 2 | 15 | 0 | 1 | 192 |
| 144 | 90 | 64 | 40 | 2 | 16 | 611900 | 4895196 | 2 | 16 | 0 | 200 | 5027508 | 2 | 16 | 0 | 1 | 208 |
| 145 | 91 | 64 | 40 | 2 | 17 | 611925 | 4895396 | 2 | 17 | 0 | 200 | 5027716 | 2 | 17 | 0 | 1 | 208 |
| 146 | 92 | 64 | 40 | 2 | 18 | 611950 | 4895596 | 2 | 18 | 0 | 200 | 5027924 | 2 | 18 | 0 | 1 | 208 |
| 147 | 93 | 64 | 40 | 2 | 19 | 611975 | 4895796 | 2 | 19 | 0 | 200 | 5028132 | 2 | 19 | 0 | 1 | 208 |
| 148 | 94 | 64 | 40 | 2 | 20 | 612000 | 4895996 | 2 | 20 | 0 | 200 | 5028340 | 2 | 20 | 0 | 1 | 208 |
| 149 | 95 | 64 | 40 | 2 | 21 | 612025 | 4896196 | 2 | 21 | 0 | 200 | 5028548 | 2 | 21 | 0 | 1 | 208 |
| 150 | 96 | 64 | 40 | 2 | 22 | 612050 | 4896396 | 2 | 22 | 0 | 200 | 5028756 | 2 | 22 | 0 | 1 | 208 |
| 151 | 97 | 64 | 40 | 2 | 23 | 612075 | 4896596 | 2 | 23 | 0 | 200 | 5028964 | 2 | 23 | 0 | 1 | 208 |
| 152 | 98 | 64 | 40 | 2 | 24 | 612100 | 4896796 | 2 | 24 | 0 | 200 | 5029172 | 2 | 24 | 0 | 1 | 208 |
| 153 | 99 | 64 | 40 | 2 | 25 | 612125 | 4896996 | 2 | 25 | 0 | 200 | 5029380 | 2 | 25 | 0 | 1 | 208 |
| 154 | 9A | 64 | 40 | 2 | 26 | 612150 | 4897196 | 2 | 26 | 0 | 200 | 5029588 | 2 | 26 | 0 | 1 | 208 |
| 155 | 9B | 64 | 40 | 2 | 27 | 612175 | 4897396 | 2 | 27 | 0 | 200 | 5029796 | 2 | 27 | 0 | 1 | 208 |
| 156 | 9C | 64 | 40 | 2 | 28 | 612200 | 4897596 | 2 | 28 | 0 | 200 | 5030004 | 2 | 28 | 0 | 1 | 208 |
| 157 | 9D | 64 | 40 | 2 | 29 | 612225 | 4897796 | 2 | 29 | 0 | 200 | 5030212 | 2 | 29 | 0 | 1 | 208 |
| 158 | 9E | 64 | 40 | 2 | 30 | 612250 | 4897996 | 2 | 30 | 0 | 200 | 5030420 | 2 | 30 | 0 | 1 | 208 |
| 159 | 9F | 64 | 40 | 2 | 31 | 612275 | 4898196 | 2 | 31 | 0 | 200 | 5030628 | 2 | 31 | 0 | 1 | 208 |
| 160 | A0 | 64 | 40 | 2 | 32 | 612300 | 4898396 | 2 | 32 | 0 | 200 | 5030836 | 2 | 32 | 0 | 1 | 208 |
| 161 | A1 | 64 | 40 | 2 | 33 | 612325 | 4898596 | 2 | 33 | 0 | 200 | 5031044 | 2 | 33 | 0 | 1 | 208 |
| 162 | A2 | 64 | 40 | 2 | 34 | 612350 | 4898796 | 2 | 34 | 0 | 200 | 5031252 | 2 | 34 | 0 | 1 | 208 |
| 163 | A3 | 64 | 40 | 2 | 35 | 612375 | 4898996 | 2 | 35 | 0 | 200 | 5031460 | 2 | 35 | 0 | 1 | 208 |
| 164 | A4 | 64 | 40 | 2 | 36 | 612400 | 4899196 | 2 | 36 | 0 | 200 | 5031668 | 2 | 36 | 0 | 1 | 208 |
| 165 | A5 | 64 | 40 | 2 | 37 | 612425 | 4899396 | 2 | 37 | 0 | 200 | 5031876 | 2 | 37 | 0 | 1 | 208 |
| 166 | A6 | 64 | 40 | 2 | 38 | 612450 | 4899596 | 2 | 38 | 0 | 200 | 5032084 | 2 | 38 | 0 | 1 | 208 |
| 167 | A7 | 64 | 40 | 2 | 39 | 612475 | 4899796 | 2 | 39 | 0 | 200 | 5032292 | 2 | 39 | 0 | 1 | 208 |
| 168 | A8 | 64 | 40 | 2 | 40 | 612500 | 4899996 | 2 | 40 | 0 | 200 | 5032500 | 2 | 40 | 0 | 1 | 208 |
| 169 | A9 | 64 | 40 | 2 | 41 | 612525 | 4900196 | 2 | 41 | 0 | 200 | 5032708 | 2 | 41 | 0 | 1 | 208 |
| 170 | AA | 64 | 40 | 2 | 42 | 612550 | 4900396 | 2 | 42 | 0 | 200 | 5032916 | 2 | 42 | 0 | 1 | 208 |
| 171 | AB | 64 | 40 | 2 | 43 | 612575 | 4900596 | 2 | 43 | 0 | 200 | 5033124 | 2 | 43 | 0 | 1 | 208 |
| 172 | AC | 64 | 40 | 2 | 44 | 612600 | 4900796 | 2 | 44 | 0 | 200 | 5033332 | 2 | 44 | 0 | 1 | 208 |
| 173 | AD | 64 | 40 | 2 | 45 | 612625 | 4900996 | 2 | 45 | 0 | 200 | 5033540 | 2 | 45 | 0 | 1 | 208 |
| 174 | AE | 64 | 40 | 2 | 46 | 612650 | 4901196 | 2 | 46 | 0 | 200 | 5033748 | 2 | 46 | 0 | 1 | 208 |
| 175 | AF | 64 | 40 | 2 | 47 | 612675 | 4901396 | 2 | 47 | 0 | 200 | 5033956 | 2 | 47 | 0 | 1 | 208 |
| 176 | B0 | 64 | 40 | 2 | 48 | 612700 | 4901596 | 2 | 48 | 0 | 200 | 5034164 | 2 | 48 | 0 | 1 | 208 |
| 177 | B1 | 64 | 40 | 2 | 49 | 612725 | 4901796 | 2 | 49 | 0 | 200 | 5034372 | 2 | 49 | 0 | 1 | 208 |
| 178 | B2 | 64 | 40 | 2 | 50 | 612750 | 4901996 | 2 | 50 | 0 | 200 | 5034580 | 2 | 50 | 0 | 1 | 208 |
| 179 | B3 | 64 | 40 | 2 | 51 | 612775 | 4902196 | 2 | 51 | 0 | 200 | 5034788 | 2 | 51 | 0 | 1 | 208 |
| 180 | B4 | 64 | 40 | 2 | 52 | 612800 | 4902396 | 2 | 52 | 0 | 200 | 5034996 | 2 | 52 | 0 | 1 | 208 |
| 181 | B5 | 64 | 40 | 2 | 53 | 612825 | 4902596 | 2 | 53 | 0 | 200 | 5035204 | 2 | 53 | 0 | 1 | 208 |
| 182 | B6 | 64 | 40 | 2 | 54 | 612850 | 4902796 | 2 | 54 | 0 | 200 | 5035412 | 2 | 54 | 0 | 1 | 208 |
| 183 | B7 | 64 | 40 | 2 | 55 | 612875 | 4902996 | 2 | 55 | 0 | 200 | 5035620 | 2 | 55 | 0 | 1 | 208 |
| 184 | B8 | 64 | 40 | 2 | 56 | 612900 | 4903196 | 2 | 56 | 0 | 200 | 5035828 | 2 | 56 | 0 | 1 | 208 |
| 185 | B9 | 64 | 40 | 2 | 57 | 612925 | 4903396 | 2 | 57 | 0 | 200 | 5036036 | 2 | 57 | 0 | 1 | 208 |
| 186 | BA | 64 | 40 | 2 | 58 | 612950 | 4903596 | 2 | 58 | 0 | 200 | 5036244 | 2 | 58 | 0 | 1 | 208 |
| 187 | BB | 64 | 40 | 2 | 59 | 612975 | 4903796 | 2 | 59 | 0 | 200 | 5036452 | 2 | 59 | 0 | 1 | 208 |
| 188 | BC | 64 | 40 | 2 | 60 | 613000 | 4903996 | 2 | 60 | 0 | 200 | 5036660 | 2 | 60 | 0 | 1 | 208 |
| 189 | BD | 64 | 40 | 2 | 61 | 613025 | 4904196 | 2 | 61 | 0 | 200 | 5036868 | 2 | 61 | 0 | 1 | 208 |
| 190 | BE | 64 | 40 | 2 | 62 | 613050 | 4904396 | 2 | 62 | 0 | 200 | 5037076 | 2 | 62 | 0 | 1 | 208 |
| 191 | BF | 64 | 40 | 2 | 63 | 613075 | 4904596 | 2 | 63 | 0 | 200 | 5037284 | 2 | 63 | 0 | 1 | 208 |
| 192 | C0 | 64 | 40 | 3 | 0 | 613098 | 4904780 | 3 | 0 | 0 | 184 | 5037476 | 3 | 0 | 0 | 1 | 192 |
| 193 | C1 | 64 | 40 | 3 | 1 | 613123 | 4904980 | 3 | 1 | 0 | 200 | 5037684 | 3 | 1 | 0 | 1 | 208 |
| 194 | C2 | 64 | 40 | 3 | 2 | 613148 | 4905180 | 3 | 2 | 0 | 200 | 5037892 | 3 | 2 | 0 | 1 | 208 |
| 195 | C3 | 64 | 40 | 3 | 3 | 613173 | 4905380 | 3 | 3 | 0 | 200 | 5038100 | 3 | 3 | 0 | 1 | 208 |

| | | | | | | | | | | | | | | | | | |
|---|---|---|---|---|---|---|---|---|---|---|---|---|---|---|---|---|---|
| 196 | C4 | 64 | 40 | 3 | 4 | 613198 | 4905580 | 3 | 4 | 0 | 200 | 5038308 | 3 | 4 | 0 | 1 | 208 |
| 197 | C5 | 64 | 40 | 3 | 5 | 613223 | 4905780 | 3 | 5 | 0 | 200 | 5038516 | 3 | 5 | 0 | 1 | 208 |
| 198 | C6 | 64 | 40 | 3 | 6 | 613248 | 4905980 | 3 | 6 | 0 | 200 | 5038724 | 3 | 6 | 0 | 1 | 208 |
| 199 | C7 | 64 | 40 | 3 | 7 | 613273 | 4906180 | 3 | 7 | 0 | 200 | 5038932 | 3 | 7 | 0 | 1 | 208 |
| 200 | C8 | 64 | 40 | 3 | 8 | 613298 | 4906380 | 3 | 8 | 0 | 200 | 5039140 | 3 | 8 | 0 | 1 | 208 |
| 201 | C9 | 64 | 40 | 3 | 9 | 613323 | 4906580 | 3 | 9 | 0 | 200 | 5039348 | 3 | 9 | 0 | 1 | 208 |
| 202 | CA | 64 | 40 | 3 | 10 | 613348 | 4906780 | 3 | 10 | 0 | 200 | 5039556 | 3 | 10 | 0 | 1 | 208 |
| 203 | CB | 64 | 40 | 3 | 11 | 613373 | 4906980 | 3 | 11 | 0 | 200 | 5039764 | 3 | 11 | 0 | 1 | 208 |
| 204 | CC | 64 | 40 | 3 | 12 | 613398 | 4907180 | 3 | 12 | 0 | 200 | 5039972 | 3 | 12 | 0 | 1 | 208 |
| 205 | CD | 64 | 40 | 3 | 13 | 613423 | 4907380 | 3 | 13 | 0 | 200 | 5040180 | 3 | 13 | 0 | 1 | 208 |
| 206 | CE | 64 | 40 | 3 | 14 | 613448 | 4907580 | 3 | 14 | 0 | 200 | 5040388 | 3 | 14 | 0 | 1 | 208 |
| 207 | CF | 64 | 40 | 3 | 15 | 613473 | 4907780 | 3 | 15 | 0 | 200 | 5040596 | 3 | 15 | 0 | 1 | 208 |
| 208 | D0 | 64 | 40 | 3 | 16 | 613500 | 4907996 | 3 | 16 | 0 | 216 | 5040820 | 3 | 16 | 0 | 1 | 224 |
| 209 | D1 | 64 | 40 | 3 | 17 | 613527 | 4908212 | 3 | 17 | 0 | 216 | 5041044 | 3 | 17 | 0 | 1 | 224 |
| 210 | D2 | 64 | 40 | 3 | 18 | 613554 | 4908428 | 3 | 18 | 0 | 216 | 5041268 | 3 | 18 | 0 | 1 | 224 |
| 211 | D3 | 64 | 40 | 3 | 19 | 613581 | 4908644 | 3 | 19 | 0 | 216 | 5041492 | 3 | 19 | 0 | 1 | 224 |
| 212 | D4 | 64 | 40 | 3 | 20 | 613608 | 4908860 | 3 | 20 | 0 | 216 | 5041716 | 3 | 20 | 0 | 1 | 224 |
| 213 | D5 | 64 | 40 | 3 | 21 | 613635 | 4909076 | 3 | 21 | 0 | 216 | 5041940 | 3 | 21 | 0 | 1 | 224 |
| 214 | D6 | 64 | 40 | 3 | 22 | 613662 | 4909292 | 3 | 22 | 0 | 216 | 5042164 | 3 | 22 | 0 | 1 | 224 |
| 215 | D7 | 64 | 40 | 3 | 23 | 613689 | 4909508 | 3 | 23 | 0 | 216 | 5042388 | 3 | 23 | 0 | 1 | 224 |
| 216 | D8 | 64 | 40 | 3 | 24 | 613716 | 4909724 | 3 | 24 | 0 | 216 | 5042612 | 3 | 24 | 0 | 1 | 224 |
| 217 | D9 | 64 | 40 | 3 | 25 | 613743 | 4909940 | 3 | 25 | 0 | 216 | 5042836 | 3 | 25 | 0 | 1 | 224 |
| 218 | DA | 64 | 40 | 3 | 26 | 613770 | 4910156 | 3 | 26 | 0 | 216 | 5043060 | 3 | 26 | 0 | 1 | 224 |
| 219 | DB | 64 | 40 | 3 | 27 | 613797 | 4910372 | 3 | 27 | 0 | 216 | 5043284 | 3 | 27 | 0 | 1 | 224 |
| 220 | DC | 64 | 40 | 3 | 28 | 613824 | 4910588 | 3 | 28 | 0 | 216 | 5043508 | 3 | 28 | 0 | 1 | 224 |
| 221 | DD | 64 | 40 | 3 | 29 | 613851 | 4910804 | 3 | 29 | 0 | 216 | 5043732 | 3 | 29 | 0 | 1 | 224 |
| 222 | DE | 64 | 40 | 3 | 30 | 613878 | 4911020 | 3 | 30 | 0 | 216 | 5043956 | 3 | 30 | 0 | 1 | 224 |
| 223 | DF | 64 | 40 | 3 | 31 | 613905 | 4911236 | 3 | 31 | 0 | 216 | 5044180 | 3 | 31 | 0 | 1 | 224 |
| 224 | E0 | 64 | 40 | 3 | 32 | 613932 | 4911452 | 3 | 32 | 0 | 216 | 5044404 | 3 | 32 | 0 | 1 | 224 |
| 225 | E1 | 64 | 40 | 3 | 33 | 613959 | 4911668 | 3 | 33 | 0 | 216 | 5044628 | 3 | 33 | 0 | 1 | 224 |
| 226 | E2 | 64 | 40 | 3 | 34 | 613986 | 4911884 | 3 | 34 | 0 | 216 | 5044852 | 3 | 34 | 0 | 1 | 224 |
| 227 | E3 | 64 | 40 | 3 | 35 | 614013 | 4912100 | 3 | 35 | 0 | 216 | 5045076 | 3 | 35 | 0 | 1 | 224 |
| 228 | E4 | 64 | 40 | 3 | 36 | 614040 | 4912316 | 3 | 36 | 0 | 216 | 5045300 | 3 | 36 | 0 | 1 | 224 |
| 229 | E5 | 64 | 40 | 3 | 37 | 614067 | 4912532 | 3 | 37 | 0 | 216 | 5045524 | 3 | 37 | 0 | 1 | 224 |
| 230 | E6 | 64 | 40 | 3 | 38 | 614094 | 4912748 | 3 | 38 | 0 | 216 | 5045748 | 3 | 38 | 0 | 1 | 224 |
| 231 | E7 | 64 | 40 | 3 | 39 | 614121 | 4912964 | 3 | 39 | 0 | 216 | 5045972 | 3 | 39 | 0 | 1 | 224 |
| 232 | E8 | 64 | 40 | 3 | 40 | 614148 | 4913180 | 3 | 40 | 0 | 216 | 5046196 | 3 | 40 | 0 | 1 | 224 |
| 233 | E9 | 64 | 40 | 3 | 41 | 614175 | 4913396 | 3 | 41 | 0 | 216 | 5046420 | 3 | 41 | 0 | 1 | 224 |
| 234 | EA | 64 | 40 | 3 | 42 | 614202 | 4913612 | 3 | 42 | 0 | 216 | 5046644 | 3 | 42 | 0 | 1 | 224 |
| 235 | EB | 64 | 40 | 3 | 43 | 614229 | 4913828 | 3 | 43 | 0 | 216 | 5046868 | 3 | 43 | 0 | 1 | 224 |
| 236 | EC | 64 | 40 | 3 | 44 | 614256 | 4914044 | 3 | 44 | 0 | 216 | 5047092 | 3 | 44 | 0 | 1 | 224 |
| 237 | ED | 64 | 40 | 3 | 45 | 614283 | 4914260 | 3 | 45 | 0 | 216 | 5047316 | 3 | 45 | 0 | 1 | 224 |
| 238 | EE | 64 | 40 | 3 | 46 | 614310 | 4914476 | 3 | 46 | 0 | 216 | 5047540 | 3 | 46 | 0 | 1 | 224 |
| 239 | EF | 64 | 40 | 3 | 47 | 614337 | 4914692 | 3 | 47 | 0 | 216 | 5047764 | 3 | 47 | 0 | 1 | 224 |
| 240 | F0 | 64 | 40 | 3 | 48 | 614364 | 4914908 | 3 | 48 | 0 | 216 | 5047988 | 3 | 48 | 0 | 1 | 224 |
| 241 | F1 | 64 | 40 | 3 | 49 | 614391 | 4915124 | 3 | 49 | 0 | 216 | 5048212 | 3 | 49 | 0 | 1 | 224 |
| 242 | F2 | 64 | 40 | 3 | 50 | 614418 | 4915340 | 3 | 50 | 0 | 216 | 5048436 | 3 | 50 | 0 | 1 | 224 |
| 243 | F3 | 64 | 40 | 3 | 51 | 614445 | 4915556 | 3 | 51 | 0 | 216 | 5048660 | 3 | 51 | 0 | 1 | 224 |
| 244 | F4 | 64 | 40 | 3 | 52 | 614472 | 4915772 | 3 | 52 | 0 | 216 | 5048884 | 3 | 52 | 0 | 1 | 224 |
| 245 | F5 | 64 | 40 | 3 | 53 | 614499 | 4915988 | 3 | 53 | 0 | 216 | 5049108 | 3 | 53 | 0 | 1 | 224 |
| 246 | F6 | 64 | 40 | 3 | 54 | 614526 | 4916204 | 3 | 54 | 0 | 216 | 5049332 | 3 | 54 | 0 | 1 | 224 |
| 247 | F7 | 64 | 40 | 3 | 55 | 614553 | 4916420 | 3 | 55 | 0 | 216 | 5049556 | 3 | 55 | 0 | 1 | 224 |
| 248 | F8 | 64 | 40 | 3 | 56 | 614580 | 4916636 | 3 | 56 | 0 | 216 | 5049780 | 3 | 56 | 0 | 1 | 224 |
| 249 | F9 | 64 | 40 | 3 | 57 | 614607 | 4916852 | 3 | 57 | 0 | 216 | 5050004 | 3 | 57 | 0 | 1 | 224 |
| 250 | FA | 64 | 40 | 3 | 58 | 614634 | 4917068 | 3 | 58 | 0 | 216 | 5050228 | 3 | 58 | 0 | 1 | 224 |
| 251 | FB | 64 | 40 | 3 | 59 | 614661 | 4917284 | 3 | 59 | 0 | 216 | 5050452 | 3 | 59 | 0 | 1 | 224 |
| 252 | FC | 64 | 40 | 3 | 60 | 614688 | 4917500 | 3 | 60 | 0 | 216 | 5050676 | 3 | 60 | 0 | 1 | 224 |
| 253 | FD | 64 | 40 | 3 | 61 | 614715 | 4917716 | 3 | 61 | 0 | 216 | 5050900 | 3 | 61 | 0 | 1 | 224 |
| 254 | FE | 64 | 40 | 3 | 62 | 614742 | 4917932 | 3 | 62 | 0 | 216 | 5051124 | 3 | 62 | 0 | 1 | 224 |
| 255 | FF | 64 | 40 | 3 | 63 | 614769 | 4918148 | 3 | 63 | 0 | 216 | 5051348 | 3 | 63 | 0 | 1 | 224 |
| 0 | 00 | 65 | 41 | 0 | 0 | 614781 | 4918244 | 0 | 0 | 0 | 96 | 5051452 | 0 | 0 | 0 | 1 | 104 |
| 1 | 01 | 65 | 41 | 0 | 1 | 614795 | 4918356 | 0 | 1 | 0 | 112 | 5051572 | 0 | 1 | 0 | 1 | 120 |
| 2 | 02 | 65 | 41 | 0 | 2 | 614809 | 4918468 | 0 | 2 | 0 | 112 | 5051692 | 0 | 2 | 0 | 1 | 120 |
| 3 | 03 | 65 | 41 | 0 | 3 | 614823 | 4918580 | 0 | 3 | 0 | 112 | 5051812 | 0 | 3 | 0 | 1 | 120 |
| 4 | 04 | 65 | 41 | 0 | 4 | 614837 | 4918692 | 0 | 4 | 0 | 112 | 5051932 | 0 | 4 | 0 | 1 | 120 |
| 5 | 05 | 65 | 41 | 0 | 5 | 614851 | 4918804 | 0 | 5 | 0 | 112 | 5052052 | 0 | 5 | 0 | 1 | 120 |
| 6 | 06 | 65 | 41 | 0 | 6 | 614865 | 4918916 | 0 | 6 | 0 | 112 | 5052172 | 0 | 6 | 0 | 1 | 120 |
| 7 | 07 | 65 | 41 | 0 | 7 | 614879 | 4919028 | 0 | 7 | 0 | 112 | 5052292 | 0 | 7 | 0 | 1 | 120 |
| 8 | 08 | 65 | 41 | 0 | 8 | 614893 | 4919140 | 0 | 8 | 0 | 112 | 5052412 | 0 | 8 | 0 | 1 | 120 |
| 9 | 09 | 65 | 41 | 0 | 9 | 614907 | 4919252 | 0 | 9 | 0 | 112 | 5052532 | 0 | 9 | 0 | 1 | 120 |
| 10 | 0A | 65 | 41 | 0 | 10 | 614921 | 4919364 | 0 | 10 | 0 | 112 | 5052652 | 0 | 10 | 0 | 1 | 120 |
| 11 | 0B | 65 | 41 | 0 | 11 | 614935 | 4919476 | 0 | 11 | 0 | 112 | 5052772 | 0 | 11 | 0 | 1 | 120 |
| 12 | 0C | 65 | 41 | 0 | 12 | 614949 | 4919588 | 0 | 12 | 0 | 112 | 5052892 | 0 | 12 | 0 | 1 | 120 |
| 13 | 0D | 65 | 41 | 0 | 13 | 614963 | 4919700 | 0 | 13 | 0 | 112 | 5053012 | 0 | 13 | 0 | 1 | 120 |
| 14 | 0E | 65 | 41 | 0 | 14 | 614977 | 4919812 | 0 | 14 | 0 | 112 | 5053132 | 0 | 14 | 0 | 1 | 120 |
| 15 | 0F | 65 | 41 | 0 | 15 | 614991 | 4919924 | 0 | 15 | 0 | 112 | 5053252 | 0 | 15 | 0 | 1 | 120 |
| 16 | 10 | 65 | 41 | 0 | 16 | 615005 | 4920036 | 0 | 16 | 0 | 112 | 5053372 | 0 | 16 | 0 | 1 | 120 |
| 17 | 11 | 65 | 41 | 0 | 17 | 615019 | 4920148 | 0 | 17 | 0 | 112 | 5053492 | 0 | 17 | 0 | 1 | 120 |
| 18 | 12 | 65 | 41 | 0 | 18 | 615033 | 4920260 | 0 | 18 | 0 | 112 | 5053612 | 0 | 18 | 0 | 1 | 120 |
| 19 | 13 | 65 | 41 | 0 | 19 | 615047 | 4920372 | 0 | 19 | 0 | 112 | 5053732 | 0 | 19 | 0 | 1 | 120 |
| 20 | 14 | 65 | 41 | 0 | 20 | 615061 | 4920484 | 0 | 20 | 0 | 112 | 5053852 | 0 | 20 | 0 | 1 | 120 |
| 21 | 15 | 65 | 41 | 0 | 21 | 615075 | 4920596 | 0 | 21 | 0 | 112 | 5053972 | 0 | 21 | 0 | 1 | 120 |
| 22 | 16 | 65 | 41 | 0 | 22 | 615089 | 4920708 | 0 | 22 | 0 | 112 | 5054092 | 0 | 22 | 0 | 1 | 120 |
| 23 | 17 | 65 | 41 | 0 | 23 | 615103 | 4920820 | 0 | 23 | 0 | 112 | 5054212 | 0 | 23 | 0 | 1 | 120 |
| 24 | 18 | 65 | 41 | 0 | 24 | 615117 | 4920932 | 0 | 24 | 0 | 112 | 5054332 | 0 | 24 | 0 | 1 | 120 |
| 25 | 19 | 65 | 41 | 0 | 25 | 615131 | 4921044 | 0 | 25 | 0 | 112 | 5054452 | 0 | 25 | 0 | 1 | 120 |
| 26 | 1A | 65 | 41 | 0 | 26 | 615145 | 4921156 | 0 | 26 | 0 | 112 | 5054572 | 0 | 26 | 0 | 1 | 120 |
| 27 | 1B | 65 | 41 | 0 | 27 | 615159 | 4921268 | 0 | 27 | 0 | 112 | 5054692 | 0 | 27 | 0 | 1 | 120 |
| 28 | 1C | 65 | 41 | 0 | 28 | 615173 | 4921380 | 0 | 28 | 0 | 112 | 5054812 | 0 | 28 | 0 | 1 | 120 |
| 29 | 1D | 65 | 41 | 0 | 29 | 615187 | 4921492 | 0 | 29 | 0 | 112 | 5054932 | 0 | 29 | 0 | 1 | 120 |
| 30 | 1E | 65 | 41 | 0 | 30 | 615201 | 4921604 | 0 | 30 | 0 | 112 | 5055052 | 0 | 30 | 0 | 1 | 120 |
| 31 | 1F | 65 | 41 | 0 | 31 | 615215 | 4921716 | 0 | 31 | 0 | 112 | 5055172 | 0 | 31 | 0 | 1 | 120 |
| 32 | 20 | 65 | 41 | 0 | 32 | 615229 | 4921828 | 0 | 32 | 0 | 112 | 5055292 | 0 | 32 | 0 | 1 | 120 |
| 33 | 21 | 65 | 41 | 0 | 33 | 615243 | 4921940 | 0 | 33 | 0 | 112 | 5055412 | 0 | 33 | 0 | 1 | 120 |
| 34 | 22 | 65 | 41 | 0 | 34 | 615257 | 4922052 | 0 | 34 | 0 | 112 | 5055532 | 0 | 34 | 0 | 1 | 120 |
| 35 | 23 | 65 | 41 | 0 | 35 | 615271 | 4922164 | 0 | 35 | 0 | 112 | 5055652 | 0 | 35 | 0 | 1 | 120 |
| 36 | 24 | 65 | 41 | 0 | 36 | 615285 | 4922276 | 0 | 36 | 0 | 112 | 5055772 | 0 | 36 | 0 | 1 | 120 |
| 37 | 25 | 65 | 41 | 0 | 37 | 615299 | 4922388 | 0 | 37 | 0 | 112 | 5055892 | 0 | 37 | 0 | 1 | 120 |
| 38 | 26 | 65 | 41 | 0 | 38 | 615313 | 4922500 | 0 | 38 | 0 | 112 | 5056012 | 0 | 38 | 0 | 1 | 120 |
| 39 | 27 | 65 | 41 | 0 | 39 | 615327 | 4922612 | 0 | 39 | 0 | 112 | 5056132 | 0 | 39 | 0 | 1 | 120 |
| 40 | 28 | 65 | 41 | 0 | 40 | 615341 | 4922724 | 0 | 40 | 0 | 112 | 5056252 | 0 | 40 | 0 | 1 | 120 |
| 41 | 29 | 65 | 41 | 0 | 41 | 615355 | 4922836 | 0 | 41 | 0 | 112 | 5056372 | 0 | 41 | 0 | 1 | 120 |
| 42 | 2A | 65 | 41 | 0 | 42 | 615369 | 4922948 | 0 | 42 | 0 | 112 | 5056492 | 0 | 42 | 0 | 1 | 120 |

| | | | | | | | | | | | | | | | | | |
|---|---|---|---|---|---|---|---|---|---|---|---|---|---|---|---|---|---|
| 43 | 2B | 65 | 41 | 0 | 43 | 615383 | 4923060 | 0 | 43 | 0 | 112 | 5056612 | 0 | 43 | 0 | 1 | 120 |
| 44 | 2C | 65 | 41 | 0 | 44 | 615397 | 4923172 | 0 | 44 | 0 | 112 | 5056732 | 0 | 44 | 0 | 1 | 120 |
| 45 | 2D | 65 | 41 | 0 | 45 | 615411 | 4923284 | 0 | 45 | 0 | 112 | 5056852 | 0 | 45 | 0 | 1 | 120 |
| 46 | 2E | 65 | 41 | 0 | 46 | 615425 | 4923396 | 0 | 46 | 0 | 112 | 5056972 | 0 | 46 | 0 | 1 | 120 |
| 47 | 2F | 65 | 41 | 0 | 47 | 615439 | 4923508 | 0 | 47 | 0 | 112 | 5057092 | 0 | 47 | 0 | 1 | 120 |
| 48 | 30 | 65 | 41 | 0 | 48 | 615453 | 4923620 | 0 | 48 | 0 | 112 | 5057212 | 0 | 48 | 0 | 1 | 120 |
| 49 | 31 | 65 | 41 | 0 | 49 | 615467 | 4923732 | 0 | 49 | 0 | 112 | 5057332 | 0 | 49 | 0 | 1 | 120 |
| 50 | 32 | 65 | 41 | 0 | 50 | 615481 | 4923844 | 0 | 50 | 0 | 112 | 5057452 | 0 | 50 | 0 | 1 | 120 |
| 51 | 33 | 65 | 41 | 0 | 51 | 615495 | 4923956 | 0 | 51 | 0 | 112 | 5057572 | 0 | 51 | 0 | 1 | 120 |
| 52 | 34 | 65 | 41 | 0 | 52 | 615509 | 4924068 | 0 | 52 | 0 | 112 | 5057692 | 0 | 52 | 0 | 1 | 120 |
| 53 | 35 | 65 | 41 | 0 | 53 | 615523 | 4924180 | 0 | 53 | 0 | 112 | 5057812 | 0 | 53 | 0 | 1 | 120 |
| 54 | 36 | 65 | 41 | 0 | 54 | 615537 | 4924292 | 0 | 54 | 0 | 112 | 5057932 | 0 | 54 | 0 | 1 | 120 |
| 55 | 37 | 65 | 41 | 0 | 55 | 615551 | 4924404 | 0 | 55 | 0 | 112 | 5058052 | 0 | 55 | 0 | 1 | 120 |
| 56 | 38 | 65 | 41 | 0 | 56 | 615565 | 4924516 | 0 | 56 | 0 | 112 | 5058172 | 0 | 56 | 0 | 1 | 120 |
| 57 | 39 | 65 | 41 | 0 | 57 | 615579 | 4924628 | 0 | 57 | 0 | 112 | 5058292 | 0 | 57 | 0 | 1 | 120 |
| 58 | 3A | 65 | 41 | 0 | 58 | 615593 | 4924740 | 0 | 58 | 0 | 112 | 5058412 | 0 | 58 | 0 | 1 | 120 |
| 59 | 3B | 65 | 41 | 0 | 59 | 615607 | 4924852 | 0 | 59 | 0 | 112 | 5058532 | 0 | 59 | 0 | 1 | 120 |
| 60 | 3C | 65 | 41 | 0 | 60 | 615621 | 4924964 | 0 | 60 | 0 | 112 | 5058652 | 0 | 60 | 0 | 1 | 120 |
| 61 | 3D | 65 | 41 | 0 | 61 | 615635 | 4925076 | 0 | 61 | 0 | 112 | 5058772 | 0 | 61 | 0 | 1 | 120 |
| 62 | 3E | 65 | 41 | 0 | 62 | 615649 | 4925188 | 0 | 62 | 0 | 112 | 5058892 | 0 | 62 | 0 | 1 | 120 |
| 63 | 3F | 65 | 41 | 0 | 63 | 615663 | 4925300 | 0 | 63 | 0 | 112 | 5059012 | 0 | 63 | 0 | 1 | 120 |
| 64 | 40 | 65 | 41 | 0 | 64 | 615677 | 4925412 | 0 | 64 | 0 | 112 | 5059132 | 0 | 64 | 0 | 1 | 120 |
| 65 | 41 | 65 | 41 | 1 | 0 | 615689 | 4925508 | 1 | 0 | 0 | 96 | 5059236 | 1 | 0 | 0 | 1 | 104 |
| 66 | 42 | 65 | 41 | 1 | 1 | 615706 | 4925644 | 1 | 1 | 0 | 136 | 5059380 | 1 | 1 | 0 | 1 | 144 |
| 67 | 43 | 65 | 41 | 1 | 2 | 615723 | 4925780 | 1 | 2 | 0 | 136 | 5059524 | 1 | 2 | 0 | 1 | 144 |
| 68 | 44 | 65 | 41 | 1 | 3 | 615740 | 4925916 | 1 | 3 | 0 | 136 | 5059668 | 1 | 3 | 0 | 1 | 144 |
| 69 | 45 | 65 | 41 | 1 | 4 | 615757 | 4926052 | 1 | 4 | 0 | 136 | 5059812 | 1 | 4 | 0 | 1 | 144 |
| 70 | 46 | 65 | 41 | 1 | 5 | 615774 | 4926188 | 1 | 5 | 0 | 136 | 5059956 | 1 | 5 | 0 | 1 | 144 |
| 71 | 47 | 65 | 41 | 1 | 6 | 615791 | 4926324 | 1 | 6 | 0 | 136 | 5060100 | 1 | 6 | 0 | 1 | 144 |
| 72 | 48 | 65 | 41 | 1 | 7 | 615808 | 4926460 | 1 | 7 | 0 | 136 | 5060244 | 1 | 7 | 0 | 1 | 144 |
| 73 | 49 | 65 | 41 | 1 | 8 | 615825 | 4926596 | 1 | 8 | 0 | 136 | 5060388 | 1 | 8 | 0 | 1 | 144 |
| 74 | 4A | 65 | 41 | 1 | 9 | 615842 | 4926732 | 1 | 9 | 0 | 136 | 5060532 | 1 | 9 | 0 | 1 | 144 |
| 75 | 4B | 65 | 41 | 1 | 10 | 615859 | 4926868 | 1 | 10 | 0 | 136 | 5060676 | 1 | 10 | 0 | 1 | 144 |
| 76 | 4C | 65 | 41 | 1 | 11 | 615876 | 4927004 | 1 | 11 | 0 | 136 | 5060820 | 1 | 11 | 0 | 1 | 144 |
| 77 | 4D | 65 | 41 | 1 | 12 | 615893 | 4927140 | 1 | 12 | 0 | 136 | 5060964 | 1 | 12 | 0 | 1 | 144 |
| 78 | 4E | 65 | 41 | 1 | 13 | 615910 | 4927276 | 1 | 13 | 0 | 136 | 5061108 | 1 | 13 | 0 | 1 | 144 |
| 79 | 4F | 65 | 41 | 1 | 14 | 615927 | 4927412 | 1 | 14 | 0 | 136 | 5061252 | 1 | 14 | 0 | 1 | 144 |
| 80 | 50 | 65 | 41 | 1 | 15 | 615948 | 4927580 | 1 | 15 | 0 | 168 | 5061428 | 1 | 15 | 0 | 1 | 176 |
| 81 | 51 | 65 | 41 | 1 | 16 | 615969 | 4927748 | 1 | 16 | 0 | 168 | 5061604 | 1 | 16 | 0 | 1 | 176 |
| 82 | 52 | 65 | 41 | 1 | 17 | 615990 | 4927916 | 1 | 17 | 0 | 168 | 5061780 | 1 | 17 | 0 | 1 | 176 |
| 83 | 53 | 65 | 41 | 1 | 18 | 616011 | 4928084 | 1 | 18 | 0 | 168 | 5061956 | 1 | 18 | 0 | 1 | 176 |
| 84 | 54 | 65 | 41 | 1 | 19 | 616032 | 4928252 | 1 | 19 | 0 | 168 | 5062132 | 1 | 19 | 0 | 1 | 176 |
| 85 | 55 | 65 | 41 | 1 | 20 | 616053 | 4928420 | 1 | 20 | 0 | 168 | 5062308 | 1 | 20 | 0 | 1 | 176 |
| 86 | 56 | 65 | 41 | 1 | 21 | 616074 | 4928588 | 1 | 21 | 0 | 168 | 5062484 | 1 | 21 | 0 | 1 | 176 |
| 87 | 57 | 65 | 41 | 1 | 22 | 616095 | 4928756 | 1 | 22 | 0 | 168 | 5062660 | 1 | 22 | 0 | 1 | 176 |
| 88 | 58 | 65 | 41 | 1 | 23 | 616116 | 4928924 | 1 | 23 | 0 | 168 | 5062836 | 1 | 23 | 0 | 1 | 176 |
| 89 | 59 | 65 | 41 | 1 | 24 | 616137 | 4929092 | 1 | 24 | 0 | 168 | 5063012 | 1 | 24 | 0 | 1 | 176 |
| 90 | 5A | 65 | 41 | 1 | 25 | 616158 | 4929260 | 1 | 25 | 0 | 168 | 5063188 | 1 | 25 | 0 | 1 | 176 |
| 91 | 5B | 65 | 41 | 1 | 26 | 616179 | 4929428 | 1 | 26 | 0 | 168 | 5063364 | 1 | 26 | 0 | 1 | 176 |
| 92 | 5C | 65 | 41 | 1 | 27 | 616200 | 4929596 | 1 | 27 | 0 | 168 | 5063540 | 1 | 27 | 0 | 1 | 176 |
| 93 | 5D | 65 | 41 | 1 | 28 | 616221 | 4929764 | 1 | 28 | 0 | 168 | 5063716 | 1 | 28 | 0 | 1 | 176 |
| 94 | 5E | 65 | 41 | 1 | 29 | 616242 | 4929932 | 1 | 29 | 0 | 168 | 5063892 | 1 | 29 | 0 | 1 | 176 |
| 95 | 5F | 65 | 41 | 1 | 30 | 616263 | 4930100 | 1 | 30 | 0 | 168 | 5064068 | 1 | 30 | 0 | 1 | 176 |
| 96 | 60 | 65 | 41 | 1 | 31 | 616286 | 4930284 | 1 | 31 | 0 | 184 | 5064260 | 1 | 31 | 0 | 1 | 192 |
| 97 | 61 | 65 | 41 | 1 | 32 | 616309 | 4930468 | 1 | 32 | 0 | 184 | 5064452 | 1 | 32 | 0 | 1 | 192 |
| 98 | 62 | 65 | 41 | 1 | 33 | 616332 | 4930652 | 1 | 33 | 0 | 184 | 5064644 | 1 | 33 | 0 | 1 | 192 |
| 99 | 63 | 65 | 41 | 1 | 34 | 616355 | 4930836 | 1 | 34 | 0 | 184 | 5064836 | 1 | 34 | 0 | 1 | 192 |
| 100 | 64 | 65 | 41 | 1 | 35 | 616378 | 4931020 | 1 | 35 | 0 | 184 | 5065028 | 1 | 35 | 0 | 1 | 192 |
| 101 | 65 | 65 | 41 | 1 | 36 | 616401 | 4931204 | 1 | 36 | 0 | 184 | 5065220 | 1 | 36 | 0 | 1 | 192 |
| 102 | 66 | 65 | 41 | 1 | 37 | 616424 | 4931388 | 1 | 37 | 0 | 184 | 5065412 | 1 | 37 | 0 | 1 | 192 |
| 103 | 67 | 65 | 41 | 1 | 38 | 616447 | 4931572 | 1 | 38 | 0 | 184 | 5065604 | 1 | 38 | 0 | 1 | 192 |
| 104 | 68 | 65 | 41 | 1 | 39 | 616470 | 4931756 | 1 | 39 | 0 | 184 | 5065796 | 1 | 39 | 0 | 1 | 192 |
| 105 | 69 | 65 | 41 | 1 | 40 | 616493 | 4931940 | 1 | 40 | 0 | 184 | 5065988 | 1 | 40 | 0 | 1 | 192 |
| 106 | 6A | 65 | 41 | 1 | 41 | 616516 | 4932124 | 1 | 41 | 0 | 184 | 5066180 | 1 | 41 | 0 | 1 | 192 |
| 107 | 6B | 65 | 41 | 1 | 42 | 616539 | 4932308 | 1 | 42 | 0 | 184 | 5066372 | 1 | 42 | 0 | 1 | 192 |
| 108 | 6C | 65 | 41 | 1 | 43 | 616562 | 4932492 | 1 | 43 | 0 | 184 | 5066564 | 1 | 43 | 0 | 1 | 192 |
| 109 | 6D | 65 | 41 | 1 | 44 | 616585 | 4932676 | 1 | 44 | 0 | 184 | 5066756 | 1 | 44 | 0 | 1 | 192 |
| 110 | 6E | 65 | 41 | 1 | 45 | 616608 | 4932860 | 1 | 45 | 0 | 184 | 5066948 | 1 | 45 | 0 | 1 | 192 |
| 111 | 6F | 65 | 41 | 1 | 46 | 616631 | 4933044 | 1 | 46 | 0 | 184 | 5067140 | 1 | 46 | 0 | 1 | 192 |
| 112 | 70 | 65 | 41 | 1 | 47 | 616654 | 4933228 | 1 | 47 | 0 | 184 | 5067332 | 1 | 47 | 0 | 1 | 192 |
| 113 | 71 | 65 | 41 | 1 | 48 | 616677 | 4933412 | 1 | 48 | 0 | 184 | 5067524 | 1 | 48 | 0 | 1 | 192 |
| 114 | 72 | 65 | 41 | 1 | 49 | 616700 | 4933596 | 1 | 49 | 0 | 184 | 5067716 | 1 | 49 | 0 | 1 | 192 |
| 115 | 73 | 65 | 41 | 1 | 50 | 616723 | 4933780 | 1 | 50 | 0 | 184 | 5067908 | 1 | 50 | 0 | 1 | 192 |
| 116 | 74 | 65 | 41 | 1 | 51 | 616746 | 4933964 | 1 | 51 | 0 | 184 | 5068100 | 1 | 51 | 0 | 1 | 192 |
| 117 | 75 | 65 | 41 | 1 | 52 | 616769 | 4934148 | 1 | 52 | 0 | 184 | 5068292 | 1 | 52 | 0 | 1 | 192 |
| 118 | 76 | 65 | 41 | 1 | 53 | 616792 | 4934332 | 1 | 53 | 0 | 184 | 5068484 | 1 | 53 | 0 | 1 | 192 |
| 119 | 77 | 65 | 41 | 1 | 54 | 616815 | 4934516 | 1 | 54 | 0 | 184 | 5068676 | 1 | 54 | 0 | 1 | 192 |
| 120 | 78 | 65 | 41 | 1 | 55 | 616838 | 4934700 | 1 | 55 | 0 | 184 | 5068868 | 1 | 55 | 0 | 1 | 192 |
| 121 | 79 | 65 | 41 | 1 | 56 | 616861 | 4934884 | 1 | 56 | 0 | 184 | 5069060 | 1 | 56 | 0 | 1 | 192 |
| 122 | 7A | 65 | 41 | 1 | 57 | 616884 | 4935068 | 1 | 57 | 0 | 184 | 5069252 | 1 | 57 | 0 | 1 | 192 |
| 123 | 7B | 65 | 41 | 1 | 58 | 616907 | 4935252 | 1 | 58 | 0 | 184 | 5069444 | 1 | 58 | 0 | 1 | 192 |
| 124 | 7C | 65 | 41 | 1 | 59 | 616930 | 4935436 | 1 | 59 | 0 | 184 | 5069636 | 1 | 59 | 0 | 1 | 192 |
| 125 | 7D | 65 | 41 | 1 | 60 | 616953 | 4935620 | 1 | 60 | 0 | 184 | 5069828 | 1 | 60 | 0 | 1 | 192 |
| 126 | 7E | 65 | 41 | 1 | 61 | 616976 | 4935804 | 1 | 61 | 0 | 184 | 5070020 | 1 | 61 | 0 | 1 | 192 |
| 127 | 7F | 65 | 41 | 1 | 62 | 616999 | 4935988 | 1 | 62 | 0 | 184 | 5070212 | 1 | 62 | 0 | 1 | 192 |
| 128 | 80 | 65 | 41 | 1 | 63 | 617022 | 4936172 | 1 | 63 | 0 | 184 | 5070404 | 1 | 63 | 0 | 1 | 192 |
| 129 | 81 | 65 | 41 | 1 | 64 | 617045 | 4936356 | 1 | 64 | 0 | 184 | 5070596 | 1 | 64 | 0 | 1 | 192 |
| 130 | 82 | 65 | 41 | 2 | 0 | 617066 | 4936524 | 2 | 0 | 0 | 168 | 5070772 | 2 | 0 | 0 | 1 | 176 |
| 131 | 83 | 65 | 41 | 2 | 1 | 617092 | 4936732 | 2 | 1 | 0 | 208 | 5070988 | 2 | 1 | 0 | 1 | 216 |
| 132 | 84 | 65 | 41 | 2 | 2 | 617118 | 4936940 | 2 | 2 | 0 | 208 | 5071204 | 2 | 2 | 0 | 1 | 216 |
| 133 | 85 | 65 | 41 | 2 | 3 | 617144 | 4937148 | 2 | 3 | 0 | 208 | 5071420 | 2 | 3 | 0 | 1 | 216 |
| 134 | 86 | 65 | 41 | 2 | 4 | 617170 | 4937356 | 2 | 4 | 0 | 208 | 5071636 | 2 | 4 | 0 | 1 | 216 |
| 135 | 87 | 65 | 41 | 2 | 5 | 617196 | 4937564 | 2 | 5 | 0 | 208 | 5071852 | 2 | 5 | 0 | 1 | 216 |
| 136 | 88 | 65 | 41 | 2 | 6 | 617222 | 4937772 | 2 | 6 | 0 | 208 | 5072068 | 2 | 6 | 0 | 1 | 216 |
| 137 | 89 | 65 | 41 | 2 | 7 | 617248 | 4937980 | 2 | 7 | 0 | 208 | 5072284 | 2 | 7 | 0 | 1 | 216 |
| 138 | 8A | 65 | 41 | 2 | 8 | 617274 | 4938188 | 2 | 8 | 0 | 208 | 5072500 | 2 | 8 | 0 | 1 | 216 |
| 139 | 8B | 65 | 41 | 2 | 9 | 617300 | 4938396 | 2 | 9 | 0 | 208 | 5072716 | 2 | 9 | 0 | 1 | 216 |
| 140 | 8C | 65 | 41 | 2 | 10 | 617326 | 4938604 | 2 | 10 | 0 | 208 | 5072932 | 2 | 10 | 0 | 1 | 216 |
| 141 | 8D | 65 | 41 | 2 | 11 | 617352 | 4938812 | 2 | 11 | 0 | 208 | 5073148 | 2 | 11 | 0 | 1 | 216 |
| 142 | 8E | 65 | 41 | 2 | 12 | 617378 | 4939020 | 2 | 12 | 0 | 208 | 5073364 | 2 | 12 | 0 | 1 | 216 |
| 143 | 8F | 65 | 41 | 2 | 13 | 617404 | 4939228 | 2 | 13 | 0 | 208 | 5073580 | 2 | 13 | 0 | 1 | 216 |
| 144 | 90 | 65 | 41 | 2 | 14 | 617430 | 4939436 | 2 | 14 | 0 | 208 | 5073796 | 2 | 14 | 0 | 1 | 216 |
| 145 | 91 | 65 | 41 | 2 | 15 | 617456 | 4939644 | 2 | 15 | 0 | 208 | 5074012 | 2 | 15 | 0 | 1 | 216 |

| | | | | | | | | | | | | | | | | | |
|---|---|---|---|---|---|---|---|---|---|---|---|---|---|---|---|---|---|
| 146 | 92 | 65 | 41 | 2 | 16 | 617482 | 4939852 | 2 | 16 | 0 | 208 | 5074228 | 2 | 16 | 0 | 1 | 216 |
| 147 | 93 | 65 | 41 | 2 | 17 | 617508 | 4940060 | 2 | 17 | 0 | 208 | 5074444 | 2 | 17 | 0 | 1 | 216 |
| 148 | 94 | 65 | 41 | 2 | 18 | 617534 | 4940268 | 2 | 18 | 0 | 208 | 5074660 | 2 | 18 | 0 | 1 | 216 |
| 149 | 95 | 65 | 41 | 2 | 19 | 617560 | 4940476 | 2 | 19 | 0 | 208 | 5074876 | 2 | 19 | 0 | 1 | 216 |
| 150 | 96 | 65 | 41 | 2 | 20 | 617586 | 4940684 | 2 | 20 | 0 | 208 | 5075092 | 2 | 20 | 0 | 1 | 216 |
| 151 | 97 | 65 | 41 | 2 | 21 | 617612 | 4940892 | 2 | 21 | 0 | 208 | 5075308 | 2 | 21 | 0 | 1 | 216 |
| 152 | 98 | 65 | 41 | 2 | 22 | 617638 | 4941100 | 2 | 22 | 0 | 208 | 5075524 | 2 | 22 | 0 | 1 | 216 |
| 153 | 99 | 65 | 41 | 2 | 23 | 617664 | 4941308 | 2 | 23 | 0 | 208 | 5075740 | 2 | 23 | 0 | 1 | 216 |
| 154 | 9A | 65 | 41 | 2 | 24 | 617690 | 4941516 | 2 | 24 | 0 | 208 | 5075956 | 2 | 24 | 0 | 1 | 216 |
| 155 | 9B | 65 | 41 | 2 | 25 | 617716 | 4941724 | 2 | 25 | 0 | 208 | 5076172 | 2 | 25 | 0 | 1 | 216 |
| 156 | 9C | 65 | 41 | 2 | 26 | 617742 | 4941932 | 2 | 26 | 0 | 208 | 5076388 | 2 | 26 | 0 | 1 | 216 |
| 157 | 9D | 65 | 41 | 2 | 27 | 617768 | 4942140 | 2 | 27 | 0 | 208 | 5076604 | 2 | 27 | 0 | 1 | 216 |
| 158 | 9E | 65 | 41 | 2 | 28 | 617794 | 4942348 | 2 | 28 | 0 | 208 | 5076820 | 2 | 28 | 0 | 1 | 216 |
| 159 | 9F | 65 | 41 | 2 | 29 | 617820 | 4942556 | 2 | 29 | 0 | 208 | 5077036 | 2 | 29 | 0 | 1 | 216 |
| 160 | A0 | 65 | 41 | 2 | 30 | 617843 | 4942740 | 2 | 30 | 0 | 184 | 5077228 | 2 | 30 | 0 | 1 | 192 |
| 161 | A1 | 65 | 41 | 2 | 31 | 617866 | 4942924 | 2 | 31 | 0 | 184 | 5077420 | 2 | 31 | 0 | 1 | 192 |
| 162 | A2 | 65 | 41 | 2 | 32 | 617889 | 4943108 | 2 | 32 | 0 | 184 | 5077612 | 2 | 32 | 0 | 1 | 192 |
| 163 | A3 | 65 | 41 | 2 | 33 | 617912 | 4943292 | 2 | 33 | 0 | 184 | 5077804 | 2 | 33 | 0 | 1 | 192 |
| 164 | A4 | 65 | 41 | 2 | 34 | 617935 | 4943476 | 2 | 34 | 0 | 184 | 5077996 | 2 | 34 | 0 | 1 | 192 |
| 165 | A5 | 65 | 41 | 2 | 35 | 617958 | 4943660 | 2 | 35 | 0 | 184 | 5078188 | 2 | 35 | 0 | 1 | 192 |
| 166 | A6 | 65 | 41 | 2 | 36 | 617981 | 4943844 | 2 | 36 | 0 | 184 | 5078380 | 2 | 36 | 0 | 1 | 192 |
| 167 | A7 | 65 | 41 | 2 | 37 | 618004 | 4944028 | 2 | 37 | 0 | 184 | 5078572 | 2 | 37 | 0 | 1 | 192 |
| 168 | A8 | 65 | 41 | 2 | 38 | 618027 | 4944212 | 2 | 38 | 0 | 184 | 5078764 | 2 | 38 | 0 | 1 | 192 |
| 169 | A9 | 65 | 41 | 2 | 39 | 618050 | 4944396 | 2 | 39 | 0 | 184 | 5078956 | 2 | 39 | 0 | 1 | 192 |
| 170 | AA | 65 | 41 | 2 | 40 | 618073 | 4944580 | 2 | 40 | 0 | 184 | 5079148 | 2 | 40 | 0 | 1 | 192 |
| 171 | AB | 65 | 41 | 2 | 41 | 618096 | 4944764 | 2 | 41 | 0 | 184 | 5079340 | 2 | 41 | 0 | 1 | 192 |
| 172 | AC | 65 | 41 | 2 | 42 | 618119 | 4944948 | 2 | 42 | 0 | 184 | 5079532 | 2 | 42 | 0 | 1 | 192 |
| 173 | AD | 65 | 41 | 2 | 43 | 618142 | 4945132 | 2 | 43 | 0 | 184 | 5079724 | 2 | 43 | 0 | 1 | 192 |
| 174 | AE | 65 | 41 | 2 | 44 | 618165 | 4945316 | 2 | 44 | 0 | 184 | 5079916 | 2 | 44 | 0 | 1 | 192 |
| 175 | AF | 65 | 41 | 2 | 45 | 618188 | 4945500 | 2 | 45 | 0 | 184 | 5080108 | 2 | 45 | 0 | 1 | 192 |
| 176 | B0 | 65 | 41 | 2 | 46 | 618213 | 4945700 | 2 | 46 | 0 | 200 | 5080316 | 2 | 46 | 0 | 1 | 208 |
| 177 | B1 | 65 | 41 | 2 | 47 | 618238 | 4945900 | 2 | 47 | 0 | 200 | 5080524 | 2 | 47 | 0 | 1 | 208 |
| 178 | B2 | 65 | 41 | 2 | 48 | 618263 | 4946100 | 2 | 48 | 0 | 200 | 5080732 | 2 | 48 | 0 | 1 | 208 |
| 179 | B3 | 65 | 41 | 2 | 49 | 618288 | 4946300 | 2 | 49 | 0 | 200 | 5080940 | 2 | 49 | 0 | 1 | 208 |
| 180 | B4 | 65 | 41 | 2 | 50 | 618313 | 4946500 | 2 | 50 | 0 | 200 | 5081148 | 2 | 50 | 0 | 1 | 208 |
| 181 | B5 | 65 | 41 | 2 | 51 | 618338 | 4946700 | 2 | 51 | 0 | 200 | 5081356 | 2 | 51 | 0 | 1 | 208 |
| 182 | B6 | 65 | 41 | 2 | 52 | 618363 | 4946900 | 2 | 52 | 0 | 200 | 5081564 | 2 | 52 | 0 | 1 | 208 |
| 183 | B7 | 65 | 41 | 2 | 53 | 618388 | 4947100 | 2 | 53 | 0 | 200 | 5081772 | 2 | 53 | 0 | 1 | 208 |
| 184 | B8 | 65 | 41 | 2 | 54 | 618413 | 4947300 | 2 | 54 | 0 | 200 | 5081980 | 2 | 54 | 0 | 1 | 208 |
| 185 | B9 | 65 | 41 | 2 | 55 | 618438 | 4947500 | 2 | 55 | 0 | 200 | 5082188 | 2 | 55 | 0 | 1 | 208 |
| 186 | BA | 65 | 41 | 2 | 56 | 618463 | 4947700 | 2 | 56 | 0 | 200 | 5082396 | 2 | 56 | 0 | 1 | 208 |
| 187 | BB | 65 | 41 | 2 | 57 | 618488 | 4947900 | 2 | 57 | 0 | 200 | 5082604 | 2 | 57 | 0 | 1 | 208 |
| 188 | BC | 65 | 41 | 2 | 58 | 618513 | 4948100 | 2 | 58 | 0 | 200 | 5082812 | 2 | 58 | 0 | 1 | 208 |
| 189 | BD | 65 | 41 | 2 | 59 | 618538 | 4948300 | 2 | 59 | 0 | 200 | 5083020 | 2 | 59 | 0 | 1 | 208 |
| 190 | BE | 65 | 41 | 2 | 60 | 618563 | 4948500 | 2 | 60 | 0 | 200 | 5083228 | 2 | 60 | 0 | 1 | 208 |
| 191 | BF | 65 | 41 | 2 | 61 | 618588 | 4948700 | 2 | 61 | 0 | 200 | 5083436 | 2 | 61 | 0 | 1 | 208 |
| 192 | C0 | 65 | 41 | 2 | 62 | 618613 | 4948900 | 2 | 62 | 0 | 200 | 5083644 | 2 | 62 | 0 | 1 | 208 |
| 193 | C1 | 65 | 41 | 2 | 63 | 618638 | 4949100 | 2 | 63 | 0 | 200 | 5083852 | 2 | 63 | 0 | 1 | 208 |
| 194 | C2 | 65 | 41 | 2 | 64 | 618663 | 4949300 | 2 | 64 | 0 | 200 | 5084060 | 2 | 64 | 0 | 1 | 208 |
| 195 | C3 | 65 | 41 | 3 | 0 | 618686 | 4949484 | 3 | 0 | 0 | 184 | 5084252 | 3 | 0 | 0 | 1 | 192 |
| 196 | C4 | 65 | 41 | 3 | 1 | 618714 | 4949708 | 3 | 1 | 0 | 224 | 5084484 | 3 | 1 | 0 | 1 | 232 |
| 197 | C5 | 65 | 41 | 3 | 2 | 618742 | 4949932 | 3 | 2 | 0 | 224 | 5084716 | 3 | 2 | 0 | 1 | 232 |
| 198 | C6 | 65 | 41 | 3 | 3 | 618770 | 4950156 | 3 | 3 | 0 | 224 | 5084948 | 3 | 3 | 0 | 1 | 232 |
| 199 | C7 | 65 | 41 | 3 | 4 | 618798 | 4950380 | 3 | 4 | 0 | 224 | 5085180 | 3 | 4 | 0 | 1 | 232 |
| 200 | C8 | 65 | 41 | 3 | 5 | 618826 | 4950604 | 3 | 5 | 0 | 224 | 5085412 | 3 | 5 | 0 | 1 | 232 |
| 201 | C9 | 65 | 41 | 3 | 6 | 618854 | 4950828 | 3 | 6 | 0 | 224 | 5085644 | 3 | 6 | 0 | 1 | 232 |
| 202 | CA | 65 | 41 | 3 | 7 | 618882 | 4951052 | 3 | 7 | 0 | 224 | 5085876 | 3 | 7 | 0 | 1 | 232 |
| 203 | CB | 65 | 41 | 3 | 8 | 618910 | 4951276 | 3 | 8 | 0 | 224 | 5086108 | 3 | 8 | 0 | 1 | 232 |
| 204 | CC | 65 | 41 | 3 | 9 | 618938 | 4951500 | 3 | 9 | 0 | 224 | 5086340 | 3 | 9 | 0 | 1 | 232 |
| 205 | CD | 65 | 41 | 3 | 10 | 618966 | 4951724 | 3 | 10 | 0 | 224 | 5086572 | 3 | 10 | 0 | 1 | 232 |
| 206 | CE | 65 | 41 | 3 | 11 | 618994 | 4951948 | 3 | 11 | 0 | 224 | 5086804 | 3 | 11 | 0 | 1 | 232 |
| 207 | CF | 65 | 41 | 3 | 12 | 619022 | 4952172 | 3 | 12 | 0 | 224 | 5087036 | 3 | 12 | 0 | 1 | 232 |
| 208 | D0 | 65 | 41 | 3 | 13 | 619050 | 4952396 | 3 | 13 | 0 | 224 | 5087268 | 3 | 13 | 0 | 1 | 232 |
| 209 | D1 | 65 | 41 | 3 | 14 | 619078 | 4952620 | 3 | 14 | 0 | 224 | 5087500 | 3 | 14 | 0 | 1 | 232 |
| 210 | D2 | 65 | 41 | 3 | 15 | 619106 | 4952844 | 3 | 15 | 0 | 224 | 5087732 | 3 | 15 | 0 | 1 | 232 |
| 211 | D3 | 65 | 41 | 3 | 16 | 619134 | 4953068 | 3 | 16 | 0 | 224 | 5087964 | 3 | 16 | 0 | 1 | 232 |
| 212 | D4 | 65 | 41 | 3 | 17 | 619162 | 4953292 | 3 | 17 | 0 | 224 | 5088196 | 3 | 17 | 0 | 1 | 232 |
| 213 | D5 | 65 | 41 | 3 | 18 | 619190 | 4953516 | 3 | 18 | 0 | 224 | 5088428 | 3 | 18 | 0 | 1 | 232 |
| 214 | D6 | 65 | 41 | 3 | 19 | 619218 | 4953740 | 3 | 19 | 0 | 224 | 5088660 | 3 | 19 | 0 | 1 | 232 |
| 215 | D7 | 65 | 41 | 3 | 20 | 619246 | 4953964 | 3 | 20 | 0 | 224 | 5088892 | 3 | 20 | 0 | 1 | 232 |
| 216 | D8 | 65 | 41 | 3 | 21 | 619274 | 4954188 | 3 | 21 | 0 | 224 | 5089124 | 3 | 21 | 0 | 1 | 232 |
| 217 | D9 | 65 | 41 | 3 | 22 | 619302 | 4954412 | 3 | 22 | 0 | 224 | 5089356 | 3 | 22 | 0 | 1 | 232 |
| 218 | DA | 65 | 41 | 3 | 23 | 619330 | 4954636 | 3 | 23 | 0 | 224 | 5089588 | 3 | 23 | 0 | 1 | 232 |
| 219 | DB | 65 | 41 | 3 | 24 | 619358 | 4954860 | 3 | 24 | 0 | 224 | 5089820 | 3 | 24 | 0 | 1 | 232 |
| 220 | DC | 65 | 41 | 3 | 25 | 619386 | 4955084 | 3 | 25 | 0 | 224 | 5090052 | 3 | 25 | 0 | 1 | 232 |
| 221 | DD | 65 | 41 | 3 | 26 | 619414 | 4955308 | 3 | 26 | 0 | 224 | 5090284 | 3 | 26 | 0 | 1 | 232 |
| 222 | DE | 65 | 41 | 3 | 27 | 619442 | 4955532 | 3 | 27 | 0 | 224 | 5090516 | 3 | 27 | 0 | 1 | 232 |
| 223 | DF | 65 | 41 | 3 | 28 | 619470 | 4955756 | 3 | 28 | 0 | 224 | 5090748 | 3 | 28 | 0 | 1 | 232 |
| 224 | E0 | 65 | 41 | 3 | 29 | 619498 | 4955980 | 3 | 29 | 0 | 224 | 5090980 | 3 | 29 | 0 | 1 | 232 |
| 225 | E1 | 65 | 41 | 3 | 30 | 619526 | 4956204 | 3 | 30 | 0 | 224 | 5091212 | 3 | 30 | 0 | 1 | 232 |
| 226 | E2 | 65 | 41 | 3 | 31 | 619554 | 4956428 | 3 | 31 | 0 | 224 | 5091444 | 3 | 31 | 0 | 1 | 232 |
| 227 | E3 | 65 | 41 | 3 | 32 | 619582 | 4956652 | 3 | 32 | 0 | 224 | 5091676 | 3 | 32 | 0 | 1 | 232 |
| 228 | E4 | 65 | 41 | 3 | 33 | 619610 | 4956876 | 3 | 33 | 0 | 224 | 5091908 | 3 | 33 | 0 | 1 | 232 |
| 229 | E5 | 65 | 41 | 3 | 34 | 619638 | 4957100 | 3 | 34 | 0 | 224 | 5092140 | 3 | 34 | 0 | 1 | 232 |
| 230 | E6 | 65 | 41 | 3 | 35 | 619666 | 4957324 | 3 | 35 | 0 | 224 | 5092372 | 3 | 35 | 0 | 1 | 232 |
| 231 | E7 | 65 | 41 | 3 | 36 | 619694 | 4957548 | 3 | 36 | 0 | 224 | 5092604 | 3 | 36 | 0 | 1 | 232 |
| 232 | E8 | 65 | 41 | 3 | 37 | 619722 | 4957772 | 3 | 37 | 0 | 224 | 5092836 | 3 | 37 | 0 | 1 | 232 |
| 233 | E9 | 65 | 41 | 3 | 38 | 619750 | 4957996 | 3 | 38 | 0 | 224 | 5093068 | 3 | 38 | 0 | 1 | 232 |
| 234 | EA | 65 | 41 | 3 | 39 | 619778 | 4958220 | 3 | 39 | 0 | 224 | 5093300 | 3 | 39 | 0 | 1 | 232 |
| 235 | EB | 65 | 41 | 3 | 40 | 619806 | 4958444 | 3 | 40 | 0 | 224 | 5093532 | 3 | 40 | 0 | 1 | 232 |
| 236 | EC | 65 | 41 | 3 | 41 | 619834 | 4958668 | 3 | 41 | 0 | 224 | 5093764 | 3 | 41 | 0 | 1 | 232 |
| 237 | ED | 65 | 41 | 3 | 42 | 619862 | 4958892 | 3 | 42 | 0 | 224 | 5093996 | 3 | 42 | 0 | 1 | 232 |
| 238 | EE | 65 | 41 | 3 | 43 | 619890 | 4959116 | 3 | 43 | 0 | 224 | 5094228 | 3 | 43 | 0 | 1 | 232 |
| 239 | EF | 65 | 41 | 3 | 44 | 619918 | 4959340 | 3 | 44 | 0 | 224 | 5094460 | 3 | 44 | 0 | 1 | 232 |
| 240 | F0 | 65 | 41 | 3 | 45 | 619943 | 4959540 | 3 | 45 | 0 | 200 | 5094668 | 3 | 45 | 0 | 1 | 208 |
| 241 | F1 | 65 | 41 | 3 | 46 | 619968 | 4959740 | 3 | 46 | 0 | 200 | 5094876 | 3 | 46 | 0 | 1 | 208 |
| 242 | F2 | 65 | 41 | 3 | 47 | 619993 | 4959940 | 3 | 47 | 0 | 200 | 5095084 | 3 | 47 | 0 | 1 | 208 |
| 243 | F3 | 65 | 41 | 3 | 48 | 620018 | 4960140 | 3 | 48 | 0 | 200 | 5095292 | 3 | 48 | 0 | 1 | 208 |
| 244 | F4 | 65 | 41 | 3 | 49 | 620043 | 4960340 | 3 | 49 | 0 | 200 | 5095500 | 3 | 49 | 0 | 1 | 208 |
| 245 | F5 | 65 | 41 | 3 | 50 | 620068 | 4960540 | 3 | 50 | 0 | 200 | 5095708 | 3 | 50 | 0 | 1 | 208 |
| 246 | F6 | 65 | 41 | 3 | 51 | 620093 | 4960740 | 3 | 51 | 0 | 200 | 5095916 | 3 | 51 | 0 | 1 | 208 |
| 247 | F7 | 65 | 41 | 3 | 52 | 620118 | 4960940 | 3 | 52 | 0 | 200 | 5096124 | 3 | 52 | 0 | 1 | 208 |
| 248 | F8 | 65 | 41 | 3 | 53 | 620143 | 4961140 | 3 | 53 | 0 | 200 | 5096332 | 3 | 53 | 0 | 1 | 208 |

| | | | | | | | | | | | | | | | | | |
|---|---|---|---|---|---|---|---|---|---|---|---|---|---|---|---|---|---|
| 249 | F9 | 65 | 41 | 3 | 54 | 620168 | 4961340 | 3 | 54 | 0 | 200 | 5096540 | 3 | 54 | 0 | 1 | 208 |
| 250 | FA | 65 | 41 | 3 | 55 | 620193 | 4961540 | 3 | 55 | 0 | 200 | 5096748 | 3 | 55 | 0 | 1 | 208 |
| 251 | FB | 65 | 41 | 3 | 56 | 620218 | 4961740 | 3 | 56 | 0 | 200 | 5096956 | 3 | 56 | 0 | 1 | 208 |
| 252 | FC | 65 | 41 | 3 | 57 | 620243 | 4961940 | 3 | 57 | 0 | 200 | 5097164 | 3 | 57 | 0 | 1 | 208 |
| 253 | FD | 65 | 41 | 3 | 58 | 620268 | 4962140 | 3 | 58 | 0 | 200 | 5097372 | 3 | 58 | 0 | 1 | 208 |
| 254 | FE | 65 | 41 | 3 | 59 | 620293 | 4962340 | 3 | 59 | 0 | 200 | 5097580 | 3 | 59 | 0 | 1 | 208 |
| 255 | FF | 65 | 41 | 3 | 60 | 620318 | 4962540 | 3 | 60 | 0 | 200 | 5097788 | 3 | 60 | 0 | 1 | 208 |
| 0 | 00 | 66 | 42 | 0 | 0 | 620330 | 4962636 | 0 | 0 | 0 | 96 | 5097892 | 0 | 0 | 0 | 1 | 104 |
| 1 | 01 | 66 | 42 | 0 | 1 | 620344 | 4962748 | 0 | 1 | 0 | 112 | 5098012 | 0 | 1 | 0 | 1 | 120 |
| 2 | 02 | 66 | 42 | 0 | 2 | 620358 | 4962860 | 0 | 2 | 0 | 112 | 5098132 | 0 | 2 | 0 | 1 | 120 |
| 3 | 03 | 66 | 42 | 0 | 3 | 620372 | 4962972 | 0 | 3 | 0 | 112 | 5098252 | 0 | 3 | 0 | 1 | 120 |
| 4 | 04 | 66 | 42 | 0 | 4 | 620386 | 4963084 | 0 | 4 | 0 | 112 | 5098372 | 0 | 4 | 0 | 1 | 120 |
| 5 | 05 | 66 | 42 | 0 | 5 | 620400 | 4963196 | 0 | 5 | 0 | 112 | 5098492 | 0 | 5 | 0 | 1 | 120 |
| 6 | 06 | 66 | 42 | 0 | 6 | 620414 | 4963308 | 0 | 6 | 0 | 112 | 5098612 | 0 | 6 | 0 | 1 | 120 |
| 7 | 07 | 66 | 42 | 0 | 7 | 620428 | 4963420 | 0 | 7 | 0 | 112 | 5098732 | 0 | 7 | 0 | 1 | 120 |
| 8 | 08 | 66 | 42 | 0 | 8 | 620442 | 4963532 | 0 | 8 | 0 | 112 | 5098852 | 0 | 8 | 0 | 1 | 120 |
| 9 | 09 | 66 | 42 | 0 | 9 | 620456 | 4963644 | 0 | 9 | 0 | 112 | 5098972 | 0 | 9 | 0 | 1 | 120 |
| 10 | 0A | 66 | 42 | 0 | 10 | 620470 | 4963756 | 0 | 10 | 0 | 112 | 5099092 | 0 | 10 | 0 | 1 | 120 |
| 11 | 0B | 66 | 42 | 0 | 11 | 620484 | 4963868 | 0 | 11 | 0 | 112 | 5099212 | 0 | 11 | 0 | 1 | 120 |
| 12 | 0C | 66 | 42 | 0 | 12 | 620498 | 4963980 | 0 | 12 | 0 | 112 | 5099332 | 0 | 12 | 0 | 1 | 120 |
| 13 | 0D | 66 | 42 | 0 | 13 | 620512 | 4964092 | 0 | 13 | 0 | 112 | 5099452 | 0 | 13 | 0 | 1 | 120 |
| 14 | 0E | 66 | 42 | 0 | 14 | 620526 | 4964204 | 0 | 14 | 0 | 112 | 5099572 | 0 | 14 | 0 | 1 | 120 |
| 15 | 0F | 66 | 42 | 0 | 15 | 620540 | 4964316 | 0 | 15 | 0 | 112 | 5099692 | 0 | 15 | 0 | 1 | 120 |
| 16 | 10 | 66 | 42 | 0 | 16 | 620554 | 4964428 | 0 | 16 | 0 | 112 | 5099812 | 0 | 16 | 0 | 1 | 120 |
| 17 | 11 | 66 | 42 | 0 | 17 | 620568 | 4964540 | 0 | 17 | 0 | 112 | 5099932 | 0 | 17 | 0 | 1 | 120 |
| 18 | 12 | 66 | 42 | 0 | 18 | 620582 | 4964652 | 0 | 18 | 0 | 112 | 5100052 | 0 | 18 | 0 | 1 | 120 |
| 19 | 13 | 66 | 42 | 0 | 19 | 620596 | 4964764 | 0 | 19 | 0 | 112 | 5100172 | 0 | 19 | 0 | 1 | 120 |
| 20 | 14 | 66 | 42 | 0 | 20 | 620610 | 4964876 | 0 | 20 | 0 | 112 | 5100292 | 0 | 20 | 0 | 1 | 120 |
| 21 | 15 | 66 | 42 | 0 | 21 | 620624 | 4964988 | 0 | 21 | 0 | 112 | 5100412 | 0 | 21 | 0 | 1 | 120 |
| 22 | 16 | 66 | 42 | 0 | 22 | 620638 | 4965100 | 0 | 22 | 0 | 112 | 5100532 | 0 | 22 | 0 | 1 | 120 |
| 23 | 17 | 66 | 42 | 0 | 23 | 620652 | 4965212 | 0 | 23 | 0 | 112 | 5100652 | 0 | 23 | 0 | 1 | 120 |
| 24 | 18 | 66 | 42 | 0 | 24 | 620666 | 4965324 | 0 | 24 | 0 | 112 | 5100772 | 0 | 24 | 0 | 1 | 120 |
| 25 | 19 | 66 | 42 | 0 | 25 | 620680 | 4965436 | 0 | 25 | 0 | 112 | 5100892 | 0 | 25 | 0 | 1 | 120 |
| 26 | 1A | 66 | 42 | 0 | 26 | 620694 | 4965548 | 0 | 26 | 0 | 112 | 5101012 | 0 | 26 | 0 | 1 | 120 |
| 27 | 1B | 66 | 42 | 0 | 27 | 620708 | 4965660 | 0 | 27 | 0 | 112 | 5101132 | 0 | 27 | 0 | 1 | 120 |
| 28 | 1C | 66 | 42 | 0 | 28 | 620722 | 4965772 | 0 | 28 | 0 | 112 | 5101252 | 0 | 28 | 0 | 1 | 120 |
| 29 | 1D | 66 | 42 | 0 | 29 | 620736 | 4965884 | 0 | 29 | 0 | 112 | 5101372 | 0 | 29 | 0 | 1 | 120 |
| 30 | 1E | 66 | 42 | 0 | 30 | 620750 | 4965996 | 0 | 30 | 0 | 112 | 5101492 | 0 | 30 | 0 | 1 | 120 |
| 31 | 1F | 66 | 42 | 0 | 31 | 620764 | 4966108 | 0 | 31 | 0 | 112 | 5101612 | 0 | 31 | 0 | 1 | 120 |
| 32 | 20 | 66 | 42 | 0 | 32 | 620778 | 4966220 | 0 | 32 | 0 | 112 | 5101732 | 0 | 32 | 0 | 1 | 120 |
| 33 | 21 | 66 | 42 | 0 | 33 | 620792 | 4966332 | 0 | 33 | 0 | 112 | 5101852 | 0 | 33 | 0 | 1 | 120 |
| 34 | 22 | 66 | 42 | 0 | 34 | 620806 | 4966444 | 0 | 34 | 0 | 112 | 5101972 | 0 | 34 | 0 | 1 | 120 |
| 35 | 23 | 66 | 42 | 0 | 35 | 620820 | 4966556 | 0 | 35 | 0 | 112 | 5102092 | 0 | 35 | 0 | 1 | 120 |
| 36 | 24 | 66 | 42 | 0 | 36 | 620834 | 4966668 | 0 | 36 | 0 | 112 | 5102212 | 0 | 36 | 0 | 1 | 120 |
| 37 | 25 | 66 | 42 | 0 | 37 | 620848 | 4966780 | 0 | 37 | 0 | 112 | 5102332 | 0 | 37 | 0 | 1 | 120 |
| 38 | 26 | 66 | 42 | 0 | 38 | 620862 | 4966892 | 0 | 38 | 0 | 112 | 5102452 | 0 | 38 | 0 | 1 | 120 |
| 39 | 27 | 66 | 42 | 0 | 39 | 620876 | 4967004 | 0 | 39 | 0 | 112 | 5102572 | 0 | 39 | 0 | 1 | 120 |
| 40 | 28 | 66 | 42 | 0 | 40 | 620890 | 4967116 | 0 | 40 | 0 | 112 | 5102692 | 0 | 40 | 0 | 1 | 120 |
| 41 | 29 | 66 | 42 | 0 | 41 | 620904 | 4967228 | 0 | 41 | 0 | 112 | 5102812 | 0 | 41 | 0 | 1 | 120 |
| 42 | 2A | 66 | 42 | 0 | 42 | 620918 | 4967340 | 0 | 42 | 0 | 112 | 5102932 | 0 | 42 | 0 | 1 | 120 |
| 43 | 2B | 66 | 42 | 0 | 43 | 620932 | 4967452 | 0 | 43 | 0 | 112 | 5103052 | 0 | 43 | 0 | 1 | 120 |
| 44 | 2C | 66 | 42 | 0 | 44 | 620946 | 4967564 | 0 | 44 | 0 | 112 | 5103172 | 0 | 44 | 0 | 1 | 120 |
| 45 | 2D | 66 | 42 | 0 | 45 | 620960 | 4967676 | 0 | 45 | 0 | 112 | 5103292 | 0 | 45 | 0 | 1 | 120 |
| 46 | 2E | 66 | 42 | 0 | 46 | 620974 | 4967788 | 0 | 46 | 0 | 112 | 5103412 | 0 | 46 | 0 | 1 | 120 |
| 47 | 2F | 66 | 42 | 0 | 47 | 620988 | 4967900 | 0 | 47 | 0 | 112 | 5103532 | 0 | 47 | 0 | 1 | 120 |
| 48 | 30 | 66 | 42 | 0 | 48 | 621002 | 4968012 | 0 | 48 | 0 | 112 | 5103652 | 0 | 48 | 0 | 1 | 120 |
| 49 | 31 | 66 | 42 | 0 | 49 | 621016 | 4968124 | 0 | 49 | 0 | 112 | 5103772 | 0 | 49 | 0 | 1 | 120 |
| 50 | 32 | 66 | 42 | 0 | 50 | 621030 | 4968236 | 0 | 50 | 0 | 112 | 5103892 | 0 | 50 | 0 | 1 | 120 |
| 51 | 33 | 66 | 42 | 0 | 51 | 621044 | 4968348 | 0 | 51 | 0 | 112 | 5104012 | 0 | 51 | 0 | 1 | 120 |
| 52 | 34 | 66 | 42 | 0 | 52 | 621058 | 4968460 | 0 | 52 | 0 | 112 | 5104132 | 0 | 52 | 0 | 1 | 120 |
| 53 | 35 | 66 | 42 | 0 | 53 | 621072 | 4968572 | 0 | 53 | 0 | 112 | 5104252 | 0 | 53 | 0 | 1 | 120 |
| 54 | 36 | 66 | 42 | 0 | 54 | 621086 | 4968684 | 0 | 54 | 0 | 112 | 5104372 | 0 | 54 | 0 | 1 | 120 |
| 55 | 37 | 66 | 42 | 0 | 55 | 621100 | 4968796 | 0 | 55 | 0 | 112 | 5104492 | 0 | 55 | 0 | 1 | 120 |
| 56 | 38 | 66 | 42 | 0 | 56 | 621114 | 4968908 | 0 | 56 | 0 | 112 | 5104612 | 0 | 56 | 0 | 1 | 120 |
| 57 | 39 | 66 | 42 | 0 | 57 | 621128 | 4969020 | 0 | 57 | 0 | 112 | 5104732 | 0 | 57 | 0 | 1 | 120 |
| 58 | 3A | 66 | 42 | 0 | 58 | 621142 | 4969132 | 0 | 58 | 0 | 112 | 5104852 | 0 | 58 | 0 | 1 | 120 |
| 59 | 3B | 66 | 42 | 0 | 59 | 621156 | 4969244 | 0 | 59 | 0 | 112 | 5104972 | 0 | 59 | 0 | 1 | 120 |
| 60 | 3C | 66 | 42 | 0 | 60 | 621170 | 4969356 | 0 | 60 | 0 | 112 | 5105092 | 0 | 60 | 0 | 1 | 120 |
| 61 | 3D | 66 | 42 | 0 | 61 | 621184 | 4969468 | 0 | 61 | 0 | 112 | 5105212 | 0 | 61 | 0 | 1 | 120 |
| 62 | 3E | 66 | 42 | 0 | 62 | 621198 | 4969580 | 0 | 62 | 0 | 112 | 5105332 | 0 | 62 | 0 | 1 | 120 |
| 63 | 3F | 66 | 42 | 0 | 63 | 621212 | 4969692 | 0 | 63 | 0 | 112 | 5105452 | 0 | 63 | 0 | 1 | 120 |
| 64 | 40 | 66 | 42 | 0 | 64 | 621226 | 4969804 | 0 | 64 | 0 | 112 | 5105572 | 0 | 64 | 0 | 1 | 120 |
| 65 | 41 | 66 | 42 | 0 | 65 | 621240 | 4969916 | 0 | 65 | 0 | 112 | 5105692 | 0 | 65 | 0 | 1 | 120 |
| 66 | 42 | 66 | 42 | 1 | 0 | 621252 | 4970012 | 1 | 0 | 0 | 96 | 5105796 | 1 | 0 | 0 | 1 | 104 |
| 67 | 43 | 66 | 42 | 1 | 1 | 621269 | 4970148 | 1 | 1 | 0 | 136 | 5105940 | 1 | 1 | 0 | 1 | 144 |
| 68 | 44 | 66 | 42 | 1 | 2 | 621286 | 4970284 | 1 | 2 | 0 | 136 | 5106084 | 1 | 2 | 0 | 1 | 144 |
| 69 | 45 | 66 | 42 | 1 | 3 | 621303 | 4970420 | 1 | 3 | 0 | 136 | 5106228 | 1 | 3 | 0 | 1 | 144 |
| 70 | 46 | 66 | 42 | 1 | 4 | 621320 | 4970556 | 1 | 4 | 0 | 136 | 5106372 | 1 | 4 | 0 | 1 | 144 |
| 71 | 47 | 66 | 42 | 1 | 5 | 621337 | 4970692 | 1 | 5 | 0 | 136 | 5106516 | 1 | 5 | 0 | 1 | 144 |
| 72 | 48 | 66 | 42 | 1 | 6 | 621354 | 4970828 | 1 | 6 | 0 | 136 | 5106660 | 1 | 6 | 0 | 1 | 144 |
| 73 | 49 | 66 | 42 | 1 | 7 | 621371 | 4970964 | 1 | 7 | 0 | 136 | 5106804 | 1 | 7 | 0 | 1 | 144 |
| 74 | 4A | 66 | 42 | 1 | 8 | 621388 | 4971100 | 1 | 8 | 0 | 136 | 5106948 | 1 | 8 | 0 | 1 | 144 |
| 75 | 4B | 66 | 42 | 1 | 9 | 621405 | 4971236 | 1 | 9 | 0 | 136 | 5107092 | 1 | 9 | 0 | 1 | 144 |
| 76 | 4C | 66 | 42 | 1 | 10 | 621422 | 4971372 | 1 | 10 | 0 | 136 | 5107236 | 1 | 10 | 0 | 1 | 144 |
| 77 | 4D | 66 | 42 | 1 | 11 | 621439 | 4971508 | 1 | 11 | 0 | 136 | 5107380 | 1 | 11 | 0 | 1 | 144 |
| 78 | 4E | 66 | 42 | 1 | 12 | 621456 | 4971644 | 1 | 12 | 0 | 136 | 5107524 | 1 | 12 | 0 | 1 | 144 |
| 79 | 4F | 66 | 42 | 1 | 13 | 621473 | 4971780 | 1 | 13 | 0 | 136 | 5107668 | 1 | 13 | 0 | 1 | 144 |
| 80 | 50 | 66 | 42 | 1 | 14 | 621494 | 4971948 | 1 | 14 | 0 | 168 | 5107844 | 1 | 14 | 0 | 1 | 176 |
| 81 | 51 | 66 | 42 | 1 | 15 | 621515 | 4972116 | 1 | 15 | 0 | 168 | 5108020 | 1 | 15 | 0 | 1 | 176 |
| 82 | 52 | 66 | 42 | 1 | 16 | 621536 | 4972284 | 1 | 16 | 0 | 168 | 5108196 | 1 | 16 | 0 | 1 | 176 |
| 83 | 53 | 66 | 42 | 1 | 17 | 621557 | 4972452 | 1 | 17 | 0 | 168 | 5108372 | 1 | 17 | 0 | 1 | 176 |
| 84 | 54 | 66 | 42 | 1 | 18 | 621578 | 4972620 | 1 | 18 | 0 | 168 | 5108548 | 1 | 18 | 0 | 1 | 176 |
| 85 | 55 | 66 | 42 | 1 | 19 | 621599 | 4972788 | 1 | 19 | 0 | 168 | 5108724 | 1 | 19 | 0 | 1 | 176 |
| 86 | 56 | 66 | 42 | 1 | 20 | 621620 | 4972956 | 1 | 20 | 0 | 168 | 5108900 | 1 | 20 | 0 | 1 | 176 |
| 87 | 57 | 66 | 42 | 1 | 21 | 621641 | 4973124 | 1 | 21 | 0 | 168 | 5109076 | 1 | 21 | 0 | 1 | 176 |
| 88 | 58 | 66 | 42 | 1 | 22 | 621662 | 4973292 | 1 | 22 | 0 | 168 | 5109252 | 1 | 22 | 0 | 1 | 176 |
| 89 | 59 | 66 | 42 | 1 | 23 | 621683 | 4973460 | 1 | 23 | 0 | 168 | 5109428 | 1 | 23 | 0 | 1 | 176 |
| 90 | 5A | 66 | 42 | 1 | 24 | 621704 | 4973628 | 1 | 24 | 0 | 168 | 5109604 | 1 | 24 | 0 | 1 | 176 |
| 91 | 5B | 66 | 42 | 1 | 25 | 621725 | 4973796 | 1 | 25 | 0 | 168 | 5109780 | 1 | 25 | 0 | 1 | 176 |
| 92 | 5C | 66 | 42 | 1 | 26 | 621746 | 4973964 | 1 | 26 | 0 | 168 | 5109956 | 1 | 26 | 0 | 1 | 176 |
| 93 | 5D | 66 | 42 | 1 | 27 | 621767 | 4974132 | 1 | 27 | 0 | 168 | 5110132 | 1 | 27 | 0 | 1 | 176 |
| 94 | 5E | 66 | 42 | 1 | 28 | 621788 | 4974300 | 1 | 28 | 0 | 168 | 5110308 | 1 | 28 | 0 | 1 | 176 |
| 95 | 5F | 66 | 42 | 1 | 29 | 621809 | 4974468 | 1 | 29 | 0 | 168 | 5110484 | 1 | 29 | 0 | 1 | 176 |

| | | | | | | | | | | | | | | | | | |
|---|---|---|---|---|---|---|---|---|---|---|---|---|---|---|---|---|---|
| 96 | 60 | 66 | 42 | 1 | 30 | 621832 | 4974652 | 1 | 30 | 0 | 184 | 5110676 | 1 | 30 | 0 | 1 | 192 |
| 97 | 61 | 66 | 42 | 1 | 31 | 621855 | 4974836 | 1 | 31 | 0 | 184 | 5110868 | 1 | 31 | 0 | 1 | 192 |
| 98 | 62 | 66 | 42 | 1 | 32 | 621878 | 4975020 | 1 | 32 | 0 | 184 | 5111060 | 1 | 32 | 0 | 1 | 192 |
| 99 | 63 | 66 | 42 | 1 | 33 | 621901 | 4975204 | 1 | 33 | 0 | 184 | 5111252 | 1 | 33 | 0 | 1 | 192 |
| 100 | 64 | 66 | 42 | 1 | 34 | 621924 | 4975388 | 1 | 34 | 0 | 184 | 5111444 | 1 | 34 | 0 | 1 | 192 |
| 101 | 65 | 66 | 42 | 1 | 35 | 621947 | 4975572 | 1 | 35 | 0 | 184 | 5111636 | 1 | 35 | 0 | 1 | 192 |
| 102 | 66 | 66 | 42 | 1 | 36 | 621970 | 4975756 | 1 | 36 | 0 | 184 | 5111828 | 1 | 36 | 0 | 1 | 192 |
| 103 | 67 | 66 | 42 | 1 | 37 | 621993 | 4975940 | 1 | 37 | 0 | 184 | 5112020 | 1 | 37 | 0 | 1 | 192 |
| 104 | 68 | 66 | 42 | 1 | 38 | 622016 | 4976124 | 1 | 38 | 0 | 184 | 5112212 | 1 | 38 | 0 | 1 | 192 |
| 105 | 69 | 66 | 42 | 1 | 39 | 622039 | 4976308 | 1 | 39 | 0 | 184 | 5112404 | 1 | 39 | 0 | 1 | 192 |
| 106 | 6A | 66 | 42 | 1 | 40 | 622062 | 4976492 | 1 | 40 | 0 | 184 | 5112596 | 1 | 40 | 0 | 1 | 192 |
| 107 | 6B | 66 | 42 | 1 | 41 | 622085 | 4976676 | 1 | 41 | 0 | 184 | 5112788 | 1 | 41 | 0 | 1 | 192 |
| 108 | 6C | 66 | 42 | 1 | 42 | 622108 | 4976860 | 1 | 42 | 0 | 184 | 5112980 | 1 | 42 | 0 | 1 | 192 |
| 109 | 6D | 66 | 42 | 1 | 43 | 622131 | 4977044 | 1 | 43 | 0 | 184 | 5113172 | 1 | 43 | 0 | 1 | 192 |
| 110 | 6E | 66 | 42 | 1 | 44 | 622154 | 4977228 | 1 | 44 | 0 | 184 | 5113364 | 1 | 44 | 0 | 1 | 192 |
| 111 | 6F | 66 | 42 | 1 | 45 | 622177 | 4977412 | 1 | 45 | 0 | 184 | 5113556 | 1 | 45 | 0 | 1 | 192 |
| 112 | 70 | 66 | 42 | 1 | 46 | 622200 | 4977596 | 1 | 46 | 0 | 184 | 5113748 | 1 | 46 | 0 | 1 | 192 |
| 113 | 71 | 66 | 42 | 1 | 47 | 622223 | 4977780 | 1 | 47 | 0 | 184 | 5113940 | 1 | 47 | 0 | 1 | 192 |
| 114 | 72 | 66 | 42 | 1 | 48 | 622246 | 4977964 | 1 | 48 | 0 | 184 | 5114132 | 1 | 48 | 0 | 1 | 192 |
| 115 | 73 | 66 | 42 | 1 | 49 | 622269 | 4978148 | 1 | 49 | 0 | 184 | 5114324 | 1 | 49 | 0 | 1 | 192 |
| 116 | 74 | 66 | 42 | 1 | 50 | 622292 | 4978332 | 1 | 50 | 0 | 184 | 5114516 | 1 | 50 | 0 | 1 | 192 |
| 117 | 75 | 66 | 42 | 1 | 51 | 622315 | 4978516 | 1 | 51 | 0 | 184 | 5114708 | 1 | 51 | 0 | 1 | 192 |
| 118 | 76 | 66 | 42 | 1 | 52 | 622338 | 4978700 | 1 | 52 | 0 | 184 | 5114900 | 1 | 52 | 0 | 1 | 192 |
| 119 | 77 | 66 | 42 | 1 | 53 | 622361 | 4978884 | 1 | 53 | 0 | 184 | 5115092 | 1 | 53 | 0 | 1 | 192 |
| 120 | 78 | 66 | 42 | 1 | 54 | 622384 | 4979068 | 1 | 54 | 0 | 184 | 5115284 | 1 | 54 | 0 | 1 | 192 |
| 121 | 79 | 66 | 42 | 1 | 55 | 622407 | 4979252 | 1 | 55 | 0 | 184 | 5115476 | 1 | 55 | 0 | 1 | 192 |
| 122 | 7A | 66 | 42 | 1 | 56 | 622430 | 4979436 | 1 | 56 | 0 | 184 | 5115668 | 1 | 56 | 0 | 1 | 192 |
| 123 | 7B | 66 | 42 | 1 | 57 | 622453 | 4979620 | 1 | 57 | 0 | 184 | 5115860 | 1 | 57 | 0 | 1 | 192 |
| 124 | 7C | 66 | 42 | 1 | 58 | 622476 | 4979804 | 1 | 58 | 0 | 184 | 5116052 | 1 | 58 | 0 | 1 | 192 |
| 125 | 7D | 66 | 42 | 1 | 59 | 622499 | 4979988 | 1 | 59 | 0 | 184 | 5116244 | 1 | 59 | 0 | 1 | 192 |
| 126 | 7E | 66 | 42 | 1 | 60 | 622522 | 4980172 | 1 | 60 | 0 | 184 | 5116436 | 1 | 60 | 0 | 1 | 192 |
| 127 | 7F | 66 | 42 | 1 | 61 | 622545 | 4980356 | 1 | 61 | 0 | 184 | 5116628 | 1 | 61 | 0 | 1 | 192 |
| 128 | 80 | 66 | 42 | 1 | 62 | 622568 | 4980540 | 1 | 62 | 0 | 184 | 5116820 | 1 | 62 | 0 | 1 | 192 |
| 129 | 81 | 66 | 42 | 1 | 63 | 622591 | 4980724 | 1 | 63 | 0 | 184 | 5117012 | 1 | 63 | 0 | 1 | 192 |
| 130 | 82 | 66 | 42 | 1 | 64 | 622614 | 4980908 | 1 | 64 | 0 | 184 | 5117204 | 1 | 64 | 0 | 1 | 192 |
| 131 | 83 | 66 | 42 | 1 | 65 | 622637 | 4981092 | 1 | 65 | 0 | 184 | 5117396 | 1 | 65 | 0 | 1 | 192 |
| 132 | 84 | 66 | 42 | 2 | 0 | 622658 | 4981260 | 2 | 0 | 0 | 168 | 5117572 | 2 | 0 | 0 | 1 | 176 |
| 133 | 85 | 66 | 42 | 2 | 1 | 622684 | 4981468 | 2 | 1 | 0 | 208 | 5117788 | 2 | 1 | 0 | 1 | 216 |
| 134 | 86 | 66 | 42 | 2 | 2 | 622710 | 4981676 | 2 | 2 | 0 | 208 | 5118004 | 2 | 2 | 0 | 1 | 216 |
| 135 | 87 | 66 | 42 | 2 | 3 | 622736 | 4981884 | 2 | 3 | 0 | 208 | 5118220 | 2 | 3 | 0 | 1 | 216 |
| 136 | 88 | 66 | 42 | 2 | 4 | 622762 | 4982092 | 2 | 4 | 0 | 208 | 5118436 | 2 | 4 | 0 | 1 | 216 |
| 137 | 89 | 66 | 42 | 2 | 5 | 622788 | 4982300 | 2 | 5 | 0 | 208 | 5118652 | 2 | 5 | 0 | 1 | 216 |
| 138 | 8A | 66 | 42 | 2 | 6 | 622814 | 4982508 | 2 | 6 | 0 | 208 | 5118868 | 2 | 6 | 0 | 1 | 216 |
| 139 | 8B | 66 | 42 | 2 | 7 | 622840 | 4982716 | 2 | 7 | 0 | 208 | 5119084 | 2 | 7 | 0 | 1 | 216 |
| 140 | 8C | 66 | 42 | 2 | 8 | 622866 | 4982924 | 2 | 8 | 0 | 208 | 5119300 | 2 | 8 | 0 | 1 | 216 |
| 141 | 8D | 66 | 42 | 2 | 9 | 622892 | 4983132 | 2 | 9 | 0 | 208 | 5119516 | 2 | 9 | 0 | 1 | 216 |
| 142 | 8E | 66 | 42 | 2 | 10 | 622918 | 4983340 | 2 | 10 | 0 | 208 | 5119732 | 2 | 10 | 0 | 1 | 216 |
| 143 | 8F | 66 | 42 | 2 | 11 | 622944 | 4983548 | 2 | 11 | 0 | 208 | 5119948 | 2 | 11 | 0 | 1 | 216 |
| 144 | 90 | 66 | 42 | 2 | 12 | 622970 | 4983756 | 2 | 12 | 0 | 208 | 5120164 | 2 | 12 | 0 | 1 | 216 |
| 145 | 91 | 66 | 42 | 2 | 13 | 622996 | 4983964 | 2 | 13 | 0 | 208 | 5120380 | 2 | 13 | 0 | 1 | 216 |
| 146 | 92 | 66 | 42 | 2 | 14 | 623022 | 4984172 | 2 | 14 | 0 | 208 | 5120596 | 2 | 14 | 0 | 1 | 216 |
| 147 | 93 | 66 | 42 | 2 | 15 | 623048 | 4984380 | 2 | 15 | 0 | 208 | 5120812 | 2 | 15 | 0 | 1 | 216 |
| 148 | 94 | 66 | 42 | 2 | 16 | 623074 | 4984588 | 2 | 16 | 0 | 208 | 5121028 | 2 | 16 | 0 | 1 | 216 |
| 149 | 95 | 66 | 42 | 2 | 17 | 623100 | 4984796 | 2 | 17 | 0 | 208 | 5121244 | 2 | 17 | 0 | 1 | 216 |
| 150 | 96 | 66 | 42 | 2 | 18 | 623126 | 4985004 | 2 | 18 | 0 | 208 | 5121460 | 2 | 18 | 0 | 1 | 216 |
| 151 | 97 | 66 | 42 | 2 | 19 | 623152 | 4985212 | 2 | 19 | 0 | 208 | 5121676 | 2 | 19 | 0 | 1 | 216 |
| 152 | 98 | 66 | 42 | 2 | 20 | 623178 | 4985420 | 2 | 20 | 0 | 208 | 5121892 | 2 | 20 | 0 | 1 | 216 |
| 153 | 99 | 66 | 42 | 2 | 21 | 623204 | 4985628 | 2 | 21 | 0 | 208 | 5122108 | 2 | 21 | 0 | 1 | 216 |
| 154 | 9A | 66 | 42 | 2 | 22 | 623230 | 4985836 | 2 | 22 | 0 | 208 | 5122324 | 2 | 22 | 0 | 1 | 216 |
| 155 | 9B | 66 | 42 | 2 | 23 | 623256 | 4986044 | 2 | 23 | 0 | 208 | 5122540 | 2 | 23 | 0 | 1 | 216 |
| 156 | 9C | 66 | 42 | 2 | 24 | 623282 | 4986252 | 2 | 24 | 0 | 208 | 5122756 | 2 | 24 | 0 | 1 | 216 |
| 157 | 9D | 66 | 42 | 2 | 25 | 623308 | 4986460 | 2 | 25 | 0 | 208 | 5122972 | 2 | 25 | 0 | 1 | 216 |
| 158 | 9E | 66 | 42 | 2 | 26 | 623334 | 4986668 | 2 | 26 | 0 | 208 | 5123188 | 2 | 26 | 0 | 1 | 216 |
| 159 | 9F | 66 | 42 | 2 | 27 | 623360 | 4986876 | 2 | 27 | 0 | 208 | 5123404 | 2 | 27 | 0 | 1 | 216 |
| 160 | A0 | 66 | 42 | 2 | 28 | 623383 | 4987060 | 2 | 28 | 0 | 184 | 5123596 | 2 | 28 | 0 | 1 | 192 |
| 161 | A1 | 66 | 42 | 2 | 29 | 623406 | 4987244 | 2 | 29 | 0 | 184 | 5123788 | 2 | 29 | 0 | 1 | 192 |
| 162 | A2 | 66 | 42 | 2 | 30 | 623429 | 4987428 | 2 | 30 | 0 | 184 | 5123980 | 2 | 30 | 0 | 1 | 192 |
| 163 | A3 | 66 | 42 | 2 | 31 | 623452 | 4987612 | 2 | 31 | 0 | 184 | 5124172 | 2 | 31 | 0 | 1 | 192 |
| 164 | A4 | 66 | 42 | 2 | 32 | 623475 | 4987796 | 2 | 32 | 0 | 184 | 5124364 | 2 | 32 | 0 | 1 | 192 |
| 165 | A5 | 66 | 42 | 2 | 33 | 623498 | 4987980 | 2 | 33 | 0 | 184 | 5124556 | 2 | 33 | 0 | 1 | 192 |
| 166 | A6 | 66 | 42 | 2 | 34 | 623521 | 4988164 | 2 | 34 | 0 | 184 | 5124748 | 2 | 34 | 0 | 1 | 192 |
| 167 | A7 | 66 | 42 | 2 | 35 | 623544 | 4988348 | 2 | 35 | 0 | 184 | 5124940 | 2 | 35 | 0 | 1 | 192 |
| 168 | A8 | 66 | 42 | 2 | 36 | 623567 | 4988532 | 2 | 36 | 0 | 184 | 5125132 | 2 | 36 | 0 | 1 | 192 |
| 169 | A9 | 66 | 42 | 2 | 37 | 623590 | 4988716 | 2 | 37 | 0 | 184 | 5125324 | 2 | 37 | 0 | 1 | 192 |
| 170 | AA | 66 | 42 | 2 | 38 | 623613 | 4988900 | 2 | 38 | 0 | 184 | 5125516 | 2 | 38 | 0 | 1 | 192 |
| 171 | AB | 66 | 42 | 2 | 39 | 623636 | 4989084 | 2 | 39 | 0 | 184 | 5125708 | 2 | 39 | 0 | 1 | 192 |
| 172 | AC | 66 | 42 | 2 | 40 | 623659 | 4989268 | 2 | 40 | 0 | 184 | 5125900 | 2 | 40 | 0 | 1 | 192 |
| 173 | AD | 66 | 42 | 2 | 41 | 623682 | 4989452 | 2 | 41 | 0 | 184 | 5126092 | 2 | 41 | 0 | 1 | 192 |
| 174 | AE | 66 | 42 | 2 | 42 | 623705 | 4989636 | 2 | 42 | 0 | 184 | 5126284 | 2 | 42 | 0 | 1 | 192 |
| 175 | AF | 66 | 42 | 2 | 43 | 623728 | 4989820 | 2 | 43 | 0 | 184 | 5126476 | 2 | 43 | 0 | 1 | 192 |
| 176 | B0 | 66 | 42 | 2 | 44 | 623753 | 4990020 | 2 | 44 | 0 | 200 | 5126684 | 2 | 44 | 0 | 1 | 208 |
| 177 | B1 | 66 | 42 | 2 | 45 | 623778 | 4990220 | 2 | 45 | 0 | 200 | 5126892 | 2 | 45 | 0 | 1 | 208 |
| 178 | B2 | 66 | 42 | 2 | 46 | 623803 | 4990420 | 2 | 46 | 0 | 200 | 5127100 | 2 | 46 | 0 | 1 | 208 |
| 179 | B3 | 66 | 42 | 2 | 47 | 623828 | 4990620 | 2 | 47 | 0 | 200 | 5127308 | 2 | 47 | 0 | 1 | 208 |
| 180 | B4 | 66 | 42 | 2 | 48 | 623853 | 4990820 | 2 | 48 | 0 | 200 | 5127516 | 2 | 48 | 0 | 1 | 208 |
| 181 | B5 | 66 | 42 | 2 | 49 | 623878 | 4991020 | 2 | 49 | 0 | 200 | 5127724 | 2 | 49 | 0 | 1 | 208 |
| 182 | B6 | 66 | 42 | 2 | 50 | 623903 | 4991220 | 2 | 50 | 0 | 200 | 5127932 | 2 | 50 | 0 | 1 | 208 |
| 183 | B7 | 66 | 42 | 2 | 51 | 623928 | 4991420 | 2 | 51 | 0 | 200 | 5128140 | 2 | 51 | 0 | 1 | 208 |
| 184 | B8 | 66 | 42 | 2 | 52 | 623953 | 4991620 | 2 | 52 | 0 | 200 | 5128348 | 2 | 52 | 0 | 1 | 208 |
| 185 | B9 | 66 | 42 | 2 | 53 | 623978 | 4991820 | 2 | 53 | 0 | 200 | 5128556 | 2 | 53 | 0 | 1 | 208 |
| 186 | BA | 66 | 42 | 2 | 54 | 624003 | 4992020 | 2 | 54 | 0 | 200 | 5128764 | 2 | 54 | 0 | 1 | 208 |
| 187 | BB | 66 | 42 | 2 | 55 | 624028 | 4992220 | 2 | 55 | 0 | 200 | 5128972 | 2 | 55 | 0 | 1 | 208 |
| 188 | BC | 66 | 42 | 2 | 56 | 624053 | 4992420 | 2 | 56 | 0 | 200 | 5129180 | 2 | 56 | 0 | 1 | 208 |
| 189 | BD | 66 | 42 | 2 | 57 | 624078 | 4992620 | 2 | 57 | 0 | 200 | 5129388 | 2 | 57 | 0 | 1 | 208 |
| 190 | BE | 66 | 42 | 2 | 58 | 624103 | 4992820 | 2 | 58 | 0 | 200 | 5129596 | 2 | 58 | 0 | 1 | 208 |
| 191 | BF | 66 | 42 | 2 | 59 | 624128 | 4993020 | 2 | 59 | 0 | 200 | 5129804 | 2 | 59 | 0 | 1 | 208 |
| 192 | C0 | 66 | 42 | 2 | 60 | 624153 | 4993220 | 2 | 60 | 0 | 200 | 5130012 | 2 | 60 | 0 | 1 | 208 |
| 193 | C1 | 66 | 42 | 2 | 61 | 624178 | 4993420 | 2 | 61 | 0 | 200 | 5130220 | 2 | 61 | 0 | 1 | 208 |
| 194 | C2 | 66 | 42 | 2 | 62 | 624203 | 4993620 | 2 | 62 | 0 | 200 | 5130428 | 2 | 62 | 0 | 1 | 208 |
| 195 | C3 | 66 | 42 | 2 | 63 | 624228 | 4993820 | 2 | 63 | 0 | 200 | 5130636 | 2 | 63 | 0 | 1 | 208 |
| 196 | C4 | 66 | 42 | 2 | 64 | 624253 | 4994020 | 2 | 64 | 0 | 200 | 5130844 | 2 | 64 | 0 | 1 | 208 |
| 197 | C5 | 66 | 42 | 2 | 65 | 624278 | 4994220 | 2 | 65 | 0 | 200 | 5131052 | 2 | 65 | 0 | 1 | 208 |
| 198 | C6 | 66 | 42 | 3 | 0 | 624301 | 4994404 | 3 | 0 | 0 | 184 | 5131244 | 3 | 0 | 0 | 1 | 192 |

| | | | | | | | | | | | | | | | | | |
|---|---|---|---|---|---|---|---|---|---|---|---|---|---|---|---|---|---|
| 199 | C7 | 66 | 42 | 3 | 1 | 624329 | 4994628 | 3 | 1 | 0 | 224 | 5131476 | 3 | 1 | 0 | 1 | 232 |
| 200 | C8 | 66 | 42 | 3 | 2 | 624357 | 4994852 | 3 | 2 | 0 | 224 | 5131708 | 3 | 2 | 0 | 1 | 232 |
| 201 | C9 | 66 | 42 | 3 | 3 | 624385 | 4995076 | 3 | 3 | 0 | 224 | 5131940 | 3 | 3 | 0 | 1 | 232 |
| 202 | CA | 66 | 42 | 3 | 4 | 624413 | 4995300 | 3 | 4 | 0 | 224 | 5132172 | 3 | 4 | 0 | 1 | 232 |
| 203 | CB | 66 | 42 | 3 | 5 | 624441 | 4995524 | 3 | 5 | 0 | 224 | 5132404 | 3 | 5 | 0 | 1 | 232 |
| 204 | CC | 66 | 42 | 3 | 6 | 624469 | 4995748 | 3 | 6 | 0 | 224 | 5132636 | 3 | 6 | 0 | 1 | 232 |
| 205 | CD | 66 | 42 | 3 | 7 | 624497 | 4995972 | 3 | 7 | 0 | 224 | 5132868 | 3 | 7 | 0 | 1 | 232 |
| 206 | CE | 66 | 42 | 3 | 8 | 624525 | 4996196 | 3 | 8 | 0 | 224 | 5133100 | 3 | 8 | 0 | 1 | 232 |
| 207 | CF | 66 | 42 | 3 | 9 | 624553 | 4996420 | 3 | 9 | 0 | 224 | 5133332 | 3 | 9 | 0 | 1 | 232 |
| 208 | D0 | 66 | 42 | 3 | 10 | 624581 | 4996644 | 3 | 10 | 0 | 224 | 5133564 | 3 | 10 | 0 | 1 | 232 |
| 209 | D1 | 66 | 42 | 3 | 11 | 624609 | 4996868 | 3 | 11 | 0 | 224 | 5133796 | 3 | 11 | 0 | 1 | 232 |
| 210 | D2 | 66 | 42 | 3 | 12 | 624637 | 4997092 | 3 | 12 | 0 | 224 | 5134028 | 3 | 12 | 0 | 1 | 232 |
| 211 | D3 | 66 | 42 | 3 | 13 | 624665 | 4997316 | 3 | 13 | 0 | 224 | 5134260 | 3 | 13 | 0 | 1 | 232 |
| 212 | D4 | 66 | 42 | 3 | 14 | 624693 | 4997540 | 3 | 14 | 0 | 224 | 5134492 | 3 | 14 | 0 | 1 | 232 |
| 213 | D5 | 66 | 42 | 3 | 15 | 624721 | 4997764 | 3 | 15 | 0 | 224 | 5134724 | 3 | 15 | 0 | 1 | 232 |
| 214 | D6 | 66 | 42 | 3 | 16 | 624749 | 4997988 | 3 | 16 | 0 | 224 | 5134956 | 3 | 16 | 0 | 1 | 232 |
| 215 | D7 | 66 | 42 | 3 | 17 | 624777 | 4998212 | 3 | 17 | 0 | 224 | 5135188 | 3 | 17 | 0 | 1 | 232 |
| 216 | D8 | 66 | 42 | 3 | 18 | 624805 | 4998436 | 3 | 18 | 0 | 224 | 5135420 | 3 | 18 | 0 | 1 | 232 |
| 217 | D9 | 66 | 42 | 3 | 19 | 624833 | 4998660 | 3 | 19 | 0 | 224 | 5135652 | 3 | 19 | 0 | 1 | 232 |
| 218 | DA | 66 | 42 | 3 | 20 | 624861 | 4998884 | 3 | 20 | 0 | 224 | 5135884 | 3 | 20 | 0 | 1 | 232 |
| 219 | DB | 66 | 42 | 3 | 21 | 624889 | 4999108 | 3 | 21 | 0 | 224 | 5136116 | 3 | 21 | 0 | 1 | 232 |
| 220 | DC | 66 | 42 | 3 | 22 | 624917 | 4999332 | 3 | 22 | 0 | 224 | 5136348 | 3 | 22 | 0 | 1 | 232 |
| 221 | DD | 66 | 42 | 3 | 23 | 624945 | 4999556 | 3 | 23 | 0 | 224 | 5136580 | 3 | 23 | 0 | 1 | 232 |
| 222 | DE | 66 | 42 | 3 | 24 | 624973 | 4999780 | 3 | 24 | 0 | 224 | 5136812 | 3 | 24 | 0 | 1 | 232 |
| 223 | DF | 66 | 42 | 3 | 25 | 625001 | 5000004 | 3 | 25 | 0 | 224 | 5137044 | 3 | 25 | 0 | 1 | 232 |
| 224 | E0 | 66 | 42 | 3 | 26 | 625029 | 5000228 | 3 | 26 | 0 | 224 | 5137276 | 3 | 26 | 0 | 1 | 232 |
| 225 | E1 | 66 | 42 | 3 | 27 | 625057 | 5000452 | 3 | 27 | 0 | 224 | 5137508 | 3 | 27 | 0 | 1 | 232 |
| 226 | E2 | 66 | 42 | 3 | 28 | 625085 | 5000676 | 3 | 28 | 0 | 224 | 5137740 | 3 | 28 | 0 | 1 | 232 |
| 227 | E3 | 66 | 42 | 3 | 29 | 625113 | 5000900 | 3 | 29 | 0 | 224 | 5137972 | 3 | 29 | 0 | 1 | 232 |
| 228 | E4 | 66 | 42 | 3 | 30 | 625141 | 5001124 | 3 | 30 | 0 | 224 | 5138204 | 3 | 30 | 0 | 1 | 232 |
| 229 | E5 | 66 | 42 | 3 | 31 | 625169 | 5001348 | 3 | 31 | 0 | 224 | 5138436 | 3 | 31 | 0 | 1 | 232 |
| 230 | E6 | 66 | 42 | 3 | 32 | 625197 | 5001572 | 3 | 32 | 0 | 224 | 5138668 | 3 | 32 | 0 | 1 | 232 |
| 231 | E7 | 66 | 42 | 3 | 33 | 625225 | 5001796 | 3 | 33 | 0 | 224 | 5138900 | 3 | 33 | 0 | 1 | 232 |
| 232 | E8 | 66 | 42 | 3 | 34 | 625253 | 5002020 | 3 | 34 | 0 | 224 | 5139132 | 3 | 34 | 0 | 1 | 232 |
| 233 | E9 | 66 | 42 | 3 | 35 | 625281 | 5002244 | 3 | 35 | 0 | 224 | 5139364 | 3 | 35 | 0 | 1 | 232 |
| 234 | EA | 66 | 42 | 3 | 36 | 625309 | 5002468 | 3 | 36 | 0 | 224 | 5139596 | 3 | 36 | 0 | 1 | 232 |
| 235 | EB | 66 | 42 | 3 | 37 | 625337 | 5002692 | 3 | 37 | 0 | 224 | 5139828 | 3 | 37 | 0 | 1 | 232 |
| 236 | EC | 66 | 42 | 3 | 38 | 625365 | 5002916 | 3 | 38 | 0 | 224 | 5140060 | 3 | 38 | 0 | 1 | 232 |
| 237 | ED | 66 | 42 | 3 | 39 | 625393 | 5003140 | 3 | 39 | 0 | 224 | 5140292 | 3 | 39 | 0 | 1 | 232 |
| 238 | EE | 66 | 42 | 3 | 40 | 625421 | 5003364 | 3 | 40 | 0 | 224 | 5140524 | 3 | 40 | 0 | 1 | 232 |
| 239 | EF | 66 | 42 | 3 | 41 | 625449 | 5003588 | 3 | 41 | 0 | 224 | 5140756 | 3 | 41 | 0 | 1 | 232 |
| 240 | F0 | 66 | 42 | 3 | 42 | 625474 | 5003788 | 3 | 42 | 0 | 200 | 5140964 | 3 | 42 | 0 | 1 | 208 |
| 241 | F1 | 66 | 42 | 3 | 43 | 625499 | 5003988 | 3 | 43 | 0 | 200 | 5141172 | 3 | 43 | 0 | 1 | 208 |
| 242 | F2 | 66 | 42 | 3 | 44 | 625524 | 5004188 | 3 | 44 | 0 | 200 | 5141380 | 3 | 44 | 0 | 1 | 208 |
| 243 | F3 | 66 | 42 | 3 | 45 | 625549 | 5004388 | 3 | 45 | 0 | 200 | 5141588 | 3 | 45 | 0 | 1 | 208 |
| 244 | F4 | 66 | 42 | 3 | 46 | 625574 | 5004588 | 3 | 46 | 0 | 200 | 5141796 | 3 | 46 | 0 | 1 | 208 |
| 245 | F5 | 66 | 42 | 3 | 47 | 625599 | 5004788 | 3 | 47 | 0 | 200 | 5142004 | 3 | 47 | 0 | 1 | 208 |
| 246 | F6 | 66 | 42 | 3 | 48 | 625624 | 5004988 | 3 | 48 | 0 | 200 | 5142212 | 3 | 48 | 0 | 1 | 208 |
| 247 | F7 | 66 | 42 | 3 | 49 | 625649 | 5005188 | 3 | 49 | 0 | 200 | 5142420 | 3 | 49 | 0 | 1 | 208 |
| 248 | F8 | 66 | 42 | 3 | 50 | 625674 | 5005388 | 3 | 50 | 0 | 200 | 5142628 | 3 | 50 | 0 | 1 | 208 |
| 249 | F9 | 66 | 42 | 3 | 51 | 625699 | 5005588 | 3 | 51 | 0 | 200 | 5142836 | 3 | 51 | 0 | 1 | 208 |
| 250 | FA | 66 | 42 | 3 | 52 | 625724 | 5005788 | 3 | 52 | 0 | 200 | 5143044 | 3 | 52 | 0 | 1 | 208 |
| 251 | FB | 66 | 42 | 3 | 53 | 625749 | 5005988 | 3 | 53 | 0 | 200 | 5143252 | 3 | 53 | 0 | 1 | 208 |
| 252 | FC | 66 | 42 | 3 | 54 | 625774 | 5006188 | 3 | 54 | 0 | 200 | 5143460 | 3 | 54 | 0 | 1 | 208 |
| 253 | FD | 66 | 42 | 3 | 55 | 625799 | 5006388 | 3 | 55 | 0 | 200 | 5143668 | 3 | 55 | 0 | 1 | 208 |
| 254 | FE | 66 | 42 | 3 | 56 | 625824 | 5006588 | 3 | 56 | 0 | 200 | 5143876 | 3 | 56 | 0 | 1 | 208 |
| 255 | FF | 66 | 42 | 3 | 57 | 625849 | 5006788 | 3 | 57 | 0 | 200 | 5144084 | 3 | 57 | 0 | 1 | 208 |
| 0 | 00 | 67 | 43 | 0 | 0 | 625861 | 5006884 | 0 | 0 | 0 | 96 | 5144188 | 0 | 0 | 0 | 1 | 104 |
| 1 | 01 | 67 | 43 | 0 | 1 | 625875 | 5006996 | 0 | 1 | 0 | 112 | 5144308 | 0 | 1 | 0 | 1 | 120 |
| 2 | 02 | 67 | 43 | 0 | 2 | 625889 | 5007108 | 0 | 2 | 0 | 112 | 5144428 | 0 | 2 | 0 | 1 | 120 |
| 3 | 03 | 67 | 43 | 0 | 3 | 625903 | 5007220 | 0 | 3 | 0 | 112 | 5144548 | 0 | 3 | 0 | 1 | 120 |
| 4 | 04 | 67 | 43 | 0 | 4 | 625917 | 5007332 | 0 | 4 | 0 | 112 | 5144668 | 0 | 4 | 0 | 1 | 120 |
| 5 | 05 | 67 | 43 | 0 | 5 | 625931 | 5007444 | 0 | 5 | 0 | 112 | 5144788 | 0 | 5 | 0 | 1 | 120 |
| 6 | 06 | 67 | 43 | 0 | 6 | 625945 | 5007556 | 0 | 6 | 0 | 112 | 5144908 | 0 | 6 | 0 | 1 | 120 |
| 7 | 07 | 67 | 43 | 0 | 7 | 625959 | 5007668 | 0 | 7 | 0 | 112 | 5145028 | 0 | 7 | 0 | 1 | 120 |
| 8 | 08 | 67 | 43 | 0 | 8 | 625973 | 5007780 | 0 | 8 | 0 | 112 | 5145148 | 0 | 8 | 0 | 1 | 120 |
| 9 | 09 | 67 | 43 | 0 | 9 | 625987 | 5007892 | 0 | 9 | 0 | 112 | 5145268 | 0 | 9 | 0 | 1 | 120 |
| 10 | 0A | 67 | 43 | 0 | 10 | 626001 | 5008004 | 0 | 10 | 0 | 112 | 5145388 | 0 | 10 | 0 | 1 | 120 |
| 11 | 0B | 67 | 43 | 0 | 11 | 626015 | 5008116 | 0 | 11 | 0 | 112 | 5145508 | 0 | 11 | 0 | 1 | 120 |
| 12 | 0C | 67 | 43 | 0 | 12 | 626029 | 5008228 | 0 | 12 | 0 | 112 | 5145628 | 0 | 12 | 0 | 1 | 120 |
| 13 | 0D | 67 | 43 | 0 | 13 | 626043 | 5008340 | 0 | 13 | 0 | 112 | 5145748 | 0 | 13 | 0 | 1 | 120 |
| 14 | 0E | 67 | 43 | 0 | 14 | 626057 | 5008452 | 0 | 14 | 0 | 112 | 5145868 | 0 | 14 | 0 | 1 | 120 |
| 15 | 0F | 67 | 43 | 0 | 15 | 626071 | 5008564 | 0 | 15 | 0 | 112 | 5145988 | 0 | 15 | 0 | 1 | 120 |
| 16 | 10 | 67 | 43 | 0 | 16 | 626085 | 5008676 | 0 | 16 | 0 | 112 | 5146108 | 0 | 16 | 0 | 1 | 120 |
| 17 | 11 | 67 | 43 | 0 | 17 | 626099 | 5008788 | 0 | 17 | 0 | 112 | 5146228 | 0 | 17 | 0 | 1 | 120 |
| 18 | 12 | 67 | 43 | 0 | 18 | 626113 | 5008900 | 0 | 18 | 0 | 112 | 5146348 | 0 | 18 | 0 | 1 | 120 |
| 19 | 13 | 67 | 43 | 0 | 19 | 626127 | 5009012 | 0 | 19 | 0 | 112 | 5146468 | 0 | 19 | 0 | 1 | 120 |
| 20 | 14 | 67 | 43 | 0 | 20 | 626141 | 5009124 | 0 | 20 | 0 | 112 | 5146588 | 0 | 20 | 0 | 1 | 120 |
| 21 | 15 | 67 | 43 | 0 | 21 | 626155 | 5009236 | 0 | 21 | 0 | 112 | 5146708 | 0 | 21 | 0 | 1 | 120 |
| 22 | 16 | 67 | 43 | 0 | 22 | 626169 | 5009348 | 0 | 22 | 0 | 112 | 5146828 | 0 | 22 | 0 | 1 | 120 |
| 23 | 17 | 67 | 43 | 0 | 23 | 626183 | 5009460 | 0 | 23 | 0 | 112 | 5146948 | 0 | 23 | 0 | 1 | 120 |
| 24 | 18 | 67 | 43 | 0 | 24 | 626197 | 5009572 | 0 | 24 | 0 | 112 | 5147068 | 0 | 24 | 0 | 1 | 120 |
| 25 | 19 | 67 | 43 | 0 | 25 | 626211 | 5009684 | 0 | 25 | 0 | 112 | 5147188 | 0 | 25 | 0 | 1 | 120 |
| 26 | 1A | 67 | 43 | 0 | 26 | 626225 | 5009796 | 0 | 26 | 0 | 112 | 5147308 | 0 | 26 | 0 | 1 | 120 |
| 27 | 1B | 67 | 43 | 0 | 27 | 626239 | 5009908 | 0 | 27 | 0 | 112 | 5147428 | 0 | 27 | 0 | 1 | 120 |
| 28 | 1C | 67 | 43 | 0 | 28 | 626253 | 5010020 | 0 | 28 | 0 | 112 | 5147548 | 0 | 28 | 0 | 1 | 120 |
| 29 | 1D | 67 | 43 | 0 | 29 | 626267 | 5010132 | 0 | 29 | 0 | 112 | 5147668 | 0 | 29 | 0 | 1 | 120 |
| 30 | 1E | 67 | 43 | 0 | 30 | 626281 | 5010244 | 0 | 30 | 0 | 112 | 5147788 | 0 | 30 | 0 | 1 | 120 |
| 31 | 1F | 67 | 43 | 0 | 31 | 626295 | 5010356 | 0 | 31 | 0 | 112 | 5147908 | 0 | 31 | 0 | 1 | 120 |
| 32 | 20 | 67 | 43 | 0 | 32 | 626309 | 5010468 | 0 | 32 | 0 | 112 | 5148028 | 0 | 32 | 0 | 1 | 120 |
| 33 | 21 | 67 | 43 | 0 | 33 | 626323 | 5010580 | 0 | 33 | 0 | 112 | 5148148 | 0 | 33 | 0 | 1 | 120 |
| 34 | 22 | 67 | 43 | 0 | 34 | 626337 | 5010692 | 0 | 34 | 0 | 112 | 5148268 | 0 | 34 | 0 | 1 | 120 |
| 35 | 23 | 67 | 43 | 0 | 35 | 626351 | 5010804 | 0 | 35 | 0 | 112 | 5148388 | 0 | 35 | 0 | 1 | 120 |
| 36 | 24 | 67 | 43 | 0 | 36 | 626365 | 5010916 | 0 | 36 | 0 | 112 | 5148508 | 0 | 36 | 0 | 1 | 120 |
| 37 | 25 | 67 | 43 | 0 | 37 | 626379 | 5011028 | 0 | 37 | 0 | 112 | 5148628 | 0 | 37 | 0 | 1 | 120 |
| 38 | 26 | 67 | 43 | 0 | 38 | 626393 | 5011140 | 0 | 38 | 0 | 112 | 5148748 | 0 | 38 | 0 | 1 | 120 |
| 39 | 27 | 67 | 43 | 0 | 39 | 626407 | 5011252 | 0 | 39 | 0 | 112 | 5148868 | 0 | 39 | 0 | 1 | 120 |
| 40 | 28 | 67 | 43 | 0 | 40 | 626421 | 5011364 | 0 | 40 | 0 | 112 | 5148988 | 0 | 40 | 0 | 1 | 120 |
| 41 | 29 | 67 | 43 | 0 | 41 | 626435 | 5011476 | 0 | 41 | 0 | 112 | 5149108 | 0 | 41 | 0 | 1 | 120 |
| 42 | 2A | 67 | 43 | 0 | 42 | 626449 | 5011588 | 0 | 42 | 0 | 112 | 5149228 | 0 | 42 | 0 | 1 | 120 |
| 43 | 2B | 67 | 43 | 0 | 43 | 626463 | 5011700 | 0 | 43 | 0 | 112 | 5149348 | 0 | 43 | 0 | 1 | 120 |
| 44 | 2C | 67 | 43 | 0 | 44 | 626477 | 5011812 | 0 | 44 | 0 | 112 | 5149468 | 0 | 44 | 0 | 1 | 120 |
| 45 | 2D | 67 | 43 | 0 | 45 | 626491 | 5011924 | 0 | 45 | 0 | 112 | 5149588 | 0 | 45 | 0 | 1 | 120 |

| | | | | | | | | | | | | | | | | | |
|---|---|---|---|---|---|---|---|---|---|---|---|---|---|---|---|---|---|
| 46 | 2E | 67 | 43 | 0 | 46 | 626505 | 5012036 | 0 | 46 | 0 | 112 | 5149708 | 0 | 46 | 0 | 1 | 120 |
| 47 | 2F | 67 | 43 | 0 | 47 | 626519 | 5012148 | 0 | 47 | 0 | 112 | 5149828 | 0 | 47 | 0 | 1 | 120 |
| 48 | 30 | 67 | 43 | 0 | 48 | 626533 | 5012260 | 0 | 48 | 0 | 112 | 5149948 | 0 | 48 | 0 | 1 | 120 |
| 49 | 31 | 67 | 43 | 0 | 49 | 626547 | 5012372 | 0 | 49 | 0 | 112 | 5150068 | 0 | 49 | 0 | 1 | 120 |
| 50 | 32 | 67 | 43 | 0 | 50 | 626561 | 5012484 | 0 | 50 | 0 | 112 | 5150188 | 0 | 50 | 0 | 1 | 120 |
| 51 | 33 | 67 | 43 | 0 | 51 | 626575 | 5012596 | 0 | 51 | 0 | 112 | 5150308 | 0 | 51 | 0 | 1 | 120 |
| 52 | 34 | 67 | 43 | 0 | 52 | 626589 | 5012708 | 0 | 52 | 0 | 112 | 5150428 | 0 | 52 | 0 | 1 | 120 |
| 53 | 35 | 67 | 43 | 0 | 53 | 626603 | 5012820 | 0 | 53 | 0 | 112 | 5150548 | 0 | 53 | 0 | 1 | 120 |
| 54 | 36 | 67 | 43 | 0 | 54 | 626617 | 5012932 | 0 | 54 | 0 | 112 | 5150668 | 0 | 54 | 0 | 1 | 120 |
| 55 | 37 | 67 | 43 | 0 | 55 | 626631 | 5013044 | 0 | 55 | 0 | 112 | 5150788 | 0 | 55 | 0 | 1 | 120 |
| 56 | 38 | 67 | 43 | 0 | 56 | 626645 | 5013156 | 0 | 56 | 0 | 112 | 5150908 | 0 | 56 | 0 | 1 | 120 |
| 57 | 39 | 67 | 43 | 0 | 57 | 626659 | 5013268 | 0 | 57 | 0 | 112 | 5151028 | 0 | 57 | 0 | 1 | 120 |
| 58 | 3A | 67 | 43 | 0 | 58 | 626673 | 5013380 | 0 | 58 | 0 | 112 | 5151148 | 0 | 58 | 0 | 1 | 120 |
| 59 | 3B | 67 | 43 | 0 | 59 | 626687 | 5013492 | 0 | 59 | 0 | 112 | 5151268 | 0 | 59 | 0 | 1 | 120 |
| 60 | 3C | 67 | 43 | 0 | 60 | 626701 | 5013604 | 0 | 60 | 0 | 112 | 5151388 | 0 | 60 | 0 | 1 | 120 |
| 61 | 3D | 67 | 43 | 0 | 61 | 626715 | 5013716 | 0 | 61 | 0 | 112 | 5151508 | 0 | 61 | 0 | 1 | 120 |
| 62 | 3E | 67 | 43 | 0 | 62 | 626729 | 5013828 | 0 | 62 | 0 | 112 | 5151628 | 0 | 62 | 0 | 1 | 120 |
| 63 | 3F | 67 | 43 | 0 | 63 | 626743 | 5013940 | 0 | 63 | 0 | 112 | 5151748 | 0 | 63 | 0 | 1 | 120 |
| 64 | 40 | 67 | 43 | 0 | 64 | 626757 | 5014052 | 0 | 64 | 0 | 112 | 5151868 | 0 | 64 | 0 | 1 | 120 |
| 65 | 41 | 67 | 43 | 0 | 65 | 626771 | 5014164 | 0 | 65 | 0 | 112 | 5151988 | 0 | 65 | 0 | 1 | 120 |
| 66 | 42 | 67 | 43 | 0 | 66 | 626785 | 5014276 | 0 | 66 | 0 | 112 | 5152108 | 0 | 66 | 0 | 1 | 120 |
| 67 | 43 | 67 | 43 | 1 | 0 | 626797 | 5014372 | 1 | 0 | 0 | 96 | 5152212 | 1 | 0 | 0 | 1 | 104 |
| 68 | 44 | 67 | 43 | 1 | 1 | 626814 | 5014508 | 1 | 1 | 0 | 136 | 5152356 | 1 | 1 | 0 | 1 | 144 |
| 69 | 45 | 67 | 43 | 1 | 2 | 626831 | 5014644 | 1 | 2 | 0 | 136 | 5152500 | 1 | 2 | 0 | 1 | 144 |
| 70 | 46 | 67 | 43 | 1 | 3 | 626848 | 5014780 | 1 | 3 | 0 | 136 | 5152644 | 1 | 3 | 0 | 1 | 144 |
| 71 | 47 | 67 | 43 | 1 | 4 | 626865 | 5014916 | 1 | 4 | 0 | 136 | 5152788 | 1 | 4 | 0 | 1 | 144 |
| 72 | 48 | 67 | 43 | 1 | 5 | 626882 | 5015052 | 1 | 5 | 0 | 136 | 5152932 | 1 | 5 | 0 | 1 | 144 |
| 73 | 49 | 67 | 43 | 1 | 6 | 626899 | 5015188 | 1 | 6 | 0 | 136 | 5153076 | 1 | 6 | 0 | 1 | 144 |
| 74 | 4A | 67 | 43 | 1 | 7 | 626916 | 5015324 | 1 | 7 | 0 | 136 | 5153220 | 1 | 7 | 0 | 1 | 144 |
| 75 | 4B | 67 | 43 | 1 | 8 | 626933 | 5015460 | 1 | 8 | 0 | 136 | 5153364 | 1 | 8 | 0 | 1 | 144 |
| 76 | 4C | 67 | 43 | 1 | 9 | 626950 | 5015596 | 1 | 9 | 0 | 136 | 5153508 | 1 | 9 | 0 | 1 | 144 |
| 77 | 4D | 67 | 43 | 1 | 10 | 626967 | 5015732 | 1 | 10 | 0 | 136 | 5153652 | 1 | 10 | 0 | 1 | 144 |
| 78 | 4E | 67 | 43 | 1 | 11 | 626984 | 5015868 | 1 | 11 | 0 | 136 | 5153796 | 1 | 11 | 0 | 1 | 144 |
| 79 | 4F | 67 | 43 | 1 | 12 | 627001 | 5016004 | 1 | 12 | 0 | 136 | 5153940 | 1 | 12 | 0 | 1 | 144 |
| 80 | 50 | 67 | 43 | 1 | 13 | 627022 | 5016172 | 1 | 13 | 0 | 168 | 5154116 | 1 | 13 | 0 | 1 | 176 |
| 81 | 51 | 67 | 43 | 1 | 14 | 627043 | 5016340 | 1 | 14 | 0 | 168 | 5154292 | 1 | 14 | 0 | 1 | 176 |
| 82 | 52 | 67 | 43 | 1 | 15 | 627064 | 5016508 | 1 | 15 | 0 | 168 | 5154468 | 1 | 15 | 0 | 1 | 176 |
| 83 | 53 | 67 | 43 | 1 | 16 | 627085 | 5016676 | 1 | 16 | 0 | 168 | 5154644 | 1 | 16 | 0 | 1 | 176 |
| 84 | 54 | 67 | 43 | 1 | 17 | 627106 | 5016844 | 1 | 17 | 0 | 168 | 5154820 | 1 | 17 | 0 | 1 | 176 |
| 85 | 55 | 67 | 43 | 1 | 18 | 627127 | 5017012 | 1 | 18 | 0 | 168 | 5154996 | 1 | 18 | 0 | 1 | 176 |
| 86 | 56 | 67 | 43 | 1 | 19 | 627148 | 5017180 | 1 | 19 | 0 | 168 | 5155172 | 1 | 19 | 0 | 1 | 176 |
| 87 | 57 | 67 | 43 | 1 | 20 | 627169 | 5017348 | 1 | 20 | 0 | 168 | 5155348 | 1 | 20 | 0 | 1 | 176 |
| 88 | 58 | 67 | 43 | 1 | 21 | 627190 | 5017516 | 1 | 21 | 0 | 168 | 5155524 | 1 | 21 | 0 | 1 | 176 |
| 89 | 59 | 67 | 43 | 1 | 22 | 627211 | 5017684 | 1 | 22 | 0 | 168 | 5155700 | 1 | 22 | 0 | 1 | 176 |
| 90 | 5A | 67 | 43 | 1 | 23 | 627232 | 5017852 | 1 | 23 | 0 | 168 | 5155876 | 1 | 23 | 0 | 1 | 176 |
| 91 | 5B | 67 | 43 | 1 | 24 | 627253 | 5018020 | 1 | 24 | 0 | 168 | 5156052 | 1 | 24 | 0 | 1 | 176 |
| 92 | 5C | 67 | 43 | 1 | 25 | 627274 | 5018188 | 1 | 25 | 0 | 168 | 5156228 | 1 | 25 | 0 | 1 | 176 |
| 93 | 5D | 67 | 43 | 1 | 26 | 627295 | 5018356 | 1 | 26 | 0 | 168 | 5156404 | 1 | 26 | 0 | 1 | 176 |
| 94 | 5E | 67 | 43 | 1 | 27 | 627316 | 5018524 | 1 | 27 | 0 | 168 | 5156580 | 1 | 27 | 0 | 1 | 176 |
| 95 | 5F | 67 | 43 | 1 | 28 | 627337 | 5018692 | 1 | 28 | 0 | 168 | 5156756 | 1 | 28 | 0 | 1 | 176 |
| 96 | 60 | 67 | 43 | 1 | 29 | 627360 | 5018876 | 1 | 29 | 0 | 184 | 5156948 | 1 | 29 | 0 | 1 | 192 |
| 97 | 61 | 67 | 43 | 1 | 30 | 627383 | 5019060 | 1 | 30 | 0 | 184 | 5157140 | 1 | 30 | 0 | 1 | 192 |
| 98 | 62 | 67 | 43 | 1 | 31 | 627406 | 5019244 | 1 | 31 | 0 | 184 | 5157332 | 1 | 31 | 0 | 1 | 192 |
| 99 | 63 | 67 | 43 | 1 | 32 | 627429 | 5019428 | 1 | 32 | 0 | 184 | 5157524 | 1 | 32 | 0 | 1 | 192 |
| 100 | 64 | 67 | 43 | 1 | 33 | 627452 | 5019612 | 1 | 33 | 0 | 184 | 5157716 | 1 | 33 | 0 | 1 | 192 |
| 101 | 65 | 67 | 43 | 1 | 34 | 627475 | 5019796 | 1 | 34 | 0 | 184 | 5157908 | 1 | 34 | 0 | 1 | 192 |
| 102 | 66 | 67 | 43 | 1 | 35 | 627498 | 5019980 | 1 | 35 | 0 | 184 | 5158100 | 1 | 35 | 0 | 1 | 192 |
| 103 | 67 | 67 | 43 | 1 | 36 | 627521 | 5020164 | 1 | 36 | 0 | 184 | 5158292 | 1 | 36 | 0 | 1 | 192 |
| 104 | 68 | 67 | 43 | 1 | 37 | 627544 | 5020348 | 1 | 37 | 0 | 184 | 5158484 | 1 | 37 | 0 | 1 | 192 |
| 105 | 69 | 67 | 43 | 1 | 38 | 627567 | 5020532 | 1 | 38 | 0 | 184 | 5158676 | 1 | 38 | 0 | 1 | 192 |
| 106 | 6A | 67 | 43 | 1 | 39 | 627590 | 5020716 | 1 | 39 | 0 | 184 | 5158868 | 1 | 39 | 0 | 1 | 192 |
| 107 | 6B | 67 | 43 | 1 | 40 | 627613 | 5020900 | 1 | 40 | 0 | 184 | 5159060 | 1 | 40 | 0 | 1 | 192 |
| 108 | 6C | 67 | 43 | 1 | 41 | 627636 | 5021084 | 1 | 41 | 0 | 184 | 5159252 | 1 | 41 | 0 | 1 | 192 |
| 109 | 6D | 67 | 43 | 1 | 42 | 627659 | 5021268 | 1 | 42 | 0 | 184 | 5159444 | 1 | 42 | 0 | 1 | 192 |
| 110 | 6E | 67 | 43 | 1 | 43 | 627682 | 5021452 | 1 | 43 | 0 | 184 | 5159636 | 1 | 43 | 0 | 1 | 192 |
| 111 | 6F | 67 | 43 | 1 | 44 | 627705 | 5021636 | 1 | 44 | 0 | 184 | 5159828 | 1 | 44 | 0 | 1 | 192 |
| 112 | 70 | 67 | 43 | 1 | 45 | 627728 | 5021820 | 1 | 45 | 0 | 184 | 5160020 | 1 | 45 | 0 | 1 | 192 |
| 113 | 71 | 67 | 43 | 1 | 46 | 627751 | 5022004 | 1 | 46 | 0 | 184 | 5160212 | 1 | 46 | 0 | 1 | 192 |
| 114 | 72 | 67 | 43 | 1 | 47 | 627774 | 5022188 | 1 | 47 | 0 | 184 | 5160404 | 1 | 47 | 0 | 1 | 192 |
| 115 | 73 | 67 | 43 | 1 | 48 | 627797 | 5022372 | 1 | 48 | 0 | 184 | 5160596 | 1 | 48 | 0 | 1 | 192 |
| 116 | 74 | 67 | 43 | 1 | 49 | 627820 | 5022556 | 1 | 49 | 0 | 184 | 5160788 | 1 | 49 | 0 | 1 | 192 |
| 117 | 75 | 67 | 43 | 1 | 50 | 627843 | 5022740 | 1 | 50 | 0 | 184 | 5160980 | 1 | 50 | 0 | 1 | 192 |
| 118 | 76 | 67 | 43 | 1 | 51 | 627866 | 5022924 | 1 | 51 | 0 | 184 | 5161172 | 1 | 51 | 0 | 1 | 192 |
| 119 | 77 | 67 | 43 | 1 | 52 | 627889 | 5023108 | 1 | 52 | 0 | 184 | 5161364 | 1 | 52 | 0 | 1 | 192 |
| 120 | 78 | 67 | 43 | 1 | 53 | 627912 | 5023292 | 1 | 53 | 0 | 184 | 5161556 | 1 | 53 | 0 | 1 | 192 |
| 121 | 79 | 67 | 43 | 1 | 54 | 627935 | 5023476 | 1 | 54 | 0 | 184 | 5161748 | 1 | 54 | 0 | 1 | 192 |
| 122 | 7A | 67 | 43 | 1 | 55 | 627958 | 5023660 | 1 | 55 | 0 | 184 | 5161940 | 1 | 55 | 0 | 1 | 192 |
| 123 | 7B | 67 | 43 | 1 | 56 | 627981 | 5023844 | 1 | 56 | 0 | 184 | 5162132 | 1 | 56 | 0 | 1 | 192 |
| 124 | 7C | 67 | 43 | 1 | 57 | 628004 | 5024028 | 1 | 57 | 0 | 184 | 5162324 | 1 | 57 | 0 | 1 | 192 |
| 125 | 7D | 67 | 43 | 1 | 58 | 628027 | 5024212 | 1 | 58 | 0 | 184 | 5162516 | 1 | 58 | 0 | 1 | 192 |
| 126 | 7E | 67 | 43 | 1 | 59 | 628050 | 5024396 | 1 | 59 | 0 | 184 | 5162708 | 1 | 59 | 0 | 1 | 192 |
| 127 | 7F | 67 | 43 | 1 | 60 | 628073 | 5024580 | 1 | 60 | 0 | 184 | 5162900 | 1 | 60 | 0 | 1 | 192 |
| 128 | 80 | 67 | 43 | 1 | 61 | 628096 | 5024764 | 1 | 61 | 0 | 184 | 5163092 | 1 | 61 | 0 | 1 | 192 |
| 129 | 81 | 67 | 43 | 1 | 62 | 628119 | 5024948 | 1 | 62 | 0 | 184 | 5163284 | 1 | 62 | 0 | 1 | 192 |
| 130 | 82 | 67 | 43 | 1 | 63 | 628142 | 5025132 | 1 | 63 | 0 | 184 | 5163476 | 1 | 63 | 0 | 1 | 192 |
| 131 | 83 | 67 | 43 | 1 | 64 | 628165 | 5025316 | 1 | 64 | 0 | 184 | 5163668 | 1 | 64 | 0 | 1 | 192 |
| 132 | 84 | 67 | 43 | 1 | 65 | 628188 | 5025500 | 1 | 65 | 0 | 184 | 5163860 | 1 | 65 | 0 | 1 | 192 |
| 133 | 85 | 67 | 43 | 1 | 66 | 628211 | 5025684 | 1 | 66 | 0 | 184 | 5164052 | 1 | 66 | 0 | 1 | 192 |
| 134 | 86 | 67 | 43 | 2 | 0 | 628232 | 5025852 | 2 | 0 | 0 | 168 | 5164228 | 2 | 0 | 0 | 1 | 176 |
| 135 | 87 | 67 | 43 | 2 | 1 | 628258 | 5026060 | 2 | 1 | 0 | 208 | 5164444 | 2 | 1 | 0 | 1 | 216 |
| 136 | 88 | 67 | 43 | 2 | 2 | 628284 | 5026268 | 2 | 2 | 0 | 208 | 5164660 | 2 | 2 | 0 | 1 | 216 |
| 137 | 89 | 67 | 43 | 2 | 3 | 628310 | 5026476 | 2 | 3 | 0 | 208 | 5164876 | 2 | 3 | 0 | 1 | 216 |
| 138 | 8A | 67 | 43 | 2 | 4 | 628336 | 5026684 | 2 | 4 | 0 | 208 | 5165092 | 2 | 4 | 0 | 1 | 216 |
| 139 | 8B | 67 | 43 | 2 | 5 | 628362 | 5026892 | 2 | 5 | 0 | 208 | 5165308 | 2 | 5 | 0 | 1 | 216 |
| 140 | 8C | 67 | 43 | 2 | 6 | 628388 | 5027100 | 2 | 6 | 0 | 208 | 5165524 | 2 | 6 | 0 | 1 | 216 |
| 141 | 8D | 67 | 43 | 2 | 7 | 628414 | 5027308 | 2 | 7 | 0 | 208 | 5165740 | 2 | 7 | 0 | 1 | 216 |
| 142 | 8E | 67 | 43 | 2 | 8 | 628440 | 5027516 | 2 | 8 | 0 | 208 | 5165956 | 2 | 8 | 0 | 1 | 216 |
| 143 | 8F | 67 | 43 | 2 | 9 | 628466 | 5027724 | 2 | 9 | 0 | 208 | 5166172 | 2 | 9 | 0 | 1 | 216 |
| 144 | 90 | 67 | 43 | 2 | 10 | 628492 | 5027932 | 2 | 10 | 0 | 208 | 5166388 | 2 | 10 | 0 | 1 | 216 |
| 145 | 91 | 67 | 43 | 2 | 11 | 628518 | 5028140 | 2 | 11 | 0 | 208 | 5166604 | 2 | 11 | 0 | 1 | 216 |
| 146 | 92 | 67 | 43 | 2 | 12 | 628544 | 5028348 | 2 | 12 | 0 | 208 | 5166820 | 2 | 12 | 0 | 1 | 216 |
| 147 | 93 | 67 | 43 | 2 | 13 | 628570 | 5028556 | 2 | 13 | 0 | 208 | 5167036 | 2 | 13 | 0 | 1 | 216 |
| 148 | 94 | 67 | 43 | 2 | 14 | 628596 | 5028764 | 2 | 14 | 0 | 208 | 5167252 | 2 | 14 | 0 | 1 | 216 |

| | | | | | | | | | | | | | | | | | |
|---|---|---|---|---|---|---|---|---|---|---|---|---|---|---|---|---|---|
| 149 | 95 | 67 | 43 | 2 | 15 | 628622 | 5028972 | 2 | 15 | 0 | 208 | 5167468 | 2 | 15 | 0 | 1 | 216 |
| 150 | 96 | 67 | 43 | 2 | 16 | 628648 | 5029180 | 2 | 16 | 0 | 208 | 5167684 | 2 | 16 | 0 | 1 | 216 |
| 151 | 97 | 67 | 43 | 2 | 17 | 628674 | 5029388 | 2 | 17 | 0 | 208 | 5167900 | 2 | 17 | 0 | 1 | 216 |
| 152 | 98 | 67 | 43 | 2 | 18 | 628700 | 5029596 | 2 | 18 | 0 | 208 | 5168116 | 2 | 18 | 0 | 1 | 216 |
| 153 | 99 | 67 | 43 | 2 | 19 | 628726 | 5029804 | 2 | 19 | 0 | 208 | 5168332 | 2 | 19 | 0 | 1 | 216 |
| 154 | 9A | 67 | 43 | 2 | 20 | 628752 | 5030012 | 2 | 20 | 0 | 208 | 5168548 | 2 | 20 | 0 | 1 | 216 |
| 155 | 9B | 67 | 43 | 2 | 21 | 628778 | 5030220 | 2 | 21 | 0 | 208 | 5168764 | 2 | 21 | 0 | 1 | 216 |
| 156 | 9C | 67 | 43 | 2 | 22 | 628804 | 5030428 | 2 | 22 | 0 | 208 | 5168980 | 2 | 22 | 0 | 1 | 216 |
| 157 | 9D | 67 | 43 | 2 | 23 | 628830 | 5030636 | 2 | 23 | 0 | 208 | 5169196 | 2 | 23 | 0 | 1 | 216 |
| 158 | 9E | 67 | 43 | 2 | 24 | 628856 | 5030844 | 2 | 24 | 0 | 208 | 5169412 | 2 | 24 | 0 | 1 | 216 |
| 159 | 9F | 67 | 43 | 2 | 25 | 628882 | 5031052 | 2 | 25 | 0 | 208 | 5169628 | 2 | 25 | 0 | 1 | 216 |
| 160 | A0 | 67 | 43 | 2 | 26 | 628905 | 5031236 | 2 | 26 | 0 | 184 | 5169820 | 2 | 26 | 0 | 1 | 192 |
| 161 | A1 | 67 | 43 | 2 | 27 | 628928 | 5031420 | 2 | 27 | 0 | 184 | 5170012 | 2 | 27 | 0 | 1 | 192 |
| 162 | A2 | 67 | 43 | 2 | 28 | 628951 | 5031604 | 2 | 28 | 0 | 184 | 5170204 | 2 | 28 | 0 | 1 | 192 |
| 163 | A3 | 67 | 43 | 2 | 29 | 628974 | 5031788 | 2 | 29 | 0 | 184 | 5170396 | 2 | 29 | 0 | 1 | 192 |
| 164 | A4 | 67 | 43 | 2 | 30 | 628997 | 5031972 | 2 | 30 | 0 | 184 | 5170588 | 2 | 30 | 0 | 1 | 192 |
| 165 | A5 | 67 | 43 | 2 | 31 | 629020 | 5032156 | 2 | 31 | 0 | 184 | 5170780 | 2 | 31 | 0 | 1 | 192 |
| 166 | A6 | 67 | 43 | 2 | 32 | 629043 | 5032340 | 2 | 32 | 0 | 184 | 5170972 | 2 | 32 | 0 | 1 | 192 |
| 167 | A7 | 67 | 43 | 2 | 33 | 629066 | 5032524 | 2 | 33 | 0 | 184 | 5171164 | 2 | 33 | 0 | 1 | 192 |
| 168 | A8 | 67 | 43 | 2 | 34 | 629089 | 5032708 | 2 | 34 | 0 | 184 | 5171356 | 2 | 34 | 0 | 1 | 192 |
| 169 | A9 | 67 | 43 | 2 | 35 | 629112 | 5032892 | 2 | 35 | 0 | 184 | 5171548 | 2 | 35 | 0 | 1 | 192 |
| 170 | AA | 67 | 43 | 2 | 36 | 629135 | 5033076 | 2 | 36 | 0 | 184 | 5171740 | 2 | 36 | 0 | 1 | 192 |
| 171 | AB | 67 | 43 | 2 | 37 | 629158 | 5033260 | 2 | 37 | 0 | 184 | 5171932 | 2 | 37 | 0 | 1 | 192 |
| 172 | AC | 67 | 43 | 2 | 38 | 629181 | 5033444 | 2 | 38 | 0 | 184 | 5172124 | 2 | 38 | 0 | 1 | 192 |
| 173 | AD | 67 | 43 | 2 | 39 | 629204 | 5033628 | 2 | 39 | 0 | 184 | 5172316 | 2 | 39 | 0 | 1 | 192 |
| 174 | AE | 67 | 43 | 2 | 40 | 629227 | 5033812 | 2 | 40 | 0 | 184 | 5172508 | 2 | 40 | 0 | 1 | 192 |
| 175 | AF | 67 | 43 | 2 | 41 | 629250 | 5033996 | 2 | 41 | 0 | 184 | 5172700 | 2 | 41 | 0 | 1 | 192 |
| 176 | B0 | 67 | 43 | 2 | 42 | 629275 | 5034196 | 2 | 42 | 0 | 200 | 5172908 | 2 | 42 | 0 | 1 | 208 |
| 177 | B1 | 67 | 43 | 2 | 43 | 629300 | 5034396 | 2 | 43 | 0 | 200 | 5173116 | 2 | 43 | 0 | 1 | 208 |
| 178 | B2 | 67 | 43 | 2 | 44 | 629325 | 5034596 | 2 | 44 | 0 | 200 | 5173324 | 2 | 44 | 0 | 1 | 208 |
| 179 | B3 | 67 | 43 | 2 | 45 | 629350 | 5034796 | 2 | 45 | 0 | 200 | 5173532 | 2 | 45 | 0 | 1 | 208 |
| 180 | B4 | 67 | 43 | 2 | 46 | 629375 | 5034996 | 2 | 46 | 0 | 200 | 5173740 | 2 | 46 | 0 | 1 | 208 |
| 181 | B5 | 67 | 43 | 2 | 47 | 629400 | 5035196 | 2 | 47 | 0 | 200 | 5173948 | 2 | 47 | 0 | 1 | 208 |
| 182 | B6 | 67 | 43 | 2 | 48 | 629425 | 5035396 | 2 | 48 | 0 | 200 | 5174156 | 2 | 48 | 0 | 1 | 208 |
| 183 | B7 | 67 | 43 | 2 | 49 | 629450 | 5035596 | 2 | 49 | 0 | 200 | 5174364 | 2 | 49 | 0 | 1 | 208 |
| 184 | B8 | 67 | 43 | 2 | 50 | 629475 | 5035796 | 2 | 50 | 0 | 200 | 5174572 | 2 | 50 | 0 | 1 | 208 |
| 185 | B9 | 67 | 43 | 2 | 51 | 629500 | 5035996 | 2 | 51 | 0 | 200 | 5174780 | 2 | 51 | 0 | 1 | 208 |
| 186 | BA | 67 | 43 | 2 | 52 | 629525 | 5036196 | 2 | 52 | 0 | 200 | 5174988 | 2 | 52 | 0 | 1 | 208 |
| 187 | BB | 67 | 43 | 2 | 53 | 629550 | 5036396 | 2 | 53 | 0 | 200 | 5175196 | 2 | 53 | 0 | 1 | 208 |
| 188 | BC | 67 | 43 | 2 | 54 | 629575 | 5036596 | 2 | 54 | 0 | 200 | 5175404 | 2 | 54 | 0 | 1 | 208 |
| 189 | BD | 67 | 43 | 2 | 55 | 629600 | 5036796 | 2 | 55 | 0 | 200 | 5175612 | 2 | 55 | 0 | 1 | 208 |
| 190 | BE | 67 | 43 | 2 | 56 | 629625 | 5036996 | 2 | 56 | 0 | 200 | 5175820 | 2 | 56 | 0 | 1 | 208 |
| 191 | BF | 67 | 43 | 2 | 57 | 629650 | 5037196 | 2 | 57 | 0 | 200 | 5176028 | 2 | 57 | 0 | 1 | 208 |
| 192 | C0 | 67 | 43 | 2 | 58 | 629675 | 5037396 | 2 | 58 | 0 | 200 | 5176236 | 2 | 58 | 0 | 1 | 208 |
| 193 | C1 | 67 | 43 | 2 | 59 | 629700 | 5037596 | 2 | 59 | 0 | 200 | 5176444 | 2 | 59 | 0 | 1 | 208 |
| 194 | C2 | 67 | 43 | 2 | 60 | 629725 | 5037796 | 2 | 60 | 0 | 200 | 5176652 | 2 | 60 | 0 | 1 | 208 |
| 195 | C3 | 67 | 43 | 2 | 61 | 629750 | 5037996 | 2 | 61 | 0 | 200 | 5176860 | 2 | 61 | 0 | 1 | 208 |
| 196 | C4 | 67 | 43 | 2 | 62 | 629775 | 5038196 | 2 | 62 | 0 | 200 | 5177068 | 2 | 62 | 0 | 1 | 208 |
| 197 | C5 | 67 | 43 | 2 | 63 | 629800 | 5038396 | 2 | 63 | 0 | 200 | 5177276 | 2 | 63 | 0 | 1 | 208 |
| 198 | C6 | 67 | 43 | 2 | 64 | 629825 | 5038596 | 2 | 64 | 0 | 200 | 5177484 | 2 | 64 | 0 | 1 | 208 |
| 199 | C7 | 67 | 43 | 2 | 65 | 629850 | 5038796 | 2 | 65 | 0 | 200 | 5177692 | 2 | 65 | 0 | 1 | 208 |
| 200 | C8 | 67 | 43 | 2 | 66 | 629875 | 5038996 | 2 | 66 | 0 | 200 | 5177900 | 2 | 66 | 0 | 1 | 208 |
| 201 | C9 | 67 | 43 | 3 | 0 | 629898 | 5039180 | 3 | 0 | 0 | 184 | 5178092 | 3 | 0 | 0 | 1 | 192 |
| 202 | CA | 67 | 43 | 3 | 1 | 629926 | 5039404 | 3 | 1 | 0 | 224 | 5178324 | 3 | 1 | 0 | 1 | 232 |
| 203 | CB | 67 | 43 | 3 | 2 | 629954 | 5039628 | 3 | 2 | 0 | 224 | 5178556 | 3 | 2 | 0 | 1 | 232 |
| 204 | CC | 67 | 43 | 3 | 3 | 629982 | 5039852 | 3 | 3 | 0 | 224 | 5178788 | 3 | 3 | 0 | 1 | 232 |
| 205 | CD | 67 | 43 | 3 | 4 | 630010 | 5040076 | 3 | 4 | 0 | 224 | 5179020 | 3 | 4 | 0 | 1 | 232 |
| 206 | CE | 67 | 43 | 3 | 5 | 630038 | 5040300 | 3 | 5 | 0 | 224 | 5179252 | 3 | 5 | 0 | 1 | 232 |
| 207 | CF | 67 | 43 | 3 | 6 | 630066 | 5040524 | 3 | 6 | 0 | 224 | 5179484 | 3 | 6 | 0 | 1 | 232 |
| 208 | D0 | 67 | 43 | 3 | 7 | 630094 | 5040748 | 3 | 7 | 0 | 224 | 5179716 | 3 | 7 | 0 | 1 | 232 |
| 209 | D1 | 67 | 43 | 3 | 8 | 630122 | 5040972 | 3 | 8 | 0 | 224 | 5179948 | 3 | 8 | 0 | 1 | 232 |
| 210 | D2 | 67 | 43 | 3 | 9 | 630150 | 5041196 | 3 | 9 | 0 | 224 | 5180180 | 3 | 9 | 0 | 1 | 232 |
| 211 | D3 | 67 | 43 | 3 | 10 | 630178 | 5041420 | 3 | 10 | 0 | 224 | 5180412 | 3 | 10 | 0 | 1 | 232 |
| 212 | D4 | 67 | 43 | 3 | 11 | 630206 | 5041644 | 3 | 11 | 0 | 224 | 5180644 | 3 | 11 | 0 | 1 | 232 |
| 213 | D5 | 67 | 43 | 3 | 12 | 630234 | 5041868 | 3 | 12 | 0 | 224 | 5180876 | 3 | 12 | 0 | 1 | 232 |
| 214 | D6 | 67 | 43 | 3 | 13 | 630262 | 5042092 | 3 | 13 | 0 | 224 | 5181108 | 3 | 13 | 0 | 1 | 232 |
| 215 | D7 | 67 | 43 | 3 | 14 | 630290 | 5042316 | 3 | 14 | 0 | 224 | 5181340 | 3 | 14 | 0 | 1 | 232 |
| 216 | D8 | 67 | 43 | 3 | 15 | 630318 | 5042540 | 3 | 15 | 0 | 224 | 5181572 | 3 | 15 | 0 | 1 | 232 |
| 217 | D9 | 67 | 43 | 3 | 16 | 630346 | 5042764 | 3 | 16 | 0 | 224 | 5181804 | 3 | 16 | 0 | 1 | 232 |
| 218 | DA | 67 | 43 | 3 | 17 | 630374 | 5042988 | 3 | 17 | 0 | 224 | 5182036 | 3 | 17 | 0 | 1 | 232 |
| 219 | DB | 67 | 43 | 3 | 18 | 630402 | 5043212 | 3 | 18 | 0 | 224 | 5182268 | 3 | 18 | 0 | 1 | 232 |
| 220 | DC | 67 | 43 | 3 | 19 | 630430 | 5043436 | 3 | 19 | 0 | 224 | 5182500 | 3 | 19 | 0 | 1 | 232 |
| 221 | DD | 67 | 43 | 3 | 20 | 630458 | 5043660 | 3 | 20 | 0 | 224 | 5182732 | 3 | 20 | 0 | 1 | 232 |
| 222 | DE | 67 | 43 | 3 | 21 | 630486 | 5043884 | 3 | 21 | 0 | 224 | 5182964 | 3 | 21 | 0 | 1 | 232 |
| 223 | DF | 67 | 43 | 3 | 22 | 630514 | 5044108 | 3 | 22 | 0 | 224 | 5183196 | 3 | 22 | 0 | 1 | 232 |
| 224 | E0 | 67 | 43 | 3 | 23 | 630542 | 5044332 | 3 | 23 | 0 | 224 | 5183428 | 3 | 23 | 0 | 1 | 232 |
| 225 | E1 | 67 | 43 | 3 | 24 | 630570 | 5044556 | 3 | 24 | 0 | 224 | 5183660 | 3 | 24 | 0 | 1 | 232 |
| 226 | E2 | 67 | 43 | 3 | 25 | 630598 | 5044780 | 3 | 25 | 0 | 224 | 5183892 | 3 | 25 | 0 | 1 | 232 |
| 227 | E3 | 67 | 43 | 3 | 26 | 630626 | 5045004 | 3 | 26 | 0 | 224 | 5184124 | 3 | 26 | 0 | 1 | 232 |
| 228 | E4 | 67 | 43 | 3 | 27 | 630654 | 5045228 | 3 | 27 | 0 | 224 | 5184356 | 3 | 27 | 0 | 1 | 232 |
| 229 | E5 | 67 | 43 | 3 | 28 | 630682 | 5045452 | 3 | 28 | 0 | 224 | 5184588 | 3 | 28 | 0 | 1 | 232 |
| 230 | E6 | 67 | 43 | 3 | 29 | 630710 | 5045676 | 3 | 29 | 0 | 224 | 5184820 | 3 | 29 | 0 | 1 | 232 |
| 231 | E7 | 67 | 43 | 3 | 30 | 630738 | 5045900 | 3 | 30 | 0 | 224 | 5185052 | 3 | 30 | 0 | 1 | 232 |
| 232 | E8 | 67 | 43 | 3 | 31 | 630766 | 5046124 | 3 | 31 | 0 | 224 | 5185284 | 3 | 31 | 0 | 1 | 232 |
| 233 | E9 | 67 | 43 | 3 | 32 | 630794 | 5046348 | 3 | 32 | 0 | 224 | 5185516 | 3 | 32 | 0 | 1 | 232 |
| 234 | EA | 67 | 43 | 3 | 33 | 630822 | 5046572 | 3 | 33 | 0 | 224 | 5185748 | 3 | 33 | 0 | 1 | 232 |
| 235 | EB | 67 | 43 | 3 | 34 | 630850 | 5046796 | 3 | 34 | 0 | 224 | 5185980 | 3 | 34 | 0 | 1 | 232 |
| 236 | EC | 67 | 43 | 3 | 35 | 630878 | 5047020 | 3 | 35 | 0 | 224 | 5186212 | 3 | 35 | 0 | 1 | 232 |
| 237 | ED | 67 | 43 | 3 | 36 | 630906 | 5047244 | 3 | 36 | 0 | 224 | 5186444 | 3 | 36 | 0 | 1 | 232 |
| 238 | EE | 67 | 43 | 3 | 37 | 630934 | 5047468 | 3 | 37 | 0 | 224 | 5186676 | 3 | 37 | 0 | 1 | 232 |
| 239 | EF | 67 | 43 | 3 | 38 | 630962 | 5047692 | 3 | 38 | 0 | 224 | 5186908 | 3 | 38 | 0 | 1 | 232 |
| 240 | F0 | 67 | 43 | 3 | 39 | 630987 | 5047892 | 3 | 39 | 0 | 200 | 5187116 | 3 | 39 | 0 | 1 | 208 |
| 241 | F1 | 67 | 43 | 3 | 40 | 631012 | 5048092 | 3 | 40 | 0 | 200 | 5187324 | 3 | 40 | 0 | 1 | 208 |
| 242 | F2 | 67 | 43 | 3 | 41 | 631037 | 5048292 | 3 | 41 | 0 | 200 | 5187532 | 3 | 41 | 0 | 1 | 208 |
| 243 | F3 | 67 | 43 | 3 | 42 | 631062 | 5048492 | 3 | 42 | 0 | 200 | 5187740 | 3 | 42 | 0 | 1 | 208 |
| 244 | F4 | 67 | 43 | 3 | 43 | 631087 | 5048692 | 3 | 43 | 0 | 200 | 5187948 | 3 | 43 | 0 | 1 | 208 |
| 245 | F5 | 67 | 43 | 3 | 44 | 631112 | 5048892 | 3 | 44 | 0 | 200 | 5188156 | 3 | 44 | 0 | 1 | 208 |
| 246 | F6 | 67 | 43 | 3 | 45 | 631137 | 5049092 | 3 | 45 | 0 | 200 | 5188364 | 3 | 45 | 0 | 1 | 208 |
| 247 | F7 | 67 | 43 | 3 | 46 | 631162 | 5049292 | 3 | 46 | 0 | 200 | 5188572 | 3 | 46 | 0 | 1 | 208 |
| 248 | F8 | 67 | 43 | 3 | 47 | 631187 | 5049492 | 3 | 47 | 0 | 200 | 5188780 | 3 | 47 | 0 | 1 | 208 |
| 249 | F9 | 67 | 43 | 3 | 48 | 631212 | 5049692 | 3 | 48 | 0 | 200 | 5188988 | 3 | 48 | 0 | 1 | 208 |
| 250 | FA | 67 | 43 | 3 | 49 | 631237 | 5049892 | 3 | 49 | 0 | 200 | 5189196 | 3 | 49 | 0 | 1 | 208 |
| 251 | FB | 67 | 43 | 3 | 50 | 631262 | 5050092 | 3 | 50 | 0 | 200 | 5189404 | 3 | 50 | 0 | 1 | 208 |

| | | | | | | | | | | | | | | | | | |
|---|---|---|---|---|---|---|---|---|---|---|---|---|---|---|---|---|---|
| 252 | FC | 67 | 43 | 3 | 51 | 631287 | 5050292 | 3 | 51 | 0 | 200 | 5189612 | 3 | 51 | 0 | 1 | 208 |
| 253 | FD | 67 | 43 | 3 | 52 | 631312 | 5050492 | 3 | 52 | 0 | 200 | 5189820 | 3 | 52 | 0 | 1 | 208 |
| 254 | FE | 67 | 43 | 3 | 53 | 631337 | 5050692 | 3 | 53 | 0 | 200 | 5190028 | 3 | 53 | 0 | 1 | 208 |
| 255 | FF | 67 | 43 | 3 | 54 | 631362 | 5050892 | 3 | 54 | 0 | 200 | 5190236 | 3 | 54 | 0 | 1 | 208 |
| 0 | 00 | 68 | 44 | 0 | 0 | 631374 | 5050988 | 0 | 0 | 0 | 96 | 5190340 | 0 | 0 | 0 | 1 | 104 |
| 1 | 01 | 68 | 44 | 0 | 1 | 631388 | 5051100 | 0 | 1 | 0 | 112 | 5190460 | 0 | 1 | 0 | 1 | 120 |
| 2 | 02 | 68 | 44 | 0 | 2 | 631402 | 5051212 | 0 | 2 | 0 | 112 | 5190580 | 0 | 2 | 0 | 1 | 120 |
| 3 | 03 | 68 | 44 | 0 | 3 | 631416 | 5051324 | 0 | 3 | 0 | 112 | 5190700 | 0 | 3 | 0 | 1 | 120 |
| 4 | 04 | 68 | 44 | 0 | 4 | 631430 | 5051436 | 0 | 4 | 0 | 112 | 5190820 | 0 | 4 | 0 | 1 | 120 |
| 5 | 05 | 68 | 44 | 0 | 5 | 631444 | 5051548 | 0 | 5 | 0 | 112 | 5190940 | 0 | 5 | 0 | 1 | 120 |
| 6 | 06 | 68 | 44 | 0 | 6 | 631458 | 5051660 | 0 | 6 | 0 | 112 | 5191060 | 0 | 6 | 0 | 1 | 120 |
| 7 | 07 | 68 | 44 | 0 | 7 | 631472 | 5051772 | 0 | 7 | 0 | 112 | 5191180 | 0 | 7 | 0 | 1 | 120 |
| 8 | 08 | 68 | 44 | 0 | 8 | 631486 | 5051884 | 0 | 8 | 0 | 112 | 5191300 | 0 | 8 | 0 | 1 | 120 |
| 9 | 09 | 68 | 44 | 0 | 9 | 631500 | 5051996 | 0 | 9 | 0 | 112 | 5191420 | 0 | 9 | 0 | 1 | 120 |
| 10 | 0A | 68 | 44 | 0 | 10 | 631514 | 5052108 | 0 | 10 | 0 | 112 | 5191540 | 0 | 10 | 0 | 1 | 120 |
| 11 | 0B | 68 | 44 | 0 | 11 | 631528 | 5052220 | 0 | 11 | 0 | 112 | 5191660 | 0 | 11 | 0 | 1 | 120 |
| 12 | 0C | 68 | 44 | 0 | 12 | 631542 | 5052332 | 0 | 12 | 0 | 112 | 5191780 | 0 | 12 | 0 | 1 | 120 |
| 13 | 0D | 68 | 44 | 0 | 13 | 631556 | 5052444 | 0 | 13 | 0 | 112 | 5191900 | 0 | 13 | 0 | 1 | 120 |
| 14 | 0E | 68 | 44 | 0 | 14 | 631570 | 5052556 | 0 | 14 | 0 | 112 | 5192020 | 0 | 14 | 0 | 1 | 120 |
| 15 | 0F | 68 | 44 | 0 | 15 | 631584 | 5052668 | 0 | 15 | 0 | 112 | 5192140 | 0 | 15 | 0 | 1 | 120 |
| 16 | 10 | 68 | 44 | 0 | 16 | 631598 | 5052780 | 0 | 16 | 0 | 112 | 5192260 | 0 | 16 | 0 | 1 | 120 |
| 17 | 11 | 68 | 44 | 0 | 17 | 631612 | 5052892 | 0 | 17 | 0 | 112 | 5192380 | 0 | 17 | 0 | 1 | 120 |
| 18 | 12 | 68 | 44 | 0 | 18 | 631626 | 5053004 | 0 | 18 | 0 | 112 | 5192500 | 0 | 18 | 0 | 1 | 120 |
| 19 | 13 | 68 | 44 | 0 | 19 | 631640 | 5053116 | 0 | 19 | 0 | 112 | 5192620 | 0 | 19 | 0 | 1 | 120 |
| 20 | 14 | 68 | 44 | 0 | 20 | 631654 | 5053228 | 0 | 20 | 0 | 112 | 5192740 | 0 | 20 | 0 | 1 | 120 |
| 21 | 15 | 68 | 44 | 0 | 21 | 631668 | 5053340 | 0 | 21 | 0 | 112 | 5192860 | 0 | 21 | 0 | 1 | 120 |
| 22 | 16 | 68 | 44 | 0 | 22 | 631682 | 5053452 | 0 | 22 | 0 | 112 | 5192980 | 0 | 22 | 0 | 1 | 120 |
| 23 | 17 | 68 | 44 | 0 | 23 | 631696 | 5053564 | 0 | 23 | 0 | 112 | 5193100 | 0 | 23 | 0 | 1 | 120 |
| 24 | 18 | 68 | 44 | 0 | 24 | 631710 | 5053676 | 0 | 24 | 0 | 112 | 5193220 | 0 | 24 | 0 | 1 | 120 |
| 25 | 19 | 68 | 44 | 0 | 25 | 631724 | 5053788 | 0 | 25 | 0 | 112 | 5193340 | 0 | 25 | 0 | 1 | 120 |
| 26 | 1A | 68 | 44 | 0 | 26 | 631738 | 5053900 | 0 | 26 | 0 | 112 | 5193460 | 0 | 26 | 0 | 1 | 120 |
| 27 | 1B | 68 | 44 | 0 | 27 | 631752 | 5054012 | 0 | 27 | 0 | 112 | 5193580 | 0 | 27 | 0 | 1 | 120 |
| 28 | 1C | 68 | 44 | 0 | 28 | 631766 | 5054124 | 0 | 28 | 0 | 112 | 5193700 | 0 | 28 | 0 | 1 | 120 |
| 29 | 1D | 68 | 44 | 0 | 29 | 631780 | 5054236 | 0 | 29 | 0 | 112 | 5193820 | 0 | 29 | 0 | 1 | 120 |
| 30 | 1E | 68 | 44 | 0 | 30 | 631794 | 5054348 | 0 | 30 | 0 | 112 | 5193940 | 0 | 30 | 0 | 1 | 120 |
| 31 | 1F | 68 | 44 | 0 | 31 | 631808 | 5054460 | 0 | 31 | 0 | 112 | 5194060 | 0 | 31 | 0 | 1 | 120 |
| 32 | 20 | 68 | 44 | 0 | 32 | 631822 | 5054572 | 0 | 32 | 0 | 112 | 5194180 | 0 | 32 | 0 | 1 | 120 |
| 33 | 21 | 68 | 44 | 0 | 33 | 631836 | 5054684 | 0 | 33 | 0 | 112 | 5194300 | 0 | 33 | 0 | 1 | 120 |
| 34 | 22 | 68 | 44 | 0 | 34 | 631850 | 5054796 | 0 | 34 | 0 | 112 | 5194420 | 0 | 34 | 0 | 1 | 120 |
| 35 | 23 | 68 | 44 | 0 | 35 | 631864 | 5054908 | 0 | 35 | 0 | 112 | 5194540 | 0 | 35 | 0 | 1 | 120 |
| 36 | 24 | 68 | 44 | 0 | 36 | 631878 | 5055020 | 0 | 36 | 0 | 112 | 5194660 | 0 | 36 | 0 | 1 | 120 |
| 37 | 25 | 68 | 44 | 0 | 37 | 631892 | 5055132 | 0 | 37 | 0 | 112 | 5194780 | 0 | 37 | 0 | 1 | 120 |
| 38 | 26 | 68 | 44 | 0 | 38 | 631906 | 5055244 | 0 | 38 | 0 | 112 | 5194900 | 0 | 38 | 0 | 1 | 120 |
| 39 | 27 | 68 | 44 | 0 | 39 | 631920 | 5055356 | 0 | 39 | 0 | 112 | 5195020 | 0 | 39 | 0 | 1 | 120 |
| 40 | 28 | 68 | 44 | 0 | 40 | 631934 | 5055468 | 0 | 40 | 0 | 112 | 5195140 | 0 | 40 | 0 | 1 | 120 |
| 41 | 29 | 68 | 44 | 0 | 41 | 631948 | 5055580 | 0 | 41 | 0 | 112 | 5195260 | 0 | 41 | 0 | 1 | 120 |
| 42 | 2A | 68 | 44 | 0 | 42 | 631962 | 5055692 | 0 | 42 | 0 | 112 | 5195380 | 0 | 42 | 0 | 1 | 120 |
| 43 | 2B | 68 | 44 | 0 | 43 | 631976 | 5055804 | 0 | 43 | 0 | 112 | 5195500 | 0 | 43 | 0 | 1 | 120 |
| 44 | 2C | 68 | 44 | 0 | 44 | 631990 | 5055916 | 0 | 44 | 0 | 112 | 5195620 | 0 | 44 | 0 | 1 | 120 |
| 45 | 2D | 68 | 44 | 0 | 45 | 632004 | 5056028 | 0 | 45 | 0 | 112 | 5195740 | 0 | 45 | 0 | 1 | 120 |
| 46 | 2E | 68 | 44 | 0 | 46 | 632018 | 5056140 | 0 | 46 | 0 | 112 | 5195860 | 0 | 46 | 0 | 1 | 120 |
| 47 | 2F | 68 | 44 | 0 | 47 | 632032 | 5056252 | 0 | 47 | 0 | 112 | 5195980 | 0 | 47 | 0 | 1 | 120 |
| 48 | 30 | 68 | 44 | 0 | 48 | 632046 | 5056364 | 0 | 48 | 0 | 112 | 5196100 | 0 | 48 | 0 | 1 | 120 |
| 49 | 31 | 68 | 44 | 0 | 49 | 632060 | 5056476 | 0 | 49 | 0 | 112 | 5196220 | 0 | 49 | 0 | 1 | 120 |
| 50 | 32 | 68 | 44 | 0 | 50 | 632074 | 5056588 | 0 | 50 | 0 | 112 | 5196340 | 0 | 50 | 0 | 1 | 120 |
| 51 | 33 | 68 | 44 | 0 | 51 | 632088 | 5056700 | 0 | 51 | 0 | 112 | 5196460 | 0 | 51 | 0 | 1 | 120 |
| 52 | 34 | 68 | 44 | 0 | 52 | 632102 | 5056812 | 0 | 52 | 0 | 112 | 5196580 | 0 | 52 | 0 | 1 | 120 |
| 53 | 35 | 68 | 44 | 0 | 53 | 632116 | 5056924 | 0 | 53 | 0 | 112 | 5196700 | 0 | 53 | 0 | 1 | 120 |
| 54 | 36 | 68 | 44 | 0 | 54 | 632130 | 5057036 | 0 | 54 | 0 | 112 | 5196820 | 0 | 54 | 0 | 1 | 120 |
| 55 | 37 | 68 | 44 | 0 | 55 | 632144 | 5057148 | 0 | 55 | 0 | 112 | 5196940 | 0 | 55 | 0 | 1 | 120 |
| 56 | 38 | 68 | 44 | 0 | 56 | 632158 | 5057260 | 0 | 56 | 0 | 112 | 5197060 | 0 | 56 | 0 | 1 | 120 |
| 57 | 39 | 68 | 44 | 0 | 57 | 632172 | 5057372 | 0 | 57 | 0 | 112 | 5197180 | 0 | 57 | 0 | 1 | 120 |
| 58 | 3A | 68 | 44 | 0 | 58 | 632186 | 5057484 | 0 | 58 | 0 | 112 | 5197300 | 0 | 58 | 0 | 1 | 120 |
| 59 | 3B | 68 | 44 | 0 | 59 | 632200 | 5057596 | 0 | 59 | 0 | 112 | 5197420 | 0 | 59 | 0 | 1 | 120 |
| 60 | 3C | 68 | 44 | 0 | 60 | 632214 | 5057708 | 0 | 60 | 0 | 112 | 5197540 | 0 | 60 | 0 | 1 | 120 |
| 61 | 3D | 68 | 44 | 0 | 61 | 632228 | 5057820 | 0 | 61 | 0 | 112 | 5197660 | 0 | 61 | 0 | 1 | 120 |
| 62 | 3E | 68 | 44 | 0 | 62 | 632242 | 5057932 | 0 | 62 | 0 | 112 | 5197780 | 0 | 62 | 0 | 1 | 120 |
| 63 | 3F | 68 | 44 | 0 | 63 | 632256 | 5058044 | 0 | 63 | 0 | 112 | 5197900 | 0 | 63 | 0 | 1 | 120 |
| 64 | 40 | 68 | 44 | 0 | 64 | 632270 | 5058156 | 0 | 64 | 0 | 112 | 5198020 | 0 | 64 | 0 | 1 | 120 |
| 65 | 41 | 68 | 44 | 0 | 65 | 632284 | 5058268 | 0 | 65 | 0 | 112 | 5198140 | 0 | 65 | 0 | 1 | 120 |
| 66 | 42 | 68 | 44 | 0 | 66 | 632298 | 5058380 | 0 | 66 | 0 | 112 | 5198260 | 0 | 66 | 0 | 1 | 120 |
| 67 | 43 | 68 | 44 | 0 | 67 | 632312 | 5058492 | 0 | 67 | 0 | 112 | 5198380 | 0 | 67 | 0 | 1 | 120 |
| 68 | 44 | 68 | 44 | 1 | 0 | 632324 | 5058588 | 1 | 0 | 0 | 96 | 5198484 | 1 | 0 | 0 | 1 | 104 |
| 69 | 45 | 68 | 44 | 1 | 1 | 632341 | 5058724 | 1 | 1 | 0 | 136 | 5198628 | 1 | 1 | 0 | 1 | 144 |
| 70 | 46 | 68 | 44 | 1 | 2 | 632358 | 5058860 | 1 | 2 | 0 | 136 | 5198772 | 1 | 2 | 0 | 1 | 144 |
| 71 | 47 | 68 | 44 | 1 | 3 | 632375 | 5058996 | 1 | 3 | 0 | 136 | 5198916 | 1 | 3 | 0 | 1 | 144 |
| 72 | 48 | 68 | 44 | 1 | 4 | 632392 | 5059132 | 1 | 4 | 0 | 136 | 5199060 | 1 | 4 | 0 | 1 | 144 |
| 73 | 49 | 68 | 44 | 1 | 5 | 632409 | 5059268 | 1 | 5 | 0 | 136 | 5199204 | 1 | 5 | 0 | 1 | 144 |
| 74 | 4A | 68 | 44 | 1 | 6 | 632426 | 5059404 | 1 | 6 | 0 | 136 | 5199348 | 1 | 6 | 0 | 1 | 144 |
| 75 | 4B | 68 | 44 | 1 | 7 | 632443 | 5059540 | 1 | 7 | 0 | 136 | 5199492 | 1 | 7 | 0 | 1 | 144 |
| 76 | 4C | 68 | 44 | 1 | 8 | 632460 | 5059676 | 1 | 8 | 0 | 136 | 5199636 | 1 | 8 | 0 | 1 | 144 |
| 77 | 4D | 68 | 44 | 1 | 9 | 632477 | 5059812 | 1 | 9 | 0 | 136 | 5199780 | 1 | 9 | 0 | 1 | 144 |
| 78 | 4E | 68 | 44 | 1 | 10 | 632494 | 5059948 | 1 | 10 | 0 | 136 | 5199924 | 1 | 10 | 0 | 1 | 144 |
| 79 | 4F | 68 | 44 | 1 | 11 | 632511 | 5060084 | 1 | 11 | 0 | 136 | 5200068 | 1 | 11 | 0 | 1 | 144 |
| 80 | 50 | 68 | 44 | 1 | 12 | 632532 | 5060252 | 1 | 12 | 0 | 168 | 5200244 | 1 | 12 | 0 | 1 | 176 |
| 81 | 51 | 68 | 44 | 1 | 13 | 632553 | 5060420 | 1 | 13 | 0 | 168 | 5200420 | 1 | 13 | 0 | 1 | 176 |
| 82 | 52 | 68 | 44 | 1 | 14 | 632574 | 5060588 | 1 | 14 | 0 | 168 | 5200596 | 1 | 14 | 0 | 1 | 176 |
| 83 | 53 | 68 | 44 | 1 | 15 | 632595 | 5060756 | 1 | 15 | 0 | 168 | 5200772 | 1 | 15 | 0 | 1 | 176 |
| 84 | 54 | 68 | 44 | 1 | 16 | 632616 | 5060924 | 1 | 16 | 0 | 168 | 5200948 | 1 | 16 | 0 | 1 | 176 |
| 85 | 55 | 68 | 44 | 1 | 17 | 632637 | 5061092 | 1 | 17 | 0 | 168 | 5201124 | 1 | 17 | 0 | 1 | 176 |
| 86 | 56 | 68 | 44 | 1 | 18 | 632658 | 5061260 | 1 | 18 | 0 | 168 | 5201300 | 1 | 18 | 0 | 1 | 176 |
| 87 | 57 | 68 | 44 | 1 | 19 | 632679 | 5061428 | 1 | 19 | 0 | 168 | 5201476 | 1 | 19 | 0 | 1 | 176 |
| 88 | 58 | 68 | 44 | 1 | 20 | 632700 | 5061596 | 1 | 20 | 0 | 168 | 5201652 | 1 | 20 | 0 | 1 | 176 |
| 89 | 59 | 68 | 44 | 1 | 21 | 632721 | 5061764 | 1 | 21 | 0 | 168 | 5201828 | 1 | 21 | 0 | 1 | 176 |
| 90 | 5A | 68 | 44 | 1 | 22 | 632742 | 5061932 | 1 | 22 | 0 | 168 | 5202004 | 1 | 22 | 0 | 1 | 176 |
| 91 | 5B | 68 | 44 | 1 | 23 | 632763 | 5062100 | 1 | 23 | 0 | 168 | 5202180 | 1 | 23 | 0 | 1 | 176 |
| 92 | 5C | 68 | 44 | 1 | 24 | 632784 | 5062268 | 1 | 24 | 0 | 168 | 5202356 | 1 | 24 | 0 | 1 | 176 |
| 93 | 5D | 68 | 44 | 1 | 25 | 632805 | 5062436 | 1 | 25 | 0 | 168 | 5202532 | 1 | 25 | 0 | 1 | 176 |
| 94 | 5E | 68 | 44 | 1 | 26 | 632826 | 5062604 | 1 | 26 | 0 | 168 | 5202708 | 1 | 26 | 0 | 1 | 176 |
| 95 | 5F | 68 | 44 | 1 | 27 | 632847 | 5062772 | 1 | 27 | 0 | 168 | 5202884 | 1 | 27 | 0 | 1 | 176 |
| 96 | 60 | 68 | 44 | 1 | 28 | 632870 | 5062956 | 1 | 28 | 0 | 184 | 5203076 | 1 | 28 | 0 | 1 | 192 |
| 97 | 61 | 68 | 44 | 1 | 29 | 632893 | 5063140 | 1 | 29 | 0 | 184 | 5203268 | 1 | 29 | 0 | 1 | 192 |
| 98 | 62 | 68 | 44 | 1 | 30 | 632916 | 5063324 | 1 | 30 | 0 | 184 | 5203460 | 1 | 30 | 0 | 1 | 192 |

| | | | | | | | | | | | | | | | | | |
|---|---|---|---|---|---|---|---|---|---|---|---|---|---|---|---|---|---|
| 99 | 63 | 68 | 44 | 1 | 31 | 632939 | 5063508 | 1 | 31 | 0 | 184 | 5203652 | 1 | 31 | 0 | 1 | 192 |
| 100 | 64 | 68 | 44 | 1 | 32 | 632962 | 5063692 | 1 | 32 | 0 | 184 | 5203844 | 1 | 32 | 0 | 1 | 192 |
| 101 | 65 | 68 | 44 | 1 | 33 | 632985 | 5063876 | 1 | 33 | 0 | 184 | 5204036 | 1 | 33 | 0 | 1 | 192 |
| 102 | 66 | 68 | 44 | 1 | 34 | 633008 | 5064060 | 1 | 34 | 0 | 184 | 5204228 | 1 | 34 | 0 | 1 | 192 |
| 103 | 67 | 68 | 44 | 1 | 35 | 633031 | 5064244 | 1 | 35 | 0 | 184 | 5204420 | 1 | 35 | 0 | 1 | 192 |
| 104 | 68 | 68 | 44 | 1 | 36 | 633054 | 5064428 | 1 | 36 | 0 | 184 | 5204612 | 1 | 36 | 0 | 1 | 192 |
| 105 | 69 | 68 | 44 | 1 | 37 | 633077 | 5064612 | 1 | 37 | 0 | 184 | 5204804 | 1 | 37 | 0 | 1 | 192 |
| 106 | 6A | 68 | 44 | 1 | 38 | 633100 | 5064796 | 1 | 38 | 0 | 184 | 5204996 | 1 | 38 | 0 | 1 | 192 |
| 107 | 6B | 68 | 44 | 1 | 39 | 633123 | 5064980 | 1 | 39 | 0 | 184 | 5205188 | 1 | 39 | 0 | 1 | 192 |
| 108 | 6C | 68 | 44 | 1 | 40 | 633146 | 5065164 | 1 | 40 | 0 | 184 | 5205380 | 1 | 40 | 0 | 1 | 192 |
| 109 | 6D | 68 | 44 | 1 | 41 | 633169 | 5065348 | 1 | 41 | 0 | 184 | 5205572 | 1 | 41 | 0 | 1 | 192 |
| 110 | 6E | 68 | 44 | 1 | 42 | 633192 | 5065532 | 1 | 42 | 0 | 184 | 5205764 | 1 | 42 | 0 | 1 | 192 |
| 111 | 6F | 68 | 44 | 1 | 43 | 633215 | 5065716 | 1 | 43 | 0 | 184 | 5205956 | 1 | 43 | 0 | 1 | 192 |
| 112 | 70 | 68 | 44 | 1 | 44 | 633238 | 5065900 | 1 | 44 | 0 | 184 | 5206148 | 1 | 44 | 0 | 1 | 192 |
| 113 | 71 | 68 | 44 | 1 | 45 | 633261 | 5066084 | 1 | 45 | 0 | 184 | 5206340 | 1 | 45 | 0 | 1 | 192 |
| 114 | 72 | 68 | 44 | 1 | 46 | 633284 | 5066268 | 1 | 46 | 0 | 184 | 5206532 | 1 | 46 | 0 | 1 | 192 |
| 115 | 73 | 68 | 44 | 1 | 47 | 633307 | 5066452 | 1 | 47 | 0 | 184 | 5206724 | 1 | 47 | 0 | 1 | 192 |
| 116 | 74 | 68 | 44 | 1 | 48 | 633330 | 5066636 | 1 | 48 | 0 | 184 | 5206916 | 1 | 48 | 0 | 1 | 192 |
| 117 | 75 | 68 | 44 | 1 | 49 | 633353 | 5066820 | 1 | 49 | 0 | 184 | 5207108 | 1 | 49 | 0 | 1 | 192 |
| 118 | 76 | 68 | 44 | 1 | 50 | 633376 | 5067004 | 1 | 50 | 0 | 184 | 5207300 | 1 | 50 | 0 | 1 | 192 |
| 119 | 77 | 68 | 44 | 1 | 51 | 633399 | 5067188 | 1 | 51 | 0 | 184 | 5207492 | 1 | 51 | 0 | 1 | 192 |
| 120 | 78 | 68 | 44 | 1 | 52 | 633422 | 5067372 | 1 | 52 | 0 | 184 | 5207684 | 1 | 52 | 0 | 1 | 192 |
| 121 | 79 | 68 | 44 | 1 | 53 | 633445 | 5067556 | 1 | 53 | 0 | 184 | 5207876 | 1 | 53 | 0 | 1 | 192 |
| 122 | 7A | 68 | 44 | 1 | 54 | 633468 | 5067740 | 1 | 54 | 0 | 184 | 5208068 | 1 | 54 | 0 | 1 | 192 |
| 123 | 7B | 68 | 44 | 1 | 55 | 633491 | 5067924 | 1 | 55 | 0 | 184 | 5208260 | 1 | 55 | 0 | 1 | 192 |
| 124 | 7C | 68 | 44 | 1 | 56 | 633514 | 5068108 | 1 | 56 | 0 | 184 | 5208452 | 1 | 56 | 0 | 1 | 192 |
| 125 | 7D | 68 | 44 | 1 | 57 | 633537 | 5068292 | 1 | 57 | 0 | 184 | 5208644 | 1 | 57 | 0 | 1 | 192 |
| 126 | 7E | 68 | 44 | 1 | 58 | 633560 | 5068476 | 1 | 58 | 0 | 184 | 5208836 | 1 | 58 | 0 | 1 | 192 |
| 127 | 7F | 68 | 44 | 1 | 59 | 633583 | 5068660 | 1 | 59 | 0 | 184 | 5209028 | 1 | 59 | 0 | 1 | 192 |
| 128 | 80 | 68 | 44 | 1 | 60 | 633606 | 5068844 | 1 | 60 | 0 | 184 | 5209220 | 1 | 60 | 0 | 1 | 192 |
| 129 | 81 | 68 | 44 | 1 | 61 | 633629 | 5069028 | 1 | 61 | 0 | 184 | 5209412 | 1 | 61 | 0 | 1 | 192 |
| 130 | 82 | 68 | 44 | 1 | 62 | 633652 | 5069212 | 1 | 62 | 0 | 184 | 5209604 | 1 | 62 | 0 | 1 | 192 |
| 131 | 83 | 68 | 44 | 1 | 63 | 633675 | 5069396 | 1 | 63 | 0 | 184 | 5209796 | 1 | 63 | 0 | 1 | 192 |
| 132 | 84 | 68 | 44 | 1 | 64 | 633698 | 5069580 | 1 | 64 | 0 | 184 | 5209988 | 1 | 64 | 0 | 1 | 192 |
| 133 | 85 | 68 | 44 | 1 | 65 | 633721 | 5069764 | 1 | 65 | 0 | 184 | 5210180 | 1 | 65 | 0 | 1 | 192 |
| 134 | 86 | 68 | 44 | 1 | 66 | 633744 | 5069948 | 1 | 66 | 0 | 184 | 5210372 | 1 | 66 | 0 | 1 | 192 |
| 135 | 87 | 68 | 44 | 1 | 67 | 633767 | 5070132 | 1 | 67 | 0 | 184 | 5210564 | 1 | 67 | 0 | 1 | 192 |
| 136 | 88 | 68 | 44 | 2 | 0 | 633788 | 5070300 | 2 | 0 | 0 | 168 | 5210740 | 2 | 0 | 0 | 1 | 176 |
| 137 | 89 | 68 | 44 | 2 | 1 | 633814 | 5070508 | 2 | 1 | 0 | 208 | 5210956 | 2 | 1 | 0 | 1 | 216 |
| 138 | 8A | 68 | 44 | 2 | 2 | 633840 | 5070716 | 2 | 2 | 0 | 208 | 5211172 | 2 | 2 | 0 | 1 | 216 |
| 139 | 8B | 68 | 44 | 2 | 3 | 633866 | 5070924 | 2 | 3 | 0 | 208 | 5211388 | 2 | 3 | 0 | 1 | 216 |
| 140 | 8C | 68 | 44 | 2 | 4 | 633892 | 5071132 | 2 | 4 | 0 | 208 | 5211604 | 2 | 4 | 0 | 1 | 216 |
| 141 | 8D | 68 | 44 | 2 | 5 | 633918 | 5071340 | 2 | 5 | 0 | 208 | 5211820 | 2 | 5 | 0 | 1 | 216 |
| 142 | 8E | 68 | 44 | 2 | 6 | 633944 | 5071548 | 2 | 6 | 0 | 208 | 5212036 | 2 | 6 | 0 | 1 | 216 |
| 143 | 8F | 68 | 44 | 2 | 7 | 633970 | 5071756 | 2 | 7 | 0 | 208 | 5212252 | 2 | 7 | 0 | 1 | 216 |
| 144 | 90 | 68 | 44 | 2 | 8 | 633996 | 5071964 | 2 | 8 | 0 | 208 | 5212468 | 2 | 8 | 0 | 1 | 216 |
| 145 | 91 | 68 | 44 | 2 | 9 | 634022 | 5072172 | 2 | 9 | 0 | 208 | 5212684 | 2 | 9 | 0 | 1 | 216 |
| 146 | 92 | 68 | 44 | 2 | 10 | 634048 | 5072380 | 2 | 10 | 0 | 208 | 5212900 | 2 | 10 | 0 | 1 | 216 |
| 147 | 93 | 68 | 44 | 2 | 11 | 634074 | 5072588 | 2 | 11 | 0 | 208 | 5213116 | 2 | 11 | 0 | 1 | 216 |
| 148 | 94 | 68 | 44 | 2 | 12 | 634100 | 5072796 | 2 | 12 | 0 | 208 | 5213332 | 2 | 12 | 0 | 1 | 216 |
| 149 | 95 | 68 | 44 | 2 | 13 | 634126 | 5073004 | 2 | 13 | 0 | 208 | 5213548 | 2 | 13 | 0 | 1 | 216 |
| 150 | 96 | 68 | 44 | 2 | 14 | 634152 | 5073212 | 2 | 14 | 0 | 208 | 5213764 | 2 | 14 | 0 | 1 | 216 |
| 151 | 97 | 68 | 44 | 2 | 15 | 634178 | 5073420 | 2 | 15 | 0 | 208 | 5213980 | 2 | 15 | 0 | 1 | 216 |
| 152 | 98 | 68 | 44 | 2 | 16 | 634204 | 5073628 | 2 | 16 | 0 | 208 | 5214196 | 2 | 16 | 0 | 1 | 216 |
| 153 | 99 | 68 | 44 | 2 | 17 | 634230 | 5073836 | 2 | 17 | 0 | 208 | 5214412 | 2 | 17 | 0 | 1 | 216 |
| 154 | 9A | 68 | 44 | 2 | 18 | 634256 | 5074044 | 2 | 18 | 0 | 208 | 5214628 | 2 | 18 | 0 | 1 | 216 |
| 155 | 9B | 68 | 44 | 2 | 19 | 634282 | 5074252 | 2 | 19 | 0 | 208 | 5214844 | 2 | 19 | 0 | 1 | 216 |
| 156 | 9C | 68 | 44 | 2 | 20 | 634308 | 5074460 | 2 | 20 | 0 | 208 | 5215060 | 2 | 20 | 0 | 1 | 216 |
| 157 | 9D | 68 | 44 | 2 | 21 | 634334 | 5074668 | 2 | 21 | 0 | 208 | 5215276 | 2 | 21 | 0 | 1 | 216 |
| 158 | 9E | 68 | 44 | 2 | 22 | 634360 | 5074876 | 2 | 22 | 0 | 208 | 5215492 | 2 | 22 | 0 | 1 | 216 |
| 159 | 9F | 68 | 44 | 2 | 23 | 634386 | 5075084 | 2 | 23 | 0 | 208 | 5215708 | 2 | 23 | 0 | 1 | 216 |
| 160 | A0 | 68 | 44 | 2 | 24 | 634409 | 5075268 | 2 | 24 | 0 | 184 | 5215900 | 2 | 24 | 0 | 1 | 192 |
| 161 | A1 | 68 | 44 | 2 | 25 | 634432 | 5075452 | 2 | 25 | 0 | 184 | 5216092 | 2 | 25 | 0 | 1 | 192 |
| 162 | A2 | 68 | 44 | 2 | 26 | 634455 | 5075636 | 2 | 26 | 0 | 184 | 5216284 | 2 | 26 | 0 | 1 | 192 |
| 163 | A3 | 68 | 44 | 2 | 27 | 634478 | 5075820 | 2 | 27 | 0 | 184 | 5216476 | 2 | 27 | 0 | 1 | 192 |
| 164 | A4 | 68 | 44 | 2 | 28 | 634501 | 5076004 | 2 | 28 | 0 | 184 | 5216668 | 2 | 28 | 0 | 1 | 192 |
| 165 | A5 | 68 | 44 | 2 | 29 | 634524 | 5076188 | 2 | 29 | 0 | 184 | 5216860 | 2 | 29 | 0 | 1 | 192 |
| 166 | A6 | 68 | 44 | 2 | 30 | 634547 | 5076372 | 2 | 30 | 0 | 184 | 5217052 | 2 | 30 | 0 | 1 | 192 |
| 167 | A7 | 68 | 44 | 2 | 31 | 634570 | 5076556 | 2 | 31 | 0 | 184 | 5217244 | 2 | 31 | 0 | 1 | 192 |
| 168 | A8 | 68 | 44 | 2 | 32 | 634593 | 5076740 | 2 | 32 | 0 | 184 | 5217436 | 2 | 32 | 0 | 1 | 192 |
| 169 | A9 | 68 | 44 | 2 | 33 | 634616 | 5076924 | 2 | 33 | 0 | 184 | 5217628 | 2 | 33 | 0 | 1 | 192 |
| 170 | AA | 68 | 44 | 2 | 34 | 634639 | 5077108 | 2 | 34 | 0 | 184 | 5217820 | 2 | 34 | 0 | 1 | 192 |
| 171 | AB | 68 | 44 | 2 | 35 | 634662 | 5077292 | 2 | 35 | 0 | 184 | 5218012 | 2 | 35 | 0 | 1 | 192 |
| 172 | AC | 68 | 44 | 2 | 36 | 634685 | 5077476 | 2 | 36 | 0 | 184 | 5218204 | 2 | 36 | 0 | 1 | 192 |
| 173 | AD | 68 | 44 | 2 | 37 | 634708 | 5077660 | 2 | 37 | 0 | 184 | 5218396 | 2 | 37 | 0 | 1 | 192 |
| 174 | AE | 68 | 44 | 2 | 38 | 634731 | 5077844 | 2 | 38 | 0 | 184 | 5218588 | 2 | 38 | 0 | 1 | 192 |
| 175 | AF | 68 | 44 | 2 | 39 | 634754 | 5078028 | 2 | 39 | 0 | 184 | 5218780 | 2 | 39 | 0 | 1 | 192 |
| 176 | B0 | 68 | 44 | 2 | 40 | 634779 | 5078228 | 2 | 40 | 0 | 200 | 5218988 | 2 | 40 | 0 | 1 | 208 |
| 177 | B1 | 68 | 44 | 2 | 41 | 634804 | 5078428 | 2 | 41 | 0 | 200 | 5219196 | 2 | 41 | 0 | 1 | 208 |
| 178 | B2 | 68 | 44 | 2 | 42 | 634829 | 5078628 | 2 | 42 | 0 | 200 | 5219404 | 2 | 42 | 0 | 1 | 208 |
| 179 | B3 | 68 | 44 | 2 | 43 | 634854 | 5078828 | 2 | 43 | 0 | 200 | 5219612 | 2 | 43 | 0 | 1 | 208 |
| 180 | B4 | 68 | 44 | 2 | 44 | 634879 | 5079028 | 2 | 44 | 0 | 200 | 5219820 | 2 | 44 | 0 | 1 | 208 |
| 181 | B5 | 68 | 44 | 2 | 45 | 634904 | 5079228 | 2 | 45 | 0 | 200 | 5220028 | 2 | 45 | 0 | 1 | 208 |
| 182 | B6 | 68 | 44 | 2 | 46 | 634929 | 5079428 | 2 | 46 | 0 | 200 | 5220236 | 2 | 46 | 0 | 1 | 208 |
| 183 | B7 | 68 | 44 | 2 | 47 | 634954 | 5079628 | 2 | 47 | 0 | 200 | 5220444 | 2 | 47 | 0 | 1 | 208 |
| 184 | B8 | 68 | 44 | 2 | 48 | 634979 | 5079828 | 2 | 48 | 0 | 200 | 5220652 | 2 | 48 | 0 | 1 | 208 |
| 185 | B9 | 68 | 44 | 2 | 49 | 635004 | 5080028 | 2 | 49 | 0 | 200 | 5220860 | 2 | 49 | 0 | 1 | 208 |
| 186 | BA | 68 | 44 | 2 | 50 | 635029 | 5080228 | 2 | 50 | 0 | 200 | 5221068 | 2 | 50 | 0 | 1 | 208 |
| 187 | BB | 68 | 44 | 2 | 51 | 635054 | 5080428 | 2 | 51 | 0 | 200 | 5221276 | 2 | 51 | 0 | 1 | 208 |
| 188 | BC | 68 | 44 | 2 | 52 | 635079 | 5080628 | 2 | 52 | 0 | 200 | 5221484 | 2 | 52 | 0 | 1 | 208 |
| 189 | BD | 68 | 44 | 2 | 53 | 635104 | 5080828 | 2 | 53 | 0 | 200 | 5221692 | 2 | 53 | 0 | 1 | 208 |
| 190 | BE | 68 | 44 | 2 | 54 | 635129 | 5081028 | 2 | 54 | 0 | 200 | 5221900 | 2 | 54 | 0 | 1 | 208 |
| 191 | BF | 68 | 44 | 2 | 55 | 635154 | 5081228 | 2 | 55 | 0 | 200 | 5222108 | 2 | 55 | 0 | 1 | 208 |
| 192 | C0 | 68 | 44 | 2 | 56 | 635179 | 5081428 | 2 | 56 | 0 | 200 | 5222316 | 2 | 56 | 0 | 1 | 208 |
| 193 | C1 | 68 | 44 | 2 | 57 | 635204 | 5081628 | 2 | 57 | 0 | 200 | 5222524 | 2 | 57 | 0 | 1 | 208 |
| 194 | C2 | 68 | 44 | 2 | 58 | 635229 | 5081828 | 2 | 58 | 0 | 200 | 5222732 | 2 | 58 | 0 | 1 | 208 |
| 195 | C3 | 68 | 44 | 2 | 59 | 635254 | 5082028 | 2 | 59 | 0 | 200 | 5222940 | 2 | 59 | 0 | 1 | 208 |
| 196 | C4 | 68 | 44 | 2 | 60 | 635279 | 5082228 | 2 | 60 | 0 | 200 | 5223148 | 2 | 60 | 0 | 1 | 208 |
| 197 | C5 | 68 | 44 | 2 | 61 | 635304 | 5082428 | 2 | 61 | 0 | 200 | 5223356 | 2 | 61 | 0 | 1 | 208 |
| 198 | C6 | 68 | 44 | 2 | 62 | 635329 | 5082628 | 2 | 62 | 0 | 200 | 5223564 | 2 | 62 | 0 | 1 | 208 |
| 199 | C7 | 68 | 44 | 2 | 63 | 635354 | 5082828 | 2 | 63 | 0 | 200 | 5223772 | 2 | 63 | 0 | 1 | 208 |
| 200 | C8 | 68 | 44 | 2 | 64 | 635379 | 5083028 | 2 | 64 | 0 | 200 | 5223980 | 2 | 64 | 0 | 1 | 208 |
| 201 | C9 | 68 | 44 | 2 | 65 | 635404 | 5083228 | 2 | 65 | 0 | 200 | 5224188 | 2 | 65 | 0 | 1 | 208 |

| | | | | | | | | | | | | | | | | | |
|---|---|---|---|---|---|---|---|---|---|---|---|---|---|---|---|---|---|
| 202 | CA | 68 | 44 | 2 | 66 | 635429 | 5083428 | 2 | 66 | 0 | 200 | 5224396 | 2 | 66 | 0 | 1 | 208 |
| 203 | CB | 68 | 44 | 2 | 67 | 635454 | 5083628 | 2 | 67 | 0 | 200 | 5224604 | 2 | 67 | 0 | 1 | 208 |
| 204 | CC | 68 | 44 | 3 | 0 | 635477 | 5083812 | 3 | 0 | 0 | 184 | 5224796 | 3 | 0 | 0 | 1 | 192 |
| 205 | CD | 68 | 44 | 3 | 1 | 635505 | 5084036 | 3 | 1 | 0 | 224 | 5225028 | 3 | 1 | 0 | 1 | 232 |
| 206 | CE | 68 | 44 | 3 | 2 | 635533 | 5084260 | 3 | 2 | 0 | 224 | 5225260 | 3 | 2 | 0 | 1 | 232 |
| 207 | CF | 68 | 44 | 3 | 3 | 635561 | 5084484 | 3 | 3 | 0 | 224 | 5225492 | 3 | 3 | 0 | 1 | 232 |
| 208 | D0 | 68 | 44 | 3 | 4 | 635589 | 5084708 | 3 | 4 | 0 | 224 | 5225724 | 3 | 4 | 0 | 1 | 232 |
| 209 | D1 | 68 | 44 | 3 | 5 | 635617 | 5084932 | 3 | 5 | 0 | 224 | 5225956 | 3 | 5 | 0 | 1 | 232 |
| 210 | D2 | 68 | 44 | 3 | 6 | 635645 | 5085156 | 3 | 6 | 0 | 224 | 5226188 | 3 | 6 | 0 | 1 | 232 |
| 211 | D3 | 68 | 44 | 3 | 7 | 635673 | 5085380 | 3 | 7 | 0 | 224 | 5226420 | 3 | 7 | 0 | 1 | 232 |
| 212 | D4 | 68 | 44 | 3 | 8 | 635701 | 5085604 | 3 | 8 | 0 | 224 | 5226652 | 3 | 8 | 0 | 1 | 232 |
| 213 | D5 | 68 | 44 | 3 | 9 | 635729 | 5085828 | 3 | 9 | 0 | 224 | 5226884 | 3 | 9 | 0 | 1 | 232 |
| 214 | D6 | 68 | 44 | 3 | 10 | 635757 | 5086052 | 3 | 10 | 0 | 224 | 5227116 | 3 | 10 | 0 | 1 | 232 |
| 215 | D7 | 68 | 44 | 3 | 11 | 635785 | 5086276 | 3 | 11 | 0 | 224 | 5227348 | 3 | 11 | 0 | 1 | 232 |
| 216 | D8 | 68 | 44 | 3 | 12 | 635813 | 5086500 | 3 | 12 | 0 | 224 | 5227580 | 3 | 12 | 0 | 1 | 232 |
| 217 | D9 | 68 | 44 | 3 | 13 | 635841 | 5086724 | 3 | 13 | 0 | 224 | 5227812 | 3 | 13 | 0 | 1 | 232 |
| 218 | DA | 68 | 44 | 3 | 14 | 635869 | 5086948 | 3 | 14 | 0 | 224 | 5228044 | 3 | 14 | 0 | 1 | 232 |
| 219 | DB | 68 | 44 | 3 | 15 | 635897 | 5087172 | 3 | 15 | 0 | 224 | 5228276 | 3 | 15 | 0 | 1 | 232 |
| 220 | DC | 68 | 44 | 3 | 16 | 635925 | 5087396 | 3 | 16 | 0 | 224 | 5228508 | 3 | 16 | 0 | 1 | 232 |
| 221 | DD | 68 | 44 | 3 | 17 | 635953 | 5087620 | 3 | 17 | 0 | 224 | 5228740 | 3 | 17 | 0 | 1 | 232 |
| 222 | DE | 68 | 44 | 3 | 18 | 635981 | 5087844 | 3 | 18 | 0 | 224 | 5228972 | 3 | 18 | 0 | 1 | 232 |
| 223 | DF | 68 | 44 | 3 | 19 | 636009 | 5088068 | 3 | 19 | 0 | 224 | 5229204 | 3 | 19 | 0 | 1 | 232 |
| 224 | E0 | 68 | 44 | 3 | 20 | 636037 | 5088292 | 3 | 20 | 0 | 224 | 5229436 | 3 | 20 | 0 | 1 | 232 |
| 225 | E1 | 68 | 44 | 3 | 21 | 636065 | 5088516 | 3 | 21 | 0 | 224 | 5229668 | 3 | 21 | 0 | 1 | 232 |
| 226 | E2 | 68 | 44 | 3 | 22 | 636093 | 5088740 | 3 | 22 | 0 | 224 | 5229900 | 3 | 22 | 0 | 1 | 232 |
| 227 | E3 | 68 | 44 | 3 | 23 | 636121 | 5088964 | 3 | 23 | 0 | 224 | 5230132 | 3 | 23 | 0 | 1 | 232 |
| 228 | E4 | 68 | 44 | 3 | 24 | 636149 | 5089188 | 3 | 24 | 0 | 224 | 5230364 | 3 | 24 | 0 | 1 | 232 |
| 229 | E5 | 68 | 44 | 3 | 25 | 636177 | 5089412 | 3 | 25 | 0 | 224 | 5230596 | 3 | 25 | 0 | 1 | 232 |
| 230 | E6 | 68 | 44 | 3 | 26 | 636205 | 5089636 | 3 | 26 | 0 | 224 | 5230828 | 3 | 26 | 0 | 1 | 232 |
| 231 | E7 | 68 | 44 | 3 | 27 | 636233 | 5089860 | 3 | 27 | 0 | 224 | 5231060 | 3 | 27 | 0 | 1 | 232 |
| 232 | E8 | 68 | 44 | 3 | 28 | 636261 | 5090084 | 3 | 28 | 0 | 224 | 5231292 | 3 | 28 | 0 | 1 | 232 |
| 233 | E9 | 68 | 44 | 3 | 29 | 636289 | 5090308 | 3 | 29 | 0 | 224 | 5231524 | 3 | 29 | 0 | 1 | 232 |
| 234 | EA | 68 | 44 | 3 | 30 | 636317 | 5090532 | 3 | 30 | 0 | 224 | 5231756 | 3 | 30 | 0 | 1 | 232 |
| 235 | EB | 68 | 44 | 3 | 31 | 636345 | 5090756 | 3 | 31 | 0 | 224 | 5231988 | 3 | 31 | 0 | 1 | 232 |
| 236 | EC | 68 | 44 | 3 | 32 | 636373 | 5090980 | 3 | 32 | 0 | 224 | 5232220 | 3 | 32 | 0 | 1 | 232 |
| 237 | ED | 68 | 44 | 3 | 33 | 636401 | 5091204 | 3 | 33 | 0 | 224 | 5232452 | 3 | 33 | 0 | 1 | 232 |
| 238 | EE | 68 | 44 | 3 | 34 | 636429 | 5091428 | 3 | 34 | 0 | 224 | 5232684 | 3 | 34 | 0 | 1 | 232 |
| 239 | EF | 68 | 44 | 3 | 35 | 636457 | 5091652 | 3 | 35 | 0 | 224 | 5232916 | 3 | 35 | 0 | 1 | 232 |
| 240 | F0 | 68 | 44 | 3 | 36 | 636482 | 5091852 | 3 | 36 | 0 | 200 | 5233124 | 3 | 36 | 0 | 1 | 208 |
| 241 | F1 | 68 | 44 | 3 | 37 | 636507 | 5092052 | 3 | 37 | 0 | 200 | 5233332 | 3 | 37 | 0 | 1 | 208 |
| 242 | F2 | 68 | 44 | 3 | 38 | 636532 | 5092252 | 3 | 38 | 0 | 200 | 5233540 | 3 | 38 | 0 | 1 | 208 |
| 243 | F3 | 68 | 44 | 3 | 39 | 636557 | 5092452 | 3 | 39 | 0 | 200 | 5233748 | 3 | 39 | 0 | 1 | 208 |
| 244 | F4 | 68 | 44 | 3 | 40 | 636582 | 5092652 | 3 | 40 | 0 | 200 | 5233956 | 3 | 40 | 0 | 1 | 208 |
| 245 | F5 | 68 | 44 | 3 | 41 | 636607 | 5092852 | 3 | 41 | 0 | 200 | 5234164 | 3 | 41 | 0 | 1 | 208 |
| 246 | F6 | 68 | 44 | 3 | 42 | 636632 | 5093052 | 3 | 42 | 0 | 200 | 5234372 | 3 | 42 | 0 | 1 | 208 |
| 247 | F7 | 68 | 44 | 3 | 43 | 636657 | 5093252 | 3 | 43 | 0 | 200 | 5234580 | 3 | 43 | 0 | 1 | 208 |
| 248 | F8 | 68 | 44 | 3 | 44 | 636682 | 5093452 | 3 | 44 | 0 | 200 | 5234788 | 3 | 44 | 0 | 1 | 208 |
| 249 | F9 | 68 | 44 | 3 | 45 | 636707 | 5093652 | 3 | 45 | 0 | 200 | 5234996 | 3 | 45 | 0 | 1 | 208 |
| 250 | FA | 68 | 44 | 3 | 46 | 636732 | 5093852 | 3 | 46 | 0 | 200 | 5235204 | 3 | 46 | 0 | 1 | 208 |
| 251 | FB | 68 | 44 | 3 | 47 | 636757 | 5094052 | 3 | 47 | 0 | 200 | 5235412 | 3 | 47 | 0 | 1 | 208 |
| 252 | FC | 68 | 44 | 3 | 48 | 636782 | 5094252 | 3 | 48 | 0 | 200 | 5235620 | 3 | 48 | 0 | 1 | 208 |
| 253 | FD | 68 | 44 | 3 | 49 | 636807 | 5094452 | 3 | 49 | 0 | 200 | 5235828 | 3 | 49 | 0 | 1 | 208 |
| 254 | FE | 68 | 44 | 3 | 50 | 636832 | 5094652 | 3 | 50 | 0 | 200 | 5236036 | 3 | 50 | 0 | 1 | 208 |
| 255 | FF | 68 | 44 | 3 | 51 | 636857 | 5094852 | 3 | 51 | 0 | 200 | 5236244 | 3 | 51 | 0 | 1 | 208 |
| 0 | 00 | 69 | 45 | 0 | 0 | 636869 | 5094948 | 0 | 0 | 0 | 96 | 5236348 | 0 | 0 | 0 | 1 | 104 |
| 1 | 01 | 69 | 45 | 0 | 1 | 636883 | 5095060 | 0 | 1 | 0 | 112 | 5236468 | 0 | 1 | 0 | 1 | 120 |
| 2 | 02 | 69 | 45 | 0 | 2 | 636897 | 5095172 | 0 | 2 | 0 | 112 | 5236588 | 0 | 2 | 0 | 1 | 120 |
| 3 | 03 | 69 | 45 | 0 | 3 | 636911 | 5095284 | 0 | 3 | 0 | 112 | 5236708 | 0 | 3 | 0 | 1 | 120 |
| 4 | 04 | 69 | 45 | 0 | 4 | 636925 | 5095396 | 0 | 4 | 0 | 112 | 5236828 | 0 | 4 | 0 | 1 | 120 |
| 5 | 05 | 69 | 45 | 0 | 5 | 636939 | 5095508 | 0 | 5 | 0 | 112 | 5236948 | 0 | 5 | 0 | 1 | 120 |
| 6 | 06 | 69 | 45 | 0 | 6 | 636953 | 5095620 | 0 | 6 | 0 | 112 | 5237068 | 0 | 6 | 0 | 1 | 120 |
| 7 | 07 | 69 | 45 | 0 | 7 | 636967 | 5095732 | 0 | 7 | 0 | 112 | 5237188 | 0 | 7 | 0 | 1 | 120 |
| 8 | 08 | 69 | 45 | 0 | 8 | 636981 | 5095844 | 0 | 8 | 0 | 112 | 5237308 | 0 | 8 | 0 | 1 | 120 |
| 9 | 09 | 69 | 45 | 0 | 9 | 636995 | 5095956 | 0 | 9 | 0 | 112 | 5237428 | 0 | 9 | 0 | 1 | 120 |
| 10 | 0A | 69 | 45 | 0 | 10 | 637009 | 5096068 | 0 | 10 | 0 | 112 | 5237548 | 0 | 10 | 0 | 1 | 120 |
| 11 | 0B | 69 | 45 | 0 | 11 | 637023 | 5096180 | 0 | 11 | 0 | 112 | 5237668 | 0 | 11 | 0 | 1 | 120 |
| 12 | 0C | 69 | 45 | 0 | 12 | 637037 | 5096292 | 0 | 12 | 0 | 112 | 5237788 | 0 | 12 | 0 | 1 | 120 |
| 13 | 0D | 69 | 45 | 0 | 13 | 637051 | 5096404 | 0 | 13 | 0 | 112 | 5237908 | 0 | 13 | 0 | 1 | 120 |
| 14 | 0E | 69 | 45 | 0 | 14 | 637065 | 5096516 | 0 | 14 | 0 | 112 | 5238028 | 0 | 14 | 0 | 1 | 120 |
| 15 | 0F | 69 | 45 | 0 | 15 | 637079 | 5096628 | 0 | 15 | 0 | 112 | 5238148 | 0 | 15 | 0 | 1 | 120 |
| 16 | 10 | 69 | 45 | 0 | 16 | 637093 | 5096740 | 0 | 16 | 0 | 112 | 5238268 | 0 | 16 | 0 | 1 | 120 |
| 17 | 11 | 69 | 45 | 0 | 17 | 637107 | 5096852 | 0 | 17 | 0 | 112 | 5238388 | 0 | 17 | 0 | 1 | 120 |
| 18 | 12 | 69 | 45 | 0 | 18 | 637121 | 5096964 | 0 | 18 | 0 | 112 | 5238508 | 0 | 18 | 0 | 1 | 120 |
| 19 | 13 | 69 | 45 | 0 | 19 | 637135 | 5097076 | 0 | 19 | 0 | 112 | 5238628 | 0 | 19 | 0 | 1 | 120 |
| 20 | 14 | 69 | 45 | 0 | 20 | 637149 | 5097188 | 0 | 20 | 0 | 112 | 5238748 | 0 | 20 | 0 | 1 | 120 |
| 21 | 15 | 69 | 45 | 0 | 21 | 637163 | 5097300 | 0 | 21 | 0 | 112 | 5238868 | 0 | 21 | 0 | 1 | 120 |
| 22 | 16 | 69 | 45 | 0 | 22 | 637177 | 5097412 | 0 | 22 | 0 | 112 | 5238988 | 0 | 22 | 0 | 1 | 120 |
| 23 | 17 | 69 | 45 | 0 | 23 | 637191 | 5097524 | 0 | 23 | 0 | 112 | 5239108 | 0 | 23 | 0 | 1 | 120 |
| 24 | 18 | 69 | 45 | 0 | 24 | 637205 | 5097636 | 0 | 24 | 0 | 112 | 5239228 | 0 | 24 | 0 | 1 | 120 |
| 25 | 19 | 69 | 45 | 0 | 25 | 637219 | 5097748 | 0 | 25 | 0 | 112 | 5239348 | 0 | 25 | 0 | 1 | 120 |
| 26 | 1A | 69 | 45 | 0 | 26 | 637233 | 5097860 | 0 | 26 | 0 | 112 | 5239468 | 0 | 26 | 0 | 1 | 120 |
| 27 | 1B | 69 | 45 | 0 | 27 | 637247 | 5097972 | 0 | 27 | 0 | 112 | 5239588 | 0 | 27 | 0 | 1 | 120 |
| 28 | 1C | 69 | 45 | 0 | 28 | 637261 | 5098084 | 0 | 28 | 0 | 112 | 5239708 | 0 | 28 | 0 | 1 | 120 |
| 29 | 1D | 69 | 45 | 0 | 29 | 637275 | 5098196 | 0 | 29 | 0 | 112 | 5239828 | 0 | 29 | 0 | 1 | 120 |
| 30 | 1E | 69 | 45 | 0 | 30 | 637289 | 5098308 | 0 | 30 | 0 | 112 | 5239948 | 0 | 30 | 0 | 1 | 120 |
| 31 | 1F | 69 | 45 | 0 | 31 | 637303 | 5098420 | 0 | 31 | 0 | 112 | 5240068 | 0 | 31 | 0 | 1 | 120 |
| 32 | 20 | 69 | 45 | 0 | 32 | 637317 | 5098532 | 0 | 32 | 0 | 112 | 5240188 | 0 | 32 | 0 | 1 | 120 |
| 33 | 21 | 69 | 45 | 0 | 33 | 637331 | 5098644 | 0 | 33 | 0 | 112 | 5240308 | 0 | 33 | 0 | 1 | 120 |
| 34 | 22 | 69 | 45 | 0 | 34 | 637345 | 5098756 | 0 | 34 | 0 | 112 | 5240428 | 0 | 34 | 0 | 1 | 120 |
| 35 | 23 | 69 | 45 | 0 | 35 | 637359 | 5098868 | 0 | 35 | 0 | 112 | 5240548 | 0 | 35 | 0 | 1 | 120 |
| 36 | 24 | 69 | 45 | 0 | 36 | 637373 | 5098980 | 0 | 36 | 0 | 112 | 5240668 | 0 | 36 | 0 | 1 | 120 |
| 37 | 25 | 69 | 45 | 0 | 37 | 637387 | 5099092 | 0 | 37 | 0 | 112 | 5240788 | 0 | 37 | 0 | 1 | 120 |
| 38 | 26 | 69 | 45 | 0 | 38 | 637401 | 5099204 | 0 | 38 | 0 | 112 | 5240908 | 0 | 38 | 0 | 1 | 120 |
| 39 | 27 | 69 | 45 | 0 | 39 | 637415 | 5099316 | 0 | 39 | 0 | 112 | 5241028 | 0 | 39 | 0 | 1 | 120 |
| 40 | 28 | 69 | 45 | 0 | 40 | 637429 | 5099428 | 0 | 40 | 0 | 112 | 5241148 | 0 | 40 | 0 | 1 | 120 |
| 41 | 29 | 69 | 45 | 0 | 41 | 637443 | 5099540 | 0 | 41 | 0 | 112 | 5241268 | 0 | 41 | 0 | 1 | 120 |
| 42 | 2A | 69 | 45 | 0 | 42 | 637457 | 5099652 | 0 | 42 | 0 | 112 | 5241388 | 0 | 42 | 0 | 1 | 120 |
| 43 | 2B | 69 | 45 | 0 | 43 | 637471 | 5099764 | 0 | 43 | 0 | 112 | 5241508 | 0 | 43 | 0 | 1 | 120 |
| 44 | 2C | 69 | 45 | 0 | 44 | 637485 | 5099876 | 0 | 44 | 0 | 112 | 5241628 | 0 | 44 | 0 | 1 | 120 |
| 45 | 2D | 69 | 45 | 0 | 45 | 637499 | 5099988 | 0 | 45 | 0 | 112 | 5241748 | 0 | 45 | 0 | 1 | 120 |
| 46 | 2E | 69 | 45 | 0 | 46 | 637513 | 5100100 | 0 | 46 | 0 | 112 | 5241868 | 0 | 46 | 0 | 1 | 120 |
| 47 | 2F | 69 | 45 | 0 | 47 | 637527 | 5100212 | 0 | 47 | 0 | 112 | 5241988 | 0 | 47 | 0 | 1 | 120 |
| 48 | 30 | 69 | 45 | 0 | 48 | 637541 | 5100324 | 0 | 48 | 0 | 112 | 5242108 | 0 | 48 | 0 | 1 | 120 |

| | | | | | | | | | | | | | | | | |
|---|---|---|---|---|---|---|---|---|---|---|---|---|---|---|---|---|
| 49 | 31 | 69 | 45 | 0 | 49 | 637555 | 5100436 | 0 | 49 | 0 | 112 | 5242228 | 0 | 49 | 0 | 1 | 120 |
| 50 | 32 | 69 | 45 | 0 | 50 | 637569 | 5100548 | 0 | 50 | 0 | 112 | 5242348 | 0 | 50 | 0 | 1 | 120 |
| 51 | 33 | 69 | 45 | 0 | 51 | 637583 | 5100660 | 0 | 51 | 0 | 112 | 5242468 | 0 | 51 | 0 | 1 | 120 |
| 52 | 34 | 69 | 45 | 0 | 52 | 637597 | 5100772 | 0 | 52 | 0 | 112 | 5242588 | 0 | 52 | 0 | 1 | 120 |
| 53 | 35 | 69 | 45 | 0 | 53 | 637611 | 5100884 | 0 | 53 | 0 | 112 | 5242708 | 0 | 53 | 0 | 1 | 120 |
| 54 | 36 | 69 | 45 | 0 | 54 | 637625 | 5100996 | 0 | 54 | 0 | 112 | 5242828 | 0 | 54 | 0 | 1 | 120 |
| 55 | 37 | 69 | 45 | 0 | 55 | 637639 | 5101108 | 0 | 55 | 0 | 112 | 5242948 | 0 | 55 | 0 | 1 | 120 |
| 56 | 38 | 69 | 45 | 0 | 56 | 637653 | 5101220 | 0 | 56 | 0 | 112 | 5243068 | 0 | 56 | 0 | 1 | 120 |
| 57 | 39 | 69 | 45 | 0 | 57 | 637667 | 5101332 | 0 | 57 | 0 | 112 | 5243188 | 0 | 57 | 0 | 1 | 120 |
| 58 | 3A | 69 | 45 | 0 | 58 | 637681 | 5101444 | 0 | 58 | 0 | 112 | 5243308 | 0 | 58 | 0 | 1 | 120 |
| 59 | 3B | 69 | 45 | 0 | 59 | 637695 | 5101556 | 0 | 59 | 0 | 112 | 5243428 | 0 | 59 | 0 | 1 | 120 |
| 60 | 3C | 69 | 45 | 0 | 60 | 637709 | 5101668 | 0 | 60 | 0 | 112 | 5243548 | 0 | 60 | 0 | 1 | 120 |
| 61 | 3D | 69 | 45 | 0 | 61 | 637723 | 5101780 | 0 | 61 | 0 | 112 | 5243668 | 0 | 61 | 0 | 1 | 120 |
| 62 | 3E | 69 | 45 | 0 | 62 | 637737 | 5101892 | 0 | 62 | 0 | 112 | 5243788 | 0 | 62 | 0 | 1 | 120 |
| 63 | 3F | 69 | 45 | 0 | 63 | 637751 | 5102004 | 0 | 63 | 0 | 112 | 5243908 | 0 | 63 | 0 | 1 | 120 |
| 64 | 40 | 69 | 45 | 0 | 64 | 637765 | 5102116 | 0 | 64 | 0 | 112 | 5244028 | 0 | 64 | 0 | 1 | 120 |
| 65 | 41 | 69 | 45 | 0 | 65 | 637779 | 5102228 | 0 | 65 | 0 | 112 | 5244148 | 0 | 65 | 0 | 1 | 120 |
| 66 | 42 | 69 | 45 | 0 | 66 | 637793 | 5102340 | 0 | 66 | 0 | 112 | 5244268 | 0 | 66 | 0 | 1 | 120 |
| 67 | 43 | 69 | 45 | 0 | 67 | 637807 | 5102452 | 0 | 67 | 0 | 112 | 5244388 | 0 | 67 | 0 | 1 | 120 |
| 68 | 44 | 69 | 45 | 0 | 68 | 637821 | 5102564 | 0 | 68 | 0 | 112 | 5244508 | 0 | 68 | 0 | 1 | 120 |
| 69 | 45 | 69 | 45 | 1 | 0 | 637833 | 5102660 | 1 | 0 | 0 | 96 | 5244612 | 1 | 0 | 0 | 1 | 104 |
| 70 | 46 | 69 | 45 | 1 | 1 | 637850 | 5102796 | 1 | 1 | 0 | 136 | 5244756 | 1 | 1 | 0 | 1 | 144 |
| 71 | 47 | 69 | 45 | 1 | 2 | 637867 | 5102932 | 1 | 2 | 0 | 136 | 5244900 | 1 | 2 | 0 | 1 | 144 |
| 72 | 48 | 69 | 45 | 1 | 3 | 637884 | 5103068 | 1 | 3 | 0 | 136 | 5245044 | 1 | 3 | 0 | 1 | 144 |
| 73 | 49 | 69 | 45 | 1 | 4 | 637901 | 5103204 | 1 | 4 | 0 | 136 | 5245188 | 1 | 4 | 0 | 1 | 144 |
| 74 | 4A | 69 | 45 | 1 | 5 | 637918 | 5103340 | 1 | 5 | 0 | 136 | 5245332 | 1 | 5 | 0 | 1 | 144 |
| 75 | 4B | 69 | 45 | 1 | 6 | 637935 | 5103476 | 1 | 6 | 0 | 136 | 5245476 | 1 | 6 | 0 | 1 | 144 |
| 76 | 4C | 69 | 45 | 1 | 7 | 637952 | 5103612 | 1 | 7 | 0 | 136 | 5245620 | 1 | 7 | 0 | 1 | 144 |
| 77 | 4D | 69 | 45 | 1 | 8 | 637969 | 5103748 | 1 | 8 | 0 | 136 | 5245764 | 1 | 8 | 0 | 1 | 144 |
| 78 | 4E | 69 | 45 | 1 | 9 | 637986 | 5103884 | 1 | 9 | 0 | 136 | 5245908 | 1 | 9 | 0 | 1 | 144 |
| 79 | 4F | 69 | 45 | 1 | 10 | 638003 | 5104020 | 1 | 10 | 0 | 136 | 5246052 | 1 | 10 | 0 | 1 | 144 |
| 80 | 50 | 69 | 45 | 1 | 11 | 638024 | 5104188 | 1 | 11 | 0 | 168 | 5246228 | 1 | 11 | 0 | 1 | 176 |
| 81 | 51 | 69 | 45 | 1 | 12 | 638045 | 5104356 | 1 | 12 | 0 | 168 | 5246404 | 1 | 12 | 0 | 1 | 176 |
| 82 | 52 | 69 | 45 | 1 | 13 | 638066 | 5104524 | 1 | 13 | 0 | 168 | 5246580 | 1 | 13 | 0 | 1 | 176 |
| 83 | 53 | 69 | 45 | 1 | 14 | 638087 | 5104692 | 1 | 14 | 0 | 168 | 5246756 | 1 | 14 | 0 | 1 | 176 |
| 84 | 54 | 69 | 45 | 1 | 15 | 638108 | 5104860 | 1 | 15 | 0 | 168 | 5246932 | 1 | 15 | 0 | 1 | 176 |
| 85 | 55 | 69 | 45 | 1 | 16 | 638129 | 5105028 | 1 | 16 | 0 | 168 | 5247108 | 1 | 16 | 0 | 1 | 176 |
| 86 | 56 | 69 | 45 | 1 | 17 | 638150 | 5105196 | 1 | 17 | 0 | 168 | 5247284 | 1 | 17 | 0 | 1 | 176 |
| 87 | 57 | 69 | 45 | 1 | 18 | 638171 | 5105364 | 1 | 18 | 0 | 168 | 5247460 | 1 | 18 | 0 | 1 | 176 |
| 88 | 58 | 69 | 45 | 1 | 19 | 638192 | 5105532 | 1 | 19 | 0 | 168 | 5247636 | 1 | 19 | 0 | 1 | 176 |
| 89 | 59 | 69 | 45 | 1 | 20 | 638213 | 5105700 | 1 | 20 | 0 | 168 | 5247812 | 1 | 20 | 0 | 1 | 176 |
| 90 | 5A | 69 | 45 | 1 | 21 | 638234 | 5105868 | 1 | 21 | 0 | 168 | 5247988 | 1 | 21 | 0 | 1 | 176 |
| 91 | 5B | 69 | 45 | 1 | 22 | 638255 | 5106036 | 1 | 22 | 0 | 168 | 5248164 | 1 | 22 | 0 | 1 | 176 |
| 92 | 5C | 69 | 45 | 1 | 23 | 638276 | 5106204 | 1 | 23 | 0 | 168 | 5248340 | 1 | 23 | 0 | 1 | 176 |
| 93 | 5D | 69 | 45 | 1 | 24 | 638297 | 5106372 | 1 | 24 | 0 | 168 | 5248516 | 1 | 24 | 0 | 1 | 176 |
| 94 | 5E | 69 | 45 | 1 | 25 | 638318 | 5106540 | 1 | 25 | 0 | 168 | 5248692 | 1 | 25 | 0 | 1 | 176 |
| 95 | 5F | 69 | 45 | 1 | 26 | 638339 | 5106708 | 1 | 26 | 0 | 168 | 5248868 | 1 | 26 | 0 | 1 | 176 |
| 96 | 60 | 69 | 45 | 1 | 27 | 638362 | 5106892 | 1 | 27 | 0 | 184 | 5249060 | 1 | 27 | 0 | 1 | 192 |
| 97 | 61 | 69 | 45 | 1 | 28 | 638385 | 5107076 | 1 | 28 | 0 | 184 | 5249252 | 1 | 28 | 0 | 1 | 192 |
| 98 | 62 | 69 | 45 | 1 | 29 | 638408 | 5107260 | 1 | 29 | 0 | 184 | 5249444 | 1 | 29 | 0 | 1 | 192 |
| 99 | 63 | 69 | 45 | 1 | 30 | 638431 | 5107444 | 1 | 30 | 0 | 184 | 5249636 | 1 | 30 | 0 | 1 | 192 |
| 100 | 64 | 69 | 45 | 1 | 31 | 638454 | 5107628 | 1 | 31 | 0 | 184 | 5249828 | 1 | 31 | 0 | 1 | 192 |
| 101 | 65 | 69 | 45 | 1 | 32 | 638477 | 5107812 | 1 | 32 | 0 | 184 | 5250020 | 1 | 32 | 0 | 1 | 192 |
| 102 | 66 | 69 | 45 | 1 | 33 | 638500 | 5107996 | 1 | 33 | 0 | 184 | 5250212 | 1 | 33 | 0 | 1 | 192 |
| 103 | 67 | 69 | 45 | 1 | 34 | 638523 | 5108180 | 1 | 34 | 0 | 184 | 5250404 | 1 | 34 | 0 | 1 | 192 |
| 104 | 68 | 69 | 45 | 1 | 35 | 638546 | 5108364 | 1 | 35 | 0 | 184 | 5250596 | 1 | 35 | 0 | 1 | 192 |
| 105 | 69 | 69 | 45 | 1 | 36 | 638569 | 5108548 | 1 | 36 | 0 | 184 | 5250788 | 1 | 36 | 0 | 1 | 192 |
| 106 | 6A | 69 | 45 | 1 | 37 | 638592 | 5108732 | 1 | 37 | 0 | 184 | 5250980 | 1 | 37 | 0 | 1 | 192 |
| 107 | 6B | 69 | 45 | 1 | 38 | 638615 | 5108916 | 1 | 38 | 0 | 184 | 5251172 | 1 | 38 | 0 | 1 | 192 |
| 108 | 6C | 69 | 45 | 1 | 39 | 638638 | 5109100 | 1 | 39 | 0 | 184 | 5251364 | 1 | 39 | 0 | 1 | 192 |
| 109 | 6D | 69 | 45 | 1 | 40 | 638661 | 5109284 | 1 | 40 | 0 | 184 | 5251556 | 1 | 40 | 0 | 1 | 192 |
| 110 | 6E | 69 | 45 | 1 | 41 | 638684 | 5109468 | 1 | 41 | 0 | 184 | 5251748 | 1 | 41 | 0 | 1 | 192 |
| 111 | 6F | 69 | 45 | 1 | 42 | 638707 | 5109652 | 1 | 42 | 0 | 184 | 5251940 | 1 | 42 | 0 | 1 | 192 |
| 112 | 70 | 69 | 45 | 1 | 43 | 638730 | 5109836 | 1 | 43 | 0 | 184 | 5252132 | 1 | 43 | 0 | 1 | 192 |
| 113 | 71 | 69 | 45 | 1 | 44 | 638753 | 5110020 | 1 | 44 | 0 | 184 | 5252324 | 1 | 44 | 0 | 1 | 192 |
| 114 | 72 | 69 | 45 | 1 | 45 | 638776 | 5110204 | 1 | 45 | 0 | 184 | 5252516 | 1 | 45 | 0 | 1 | 192 |
| 115 | 73 | 69 | 45 | 1 | 46 | 638799 | 5110388 | 1 | 46 | 0 | 184 | 5252708 | 1 | 46 | 0 | 1 | 192 |
| 116 | 74 | 69 | 45 | 1 | 47 | 638822 | 5110572 | 1 | 47 | 0 | 184 | 5252900 | 1 | 47 | 0 | 1 | 192 |
| 117 | 75 | 69 | 45 | 1 | 48 | 638845 | 5110756 | 1 | 48 | 0 | 184 | 5253092 | 1 | 48 | 0 | 1 | 192 |
| 118 | 76 | 69 | 45 | 1 | 49 | 638868 | 5110940 | 1 | 49 | 0 | 184 | 5253284 | 1 | 49 | 0 | 1 | 192 |
| 119 | 77 | 69 | 45 | 1 | 50 | 638891 | 5111124 | 1 | 50 | 0 | 184 | 5253476 | 1 | 50 | 0 | 1 | 192 |
| 120 | 78 | 69 | 45 | 1 | 51 | 638914 | 5111308 | 1 | 51 | 0 | 184 | 5253668 | 1 | 51 | 0 | 1 | 192 |
| 121 | 79 | 69 | 45 | 1 | 52 | 638937 | 5111492 | 1 | 52 | 0 | 184 | 5253860 | 1 | 52 | 0 | 1 | 192 |
| 122 | 7A | 69 | 45 | 1 | 53 | 638960 | 5111676 | 1 | 53 | 0 | 184 | 5254052 | 1 | 53 | 0 | 1 | 192 |
| 123 | 7B | 69 | 45 | 1 | 54 | 638983 | 5111860 | 1 | 54 | 0 | 184 | 5254244 | 1 | 54 | 0 | 1 | 192 |
| 124 | 7C | 69 | 45 | 1 | 55 | 639006 | 5112044 | 1 | 55 | 0 | 184 | 5254436 | 1 | 55 | 0 | 1 | 192 |
| 125 | 7D | 69 | 45 | 1 | 56 | 639029 | 5112228 | 1 | 56 | 0 | 184 | 5254628 | 1 | 56 | 0 | 1 | 192 |
| 126 | 7E | 69 | 45 | 1 | 57 | 639052 | 5112412 | 1 | 57 | 0 | 184 | 5254820 | 1 | 57 | 0 | 1 | 192 |
| 127 | 7F | 69 | 45 | 1 | 58 | 639075 | 5112596 | 1 | 58 | 0 | 184 | 5255012 | 1 | 58 | 0 | 1 | 192 |
| 128 | 80 | 69 | 45 | 1 | 59 | 639098 | 5112780 | 1 | 59 | 0 | 184 | 5255204 | 1 | 59 | 0 | 1 | 192 |
| 129 | 81 | 69 | 45 | 1 | 60 | 639121 | 5112964 | 1 | 60 | 0 | 184 | 5255396 | 1 | 60 | 0 | 1 | 192 |
| 130 | 82 | 69 | 45 | 1 | 61 | 639144 | 5113148 | 1 | 61 | 0 | 184 | 5255588 | 1 | 61 | 0 | 1 | 192 |
| 131 | 83 | 69 | 45 | 1 | 62 | 639167 | 5113332 | 1 | 62 | 0 | 184 | 5255780 | 1 | 62 | 0 | 1 | 192 |
| 132 | 84 | 69 | 45 | 1 | 63 | 639190 | 5113516 | 1 | 63 | 0 | 184 | 5255972 | 1 | 63 | 0 | 1 | 192 |
| 133 | 85 | 69 | 45 | 1 | 64 | 639213 | 5113700 | 1 | 64 | 0 | 184 | 5256164 | 1 | 64 | 0 | 1 | 192 |
| 134 | 86 | 69 | 45 | 1 | 65 | 639236 | 5113884 | 1 | 65 | 0 | 184 | 5256356 | 1 | 65 | 0 | 1 | 192 |
| 135 | 87 | 69 | 45 | 1 | 66 | 639259 | 5114068 | 1 | 66 | 0 | 184 | 5256548 | 1 | 66 | 0 | 1 | 192 |
| 136 | 88 | 69 | 45 | 1 | 67 | 639282 | 5114252 | 1 | 67 | 0 | 184 | 5256740 | 1 | 67 | 0 | 1 | 192 |
| 137 | 89 | 69 | 45 | 1 | 68 | 639305 | 5114436 | 1 | 68 | 0 | 184 | 5256932 | 1 | 68 | 0 | 1 | 192 |
| 138 | 8A | 69 | 45 | 2 | 0 | 639326 | 5114604 | 2 | 0 | 0 | 168 | 5257108 | 2 | 0 | 0 | 1 | 176 |
| 139 | 8B | 69 | 45 | 2 | 1 | 639352 | 5114812 | 2 | 1 | 0 | 208 | 5257324 | 2 | 1 | 0 | 1 | 216 |
| 140 | 8C | 69 | 45 | 2 | 2 | 639378 | 5115020 | 2 | 2 | 0 | 208 | 5257540 | 2 | 2 | 0 | 1 | 216 |
| 141 | 8D | 69 | 45 | 2 | 3 | 639404 | 5115228 | 2 | 3 | 0 | 208 | 5257756 | 2 | 3 | 0 | 1 | 216 |
| 142 | 8E | 69 | 45 | 2 | 4 | 639430 | 5115436 | 2 | 4 | 0 | 208 | 5257972 | 2 | 4 | 0 | 1 | 216 |
| 143 | 8F | 69 | 45 | 2 | 5 | 639456 | 5115644 | 2 | 5 | 0 | 208 | 5258188 | 2 | 5 | 0 | 1 | 216 |
| 144 | 90 | 69 | 45 | 2 | 6 | 639482 | 5115852 | 2 | 6 | 0 | 208 | 5258404 | 2 | 6 | 0 | 1 | 216 |
| 145 | 91 | 69 | 45 | 2 | 7 | 639508 | 5116060 | 2 | 7 | 0 | 208 | 5258620 | 2 | 7 | 0 | 1 | 216 |
| 146 | 92 | 69 | 45 | 2 | 8 | 639534 | 5116268 | 2 | 8 | 0 | 208 | 5258836 | 2 | 8 | 0 | 1 | 216 |
| 147 | 93 | 69 | 45 | 2 | 9 | 639560 | 5116476 | 2 | 9 | 0 | 208 | 5259052 | 2 | 9 | 0 | 1 | 216 |
| 148 | 94 | 69 | 45 | 2 | 10 | 639586 | 5116684 | 2 | 10 | 0 | 208 | 5259268 | 2 | 10 | 0 | 1 | 216 |
| 149 | 95 | 69 | 45 | 2 | 11 | 639612 | 5116892 | 2 | 11 | 0 | 208 | 5259484 | 2 | 11 | 0 | 1 | 216 |
| 150 | 96 | 69 | 45 | 2 | 12 | 639638 | 5117100 | 2 | 12 | 0 | 208 | 5259700 | 2 | 12 | 0 | 1 | 216 |
| 151 | 97 | 69 | 45 | 2 | 13 | 639664 | 5117308 | 2 | 13 | 0 | 208 | 5259916 | 2 | 13 | 0 | 1 | 216 |

| | | | | | | | | | | | | | | | | | |
|---|---|---|---|---|---|---|---|---|---|---|---|---|---|---|---|---|---|
| 152 | 98 | 69 | 45 | 2 | 14 | 639690 | 5117516 | 2 | 14 | 0 | 208 | 5260132 | 2 | 14 | 0 | 1 | 216 |
| 153 | 99 | 69 | 45 | 2 | 15 | 639716 | 5117724 | 2 | 15 | 0 | 208 | 5260348 | 2 | 15 | 0 | 1 | 216 |
| 154 | 9A | 69 | 45 | 2 | 16 | 639742 | 5117932 | 2 | 16 | 0 | 208 | 5260564 | 2 | 16 | 0 | 1 | 216 |
| 155 | 9B | 69 | 45 | 2 | 17 | 639768 | 5118140 | 2 | 17 | 0 | 208 | 5260780 | 2 | 17 | 0 | 1 | 216 |
| 156 | 9C | 69 | 45 | 2 | 18 | 639794 | 5118348 | 2 | 18 | 0 | 208 | 5260996 | 2 | 18 | 0 | 1 | 216 |
| 157 | 9D | 69 | 45 | 2 | 19 | 639820 | 5118556 | 2 | 19 | 0 | 208 | 5261212 | 2 | 19 | 0 | 1 | 216 |
| 158 | 9E | 69 | 45 | 2 | 20 | 639846 | 5118764 | 2 | 20 | 0 | 208 | 5261428 | 2 | 20 | 0 | 1 | 216 |
| 159 | 9F | 69 | 45 | 2 | 21 | 639872 | 5118972 | 2 | 21 | 0 | 208 | 5261644 | 2 | 21 | 0 | 1 | 216 |
| 160 | A0 | 69 | 45 | 2 | 22 | 639895 | 5119156 | 2 | 22 | 0 | 184 | 5261836 | 2 | 22 | 0 | 1 | 192 |
| 161 | A1 | 69 | 45 | 2 | 23 | 639918 | 5119340 | 2 | 23 | 0 | 184 | 5262028 | 2 | 23 | 0 | 1 | 192 |
| 162 | A2 | 69 | 45 | 2 | 24 | 639941 | 5119524 | 2 | 24 | 0 | 184 | 5262220 | 2 | 24 | 0 | 1 | 192 |
| 163 | A3 | 69 | 45 | 2 | 25 | 639964 | 5119708 | 2 | 25 | 0 | 184 | 5262412 | 2 | 25 | 0 | 1 | 192 |
| 164 | A4 | 69 | 45 | 2 | 26 | 639987 | 5119892 | 2 | 26 | 0 | 184 | 5262604 | 2 | 26 | 0 | 1 | 192 |
| 165 | A5 | 69 | 45 | 2 | 27 | 640010 | 5120076 | 2 | 27 | 0 | 184 | 5262796 | 2 | 27 | 0 | 1 | 192 |
| 166 | A6 | 69 | 45 | 2 | 28 | 640033 | 5120260 | 2 | 28 | 0 | 184 | 5262988 | 2 | 28 | 0 | 1 | 192 |
| 167 | A7 | 69 | 45 | 2 | 29 | 640056 | 5120444 | 2 | 29 | 0 | 184 | 5263180 | 2 | 29 | 0 | 1 | 192 |
| 168 | A8 | 69 | 45 | 2 | 30 | 640079 | 5120628 | 2 | 30 | 0 | 184 | 5263372 | 2 | 30 | 0 | 1 | 192 |
| 169 | A9 | 69 | 45 | 2 | 31 | 640102 | 5120812 | 2 | 31 | 0 | 184 | 5263564 | 2 | 31 | 0 | 1 | 192 |
| 170 | AA | 69 | 45 | 2 | 32 | 640125 | 5120996 | 2 | 32 | 0 | 184 | 5263756 | 2 | 32 | 0 | 1 | 192 |
| 171 | AB | 69 | 45 | 2 | 33 | 640148 | 5121180 | 2 | 33 | 0 | 184 | 5263948 | 2 | 33 | 0 | 1 | 192 |
| 172 | AC | 69 | 45 | 2 | 34 | 640171 | 5121364 | 2 | 34 | 0 | 184 | 5264140 | 2 | 34 | 0 | 1 | 192 |
| 173 | AD | 69 | 45 | 2 | 35 | 640194 | 5121548 | 2 | 35 | 0 | 184 | 5264332 | 2 | 35 | 0 | 1 | 192 |
| 174 | AE | 69 | 45 | 2 | 36 | 640217 | 5121732 | 2 | 36 | 0 | 184 | 5264524 | 2 | 36 | 0 | 1 | 192 |
| 175 | AF | 69 | 45 | 2 | 37 | 640240 | 5121916 | 2 | 37 | 0 | 184 | 5264716 | 2 | 37 | 0 | 1 | 192 |
| 176 | B0 | 69 | 45 | 2 | 38 | 640265 | 5122116 | 2 | 38 | 0 | 200 | 5264924 | 2 | 38 | 0 | 1 | 208 |
| 177 | B1 | 69 | 45 | 2 | 39 | 640290 | 5122316 | 2 | 39 | 0 | 200 | 5265132 | 2 | 39 | 0 | 1 | 208 |
| 178 | B2 | 69 | 45 | 2 | 40 | 640315 | 5122516 | 2 | 40 | 0 | 200 | 5265340 | 2 | 40 | 0 | 1 | 208 |
| 179 | B3 | 69 | 45 | 2 | 41 | 640340 | 5122716 | 2 | 41 | 0 | 200 | 5265548 | 2 | 41 | 0 | 1 | 208 |
| 180 | B4 | 69 | 45 | 2 | 42 | 640365 | 5122916 | 2 | 42 | 0 | 200 | 5265756 | 2 | 42 | 0 | 1 | 208 |
| 181 | B5 | 69 | 45 | 2 | 43 | 640390 | 5123116 | 2 | 43 | 0 | 200 | 5265964 | 2 | 43 | 0 | 1 | 208 |
| 182 | B6 | 69 | 45 | 2 | 44 | 640415 | 5123316 | 2 | 44 | 0 | 200 | 5266172 | 2 | 44 | 0 | 1 | 208 |
| 183 | B7 | 69 | 45 | 2 | 45 | 640440 | 5123516 | 2 | 45 | 0 | 200 | 5266380 | 2 | 45 | 0 | 1 | 208 |
| 184 | B8 | 69 | 45 | 2 | 46 | 640465 | 5123716 | 2 | 46 | 0 | 200 | 5266588 | 2 | 46 | 0 | 1 | 208 |
| 185 | B9 | 69 | 45 | 2 | 47 | 640490 | 5123916 | 2 | 47 | 0 | 200 | 5266796 | 2 | 47 | 0 | 1 | 208 |
| 186 | BA | 69 | 45 | 2 | 48 | 640515 | 5124116 | 2 | 48 | 0 | 200 | 5267004 | 2 | 48 | 0 | 1 | 208 |
| 187 | BB | 69 | 45 | 2 | 49 | 640540 | 5124316 | 2 | 49 | 0 | 200 | 5267212 | 2 | 49 | 0 | 1 | 208 |
| 188 | BC | 69 | 45 | 2 | 50 | 640565 | 5124516 | 2 | 50 | 0 | 200 | 5267420 | 2 | 50 | 0 | 1 | 208 |
| 189 | BD | 69 | 45 | 2 | 51 | 640590 | 5124716 | 2 | 51 | 0 | 200 | 5267628 | 2 | 51 | 0 | 1 | 208 |
| 190 | BE | 69 | 45 | 2 | 52 | 640615 | 5124916 | 2 | 52 | 0 | 200 | 5267836 | 2 | 52 | 0 | 1 | 208 |
| 191 | BF | 69 | 45 | 2 | 53 | 640640 | 5125116 | 2 | 53 | 0 | 200 | 5268044 | 2 | 53 | 0 | 1 | 208 |
| 192 | C0 | 69 | 45 | 2 | 54 | 640665 | 5125316 | 2 | 54 | 0 | 200 | 5268252 | 2 | 54 | 0 | 1 | 208 |
| 193 | C1 | 69 | 45 | 2 | 55 | 640690 | 5125516 | 2 | 55 | 0 | 200 | 5268460 | 2 | 55 | 0 | 1 | 208 |
| 194 | C2 | 69 | 45 | 2 | 56 | 640715 | 5125716 | 2 | 56 | 0 | 200 | 5268668 | 2 | 56 | 0 | 1 | 208 |
| 195 | C3 | 69 | 45 | 2 | 57 | 640740 | 5125916 | 2 | 57 | 0 | 200 | 5268876 | 2 | 57 | 0 | 1 | 208 |
| 196 | C4 | 69 | 45 | 2 | 58 | 640765 | 5126116 | 2 | 58 | 0 | 200 | 5269084 | 2 | 58 | 0 | 1 | 208 |
| 197 | C5 | 69 | 45 | 2 | 59 | 640790 | 5126316 | 2 | 59 | 0 | 200 | 5269292 | 2 | 59 | 0 | 1 | 208 |
| 198 | C6 | 69 | 45 | 2 | 60 | 640815 | 5126516 | 2 | 60 | 0 | 200 | 5269500 | 2 | 60 | 0 | 1 | 208 |
| 199 | C7 | 69 | 45 | 2 | 61 | 640840 | 5126716 | 2 | 61 | 0 | 200 | 5269708 | 2 | 61 | 0 | 1 | 208 |
| 200 | C8 | 69 | 45 | 2 | 62 | 640865 | 5126916 | 2 | 62 | 0 | 200 | 5269916 | 2 | 62 | 0 | 1 | 208 |
| 201 | C9 | 69 | 45 | 2 | 63 | 640890 | 5127116 | 2 | 63 | 0 | 200 | 5270124 | 2 | 63 | 0 | 1 | 208 |
| 202 | CA | 69 | 45 | 2 | 64 | 640915 | 5127316 | 2 | 64 | 0 | 200 | 5270332 | 2 | 64 | 0 | 1 | 208 |
| 203 | CB | 69 | 45 | 2 | 65 | 640940 | 5127516 | 2 | 65 | 0 | 200 | 5270540 | 2 | 65 | 0 | 1 | 208 |
| 204 | CC | 69 | 45 | 2 | 66 | 640965 | 5127716 | 2 | 66 | 0 | 200 | 5270748 | 2 | 66 | 0 | 1 | 208 |
| 205 | CD | 69 | 45 | 2 | 67 | 640990 | 5127916 | 2 | 67 | 0 | 200 | 5270956 | 2 | 67 | 0 | 1 | 208 |
| 206 | CE | 69 | 45 | 2 | 68 | 641015 | 5128116 | 2 | 68 | 0 | 200 | 5271164 | 2 | 68 | 0 | 1 | 208 |
| 207 | CF | 69 | 45 | 3 | 0 | 641038 | 5128300 | 3 | 0 | 0 | 184 | 5271356 | 3 | 0 | 0 | 1 | 192 |
| 208 | D0 | 69 | 45 | 3 | 1 | 641066 | 5128524 | 3 | 1 | 0 | 224 | 5271588 | 3 | 1 | 0 | 1 | 232 |
| 209 | D1 | 69 | 45 | 3 | 2 | 641094 | 5128748 | 3 | 2 | 0 | 224 | 5271820 | 3 | 2 | 0 | 1 | 232 |
| 210 | D2 | 69 | 45 | 3 | 3 | 641122 | 5128972 | 3 | 3 | 0 | 224 | 5272052 | 3 | 3 | 0 | 1 | 232 |
| 211 | D3 | 69 | 45 | 3 | 4 | 641150 | 5129196 | 3 | 4 | 0 | 224 | 5272284 | 3 | 4 | 0 | 1 | 232 |
| 212 | D4 | 69 | 45 | 3 | 5 | 641178 | 5129420 | 3 | 5 | 0 | 224 | 5272516 | 3 | 5 | 0 | 1 | 232 |
| 213 | D5 | 69 | 45 | 3 | 6 | 641206 | 5129644 | 3 | 6 | 0 | 224 | 5272748 | 3 | 6 | 0 | 1 | 232 |
| 214 | D6 | 69 | 45 | 3 | 7 | 641234 | 5129868 | 3 | 7 | 0 | 224 | 5272980 | 3 | 7 | 0 | 1 | 232 |
| 215 | D7 | 69 | 45 | 3 | 8 | 641262 | 5130092 | 3 | 8 | 0 | 224 | 5273212 | 3 | 8 | 0 | 1 | 232 |
| 216 | D8 | 69 | 45 | 3 | 9 | 641290 | 5130316 | 3 | 9 | 0 | 224 | 5273444 | 3 | 9 | 0 | 1 | 232 |
| 217 | D9 | 69 | 45 | 3 | 10 | 641318 | 5130540 | 3 | 10 | 0 | 224 | 5273676 | 3 | 10 | 0 | 1 | 232 |
| 218 | DA | 69 | 45 | 3 | 11 | 641346 | 5130764 | 3 | 11 | 0 | 224 | 5273908 | 3 | 11 | 0 | 1 | 232 |
| 219 | DB | 69 | 45 | 3 | 12 | 641374 | 5130988 | 3 | 12 | 0 | 224 | 5274140 | 3 | 12 | 0 | 1 | 232 |
| 220 | DC | 69 | 45 | 3 | 13 | 641402 | 5131212 | 3 | 13 | 0 | 224 | 5274372 | 3 | 13 | 0 | 1 | 232 |
| 221 | DD | 69 | 45 | 3 | 14 | 641430 | 5131436 | 3 | 14 | 0 | 224 | 5274604 | 3 | 14 | 0 | 1 | 232 |
| 222 | DE | 69 | 45 | 3 | 15 | 641458 | 5131660 | 3 | 15 | 0 | 224 | 5274836 | 3 | 15 | 0 | 1 | 232 |
| 223 | DF | 69 | 45 | 3 | 16 | 641486 | 5131884 | 3 | 16 | 0 | 224 | 5275068 | 3 | 16 | 0 | 1 | 232 |
| 224 | E0 | 69 | 45 | 3 | 17 | 641514 | 5132108 | 3 | 17 | 0 | 224 | 5275300 | 3 | 17 | 0 | 1 | 232 |
| 225 | E1 | 69 | 45 | 3 | 18 | 641542 | 5132332 | 3 | 18 | 0 | 224 | 5275532 | 3 | 18 | 0 | 1 | 232 |
| 226 | E2 | 69 | 45 | 3 | 19 | 641570 | 5132556 | 3 | 19 | 0 | 224 | 5275764 | 3 | 19 | 0 | 1 | 232 |
| 227 | E3 | 69 | 45 | 3 | 20 | 641598 | 5132780 | 3 | 20 | 0 | 224 | 5275996 | 3 | 20 | 0 | 1 | 232 |
| 228 | E4 | 69 | 45 | 3 | 21 | 641626 | 5133004 | 3 | 21 | 0 | 224 | 5276228 | 3 | 21 | 0 | 1 | 232 |
| 229 | E5 | 69 | 45 | 3 | 22 | 641654 | 5133228 | 3 | 22 | 0 | 224 | 5276460 | 3 | 22 | 0 | 1 | 232 |
| 230 | E6 | 69 | 45 | 3 | 23 | 641682 | 5133452 | 3 | 23 | 0 | 224 | 5276692 | 3 | 23 | 0 | 1 | 232 |
| 231 | E7 | 69 | 45 | 3 | 24 | 641710 | 5133676 | 3 | 24 | 0 | 224 | 5276924 | 3 | 24 | 0 | 1 | 232 |
| 232 | E8 | 69 | 45 | 3 | 25 | 641738 | 5133900 | 3 | 25 | 0 | 224 | 5277156 | 3 | 25 | 0 | 1 | 232 |
| 233 | E9 | 69 | 45 | 3 | 26 | 641766 | 5134124 | 3 | 26 | 0 | 224 | 5277388 | 3 | 26 | 0 | 1 | 232 |
| 234 | EA | 69 | 45 | 3 | 27 | 641794 | 5134348 | 3 | 27 | 0 | 224 | 5277620 | 3 | 27 | 0 | 1 | 232 |
| 235 | EB | 69 | 45 | 3 | 28 | 641822 | 5134572 | 3 | 28 | 0 | 224 | 5277852 | 3 | 28 | 0 | 1 | 232 |
| 236 | EC | 69 | 45 | 3 | 29 | 641850 | 5134796 | 3 | 29 | 0 | 224 | 5278084 | 3 | 29 | 0 | 1 | 232 |
| 237 | ED | 69 | 45 | 3 | 30 | 641878 | 5135020 | 3 | 30 | 0 | 224 | 5278316 | 3 | 30 | 0 | 1 | 232 |
| 238 | EE | 69 | 45 | 3 | 31 | 641906 | 5135244 | 3 | 31 | 0 | 224 | 5278548 | 3 | 31 | 0 | 1 | 232 |
| 239 | EF | 69 | 45 | 3 | 32 | 641934 | 5135468 | 3 | 32 | 0 | 224 | 5278780 | 3 | 32 | 0 | 1 | 232 |
| 240 | F0 | 69 | 45 | 3 | 33 | 641959 | 5135668 | 3 | 33 | 0 | 200 | 5278988 | 3 | 33 | 0 | 1 | 208 |
| 241 | F1 | 69 | 45 | 3 | 34 | 641984 | 5135868 | 3 | 34 | 0 | 200 | 5279196 | 3 | 34 | 0 | 1 | 208 |
| 242 | F2 | 69 | 45 | 3 | 35 | 642009 | 5136068 | 3 | 35 | 0 | 200 | 5279404 | 3 | 35 | 0 | 1 | 208 |
| 243 | F3 | 69 | 45 | 3 | 36 | 642034 | 5136268 | 3 | 36 | 0 | 200 | 5279612 | 3 | 36 | 0 | 1 | 208 |
| 244 | F4 | 69 | 45 | 3 | 37 | 642059 | 5136468 | 3 | 37 | 0 | 200 | 5279820 | 3 | 37 | 0 | 1 | 208 |
| 245 | F5 | 69 | 45 | 3 | 38 | 642084 | 5136668 | 3 | 38 | 0 | 200 | 5280028 | 3 | 38 | 0 | 1 | 208 |
| 246 | F6 | 69 | 45 | 3 | 39 | 642109 | 5136868 | 3 | 39 | 0 | 200 | 5280236 | 3 | 39 | 0 | 1 | 208 |
| 247 | F7 | 69 | 45 | 3 | 40 | 642134 | 5137068 | 3 | 40 | 0 | 200 | 5280444 | 3 | 40 | 0 | 1 | 208 |
| 248 | F8 | 69 | 45 | 3 | 41 | 642159 | 5137268 | 3 | 41 | 0 | 200 | 5280652 | 3 | 41 | 0 | 1 | 208 |
| 249 | F9 | 69 | 45 | 3 | 42 | 642184 | 5137468 | 3 | 42 | 0 | 200 | 5280860 | 3 | 42 | 0 | 1 | 208 |
| 250 | FA | 69 | 45 | 3 | 43 | 642209 | 5137668 | 3 | 43 | 0 | 200 | 5281068 | 3 | 43 | 0 | 1 | 208 |
| 251 | FB | 69 | 45 | 3 | 44 | 642234 | 5137868 | 3 | 44 | 0 | 200 | 5281276 | 3 | 44 | 0 | 1 | 208 |
| 252 | FC | 69 | 45 | 3 | 45 | 642259 | 5138068 | 3 | 45 | 0 | 200 | 5281484 | 3 | 45 | 0 | 1 | 208 |
| 253 | FD | 69 | 45 | 3 | 46 | 642284 | 5138268 | 3 | 46 | 0 | 200 | 5281692 | 3 | 46 | 0 | 1 | 208 |
| 254 | FE | 69 | 45 | 3 | 47 | 642309 | 5138468 | 3 | 47 | 0 | 200 | 5281900 | 3 | 47 | 0 | 1 | 208 |

| | | | | | | | | | | | | | | | | | |
|---|---|---|---|---|---|---|---|---|---|---|---|---|---|---|---|---|---|
| 255 | FF | 69 | 45 | 3 | 48 | 642334 | 5138668 | 3 | 48 | 0 | 200 | 5282108 | 3 | 48 | 0 | 1 | 208 |
| 0 | 00 | 70 | 46 | 0 | 0 | 642346 | 5138764 | 0 | 0 | 0 | 96 | 5282212 | 0 | 0 | 0 | 1 | 104 |
| 1 | 01 | 70 | 46 | 0 | 1 | 642360 | 5138876 | 0 | 1 | 0 | 112 | 5282332 | 0 | 1 | 0 | 1 | 120 |
| 2 | 02 | 70 | 46 | 0 | 2 | 642374 | 5138988 | 0 | 2 | 0 | 112 | 5282452 | 0 | 2 | 0 | 1 | 120 |
| 3 | 03 | 70 | 46 | 0 | 3 | 642388 | 5139100 | 0 | 3 | 0 | 112 | 5282572 | 0 | 3 | 0 | 1 | 120 |
| 4 | 04 | 70 | 46 | 0 | 4 | 642402 | 5139212 | 0 | 4 | 0 | 112 | 5282692 | 0 | 4 | 0 | 1 | 120 |
| 5 | 05 | 70 | 46 | 0 | 5 | 642416 | 5139324 | 0 | 5 | 0 | 112 | 5282812 | 0 | 5 | 0 | 1 | 120 |
| 6 | 06 | 70 | 46 | 0 | 6 | 642430 | 5139436 | 0 | 6 | 0 | 112 | 5282932 | 0 | 6 | 0 | 1 | 120 |
| 7 | 07 | 70 | 46 | 0 | 7 | 642444 | 5139548 | 0 | 7 | 0 | 112 | 5283052 | 0 | 7 | 0 | 1 | 120 |
| 8 | 08 | 70 | 46 | 0 | 8 | 642458 | 5139660 | 0 | 8 | 0 | 112 | 5283172 | 0 | 8 | 0 | 1 | 120 |
| 9 | 09 | 70 | 46 | 0 | 9 | 642472 | 5139772 | 0 | 9 | 0 | 112 | 5283292 | 0 | 9 | 0 | 1 | 120 |
| 10 | 0A | 70 | 46 | 0 | 10 | 642486 | 5139884 | 0 | 10 | 0 | 112 | 5283412 | 0 | 10 | 0 | 1 | 120 |
| 11 | 0B | 70 | 46 | 0 | 11 | 642500 | 5139996 | 0 | 11 | 0 | 112 | 5283532 | 0 | 11 | 0 | 1 | 120 |
| 12 | 0C | 70 | 46 | 0 | 12 | 642514 | 5140108 | 0 | 12 | 0 | 112 | 5283652 | 0 | 12 | 0 | 1 | 120 |
| 13 | 0D | 70 | 46 | 0 | 13 | 642528 | 5140220 | 0 | 13 | 0 | 112 | 5283772 | 0 | 13 | 0 | 1 | 120 |
| 14 | 0E | 70 | 46 | 0 | 14 | 642542 | 5140332 | 0 | 14 | 0 | 112 | 5283892 | 0 | 14 | 0 | 1 | 120 |
| 15 | 0F | 70 | 46 | 0 | 15 | 642556 | 5140444 | 0 | 15 | 0 | 112 | 5284012 | 0 | 15 | 0 | 1 | 120 |
| 16 | 10 | 70 | 46 | 0 | 16 | 642570 | 5140556 | 0 | 16 | 0 | 112 | 5284132 | 0 | 16 | 0 | 1 | 120 |
| 17 | 11 | 70 | 46 | 0 | 17 | 642584 | 5140668 | 0 | 17 | 0 | 112 | 5284252 | 0 | 17 | 0 | 1 | 120 |
| 18 | 12 | 70 | 46 | 0 | 18 | 642598 | 5140780 | 0 | 18 | 0 | 112 | 5284372 | 0 | 18 | 0 | 1 | 120 |
| 19 | 13 | 70 | 46 | 0 | 19 | 642612 | 5140892 | 0 | 19 | 0 | 112 | 5284492 | 0 | 19 | 0 | 1 | 120 |
| 20 | 14 | 70 | 46 | 0 | 20 | 642626 | 5141004 | 0 | 20 | 0 | 112 | 5284612 | 0 | 20 | 0 | 1 | 120 |
| 21 | 15 | 70 | 46 | 0 | 21 | 642640 | 5141116 | 0 | 21 | 0 | 112 | 5284732 | 0 | 21 | 0 | 1 | 120 |
| 22 | 16 | 70 | 46 | 0 | 22 | 642654 | 5141228 | 0 | 22 | 0 | 112 | 5284852 | 0 | 22 | 0 | 1 | 120 |
| 23 | 17 | 70 | 46 | 0 | 23 | 642668 | 5141340 | 0 | 23 | 0 | 112 | 5284972 | 0 | 23 | 0 | 1 | 120 |
| 24 | 18 | 70 | 46 | 0 | 24 | 642682 | 5141452 | 0 | 24 | 0 | 112 | 5285092 | 0 | 24 | 0 | 1 | 120 |
| 25 | 19 | 70 | 46 | 0 | 25 | 642696 | 5141564 | 0 | 25 | 0 | 112 | 5285212 | 0 | 25 | 0 | 1 | 120 |
| 26 | 1A | 70 | 46 | 0 | 26 | 642710 | 5141676 | 0 | 26 | 0 | 112 | 5285332 | 0 | 26 | 0 | 1 | 120 |
| 27 | 1B | 70 | 46 | 0 | 27 | 642724 | 5141788 | 0 | 27 | 0 | 112 | 5285452 | 0 | 27 | 0 | 1 | 120 |
| 28 | 1C | 70 | 46 | 0 | 28 | 642738 | 5141900 | 0 | 28 | 0 | 112 | 5285572 | 0 | 28 | 0 | 1 | 120 |
| 29 | 1D | 70 | 46 | 0 | 29 | 642752 | 5142012 | 0 | 29 | 0 | 112 | 5285692 | 0 | 29 | 0 | 1 | 120 |
| 30 | 1E | 70 | 46 | 0 | 30 | 642766 | 5142124 | 0 | 30 | 0 | 112 | 5285812 | 0 | 30 | 0 | 1 | 120 |
| 31 | 1F | 70 | 46 | 0 | 31 | 642780 | 5142236 | 0 | 31 | 0 | 112 | 5285932 | 0 | 31 | 0 | 1 | 120 |
| 32 | 20 | 70 | 46 | 0 | 32 | 642794 | 5142348 | 0 | 32 | 0 | 112 | 5286052 | 0 | 32 | 0 | 1 | 120 |
| 33 | 21 | 70 | 46 | 0 | 33 | 642808 | 5142460 | 0 | 33 | 0 | 112 | 5286172 | 0 | 33 | 0 | 1 | 120 |
| 34 | 22 | 70 | 46 | 0 | 34 | 642822 | 5142572 | 0 | 34 | 0 | 112 | 5286292 | 0 | 34 | 0 | 1 | 120 |
| 35 | 23 | 70 | 46 | 0 | 35 | 642836 | 5142684 | 0 | 35 | 0 | 112 | 5286412 | 0 | 35 | 0 | 1 | 120 |
| 36 | 24 | 70 | 46 | 0 | 36 | 642850 | 5142796 | 0 | 36 | 0 | 112 | 5286532 | 0 | 36 | 0 | 1 | 120 |
| 37 | 25 | 70 | 46 | 0 | 37 | 642864 | 5142908 | 0 | 37 | 0 | 112 | 5286652 | 0 | 37 | 0 | 1 | 120 |
| 38 | 26 | 70 | 46 | 0 | 38 | 642878 | 5143020 | 0 | 38 | 0 | 112 | 5286772 | 0 | 38 | 0 | 1 | 120 |
| 39 | 27 | 70 | 46 | 0 | 39 | 642892 | 5143132 | 0 | 39 | 0 | 112 | 5286892 | 0 | 39 | 0 | 1 | 120 |
| 40 | 28 | 70 | 46 | 0 | 40 | 642906 | 5143244 | 0 | 40 | 0 | 112 | 5287012 | 0 | 40 | 0 | 1 | 120 |
| 41 | 29 | 70 | 46 | 0 | 41 | 642920 | 5143356 | 0 | 41 | 0 | 112 | 5287132 | 0 | 41 | 0 | 1 | 120 |
| 42 | 2A | 70 | 46 | 0 | 42 | 642934 | 5143468 | 0 | 42 | 0 | 112 | 5287252 | 0 | 42 | 0 | 1 | 120 |
| 43 | 2B | 70 | 46 | 0 | 43 | 642948 | 5143580 | 0 | 43 | 0 | 112 | 5287372 | 0 | 43 | 0 | 1 | 120 |
| 44 | 2C | 70 | 46 | 0 | 44 | 642962 | 5143692 | 0 | 44 | 0 | 112 | 5287492 | 0 | 44 | 0 | 1 | 120 |
| 45 | 2D | 70 | 46 | 0 | 45 | 642976 | 5143804 | 0 | 45 | 0 | 112 | 5287612 | 0 | 45 | 0 | 1 | 120 |
| 46 | 2E | 70 | 46 | 0 | 46 | 642990 | 5143916 | 0 | 46 | 0 | 112 | 5287732 | 0 | 46 | 0 | 1 | 120 |
| 47 | 2F | 70 | 46 | 0 | 47 | 643004 | 5144028 | 0 | 47 | 0 | 112 | 5287852 | 0 | 47 | 0 | 1 | 120 |
| 48 | 30 | 70 | 46 | 0 | 48 | 643018 | 5144140 | 0 | 48 | 0 | 112 | 5287972 | 0 | 48 | 0 | 1 | 120 |
| 49 | 31 | 70 | 46 | 0 | 49 | 643032 | 5144252 | 0 | 49 | 0 | 112 | 5288092 | 0 | 49 | 0 | 1 | 120 |
| 50 | 32 | 70 | 46 | 0 | 50 | 643046 | 5144364 | 0 | 50 | 0 | 112 | 5288212 | 0 | 50 | 0 | 1 | 120 |
| 51 | 33 | 70 | 46 | 0 | 51 | 643060 | 5144476 | 0 | 51 | 0 | 112 | 5288332 | 0 | 51 | 0 | 1 | 120 |
| 52 | 34 | 70 | 46 | 0 | 52 | 643074 | 5144588 | 0 | 52 | 0 | 112 | 5288452 | 0 | 52 | 0 | 1 | 120 |
| 53 | 35 | 70 | 46 | 0 | 53 | 643088 | 5144700 | 0 | 53 | 0 | 112 | 5288572 | 0 | 53 | 0 | 1 | 120 |
| 54 | 36 | 70 | 46 | 0 | 54 | 643102 | 5144812 | 0 | 54 | 0 | 112 | 5288692 | 0 | 54 | 0 | 1 | 120 |
| 55 | 37 | 70 | 46 | 0 | 55 | 643116 | 5144924 | 0 | 55 | 0 | 112 | 5288812 | 0 | 55 | 0 | 1 | 120 |
| 56 | 38 | 70 | 46 | 0 | 56 | 643130 | 5145036 | 0 | 56 | 0 | 112 | 5288932 | 0 | 56 | 0 | 1 | 120 |
| 57 | 39 | 70 | 46 | 0 | 57 | 643144 | 5145148 | 0 | 57 | 0 | 112 | 5289052 | 0 | 57 | 0 | 1 | 120 |
| 58 | 3A | 70 | 46 | 0 | 58 | 643158 | 5145260 | 0 | 58 | 0 | 112 | 5289172 | 0 | 58 | 0 | 1 | 120 |
| 59 | 3B | 70 | 46 | 0 | 59 | 643172 | 5145372 | 0 | 59 | 0 | 112 | 5289292 | 0 | 59 | 0 | 1 | 120 |
| 60 | 3C | 70 | 46 | 0 | 60 | 643186 | 5145484 | 0 | 60 | 0 | 112 | 5289412 | 0 | 60 | 0 | 1 | 120 |
| 61 | 3D | 70 | 46 | 0 | 61 | 643200 | 5145596 | 0 | 61 | 0 | 112 | 5289532 | 0 | 61 | 0 | 1 | 120 |
| 62 | 3E | 70 | 46 | 0 | 62 | 643214 | 5145708 | 0 | 62 | 0 | 112 | 5289652 | 0 | 62 | 0 | 1 | 120 |
| 63 | 3F | 70 | 46 | 0 | 63 | 643228 | 5145820 | 0 | 63 | 0 | 112 | 5289772 | 0 | 63 | 0 | 1 | 120 |
| 64 | 40 | 70 | 46 | 0 | 64 | 643242 | 5145932 | 0 | 64 | 0 | 112 | 5289892 | 0 | 64 | 0 | 1 | 120 |
| 65 | 41 | 70 | 46 | 0 | 65 | 643256 | 5146044 | 0 | 65 | 0 | 112 | 5290012 | 0 | 65 | 0 | 1 | 120 |
| 66 | 42 | 70 | 46 | 0 | 66 | 643270 | 5146156 | 0 | 66 | 0 | 112 | 5290132 | 0 | 66 | 0 | 1 | 120 |
| 67 | 43 | 70 | 46 | 0 | 67 | 643284 | 5146268 | 0 | 67 | 0 | 112 | 5290252 | 0 | 67 | 0 | 1 | 120 |
| 68 | 44 | 70 | 46 | 0 | 68 | 643298 | 5146380 | 0 | 68 | 0 | 112 | 5290372 | 0 | 68 | 0 | 1 | 120 |
| 69 | 45 | 70 | 46 | 0 | 69 | 643312 | 5146492 | 0 | 69 | 0 | 112 | 5290492 | 0 | 69 | 0 | 1 | 120 |
| 70 | 46 | 70 | 46 | 1 | 0 | 643324 | 5146588 | 1 | 0 | 0 | 96 | 5290596 | 1 | 0 | 0 | 1 | 104 |
| 71 | 47 | 70 | 46 | 1 | 1 | 643341 | 5146724 | 1 | 1 | 0 | 136 | 5290740 | 1 | 1 | 0 | 1 | 144 |
| 72 | 48 | 70 | 46 | 1 | 2 | 643358 | 5146860 | 1 | 2 | 0 | 136 | 5290884 | 1 | 2 | 0 | 1 | 144 |
| 73 | 49 | 70 | 46 | 1 | 3 | 643375 | 5146996 | 1 | 3 | 0 | 136 | 5291028 | 1 | 3 | 0 | 1 | 144 |
| 74 | 4A | 70 | 46 | 1 | 4 | 643392 | 5147132 | 1 | 4 | 0 | 136 | 5291172 | 1 | 4 | 0 | 1 | 144 |
| 75 | 4B | 70 | 46 | 1 | 5 | 643409 | 5147268 | 1 | 5 | 0 | 136 | 5291316 | 1 | 5 | 0 | 1 | 144 |
| 76 | 4C | 70 | 46 | 1 | 6 | 643426 | 5147404 | 1 | 6 | 0 | 136 | 5291460 | 1 | 6 | 0 | 1 | 144 |
| 77 | 4D | 70 | 46 | 1 | 7 | 643443 | 5147540 | 1 | 7 | 0 | 136 | 5291604 | 1 | 7 | 0 | 1 | 144 |
| 78 | 4E | 70 | 46 | 1 | 8 | 643460 | 5147676 | 1 | 8 | 0 | 136 | 5291748 | 1 | 8 | 0 | 1 | 144 |
| 79 | 4F | 70 | 46 | 1 | 9 | 643477 | 5147812 | 1 | 9 | 0 | 136 | 5291892 | 1 | 9 | 0 | 1 | 144 |
| 80 | 50 | 70 | 46 | 1 | 10 | 643498 | 5147980 | 1 | 10 | 0 | 168 | 5292068 | 1 | 10 | 0 | 1 | 176 |
| 81 | 51 | 70 | 46 | 1 | 11 | 643519 | 5148148 | 1 | 11 | 0 | 168 | 5292244 | 1 | 11 | 0 | 1 | 176 |
| 82 | 52 | 70 | 46 | 1 | 12 | 643540 | 5148316 | 1 | 12 | 0 | 168 | 5292420 | 1 | 12 | 0 | 1 | 176 |
| 83 | 53 | 70 | 46 | 1 | 13 | 643561 | 5148484 | 1 | 13 | 0 | 168 | 5292596 | 1 | 13 | 0 | 1 | 176 |
| 84 | 54 | 70 | 46 | 1 | 14 | 643582 | 5148652 | 1 | 14 | 0 | 168 | 5292772 | 1 | 14 | 0 | 1 | 176 |
| 85 | 55 | 70 | 46 | 1 | 15 | 643603 | 5148820 | 1 | 15 | 0 | 168 | 5292948 | 1 | 15 | 0 | 1 | 176 |
| 86 | 56 | 70 | 46 | 1 | 16 | 643624 | 5148988 | 1 | 16 | 0 | 168 | 5293124 | 1 | 16 | 0 | 1 | 176 |
| 87 | 57 | 70 | 46 | 1 | 17 | 643645 | 5149156 | 1 | 17 | 0 | 168 | 5293300 | 1 | 17 | 0 | 1 | 176 |
| 88 | 58 | 70 | 46 | 1 | 18 | 643666 | 5149324 | 1 | 18 | 0 | 168 | 5293476 | 1 | 18 | 0 | 1 | 176 |
| 89 | 59 | 70 | 46 | 1 | 19 | 643687 | 5149492 | 1 | 19 | 0 | 168 | 5293652 | 1 | 19 | 0 | 1 | 176 |
| 90 | 5A | 70 | 46 | 1 | 20 | 643708 | 5149660 | 1 | 20 | 0 | 168 | 5293828 | 1 | 20 | 0 | 1 | 176 |
| 91 | 5B | 70 | 46 | 1 | 21 | 643729 | 5149828 | 1 | 21 | 0 | 168 | 5294004 | 1 | 21 | 0 | 1 | 176 |
| 92 | 5C | 70 | 46 | 1 | 22 | 643750 | 5149996 | 1 | 22 | 0 | 168 | 5294180 | 1 | 22 | 0 | 1 | 176 |
| 93 | 5D | 70 | 46 | 1 | 23 | 643771 | 5150164 | 1 | 23 | 0 | 168 | 5294356 | 1 | 23 | 0 | 1 | 176 |
| 94 | 5E | 70 | 46 | 1 | 24 | 643792 | 5150332 | 1 | 24 | 0 | 168 | 5294532 | 1 | 24 | 0 | 1 | 176 |
| 95 | 5F | 70 | 46 | 1 | 25 | 643813 | 5150500 | 1 | 25 | 0 | 168 | 5294708 | 1 | 25 | 0 | 1 | 176 |
| 96 | 60 | 70 | 46 | 1 | 26 | 643836 | 5150684 | 1 | 26 | 0 | 184 | 5294900 | 1 | 26 | 0 | 1 | 192 |
| 97 | 61 | 70 | 46 | 1 | 27 | 643859 | 5150868 | 1 | 27 | 0 | 184 | 5295092 | 1 | 27 | 0 | 1 | 192 |
| 98 | 62 | 70 | 46 | 1 | 28 | 643882 | 5151052 | 1 | 28 | 0 | 184 | 5295284 | 1 | 28 | 0 | 1 | 192 |
| 99 | 63 | 70 | 46 | 1 | 29 | 643905 | 5151236 | 1 | 29 | 0 | 184 | 5295476 | 1 | 29 | 0 | 1 | 192 |
| 100 | 64 | 70 | 46 | 1 | 30 | 643928 | 5151420 | 1 | 30 | 0 | 184 | 5295668 | 1 | 30 | 0 | 1 | 192 |
| 101 | 65 | 70 | 46 | 1 | 31 | 643951 | 5151604 | 1 | 31 | 0 | 184 | 5295860 | 1 | 31 | 0 | 1 | 192 |

| | | | | | | | | | | | | | | | | | |
|---|---|---|---|---|---|---|---|---|---|---|---|---|---|---|---|---|---|
| 102 | 66 | 70 | 46 | 1 | 32 | 643974 | 5151788 | 1 | 32 | 0 | 184 | 5296052 | 1 | 32 | 0 | 1 | 192 |
| 103 | 67 | 70 | 46 | 1 | 33 | 643997 | 5151972 | 1 | 33 | 0 | 184 | 5296244 | 1 | 33 | 0 | 1 | 192 |
| 104 | 68 | 70 | 46 | 1 | 34 | 644020 | 5152156 | 1 | 34 | 0 | 184 | 5296436 | 1 | 34 | 0 | 1 | 192 |
| 105 | 69 | 70 | 46 | 1 | 35 | 644043 | 5152340 | 1 | 35 | 0 | 184 | 5296628 | 1 | 35 | 0 | 1 | 192 |
| 106 | 6A | 70 | 46 | 1 | 36 | 644066 | 5152524 | 1 | 36 | 0 | 184 | 5296820 | 1 | 36 | 0 | 1 | 192 |
| 107 | 6B | 70 | 46 | 1 | 37 | 644089 | 5152708 | 1 | 37 | 0 | 184 | 5297012 | 1 | 37 | 0 | 1 | 192 |
| 108 | 6C | 70 | 46 | 1 | 38 | 644112 | 5152892 | 1 | 38 | 0 | 184 | 5297204 | 1 | 38 | 0 | 1 | 192 |
| 109 | 6D | 70 | 46 | 1 | 39 | 644135 | 5153076 | 1 | 39 | 0 | 184 | 5297396 | 1 | 39 | 0 | 1 | 192 |
| 110 | 6E | 70 | 46 | 1 | 40 | 644158 | 5153260 | 1 | 40 | 0 | 184 | 5297588 | 1 | 40 | 0 | 1 | 192 |
| 111 | 6F | 70 | 46 | 1 | 41 | 644181 | 5153444 | 1 | 41 | 0 | 184 | 5297780 | 1 | 41 | 0 | 1 | 192 |
| 112 | 70 | 70 | 46 | 1 | 42 | 644204 | 5153628 | 1 | 42 | 0 | 184 | 5297972 | 1 | 42 | 0 | 1 | 192 |
| 113 | 71 | 70 | 46 | 1 | 43 | 644227 | 5153812 | 1 | 43 | 0 | 184 | 5298164 | 1 | 43 | 0 | 1 | 192 |
| 114 | 72 | 70 | 46 | 1 | 44 | 644250 | 5153996 | 1 | 44 | 0 | 184 | 5298356 | 1 | 44 | 0 | 1 | 192 |
| 115 | 73 | 70 | 46 | 1 | 45 | 644273 | 5154180 | 1 | 45 | 0 | 184 | 5298548 | 1 | 45 | 0 | 1 | 192 |
| 116 | 74 | 70 | 46 | 1 | 46 | 644296 | 5154364 | 1 | 46 | 0 | 184 | 5298740 | 1 | 46 | 0 | 1 | 192 |
| 117 | 75 | 70 | 46 | 1 | 47 | 644319 | 5154548 | 1 | 47 | 0 | 184 | 5298932 | 1 | 47 | 0 | 1 | 192 |
| 118 | 76 | 70 | 46 | 1 | 48 | 644342 | 5154732 | 1 | 48 | 0 | 184 | 5299124 | 1 | 48 | 0 | 1 | 192 |
| 119 | 77 | 70 | 46 | 1 | 49 | 644365 | 5154916 | 1 | 49 | 0 | 184 | 5299316 | 1 | 49 | 0 | 1 | 192 |
| 120 | 78 | 70 | 46 | 1 | 50 | 644388 | 5155100 | 1 | 50 | 0 | 184 | 5299508 | 1 | 50 | 0 | 1 | 192 |
| 121 | 79 | 70 | 46 | 1 | 51 | 644411 | 5155284 | 1 | 51 | 0 | 184 | 5299700 | 1 | 51 | 0 | 1 | 192 |
| 122 | 7A | 70 | 46 | 1 | 52 | 644434 | 5155468 | 1 | 52 | 0 | 184 | 5299892 | 1 | 52 | 0 | 1 | 192 |
| 123 | 7B | 70 | 46 | 1 | 53 | 644457 | 5155652 | 1 | 53 | 0 | 184 | 5300084 | 1 | 53 | 0 | 1 | 192 |
| 124 | 7C | 70 | 46 | 1 | 54 | 644480 | 5155836 | 1 | 54 | 0 | 184 | 5300276 | 1 | 54 | 0 | 1 | 192 |
| 125 | 7D | 70 | 46 | 1 | 55 | 644503 | 5156020 | 1 | 55 | 0 | 184 | 5300468 | 1 | 55 | 0 | 1 | 192 |
| 126 | 7E | 70 | 46 | 1 | 56 | 644526 | 5156204 | 1 | 56 | 0 | 184 | 5300660 | 1 | 56 | 0 | 1 | 192 |
| 127 | 7F | 70 | 46 | 1 | 57 | 644549 | 5156388 | 1 | 57 | 0 | 184 | 5300852 | 1 | 57 | 0 | 1 | 192 |
| 128 | 80 | 70 | 46 | 1 | 58 | 644572 | 5156572 | 1 | 58 | 0 | 184 | 5301044 | 1 | 58 | 0 | 1 | 192 |
| 129 | 81 | 70 | 46 | 1 | 59 | 644595 | 5156756 | 1 | 59 | 0 | 184 | 5301236 | 1 | 59 | 0 | 1 | 192 |
| 130 | 82 | 70 | 46 | 1 | 60 | 644618 | 5156940 | 1 | 60 | 0 | 184 | 5301428 | 1 | 60 | 0 | 1 | 192 |
| 131 | 83 | 70 | 46 | 1 | 61 | 644641 | 5157124 | 1 | 61 | 0 | 184 | 5301620 | 1 | 61 | 0 | 1 | 192 |
| 132 | 84 | 70 | 46 | 1 | 62 | 644664 | 5157308 | 1 | 62 | 0 | 184 | 5301812 | 1 | 62 | 0 | 1 | 192 |
| 133 | 85 | 70 | 46 | 1 | 63 | 644687 | 5157492 | 1 | 63 | 0 | 184 | 5302004 | 1 | 63 | 0 | 1 | 192 |
| 134 | 86 | 70 | 46 | 1 | 64 | 644710 | 5157676 | 1 | 64 | 0 | 184 | 5302196 | 1 | 64 | 0 | 1 | 192 |
| 135 | 87 | 70 | 46 | 1 | 65 | 644733 | 5157860 | 1 | 65 | 0 | 184 | 5302388 | 1 | 65 | 0 | 1 | 192 |
| 136 | 88 | 70 | 46 | 1 | 66 | 644756 | 5158044 | 1 | 66 | 0 | 184 | 5302580 | 1 | 66 | 0 | 1 | 192 |
| 137 | 89 | 70 | 46 | 1 | 67 | 644779 | 5158228 | 1 | 67 | 0 | 184 | 5302772 | 1 | 67 | 0 | 1 | 192 |
| 138 | 8A | 70 | 46 | 1 | 68 | 644802 | 5158412 | 1 | 68 | 0 | 184 | 5302964 | 1 | 68 | 0 | 1 | 192 |
| 139 | 8B | 70 | 46 | 1 | 69 | 644825 | 5158596 | 1 | 69 | 0 | 184 | 5303156 | 1 | 69 | 0 | 1 | 192 |
| 140 | 8C | 70 | 46 | 2 | 0 | 644846 | 5158764 | 2 | 0 | 0 | 168 | 5303332 | 2 | 0 | 0 | 1 | 176 |
| 141 | 8D | 70 | 46 | 2 | 1 | 644872 | 5158972 | 2 | 1 | 0 | 208 | 5303548 | 2 | 1 | 0 | 1 | 216 |
| 142 | 8E | 70 | 46 | 2 | 2 | 644898 | 5159180 | 2 | 2 | 0 | 208 | 5303764 | 2 | 2 | 0 | 1 | 216 |
| 143 | 8F | 70 | 46 | 2 | 3 | 644924 | 5159388 | 2 | 3 | 0 | 208 | 5303980 | 2 | 3 | 0 | 1 | 216 |
| 144 | 90 | 70 | 46 | 2 | 4 | 644950 | 5159596 | 2 | 4 | 0 | 208 | 5304196 | 2 | 4 | 0 | 1 | 216 |
| 145 | 91 | 70 | 46 | 2 | 5 | 644976 | 5159804 | 2 | 5 | 0 | 208 | 5304412 | 2 | 5 | 0 | 1 | 216 |
| 146 | 92 | 70 | 46 | 2 | 6 | 645002 | 5160012 | 2 | 6 | 0 | 208 | 5304628 | 2 | 6 | 0 | 1 | 216 |
| 147 | 93 | 70 | 46 | 2 | 7 | 645028 | 5160220 | 2 | 7 | 0 | 208 | 5304844 | 2 | 7 | 0 | 1 | 216 |
| 148 | 94 | 70 | 46 | 2 | 8 | 645054 | 5160428 | 2 | 8 | 0 | 208 | 5305060 | 2 | 8 | 0 | 1 | 216 |
| 149 | 95 | 70 | 46 | 2 | 9 | 645080 | 5160636 | 2 | 9 | 0 | 208 | 5305276 | 2 | 9 | 0 | 1 | 216 |
| 150 | 96 | 70 | 46 | 2 | 10 | 645106 | 5160844 | 2 | 10 | 0 | 208 | 5305492 | 2 | 10 | 0 | 1 | 216 |
| 151 | 97 | 70 | 46 | 2 | 11 | 645132 | 5161052 | 2 | 11 | 0 | 208 | 5305708 | 2 | 11 | 0 | 1 | 216 |
| 152 | 98 | 70 | 46 | 2 | 12 | 645158 | 5161260 | 2 | 12 | 0 | 208 | 5305924 | 2 | 12 | 0 | 1 | 216 |
| 153 | 99 | 70 | 46 | 2 | 13 | 645184 | 5161468 | 2 | 13 | 0 | 208 | 5306140 | 2 | 13 | 0 | 1 | 216 |
| 154 | 9A | 70 | 46 | 2 | 14 | 645210 | 5161676 | 2 | 14 | 0 | 208 | 5306356 | 2 | 14 | 0 | 1 | 216 |
| 155 | 9B | 70 | 46 | 2 | 15 | 645236 | 5161884 | 2 | 15 | 0 | 208 | 5306572 | 2 | 15 | 0 | 1 | 216 |
| 156 | 9C | 70 | 46 | 2 | 16 | 645262 | 5162092 | 2 | 16 | 0 | 208 | 5306788 | 2 | 16 | 0 | 1 | 216 |
| 157 | 9D | 70 | 46 | 2 | 17 | 645288 | 5162300 | 2 | 17 | 0 | 208 | 5307004 | 2 | 17 | 0 | 1 | 216 |
| 158 | 9E | 70 | 46 | 2 | 18 | 645314 | 5162508 | 2 | 18 | 0 | 208 | 5307220 | 2 | 18 | 0 | 1 | 216 |
| 159 | 9F | 70 | 46 | 2 | 19 | 645340 | 5162716 | 2 | 19 | 0 | 208 | 5307436 | 2 | 19 | 0 | 1 | 216 |
| 160 | A0 | 70 | 46 | 2 | 20 | 645363 | 5162900 | 2 | 20 | 0 | 184 | 5307628 | 2 | 20 | 0 | 1 | 192 |
| 161 | A1 | 70 | 46 | 2 | 21 | 645386 | 5163084 | 2 | 21 | 0 | 184 | 5307820 | 2 | 21 | 0 | 1 | 192 |
| 162 | A2 | 70 | 46 | 2 | 22 | 645409 | 5163268 | 2 | 22 | 0 | 184 | 5308012 | 2 | 22 | 0 | 1 | 192 |
| 163 | A3 | 70 | 46 | 2 | 23 | 645432 | 5163452 | 2 | 23 | 0 | 184 | 5308204 | 2 | 23 | 0 | 1 | 192 |
| 164 | A4 | 70 | 46 | 2 | 24 | 645455 | 5163636 | 2 | 24 | 0 | 184 | 5308396 | 2 | 24 | 0 | 1 | 192 |
| 165 | A5 | 70 | 46 | 2 | 25 | 645478 | 5163820 | 2 | 25 | 0 | 184 | 5308588 | 2 | 25 | 0 | 1 | 192 |
| 166 | A6 | 70 | 46 | 2 | 26 | 645501 | 5164004 | 2 | 26 | 0 | 184 | 5308780 | 2 | 26 | 0 | 1 | 192 |
| 167 | A7 | 70 | 46 | 2 | 27 | 645524 | 5164188 | 2 | 27 | 0 | 184 | 5308972 | 2 | 27 | 0 | 1 | 192 |
| 168 | A8 | 70 | 46 | 2 | 28 | 645547 | 5164372 | 2 | 28 | 0 | 184 | 5309164 | 2 | 28 | 0 | 1 | 192 |
| 169 | A9 | 70 | 46 | 2 | 29 | 645570 | 5164556 | 2 | 29 | 0 | 184 | 5309356 | 2 | 29 | 0 | 1 | 192 |
| 170 | AA | 70 | 46 | 2 | 30 | 645593 | 5164740 | 2 | 30 | 0 | 184 | 5309548 | 2 | 30 | 0 | 1 | 192 |
| 171 | AB | 70 | 46 | 2 | 31 | 645616 | 5164924 | 2 | 31 | 0 | 184 | 5309740 | 2 | 31 | 0 | 1 | 192 |
| 172 | AC | 70 | 46 | 2 | 32 | 645639 | 5165108 | 2 | 32 | 0 | 184 | 5309932 | 2 | 32 | 0 | 1 | 192 |
| 173 | AD | 70 | 46 | 2 | 33 | 645662 | 5165292 | 2 | 33 | 0 | 184 | 5310124 | 2 | 33 | 0 | 1 | 192 |
| 174 | AE | 70 | 46 | 2 | 34 | 645685 | 5165476 | 2 | 34 | 0 | 184 | 5310316 | 2 | 34 | 0 | 1 | 192 |
| 175 | AF | 70 | 46 | 2 | 35 | 645708 | 5165660 | 2 | 35 | 0 | 184 | 5310508 | 2 | 35 | 0 | 1 | 192 |
| 176 | B0 | 70 | 46 | 2 | 36 | 645733 | 5165860 | 2 | 36 | 0 | 200 | 5310716 | 2 | 36 | 0 | 1 | 208 |
| 177 | B1 | 70 | 46 | 2 | 37 | 645758 | 5166060 | 2 | 37 | 0 | 200 | 5310924 | 2 | 37 | 0 | 1 | 208 |
| 178 | B2 | 70 | 46 | 2 | 38 | 645783 | 5166260 | 2 | 38 | 0 | 200 | 5311132 | 2 | 38 | 0 | 1 | 208 |
| 179 | B3 | 70 | 46 | 2 | 39 | 645808 | 5166460 | 2 | 39 | 0 | 200 | 5311340 | 2 | 39 | 0 | 1 | 208 |
| 180 | B4 | 70 | 46 | 2 | 40 | 645833 | 5166660 | 2 | 40 | 0 | 200 | 5311548 | 2 | 40 | 0 | 1 | 208 |
| 181 | B5 | 70 | 46 | 2 | 41 | 645858 | 5166860 | 2 | 41 | 0 | 200 | 5311756 | 2 | 41 | 0 | 1 | 208 |
| 182 | B6 | 70 | 46 | 2 | 42 | 645883 | 5167060 | 2 | 42 | 0 | 200 | 5311964 | 2 | 42 | 0 | 1 | 208 |
| 183 | B7 | 70 | 46 | 2 | 43 | 645908 | 5167260 | 2 | 43 | 0 | 200 | 5312172 | 2 | 43 | 0 | 1 | 208 |
| 184 | B8 | 70 | 46 | 2 | 44 | 645933 | 5167460 | 2 | 44 | 0 | 200 | 5312380 | 2 | 44 | 0 | 1 | 208 |
| 185 | B9 | 70 | 46 | 2 | 45 | 645958 | 5167660 | 2 | 45 | 0 | 200 | 5312588 | 2 | 45 | 0 | 1 | 208 |
| 186 | BA | 70 | 46 | 2 | 46 | 645983 | 5167860 | 2 | 46 | 0 | 200 | 5312796 | 2 | 46 | 0 | 1 | 208 |
| 187 | BB | 70 | 46 | 2 | 47 | 646008 | 5168060 | 2 | 47 | 0 | 200 | 5313004 | 2 | 47 | 0 | 1 | 208 |
| 188 | BC | 70 | 46 | 2 | 48 | 646033 | 5168260 | 2 | 48 | 0 | 200 | 5313212 | 2 | 48 | 0 | 1 | 208 |
| 189 | BD | 70 | 46 | 2 | 49 | 646058 | 5168460 | 2 | 49 | 0 | 200 | 5313420 | 2 | 49 | 0 | 1 | 208 |
| 190 | BE | 70 | 46 | 2 | 50 | 646083 | 5168660 | 2 | 50 | 0 | 200 | 5313628 | 2 | 50 | 0 | 1 | 208 |
| 191 | BF | 70 | 46 | 2 | 51 | 646108 | 5168860 | 2 | 51 | 0 | 200 | 5313836 | 2 | 51 | 0 | 1 | 208 |
| 192 | C0 | 70 | 46 | 2 | 52 | 646133 | 5169060 | 2 | 52 | 0 | 200 | 5314044 | 2 | 52 | 0 | 1 | 208 |
| 193 | C1 | 70 | 46 | 2 | 53 | 646158 | 5169260 | 2 | 53 | 0 | 200 | 5314252 | 2 | 53 | 0 | 1 | 208 |
| 194 | C2 | 70 | 46 | 2 | 54 | 646183 | 5169460 | 2 | 54 | 0 | 200 | 5314460 | 2 | 54 | 0 | 1 | 208 |
| 195 | C3 | 70 | 46 | 2 | 55 | 646208 | 5169660 | 2 | 55 | 0 | 200 | 5314668 | 2 | 55 | 0 | 1 | 208 |
| 196 | C4 | 70 | 46 | 2 | 56 | 646233 | 5169860 | 2 | 56 | 0 | 200 | 5314876 | 2 | 56 | 0 | 1 | 208 |
| 197 | C5 | 70 | 46 | 2 | 57 | 646258 | 5170060 | 2 | 57 | 0 | 200 | 5315084 | 2 | 57 | 0 | 1 | 208 |
| 198 | C6 | 70 | 46 | 2 | 58 | 646283 | 5170260 | 2 | 58 | 0 | 200 | 5315292 | 2 | 58 | 0 | 1 | 208 |
| 199 | C7 | 70 | 46 | 2 | 59 | 646308 | 5170460 | 2 | 59 | 0 | 200 | 5315500 | 2 | 59 | 0 | 1 | 208 |
| 200 | C8 | 70 | 46 | 2 | 60 | 646333 | 5170660 | 2 | 60 | 0 | 200 | 5315708 | 2 | 60 | 0 | 1 | 208 |
| 201 | C9 | 70 | 46 | 2 | 61 | 646358 | 5170860 | 2 | 61 | 0 | 200 | 5315916 | 2 | 61 | 0 | 1 | 208 |
| 202 | CA | 70 | 46 | 2 | 62 | 646383 | 5171060 | 2 | 62 | 0 | 200 | 5316124 | 2 | 62 | 0 | 1 | 208 |
| 203 | CB | 70 | 46 | 2 | 63 | 646408 | 5171260 | 2 | 63 | 0 | 200 | 5316332 | 2 | 63 | 0 | 1 | 208 |
| 204 | CC | 70 | 46 | 2 | 64 | 646433 | 5171460 | 2 | 64 | 0 | 200 | 5316540 | 2 | 64 | 0 | 1 | 208 |

| | | | | | | | | | | | | | | | | | |
|---|---|---|---|---|---|---|---|---|---|---|---|---|---|---|---|---|---|
| 205 | CD | 70 | 46 | 2 | 65 | 646458 | 5171660 | 2 | 65 | 0 | 200 | 5316748 | 2 | 65 | 0 | 1 | 208 |
| 206 | CE | 70 | 46 | 2 | 66 | 646483 | 5171860 | 2 | 66 | 0 | 200 | 5316956 | 2 | 66 | 0 | 1 | 208 |
| 207 | CF | 70 | 46 | 2 | 67 | 646508 | 5172060 | 2 | 67 | 0 | 200 | 5317164 | 2 | 67 | 0 | 1 | 208 |
| 208 | D0 | 70 | 46 | 2 | 68 | 646533 | 5172260 | 2 | 68 | 0 | 200 | 5317372 | 2 | 68 | 0 | 1 | 208 |
| 209 | D1 | 70 | 46 | 2 | 69 | 646558 | 5172460 | 2 | 69 | 0 | 200 | 5317580 | 2 | 69 | 0 | 1 | 208 |
| 210 | D2 | 70 | 46 | 3 | 0 | 646581 | 5172644 | 3 | 0 | 0 | 184 | 5317772 | 3 | 0 | 0 | 1 | 192 |
| 211 | D3 | 70 | 46 | 3 | 1 | 646609 | 5172868 | 3 | 1 | 0 | 224 | 5318004 | 3 | 1 | 0 | 1 | 232 |
| 212 | D4 | 70 | 46 | 3 | 2 | 646637 | 5173092 | 3 | 2 | 0 | 224 | 5318236 | 3 | 2 | 0 | 1 | 232 |
| 213 | D5 | 70 | 46 | 3 | 3 | 646665 | 5173316 | 3 | 3 | 0 | 224 | 5318468 | 3 | 3 | 0 | 1 | 232 |
| 214 | D6 | 70 | 46 | 3 | 4 | 646693 | 5173540 | 3 | 4 | 0 | 224 | 5318700 | 3 | 4 | 0 | 1 | 232 |
| 215 | D7 | 70 | 46 | 3 | 5 | 646721 | 5173764 | 3 | 5 | 0 | 224 | 5318932 | 3 | 5 | 0 | 1 | 232 |
| 216 | D8 | 70 | 46 | 3 | 6 | 646749 | 5173988 | 3 | 6 | 0 | 224 | 5319164 | 3 | 6 | 0 | 1 | 232 |
| 217 | D9 | 70 | 46 | 3 | 7 | 646777 | 5174212 | 3 | 7 | 0 | 224 | 5319396 | 3 | 7 | 0 | 1 | 232 |
| 218 | DA | 70 | 46 | 3 | 8 | 646805 | 5174436 | 3 | 8 | 0 | 224 | 5319628 | 3 | 8 | 0 | 1 | 232 |
| 219 | DB | 70 | 46 | 3 | 9 | 646833 | 5174660 | 3 | 9 | 0 | 224 | 5319860 | 3 | 9 | 0 | 1 | 232 |
| 220 | DC | 70 | 46 | 3 | 10 | 646861 | 5174884 | 3 | 10 | 0 | 224 | 5320092 | 3 | 10 | 0 | 1 | 232 |
| 221 | DD | 70 | 46 | 3 | 11 | 646889 | 5175108 | 3 | 11 | 0 | 224 | 5320324 | 3 | 11 | 0 | 1 | 232 |
| 222 | DE | 70 | 46 | 3 | 12 | 646917 | 5175332 | 3 | 12 | 0 | 224 | 5320556 | 3 | 12 | 0 | 1 | 232 |
| 223 | DF | 70 | 46 | 3 | 13 | 646945 | 5175556 | 3 | 13 | 0 | 224 | 5320788 | 3 | 13 | 0 | 1 | 232 |
| 224 | E0 | 70 | 46 | 3 | 14 | 646973 | 5175780 | 3 | 14 | 0 | 224 | 5321020 | 3 | 14 | 0 | 1 | 232 |
| 225 | E1 | 70 | 46 | 3 | 15 | 647001 | 5176004 | 3 | 15 | 0 | 224 | 5321252 | 3 | 15 | 0 | 1 | 232 |
| 226 | E2 | 70 | 46 | 3 | 16 | 647029 | 5176228 | 3 | 16 | 0 | 224 | 5321484 | 3 | 16 | 0 | 1 | 232 |
| 227 | E3 | 70 | 46 | 3 | 17 | 647057 | 5176452 | 3 | 17 | 0 | 224 | 5321716 | 3 | 17 | 0 | 1 | 232 |
| 228 | E4 | 70 | 46 | 3 | 18 | 647085 | 5176676 | 3 | 18 | 0 | 224 | 5321948 | 3 | 18 | 0 | 1 | 232 |
| 229 | E5 | 70 | 46 | 3 | 19 | 647113 | 5176900 | 3 | 19 | 0 | 224 | 5322180 | 3 | 19 | 0 | 1 | 232 |
| 230 | E6 | 70 | 46 | 3 | 20 | 647141 | 5177124 | 3 | 20 | 0 | 224 | 5322412 | 3 | 20 | 0 | 1 | 232 |
| 231 | E7 | 70 | 46 | 3 | 21 | 647169 | 5177348 | 3 | 21 | 0 | 224 | 5322644 | 3 | 21 | 0 | 1 | 232 |
| 232 | E8 | 70 | 46 | 3 | 22 | 647197 | 5177572 | 3 | 22 | 0 | 224 | 5322876 | 3 | 22 | 0 | 1 | 232 |
| 233 | E9 | 70 | 46 | 3 | 23 | 647225 | 5177796 | 3 | 23 | 0 | 224 | 5323108 | 3 | 23 | 0 | 1 | 232 |
| 234 | EA | 70 | 46 | 3 | 24 | 647253 | 5178020 | 3 | 24 | 0 | 224 | 5323340 | 3 | 24 | 0 | 1 | 232 |
| 235 | EB | 70 | 46 | 3 | 25 | 647281 | 5178244 | 3 | 25 | 0 | 224 | 5323572 | 3 | 25 | 0 | 1 | 232 |
| 236 | EC | 70 | 46 | 3 | 26 | 647309 | 5178468 | 3 | 26 | 0 | 224 | 5323804 | 3 | 26 | 0 | 1 | 232 |
| 237 | ED | 70 | 46 | 3 | 27 | 647337 | 5178692 | 3 | 27 | 0 | 224 | 5324036 | 3 | 27 | 0 | 1 | 232 |
| 238 | EE | 70 | 46 | 3 | 28 | 647365 | 5178916 | 3 | 28 | 0 | 224 | 5324268 | 3 | 28 | 0 | 1 | 232 |
| 239 | EF | 70 | 46 | 3 | 29 | 647393 | 5179140 | 3 | 29 | 0 | 224 | 5324500 | 3 | 29 | 0 | 1 | 232 |
| 240 | F0 | 70 | 46 | 3 | 30 | 647418 | 5179340 | 3 | 30 | 0 | 200 | 5324708 | 3 | 30 | 0 | 1 | 208 |
| 241 | F1 | 70 | 46 | 3 | 31 | 647443 | 5179540 | 3 | 31 | 0 | 200 | 5324916 | 3 | 31 | 0 | 1 | 208 |
| 242 | F2 | 70 | 46 | 3 | 32 | 647468 | 5179740 | 3 | 32 | 0 | 200 | 5325124 | 3 | 32 | 0 | 1 | 208 |
| 243 | F3 | 70 | 46 | 3 | 33 | 647493 | 5179940 | 3 | 33 | 0 | 200 | 5325332 | 3 | 33 | 0 | 1 | 208 |
| 244 | F4 | 70 | 46 | 3 | 34 | 647518 | 5180140 | 3 | 34 | 0 | 200 | 5325540 | 3 | 34 | 0 | 1 | 208 |
| 245 | F5 | 70 | 46 | 3 | 35 | 647543 | 5180340 | 3 | 35 | 0 | 200 | 5325748 | 3 | 35 | 0 | 1 | 208 |
| 246 | F6 | 70 | 46 | 3 | 36 | 647568 | 5180540 | 3 | 36 | 0 | 200 | 5325956 | 3 | 36 | 0 | 1 | 208 |
| 247 | F7 | 70 | 46 | 3 | 37 | 647593 | 5180740 | 3 | 37 | 0 | 200 | 5326164 | 3 | 37 | 0 | 1 | 208 |
| 248 | F8 | 70 | 46 | 3 | 38 | 647618 | 5180940 | 3 | 38 | 0 | 200 | 5326372 | 3 | 38 | 0 | 1 | 208 |
| 249 | F9 | 70 | 46 | 3 | 39 | 647643 | 5181140 | 3 | 39 | 0 | 200 | 5326580 | 3 | 39 | 0 | 1 | 208 |
| 250 | FA | 70 | 46 | 3 | 40 | 647668 | 5181340 | 3 | 40 | 0 | 200 | 5326788 | 3 | 40 | 0 | 1 | 208 |
| 251 | FB | 70 | 46 | 3 | 41 | 647693 | 5181540 | 3 | 41 | 0 | 200 | 5326996 | 3 | 41 | 0 | 1 | 208 |
| 252 | FC | 70 | 46 | 3 | 42 | 647718 | 5181740 | 3 | 42 | 0 | 200 | 5327204 | 3 | 42 | 0 | 1 | 208 |
| 253 | FD | 70 | 46 | 3 | 43 | 647743 | 5181940 | 3 | 43 | 0 | 200 | 5327412 | 3 | 43 | 0 | 1 | 208 |
| 254 | FE | 70 | 46 | 3 | 44 | 647768 | 5182140 | 3 | 44 | 0 | 200 | 5327620 | 3 | 44 | 0 | 1 | 208 |
| 255 | FF | 70 | 46 | 3 | 45 | 647793 | 5182340 | 3 | 45 | 0 | 200 | 5327828 | 3 | 45 | 0 | 1 | 208 |
| 0 | 00 | 71 | 47 | 0 | 0 | 647805 | 5182436 | 0 | 0 | 0 | 96 | 5327932 | 0 | 0 | 0 | 1 | 104 |
| 1 | 01 | 71 | 47 | 0 | 1 | 647819 | 5182548 | 0 | 1 | 0 | 112 | 5328052 | 0 | 1 | 0 | 1 | 120 |
| 2 | 02 | 71 | 47 | 0 | 2 | 647833 | 5182660 | 0 | 2 | 0 | 112 | 5328172 | 0 | 2 | 0 | 1 | 120 |
| 3 | 03 | 71 | 47 | 0 | 3 | 647847 | 5182772 | 0 | 3 | 0 | 112 | 5328292 | 0 | 3 | 0 | 1 | 120 |
| 4 | 04 | 71 | 47 | 0 | 4 | 647861 | 5182884 | 0 | 4 | 0 | 112 | 5328412 | 0 | 4 | 0 | 1 | 120 |
| 5 | 05 | 71 | 47 | 0 | 5 | 647875 | 5182996 | 0 | 5 | 0 | 112 | 5328532 | 0 | 5 | 0 | 1 | 120 |
| 6 | 06 | 71 | 47 | 0 | 6 | 647889 | 5183108 | 0 | 6 | 0 | 112 | 5328652 | 0 | 6 | 0 | 1 | 120 |
| 7 | 07 | 71 | 47 | 0 | 7 | 647903 | 5183220 | 0 | 7 | 0 | 112 | 5328772 | 0 | 7 | 0 | 1 | 120 |
| 8 | 08 | 71 | 47 | 0 | 8 | 647917 | 5183332 | 0 | 8 | 0 | 112 | 5328892 | 0 | 8 | 0 | 1 | 120 |
| 9 | 09 | 71 | 47 | 0 | 9 | 647931 | 5183444 | 0 | 9 | 0 | 112 | 5329012 | 0 | 9 | 0 | 1 | 120 |
| 10 | 0A | 71 | 47 | 0 | 10 | 647945 | 5183556 | 0 | 10 | 0 | 112 | 5329132 | 0 | 10 | 0 | 1 | 120 |
| 11 | 0B | 71 | 47 | 0 | 11 | 647959 | 5183668 | 0 | 11 | 0 | 112 | 5329252 | 0 | 11 | 0 | 1 | 120 |
| 12 | 0C | 71 | 47 | 0 | 12 | 647973 | 5183780 | 0 | 12 | 0 | 112 | 5329372 | 0 | 12 | 0 | 1 | 120 |
| 13 | 0D | 71 | 47 | 0 | 13 | 647987 | 5183892 | 0 | 13 | 0 | 112 | 5329492 | 0 | 13 | 0 | 1 | 120 |
| 14 | 0E | 71 | 47 | 0 | 14 | 648001 | 5184004 | 0 | 14 | 0 | 112 | 5329612 | 0 | 14 | 0 | 1 | 120 |
| 15 | 0F | 71 | 47 | 0 | 15 | 648015 | 5184116 | 0 | 15 | 0 | 112 | 5329732 | 0 | 15 | 0 | 1 | 120 |
| 16 | 10 | 71 | 47 | 0 | 16 | 648029 | 5184228 | 0 | 16 | 0 | 112 | 5329852 | 0 | 16 | 0 | 1 | 120 |
| 17 | 11 | 71 | 47 | 0 | 17 | 648043 | 5184340 | 0 | 17 | 0 | 112 | 5329972 | 0 | 17 | 0 | 1 | 120 |
| 18 | 12 | 71 | 47 | 0 | 18 | 648057 | 5184452 | 0 | 18 | 0 | 112 | 5330092 | 0 | 18 | 0 | 1 | 120 |
| 19 | 13 | 71 | 47 | 0 | 19 | 648071 | 5184564 | 0 | 19 | 0 | 112 | 5330212 | 0 | 19 | 0 | 1 | 120 |
| 20 | 14 | 71 | 47 | 0 | 20 | 648085 | 5184676 | 0 | 20 | 0 | 112 | 5330332 | 0 | 20 | 0 | 1 | 120 |
| 21 | 15 | 71 | 47 | 0 | 21 | 648099 | 5184788 | 0 | 21 | 0 | 112 | 5330452 | 0 | 21 | 0 | 1 | 120 |
| 22 | 16 | 71 | 47 | 0 | 22 | 648113 | 5184900 | 0 | 22 | 0 | 112 | 5330572 | 0 | 22 | 0 | 1 | 120 |
| 23 | 17 | 71 | 47 | 0 | 23 | 648127 | 5185012 | 0 | 23 | 0 | 112 | 5330692 | 0 | 23 | 0 | 1 | 120 |
| 24 | 18 | 71 | 47 | 0 | 24 | 648141 | 5185124 | 0 | 24 | 0 | 112 | 5330812 | 0 | 24 | 0 | 1 | 120 |
| 25 | 19 | 71 | 47 | 0 | 25 | 648155 | 5185236 | 0 | 25 | 0 | 112 | 5330932 | 0 | 25 | 0 | 1 | 120 |
| 26 | 1A | 71 | 47 | 0 | 26 | 648169 | 5185348 | 0 | 26 | 0 | 112 | 5331052 | 0 | 26 | 0 | 1 | 120 |
| 27 | 1B | 71 | 47 | 0 | 27 | 648183 | 5185460 | 0 | 27 | 0 | 112 | 5331172 | 0 | 27 | 0 | 1 | 120 |
| 28 | 1C | 71 | 47 | 0 | 28 | 648197 | 5185572 | 0 | 28 | 0 | 112 | 5331292 | 0 | 28 | 0 | 1 | 120 |
| 29 | 1D | 71 | 47 | 0 | 29 | 648211 | 5185684 | 0 | 29 | 0 | 112 | 5331412 | 0 | 29 | 0 | 1 | 120 |
| 30 | 1E | 71 | 47 | 0 | 30 | 648225 | 5185796 | 0 | 30 | 0 | 112 | 5331532 | 0 | 30 | 0 | 1 | 120 |
| 31 | 1F | 71 | 47 | 0 | 31 | 648239 | 5185908 | 0 | 31 | 0 | 112 | 5331652 | 0 | 31 | 0 | 1 | 120 |
| 32 | 20 | 71 | 47 | 0 | 32 | 648253 | 5186020 | 0 | 32 | 0 | 112 | 5331772 | 0 | 32 | 0 | 1 | 120 |
| 33 | 21 | 71 | 47 | 0 | 33 | 648267 | 5186132 | 0 | 33 | 0 | 112 | 5331892 | 0 | 33 | 0 | 1 | 120 |
| 34 | 22 | 71 | 47 | 0 | 34 | 648281 | 5186244 | 0 | 34 | 0 | 112 | 5332012 | 0 | 34 | 0 | 1 | 120 |
| 35 | 23 | 71 | 47 | 0 | 35 | 648295 | 5186356 | 0 | 35 | 0 | 112 | 5332132 | 0 | 35 | 0 | 1 | 120 |
| 36 | 24 | 71 | 47 | 0 | 36 | 648309 | 5186468 | 0 | 36 | 0 | 112 | 5332252 | 0 | 36 | 0 | 1 | 120 |
| 37 | 25 | 71 | 47 | 0 | 37 | 648323 | 5186580 | 0 | 37 | 0 | 112 | 5332372 | 0 | 37 | 0 | 1 | 120 |
| 38 | 26 | 71 | 47 | 0 | 38 | 648337 | 5186692 | 0 | 38 | 0 | 112 | 5332492 | 0 | 38 | 0 | 1 | 120 |
| 39 | 27 | 71 | 47 | 0 | 39 | 648351 | 5186804 | 0 | 39 | 0 | 112 | 5332612 | 0 | 39 | 0 | 1 | 120 |
| 40 | 28 | 71 | 47 | 0 | 40 | 648365 | 5186916 | 0 | 40 | 0 | 112 | 5332732 | 0 | 40 | 0 | 1 | 120 |
| 41 | 29 | 71 | 47 | 0 | 41 | 648379 | 5187028 | 0 | 41 | 0 | 112 | 5332852 | 0 | 41 | 0 | 1 | 120 |
| 42 | 2A | 71 | 47 | 0 | 42 | 648393 | 5187140 | 0 | 42 | 0 | 112 | 5332972 | 0 | 42 | 0 | 1 | 120 |
| 43 | 2B | 71 | 47 | 0 | 43 | 648407 | 5187252 | 0 | 43 | 0 | 112 | 5333092 | 0 | 43 | 0 | 1 | 120 |
| 44 | 2C | 71 | 47 | 0 | 44 | 648421 | 5187364 | 0 | 44 | 0 | 112 | 5333212 | 0 | 44 | 0 | 1 | 120 |
| 45 | 2D | 71 | 47 | 0 | 45 | 648435 | 5187476 | 0 | 45 | 0 | 112 | 5333332 | 0 | 45 | 0 | 1 | 120 |
| 46 | 2E | 71 | 47 | 0 | 46 | 648449 | 5187588 | 0 | 46 | 0 | 112 | 5333452 | 0 | 46 | 0 | 1 | 120 |
| 47 | 2F | 71 | 47 | 0 | 47 | 648463 | 5187700 | 0 | 47 | 0 | 112 | 5333572 | 0 | 47 | 0 | 1 | 120 |
| 48 | 30 | 71 | 47 | 0 | 48 | 648477 | 5187812 | 0 | 48 | 0 | 112 | 5333692 | 0 | 48 | 0 | 1 | 120 |
| 49 | 31 | 71 | 47 | 0 | 49 | 648491 | 5187924 | 0 | 49 | 0 | 112 | 5333812 | 0 | 49 | 0 | 1 | 120 |
| 50 | 32 | 71 | 47 | 0 | 50 | 648505 | 5188036 | 0 | 50 | 0 | 112 | 5333932 | 0 | 50 | 0 | 1 | 120 |
| 51 | 33 | 71 | 47 | 0 | 51 | 648519 | 5188148 | 0 | 51 | 0 | 112 | 5334052 | 0 | 51 | 0 | 1 | 120 |

| | | | | | | | | | | | | | | | | | |
|---|---|---|---|---|---|---|---|---|---|---|---|---|---|---|---|---|---|
| 52 | 34 | 71 | 47 | 0 | 52 | 648533 | 5188260 | 0 | 52 | 0 | 112 | 5334172 | 0 | 52 | 0 | 1 | 120 |
| 53 | 35 | 71 | 47 | 0 | 53 | 648547 | 5188372 | 0 | 53 | 0 | 112 | 5334292 | 0 | 53 | 0 | 1 | 120 |
| 54 | 36 | 71 | 47 | 0 | 54 | 648561 | 5188484 | 0 | 54 | 0 | 112 | 5334412 | 0 | 54 | 0 | 1 | 120 |
| 55 | 37 | 71 | 47 | 0 | 55 | 648575 | 5188596 | 0 | 55 | 0 | 112 | 5334532 | 0 | 55 | 0 | 1 | 120 |
| 56 | 38 | 71 | 47 | 0 | 56 | 648589 | 5188708 | 0 | 56 | 0 | 112 | 5334652 | 0 | 56 | 0 | 1 | 120 |
| 57 | 39 | 71 | 47 | 0 | 57 | 648603 | 5188820 | 0 | 57 | 0 | 112 | 5334772 | 0 | 57 | 0 | 1 | 120 |
| 58 | 3A | 71 | 47 | 0 | 58 | 648617 | 5188932 | 0 | 58 | 0 | 112 | 5334892 | 0 | 58 | 0 | 1 | 120 |
| 59 | 3B | 71 | 47 | 0 | 59 | 648631 | 5189044 | 0 | 59 | 0 | 112 | 5335012 | 0 | 59 | 0 | 1 | 120 |
| 60 | 3C | 71 | 47 | 0 | 60 | 648645 | 5189156 | 0 | 60 | 0 | 112 | 5335132 | 0 | 60 | 0 | 1 | 120 |
| 61 | 3D | 71 | 47 | 0 | 61 | 648659 | 5189268 | 0 | 61 | 0 | 112 | 5335252 | 0 | 61 | 0 | 1 | 120 |
| 62 | 3E | 71 | 47 | 0 | 62 | 648673 | 5189380 | 0 | 62 | 0 | 112 | 5335372 | 0 | 62 | 0 | 1 | 120 |
| 63 | 3F | 71 | 47 | 0 | 63 | 648687 | 5189492 | 0 | 63 | 0 | 112 | 5335492 | 0 | 63 | 0 | 1 | 120 |
| 64 | 40 | 71 | 47 | 0 | 64 | 648701 | 5189604 | 0 | 64 | 0 | 112 | 5335612 | 0 | 64 | 0 | 1 | 120 |
| 65 | 41 | 71 | 47 | 0 | 65 | 648715 | 5189716 | 0 | 65 | 0 | 112 | 5335732 | 0 | 65 | 0 | 1 | 120 |
| 66 | 42 | 71 | 47 | 0 | 66 | 648729 | 5189828 | 0 | 66 | 0 | 112 | 5335852 | 0 | 66 | 0 | 1 | 120 |
| 67 | 43 | 71 | 47 | 0 | 67 | 648743 | 5189940 | 0 | 67 | 0 | 112 | 5335972 | 0 | 67 | 0 | 1 | 120 |
| 68 | 44 | 71 | 47 | 0 | 68 | 648757 | 5190052 | 0 | 68 | 0 | 112 | 5336092 | 0 | 68 | 0 | 1 | 120 |
| 69 | 45 | 71 | 47 | 0 | 69 | 648771 | 5190164 | 0 | 69 | 0 | 112 | 5336212 | 0 | 69 | 0 | 1 | 120 |
| 70 | 46 | 71 | 47 | 0 | 70 | 648785 | 5190276 | 0 | 70 | 0 | 112 | 5336332 | 0 | 70 | 0 | 1 | 120 |
| 71 | 47 | 71 | 47 | 1 | 0 | 648797 | 5190372 | 1 | 0 | 0 | 96 | 5336436 | 1 | 0 | 0 | 1 | 104 |
| 72 | 48 | 71 | 47 | 1 | 1 | 648814 | 5190508 | 1 | 1 | 0 | 136 | 5336580 | 1 | 1 | 0 | 1 | 144 |
| 73 | 49 | 71 | 47 | 1 | 2 | 648831 | 5190644 | 1 | 2 | 0 | 136 | 5336724 | 1 | 2 | 0 | 1 | 144 |
| 74 | 4A | 71 | 47 | 1 | 3 | 648848 | 5190780 | 1 | 3 | 0 | 136 | 5336868 | 1 | 3 | 0 | 1 | 144 |
| 75 | 4B | 71 | 47 | 1 | 4 | 648865 | 5190916 | 1 | 4 | 0 | 136 | 5337012 | 1 | 4 | 0 | 1 | 144 |
| 76 | 4C | 71 | 47 | 1 | 5 | 648882 | 5191052 | 1 | 5 | 0 | 136 | 5337156 | 1 | 5 | 0 | 1 | 144 |
| 77 | 4D | 71 | 47 | 1 | 6 | 648899 | 5191188 | 1 | 6 | 0 | 136 | 5337300 | 1 | 6 | 0 | 1 | 144 |
| 78 | 4E | 71 | 47 | 1 | 7 | 648916 | 5191324 | 1 | 7 | 0 | 136 | 5337444 | 1 | 7 | 0 | 1 | 144 |
| 79 | 4F | 71 | 47 | 1 | 8 | 648933 | 5191460 | 1 | 8 | 0 | 136 | 5337588 | 1 | 8 | 0 | 1 | 144 |
| 80 | 50 | 71 | 47 | 1 | 9 | 648954 | 5191628 | 1 | 9 | 0 | 168 | 5337764 | 1 | 9 | 0 | 1 | 176 |
| 81 | 51 | 71 | 47 | 1 | 10 | 648975 | 5191796 | 1 | 10 | 0 | 168 | 5337940 | 1 | 10 | 0 | 1 | 176 |
| 82 | 52 | 71 | 47 | 1 | 11 | 648996 | 5191964 | 1 | 11 | 0 | 168 | 5338116 | 1 | 11 | 0 | 1 | 176 |
| 83 | 53 | 71 | 47 | 1 | 12 | 649017 | 5192132 | 1 | 12 | 0 | 168 | 5338292 | 1 | 12 | 0 | 1 | 176 |
| 84 | 54 | 71 | 47 | 1 | 13 | 649038 | 5192300 | 1 | 13 | 0 | 168 | 5338468 | 1 | 13 | 0 | 1 | 176 |
| 85 | 55 | 71 | 47 | 1 | 14 | 649059 | 5192468 | 1 | 14 | 0 | 168 | 5338644 | 1 | 14 | 0 | 1 | 176 |
| 86 | 56 | 71 | 47 | 1 | 15 | 649080 | 5192636 | 1 | 15 | 0 | 168 | 5338820 | 1 | 15 | 0 | 1 | 176 |
| 87 | 57 | 71 | 47 | 1 | 16 | 649101 | 5192804 | 1 | 16 | 0 | 168 | 5338996 | 1 | 16 | 0 | 1 | 176 |
| 88 | 58 | 71 | 47 | 1 | 17 | 649122 | 5192972 | 1 | 17 | 0 | 168 | 5339172 | 1 | 17 | 0 | 1 | 176 |
| 89 | 59 | 71 | 47 | 1 | 18 | 649143 | 5193140 | 1 | 18 | 0 | 168 | 5339348 | 1 | 18 | 0 | 1 | 176 |
| 90 | 5A | 71 | 47 | 1 | 19 | 649164 | 5193308 | 1 | 19 | 0 | 168 | 5339524 | 1 | 19 | 0 | 1 | 176 |
| 91 | 5B | 71 | 47 | 1 | 20 | 649185 | 5193476 | 1 | 20 | 0 | 168 | 5339700 | 1 | 20 | 0 | 1 | 176 |
| 92 | 5C | 71 | 47 | 1 | 21 | 649206 | 5193644 | 1 | 21 | 0 | 168 | 5339876 | 1 | 21 | 0 | 1 | 176 |
| 93 | 5D | 71 | 47 | 1 | 22 | 649227 | 5193812 | 1 | 22 | 0 | 168 | 5340052 | 1 | 22 | 0 | 1 | 176 |
| 94 | 5E | 71 | 47 | 1 | 23 | 649248 | 5193980 | 1 | 23 | 0 | 168 | 5340228 | 1 | 23 | 0 | 1 | 176 |
| 95 | 5F | 71 | 47 | 1 | 24 | 649269 | 5194148 | 1 | 24 | 0 | 168 | 5340404 | 1 | 24 | 0 | 1 | 176 |
| 96 | 60 | 71 | 47 | 1 | 25 | 649292 | 5194332 | 1 | 25 | 0 | 184 | 5340596 | 1 | 25 | 0 | 1 | 192 |
| 97 | 61 | 71 | 47 | 1 | 26 | 649315 | 5194516 | 1 | 26 | 0 | 184 | 5340788 | 1 | 26 | 0 | 1 | 192 |
| 98 | 62 | 71 | 47 | 1 | 27 | 649338 | 5194700 | 1 | 27 | 0 | 184 | 5340980 | 1 | 27 | 0 | 1 | 192 |
| 99 | 63 | 71 | 47 | 1 | 28 | 649361 | 5194884 | 1 | 28 | 0 | 184 | 5341172 | 1 | 28 | 0 | 1 | 192 |
| 100 | 64 | 71 | 47 | 1 | 29 | 649384 | 5195068 | 1 | 29 | 0 | 184 | 5341364 | 1 | 29 | 0 | 1 | 192 |
| 101 | 65 | 71 | 47 | 1 | 30 | 649407 | 5195252 | 1 | 30 | 0 | 184 | 5341556 | 1 | 30 | 0 | 1 | 192 |
| 102 | 66 | 71 | 47 | 1 | 31 | 649430 | 5195436 | 1 | 31 | 0 | 184 | 5341748 | 1 | 31 | 0 | 1 | 192 |
| 103 | 67 | 71 | 47 | 1 | 32 | 649453 | 5195620 | 1 | 32 | 0 | 184 | 5341940 | 1 | 32 | 0 | 1 | 192 |
| 104 | 68 | 71 | 47 | 1 | 33 | 649476 | 5195804 | 1 | 33 | 0 | 184 | 5342132 | 1 | 33 | 0 | 1 | 192 |
| 105 | 69 | 71 | 47 | 1 | 34 | 649499 | 5195988 | 1 | 34 | 0 | 184 | 5342324 | 1 | 34 | 0 | 1 | 192 |
| 106 | 6A | 71 | 47 | 1 | 35 | 649522 | 5196172 | 1 | 35 | 0 | 184 | 5342516 | 1 | 35 | 0 | 1 | 192 |
| 107 | 6B | 71 | 47 | 1 | 36 | 649545 | 5196356 | 1 | 36 | 0 | 184 | 5342708 | 1 | 36 | 0 | 1 | 192 |
| 108 | 6C | 71 | 47 | 1 | 37 | 649568 | 5196540 | 1 | 37 | 0 | 184 | 5342900 | 1 | 37 | 0 | 1 | 192 |
| 109 | 6D | 71 | 47 | 1 | 38 | 649591 | 5196724 | 1 | 38 | 0 | 184 | 5343092 | 1 | 38 | 0 | 1 | 192 |
| 110 | 6E | 71 | 47 | 1 | 39 | 649614 | 5196908 | 1 | 39 | 0 | 184 | 5343284 | 1 | 39 | 0 | 1 | 192 |
| 111 | 6F | 71 | 47 | 1 | 40 | 649637 | 5197092 | 1 | 40 | 0 | 184 | 5343476 | 1 | 40 | 0 | 1 | 192 |
| 112 | 70 | 71 | 47 | 1 | 41 | 649660 | 5197276 | 1 | 41 | 0 | 184 | 5343668 | 1 | 41 | 0 | 1 | 192 |
| 113 | 71 | 71 | 47 | 1 | 42 | 649683 | 5197460 | 1 | 42 | 0 | 184 | 5343860 | 1 | 42 | 0 | 1 | 192 |
| 114 | 72 | 71 | 47 | 1 | 43 | 649706 | 5197644 | 1 | 43 | 0 | 184 | 5344052 | 1 | 43 | 0 | 1 | 192 |
| 115 | 73 | 71 | 47 | 1 | 44 | 649729 | 5197828 | 1 | 44 | 0 | 184 | 5344244 | 1 | 44 | 0 | 1 | 192 |
| 116 | 74 | 71 | 47 | 1 | 45 | 649752 | 5198012 | 1 | 45 | 0 | 184 | 5344436 | 1 | 45 | 0 | 1 | 192 |
| 117 | 75 | 71 | 47 | 1 | 46 | 649775 | 5198196 | 1 | 46 | 0 | 184 | 5344628 | 1 | 46 | 0 | 1 | 192 |
| 118 | 76 | 71 | 47 | 1 | 47 | 649798 | 5198380 | 1 | 47 | 0 | 184 | 5344820 | 1 | 47 | 0 | 1 | 192 |
| 119 | 77 | 71 | 47 | 1 | 48 | 649821 | 5198564 | 1 | 48 | 0 | 184 | 5345012 | 1 | 48 | 0 | 1 | 192 |
| 120 | 78 | 71 | 47 | 1 | 49 | 649844 | 5198748 | 1 | 49 | 0 | 184 | 5345204 | 1 | 49 | 0 | 1 | 192 |
| 121 | 79 | 71 | 47 | 1 | 50 | 649867 | 5198932 | 1 | 50 | 0 | 184 | 5345396 | 1 | 50 | 0 | 1 | 192 |
| 122 | 7A | 71 | 47 | 1 | 51 | 649890 | 5199116 | 1 | 51 | 0 | 184 | 5345588 | 1 | 51 | 0 | 1 | 192 |
| 123 | 7B | 71 | 47 | 1 | 52 | 649913 | 5199300 | 1 | 52 | 0 | 184 | 5345780 | 1 | 52 | 0 | 1 | 192 |
| 124 | 7C | 71 | 47 | 1 | 53 | 649936 | 5199484 | 1 | 53 | 0 | 184 | 5345972 | 1 | 53 | 0 | 1 | 192 |
| 125 | 7D | 71 | 47 | 1 | 54 | 649959 | 5199668 | 1 | 54 | 0 | 184 | 5346164 | 1 | 54 | 0 | 1 | 192 |
| 126 | 7E | 71 | 47 | 1 | 55 | 649982 | 5199852 | 1 | 55 | 0 | 184 | 5346356 | 1 | 55 | 0 | 1 | 192 |
| 127 | 7F | 71 | 47 | 1 | 56 | 650005 | 5200036 | 1 | 56 | 0 | 184 | 5346548 | 1 | 56 | 0 | 1 | 192 |
| 128 | 80 | 71 | 47 | 1 | 57 | 650028 | 5200220 | 1 | 57 | 0 | 184 | 5346740 | 1 | 57 | 0 | 1 | 192 |
| 129 | 81 | 71 | 47 | 1 | 58 | 650051 | 5200404 | 1 | 58 | 0 | 184 | 5346932 | 1 | 58 | 0 | 1 | 192 |
| 130 | 82 | 71 | 47 | 1 | 59 | 650074 | 5200588 | 1 | 59 | 0 | 184 | 5347124 | 1 | 59 | 0 | 1 | 192 |
| 131 | 83 | 71 | 47 | 1 | 60 | 650097 | 5200772 | 1 | 60 | 0 | 184 | 5347316 | 1 | 60 | 0 | 1 | 192 |
| 132 | 84 | 71 | 47 | 1 | 61 | 650120 | 5200956 | 1 | 61 | 0 | 184 | 5347508 | 1 | 61 | 0 | 1 | 192 |
| 133 | 85 | 71 | 47 | 1 | 62 | 650143 | 5201140 | 1 | 62 | 0 | 184 | 5347700 | 1 | 62 | 0 | 1 | 192 |
| 134 | 86 | 71 | 47 | 1 | 63 | 650166 | 5201324 | 1 | 63 | 0 | 184 | 5347892 | 1 | 63 | 0 | 1 | 192 |
| 135 | 87 | 71 | 47 | 1 | 64 | 650189 | 5201508 | 1 | 64 | 0 | 184 | 5348084 | 1 | 64 | 0 | 1 | 192 |
| 136 | 88 | 71 | 47 | 1 | 65 | 650212 | 5201692 | 1 | 65 | 0 | 184 | 5348276 | 1 | 65 | 0 | 1 | 192 |
| 137 | 89 | 71 | 47 | 1 | 66 | 650235 | 5201876 | 1 | 66 | 0 | 184 | 5348468 | 1 | 66 | 0 | 1 | 192 |
| 138 | 8A | 71 | 47 | 1 | 67 | 650258 | 5202060 | 1 | 67 | 0 | 184 | 5348660 | 1 | 67 | 0 | 1 | 192 |
| 139 | 8B | 71 | 47 | 1 | 68 | 650281 | 5202244 | 1 | 68 | 0 | 184 | 5348852 | 1 | 68 | 0 | 1 | 192 |
| 140 | 8C | 71 | 47 | 1 | 69 | 650304 | 5202428 | 1 | 69 | 0 | 184 | 5349044 | 1 | 69 | 0 | 1 | 192 |
| 141 | 8D | 71 | 47 | 1 | 70 | 650327 | 5202612 | 1 | 70 | 0 | 184 | 5349236 | 1 | 70 | 0 | 1 | 192 |
| 142 | 8E | 71 | 47 | 2 | 0 | 650348 | 5202780 | 2 | 0 | 0 | 168 | 5349412 | 2 | 0 | 0 | 1 | 176 |
| 143 | 8F | 71 | 47 | 2 | 1 | 650374 | 5202988 | 2 | 1 | 0 | 208 | 5349628 | 2 | 1 | 0 | 1 | 216 |
| 144 | 90 | 71 | 47 | 2 | 2 | 650400 | 5203196 | 2 | 2 | 0 | 208 | 5349844 | 2 | 2 | 0 | 1 | 216 |
| 145 | 91 | 71 | 47 | 2 | 3 | 650426 | 5203404 | 2 | 3 | 0 | 208 | 5350060 | 2 | 3 | 0 | 1 | 216 |
| 146 | 92 | 71 | 47 | 2 | 4 | 650452 | 5203612 | 2 | 4 | 0 | 208 | 5350276 | 2 | 4 | 0 | 1 | 216 |
| 147 | 93 | 71 | 47 | 2 | 5 | 650478 | 5203820 | 2 | 5 | 0 | 208 | 5350492 | 2 | 5 | 0 | 1 | 216 |
| 148 | 94 | 71 | 47 | 2 | 6 | 650504 | 5204028 | 2 | 6 | 0 | 208 | 5350708 | 2 | 6 | 0 | 1 | 216 |
| 149 | 95 | 71 | 47 | 2 | 7 | 650530 | 5204236 | 2 | 7 | 0 | 208 | 5350924 | 2 | 7 | 0 | 1 | 216 |
| 150 | 96 | 71 | 47 | 2 | 8 | 650556 | 5204444 | 2 | 8 | 0 | 208 | 5351140 | 2 | 8 | 0 | 1 | 216 |
| 151 | 97 | 71 | 47 | 2 | 9 | 650582 | 5204652 | 2 | 9 | 0 | 208 | 5351356 | 2 | 9 | 0 | 1 | 216 |
| 152 | 98 | 71 | 47 | 2 | 10 | 650608 | 5204860 | 2 | 10 | 0 | 208 | 5351572 | 2 | 10 | 0 | 1 | 216 |
| 153 | 99 | 71 | 47 | 2 | 11 | 650634 | 5205068 | 2 | 11 | 0 | 208 | 5351788 | 2 | 11 | 0 | 1 | 216 |
| 154 | 9A | 71 | 47 | 2 | 12 | 650660 | 5205276 | 2 | 12 | 0 | 208 | 5352004 | 2 | 12 | 0 | 1 | 216 |

| | | | | | | | | | | | | | | | | | |
|---|---|---|---|---|---|---|---|---|---|---|---|---|---|---|---|---|---|
| 155 | 9B | 71 | 47 | 2 | 13 | 650686 | 5205484 | 2 | 13 | 0 | 208 | 5352220 | 2 | 13 | 0 | 1 | 216 |
| 156 | 9C | 71 | 47 | 2 | 14 | 650712 | 5205692 | 2 | 14 | 0 | 208 | 5352436 | 2 | 14 | 0 | 1 | 216 |
| 157 | 9D | 71 | 47 | 2 | 15 | 650738 | 5205900 | 2 | 15 | 0 | 208 | 5352652 | 2 | 15 | 0 | 1 | 216 |
| 158 | 9E | 71 | 47 | 2 | 16 | 650764 | 5206108 | 2 | 16 | 0 | 208 | 5352868 | 2 | 16 | 0 | 1 | 216 |
| 159 | 9F | 71 | 47 | 2 | 17 | 650790 | 5206316 | 2 | 17 | 0 | 208 | 5353084 | 2 | 17 | 0 | 1 | 216 |
| 160 | A0 | 71 | 47 | 2 | 18 | 650813 | 5206500 | 2 | 18 | 0 | 184 | 5353276 | 2 | 18 | 0 | 1 | 192 |
| 161 | A1 | 71 | 47 | 2 | 19 | 650836 | 5206684 | 2 | 19 | 0 | 184 | 5353468 | 2 | 19 | 0 | 1 | 192 |
| 162 | A2 | 71 | 47 | 2 | 20 | 650859 | 5206868 | 2 | 20 | 0 | 184 | 5353660 | 2 | 20 | 0 | 1 | 192 |
| 163 | A3 | 71 | 47 | 2 | 21 | 650882 | 5207052 | 2 | 21 | 0 | 184 | 5353852 | 2 | 21 | 0 | 1 | 192 |
| 164 | A4 | 71 | 47 | 2 | 22 | 650905 | 5207236 | 2 | 22 | 0 | 184 | 5354044 | 2 | 22 | 0 | 1 | 192 |
| 165 | A5 | 71 | 47 | 2 | 23 | 650928 | 5207420 | 2 | 23 | 0 | 184 | 5354236 | 2 | 23 | 0 | 1 | 192 |
| 166 | A6 | 71 | 47 | 2 | 24 | 650951 | 5207604 | 2 | 24 | 0 | 184 | 5354428 | 2 | 24 | 0 | 1 | 192 |
| 167 | A7 | 71 | 47 | 2 | 25 | 650974 | 5207788 | 2 | 25 | 0 | 184 | 5354620 | 2 | 25 | 0 | 1 | 192 |
| 168 | A8 | 71 | 47 | 2 | 26 | 650997 | 5207972 | 2 | 26 | 0 | 184 | 5354812 | 2 | 26 | 0 | 1 | 192 |
| 169 | A9 | 71 | 47 | 2 | 27 | 651020 | 5208156 | 2 | 27 | 0 | 184 | 5355004 | 2 | 27 | 0 | 1 | 192 |
| 170 | AA | 71 | 47 | 2 | 28 | 651043 | 5208340 | 2 | 28 | 0 | 184 | 5355196 | 2 | 28 | 0 | 1 | 192 |
| 171 | AB | 71 | 47 | 2 | 29 | 651066 | 5208524 | 2 | 29 | 0 | 184 | 5355388 | 2 | 29 | 0 | 1 | 192 |
| 172 | AC | 71 | 47 | 2 | 30 | 651089 | 5208708 | 2 | 30 | 0 | 184 | 5355580 | 2 | 30 | 0 | 1 | 192 |
| 173 | AD | 71 | 47 | 2 | 31 | 651112 | 5208892 | 2 | 31 | 0 | 184 | 5355772 | 2 | 31 | 0 | 1 | 192 |
| 174 | AE | 71 | 47 | 2 | 32 | 651135 | 5209076 | 2 | 32 | 0 | 184 | 5355964 | 2 | 32 | 0 | 1 | 192 |
| 175 | AF | 71 | 47 | 2 | 33 | 651158 | 5209260 | 2 | 33 | 0 | 184 | 5356156 | 2 | 33 | 0 | 1 | 192 |
| 176 | B0 | 71 | 47 | 2 | 34 | 651183 | 5209460 | 2 | 34 | 0 | 200 | 5356364 | 2 | 34 | 0 | 1 | 208 |
| 177 | B1 | 71 | 47 | 2 | 35 | 651208 | 5209660 | 2 | 35 | 0 | 200 | 5356572 | 2 | 35 | 0 | 1 | 208 |
| 178 | B2 | 71 | 47 | 2 | 36 | 651233 | 5209860 | 2 | 36 | 0 | 200 | 5356780 | 2 | 36 | 0 | 1 | 208 |
| 179 | B3 | 71 | 47 | 2 | 37 | 651258 | 5210060 | 2 | 37 | 0 | 200 | 5356988 | 2 | 37 | 0 | 1 | 208 |
| 180 | B4 | 71 | 47 | 2 | 38 | 651283 | 5210260 | 2 | 38 | 0 | 200 | 5357196 | 2 | 38 | 0 | 1 | 208 |
| 181 | B5 | 71 | 47 | 2 | 39 | 651308 | 5210460 | 2 | 39 | 0 | 200 | 5357404 | 2 | 39 | 0 | 1 | 208 |
| 182 | B6 | 71 | 47 | 2 | 40 | 651333 | 5210660 | 2 | 40 | 0 | 200 | 5357612 | 2 | 40 | 0 | 1 | 208 |
| 183 | B7 | 71 | 47 | 2 | 41 | 651358 | 5210860 | 2 | 41 | 0 | 200 | 5357820 | 2 | 41 | 0 | 1 | 208 |
| 184 | B8 | 71 | 47 | 2 | 42 | 651383 | 5211060 | 2 | 42 | 0 | 200 | 5358028 | 2 | 42 | 0 | 1 | 208 |
| 185 | B9 | 71 | 47 | 2 | 43 | 651408 | 5211260 | 2 | 43 | 0 | 200 | 5358236 | 2 | 43 | 0 | 1 | 208 |
| 186 | BA | 71 | 47 | 2 | 44 | 651433 | 5211460 | 2 | 44 | 0 | 200 | 5358444 | 2 | 44 | 0 | 1 | 208 |
| 187 | BB | 71 | 47 | 2 | 45 | 651458 | 5211660 | 2 | 45 | 0 | 200 | 5358652 | 2 | 45 | 0 | 1 | 208 |
| 188 | BC | 71 | 47 | 2 | 46 | 651483 | 5211860 | 2 | 46 | 0 | 200 | 5358860 | 2 | 46 | 0 | 1 | 208 |
| 189 | BD | 71 | 47 | 2 | 47 | 651508 | 5212060 | 2 | 47 | 0 | 200 | 5359068 | 2 | 47 | 0 | 1 | 208 |
| 190 | BE | 71 | 47 | 2 | 48 | 651533 | 5212260 | 2 | 48 | 0 | 200 | 5359276 | 2 | 48 | 0 | 1 | 208 |
| 191 | BF | 71 | 47 | 2 | 49 | 651558 | 5212460 | 2 | 49 | 0 | 200 | 5359484 | 2 | 49 | 0 | 1 | 208 |
| 192 | C0 | 71 | 47 | 2 | 50 | 651583 | 5212660 | 2 | 50 | 0 | 200 | 5359692 | 2 | 50 | 0 | 1 | 208 |
| 193 | C1 | 71 | 47 | 2 | 51 | 651608 | 5212860 | 2 | 51 | 0 | 200 | 5359900 | 2 | 51 | 0 | 1 | 208 |
| 194 | C2 | 71 | 47 | 2 | 52 | 651633 | 5213060 | 2 | 52 | 0 | 200 | 5360108 | 2 | 52 | 0 | 1 | 208 |
| 195 | C3 | 71 | 47 | 2 | 53 | 651658 | 5213260 | 2 | 53 | 0 | 200 | 5360316 | 2 | 53 | 0 | 1 | 208 |
| 196 | C4 | 71 | 47 | 2 | 54 | 651683 | 5213460 | 2 | 54 | 0 | 200 | 5360524 | 2 | 54 | 0 | 1 | 208 |
| 197 | C5 | 71 | 47 | 2 | 55 | 651708 | 5213660 | 2 | 55 | 0 | 200 | 5360732 | 2 | 55 | 0 | 1 | 208 |
| 198 | C6 | 71 | 47 | 2 | 56 | 651733 | 5213860 | 2 | 56 | 0 | 200 | 5360940 | 2 | 56 | 0 | 1 | 208 |
| 199 | C7 | 71 | 47 | 2 | 57 | 651758 | 5214060 | 2 | 57 | 0 | 200 | 5361148 | 2 | 57 | 0 | 1 | 208 |
| 200 | C8 | 71 | 47 | 2 | 58 | 651783 | 5214260 | 2 | 58 | 0 | 200 | 5361356 | 2 | 58 | 0 | 1 | 208 |
| 201 | C9 | 71 | 47 | 2 | 59 | 651808 | 5214460 | 2 | 59 | 0 | 200 | 5361564 | 2 | 59 | 0 | 1 | 208 |
| 202 | CA | 71 | 47 | 2 | 60 | 651833 | 5214660 | 2 | 60 | 0 | 200 | 5361772 | 2 | 60 | 0 | 1 | 208 |
| 203 | CB | 71 | 47 | 2 | 61 | 651858 | 5214860 | 2 | 61 | 0 | 200 | 5361980 | 2 | 61 | 0 | 1 | 208 |
| 204 | CC | 71 | 47 | 2 | 62 | 651883 | 5215060 | 2 | 62 | 0 | 200 | 5362188 | 2 | 62 | 0 | 1 | 208 |
| 205 | CD | 71 | 47 | 2 | 63 | 651908 | 5215260 | 2 | 63 | 0 | 200 | 5362396 | 2 | 63 | 0 | 1 | 208 |
| 206 | CE | 71 | 47 | 2 | 64 | 651933 | 5215460 | 2 | 64 | 0 | 200 | 5362604 | 2 | 64 | 0 | 1 | 208 |
| 207 | CF | 71 | 47 | 2 | 65 | 651958 | 5215660 | 2 | 65 | 0 | 200 | 5362812 | 2 | 65 | 0 | 1 | 208 |
| 208 | D0 | 71 | 47 | 2 | 66 | 651983 | 5215860 | 2 | 66 | 0 | 200 | 5363020 | 2 | 66 | 0 | 1 | 208 |
| 209 | D1 | 71 | 47 | 2 | 67 | 652008 | 5216060 | 2 | 67 | 0 | 200 | 5363228 | 2 | 67 | 0 | 1 | 208 |
| 210 | D2 | 71 | 47 | 2 | 68 | 652033 | 5216260 | 2 | 68 | 0 | 200 | 5363436 | 2 | 68 | 0 | 1 | 208 |
| 211 | D3 | 71 | 47 | 2 | 69 | 652058 | 5216460 | 2 | 69 | 0 | 200 | 5363644 | 2 | 69 | 0 | 1 | 208 |
| 212 | D4 | 71 | 47 | 2 | 70 | 652083 | 5216660 | 2 | 70 | 0 | 200 | 5363852 | 2 | 70 | 0 | 1 | 208 |
| 213 | D5 | 71 | 47 | 3 | 0 | 652106 | 5216844 | 3 | 0 | 0 | 184 | 5364044 | 3 | 0 | 0 | 1 | 192 |
| 214 | D6 | 71 | 47 | 3 | 1 | 652134 | 5217068 | 3 | 1 | 0 | 224 | 5364276 | 3 | 1 | 0 | 1 | 232 |
| 215 | D7 | 71 | 47 | 3 | 2 | 652162 | 5217292 | 3 | 2 | 0 | 224 | 5364508 | 3 | 2 | 0 | 1 | 232 |
| 216 | D8 | 71 | 47 | 3 | 3 | 652190 | 5217516 | 3 | 3 | 0 | 224 | 5364740 | 3 | 3 | 0 | 1 | 232 |
| 217 | D9 | 71 | 47 | 3 | 4 | 652218 | 5217740 | 3 | 4 | 0 | 224 | 5364972 | 3 | 4 | 0 | 1 | 232 |
| 218 | DA | 71 | 47 | 3 | 5 | 652246 | 5217964 | 3 | 5 | 0 | 224 | 5365204 | 3 | 5 | 0 | 1 | 232 |
| 219 | DB | 71 | 47 | 3 | 6 | 652274 | 5218188 | 3 | 6 | 0 | 224 | 5365436 | 3 | 6 | 0 | 1 | 232 |
| 220 | DC | 71 | 47 | 3 | 7 | 652302 | 5218412 | 3 | 7 | 0 | 224 | 5365668 | 3 | 7 | 0 | 1 | 232 |
| 221 | DD | 71 | 47 | 3 | 8 | 652330 | 5218636 | 3 | 8 | 0 | 224 | 5365900 | 3 | 8 | 0 | 1 | 232 |
| 222 | DE | 71 | 47 | 3 | 9 | 652358 | 5218860 | 3 | 9 | 0 | 224 | 5366132 | 3 | 9 | 0 | 1 | 232 |
| 223 | DF | 71 | 47 | 3 | 10 | 652386 | 5219084 | 3 | 10 | 0 | 224 | 5366364 | 3 | 10 | 0 | 1 | 232 |
| 224 | E0 | 71 | 47 | 3 | 11 | 652414 | 5219308 | 3 | 11 | 0 | 224 | 5366596 | 3 | 11 | 0 | 1 | 232 |
| 225 | E1 | 71 | 47 | 3 | 12 | 652442 | 5219532 | 3 | 12 | 0 | 224 | 5366828 | 3 | 12 | 0 | 1 | 232 |
| 226 | E2 | 71 | 47 | 3 | 13 | 652470 | 5219756 | 3 | 13 | 0 | 224 | 5367060 | 3 | 13 | 0 | 1 | 232 |
| 227 | E3 | 71 | 47 | 3 | 14 | 652498 | 5219980 | 3 | 14 | 0 | 224 | 5367292 | 3 | 14 | 0 | 1 | 232 |
| 228 | E4 | 71 | 47 | 3 | 15 | 652526 | 5220204 | 3 | 15 | 0 | 224 | 5367524 | 3 | 15 | 0 | 1 | 232 |
| 229 | E5 | 71 | 47 | 3 | 16 | 652554 | 5220428 | 3 | 16 | 0 | 224 | 5367756 | 3 | 16 | 0 | 1 | 232 |
| 230 | E6 | 71 | 47 | 3 | 17 | 652582 | 5220652 | 3 | 17 | 0 | 224 | 5367988 | 3 | 17 | 0 | 1 | 232 |
| 231 | E7 | 71 | 47 | 3 | 18 | 652610 | 5220876 | 3 | 18 | 0 | 224 | 5368220 | 3 | 18 | 0 | 1 | 232 |
| 232 | E8 | 71 | 47 | 3 | 19 | 652638 | 5221100 | 3 | 19 | 0 | 224 | 5368452 | 3 | 19 | 0 | 1 | 232 |
| 233 | E9 | 71 | 47 | 3 | 20 | 652666 | 5221324 | 3 | 20 | 0 | 224 | 5368684 | 3 | 20 | 0 | 1 | 232 |
| 234 | EA | 71 | 47 | 3 | 21 | 652694 | 5221548 | 3 | 21 | 0 | 224 | 5368916 | 3 | 21 | 0 | 1 | 232 |
| 235 | EB | 71 | 47 | 3 | 22 | 652722 | 5221772 | 3 | 22 | 0 | 224 | 5369148 | 3 | 22 | 0 | 1 | 232 |
| 236 | EC | 71 | 47 | 3 | 23 | 652750 | 5221996 | 3 | 23 | 0 | 224 | 5369380 | 3 | 23 | 0 | 1 | 232 |
| 237 | ED | 71 | 47 | 3 | 24 | 652778 | 5222220 | 3 | 24 | 0 | 224 | 5369612 | 3 | 24 | 0 | 1 | 232 |
| 238 | EE | 71 | 47 | 3 | 25 | 652806 | 5222444 | 3 | 25 | 0 | 224 | 5369844 | 3 | 25 | 0 | 1 | 232 |
| 239 | EF | 71 | 47 | 3 | 26 | 652834 | 5222668 | 3 | 26 | 0 | 224 | 5370076 | 3 | 26 | 0 | 1 | 232 |
| 240 | F0 | 71 | 47 | 3 | 27 | 652859 | 5222868 | 3 | 27 | 0 | 200 | 5370284 | 3 | 27 | 0 | 1 | 208 |
| 241 | F1 | 71 | 47 | 3 | 28 | 652884 | 5223068 | 3 | 28 | 0 | 200 | 5370492 | 3 | 28 | 0 | 1 | 208 |
| 242 | F2 | 71 | 47 | 3 | 29 | 652909 | 5223268 | 3 | 29 | 0 | 200 | 5370700 | 3 | 29 | 0 | 1 | 208 |
| 243 | F3 | 71 | 47 | 3 | 30 | 652934 | 5223468 | 3 | 30 | 0 | 200 | 5370908 | 3 | 30 | 0 | 1 | 208 |
| 244 | F4 | 71 | 47 | 3 | 31 | 652959 | 5223668 | 3 | 31 | 0 | 200 | 5371116 | 3 | 31 | 0 | 1 | 208 |
| 245 | F5 | 71 | 47 | 3 | 32 | 652984 | 5223868 | 3 | 32 | 0 | 200 | 5371324 | 3 | 32 | 0 | 1 | 208 |
| 246 | F6 | 71 | 47 | 3 | 33 | 653009 | 5224068 | 3 | 33 | 0 | 200 | 5371532 | 3 | 33 | 0 | 1 | 208 |
| 247 | F7 | 71 | 47 | 3 | 34 | 653034 | 5224268 | 3 | 34 | 0 | 200 | 5371740 | 3 | 34 | 0 | 1 | 208 |
| 248 | F8 | 71 | 47 | 3 | 35 | 653059 | 5224468 | 3 | 35 | 0 | 200 | 5371948 | 3 | 35 | 0 | 1 | 208 |
| 249 | F9 | 71 | 47 | 3 | 36 | 653084 | 5224668 | 3 | 36 | 0 | 200 | 5372156 | 3 | 36 | 0 | 1 | 208 |
| 250 | FA | 71 | 47 | 3 | 37 | 653109 | 5224868 | 3 | 37 | 0 | 200 | 5372364 | 3 | 37 | 0 | 1 | 208 |
| 251 | FB | 71 | 47 | 3 | 38 | 653134 | 5225068 | 3 | 38 | 0 | 200 | 5372572 | 3 | 38 | 0 | 1 | 208 |
| 252 | FC | 71 | 47 | 3 | 39 | 653159 | 5225268 | 3 | 39 | 0 | 200 | 5372780 | 3 | 39 | 0 | 1 | 208 |
| 253 | FD | 71 | 47 | 3 | 40 | 653184 | 5225468 | 3 | 40 | 0 | 200 | 5372988 | 3 | 40 | 0 | 1 | 208 |
| 254 | FE | 71 | 47 | 3 | 41 | 653209 | 5225668 | 3 | 41 | 0 | 200 | 5373196 | 3 | 41 | 0 | 1 | 208 |
| 255 | FF | 71 | 47 | 3 | 42 | 653234 | 5225868 | 3 | 42 | 0 | 200 | 5373404 | 3 | 42 | 0 | 1 | 208 |
| 0 | 00 | 72 | 48 | 0 | 0 | 653246 | 5225964 | 0 | 0 | 0 | 96 | 5373508 | 0 | 0 | 0 | 1 | 104 |
| 1 | 01 | 72 | 48 | 0 | 1 | 653260 | 5226076 | 0 | 1 | 0 | 112 | 5373628 | 0 | 1 | 0 | 1 | 120 |

| | | | | | | | | | | | | | | | | | |
|---|---|---|---|---|---|---|---|---|---|---|---|---|---|---|---|---|---|
| 2 | 02 | 72 | 48 | 0 | 2 | 653274 | 5226188 | 0 | 2 | 0 | 112 | 5373748 | 0 | 2 | 0 | 1 | 120 |
| 3 | 03 | 72 | 48 | 0 | 3 | 653288 | 5226300 | 0 | 3 | 0 | 112 | 5373868 | 0 | 3 | 0 | 1 | 120 |
| 4 | 04 | 72 | 48 | 0 | 4 | 653302 | 5226412 | 0 | 4 | 0 | 112 | 5373988 | 0 | 4 | 0 | 1 | 120 |
| 5 | 05 | 72 | 48 | 0 | 5 | 653316 | 5226524 | 0 | 5 | 0 | 112 | 5374108 | 0 | 5 | 0 | 1 | 120 |
| 6 | 06 | 72 | 48 | 0 | 6 | 653330 | 5226636 | 0 | 6 | 0 | 112 | 5374228 | 0 | 6 | 0 | 1 | 120 |
| 7 | 07 | 72 | 48 | 0 | 7 | 653344 | 5226748 | 0 | 7 | 0 | 112 | 5374348 | 0 | 7 | 0 | 1 | 120 |
| 8 | 08 | 72 | 48 | 0 | 8 | 653358 | 5226860 | 0 | 8 | 0 | 112 | 5374468 | 0 | 8 | 0 | 1 | 120 |
| 9 | 09 | 72 | 48 | 0 | 9 | 653372 | 5226972 | 0 | 9 | 0 | 112 | 5374588 | 0 | 9 | 0 | 1 | 120 |
| 10 | 0A | 72 | 48 | 0 | 10 | 653386 | 5227084 | 0 | 10 | 0 | 112 | 5374708 | 0 | 10 | 0 | 1 | 120 |
| 11 | 0B | 72 | 48 | 0 | 11 | 653400 | 5227196 | 0 | 11 | 0 | 112 | 5374828 | 0 | 11 | 0 | 1 | 120 |
| 12 | 0C | 72 | 48 | 0 | 12 | 653414 | 5227308 | 0 | 12 | 0 | 112 | 5374948 | 0 | 12 | 0 | 1 | 120 |
| 13 | 0D | 72 | 48 | 0 | 13 | 653428 | 5227420 | 0 | 13 | 0 | 112 | 5375068 | 0 | 13 | 0 | 1 | 120 |
| 14 | 0E | 72 | 48 | 0 | 14 | 653442 | 5227532 | 0 | 14 | 0 | 112 | 5375188 | 0 | 14 | 0 | 1 | 120 |
| 15 | 0F | 72 | 48 | 0 | 15 | 653456 | 5227644 | 0 | 15 | 0 | 112 | 5375308 | 0 | 15 | 0 | 1 | 120 |
| 16 | 10 | 72 | 48 | 0 | 16 | 653470 | 5227756 | 0 | 16 | 0 | 112 | 5375428 | 0 | 16 | 0 | 1 | 120 |
| 17 | 11 | 72 | 48 | 0 | 17 | 653484 | 5227868 | 0 | 17 | 0 | 112 | 5375548 | 0 | 17 | 0 | 1 | 120 |
| 18 | 12 | 72 | 48 | 0 | 18 | 653498 | 5227980 | 0 | 18 | 0 | 112 | 5375668 | 0 | 18 | 0 | 1 | 120 |
| 19 | 13 | 72 | 48 | 0 | 19 | 653512 | 5228092 | 0 | 19 | 0 | 112 | 5375788 | 0 | 19 | 0 | 1 | 120 |
| 20 | 14 | 72 | 48 | 0 | 20 | 653526 | 5228204 | 0 | 20 | 0 | 112 | 5375908 | 0 | 20 | 0 | 1 | 120 |
| 21 | 15 | 72 | 48 | 0 | 21 | 653540 | 5228316 | 0 | 21 | 0 | 112 | 5376028 | 0 | 21 | 0 | 1 | 120 |
| 22 | 16 | 72 | 48 | 0 | 22 | 653554 | 5228428 | 0 | 22 | 0 | 112 | 5376148 | 0 | 22 | 0 | 1 | 120 |
| 23 | 17 | 72 | 48 | 0 | 23 | 653568 | 5228540 | 0 | 23 | 0 | 112 | 5376268 | 0 | 23 | 0 | 1 | 120 |
| 24 | 18 | 72 | 48 | 0 | 24 | 653582 | 5228652 | 0 | 24 | 0 | 112 | 5376388 | 0 | 24 | 0 | 1 | 120 |
| 25 | 19 | 72 | 48 | 0 | 25 | 653596 | 5228764 | 0 | 25 | 0 | 112 | 5376508 | 0 | 25 | 0 | 1 | 120 |
| 26 | 1A | 72 | 48 | 0 | 26 | 653610 | 5228876 | 0 | 26 | 0 | 112 | 5376628 | 0 | 26 | 0 | 1 | 120 |
| 27 | 1B | 72 | 48 | 0 | 27 | 653624 | 5228988 | 0 | 27 | 0 | 112 | 5376748 | 0 | 27 | 0 | 1 | 120 |
| 28 | 1C | 72 | 48 | 0 | 28 | 653638 | 5229100 | 0 | 28 | 0 | 112 | 5376868 | 0 | 28 | 0 | 1 | 120 |
| 29 | 1D | 72 | 48 | 0 | 29 | 653652 | 5229212 | 0 | 29 | 0 | 112 | 5376988 | 0 | 29 | 0 | 1 | 120 |
| 30 | 1E | 72 | 48 | 0 | 30 | 653666 | 5229324 | 0 | 30 | 0 | 112 | 5377108 | 0 | 30 | 0 | 1 | 120 |
| 31 | 1F | 72 | 48 | 0 | 31 | 653680 | 5229436 | 0 | 31 | 0 | 112 | 5377228 | 0 | 31 | 0 | 1 | 120 |
| 32 | 20 | 72 | 48 | 0 | 32 | 653694 | 5229548 | 0 | 32 | 0 | 112 | 5377348 | 0 | 32 | 0 | 1 | 120 |
| 33 | 21 | 72 | 48 | 0 | 33 | 653708 | 5229660 | 0 | 33 | 0 | 112 | 5377468 | 0 | 33 | 0 | 1 | 120 |
| 34 | 22 | 72 | 48 | 0 | 34 | 653722 | 5229772 | 0 | 34 | 0 | 112 | 5377588 | 0 | 34 | 0 | 1 | 120 |
| 35 | 23 | 72 | 48 | 0 | 35 | 653736 | 5229884 | 0 | 35 | 0 | 112 | 5377708 | 0 | 35 | 0 | 1 | 120 |
| 36 | 24 | 72 | 48 | 0 | 36 | 653750 | 5229996 | 0 | 36 | 0 | 112 | 5377828 | 0 | 36 | 0 | 1 | 120 |
| 37 | 25 | 72 | 48 | 0 | 37 | 653764 | 5230108 | 0 | 37 | 0 | 112 | 5377948 | 0 | 37 | 0 | 1 | 120 |
| 38 | 26 | 72 | 48 | 0 | 38 | 653778 | 5230220 | 0 | 38 | 0 | 112 | 5378068 | 0 | 38 | 0 | 1 | 120 |
| 39 | 27 | 72 | 48 | 0 | 39 | 653792 | 5230332 | 0 | 39 | 0 | 112 | 5378188 | 0 | 39 | 0 | 1 | 120 |
| 40 | 28 | 72 | 48 | 0 | 40 | 653806 | 5230444 | 0 | 40 | 0 | 112 | 5378308 | 0 | 40 | 0 | 1 | 120 |
| 41 | 29 | 72 | 48 | 0 | 41 | 653820 | 5230556 | 0 | 41 | 0 | 112 | 5378428 | 0 | 41 | 0 | 1 | 120 |
| 42 | 2A | 72 | 48 | 0 | 42 | 653834 | 5230668 | 0 | 42 | 0 | 112 | 5378548 | 0 | 42 | 0 | 1 | 120 |
| 43 | 2B | 72 | 48 | 0 | 43 | 653848 | 5230780 | 0 | 43 | 0 | 112 | 5378668 | 0 | 43 | 0 | 1 | 120 |
| 44 | 2C | 72 | 48 | 0 | 44 | 653862 | 5230892 | 0 | 44 | 0 | 112 | 5378788 | 0 | 44 | 0 | 1 | 120 |
| 45 | 2D | 72 | 48 | 0 | 45 | 653876 | 5231004 | 0 | 45 | 0 | 112 | 5378908 | 0 | 45 | 0 | 1 | 120 |
| 46 | 2E | 72 | 48 | 0 | 46 | 653890 | 5231116 | 0 | 46 | 0 | 112 | 5379028 | 0 | 46 | 0 | 1 | 120 |
| 47 | 2F | 72 | 48 | 0 | 47 | 653904 | 5231228 | 0 | 47 | 0 | 112 | 5379148 | 0 | 47 | 0 | 1 | 120 |
| 48 | 30 | 72 | 48 | 0 | 48 | 653918 | 5231340 | 0 | 48 | 0 | 112 | 5379268 | 0 | 48 | 0 | 1 | 120 |
| 49 | 31 | 72 | 48 | 0 | 49 | 653932 | 5231452 | 0 | 49 | 0 | 112 | 5379388 | 0 | 49 | 0 | 1 | 120 |
| 50 | 32 | 72 | 48 | 0 | 50 | 653946 | 5231564 | 0 | 50 | 0 | 112 | 5379508 | 0 | 50 | 0 | 1 | 120 |
| 51 | 33 | 72 | 48 | 0 | 51 | 653960 | 5231676 | 0 | 51 | 0 | 112 | 5379628 | 0 | 51 | 0 | 1 | 120 |
| 52 | 34 | 72 | 48 | 0 | 52 | 653974 | 5231788 | 0 | 52 | 0 | 112 | 5379748 | 0 | 52 | 0 | 1 | 120 |
| 53 | 35 | 72 | 48 | 0 | 53 | 653988 | 5231900 | 0 | 53 | 0 | 112 | 5379868 | 0 | 53 | 0 | 1 | 120 |
| 54 | 36 | 72 | 48 | 0 | 54 | 654002 | 5232012 | 0 | 54 | 0 | 112 | 5379988 | 0 | 54 | 0 | 1 | 120 |
| 55 | 37 | 72 | 48 | 0 | 55 | 654016 | 5232124 | 0 | 55 | 0 | 112 | 5380108 | 0 | 55 | 0 | 1 | 120 |
| 56 | 38 | 72 | 48 | 0 | 56 | 654030 | 5232236 | 0 | 56 | 0 | 112 | 5380228 | 0 | 56 | 0 | 1 | 120 |
| 57 | 39 | 72 | 48 | 0 | 57 | 654044 | 5232348 | 0 | 57 | 0 | 112 | 5380348 | 0 | 57 | 0 | 1 | 120 |
| 58 | 3A | 72 | 48 | 0 | 58 | 654058 | 5232460 | 0 | 58 | 0 | 112 | 5380468 | 0 | 58 | 0 | 1 | 120 |
| 59 | 3B | 72 | 48 | 0 | 59 | 654072 | 5232572 | 0 | 59 | 0 | 112 | 5380588 | 0 | 59 | 0 | 1 | 120 |
| 60 | 3C | 72 | 48 | 0 | 60 | 654086 | 5232684 | 0 | 60 | 0 | 112 | 5380708 | 0 | 60 | 0 | 1 | 120 |
| 61 | 3D | 72 | 48 | 0 | 61 | 654100 | 5232796 | 0 | 61 | 0 | 112 | 5380828 | 0 | 61 | 0 | 1 | 120 |
| 62 | 3E | 72 | 48 | 0 | 62 | 654114 | 5232908 | 0 | 62 | 0 | 112 | 5380948 | 0 | 62 | 0 | 1 | 120 |
| 63 | 3F | 72 | 48 | 0 | 63 | 654128 | 5233020 | 0 | 63 | 0 | 112 | 5381068 | 0 | 63 | 0 | 1 | 120 |
| 64 | 40 | 72 | 48 | 0 | 64 | 654142 | 5233132 | 0 | 64 | 0 | 112 | 5381188 | 0 | 64 | 0 | 1 | 120 |
| 65 | 41 | 72 | 48 | 0 | 65 | 654156 | 5233244 | 0 | 65 | 0 | 112 | 5381308 | 0 | 65 | 0 | 1 | 120 |
| 66 | 42 | 72 | 48 | 0 | 66 | 654170 | 5233356 | 0 | 66 | 0 | 112 | 5381428 | 0 | 66 | 0 | 1 | 120 |
| 67 | 43 | 72 | 48 | 0 | 67 | 654184 | 5233468 | 0 | 67 | 0 | 112 | 5381548 | 0 | 67 | 0 | 1 | 120 |
| 68 | 44 | 72 | 48 | 0 | 68 | 654198 | 5233580 | 0 | 68 | 0 | 112 | 5381668 | 0 | 68 | 0 | 1 | 120 |
| 69 | 45 | 72 | 48 | 0 | 69 | 654212 | 5233692 | 0 | 69 | 0 | 112 | 5381788 | 0 | 69 | 0 | 1 | 120 |
| 70 | 46 | 72 | 48 | 0 | 70 | 654226 | 5233804 | 0 | 70 | 0 | 112 | 5381908 | 0 | 70 | 0 | 1 | 120 |
| 71 | 47 | 72 | 48 | 0 | 71 | 654240 | 5233916 | 0 | 71 | 0 | 112 | 5382028 | 0 | 71 | 0 | 1 | 120 |
| 72 | 48 | 72 | 48 | 1 | 0 | 654252 | 5234012 | 1 | 0 | 0 | 96 | 5382132 | 1 | 0 | 0 | 1 | 104 |
| 73 | 49 | 72 | 48 | 1 | 1 | 654269 | 5234148 | 1 | 1 | 0 | 136 | 5382276 | 1 | 1 | 0 | 1 | 144 |
| 74 | 4A | 72 | 48 | 1 | 2 | 654286 | 5234284 | 1 | 2 | 0 | 136 | 5382420 | 1 | 2 | 0 | 1 | 144 |
| 75 | 4B | 72 | 48 | 1 | 3 | 654303 | 5234420 | 1 | 3 | 0 | 136 | 5382564 | 1 | 3 | 0 | 1 | 144 |
| 76 | 4C | 72 | 48 | 1 | 4 | 654320 | 5234556 | 1 | 4 | 0 | 136 | 5382708 | 1 | 4 | 0 | 1 | 144 |
| 77 | 4D | 72 | 48 | 1 | 5 | 654337 | 5234692 | 1 | 5 | 0 | 136 | 5382852 | 1 | 5 | 0 | 1 | 144 |
| 78 | 4E | 72 | 48 | 1 | 6 | 654354 | 5234828 | 1 | 6 | 0 | 136 | 5382996 | 1 | 6 | 0 | 1 | 144 |
| 79 | 4F | 72 | 48 | 1 | 7 | 654371 | 5234964 | 1 | 7 | 0 | 136 | 5383140 | 1 | 7 | 0 | 1 | 144 |
| 80 | 50 | 72 | 48 | 1 | 8 | 654392 | 5235132 | 1 | 8 | 0 | 168 | 5383316 | 1 | 8 | 0 | 1 | 176 |
| 81 | 51 | 72 | 48 | 1 | 9 | 654413 | 5235300 | 1 | 9 | 0 | 168 | 5383492 | 1 | 9 | 0 | 1 | 176 |
| 82 | 52 | 72 | 48 | 1 | 10 | 654434 | 5235468 | 1 | 10 | 0 | 168 | 5383668 | 1 | 10 | 0 | 1 | 176 |
| 83 | 53 | 72 | 48 | 1 | 11 | 654455 | 5235636 | 1 | 11 | 0 | 168 | 5383844 | 1 | 11 | 0 | 1 | 176 |
| 84 | 54 | 72 | 48 | 1 | 12 | 654476 | 5235804 | 1 | 12 | 0 | 168 | 5384020 | 1 | 12 | 0 | 1 | 176 |
| 85 | 55 | 72 | 48 | 1 | 13 | 654497 | 5235972 | 1 | 13 | 0 | 168 | 5384196 | 1 | 13 | 0 | 1 | 176 |
| 86 | 56 | 72 | 48 | 1 | 14 | 654518 | 5236140 | 1 | 14 | 0 | 168 | 5384372 | 1 | 14 | 0 | 1 | 176 |
| 87 | 57 | 72 | 48 | 1 | 15 | 654539 | 5236308 | 1 | 15 | 0 | 168 | 5384548 | 1 | 15 | 0 | 1 | 176 |
| 88 | 58 | 72 | 48 | 1 | 16 | 654560 | 5236476 | 1 | 16 | 0 | 168 | 5384724 | 1 | 16 | 0 | 1 | 176 |
| 89 | 59 | 72 | 48 | 1 | 17 | 654581 | 5236644 | 1 | 17 | 0 | 168 | 5384900 | 1 | 17 | 0 | 1 | 176 |
| 90 | 5A | 72 | 48 | 1 | 18 | 654602 | 5236812 | 1 | 18 | 0 | 168 | 5385076 | 1 | 18 | 0 | 1 | 176 |
| 91 | 5B | 72 | 48 | 1 | 19 | 654623 | 5236980 | 1 | 19 | 0 | 168 | 5385252 | 1 | 19 | 0 | 1 | 176 |
| 92 | 5C | 72 | 48 | 1 | 20 | 654644 | 5237148 | 1 | 20 | 0 | 168 | 5385428 | 1 | 20 | 0 | 1 | 176 |
| 93 | 5D | 72 | 48 | 1 | 21 | 654665 | 5237316 | 1 | 21 | 0 | 168 | 5385604 | 1 | 21 | 0 | 1 | 176 |
| 94 | 5E | 72 | 48 | 1 | 22 | 654686 | 5237484 | 1 | 22 | 0 | 168 | 5385780 | 1 | 22 | 0 | 1 | 176 |
| 95 | 5F | 72 | 48 | 1 | 23 | 654707 | 5237652 | 1 | 23 | 0 | 168 | 5385956 | 1 | 23 | 0 | 1 | 176 |
| 96 | 60 | 72 | 48 | 1 | 24 | 654730 | 5237836 | 1 | 24 | 0 | 184 | 5386148 | 1 | 24 | 0 | 1 | 192 |
| 97 | 61 | 72 | 48 | 1 | 25 | 654753 | 5238020 | 1 | 25 | 0 | 184 | 5386340 | 1 | 25 | 0 | 1 | 192 |
| 98 | 62 | 72 | 48 | 1 | 26 | 654776 | 5238204 | 1 | 26 | 0 | 184 | 5386532 | 1 | 26 | 0 | 1 | 192 |
| 99 | 63 | 72 | 48 | 1 | 27 | 654799 | 5238388 | 1 | 27 | 0 | 184 | 5386724 | 1 | 27 | 0 | 1 | 192 |
| 100 | 64 | 72 | 48 | 1 | 28 | 654822 | 5238572 | 1 | 28 | 0 | 184 | 5386916 | 1 | 28 | 0 | 1 | 192 |
| 101 | 65 | 72 | 48 | 1 | 29 | 654845 | 5238756 | 1 | 29 | 0 | 184 | 5387108 | 1 | 29 | 0 | 1 | 192 |
| 102 | 66 | 72 | 48 | 1 | 30 | 654868 | 5238940 | 1 | 30 | 0 | 184 | 5387300 | 1 | 30 | 0 | 1 | 192 |
| 103 | 67 | 72 | 48 | 1 | 31 | 654891 | 5239124 | 1 | 31 | 0 | 184 | 5387492 | 1 | 31 | 0 | 1 | 192 |
| 104 | 68 | 72 | 48 | 1 | 32 | 654914 | 5239308 | 1 | 32 | 0 | 184 | 5387684 | 1 | 32 | 0 | 1 | 192 |

| | | | | | | | | | | | | | | | | | |
|---|---|---|---|---|---|---|---|---|---|---|---|---|---|---|---|---|---|
| 105 | 69 | 72 | 48 | 1 | 33 | 654937 | 5239492 | 1 | 33 | 0 | 184 | 5387876 | 1 | 33 | 0 | 1 | 192 |
| 106 | 6A | 72 | 48 | 1 | 34 | 654960 | 5239676 | 1 | 34 | 0 | 184 | 5388068 | 1 | 34 | 0 | 1 | 192 |
| 107 | 6B | 72 | 48 | 1 | 35 | 654983 | 5239860 | 1 | 35 | 0 | 184 | 5388260 | 1 | 35 | 0 | 1 | 192 |
| 108 | 6C | 72 | 48 | 1 | 36 | 655006 | 5240044 | 1 | 36 | 0 | 184 | 5388452 | 1 | 36 | 0 | 1 | 192 |
| 109 | 6D | 72 | 48 | 1 | 37 | 655029 | 5240228 | 1 | 37 | 0 | 184 | 5388644 | 1 | 37 | 0 | 1 | 192 |
| 110 | 6E | 72 | 48 | 1 | 38 | 655052 | 5240412 | 1 | 38 | 0 | 184 | 5388836 | 1 | 38 | 0 | 1 | 192 |
| 111 | 6F | 72 | 48 | 1 | 39 | 655075 | 5240596 | 1 | 39 | 0 | 184 | 5389028 | 1 | 39 | 0 | 1 | 192 |
| 112 | 70 | 72 | 48 | 1 | 40 | 655098 | 5240780 | 1 | 40 | 0 | 184 | 5389220 | 1 | 40 | 0 | 1 | 192 |
| 113 | 71 | 72 | 48 | 1 | 41 | 655121 | 5240964 | 1 | 41 | 0 | 184 | 5389412 | 1 | 41 | 0 | 1 | 192 |
| 114 | 72 | 72 | 48 | 1 | 42 | 655144 | 5241148 | 1 | 42 | 0 | 184 | 5389604 | 1 | 42 | 0 | 1 | 192 |
| 115 | 73 | 72 | 48 | 1 | 43 | 655167 | 5241332 | 1 | 43 | 0 | 184 | 5389796 | 1 | 43 | 0 | 1 | 192 |
| 116 | 74 | 72 | 48 | 1 | 44 | 655190 | 5241516 | 1 | 44 | 0 | 184 | 5389988 | 1 | 44 | 0 | 1 | 192 |
| 117 | 75 | 72 | 48 | 1 | 45 | 655213 | 5241700 | 1 | 45 | 0 | 184 | 5390180 | 1 | 45 | 0 | 1 | 192 |
| 118 | 76 | 72 | 48 | 1 | 46 | 655236 | 5241884 | 1 | 46 | 0 | 184 | 5390372 | 1 | 46 | 0 | 1 | 192 |
| 119 | 77 | 72 | 48 | 1 | 47 | 655259 | 5242068 | 1 | 47 | 0 | 184 | 5390564 | 1 | 47 | 0 | 1 | 192 |
| 120 | 78 | 72 | 48 | 1 | 48 | 655282 | 5242252 | 1 | 48 | 0 | 184 | 5390756 | 1 | 48 | 0 | 1 | 192 |
| 121 | 79 | 72 | 48 | 1 | 49 | 655305 | 5242436 | 1 | 49 | 0 | 184 | 5390948 | 1 | 49 | 0 | 1 | 192 |
| 122 | 7A | 72 | 48 | 1 | 50 | 655328 | 5242620 | 1 | 50 | 0 | 184 | 5391140 | 1 | 50 | 0 | 1 | 192 |
| 123 | 7B | 72 | 48 | 1 | 51 | 655351 | 5242804 | 1 | 51 | 0 | 184 | 5391332 | 1 | 51 | 0 | 1 | 192 |
| 124 | 7C | 72 | 48 | 1 | 52 | 655374 | 5242988 | 1 | 52 | 0 | 184 | 5391524 | 1 | 52 | 0 | 1 | 192 |
| 125 | 7D | 72 | 48 | 1 | 53 | 655397 | 5243172 | 1 | 53 | 0 | 184 | 5391716 | 1 | 53 | 0 | 1 | 192 |
| 126 | 7E | 72 | 48 | 1 | 54 | 655420 | 5243356 | 1 | 54 | 0 | 184 | 5391908 | 1 | 54 | 0 | 1 | 192 |
| 127 | 7F | 72 | 48 | 1 | 55 | 655443 | 5243540 | 1 | 55 | 0 | 184 | 5392100 | 1 | 55 | 0 | 1 | 192 |
| 128 | 80 | 72 | 48 | 1 | 56 | 655466 | 5243724 | 1 | 56 | 0 | 184 | 5392292 | 1 | 56 | 0 | 1 | 192 |
| 129 | 81 | 72 | 48 | 1 | 57 | 655489 | 5243908 | 1 | 57 | 0 | 184 | 5392484 | 1 | 57 | 0 | 1 | 192 |
| 130 | 82 | 72 | 48 | 1 | 58 | 655512 | 5244092 | 1 | 58 | 0 | 184 | 5392676 | 1 | 58 | 0 | 1 | 192 |
| 131 | 83 | 72 | 48 | 1 | 59 | 655535 | 5244276 | 1 | 59 | 0 | 184 | 5392868 | 1 | 59 | 0 | 1 | 192 |
| 132 | 84 | 72 | 48 | 1 | 60 | 655558 | 5244460 | 1 | 60 | 0 | 184 | 5393060 | 1 | 60 | 0 | 1 | 192 |
| 133 | 85 | 72 | 48 | 1 | 61 | 655581 | 5244644 | 1 | 61 | 0 | 184 | 5393252 | 1 | 61 | 0 | 1 | 192 |
| 134 | 86 | 72 | 48 | 1 | 62 | 655604 | 5244828 | 1 | 62 | 0 | 184 | 5393444 | 1 | 62 | 0 | 1 | 192 |
| 135 | 87 | 72 | 48 | 1 | 63 | 655627 | 5245012 | 1 | 63 | 0 | 184 | 5393636 | 1 | 63 | 0 | 1 | 192 |
| 136 | 88 | 72 | 48 | 1 | 64 | 655650 | 5245196 | 1 | 64 | 0 | 184 | 5393828 | 1 | 64 | 0 | 1 | 192 |
| 137 | 89 | 72 | 48 | 1 | 65 | 655673 | 5245380 | 1 | 65 | 0 | 184 | 5394020 | 1 | 65 | 0 | 1 | 192 |
| 138 | 8A | 72 | 48 | 1 | 66 | 655696 | 5245564 | 1 | 66 | 0 | 184 | 5394212 | 1 | 66 | 0 | 1 | 192 |
| 139 | 8B | 72 | 48 | 1 | 67 | 655719 | 5245748 | 1 | 67 | 0 | 184 | 5394404 | 1 | 67 | 0 | 1 | 192 |
| 140 | 8C | 72 | 48 | 1 | 68 | 655742 | 5245932 | 1 | 68 | 0 | 184 | 5394596 | 1 | 68 | 0 | 1 | 192 |
| 141 | 8D | 72 | 48 | 1 | 69 | 655765 | 5246116 | 1 | 69 | 0 | 184 | 5394788 | 1 | 69 | 0 | 1 | 192 |
| 142 | 8E | 72 | 48 | 1 | 70 | 655788 | 5246300 | 1 | 70 | 0 | 184 | 5394980 | 1 | 70 | 0 | 1 | 192 |
| 143 | 8F | 72 | 48 | 1 | 71 | 655811 | 5246484 | 1 | 71 | 0 | 184 | 5395172 | 1 | 71 | 0 | 1 | 192 |
| 144 | 90 | 72 | 48 | 2 | 0 | 655832 | 5246652 | 2 | 0 | 0 | 168 | 5395348 | 2 | 0 | 0 | 1 | 176 |
| 145 | 91 | 72 | 48 | 2 | 1 | 655858 | 5246860 | 2 | 1 | 0 | 208 | 5395564 | 2 | 1 | 0 | 1 | 216 |
| 146 | 92 | 72 | 48 | 2 | 2 | 655884 | 5247068 | 2 | 2 | 0 | 208 | 5395780 | 2 | 2 | 0 | 1 | 216 |
| 147 | 93 | 72 | 48 | 2 | 3 | 655910 | 5247276 | 2 | 3 | 0 | 208 | 5395996 | 2 | 3 | 0 | 1 | 216 |
| 148 | 94 | 72 | 48 | 2 | 4 | 655936 | 5247484 | 2 | 4 | 0 | 208 | 5396212 | 2 | 4 | 0 | 1 | 216 |
| 149 | 95 | 72 | 48 | 2 | 5 | 655962 | 5247692 | 2 | 5 | 0 | 208 | 5396428 | 2 | 5 | 0 | 1 | 216 |
| 150 | 96 | 72 | 48 | 2 | 6 | 655988 | 5247900 | 2 | 6 | 0 | 208 | 5396644 | 2 | 6 | 0 | 1 | 216 |
| 151 | 97 | 72 | 48 | 2 | 7 | 656014 | 5248108 | 2 | 7 | 0 | 208 | 5396860 | 2 | 7 | 0 | 1 | 216 |
| 152 | 98 | 72 | 48 | 2 | 8 | 656040 | 5248316 | 2 | 8 | 0 | 208 | 5397076 | 2 | 8 | 0 | 1 | 216 |
| 153 | 99 | 72 | 48 | 2 | 9 | 656066 | 5248524 | 2 | 9 | 0 | 208 | 5397292 | 2 | 9 | 0 | 1 | 216 |
| 154 | 9A | 72 | 48 | 2 | 10 | 656092 | 5248732 | 2 | 10 | 0 | 208 | 5397508 | 2 | 10 | 0 | 1 | 216 |
| 155 | 9B | 72 | 48 | 2 | 11 | 656118 | 5248940 | 2 | 11 | 0 | 208 | 5397724 | 2 | 11 | 0 | 1 | 216 |
| 156 | 9C | 72 | 48 | 2 | 12 | 656144 | 5249148 | 2 | 12 | 0 | 208 | 5397940 | 2 | 12 | 0 | 1 | 216 |
| 157 | 9D | 72 | 48 | 2 | 13 | 656170 | 5249356 | 2 | 13 | 0 | 208 | 5398156 | 2 | 13 | 0 | 1 | 216 |
| 158 | 9E | 72 | 48 | 2 | 14 | 656196 | 5249564 | 2 | 14 | 0 | 208 | 5398372 | 2 | 14 | 0 | 1 | 216 |
| 159 | 9F | 72 | 48 | 2 | 15 | 656222 | 5249772 | 2 | 15 | 0 | 208 | 5398588 | 2 | 15 | 0 | 1 | 216 |
| 160 | A0 | 72 | 48 | 2 | 16 | 656245 | 5249956 | 2 | 16 | 0 | 184 | 5398780 | 2 | 16 | 0 | 1 | 192 |
| 161 | A1 | 72 | 48 | 2 | 17 | 656268 | 5250140 | 2 | 17 | 0 | 184 | 5398972 | 2 | 17 | 0 | 1 | 192 |
| 162 | A2 | 72 | 48 | 2 | 18 | 656291 | 5250324 | 2 | 18 | 0 | 184 | 5399164 | 2 | 18 | 0 | 1 | 192 |
| 163 | A3 | 72 | 48 | 2 | 19 | 656314 | 5250508 | 2 | 19 | 0 | 184 | 5399356 | 2 | 19 | 0 | 1 | 192 |
| 164 | A4 | 72 | 48 | 2 | 20 | 656337 | 5250692 | 2 | 20 | 0 | 184 | 5399548 | 2 | 20 | 0 | 1 | 192 |
| 165 | A5 | 72 | 48 | 2 | 21 | 656360 | 5250876 | 2 | 21 | 0 | 184 | 5399740 | 2 | 21 | 0 | 1 | 192 |
| 166 | A6 | 72 | 48 | 2 | 22 | 656383 | 5251060 | 2 | 22 | 0 | 184 | 5399932 | 2 | 22 | 0 | 1 | 192 |
| 167 | A7 | 72 | 48 | 2 | 23 | 656406 | 5251244 | 2 | 23 | 0 | 184 | 5400124 | 2 | 23 | 0 | 1 | 192 |
| 168 | A8 | 72 | 48 | 2 | 24 | 656429 | 5251428 | 2 | 24 | 0 | 184 | 5400316 | 2 | 24 | 0 | 1 | 192 |
| 169 | A9 | 72 | 48 | 2 | 25 | 656452 | 5251612 | 2 | 25 | 0 | 184 | 5400508 | 2 | 25 | 0 | 1 | 192 |
| 170 | AA | 72 | 48 | 2 | 26 | 656475 | 5251796 | 2 | 26 | 0 | 184 | 5400700 | 2 | 26 | 0 | 1 | 192 |
| 171 | AB | 72 | 48 | 2 | 27 | 656498 | 5251980 | 2 | 27 | 0 | 184 | 5400892 | 2 | 27 | 0 | 1 | 192 |
| 172 | AC | 72 | 48 | 2 | 28 | 656521 | 5252164 | 2 | 28 | 0 | 184 | 5401084 | 2 | 28 | 0 | 1 | 192 |
| 173 | AD | 72 | 48 | 2 | 29 | 656544 | 5252348 | 2 | 29 | 0 | 184 | 5401276 | 2 | 29 | 0 | 1 | 192 |
| 174 | AE | 72 | 48 | 2 | 30 | 656567 | 5252532 | 2 | 30 | 0 | 184 | 5401468 | 2 | 30 | 0 | 1 | 192 |
| 175 | AF | 72 | 48 | 2 | 31 | 656590 | 5252716 | 2 | 31 | 0 | 184 | 5401660 | 2 | 31 | 0 | 1 | 192 |
| 176 | B0 | 72 | 48 | 2 | 32 | 656615 | 5252916 | 2 | 32 | 0 | 200 | 5401868 | 2 | 32 | 0 | 1 | 208 |
| 177 | B1 | 72 | 48 | 2 | 33 | 656640 | 5253116 | 2 | 33 | 0 | 200 | 5402076 | 2 | 33 | 0 | 1 | 208 |
| 178 | B2 | 72 | 48 | 2 | 34 | 656665 | 5253316 | 2 | 34 | 0 | 200 | 5402284 | 2 | 34 | 0 | 1 | 208 |
| 179 | B3 | 72 | 48 | 2 | 35 | 656690 | 5253516 | 2 | 35 | 0 | 200 | 5402492 | 2 | 35 | 0 | 1 | 208 |
| 180 | B4 | 72 | 48 | 2 | 36 | 656715 | 5253716 | 2 | 36 | 0 | 200 | 5402700 | 2 | 36 | 0 | 1 | 208 |
| 181 | B5 | 72 | 48 | 2 | 37 | 656740 | 5253916 | 2 | 37 | 0 | 200 | 5402908 | 2 | 37 | 0 | 1 | 208 |
| 182 | B6 | 72 | 48 | 2 | 38 | 656765 | 5254116 | 2 | 38 | 0 | 200 | 5403116 | 2 | 38 | 0 | 1 | 208 |
| 183 | B7 | 72 | 48 | 2 | 39 | 656790 | 5254316 | 2 | 39 | 0 | 200 | 5403324 | 2 | 39 | 0 | 1 | 208 |
| 184 | B8 | 72 | 48 | 2 | 40 | 656815 | 5254516 | 2 | 40 | 0 | 200 | 5403532 | 2 | 40 | 0 | 1 | 208 |
| 185 | B9 | 72 | 48 | 2 | 41 | 656840 | 5254716 | 2 | 41 | 0 | 200 | 5403740 | 2 | 41 | 0 | 1 | 208 |
| 186 | BA | 72 | 48 | 2 | 42 | 656865 | 5254916 | 2 | 42 | 0 | 200 | 5403948 | 2 | 42 | 0 | 1 | 208 |
| 187 | BB | 72 | 48 | 2 | 43 | 656890 | 5255116 | 2 | 43 | 0 | 200 | 5404156 | 2 | 43 | 0 | 1 | 208 |
| 188 | BC | 72 | 48 | 2 | 44 | 656915 | 5255316 | 2 | 44 | 0 | 200 | 5404364 | 2 | 44 | 0 | 1 | 208 |
| 189 | BD | 72 | 48 | 2 | 45 | 656940 | 5255516 | 2 | 45 | 0 | 200 | 5404572 | 2 | 45 | 0 | 1 | 208 |
| 190 | BE | 72 | 48 | 2 | 46 | 656965 | 5255716 | 2 | 46 | 0 | 200 | 5404780 | 2 | 46 | 0 | 1 | 208 |
| 191 | BF | 72 | 48 | 2 | 47 | 656990 | 5255916 | 2 | 47 | 0 | 200 | 5404988 | 2 | 47 | 0 | 1 | 208 |
| 192 | C0 | 72 | 48 | 2 | 48 | 657015 | 5256116 | 2 | 48 | 0 | 200 | 5405196 | 2 | 48 | 0 | 1 | 208 |
| 193 | C1 | 72 | 48 | 2 | 49 | 657040 | 5256316 | 2 | 49 | 0 | 200 | 5405404 | 2 | 49 | 0 | 1 | 208 |
| 194 | C2 | 72 | 48 | 2 | 50 | 657065 | 5256516 | 2 | 50 | 0 | 200 | 5405612 | 2 | 50 | 0 | 1 | 208 |
| 195 | C3 | 72 | 48 | 2 | 51 | 657090 | 5256716 | 2 | 51 | 0 | 200 | 5405820 | 2 | 51 | 0 | 1 | 208 |
| 196 | C4 | 72 | 48 | 2 | 52 | 657115 | 5256916 | 2 | 52 | 0 | 200 | 5406028 | 2 | 52 | 0 | 1 | 208 |
| 197 | C5 | 72 | 48 | 2 | 53 | 657140 | 5257116 | 2 | 53 | 0 | 200 | 5406236 | 2 | 53 | 0 | 1 | 208 |
| 198 | C6 | 72 | 48 | 2 | 54 | 657165 | 5257316 | 2 | 54 | 0 | 200 | 5406444 | 2 | 54 | 0 | 1 | 208 |
| 199 | C7 | 72 | 48 | 2 | 55 | 657190 | 5257516 | 2 | 55 | 0 | 200 | 5406652 | 2 | 55 | 0 | 1 | 208 |
| 200 | C8 | 72 | 48 | 2 | 56 | 657215 | 5257716 | 2 | 56 | 0 | 200 | 5406860 | 2 | 56 | 0 | 1 | 208 |
| 201 | C9 | 72 | 48 | 2 | 57 | 657240 | 5257916 | 2 | 57 | 0 | 200 | 5407068 | 2 | 57 | 0 | 1 | 208 |
| 202 | CA | 72 | 48 | 2 | 58 | 657265 | 5258116 | 2 | 58 | 0 | 200 | 5407276 | 2 | 58 | 0 | 1 | 208 |
| 203 | CB | 72 | 48 | 2 | 59 | 657290 | 5258316 | 2 | 59 | 0 | 200 | 5407484 | 2 | 59 | 0 | 1 | 208 |
| 204 | CC | 72 | 48 | 2 | 60 | 657315 | 5258516 | 2 | 60 | 0 | 200 | 5407692 | 2 | 60 | 0 | 1 | 208 |
| 205 | CD | 72 | 48 | 2 | 61 | 657340 | 5258716 | 2 | 61 | 0 | 200 | 5407900 | 2 | 61 | 0 | 1 | 208 |
| 206 | CE | 72 | 48 | 2 | 62 | 657365 | 5258916 | 2 | 62 | 0 | 200 | 5408108 | 2 | 62 | 0 | 1 | 208 |
| 207 | CF | 72 | 48 | 2 | 63 | 657390 | 5259116 | 2 | 63 | 0 | 200 | 5408316 | 2 | 63 | 0 | 1 | 208 |

| | | | | | | | | | | | | | | | | | |
|---|---|---|---|---|---|---|---|---|---|---|---|---|---|---|---|---|---|
| 208 | D0 | 72 | 48 | 2 | 64 | 657415 | 5259316 | 2 | 64 | 0 | 200 | 5408524 | 2 | 64 | 0 | 1 | 208 |
| 209 | D1 | 72 | 48 | 2 | 65 | 657440 | 5259516 | 2 | 65 | 0 | 200 | 5408732 | 2 | 65 | 0 | 1 | 208 |
| 210 | D2 | 72 | 48 | 2 | 66 | 657465 | 5259716 | 2 | 66 | 0 | 200 | 5408940 | 2 | 66 | 0 | 1 | 208 |
| 211 | D3 | 72 | 48 | 2 | 67 | 657490 | 5259916 | 2 | 67 | 0 | 200 | 5409148 | 2 | 67 | 0 | 1 | 208 |
| 212 | D4 | 72 | 48 | 2 | 68 | 657515 | 5260116 | 2 | 68 | 0 | 200 | 5409356 | 2 | 68 | 0 | 1 | 208 |
| 213 | D5 | 72 | 48 | 2 | 69 | 657540 | 5260316 | 2 | 69 | 0 | 200 | 5409564 | 2 | 69 | 0 | 1 | 208 |
| 214 | D6 | 72 | 48 | 2 | 70 | 657565 | 5260516 | 2 | 70 | 0 | 200 | 5409772 | 2 | 70 | 0 | 1 | 208 |
| 215 | D7 | 72 | 48 | 2 | 71 | 657590 | 5260716 | 2 | 71 | 0 | 200 | 5409980 | 2 | 71 | 0 | 1 | 208 |
| 216 | D8 | 72 | 48 | 3 | 0 | 657613 | 5260900 | 3 | 0 | 0 | 184 | 5410172 | 3 | 0 | 0 | 1 | 192 |
| 217 | D9 | 72 | 48 | 3 | 1 | 657641 | 5261124 | 3 | 1 | 0 | 224 | 5410404 | 3 | 1 | 0 | 1 | 232 |
| 218 | DA | 72 | 48 | 3 | 2 | 657669 | 5261348 | 3 | 2 | 0 | 224 | 5410636 | 3 | 2 | 0 | 1 | 232 |
| 219 | DB | 72 | 48 | 3 | 3 | 657697 | 5261572 | 3 | 3 | 0 | 224 | 5410868 | 3 | 3 | 0 | 1 | 232 |
| 220 | DC | 72 | 48 | 3 | 4 | 657725 | 5261796 | 3 | 4 | 0 | 224 | 5411100 | 3 | 4 | 0 | 1 | 232 |
| 221 | DD | 72 | 48 | 3 | 5 | 657753 | 5262020 | 3 | 5 | 0 | 224 | 5411332 | 3 | 5 | 0 | 1 | 232 |
| 222 | DE | 72 | 48 | 3 | 6 | 657781 | 5262244 | 3 | 6 | 0 | 224 | 5411564 | 3 | 6 | 0 | 1 | 232 |
| 223 | DF | 72 | 48 | 3 | 7 | 657809 | 5262468 | 3 | 7 | 0 | 224 | 5411796 | 3 | 7 | 0 | 1 | 232 |
| 224 | E0 | 72 | 48 | 3 | 8 | 657837 | 5262692 | 3 | 8 | 0 | 224 | 5412028 | 3 | 8 | 0 | 1 | 232 |
| 225 | E1 | 72 | 48 | 3 | 9 | 657865 | 5262916 | 3 | 9 | 0 | 224 | 5412260 | 3 | 9 | 0 | 1 | 232 |
| 226 | E2 | 72 | 48 | 3 | 10 | 657893 | 5263140 | 3 | 10 | 0 | 224 | 5412492 | 3 | 10 | 0 | 1 | 232 |
| 227 | E3 | 72 | 48 | 3 | 11 | 657921 | 5263364 | 3 | 11 | 0 | 224 | 5412724 | 3 | 11 | 0 | 1 | 232 |
| 228 | E4 | 72 | 48 | 3 | 12 | 657949 | 5263588 | 3 | 12 | 0 | 224 | 5412956 | 3 | 12 | 0 | 1 | 232 |
| 229 | E5 | 72 | 48 | 3 | 13 | 657977 | 5263812 | 3 | 13 | 0 | 224 | 5413188 | 3 | 13 | 0 | 1 | 232 |
| 230 | E6 | 72 | 48 | 3 | 14 | 658005 | 5264036 | 3 | 14 | 0 | 224 | 5413420 | 3 | 14 | 0 | 1 | 232 |
| 231 | E7 | 72 | 48 | 3 | 15 | 658033 | 5264260 | 3 | 15 | 0 | 224 | 5413652 | 3 | 15 | 0 | 1 | 232 |
| 232 | E8 | 72 | 48 | 3 | 16 | 658061 | 5264484 | 3 | 16 | 0 | 224 | 5413884 | 3 | 16 | 0 | 1 | 232 |
| 233 | E9 | 72 | 48 | 3 | 17 | 658089 | 5264708 | 3 | 17 | 0 | 224 | 5414116 | 3 | 17 | 0 | 1 | 232 |
| 234 | EA | 72 | 48 | 3 | 18 | 658117 | 5264932 | 3 | 18 | 0 | 224 | 5414348 | 3 | 18 | 0 | 1 | 232 |
| 235 | EB | 72 | 48 | 3 | 19 | 658145 | 5265156 | 3 | 19 | 0 | 224 | 5414580 | 3 | 19 | 0 | 1 | 232 |
| 236 | EC | 72 | 48 | 3 | 20 | 658173 | 5265380 | 3 | 20 | 0 | 224 | 5414812 | 3 | 20 | 0 | 1 | 232 |
| 237 | ED | 72 | 48 | 3 | 21 | 658201 | 5265604 | 3 | 21 | 0 | 224 | 5415044 | 3 | 21 | 0 | 1 | 232 |
| 238 | EE | 72 | 48 | 3 | 22 | 658229 | 5265828 | 3 | 22 | 0 | 224 | 5415276 | 3 | 22 | 0 | 1 | 232 |
| 239 | EF | 72 | 48 | 3 | 23 | 658257 | 5266052 | 3 | 23 | 0 | 224 | 5415508 | 3 | 23 | 0 | 1 | 232 |
| 240 | F0 | 72 | 48 | 3 | 24 | 658282 | 5266252 | 3 | 24 | 0 | 200 | 5415716 | 3 | 24 | 0 | 1 | 208 |
| 241 | F1 | 72 | 48 | 3 | 25 | 658307 | 5266452 | 3 | 25 | 0 | 200 | 5415924 | 3 | 25 | 0 | 1 | 208 |
| 242 | F2 | 72 | 48 | 3 | 26 | 658332 | 5266652 | 3 | 26 | 0 | 200 | 5416132 | 3 | 26 | 0 | 1 | 208 |
| 243 | F3 | 72 | 48 | 3 | 27 | 658357 | 5266852 | 3 | 27 | 0 | 200 | 5416340 | 3 | 27 | 0 | 1 | 208 |
| 244 | F4 | 72 | 48 | 3 | 28 | 658382 | 5267052 | 3 | 28 | 0 | 200 | 5416548 | 3 | 28 | 0 | 1 | 208 |
| 245 | F5 | 72 | 48 | 3 | 29 | 658407 | 5267252 | 3 | 29 | 0 | 200 | 5416756 | 3 | 29 | 0 | 1 | 208 |
| 246 | F6 | 72 | 48 | 3 | 30 | 658432 | 5267452 | 3 | 30 | 0 | 200 | 5416964 | 3 | 30 | 0 | 1 | 208 |
| 247 | F7 | 72 | 48 | 3 | 31 | 658457 | 5267652 | 3 | 31 | 0 | 200 | 5417172 | 3 | 31 | 0 | 1 | 208 |
| 248 | F8 | 72 | 48 | 3 | 32 | 658482 | 5267852 | 3 | 32 | 0 | 200 | 5417380 | 3 | 32 | 0 | 1 | 208 |
| 249 | F9 | 72 | 48 | 3 | 33 | 658507 | 5268052 | 3 | 33 | 0 | 200 | 5417588 | 3 | 33 | 0 | 1 | 208 |
| 250 | FA | 72 | 48 | 3 | 34 | 658532 | 5268252 | 3 | 34 | 0 | 200 | 5417796 | 3 | 34 | 0 | 1 | 208 |
| 251 | FB | 72 | 48 | 3 | 35 | 658557 | 5268452 | 3 | 35 | 0 | 200 | 5418004 | 3 | 35 | 0 | 1 | 208 |
| 252 | FC | 72 | 48 | 3 | 36 | 658582 | 5268652 | 3 | 36 | 0 | 200 | 5418212 | 3 | 36 | 0 | 1 | 208 |
| 253 | FD | 72 | 48 | 3 | 37 | 658607 | 5268852 | 3 | 37 | 0 | 200 | 5418420 | 3 | 37 | 0 | 1 | 208 |
| 254 | FE | 72 | 48 | 3 | 38 | 658632 | 5269052 | 3 | 38 | 0 | 200 | 5418628 | 3 | 38 | 0 | 1 | 208 |
| 255 | FF | 72 | 48 | 3 | 39 | 658657 | 5269252 | 3 | 39 | 0 | 200 | 5418836 | 3 | 39 | 0 | 1 | 208 |
| 0 | 00 | 73 | 49 | 0 | 0 | 658669 | 5269348 | 0 | 0 | 0 | 96 | 5418940 | 0 | 0 | 0 | 1 | 104 |
| 1 | 01 | 73 | 49 | 0 | 1 | 658683 | 5269460 | 0 | 1 | 0 | 112 | 5419060 | 0 | 1 | 0 | 1 | 120 |
| 2 | 02 | 73 | 49 | 0 | 2 | 658697 | 5269572 | 0 | 2 | 0 | 112 | 5419180 | 0 | 2 | 0 | 1 | 120 |
| 3 | 03 | 73 | 49 | 0 | 3 | 658711 | 5269684 | 0 | 3 | 0 | 112 | 5419300 | 0 | 3 | 0 | 1 | 120 |
| 4 | 04 | 73 | 49 | 0 | 4 | 658725 | 5269796 | 0 | 4 | 0 | 112 | 5419420 | 0 | 4 | 0 | 1 | 120 |
| 5 | 05 | 73 | 49 | 0 | 5 | 658739 | 5269908 | 0 | 5 | 0 | 112 | 5419540 | 0 | 5 | 0 | 1 | 120 |
| 6 | 06 | 73 | 49 | 0 | 6 | 658753 | 5270020 | 0 | 6 | 0 | 112 | 5419660 | 0 | 6 | 0 | 1 | 120 |
| 7 | 07 | 73 | 49 | 0 | 7 | 658767 | 5270132 | 0 | 7 | 0 | 112 | 5419780 | 0 | 7 | 0 | 1 | 120 |
| 8 | 08 | 73 | 49 | 0 | 8 | 658781 | 5270244 | 0 | 8 | 0 | 112 | 5419900 | 0 | 8 | 0 | 1 | 120 |
| 9 | 09 | 73 | 49 | 0 | 9 | 658795 | 5270356 | 0 | 9 | 0 | 112 | 5420020 | 0 | 9 | 0 | 1 | 120 |
| 10 | 0A | 73 | 49 | 0 | 10 | 658809 | 5270468 | 0 | 10 | 0 | 112 | 5420140 | 0 | 10 | 0 | 1 | 120 |
| 11 | 0B | 73 | 49 | 0 | 11 | 658823 | 5270580 | 0 | 11 | 0 | 112 | 5420260 | 0 | 11 | 0 | 1 | 120 |
| 12 | 0C | 73 | 49 | 0 | 12 | 658837 | 5270692 | 0 | 12 | 0 | 112 | 5420380 | 0 | 12 | 0 | 1 | 120 |
| 13 | 0D | 73 | 49 | 0 | 13 | 658851 | 5270804 | 0 | 13 | 0 | 112 | 5420500 | 0 | 13 | 0 | 1 | 120 |
| 14 | 0E | 73 | 49 | 0 | 14 | 658865 | 5270916 | 0 | 14 | 0 | 112 | 5420620 | 0 | 14 | 0 | 1 | 120 |
| 15 | 0F | 73 | 49 | 0 | 15 | 658879 | 5271028 | 0 | 15 | 0 | 112 | 5420740 | 0 | 15 | 0 | 1 | 120 |
| 16 | 10 | 73 | 49 | 0 | 16 | 658893 | 5271140 | 0 | 16 | 0 | 112 | 5420860 | 0 | 16 | 0 | 1 | 120 |
| 17 | 11 | 73 | 49 | 0 | 17 | 658907 | 5271252 | 0 | 17 | 0 | 112 | 5420980 | 0 | 17 | 0 | 1 | 120 |
| 18 | 12 | 73 | 49 | 0 | 18 | 658921 | 5271364 | 0 | 18 | 0 | 112 | 5421100 | 0 | 18 | 0 | 1 | 120 |
| 19 | 13 | 73 | 49 | 0 | 19 | 658935 | 5271476 | 0 | 19 | 0 | 112 | 5421220 | 0 | 19 | 0 | 1 | 120 |
| 20 | 14 | 73 | 49 | 0 | 20 | 658949 | 5271588 | 0 | 20 | 0 | 112 | 5421340 | 0 | 20 | 0 | 1 | 120 |
| 21 | 15 | 73 | 49 | 0 | 21 | 658963 | 5271700 | 0 | 21 | 0 | 112 | 5421460 | 0 | 21 | 0 | 1 | 120 |
| 22 | 16 | 73 | 49 | 0 | 22 | 658977 | 5271812 | 0 | 22 | 0 | 112 | 5421580 | 0 | 22 | 0 | 1 | 120 |
| 23 | 17 | 73 | 49 | 0 | 23 | 658991 | 5271924 | 0 | 23 | 0 | 112 | 5421700 | 0 | 23 | 0 | 1 | 120 |
| 24 | 18 | 73 | 49 | 0 | 24 | 659005 | 5272036 | 0 | 24 | 0 | 112 | 5421820 | 0 | 24 | 0 | 1 | 120 |
| 25 | 19 | 73 | 49 | 0 | 25 | 659019 | 5272148 | 0 | 25 | 0 | 112 | 5421940 | 0 | 25 | 0 | 1 | 120 |
| 26 | 1A | 73 | 49 | 0 | 26 | 659033 | 5272260 | 0 | 26 | 0 | 112 | 5422060 | 0 | 26 | 0 | 1 | 120 |
| 27 | 1B | 73 | 49 | 0 | 27 | 659047 | 5272372 | 0 | 27 | 0 | 112 | 5422180 | 0 | 27 | 0 | 1 | 120 |
| 28 | 1C | 73 | 49 | 0 | 28 | 659061 | 5272484 | 0 | 28 | 0 | 112 | 5422300 | 0 | 28 | 0 | 1 | 120 |
| 29 | 1D | 73 | 49 | 0 | 29 | 659075 | 5272596 | 0 | 29 | 0 | 112 | 5422420 | 0 | 29 | 0 | 1 | 120 |
| 30 | 1E | 73 | 49 | 0 | 30 | 659089 | 5272708 | 0 | 30 | 0 | 112 | 5422540 | 0 | 30 | 0 | 1 | 120 |
| 31 | 1F | 73 | 49 | 0 | 31 | 659103 | 5272820 | 0 | 31 | 0 | 112 | 5422660 | 0 | 31 | 0 | 1 | 120 |
| 32 | 20 | 73 | 49 | 0 | 32 | 659117 | 5272932 | 0 | 32 | 0 | 112 | 5422780 | 0 | 32 | 0 | 1 | 120 |
| 33 | 21 | 73 | 49 | 0 | 33 | 659131 | 5273044 | 0 | 33 | 0 | 112 | 5422900 | 0 | 33 | 0 | 1 | 120 |
| 34 | 22 | 73 | 49 | 0 | 34 | 659145 | 5273156 | 0 | 34 | 0 | 112 | 5423020 | 0 | 34 | 0 | 1 | 120 |
| 35 | 23 | 73 | 49 | 0 | 35 | 659159 | 5273268 | 0 | 35 | 0 | 112 | 5423140 | 0 | 35 | 0 | 1 | 120 |
| 36 | 24 | 73 | 49 | 0 | 36 | 659173 | 5273380 | 0 | 36 | 0 | 112 | 5423260 | 0 | 36 | 0 | 1 | 120 |
| 37 | 25 | 73 | 49 | 0 | 37 | 659187 | 5273492 | 0 | 37 | 0 | 112 | 5423380 | 0 | 37 | 0 | 1 | 120 |
| 38 | 26 | 73 | 49 | 0 | 38 | 659201 | 5273604 | 0 | 38 | 0 | 112 | 5423500 | 0 | 38 | 0 | 1 | 120 |
| 39 | 27 | 73 | 49 | 0 | 39 | 659215 | 5273716 | 0 | 39 | 0 | 112 | 5423620 | 0 | 39 | 0 | 1 | 120 |
| 40 | 28 | 73 | 49 | 0 | 40 | 659229 | 5273828 | 0 | 40 | 0 | 112 | 5423740 | 0 | 40 | 0 | 1 | 120 |
| 41 | 29 | 73 | 49 | 0 | 41 | 659243 | 5273940 | 0 | 41 | 0 | 112 | 5423860 | 0 | 41 | 0 | 1 | 120 |
| 42 | 2A | 73 | 49 | 0 | 42 | 659257 | 5274052 | 0 | 42 | 0 | 112 | 5423980 | 0 | 42 | 0 | 1 | 120 |
| 43 | 2B | 73 | 49 | 0 | 43 | 659271 | 5274164 | 0 | 43 | 0 | 112 | 5424100 | 0 | 43 | 0 | 1 | 120 |
| 44 | 2C | 73 | 49 | 0 | 44 | 659285 | 5274276 | 0 | 44 | 0 | 112 | 5424220 | 0 | 44 | 0 | 1 | 120 |
| 45 | 2D | 73 | 49 | 0 | 45 | 659299 | 5274388 | 0 | 45 | 0 | 112 | 5424340 | 0 | 45 | 0 | 1 | 120 |
| 46 | 2E | 73 | 49 | 0 | 46 | 659313 | 5274500 | 0 | 46 | 0 | 112 | 5424460 | 0 | 46 | 0 | 1 | 120 |
| 47 | 2F | 73 | 49 | 0 | 47 | 659327 | 5274612 | 0 | 47 | 0 | 112 | 5424580 | 0 | 47 | 0 | 1 | 120 |
| 48 | 30 | 73 | 49 | 0 | 48 | 659341 | 5274724 | 0 | 48 | 0 | 112 | 5424700 | 0 | 48 | 0 | 1 | 120 |
| 49 | 31 | 73 | 49 | 0 | 49 | 659355 | 5274836 | 0 | 49 | 0 | 112 | 5424820 | 0 | 49 | 0 | 1 | 120 |
| 50 | 32 | 73 | 49 | 0 | 50 | 659369 | 5274948 | 0 | 50 | 0 | 112 | 5424940 | 0 | 50 | 0 | 1 | 120 |
| 51 | 33 | 73 | 49 | 0 | 51 | 659383 | 5275060 | 0 | 51 | 0 | 112 | 5425060 | 0 | 51 | 0 | 1 | 120 |
| 52 | 34 | 73 | 49 | 0 | 52 | 659397 | 5275172 | 0 | 52 | 0 | 112 | 5425180 | 0 | 52 | 0 | 1 | 120 |
| 53 | 35 | 73 | 49 | 0 | 53 | 659411 | 5275284 | 0 | 53 | 0 | 112 | 5425300 | 0 | 53 | 0 | 1 | 120 |
| 54 | 36 | 73 | 49 | 0 | 54 | 659425 | 5275396 | 0 | 54 | 0 | 112 | 5425420 | 0 | 54 | 0 | 1 | 120 |

| | | | | | | | | | | | | | | | | | |
|---|---|---|---|---|---|---|---|---|---|---|---|---|---|---|---|---|---|
| 55 | 37 | 73 | 49 | 0 | 55 | 659439 | 5275508 | 0 | 55 | 0 | 112 | 5425540 | 0 | 55 | 0 | 1 | 120 |
| 56 | 38 | 73 | 49 | 0 | 56 | 659453 | 5275620 | 0 | 56 | 0 | 112 | 5425660 | 0 | 56 | 0 | 1 | 120 |
| 57 | 39 | 73 | 49 | 0 | 57 | 659467 | 5275732 | 0 | 57 | 0 | 112 | 5425780 | 0 | 57 | 0 | 1 | 120 |
| 58 | 3A | 73 | 49 | 0 | 58 | 659481 | 5275844 | 0 | 58 | 0 | 112 | 5425900 | 0 | 58 | 0 | 1 | 120 |
| 59 | 3B | 73 | 49 | 0 | 59 | 659495 | 5275956 | 0 | 59 | 0 | 112 | 5426020 | 0 | 59 | 0 | 1 | 120 |
| 60 | 3C | 73 | 49 | 0 | 60 | 659509 | 5276068 | 0 | 60 | 0 | 112 | 5426140 | 0 | 60 | 0 | 1 | 120 |
| 61 | 3D | 73 | 49 | 0 | 61 | 659523 | 5276180 | 0 | 61 | 0 | 112 | 5426260 | 0 | 61 | 0 | 1 | 120 |
| 62 | 3E | 73 | 49 | 0 | 62 | 659537 | 5276292 | 0 | 62 | 0 | 112 | 5426380 | 0 | 62 | 0 | 1 | 120 |
| 63 | 3F | 73 | 49 | 0 | 63 | 659551 | 5276404 | 0 | 63 | 0 | 112 | 5426500 | 0 | 63 | 0 | 1 | 120 |
| 64 | 40 | 73 | 49 | 0 | 64 | 659565 | 5276516 | 0 | 64 | 0 | 112 | 5426620 | 0 | 64 | 0 | 1 | 120 |
| 65 | 41 | 73 | 49 | 0 | 65 | 659579 | 5276628 | 0 | 65 | 0 | 112 | 5426740 | 0 | 65 | 0 | 1 | 120 |
| 66 | 42 | 73 | 49 | 0 | 66 | 659593 | 5276740 | 0 | 66 | 0 | 112 | 5426860 | 0 | 66 | 0 | 1 | 120 |
| 67 | 43 | 73 | 49 | 0 | 67 | 659607 | 5276852 | 0 | 67 | 0 | 112 | 5426980 | 0 | 67 | 0 | 1 | 120 |
| 68 | 44 | 73 | 49 | 0 | 68 | 659621 | 5276964 | 0 | 68 | 0 | 112 | 5427100 | 0 | 68 | 0 | 1 | 120 |
| 69 | 45 | 73 | 49 | 0 | 69 | 659635 | 5277076 | 0 | 69 | 0 | 112 | 5427220 | 0 | 69 | 0 | 1 | 120 |
| 70 | 46 | 73 | 49 | 0 | 70 | 659649 | 5277188 | 0 | 70 | 0 | 112 | 5427340 | 0 | 70 | 0 | 1 | 120 |
| 71 | 47 | 73 | 49 | 0 | 71 | 659663 | 5277300 | 0 | 71 | 0 | 112 | 5427460 | 0 | 71 | 0 | 1 | 120 |
| 72 | 48 | 73 | 49 | 0 | 72 | 659677 | 5277412 | 0 | 72 | 0 | 112 | 5427580 | 0 | 72 | 0 | 1 | 120 |
| 73 | 49 | 73 | 49 | 1 | 0 | 659689 | 5277508 | 1 | 0 | 0 | 96 | 5427684 | 1 | 0 | 0 | 1 | 104 |
| 74 | 4A | 73 | 49 | 1 | 1 | 659706 | 5277644 | 1 | 1 | 0 | 136 | 5427828 | 1 | 1 | 0 | 1 | 144 |
| 75 | 4B | 73 | 49 | 1 | 2 | 659723 | 5277780 | 1 | 2 | 0 | 136 | 5427972 | 1 | 2 | 0 | 1 | 144 |
| 76 | 4C | 73 | 49 | 1 | 3 | 659740 | 5277916 | 1 | 3 | 0 | 136 | 5428116 | 1 | 3 | 0 | 1 | 144 |
| 77 | 4D | 73 | 49 | 1 | 4 | 659757 | 5278052 | 1 | 4 | 0 | 136 | 5428260 | 1 | 4 | 0 | 1 | 144 |
| 78 | 4E | 73 | 49 | 1 | 5 | 659774 | 5278188 | 1 | 5 | 0 | 136 | 5428404 | 1 | 5 | 0 | 1 | 144 |
| 79 | 4F | 73 | 49 | 1 | 6 | 659791 | 5278324 | 1 | 6 | 0 | 136 | 5428548 | 1 | 6 | 0 | 1 | 144 |
| 80 | 50 | 73 | 49 | 1 | 7 | 659812 | 5278492 | 1 | 7 | 0 | 168 | 5428724 | 1 | 7 | 0 | 1 | 176 |
| 81 | 51 | 73 | 49 | 1 | 8 | 659833 | 5278660 | 1 | 8 | 0 | 168 | 5428900 | 1 | 8 | 0 | 1 | 176 |
| 82 | 52 | 73 | 49 | 1 | 9 | 659854 | 5278828 | 1 | 9 | 0 | 168 | 5429076 | 1 | 9 | 0 | 1 | 176 |
| 83 | 53 | 73 | 49 | 1 | 10 | 659875 | 5278996 | 1 | 10 | 0 | 168 | 5429252 | 1 | 10 | 0 | 1 | 176 |
| 84 | 54 | 73 | 49 | 1 | 11 | 659896 | 5279164 | 1 | 11 | 0 | 168 | 5429428 | 1 | 11 | 0 | 1 | 176 |
| 85 | 55 | 73 | 49 | 1 | 12 | 659917 | 5279332 | 1 | 12 | 0 | 168 | 5429604 | 1 | 12 | 0 | 1 | 176 |
| 86 | 56 | 73 | 49 | 1 | 13 | 659938 | 5279500 | 1 | 13 | 0 | 168 | 5429780 | 1 | 13 | 0 | 1 | 176 |
| 87 | 57 | 73 | 49 | 1 | 14 | 659959 | 5279668 | 1 | 14 | 0 | 168 | 5429956 | 1 | 14 | 0 | 1 | 176 |
| 88 | 58 | 73 | 49 | 1 | 15 | 659980 | 5279836 | 1 | 15 | 0 | 168 | 5430132 | 1 | 15 | 0 | 1 | 176 |
| 89 | 59 | 73 | 49 | 1 | 16 | 660001 | 5280004 | 1 | 16 | 0 | 168 | 5430308 | 1 | 16 | 0 | 1 | 176 |
| 90 | 5A | 73 | 49 | 1 | 17 | 660022 | 5280172 | 1 | 17 | 0 | 168 | 5430484 | 1 | 17 | 0 | 1 | 176 |
| 91 | 5B | 73 | 49 | 1 | 18 | 660043 | 5280340 | 1 | 18 | 0 | 168 | 5430660 | 1 | 18 | 0 | 1 | 176 |
| 92 | 5C | 73 | 49 | 1 | 19 | 660064 | 5280508 | 1 | 19 | 0 | 168 | 5430836 | 1 | 19 | 0 | 1 | 176 |
| 93 | 5D | 73 | 49 | 1 | 20 | 660085 | 5280676 | 1 | 20 | 0 | 168 | 5431012 | 1 | 20 | 0 | 1 | 176 |
| 94 | 5E | 73 | 49 | 1 | 21 | 660106 | 5280844 | 1 | 21 | 0 | 168 | 5431188 | 1 | 21 | 0 | 1 | 176 |
| 95 | 5F | 73 | 49 | 1 | 22 | 660127 | 5281012 | 1 | 22 | 0 | 168 | 5431364 | 1 | 22 | 0 | 1 | 176 |
| 96 | 60 | 73 | 49 | 1 | 23 | 660150 | 5281196 | 1 | 23 | 0 | 184 | 5431556 | 1 | 23 | 0 | 1 | 192 |
| 97 | 61 | 73 | 49 | 1 | 24 | 660173 | 5281380 | 1 | 24 | 0 | 184 | 5431748 | 1 | 24 | 0 | 1 | 192 |
| 98 | 62 | 73 | 49 | 1 | 25 | 660196 | 5281564 | 1 | 25 | 0 | 184 | 5431940 | 1 | 25 | 0 | 1 | 192 |
| 99 | 63 | 73 | 49 | 1 | 26 | 660219 | 5281748 | 1 | 26 | 0 | 184 | 5432132 | 1 | 26 | 0 | 1 | 192 |
| 100 | 64 | 73 | 49 | 1 | 27 | 660242 | 5281932 | 1 | 27 | 0 | 184 | 5432324 | 1 | 27 | 0 | 1 | 192 |
| 101 | 65 | 73 | 49 | 1 | 28 | 660265 | 5282116 | 1 | 28 | 0 | 184 | 5432516 | 1 | 28 | 0 | 1 | 192 |
| 102 | 66 | 73 | 49 | 1 | 29 | 660288 | 5282300 | 1 | 29 | 0 | 184 | 5432708 | 1 | 29 | 0 | 1 | 192 |
| 103 | 67 | 73 | 49 | 1 | 30 | 660311 | 5282484 | 1 | 30 | 0 | 184 | 5432900 | 1 | 30 | 0 | 1 | 192 |
| 104 | 68 | 73 | 49 | 1 | 31 | 660334 | 5282668 | 1 | 31 | 0 | 184 | 5433092 | 1 | 31 | 0 | 1 | 192 |
| 105 | 69 | 73 | 49 | 1 | 32 | 660357 | 5282852 | 1 | 32 | 0 | 184 | 5433284 | 1 | 32 | 0 | 1 | 192 |
| 106 | 6A | 73 | 49 | 1 | 33 | 660380 | 5283036 | 1 | 33 | 0 | 184 | 5433476 | 1 | 33 | 0 | 1 | 192 |
| 107 | 6B | 73 | 49 | 1 | 34 | 660403 | 5283220 | 1 | 34 | 0 | 184 | 5433668 | 1 | 34 | 0 | 1 | 192 |
| 108 | 6C | 73 | 49 | 1 | 35 | 660426 | 5283404 | 1 | 35 | 0 | 184 | 5433860 | 1 | 35 | 0 | 1 | 192 |
| 109 | 6D | 73 | 49 | 1 | 36 | 660449 | 5283588 | 1 | 36 | 0 | 184 | 5434052 | 1 | 36 | 0 | 1 | 192 |
| 110 | 6E | 73 | 49 | 1 | 37 | 660472 | 5283772 | 1 | 37 | 0 | 184 | 5434244 | 1 | 37 | 0 | 1 | 192 |
| 111 | 6F | 73 | 49 | 1 | 38 | 660495 | 5283956 | 1 | 38 | 0 | 184 | 5434436 | 1 | 38 | 0 | 1 | 192 |
| 112 | 70 | 73 | 49 | 1 | 39 | 660518 | 5284140 | 1 | 39 | 0 | 184 | 5434628 | 1 | 39 | 0 | 1 | 192 |
| 113 | 71 | 73 | 49 | 1 | 40 | 660541 | 5284324 | 1 | 40 | 0 | 184 | 5434820 | 1 | 40 | 0 | 1 | 192 |
| 114 | 72 | 73 | 49 | 1 | 41 | 660564 | 5284508 | 1 | 41 | 0 | 184 | 5435012 | 1 | 41 | 0 | 1 | 192 |
| 115 | 73 | 73 | 49 | 1 | 42 | 660587 | 5284692 | 1 | 42 | 0 | 184 | 5435204 | 1 | 42 | 0 | 1 | 192 |
| 116 | 74 | 73 | 49 | 1 | 43 | 660610 | 5284876 | 1 | 43 | 0 | 184 | 5435396 | 1 | 43 | 0 | 1 | 192 |
| 117 | 75 | 73 | 49 | 1 | 44 | 660633 | 5285060 | 1 | 44 | 0 | 184 | 5435588 | 1 | 44 | 0 | 1 | 192 |
| 118 | 76 | 73 | 49 | 1 | 45 | 660656 | 5285244 | 1 | 45 | 0 | 184 | 5435780 | 1 | 45 | 0 | 1 | 192 |
| 119 | 77 | 73 | 49 | 1 | 46 | 660679 | 5285428 | 1 | 46 | 0 | 184 | 5435972 | 1 | 46 | 0 | 1 | 192 |
| 120 | 78 | 73 | 49 | 1 | 47 | 660702 | 5285612 | 1 | 47 | 0 | 184 | 5436164 | 1 | 47 | 0 | 1 | 192 |
| 121 | 79 | 73 | 49 | 1 | 48 | 660725 | 5285796 | 1 | 48 | 0 | 184 | 5436356 | 1 | 48 | 0 | 1 | 192 |
| 122 | 7A | 73 | 49 | 1 | 49 | 660748 | 5285980 | 1 | 49 | 0 | 184 | 5436548 | 1 | 49 | 0 | 1 | 192 |
| 123 | 7B | 73 | 49 | 1 | 50 | 660771 | 5286164 | 1 | 50 | 0 | 184 | 5436740 | 1 | 50 | 0 | 1 | 192 |
| 124 | 7C | 73 | 49 | 1 | 51 | 660794 | 5286348 | 1 | 51 | 0 | 184 | 5436932 | 1 | 51 | 0 | 1 | 192 |
| 125 | 7D | 73 | 49 | 1 | 52 | 660817 | 5286532 | 1 | 52 | 0 | 184 | 5437124 | 1 | 52 | 0 | 1 | 192 |
| 126 | 7E | 73 | 49 | 1 | 53 | 660840 | 5286716 | 1 | 53 | 0 | 184 | 5437316 | 1 | 53 | 0 | 1 | 192 |
| 127 | 7F | 73 | 49 | 1 | 54 | 660863 | 5286900 | 1 | 54 | 0 | 184 | 5437508 | 1 | 54 | 0 | 1 | 192 |
| 128 | 80 | 73 | 49 | 1 | 55 | 660886 | 5287084 | 1 | 55 | 0 | 184 | 5437700 | 1 | 55 | 0 | 1 | 192 |
| 129 | 81 | 73 | 49 | 1 | 56 | 660909 | 5287268 | 1 | 56 | 0 | 184 | 5437892 | 1 | 56 | 0 | 1 | 192 |
| 130 | 82 | 73 | 49 | 1 | 57 | 660932 | 5287452 | 1 | 57 | 0 | 184 | 5438084 | 1 | 57 | 0 | 1 | 192 |
| 131 | 83 | 73 | 49 | 1 | 58 | 660955 | 5287636 | 1 | 58 | 0 | 184 | 5438276 | 1 | 58 | 0 | 1 | 192 |
| 132 | 84 | 73 | 49 | 1 | 59 | 660978 | 5287820 | 1 | 59 | 0 | 184 | 5438468 | 1 | 59 | 0 | 1 | 192 |
| 133 | 85 | 73 | 49 | 1 | 60 | 661001 | 5288004 | 1 | 60 | 0 | 184 | 5438660 | 1 | 60 | 0 | 1 | 192 |
| 134 | 86 | 73 | 49 | 1 | 61 | 661024 | 5288188 | 1 | 61 | 0 | 184 | 5438852 | 1 | 61 | 0 | 1 | 192 |
| 135 | 87 | 73 | 49 | 1 | 62 | 661047 | 5288372 | 1 | 62 | 0 | 184 | 5439044 | 1 | 62 | 0 | 1 | 192 |
| 136 | 88 | 73 | 49 | 1 | 63 | 661070 | 5288556 | 1 | 63 | 0 | 184 | 5439236 | 1 | 63 | 0 | 1 | 192 |
| 137 | 89 | 73 | 49 | 1 | 64 | 661093 | 5288740 | 1 | 64 | 0 | 184 | 5439428 | 1 | 64 | 0 | 1 | 192 |
| 138 | 8A | 73 | 49 | 1 | 65 | 661116 | 5288924 | 1 | 65 | 0 | 184 | 5439620 | 1 | 65 | 0 | 1 | 192 |
| 139 | 8B | 73 | 49 | 1 | 66 | 661139 | 5289108 | 1 | 66 | 0 | 184 | 5439812 | 1 | 66 | 0 | 1 | 192 |
| 140 | 8C | 73 | 49 | 1 | 67 | 661162 | 5289292 | 1 | 67 | 0 | 184 | 5440004 | 1 | 67 | 0 | 1 | 192 |
| 141 | 8D | 73 | 49 | 1 | 68 | 661185 | 5289476 | 1 | 68 | 0 | 184 | 5440196 | 1 | 68 | 0 | 1 | 192 |
| 142 | 8E | 73 | 49 | 1 | 69 | 661208 | 5289660 | 1 | 69 | 0 | 184 | 5440388 | 1 | 69 | 0 | 1 | 192 |
| 143 | 8F | 73 | 49 | 1 | 70 | 661231 | 5289844 | 1 | 70 | 0 | 184 | 5440580 | 1 | 70 | 0 | 1 | 192 |
| 144 | 90 | 73 | 49 | 1 | 71 | 661254 | 5290028 | 1 | 71 | 0 | 184 | 5440772 | 1 | 71 | 0 | 1 | 192 |
| 145 | 91 | 73 | 49 | 1 | 72 | 661277 | 5290212 | 1 | 72 | 0 | 184 | 5440964 | 1 | 72 | 0 | 1 | 192 |
| 146 | 92 | 73 | 49 | 2 | 0 | 661298 | 5290380 | 2 | 0 | 0 | 168 | 5441140 | 2 | 0 | 0 | 1 | 176 |
| 147 | 93 | 73 | 49 | 2 | 1 | 661324 | 5290588 | 2 | 1 | 0 | 208 | 5441356 | 2 | 1 | 0 | 1 | 216 |
| 148 | 94 | 73 | 49 | 2 | 2 | 661350 | 5290796 | 2 | 2 | 0 | 208 | 5441572 | 2 | 2 | 0 | 1 | 216 |
| 149 | 95 | 73 | 49 | 2 | 3 | 661376 | 5291004 | 2 | 3 | 0 | 208 | 5441788 | 2 | 3 | 0 | 1 | 216 |
| 150 | 96 | 73 | 49 | 2 | 4 | 661402 | 5291212 | 2 | 4 | 0 | 208 | 5442004 | 2 | 4 | 0 | 1 | 216 |
| 151 | 97 | 73 | 49 | 2 | 5 | 661428 | 5291420 | 2 | 5 | 0 | 208 | 5442220 | 2 | 5 | 0 | 1 | 216 |
| 152 | 98 | 73 | 49 | 2 | 6 | 661454 | 5291628 | 2 | 6 | 0 | 208 | 5442436 | 2 | 6 | 0 | 1 | 216 |
| 153 | 99 | 73 | 49 | 2 | 7 | 661480 | 5291836 | 2 | 7 | 0 | 208 | 5442652 | 2 | 7 | 0 | 1 | 216 |
| 154 | 9A | 73 | 49 | 2 | 8 | 661506 | 5292044 | 2 | 8 | 0 | 208 | 5442868 | 2 | 8 | 0 | 1 | 216 |
| 155 | 9B | 73 | 49 | 2 | 9 | 661532 | 5292252 | 2 | 9 | 0 | 208 | 5443084 | 2 | 9 | 0 | 1 | 216 |
| 156 | 9C | 73 | 49 | 2 | 10 | 661558 | 5292460 | 2 | 10 | 0 | 208 | 5443300 | 2 | 10 | 0 | 1 | 216 |
| 157 | 9D | 73 | 49 | 2 | 11 | 661584 | 5292668 | 2 | 11 | 0 | 208 | 5443516 | 2 | 11 | 0 | 1 | 216 |

| | | | | | | | | | | | | | | | | | |
|---|---|---|---|---|---|---|---|---|---|---|---|---|---|---|---|---|---|
| 158 | 9E | 73 | 49 | 2 | 12 | 661610 | 5292876 | 2 | 12 | 0 | 208 | 5443732 | 2 | 12 | 0 | 1 | 216 |
| 159 | 9F | 73 | 49 | 2 | 13 | 661636 | 5293084 | 2 | 13 | 0 | 208 | 5443948 | 2 | 13 | 0 | 1 | 216 |
| 160 | A0 | 73 | 49 | 2 | 14 | 661659 | 5293268 | 2 | 14 | 0 | 184 | 5444140 | 2 | 14 | 0 | 1 | 192 |
| 161 | A1 | 73 | 49 | 2 | 15 | 661682 | 5293452 | 2 | 15 | 0 | 184 | 5444332 | 2 | 15 | 0 | 1 | 192 |
| 162 | A2 | 73 | 49 | 2 | 16 | 661705 | 5293636 | 2 | 16 | 0 | 184 | 5444524 | 2 | 16 | 0 | 1 | 192 |
| 163 | A3 | 73 | 49 | 2 | 17 | 661728 | 5293820 | 2 | 17 | 0 | 184 | 5444716 | 2 | 17 | 0 | 1 | 192 |
| 164 | A4 | 73 | 49 | 2 | 18 | 661751 | 5294004 | 2 | 18 | 0 | 184 | 5444908 | 2 | 18 | 0 | 1 | 192 |
| 165 | A5 | 73 | 49 | 2 | 19 | 661774 | 5294188 | 2 | 19 | 0 | 184 | 5445100 | 2 | 19 | 0 | 1 | 192 |
| 166 | A6 | 73 | 49 | 2 | 20 | 661797 | 5294372 | 2 | 20 | 0 | 184 | 5445292 | 2 | 20 | 0 | 1 | 192 |
| 167 | A7 | 73 | 49 | 2 | 21 | 661820 | 5294556 | 2 | 21 | 0 | 184 | 5445484 | 2 | 21 | 0 | 1 | 192 |
| 168 | A8 | 73 | 49 | 2 | 22 | 661843 | 5294740 | 2 | 22 | 0 | 184 | 5445676 | 2 | 22 | 0 | 1 | 192 |
| 169 | A9 | 73 | 49 | 2 | 23 | 661866 | 5294924 | 2 | 23 | 0 | 184 | 5445868 | 2 | 23 | 0 | 1 | 192 |
| 170 | AA | 73 | 49 | 2 | 24 | 661889 | 5295108 | 2 | 24 | 0 | 184 | 5446060 | 2 | 24 | 0 | 1 | 192 |
| 171 | AB | 73 | 49 | 2 | 25 | 661912 | 5295292 | 2 | 25 | 0 | 184 | 5446252 | 2 | 25 | 0 | 1 | 192 |
| 172 | AC | 73 | 49 | 2 | 26 | 661935 | 5295476 | 2 | 26 | 0 | 184 | 5446444 | 2 | 26 | 0 | 1 | 192 |
| 173 | AD | 73 | 49 | 2 | 27 | 661958 | 5295660 | 2 | 27 | 0 | 184 | 5446636 | 2 | 27 | 0 | 1 | 192 |
| 174 | AE | 73 | 49 | 2 | 28 | 661981 | 5295844 | 2 | 28 | 0 | 184 | 5446828 | 2 | 28 | 0 | 1 | 192 |
| 175 | AF | 73 | 49 | 2 | 29 | 662004 | 5296028 | 2 | 29 | 0 | 184 | 5447020 | 2 | 29 | 0 | 1 | 192 |
| 176 | B0 | 73 | 49 | 2 | 30 | 662029 | 5296228 | 2 | 30 | 0 | 200 | 5447228 | 2 | 30 | 0 | 1 | 208 |
| 177 | B1 | 73 | 49 | 2 | 31 | 662054 | 5296428 | 2 | 31 | 0 | 200 | 5447436 | 2 | 31 | 0 | 1 | 208 |
| 178 | B2 | 73 | 49 | 2 | 32 | 662079 | 5296628 | 2 | 32 | 0 | 200 | 5447644 | 2 | 32 | 0 | 1 | 208 |
| 179 | B3 | 73 | 49 | 2 | 33 | 662104 | 5296828 | 2 | 33 | 0 | 200 | 5447852 | 2 | 33 | 0 | 1 | 208 |
| 180 | B4 | 73 | 49 | 2 | 34 | 662129 | 5297028 | 2 | 34 | 0 | 200 | 5448060 | 2 | 34 | 0 | 1 | 208 |
| 181 | B5 | 73 | 49 | 2 | 35 | 662154 | 5297228 | 2 | 35 | 0 | 200 | 5448268 | 2 | 35 | 0 | 1 | 208 |
| 182 | B6 | 73 | 49 | 2 | 36 | 662179 | 5297428 | 2 | 36 | 0 | 200 | 5448476 | 2 | 36 | 0 | 1 | 208 |
| 183 | B7 | 73 | 49 | 2 | 37 | 662204 | 5297628 | 2 | 37 | 0 | 200 | 5448684 | 2 | 37 | 0 | 1 | 208 |
| 184 | B8 | 73 | 49 | 2 | 38 | 662229 | 5297828 | 2 | 38 | 0 | 200 | 5448892 | 2 | 38 | 0 | 1 | 208 |
| 185 | B9 | 73 | 49 | 2 | 39 | 662254 | 5298028 | 2 | 39 | 0 | 200 | 5449100 | 2 | 39 | 0 | 1 | 208 |
| 186 | BA | 73 | 49 | 2 | 40 | 662279 | 5298228 | 2 | 40 | 0 | 200 | 5449308 | 2 | 40 | 0 | 1 | 208 |
| 187 | BB | 73 | 49 | 2 | 41 | 662304 | 5298428 | 2 | 41 | 0 | 200 | 5449516 | 2 | 41 | 0 | 1 | 208 |
| 188 | BC | 73 | 49 | 2 | 42 | 662329 | 5298628 | 2 | 42 | 0 | 200 | 5449724 | 2 | 42 | 0 | 1 | 208 |
| 189 | BD | 73 | 49 | 2 | 43 | 662354 | 5298828 | 2 | 43 | 0 | 200 | 5449932 | 2 | 43 | 0 | 1 | 208 |
| 190 | BE | 73 | 49 | 2 | 44 | 662379 | 5299028 | 2 | 44 | 0 | 200 | 5450140 | 2 | 44 | 0 | 1 | 208 |
| 191 | BF | 73 | 49 | 2 | 45 | 662404 | 5299228 | 2 | 45 | 0 | 200 | 5450348 | 2 | 45 | 0 | 1 | 208 |
| 192 | C0 | 73 | 49 | 2 | 46 | 662429 | 5299428 | 2 | 46 | 0 | 200 | 5450556 | 2 | 46 | 0 | 1 | 208 |
| 193 | C1 | 73 | 49 | 2 | 47 | 662454 | 5299628 | 2 | 47 | 0 | 200 | 5450764 | 2 | 47 | 0 | 1 | 208 |
| 194 | C2 | 73 | 49 | 2 | 48 | 662479 | 5299828 | 2 | 48 | 0 | 200 | 5450972 | 2 | 48 | 0 | 1 | 208 |
| 195 | C3 | 73 | 49 | 2 | 49 | 662504 | 5300028 | 2 | 49 | 0 | 200 | 5451180 | 2 | 49 | 0 | 1 | 208 |
| 196 | C4 | 73 | 49 | 2 | 50 | 662529 | 5300228 | 2 | 50 | 0 | 200 | 5451388 | 2 | 50 | 0 | 1 | 208 |
| 197 | C5 | 73 | 49 | 2 | 51 | 662554 | 5300428 | 2 | 51 | 0 | 200 | 5451596 | 2 | 51 | 0 | 1 | 208 |
| 198 | C6 | 73 | 49 | 2 | 52 | 662579 | 5300628 | 2 | 52 | 0 | 200 | 5451804 | 2 | 52 | 0 | 1 | 208 |
| 199 | C7 | 73 | 49 | 2 | 53 | 662604 | 5300828 | 2 | 53 | 0 | 200 | 5452012 | 2 | 53 | 0 | 1 | 208 |
| 200 | C8 | 73 | 49 | 2 | 54 | 662629 | 5301028 | 2 | 54 | 0 | 200 | 5452220 | 2 | 54 | 0 | 1 | 208 |
| 201 | C9 | 73 | 49 | 2 | 55 | 662654 | 5301228 | 2 | 55 | 0 | 200 | 5452428 | 2 | 55 | 0 | 1 | 208 |
| 202 | CA | 73 | 49 | 2 | 56 | 662679 | 5301428 | 2 | 56 | 0 | 200 | 5452636 | 2 | 56 | 0 | 1 | 208 |
| 203 | CB | 73 | 49 | 2 | 57 | 662704 | 5301628 | 2 | 57 | 0 | 200 | 5452844 | 2 | 57 | 0 | 1 | 208 |
| 204 | CC | 73 | 49 | 2 | 58 | 662729 | 5301828 | 2 | 58 | 0 | 200 | 5453052 | 2 | 58 | 0 | 1 | 208 |
| 205 | CD | 73 | 49 | 2 | 59 | 662754 | 5302028 | 2 | 59 | 0 | 200 | 5453260 | 2 | 59 | 0 | 1 | 208 |
| 206 | CE | 73 | 49 | 2 | 60 | 662779 | 5302228 | 2 | 60 | 0 | 200 | 5453468 | 2 | 60 | 0 | 1 | 208 |
| 207 | CF | 73 | 49 | 2 | 61 | 662804 | 5302428 | 2 | 61 | 0 | 200 | 5453676 | 2 | 61 | 0 | 1 | 208 |
| 208 | D0 | 73 | 49 | 2 | 62 | 662829 | 5302628 | 2 | 62 | 0 | 200 | 5453884 | 2 | 62 | 0 | 1 | 208 |
| 209 | D1 | 73 | 49 | 2 | 63 | 662854 | 5302828 | 2 | 63 | 0 | 200 | 5454092 | 2 | 63 | 0 | 1 | 208 |
| 210 | D2 | 73 | 49 | 2 | 64 | 662879 | 5303028 | 2 | 64 | 0 | 200 | 5454300 | 2 | 64 | 0 | 1 | 208 |
| 211 | D3 | 73 | 49 | 2 | 65 | 662904 | 5303228 | 2 | 65 | 0 | 200 | 5454508 | 2 | 65 | 0 | 1 | 208 |
| 212 | D4 | 73 | 49 | 2 | 66 | 662929 | 5303428 | 2 | 66 | 0 | 200 | 5454716 | 2 | 66 | 0 | 1 | 208 |
| 213 | D5 | 73 | 49 | 2 | 67 | 662954 | 5303628 | 2 | 67 | 0 | 200 | 5454924 | 2 | 67 | 0 | 1 | 208 |
| 214 | D6 | 73 | 49 | 2 | 68 | 662979 | 5303828 | 2 | 68 | 0 | 200 | 5455132 | 2 | 68 | 0 | 1 | 208 |
| 215 | D7 | 73 | 49 | 2 | 69 | 663004 | 5304028 | 2 | 69 | 0 | 200 | 5455340 | 2 | 69 | 0 | 1 | 208 |
| 216 | D8 | 73 | 49 | 2 | 70 | 663029 | 5304228 | 2 | 70 | 0 | 200 | 5455548 | 2 | 70 | 0 | 1 | 208 |
| 217 | D9 | 73 | 49 | 2 | 71 | 663054 | 5304428 | 2 | 71 | 0 | 200 | 5455756 | 2 | 71 | 0 | 1 | 208 |
| 218 | DA | 73 | 49 | 2 | 72 | 663079 | 5304628 | 2 | 72 | 0 | 200 | 5455964 | 2 | 72 | 0 | 1 | 208 |
| 219 | DB | 73 | 49 | 3 | 0 | 663102 | 5304812 | 3 | 0 | 0 | 184 | 5456156 | 3 | 0 | 0 | 1 | 192 |
| 220 | DC | 73 | 49 | 3 | 1 | 663130 | 5305036 | 3 | 1 | 0 | 224 | 5456388 | 3 | 1 | 0 | 1 | 232 |
| 221 | DD | 73 | 49 | 3 | 2 | 663158 | 5305260 | 3 | 2 | 0 | 224 | 5456620 | 3 | 2 | 0 | 1 | 232 |
| 222 | DE | 73 | 49 | 3 | 3 | 663186 | 5305484 | 3 | 3 | 0 | 224 | 5456852 | 3 | 3 | 0 | 1 | 232 |
| 223 | DF | 73 | 49 | 3 | 4 | 663214 | 5305708 | 3 | 4 | 0 | 224 | 5457084 | 3 | 4 | 0 | 1 | 232 |
| 224 | E0 | 73 | 49 | 3 | 5 | 663242 | 5305932 | 3 | 5 | 0 | 224 | 5457316 | 3 | 5 | 0 | 1 | 232 |
| 225 | E1 | 73 | 49 | 3 | 6 | 663270 | 5306156 | 3 | 6 | 0 | 224 | 5457548 | 3 | 6 | 0 | 1 | 232 |
| 226 | E2 | 73 | 49 | 3 | 7 | 663298 | 5306380 | 3 | 7 | 0 | 224 | 5457780 | 3 | 7 | 0 | 1 | 232 |
| 227 | E3 | 73 | 49 | 3 | 8 | 663326 | 5306604 | 3 | 8 | 0 | 224 | 5458012 | 3 | 8 | 0 | 1 | 232 |
| 228 | E4 | 73 | 49 | 3 | 9 | 663354 | 5306828 | 3 | 9 | 0 | 224 | 5458244 | 3 | 9 | 0 | 1 | 232 |
| 229 | E5 | 73 | 49 | 3 | 10 | 663382 | 5307052 | 3 | 10 | 0 | 224 | 5458476 | 3 | 10 | 0 | 1 | 232 |
| 230 | E6 | 73 | 49 | 3 | 11 | 663410 | 5307276 | 3 | 11 | 0 | 224 | 5458708 | 3 | 11 | 0 | 1 | 232 |
| 231 | E7 | 73 | 49 | 3 | 12 | 663438 | 5307500 | 3 | 12 | 0 | 224 | 5458940 | 3 | 12 | 0 | 1 | 232 |
| 232 | E8 | 73 | 49 | 3 | 13 | 663466 | 5307724 | 3 | 13 | 0 | 224 | 5459172 | 3 | 13 | 0 | 1 | 232 |
| 233 | E9 | 73 | 49 | 3 | 14 | 663494 | 5307948 | 3 | 14 | 0 | 224 | 5459404 | 3 | 14 | 0 | 1 | 232 |
| 234 | EA | 73 | 49 | 3 | 15 | 663522 | 5308172 | 3 | 15 | 0 | 224 | 5459636 | 3 | 15 | 0 | 1 | 232 |
| 235 | EB | 73 | 49 | 3 | 16 | 663550 | 5308396 | 3 | 16 | 0 | 224 | 5459868 | 3 | 16 | 0 | 1 | 232 |
| 236 | EC | 73 | 49 | 3 | 17 | 663578 | 5308620 | 3 | 17 | 0 | 224 | 5460100 | 3 | 17 | 0 | 1 | 232 |
| 237 | ED | 73 | 49 | 3 | 18 | 663606 | 5308844 | 3 | 18 | 0 | 224 | 5460332 | 3 | 18 | 0 | 1 | 232 |
| 238 | EE | 73 | 49 | 3 | 19 | 663634 | 5309068 | 3 | 19 | 0 | 224 | 5460564 | 3 | 19 | 0 | 1 | 232 |
| 239 | EF | 73 | 49 | 3 | 20 | 663662 | 5309292 | 3 | 20 | 0 | 224 | 5460796 | 3 | 20 | 0 | 1 | 232 |
| 240 | F0 | 73 | 49 | 3 | 21 | 663687 | 5309492 | 3 | 21 | 0 | 200 | 5461004 | 3 | 21 | 0 | 1 | 208 |
| 241 | F1 | 73 | 49 | 3 | 22 | 663712 | 5309692 | 3 | 22 | 0 | 200 | 5461212 | 3 | 22 | 0 | 1 | 208 |
| 242 | F2 | 73 | 49 | 3 | 23 | 663737 | 5309892 | 3 | 23 | 0 | 200 | 5461420 | 3 | 23 | 0 | 1 | 208 |
| 243 | F3 | 73 | 49 | 3 | 24 | 663762 | 5310092 | 3 | 24 | 0 | 200 | 5461628 | 3 | 24 | 0 | 1 | 208 |
| 244 | F4 | 73 | 49 | 3 | 25 | 663787 | 5310292 | 3 | 25 | 0 | 200 | 5461836 | 3 | 25 | 0 | 1 | 208 |
| 245 | F5 | 73 | 49 | 3 | 26 | 663812 | 5310492 | 3 | 26 | 0 | 200 | 5462044 | 3 | 26 | 0 | 1 | 208 |
| 246 | F6 | 73 | 49 | 3 | 27 | 663837 | 5310692 | 3 | 27 | 0 | 200 | 5462252 | 3 | 27 | 0 | 1 | 208 |
| 247 | F7 | 73 | 49 | 3 | 28 | 663862 | 5310892 | 3 | 28 | 0 | 200 | 5462460 | 3 | 28 | 0 | 1 | 208 |
| 248 | F8 | 73 | 49 | 3 | 29 | 663887 | 5311092 | 3 | 29 | 0 | 200 | 5462668 | 3 | 29 | 0 | 1 | 208 |
| 249 | F9 | 73 | 49 | 3 | 30 | 663912 | 5311292 | 3 | 30 | 0 | 200 | 5462876 | 3 | 30 | 0 | 1 | 208 |
| 250 | FA | 73 | 49 | 3 | 31 | 663937 | 5311492 | 3 | 31 | 0 | 200 | 5463084 | 3 | 31 | 0 | 1 | 208 |
| 251 | FB | 73 | 49 | 3 | 32 | 663962 | 5311692 | 3 | 32 | 0 | 200 | 5463292 | 3 | 32 | 0 | 1 | 208 |
| 252 | FC | 73 | 49 | 3 | 33 | 663987 | 5311892 | 3 | 33 | 0 | 200 | 5463500 | 3 | 33 | 0 | 1 | 208 |
| 253 | FD | 73 | 49 | 3 | 34 | 664012 | 5312092 | 3 | 34 | 0 | 200 | 5463708 | 3 | 34 | 0 | 1 | 208 |
| 254 | FE | 73 | 49 | 3 | 35 | 664037 | 5312292 | 3 | 35 | 0 | 200 | 5463916 | 3 | 35 | 0 | 1 | 208 |
| 255 | FF | 73 | 49 | 3 | 36 | 664062 | 5312492 | 3 | 36 | 0 | 200 | 5464124 | 3 | 36 | 0 | 1 | 208 |
| 0 | 00 | 74 | 4A | 0 | 0 | 664074 | 5312588 | 0 | 0 | 0 | 96 | 5464228 | 0 | 0 | 0 | 1 | 104 |
| 1 | 01 | 74 | 4A | 0 | 1 | 664088 | 5312700 | 0 | 1 | 0 | 112 | 5464348 | 0 | 1 | 0 | 1 | 120 |
| 2 | 02 | 74 | 4A | 0 | 2 | 664102 | 5312812 | 0 | 2 | 0 | 112 | 5464468 | 0 | 2 | 0 | 1 | 120 |
| 3 | 03 | 74 | 4A | 0 | 3 | 664116 | 5312924 | 0 | 3 | 0 | 112 | 5464588 | 0 | 3 | 0 | 1 | 120 |
| 4 | 04 | 74 | 4A | 0 | 4 | 664130 | 5313036 | 0 | 4 | 0 | 112 | 5464708 | 0 | 4 | 0 | 1 | 120 |

| | | | | | | | | | | | | | | | | |
|---|---|---|---|---|---|---|---|---|---|---|---|---|---|---|---|---|
| 5 | 05 | 74 | 4A | 0 | 5 | 664144 | 5313148 | 0 | 5 | 0 | 112 | 5464828 | 0 | 5 | 0 | 1 | 120 |
| 6 | 06 | 74 | 4A | 0 | 6 | 664158 | 5313260 | 0 | 6 | 0 | 112 | 5464948 | 0 | 6 | 0 | 1 | 120 |
| 7 | 07 | 74 | 4A | 0 | 7 | 664172 | 5313372 | 0 | 7 | 0 | 112 | 5465068 | 0 | 7 | 0 | 1 | 120 |
| 8 | 08 | 74 | 4A | 0 | 8 | 664186 | 5313484 | 0 | 8 | 0 | 112 | 5465188 | 0 | 8 | 0 | 1 | 120 |
| 9 | 09 | 74 | 4A | 0 | 9 | 664200 | 5313596 | 0 | 9 | 0 | 112 | 5465308 | 0 | 9 | 0 | 1 | 120 |
| 10 | 0A | 74 | 4A | 0 | 10 | 664214 | 5313708 | 0 | 10 | 0 | 112 | 5465428 | 0 | 10 | 0 | 1 | 120 |
| 11 | 0B | 74 | 4A | 0 | 11 | 664228 | 5313820 | 0 | 11 | 0 | 112 | 5465548 | 0 | 11 | 0 | 1 | 120 |
| 12 | 0C | 74 | 4A | 0 | 12 | 664242 | 5313932 | 0 | 12 | 0 | 112 | 5465668 | 0 | 12 | 0 | 1 | 120 |
| 13 | 0D | 74 | 4A | 0 | 13 | 664256 | 5314044 | 0 | 13 | 0 | 112 | 5465788 | 0 | 13 | 0 | 1 | 120 |
| 14 | 0E | 74 | 4A | 0 | 14 | 664270 | 5314156 | 0 | 14 | 0 | 112 | 5465908 | 0 | 14 | 0 | 1 | 120 |
| 15 | 0F | 74 | 4A | 0 | 15 | 664284 | 5314268 | 0 | 15 | 0 | 112 | 5466028 | 0 | 15 | 0 | 1 | 120 |
| 16 | 10 | 74 | 4A | 0 | 16 | 664298 | 5314380 | 0 | 16 | 0 | 112 | 5466148 | 0 | 16 | 0 | 1 | 120 |
| 17 | 11 | 74 | 4A | 0 | 17 | 664312 | 5314492 | 0 | 17 | 0 | 112 | 5466268 | 0 | 17 | 0 | 1 | 120 |
| 18 | 12 | 74 | 4A | 0 | 18 | 664326 | 5314604 | 0 | 18 | 0 | 112 | 5466388 | 0 | 18 | 0 | 1 | 120 |
| 19 | 13 | 74 | 4A | 0 | 19 | 664340 | 5314716 | 0 | 19 | 0 | 112 | 5466508 | 0 | 19 | 0 | 1 | 120 |
| 20 | 14 | 74 | 4A | 0 | 20 | 664354 | 5314828 | 0 | 20 | 0 | 112 | 5466628 | 0 | 20 | 0 | 1 | 120 |
| 21 | 15 | 74 | 4A | 0 | 21 | 664368 | 5314940 | 0 | 21 | 0 | 112 | 5466748 | 0 | 21 | 0 | 1 | 120 |
| 22 | 16 | 74 | 4A | 0 | 22 | 664382 | 5315052 | 0 | 22 | 0 | 112 | 5466868 | 0 | 22 | 0 | 1 | 120 |
| 23 | 17 | 74 | 4A | 0 | 23 | 664396 | 5315164 | 0 | 23 | 0 | 112 | 5466988 | 0 | 23 | 0 | 1 | 120 |
| 24 | 18 | 74 | 4A | 0 | 24 | 664410 | 5315276 | 0 | 24 | 0 | 112 | 5467108 | 0 | 24 | 0 | 1 | 120 |
| 25 | 19 | 74 | 4A | 0 | 25 | 664424 | 5315388 | 0 | 25 | 0 | 112 | 5467228 | 0 | 25 | 0 | 1 | 120 |
| 26 | 1A | 74 | 4A | 0 | 26 | 664438 | 5315500 | 0 | 26 | 0 | 112 | 5467348 | 0 | 26 | 0 | 1 | 120 |
| 27 | 1B | 74 | 4A | 0 | 27 | 664452 | 5315612 | 0 | 27 | 0 | 112 | 5467468 | 0 | 27 | 0 | 1 | 120 |
| 28 | 1C | 74 | 4A | 0 | 28 | 664466 | 5315724 | 0 | 28 | 0 | 112 | 5467588 | 0 | 28 | 0 | 1 | 120 |
| 29 | 1D | 74 | 4A | 0 | 29 | 664480 | 5315836 | 0 | 29 | 0 | 112 | 5467708 | 0 | 29 | 0 | 1 | 120 |
| 30 | 1E | 74 | 4A | 0 | 30 | 664494 | 5315948 | 0 | 30 | 0 | 112 | 5467828 | 0 | 30 | 0 | 1 | 120 |
| 31 | 1F | 74 | 4A | 0 | 31 | 664508 | 5316060 | 0 | 31 | 0 | 112 | 5467948 | 0 | 31 | 0 | 1 | 120 |
| 32 | 20 | 74 | 4A | 0 | 32 | 664522 | 5316172 | 0 | 32 | 0 | 112 | 5468068 | 0 | 32 | 0 | 1 | 120 |
| 33 | 21 | 74 | 4A | 0 | 33 | 664536 | 5316284 | 0 | 33 | 0 | 112 | 5468188 | 0 | 33 | 0 | 1 | 120 |
| 34 | 22 | 74 | 4A | 0 | 34 | 664550 | 5316396 | 0 | 34 | 0 | 112 | 5468308 | 0 | 34 | 0 | 1 | 120 |
| 35 | 23 | 74 | 4A | 0 | 35 | 664564 | 5316508 | 0 | 35 | 0 | 112 | 5468428 | 0 | 35 | 0 | 1 | 120 |
| 36 | 24 | 74 | 4A | 0 | 36 | 664578 | 5316620 | 0 | 36 | 0 | 112 | 5468548 | 0 | 36 | 0 | 1 | 120 |
| 37 | 25 | 74 | 4A | 0 | 37 | 664592 | 5316732 | 0 | 37 | 0 | 112 | 5468668 | 0 | 37 | 0 | 1 | 120 |
| 38 | 26 | 74 | 4A | 0 | 38 | 664606 | 5316844 | 0 | 38 | 0 | 112 | 5468788 | 0 | 38 | 0 | 1 | 120 |
| 39 | 27 | 74 | 4A | 0 | 39 | 664620 | 5316956 | 0 | 39 | 0 | 112 | 5468908 | 0 | 39 | 0 | 1 | 120 |
| 40 | 28 | 74 | 4A | 0 | 40 | 664634 | 5317068 | 0 | 40 | 0 | 112 | 5469028 | 0 | 40 | 0 | 1 | 120 |
| 41 | 29 | 74 | 4A | 0 | 41 | 664648 | 5317180 | 0 | 41 | 0 | 112 | 5469148 | 0 | 41 | 0 | 1 | 120 |
| 42 | 2A | 74 | 4A | 0 | 42 | 664662 | 5317292 | 0 | 42 | 0 | 112 | 5469268 | 0 | 42 | 0 | 1 | 120 |
| 43 | 2B | 74 | 4A | 0 | 43 | 664676 | 5317404 | 0 | 43 | 0 | 112 | 5469388 | 0 | 43 | 0 | 1 | 120 |
| 44 | 2C | 74 | 4A | 0 | 44 | 664690 | 5317516 | 0 | 44 | 0 | 112 | 5469508 | 0 | 44 | 0 | 1 | 120 |
| 45 | 2D | 74 | 4A | 0 | 45 | 664704 | 5317628 | 0 | 45 | 0 | 112 | 5469628 | 0 | 45 | 0 | 1 | 120 |
| 46 | 2E | 74 | 4A | 0 | 46 | 664718 | 5317740 | 0 | 46 | 0 | 112 | 5469748 | 0 | 46 | 0 | 1 | 120 |
| 47 | 2F | 74 | 4A | 0 | 47 | 664732 | 5317852 | 0 | 47 | 0 | 112 | 5469868 | 0 | 47 | 0 | 1 | 120 |
| 48 | 30 | 74 | 4A | 0 | 48 | 664746 | 5317964 | 0 | 48 | 0 | 112 | 5469988 | 0 | 48 | 0 | 1 | 120 |
| 49 | 31 | 74 | 4A | 0 | 49 | 664760 | 5318076 | 0 | 49 | 0 | 112 | 5470108 | 0 | 49 | 0 | 1 | 120 |
| 50 | 32 | 74 | 4A | 0 | 50 | 664774 | 5318188 | 0 | 50 | 0 | 112 | 5470228 | 0 | 50 | 0 | 1 | 120 |
| 51 | 33 | 74 | 4A | 0 | 51 | 664788 | 5318300 | 0 | 51 | 0 | 112 | 5470348 | 0 | 51 | 0 | 1 | 120 |
| 52 | 34 | 74 | 4A | 0 | 52 | 664802 | 5318412 | 0 | 52 | 0 | 112 | 5470468 | 0 | 52 | 0 | 1 | 120 |
| 53 | 35 | 74 | 4A | 0 | 53 | 664816 | 5318524 | 0 | 53 | 0 | 112 | 5470588 | 0 | 53 | 0 | 1 | 120 |
| 54 | 36 | 74 | 4A | 0 | 54 | 664830 | 5318636 | 0 | 54 | 0 | 112 | 5470708 | 0 | 54 | 0 | 1 | 120 |
| 55 | 37 | 74 | 4A | 0 | 55 | 664844 | 5318748 | 0 | 55 | 0 | 112 | 5470828 | 0 | 55 | 0 | 1 | 120 |
| 56 | 38 | 74 | 4A | 0 | 56 | 664858 | 5318860 | 0 | 56 | 0 | 112 | 5470948 | 0 | 56 | 0 | 1 | 120 |
| 57 | 39 | 74 | 4A | 0 | 57 | 664872 | 5318972 | 0 | 57 | 0 | 112 | 5471068 | 0 | 57 | 0 | 1 | 120 |
| 58 | 3A | 74 | 4A | 0 | 58 | 664886 | 5319084 | 0 | 58 | 0 | 112 | 5471188 | 0 | 58 | 0 | 1 | 120 |
| 59 | 3B | 74 | 4A | 0 | 59 | 664900 | 5319196 | 0 | 59 | 0 | 112 | 5471308 | 0 | 59 | 0 | 1 | 120 |
| 60 | 3C | 74 | 4A | 0 | 60 | 664914 | 5319308 | 0 | 60 | 0 | 112 | 5471428 | 0 | 60 | 0 | 1 | 120 |
| 61 | 3D | 74 | 4A | 0 | 61 | 664928 | 5319420 | 0 | 61 | 0 | 112 | 5471548 | 0 | 61 | 0 | 1 | 120 |
| 62 | 3E | 74 | 4A | 0 | 62 | 664942 | 5319532 | 0 | 62 | 0 | 112 | 5471668 | 0 | 62 | 0 | 1 | 120 |
| 63 | 3F | 74 | 4A | 0 | 63 | 664956 | 5319644 | 0 | 63 | 0 | 112 | 5471788 | 0 | 63 | 0 | 1 | 120 |
| 64 | 40 | 74 | 4A | 0 | 64 | 664970 | 5319756 | 0 | 64 | 0 | 112 | 5471908 | 0 | 64 | 0 | 1 | 120 |
| 65 | 41 | 74 | 4A | 0 | 65 | 664984 | 5319868 | 0 | 65 | 0 | 112 | 5472028 | 0 | 65 | 0 | 1 | 120 |
| 66 | 42 | 74 | 4A | 0 | 66 | 664998 | 5319980 | 0 | 66 | 0 | 112 | 5472148 | 0 | 66 | 0 | 1 | 120 |
| 67 | 43 | 74 | 4A | 0 | 67 | 665012 | 5320092 | 0 | 67 | 0 | 112 | 5472268 | 0 | 67 | 0 | 1 | 120 |
| 68 | 44 | 74 | 4A | 0 | 68 | 665026 | 5320204 | 0 | 68 | 0 | 112 | 5472388 | 0 | 68 | 0 | 1 | 120 |
| 69 | 45 | 74 | 4A | 0 | 69 | 665040 | 5320316 | 0 | 69 | 0 | 112 | 5472508 | 0 | 69 | 0 | 1 | 120 |
| 70 | 46 | 74 | 4A | 0 | 70 | 665054 | 5320428 | 0 | 70 | 0 | 112 | 5472628 | 0 | 70 | 0 | 1 | 120 |
| 71 | 47 | 74 | 4A | 0 | 71 | 665068 | 5320540 | 0 | 71 | 0 | 112 | 5472748 | 0 | 71 | 0 | 1 | 120 |
| 72 | 48 | 74 | 4A | 0 | 72 | 665082 | 5320652 | 0 | 72 | 0 | 112 | 5472868 | 0 | 72 | 0 | 1 | 120 |
| 73 | 49 | 74 | 4A | 0 | 73 | 665096 | 5320764 | 0 | 73 | 0 | 112 | 5472988 | 0 | 73 | 0 | 1 | 120 |
| 74 | 4A | 74 | 4A | 1 | 0 | 665108 | 5320860 | 1 | 0 | 0 | 96 | 5473092 | 1 | 0 | 0 | 1 | 104 |
| 75 | 4B | 74 | 4A | 1 | 1 | 665125 | 5320996 | 1 | 1 | 0 | 136 | 5473236 | 1 | 1 | 0 | 1 | 144 |
| 76 | 4C | 74 | 4A | 1 | 2 | 665142 | 5321132 | 1 | 2 | 0 | 136 | 5473380 | 1 | 2 | 0 | 1 | 144 |
| 77 | 4D | 74 | 4A | 1 | 3 | 665159 | 5321268 | 1 | 3 | 0 | 136 | 5473524 | 1 | 3 | 0 | 1 | 144 |
| 78 | 4E | 74 | 4A | 1 | 4 | 665176 | 5321404 | 1 | 4 | 0 | 136 | 5473668 | 1 | 4 | 0 | 1 | 144 |
| 79 | 4F | 74 | 4A | 1 | 5 | 665193 | 5321540 | 1 | 5 | 0 | 136 | 5473812 | 1 | 5 | 0 | 1 | 144 |
| 80 | 50 | 74 | 4A | 1 | 6 | 665214 | 5321708 | 1 | 6 | 0 | 168 | 5473988 | 1 | 6 | 0 | 1 | 176 |
| 81 | 51 | 74 | 4A | 1 | 7 | 665235 | 5321876 | 1 | 7 | 0 | 168 | 5474164 | 1 | 7 | 0 | 1 | 176 |
| 82 | 52 | 74 | 4A | 1 | 8 | 665256 | 5322044 | 1 | 8 | 0 | 168 | 5474340 | 1 | 8 | 0 | 1 | 176 |
| 83 | 53 | 74 | 4A | 1 | 9 | 665277 | 5322212 | 1 | 9 | 0 | 168 | 5474516 | 1 | 9 | 0 | 1 | 176 |
| 84 | 54 | 74 | 4A | 1 | 10 | 665298 | 5322380 | 1 | 10 | 0 | 168 | 5474692 | 1 | 10 | 0 | 1 | 176 |
| 85 | 55 | 74 | 4A | 1 | 11 | 665319 | 5322548 | 1 | 11 | 0 | 168 | 5474868 | 1 | 11 | 0 | 1 | 176 |
| 86 | 56 | 74 | 4A | 1 | 12 | 665340 | 5322716 | 1 | 12 | 0 | 168 | 5475044 | 1 | 12 | 0 | 1 | 176 |
| 87 | 57 | 74 | 4A | 1 | 13 | 665361 | 5322884 | 1 | 13 | 0 | 168 | 5475220 | 1 | 13 | 0 | 1 | 176 |
| 88 | 58 | 74 | 4A | 1 | 14 | 665382 | 5323052 | 1 | 14 | 0 | 168 | 5475396 | 1 | 14 | 0 | 1 | 176 |
| 89 | 59 | 74 | 4A | 1 | 15 | 665403 | 5323220 | 1 | 15 | 0 | 168 | 5475572 | 1 | 15 | 0 | 1 | 176 |
| 90 | 5A | 74 | 4A | 1 | 16 | 665424 | 5323388 | 1 | 16 | 0 | 168 | 5475748 | 1 | 16 | 0 | 1 | 176 |
| 91 | 5B | 74 | 4A | 1 | 17 | 665445 | 5323556 | 1 | 17 | 0 | 168 | 5475924 | 1 | 17 | 0 | 1 | 176 |
| 92 | 5C | 74 | 4A | 1 | 18 | 665466 | 5323724 | 1 | 18 | 0 | 168 | 5476100 | 1 | 18 | 0 | 1 | 176 |
| 93 | 5D | 74 | 4A | 1 | 19 | 665487 | 5323892 | 1 | 19 | 0 | 168 | 5476276 | 1 | 19 | 0 | 1 | 176 |
| 94 | 5E | 74 | 4A | 1 | 20 | 665508 | 5324060 | 1 | 20 | 0 | 168 | 5476452 | 1 | 20 | 0 | 1 | 176 |
| 95 | 5F | 74 | 4A | 1 | 21 | 665529 | 5324228 | 1 | 21 | 0 | 168 | 5476628 | 1 | 21 | 0 | 1 | 176 |
| 96 | 60 | 74 | 4A | 1 | 22 | 665552 | 5324412 | 1 | 22 | 0 | 184 | 5476820 | 1 | 22 | 0 | 1 | 192 |
| 97 | 61 | 74 | 4A | 1 | 23 | 665575 | 5324596 | 1 | 23 | 0 | 184 | 5477012 | 1 | 23 | 0 | 1 | 192 |
| 98 | 62 | 74 | 4A | 1 | 24 | 665598 | 5324780 | 1 | 24 | 0 | 184 | 5477204 | 1 | 24 | 0 | 1 | 192 |
| 99 | 63 | 74 | 4A | 1 | 25 | 665621 | 5324964 | 1 | 25 | 0 | 184 | 5477396 | 1 | 25 | 0 | 1 | 192 |
| 100 | 64 | 74 | 4A | 1 | 26 | 665644 | 5325148 | 1 | 26 | 0 | 184 | 5477588 | 1 | 26 | 0 | 1 | 192 |
| 101 | 65 | 74 | 4A | 1 | 27 | 665667 | 5325332 | 1 | 27 | 0 | 184 | 5477780 | 1 | 27 | 0 | 1 | 192 |
| 102 | 66 | 74 | 4A | 1 | 28 | 665690 | 5325516 | 1 | 28 | 0 | 184 | 5477972 | 1 | 28 | 0 | 1 | 192 |
| 103 | 67 | 74 | 4A | 1 | 29 | 665713 | 5325700 | 1 | 29 | 0 | 184 | 5478164 | 1 | 29 | 0 | 1 | 192 |
| 104 | 68 | 74 | 4A | 1 | 30 | 665736 | 5325884 | 1 | 30 | 0 | 184 | 5478356 | 1 | 30 | 0 | 1 | 192 |
| 105 | 69 | 74 | 4A | 1 | 31 | 665759 | 5326068 | 1 | 31 | 0 | 184 | 5478548 | 1 | 31 | 0 | 1 | 192 |
| 106 | 6A | 74 | 4A | 1 | 32 | 665782 | 5326252 | 1 | 32 | 0 | 184 | 5478740 | 1 | 32 | 0 | 1 | 192 |
| 107 | 6B | 74 | 4A | 1 | 33 | 665805 | 5326436 | 1 | 33 | 0 | 184 | 5478932 | 1 | 33 | 0 | 1 | 192 |

| | | | | | | | | | | | | | | | | | |
|---|---|---|---|---|---|---|---|---|---|---|---|---|---|---|---|---|---|
| 108 | 6C | 74 | 4A | 1 | 34 | 665828 | 5326620 | 1 | 34 | 0 | 184 | 5479124 | 1 | 34 | 0 | 1 | 192 |
| 109 | 6D | 74 | 4A | 1 | 35 | 665851 | 5326804 | 1 | 35 | 0 | 184 | 5479316 | 1 | 35 | 0 | 1 | 192 |
| 110 | 6E | 74 | 4A | 1 | 36 | 665874 | 5326988 | 1 | 36 | 0 | 184 | 5479508 | 1 | 36 | 0 | 1 | 192 |
| 111 | 6F | 74 | 4A | 1 | 37 | 665897 | 5327172 | 1 | 37 | 0 | 184 | 5479700 | 1 | 37 | 0 | 1 | 192 |
| 112 | 70 | 74 | 4A | 1 | 38 | 665920 | 5327356 | 1 | 38 | 0 | 184 | 5479892 | 1 | 38 | 0 | 1 | 192 |
| 113 | 71 | 74 | 4A | 1 | 39 | 665943 | 5327540 | 1 | 39 | 0 | 184 | 5480084 | 1 | 39 | 0 | 1 | 192 |
| 114 | 72 | 74 | 4A | 1 | 40 | 665966 | 5327724 | 1 | 40 | 0 | 184 | 5480276 | 1 | 40 | 0 | 1 | 192 |
| 115 | 73 | 74 | 4A | 1 | 41 | 665989 | 5327908 | 1 | 41 | 0 | 184 | 5480468 | 1 | 41 | 0 | 1 | 192 |
| 116 | 74 | 74 | 4A | 1 | 42 | 666012 | 5328092 | 1 | 42 | 0 | 184 | 5480660 | 1 | 42 | 0 | 1 | 192 |
| 117 | 75 | 74 | 4A | 1 | 43 | 666035 | 5328276 | 1 | 43 | 0 | 184 | 5480852 | 1 | 43 | 0 | 1 | 192 |
| 118 | 76 | 74 | 4A | 1 | 44 | 666058 | 5328460 | 1 | 44 | 0 | 184 | 5481044 | 1 | 44 | 0 | 1 | 192 |
| 119 | 77 | 74 | 4A | 1 | 45 | 666081 | 5328644 | 1 | 45 | 0 | 184 | 5481236 | 1 | 45 | 0 | 1 | 192 |
| 120 | 78 | 74 | 4A | 1 | 46 | 666104 | 5328828 | 1 | 46 | 0 | 184 | 5481428 | 1 | 46 | 0 | 1 | 192 |
| 121 | 79 | 74 | 4A | 1 | 47 | 666127 | 5329012 | 1 | 47 | 0 | 184 | 5481620 | 1 | 47 | 0 | 1 | 192 |
| 122 | 7A | 74 | 4A | 1 | 48 | 666150 | 5329196 | 1 | 48 | 0 | 184 | 5481812 | 1 | 48 | 0 | 1 | 192 |
| 123 | 7B | 74 | 4A | 1 | 49 | 666173 | 5329380 | 1 | 49 | 0 | 184 | 5482004 | 1 | 49 | 0 | 1 | 192 |
| 124 | 7C | 74 | 4A | 1 | 50 | 666196 | 5329564 | 1 | 50 | 0 | 184 | 5482196 | 1 | 50 | 0 | 1 | 192 |
| 125 | 7D | 74 | 4A | 1 | 51 | 666219 | 5329748 | 1 | 51 | 0 | 184 | 5482388 | 1 | 51 | 0 | 1 | 192 |
| 126 | 7E | 74 | 4A | 1 | 52 | 666242 | 5329932 | 1 | 52 | 0 | 184 | 5482580 | 1 | 52 | 0 | 1 | 192 |
| 127 | 7F | 74 | 4A | 1 | 53 | 666265 | 5330116 | 1 | 53 | 0 | 184 | 5482772 | 1 | 53 | 0 | 1 | 192 |
| 128 | 80 | 74 | 4A | 1 | 54 | 666288 | 5330300 | 1 | 54 | 0 | 184 | 5482964 | 1 | 54 | 0 | 1 | 192 |
| 129 | 81 | 74 | 4A | 1 | 55 | 666311 | 5330484 | 1 | 55 | 0 | 184 | 5483156 | 1 | 55 | 0 | 1 | 192 |
| 130 | 82 | 74 | 4A | 1 | 56 | 666334 | 5330668 | 1 | 56 | 0 | 184 | 5483348 | 1 | 56 | 0 | 1 | 192 |
| 131 | 83 | 74 | 4A | 1 | 57 | 666357 | 5330852 | 1 | 57 | 0 | 184 | 5483540 | 1 | 57 | 0 | 1 | 192 |
| 132 | 84 | 74 | 4A | 1 | 58 | 666380 | 5331036 | 1 | 58 | 0 | 184 | 5483732 | 1 | 58 | 0 | 1 | 192 |
| 133 | 85 | 74 | 4A | 1 | 59 | 666403 | 5331220 | 1 | 59 | 0 | 184 | 5483924 | 1 | 59 | 0 | 1 | 192 |
| 134 | 86 | 74 | 4A | 1 | 60 | 666426 | 5331404 | 1 | 60 | 0 | 184 | 5484116 | 1 | 60 | 0 | 1 | 192 |
| 135 | 87 | 74 | 4A | 1 | 61 | 666449 | 5331588 | 1 | 61 | 0 | 184 | 5484308 | 1 | 61 | 0 | 1 | 192 |
| 136 | 88 | 74 | 4A | 1 | 62 | 666472 | 5331772 | 1 | 62 | 0 | 184 | 5484500 | 1 | 62 | 0 | 1 | 192 |
| 137 | 89 | 74 | 4A | 1 | 63 | 666495 | 5331956 | 1 | 63 | 0 | 184 | 5484692 | 1 | 63 | 0 | 1 | 192 |
| 138 | 8A | 74 | 4A | 1 | 64 | 666518 | 5332140 | 1 | 64 | 0 | 184 | 5484884 | 1 | 64 | 0 | 1 | 192 |
| 139 | 8B | 74 | 4A | 1 | 65 | 666541 | 5332324 | 1 | 65 | 0 | 184 | 5485076 | 1 | 65 | 0 | 1 | 192 |
| 140 | 8C | 74 | 4A | 1 | 66 | 666564 | 5332508 | 1 | 66 | 0 | 184 | 5485268 | 1 | 66 | 0 | 1 | 192 |
| 141 | 8D | 74 | 4A | 1 | 67 | 666587 | 5332692 | 1 | 67 | 0 | 184 | 5485460 | 1 | 67 | 0 | 1 | 192 |
| 142 | 8E | 74 | 4A | 1 | 68 | 666610 | 5332876 | 1 | 68 | 0 | 184 | 5485652 | 1 | 68 | 0 | 1 | 192 |
| 143 | 8F | 74 | 4A | 1 | 69 | 666633 | 5333060 | 1 | 69 | 0 | 184 | 5485844 | 1 | 69 | 0 | 1 | 192 |
| 144 | 90 | 74 | 4A | 1 | 70 | 666656 | 5333244 | 1 | 70 | 0 | 184 | 5486036 | 1 | 70 | 0 | 1 | 192 |
| 145 | 91 | 74 | 4A | 1 | 71 | 666679 | 5333428 | 1 | 71 | 0 | 184 | 5486228 | 1 | 71 | 0 | 1 | 192 |
| 146 | 92 | 74 | 4A | 1 | 72 | 666702 | 5333612 | 1 | 72 | 0 | 184 | 5486420 | 1 | 72 | 0 | 1 | 192 |
| 147 | 93 | 74 | 4A | 1 | 73 | 666725 | 5333796 | 1 | 73 | 0 | 184 | 5486612 | 1 | 73 | 0 | 1 | 192 |
| 148 | 94 | 74 | 4A | 2 | 0 | 666746 | 5333964 | 2 | 0 | 0 | 168 | 5486788 | 2 | 0 | 0 | 1 | 176 |
| 149 | 95 | 74 | 4A | 2 | 1 | 666772 | 5334172 | 2 | 1 | 0 | 208 | 5487004 | 2 | 1 | 0 | 1 | 216 |
| 150 | 96 | 74 | 4A | 2 | 2 | 666798 | 5334380 | 2 | 2 | 0 | 208 | 5487220 | 2 | 2 | 0 | 1 | 216 |
| 151 | 97 | 74 | 4A | 2 | 3 | 666824 | 5334588 | 2 | 3 | 0 | 208 | 5487436 | 2 | 3 | 0 | 1 | 216 |
| 152 | 98 | 74 | 4A | 2 | 4 | 666850 | 5334796 | 2 | 4 | 0 | 208 | 5487652 | 2 | 4 | 0 | 1 | 216 |
| 153 | 99 | 74 | 4A | 2 | 5 | 666876 | 5335004 | 2 | 5 | 0 | 208 | 5487868 | 2 | 5 | 0 | 1 | 216 |
| 154 | 9A | 74 | 4A | 2 | 6 | 666902 | 5335212 | 2 | 6 | 0 | 208 | 5488084 | 2 | 6 | 0 | 1 | 216 |
| 155 | 9B | 74 | 4A | 2 | 7 | 666928 | 5335420 | 2 | 7 | 0 | 208 | 5488300 | 2 | 7 | 0 | 1 | 216 |
| 156 | 9C | 74 | 4A | 2 | 8 | 666954 | 5335628 | 2 | 8 | 0 | 208 | 5488516 | 2 | 8 | 0 | 1 | 216 |
| 157 | 9D | 74 | 4A | 2 | 9 | 666980 | 5335836 | 2 | 9 | 0 | 208 | 5488732 | 2 | 9 | 0 | 1 | 216 |
| 158 | 9E | 74 | 4A | 2 | 10 | 667006 | 5336044 | 2 | 10 | 0 | 208 | 5488948 | 2 | 10 | 0 | 1 | 216 |
| 159 | 9F | 74 | 4A | 2 | 11 | 667032 | 5336252 | 2 | 11 | 0 | 208 | 5489164 | 2 | 11 | 0 | 1 | 216 |
| 160 | A0 | 74 | 4A | 2 | 12 | 667055 | 5336436 | 2 | 12 | 0 | 184 | 5489356 | 2 | 12 | 0 | 1 | 192 |
| 161 | A1 | 74 | 4A | 2 | 13 | 667078 | 5336620 | 2 | 13 | 0 | 184 | 5489548 | 2 | 13 | 0 | 1 | 192 |
| 162 | A2 | 74 | 4A | 2 | 14 | 667101 | 5336804 | 2 | 14 | 0 | 184 | 5489740 | 2 | 14 | 0 | 1 | 192 |
| 163 | A3 | 74 | 4A | 2 | 15 | 667124 | 5336988 | 2 | 15 | 0 | 184 | 5489932 | 2 | 15 | 0 | 1 | 192 |
| 164 | A4 | 74 | 4A | 2 | 16 | 667147 | 5337172 | 2 | 16 | 0 | 184 | 5490124 | 2 | 16 | 0 | 1 | 192 |
| 165 | A5 | 74 | 4A | 2 | 17 | 667170 | 5337356 | 2 | 17 | 0 | 184 | 5490316 | 2 | 17 | 0 | 1 | 192 |
| 166 | A6 | 74 | 4A | 2 | 18 | 667193 | 5337540 | 2 | 18 | 0 | 184 | 5490508 | 2 | 18 | 0 | 1 | 192 |
| 167 | A7 | 74 | 4A | 2 | 19 | 667216 | 5337724 | 2 | 19 | 0 | 184 | 5490700 | 2 | 19 | 0 | 1 | 192 |
| 168 | A8 | 74 | 4A | 2 | 20 | 667239 | 5337908 | 2 | 20 | 0 | 184 | 5490892 | 2 | 20 | 0 | 1 | 192 |
| 169 | A9 | 74 | 4A | 2 | 21 | 667262 | 5338092 | 2 | 21 | 0 | 184 | 5491084 | 2 | 21 | 0 | 1 | 192 |
| 170 | AA | 74 | 4A | 2 | 22 | 667285 | 5338276 | 2 | 22 | 0 | 184 | 5491276 | 2 | 22 | 0 | 1 | 192 |
| 171 | AB | 74 | 4A | 2 | 23 | 667308 | 5338460 | 2 | 23 | 0 | 184 | 5491468 | 2 | 23 | 0 | 1 | 192 |
| 172 | AC | 74 | 4A | 2 | 24 | 667331 | 5338644 | 2 | 24 | 0 | 184 | 5491660 | 2 | 24 | 0 | 1 | 192 |
| 173 | AD | 74 | 4A | 2 | 25 | 667354 | 5338828 | 2 | 25 | 0 | 184 | 5491852 | 2 | 25 | 0 | 1 | 192 |
| 174 | AE | 74 | 4A | 2 | 26 | 667377 | 5339012 | 2 | 26 | 0 | 184 | 5492044 | 2 | 26 | 0 | 1 | 192 |
| 175 | AF | 74 | 4A | 2 | 27 | 667400 | 5339196 | 2 | 27 | 0 | 184 | 5492236 | 2 | 27 | 0 | 1 | 192 |
| 176 | B0 | 74 | 4A | 2 | 28 | 667425 | 5339396 | 2 | 28 | 0 | 200 | 5492444 | 2 | 28 | 0 | 1 | 208 |
| 177 | B1 | 74 | 4A | 2 | 29 | 667450 | 5339596 | 2 | 29 | 0 | 200 | 5492652 | 2 | 29 | 0 | 1 | 208 |
| 178 | B2 | 74 | 4A | 2 | 30 | 667475 | 5339796 | 2 | 30 | 0 | 200 | 5492860 | 2 | 30 | 0 | 1 | 208 |
| 179 | B3 | 74 | 4A | 2 | 31 | 667500 | 5339996 | 2 | 31 | 0 | 200 | 5493068 | 2 | 31 | 0 | 1 | 208 |
| 180 | B4 | 74 | 4A | 2 | 32 | 667525 | 5340196 | 2 | 32 | 0 | 200 | 5493276 | 2 | 32 | 0 | 1 | 208 |
| 181 | B5 | 74 | 4A | 2 | 33 | 667550 | 5340396 | 2 | 33 | 0 | 200 | 5493484 | 2 | 33 | 0 | 1 | 208 |
| 182 | B6 | 74 | 4A | 2 | 34 | 667575 | 5340596 | 2 | 34 | 0 | 200 | 5493692 | 2 | 34 | 0 | 1 | 208 |
| 183 | B7 | 74 | 4A | 2 | 35 | 667600 | 5340796 | 2 | 35 | 0 | 200 | 5493900 | 2 | 35 | 0 | 1 | 208 |
| 184 | B8 | 74 | 4A | 2 | 36 | 667625 | 5340996 | 2 | 36 | 0 | 200 | 5494108 | 2 | 36 | 0 | 1 | 208 |
| 185 | B9 | 74 | 4A | 2 | 37 | 667650 | 5341196 | 2 | 37 | 0 | 200 | 5494316 | 2 | 37 | 0 | 1 | 208 |
| 186 | BA | 74 | 4A | 2 | 38 | 667675 | 5341396 | 2 | 38 | 0 | 200 | 5494524 | 2 | 38 | 0 | 1 | 208 |
| 187 | BB | 74 | 4A | 2 | 39 | 667700 | 5341596 | 2 | 39 | 0 | 200 | 5494732 | 2 | 39 | 0 | 1 | 208 |
| 188 | BC | 74 | 4A | 2 | 40 | 667725 | 5341796 | 2 | 40 | 0 | 200 | 5494940 | 2 | 40 | 0 | 1 | 208 |
| 189 | BD | 74 | 4A | 2 | 41 | 667750 | 5341996 | 2 | 41 | 0 | 200 | 5495148 | 2 | 41 | 0 | 1 | 208 |
| 190 | BE | 74 | 4A | 2 | 42 | 667775 | 5342196 | 2 | 42 | 0 | 200 | 5495356 | 2 | 42 | 0 | 1 | 208 |
| 191 | BF | 74 | 4A | 2 | 43 | 667800 | 5342396 | 2 | 43 | 0 | 200 | 5495564 | 2 | 43 | 0 | 1 | 208 |
| 192 | C0 | 74 | 4A | 2 | 44 | 667825 | 5342596 | 2 | 44 | 0 | 200 | 5495772 | 2 | 44 | 0 | 1 | 208 |
| 193 | C1 | 74 | 4A | 2 | 45 | 667850 | 5342796 | 2 | 45 | 0 | 200 | 5495980 | 2 | 45 | 0 | 1 | 208 |
| 194 | C2 | 74 | 4A | 2 | 46 | 667875 | 5342996 | 2 | 46 | 0 | 200 | 5496188 | 2 | 46 | 0 | 1 | 208 |
| 195 | C3 | 74 | 4A | 2 | 47 | 667900 | 5343196 | 2 | 47 | 0 | 200 | 5496396 | 2 | 47 | 0 | 1 | 208 |
| 196 | C4 | 74 | 4A | 2 | 48 | 667925 | 5343396 | 2 | 48 | 0 | 200 | 5496604 | 2 | 48 | 0 | 1 | 208 |
| 197 | C5 | 74 | 4A | 2 | 49 | 667950 | 5343596 | 2 | 49 | 0 | 200 | 5496812 | 2 | 49 | 0 | 1 | 208 |
| 198 | C6 | 74 | 4A | 2 | 50 | 667975 | 5343796 | 2 | 50 | 0 | 200 | 5497020 | 2 | 50 | 0 | 1 | 208 |
| 199 | C7 | 74 | 4A | 2 | 51 | 668000 | 5343996 | 2 | 51 | 0 | 200 | 5497228 | 2 | 51 | 0 | 1 | 208 |
| 200 | C8 | 74 | 4A | 2 | 52 | 668025 | 5344196 | 2 | 52 | 0 | 200 | 5497436 | 2 | 52 | 0 | 1 | 208 |
| 201 | C9 | 74 | 4A | 2 | 53 | 668050 | 5344396 | 2 | 53 | 0 | 200 | 5497644 | 2 | 53 | 0 | 1 | 208 |
| 202 | CA | 74 | 4A | 2 | 54 | 668075 | 5344596 | 2 | 54 | 0 | 200 | 5497852 | 2 | 54 | 0 | 1 | 208 |
| 203 | CB | 74 | 4A | 2 | 55 | 668100 | 5344796 | 2 | 55 | 0 | 200 | 5498060 | 2 | 55 | 0 | 1 | 208 |
| 204 | CC | 74 | 4A | 2 | 56 | 668125 | 5344996 | 2 | 56 | 0 | 200 | 5498268 | 2 | 56 | 0 | 1 | 208 |
| 205 | CD | 74 | 4A | 2 | 57 | 668150 | 5345196 | 2 | 57 | 0 | 200 | 5498476 | 2 | 57 | 0 | 1 | 208 |
| 206 | CE | 74 | 4A | 2 | 58 | 668175 | 5345396 | 2 | 58 | 0 | 200 | 5498684 | 2 | 58 | 0 | 1 | 208 |
| 207 | CF | 74 | 4A | 2 | 59 | 668200 | 5345596 | 2 | 59 | 0 | 200 | 5498892 | 2 | 59 | 0 | 1 | 208 |
| 208 | D0 | 74 | 4A | 2 | 60 | 668225 | 5345796 | 2 | 60 | 0 | 200 | 5499100 | 2 | 60 | 0 | 1 | 208 |
| 209 | D1 | 74 | 4A | 2 | 61 | 668250 | 5345996 | 2 | 61 | 0 | 200 | 5499308 | 2 | 61 | 0 | 1 | 208 |
| 210 | D2 | 74 | 4A | 2 | 62 | 668275 | 5346196 | 2 | 62 | 0 | 200 | 5499516 | 2 | 62 | 0 | 1 | 208 |

| | | | | | | | | | | | | | | | | | |
|---|---|---|---|---|---|---|---|---|---|---|---|---|---|---|---|---|---|
| 211 | D3 | 74 | 4A | 2 | 63 | 668300 | 5346396 | 2 | 63 | 0 | 200 | 5499724 | 2 | 63 | 0 | 1 | 208 |
| 212 | D4 | 74 | 4A | 2 | 64 | 668325 | 5346596 | 2 | 64 | 0 | 200 | 5499932 | 2 | 64 | 0 | 1 | 208 |
| 213 | D5 | 74 | 4A | 2 | 65 | 668350 | 5346796 | 2 | 65 | 0 | 200 | 5500140 | 2 | 65 | 0 | 1 | 208 |
| 214 | D6 | 74 | 4A | 2 | 66 | 668375 | 5346996 | 2 | 66 | 0 | 200 | 5500348 | 2 | 66 | 0 | 1 | 208 |
| 215 | D7 | 74 | 4A | 2 | 67 | 668400 | 5347196 | 2 | 67 | 0 | 200 | 5500556 | 2 | 67 | 0 | 1 | 208 |
| 216 | D8 | 74 | 4A | 2 | 68 | 668425 | 5347396 | 2 | 68 | 0 | 200 | 5500764 | 2 | 68 | 0 | 1 | 208 |
| 217 | D9 | 74 | 4A | 2 | 69 | 668450 | 5347596 | 2 | 69 | 0 | 200 | 5500972 | 2 | 69 | 0 | 1 | 208 |
| 218 | DA | 74 | 4A | 2 | 70 | 668475 | 5347796 | 2 | 70 | 0 | 200 | 5501180 | 2 | 70 | 0 | 1 | 208 |
| 219 | DB | 74 | 4A | 2 | 71 | 668500 | 5347996 | 2 | 71 | 0 | 200 | 5501388 | 2 | 71 | 0 | 1 | 208 |
| 220 | DC | 74 | 4A | 2 | 72 | 668525 | 5348196 | 2 | 72 | 0 | 200 | 5501596 | 2 | 72 | 0 | 1 | 208 |
| 221 | DD | 74 | 4A | 2 | 73 | 668550 | 5348396 | 2 | 73 | 0 | 200 | 5501804 | 2 | 73 | 0 | 1 | 208 |
| 222 | DE | 74 | 4A | 3 | 0 | 668573 | 5348580 | 3 | 0 | 0 | 184 | 5501996 | 3 | 0 | 0 | 1 | 192 |
| 223 | DF | 74 | 4A | 3 | 1 | 668601 | 5348804 | 3 | 1 | 0 | 224 | 5502228 | 3 | 1 | 0 | 1 | 232 |
| 224 | E0 | 74 | 4A | 3 | 2 | 668629 | 5349028 | 3 | 2 | 0 | 224 | 5502460 | 3 | 2 | 0 | 1 | 232 |
| 225 | E1 | 74 | 4A | 3 | 3 | 668657 | 5349252 | 3 | 3 | 0 | 224 | 5502692 | 3 | 3 | 0 | 1 | 232 |
| 226 | E2 | 74 | 4A | 3 | 4 | 668685 | 5349476 | 3 | 4 | 0 | 224 | 5502924 | 3 | 4 | 0 | 1 | 232 |
| 227 | E3 | 74 | 4A | 3 | 5 | 668713 | 5349700 | 3 | 5 | 0 | 224 | 5503156 | 3 | 5 | 0 | 1 | 232 |
| 228 | E4 | 74 | 4A | 3 | 6 | 668741 | 5349924 | 3 | 6 | 0 | 224 | 5503388 | 3 | 6 | 0 | 1 | 232 |
| 229 | E5 | 74 | 4A | 3 | 7 | 668769 | 5350148 | 3 | 7 | 0 | 224 | 5503620 | 3 | 7 | 0 | 1 | 232 |
| 230 | E6 | 74 | 4A | 3 | 8 | 668797 | 5350372 | 3 | 8 | 0 | 224 | 5503852 | 3 | 8 | 0 | 1 | 232 |
| 231 | E7 | 74 | 4A | 3 | 9 | 668825 | 5350596 | 3 | 9 | 0 | 224 | 5504084 | 3 | 9 | 0 | 1 | 232 |
| 232 | E8 | 74 | 4A | 3 | 10 | 668853 | 5350820 | 3 | 10 | 0 | 224 | 5504316 | 3 | 10 | 0 | 1 | 232 |
| 233 | E9 | 74 | 4A | 3 | 11 | 668881 | 5351044 | 3 | 11 | 0 | 224 | 5504548 | 3 | 11 | 0 | 1 | 232 |
| 234 | EA | 74 | 4A | 3 | 12 | 668909 | 5351268 | 3 | 12 | 0 | 224 | 5504780 | 3 | 12 | 0 | 1 | 232 |
| 235 | EB | 74 | 4A | 3 | 13 | 668937 | 5351492 | 3 | 13 | 0 | 224 | 5505012 | 3 | 13 | 0 | 1 | 232 |
| 236 | EC | 74 | 4A | 3 | 14 | 668965 | 5351716 | 3 | 14 | 0 | 224 | 5505244 | 3 | 14 | 0 | 1 | 232 |
| 237 | ED | 74 | 4A | 3 | 15 | 668993 | 5351940 | 3 | 15 | 0 | 224 | 5505476 | 3 | 15 | 0 | 1 | 232 |
| 238 | EE | 74 | 4A | 3 | 16 | 669021 | 5352164 | 3 | 16 | 0 | 224 | 5505708 | 3 | 16 | 0 | 1 | 232 |
| 239 | EF | 74 | 4A | 3 | 17 | 669049 | 5352388 | 3 | 17 | 0 | 224 | 5505940 | 3 | 17 | 0 | 1 | 232 |
| 240 | F0 | 74 | 4A | 3 | 18 | 669074 | 5352588 | 3 | 18 | 0 | 200 | 5506148 | 3 | 18 | 0 | 1 | 208 |
| 241 | F1 | 74 | 4A | 3 | 19 | 669099 | 5352788 | 3 | 19 | 0 | 200 | 5506356 | 3 | 19 | 0 | 1 | 208 |
| 242 | F2 | 74 | 4A | 3 | 20 | 669124 | 5352988 | 3 | 20 | 0 | 200 | 5506564 | 3 | 20 | 0 | 1 | 208 |
| 243 | F3 | 74 | 4A | 3 | 21 | 669149 | 5353188 | 3 | 21 | 0 | 200 | 5506772 | 3 | 21 | 0 | 1 | 208 |
| 244 | F4 | 74 | 4A | 3 | 22 | 669174 | 5353388 | 3 | 22 | 0 | 200 | 5506980 | 3 | 22 | 0 | 1 | 208 |
| 245 | F5 | 74 | 4A | 3 | 23 | 669199 | 5353588 | 3 | 23 | 0 | 200 | 5507188 | 3 | 23 | 0 | 1 | 208 |
| 246 | F6 | 74 | 4A | 3 | 24 | 669224 | 5353788 | 3 | 24 | 0 | 200 | 5507396 | 3 | 24 | 0 | 1 | 208 |
| 247 | F7 | 74 | 4A | 3 | 25 | 669249 | 5353988 | 3 | 25 | 0 | 200 | 5507604 | 3 | 25 | 0 | 1 | 208 |
| 248 | F8 | 74 | 4A | 3 | 26 | 669274 | 5354188 | 3 | 26 | 0 | 200 | 5507812 | 3 | 26 | 0 | 1 | 208 |
| 249 | F9 | 74 | 4A | 3 | 27 | 669299 | 5354388 | 3 | 27 | 0 | 200 | 5508020 | 3 | 27 | 0 | 1 | 208 |
| 250 | FA | 74 | 4A | 3 | 28 | 669324 | 5354588 | 3 | 28 | 0 | 200 | 5508228 | 3 | 28 | 0 | 1 | 208 |
| 251 | FB | 74 | 4A | 3 | 29 | 669349 | 5354788 | 3 | 29 | 0 | 200 | 5508436 | 3 | 29 | 0 | 1 | 208 |
| 252 | FC | 74 | 4A | 3 | 30 | 669374 | 5354988 | 3 | 30 | 0 | 200 | 5508644 | 3 | 30 | 0 | 1 | 208 |
| 253 | FD | 74 | 4A | 3 | 31 | 669399 | 5355188 | 3 | 31 | 0 | 200 | 5508852 | 3 | 31 | 0 | 1 | 208 |
| 254 | FE | 74 | 4A | 3 | 32 | 669424 | 5355388 | 3 | 32 | 0 | 200 | 5509060 | 3 | 32 | 0 | 1 | 208 |
| 255 | FF | 74 | 4A | 3 | 33 | 669449 | 5355588 | 3 | 33 | 0 | 200 | 5509268 | 3 | 33 | 0 | 1 | 208 |
| 0 | 00 | 75 | 4B | 0 | 0 | 669461 | 5355684 | 0 | 0 | 0 | 96 | 5509372 | 0 | 0 | 0 | 1 | 104 |
| 1 | 01 | 75 | 4B | 0 | 1 | 669475 | 5355796 | 0 | 1 | 0 | 112 | 5509492 | 0 | 1 | 0 | 1 | 120 |
| 2 | 02 | 75 | 4B | 0 | 2 | 669489 | 5355908 | 0 | 2 | 0 | 112 | 5509612 | 0 | 2 | 0 | 1 | 120 |
| 3 | 03 | 75 | 4B | 0 | 3 | 669503 | 5356020 | 0 | 3 | 0 | 112 | 5509732 | 0 | 3 | 0 | 1 | 120 |
| 4 | 04 | 75 | 4B | 0 | 4 | 669517 | 5356132 | 0 | 4 | 0 | 112 | 5509852 | 0 | 4 | 0 | 1 | 120 |
| 5 | 05 | 75 | 4B | 0 | 5 | 669531 | 5356244 | 0 | 5 | 0 | 112 | 5509972 | 0 | 5 | 0 | 1 | 120 |
| 6 | 06 | 75 | 4B | 0 | 6 | 669545 | 5356356 | 0 | 6 | 0 | 112 | 5510092 | 0 | 6 | 0 | 1 | 120 |
| 7 | 07 | 75 | 4B | 0 | 7 | 669559 | 5356468 | 0 | 7 | 0 | 112 | 5510212 | 0 | 7 | 0 | 1 | 120 |
| 8 | 08 | 75 | 4B | 0 | 8 | 669573 | 5356580 | 0 | 8 | 0 | 112 | 5510332 | 0 | 8 | 0 | 1 | 120 |
| 9 | 09 | 75 | 4B | 0 | 9 | 669587 | 5356692 | 0 | 9 | 0 | 112 | 5510452 | 0 | 9 | 0 | 1 | 120 |
| 10 | 0A | 75 | 4B | 0 | 10 | 669601 | 5356804 | 0 | 10 | 0 | 112 | 5510572 | 0 | 10 | 0 | 1 | 120 |
| 11 | 0B | 75 | 4B | 0 | 11 | 669615 | 5356916 | 0 | 11 | 0 | 112 | 5510692 | 0 | 11 | 0 | 1 | 120 |
| 12 | 0C | 75 | 4B | 0 | 12 | 669629 | 5357028 | 0 | 12 | 0 | 112 | 5510812 | 0 | 12 | 0 | 1 | 120 |
| 13 | 0D | 75 | 4B | 0 | 13 | 669643 | 5357140 | 0 | 13 | 0 | 112 | 5510932 | 0 | 13 | 0 | 1 | 120 |
| 14 | 0E | 75 | 4B | 0 | 14 | 669657 | 5357252 | 0 | 14 | 0 | 112 | 5511052 | 0 | 14 | 0 | 1 | 120 |
| 15 | 0F | 75 | 4B | 0 | 15 | 669671 | 5357364 | 0 | 15 | 0 | 112 | 5511172 | 0 | 15 | 0 | 1 | 120 |
| 16 | 10 | 75 | 4B | 0 | 16 | 669685 | 5357476 | 0 | 16 | 0 | 112 | 5511292 | 0 | 16 | 0 | 1 | 120 |
| 17 | 11 | 75 | 4B | 0 | 17 | 669699 | 5357588 | 0 | 17 | 0 | 112 | 5511412 | 0 | 17 | 0 | 1 | 120 |
| 18 | 12 | 75 | 4B | 0 | 18 | 669713 | 5357700 | 0 | 18 | 0 | 112 | 5511532 | 0 | 18 | 0 | 1 | 120 |
| 19 | 13 | 75 | 4B | 0 | 19 | 669727 | 5357812 | 0 | 19 | 0 | 112 | 5511652 | 0 | 19 | 0 | 1 | 120 |
| 20 | 14 | 75 | 4B | 0 | 20 | 669741 | 5357924 | 0 | 20 | 0 | 112 | 5511772 | 0 | 20 | 0 | 1 | 120 |
| 21 | 15 | 75 | 4B | 0 | 21 | 669755 | 5358036 | 0 | 21 | 0 | 112 | 5511892 | 0 | 21 | 0 | 1 | 120 |
| 22 | 16 | 75 | 4B | 0 | 22 | 669769 | 5358148 | 0 | 22 | 0 | 112 | 5512012 | 0 | 22 | 0 | 1 | 120 |
| 23 | 17 | 75 | 4B | 0 | 23 | 669783 | 5358260 | 0 | 23 | 0 | 112 | 5512132 | 0 | 23 | 0 | 1 | 120 |
| 24 | 18 | 75 | 4B | 0 | 24 | 669797 | 5358372 | 0 | 24 | 0 | 112 | 5512252 | 0 | 24 | 0 | 1 | 120 |
| 25 | 19 | 75 | 4B | 0 | 25 | 669811 | 5358484 | 0 | 25 | 0 | 112 | 5512372 | 0 | 25 | 0 | 1 | 120 |
| 26 | 1A | 75 | 4B | 0 | 26 | 669825 | 5358596 | 0 | 26 | 0 | 112 | 5512492 | 0 | 26 | 0 | 1 | 120 |
| 27 | 1B | 75 | 4B | 0 | 27 | 669839 | 5358708 | 0 | 27 | 0 | 112 | 5512612 | 0 | 27 | 0 | 1 | 120 |
| 28 | 1C | 75 | 4B | 0 | 28 | 669853 | 5358820 | 0 | 28 | 0 | 112 | 5512732 | 0 | 28 | 0 | 1 | 120 |
| 29 | 1D | 75 | 4B | 0 | 29 | 669867 | 5358932 | 0 | 29 | 0 | 112 | 5512852 | 0 | 29 | 0 | 1 | 120 |
| 30 | 1E | 75 | 4B | 0 | 30 | 669881 | 5359044 | 0 | 30 | 0 | 112 | 5512972 | 0 | 30 | 0 | 1 | 120 |
| 31 | 1F | 75 | 4B | 0 | 31 | 669895 | 5359156 | 0 | 31 | 0 | 112 | 5513092 | 0 | 31 | 0 | 1 | 120 |
| 32 | 20 | 75 | 4B | 0 | 32 | 669909 | 5359268 | 0 | 32 | 0 | 112 | 5513212 | 0 | 32 | 0 | 1 | 120 |
| 33 | 21 | 75 | 4B | 0 | 33 | 669923 | 5359380 | 0 | 33 | 0 | 112 | 5513332 | 0 | 33 | 0 | 1 | 120 |
| 34 | 22 | 75 | 4B | 0 | 34 | 669937 | 5359492 | 0 | 34 | 0 | 112 | 5513452 | 0 | 34 | 0 | 1 | 120 |
| 35 | 23 | 75 | 4B | 0 | 35 | 669951 | 5359604 | 0 | 35 | 0 | 112 | 5513572 | 0 | 35 | 0 | 1 | 120 |
| 36 | 24 | 75 | 4B | 0 | 36 | 669965 | 5359716 | 0 | 36 | 0 | 112 | 5513692 | 0 | 36 | 0 | 1 | 120 |
| 37 | 25 | 75 | 4B | 0 | 37 | 669979 | 5359828 | 0 | 37 | 0 | 112 | 5513812 | 0 | 37 | 0 | 1 | 120 |
| 38 | 26 | 75 | 4B | 0 | 38 | 669993 | 5359940 | 0 | 38 | 0 | 112 | 5513932 | 0 | 38 | 0 | 1 | 120 |
| 39 | 27 | 75 | 4B | 0 | 39 | 670007 | 5360052 | 0 | 39 | 0 | 112 | 5514052 | 0 | 39 | 0 | 1 | 120 |
| 40 | 28 | 75 | 4B | 0 | 40 | 670021 | 5360164 | 0 | 40 | 0 | 112 | 5514172 | 0 | 40 | 0 | 1 | 120 |
| 41 | 29 | 75 | 4B | 0 | 41 | 670035 | 5360276 | 0 | 41 | 0 | 112 | 5514292 | 0 | 41 | 0 | 1 | 120 |
| 42 | 2A | 75 | 4B | 0 | 42 | 670049 | 5360388 | 0 | 42 | 0 | 112 | 5514412 | 0 | 42 | 0 | 1 | 120 |
| 43 | 2B | 75 | 4B | 0 | 43 | 670063 | 5360500 | 0 | 43 | 0 | 112 | 5514532 | 0 | 43 | 0 | 1 | 120 |
| 44 | 2C | 75 | 4B | 0 | 44 | 670077 | 5360612 | 0 | 44 | 0 | 112 | 5514652 | 0 | 44 | 0 | 1 | 120 |
| 45 | 2D | 75 | 4B | 0 | 45 | 670091 | 5360724 | 0 | 45 | 0 | 112 | 5514772 | 0 | 45 | 0 | 1 | 120 |
| 46 | 2E | 75 | 4B | 0 | 46 | 670105 | 5360836 | 0 | 46 | 0 | 112 | 5514892 | 0 | 46 | 0 | 1 | 120 |
| 47 | 2F | 75 | 4B | 0 | 47 | 670119 | 5360948 | 0 | 47 | 0 | 112 | 5515012 | 0 | 47 | 0 | 1 | 120 |
| 48 | 30 | 75 | 4B | 0 | 48 | 670133 | 5361060 | 0 | 48 | 0 | 112 | 5515132 | 0 | 48 | 0 | 1 | 120 |
| 49 | 31 | 75 | 4B | 0 | 49 | 670147 | 5361172 | 0 | 49 | 0 | 112 | 5515252 | 0 | 49 | 0 | 1 | 120 |
| 50 | 32 | 75 | 4B | 0 | 50 | 670161 | 5361284 | 0 | 50 | 0 | 112 | 5515372 | 0 | 50 | 0 | 1 | 120 |
| 51 | 33 | 75 | 4B | 0 | 51 | 670175 | 5361396 | 0 | 51 | 0 | 112 | 5515492 | 0 | 51 | 0 | 1 | 120 |
| 52 | 34 | 75 | 4B | 0 | 52 | 670189 | 5361508 | 0 | 52 | 0 | 112 | 5515612 | 0 | 52 | 0 | 1 | 120 |
| 53 | 35 | 75 | 4B | 0 | 53 | 670203 | 5361620 | 0 | 53 | 0 | 112 | 5515732 | 0 | 53 | 0 | 1 | 120 |
| 54 | 36 | 75 | 4B | 0 | 54 | 670217 | 5361732 | 0 | 54 | 0 | 112 | 5515852 | 0 | 54 | 0 | 1 | 120 |
| 55 | 37 | 75 | 4B | 0 | 55 | 670231 | 5361844 | 0 | 55 | 0 | 112 | 5515972 | 0 | 55 | 0 | 1 | 120 |
| 56 | 38 | 75 | 4B | 0 | 56 | 670245 | 5361956 | 0 | 56 | 0 | 112 | 5516092 | 0 | 56 | 0 | 1 | 120 |
| 57 | 39 | 75 | 4B | 0 | 57 | 670259 | 5362068 | 0 | 57 | 0 | 112 | 5516212 | 0 | 57 | 0 | 1 | 120 |

| | | | | | | | | | | | | | | | | | |
|---|---|---|---|---|---|---|---|---|---|---|---|---|---|---|---|---|---|
| 58 | 3A | 75 | 4B | 0 | 58 | 670273 | 5362180 | 0 | 58 | 0 | 112 | 5516332 | 0 | 58 | 0 | 1 | 120 |
| 59 | 3B | 75 | 4B | 0 | 59 | 670287 | 5362292 | 0 | 59 | 0 | 112 | 5516452 | 0 | 59 | 0 | 1 | 120 |
| 60 | 3C | 75 | 4B | 0 | 60 | 670301 | 5362404 | 0 | 60 | 0 | 112 | 5516572 | 0 | 60 | 0 | 1 | 120 |
| 61 | 3D | 75 | 4B | 0 | 61 | 670315 | 5362516 | 0 | 61 | 0 | 112 | 5516692 | 0 | 61 | 0 | 1 | 120 |
| 62 | 3E | 75 | 4B | 0 | 62 | 670329 | 5362628 | 0 | 62 | 0 | 112 | 5516812 | 0 | 62 | 0 | 1 | 120 |
| 63 | 3F | 75 | 4B | 0 | 63 | 670343 | 5362740 | 0 | 63 | 0 | 112 | 5516932 | 0 | 63 | 0 | 1 | 120 |
| 64 | 40 | 75 | 4B | 0 | 64 | 670357 | 5362852 | 0 | 64 | 0 | 112 | 5517052 | 0 | 64 | 0 | 1 | 120 |
| 65 | 41 | 75 | 4B | 0 | 65 | 670371 | 5362964 | 0 | 65 | 0 | 112 | 5517172 | 0 | 65 | 0 | 1 | 120 |
| 66 | 42 | 75 | 4B | 0 | 66 | 670385 | 5363076 | 0 | 66 | 0 | 112 | 5517292 | 0 | 66 | 0 | 1 | 120 |
| 67 | 43 | 75 | 4B | 0 | 67 | 670399 | 5363188 | 0 | 67 | 0 | 112 | 5517412 | 0 | 67 | 0 | 1 | 120 |
| 68 | 44 | 75 | 4B | 0 | 68 | 670413 | 5363300 | 0 | 68 | 0 | 112 | 5517532 | 0 | 68 | 0 | 1 | 120 |
| 69 | 45 | 75 | 4B | 0 | 69 | 670427 | 5363412 | 0 | 69 | 0 | 112 | 5517652 | 0 | 69 | 0 | 1 | 120 |
| 70 | 46 | 75 | 4B | 0 | 70 | 670441 | 5363524 | 0 | 70 | 0 | 112 | 5517772 | 0 | 70 | 0 | 1 | 120 |
| 71 | 47 | 75 | 4B | 0 | 71 | 670455 | 5363636 | 0 | 71 | 0 | 112 | 5517892 | 0 | 71 | 0 | 1 | 120 |
| 72 | 48 | 75 | 4B | 0 | 72 | 670469 | 5363748 | 0 | 72 | 0 | 112 | 5518012 | 0 | 72 | 0 | 1 | 120 |
| 73 | 49 | 75 | 4B | 0 | 73 | 670483 | 5363860 | 0 | 73 | 0 | 112 | 5518132 | 0 | 73 | 0 | 1 | 120 |
| 74 | 4A | 75 | 4B | 0 | 74 | 670497 | 5363972 | 0 | 74 | 0 | 112 | 5518252 | 0 | 74 | 0 | 1 | 120 |
| 75 | 4B | 75 | 4B | 1 | 0 | 670509 | 5364068 | 1 | 0 | 0 | 96 | 5518356 | 1 | 0 | 0 | 1 | 104 |
| 76 | 4C | 75 | 4B | 1 | 1 | 670526 | 5364204 | 1 | 1 | 0 | 136 | 5518500 | 1 | 1 | 0 | 1 | 144 |
| 77 | 4D | 75 | 4B | 1 | 2 | 670543 | 5364340 | 1 | 2 | 0 | 136 | 5518644 | 1 | 2 | 0 | 1 | 144 |
| 78 | 4E | 75 | 4B | 1 | 3 | 670560 | 5364476 | 1 | 3 | 0 | 136 | 5518788 | 1 | 3 | 0 | 1 | 144 |
| 79 | 4F | 75 | 4B | 1 | 4 | 670577 | 5364612 | 1 | 4 | 0 | 136 | 5518932 | 1 | 4 | 0 | 1 | 144 |
| 80 | 50 | 75 | 4B | 1 | 5 | 670598 | 5364780 | 1 | 5 | 0 | 168 | 5519108 | 1 | 5 | 0 | 1 | 176 |
| 81 | 51 | 75 | 4B | 1 | 6 | 670619 | 5364948 | 1 | 6 | 0 | 168 | 5519284 | 1 | 6 | 0 | 1 | 176 |
| 82 | 52 | 75 | 4B | 1 | 7 | 670640 | 5365116 | 1 | 7 | 0 | 168 | 5519460 | 1 | 7 | 0 | 1 | 176 |
| 83 | 53 | 75 | 4B | 1 | 8 | 670661 | 5365284 | 1 | 8 | 0 | 168 | 5519636 | 1 | 8 | 0 | 1 | 176 |
| 84 | 54 | 75 | 4B | 1 | 9 | 670682 | 5365452 | 1 | 9 | 0 | 168 | 5519812 | 1 | 9 | 0 | 1 | 176 |
| 85 | 55 | 75 | 4B | 1 | 10 | 670703 | 5365620 | 1 | 10 | 0 | 168 | 5519988 | 1 | 10 | 0 | 1 | 176 |
| 86 | 56 | 75 | 4B | 1 | 11 | 670724 | 5365788 | 1 | 11 | 0 | 168 | 5520164 | 1 | 11 | 0 | 1 | 176 |
| 87 | 57 | 75 | 4B | 1 | 12 | 670745 | 5365956 | 1 | 12 | 0 | 168 | 5520340 | 1 | 12 | 0 | 1 | 176 |
| 88 | 58 | 75 | 4B | 1 | 13 | 670766 | 5366124 | 1 | 13 | 0 | 168 | 5520516 | 1 | 13 | 0 | 1 | 176 |
| 89 | 59 | 75 | 4B | 1 | 14 | 670787 | 5366292 | 1 | 14 | 0 | 168 | 5520692 | 1 | 14 | 0 | 1 | 176 |
| 90 | 5A | 75 | 4B | 1 | 15 | 670808 | 5366460 | 1 | 15 | 0 | 168 | 5520868 | 1 | 15 | 0 | 1 | 176 |
| 91 | 5B | 75 | 4B | 1 | 16 | 670829 | 5366628 | 1 | 16 | 0 | 168 | 5521044 | 1 | 16 | 0 | 1 | 176 |
| 92 | 5C | 75 | 4B | 1 | 17 | 670850 | 5366796 | 1 | 17 | 0 | 168 | 5521220 | 1 | 17 | 0 | 1 | 176 |
| 93 | 5D | 75 | 4B | 1 | 18 | 670871 | 5366964 | 1 | 18 | 0 | 168 | 5521396 | 1 | 18 | 0 | 1 | 176 |
| 94 | 5E | 75 | 4B | 1 | 19 | 670892 | 5367132 | 1 | 19 | 0 | 168 | 5521572 | 1 | 19 | 0 | 1 | 176 |
| 95 | 5F | 75 | 4B | 1 | 20 | 670913 | 5367300 | 1 | 20 | 0 | 168 | 5521748 | 1 | 20 | 0 | 1 | 176 |
| 96 | 60 | 75 | 4B | 1 | 21 | 670936 | 5367484 | 1 | 21 | 0 | 184 | 5521940 | 1 | 21 | 0 | 1 | 192 |
| 97 | 61 | 75 | 4B | 1 | 22 | 670959 | 5367668 | 1 | 22 | 0 | 184 | 5522132 | 1 | 22 | 0 | 1 | 192 |
| 98 | 62 | 75 | 4B | 1 | 23 | 670982 | 5367852 | 1 | 23 | 0 | 184 | 5522324 | 1 | 23 | 0 | 1 | 192 |
| 99 | 63 | 75 | 4B | 1 | 24 | 671005 | 5368036 | 1 | 24 | 0 | 184 | 5522516 | 1 | 24 | 0 | 1 | 192 |
| 100 | 64 | 75 | 4B | 1 | 25 | 671028 | 5368220 | 1 | 25 | 0 | 184 | 5522708 | 1 | 25 | 0 | 1 | 192 |
| 101 | 65 | 75 | 4B | 1 | 26 | 671051 | 5368404 | 1 | 26 | 0 | 184 | 5522900 | 1 | 26 | 0 | 1 | 192 |
| 102 | 66 | 75 | 4B | 1 | 27 | 671074 | 5368588 | 1 | 27 | 0 | 184 | 5523092 | 1 | 27 | 0 | 1 | 192 |
| 103 | 67 | 75 | 4B | 1 | 28 | 671097 | 5368772 | 1 | 28 | 0 | 184 | 5523284 | 1 | 28 | 0 | 1 | 192 |
| 104 | 68 | 75 | 4B | 1 | 29 | 671120 | 5368956 | 1 | 29 | 0 | 184 | 5523476 | 1 | 29 | 0 | 1 | 192 |
| 105 | 69 | 75 | 4B | 1 | 30 | 671143 | 5369140 | 1 | 30 | 0 | 184 | 5523668 | 1 | 30 | 0 | 1 | 192 |
| 106 | 6A | 75 | 4B | 1 | 31 | 671166 | 5369324 | 1 | 31 | 0 | 184 | 5523860 | 1 | 31 | 0 | 1 | 192 |
| 107 | 6B | 75 | 4B | 1 | 32 | 671189 | 5369508 | 1 | 32 | 0 | 184 | 5524052 | 1 | 32 | 0 | 1 | 192 |
| 108 | 6C | 75 | 4B | 1 | 33 | 671212 | 5369692 | 1 | 33 | 0 | 184 | 5524244 | 1 | 33 | 0 | 1 | 192 |
| 109 | 6D | 75 | 4B | 1 | 34 | 671235 | 5369876 | 1 | 34 | 0 | 184 | 5524436 | 1 | 34 | 0 | 1 | 192 |
| 110 | 6E | 75 | 4B | 1 | 35 | 671258 | 5370060 | 1 | 35 | 0 | 184 | 5524628 | 1 | 35 | 0 | 1 | 192 |
| 111 | 6F | 75 | 4B | 1 | 36 | 671281 | 5370244 | 1 | 36 | 0 | 184 | 5524820 | 1 | 36 | 0 | 1 | 192 |
| 112 | 70 | 75 | 4B | 1 | 37 | 671304 | 5370428 | 1 | 37 | 0 | 184 | 5525012 | 1 | 37 | 0 | 1 | 192 |
| 113 | 71 | 75 | 4B | 1 | 38 | 671327 | 5370612 | 1 | 38 | 0 | 184 | 5525204 | 1 | 38 | 0 | 1 | 192 |
| 114 | 72 | 75 | 4B | 1 | 39 | 671350 | 5370796 | 1 | 39 | 0 | 184 | 5525396 | 1 | 39 | 0 | 1 | 192 |
| 115 | 73 | 75 | 4B | 1 | 40 | 671373 | 5370980 | 1 | 40 | 0 | 184 | 5525588 | 1 | 40 | 0 | 1 | 192 |
| 116 | 74 | 75 | 4B | 1 | 41 | 671396 | 5371164 | 1 | 41 | 0 | 184 | 5525780 | 1 | 41 | 0 | 1 | 192 |
| 117 | 75 | 75 | 4B | 1 | 42 | 671419 | 5371348 | 1 | 42 | 0 | 184 | 5525972 | 1 | 42 | 0 | 1 | 192 |
| 118 | 76 | 75 | 4B | 1 | 43 | 671442 | 5371532 | 1 | 43 | 0 | 184 | 5526164 | 1 | 43 | 0 | 1 | 192 |
| 119 | 77 | 75 | 4B | 1 | 44 | 671465 | 5371716 | 1 | 44 | 0 | 184 | 5526356 | 1 | 44 | 0 | 1 | 192 |
| 120 | 78 | 75 | 4B | 1 | 45 | 671488 | 5371900 | 1 | 45 | 0 | 184 | 5526548 | 1 | 45 | 0 | 1 | 192 |
| 121 | 79 | 75 | 4B | 1 | 46 | 671511 | 5372084 | 1 | 46 | 0 | 184 | 5526740 | 1 | 46 | 0 | 1 | 192 |
| 122 | 7A | 75 | 4B | 1 | 47 | 671534 | 5372268 | 1 | 47 | 0 | 184 | 5526932 | 1 | 47 | 0 | 1 | 192 |
| 123 | 7B | 75 | 4B | 1 | 48 | 671557 | 5372452 | 1 | 48 | 0 | 184 | 5527124 | 1 | 48 | 0 | 1 | 192 |
| 124 | 7C | 75 | 4B | 1 | 49 | 671580 | 5372636 | 1 | 49 | 0 | 184 | 5527316 | 1 | 49 | 0 | 1 | 192 |
| 125 | 7D | 75 | 4B | 1 | 50 | 671603 | 5372820 | 1 | 50 | 0 | 184 | 5527508 | 1 | 50 | 0 | 1 | 192 |
| 126 | 7E | 75 | 4B | 1 | 51 | 671626 | 5373004 | 1 | 51 | 0 | 184 | 5527700 | 1 | 51 | 0 | 1 | 192 |
| 127 | 7F | 75 | 4B | 1 | 52 | 671649 | 5373188 | 1 | 52 | 0 | 184 | 5527892 | 1 | 52 | 0 | 1 | 192 |
| 128 | 80 | 75 | 4B | 1 | 53 | 671672 | 5373372 | 1 | 53 | 0 | 184 | 5528084 | 1 | 53 | 0 | 1 | 192 |
| 129 | 81 | 75 | 4B | 1 | 54 | 671695 | 5373556 | 1 | 54 | 0 | 184 | 5528276 | 1 | 54 | 0 | 1 | 192 |
| 130 | 82 | 75 | 4B | 1 | 55 | 671718 | 5373740 | 1 | 55 | 0 | 184 | 5528468 | 1 | 55 | 0 | 1 | 192 |
| 131 | 83 | 75 | 4B | 1 | 56 | 671741 | 5373924 | 1 | 56 | 0 | 184 | 5528660 | 1 | 56 | 0 | 1 | 192 |
| 132 | 84 | 75 | 4B | 1 | 57 | 671764 | 5374108 | 1 | 57 | 0 | 184 | 5528852 | 1 | 57 | 0 | 1 | 192 |
| 133 | 85 | 75 | 4B | 1 | 58 | 671787 | 5374292 | 1 | 58 | 0 | 184 | 5529044 | 1 | 58 | 0 | 1 | 192 |
| 134 | 86 | 75 | 4B | 1 | 59 | 671810 | 5374476 | 1 | 59 | 0 | 184 | 5529236 | 1 | 59 | 0 | 1 | 192 |
| 135 | 87 | 75 | 4B | 1 | 60 | 671833 | 5374660 | 1 | 60 | 0 | 184 | 5529428 | 1 | 60 | 0 | 1 | 192 |
| 136 | 88 | 75 | 4B | 1 | 61 | 671856 | 5374844 | 1 | 61 | 0 | 184 | 5529620 | 1 | 61 | 0 | 1 | 192 |
| 137 | 89 | 75 | 4B | 1 | 62 | 671879 | 5375028 | 1 | 62 | 0 | 184 | 5529812 | 1 | 62 | 0 | 1 | 192 |
| 138 | 8A | 75 | 4B | 1 | 63 | 671902 | 5375212 | 1 | 63 | 0 | 184 | 5530004 | 1 | 63 | 0 | 1 | 192 |
| 139 | 8B | 75 | 4B | 1 | 64 | 671925 | 5375396 | 1 | 64 | 0 | 184 | 5530196 | 1 | 64 | 0 | 1 | 192 |
| 140 | 8C | 75 | 4B | 1 | 65 | 671948 | 5375580 | 1 | 65 | 0 | 184 | 5530388 | 1 | 65 | 0 | 1 | 192 |
| 141 | 8D | 75 | 4B | 1 | 66 | 671971 | 5375764 | 1 | 66 | 0 | 184 | 5530580 | 1 | 66 | 0 | 1 | 192 |
| 142 | 8E | 75 | 4B | 1 | 67 | 671994 | 5375948 | 1 | 67 | 0 | 184 | 5530772 | 1 | 67 | 0 | 1 | 192 |
| 143 | 8F | 75 | 4B | 1 | 68 | 672017 | 5376132 | 1 | 68 | 0 | 184 | 5530964 | 1 | 68 | 0 | 1 | 192 |
| 144 | 90 | 75 | 4B | 1 | 69 | 672040 | 5376316 | 1 | 69 | 0 | 184 | 5531156 | 1 | 69 | 0 | 1 | 192 |
| 145 | 91 | 75 | 4B | 1 | 70 | 672063 | 5376500 | 1 | 70 | 0 | 184 | 5531348 | 1 | 70 | 0 | 1 | 192 |
| 146 | 92 | 75 | 4B | 1 | 71 | 672086 | 5376684 | 1 | 71 | 0 | 184 | 5531540 | 1 | 71 | 0 | 1 | 192 |
| 147 | 93 | 75 | 4B | 1 | 72 | 672109 | 5376868 | 1 | 72 | 0 | 184 | 5531732 | 1 | 72 | 0 | 1 | 192 |
| 148 | 94 | 75 | 4B | 1 | 73 | 672132 | 5377052 | 1 | 73 | 0 | 184 | 5531924 | 1 | 73 | 0 | 1 | 192 |
| 149 | 95 | 75 | 4B | 1 | 74 | 672155 | 5377236 | 1 | 74 | 0 | 184 | 5532116 | 1 | 74 | 0 | 1 | 192 |
| 150 | 96 | 75 | 4B | 2 | 0 | 672176 | 5377404 | 2 | 0 | 0 | 168 | 5532292 | 2 | 0 | 0 | 1 | 176 |
| 151 | 97 | 75 | 4B | 2 | 1 | 672202 | 5377612 | 2 | 1 | 0 | 208 | 5532508 | 2 | 1 | 0 | 1 | 216 |
| 152 | 98 | 75 | 4B | 2 | 2 | 672228 | 5377820 | 2 | 2 | 0 | 208 | 5532724 | 2 | 2 | 0 | 1 | 216 |
| 153 | 99 | 75 | 4B | 2 | 3 | 672254 | 5378028 | 2 | 3 | 0 | 208 | 5532940 | 2 | 3 | 0 | 1 | 216 |
| 154 | 9A | 75 | 4B | 2 | 4 | 672280 | 5378236 | 2 | 4 | 0 | 208 | 5533156 | 2 | 4 | 0 | 1 | 216 |
| 155 | 9B | 75 | 4B | 2 | 5 | 672306 | 5378444 | 2 | 5 | 0 | 208 | 5533372 | 2 | 5 | 0 | 1 | 216 |
| 156 | 9C | 75 | 4B | 2 | 6 | 672332 | 5378652 | 2 | 6 | 0 | 208 | 5533588 | 2 | 6 | 0 | 1 | 216 |
| 157 | 9D | 75 | 4B | 2 | 7 | 672358 | 5378860 | 2 | 7 | 0 | 208 | 5533804 | 2 | 7 | 0 | 1 | 216 |
| 158 | 9E | 75 | 4B | 2 | 8 | 672384 | 5379068 | 2 | 8 | 0 | 208 | 5534020 | 2 | 8 | 0 | 1 | 216 |
| 159 | 9F | 75 | 4B | 2 | 9 | 672410 | 5379276 | 2 | 9 | 0 | 208 | 5534236 | 2 | 9 | 0 | 1 | 216 |
| 160 | A0 | 75 | 4B | 2 | 10 | 672433 | 5379460 | 2 | 10 | 0 | 184 | 5534428 | 2 | 10 | 0 | 1 | 192 |

| | | | | | | | | | | | | | | | | | |
|---|---|---|---|---|---|---|---|---|---|---|---|---|---|---|---|---|---|
| 161 | A1 | 75 | 4B | 2 | 11 | 672456 | 5379644 | 2 | 11 | 0 | 184 | 5534620 | 2 | 11 | 0 | 1 | 192 |
| 162 | A2 | 75 | 4B | 2 | 12 | 672479 | 5379828 | 2 | 12 | 0 | 184 | 5534812 | 2 | 12 | 0 | 1 | 192 |
| 163 | A3 | 75 | 4B | 2 | 13 | 672502 | 5380012 | 2 | 13 | 0 | 184 | 5535004 | 2 | 13 | 0 | 1 | 192 |
| 164 | A4 | 75 | 4B | 2 | 14 | 672525 | 5380196 | 2 | 14 | 0 | 184 | 5535196 | 2 | 14 | 0 | 1 | 192 |
| 165 | A5 | 75 | 4B | 2 | 15 | 672548 | 5380380 | 2 | 15 | 0 | 184 | 5535388 | 2 | 15 | 0 | 1 | 192 |
| 166 | A6 | 75 | 4B | 2 | 16 | 672571 | 5380564 | 2 | 16 | 0 | 184 | 5535580 | 2 | 16 | 0 | 1 | 192 |
| 167 | A7 | 75 | 4B | 2 | 17 | 672594 | 5380748 | 2 | 17 | 0 | 184 | 5535772 | 2 | 17 | 0 | 1 | 192 |
| 168 | A8 | 75 | 4B | 2 | 18 | 672617 | 5380932 | 2 | 18 | 0 | 184 | 5535964 | 2 | 18 | 0 | 1 | 192 |
| 169 | A9 | 75 | 4B | 2 | 19 | 672640 | 5381116 | 2 | 19 | 0 | 184 | 5536156 | 2 | 19 | 0 | 1 | 192 |
| 170 | AA | 75 | 4B | 2 | 20 | 672663 | 5381300 | 2 | 20 | 0 | 184 | 5536348 | 2 | 20 | 0 | 1 | 192 |
| 171 | AB | 75 | 4B | 2 | 21 | 672686 | 5381484 | 2 | 21 | 0 | 184 | 5536540 | 2 | 21 | 0 | 1 | 192 |
| 172 | AC | 75 | 4B | 2 | 22 | 672709 | 5381668 | 2 | 22 | 0 | 184 | 5536732 | 2 | 22 | 0 | 1 | 192 |
| 173 | AD | 75 | 4B | 2 | 23 | 672732 | 5381852 | 2 | 23 | 0 | 184 | 5536924 | 2 | 23 | 0 | 1 | 192 |
| 174 | AE | 75 | 4B | 2 | 24 | 672755 | 5382036 | 2 | 24 | 0 | 184 | 5537116 | 2 | 24 | 0 | 1 | 192 |
| 175 | AF | 75 | 4B | 2 | 25 | 672778 | 5382220 | 2 | 25 | 0 | 184 | 5537308 | 2 | 25 | 0 | 1 | 192 |
| 176 | B0 | 75 | 4B | 2 | 26 | 672803 | 5382420 | 2 | 26 | 0 | 200 | 5537516 | 2 | 26 | 0 | 1 | 208 |
| 177 | B1 | 75 | 4B | 2 | 27 | 672828 | 5382620 | 2 | 27 | 0 | 200 | 5537724 | 2 | 27 | 0 | 1 | 208 |
| 178 | B2 | 75 | 4B | 2 | 28 | 672853 | 5382820 | 2 | 28 | 0 | 200 | 5537932 | 2 | 28 | 0 | 1 | 208 |
| 179 | B3 | 75 | 4B | 2 | 29 | 672878 | 5383020 | 2 | 29 | 0 | 200 | 5538140 | 2 | 29 | 0 | 1 | 208 |
| 180 | B4 | 75 | 4B | 2 | 30 | 672903 | 5383220 | 2 | 30 | 0 | 200 | 5538348 | 2 | 30 | 0 | 1 | 208 |
| 181 | B5 | 75 | 4B | 2 | 31 | 672928 | 5383420 | 2 | 31 | 0 | 200 | 5538556 | 2 | 31 | 0 | 1 | 208 |
| 182 | B6 | 75 | 4B | 2 | 32 | 672953 | 5383620 | 2 | 32 | 0 | 200 | 5538764 | 2 | 32 | 0 | 1 | 208 |
| 183 | B7 | 75 | 4B | 2 | 33 | 672978 | 5383820 | 2 | 33 | 0 | 200 | 5538972 | 2 | 33 | 0 | 1 | 208 |
| 184 | B8 | 75 | 4B | 2 | 34 | 673003 | 5384020 | 2 | 34 | 0 | 200 | 5539180 | 2 | 34 | 0 | 1 | 208 |
| 185 | B9 | 75 | 4B | 2 | 35 | 673028 | 5384220 | 2 | 35 | 0 | 200 | 5539388 | 2 | 35 | 0 | 1 | 208 |
| 186 | BA | 75 | 4B | 2 | 36 | 673053 | 5384420 | 2 | 36 | 0 | 200 | 5539596 | 2 | 36 | 0 | 1 | 208 |
| 187 | BB | 75 | 4B | 2 | 37 | 673078 | 5384620 | 2 | 37 | 0 | 200 | 5539804 | 2 | 37 | 0 | 1 | 208 |
| 188 | BC | 75 | 4B | 2 | 38 | 673103 | 5384820 | 2 | 38 | 0 | 200 | 5540012 | 2 | 38 | 0 | 1 | 208 |
| 189 | BD | 75 | 4B | 2 | 39 | 673128 | 5385020 | 2 | 39 | 0 | 200 | 5540220 | 2 | 39 | 0 | 1 | 208 |
| 190 | BE | 75 | 4B | 2 | 40 | 673153 | 5385220 | 2 | 40 | 0 | 200 | 5540428 | 2 | 40 | 0 | 1 | 208 |
| 191 | BF | 75 | 4B | 2 | 41 | 673178 | 5385420 | 2 | 41 | 0 | 200 | 5540636 | 2 | 41 | 0 | 1 | 208 |
| 192 | C0 | 75 | 4B | 2 | 42 | 673203 | 5385620 | 2 | 42 | 0 | 200 | 5540844 | 2 | 42 | 0 | 1 | 208 |
| 193 | C1 | 75 | 4B | 2 | 43 | 673228 | 5385820 | 2 | 43 | 0 | 200 | 5541052 | 2 | 43 | 0 | 1 | 208 |
| 194 | C2 | 75 | 4B | 2 | 44 | 673253 | 5386020 | 2 | 44 | 0 | 200 | 5541260 | 2 | 44 | 0 | 1 | 208 |
| 195 | C3 | 75 | 4B | 2 | 45 | 673278 | 5386220 | 2 | 45 | 0 | 200 | 5541468 | 2 | 45 | 0 | 1 | 208 |
| 196 | C4 | 75 | 4B | 2 | 46 | 673303 | 5386420 | 2 | 46 | 0 | 200 | 5541676 | 2 | 46 | 0 | 1 | 208 |
| 197 | C5 | 75 | 4B | 2 | 47 | 673328 | 5386620 | 2 | 47 | 0 | 200 | 5541884 | 2 | 47 | 0 | 1 | 208 |
| 198 | C6 | 75 | 4B | 2 | 48 | 673353 | 5386820 | 2 | 48 | 0 | 200 | 5542092 | 2 | 48 | 0 | 1 | 208 |
| 199 | C7 | 75 | 4B | 2 | 49 | 673378 | 5387020 | 2 | 49 | 0 | 200 | 5542300 | 2 | 49 | 0 | 1 | 208 |
| 200 | C8 | 75 | 4B | 2 | 50 | 673403 | 5387220 | 2 | 50 | 0 | 200 | 5542508 | 2 | 50 | 0 | 1 | 208 |
| 201 | C9 | 75 | 4B | 2 | 51 | 673428 | 5387420 | 2 | 51 | 0 | 200 | 5542716 | 2 | 51 | 0 | 1 | 208 |
| 202 | CA | 75 | 4B | 2 | 52 | 673453 | 5387620 | 2 | 52 | 0 | 200 | 5542924 | 2 | 52 | 0 | 1 | 208 |
| 203 | CB | 75 | 4B | 2 | 53 | 673478 | 5387820 | 2 | 53 | 0 | 200 | 5543132 | 2 | 53 | 0 | 1 | 208 |
| 204 | CC | 75 | 4B | 2 | 54 | 673503 | 5388020 | 2 | 54 | 0 | 200 | 5543340 | 2 | 54 | 0 | 1 | 208 |
| 205 | CD | 75 | 4B | 2 | 55 | 673528 | 5388220 | 2 | 55 | 0 | 200 | 5543548 | 2 | 55 | 0 | 1 | 208 |
| 206 | CE | 75 | 4B | 2 | 56 | 673553 | 5388420 | 2 | 56 | 0 | 200 | 5543756 | 2 | 56 | 0 | 1 | 208 |
| 207 | CF | 75 | 4B | 2 | 57 | 673578 | 5388620 | 2 | 57 | 0 | 200 | 5543964 | 2 | 57 | 0 | 1 | 208 |
| 208 | D0 | 75 | 4B | 2 | 58 | 673603 | 5388820 | 2 | 58 | 0 | 200 | 5544172 | 2 | 58 | 0 | 1 | 208 |
| 209 | D1 | 75 | 4B | 2 | 59 | 673628 | 5389020 | 2 | 59 | 0 | 200 | 5544380 | 2 | 59 | 0 | 1 | 208 |
| 210 | D2 | 75 | 4B | 2 | 60 | 673653 | 5389220 | 2 | 60 | 0 | 200 | 5544588 | 2 | 60 | 0 | 1 | 208 |
| 211 | D3 | 75 | 4B | 2 | 61 | 673678 | 5389420 | 2 | 61 | 0 | 200 | 5544796 | 2 | 61 | 0 | 1 | 208 |
| 212 | D4 | 75 | 4B | 2 | 62 | 673703 | 5389620 | 2 | 62 | 0 | 200 | 5545004 | 2 | 62 | 0 | 1 | 208 |
| 213 | D5 | 75 | 4B | 2 | 63 | 673728 | 5389820 | 2 | 63 | 0 | 200 | 5545212 | 2 | 63 | 0 | 1 | 208 |
| 214 | D6 | 75 | 4B | 2 | 64 | 673753 | 5390020 | 2 | 64 | 0 | 200 | 5545420 | 2 | 64 | 0 | 1 | 208 |
| 215 | D7 | 75 | 4B | 2 | 65 | 673778 | 5390220 | 2 | 65 | 0 | 200 | 5545628 | 2 | 65 | 0 | 1 | 208 |
| 216 | D8 | 75 | 4B | 2 | 66 | 673803 | 5390420 | 2 | 66 | 0 | 200 | 5545836 | 2 | 66 | 0 | 1 | 208 |
| 217 | D9 | 75 | 4B | 2 | 67 | 673828 | 5390620 | 2 | 67 | 0 | 200 | 5546044 | 2 | 67 | 0 | 1 | 208 |
| 218 | DA | 75 | 4B | 2 | 68 | 673853 | 5390820 | 2 | 68 | 0 | 200 | 5546252 | 2 | 68 | 0 | 1 | 208 |
| 219 | DB | 75 | 4B | 2 | 69 | 673878 | 5391020 | 2 | 69 | 0 | 200 | 5546460 | 2 | 69 | 0 | 1 | 208 |
| 220 | DC | 75 | 4B | 2 | 70 | 673903 | 5391220 | 2 | 70 | 0 | 200 | 5546668 | 2 | 70 | 0 | 1 | 208 |
| 221 | DD | 75 | 4B | 2 | 71 | 673928 | 5391420 | 2 | 71 | 0 | 200 | 5546876 | 2 | 71 | 0 | 1 | 208 |
| 222 | DE | 75 | 4B | 2 | 72 | 673953 | 5391620 | 2 | 72 | 0 | 200 | 5547084 | 2 | 72 | 0 | 1 | 208 |
| 223 | DF | 75 | 4B | 2 | 73 | 673978 | 5391820 | 2 | 73 | 0 | 200 | 5547292 | 2 | 73 | 0 | 1 | 208 |
| 224 | E0 | 75 | 4B | 2 | 74 | 674003 | 5392020 | 2 | 74 | 0 | 200 | 5547500 | 2 | 74 | 0 | 1 | 208 |
| 225 | E1 | 75 | 4B | 3 | 0 | 674026 | 5392204 | 3 | 0 | 0 | 184 | 5547692 | 3 | 0 | 0 | 1 | 192 |
| 226 | E2 | 75 | 4B | 3 | 1 | 674054 | 5392428 | 3 | 1 | 0 | 224 | 5547924 | 3 | 1 | 0 | 1 | 232 |
| 227 | E3 | 75 | 4B | 3 | 2 | 674082 | 5392652 | 3 | 2 | 0 | 224 | 5548156 | 3 | 2 | 0 | 1 | 232 |
| 228 | E4 | 75 | 4B | 3 | 3 | 674110 | 5392876 | 3 | 3 | 0 | 224 | 5548388 | 3 | 3 | 0 | 1 | 232 |
| 229 | E5 | 75 | 4B | 3 | 4 | 674138 | 5393100 | 3 | 4 | 0 | 224 | 5548620 | 3 | 4 | 0 | 1 | 232 |
| 230 | E6 | 75 | 4B | 3 | 5 | 674166 | 5393324 | 3 | 5 | 0 | 224 | 5548852 | 3 | 5 | 0 | 1 | 232 |
| 231 | E7 | 75 | 4B | 3 | 6 | 674194 | 5393548 | 3 | 6 | 0 | 224 | 5549084 | 3 | 6 | 0 | 1 | 232 |
| 232 | E8 | 75 | 4B | 3 | 7 | 674222 | 5393772 | 3 | 7 | 0 | 224 | 5549316 | 3 | 7 | 0 | 1 | 232 |
| 233 | E9 | 75 | 4B | 3 | 8 | 674250 | 5393996 | 3 | 8 | 0 | 224 | 5549548 | 3 | 8 | 0 | 1 | 232 |
| 234 | EA | 75 | 4B | 3 | 9 | 674278 | 5394220 | 3 | 9 | 0 | 224 | 5549780 | 3 | 9 | 0 | 1 | 232 |
| 235 | EB | 75 | 4B | 3 | 10 | 674306 | 5394444 | 3 | 10 | 0 | 224 | 5550012 | 3 | 10 | 0 | 1 | 232 |
| 236 | EC | 75 | 4B | 3 | 11 | 674334 | 5394668 | 3 | 11 | 0 | 224 | 5550244 | 3 | 11 | 0 | 1 | 232 |
| 237 | ED | 75 | 4B | 3 | 12 | 674362 | 5394892 | 3 | 12 | 0 | 224 | 5550476 | 3 | 12 | 0 | 1 | 232 |
| 238 | EE | 75 | 4B | 3 | 13 | 674390 | 5395116 | 3 | 13 | 0 | 224 | 5550708 | 3 | 13 | 0 | 1 | 232 |
| 239 | EF | 75 | 4B | 3 | 14 | 674418 | 5395340 | 3 | 14 | 0 | 224 | 5550940 | 3 | 14 | 0 | 1 | 232 |
| 240 | F0 | 75 | 4B | 3 | 15 | 674443 | 5395540 | 3 | 15 | 0 | 200 | 5551148 | 3 | 15 | 0 | 1 | 208 |
| 241 | F1 | 75 | 4B | 3 | 16 | 674468 | 5395740 | 3 | 16 | 0 | 200 | 5551356 | 3 | 16 | 0 | 1 | 208 |
| 242 | F2 | 75 | 4B | 3 | 17 | 674493 | 5395940 | 3 | 17 | 0 | 200 | 5551564 | 3 | 17 | 0 | 1 | 208 |
| 243 | F3 | 75 | 4B | 3 | 18 | 674518 | 5396140 | 3 | 18 | 0 | 200 | 5551772 | 3 | 18 | 0 | 1 | 208 |
| 244 | F4 | 75 | 4B | 3 | 19 | 674543 | 5396340 | 3 | 19 | 0 | 200 | 5551980 | 3 | 19 | 0 | 1 | 208 |
| 245 | F5 | 75 | 4B | 3 | 20 | 674568 | 5396540 | 3 | 20 | 0 | 200 | 5552188 | 3 | 20 | 0 | 1 | 208 |
| 246 | F6 | 75 | 4B | 3 | 21 | 674593 | 5396740 | 3 | 21 | 0 | 200 | 5552396 | 3 | 21 | 0 | 1 | 208 |
| 247 | F7 | 75 | 4B | 3 | 22 | 674618 | 5396940 | 3 | 22 | 0 | 200 | 5552604 | 3 | 22 | 0 | 1 | 208 |
| 248 | F8 | 75 | 4B | 3 | 23 | 674643 | 5397140 | 3 | 23 | 0 | 200 | 5552812 | 3 | 23 | 0 | 1 | 208 |
| 249 | F9 | 75 | 4B | 3 | 24 | 674668 | 5397340 | 3 | 24 | 0 | 200 | 5553020 | 3 | 24 | 0 | 1 | 208 |
| 250 | FA | 75 | 4B | 3 | 25 | 674693 | 5397540 | 3 | 25 | 0 | 200 | 5553228 | 3 | 25 | 0 | 1 | 208 |
| 251 | FB | 75 | 4B | 3 | 26 | 674718 | 5397740 | 3 | 26 | 0 | 200 | 5553436 | 3 | 26 | 0 | 1 | 208 |
| 252 | FC | 75 | 4B | 3 | 27 | 674743 | 5397940 | 3 | 27 | 0 | 200 | 5553644 | 3 | 27 | 0 | 1 | 208 |
| 253 | FD | 75 | 4B | 3 | 28 | 674768 | 5398140 | 3 | 28 | 0 | 200 | 5553852 | 3 | 28 | 0 | 1 | 208 |
| 254 | FE | 75 | 4B | 3 | 29 | 674793 | 5398340 | 3 | 29 | 0 | 200 | 5554060 | 3 | 29 | 0 | 1 | 208 |
| 255 | FF | 75 | 4B | 3 | 30 | 674818 | 5398540 | 3 | 30 | 0 | 200 | 5554268 | 3 | 30 | 0 | 1 | 208 |
| 0 | 00 | 76 | 4C | 0 | 0 | 674830 | 5398636 | 0 | 0 | 0 | 96 | 5554372 | 0 | 0 | 0 | 1 | 104 |
| 1 | 01 | 76 | 4C | 0 | 1 | 674844 | 5398748 | 0 | 1 | 0 | 112 | 5554492 | 0 | 1 | 0 | 1 | 120 |
| 2 | 02 | 76 | 4C | 0 | 2 | 674858 | 5398860 | 0 | 2 | 0 | 112 | 5554612 | 0 | 2 | 0 | 1 | 120 |
| 3 | 03 | 76 | 4C | 0 | 3 | 674872 | 5398972 | 0 | 3 | 0 | 112 | 5554732 | 0 | 3 | 0 | 1 | 120 |
| 4 | 04 | 76 | 4C | 0 | 4 | 674886 | 5399084 | 0 | 4 | 0 | 112 | 5554852 | 0 | 4 | 0 | 1 | 120 |
| 5 | 05 | 76 | 4C | 0 | 5 | 674900 | 5399196 | 0 | 5 | 0 | 112 | 5554972 | 0 | 5 | 0 | 1 | 120 |
| 6 | 06 | 76 | 4C | 0 | 6 | 674914 | 5399308 | 0 | 6 | 0 | 112 | 5555092 | 0 | 6 | 0 | 1 | 120 |
| 7 | 07 | 76 | 4C | 0 | 7 | 674928 | 5399420 | 0 | 7 | 0 | 112 | 5555212 | 0 | 7 | 0 | 1 | 120 |
[truncated: 4,902,288 more chars]
